# Sentiment Analysis of Conservation Studies Captures Successes of Species Reintroductions

**DOI:** 10.1016/j.patter.2020.100005

**Published:** 2020-03-20

**Authors:** Kyle S. Van Houtan, Tyler Gagne, Clinton N. Jenkins, Lucas Joppa

**Affiliations:** 1 Monterey Bay Aquarium, 886 Cannery Row, Monterey, CA 93940, USA; 2 Nicholas School of the Environment, Duke University, Box 90328, Durham, NC 27708, USA; 3 IPÊ - Instituto de Pesquisas Ecológicas, Rod. Dom Pedro I, km 47, Caixa Postal 47, Nazaré Paulista, São Paulo 12960-000, Brazil; 4 Microsoft, One Microsoft Way, Redmond, WA 98075, USA

**Keywords:** natural language processing, deep learning, effectiveness, evidence-based conservation, conservation biology, reintroduction, endangered species, sentiment analysis

## Abstract

Learning from the rapidly growing body of scientific articles is constrained by human bandwidth. Existing methods in machine learning have been developed to extract knowledge from human language and may automate this process. Here, we apply sentiment analysis, a type of natural language processing, to facilitate a literature review in reintroduction biology. We analyzed 1,030,558 words from 4,313 scientific abstracts published over four decades using four previously trained lexicon-based models and one recursive neural tensor network model. We find frequently used terms share both a general and a domain-specific value, with either positive (success, protect, growth) or negative (threaten, loss, risk) sentiment. Sentiment trends suggest that reintroduction studies have become less variable and increasingly successful over time and seem to capture known successes and challenges for conservation biology. This approach offers promise for rapidly extracting explicit and latent information from a large corpus of scientific texts.

## Highlights

• The volume of science is rapidly growing, challenging learning within the community
• Machine learning can automate the process of extracting data from human language
• We applied five natural language processing models to over 4,300 scientific abstracts
• The approach captured broad trends and known lessons, offering future promise

## The Bigger Picture

The volume of peer-reviewed published science is increasingly growing, presenting new opportunities for growth in research on research itself, also known as meta-analysis. Such research operates by (1) acquiring a body of scientific texts from public archives, (2) extracting the desired information from the texts, and (3) performing analyses on the extracted data. While such analyses hold great value, they may require substantial resources and manual effort throughout the project pipeline. Here, we detail how much of the process of scientific meta-analysis may be automated using a type of machine learning known as natural language processing (NLP). We apply this technique to a specific problem in environmental conservation, show how off-the-shelf NLP models perform, and offer recommendations for future improvements to the process. Such investments may be critical for advances from research, perhaps especially to ensure that scientific productivity meets practical progress.

## Graphical Abstract

**Figure:**
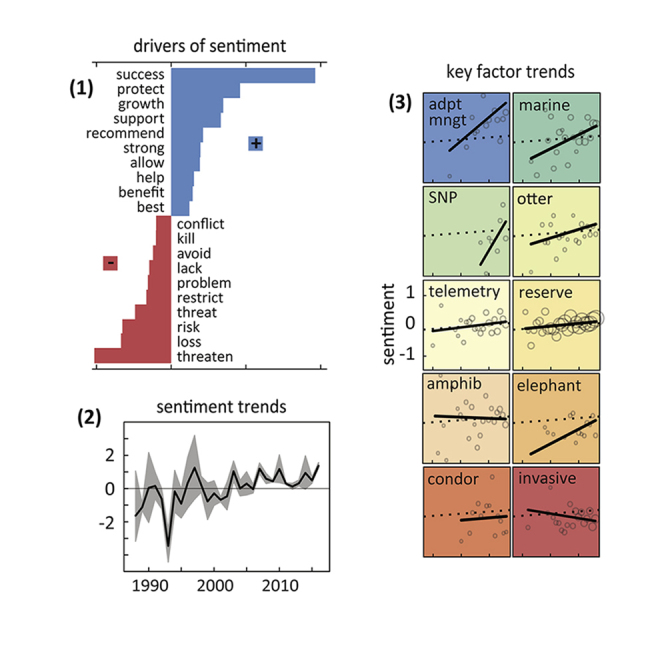

## Teaser

Science and technology are increasingly integrated into our everyday lives. A key aspect of science is that the community learns through verified, published findings. Online archives and publications have vastly increased the volume of published science, affording greater access to research results while also presenting new challenges. This study uses established methods in artificial intelligence to assess whether reading scientific papers can be automated. The results are promising, although technical disciplines with specific vocabulary will require special considerations.

## Introduction

The sheer volume of scientific literature challenges the goal of capturing knowledge from the published body of peer-reviewed science. A recent review^1^ of species reintroductions, for example, manually extracted information from 361 published articles. While this was admittedly a small fraction of the total literature on the topic, it still required months of effort from highly trained experts just to obtain the raw data, which they then had to analyze. However, because population reintroductions are an effective means to accomplish an elusive task—to recover species and restore ecosystems^2^, ^3^, ^4^—understanding what determines their success or failure is considered broadly important (S.L. Becker, T.E. Nicholson, K.A. Mayer, M.J. Murray, and K.S.V.H., unpublished data).^5^ Therefore, such narrated lessons from established evidence are critical for conservation practices and management decisions.^1^^,^^6^ How can we lower the barrier to learning them?

Natural language processing (NLP) is a branch of artificial intelligence, or machine learning, which analyzes strings of human language to extract usable information. One goal of NLP is to automate the processing of large volumes of text with minimal human supervision,^7^^,^^8^ yet crucially in a manner that approximates the performance of a human reader. As applied here, sentiment analysis (SA) parses different affective states of sentiment to capture either single or combined emotions, attitudes, or traits.^9^^,^^10^ While an array of methods exists, the basic principle of SA is to use a trained set of text that has previously been attributed with a sentiment score to define the sentiment for a separate body of unlabeled text. Although SA has been developed to describe more complex and sophisticated human emotions (e.g., empathy, greed, trust, and fear), such results are, perhaps expectedly, variable.^11^ By comparison, a more simplistic score of negative to positive sentiment, in the form of a weighted polarity, is far more robust in capturing basic attitudes across various types of texts and fields of study.^11^^,^^12^ Aside from labeling raw texts, the lexicon-based and machine learning-based SA models have additional value as they are resilient to various structures of text strings (e.g., letter case, punctuation, and stop words) and require little to no text preprocessing. Although NLP is a dynamic and rapidly advancing research area with extensive scientific and commercial applications, and there are more sophisticated approaches to NLP than SA,^13^, ^14^, ^15^, ^16^, ^17^ the SA approach we deploy here can produce straightforward and robust results with broad and intuitive interpretative value.

In this study, we explore the use of supervised SA to facilitate a new meta-analysis of the species reintroduction literature with a goal of understanding effectiveness and identifying what determines success. We query public databases to build a robust corpus, numbering in the thousands of scientific abstracts and use existing or “off-the-shelf” lexicons and NLP models to identify the terms that drive sentiment and the domain concepts associated with success. This basic yet novel application shows the potential to enable a more rapid understanding of the growing volume of scientific literature. Such research on research, or meta-analyses, can produce results important for research journal practices^18^ as well as the topical domain itself.^19^

## Results and Discussion

**Figure 1:**
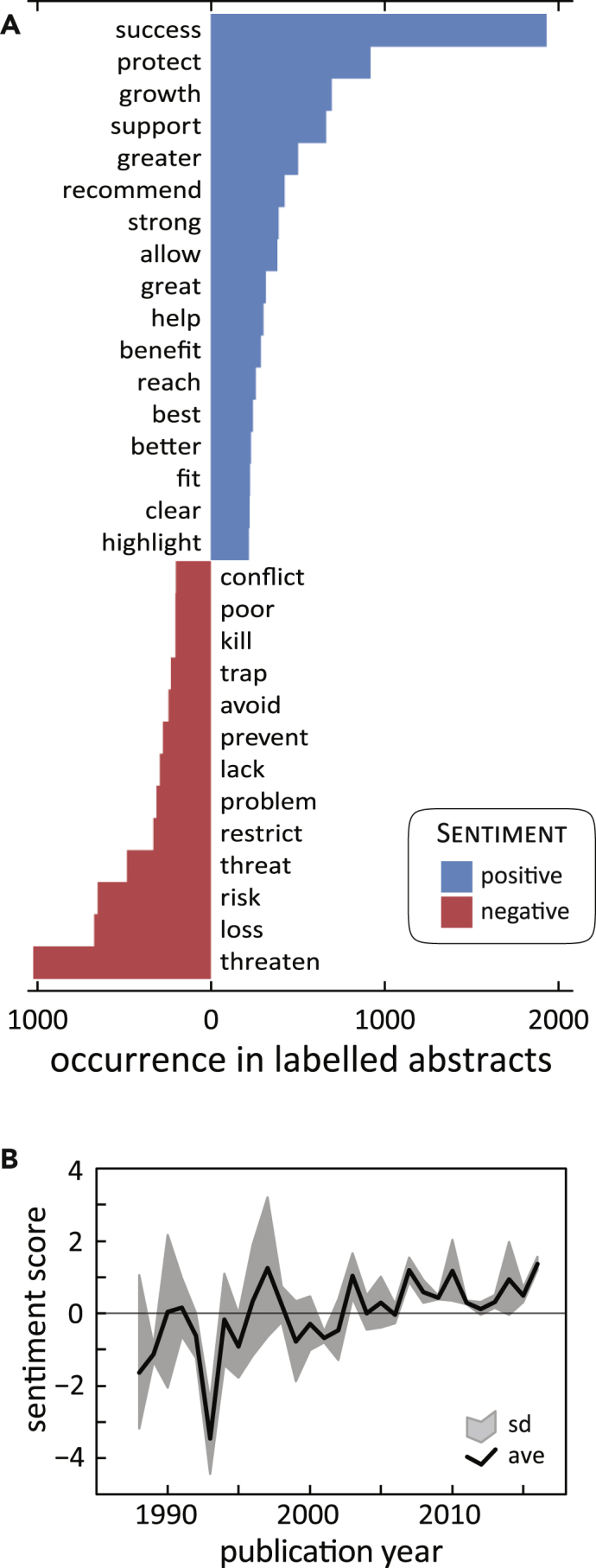
The Estimated Sentiment of Reintroduction Abstracts Is Associated with Meaningful Terms (A) The most frequent terms occurring in 4,313 labeled scientific abstracts, associated with either positive (blue) or negative (red) sentiment as determined from the AFINN lexicon. “Success” drives positive sentiment, and words challenging reintroduction successes (e.g., “threaten,” “loss,” and “risk,” among others) occur with negative sentiment. (B) Ensemble (black line) of five independent models, pooled annually, of annual sentiment shows a robust increase in sentiment with an accompanying decrease in uncertainty (gray confidence interval) over three decades. This indicates that words and sentences associated with positive sentiment are increasing over time, which, given (A), suggests that reintroduction science is achieving positive results and becoming more successful over the last 30 years. ave, ensemble model average; sd, standard deviation.

Since we are applying language models created for general use to extract information specifically from scientific abstracts, it is important to evaluate how the terms contained in our abstracts correlate with sentiment scores. Figure 1A shows the contribution of frequent terms from our reintroduction abstracts to sentiment scores using the AFINN lexicon.^20^ The most common terms associated with positive sentiment are success, protect, growth, support, help, benefit, and others. The most negative influences are from threaten, loss, risk, threat, problem, and kill. As the term success drives sentiment more than any other term (either positively or negatively), and as this list seems to capture terms that genuinely reflect how authors communicate successes and failures of population metrics as well as reintroduction programs,^2^ the abstract-level sentiment score serves as a rational proxy to capture the lessons learned from the studies. Figures S5 and S6 provide both sentence- and abstract-level sentiment scores from a range of abstracts highlighting a range of polarity values across the collected corpus.

Figure 1B summarizes the annual multi-model ensemble of sentiment of reintroduction abstracts over three decades. Though there is some variation between the five models (see Results and Discussion in Figure S4), the models converge on a general trend of decreasing variation and increasingly positive sentiment through time. To this point, uniquely over the study period, the ensemble, including the confidence interval, is positive from 2007 to 2016, encapsulating the final 10 years of the study. Taken with Figure 1A, this suggests that reintroduction studies have become more positively framed, having emerged from an earlier period of negative sentiment and significant challenges when the methods and the discipline itself were just beginning.

**Figure 2:**
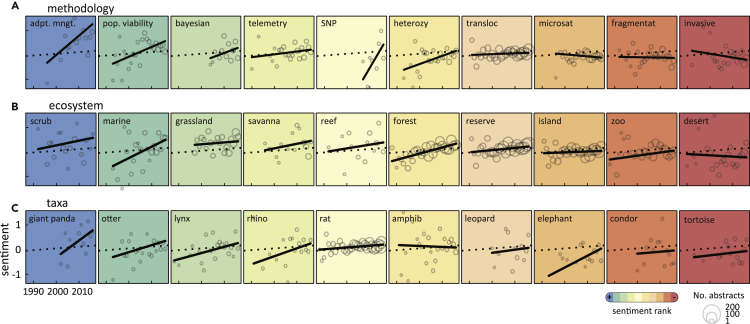
Sentiment Analysis Captures the Known Successes and Failures of Key Reintroduction Factors Sentiment of scientific abstracts associated with reintroduction (A) methods, (B) ecosystems, and (C) taxonomic groups. Panels describe a linear model (black line) of sentiment from the annual sample of abstracts (open circles) for each extracted term. Panels are color coded and ranked left to right by the final model value. The linear model (dotted line) for all abstracts throughout the study is a baseline reference in all panels. Studies using adaptive management or population viability analyses, studies set in scrub, marine, and grassland ecosystems, and studies reintroducing giant panda, otters, and lynx have increasingly positive sentiment. By contrast, our models suggest that reintroduction studies dealing with habitat fragmentation and invasive species, set in islands or deserts, or reintroducing condor or tortoises seem to have persistent challenges.

Our results seem to capture the terms and broad trends of successful reintroductions;^1^^,^^3^^,^^21^ whether it captures the success associated with specific settings, however, is of interest. Figure 2A shows the frequency and sentiment trends of ten issues in reintroductions, ranked by their final modeled sentiment score. Abstracts containing terms that reference methods known to be effective, such as adaptive management and population viability analysis,^2^^,^^3^^,^^22^ have increasing sentiment throughout the study period. More recently deployed yet successful methods, such as Bayesian statistics and SNPs (single-nucleotide polymorphisms), also unsurprisingly demonstrate positive sentiment.^23^^,^^24^ Conversely, reintroduction studies dealing with fragmented populations^25^^,^^26^ and invasive species^2^^,^^27^^,^^28^ have negative sentiment, indicating that barriers to success are likely impactful.

Figures 2B and 2C repeat this approach but for ecosystem and taxa contexts. Although many studies address rat depredation and island environments, neither set shows a clear signal of success, likely owing to the many conservation challenges associated with both.^2^^,^^29^ Likewise, the management of small populations (such as with condors and tortoises) are challenging^30^, ^31^, ^32^ while other heavily studied species with improving conservation status (such as giant pandas and river otters)^29^^,^^33^^,^^34^ have increasing sentiment. Although there is variability between years, reintroduction studies in scrub, grassland, and savanna habitat, in the ocean and in coral reefs, and in forests reflect a positive sentiment that is either stable or increasing through the study. By contrast, studies involving zoo collections have persistently negative sentiment. This result may simply reflect that zoo-based captive breeding programs are often supporting species that are critically endangered in the wild^2^^,^^35^^,^^36^ and face extreme challenges. Lastly, commonly applied techniques such as telemetry^37^^,^^38^ and translocation may not depart from the overall trend simply because they are so widespread, and thus their trend dominates the overall corpus.

### Conclusions

While text-mining algorithms are broadly applied in a range of powerful applications,^7^^,^^8^ they have yet to find a regular use in conservation science. Here, we developed a novel use of NLP by using SA to review and extract knowledge from a large body of scientific literature on population reintroductions. Our approach provided several lessons and recommendations. To begin, although the underlying models are trained on words and sentences from a general domain,^20^^,^^39^^,^^40^ our application here captures the specific terms (Figure 1A) as well as the broad success and failures associated with specific management settings (Figure 2) for conservation biology more generally and reintroductions more specifically.

Next, this application is a modest proof of concept toward automating meta-analyses of the scientific literature. Future analyses will achieve greater success by using lexicons and other NLP models that are trained for the specific domain, that compare word- versus sentence-level methods of scoring, and when possible are benchmarked and validated against specific empirical metrics of interest (see Supplemental Information). Such investments may be particularly important for recursive neural tensor network (RNN) models that incorporate syntax on top of word significance, as syntax (more so than individual words) may transfer less easily from a general to an applied domain (see Figure S4). Therefore, while the significance of individual words (see Figure 1A) may retain significance across applications and word-based lexicons may find transdisciplinary applications apt, RNN models may more require domain-based treatments.

These improvements need to be weighed against the logistical cost of model training and validation, but the proposed value of automation and generation of latent information and novel syntheses^41^ is well known from other scientific disciplines. For reintroduction biology, for example, such approaches may pinpoint whether polarity from SA models correlates with quantitative data on reintroduction successes reported within the studies themselves, or more reflects the overall conservation status of the population in question. However, popular metrics within information theory, such as TF∗IDF scores,^42^ though useful in locating clusters of uncommon terms and key phrases, may have limitations for the purposes of training domain-specific lexicons for SA (see Figure S3).

Lastly, aside from innovating the analytical methods, greater access and data sharing from publishing groups and popular indexing services^43^ will provide significant advances. Here, we manually accessed and extracted abstracts from a single repository of published literature. Although this presented a substantial body of information, future analyses will be improved by the development of public application programming interfaces and automated and open access to entire journal articles, not simply the condensed abstracts. Such advances will facilitate the intended greater good of science^1^^,^^6^ by making more scientific articles, and more of each article, freely available to the public, which may advance future analyses through facilitating increased meta-analyses.

## Experimental Procedures

We developed our corpus by searching the Web of Science indexing service, a representative database^43^ of the peer-reviewed scientific literature. We used the Boolean operators AND, OR to retrieve studies containing all of the terms species, conservation, population, and either reintroduction or translocation in their abstracts. As translocation also indicates chromosomal transfer, we used the NOT operator to remove studies with the terms protein, yeast, *Arabidopsis*, *Drosophila*, *Saccharomyces*, and *Escherichia* most associated with the non-target term use and thus generating false positives. We removed studies lacking abstracts, and excluded studies without English abstracts.^44^ This resulted in a corpus of 4,313 studies (Figure S1) published from 1987 to 2016 with searchable abstracts containing 1,030,558 words.

From this body of text, we estimated abstract-level sentiment with an ensemble model. We built sentiment scores from four lexicon-based models (AFINN, Bing, NRC, and Syuzhet) and one trained RNN model, the Stanford coreNLP.^45^ The lexicons classify sentiment in text strings from the accumulation of sentiment scores of individual words, which each lexicon labels via some form of crowdsourcing. The RNN model is also derived from labeled training text (in this case over 10,000 scored film reviews) and models sentiment not of each individual word but rather by considering the syntax at the sentence level. We used the R packages Syuzhet, coreNLP, and NLP to generate a polar score (negative to positive) of mean sentiment for each abstract for each model. From these single polarity scores of each model for each abstract, we derived a multi-model ensemble average. We employ this approach, as NLP^46^ and climate studies^47^^,^^48^ show that such ensembles are reliable and perform better than individual models alone. We, however, also calculate the standard deviation between model outputs to inform on the uncertainty of the ensemble. Published studies^9^^,^^20^^,^^39^^,^^40^^,^^45^ provide more information on the individual lexicons, classification methods, crowdsourcing, and R packages. An additional Supplemental Information file with annotated code (R markdown at https://osf.io/f4dc7/) also provides further details.

Now possessing a corpus of domain-specific abstracts with modeled sentiment, we performed a series of analyses to explore the patterns associated with positive and negative sentiment. We first plot the broad sentiment trends over time and then extract the terms most associated with sentiment polarity. This is a key step, as we are transferring lexicons trained from a more general domain in order to understand a particular domain—this scientific corpus. As a result, combining term frequency with sentiment polarity will reveal sentiment drivers relative to the previously unlabeled corpus, and perhaps the new domain more broadly. To understand the various factors broadly associated with successes and failures of reintroduction programs, our core aim in this study, we curated a list of methods, ecosystems, and taxonomic groupings. We extracted the abstracts containing these terms, averaged their sentiment scores in each calendar year, plotted the results across the duration of the study, and fitted simple trend models to the series. All analyses and visualizations were generated in the R environment.^49^

## Ethics Statement

Our study did not require human or animal subjects.

## Data and Code Availability

All the raw data used in this project are available at the Web of Science in a third-party project repository (https://osf.io/f4dc7/), at Github (https://bit.ly/2S6dahD), or included in Supplemental Information.

## Supplemental Information

Document S1. Supplemental Experimental Procedures and Figures S1–S6

Data S1. Raw Data File of the Text Corpus This data file provides the raw information obtained from the scientific abstract indexing service, Web of Science. We add an abstract number (ARTICLE), line identification number (ID), and publication year (YEAR). Here we also retain many of the field codes (WoS_CODE) from the native database and the contents of that field (WoS_FIELD). A full list of these fields, tags, and explanation can be found at https://bit.ly/2N88xAx. Although we retain all fields reported by the database, here we provide the primary tags of interest. “PT” is the publication type (J = Journal, B = Book), “AU” is the author list, “TI” is the work's title, “SO” is the publication name or source, “PY” is the publication year, and “AB” is the full abstract.

Data S2. Raw File of the Model Results This file provides the raw sentiment scores for each article (ARTICLE#) from each model (AFINN, BING, NRC, coreNLP, SYUZHET). This also provides the multi-model average scores (ENSEMBLE). The abstracts are listed in the order of publication year (YEAR_PUB), and the full abstract text (ABSTRACT) is provided.

Data S3. Sentence- and Abstract-Level Sentiment Scores for Each Abstract Analyzed in This Study All sentiment scores here are determined from the Syuzhet lexicon. The abstract-level score matches the Syuzhet model score for each abstract in Data S2. Light-green sentences are encoded positive, pink sentences are negative.

Document S2. Article plus Supplemental Information
