# Supplementary material for: Sentiment Analysis of Conservation Studies Captures Successes of Species Reintroductions
Source: Patterns (N Y). 2020 Mar 20;1(1):100005. doi: 10.1016/j.patter.2020.100005 (PMC7660424; doi:10.1016/j.patter.2020.100005)
Supplement: Data S3. Sentence- and Abstract-Level Sentiment Scores for Each Abstract Analyzed in This Study — All sentiment scores here are determined from the Syuzhet lexicon. The abstract-level score matches the Syuzhet model score for each abstract in Data S2. Light-green sentences are encoded positive, pink sentences are negative. [file mmc4.pdf]

99: *-.106*

In the framework of a project to reintroduce the European lynx, the criteria for the reintroduction of locally extinct animal species, the historical and current distribution of the lynx, its ecological requirements and population genetic aspects of a reintroduction were evaluated. The supply of the animals and a possible inclusion of zoo animals bred in captivity via adoption techniques were discussed.

109: *+.171*

After a sharp decline in numbers during the early decades of the 20th century, the last raven was found breeding in 1944 (fig. 1). In 1966, a re-introduction project was started. During 1966-78 ravens were imported from FRG (39), GDR (75), and Switzerland (2) (appendix 1). The imported ravens were partly kept in cages and partly released (appendix 1). Most young hatched in the cages were released (appendix 1). In 1976, the first free-living breeding pairs were observed (fig. 2). After a few years of increasing numbers, the population crashed (fig. 2). Since 1983-84, a strong increase takes place up to 17 breeding pairs in 1988. Most ravens breed on the Veluwe, a large area with coniferous and deciduous forests, and heather fields. One pair was recorded in the dunes (1979) and one on the Utrechtse Heuvelrug (1976-88). Since 1980, solitary ravens are recorded elsewhere in the country. In the first years, the breeding success was quite low, but improved with time (appendix 2). In 1976-88, 87 pairs raised 164 young, average 1.89/pair (s.d. 1.57). For the successful pairs (68%), the average was 2.78 young (s.d. 1.07). At present, the population comprises nearly exclusively of birds born in the wild. The decline in 1980-81 probably marks the extinction of introduced birds at a moment the numbers of free-born ravens were not yet large enough to fill the gap (fig. 3, appendix 1). In the Netherlands the Raven feeds, among other things as afterbirths of sheep and deer, and artificial feeding sites for game, on insects and their larvae, carrion, and rubbish. Around 1945, ravens only remained in Schleswig-Holstein, Poland, eastern Czechoslovakia, in the higher Alps, and along the western coast of France. After strong protective measures were taken, the species re-occupied several parts of western Europe (fig. 4). The re-introduction in the Netherlands was the first in western Europe, and was followed by successful ones in Belgium and FRG.

113: *+.116*

Lathyrus pisiformis was recently found at 14 localities in Poland (known from 21 localities altogether). 9 of them occur at the Kielce-Sandomierz Upland (central Poland), the other five are scattered in the North Poland. The latter ones are marginal, at the NW outskirts of the general distribution of this species. The distribution center of L. pisiformis occurs in the South Siberian province, in Europe it grows only at insular localities. The habitats of L. pisiformis are dry or fresh, moderately fertile, neutral of alkaline rendzinas or brown soils. It prefers places with some little shade. Its phytocoenotic optimum is in open thermophilous forests of Querco-Fagetea class and in shrub communities, on their margins, and exceptionally it can be found on secondary habitats e.g. in ditches or on road escarpments. THe loss of localities found recently (4 disappeared for certain, the other 3 probably), is restricted to the North Poland, and its direct reason is growing shadiness caused by the development of shrubs and trees. It is why this species requires the active protection on the localities still existing, and the reintroduction on a part of historical ones.

122: *-.121*

By help of questionnaires sent to the respective organisations maps on the present distribution of roe deer, follow deer and red deer in Spain were elaborated. At the beginning of this century most of these species were extinguished because of bad hunting politics. They were reintroduced in the sixties and, together with rural depopulation and decrease of predators, spread widely.

126: ***+.171***

In vitro seed germination of Bletia urbana was achieved on two basal media (KC and MS) with organic supplements. Cultures were maintained in three environmental conditions. Germination, defined as the presence of protocorms with one leaf primordium one month after culture, occurred under all the conditions levels, varying from 21-100%. Not all germinated seeds reached the adult stage (defined as plantlets with leaves at least 30 mm long, pseudobulbs, and roots). The best germination occurred on KC with 3% sucrose and 10% coconut water (CW) at 25 C .+-. 2 and 16-hr photoperiods (1200 lux). Under these conditions all seedlings developed to the adult stage, forming leaves, pseudobulbs, and roots after 90 days of in vitro culture. The seedlings obtained were preadapted in a greenhouse where survival was 100% before transplanting them into their natural environment. One hundred and thirty-three plants were placed in the wild, protected with cages, and monitored for 90 days. Of the original seedlings, 56% survived in the field during this period. One year later, 15% of the seedlings started to grow and form new leaves and bulbs, suggesting that the reestablished plants are able to complete at least part of their life cycle.

127: ***+.088***

Cupressus dupreziana has been proclaimed by IUCN as one of the twelve mostly endangered plant species on a world scale. Information is presented on the relict population in the Tassili Range in the extreme south-east of Algeria. Successful reintroduction of six-year-old trees from Czechoslovakia to Tassili is described. Dendrochronological investigation showed a very complex tree-ring structure. A xylotomical study proved that by means of quantitative xylotomy it is possible to distinguish the wood of C. dupreziana from the wood of other cypress species and several related taxa. Perspectives are mentioned of C. dupreziana further research and conservation.

133: ***+.316***

Between 1967 and 1978, 145 Ibexes were reintroduced in Haute-Savoie. In 10 different sites. The choice of the massifs was governed by the ecological knowledge available about the species, but without any thorough study. In 1986, the National Research Program on the Ibex made it possible to evaluate this project. At present, the population is estimated to consist of 700 individuals, i.e. 36% of the national numbers. The animals were released on sites offering quite varied environmental conditions. This diversity of situation evidently resulted in variable "success rate", the analysis of which enables us to derive some experience for future prospects of reintroduction in the Alps.

141: ***+.166***

Hawaii Volcanoes National Park has supported a Nene Reintroduction Programme since 1974. The Resources Management Division is responsible for the management and monitoring of birds within and adjacent to the Park boundaries. The management aim has been to work toward the production of a wild, self-sustaining population. The method employed in order to achieve this has been to release captive-bred Nene into the wild. To date, a total of 177 birds has been released and

monitored in and near the Park. This report summarises progress over the last 15 years.

142: +.122

Breeding results and recolonization data are reported for a ten-year project of the German nature conservation association, Bund Naturschutz, aimed at reintroducing the European wildcat (Felis silvestris silvestris) in Bavaria. Criteria for successful reintroduction of endangered animal species are discussed and their fulfillment in the wildcat reintroduction program is evaluated.

144: +.108

A survey of ibex (Capra ibex ibex L) habitat in the European Alps reveals for 1983 a total number of c. 18,000 animals. Of these, Switzerland and Italy account for respectively 56% and 25%, followed by Austria with 10% and France with 7%, while the remaining numbers occur in Germany and Yugoslavia. There is still a potential for further re-introduction of the ibex in five major mountain massifs in the franco-italian Alps and in South Tyrol. New insights in re-introduction techniques are discussed against the background of landscape ecology and behavioural ecology. In thriving populations, ibex numbers increase at an estimated annual rate of 11%. Hunting in Switzerland and in Austria, together with poaching are major man-induced regulating factors of ibex numbers. Nowadays the emphasis in ibex conservation and management has shifted towards population management and problems of local abundancy. It is argued that management orientated research should be undertaken, and especially the monitoring of the impact of the ibex on habitat condition. Recent pressure to undertake culling in the largest ibex population of the Alps, the Gran Paradiso-Vanoise massif, is critically discussed.

148: +.020

The lynx (Lynx lynx, L.1758) was indigenous to the Black Forest until the 17th century. At the end of the 30-Year War, an increased persecution of the lynx by means of hunting began. This subsequently caused an almost complete disappearance by the end of that century and finally lead to a total extinction during the 18th century. Derived from Wuerttemberg hunting records from the beginning of the reduction period, the former lynx density was 1.5 lynx/100 km2. The present day suitability of the Black Forest as an appropriate habitat for the lynx has been concluded based on experiences made in neighboring countries with lynx populations. Accordingly, the Black Forest with its expansive forests (percentage of entire forest area is 66%) offers sufficient shelter and retreat space. Under such conditions, heavy tourism proves not to be problematic. Several east/west bound highways cut through the Black Forest, however, there is not enough traffic during the night to present a serious barrier to migrations, since the lynx roams mainly during twilight and at night. Nevertheless, roads and train tracks pose a latent threat. Thus, occasional acoidental deaths can be expected. Roe deer (Capreolus capreolus, L.) (in high density) and brown hare (Lepus europaeus) (in lesser density) as well as the locally occurring chamois (Rupicopra rupicapra, L.), red deer (Cervus elaphus, L.) and wild boar (Sus srofa, L.) are all suitable prey for the lynx. In the Black Forest, the population of the roe deer alone would be enough to satisfy the nutritional needs of a lynx population. In order to estimate the possible size of a lynx population in the Black Forest, a density of 1 lynx/100 km2 was assumed. The Black forest area was divided into unsuitable regions (settlements, inclusive of a buffer zone, and non-wooded areas with widths of more than 1 km), partially suitable areas (regions with scattered forests), and suitable areas. Based on these criteria, 4100 km2 are suitable habitat, and 1000 km2 can be used for passage, respectively for hunting. The forest area of these two categories combined is 4500 km2. Consequently, the Black Forest can accomodate at least 40 lynx. A population of this size can be

expected to survive, even without contact to neighboring populations. The impact of these lynx on their prey species would be small. The calculated lynx population needs 0,7 roe deer/km2 anually or an equivalent amount of other prey. Such a loss of roe deer would probably fall into the range of compensatory mortality, thus reducing natural deaths, and therefore not affect the hunting yield. While roe deer are caught by the lynx more or less without selection, the hunting success of the lynx in the larger and stronger ungulates is shifting increasingly towards juvenile, overaged, weak and ill animals, which are of lower interest for hunters. The capercaillie (Tetrao urogallus, L.) and the hazel hen (Bonasa bonasia, L.) are not jeopardized by the lynx due to low population densities and a rich supply of altnerative prey. The only danger for domestic animals is with sheep which are left unwatched overnight in enclosures adjacent to forest. Based on experience, a negative influence of the lynx on tourism is not to be expected. Since wild lynx are absolutely harmless towards people, their presence even might become a tourist attraction. The Black Forest offers favorable habitat conditions to lynx. Damage caused by these animals will be small. Therefore their reintroduction to the Black Forest is recommended. For release, a site in the northern part of the area should be considered.

151: *+.202*

Twenty-seven ponds were restored or created at Feyent Country Park, Middlesex, in the London Borough of Brent during the 1980s. The ponds have been colonised by 68 plant species, the distribution of which is described. Since the start of the pond restoration programme, several new species have been recorded in the ponds and many species have increased their range. About 22 species have been introduced. The results are discussed in relation to the conservation of pond flora.

162: *+.124*

Mountain sheep (Ovis canadensis) are closely associated with steep, mountainous, open terrain. Their habitat consequently occurs in a naturally fragmented pattern, often with substantial expanses of unsuitable habitat between suitable patches; the sheep have been noted to be slow colonizers of vacant suitable habitat. As a result, resource managers have focused on (1) conserving "traditional" mountainous habitats, and (2) forced colonization through reintroduction. Telemetry studies in desert habitats have recorded more intermountain movement by desert sheep than was previously thought to occur. Given the heretofore unrecognized vagility of mountain sheep, we argue that existing corridors of "nontraditional" habitat connecting mountain ranges be given adequate conservation consideration. Additionally, small areas of mountainous habitat that are not permanently occupied but that may serve as "stepping stones" within such corridors must be recognized for their potential importance to relatively isolated populations of mountain sheep. We discuss the potential importance of such corridors to other large, vagile species.

163: *+.171*

Efforts to restore the endangered masked bobwhite (Colinus virginianus ridgwayi) to its former range have required 1) habitat acquisition, restoration, and preservation; 2) captive propagation; and 3) reintroduction of captive-bred stock. In its role to recover the masked bobwhite, the Patuxent Wildlife Research Center (U.S. Fish and Wildlife Service) has refined captive breeding techniques; provided captive-produced stock for release; conducted field research on the distribution, limiting factors, and habitat characteristics of this species; and developed release methods. Techniques for the husbandry and captive management, breeding, artificial incubation and hatching of eggs, and rearing of young of the masked bobwhite have been developed.

Successful reintroduction techniques for the masked bobwhite have included prerelease conditioning and/or cross-fostering of captive-reared masked bobwhite chicks to a wild-caught, related, vasectomized bobwhite species and their release to the wild as family units. In addition, the establishment by the U.S. Fish and Wildlife Service of the Buenos Aires National Wildlife Refuge in 1985 has further enhanced the potential for establishing a self-sustaining population of the masked bobwhite in the U.S. Through continued releases and active management of habitat, therefore, it is believed that the masked bobwhite can become permanently established at the refuge to ensure its continued survival in the wild.

173: ***-.063***

The cytogenetic survey of a captive population of Arabian Oryx (Oryx leucoryx) in Saudi Arabia has permitted the detection of a Robertsonian translocation involving chromosome pairs 17 and 19. This population has been constituted with animals from various arabian peninsula countries. The origin of this translocation is uncertain. Our results suggest that it originated from Qatar.

176: ***+.085***

On July 11th 1990 beaver Castor fiber L., 1758 was seen in the river Rur, near Kuechelscheid in the Hautes Fagnes. To our knowledge this was the first beaver seen in Belgium since 1848. It seems very likely that the animal orignates from a German reintroduction project in the northern Eifel.

177: ***+.054***

The continuing loss of isolated headwater populations of brook trout (Salvelinus fontinalis) in the southern Appalachians [USA] cannot be attributed solely to competition from invading rainbow trout (Oncorchynhus mykiss). Brook trout in headwater habitats may even enjoy a competitive advantage over rainbows, yet they continue to experience local extinctions due to demographic stochasticity and natural catastrophes. Reintroduction of brook trout into headwater habitats currently occupied by rainbow trout may be a useful addition to current management policies designed to arrest the decline of brook trout.

178: ***+.094***

Fishes of seven North Carolina and South Carolina [USA] river systems were sampled to determine the distribution and current status of two fishes, the sandhills chub Semotilus lumbee and the pinewoods darter Etheostoma mariae both endemic to the Carolina Sandhills area. A total of 261 localities in 15 counties was sampled. The chub was found at 53 of these localities, and the darter at 37. The chub could not be located at 11 (42%), and the darter at 9 (31%), of previously known localities. In many cases this was due to habitat deterioration caused man and the recent and highly successful reintroduction of the beaver. We found the chub at 38 and the darter at 17 previously undocumented localities. We took the chub in the Wateree River system, South Carolina, in which it was previously not known. Both species are present are doing well, but, because of their limited distributions, their status should be monitored.

180: ***+.228***

The inland waters of Australia have been colonized successfully by 20 species of freshwater fishes introduced to the continent, including 6 poeciliids, 3 salmonids, 4 cyprinids, 5 cichlids, 1 percid

and 1 cobitid, all of which reproduce there in the wild. The ecological and genetic consequences of fish introduction and translocations have concerned scientists and water authorities since the late 1960's, although the earliest introductions of salmonids for sport were made towards the end of the 19th century. Impacts of introduced fishes, including hybridization problems, habitat and water quality alterations, competition for space and food, predation and the introduction of exotic parasites and diseases are reviewed. Emphasis is placed on sub-tropical and tropical species, since they appear to present the greatest threat to natural ecosystems. Problems in the translocation of indigenous Australian fishes between drainage systems to supplement recreational fishing are noted; a review of the Lake Eacham rainbowfish, supposedly brought close, to extinction in its natural habitat by translocated Australian piscivores, demonstrates that unanticipated predator-prey interactions may result from the thoughtless addition of piscivores to an endomic community. A theoretical perspective on the assessment of candidate species for introduction and translocation is given, and areas of theoretical ecology that may assist in predicting future impacts of introduced species are discussed.

183: *+.009*

Beginning in the 1500s, over-exploitation and poaching led to a steady decline of ibex (Capra ibex ibex) numbers in the European Alps. The use of ibex products in many folk remedies guaranteed high financial returns for the hunter and resulted in the relentless pursuit of this species. By the early 1800s, < 100 animals survived in a single population in the Italian Gran Paradiso mountain massif. The recovery to more than 20 000 animals today is the result of a four-stage conservation effort which returned alpine ibex to almost their entire original range of distribution: (1) effective protection of the last remaining population, (2) captive breeding of animals caught in the recovered last population, (3) reintroduction of captive-bred animals into protected original habitat, (4) translocation of animals from successfully established "reservoir" populations to uninhabited sites. Alpine ibex management faces two challenges today: (1) habitat destruction in areas of high population densities, and (2) low genetic variability possibly a result of inbreeding during a succession of four potential population bottleneck situations.

184: *-.007*

Przewalski's horses (Equus przewalskii) are believed to be extinct in the wild; the current known population of 797 animals exists wholly in zoos. The Species Survival Commission of the International Union for Conservation of Nature and Natural Resources is proposing to reintroduce this endangered species into its former Mongolian habitat within the next decade. Knowledge of the behavior of harem-forming equids in general and of Przewalski's horses in particular, is of great importance to the captive propagation and eventual reintroduction of this species. Horses are rarely solitary by nature. Solitary captive animals are prone to pacing. Juvenile male feral horses (Equus caballus) form bachelor herds upon dispersal from their natal band. Zoos can set up bachelor herds as a way of managing surplus males. The older, more dominant feral horse bachelors are the first to acquire mares. Bachelors do not generally obtain females until they are 4 or 5 years of age. The first females acquired are usually 1- and 2-year-old fillies dispersing from their natal band. Because of the age differential, the stallions are generally dominant to their mares. Behavioral impotence may result if captive stallions are given a harem at too young an age, especially if the harem contains older, more dominant, females. Typical harem sizes in the wild are 3-5 mares. Captive stallions with too large a harem may become either apathetic or agressive toward their mares. Wild horses spend 60-70% of their time foraging. Captive animals may quickly consume their limited amounts of food and develop vices out of boredom. Provision of hay ad libitum reduces the amount of pacing seen in captive animals, and virtually eliminates

coprophagy.

185: ***+.189***

Natural populations of woody perennials on lead-mining sites in the Mechernich area of the Eifel Mountains were investigated with respect to soil factors determining the degree and type of heavy metal tolerance. Salix caprea L. (Goat Willow) grew on soils with up to 17000 mg kg-1 total lead (ca. 4000 mg kg-1 ammonium acetate-exchangeable Pb). Betula pendula Roth (Silver Birch) was found on soils containing as much as 29000 mg kg-1 total lead (7000 mg kg-1 ammonium acetate-exchangeable Pb). Other woody perennials, with the exception of the dwarf shrub Calluna vulgaris, were not found in the contaminated area even though they did occur in the immediate vicinity. The two lead-tolerant tree species did not form mixed populations. Because of a significantly lower Pb/Ca ratio in Salix soils (2.2) compared with Betula soils (7.4), a calcium-dependent mechanism of lead tolerance is suggested for Salix, but not for Betula. The Betula population could be divided into two groups, each showing a highly significant correlation between root-lead content and exchangeable lead amounts in the soil, but with different levels of lead uptake. The only soil factor distinguishing the two groups was found to be the level of soluble phosphate. A distinctly low level of soil phosphate correlated with a high lead concentration in roots of the one group (30000 mg Pb kg-1 DW), whereas high phosphate amounts corresponded with a much lower lead concentration in roots of the other (12000 mg Pb kg-1 DW-1). Since the correlation between lead in the soil and in plants was similar for the two groups, it is concluded that the type of lead tolerance in Betula is determined by the status of plant phosphate nutrition, rather than by simple phosphate precipitation in the soil. A comparison of growth between different populations of Betula seedlings on homogenized soils from the mining area revealed the Mechernich population to be a distinct ecotype with respect to lead tolerance. The control population obtained from a non-contaminated area exhibited a lower degree of lead tolerance coupled with a two-step strategy of adaptation to lead.

186: ***-.019***

Jordan lies at a meeting-point of three zoogeographical areas: the Palaearctic, the Afrotropical, and the Oriental - circumstances which were conductive to habitat diversity in Jordan and hence the diversity and often abundant occurrence of different floral and faunal elements. The present paper gives information about the history and past occurrence of large mammalian species and their populations in Jordan, while also focusing on the present status of the large mammalian species, and gives the reasons for extinctions and the preventive measures that are being taken to conserve the populations remaining. In addition, this study gives a future prospective outlook for habitat and mammalian conservation, and the reintroduction programme that is being implemented in Jordan. The first part of the paper sheds light on the past existence of large mammals in Jordan. The historical sequence of events starting from the Upper Palaeolithic and continuing right up to the present, and the succession of civilizations, had a negative impact on the large mammalian populations. This section gives tabulated data on the past occurrence of 26 species, supported by evidence of their past existence such as bone remains, rock drawings, engravings, mosaics, and frescos, found in various archaeological sites in Jordan. The second part of the paper deals with the present status of large mammalian populations, their distribution being reflected through maps indicating recent sightings in some outstanding cases. Among those are reintroduced species such as the Arabian Oryx and the Syrian Wild Ass, and highly threatened species such as the Nubian Ibex. This part also investigates the reasons for extinction, which mainly link the destruction of habitats with the direct deterioration of mammalian populations. Habitat destruction was chiefly due to the rapid decline of the natural pasture areas, overgrazing, urban expansions, unorganized

construction plans, human impact, mismanagement of the highlands, fire, and hunting. All of these factors led to deforestation and soil erosion, resulting in a patchy, fragile ecocomplex. The decrease of rainfall assisted the severe deterioration of natural forests. The future outlook of this study includes plans to restore ecosystems and, with them, much of the overall ecocomplex. For this and other reasons it also stresses the urgent need to establish more reserves, both grazing and natural. This concluding part of the paper encourages the improvement of national parks, and urges the authorities to look seriously at the often disastrous effects of mismanagement. The importance of collaboration between Jordan and the various international agencies is emphasized, as well as the need to speed up work on the National Conservation Strategy of Jordan.

187: ***-.079***

The proposition is examined that cytogenetic studies are needed in the conservation of wild captive animals. Several cases of polymorphic species have hybridized to produce infertile offspring. In several gazelle species, this accidental hybridization among look-alike animals has led to the extinction of zoo populations. The suggestion that this is always the result of inbreeding is thus erroneous. Cytogenetic study is also needed in animals that are destined for reintroduction, as accidental release of chromosomally different species is counterproductive to the reestablishment of wild stock. Several examples of mammalian species that have flourished from an extremely small founder stock are here examined to draw attention to the possibility that "inbreeding" is not invariably disadvantageous. The karyotypes of two hybridizing Kobus species with divergent chromosomal numbers are described.

188: ***+.091***

The little spotted kiwi (Apteryx owenii) is probably extinct on both main islands of New Zealand. Its only known surviving population is on Kapiti Island (1,970 ha). Translocations to other offshore islands are seen as essential to the survival of the species. In this paper we discuss the criteria for assessment of islands for translocations, and the early results of two transfers. Only five of > 700 known islands were identified as potentially suitable. None was greater than 500 ha in size. Between 1982 and 1989 little spotted kiwis were transferred to three islands, Long, Red Mercury, and Hen Is. All, or nearly all, of the transferred birds survived on Long and Red Mercury Islands and have since bred on both. The result of the transfer to Hen Island has yet to be determined. The limited potential carrying capacity of these islands for little spotted kiwis, and the need for a new transfer to a larger island, are discussed.

189: ***+.029***

The possible occurrence of low molecular weight, soluble ligands binding copper, zinc, cadmium and lead was investigated in Rhynchostegium riparioides, a widespread moss in flowing waters, which occurs at sites influenced by "heavy" metal contamination as well as ones free of contamination. In order to identify metal complexes with any such ligands, Rhynchostegium plants from an uncontaminated site were first incubated for 48 hr in medium including the metal (10 or 100-mu-M) in question. Fractionation of cell extracts of this material by gel permeation HPLC revealed the presence of complexes with a similar retention time to poly(gamma-glutamylcysteinyl)glycine metal complexes extracted from cell suspension cultures of Datura innoxia. However, the peaks corresponding to metal associated with these complexes in HPLC profiles of extracts from Rhynchostegium were low, so an acetone precipitation step was introduced to concentrate the bound metal. The estimated yield of Cu associated with low M(r) ligands following acetone precipitation was 65% for Rhynchostegium and 106% for D. innoxia;

the equivalent values for Cd were 25 and 40%, respectively. A Zn complex with a different apparent retention time was also present; the possibility that this might be an artifact associated with the extraction procedure is discussed. There was no evidence for intracellular binding of Pb by low M(r) ligands. Estimates of the amount of Cu and Cd associated with low M(r) ligands increased following incubation of Rhynchostegium from an uncontaminated site in media containing increasing concentrations (10, 50, 100-mu-M) of metal. Similar complexes were also detected in populations of the same species taken directly from a contaminated site, i.e. without laboratory incubation in the presence of metal. Estimates of the proportions of Cu and Cd in Rhynchostegium from contaminated sites which were bound by these complexes, expressed as a percentage of the total metal accumulated by the moss, ranged from 0.06 to 0.65% for Cu and 0.24 to 2.08% for Cd.

190: *+.223*

The black-footed ferret (Mustela nigripes; Mustelidae) is a weasel-like carnivore that preys almost exclusively upon prairie dogs (Cynomys spp.; Rodentia) in central North America. This tightly coupled system and the endangered status of ferrets motivated our predation model. An age-dependent model is formulated, clearly stating and comparing assumptions with the actual biological system. Tenable parameters and values are built into the model in order to make predictions based on "real" data. Species-specific population data and energetic models, as well as general mustelid features, are the basis of ferret inputs. Extensive population data from several prairie dog species are used to create a "generic" prairie dog. A Holling type III functional response and sigmoidal birth and survival functions describe ferrets living in a single, very large colony of prairie dogs. The model consists of discrete-time equations for the annual birth pulse for each species, with the remaining year governed by a system of seven differential equations that allow both populations to decrease from predation and other mortality factors. The model is normalized, and it is shown that age dependence is destabilizing, except within a narrow range of values for ferret survival and birth functions. These values are close to but greater than the initial values. Sensitivity analysis reveals that general stability can be achieved by slightly increasing the hunting efficiency for the ferrets. It is suggested that higher prairie dog densities than previously predicted from energetic-based models may be necessary to ensure a stable ferret/prairie dog system. Finally, the results indicate that system stability is likely to be enhanced if patch dynamics are accounted for. Because wild black-footed ferrets are virtually extinct, these results may be a useful tool for black-footed ferret recovery efforts, particularly for planning reintroduction of captive-reared specimens.

191: *+.116*

Conservation strategies involving relocations, repatriations, and translocations (RRT) have been carried out, are underway, or are advocated for a number of endangered and threatened amphibians and reptiles. However, recent reviews of RRT projects involving birds and mammals suggest that the success rate is low and that the factors that lead to endangerment operate to impede effective RRT results. In this paper, we review available information on RRT projects involving amphibians and reptiles, examine the motives for advocating RRT strategies, and recommend biological and management criteria that should be considered prior to undertaking RRT projects. Most RRT projects involving amphibians and reptiles have not demonstrated success as conservation techniques and should not be advocated as if they are acceptable management and mitigation practices. We urge caution in accepting claims of success and urge colleagues to publish detailed methods and results of past and ongoing RRT projects.

192: *-.218*

Adult populations of water beetles were sampled in an upland loch subject to liming of its catchment prior to the reintroduction of brown trout. Populations generally declined and the commonest species, Hydroporus palustris, was temporarily displaced by Potamonectes depressus elegans before it too declined. The reasons for these changes are discussed.

193: *+.199*

During the past several millennia, hundreds if not thousands of Polynesian land bird populations have been reduced or eliminated by human activities-direct predation, habitat alteration, and introduction of predators and pathogens. Many of the surviving populations and species of Polynesian land birds, particularly pigeons, doves, parrots, kingfishers, and passerines, are likely to become extinct within the next few decades unless we intervene to save them. One strategy is to translocate species onto previously occupied islands, if the habitat is suitable and current human activities are compatible. In this study we evaluate a procedure for analyzing terrestrial habitats in a geographical information system (GIS) using aerial photographs, satellite imagery, topographic maps, and thematic maps. In the relatively simple island ecosystems of Atiu and Mitiaro (southern Cook Islands), mapping land cover and evaluating habitat suitability of land birds is straightforward; measures of the shape and spatial relationships of land cover patches are of limited value. Although the rate of land cover disturbance by people has not increased in recent times, even small disturbances can eliminate a significant proportion of preferred habitat for land birds. We suggest that, whenever possible, translocation efforts should focus on islands uninhabited by humans. The potential for successful translocation in the Cook Islands is limited to a few species. In Tonga, where more species of birds have been extirpated and where there are more uninhabited islands, the potential to reestablish viable land bird populations is much greater.

194: *+.289*

Over the past 30 years the black rhinoceros (Diceros bicornis) populations in Africa have dwindled dramatically. To enhance the survival prospects of the species, a national conservation strategy has been developed in South Africa. Its main goal is to formulate and implement policies to increase the southern African rhino population as rapidly as possible. This involves translocating animals from areas where the population is approaching the ecological carrying capacity and establishing new viable populations in other suitable reserves. A non-linear differential equation model for a population of black rhino was developed. The model is used with a combination of analytical and numerical techniques to investigate a number of issues relating to the translocation of rhino from well-stocked, high-density areas to low-density areas with small herds or no herds. Firstly, the model is used to determine the maximum sustainable yield from the well-stocked reserves. The model is then applied to a newly established population to determine optimal import policies. Finally, the model is extended to include both an established exporting population and a new understocked importing population. Simulations are performed to give an indication of the number and age of animals which should be translocated to maximise the growth rate of the total rhino population in southern Africa.

196: *-.032*

Northern Bald Ibis (Geronticus eremita) is a threatened species, one of the two remaining populations of which went extinct in Birecik in 1989. One of the main reasons for the extinction of this bald ibis population is DDT spraying carried out simultaneously by two government offices in

the 1950s. As a result of this insecticide application, 70% of the bald ibis population died because of acute poisoning and the reproductive success of the population fell to very low levels for the next 10-12 years. A conservation project initiated in 1973 its first goal of attracting the population away from the town, but failed in its more important aim of captive breeding and reintroduction. The main reasons of this failure are insufficient and inadequate nutrition, overcrowding in the captive population, and the indifferent attitude of the Forestry General Directory towards conservation efforts. In this article, history of the Birecik population and the ecology of the Bald Ibis are summarized, the changes in this population in the last 12 years are described, the reasons for the extinction of the population and for the failure of the conservation program are evaluated.

197: ***+.052***

Species-rich meadows (Calthion communities) of the Oste valley in northwestern Germany (mowed twice a year) developed into stands of reeds and tall forbs (such as Filipendula ulmaria, Glyceria maxima, Phalaris arundinacea and Urtica dioica) with low species diversity when the management was abandoned. Changes in species composition and dominance, as well as a marked decline in the species diversity per plot are distinct. The change from marsh-marigold meadows (Senecioni-Brometum) to tall grass-reeds and/or tall forb communities proceeds rapidly: the expansion of some of the component species of the "initial floristic composition" occurred in the first years after abandonment. Dominant populations of the taller species inhibit succession both shortly after abandonment and over the long run. The major constraint on succession is shade: from the dominant plant species themselves as well as from a dense litter layer. These stands become slowly enriched, because environmental inputs are stored in the living plant material. Over a period of 3-5 years, a re-establishment of a mowing routine results in a rapid reduction of aboveground biomass, combined with a rapid increase in species richness. Decreases in available resources due to interruption of internal translocation inhibit the growth of the formerly dominant plant species. As result species of the Senecioni-Brometum meadows are able to become established. The ability to grow with fewer resources and the removal of the aboveground biomass (2x/year, fulfilling a demand for light) favour coexistence. The regeneration experiment has already been quite successful. Management costs may be reduced for some years by restricting the mowing frequency, without an ultimate loss in species diversity.

198: ***+.083***

Because of their small size and limited mobiity aquatic insects are seldom recognized as invasive species and only where verifiable historical data exist can introductions of exotic species be confirmed. Records of some generally considered cosmopolitan species could in fact be undocumented introductions. Of the entirely aquatic orders of insects only the Odonata are noted as regular invaders. Trichoptera, Ephemeroptera, Plecoptera and Megaloptera are all poor colonizers. In contrast, the Hemiptera, Diptera and Coleoptera have many species which show excellent colonizer and invader qualities. Diptera are recognized as the most deleterious of introduced aquatic insects because many of the species are pests and disease vectors of man and his livestock. Although no alien aquatic insect pests have been recorded in South Africa, the potential for the introduction of problem species exists. There are certain life history styles which make an aquatic insect predisposed to colonize a new region successfully. There are also qualities of the habitat which enable successful colonization of the region. Both of these aspects are discussed and demonstrated with case histories. Streams in the southern Cape contain a large number of biogeographically important palaeo-endemic aquatic insects. These animals have evolved without the presence of fish predators and their behaviour and life history styles would make them highly vulnerable to extinction by the introduction of alien fish predators or even

translocation of indigenous species. It is important to conserve this biologically important stock of invertebrates in as near a pristine state as possible and prevent all introductions or translocations of fauna alien to their streams or section of streams where natural barriers keep out fish.

199: *-.020*

The Cape Floristic Region (CFR) contains a disproportionate number of southern Africa's Red Data Book plants (70%), freshwater fish (57%), amphibians (43%), butterflies (38%), and reptiles (35%), relative to the small area (4%) of the subcontinent which it occupies. With the exception of reptiles, the vast majority of these species are endemic to the CFR, occurring mainly in mountain and lowland fynbos vegetation. Among the threats to the continued survival of these species, agriculture and alien invasive plants rank highest overall, although many scavenging birds and large mammals have been hunted and poisoned to extinction within the CFR. An active reintroduction of these large mammals from outside the CFR has been the dominant strategy of local conservation agencies. The focus on large mammals as target species for conservation strategies has been to the detriment of floral preservation. The geographical distribution of threatened species is not related to the extent to which vegetation types have been transformed. Thus renoster shrubland, which has been reduced to 6 per cent of its original lowland extent, contains few Red Data Book taxa relative to fynbos. By far the largest concentration of Red Data Book species of plants, butterflies, reptiles and amphibians occurs in the greater Cape Town metropolitan area. With 15.1 Red Data Book plant species per km2, this area ranks as one of the most urgent conservation priorities in the world.

200: *+.084*

The so-called "Lage Fronten", situated in the middle of the city of Maastricht, consist of a few remains of 19th century fortifications (a moat, old walls and an earth ridge) and a deserted railway yard situated on the former main earth ridge. This small area (approx. 4.5 ha) is of particular biological interest, since it is one of the two remaining sites in the Netherlands where a small population of Wall lizards (Podarcis muralis) survives. A preliminary and hence incomplete survey of plant and animal species is presented. Table I lists about 200 plant species, 24 of which are under threat. The well developed mural vegetation is of particular interest. Furthermore, details are given about some invertebrate groups (aculeate Hymenoptera table II, and Lepidoptora), fish, herpetofauna, birds and small mammals. Protection of this site is extremely urgent, since the population of Wall lizards consists of only a few dozen individuals (table III). Due to the very low numbers in some of the colonies, there is a serious danger of inbreeding. Moreover, the population of Wall lizards is under serious threat, as is the entire biocoenosis, as a result of plans of the local authorities to develop the area for tourist, recreational, industrial and housing purposes. It is suggested, therefore, that the terrain should be given the legal status of "Protected Nature Reserve" immediately. Conservation measures proposed in this paper include the reintroduction of Wall lizards, the maintenance of the old walls as sunny, dry habitats and the (re-)creation of a fairly low, varied vegetational structure. Furthermore, if restoration of the walls is found to be necessary, this should be done in such a way that the crevice systems between and behind the bricks are preserved. Further conservation measures should be directed towards the preservation of other biological elements, including mural plants, Edible frog (Rana kl. esculenta) and Slow worm (Anguis fragilis). Habitats of both the Wall lizard and Slow worm ought to be protected against recreational activities.

201: *+.265*

Seventy-six greater prairie-chickens (Tympanuchus cupido) were trapped on leks during 1984 (36) and 1985 (40) and released on the Tamarack State Wildlife Area in northeast Colorado in an effort to establish a second self-sustaining population within the state. Leks were established, and reproduction and recruitment were documented. Fifteen different leks were located between 1984 and 1991; 13 were more than 4 km from the release site. The number of active leks located per year increased from two in 1984 to 11 in 1990. Since 1987, lek counts have averaged 5.0-6.4 males/lek. Plains sharp-tailed grouse (Tympanuchus phasianellus jamesii) were first observed on leks in 1989. Their numbers have slowly increased, but they are not considered a threat to the prairie-chicken population. Spring releases are recommended over summer releases based on trapping success, post-release dispersal patterns and survival, and genetic considerations.

202: ***+.143***

During a 4-month study in French Polynesia, Pacific Ocean the authors visited 28 islands, seven of which had never been explored by ornithologists. They collected ecological data on endemic land birds, introduced animals and habitats, focusing particularly on the factors involved in population declines and extinctions. As well as hunting and habitat destruction, it appears that introduced predators play a major role, with the roof rat Rattus rattus being the most dangerous. Rapid action to eradicate introduced predators, coupled with translocations, would be the most effective way to ensure the survival of the remaining bird species.

203: ***+.092***

Four 'rehabilitated' hedgehogs from an animal hospital were taken to Yorkshire (England, UK) and radio tracked for two weeks to determine their fate after reintroduction to the wild. One died for reasons not apparently related to the study. The other three (1 male and 2 females) successfully built natural nests, and soon learned the geography of their new environment. They integrated well with the wild resident hedgehogs and developed home ranges similar to what would be expected of wild hedgehogs in the same habitat. However, only one managed to maintain its body weight, the others lost significant mass. A longer study, with more animals is needed to confirm the success of hedgehogs released into the wild after a period of veterinary care.

204: ***+.082***

In 1978, the re-introduction-project of the Bearded Vulture into the Alps was started. At that time, the principal purpose was to establish a breeding stock in European zoos as well as a central breeding station (Vienna Breeding Unit). Both WWF (WWF/IUCN-Project 1657/78) and Frankfurter Zoological Society (Project No. 832/78) support this project. The "hacking practice" proved to be the most suitable method to release young vultures. In this case, the birds are placed into an artificial nest-site, some weeks before they are able to fly. The young vultures are fed until they attain independence (at an age of about 6 months). Afterwards, addition feeding is stopped and the vultures are able to exploit the rich, natural food supply without any help. In 1986 the first release took place in the Austrian Alps (Hohe Tauern, Rauris), using 4 birds, bred in captivity. Until 1991, 35 Bearded Vultures have been set free in 3 different locations (Hohe Tauern, Austria; Hoch-Savoyen, France; national park of Switzerland). It was necessary to recapture 2 of them, 3 additional vultures died (disease, electric wire, avelanche) and 2 more birds disappeared. The released birds spread all over the Alps. Formation of pairs was observed in Austria and France and reproduction is expected in some years. The national park Argentera/Mercantour is designated as 4-th location for releasing.

209: ***+.067***

A comparison between forest structure and use of forest by Brachyteles at Fazenda Montes Claros and Fazenda Esmeralda in Minas Gerais, Brazil indicated differences in the proportion of large trees available at the two sites. However, despite these differences in forest structure, Brachyteles at both sites showed similar preferences for feeding in trees greater than 25 cm in DBH. The results of this study suggest that forest structure, as well as forest species composition, must be evaluated before initiating efforts to translocate threatened populations of this endangered primate.

210: ***+.155***

The cheer pheasant Catreus wallichii is an endangered species frequenting open habitats in the Himalayan foothills of Pakistan, India and Nepal. Part of this paper describes how early successional habitats used by cheer at one intensively studied site are maintained by a traditional form of scrub clearance and single-season crop growing on a long rotation, together with seasonal grazing. A review of the habitat characteristics and land-use patterns at a series of other present-day cheer sites reveals that, with the exception of a few small refuges, all component habitats are heavily disturbed. The history of a continuing reintroduction attempt for cheer in the Margalla Hills National Park in Pakistan is then reviewed in the light of these findings. We conclude that habitat management involving the rotational burning of a dense successional scrub is necessary if the released population is to have any prospects of becoming self-sustaining. A similar type of management needs to be maintained at sites still holding wild cheer in India and Nepal.

211: ***+.020***

In 1977-78 some 40 Seychelles magpie robins Copsychus sechellarum, the entire world population, survived on Fregate island. These lived in 12 territorial groups of up to six individuals. Their range on Fregate was limited by the amount of feeding habitat, specifically bare earth and leaf litter which occurred under mature shady woodland and in cultivated vegetable gardens. Two attempts were made to reintroduce the species to Aride Island in 1978 and 1979. These were unsuccessful and the translocations had to be abandoned when a new threat impinged on the parent population of Fregate in 1980. By 1981 numbers there had declined to 18, with virtually no recruitment, and an increase in the feral cat population was implicated. A successful cat eradication programme by trapping and poisoning was carried out in 1981-82. By 1983-84 the population showed a recovery with recruitment again healthy, although the abandonment of agriculture on Fregate between 1979 and 1983 had caused a reduction in the amount of feeding habitat and in the carrying capacity of the island to around 25 individuals in eight territorial groups. A range of management options is discussed.

212: ***+.052***

New data are presented on the foraging ecology, behavior, vocalizations, body masses, and soft part colors of the endemic land birds of Henderson Island (128-degrees-20'W, 24-degrees-20'S), southeastern Polynesia. Population estimates of species range from 720-1820 for Stephen's Lorikeet (Vini stepheni), 3240 +/- for Henderson Island Crake (Porzana atra), 3420 +/- for Henderson Island Fruit-Dove (Ptilonopus insularis), to 10,800 +/- for Henderson Island Reed-Warbler (Acrocephalus taiti). Conservation issues and translocation of species are discussed.

213: ***+.192***

The reintroduction of plants will become an increasingly utilized strategy in plant conservation and protected area management. Reintroduction is the deliberate establishment of individuals of a species into an area and/or habitat where it has become extirpated with the specific aim of establishing a viable self-sustaining population for conservation purposes. Plant reintroduction can involve the establishment of an extirpated species into a relatively intact habitat or it can be part of the restoration of a degraded habitat. This will be performed as species become extinct for a number of reasons, such as collecting, introduced herbivores or pathogens and potentially climate change. Although plant reintroductions have the potential to play an important role in species' conservation the long term viability of many reintroductions has yet to be assessed. For the technique to reach it's full potential it requires greater integration with habitat management, restoration and increased international coordination between both the ex situ and in situ agencies. In addition the value of introducing stocks of endangered species lacking viable sites for reintroduction to non native sites is discussed.

214: *-.023*

During September 1989, 700 000 e of black liquor from a paper mill was spilled into the Elands and Crocodile Rivers on the Eastern Transvaal escarpment. The impact of this effluent on the fish populations of these two rivers was monitored. Observations three days after the spill indicated massive fish mortalities in both rivers for a distance of 40 km downstream from the point of discharge down to the town of Nelspruit. A detailed survey in October 1989 indicated that the fish in the River below the spill were largely decimated. Mortalities in the Crocodile River downstream to Nelspruit were lower. Downstream between Nelspruit and the Kruger National Park boundary, mortalities were observed in one species only. In total 14 fish species were affected in the river from die paper mill down to Nelspruit. One threatened species (Chiloglanis bifurcus) suffered serious mortalities while another (Opsaridium zambezense) was only marginally influenced. During November 1989 and March and June 1990, surveys were undertaken to assess the success of the natural recolonisation of fish in the affected areas. Surveys were primarily undertaken in fast-flowing water and indicated that, although recolonisation is taking place, the species richness and population density in the Elands River in particular is still considerably lower than the numbers found during surveys since 1978. An intensive survey, which duplicated that done in October 1989, was undertaken in September 1990. This survey confirmed that recolonisation of the affected area is taking place. A survey during October 1991 which was limited to the affected part of the Elands River, confirmed the results of the September 1990 survey in this section. It is estimated that the river will probably only recover after several years. Translocation of fish to the most damaged section of the Elands River is an option which will be considered if future surveys do not indicate improved recolonisation rates. The importance of conserving fish refugia as centres from which recolonisation can occur is emphasised by this disaster.

215: *+.012*

Three ecologically dominant coral species in a northern Philippine reef were compared in terms of growth and mortality, and responses to transplantation. The purpose of this study was to examine the feasibility of using the species concerned in establishing new coral populations through deliberate fragmentation. The species, Acropora hyacinthus, Pocillopora damicornis and Pavona frondifera, displayed distinct differences which could be related to their respective life-history strategies. A. hyacinthus showed tendencies towards an r-mode, with rapid linear growth but also high mortality rates. Response to transplantation was poor. Pocillopora damicornis had intermediate linear growth rates and relatively high mortality. Transplants fared poorly in the

initial part of the experiment, though they showed successful adaptation after a year. Mortality rates of both A. hyacinthus and P. damicornis were increased by high temperatures during certain times of the year. Pavona frondifera had the highest linear growth rates and no mortality, tending towards a K-mode of life history strategy. It showed the best response to transplantation. This species is thus a suitable candidate for large-scale reef restoration.

216: *+.029*

To help the recovery of a remnant grizzly (Ursus arctos horribilis) population in the Cabinet-Yaak Ecosystem in northwestern Montana, wildlife managers have proposed augmenting the population with bears translocated from a larger population. This proposal has raised fears that translocated bears might endanger human lives and livelihoods. To reconcile the biological needs of this grizzly population with the socioeconomic needs of the human population, we used a combination of decision analysis, consensus of expert opinion, and tradeoff analysis to design a pilot scale augmentation program to enhance grizzly viability while minimizing bear-human conflicts. The analysis incorporated objective and subjective information on the movements, behavior and reproductive potential of different age-sex classes elicited from a group of experts on grizzly biology and management This information was used to rank the different age-sex classes according to three criteria retention of translocated bears in the Cabinet-Yaak Ecosystem, conflict with human activities, and relative contribution to grizzly population growth. Subadult females were high in reproductive contribution and moderate in conflict, adult females were moderate in reproductive contribution and low in conflict, adult males were low in reproductive contribution and low in conflict, and subadult males were very low in reproductive contribution and high in conflict These rankings were insensitive to minor changes in the expert judgments used in the analysis. Because the goals of maximizing population growth and minimizing conflict could not be met with any age-sex class, we used tradeoff analysis to establish minimum thresholds for retention and maximum thresholds for conflict and to characterize the experts' preferences among different combinations of reproductive contribution and conflict. A reranking of the age-sex classes based on the tradeoff analysis showed that 4- to 8-year-old females were most preferred. The analysis revealed that no more than one out of three translocated bears was likely to remain within the Cabinet-Yaak Ecosystem and that as many as one out of four might seriously conflict with human activities. These results prompted an intensive public education program to muster support for the augmentation program before any translocations were attempted.

217: *+.303*

Given the inconsistent history of successful reintroductions, and the likelihood that the use of reintroductions as a management tool will increase, the need for an accepted conceptual model for reintroduction planning seems apparent. This paper proposes the use of social theory for reintroduction planning. The social theory presented by Firey in Man, Mind, and Land: A Theory of Resource Use (1960) offers an appropriate conceptual framework relevant to reintroductions. Application of this theory to an existing reintroduction project demonstrates its usefulness. It is suggested that use of this theory in the planning stages of reintroductions may help to improve reintroduction success rates.

218: *-.146*

Translocation of wild ungulates is a common wildlife management tool and an integral component of game farming activities throughout the world, Elk (Cervus elaphus) are a primary species being moved, and the possibility of translocating a hazardous parasite led us to examine the potential for

elk to survive infections of meningeal worm (Parelaphostrongylus tenuis), to shed larvae in feces, and thus, to translocate the parasite. Twenty-six elk calves were exposed to 15-300 infective larvae of meningeal worm. All 13 elk exposed to greater-than-or-equal-to 125 larvae developed neurologic disease and died; 2 shed larvae in feces prior to death. At necropsy, numerous adult meningeal worms were found throughout the central nervous system of each elk. Meningeal worm established infections in all 8 elk exposed to 25 or 75 larvae: 6 calves developed neurological signs (4 recovered, 2 died), the remaining 2 elk did not exhibit any clinical signs. Seven elk given 25 or 75 larvae shed larvae in feces. None of 5 elk exposed to 15 larvae developed clinical neurological signs or shed larvae; meningeal worms were recovered from the central nervous system of only 2. The ability to survive exposure to small numbers of meningeal worms may explain the persistence of the few remnant populations of elk in eastern North America. The potential for larvae to be present in the feces of these animals (or captive elk in the same areas) leads us to recommend that elk not be translocated from eastern to western North America until a reliable diagnostic test or treatment for meningeal worm is available.

219: *+.116*

A nucleus population is a small captive population genetically supported by periodic importation of wild caught animals. Periodic importation will allow nucleus populations to maintain the same amount of gene diversity as larger captive populations that do not import wild caught animals. The function of nucleus populations as envisioned by the IUCN/SSC Captive Breeding Specialist Group (CBSG) is to make additional captive space available for endangered taxa not currently maintained in captivity. In this article, mathematical models are developed to assess the effectiveness of the nucleus population concept in reducing the population sizes necessary to maintain appreciable amounts of gene diversity in captive populations. It is shown that the Nucleus I population concept, as defined and promoted by the CBSG, requires an importation rate 10-20 times greater than they have indicated. Whereas nucleus populations are not appropriate for maintenance of significant amounts of gene diversity in long-term breeding programs, small populations can be valuable for research, education, and reintroduction projects with short-term goals. Decisions have to be made on which of the many endangered taxa will be maintained and for what purposes, if captive breeding is to be an effective component of species conservation. (C) 1993 Wiley-Liss, Inc.

220: *-.427*

Disease may play an important role in the decline or extinction of small, isolated animal populations. Disease also has thwarted attempts to reintroduce some endangered captive-bred species. Despite this, the impacts of disease rarely have been considered in the planning and design of reintroduction programmes. A remnant wild population could be decimated by a disease cointroduced with reintroduced animals. Alternatively, diseases that are endemic in wild animal populations could be fatal for those immunologically naive individuals that are reintroduced. We contend that the planning of reintroduction programmes should include an examination of the potential impacts of disease on extant populations and on animals targeted for release. A number of steps are outlined to reduce disease risk and to minimise the probability of failure of reintroductions because of disease.

222: *+.066*

The Raven became extinct in The Netherlands in the First half of this century, mainly as a result of human persecution. Regular breeding ended in most areas in the course of the 1920s. In 1966, a

reintroduction project was started that appeared to be successful. Since 1976 the Raven has bred annually on the Veluwe, a large wood- and heathland area (1240 km-2). After a period of fluctuating low numbers, a marked increase took place from 1984 onwards (fig. 1, 2). In 1992, 50 breeding pairs and at least 31 territorial pairs were found, most of them on the Veluwe. Here, the species occurs in 65% of the 5 times 5 km squares. The spacing of territories is rather regular on the Veluwe, but highest densities occur in the central part of the area. Almost all breeding pairs are located in old pine forests Pinus sylvestris near heathland or drift sands. Since 1990 some pairs are breeding in fir-woods Pseudotsuga menziesii, damaged by gales. Breeding success is slightly lower than elsewhere in Europe, 2.1 young reared per breeding pair (calculated for pairs that started egg-laying only). It is expected that the Dutch breeding area will expand in the next decades, and that Dutch and German populations will interchange before the year 2000 (fig. 3). Territorial Ravens probably stay within their territories during the whole year. Non-breeders often birds occur in wandering groups. Non-breeding birds have been studied systematically since 1989. About 95% of all observations were made on the Veluwe (fig. 4). Since the winter of 1986/87 flocks of 30-50 non-breeding birds are found here each winter. Groups of 6-8 non-breeding birds are observed in the woodland area of the Utrechtse Heuvelrug (240 km-2) since spring 1992. Despite the strong increase, Ravens are seldom seen in agricultural areas. The size and dispersion of the flocks of non-breeding birds on the Veluwe probably depends on the abundance of food. In early spring, flocks of up to 30 birds are regularly observed on heathland in the SE-Veluwe, often in the vicinity of sheep and feeding on still-born lambs and afterbirths (in this, they differ from the breeding birds that stay in pine forests and heathland in early spring, foraging on carrion and beetle-larvae, taken by slashing at putrefied trees). In April and May non-territorial Ravens occur all-over the Veluwe in flocks of 5-15 birds (up to 42 birds). Important food sources in late spring and summer are probably invertebrates, garbage left by tourists and carrion including animals killed by traffic. In autumn and winter up to 50 Ravens may occur near feeding places of wild boar and red deer, foraging on entrails left after hunting. In the course of winter the main groups seem to move from the northeast to the southeast of the Veluwe (fig. 5). In February, groups of 50 Ravens have been observed. In winter 1990/91 a roost (max. 38 birds) was studied on the SE-Veluwe in coniferous woodland, close to major feeding-grounds. Highest numbers were present in February (figure 6). Small roosts are likely to occur elsewhere on the Veluwe as well. In autumn 1992, the Dutch population was estimated at 400-475 individuals, about 95% of which on the Veluwe.

223: ***+.084***

We removed eggs from bald eagle (Haliaeetus leucocephalus) nests in northern Florida from 1985 through 1988 to determine if pairs would lay again and to evaluate how egg removal affected subsequent productivity. Of 58 pairs that had first clutches removed, 45 (78%) laid a second clutch within an average of 29.4 days. In 1 study area, productivity of pairs that had their first clutch removed (1.00 young fledged/breeding attempt) was less ($P = 0.02$) than control pairs (1.47) that produced their clutches during the same time period. In contrast, no difference ($P = 0.75$) in productivity occurred between donor (1.17) and control pairs (1.09) produced during the same period in a second study area. Productivity of donor nests 1 year prior to egg removal was greater ($P = 0.03$) than 1 year after egg removal. However, a simple age-structured demographic model (RAMAS) revealed that population size after 25 years was only slightly higher for the control population. Consequently, egg removals over a limited number of seasons and nests were effective in providing large numbers of eagles for release, with limited adverse effects on Florida's donor population. An egg-removal program may be an effective alternative strategy to captive breeding and translocation of young in recovery actions among raptor populations requiring active management, particularly for tropical species that have long breeding seasons.

224: ***-.017***

One hundred twenty-eight fecal examinations from 57 muriquis or woolly spider monkeys, Brachyteles arachnoides, and 62 fecal samples from 9 brown howling monkeys, Alouatta fusca, that coexist in the Atlantic forest in southeastern Brazil were examined for evidence of parasite infections. Hosts from 4 sites, 2 in Minas Gerais (EBC and FE) and 2 in Sao Paulo (FBR and PECB), showed differences in parasite prevalence and diversity. Neither muriquis nor brown howling monkeys from site 1 (EBC) revealed parasites. Monkeys from site 2 (FE) had only eggs presumed to be Strongyloides cebus and those from site 3 (FBR) had only Trypanoxyuris brachytelesi. Eggs and larvae presumed to be Strongyloides cebus, Trypanoxyuris brachytelesi, Graphidiodes berlai, and an unidentified digenean were found at site 4 (PECB). The 4 study sites differ substantially in size (40-37,000 ha), degree of human disturbance, climate, plant species composition, and muriqui densities. Contrary to expectations, muriquis from the largest and least-disturbed forest (with the lowest population density) had the highest prevalence and diversity of parasitic infection. A variety of factors, including vegetation and climatic differences, could explain this paradoxical pattern. However, the fact that PECB is the site least affected by human disturbance also suggests that the complex ecological web involved in parasitic transmission has been disrupted at the other sites. Our findings reaffirm the importance of using parasites as ecological indicator species in studies of wild primates and suggest that management plans which involve translocations must be sensitive to the potential effects of parasites on naive hosts.

226: ***-.046***

Isolated and long established communities of plants and animals serve as natural laboratories for evolutionary studies. Snails of the genus Partula, indigenous to the tiny island of Moorea in the Society Islands, have been studied for over a century. Research on the separation of species by natural barriers, and on the effects of shell chirality, has provided the best information for understanding the evolution of the genus on Moorea. Unfortunately this field laboratory has been destroyed recently by the ill-advised introduction of a voracious predator.

227: ***+.025***

A cytotaxonomic study was made of Diplodus annularis (Sparidae), Serranus cabrilla and S. scriba (Serranidae) and Gambusia affinis holbrooki (Poecilidae). Inter-individual Robertsonian translocation occurred in D. annularis along with two other chromosome polymorphisms, one being attributed to pericentric inversion, the other involving both the number and location of nucleolus organizer regions (NORs). Polymorphism due to pericentric inversion occurred in the G. affinis holbrooki karyotype. S. cabrilla and S. scriba both showed heteromorphism in the size of the NOR region. Non-differential Giemsa staining, together with NOR-, C- and G-banding techniques in S. cabrilla indicated that this polymorphism was structural rather than functional.

228: ***-.071***

In the last fifty years, 91 reintroductions and reinforcements of Alpine marmots have occurred in is mountainous areas. Most of the reintroductions failed except those attempted in the Alps and Pyrenees, probably because of no formal planning. Increase of species territory and lack of data on population dynamics suggest a limit to reintroductions and the maintainance of well-planned reinforcements.

229: ***-.127***

The Anjouan Scops Owl Otus capnodes, not recorded with certainty since about 1886, was rediscovered in June 1992. It has usually been treated as a race of Madagascar Scops Owl Otus rutilus but, judging from plumage and especially voice, it is a distinct species. It is found only in the remaining patches of upland forest (above 800 m) on Anjouan (Ndzuani), Comoro Islands, where the population is estimated to be at least several tens of pairs, probably between 100 and 200 pairs. The species is highly endangered by accelerating forest clearance and capture for food by the huge and increasing human population. Long-term environmental programmes are essential but may be too late to save this and other endangered species on Anjouan. Species-specific actions are needed: for the owl, a translocation to the neighbouring island of Moheli should be investigated.

231: *-.030*

Geographic chromosomal variation, which in many instances does not correlate with variation in phenotype, is increasingly being detected within both large and small species of mammals. We argue that this cryptic chromosome variation can pose a significant threat to translocation practices involving the admixture of specimens between geographically distant populations. Matings between individuals characterized by different cytotypes can result in perinatal mortality or, at a later stage, in reduced fertility of offspring heterozygous for chromosomal rearrangements. This can thus impact heavily on the species, or population, that management is trying to conserve. Some of the more frequently encountered structural rearrangements in mammals and the possible deleterious effects that these can have in the heterozygous condition are reviewed and guidelines proposed for the use of cytogenetics as a conservation tool in wildlife management.

232: *+.100*

The Western Division of New South Wales is an administrative region of 325000 km2 on the eastern fringe of the Australian arid zone. Since European settlement in 1788, 71 species of native mammals have been recorded in the Division, seven more have been documented only as sub-fossils, and a further 15 species occur within 100 km of the Division boundary. At least 27 of the original species have become regionally extinct, and a further 11 have declined in distribution. As in other regions of Australia, species losses have been greatest for rodents and marsupials in a critical weight range of 35 5500 g, and least for bats. However, percentage losses among the terrestrial fauna are high relative to other regions, and probably reflect both the early settlement of New South Wales and the marginal distribution in the Division of 49% of the original fauna. Feral cats are implicated in the regional extinction of up to ten species of native mammals prior to 1857. Subsequent extinctions and range reductions are attributed to combinations of causes, including predation from cats Felis catus and red foxes Vulpes vulpes, competition and habitat degradation from rabbits Oryctolagus cuniculus, stock and other introduced herbivores, clearing of trees, changes in fire regimes and human persecution. We identified 28 species of particular conservation concern in the Division based on low abundance, distribution or survival prognosis. Nine species are of national significance, four of state, and 15 of regional, significance. The major current threats to these species are from grazing by stock, interference from feral mammals and clearing. Further land reservation is an important conservation measure, but must be complemented by more effective management of non-reserved land and by broad-scale management of feral species and other threatening processes if the current species diversity is to be maintained. Long-term fauna surveys should be initiated throughout the Western Division to provide feedback on the effectiveness of management measures, and species reintroductions should be considered in situations where threats have been removed.

233: ***+.031***

Population characteristics, breeding structure, and sources of mortality of wild water buffalo Bubalus bubalis were studied in Kosi Tappu Wildlife Reserve, a small reserve located in the southeastern lowlands of Nepal, during 1987 and 1988. Comparisons with an earlier census indicated that the population was growing over a 12-year time interval. However, observations on the physical nature of the reserve and known seasonal movements of buffalo suggested that the population is not viable for several reasons. Most morality observed during the study took place over the rainy season, suggesting that flooding was the major source; half of the first-year calves disappeared during that time. Because the reserve was subject to extensive flooding during the rains, buffalo regularly left to reside in cane and sugar fields. Hence the reserve did not include the annual home range of any buffalo herds. In addition, thousands of domestic cattle and hundreds of domestic buffalo used the reserve illegally, which posed a risk of disease to wild water buffalo. Management recommendations, including a reintroduction of the species into Royal Chitwan National Park, are made in light of the analysis.

234: ***+.081***

During the last decade some fanciful hypotheses about a presumed trend of Marsican Brown Bears spreading largely around, and abandoning Abruzzo National Park, have been suggested or admitted by some authors. To explain this alleged phenomenon various explanations, more or less synergic, have been developed: excessive and uncontrolled tourism and/or massive Wild Boars invasion, as a result of unsound reintroduction of these animals from Hunting Organisations in areas surrounding the Park. The authors doubt of these explanations, but on the other hand they collected many bear evidence signs outside of the Park. Cooperating with many other members of Italian Bear Group (Gruppo Orso Italia), a wide monitoring program was developed about bear signs outside of Abruzzo National Park. The aim of the project was to assess how consistent with the evidence might be the hypothesis of the periferic individuals and/or small populations still existing but remained unknown in the past time, depending on scarce interest of local authorities, limited availability of financial resources and so on. On the other hand this idea had been suggested by the founder of Abruzzo National Park Erminio Sipari already in the year 1926. The monitoring program covers the period from 1900 to the present time. More than 310 data have been collected with a good reliability. The percentages of those: 38.3% in the Maiella Massif, 18.8% in the Emici-Simbruini Chains, 8.9% in the Genzana Massif, 18.2% in the Velino-Sirente Mountains, 4.5% in the N-W of Molise Regione and further 8.3% in other spotted areas. The continuous evidence may confirm the original idea of several bear nucleons, which remained virtually unknown in the Park's surroundings. In the authors' opinion the hypothesis of bears "run away" from the Park may consequently be definitely dismissed. The results of the present research strongly support the ideas stressed in many occasions by the Park's Superintendent Franco Tassi, as well as all the conceptions that inspired the Park's policy during the past years: creation of a large Buffer Area around the Park, progressive inclusion of the most sensitive and pristine areas in the Park, development of a system of Protected Areas in the Central Apennines. In other words, the results of this report offer further scientific and ecological support to the project "Abruzzo South European Park": more than 500,000 hectares of magnificent wild areas in which all or most of the territories inhabited not only by bears, but also Abruzzo Chamois, Apennine Wolf and itself as the first wide wildlife management operation in Italy: a conservation scheme strictly connected with the ecological needs of some of the most significant species (from scientific and cultural point of view) of Italian big mammals.

235: ***+.191***

The authors review the present knowledge on the distribution of Pelobates fuscus insubricus in north-eastern Italy and report the first results of a reintroduction programme of this species in the same area.

236: ***+.253***

The value of the preservation of biodiversity, methods of conservation of rare or endangered plants and animals, the role of humans in the distribution of species through introduction (voluntary or involuntary) and reintroduction, and the paradox between natural isolation of species and ecological meshing are wildlife policy is the protection of endangered species in situ or the reimplantation of a plant or animal into an ecosystem regardless of several important factors (role in the ecosystem, chance of recovery, risk of genetic pollution, probability of success, etc.). The ethical aspects of reintroduction strategies are illustrated with examples such as the creation of "natural gardens," or of plant populations devoted to apiculture or to the biological control of agricultural pests. Such operations may seem legitimate from a human point of but their objectives should not be confused with strategies of biodiversity restoration. Introduction and reintroduction no longer appear definitively as recommendable, acceptable or condemnable scientific techniques, but as decisions society must face at the intersection of conservation of original biodiversity and the "artificialization" of nature.

238: ***-.049***

Data from a Badger sett survey undertaken in the mid-1980s were used to calculate the effects of past persecution and land-use changes on Badger numbers. The current British Badger population was estimated to be 41,894 +/- 4404 social groups; if the effects of past persecution were eliminated, the population could be 43,437 +/- 4731 social groups, an increase of 3.7%. Most of this increase relates to persecution in Norfolk and Suffolk last century. In Britain the Badger population is largely confined to those areas which are intensively managed, and the numbers and distribution of Badgers reflect patterns of agricultural activity. However, over-intensive use of the landscape is detrimental to Badger numbers. To quantify the impact of land-use changes on the British Badger population, a number of habitat features favourable to Badgers were identified; 1-km squares that contained five or more such features had significantly higher mean Badger densities. If all the 1-km squares were managed to include five or more of the habitat features favoured by Badgers, and the effects of persecution were eliminated, the Badger population could be 58,284 +/- 5640 social groups, an increase of 39%. The absence of semi-natural broadleaved woodlands in a 1-km square had the greatest effect in reducing Badger numbers, and tree-planting schemes may be beneficial in providing potential new sett sites. However, Badgers are poor colonists, and the construction of artificial setts and the translocation of displaced social groups of Badgers will greatly facilitate the colonization process. The value of such an approach for predicting the effects of future land-use policy on Badgers and other species, and for managing the British wildlife resources, is discussed.

239: ***-.073***

The red swamp crawfish Procambarus clarkii (Girard) has been found in the sponge area of the headwaters of the Crocodile River in the Transvaal. Indications are that it occurs in relatively low numbers despite potentially favourable environmental conditions. Predators such as otters may play a role in its control. The danger exists that it may spread further downstream with time or be translocated to other localities by anglers visiting the site.

240: *+.198*

Germination of Agrostis castellana caryopses is not affected by arsenate. The viability of caryopses of tolerant Agrostis castellana is lower than that of sensitive populations. The possible relation with 'cost' of tolerance is discussed. Tolerant populations of Agrostis castellana and Agrostis delicatula are clearly distinguished by their maximum root growth (MRG) from sensitive populations. The sensitive population of Agrostis castellana reaches 50% MRG reduction at 25 muM As, that of Agrostis delicatula at 60 muM As. The tolerant populations of both species do not reach 100% MRG reduction below 1280 muM As. Among the tolerant populations of Agrostis castellana we distinguish a more tolerant population with no significant MRG reduction in the range of 0-40 muM As from two tolerant populations with 10-25% MRG reduction in the same range. 50% MRG reduction occurs finally in the more tolerant population at 580 muM As, in the other tolerant populations at 320 and 230 muM As, respectively. Tolerant and sensitive populations grown without arsenate do not show significant differences in MRG. The relation between MRG and tolerance mechanisms is discussed.

242: *+.046*

The Shoshone sculpin (Cottus greenei) is found only in springs of the Thousand Springs formation along the Snake River in Idaho. In 1983 a small population of Shoshone sculpin was introduced into an unnamed spring in the Thousand Springs formation in an attempt to increase the range of the species. Previously, the only sculpin in that spring was the mottled sculpin (Cottus bairdi). The Shoshone sculpin was able to establish itself and become the predominant fish within S years.

243: *+.072*

Loss of genetic variability in isolated populations is an important issue for conservation biology. Most studies involve only a single population of a given species and a single method of estimating rate of loss. Here we present analyses for three different Red-cockaded Woodpecker (Picoides borealis) populations from different geographic regions. We compare two different models for estimating the expected rate of loss of genetic variability, and test their sensitivity to model parameters. We found that the simpler model (Reed et al. 1988) consistently estimated a greater rate of loss of genetic variability from a population than did the Emigh and Pollak (1979) model. The ratio of effective population size (which describes the expected rate of loss of genetic variability) to breeder population size varied widely among Red-cockaded Woodpecker populations due to geographic variation in demography. For this species, estimates of effective size were extremely sensitive to survival parameters, but not to the probability of breeding or reproductive success. Sensitivity was sufficient that error in estimating survival rates in the field could easily mask true population differences in effective size. Our results indicate that accurate and precise demographic data are prerequisites to determining effective population size for this species using genetic models, and that a single estimate of rate of loss of genetic variability is not valid across populations.

244: *+.117*

The Insecta is the most speciose class in the Animal Kingdom. The insect-plant relationship is the dominant biotic interaction, yet plants have many times the biomass of all animals together. The functional significance of insects is enormous, owing to the large numbers of individuals and great intra- and interspecific variety. Lack of human appreciation of importance, coupled with the general disregard and dislike of insects, is an enormous perception impediment to their

conservation. This impediment coupled with the taxonomic impediment (at most only about 7-10% of insects are scientifically described) must be overcome for realistic biodiversity conservation. As it is not possible to know all the species relative to the rate at which they are becoming extinct, it is essential to conserve as many biotopes and landscapes as possible. These would be for typical species and communities, as well as for endemic sinks. It is also essential to preserve species-dynamo areas as an insurance for future biodiversity. Preserved areas must also be linked by movement and gene-flow corridors as much as possible. Recognition, functional importance, taxic uniqueness, typicalness, genetic variation and important behavioural traits place much more emphasis on qualitative biodiversity conservation than on quantitative approaches. Ecological entomologists play a significant double role, suppressing noxious populations on crops, livestock and other products, while at the same time identifying and using beneficial species. There are well-known inherent and environmental risks with many traditional control methods and high risks with the use of genetically engineered biopesticide baculoviruses. Preservation technologies, where individuals are held in suspended animation, must be developed soon. However, such technologies, as with restoration activities such as site restoration, captive breeding, reintroductions and translocations, all require considerable knowledge and economic input to be predictably successful. Ecological restoration involves so many biotic and abiotic interactions in even the simplest of communities, that predictiveness under all potential conditions is virtually unattainable. Instead, there should be strong focus on the preservation and conservation of as many, and as large as possible, pristine and near-pristine unique and typical landscapes as soon as possible.

245: *+.093*

Less of genetic variation due to population bottlenecks may be a severe threat for the survival of endangered species. Assessment and maintenance of genetic variability are thus crucial for conservation programs related to endangered populations. Scandinavian beavers went through an extensive bottleneck during the last century due to over-hunting. In Sweden the species became extirpated but in Norway extinction was avoided by legal protection. Following reintroductions of small numbers of remaining Norwegian animals in 1922-1939, the Swedish population has increased tremendously, now harboring 100,000 animals. We show here that this viable population of beavers possesses extremely low levels of genetic variability at DNA fingerprinting loci and monomorphism at major histocompatibility complex (MHC) class I and class II loci. A similar pattern was also evident among Norwegian beavers but low levels of genetic variability were not a characteristic of the species since Russian conspecifics displayed substantial DNA fingerprinting polymorphism. However, the Russian animals were monomorphic at MHC loci, indicating that the European beaver is exceptional in its low level of MHC variability. The results demonstrate that a conservation program can be successful despite low levels of genetic variation in the founder population.

246: *-.066*

Disease risks are an inherent part of captive breeding, translocation, and reintroduction Programs and of research involving contact between researchers and animals. Thus, quantitative assessment of the risks of disease should be an important part of developing conservation strategies. Population viability analysis (PVA) and the use of computer modeling allows conservation planners to examine the potential effects of both the catastrophic and subcatastrophic effects of disease on long-term population viability, measured as probability of extinction, and to explore strategies to mitigate unacceptably high risks. The effects of diseases on the demographic, environmental, and genetic aspects of a population should be examined. Metapopulation

management strategies that establish geographically separate populations are likely to provide protection against total population extinction due to single disease epidemics. However, to offset the genetic costs of smaller population size, movement of animals (under quarantined supervision) may be required.

247: *-.116*

Outbreaks of infectious disease can ruin conservation programs. In order to minimize catastrophic disease outbreaks, it is important to understand the dynamic relationship between populations of threatened hosts and their parasites. The dynamics of the host-parasite relationship are ineluctably linked to host-population density. Intensive conservation management programs are often characterized by small population size. In small host populations, the dynamics of host-parasite relationships are particularly fragile and disease outbreaks will tend to die out rapidly. The most significant disease risks for intensive management programs will probably occur when hosts are chronically stressed, when they are exposed to novel pathogens, and when herd immunity is lost. Biologically diverse parasites will never be entirely eliminated from intensively managed wildlife. Rather, one of the great challenges facing conservation biologists is to learn how to manage the natural and healthy relationship between parasites and their hosts. The population dynamics of host-parasite relationships must be integrated into species survival and recovery plans.

248: *-.333*

Conservation programs for captive breeding and reintroduction of threatened and endangered species need a means to assess the risk of introducing infectious diseases into or obtaining diseases from the reintroduction environment. Risk assessment is seriously hampered by insufficient knowledge of the diseases of these species. Acquisition of data on the infectious diseases of threatened and endangered species can be expedited through cooperative disease surveys of captive and free-ranging animals. Integrating information from various sources will be greatly facilitated by setting standards for data collection. Standard guidelines for monitoring, investigating, and surveillance of infectious diseases in captive animals are presented that will provide essential information for an infectious disease data base. A method for prioritizing infectious diseases by the degree of threat to a species or environment is also proposed, so that the limited resources for disease investigations can be appropriately allocated. Application of these methods to species targeted for reintroduction will reduce the risk of human-caused catastrophic epidemics in endangered species. Furthermore, these methods will significantly increase our knowledge of disease epidemiology in nondomestic species.

249: *+.212*

Diseases in free-ranging wildlife, other than those in species of economic importance or diseases of public health significance. have long been overlooked and thus are rarely monitored. Recent findings, however, demonstrate that disease agents must be considered as integral in shaping many aspects of wildlife behavior and ecology. Very few scientists are trained in or function specifically with the study of diseases of free-ranging wildlife, and funding for such projects is difficult to obtain. The application of new biotechnological tools will no doubt contribute considerably to the rapid advancement of this field. The consideration of disease in all stages of species conservation projects is especially important because of the possibilities of genetic depression of immunity and the introduction of diseases into wild of populations.

250: *+.066*

The relocation of wild mammals and birds for the reinforcement of a population or for the reintroduction of a species that has become extinct in its natural range has become a popular wildlife management technique. Concern has been expressed that these translocated wild animals, which often originate in captive-breeding facilities in a distant country, may bring with them diseases and parasites that could threaten the health of the valuable domestic livestock and wildlife resources of the recipient country. At present, the International Zoo-Sanitary Code of the International Office of Epizootics (OIE) in Paris, which is implemented by all 126 nation members of OIE, applies only to the important diseases of domestic livestock that may affect wild animals. However, proposals are now being made for the collection, through the OIE global network, of data on the worldwide occurrence of wildlife diseases. Following this initiative, regulations for the international movement of wildlife will be reviewed, as will proposals for the standardization of both diagnostic procedures for wildlife diseases and vaccination protocols for the immunization of wildlife. All veterinarians involved in wildlife translocation projects must cooperate closely with national and international animal disease control agencies so that regulations will be both effective and realistic.

251: ***-.144***

Many organisms, from viruses to metazoan parasites, can be spread when domesticated or nondomesticated birds are moved from one locality to another. Theoretically, potential pathogens might establish themselves in released wild birds or be spread from them to other species. There are very few fully substantiated accounts of disease problems associated with translocation or releases of wild birds, and much of the "evidence" has been circumstantial. Serious study of the subject has been confined to Hawaii, Mauritius, and a few other locations. Although such research has added weight to the thesis that infectious disease may contribute to a decline in free-living populations, dissemination of organisms by released birds has generally not been confirmed. The paucity of sound data on this subject is probably attributable to the failure to adequately monitor pathogens and disease in birds prior to and following release. More rigorous research is needed to evaluate the true role of released birds in the introduction and dissemination of pathogenic organisms.

252: ***-.038***

Health and disease are becoming extremely relevant issues for the conservation biology of members of all major groups of vertebrates. The importance of disease in captive propaption, relocation, repatriation, and translocation (RRT) programs is just being appreciated. All of us experience multiple diseases in our lifetimes. Reptiles are not different. They are susceptible to the range of infectious agents known to occur in other vertebrates. The causes of disease in captive reptiles are better understood than those in their wild counterparts. However, several important diseases have recently been documented in wild chelonians. One of these, upper respiratory tract disease in the desert tortoise (Gopherus agassizii) is thought to have been introduced into populations of tortoises in the Mojave Desert in the southwestern USA by release of ill captive desert tortoises. A similar situation appears to exist for certain populations of the gopher tortoise (Gopherus polyphemus) in Florida, USA. Although conservation strategies such as RRT programs have been implemented for a number of threatened/endangered reptiles, results indicate that the success rate is rather low. Because of this low success rate and the recent awareness of the possible introduction of exotic pathogens acquired in captivity, release programs should be scrutinized more closely.

253: ***-.262***

The impact of some diseases on the translocation of African wildlife has been wide-spread. Rinderpest has remained in Africa since its introduction in 1888. The rigid control measures adopted to prevent the spread of rinderpest. foot-and-mouth disease, and theileriosis to domestic animals in Africa restrict the possible translocation of even endangered species. In some cases, the translocation of wild animals has resulted in the transmission of the disease. African horse sickness was carried to Spain in 1987 when zebras were translocated there from Namibia. When springbok (Antidorcas marsupialis) were moved to the Bontebok National Park, Republic of South Africa (RSA), they introduced lungworm (Bronchonema magna), which then infested the bontebok (Damaliscus dorcas dorcas). Translocated wild animals also succumb to endemic disease: heartwater in the Transvaal bushveld prevents the translocation of springbok into this area because this species is susceptible to this disease, and babesiosis has been reported in naive sable antelope (Hippotragus niger) imported into the RSA from Europe. Animals translocated into unsuitable habitats, e.g., roan antelope (Hippotragus equinus) translocated to the Percy Fyfe Nature Reserve, RSA, and gemsbok (Oryx gazella) translocated to the Orange Free State, RSA, do not thrive. Salmonellosis, apparently acquired while being held in enclosures, has been reported in African elephants (Loxodonta africana) and a black rhinoceros (Diceros bicornis). Feline infectious peritonitis has been described in captive wild-caught cheetahs (Acinonyx jubatus).

255: ***+.052***

The farming of freshwater crayfish (astaciculture) is mainly carried out in the southern states of the USA, and in Australia and Europe. Production levels vary with climate but are in the region of 40000 to 60000 tonnes per annum. In addition, at least an equivalent amounts is harvested from the wild, particularly in North America, China, Australia, Kenya, Turkey and Europe. Crayfish farming is usually either of an extensive (ranching) or semi-intensive nature, intensive methods being infrequent, except for the rearing of juveniles for stocking (or restocking of natural waters depleted of crayfish). As crayfish do not have larvae and are polytrophic, they are relatively easy to rear, although fecundity is much lower when compared with other cultured crustaceans. At least 85% of world production is based on the red swamp crayfish, Procambarus clarkii, mainly from Louisiana and other southern USA states, but also from China, Kenya and Spain, where it has been introduced. In Australia, three species are of aquacultural importance, the yabbie, Cherax destructor; the marron, C. tenuimanus; and the red claw, C. quadricarinatus. Some very large production units have been built but none have lived up to their promise. The red claw is thought to have considerable aquacultural potential, but, being a tropical species, needs warm water for good growth. In Europe, the only endemic species cultured to any extent is the noble crayfish, Astacus astacus, mainly as juveniles for restocking. It fetches a higher price than other crayfish. The North American signal crayfish, Pacifastacus leniusculus, has been introduced to most European countries, but farmed production is relatively low. About 98% of crayfish consumed in Europe come from extensive systems or the wild harvest. European crayfish markets were upset by the collapse of the Turkish crayfishery (based on Astacus leptoductylus) due to overfishing and disease in the mid-1980s. The environmental impact of crayfish farming is most noticeable in Europe. Crayfish plague, introduced from North America last century, has devastated populations of the native species in many countries. Its spread has been exasperated by the translocation of foreign crayfish (and probably by fish) for aquacultural purposes. In addition, introduced crayfish frequently escape into the wild and form large populations, often in direct competition with native species. Burrowing and prolific species, such as P. clarkii, can also do considerable environmental damage

256: ***+.065***

Strong Site tenacity might prevent Palila (Loxioides bailleui), an endangered Hawaiian honeycreeper, from repopulating favorable habitats in their former range. We used radio telemetry during the nonbreeding and breeding seasons to study movements and dispersal rates of 57 Palila. All Palila remained in the study area, and home range sizes and movement distances were small relative to the potential mobility of the species. Banding and nesting studies revealed that Palila show strong site tenacity. An inverse correlation between movements and elevation was related to an elevational gradient in food supply. Translocations of Palila into presently unoccupied areas in their range might speed the recovery of this endangered species.

257: ***+.125***

An ecological collapse has precipitated pioneering conservation initiatives in New Zealand. Many terrestrial communities in the New Zealand archipelago have been devastated by over-exploitation, introduced mammals and habitat destruction. More recently, marine ecosystems have been depleted by over-harvesting. To mitigate against these losses, conservation in terrestrial environments has focused on protection of species and habitats. A similar approach is now under way in marine environments with the establishment of 'no-take' marine reserves. On land, conservation is now reaching beyond protection to the eradication of pests from islands and restoration of their terrestrial ecosystems. Restoration on islands not only reduces threats to rare species; it also raises opportunities to investigate how species interact. In the sea, marine reserves not only enhance the diversity of depleted marine communities; they may also augment stocks of commercially harvested species. These initiatives provide many lessons that could be applied to degraded habitats elsewhere.

258: ***+.215***

Only over the last few decades has there been a concerted effort to develop and refine release methods for threatened species. With the goal of achieving self-sustaining wild populations, three main techniques have been employed: parent-rearing, cross-fostering, and isolation-rearing. Although there are many considerations in developing or selecting the most efficient method for any given species, the behavioral aspects of preparing birds for release are important. The concept of different life history strategies may also help in designing a preparation and release methodology. The degree of interspecific and intraspecific sociality also is important in the development of effective behavioral preparation of individuals for release. (C) 1994 Wiley-Liss, Inc.

259: ***+.051***

The wild 'Alala (Corvus hawaiiensis) population has been declining for many years, and only a few pairs of birds are currently reproductively active on the island of Hawaii. A recovery program was initiated in 1993 which included removing eggs from wild nesting birds for artificial rearing and reintroduction. This paper describes the artificial incubation and hand-rearing techniques. Eleven eggs were removed from three nesting pairs; eight were fertile, and seven hatched and were hand-reared (fertility, 72.7%; hatchability, 87.5%; survivability, 100%). Eggs were incubated in a forced-air incubator at 99.5-degrees-F (dry bulb), 80.0-86.0-degrees-F (wet bulb), and hatched under still-air conditions at 99.0-degrees-F (dry bulb) and 88.0-90-degrees-F (wet bulb). Hatched chicks were hand-fed a diet of fruit, insects, and mouse pups. (C) 1994 Wiley-Liss, Inc.

260: ***+.021***

In former times capercaillie occurred throughout the Sauerland, while today areas with suitable habitats still exist for this species. In light of this the Research Station for Hunting and Game Damage Prevention of North Rhine/Westphalia conducted reintroduction trials of this species from 1980 to 1992. These trials were located in the area around Hunau a secondary chain of mountains between Fredeburg, Bodefeld, and Altastenberg. The birds were first hatched and raised in cages, and at the age of 16 weeks transferred to large flight cages for four weeks to adapt to free land conditions before being released. The goal was to release 50 individuals/year in this manner over a period of 10-15 years. For this purpose a breeding station was established at the research station. The planned number of capercaillie (15/year) released annually could only be attained in the last 4 years of the trial. A total of 393 capercaillie (226 cocks, 167 hens) were released during the trial period. A large proportion of the released birds succumbed primarily to predation; a few were killed by accidents. Sick or lost animals were not observed. Some of the cocks lived 5-7 years as could be ascertained by individual ringing. In 1986 a nest with 7 eggs at the foot of a spruce tree was found in the trial area, and repeated observations were made of free land raised young birds. In the spring of 1992 a population of 30-40 capercaillie over an area of 20,000 ha were present in the Sauerland due to the re-introduction trials. The establishment of a population of 30-40 capercaillie is a successful result. However, this population is still insufficient to perpetuate itself. The re-introduction of more birds is necessary. As of 1993 the Association for the Preservation of Capercaillie in the Sauerland, Siegerland and in Wittgensteinerland will continue the re-introduction trials.

261: *+.132*

The results of rarity species investigation of Kryvyi Rih urban flora are presented. The list of rare, endemic and vanishing plants of Kryvyi Rih urban flora numbers 82 species of 59 general 33 families and 3 sections. There are 16 species from the Red Book of the Ukraine (second edition) among them. Rarity of the species majority depends on the anthropogenous and technogenous factors first of all and on natural and historical ones at least. The analysis of rarity species for Kryvyi Rih urban flora, demonstrated the location of the third part of these species in the reserved territories and their protection from extraction. However, the species from the Red Book of he Ukraine Cymbochasma borysthenica, Caragana scythica, Genista scythica, Chamaecytisus skrobiszewskii, Bulbocodium versicolor occurred outside the reserved territories. The registration and certification of these species localities have been carried out for the Purposes of their preservation. Necessity to decree the moratorium on economic activity for such a territories before the solution of problem have been decided. The question is raised about foundation of townspeople's reserve placed under the authority of Kryvyi Rih Botanical Garden, Academy of Sciences of tile Ukraine. Flora and vegetation of the reserve will be the unique model for investigation of theoretical and practical problems of phytosozology under anthropogenous stress, industrialization and urbanization. Introduction of rare species in botanical gardens and re-introduction in natural and artificial phytocenoses, renewal of natural vegetation in anthropogenously transformed territories, organization of the bank for seeds of rare, endemic and vanishing species are the complementary measure.

262: *+.034*

The florosociological role of a small river basin under the conditions of anthropogenous transformation of the landscape is considered at the example of the Kazenny Torets (the north-western macroslope of the Donetsky ridge, the Don's system). The flora of the Kazenny Torets basin comprises 964 species which are divided into seven groups according to a degree of their modern distribution: 243 species occur everywhere (25%), 407 species are usual in specific

habitats (42%), 200 species (21%) are sporadic, rare species (up to 10 habitats) - 57 species (6%), 21 species (2%) occur in 2-3 habitats, 28 species (3%) are in 1 habitat, 8 species might have disappeared. 10 species are registered in the "European list" and 33 - in the Red book of the Ukraine. Methods of the comparative analysis have shown considerable anthropogenous transformation of the Kazenny Torets basin's flora, which reflected in impoverishment of the autochthonous nuclear, increase of the apophyte fraction share and significant perturbations of partial and florocenotic structures. It is dangerous that under the present conditions it is impossible to conserve the flora of the Kazenny Torets basin, because a share of species preserved on the territories of the nature-reserve fund (existing and which will be) does not exceed 40% and the rest unploughed plots do not meet the requirements of nature-protected territories. Revitalization of the vegetative cover of such plots as the Kazenny Torets basin requires considerable time and expenditure for reintroduction measures.

267: ***+.037***

The Northern Hairy-nosed Wombat Lasiorhinus krefftii now survives only in Epping Forest National Park, central Queensland. The species was formerly more widespread in the Epping Forest region than at present. Its decline appears to have occurred in three distinct episodes of contraction, two of which were associated with prolonged drought. Indirect monitoring of abundance suggests that the population was stable from 1974 to 1981, when cattle were excluded from the Park, but has increased since 1983. A trapping programme between 1985 and 1989 suggests that the absolute size of the population may be about 70. The population contains approximately equal numbers of males and females; almost all females breed; and a large and apparently increasing proportion of the population consists of young animals. Further increases in density and range within Epping Forest National Park appear possible. Removal of some animals for translocation should be attempted when it is clear that such removals will not jeopardize the stability and vigour of the Epping Forest population.

268: ***+.190***

A programme to improve the conservation status of Whitaker's skink (Cyclodina whitakeri), previously confined to <20 ha of usable habitat, is described. The programme involved eradication of introduced Pacific rats or kiore (Rattus exulans) from 18 ha Korapuki Island (Mercury Islands, north-eastern New Zealand), documentation of the response of five species of resident lizards to release from the effects of rats, and transfer of 28 Whitaker's skinks from nearby Middle Island between 1988 and 1990. Following removal of rats from Korapuki, resident lizard numbers at some coastal sites increased within 12 months and rose 30-fold over 5 years, but measurable increases of numbers of lizards in forest areas took up to 6 years. Fifteen of the founding Whitaker's skinks on Korapuki Island have been recaptured 36 times since their release, and five Korapuki-born young have also been caught. The population is now estimated as 33. The increase in number of resident lizards and the success of the introduction of Whitaker's skinks demonstrate that predation rather than habitat deficiencies were responsible for the depleted resident lizard fauna on Korapuki Island. Repatriation strategies for species with low intrinsic rates of increase are proposed.

269: ***+.157***

Six species of Leiopelma frog endemic to New Zealand have been described, but three are extinct. Field surveys have extended the known contemporary ranges of L. archeyi and L. hochstetteri, though sub-fossils reveal that both L. hochstetteri and L. archeyi/hamiltoni were formerly more

widespread in New Zealand than they are now. A new North Island population of terrestrial Leiopelma resembling L. archeyi has recently been found. Introduced predators and food competitors, especially Rattus, have probably had a major detrimental impact on Leiopelma. No extant species is immediately at risk of extinction, but L. hamiltoni on Stephens and Maud Islands is very restricted in range and/or numbers. Leiopelma reaches high densities (up to 8 frogs/m(2)) in suitable rock-strewn habitats and can be relatively long-lived (L. archeyi 17+ years, L. hamiltoni 23+ years). Population levels of L. archeyi have fluctuated in a Coromandel study plot sampled approximately annually over 1982-93, but on Maud Island L. hamiltoni numbers were more stable or increased slightly over the years 1983-93. Experimental translocations of L. hamiltoni were made on Maud Island (1984-85) and on Stephens Island (1999). The new colony on Maud Island has bred successfully, and locally bred young have been recruited into the population.

270: *+.277*

Few guidelines address the considerations and criteria necessary for judging the appropriateness of translocating species onto designated conservation areas. This paper presents a dichotomous key that will assist natural resource decision-makers in assessing the biological and genetic needs and impacts of introducing, reintroducing, or augmenting species. I argue that each translocation decision should be well documented prior to action in a format that includes current and proposed site management information, consideration of the factors addressed in the key, and monitoring of translocated individuals or populations. Although translocation should not be viewed as an alternative to in situ conservation of species, this tactic may be necessary for conservation of species or processes in natural areas.

271: *+.090*

In the upper Great Lakes region, Myriophyllum spicatum, Elodea canadensis, Potamogeton crispus, and Ceratophyllum demersum are the most significant nuisances. M. spicatwn and P. crispus have generally invaded lakes with hard water, abundant nutrients,and a history of disturbance or heavy human use. M. spicatum has declined in some Wisconsin lakes, but not in all that it invaded. The reason for declines was never satisfactorily explained. Following its decline in Madison, WI, lakes, the M. spicatum population never regained its former predominance. There are no long-term data to determine whether populations of native macrophyte species are expanding, stable, or declining on a regional basis, although diversity of the aquatic plant communities of Madison-area lakes has declined over the last 80 years. Most aquatic plant management in the upper Great Lakes region involves controlling nuisance species. Herbicides and mechanical harvesting are the primary means of management, although drawdowns, bottom blanketing, and other methods are also used. Minnesota and Wisconsin have public education programs intended to promote early detection of nuisance species and to minimize their spread. In Wisconsin, reintroduction of aquatic vegetation to lakes is being attempted on an experimental basis for a variety of reasons, including fish and wildlife habitat and water-quality improvement.

272: *+.061*

Zoos are becoming increasingly important as sites for captive breeding programs for endangered animals. The ultimate goal of many of these programs is reintroduction of these species into their natural or restored native habitats. This text presents a multidisciplinary review of captive breeding efforts, including philosophical, biological, and economic aspects. Thirty-one chapters are divided into four sections, covering general issues, reintroduction, case studies, and regional

approaches. Chapter topics include the role of flagship species, meta-populations, reproductive technologies, criteria for reintroduction, and conservation assessment and management plans. Much of the information contained herein was originally presented at an international conference on breeding endangered species held on the Isle of Jersey in 1992. The text is illustrated and indexed.

275: *+.045*

The Seychelles warbler Acrocephalus sechellensis was once a highly threatened single-island endemic species with a population of 26 individuals confined to Cousin Island in the inner Seychelles. Following long-term management of Cousin, the population steadily recovered to around 300-360 birds. Given the vulnerability of one small island in the Indian Ocean, the possibility of establishing the species on additional islands had been proposed as a priority conservation measure, in ol del to give the species the security of additional breeding populations, lest some ecological disaster should befall the parent population. Successful translocations of warblers to the islands of Aride and Cousine took place in September 1988 and June 1990 respectively. Given the presence now of thr ee healthy breeding populations, it is considered that the Seychelles warbler will soon no longer be a globally threatened species. It is not often that people are allowed to pull a species so dramatically backfrom the brink of extinction.

276: *+.175*

The number of known sites (populations) documented by conservationists for the natterjack toad Bufo calamita between 1970 and 1990 in Britain rose from 21 to 40, excluding those resulting from translocations. The progressive reduction in rate of discovery of undocumented sites suggests that few further populations will be located. The real overall number of natterjack sites has probably changed little over this period, with successful translocations approximately compensating for the few (three to five) concurrent extinctions. More than 90% of natterjacks in Britain now live in the vicinity of just five river estuaries. Habitat protection, mainly the result of scheduling land as Sites of Special Scientific Interest (SSSI), has been increasingly effective in safeguarding natterjacks, the proportion of safeguarded sites rising from 60% in 1970 to 83% by 1990. However, the legislation for species protection has proved much less effective. Much of the recent damage on unscheduled sites was a result of agricultural improvements by landowners ignorant of the presence of natterjacks on their land. The breeding records from natterjack sites are wed to examine the possible impacts of radioactivity and climate change, against which site protection alone is no safeguard. Recommendations are made for conserving natterjack populations in the future.

277: *-.080*

Examining the dynamics of naturally declining populations can be useful in understanding some of the problems encountered by endangered populations. A study of a declining population of vervet monkeys Cercopithecus aethiops provides a unique opportunity to examine factors associated with minimum social group size, a potentially key variable in population viability analysis. The minimum group size in vervets ranged from three to six individuals, but always with a minimum of two adults. Groups appeared to be stable as long as there were at least two adults, but ceased to exist as independent groups within days of losing their penultimate adult. For species with discrete group structure, population extinction could occur if all individual groups fall below a minimum social group size, even if the overall population is above a 'minimum' size. However, group fusions may minimum population losses. Group fusion in vervets is rare, and appears to

occur only as a behavioural response to minimum group size. We also review evidence of the costs vervets incur through movement into unfamiliar places or groups (analogous to translocation and reintroduction), and the benefits they can receive from living in proximity to humans.

278: *+.038*

One potential measure to counteract road mortality of common toads Bufo bufo is the establishment of populations at artificially created ponds by translocating adults' This is thought to be difficult because of this species' strong site fidelity. We describe here a successful resettlement of a substitute breeding pond in Northern Germany (Lower Saxony). We first created a barrier system made of used metal vehicle crash barriers which successfully prevented toads from crossing a road to reach a breeding pond. The toads were collected annually in pitfall traps and transferred to the substitute breeding pond. A majority of the adult population of common toads became attached to the substitute breeding pond after only two to three years. The migration directed at the old breeding site dropped to approximately 15% after four years and subsequently to less than 1%. Our findings show that the provision of substitute breeding ponds can be a useful measure for the protection of amphibians.

279: *+.094*

A survey of historical records revealed that the disappearance of characteristic rich-fen bryophytes such as Scorpidium scorpioides, S. revolvens and Campylium stellatum from the springs of De Mosbeek, the Netherlands, has taken place between 1951 and 1956. A study of the present ecohydrological situation suggests that the present water chemistry (pH, EC and Ionic Ratio) and trophic status (P concentrations) fall within the range suitable for S. scorpioides and that the water is well-buffered. To test the hypothesis that the present absence of rich-fen bryophytes is due to past habitat change rather than ecohydrological conditions, a transplantation experiment with S. scorpioides was carried out. Reintroduction of the species resulted in establishment of new shoots throughout the spring channel up to a distance of 2 m from the initial transplants within three years, which confirmed that the water composition is not detrimental to the species. It is unknown what caused the disappearance of the species in the 1951-1956 period An unusual rainfall pattern, increased atmospheric deposition and changes in management all could have played a role. Historical data suggest a strong expansion of Sphagnum spp. and, via this, the disappearance of S. scorpioides and associated species. The present absence of S. scorpioides seems to be due to poor dispersal and establishment rather than habitat conditions.

280: *+.225*

We investigated the effect of five plant and habitat characteristics on the national distribution of scar ce British plants defined as those occurring in 16-50 10 x 10 km squares ('loci'). rn all 139 species considered loci were more aggregated than expected by chance. Habitat, dispersal ability and pollination type were significantly correlated with pattern of locus distribution. Seed size and life history were unrelated to the degree of aggregation of loci. The most important result from a conservation perspective is the tendency for plants with pool dispersal ability to have more aggregated distributions (i.e. they have loci closer together) than plants with good dispersal ability. One implication of this result may be that reintroduction or translocation could be useful techniques for supplementing natural dispersal in the management of such species.

282: *-.010*

The article briefly reviews one field of plant biotechnology, which can be used in conserving endangered plant species. Micropropagation could be applied to plant species which can not be efficiently propagated by conventional methods. Rubus humulifolius C.A. Meyer is a Rosaceae plant species, which became extinct in 1957 in its only natural habitat in Finland. In 1986 one specimen was taken from a forest, where about 10 individuals had been preserved. At 1500 plants were propagated from this individual through tissue culture. They were successfully reintroduced in the vicinity of the original habitat in 1988. This is probably the first reported case of large scale micropropagation of an endangered plant species for reintroduction.

283: *+.110*

In Finland, Pseudophilotes baton schiffermuelleri is an endangered blue butterfly associated with open and dry esker habitats. During this century P. baton has been recorded from about twenty sites, but only one population is known to have survived, at Sakyla, SW Finland. Forest fires and from 1963 onwards the military rifle and grenade shooting ranges have kept the southwestern slopes of the sandy esker open and suitable for the butterfly. The population size and movements of P. baton were investigated by mark-recapture method and its habitat preferences were studied. The total adult population size was estimated in 1990 at about 850 individuals. The number of males increased as the tree cover of the habitat decreased. The proportion of bare mineral soil and the coverage of the host plant Thymus serpyllum also contributed to the habitat preferences of the butterfly. Individuals flew on average more than 100 m, and females flew longer distances than males. A conservation programme is proposed for P. baton, including active habitat management and a reintroduction plan.

284: *+.121*

Gray wolves were systematically and fervently eliminated from the northwestern United States between the mid-1800s and early 1900s. Wolves disappeared from lower elevations first and generally persisted longer in more remote, mountainous areas. Preservation of large tracts of public land, primarily for commodity use, at the tum of the century, had the unforeseen effect of allowing conditions for wolf recovery to occur later. Improving attitudes toward the species and the recovery of ungulate prey populations from their tum of the century lows are the proximate factors making wolf recovery possible in areas with vast public lands. Planners for wolf recovery in the Northern Rockies identified three areas for wolf recovery, northwestern Montana, central Idaho, and the Greater Yellowstone area, because they consisted primarily of national parks, designated wilderness, and national forests. Those areas had previously been designated as public lands largely because they were too unproductive for agriculture; they consist in part of high elevation habitat that supports relatively few prey for wolves in winter. So far, recolonizing wolves have settled in lower elevation habitats where deer and elk are most abundant. Since private lands are most often in these lower elevations, they may be more important to the recovery and maintenance of viable wolf populations than was earlier envisioned. The negative symbolic nature of the wolf was a major factor in its eradication and continues to be a major factor in considerations of reintroduction and natural recolonization; the newer positive symbolic nature of the animal will ultimately facilitate its return and contribute indirectly toward long-term conservation of wild spaces and biodiversity in North America.

287: *+.097*

Some fem species form spore banks - reservoirs in the soil of viable spores which remain dormant while buried but germinate in light if brought to the surface. The recently discovered

characteristics of these spore banks are described. Enough is now known to suggest that they might have a role in the conservation of endangered fem species as alternatives to ex situ collections of sporophytes, gametophytes and spores, the relative merits of which are also considered. Mature sporophytes of several British species have now been raised from natural spore banks in soil samples; if this proves to be possible also for endangered species, some interesting options become available. The possibilities are discussed of augmenting surviving populations and increasing their genetic diversity, even perhaps of retrieving lost populations, by reintroduction of spore bank-derived plants or by stimulating regeneration from spore banks in situ. Botanic gardens are well placed to provide the further research, the regular monitoring of endangered populations, and the taxonomic and horticultural support required to realise these possibilities.

288: *+.113*

Fifty percent of all the mammal species to have become extinct worldwide in the past 200 years have been lost from the Australian fauna giving Australia the worst record for mammal conservation of any country or continent. Sixteen species from a mammal fauna of 245 are believed to be extinct, 26 species now occur only as remnant populations occupying <20% of their former ranges. Offshore islands, tropical Australia, and the mesic fringe of the continent have provided refuges where mammal communities have survived. relatively intact. The drier interior regions, which include cereal growing areas, intensive and extensive pastoral areas, and the little-used Triodia deserts, have lost many species. Extinctions and declines have not occurred equally throughout the Australian fauna, but have occurred at a disproportionately higher rate among medium-sized ground-dwelling mammals in the weight range 0.035-5.500 kg. Historically, conservation of mammals has concentrated on protection from hunting and trade, reservation of land as national parks and nature reserves, and faunal surveys to map distribution and abundance. These approaches have been necessary but insufficient to either stabilize the decline of endangered mammals or to promote their recovery. The past 20 years have seen many attempts to conserve endangered mammals, either by evading the presumed cause(s) of extinction or decline by translocation to islands, or by managing these causes within reserves by controlling exotic predators, controlling or excluding exotic herbivores, or applying a particular fire pattern or regime. The most significant successes have come with effective control of exotic predators, either by establishing populations of endangered species on predator-free islands or by the intensive use of 1080 poison to control exotic predators at mainland sites.

291: *+.292*

Re-introductions are increasingly used conservation tools. Often, criteria for re-introducing species are based on policies or politics and little attention paid, albeit theoretical, to understanding what ecological possibilities habitats may have in sustaining introduced animals. Assessing potential carrying capacities is complex but easier for grazers, since biomass of these herbivores is empirically correlated with habitat primary productivity. The case is made here that the Barbary macaque, Macaca sylvanus, a vulnerable North African primate with a large surplus captive stock, can be viewed as a grazer. Because of this attribute, and unlike congenerics, it is possible to estimate potential densities in extant habitats in a fashion similar to predicting stocking levels for domestic herbivores. Thus, from values of consumable primary productivity for domestic stock in Mediterranean countries and the monkey's energy requirements, attainable macaque populations in studied habitats could be much higher than actual. Though these numbers may be unreachable in nature, this study shows that present macaque populations could increase after restorative management of habitats in which re-stocking with captive-born animals may play a part. However, since only 10% of potential monkey habitat in Morocco and Algeria is occupied by the species,

finding areas for releasing captive-born macaques is more advisable.

292: ***+.019***

In Thailand, a predominantly Buddhist country, isolated populations of long-tailed macaques (Macaca fascicularis) especially may be found at temples in both rural and urban areas where they are provisioned by people making merit although the monkeys may be shot as agricultural pests in adjacent rice or corn fields in rural areas. At the same time, macaques and gibbons, especially the lar gibbon (Hylobates lar), have been kept as pets in both rural and urban areas. Gibbons even are reported to be the <>. The number of primates being offered for sale has risen dramatically as legal and illegal timbering have reduced Thailand's forest cover to, perhaps no more than 12 %, peripheralizing primate populations and making them more vulnerable to hunting. A provision in Thai law permitted individuals to possess as many as two primates or other wild animals of the same species - <> - even though their capture and sale was prohibited. Changes in Thailand's wildlife laws governing private possession of wild animals have caused owners to abandon pets to government agencies and temples. Private rescue centers offer acute care and permanent shelter for some of these primates but cannot cope with the numbers involved. Pilot studies suggest that rehabilitation and reintroduction may be feasible alternatives for some former captive gibbons.

293: ***-.094***

The European wildcat, Felis silvestris, faces a number of threats to its population in Scotland and continental Europe. Population minimums probably occurred in Europe at the beginning of the 20th century. Several populations have recovered since, due to some restoration of preferred habitat and a reduction in persecution. Our study of European wildcats in Scotland found that direct killing because of alleged attacks on gamebirds and livestock still occurred. Accidental killing by dogs, snares or poison baits is probably still common. Viral diseases, such as feline leukaemia have recently been identified. Low levels of dieldrin were detected in two wildcats, but few other toxic agrochemicals were found. A major threat to European wildcats is the loss of speciation due to hybridization with domestic cats. Many introgressive hybrids are now reported. These can be readily detected by skull measurements and coat markings. Major reintroduction programs are probably not necessary to "save" the wildcat, but local education of hunters, reductions in feral domestic cats and instigation of wildlife management procedures would greatly enhance the outlook for the species.

294: ***+.013***

We conducted DNA fingerprinting analyses to ascertain the mating system and population genetic structure of the palila, an endangered Hawaiian honeycreeper, which occupies a fragmented range on the Mauna Kea volcano of the island of Hawai'i. DNA fingerprinting of twelve complete families from the Pu'u La'au population revealed no evidence of extrapair fertilization or intraspecific brood parasitism. Band-sharing coefficients from fingerprints produced with two probes revealed that the large Pu'u La'au population on the southwest slope of Mauna Kea, and a smaller, geographically separate population on the east slope (at Kanakaleonui) had relatively high and virtually identical levels of minisatellite variability (mean S of 0.27 for each population based on combined data of M13 and Jeffreys 33.15 probes). The two populations also had nearly identical allele frequencies based on their mean corrected similarity, S-ij. of 0,98. These data suggest that the two populations have not been fragmented long and/or have sufficient current gene flow to ameliorate any affects of genetic drift, We conclude that present levels of inbreeding are low within both populations, and that proposed translocations of individuals from Pu'u La'au to

Kanakaleonui appear appropriate from a genetic standpoint.

295: *+.173*

Deliberate reintroductions of locally exterminated animal species to areas within their former ranges is an increasingly important conservation tool. Most reintroductions are fairly recent and still in an initial phase of population development. There are few long-term studies of reintroduced populations. The aim of this study was to see if the population development of the reintroduced European beaver (Castor fiber) population in Sweden exhibits the same pattern of population development as other introductions, in accordance with general theories, and to discuss possible management consequences. Since the European beaver was reintroduced to Sweden 70 years ago, the population has developed in a way predicted by the Riney-Caughley model for introduced ungulates, exhibiting an irruption and a subsequent decline. In two study areas, the rate of population increase (r) turned negative after 34 and 25 years and at densities of 0.25 and 0.20 colonies/km2, respectively. The data suggest that management policy for an irruptive species should allow hunting during the rapid-increase phase, thus maintaining food resources and avoiding uncontrolled population decline.

296: *+.108*

The dispersal patterns of radio-collared Corsican mouflon (Ovis musimon) ewes inhabiting a low Mediterranean massif in the south of France were followed. Despite spatial instability being more marked in winter and spring, females (n = 17) remained on a single home range. The changes in spatial behaviour which appeared in March did not seem to be linked solely to ecological parameters. The use of the spring range was particularly noticeable among the different seasonal ranges since it was characterised by long-distance movements (n = 16, x = 740 +/- 320 m) and the large overall area used (x = 330 +/- 90 ha). The age of individuals had an important influence on space use. With increasing age, spatial patterns became more firmly fixed, which appeared to be linked to a better understanding of the different ecological and social contexts occurring in the population. Some characteristics of spatial behaviour may create certain management difficulties in the introduction or reintroduction of the species. Age and season seem to have a strong effect on the organization of spatial patterns. Managers and hunters should therefore consider the implications of the age of individuals and thus their stage of development, and also of the season, to guide management decisions. For example, disturbance of young individuals must be strictly limited, particularly during the spring and the rut which appear to be important periods in the organization of spatial behaviour patterns.

297: *+.021*

In former times capercaillie occurred throughout the Sauerland, while today areas with suitable habitats still exist for this species. In light of this the Research Station for Hunting and Game Damage Prevention of North Rhine/Westphalia conducted reintroduction trials of this species from 1980 to 1992. These trials were located in the area around Hunau a secondary chain of mountains between Fredeburg, Bodefeld, and Altastenberg. The birds were first hatched and raised in cages, and at the age of 16 weeks transferred to large flight cages for four weeks to adapt to free land conditions before being released. The goal was to release 50 individuals/year in this manner over a period of 10-15 years. For this purpose a breeding station was established at the research station. The planned number of capercaillie (15/year) released annually could only be attained in the last 4 years of the trial. A total of 393 capercaillie (226 cocks, 167 hens) were released during the trial period. A large proportion of the released birds succumbed primarily to predation; a few were

killed by accidents. Sick or lost animals were not observed. Some of the cocks lived 5-7 years as could be ascertained by individual ringing. In 1986 a nest with 7 eggs at the foot of a spruce tree was found in the trial area, and repeated observations were made of free land raised young birds. In the spring of 1992 a population of 30-40 capercaillie over an area of 20,000 ha were present in the Sauerland due to the re-introduction trials. The establishment of a population of 30-40 capercaillie is a successful result. However, this population is still insufficient to perpetuate itself. The re-introduction of more birds is necessary. As of 1993 the Association for the Preservation of Capercaillie in the Sauerland, Siegerland and in Wittgensteiner-land will continue the re-introduction trials.

298: *+.046*

Little is known of the life history of vultures. The reintroduction program of Griffon Vultures (Gyps fulvus fulvus) in the Causses (south of the Massif Central, France) and extensive monitoring by capture-mark-resighting of the released birds allowed us to obtain the first estimates of their survival. Adult survival rates are high ((x) over bar = 0.987 +/- SE of 0.006). A release effect on adult survival was detected (only 0.743 +/- 0.006 survival during the first year after release). Young born in the wild (less than three years old) had an annual survival rate of 0.858 +/- 0.039. Mortality causes and erratic behavior of immature birds are considered in order to assess the effectiveness of this reintroduction program. Our results indicate that reintroductions of vultures and similar species should use adults that have bred in captivity within the target area rather than juveniles or immatures.

301: *+.117*

Predation by introduced foxes and cars is generally thought to be the main reason for the poor success rate of macropod reintroductions on the Australian mainland. Predator-prey theory suggests that predation may have particularly severe impacts on very small populations, especially if a more common primary prey species is present (such as the rabbit). Thus, a sufficiently large reintroduction may overcome predation and succeed where a smaller one would fail. The minimum viable population would, however, be much larger than that predicted by standard population-viability analysis. We use a simple stochastic model based upon the bridled nailtail wallaby to explore this possibility. Even very small amounts of predation (2-4 individuals per six months) can be sufficient to cause reintroductions of up to 50 animals to fail. No clear threshold population size beyond which reintroductions will succeed is evident and, for a given mean, the probability distribution of predation has a very limited impact on the success of reintroductions. In almost all circumstances, a single reintroduction of a given size is preferable to multiple reintroductions of the same total number of individuals.

302: *-.087*

Physiological responses of conserved plants (transpiration, irrigation, water deficiency in leaves) to the change of environmental conditions (illumination intensity, temperature, air humidity and soil moisture) caused by anthropogenic influence on phytocenosis were studied.

303: *+.101*

Ospreys historically nested throughout the wooded portion of Minnesota including the Minneapolis-St. Paul metropolitan area. They were eliminated as a nesting species in the southern two-thirds of the state before the turn of the century, primarily due to uncontrolled shooting. In

1984 a reintroduction effort was begun to restore breeding Ospreys to wildlife preserves in the western portions of the Twin Cities. In 1992, the program entered a new phase when efforts began to return Ospreys to urban metropolitan lakes. Osprey chicks were translocated from nests in north-central Minnesota at 5 1/2-7 weeks of age and released when they reached flight stage using modified raptor hacking techniques. A corps of over 75 volunteers was enlisted to monitor the fledglings from dawn to dusk for 4 weeks after their release. Between 1984 and 1993, we released 119 wild-caught Osprey nestlings at 5 sites in the Minneapolis-St. Paul Metropolitan area. The first nesting attempt took place in 1986. In 1993, 9 nesting attempts involving released birds were recorded. These resulted in 15 fledglings. We have recorded mortality of six birds in the vicinity of the hack box. An additional three post-fledgling birds have been reported dead from Central and South America. Ospreys learn to fly and fish without adult supervision. The Twin Cities Ospreys have become tolerant of human activity and the birds use perches in areas of moderate human activity, as well as in more secluded areas. Reintroducing Ospreys to a metropolitan area is an effective contribution to restoring an ecosystem, as well as educating the general public about raptors, wildlife, general ecological principles, and the human role in natural resources conservation.

304: ***+.175***

After their extinction as a breeding species in 1916 Ospreys returned to Scotland in 1954 to breed with one pair. In 1959 the Royal Society for the Protection of Birds established a public observation post at this eyrie at Loch Garten and over 1.7 million visitors have viewed the nest since. Protection of the birds through guarding of eyries and the construction of artificial nests made the population grow to 94 pairs in 1994. Over the last 40 years at least 1281 young have been reared from a total of 995 nesting attempts, giving a productivity of 1.29 young per occupied nest. The population has been monitored annually, in 1967 a ringing program was started and since 1972 an annual newsletter has been produced. Ospreys in Scotland are breeding in small and loose 'colonies' with a mean distance of 39.7 km between each other (min. 12.5 km, max. 67.5 km). Over 90% of the new eyries are located in the East, which has a higher human population, although above 90% of the historical sites were in the western half of the country. The faithfulness of Ospreys to their natal areas results in a slow extension of the breeding range. Means for the restoration of the natural breeding range such as the provision of artificial nests and the translocation of young to potential nesting areas are discussed.

305: ***+.287***

Brown trout population maintained by annual restocking were studied in Siena area. This species, introduced 30 years ago, showed a satisfactory growth even if, its reproductive capability is still unknown. However, according to previous evidences, the native trout in the Tyrrhenian regions probably is Salmo (trutta) macrostigma. This study indicates that Salmo (trutta) macrostigma is more suitable for environmental conditions of these streams and its re-introduction is suggested.

310: ***-.006***

The biology of most of the indigenous parastacid crayfishes inhabiting the highlands of New South Wales is poorly known, but many species have very limited ranges. Analysis of these distributions in relation to the National Parks and State Forests shows that most species are protected in reserves; however, around and within these areas a large number of potential polluted sites have been identified. The effects of clearing, longstanding salmonid stocking and the potential problems of recent widespread introductions of non-indigenous Cherax species for

aquaculture are discussed. Recommendations for conservation and future management include: biological programmes to provide data on environmental preferences, interactions between indigenous and non-indigenous crays and influences of introduced salmonids; restoration and maintenance of riparian zones; surveys of polluted sites in, or adjacent to, very small ranges with initiation of remedial measures where necessary; more active policing of quarantine measures at aquaculture facilities; development of comprehensive, workable and enforceable policy on translocations; implementation of eel control measures at large impoundments on eastern drainages.

312: *+.139*

One of the most common tools in New Zealand conservation is to translocate species to new locations. There have now been over 400 translocations done for conservation reasons, mainly involving terrestrial birds. Most translocations have been done strictly as management exercises, with little or no reference to theory. Nevertheless, translocations always involve some underlying theory, given that people must inevitably choose among a range of potential translocation strategies. We review theory relevant to translocations in the following areas: habitat requirements, susceptibility to predation, behavioural adaptation, population dynamics, genetics, metapopulation dynamics, and community ecology. For each area we review and evaluate the models that seem to underpin translocation strategies used in New Zealand. We report experiments testing some of these models, but note that theory underlying translocation strategies is largely untested despite a long history of translocations. We conclude by suggesting key areas for research, both theoretical and empirical. We particularly recommend that translocations be designed as experimental tests of hypotheses whenever possible.

313: *+.020*

The wild alala Corvus hawaiiensis population has been declining for many years and only three pairs of birds are currently reproductively active on the island of Hawaii. At the recommendation of a committee formed by the National Academy of Sciences, a restoration programme was initiated in 1993 by The Peregrine Fund in collaboration with private land-owners, the US Fish and Wildlife Service, National Biological Service and the State of Hawaii. The restoration programme includes removing eggs from wild nesting birds for artificial incubation, handrearing and reintroduction. In two breeding seasons (1993, 1994), 17 eggs were removed from alala nests in the wild. Three eggs were infertile, 13 chicks hatched and 12 alala were successfully reared (hatchability: 93 per cent, survivability: 92 per cent). Four of these chicks were sent to the State of Hawaii's Olinda Endangered Species Propagation Facility, while four chicks from this facility were sent to the reintroduction programme. Twelve alala have been released by The Peregrine Fund:five in 1993 and seven in 1994. Three of the five birds released in 1993 and all seven of the birds released in 1994 are currently surviving in the wild.

314: *+.093*

The community status of 5,666 stocks of Arctic char (Salvelinus alpinus) in Norwegian lakes was monitored using interviews. A total of 228 stocks are extinct, and an additional 268 stocks are in differing stages of decline. Acidification is recognized as the most severe threat to Arctic char in Norway, and areas in southernmost Norway are seriously affected. The majority of the stocks in this region were affected after 1960, but the earliest indications of decline occurred in the early 1900s. Stocks in western Norway have experienced the most recent changes. Analyses of principle components, variance, discriminant and regression, indicated that pH and monomeric inorganic

aluminium were the water quality parameters most strongly related to char status. However, acid-neutralizing capacity (ANC), alkalinity and calcium were also correlated to char status. Arctic char appear to be more sensitive to acidification than brown trout (Salmo trutta) and perch (Perca fluviatilis), which occur most commonly in sympatry with Arctic char. The natural distribution of Arctic char in Norway extends along coastal areas throughout the country. However, translocations and introductions over the past 100-120 years have increased the distribution of the species significantly. This is particularly the case in some interior areas in southern Norway and in northern Norway, although exact data from the latter mentioned region are scarce. Most of the introduced stocks cited by Huitfeldt-Kaas (1918), have established self-sustaining populations. A conservation programme for Arctic char in Norwegian lakes is suggested. Unique stocks should be recognized and given priority for conservation. In acidified areas liming may save threatened stocks.

315: *+.369*

The Arctic Charr Salvelinus alpinus (L.) is the most northerly freshwater fish and occurs in many countries in the northern hemisphere - in Asia, Europe and North America. Worldwide, the status of the species is satisfactory but in a number of countries, especially those on the southern fringe of its distribution, many individual populations have been lost. The main threats to Arctic Charr are from acidification, eutrophication, pollution, hydropower developments, overfishing, genetic degradation from alien translocated Salvelinus, introductions of other, disruptive, fish species and possibly global warming. The Arctic Charr is an important species in the northern hemisphere and merits serious international conservation efforts to maintain its integrity and status. It is well known in some countries, but less well known in others. It is important to many native peoples in the arctic and more generally of value to anglers, to commercial netsmen and, more recently, to fish farmers. Conservation management for this species varies widely and more programmes are needed at both national and international levels. Reliable inventories of individual populations in each geographic area are highly desirable. The main methods proposed for conservation include improved legislation, habitat management and restoration, and in some cases, careful translocation, captive breeding and cryopreservation. Improved education is also greatly needed in order to increase public awareness of this most attractive and valuable fish species.

316: *+.028*

Beavers were recently re-introduced in the Netherlands after the species had become extinct in 1826. Between 1988 and 1991, 42 beavers were released in the Biesbos (Rhine-Meuse estuary). In 1994, another 15 beavers were released in the Gelderse Poort, along the Rhine between Kleve-Nijmegen-Arnhem. Between 1990 and 1992, some beavers escaped from captivity in the Biesbos and in the provinces of Flevoland and Limburg. One animal found dead in Roermond (Limburg) in 1993 had probably dispersed from the Eifel (Germany), another re-introduction site. By the end of 1994, c. 70 free-ranging beavers were present in the Netherlands. As far as known, at present all free-ranging beavers in the Netherlands originate from the Elbe population in Germany.

317: *+.254*

We developed a spatially explicit modeling approach, using a county-scaled remote forest (i.e., forested area reserved from or having no direct human interference) assessment derived from 1984-90 forest resource inventory data and a 1984 black bear (Ursus americanus) range map for 12 states in the southern United States. We defined minimum suitable and optimal black bear habitat criteria and gee-referenced remote forest classification with existing black bear range.

Using a suitable habitat criterion, we classified 97.2% of occupied and 9.7% of unoccupied range (38.9% of the south. U.S. region's area). Using optimal habitat criteria, we classified 69.8% of occupied and 60.1% of unoccupied range (63.3% of the region's area), interpreted occupied range without optimal habitat and suboptimal areas (9.9% of the region's area), and unoccupied range with optimal habitat as areas with repopulation potential (26.8% of the region's area). There was a lack of high-density (greater than or equal to 34%) optimal habitat linkages among existing black bear populations, which we construed as a limitation on interpopulation gene now. We recommend expansion of future regional land surveys to (1) address large carnivore mammal habitat and broad home ranges of other species that may conflict with humans or domestic animals, (2) include held inventories of woodland and reserved areas, (3) use standard measures to assess remote forests, and (4) organize available data in a geographic information system.

318: *+.055*

The butterfly Euphydryas gillettii (Barnes) lives in moist mountain meadows connected by riparian corridors, thus forming metapopulations in which local extinctions and recolonizations occur infrequently. Following the 1988 fires in the Greater Yellowstone Ecosystem, I chose 8 unoccupied patches of suitable habitat, 4 of which had been burned, and introduced a single eggmass into each. Larvae survived to diapause in at least 4 of the 8 sites, but only one introduction led to the establishment of a new colony the next year. This was at a burned site. The new population increased rapidly for 2 years but then declined and disappeared. These results suggest that: (1) a single, isolated eggmass is sufficient for colonization of open habitat; (2) most single, isolated eggmasses do not survive to produce adults the following year; and (3) recently burned sites provide acceptable habitat for this scarce butterfly.

319: *+.250*

We reviewed key features of the evolutionary biology of lake trout (Salvelinus namaycush) and their significance for rehabilitation programs in the Great Lakes. Despite repeated translocation. by glacial advances during the Ice Age (the Pleistocene) that eliminated most populations, lake trout have genetic diversity comparable with other North American salmonines. Various embryological and adult features suggest lake trout had a long reproductive history in lakes, although river spawning may be a primitive feature of the species and may have been important in glacial refugia. Observations that hatchery-reared lake trout select mostly mainland shoals for spawning in the Great Lakes are interpreted by us to be a result of evolution in smaller lakes where the main source of spawning gravels is shoreline erosion. We hypothesize that longevity in lake trout (a record among chars) may have evolved because of a near absence of predation on adults in contrast to predation on juveniles that survived less well, in parr, because of cannibalism. Longevity, a physiological ability to colonize the coldest of waters during deglaciation, and an ecological role as a dominant piscivore in unperturbed systems all indicate that lake trout should fare best under conditions of low adult mortality and high biomass. Although the Great Lakes fish community is enriched compared with when lake trout populations were abundant or with where lake trout evolved, the species has the potential to suppress other fishes to its benefit. We provide ecological and ethical reasons why lake trout rehabilitation should be a priority for the Great Lakes: lake trout are particularly suited for the deepwater food chain, they are the only salmonine (among those currently stocked in the lakes) that have the potential to become self-sustaining at their current levels of abundance, and emphasis on stocked exotics reflects adherence to a scientifically obsolete philosophy of "wise use" that ignores evolutionary-ecological relationships. For fishery management we recommend greater use of genetic diversity and of life stages capable of being imprinted, maintenance of high adult survivorship and biomass, and expanded

communication with a wider array of clients. We also advocate lines of research that will test our management recommendations, including assessing the implications of attempting to keep the Great Lakes fish community in an early stage of succession.

320: *+.158*

Complete restoration of the Great Lakes is unlikely, due to naturalization of exotic species, habitat degradation and destruction, heavy fishing mortality, lack of native gene pools, and complicated political jurisdictions that rarely work toward a common vision. A more realistic goal is rehabilitation, a movement along the trajectory toward complete restoration. Proper rehabilitation employs an evolutionary-genetic perspective, which protects and works with the remaining genetic variation available in lake trout or other species of concern. A difficult question is how to define the units of genetic conservation; one answer is to determine Evolutionarily Significant Units. To do this, population structure must be defined, and various conceptual models are here presented that determine genetic population structure as a function of geographic structure of the habitat. Several concepts from the developing field of conservation biology should also be incorporated into lake trout rehabilitation. These include metapopulations-groups of populations that experience some degree of regular or intermittent gene flow, and that serve as recolonization sources after local extinctions. Related to this is "source-sink dynamics," a recognition that habitats typically are unequal in quality. Habitat fragmentation is also relevant because it can stop inter-population movement and disrupt metapopulation structure and source-sink dynamics. Finally, hatcheries should be used in rehabilitation only with great caution and skepticism. Hatcheries tend to address the symptoms, rather than the causes, of fish declines, and may mask the underlying problems by continually replacing declining fish. Improved habitat quality, rather than artificial production, is the key To rehabilitation of the Great Lakes for lake trout and other native species, and hatcheries should only be used as a stopgap measure to prevent further losses of genetic diversity.

321: *+.137*

The current status of Kakapo Strigops habroptilus and Takahe Porphyrio mantelli is described along with recent developments in programmes for their conservation. Both species were (at different times) thought to be effectively extinct, and both have been temporarily reprieved by the discovery of new populations. Population declines have continued, with Kakapo now reduced to less than 50 individuals and Takahe to about 150. Kakapo are especially at risk; 87% of the remaining birds are over 14 years old and only 17 females are known. Research on relict populations of both species has identified predation and competition from introduced mammals as major threats. Both species have high rates of egg infertility and low survival of young. Increasingly intensive management of both Kakapo and Takahe over recent years has included translocation to predator-free island refuges, supplementary feeding to encourage breeding, clutch manipulation, captive rearing and predator control. All known Kakapo have now been transferred to three island refuges, where the overall rate of population decline has slowed and supplementary feeding has apparently encouraged more frequent breeding attempts, Takahe conservation has concentrated largely on attempts to increase the population in Fiordland, New Zealand, through clutch manipulation and release of captive-reared young, but birds have also been released on four islands, which now hold 19% of the total population. The relict Fiordland populations of both Kakapo and Takahe were confined to apparently suboptimal habitat. Both species have successfully adapted to novel environments and foods when translocated, and the populations which now exist present improved opportunities for intensive management using a range of conservation techniques to enhance productivity and survival. Recent population trends of Kakapo and Takahe are reconstructed, and the contribution of research to their conservation is reviewed.

322: *+.016*

The establishment of large populations of common frogs Rana temporaria and common toads Bufo bufo was monitored for six years in a newly-created reserve, following stocking with spawn of both species and with toads rescued from a site to be destroyed. Frog spawn output reached a peak three years after the initial introduction. There was high mortality and/or emigration (64% loss) during the first year following translocation of over 5,000 adult male toads. Thereafter male mortality decreased and/or site fidelity increased: 39% loss year 2 to 3, 42% loss year 3 to 4. Comparable data were not available for the 795 females translocated, but there is no reason to believe their mortality was lower. Transfer of spawn is probably more effective as a means of establishing a new population of toads than transfer of adults. Counts of male toads peaked after three years, but delayed sexual maturity of females meant that counts of pairs and estimates of spawn output continued to increase for about six years. Age determination in a sample of 43 male toads showed that length was significantly related to age, but there were some notably disparate rates of growth. A sample of 16 females showed no correlation between age and length. The greatest recorded losses of naturally-laid spawn of both species (up to 16% in one year for the frog and 39% for the toad) were due to desiccation, presumed collection by people and fungal infestation.

323: *+.235*

The dominance of Polygonum bistorta in abandoned mountain meadows was studied. Successional and seasonal changes in biomass, nitrogen concentration and content were studied at the community level. During the succession, P. bistorta increases its biomass both in absolute and mainly in relative values compared to the surrounding species. Nitrogen stored in its rhizomes seems to be quickly translocated into above-ground parts at the beginning of the growing season. High amounts of nitrogen allow rapid growth of both vegetative and generative plant parts. The success of P. bistorta is probably the result of efficient use of stored nitrogen, suppression of surrounding vegetation by above-ground cover of leaves and by decrease in the level of soil nitrogen, and the effect of a large amount of litter. Management consisting in the combination of mowing and fertilization is proposed.

325: *+.204*

The activities of conservation of the flora of the Columbretes Islands Natural Park (UTM 31S CE 01, Castellon, Valencian Community, Spain) are exposed. Since 1987 it is being developed a conservation project of endemic species (Medicago arborea subsp. citrina and Lobularia maritima subsp. columbretensis) by means of "ex situ" conservation techniques (seed-banks) and also "in situ" ones (propagation and reintroduction of M. a. subsp. citrina in one of the islands of the archipelago, as well as recovery of the most important and threatened climactic arbustive species of these islands (Chamaerops humilis, Pistacia lentiscas, Withania frutescens, Smilax aspera and Lycium intricatum).

326: *+.081*

The development of a collaborative conservation project on Mauritius and Rodrigues (Mascarene islands, south-west Indian Ocean) is followed from its inception in 1972 to early 1995. The project has a tripartite structure, being run jointly by the Government of Mauritius, a local non-government organisation - the Mauritian Wildlife Fund, and international organisations including the Wildlife Preservation Trusts. Work has focused on the endangered endemic birds, bats, reptiles

and plants. Intensive management techniques, including in situ plant propagation, captive breeding, reintroductions of plants and animals, supplemental feeding and manipulation of the nesting biology of the birds to increase productivity, have proved to be very successful. These, together with pest control and the protection of native habitat, resulted in the increase of some of the rarest species and the restoration of small islands and areas of native vegetation. This work convinced the Government to establish Mauritius' First National Park. The interface between captive breeding and the management of wild populations is one that offers many possibilities. During the 1970s the future for many of the endemic species seemed hopeless; two decades later the long term conservation of many of the surviving endemic species is within our grasp. A bibliography of 447 titles of articles, papers and books that that directly influenced the project or have been generated or inspired by it, is included.

327: *+.089*

During the Kemp's ridley sea turtle (Lepidochelys kempii) head-start experiment, the National Marine Fisheries Service's Galveston Laboratory reared, tagged, and released 22,255 yearlings of the 1978 to 1992 year classes along the coasts of Texas, west Florida, and Campeche, Mexico. A total of 805 recaptures were recorded (3.6% of the yearlings released). Annual survival rate, S, was estimated from recaptures of successive age groups. Values of S-2-3 (annual survival rate estimated from recaptures of age groups 2 and 3 years) were probably more reliable than those of S-3-4 and S-4-5, because they were based on larger numbers of recaptures. For Texas releases S-2-3 ranged from 0.10 in the 1980 year class to 0.43 in the 1986 year class. For Florida releases S-2-3 ranged from 0.36 in the 1978 year class to 0.50 in the 1979 year class. With recaptures from year classes combined, S-2-3 was lower for Texas releases (0.15) than for Florida releases (0.39). Because of tag loss and uncontrollable factors affecting the reporting of recaptures, S estimated from recaptures of foreflipper-tagged turtles are crude approximations which underestimate true survival at sea. A constant S of 0.45 would be required to produce one survivor at age 10 yr (assumed age at maturity) from the average Texas release of 1437 yearlings per year. If S were higher than 0.45 or increased with age, then more head-started ridleys could have survived. It remains to be determined whether head-started Kemp's ridleys survive to maturity and nest.

328: *+.215*

Research on birds has shown that familiarity between mates and neighbours leads to lower aggression and higher reproductive success. This study addresses the hypothesis that founder groups used for translocations will do better if made up of individuals that are familiar with one another. The study involved a translocation of a territorial forest bird, the North Island robin Petroica australis longipes to an offshore island. I created both 'familiar groups' (made up of birds that had been adjacent to one another at the source location) and 'unfamiliar groups' (made up of birds that had been widely separated). I released the groups in separate forest patches on the island, and assessed the effects of familiarity on aggression, dispersal, survival and pair bonding. While the study was limited by the sample sizes possible, there was no indication that 'familiar groups' performed differently than 'unfamiliar groups', or that familiarity at the source location affected behavioural interactions following translocation. Post-translocation familiarity was clearly important, for agression declined according to how long birds had been neighbours at the new location. There was little aggression in general over the first two to five weeks, the period when an effect of prior familiarity on aggression may have been most important. In addition, breakup of 'familiar' groups by dispersal meant that there was limited opportunity for interaction among familiar birds. These results suggest that familiarity within founder groups is unlikely to have strong effects following translocation.

329: ***+.120***

While ecological and conservation consequences of combining animals of varied genetic backgrounds have been widely discussed the demonstration of effects that stem from lineage mixing remains elusive. Since management agencies relocate populations or supplement them with individuals regularly, the opportunity for either inbreeding or outbreeding depression may be high; still, any putative effects will go unnoticed without detailed knowledge of life-history and behaviour. Here, we report potential consequences of lineage mixing in a restored population of North American Bos bison studied for five years. In 1984 two allopatric lineages became sympatric in Badlands National Park, South Dakota, they differed in both founding population size and the number of demographic bottlenecks experienced since 1907. Measures of reproductive variance in both sexes were employed to estimate effective population size based on 261 copulations and the survivorship of calves between 1985 and 1989. We assumed that the reproductive variance and mortality documented in this study are representative of the bison's recent past and based on this assumption we calculated N-e separately for each generation for which the lineages were allopatric. Four potential correlates of fitness were studied in the new sympatric population: (1) female fecundity; (2) juvenile survival; (3) growth rates, and (4) female age at puberty. Of these, neither female fecundity nov juvenile survival was associated with lineage but growth rates were more rapid and ages at puberty were lower for F-1 purebred (inbred) juveniles than for F-1 hybrid (outbred) juveniles. Possible consequences of this variation in the F-1 generation include (1) higher winter mortality in the slower growing line as well as (2) decreased lifetime production of young; both are life-history parameters that could be interpreted as long-term selection against outbreeding. However, these data by themselves do not constitute support for an outbreeding depression hypothesis. The failure of males from one lineage to mate at all prevented the possible combinations of the F-1 generation needed for the appropriate statistical contrasts. Nevertheless, these interpretations (1) substantiate a level of variation in life-history parameters stemming from lineage mixing; and (2) suggest that advice regarding prudent conservation strategies must be sought concerning the generic histories of individuals and populations to be selected for re-introduction. This applies to both wild and captive populations. The prevalence of outbreeding and inbreeding tolerances within populations of managed and protected species needs verifiable documentation.

330: ***+.337***

Conservation efforts for imperiled fishes in western United Stares have included numerous translocations, either among natural al localities or from nature to propagation facilities then back into nature. The goal has been to increase population size and dispersion while maintaining genetic diversity, thus increasing probability of survival. Environmental laws governing translocations of fishes for conservation purposes involve complexities often equally as difficult to cope with as the biological problems of species' endangerment. Translocations perceived not to impinge on resource use or proprietary rights may be readily approved, while those which interfere with actual or projected development may meet strong resistance. Major biological considerations include the suitability and security of transplant sites (assurances that each meets a taxon's life-history and other requirements) and appropriateness of transplanted individuals (genetic and population structure, sufficient numbers of individuals, freedom from disease, etc.) for establishing new populations. Success of translocation is difficult to define and major inadequacies exist in information exchange - the latter can be remedied by publication in the peel-reviewed literature. It is anticipated that fish translocations and the technology required to support them will expand along with future needs and desires to re-establish native biotic elements in depleted communities and ecosystems.

331: *-.009*

Captive breeding and the release of captive-bred individuals into the wild are among the techniques used for the conservation of rare and endangered fish species. After a brief description of the methods of captive breeding and the establishment of breeding stocks, this paper provides examples of the application of these techniques to endemic fish species of arid regions in south-western USA and examines some current cases and the future possibilities for their use in the Mediterranean region. Special attention is given to the analysis of the strict constraints imposed on fish breeding for conservation purposes, in which the aim is to produce fish with all the morphological, behavioural and genetic characteristics of the taxa to be conserved, and which are capable of effectively adapting to the natural environment when introduced. In terms of genetic management of captive populations, the fundamental problems which are faced involve the categorization of the species-resources to be conserved (identification of cases of inter- and intra-specific introgression), the establishment of founder stocks that contain the maximum genetic diversity depending on the genetic structure of the species (strong intra- or interpopulation variability), and the retention of genetic variability during captive breeding (the need to reduce to the minimum the phenomena of genetic drift, inbreeding and unintentional selection of non-adapted genotypes). Because of these difficulties and risks in terms of genetic conservation, captive breeding should remain a temporary safeguard measure, while awaiting the implementation of measures for protecting species in their restored original habitat or translocation to strictly protected substitute habitats. With this aim in view and in conclusion, the paper suggests methods for organizing a critical plan to safeguard the most endangered species or subspecies in the Mediterranean region by captive breeding.

332: *-.055*

There are 35 species and subspecies of fish and two lamprey species in the Adriatic catchment of Slovenia. Five have been introduced and five transplanted None of the regionally endemic fishes nor the lampreys live exclusively in Slovenia. Of these, Salmo marmoratus, and Lethenteron zanandreai are threatened while Chondrosoma genei became extinct after the introduction of Chondrostoma nasus nasus into the area around 1960.

333: *-.010*

In Italy, as in several other circum-Mediterranean countries, there is a unique assemblage of endemic freshwater fishes. Speciation processes, especially of endemic primary or near-primary freshwater fish, most probably occurred during the Lago Mare era of the Mediterranean (about 5 million years BP). However, the present-day distribution patterns are surely the result of events that occurred more recently, from the Pleistocene until historical times. However, natural events are now being superseded by anthropogenic interference with fish faunas. Among the various negative human factors the most harmful is the introduction of non-native species, which in Italy are responsible for the present catastrophic situation. Of the 71 species currently with natural self-sustaining populations in Italian waters, only 45 are natives. Of the latter, only 16 (or fewer) have not been subjected to deliberate human transfers. In Italy there about 28 endemic Mediterranean taxa, but most have either been deliberately or accidentally introduced to areas outside their natural range. The result of this persistent practice on a little known and unique fish fauna is zoogeographic pollution, massive cases of hybridization and loss of genetic identity by local native populations. The problem of freshwater fish conservation is not a matter of public concern in Italy. Sport fishing has a political and economic value and introductions are legally carried out and 'welcomed' by most fishermen; most biologists and conservationists ave either unaware or

unconcerned.

334: ***+.081***

Habitats used by most species are becoming increasingly fragmented requiring a metapopulation modelling approach to population viability analysis (PVA). Recognising habitat patchiness from an endangered species' point of view requires utilisation of spatial information on habitat suitability. Both of these requirements mag, be met by linking metapopulation modelling with landscape data using GIS technology. We present a PVA model that links spatial data directly to a metapopulation model for extinction risk assessment, viability analysis, reserve design and wildlife management. The use of the model is demonstrated by an application to the spatial dynamics of the helmeted honeyeater Lichenostomus melanops cassidix, an endangered bird species endemic to Victoria, Australia. We use spatial data, organised by a GIS, on the habitat requirements of the helmeted honeyeater to define the patch structure. We then combine this patch structure with demographic data to build a metapopulation model, and use the model to analyse the effectiveness of translocations as a conservation strategy for the helmeted honeyeater.

336: ***-.016***

On mainland Australia the eastern barred bandicoot, Perameles gunnii, is confined to a relic wild population numbering less than 100 individuals in the city of Hamilton, Animals derived from this population are being bred in captivity in order to promote their recovery The species also exists in Tasmania, where little is known of its conservation and taxonomic status Mitochondrial DNA variability was compared within and between populations of p. gunnii using restriction fragment length polymorphisms. Genetic variability was found to be high among P. gunnii in Hamilton compared to those in Tasmania (higher diversity index, nucleotide sequence divergence and greater number of haplotypes), despite the known decline and subdivision of the Hamilton population Restriction fragment length polymorphisms distinguished animals from the east and the west of Hamilton and from the north and south of Tasmania Nucleotide sequence divergence was substantial (2.2-2.5%) between Hamilton and Tasmania Implications are that captive breeding and reintroduction should be designed to genetically, represent the structure within Hamilton in order to minimize inbreeding and that the introduction of Tasmanian P. gunnii would not benefit the Hamilton population It is concluded that mitochondrial DNA markers clearly can provide useful information about the history and current status of endangered marsupial populations, to the benefit of conservation management.

337: ***+.008***

On tropical Pacific islands, a human-caused "biodiversity crisis" began thousands of years ago and has nearly run its course. Bones identified from archaeological sites show that most species of land birds and populations of seabirds on those islands were exterminated by prehistoric human activities. The loss of birdlife in the tropical Pacific may exceed 2000 species (a majority of which were species of flightless rails) and thus represents a 20 percent worldwide reduction in the number of species of birds. The current global extinction crisis therefore has historic precedent.

338: ***+.109***

At least 21 species of exotic arthropods have successfully established breeding populations in the Kruger National Park, South Africa. Of these, seven species were intentionally introduced as biological control agents of alien weeds, four species inadvertently accompanied vertebrate hosts

during game translocation, and the remainder are presumed to have gained entry through packing crates, human activities, or natural dispersal in some instances. The modes of entry of exotic species are discussed and, where known, the time of first appearance in the Kruger National Park recorded. The rapidly escalating practice of wildlife translocation within and between countries is discussed in relation to passive transfer of diseases and parasites, and reasons are listed why routine precautionary steps should be instituted to avoid transfer of non-target organisms during translocation operations.

339: *+.010*

Seasonal distribution, relative abundance, biomass production, population consumption, and energy storage in bay anchovy Anchoa mitchilli were estimated from midwater trawl surveys in the upper and mid Chesapeake Bay from April 1990 to October 1991. Abundance and biomass both peaked in late summer and fall before declining significantly in winter, a result of southward migration to lower Bay areas that were not sampled. Production also peaked between summer and fall, a consequence of rapid growth and recruitment of larval and juvenile anchovies. Annual production of young-of-the-year (YOY) anchovy was 856.69 g 100 m(-3), 87.9% of which was produced in the first 3 mo of life. Production by YOY anchovy accounted for nearly all annual production (92.6%) in this short-lived species. Total annual production was estimated to be 233014 t wet wt in upper to mid-Bay regions. The production/biomass (P/($) over bar B) ratio for YOY anchovy was 8.07 when larval and early juvenile stages were included but only 0.97 without those stages. The estimated translocation of nitrogen biomass from the upper and mid Bay to the lower Bay via anchovy migration was 1027 t N during fall 1990, which is approximately 0.8% of the annual N input to Chesapeake Bay. Estimated population consumption (primarily zooplankton) by bay anchovy ranged from 5.29 to 12.81 g dry wt 100 m(-3) d(-1) in August and from 5.35 to 6.78 g dry wt 100 m(-3) d(-1) in October, suggesting that consumption by larvae and juveniles of this species could significantly impact populations of its plankton prey.

340: *-.095*

Population densities of terrestrial animals tend to be higher and less variable near the center versus along the periphery of a species' geographic range. If extinctions are tied to local population dynamics, geographic ranges of endangered species should collapse inward, with remnant populations persisting near the center of a species' historic range. Geographic-Information-System analysis of range collapse in nonvolant, terrestrial mammals reveals, however, that extant populations of 23 of 31 species were located along the periphery, not the center, of their historic range. In addition, range collapse appears to be independent of fragment area and has a directional bias from east to west. Persistence of endangered populations also appears to be greater on islands than on continents. These results contradict conventional wisdom in biogeography and macroecology and have important implications for conserving biodiversity. Because of their relative isolation from central populations and from a suite of anthropogenic disturbances, islands and other sites along the periphery of a species' historic range represent critical refugia for many endangered species.

341: *+.074*

Present knowledge about chromosomes and nuclear DNA of Crustacea is reviewed. Haploid chromosome numbers range from 3 (Acanthocyclops) to 188 (Astacus). Chromosomes are generally small to medium in size (1-5 mu m) and punctiform or rod-shape. Nuclear DNA amounts show a large range, lying between 0.37 (Daphnia) and 22.6 pg (Decapoda) per haploid

genome, 5.5 x 10(8) nt to 1.8 x 10(10) nt. Highly repetitive sequences may represent as much as 30% of the genome and show a high degree of conservation in Brachyura, while some intermediate repetitive sequences are under-represented. The relationship between taxonomy and cytogenetics appears very complex in some taxa, such as Artemia (Branchiopoda). This genus includes bisexual sibling species with female heterogamety and parthenogenetic populations with different levels of ploidy, up to triploid and pentaploid. Daphnia presents diploid and polyploid strains, and parthenogenetic reproduction. Chromatin diminution occurs in Copepoda; in this group male and female heterogamety coexist with a ZO-ZZ type, unique among animals. Evolution in Copepoda tends to the reduction of chromosome number. Some parthenogenetic, freshwater Ostracoda have supernumerary chromosomes; Ostracoda also present complex sex chromosomes mechanisms (XO and XY, with cases of multiple X's and multiple Y's). Within Rhizocephala, chromosome numbers can be used to distinguish species of parasitic Crustacea, such as Sacculina. Robertsonian translocations are known in some Isopoda. Jaera albifrons albifrons presents a dine, diminishing from north (n=13) to south (n=9) along N-E European coasts. As in Copepoda, female and male heterogamety are present. Cytogenetics of Amphipoda is not well known, and many species have the same number; as much as 9 supernumerary (B) chromosomes have been reported. One of the highest numbers of chromosomes known for animals occurs in crayfish Astacus (2n=376). DNA amount in shrimps has the highest range in Crustacea (3-22 pg/c) and one of the highest for animals. Within several Decapoda groups, viz. lobsters, crayfish, scyllarids and hermit crabs, polyploidy may have acted as an evolutionary factor. Chromosome evolution within Decapoda is difficult to understand because the chromosomes are numerous, very small and punctiform; differentiated sex chromosomes have sometimes been reported, the male being the heterogametic sex. Somatic endopolyploidy is present in many groups mainly in the digestive tract; polyploidy in germ cells might have occurred in different groups during evolution.

342: *+.229*

Reintroduction is the release of animals into an area where they were extirpated or have significantly declined. Little is known about the factors that determine the success or failure of ungulate reintroduction. We studied the dynamics of a reintroduced Asiatic wild ass (Equus hemionus) population for 10 yr (1983-1993) following the first successful release into the wild. A total of 14 adult females and 14 adult males were released into a nature reserve in the Negev Desert of southern Israel. Over this 10-yr span the female population has grown to only 16 adults. Reproductive success of reintroduced females was low in the first 5 yr following release (0.0-0.8 foals . female(-1). yr(-1)), but increased to 0.5-1.0 foals . female(-1). yr(-1) in the last 5 yr. Reproductive success of wild-born females greater than or equal to 3 yr old was higher than that of reintroduced females of similar ages, and ranged from 0.5-1.0 foals . female .(-1)yr(-1). Our study and data from the E. hemionus studbook suggest that young nonprimiparous females produced primarily males, while primiparous and old females produced primarily females. We attribute the low reproductive success following reintroduction to the stress caused by capture, transport, and release procedures; we consider the age-dependent progeny sex ratio within the framework of Trivers and Willard's (1973) maternal allocation hypothesis. We conclude that the slow growth of the female population was due to: (a) low reproductive success of females in the early years following reintroduction, and (b) a male-skewed progeny sex ratio among prime-aged reintroduced females. A simple stochastic Leslie matrix model suggests that high survival and improved reproductive success of reintroduced females at later stages of the study, and the reproductive success of wild-born females, make the population relatively unsusceptible to extinction from random demographic processes. In-depth knowledge of the dynamics of reintroduced populations is vital for the correct assessment of their viability. We offer suggestions

for increasing the efficacy of future wild ass reintroductions.

343: ***+.101***

Robertsonian polymorphism has been analysed in samples of successive ages of Atlantic salmon (Salmo salar L.) and brown trout (Salmo trutta L.). Polymorphism changed significantly with ageing, the standard 2n class increasing for each species (2n = 58 for Atlantic salmon and 2n = 80 for brown trout), No significant within-population changes were found among same-aged samples from different generations. The results support the notion that selection favours a standard karyotype. On the other hand, permanence of polymorphic patterns within age groups in successive generations also was demonstrated.

344: ***+.127***

Seven individuals of captive Gazella bennetti were found to have chromosomal complements of 2n=49-52, and seven captive G. saudiya had complements of 2n=46-53. G-banded karyotypes revealed that variation in diploid number was the result of an autosome-to-X chromosome translocation and four independent Robertsonian translocations. There were no fixed chromosomal differences between G. bennetti and G. saudiya, but two pericentric inversions distinguished Pakistani G. bennetti from Iranian G. bennetti and G. sandiya. Several pairs of metacentric chromosomes of both species were monobrachially homologous with metacentrics of G. dorcas and G. gazella, indicating G. bennetti and G. saudiya are reproductively isolated from G. dorcas and G. gazella. As with other species of gazelles, chromosomal studies of natural populations are needed for these species.

345: ***-.158***

Crop damage and livestock predation were a serious problem in three Village Development Committees (VDCs) situated adjacent to the Southwestern Part of the Royal Bardia National Park. The seriousness of crop and livestock losses varied considerably with the distance from the Park's border and the specific location of farms. This was explained by the variations in the distribution of animal wildlife inside the Park, the presence of natural and Man-made barriers, the availability of forested areas outside the Park, and the agricultural cropping pattern. Adjacent to the section of the Park with the highest animal densities, crop losses varied from 47% for Lentil to 24% for Wheat. Farther away, the extent of crop losses was reduced. In the 'Far' zone situated 2-8 km from the Park, only 3% of the Paddy was reported lost. Two wildlife species, Chital and Wild Boar, were responsible for roughly half of the total damage to crops by animals. Damage from a newly-introduced population of Rhinoceros unicornis was increasing, with Paddy (Rice) and Lentil the crops most affected by that species. The economic value of livestock li,ss to wild predators was estimated to be two percent of the value of total crop losses. The estimated value of grasses harvested by local villagers inside the Park, seen as 'compensation' for crop losses and denial of access to traditional resources, constituted only 10% of the total economic loss from crop losses and livestock predation. Due to rapid increase in the population of wild ungulates since the establishment of the National Park, and to the lack of functioning protective measures, the farmers' problems were increasing. The reintroduction of Rhinos, translocated from Royal Chitwan National Park, has further increased the problem. Local villagers so far have a positive attitude towards the Park, but this positive attitude may erode soon unless serious action is taken to reduce the problems of crop damage and livestock predation in the area.

346: ***-.021***

Reintroduction to the wild of threatened species has become a modern additional justification for captive propagation. This conservation procedure is costly, and both economic resources and the abscence of optimal conditions in the field limit the IUCN recommendations for reintroduction to a small proportion of potential candidate species. Furthermore reintroduction attempts often fail. In carnivores, reintroduction failure is attributed to unsuitable adaptation in the field by captive-reared animals, due to their lack of hunting skills, their tendency to leave the target area, their inadequate interaction with conspecifics or their excessive confidence in humans. This list of causes is based on very few studies of carnivore adaptation after reintroduction. In very rare and endangered species, monitoring individual case-histories is the only way to evaluate reintroduction success. We report a successful experimental release of an Iberian lynx (Lynx pardinus) which grew up in captivity. Careful feeding-training and avoidance of human contact during the captive phase, as well as good habitat quality and correct interaction with other wild lynx in the release site, seem to account for the observed success. Permanence of the lynx within the release area might be related to the availability of territory vacancies in the receiving population. Our results allow some optimism for future reintroductions of this endangered species in areas where it has become extinct recently.

348: *-.006*

Zoological parks are playing an increasingly important role in the management of threatened species through education, the maintenance of captive gene banks and the reintroduction of captive-bred animals into the wild. This paper discusses the suitability of amphibian species for reintroduction. The important features of amphibians for these programmes are highlighted, i.e., their high fecundity to allow rapid build-up of captive populations coupled with few behavioural problems with captive-bred animals, as well as the low cost of maintenance. The potential problems of inbreeding, poor retention of innate behavioural repertoires after multiple generation captive breeding, and the introduction of alien pathogens into wild populations are considered. Release programmes for two species, the Mallorcan midwife toad Alytes muletensis and the Puerto Rican crested toad Peltophryne lemur, are reviewed.

349: *+.317*

Growing deterministic and stochastic threats to many wild populations of large vertebrates have focused attention on the conservation significance of captive breeding and subsequent reintroduction. However, work on both gorillas and black rhinos questions this shift in emphasis. In these species, field-based conservation can be effective if properly supported and, although this is not cheap, per capita costs may still be considerably lower than for ex situ propagation in captivity. Here we attempt to broaden the scope of this debate by contrasting the breeding success and costs of in situ and captive programmes for a range of threatened mammals. Data are scarce, but we find that across nine large-bodied genera, in situ conservation achieves comparable rates of population growth to those seen in established captive breeding programmes. Moreover, comparing budgets of well-protected reserves with toes' own estimates of maintenance costs and the costs of zoo adoption schemes, we find that per capita costs for effective in situ conservation are consistently lower than those of maintenance in captivity. Captive breeding may be more cost-effective for smaller-bodied tars and will often remain desirable for large mammals restricted to one or two vulnerable wild populations. However, our results, coupled with the fact that effective in situ conservation protects intact ecosystems rather than single species, lead us to suggest that toes might maximize their contribution to large mammal conservation by investing where possible in well-managed field-based initiatives, rather than establishing additional ex situ breeding programmes.

350: *+.093*

The relocation of unionacean mussels is commonly used as a conservation and management tool in large rivers and streams. Relocation has been used to recolonize areas where mussel populations have been eliminated by prior pollution events, to remove mussels from construction zones and to re-establish populations of endangered species. More recently, relocation has been used to protect native freshwater mussels from colonization by the exotic zebra mussel Dreissena polymorpha. We conducted a literature review of mussel relocations and evaluated their relative success as a conservation and management strategy. We found that 43% of all relocations were conducted because of construction projects that were forced to comply with the Endangered Species Act 1973 and that only 16% were monitored for five or more consecutive years. Most (43%) relocation projects were conducted from July to September, presumably a period when reproductive stress is relatively low for most species and the metabolic rate is sufficient for reburrowing in the substrate. The mortality of relocated mussels was unreported in 27% of projects; reported mortality varied widely among projects and species and was difficult to assess. The mean mortality of relocated mussels was 49% based on an average recovery rate of 43%. There is little guidance on the methods for relocation or for monitoring the subsequent long-term status of relocated mussels. Based on this evaluation, research is needed to develop criteria for selecting a suitable relocation site and to establish appropriate methods and guidelines for conducting relocation projects.

351: *+.096*

We assessed the genetic structure of two subspecies of endangered Clapper Rails (Rallus longirostris) in Southern California using DNA fingerprinting to uncover variation in minisatellite DNA. Minisatellite DNA variation in the Salton Sen population of the R. l. yumanensis subspecies was at a level typical of outbred avian species (average proportion of fragments shared, or S, was 0.33). Variation was extremely low (S from 0.63 to 0.77), however, within four coastal, salt-marsh populations of the subspecies R. l. levipes located along a transect extending about 260 km northwest from the Mexican border. Between-population similarity (S-ij) was also high for the four levipes populations, although individuals of the small, isolated population at Mugu Lagoon consistently clustered separately in phenograms constructed using neighbor-joining or other algorithms. Individuals of yumanensis always clustered as a sister group to all levipes individuals. The minisatellite data were contrasted with the extremely low mtDNA and RAPD variation we found in both subspecies. We propose that variation in these less-mutable markers was lost in a bottleneck that occurred at least 1000 years ago, thus allowing sufficient time for recovery of variation in the rapidly mutating (mu similar to 0.001/gamete/generation) minisatellites (t = 1/mu, or 1000 generations). A second more-recent bottleneck or series of bottlenecks within a metapopulation structure, likely resulted in the depauparate variation seen in levipes today. We suggest that translocations from large to small levipes populations could restore important genetic variation to the small populations and would not compromise genetic boundaries.

352: *-.138*

The process of ecological risk assessment should involve the ability to predict adverse outcomes of particular environmental contaminants or human intrusions. Ecological risk assessment generally focuses on populations, communities, and ecosystems, rather than on individual health. We explore the importance of life history strategies of aquatic turtles to their risk from environmental contaminants and other human activities using three examples: the wood turtle Clemmys insculpta, a freshwater species; the diamondback terrapin Malaclemys terrapin, a littoral species; and marine turtles as a group. These turtles are partly herbivorous and are at low or

intermediate levels on the food chain, yet are particularly vulnerable due to their life history strategies of being long-lived with relatively low survival of young. They suffer a variety of natural mortality factors that include predation, starvation, and disease, as well as inundation and destruction of nesting beaches and their eggs by storms. Yet they also face a number of anthropogenic hazards, including toxic chemicals and floatables (plastics); capture for food, other products, and pets; incidental mortality in fishing gear; disturbance while nesting or moving on land; injuries or death by collision with boats; and increased predator exposure because of humans. The three turtle species (or groups of species) examined have experienced these natural and anthropogenic pressures differentially, with resultant differences in the rates of population declines. Because they are lower on the food chain than other obligate carnivores, they are less vulnerable to toxics, and to date, toxics seem a relatively inconsequential environmental risk to turtles.

353: *+.179*

A small number of ancient lakes (mostly >10(6) y old) scattered around the globe contain an extraordinary percentage of the world's documented freshwater biodiversity. Endemic benthic invertebrates and fish in most of these lakes today face a variety of anthropogenic threats, including damaging fishing activities, water pollution, species introductions and translocations, climate change, and watershed disturbances. Lake Tanganyika, one of the oldest and most diverse of the ancient lakes, provides a model for studying the effects on endemic faunal diversity from watershed disturbances caused by deforestation. Increased erosion rates around the northern portion of the lake are associated with substantially lower diversity levels for both benthic invertebrates and fish. Disturbance processes in the biologically complex littoral region of this lake that are related to this excess siltation include reductions in light penetration (affecting algal habitat and herbivory), reductions in habitat heterogeneity and reduced connectivity between benthic habitat patches. Paleoecological data for readily fossilized taxa may be used in Lake Tanganyika to determine background levels and variability in diversity and the historical relationship between human activities in the watershed and changes in benthic community structure. Biogeographic and paleoecologic studies of endemic ostracodes demonstrate that many taxa have extremely patchy distributions which are consistent with metapopulation models of organization. The extremely high diversities encountered in Lake Tanaganyika and other ancient lakes may be dependent on nonequilibrium interactions between patchily distributed species, which undergo periodic but uncorrelated extinctions and recolonizations of appropriate habitat patches. Conversely, excess sedimentation along rocky coastlines may reduce habitat patch interconnections and lead to local extinctions, even in areas that are not directly affected by damaging siltation Paleoecological data may be used by aquatic conservation biologists to determine the history of this and similar problems and, more generally, to obtain a dynamic view of diversity change in lakes through time.

354: *+.106*

Red-cockaded Woodpecker (Picoides borealis) population trends and con curre nt management on four national forests in eastern Texas were evaluated from 1983 through 1993. Following years of decline, populations stabilized and began to increase after intensive management efforts were initiated. Management activities included control of hardwood midstory and understory thinning pines within woodpecker cavity-tree cluster areas, use of cavity restrictors and artificial cavities, translocation of first-year woodpeckers to replace lost breeders, and reintroductions of male and female first-year woodpeckers to form totally new breeding pairs. Most newly formed woodpecker groups were associated with midstory removal (30) and installation of artificial cavities (22).

Reversal of severe declines on the three small populations in eastern Texas suggests that recovery of other small populations throughout the south is an achievable goal if management is committed to recovery of the species.

355: ***+.224***

This paper presents the results of a long-term study of changing predator densities and cascading effects in a Michigan lake in which the top carnivore, the largemouth bass (Micropterus salmoides), was eliminated in 1978 and then reintroduced in 1986. The elimination of the bass was followed by a dramatic increase in the density of planktivorous fish, the disappearance of large zooplankton (e.g., two species of Daphnia that had historically dominated the zooplankton community), and the appearance of a suite of small bodied cladoceran (zooplankton) species. The system remained in this state until bass were reintroduced. As the bass population increased, the system showed a steady and predictable return to its previous state; planktivore numbers declined by two orders of magnitude, large-bodied Daphnia reappeared and again dominated the zooplankton, and the suite of small-bodied cladocerans disappeared. Within each cladoceran species there was a steady increase in mean adult body size as planktivore numbers declined. Total zooplankton biomass increased approximate to 10-fold following the return of large-bodied Daphnia, and water clarity increased significantly with increases in Daphnia biomass and total cladoceran biomass. These changes in community structure and trophic-level biomasses demonstrate the strong impact of removing a single, keystone species, and the capacity of the community to return to its previous state after the species is reintroduced.

357: ***+.177***

The lion tamarin was first described in the wild over 475 years ago, and there are records of the species being kept as pets as early as the mid-1750s. With such a long captive history, it is a sad reflection on our knowledge that, as recently as the early 1970s, so little success had been recorded with the maintenance and husbandry of Leontopithecus in captivity and the species seemed certain to disappear from zoos as well as from the wild. After a ban on exportation of the species from Brazil, the captive population of L. rosalia outside of Brazil decreased from a total of 102 in 1968 to 82 in 1976. Zoos rarely bred a second generation, and with no prospects of further imports from the wild, by 1977, the likelihood of the captive population becoming genetically unviable and going extinct was high. Fortunately, thanks to the development of a professionally coordinated and scientifically managed captive population and the research efforts of many individuals and institutions, both in Brazil and elsewhere, the future of the captive population has been ensured. The international efforts to save the golden lion tamarin through the collaborative work of the GLTCP represents a classic example of what can be achieved when various disciplines working for the conservation of a species cooperate and coordinate their activities in pursuit of a common goal. The majority of authorities now recognize that the future of animals in captivity will increasingly rely on national and international cooperation; much of this will depend on the integrity and goodwill of the people concerned. The development of international strategies to safeguard species survival increasingly relies on field workers, conservationists, educationists, academics and zoo professionals cooperating wholeheartedly. As KLEIMAN et al. (1986) have suggested, such cross-institutional collaborative efforts with L. rosalia represent a model for international conservation projects in the future. Since the June, 1990 Leontopithecus Population Viability Analysis (PVA) Workshop held in Belo Horizonte, Minas Gerais, Brazil (IUCN/SSC/CBSG 1991), the four International Management Committees for the Lion Tamarin genus have been recognised by Federal Brazilian law as technical advisors to IBAMA on conservation, research, and management of both the captive and wild populations of

Leontopithecus. Nearly 100 per cent of the tamarins that come under the committees' jurisdiction are the property of the Brazilian people through IBAMA; this transfer of title to IBAMA involved a renunciation of ownership of specimens housed at zoos internationally. For L. rosalia, ownership was turned over in 1991. We believe that this may be the first case of the international zoo community returning ownership of legally-held animals to the country of origin while continuing to maintain responsibility for their management. With the reintroduction programme for L. rosalia having resulted in an increase of protected Atlantic coastal forest, in the State of Rio de Janeiro, by about 38 per cent (2,300 ha). And with our growing understanding of the science of conservation today, the significance of the GLTCP's work with this endangered species (RYLANDS) highlights how well the adoption of 'flagship' species and publication of the plight of remnant populations in depleted environments can promote public attention and associated habitat.

358: *+.151*

The eastern barred bandicoot, Perameles gunnii, is functionally extinct on mainland Australia. Conservation of this unique taxon is dependent on reintroduction, based on a managed captive-breeding programme that provides founder animals. Existing reserves at which reintroduction has occurred are too small to support long-term genetically viable populations. Therefore, reintroductions must be made at a number of sites and the resulting populations managed as a metapopulation. A habitat-suitability model has been developed to assess and compare reintroduction sites. This is the first application of this concept to an Australian species. The model is composed of five variables-size, habitat structure, predation, shape and security-values of which are combined in a simple relationship to produce comparable mathematical statements for proposed reintroduction sites. The model has been applied to existing reserves to test their contribution to the recovery programme.

359: *+.135*

Introduced populations of birds and mammals may have genetic characteristics differing from those of naturally occurring populations. Such populations are often created by translocation of small numbers of individuals. This leads to founder effects and subsequent genetic drift, often resulting in larger differences in allozyme patterns between introduced populations than between naturally established populations. In many cases, a large proportion of alleles has been lost a few generations after the introduction. Under certain conditions, the mean level of heterozygosity is also severely reduced. Theoretically, a reduction in the number of alleles in a population will result in a lowered potential to track environmental changes, but there is scant evidence for this in introduced wildlife. Likewise, evidence is lacking for inbreeding depression occurring in introduced populations of birds and mammals in the wild. Finally, some conclusions are drawn concerning management strategies for wildlife introductions with respect to genetic considerations.

360: *+.008*

Molecular techniques show that hybridization can be a major conservation problem. Introgression can lessen genotypic variety and break up gene complexes coadapted to local environments. Gamete wastage can be a problem for hybridizing species even when gene flow does not occur. Birds and mammals introduced for game purposes have hybridized with native populations, a process often facilitated by habitat modification. Further, translocation and stock enhancement programs can lead to loss of well-adapted genotypes. These problems increase as populations become fragmented and isolated, because finding mates is harder and habitat differences that bar

reproduction are obliterated. What to do about hybridization is sometimes not obvious. Even if the problem is seen as important, often little can be done. Culling may be impossible because of difficulties in recognizing introgressed individuals. Wildlife biologists and managers should work to prevent introductions and translocations if hybridization problems cannot be ruled out. Further, wildlife professionals should educate the public about evolutionary aspects of introductions and translocations, including hybridization and introgression.

361: *-.030*

In the Bialowieza Forest, thermophilous oak forest, Potentillo albae-Quercetum, has its northern limit, while generally in Poland the community reaches the northwestern limit of its distribution. For last 30 years, the habitats of oak forest have been invaded by hornbeam; at the same time, European bison has returned to the forest (Kwiatkowska and Wyszomirski 1988). A proximate cause of the decline of Potentillo albae-Quercetum is hornbeam invasion (Kwiatkowska and Wyszomirski 1990). An increase in the proportion of hornbeam in the shrub layer of oak forests has also been recorded in other regions of Poland (Jakubowska - Gabara 1993). The Bialowieza Forest is a unique model object allowing the analysis of the causes of hornbeam invasion since (1) the direct and indirect human impact is small as compared to other regions of Poland, (2) the communities of oak forest and adjacent lime-hornbeam forest are natural to a high degree, (3) since the 15th century the area has been used for game hunting while logging has been restricted, (4) there are detailed records of the number of dominant herbivores covering the last two centuries, (5) long-term investigations have been carried here on a permanent plot for 25 years (Kwiatkowska 1994a,1994b). The main factor disrupting the stability of phytocoenoses in the Bialowieza Forest has been the changes in the number and proportion of the dominant herbivores, European bisons and red deer (Fig. 5). At the end of the 19th century, the dominant herbivore was the bison, the diet of which includes 90% of herbs; the pressure on the perennials was therefore strong. Intense grazing might have created gaps in a dense ground layer while trampling produced hollows in a thick oak-leaf litter. In undisturbed patches of Potentillo albae-Quercetum, the microsites with a thick litter (more than 2.5 cm) and a dense ground-layer prevail (Table 1, Figs. 2 and 3). They may constitute more than 75% of all available microsites. The dense ground-layer and thick litter are mechanical barriers which make it difficult for light hornbeam seeds to reach the ground and for their short-rooted seedlings to get to the soil. Such places are, however, safe sites forjuvenile oaks (Fig. 4), while the "demand" of hornbeam seedlings is oriented towards the sites with a thin litter and a loose cover of the ground-layer (Figs. 2 and 3). Grazing by bisons enlarged the "supply" of safe sites for hornbeam, and effectively increased the survivorship in the juvenile phase thus increasing the density of seedlings and saplings. This is confirmed by the age structure of hornbeam in the Bialowieza Forest. where 90-120 years old trees predominate at present (Fig. 6). Higher density of juvenile hornbeams on the turn of the 19th century meant more food for deer. The population of red deer grew rapidly and reached 6800 individuals in 1914 (Fig. 5a) changing the direction of the herbivore pressure', grazing by roe deer has never been more than 20% of the total pressure. The diet of red deer includes 66% of woody plants, their high number therefore helped to control the density of juvenile hornbeams and to limit the efficiency of colonization of new safe sites. After the reduction of game by poachers at the end of World War 1, the density of juvenile hornbeams increased again, bringing about a consecutive growth in the number of red deer. Between the two Word Wars, the red deer is the dominant herbivore and the pressure on woody plants is twice as much as that on perennials (Fig. 5b). Successive drop in the number of game following World War II, subsequent control of the deer population by man and a lower pressure on woody plants, and the reintroduction of the European bison contributed to the successful recruitment of the previously grazed juvenile hornbeams to the shrub-layer and to the creation of new safe sites for the seedlings. Between the years 1960 and 1990, due to a low and

similar pressure of the herbivores on perennials and woody plants, grazing did not control the density of hornbeam juveniles but continued to supply new safe sites for the species. Immediate recruitment of the previously grazed juveniles to the shrub-layer additionally contributes to the decline of the oak forest communities. The saplings shade the surrounding ground causing a withdrawal of the heliophilous perennials and a drop in the density of the ground-layer. Autumn mass fall of hornbeam leaves accelerates the decomposition of the litter, which effectively becomes thinner. In the patches of oak forest with high proportion of hornbeam in the shrub-layer (which is a phase of the decline of Potentillo albae-Quercetum), the litter in much thinner while the density of the ground-layer is lower (Table I, Figs. 2, 3, and 4). Eventually, the "supply" of safe sites is higher and higher and the process of hornbeam invasion progresses due to a positive feed-back. The models explaining the effects of the differential pressure of herbivores on the oak forest communities have been proposed (Figs. 7 and 8). However, the mechanism initiating the invasion of hornbeam does not have to be set in motion exclusively by the action of animals. Every direct and indirect human activity leading to the creation of gaps in the ground-layer and the destruction of the litter of oak forests, such as raking of mulch, intense grazing by cattle, logging and the accompanying damage, may provoke similar effects as in the model in the Fig. 8.

363: ***+.206***

This report is published under the auspices of the Cat Specialist Group of the International Union for Conservation of Nature and Natural Resources/Species Survival Commission. The report contains a review and analysis of the conservation status of the wild cats and a comprehensive strategic plan for increasing the effectiveness of conservation methods. The work is divided into 3 parts, an introductory section and 7 appendices. The introductory section consists of an executive summary plus 3 chapters covering the taxonomy of Felidae. Part I contains species accounts of the wild cats from sub-Saharan Africa, North Africa and Southwest Asia, Tropical Asia, Eurasia, and the Americas. Par II covers the major issues in cat conservation: cats and habitat loss, management of big cats near people, research, trade, cats in captivity, and reintroduction. The third part is an action plan for cat conservation. The 7 appendices are classically described cat subspecies, "Scientific Postmortem: A Protocol for Collection of Data and Specimens", scientific names of species mentioned in the text, species-habitat associations, cat specialist group members, a statement by the International Fur Trade Federation, and a list of maps, figures, and tables. The report is illustrated by color plates of each species, photographs, cladograms, and graphs.

366: ***+.036***

The progress of studies on the minks in Poland resulted in an increased interest in these animals, both among professional zoologists, and hunters and anglers. The spontaneous expansion of the American mink in our country, signalled in various publications, still continues and appears to get out of any rational control. Moreover, our earlier postulates (Ruprecht et al., 1983) regarding the legal status of the American mink in Poland have never been executed in practice. First cases of killing the American mink in the wild in Poland were noted as early as 1954, and still happen at present. They are especially frequent in NE Poland, and no doubt associated with the natural expansion of M. vison which was introduced among others in Belarus in 1953-1958. Most of these animals, are however, killed illegally and the cases are prosecuted by law as poaching. It is impossible for the Police to adopt an unambiguous attitude because of the uncertain legal status of the American mink in Poland. Our postulates regarding the mink in Poland are the following: 1. Including the American mink in the list of game species with the whole-year protection period. Here we somewhat depart from our original suggestion which included a shorter protection period (March-September). 2. Maintaining the total protection of the European mink Mustela lutreola (L.,

1761) whose present occurrence in Poland cannot be excluded (it occurs in Kaliningrad district adjacent to Olsztyn and Suwalki voyvodeships). Systematic research should be aimed at explaining the problem, especially in NE Poland. 3. The whole-year protection of the American mink remain stay in force till an unequivocal explanation becomes available whether or not the native European mink became extinct in Poland. In case of official hunting for American mink (attractive fur animal) a danger may appear of extermination of the last living individuals of the European mink, already on the brink of extinction. The danger will be all the more real, if reintroduction of M. lutreola based on young born in zoological gardens is accomplished (Ratajszczak and Smielowski, 1992).

368: ***+.104***

Populations of the Carpathian lynx (Lynx lynx carpathica Kratochvil et Stollmann, 1963) became almost extinct in Slovakia, by shooting, 75 years ago. Subsequently, numbers of lynx in Slovakia have increased again and the distribution range extended, owing, to the introduction of legal protection. Protection of the lynx had been discontinued in 1955 and it was introduced again in 1975, as a consequence of the observed decline in numbers. The lynx is now protected from the 1st of March to the 15th of September. This period of protection, however, does not seem to be appropriate, and the period from the 16th of March to the 31st of December is now proposed. The present range of the-lynx in Slovakia extends over an area of woodland of 13 700 km-2 and the total population size is estimated to range between 400 and 500 individuals. In 1955 to 1994, the annual mean harvest rate was 74.83 individuals i.e. one individual per 183 km-2 of woodland area on average. The total official bag was 2993 lynx during the whole period. Furthermore, certain individuals dispersed from Slovakia to Moravia and even to Hungary and Austria. Successful hunting management of this predator is believed to be feasible at this time. This is backed up by the fact that the bag of lynx increased 5.2 times during the period from the late 1920's to 1991 and, in the same time interval, production of hunted game ungulates increased 13.5 times. Roe deer (Capreolus capreolus) appears to be the preferred prey species of lynx and the losses caused by lynx to sheep breeding are low in Slovakia at present. Numbers of lynx now appear to have declined again in Slovakia, the annual harvest having decreased from 105 individuals in 1991 to 44 individuals in 1994. This decline is particularly caused by increasing hunting pressure, stimulated by the attractiveness of the rare and valuable trophy of lynx, and also by the rapid reduction in the food base, resulting from a decrease in numbers of grazing sheep. The main causes of the decrease in numbers of prey species are both poaching and the economic recession in agriculture. In this contribution, more effective protection and hunting management of the lynx is proposed. It is emphasised that were the source of individuals used in re-introduction efforts performed in Slovenia, Germany, France, and the Czech Republic (the Sumava Mts.).

370: ***-.008***

Human persecution may have been the major cause of extirpation of the bearded vulture Gypaetus barbatus in the western Alps around 1920-1930. However, as shown in a previous work (Mingozzi & Esteve, 1996), historical information by itself is insufficient to uphold this hypothesis because the real extent of the shooting pressure endured by the species is not ascertainable. The aim of this paper was to test whether the rapid population decline (30-40 years) shown by historical data is consistent with human persecution. By making assumptions about hypothetical population size and natural survival rates, we tried to assess what rate of additional adult mortality caused by human impact would eventually hal tt led to extirpation. The size of our hypothetical population was estimated by comparison between the current population size (number of breeding pairs) of the golden eagle Aquila chrysaetos in the Pyrenees and in the western Alps. All the life-history

parameters available in the recent European literature were used as reference data for a Population Vulnerability Analysis (PVA). A series of computer simulations was performed with R.C. Lacy's programme, VORTEX. Three different scenarios were explored: one without hunting persecution and two with different killing rates. The results suggest that the vulnerability of G. barbatus was extremely high and even a moderate rate of killing would have led to extirpation within a very few decades, as suggested by the historical data. Moreover, the species vulnerability shown by the simulations has led to cautious conclusions about the success rate of the current reintroduction project of the bearded vulture in the Alps.

371: *+.019*

The golden lion tamarin Leontopithecus rosalia is one of the most endangered primates in the New World. In order to assess the status of golden lion tamarin habitat, satellite images covering the original distribution of the species were analysed and the size of all forests measured. Effective size (Ne) of remaining populations was calculated and the time of isolation estimated for all subpopulations, Current threats were identified in each forest the species was found. Most of the remaining forests within the species' original distribution were in fragments smaller than 10km(2). The majority of the golden lion tamarins found were in forest fragments which have been isolated for more than 15 years. Today, the principal threats to the survival of the species are the small size and isolation of forest patches, and the small size of subpopulations. Threats such as deforestation and hunting have also been observed in all forests. The small number of remaining animals and their disjunct distribution will likely result in the loss of genetic variability and in inbreeding depression. Conservation strategies, such as translocation of isolated groups, reintroduction of more captive animals, protection of the forests, reforestation, and management of the subpopulations as a metapopulation, will be essential for the maintenance of the species in the wild. The larger and better-preserved forests without tamarins were suggested as potential areas for reintroduction and translocation.

373: *+.242*

The European beaver eliminated in Western Siberia in the XVIII-XIX centuries has survived only in the upper reaches of the Konda and Northern Sos'va rivers, where it is now under special protection. In the thirtieth-seventieth (the XX century), in the southern part of the region, there was conducted wide reacclimation work (beavers were brought from the European part of the country). The work has not affected the Khanty-Mansiisk Region, where there is also a possibility to restore the area. It is recommended to distribute beavers living in southern parts of the Tyumen' Region to the north. It also appears possible to use aborigen population (the Konda river), but it should be done with extreme care.

374: *+.099*

Primula vulgaris is one of the endangered species in Poland. At present Debowka near Lublin is its unique natural locality in Poland. The adopted programme of active protection includes: reproduction in ex situ conditions (The Botanical Garden UMCS), reintroduction of the population in Debowka, and restoration of an extinct locality in Lancuchow (re-introduction of the species).

377: *+.117*

Fossils and other evidence from the Southern Cook Islands show that the Rimatara Lorikeet Vini kuhlii, known as the Kura, was widespread in the group during prehistoric times and, it was

probably extirpated due to exploitation for its red feathers. Today, it survives only on Rimatara in the Austral Islands, where it is known as the 'Ura. On Rimatara during 5-11 August 1992 we saw/heard 263 'Ura, and estimated the total population at 900 birds. The mixed horticultural belt, about 32% of the island, was the most favoured habitat at 2.2 birds ha-1 and it supported about 61% of the total population. The species was uncommon in the coastal coconut plantations and central hills, and rare in the makatea/feo. Rats, especially Rattus rattus, have often been associated with the loss of forest birds on oceanic islands and R. rattus is thought to be responsible for the decline in other lorikeets of French Polynesia. A preliminary trapping study located R. norvegicus and R. exulans, but not R. rattus. The highest conservation priority should be given to confirming the absence of R. rattus on Rimatara and the implementation of a major quarantine programme to ensure that it is not accidentally introduced. We recommend reintroducing the lorikeet to islands within its former natural range.

378: ***+.160***

After European settlement, the range of the Noisy Scrub-bird Atrichornis clamosus contracted and only the population at Two Peoples Bay survived. Management policy at Two Peoples Bay Nature Reserve has been one of wildfire prevention, which has created increased areas of suitable habitat for the species. Territories rose from 45 in 1970 to 189 in 1994. Habitat use was assessed by grouping the dominant vegetation associations in each territory in five categories, in descending order of suitability (assessed on breeding and feeding resources): low eucalypt forest, Agonis forest, tall thicket, low thicket and heath. Turnover rates in territory use gave the same habitat rankings. A range of values of N-e (effective population size) was calculated using assumptions about breeding potential of each habitat category. There may be insufficient habitat at Two Peoples Bay for the population to reach an N-e of 500. Successful translocation to Mt Manypeaks, and the establishment of three small populations by birds dispersing from Two Peoples Bay, suggest that an integrated regional population can develop with an N-e well in excess of 500. The management of areas with populations and the corridors linking them must include minimizing the incidence and extent of wildfires.

379: ***-.011***

We monitored survival, reproduction and emigration of a translocated beaver Castor fiber population in the Netherlands for five years and used a stochastic model to assess its viability. Between 1988 and 1991, 42 beavers were released in the Biesbosch National Park. The mortality was initially high but gradually fell to normal rates. However, the breeding success was low, and we hypothesized that this was either a temporary phenomenon (the translocation hypothesis) or a permanent feature (the poor habitat hypothesis). According to the computer simulations, the isolated population was viable under the first but not under the second hypothesis. In the latter case, the prospects generally improved by the foundation of another population in the Gelderse Poort (100 km from the Biesbosch), However, this second habitat should be optimal for beavers in order to reduce the extinction probability of the Biesbosch population to below 10% in 100 years,' the loss of genetic variability (1-2% per generation) was just above the applied tolerable risk (1%), but the effects of inbreeding are unknown in beavers. We conclude that the beaver population in the Biesbosch is not viable unless the reproductive success increases, either in the Biesbosch itself or in a nearby population. We recommend applying such viability analyses to evaluate the likely success of any translocation.

380: ***-.013***

This paper reports the results of an aerial survey of a marsh deer Blastocerus dichotomus population occupying an area to be flooded by the construction of a hydroelectric power plant (Porto Primavera) in the Parana River, Brazil. Forty transects totalling 143 km were flown during the dry and flood seasons. Population size was estimated using the modified Petersen estimator, the line transect method, and two variations of the strip transect method. The results revealed mean densities of 0.50-0.54 deer/km(2) and a population of approximately 950 individuals, the second largest population reported for this vulnerable species in Brazil. Individuals were most commonly alone, but group sizes of up to Jive deer were observed. Newborn fawns were observed from September to November. The effectiveness of a rescue operation is discussed relative to the possible benefits for the species' conservation. Deer translocations have a high failure rate. Public pressure to translocate marsh deer is not likely to aid in their conservation but may actually assist to precipitate their decline locally. Instead, acquisition of suitable marsh habitat in which the deer already reside could be much more successful and less costly.

381: ***+.274***

The Mala was once one of the most abundant and widespread macropods of central Australia but has declined to the point where it is now one of the rarest and most limited in distribution; Efforts to recover and conserve the Mala have been considerable and predate the national Endangered Species Protection Act 1992 which provides a framework for preparing and implementing recovery plans for threatened species and communities, The initial recovery plan for the Mala was developed in 1992 and focused on research and management actions related to the central Australian form of Mala. Implementation of this plan commenced in 1992 with ANCA funding to support major actions related to management and translocation of mainland populations, The formation of a recovery team in 1993 was the initial stage in the more formal review of the program. This review examined both progress towards implementing the plan and any need to refine existing actions and define new ones. Several program highlights were identified including establishment of captive colonies, implementation of translocation programs, co-operative programs with Aboriginal landholders and an enhanced understanding of the ecology of the Mala. A revised plan was finalized during 1995 providing a more national focus to the recovery effort, the critically endangered status of the Mda ensures that this effort will be ongoing. The development, implementation and revision of the Mala Recovery Plan has provided a useful insight into the potential achievements and shortcomings of the recovery process for conservation of threatened species.

382: ***+.302***

With the aid of case studies, priorities for achieving practical conservation management outcomes from the genetic studies of rare plants are defined. In Acronychia littoralis, genetic analysis did not support the hypothesis that the species was a hybrid. Rather A. littoralis was found to be generically very similar to another species, highlighting the need for further taxonomic research. It is now apparent from a genetic study of Haloragodendron lucasii, that clonality is extensive with fewer than 10 genetic individuals represented among thousands of stems. Thus the location of new populations is now the highest management priority and is currently being attempted by an integrated conservation initiative. Zieria prostrata has been the subject of an ex situ and translocation program. Genetic study has revealed that the initial ex: situ collection may fail to adequately conserve generic diversity and has raised the possibility of mislabelled plants. Consequently, a postponement of the translocation program has been recommended. With respect to achieving practical conservation outcomes in future studies, we rank the genetic study of clonal plants, plants targeted for translocation, and plants of uncertain taxonomic status a high priority,

particularly when conducted within a hypothesis testing framework. In contrast, we rank other areas, including many of those proposed for genetic study in existing recovery plans, a lower priority. Clearly a more critical evaluation and justification of the need for conservation generic research on a case by case basis is essential for more effective recovery plans.

383: ***-.031***

The discovery of the Wollemi Pine Wollemia nobilis in 1994 not only brought to light a new genus in the Araucariaceae and a conifer with at least a 91 million year old Gondwanan history, it also increased the threat to the two known wild populations of 40 adults and about 130 seedlings. Although growing in an inaccessible, warm temperate rainforest-lined gorge in a large national park, the impacts of visitation, and indeed researchers, could prove costly to the species. The main threats from people are trampling of seedlings, compaction of the ground and introduction of pathogens. Another threat is wildfire which has the potential to destroy much of the population in one catastrophic event. A range of hz situ ecological research and ex situ botanical and horticultural research is being conducted on the species to aid its conservation. A species recovery plan has also been prepared. In the short term, a key research program aims to discover the most efficient way to propagate and cultivate the species to meet marker demand for garden plants. This would remove the pressure of illegal seed collection from the fragile wild populations. Since the Wollemi Pine is a relic species, "recovery" is not the question. Management should aim to maintain the current population and genetic variation. Translocation may arise as an issue in the long run, but there would need to be sound reasons for it to be undertaken.

384: ***+.053***

A species may be considered threatened if it (1) is represented by one or a few stable but small populations, or (2) was formerly widespread or abundant but is now in decline. In the first case, it may not be appropriate to attempt recovery of the species if it has not declined; monitoring and perhaps captive breeding should be the only management needed. In the second case, research is important to identify the processes causing population decline and to ensure that management for recovery is informed and effective. A rigorous scientific methodology is advocated to identify causes of declines and is applied in case studies of the Lord Howe Island Woodhen and a small dasyurid marsupial, the Mulgara. Four steps are involved: (1) establish the magnitude and rate of population decline, and list possible explanations for it (observations); (2) identify the most plausible threatening process or processes that may de causing the decline (model); (3) derive predictions from the models (hypothesis); and (4) lest critically, using null hypotheses, whether the putative threatening processes identified in the second step are causing the observed population decline, by using mensurative or manipulative experiments. When one or more processes causing population decline have been identified, management should be initiated to alleviate them and subsequent population changes monitored. It is proposed further that scientific methodology be used to identify effective management techniques, evaluate contentious practices, and guide reintroduction programs. I discuss objections that science is sometimes irrelevant for recovery of threatened species, and propose that strengthened links between research and management would increase the effectiveness of recovery planning.

385: ***+.174***

Species and populations are, and always have been, tied intimately to a geographical place. Much of what humans value in species/populations arises from their natural association with place. Translocation breaks forever the historical natural link between the organism and place. What then

are we really "saving" when we translocate them?

386: ***+.064***

Microplanktonic red tide blooms (dominated by dinoflagellates) were observed in brackish water fish ponds of Terengganu between March 1992 to January 1993. The first short-lived bloom (2-3 days) occurred in October 1992 while the second long-lived bloom (6-7 days) occurred in January 1993. The dominant dinoflagellate species comprised of Peridinium quinquecorne (>90% total cell count) with considerable proportion of Protoperidinium excentricum. Ciliophora consisting of Tintinopsis sp. and Favella sp. were also present during the bloom period. The total ash, chlorophyll, phaeopigment, lipid and fatty acid content of the microplankton were studied. Considerable amounts (6-11% of the total fatty acid) of the polyunsaturated fatty acid 18:3w3 (linolenic acid) were present in the microplankton. However, high amounts of 20:5w3 (eicosapentanoic acid) and 22:6w3 (docosahexaenoic acid) were present with variable but usually high amounts of 22:4w6 and 22:5w6 acids. The latter microplankton bloom contained higher amounts of 20:5w3 and 22:6w3 acids than the earlier bloom. Lipid content were three to five times higher than chlorophyll a. There was an increase with successive day after bloom outbreak in the relative proportion of total C18, C20, and C22 fatty acid components. The algae microplankton contained the w3-polyunsaturated fatty acids (PUFAs) probably needed for the growth and survival rate of grazing pond animals.

387: ***+.241***

Of 20 groups of Cape mountain zebras re-introduced into new conservation areas 12 have increased, 6 have remained stable or decreased, and 2 went extinct. One of the main reasons for the poor performance of some of the groups was that insufficient numbers were re-introduced. In those cases where the population increased the mean number originally introduced was 15.1. This is significantly higher ($P < 0.05$) than the number introduced in the cases that failed to increase (8.1). This is in accord with other studies that have shown that the success of re-introductions is closely linked to the numbers of animals re-introduced. The mean percentage annual increase of the populations over the first three to five years after re-introduction (0.4%) was significantly ($P < 0.05$) lower than that over the subsequent 3 to 5 years (9.3%). This probably reflects the effects of capture stress, break up of family groups and adaptation to a-new environment. There was no significant relationship between maternal age and progeny sex ratio in Cape mountain zebra. A simulation modelling package, VORTEX was used as an aid to the management of Cape mountain zebra. Rates of population growth and sustainable harvests derived by means of the model were similar to those observed in the real population. The model is useful in analysing the poor performance of small populations re-introduced into new conservation areas, which may help to avoid unrealistic expectations on the part of new owners of mountain zebra herds and to underline the necessity of starting with a reasonable number of animals.

388: ***-.049***

Previous studies demonstrated strong fertility selection for a self-fertile, homostyle morph due to pollinator loss in an isolated population of Primula sieboldii, an endangered heterostylous species. To predict genetic consequences of the selection we developed a deterministic genetic model based on a classical "super-gene" model and we studied the effects of pollinator availability and inbreeding depression on temporal changes of morph frequencies through model simulation. Because of the severe pollinator limitation experienced by the population fast, irreversible loss of the thrum morph from the population was predicted even if high inbreeding depression was

assumed To prevent the breakdown of the normal breeding system of the species, morph frequency monitoring for timely active management should be implemented Active management should include hand pollinations and pollinator therapy-reintroduction and reestablishment of suitable pollinator populations The method we adopted in this study to parametrize pollinator availability can be used widely in conservation modeling for a range of plant species that have multiple mating types with different degrees of self-incompatibility.

389: ***+.015***

Transposition of diagnostic tests used in domestic livestock species to free-ranging and captive wildlife species has two problems. First, most existing tests have not been adequately validated in domestic livestock; Second, assumptions that a serological test will perform identically in wildlife and livestock species may not be correct, due to differences in pathogenic strains and serovars, host serological responses, and exposure to organisms of similar antigenic structure which produce cross-reacting;antibodies. Some assays require species-specific reagents/test components which might not be commercially available, and most assays have not been standardized The authors outline the principles involved in the evaluation of a serological diagnostic test, and provide examples of how knowledge of test sensitivity and specificity can be used to estimate true prevalence, to determine whether a population is infected, and to facilitate management decisions with regard to animal translocations.

390: ***-.065***

In the second half of the 20th century, populations of many wild animal species have been established in captivity for various purposes (e. g education, conservation, research, farming) and many are kept as companion animals. With continuing human pressure on the environment, captive or semi-captive management is likely to become a component in the conservation of an increasing range of species throughout Europe and Asia. The management of small and divided populations (some of which may be in toes while some are free-ranging) requires careful control to minimise loss of genetic diversity. This, in turn, may require movements of animals or germplasm between sub-populations. A potentially serious hazard associated with these translocations is the accidental introduction of infectious agents into populations which have hitherto been geographically or ecologically isolated from these agents. When planning translocations, the state veterinary authorities of both importing and exporting countries should be contacted at an early stage for details of legal requirements in relation to animal health, welfare and conservation. Animal health legislation is mostly aimed at preventing disease in man and domestic animals, and further disease control measures may be required when translocating wild animals. In addition to the need for efforts in ensuring genetic diversity and disease control, good husbandry and welfare can often be challenging in species about which relatively little is known. A variety of organisations are involved in coordinating captive breeding programmes in Europe and Asia, and some of these organisations and the routes through which they can be identified are mentioned here.

391: ***-.166***

Species Survival Plans in North America - and comparable programmes in other parts of the world - address the management issues related to maintaining populations of endangered species in captivity. The need has been recognized for universal methods of assessing, evaluating, monitoring and preventing the transmission of disease to naive wildlife by reintroduced species. Standardized protocols are presented for evaluation of the health status of captive animals

intended for release.

392: *+.229*

The eastern barred bandicoot, Perameles gunnii, formerly widespread on the volcanic plains of western Victoria, has been reduced to a single, rapidly-declining, remnant population at Hamilton, Recovery of this critically endangered species has included local management, in an attempt to stabilise the wild population, captive breeding and reintroduction to selected sites. Veterinary advice and assistance have been an integral part of the investigation, planning and implementation phases of the program. The development of appropriate, standardised techniques has enabled successful treatment of problems in the captive colony, Husbandry, including the hand-rearing of pouch young has been refined, Parasitism, identified as a contributor to poor health or death, has been investigated, Experimental development of techniques for the attachment of radio-transmitters to bandicoots has enabled improved field research to take place, Fox predation, a major limiting factor in the recovery program, has been studied in detail, in order to refine control protocols.

393: *-.074*

The Mexican wolf (Canis lupus baileyi), and endangered subspecies of gray wolf, was native to parts of Mexico and the southwestern United States. Currently, only a few individuals, if any, exist in the wild, so planned reintroduction programs must use captive-raised wolves. In only one captive population, however, designated the certified lineage, are all the founders (n = 4) known to be obtained from a wild population of Mexican wolves. Two captive populations were founded from individuals of uncertain ancestry and have not been included in the species survival plan. To preserve genetic diversity and reduce inbreeding so that fitness will be maintained, it would be desirable to include these two captive populations in the breeding program if it could be shown that they were derived from a wild population similar to the certified lineage. We compared allele frequencies of 10 hypervariable microsatellite loci in Mexican gray wolves with those found in a sample of 42 domestic dogs. 151 northern gray wolves, and 142 coyotes to determine if uncertified Mexican wolves had specific markers from these animals. We analyzed pairwise genetic distance measures to demonstrate that the three captive populations of Mexican gray wolves were closely related to each other and distinct from dogs and northern gray wolves. The three captive populations are genetically more similar to each other than to any other population of dog or wolf-like canid, and they shared alleles that were rare in other canids. The genetic distance between them is similar to that between closely spaced populations of northern gray wolves. As a group, moreover, they are the most genetically distinct population of North American gray wolf. Therefor, the three captive populations could potentially be interbred to augment the genetic diversity of the certified lineage. Source individuals for reintroduction should be derived from the captive Mexican wolf population rather than populations of captive or wild northern gray wolves.

394: *+.176*

The use of captive breeding in species recovery has grown enormously in recent years, but without a concurrent growth in appreciation of its limitations. Problems with (1) establishing self-sufficient captive populations, (2) poor success in reintroductions, (3) high costs, (4) domestication, (5) preemption of other recovery techniques, (6) disease outbreaks, and (7) maintaining administrative continuity have all been significant. The technique has often been invoked prematurely and should not normally be employed before a careful field evaluation of costs and benefits of all conservation alternatives has been accomplished and a determination made that captive breeding is essential for

species survival. Merely demonstrating that a species' population is declining or has fallen below what may be a minimum viable size does not constitute enough analysis to justify captive breeding as a recovery measure. Captive breeding should be viewed as a last resort in species recovery and not a prophylactic or long-term solution because of the inexorable genetic and phenotypic changes that occur in captive environments. Captive breeding can play a crucial role in recovery of some species for which effective alternatives are unavailable in the short term. However, it should not displace habitat and ecosystem protection nor should it be invoked in the absence of comprehensive efforts to maintain or restore populations in wild habitats. Zoological institutions with captive breeding programs should operate under carefully defined conditions of disease prevention and genetic/behavioral management. More important, these institutions should help preserve biodiversity through their capacities for public education, professional training, research, and support of in situ conservation efforts.

395: ***+.245***

Whether or not a bird species will establish a new population after invasion of uncolonized habitat depends, from theory, on its life-history attributes and initial population size. Data about initial population sizes are often unobtainable for natural and deliberate avian invasions. In New Zealand, however, contemporary documentation of introduction efforts allowed us to systematically compare unsuccessful and successful invaders without bias. We obtained data for 79 species involved in 496 introduction events and used the present-day status of each species as the dependent variable in fitting multiple logistic regression models. We found that introduction efforts for species that migrated within their endemic ranges were significantly less likely to be successful than those for nonmigratory species with similar introduction efforts, Initial population size, measured as number of releases and as the minimum number of propagules liberated in New Zealand, significantly increased the probability of translocation success. A null model showed that species released more times had a higher probability per release of successful establishment. Among 36 species for which data were available, successful invaders had significantly higher natality/mortality ratios. Successful invaders were also liberated at significantly more sites. Invasion of New Zealand by exotic birds was therefore primarily related to management, an outcome that has implications for conservation biology.

396: ***+.130***

The Karner blue butterfly (Lycaeides melissa samuelis) is listed as a federally endangered species in the United States. It occurs in oak savanna and pine barren habitats from eastern Minnesota to New Hampshire. In 1994, we successfully reared Karner blue larvae under controlled laboratory conditions for experimental purposes, and report on those rearing methods here. We collected 20 female Karner blue adults of the spring generation from two areas in Michigan, and housed them in cages in an environmental chamber at 24 degrees-26 degrees C for 5 days. The female butterflies produced 154 eggs, of which 72 hatched in an average of 4.5 days, and 68 first instars survived. Eggs, larvae and pupae were kept in a growth chamber at 24 degrees C. Developmental time from egg to adult averaged 26 days; the average duration of each instar ranged from 3 to 4 days, and the average pupal duration was 8 days. Thirty three laboratory-reared Karner blue larvae successfully completed the 4 instars, and were released as adults into maternal collection sites. Laboratory rearing may be a viable means of providing Karner blue individuals for reintroduction into areas where the species is extirpated, for supplementation of small populations, or for research. Ultimately, such methods may become an integral part in the recovery of this and other rare invertebrate species.

397: *+.253*

Experiences with the reintroduction of the takhi, or Przewalski horse (Equus ferus przewalskii), in Mongolia can serve as valuable lessons for reintroduction of ungulates in general. We discuss the present taxonomic, historical, and biological evidence and conclude that takhi should be viewed as a typical steppe herbivore. Its last refuge, the Dzungarian Gobi, should therefore be seen as a marginal habitat because it consists mainly of desert and semidesert. Since 1992 two reintroduction projects have been in the acclimatization phase in Mongolia. Despite promising developments, problems with cooperation, management, habitat choice, insufficient knowledge of the ethology of the species, and current land use within the different project areas could jeopardize the successful reintroduction of takhi. We review the conditions required for a potentially successful ungulate reintroduction. The planning of a reintroduction within the framework of safe-guarding an entire ecosystem with an integrated management plan appears essential. Each potential reintroduction site should be assessed thoroughly for its suitability, including size, habitat types, current land use, socioeconomics, legislation, and potential problems. Each site should be provided with one or more acclimatization facilities to harbor genetically and physically healthy, socially adapted animals in biologically should groups. An organization structure should be established for each reintroduction site. Its objective should be to develop an effective management plan and to carefully monitor the population and its surrounding ecosystem. Special attention should be given to local socioeconomic situations, community participation, and training of staff for management, research, and ranger and warden activities.

398: *-.115*

Fire suppression and habitat fragmentation during the twentieth century have generated many long-unburned stands of relatively isolated sand pine scrub. Such stands often lose most of their endemic herbs as woody species come to dominate. A sand pine scrub stand last burned in 1926 or 1927 was prescription burned in February 1986 after a 1985 vegetation census and then was resampled five times over a 7-year recovery period. In spite of a wide variety of recovery strategies to the low-intensity burn, there was little change in the floristic makeup of postburn stands compared to the preburn stand. Detrended correspondence analyses suggested that floristic composition and community structure of postburn stands shifted away from those of the preburn stand. However, this shift did not restore the populations of narrowly endemic scrub herbs. Instead, the change was toward xeric hammock, characterized by the persistence of woody understory species, the unsuccessful restoration of sand pine (Pinus clausa) as a canopy dominant, and the near absence of most herbs. These results suggest that long-unburned sand pine scrub will not return to typical scrub through the reintroduction of a single low-intensity, winter burn. This study demonstrates the necessity of monitoring the consequences of the reintroduction of fire to long-unburned vegetation associations and of recognizing variation in vegetative responses to different fire regimes.

399: *+.157*

The total population of the Seychelles Magpie Robin Copsychus sechellarum declined from 38-41 birds in 12-13 territories in 1977-1978 to 17-21 birds in eight to nine territories in 1988-1990 and was entirely confined to Fregate Island (210 ha) in the Seychelles. After a successful cat eradication program in 1981-1982, recruitment improved, although the abandonment of agriculture had caused a reduction in the amount of feeding habitat and hence in the carrying capacity of the island, The population declined because of the failure of recruitment to compensate for the annual adult mortality, Foraging activity of the Magpie Robin was greater in high-quality

territories (measured by soil invertebrates available), leading to increased reproductive success, Through supplementary feeding, five times as many recruits were produced, Of the 11.5 potential annual breeding recruits, 5.3 are required to compensate for adult mortality, and the other 6.2 recruits can be regarded as "surplus" contributing to an increase, Magpie Robins prefer to breed in rotten trees, which are a scarce resource, The greater the distance between the nest site and feeding areas, the less time was spent in incubation and nest guarding, resulting in greater egg loss. Because of lack of suitable areas for establishing territories, many young Magpie Robins became "floaters". Nest disturbance, both by these floaters and by the introduced Indian Mynah Acridotheres tristis, had adverse effects on the breeding success of robins. A recovery plan has been designed to save the Magpie Robin. Territories have been improved for feeding (by tree planting) and for breeding (by providing nestboxes and reducing nest disturbances). Given the vulnerability of one small island, the presence of surplus birds (supported mainly by supplementary food) and the suitability of neighbouring Aride Island (68 ha), successful translocations to this island took place in 1992 and 1994. Given the presence now of 47 individuals on two islands, it is hoped that the species will pull back from the brink of extinction.

400: *+.046*

Several conservation measures are intended to enhance the movement of individuals among populations. These include the establishment of wildlife corridors, captive breeding and release programs, and translocation of individuals among populations. Many metapopulation models show that increasing movement among populations reduces the chance of metapopulation extinction. However, epidemiological models indicate that increased contact among populations enhances the spread of disease and can trigger epidemics. I have synthesized elements of mathematical epidemiology with metapopulation models. An analytic model showed that highly contagious diseases of moderate severity spread widely, increasing the probability of metapopulation extinction. I also used a simulation model to examine four spatial arrangements of populations: island, necklace, loop, and spider. When infected individuals were allowed to move freely among populations, all of the arrangements exhibited qualitative behavior similar to that exhibited by the analytic models. The most dangerous diseases were those for which infected populations grew large enough to produce dispersers that infected other populations, but which also reduced the geometric rate of increase for infected populations to near unity. Under those conditions, random demographic and environmental events caused metapopulation extinction. Major differences among the spatial arrangements emerged when a quarantine population was established. A centralized quarantine in the spider and necklace arrangement yielded the most dramatic reductions in metapopulation extinction probability. A single quarantine patch was of little value in an island arrangement. These results have several implications for managing metapopulations. Most notably, some spatial arrangements of populations are more amenable to disease control than others, and establishing a quarantine population can increase the probability of detecting new diseases and reduce the impact of diseases that do appear.

401: *-.000*

Apalachicola rosemary Conradina glabra Shinners is an endangered woody mint, endemic to the northwestern portion of Liberty County, Florida, USA. Because the few remaining populations of this species ave on private silvicultural lands and subject to disturbance, C. glabra was reintroduced within its original range onto The Nature Conservancy's Apalachicola Bluffs and Ratlines Preserve. Forty-eight plots of nine rooted cuttings were planted in each of three sites at the preserve in 1991. All above-ground herbaceous and small woody vegetation was clipped monthly during the growing season around 24 of the plots at each site in 1991 and 1992. Removal

of competition resulted in significantly taller plants with move branches and flowers than in control plots across all sites. By the end of the second growing season, however, treatment effects diminished Survival of planted cuttings was 94%, but was reduced by the prescribed fire management applied to two of the sites. Roughly 25% of the plants which had 50-100% tissue scorch in the fires resprouted. Seedling establishment was highest in the burned sites, and almost entirely limited to clipped plots. Evaluation of the success of this translocation effort will require long-term monitoring of these C. glabra populations and some reduction of fuel around plants prior to prescribed burning. Copyright (C) 1996 Elsevier Science Ltd

402: ***+.284***

In 1993 we conducted a follow-up study of the 1987 survey by Griffith et al. (1989) of 421 avian ans mammalian translocation programs in North America, Australia, and New Zealand to reassess the programs' status and the biological and methodological factors associated with success. Our survey response rate was 81%. Approximately 38% of usable programs in 1993 reported a change in outcome from 1987 (e.g., a translocated population was "declining" but now is "self-sustaining"), but the difference between the overall success rates was not statistically significant (66% in 1987 and 67% in 1993). Since 1987, and increase was observed in the median number of animals translocated per program (31.5 to 50.5), median duration of releases (2 to 3 years), and proportion of programs releasing more than 30 animals (46% to 68%). Multiple logistic regression analyses indicated that release into the core of the historical range, good-to-excellent habitat quality, native game species, greater numbers of released animals, and an omnivorous diet were positively associated with translocation success. Moreover, our results indicate that translocated birds were less successful at establishing self-sustaining populations than translocated mammals. Our findings using comparable logistic analyses, generally corroborate the results of Griffith et al. (1989). Variables not found to be significantly correlated with translocation success include species' reproductive potential (number of offspring and first age of reproduction), number and duration of the releases, and source of the translocated animals (wild-caught versus captive-reared).

404: ***+.025***

For a number of decades, the lesser white-fronted goose (Anser erythropus) has been almost-absent from the Fennoscandian fauna and has a current population size of only about 60 breeding pairs, with fewer than 10 pairs in Sweden. During the period 1981-1991 more than 200 young have been reintroduced in northern Sweden. However, the origin and possible relatedness of lesser white-fronted individuals were unknown when the breeding program started. We have used DNA fingerprinting to assess the similarity of 18 individuals, ie., the entire captive population used for breeding in 1991 and about 60% of the captive population used in 1981-1991. Minisatellite probe 33.15 provided an index for an average similarity of 0.39 between the mates of the 12 breeding pairs used for producing offspring for reintroduction. This is a higher similarity than in natural populations of birds in general but lower than in populations that have passed through serious population bottlenecks. Individuals originating from different breeders are more dissimilar than those from the same breeder. However, the close relationships (similarity, 0.5-0.6) found in a group of five individuals from different breeders show that selecting individuals from different breeding groups is not sufficient to prevent mating between closely related individuals.

405: ***+.086***

The U.S. Fish and Wildlife Service (USFWS) was directed by Congress to study the feasibility of

including Colorado in the Northern Rocky Mountain Wolf Recovery Plan (U.S. Fish and Wildl. Serv. 1987). A mail survey of Colorado residents was conducted during the summer of 1994 to determine the social acceptability of gray wolf (Canis lupus) reintroduction. Results showed strong support for reintroducing wolves into Colorado, as well as generally positive attitudes toward wolves. As expected, attitudes toward wolf reintroduction were predictive of how an individual would vote on this issue. Those with negative attitudes toward wolf reintroduction considered it undesirable based on beliefs it would likely result in wolf attacks on livestock, financial loss to ranchers, wolves wandering into residential areas, and large losses in deer and elk. Those with positive attitudes considered reintroduction desirable based on beliefs it would keep deer and elk populations in balance, preserve the wolf as a wildlife species, return the environment back to the way it once was, help people understand the importance of wilderness, and lead to greater control of rodents. Respondents in densely populated areas east of the continental divide were more supportive and positive toward wolf reintroduction than those in the predominately rural areas west of the continental divide.

406: ***+.029***

From the study of parasitic results, concerning 236 digestive tracts and 262 pulmonary tracts of Chamois (Rupicapra rupicapra), we can classify helminths on the basis of their geographic distribution and host specificity. The distribution of the 53 identified species is apparently closely correlated to biogeographic and anthropic factors. The particular role of domestic ruminants in widespreading parasites on mountain grazing pastures is pointed out. As parasites are concerned in regulating ecosystems, since transhumance in protected areas and wildlife reintroductions are likely to induce artificial migrations of pathogens, we insist about taking pathology into account, in wildlife and grazing livestock management.

408: ***-.007***

Conservation of the endangered red wolf (Canis rufus) has become a controversial issue because its genetic and morphological composition has been altered by hybridization with coyotes (C. latrans) and possibly gray wolves (C. lupus) making its evolutionary origins difficult to ascertain. The evolutionary hypothesis based on morphological data is that the red wolf had an Early Pleistocene origin and was the predecessor of both modern coyotes and gray wolves. After 1940 red wolves hybridized with coyotes as the species vanished from the wild. In contrast to this ancient origin-recent introgression hypothesis, molecular data are more consistent with an origin through hybridization between gray wolves and coyotes. Interspecific hybridization may have occurred repeatedly over time prior to European settlement in the southcentral United States or may have been induced recently by anthropogenic changes. We review recent molecular evidence and present new results from the analysis of mitochondrial and nuclear DNA markers in pre-1940 populations of red wolves. Our results are inconsistent with an ancient origin of the red wolf and support the hybridization model. We discuss possible hybridization scenarios and reasons for the red wolf reintroduction program to be concerned with the effects of genetic introgression from coyotes.

409: ***+.042***

Ecology is a subject where theoretical predictions are often difficult to test experimentally in the field. To address this challenge, the Ecological Society of America suggested exploiting large-scale environmental management decisions in a scientific way. This 'adaptive management' constitutes one of the purposes of the Sustainable Biosphere Initiative. Meanwhile, in the current

context of the biodiversity crisis, translocations and particularly reintroductions of threatened species are becoming more numerous. It is time for ecologists and wildlife managers to collaborate on these unique opportunities for large-scale studies.

410: *+.187*

Numerous methods have been proposed for enhancing species viability. Much attention has been given to the minimum required number of individuals, size and number of nature reserves, and value of habitat corridors. Surprisingly, however, the potential value of active management of a population through a program of translocations has only rarely been suggested, and explicit formulations of a theoretical basis for such a program are nonexistent. By drawing on the mathematical optimization technique known as dynamic programming, I develop an optimal dynamic strategy for translocation in a model population. I demonstrate this approach using a simple model for a hypothetical remnant population with two available habitat reserves incorporating demographic, environmental. and catastrophic forms of stochasticity. I then generalize the results by examining the effect on the cost and effectiveness of optimal translocation management of reserve size, population growth rate, environmental stochasticity, catastrophe size and frequency, translocation mortality rate, spatial correlation of population dynamics, and reserve size asymmetry. Simulated application of the optimal strategies, under a wide range of conditions, demonstrates that managed translocations averaging between 1 and 6 individuals/yr might dramatically enhance the probability of species persistence and reduce the required size of nature reserves, potentially by >1 order of magnitude. Given prohibitive financial and political costs of land acquisition for nature reserves, this technique could provide an important alternative for saving many endangered species.

411: *+.156*

During 1992 and 1993, 14 reintroduced Przewalski Horses or Takhi (Equus ferus przewalskii) were studied in the Hustain Nuruu Mountain Steppe reserve in Mongolia. Most of the individuals did not know each other before reintroduction. These Takhi were the first of five groups due to be released in the reserve after an acclimatisation period of at least 1 year. During acclimatisation the Takhi, lived visually and acoustically separately, in fenced enclosures of approximately 45 ha each. The observations, mostly scan-sampling, were carried out in each season. The observation bouts were divided over six periods and over two harem herds. Two of the periods were in the Same consecutive seasons, so comparison over the years was possible. Social integration within the Takhi herds was very high from the beginning, as described by the spatial relation and synchronisation data. Between 50 and 89% of the observation time, the behaviour of all herd members was synchronised, The amount of time spent grazing by the Takhi (30-68% of the daylight period) was similar to that of feral horses and Takhi in captivity and semi-reserves. The Takhi tended to rest in the morning and have a bimodal period of grazing at dawn and in the afternoon. The Takhi displayed clear habitat preferences for certain activities, They had a strong preference to rest at the highest point in their enclosure. They fed preferably on two or three different vegetation types (with five types available in each enclosure), The amount of time spent grazing during the non-growing seasons (49 +/- 15%) indicates that the feeding value and availability of food were sufficient. Health changes were detected adequately using condition scoring sheets. No supplementary food or water was supplied during the harsh winters. Moreover, low mortality rates and high reproductive success show that the mountain steppe is a habitat which is potentially suitable for establishing a healthy Takhi population. Takhi is the first species to return to its native habitat after living only in toes for so many generations.

412: ***+.188***

We assessed the potential for reestablishment of endangered piping plovers (Charadrius melodus) at selected Great Lakes beaches by comparing invertebrate biomass and abundance with that at alkali wetland beaches occupied by breeding plovers in the Great Plains. We measured invertebrate occurrence using sticky traps secured to beach foraging substrates. Diptera (especially Chironomidae and Ephydridae) comprised from 45 to 99% of invertebrate abundance and 24 to 53% of the biomass at six separate sites in the Great Lakes. Coleoptera (< 1-17% abundance; 28-54% biomass) and Hymenoptera (< 1-10% abundance; < 1-9% biomass) also were frequently detected on beaches at the Great Lakes sites. At alkali wetland beaches in North Dakota, Diptera (Ephydridae and Chironomidae) comprised 95% of the invertebrate fauna numerically and made up more than 80% of the biomass. The mean invertebrate biomass at two Great Lakes beaches (Platte Bay, MI = 0.16 g/m(2)/hr; Long Island, WI = 0.11 g/m(2)/hr) was similar ($P > 0.05$) to that at North Dakota alkali beaches (0.09 g/m(2)/1 hr) occupied by breeding piping plovers. However, at four other Great Lakes beaches, we measured 3 to 6 times less (0.01-0.02 g/m(2)/hr; $P < 0.01$) invertebrate biomass than at the alkali wetland beaches. Mean invertebrate abundance showed similar patterns among the sites. Food availability may limit the reestablishment of piping plovers at some Great Lakes beaches. We recommend that invertebrate abundance and biomass be measured at currently occupied piping plover nesting areas in the Great Lakes and be assessed at any sites proposed for reintroduction of piping plovers.

413: ***-.027***

Four native fish species exist in the spring-fed, perennial lower 21 km of the Little Colorado River (LCR), Gland Canyon, Arizona, but only speckled dace (Rhinichthys osculus) is present in the entire reach. The other three species, humpback chub (Gila cypha), bluehead sucker (Pantosteus discobolus) and flannelmouth sucker (Catostomus latipinnis), are present only in the lower 14.2 km below Chute Falls. During base flow, unfavorable water chemistry (i.e., high CO2) and the physical obstacle of Chute Falls may limit the distributions of the three large-bodied native fishes to below Chute Falls. We sampled benthic algae and invertebrates and analyzed water chemistry downstream (Reach 1) and upstream (Reach 2) of Chute Falls to determine whether the disparity in fist distributions could he attributed to these factors. Algal biomass (chlorophyll a) and density and biomass of invertebrates used as food by fishes were greater in Reach 2 than Reach 1. Free carbon dioxide (CO2) and alkalinity generally decreased from Reach 2 to Reach 1, whereas dissolved oxygen, pH and specific conductance generally increased. To determine if water chemistry above Chute Falls was unsuitable to native fishes other than speckled dace, we relocated age-0 (post-larval) and age-1 humpback chub and age-1 bluehead suckers from Reach 1 to three sites in Reach 2 and held them in cases for 3 days. All age-1 fish survived the experiment, although some individuals of both species experienced short-term respiratory stress. Age-0 humpback chub experienced significantly higher mortalities, and exhibited more stress behaviors at the most upstream relocation site than at other sites. We conclude that neither food resources nor water chemistry limit these native fishes to Reach 1. Translocation of fish to the reach above Chute Falls, or breaching that barrier, may be feasible management actions to increase available habitat for the endangered humpback chub and other native fishes in the LCR.

414: ***+.161***

Six south Florida populations of the endangered red-cockaded woodpecker (Picoides borealis) were sampled to examine genetic diversity and population structure in the southernmost portion of the species' range relative to 14 previously sampled populations from throughout the species

range. Random amplified polymorphic DNA (RAPD) anafyses were used to evaluate the populations (n = 161 individuals, 13 primers, one band/primer). Results suggested that south Florida populations have significant among population genetic differentiation (F-ST = 0.17, P < 0.000), although gene flow may be adequate to offset drift (N-m = 1.26). Comparison of Florida populations with others sampled indicated differentiation was less in Florida (F-ST for all populations = 0.21). Cluster analyses of all 20 populations did not reflect complete geographical predictions, although clustering of distant populations resulted in a significant correlation between genetic distance and geographical distance. Overall, results suggest populations in south Florida, similar to the remainder of the species, have low genetic diversity and high population fragmentation. Exact clustering of distant populations supports the ability of RAPDs to differentiate populations accurately. Our results further support past management recommendations that translocations of birds among geographically proximate populations is preferable to movement of birds between distant populations.

415: *-.013*

1. The recent recovery of the wolf in southern Europe has not yet removed the risk of local extinction. Wolf populations are fragmented and often comprise fewer than 500 individuals. In North America, northern and eastern Europe, wolves feed mainly on wild herbivores. In southern Europe, this canid has apparently adapted to feed also on fruit, rubbish, livestock, small and medium-size mammals.2. The main conservation problem lies with predation on domestic ungulates, which leads to extensive killing of wolves. The reintroduction of wild large herbivores has been advocated as a means of reducing attacks on livestock, but predation on the latter may remain high if domestic ungulates are locally abundant.3. Our synthesis of 15 studies, published in the last 15 years, on food habits of the wolf in southern Europe, has shown that ungulates have been the main diet component overall. A significant inverse correlation was found between the occurrence (%) of wild and domestic ungulates in the diet. The presence of relatively few wild ungulate species was necessary to reduce predation on livestock.4. Selection of wild and domestic ungulate prey was influenced mainly by their local abundance, but also by their accessibility. Feeding dependence on rubbish was local and rare. In Italy, the consumption of rubbish/fruit and that of ungulates was significantly negatively correlated. Diet breadth increased as the presence of large prey in the diet decreased.5. The simultaneous reintroduction of several wild ungulate species is likely to reduce predation on livestock and may prove to be one of the most effective conservation measures.

416: *+.229*

Samango monkeys inhabit the highly fragmented forests of the midlands of Kwazulu-Natal, South Africa. Nor all forest patches are occupied and the survival of monkey populations in the face of further fragmentation provides the motivation for a theoretical consideration of metapopulation persistence under different management policy conditions. The primary aims of the model are to examine the influence of connectivity on metapopulation persistence and also to provide a temporal scale for metapopulation persistence in the case of this species. We develop a stochastic, spatial population model, in which we model edge and core troops in a patch separately, and take into account the effect of demographic and environmental stochasticity, as well as the impact of natural catastrophes. The effects of different levels of connectivity between forest patches are investigated, and we allow for a variable number of patches in the metapopulation. We compare the management options of reintroduction of troops and of providing corridor links between patches in a metapopulation. The model shows that neither management option has any meaningful effect on short-term(<200 years) survival. In the long-term, however, corridors

significantly improve metapopulation persistence. In the absence of interventive management to improve connectivity, samango monkey metapopulations (typically 8 forest patches) show poor persistence probabilities, less than 0.75 to 800 pears and less than 0.34 to 1500 years, In contrast these survival probabilities are 0.9 to 800 years and 0.8 to 1500 years when connectivity is provided. The creation of corridors are of the utmost importance for the long term survival of the species.

417: *+.082*

Techniques for captive-rearing and releasing piping plovers (Charadrius melodus) were developed using a surrogate species, killdeer (Charadrius vociferus). We compared captive-and parent-reared killdeer, and parent-reared piping plovers and determined that growth and behavior were similar. After surrogate trials determined that captive-rearing was feasible, we used the same methods to raise piping plover chicks from salvaged eggs. For captive-reared chick of both species, survival to fledging was higher than and behaviors similar to parent-reared chicks in the wild. Rearing techniques were fine-tuned, and ten piping plover fledglings were released to the wild. Based on our results, we developed recommendations for captive-rearing piping plovers using salvaged eggs to enhance productivity of small populations. (C) 1997 Wlley-Liss, Inc.

418: *+.041*

The otter has gone extinct in the Netherlands (1989). Reintroduction of the otter is considered. In this study the feasibility of a reintroduction is assessed using the stochastic computer program VORTEX. Various scenarios have been tested applying feasibility criteria of a minimum of 90% survival over 100 years and a minimum of 90% retained genetic variation over 100 years. Reintroduction in the present situation (scenario 1), in a situation with increase in carrying capacity (scenario 2) or when restocking after an initial release (scenario 3) is not feasible.

419: *+.129*

Mounds of the western pebble-mound mouse, Pseudomys chapmani, are found throughout the species' Pilbara range in areas with iron-ore deposits of economic significance. Translocation techniques are being examined as a means of minimising the impact of mining on this species. In the absence of detailed information on the biology of P. chapmani, translocation is inadvisable. To provide such basic information, animal densities, mound demographics and population sizes, and home-range end core-area sizes were obtained by a combination of trapping and radio-tracking. Mounds of P. chapmani were found to be inhabited by social groups of up to 12 animals. Estimates of home-range size gave mean (+/- s.e.) values of 14.4 +/- 6.7 ha and 4.6 +/- 2.7 ha for males and females, respectively; core areas were recorded at 0.93 +/- 0.29 ha sor males and 0.29 +/- 0.16 ha for females. Considerable overlap of home ranges was recorded between individuals from the same and different mounds. Overlap at the core-area level occurred only between individuals from the same mound. The high level of social complexity and mound fidelity indicates that translocations should be directed at the level of the social group rather than at the level of the individual.

420: *+.091*

Nesting in trees is an ecological peculiarity of certain Peregrine Falcon populations, a species which primarily nests in cliffs or on the ground. Worldwide there were only three regions where breeding in trees regularly occurred. The largest such population was in Europe, from northern

Germany to western Russia. It became extinct in the mid 1970s, partly as a consequence of excessive pesticide use. A recovery project started in 1990 by the "Arbeitskreis Wanderfalkenschutz" and "Deutscher Falkenorden" resulted in the first pair of Peregrines breeding in a tree nest in northern Brandenburg in 1996. Both adults were colour-ringed and had been released in 1994 as part of a reintroduction program. They bred successfully in an old kite Milvus spec. nest in a pine tree. The nest site resembled the classic type used by the extinct population, 580 hours of nest observation enable us to give a detailed account of the activities of the pair and its young. The key points were: adults first seen at breeding site on 1st March, incubation started 18th March, first chick hatched 19th April (incubation period about 33 days); three young hatched; one chick died (probably fell off the nest); the young male left the nest at the age of 42 days (31st May) and first flew at 45 days; the young female also first took flight three days after leaving the nest (7th June); last sighting of a juvenile was on 3rd August; adults returned regularly to nest site from beginning of September. Peculiarities of the breeding record are discussed, e.g. the young age of adults (both two years old), early start to age-laying despite winter weather conditions, special hunting strategy (largely hunting from perches) with high success rate, prey including bats and insects. There were suggestions of potential tree-breeding pairs at four other sites in north-eastern Germany. The situation in 1996 gives cause for optimism regarding the potential for recovery of a tree-nesting population of Peregrines within its former range.

421: ***+.009***

The Yellow Eyebright Euphrasia scabra was widespread in northern and eastern Tasmania in the nineteenth century and the first half of the twentieth century. Today, only two populations are known, and both have declining numbers. This annual species tends to occur patchily and returns each year to approximately the same locations, which have a distinct species composition in contrast to the closely cropped herbfield and grassland that is its normal habitat. Recent apparent extinctions and the current decline in the numbers of the species may relate to increasing shrub cover. Reservation, fire management and ex situ cultivation, including reintroductions, may be necessary to maintain the species in Tasmania.

422: ***+.145***

In 1935 the Juan Fernandez Islands in the Pacific Ocean were declared a Chilean National Park to protect the unique flora and fauna, and later given status as a Biosphere Reserve by IUCN. Exotic plants deliberately and inadvertently introduced are threats to the natural vegetation. We review the introduced and/or adventitious flora of the archipelago in this paper. We report 21 recent arrivals, eight earlier introductions from the mid-1700s to the 1900s that have not been recognized before in the flora, six misidentified taxa, and five taxa present earlier but now reported from another island, resulting in a total of 227 introduced and naturalized species. Each species is discussed briefly with its native distribution, uses, first arrival in Chile and/or the archipelago, and comments on conservation impact. Identities of some previously cited taxa are clarified (e.g., Cupressus macrocarpa appears to be a misidentification for two other species of the same genus). Other noxious weeds known worldwide have recently been deliberately introduced, especially as garden ornamentals. Two of the most serious potential pests are the bird-dispersed Lantana camara and Lonicerca japonica. We recommend immediate eradication of these two taxa and restriction on reintroduction. A conservation program emphasizing strong physical and biological methods is urgently needed to control the introduced species.

423: ***+.309***

In 1991 the British Population of the field cricket Gryllus campestris was close to extinction with fewer than a hundred individuals remaining in the single surviving colony. A key component of English Nature's recovery programme for the species involved the creation of six new colonies in areas of the cricket's former UK range. This objective required the establishment of a captive-breeding programme to provide the large number of animals needed to found the new colonies. Between 1992 and 1995 gt 4000 captive-bred nymphs were released into five new sites. Monitoring confirmed that the release populations are becoming successfully established.

424: *+.162*

The appearance of new species in nature development areas may be indicative for the success of nature management. The absence of species, however, can be due to both unsuitable environmental conditions or shortage of dispersules. Specific knowledge of the chance that species are present in the seed bank or able to enter an area is therefore necessary for evaluating management measures. This knowledge may also be important when reintroductions are considered. In this paper the methods and the practical implications of research on seed dispersal, seed persistence, seed rain and seed banks are discussed. It is emphasized that it is only possible to generate roughly estimated chances of establishment on a landscape scale. In many situations this will give enough information to answer questions about the possibility of establishment of a given species, when combined with knowledge of the landscape and data on population dynamics of the species. An example is given of a hypothetical situation in which persistence and dispersal data are used in estimating the chance of establishment.

425: *+.138*

To develop successful restoration programs for wildlife, conservation biologists should consider features of animal behavior to help design restoration plans. This paper presents case studies of the first restored colonies of Arctic Terns (Sterna paradisaea), Common Terns (S. hirundo) and Roseate Terns (S. dougallii), Atlantic Puffins (Fratercula arctica) and Leach's Storm-Petrels (Oceanodroma leucorhoa). These restoration projects are based on the use of two fundamental and commonly occurring features of colonial waterbird behavior: social facilitation and philopatry. Social facilitation, the influence of one animal's behavior on that of its associates, was adapted for conservation purposes by placing decoys and sound recordings at historic nesting islands to simulate established colonies. The use of decoys and audio recordings to simulate and encourage social facilitation is referred to as social attraction. Philopatry, the tendency for young birds to recruit to nesting populations at their natal home, was adapted for conservation purposes by translocating puffin chicks from a large colony at the center of the range to historic nesting islands at the southern, historic limit of the puffin's range where they were extirpated nearly a century, earlier by excessive hunting for food and feathers. Young puffins, reared and released from artificial burrows at two historic sites learned the location of the release sites and eventually returned to the long-extinct colonies rather than their hatching place. To restore colonies of terns and puffins that were displaced by Herring and Great Black-backed Gulls, nesting population of these gulls were reduced at Eastern Egg Rock and Seal Island National Wildlife Refuge (NWR) to encourage recolonization by terns and puffins. Common, Arctic and Roseate Terns were attracted to island recolonization sites off mid-coast Maine, USA using decoys and 24-hour playback recordings. By 1996, Seal Island NWR was the largest Arctic Tern colony in Maine with 956 pairs and Eastern Egg Rock was the largest Roseate Tern colony in the state with 126 nesting pairs of this endangered species. These successes using social attraction are compared to tern restoration on Petit Manan Island, Maine where gull control alone resulted in recolonization. In this example, terns were absent for only 4 years and many were still living with a memory of nesting on the

island. After gull removal, these birds quickly recolonized in the same year that gull control began. In contrast, using gull control and social attraction, it took 3 and 5 years respectively for terns to re-colonize Eastern Egg Rock and Seal Island NWR (where terns had not nested for 44 and 36 years). In these cases, no living terns with a memory of nesting on these islands were available for recolonization. Puffin colonies at Eastern Egg Rock and Seal Island NWR were restored by translocating 3-40 (average 17) day-old puffin chicks from Newfoundland, Canada (approx. 1610 km distant). A total of 954 and 950 chicks were translocated to Eastern Egg Rock and Seal Island NWR respectively. These were captive-reared in individual sod burrows which were opened at their entrance after the first week. The chicks were banded and permitted to fledge into the adjacent ocean. Translocated puffins began to return when two years old; many also visited nearby puffin colonies where some eventually nested. Re- colonization at the historic nesting sites occurred in 1981 and 1992 respectively at Eastern Egg Rock and Seal Island NWR- in both cases 8 years after initiation of translocations. Four pairs nested in 1981, a number that increased to 19 pairs by 1985 and has remained constant at 16-19 pairs, due to recruitment of native-hatched chicks. At Seal Island NWR, 7 pairs nested in 1992 and increased to 40 pairs by 1996. The rapid increase at Seal Island was due largely to recruitment of native-hatched puffins from nearby colonies which comprised at least 68% of breeders by 1996. Social attraction, consisting of playback of recorded Leach's Storm-Petrel calls and artificial burrows were used to encourage Storm-petrels to establish new colonies. A colony of Leach's Storm-Petrels was established in the first year using playback burrow calls near artificial burrows on Ross Island and a second colony was established in the second year of playing burrow calls at Old Hump Ledge, an historic nesting island. Fifteen years after suspension of the attraction program, at least three pairs of storm-petrels were nesting in natural burrows near the site of artificial burrows at Old Hump Ledge.

426: ***+.209***

1. Information on the approximate number of individuals released is available for 47 of the 133 exotic bird species introduced to New Zealand in the late 19th and early 20th centuries. Of these, 21 species had populations surviving in the wild in 1969-79. The long interval between introduction and assessment of outcome provides a rare opportunity to examine the factors correlated with successful establishment without the uncertainty of long-term population persistence associated with studies of short duration.2. The probability of successful establishment was strongly influenced by the number of individuals released during the main period of introductions. Eight-three per cent of species that had more than 100 individuals released within a 10-year period became established, compared with 21% of species that had less than 100 birds released. The relationship between the probability of establishment and number of birds released was similar to that found in a previous study of introductions of exotic birds to Australia.3. It was possible to look for a within-family influence on the success of introduction of the number of birds released in nine bird families. A positive influence was found within seven families and nd effect in two families. This preponderance of families with a positive effect was statistically significant.4. A significant effect of body weight on the probability of successful establishment was found, and negative effects of clutch size and latitude of origin. However, the statistical significance of these effects varied according to whether comparison was or was not restricted to within-family variation. After applying the Bonferroni adjustment to significance levels, to allow for the large number of variables and factors being considered, only the effect of the number of birds released was statistically significant.5. No significant effects on the probability of successful establishment were apparent for the mean date of release, the minimum number of years in which birds were released, the hemisphere of origin (northern or southern) and the size and diversity of latitudinal distribution of the natural geographical range.

427: ***+.052***

The methods used to assess a red squirrel Sciurus vulgaris reintroduction experiment at Parco Groane, northern Italy, are described. Population size at the removal site was estimated (c. 2400 squirrels) using drey counts, to evaluate the effect of removing animals for reintroduction. suitability of the release site was investigated in two ways: (1) beforehand, by comparing tree seed abundance with that at the removal site, (2) retrospectively, by comparing drey-site choice between removal and release site. Increase and dispersion of the reintroduced population were monitored using drey counts and searching for species-specific feeding signs. Although only eight squirrels could be released (four males, four females), the experiment was successful and by 1996 all wooded habitats at Parco Groane were inhabited by squirrels.

432: ***-.098***

The Mallorcan midwife toad Alytes muletensis is a species with which the Jersey Wildlife Preservation Trust (JWPT) has a long standing involvement, The month of July 1997 was spent in the Sierra de Tramuntana mountains of Mallorca carrying out fieldwork with essentially three aims: 1) release of captive-bred toads into the wild, 2) a survey and census of all current toad sites in the wild, and 3) capture of wild toads for further captive breeding. This paper describes the details of this fieldwork and discusses the history, current status and possible future of the Mallorcan midwife toad recovery programme.

433: ***+.104***

After having been absent from the Danish fauna for exactly 100 years, the Eagle Owl began breeding again in 1984 when a pair was discovered in southern Jutland. Since then, the species has become established in most of Jutland south of Limfjorden, with an estimated population of 25-30 pairs. The recolonization of Denmark was a direct result of a reintroduction program in Schleswig-Holstein, northern Germany, where a total of 563 young birds were released into the wild between 1981 and 1995. Eagle Owls are doing surprisingly well in the modern Danish landscape and are quite capable of rearing young in populated areas (Tab. 1). Six of nine nesting areas in southern Jutland were in gravel pits, with the nest typically placed on a ledge on the bank of a disused and partly revegetated part of the pit. Another nest was on a natural steep slope in a wood clearing, and three (in two different territories) were placed in old Goshawk nests. The owls are opportunistic feeders, taking a variety of mammals and birds weighing up to 1.5 kg. Samples from five territories included the remains of 13 species of mammals and 21 species of birds; the most important among the mammals were hedgehog Erinaceus europaeus, brown rat Rattus norvegicus, water vole Arvicola terrestris, and small Apodemus and Microtus rodents, while the commonest bird species were Mallard Anas platyrhynchos, Coot Fulica atra, Woodpigeon Columba palumbus, and Crow Corvus corone.

434: ***+.168***

The Berry Botanic Garden is a small, non-profit organization in Portland, Oregon, that is strongly committed to conserving rare and endangered plants of the Pacific Northwest. Toward that end, the Garden has operated a regional seed bank for rare and endangered plant species since 1983. The Garden is the participating institution in the Pacific Northwest of the Center for Plant Conservation. Garden staff also conduct a wide range of conservation related scientific research on rare plants, a small portion of which will be described in the context of how stored seed might be used for reintroduction. Banking seed begins with the choice of species, and proceeds through

collection and transportation to the seed bank, where initial drying, cleaning and seed counting are done. It culminates when seeds are packaged and placed in frozen storage. Germination and growth trials follow. Stored seed are a means to an end-the conservation of rare and endangered plant taxa and populations in the wild. Our work is intended to offer a complementary tool to increase the chances that irreplaceable on-site efforts by land managers will succeed. The conservation value of stored seed can be realized in a variety of ways. Only if appropriate collections exist can stored seed be used to reintroduce extirpated populations or extinct species. Stored seed can also be used to augment (i.e., increase population size and growth rate) of extant populations. Reintroduction may have considerable conservation value in recovery, but is less likely to be of benefit and may even be harmful if used to mitigate and thus facilitate habitat destruction. A framework for getting the most out of reintroduction is discussed, as are several means stored seed might be used to intervene demographically.

437: ***-.110***

The Ultramarine Lory (Ultramarine Lorikeet, Marquesas Lorikeet, Pihiti) Vini ultramarina is one of the most threatened insular Lory species. Endemic to the Marquesas Islands, where it probably once ranged throughout the archipelago, it has been nearly extirpated from all but the tiny island of Ua Huka. Given the vulnerability of a single population inhabiting one small island, and the decline of the species in recent years, establishment of the Ultramarine Lory on another less disturbed island within its historic range has been proposed as a conservation strategy. This paper describes a 1991 survey evaluating the status of the Ultramarine Lory in the Marquesas Islands, and three subsequent translocations of birds from the island of Ua Huka to the island of Fatu Hiva. Twenty-nine birds were relocated during the period from 1992 to 1994 at the request of the Delegation de L'Environnement, French Polynesia. A preliminary survey, prior to the third translocation, indicates that previously transferred birds are surviving and may be reproducing; an intensive survey is planned in 1997.

438: ***+.084***

We reintroduced the endangered Baton Blue butterfly (Pseudophilotes baton schiffermuelleri, Bergstrasser) from its last known Finnish population to a site where it had become extinct. The butterfly is associated with dry and open eskers, but because of drastic habitat degradation only one population is known to have survived in Finland. The main reason for habitat loss is succession of pine forests. The last verified records of the species at the site of reintroduction date from 1984. The habitat was managed by the selective logging of pines in 1992. In 1994, after vegetation followups, 10 female baron blues were translocated. The introduced population was studied with the mark-recapture method in 1995-1996. Its size increased during this period to ca. 50 butterflies in 1996. Reintroduction, accomplished in close cooperation with forest industry, administration authorities and environmentalists, has diminished the probability of extinction of the baton blue in Finland.

439: ***-.275***

The Lord Howe island Woodhen is a flightless rail endemic to Lord Howe Island that became endangered due to human over-exploitation and predation from wild pigs. It has recently recovered from a population size of 20-30 to around 200 as a result of a captive breeding and reintroduction programme. Its classification has been downgraded from endangered to vulnerable, but no quantitative assessment of its future prospects had been undertaken. A population viability analysis (PVA) was performed on the Lord Howe Island Woodhen to project its possible fate using

VORTEX, a package that realistically reflects the woodhen's recent history. Prospective analyses showed the woodhen to be acutely sensitive to minor changes in mortality and fecundity, and to catastrophes, due to exotic species, inbreeding, or disease. A remote population needs to be established if the likelihood of the woodhen's extinction is to be minimized. According to the most recent IUCN Red List categories, the woodhen satisfies the criteria for endangered status.

440: *+.130*

Introduced species of mammals have now been removed from many islands around New Zealand, thus providing singular opportunities for ecological restoration. If island restoration is to be attempted, the way island biota originate and the precise effects of introduced organisms must be identified. Plants introduced to the New Zealand archipelago may have transitory effects, but others may modify forest structure and disrupt succession. Goats have been the most destructive introduced herbivore on islands. Among introduced predators, cats have extirpated colonies of seabirds, and rats (depending on species) affect invertebrates, lizards, and birds. Ecological theories and concepts that may help with island restoration projects include: the keystone species concept, in which the effects of one species on others is disproportionate relative to its abundance; the "intermediate predator" hypothesis, where removal of the top introduced predator may lead to rebound effects of intermediate predators; and ecological chain reactions, where local extinction of some species can cause complicated multiple effects. Problems with restoration of islands may be encountered because of meager data on the previous effects of pests (such as predators), use of non-seral species in revegetation projects, proliferations of indigenous or introduced species that have unforeseen community effects, and inexplicable difficulties with some translocations. A restoration case study in the continental Mercury Islands and on Cuvier Island showed success with removal of introduced mammals and demonstrates the various effects of introduced browsers, grazers, and predators. A contrasting case study is provided by oceanic Mangere Island in the Chatham Islands where 22 species of avifauna have been lost, seven as permanent extinctions. Restoration targets for some New Zealand islands can be clarified by palaeoecological studies of Maori (Polynesian) middens and natural deposits. Understanding the role of disturbance in island systems may also help clarify restoration targets. When exotic keystone species are introduced, physical disturbance may be overridden by biotic disturbance. This replacement in turn has implications for trophic structure. With high levels of biotic disturbance, continental islands may be changed from relatively species-rich bottom-up food webs to species-poor top-down trophic cascades. These possibilities can be tested with an experimental approach to restoration, although such experiments may be hard to interpret because of difficulties with replicates and controls. Ecological restoration on New Zealand islands has potential to replace damaged or lost communities, expand the ranges of relict populations, reduce the selective influence of exotic (keystone) species on indigenous species, help in understanding how the systems are formed, provide opportunities for educational and scientific investigation, and act as a testing ground for new technologies against pests.

441: *-.050*

Koala (Phascolarctos cinereus) populations in southern Australia have a history of bottlenecks earlier this century the species became extinct in South Australia, and almost so in Victoria. Subsequently large numbers of animals from island populations (founded from very few animals) have been translocated back to mainland sites and to other islands in the region. As part of a larger study of the genetic structure of koala populations in southern Australia, we have undertaken a survey of mitochondrial DNA restriction fragment length polymorphism (mtDNA-RFLP) variability. Genomic DNA from 91 koalas from five populations was examined using 23 restriction

enzymes, and mtDNA fragments were detected using a domestic cat full-length mtDNA clone. Only one of the enzymes, TaqI, revealed polymorphism - a relatively low amount of variation compared with other mammals, although low mtDNA-RFLP variation has also been reported in Queensland koalas. French Island and populations established predominantly from French Island immigrant koalas, either directly or via other island populations, were indistinguishable by haplotype frequencies. The mtDNA data are thus consistent with the interpretation that the koala translocation programme has homogenized gene frequencies amongst those populations involved. South Gippsland is not recorded as having received translocated koalas directly, and has significantly different mtDNA-RFLP haplotype frequencies from all other populations examined. The fact that this distinction was not previously observed in nuclear gene frequencies may reflect predominantly male-mediated dispersal in koalas.

442: *+.181*

We asked whether the reproductive capacity of the endangered Cordylanthus maritimus ssp. maritimus (salt marsh bird's-beak) at Sweetwater Marsh (San Diego Bay, California) was sufficient to provide a self-sustaining population, as required by a mitigation agreement under the Endangered Species Act. After seeds were sown for three consecutive years (1990-1992) to an upper intertidal marsh where the historical population was last seen in 1987, the number of plants censused rose from approximate to 5000 in 1992 to approximate to 14 000 in 1995. The transplanted population initiated fewer seed capsules per flower (mean 0.2-0.4 capsules in 1992-1994) than the donor population (up to 0.9 capsules in 1991). Pollen supplementation (hand-pollination) treatments increased the number of capsules by 89% in 1992, and by 52% in 1993. Abundance of pollinators appeared to be less important than the relative abundance of particular genera (i.e., Anthidium and Bombus). However, in areas where pollen supply was sufficient, nitrogen availability seemed to limit capsule set. Plant growth rates of the transplanted population compared favorably with those of the donor population (mean height 12-13 cm). At Sweetwater Marsh, larger plants produced more flowers, and aboveground dry mass of plants correlated strongly with inorganic nitrogen in the soil, which was $> 71\%$ sand. Nitrogen fertilization treatments increased plant size, foliar nitrogen content, and mean seed mass. Even though the population produced $> 1.5 \times 10^{6}$ seeds from 1992 to 1994, it is not clear that C. m. maritimus will be able to sustain itself at Sweetwater Marsh. The number of plants in 1994 was $< 3\%$ of the estimated seed production in 1992-1994. Opportunities for seed germination and seedling establishment may be more limiting than reproductive capacity. The most difficult challenge to habitat managers may be maintaining ecological relationships that are dependent on external factors, i.e., pollinators, nutrient inflows, and canopy disturbances (from debris or mammals) that create the necessary openings for seedling recruitment.

443: *+.083*

Between May 1989 and October 1990 a highway was constructed through the core area of one of the largest French populations of Testudo hermanni, a species seriously threatened by habitat loss and habitat fragmentation, Two general strategies were used to alleviate negative impacts of the highway on the tortoise population: first, 300 tortoises, that were directly, affected by the highway, construction, were temporarily maintained in outdoor enclosures and relocated after construction was completed; secondly, attempts were made to reduce traffic impacts on the tortoise population, including the use of fences to keep tortoises off the road and the construction of culverts and a tunnel under the road to provide for movements of animals between the separated habitat areas. The short-term outcome of this conservation exercise was a success: the annual survival rate of 78% observed in reintroduced tortoises was comparable to published results of translocation of

other tortoise species into unfamiliar habitat, traffic mortality of tortoises in the 4 years following highway, construction was low and mark-recapture results indicate a stable adult population and the use of the culverts and the tunnel by tortoises to cross the road. Copyright (C) 1996 Elsevier Science Limited

444: *-.037*

Translocations of rare and endangered species face significant valuational and organizational challenges; however, these dimensions are rarely discussed in the translocation literature. We employed a 98 question sample mail survey to assess these variables and received 131 responses from 110 individuals in 10 nations. In contrast to the literature which suggests that most translocations fail, a large proportion of the programs surveyed were perceived as being successful and most respondents identified relatively few valuational or organizational problems or difficulties in their translocation programs. While perceived local support was correlated with perceived translocation success, the presence of public relations/education programs was not. Therefore, simply having a public relations/education program is no guarantee that public support will increase or that a translocation will be more successful. Organizationally, translocations which established special groups or teams were perceived as increasing innovation, but not in improving program organization, nor decreasing conflict Further, the presence of special groups or teams was not correlated with translocation success. Finally, translocations with the objective of augmenting populations were perceived to suffer from more problems, including greater local opposition, poorer public understanding, less money, and poorer leadership, than were translocations aimed at establishing or re-establishing populations. The results illustrated the difficulty of analyzing valuational and organizational aspects of conservation programs using questionnaires. Copyright (C) 1996 Elsevier Science Limited

445: *+.028*

Propagation of the endangered hemiparasite Schwalbea americana was conducted in the presence of several species of potential host plants. Seeds were germinated and the seedlings transferred to pots containing potential host plants. Potted seedlings and potential hosts then were transplanted to field sites adjacent to a population of S. americana from which the seed had been collected. Germination of seedlings was >90% after a wet-cold treatment; a dry-cold treatment resulted in low germination. Seedlings grown in association with seedlings of the grass Schizachyrium scoparium did not differ in size or longevity from seedlings grown alone. Growth of seedlings was slightly greater in the presence of host plants than without, but seedlings still achieved only limited size in greenhouse conditions before transplantation (2 to 4 mm in width and height). After transplantation to the field the majority of seedlings died rapidly, but several persisted for over 45 days without showing appreciable growth. Poor seedling establishment and survival are believed to contribute to the rarity of this species. The development of improved methods for the propagation and transplantation of S. americana, and the greater understanding of the causes for the rarity of this endangered species, will increase options for recovery strategies.

446: *+.117*

The Hawaiian monk seal (Monachus schauinslandi) is a critically endangered species that has failed to recover from human exploitation despite decades of protection and ongoing management efforts designed to increase population growth. The seals breed at five principal locations in the northwestern Hawaiian islands, and inter-island migration is limited. Genetic variation in this species is expected to be low due to a recent population bottleneck and probable inbreeding within

small subpopulations. To test the hypothesis that small population size and strong site fidelity has led to low within-island genetic variability and significant between-island differentiation, we used two independent approaches to quantify genetic variation both within and among the principal subpopulations. Mitochondrial control region and tRNA gene sequences (359 base pairs) were obtained from 50 seals and revealed very low genetic diversity (0.6% variable sites), with no evidence of subpopulation differentiation. Multilocus DNA fingerprints from 22 individuals also indicated low genetic variation in at least some subpopulations (band-sharing values for "unrelated" seals from the same island ranged from 49 to 73%). This method also provided preliminary evidence of population subdivision (F'(st) estimates of 0.20 and 0.13 for two adjacent island pairs). Translocations of seals among islands may therefore have the potential to relieve local inbreeding and possibly to reduce the total amount of variation preserved in the population. Genetic variation is only one of many factors that determine the ability of an endangered species to recover. Maintenance of existing genetic diversity, however, remains an important priority for conservation programs because of the possibility of increased disease resistance in more variable populations and the chance that inbreeding depression may only be manifest under adverse environmental conditions.

447: *+.087*

Sea Otters (Enhydra lutris) ranged historically across the north Pacific from the central Pacific coast of Baja California, to northern Japan. An intensive fur hunt in the 18th and 19th centuries extirpated the Sea Otter from much of its range. The species was protected in 1911, and now occupies approximately half of its historic range. Presently, the worldwide population of Sea Otters is thought to be about 150 000 animals. The British Columbia Sea Otter population, originating from 89 animals reintroduced to Vancouver Island from 1969 to 1972, has increased at a finite rate of 18.6% per year, a rate similar to reintroduced populations in Washington and southeast Alaska. In 1995, a minimum of 1522 Sea Otters were found in British Columbia. Most of these animals occurred off Vancouver Island, but at least 135 Sea Otters were found near Goose Island, 125 km north of Vancouver Island. The origin of this latter group is uncertain. Sea Otters are presently classed as threatened in British Columbia and are protected under Federal and Provincial regulations.

448: *+.103*

During the last century, the decline of the apollo butterfly in the Pieniny mountains has been observed. This decline is attributed to environmental pollution, shrinkage of the area of open habitats inhabited by the species (as a result of introducing new, more expansive varieties of spruce in the area of Pieniny mountains), genetic erosion affecting small populations, and decimating this butterfly by collectors. The programme of the reintroduction of the apollo butterfly in the Pieniny National Park includes three main areas of activity: captive breeding of specimens to reinforce wild populations, preparation of habitats once inhabited by the apollo butterfly, including the removal of trees and shrubs and implanting the host plant, and research work. This was concerned with the ecology and dynamics of wild and reintroduced populations of the apollo butterfly, description of the decline process and explanation of its causal factors, and evaluation of the state of their habitat with special regard to the abundance and ecology of their host plant - Sedum maximum The five-years' reintroduction work has resulted in the triple growth of the native population inhabiting the massive of Trzy Korony (from c. 20 in 1991 to c. 60 in 1995); moreover, butterflies raised in captivity were released in four new, reconstructed localities. In 1995 migrations of specimens between all but one localities were observed.

450: *+.222*

A programme for the re-introduction of the European beaver in to the Litovelske Pomoravi, Danube (Morava) river basin is described. Beavers were released in to prepared artificial lodges in 1991 and 1992. the programme appears to have been successful to date, population growth has started and new sites have been colonized.

451: *-.045*

The aims of this study were to determine whether translocated noisy miners Manorina melanocephala were assimilated readily into noisy miner populations resident at release sites and, secondly, whether the number of birds in a release group influenced the subsequent behaviour, movement patterns and fate of translocated individuals. Birds were released in dues (n=5) or in groups of six (n=5). One bird in each due or group was fitted with a radio transmitter. Although most translocated birds survived at least a month following translocation, they were not assimilated readily into resident populations of miners, but instead wandered much further (mean maximum distance from release site=4.2 km) than the average diameter of the species' home range (similar to 200 m). At least two of the 40 translocated birds returned to their capture site; a distance of 18 km. The distances travelled by radio-tagged birds from their release sites rendered invalid the assumption that one release group/duo did not influence how another group/duo behaved. The difficulties such widespread dispersal pose to attempts at experimental examination of factors influencing the success of translocations are discussed. (C) 1997 Elsevier Science Ltd.

452: *+.120*

1. To determine the effect of grassland gap size on patterns of seedling recruitment and juvenile establishment of the endangered composite Rutidosis leptorrhynchoides, seed and transplants were introduced irate artificially created canopy gaps in both a 'short' and 'tail' temperate Themeda triandra tussock grassland in southern Victoria, Australia. Square gaps of 0 sm (undisturbed control), 15 cm, 30 cm, 50 sm and 100 cm width were created by removing the dominant grass, and the emergence, survival and growth of R. leptorrhynchoides were followed for 1 year. Survival and performance were compared with light quantity at ground level and soil moisture differences between the gags.2. Emergence of seedlings was greatest in large gaps (30 cm, 50 cm and 100 cm) but survival was restricted primarily to the 100-cm gaps in both grasslands.3. Survival of transplants to 1 year occurred in 30-cm, 50-cm and 100-cm gaps, suggesting that juvenile plants tolerate competition for resources better than germinating seedlings do. Survival, rate of growth and total number of inflorescences produced, however, was significantly greater in 100-cm gaps.4. There were few differences in the seasonal pattern of soi moisture between gaps in bath grasslands. The amount of light at ground level was significantly greater in the short-grasslands. and in large gaps (30 cm, 50 cm and 100 cm) at most times during the year. Differences between gaps in total soil moisture and light levels, however, only partly explain the patterns of transplant survival and growth observed. Sail moisture variability is suggested to have been an important factor influencing transplant survival over summer.5. These results confirm that R. leptorrhynchoides is a gap-sensitive species, with recruitment and survival unlikely to occur in canopy Sags less than 30-50 cm in diameter. Management of remnant populations needs to ensure that large canopy gaps are regularly maintained to maximize successful seedling recruitment and maintain the standing population. This may be achieved by burning the grassland at 3-year intervals. In the absence of the frequent burning of productive grasslands, localized extinction is likely.6. Reintroduction of the species into secure grassland reserves is likely to be problematic given the paucity of large Themeda-free gaps in these grasslands. Alternative stategies for

conserving the species include its introduction into grassy woodlands where gap closure rates are likely to be slower.

453: *+.256*

1. Native species are translocated for conservation, commercial, amenity and research purposes. These activities are related, and need to be considered and planned in terms of their effects on biodiversity.2. Procedures for assessing, implementing and regulating translocations have, however, been subject to largely uncoordinated development; recommendations or guidelines being produced separately for each area of expertise. There is a need to pull together the profuse information concerning specific translocations in order to present a broad approach to the general problems and concerns.3. Here, the extent and nature of translocations of native species within the UK are reviewed, and recommendations for policy and legislation are made in the context of those currently in use in the UK. The recommendations include the following: (i) improvement of the ways in which relevant information is disseminated; (ii) ease of implementation should be a prime consideration; and (iii) formation of new policy and guidelines should include all UK and international organizations involved in carrying out, advising on, or licensing translocations.

454: *+.097*

A discrete-time reaction-diffusion model for black-footed ferret release, population growth, and dispersal is combined with ferret carrying capacity constraints based on prairie dog population management decisions to form a spatial optimization model. Spatial arrangement of active prairie dog colonies within a ferret reintroduction area is optimized over time for maximum expected adult ferret population. This modeling approach is applied in an exploratory case study to a black-footed ferret reintroduction program in Badlands National Park and Buffalo Gap National Grassland, South Dakota. The model is currently being used to evaluate prairie dog population management alternatives and captive-bred ferret release locations for the Buffalo Gap National Grassland. This approach is also being adapted for use on other grasslands and with other species in the northern Great Plains.

455: *-.213*

A fungal infection was identified as the cause of a high mortality rate of captive wartbiter cricket (Decticus verrucivorus) nymphs at the London Zoo (United Kingdom) in 1994. This species is threatened with extinction in the United Kingdom and the animals concerned were part of a captive breeding and reintroduction program. Following these findings, reintroductions were postponed and release sites were used only where there were no extant wartbiter crickets.

457: *+.224*

1, Establishment patterns of reintroduced European otters Lutra lutra are described from a behavioural and a conservation perspective.2, Thirty-six 1-year-old otters, 11 wild-caught and 25 captive-bred, were released and radio-tracked in Sweden between 1989 and 1992.3, Movements after release depended on whether the release area was already occupied by other otters.4: Establishment occurred in areas that were unoccupied or were occupied by otters of the opposite sex,5, Females established exclusive home ranges and remained in these areas on attaining maturity,6, There was a tendency among males to expand their home ranges coincident with sexual maturity; no such range expansion was observed among females,7, Competition for resources largely explains the movement and establishment pattern in released otters, Competition

for mates over-rides competition for other resources in adult males,8, A distance of c. 10 km between the release site and areas occupied by other otters is recommended.9, Areas occupied by otters were associated with lakes and rivers with high biomass production.

459: *+.031*

Because populations in zoological parks and nature reserves often are derived from only a few individuals, conservationists have attempted to minimize founder effects by equalizing family groups sizes and increasing the reproductive contributions of all individuals. Although such programs reduce potential losses of genetic diversity, information is rarely available about the actual persistence of family groups or genetic lineages in natural populations. In the absence of such data, it can be difficult to weight the importance of human intervention in the conservation of small populations. Separate long-term studies of two mammals, the North American bison (Bison bison) and the white-nosed coati (Nasua narica), and a bird, the Acorn Woodpecker (Melanerpes formicivorus), demonstrate differential extinction of genetic lineages. Irrespective of the mechanisms affecting population structure, which may range from stochastic environmental events to such behavioral phenomena as poor intrasexual competitive abilities, our results show that lineages can be lost at rapid rates from natural populations. A survey of comparable studies from the literature indicates that the loss of matrilines over the course of the study varies from 3% to 87% in wild mammals and from 30% to 80% in birds, with several small mammals losing approximately 20% of matrilines per year of study. These lineage extinctions were not an artifact of the length of the study or the generation time of the species. Such rapid loses of lineages in less than 20-year periods in natural populations suggest that efforts to maintain maximal genetic diversity within populations may not always reflect processes that occur in the wild. Conservation biologists need to give further thought to the extent to which parity among genetic lines should be a primary goal of management of captive and small wild populations.

460: *-.107*

This study evaluated the genetic consequences of a reintroduction of the endangered annual plant Cordylanthus maritimus ssp. maritimus to Sweetwater Marsh (San Diego County, California). A survey of 21 enzyme loci in natural populations revealed that genetic diversity is very low and is primarily found as rare alleles at a few loci, making this species especially susceptible to the loss of alleles and heterozygosity through genetic drift. The reintroduction was performed in 1991 and 1992 by sowing seeds (collected from Tijuana Estuary) in numerous small patches of suitable habitat. For this study, leaf tissue was collected from all plants in all patches during flowering in 1995 and surveyed for genotype at the three enzyme loci that are polymorphic at Tijuana Estuary. Rare alleles were absent in 27 out of 30 patches for Pgm-l, in 17 out of 30 patches for Pgm-2, and in 10 out of 11 patches for Mdh-1. In all, half of the patches lacked any rare allele. Rare alleles tended to occur in patches with few individuals. Overall rare allele frequency was lower than in the colonies from which seeds were collected at two of the three loci, and heterozygosity was reduced. The Sweetwater Marsh population is at risk of losing most of its genetic variation at enzyme loci through the extinction of patches with few individuals. Future reintroduction attempts should attempt to create contiguous sets of patches or to periodically reseed existing patches to reduce the loss of genetic variation.

461: *+.242*

To identify populations with the ability to accumulate heavy metals, approximately 300 accessions pertaining to 30 plant species were grown for 4 wk in a hydroponic media that approximated the

nutrient and heavy metal composition of a soil contaminated with moderate levels of cadmium (Cd), copper (Cu), and zinc (Zn), The results indicated that several Brassica spp, exhibited moderately enhanced Zn and Cd accumulation, Selected accessions of Brassica juncea (L,) Czern, B. napus L,, and B, rapa L, were then grown in pots with heavy metal-contaminated soil to compare the Zn and Cd phytoextraction by these species to that of Thlaspi caerulescens J. & C, Presl, a known Zn and Cd hyperaccumulator, and two grass species, Agrostis capillaris L, and Festuca rubra L, The three Brassica spp, were the most effective in removing Zn from the contaminated soil, primarily because they produced more than 10 times the shoot biomass produced by T. caerulescens, When the soil was amended with Gro-Power, a commercial soil amendment that improves soil structure and fertility, removal of Zn by plant shoots doubled to more than 30 000 mg Zn pot(-1) (4.5 kg). The results suggest that for phytoremediation of metal-polluted soils to be successful, a strategy should be considered that combines rapid screening of plant species possessing the ability to tolerate and accumulate heavy metals with agronomic practices that enhance shoot biomass production and/or increase metal bioavailability in the rhizosphere.

463: *+.120*

Captive-born mountain gazelles were reintroduced to Hawtah Reserve in central Saudi Arabia from 1991 to 1995. This study describes the survival, reproduction and changing number of gazelles within the population during the first four years of the programme. The mortality rate was highest during the first month after release, bur 69-73% of gazelles survived for greater than or equal to 1 year and 58-59% survived for greater than or equal to 2 years. Weaned gazelles <2 years-of-age had the highest survival rate during the first year after release. The mean annual survival rate for gazelles which survived their first month of freedom was 78%. Predation was a major mortality factor and older gazelles were particularly vulnerable. Rainfall was seasonal, but calves were born throughout the year, with peaks in the number of births during spring and autumn. Females often produced two calves annually. All calves were singletons. The exponential rate of increase (r) of a subpopulation during two years after the last release was 0.275 on a yearly basis. In a wadi protected from domestic livestock, the density of gazelles remained constant after reaching ca. 15 gazelles km(-2). By the end of 1994, the estimated number of gazelles in the reserve was 165, more than twice the number released during the previous four years. An important lesson for all reintroductions is that predators can be a major problem even when uncommon. Recommendations are given for selecting gazelles for release during future reintroductions. (C) 1997 Published by Elsevier Science Ltd.

465: *-.078*

The status of Yellow-footed Rock-wallabies (Petrogale xanthopus) and Black-footed Rock-wallabies (P. lateralis) in South Australia was assessed by comparing recent survey and census data with previously collated information about the distribution and relative abundance of each taxon. Petrogale xanthopus has maintained most of its known geographic range within the state; however its relative abundance has declined significantly and 35 (or 15%) of a total of 229 recorded colonies have become extinct since European settlement. Eight of these colony extinctions have occurred over the past 25 years; three of them since 1981. As this species is continuing to decline it should be regarded as threatened within the state. Petrogale lateralis has at least two sub-species which occur in South Australia. Petrogale lateralis pearsoni is endemic to the state and occurs on offshore islands. Since 1960 its natural occurrence of about 3-500 individuals on North Pearson Island has been expanded to four other islands through translocations and the total population is now about 700-1100 animals. This subspecies, while not occurring in large

numbers, is nonetheless relatively secure due to the additional populations established and the fact that these are on islands isolated from most mainland threats. The mainland subspecies, Petrogale lateralis MacDonnell Ranges race, by comparison has suffered a drastic reduction in both geographic range and abundance to the point where it is South Australia's most critically endangered vertebrate taxon. It has declined from being a very common species in the state's far northwest to only two known, widely separated, colonies which total less than 100 animals between them. Management and research recommendations are provided.

466: *+.061*

In 1989, 13 Sugar Gliders Petaurus breviceps were introduced into the Organ Pipes National Park, a re-afforested valley, 26 km north-west of Melbourne, Victoria. These Sugar Gliders were relocated from a nearby (20 km) State Forest at Pyrete Range, Toolern Vale. This initial release was followed up in 1990 with the release of a further 24 animals. Due to the absence of natural tree hollows, 24 nesting boxes were installed prior to the release, spread over a 10 ha area. Introduced glider population densities varied from 0.8 ha-1 to 5 ha-1 from February 1989 to October 1992. In June 1993, 43 Sugar Gliders were trapped and nesting boxes were installed over a wider area. By May 1995, 31 Sugar Gliders were trapped, marked with 'electronic chips' and some were found nesting in boxes specifically designed for bats. Although there had been a number of reintroduction's at other locations in Victoria, namely Tower Hill, Blackburn Lake and Coolart, this was the first time wild Sugar Gliders had been trapped and relocated. Due to senescence amongst Black Wattles Acacia mearnsii at Organ Pipes National Park, food sources may have reduced during the study period. Despite this, the data indicate that the population may have been successfully established.

467: *-.014*

The Hawaiian goose or nene (Branta sandvicensts) restoration program, initiated in 1949, and with the release of about 2,150 captive-bred birds, has not resulted in a self-sustaining wild population. Results from a capture-recapture analysis indicate that 3 factors affected mortality rates: the year of release, age-class and method of release. Estimated annual mortality ranged from 0 to 87%. Comparisons between sites revealed the unsuitability of some upland sanctuaries as release sites. Hawaiian geese released in the 1960s survived at levels comparable to other wild, healthy goose populations (i.e., between 5 and 16% mortality) until the drought years 1976-83. During this period the majority of captive-reared and released Hawaiian geese (about 1,200) perished. Previously released adults generally survived better than newly released goslings. Birds made temporarily flightless initially to contain them in release pens survived less well than those released before fledging. The upland Hawaiian geese that did survive, did so at areas other than their release site; if they emigrated they survived better, especially during drought years. In all cases movement was away from habitats with dry upper montane-volcanic scrubland to areas with managed grasslands, managed ranch land, or supplemental feed and water. More movements occurred in drought years. Once the geese moved they rarely returned to their release sites. The lowest mortality and highest nest success rates were achieved by geese living at Hawaii Volcanoes and Haleakala National Parks. Hawaiian geese in these areas survived at levels similar to other nonhunted goose populations.

468: *+.210*

Gray wolf (Canis lupus) recovery in the Rocky Mountains of the U.S. is proceeding by both natural recolonization and managed reintroduction. We used DNA microsatellite analysis of

wolves transplanted from Canada to two reintroduction sites in the U.S. to study population structure in native and reintroduced wolf populations. Gene flow due to migration between regions in Canada is substantial, and all three recovery populations in the U.S. had high genetic variation. The reintroduced founders were moderately genetically divergent from the naturally colonizing U.S. population. These findings corroborate that the reintroduction more than meets generally accepted genetic guidelines. Maintaining this variation, however, will depend on ample reproduction in the first few generations. In the long term genetic variation will best be retained if migration occurs among the recolonizing and the two transplanted populations. Evidence from field observation and genetic studies shows extensive dispersal by wolves, and we conclude that exchange among these groups due to natural dispersal is likely if public tolerance and legal protection are adequate outside lands designated for wolf recovery.

469: *+.093*

the endangered Mauna Kea silversword (Argyroxiphium sandwicense ssp. sandwicense) has experienced a severe decline in distribution and abundance because of predation by alien ungulates. By the late 1970s only a small remnant natural population persisted on the Mauna Kea volcano on the Island of Hawaii. The Hawaii Department of Land and Natural Resources, Division of Forestry and Wildlife, initiated an outplanting program in the 1970s to promote recovery of A. sandwicense ssp. sandwicense. Intermittent outplanting since 1973 has generated an outplanted population of over 450 plants on Mauna Kea, but the program has unintentionally resulted in a major population bottleneck. All plants in the outplanted population appear to be first- or subsequent-generation offspring of only two maternal founders from the natural population. Genetic variation in the natural and outplanted populations was assessed for 90 random amplified polymorphic DNA loci. Eleven loci were detectably polymorphic in the natural population, whereas only three loci were detectably polymorphic in the outplanted population. Thus, the population bottleneck has been accompanied by a 73% reduction in the level of detectable polymorphism. In addition, for eight loci, the population bottleneck has resulted in the loss of the marker allele in the outplanted population. A management strategy involving manual pollen transfer has recently been implemented to incorporate additional founders from the natural population into the outplanting program. As a supplement to the outplanting program, the strategy also includes a program promoting direct seedling establishment following manual pollen transfer. Incorporating additional founders may serve to overcome the legacy of the population bottleneck, especially if founder representation can be equalized.

470: *+.241*

The demography of red squirrels Sciurus vulgaris removed from three different source areas and reintroduced between February 1987 and January 1988 in an urban park near Antwerp, Belgium was monitored for six years. Animals that were used to human presence had a higher initial survival than those not accustomed to human disturbance. Contrary to our predictions, woodland structure at the removal site did not affect a squirrel's settlement success, and post-release survival of subadults was not higher than that of adults. Only three (33%) introduced males, and five (50%) females survived to produce offspring. Reproductive rare was density-dependent and tended to increase with food abundance (tree seed crop of beech and oak). Consequently, population growth was high the first three years (low density) and decreased after 1990 (high density), when both adult and post-breeding densities (0.8-0.9 squirrels/ha) remained stable, suggesting the reintroduced population had reached its equilibrium density. We discuss some guidelines to increase the success of red squirrel reintroduction projects. (C) 1997 Elsevier Science Ltd.

471: *-.002*

Fire history and stand structure was examined in twelve virgin forest stands situated within forest reserves in northern Sweden. The selected stands represented fire refuges as well as different successional stages after fire. Six of the stands were dominated by Norway spruce (Picea abies L. Karat.), three were dominated by Scots pine (Pinus sylvestris L.), and three were dominated by hairy birch (Betula pubescens Ehrh.) or aspen (Populus tremula L.). In 3 of the southernmost stands, the average fire interval was 34 to 65 years during the late 1600s to late 1800s, but since 1888 no fires had occurred in any of the stands. The absence of fire disturbance since 1888 is probably caused by the fire suppression in the overall landscape. The standing volume of living trees ranged between 87 and 511 m(3) ha(-1) while the volume of dead trees, including both snags and logs, ranged between 27 and 201 m(3) ha(-1). The volume of dead trees constituted ca. 30% of the total stem volume. In the conifer dominated stands, there was a statistically significant relationship between total stem volume, including both living and dead trees, and site productivity. A comparison between the amount of dead and living trees indicated substantial changes in tree species composition in several stands. It is suggested that data on the amount of dead trees, especially logs, and its distribution over decay classes could be used to examine the continuity of certain tree species. All stands had a multi-sized tree diameter distribution, which in most cases was similar to a reversed J-shaped distribution. In general spruce was numerous in the seedling cohort and in small diameter classes, indicating that its proportion in the stands was stable, or was increasing at the expense of pioneer tree species such as pine, aspen and silver birch (Betula pendula Roth.). The most numerous species in the seedling cohort, rowan (Sorbus aucuparia L.), was almost totally missing in the tree layer, indicating a high browsing pressure preventing rowan seedlings from growing into trees. The general increase of spruce and the sparse regeneration of pioneer species, in the stands previously affected by fire, are discussed in relation to natural disturbance regimes, biological diversity and nature conservation policies. It is proposed that reintroduction of fire disturbance is a necessity for future management plans of forest reserves. Other management practices to increase species diversity within forest reserves are also discussed.
(C) 1997 Elsevier Science B.V.

472: *+.318*

There is a great need for developing new approaches to monitoring and recovering rare plants. We have only begun to adopt or invent the perspectives, tools, and institutions to provide better data for making management decisions regarding populations in the wild. Monitoring approaches and tools are best applied when they provide clear answers to specific, management-oriented questions. Recovery of rare plants through reintroduction or enhancement of populations is an experiment, supported by ex situ conservation techniques and informed by the results of modeling the dynamics of surrogate populations. There is a larger role for botanical gardens and arboreta to play in conservation. That larger role must extend into the realm of offsite projects, resource management, and other applied aspects of plant conservation. By combining ex situ and in situ approaches, botanic gardens will thus develop expertise in managing wild-lands as potential habitat for rare and endangered species. However, creating and managing plant populations in the wild is but one facet of restoration ecology, a fledgling science in need of an institutional home that promotes a full exchange between theory and practice.

473: *+.000*

Euphorbia clivicola R.A. Dyer, an endangered Northern Province endemic, is restricted to only two known populations, both of which are threatened with extinction. The area between and

surrounding the known populations, totalling 8874 km(2), was therefore searched for sites where either unknown populations could exist, or which could represent a suitable habitat for the introduction of a new population. A raster-based Geographical Information Systems package, IDRISI for Windows, was utilized to manipulate two point vector files, representing rainfall and altitude of the searched area, digitized within the TOSCANELLI system. The lNTERPOL module produced two digital elevation models, from which the SURFACE module created slope and aspect images representative of the searched area. RECLASS and OVERLAY modules were finally utilized to identify 12 potential sites, covering only 1.4% of the total searched area. Ground-truthing is now needed to determine the presence or absence of further populations and to assess habitat suitability. This study emphasizes an additional application of GIS techniques to investigations with relevance to the biological sciences, specifically to problems of rare and threatened species. GIS represents a cost-effective alternative method for addressing these problems by focusing ground searching activities to only a small number of potential sites.

474: ***+.136***

In the 1980s, Little Spotted Kiwi (Apteryx owenii) were transferred to Long, Red Mercury and Hen Islands to establish new populations beyond their strong hold on Kapiti Island. Recent surveys indicate that all three populations are increasing at between 4% and 8% per annum. Recently, a fifth population has been established successfully on Tiritiri Matangi Island in the Hauraki Gulf, and has grown at a rate of about 11% per annum. Little Spotted Kiwi now appear to be secure with five viable populations on predator-free islands. Aerial poisoning of rats using brodifacoum baits does not appear to have had any long-term adverse effect on the populations of Little Spotted Kiwi on Red Mercury and Tiritiri Matangi Islands.

475: ***+.161***

One of the oldest forms of tillage in the world is the digging of subterranean organs of wild plants for food and other purposes. Many areas were managed for increased densities and abundances of wild plants with edible corms, bulbs, tubers, and rhizomes. The horticultural techniques of digging, replanting, and sparing, in conjunction with larger-scale habitat management, created ecological effects at the species, population, community, and landscape levels. California provides a vivid example of an area where tillage was an important element in a comprehensive land management system that was in place for millennia. It is hypothesized that native California tillage activities mimicked natural disturbances with which plants coevolved, and played an ecological role that is now vacant in many wildlands, where Native Americans can no longer harvest and manage plants. Their land management system needs to be studied, described, interpreted, and experimentally mimicked to better understand indigenous disturbance regimes. It is suggested that some wildland areas would benefit from the reintroduction of management and harvesting regimes that authentically mimic indigenous techniques.

476: ***+.024***

We conducted a trail survey of De Brazza's monkey (Cercopithecus neglectus) on Mt. Elgon and the Cherangani Hills between October 1994 and February 1995. The objectives were to assess the status and distribution of the species and its habitats and to formulate recommendations on its conservation. We counted a total of 49 monkeys: 3 groups and 6 lone individuals near Kimothon River (Mt. Elgon) and 4 groups and 6 lone individuals at Kapolet Forest Reserve and its environs (Cherangani Hills). The mean group size is 6.6. Wanton habitat destruction was evident in all the forest habitats. The species now inhabits unprotected remnant strips of riverine forest.

Furthermore, the Kapolet Forest Reserve offers little or no protection to De Brazza's monkeys or their habitat. Translocation of the monkeys from unprotected areas to a protected habitat is recommended as an urgent conservation measure to save the De Brazza's population in Mt. Elgon and the Cherangani areas of Kenya.

477: ***-.025***

African wild dogs (Lycaon pictus) are a seriously endangered species with only around 5000 individuals left. We examined the factors likely to be affecting the distribution and density of wild dogs living in the Kruger National Park, South Africa. We collected data, by traditional and satellite radio-tracking, on diet selection, sizes of packs, dispersion and sizes of territories, and patterns of habitat selection for eight packs of wild dogs In Kruger wild dogs specialize on impala (Aepyceros melampus) and kudu (Tragelaphus strepsiceros). They live in packs averaging about nine dogs and inhabit territories of 350-950 km(2). Neighboring territories overlap to some extent but packs meet very rarely. The dispersion pattern of food plays only a minor role in determining the density and distribution of dogs in the Kruger; dogs exist at their lowest density where their food is most abundant This apparent paradox comes about because the dogs seem to avoid areas that, although prey-rich, are heavily used by lions. This is a sensible strategy because lions are a major cause of dog mortality, accounting for some 39% of natural pup deaths and at least 43% of natural adult deaths. Wild dogs may be conserved most successfully in areas with moderate to low densities of lions and spotted hyenas, and management strategies should be careful not to promote these species to the detriment of wild dogs and other carnivores The densities of lions and spotted hyenas should be important criteria when considering an area for the reintroduction of wild dogs and other similar sized carnivores.

478: ***+.313***

The natterjack toad (Bufo calamita) is an endangered species in Britain and has been legally protected since 1975. This amphibian suffered a major decline during the first half of the twentieth century, due partly to habitat destruction but mostly to successional changes in its specialized biotopes and anthropogenic acidification of breeding sites. In addition to site and species protection, extensive autecological research over the past 25 years has provided the foundations for an intensive, 3-year species recovery program funded by the statutory nature conservation organizations (English Nature and the countryside Council for Wales). This program was based on habitat management and reintroductions to restored sites and followed similar but less intensive efforts. Management of heath and dune habitats focused on restoration and maintenance of early stages of seral succession, initially through physical clearance of invasive scrub and woodland vegetation and later by the reestablishment of grazing regimes similar to those prevalent in earlier centuries. In some cases extra breeding pools wee provided to either increase or stabilize natterjack toad populations that had become reliant on one or very few pools at small sites or to promote range expansion within large habitat areas. By 1995 proactive conservation work had been carried out at 29 (69%) of the 39 sites with extant native populations, including 8 during the recovery program. Twenty reintroductions also had been attempted, including nine during the program. At least six reintroductions resulted in the foundation of expanding new populations, and an additional eight have shown initial signs of success. Conservation methods developed for Bufo calamita should provide a useful precedent for long-term conservation of early successional habitats and species.

479: ***+.364***

This work was directed to the repopulation of the pearl oyster Pteria sterna through artificial seeding techniques. Four different seeding conditions were tested on 658 young cultured pearl oysters: protected substrate, unprotected substrate, natural substrate, and artificial substrate. Two other bioecological variables were tested: depth and specimen size. We assessed survival, settlement speed acid strength, and the in situ growth of specimens, With the best results obtained, we attempted to define the optimum depth, size, and seeding materials to be used for further extensive repopulation actions, Protection was a very important factor for the survival of individuals; all oysters seeded on unprotected substrates were killed in few days. Natural substrates enhanced settlement speed and the strength and growth of individuals, whereas artificial substrates did not. A depth of 9 m and small-medium sizes gave the best results in terms of survival, settlement speed, and strength, and growth.

480: ***-.146***

The Pall mynah (Leucopsar rothschildi) is an endangered bird endemic to the island of Pall, Indonesia. The wild population of approximately 17 birds is confined to West Ball National Park (Taman Nasional Ball Barat). The species was intensively collected for the bird trade in the 1960s and 1970s, and poaching for the internal trade in Indonesia remains the primary threat to the wild population. We conducted an experimental release of one captive-bred and five confiscated, wild origin Ball mynahs on the small offshore island Pulau Menjangan. Released birds readily developed flight and foraging skills. Of the six, radio-tagged birds released, one was stolen, three were likely killed by a raptor, and two were returned successfully to the wild. Many challenges remain for the conservation of this species, including poaching in the national park, disease in the captive population, predation by raptors on newly released birds, and destruction of essential habitat. Results suggest that the Pall mynah is a suitable species for an expanded propagation and release program, provided that poaching is curtailed and disease in birds intended for release can be eliminated. (C) 1998 Wiley-Liss, Inc.

482: ***+.153***

In their biogeography model, MacArthur and Wilson (1967) assumed that populations occurring in isolated patches of suitable habitat differ in that some of them, called continental, are large and persistent, whereas the others, called insular, are small and submitted to periodic extinction and thus making some patches vacant. The former populations are the source of immigrants which colonise patches in which local populations have become extinct. The constant process of extinction and colonisation is called turnover. Another attempt of describing multi-population system was metapopulation model proposed by Levins (1970). Each population here, its size notwithstanding, has equal chances of becoming extinct or turning into the source of emigrants colonising vacant patches of habitat. Pulliam (1988) was the first to incorporate habitat quality in multi-population models. Assuming that populations occupy a mosaic of habitats that vary in quality, he divided them into two types: sink habitats, where reproduction fails to balance mortality, and very productive source habitats, where reproduction exceeds mortality. Decisions about species conservation made without distinguishing between sink and source populations, may lead to undesirable results. If the number of specimens is the only criterion taken into account, which is often the case, large sink populations may be protected, despite the fact that they may be strictly dependent upon small but very productive source populations which are far more worth protecting (Pulliam 1988). If non-protected source habitat is destroyed, extinction of the whole metapopulation may occur. Attempts to restore species by reintroductions should be carried out into former source habitat, i.e. into the core of the former range, rather than into sink ones, i.e. periphery of the former range (Lawton 1993). There is a considerable deal of discussion about the

ways of distinguishing sink and source populations in practice. A comparison of demographic parameters in populations occurring in different habitats was primarily suggested as yielding the strongest evidence for sinks and sources (Bergerud 1988, Pulliam 1988, Pulliam and Danielson 1991). The studies required are long and estimation of immigration and emigration is particularly difficult (Jarvinen and Vaisanen 1984). Questioning the previous criteria of distinguishing sources and sinks, Watkinson and Sutherland (1995) defined "pseudo-sink" populations: viable as sources, yet appearing as sinks due to intensive dispersal into them. According to the authors, not only simple comparison of demographic parameters between populations, but also determination of the nature of density-dependence acting upon them is necessary to identify sources and sinks. These methods are in practice impossible for most organisms.

486: ***+.131***

Vast literature, was analysed and data bank on rare and vanishing plants of the Russian flora has been compiled on this basis. It allows to compile a list of rare and vanishing plant species in their habitats, one of the main limiting factor for which is pasture of farm animals. Recommendations for investigation, conservation and possible restoration of rare and vanishing plants on the territory of Russia, growing in wild nature as well as introduced in botanical gardens, are given.

487: ***-.072***

In 1992 and 1993, 411 live reindeer were shipped by air from Hagemeister Island to the Anchorage area, Alaska. Reindeer were either rounded up by helicopter and then corralled or captured by net-gun fired from a helicopter. Outcome of both capture events showed that helicopter corralling of reindeer was more successful than catching them with a net-gun and that post-rut rounding up was more successful than rounding up during the rut itself.

488: ***+.152***

The Noisy Scrub-bird is a small, semi-flightless insectivore which forages in leaf litter and the lower stratum of dense vegetation. More widespread when discovered last century the species declined rapidly after European settlement of Western Australia due to habitat clearing and large scale, uncontrolled wildfires. A small, remnant population survived in dense low forest and thickets on Mt Gardner at Two Peoples Bay. Exclusion of fire from scrub-bird habitat allowed the population at Two Peoples Bay to increase through natural breeding. Translocations to new sites, where successful, have provided more habitat for the scrub-bird resulting in seven sub-populations in the area between Oyster Harbour and Cheyne Beach east of Albany. Rapid increases in numbers have occurred in the Mt Manypeaks area in recent years. The whole population has now increased to 10.5 times its size at rediscovery. This paper reviews Noisy Scrub-bird conservation efforts during the 35 years since its rediscovery and focuses on recent progress, population trends, and the current status of the species. Successful conservation management has been based on research into the scrub-bird's biology and ecology and has involved habitat reservation and protection, fire management, population monitoring, and translocation. Scrub-bird conservation has also provided benefits for other threatened species.

489: ***+.172***

The Pampas deer (Ozotoceros bezoarticus L. 1758) is the most endangered neotropical cervid, and in the past occupied a wide range of open habitats including grassland, pampas, savanna, and cerrado (Brazil) from 5 degrees to 41 degrees S. To better understand the effect of habitat

fragmentation on gene flow and genetic variation, and to uncover genetic units for conservation, we examined DNA sequences from the mitochondrial control region of 54 individuals from six localities distributed throughout the present geographical range of the Pampas deer. Our results suggest that the control region of the Pampas deer is one of the most polymorphic of any mammal. This remarkably high variability probably reflects large historic population sizes of millions of individuals in contrast to numbers of fewer than 80 000 today. Gene flow between populations is generally close to one migrant per generation and, with the exception of two populations from Argentina, all populations are significantly differentiated. The degree of gene flow was correlated with geographical distance between populations, a result consistent with limited dispersal being the primary determinant of genetic differentiation between populations. The molecular genetic results provide a mandate for habitat restoration and reintroduction of Pampas deer so that levels of genetic variation can be preserved and historic patterns of abundance can be reconstructed. However, the source of individuals for reintroduction generally should be from populations geographically closest to those now in danger of extinction.

490: ***+.100***

The Asp viper (Vipera aspis) went extinct at several localities in northwestern Switzerland during the past decades. The present study shows that localities where the species went extinct are overgrown by forest, whereas localities where V. aspis still occurs are free of forest. The genetic variability of the extant viper populations in the study area does not follow any geographic pattern. This suggests that gene flow may have occurred until recently within a large area of the snake's distribution area. The present study also shows that small populations have a smaller genetic variability than large ones. To preserve the genetic variability it is important to protect not only the small, but also the large populations. Translocations of vipers from large into small populations are accompanied by many problems, e.g. the introduction of pathogens or locally maladaptive animals. Therefore, it is better to maintain the habitat suitable for the viper by regularly cutting the new-growing bushes and trees. Large populations can be promoted by enlarging the area of their habitat and connecting them with neighbouring populations.

491: ***-.097***

Fourteen radio-tagged red squirrels were released in pine woodland containing grey squirrels. Movements of the squirrels were related to the tree species of the donor site. Survival after release was lower than for the grey squirrels: of 11 red squirrels that survived at least a week, only three survived more than three months and none for four months. More than half were eaten or cached by predators, mainly foxes; an experiment with grey squirrel carcasses indicated that they had been killed, not scavenged after death. Hypertrophied adrenals, disease and loss of weight indicated stress as another factor in the deaths. Data on overlap of core ranges, and reluctance of red squirrels to enter traps used by grey squirrels in the mixed population, indicated interference competition between the two species, with grey squirrels possibly dominant. We recommend: (i) that care should be taken to release translocated animals in similar habitat to their origin; (ii) that grey squirrels should be excluded from future release areas until red squirrels have settled and, before biodiversity is reduced by landscape management for red squirrels; (iii) more research to determine whether interactions with grey squirrels or differential predation will ultimately displace red squirrels in conifers.

492: ***+.154***

The responses of ruffe (Gymnocephalus cernuus (L.)) abundance to eutrophication were studied in

the brackish water of the Baltic Sea off Helsinki. The productivity gradients in the area permitted an analysis of the dependence of ruffe catches taken with gill nets in 1969 to 1972 on spatial differences in eutrophication level. There appeared to be a relationship between the level of primary productivity and the catch per unit effort of ruffe. The gill net catches showed that ruffe were more abundant in eutrophic than oligotrophic areas bur less abundant in the most eutrophic areas. Due to the better purification of municipal sewage and the relocation of effluent discharge sites, primary production has declined in the most eutrophicated inner bays during recent decades. The responses of ruffe reproduction to these changes were studied on the basis of catches taken with small bench seines in 1982 to 1996. The number of zero-catches of 0+ ruffe increased significantly after oligotrophication, bur decreased again in 1996 after the accidental reintroduction of waste waters.

493: ***+.146***

Since 1989 the orang-utan Pongo pygmaeus has been a protected species in Taiwan. Between July 1995 and September 1996 a survey was carried out of 16 facilities maintaining orangutans in Taiwan. Data on the management, age, sex and life history were collected for 72 orangutans housed at three public zoos, nine private parks, two animal breeding farms, one rescue centre and a temple. Information on animals registered as privately owned was also examined. This paper presents data on the status of orangutans maintained in Taiwan and gives recommendations for their future management. Reintroduction strategies, developed at a workshop organized by the Pingtung Rescue Center for Endangered Wild Animals, are also presented.

494: ***+.047***

Effective live-trapping of beaver (Castor spp.) has been and will continue to be necessary for introductions, re-introductions, translocation for the preservation of populations, removal of nuisance beaver, and to obtain animals for fur breeding, zoological gardens and research. The aim of this study was therefore to review the existing knowledge about live-trapping through a literature study, contact with individuals involved in live-trapping, and personal experience. Before starting live-trapping, the area used by the beaver should be determined by careful reconnaissance. Beaver are usually captured either in unbaited traps placed at sites frequently visited by beaver, or in traps baited with castoreum or an aspen twig. Human scent on traps and around the trap site may be reduced by adding mud, water or scent. Usually Bailey or Hancock traps are used for live-trapping beaver. The Hancock trap is preferable because of its greater catch efficiency and versatility. Other traps reviewedare the Breathe Easy trap, Scheffer-Couch trap and different types of cage traps. Other methods used for live-trapping of beaver are snares (often considered highly effective), the Byelorussian trapping method, nets and seines, landing nets, pits and enclosures. Capture methods involving the destruction of lodges/dens and dams have often been employed in the past. Such methods require special permission in most countries and are usually not to be recommended. The Eurasian beaver (C. fiber Linnaeus, 1758) is reportedly more difficult to live-trap than its North American counterpart (C. canadensis Kuhl, 1820). New, effective methods for live-trapping of beaver also need to be developed.

495: ***+.087***

Black-footed ferrets Mustela nigripes are considered one of the world's most endangered mammals. The last known wild population was found in Meeteetse, Wyoming, and it collapsed due to an epizootic of canine distemper, but 18 free-ranging ferrets were successfully raptured to initiate a breeding programme. Captive propagation has been successful and, throughout the last

12 years, more than 2,600 kits have been born in captivity. Reintroduction began in 1991. Since then, ferrets have been released in Wyoming, Montana, South Dakota, and Arizona, USA. In 1995, reintroduction efforts were discontinued in Wyoming due to an epizootic of sylvatic plague. Montana and South Dakota first received ferrets in 1994. Reproduction in the wild was consistently successful and by 1998 there were approximately 100 wild-horn young between both sites. Expectations are high that a viable wild population can be established in one or both of these states. Reintroduction began in Arizona in 1996 and current efforts are aimed at developing on-site breeding techniques in outdoor naturalistic enclosures. A new reintroduction/on-site breeding area was established in the Colorado-Utah border area in 1998. The ferret programme has been using scientific research as a tool to direct recovery efforts. Experimental work has demonstrated that a naturalistic captive environment helps "precondition" black-footed ferrets for life in the wild and significantly increases survival after release. Results from captive and field research have been critical in shaping current recovery efforts.

498: ***-.035***

Created in 1934 in Eastern Rwanda, the Akagera National Park (ANP) has been the scene of two introductions and one reintroduction of large ungulates. Two male and four female black rhinoceros, a species which never lived before in the ANP but was present in Tanzania, beyond the river Akagera matching approximately the international border and working as a biogeographical barrier, were introduced in 1957 ; these animals reproduced and spread over the whole savanna. Their real number was never accurately known as they were regularly poached by Rwandeses and Tanzanians. About ten specimens were still living before the Rwandese war and at least four at the end of 1996. The last elephants living in the ANP were noticed in 1960-61 before the species was reintroduced in 1975. The government had decided to eradicate a population of some 150 individuals causing trouble to peasants in the Bugesera ; all large, aged animals were shot whereas 25 young ones were transferred into the ANP, where theysettled in the lakes border zone. There were 45 individuals before the war and these seem to have escaped from damages. The giraffe is considered a species never living for historical times in the area presently known as ANP. A personal deal between the president of Kenya and the former president of Rwanda resulted in the introduction in 1986 of six young animals - two males and four females - in order to improve the park diversity and attractiveness. Scientists were reluctant. The animals reproduced, extended their home range, and their numbers grew up ; they resisted the war and some 20 individuals were still living in December 1997. The reintroduction in the ANP of the elephant from a Rwandese population was legitimous. The introduction of the black rhinoceros was questionable. The introduction of giraffes had no biological justification and was opposed by scientists : they were sure that the operation would be a wreckage. Paradoxically, it is thanks to the presence of giraffes that the new governmental authorities decided it was worth preserving 100 000 ha of the park, considering that the giraffe investment had to be saved. Non-scientific arguments succeeded where biological ones had not...

499: ***-.010***

The disappearance of the Otter, linked with anthropological factors, is also due to contamination of its food by PCB and heavy metals (cadmium, mercury, lead). With a view to reintroducing or strengthening a population, it was suitable to determine which rivers in the Rhone-Alpes area could offer feeding possibilities of satisfactorily quality. Seven rivers were selected as they all offered favourible possibilities for otters to settle and as more or less old observations had been made on their courses. The Allier river, which still shelters important otter populations, was choosen as reference. The PCB and heavy metals accumulate in the fats in different ways

according to the species and diet, so that five species of fish (Gudjeon -Gobio gobio-, Trout -Salmo trutta-, Barbel -Barbus barbus, Bladgeon -Leuciscus souffia-, Chub -Leuciscus cephalus-) among the most abundant were choosen. The analyses for PCB show generalized contamination. The Bladgeons of the Isere river are definitely more contaminated than those of the Drome or the Buech rivers. The Chub of the Rhone and Guiers Rivers are less contaminated than those of the lower part of the Rhone, where human and industrial concentration is important. The Gudgeon and Barbel have relatively weak amounts, whereas the Trout of the Arve river have average ones. The Buech, the Drome, and the Allier are the least polluted rivers, while the most polluted by PCB are the Arve, the Isere and the lower Rhone. Lead was not traceable in any of the samples. The highest rates of cadmium and mercury are to be found in the upper Rhone; but they are relatively low and the concentrations in heavy metals do not seem to be an essential limiting factor. At present, a reintroduction on the Drac, the Isere, the Arve, and the lower Rhone cannot be contemplated. The Ain and the upper Rhone are in an intermediate position. The Drome and the Buech are comparatively favourable.

500: *+.164*

The pollination of flowering plants by animals represents a critical ecosystem service of great value to humanity, both monetary and otherwise. However, the need for active conservation of pollination interactions is only now being appreciated. Pollination systems are under increasing threat from anthropogenic sources, including fragmentation of habitat, changes in land use, modern agricultural practices, use of chemicals such as pesticides and herbicides, and invasions of non-native plants and animals. Honeybees, which themselves are non-native pollinators on most continents, and which may harm native bees and other pollinators, are nonetheless critically important for crop pollination. Recent declines in honeybee numbers in the United States and Europe bring home the importance of healthy pollination systems, and the need to further develop native bees and other animals as crop pollinators. The "pollination crisis" that is evident in declines of honeybees and native bees, and in damage to webs of plant-pollinator interaction, may be ameliorated not only by cultivation of a diversity of crop pollinators, but also by changes in habitat use and agricultural practices, species reintroductions and removals, and other means. In addition, ecologists must redouble efforts to study basic aspects of plant-pollinator interactions if optimal management decisions are to be made for conservation of these interactions in natural acid agricultural ecosystems.

501: *+.022*

The Lake Eacham rainbowfish (Melanotaenia eachamensis) was declared extinct in the wild in the late 1980s after it disappeared from its only known locality, an isolated crater lake in northeast Queensland. Doubts have been raised about whether this taxon is distinct from surrounding populations of the eastern rainbowfish (Melanotaenia splendida splendida). We examined the evolutionary distinctiveness of M. eachamensis, obtained from captive stocks, relative to M. s. splendida through analysis of variation in mtDNA sequences, nuclear microsatellites, and morphometric characters Captive M. eachamensis had mtDNAs that were highly divergent from those in most populations of M. s. splendida. A broader geographic survey using RFLPs revealed some populations initially identified as M. s. splendida, that carried eachamensis mtDNA, whereas some others had mixtures of eachamensis and splendida mtDNA. The presence of eachamensis-like mtDNA in these populations could in principle be due to (1) sorting of ancestral polymorphisms, (2) introgression of M. eachamensis mtDNA into M. s. splendida, or (3) incorrect species boundaries, such that some populations currently assigned to M. s. splendida are M. eachamensis or are mixtures of the two species. These alternatives hypotheses were evaluated

through comparisons of four nuclear microsatellite loci and morphometrics and meristics. In analyses of both data sets, populations of M. s. splendida with eachamensis mtDNA were more similar to captive M. eachamensis than to M. s. splendida with splendida mtDNA, supporting hypothesis 3. These results are significant for the management of M. eachamensis in several respects. First the combined molecular and morphological evidence indicates that M. eachamensis is a distinct species and a discrete evolutionarily significant unit worthy of conservation effort. Second it appears that the species boundary between M. eachamensis and M. s. splendida has been misdiagnosed such that there are extant populations on the Atherton Tableland as well as areas where both forms coexist. Accordingly we suggest that M. eachamensis be listed as vulnerable, rather than critical (or extinct in the wild). Third, the discovery of extant but genetically divergent populations of M. eachamensis on the Atherton Tableland broadens the options for future reintroductions to Lake Eacham.

502: *+.331*

Following reports of dramatic declines in managed and feral honey bees from nearly every region of North America, scientists and resource managers from the U.S., Mexico, and Canada come together to review the quality of the evidence that honey bees as well as other pollinators are in long-term decline and to consider the potential consequences of these losses on the conservation of biodiversity and the stability of the yield of food crops. These experts in pollination ecology confirmed that the last 5 years of losses of honeybee colonies in North America leaves us with fewer managed pollinators than at any time in the last 50 years and that the management and protection of wild pollinators is an issue of paramount importance to our food supply system. Although there are conclusive data that indicate 1200 wild vertebrate pollination may be at risk, data on the status of most invertebrate species that act as pollination agents is lacking. The recommendations from a working group of over 20 field scientists, presented here, have been endorsed by 14 conservation and sustainable agriculture organizations, research institutes, and professional societies including the Society for Conservation Biology. Among the most critical priorities for future research and conservation of pollinator species are (1) increased attention to invertebrate systematics, monitoring, and reintroduction as part of critical habitat management and restoration plans. (2) multi-year assessments of the lethal and sublethal effects of pesticides, herbicides, and habitat fragmentation on the wild pollinator populations in and near croplands; (3) inclusion of the monitoring of seed and fruit set and floral visitation rates in endangered plant management and recovery plans; (4) inclusion of habitat needs for critically-important pollinators in the critical habitat designations for endangered plants; (5) identification and protection of floral reserves near roost sites along the "nectar corridors" of threatened migratory pollinators; and (6) investment in the restoration and management of a diversity of pollinators and their habitats adjacent to croplands in order to stabilize or improve crop yields The work group encourages increased education and training to ensure that both the lay, public and resource managers understand that pollination is one of the most important ecological services provided to agriculture through the responsible management and protection of wildland habitats and their populations of pollen-vectoring animals and nectar-producing plants.

503: *+.061*

Due to over-hunting c. 1200 Eurasian beavers Castor fiber survived in eight relict populations in Europe and Asia at the beginning of the 20th century. Following hunting restrictions and translocation programmes in IS countries, the Eurasian beaver became re-established over much of its former range, and presently numbers c. 430 000. The translocated populations often consist of a mixture of geographical forms. Preservation of the original, unmixed populations has therefore top

priority. all five in Europe have reached the assumed minimum viable population size of c. 1880 animals each, but the three in Asia are still endangered. Their protection should be carried out at the level of river catchments. Nowadays the main threats for beavers are habitat destruction and introduced North American beavers Castor canadensis. On the other hand, gr owing beaver populations cause increasing conflicts with man, and population and/or damage control may therefore be required. In view of these two very different problems, we conclude that the conservation of beavers is best served by preservation and restoration of riparian woods with intact natural water regimes. (C) 1998 Elsevier Science Ltd.

504: *+.031*

The lycaenid butterfly, Maculinea rebeli, and its specialist parasitoid, Ichneumon eumerus, live in small, closed populations. Given the threatened status of the butterfly, it is reasonable to assume that its specialist parasitoid is even more vulnerable to local extinction than the butterfly host. Based on a mechanistic model recently developed for the tightly-woven community surrounding M. rebeli at a site in the Spanish Pyrenees, we investigate how the removal of habitat, and more particularly, specific habitat promoting the persistence of the butterfly, affects the population persistence of the parasitoid. Because of the relatively small impact of the parasitoid on the butterfly population in the Spanish Pyrenees, guidelines for conserving the parasitoid are only slightly more restrictive than those for its host. It is argued that at sites of more marginal quality for the butterfly than the reference site, achieving the dual aims of conserving both species will be more problematic.

505: *+.040*

Details of a reintroduction of Maculinea teleius (scarce large blue butterfly) and Maculinea nausithous (dusky large blue butterfly) into a nature reserve in the Netherlands are given. The introduced population of M. teleius expanded during the first three years. In contrast, the newly established population of M. nausithous declined in the year after the reintroduction but expanded later. After a considerable increase in numbers, this species can nowadays be seen in two subpopulations: one in and one outside the nature reserve. Although the results are quite positive with respect to the numbers of butterflies, some alarming features have also been found. Both species, but especially M. teleius, have a very low mean minimal lifetime, which makes them more susceptible to stochastic catastrophes. While the area populated by M. nausithous is still expanding, M. teleius can only be found on the meadow where it was released and has not dispersed at all. The possible consequences of reintroductions are discussed.

506: *+.103*

1, Reintroduction of plants and animals has become a common practice in the attempt to establish, re-establish or augment endangered populations. The fate of these translocation programmes is likely to depend, on the one hand, on several ecological attributes of the introduced species, on the other hand on introduction effort.2, In a recent paper, Veltman, Nee & Crawley (1996) reported the correlates of introduction success of exotic birds released by humans in New Zealand before 1907. introduction effort (minimum number of release events and minimum number of released propagules) accounted for most of the variance in introduction success.3, One of the factors potentially affecting the establishment of new populations concerns the intensity of sexual selection. Sexually selected species may be more vulnerable to extinction risks for several reasons (they may be more sensitive to both environmental and demographic stochasticity) and therefore have lower introduction success when compared to nonsexually selected species.4. In the present

study the data set reported by Veltman et al. (1996) was used to address the particular issue concerning sexual selection and introduction success. Each bird species was scored as monochromatic or dichromatic; plumage dichromatism is supposed to have evolved under sexual selection pressures. These scores were then correlated to the fate of introduction.5. Plumage dichromatism was found to be, as expected, a significant predictor of introduction success, after removing the potential confounding effect of introduction effort. Dichromatic species had lower introduction success than monochromatic species. This pattern holds true for nonpasserines and passerines, although the correlation between plumage dichromatism and introduction success was not significant for the latter.6, These results indicate that dichromatic species may experience reduced chances of establishing new populations when compared to monochromatic species. The presumed mechanisms involved in this phenomenon range from a reduced ability to adapt to a novel environment, to an increase in the risks of extinction through environmental and demographic stochasticity.

507: ***-.018***

A literature search on hazel grouse, Bonasa bonasia, biotopes has shown that this bird most often frequented "heterogeneous" woodlands, i.e. those in which it will find high trees, understoreys and edges. A satellite image (LANDSAT August 1991) was analyzed to detect habitats potentially suitable for a theoretical reintroduction of the hazel grouse in the Cevennes National Park (40,000 ha). A preliminary treatment of the image allowed to locate wooded habitats and to identify the heterogeneous sectors. Then, based on a selection in the field of biotopes that are "favourable" to the species, a classification of the vegetation cover, called "supervised classification", was made for the whole park to locate favourable sectors, which were arranged hierarchically. The method was validated by a survey among the agents of the "Office National des Forets", and by several field trips. On condition that the selected criteria are the most relevant ones and that biases are minimized, an analysis of the image thus carried out has proven to be significant. In the Cevennes National Park, 30% of the woodlands would be favourable for a reintroduction of the hazel grouse. However, because of the dispersion of these favourable sectors, a "viable" population could only be established in the Aigoual sector. The less extensive, more broken up but not isolated sectors of Bouges, Monts Lozere and Goulet, could be used in case of a hypothetical expansion of the population of Aigoual.

508: ***+.378***

Long-overdue concerns about the genetic effects of releases of hatchery-reared progeny on wild populations have been increasingly addressed during the past 20 yrs. Because of an extensive history of translocation and culture, much attention in both of these categories has focused on salmonid species. Native salmonids have been indirectly affected by translocations through such activities as induced overharvest in stock mixtures, disease introductions, and displacements resulting in fragmented populations with reduced numbers and, commonly, localized extinctions. Direct genetic effects through introgressive hybridizations of translocated salmonids have resulted in replacement of many native populations by hybrid swarms. Genetic effects from salmonid culture have led to losses of variability, both within the cultured populations and between the total cultured and wild populations, and to adaptive divergences distinguishing cultured and wild populations. Examples are provided that show that the overall salmonid experience is directly relevant to marine culture as well, although special considerations deal with the lower among-population diversity and greater fecundity often found in marine species. As marine enhancement programs inevitably expand identifying and preserving natural populations are concerns common to harvest, management, and conservation interests. Coordinated rather than polarized approaches

to these concerns best serve all parties as well as the irreplaceable resources.

509: *+.176*

Capercaillie (Tetrao urogallus) populations are declining in western parts of their range, including Scotland. It has been proposed that their numbers, and the extent of their range in Scotland be increased to reduce the risk of a second extinction in the UK. The feasibility of a reintroduction of capercaillie to coniferous plantations in southern Scotland was assessed by undertaking a population viability analysis. Following a review of capercaillie ecology and habitat requirements, VORTEX population simulation software was used to identify a minimum viable population (MVP). From this the minimum dynamic area of suitable habitat required in order to support such a MVP was then calculated. It was estimated that a minimum of 60 individuals would be required in approximately 5000 ha of habitat in order for the population to have a >0.95 probability of surviving for 50 years. Supplementation of populations with two unrelated individuals every five years reduced the MVP to ten individuals. Further simulations were run in order to establish the sensitivity of the model to changes in three key parameters. Assessment of areas of suitable habitat identified two potential release sites, Wauchope and Newcastleton forests, in southern Scotland. Some practical considerations relating to management of a release population are outlined. It was concluded that an appropriately planned and resourced reintroduction was feasible.

514: *+.145*

The Yellow-shouldered Amazon (Amazona barbadensis) is one of the most endangered species of parrots in Venezuela. An integrated conservation program has focused on reversing the causes of parrot population decline on the Macanao Peninsula in Margarita Island. As a result, the parrot population on the island has increased to about 1900 individuals in 1996 from an estimated population of 750 in 1989, when the project started. Cooperation from national and local authorities and the project's community outreach have resulted in several confiscated chicks. Whereas most confiscated chicks were successfully reintroduced in a cross-fostering nest program, some had to be kept in captivity for later release. We hand-reared 14 A. barbadensis and housed the for a year in a large outdoor aviary. Before release the birds were screened to determine their general health. Four parrots were fitted with radio transmitters and monitored for a minimum of 11 months. All 4 birds with radio transmitters survived and adapted successfully to their natural environment, 10 of the 12 released parrots survived at least 1 year, and 1 was seen alive 34 months after release. Integration into wild groups varied from 5 to 9 months, with the two youngest parrots showing a slower integration process. None of the parrots reproduced the first year after release. Later three were seen scouting nesting holes with their partners, and one of the parrots was confirmed attending a nest with three eggs 28 months after release. Two chicks fledged from this nest. A substantial portion of the success orf this program rests on 5 years of previous work on environmental education, public awareness, and studies on the parrot's biology. To provide some guidance on the costs of reintroduction projects, we estimated an overall expenditure of about U.S. $2800 per parrot. Previous attempts to reintroduce captive-raised parrots are introduced to an area with a resident population. Although reintroduction can significantly reduce the chances of extinction, it also involves some risks. The long-term solution against extinction of A. barbadensis will be a combination of scientific understanding of their biology and habitat, awareness by local human communities, reduction in the wild bird trade, and continued commitment by conservation enforcement agencies.

516: *+.193*

. Species richness in calcareous grassland is discussed against the background of historical dispersal processes associated with traditional land-use management such as grazing, but also the artificial establishment by hayseed. Important vectors in the traditionally man-made landscape were sheep and cattle or other livestock such as goats. Calcareous grasslands were not only connected to each other but also to other habitats such as villages, forests, arable fields and heathlands by these vectors which could cover large distances (e.g. transhumance shepherding), which is not the case in the current man-made landscape. Species richness after restoration management of abandoned and afforested calcareous grasslands was predicted by using characters of dispersability in space and time. These were the persistence of the species in the vegetation and the diaspore bank after abandonment or afforestation and the dispersal capacity through wind and sheep. The results reveal that reintroduction of sheep grazing is necessary to reestablish the original species richness. The first validation of the prediction of the succession on clear-cut sites and a comparison with data of species composition in abandoned quarries and the surroundings made it obvious that a species' own dispersal capacity in space is very low except for some well wind-dispersed species. Therefore, it is recommended to include and to simulate dispersal processes in future management to be able to restore the original species richness.

517: ***-.012***

. Rare vascular plant species are endangered worldwide. Population losses are most commonly caused by human-related factors. Conservation management seeks to halt this adverse trend and if possible, to enhance long-lasting self-sustainable populations. In general, rare species are poorly recruited from seed banks, or disperse themselves very poorly. It may be a management option to translocate such plants by seeds and/or transplants. This paper asks which problems may be faced. It is argued that translocation is only acceptable if it is based on knowledge of species biology and ecology and the size and structure of its geographic range through time. Such knowledge of rare species is often lacking. The finite management goal can only be achieved if conservationists closely cooperate with both ecologists and geneticists.

518: ***+.232***

The natterjack toad Bufo calamita is rare in Britain, which is at the northwestern edge of its biogeographical range. We investigated the level of genetic differentiation amongst almost all (34 out of 38) of the surviving British populations of this species, and among six new populations established by translocations during the 1980s. For eight microsatellite loci, allele sizes and frequencies were analysed using samples from each of these populations. The populations clustered into three robustly differentiated groups, each of which corresponded with a geographical region (east/southeast England, Merseyside and Cumbria). The Cumbrian populations showed a further weak geographical substructuring into northern and southern clades. The populations in south Cumbria were genetically more diverse than those in any of the other regions, as judged by the mean numbers of alleles per locus and the mean heterozygosity estimates. The translocated populations clustered close to their founders and, with one exception, did not differ significantly with respect to mean allele numbers, heterozygosity or polymorphism level. However, significant genetic differentiation las measured by unbiased R-ST was found between all but one of the founder-translocation pairs. The implications of this phylogeographic study for the future conservation of B. calamita in Britain are discussed.

519: ***+.202***

Historically documented founder events provide opportunities to assess the effects of population

size reductions on genetic variation, but the actual magnitude of genetic change can be measured only when direct comparisons can be made to the source or ancestral population. We assayed variation at nine microsatellite loci in the translocated population of the Laysan finch (Telespiza cantans) at Pearl and Hermes reef (PHR), and compared the level of variation to that in the source population pn Laysan Island. Heterogeneity in allele frequencies was highly significant at eight of the nine loci, primarily as a result of fluctuations in allele frequencies in the three PHR populations. Intra- and interpopulational measures of genetic diversity generally matched predictions based on the well-documented history of three islet populations at PHR: significantly lower numbers of alleles and polymorphic loci, as well as higher pairwise F(ST) values and genetic distance, were observed for the two populations that underwent severe size reductions. Changes in heterozygosity at single loci were unpredictable, as both significant increases and decreases were observed in founder populations. A significant excess of heterozygotes was found in two populations and was highly significant over all four finch populations ($P < 0.003$). Estimates of effective population size from temporal changes in heterozygosity and allele frequencies were very small (N(e) less than or equal to 30) as a result of the founding events and the constraints of islet area on population numbers. We concluded that the PHR population is not adequate as a secondary genetic reserve for T. cantans, and an alternative refuge needs to be established.

520: *-.016*

Sandhill Cranes are large, gray birds that are widely distributed in North America. Although Sandhills usually nest in water, they spend most of their time in uplands. Most Sandhill Cranes are migratory, but Florida has a substantial population of resident cranes. Because Sandhills are common in some areas, sick or injured individuals are frequently brought to rehabilitators. This article summarizes the species' distribution and biology, and treatment of its most important health problems. In addition, we describe techniques for raising chicks and strategies for releasing chicks or adults then back to the wild.

521: *+.069*

During a recent land drainage scheme, a population of the Freshwater Pearl Mussel Margaritifera margaritifera (L., 1758) was discovered in a small stream in north-west Wales. The dredging operations effectively destroyed the population which was estimated to have been as high as 5000 individuals. The presence of juvenile shells under 20 mm in the spoil tips suggests this was a viable, recruiting population. An attempt to translocate the remaining mussels to an unaffected downstream site was unsuccessful. The loss of this nationally important population demonstrates the urgent need for comprehensive distributional data upon which an effective conservation policy can be based.

522: *+.345*

Discovery of populations of the marsh snail Vertigo moulinsiana near and within the route of a new road in southern England whilst site clearance was in progress, caused the urgent development of a strategy to protect the animals. The snail's inclusion on Annex II of the European Habitats & Species Directive made it vital for appropriate action to be implemented to protect the snail along the proposed road. As it was not feasible to change the route it was decided to translocate the wetland habitat supporting the Vertigo moulinsiana populations to places off-route. As a further safeguard, new marsh habitat was created to provide the opportunity for V. moulinsiana colonisation. The methods of translocation and the subsequent monitoring

programme are described. Early results indicate that the translocation of snail rich vegetation has been successful and that the snails are breeding.

523: ***+.343***

Discovery of populations of the marsh snail Vertigo moulinsiana near and within the route of a new road in southern England whilst site clearance was in progress, caused the urgent development of a strategy to protect the animals. The snail's inclusion on Annex II of the European Habitats and Species Directive made it vital for appropriate action to be implemented to protect the snail along the proposed road. As it was not feasible to change the route it was decided to translocate the wetland habitat supporting the Vertigo moulinsiana populations to places off-route. As a further safeguard, new marsh habitat was created to provide the opportunity for V. moulinsiana colonisation. The methods of translocation and the subsequent monitoring programme are described. Early results indicate that the translocation of snail rich vegetation has been successful and that the snails are breeding.

524: ***+.220***

All species of howlers are at risk due to habitat destruction. I review the three most serious threats to howler survival-agricultural disturbance, logging disturbance, and hunting-both in a general context and in species accounts. Withal, the adaptability of howlers has allowed them to be a widespread genus throughout Central and South America. Alouatta is especially adaptable due to its generalized folivorous diet. I discuss how this adaptability relates to survival in situations of habitat destruction. In addition, I examine howler preference for riverine habitats and their ability to capitalize on secondary forests and secondary growth. I take a practical approach, suggesting some solutions for species survival, which include habitat management and reintroductions. Successful conservation models highlighted are the Community Baboon Sanctuary in Belize, in which villagers manage their lands for the benefit of the black howler and for their own economic benefit from ecotourism, and a village-sustainable logging system in Quintana Roo, Mexico, which has reduced levels of deforestation while benefiting both villagers and howlers, as well as other wildlife. A successful translocation of black howlers in Belize in order to reestablish a viable population is also described. The paper concludes with further suggestions to encourage howler conservation.

525: ***-.087***

As assessed by the IUCN Mace-Lande system, seven (35%) of the 20 Alouatta species and subspecies with adequate data are classified as "threatened," i.e., critically endangered, endangered, or vulnerable (Rylands et al, 1995). This percentage is much lower than the 75 to 100% threatened taxa for, the other large-bodied genera: Ateles, Lagothrix, and Brachyteles. Only 5 of the 16 Neotropical genera have lower percentages of threatened taxa than that of Alouatta: Cebuella, Pithecia, Saguinus, Saimiri, and Cebus The threatened howler taxa occupy small distributions in areas of forest fragmentation. In general, populations are most affected by major habitat disturbance, such as total deforestation and flooding from dam construction, and by human hunters. Facilitated by their ability to exploit folivorous diets in small home ranges, howlers can tolerate considerable habitat fragmentation but not the increased exposure to hunting that may accompany it. Howlers seem particularly vulnerable to yellow fever and bot fly parasitism. Although the former threat may decrease by increasing fragmentation of the habitat, other sorts of parasitism may ino ease in disturbed habitats. The Low genetic variability of the Central American howlers suggests a resistance to inbreeding depression potentially experienced during population

bottlenecks. Greater between-population variability may still exist. Although howlers are not readily bred in captivity, they respond well to translocation. Translocation has been successfully achieved for greater than or equal to 4 howler species and is a viable option for introducing new genetic variability into population fragments and repopulating areas from which howlers are extinct. Their pattern of bisexual dispersal facilitates colonization of regenerating habitats, and in suitable, protected habitats they have shown the capacity for strong population recovery.

526: *+.097*

There is considerable interest in restoring stocks of Atlantic sturgeon (Acipenser oxyrinchus), one of only two dozen extant sturgeons worldwide. Populations of its two subspecies are found in large rivers along the North American coasts of the Atlantic (A. o. oxyrinchus) and the Gulf of Mexico (A. o. desotoi). Although these populations were severely reduced because of overfishing and, in some instances, blockage of spawning runs, their present statuses, vary from those that still support limited fisheries (St. John River, new Brunswick), to a large but apparently declining population (Hudson River), to relict-sized (Delaware River, Mobile River), to apparently extirpated (Maryland tributaries of Chesapeake Bay; St. Johns, river, Florida). There are two primary alternatives for restoring these populations: (1) eliminating harvest and allowing natural recolonization and increase and (2) hatchery-based stocking (reintroduction or supplementation). We performed genetic analyses aimed at guiding restoration efforts. Mitochondrial DNA analysis revealed strong stock structure along both coasts at eh regional and, in some instances, population levels. Estimates of gene flow rates were low and suggested slow natural recolonization rates. Thus, the first alternative avoids the genetic risks of interstock transfer and inbreeding depression that may result from hatchery-based programs, but given the low intrinsic rates of increases and recolonization of Atlantic sturgeon, recovery may take decades to centuries, if it occurs at all. Therefore,it is imperative that the restoration approach be tailored to the particular circumstances and demographics of each population; however, the characteristics of this species and poor funding prospects make this information difficult to obtain. It appears likely that initial Atlantic sturgeon stocking efforts will target systems where they are extinct, as recently occurred in the Nanticoke River of Chesapeake Bay.

527: *-.026*

In parts of the world such as the Pacific Islands, Australia, and New Zealand,introduced vertebrate predators have caused the demise of indigenous mammal and bird species. A number of releases for reestablishment of these mammal species in mainland Australia have failed because predators extirpated the new populations. The nature of the decline of both extant populations and reintroduced colonies provides information on the dynamics of predation. Predator-prey theory suggests that the effects of predation are usually inversely dependent on density (depensatory) when the prey are not the primary food supply of exotic predators. Thus, such predators can cause extinction of endemic prey species. Three types of evidence can be deduced from the predator-prey interactions that allow predictions for conservation: (1) whether per capital rates of change for prey increase or decrease with declining prey densities, (2) whether predation is depensatory or density-dependent, and (3) the overall magnitude of predation. If this magnitude is too high for coexistence, then the degree of predator removal required can be predicted. If the magnitude of predation is sufficiently low, then the threshold density of prey that management must achieve to allow predator and prey to coexist can also be predicted. We analyzed published reports of both declining populations and reintroduced colonies of endangered marsupial populations in Australia. The observed predation curves conformed to the predictions of predator-prey theory. Some, such as the black-footed rock-wallaby (Petrogale lateralis), were classic alternate prey and were

vulnerable below a threshold population size. Others, such as the brush-tailed bettong (Bettongia penicillata), have a refuge at low numbers and thus offer the best chance for reintroduction. Our predictions suggest a protocol for an experimental management program for the conservation of sensitive prey species: (1) determination of net rates of change of prey with declining population, (2) improvement of survivorship through habitat manipulation, (3) improvement of survivorship through predator removal, (4) determination of the threshold density above which reintroductions can succeed, and (5) manipulations to change interactions form Type II to Type III. The task in the future is to determine how to change the vulnerability of the prey so that they can have a refuge at low numbers.

528: ***+.109***

A Recovery Team oversaw implementation of a Recovery Plan for the woylie (Bettongia penicillata ogilbyi (Waterhouse 1841)) from 1990 to 1995, and then reviewed its conservation status. Using the 1994 IUCN Red List criteria, the team showed that the species had been recovered from Vulnerable to Lower Risk (Conservation Dependent). Furthermore, it no longer met the requirements for listing as a threatened species under Western Australian or Commonwealth legislation and all but one of the specific targets of the Recovery Plan had been achieved. The exception, at least 7.5 percent trap success in a new population at Julimar Conservation Park, had not been met at that time because translocation to Julimar had been delayed. However, a population had been established and was increasing. Criteria for listing threatened species under Western Australian and Commonwealth legislation are based on the subject's status nationally. South Australian legislation requires consideration of its status within the State. The Recovery Team recommended that the authorities responsible for advising the Western Australian and Commonwealth conservation Ministers should convey to them the Recovery Team's conclusions. The woylie has since been removed from categories of threatened taxa established under those jurisdictions. The team recommended to the South Australian authorities that they should review its status within that State. The review is an unpublished report addressed to those authorities who would have to progress the Recovery Team's recommendations. However, as it was the first to use the 1994 IUCN criteria to assess the outcome of an Australian Recovery Plan, the issues encountered by the recovery team provide a useful case study for other people preparing Recovery Plans and reviewing the outcomes. Therefore it is published here.

529: ***+.081***

Since 1960 107 translocations of wild-caught Weka (genus Gallirallus) have occurred in New Zealand. Only four of these Weka liberations resulted in a population that persisted for more than 10 years and only one was successful on the North Island (the resultant population is now believed extinct). The reason for these failures was not known. In 1991 members of the Royal Forest and Bird Protection Society commenced breeding North Island Weka Gallirallus australis greyi in captivity for another liberation. Between 1992 and 1996 101 weka were released. We used radio telemetry to follow the fates of the first 17 Weka released in the Karangahake Gorge, near Paeroa, North Island, New Zealand to determine possible outcomes of the liberation. Only one of the 17 birds released survived until 242 days post release. Most newly released Weka were killed by predators, mainly dogs. Future Weka and flightless rail introductions should occur only in areas where predators are being removed to allow survival of released birds and production of young to exceed mortality.

530: ***+.052***

The National Commission for Wildlife Conservation and Development was established in 1986 to oversee all wildlife conservation programmes in Saudi Arabia. The Arabian oryx Oryx leucoryx is one of the flagship species of the Saudi Arabian reintroduction policy. It has been captive-bred since 1986 at the National Wildlife Research Center near Taif. With the creation of a network of protected areas in the former distribution range of the species, attention has shifted to the release of captive-bred oryx into Mahazat as-Sayd and 'Uruq Bani Ma' arid reserves. Similar programmes carried out in other countries of the Arabian Peninsula underline the need for regional co-operation and pan-Arabic public awareness programmes, in addition to captive-breeding and reintroduction projects.

531: ***+.231***

Tidal marshes have been actively restored in Connecticut for nearly 20 years, but evaluations of these projects are typically based solely on observations of vegetation change. A formerly impounded valley marsh at the Barn Island Wildlife Management Area is a notable exception; previous research at this site has also included assessments of primary productivity macroinvertebrates, and use by fishes. To determine the effects of marsh restoration on higher trophic levels, we monitored bird use at five sites within the Barn Island complex, including both restoration and reference marshes. Use by summer bird populations within fixed plots was monitored over two years at all sites. Our principal focus was Impoundment One, a previously impounded valley marsh reopened to full tidal exchange in 1982. This restoration site supported a greater abundance of wetland birds than our other sites, indicating that it is at least equivalent to reference marshes within the same system for this ecological function. Moreover, the species richness of birds and their frequency of occurrence at impoundment One was greater than at 11 other estuarine marshes in southeastern Connecticut surveyed in a related investigation. A second marsh, under restoration for approximately ten years, appears to be developing in a similar fashion. These results complement previous studies on vegetation, macroinvertebrates, and fish use in this system to show that, over time, the reintroduction of tidal flooding can effectively restore important ecological functions to previously impounded tidal marshes.

532: ***+.281***

The Sword-grass Brown Butterfly Project (SBBP) is a community driven project initiated by the Knox Environment Society. We are planting habitat at sites between known populations of the butterflies in the hope of increasing their numbers. Project activities include revegetation and propagation, community art, monitoring and translocation. SBBP is also raising public awareness of the needs of Sword-grass Brown Butterfly and other butterfly species within The Basin-Boronia area.

533: ***-.268***

Reintroductions of the endangered razorback sucker Xyrauchen texanus and Colorado squawfish Ptychocheilus lucius into the Verde River, Arizona, have failed to establish self-sustaining populations. The failure of these reintroductions is often attributed to predation and competition by normative fishes and habitat loss, but parasite infestations (particularly a copepod, Lernaea cyprinacea) also have been implicated. We assessed the relationships among fish health, eukaryotic parasites, and bacterial and viral infections of these endangered fishes collected from two locations (Perkinsville and Childs) on the Verde River, Arizona, during February and June 1996. We also examined surrogate nonendangered native fishes (Sonora sucker Catostomus insignis, desert sucker Catostomus clarki, and roundtail chub Gila robusta) and normative species

(common carp Cyprinus carpio and smallmouth bass Micropterus dolomieu) to determine if there were any systemwide patterns in parasite infestation and health of fishes. All tests for viruses were negative. We identified 19 bacteria (7 known fish pathogens) and 14 parasites from examined fishes. Six of the seven dominant (>10% prevalence) parasite taxa-were more prevalent at Perkinsville than at Childs; Lernaea occurrence was not related to site. Time of year did not have a consistent effect on parasite prevalence across fish species, and Lernaea occurrence was not related to time of year. Lernaea cyprinacea were present on 17.3% of the 313 fish examined; 67% of the parasitized fish were infected with a single L. cyprinacea. Regression analyses on the calculated health assessment index, hematocrit, and leukocrit indicated that month, site, Trichodina, Ichthyophthirius (ich), Ornithodiplostomum and Posthodiplostomum (white grubs), and the cestode Isoglaridacris hexacotyle affected the health of fishes. However, all fishes examined were considered "healthy," suggesting that parasites were not seriously affecting Verde River fishes. A suite of factors is likely responsible for the failure of these endangered species to reestablish.

534: *+.019*

The results of two reintroduction programmes of Badgers are discussed, one with repeated releases of a few individuals at time, and the other with a more or less simultaneous release of eight animals. It turned out that the latter attempt was much more successful. in the former attempt more animals left the study area and especially (but not always) the last introduced animal was chased away by Badgers that had already settled. Most of the straying animals became victim of traffic or drowned in a sheetpiled watercourse. As mustelids can disperse over rather long distances, reintroduction is only relevant if artificial barriers can be removed and the habitat quality is restored. Reintroduction to overcome natural barriers is quite out of the question.

535: *+.028*

1. We examined the effects of phosphorus. an important pollutant in fresh waters, on mussels Anodonta anatina and embryos of European bitterling Rhodeus sericeus amarus. These species are involved in an unusual symbiosis, with the fish depositing eggs inside the mussels' gills, where the embryos develop for 3-6 weeks until the larvae leave the mussel,2. High concentrations of phosphate (500 mu g L(-1) and higher) caused a significant increase in the rate of premature expulsion and emergence of bitterling embryos from the mussels, with a concomitant increase in fish mortality.3. At concentrations of 750 mu g L(-1) and higher, mussels reduced their frequency of ventilation and movements through the substratum.4. These results have implications for understanding the nature of the symbiosis between the fish and their mussel hosts, showing that mussels have control over bitterling developing in their gills. The premature expulsion of bitterling embryos at high phosphate levels is detrimental to the fish that they harbour.5. Recommendations for the conservation of bitterling and species of mussels, which are threatened or endangered over parts of their ranges, include reductions in levels of phosphorus in natural habitats and the need for low levels in reintroduction sites for the fish and their mussel hosts.

536: *+.302*

As effective population size (N-e) decreases, genetic factors may become relatively important to a population's or species' persistence. Conservation biologists should be aware of anything that can potentially cause a sudden reduction in N-e. I used simple models to illustrate how certain types of female mating preferences combined with certain types of male traits may lead to a sudden and substantial decrease in Ne. Specifically, if and when there is a sudden 'downward' shift in the

expression of condition-dependent male traits, females using fixed-threshold mate choice criteria might find fewer acceptable males. While mechanisms of female choice remain elusive, a variety of sexually selected traits may be condition dependent. Because the expression of condition-dependent traits is likely to be impacted by natural or human-induced environmental changes, behavioral and conservation biologists should pay special attention to them around the mating season. Armed with knowledge of condition-dependent male traits, it may be possible to minimize the impact on condition-dependent traits while planning translocations or reintroductions.

537: ***-.203***

The present status and distribution of Cape buffalo Syncerus caffer caffer in southern Africa is reviewed. The present estimate of southern Africa's buffalo populations are Botswana 29 300 (+/-9000), Zimbabwe 48 200 (+/-21000), Namibia 2 800 (+/-1000) and South Africa 31 500 (+/-10000). Buffalo numbers for Mozambique are incomplete, but a decline is estimated at 79% in the buffalo population in the Marromeu Complex. The total number of buffalo in the six largest excluding Mozambique, has decreased by more than 50% between 1991 and 1996. The main factor influencing buffalo numbers and distribution in the sub-region is the development of the cattle industry, followed by drought and disease. Only Sough Africa and Zimbabwe activity produce surplus buffalo for translocation or sale. However, the cumulative loss of genetic diversity in these small, isolated populations may have potentially serious consequences.

538: ***+.091***

Reintroduction programs are a high-risk conservation strategy for restoring populations of endangered species. The success of these programs often depends on the ability to identify suitable habitat within the species' former range. Bioclimatic analysis offers an empirical, explicit, robust, and repeatable method to analyze large areas rapidly using a small number of locality records, and in turn predicting (and/or reconstructing) its potential distribution limits. This approach therefore can estimate the broad limits of the distribution of a taxon, using data that may be inadequate for standard forms of statistical analysis. We illustrate the potential value of bioclimatic modeling for reintroduction biology using a case study of the highly endangered Helmeted Honeyeater (Lichenostomus melanops cassidix) from Victoria, southeastern Australia. The results of our analyses assisted us to both predict the former range limits of the Helmeted Honeyeater and determine the broad limits of those areas that may contain potentially suitable sites for future reintroduction programs for the subspecies. The analysis predicted that the range of the Helmeted Honeyeater extends from the Yarra River district east of Melbourne, south to the Western Port Bay and east as far as the Morwell area of Victoria. The climatic characteristics of habitat occupied by the extant population of the Helmeted Honeyeater were found to be unique within its predicted range. We recommend that reintroduction efforts therefore be concentrated within this small area, as has occurred to date.

539: ***+.003***

This review of Iowa's mammal fauna at the close of the 20th century summarizes changes in distributional patterns following Euroamerican settlement in the early 1800s. Data from historical records, museum specimens, and presettlement late Holocene fossils indicate presence of 69 resident mammals at that time. Hunting pressures and the conversion of prairie and forest to agricultural fields reduced the populations and ranges of many state mammals, and 14 species were extirpated by 1900. An additional 15 species are either uncommon or rare today, and the Iowa Department of Natural Resources lists four species as endangered, three as threatened and

one as of special concern. Species dependent upon either forest of prairie have suffered most; forest-edge species generally have thrived and even increased. River otters and a captive bison herd were reintroduced recently, and several (like the spotted skunk and the bobcat) have benefitted from setaside acreage, flood-plain and wetland reclamation, and roadside management initiatives. Species distribution and relative abundance vary slightly among the state's five geographic regions.

540: *+.143*

Repeated liming of Hovvatn during the 1981-1995 period assured successful reintroduction of lake spawning brown trout, Salmo trutta. Poor natural recruitment to the population was associated with low survival during early life stages (before hatching) as shown by the 0.5, 3.5, 0.9 and 1.0% of live embryos found in natural redds during the 1992-1995 period, respectively. The low survival was most likely caused by the combination of shallow spawning areas (<2.0 m) and acidic runoff (pH 4.0-4.8) which overlayed the limed part of the water body during the ice covered period. It is therefore concluded that this type of episodic acidification poses a major threat to lake spawning salmonids, and that it can retard or inhibit biotic recovery towards preacidified conditions expected as a result of liming. Addition of limestone gravel (8-32 mm) onto spawning grounds was an efficient alternative liming strategy as 33-36% live embryos were found in this substrate. Conversely, the trout actively avoided additions of shellsand, a behaviour most likely caused by the small particle size of shellsand (3-7 mm) relative to natural spawning gravel.

541: *+.124*

Flying foxes (Ptero us spp.) are important pollinators and seed dispersers in many island ecosystems. Populations of flying foxes have declined markedly on most islands in the South Pacific since the 1940's. One potential conservation strategy is to reintroduce bats on islands where they historically occurred. In this manner, the risk of species extinction due to local catastrophic events can be reduced. Ideally the source population for such reintroductions must be robust. One such population might be in the Kingdom of Tonga, where flying foxes have been protected by local custom for a long time. However, virtually nothing has been published on the numbers, locations, and reproductive biology of Pteropus tonganus in Tonga. I therefore censused flying fox populations on as many islands as possible from 28 June to 26 July 1995. I found seven major roosts containing a total of 3,582 bats on Tongatapu, one roost with 30 bats in the Ha'apai group, and 27 roosts with a total of 5,925 bats on 14 islands within the Vava'u group. Thus, the flying fox population in Tonga seems robust and dispersed on many islands and is therefore a valuable potential source population.

542: *+.111*

The Zanzibar red colobus Procolobus kirkii is one of Africa's most endangered primates, with only c. 1500-2000 individuals remaining in the wild. The authors made preliminary surveys of three areas where this monkey was translocated or introduced in the 1970s and 1980s. It appears that only one of these releases was successful. A total of 67 animals were caught and translocated or introduced (including four that died during the process). Thirteen to 20 years later, 62-70 red colobus were located at the three sites; i.e. there had been Mo Met gain in colobus numbers as a result of the translocations and introduction. One attempt apparently failed because of insufficient habitat, and another perhaps because of insufficient numbers of colobus or an imbalance in the age and sex composition of animals released. The one successful translocation involved moving a fairly large number of monkeys (36) into a forest with a relatively high diversity of tree species. In

light of these findings, the authors recommend that far more attention be given to protecting the monkeys and their habitat where they currently exist, rather than spend time and money on translocations that are as likely to fail as to succeed.

543: *+.165*

Captive propagation has become increasingly important in preventing extinction in many avian species, including the Hawaiian crow (Corvus hawaiiensis) and Mariana crow (C. kubaryi). We used 3 surrogate species, common raven (C. corax), American crow (C. brachyrynchos), and black-billed magpie (Pica pica), to develop captive-rearing protocols for endangered corvids. Here we; compare the health, growth, and survival among nestlings hand-reared on 14 feeding regimes. Frequency of feeding chicks for the first 2 weeks after hatch varied from once every 30 min to once every 2 hr. From 2 weeks until near fledging age, frequency of feeding varied from once every hour to once every 3 hr. Initial amounts of food fed varied from unlimited (ad libitum) to a restricted amount (a total of 15, 25, or 40% of a chick's body mass). Combinations of frequent feeding and large amounts of food produced fast-growing, heavy birds with few fault bars in their feathers, but all hand-reared nestlings grew more slowly than wild-reared chicks. Initial amount of food offered to day-old chicks affected growth and survival more than frequency of feeding in very large passerines (e.g., common ravens). In small- to medium-sized passerines (e.g., black-billed magpies, American crows), frequency of feeding affected growth and survival more than initial amount of food.

544: *+.075*

Methods for trawl-acoustic surveys were developed and used on the basis of visual registration of the state of acoustic recording with simultaneous recording of the size of catches, using T. chalcograma as an example. Five surveys of the resources of the species were carried out near western Kamchatka during the prespawning and spawning periods in February-April 1996. Main concentrations of the wall-eye pollock were shown as were their appearance, translocations and disappearance. A wave of winter spawning predominated. The second spawning wave taking place in spring was observed in southern areas. The total potential catch was evaluated on the basis of the results of a series of surveys. Five groups were identified based on the sizes of individuals, and the location and time of spawning; spawning in two of the groups took place in spring. These groups regardless of their smaller size have higher commercial value and require further investigations and immediate protection measures.

547: *-.147*

Eight of 11 native forest bird species on Guam were extirpated by the introduction of the Brown Tree Snake Boiga irregularis. Emergency measures necessary to rescue the Guam subspecies of Micronesian Kingfisher Halcyon cinnamomina cinnamomina from extinction involved translocation and captive breeding in American mainland zoos. Soon after the establishment of a captive breeding population, the kingfisher demonstrated a high degree of susceptibility to avian tuberculosis (ATB), a disease that proved to be a major threat to the preservation of the species. The cause of ATB is Mycobacterium avium which produces a prolonged course of infection in kingfishers and other birds. Kingfishers infected with M. avium are difficult to detect until late in the course of the disease, thereby potentially posing a risk of transmitting ATB to the Guam captive population of Guam Rails Gallirallus owstoni, if kingfishers are repatriated. M. avium is considered to be ubiquitous in nature. However, there are no reported mortalities due to ATB in any bird species on Guam. In this study, six of twenty-one cultures yielded Mycobacterium spp.,

two of which were further identified as M. avium. Since this study demonstrates that M. avium already exists on Guam, repatriating kingfishers to Guam poses no threat of introducing a new pathological agent to the island's ecosystem. Strict quarantine procedures along with rigorous animal husbandry protocols should minimize risks of repatriating infected kingfishers to Guam, and prevent transmission of ATB to the captive population of Guam Rails and other bird populations on Guam.

548: *+.060*

Ecologically sound efforts to manage or reintroduce populations of rare species require detailed knowledge of species habitat requirements. However, the fact that such species are rare implies that the data needed for habitat characterization are sparse and that species might well be absent from favorable sites due to chance aspects of dispersal or mortality. We use four rare plant species endemic to southern Appalachian high-elevation rock outcrops, to illustrate how nonparametric and parametric logistic regression can yield predictive models of the probability that a species will occur, given certain site conditions. Models were constructed for each species at two scales: 100-m(2) plots and 1-m(2) subplots. At the 100-m(2) plot scale, absences beyond the current geographic range were excluded. At the 1-m(2) subplot scale, absences from subplots were only included if the species occurred elsewhere on the 100-m(2) plot. Six significant models resulted; no significant model could be constructed for Solidago spithamaea or Calamagrostis cainii on 1-m(2) subplots. For 100-m(2) plots, the most valuable predictors were potential solar radiation, a soils gradient related to available soil iron, boron, and copper, and coarse-scale rock surface texture, although Geum radiatum occurrences were difficult to predict at this scale. For 1-m(2) subplots the best predictors were available soil cations, potential solar radiation, the proportion of exposed bedrock, and vegetation height. Along individual gradients response curves were often similar, but no two species were predicted by identical sets of site parameters. Beyond current range limits, existence of suitable habitat on 100-m(2) plots was demonstrated for Solidago spithamaea, supporting a view that the range limits of this species are not necessarily set by availability of suitable habitat. Habitat-based models have numerous management applications (such as to guide restoration and reintroduction efforts as well as to direct searches for additional populations) and provide a framework for future work on species-specific physiological requirements.

549: *+.203*

We use the phylogenetically based statistical method of independent contrasts to reanalyze the Wolf, C.M., Griffith, B., Reed, C., Temple, S.A. (1996. Avian and mammalian translocations: update and reanalysis of 1987 survey data. Conservation Biology 10, 1142-1154). translocation data set for 181 programs involving 17 mammalian and 28 avian species. Although still novel in conservation and wildlife biology, the incorporation of phylogenetic information into analyses of interspecific comparative data is widely accepted and routinely used in several fields. To facilitate application of independent contrasts, we converted the dichotomous (success/failure) dependent variable (Wolf et al., 1996; Griffith, B., Scott, J.M. Carpenter, J.W., Reed, C., 1989. Translocations as a species conservation tool: status and strategy. Science 245, 477-480) into a more descriptive, continuous variable with the incorporation of persistence of the translocated population beyond the last release year, relative to the species' longevity. For comparison, we present three models: nonphylogenetic multiple logistic regression with the dichotomous dependent variable (the method used by Wolf et al. 1996 and Griffith et al. 1989), nonphylogenetic multiple regression with the continuous dependent variable, and multiple regression using phylogenetically independent contrasts with the continuous dependent variable. Results of the phylogenetically

based multiple regression analysis indicate statistical significance of three independent variables: habitat quality of the release area, range of the release site relative to the historical distribution of the translocated species, and number of individuals released. Evidence that omnivorous species are more successful than either herbivores or carnivores is also presented. The results of our reanalysis support several of the more important conclusions of the Wolf et al. (1996) and Griffith et al. (1989) studies and increase our confidence that the foregoing variables should be considered carefully when designing a translocation program. However, the phylogenetically based analysis does not support either the Wolf et al. (1996) or Griffth et al. (1989) findings with respect to the statistical significance of taxonomic class (bird vs mammal) and status (game vs threatened, endangered, or sensitive), or the Griffith et al. (1989) findings with respect to the significance of reproductive potential of the species and program length. (C) 1998 Elsevier Science Ltd. All rights reserved.

550: ***+.080***

This study assesses the impact of Pacific Rat Rattus exulans predation on the endangered Ouvea Parakeet Eunymphicus cornutus uvaeensis and investigates the feasibility of translocating this bird to the island of Lifou (despite the presence of Ship Rat R. rattus). A rat trapping campaign, conducted from July 1995 to October 1996 in the forest of Ouvea and Lifou (Loyalty Islands, New Caledonia, South-west Pacific), confirmed the presence of Pacific Rats in Ouvea and both Pacific and Ship Rats in Lifou. Population dynamics, sex ratio, age structure and diet of both rats were investigated. An experiment, using quail eggs in natural tree holes during the main bird breeding season, in Ouvea in 1994 and in Ouvea and Lifou in 1996, compares the nest predation rate between these islands. Mayfield's estimator for a 21 day theoretical incubation period gives a survival rate of 0.80 and 0.99 in the two experiments in Ouvea but only 0.22 in Lifou. Most of the difference in egg survival observed between the islands appears to be related to the presence of Ship Rat in Lifou, while the difference in the two experiments in Ouvea may estimate the effects of trapping. The study shows that Lifou is not a suitable place for translocating Ouvea Parakeets, unless active habitat management is carried out to protect this bird against Ship Rats. The value of low intensity rat control in Ouvea immediately prior to the parakeet breeding period is also suggested.

551: ***+.047***

I used VORTEX to make a prior evaluation of the planned reintroduction project of the endangered bearded vulture (Gypaetus barbatus) in the mountains of southern Spain. The minimum size of a captive population needed to ensure the release of two bearded vulture fledglings per year is 30 individuals. If breeding success was improved, this minimum captive population could be reduced to 10 individuals. As the cost of the project is proportional to the size of the captive population, some research effort should be devoted to improving breeding success in captivity. The current size of the captive population - four individuals - is not enough to start the releases, even if the vultures were already breeding. The first step for the project should be to increase the size of the captive population. If on average one individual per year was added to the captive population, it would take 15 years to reach 30 individuals. If breeding in captivity was improved, it would take only three to seven years to reach the minimum captive population of 10 individuals. Once releases were started, the project would need on average 20 years to reach a goal of 15 adult pairs in the Cazorla mountains. Both the success of the project and the time necessary to reach this stated goal are very sensitive to the mortality rates in the wild. There is little known about these rates, and better estimates are urgently needed to improve the predictions of the models.

552: ***+.147***

Recent reintroduction of the black-footed ferret (Mustela nigripes) in west-central South Dakota has focused new attention on black-railed prairie dogs (Cynomys ludovicianus), because prairie dog colonies provide essential habitat for ferrets. Currently, management agencies are assessing prairie dog populations by counting active burrows, a technique that is attracting criticism. We correlated active and total burrow counts with prairie dog mark-recapture population estimates from 12 colonies located in Badlands National Park and adjacent Buffalo Cap National Grassland, We also correlated visual counts of prairie dogs and counts of mounds from aerial photographs with mark-recapture estimates to assess an alternative method to index populations. We found no significant relationships ($P > 0.05$) with any form of active burrow or total burrow counts (ground or aerial) using the linear model $Y = a + bX$. However, visual counts of prairie dogs, using maximum rather than mean values, on 4-ha plots were significantly related ($P < 0.0138$). The best model was $Y = 3.04 + 0.40X$, where Y is the maximum visual count and X is the estimated population density. The inverse of this equation $X = (Y - 3.04)/(0.40)$, could be used to index numbers of black-tailed prairie dogs from visual counts under conditions similar to those encountered in this study. An 8-point protocol for making visual counts is provided.

554: ***+.030***

Riparian plants have been classified as "drought avoiders" due to their access to an abundant subsurface water supply. Recent water-relations research that tracks water sources of riparian plants using the stable isotopes of water suggests that many plants of the riparian zone use ground water rather than stream water and not all riparian plants are obligate phreatophytes (dependent on ground water as a moisture source) but may occasionally be dependent on unsaturated soil moisture sources. A more thorough understanding of riparian plant-water relations must include water-source dynamics and how those dynamics vary over both space and time. Many rivers in the desert Southwest have been invaded by the exotic shrub Tamarix ramosissima (saltcedar. Our studies of Tamarix invasion into habitats formerly dominated by native riparian forests of primarily Populus and Sallix have shown that Tamarix successfully invades these habitats because of its (I) greater tolerance to water stress and salinity, (2) status as a facultative, rather than obligate, phreatophyte and, therefore, its ability to recover from droughts and periods of ground-water drawdown, and (3! superior regrowth after fire. Analysis of water-loss rates indicate that Tamarix-dominated stands can have extremely high evapotranspiration rates when water tables are high but not necessarily when water tables are lower. Tamarix has leaf-level transpiration rates that are comparable to native species, whereas sap-flow rates per unit sapwood area are higher than in natives, suggesting that Tamarix maintains higher leaf area than can natives, probably due to its greater water stress tolerance. Tamarix desiccates rind salinizes floodplains, due to its salt exudation and high transpiration rates, and may also accelerate fire cycles, thus predisposing these ecosystems to further loss of native taxa. Riparian species on regulated rivers can be exposed to seasonal water stress due to depression of floodplain water tables and elimination of annual hoods. This can potentially result in a community shift toward more stress-tolerant taxa, such as Tamarix, due to the inability of other riparian specie!, to germinate and establish in the desiccated floodplain environment. Management efforts aimed at maintaining native forests on regulated rivers and slowing the spread of Tamarix invasion must include at least partial reintroduction of historical flow regimes, which favor the recruitment of native riparian species and reverse long-term desiccation of desert floodplain environments.

555: ***+.178***

The development of ex situ conservation work for Orthoptera is demonstrated by examples of two captive-breeding programmes at the Zoological Society of London. The field cricket Gryllus campestris and wart-biter bush cricket Decticus verrucivorus are both species that have been bred in captivity and reintroduced to field sites in attempts to strengthen dwindling wild populations. Despite a similar approach to both programmes, we have encountered significant differences in the practical applications of the captive management process for the two species. By reviewing these breeding programmes, we examine some of the practical considerations associated with ex situ orthopteran programmes and compare some of the different elements that can influence programme success.

556: *+.113*

Up to 30 species of fish were generally found in medium-sized rainforest streams in Sabah, Malaysia. However, depauperate fish communities were found in two streams above large (>8 m) waterfalls (five and nine species) and in two very small streams located in deep forest (five and six species). A fifth stream with small cascades and waterfalls had a faunal fist of 12 species. Species resident above waterfalls were predominantly herbivores while fishes in the two forest streams were from a variety of trophic groups. Abundance and biomass of fishes above waterfalls were significantly lower than all other sites. A translocation experiment was performed in one stream to distinguish between the hypotheses that fish communities above waterfalls were determined solely by colonization ability and were unsaturated with species or that they were limited by habitat quality or food. Seven species (775 individuals) were translocated into one isolated section and four species (570 individuals) into another. Species that were translocated included trophic groups that were not represented by resident species. Twelve months after translocation, only four and one translocated species were collected in the two sections, all at greatly reduced densities. After 20 months, the number of species were three and two, respectively. One translocated species, Rasbora sumatrana, had increased in abundance from 12 months and juveniles were present in the population. Individuals of other translocated species appeared to be remnants of stocked populations. Abundance and biomass of resident species fluctuated widely between years. Interpretation of results was complicated by a large flood which substantially changed habitat conditions about a month after translocations were performed. It is suggested that two different factors were responsible for depauperate communities: movement barriers for waterfall sites and physico-chemical conditions and/or habitat availability at other sites. (C) 1998 The Fisheries Society of the British Isles.

557: *+.073*

The effects of deer hunting by humans on deer population dynamics and behavior may indirectly affect the population dynamics and behavior of deer predators We present data on the effects of hunting on the behavior of white-tailed deer (Odocoileus virginianus) on the Osceola National Forest, a potential reintroduction site for the endangered Florida panther (Felis concolor coryi). We then use this information to formulate and recommend testable hypotheses to investigate whether these changes in deer behavior influence panther movements, mortality: and hunting success. We monitored 14 radio-collared deer front June 1990 through July 1991 to compare movement activity, and habitat-use patterns between the hunting and non-hunting seasons. Mean distance of deer to the nearest road, mean distance of activity centers of diel home ranges to the nearest road, and mean nocturnal rate of activity were gr eater during the hunting than the nonhunting seasons. During the hunting season, deer avoided clearcuts, young pine plantations (4-10 years old), and other open habitats and preferred su,amp and mature pine forests, both of which provided cover. These results suggest that deer responded to hunter disturbance by moving away

from roads and increasing nocturnal activity Although recreational deer hunting may reduce the prey base for panthers the changes we observed in deer behavior during the hunting season may benefit panthers in the following ways: (I) an increase in nocturnal activity and movement away from roads by deer into areas frequented by panthers may increase prey availability for panther's; (2) the movement of deer away from roads may in turn draw panthers away from om roads, which may decrease the chance of panthers being killed by vehicular traffic or poachers.

558: ***-.070***

The single surviving wild population of Campbell Island Teal, restricted to Dent Island in the New Zealand subantarctic, is unlikely to exceed 25 pairs. Twelve Campbell Island Teal were released in 1999 onto Codfish Island in southern New Zealand to establish, temporarily, a second wild population. These birds originated from a captive breeding programme that has produced 40 fledglings during 1994 - 1998 from an initial wild-caught stock of 11 birds. Wild-raised and wild-acclimatised teal from Codfish Island are intended as the source for eventual re-establishment on Campbell Island. Planning for the removal of Norway rats, the sole remaining alien mammal there, has commenced.

559: ***-.214***

The Siberian Crane is the third rarest of the world's 15 species of cranes and considered by the International Crane Foundation to be the most endangered. Field surveys suggest that only 2900-3000 birds in three populations remain. While potentially encouraging, these numbers can be misleading due to the significant threats faced by this species.

560: ***+.091***

Dipodomys merriami parvus was listed as endangered by the U. S. Fish and Wildlife Service (USFWS) in September 1998 following its emergency listing of January 1998. A Biological Opinion issued by USFWS on a sand and gravel mining within Cajon Wash, an area of critical habitat, included translocation as a minimization measure. Preliminary to translocation, a salvage trapping effort was initiated in May 1998 to remove animals from a 32.5-ha site scheduled for mining operations. Fifteen D. m. parvus (11 males and 4 females) were captured and released on a re-vegetated reclamation site that contained a complement of native rodents including three resident D. m. parvus. All translocated animals were reproductively active. Translocation success was assessed in August 1998. Thirty-three percent of the original resident D. m. parvus and 30% of the translocated individuals were: still present on the site. In contrast, among resident animals of other species, 57.1% of Dipodomys simulans and 15.4% of Peromyscus maniculatus remained on the site. Three juvenile D. m. parvus, apparently offspring from released animals, were captured in the release area. All adult D. m. parvus captured in August showed signs of reproductive activity, including evidence of a second litter in progress. Continued monitoring will verify the apparent short-term success of the translocation and suggest refinements for future efforts.

562: ***-.010***

We question the rationale and methodology behind two recent amphibian translocations into west-central Illinois. In both cases, species were translocated outside their native ranges and no rigorous criteria for gauging success were utilized. We are concerned that translocations such as these will give the mistaken impression that new populations of amphibians can be easily established, even

in marginal habitats, and that any translocation effort will result in successful amphibian conservation.

563: *-.082*

Although river otters (Lutra canadensis) were common in Illinois during early European settlement, habitat degradation and unregulated harvests caused populations to decline dramatically by the mid-1800s. Otters were considered rare in the state by the early 1900s, and were listed as a state endangered species in 1989. Recovery strategies developed by the Illinois Department of Natural Resources included reintroducing otters in suitable but unoccupied habitats. Wild otters obtained from Louisiana were released in the Wabash (n = 137), Kaskaskia (n = 72) and Illinois (n = 137) River basins from January 1994 through March 1997. Dead otters recovered from 1994 through 1997 included more males (n = 20) than females (n = 9). Losses were attributed to hoop nets (n = 8), vehicles (n = 7), traps (n = 7), stress (n = 6), and domestic dogs (n = 1). Surveys and sightings indicated that otters were persisting and reproducing in release areas. Reports from outside of release areas suggested that native populations along the Mississippi and Cache Rivers were increasing and expanding their ranges. Otters had colonized the Central Mississippi Landscape Management Unit, probably as the result of releases in Missouri during the 1980s. Criteria for changing this species' status from endangered to threatened have been met.

564: *+.043*

Reports suggest: that there is widespread reproductive failure in Linnaea borealis in Britain, Scandinavia, and North America. Our investigations of Scottish populations of this clonal dwarf shrub indicate that, although visited by a number of different insects, pollen transfer in this species is highly effective and principally occurs by small flies (Muscidae). However, natural levels of fruit set varied between populations (from 0% to 25.1%) and reproductive failure was most severe in populations which were composed of single clones. Microscopic examination of stigmas showed that there is no barrier to pollen flow since at least 85% of stigmas have sufficient germinating pollen to effect seed set. fluorescence microscopy of germinating pollen grains indicates high levels of pollen rejection in the style and only a small proportion of the pollen tubes were able to reach the ovary. It was concluded that lack. of xenogamous pollination limits fruit formation in populations of L. borealis. Reproductive success in an isolated population with extreme reproductive failure was restored by experimental held pollination with viable pollen imported from plants from another population. In Scotland, L. borealis occurs in small, isolated populations and restoration of reproduction can be achieved by the reintroduction of compatible mating partners. This is an important consideration for the conservation management of isolated populations since their long-term recovery may only be possible by translocation of different genotypes from elsewhere into the population. Habitat fragmentation in any part of the species range may impose a potential reproductive bottleneck by causing loss of population diversity and this could explain the low levels of seed set recorded for this species in other parts of its range.

565: *+.227*

In 1989 an experiment was started at the Bukhara Breeding Centre, Kyzylkum Desert, Uzbekistan, to discover whether zoo-bred Przewalski's horses Equus przewalskii could adapt to semi-wild desert conditions. One stallion and four mares of different ages were released into a 5126-ha fenced area and monitored over a period of 17 years. This paper presents the information gained from the studies concerning home ranges, interactions with other ungulate species, adaptation to new food resources and comparative changes in daily activity. The results of breeding and changes

in the structure of the group are also presented. The horses appeared to adapt well to the new conditions and there appeared to be no negative effects of interspecific interaction with introduced kulans Equus hemionus kulan in the release area.

566: *-.086*

The recovery of dead animals and incidental captures of live animals (= without looking for the animals) are reliable proofs of presence which may be collected on a large scale and at low cost when studying the distribution of rare and secretive species of large carnivores. We used the number of data collected per five-year period to study the long term population trend of re-introduced lynx (Lynx lynx) populations in France. Causes of death and sex-ratio of the juveniles and adults-subadults found dead in the wild were also examined. From 1974 to 1998, 52 lynx were found dead or were wounded in France and 5 lynx were caught incidentally. In the Jura mountains, the number of data collected by five-year period increased regularly, from one in 1974-1978 to 20 in 1994-1998. This trend confirmed the settlement of the lynx in the Jura mountains concurrently with the geographical expansion of the species. In the Alps, a maximum of two data were collected per period. The last data was collected in 1990, which suggests that there is no well-established population. In the Vosges mountains, two to four data were collected per five-year period since the first re-introductions of lynx in 1983. This may well reflect the onset of colonisation. In the Pyrenees, the absence of data for decades is linked with the extinction of the species. Causes of death were undetermined in six cases. Mortality was man-related, either directly or indirectly, in 83 % of the cases (n = 38). Mortality factors were road or train traffic (n = 26), illegal killing (n = 10) or secondary poisoning by anticoagulants (n = 2). Natural mortality factors (rabies, starving, etc.) were at the origin of 17 % of the cases (n = 8). The juvenile (less than or equal to 12 months) to adult-subadult (> 12 months) ratio varied with the causes of death and captures. Juveniles were more frequent (75 %) than adults-subadults in the cases of natural mortality. Adults-subadults were more frequent (60.5 %) in cases of man-related mortality. The sex-ratio did not vary with the causes of death and captures and did not differ from 1 : 1 despite a trend in an over-representation of females (28 : 20).

567: *-.060*

Trout stocking in the mid-1960s eliminated the calanoid copepod Hesperodiaptomus arcticus and other large-bodied crustaceans such as Gammarus lacustris, Daphnia middendorffiana, and Daphnia pulex from many alpine lakes in the Rocky Mountain Parks of Canada. H. arcticus frequently dominates the plankton communities of fishless lakes, preying on rotifers and nauplius larvae. Following the extirpation of H. arcticus, rotifers and small-bodied cyclopoid copepods dominate the zooplankton assemblages of alpine lakes. We studied the zooplankton community of Snowflake Lake, Banff National Park, from 1966 to 1995. H. arcticus was eliminated following stocking of the lake with trout in the 1960s. It failed to become reestablished after the disappearance of the fish population in the mid-1980s. Several species of rotifers and small-bodied crustaceans, species originally rare or absent from the plankton, became abundant following fish stocking and remained so after the fish population declined. In 1992, we reintroduced H. arcticus to Snowflake Lake. The H. arcticus population grew exponentially for 4 yr, but had not reached stable densities typical of unmanipulated alpine lakes by 1995. By 1994, however, even the small population of Hesperodiaptomus was beginning to suppress populations of rotifers, copepod nauplii, and large diatoms. Because H. arcticus is omnivorous, a simple model of cascading trophic interactions did not predict the outcome of trophic manipulations in this alpine lake.

568: *+.089*

Translocation of nuisance raccoons (Procyon lotor) is a common practice, but the fates of translocated animals after release are not known. We monitored postrelease survival rates and dispersal of radiocollared raccoons that were trapped as nuisance wildlife in suburban Chicago and translocated to a rural forest preserve (translocated urban), trapped in another wooded area and translocated to the forest preserve (translocated rural), and trapped and released in the forest preserve that served as the release site for the translocations (resident). Thirty-one raccoons were radiotracked in autumn 1993, and 45 were radiotracked in autumn 1994. We detected no differences in survival rates among the 3 treatment groups ($P > 0.05$). Resident raccoons tended to remain in the vicinity of the release site, whereas translocated raccoons left the release site within hours to days and dispersed into the surrounding area. Dispersing raccoons had high daily movement rates for the first 2 weeks postrelease but then seemed to establish new home ranges. Translocated raccoons frequently denned near human residences and in agricultural fields, whereas resident raccoons denned primarily in the forest preserve. Because translocated raccoons survived well, translocation could be an effective way to supplement depleted or reestablish extirpated populations of this species. However, translocating large numbers of raccoons for animal damage control could cause problems for other wildlife and human residents near release sites, and translocated animals could serve as vectors for wildlife diseases during zoonotic outbreaks.

569: ***+.066***

The cervids represent a complex assemblage of taxa characterized by extreme diversity in morphology, physiology, ecology and geographical distribution. Farmed species (for example red deer and fallow deer) are usually the common larger-bodied, gregarious and monotocous species that express marked reproductive seasonality in their temperate environment. Their commercial importance has facilitated considerable research into reproductive physiology and the development of assisted reproductive technologies (ART). In contrast, the remaining species, including many of tropical origin, show wide diversity in reproductive patterns, have generally received little scientific scrutiny, and include a number of endangered taxa that are reliant on ex situ conservation efforts (such as captive breeding) to ensure their survival. Domestication and ex situ management programmes have been associated with widespread translocation of various cervid species around the world, often placing the animals in environments that are not compatible with their evolved reproductive patterns. For example, the summer calving/lactation pattern of red deer, attuned to northern continental climatic patterns, is frequently misaligned with seasonal changes in feed availability in the Australasian pastoral environment. Similarly, seasonal or aseasonal calving patterns of tropical species translocated to temperate regions are usually associated with increased perinatal mortality of calves born in cool seasons. Conversely, temperate species in tropical zones may exhibit aberrant reproductive patterns in the absence of biologically significant photoperiod fluctuations. ARTs, which presently include artificial insemination, embryo transfer and in vitro embryo production, have potential application to the genetic management and population growth of various cervid species. Although application to some farmed cervid species is widespread, these technologies are rarely directly transferable from farmed to endangered species. Even within species, ART protocols developed successfully for one genotype (i.e. subspecies) may be ineffective in another (for example superovulation of red deer and wapiti). Therefore, application to genetic management of endangered species necessitates prior research into their reproductive patterns. This is often difficult because of the rarity of the animals, a lack of suitable handling facilities for the particular species, and the timid nature of the deer. More recently, however, non-invasive reproductive profiling, based on remote collection and monitoring of excreted steroid metabolites, has facilitated such research.

570: ***+.088***

The cervids represent a complex assemblage of taxa characterized by extreme diversity in morphology, physiology, ecology and geographical distribution. Farmed species (for example red deer and fellow deer) are usually the common larger-bodied, gregarious and monotocous species that express marked reproductive seasonality in their temperate environment. Their commercial importance has facilitated considerable research into reproductive physiology and the development of assisted reproductive technologies (ART). In contrast, the remaining species, including many of tropical origin, show wide diversity in reproductive patterns, have generally received little scientific scrutiny, and include a number of endangered taxa that are reliant on ex situ conservation efforts (such as captive breeding) to ensure their survival. Domestication and ex situ management programmes have been associated with widespread translocation of various cervid species around the world, often placing the animals in environments that are not compatible with their evolved reproductive patterns. For example, the summer calving/lactation pattern of red deer, attuned to northern continental climatic patterns, is frequently misaligned with seasonal changes in feed availability in the Australasian pastoral environment. Similarly, seasonal or aseasonal calving patterns of tropical species translocated to temperate regions are usually associated with increased perinatal mortality of calves born in cool seasons. Conversely, temperate species in tropical zones may exhibit aberrant reproductive patterns in the absence of biologically significant photoperiod fluctuations. ARTs, which presently include artificial insemination, embryo transfer and in vitro embryo production, have potential application to the genetic management and population growth of various cervid species. Although application to some farmed cervid species is widespread, these technologies are rarely directly transferable from farmed to endangered species. Even within species, ART protocols developed successfully for one genotype (i.e. subspecies) may be ineffective in another (for example superovulation of red deer and wapiti). Therefore, application to genetic management of endangered species necessitates prior research into their reproductive patterns. This is often difficult because of the rarity of the animals, a lack of suitable handling facilities for the particular species, and the timid nature of the deer More recently, however, non-invasive reproductive profiling, based on remote collection and monitoring of excreted steroid metabolites, has facilitated such research.

571: ***+.161***

The setting of conservation priorities within species requires explicit goals and identification of the appropriate targets for conservation. I suggest that the conservation goal should be to conserve ecological and evolutionary processes; rather than to preserve specific phenotypic variants - the products of those processes. From this perspective, we should seek to conserve historically isolated, and thus independently evolving, sets of populations (i.e., Evolutionarily Significant Units, ESU). This can require manipulation of the component Management Units (MUs), some of which may be phenotypically distinct. Here I explore the application of this approach to the design of translocations for conservation management. A process-oriented approach suggests that individuals should not be translocated between ESUs, but under some circumstances it is appropriate to mix individuals from different MUs within an ESU. These circumstances include augmentation of remnant populations that are showing signs of inbreeding depression or increased fragmentation and the use of mixed stocks for reintroductions into modified or changing environments or for introductions into novel environments. These actions are consistent with the goal of maintaining processes, but the extent to which differences in adaptation or coadaptation constrain the viability of populations subject to translocation needs further exploration. Both theory and limited experimental evidence suggests that these constraints can be overcome if sufficient genetic variation is present and evolutionary processes can operate without substantially reducing population viability. One remaining question is the extent to which genetic coadaptation, and the resulting outbreeding depression, develops along environmental gradients and how this

compares to the build-up of genetic incompatibility between historically isolated populations.

572: ***+.089***

Three new microsatellite loci were isolated and characterized in the Alpine marmot (Marmota marmota). Using these markers together with the previously published L-I microsatellite locus eight populations from Austria, Switzerland and Spain were analysed. In the autochthonous populations the expected heterozygosity values (H-c) are rather similar (from 23.1 to 30.2), whereas the introduced populations show a much broader range (from 3.4 to 60.1). Only two of the introduced populations from Austria appear to be genetically deprived. Some of the introduced populations are even highly variable, e.g., the populations from the Pyrenees and from Turracher Hohe. The allele frequencies and the occurrence of particular alleles allow for the estimation of the origins of the introduced populations. The three primer pairs designed for M. marmota proved to be also applicable for cross species amplification of the microsatellites in the North American species Marmota vancouverensis. For the study of this highly endangered species, hair samples were used for DNA-extraction and microsatellite analysis. The results obtained for the three loci reveal a severe reduction of genetic variability.

573: ***+.090***

Five microsites in shallow dystrophic waters were selected for the introduction of a critically endangered aquatic carnivorous plant Aldrovanda vesiculosa in the Trebon region in S Bohemia, the Czech Republic. The selected sites were fen pools close to two hypertrophic fishponds, Ptaci blato (four microsites) and Domaninsky tone microsite). In June 1995, 30 plants from an allochthonous population in E Poland were introduced in each of the microsites. Water chemistry and plant growth dynamics were investigated at the microsites throughout the 1995-1996 seasons, and the maximum size of the subpopulations were estimated during the 1997-1999 seasons. The selected waters may be characterized as oligo-mesotrophic. The warm and dry 1995 season resulted in a low water level (ca. 10 cm below average) and plant propagation was poor at all but one microsite. However, the rainy and colder 1996 season resulted in a high water level, and thus rapid plant propagation occurred at all four microsites at the Ptaci blato fishpond and between 841 and 2669 turions were formed. Here, the doubling time of the apices was between 13.5-19.3 days in the summer. At the Domaninsky fishpond, however, the growth was relatively poor in 1996. The overwintering rate of turions (19-100%) was found as being high enough to keep the introduced plant populations stable. At each of the four microsites at Ptaci blato, the maximum numbers of shoot apices were estimated to be between 1,000-20,000 in the 1997-1999 seasons. Depth of free water appears to be the crucial factor for the rapid growth of Aldrovanda at some selected sites; water depth below 5 cm is unfavourable. Overall, Aldrovanda was successfully established in an intensively agricultural landscape in the Trebon region.

574: ***+.118***

The Norwegian Directorate for nature management has begun planning a management strategy for the large freshwater mussels in Norway. The present paper reviews the biology of the freshwater pearl mussel Margaritifera margaritifera (L., 1758). This is a contribution to increase the knowledge of this species in Norway. The freshwater pearl mussel is an interesting species with a particular biology. It is long-lived (150-200 years), has a parasitic larval stage on fish, is an efficient filterer and stores environmental information in the shell. Despite mussel has not been subject to indepth studies in Norway. The populations of the mussel have shown a steady decrease over a long period. In many localities the populations have become extinct or the recruitment has

stopped resulting in populations totally dominated by old individuals. The explanation for the decrease earlier could be overexploitation in search of pearls in certain localities. More importantly today, however, are environmental disturbances and destruction of biotopes through acidification, extinction of fish, hydroelectric activity, eutrofication, pollution, construction of canals and closing of streams, drainage of mires, erosion and clear-cutting of forest. The net result of this influence is that the freshwater pearl mussel is listed as threatened in the Norwegian red list. Surveys of the freshwater pearl mussel have shown that the species has declined dramatically in occurrence and distribution within its zoogeographic range since the turn of the century. It has been indicated that the population has decreased by 95% in central Europe, and absence of young individuals has been observed in most of the European populations. In many cases the recruitment stopped 50-60 years ago, and the mussel may become extinct in many areas. In Sweden the mussel has been found all over its geographic range, but individual populations have declined and the species has disappeared from one third of all watercourses where it was recorded early this century. Such data are not available for Norway, but we believe the situation is very much the same only in approximately one third of the watercourses where the mussel still can be found, and the remaining populations have become fragmented. Healthy populations are only found in watercourses which are relatively undisturbed by human activity. The normal length of the freshwater pearl mussel is 10-13 cm with a maximum size of 15-16 cm. The species is normally dioecious with male and female individuals. Sperm and eggs mature in the gonads normally in summer. Sperm is released into the water by the males and reach the females' gills with the water current. The eggs migrate from the gonad to the gills where they are fertilised. After fertilisation the zygotes develop into glochidia which are stored by the female in all the four demibranchs. They develop into sessile mussels only after an obligatory parasitic larval stage on the gills of a suitable fish (salmon or trout). The water temperature determines the length of the parasitic stage, which normally lasts 8-11 months. The glochidia grow during this period (from 0.05 to 0.45-0.50 mm), and go through a metamorphosis. After the glochidia have left the fish they live submerged in the gravel the first 4-5 years after which they emerge. They can live until they reach an age of 70-150 years. A freshwater pearl mussel can filter 50 litres of water over the gills within 24 hours. Food particles are filtered out, while organic compounds are released into the environment and fall to the bottom. In this way the mussel may filter out 92-100% of the particles in the water, and act as very important natural water cleansers. This is important for may contribute to maintain large populations of freshwater fish. Since the shell of the mussel is compact, chemical compounds may be stored in the shell and may be traced even after the mussel is dead. The shell may therefore be used as an environmental archive, and element analyses may describe a watercourse over a timespan of more than a hundred years. Swedish scientists have, by this method, found radioactive isotopes from nuclear tests, mercury from industry, lead from combustion engines, pollution from mining, development of eutrofication and the industrialisation as well as acid rain. The freshwater pearl mussel lives mainly in running water and is normally found at the depth of 0.5-2.0 m, but occasionally occurs in deeper stretches. The mussel is normally present at water velocities of 0.1-0.8 m/s, but it tolerates up to 2 m/s. The number of young mussels decreases with increasing water velocity, and at 0.3 m/s the number of young mussels is close to zero. The mussel has a relatively wide temperature tolerance, and survives in temperatures close to zero as long as it does not freeze, and may tolerate up to 20degreeC for shorter periods. The freshwater pearl mussel is considered to be stenohaline and can only be found in waters with < 0.5 0/00 salt content. It avoids localities with high turbidity, and tends not to thrive in areas with high levels of humus. In adult mussels there is a clear connection between survival and pH; when pH falls below 5 a negative effect is manifest. Young individuals are even more sensitive to acidification than mussel occurs naturally in areas with low calcium content in the soil, but it may also occur in more calcareous areas. In acidified areas where liming has been carried out, positive changes has been observed with an increase in recruitment and improved growth. Added contents of phosphor and nitrogen,

as well as organic compounds, lead to poorer water quality due to an increase in eutrofication. This results in an increase in sedimentation, as well as increased oxygen consumption in the sediment. This may be detrimental to young mussels in the first few years when they live submerged in the sediment. The freshwater pearl mussel has been found to survive in water containing 4.5-14.0 mg O2/l, but it has an optimum growth at 5.5-8.5 mg O2/l. Young mussels have higher oxygen requirements than older ones. To protect the remaining populations of pearl mussel, a ban on catching and increased knowledge about its biology alongside a management strategy is necessary. A law protecting the species is not sufficient. It has to be followed by protection of remaining habitats, as well as an identification of key factors responsible for the decline in the populations. Measures to reduce erosion and securing areas with erosion will be important. Clear-cutting, removal of riparian vegetation and various other man-made alterations along rivers and streams containing mussel should be prevented. Adding of organic compounds, phosphor and nitrogen should also be considered detrimental and therefore reduced. Other measurements to protect and mussels could include introducing adult mussels or infected fish, alongside hatching and release of young mussels in suitable localities. In acidified areas liming is important to secure remaining populations or create an environment for reintroduction of mussels.

575: *+.068*

Native fish stocks in the high-altitude saline Lake Issyk-kul (altitude 1608 m) in Kyrgyzstan have been subjected to predatory pressure from the introduced Sevan salmon (Salmo ischchan gegarkuni). Due to its oligotrophic character, the lake has low fish yields. The rapid growth in human population, development of tourism around the lake, increasing eutrophication and high demand for fish have necessitated a new assessment of the existing fishery and formulation of new fishery strategies. The best response to the current trends in the changing environment is to concentrate on the development of fish stocks to satisfy recreational fishery, while gradually reducing the commercial fishery. South of Lake Issyk-kul are the high mountains of the Pamir. Four of the existing several hundred lakes, all situated from 3220 m to 4260 m altitude, have a medium productivity. Their fish yield estimates range from 20.4 to 39.6 kg ha-1. Such lakes offer good conditions for fish feeding on zooplankt on, and in some lakes the northern whitefish (Coregonus peled) have been stocked. Few of these lakes are fished at present. It has been suggested that some of the Pamir lakes serve as sanctuaries for the preservation of coldwater fish species which are threatened in their original habitat. In the River Chu (Kyrgyzstan/Kazakhstan) damming, water diversion for irrigation and increased water salinity in its lower course as a result of the reintroduction of drainage waters from irrigation, have had a negative impact on the indigenous coldwater fish stocks. The present fish production in reservoirs and fish ponds, achieved through management efforts, produces about the same quantity as before the indigenous fish loss, but at a much higher cost.

576: *+.026*

The distribution of native and alien crayfish inhabiting the British Isles is described separately for England, Wales, Scotland, Northern Ireland, Irish Republic, and some associated islands. Distribution of the single native species, Austropotamobius pallipes, is largely determined by geology, water quality and human intervention. Very large populations exist in England, Wales, and the Irish Republic, but in recent years some of these have been eliminated, particularly by crayfish plague in the 1980s. Active measures are being taken to conserve the remaining stocks of this species in line with the terms of the Rio Convention and European Directives. Since the 1970s, a number of alien crayfish have been introduced into England, Wales, and Scotland. Pacifastacus leniusculus is cultivated in a number of extensive and semi-intensive facilities.

Astacus leptodactylus is not cultivated to any extent but those caught from the wild are used for culinary purposes. Large populations of both species now exist in the wild as a result of escapes and implants, particularly in central and southern England. The wild harvest of these two species is now probably greater than that derived from cultivation. Other alien crayfish such as Astacus astacus, Procambarus clarkii, and Orconectes limosus are also present in the wild in England, and Cherax quadricarinatus is kept by aquarists. Due to the environmental impact that wild populations of alien crayfish are having in Great Britain, stringent legislation has recently been introduced to try and stem their spread and that of crayfish plague. Northern Ireland and the Irish Republic ban the import of alien crayfish, although Great Britain does not. Reintroduction programs for A. pallipes are being initiated and monitoring of populations of both native and alien crayfish are being carried out at national and local level.

577: ***+.169***

Conservation ecology is a new field which provides an interdisciplinary approach to the study of the problems of biodiversity and threats to biodiversity. This textbook is intended for ecological or environmental laboratory courses at the undergraduate level. The work contains fourteen laboratory exercises based on the RAMAS EcoLab software covering biodiversity, population growth, population parameters, density dependence, extinction, conservation of threatened and endangered species, sustainable harvesting, management of wildlife, reintroduction of endangered species, island biogeography, metapopulation biology, human population growth and consumption, and conservation decision making. The manual may also be used in conjunction with standard textbooks in the field. A CD-ROM containing the RAMAS EcoLab software program and an appendix containing the EcoLab software manual are included. EcoLab 2.0: SPECIFICATIONS: IBM-compatible computer with Windows 95, Windows, 98, Windows NT 4.0, or later. 16MB memory. Pentium or faster processor. 2MB hard disk space. DESCRIPTION: RAMAS EcoLab contains four main programs: Random Numbers; Population Growth; Age and Stage Structure; and Multiple Populations. The Random Numbers program gives a pair of uniform random numberes whenever a button is pushed. The Population Growth program allows a single population model to be constructed with no age or stage structure. The Age and Stage Structure program constructs a single population model with age and stage structure, such as Leslie matrix models. The Multiple Population program creates metapopulation models with spatial structure. Simulations using any of these programs may be run. Results of all simulations may be viewed, saved and printed. The software is user-friendly with on-line context-sensitive help from any screen. Technical support for RAMAS EcoLab is available at http://www.ramas.com; ecolab@ramas.com; and RAMAS, 100 North Country Road, Setauket, NY, 11733 USA.

580: ***-.024***

We used DNA fingerprinting in order to assess 'a posteriori' the outcome of a captive breeding and reintroduction program of guans Penelope obscura bronzina and P. superciliaris jacupemba. The reintroductions started after the reforestation of part of the area that was damaged during the construction of a hydroelectric power dam in Sao Paulo, Brazil, in a region belonging to the Atlantic Forest. Our results showed that the free-living populations that became established in the reforested areas were related to the birds of the captive breeding and reintroduction programs. The segregation pattern of paternal and maternal DNA fragments of a captive family show that at least nine independent loci were detected by each of the human minisatellite multilocus probes used in the present work (33.6 and 33.15). Even though we detected a reduction in the genetic variability of these free-living populations compared to that of the captive stocks as a consequence of their

small number of founders, the birds of both species have been breeding adequately in the reforested areas. (C) 1998 Elsevier Science Ltd. All rights reserved.

581: ***+.138***

Lumholtz's tree-kangaroo (Dendrolagus lumholtzi) and Bennett's tree-kangaroo (Dendrolagus bennettianus) are the two largest arboreal folivores in Australia and are both restricted to the tropical rainforests and adjacent forest communities in North Queensland. Both species display cryptic and secretive behaviour, and consequently are poorly studied. Bennett's tree-kangaroos are found within a relatively small area (similar to 2000 km(2)) of the Wet Tropics, however the majority of this area is within a protected 'World Heritage Area', and consequently the conservation status of this species is considered moderately safe despite its rarity. The overall distribution of Lumholtz's tree-kangaroo, which is also considered rare, covers a larger area (similar to 5500 km(2)), which also has considerable overlap with the 'World Heritage Area' (WHA). Despite this, the species appears to maintain its highest densities in forest outside the WHA conservation zone. Lumholtz's tree-kangaroos are more commonly found in the fragmented forests on the Atherton Tablelands, and are often associated with remnant and secondary rainforests on basalt soils. These forest communities are considered rare and are poorly represented in existing reserves. This paper considers the conservation issues for both of these species, but focuses particularly on Lumholtz's tree-kangaroos for which more data is available. Issues discussed include the loss of habitat outside the World Heritage Area, road deaths of animals, predation by canids, traditional hunting, and fecundity and recruitment. Measures for the conservation of these species are suggested, including measures for the retention-of habitat on private land, public education, translocation and captive breeding. The applicability of-research on Australian tree-kangaroos to the conservation of the eight species of tree-kangaroos in Papua New Guinea is also considered. (C) 1998 Published by Elsevier Science Ltd. All rights reserved.

583: ***+.098***

Reproduction of Megalonaias nervosa (Rafinesque, 1820) has not been documented for over 20 y in much of the Cumberland River, where water temperatures have decreased and flow regimes have been greatly altered by hypolimnetic discharges from impoundments. Studies in other streams have implicated low temperatures or changes in discharge patterns as causative factors inhibiting reproduction, Megalonaias nervosa were collected from the Cumberland River, translocated to the Tennessee River, and held in an embayment of Kentucky Lake. After the first and second y samples of M. nervosa were taken from the Cumberland River, an existing population in Kentucky Lake, and the translocated group. Histological examination indicated that translocated mussels had a high incidence of hermaphroditism, and like mussels originating in Kentucky Lake, had undergone an otherwise normal reproductive development. Individuals functioning successfully as females from the translocated group had mature glochidia in their marsupia. Females from the Kentucky Lake sample also had mature glochidia present. In contrast, there was no indication of reproductive activity in gonads or marsupia of individuals collected from the Cumberland River. Our results indicate that a return to a more natural temperature regime in the Cumberland River would reinstate a normal reproductive cycle. We suspect that the altered temperature regime is also disrupting the gametogenic cycle of all mussels, including at least six federally listed endangered species occurring in the Cumberland River. These relic populations will disappear unless they are translocated or the thermal regime returned to normal.

584: ***+.092***

Reproduction of Megalonaias nervosa (Rafinesque, 1820) has not been documented for over 20 y in much of the Cumberland River, where water temperatures have decreased and flow regimes have been greatly altered by hypolimnetic discharges from impoundments. Studies in other streams have implicated low temperatures or changes in discharge patterns as causative factors inhibiting reproduction. Megalonaias nervosa were collected from the Cumberland River, translocated to the Tennessee River, and held in an embayment of Kentucky Lake. After the first and second y, samples of M. nervosa were taken from the Cumberland River, an existing population in Kentucky Lake, and the translocated group. Histological examination indicated that translocated mussels had a high incidence of hermaphroditism, and like mussels originating in Kentucky Lake, had undergone an otherwise normal reproductive development. Individuals functioning successfully as females from the translocated group had mature glochidia in their marsupia. Females from the Kentucky Lake sample also had mature glochidia present. In contrast, there was no indication of reproductive activity in gonads or marsupia of individuals collected from the Cumberland River. Our results indicate that a return to a more natural temperature regime in the Cumberland River would reinstate a normal reproductive cycle. We suspect that the altered temperature regime is also disrupting the gametogenic cycle of all mussels, including at least six federally listed endangered species occurring in the Cumberland River. These relic populations will disappear unless they are translocated or the thermal regime returned to normal.

585: *+.021*

The adjustment of captive-reared and developmentally deprived ringtailed lemurs (Lemur catta) to supported release on St. Catherine's Island, Georgia, was studied over 7 years to examine if these animals developed behavior comparable to wild populations. Initial changes after release included decreased obesity and increased agility as well as foraging for appropriate novel plants. Ranging, daily behavior cycles, and vocalizations developed more slowly over 1-3 years, but eventually the behavior resembled that of wild groups. Group composition and social structure changed through conflict to resemble wild and captive troops in social organization, including the emergence of matrilineal dominance and male emigration. Since behavior eventually resembled that seen in the wild, some resilience of species-typical wild behavior in captivity is supported. (C) 1999 Wiley-Liss, Inc.

586: *+.094*

The introduction of rare and threatened species on new or previously occupied sites was examined. Results point to both the success (Gentiana pneumonanthe, Littorella uniflora, Polygonum bistorta, Senecio aquaticus), uncertainty (Pedicularis palustris) and failure (Alopecurus bulbosus) of establishment. Population dynamics, niche requirements and life properties of species are sensitive indicators of ecological prerequisites of several communities e.g. site conditions and management practices. Rarity and limiting dispersal of target species require the systematic (re-) introduction by man to preserve local populations even under improved environmental conditions.

587: *+.114*

Mitochondrial DNA (mtDNA) control region sequences were analysed to determine the geographical genetic structure of Hippotragus niger (sable antelope) and H. equinus (roan antelope). Analyses by AMOVA, minimum evolution networks, parsimony, neighbour joining and maximum likelihood show that the roan antelope populations are geographically partitioned and that their phylogeography corresponds well with subspecific boundaries. In sharp contrast,

however, our sequence data group all the sable antelope into two well-delineated maternal clades. One comprising specimens from the strictly east African H.n. roosevelti, and a second geographically diverse group which includes H.n. niger (South Africa, Zimbabwe), H.n. variani (Angola), and H.n. kirkii (Zambia, Malawi). The differences in the genetic population structure suggest that distinct extrinsic and intrinsic factors have shaped the phylogeography of these two closely related antelope species. In the absence of obvious extrinsic barriers to gene flow, we suggest that behavioural differences may be responsible, at least in part, for the contrasting spatial patterns detected among geographical localities. Although our data reflect only the maternal phylogeny of these important game species, they nonetheless suggest that different management strategies should be applied. We recommend that roan antelope be translocated only within the currently defined subspecies boundaries, while in the case of sable antelope, conservation measures should focus on preserving the genetic integrity of the east African and southern African sable populations. Implicit in this is that translocations of animals between populations comprising these two distinct maternal genetic clades should be actively discouraged.

588: *+.187*

Reintroduction of animals is becoming increasingly popular as a means of restoring populations of threatened species. Sometimes depletion of wild populations leaves only captive populations from which reintroduction projects can obtain founders for releases. The World Conservation Union guidelines on reintroductions recommend that the individuals to be reintroduced should be of the same subspecies as those that were extirpated. In some cases, however, a subspecies may have become extinct in the wild and in captivity A substitute form may then be chosen for possible release. Such substitutions are actually a form of benign introduction. Considerations include assessment of the value of a substitution project and the selection of a suitable substitute. Species substitutions increase biodiversity, conserve related forms, improve public awareness of conservation issues, educate the public, and may be implemented for aesthetic or economic reasons Selection of a suitable substitute should focus on extant subspecies and consider genetic relatedness, phenotype, ecological compatibility, and conservation value of potential candidates. An example of a substitution project is the reintroduction of the North African Red-necked Ostrich (Struthio camelus camelus) into areas once occupied by the now extinct Arabian Ostrich (Struthio camelus syriacus). S. c. camelus was chosen as a substitute because of its geographic proximity, phenotypic similarity, and conservation value The World Conservation Union's reintroduction guidelines should be consulted before a project is begun.

589: *+.196*

An early motivation for this study was the problem of relocation of scarce or endangered species of animals for breeding and/or reintroduction to establish new populations in the wild. In this paper, we introduce single and multiple objective optimization models which are designed to comprehend a wide variety of objectives which are of interest to conservation and wildlife managers. We present the models in a general way and point out special features relative to ecology as they arise. Thus, the models may be used for relocation decisions analysis in diverse fields, not only in conservation and ecology. After presentation of the model in such a general way, we reformulate the models to make use of the special structure present. Such reformulation reduces the number of decision variables and constraints and, in general, makes solutions easy to obtain. By easy to obtain, we mean that tools from linear and mixed-integer programming together with elementary sorting procedures provide the basis for solving the models. In order to illustrate the capabilities of the models and solution techniques developed, we present the results of their application to the real-life relocation problem arising while analyzing restoration of the globally

endangered Przewalski's horse population. (C) 1999 Elsevier Science Ltd. All rights reserved.

590: ***+.112***

Understanding the evolutionary role of gene flow is pivotal to the conservation of endangered populations. Gene flow can be enhanced through population translocations that are conducted to maintain genetic variation and combat the negative consequences of inbreeding depression (two of the major concerns in the conservation of subdivided or isolated populations). While researchers have given extensive consideration to the idea that gene flow can act as a creative evolutionary force by maintaining genetic variation or spreading adaptive gene complexes, the focus of this paper is to investigate gene flow as a force that constrains local adaptation. I briefly review the theoretical basis of and summarize empirical studies that indicate gene flow can constrain local adaptation, and may thereby lower short-term population fitness. This review suggests that knowledge of gene flow rates and understanding ecological differences among populations is necessary before embarking on a program to artificially enhance gene flow. (C) 1998 Elsevier Science Ltd. All rights reserved.

591: ***+.032***

The range of the tammar wallaby (Macropus eugenii) has decreased substantially in Australia in recent times. Because two introduced populations in New Zealand may have descended from a now-extinct Australian population(s), we investigated their origins using a novel approach. We combined data from seven highly polymorphic microsatellite markers with simulation modelling to infer the history and conservation value of the New Zealand populations. The microsatellite alleles in one New Zealand population (Rotorua) are merely a subset of those in the other (Kawau Is.), suggesting Rotorua had no immigrants from an independent source. However, in the Kawau Is. population (sample size 33-37), 37 alleles were absent that were present in the Kangaroo Is. (South Australia) population at frequencies ranging from 1-40%. The probability of those alleles being lost during a founding event from Kangaroo Is. was investigated using a program that simulates the genetic consequences of population size bottlenecks. Use of realistic bottleneck parameters resulted in probabilities approaching zero that the observed allele deficiencies in the Kawau population could have occurred randomly, identifying the New Zealand tammar wallabies as descendants of a distinct taxon that is no longer extant in Australia.

592: ***+.136***

Habitat characteristics of wetlands that contain the federally endangered sedge, Scirpus ancistrochaetus, were investigated in a one-point-in-time held survey. Sixteen adjacent seasonal ponds, four of which supported populations of S. ancistrochaetus, were sampled for 26 habitat variables. Using only five of these variables, we were able to separate ponds with and without S. ancistrochaetus in a linear discriminant analysis. These five variables are wetland area, percent cover of overlying forest canopy, soil percent organic matter, soil exchangeable [Na], and water [H-]. Ponds containing S. ancistrochaetus had higher soil organic matter content, higher soil [Na], greater area, lower water [H+], and lower percent forest canopy cover compared to ponds that did not support this species. This information can be used toward understanding factors that control the distribution of this species and also toward evaluating possible habitat fur reintroduction of this endangered species.

593: ***+.176***

Fifty-three captive-bred New Zealand Shore Plover (Thinornis novaeseelandiae) were released on Motuora Island in the Hauraki Gulf, New Zealand in an attempt to establish a second population of this endangered shorebird in the wild. The birds were liberated in four releases between September 1994 and February 1997. In September 1997, eight (15%) of the released birds were still resident on Motuora Island. Dispersal to the mainland was the principal known cause of loss of birds from the island, with predation being the next most important cause. Differences were found between the use of adult and juvenile birds for release but there did not seem to be any difference between using hand- or parent-reared birds. Possible seasonal patterns of disappearance may become clearer once more birds have been released on the island. Recommendations for future management and research include continuing the transfer programme to Motuora Island with intensive monitoring during the first month after release, inclusion of more adult birds in releases, release of both hand- and parent-reared captive birds and conducting more research into Morepork predation of Shore Plover.

594: *-.011*

The return of the Arabian oryx Oryx leucoryx to Oman symbolized the success of a new approach to species conservation and established reintroduction as a conservation tool. Ten years after the species had been exterminated in the wild by poaching, the first 10 founder oryx, descendants of the 'World Herd', were reintroduced to the desert,in central Oman in January 1982. A second release followed in 1984 and the population grew slowly through a 3-year drought that was broken by rain in. June 1986. Further years of good rainfall and more founders meant that by April 1990 there were over 100 oryx in the wild, independent of supplementary feed and water, and using a range of over 11,000 sq km. At that time a new monitoring programme was implemented that allowed the transition from individual- to population-based monitoring and management. The population continued to grow and by October 1995 numbered approximately 280 in the wild (of which 22 were surviving founders) and used over 16,000 sq km of the Arabian Oryx Sanctuary. However; in February 1996 poaching resumed and oryx were captured for sale as live animals outside the country. Despite the poaching the population continued to increase and by October 1996 was estimated to be just over 400. However, poaching intensified and continued through late 1996 and 1997. By September 1998 it had reduced the wild population to an estimated 138 animals, of which just 28 were females. The wild population was no longer considered viable and action was taken to rescue some of the remaining animals from the wild to form a captive herd.

595: *-.267*

The Chinese alligator Alligator sinensis is one of the world's most endangered reptiles. At one-time widespread throughout much of the lower Yangzi River basin, the remaining wild individuals are now restricted to a-small area in Southern Anhui Province and perhaps:in adjacent Zhejiang Province. Population estimates conducted in the 1980s suggested that only 500-735 wild individuals remained at that time. Current figures suggest that the wild population is c. 400 individuals and continues-to decline. The principal. factor contributing to historic population decline has been habitat loss, but deliberate killing of alligators and the heavy use of-pesticides have also had significant negative effects; The current conservation programme in Anhui Province is based on captive breeding and the establishment of a reserve for small groups of wild alligators. However, the inferred recent decline in the size of the wild population suggests that the reserve design is inadequate for the long-term survival of alligators. Programmes to survey the status of the remaining wild populations and evaluate the feasibility of establishing new wild populations by reintroducing captive-bred animals are currently being developed.

596: ***+.022***

African wild dogs Lycaon pictus have been extirpated across most of West and central Africa, and greatly depleted in eastern and southern Africa. Given an urgent need for population recovery, especially in West and central Africa, this paper discusses the possibilities for using reintroduction to re-establish wild dog populations. Reintroduction is probably now technically possible, as long as release groups include wild-caught animals; several past attempts failed because captive-reared animals lacked skills needed to survive in the wild. However, reintroduction has only a limited role to play in wild dog conservation. ideally, it should involve animals of the appropriate local genotype. Limited genetic data indicate that wild dogs from West and central Africa may be distinct from those in eastern and southern Africa. Because there are no wild dogs with West or central African genotypes in captivity, and no wild populations in the region large enough to be harvested for translocation, future reintroductions might have to use animals with non-native genotypes. In addition, there appear to be no suitable sites for wild dog reintroduction in West or central Africa, and few in eastern and southern Africa. Releases currently planned in the Republic of South Africa will be locally valuable, but will not establish a population likely to remain viable without intensive management in perpetuity. For these reasons, protecting remaining wild dog populations currently represents a better investment than any attempt at reintroduction.

597: ***-.003***

Before-and-after surveys at several southern California sites indicated that populations of endangered tidewater goby Eucyclogobius newberryi persisted through heavy flooding in 1995. This was contrary to our expectations that flooding might have led to extirpation in some smaller wetlands. There was also no significant change in tidewater goby density before and after the flooding. Several apparent recolonization events coincided with the flood, suggesting that flooding may be important for the long-term persistence of the species.

598: ***+.103***

Sophora toromiro was endemic to the Pacific island of Papa Nui (Easter Island) and is extinct in the wild. The species has survived in private and botanic gardens. The species is the subject of an international study to support its conservation ex situ and eventual reintroduction. As a contribution to this study the genetic diversity of the surviving stocks and herbarium samples has been assessed by means of random amplified polymorphic DNA (RAPD) and inter-simple sequence repeat (inter-SSR) analysis. The analysis indicated low levels of genetic diversity suggesting a very small number of founders. In addition, misidentified trees were identified allowing their removal from the conservation project.

599: ***-.132***

Metapopulation structure of species in fragmented landscapes is ultimately the result of spatial variability in demographic processes. While specific information on demographic parameters is desirable, a more practical approach to studying metapopulations in fragmented landscapes may begin with analyses of species' occurrence in relation to large-scale habitat variability. Here, we analyzed occurrence of stream-living bull trout (Salvelinus confluentus) in relation to physical, biotic, and geometrical characteristics of habitats. Bull trout occurrence was analyzed at several spatial (10(chi) m) scales. Data were from nested sampling of 720 sites (10 m), 179 reaches (10(2) m), and 81 patches (greater than or equal to 10(3) m) of stream habitats within the Boise River basin of central Idaho. Based on previous findings, patches were defined as stream catchments

with suitable conditions for spawning and rearing of bull trout (>1600 m elevation). Patch-scale bull trout occurrence was significantly related to patch area and isolation (stream distance between occupied patches). Lack of spatial autocorrelation between patches indicated that isolation effects were more likely the result of limited interaction among habitats (such as dispersal), rather than of correlated environmental conditions. A third factor, human disturbance in the form of roads, was associated with reduced bull trout occurrence at the patch-scale. Analyses of occurrence among reaches within occupied patches showed bull trout may select larger (>2 m width) stream habitats. Occurrence of bull trout was not associated with nonnative brook trout (Salvelinus fontinalis) at large (patch), intermediate (reach), or small (site) spatial scales. Definition of a metapopulation structure for bull trout in the Boise River basin was complicated by uncertainties in the frequency and magnitude of dispersal. From the distribution of patch sizes and isolation among occupied patches, we suggest that the metapopulation is a complex mosaic of several elements found in conceptual models. This complexity poses a challenge to empirical and theoretical attempts to study stream-living bull trout. Future work to define the structure of bull trout metapopulations must relate temporal and spatial patterns of patch occupancy with complex patterns of dispersal that likely interact with habitat spatial structure, life history variability, and the historical context of regional climate changes. Results of this work suggest that conservation of bull trout should involve protection of larger, less isolated, and less disturbed (as indexed by road densities) habitats that may serve as important refugia or sources of recolonization. Bull trout populations in smaller, isolated, and more disturbed habitats may be at risk of extinction. Finally, metapopulation structure implies the existence of suitable, but presently unoccupied habitat, which should be managed carefully to facilitate potential natural recolonization or reintroductions of bull trout.

600: *+.159*

Indigenous populations of the North America river otter (Lontra canadensis) were extirpated from Indiana by 1942. To initiate a statewide restoration program, 25 otters (15M:10F) from Louisiana were released at Muscatatuck National Wildlife Refuge in southern Indiana. We evaluated their post-release survival, movements, and reproductive activity using radiotelemetry with 15 animals (9M:6F), field surveys, and observations for >1 year. Cause of death for 4 radiotransmittered otters (2M:2F) and 1 non-radiotransmittered male included collisions with vehicles (n=3), research-related factors (n=1), and unknown causes (n=1). Survival for radiotransmittered otters was 71% during the first year following release. We monitored 14 otters (8M:6F) for 235.1+/-112.9 days and obtained 1,218 radio locations ((x) over bar=87.0/otter; range 24-144). Adaptive kernel home ranges averaged 35.1 (SD=26.4), 10.9 (SD=8.0), and 3.0 (SD=2.6) km(2) for the 95%, 75%, and 50% utilization distributions, respectively. Otters dispersed an average of 2.8+/-1.5 km, but distance traveled did not differ between sexes. Twelve of 15 otters known to have survived >100 days established 50% core areas on the refuge or adjacent waterways. Extensive intrasexual ((x) over bar=59% between males; (x) over bar=36% between females) and intersexual overlap ((x) over bar=48%) of these areas, sightings of otter groups, and concurrent use of common den sites suggest a high degree of post-release interaction among radiotransmittered animals. Recruitment was not confirmed in the first year, but 3 litters were documented in the second reproductive season after release. Data obtained since this study indicate that otters persist in the release area and have expanded to adjacent portions of the watershed.

602: *+.162*

A genetic map was constructed from an F-2 population of 76 individuals for the purpose of comparing the arrangement of loci in the A and E Solanum genomes. This progeny was derived from an interspecific cross between the species Solanum palustre x Solanum etuberosum, both of

which are E-genome species. Two hundred and eighty one probes previously mapped in tomato and potato (A-genome, as postulated for diploid cultivated potato species by Matsubayashi 1991) disclosed 109 segregating loci in this population. Of these, 80 loci were linked in 19 linkage groups covering a total of 720.4 cM, with an average of 9 cM between markers. Although the genetic map of the E-genome showed conservation for most linkage groups with those of tomato and the A-genome, various translocations and possible inversions and transpositions were detected. It is evident that the accumulation of these structural changes in the E-genome is sufficient to cause the observed hybrid sterility. The major rearrangements in the E-genome included multiple translocations involving mosly linkage groups 2 and 8. Also a transposition was detected on group 9, with the same group-10 inversion distinguishing potato from tomato. Definitively groups 2, 8, 9 and 10, and possibly groups 1, 4 and 12, in the E-genome are structurally different from their homologues in the A-genome. In general, recombination values were larger in the E- than in the A-genome. The extensive structural differentiation of the E-genome with respect to that of potato and tomato justifies its present designation as a different genome, which is supported by previous chromosome-pairing studies. The difficult introgression of desirable traits from the Etuberosum species into potato can be explained by these structural differences.

603: *+.081*

The Mariana crow (Corvus kubaryi) is an endangered species that is restricted to the islands of Guam and Rota in the Mariana archipelago. Predation by the introduced brown tree snake (Boiga irregularis) has decimated bird populations on Guam, and the crow population there is the last wild remnant of the endemic forest avifauna. The population on Guam is critically endangered and, despite intensive management, the population has continued to decline. Additional management options include intermixing the Guam and Rota populations, but such options are best evaluated within a population genetics framework. We used three types of molecular markers to assay genetic variation in the Mariana crow: mitochondrial DNA (mtDNA) sequences, minisatellites and microsatellites. The two populations could be differentiated by mtDNA sequencing and they differed in allele frequencies at nuclear markers. Thus, the populations could be designated as evolutionarily significant units. However, the Guam population is genetically more diverse than the Rota population, and its survival probability if managed separately is very low. All markers did indicate that the two populations are closely related and separated by a shallow genealogical division. Intermixing the populations is justified by two rationales. First, the apparent population differences may result from recent human activities. Second, a greater amount of genetic information may be preserved by joint management. The translocation of birds from Rota to Guam has begun, but strategies that will ensure maintenance of the variation in the Guam population warrant further exploration.

604: *+.123*

Behavioral theories, insights, and techniques are too frequently ignored by conservation biologists. Yet an animal's survival and reproductive success clearly depend on its behavior. Using examples from marine, freshwater, and terrestrial realms, I assert that behavioral information is invaluable in five conservation areas: (1) managing wild species (e.g., designing marine reserves; reducing animal-human conflicts; understanding and managing species' responses to human-induced environmental stress such as fishing, introduced species, and chemical, visual, and acoustic pollution); (2) actively reversing the decline of imperiled wild species (e.g., reducing by catch by improving selectivity of fishing gear; re-establishing breeding populations and boosting reproduction); (3) assessing biodiversity (e.g., modeling population viability; censusing and

monitoring populations and species); (4) captive breeding and reintroduction programs (e.g., minimizing loss of valuable phenotypes; teaching or maintaining valuable survival skills); and (5) changing human behavior in resource exploitation (e.g., using principles from social psychology). Both realized and potential applications to fishes are stressed. Finally, behavioral diversity, a valuable but neglected element of biodiversity, needs to be explicitly conserved to maintain diverse populations. Arguments are presented that the conservation of species diversity and genetic diversity alone does not necessarily protect important behavioral diversity. The maintenance of both individual and population variability may be essential for the preservation of a species.

605: *+.114*

Understanding the ecology and behavior of endangered species, such as the tidewater goby, Eucyclogobius newberryi, is important for identifying problems and formulating solutions for species recovery. The tidewater goby forms isolated populations in California's coastal lagoons, creeks, and marshes. Rapid declines in the number of populations led to its listing as an endangered species in 1994. This benthic fish prefers stillwater habitats and feeds on small invertebrates. It is an annual species with an extended breeding season. Fish are larger in marsh habitats than in lagoon or creek habitats. The male digs a spawning burrow, preferably in sand, where he provides care for a single clutch. The tidewater goby is sex-role reversed: females compete more intensely than males for access to mates. The tidewater goby is a species at risk, in part due to narrow habitat: preferences, isolation of populations, short lifespan, lack of marine dispersal, and vulnerability to introduced predatory fishes. Attributes that favor its recovery include euryhaline tolerances, rapid reproductive rate, its potential for opportunistic feeding, and the possibility of natural recolonization under certain circumstances. Potential conservation measures include protecting coastal marshes that adjoin creeks and lagoons, maintaining natural hydrologic regimes, preventing artificial breaching of the sandbar at the estuary's mouth, and preventing introductions of predatory fishes. Captive breeding and reintroduction of tidewater gobies are potential tools for recovery, provided that underlying problems of habitat availability and suitability and issues of genetic integrity and disease transmission are addressed. Further research into the tidewater goby's utilization of marsh habitats, dispersal mechanisms, response to artificial breaching events, and metapopulation genetics would provide additional information for management.

606: *+.167*

Species recovery efforts generally focus on in situ actions such as habitat protection. However, captive breeding can also provide critical life history information, as well as helping supplement existing or restoring extirpated populations. We have successfully propagated nine species in captivity, including blackside dace, spotfin chubs, bloodfin darters, and boulder darters. Threatened blackside dace, Phoxinus cumberlandensis, were induced to spawn in laboratory aquaria by exposing them to milt from a reproductively mature male stoneroller, Campostoma anomalum or river chub, Nocomis micropogon. The latter are nest-building minnows, with which Phoxinus may spawn in nature. Eggs are broadcast among gravel and pebbles. Blackside dace individuals reared in captivity were used for translocation. Threatened spotfin chubs, Cyprinella monacha, fractional crevice spawners, deposited eggs in laboratory aquaria in the spaces created between stacks of ceramic tiles. Captively produced spotfin chubs were used as part of a larger stream restoration and fish reintroduction project in the Great Smoky Mountains National Park. The bloodfin darter, Etheostoma sanguifluum, was first used as a surrogate to develop techniques for spawning a closely related species, the endangered boulder darter, E. wapiti. Both darter

species mated in a wedge created between two ceramic tiles. Our efforts have had variable but generally high success, with survival rates of 50-90% of eggs deposited. Captive production of nongame fishes can aid recovery of rare species or populations, aid in watershed restoration, and can help to refine water quality standards. In addition, captive breeding allows discovery of important behavioral or life history characteristics that may constrain reproduction of rare species in altered natural habitats.

607: ***-.058***

Captive propagation and reintroduction programs for rare and endangered species rarely include testing of techniques prior to initiation of recovery efforts. To experimentally test tbe effects of rearing social altricial birds with or without a conspecific parental model (a puppet), we used Common Ravens (Corvus corax) in southwestern Idaho ns surrogates for tbe endangered Hawalian Crow (Corvus hawaiiensis) and Mariana Crow (Corvus kubaryi). A puppet is believed to reduce sexual and filial imprinting on human caretakers. We raised 49 ravens without a puppet and 25 ravens with a puppet. We initiated puppet-rearing when birds were 7 days of age and continued until 60 days of age when nestlings could ent well on their own. The influence of puppet-rearing was tested as part of a factorial design that also tested for the effects of conspecific tutoring and age at release. Rearing ravens with a puppet did nor affect social behaviors prior to release dispersal from the release area, or integration with wild birds after release. Ravens raised with a puppet, however, were more fearful of caretakers and more vigilant prior to release, characteristics that may have improved their chances for survival after release. If is important for, researchers to consider the species and the desired pre- and post-release behaviors to determine if rearing with a puppet is appropriate for their recovery efforts. These considerations can be taken into account by using a surrogate species prior to recovery efforts and creative management approaches in the initial phases of an endangered species recovery program.

608: ***+.189***

Zieria prostrata (Rutaceae) is known from only four headlands within a 3-km stretch of coastline in New South Wales, Australia. The species was presumed to have occurred at a headland 24 km south of its present range. We used random amplified polymorphic DNA analysis to assess patterns of genetic variation within and among the extant populations. The analysis also included an individual reputedly rescued from the now extinct population. A high level of population divergence was revealed by principal coordinate analysis and an analysis of molecular variance (AMOVA; 37% among populations). Our genetic findings provide implications for the conservation management of the species. First, the loss of any one population would lead to a severe loss of genetic variation. Second, an adequate ex situ collection must sample the full range of genetic diversity from all populations. Third, the consequences of mixing populations may be an important conservation consideration if further translocations proceed. Fourth, the individual apparently sampled prior to its population extinction is genetically similar to individuals from one of the extent sites. This degree of similarity was unexpected and, after further investigation, led to the conclusion that prior existence of the species at the site is doubtful. Subsequently, a planned reintroduction program was abandoned. So far, of these four management implications, only the last has had a direct management outcome. These implications that failed to lead to practical management outcomes did so because the same management recommendations could be obtained without genetic research. Clearly, the challenge for more effective conservation managers. This may be best achieved by assessing the outcomes of genetic studies already conducted.

610: ***+.105***

We evaluated translocation as a technique for restoring the Florida scrub-jay (Aphelocoma coerulescens) to unoccupied portions of its historic range. Prior to the breeding seasons of 1989, 1990, and 1995, we translocated 20 jays (14 nonbreeders, 3 breeding pairs) from Archbold Biological Station in Highlands County to suitable but unoccupied habitat at Rookery Bay National Estuarine Research Reserve in Collier County. Nine of the 20 jays disappeared within the first 8 weeks following translocation; 11 jays settled at Rookery Bay. Successful nesting first occurred at Rookery Bay in 1989, and from 1991 through 1997 the population remained relatively stable with 6-10 individuals and 1-3 breeding pairs. Nesting success, juvenile survival, and adult survival of jays in the reintroduced population were all comparable to those of jays at Archbold Biological Station. The sex ratio of the reintroduced population was biased toward males, apparently because of high rates of mortality or emigration by females fledged at Rookery Bay Overall, our results suggest translocation is a useful management technique for die Florida scrub-jay. However, because of the scarcity of properly managed translocation sites, the initially high rates at which jays emigrate or die following translocation, and the potential effect of translocation on source populations, translocation should not be viewed as an acceptable substitute for the management of existing populations of this threatened species.

611: ***+.084***

The effects of demographic isolation may be particularly severe in small, isolated populations of the endangered red-cockaded woodpecker (Picoides borealis). Augmentation of single adult woodpeckers with subadult birds of the opposite sex allows managers to stabilize small, isolated populations but does not provide a means to significantly increase populations. The reintroduction of pairs of subadult red-cockaded woodpeckers into unoccupied habitat provides a technique to bolster small populations. We report the results of such efforts to increase a small, isolated red-cockaded woodpecker population in eastern Texas, and we describe postrelease movements of translocated red-cockaded woodpeckers. Seventeen red-cockaded woodpeckers (9 M, 8 F) were translocated to the Sabine National Forest in eastern Texas between December 1994 and March 1995. Prior to translocations, this forest contained 13 groups of red-cockaded woodpeckers. Five pairs, consisting of a subadult male and female, were released to attempt to establish new breeding pairs. Seven additional subadult woodpeckers were translocated to provide mates to solitary individuals. Nine previously unoccupied sites were occupied. Of the 17 woodpeckers translocated, 12 (71% 6 M, 6 F) were established in territories following the 1995 or 1996 breeding seasons. The remaining 5 woodpeckers were unaccounted for. Of the 12 woodpeckers resighted, 3 (18%) established territories at their release sites. Woodpeckers that dispersed from their release site were relocated in sites an average of 2.8 km (range = 0.5-9.6 km) away. One (20%) of the 5 pairs released remained together into the 1995 breeding season. Eight (89%) of the 9 new pairs found during 1995 and 1996 included at least 1 translocated red-cockaded woodpecker and bred during 1995 or 1996. Our results demonstrate that the direct reintroduction of multiple pairs is an effective technique for reestablishing breeding units in formerly vacant habitat. Our results also suggest the reintroduction of pairs in a spatial array dense enough to allow social contact between adjacent pairs and with preexisting clusters substantially increases the formation of new pairs.

612: ***-.064***

Ecology of the endangered Gila topminnow is reviewed, described, and reconstructed; natural conditions are placed in perspective with human-altered habitats of today. In the natural state of waxing and waning of habitat size as a function of precipitation patterns and, catastrophic events such as severe winter cold, the species in the past likely underwent dramatic expansions and contractions in population size and geographic range. Today, population expansions are unlikely

because of constraints imposed by human activities. The original patterns of dispersal from refugia in "good" times and retreat in "bad" times, if they are to occur, must be re-created through human translocation. Further, most refugia now are destroyed. or inaccessible to recolonization, so remnant, natural populations, along with established, transplanted stocks of appropriate size and genetic quality need protection. Populations also must he established and maintained in artificial refugia. Last, even if connectedness were to Le reestablished and refugia provided, intervening habitats harbor introduced piscivores such as western mosquitofish that: interdict and eat dispersing topminnows and their progeny. Elimination, exclusion, or management against such offending species is mandatory to prevent topminnow extinction and achieve recovery.

613: ***+.049***

Social system variability in a population of reintroduced Arabian oryx Oryx leucoryx in Oman was investigated from 1982 to 1989 by comparing two time periods on either side of June 1986, when widespread rain ended a prolonged period of drought and supplemental feeding. Variation in three social subsystems of resource exploitation, mating, and calf-rearing were observed. From the first release of 10 oryx in 1982 until June 1986, median herd size increased over time, while average group size increased as forage conditions deteriorated after rain. In addition, within each herd subordinate adult males were tolerated by dominant males, only a few dominant males sired the majority of calves, and the proportion of mother/calf pairs involving older, weaned calves progressively increased. After June 1986 until the end of the study period in December 1989, these trends reversed. Median herd size decreased over time, and average group size decreased as forage conditions deteriorated after rain. Subordinate adult males were no longer tolerated within herds, but instead existed as solitary males. The concurrent increase in solitary males and the number of herds resulted in more breeding opportunities for the expanding adult male population. The mating system became more dynamic, and involved the emergence of previously unrecorded territorial behaviors. In addition? the proportion of mother/calf pairs including weaned calves decreased. As the transition from captivity to the wild may present special difficulties in the development of social systems specifically suited to local conditions,species with more adaptable social systems, such as the oryx, are likely better candidates for reintroduction. (C) 1999 Elsevier Science Ltd. All rights reserved.

614: ***+.293***

Translocations have been widely used to conserve threatened fauna worldwide, and although the benefits of monitoring newly translocated animals are widely recognised, post-release monitoring seldom occurs. This paper reports the dispersal patterns, social organisation and survival of a newly translocated population of 26 South Island saddlebacks (Philesturunus carunculatus carunculatus), on Motuara Island, New Zealand. South Island saddlebacks dispersed widely through forest on Motuara Island after release and 8-10 months after release, territory sizes ranged from 1.9-8.8 ha ((x) over bar = 4.21 ha, s = 2.42). Saddlebacks in both subadult and adult plumage attempted to breed in the first breeding season after release and an estimated 10 fledglings were produced. Maximum mortality of the translocated group was 50% 8-10 months after release. Translocations of 15-59 South Island saddlebacks have been successful in the past, suggesting the success of the transfer to Motuara Island is not jeopardised by the relatively small size of the founder group. Saddlebacks appear relatively flexible in habitat requirements and are capable of high reproductive output, enhancing the success of translocations of this species. (C) 1999 Elsevier Science Ltd. All rights reserved.

615: ***+.191***

The Mauna Kea silversword Argyroxiphium sandwicense is an endangered plant endemic to the high elevation cinder deserts of Mauna Kea on the Island of Hawaii. Restoration efforts have increased the total naturally occurring population of ca. 50 adult plants by ca. 500 individuals, primarily by the outplanting of silverswords initially grown under controlled conditions. We evaluated the direct sowing of seeds into field sites as an alternative to outplanting. Gravel-covered surfaces enhanced germination and growth of seedlings and increased soil moisture. The presence of shrubs and trees was associated with increased silversword germination but decreased silversword growth and reproduction. The best environment for silverswords varied throughout the life cycle of the plant, but critical stages are pollination, seed production, germination, and seedling survival. Sowing and outplanting each have unique advantages. High elevation, moist, gravel surfaces that can be protected from ungulate browsing should be the focus of future sowing and outplanting efforts to maximize restoration success. (C) 1999 Elsevier Science Ltd. All rights reserved.

616: *+.210*

Lake sturgeon Acipenser fulvescens declined in abundance in Lake Superior's St. Louis River during the late 1800s and were eliminated from the river during the early 1900s because of the combined effects of exploitation, pollution, and habitat alteration. Since then, exploitation in the river and in Lake Superior has been reduced. Furthermore, water quality in the St. Louis River has improved, and its upper-estuary spawning habitat has remained relatively unchanged and adequate. Lake sturgeon have been stocked annually in the St. Louis River since 1983; from 1983 to 1994 stockings included 736,000 fry, 128,000 fingerlings, and 500 yearlings of the Lake Winnebago strain. Relative abundance, distribution, and growth were determined by sampling marked fish in the St. Louis River estuary and western Lake Superior with graded-mesh gill nets and bottom trawls. During 1983-1998, 644 lake sturgeon were caught in 15,486 m of gill net, and 196 were caught in 1,200 trawl tows. Lake sturgeon wer e sampled most frequently near channelized portions of the St. Louis River and stayed in the estuary up to 5 years before entering Lake Superior. Lake sturgeon were not captured in western Lake Superior prior to stocking, but abundance increased dramatically after 1985. Of 582 lake sturgeon sampled along the Wisconsin shore of Lake Superior from 1985 through 1998 (347,000 m of gill nets), 93% were captured in less than 30 m of water. A total of 93 lake sturgeon were reported from assessment netting conducted along the Minnesota shore of Lake Superior from 1992 through 1997. The current range of stocked lake sturgeon extends from the St. Louis River 145 km east to the Apostle Islands in Wisconsin and 110 km northeast to Little Marais in Minnesota. Increases in lake sturgeon abundance were directly attributed to the stocking program. We recommend stocking a minimum of 20 year-classes and the use of a Lake Superior egg source, if possible. Final evaluation of the project will be detection of tagged lake sturgeon successfully spawning at historical spawning areas.

617: *-.080*

The small population of wild dog Lycaon pictus (n = 3 to 30) in Hluhluwe-Umfolozi Park (HUP) has declined since 1992. The survival of dogs in HUP is dependent on the reintroduction of more dogs; however, wild dog reintroduction programmes are fraught with problems and many have failed. In this paper the diet and capture success of the wild dog pack in the Hluhluwe Section, and the influence of dietary considerations on the success of the future reintroduction of wild dogs in this reserve, are investigated. Diet choice was determined from seat analysis, personal observation and field staff records. Eight ungulate prey species were identified from seat analysis: nyala Tragelaphus angasi and impala Aepyceros melampus were the most abundant ungulate species in

HUP and accounted for 77% of the diet. On the whole, wild dogs included prey types in the diet consistent with a rate-maximizing foraging approach, although some prey were dearly taken opportunistically. The dogs preyed mostly on small (<25 kg) to medium-sized (40-90 kg) prey, while the young of large (> 90 kg) prey species or scavenged carcasses supplemented the diet during the dry season. Adult nyala were taken more frequently than other age classes, but wild dog preyed on juvenile impala more than expected. Female prey were taken more frequently than males but selection did not differ from prey population sex ratios. Prey capture success was similar to that of previous studies from both open and densely wooded habitats and the wild dogs successfully caught 48% of all prey species pursued. Results suggest that wild dogs are quite capable of adapting both their diet choice and foraging technique to the dense vegetation in HUP. We conclude that prey type, prey availability and habitat constraints on prey capture success, will not affect the reintroduction of more wild dogs into HUP.

618: *-.032*

Asiatic wild asses, Equus hemionus, were driven to extinction in Israel in the early 20th century. In 1983, a herd of these animals was re-introduced to the wild in Makhtesh Ramon, a large erosion cirque in the central Negev desert, Israel. The population has grown steadily ever since and now numbers some 100 animals. In order to determine whether the wild asses are having a significant impact on the vegetation, we have monitored the plant communities in Makhtesh Ramon since 1992, using McAuliffe's log-series survey method. Our study involves 11 pairs of plots along the length of the altitudinal gradient in Makhtesh Ramon. The altitudinal gradient results in a rainfall gradient from an average of 95 mm rain per year to an average of about 40 mm per year. Each pair of plots consists of: (1) an unfenced plot, and (2) a fenced plot that excludes wild asses but not the other large mammalian herbivore, the dorcas gazelle Gazella dorcas. The wild asses have not had a significant impact on vegetation cover, species richness, diversity or dominance. Three plant species showed significant increases in percentage cover in fenced plots, while one species showed a significant increase in percentage cover in unfenced plots. Furthermore, eight plant species invaded fenced plots, three species invaded unfenced plots and one species disappeared from unfenced plots during the study. Using Detrended Correspondence Analysis, we found that the major differences among plots are due to position along the altitudinal gradient. The Detrended Correspondence Analyses indicated that the wild asses have had no significant effect on vegetation community structure.

619: *-.080*

Management practices favoring conifers at the expense of deciduous tree species, and the eradication of deciduous trees, especially aspen Populus tremula, from managed forests have resulted in population declines in several species in Fennoscandia. In addition to species depending on decaying wood of deciduous trees, earlier evidence suggests that leaf litter, especially that of aspen, is favored by many carabid species. We ran a four-year experiment in order to compare carabid assemblages of unchanged forest Boor with artificially created leaf-litter plots id central Finland. A total of 18 plots (5 m in diameter) were established in three forest stands without aspen a few kilometers apart. Each stand had 3 litter plots (litter added) and 3 control plots. Pre-treatment samples were compared with those collected after litter addition. The litter addition affected the carabid-assemblage structure by increasing the catches of some species and decreasing the catch of one species. The number of carabid species was similar in control and litter plots. The litter effect was smaller than variation among forest stands and year-to-year fluctuations. There was a strong temporal constancy among the plots: "rich" plots remained "rich" from year to year and similarly "poor" plots remained "poor". The significant influence of leaf

litter on carabid abundance can be attributable to both abiotic factors (microenvironmental conditions, especially humidity and temperature), and biotic ones (changes in niche structure, improved food supply). Leaf litter seems to have an effect on carabid distribution patterns, and deciduous trees scattered among conifers are likely to be of importance on carabid fauna in boreal forests.

620: ***+.020***

A form of lowland, sandplain Fynbos restricted to the Cape Flats near the city of Cape Town is the South African vegetation type most threatened by urban and agricultural development. Cape Flats Fynbos remnants, totaling 4.8 km(2), contain 14 plant species endemic to the Cape Flats scattered through four protected areas and 11 unprotected vegetation fragments on public land. Despite their small size and management problems, the remaining protected areas are underpressure from the public and developers to function as "lifeboats," or places of safety, for attractive indigenous plant species from the unprotected areas destined for development because the popular perception is that any indigenous plant population threatened by habitat destruction should be saved by being transplanted into a protected area. The urgency for dealing with the issue of plant rescue in the Cape Town metropolitan area was the catalyst for a workshop held at the University of Cape Town in 1998 to develop protocols for Fynbos plant species translocations. We report on points raised by managers and academics at the workshop including the selection of target taxa, individuals and establishment sites, and methods for introduction, and present case histories illustrating possible solutions to the problems encountered.

622: ***-.026***

We analysed mortality and behaviour of hihi, an endangered New Zealand honeyeater, in the first three months after translocation to 135 ha Mokoia island. Our aims were to assess: (1) whether mortality and behaviour were affected by the translocation process or post-release management, and (2) whether the fate of birds during this establishment phase affected the viability of the population. Forty hihi were translocated from the wild population on Little Barrier Island, released immediately in three different locations, and provided with sugar water feeders. Many of the birds suffered leg injuries due to the bands initially used, and up to 7 birds may have died from these injuries. Nevertheless, the mortality rate over the first three months was similar to the average rate over the first 3 years. Therefore, except for the bands used, there was no evidence of post-release mortality associated with translocation stress. Most hihi discovered the feeders quickly. However, feeder use varied greatly among birds and there was no evidence that access to feeders reduced mortality. Access to feeders also did not affect overall time spent foraging. However, birds using feeders allocated most of their foraging effort to invertebrate feeding, whereas birds not using feeders foraged mostly on flowers and fruits. Hihi dispersed quickly after release, and moved ail over the island. Transmitters increased re-sighting rates over the first 3 weeks, but intensive observation during that period provided no useful information relevant to subsequent survival and reproduction. There was a slight tendency for birds to settle closer to their release sites than expected by chance, but there was no tendency for birds released together to form breeding pairs. We conclude that the viability of this population was not affected by any problems in the establishment phase. However, the population has had a consistently high mortality rate over the first 3 years, and its long-term viability appears poor. Our subsequent research is, therefore, addressing the factors that might be limiting the population in the long-term. (C) 1999 Elsevier Science Ltd. All rights reserved.

623: ***+.171***

Lacerta schreiberi is an endemic lizard in the northwest of the Iberian Peninsula, with some isolated populations in the south. The aims of this work are to select priority areas for the conservation of the species, identify extinction risk areas, evaluate the current degree of protection and define a strategy for the conservation of this species in Portugal. We used an evaluation model of areas in a GIS environment. Conservation priority areas represent 15% of the distribution area of the species in Portugal. Populations with high risk of extinction are located primarily along the littoral strip. About 50% of the conservation priority areas are already included in the Portuguese protected areas (PPA). The conservation strategy for the species needs efficient protection of habitats, increase of PPA and creation of Regional Reserves for the littoral populations. For the isolated populations we propose five types of management actions: habitat restoration, supplementation of populations, reintroduction of individuals, captive breeding and population monitoring. (C) 1999 Elsevier Science Ltd. All rights reserved.

624: *+.090*

The introduction of plants into secure, managed reserves is seen as vital to the conservation of many grassland species that are now confined to small, fragmented remnants in western Victoria, Australia. The survival (> 5 yrs after planting) of reintroduction plantings conducted at three sites were assessed and success related to plant and site attributes. Of the 151 plant populations, 48 species and 10 699 plants introduced by tubestock plantings to these sites over the period 1985-1992, 48 plant populations, 19 species and 1285 plants remained at November 1997. No single plant or site attribute explained success or failure across the sites, suggesting that reintroductions will present many novel challenges to practitioners. Interestingly, threatened species proved no more difficult to establish than non-threatened species. Second generation seedling recruitment was exceedingly rare: only five species in a total of 16 populations produced successful seedling cohorts and only three species (Acaena echinata, Caesia calliantha and Podolepis sp. aff. jaceoides) are considered to be established in at least one site. Failure of most populations to recruit threatens the longterm persistence of reintroduced plantings and suggests that the conservation and management of remnant populations in situ remains crucial to the survival of these species in natural habitats. Determining the requirements for seedling recruitment of reintroduced populations presents a major challenge to ecologists. Without this knowledge, plant reintroductions in western Victorian grasslands, and their implicit role as a conservation tool, are likely to remain problematic. (C) 1999 Elsevier Science Ltd. All rights reserved.

625: *+.253*

In their native Australia, tammar (Macropus eugenii) and parma (M. parma) wallabies are allopatric and differ markedly in habitat use, social structure and degree of reproductive synchrony. They occur sympatrically only on Kawau Is., New Zealand, as a result of introductions last century. Reproductive data collected from wallabies on Kawau Is. in April 1996 show that M. eugenii maintains high reproductive synchrony while M. parma remains asynchronous. Both species show sexual dimorphism and differences in habitat use as seen in Australia. In addition M. parma is more solitary, and is frequently found in closed-canopy woodland, while M. eugenii tends to aggregate in open grassy clearings. Thus the species maintain broad reproductive and biological distinctions, despite enforced sympatry in a novel environment. Highly-resolving genetic (microsatellite) data reveal no evidence for hybridization. This is essential information given the known (M. eugenii) or likely (M. parma) high conservation value of New Zealand stocks. Kawau Is. is the only predator-free site with M. parma, a species whose persistence in Australia is threatened by fox predation. Finally, we use data on mitochondrial cytochrome b sequence divergence amongst several Macropus taxa to demonstrate close agreement with degree

of reproductive isolation.

626: *+.315*

The success of captive-breeding and release programmes is often compromised by predation of released individuals, which are naive about predators. Pre-release behavioural preparation of release candidates in the form of anti-predator training has been attempted infrequently, usually using models of predators, but success was most often measured in terms of improved behavioural responses rather than survival to breeding age after release. Here we report that post-release survival of captive-reared houbara bustards (Chlamydotis [undulata] macqueenii) was improved through exposure to a live predator before release: a result with possible applications for a wide range of species currently the focus of reintroduction projects. We also show that rearing houbara with minimal human contact and training with a model of a predator had no effect on post- release survival. Moreover, neither pre-release behavioural responses to a model predator nor the degree of tonic immobility were predictors of post- release survival.

627: *-.000*

Niuafo'ou lies very isolated in the Pacific, is well forested and not densely populated by humans. These facts as well as the lack of larger rat species make it a refuge for birds rare elsewhere in the region. This paper covers all 17 breeding species and gives breeding data for 14 of them, collected from October 1991 to December 1992. Ten species had a well-defined breeding season of 2-7 months somewhere between September and April, which often differed from other adjacent islands: Audubon's Shearwater Puffinus Iherminieri, Pacific Black Duck Anas superciliosa, Banded Rail Rallus phillippensis, Spotless Crake Porzana tabuensis, Purple Swamphen Porphyrio porphyrio, Barn Owl Tyto alba, Red-vented Bulbul Pycnonotus cafer, Polynesian Starling Aplonis tabuensis nesiotes, and Jungle Myna Acridotheres fuscus. The Blue-crowned Lorikeet Vini australis nested in October, November and July. It did not only breed in tree hollows, but also inside a rotten log on the ground. Other observations suggest that it visits ground holes as well, either for nesting or resting. A breeding colony of Audubon's Shearwater is the first one confirmed for Tonga. Four species nested in the wet and dry season: White-tailed Tropicbird Phaethon lepturus, Pacific Reef-heron Egretta sacra, Pacific Pigeon Ducula pacifica and Polynesian Megapode Megapodius pritchardii. Since September to March is the main breeding season for birds on Niuafo'ou, it is proposed that hunting and egg collecting, both important parts of the local tradition, are restricted to the other months of the year. In case of the endangered Polynesian Megapode we suggest a restriction of egg collecting and propose a translocation programme to another island. Additionally, we suggest that the islets in the crater lake become protected as they are free of feral cats, and some birds occur in higher densities there. Niuafo'ou also deserves attention as resting place for six vagrant and migrant species; large numbers of Wedge-tailed Shearwaters Puffinus pacificus are hunted when they visit between October and June.

628: *+.029*

The numbat has been reduced to two populations in Western Australia. To better understand the effects of range reduction on gene now and genetic variation, and to address questions crucial for the species' management, we analysed mitochondrial DNA (mtDNA) sequences of free-ranging individuals and museum specimens. The results suggest recent connectivity between the remnant populations, although one of those may have lost significant amounts of genetic diversity during the recent population size reduction. We propose that for management purposes the remnant populations should be treated as a single historical lineage and that, subject to certain caveats,

consideration should be given to population augmentation by translocation.

629: *+.238*

Corn and rice genetic linkage map alignments were extended and refined by the addition of 262 new, reciprocally mapped maize cDNA loci. Twenty chromosomal rearrangements were identified in maize relative to rice and these included telomeric fusions between rice linkage groups, nested insertion of rice linkage groups, intrachromosomal inversions, and a nonreciprocal translocation. Maize genome evolution was inferred relative to other species within the Panicoideae and a progenitor maize genome with eight linkage groups was proposed. Conservation of composite linkage groups indicates that the tetrasomic state arose during maize evolution either from duplication of one progenitor corn genome (autoploidy) or from a cross between species that shared the composite linkages observed in modern maize (alloploidy). New evidence of a quadruplicated homeologous segment on maize chromosomes 2 and 10, and 3 and 4, corresponded to the internally duplicated region on rice chromosomes 11 and 12 and suggested that this duplication in the rice genome predated the divergence of the Panicoideae and Oryzoideae subfamilies. Charting of the macroevolutionary steps leading to the modern maize genome clarifies the interpretation of intercladal comparative maps and facilitates alignments and genomic cross-referencing of genes and phenotypes among grass family members.

630: *+.238*

The eight papers resulting from the Ecological Genetics session of the symposium Ecology of Stream Fish: State of the Art and Future Prospects (Luarca, Spain, April 1998) describe the use of molecular genetic markers to investigate questions of population distinctness, interbreeding, and adaptation in five salmonid species. Widely contrasting ecological distinctions described in four species indigenous to western North America [steelhead (rainbow) trout, sockeye salmon, pink salmon and bull trout] reflect past natural and human-induced activities, serving to guide future management and conservation actions through optimizing adaptive opportunities, and preventing genetic losses through localized extinctions. Studies of brown trout contrast widespread introgressions from exogenous hatchery introductions that threaten the integrity of native Spanish populations with a reduced threat based on use of indigenous fish in Portuguese hatcheries. A 9-year study of natural progeny of two genetically distinct Swedish brown trout populations introduced to a previously trout-free area identifies apparent local adaptations as a model to guide other translocations.

631: *+.002*

Helianthus schweinitzii (Asteraceae) is a federally endangered, endemic sunflower that only occurs in the Piedmont of North and South Carolina. This species' habitat is relegated to disturbed sites such as roadsides, utility rights-of way, edges of woods, and other open sites. We used allozyme electrophoresis to investigate the genetic variation among 25 populations throughout the range of the species. Data collected from 11 enzyme systems representing 14 loci indicated that most populations are genetically similar and that the species, as a whole, exhibits moderate variation. Genetic distances among populations were not correlated with geographic distances. This may be due to recent fragmentation of large, contiguous populations of the sunflower into isolated groups. Conservation strategies for H. schweinitzii should include preservation and/or creation of suitable habitat and translocation of threatened populations into protected areas. Our study suggests that mitigation programsare unlikely to disturb the genetic architecture of the species or result in outbreeding depression.

632: *-.041*

Translocation is often used as mitigation in cases where sites containing protected species are threatened by development. However, the conservation value of such exercises is unproven for many species. This paper describes a case study in which translocation was used as a mitigation measure for the slow-worm (Anguis fragilis) a cryptic legless lizard. At a site scheduled for development, the removal of 103 lizards over a three-month period resulted in no significant depletion of the population. At the receptor site, which had no previous slow-worm population, recaptures of translocated slow-worms declined during 2 years of subsequent monitoring; recaptured lizards were in poorer condition than those in a nearby natural population; and there was little evidence of successful reproduction. As the exercise may have prevented the inadvertent killing of a number of slow-worms, it may have been successful in terms of meeting the statutory obligations for this species. As an exercise in conserving the population in the longterm, however, the value of the translocation was questionable. (C) 1999 Elsevier Science Ltd. All rights reserved.

633: *+.085*

History and pre-history tells us that Australian mammals have fared poorly through their association with humans. First there were the Pleistocene megafauna extinctions as Aborigines colonised the continent followed later by extinction of the large marsupial carnivores after introduction of the Dingo. European colonisation first appeared to favour many native mammals but later led to declines and extinctions at an unprecedented rate. The shock waves of these massive changes are most probably still reverberating across the landscape and need consideration in our approach to species conservation. Species recovery is a new and important science. Current management efforts focus on broad scale baiting of Foxes, translocation of endangered species to offshore predator-free islands, and their protection in predator enclosures. A national and more corporate approach to species conservation has arisen from national species recovery teams. Sharing of resources, particularly technical information on threat management, is critical to effective species recovery. This is especially important in forging the desperately needed breakthrough in control of Feral Cats. History tells us that disturbance of one component of the ecosystem will often provoke changes in other areas. The current intensive focus on Foxes demands careful monitoring of the general system as an early warning of developing problems. Study of the interactions between Foxes, Dingoes, Feral Cats and Rabbits are needed to discover the likely outcomes of control programs, particularly in the more arid regions where all four species coexist. Experimental reintroduction of yet untried species occupying niches yet to be examined by this technique may expose additional but more subtle threatening processes.

634: *+.018*

At least 16 species of Australian mammals have become extinct over the past 200 years. Without islands, however, this figure would be even worse as nine species that were formerly widespread on mainland Australia were or are restricted to land-bridge islands. In addition, 13 species and subspecies of endangered and vulnerable mainland mammals that still occur on the mainland have island populations, reducing their chance of extinction. In all, 43 islands protect 29 taxa of Australian threatened mammals. Since European settlement some island mammal populations have become extinct, while many new populations, of both Australian and exotic mammals, have been established. The extinction of island native mammal populations is significantly correlated with the introduction of exotic mammals. Management of islands needs to concentrate on four areas: quarantine, monitoring (of both native mammals and possible introduction of exotics), eradication of exotics and translocations of native species. Prevention of introduction and

establishment of further exotics to important islands through quarantine procedures is vital, especially for islands with permanent or temporary human habitation. Eradication or control of existing exotics is required for many islands and eradication of further introductions, as soon after detection as possible, should be a high priority action for nature conservation agencies. Past exotic mammal eradications and needs for the future are discussed. Translocations of island mammal populations to the mainland should take place only where the species is extinct on the mainland. Translocation to islands, where translocation to or on the mainland is not feasible, is an important conservation technique. Islands with exotics can be of value for re-introduction of locally extinct mammals or introductions (marooning) of threatened species that are at risk from feral predators on the mainland once the exotics have been eliminated.

635: *+.368*

The high biodiversity and physical heterogeneity of the Klamath-Siskiyou Ecoregion of the Pacific Northwest (USA) suggest the need for an ambitious and multifaceted approach to conservation research and planning. We developed a process of reserve selection and design that proceeds along three parallel tracks: (1) protection of special elements, such as rare species hotspots, old-growth forests, and key watersheds; (2) representation of physical and vegetative habitat types; and (5) maintenance of viable populations of focal species (e.g., fisher, Martes pennanti Erxleben). Each of these complementary research tracks identified important conservation opportunities in the region. In combination they provide a basis for a reserve design and management plan that meets conservation goals better than the existing management situation established by the federal Northwest Forest Plan. Our proposed Phase I reserve design begins with protection of roadless areas on public lands that score high under the criteria of our three-track approach. A relatively small area of additional public and private land is necessary to provide habitat contiguity among roadless areas and capture remaining biological hotspots. This design would place approximately 34% of the region into the strictest category of protected areas, compared to 13% under current management, and would place an additional 19% of the region into moderate protection. A second, proposed phase of conservation would include protection of additional private lands to meet representation objectives. Also included in Phase II would be protection of linkages to other regions, necessary for long-term persistence of wide-ranging animals such as large carnivores (e.g., gray wolf, Canis lupus L.), which are being considered for reintroduction to the region. When implemented, Phase II would bring approximately 60-65% of the region into strict and moderate protection. Linkage design and ecological management (e.g., fire regimes) are among the critical topics for further research.

636: *+.010*

The elephant population of the Yala Protected Area Complex (YPC) was studied by indirect dung count method. The YPC consists of one Strict Natural Reserve, two National Parks (NP) and three sanctuaries, and occupies an area of 1,518 km2. The elephant densities in different habitats were estimated using the dung pile densities in belt transects and plots. Twelve distinct habitats were recognised. The highest density occurred in grass-scrub (1.03 km-2) and the lowest in the riparian forest (0.08 km-2). No dung piles were found in the moist semi-evergreen forest, which, however formed only a small part (about 2.2% by area) of the complex. The average elephant density of the entire YPC is estimated to be 0.51 km-2. The component reserves of the YPC had different elephant densities, the highest observed being in the Block I of the Ruhuna NP (0.80 km-2) and the lowest (0.31 km-2) in the Block IV of the Ruhuna NP, the differences being attributable to their different habitats. Therecent translocation of a large group of elephants may have increased the elephant density of the YPC to 0.66 km-2. When the habitat differences are not taken into

account, the study indicated a higher density of 0.61 km-2 than when the particular habitats were treated separately (0.51 km-2).

637: *+.012*

Predation on endangered species by nonnative red foxes (Vulpes vulpes) and the resulting controversy over red fox control efforts in California prompted our investigation of the introduction and range expansion of the red fox in California. Since the late-1800s, nonnative red foxes have been introduced into California by escaping from fur farms and fox hunters, through intentional releases by pet owners and fur-farm owners and translocations of previously introduced foxes. From 1990-1993 we conducted telephone interviews of wildlife professionals to obtain observations of nonnative red foxes outside the historical range of the native Sierra Nel ada red fox (V. v. necator). Nonnative red foxes now occur throughout lowland areas of California including the Sacramento and San Joaquin valleys, San Francisco Bay-Delta area, the southern California Coast Range and Coastal Plain and most major urban areas. Their range expansion over the last 100 y was the result of population growth from numerous points of introduction and exhibited by the exponential growth typical of invading species. Fox predation on endangered species and opposition to red fox management have been the: two largest management issues associated with this range expansion.

638: *+.004*

Karyotypic variation in five gibbon species of the subgenus Hylobates (2n = 44) was assessed in 63 animals, 23 of them wild born. Acquisition of key specimens of Hylobates agilis (agile gibbon), whose karyotype had been problematic due to unresolved structural polymorphisms, led to disclosure of a compound inversion/translocation polymorphism. A polymorphic region of chromosome 8 harboring two pericentric inversions, one nested within the other, was in turn bissected by one breakpoint of a reciprocal translocation. In double-inversion + translocation heterozygotes, the theoretical meiotic pairing configuration is a double inversion loop, with four arms of a translocation quadrivalent radiating from the loop. Electronmicroscopic analysis of synaptonemal complex configurations consistently revealed translocation quadrivalents but no inversion loops. Rather, nonhomologous pairing was evident in the inverted region, a condition that should preclude crossing over and the subsequent production of duplication-deficiency gametes. This is corroborated by the existence of normal offspring of compound heterozygotes, indicating that fertility may not be reduced despite the topological complexity of this polymorphic system. The distribution of inversion and translocation morphs in these taxa suggests application of cytogenetics in identifying gibbon specimens and avoiding undesirable hybridization in captive breeding efforts. (C) 1999 Wiley-Liss, Inc.

639: *+.133*

The prospective for using molecular approaches in conservation and traditional biological studies is reviewed. The focus is on the methods of allozyme analysis and the polymerase chain reaction with random primers (randomly amplified polymorphisms of DNA) used to study genome polymorphism and genetic structure in the populations of Panax ginseng C.A. Meyer and to tag the species in the genus Iris. The feasibility of these approaches; is discussed, in the context of developing a strategy for conservation and the reintroduction of the rare and vanishing plant species.

640: *+.189*

This study addressed the initial effects of a reintroduction of Gunnison's prairie dogs (Cynomys gunnisoni) on resident small mammal and plant communities on the Sevilleta. National Wildlife Refuge (SNWR), New Mexico. In spring 1997, 60 prairie dogs (36.8 kg live mass) were introduced onto a former prairie dog colony in a desert grassland site. Small mammals and vegetation were sampled on both a treatment (reintroduction site) and a control site (without prairie dogs) before and after the prairie dogs were reintroduced. We tested for differences in small mammal and plant community change during the 1st year of the colony's existence using repeated measures analysis of variance. Although prairie dog biomass was ca. 32 times greater than that of the resident rodent community (1.2 kg), reintroduction of prairie dogs had no significant effect on the resident small mammal and plant communities. Total biomass and abundance of rodents, and percent cover and species richness of plants did not change during the 1st year following reintroduction of prairie dogs. However, two rodent species showed significant differences in abundance between the prairie dog colony and the control site. The banner-tailed kangaroo rat (Dipodomys spectabilis) was significantly more abundant on the treatment site before and after reintroduction of prairie dogs. In contrast, the white-footed mouse (Peromyscus leucopus) was significantly more abundant on the control site following reintroduction of prairie dogs. Habitat modifications made by the former prairie dog colony may be responsible for the habitat preferences observed by D. spectabilis and P. leucopus. Although reintroduction of prairie dogs had no observable influence on the resident small mammal and plant communities in the short-term, their influences may be more evident on a long-term time scale.

641: *+.153*

The possibility of using molecular approaches to the problems of nature conservation and classical biology is considered. Special attention is paid to the use of allozyme analysis and polymerase chain reaction with arbitrary primers (RAPD) for the studies of genome polymorphism and genetic structure of Panax ginseng populations, as well as genotyping of the genus Iris. Expediency of such studies for the development of the policy of preservation and reintroduction of rare and disappearing plants is discussed.

642: *+.197*

In both conservation and biological control, translocation (the human-aided release or redistribution of individuals within a landscape) is an important management tool aimed at enhancing species persistence. Metapopulation theory, which has become a focus for understanding species' dynamics in patchy landscapes, has traditionally emphasized long-term and equilibrial dynamics rather than the consequences of a species' initial dispersion. Here, I examine a metapopulation model with stochastic catastrophes to assess the relative influence of a species' initial dispersion on metapopulation persistence times. My analyses reveal that, in contrast to the strong influences of patch number, reproductive rate, and dispersal rate, variation in initial dispersion often has minimal effects on metapopulation persistence. However, initial dispersion appears to be a more important factor in metapopulation persistence for systems facing extra threats at low density, such as Allee effects or demographic stochasticity. If proven to be more generally true, such surprisingly negligible effects of initial dispersion on metapopulation persistence times would suggest that, for some systems, the micromanagement of individuals' distributions among patches may not be a high priority.

643: *-.040*

Reintroductions of captive-managed animals are a vital tool for the conservation of populations

and species extinct in the wild. Because genetic diversity may impact population persistence, the genetic composition of reintroduced and captive populations is critical. For captive-managed species with known pedigrees, individual animals can be selected to create reintroduced populations with specific genetic compositions. Five genetic and demographic strategies for selecting animals were tested on four species. Selection strategies were based on criteria identified in field and theoretical studies. Simulated reintroductions of animals from captive-breeding programmes created a genetic conflict. Among the different strategies, increases in the genetic diversity of one subpopulation were negatively correlated with changes in the genetic diversity of the other subpopulation. For example, the release of genetically over-represented animals was the most beneficial strategy for a captive-breeding programme, but provided the least genetic diversity for the reintroduced population. However, gains and losses in genetic diversity between populations varied with different selection criteria and different population structures. Because captive-breeding histories vary for each species, changes in genetic composition cannot be accurately predicted and no one strategy is universally optimal. Thus, genetic trade-offs must be assessed for each population relative to specific programme goals.

644: *+.252*

Effective management of our National Park Service lands requires information about the social aspects or human dimensions of wildlife. Understanding attitudes aids fish and wildlife professionals to predict public responses to management strategies like species restorations. We documented visitor attitudes toward and knowledge of restored bobcats (Lynx rufus) on Cumberland Island National Seashore (CINS). Bobcats were restored on CINS in 1988 and 1989. During fall 1997, we compared 4 visitor user-groups (white-tailed deer [Odocoileus virginianus] hunters [DH], day-only [DO] visitors, developed-site [DS] campers, and back-country [BC] campers) concerning their attitudes and knowledge, using a self-administered, drop-off questionnaire distributed on return ferries and at island campsites. We contacted 1,138 individuals. Overall response rate was 82.6%. Across 4 visitor user-groups, the mean attitude-toward-restored-bobcat score was 0.8, with a range of -18 to 16. A positive score represented a positive attitude, and a negative score represented a negative attitude. Zero represented neutrality. Deer hunters had a statistically less positive mean attitude score (-0.1) than the 3 other visitor user-groups. Overall mean score for knowledge-of-bobcats was 3.8 out of a perfect score of 10.0. Deer hunters had a statistically greater mean knowledge score (5.1) than the 3 other visitor user-groups. Thus, our results indicated that visitor altitudes toward and knowledge of bobcats on CINS differed among the 4 visitor user-groups. Wildlife interpretive and education programs should be specifically targeted to address the differences in attitudes and knowledge among visitor user-groups.

645: *+.103*

A character-based phylogenetic species concept approach was used to examine conservation unit status for three wild populations of black and white ruffed lemurs, Varecia vareigata variegata, from Betampona (N = 3), Manombo (N = 6), and Ranomafana (N = 14), Madagascar. Population aggregation analysis was performed on 548 bp from the control region (D-loop) of the mitochondrial DNA (mtDNA). Twenty-one diagnostic sites were found to differentiate the Betampona (northern) population from the Manombo/Ranomafana (southern) populations. Additionally, individuals from the North American captive population (N = 11) and from Parc Ivoloina, Madagascar (N = 6) were examined for the same mtDNA fragment. The captive animals more closely resembled the southern populations and the Parc Ivoloina animals were more similar to the northern population. However, the inclusion of these ex situ animals reduced the number of diagnostic sites differentiating the northern and southern populations. Our genetic data were used

to assess the ongoing management strategy for reintroducing individuals into the Betampona population and for introducing new founders into the ex situ population. This study demonstrates the utility of combining genetic information with a consideration of conservation priorities in evaluating the implementation of management strategies.

646: *+.200*

A monitoring system has been established to evaluate survival rate and dispersal behaviour of reintroduced Bearded Vultures (Gypaetus barbatus) in Austria. In 1998 the number of observations increased by about 400 %. Nevertheless there was still a severe shortage of observations from some regions of the country even though it is likely that Bearded Vultures are present. After several years of collecting basic information we are now focussing on specific information exchange between voluntary collaborators and a central coordination. To enable individual released birds to be recognized a system of feather bleaching using hydrogen peroxide was introduced and has proved effective. An extensive network of observers has been established now. In 1996/97 the first pair of reintroduced Bearded Vultures started to breed successfully in Haute Savoie/France. Since then, additional pairs have formed in northern Italy and further pair formations may take place soon in other alpine regions. Preliminary results indicate some possible preferences in habitat selection.

647: *+.198*

The effects of small population size on genetic diversity and subsequent population recovery are theoretically predicted, but few empirical data are available to describe those relations. We use data from four remnant and three translocated sea otter (Enhydra lutris) populations to examine relations among magnitude and duration of minimum population size, population growth rates, and genetic variation. Mitochondrial (mt)DNA haplotype diversity was correlated with the number of years at minimum population size ($r(s) = -0.741$, $p = 0.038$) and minimum population size, ($r(s) = 0.709$, $p = 0.054$). We found no relation between population growth and haplotype diversity, although growth was significantly greater in translocated than in remnant populations. Haplotype diversity in populations established from two sources was higher than in a population established from a single source and was higher than in the respective source populations. Haplotype frequencies in translocated populations of foundling sizes of 4 and 28 differed from expected, indicating genetic drift and differential reproduction between source populations, whereas haplotype frequencies in a translocated population with a founding size of 150 did not. Relations between population demographics and genetic characteristics suggest that genetic sampling of source and translocated populations can provide valuable inferences about translocations.

648: *+.188*

The Green and Golden Bell Frog Litoria aurea is recognized in New South Wales and nationally as a species in serious decline. We surveyed its current distribution and abundance in the Illawara region of New South Wales in order to devise a regional conservation plan for this species, based on the objective of establishing at least three isolated, viable populations. The survey was conducted during 1997-1999 across 18 sites based on historic and current records of the species, and habitat suitability. Bell frogs were detected at eight sites, four of which were on public land. The maximum number of adult frogs varied from 11 to 78 across seven sites in Port Kembla, suggesting a population of > 100 adult frogs. Breeding was poor at most sites during this study but a moderate number (ca 50) of juvenile frogs was observed at the BHP Steelworks site in January 1998 and at Coomaditchy Lagoon in February 1999. Bell frogs were recorded previously at

Shellharbour, where a large population occurred, and at Bulli. These areas are proposed as the other regional sites for bell frog conservation because they are well isolated (> 10 km away) from Port Kembla. A small number of bell frogs was present at Bulli, while none was found at Shellharbour. The apparent stability of bell frogs in the Port Kembla area suggests that the presence of multiple breeding sites within a relatively small area (2.5 km radius) is very important and the establishment of viable populations in other areas should consider this situation as a model. This will require the creation of additional breeding sites at Bulli and Shellharbour, and may require translocation to Shellharbour if no occupied sites are found in the next few years. The presence of predatory fish at nine sites demonstrates the need for the conservation strategy to include activities that improve the quality of the habitat currently available to bell frogs.

649: *-.046*

From 1995 to 1999, two species of endemic Hawaiian thrushes,'Oma'o (Myadestes obscurus) and Puaiohi (M. palmeri), were captive-reared and re-introduced into their historic range in Hawai'i by The Peregrine Fund, in collaboration with the U.S. Geological Survey-Biological Resources Division (BRD) and the Hawai'i State Department of Land and Natural Resources. This paper describes the management techniques that were developed (collection of wild eggs, artificial incubation, hand-rearing, captive propagation, and release) with the non-endangered surrogate species, the 'Oma'o; techniques that are now being used for recovery of the endangered Puaiohi. In 1995 and 1996, 29 viable 'Oma'o eggs were collected from the wild. Of 27 chicks hatched, 25 were hand-reared and released into Pu'u Wa'awa'a Wildlife Reserve. Using the techniques developed for the 'Oma'o, a captive propagation and release program was initiated in 1996 to aid the recovery of the endangered Puaiohi. Fifteen viable Puaiohi eggs were collected from the wild (1996-1997) to establish a captive breeding flock to produce birds for re-introduction. These Puaiohi reproduced for the first time in captivity in 1998 (total Puaiohi chicks reared in captivity 1996-1998 = 41). In 1999, 14 captive-bred Puaiohi were re-introduced into the Alakdi Swamp, Kaua'i.

650: *+.129*

Twelve wild adult Colorado pikeminnow (Ptychocheilus lucius), captured in the tailwaters of Taylor Draw Dam on the White River, Colorado, were implanted with radio transmitters and their movement patterns monitored from 1992 to 1994. The spawning migration of these fish was extensive. In 1993, the only full year of the study, the fish migrated an average of 658 km from the White River to spawning sites in the Yampa or Green rivers and back to the White River. Eight of these fish were translocated in the river upstream of the dam in April 1993. These fish and the 4 others below the dam remained in the river until May 1993. All 12 had migrated down the White River to spawning sites in the Green and Yampa rivers by July 1993. The fish that were located above the dam successfully passed over the dam during their downstream migration. Seven fish migrated upstream toward the Yampa River Canyon spawning site and 5 migrated downstream toward the Green River Desolation/ Gray Canyon spawning site. Five of 7 Yampa River fish were found at the spawning site. The other 2 were found 5-8 km downstream of the site. One of 5 Green River fish was found at the spawning site, the other 4 between 16 and 62 km upstream of the site. All fish migrated back to the White River by August 1993 and were found near the dam by October 1993. Two fish were recaptured and translocated above the dam in September 1993. Five fish were located below the dam and 2 above the dam in April 1994. By July 1994 seven of the same fish that had migrated toward the Yampa River in 1993 were found at the Yampa Canyon spawning site. At the same time, 3 of 5 fish that migrated toward the Green River in 1993 were found at the Desolation/Gray Canyon spawning site. This included 2 fish that had been found

upstream of the site in 1993. The 12 fish traveled an average of 6 km d(-1) (range: 4-10 km d(-1)) during the migration period from May through October 1993. Generally, fish moved faster to the spawning site than back from the site to the White River. These fish moved very little within their home ranges in the White River. Six fish tagged in 1992 moved only 0.1-2.3 km in the tailwater reach below Taylor Draw Dam from September 1992 through April 1993. All fish, after their spawning runs, had moved up to or near the dam by October 1993. These fish were not tracked again until April 1994. Their movement patterns in April 1994 were similar to those observed in April 1993. The greatest amount of fish movement in the White River was displayed by the 8 fish placed above Taylor Draw Dam in April 1993 and the 2 placed in Kenney Reservoir in September 1993. They moved 1.1-40.6 km in the river before and after their spawning migration in spring and autumn 1993. These spawning migrations suggest that adult Colorado pikeminnow in the White River were recruited from both Green and Yampa river spawning populations and were presumably imprinted to these respective spawning sites. Those fish placed above Taylor Draw Dam established home ranges in habitats previously occupied by Colorado pikeminnow before the dam was completed. They remained there until they migrated downstream during the spawning period. Although we did not study fish passage, our study demonstrates that adult Colorado pikeminnow will use habitat if access is provided. Translocation of wild adult fish into historic but unoccupied habitats may be a valuable recovery option.

651: *-.004*

Genetic variation within and among wild Turkey, Meleagris gallopavo, subspecies was measured using DNA sequencing and PCR-RFLP analysis of a 655 bp portion of the mtDNA D-loop region. DNA sequencing from 25 individual Eastern, Merriam, Rio Grande and domestic Turkeys revealed 16 polymorphic sites. Parsimony and neighbor-joining analysis did not show support for the four wild Turkey subspecies studied. PCR-RFLP analysis of 118 individuals revealed 13 distinct haplotypes. Haplotype variation was detected in all wild Turkey populations, and Wright's F-statistics revealed pronounced differentiation (FST = 0.302) among populations. Based on these data, non-native wild Turkey populations do not appear to have suffered a genetic bottle-neck since their reintroduction.

652: *-.070*

Since late April 2000, a Turkey Vulture Cathartes aura has been observed at several sites in The Netherlands, i.e. Monster, Zwolle, Texel and Wieringen. At two such sites, it was noted that the bird did not wear rings or other artificial markings. The bird at Wieringen was seen on 13 May 2000, while foraging on a freshly dead sheep; it tried to penetrate the head through the eye sockets. At Mallnitz, in the Austrian Alps, two juvenile Bearded Vultures Gypaetus barbatus were released at an artificial nest in May 2000. These birds are part of a WWF-project to re-establish this species, started in 1986. Since 1997, several Bearded Vultures turned up in the lowlands of The Netherlands; these birds originated from similar reintroduction projects in the Alps and have a small chance of survival. The colonization of Austria by White-tailed Eagles Haliaeetus albicilla was frustrated in 2000 by poisoning incidents in winter 1999/2000 (involving three birds) and road accidents (two).

653: *+.154*

The first records of exotic Nile tilapia, Oreochromis niloticus (Linnaeus, 1758),from the Limpopo River system are reported, and differences between native Mozambique tilapia, Oreochromis mossambicus (Peters, 1852), and O. niloticus are described. Conservation implications, such as the

translocation of fish and possible hybridization with indigenous Oreochromis, are discussed.

655: ***+.168***

The growing human population presents threats to the viability of many species of vertebrates through land use changes, environmental pollution, killing or disturbance, and the introduction of non-indigenous species. The development of appropriate responses depends on accurate identification of threats. and this has involved studies on captive animals. Furthermore, conservation programmes increasingly include active interventions such as translocation, captive breeding and reintroduction. In addition, animals are used in research to underpin these and other conservation technologies. A high standard of husbandry, in terms of welfare, of wild animals in these programmes can present considerable challenges. because, for many species, knowledge of biological requirements and how these can best be met is incomplete. Actions that are good for species conservation might not be in the best interests of the individual animals involved, and balancing conservation benefits and welfare costs in proposed research or intervention for conservation is not easy. Environmentalists tend to give priority to species conservation, while proponents of animal rights give priority to individual animal welfare. It is important that an ethical review process takes place in which conservation objectives and potential welfare costs are clearly identified and considered. The involvement of animals often cannot be replaced in this species-specific work and usually. therefore, only two Rs apply, and refinement warrants particular attention. Knowledge of the biology of the species in the wild, and previous experience of its maintenance in captivity, provide a basis for husbandry practices. Close health monitoring, observation of behaviour, and controlled studies, provide information on which to base further improvements.

656: ***+.158***

When reindeer (Rangifer tarandus tarandus) were introduced to Alaska's Seward Peninsula between 1892 and 1902, other ungulates were not present and large predators were either absent or less abundant than after reindeer were established. During the next 100 years reindeer numbers and distribution increased and declined precipitously on the Seward Peninsula: wildlife species repopulated this region through natural processes or translocations by man; the non-Native human population of Alaska increased dramatically and wildlife management became an issue of national concern creating diverse public desires regarding resource use; and both range and wildlife became intensively managed through complex, politicized processes. This paper provides an historical overview of reindeer and wildlife abundance on the Seward Peninsula during the 20th century and describes the effects of wildlife on the reindeer industry. Cooperative public processes have been initiated to bring diverse public interests together; meld indigenous, scientific and local knowledge of resources; and supplement governmental wildlife management programmes. Even so, the Seward Peninsula reindeer industry has been severely impacted by wildlife, especially caribou (R. t. granti).

657: ***+.290***

A period without management had transformed the vegetation of a species-rich fenmeadow, with no external change in the hydrological regime, into three communities: alder thicket (unmanaged 15 years), tall herb and sedge dominated communities (unmanaged 11 years). Following reintroduction of management (felling of alder thicket, mowing and grazing) the vegetation development was monitored and species cover was measured along a permanent transect before and during 12 years of restoration succession. Management increased the density of fenmeadow

species and made the three communities more similar. The appearance of new fen species and the increase in species density followed immediately after the introduction of management. Thereafter, only a few new species appeared and the turnover index stabilised. Management by mowing and grazing both increased species density of herbs and promoted establishment of biennials and hemicryptophytes. Other plant groups responded differently to management: mowing increased total cover, the cover of grasses, and promoted phanerophytes and chamaephytes; grazing reduced the influence of these groups and promoted 'sedge & rush' and geophytes. Restoration was particularly successful in the felled alder thicket, but the success was caused by spreading of species from the pool of species within the site and establishment of species having persistent seed bank, i.e. inherent good potential for restoration. The results are discussed in relation to use of functional plant groups and Ellenberg N- and L-indices as response indicators for monitoring restoration progress.

658: *+.032*

The diffuse presence of free ranging dogs (non-controlled, stray, and feral) in Italy is considered a severe conservation threat because of the potential impact on the wolf and on other wildlife species. In particular, it is generally believed that non-controlled owned dogs are rapidly increasing their number, representing a major part of the problem. The present legal framework does not allow destruction of dogs and cats, and the management of these pet species is based on mandatory marking, and on capture of free ranging animals for perpetual captivation in public kennels. The present research was aimed to: 1) collect and analyze available information on the impact of free ranging dogs on the wolf and on wildlife; 2) census owned dogs in rural areas of Italy (including urban centers with less than 30,000 inhabitants); 3) estimate the proportion of owned dogs that are free to range; 4) assess the public perception of problems posed by free ranging dogs; 5) assess the public attitude toward management alternatives; and 6) define management guidelines. Free ranging dogs resulted to prey upon all ungulate species and colonial ground-nesting birds. Dogs are the main limiting factor in translocation projects involving roe and red-deer, and represent a key obstacle to the recolonization of central and southern Italy by these species. Impact on the wolf is also discussed. Dogs were censused through direct interviews to 2,903 Italian families, randomly selected by the electoral lists. The sample was homogeneously distributed in the country, in order to test for differences among areas (n=4 sub-regions: northeast, northwest, center, south and islands). The total number of dogs is estimated at 6,099,011 +/- 307,.234. Of these, 19.7% (n=1,209,973 151,280) are free to range at least part of their time. Despite the lack of reliable data on the past dog numbers, we estimated an average 5 % year increase of the total population of dogs. The high increase rate is explained by the limited number of sterilized females, and the consequent high percentage of females reproducing every year. Number of dogs is negatively correlated to the size of urban areas, and increases from north to south. Control by owners follows opposite patterns. Despite the increasing number of non-controlled dogs, Italians have a limited perception of the social, sanitary, and conservation risks caused by dogs: 51.1 % of Italians consider that dogs do not represent a problem at all, and only 3.8% of the population considers the destruction of dogs an acceptable alternative to perpetual captivity.

659: *+.105*

Hihi (or stitchbird, Notiomystis cincta) is a rare honeyeater endemic to the North Island of New Zealand. Hihi were translocated from Little Barrier Island to Mokoia Island, Lake Rotorua, in 1994. Mokoia is a small (135 ha) island with secondary vegetation, so there was some doubt as to whether the island had sufficient diversity of fruit and nectar sources to support a hihi population.

This paper reports data collected in the year after the translocation on the density, distribution and phenology of plants likely to be used by hihi. We address the following questions. (1) How many hihi food plant species are on Mokoia? (2) How are the food plant species distributed over the island? (3) Are there periods when flower and fruit sources are scarce and/or spatially confined? (4) How might the availability of fruit and nectar change with succession or additional planting? There was always a minimum of 2-3 species providing nectar or fruit used by hihi. Most (16/21) of the species providing nectar flowered during the hihi breeding period, from October-February, and most (9/16) of these were canopy tree species. The greatest diversity of fruit sources was from March-May. August-September stood out as the period with the lowest diversity of fruit and flower sources, followed by June-July. While there was no time of year when hihi clearly suffered from shortage of fruit and nectar, we suggest that they may be susceptible to shortages in future years at times when diversity of food sources is low. We recommend further planting that could make the island more suitable for hihi in the long term.

660: *+.175*

It is well known that many animal species are forced into extinction by causes related to human activity, and that their possibilities to survive in the future are seriously compromised due to the great loss of genetic diversity that characterizes their populations. The use of the assisted reproduction technology in threatened wild animals may help to improve their genetic status as it has been done with livestock species. Sperm, oocyte, and embryo freezing would make possible the establishment of a Genetic Resource Bank, wick, in turn, would be of use in conservation programmes since Artificial Insemination, in Vitro Fertilization, and Embryo Transfer would allow this genetic material to be used so as to enrich the genetic diversity of both wild animals in natures (in situ conservation programmes) and captive populations that are being managed for a reintroduction plan (ex situ conservation programmes). However, the application of this technology involves overcoming a series of difficulties: there is a lack of basic knowledge on reproductive cycles to time artificial insemination or embryo transfer, genetic material used must be relatively abundant and of good quality, and, finally, protocols have to be designed for sperm and embryo freezing, for in vitro culture of oocytes and embryos, or for managing the female oestrus unless the methods used in their phylogenetically related domestic species can be succesfully adapted.

661: *+.055*

During the last century, human activities have promoted large changes in habitats, leading to changes in the abundance and composition of mammal communities. Some species have been favoured intentionally (specially those with high economic importance), or unintentionally (i.e. opportunistic species), introduced las some livestock species), or persecuted (i.e. species that affect livestock or game). In this paper, we show and explain the changes in the composition of the large mammal community in Aragon, a northeastern Spanish area where there have been extinctions (lynx, Lynx sp.); introductions (moufflon, Ovis ammon; fallow deer, Dama dame); reintroductions (red deer, Cervus elaphus); and particularly natural expansions (wild boar, Sus scrofa; roe deer, Capreolus capreolus; red deer; Pyrenean chamois, Rupicapra pyrenaica pyrenaica). Some taxa are currently almost extinct (brown bear, Ursus arctos; Pyrenean ibex, Capra pyrenaica pyrenaica) and others are recovering after getting close to extinction (wolf, Canis lupus). Three interconnected causes may explain the clear increment of ungulates in our study area: (1) the abandonment of rural activities, (2) the subsequent decrease of livestock, and (3) the increase of forest habitats. None of those species are currently below 150 % of their range in the 19(th) century. The setting up of hunting reservations and the design of a hunting legislation have

also contributed to the observed changes. Traditionally, large predators have not been favoured by human activities, while compensatory protection measures carried out have been scarce or came too late. While some of these species are probably extinct in the area, the remaining occupy currently less than one fourth of their former range and need urgent conservation measures for to recover.

662: ***+.055***

One hundred years passed since the time when the first transport of Przewalski's horses captured in China and Mongolia by hunting expedition arrived to Europe. Its existence came through dramatic breaks in the course of 20th century. Przewalski's horse, the last living wild horse became the symbol of the all endangered species on Earth. In several transports were imported in 1900-1902 to Europe 54 wild horses but only 12 of them gave offspring. Today world-wide population numbering over 1590 horses bred in zoological gardens and breeding centers of five continents. Observations of the wild-living Przewalski's horse from the May 1968 is considered to be the last credible report. No one later report about observation of wild horses in northern Xinjiang and Mongolian Altai Gobi was verified. An airplane survey of the Djungarian Gobi desert including Baytag Bodgo mountains and areas next to the Mongolian border made by the Chinese in 1986-1987 did not confirm the existence on any free living Przewalski's horses. On 1st Symposium on preservation of the Przewalski's horses in Prague in 1959 has been charged the Prague zoo due to its creditable work in breeding of this species with keeping of the International studbook. Since 1960, the population of Przewalski's horses grew permanently, which increased hope for their return back to the wild. Possibility of reintroduction started to be discussed after year 1980 when the worldwide population reached the number of 500 individuals and the annual growth amounted to approximately 10%. In May 1985, an international symposium was held in Moscow under the auspice of UNO, FAO and UNEP, where experts agreed on the way and time schedule of transports of first chosen Przewalski's horses to Mongolian semireserves. However it remained only in the stage of intentions. The world conservation organisation like a IUCN or WWF failed at the historical moment, instead taking supervision of the whole project, they rather supported some regional and fragmentary actions and concentrated for the common and theoretical recommendation. On the end of eighties and nineties, the initive devolved upon the side of privat foundation in Germany and Netherlands. These foundation set acclimatization centers in China (Jimsar in northern Xinjiang, Wuwei in Gansu province) and in Mongolia (Hustain Nuruu National Park near Ulan Bator and Tachin Tal in Altai Gobi, in the last known refugium of the species). In this phase, mutual relationships between concerned organisations as well as persons complexified, and resulted in open indictment, which cast an unfavourable on the whole effort to return of last species of wild horse to its homeland. Hoping and expectations of zoological gardens were not met and zoos had no possibility and often no interest to participate on the reintroduction. But in last time seems that almost of contraversian position fall off and there is hope for new era of cooperation. Despite all problems, although the reintroduction has not been as successful as the Przewalski's horses returned to its former native country and today there are living individuals born here in the second generation. In Mongolia in both acclimatization centres (Hustain Nuruu and Tachin Tal) several small herds are living in the free. In China, despite significant breeding success in the acclimatization centers, the problem has not yet been solved - where to release the acclimatizated horses, and they remain entirely dependent on man help and care. The only choiced place - basin along river Ulungur in the Kalameili Mountain reserve is in traditional wintering place of 8000 herdsmen with more than 40.000 domestic animals including feral horses. Progress in reintroduction is therefore being made only in Mongolia. Evidence of losses of horses showed that most of falls can be attributed to injury caused by transport or by traumas from mutual attacks of horses (namely stallions in unsuitably located enclosures). In the harsh climate of the Gobi

desert, the biggest problems the horses face when getting acclimazed are respiratory deseases. In all the reintroduction centres, horses suffer from blood parasites, and as regard afted by predators, especially wolves. It has become apparent that those that find it most difficult to accommodate to new conditions are the older horses. What cause problems are for Mongolia too early births of foals (April, May), which are due to the shift in the reproduction pattern from living in quite different condition in of Europe and Northern America for more than 14 generation. On April 24, 1999, for example, the temperature of -28 degreeC was recorded in the Tachin Tal in the Gobi, and one of born foal froze to death. The optimal period for giving birth in this area would be end of May to July. Despite all these problems, the number of the Przewalski's horses in the wild is growing. In 2000 transports have been completed to the NP Hustain Nuruu near Ulan Bator, where there is a stable population almost 120 horses, now with an annual growth of some 10%. The year 2000 has been a turning year in the hard condition Tachin Tal centre, where 14 foals was born in the spring, half of these in free living herds. When predicting future development of reintroduction, we have to bear in mind that the factors are still there, that in the 1960's led to extinction of the Przewalski's horse from the wild, especially in the Gobi: desertification and aridisation of semi-desert areas and steppes, limited number of water resources used part of the year by herds of domestic animal, grazing-out of vulnerable semi-desert ecosystems and the risk of cross-breeding with omnipresent feral horses. Jiri Volf, the former stud-book-keeper, on ground of his stay in the Tachin Tal region in 1997, believes that the capacity of water resources is sufficient for all large mammal species, but the question remains whether these sources are available all year, especially in hard winter time without snow.

663: ***-.008***

Freshwater ecosystems in Asia are under grave threat. Large and growing human populations and the rapid pace of development have led to the degradation of natural environments throughout the region. Conservation of freshwater biodiversity faces particular challenges because of a lack of public awareness of its magnitude and importance. Even taxa with high public-relations value, such as fishes, are rather poorly known, and the variety of other animals associated with lakes and riverine wetlands, including charismatic and endangered megafauna, seems to have escaped wide attention. The rate and extent of environmental change in Asia are having impacts on the aquatic biota that may be greater than anywhere else on the planet. Particular threats include water pollution, from point and nonpoint sources, which is almost ubiquitous; overharvest of fishes, turtles, and crocodilians; flow regulation and impoundment of rivers; as well as drainage basin degradation and climate change. Many lakes have been so modified by human activities that they function as enormous fishponds, and the introduction of exotic species (especially, but not only, fishes) or the translocation of native taxa has contributed to the extinction of endemic species in isolated drainage basins. The prognosis is grim, and we can anticipate a loss in biodiversity and homogenization of the regional biota. Reversal of these trends will require a change in focus by limnologists and water-resource managers, and the urgent adoption of a conservation agenda for freshwater science in Asia.

664: ***+.175***

Neonatal reptiles are here defined as an age class of young eureptilian amniotes (excluding birds) that express attributes most influenced by the pre-paritive development environment (oviduct, egg, and egg nest) and by the demands of parition and first dispersal. Neonatal character states are typically transformed, reduced, or eliminated during the first 10% of their prereproductive development. Traditionally, neonates have not been distinguished from juvenile reptiles. As a result the neonatology of reptiles has rarely been addressed in past literature. Recent studies reveal

a complex array of developmental scenarios involving character state transformations, heterochrony, unique character states in morphology, behavior, physiology, nutrition, dispersion and health. Unique morphological features (such as egg teeth) and limited skeletal ossification characterize many neonates. Distinguishing behaviors include "reversal" movements, utilization of bright color patterns, and startling movements with both serving as anti-predation mechanisms. Prolonged association with protective parents, group migration, unique agonistic behavior, and tendencies toward rapid dispersion characterize the neonates of individual species. Neonatal physiological attributes include: a special availability to inoculation by symbiont fermenting anaerobes in herbivores, rapid conforming responses to their external environments in thermal and hydric exchanges, and in the case of some turtles, extraordinary capacities for supercooling (8.9 C). Post-paritive lecithotrophy (nutrition from residual yolk) sustain both the ovenwintering of nestlings and the dispersion of nonfeeding young for as long as several months. Resistance to infections (such as mycoplasmas) from their maternal parents,combine with nutritive reserves of residual yolk and a common tendency for rapid dispersion to make neonates attractive candidates for augmentation and translocation programs. Coupled with the practical advantages of maintaining and manipulating small animals in a laboratory environment, these qualities distinguish neonates as particularly useful models for experimentally evaluating the relative apportionment of reproductive resources into greater numbers of offspring or into improved quality/survivorship of individual offspring.

665: ***+.031***

Limitations for in situ conservation are discussed in the light of variable environmental conditions and population characteristics. Environmental influences like clear cutting, over utilisation, immission load, climatic change, catastrophes and diseases can set limitations for in situ conservation. Genetic limitations can be set by narrow genetic base, badly adapted populations or hybridization with other species and/or cultural varieties. Necessity for double conservation measures, reintroduction of rare species from reestablished adaptable populations and limitations for natural regeneration are discussed. The problem of species integrity versus hybridization and enlarged genetic base is mentioned.

666: ***-.005***

Ankarafantsika is the only protected area in Madagascar with good habitat for the endangered, endemic freshwater turtle Erymnochelys madagascariensis, but due to exploitation many populations are already depleted. A conservation strategy for the species at Ankarafantsika was developed in 1997 and includes a captive breeding and rearing operation to produce juvenile turtles for reintroduction and population augmentation. A facility comprising 22 spacious concrete ponds and a laboratory was built in Ampijoroa and is operated by Durrell Wildlife Conservation Trust. A motor pump is used to fill the ponds with water from a nearby lake and all ponds have a drainage system. Ponds are designed differently for hatchlings, juveniles, adult females and males. Females have access to beach areas where eggs will be incubated naturally. The facility can hold at least six adult females and males and provides for at least 200 hatchlings to be raised for two to three years.

667: ***+.014***

The lesser white-fronted goose (Anser erythropus) is the most threatened of the Palearctic goose species with a declining population trend throughout its distributional range. The current estimate of the Fennoscandian subpopulation size is 30-50 breeding pairs, whereas it still numbered more

than 10 000 individuals at the beginning of the last century. Reintroduction and restocking have been carried out in Sweden and Finland using captive lesser white-fronted goose stock with unknown origins. We have carried out a study of the genetic composition of captive-bred stock by sequencing a 221 bp hypervariable fragment of the mitochondrial DNA (mtDNA) control region from 15 individuals from the Hailuoto farm, Finland. Two out of the three maternal lineages detected in the captive stock are also present in wild populations. The third maternal lineage among the captive lesser white-fronted geese originates from the closely related greater white-fronted goose (Anser albifrons). None of the investigated wild lesser white-fronted goose individuals carried the mtDNA of the greater white-fronted goose. The presence of greater white-fronted goose mtDNA in the lesser white-fronted goose captive stock suggests that hybridization has occurred during captive propagation.

668: ***+.398***

Bighorn sheep populations experienced a drastic reduction in both distribution and abundance until the advent of modern wildlife management, where improving viability of extant populations and translocating animals into historical habitat range have been the most important management policies. The fact that subspecies relationships among bighorn are ambiguous, together with the importance of selecting appropriate source stock and the expense of translocation projects, makes an understanding of subspecies relationships and genetic variation, within and between populations, important for the management and conservation of this species. In this study, genetic variation in 279 bighorn sheep from 13 study sites in Arizona, California, New Mexico and Alberta, Canada were examined by analyzing ten microsatellite loci to determine interpopulation differentiation and relationships between closely related taxa. All populations contained a substantial amount of genetic variation. Genetic differences between populations were large and roughly proportional to geographic distance. The significance of this to desert subspecies relationships and management is discussed.

669: ***-.022***

Modern fishery management strategies, based on species biology and monitored with landings data, failed to sustain either exploited populations of abalone, Haliotis spp., or fishery landings in southern California. California law and policy require that the State restore depleted populations and explore new management strategies to sustain abalones and the fisheries they could produce. Restoration strategies will vary by species and by the causes of depletion. Fishing depleted white abalone, H. sorenseni, to critically low densities (apprx100cntdotha-1) in the 1970s. By the 1990s, natural mortality had further reduced the population density to 1cntdotha-1. Captive breeding and rearing, with adult out-planting, may be the only way to restore white abalone with sufficient certainty to avoid extinction. Fishing also depleted pink abalone, H. corrugata, but sufficient numbers of adults exist to artificially aggregate critical spawning densities (2000-4000cntdotha-1) in the wild with little risk of extinction at this time. A withering syndrome of uncertain etiology reduced isolated remnant populations of black abalone, H. cracherodii, to extremely low densities. Restoration of this species will require finding and identifying disease-resistant survivors to use for captive breeding and rearing. Persistence of red abalone, H. rufescens, in northern California and experimental success of refugia created by translocating adult pink abalone and green abalone, H. fulgens, in southern California, indicate that refugia-based management could sustain exploited populations and fisheries. Refugia-based strategies are being explored to restore and sustain southern. California pink and white abalone populations.

671: ***+.028***

The white-clawed crayfish, Austropotamobius pallipes, is the only native species of freshwater crayfish in Spain. This species sustained a first-magnitude inland fishery up to the end of 1970's, when the crayfish plague struck the Spanish waters. It is detected an overall loss of distribution area of populations inhabiting the medium and lower reaches of the main river catchments, especially in Southern and Central Spain. The number of surviving populations can be estimated in ca. 700. Remaining native crayfish populations currently inhabit marginal areas. The populations are very fragmented, occupy short stretches, and are frequently isolated from the main river system. The current distribution is the result of a sum of different factors, ie. crayfish plague, habitat alterations, extreme climatic drought, etc. The incidence and relative importance of each one varied during the last 15 years, with crayfish plague being a predominant negative factor in the seventies and eighties, and climatic drought in the nineties. The current trend of disappearance is a sufficient reason to consider the native crayfish as at risk of extinction. However neither the national legislation nor most of the regional government legislations have listed this species as endangered. An analysis of the legislation dealing with freshwater crayfish in Spain shows : (a) an uneven and generally low level of protection given to the native species and (b) a very complex fishing and commercialization regulations for crayfish that show significant changes from one region to another. This complex legislation, complemented with a general lack of data on crayfish populations, low levels of management and public involvement, and different strategies in regional governments regarding restocking programs with exotic species, makes a confusing situation. In order to decrease the general tendency of regression of A. pallipes a conservation program for this species needs to be implemented in Spain. This program should include a number of regulations to be followed for the particular autonomous regions of Spain. This program should include measures : (a) to restore native crayfish populations, i.e. by translocations or stocking with farm raised of summerlings, in designated areas where the risk for crayfish plague outbreaks is low, (b) to control the spread of introduced species, by controlling their means of dispersal which usually occurs by human activities, i.e. trade of live crayfish, exotic crayfish stocking programs, crayfishing, aquaculture purposes, etc., and by developing eradication methods for unwanted crayfish populations; (c) to increase the surveillance of remaining native populations by providing the areas with native crayfish the protection level required by the European Community Directives for the Conservation of Natural Habitats and Wild Flora and Fauna (92/43/EEC and 97/62/EU); and finally (d) to improve public and administration awareness.

672: ***+.125***

The transplantation of 0.4 ha of montane grassland in the Hart Mountains was accompanied by evaluating the achievement of declared objectives for 5 years. The project is unique in central Europe with respect to the transplantation technique employed, size of translocated area and intensity of monitoring. The meadow was transplanted to a nearby location using a bulldozer with a special excavating shovel. The preservation of the flora of the whole transplanted area was successful. Disturbance indicator species appeared only to a negligible degree. Of 18 regionally threatened species present, the majority responded indifferently or positively to the transplantation; only two showed a distinct decline in population size, which was probably not caused by the transplantation alone. The preservation of the species composition of the four vegetation types occurring in the study area after a period of 4 years was also successful with all types reverting toward their pre-transplantation state. In contrast, the maintainance of the spatial vegetation mosaic failed completely. Dissection of previously coherent vegetation types and levelling of the ground surface resulted in an artificial vegetation pattern with predominantly geometric patterns. (C) 1999 Elsevier Science Ltd. All rights reserved.

673: ***+.043***

The range of the Brown bear in Italy is limited to the population of central Apennines, and to a residual population of few individuals in the Adamello Brenta Natural Park (Central Alps). Moreover, some individuals are occasionally recorded on the eastern Alps, arriving from Slovenja and Austria. A translocation project aimed to re-establish a viable population of bears in the Central Alps, proposed by the Adamello-Brenta Natural Park administration, has been funded by CEE through a "LIFE" program. The project is carried out in co-operation with the Province of Trento, with the National Wildlife Institute, and with the authorities of Slovenja, where bears will be captured. Accordingly to the national and international guide lines for the translocation of wildlife (AA.VV. 1997b, IUCN 1998), aim of the translocation is the establishment of a viable population of brown bears in the Italian Central Alps. Objectives of the feasibility study are to 1) verify that there is no possibility to recover the residual population without a translocation; 2) assess if the region can sustain a viable population of bears; 3) assess if conflicts that will arise between bears and human activities are sustainable; 4) identify the main factors that could negatively affect the establishment of a population in the area. The feasibility analyses were performed in a 6,495 km2 area, defined considering several parameters; 1) large enough to ensure good probability of comprehending a Minimum Viable Population (MVP); 2) centred in the Adamello-Brenta Natural Park; 3) larger than dispersal movements of released bears; 4) borders defined in higher human disturbance areas (densely inhabited valleys). The residual population has been censused through a DNA analysis of samples collected from faeces and hair. Results of this analysis confirm that only 3 individuals still inhabit the area. As no reproduction has occurred in the area since 1989, we consider that the remaining individuals are probably not reproductive, and the population can thus be considered ecologically extinct. In this respect, weit has decided to define the project as a re-introduction, instead of a reinforcement. The decrease occurred in historic time was analysed by collecting all available information on bear presence and kills in the last centuries. The data collected indicate that in the XVII century, bears were still abundant and widely distributed over most of the Alps; only after the XVIII century, distribution of the species decreased in the valleys, in relation to progressive timber and agricultural activities. After 1850, a more intensive direct persecution started, adding its effects to the increasing habitat fragmentation also caused by the increased exploitation of the high altitude areas for agriculture. In the Trento province, 192 kills are reported for the last 150 years, of which 84 only in the Brenta massif; before the bear became protected (in 1939), a decrease in the number of kills had already occurred, indicating that the bear population was already in critical conditions, and that legal protection arrived too late. From these data, we estimate that the Brenta population went below a critical threshold already in the early '70ies, when probably only 15 animals still inhabited the area. In conclusion, historical data indicate that direct persecution by humans was the main factor of dramatic bears decrease recorded until the first half of XX century, but in the last decades the population failed to naturally recover probably for demographic and stochastic factors, that can be remedied through a translocation. To assess if the area can still sustain a bear population, a habitat suitability model has been implemented by logistic regression analysis on bears presence data collected in central Alps during the last 20 years (n = 1,777), versus more than 60 parameters, including habitat characteristics and human pressure indexes, were considered. Results indicate that there are about 1,700 km2 of suitable area for the bear; assuming that bears' densities can range from 2 to 3 individuals / 100 km2, the area could thus support a populations of 34-51 bears, or 79-118 if including scarcely suitable areas. Such numbers are above the value indicated for a minimum viable population. Several decades will be necessary to reach a viable population in the area (18-41 years needed for a population of 70 individuals, with an year increase rate of 10-5%). In the medium term, a connection with the bears' population of the eastern Alps is possible, enhancing the probability of survival of the Central Alps bears' population in the long term. The cost of the project has been estimated, in co-operation with private professional analysts (Agri-consulting S.p.a), considering direct costs and potential impact on human activities for the total

period needed to establish a population of 50 individuals. Potential damages caused by bears to economical activities were estimated on the basis of data gathered in similar translocation projects in Austria and France; a probabilistic prediction of the impact of "problem bears" was also stated. Cost of average bear damages was estimated in 775 Euro/year per bear; to evaluate the risk related to problem bears causing very high damages, we assigned to each bear a 0.20 probability to be a "problem bear" (French and Spanish data: 3 problem bears out of 15 monitored), and a much higher level of damages was predicted for these individuals (25,826 Euro/year). The cost of the project is potentially very high, ranging from 1,422,000 to 5,482,000 euros mainly in respect to the frequency of "problem bears" causing severe damages to livestock, and to the direct (poaching) or indirect impact of human pressure on the increase of bear population, that may extend significantly the time needed to achieve a minimum viable population of bears. Human dimension aspects have been considered with care; in fact human pressure soundly represents the main limiting factor for the future population dynamics in the area, as Central Alps are presumably the area with the highest tourist pressure in the total range of the Brown bear. A survey on the attitudes of the local population towards the Brown bear, carried out through 1,500 telephone interviews by the DOXA S.r.l., indicated a positive opinion of 75% of the residents, raising to over 80% when stating that bears will be constantly monitored, and that problem individuals will be removed or destroyed. In this respect, an emergency team has been created, training rangers to aversion and trapping techniques. The telephone survey also permitted to estimate the value that people assign to the bear, through a simplified contingent valuation. The total sample was divided into 10 random sub-samples; a different fine for bears poaching was indicated to the 10 sub-samples, and it was asked if they considered the fine reasonable. Values ranged from 258 to 25,826 Euros. Results indicate that the perceived value of a bear ranges between 6,198 and 10,331 Euros, with significant differences among areas. In conclusion, the feasibility study indicates that the translocation has good probabilities to be successful in the medium-long term. The measures planned for the translocation can significantly increase also the chances of survival for bears naturally arriving to the area. The legal framework has already been updated. The predicted cost of the project is high, but it has been considered sustainable by the administrations funding the translocation, and funds for damage prevention and compensation have thus been approved. Human disturbance is a critical factor, and in this respect a risk of failure cannot be excluded; therefore, all measures aimed to solve or reduce conflicts between bears and humans are a priority, and a correct information on the project should be ensured. The possibility of attacks to humans, although very limited, makes important to plan every possible measure (selection of bears to be released, emergency team, correct information on how to behave when encountering a bear, etc) to reduce such risk. Operative guidelines include a minimum number of 9 bears to be released in four years, an intensive post-release monitoring program, comprehending the radio-tagging of all released bears. In order to assess the success of the re-introduction, the dynamic of the population will be carefully monitored. In case of low rate of increase (<2%/year), or loss of bears for poaching, a re-evaluation of the project is planned.

674: ***+.149***

Isoodon obesulus exhibits geographic variation in body size and shape, which appears to be adaptive. The geographic range of this species is declining, so the presence of adaptive divergence is of concern for the conservation of this species, both in the long term (loss of diversity decreasing evolutionary potential) and short term (choice of source populations for translocations). In this study, skulls of I. obesulus, both recent (animals alive within the last 100 years) and fossil (a few thousand years old), were examined and a range of measurements obtained. Comparisons were made between the two data sets to see whether skull morphology has changed over this relatively short period. Such a change may indicate rapid evolution of these characters and

therefore the potential for fast regeneration of any lost geographic variation. Fossil skulls were smaller than their recent counterparts, had shorter 'snouts' relative to skull width and depth, and displayed no geographic variation in size and shape, whereas recent skulls were geographically differentiated. Because of the apparent rapid evolution in these characters, the implications of adaptive variation in size and shape in I. obesulus with regard to its conservation may be strictly short term, since any geographic variation lost may be quickly recovered if suitable conditions exist.

675: *+.001*

The white-clawed crayfish, Austropotamobius pallipes, an endangered species in Europe, was surveyed for genetic and morphological variation as part of a larger project aimed at clarifying its French stock structure. Analysis from four morphological characters discriminated two groups, one including the 8 stream populations and the other comprising a single pond population. Total mitochondrial DNA variation was examined by RFLP analysis using 11 restriction enzymes for 120 animals sampled from these 9 populations located in Poitou-Charentes region. Among the three haplotypes revealed, two were found in animals sampled from brook populations and the third haplotype was only found in individuals from the pond population. Mitochondrial DNA nucleotide diversity values within species ranged from 0.57 to 1.31%. The analysis of genetic variance showed no structuring of genetic variation by hydrographic basins and could reflect the impact of translocations by man although other explanations are possible.

676: *+.035*

The translocation of aquatic organisms is an issue of increasing concern. In assessing the genetic risks from translocations, a primary distinction should be made between translocation for the purpose of aquaculture and translocation for the purpose of stock enhancement. When translocation is for aquaculture, the question of interest is: what is the maximum level of escapes that should be permitted, beyond which there is an unacceptable probability of adverse genetic effects upon the natural population? Risk minimization should concentrate on management solutions that reduce escapes from aquaculture facilities. When translocation is for stock enhancement, the question of interest is: what is the maximum level of genetic differences between hatchery and wild stock that should be permitted, beyond which there is an unacceptable probability of adverse genetic effects upon the natural population? Risk minimization should concentrate on hatchery management procedures that reduce genetic differences in fitness traits between hatchery stock and wild stock from the proposed recipient population. Where translocation poses a significant risk of adverse genetic changes, then a monitoring programme should be put in place, linked to a policy that prescribes management actions for the range of possible outcomes from the monitoring. The main limitation to our ability to develop an effective risk assessment and monitoring process is our lack of understanding of how the interaction between genetically different stocks affects the genetic basis of quantitative fitness traits that adapt organisms to their local environment.

677: *-.016*

Genetic subdivision of a species indicates the potential for local adaptation, and the genetic differences among populations are a key component of genetic diversity. Molecular genetic markers are generally used to assess the extent and pattern of subdivision. These traits provide an abundance of simple genetic markers, and they allow comparisons across studies. However, the connection of molecular genetic variation to local adaptation and, hence, to possible genetic

problems of translocation, is weak. In the extreme case of no genetic subdivision, there is no reason to expect genetic problems with translocation. Where there is deep genetic structure, indicating substantial evolutionary independence of sets of populations, translocations may threaten basic components of genetic diversity. Between these extremes, however, predicting genetic problems of translocations is extremely difficult. The molecular markers used to measure genetic structure indicate where there has been opportunity for local adaptation, but they are not directly related to such adaptation. The relationship of the level of genetic divergence to genetic incompatibilities is very loose, although quantitative tests are scarce. However, studies of reproductive isolation between species illustrate the fundamental inadequacy of using measures of genetic divergence to predict interactions between populations. Although it is tempting to use simple measures as predictors, such use may provide a false sense of scientific rigour. There is no substitute for direct tests for variation in ecologically relevant traits and possible genetic incompatibilities among populations.

678: *+.161*

Some biologists have expressed concerns about the possible genetic impacts of translocation between stocks of barramundi Lates calcarifer (Bloch) in Australia. Recent genetic, biogeographical studies have provided an understanding of the evolution of the currently observed population structure in Australian barramundi by assessing the impacts of ice-age, sea-level changes on their distribution. These studies found that genetic differences between most barramundi populations are extremely small, have arisen in the past 17 000 years, and substantial migration and hybridization between eastern and western populations, isolated for at least 110 000 years, has occurred naturally. Some phenotypic support for these minor genetic differences can be inferred from the lack of adaptation to temperature in growth and survival responses of widely separated stocks (tropical and temperate). Based on a low level of genetic differentiation and high levels of gene flow between populations, with little evidence of local adaptation, translocation between populations should not pose a significant risk or problem.

679: *+.062*

Species or strains of fish may be translocated for farming, where the only access to the wild is via inadvertent escapes, or for stocking, where deliberate releases are undertaken. In either case, it is important that the translocated animals are representative of the donor population(s) in terms of genetic composition and level of variability. Many studies have shown that this ideal is difficult to achieve, the major reason being the use of inadequate numbers or composition of broodstock as founders of a strain. Also, where more than one conspecific population is involved, there may be outbreeding depression problems. In the case of farming, measures to improve the introduced strain genetically are likely to be undertaken, e.g. breeding programmes, manipulation of sex and ploidy, transgenic techniques. Such approaches are necessary economically, but can alter genetic make-up. Thus, stringent attempts must be made to minimize escapes or reduce their impact should they occur. With stocking, genetic change during captive rearing should be avoided. No strain manipulation should be undertaken, and other agents of change should be minimized. Stocking may result in hybridization with related species or with endemic populations of the same species. In either case, there can be detrimental genetic effects on the native forms. To be able to identify subsequently any genetic changes in reared strains, whether intended for farming or stocking, wild population composition should be determined, using appropriate molecular techniques. Such molecular methods will demonstrate the degree of interpopulation differentiation and, thus, reproductive isolation. The same markers should then be used in each subsequent generation (in the hatchery and after escape or reintroduction to the wild) to monitor any changes

in genetic composition or variability. Markers should include microsatellite DNA loci, but the inclusion of more than one type of marker is recommended. However, as the aforementioned markers are not considered to be influenced by natural selection, they give no information on the adaptive nature of such differences. For this reason, it is suggested that markers influenced by selection should be investigated. Monitoring a strain subsequent to deliberate or inadvertent release can be undertaken using genetic markers, either deliberately enhanced by breeding or occurring naturally. Highly variable minisatellite DNA loci have been used as family markers in farmed escape studies with Atlantic salmon. These investigations have demonstrated significantly superior survival of native strains compared with farmed salmon in natural stream conditions. These latter results, demonstrating fitness differences, were strongly indicative of local adaptation. Thus, methods exist to monitor the genetic effects of translocation and stocking. However, a holistic approach should be taken to such exercises, where genetics forms part of a wider suite of considerations.

680: *+.274*

Genetic improvement of aquaculture species offers a substantial opportunity for increased production efficiency, health, product quality and, ultimately, profitability in aquacultural enterprises. Technolo-gies exist that can be implemented immediately to improve multiple traits that have economic value, while simultaneously accounting for inbreeding effects. Genetic improvement techniques for delivering genetic gain include formal definition of the breeding objective, estimation of genetic parameters that describe populations and their differences, evaluation of additive and non-additive genetic merit of individuals or families and defining the structure of a breeding programme in terms of mating plans. Novel genetic technologies involving the use of DNA-based tools are also under development for a range of aquaculture species. These gene marker technologies can be used for identification and monitoring of lines, families and individuals, monitoring and control of inbreeding, diagnosis of simply inherited traits and genetic improvement through selection for favourable genes and gene combinations. The identification of quantitative trait loci (QTL), and direct or linked markers for them, will facilitate marker-assisted selection in aquaculture species, enabling improvement in economically important traits, particularly those that are difficult to breed for, such as food conversion efficiency and disease resistance.

681: *+.073*

Throughout the first 90 years after their discovery, sulfate-reducing bacteria were thought to be strict anaerobes. During the last 15 years, however, it has turned out that they have manifold properties that enable them to cope with oxygen. Sulfate-reducing bacteria not only survive oxygen exposure for at least days, but many of them even reduce oxygen to water. This process can be a true respiration process when it is coupled to energy conservation. Various oxygen-reducing systems are present in Desulfovibrio species. In Desulfovibrio vulgaris and Desulfovibrio desulfuricans, oxygen reduction was coupled to proton translocation and ATP conservation. In these species, the periplasmic fraction, which contains hydrogenase and cytochrome c(3), was found to catalyze oxygen reduction with high rates. In Desulfovibrio gigas, a cytoplasmic rubredoxin oxidase was identified as an oxygen-reducing terminal oxidase. Generally, the same substrates as with sulfate are oxidized with oxygen. As additional electron donors, reduced sulfur compounds can be oxidized to sulfate. Sulfate-reducing bacteria are thus able to catalyze all reactions of a complete sulfur cycle. Despite a high respiration rate and energy coupling, aerobic growth of pure cultures is poor or absent. Instead, the respiration capacity appears to have a protective function. High numbers of sulfate-reducing bacteria are present in the

oxic zones and near the oxic-anoxic boundaries of sediments and in stratified water bodies, microbial mats and termite guts. Community structure analyses and microbiological studies have shown that the populations in those zones are especially adapted to oxygen. How dissimilatory sulfate reduction can occur in the presence of oxygen is still enigmatic, because in pure culture oxygen blocks sulfate reduction. Behavioral responses to oxygen include aggregation, migration to anoxic zones, and aerotaxis. The latter leads to band formation in oxygen-containing zones at concentrations of less than or equal to 20% air saturation.

682: ***+.154***

State Forest Service has carried out a reintroduction project of Griffon Vulture Gyps fulvus in the Monte Velino Montagna della Duchessa Natural Reserve in the mountain of central Italy. Preliminary feasibility study was carried out to evaluate present ecological conditions in the area for the species. Reintroduction method consists on releasing adult and sub-adult individuals to fix a breeding group. The release stock was provided by spanish raptors rehabilitation centers. On July 1994 first six Griffons were released equipped with tail mounted transmitters to monitor their dispersal movements and some feathers of the griffons were decoloured before beeing released. Two feeding stations have been set to supply the necessary food to the griffons and to keep them in the area. In the 1997 first breeding occurs and at present the griffon colony is stable nevertheless 10 animals in the 1998 died for poisoning. Data on reproductive parameters and population dynamic are reported.

685: ***-.087***

As one moves from the core to the periphery of a species' geographical range, populations occupy less favourable habitats and exhibit lower and more variable densities(1-4). Populations along the periphery of the range tend to be more fragmented and, as a result, are less likely to receive immigrants from other populations. A population's probability of extinction is directly correlated with its variability and inversely correlated with density and immigration rate(5-9). This has led to the prediction that, when a species becomes endangered, its geographical range should contract inwards, with the core populations persisting until the final stages of decline(2,10). Convinced by these logical but untested deductions, conservation biologists and wildlife managers have been instructed to avoid the range periphery when planning conservation strategies or allocating resources for endangered species(11-13). We have analysed range contraction in 245 species from a broad range of taxonomic groups and geographical regions, Here we report that observed patterns of range contraction do not support the above predictions and that most species examined persist in the periphery of their historical geographical ranges.

686: ***-.063***

Emerging infectious diseases (EIDs) of free-living wild animals can be classified into three major groups on the basis of key epizootiological criteria: (i) EIDs associated with "spill-over" from domestic animals to wildlife populations living in proximity; (ii) EIDs related directly to human intervention, via host or parasite translocations; and (iii) EIDs with no overt human or domestic animal involvement. These phenomena have two major biological implications: first, many wildlife species are reservoirs of pathogens that threaten domestic animal and human health; second, wildlife EIDs pose a substantial threat to the conservation of global biodiversity.

687: ***-.033***

Swayne's hartebeest (Alcelaphus buselaphus swaynei) is an endangered antelope that survives in four or five relict populations in Ethiopia. We examined the two main populations (Senkele and Nechisar) for mitochondrial (D-loop) and nuclear (microsatellite) variability in order to measure levels of genetic variation within the subspecies and degree of differentiation between populations. For comparison, we examined samples from a large population of red hartebeest (Alcelaphus buselaphus caama). Both swaynei and caama exhibited high levels of variation. There was significant differentiation between the populations of swaynei at Senkele and Nechisar, and gene diversity in Nechisar, the smaller of the two populations, was significantly lower than that in Senkele. Many mitochondrial haplotype and microsatellite alleles present at high frequencies among the Senekele individuals were missing in Nechisar, suggesting that the translocation of animals from Senekele undertaken in 1974 did not contribute notably to the gene pool in Nechisar. Subsamples taken from Senekele in 1988 and 1995 showed a significant change in allele frequencies, a change that probably can be attributed to a massive population decline this period. We recommended that both populations be protected in situ to maintain as much as possible of the diversity that exists within the taxon and that, a breeding program be established. In spite of the earlier unsuccessful attempt, we argue that translocation of animals for enhancement of population size as well as genetic variation in Nechisar should be considered.

688: ***+.102***

The South Island saddleback (Philesturnus carunculatus carunculatus) is one of two subspecies of the New Zealand saddleback. Despite the endangered status of this subspecies, it was not studied in detail until 1994, when 26 birds were released Onto Motuara Island in the Marlborough Sounds of New Zealand. I report the foraging behaviour and diet of this reintroduced population during the first breeding season after release. South Island saddlebacks used their bills in a variety of ways when foraging, and were predominantly insectivorous. They obtained most food from the ground and five-finger (Pseudopanax arboreus), and the number of prey captured generally reflected the amount of time saddlebacks spent on foraging substrates. North and South Island saddlebacks are very similar in terms of foraging behaviour, prey handling techniques and types of invertebrate prey consumed. The foraging patterns and diet of South Island saddlebacks on Motuara Island differed from all potential competitors. I conclude that the success of the South Island saddleback transfer to Motuara Island should not be threatened by a lack of food or foraging opportunities.

689: ***+.136***

We present a comprehensive survey of genetic variation across the range of the narrowly distributed endemic Yosemite toad Bufo canorus, a declining amphibian restricted to the Sierra Nevada of California. Based on 322 bp of mitochondrial cytochrome b sequence data, we found limited support for the monophyly of B. canorus and its closely related congener B. exsul to the exclusion of the widespread western toad B. boreas. However, B. exsul was always phylogenetically nested within B. canorus, suggesting that the latter may not be monophyletic. SSCP (single-strand conformation polymorphism) analysis of 372 individual B. canorus from 28 localities in Yosemite and Kings Canyon National Parks revealed no shared haplotypes among these two regions and lead us to interpret these two parks as distinct management units for B. canorus. Within Yosemite, we found significant genetic substructure both at the level of major drainages and among breeding ponds. Kings Canyon samples show a different pattern, with substantial variation among breeding sites, but no substructure among drainages. Across the range of B. canorus as well as among Yosemite ponds, we found an isolation-by-distance pattern suggestive of a stepping stone model of migration. However, in Kings Canyon we found no hint of

such a pattern, suggesting that movement patterns of toads may be quite different in these nearby parklands. Our data imply that management for B. canorus should focus at the individual pond level, and effective management may necessitate reintroductions if local extirpations occur. A brief review of other pond-breeding anurans suggests that highly structured populations are often the case, and thus that our results for B. canorus may be general for other species of frogs and toads.

690: ***+.136***

We present a comprehensive survey of genetic variation across the range of the narrowly distributed endemic Yosemite toad Bufo canorus, a declining amphibian restricted to the Sierra Nevada of California. Based on 322 bp of mitochondrial cytochrome b sequence data, we found limited support for the monophyly of B. canorus and its closely related congener B. exsul to the exclusion of the widespread western toad B. boreas. However, B. exsul was always phylogenetically nested within B. canorus, suggesting that the latter may not be monophyletic. SSCP (single-strand conformation polymorphism) analysis of 372 individual B. canorus from 28 localities in Yosemite and Kings Canyon National Parks revealed no shared haplotypes among these two regions and lead us to interpret these two parks as distinct management units for B. canorus. Within Yosemite, we found significant genetic substructure both at the level of major drainages and among breeding ponds. Kings Canyon samples show a different pattern, with substantial variation among breeding sites, but no substructure among drainages. Across the range of B. canorus as well as among Yosemite ponds, we found an isolation-by-distance pattern suggestive of a stepping stone model of migration. However, in Kings Canyon we found no hint of such a pattern, suggesting that movement patterns of toads may be quite different in these nearby parklands. Our data imply that management for B. canorus should focus at the individual pond level, and effective management may necessitate reintroductions if local extirpations occur. A brief review of other pond-breeding anurans suggests that highly structured populations are often the case, and thus that our results for B. canorus may be general for other species of frogs and toads.

691: ***-.086***

Through the Red Wolf Species Survival Plan. the captive red wolf (Canis rufus) population was developed with the Intent of reestablishing wild populations. One part of the plan was a survey for diseases that might occur as a result of population homogeneity or that might impede breeding success and reintroduction. For this survey, complete necropsies and histopathologic analyses were performed on 62 red wolves from 1992 to 1996. Major causes of 22 neonatal deaths were parental trauma, parasitic pneumonia. and septicemia. Common neonatal lesions included pododermatitis and systemic ascariasis. Cardiovascular anomalies and systemic parasitism were found in two juveniles. Causes of death in the 38 adults included conspecific trauma, neoplasia, or gastrointestinal diseases such as necrotizing enteritis. intestinal perforation, and gastric volvulus. Lymphosarcoma represented 50% of the fatal neoplasms. Three adults died from cardiovascular failure or hyperthermia during handling, and several adults were euthanized for suspected genetic diseases. Overall. the captive population had few significant health problems, but population fitness might be improved by continued removal of potentially deleterious genes from the breeding population and by modifying the husbandry of neonates and adults.

692: ***+.145***

The threatened status of redside dace, Clinostomus elongatus, in Michigan inhibits study and management of remnant populations of the species. We present a phenotypic approach to evaluate the use of redside dace from New York as behavioral and physiological models for Michigan

populations. We evaluated behavioral similarity by comparing patterns of microhabitat use and physiological similarity by comparing resting routine metabolic rates measured in the field. Variation between sites in available microhabitat made direct comparisons difficult; however, redside dace in Michigan and New York showed a common preference for mid-water positions in the deepest parts of pools under overhanging structure. Field measurements at 10 degrees C showed that Michigan fish had higher metabolic rates than rates predicted for New York fish at the same temperature, though biological significance of this difference is questionable. In laboratory experiments, we measured metabolic rate and upper thermal tolerance in relation to acclimation temperatures of 6-20 degrees C using redside dace collected from four streams in New York. Redside dace showed a significant increase in metabolic rate as acclimation temperature increased (Q(10) = 2.3). Critical thermal maxima (CTM) of New York redside dace also increased with acclimation temperature. Obstacles related to the transferability of habitat use data and variation in physiology due to uncontrolled and unmeasured environmental factors in the field lead us to urge caution when extrapolating behavioral and physiological characteristics between widely-separated populations of a species. Despite these obstacles, we described useful patterns of microhabitat use and provided estimates of physiological tolerances that will assist resource managers in the recovery of Michigan redside dace.

693: ***-.059***

Przewalski's gazelle Procapra przewalskii is endemic to China and is classified as Critically Endangered by IUCN-The World Conservation Union. Historically, the species occurred in parts of the provinces of Gansu, Inner Mongolia, Ningxia and Qinghai but now appears to be restricted to three populations around Qinghai Lake. These three populations-Bird Island, Hudong-Ketu and Yuanzhe-have all declined since 1988. The populations have been monitored since 1994 and the smallest, on Bird Island, appears to be on the brink of extinction, with only seven individuals being recorded in 1998. In the same year, the Hudong-Ketu population comprised 56 individuals (29.4 per cent males, 50 per cent females and 21 per cent juveniles) and the Yuanzhe population 51 individuals (29.4 per cent males, 43.1 per cent females and 27.5 per cent juveniles). The causes of the declines vary for each population but include loss of habitat as a result of desertification, poaching and, possibly, wolf predation. Human activity and high juvenile mortality are major threats to the continued survival of the gazelle. Conservation measures proposed are: (i) the establishment of a special reserve for Przewalski's gazelle; (ii) a study of the wolf-gazelle relationship and control of the number of wolves if necessary; (iii) a search for remnant populations of Przewalski's gazelle in other regions in their historical range and the identification of suitable sites for translocation and establishment of new populations.

694: ***-.059***

Przewalski's gazelle Procapra przewalskii is endemic to China and is classified as Critically Endangered by IUCN-The World Conservation Union. Historically, the species occurred in parts of the provinces of Gansu, Inner Mongolia, Ningxia and Qinghai but now appears to be restricted to three populations around Qinghai Lake. These three populations-Bird Island, Hudong-Ketu and Yuanzhe-have all declined since 1988. The populations have been monitored since 1994 and the smallest, on Bird Island, appears to be on the brink of extinction, with only seven individuals being recorded in 1998. In the same year, the Hudong-Ketu population comprised 56 individuals (29.4 per cent males, 50 per cent females and 21 per cent juveniles) and the Yuanzhe population 51 individuals (29.4 per cent males, 43.1 per cent females and 27.5 per cent juveniles). The causes of the declines vary for each population but include loss of habitat as a result of desertification, poaching and, possibly, wolf predation. Human activity and high juvenile mortality are major

threats to the continued survival of the gazelle. Conservation measures proposed are: (i) the establishment of a special reserve for Przewalski's gazelle; (ii) a study of the wolf-gazelle relationship and control of the number of wolves if necessary; (iii) a search for remnant populations of Przewalski's gazelle in other regions in their historical range and the identification of suitable sites for translocation and establishment of new populations.

695: *-.002*

Over the last 7 decades, 23 exotic fish species have been introduced into the inland waters of Greece. Some introductions were deliberately planned to take advantage of particular ecological or economic qualities of the species concerned. These include rainbow trout, Oncorhynchus mykiss (Walbaum), Pacific salmon, Oncorhynchus sp., vendace, Coregonus lavaretus (L.), grass carp, Ctenopharyngodon idella (Valenciennes), and silver carp, Hypophthalmichthys molitrix (Valenciennes). Other introductions, such as that of pumpkinseed, Lepomis gibbosus (L.), and false rasbora, Pseudorasbora parva (Temminck & Schlegel), were unintentional. Further transfers were made of species between various basins within the country, including common carp, Cyprinus carpio L., tench, Tinca tinca (L.), crucian carp, Carassius auratus gibelio (Bloch), bitterling, Rhodeus amarus (Bloch), and Aristotle's catfish, Silurus aristotelis Garman. Some species have become fully acclimatized and have built up important populations. In other cases, the transfers and introductions have had considerable negative impacts, particularly where introduced species have outcompeted native forms, as in the cases of the mosquito fish, Gambusia affinis (Baird & Girard), versus Greek toothcarp, Valencia letourneuxi (Sauvage), in the western Greek marshes, and of Aristotle's catfish versus the wels, Silurus glanis L., in Lake Volvi.

696: *+.134*

Reintroductions are often used for reestablishment of animal populations. The choice of age-class of released individuals, however, is often arbitrary or based on expediency. We developed a demographic model that predicts the relative efficiency of releasing juveniles or adults for a given life cycle. The model accounts for possible reduction of survival and fertility of released adults. It also incorporates demographic and environmental stochasticity to compare release strategies of varying duration and number of releases. We applied the model to the case of reintroduction of Griffon Vultures (Gyps fulvus) in southern France, for which accurate monitoring allowed us to estimate demographic rates and consequences of releases. Overall, for Griffon Vultures, it is more efficient to release adults than juveniles, despite the observed reduction of demographic parameters following release. This approach could be used for the reintroduction of other species.

697: *+.190*

A wide translocation program was conducted on neotropical fauna in French Guiana during the filling of a hydroelectric reservoir. Red howler monkeys (Alouatta seniculus) were studied because available data on their behavior in undisturbed conditions provided the basis for behavioral comparison with translocated animals. A resident howler population was present in the selected release area, but population densities were severely reduced by previous strong hunting pressure. A post-release survey was conducted during 18 months using radio-tracking and visual observations of 16 females. Most radio-tagged females settled in a stable home range. All but one of the released troops split up but all the monitored females were thereafter re-observed with resident congeners, sometimes after a period of solitary living. Most of the behavioral characteristics of the translocated individuals corresponded to previous observations on undisturbed animals. It is concluded that the behavioral abilities of this species helped them to

survive and settle in a new area. Population reinforcements or re-introduction of red howlers may therefore be conducted with a good probability of success. (C) 2000 Elsevier Science Ltd. All rights reserved.

698: *+.129*

Systematic study of the islands of Western Australia indicates the occurrence of 254 islands 100 ha or larger in area, with 90% in tropical seas. All but eight and 16 of these islands are free of the exotic carnivores, red fox Vulpes vulpes and cat Felis catus respectively. The few inshore islands to which these predators have spread naturally are instructive in deducing the swimming capability of the fox and cat. When distance from mainland is considered (greater than or equal to 1 km or greater than or equal to 2 km), 147 or 122 islands offer potential as sites for establishing populations of mammal species not otherwise represented on islands and with restricted distribution on mainland Western Australia. A procedure for selecting appropriate islands for translocation is outlined, emphasizing those islands without significant biological characteristics such as large colonies of nesting seabirds and the occurrence of native mammal species, but also considering island area, degree of isolation and habitats present. A prioritized list of candidate species of native terrestrial mammals, based on size of geographical range, natural absence from any Western Australian island, and the degree to which current threatening processes are not being addressed successfully, is provided for more detailed consideration. Because the cat and fox have occurred on the mainland coast of Western Australia for > 100 years and ca. 70 years respectively, it is suggested that islands within swimming distance and not yet occupied by these predators are unlikely to be colonized. These islands should therefore be considered as possible translocation sites. (C) 2000 Elsevier Science Ltd. All rights reserved.

699: *+.117*

Given that small populations are susceptible to extinction and inbreeding, a primary goal of re-introductions should be to maximize the initial rate of increase of a re-introduced population. We investigated how the rate of increase of newly re-introduced populations of artiodactyl species was affected by population characteristics, such as their size, sex-, and age-structure. While selecting the data of past re-introductions for our analysis, we controlled for factors that are known to affect success. The growth rate r of the populations increased with the number of animals released, up to about a population size of 20, at which point an asymptote was reached. All larger populations grew at a median r = 0.17, while several smaller populations declined. Small populations grew faster if they contained more mature individuals. Small, heavily female-biased populations were more variable in r than those of a more equal sex ratio. These female-biased populations also grew on average less well. Generally, populations of <20 were more variable in r than larger ones and both age and sex structure explained a significant portion of this variation. (C) 2000 Elsevier Science Ltd. All rights reserved.

700: *+.161*

Diet, prey availability, and breeding success were studied in a population of Bonelli's eagle (Hieraaetus fasciatus) in the province of Granada, SE Spain. The densities of the main prey species, European wild rabbit (Oryctolagus cuniculus), red-legged partridge (Alectoris rufa), wood pigeon (Columba palumbus), and rock dove (Columba livia), representing 90.2% of the biomass, were analysed in 19 territories occupied by pairs of Bonelli's eagles and 15 potential territories that were unoccupied but appropriate for nesting. We found no significant differences in prey density between the two types of territories. Contrary to other raptor species, we found no significant

relationship between the density of their main prey species and the distance of the neighbouring pairs, either between that density and breeding success. These findings, together with the prior knowledge of nest-site selection of this eagle in the study area, can be used for proper planning of conservation strategies. These should avoid unnecessary effort to increase the eagle's food supply, and should promote the reintroduction of individuals to suitable sites for Bonelli's eagle. (C) 2000 Elsevier Science Ltd. All rights reserved.

701: *+.099*

A survey of mammals and birds was carried out in a semi-deciduous forest fragment of 150 ha located in a zone of intensive agriculture in Ribeirao Preto, State of Sao Paulo, south-eastern Brazil. Line transect sampling was used to census mammals and birds during six days, totalling 27.8 km of trails and 27.8 hours of observation. Twenty mammal species were confirmed in the area (except bats and small mammals), including rare or endangered species, such as the mountain lion (Puma concolor), the maned wolf (Chrysocyon brachyurus), and the ocelot (Leopardus pardalis). The brown capuchin monkey (Cebus apella) and the black-tufted-ear marmoset (Callithrix penicillata) were found frequently, suggesting high population density in the fragment. Regarding the avifauna, 49 bird species were recorded, most of them typical of open areas or forest edges. Some confirmed species, however, are becoming increasingly rare in the region, as for example the muscovy duck (Cairina moschata) and the toco toucan (Ramphastos toco). The results demonstrate that forest fragment of this size are refuges for native fauna in a region dominated almost exclusively by sugar-cane plantations. Besides faunal aspects, the conservation of these fragments is of great importance for the establishment of studies related to species preservation in the long term, including reintroduction and translocation projects, as well as studies related to genetic health of isolated populations.

702: *-.046*

A critical aspect in the viability of large copper (Lycaena dispar) populations in Britain is the period of winter hibernation (diapause). Studies on captive-bred larvae, overwintering in England, showed that they suffered substantially greater mortality than did a Dutch population in its native habitat. Inter-habitat variance in survivorship, at both English and Dutch sites, was only marginally influenced by habitat features per se, although current management practices in the Netherlands may contribute significantly towards overwinter losses. Under identical field conditions in England, hibernal survival of wild-caught larvae, collected in the Netherlands, was superior to that of captive-reared British stock. Conversely, when larvae from both sources were 'overwintered' in captivity differences in recorded survivorship were not significant. From these two strands of evidence we tentatively infer a genetic divergence of the two stocks. Future re-introduction of this species should therefore involve direct translocation from wild populations rather than using captive-bred populations. (C) 2000 Elsevier Science Ltd. All rights reserved.

703: *-.029*

The burying beetle Nicrophorus americanus Olivier is an endangered species known to occur in disjunct populations in 6 states. Parsimony and maximum likelihood analysis of the nuclear ribosomal DNA first internal transcribed spacer (ITS1) sequences from 10 Nicrophorinae species revealed N. americanus to form a distinct clade with N. orbicollis Say. Genetic variation within and among 5 N. americanus populations, collected from South Dakota, Nebraska, Oklahoma, Arkansas, and Rhode Island, was studied. Ribosomal DNA ITS1 sequences from 14 beetles revealed 48 polymorphic and 20 informative nucleotide sites. N. americanus genetic divergence

was between 0.16 and 4.76%. We found little evidence that these 5 populations have maintained unique genetic variation. No nucleotide sites were found that were diagnostic for any of the 5 populations examined, indicating that these populations may not be necessarily treated as separate, independent objects of conservation. However, further genetic investigation is warranted before translocations are attempted among the remaining populations of the American burying beetle.

705: *+.183*

To prevent species from going extinct and to restore locally extinct species to conservation areas, conservationists have been attempting to create new populations of rue and endangered species. Such efforts are still at an early stage, with the basic methodology still being developed and many efforts resulting in failures or only modest success. The purpose of this work was to develop some general rules about how to carry out reintroduction efforts using four methods to create many new populations of eight perennial species. Our results demonstrate that the chances of success were greater when planting seedling and adult material rather than sowing seeds on the sites. Using larger adult material was more successful than using seedlings. Adult transplants also flowered and fruited right away, in contrast to plants derived from seeds, which rarely flowered even after several years. Digging up the site to expose the soil and reduce competition prior to sowing seed did not result in a greater establishment of seedlings. At many sites no plants survived at all, or success was low. These results emphasize the difficulties of establishing new plant populations. To increase the rate of success, attempts should utilize many sites, numerous seeds or plants, and various methods in order to develop a workable methodology for the species in question. Because of the difficulties of establishing new populations, conservation of rare and endangered species should first protect existing populations and only secondarily rely on reintroductions to ensure species survival.

706: *-.014*

As a restoration strategy, translocation of endangered plant populations may be a risky procedure with uncertain outcomes. Often, very little ecological information is known about these populations before, as well as after, translocation. The endangered vernal pool plant, Blennosperma bakeri, is a case in point. As a consequence of vernal pool habitat destruction, many populations of B. bakeri have been transplanted or translocated to various receptor sites. In this study, we examine the incidence of a thrips herbivore, Frankliniella minuta, in natural and translocated populations of B. bakeri in relation to floral patch size and degree of isolation and present implications for B. bakeri restoration and management. At a vernal pool mitigation site in California, U.S.A., thrips were present in both kinds of B. bakeri populations, and the range of thrips densities in both kinds of populations was similar for adults and immatures. Significant negative relationships between patch size, patch isolation, and numbers of flower heads infested with adult or immature thrips were found only among natural patches. Natural patches tended to be smaller in size than translocated patches, but patch isolation distances were similar. Some evidence suggests that B. bakeri is a F. minuta host plant, but the impact of these herbivores on B. bakeri remains unclear. Our findings, although preliminary, suggest that the distribution and abundance of F. minuta varied with floral patchiness, thrips stage, and the natural or translocated status of B. bakeri populations. In addition, our results provide a starting point for understanding the spatial context of plant-herbivore interactions in artificially altered vernal pool landscapes.

707: *+.184*

The application of molecular techniques to conservation genetics issues can provide important

guidance criteria for management of endangered species. The results from this study establish that PCR-based approaches for sex determination developed in other bird species (Griffiths and Tiwari 1995; Griffiths et al. 1996, 1998; Ellegren 1996) can be applied with a high degree of confidence to at least four species of Hawaiian honeycreepers. This provides a rapid, reliable method with which population managers can optimize sex ratios within populations of endangered species that are subject to artificial manipulation through captive breeding programmes or geographic translocation.

708: *+.146*

The whiptail lizard Cnemidophorus vanzoi is a large-bodied teiid found only on two islands (Maria Major and Maria Minor), off St Lucia, West Indies. In May 1995, 42 lizards from Maria Major were introduced to the smaller uninhabited Praslin Island on the same coastline. Three years post-release, we studied abundance, demography and morphometrics of the translocated lizard population, during a 6-month period covering wet and dry seasons. Age, sex, snout-vent length (SVL), body mass (BM), tail length, tail regeneration, and overall condition (moulting, reproductive condition, cuts, external parasites) of 107 animals caught during the study are analysed in the present paper. Comparisons are also made with the source population. A body condition index (CI (BM/SVL)), sex ratio (adult males : adult females), age ratio (adults :juveniles), and sexual size dimorphism ratio (SVL adult male:SVL adult females) were calculated for the study population. Distance sampling and mark-re-sight surveys were used to estimate population size and lizard density. A total of 155 +/- 26 individuals were estimated. The lizard population was found to have a high growth rate (r = 0.97-3.95). There were significant seasonal changes in lizard abundance. Seasonal differences in lizard numbers, BM and CI suggest either severe resource limitation during the dry season, or selective aestivation. A high frequency of tail autotomy may point to intense intraspecific competition as the island is relatively free from main predators such as the black rat Rattus rattles. Sex ratio, sexual size dimorphism and sexual dichromatism all indicate a territorial species in a generally non-territorial family (Teiidae). Some adult males seem to maintain juvenile colours. It is suggested that the introduced population has successfully colonized its new environment and that no significant change in the animals condition or size has occurred during the 3 years since translocation.

709: *+.155*

1 Nutrient dynamics and growth of the feathermoss Hylocomium splendens were studied in a subarctic birch woodland. My aims were to estimate the species' nutrient use strategy (and its determinants: mean residence time (MRT) and annual nutrient productivity (aNP)) and to unravel possible traits related to nutrient conservation and their implications for ecosystem nitrogen flux. Three methods to estimate nutrient losses in bryophytes were evaluated: a conventional growth analysis technique, a retrospective analysis and a N-15-tracer approach.2 Estimates for nitrogen retention varied between 3 and 10 years as a result of the different N pools considered by the three methods. Growth analysis results depended on the distinction between live and dead tissues, whereas retrospective analysis gave valuable information on N release from decaying segments but did not measure MRT within the living segments. Valid estimates of MRT were obtained by a N-15-tracer approach.3 MRT and aNP of H. splendens were similar to values typically found in woody evergreen vascular plants. Efficient nutrient recycling and a relatively long segment life span were responsible for the long residence time of nitrogen. Feathermosses show efficient nutrient acquisition, nutrient recycling and acropetal transport; nutrient losses will therefore be small.4 Dominant bryophytes may retard the nutrient turnover at the forest floor through their production of acidic nutrient-poor organic matter and their negative effect on soil temperature, and

they may therefore function as autogenic ecosystem engineers.

710: *+.136*

In 1990, the myrmecophilous butterfly species Maculinea teleius and Maculinea nausithous have been reintroduced into the nature reserve "Moerputten" in The Netherlands. The introduced population of M. teleius expanded during the first three years. In contrast, the newly established population of M. nausithous declined in the year after the re-introduction but expanded later. After a considerable increase in numbers, this species can nowadays be seen in three subpopulations in and outside the nature reserve. While the area populated by M. nausithous is still expanding, M. teleius can only be found on the meadow where it was released and has not dispersed at all. To enable further expansion of both species on road verges and canal borders, the management of these landscape elements had to be changed to enhance the development of rough vegetation where the specific host ant species occur. This was realized by an agreement ("Convenant Pimpernelblauwtjes") in which the province, local communities, water and nature organizations, farmers and Dutch Butterfly Conservation participate.

711: *+.182*

Wilful introductions of insects already have a long history. Predacious and parasitic insect species have been introduced in attempts to control pest insects and weeds in agriculture, horticulture and forestry. Nature conservation bodies also practise insect introductions, but now in order to safeguard endangered species or to restore depaupered communities. However, a very large number of insect releases were done surreptitiously by butterfly lovers. The average rate of success in establishing new populations is very low, about 10%, and similar to that of establishment by hazardous introductions. From these cases we can learn that: 1) the quality of the site as a habitat of the species involved is often overestimated; 2) success depends on the stage in development at the time of the release and this, on its turn, depends upon the species; 3) geographical differences between areas of origin of the donor population and the site of introduction may also frustrate the success because of the incompatibility of diapause and bet-hedging adjustment, and 4) the migration behaviour of the species has to be taken into account. The current paper stresses the importance of assessing the ecological potential of the species and their habitat requirements. An approach is proposed, based on analyses of biological features of the species, and some examples are presented as an illustration. Nowadays most nature conservation bodies have accepted the release of insects as a management practise. One of the conditions for release is that the species involved should have inhabited the site or its surroundings in the past, and that it has disappeared due to human activities. The Dutch government intends to subsidise management and development of nature reserves only when well-defined results are obtained, such as the conservation of a number of so-called target species. The presence of these target species in nature reserves is evidence for successful management and may thus have financial consequences. This will undoubtedly increase the demand for re-introductions.

712: *+.121*

We investigated the genetic and morphological status of an endemic subspecies of deer mice (Peromyscus maniculatus anacapae) on Anacapa Island of California through mitochondrial DNA (mtDNA) analysis, morphometric discriminant function analysis, and population viability analysis. We sought to assist the development of a management plan that may include captive breeding, reintroduction, or translocation of mice following eradication of introduced rats. The genetic and morphological data were used to investigate whether the subspecies or populations on

each of the three islets of Anacapa represent evolutionarily significant units for conservation. The status of the East Anacapa population was of particular concern because deer mice have recently been caught there following more than 15 years of no records of deer mice. Sequences of the mtDNA cytochrome oxidase c subunit II gene (COII) indicated that the Anacapa subspecies had unique haplotypes not found on neighboring islands or the mainland and thus represents a distinct unit for conservation. Further, one of these haplotypes was shared among the islets, including most of the East Anacapa mice, suggesting that the East Anacapa population had either recovered from a severe bottleneck or had been recolonized by P. m. anacapae, but that it was not derived from other subspecies. Discriminant function analysis of morphological data also supported classification of the East Anacapa mice as P. m. anacapae. The mitochondrial mtDNA sequence data yielded estimates of two to seven migrants per generation among the Anacapa islets, suggesting a functioning metapopulation. Incorporating these data and information available on the life history and demographics of deer mice, we used a novel type of population viability analysis to develop a captive breeding and reintroduction plan for Anacapa deer mice should they be eradicated along with the rats. A sine wave was incorporated into the population viability analysis to simulate population size cyclicity. Our study provides baseline information needed for developing a comprehensive conservation and management plan for a threatened island endemic.

713: ***+.102***

Populations of the endangered Rutidosis leptorrhynchoides (Asteraceae) have been reestablished in conservation reserves to reduce their risk of extinction in the wild. The reproductive success (Number of seeds per inflorescence, percent seed set, germinability) of five small reestablished populations (at 5-10 years after establishment) was compared to that of two large natural remnant populations from which they were derived. Initial growth rates under glasshouse conditions were compared for seedlings derived from seed from remnant and reestablished populations. Seed set per inflorescence in all five reestablished populations was equal to or greater than seed set in remnant population plants. The resulting seed was as germinable, and in some cases more germinable, than seed derived from remnant populations; the seedlings then grew as large or larger than remnant population seedlings frown under glasshouse conditions. Fitness reductions in small reestablished populations of R. leptorrhynchoides are not evident at the reproductive stage. Hence, these populations have some potential to maintain natural regeneration processes and might therefore positively contribute to the conservation of this species.

714: ***+.240***

Koalas are the only living member of their family and therefore deserve serious conservation consideration. Koalas have low levels of genetic variation within and among populations in the southern part of their range, where they have experienced many relocations and population crashes since European colonization of Australia. The importance of this change in variation is underlined by preliminary indications that levels of genetic variation may affect fitness in koalas. Techniques have been developed to help identify and monitor genetic problems in koalas and to provide the information and tools to make genetic management an integral part of koala conservation. The koala is currently at an appropriate point for conservation intervention: there is clear evidence of decline in some populations, but the existence of other robust populations offers the possibility of a variety of creative solutions to their conservation problems. Managers should aim to maintain this species' current ecological amplitude (the range of environments in which populations are found) and minimize the loss, fragmentation, or decline of populations. There are no data to suggest that any population requires genetic supplementation. The concepts of evolutionarily significant unit (ESU) and management unit (MU) can be useful in the genetic management of

koalas, including monitoring and management regimes. But ESUs and MUs can also be misleading if they are not interpreted carefully in terms of population history and the ultimate goal of management. Translocations should not involve extensive use of stock from a single source, especially those with low grade variations, and they require careful management to avoid possible problems when individuals encounter novel strains of the pathogen Chlamydia pecorum, because several genetically distinct strains have been found in koalas, some of which may derive from introduced species. Genetic indicators can and must make considerable contributions to koala management, but they require careful interpretation.

715: *+.048*

The takahe (Porphyria hochstetteri) is a highly endangered flightless rail, endemic to New Zealand. Since 1984, a total of 24 takahe have been translocated from a small, alpine population of approximately 120 in Fiordland and successfully established on four predator-free lowland islands with introduced pasture grasslands. However, island takahe produce fewer juveniles per egg due to hatching failure being twice as high as it is in the natural population in Fiordland. A comparison among several small, inbred populations of New Zealand birds indicated those that are the result of translocations to new habitat types (takahe and kakapo Strigops habroptilus), suffered much higher rates of egg infertility than birds that have remained within their natural habitat range. For takahe, whether breeders had been translocated as juveniles or had been raised on the islands was a poor predictor of reproductive success. The coefficient of inbreeding was high for island takahe but high infertility and low juvenile productivity were features of breeding pairs whether parents were related or not. However, this result might be expected if takahe were already inbred before they were translocated to the islands. If high egg infertility is a consequence of an inbred population being translocated to a new or different habitat, then poor breeding success is something that managers may have to live with in the short term until there is local selection for better breeders. In more general terms, the results of the study have implications for the successful translocation of individuals from inbred populations. (C) 2000 Elsevier Science Ltd. All rights reserved.

716: *+.086*

We examined the genetic variability and structure in 20 subpopulations belonging to seven populations of the threatened snake Vipera aspis in the Swiss Jura mountains (n = 114) and in two subpopulations from central France (n = 12) using RAPDs (seven primers produced 70 polymorphic bands). The analysis of molecular variance (AMOVA) showed that the within-populations genetic variability accounted for 87.0% and the among-populations variability for 13.0% of the total variability. The within-subpopulation genetic variability accounted for 74.3%, the among-subpopulation variability within populations for 21.5% and the among-population variability for 4.2% of the total variability. An unweighted pair group method with arithmetic means (UPGMA)-cluster analysis based on mean Jaccard distances did not assemble the subpopulations according to their populations. The combined results of the AMOVA and the cluster analysis suggest that gene flow may have occurred over large parts of the snake's distribution area. In large subpopulations the genetic variability was larger than in small subpopulations. There was no difference in the genetic variability between connected and isolated subpopulations. We suggest that habitat management may prove to be more effective in maintaining the genetic variability of V. aspis in the north-western Swiss Jura mountains than any translocation of individuals. (C) 2000 Elsevier Science Ltd. All rights reserved.

717: *+.007*

Brachypelma, a genus of nine endangered tarantula species in Mexico, is the only group of spiders included in Appendix II of CITES, owing to habitat degradation and illegal trafficking. However, while the majority of the nine species of Brachypelma are thought to be threatened, little is known of their ecology and distribution. Brachypelma klaasi is the rarest species, occurring in a few isolated populations on the Pacific coast of Mexico. We present an analysis of population distribution and micro-habitat requirements of B. klaasi over different spatial scales within the biological reserve at Chamela, Jalisco as part of a wider ecological study of the endangered Brachypelma group. Burrows and dispersing spiders were confined to a southern area of the reserve covering approximately 0.5 km(2). Within this area, burrows were not aggregated at lower spatial scales (2(4)-2(16) m(2)), unlike other related species. Also, there was no evidence that intra-specific interactions (either positive or negative interactions) influenced the distribution of burrows. Distribution of burrows at low spatial scales was related to low afternoon temperatures and high humidity in mid-summer. These abiotic factors may influence the survival and development of eggs and spiderlings, and appear to be more important in governing the distribution of B. klaasi than are food resources or intra-specific interactions. We discuss how these findings may facilitate the re-introduction of captive-bred individuals of B. klaasi and other Brachypelma species.

718: *-.192*

The Orinoco crocodile Crocodylus intermedius historically habited all the Orinoco river basin in Venezuela and Colombia. The American crocodile Crocodylus acutus range was all the Venezuelan coast, lakes, lagoons and tributary rivers of the Caribbean sea. After the commercial indiscriminate hunting of the first half of the XX century the Orinoco crocodile was left in critical levels and in danger of extinction. The American crocodile is considered a threatened specie. In the eighties recovery programs of both species started. Census and monitoring of the wild populations, protection of its habitats and a captive breeding program for future reintroduction and recovery of its populations. For that purpose on 1998 there were four crocodile farms of the Orinoco crocodile and one of the American crocodile. The diseases observed in the farms were: NEONATES (omphalitis, congenital anomalies and intoxications); HATCHLINGS AND JUVENILES (nutritional deficiencies such as vitamins A, E and B 1 deficiencies, scoliosis, osteodystrophia, bacterial, viral and mycotic diseases, hatchling alligator syndrome. Parasitary casuistic of protozoa, nematodes and trematodes. Trauma and shock) and ADULTS (nutritional deficiencies, xiphosis, osteodystrophia, nutritional secondary hyperparatyroidism, trauma, wounds and shock). In hatchlings and juveniles annual mortality rates reached until 70% per crocodile farm. This data was collected between 1985 and 1998.

719: *+.064*

Restoration of formerly nutrient-poor and species-rich grasslands generally leads to an increase in species diversity. However, species without a persistent seed bank and with poor dispersal ability often do not re-establish spontaneously. Here, reintroduction is an option. If existing populations are comparable in their genetic composition, any population will do. This is not the case if populations have local adaptations. Unfortunately, whether populations are adapted locally is not easily determined, in contrast to assessing differentiation using neutral genetic markers. We used AFLP to study genetic diversity of Cirsium dissectum and Succisa pratensis within and among several Junco-Molinion plant communities in the Netherlands (up to 200 km apart) that were potential source populations, and followed the reintroduction using seeds from these populations. Also, vegetative growth phase characteristics of three populations of C. dissectum were analyzed under controlled conditions. Most of the genetic variation in these cross-fertilizing species was

found within populations. Small but significant genetic differences in band frequencies were found among populations (F-st 0.100-0.135). The first generation of reintroduced plants contained less polymorphic bands than the source populations. The genetic differences caused by reintroduction using a limited number of seeds (founder effects) were significant in all except one case (F-st 0.012-0.101 between source and corresponding reintroduced population), but the magnitude was smaller than the source population differentiation. In assignment tests, reintroduced populations resembled their source population more than any other population, but all populations contained sizeable proportions of plants that were assigned to most similar plants from other populations, indicating that the populations are only marginally distinct. Calculations show that reintroduction from more than one source population introduces significantly more polymorphic bands into the new population, capitalizing on the existence of band frequency differences among populations.

720: ***-.189***

The Siamese crocodile Crocodylus siamensis, now regarded as one of the world's most endangered crocodilians, was formerly common in the wetlands of southern Vietnam. Populations are thought to have declined in recent years, although quantitative status assessments are unavailable. We surveyed five areas previously believed to harbour he only remaining Siamese crocodile populations in Vietnam, and our results strongly suggest that viable populations are no longer extant. Crocodiles were not observed during spotlight counts of Crocodile Swamp (Nam Cat Tien National Park) or Lac Lake. Crocodiles do not occur in Upper or Lower Krong Pach Reservoirs, and these degraded sites no longer constitute suitable habitat. Remnant populations may exist in the Sere Pok River and Tay Son Lake, but these crocodiles remain subject to persecution. Population declines are attributed to a combination of hunting, habitat destruction, incidental capture in fishing nets and collecting for crocodile farms. Reintroduction to Nam Cat Tien National Park is recommended.

721: ***+.199***

The Canadian Swift Fox Reintroduction Programme lasted from 1972 to 1997. From 1983 to 1997, a total of 841 captive-raised swift foxes and 91 translocated swift foxes were released in the Canadian Prairies. In South Central Saskatchewan, 406 captive-raised animals and 14 translocated animals were released from 1990 to 1997. This area was used to develop new release methods (in particular, portable protective shelters (PPS)) and saw the co-operation of a nongovernmental organization (NGO) (Cochrane Ecological Institute (CEI)) with two levels of government (Heritage Canada (Grasslands National Park) and the Government of Saskatchewan). A 1996-97 survey of swift foxes in South Central Saskatchewan estimated the population to be 87 animals. No attempt has been made to establish if this population level is sustainable.

722: ***+.022***

The Red Kite Milvus milvus was formerly common and widespread throughout Britain, but by the end of the nineteenth century, as a direct result of human persecution, it had been reduced to a handful of pairs in mid Wales. In 1989, a re-establishment programme began in England and Scotland, and breeding populations have already become established in southern England, the English Midlands and northern Scotland. Intensive monitoring of released birds, and studies of the populations re-establishing themselves in England, have shown that the Red Kite is well adapted to our lowland landscapes and is able to thrive at high densities in the release areas. The Red Kite's generalist diet and lack of specialist habitat requirements should enable it to regain its former status in Britain as one of our most widespread and familiar birds, provided that certain threats

resulting from human activity can be reduced. In particular, the Red Kite is vulnerable to the use of illegal poison baits, secondary rodenticide poisoning, and shooting. The Red Kite is generally very popular with local people, and is proving to be an extremely useful 'ambassador species' for raptor conservation at a time when some specialist-interest groups view increasing raptor populations as a problem.

723: *+.005*

The Hihi (Stitchbird, Notiomystis cincta) is an endemic New Zealand honeyeater that after European colonization survived on only one offshore island. Attempts to reintroduce Hihi to other islands have been unsuccessful, with populations slowly declining. The main hypothesis for these declines was an inadequate year-round supply of carbohydrate food (nectar and fruit) due to human impacts on the forest habitat. When Hihi were reintroduced to Mokoia, another island with regenerating forest, we tested whether survival was limited by carbohydrate food in the year after release. We conducted an "on-off" experiment in which ad libitum sugar water was available to birds for 2 out of every 4 weeks. We compared the masses of individual birds at the end of on and off periods to identify times when birds lost condition with no supplementary food and would be likely to starve. We also used mark-recapture analysis of resighting data to test whether mortality rates were higher when supplementary food was unavailable. The only effect of the supplementary food was that Hihi spent less time foraging for nectar and fruit and more time foraging for invertebrates. There was no time of year when birds lost mass when the food was taken away, and survival rates were not significantly lower when supplementary food was absent. The low (38%) annual survival rate could not be accounted for by shortage of carbohydrate food, and population viability analysis suggests that the population is likely to slowly decline. Our experiment shows that declines of reintroduced Hihi populations may be unrelated to food supply and that alternative hypotheses should be developed and tested. Our results also show the importance of using experimental methods, when possible, to test hypotheses about factors thought to be limiting reintroduced populations.

724: *+.016*

The remnant wild population of California Condors (Gymnogyps californianus) of the 1980s exhibited a rapid population decline caused by high mortality rates among adult and immature birds. The most prominent mortality factor was lead poisoning resulting from ingestion of bullet fragments in carcasses. Successful captive breeding has allowed many birds to be released to the wild since 1992, based originally on an assumption that exposure to lead could be prevented by food subsidy. The mortality of released birds, however, has generally exceeded levels needed for population stability calculated from simple population models. Collision with overhead wires was the most frequent cause of death in releases before 1994. Lead poisoning again surfaced as a problem starting in 1997 as older birds began feeding on carcasses outside the subsidy program. Although poisonings have been treated successfully by chelation therapy in recaptured birds, food subsidy is proving an ineffective solution to lead exposure. The best long-term solution appears to be either the creation of large reserves where hunting is prohibited or the restriction of hunting to nontoxic ammunition in release areas. Until sources of lead contamination are effectively countered, releases cannot be expected to result in viable populations. In addition, problems involving human-oriented behavior have resulted in the permanent removal of many released birds from the wild. The most promising reduction in human-oriented behavior has been achieved in one release of aversively conditioned, parent-reared birds. Rigorous evaluation of the factors reducing attraction to humans and human structures has been hampered by confounding of techniques in releases. Behavioral problems could be more quickly overcome by adoption of a

comprehensive experimental approach.

725: *+.159*

Translocation is an important tool in conservation biology. However, translocation success is generally low for numerous animal species, therefore experiments are required for improvement. We carried out an experimental translocation of European wild rabbits (Oryctolagus cuniculus). The results may be of great interest for conservation biology, although we used a common species. As rabbits are known to experience a high mortality during the first days following release, it was necessary to assess the influence of handling trauma and environment novelty, respectively, and ways of suppressing them. Both tranquillization treatment during handling and a 'soft' release protocol (acclimatization pens in the new territory) were tested. Tranquillization did not increase survival, while the effect of acclimatization depended on sex. Females survived better when acclimatized, while males showed the opposite tendency. This difference is discussed in terms of sex-specific social behaviour, which is possibly an important correlate of translocation success. Finally, environmental stress seemed Lo override handling stress in determining the level of early survival for translocated wild rabbits.

726: *+.127*

Pere David's deer (Elaphurus davidianus) was reintroduced to China after its extirpation in the country in the nineteenth century. The first reintroduction was made to the Beijing Milu Park in 1985 and the second to the Dafeng Milu Natural Reserve in 1986. Those reintroduction sites have different characteristics. The Beijing Milu Park is near a metropolitan region and has limited size, whereas the Dafeng Natural Reserve is located in a coastal region with little human settlement. Except for a nuclear breeding herd, the Pere David's deer of the Beijing Milu Park were shipped to other sites in eastern China to reduce the grazing pressure on the park vegetation and expand the distribution of Pere David's deer. The Dafeng Milu Natural Reserve has the potential to hold a large population of Pere David's deer ii more land were acquired to expand the natural reserve. By the end of 1997, total number of Pere David's deer in China reached 671, half of which were offspring from animals in Beijing Milu Park. The Beijing and Dafeng herds remain as 2 of the largest Pere David's deer populations in China, the Beijing population accounting for 19% and the Dafeng population for 45% of the total population. Pi re David's deer of Beijing Milu Park have been transferred to 12 other locations. During 1987-1997, birth rates and net growth rates did not differ between populations (P=0.09 and P=0.74); however, death rates differed (P<0.01). After the period of adaptation, there was a period of population expansion, during which the birth rate in the Beijing and Dafeng populations declined as the population continued to grow. However, population density explained little about variation in birth rates in the Beijing and Dafeng populations; a longer-term monitoring of those populations is needed to assess plausible density-dependent population growth.

727: *+.163*

Wilderness in the United States has been controversial since its establishment in 1964. One school Of thought argues that wilderness policy should prescribe wilderness boundaries and prevent management. Another believes that management practices must be used to maintain wilderness values. We described and tabulated wildlife management activities practiced in all 273 designated wilderness areas in the southwestern United States. We conducted a survey of personnel involved with wildlife management in wilderness including 60 employed by the United States Forest Service (USFS), 19 by the Bureau of Land Management (BLM), 14 by the National Park Service

(NPS), 7 by the United States Fish and Wildlife Service (USFWS), and 10 by state game and fish agencies. Wildlife management activities (not including population surveys and research) occurred in 174 wilderness areas. The most frequently mentioned wildlife management activities were maintaining artificial water developments, conservation education efforts designed to protect wildlife, and removing or controlling non-native animal species.

728: ***+.113***

The eastern wild turkey ((Meleagris gallopavo silvestris) experienced population declines in the United States beginning in the 19th century and continuing through the early 20th century. Transgeographic reintroduction efforts have reestablished eastern wild turkeys throughout most of their historic range but effects of reintroductions on genetic composition of resulting populations are unclear. Most reintroduced populations are maintaining self-sustainable populations. However, for unknown reasons, the population in southeastern Oklahoma decreased by 77% from 1980 to 1994. We used multi-focus DNA fingerprinting to evaluate levels of similarity, heterozygosity, and allelic diversity within and among wintering flocks occurring on the Pushmataha Wildlife Management Area in southeastern Oklahoma. Results from two restriction enzyme-probe combinations revealed mean similarity, heterozygosity, and allelic diversity similar to those in other outbred avian populations. Therefore, the decline of wild turkeys in southeastern Oklahoma is not related to reduced genetic variability, but may be due to biotic and abiotic factors not accounted for in this study.

729: ***+.126***

Populations of lesser prairie-chicken (Tympanuchus pallidicinctus), apparently abundant in southeastern Colorado prior to EuroAmerican settlement, reached a low during the "Dust Bowl" years in the 1930's. Restoration of native sand sagebrush (Artemisia filifolia) grasslands and management of grazing on the Comanche National Grasslands by the United States Forest Service have resulted in modest population increases of lesser prairie-chicken since the 1960's. Past translocation efforts to expand distribution in Colorado and increase population size were not successful because too few birds were released and habitats may not have been suitable. Continued restoration and management of degraded habitats, as well as successful transplants into suitable habitats, could result in substantial increases in both distribution and population size. Precipitation appears to be a major factor affecting population changes as reflected in annual counts of active leks and males. Present populations in Colorado are estimated at less than 1,500 breeding individuals.

730: ***+.445***

This meeting contains abstracts of 15 papers, written in English, covering topics in ornithology, including endemism, seabird translocations, conservation biology, trophic interactions, species interactions, molecular ecology, nesting behavior, breeding success, population studies, and bird pest management in various New Zealand bird species.

733: ***+.164***

What features of the mustelids might yield insight into issues of general importance for conservation and ecology? This review focuses attention on correlates of threat, intra-guild competition and reintroductions. Using the IUCN classification of the status of mustelid species, I constructed a statistical model of correlates of threat. This suggested that otters and martens, and

mustelids with small distributional range sizes, were the most threatened. Whether or not a species was hunted and its habitat specificity did not correlate with threat. This analysis should be treated as indicative only, because the response variable (threat) was not totally independent of the explanatory variables. There is growing evidence that intraguild predation influences mustelids. In Scotland, there is some evidence for spatial segregation between Pine Martens and Red Foxes (a potential predator), Pine Marten relative abundance was higher in woodland interior than woodland edge. Red Foxes were equally abundant in woodland edge and interior. However. habitat fragmentation (woodland patch size) was the strongest correlate of relative abundance of both species. Research on mustelids has demonstrated the importance of appropriate release techniques during reintroductions. It has also shown that reintroductions of small numbers of animals can be successful, provided that they are conducted in high quality habitats, The implication is that habitat suitability assessments need to be given high priority. In this: respect, research in Britain suggests that inferring habitat needs from areas of relict distribution may lead to highly misleading conclusions.

734: ***+.508***

Granite outcrops in Western Australia contribute significantly to the conservation of several species of mammal. In the arid zone and wheatbelt, granite outcrops are particularly important for Petrogale spp and the euro Macropus robustus. Other species such as brushtail possums and chuditch also take advantage of the extra shelter provided by granite outcrops. The many granite islands off the south coast support remnant populations of threatened terrestrial and marine mammals. Granite rocks provide mammals with refuge from heat and introduced predators, and they are capable of modifying fire and moisture regimes to the benefit of some species. Many of the larger wheatbelt nature reserves contain granite outcrops and these will be important as mammal reintroduction sites for the Western Shield fauna recovery program. Fox control has been shown to be important for medium-sized mammal conservation on granite outcrops. Rock-wallabies in particular increase in abundance and this offers opportunities for nature based tourism. Appropriate management of granite outcrops, including islands, will contribute significantly towards the enhancement of mammal diversity in Western Australia.

736: ***+.073***

Between the second half of the 19th century and the late 1970s beavers, Castor fiber, became extinct in Slovakia. Between 1976 and 1988, 42 beavers were released in Austria in the area neighbouring southwestern Slovakia. On the basis of direct or literary records of beaver observations (dead or alive) and activities (tree cuttings, huts and dams), we describe the recent and present status of the beavers in southwestern Slovakia. Austrian beavers and their offspring immigrated into the Zahorska lowland and the Morava-river basin. In this area of developed and cultivated landscape, the habitat conditions are favourable to beavers. In 2000, we here found some 60-65 beavers, including 7-8 families. The Danube stream, including the still-water system, beneath the city of Bratislava has also been colonized by beavers between 1984 and 1992. In spring 1993, the construction of the hydroelectric power station near Gabcikovo and Cunovo was completed. Since then it prevents the beavers from migrating to the still water downstream the reservoir created by the barrages. At present, there are only 3 inhabited beaver sites (6-8 beavers) here, including one with reproduction. Upstream the reservoir and barrages we found 9 beaver sites with 20-25 beavers along a 4-km-long bank. Some beavers moved from west to east into the derivation-canal and were here in danger. Therefore the colonization of the Danube still-water system downstream the barrages is now possible only from the north east via the long way of the Male Karpaty mountains (watershed) and the Vah and Dudvah rivers into the Danube. This is

assessed by the presence of beavers in the localities of Breza pod Bradlom, Mostova, Horne Saliby and Tomazikovo. The total number of beavers in the area of Lower Austria and southwestern Slovakia (Danube, tributaries and brooks) presently amounts to about 540 beavers (respectively about 450 and 90), so that it is justified to speak about a genetically viable population. To maintain and increase this number it is imperative to build an ecological bypass allowing beavers and other aquatic animals to escape the barrages. The still-water systems of the former Danube riverbed are offering several beaver sites and may be considered a potential settlement area. The beaver reintroduction and immigration from Poland into the eastern part of the Slovakia (Beskydy Mountains) is presently insignificant.

737: ***+.046***

At least 31 species of marine mammals, marine and coastal birds, and marine and anadromous fish have disappeared temporarily or permanently from the coasts of The Netherlands and in most cases also from the south-eastern North Sea (south of 54 degrees N) during the past 2000 years-In 18-22 cases, the disappearance was probably due to overexploitation. For 9-12 species, physical destruction of their habitat was involved and, for 3-5 species, pollution probably played a part. Five species have returned to the area; these are doing very well. Three species may return through expansion of populations elsewhere. Anadromous fish and demersal fish species that have disappeared because of bottom-trawling in the North Sea have little chance of returning under the present conditions. For the gray whale (Eschrichtius robustus) and the Dalmatian pelican (Pelecanus crispus) suggestions are made to investigate the possibilities for re-introduction. (C) 2000 Elsevier Science Ltd. All rights reserved.

738: ***+.136***

The importance of structure in grasslands to arthropods is emphasised. Community dynamics below ground are briefly described. The characteristics of stenophagous, polyphagous, predacious and parasitoid arthropods in relation to structure are outlined. Tall grassland supports more species, individuals and a greater diversity of arthropods than short swards, but some species are characteristic of the latter. The classification of structure in grassland is briefly reviewed. Change in grassland structure occurs through the opposing forces of succession and, in most cases, management. The commonest method of management is grazing, which is characterised by selectivity in the foliage eaten, treading of the sward and deposition of dung. Cutting is sudden, but its effects of defoliation are similar to those of grazing. Burning is considered in less detail. Ploughing, rotovating, re-seeding, fertiliser application, translocation and set-aside are generally forms of agricultural or 'creative' management. Human treading can have severe effects on invertebrate diversity and abundance. Management interacts with other factors, such as seasonality, timing, topography, site-specific characteristics and connectivity to produce varied effects on arthropods. The various types of management systems, reclamation, maintenance, agricultural, rotational and others are discussed in relation to management plans and objectives in conservation. It is concluded that conservationists need to be more aware of grassland dynamics, that management by reference to past land-use must be tempered by full consideration of ecological factors and conservation aims and that 'practical' and 'theoretical' conservationists need to interact more effectively. (C) 2000 Elsevier Science Ltd. All rights reserved.

739: ***-.030***

During a 7 year period 30 captive born lynxes were released after spending an average of 192 days in an in situ adaptation enclosure. 13 individuals were found dead after a mean post release

lifespan of 271.5 days due to traffic accidents, poaching or unknown disease. Impact of terrestrial traffic and potential danger from contagious viral diseases of domestic dogs and cats as well as poaching are presumed to be the main anthropogenic factors negatively influencing desired lynx population growth. There is a demand for future investigation regarding canine and viral disease seroprevalence in domestic carnivores and lynxes in the Kampinoski National Park area. 5 lynxes repeatedly preyed on domestic lifestock and had to be recaptured. 4 lynxes released for a second time succeeded in hunting wild prey species. Damages on domestic lifestock could not simply be deduced to the use of captive born predators at all since predator opportunism by switching from hunting game to poultry can be observed in wildborn lynxes too. Even among siblings individual variances of adaptation to the wild environment were mainly a result of differences in inborn factors influencing learning of certain behavioural skills rather than being dependant on maintenance techniques at their birth enclosures or certain ways of pre-release management. 6 adult females reared pups during one year or repeatedly. 12 distinct observations of independant juvenile individuals were reported from all parts of the park. 7 out of 8 recorded dislocations outside the National Park area over distances between 10-50 km directed toward neighbouring nature reserves and were recorded during the past two years. Descending from these reintroduction activities a reliable minimum population of 19 lynxes of which 9 individuals represent the wildborn filial generation exists in central Poland at present. During the last 5 years of the project the National Park management improved lynx habitat by acquiring former farmland increasing total area of natural succession and renaturalization of drainage canals. Thus potential lynx pup rearing areas increase. Due to the numbers of sight reports of wildborn individuals in the National Park area and due to recent lynx dislocation in two neighbouring nature reserves the projects next phase favours ceasure of further releases and monitoring natural population development.

740: *+.280*

We reviewed the history and conservation of Sophora toromiro, a species that has been extinct in the wild since 1960 but has survived as scattered individuals in botanic gardens and private collections. The short-term conservation of S. toromiro is dependent on the management of surviving ex situ stocks. Ibis is being achieved through international collaboration by a working group established to coordinate the conservation management of the species. Molecular evidence indicates that the species retains greater genetic variability than expected. The greatest amount of genetic variability was located in specimens outside botanic garden collections. No unmodified natural habitat survives on Rapa Nui (Easter Island); so opportunities to establish a viable wild population are limited. Evidence from past reintroductions indicates that the best shortterm opportunity for the species is through conventional horticultural management in botanic gardens and traditional farm plots on Rapa Nui. Some extinct-in-the-wild taxa (sensu World Conservation Union 1994), such as the Toromiro, retain genetic variability and appropriate reintroduction sites exist These taxa represent valid priorities for conservation management.

741: *+.236*

When translocating individuals to found new populations, managers must allocate limited funds among release and monitoring activities that differ in method, cost, and probable result. In addition, managers are increasingly expected to justify the funding decisions they have made. Within the framework of decision analysis, we used robust optimization to formulate and solve different translocation problems in which both population growth and future funding were uncertain. Performance criteria included maximizing: mean population size and minimizing the risk (1) of undesirable population-size outcomes. Robust optimization provided several insights into the design of translocation strategies: (1) risk reduction is obtained at the expense of mean

population size; (2) as survival of released animals becomes more important, funds should be allocated to release methods with lower risks of failure, regardless of costs; (3) the performance gain from monitoring drops as the proportion of a fixed budget required to pay for monitoring increases; and (4) as the likelihood of obtaining future funding increases, more of the existing budget should be spent on building release capacity rather than saved for future operating costs. These relationships highlight the importance of performance criteria and economic costs in determining optimal release and monitoring strategies.

742: *+.143*

Native subspecies of cutthroat trout Oncorhynchus clarki have declined drastically because of the introduction of nonnative salmonids, overharvesting, and habitat degradation. Conservation of most declining subspecies will include establishing new populations through translocation of genetically pure fish. Recovery of greenback cutthroat trout O. clarki stomias has been ongoing for 25 years, so the attempted translocations of this subspecies provide unique empirical information to guide recovery of other nonanadromous salmonids. We compared 14 translocations that successfully established populations of greenback cutthroat trout to 23 that failed to determine the factors that influenced translocation success. Of the translocations that failed, 48% were reinvaded by nonnative salmonids, 43% apparently had unsuitable habitat, and 9% experienced suppression by other factors. Reinvasion occurred most often because of failed artificial barriers or incomplete removal of nonnative salmonids in complex habitats. Of those areas that were not reinvaded, success was highest in receiving waters with at least 2 ha of habitat that had previously supported reproducing trout populations.

743: *+.054*

The endangered kokako Callaeas cinerea wilsoni (Callaeidae), an endemic forest-dwelling passerine of New Zealand, has declined over the last century to a number of small isolated populations due to widespread habitat clearance and predation by introduced predators. To evaluate the genetic consequences of inbreeding and genetic drift, we examined genetic variability within and among 3 of the major remaining kokako populations using 4 polymorphic microsatellite loci. The largest remaining kokako population in the Te Ureweras was shown to have greater variability than the smaller Mapara and Rotoehu populations. Differentiation among the populations was low to moderate. We suggest there is no genetic barrier to translocations between the populations and that translocations of some individuals between the remaining kokako populations could enhance genetic variability of small populations to levels found in larger populations (e.g. Te Ureweras). (C) 2000 Elsevier Science Ltd. All rights reserved.

744: *+.186*

We document the recolonization of an indigenous large herbivore into its historic range. Eighteen wood bison (Bison bison athabascae) were reintroduced into the Mackenzie Bison Sanctuary of the Northwest Territories, Canada, in 1963. The population subsequently increased in number and range, peaking at about 2400 in 1989; numbers were estimated at about 1900 in 1998. Recolonization occurred through a series of increases in local areas followed by pulses of dispersal and range expansion. This pattern was originally described for exotic species' introductions. Differences in diet and overwinter survival of calves over the bison's range suggest that intraspecific competition for food provided the stimulus for range expansion. For a conservation strategy, the reintroduction of animals into several independent sites in their historic range would facilitate recolonization and achieve a faster spread than a reintroduction into one site followed by

waiting for the population to spread as a result of its own density dependent responses.

745: ***-.015***

The "Pepperbark" tree, Warburgia salutaris (Bertol.f.) Chiov., is a small to medium tree (5-10 m) facing a high risk of extinction in the wild. It is now a critically endangered species in Zimbabwe. It has undergone a considerable decline throughout its natural habitat (range) in Zimbabwe mainly due to over-exploitation for medicinal purposes. Any part of the plant can be used for treating various ailments, fetching a very high price on the market. A full botanical description, of the species, its significance for economic use, past distribution and historical background are discussed in this paper. The options available for its cultivation and successful reintroduction to its historic range are described.

746: ***-.127***

Using the example of a reintroduced bighorn sheep population in Badlands National Park, South Dakota we demonstrate the usefulness of neutrality tests and demographic data for detecting a severe genetic bottleneck ($Ne < 10$). From demographic data the effective population size of the founding population at Badlands was estimated to be six, and a heterozygosity excess test revealed evidence of a severe population bottleneck. We discuss the criteria for intervention when there is evidence of a severe bottleneck, and propose methods of mitigating the potentially deleterious long-term consequences of such bottlenecks. These issues are presented in the context of bighorn sheep reintroductions, but the issues are also of general importance to restoration efforts involving other large vertebrates.

747: ***+.118***

By 1950, bighorn sheep were extirpated from large areas of their range. Most extant populations of bighorn sheep (Ovis canadensis) in the Intermountain West consist of <100 individuals occurring in a fragmented distribution across the landscape. Dispersal and successful colonizations of unoccupied habitat patches has been rarely reported, and, in particular, translocated populations have been characterized by limited population growth and limited dispersal rates. Restoration of the species is greatly assisted by dispersal and successful colonization of new patches within a metapopulation structure versus the existing scenario of negligible dispersal and fragmented, small populations. We investigated the correlates for the rate of colonizations of 79 suitable, but unoccupied, patches by 31 translocated populations of bighorn sheep released into nearby patches of habitat. Population growth rates of bighorn sheep in the release patches were correlated to N-e of the founder group, and early contact with a second released population in a nearby release patch (logistic regression, $p = 0.08$). Largest population size of all extant released populations in 1994 was correlated to potential N-e of the founder group, the number of different source populations represented in the founder, and early contact with a second released population ($p = 0.016$). Dispersal rates were 100% higher in rams than ewes ($p = 0.001$). Successful colonizations of unoccupied patches ($n = 24$ of 79 were colonized) were associated with rapid growth rates in the released population, years since release, larger area of suitable habitat in the release patch, larger population sizes, and a seasonal migratory tendency in the released population ($p = 0.05$). Fewer water barriers, more open vegetation and more rugged, broken terrain in the intervening habitat were also associated with colonizations (p = (0.05). We concluded that high dispersal rates and rapid reoccupation of large areas could occur if bighorn sheep are placed in large patches of habitat with few barriers to movements to other patches and with no domestic sheep present. Many restorations in the past that did not meet these criteria may have contributed to an insular

population structure of bighorn sheep with limited observations of dispersal.

748: ***+.091***

Restoration of desert bighorn sheep (Ovis canadensis) is proceeding in several western states. Measurement of nutritional resources (quality and quantity) is not normally conducted to assess future and present translocation sites, although it has been recommended generally for evaluation of ungulate habitat. We estimated nutritional carrying capacity (based on nitrogen in forage species) of three mountain ranges in Trans-Pecos Texas where desert bighorn sheep have been translocated. We used an explicit nutritional constraint model to estimate carrying capacity on a seasonal basis. Although drought conditions prevailed during the study period, bighorns were not limited by quantity of vegetation. However, forage biomass of high nutritional quality (>1.5% N) was very low (<9 kg/ha in spring; <1 kg/ha in all other seasons). Consequently, there appeared to be inadequate forage of high N content for bighorns to meet reproductive requirements in some seasons. Estimates generated using the explicit nutritional constraint carrying capacity model appeared to be realistic relative to observed densities. Nutritional sampling and modeling can predict potential carrying capacity on areas scheduled for restoration or on ranges already stocked, predict effects of management on carrying capacity, and allow managers to compare among restoration sites. We recommend that desert bighorn managers focus on monitoring the abundance of high-quality (%N) forage species, especially during periods of drought or higher nutritional requirements (e.g., lactation, post-weanling growth).

750: ***+.071***

The Mauna Kea silversword, Augyroxiphium sandwicense ssp. sandwicense, has experienced both a severe population crash associated with an increase in alien ungulate populations on Mauna Kea, and a population bottleneck associated with reintroduction. In this paper, we address the genetic consequences of both demographic events using eight microsatellite loci. The population crash was not accompanied by a significant reduction in number of alleles or heterozygosity. However, the population bottleneck was accompanied by significant reductions in observed number of alleles, effective number of alleles, and expected heterozygosity, though not in observed heterozygosity. The effective size of the population bottleneck was calculated using both observed heterozygosities and allele frequency variances. Both methods corroborated the historical census size of the population bottleneck of at most three individuals. The results suggest that: (i) small populations, even those that result from severe reductions in historical population size and extent, are not necessarily genetically depauperate; and (ii) species reintroduction plans need to be conceived and implemented carefully, with due consideration to the genetic impact of sampling for reintroduction.

751: ***+.067***

The aquatic North American river otter (Lontra canadensis), a charismatic nonendangered mustelid, presently occupies a greatly reduced range. The species is common in toes and has economic value as a furbearer. At least 17 states and one Canadian province have undertaken recent reintroduction programs. Nevertheless, little has been published on nonparasitic diseases of river otters, and little is known of the clinical significance of most of their parasitic infections. Records of environmental contaminant-related diseases and traumatic injuries in free-ranging North American river otters are also rare. This paper reviews present knowledge of North American river otter diseases, especially those with the greatest potential for impacting reintroduction programs.

752: +.115

The Chamois of the Chartreuse mountains, Rupicapra rupicapra cartusiana, is a sub-species which, apparently, is well-adapted to the Grande Chartreuse mountain massif (Isere). In spite of that its population decreased in number, and between 1960 and 1970 reduced the amount of area used. As a remedy to this situation, in 1990 and 1992 an attempt was made to reintroduce the species by translocation. Using radiotelemetry we monitored the dynamics of space occupation by 9 male and 17 female Chartreuse chamois, translocated in April between 1990 and 1992 from the western to the eastern slopes of this range. Before translocation the animals were kept together in an enclosure on the site of release, and set free as a group. The average home range sizes used throughout this study, as measured by the minimum convex polygon method, were 8,244 ha for males and 2,244 ha for females, by the "100%" cluster method respectively 6,201 ha and 1,789 ha, and by the 90% cluster method, respectively 437 ha and 333 ha. Distance between home range center and point of release was 3,400 m in males and 1,449 m in females. If its home range center was more than 2 km away from the point of release we considered an individual to have left the release site. The reintroduction campaign was a success since 85% of the animals (n=26) settled down in their release site. Three of the four released animals (two males and two females) left the reintroduction site and returned to their range of origin. The translocated animals had a tendency to explore their new range progressively. In fact, the areas occupied during the first three months after release were smaller than later on. Apart from 3 males with a very large home range (16,000 ha, 18,000 ha and 26,000 ha, minimum convex polygon) the home ranges of all other males and females were more or less of the same size (about 2,200 ha). Nevertheless, the oldest animals had a tendency to occupy larger home ranges than younger ones. Two of the three chamois which returned to their site of origin were among the oldests ones. This is why for reintroductions by translocation we recommend to take relatively young animals, to set the date of release in early April, to release many animals (between 20 and 30) together, preferably in groups, with an unbalanced sex ratio in favour of females.

753: +.083

Empirical data on behavior such as space-use patterns, are important to the success of animal reintroductions. We studied space-use patterns in a growing population of Asiatic wild ass (Equus hemionus) reintroduced into the Ramon erosion cirque in the Negev desert, Israel. Between 1988 and 1995 we used direct observation to determine the location and association of males and females. All adult females and dominant males were individually recognized. Home ranges of dominant males overlapped little, suggesting that in this population males are territorial. After the first release of males and females into the wild, only one territory was established and it covered most of the 20,000 ha of the cirque. After 6 years the number of male territories increased as the number of males in the population increased, and average territory size decreased. Male territories were near permanent and ephemeral water sources, but the water sources were at the peripheries of the territories and were not centers of activity. When there was only one territorial male, female home ranges were almost entirely within the territory. As male territory size decreased, so did the spatial association of females with a single male. During the breeding season, males spent more time in close association with female groups, adopting what may temporarily appear to be a harem breeding strategy. Although demographic and environmental factors pose a greater threat to small populations, our data support the hypothesis that in small, reintroduced populations of territorial, polygynous species, effective population size (N-e) may be dangerously small. Our data suggest that this situation may last for several years until new males are recruited into the population. Thereafter, rapid male turnover and female use of several male territories may ameliorate this problem. We found no relationship between male turnover rate and female reproductive success.

The establishment of more male territories is key to increasing N-e and should be the basis for planning reserves for territorial, polygynous species.

754: ***+.235***

Changes in historical disturbance regimes have been shown to facilitate non-native invasions, but reinstatement of disturbance can be successful only if native colonizers are able to outcompete colonizing invasives. Reintroduction of flooding in the southwestern United States is being promoted as a means of reestablishing Populus deltoides subsp. wislizenii, but flooding can also promote establishment of an introduced, invasive species, Tamarix ramosissima. We investigated competition between Populus and Tamarix at the seedling stage to aid in characterizing the process by which Tamarix may invade and to determine the potential ability of Populus to establish itself with competitive pressure from Tamarix. We planted seedlings of Tamarix and Populus in five ratios at three densities for a total of 15 treatments. The growth response of each species was measured in terms of height, above-ground biomass, and tissue concentrations of nitrogen and phosphorous. These measurements across treatments were modeled as three-dimensional response surfaces. For both species, Populus density was more important than Tamarix density for determining growth response. Both species were negatively affected by increasing numbers of Populus seedlings. Due to the large size of the native Populus, we predict that its superior competitive ability can lead to its dominance when conditions allow native establishment. Our results suggest that even in the presence of an invader that positively responds to disturbance, reestablishment of historical flooding regimes and post-flood hydrology can restore this ecosystem by promoting its dominant plant species.

755: ***+.237***

The burrowing bettong was successfully reintroduced to the Australian mainland in 1992 after an absence of 50 years. The population, derived from 42 individuals translocated from a remnant population on an offshore island, has persisted for over seven years on the Heirisson Prong peninsula at Shark Bay in Western Australia. It has grown to over 260 individuals that are distributed widely through available habitat. The successful management of exotic European foxes and feral cats proved crucial to the outcome of the reintroduction. Factors contributing to the successful management of predators and to a reduction in their impact included: the choice of a narrow peninsula as the site for reintroduction (permitting cost-effective use of predator-proof fencing); effective baiting (fox and cat) and trapping (cat) strategies; the maintenance of an in situ breeding colony (to provide insurance against major loss of free-range animals to predators); choice of high quality habitat (providing reasonable cover and promoting a high and relatively stable rate of increase for bettongs, even during dry years), and choice of a site that was accessible for regular management visits. An abundance of European rabbits at the reintroduction site appeared not to be a limiting factor. The success of this reintroduction has stimulated a range of other reintroductions of endangered mammals to arid and semi-arid sites across Australia, particularly to peninsulas or other sites where exotic predators can be controlled effectively. (C) 2000 Elsevier Science Ltd. All rights resented.

756: ***+.005***

According to literature, toponymy and personal communication, Ospreys were common along the rocky coast of Portugal until the beginning of the 20th century. Thereafter, increasing human encroachment on the coastline, especially through persecution, caused a severe and steady decline that led to the verge extinction as the breeding population became reduced to one single pair.

Considering the critical demographic situation and geographic isolation of the population, natural recovery is unlikely. Precipitous sea-cliffs, exposed coastline and moderate to low human presence are the dominant features of Osprey breeding habitat in Portugal. Nests are built atop rock pinnacles and on cliff ledges. During periods of rough water surface at sea, fresh and brackish water fish from inland dams and coastal lagoons predominate over marine fish in the diet. Freshwater prey is often taken at long distances from nest sites, presumably involving high energetic expenditure. Planned conservation actions aim at reducing disturbance and conflict with human interests through access management, building of artificial nests in areas of low angling quality, education and negotiation with local representatives; and at reducing foraging costs by providing new freshwater foraging sites closer to the coastline. As the management of habitat and its use by humans alone will predictably be insufficient, restocking the population with young from donor populations by hacking techniques is fundamental.

757: ***+.116***

The Osprey has been absent as a breeding species in England for about 150 years but birds occur regularly on migration. The Scottish population has increased from one pair in the 1950s to at least 130 pairs in 1998. It has been subject to an intensive programme of conservation, management and research. In 1996, an experimental project was started to reintroduce Ospreys to England. A detailed project appraisal was undertaken before the necessary government licences were approved. Eight young from Scotland were translocated in 1996 and 1997, and twelve in 1998 to Rutland water Nature Reserve, a very large man-made reservoir in central England. In order to minimise effects on the wild population, attempts were made in 1996 to take 'runt chicks' which were likely to die but this was not very successful because of problems associated with salmonella infections. Out of eight young, six were released from specially built release cages on towers and four of them migrated. In the following two years, a total of 20 young were translocated and successfully released. Chicks were kept in captivity in broods of three and fed fresh fish in secluded cages over-looking the lake. After release young birds were fed at artificial nest platforms, to mimic food supply by adult male Ospreys, for over a month and all young set off on migration in a manner similar to wild birds in Scotland. Two of the young have subsequently been recovered dead in West Africa, one in Senegal in February and the other in Guinea in August, and one was observed on migration 200 km due south of the release site. The project team have refined the techniques of Osprey translocation and intend to release 12 young in 1999 and 2000, by which time it is hoped the first birds will have returned to breed.

758: ***+.155***

Within the framework of a European program of brown bear (Ursus arctos) restoration, 2 adult female brown bears were captured in May and June of 1996 in Medved Reserve, Slovenia, and translocated to the central Pyrenees, France. The 2 females (Ziva and Mellba) were fitted with radiotransmitters and monitored from release to den entrance. After release from the same site, the bears were highly mobile and were located up to 55 km (Ziva) and 52 km (Mellba) from the release site. Their home ranges encompassed 1,233 km(2) (Ziva) and 796 km2 (Mellba) during this period. The 2 females used their home range in a multimodal fashion with areas of concentrated utilization. Ziva and Mellba. denned on 27 and 23 November 1996 and emerged on 19 and 4 April 1997, respectively. Denning sites were near the release site. These preliminary results suggest that translocation can be an effective technique to restore bear populations or to save small bear populations.

760: ***+.345***

This recovery plan succeeds the earlier plan (Cree and Butler 1993) which guided tuatara conservation during the past decade and saw major achievements that have placed the species in a far less vulnerable position. Rats have been eradicated from several island habitats, and tuatara have been translocated to other islands in the hope of establishing new wild populations. Research and enhanced animal husbandry techniques have led to successful incubation of eggs and raising of juveniles in captivity. This increased productivity has been used to augment relict populations and provide animals for new wild populations. Future work will focus on advocating for the eradication of rats from Little Barrier (Hauturu), Mauitaha and Hen (Taranga) Islands and in maintaining the pest-free status of other island habitats. Further translocations are planned as part of the long-term objective of establishing wild populations of tuatara throughout their pre-human range. The potential to use tuatara in education, to enhance public awareness of conservation issues, and for research are all promoted within the plan. All are seen as essential for long-term conservation of the species. The increased involvement of iwi is essential if the plan is to succeed.

761: *-.006*

Bombina vuriegata is included in Enclosure II of Directive CEE 92/43 owing to the decline of its populations in some European Countries (Austria, Belgium, Italy, Germany, Holland, Switzerland, ex Sovietic Union), in recent years. For this reason, apart from the real situation of its populations, this species and Bombina pachypus have been given particular attention during the identification of regional S.I.C. (Sites of Community Interest-Nature 2000) for the BioItaly programme in Italy. Where Bombina's populations were present in the past, some activities involving in their reintroduction or the development of their habitats were proposed. Before carrying out these activities, it's necessary to study the causes of the disappearance. Moreover, during the planning and carring out of these projects, it is necessary to consider the main ecological requirements of these Amphibians. The types of habitat identified for Bombina v. variegata and/or Bombina pachypus are: torrents (with calcareous or marly substratum); 2. troughs; 3. drinking troughs and irrigation tanks in stone or cement; 4. karst ponds; 5. corrosion basins; 6. quagmires and furrows cut by tractors or cars wheels.

762: *+.007*

Moving of groups or whole populations of amphibians are widespread conservation tools. Unfortunately exhaustive reports and publications about translocations are not so common. Even more rare are data about long-term monitoring programs. The present work proposes and defines the following terminology about translocations: Translocation=moving living organisms from one area to another; Introduction=release of species in areas or in biotopes where the same species was historically absent (inside=intrapatric introduction, or outside=extrapatric introduction, the species'native range); Re-introduction=release of a species in areas or in biotopes where the same species is now extinct but it was historically present; Re-stocking=release of a species in areas or in biotopes where the same species is still present; Direct translocation=release of specimens immediately after catching and moving them; Indirect translocation=release of specimens born and/or raised in captivity. Different experiences of translocation of amphibians are reported and guidelines about amphibians translocations are consequently provided. Finally it is underlined that every translocation program should be carefully planned, its appropiateness carefully considered, and the results monitored in the long-term and reported in detaill, irrespective of its final succes or failure.

763: *-.031*

In 1995 and 1998/99 single species mass mortalities of sardine/pilchard Sardinops sagax (Clupeidae) spread rapidly throughout this species' range in Australia from the central coast of South Australia, dramatically decreasing the population size and representing the two most extensive mass mortalities recorded for marine organisms. The behavior of each epizootic indicated that an exotic pathogen was responsible, with the fatal agent shown to be a previously unknown herpesvirus. The focal origin of both events from a limited region within the extensive Australian range of S. sagax was not random. Tuna (Thunnus maccoyii) feedlots located in the same region as the epizootics' origins are responsible for delivering to the marine environment the largest quantities of S. sagax imported into Australia, which provides qualitative evidence of a link between the two events and imported S. sagax. This link provides an example of the need to undertake a review of the current international standards for import risk analysis (IRA) that requires a disease to be identified before it can be considered a risk. Regardless of the identity of the disease agents responsible, case histories of mass mortalities need to be given due consideration in both deciding whether to instigate an IRA and also form part of the IRA. Identification of a hazard should thus not be restricted to dealing only with identifiable diseases, but should also embrace case histories of epizootic events linked to (a) previously unidentified pathogens and (b) translocation of particular species between geographically separate populations.

764: ***-.035***

Wildlife management involves regulation of population numbers of wild vertebrate species. in some cases there are too many animals and in others there are too few. Genetic issues arise in both instances. The historical and genetic evidence for the number of mammals that were in the founder populations of successful colonizing species in Australia and New Zealand is reviewed here. Small numbers have often given rise to large populations, despite the concomitant loss of genetic variability. Restriction of the number of over-abundant and pest species by either physical or chemical methods frequently constitutes very strong artificial selection, which leads to rapid genetic change; an example of major importance in the two countries is sodium monofluoroacetate (compound 1080). Pathogenic agents, surgical sterilization, hormonal contraceptives and translocation have all been used with varying degrees of success. The strengths and weaknesses of these techniques are assessed. A method that has received much attention is immunocontraception. We argue that this attempt to use the animals' own immune system to modulate reproduction is incompatible with the basic biological function of protection against infectious disease. Immune function genes are highly variable in vertebrates, and so often genetic change in the population subjected to immunocontraception is likely to be even more rapid than is the case with lethal agents. Selection for failure to respond to the immunocontraceptive will occur, and will change immune function in general. Poor scientific description of ecosystem complexity makes it difficult to predict the consequences of immunocontraception on wildlife populations.

766: ***+.167***

The Chatham petrel (Pterodroma axillaris) is an endangered species restricted to a single population on South East Island, Chatham Islands, New Zealand. The key threat to Chatham petrel breeding success is interference with chicks by broad-billed prions (Pachyptila vittata) prospecting for burrows for their oncoming breeding season. This burrow competition has resulted from alteration to breeding habitat by humans throughout the Chatham Islands. Understanding habitat preferences may enable managers to manipulate habitat to reduce burrow competition and will be essential in order to translocate Chatham petrels to a proposed second colony. Habitat characteristics surrounding both Chatham petrel and broad-billed prion burrows were quantified and selection ratios compared. Both Chatham petrels and broad-billed prions selected habitat

factors associated with mature forest. Chatham petrels avoided a large number of habitat characteristics, which suggests they were habitat specific, and their preferred habitat is now limited. Broad-billed prions used a wide range of habitat characteristics, which suggests they are not habitat specific. This study recommends that selection values be used when deciding on the best location to establish a second Chatham petrel colony.

767: *+.119*

Hihi (Notiomystis cincta) were reintroduced to Mokoia Island, Lake Rotorua, New Zealand, in September 1994, and two years later there was an aerial drop of brodifacoum cereal pellets aimed to eradicate mice (Mus musculus). Using Program MARK, we analyzed data from resighting surveys to assess whether hihi had lower than normal survival in the 6-week interval following the drop. The resighting data were collected on a regular basis over a 3-year period, from 1994-97, allowing us to control for yearly and seasonal variation in resighting and survival probabilities. We initially established that the Cormack-Jolly-Seber model had a good fit to the data and could therefore be used as the global model for our analysis. We then compared a range of simpler candidate models, some of which included a poison effect (an unusual survival rate for the interval after the poison drop). Under the best model (that with the lowest AIC), the survival probability was constant over time and there was no poison effect. The estimated survival probability for the 6 weeks after the poison drop was 0.95, which is slightly higher than the value of 0.89 expected based on pre- and post-poison intervals. The approximate 95% confidence interval for the probability of a bird dying due to poison ranged from -0.17 (i.e., a decrease in mortality rate due to the poison) to +0.04. We therefore concluded that the poison caused at most a negligible increase in mortality, and that mark-recapture analysis on resighting data provided a powerful method for assessing the impact of the poison drop. We discuss the relative costs and benefits of radio tagging versus resighting surveys of banded birds for estimating impacts of poison operations. For species with relatively high resighting rates, such as hihi, analysis of resighting surveys is a much more reliable and cost-efficient methodology.

768: *+.063*

Several recent studies have used "roll calls" - searches for individually-marked birds - to assess impacts of aerial poison operations on non-target species. Roll calls have advantages over methods such as 5-minute bird counts, call counts, and dead body counts, but roll calls are based on the assumption that detection rates are 100%, or that detection rates are constant over time and space. They also require more than one group of birds, at a poisoned and unpoisoned site for example, for valid statistical comparisons. With minor adjustment of field methods, however, a series of surveys can be treated as a mark-recapture experiment, allowing powerful analysis without such restrictive assumptions. Survival and detection rates can be estimated independently for each time interval, and alternative models fitted to the data for factors affecting those rates. Using the software package MARK, we analyzed data for New Zealand robins (Petroica australis) on Tiritiri Matangi Island to estimate impact of an aerial brodifacoum operation conducted in September 1993 to eradicate kiore (Rattus exulans). The population was established by translocations in 1992 and 1993, and consisted of 40 birds at the time of the operation. Taking seasonal and yearly Variation in detection and survival rates into account, we estimate that 11% of robins (4-5 birds) died as a result of the poison. There was strong evidence that survival probability was lower immediately after the operation, despite the small number of birds disappearing. The poison effect was confined to the interval from September-October 1993, including the first two weeks after the poison drop, and survival for subsequent monthly intervals was consistent with the normal summer rate. Using simulation modelling based on our data from the population, we predict that the operation set

population growth back by about one year but had no longer-term impact.

769: ***+.287***

Southeastern United States habitats dominated by longleaf pine (Pinus palustris Miller) and associated plant species have declined dangerously. Conservation of rare and common plants of longleaf pine habitats may be aided by starting new populations in the field. We review methods for initiating plant populations and integrate information from our studies of rare and common longleaf pine ground-layer plants of the outer South Carolina Coastal Plain. In our experience it is possible to start new populations of most longleaf pine ground-layer plants, including rare species if (1) seeds are collected from frequently burned sites with reasonably large populations of desired species; (2) appropriate media are used for seedling propagation; (3) outplanting of nursery grown seedlings or direct seeding is done during periods of sufficient soil moisture; and (4) introduction sites properly match habitat requirements (inferred from indicator plants) of desired species, and the sites can be managed with frequent prescribed fire.

770: ***+.021***

Mexican wolf (Canis lupus baileyi) recovery is one of three gray wolf restoration efforts in the United States conducted by the US. Fish and Wildlife Service (USFWS). The Endangered Species Act (ESA) of 1973 (16 USC 1531-1544) saved the Mexican wolf from almost certain extinction. All known Mexican wolves descend from seven founders that were captured from the wild and bred in captivity (Parsons 1996). Two challenges faced by Mexican wolf recovery include the use of naive, captive-reared wolves for release stock and sociopolitical opposition to restoring predators. Following years of planning and public involvement, reintroduction of Mexican wolves began in March 1998 (Parsons 1998). In this chapter we describe the history and ecology of the Mexican wolf and its narrow escape from extinction. Details and early results of ongoing efforts to restore this extirpated predator to the American Southwest are presented. We hope this information will be of interest and value to others pursuing carnivore restoration efforts.

771: ***-.076***

We describe white rhinoceros (Ceratotherium simum) mortality at Pilanesberg National Park, South Africa, focussing on mortality caused by African elephant (Loxodonta africana). We reconstructed records from a range of historical sources, and estimated that up to 49 rhino were killed by elephant. There was confirmed mortality in 1994 and 1996, and based on patterns, we suggest a set of rhino mortality from elephant in 1992. Both sexes and all age classes were victims. There was no significant bias to older animals, but given the rhino population structure, there was a significant bias towards males in adult deaths. The culprits were identified as young male elephants that entered musth about 10 years younger than expected, and maintained musth for a full term at first occurrence. We attributed this to the lack of a mature bull hierarchy in the park, because these elephants were the product of translocation of young animals (<10 years old) remaining from culls in Kruger National Park. We emphasise the need for accurate monitoring and record keeping, and a focus on individual identification of key species in small reserves.

772: ***-.012***

By the beginning of the 20th century the lynx, wolf and bearded vulture had disappeared from Switzerland and the whole of the Alps. Only brown bears survived as a relict population in the Italian Alps. The recent increase in the populations of large carnivore all over Europe has also

affected the Alps and Switzerland. The four species discussed here are coming back: the lynx and the bearded vulture through re-introductions, the wolf and the brown bear by natural immigration. Their reappearance also revives many of the potential conflicts between humans and large carnivores. We describe briefly the history of the extermination of the four species in the Alps and in Switzerland and their recovery at the end of the 20th century, and define the most prominent reasons for conflicts. The red fox has never disappeared from Switzerland. However, the population developed very dynamically during the 20th century due to a rabies epizootic, on the one hand, and an increase in the carrying capacity of the habitat, on the other.

776: *+.315*

Assessing the colonizing ability of a species is important for predicting its future distribution or for planning the introduction or reintroduction of that species for conservation purposes. The best way to assess colonizing ability is by making experimental introductions of the species and monitoring the outcome. In this study, different-sized propagules of Roesel's bush-cricket, Metrioptera roeseli, were experimentally introduced into 70 habitat islands, previously uninhabited by the species, in farmland fields in southeastern Sweden. The areas of introduction were carefully monitored for 2-3 yr to determine whether the propagules had successfully colonized the patches. The study showed that large propagules resulted in larger local populations during the years following introduction. Probability of colonization for each propagule size was measured and showed that propagule size had a significant effect on colonization success, i.e., large propagules were more successful in colonizing new patches. If future introductions were to be made with this or a similar species, a propagule size of at least 32 individuals would be required to establish a viable population with a high degree of certainty.

777: *+.033*

Deteriorating environmental conditions and overexploitation in situations of insufficient fish stocking or a complete lack of it led to the extinction of salmon in Polish waters. The last population of salmon in the Drawa River disappeared in the mid-1980s. The aim of this work was to re-establish salmon in Polish waters. The salmon for this purpose came from the Daugava River, when in 1985 and 1987, 50 000 and 30 000, eyed salmon eggs were bought, respectively. Up to 1996, salmon spawners were reared in the floating net cages in of the Puck Bay. Since 1994, spawns of salmon have been reared in freshwater at the fish farm "Aquamar" at Miastko. Stocking of smolt started in 1994 and by the year 2001, 2 238 653 one- and two-year-old smolts had been released. Smolts were released into rivers in the Pomeranian region, the Drawa, and the Vistula and its tributaries. Between 1994 and 2000, 78912 one- and two-year-old tagged smolts were released together with untagged ones. The first salmon in the Vistula and Drweca rivers were noted in 1996. From 1997 to 2000, salmon catches in rivers fluctuated between 413 and 9714 kg. From 1997 salmon spawners were captured for artificial spawning. The largest spawners were 120-cm long and weighed 17.5 kg. Alongside untagged spawners, single tagged salmon were also used for spawning purposes. In 1997, a total of 382 000 eggs were obtained. In later years the number of spawns obtained grew, and in 2000 the number of eggs collected was 2 260 000. In 1996, spawns from salmon raised in pools at Swierzenko were collected. In 2000, there were 1168 females (length 30-70 cm) from which 3 300 000 eggs were collected. From 1997, large nests were observed at the spawning grounds in the Drawa River. In next years salmon nests were observed in the Drawa River and also in Parseta and Wieprza rivers. Percentages of recovers from tagged salmon were very low and varied from zero to 7.35. Tagged salmon migrated all over the Baltic Sea. The most often they were caught in the Gulf of Gdansk area, near Bornholm Island and in the western part of the Gulf of Finland. Salmon entered rivers mainly into Vistula, Drweca and

Wieprza rivers for spawning purposes. Some spawners strayed and entered rivers to which they were not released as smolt. Higher percentage of homing were observed among smolt released into Vistula i Drweca rivers than among smolt released into Pomeranian rivers. Tagged salmon after the first year reached average length of 59.4 cm and average weight of 2583 g, after the second year 81.8 and 6255 g, after the third year 102.4 cm and 11800 g respectively.

779: *+.165*

The Louisiana Department of Wildlife and Fisheries regulates an experimental alligator (Alligator mississippiensis) egg collection programme which requires the return of a portion of juvenile alligators to the wild to ensure recruitment and maintain populations. Numerous studies have been and are currently being conducted by Department personnel to evaluate the success of this programme. Farm-released juvenile alligators are able to forage for food after release to the wild. Growth rates are equal to or higher in juveniles released from farms than native wild alligators matched for size and sex. The accelerated growth is maintained for several years after release. An extensive tag and release programme of farm-released juvenile alligators was evaluated by analysis of later harvest of recaptured sub-adult and adult alligators. Results suggest fairly high survival rates of farm-released alligators. No differences were found in stress hormone levels between farm-releases later caught in the wild and native-wild alligators. Farm-released juvenile alligators have attained sexual maturity and successfully nested in the wild. Numerous parameters suggest egg ranching and subsequent return of a portion of juvenile alligators to those wetlands is a successful management tool that uses a resource that would otherwise be lost to high natural mortality. A brief review of release programmes for other crocodilians is provided.

781: *+.235*

Large fruit-eating pigeons of New Caledonia and French Polynesia provide an excellent model to illustrate the hypothesis that sustainable management of biodiversity resources is essential under high human pressure conditions. An adult giant imperial pigeon (Ducula goliath) can weigh more than 800 g, making this endemic New Caledonian bird the largest pigeon in the world and of considerable interest in terms of game resources. In French Polynesia, the habitat of the Marquesas imperial pigeon (Ducula galeata) is limited to Nuku Hiva island, with a maximum of 200 birds present. The Polynesian imperial pigeon (Ducula aurorea) is now very rare in Tahiti, with a maximum population of around 500 birds present only on the island of Makatea (Tuamotu). Due to the small populations and their isolation, these latter two imperial pigeons must be carefully managed to avoid their extinction. By contrast, the populations of giant fruit-eating pigeons found solely on Grande-Terre island can only be maintained by hunting regulations drawn up on the basis of a better understanding and monitoring of their ecology and by a perception of the conflictual interests of hunters and the different communities. After reviewing available data, this article analyses possible methods for monitoring and active management of these species, e.g. rational sampling strategies, awareness campaigns, translocation operations and methods for founding new populations.

783: *+.168*

Blue-and-gold macaws (Ara ararauna), once found in the Nariva Swamp in Trinidad, were extirpated in the 1960's because of nest poaching for the pet trade and habitat alteration. In 1993, the Forestry Division of Trinidad and Tobago, The Centre for the Rescue of Endangered Species of Trinidad and Tobago (CRESTT) and the Cincinnati Zoo and Botanical Garden embarked on a mission to restore this species to its island habitat. Food source and nest site surveys indicated that

this now protected wetland could still support a population of reintroduced Blue-and-gold macaws. After several unsuccessful attempts to obtain chicks for release through captive breeding, a pilot study on the reintroduction of wild-caught birds was implemented. In 1999, nine potential pairs (18 birds) were imported from stable wild populations in Guyana. After quarantines, testing and physical examinations, the birds spent four weeks in a pre-release flight cage located in a protected wildlife sanctuary within the Nariva Swamp. They were monitored by local villagers and introduced to natural foods of the area. Over a period of three months, 14 birds were released (eight males and six females). More than one-year later, nine macaws continue to be sighted in the wild. Behavioral observations of six of these birds during February to May 2001 suggest nesting activity. Trained local villagers in the communities bordering the swamp have been monitoring flight patterns and potential nesting sites of the released birds. Education programs have helped rural communities and schools to learn more about the sustainable use of the Nariva wetlands. Private and corporate sponsors and the media have raised public awareness island-wide to the significance of this conservation effort.

784: ***+.362***

Wildlife conservationists and managers must address a wide range of issues relating to bird populations including sustainable management of the wider countryside, the protection of individual sites and site networks and proper ly planned species recovery. Sound approaches to these problems need to be based on a good understanding of the population processes involved. Much of the research that provides such understanding is based on ringing, which is used to measure many aspects of avian demography, particularly survival rates, recruitment and dispersal. Ringing studies have allowed the demographic causes of population declines to be identified, providing indications and tests of their environmental causes. Spatial population modelling, supported by good empirical data on dispersal, has the potential to contribute to both species and landscape conservation. The success of rehabilitation and reintroduction programmes can often be evaluated from studies of marked birds. Descriptions of patterns of movements form an essential part of the information required to manage flyway populations and site networks. Ringing is also a key component of research into the effects of hunting because it can be used to measure both harvest and survival rates. The control of gull populations illustrates the application of population dynamics principles to reduce population size. The contribution of ringing to conservation science during the 21(st) century can be enhanced by using information technology to improve the use of existing information and data, by undertaking more long-term monitoring and through giving careful attention to the design of research projects.

786: ***+.071***

Analyses of mitochondrial control region sequences were used to infer phylogeny of Anser species, phylogeography of the lesser white-fronted goose, and genetic background of a captive stock. The genetic distances among the Anser species ranged from 0.9 to 5.5% in the complete control region sequences and supported the view of close relatedness of these species. Among the four most closely related species, the bean, pink-footed, white-fronted and lesser white-fronted goose, the branching order is uncertain. The short internal branches and low support for the branching order suggest that the species have diverged recently within short time-intervals. The mtDNA tree obtained is incongruent with the traditional view of the species relationships, but the reasons for this remain to be clarified. Two diverged mitochondrial lineages were found in the lesser white-fronted goose and a refugial origin was proposed. Basal haplotypes are geographically widespread and indicate a recent common ancestry for populations. The derived haplotypes are confined to singular breeding populations and suggest restrictions to the present

female gene flow. A shift in the frequency of the mDNA lineages approximately coincides with a migratory divide in the Taimyr Peninsula. Low mtDNA diversity and significant difference in the haplotype frequencies observed in Fennoscandian subpopulation suggested that it should be considered as a management unit. The fossil record was examined to gain additional information about the colonisation history of the species, but was found to be of limited use. The captive lesser white-fronted goose stock used for reintroduction/restocking was shown to be incompatible with the Fennoscandian wild population. Some captive individuals carried the mtDNA of the white-fronted goose suggesting a hybrid origin. Hybridisation has probably occurred during captive propagation, but to clarify further the extent of introgression, nuclear markers should be applied. The structure and evolution of the control region were studied by comparing complete avian sequences. Saturation was found to occur at pairwise divergences of 10% as shown for third codon positions of the mitochondrial genes previously. In pairwise comparisons of the control region and cytochrome b sequences, the rate of divergence varied among the lineages. Two conserved sequence blocks showed considerable sequence conservation when compared to mammalian sequences.

787: ***+.312***

The fossil record, particularly from the early Holocene and Pleistocene, can provide an historical basis for understanding long-term ecological change. Available data on the rate and types of environmental change in modern ecosystems is often limited to a few decades, whereas similar information provided by paleontological evidence can provide a record spanning thousands of years. This perspective becomes especially important in recognizing naturally occurring events that have a periodicity exceeding human observation. Thus the fossil record establishes a useful baseline for ascertaining whether changes impacting habitat have natural or anthropogenic causes. Fossil remains can also provide reliable documentation on the paleo-distribution of endangered species and indicate areas favorable for reintroduction. Occasionally fossil preservation permits the recovery of DNA, allowing a very precise match between fossil and extant populations. More importantly, the paleoecological reconstruction of the former habitat has the potential for identifying new ranges where endangered species may be successfully introduced. We do not advocate that the entire paleontological record will always be applicable to modern ecosystem management However, we do maintain that part of the record from .semi-deep time. extending tens to hundreds of thousands of years in the past is certainly relevant. Therefore, proper management of fossil resources is essential in preserving an irreplaceable record of environmental change. In turn, documentation of long term ecological history represents a potentially critical input in the decision-making process with regard to current ecological problems.

788: ***+.166***

A direct comparison of the genetic linkage maps of sorghum and rice is proposed. It is based on the mapping of a common set of 123 RFLP probes scattered on the genomes of both species. For each species a composite map was established by merging two individual maps comprising many common loci. This enabled us to confirm the global correspondence scheme that had previously been established between the chromosomes of sorghum and rice. It also provided a more detailed insight into the conservation of synteny and colinearity: 69% of the loci mapped on a given rice chromosome mapped to the corresponding homoeologous chromosome in sorghum; among them, 84% formed a colinear arrangement between the two species. Local inversions and translocations were detected.

789: ***+.053***

The genetic integrity of many salmonid fish populations is threatened by stocking of domesticated conspecifics. The purpose of this study was to assess the utility of microsatellite DNA markers for detecting loss of genetic diversity in hatchery strains, for estimating their genetic relationships, and for monitoring the genetic impact of stocking activity on wild populations of salmonid fishes. Brown trout from ten hatchery strains, one supportive breeding "strain," and five wild populations were screened for variation at eight loci. In most hatchery strains, genetic variation was comparable to that of wild populations, but three strains showed loss of allelic variation. In six of the hatchery strains, significant differentiation was observed between age classes. Genetic differentiation among all populations was moderate ($F_{ST} = 0.065$, $\rho_{ST} = 0.076$), and only a minor part of genetic diversity was distributed between the wild and hatchery populations. We assessed whether wild populations were introgressed by stocked hatchery trout by performing assignment tests to determine population of origin and estimating maximum potential introgression rates. The results suggested that genetic introgression by hatchery trout had occurred for only two of the five populations potentially influenced by stocking. In one of these two rivers, microsatellite data obtained from a limited number of old scale samples indicated that individuals from the original population were genetically divergent from these of the present population, suggesting that extinction of the original population had taken place. The study demonstrates that microsatellite analysis provides a useful tool for distinguishing heavily introgressed populations from those unaffected by stocking. The information can be used to assist in (1) prioritizing populations for conservation and (2) the choice of wild populations to be used as sources for the reintroduction of salmonids in areas where local, indigenous gene pools have been extirpated.

790: ***+.290***

In many respects, amphibian spatial dynamics resemble classical metapopulation models, in which subpopulations in breeding ponds blink in and out of existence and extinction and colonization rates are functions of pond spatial arrangement. This "ponds-as-patches" view of amphibian spatial dynamics is useful in several respects. First, it highlights the importance of regional and landscape processes in determining local patterns of abundance. Second, it offers a straightforward, pond-based approach to monitoring and managing amphibian populations. For many species, however, the ponds-as-patches view may be an over-simplification and metapopulation structure may be more apparent than real. Changes in distribution may be caused by processes other than extinction and recolonization, and most extinctions probably result from deterministic factors, not stochastic processes. In addition, the effects of pond isolation appear to be important primarily in disturbed environments, and in many cases these isolation effects may be better explained by the distribution of terrestrial habitats than by the distribution of breeding ponds. These complications have important implications for both researchers and managers. For researchers, future efforts need to determine the mechanisms underlying patterns of abundance and distributional change and patterns in amphibian populations. For managers, effective conservation strategies must successfully balance metapopulation considerations with careful attention to local habitat quality. Furthermore, translocations and active management may be indispensable tools for conserving amphibians in landscapes containing multiple breeding ponds.

791: ***+.057***

A fundamental role of government conservation agencies is to set priorities for rhs conservation and management of biodiversity. This is particulary important in an area such as Western Australia which has a rich and highly endemic flora with over 11,000 listed native vascular plants. Legislation provides an initial focus for priority setting through the listing and protection of threatened flora, although this excludes over 1900 Western Australian plant taxa which are poorly

known but may be of conservation significance. The priority setting process for the conservation of this flora, discussed herein, focuses on single taxa, groups of taxa within geographic regions, populations and threatening processes. This process is particularly applicable to the highly diverse and endemic flora of the south-west Botanical Province. Within this region there has been extensive habitat loss and degradation over the last 100 years. Currently the prioritisation process has identified 95 critically endangered taxa in the southwest which require immediate remedial action to prevent extinction. Actions such as translocations are already showing promise, but with this number of critically endangered taxa and limited resources there may also need to be some ranking of taxa for immediate translocation. Although the priority setting process outlined here relates primarily to taxa, we emphasise that the conservation of this flora is also addressed at ecosystem and ecological community levels, and that each approach has its merits depending upon land tenure, location within the State and, in particular, the level of land degradation. Crown Copyright (C) 2000 Published by Elsevier Science Ltd. All rights reserved.

792: ***-.015***

Translocation is increasingly used in conservation to re-establish or augment populations of threatened species or to remove individual animals from areas of human-wildlife conflict. We assess the feasibility and utility of translocating hen harriers (Circus cyaneus) in the UK to enhance their distribution and abundance whilst simultaneously reducing the impact of harrier predation on red grouse (Lagopus lagopus scoticus) populations and shooting bags. Current knowledge of hen harrier feeding ecology? dispersal, survival and recruitment suggests that they would be suitable subjects for translocation with the aim of increasing their distribution in the UK. Assessment of habitat and fond availability suggest that there are suitable recipient sites beyond the current range of the hen harrier in the UK. However, translocation would not be a sustainable method of reducing predation on grouse moors because it would have to continue indefinitely as long as grouse moors attracted harriers. Translocation of harriers to grouse moors when they have been locally extirpated would not be appropriate until levels of illegal control are reduced. Establishing new harrier populations through translocation away from grouse moors may become desirable if initiatives to reduce human-raptor conflicts on grouse moors are unsuccessful, or as an interim measure to accelerate the recovery of hen harriers in the UK.

793: ***+.126***

Since the reintroduction of the Pere david's deer in China in mid 1980's, the Beijing and Dafeng Pere david's deer breeding populations have been established. During the past 14 years, the Beijing and Dafeng populations all experienced the periods of acclimatization and population growth. By the end of 1997, the total number of the Pere david's deer reached 671 in China. The sex ratios in both Beijing and Dafeng Pere david's deer reached balanced sex ratios and the effective population sizes also approached to sizes of the actual population in both populations. However, the carrying capacity of the Beijing Milu Park is limited, the Pere david's deer in the Dafeng Pere david's Deer Natural Reserve are mainly living in three paddocks of 1km2, thus, population densities increased as the populations grew. In the 1990's, the growths of both Beijing and Dafeng populations showed density-dependent patterns, especially in the Beijing population. Hence, population regulation measures such as artificial dispersal and controlling of birth rate must be taken in order to slow down the rapid growth in the Beijing population. There is vast seashore in the Dafeng Pere david's Deer Natural Reserve. Captive-bred Pere david's deer can be released into open coast area and to reestablish wild Pere david's deer population, thus, to reduce grazing pressure by Pere david's deer in the paddocks. The relocation of Pere david's deer to Tianezhou, Shishou, Hubei is met with a success. The relocated Pere david's deer adopted the

climate of the lower reach of the Yangtze river, now the population of the Tianezhou reserve is over 200, the population growth rate is 35%. That means the lower reach of Yangtze river is suitable for further field release of the Pere david's deer. When considering manipulating population structure and releasing captive-bred individuals to the field, we should also keep a group of Pere david's deer in captivity, to continue the process of domestication of the deer. Therefore, while restoring the Pere david's deer in the nature, we will also produce a domestic Pere david's deer breed, which can be farmed in the future.

794: ***-.437***

The current extinction of many of Earth's Large terrestrial carnivores has Left some extant prey species Lacking knowledge about contemporary predators, a situation roughly parallel to that 10,000 to 50,000 years ago, when naive animals first encountered colonizing human hunters. Along present-day carnivore recolonization fronts, brown (also called grizzly) bears killed predator-naive adult moose at disproportionately high rates in Scandinavia, and moose mothers who Lost juveniles to recolonizing wolves in North America's Yellowstone region developed hypersensitivity to wolf howls. Although prey that had been unfamiliar with dangerous predators for as few as 50 to 130 years were highly vulnerable to initial encounters, behavioral adjustments to reduce predation transpired within a single generation. The fact that at Least one prey species quickly Learns to be wary of restored carnivores should negate fears about Localized prey extinction.

797: ***-.170***

Using radio-telemetry we evaluate and discuss effects of a prescribed fire in a Madrean community on mortality, behavior, and habitat of 3 rattlesnake species. Eight snakes exposed to low intensity fire survived, whereas a snake exposed to intense fire died. Spatial descriptors of activity did not significantly differ before versus after the fire; however, individuals moved significantly less frequently and were found in subterranean retreats more frequently after the fire than before the fire. Wooded canyons and wooded steep slopes burned intensely because of high fuel accumulation, resulting in habitat loss for Crotalus willardi obscurus. Reintroduction of fire is essential in maintaining a mosaic of habitats and ecosystem function. Prior to reintroduction of large-scale summer fires, consideration should be given to reducing artificially high fuel loads to preserve C. w. obscurus habitat and reduce mortality.

798: ***+.068***

Asiatic wild asses and Przewalski horses initially inhabited steppe, semi-desert and desert areas, but Przewalski horses became extinct in the wild, and Mans disappeared at the beginning of the 20th century, except for a small population in Turkmenistan. The Bukhara Breeding Centre (Uzbekistan) was created in 1976 for reintroduction and conservation of wild ungulate species. In 1977-1978, Rye Mans (two males and three females), from Barsa-Kelmes island on the Aral sea, were introduced into the reserve. The group increased to 25-30 animals in 1989-1990, when eight Przewalski horses from Moscow and St Petersburg toes were introduced. We analysed the home ranges, preferred habitats and social interactions of these closely related species during 1995-1998 by seasonal and group composition. Horses and asses formed a reproductive group and a secondary non-reproductive group. The home range of the secondary group was larger than the reproductive group and seemed to be less dependent from the watering places. Przewalski horses were less adapted to semi-desert conditions (both water and vegetation needs) than kulan. (C) 2001 Academic Press.

799: *+.075*

We studied the thermal sensitivity of sprint speed in the captive population of the endangered lizard Gallotia simonyi (Lacertidae) on El Hierro island and in C. stehlini, an abundant, large-sized lizard from the nearby island of Gran Canaria (Canary Islands, Spain). Among adults, lizards of both species were faster than adults of other lacertids. The sprint performance curves for both species had a flat upper plateau, and the obtained values for performance breadth were high, indicating that species attained near maximum sprint speeds over a wide range of body temperatures. Gallotia stehlini was significantly faster than G. simonyi both in absolute (cm/s) and relative speed (SVL/s). Juveniles of G, simonyi were significantly slower than juveniles of G. stehlini only in absolute speed. The lower sprint speed of G. simonyi is consistent with the hypothesis that higher vulnerability. of this species to introduced predators may have played a role in its near extinction. Juveniles rather than adults from the captive population of G. simonyi should be used for reintroduction to establish wild populations, because the sprint performance of adults may have been compromised by prolonged captivity.

800: *+.030*

We analyzed genetic variability among the four naturally-occurring populations of the endangered plant Cordylanthus palmatus to test whether a central tenet of conservation genetics - large populations are more genetically diverse than small populations held true in our study system and to guide long-term conservation planning for the species. Genetic variability in C. palmatus was moderate at the species level but relatively low in several populations. About 2% of the measured genetic variation was attributable to variation between populations. Genetic variability in C. palmatus did not increase with population size. The two largest populations were relatively invariate and genetically similar, and neither contained any unique alleles. (C) 2001 Elsevier Science Ltd. All rights reserved.

801: *+.129*

The red-legged partridge (Alectoris rufa) is the only indigenous Alectoris species in the Iberian Peninsula. Local populations are often reinforced with captive-bred individuals, sometimes including hybrids between the red-legged partridge and the exotic rock partridge (A. graeca). Hunters and wildlife managers oppose releases of hybrids, but their identification by visual inspection is difficult beyond the first hybrid generation. Here we report the development of a set of RAPD markers to identify hybrid partridges using blood samples. We initially screened 46 RAPD primers on a subsample of pure red-legged and rock partridges, and finally selected six primers that produced 11 markers specific of the rock partridge. The selected primers were tested on hybrids of different generations bred in captivity. This set of loci permitted the detection of 100% (n = 31) Fl hybrids, 100% (n = 14) backcrosses of Fl to red-legged partridge, all but one (95%, n = 18) hybrid of the second backcross, and 18 out of 27 (67%, n = 27) hybrids of the third backcross. Efficient detection of backcross 1 and 2 individuals is essential, as these are the ones released for re-stocking purposes in hunting states. Although we have only used blood samples, other sources of high-quality DNA, such as muscle, should provide the same results. Therefore, it would be feasible to monitor the genetic purity of partridges in farms and hunting states at different stages of production, including embryos, chicks or hunted specimens. (C) 2001 Elsevier Science Ltd. All rights reserved.

802: *+.111*

The Critically Endangered Antiguan racer Alsophis antiguae is confined to Great Bird Island, a 9.9-ha (24.5-acre) islet off the north-east coast of Antigua in the Lesser Antilles. This island represents weil under 0.1 per cent of the species's historical distribution range. During the past 5 years, the total number of: racers aged 1 year or more has fluctuated between 51 and 114, and currently stands at approximately 80. Since 1995, the Antiguan Racer Conservation Project (ARCP) has endeavoured to save this harmless snake from extinction by using a combination of education, conservation breeding, habitat restoration, local capacity building and applied research. The Antiguan racer's ecology and population dynamics have become well understood after 5 years of intensive study, and the species has evidently benefited from the project's rat eradication programme. The snakes are still seriously threatened by other intrinsic and extrinsic factors, however, including inbreeding depression, frequent hurricanes, invasive predators and deliberate killing by toruists, as well as the problem that Great Bird island is too small to support more than about 100 individuals. This paper describes the activities and impact of this project to date, and outlines a series of conservation activities to safeguard the long-term future of the species, which include reintroduction of the Antiguan racer to restored islands within its former distribution range.

803: *+.092*

Mountain gazelles were reintroduced to central Arabia during 1991-95. Hawtah reserve was searched for gazelles during the 1998-99 winter. Gazelles were seen in one wadi system and their signs were found in several others and on the plateau. sightings were used to calculate the minimum number of gazelles in the Matham wadi system, which previously held most of the population. During October-November 1998, the minimum number was 64 per cent less than 4 years earlier. Frequent observation of recent signs in areas where no gazelles were seen suggested that daytime sightings alone were no longer adequate for monitoring this population The decline in the number of gazelles seen, an increase in their flight distance and an apparent change in their activity patterns were consistent with the rangers' claim that poaching had commonly occurred. Poaching started after reserve management built, without adequate consultation, a new fence that was intended to bar local people from part of the reserve. Management lessons include the need for the following: continued monitoring of reintroduced populations after the initial postrelease phase; long-term dialogue with local people; effective law enforcement; and the management of aridland domestic livestock in ways that prevent inter specific competition for food causing the elimination of wild ungulates.

804: *+.041*

The endangered red-cockaded woodpecker (Picoides borealis), a nonmigratory, cooperatively breeding species limited to the older-growth pine forests of the southeastern United States, requires living pine trees for cavity excavation. Since the 1950s, alteration and destruction of critical woodpecker habitat have caused a decline in red-cockaded woodpecker populations. The red-cockaded woodpecker now exists mostly in small, scattered populations whose respective members have little opportunity to interbreed. In general, as populations become smaller and more isolated, the frequency of inbreeding increases and genetic diversity and population viability may decrease. The introduction of unrelated individuals into disjunct red-cockaded woodpecker populations may reduce inbreeding, maintain genetic variability, and reduce local extinction. We developed a technique for selecting red-cockaded woodpecker nestlings and transferring them into the nests of unrelated conspecifics. For each translocation, we paired nests ht age and exchanged 1 nestling From each, so that 2 introductions were made with reciprocal translocation event. Nest visitation by the parents and fledging success of nestlings were monitored. Fostered nestlings were

accepted by their new parents, and the rate of successful fledging hy fostered nestlings was not different from that of nonfostered control nestlings. We concluded that reciprocal intraspecific Fostering of similarly aged nestlings can be used safely and effectively to translocate red-cockaded woodpeckers and may have advantages over the translocation of adults and juveniles under certain conditions.

805: *+.010*

Variation in mitochondrial DNA (mtDNA) was surveyed, using restriction endonucleases, in the white-clawed crayfish, Austropotamobius pallipes lusitanicus, from 14 populations sampled in Spain. Four additional samples from France (1), Slovenia (1) and Italy (2) were also analysed. Among the 11 haplotypes listed, only one was detected from the 154 animals sampled from Spanish populations. This haplotype was also recorded in the Fosso di Ferfereta population (Italy). Estimates of nucleotide sequence divergence among haplotypes ranged from 0.45% to 17.4%. Interpopulational genetic relationships showed that Spanish populations were closely related to those of Fosso di Ferfereta with a small genetic distance (0.0003) found between them. AMOVA revealed that most of the genetic variance (71.97%) was attributed to variation between European regions. These results are in accordance with a drastic bottleneck event during the history of the Spanish populations. Four suggestions, based on human introduction, selection and recent or ancient historical events are discussed in relation to the lack of genetic variation in the Spanish crayfish stock.

806: *+.252*

Peregrine Falcons (Falco peregrinus) of seven subspecies from four continents were bred in captivity, and approximately 1173 of their progeny were released in the midwestern United States and adjacent regions of Ontario and Manitoba in an attempt to replace the original population that was extirpated by chlorinated hydrocarbon poisoning in the 1950s. We analyzed the success of individuals of the different subspecies introduced to the Midwest. Five of the seven subspecies released have contributed to the current breeding population. Subspecies of breeding Peregrine Falcons were equally represented when breeding birds of high productivity were compared with less prolific breeders. The subspecific makeup of the breeding population did not differ significantly from that of the released population, suggesting that adaptability in this species was sufficient to override genetic differences between subspecies. Peregrines of widely different genetic stocks have thrived after release, making substantial genetic contributions to the new population.

807: *+.085*

The endangered Hawaiian monk seal breeds at six locations in the northwestern Hawaiian Islands. To determine whether significant genetic differentiation exists among these sites, we used microsatellite loci to examine the monk seal population structure at the five largest breeding colonies. Of 27 loci isolated from other seal species, only 3 were polymorphic in an initial screening of one individual from each breeding site. Only two alleles were found at each of these 3 loci in samples of 46-108 individuals. This extremely low variation is consistent with other measures of genetic variability in this species and is probably the result of a recent severe population bottleneck, combined with a long-term history of small population sizes. Although the smallest monk seal subpopulation in this study (Kure Atoll) showed some evidence of heterozygote deficit, possibly indicative of inbreeding, the next smallest (Pearl and Hermes Reef) had an apparent excess of heterozygous individuals. Genetic differentiation was detected between

the two subpopulations at extreme ends of the range (Kure and French Frigate Shoals). This trend was significant only at the microsatellite locus for which we had the largest sample size (Hg6.3: R-ST = 0.206, p = 0.002; allelic goodness of fit G(h) = 15.412, p < 0.005). French Frigate Shoals is the source population for translocated animals that have been released primarily at Kure Atoll. Differentiation between these sites consisted of allele frequency differences (with the same allele predominant in each location at all three loci), rather than the preservation of alternative alleles. Although the translocations have had positive demographic effects, we recommend continued genetic monitoring of both the source and recipient populations because translocated individuals are now entering the breeding population.

808: *+.161*

Global climate change is frequently considered a major conservation threat. The Earth's climate has already warmed by 0.5 degrees C over the past century, and recent studies show that it is possible to detect the effects of a changing climate on ecological systems. This suggests that global change may be a current and future conservation threat. Changes in recent decades are apparent at all levels of ecological organizations: population and life-history changes, shifts in geographic range, changes in species composition of communities, and changes in the structure and functioning of ecosystems. These ecological effects can be linked to recent population declines adn to both local and global extinctions of species. Although it is impossible to prove that climate change is the cause of these ecological effects, these findings have important implications for conservation biology. It is no longer safe to assume that all of a species' historic range remains suitable. In drawing attention to the importance of climate change as a current threat to species, these studies emphasize the need for current conservation efforts to consider climate change in both in situ conservation and reintroduction efforts. Additional threats will emerge as climate continues to change, especially as climate interacts with other stressors such as habitat fragmentation. These studies can contribute to preparations for future challenges by providing valuable input to models and direct examples of how species respond to climate change.

809: *+.143*

Symphyotrichum laurentianum, the Gulf of St. Lawrence Aster, is an endemic aster of Prince Edward Island, New Brunswick, and the Magdalene Islands. It is considered by the Committee on the Status of Endangered Wildlife in Canada (COSEWIC) to be of special concern in Canada and to be critically imperiled in Prince Edward Island. One goal of this study was to test germination to clarify the relationship between dormancy and germination in this species. Seeds germinated in culture media had an overall mean percent germination of 68.37% whereas seeds germinated in soil had an overall mean percent germination of 17.38%. Kinetin supplements positively affected germination rates. However, these kinetin supplements eventually led to abnormal morphological development in S. laurentianum seedlings. Cold treatments had no significant effects on the percent germination of S. laurentianum seeds. Micropropagation of this type of explant and reintroduction should therefore be considered by Prince Edward Island as potential techniques for the conservation of S. laurentianum on Prince Edward Island. A complementary goal of this study was to survey the Blooming Point and Covehead sites to update the population statuses of S. laurentianum within the Prince Edward Island National Park. The size of the S. laurentianum population at Covehead Pond during 1999 was very similar to the population size at this site in 1993. Population sizes showed more fluctuation at Blooming Point between 1993 and 1999. No asters were found in the East Marsh in 1999; however, there was a large increase in population numbers at the Dune Slack site, Seeds of S. laurentianum appear to respond well to tissue culture. Annual monitoring of S. laurentianum populations should also occur to ensure appropriate

management of this species on Prince Edward Island.

810: ***+.324***

We present a case study on the differing roles of temperate and tropical ex situ facilities in supporting the conservation of a major tropical plant group. The conservation value of botanic garden palm collections is reviewed by using (1) survey data from 35 collections in 20 countries, and (2) case studies examining the conservation value and utility of botanic garden palm collections for selected species. The collections surveyed hold a total of 902 palm species from 169 genera, representing 33% of global species diversity and 84% of generic diversity. The collections hold 77 (34%) of the 222 IUCN/Species Survival Commission (SSC) listed threatened palms. Recommendations are presented on the effective role of botanic garden palm collections in light of both the Convention on Biological Diversity and the IUCN/SSC Palm Action Plan. It is concluded that temperate botanic garden collections do not adequately reflect palm conservation priorities and that a range of genetic and logistical constraints precludes repatriation and reintroduction, and accordingly, reduces their direct contribution to species recovery. The most effective application of ex situ conservation is in-country and linked with habitat conservation. We, therefore, recommend that temperate palm collections direct investments, in terms of displays and fund-raising, towards supporting in-country conservation activities in the tropics. (C) 2001 Elsevier Science Ltd. All rights reserved.

811: ***+.119***

The successful removal of rodents from islands around New Zealand has enabled translocation of rare species of lizards to new sites within their former range. Four species of skinks were translocated to Korapuki Island, Mercury Islands, New Zealand: Cyclodina alani, C. oliveri, C. whitakeri and Oligosoma suteri. Responses of three of the lizard species to release were predicted using a deterministic population model then compared with the actual performance of wild populations. The population models predicted that if populations of about 30 C. whitakeri and O. suteri were released, C. whitakeri would be increasing by 7% p.a. and O. suteri would be increasing by over 35% p.a, during year five. If 20 C. alani were released, the population could be increasing by 70% p.a. during year five. Population expansions are being recorded for all three species, but observed population increases were lower than those predicted for two species, partly because of low catchability of immature lizards. The lowest rate of increase (up to 7% p.a.) was for C. whitakeri. Low rates of population expansion found in species such as C. whitakeri raise a conundrum: the rarer a species is, and the lower its annual reproductive output, the larger the number of individuals that may be required for translocations to succeed. Translocations may be regarded as successful when new populations are self-sustaining and comprised only of locally born animals. Despite the populations increasing, it may not be possible to claim success far the three intensively studied species on Korapuki Island until at least 20 years after release. This is because of longevity of the founders, which are still being caught up to 12 years after release. Extreme longevity, and hence prolonged post-release monitoring, is likely to be a recurrent problem for translocations of rare lizards. O 2001 Elsevier Science Ltd. All rights reserved.

812: ***+.055***

Desert bighorn sheep (Ovis canadensis mexicana) became extinct in Texas, U.S.A., during the 1960s and reintroduction efforts have resulted in an extant population of about 300 free-ranging animals. We studied diet preferences of reintroduced desert bighorn sheep in three mountain ranges in western Texas, U.S.A., during 1994-1995. We predicted that botanical composition and

species richness of diets of reintroduced male and female desert bighorns differ when sexes are segregated, but are similar during portions of the year when sexes are aggregated. There were few consistent differences in diet composition between sexes. Species richness of diets of male bighorns was greater than for females. Forbs were highly preferred by both sexes but forb biomass was less than 1 kg ha (-1) in all seasons. Drought and impacts of historical livestock grazing on vegetation may have masked diet partitioning by sex. (C) 2001 Academic Press.

813: *+.303*

1. The Mauritius kestrel Falco punctatus (Temminck 1823) has recovered from very low numbers. In order to evaluate the severity of the population bottleneck that it experienced, we have developed a method for estimating the productivity of the nests that escaped detection. This method uses ringing records for MCMC estimation of parameters describing the recruitment of adults to the breeding population and the growth in productivity of undiscovered nests.2. Comparison of the estimates for the two restored populations (eastern and western) showed a far lower proportion of undiscovered nests in the former, as predicted because of widespread use of nestboxes. This served to verify the method of estimation. The estimates show a steady increase in population size, in contrast with field estimates indicating a recent reduction in growth.3. The results suggest that the alarmingly low estimates of population size in 1974 (two breeding pairs) were accurate, and that few undiscovered nests existed during the bottleneck.4. The recovery of the population seems to have been initiated by the intensive conservation effort. The most rapid period of population growth coincides with the reintroduction programme. The results imply that the eastern population is much more reliant on intensive management for its future growth.

814: *+.183*

Plant diversity in Indian subcontinent is under threat mainly due to human activities and reproductive isolation of the species. It is estimated that 15-20 per cent of our vascular flora i.e. about 2,500 species of plants are under various degrees of threat of which about 1,000 species are native to tropical and subtropical regions of the country. The increasing loss of plant diversity has turned the attention of various agencies to take the immediate conservation measures and save it from further depletion and extinction. Besides protection of species in-situ, the off-site (ex-situ) conservation of species particularly of those which are rare and endangered, has become an important tool for conservation of biological diversity. Considering the urgency for conservation of plant diversity we have taken up the task of ex-situ conservation of some tropical and subtropical threatened plant species of India in the N.B.R.I. Botanic Garden. The main aim of this work is to acclimatise, cultivate, propagate and multiply such taxa under ex-situ condition and subsequently reintroduce them to natural or semi-natural habitats together with maintenance of the species ex-situ in the botanic garden.

815: *+.165*

Since a review of the management of the critically endangered kakapo (Strigops habroptilus) in 1994, management of this species has become more intensive. This paper reports on new developments in the supplementary feeding of kakapo, continuous monitoring and protection of nests from predators, translocations of kakapo between islands, and artificial incubation of eggs and hand-raising of chicks. Supplementary feeding probably increases chick survival especially when natural food supplies fail. It may also increase breeding frequency, either by triggering breeding, or raising kakapo above a nutritional threshold. Protection of nests from predators has eliminated rat predation of eggs and chicks, and continuous monitoring and rebuilding of poor nest

sites has improved kakapo nesting success. Recent translocations of kakapo between islands have aimed to prevent possibly infertile males from mating, facilitate supplementary feeding, improve the breeding prospects of some birds, test the suitability of a new island for kakapo, and facilitate the eradication of rats. Eggs have been artificially incubated and chicks hand-raised in an attempt to establish a captive population of hand-raised birds, to rescue sickly and unthrifty chicks, and to forestall likely nest failures. (C) 2001 Elsevier Science Ltd. All rights reserved.

816: *+.116*

It is in the nature of today's world that interventionist, rather than protectionist, management strategies are likely to be in greater demand, because an enormous area of the earth's habitable surface has already been transformed by human action. [Holdgate, M.W., 1986. Summary and conclusions: characteristics and consequences of biological invasions. In: Kornberg, H., Williamson, M.H. (Eds), Quantitative aspects of the ecology of biological invasions. The Royal Society, London, pp. 733-742]Progress with ecological restoration in New Zealand is reviewed. A useful goal for restoration is that of rebuilding, as far as possible, the evolutionary and ecological context of species in the system, i.e. reinstating earlier selection regimes. Opportunities for restoring biological components of these regimes are greater than those available for restoring physical conditions. In this country, effective ecological restoration is not possible without control or eradication of introduced mammals. Descriptive models of systems to be restored are also a necessity for achieving goals. A particular problem is that caused by past extinctions of animal species. Replacement of some extinct species, within particular trophic guilds, with ecologically appropriate and related extant species, is suggested as a possible response to this problem. (C) 2001 Elsevier Science Ltd. All rights reserved.

817: *+.047*

The avifauna of New Zealand has been severely depleted since human colonisation and currently contains a disproportionately high number of threatened species. Of the 23 threatened shorebird species worldwide, six are endemic to New Zealand. We review the status of New Zealand's endemic shorebirds and examine the impact on them of various threats, particularly predation by introduced mammals. The conservation status of the 10 extant species (three oystercatchers, one stilt, four plovers and two snipe) is outlined and the factors that predisposed them to predation by introduced mammals are summarised. Individual species accounts are presented, including data on population trends, known or suspected impacts of predation, identification of important predator species, other threats, and conservation measures currently in place or required. One species and two subspecies are extinct, three species are confined to predator-free islands and another is found only on the Chatham Islands group. Six survive on the mainland but three have declined to varying degrees and are assigned threatened status by Collar et al. (1994). Only one plover and two oystercatehers are still relatively numerous and/or widespread. Rats, cats and mustelids have had the greatest overall impacts. Conservation measures in place to mitigate the effects of introduced predators include the formulation of recovery plans, predator control around breeding areas, captive breeding and rearing programmes and the founding of new populations by translocation. There are often substantial differences in susceptibility to predation of closely related or ecologically similar taxa, and we stress the importance of basing conservation management decisions on relevant and detailed demographic and ecological studies. The main threat to threatened shorebirds elsewhere in the world is loss or degradation of habitat; the disproportionate impact of mammalian predators on New Zealand shorebirds is unusual but not unique. (C) 2001 Elsevier Science Ltd. All rights reserved.

818: ***+.160***

Microhabitat use studies of translocated species are crucial to assess their adaptation to the new environment. A total of 42 St Lucia whiptail lizards (Cnemidophorus vanzoi), an endemic to two islets (Maria Major, 10.2 ha acid Maria Minor, 1.8 ha) off St Lucia in the Lesser Antilles, were translocated from Maria Major to Praslin Island (1.1 ha) in 1995. Three years after the release, we studied the abundance, density and distribution of the translocated lizard population in the prevailing five habitat types on the island. Habitats varied from more exposed open grasslands to tall, continuously canopied woodlands. Although lizards were distributed throughout the island, some habitats were used more than predicted with respect to availability. Habitat utilization showed a seasonal shift in distribution. Line transect sampling estimated a population of 145 +/- 23 lizards on Praslin Island. There were significant seasonal differences in estimated lizard abundance indicating a change in activity during the dry season. Lizard density was significantly correlated with general habitat characteristics, rather than with any specific microhabitat variable measured. Factors affecting distribution (thermoregulation, food resources and competitive exclusion) are discussed and it is suggested that habitat use during the dry season may be best explained by thermal constraint. These results will help managers choose future translocation sites.

819: ***+.212***

The benefits of monitoring habitat use patterns of translocated populations are widely acknowledged. However, this monitoring seldom occurs. Here, I report the habitat use and foraging patterns of a newly translocated population of South Island saddlebacks (Philesturnus carunculatus carunculatus) on Motuara Island, New Zealand, during the 1st breeding season after release. Reintroduced South Island saddlebacks spent most foraging time on the ground and in Pseudopanax arboreus. Foraging substrates used by male and female saddlebacks differed significantly. Saddlebacks focused foraging activities at 0-4 m above ground, and appeared to prefer to forage in larger trees, although the species composition of forested areas did not seem to influence the birds' choices of places to settle. With increasing population density, saddlebacks on Motuara Island may increase their areal foraging efficiency by using a wider range of plant species, vertically stratifying foraging locations within pairs, increasing use of smaller trees for foraging, and possibly by using scrub habitats more extensively. South Island saddlebacks appear to be highly adaptable in their choice of foraging sites and this plasticity may enhance the success of translocations.

820: ***+.304***

Native unionid mussel populations have recently declined throughout North America as a result of zebra mussel (Dreissena polymorpha) fouling. Periodic cleaning of fouled unionids and cleaning followed by translocation have been suggested as methods for reducing mortality. Leptodea fragilis and Potamilus alatus were used to determine survival, recovery of energetic stores, and accumulation of newly settled zebra mussels after cleaning and replacement in situ. Both species had high survival, and L. fragilis increased energetic stores after cleaning. Elliptio complanata and Lampsilis radiata were used to compare conservation strategies for unionids fouled by zebra mussels. Survival and glycogen content were used to evaluate stress induced by cleaning and replacement in situ, cleaning and translocation, and cleaning, quarantine, and translocation, relative to the stress in fouled unionids and control (never fouled) unionids. New zebra mussel settlement was assessed to estimate the frequency of cleanings needed. Cleaned E. complanata and L. radiata maintained significantly higher glycogen levels and had higher survival than fouled unionids in all treatments; however, 30% of L. radiata died while in quarantine but no E.

complanata died. Translocated unionids were difficult to relocate in the riverine refugium The inability to find translocated unionids, coupled with high survival and energetic stores in cleaned and replaced unionids, indicate that cleaning and replacement is an effective conservation strategy. Cleaning and replacement may be used as the Ist step to conserve small populations of fouled unionids Living in environments where food is not Limiting and where collection and cleaning are logistically feasible.

821: ***-.028***

Reintroduced houbara bustards (Chlamydotis [undulata] macqueenii) in a large, fenced reserve in central Saudi Arabia are subject to predation pressures from mammalian carnivores. From 1993 to 1997 a total of 98 Arabian red foxes (Vulpes vulpes arabica) and 56 Ruppell's foxes (Vulpes ruppelli sabea) were translocated away from houbara reintroduction sites in a predator control exercise. Translocation distances varied from 15 to 150 km and both species showed the same likelihood of return and recapture (ca. 12%). No fox of either species was recorded as having returned from translocation distances in excess of 150 km. Red fox return appeared to be related to distance translocated up to 150 km, but this was not the case for Ruppell's fox. In this trial, translocation was not found to be a time-effective tool to reduce predation. (C) Academic Press.

822: ***-.134***

1. In recent decades, the Eurasian lynx Lynx lynx has recolonized former habitat, bringing it into potential conflict with livestock. We studied the spatial and temporal distribution of lynx attacks on sheep in the French Jura between 1984 and 1998, during and after its population expansion. We estimated the local and regional impact of lynx predation on livestock.2. The number of attacks increased from three in 1984 to 188 in 1989, concurrently with the colonization of the main sheep range by lynx. During subsequent years, 66-131 attacks were recorded annually (92-194 sheep killed per year).3. On average, 1.6 sheep were killed per attack. Lynx preyed disproportionately on lambs and subadult sheep. A small percentage of flocks (9.5-22.9%) were attacked, most of which (75.2%) were attacked once or twice a year. At the regional level, annual sheep losses to lynx were 0.14-0.59% of the total number of sheep.4. The major lynx-livestock problem was due to clustered attacks in a few small areas. Each year, two to six 'hot spots' (33-69% of the attacks) were identified. Hot spots covered 0.3-4.5% of the total area where attacks occurred (1835-4061 km(2)). Roe deer abundance was higher in hot spots and, even here, sheep only made up 3.1% of the lynx diet. These data show that lynx were not killing sheep due to shortages of alternative prey or in response to an increased need for food when rearing young.5. The concentration of hot spots in only nine small areas between 1984 and 1998 indicated that only a few individual lynx were involved. The reappearance of hot spots at the same sites, after years of interruption and despite the removal of lynx, suggested that the ultimate factors causing hot spots were factors inherent to those sites. Further investigation is needed to identify causal factors with a view to eliminating them. These may relate to landscapes features, animal husbandry practices or the behavioural ecology of lynx.6. In future, where large predator reintroductions are planned, the potential for concentrated, localized, impact should be evaluated and mitigation measures put in place. For scattered and episodic lynx damage, financial compensation is the only realistic option at present. In hot spots, the cost-effectiveness of guard-dogs or the selective removal of some individual lynx should be evaluated.

823: ***+.107***

Regulated river restoration through planned flooding involves trade-offs between aquatic and

terrestrial components, between relict pre-dam and novel post-dam resources and processes, and between management of individual resources and ecosystem characteristics. We review the terrestrial;(wetland and riparian) impacts of a 1274 m(3)/s test flood conducted by the U.S. Bureau of Reclamation in March/April 1996, which was designed to improve understanding of sediment transport and management downstream from Glen Canyon Dam in the Colorado River ecosystem. The test flood successfully restored sandbars throughout the river corridor and was timed to prevent direct impacts to species of concern. A total of 1275 endangered Kanab ambersnail (Oxyloma haydeni kanabensis) were translocated above the flood zone at Vaseys Paradise spring, and an estimated 10.7% of the total snail habitat and 7.7% of the total snail population were lost to the flood. The test flood scoured channel margin wetlands, including potential foraging habitats of endangered Southwestern Willow Flycatcher (Empidonax traillii extimus). It also buried ground-covering riparian vegetation under >1 m of fine sand but only slightly altered woody sandbar vegetation and some return-current channel marshes. Pre-flood control efforts and appropriate flood timing limited recruitment of four common nonnative perennial plant species. Slight impacts on ethnobotanical resources were detected > 430 km downstream, but those plant assemblages recovered rapidly. Careful design of planned flood hydrograph shape and seasonal timing is required to mitigate terrestrial impacts during efforts to restore essential fluvial geomorphic and aquatic habitats in regulated river ecosystems.

824: ***+.020***

Captive breeding and reintroduction programs are rarely evaluated, and assessment criteria vary widely. We used the following criteria to evaluate a bighorn sheep (Ovis canadensis) augmentation program: (1) survival and recruitment rates in the captive population, (2) survival of released animals, (3) recruitment of released animals (4) growth rate of the reintroduced or augmented population, and (5) establishment of a viable wild population. Captive bighorn survival and recruitment was high, averaging 0.98 (SD = 0.05) and 71.0% (SD = 19.4), respectively. Annual survival of free-ranging captive-reared bighorn (n = 73, (x) over bar = 0.80 SD = 0.11) did not differ (Z = -0.85, p = 0.40; n = 14) from survival of wild-reared bighorn (n = 43, (x) over bar = 0.81, SD = 0.12). Recruitment was unusually low for both captive-reared ((x) over bar = 13.7%, SD = 0.24) and wild-reared ewes ((x) over bar = 13.7%, SD = 0.20). Although reintroduction did not result in population growth or establishment of a viable population, it helped prevent extirpation of the reinforced deme, preserved metapopulation linkage, and aided habitat preservation. Chronic low recruitment and low adult survivorship precluded achievement of criteria 3-5. Environmental conditions in the release area also appeared to hinder program success. Standard evaluation criteria for ongoing reintroductions allow for informative assessments and facilitate comparisons needed to refine reintroduction science as a recovery tool for threatened or endangered populations.

825: ***+.114***

New World parrots are declining throughout their range. Blue and Gold Macaw Ara ararauna was extirpated from the island of Trinidad in the late 1960s through anthropogenic processes. The Cincinnati Zoo & Botanical Garden is assisting the Wildlife Section of the Forestry Division, Trinidad on a mission to restore the species into its historical range within the Bush-Bush Sanctuary in the Nariva Swamp. The Sanctuary has been protected by the government of Trinidad and is the home of the endangered Red-bellied Macaw Ara manilata and West Indian Manatee Trichechus manatus. Zoo and Wildlife personnel participated in three separate releases, beginning in December 1999 through to March 2000. The initial release consisted of an identical sex group of macaws followed by two additional releases of pairs/groups of birds. Of 18 Blue and Gold

Macaws, 14 were released into the Nariva Swamp and are currently being monitored by the Wildlife Section, Zoo personnel and surrounding community. Translocation of wild populations of parrots has proven to be a viable solution to restocking wild populations that have been regionally extirpated.

826: ***-.065***

River engineering work has historically been responsible for the decline and extinction of a number of endangered freshwater pearl mussel [Margaritifera margaritifera (L.)] populations and potentially remains a significant threat. The impact of different types of river engineering work on a number of Scottish M. margaritifera populations is examined and discussed. M. margaritifera is now fully protected under law in most countries and guidance is urgently needed so that river managers can integrate ecological and socio-economic factors when considering the impact of proposed activities on M. margaritifera populations. To safeguard the remaining important populations, a simple conflict resolution framework is suggested for the appraisal of proposed developments in rivers with pearl mussel populations. Operations likely to harm mussels and permanently damage their river bed habitat should not proceed. In exceptional circumstances (outlined), the translocation of small numbers of adult mussels may possibly be considered as a potential management tool, but mussel translocation has been little used and should be considered experimental and last resort. (C) 2001 Elsevier Science Ltd. All rights reserved.

827: ***+.286***

Instances of range contraction and population decline in bird species in Europe are more common than cases of recovery following decline. Here we report on the recovery and expansion processes of an endangered bird species in Europe, the purple swamphen (Porphyrio porphyrio L., Rallidae), with special reference to the Iberian Peninsula, its main distribution area in Western Europe. After a drastic decline during the first half of the 20th century, which restricted its range to a few areas in southern Spain, the Iberian population has recovered. Currently, the species occurs in a range similar to the one it had at the beginning of the 20th century, and has even colonized new areas. These processes seem to be the result of both human-mediated (effective protection of the species and suitable habitats; success of reintroduction programs as expansion focuses of birds colonizing nearby and distant regions [> 300 km] wetlands) and natural factors (inter- and intra-seasonal cycles of wet and dry years). Thus, we found a significant relationship between rainfall and bird abundance and productivity, suggesting that such inter- and intra-seasonal cycles may have significant effects on demographic parameters that could be related to the expansion process. Finally the species shows remarkable adaptability, as proven by data on breeding success in recently colonized areas and the ability to breed shortly after reintroduction. We hypothesize that saturation of breeding habitats in established areas, a reasonable habitat continuity and favorable environmental conditions, and protection have facilitated the dispersion of birds.

828: ***+.041***

In the XIX century the Ruddy Shelduck was regarded as a common species in Ukraine. Now it is in the Red Data Book of Ukraine and on the Berne Convention List. In 1885 it appeared in the Ascanian Zoo, and a year later there were 16 birds. Nowadays, in the summer and autumn their numbers range from 700 to 1200. The territorial centre of the population are the zoo ponds and the botanical garden, where 48-65 pairs nest. This semi-wild population has been formed as a result of successive acclimatization measures: forming broods with foster parents (Tadorna ferruginea, Cairina moschata, Anas platyrhynchos), creating artificial nests, regular feeding, and the

availability of unfrozen water in the pond. Free-living birds migrate from Ascania Nova to other places, even to distant regions.

829: *+.039*

Reintroduction of populations of endangered species is a challenging task, involving a number of environmental, demographic and genetic factors. Genetic parameters of interest include historical patterns of genetic structure and gene flow. Care must be taken during reintroduction to balance the contrasting risks of inbreeding and outbreeding depression. The Mauna Loa silversword, Argyroxiphium kauense, has experienced a severe decline in population size and distribution in the recent past. Currently three populations with a total of fewer than 1000 individuals remain. We measured genetic variation within and among the remnant populations using seven microsatellite loci. We found significant genetic variation remaining within all populations, probably related to the recent nature of the population impact, the longevity of the plants, and their apparent self-incompatibility. We also found significant genetic differentiation among the populations, reinforcing previous observations of ecological and morphological differentiation. With respect to reintroduction, the results suggest that, in the absence of additional data to the contrary, inbreeding depression may not be a substantial risk as long as propagules for the founding of new populations are adequately sampled from within each source population before additional inbreeding takes place. The results further suggest that if mixing of propagules from different source populations is not required to increase within-population genetic variation in the reintroduced populations, it may best be avoided.

830: *-.041*

Habitat loss and fragmentation affect forest birds through direct loss of breeding habitats, detrimental edge effects such as increased nest predation and brood parasitism, and possibly by limiting movements among remaining forest patches. Despite indirect evidence suggesting that landscape-scale bird movements are constrained by open areas, skepticism remains because birds routinely cross inhospitable terrain during migration. Here, we report evidence from 201 independent homing trials showing that landscape composition and configuration influence the movements (1-4 km) of two neotropical migrant (Black-throated Blue Warbler, Dendroica caerulescens and the Ovenbird, Seiurus aurocapillus) and one resident (Black-capped Chickadee, Poecile atricapillus) forest bird species in Quebec, Canada. Trials consisted of translocating territorial, mated males and measuring the time they needed to return to their territories (homing time), as well as the probability with which they returned to their territories within 30 h (homing success). Birds took more time and were less likely to return to their territories as forest cover decreased in the landscape. Once the simple linear variation due to forest cover was removed from landscape configuration variables, their influence on homing time and success was nonexistent or subtle, suggesting that landscape composition has greater predictive value than landscape configuration to infer constraints on forest bird movements. Indeed, and contrary to our expectation, mean nearest-neighbor distance between forest patches had no impact on homing time or success, but its coefficient of variation was positively correlated with homing time and negatively correlated with homing success. On the other hand, homing time and success were not influenced by the number of forest patches or the amount of edge per unit of forest cover. These results were consistent for all three bird species that we studied. Our data support the hypothesis that movements are constrained when forest birds travel in deforested and fragmented landscapes outside migratory periods. Such an impediment is likely to disrupt habitat selection processes. reduce the colonization of isolated forest patches. and ultimately, alter population structure and dynamics.

831: *-.036*

In order to detect the evolutionary potential of two endangered species, Brassica insularis (Brassicaceae) and Centaurea corymbosa (Asteraceae), within and among-population genetic variation for both quantitative traits and allozymic markers was examined. Four populations of each species were studied, representing a large proportion of extant populations. High values of theta (ST) (0.213 and 0.364 for B. insularis and C. corymbosa respectively) suggested that low amounts of gene flow occur among the study populations. In each species, the genetic distance based on allozymes (estimated by the ratio (theta (ST)/1-theta (ST))) was positively correlated with the geographical distance, indicating isolation by distance. In contrast to previous studies in either outcrossing or selfing plant species, and especially for B. insularis, population differentiation for quantitative traits (theta (ST)) was generally found lower than differentiation for allozymes (theta (ST)), suggesting that the populations studied were experiencing similar selective forces acting upon the quantitative traits measured. Such forces would be strong enough to counteract local genetic drift. Interestingly, for both species theta (ST)'s were statistically independent of geographical distance, in contrast to the marginally significant positive isolation by distance shown by theta (ST). Altogether, these results suggest that theta (ST)'s might not always be used as conservative estimates of Q(ST')s, and might instead overestimate the evolutionary potential of endangered species. This would be especially expected in narrow-endemic species, whose ecological niche is often so restricted that indeed homogeneous selective forces are likely to occur, whereas small population sizes and restricted dispersal are likely to produce strong differentiation for :neutral variation. In fact, knowledge of both neutral and quantitative diversity patterns allows identification of those traits undergoing natural selection, and could be useful in designing reinforcement or reintroduction programs. However, this approach might have limitations too, in the presence of outbreeding depression due to locally coevolved gene complexes, (C) 2001 Elsevier Science Ltd. All rights reserved.

832: *-.091*

The freshwater fish Banded Grunter Amniataba percoides is endemic to northern Australia, and this paper is the first record of the species from New South Wales waters. Six specimens (range total length, 43.5-144.6 mm) were collected from the Clarence River near Grafton. The identification of a Banded Grunter in a consignment of Silver Perch Bidyanus bidyanus fingerlings from a commercial hatchery in Queensland implicates aquaculture in the translocation of this species. Banded Grunter is a hardy, aggressive, omnivorous fish that is known to disperse rapidly over large distances to a wide range of habitats, and the establishment of a population in the Clarence river system may pose a serious threat to the endemic fauna, including the endangered Eastern Freshwater Cod Maccullochella ikei. Because of the potential threat of Banded Grunter, it has been declared a noxious fish under the New South Wales Fisheries Management Act.

833: *-.055*

The Stock Island tree snail, Orthalicus reses reses, went extinct in its native range in the Florida Keys in 1992. Fortunately, O. r. reses has been introduced elsewhere and further reintroductions are currently planned. Before these reintroductions are implemented, it is important to try and determine which factors were most likely to have caused the decline and extinction. While habitat destruction was probably the ultimate reason why there were so few tree snails, it is likely that an interaction of habitat fragmentation and the invasion of an exotic predator caused the final decline that lead to the extinction in 1992. We examined the last 93 O. r. reses shells to infer cause of death. In addition, using surrogate Florida tree snails, Liguus fasciatus, we conducted experiments

on two previously unstudied causes of mortality: predation by red imported fire ants, Solenopsis invicta, and mortality from falls caused by wind. We found that the majority of the last O. r. reses shells were intact, indicating that mammalian and bird predation were not the greatest causes of mortality. Mortality caused by wind knocking tree snails onto the rocky hammock substrate appeared to be a potential source of mortality, but few of the tree snails exhibited signs of breakage. Mortality from fire ants appears to be one of the most likely causes of the recent decline and extinction of O. r. reses. Experiments indicated that in a semi-natural enclosure, fire ants were capable of killing all ages of L. fasciatus, even during aestivation. Fire ants are currently found throughout the last known habitat of O. r. reses and were first discovered in this area at the time of the decline.

834: *-.152*

The identification of taxonomically appropriate Populations of endangered species for captive breeding and reintroduction programs is fundamental to the success of those programs. The Saudi gazelle (Gazella saudiya) was endemic to the Arabian peninsula but is now considered extinct in the wild and is potentially a candidate for captive breeding and reintroduction. Using 375 base pairs of mitochondrial DNA (mtDNA) cytochrome b gene derived from museum samples collected from the wild prior to the presumed extinction of this species, we show that G. saudiya is the sister taxon of the African dorcas gazelle (G. dorcas). Reciprocal monophyly of G. saudiya mtDNA haplotypes with G. dorcas, coupled with morphological distinctiveness, suggests that it is an evolutionarily significant unit. These data indicate that captive populations identified previously as potential sources of G. saudiya for captive breeding appear incorrectly designated and are irrelevant to the conservation of G. saudiya. The polymerase chain reaction-restriction fragment length polymorphism (PCR-RFLP) analysis of several private collections of living gazelles in Saudi Arabia provides no evidence for the survival of G. saudiya. We recommend that field surveys be undertaken to establish whether G. saudiya is indeed extinct in the wild and that other private collections within the Arabian peninsula be screened genetically. We urge caution when captive animals of unknown provenance are used to investigate the phylogenetics of cryptic species groups.

835: *+.164*

Since 1954, several southern African institutions have established captive breeding programs to ensure the long-term survival of the African wild dog (Lycaon pictus). To aid this, a studbook was assembled to provide genetic and demographic information for the southern African captive populations, comprising the largest existing regional population of captive African wild dogs. These populations were investigated over three time frames: during 1985-1990, during 1991-1996 and populations alive in January 1997. The captive-breeding programme is successful with a positive population growth, a significant lowering of inbreeding and mean kinship and an increased genetic diversity. However, genetic variability levels appear lower and levels of inbreeding appear higher compared with wild populations. In addition, there have been no successful long-term re-introductions into the southern African wild using captive-bred dogs, mainly due to the lack of close collaboration between captive breeding and nature conservation institutions. The ultimate success of a conservation programme not only depends on proper demographic and genetic management of the captive population, but primarily on the successful collaboration of all scientific, captive breeding and conservation agencies involved. (C) 2001 Elsevier Science Ltd. All rights reserved.

836: *-.014*

Although the rarity of an endangered plant species can seldom be ascribed to its breeding system, knowledge of its breeding system may be critical to its recovery. The federally listed endangered Ziziphus celata (Florida ziziphus), a woody clonal shrub narrowly endemic to xeric upland habitats of the Lake Wales Ridge in central Florida, USA, is known from only five populations, four of which are sterile and perhaps uniclonal. Altogether only 11 genotypes of Florida ziziphus have been identified by allozyme electrophoresis. We studied the breeding system of Florida ziziphus and found that it is an obligate outcrosser and that some genotypes are cross-incompatible. We have been able to demonstrate the cross-compatibility of only 11 of the 44 test crosses performed to date (25%), as measured by fruit yield. Cross-incompatibility is most likely due to shared self-incompatibility (S) alleles among the few remaining genotypes of these fragmented populations. The identification and translocation of compatible mating types to create reproductively viable populations is essential for the recovery of Florida ziziphus. (C) 2001 Elsevier Science Ltd. All rights reserved.

837: *-.020*

We performed a line transect survey (352.4 km) of primates in the Serra de Paranapiacaba, at one of the largest relatively undisturbed fragments of the Atlantic rainforest of Southeastern Brazil (ca. 1400 km(2)), in August 1998. The brown capuchin, Cebus apella nigritus, was the most common species found in the area (20 groups, density estimate: 5.31 +/- 2.05 individuals per km(2), mean +/- SE). Nine groups of the brown howler monkey, Alouatta guariba clamitans, and eight of the woolly spider monkey, Brachyteles arachnoides arachnoides, were also recorded, with preliminary density estimates of 0.79 +/- 0.40 and 2.33 +/- 1.37 individuals per km(2), respectively. Density estimates for these species in other fragments of Atlantic rainforest are reviewed, showing that densities in Paranapiacaba are among the lowest reported. It is suggested that the higher densities reported for isolated populations in small forest patches (< 50 km(2)) is related to the absence of main primate predators, the density compensation phenomenon and the ecological plasticity of some primate species. In contrast, local extinction in many small patches is probably related to hunting pressure. Given the important primate populations found in the Paranapiacaba fragment, conservation strategies for the studied species should give priority to effective protection of the largest remnant fragments from illegal hunting and deforestation, rather than translocation of individuals or captive breeding programs to introduce monkeys in small forest fragments vulnerable to hunting and of uncertain future.

838: *-.028*

We suggest that reintroductions, like biological invasions, have two phases: establishment when a new population becomes self-sustaining, and spread when a population increases its distribution. Stochastic effects on mortality and sex ratios are most likely to determine whether a population becomes established, while factors influencing birth rates will probably most influence spread. Using this establishment-spread structure, we evaluate the autecological suitability of regions in England for pine marten Martes martes reintroductions. Risks of mortality from predator control, traffic accidents and predation by foxes were used to evaluate suitability for establishment. Mortality risk was higher in all potential release regions in England (selected as having 25% or more woodland cover) than in regions of current pine marten distribution in Scotland; risk of predation was higher in the latter. Indices of prey abundance were used to evaluate suitability for populations to spread. Prey indices in potential release regions were generally higher than in regions of current distribution. A relation between prey, woodland cover, and known pine marten densities suggested that potential release regions are capable of supporting relatively high densities of pine martens, though these might be reduced by higher mortality. We concluded that all

potential release regions are suitable for pine marten populations to spread. However, reintroductions should first be to regions with lower risk of mortality, in case higher levels of the latter prevent establishment. The suitability of relict regions of distribution in northern England was low until post-war afforestation, suggesting that habitat suitability constrained recovery of relict populations. These regions remain less suitable for reintroductions.

839: *+.044*

Koalas have undergone a series of sequential founding events on islands in south-eastern Australia in recent times, Populations in South Australia at the Eyre Peninsula and Mt Lofty Ranges were founded in the 1960s from a colony on Kangaroo Island. The Kangaroo Is. colony was derived from animals introduced to French Island from mainland Victoria over a century ago. In this study, we first use microsatellite markers to quantify levels of genetic variation within the South Australian koala populations and the relatively unperturbed Strzelecki Ranges population from mainland Victoria. This analysis revealed low levels of allelic diversity (1.7 +/- 0.2 to 2.7 +/- 0.5) and heterozygosity (0.208 +/- 0.088 to 0.340 +/- 0.110) in the three South Australian koala populations relative to the Strzelecki Ranges population, which has the highest levels of allelic diversity (4.7 +/- 1.1) and heterozygosity (0.476 +/- 0.122) in Victoria. Second, we measured the incidence of testicular aplasia, a unilateral or bilateral failure in testicular development, in the Eyre Peninsula and Kangaroo Is. populations, and in the ultimate founding population at French Is. Testicular aplasia was present at a frequency of 4.3% in French Is., 12.8% in Kangaroo is. and 23.9% in the Eyre Peninsula, but was undetectable in the non-bottle necked Pilliga State Forest population of New South Wales. The incidence of testicular aplasia correlated positively with effective inbreeding coefficients derived from heterozygosity values (0.13 +/- 0.06 in the Pilliga State Forest, 0.57 +/- 0.17 in French Is., 0.63 +/- 0.12 on Kangaroo Is. and 0.77 +/- 0.12 in the Eyre Peninsula), which may indicate inbreeding depression. These findings are of concern when evaluating the long-term conservation and viability of the South Australian koala populations, which may benefit from genetic augmentation in the future. Finally, unconfirmed reports suggested that animals from other states in Australia were introduced into the Mt Lofty Ranges population. Therefore, we quantified differentiation between the three South Australian populations and the Strzelecki Ranges and French Is. populations, based on microsatellites and mtDNA d-loop region variation. R-statistics and Goldstein's delta mu square distance revealed that differentiation at nuclear loci between populations paralleled known recent population history, except for the close relationship between Mt Lofty Ranges and French Is. This suggested a recent contribution to the Mt Lofty Ranges populations of animals derived from the French Is. translocation program. Furthermore, mtDNA d-loop analysis found no evidence of contributions to the gene pool from animals of New South Wales or Queensland stock, implying that the population was derived exclusively from Victorian stock.

840: *+.044*

Conservation breeding for reintroduction is becoming a popular option for restoring threatened populations. Whereas post-release results are widely reported in the conservation literature, little empirical information is presented on the captive populations that often make such releases possible, even though fecundity, fertility and survival rates can have an impact on the outcome and cost of wild population re-establishment. We present results of survival analyses carried out on a captive population of houbara bustards to determine peak periods of mortality, and by identifying sources of variations in mortality to recognize the potential impact of management practices on productivity. There were two main mortality peaks: during incubation (53% survival of fertile eggs), and by 6 months posthatching (75% survival of hatchlings). Management-related variables

influencing survival of eggs and chicks included the year when laid, the cohort of females laying them, and possibly sex. Most posthatching deaths were due to trauma and infectious diseases. Trauma-related deaths usually follow collisions with cages, and imply selection for the captive environment, an undesirable trend in populations maintained for reintroductions. Reducing losses during incubation would have the largest impact on production of birds for reintroduction, enabling the release of more birds, and reducing the overall costs of the project.

841: ***+.174***

The swift fox (Vulpes velox) is a rare, house-cat-sized carnivore that can race across native prairie at speeds of up to 60 km/hr. Although swift foxes were once so abundant in Canada that 117 025 were trapped between 1853 and 1877, this species was extirpated from Canada and northern Montana by the late 1930s. Since 1983, a reintroduction program has been underway to restore this species to Canada and the most recent releases were made in Grasslands National Park, Saskatchewan in 1997. A Canadian swift fox census during the winter of 1996-1997 revealed that the reintroduced population was located within two regions: 1) approximately 192 foxes were estimated to span the Alberta/Saskatchewan border south of the Cypress Hills; and 2) approximately 89 foxes were thought to exist along the United States border in and around Grasslands National Park, Saskatchewan. Concurrent with the Canadian swift fox reintroduction program, mounting evidence suggested that Canadian fox releases had also established a small swift fox population in north-central Montana. However, a coordinated international effort has not been previously conducted to assess the extent and composition of the shared swift fox population in Canada and Montana. The focus of the 2000-2001 census was: 1) to estimate changes in the distribution and abundance of swift foxes within Canada since the 1996-1997 census; 2) to estimate the distribution and abundance of swift foxes in adjacent areas of Montana. The 1996-1997 census area of 108 Canadian townships was resurveyed and supplemented with 80 Montana townships to form a total study area of 17 326.1 km2. Following training at the Calgary Zoo, six field teams conducted catch-and-release surveys in 80.3% of the study area townships from November 4, 2000 until February 15, 2001. Significant results were as follows: 1. In total, 149 swift foxes were live-trapped: 97 in the Alberta/Saskatchewan border area population, 14 in the Grasslands National Park region, and 38 in adjacent Montana areas. By comparison, 32 swift foxes were caught during catch-and-release efforts during the Canadian 1996-1997 swift fox census. In 2000-2001, 98.6% of captured foxes were unmarked, which means that they were wild-born in the Canadian/Montana population. This is a greater proportion of wild-born foxes than that recorded in 1996-1997, when 81.3% were unmarked. 2. The known distribution of swift foxes in Canada and Montana has substantially increased through the results of this census. In the Alberta/Saskatchewan border area swift foxes were found in 18 townships in 1996-1997 and during the 2000- 2001 census they were found in 38 townships. In the Grasslands National Park region, swift foxes were found in 7 townships during the previous census, whereas they have now been located in 13 townships. In Montana, where a previous census of this kind had not been conducted, swift foxes were found in 25 townships. Hence, the 1996-1997 census found swift foxes in 25 townships whereas the current survey yielded evidence of swift foxes in 76 townships; this represents a three-fold increase in the known swift fox distribution. 3. The number of swift fox captures in Canada has tripled since 1996-1997, in areas that were previously surveyed at the same time of year. Similar recapture rates between the 2000-2001 census and the 1996-1997 census indicate that the threefold increase of the Canadian swift fox population in replicated areas is not due to higher trapability of foxes during this census but, in fact, that this represents a statistically significant, three-fold increase in the fox population in these areas. 4. Fox body condition and age ratios are similar to those of the 1996-1997 census, but there has been a significant shift from a male-biased sex ratio previously to a female-biased population during this census. 5. The increase

in swift fox population size since 1996-1997 differed significantly between the Canadian swift fox subpopulations. Captures in the replicated regions of the Alberta/Saskatchewan border subpopulation have significantly increased by a factor of 3.5. By comparison, the 1.6-fold increase in the Grasslands National Park region is not statistically significant. 6. In newly surveyed areas, 50% of 16 townships in the Alberta/Saskatchewan border area had successful captures for a total of 22 foxes. By comparison, only 9% of 11 newly surveyed townships in the Grasslands National Park area had successful captures totalling one fox. In Montana, which had not been previously surveyed, 31.8% of the 66 townships had swift foxes totalling captures of 38 individuals. 7. Capture success for replicated and new areas combined was highest in the Alberta/Saskatchewan border area with 1 new capture every 10.0 trapnights, intermediate in Montana with 1 new capture/33.2 trapnights, and lowest for the Grasslands National Park region with 1 new capture/38.6 trapnights. 8. Application of the same population estimation technique utilized in 1996-1997 suggests that the Alberta/Saskatchewan border population consists of 560 individuals (compared to 192 previously), the Grasslands National Park area contains 96 individuals (compared to 87 previously), and the sampled Montana area contains 221 foxes. This suggests a total population size of 877 foxes. 9. Previously the Canadian swift fox population has been thought to consist of two isolated subpopulations. The comparative results in Canada suggest that thispopulation has experienced a significant increase since 1996-1997. Furthermore, the present census suggests that the foxes in Canada and Montana now form one loosely-connected population.

842: ***+.112***

More than 1000 pairs of tree-nesting Peregrines once occupied the European lowlands from Germany to Russia. In northern and eastern Germany a population of about 500 pairs of pure tree-nesters existed among which only a very few occasionally nested on buildings and cliffs. This population which was definitely affected by toxic agro-chemicals in its nutritional chain became completely extinct after 1972 throughout the whole tree-nesting area. The habit of tree-nesting is definitely based on imprinting and tradition. On this premise a reintroduction project was started in 1990 in north eastern Germany in which young Peregrines reared in captivity were imprinted to tree-nesting habits before and after their release. This will be continued until 20 sites are occupied by tree-nesters in Germany. Up to now (2000) 201 young Peregrines were released successfully; an additional 29 fledglings came from 3 wild broods found established in old pine woods since 1996. All Peregrines of the new eastern German population which has been established since 1981 are individually marked by ring combinations which permit the reading by telescopic means of whether the birds are coming from trees, buildings, or cliffs and are released or fledged from wild broods. Also the year and site they came from is identifiable. To monitor the success of the project, a table of survival was calculated (Tab. 1) which shows that 22 adult Peregrines may exist from all released and wild fledged birds together in the year 2000. All the falcons at the 3 tree-nesting sites were found to be released from the project, thus confirming the effectiveness of imprinting to this type of nesting. Another 11 falcons from the project group were identified as mates at buildings and cliffs. This indicates a 50% return to the inborn nesting type. However, up to now not a single falcon fledged from cliffs or buildings changed to tree-nesting. The exchange between falcons of different nesting types corresponds to a one way road with the blocked direction toward tree-nesting. As a consequence of this new finding a tree-nesting population can only become established and remain self-sustained in areas without or with very scarce nesting sites on buildings or cliffs. Nesting sites on buildings should not be offered in tree-nesting areas! This initial success can only be maintained if more young Peregrines fledge from trees than from buildings in the tree-nesting areas. On all accounts an early protection against losses of species should be preferred to expensive and not always practicable reintroduction.

843: ***+.067***

The endangered red-cockaded woodpecker (Picoides borealis) is well adapted to fire-maintained pine ecosystems of the southeastern United States. Management practices vary greatly among land ownerships. In some wilderness areas and state parks, a "no management" policy has eliminated use of prescribed fire, artificial cavities, and woodpecker translocation, tools that have proved effective elsewhere in recovering woodpecker populations. We compared forests with essentially "no management" to actively managed forests of similar tree ages and similar red-cockaded woodpecker population demographics. We also compared sites that had received no management in the past to the same sites after management. In every case, populations in forests that did riot use state-of-the-art management for woodpeckers declined severely compared to those in managed forests. Because managed forests typically used all available management techniques concurrently, it was not possible to separate and rank effectiveness of specific management activities. One exception was the Wade Tract in Georgia, where prescribed fire was the primary activity for herbaceous layer and hardwood management in a high-density, stable woodpecker population. Wilderness areas, which are intended to be pristine places that preserve biodiversity, are losing red-cockaded woodpeckers, a keystone species in the ecosystem, at an alarming rate. Collectively, 9 groups of red-cockaded woodpeckers were present in 4 wilderness areas in Texas national forests in 1983. At the close of the millennium, only one woodpecker group remained and its continued existence is unlikely without management. The very fragmented features of present-day landscapes and intervention by humans impair the effectiveness of natural disturbance processes, primarily growing-season fire, that historically produced and maintained open pine savannas with grass-forb herbaceous layers in the pre-Columbian forests of the southeastern U.S. therefore, active management must be used if the red-cockaded woodpecker is to persist.

844: ***+.109***

Prairie dogs (Cynomys spp.) have declined greatly in abundance during the past century, and this warrants efforts to restore populations. Restoration often requires translocating animals to previously occupied areas. Workers should follow standard protocols for animal handling and care. Translocation involves selecting source populations and release sites, capturing and transporting animals, preparing release sites with attendant soft-release infrastructure, and monitoring and managing animals. Source populations should be free of plague and genetically appropriate for the translocation strategy. Release sites ideally have physical or historical evidence of previous occupancy, but also may be selected based on soils, slope, and vegetation. Capture, transport, and release of animals must comply with federal, state, and local regulations. Animals have been commonly captured with live traps, by flooding their burrows, or by using a specially adapted vacuum truck; those captured in plague-prone areas are treated to control fleas. Captives usually are hauled to release sites in covered pickup truck beds or trailers. High-quality release sites have short vegetation (< 12 cm tall) and pre-existing burrows; sites without these qualities may need modification. Retention baskets or fenced enclosures, sometimes combined with artificial underground nest chambers, have been used to reduce dispersal and predation. Control of predators may be needed prior to or following release. Post-release monitoring to detect and remedy potential problems such as dispersal and predation is recommended, and providing a food subsidy may reduce dispersal and elevate survival.

845: ***+.086***

The river otter (Lontra canadensis) is an important North American furbearer species that was eliminated from most of its range by the early 1900s. Many state agencies have undertaken

restoration efforts, although no comprehensive study on the extent or type of management exists. I conducted telephone interviews with wildlife agency biologists in the 49 continental states (USA) to update status and management of river otters, with emphasis on reviewing the use of reintroduction as a management tool to restore extirpated otter populations. As of 1998, river otters occupied at least portions of their historic range in every state except New Mexico. Between 1976 and 1998, 21 states and 1 national park implemented reintroduction projects, releasing 4,018 river otters. States conducting reintroduction projects obtained otters from a variety of sources, but 14 (64%) used at least some otters obtained from coastal Louisiana. All states implemented postrelease evaluations, including radiotelemetry studies by 15 projects. Based on various forms of direct and circumstantial evidence, most biologists stated that reintroductions were successful in restoring extirpated otter populations.

847: *+.181*

The Hihi Notiomystis cincta, a New Zealand honeyeater (Aves: Meliphagidae), became extinct everywhere except one offshore island following European colonization. Attempts to establish Hihi on additional islands in the 1980s had poor success, and this was attributed to food limitation. These islands had all been modified by human use, and had a lower diversity of natural carbohydrate (fruit and nectar) sources than the source island, particularly in winter. When Hihi were released on two additional islands, Mokoia and Tiritiri Matangi, we used supplementation experiments to test whether condition and survival of birds were limited by availability of carbohydrate food. Sugar water was provided on an on-off basis from autumn through spring in the year after the release. Birds were weighed at the beginning and end of fed periods, and survival for fed and unfed periods was estimated using mark-recapture analysis on sighting data. Armstrong and Perrott (2000) reported that supplementary feeding had no effect on condition or survival on Mokoia, and annual survival was about 40%, both in the year of the experiment and in subsequent years when food was supplied continuously. This paper reports contrasting results for Tiritiri Matangi. Supplementary feeding on Tiritiri Matangi increased both condition and survival, and overall survival was substantially higher than on Mokoia-66% in the year of the experiment and 76% the following year when food was supplied continuously. It therefore appears that supplementary feeding can be used to improve survival of Hihi on Tiritiri Matangi, whereas survival is constrained to a low level by other factors on Mokoia. These results emphasize the value of habitat manipulation experiments for developing appropriate management strategies for reintroduced populations.

848: *-.049*

This study investigates the reproductive potential of 22 rare and threatened Western Australian taxa in the genus Verticordia (Myrtaceae) over a 5-year period. Considerable inter- and intra-specific variation in both seed production and germinability was demonstrated for the majority of taxa. The seed to flower ratio, or "seed set", ranged from 0% to 68% with an overall mean of 21% in 82 accessions representing seed from 48 populations of the 22 taxa. Percentage germination ranged from 7% to 100% with an average of 49% for 68 accessions. The precariously low annual reproductive capacity of some of the more restricted and critically endangered taxa threatens their survival and unexpected disturbance events may result in population decline or even localised extinction. Mitigation measures such as the reintroduction of plant material into new sites and the enhancement of existing populations through additional plantings may be warranted for many of Western Australia's rare and threatened Verticordia.

849: *-.002*

An optimal control model of wildlife management is developed to analyze, the transitory dynamics set forth by the reintroduction of animals that provide benefits but can also come into conflict with humans. The shadow price of these resources can be positive or negative, potentially creating a nonconvexity. The conditions under which reintroduction is not desirable are determined, and those characterizing the initiation of costly population control are investigated. An application to deer management illustrates the relevance of the nonconvexity and the magnitude of losses stemming from delays in implementing optimal management. The impact of distributional concerns on efficiency is discussed. (C) 2001 Academic Press.

850: ***+.110***

The reconstruction of recent historical population sizes allowed us to investigate the influence of random evolutionary processes on present-day genetic diversity in populations of Dryopteris cristata. This long-lived, allotetraploid fern is, rare and endangered in the study area at the southwestern border of its European distribution. Random amplified polymorphic DNA (RAPD) diversity of 280 individuals from 14 populations of D. cristata was extraordinarily low, suggesting an ancient bottleneck in the species.' history. Analysis of molecular variance (AMOVA) of 25 different RAPD multiband phenotypes revealed significant genetic variation among three geographical regions (15%) and among populations within regions (34%); 51% of total variance was attributed to variation within populations. High population differentiation indicated limited gene flow among populations, and genetic divergence was not correlated with geographical distance. There was no relationship between genetic variation within population, estimated as molecular variance, and present-day population size. Populations with recent historical bottlenecks of fewer than 25 individuals showed a substantial and significant reduction in genetic variation, compared with populations without bottlenecks. Comparatively high levels of genetic variation were still maintained in small remnants, (60-110 individuals), of formerly large populations. Average deviations of frequencies of widespread polymorphic markers within populations from their frequencies in the whole dataset were significantly higher in small or recently bottlenecked populations than in constantly large populations, thus providing evidence for random sampling effects during genetic bottlenecks and drift in small populations. The present investigation demonstrates the importance of population history for understanding present-day genetic diversity within natural populations, as well as for conservation biology.

851: ***+.082***

We present analyses of our transect survey data from consecutive years at 11 sites in Wisconsin during 1990-99 for the regal fritillary (Speyeria idalia), which is listed under state law as endangered, and the closely related but more widespread and abundant Aphrodite fritillary (Speyeria aphrodite). Within year, the date of peak survey numbers at each site ranged over a period of several weeks or more for each fritillary. Within year and site, the Aphrodite fritillary peak was typically a few days to a week prior to the regal fritillary's peak. Both fritillaries exhibited large annual fluctuations which were significantly correlated between the two species. Relatively larger regal fritillary densities were consistently associated with active non-fire managements (grazing, cutting), relatively lower densities with burning, and widely varying densities with non-management. More unfavorable outcomes from burning occurred at sites where the entire habitat patch was fire-managed. Similar but less sensitive was the Aphrodite fritillary, which did not respond as strongly or clearly to burning, although higher densities were associated with unintensive non-fire managements. In Wisconsin and adjoining areas, the Aphrodite fritillary appears useful as a substitute in tests of techniques for habitat restoration or reintroduction for the regal fritillary. Since the Aphrodite fritillary may be less sensitive than the regal fritillary, success

with the former certainly doesn't prove suitability for the latter. But unless and until the method works for the Aphrodite fritillary, it is almost certainly unsuitable for the regal fritillary.

853: *-.057*

In June 1998, 30 mala (Lagorchestes hirsutus undescribed central Australian subspecies) were translocated from a semi-captive colony in the Tanami Desert, Northern Territory to Trimouille Island, part of the Montebello Islands Conservation Park, off the Pilbara coast of Western Australia. Mala are 'Extinct in the Wild' according to IUCN (1994, 2000) Red List Categories and Criteria. The translocation was made possible by the eradication of black rats (Rattus rattus) and confirmation of the absence of feral cats (Felis catus), which were recorded on the island in the 1970s. Post-release monitoring up to October 2001 showed that mala were breeding and expanding the area occupied.

854: *+.030*

The northern leopard frog (Rana pipiens) is designated as Threatened in the province under the Alberta Wildlife Act (Alberta Environment 1996) and is nationally listed by the Committee on the Status of Endangered Wildlife in Canada (COSEWIC) as a Special concern species (COSEWIC 2000). The leopard frog has exhibited population declines and is currently extirpated from much of its former range in Alberta. The historic distribution of the leopard frog in Alberta is closely associated with major river drainages that may reflect optimal breeding and hibernation sites. Information on current known leopard frog populations indicate that it is largely extirpated from the central parkland and has virtually vanished from the North Saskatchewan River drainage basin and is completely absent from the upper Red Deer River drainage basin. Isolated and fragmented, remnant leopard frog breeding populations in southern Alberta are vulnerable to disturbance and degradation, potentially leading to further local loss of the species. The northern leopard frog has exhibited little ability for natural dispersal back into historic parts of its range. A management decision was made in 1998 to begin a reintroduction project for the leopard frog. The goal of the project is to re-establish the leopard frog in historically occupied habitats in the headwaters of the upper Red Deer and North Saskatchewan River drainage basins, consequently allowing natural downstream dispersal along these watersheds. The northern leopard frog conforms to many of the requirements proposed in conservation literature for successful translocations. In April 1999, a pilot captive rearing program was initiated at the Raven Brood Trout Station (Sustainable Resource Development) located southeast of Caroline, Alberta and in the upper Red Deer River drainage. The facility offers infrastructure, managed access, and two rearing ponds that provide a controlled environment in which leopard frogs can be reared and temporarily confined. Several release sites were chosen in the area proximal to the Raven Brood Trout Station based on historic leopard frog records and the presence of potential suitable breeding, summering and over-wintering habitat. A second year of managed captive rearing of leopard frogs was undertaken in 2000. To date, approximately 2500 juvenile leopard frogs have been captive reared and released into the wild near Caroline, Alberta. However, apart from a single observation in the spring of 2000 of a 1999 released frog, no released leopard frogs have been capture or observed since. During the captive rearing process, water quality, growth and development of the tadpoles, natural history observations, and population numbers were recorded and monitored. Prior to release into the wild, a percentage of the captive reared juvenile leopard frogs were randomly weighed and measured. All captive reared leopard frogs were marked using a Visible Implant Elastomer (VIE) tagging system. The bio-compatible marker was located in the webbing between the toes of one of the hind feet of each young-of-the-year leopard frog, producing an unique color and foot combination. The marking technique allows for the long-term monitoring of released frogs and the

evaluation of the survival success at each release site and in each year of release. The following report details the results of the 2000 captive-rearing program of the northern leopard frog reintroduction project.

855: *+.074*

Lions (Panthera leo) resident around the shores of Lake Kariba in Zimbabwe are vulnerable if their home ranges include communal lands inhabited by subsistence farmers. The primary cause of death is strangulation in wire snares set by game-meat poachers but illegal poisoning of carcasses, legal safari hunting and natural predation also contribute to mortality. A protected enclave of land on the lakeshore supported a resident pride of lions which was gradually eliminated in 1995. Tourist demand warranted an attempt to reintroduce lions which was assisted and probably accelerated by the capture and temporary confinement of an adult female lion and her three cubs in a stoutly constructed boma. The female came into oestrus and attracted several itinerant young male lions back to the area. The subsequently released lion family and five immigrant males formed the basis of a new resident pride which reproduced and behaved normally, attracting other lions who challenged the social hierarchy.

856: *+.148*

The restoration of wildlife populations to historically occupied ranges is an important goal of modern wildlife management. In Arizona, USA, efforts have begun to reintroduce the Gould's subspecies of wild turkey (Meleagris gallopavo mexicana) into its former range in the southeastern part of the state. However, individuals or descendants of the Merriam's subspecies (M. g. merriami) may remain in the reintroduction area from earlier releases and could potentially interbreed with reintroduced Gould's turkeys. We used 3 fundamentally different genetic markers to determine whether the wild turkeys currently occupying the Huachuca Mountains in southeastern Arizona were descended From the Gould's turkeys translocated there during the 1980s, or whether interbreeding had occurred with descendants of Merriam's turkeys from a translocation during 1950. We found consistent genetic differences between relict Populations of the Merriam's turkey in Arizona and the Gould's turkey in northern Mexico. The Huachuca Mountains wild turkey population consistently grouped with the relict Gould's populations and showed no evidence of interbreeding with the Merriam's subspecies. In addition, we found evidence that the Huachuca Mountains population was less genetically diverse than the relict populations, and we recommend that this population be monitored for signs of inbreeding depression. The molecular markers developed for this study are important tools for future management of wild turkeys.

857: *+.045*

Fire is being experimentally reintroduced to the forests of southern Ohio to determine its effectiveness in restoring and maintaining mixed-oak (Quercus spp.) forest communities. We studied the effects of repeated burning (1-4 years of annual burning) and recovery (1 year after burning) on the breeding bird community. Burning resulted in incremental but temporary reductions in the availability of leaf litter, shrubs, and saplings, but it did not affect trees, snags, or understory vegetation cover. Of 30 bird species monitored, 4 were affected negatively and 2 were affected positively by burning. Population densities of Ovenbirds (Seiurus aurocapillus), Worm-eating Warblers (Helmitheros vermivorus), and Hooded Warblers (Wilsonia citrina) declined incrementally in response to repeated burning and did not recover within 1 year after burning, suggesting a lag time in response to the changes in habitat conditions. Densities of Northern

Cardinals (Cardinalis cardinalis) fluctuated among years in the control units, but remained low in the burned units. Densities of American Robins (Turdus migratorius) and Eastern Wood-Pewees (Contopus virens) increased in response to burning, but these increases were apparent only after several years of repeated burning. In general, burning resulted in short-term reductions in the suitability of habitat for ground- and low-shrub-nesting birds, but it improved habitat for ground- and aerial-foraging birds. Overall, there were no changes in the composition of the breeding-bird community. Total breeding bird population levels were also unaffected by burning. Our results suggest that prescribed burning applied on a long-term basis or across large spatial scales is likely to have adverse effects on ground- and low-shrub-nesting bird species, but other changes in the composition of the breeding-bird community are likely to be minimal as long as the closed-canopy forest structure is maintained within the context of prescribed burning.

858: ***+.141***

Returning confiscated animals to their native habitats is desirable when it makes a positive contribution to the conservation of the species. Release of captive Individuals is complex and controversial, however, particularly when risks are potentially high, as In the case of orphaned apes. We describe the decision-making process that led to the successive release of 20 wild-born orphan chimpanzees (Pan troglodytes troglodytes) into the Conkouati Reserve in the Republic of Congo. Recommendations of the Reintroduction Specialist Group of the World Conservation Union's Species Survival Commission were followed closely The conservation status, ecology, and behavior of wild chimpanzees, the biological, social, economic and political context of the release site; and the health and genetic status of the candidates for release were all taken into account in the planning and execution of the project. Rigorous post-release monitoring of behavior and health allowed documentation of the outcome. ne project was of benefit to the chimpanzees that were released but also brought broad benefits to the site through effective protection from poaching and deforestation, and direct and indirect benefits to local people. ne genetic and behavioral diversity of chimpanzees require a variety of conservation strategies to reduce threats and maintain as many viable wild populations as possible.

859: ***+.000***

The capercaillie (Tetrao urogallus L.), a large forest gamebird reintroduced into Scotland in the 1830s, has been declining since the 1970s. This has been attributed to a reduced reproductive rate associated with climate change, and deaths of full-grown birds flying into forest fences. Here, three independent estimates are combined to show that in the 1990s the mean annual rate of decline for adult hens was 18% (S.E. 5%). Without fence deaths, it is calculated that the hen population could have increased at an annual rate of 6% (S.E. 10%), If recent trends persist, the bird will soon be extinct again in Scotland, but without forest fences it would probably survive.
(C) 2001 Elsevier Science Ltd. All rights reserved.

860: ***+.227***

A model of the joint dynamics of change in population size N and evolution in a quantitative trait z, as a result of a general form of density dependence, local stabilizing selection, and immigration of individuals deviating from the local optimum, is analyzed. For weak selection and migration, a reduction in total equilibrium population size below the initial level without immigration, K, is shown to occur if the immigrants deviates more than root8 p 2.83 genetic standard deviations from the optimum and if the rate of migration m is sufficiently large relative to the strength of stabilizing selection s. For the Lotka-Volterra form of density dependence, two additional

equilibria are shown to exist below K, provided that the strength of selection is large relative to the strength of density dependence. Reintroduction of an initially extinct population is possible if the immigrants are not too maladapted and if the genetic variance is sufficiently large. For a simplified version of the model corresponding to competition between similar species or different haplotypes, the equilibrium population size is always exactly at K if m

861: ***+.312***

Rates of hybridization and introgression are increasing dramatically worldwide because of translocations of organisms and habitat modifications by humans. Hybridization has contributed to the extinction of many species through direct and indirect means. However, recent studies have found that natural hybridization has played an important role in the evolution of many plant and animal taxa. Determining whether hybridization is natural or anthropogenic is crucial for conservation, but is often difficult to achieve. Controversy has surrounded the setting of appropriate conservation policies to deal with hybridization and introgression. Any policy th at deals with hybrids must be flexible and must recognize that nearly every situation involving hybridization is different enough that general rules are not likely to be effective. We provide a categorization of hybridization to help guide management decisions.

862: ***-.061***

The macroinvertebrate benthos of Scott Creek, an intermittent stream in the Mt Lofty Ranges, was assessed to determine whether it could sustain a population of platypus (Omithorhynchus anatinus), a species that has been locally extinct for about 100 years. The benthic fauna was compared to that of Rocky River and Breakneck River, two streams on Kangaroo Island where platypus have been introduced. Little difference was observed in the abundance and biomass of macroinvertebrates in the three streams, suggesting that Scott Creek does produce a sufficient food supply. The resource may be marginal, however, as Scott Creek in common with the two island streams contracts to pools in late summer/early autumn where the platypus populations are limited by this habitat truncation. Further assessment is needed of the physical suitability of Scott Creek for platypus reintroduction (e.g. consolidated banks, over-hanging plants, permanent pools) and the risk of predation by foxes.

863: ***+.092***

Black bears (Ursus americanus) were extirpated from most of their range by the early 1900s by habitat destruction and unregulated hunting. Since then, bear habitat has recovered in many areas, but isolation may prevent natural recolonization. Black bear translocations often have limited success because of high mortality rates and low site fidelity. We tested 2 reintroduction techniques designed to overcome those problems. The first technique used a winter release whereby pre- or post-parturient female bears were removed from their dens and placed in new dens at the release area. The second technique involved translocating female bears to the reintroduction area during summer and holding them in pens for a 2-week acclimation period before release. We translocated 8 female bears with cubs with the winter-release technique and 6 female bears with the summer-release technique. After release, total distance moved, net distance moved, mean daily distance moved, and circuity for winter-released bears ((x) over bar= 18.3 km, 7.1 km, 1.4 km, and 0.36, respectively) were less than summer-released bears ((x) over bar =97.6 km, 63.4 km, 5.1 km, and 0.74; P=0.010, 0.040, 0.019, and 0.038, respectively). Also, survival of winter-released bears (0.88) was greater than that for summer-released bears (0.20, P= 0.001). Population modeling indicated that at least one additional stocking of 6 adult females with 12 cubs would greatly

increase chances of population reestablishment. The winter-release technique has distinct advantages over the summer-release technique, limiting post-release movements and increasing survival of translocated bears.

864: ***+.168***

The dependence of seeds of terrestrial orchids on specific fungi for germination provides a means of locating these fungi in the wild and to investigate the role of appropriate fungi in the germination of orchid seed and development of seedlings under natural field conditions. Seed baits, comprising orchid (Caladenia arenicola) seed enclosed in fine nylon mesh, were placed at sample points along four transects through two orchid populations in bushland in Western Australia. Seed germination was scored and compared with adult orchid plant distribution and soil factors. A small fraction of available seed (< 1 %) germinated to a stage of tuber formation where survival over the subsequent dry season would have been possible. Germination increased in the vicinity of adult C arenicola plants, but other factors, such as soil potassium levels and presence of leaf litter, were also correlated with seed germination. The measurement of the spatial variability in germination events within an orchid habitat demonstrated the availability of new recruitment sites. This information is required to assess the natural recruitment capacity and the potential for orchid reintroduction in natural habitats.

865: ***-.023***

The European otter (Lutra lutra) is considered an endangered species in Europe. Therefore European zoos started a co-operative breeding programme - a so called European Endangered species Programme (EEP) - for this species in 1990. An important goal of the European otter EEP is to maintain an ex-situ (i.e. captive) population without imports of animals from the wild. Moreover, this population will be an important resource for reintroduction projects for this species. A reintroduction project of European otters in The Netherlands started in July 2002. Genetic management to avoid inbreeding and to maintain genetic variation is a pre-requisite to maintain a viable population. Examples of management strategies are described in this paper.

866: ***+.141***

European zoos, associated in the European Association of Zoos and Aquaria (EAZA), established cooperative breeding programmes for zoo populations in 1985. European Endangered species Programmes (EEPs) and European Studbooks (ESBs) for some 250 species have been established as at March 2002. The goals of these programmes are to maintain viable zoo populations without (or with limited) import from the wild. These populations can serve, whenever feasible, as sources for reintroduction of endangered species. Retention of genetic variation in populations of endangered species is a prerequisite for their future survival. This means that conservation efforts should also include genetic management of zoo populations, especially when reintroduction is part of a conservation programme. Therefore, maintenance of sufficient levels of genetic variation and minimising inbreeding are important objectives of zoo breeding programmes. Population biologists who work for zoological gardens have developed various strategies, models and computer tools to assist zoo biologists in the co-operative management of zoo populations.

867: ***-.045***

Burrowing Owls (Athene cunicularia) are undergoing mild to relatively severe local and regional population declines throughout much of western North America. In Canada, Burrowing Owls are

declining precipitously and are listed as endangered. In the United States of America, Burrowing Owls continue to decline in many states, but they are not listed federally. In Mexico, there is little quantitative data, but the species is listed as threatened. Here, we propose a conservation plan with five major action components: status, management and conservation, education, research, and administration. Given continued declines of Burrowing Owls in many parts of western North America, we urge increased cooperation among interested agencies and organizations to implement effective conservation of this species.

868: *+.066*

British Columbia (BC), designated the Burrowing Owl (Athene cunicularia) as endangered in 1980. In 1989, non-government organizations and local resource users, under the direction of the Ministry of the Environment, Lands, and Parks, launched a cooperative, captive-breeding and release program to restore Burrowing Owl populations in BC. The first phase of this program (1992-97) emphasized refining breeding protocols and identifying critical habitat features necessary for owl survival and reproduction in the wild. Successive releases provided insight into the feasibility of re-establishing populations to the grasslands of the Thompson-Nicola region. Results indicate that 1-yr old, captive-bred owls are capable of. 1) surviving at release sites, 2) raising broods, 3) over-wintering at or near release sites, and 4) migrating south and sometimes returning to release sites the following spring. Given these general results, the potential for a successful reintroduction of Burrowing Owls in BC exists, provided that more owls are released, and key habitat is enhanced. The second phase will emphasize ecosystem restoration, taking into account historical changes in natural processes (i.e., fire, grazing, and the resulting impact on faunal and floral composition on grassland habitats). In the second phase, die number of released owls will be increased to 50 pairs/year.

869: *-.034*

Burrowing Owls (Athene cunicularia) have been recorded nesting in most of Minnesota's western counties. Considered common in the early 1920s, by the mid-1960s only 9-10 breeding pairs were known with estimates of no more than 20 pairs in the west-central part of the state. Ten breeding records exist for the period 1965-85. In 1984, Burrowing Owls were listed as Endangered by the State of Minnesota. In 1986, we began surveys and site management for nesting Burrowing Owls and experimented with a reintroduction program. From 1986-90, 13 nests were found at eight sites, with a mean reproductive success of 3.5 fledglings/pair. The maximum number of breeding pairs/yr was four. Nest burrows were found in alfalfa fields (37.5%), pastures (37.5%), roadside ditches (12.5%), and fencelines between row crop fields (12.5%). We released 105 wild, preflighted juveniles: nine in 1986, 18 in 1987, 21 in 1988, 27 in 1989, and 30 in 1990. Young owls were kept in hack pens with roofs and sides made from cotton mesh fish netting. Burrows inside each pen and in surrounding fields were available to the owls. Crippled adults were placed in each pen with the juveniles but were not released. We documented eight mortalities, all of which were fledglings recovered in the release area. No owls were found, or reported, after leaving their hack sites. No successful nestings occurred from 1992-98.

870: *-.091*

The Chinese alligator is one of the world's most critically endangered reptiles. Although there is a relatively large captive population, in the wild small groups of alligators are limited to a few small ponds in an agricultural landscape in southeastern Anhui Province. As part of an effort to develop plans for the conservation of Chinese alligators in the wild, we investigated aspects of the

reproductive ecology of wild alligators during a survey of the last remaining groups. We also compiled published and unpublished information on the reproduction of alligators in captivity and in the wild. Nesting was only reported from four sites in 1999, and we describe two of these areas. Because of the intense human use of the landscape, alligators seek small patches of relatively undisturbed vegetation for nesting, and these fall into two main categories: vegetated hillsides, usually covered with pine trees, and small islands in agricultural ponds. Our observations of one nest on a pine hillside suggest that pine needles may make a poor nest substrate leading to lethally low temperatures for developing embryos. The selection of sites for the reintroduction of alligators should take the nature of potential nesting habitat into consideration.

871: *+.141*

The ecology of four relict Irish populations of pollan (Coregonus autumnalis) is compared with that of the species elsewhere, and used to advocate conservation. The threats to these populations from introduced/invasive species, habitat degradation, climate warming and commercial exploitation are summarized and the legislation governing conservation of the stocks is reviewed. Conservation options (legislation, habitat restoration, stock translocation and stock augmentation) are outlined and their practicality and efficacy considered. A preliminary search indicates that there are a number of lakes that appear to be suitable for pollan translocation. (C) 2001 The Fisheries Society of the British Isles.

872: *-.018*

In a survey of the west-central Italian rivers Ombrone, Fiora, Albegna and Bruna, among established exotic species, the Iberian barbel Barbus graellsii was recorded in Italian fresh waters for the first time. Morphological identification was supported by comparison of cytochrome b sequences with those from related barbel species. Other exotics of particular note were Barbus barbus. Pseudorasbora parva and Leuciscus cephalus, together with the Padano-Venetian Chondrostoma genei and Padogobius bonelli. Native species still present included Leuciscus lucumonis, Telestes muticellus, Rutilus rubilio and Padogobius nigricans, but were now more restricted to upper reaches and smaller watercourses. The deleterious effect of alien species on native forms is discussed with particular reference to probable competition between C. genei and L. lucumonis, and between the two gobies. The checklist of species introduced to the fresh waters of Italy is now, updated to 34. For the Italian freshwater fish fauna in general, the conservation status of three native species (Acipenser naccari, Salmo marmoratus and Knipowitschia punctatissima) has been improved but five species are believed to he at risk (Salmo carpio, L lucumonis, Scardinius scardafa, Gobio benacensis and P. nigricans) and four anadromous species (Petromyzon murinus, Lampetra fluviatilis, Acipenser sturio and Huso huso) no longer breed in Italian fresh waters. The processes of change in the composition of the Italian freshwater fish fauna as a whole may be summarized as successively 'padanization', 'danubization' and now 'globalization'. (C) 2001 The Fisheries Society of the British Isles.

873: *+.132*

1. Wilkinson (2001) argues that we cannot assume that hybrids between local and alien genotypes will have low fitness, and therefore, as low hybrid fitness has been presented as justification for using only locally provenanced material in habitat restoration schemes. provenance is not important.2. His observations on fitness are important, correct and deserve wider recognition.3. Nevertheless, I dispute his conclusion about the importance of provenance, for two main reasons. One is that his argument is based on questionable objectives for biodiversity conservation. The

second is that, even if we accept these underlying objectives, the fitness of hybrids is only one of numerous relevant issues.4. Use of locally provenanced seed should be standard practice, except where the introduction of non-local genotypes is specifically justified in terms of conservation genetics.

874: *+.160*

1. Many habitat creation schemes specify that biological material of local provenance should be used in reintroductions. This has come to be the 'text book' approach. However, very little discussion of the theory underlying this idea has been published in the scientific literature. This paper aims to initiate this much-needed discussion.2. A major reason for the use of local provenance is the claimed importance of conserving locally adapted genotypes, which are assumed to show high fitness. Using both genetic arguments and a consideration of Quaternary environmental change I argue that this reason will seldom be important.3. I make tentative suggestions of when local provenance is likely to be important and when it can be given a low priority in habitat creation schemes.

875: *+.087*

Most of Brazil's electricity is generated by hydroelectric power plants that require the flooding of huge areas and affect the stability of local ecosystems. The area of the Porto Primavera power dam was flooded in 1998 and a rescue programme was executed to save the fauna sheltering on treetops or emerging islands. Using DNA fingerprinting we estimated the genetic variability in a sample of 23 Bare-faced Curassow Crax fasciolata rescued in this area and found that the mean heterozygosity was H = 0.89. Although similar data on other natural populations of cracids is not available, the mean heterozygosity observed in the present sample is in the range found for free-living populations of two species of reintroduced cracids whose origin was captive breeding of a limited number of founders. We suggest that a collaborative captive breeding and reintroduction programme between the facilities holding the birds caught at Porto Primavera should start immediately to avoid the loss of genetic variability due to the small number of founders in captivity. Habitat protection, responsibility in fauna management and measures that prevent or correct the isolation of habitat fragments are needed to establish an equilibrium between progress and conservation in developing countries.

876: *+.099*

Brazilian Merganser Mergus octosetaceus is considered to be one of the rarest and most threatened species in the Neotropical region, yet little is known about its distribution and life-history. We studied the population of Brazilian Merganser in and around Serra da Canastra National Park (SCNP) in Minas Gerais, Brazil, during 1996, with additional observations from 1997 to 2000. In this paper we report the sighting of previously undiscovered pairs and present some new behavioural data, including a description of calls performed by males and females, feeding behaviour, home ranges, parental care and population density. We also describe a previously unreported plumage of the young. A total of 39 individuals were recorded, comprising 12 adults (six pairs) and 27 Young. Brood size ranged from two to four (mean 2.7), being smaller than in other Mergus spp. (e.g. Goosander Mergus merganser). Two pairs were located within the SCNP, and four on unprotected stretches of river outside. Home range was large compared with other Mergus spp., each pair using on average 9 km of river. Range size was thought to be related to the availability of suitable nesting and feeding sites. SCNP and its environs contain very little mature gallery forest with trees large enough to provide nest sites (most having been selectively logged in

the past). There is also competition for nest sites with other birds and mammals. All suitable habitat surrounding the SCNP is now occupied by adult birds, leaving none for young birds. Further research is needed to determine the fate and dispersion routes of juveniles. Future conservation priorities for Brazilian Merganser in and around SCNP include the installation of nest boxes to test whether the availability of good quality nest sites limits breeding success. A captive breeding programme could be established using eggs from these nest boxes. This would help to ensure the survival of this species and to provide birds for future reintroduction programmes should they be deemed necessary. Further surveys, with subsequent establishment of reserves at key sites, are required throughout the Brazilian Merganser's range.

877: *+.071*

Despite numerous, generally unsuccessful attempts to reintroduce threatened Australian mammals, the factors leading to their failure have not been fully clarified, although predator control would appear to be of paramount importance. An experimental approach was taken in attempting to establish a population of bridled nailtail wallabies ih an area of apparently suitable habitat and low fox density, but on the edge of the species' former range. The 133 wallabies released since late 1996 comprised four groups captive-bred animals, wild caught from the single remaining wild population, animals that were captive bred and acclimatised at the translocation site in a 10 ha predator-proof enclosure, and animals which had been bred in the enclosure. Survival was highest in those bred in the enclosure and highly variable among captive-bred animals. Survival estimates for wild recruits suggested the population would maintain a positive rate of increase under prevailing environmental conditions. Spotlighting surveys suggested the population had increased to approximately 400 animals by late 1999. Above average rainfall during 1996-1999 and no apparent predation suggests caution in describing the translocation as a success. Ongoing monitoring is critical, because it A uncertain ho v the population will cope with drought and inevitable predation events, and whether the population will expand and persist outside of limited preferred habitat. (C) 2001 Elsevier Science Ltd. All rights reserved.

878: *+.080*

The free-flowing Clinch and Powell River Basin, located in southwestern Virginia, United States, historically had one of the richest assemblages of native fish and freshwater mussels in the world. Nearly half of the species once residing here are now extinct, threatened, or endangered. The United States Environmental Protection Agency's framework for conducting an ecological risk assessment was used to structure a watershed-scale analysis of human land use, in-stream habitat quality, and their relationship to native fish and mussel populations in order to develop future management strategies and prioritize areas in need of enhanced protection. Our analyses indicate that agricultural and urban land uses as well as proximity to mining activities and transportation corridors are inversely related to fish index of biotic integrity (IBI) and mussel species diversity. Forward stepwise multiple regression analyses indicated that coal mining had the most impact on fish IBI followed by percent cropland and urban area in the riparian corridor ($R-2 = 0.55$, $p = 0.02$); however, these analyses suggest that other site-specific factors are important. Habitat quality measures accounted for as much as approximately half of the variability in fish IBI values if the analysis was limited to sites within a relatively narrow elevation range. These results, in addition to other data collected in this watershed, suggest that nonhabitat-related stressors (e.g., accidental chemical spills) also have significant effects on biota in this basin. The number of cooccurring human land uses was inversely related to fish IBI ($r= -0.49$, $p < 0.01$). Sites with greater than or equal to2 co-occurring land uses had > 90% probability of having <2 mussel species present. Our findings predict that many mussel concentration sites are vulnerable to future extirpation. In

addition, our results suggest that protection and enhancement of naturally vegetated riparian corridors, better controls of mine effluents and urban runoff, and increased safeguards against accidental chemical spills, as well as reintroduction or augmentation of threatened and endangered species, may help sustain native fish and mussel populations in this watershed.

879: *+.128*

The European sturgeon, Acipenser sturio, was an important component of the fish communities of all large German rivers, in particular the River Elbe, until the end of the 19th century. At that time about 4.000 specimens were caught in the River Elbe system annually. Since 1890, a dramatic decrease of the stocks occurred. The main reasons were: overfishing, hydroconstruction, and pollution. The last subpopulation was observed in the River Eider until the end of the 1960s. Until the end of the 1980s, politicians and also ecologists did not consider restoration measures for the sturgeon feasible. In 1994, after the German unification, the joint action of both fish culturists and scientists led to the foundation of the "Society to Save the Sturgeon (Acipenser stunn L.) e.V." with the aim to initiate and coordinate activities for the restoradon of A. stuna into German rivers. A project for the remediation of the species was begun in 1996 with financial support of the Federal Ministry of Environment. At the same time juvenile A. sturia, originating from an artificial reproduction in 1995, were transferred to the Leibniz-Institut of Freshwater Ecology and Inland Fisheries in Berlin as nucleus of a brood stock. The main objective was to obtain maximum growth under safe rearing conditions in order to initiate early gonad maturation of the 27 fish of the brood stock (range of total length in April 2002: 85 cm [2.558 kg] to 118 cm [7.410 kg]). Furthermore, unsolved questions concerning feeding and gonad maturation are investigated using different sturgeon species which are more readily available such as A. rutbenus, A. baerii, and A. oxyrinchus. Genetic studies were performed to characterize various populations (Baltic, North and Mediterranean Sea) of A. stum. Thus providing an important background for sound strategic decisions concerning the selection of donor stocks for re-introduction measures. It was shown that the brood stock (originating from the French Gironde system) is genetically identical to the historical material originating from the Elbe system (based on museum samples). In addition, the genetic characterization of the intra-specific differences of the Gironde population as well as the French and German brood stocks were performed in order to develop a genetic breeding plan. Investigations on potential spawning habitats for the River Elbe and its tributaries Stor and Oste are in preparation.

880: *-.021*

We present comparative success of various trapping methods trialed during control of feral cats at a site for the reintroduction of threatened mammals at Shark Bay, Western Australia. Our results come from 31 703 trap-nights that caught 263 cats (an average of 0.83 per 100 trap-nights). Cats differed markedly in their vulnerability to trapping depending on whether they primarily scavenged at rubbish tips or around human settlement or whether they hunted for their food in the bush. Cage traps were an effective means of controlling the former, with 9.4 cats captured per 100 trap-nights. Scavenging cats included a higher proportion of sub-adults and kittens and lower proportion of adult males than hunting cats. Variation between years in capture success for hunting cats was largely explained by the abundance of rabbits relative to that of cats and whether the rabbit population was increasing or decreasing. These factors accounted for a nine-fold difference in trap success. The number of cats caught in any particular trapping session could be explained by location (rubbish tip or town versus bush), trapping effort (typically greater effort yielded higher captures), abundance of cats at the site (captures were highest when cats were abundant), and season (captures were highest in the first half of the year when the young of the year were

becoming independent). Concealed foot-hold traps, in a range of possible sets, provided effective methods for capturing cats that hunt, except where capture of non-target species was a critical limiting factor. Cage traps caught cats at a comparable rate to foot-hold traps for standard sets, but caught a significantly different cohort. Concealed foot-hold traps caught a higher percentage of adult cats, particularly males, than did cage traps. Mouse carcases and rabbit pieces were significantly more effective as lures when rabbits (the major food of cats at the site) were at low densities, whereas the success of commercial scent lures was unrelated to food availability. Significantly more cats than expected were caught using food as an attractant at times of food shortage (late summer, autumn and early winter) for both scavenging and hunting cats. In contrast, scent lures caught significantly more cats than expected in spring and summer when cats were defending access to mates and/or territory. Hence, no single trap type, trap set, or lure provided unequivocally superior performance over others. Control is likely to be best achieved by a variety of trapping methods and lure types used in combination, supplementing well timed poisoning efforts. Trap success is likely to be maximised by trapping at times when the dominant prey of cats are scarce relative to the number of cats and are decreasing in abundance.

881: *+.075*

Burrowing Owl (Athene cunicularia) populations are declining in many portions of their range, and research and management efforts into stemming declines are underway. One tool with promise is die artificial burrow, which can supplement nesting opportunities and play a role in research, mitigation, translocation, and reintroduction studies. However, few studies directly assess important burrow and surrounding topographic features upon which owls choose sites and then construct and install artificial burrows accordingly. In this study we (1) measure physical, vegetative, and topographic characteristics of Burrowing Owl nest sites in die Snake River Birds of Prey National Conservation Area (SRBPNCA); (2) compare used and unused burrows to determine features important in nest-site selection; and (3) use this information to help guide current and future construction and placement of artificial burrows in the SRBPNCA. Owls nested in abandoned American badger (Taxidea taxus) burrows in areas with more than one burrow, close to roads and irrigated agricultural fields, and characterized by sparse and low vegetation dominated by normative plant species. Only one feature studied, tunnel entrance angle, corresponded with choice by owls; odds of burrow use decreased 17% with each 1degrees increase in slope of the tunnel entrance. Owls nesting near irrigated agricultural fields also had higher productivity. We discuss applications of our results to construction and placement of artificial burrows in the SRBPNCA and similar shrub-steppe environs in western North America.

882: *+.174*

Reintroduction efforts have established small, scattered flocks of Trumpeter Swans (Cygnus buccinator) in the Midwestern United States. Management of these flocks as distinct population units is debated among wildlife managers. The purpose of this study was to quantify generic differences among three recognized populations of Trumpeter Swans. Blood and tissue samples were collected during 1999 and 2000 and analyzed using horizontal starch-gel electrophoresis. Trumpeter Swans from the High Plains Flock and the Greater Yellowstone Population, from which the High Plains Flock was derived, were genetically indistinguishable. However, both flocks differed from the Pacific Coast Population in allele frequencies at the adenosine deaminase (ADA) locus. Founder effects may have influenced both the High Plains Flock and the Greater Yellowstone Population. Results also suggest unidirectional gene flow from the Pacific Coast Population into the other populations. The High Plains Flock may be a suitable source of individuals to increase the size of the Greater Yellowstone Population while maintaining similar

allele frequencies. Additional higher resolution genetic surveys are warranted.

883: *-.065*

Restoration of Trumpeter Swans (Cygnus buccinator) to their former range in Ontario has involved many techniques, some more successful than others. Rearing methods included cross-fostering on wild Mute Swans (Cygnus olor), artificial incubation, and incubation and rearing by their own Trumpeter Swan parents. Egg hatchabilily was low ((x) over bar = 56%) for eggs laid by captive pairs and was suspected to be due to inbreeding. Few toxic contaminants were found in unhatched eggs, but 15-17% of unhatched eggs that had been artificially incubated were infertile. Eggs cross-fostered under wild Mute Swans had lower hatchability than those incubated by Trumpeter Swans, and cygnets raised by Mute Swans survived less well to fledging. Time budgets revealed that rearing methods significantly influenced behavior but differences disappeared by 26 weeks of age. However, wild-hatched swans, when adults, bred somewhat but not significantly more frequently than those hatched by their own, captive parents. Translocating adult wild-caught Trumpeter Swans did not prove to be an effective reintroduction technique; none of the six translocated adults were resighted after the first year. Annual survival rate of wild-hatched males and females more than 2 years old was 0.831; survival rates did not differ between captive- and wild-hatched swans in any age or sex category. The most common cause of death was lead poisoning. Released Trumpeter Swans bred for the first time at two to eight years of age. Four adults were taught to follow an ultralight aircraft to a predetermined wintering site. They returned on their own in the spring but did not migrate back to the wintering site during the next autumn.

884: *+.173*

Reintroduction is defined as an attempt to establish a species in an area thatwas once part of its historical range, but from which it has been extirpated or become extinct. Historically, one of the most successful programs was the reintroduction of 254 American black bears (Ursus americanus) from Minnesota tothe Interior Highlands of Arkansas in the 1960s; that population has grown to>2,500 today. More recent efforts have involved fewer but better monitored animals and have sometimes employed techniques to improve site fidelity and survival. In Pennsylvania, for example, pregnant female American black bears were successfully translocated from winter dens, the premise being that the adult females would be less likely to return because of the presence of young cubs. That winter-release technique was compared to summer trapping and release in Tennessee; winter releases resulted in greater survival and reduced post-releasemovements. Homing has not been a problem for small numbers of brown bears (Ursus arctos) reintroduced to the Cabinet-Yaak ecosystem in Montana and Idaho and to the mountains of Austria and France. Reintroduction success appears to becorrelated with translocation distance and is greater for subadults and females. As with any small population, reintroduced bear populations are susceptible to environmental variation and stochastic demographic and genetic processes. Although managers have focused on these biological barriers, sociopolitical impediments to bear reintroduction are more difficult to overcome. Poor public acceptance and understanding of beats are the main reasons some reintroductionprograms have been derailed. Consequently, the public should be involved in the reintroduction process from the outset; overcoming negative public perceptions about bear reintroduction will be our greatest challenge.

885: *+.230*

During the past 20 to 25 years, dramatic declines and disappearances of amphibian populations have been observed and documented worldwide. One of the species that has experienced such a

dramatic decline is the southern Rocky Mountain population (SRMP) of the boreal toad (Bufo boreas boreas). Although once considered fairly common in most mountainous areas in southern Wyoming, much of Colorado, and northern New Mexico, it is much less common today, and absent from some of the areas where it occurred historically. Reasons for the decline are being investigated. Although causes for the decline have not all been completely investigated or clearly identified, discovery of boreal toad die-offs due to the chytrid fungus (Batrachochytrium dendrobatidis) in 1999 point to this pathogen as the proximate cause of deaths of boreal toads in at least two populations. In general, lack of suitable habitat does not appear to be a significant limiting factor for boreal toads in the southern Rocky Mountains. As a result of the observed decline in population, the boreal toad has been listed as endangered by both New Mexico and Colorado, and the Wyoming Game and Fish Department lists the boreal toad as status NSS1 (Native Species Status 1)1. It is presently considered "warranted but precluded" for federal listing under the Endangered Species Act (ESA), and is considered a "Sensitive Species" in the Rocky Mountain Region of the USFS. An interagency recovery team was formed in late 1994,and has been working on the conservation and recovery of this species since that time. These actions led to the writing of a boreal toad recovery plan for Colorado, a draft Conservation Strategy, and a draft Conservation Agreement. The various documents resulting from the actions and agreements described above created redundancy and confusion about the purpose and intent of each document as related to the other. Therefore, it was agreed in 1997 that it would be advisable to combine the components of the various documents into one comprehensive Boreal Toad Conservation Plan and Agreement (Conservation Plan). The first edition of the Conservation Planwas produced in 1998, and this document is the first revision of that plan. The intent of this document is to provide comprehensive guidance for the recovery and management of the boreal toad and its habitat in the southern Rocky Mountains, and to provide a means for all involved and interested parties to make a formal commitment to the implementation of the actions recommended in this plan. Federal, State, and private parties wishing to commit to participating in the implementation of parts of, or the entirety of this plan, can make that commitment through Conservation Agreements, which are appended to this document. These agreements will be brief and concise statements that can be edited, deleted, or appended at any time, without the need to revise the entire plan. This approach will expedite the process of implementing the Conservation Plan, as it does not require the "up front" approval of all potentially involved agencies before implementation. If some agencies are not willing or able to sign an agreement immediately, they can do so at a later date without holding up the work of those agencies that are ready to make an immediate commitment and begin implementing the plan. Work on boreal toad recovery and management has been going on since about 1994, based on the guidance of the Recovery Team, the State of Colorado's Boreal Toad Recovery Plan, and subsequently, the Boreal Toad Conservation Plan and Agreement. Work to date has involved several state and federal resource management agencies, personnel from universities, and various other interested parties, including local land use planners and private land owners. Management activities to date have included (1) the conducting of surveys of historic and potential suitable habitats to locate toad populations, (2) the annual monitoring of known breeding populations, (3) research to identify and evaluate biotic and abiotic limiting factors to toad survival, (4) research to better define boreal toad habitat and boreal toad biology/ecology, (5) development and testing of techniques and protocols for captive breeding and rearing of boreal toads, (6) experimental reintroductions of toads to vacant historic habitat, (7) protection of boreal toads and their habitats via coordination with land management agencies - in particular with the USFS, (8) work with local land use planners and developers aimed at avoiding or minimizing potential impacts of private land development on boreal toads and their habitat, and (9) efforts to increase public awareness of this species and its plight via informational/educational activities and public involvement in searches for new populations of boreal toads. As of the summer of the year 2000, the boreal toad is known to still occur in eleven counties (Routt, Larimer, Grand, Eagle,

Summit, Clear Creek, Pitkin, Gunnison, Chaffee, Hinsdale, and Mineral) in Colorado, and one county (Albany) in southern Wyoming. Unconfirmed but reliable reports indicate that boreal toads may also still occur in Jackson, Garfield, Boulder, Lake, Park, Saguache, and Conejos counties in Colorado, and in Rio Arriba County, New Mexico. Significant progress hasbeen made with the boreal toad recovery and conservation effort in the past five years, and it is anticipated that much can be accomplished towards recovering this species in the next five to ten years, provided adequate funding and personnel are available. The discovery of the chytrid fungus and its role in the decline of boreal toads has introduced a significant new factor into the recovery and conservation effort, and will require additional research and possible modification of management approaches. The Recovery Team recognizes that both personnel and funding are in short supply, and will pursue innovative approaches to accomplish needed work, including partnerships and other cooperative efforts. However, without a significant commitment of funds and time from all the involved agencies, recovery may be difficult, if not impossible, to achieve in the foreseeable future.

886: *+.081*

We examined reproductive performance of yearling males in a recently established free-ranging elk (Cervus elaphus nelsoni, Erxleben) population in southeastern Kentucky. Sixteen of 18 (89%) adult females bred by yearling males produced a calf. The calving period, determined for 11 cows, peaked in mid-June; however, 3 females bred by yearlings did not calf until mid-July or August. It is not possible to attribute the extended calving period to breeding by yearling males, as it may have been a result of poor physical condition of females or the disruption of social bonds after translocation the previous winter.

887: *+.114*

Current Status: The Sonora tiger salamander is federally listed as endangered without critical habitat. As of this writing, the subspecies has been found in 53 ponds in the San Rafael Valley of Arizona. Habitat Requirements and Limiting Factors: This salamander requires standing water from January through June for breeding and larval growth. Adult, metamorphosed salamanders inhabit adjacent grassland and oak woodland terrestrial habitat when not in ponds. Threats to Sonora tiger salamanders include the following: 1) restricted distribution, 2) disappearance of natural standing water habitat, 3) predation by non-native fish, bullfrogs, and crayfish, 4) genetic swamping by introduced, non-native barred tiger salamanders, 5) disease, 6) low genetic diversity, and 7) collection for bait or translocation by anglers. Recovery Priority: 3 on a scale of 1 to 18. The priority is based on its being a subspecies (rather than a full species) with a high degree of threat and high recovery potential. Recovery Objectives: 1) Reclassify from endangered to threatened status. 2) Delist. Recovery Criteria: The Sonora tiger salamander may be reclassified to threatened status when approximately 90 percent of salamander's currently-occupied range and approximately 90 percent of current breeding ponds are protected and maintained to prevent habitat loss and degradation, predator introductions, barred tiger salamander introductions, and collection of salamanders for bait. Scientifically credible monitoring over a five year period must indicate that the number of Sonora tiger salamander populations is not in decline and that there are no new factors that threaten the persistence of Sonora tiger salamanders. The Sonora tiger salamander will be considered for delisting when quantitative criteria in terms of number of breeding populations and amount, distribution, and type of available habitat are defined and met. Criteria will be based on research, continued monitoring, and population viability analysis. In addition, regulatory mechanisms and land management commitments must be implemented that provide for adequate long-term protection of the Sonora tiger salamander and its habitat. These

commitments and mechanisms should address habitat maintenance and protection, management of non-native predators, disease transmission, introduction and collection of salamanders, interbreeding with non-native salamanders, and public education. Finally, the Sonora tiger salamander must be unlikely to need protection under the Endangered Species Act in the foreseeable future. Actions Needed: 1. Maintain and enhance habitat where salamanders have been found, and create new habitat, if deemed necessary. 2. Control non-native predators (fish, bullfrogs, and crayfish) by enforcing and enhancingexisting policies prohibiting the introduction and pond to pond transport of these taxa andby removing populations of non-native fish, bullfrogs, and crayfish. 3. Control introduction, transport, and collection of tiger salamanders in the San Rafael Valley by enforcing existing policies prohibiting these acts and by removing populations of barred tiger salamanders.4. Create and enforce policies to minimize frequency of die-offs. 5. Monitor salamander populations and their habitat on public and, if permitted, private land, to observe threats as they arise and fulfill research objectives. 6. Conduct research to acquire demographic and dispersal information and develop a population viability analysis, better understand salamander disease, conduct genetic analyses, investigate reports of low pH, and determine distribution of crayfish and methods of crayfish removal. 7. Develop public education and information programs. 8. Practice adaptive management. Total Cost of Recovery (minimum): $1,016,000Date of Recovery: If recovery criteria are met, reclassification to threatened status could beinitiated in 2007.

888: ***+.056***

Significant loss of rare plants and their habitats have occurred on the coastal plain of Virginia through urbanization, drainage of wetlands, fire suppression, and land use changes. Existing conservation practices such as easements and preserves have been somewhat successful in preserving biodiversity but have neglected the role that highway rights-of-way could serve as restoration areas for rare plants and their ecosystems. We propagated a number of rare plant species, many only still surviving on powerline rights-of-way, and reintroduced them in appropriate habitat on mitigation projects and cloverleafs along Virginia Department of Transportation highway rights-of-way. Key elements of our program include: utilization of indigenous plant stocks from the local area, registry of reintroductions with state authorities, management of sites through mechanical or chemical means, and monitoring of the population biology of introduced plants. Highway rights-of-way represent a potentially underutilized area for rare plant conservation and could augment species preservation and recovery efforts.

889: ***+.068***

The European Commission's LIFE-nature programme has funded a five-year programme (1997-2002) on integrated management of the ecosystems of Mediterranean evergreen oak. The programme is being applied at two calcareous sites: La Clape close to Narbonne, and Le Gardon close to Nimes and Pont du Gard. Due to the decline of traditional sheep grazing, the environment has tended to close in with the consequence that Brachypodium retusum grassland has been replaced by garrigue. Tourist activities (hiking, climbing) are threatening the survival of the Bonelli Eagle. Furthermore, the Centaurea corymbosa, which is endemic to La Clape, now numbers just six populations attached to just a few cliffs, may disappear unless there is artificial reintroduction. The programme has identified management methods that protect these species or habitats. They consist in secluding the areas inhabited by the birds by deviating paths and climbing routes, artificially clearing the environment by mechanical means or controlled fire so as to restore the grasslands, together with evergreen oak-, Aleppo pine- and riverain-based silvicultural practices. By organising active consultations between elected officials, users of the

forests and scientists, this LIFE programme has paved the way towards Natura 2000 objectives documents in anticipation of contract-based management aimed at safeguarding the threatened species and habitats.

890: *+.090*

The wolf is afundamental element of natural ecosystems. The ecological needs of this predator include large suitable habitats and abundant natural prey. The conservation of viable wolf populations is therefore beneficial to biological diversity and to the environment. The wolf almost became extinct in Italy during the second half of the last century, when the species' range was limited to a few scattered areas of the Apennines. Since the '70s, as a consequence of legal protection, the abandonment of many rural areas, and the increase in the number of wild prey, the wolf rapidly increased its range. In the early '90s it reached the western Alps, where there is now a transboundary population of about 50 wolves. In total, 400-500 wolves are estimated to live in the country, but this number should be considered only as an order of magnitude, as no reliable censuses of the Italian wolf population are available. In the medium term afurther expansion of the species to the entire Alpine region is predicted, thereby the definition of common policies among neighbouring States has become urgent. The wolf causes relevant management problems, primarily because of its impact on livestock; consequently, Italy is characterised by diffused poaching considered to be the main cause of mortality for the species. The wolf is a non-hunted species since 1971, is strictly protected under the national laws (L. 157/92, D.PR. 357/97) and several international directives. Although quantitative data is lacking on the relative impact of the different causes of mortality, leading Italian wolf experts consider main limiting factors for the species to include: poaching (accounting for a 10-20% loss of tne population yearly); conflicts with farmers and hunters (at the origin of a large proportion of poaching); competition and genetic pollution with free roaming dogs (the presence of several dog-wolf hybrids has been recorded). Other factors (habitat loss and fragmentation, human disturbance, demographic factors, range fragmentation) are considered of secondary importance. Despite the fact that the biology of the species (presenting low densities and moving over very wide areas) calls for the development and application of national or transboundary conservation policies, the present Italian legal and administrative framework (characterised by a decentralisation at decisional levels and by a complex repartition of roles and competencies) limits the possibility to develop coherent action at the national scale. Tne scope of the action plan is to maintain and recover, in coexistence with people, viable populations of wolves. In order to achieve this aim it is important that the population of the Italian peninsula is maintained at its present level of size and range; the recolonisation of the entire Alpine region is promoted; wolf-human conflicts are mitigated. The present action plan has a 5 year term of validity. The plan is based on available scientific information, however, given the lack of data on many aspects of the wolf biology and dynamics, it is also based on the guestimates of leading Italian experts on wolf biology. The plan is coherent with national and international provisions, and is based on the assumption that it is fundamental to increase apositive attitude of people towards wolves. Involvement of local communities in the decision-making process, is thus fundamental and would furthermore avoiding that a top down process take place during the definition of a policy on the issue. Thus, the present action plan does not cover all the instruments required for the implementation of a wolf conservation policy, but it does define the key elements of a strategy for wolf conservation, as well as identify priority actions, and describe the critical steps that need to be undertaken for an open and transparent consultation process. In this regard, the plan proposes the creation of a "National Wolf Committee" (open to all main stakeholders) with the mandate to discuss, develop and revise a national policy on the issue. Furthermore, the creation of a "Standing Committee for Wolf Conservation in the Alps" among Italy, Prance and Switzerland is also proposed in order to discuss

and define common positions on the wolf conservation and management in the Alpine region. It is critical to undertake studies and activities aimed at increasing present knowledge on wolf distribution, population size, dynamics, impact on human activities and efficiency of prevention measures. In particular, efficiency, reliability and standardisation of monitoring must be increased. In this regard the production of a handbook for wolf monitoring is proposed andpreliminary guidelines for wolf monitoring are reported as an appendix to the action plan. Other key elements for the conservation of the wolf include: the reduction of poaching; education and information campaigns; a revision of the policy on the control of free-roaming dogs; strict regulation of the existing non-Italian wolf and dog-wolf hybrids breeding farms; cautious control of the small stock of captive Italian wolves (15 individuals); the recovery of roe and red deer in central and southern Italy; an assessment of the role of functional connectivity areas (corridors); the development of a coherent policy on damage prevention and compensation (priority given to prevention, compensation conditioned to prevention, priority use of the available resources in the key areas for wolf conservation and potential corridors). Compensation must be based on the following principles: the entire market price of the loss is covered; all the indirect costs are covered; compensation is paid very rapidly; no distinction between dogs and wolves is applied; on-the-spot inspection is always carried out immediately after the claim. The alternative of providing incentives to farmers in key areas for wolf conservation should be explored, ifavailable resources are not sufficient to cover all losses, priority should begiven to key areas for wolf conservation and to corridors, rather than to other areas of wolf presence. No compensation shall be paid in areas where the species is not present and that are non-critical for wolf conservation and expansion. Any translocation of wolves is strictly banned and prevented; captive breeding programs (aimed at future reintroduction into the wild) are not considered a useful option. The introduction of the possibility to control the wolf for limiting damage to livestock is acceptable only if this measure (mitigating the conflicts with people and thereby reducing poaching) does not determine an increase in the overall mortality of the population. In this regard, the development of a more efficient monitoring system is a critical requisite. Wolf control is thus excluded for the term of validity of the plan. Although even the biggest national parks are not sufficiently large to allow wolf protection, protected areas can play a key role in wolf conservation by promoting education, information, monitoring, regulating tourism, etc.

891: ***+.152***

From time immemorial, though particularly in the recent two hundred years, the wolf has been persecuted and exterminated with all available means. This has brought about a significant reduction in the area of its occurrence all over Europe and in Poland as well. Rational modeling of wolf population in Poland, understood as far-reaching activities aimed at protection and conservation of the species, is the fundamental prerequisite for re-introduction of the wolf into western Europe. A noteworthy form of wolf protection are the so called Wolfs' Parks" in which the animals stay in conditions resembling nature - on a large enclosed area with look-outs erected around the fencing.

892: ***+.063***

Reintroductions of Delmarva fox squirrels (Sciurus niger cinereus) to suitable habitat have been a recovery tool used for this endangered species. In Maryland, we attempted reintroductions at 11 sites beginning in 1978. The last reintroduction was completed in 1992. At each site, 8-42 individuals were released during spring or fall over a 1-3 year period. Attempts were made to release an equal number of males and females. Monitoring at reintroduction sites by live-trapping has documented recruitment and establishment of populations at 9 sites. Criteria used for

determining population establishment follows that of the Delmarva Fox Squirrel Recovery Plan (U.S. Fish and Wildlife Service 1993). Because 7 of these populations were established with <24 individuals, supplemental releases of Delmarva fox squirrels were conducted to bolster genetic diversity. This paper summarizes the history of Delmarva fox squirrel reintroductions in Maryland, provides the results of recent live-trapping efforts at the sites, and discusses success of these efforts.

893: ***-.035***

The Seychelles kestrel Falco araea is classified as a Vulnerable species with main breeding populations restricted to two islands of the Seychelles group (Mahe and Silhouette). An attempted reintroduction to Praslin in 1977 has had poor results. It is the worlds smallest kestrel species and is adapted to preying on small lizards in forest habitats. The habitat preferences, territory size and distribution on Silhouette island was studied confirming previous reports of a preference for lowland habitats. The Silhouette population is estimated at 48 pairs, showing no significant change from the previous (1981) estimate. Exceptionally small territories (as small as 9.0ha) were found in habitat mosaics of lowland forest on bare rock, associated with abundant cliff nesting sites and high lizard population densities. The larger island of Praslin supports very few kestrels and as the size distribution of lizards on that island is skewed towards large individuals, this may result in food scarcity. Combined with nest site scarcity the paucity of small prey may cause the lack of significant population recovery on Praslin.

894: ***+.270***

For Cicindela puritana to be a viable member of New England's biota, there must be more than the current two occurrences. Assessment of the chance that the species can spread, whether on its own or through reintroduction, required identifying vacant habitat patches, which in turn, required refining the description of the species' larval habitat. Analysis of larval microhabitat variables identified sand texture as the most important determinant of habitat suitability. I then surveyed a 79 km stretch of the Connecticut River in Connecticut looking for suitable habitat patches. Of 32 beaches, none that appeared to be suitable was nearer than 12 km from currently occupied patches. Dispersal is unlikely to lead to establishment of new populations, so I recommend reintroducing C. puritana to an area in the vicinity of Windsor, CT where there are beaches on three islands that appear to be suitable larval habitat.

895: ***-.141***

Annual trends in numbers of ungulate species on a 15 km2 reserve from 1993 to 1998, were evaluated in the context of lion Panthera leo reintroduction during 1996, and subsequent predation by them. The ungulate prey base was enumerated annually by aerial counts and a road count that took place during 1998. The lion prey record was obtained from direct observations of a radio-located pride of eight lions and daily reserve management records. All ungulate species that underwent precipituous declines were also the most important prey to lions, comprising over 80% of their prey, and they were preyed upon according to their availability. Lion predation was causal for the declines in wildebeest Connochaetes taurinus, blesbok Damaliscus pygargus phillipsi and warthog Phacochoerus africana, while the decline in kudu Tragelaphus strepsiceros was only partly ascribed to lions, as other non-lion related mortality sources were identified. The only ungulate species to increase subsequent to lion reintroduction was the impala Aepyceros melampus, which was furthermore under-selected by lions. The uncontrolled population growth of impala could have elicited ecological degradation, and it was advised to either not stock impala, or

otherwise control their numbers if lions are unable to do so. Lion hunting success and kill rate, were 21% (n=63) and 1 kill/4.4 days, respectively. Three bushpigs Potamochoerus larvatus were killed but not utilised, and this finding is corroborated by an intensive study in Kwazulu-Natal, and this aversion is discussed. Predators can cause unprecedented declines of their prey where the prey are confined to small reserves that have no refuge from predation. On an annual basis, prey may need to be augmented to sustain predators on small reserves.

896: *+.029*

Translocation of wild birds is a potential conservation strategy for the endangered red-cockaded woodpecker (Picoides borealis). We developed and tested 8 large-scale translocation strategy models for a regional red cockaded woodpecker reintroduction program. The purpose of the reintroduction program is to increase the number of red-cockaded woodpeckers by moving subadult birds from large populations to smaller populations that are unlikely to increase on their own. A major problem in implementing the program is determining where birds will be moved because the larger donor populations cannot supply enough birds for all small recipient populations each year. Our goals were to develop translocation strategies and model which ones would (1) result in the most groups of wood peckers in a given amount of time, (2) most quickly reach the goal of at least 30 groups of woodpeckers in every population, and (3) result in the fewest population extinctions. We developed lump-sum strategies that moved all the translocated birds to 1 population each year, and partitioning strategies that divided the birds among several populations every year. In our simulations, the lump-sum strategies resulted in the most woodpeckers for the overall program and the highest number of population extinctions. Partitioning strategies had the lowest population extinction rate but produced the lowest rate of increase in the number of woodpecker groups. The model that partitioned birds to the 6 largest recipient populations with fewer than 30 groups was the best overall strategy for meeting our goals because it reached 30 groups in every population the fastest, produced many birds, and had only a moderate population extinction rate. We suggest that adhering to a single strategy that meets the goals of the participants should simplify the program and reduce its cost.

897: *+.114*

Wild populations of eastern grey kangaroos (Macropus giganteus) can pose serious management problems when conditions favor a high population growth rate. Contraception methods are becoming an ethically more acceptable alternative to culling or translocation when dealing with population control of large mammals. We investigated behavioral effects of levonorgestrel contraception in this species. A total of 82.4% of female eastern grey kangaroos treated with levonorgestrel. implants did not reproduce, whereas only 12.5% of control females did not reproduce. Levonorgestrel contraception had no significant effect on time spent feeding, scanning, grooming, moving, and sexual interactions with. males during the observation sessions. However, control females spent more time in open paddocks feeding than contracepted females over a 24-hr period. Males preferred to associate with groups containing more (and a higher percentage of) control females.

898: *+.095*

The paper attempts to describe Italian mammal diversity in an evolutionary context. With 122 species according latest researches, Italy holds the richest mammal assemblage among European countries. Specific taxa are often represented by clearly distinctive lineages and several of them appear restricted to the Italian peninsula. Poor knowledge of taxonomy and uncritical application

of IUCN threat categories at the national level could produce a flawed set of conservation priorities, independently from the rigorous application of the proposed guidelines for national Red List assessments. Furthermore, classical conservation assessments and protective legislation only consider traditionally named taxa, often privileging insular taxa of artificial origin but neglecting most of the results of genetic and molecular studies on intraspecific variation. The aim of the present work is to outline the need to incorporate phylogenetic and biogeographic data in the assessment of conservation priorities among mammals in Italy, in order to maximise the national contribution to biodiversity conservation in Europe. To this end, distribution, threat status, intraspecific and supraspecific taxonomy of the native mammal fauna should be analysed in a global context. Phylogeographic patterns emerging from previous studies indicate the general inadequacy of continental European populations serving as sources for re-stocking or re-introductions operations in Italy and the other European peninsulas. Thus the importance of integrating international guidelines on reintroductions with a clear understanding of national biogeographical peculiarities is highlighted.

899: *-.022*

Live wild animals are confiscated by local, regional, and national authorities for a variety of reasons. Once they have taken possession of these animals, these authorities must dispose of them responsibly, in a timely and efficient manner. Prevailing legislation, cultural practices, and economic conditions will influence decisions on appropriate disposition of confiscated animals. Within a conservation context, there are several possible options from which to choose: 1) to maintain the animals in captivity for the remainder of their natural lives; 2) to return the animals to the wild; 3) to euthanize the animals, i.e., humanely destroy them The IUCN Guidelines for the Placement of Confiscated Animals discuss the benefits and risks involved in each of these options. These Guidelines should be read in conjunction with the IUCN Guidelines for Re-introductions (IUCN 1998), annexed hereto. They should also be read with reference to the CITES Guidelines for the Disposal of Confiscated Live Species of Species Included in the Appendices (Resolution Conf. 10.7) and the IUCN Guidelines for the Prevention of Biodiversity Loss due to Biological Invasion. Returning confiscated animals to the wild is often considered the most popular option for a confiscating agency and can garner strong public support. However, such action poses real risks and problems and generally confers few benefits. These risks and problems include, but are not limited to, the following. 1. The mortality of animals released from captivity is usually high. Confiscated mammals and birds captured as juveniles have not learned the skills they need to survive in the wild. Other animals may be weakened or otherwise affected by their time in captivity and, thus, less able to survive. Finally, there is little chance of survival if the animals are released at a site that is not appropriate for the ecology or behavior of the species. 2. Animals released into the wild outside of their natural range - if they survive at all - have the potential to become pests or invasive. The effects of invasive alien species are a major cause of biodiversity loss, as such species compete with native species and in other ways compromise the ecological integrity of the habitats in which they have become established. 3. Having been in trade or a holding facility often in association with other wild animals and, in some instances, domesticated ones, confiscated wild animals are likely to have been exposed to diseases and parasites. If returned to the wild, these animals may infect other wild animals, thus causing serious, and potentially irreversible, problems. 4. In many instances, confiscated wild animals have been moved great distances from the site of capture and changed hands several times, such that their actual provenance is unknown. It may, therefore, be impossible or very difficult to establish an appropriate site for return to the wild that takes into account the ecological needs of the species, the animals' genetic make-up, and other attributes that are important to minimize risks (e.g., competition, hybridization) to wild populations at a release site. 5. in cases where the provenance

is known, the ecological niche vacated by that animal may already be filled by other individuals and replacing the animal could result in further undesired disturbance of the ecosystem 6. Responsible programs to return animals to the wild (c.f. IUCN 1998) are long-term endeavors that require substantial human and financial resources; hence, they can divert scarce resources away from other more effective conservation activities. If returning confiscated animals to the wild is to be consistent with conservation principles and practice, it should a) only be into a site outside of the species' natural range if such an action is in accordance with the IUCN Guidelines for Reintroductions for a conservation introduction; and b) only be practiced in cases where the animals are of high conservation value and/or the release is part of a management programme. Any release to the wild must include the necessary screening and monitoring to address potential negative impacts, as set forth in the IUCN Guidelines for Re-introductions (IUCN 1998). Retaining confiscated wild animals in captivity is a clear - and, in most cases, preferable - alternative to returning them to the wild. Clearly, returning animals to their owners will be required in cases of theft. There are a number of options for keeping animals in captivity; however, each of these also has costs and risks. * As confiscated animals are likely to have been exposed to diseases and parasites, if held in captivity, they may infect other captive animals, causing serious, and potentially irreversible, problems. * Finding an appropriate home for confiscated animals can be time-consuming, and caring for the animals during that time can be expensive. * Wild animals have specific nutritional requirements and require specific care. Short-term and long-term humane care of confiscated wild animals requires space, finances and expertise not readily available in many countries. * Transfer of ownership from a confiscating government authority to a private entity - individual or non-commercial or commercial care facility - can raise complicated legal and ethical issues, which are difficult - and time- consuming - to address. Sale or transfer of ownership may - or may be seen to - stimulate demand for these animals and exacerbate any threat that trade may pose to the species. It may also give the appearance that the government condones illegal or irregular trade or, in the case of actual sale, is benefiting from such trade. In addition to avoiding risks to wild populations engendered by return to the wild, keeping confiscated animals in captivity provides other benefits, for example: * Confiscated animals can be used to educate people about wildlife and conservation, as well as the consequences of trade in live wildlife. * Confiscated animals placed in captivity can provide breeding stock for zoos, aquariums, and other facilities, thus potentially reducing the demand for wild-caught animals although the opposite effect may also occur. * In specific instances where the provenance of the confiscated specimens is known, these animals can provide the nucleus, and breeding stock, for possible reintroduction programs. * Confiscated animals can be the subject of a range of non-invasive research, training and teaching programs with important potential benefits for conservation. Euthanasia must be considered a valid alternative to placing animals in captivity or returning them to the wild. Although it may appear counter-intuitive to employ euthanasia, it is by definition a humane act and can be wholly consistent with both conservation and animal welfare considerations. Further, although many confiscating authorities may be wary of criticism elicited by a decision to euthanize confiscated animals, there are a number of reasons to justify its use, including the following: * In many, if not most, circumstances, euthanasia offers the most humane alternative for dealing with confiscated wild animals. 8 * Euthanasia eliminates the genetic, ecological, and other risks that release to the wild may pose to wild populations and ecosystems. * Euthanasia eliminates the serious risk of spreading disease to wild or captive populations of animals. * Euthanasia will often be the least costly option. Establishment of an overall policy framework, with specific procedures for confiscating authorities, will facilitate consideration of the above three options for disposition, including the logistical, legal, and ethical questions that these authorities must addre

900: *+.014*

A radio-tracking study was carried out on a re-introduced population of grey partridges in a 10.6-km(2) study area located in central Italy, in order to assess mortality rate and to evaluate the feasibility of carrying out large scale re-introductions of the species, Thirty-nine grey partridges were caught by live-traps during winter 2000 and equipped with backpack radio-transmitter. A released birds were offspring of partridges re-introduced previously on the study area and no significant differences were found in survival probabilities between sexes, age classes (juveniles or adults), and weight classes (<400 g or greater than or equal to400 g), with the exception of the juveniles-adult comparison with the weight class greater than or equal to400 g. Kaplan-Meier estimates give an average survival of 73.4 days (SE = 15.5). GLM analysis showed an overall interaction effect of the sex, age, and weight (P = 0 03). Mortality increased during the covey break up, if compared to other periods, although the differences were not significant; the same was for males, for adults, and for both weight classes, while significant differences resulted for female and juvenile survival rate among periods. Predation was the main mortality source for radio-tagged grey partridges (71.0%), followed by disease (25.8%). The high mortality rate found in our study could be explained in pail with the origin of the population that was originally re-introduced using hand-reared grey partridges. Some important negative characters of reared birds, such as the poor anti-predator behaviour and the low diseases resistance, could carry over during the first and following wild generations.

901: ***+.249***

This 20-chapter textbook provides an introduction to genetic principles and practices in conservation and covers topics such as evolutionary genetics of natural populations, loss of genetic diversity in small populations, inbreeding and loss of fitness, population fragmentation, and much more. It is written for the advanced undergraduate and graduate students of conservation, as well as conservation biologists and wildlife managers. Each chapters include exercises and problems, and the answers are given at the end of the book. Bibliographical references, a glossary, and an index are also included.

902: ***+.125***

During the past two centuries, the meandering Lower Rhine has increasingly suffered under waste-water pollution, engineering projects for shipping traffic, and under settlements built on the former floodplain. However, in the past three decades the pollution has been remarkably reduced through the implementation of sewage treatment plants. This has resulted in a resettlement by both generalist and non-indigenous macrozoobenthos species (MZB) as well as by fish. Because the current species abundance pattern may change from year to year, it may be suggested that the river community (syn. biocoenosis) is continuously impacted by detrimental environmental conditions such as the residue from chemical spills, dispersal of dominating non-indigenous NIZB species, strong fluctuations in the water load of the channel (correlated with long-term exposures of the river banks during low water levels), as well as by the lack of connected backwaters in floodplain areas. In 1987, the Rhine Action Programme focused on the reintroduction of long-distance migrating fish into the ecosystem of the River Rhine, in particular of Atlantic salmon. Migrating fish and their successful reproduction are important ecological signposts that can signalise an optimal rehabilitation of a river ecosystem. Thus, when studying the health of the ecosystem of a large river, the ecological state of salmon spawning grounds in the catchment area must also be taken into consideration. This paper focuses on current ecological problems and makes suggestions for future studies on the Lower Rhine and its tributaries.

903: ***+.245***

The transplantations of Formica lugubris carried out in Italy till 1981 were to defend forests against phytophagous Insects and, following the first good results, populations of the species were transplanted in different apenninic areas. In subsequent years pine-woods have lost their economical value almost everywhere and Bacillus thuringiensis has become available. However, new studies were studied in the original environment of Formica lugubris, observing that environmentally suitable areas, only partially occupied by Formica lugubris, were slowly colonized with small populations . which break away from large strong neighbouring colonies. The observations above gave birth to a new hypothesis of transplantation, whose objective is to reintroduce the species in some alpine areas of origin, from which it has been eliminated due to human interventions. So the propagation speed of the species into biologically suitable areas would become faster and the forest environment would be enriched by an important element of biodiversity, leading towards conditions of ecological balance more quickly.

905: ***+.017***

In order to conserve the native ichthyofauna and as mitigation procedure to prevent the impact generated by the interruption of the free displacement of fish in the Laja river (Biobio Region), a translocation program for native fish was carried out. Between the years 1997 and 2000 the species composition, communitary and population structure of the ichthyofauna were studied, determining the need to transfer individuals of three native species with conservation problems: Diplomystes nahuelbutaensis, Trichomycterus areolatus and Percilia irwini. The translocation campaign was carried out in January of the 2001, capturing a total of 1835 fishes, of which 1653 was restocking. The number of survivals of the three species was high during the three phases of the campaign, being upper 90% in each one of them. 852 P. irwini were captured and 760 restocked; 643 T. areolatus were captured and 591 restocked, and 340 D. nahuelbutaensis were captured and 302 restocked. In the three species the highest mortality occurred in the juveniles. T. areolatus and P. irwini showed greater mortality during data recording (weight and body length), while D. nahuelbutaensis resulted more sensitive to the transfer. In all the cases, this mortality was lower to 7%. To enlarge the efficiency of this strategy of conservation we suggested: (a) to avoid the juvenile extractions, (b) to eliminate the phase of taking data of each specimen captured, (c) to reduce the time of fish stored in containers, and (d) to transport the fishes in low densities.

907: ***+.158***

The Reintroduction Plan of Stripless treefrog in Mt. Mendizorrotz (Gipuzkoa province, Basque Country) has started in 1998. The results and monitoring performances are exposed at the present study. Some new pools were constructed for translocation of adults, juveniles and tadpoles. A total number of 675 adults (540 males and 135 females), 40 juveniles and 10.039 bred in semicaptivity tadpoles were translocated from their emergence pools to the arranged Berio, Goienetxe, Arpita and Egioleta pools resulting in a diverse acceptance degree. In three of the reintroducted pools we confirm the presence of the species in later visits, proving its reproduction in only one of them in 2000. Likewise, 457 adults (330 males and 127 females) were translocated from one to another swimming pool in Berio for increasing population reproductive success.

910: ***-.009***

Since December 1997, 700 blood plasma samples from 31 different species of captive and free-living birds of prey from Spain were analyzed by hemagglutination inhibition (HI) test for the presence of antibodies to avian paramyxovirus (aPMV) 1,2, and 3. Out of 700 birds, 120 tested positive for aPMV-1, 10 birds had antibodies to aPMV-2, and 4 birds tested positive against

aPMV-3. Prevalence of antibodies against aPMV-1 was significantly higher in captive than in free-living birds of prey and in Falconiformes than in Strigidae and Accipitridae. Infection or exposure in captive birds may be due to the use of avian-derived food in rehabilitation and captive-breeding centers. This may be of concern at the time of reintroduction of these birds into free-living populations.

912: *+.112*

Genetic monitoring of reintroduced plant populations can allow assessment of the success in establishing new populations that genetically resemble native populations. We used a PCR-based method (Intersimple Sequence Repeats) to quantify genetic variation in four reintroduced populations of Abronia umbellata ssp. breviflora, an annual forb native to the Pacific Coast that is state-listed endangered in Oregon. The reintroduced populations ranged in size from 18 to 4, 111 individuals in the year they were sampled. Genetic variation was also quantified in the natural population that served as the seed source for the reintroduction efforts. A total of 77 loci (bands) was observed using two ISSR primers, providing 65 polymorphic loci. A significant, positive regression was observed between the log of population size for the five populations and genetic variation when measured as percent polymorphic loci (P), expected heterozygosity (H-e), and with a dissimilarity index (1 - S-xy) based on band sharing. Two of the reintroduced populations maintained approximately 90% of the genetic variation we observed in the source population. Based on these results, we predict that reintroduced populations of A. u. ssp. breviflora that have at least 1, 000 individuals should maintain 90% of the genetic variation of the source population.

913: *+.040*

Although there is a consensus of opinion that habitat fragmentation has deleterious effects on animal populations, primarily by inhibiting dispersal among remaining patches, there have been few explicit demonstrations of the ways by which degraded habitats actually constrain individual movement. Two impediments are primarily responsible for this paucity: it is difficult to separate the effects of habitat fragmentation (configuration) from habitat loss (composition), and conventional measures of fragmented habitats are assumed to be, but probably are not, isotropic. We addressed these limitations by standardizing differences in forest cover in a clearly anisotropic configuration of habitat fragmentation by conducting a homing experiment with three species of forest birds in the Bow Valley of Banff National Park, Canada. Birds were translocated (1.2-3.5 km) either parallel or perpendicular to four/five parallel barriers that are assumed to impede the cross-valley travel of forest-dependent animals. Taken together, individuals exhibited longer return times when they were translocated across these barriers, but differences among species suggest a more complex interpretation. A long-distance migrant (Yellow-rumped Warbler, Dendroica coronata) behaved as predicted, but a short-distance migrant (Golden-crowned Kinglet, Regulus satrapa) was indifferent to barrier configuration. A resident (Red-breasted Nuthatch, Sitta canadensis) exhibited longer return times when it was translocated parallel to the barriers. Our results suggest that an anisotropic arrangement of small, open areas in fragmented landscapes can have a cumulative barrier effect on the movement of forest animals, but that both modelers and managers will have to acknowledge potentially counterintuitive differences among species to predict the effect that these may have on individual movement and, ultimately, dispersal.

914: *+.104*

The method and results of monitoring between 1993 and 1995 the reintroduction of 16 beavers originating from the native Rhone population to the North of Alsace are presented. The presence

of the species is attested in 18 localities in the North of Alsace (Fr.) and Bade region (Ger.). The current strengths, which can be estimated to 50-60 individuals are distributed in 26-28 sectors of activity of which 8-10 are occupied by established couples. An important potential of colinisation exists on both sides of the Rhine. However, there are various limiting factors too : obstacles formed by the channelled Rhine, deaths caused by road traffic or by non selective methods of elimination of the coypu and muskrat. Stemming from 4-5 founding couples at the most, the very narrow genetic base of this new sub-population could be widened. Indeed there are possibili- ties of natural exchanges with those already established in the centre and the South of Alsace and in the nearby Saar basin, and besides this, projects of reintroduction in the German floodplains of the Rhine (Bade and Palatinate regions) studied since the 70's could be executed especially with beavers coming from the native Elbe population.

915: *-.015*

Two major drainages of southeastern Australia, the Snowy River and the Murray River, were artificially linked by a major hydroelectric project during the early 20th century. This development diverts Snowy River flow into tributaries of the Murray River via a series of extensive tunnels. In 1990, fish surveys of the upper Murray River system recorded Galaxias brevipinnis, an aggressive migratory species previously unrecorded from the drainage. We used genetic analysis to discriminate between alternative hypotheses for Murray River G. brevipinnis: (i) anthropogenic translocation via the Snowy River diversion or (ii) a previously undiscovered natural population. Landlocked G. brevipinnis from the Murray River (43 fish, eight control region haplotypes) and Snowy River (39 fish, 11 haplotypes) exhibit similar levels of mtDNA diversity, share six haplotypes, and are not significantly differentiated for microsatellite loci ($p = 0.0884$). Coastal samples exhibit higher haplotypic diversity (40 fish, 20 haplotypes) but share only three haplotypes with Murray River and are significantly differentiated from Murray River samples for microsatellite loci ($p = 0.0008$). Our data are consistent with the translocation hypothesis but are generally inconsistent with a natural origin for Murray River G. brevipinnis. The suggested human-mediated translocation represents a risk to native fauna.

916: *+.118*

Beaver (Castor canadensis) through their dam building activities, store water, trap sediment. subirrigate vegetation, and subsequently improve habitat for fish, wildlife, and livestock. Many landowners realize the benefits that Beaver can bring to a riparian area and are interested in using them to improve this habitat. From 1994 to 1999 we trapped and relocated 234 Beaver to 14 areas throughout Wyoming to improve riparian habitat and create natural wetlands. We attached radio transmitters to 114 Beaver and subsequently determined movements and mortality of released Beaver, and the overall success of our releases. Mortality and emigration (including transmitter failure) accounted for the loss of 30% and 51%, respectively, of telemetered Beaver within 6 months of release. Kaplan-Meier survival estimates were 0.49 (SE = 0.068) for ISO days and 0.433 (SE = 0.084) for 360 days, and did not differ significantly between age classes. On average, 17 Beaver were transplanted to each release site, and at 11 locations, in an attempt to augment single Beaver that had become established and increase transplant success, we transplanted Beaver in two or more years. Success of an individual Beaver's relocation was unrelated to any of the variables we tested, although 2-3.5 year-old Beaver had higher average success (measured in days of occupancy at the release site) than older animals. Animals < 2 years old had 100% mortality and emigration losses within 6 months of release. High predation and mortality rates of our released Beaver may be due to habitat (our streams were shallow with no ponds and provided little protection) and extensive predator communities. We established Beaver at 13/14 of our release

sites and they eventually reproduced. Our results show that Beaver can be relocated successfully but losses from mortality and emigration need to be considered and planned for.

917: ***+.168***

The present paper summarizes the outcome of a roundtable discussion on reintroductions or restocking as a management strategy for native European crayfish, which took place during the European Crayfish conference in Poitiers, France in September 2001. Aspects related to the suitability of the target habitat, the stocking material and the stocking procedure itself are important to consider during any reintroduction measure: Apart from general water quality and structural parameters, a suitable habitat is ideally geographically isolated from other surface waters and human activities such as intensive fishing pressure. However, it is of striking importance to make sure that the target habitat is free of crayfish plague, e.g. by experimental in situ exposure of native specimens. Over-exploitation should be avoided if the stocking material originates from other surface waters and genetic aspects should be considered in stocking material originating from both natural surface waters and crayfish farms. Information for the stocking procedure itself are summarized from experiences gathered in various European countries. As an overall result of the discussion, the following three key questions related to reintroductions of native crayfish arised: 1. Why are the native crayfish not present (anymore) in the target habitat? 2. Do native crayfish survive in situ exposure as a test for crayfish plague? 3. Is the reintroduction or restocking measure successful in establishing a self-sustaining stock of native crayfish?

918: ***+.265***

Irish white-clawed crayfish stocks show high genetic uniformity between systems, although individual populations usually show good heterozygosity. The inference is that they were derived from a single immigrating stock, related to those in Western France. Irish crayfish stocks occur widely and often in good numbers in lakes and streams; they are protected and rarely fished, and there are no alien species. Their use for reintroduction into depleted areas within Ireland, including Special Areas of Conservation, is examined. Irish crayfish also have potential value for restocking depleted continental locations, under stringent conditions of environmental suitability and genetic conformity.

919: ***-.110***

Freshwater crayfish are among the most endangered animals in Europe. Information on freshwater crayfish in the Italian province of South Tyrol was insufficient until the recent studies on their historical and present distribution, which showed the alarming facts that only 15 standing and running waters remained as freshwater crayfish habitats, carrying the native Austropotamobius pallipes and the allochthonous Astacus astacus, Orconectes limosus, and Pacifastacus leniusculus. Still ongoing alterations or degradation of water bodies have contributed to the high degree of population decline and loss. While the exotic crayfish O. limosus and P leniusculus could develop higher densities, most of the autochthonous A. pallipes populations were weak and consisted only of a few individuals. Three populations of A. pallipes, however, remained in a good condition, representing potential sources for recovery programs. Protection measures currently carried out in South Tyrol involve breeding, restocking and reintroduction of autochthonous A. pallipes individuals in ecological intact water bodies.

920: ***-.068***

Populations of rare clonally spreading species are increasingly being shown to be composed of one or a few genotypes. Clones have potentially unlimited life but two factors, genetic erosion due to random events and increasing accumulation of genetic load in older genotypes, combine to expose them to sexual failure and loss of fertility. Non-breeding clonal populations are at risk of extinction because they lack the ability to adapt or escape from changing environments. Twinflower (Linnaea borealis L.) has been lost from nearly 50% of its pre-1970 sites. It is a clonal self-incompatible plant and sexual failure has been reported from Canada, Britain and Scandinavia. Sexual failure is due to high levels of within-clone pollination by flies. Seed set occurs naturally in Scottish populations which have some genetic diversity. A non-breeding population in Scotland has been restored to viability by experimental cross-pollination with pollen from a distant source. Conservation action for this species should therefore be directed to the prevention of loss of habitat and restoration of population viability by translocation of compatible mates. Translocating plants into extant, but non-breeding, populations are still a matter of debate and are frequently opposed. However, experimental translocations must now be performed to establish whether intervention can secure the long-term survival of rare clonal plants without the constant need for re-introduction.

921: *+.276*

The Jersey population of the Agile Frog has been declining in both range and number since the early 1900s. At the present time, there is only one site on the island that supports a natural agile frog population. Since the 1980s, a captive breeding, reintroduction and habitat management programme involving various organisations has been trying to arrest this dramatic decline in agile frog numbers in the wild. All parties involved agreed that without a clearer understanding of the ecology and habitat requirements of the agile frog in Jersey, it was difficult to develop management techniques for the protection and improvement of key habitat areas. The objectives of the study are to (1) estimate the number of adult frogs through a mark - recapture programme; (2) monitor hatching success and recruitment; (3) study the interactions with other species present in the pond (toads, newts, invertebrates and grass snakes) and in the surrounding area (grass snakes, small mammals and birds); (4) determine the frog's overwintering preferences; (5) monitor the captive breeding programme and carry out an investigation of potential reintroduction sites; (6) develop optimal conditions for captive rearing of tadpoles and consider a health monitoring protocol prior to release of captive raised frogs; (7) compare the habitat requirements of the Agile Frog in Jersey and in northern France. Preliminary results of the study obtained from the observations collected during the first fieldwork season are presented here.

922: *+.163*

Birds species, like plants and other animals are facing an unprecedented decline. About 11% of bird species are threatened with extinction. Captive breeding as a conservation tool can be used as a substitute for wild populations in research and education, to provide demographic and genetic reservoirs for reinforcing or founding wild populations, and as a last resort for species that have no immediate opportunity for survival in nature, Candidates for captive propagation are zoos, private breeders, state agencies, conservation foundations and research centres within or outside of universities. The problems in managing endangered species have motivated conservation biologists to conduct research in many disciplines such as behaviour, physiology, endocrinology, genetics, husbandry, nutrition and veterinary medicine. In addition, an increasing number of techniques for increasing reproductive success, improving genetic management and enhancing reintroduction success have been employed. Breeding success has been improved by improved knowledge of factors that trigger reproduction and stimulate replacement clutches, as well as by

improvements in artificial incubation and artificial insemination. Assuming that the correct social behaviour group is constituted and the animals start to breed, genetic problems are likely to occur in species with only a few remaining individuals. Once numbers are low, genetic variability is reduced and this brings about diminished ability to respond to environmental change. Recent advances in molecular genetics have enabled studies in taxonomy to be carried out in order to determine species, subspecies and population boundaries. They have also contributed to the theoretical development of small population genetics and demographics aimed at improving ex-situ population management, Captive birds are often hand-reared, but since many behaviours are acquired from the parents, or by experience in the wild, artificial rearing by hand could affect the survival potential of released animals. In order to prevent imprinting on humans and other detrimental effects of hand-rearing techniques, parents, foster-parents, puppets or costumes have been used to rear chicks. Finally, recent developments in research on training programmes to find food, to fly, to avoid specific dangers, or to recognise and to avoid predators, appear to have substantially increased release success in reintroduction programmes.

923: *+.138*

In the 1930-1960s the moose Alces alces L. population increased markedly in northern (Skandinavia) and eastern Europe (the European part of the former Soviet Union), and expanded into western and southern Europe. Polish population of moose was growing in the l960-1970s (Fig. 1), mainly due to spontaneous increase of the local population inhabiting the Biebrza River valley,as well as due to reintroduction of moose into Kampinos Forest (Central Poland) and immigration of animals from the east and north. Since the early 1980s, moose population in Poland and in whole Europe began to decline (Table 1), predominantly because of severe hunting harvest. In this paper, the major migratory routes of moose in Poland have been mapped and the most imporant refuges (existing at present or in the period of the biggest densities of moose) were localised (Fig. 2). The proposed program of moose restitution includes restricted or banned hunting harvest in the refuges for several years, improvement of migratory corridors to western Poland and seleting areas for refuges there. The aim of the program is to enable migrations of moose to Germany, as the first step towards restitution of the historical range of this species in Central Europe.

924: *+.082*

Rates of increase, survival and reproduction were studied in two re-introduced populations of mountain ungulates, the Pyrenean chamois (also named isard, Rupicapra pyrenaica) and the ibex (Capra ibex) in the French Alps. The populations were monitored from 1983 to 1998 (ibex) or 1984 to 1999 (isard) using capture, marking and resighting of individuals. Population rates of increase were estimated during the colonizing phase from: (1) a time series of estimates of population size; (2) projection matrices based on rates of survival and fecundity. Both populations had exponential rates of increase, with annual multiplication rates of 1.28 for isard and 1.30 for ibex. Age of first reproduction was 2 years in both species, with 77% of females breeding at this age in isard and 43% in ibex. From 3 years of age onwards, 90% of isard and 87% of ibex females bred each year. Survival rates of adult females, estimated using capture-recapture models, equalled 0.94 for isard and 0.98 for ibex, whatever the age and year. The projection matrix led to a slightly lower asymptotic multiplication rate than the time series of population size. Despite adult body size of the ibex being about twice that of the isard, no difference was detected in the intrinsic rate of increase between the two species. This supported the hypotheses of homogeneous life-history traits among ungulates, varying little with body size.

925: ***+.013***

Predation is a common problem limiting the success of efforts to reintroduce vertebrates. Naive animals typically suffer high predation rates soon after release despite strong site fidelity in the first few days that would normally reduce predator encounter rates. However, recent theory predicts that low mobility can be associated with high concentrations of odour wastes that are attractive to predators, leading to low survival for individuals that move little. In this paper we test this model for captive-raised Microtus voles reintroduced into suitable habitat in western Finland where they are mainly hunted by chemo-sensing predators. Patterns in vole movement behaviour showed a humped relationship with time: animals moved little soon after release, then made exploratory movements from 3 to 6 days, which then subsided. Predation rates were highest in the first 3 days, with no vole mortalities occurring after voles began substantially moving beyond their release sites. Moreover, voles that survived the 3-week study period were more mobile than voles that were killed by scent-hunting predators, during the initial 3 days and during the final 3 days when voles also had lower mobility. These results suggest that the innate fear response of naive animals, which limit their movements in an unfamiliar environment, may be counterproductive when reduced mobility leads to waste accumulation that is attractive to potential predators. Consequently, pre-release conditioning of captive-raised individuals may need to consider methods to overcome initial release-site fidelity in order to enhance long-term survival prospects after reintroduction. (C) 2001 Elsevier Science Ltd. All rights reserved.

926: ***+.076***

The northern leopard frog (Rana pipiens) was once a common and widespread amphibian found throughout central and southern Alberta. During the late 1970s, the leopard frog experienced a dramatic decline in distribution and numbers over much of its historic range in Alberta. As a result, the leopard frog was designated as "Threatened" under Alberta's Wildlife Act in 1996. The Committee on the Status of Endangered Wildlife in Canada (COSEWIC) lists the prairie population of the northern leopard frog as "Special Concern" (COSEWIC 2000). In 1998, the Alberta Fish and Wildlife Division began to explore the feasibility of reintroducing leopard frogs into formerly occupied habitats in the upper Red Deer River and North Saskatchewan River drainage basins. Large areas of unsuitable habitat limit the ability of the leopard frog to disperse back into historic parts of its range. As a result, a pilot reintroduction project for the leopard frog was initiated in 1999 at the Raven Brood Trout Station near Caroline, Alberta. The project involved the captive-rearing of leopard frogs from egg stage of development to metamorphosed frog, in two man-made outdoor ponds. The primary objective of the project is to re-establish leopard frogs in the headwaters of the Red Deer River and the North Saskatchewan River drainages, consequently allowing natural downstream dispersal along these drainages. Over a three-year period, more than 4500 captive-reared leopard frogs have been released into historic habitat in the upper headwaters of the Red Deer River near Caroline, Alberta. In 2001, 750 young frogs were released at a pilot release site in the upper headwaters of the North Saskatchewan River near Rocky Mountain House, Alberta. All captive-reared and released leopard frogs were marked using a Visible Implant Elastomer (VIE) tagging system that allowed unique marking schemes to be used to assess the success at each release site and monitor the dispersal of released frogs. On 19 June 2001, three previously released leopard frogs were found within the study area near Caroline, marking the first occurrence of leopard frogs in that area in nearly 50 years. At least 10 subadult and adult leopard frogs released in previous years were observed or captured within the study area in 2001 and calling activity was recorded. Evidence of the overall success of the project at the first release site may be realized in the summer of 2002, when previously released frogs reach sexual maturity, breed and successfully produce young-of-the-year frogs. During the 2001 field season,

20 potential leopard frog release sites were investigated in the central parkland region of the province and in the upper headwaters of the Red Deer River near Caroline and the North Saskatchewan River near Rocky Mountain House. At each identified site, data were collected on the availability of breeding and summer habitat. In addition, dispersal opportunities into surrounding habitats, including the Red Deer River and North Saskatchewan River drainages, were considered.

927: ***-.115***

We conducted a literature review of coypu Wyocastor coypus) introduction and eradication efforts worldwide. The coypu (also called nutria) has been introduced from its origins in South America to every continent except Australia and Antarctica. While perceived in some regions as a valuable resource, in most regions the animals are considered a pest species. Coypus have caused damage to water control structures, crops, and marsh systems and are considered a disease host. Eradication efforts have met with varying degrees of success. For those efforts to be successful, the coypu populations must be isolated to prevent reintroduction, harsh winters are necessary to reduce their populations, and intensive trapping efforts must be sustained until the last coypu has been removed.

928: ***+.113***

We evaluated the usefulness of microsatellites and recently developed statistical methods for the conservation management of fragmented and reintroduced populations, using the alpine ibex (Capra ibex) as a model species. First, we assessed the effects of past reintroduction programmes on genetic diversity and population differentiation considering different population sizes and histories. We show that genetic variability in ibex populations (H-E approximate to 0.13) is among the lowest reported from microsatellites in mammal species, and that the Alpi Marittime-Mercantour population has suffered from a severe genetic bottleneck associated with its reintroduction. Second, using a computer-simulation approach, we provide examples and rough guidelines for translocation programmes concerning the number and origin of individuals for future reintroductions and for the reinforcement of populations with low genetic variability. Finally, we use the ibex microsatellite data to assess the usefulness of several published statistical tests for detecting population bottlenecks and assigning individuals to their population of origin. This study illustrates that microsatellites allow: M evaluation of alternative translocation scenarios by simulating different numbers and origins of 'migrants'; (ii) identification of bottlenecked. populations (especially using the Wilcoxon signed-ranks test); and (iii) population assignment with a high certainty ($P < 0.001$) of almost 100% of the individuals (or trophies or carcasses) from two distant populations (especially using STUCTURE or WHICHRUN software).

929: ***+.033***

Mitochondrial DNA (mtDNA) sequences were used to evaluate phylogeographic structure within and among populations of three endangered Hawaiian tree snail species ($n = 86$). The primary focus of this investigation was on setting conservation priorities for Achatinella mustelina. Limited data sets for two additional endangered Hawaiian tree snails, A. livida and A. sowerbyana, were also developed for comparative purposes. Pairwise genetic distance matrices and phylogenetic trees were generated, and an analysis of molecular variance was performed on 675-base pair cytochrome oxidase I gene sequences from multiple populations of Hawaiian tree snails. Sequence data were analysed under distance-based maximum-likelihood, and maximum-parsimony optimality criteria. Within the focal species, A. mustelina, numbers of variable and

parsimony informative sites were 90 and 69, respectively. Pairwise intraspecific mtDNA sequence divergence ranged from 0 to 5.3% in A. mustelina, from 0 to 1.0% in A. livida and from 0 to 1.9% in A. sowerbyana. For A. mustelina, population genetic structure and mountain topography were strongly correlated. Maximum genetic distances were observed across deep, largely deforested valleys, and steep mountain peaks, independent of geographical distance. However, in certain areas where forest cover is presently fragmented, little mtDNA sequence divergence exists despite large geographical scales (8 km). Genetic data were used to define evolutionarily significant units for conservation purposes including decisions regarding placement of predator exclusion fences, captive propagation, re-introduction and translocation.

930: *+.179*

River otters (Lontra canadensis) were extirpated from much of their historic distribution because of exposure to pollution and urbanization, resulting in expansive reintroduction programmes that continue today for this and other species of otters worldwide. Bioaccumulation of toxins negatively affects fecundity among mustelids, but high vagility and different dispersal distances between genders may permit otter populations to recover from extirpation caused by localized environmental pollution. Without understanding the influence of factors such as social structure and sex-biased dispersal on genetic variation and gene flow among populations, effects of local extirpation and the potential for natural recolonization (i.e. the need for translocations) cannot be assessed. We studied gene flow among seven study areas for river otters (n = 110 otters) inhabiting marine environments in Prince William Sound, Alaska, USA. Using nine DNA microsatellite markers and assignment tests, we calculated immigration rates and dispersal distances and tested for isolation by distance. In addition, we radiotracked 55 individuals in three areas to determine characteristics of dispersal. Gender differences in sociality and spatial relationships resulted in different dispersal distances. Male river otters had greater gene flow among close populations (within 16-30 km) mostly via breeding dispersal, but both genders exhibited an equal, low probability of natal dispersal; and some females dispersed 6090 km. These data, obtained in a coastal environment without anthropogenic barriers to dispersal (e.g. habitat fragmentation or urbanization), may serve as baseline data for predicting dispersal under optimal conditions. Our data may indicate that natural recolonization of coastal river otters following local extirpation could be a slow process because of low dispersal among females, and recolonization may be substantially delayed unless viable populations occurred nearby. Because of significant isolation by distance for male otters and low gene flow for females, translocations should be undertaken with caution to help preserve genetic diversity in this species.

931: *-.065*

Thirty-three species of landsnail were recorded during surveys of Great Island in 1996-99. Twelve of these species were endemic to Great Island, and another 17 were restricted to the Three Kings group. One previously recorded species, Cytora kiama Climo, 1973, is regarded here as a junior synonym of Cytora solitaria (Powell, 1935). For more than 300 years up to 1946, sequential anthropic disturbances severely restricted distributions and diminished populations of many landsnail species on Great Island. Some original landsnail species probably became extinct during this period. Conversely, it is possible that some present-day landsnails originated from accidental anthropic translocations from other islands in the Three Kings group and mainland Northland. Since feral goats (Capra hircus) were eradicated from the island in 1946, many formerly rare landsnail species have become widespread and abundant, primarily in response to vegetation changes.

932: *+.147*

We translocated Margaritifera hembeli in 2 streams in central Louisiana to investigate the role of stream, habitat (riffle vs pool), and population density in determining the survival, movement, and growth of transplanted mussels. Translocated animals survived well, although 1 translocation site with few resident mussels suffered high mussel mortality from a spate. Translocated animals grew at rates similar to control animals, especially if moved upstream, but moved at greater rates, especially in high-density riffles. Growth rates differed between streams, and were greater in riffles than in pools; pools, where resident mussels are rare, may be suboptimal habitats for growth. Density had variable effects on growth, and growth rates in some cases actually increased with density. Mussels at higher densities may be feeding on bacteria colonizing pseudofeces or have reduced respiration rates, explaining higher growth rates. Our results suggest that translocation is a viable short-term management strategy for endangered margaritiferids threatened by habitat alteration, and that habitat and population density affect mussel survival and growth.

933: *+.123*

Genetic diversity of the giant panda was examined by DNA fingerprinting method in order to determine genetic distances between two groups, big and small populations. Results obtained suggest that small population may induce the reduction of genetic diversity of the giant panda and result in extinction of the species. These results suggest the need for reintroduction of individuals into small population for preventing the reduction of genetic diversity in giant panda population.

934: *+.075*

Animal translocation is an important tool in wildlife management and conservation biology. However, translocation success is generally low for many species as released individuals may be particularly prone to death or fail to breed successfully. We translocated 49 European wild rabbit, Oryctolagus cuniculus, from Chevreloup (near Paris) to Fay-de-Bretagne (near Nantes, Loire-Atlantique) in January 1996, and studied their survival and movements during the first three weeks after release. All individuals were ear-tagged with colour reflective paper and monitored by the resighting survey method (nocturnal spotlighting). Twenty-nine of them were also radio-equipped. Thus, thanks to the radiotracking survey, we could ascertain the validity of the resighting survey method to estimate survival rates, despite (i) slight differences in survival rate estimates, and (ii) a partial resighting-dependence (catch-happiness in radio-equipped males). During the first two days after release, the mortality rate was 41-51% due either to stress or predation. Concurrently, an intense and early movement activity was displayed by part of the rabbits. Thereafter, the mortality rate was very low and reached the natural one for non-translocated rabbits. The rabbits finally settled down on average at about 225 m from their respective release warrens. Moreover, no individuals actually left the release area, which promotes population founding. Overall, radio-collars and early movements did not influence survival and resighting probabilities. Otherwise, the resighting rate was negatively correlated with body mass.

935: *-.099*

The Critically Endangered P re David's deer Elaphurus davidianu became extinct in the wild in China in about 1900, and the only surviving animals were held in captivity, at Woburn Abbey in the UK. During 1985-1987, individuals were returned to China, and subsequent growth of the captive population in enclosures at Dafeng Reserve necessitated a trial release of a small group of deer as a prelude to further releases. Seven individuals were released into the unfenced coastal

region of the Dafeng Reserve in 1998. Behaviour, daily activity rhythm, habitat selection, activity range and body condition were recorded for six months after release, The deer exhibited initial changes in behaviour, but returned to their pre-release patterns about four weeks after release, and by six weeks after release their body condition had improved compared to their previous condition in captivity. They left the Reserve and began to forage on farmland, causing conflict with local people. Further releases should be into areas with either a natural or artificial boundary in order to avoid unmanageable levels of conflict between the needs of the deer and those of farmers.

936: *+.190*

1. After an absence of almost 100 years, the Eurasian lynx Lynx lynx is slowly recovering in Germany along the German-Czech border. Additionally, many reintroduction schemes have been discussed, albeit controversially, for various locations. We present a habitat suitability model for lynx in Germany as a basis for further management and conservation efforts aimed at recolonization and population development.2. We developed a statistical habitat model using logistic regression to quantify the factors that describe lynx home ranges in a fragmented landscape. As no data were available for lynx distribution in Germany, we used data from the Swiss Jura Mountains for model development and validated the habitat model with telemetry data from the Czech Republic and Slovenia. We derived several variables describing land use and fragmentation, also introducing variables that described the connectivity of forested and non-forested semi-natural areas on a larger scale than the map resolution.3. We obtained a model with only one significant variable that described the connectivity of forested and non-forested semi-natural areas on a scale of about 80 km(2). This result is biologically meaningful, reflecting the absence of intensive human land use on the scale of an average female lynx home range. Model testing at a cut-off level of $P > 0.5$ correctly classified more than 80% of the Czech and Slovenian telemetry location data of resident lynx. Application of the model to Germany showed that the most suitable habitats for lynx were large-forested low mountain ranges and the large forests in east Germany.4. Our approach illustrates how information on habitat fragmentation on a large scale can be linked with local data to the potential benefit of lynx conservation in central Europe. Spatially explicit models like ours can form the basis for further assessing the population viability of species of conservation concern in suitable patches.

937: *+.119*

1. The effects of predation are among the most pervasive in ecology. As parasitoids, parasites, grazers or top carnivores, predators have large influences on the distribution, density, dynamics and evolved traits of other organisms. Effects scale-up to influence community attributes such as species coexistence and ecosystems processes such as production or trophic cascades.2. Increasingly, however, some of the largest predation issues fall clearly within the scope of applied ecology. They include instances where, due to their ecological attributes and trophic position: (i) predators are valuable to nature conservation, as biocontrol agents, as natural enemies, or as grazers used in rangeland or ecosystem management; (ii) natural or introduced predators are viewed negatively due to effects on conservation, agriculture, forestry, hunting or disease transmission; (iii) predators are affected by human activities such as, resource exploitation, or from exposure to factors such as biomagnified pollutants and disturbance; (iv) predators are controversial because different groups view them as either desirable or undesirable.3. In all these cases, ecologists have a pivotal role in facilitating appropriate management. For valued predators, this involves developing sufficient ecological understanding to optimize habitat, increase prey abundance or to reinforce, establish or reintroduce desirable species. For predators considered undesirable, management can involve direct control. In other cases, predation and its

consequences can be mitigated by deterrent, exclusion, supplementary feeding, habitat management to favour prey, predator swamping, or by compensating losses financially. These latter strategies are often used where predators are themselves considered too valuable to remove or control.4. This collection of seven papers illustrates many of these themes by examining contrasting aspects of the applied ecology of Eurasian lynx; by further probing the interaction between predatory birds and red grouse; by exploring the effects of weather on biocontrol; and by illustrating effects on plant species where grazing or seed predation play a dominant role.5. A key lesson from these and other recent papers in the Journal of Applied Ecology is that the successful management of predators depends invariably on understanding adequately the exact ecological context in which predator-prey interactions take place and in which problems arise. With predator-related issues growing rather than diminishing, ecologists will need sufficient resources to maintain current research if they are to provide the understanding required to offer and evaluate sound management.

938: *+.121*

During the last two decades, the State of Connecticut has restored tidal flow to many impounded salt marshes. One of the first of these and the one most extensively studied is Impoundment One in the Barn Island Wildlife Management Area in Stonington, Connecticut. In 1990, twelve years after the re-establishment of tidal flooding, the density of the marsh snail Melampus bidentatus, the numerically dominant macroinvertebrate of the high marsh, in Impoundment One was about half that in reference marshes below the breached impoundment dike. By 1999 the densities of Melampus above and below the dike were not significantly different, but the shell-free biomass was greater above the dike as a result of the somewhat larger number and size of the snails there. Twenty-one years after the renewal of tidal flooding, three marsh macroinvertebrates (the amphipods Orchestia grillus and Uhlorchestia spartinophila and the mussel Geukensia demissa) were significantly less abundant in the previously impounded marsh than in the reference marshes, whereas another amphipod (Gammarus palustris) was more abundant above the breached dike where conditions appeared to be somewhat wetter. In 1991 the fish assemblage in a mosquitocontrol ditch in Impoundment One was similar to that in a ditch below the breached dike; however, the common mummichog Fundulus heteroclitus appeared to be less abundant in the restoring marsh. By 1999 the number of mummichogs caught in ditches was significantly greater in Impoundment One than in the reference marsh, but the numbers of mummichogs trapped along the tidal creek were comparable above and below the dike. The results obtained in this study and those of other restoring marshes at Barn Island indicate the full recovery of certain animal populations following the reintroduction of tidal flow to impounded marshes may require up to two or more decades. Furthermore, not only do different species recover at different rates on a single marsh, but the time required for the recovery of a particular species may vary widely from marsh to marsh, often independently of other species.

939: *+.062*

Translocation is an important management strategy in conservation programs for endangered or threatened species, including native cutthroat trout (Oncorhynchus clarki) in the western United States. Most subspecies of cutthroat trout have declined to <5% of their historical range, and both historical and translocated populations now persist in small isolated fragments of habitat. Success rates for translocations of fishes are generally <50%, and habitat quality or quantity are frequently cited as the cause of failure. Therefore, we conducted field surveys of stream-scale habitat and measured basin-scale habitat using a Geographic Information System for 27 streams where two subspecies of cutthroat trout were translocated in Colorado and New Mexico, to identify specific

habitat attributes that contribute to the success of translocations. We used polytomous logistic regression to develop models that predict three categories of cutthroat trout translocation success (high, low, absent) from habitat attributes at two spatial scales. Models based on stream-scale habitat attributes indicated that cold summer water temperature, narrow stream width, and lack of deep pools limited translocations of cutthroat trout. Cold summer temperatures are known to delay spawning and prolong egg incubation, which reduces the growth of fry and likely limits their overwinter survival. Furthermore, small streams with few deep pools may lack the space necessary to permit overwinter survival of a sufficient number of individuals to sustain a population. Models based on basin-scale habitat were not as effective as stream-scale habitat models for distinguishing among translocation sites with high, low, or absent population status but indicated that a minimum watershed area of 14.7 km(2) was useful as a coarse filter for separating sites with high numbers of cutthroat trout from those with low or absent status. Watersheds larger than this are expected to encompass low-elevation habitat that provides warmer summer temperatures and to have relatively wide stream channels of sufficient length to provide an adequate number of deep pools. These results indicate that the appropriate scale of habitat measurement for predicting cutthroat trout translocation success in fragmented watersheds is at the patch rather than landscape scale, which is similar to results for other salmonids and vertebrate taxa in general.

940: *+.126*

We translocated 80 individuals of Phrynocephalus guttatus guttatus into non-occupied isolated sand dune in Southern Kalmykya in June 1998. Observations during the following two years signify to the viability of the population. The demographic structure remained stabile since 1999, signifying to the congruence of habitat quality and ecological demands of the lizard species. We evaluated differences in the rate of postnatal development of males and females. In result, the number of sexually active males prevailed over the number of receptive females during the reproductive period, increasing probability of females' pregnancy. Maximum life span of Ph.g. guttatus was estimated as up to four years.

941: *+.060*

Aim Many species find themselves isolated from the predators with which they evolved. This situation commonly occurs with island biota, and is similar to moving from the dangerous inner-city to the suburbs. Economic thinking tells us that we should expect costly antipredator behaviour to be lost if it is no longer beneficial. The loss of antipredator behaviour has important consequences for those seeking to translocate or reintroduce individuals from predator-free islands back to the predator-rich mainland, but we have neither a detailed understanding of the mechanisms of loss nor information on the time course of relaxed selection. Some antipredator behaviours are experience-dependent: experience with predators is required for their proper performance. In these cases, antipredator behaviour is lost after only a single generation of isolation, but it should be able to be regained following exposure to predators. Other behaviours may be more 'hard-wired'. The evolutionary loss of antipredator behaviour may occur over as few as several generations, but behaviours may also persist for many thousands of years of predator-free living. Location Australia and New Zealand. Methods I discuss the results of a series of studies designed to document the mechanisms and time course of relaxed selection for antipredator behaviour in macropodid marsupials. Controlled studies of visual, acoustic and olfactory predator recognition, as well as field studies of antipredator vigilance focused on several species of kangaroos and wallabies. Results Visual predator recognition may be retained following 9500 years of relaxed selection, but olfactory and acoustic predator recognition may have to be learned. Insular populations allow humans to approach closer before fleeing than mainland

animals. Insular species may retain 'group size effects' - the ability to seek safety in numbers - when they are exposed to any predators. Main conclusions I suggest that the presence of any predators may be an important factor in maintaining functional antipredator behaviour. Managers should pay particular attention as to the source and evolutionary history of their population when planning translocations or reintroductions.

942: *+.141*

Aim We studied dynamics of four populations of New Zealand forest birds for 5-9 years after reintroduction to islands. We primarily aimed to predict whether these populations were viable, and what, if any, management was needed to maintain them. However, the small scale of these islands also provided an opportunity to study density-dependent population growth over a short time frame. Location We studied New Zealand robin (toutouwai, Petroica australis) and stitchbird (hihi, Notiomystis cincta) populations reintroduced to Tiritiri Matangi, a 220-ha offshore island near Auckland, and saddleback (tieke, Philesturnus carunculatus) and stitchbird populations reintroduced to Mokoia, a 135-ha island in Lake Rotorua. These islands are free of mammalian predators, but have highly modified habitat following clearing and regeneration. Methods We closely monitored each population, individually marking all or most of the birds and in some cases experimentally manipulated population density or food supply. We used model selection procedures to understand factors affecting survival, fecundity and dispersal, and developed stochastic simulation models. Results The Tiritiri Matangi robin and Mokoia saddleback populations grew without management and appear to be viable. Both showed strong evidence of density-dependent growth, with fecundity (saddlebacks) and juvenile survival (both populations) declining with increasing density. Neither stitchbird population appears viable without management and supplementation experiments showed reproduction and/or survival to be limited by food supply. The Tiritiri Matangi population appears viable as long as supplementary feeding continues. However, the Mokoia population has a high mortality rate regardless of supplementary feeding, resulting in tenuous viability even with intensive management. Mokoia stitchbirds suffer from infection by Aspergillus fumigatus, a pathogenic fungus that is prevalent in highly modified habitats and more abundant on Mokoia than Tiritiri Matangi. Main conclusions Some forest birds can thrive in regenerating forest on islands and strong evidence of density dependence can be detected in such populations in as little as 5 years. This allows density-dependent models to be developed, providing guidance when island populations are harvested for further translocations. Other species are limited by food supply in regenerating environments, a problem potentially overcome by management. However, prevalence of A. fumigatus may render highly modified environments uninhabitable by some species regardless of management.

943: *+.071*

Aim Success with eradicating invasive species from islands around New Zealand raises the prospect of reversing the loss of species by restoring biotic communities on modified islands. I seek to identify methods that can be used to clarify restoration targets on Korapuki Island, which was modified by introduced mammals until 1987. Location Korapuki, Green and Middle islands, Mercury Islands, north-eastern New Zealand. Methods I describe methods of identifying restoration targets by using biogeographical frameworks, and benchmark systems based on neighbouring unmodified islands. Methods that can help identify directions and rates of change are also investigated. Results The benchmark islands (Middle and Green) have complex forest systems with a distinctive component of large, flightless invertebrates, dense populations of tuatara Sphenodon punctatus (Gray), dense and diverse populations of lizards and dense populations of burrowing seabirds. The restoration island (Korapuki) has simplified forest systems, few large

invertebrates, no S. punctatus, a depauperate fauna of lizards and scattered populations of seabirds. These differences are consistent with the recorded effects of rabbits Oryctolagus cuniculus (L.) and Pacific rats Rattus exulans (Peale) removed from Korapuki Island in 1986-87. Three restoration scenarios are described and restoration targets identified. Time scales for natural succession are likely to be influenced by a long-lived colonizing tree species that now predominate over much of the island. The rate of expansion of reintroduced animals is influenced by the very low productivity of many of the species apparently extirpated by introduced mammals. Main conclusions Ecological restoration of island ecosystems has been likened to reconstituting the ambiguous because of conceptual and practical difficulties. Goals for restoration of island systems are often dependent on value judgements. Biological outcomes or targets can be clarified by the use of unmodified neighbouring islands as benchmarks. However, successional pathways on the restored island may not converge with the benchmarks because of environmental differences between sites.

944: ***+.256***

We develop a stochastic model of population viability which explicitly links demography and genetics in order to examine the consequences for extinction dynamics of different levels of heritable fitness variance within a population. We particularly focus on situations in which a local small population is artificially built with individuals that were taken from several large source populations. Our results suggest that different levels of fitness variability within a population (due to partially recessive deleterious alleles rather than local adaptation) have a large influence on its viability. Moreover, the optimal level of fitness variance for maximizing population persistence is a function of the species life-cycle. Two mechanisms with opposite effects are mainly responsible for the different patterns of extinction obtained depending on the life-cycle, (1) purging of deleterious alleles, (2) demographic stochasticity. For high growth rate or long-lived species, a high fitness variance decreases short-term viability and increases long-term viability. In contrast, for other cases, a high fitness variance increases both short- and long-term viability.

945: ***+.024***

Many species modify their behaviour in response to the scents of their predators, but species or populations living without predators may lose such abilities. This loss has been suggested to be irreversible, and to constitute a significant hurdle in restoring historical ecosystems. Olfactory predator recognition was studied in two macropodid marsupials - the tammar wallaby (Macropus eugenii) and the red-necked pademelon (Thylogale thetis). Both species are in the 'critical weight range' of Australian native mammals that have been negatively affected by the introduction of novel predators since European settlement. Predator-naive animals were tested by exposing subjects simultaneously to two feeders with either a predator or a herbivore faecal or urine sample beneath the food tray. The presence of predator olfactory cues beneath the feeder did not affect foraging behaviour or feeder use when compared to control stimuli (herbivore faeces or urine). Previous studies have found that predator-experienced herbivorous marsupials modify their behaviour in the presence of predator scents. In contrast, our studies of predator-naive individuals found no evidence of such selectivity, suggesting that marsupial herbivores may have to learn to modify their behaviour in response to olfactory cues from predators. This implies that the loss of olfactory predator recognition may not be irreversible. Animals translocated from predator-free areas could potentially be trained to recognise the smells of their predators.

946: ***-.109***

Progressive diebacks of outer canopy branchlets of Ceanothus crassifolius were repeatedly observed after rainless periods up to 9 mo in duration in the Santa Monica Mountains of southern California. Mean xylem pressures of branchlets near the end of drought were as low as -11.2 MPa (N = 22) with a mean of about 60 dead branchlets per shrub. Inoculation (N = 15) with three species of fungi previously isolated from the same population of C. crassifolius did not promote dieback, suggesting that the observed decline was not fungal induced, as had been proposed. Further, at least 50% of healthy-appearing twigs, without symptoms of dieback, contained isolatible endophytic fungi. We used a centrifugal force method to determine the range of xylem pressure causing cavitation (vulnerability curves) for branchlets (N = 12) and roots (N = 16). We combined vulnerability curves with soil texture data (N = 6) into a water transport model that estimated the critical values (P-Lerit) of leaf xylem pressure associated with the loss of water from soil to foliage. Maximum P-Lerit was between -10 and -11 MPa and within the range of minimum measured xylem pressures of branchlets during drought and dieback. Branchlet dieback correlated with seasonal declines in xylem pressure in concert with declining safety margins from hydraulic failure. Symptoms of dieback were duplicated in the field by partially severing stem xylem that normally supplied branchlets with water. Taken together, these results indicate that loss of hydraulic conductance to foliage was the probable cause of the observed dieback in C. crassifolius. Partial dieback of peripheral branchlets, and its attendant reduction in evaporative surface area, may be a last-resort mechanism for whole-plant water conservation and drought survival in this species.

947: ***+.025***

Approximately three million years ago the Isthmus of Panama formed an impenetrable land barrier between the tropical eastern Pacific Ocean and the tropical western Atlantic Ocean. Since this time, isolated geminate species have evolved from once contiguous populations, either side of the barrier. One such organism whose distribution is divided by the Isthmus is the tropical brittlestar Ophiactis savignyi, once suggested to be the most common brittlestar in the world. Rather than showing a genetic pattern consistent with a history of isolation, we show that this species has recently dispersed between the Pacific Ocean and the western Atlantic Ocean. This conclusion is based upon a phylogenetic analysis using sequences of the COI mitochondrial DNA gene from these populations. Identical haplotypes between oceans, and a genetic signature of population expansion, provide compelling evidence that the western Atlantic contains at least one cluster of haplotypes recently derived from the Indo-Pacific. Inadvertent human-aided translocation of individuals, presumably in ballast water or fouling communities, is strongly implicated as a mechanism for dispersal between oceans. We believe that cryptic marine invasions are likely to be common and our awareness of them will rapidly increase as systematic and phylogeographic knowledge of marine taxa grow.

948: ***+.268***

The IUCN/SSC Re-introduction Specialist Group: Guidelines for Nonhuman Primate Re-introductions is intended as a guide to re-introduction programmes. The priority has been to develop standards that are of direct, practical assistance to those planning, approving, or implementing reintroductions. The primary audience of these guidelines is the re-introduction practitioner. Because re-introduction projects are often restricted by location, resources, and other limitations, this document is meant as a "best-practice" model, or an ideal code of conduct. Re-introduction managers are strongly encouraged to use this document as their principal guide to primate reintroductions. Each re-introduction project should develop written guidelines that apply specifically to its taxon, region, legal structure, etc. These customised documents should be

updated over time and eventually result in a re-introduction manual for the taxon of interest. They should also directly relate to this existing document, so these guidelines can be regularly updated with new information and techniques. Guidelines for Nonhuman Primate Re-introductions covers the main steps of a re-introduction effort. The steps are listed in a suggested order of execution, although some steps overlap with one another (see "Basic Principles of Reintroductions," below). It is realised that many projects have been operating for some time, so managers of these projects should attempt to integrate the guidelines as soon as possible into their current operating procedures and protocol. Before initiating any re-introduction project, managers mustclearly define why that project is needed and conduct a rapid overall assessment to ensure key requirements, such as habitat suitability, can likely be met. The main goal of any re-introduction effort should be to re-establish selfsustaining populations of primates in the wild and to maintain the viability of those populations. Although exceptions to this, such as trial re-introductions of common species and rescue/welfare releases, should also adhere to these guidelines as much as possible, such projects are notconsidered true re-introductions or conservation approaches and are not specifically covered in these guidelines. Re-introduction practitioners are strongly encouraged to contact the IUCN/SSC Re-introduction Specialist Group (RSG) and present and discuss their re-introduction proposals and results (see Annex III, Page 54). As a result, a network of contacts can be developed and information from various projects shared. Note that details regarding the care of animals held in captivity prior to release, such as enclosure enrichment, are not specifically covered in these guidelines. However, where appropriate, important points regarding these topics will be noted.

949: ***+.125***

Biodiversity provides to the humankind enormous direct economic benefits and an array of indirect essential services through natural ecosystems. Biodiversity is essential for the ecosystem function and stability; several hypotheses are being tested for the relationship between biodiversity and ecosystem function. Biodiversity at the global scale is a balance between the rate of speciation and the rate of extinction. At a regional level it is a balance between the rates of immigration/invasion and that of local/regional extinction. The current rates of extinction are 1000-10000 times higher than the background rate of 10-7 species/species year inferred from fossil record. Today we seem to be losing two to five species per hour from tropical forests alone. This amounts to a loss of 16 m populations per year or 1800 populations per hour. Biodiversity is not uniformly distributed on the earth. There are broad global patterns, and regional and local concentrations of biodiversity. About 44% of vascular plants and 35% of vertebrates are concentrated as endemic species in 25 hotspots, which account for only 1.4% of the global land area. There are several theories to explain the nonuniform distribution of biodiversity. Biodiversity can be assessed at genetic, population, species, ecosystem and landscape levels, at three spatial scales, viz. local, regional, and global. The strategies to conserve biodiversity include establishment of protected area network and corridors with emphasis on appropriate levels of management, reduction of anthropogenic pressure on natural population by cultivating them elsewhere, programmes of augmentation, reintroduction and introduction of target taxa, and in situ techniques such as establishing botanical and zoological gardens, and banks of pollen, seed, tissue culture, DNA, etc.

950: ***+.221***

Although New Zealand's native fauna shares a Gondwana origin with that of Australia, there are major differences between our countries. The near-absence of land mammals and the restricted biodiversity and habitat range of New Zealand, contrast with the species-rich fauna and habitat

variety of Australia. Both countries share an unenviable extinction record, particularly birds in New Zealand and mammals in Australia. Introduced mammals, often interacting with habitat destruction, have frequently been responsible for these losses in New Zealand. In some places, entire vertebrate foraging guilds have disappeared. On the mainland, control of introduced mammals has had limited success but a steadily increasing number of islands have been cleared of alien mammals. This has created new opportunities for translocating threatened species of native vertebrates and invertebrates to pest-free islands. It has also created options for substituting an ecologically similar species for one that is extinct, thus potentially achieving a more comprehensive restoration. Recent progress with island restoration has stimulated a "mainland island" strategy involving simultaneous intensive control of several pest mammals within a limited area that is then used to re-establish viable populations of threatened species. Examples are given to illustrate these conservation actions.

951: ***+.038***

We describe the isolation, development and application of seven microsatellite loci in the eastern wild turkey, Meleagris gallopavo silvestris, as well as their amplification and levels of polymorphism in the domestic turkey. The number of alleles per locus ranged from 5 to 15 and average heterozygosity was high for almost all loci. Domestic turkeys showed significantly reduced numbers of alleles per locus and overall heterozygosities when compared to eastern wild turkeys. The high variability in these markers should provide the level of resolution required to continue studies of wild turkey population genetics.

952: ***-.035***

Eight polymorphic microsatellite loci have been characterized for the endangered St Lucia Whiptail Lizard Cnemidophorus vanzoi. Endemic to two small islets, Maria Major and Maria Minor, off the coast of St Lucia, Lesser Antilles, the world population is estimated at < 1000 individuals. However, representatives of the systematically complex genus Cnemidophorus, containing sexual species and parthenogenetic allopolyploids, are distributed widely in North, Central and South America, and on several islands in the Caribbean Sea. These microsatellite markers are being used to monitor the genetic structure of a population of St Lucia Whiptails, recently founded through translocation, on the nearby Praslin Island.

954: ***+.179***

Artificial breeding for endangered species and subsequent reintroduction are often implemented as conservation strategies, but the reliability of the measures in avoiding the extinction of wild populations is questionable. In this study, we demonstrated reduced genetic variability in a farmed population of the Chinese sucker (Myxocyrinus asiaticus) by DNA fingerprinting. Direct parameters (genetic variability, hypervariable loci and heterozygosity) and indirect parameters (band-sharing coefficient and allelic frequency) revealed that artificial breeding of this species caused the reduction of genetic diversity below historic levels, which predicts reduced individual fitness and lower population adaptability than in historic individuals. As a result, conservation efforts, including the introduction of parent fish from natural waters into farmed populations, are needed to improve the genetic structure of farmed breeding stock and to maximize chances for persistence of the whole species after subsequent reintroduction of farmed individuals.

955: ***+.056***

Approximately 440 base pairs (bp) of the mitochondrial 16S ribosomal ribose nucleic acid (rRNA) coding region were sequenced from 13 marron, Cherax tenuimanus (Smith), samples from six locations and samples of two additional Cherax species from Western Australia. The results indicated that, with the exception of the Margaret River, no variation was found within or between marron populations. In contrast, marron from the Margaret River were found to be polymorphic for two divergent haplotypes (2.76% divergence). These findings were consistent with allozyme data that highlight the general lack of genetic variability within and between populations of this species apart from the Margaret River stocks. The genetic polymorphisms in the Margaret River stocks contrasted with earlier studies and indicated the recent translocation and mixing of genetically differentiated stocks within this river system. The implications of these findings for the conservation and management of genetic diversity within marron are discussed.

956: ***+.217***

The world's fish species are under threat from habitat degradation and over-exploitation. In many instances, attempts to bolster stocks have been made by rearing fish in hatcheries and releasing them into the wild. Fisheries restocking programmes have primarily headed these attempts. However, a substantial number of endangered species recovery programmes also rely on the release of hatchery-reared individuals to ensure long-term population viability. Fisheries scientists have known about the behavioural deficits displayed by hatchery-reared fish and the resultant poor survival rates in the wild for over a century. Whilst there remain considerable gaps in our knowledge about the exact causes of post-release mortality, or their relative contributions, it is clear that significant improvements could be made by rethinking the ways in which hatchery fish are reared, prepared for release and eventually liberated. We emphasize that the focus of fisheries research must now shift from husbandry to improving post-release behavioural performance. In this paper we take a leaf out of the conservation biology literature, paying particular attention to the recent developments in reintroduction biology. Conservation reintroduction techniques including environmental enrichment, life-skills training, and soft release protocols are reviewed and we reflect on their application to fisheries restocking programmes. It emerges that many of the methods examined could be implemented by hatcheries with relative ease and could potentially provide large increases in the probability of survival of hatchery-reared fish. Several of the necessary measures need not be time-consuming or expensive and many could be applied at the hatchery level without any further experimentation.

957: ***+.132***

The reintroduction of anadromous alewives, Alosa pseudoharengus, to their historic habitats in the inland waters of the United States and Canada, has prompted concerns about possible interactions with a popular sport fish, white perch, Morone americana. Both species are now widely distributed in northeastern North America. Diets of white perch in Lake George, Maine, U. S. A., where alewives were absent, were monitored and compared with those of white perch populations that were sympatric with anadromous alewives in two coastal Maine lakes, Biscay Pond and North Pond. In the presence of introduced alewives, the diet of adult white perch became almost exclusively juvenile alewives by late summer in ponds where both species were present. White perch that were sympatric with alewives were more piscivorus than were Lake George white perch, which primarily consumed Cladocera. Not only were alewives the principal prey item in the diet of white perch in Biscay and North ponds, but adult alewives were largely cannibalistic by August. Thus, success of reintroducing anadromous alewives in waters containing white perch may be negatively impacted by predation as well as cannibalism.

958: *-.028*

Many interior populations of cutthroat trout Oncorhynchus clarki are at increased risk for extinction because of small population size and the limited length of habitable streams. Further, many of these streams cannot be altered to provide adequate conditions for population persistence. I constructed a stage-based stochastic population matrix model to (1) evaluate reintroduction strategies for streams capable of population restoration and (2) evaluate the feasibility of maintaining populations through periodic supplemental stocking. In general, stocking adults reduced extinction risk more than did stocking comparable numbers of age-0 fish, subadults, or subadults plus adults. Moreover, the greater the numbers of fish stocked, the greater the decrease in extinction risk. When reintroducing populations for restoration, stocking at least 250 adults (10% of the carrying capacity) in a one-time reintroduction event was as effective in achieving population persistence as stocking multiple times over several years. In contrast, the use of age-0 fish or subadults required either repeated stocking over multiple years or adding large numbers of fish relative to the carrying capacity. The persistence of restored populations did not differ substantially between the multiple-year stocking strategies when fish were added during years 1, 2, and 3 or during years 1, 3, and 5. Simulations of supplemental stocking showed that even very small populations were persistent when stocked with as few as 10 adults every 10-20 years. The results of the simulation model indicate that supplementing populations may be an effective conservation strategy to achieve long-term population persistence if no other options for expanding population capacity exist.

959: *+.021*

Relocation programs have restored elk (Cervus elaphus) to portions of its vast historical range. We examine the consequences of these relocation programs by assessing variation at 10 microsatellite loci in three elk herds, a source herd (Yellowstone National Park), a large herd reintroduced from Yellowstone (Custer State Park) and a bottlenecked herd reintroduced from both Yellowstone and Custer (the Pennsylvania herd). Observed single locus heterozygosities ranged from 0.000 to 0.739. Multi-locus heterozygosities ranged from 0.222 to 0.589. Although significant differences were detected among all three herds, the Yellowstone National Park and Custer State Park herds possessed similar levels of variation and heterozygosity, and the genetic distance between these two herds was small. The Pennsylvania herd, on the other hand, experienced a 61.5% decrease in heterozygosity relative to its source herds, possessed no unique and few rare alleles, and the genetic distances between the Pennsylvania herd and its sources were large. Simulations were performed to identify bottleneck scenarios in agreement with levels of variation in the Pennsylvania herd. Our data confirm that the rate of population growth post-relocation may have important genetic consequences and indicate that theoretical predictions regarding the maintenance of genetic variation during relocation events must be viewed with caution when small numbers of a polygynous species are released.

960: *+.135*

The first hypervariable fragment (HVI) of the mitochondrial DNA control region was sequenced in 90 individuals of the European roe deer (Capreolus capreolus) from the Alps, central Italy and Spain. Pooling these data with 70 published sequences from several European regions, we were able to identify patterns of divergence within the Italian peninsula, and in Europe in general. The results we obtained can be summarized as follows. First, the genetic structure of European roe deer populations is substantial (Phi(ST) values around 0.6). Second, the divergence between some central Italian populations, the Alpine group (which is genetically close to the French, the Spanish

and the Norwegian samples) and the Eastern European populations seems to reflect Upper Pleistocene subdivisions, possibly related to three southern European refugia. Third, a different group of central Italian individuals probably diverged more recently from the Alpine group, and their attribution to the subspecies C. c. italicus does not appear justified. Fourth, the analysis of mitochondrial DNA in the roe deer can be used to identify recently reintroduced animals in the western Alps which clearly cluster within the Eastern European group, thus providing an important tool for conservation and management strategies for this species.

961: *-.039*

All remaining populations of the wild Asiatic buffalo are known to cross breed with domestic and feral forms living in the vicinity of the handful of reserves in which the species is found. Censuses of these animals done in various places in India and Nepal have been criticized because researchers have used various criteria to differentiate wild buffalo versus feral backcrosses, thus rendering population estimates unreliable. Due to the highly endangered status of the wild buffalo, there is an urgent need for a set of criteria that can be applied more broadly to distinguish wild from feral forms. This paper describes the phenotypic and behavioural characteristics used to census wild buffalo and feral backcrosses in Kosi Tappu Wildlife Reserve, Nepal, which contains the last Nepalese population of the species. It is hoped that other researchers in the region may find these field characteristics useful in identifying wild stocks in South and Southeast Asia, where they are still thought to occur. Ideally, in all cases, detailed genetic studies are needed to plan managerial interventions such as translocation projects. Given the expense of such studies, it is suggested that translocations can be planned using consistent field identification criteria, until such time as more detailed genetic work is done.

962: *+.065*

The Carpentarian rock-rat (Zyzomys palatalis) is a critically endangered endemic rodent known from only four sandstone gorges in the southeast Gulf of Carpentaria, Northern Territory, Australia. These gorges harbour thickets of monsoon rainforest and broadleaf woodland, surrounded by a Eucalypt savanna matrix. The long-term persistence of Z. palatalis is threatened by altered fire regimes, grazing by feral animals and stock, weed intrusion, and the stochastic hazards associated with small, fragmented populations. To assess the relative importance of these threats and develop practical management options, a population and habitat simulation model was developed, based on the best existing data. Population viability was predicted to be highly sensitive to the frequency of hot, late dry-season fires. Progressive habitat degradation (due predominantly to intense late dry-season fires) is likely to substantially reduce population size and lead to the probable extinction of the species within the next 100 years. The most effective management strategy to counteract this threat would be regular, controlled, fuel reduction bums in the vegetation around the gorge entrances during the early dry season. Establishing a new population (through translocation of captive-bred individuals) would not appreciably reduce extinction risk, but could provide valuable additional data on the impact of threats, if conducted as an adaptive management experiment. (C) 2002 Published by Elsevier Science Ltd.

963: *+.130*

New Zealand Robins (Petroica australis) were reintroduced to Tiritiri Matangi, a highly modified island undergoing an extensive revegetation program. The island had only 13 ha of fragmented forest likely to be suitable for robins. We wished to determine whether the population was likely to persist until additional habitat became available through maturation of the revegetation and,

therefore, whether it was sensible to reintroduce this species early in the restoration program. We used an information-theoretic approach to model survival, fecundity, sex ratio, and juvenile dispersal for this population, based on data collected in the first 6 years after reintroduction. This involved nominating a limited set of plausible models for each process and then selecting the models that best explained the data based on Akaike's information criterion. We used mark-recapture analysis on survey data to model survival and general linear modeling on fecundity data. The best models were as follows: (1) juvenile survival declined as population size increased, whereas adult survival was constant among years; (2) fecundity was substantially lower for females in their first year after translocation and varied among forest patches, but did not vary among age classes or years; (3) the expected sex ratio of recruits was 50:50; (4) the distribution of recruits depended on the number of existing residents in patches and patch sizes, but not on patch isolation. We used VORTEX to simulate the dynamics of the population based on these models and associated parameter estimates. The population was predicted to have negligible chance of extinction over several decades under current conditions, and this prediction was not sensitive to uncertainty in parameter estimates or model structure. Maturation of the planted matrix should allow the population to expand to several hundred birds over the next few decades, preventing extreme loss of genetic variation. We therefore conclude that the early reintroduction of this species was reasonable, despite the limited habitat available at the time. Our results show that it is critical to obtain data for the first few years after reintroduction if habitat is likely to be limited, because it is otherwise impossible to detect density-dependent processes critical to population viability. Results also show that the first year after reintroduction may be misleading if considered alone.

964: ***+.186***

Grevillea scapigera is one of the world's rarest plant species, currently known from only five plants in the wild. In 1995, 10 plants were selected from the 47 plants known at the time to act as genetically representative founders for translocation into secure sites. Ramets were micropropagated and introduced into one of these secure sites (Corrigin) in 1996, 1997, and 1998. By late 1998, 266 plants had been successfully translocated and were producing large numbers of seeds. With the development of an artificial seed-germination technique and because of an absence of seed germination in situ, seed was collected from these plants and germinated ex situ, and 161 seedlings were returned to the field site in winter 1999. We used the DNA fingerprinting technique of amplified fragment-length polymorphism (AFLP) to (1) assess the genetic fidelity of the clones through the propagation process, (2) contrast genetic variation and average genetic similarities of the F1s to their parents to assess genetic decline, and (3) assign paternity to the reintroduced seeds to assess the reproductive success of each clone. We found that 8 clones, not 10, were present in the translocated population, 54% of all plants were a single clone, and the F1s were on average 22% more inbred and 20% less heterozygous than their parents, largely because 85% of all seeds were the product of only 4 clones. Ultimately, effective population size (N-e) of the founding population was approximately two. Our results highlight the difficulty of maintaining genetic fidelity through a large translocation program. More generally, rapid genetic decline may be a feature of many translocated populations when N-e is small, which may ultimately threaten their long-term survival. Strategies to reverse this genetic decline include equalizing founder numbers, adding new genotypes when discovered, optimizing genetic structure and plant density to promote multiple siring and reduce kinship, promoting natural seed germination in situ rather than artificially germinating seeds ex situ, and creating a metapopulation of numerous translocated populations to restore historical distribution patterns and processes.

965: ***+.145***

Translocations of reptiles and amphibians have been questioned as a conservation tool because they generally have not been successful. However, translocation is the only method by which many species can be restored to parts of their former range, which increases their conservation security. Tuatara (Sphenodon spp.), sole surviving members of the reptilian Order Sphenodontia, are restricted to offshore islands of New Zealand. Dispersal into former habitat on the mainland or other islands, following removal of the causes of extinction, is unlikely. Captive rearing of reptile hatchlings from eggs collected in the wild to produce founders for new populations is also a conservation technique that has had mixed success. However, captive incubation and rearing are techniques that allow large numbers of tuatara to be produced to start new populations without detrimental effects on small source populations. We document the first contemporary translocation of S. guntheri , providing details on selection of release sites and founders. Release sites were chosen on a rodent-free island that provided a variety of habitats for tuatara as well as abundant food and shelter. The release propagule comprised 18 wild-caught adults and 50 captive-reared juveniles. Four separate release sites were established to separate adults from juveniles during the establishment phase. Five years of monitoring indicate that at least 57% of tuatara survived translocation and that reproduction has occurred on the new island. All tuatara increased in weight and length, demonstrating that tuatara have an indeterminate growth pattern. The 56% of recaptured juveniles represented a cross section of the sizes released. We evaluated the use of head-started juveniles as founders, based on survival and growth data. Because tuatara are long-lived, late-maturing reptiles with slow reproduction, establishment of a self-sustaining population will take decades of monitoring to confirm.

966: *+.179*

The range of the green and golden bell frog (Litoria aurea) has undergone a widespread yet unexplained contraction in southeastern Australia since the mid 1970s, and the species is now listed as endangered in the New South Wales Threatened Species Conservation Act 1995 and as vulnerable in the federal Environmental Protection and Biodiversity Conservation Act 2000. Although the cause of the range contraction is unknown, remedial action is necessary to ensure the long-term viability of remaining populations. This can include habitat creation around existing populations and reintroduction projects. However, the habitat requirements of this species are poorly understood. We examined the habitat at 43 waterbodies on Kooragang Island, Australia, with the aim of producing a set of variables which could differentiate between those occupied by L. aurea and those where the species was absent. L. aurea was found in 21 waterbodies and breeding was confirmed in four. Aspects of waterbody physiognomy and water chemistry could not explain L. altrea distribution. The plant species Juncus kraussii, Schoenoplectus litoralis and Sporobolus virginicus were significant predictors of the presence of L. aurea, as was the diversity of vegetation on the banks of waterbodies. The presence of the eastern mosquitofish (Gamhusia holbrooki) did not influence waterbody occupancy and tadpoles were found co-existing with this introduced fish. The distribution of waterbodies occupied by L. aurea was aggregated. A waterbody was more likely to be occupied if neighbouring waterbodies within 50 m were also occupied. Habitat creation projects for this species are most likely to succeed if wetland habitat is created in proximity to an existing population. (C) 2002 Elsevier Science Ltd. All rights reserved.

967: *-.067*

In 1998 the protected Population of Perameles gunnii (eastern barred bandicoot) at Woodlands Historic Park, Victoria, Australia became functionally extinct following removals of individuals for translocation between 1994 and 1996. Population viability analysis and data collected whilst the population was in decline are used to explore the impact that these removals had oil the decline

of the P. gunnii population at Woodlands. Due to some ambiguities in the life history of P. gunnii at Woodlands, two structurally different population models were used. Explicitly including the removal of animals in the analysis indicates that there may be at least a three-fold increase in the risk of quasiextinction due to removals. In some scenarios, over 17% of the trajectories decrease to a level less than or equal to 10 females within the observed time period of the collapse of the population, that is by April 1998. The modelling approach identified a number of critical factors in the decline of the population, such as variation in survival rates. By representing the life history strategy using two alternate models, quantitative statements about the impact that removals had on the population decline at Woodlands are made. While removals probably contributed to the collapse of the population, removals were not the sole cause of population decline. The real reasons for decline remain unknowns however, it is likely to be closely linked to habitat decline, difficult, environmental conditions and predation. If P. gunnii are reintroduced to Woodlands again, then the models presented here may help to develop management strategies to establish a self sustaining Population, as well as exploring translocations options. (C) 2002 Elsevier Science Ltd. All rights reserved.

968: ***+.053***

Logging and poaching have dramatically reduced chimpanzee density and distribution in the Republic of Congo. Most chimpanzee translocations attempted in the past failed because a number of biological and non-biological factors can influence success. Biological considerations include knowledge of behaviour, disease, habitat requirements and genetics. We critically review genetic considerations in pre- and post-release phases of translocations and apply them to a welfare-based chimpanzee release project in the Republic of Congo which aimed to re-establish orphan chimpanzees in the wild with a native chimpanzee population. We analyze genetic diversity and relatedness in released animals and devise a genotyping strategy for monitoring of released individuals and their future offspring. Fifty-nine confiscated chimpanzees from different areas of the Republic of Congo were typed for 20 microsatellites using plucked hair as the DNA source. Genetic diversity was high, with an average expected heterozygosity of 81%, and three to 18 alleles per locus. Between 1996 and 1998, 19 individuals were released, and genetic analysis showed that these are unrelated (mean r +/- jack-knifed SD = -0.014 +/- 0.001). Using F(ST) and population admixture analysis, we identified population structure in wild chimpanzees. For long-term genetic monitoring of released and native chimpanzees, we identified a set of six informative markers, which are easy to score using basic techniques.

969: ***-.034***

All existing sea otter (Enhydra lutris) populations have suffered at least 1, and in some cases 2, population bottlenecks. The 1st occurred during the 18th and 19th centuries as a result of commercial hunting that eliminated sea otters from much their native range and reduced surviving populations to small remnants. The 2nd bottleneck occurred when small numbers of otters were reintroduced, via translocation, to areas where the species had been eliminated. We examined genetic variation at 7 microsatellite loci and the mitochondrial DNA (mtDNA) control region in 3 remnant populations, Amchitka Island (Aleutian Islands, Alaska), central coastal California, and Prince William Sound (Alaska), and in 2 reintroduced populations, southeast Alaska and Washington, that were founded with transplants from Amchitka, and in the case of southeast Alaska, individuals from Prince William Sound as well. We found no evidence of reduced genetic diversity in translocated populations. Average expected microsatellite heterozygosities (H,) were similar in all populations (range, 0.40-0.47), and mtDNA haplotype diversities were higher in reintroduced populations (0.51 for both Washington and southeast Alaska) than in remnant

populations ((X) over bar = 0.35; range, 0.18-0.45). The levels of genetic diversity we observed within sea otter populations were relatively low when compared with other mammals and are thought to be the result of fur trade exploitation.

970: *+.129*

Tidal flow to salt marshes throughout the northeastern United States is often restricted by roads, dikes, impoundments, and inadequately sized culverts or bridge openings, resulting in altered ecological structure and function. In this study we evaluated the response of vegetation and nekton (fishes and decapod crustaceans) to restoration of full tidal flow to a portion of the Sachuest Point salt marsh, Middletown, Rhode Island. A before, after, control, impact study design was used, including evaluations of the tide-restricted marsh, the same marsh after reintroduction of tidal flow (i.e., tide-restored marsh), and an unrestricted control marsh. Before tidal restoration vegetation of the 3.7-ha tide-restricted marsh was dominated by Phragmites australis and was significantly different from the adjacent 6.3-ha Spartina -dominated unrestricted control marsh (analysis of similarities randomization test, $p < 0.001$). After one growing season vegetation of the tide-restored marsh had changed from its pre-restoration condition (analysis of similarities randomization test, $p < 0.005$). Although not similar to the unrestricted control marsh, Spartina patens and S. alterniflora abundance increased and abundance and height of Phragmites significantly declined, suggesting a convergence toward typical New England salt marsh vegetation. Before restoration shallow water habitat (creeks and pools) of the unrestricted control marsh supported a greater density of nekton compared with the tide-restricted marsh (analysis of variance, $p < 0.001$), but after one season of restored tidal flow nekton density was equivalent. A similar trend was documented for nekton species richness. Nekton density and species richness from marsh surface samples were similar between the tide-restored marsh and unrestricted control marsh. Fundulus heteroclitus and Palaemonetes pugio were the numerically dominant fish and decapod species in all sampled habitats. This study provides an example of a quantitative approach for assessing the response of vegetation and nekton to tidal restoration.

971: *+.188*

Overhunting reduced Eurasian Beaver (Castor fiber) populations to c. 1200 animals, in eight isolated populations, around the end of the 19th century. Protection, natural spread and reintroductions led to a powerful recovery in both range and populations during the 20th century, which continues at a rapid pace. The minimum population estimate is 593 000. There are also c . 12 500 North American Beaver (C. canadensis) established in Finland and Russian Karelia; however, other populations of C. canadensis introduced in Austria, Poland and France ap-pear to be extinct. Castor fiber is now established throughout Europe with the exception of the British Isles, Iberia, Italy and the southern Balkans; reintroductions are continuing. Considerable further expansion in range and population, especially in western Europe and the lower Danube basin, can be expected. If current trends continue, C. fiber will, within a few decades, be a fairly common mammal in much of Europe. Following initial recolonization, populations typically show a pattern of rapid range extension within a watershed, followed only later by rapid population growth, and a barrier effect of watershed divides, which can be strongly isolating where physical or habitat barriers (such as mountains or intensive farmland) intrude between watersheds. Management of beaver distribution should therefore operate at the watershed scale, except where large human-made dams form significant bar-riers to spread. The period of rapid population increase, if unchecked, leads to a phase of population decline as marginal habitats are occupied and exhausted. This coincides with a peak in conflicts with human land-use interests. A regulated hunting take of healthy beaver populations is recommended as the optimal management regime in managed

landscapes. Early provision of interpretation and public viewing opportunities has been a feature of several recent reintroductions. This provides a benefit to the local economy through wildlife tourism, and helps foster positive attitudes to beavers.

972: ***-.053***

In 1993 a reintroduction project for the Eurasian otter (Lutra lutra) was initiated in northeastern Spain (Girona, Catalonia) to restore extirpated populations. Between 1996 and 2000, 43 otters were captured from southwestern and northern Spain and from Portugal with modified foot-hold traps and transported to Barcelona Zoo. Lesions produced by capture were classified into four categories of increasing severity. Thirty four (79%) animals had category 1, three (7%) had category 11, five (12%) bad category 111, and only one (2%) had category IV injuries. During captivity five (11%) animals died, including one from a precapture problem. Radiotransmitter devices were implanted i.p. into 36 otters to monitor postrelease movement and survival. At least three radio-implanted otters have bred successfully in Girona province, Catalonia, after release in that area.

973: ***+.024***

The authors monitored postrelease survival rates and dispersal of radiocollared raccoons (Procyon lotor) that were trapped as nuisance wildlife in suburban Chicago and translocated to a rural forest preserve (translocated urban); trapped in another wooded area and translocated to the forest preserve (translocated rural); and trapped and released in the forest preserve that served as the release site for the translocations (resident). The authors detected no difference in survival rates among the three treatment groups ($P < 0.05$), although their proximity to the release site and their denning locations differed. Because translocated raccoons survived well, translocation could be an effective way to supplement depleted or re-establish extirpated populations of this species. However, translocating large numbers of raccoons for aimal damage control could cause problems for other wildlife and humans near release sites, and translocated animals could serve as vectors for wildlife diseases during zoonotic outbreaks.

974: ***+.008***

The fate of radio-tagged hatchery-reared salmon smolts (Salmo salar L.) was investigated in the tailrace of a hydropower station in the Danish River Gudenaa during 2 years. Seventeen and 27 smolts were tagged and released in late May 1996 and during April 1999, respectively. Out of the total of 44 smolts, only two were recorded to leave the river and enter the estuary. In both years of study, electrofishing was used to sample tagged smolts. In 1996, these attempts were unsuccessful, while in 1999, 19 (70% of total) transmitters were retrieved in the stomachs of pikeperch (Stizostedion lucioperca (L.)) and pike (Esox lucius L.), and in grey heron (Ardea cinerea L.) nests. The present study demonstrates that the physical condition of the watershed, with respect to spawning and rearing, may not be the only crucial determining factors of the success of reintroduction of Atlantic salmon. Hydropower plants not only block the free movement of migratory fish, hence preventing the utilisation of spawning and rearing areas, but also form the basis of areas with increased mortality.

976: ***+.012***

The first record of the trematode Stichorchis subtriquetrus is reported from European beaver (Castor tiber) in the Czech Republic. Detailed redescription is added and is compared by the

available findings on the morphometric variability of this taxon. The presence of this parasite in the Czech territory where the beaver became extinct some 150-200 years ago is a result of reintroduction of the definitive host. The issue of stichorchosis epizootology of beavers is discussed. As to the ecosozoological aspects, law protects the beaver and its specific parasites in the Czech Republic.

977: ***+.132***

To examine habitat conditions associated with abundance of Macrhybopsis hyostoma, 10 seine-hauls were made at each of 24 sites encompassing the major western tributaries of the Arkansas River. All seine hauls were made in habitats that, within each collection locality, were considered most likely to include the species. Each haul was characterized for mean depth, water velocity, and substratum compaction and particle size. Comparison of use versus availability within intervals of these variables demonstrated nonrandom distribution of the species for each variable measured. At locations where the species occurred, usage curves and electivity indexes indicated that both age-groups preferred moderate current velocities (20-40 cm/s) and substrata with high percentages of pea-sized gravel (60-90%) and moderate levels of compaction. A discriminant function based on stream-reaches of occurrence for the species predicted either absence (90% of the seine hauls) or rarity of the species (10% of the hauls) in stream reaches where M. hyostoma or a close relative (M. tetranema) had been extirpated. Based on these results, and in the absence of further knowledge on habitat availability and suitability it seems inadvisable to attempt reintroductions of M. hyostoma into presently unoccupied streams within its historical range in Oklahoma.

978: ***+.028***

Sanctuaries - centres that care for rescued, orphaned or confiscated animals - hold an uneasy position in the conservation community. Commonly, the problem of captive primates is seen as an unfortunate distraction from the real issue of habitat loss and over-hunting, and sanctuaries are categorized as serving welfare purposes only. Frequently, they struggle for funding and are unable to secure the large grants that are directed towards other conservation initiatives. Sanctuaries also have to face welfare problems created when capacities are exceeded and they lack safe, viable habitat for the species they try to protect. The solutions, discussed in this paper, are integrating care for rescued animals with habitat protection and education. Reintroduction efforts, which would otherwise be of conservation benefit, are constrained by the unresolved issues including disease transfer, competition with wild populations, and the interface with ecotourism activities.

979: ***-.154***

State wildlife agencies have translocated thousands of wild turkeys (Meleagris gallopavo) since the 1930s to reestablish this species. Because of threats to the domestic poultry industry and wild birds, screening for selected infectious agents has become routine since the early 1980s. One of the principal sources for Rio Grande wild turkeys (M. gallopavo intermedia) for translocation purposes was the Edwards Plateau of Texas (USA). Unfortunately, turkey abundance has declined in the southern Edwards Plateau since the late 1970s. Surprisingly few studies have addressed wild turkeys in this region, perhaps reflecting its status as the heart of Rio Grande turkey range. We surveyed 70 free-living Rio Grande wild turkeys from Bandera and Kerr counties, Texas, for evidence of exposure to Salmonella typhimurium, S. pullorum, Mycoplasma gallisepticum, M. meleagridis, M. synoviae, Chlamydophila psittaci, and the avian influenza, Newcastle disease, turkey corona, and reticuloendotheliosis viruses. Of these, 80% (56) were seropositive for both M. gallisepticum and M. synoviae on the serum plate antigen test. Ten of these individuals (14% of

total) were positive for M. synoviae by hemagglutination inhibition testing. All other serologic tests were negative. Two adult females sampled in Kerr County, whose body mass was significantly less than that of other adult females trapped in the area, tested positive for reticuloendotheliosis virus (REV) proviral DNA on polymerase chain reaction. Reticuloendotheliosis virus was isolated from one of these individuals. The pathogenesis, transmission, and/or population-level influences of M. gallisepticum, M. synoviae, and REV in Rio Grande wild turkeys deserves further study.

980: *+.297*

Conservation biologists often must make management decisions based on little empirical information. In Germany biologists are concerned that the recovery and reintroduction of Eurasian lynx (Lynx lynx) may fail because the remaining suitable habitat may be insufficient to sustain a viable population. However, no comprehensive study ad dressing this concern has been made that not only considers distribution of suitable habitat, but also connectivity to other populations. The aims of this study were (1) to quantify the amount and location of potentially suitable lynx habitat in. Germany, (2) to estimate the connectivity between patches of suitable habitat, and (3) to evaluate lynx conservation programs. Habitat preferences of lynx were described in a rule-based model based on the availability of forest cover (defined by patch size) and the spatial structure of the habitat. Rules were implemented in a geographic information system to predict locations of suitable habitat. Optimal connections among patches were modeled using a cost-path analysis based on. habitat-specific probabilities of lynx crossing patches. Results indicated wide variation in the size of patches of suitable habitat, with 10 areas each sufficiently large to sustain >20 resident lynxes. Overall, a total of 380 lynxes could be sustained by the 10 areas. Uncertainty analyses of model parameters and assumptions revealed little variation in predicted habitat, primarily because results were constrained by the actual distribution of forest habitat. Our. analyses suggest that lynx reintroduction programs should emphasize large, connected areas and consider broad-scale habitat connectivity in the landscape. Our approach also demonstrates how biologically plausible rules can be applied in conservation to identify areas in which success is most likely, even when few empirical data are available.

981: *+.076*

The spatial scale at which anthropogenic disturbance affects an animal population depends on the degree of philopatry and homing of individual organisms within that population. Even in highly vagile species, local populations may comprise ecologically separate entities if most animals display strong and consistent site attachment. We conducted a mark-recapture study on yellow-lipped sea kraits (Laticauda colubrina) on two small Fijian islands separated by 5.3 km. These snakes forage over many kilometers in the ocean, but return to land to reproduce, slough, and digest their food. Recovery of marked snakes showed that the populations on these two islands were essentially separate. Relocated snakes returned "home" almost immediately after they were released. Growth rates and mean adult body sizes of male sea snakes also differed between the two islands. This high site fidelity means that activities such as resort development or commercial harvesting for the skin trade are likely to have intense localized effects rather than diffuse broad-scale effects on sea snake populations. Our results also call into question the feasibility of plans to reintroduce snake populations to areas where a species has been eliminated by overexploitation.

982: *+.219*

A recent recovery in cirl bunting (Emberiza cirlus) numbers in the UK has been aided by

deployment of prescriptions for environmentally friendly management of farmland under the Countryside Stewardship Scheme. However, the species' range has not expanded and the population is increasingly under threat from habitat loss due to built development. The UK Biodiversity Action Plan calls for re-establishment of populations outside the current range in order to ensure a wider geographical spread of this species. This study examined territory distribution and foraging patterns of cirl buntings in relation to habitat variables in order to inform the process of site protection and site selection for a possible translocation programme. Cirl bunting territories were positively associated with rough grassland, non-linear scrub and habitat richness, and intensive permanent pasture was avoided. Resampling methods showed that rough grassland was used significantly more than improved pasture as a foraging habitat and that both rough grass and cereals were used more than other arable crops. The results emphasise the importance of protection of unimproved pastures in 'green field' sites and will help quantify the potential carrying capacity of candidate `receptor' sites. (C) 2002 Elsevier Science Ltd. All rights reserved.

983: *+.241*

In 1999, we intensively surveyed all suitable habitat on the Mascarene Island of Rodrigues and mapped 334 territories of the threatened endemic Rodrigues fody (Foudia flavicans). In addition, we recorded 58 unpaired males, 85 juveniles, and 100 greybrown-plumaged birds; for a minimum estimated population size of 911 birds. This represents a near 100-fold increase in population size since 1968, which has been achieved in the absence of translocation or taxon-specific management. Birds were generally distributed in direct proportion to the availability of various forest types, but relatively more birds were found in mature, dense forests. Fody density at 10 selected sites, where vegetation surveys were carried out, increased significantly with increasing tree height, canopy cover and tree species diversity. This suggests that habitat management aimed at enhancing Rodrigues fody populations should focus on the protection of existing wooded valleys to allow forest maturation and expansion of afforested areas. (C) 2002 Elsevier Science Ltd. All rights reserved.

984: *+.174*

Reproductive isolation can function as a mechanism to maintain locally adapted gene complexes while decreasing the heterozygosity in distinct populations. As a result, reproductive behaviour should be considered it fundamental factor influencing reproductive isolation. This is of interest to conservation biology when one desires to regulate gene flow between two populations either by creating opportunities for increased dispersal, by relocation of individuals, or by re-introduction of a species to its natural habitat. Reproductive behaviour can also influence the effective population size and the actual population size through the Allee effect. We investigated the reproductive behaviour of individuals from two isolated populations of the red winged grasshopper, Oedipoda germanica, an endangered species in Central Europe. We detail several methods to show how several aspects of the reproductive behaviour of this species interact with the conservation of this species. Foreign males were not disfavoured in mate choice and male body size was also unimportant in mating success. Heterogamic matings were as productive as homogamic matings in terms of total number of eggs per female, egg hatching rate, or nymph survival. Therefore, we suggest that cross-matings of individuals from different populations do not positively influence population size by heterosis effects nor act they negatively in the form of outbreeding depression. We found that female O. germanica were able to store viable sperm for extended periods but egg pods showed a decrease in hatching rate when these females were deprived of additional mating opportunities. Multiple mated females laid more eggs than once-mated females. Hence, females

are capable of founding new Populations even after only one mating but their reproductive output may be reduced. Present-day gene flow between populations of O. germanica probably does not occur. Consequently, there is an urgent need to pursue habitat management and release programmes that maintain current population sizes of this species. With regard to the reproductive behaviour of the red-winged grasshopper, we suggest that relocation programmes release males and female together and at an early adult stage. Also, because female reproductive output increases with mating activity, the initial release should involve excess females. Because males have it shorter lifespan than females a subsequent release of males at a later time may enhance the overall success of local conservation efforts by resulting in more offspring per female. (C) 2002 Elsevier Science Ltd. All rights reserved.

985: *+.080*

The objectives of this study were to determine the distribution and abundance of endangered Oregon chub Oregonichthys crameri, a small floodplain minnow endemic to the Willamette Valley of western Oregon; to describe the fish communities in current and historic chub habitats; and to establish new populations through introductions. Various sampling gears were used, including seines, minnow traps, dip nets, and a gill net. Oregon chub distribution was found to be restricted in comparison with their historical range, whereas nonnative fishes were widespread in the off-channel habitats preferred by Oregon chub. Oregon chub were absent, or low in abundance, when nonnative fishes were present, and several populations declined or were extirpated when their habitats were invaded by nonnative fishes. Isolated habitats with low connectivity supported larger populations of Oregon chub and were less likely to contain nonnative fish species than were habitats with high connectivity. In habitats that supported abundant Oregon chub populations, chub were often the numerically dominant fish species. Results suggest that increasing the connectivity of floodplain habitats in a system where nonnative fishes are widespread may be detrimental to the conservation and recovery of this species.

986: *+.242*

The wetlands around the Dongting Lake were inhabited by Pere David's deer before its extinction in the wild. In preparation to restore a wild Pere David's deer populations in the Dongting Lake region, we inspected the Pere David's deer populations in the Tianezhou Reserve, Beijing Milu Park and Dafeng Milu Nature Reserve. We studied the available wetlands in the Yueyang Municipality, Changde Municipality and Yiyang Municipality, and selected Julinyuan of Hanshou County, Changde Municipality and Jichengyuan of the Huarong County, Yueyang Municipality as a potential reintroduction site for Pere David's deer. We studied the vegetations and sampled above-ground biomass of plant in the two sites. We found the natural environments in Julinyuan and Jichengyuan similar to that of the Tianezhou Reserve. There are three Vegetation Type Groups and vascular plant of 75 families, 189 genera and 264 species in Jichengyuan. The habitat available for reintroduction of Pere David's deer in Jichengyuan is 2000 hectare, which can provide the reintroduced deer with 21 158.4 tons of fresh plants, enough to support 1000 Pere David's deers. The natural vegetation rapidly recovered after the lands were abandoned designated for wetland restoration. There are also three Vegetation Type Groups and vascular plantsof 75 families, 189 genera and more than 200 species in Julinyuan. The habitat available for reintroduction of Pere David's deer in Julinyuan is 1703. 1 hectare, which can provide reintroduced deer with 18 859. 0 tons of fresh plants, enough to support 850 Pere David's deers. Judged by the climate, food resources and environmental carrying capacity, Julinyuan and Jichengyuan are both suitable for reintroduction of Pere David's deer. We also discuss the human-assisted survival strategies for Pere David's deer, such as guarantee living space, diseases

prevention, habitat restoration, and population and habitat monitoring.

987: ***+.055***

Antirrhinum subbaeticum is an endangered species inhabiting fragmented limestone cliffs. In the last 3 years, a drastic population decline has been observed in three of four known populations and the estimated number of surviving individuals is now close to 400. A RAPID study was conducted to evaluate the levels of genetic variation present in this species to improve conservation guidelines. Thity-nine polymorphic products identified 66.1% of the samples by unique RAPID multilocus profiles. A cluster analysis grouped the samples into two broad groups corresponding to northern or southern provenances. AMOVA analysis showed that only 17.7% of the genetic diversity was partitioned within populations. These results are in contrast to data available for other Antirrhinum species, This genetic structure could be explained by the predominant selfing behaviour exhibited by A. subbaeticum as opposed to the allogamy of other congeners. Genetic diversity within populations does not seem to be strongly related to population size and historical factors could be responsible for the very low levels of genetic diversity found in one population. Given the low genetic diversity within populations, it is suggested that an extensive sampling of individuals be made for recovering appropriate levels of the gene pool for ex situ preservation. However, translocation of individuals to the genetically weakened Bogarra population from other sources is not recommended.

988: ***+.238***

We have studied the exotic parrot fauna of Broward County, Florida since July 1999. During this period, 31 species of parrots were observed, with 20 of these not previously known to occur in the county. Twenty-four species (77%) were documented by still or video photographs. Three species are newly reported for Florida but only one of these was photographed. Breeding of 14 species was confirmed during our study. We summarize the population status, distribution, and verifiable evidence of occurrence for all parrots that have been reported in Broward County, including seven species observed prior to, but not during, our study. Populations of Red-crowned Parrots in Broward County may be suitable for reintroduction programs into Mexico, should such an effort be undertaken. Increased monitoring of parrots and other exotic birds in Florida is encouraged.

989: ***+.086***

The Hine's emerald dragonfly (Somatochlora hineana) may be the United States' most endangered species of dragonfly. S. hineana currently inhabits Wisconsin, Michigan, Illinois, and Missouri. It is a very site-specific species, living in calcareous wetlands with slow flowing water where larvae can spend up to four years growing and developing. Human alterations of the species' limited habitat, such as filling in and fragmenting wetlands with development of roads and ditches, have contributed to the dragonfly's status. The species is federally listed as endangered, and a recovery plan was completed and approved by the U.S. Fish and Wildlife Service in September 2001. Conservation efforts include the management of extant populations, research, searches for additional populations, an education program, a reintroduction program, and tracking of the recovery progress.

990: ***+.070***

Capsule The first co-ordinated Red Kite survey across Britain since the reintroduction programme began in 1989, yields 430 breeding pairs. Aims To estimate the current size and extent of the

British breeding population. Methods A complete census of the populations in the East Midlands, Yorkshire, central and north Scotland was undertaken, while in Wales and the Chilterns, populations were surveyed using a stratified sample of tetrads. Breeding and territorial pairs were identified. Results The survey indicated that there were 430 breeding pairs in Britain (95% Cls, 372-490). There were 259 breeding pairs in Wales (95% Cls, 200-318) and 109 (95% Cis, 96-124) in the Chilterns. Elsewhere, 16 breeding pairs were located in the East Midlands, three in Yorkshire, seven in central Scotland and 33 in north Scotland. An additional three pairs were recorded in southern England, away from the main population centres. Conclusion The 2000 survey provides a baseline against which to measure future changes in Red Kite populations in Britain, using standard, repeatable methods.

991: *+.192*

Australian mammals have one of the world's worst records of recent extinctions. A number of studies have demonstrated that red foxes (Vulpes vulpes) have a profound effect on the population biology of some species. However, not all species exposed to fox predation have declined. We studied the antipredator behaviour of a species that has not declined - the red-necked pademelon (Thylogale thetis), and contrasted it with previous studies on a species that has declined - the tammar wallaby (Macropus eugenii), to try to understand behavioural factors associated with survival. We focused on two antipredator behaviours: predator recognition and the way in which antipredator vigilance is influenced by the presence of conspecifics. We found that predator-naive pademelons responded to the sight of certain predators, suggesting that they had some degree of innate recognition ability. However, pademelons responded similarly to a broad range of acoustic stimuli, including dingo howls, wedge-tailed eagle calls, foot-thumps - a predator-elicited sound - and a control sound, suggesting that they did not specifically recognize predator vocalizations. Unlike a number of other macropodid marsupials, including tammars, pademelons did not modify time allocated to antipredator vigilance as group size increased. Taken together, these results suggest that red-necked pademelons independently assessed and managed their predation risk whereas tammars relied more on conspecifics to assess and manage risk. We suggest that these factors may have been important determinants of species survival. More generally, we suggest that a fundamental understanding of antipredator behaviour can enlighten conservation efforts.

992: *+.156*

While wild populations continue to decrease, the number of orphaned primates, sanctuaries, and attempts to reintroduce primates back to the natural environment are increasing. An umbrella organization called the Pan-African Sanctuary Alliance (PASA) was formed in 2000 and recently the IUCN Reintroduction Specialist Group developed a set of specific policy guidelines for primates (2002). Data presented in this report are based upon questionnaire responses by managers from 17 African facilities that have become members of PASA (membership in PASA is defined by attendance at an annual PASA workshop). These PASA facilities house over 500 great apes. (There may be other facilities not represented here simply because their managers did not attend a PASA workshop.) The majority of the apes arrived at the sanctuaries when they were less than 4 years old and half were confiscated. Over 40% were found awaiting sale, and 30% had been previously kept as pets. Common ailments upon arrival included internal parasites, behavioral abnormalities, and malnutrition; 20% of the total sanctuary population died prematurely. Most sanctuaries use a combination of enclosures surrounded by electric fencing and cages to accommodate the apes. Sanctuaries actively participate in conservation education, habitat protection, tourism, scientific data collection, local development, and reintroduction. The median total facility operating cost was US$65,000 per annum. The median facility cost per ape was

US$2,222 per annum. Most funding comes from overseas nongovernmental agencies. Discussion focuses on evaluating the present status of sanctuaries, the problems facing them, and their potential role in African conservation issues. (C) 2002 Wiley-Liss, Inc.

993: *-.125*

A male and female Harpy Eagle (Harpia harpyja), both reintroduced captive-bred subadults, were observed on Barro Colorado Island (BCI), Panama for 89 and 205 days, respectively, between June 1999 and August 2000. The male captured 25 individuals from nine different mammalian species and the female captured 46 individuals from 8 different mammalian and one reptilian species during the period of observation. Fifty-two percent of captures by the male were of two-toed (Choloepus hoffmanni) and three-toed (Bradypus variegatus) sloths. Fifty-four percent of captures by the female were of sloths. On average, the male made a capture every 3.6 days with a daily average consumption of 888 g. The female made a capture on average every 4.4 days with a daily average consumption of 812 g. The eagles attacked solitary arboreal prey species from 1-50 m distance, sometimes with several calculated attempts from within 5 m. Social arboreal prey species were most often attacked by surprise from less than 30 m, and terrestrial prey species were attacked by surprise from less than 10 m. The female eagle was observed to capture solitary arboreal prey significantly more during sunny weather and the dry season. She was also observed to capture social arboreal prey significantly more during cloudy weather and the wet season. Capture success rates of observed predations for the male and female eagle were 38% and 49%, respectively.

994: *+.063*

Wild rabbit (Oryctolagus cuniculus) restocking is a common practice in management in France. However rabbits generally experience heavy mortality after release, which jeopardizes restocking efficiency. Release success is full only when introduced individuals produce offspring. We present a controlled release that is appraised through survival and genetic data to evaluate the subsequent breeding contribution of introduced rabbits. Preliminary genetic analyses with 9 microsatellite markers led to choose a dense population of rabbits in Lot (southwestern France) to restock a small population in Vendee (northwestern France). Both populations had comparable polymorphism and allelic variability, and unfortunately only a few specific alleles at low frequency. We translocated 55 rabbits from Lot to Vendee in late July 1999, and by radiotracking 35 rabbits, we assessed a 49% survival in December 1999, resulting in 20 introduced rabbits still alive when the breeding season began. Concurrent spotlight census indicated a resident breeding population size of about 45 individuals. Thus, there were 31% of introduced rabbits within the breeding population. Then, the population was genetically sampled by live trapping, and thereafter by hunting. We distinguished two age groups of individuals: breeders and offspring. Genotypes of young individuals (n = 20) were compared with that of adults originating from either the resident population (n = 29) or the source population. Assignment of offspring to each parental population indicated a low breeding contribution of introduced individuals as not any young fully originated from the source population, whereas only 32% of them resulted from crosses between both populations.

995: *+.209*

Traditionally the distribution of the Arabian oryx in the Kingdom of Saudi Arabia has been closely associated with that of the major sand deserts, notably the Great Nafud in the north and the Rub' al Khali in the south. Due to a successful captive breeding programme the Arabian oryx was

reintroduced in the 'Uruq Barn Ma'arid Protected Area, on the western edge of the Rub' al Khali during 1995, thereby re-establishing the southern population in the Kingdom of Saudi Arabia. Population viability analysis is used to demonstrate the re-establishment of the northern oryx population, the vulnerability of certain populations and to suggest a management approach that should enhance the survival of the free-living populations in the Kingdom of Saudi Arabia. Population models predict that the 'Uruq Bani Ma'arid population could be subject to extinction (Psurvival = 0.38) when modeled in isolation. Associated with this is a low heterozygosity. The proposed release strategy of oryxes into a northern protected area results in maximum population growth during the first 20-years and continued positive population growth during the 100-year modeling period. The latter free-living population is, however, also subject to extinction due to its relatively small size. By managing all of the Arabian oryx populations in the Kingdom of Saudi Arabia as a single metapopulation, survival of the free-living populations are enhanced, without negatively affecting the other populations.

996: ***+.091***

Nekton (fishes and decapod crustaceans) is an abundant and productive faunal component of salt marshes, yet nekton responses to tidal manipulations of New England salt marshes remain unclear. This study examined nekton use of a tidally restricted salt marsh in Narragansett, Rhode Island relative to an unrestricted marsh during summer. In addition, a before-after-control-impact design was used to examine early responses of nekton to the reintroduction of natural tidal flushing. Species richness and densities of Cyprinodon variegatus, Lucania parva, Menidia beryllina, and Palaemonetes pugio were higher in the restricted marsh compared with the unrestricted marsh. The unrestricted marsh supported higher densities of Menidia menidia and Fundulus majalis. Mean lengths of Carcinus maenas and P. pugio were greater in the restricted marsh. Tidal restoration resulted in increased tidal flushing, salinity, and water depth in the restricted marsh. Densities of Fundulus heteroclitus, F. majalis, and Callinectes sapidus were higher after 2 years of restoration. Density of L. parva decreased after restoration, probably in response to a loss of macroalgal habitat. Species richness also decreased after 2 years, from 20.9 species when the marsh was restricted to 13.0 species. Total nekton density did not change with restoration, but shifts in community composition were evident. In this study restoration induced rapid changes in the composition, density, size, and distribution of nekton species, but additional monitoring is necessary to quantify longer-term effects of salt marsh restoration on nekton.

997: ***+.233***

Populations of the marble trout (Salmo marmoratus) have declined critically due to introgression by brown trout (Salmo trutta) strains. In order to define strategies for long-term conservation, we examined the genetic structure of the 8 known pure populations using 15 microsatellite loci. The analyses reveal extraordinarily strong genetic differentiation among populations separated by < 15 km, and extremely low levels of intrapopulation genetic variability. As natural recolonization seems highly unlikely, appropriate management and conservation strategies should comprise the reintroduction of pure populations from mixed stocks (translocation) to avoid further loss of genetic diversity.

998: ***+.033***

The European beaver (Castor fiber) once was a common species in Europe. Due to hunting and increased intolerance, the species disappeared from most countries of its former range. In Belgium, the last beaver was killed in 1848. During the last decade many countries have carried

out reintroduction and translocation programmes to restore the range of the beaver in Europe. In spite of these and many other initiatives, the beaver is still threatened: it only inhabits a limited part of its former range and its populations are small and scattered. As an assignment of the department Nature of the Ministry of the Flemish Community research has been started to assess the chances and risks for a successful reintroduction of beavers in the basin of the Schelde and the Dijle. Suitable habitat was mapped, the coherence of these areas were investigated, bottlenecks for the return of the beaver were assessed and the need and feasibility of a reintroduction were evaluated. In this article, the methods and results of this study are discussed. Within the research area are enough suitable areas for a viable, coherent population of at least 40 beaver families. It is to be expected that there are also many suitable habitats in the adjacent basins outside the study area. There are no large bottlenecks for the development of a population: the largest problem is the city of Leuven, as an obstacle for dispersal. The risks of nuisance due to digging, gnawed trees and the building of dams are considered small. It is expected that at certain locations, beavers will build up a high load of cadmium and other heavy metals. The chances of a natural recolonization of Flanders by dispersal from adjacent populations in the basin of the Maas and reproduction of resident beavers in the Laan and the Dijle are estimated to be small. It is concluded that a viable beaver population can only develop when at least 20-30 individuals are reintroduced. The choice of a genetically mixed group is possibly the best option.

999: ***-.046***

The island endemic Madagascar Fish-Eagle (Haliaeetus vociferoides) is one of the most endangered birds of prey. Certain populations in west-central Madagascar sometimes exhibit a third, and sometimes a fourth, adult involved in breeding activities at a nest. We applied DNA fingerprinting to assess relatedness among 17 individuals at four nests. In all nests with young, a subordinate rather than the dominant male sired the offspring. Within-nest relatedness comparisons showed that some dominant males had an apparent first-order relationship with the female. Between-nest relatedness comparisons showed that some adults had an apparent first-order relative at another nest in the study area. Findings that subordinate males contribute to breeding, and that adults in an area may be related, may require conservation measures such as translocation to assure the species' survival.

1000: ***+.202***

The lake sturgeon (Acipenser fulvescens) is a species of cultural significance to the Menominee Nation, a Native American tribe indigenous to northeast Wisconsin. Lake sturgeon in the Lake Winnebago system historically migrated up the Wolf River to spawn at Keshena Falls, within the current boundaries of the Menominee Reservation where, each spring, the Menominee people gathered at Keshena Falls to harvest lake sturgeon. The construction of two dams on the Wolf River downstream of Keshena Falls in the late nineteenth and early twentieth centuries contributed to extirpation of this species on the Menominee Reservation. The general public has had the opportunity to harvest lake sturgeon from Lake Winnebago during a winter spear fishery since 1932. Those who participate in this fishery feel as strongly about the lake sturgeon resource as do the Menominee people. Cooperation among multiple resource management agencies and the public was necessary to address the interests of both groups and at the same time ensure protection of the Winnebago system lake sturgeon population. In 1993, tribal, state, and federal representatives developed a plan to reintroduce lake sturgeon to the Menominee Reservation. The planning process was designed to involve all the various, often competing, tribal and non-tribal interests in a constructive effort to develop and implement a Menominee Reservation sturgeon rehabilitation plan that all parties could accept and endorse. The core planning group was the

Menominee Reservation Lake Sturgeon Enhancement Committee which worked with the various sturgeon 'publics' to identify common sturgeon restoration goals, objectives and management strategies, and which has worked together since that time in the effort to re-establish viable lake sturgeon populations in Menominee Reservation waters. Since 1994, over 30 000 hatchery-raised lake sturgeon have been stocked in reservation lakes, and 87 Wolf River lake sturgeon have been radio-tagged and relocated upstream to reservation waters of the river. Enough mature lake sturgeon were present in this river reach in 2001 to permit spawning there for the first time in 50 years. The success of the co-management effort has been encouraging and participants are committed to the goal of establishing self-sustaining lake sturgeon populations in reservation waters.

1001: ***+.043***

Restoration of the crested ibis, Nipponia nippon. J. Appl. Anim. Res., 22: 193-200. In China, the crested ibis Nipponia nippon was thought to have become extinct until seven birds were rediscovered in 1981. After its rediscovery, various projects for the conservation of the species in the wild and for captive breeding were begun with the goal of managing the population. A total number of this. species increased to more than 200 birds including about 100 in the wild and 130 in captivity by 2000. Despite captive breeding inbreeding depression has not been observed. In the course of research, however, various factors were influencing the rate of increase of the crested ibis population. The factors include: habitat loss, the increased human population within the species range, environmental pollution resulting from the use of agro-chemicals in particular pesticides and also predators such as snakes and birds. The fate of the crested ibis probably depends largely upon the life style of humankind.

1002: ***-.065***

Alien species invasions have already caused substantial ecological and economic damage and will likely have even greater negative consequences in the future. Thus, it is imperative to improve our basic ecological understanding of these invasions and enhance our ability to reverse or mitigate their often devastating effects. Invasions by fire-promoting alien grasses have played a particularly important role in the destruction, of tropical dry forests and are a major reason why these ecosystems are now among the most endangered in the world. We investigated how light availability (full sun and 50% shade), alien grass control (bulldoze, herbicide, plastic mulch, and trim treatments), and native species additions (outplanting and direct-seeding) affected the establishment of native plants and the suppression of a dominant invasive bunchgrass (fountain grass, Pennisetum setaceum) within a highly degraded fenced dry forest remnant on the island of Hawaii. The percent cover of. native species increased in all light, grass control, and species addition treatments throughout the 20 mo of the experiment, and was greatest in the shade, bulldoze, and outplant treatments. Although fountain grass cover also increased over time in all grass control treatments, the three more aggressive techniques all significantly reduced grass cover relative to the more moderate trim treatment. In addition, there was a significant overall negative correlation between the final cover of fountain grass and native species, suggesting that these native species may successfully compete with fountain grass for water and/or nutrients. Outplant survival and the number of individuals established from direct-seeding differed significantly among the grass control treatments, and in each case, the response was highly species specific. Photosynthetic rates of established outplanted individuals and fountain grass did not differ significantly across most experimental environments, indicating that the local dominance of fountain grass may not be due to superior physiological attributes-The results of this experiment highlight the importance of investigating species- and treatment-specific responses before

attempting larger-scale restoration projects, particularly when using rare and endangered species. This study also suggests that relatively simple techniques may be used to simultaneously establish populations of vigorous understory native species and suppress alien grasses at relatively large spatial scales, and that remnant or newly created favorable microsites may be exploited to facilitate the establishment of rarer native overstory species.

1003: *+.183*

The successful recovery of critically endangered flora will rely increasingly on its translocation to secure sites where the amelioration of threats has been successful or where current threats such as weeds are absent. Translocations are both costly and time consuming and in many cases involve very small numbers of plants from critically endangered populations. Consequently it is important that effective, efficient methodologies are developed for the process and for monitoring success. Equally critical, as part of the same monitoring program, is the need to develop protocols for determining and predicting translocation success. Of the 28 Acacia species listed as threatened (Declared Rare Flora) 12 are critically endangered and all occur in agricultural areas where there has been extensive land clearing and habitat degradation. The Department of Conservation and Land Management has carried out 15 experimental translocations of critically endangered taxa as part of approved Interim Recovery Plans, including Acacia aprica and Acacia cochlocarpa subsp. cochlocarpa. Preliminary data are presented on the current status and success of experimental translocations of these two taxa of Acacia. Artificial watering and mulch had little effect on enhancing survival or growth of either taxon. Protection from grazing appears to be essential in ensuring translocation success. Continued monitoring is required to evaluate translocation success satisfactorily.

1004: *+.121*

The European bison, extirpated from the Carpathian Mountains over 200 years ago, was reintroduced to the Bieszczady Mountains in the 1960/1970s in two small, isolated herds, and is now threatened by high inbreeding and low genetic variability A new program of re-establishing viable populations in the Carpathians is based on the genetic analysis of formerly released animals, using the European Bison Pedigree Book. Calculating founder contributions, founder genome survival, founder genome equivalent, inbreeding coefficient and mean kinship allows the identification of under-represented or missing founders. Since genetic variability is much higher among bison in captivity and, in Poland, the Lowland-Caucasian line is represented only in those free-ranging herds, the reintroduction will be based on animals from foreign breeding centres. Bison will be released into existing herds, and planned new introduction sites, to facilitate natural gene exchange in the future. The same approach will be recommended for planned introductions in the Slovakian and Romanian Carpathians. (C) 2002 Elsevier Science Ltd. All rights reserved.

1005: *+.103*

The pattern of dispersal of burrowing bettongs (Bettongia lesueur) was studied in a population reintroduced to a peninsula protected from exotic predators at Heirisson Prong in Shark Bay, Western Australia. The reintroduced population was growing strongly in numbers and expanding in area during the study. Young were first marked in the pouch and subsequently monitored by trapping and radio-telemetry after independence to establish their movements relative to those of their mothers. B. lesueur on Heirisson Prong dispersed between the ages of 170 and 250 days, coinciding with the period from weaning to sexual maturity. Dispersal was male-biased. Young males dispersed significantly further than young females with mean dispersal distances of 4600 m

and 1100 m respectively. Male B. lesueur also frequented significantly more warrens than females, being located at a mean of 0.37 warrens per daily radio-tracking fix compared with a mean of 0.24 per fix for females. Scarring from intra-specific aggression occurred in male bettongs only. Males may disperse to the periphery of the population to escape aggressive interactions with established adult males and visit more warrens to increase mating opportunities. Dispersal in females may be related to resource quality or inbreeding avoidance.

1006: ***-.046***

Crocodylus siamensis, the Siamese crocodile, is a critically endangered species of freshwater crocodile previously distributed throughout much of SE Asia. Recovery plans call for reintroductions to the wild using founder individuals currently in captivity, mostly in commercial crocodile farms: On many farms C. siamensis has been intentionally hybridised with either Cuban crocodiles, C. rhombifer, or the estuarine crocodile, C. porosus, and hybrids may be difficult to distinguish morphologically. We report on the combined use of microsatellite and mtDNA genetic markers to determine the species status of potential founder individuals for reintroduction of C. siamensis. Genetic markers were used to characterise 103 captive and wild-caught individuals of C. siamensis; C. rhombifer and C. porosus in Vietnam and to distinguish purebred versus hybrid individuals. Although the microsatellite loci used had some overlap of allele sizes among species, assignment tests allowed differentiation. Four hybrids were identified, two of which had not been recognised morphologically as hybrids, and one of these was thought to be a C. siamensis suitable for reintroduction. Nineteen of the identified purebred C. siamensis were subsequently released into Cat Tien National Park in southern Vietnam. J. Exp. Zool. (C) 2002 Wiley-Liss, Inc.

1007: ***-.015***

Empirical support for the genetic management strategies employed by captive breeding and reintroduction programs is scarce. We evaluated the genetic management plan for the highly endangered black-footed ferret (Mustela nigripes) developed by the American Zoo and Aquarium Associations (AZA) as a part of the species survival plan (SSP). We contrasted data collected from five microsatellite loci to predictions from a pedigree-based kinship matrix analysis of the captive black-footed ferret population. We compared genetic diversity among captive populations managed for continued captive breeding or reintroduction, and among wild-born individuals from two reintroduced populations. Microsatellite data gave an accurate but only moderately precise estimate of heterozygosity. Genetic diversity was similar in captive populations maintained for breeding and release, and it appears that the recovery program will achieve its goal of maintaining 80% of the genetic diversity of the founder population over 25 years. Wild-born individuals from reintroduced populations maintained genetic diversity and avoided close inbreeding. We detected small but measurable genetic differentiation between the reintroduced populations. The model of random mating predicted only slightly lower levels of heterozygosity retention compared to the SSP strategy. The random mating strategy may be a viable alternative for managing large, stable, captive populations such as that of the black-footed ferret. (C) 2003 Wiley-Liss, Inc.

1008: ***+.186***

Nine bilbies were reintroduced to a 14-km(2) reserve free of rabbits, cats and foxes in South Australia in April 2000. The survival, growth and ecology of the population were studied for 17 months after release by means of radio-tracking and trapping. Reproduction was continuous over the study period, with juveniles successfully recruited into the population. Home-range size of female bilbies averaged 0.18 km(2) and was significantly smaller than home ranges of males,

which averaged 3.16 km(2). Wild-born subadults had smaller home ranges than adults. While male home ranges, and male and female home ranges overlapped considerably, females appeared to maintain areas discrete from other adult females. Bilbies showed a significant preference for dune habitat. As swale habitat appears too hard for burrow construction and males moved greater distances from diurnal burrows than females, males are likely to access food reserves that are under-used by females. Both males and females reused at least 30% of their burrows, and females displayed long-term site fidelity. The release was considered successful and suggests that despite historical damage from rabbits and stock, bilbies are able to successfully recolonise parts of their former range in arid South Australia once rabbits, cats and foxes are removed.

1009: *+.032*

Wild boar Sus scrofa has been extinct in Britain for several centuries. Recently, however, some conservationists have argued that it should be reintroduced. Here, we report that two populations of free-living wild boar are already present in Britain, in the south of England, ranging over areas of approximately 15 km(2) in the county of Dorset and 175 km 2 in the counties of Kent and East Sussex. Presence of the animals was indicated initially by unsolicited reports to the Department of Environment Food and Rural Affairs, and was confirmed by searching the relevant areas for field signs such as tracks, faeces, nests and rooted areas. Six carcasses of road-killed or shot animals were available for inspection from the Kent/East Sussex area and had the morphological characteristics of wild boar. Breeding was confirmed in the Kent/East Sussex area and is suspected in the Dorset population. A simple population dynamics model, based on an estimated initial population of 100 animals, suggests a growth rate, r, of between 0.016 and 0.267 for the Kent/East Sussex population. We conclude that the Kent/East Sussex population is likely to prove viable unless actively persecuted, and discuss the social, agricultural, ecological and conservational implications.

1010: *+.040*

The Eagle Owl has been considered extinct in Hesse. western Germany. since 1910. Reasons for its disappearance were mainly persecution by shooting, but also by egg collecting and 'trophy hunting' etc. As late as 1925 a pair of Eagle Owls was shot at Creuzburg near the border between Thuringia and Hesse. The main reasons for the reappearance of the Eagle Owl in Hesse are reintroduction programmes in adjacent regions like Lower Saxony and Northrhine-Westphalia. Through these successful programmes the Eagle Owl also reappeared in Hesse and bred for the first time successfully in 1977 in the district of Limburg-Weilburg. Since then numbers of breeding pairs increased slowly, but by 1985 had reached only four pairs. In the early 1990s a rapid growth started and in 1993 a total of 43 Eagle Owl territories were occupied with an average reproductive output of 1.93 young per pair. During the mid-1990s there was a decline in reproductive success in Hesse, the number of fledglings per pair dropped to 1.0-12. An exception was the Hessian Weserbergland with a rate of usually 2.0 young per pair. In the year 2000 the rate increased to 1.4-1.6 young per pair overall and the total population grew up to 58-60 pairs. Although shooting has been eliminated and pesticide contamination is of minor importance, many Eagle Owls are killed by collisions with wires, vehicles and by electrocution. Inbreeding depression - seriously discussed especially for the reintroduction project - did not play a role among captive-breeding Eagle Owls nor among the released birds. On the contrary the reintroduced Eagle Owls showed a high fitness, which was evident in a higher reproductive rate in the initial stage compared to other federal states (e.g. Baden-Wuerttemberg, Bavaria and Thuringia). Competition for nest sites with other birds does not occur in Eagle Owls. because it is the dominant, prevailing species. I predict that the number of Eagle Owls in Hesse will increase further because there are still vacant

potential breeding habitats. Eagle Owls show very high flexibility and are now by far less prone to disturbance than the founder population, plus they occupy more and more secondary breeding habitats.

1011: *+.103*

The Eagle Owl became extinct in Schleswig-Holstein in about 1850 as a direct result of human persecution. In 1981 the state government of Schleswig-Holstein decided to attempt to re-introduce the Eagle Owl as a characteristic species within the state. The species protection programme "Re-introduction of the Eagle Owl in Schleswig-Holstein" was carried out successfully by the State Society for Owl Protection in Schleswig-Holstein. In the period 1983 to 2002, 681 young Eagle Owls, reared in captivity, were released into the wild from release aviaries. Since 1984. 650 successful breeding attempts have been recorded in the wild in Schleswig-Holstein. producing 1314 young. Unsuccessful breeding attempts were recorded in a further 172 cases. The Eagle Owl is now an established part of the Schleswig-Holstein avifauna with 100 pairs breeding annually. The Eagle Owl has mainly colonised areas with a rich landscape structure including woods, rivers, lakes and meadows. These diverse habitats are particularly favourable. supplying the species with breeding sites and sufficient food. Observations have shown that the Eagle Owl has little or no detrimental effect on the populations of the rare breeding species such as White-tailed Eagle. Black Stork, Peregrine, Raven and Common Buzzard. However, the Goshawk has been displaced from traditional breeding territories by the Eagle Owl, which led to regional declines in breeding numbers. The re-establishment in Schleswig-Holstein has led to re-colonisation of the neighbouring country of Denmark by the Eagle Owl where 25-30 pairs now breed.

1013: *-.115*

Until recently, eastern woodrats (Neotoma floridana, Ord) at Pine Hills, Union County, were believed to be the only extant population in Illinois. During 1994, additional populations in adjacent Jackson County were discovered at Fountain Bluff, Horseshoe Bluff, and Little Grand Canyon. Genetic variation among Illinois woodrat populations was estimated based on microsatellite DNA at 6 loci. The four populations were separated by 2-14 km, yet all exhibited significant genetic differentiation. Fountain Bluff was the most geographically isolated population and had the lowest genetic heterozygosity. A sample of individuals from a more contiguous population in Missouri exhibited the highest heterozygosity. Populations were monitored by mark-recapture live-trapping for 39 months at Pine Hills and 19 months at Fountain Bluff. Although the Fountain Bluff population was smaller than the Pine Hills population, seasonal demographics and reproductive output were similar. Natural dispersal from all 4 populations was limited by fragmented habitat, natural barriers, and anthropogenic barriers. Reintroductions of woodrats may be necessary for the species to repopulate formerly occupied sites throughout southern Illinois.

1014: *+.171*

The irregular distribution of the White-tailed Eagle in Europe is considered to be a relict of an earlier pan-European range. It was due to intense human persecution in Western Europe in the last millennium. Although populations in northern Europe have recovered, there is no such change in the rest of the continent. Most ornithologists regard this eagle as a northern species living in wild places but changes in attitudes towards large raptors gives hope of successful re-colonisation in more inhabited lands. Migrants are becoming commoner in winter to the south of the breeding areas but experience suggests long distance natural recovery is unlikely. Sea eagles exhibit a slow

rate of range expansion, even in productive breeding populations. The re-introduced Scottish population has reached 21 breeding pairs and the techniques for re-introduction are now well proven and successful. In order to restore the white-tailed eagle to large parts of its original range, it is recommended that a program of new translocations is carried out in western and southern Europe.

1015: *+.011*

The last two pairs of White-tailed Sea Eagle were extinguished from Israel in 1957 following the drainage of the Hula swamp and the massive and uncontrolled use of Thallium sulphate. These were probably the last breeding pairs of the species in this area of the Middle East: Lebanon, Israel, Syria and Iraq. Since the extinction, only wintering, or migrating, White-tailed Sea Eagles (less than seven per year) have been observed in Israel. In 1991, a re-introduction program was started to restore the White-tailed Sea Eagle population in Israel, and hopefully in the Middle East. The source is captive bred White-tailed Sea Eagles from Israel and European zoos. Captives are released by hacking: chicks are placed in an artificial nest for acclimatization until the age of fledging ([approximately]80 d) and then released. Since 1992, 21 White-tailed Sea Eagles have been released. Nineteen by hacking: 12 in the Hula Nature Reserve and 7 in Beit Shean valley; 2 two-year old birds were released after long acclimatization in the Hula valley. Eight losses of released birds (38%) have been recorded, three in the Hula valley and five in Beit Shean valley: one drowned in a small fish tank, one was poisoned in Jordan; three males and a female were predated shortly after release and two were electrocuted. Since the releases started, White-tailed Sea Eagles have occupied the Hula Valley and the adjacent Golan Heights and Upper Galilee. A male released in 1992 and female in 1993 formed a pair in 1996 and bred from 1998 in a two-meter wide nest, 30 m above the ground in a Eucalyptus tree but as yet without success. The diet of the released birds varied from livestock carcasses to all vertebrates classes except Amphibia, apparently according to local availability.

1016: *+.147*

Archaeology, placenames and documentary evidence clearly reveals that the Sea Eagle was once a common and widespread bird in the British Isles. Habitat loss and persecution reduced it to west and north coast strongholds in the 19th century leading to its ultimate extinction as a breeding bird by 1916. The localities of both Golden and Sea Eagle clutches taken in the past indicate that the two species occupied separate habitats, but some overlap did occur in certain freshwater systems inland, and on larger, mountainous islands. Once the Sea Eagle disappeared Golden Eagles spread to occupy some of its former territories on the coast. Although the smaller of the two is the more aggressive, the Sea Eagle seems to be experiencing little difficulty in re-establishing, some Golden Eagles being ousted completely but others managing to live alongside their larger cousin. There are also areas available to Sea Eagles that are without Golden Eagles altogether. Thus the prospects are good for the continued growth and expansion of the reintroduced population.

1019: *+.190*

Current Status: The Quino checkerspot butterfly (Euphydryas editha quino), is federally listed as endangered. This taxon occurs in San Diego and Riverside Counties and several localities in Baja California Norte, Mexico. Although no long-term empirical monitoring of populations has been conducted, some conclusions may be drawn regarding the species' overall status. The Quino checkerspot butterfly has apparently undergone a limited increase in abundance and distribution following its disappearance during the prolonged 1980's drought. However, current species

abundance and distribution remain far below the pre-drought 1970's levels, and there is no evidence that the long-term decline due to human impacts has slowed (see section I.C.5 below, Metapopulation Resilience). Although large portions of occupied habitat are under public ownership, few, if any, known population distributions (preliminarily delineated in this document as occurrence complexes, further defined below) are entirely protected. There are no populations currently known to be resilient. Destruction and degradation of occupied habitat continues throughout the range of the Quino checkerspot butterfly, and some level of ongoing degradation exists in all occupied habitat. Habitat Requirements and Limiting Factors: The Quino checkerspot butterfly is found in association with topographically diverse open woody canopy landscapes containing low to moderate levels of nonnative vegetation compared to disturbed habitat. Vegetation types that support the Quino checkerspot butterfly include coastal sage scrub, open chaparral, juniper woodland, and native grassland. Soil and climatic conditions, as well as other ecological and physical factors, affect the suitability of habitat within the species' range. Urban and agricultural development, invasion of nonnative species, habitat fragmentation and degradation, and other human-caused disturbances have resulted in substantial losses of habitat and declines in habitat suitability throughout the species' historic range. Conservation needs include protection and management of landscape connectivity (habitat patches and intervening dispersal areas); habitat restoration and enhancement; and establishment of a formal Quino checkerspot butterfly captive breeding program. The recovery strategy focuses on landscape-level protection of metapopulations that experience marked fluctuations in density and geographic distribution on a scale of 5 to 10 years. This recovery plan identifies six recovery units (Northwest Riverside, Southwest Riverside, South Riverside, South Riverside/North San Diego, Southwest San Diego, and Southeast San Diego). Recovery units are the major units for managing recovery efforts. Most recovery units contain one or more existing core (relatively large) occurrence complexes. A number of factors were considered in identifying recovery units, primarily biological and genetic factors, political boundaries, and ongoing conservation efforts. In some instances, recovery unit boundaries were modified to maximize efficiency of reserves, encompass areas of common threats, or accommodate logistic concerns. Recovery units may include areas of apparently suitable networks of habitat patches and dispersal areas that are not known to be occupied, when biological evidence warrants. Biologically, Quino checkerspot butterfly recovery units include areas within which gene flow is currently possible. Recovery Priority: 6C, per criteria published in the Federal Register (U.S. Fish and Wildlife Service 1983a, 1983b). The priority is based on designation as a subspecies with a high degree of threat, a moderate to low potential for recovery, and existing conflict between the species' conservation and development. Objectives: The overall objective of this recovery plan is to reclassify the Quino checkerspot butterfly from endangered to threatened status and ensure the species' long-term conservation. Interim goals include: (1) protect and manage habitat supporting known current population distributions (occurrence complexes) and landscape connectivity between them; (2) maintain or create resilient populations; and (3) conduct research necessary to refine recovery criteria. Reclassification to threatened status is appropriate when a taxon is no longer in danger of extinction throughout a significant portion of its range. Because data upon which to base reclassification decisions are incomplete, downlisting criteria in this recovery plan are necessarily preliminary. There are insufficient data on which to base delisting criteria at this time. Recovery Criteria:The Quino checkerspot butterfly could be downlisted to threatened when the following criteria are met: 1) Permanently protect the habitat within occurrence complexes (estimated occupied areas based on habitat within 1 kilometer (0.6 mile) of recent butterflyoccurrences; see section I.D below, Distribution and Habitat Considerations), a configuration designed to support resilient populations. One or more occurrence complexes may belong to a single greater population distribution, an occurrence complex may contain more than one whole or partial population distributions. When population distributions are determined, they will replacethe occurrence complex as the protected

unit. There are currently 46 described occurrence complexes. 2) Conduct research including: determine the current short-term and potential long-term distributions of populations and associated habitat; conduct preliminary modeling of metapopulation dynamics for core occurrence complexes identified in section I.D below (Distribution and Habitat Considerations). 3) Permanently provide for and implement management of occurrence complexes (or population distributions when delineated) to restore or enhance habitat quality and population resilience. Management should be implemented as described in Recovery Action 1 (section II.C below, Recovery Action Narrative). 4) The protected, managed (conserved) population segments within core occurrence complexes (or population distributions when delineated) must demonstrate evidence of resilience. Evidence of resilience is demonstrated if a decrease in the number of occupied habitat patches over a 10- to 20-year period within an occurrence complex (or population distribution when delineated) is followed by increases of equal or greater magnitude. Monitoring must be initiated in the third of three years of favorable climate (total annual January and February precipitation within one standard error of the average total for those months over the past 30 years, based on local or proxy climate data). Populations that do not demonstrate resilience after 20 years should be augmented and monitoring reinitiated. 5) One additional population should be documented or introduced within the Lake Matthews population site (formerly occupied, not known to be currently occupied) in the Northwest Riverside Recovery Unit. At least one of the extant populations outside of current recovery units (e.g. the San Vicente Reservoir occurrence complex) must meet resilience specifications above unless an additional population is established or documented within 10 kilometers (6 miles) of the ocean (a more stable marine climate influence should minimize susceptibility to drought and reduce probability of extirpation). 6) Establish and maintain a captive propagation program for purposes of maintenance of representative refugia populations, research, and reintroduction and augmentation of wild populations as appropriate. 7) Initiate and implement a cooperative outreach program targeting areas where Quino checkerspot butterfly populations are concentrated in western Riverside and southern San Diego Counties. At present there is insufficient information about the biology of the species toestablish criteria and timeframes for delisting. Researc h needs for development of delisting criteria are described under Recovery Action 6 below.

1020: ***+.160***

The eastern Gulf Slope streams draining the Apalachicolan Region are defined as streams from the Escambia to the Suwannee River systems. Occurring in southeast Alabama, west-central and southwest Georgia, and north Florida, these river systems collectively form one of the largest drainage areas in the eastern Gulf Coastal Plain. Historically, these rivers were known for their rich freshwater mussel populations. However, the fat threeridge (Amblema neislerii), shinyrayed pocketbook (Lampsilis subangulata), Gulf moccasinshell (Medionidus penicillatus), Ochlockonee moccasinshell (Medionidus simpsonianus), oval pigtoe (Pleurobema pyriforme), Chipola slabshell (Elliptio chipolaensis), and purple bankclimber (Elliptoideus sloatianus) mussels have all undergone significant reduction in total range and abundance. Current Status: The fat threeridge, shinyrayed pocketbook, Gulf moccasinshell, Ochlockonee moccasinshell, and oval pigtoe were federally listed as endangered species; and the Chipola slabshell and purple bankclimber were federally listed as threatened species under the Endangered Species Act of 1973, as amended, on March 16, 1998 (63 FR 12664). These mussel species are vulnerable to extinction due to significant habitat loss, range restriction, and population fragmentation and size reduction. Only one (purple bankclimber) of the species remains in the Chattahoochee River main stem, having recently (2000) been rediscovered after a nearly 150-year absence from collection records. There is little evidence of recent recruitment documented during status surveys for these species except the fat threeridge. The restricted distribution of these seven species also makes localized

subpopulations susceptible to adverse habitat and water quality alterations, toxic chemical spills, and the deleterious effects of genetic isolation. Recovery Goal: Restore viable populations of the fat threeridge, shinyrayed pocketbook, Gulf moccasinshell, Ochlockonee moccasinshell, oval pigtoe, Chipola slabshell, and purple bankclimber within a significant portion of their historical ranges, and eliminate or reduce threats to their continued survival, so that their protection under the Endangered Species Act is no longer required. Recovery Strategy: Because of the extent of their decli decline and the continuing threats to their habitats, securing the viability of existing subpopulations of the seven species and their habitats is the most important and urgent recovery objective. The most efficient means of accomplishing this objective is by applying knowledge of the distribution and habitat needs of these species towards reducing and preventing threats to the existing populations and their habitats through existing regulatory mechanisms, habitat restoration programs, and partnerships with various stakeholders. That knowledge will grow as research outlined in this plan, about the species' life history, threats, and management techniques for habitat restoration, is completed. As the viability of existing subpopulations and their habitats is secured, the next objective is to increase the number of subpopulations and extend their range. This objective is necessary in order to reach our recovery goal for these mussels. Increases can occur in two basic ways, by either finding a previously unknown subpopulation, or artificially reestablishing a new subpopulation. Reestablishing new subpopulations will require close coordination and concurrence of the State(s) and other partners with interests at any potential reintroduction sites. Due to the low numbers of animals in most extant subpopulations, the propagation of laboratory- or hatchery-reared progeny is the most likely means of providing animals for new subpopulations. Priorities for recovery via artificial propagation are: (1) develop propagation technology; (2) augment and expand the ranges of extant subpopulations to ensure their viability; and (3) reestablish viable populations in other streams within their historical range that have suitable habitat and water quality. As these elements of our recovery strategy are implemented, we will also periodically monitor subpopulations and habitats in order to measure progress towards recovery and to support possible modifications to the recovery plan. Because these mussels are affected by land and water uses that occur upstream in their watersheds, an outreach program is proposed to educate the public about watershed conservation generally, and the role of mussels in their ecosystem specifically. The last element of our recovery strategy is to periodically evaluate the effectiveness of recovery plan implementation. This is always a necessary component of recovery plans, but is an especially important one in this plan because we have much to learn still about these species and we anticipate that recovery will take more than 15 years to accomplish. Recovery Criteria: The Service will consider the fat threeridge, shinyrayed pocketbook, Gulf moccasinshell, Ochlockonee moccasinshell, and oval pigtoe for reclassification to threatened status when each species has: (1) shown an increase in its current range to reflect occupation of at least 50 percent of the total historical habitat; (2) at least three viable subpopulations in each of the watersheds (listed in Table 8) that currently supports the species (e.g., Econfina Creek, lower Flint River); and (3) at least ten viable subpopulations in the large river basins (i.e., Apalachicola- Chattahoochee-Flint, Ochlockonee, Suwannee Rivers) within the historical range of the species, for at least 3 generations. The Service will consider delisting the five endangered mussels, the Chipola slabshell, and purple bankclimber when biennial monitoring shows that an increase of the current number of subpopulations/sites and extent of occurrence is enough to ensure population viability, reduce isolation among populations, and increase the potential for genetic exchange. Specific increases in subpopulations and river miles needed to delist all 7 mussels from threatened status are currently unknown and will be determined by tasks outlined in this recovery plan. To downlist and delist these seven mussels, all necessary subpopulations must be viable and secure, and all current and foreseeable threats must be identified and reduced as addressed under the Listing/Recovery Factor Criteria.

1021: *-.051*

The Peary caribou (Rangifer tarandus pearyi) was recognized as 'Threatened' by the Committee on the Status of Endangered Wildlife in Canada in 1979 and 'Endangered' in 1991. It is the only member of the deer family (Cervidae) found on the Queen Elizabeth Islands (QEI) of the Canadian High Arctic. The Peary caribou is a significant part of the region's biodiversity and a socially important and economically valuable part of Arctic Canada's natural heritage. Recent microsatellite DNA findings indicate that Peary caribou on the QEI are distinct from caribou on the other Arctic Islands beyond the QEI, including Banks Island. This fact must be kept in mind if any translocation of caribou to the QEI is proposed. The subspecies is too gross a level at which to recognize the considerable diversity that exists between Peary caribou on the QEI and divergent caribou on other Canadian Arctic Islands. The Committee on the Status of Endangered Wildlife in Canada should take this considerable diversity among these caribou at below the subspecies classification to mind when assigning conservation divisions (units) to caribou on the Canadian Arctic Islands. In summer 1961, the first and only nearly range-wide aerial survey of Peary caribou yielded a population estimate on the QEI of 25 845, including about 20% calves. There was a strong preference for range on the western QEI (WEQI), where 94% (24 363) of the estimated caribou occurred on only 24% (ca. 97 000 km2) of the collective island-landmass. By summer 1973, the overall number of Peary caribou on the QEI had decreased markedly and was estimated at about 7000 animals. The following winter and spring (1973-74), the Peary caribou population declined 49% on the WQEI. The estimated number dropping to 3000, with no calves seen by us in summer 1974. Based on estimates from several aerial surveys conducted on the WQEI from 1985 to 1987, the number of Peary caribou on the QEI as a whole was judged to be 3300-3600 or only about 13-14% of the 1961 estimate. After a partial recovery in the late 1980s and early 1990s, Peary caribou on the WQEI declined drastically between 1994 and 1997 and were estimated at an all-time known low of about 1100 animals by summer 1997. The number of Peary caribou on the QEI in summer 1997 was likely no more than 2000-2400 or only 8-9% of the 1961 estimate. The four known major die-offs of Peary caribou on the WQEI between 1973 and 1997 occurred during winter and spring periods (1 Sep-21 Jun) with significantly greater (P0.005) total snowfall, when compared to the long-term mean obtained from 55 caribou-years (1 Jul-30 Jun), 1947/48-2001/02, of weather records from Resolute Airport on Cornwallis Island. Of ecological significance is that the die-offs occurred when the caribou were at low mean overall densities and involved similar high annual rates of loss among muskoxen (Ovibos moschatus). All of the available evidence indicates that Peary caribou (and muskoxen) on the QEI experienced die-offs from prolonged, undernutrition (starvation) caused by relative unavailability of forage-the forage was there but it was inaccessible to the caribou due to snow and/or ice cover. We cannot control the severe weather that greatly restricts the forage supply but we should try to reduce the losses of Peary caribou from other sources-humans, predators and competitors.

1022: *+.032*

Currently, river otters (Lontra canadensis) range throughout Canada and northern parts of the United States, which constitutes half their historical range. River otters are a threatened species in South Dakota, although their current status and distribution are relatively unknown. We attempted to determine the status and distribution of river otters in South Dakota. To conduct river otter surveys, 14 rivers and 3 creeks were selected throughout South Dakota based on stream size (orders three to seven), water gradient, and water permanence. At each river or creek, line transects and shorelines (below high water mark) were surveyed for river otter sign. In addition, reports were collected from the Natural Heritage Database and landowners, and observation report forms were mailed to state conservation officers collecting their observations. Thirty-four verified

and three unverified sightings were recorded during our research. Eighty-nine percent of the sightings were reported in eastern South Dakota, particularly along the Big Sioux River. There may be a small population of river otters residing in the Big Sioux River watershed, which is possibly the result of reintroduction efforts by the Flandreau Santee Sioux Tribe. From our survey efforts, we found no indication of a remnant river otter population in South Dakota, though a small population of reintroduced river otters may reside in the eastern third of South Dakota. Because river otter populations do not likely exist in other areas of the state, efforts should be taken to restore this native animal to South Dakota's river systems.

1023: *+.068*

We report a case of successful reproduction in wild-released orphan chimpanzees. Using non-invasive genetic analysis, we determined the paternity of an infant born to a female chimpanzee released by HELP (Habitat Ecologique et Liberte des Primates) Congo into the Conkouati-Douli National Park, Republic of Congo. The sire was a released male, thus demonstrating successful reproduction in both male and female released chimpanzees. These results provide evidence that release into the wild may be a viable response to the plight of orphan chimpanzees in Africa, and we discuss further applications of non-invasive genetic tagging to release programs.

1024: *-.066*

Dasyurid marsupials are distributed throughout the major terrestrial environments of Australia, but since European settlement have suffered local and regional extinctions, range reductions and population decline In this paper we examine the conservation status of small dasyurids (<500 g) and the threats they face. We also evaluate recovery procedures for threatened taxa and assess their success. Twenty-four percent ofsmaller dasyurids are classified as vulnerable, endangered or data deficient. Large body size and occupancy of one or two habitat types are correlated strongly with endangerment; species currently considered as 'low risk, near threatened' group closely with vulnerable and endangered species, indicating a risk of further declines. The processes contributing most to declines include habitat loss and fragmentation, altered fire regimes and predation. As of April 2001, no Recovery Plans had been adopted by the Commonwealth Government for any small dasyurid species. There is much information on the reproduction and development of smaller dasyurids, making them suitable for captive breeding. However, captive breeding programs have been limited, the dibbler Parantechinus apicalis being the only species bred systematically for reintroductions. There is a need for integration between captive breeding programs and recovery planning, as well as for more information on the population viability and metapopulation structures of small dasyurids, genetic diversity of populations and inbreeding depression. We suggest a program of survey, research, management and education to improve conservation outcomes for all small dasyurids.

1025: *+.073*

The genetic diversity of re-established population of endangered species Allium angulosum L. was tested as a one part of rescue program. Founder individuals were picked in Chropyne - Zarici area (North Moravia, Czech Republic) and new population was set in Protected Landscape Area Litovelske Pomoravi (North Moravia, Czech Republic). The task was whether the newly founded population was made by representative individuals to cover (include) the genetic variability of source (mother) population. Items were tested with variability assay of six isozyme systems (G-6-PDH, AAT, PGM, EST, ACP, PGI) using discontinuous polyacrylamide gel electrophoresis (PAGE). The method stated relatively sufficient level of variability on condition that new

population would be raised to prevent genetic changes. Application of more tests checking the genetic diversity within population could be useful during reintroduction and management of endangered plant species.

1026: *+.155*

The aim of this work was to assess density, biomass and species composition of earthworm communities within four translocated grasslands at Runway 2, Manchester Airport. Results from this study also evaluated the availability of earthworms as a food source for displaced, protected vertebrate species. The sites studied differed in topography and method/type of soil/turf transfer Earthworms were extracted by digging and hand sorting of soil from replicated 0.1 m(2) plots, followed by vermifuge application. Monitoring from 1998-2001 produced nine species of earthworm representing all three ecological groupings. Throughout, areas supplied with turf contained significantly ($P < 0.05$) more earthworms with greater mass than adjacent areas supplied with soil only. Areas of hummocks and adjacent wet hollows, both covered with the same translocated turf, differed in species composition. Data are presented for mean earthworm community density and mass over the sampling period. Across all sites, figures for density and mass (in 2001), ranged from 50-427 m(-2) and 12-128 g m(-2) respectively. In all sampled areas, density and mass of earthworms has either increased or remained relatively stable, suggesting each translocation method to be successful after 4 years.

1027: *+.189*

The ecological communities of Quail Island (Otamahua) in Lyttelton Harbour, Banks Peninsula, New Zealand, are being restored to a more natural condition. Invertebrates were collected from the island and identified to produce an inventory. Banks Peninsula endemic species currently known from Quail Island include: five spider species Misgolas borealis, Migas saxatilis, Maniho ngaitahu, Pahora kaituna and Stanwellia sp., a tenebrionid beetle Mimopeus granulosus, a cockroach Celatoblatta sp., a ground weta Hemiandrus sp., a cicada Kikihia sp., a silverfish Heterolepisma sp., a millipede Icosidesmus schenkeli and a snail Charopa pseudocoma. A rare native aphid, Aphis cottieri, was found on Muehlenbeckia complexa and the populations represent some of the largest known for this aphid. Species absent from Quail Island but found close by in Orton Bradley Park include a tree weta (Anostostomatidae) Hemideina femorata and five ground beetle (Carabidae) species. Insects proposed for introduction are listed and habitat requirements are discussed.

1028: *+.300*

This Strategy provides a response to the key threats to native fish populations in the Murray-Darling Basin. These range from flow regulation, habitat degradation, lowered water quality, manmade barriers to fish movement, the introduction of alien fish species, fisheries exploitation, the spread of diseases and translocation and stocking of fish. Native fish populations in the Basin's rivers have declined under these threats with experts estimating that current levels are about 10 per cent compared to pre-European settlement. The vision of this Strategy is to ensure that the Basin sustains viable fish populations and communities throughout its rivers. The goal of this Strategy is to rehabilitate native fish communities in the Basin back to 60 per cent of their estimated pre-European settlement levels after 50 years of implementation. In the absence of targets that underpin accountability arrangements, the best expert advice recommends the following as examples of indications of the level of implementation needed by 2013: * an overall increase of aquatic structural habitat values of 20 per cent; and * functional processes and river floodplain

links re-established for 80 per cent of remaining wetland habitats through improved flow management. This Strategy has been developed and will be implemented within the context of the Murray- Darling Basin Commission's Integrated Catchment Management Policy. This policy reflects a commitment by the community and governments to do all that needs to be done to manage and use the resources of the Basin in an ecologically sustainable manner. A substantial reallocation of funding will be needed by governments and the community to implement this Strategy. This partnership approach depends on the commitment of individual landholders, Indigenous communities, Landcare groups, catchment management organisations, waterway managers, urban and rural community groups, local, State and Australian Government agencies, as well as the Murray-Darling Basin Commission. The Strategy will address its goal and targets through strategic actions designed to achieve 13 objectives directed at improving the status of native fish populations in the Basin. These objectives are to: 1. repair and protect key components of aquatic and riparian habitats; 2. rehabilitate and protect the natural functioning of wetlands and floodplain habitats; 3. improve key aspects of water quality that affect native fish; 4. modify flow regulation practices; 5. provide adequate passage for native fish; 6. devise and implement recovery plans for threatened native fish species; 7. create and implement management plans for other native fish species and communities; 8. control and manage alien fish species; 9. protect native fish from threats of disease and parasites; 10. manage fisheries in a sustainable manner; 11. protect native fish from the adverse effects of translocation and stocking; 12. ensure native fish populations are not threatened from aquaculture; and 13. ensure community and partner ownership and support for native fish management. These 13 objectives will be achieved by implementing six driving actions that include management, research and investigation, and community engagement interventions: * rehabilitating fish habitat - helping to achieve objectives 1-8; * protecting fish habitat - helping to achieve objectives 1-8; * managing riverine structures - helping to achieve objectives 4-8; * controlling alien fish species - helping to achieve objectives 6-9; * protecting threatened native fish species - helping to achieve objectives 6 and 10; and * managing fish translocation and stocking - helping to achieve objectives 9-12. All of the driving actions include a community engagement component designed to achieve objective 13. The establishment of effective fish passages through major barriers and the implementation of engineering and operational solutions to thermal pollution are the core actions for managing riverine structures. Alien fish species will be managed through integrated pest management approaches that use a combination of actions to reduce populations and stop the spread of alien fish species. The Strategy urges immediate investment in the development and implementation of species' recovery plans for threatened or endangered native fish species. The establishment of Habitat Management Areas will help such recovery plans. A consistent, coordinated and firm Basin-wide approach is needed for managing fish translocation and stocking. The implementation of the driving actions will not see an immediate return on investment. While the rehabilitation of fish habitat and the management of riverine structures should result in changes within the next 10 to 15 years to native fish communities, the other driving actions are likely to take considerably longer before benefits become obvious. However, if this investment is delayed it will prove more costly to rehabilitate the Basin's native fish communitiesin the future. A key feature of the driving actions, especially for rehabilitation of fish habitat, will be the establishment of Riverine Management Zones that reflect the ecological functioning of the Basin's rivers as well as management capabilities. Management plans will be developed for eachZone and will be prioritised for investment according to both their feasibility and importance. Plans will be integrated with other planning processes. Within Riverine Management Zones there may be demonstration reaches, varying from a few kilometres in length to larger sections of about 100 kilometres. The demonstration reaches will integrate all land and water programs to form comprehensive rehabilitation exercises on important and visible river reaches. The key purpose of a demonstration reach is to show the community the cumulative benefits of using a number of actions for rehabilitating native fish populations and communities.

Riverine Management Zones may also include Habitat Management Areas that aim to protect remnant areas of healthy fish habitat. The Habitat Management Areas can range from those with limited human access to multiple-use areas, such as those which allow sustainable recreational angling. Experts have agreed that the actions such as habitat restoration and improved environmental flows detailed in the Strategy must be acted upon in an integrated way, if they are to be effective. If undertaken singly, the capacity of these interventions to recover the native fish populations of the Basin beyond 25 per cent of their pre-European level is questionable. Thetargeting of investment in the actions of developing a system of Habitat Management Areas and managing other alien fish species will also ensure a greater level of success with this approach. This Strategy will be monitored against accountability indicators in conjunction with the Sustainable Rivers Audit process. Its progress will be reviewed annually, with major external reviews of the Strategy conducted in the fifth and tenth year of its operation. In 2013, development of the 2013-23 Native Fish Strategy will ensure Basin-wide approaches to native fish management into the foreseeable future. The successful implementation of this Strategy relies on knowledge generation and exchange. This will be achieved by research and investigation to fill gaps in our knowledge and understanding about fish and river ecology, and through a comprehensive communicationstrategy that focuses on partner consultation and engagement. An agreed, fully funded Native Fish Strategy will provide benefits beyond just those for native fish: * There are obvious and significant economic advantages to the recreational fishing and tourism industries through having healthy fish populations and healthy rivers; * Healthy fish populations would signal a return of cultural values and the notion of community 'connectedness' to the River; * Indigenous people across the Basin have always had a strong spiritual and physical connection to the environment, and healthy fish populations and river systems will ensure the continuation of this connection; * The holistic and integrated approach promoted in the Strategy will lead to enhanced biodiversity conservation generally, and highlight the contribution of improved native fish populations to river ecosystems;* The aesthetic value of an improved, functioning river system cannot be overstated - for example, carp-free wetlands and intact riparian vegetation; * Improved river health will enhance all recreational activities, particularly for those Australians who live in the vicinity of the Basin's rivers and wetlands. All available evidence indicates that on a Basin-wide scale, native fish populations and communities are not in good shape.However, we are not starting from scratch- there have been some success stories to date. There are a number of examples where the sort of 'interventions' mentioned in the Strategy have led to increased native fish populations. For example: * Resnagging has been conducted at 14 sites on the River Murray with the addition of over 300 large river red gum snags. The resnagging was conducted on a scientific basis which allowed thetesting of different snag pile locations and configurations. Structured monitoring has been undertaken which shows that a range of native fish species use the new snags, in the same proportions as would have been expected in natural snag piles. The snags were favoured by native species such as Murray cod, trout cod and golden perch, but were not heavily used by carp. Different locations were more successful than others, indicating designs to maximise environmental benefit. Similarly, different locations were used by different species. A cost benefit analysis of resnagging was also conducted. * Torrumbarry was the first fishway built in Australia that was directly based on experiments on the swimming ability of native fish. In 1990 it was the highest fishway at the time, at 6.5 metres. It opened up 350 km of river, to Yarrawonga Weir upstream. From the moment it was opened, native fish used it; in the first two years the fishway passed over 20 000 native fish from seven species. Above the weir, numbersof silver perch increased and juvenile golden perch were seen for the first time in decades. Tagged golden perch moved all the way to Yarrawonga Weir and also entered the Goulburn River. Shortheaded lampreys, a very uncommon native species, used the fishway-these fish migrate from the sea and have swum over 1600 km to reach Torrumbarry. * Before Googong Dam was constructed on the Queanbeyan River in 1978, therewas a small population of the threatened Macquarie perch in the

river. After the dam was built, a fish monitoring program revealed that Macquarie perch were present in the reservoir, but were not breeding. It is believed that the filling of the reservoir flooded all available Macquarie perch spawning sites, and the population was unable to access the spawning habitats in the river above the reservoir because of a natural barrier posed by a waterfall. Fifty-seven adult Macquarie perch were netted from the reservoir and transported upstream, past the waterfall, and released at two sites on the Queanbeyan River. There is now a thriving population of Macquarie perch, with regular breeding occurring. * In the lower Murray, there are several local areas where anglers have reported increased catch rates for the larger species (such as Murray cod), while at the same time carp populations seem to be in decline

1029: *+.073*

Translocation of animals to re-establish extirpated populations or to maintain declining ones has often been carried out without genetic information on source or target populations, or adequate consideration of the potential effects of mixing genetic stocks. We consider the conservation status of the fisher (Martes pennanti) and evaluate the potential genetic consequences of past and future translocations on this medium-sized carnivore by examining population variation in mitochondrial control-region sequences. We sampled populations throughout the fisher's range in North America including five populations unaffected by translocations and two western populations that had received long-distance translocations. Twelve haplotypes showed little sequence divergence. Haplotype frequencies differed significantly among subspecies and between populations within subspecies. Analysis of molecular variance (AMOVA) and neighbour-joining analyses of haplotype relationships revealed population subdivision similar to current subspecies designations, but which may reflect an isolation-by-distance pattern. Populations in Oregon and in Montana and Idaho received several translocations and each showed greater similarity to the populations where translocations originated than to adjacent populations. Additional sequences obtained from museum specimens collected prior to any translocations suggest historical gene flow among populations in British Columbia, Washington, Oregon, and California. Anthropogenic impacts in that region have greatly reduced and isolated extant populations in Oregon and California. Future translocations may be necessary to recover populations in Washington and portions of Oregon and California; our results indicate that British Columbia would be the most appropriate source population.

1031: *+.158*

Current Species Status: The Karner blue butterfly, Lycaeides melissa samuelis Nabokov (Lepidoptera: Lycaenidae), formerly occurred in a band extending across 12 states from Minnesota to Maine and in the province of Ontario, Canada, and now only occurs in the seven states of Minnesota, Wisconsin, Indiana, Michigan, New York, New Hampshire, and Ohio. Wisconsin and Michigan support the greatest number of Karner blue butterflies and butterfly sites. The majority of the populations in the remaining states are small and several are at risk of extinction from habitat degradation or loss. Based on the decline of the Karner blue across its historic range, it was listed as endangered in 1992. Since listing, two populations have been extirpated and are being reintroduced to Concord, New Hampshire, and West Gary, Indiana. A third population is being reintroduced to Ohio. Habitat Requirements and Limiting Factors: The Karner blue butterfly is dependent on wild lupine, Lupinus perennis L. (Fabaceae), its only known larval food plant, and on nectar plants. These plants historically occurred in savanna and barrens habitats typified by dry sandy soils, and now occur in remnants of these habitats, as well as other locations such as roadsides, military bases, and some forest lands. The primary limiting factors are loss of habitat through development, and canopy closure (succession) without a concomitant restoration of

habitat. A shifting geographic mosaic that provides a balance between closed and open-canopy habitats is essential for the maintenance of large viable populations of Karner blue butterflies. Recovery Objectives: The objective of this recovery plan is to restore viable metapopulations of Karner blues across the species extant range so that it can be reclassified from endangered to threatened. The long-range goal is to remove it from the Federal list of Endangered and Threatened Wildlife and Plants. Recovery Criteria: The reclassification criteria will be met when a minimum of 27 metapopulations [19 viable metapopulations (supporting 3,000 butterflies each), and 8 large viable metapopulations (supporting 6,000 butterflies each)] are established within at least 13 recovery units across the butterfly's range and are being managed consistent with the recovery objectives outlined in this plan. Delisting will be considered when a minimum of 29 metapopulations (13 viable and 16 large viable metapopulations) have been established within at least 13 recovery units and are being managed consistent with the plan. Actions Needed: 1. Protect and manage Karner blue and its habitat to perpetuate viable metapopulations. 2. Evaluate and implement translocation where appropriate. 3. Develop rangewide and regional management guidelines. 4. Develop and implement information and education program. 5. Collect important ecological data on Karner blue and associated habitats. 6. Review and track recovery progress (includes re-evaluation of recovery goals for Wisconsin).

1032: ***+.070***

Baseline data on health of free-ranging wildlife is essential to evaluate impacts of habitat transformation and wildlife translocation, rehabilitation, and reintroduction programs. Health information on many species, especially great apes, is extremely limited. Between 1996 and 1998, 84 free-ranging orangutans captured for translocation, underwent a complete health evaluation. Analogous data were gathered from 60 semi-captive orangutans in Malaysia. Baseline hematology and serology; vitamin, mineral and pesticide levels; and results of health evaluations, including physical examination, provide a baseline for future monitoring. Free-ranging and semicaptive orangutans shared exposure to 11 of 47 viruses. The semi-captive orangutans had significantly higher prevalence of antibodies to adenovirus ($P<0.0005$) and rota (SA 11) virus ($P<0.008$). More free-ranging than semi-captive animals had antibodies to Japanese encephalitis virus ($P<0.08$) and foamy virus ($P=0.05$). Exposure to parainfluenza and langat viruses was detected exclusively in semi-captive animals and exposure to sinbis virus was only found in free-ranging orangutans. There was evidence of exposure to respiratory syncytial virus, coxsackie virus, dengue virus, and zika virus in both groups. Ebstein-Barr virus was ubiquitous in both groups. Prevalence of antibodies against mumps virus changed from 0% in 1996 to 45% in 1998. No antibodies were detected to many important zoonotic viral pathogens, including herpesvirus and hepatitis virus. Prevalence of Balantidium coli and Plasmodium pitheci infections and exposure to mycobacterium was higher in the semi-captive animals. Differences in exposure to pathogens between the groups may be due to environmental factors including differences in exposures to other species, habitat quality, nutritional status, and other potential stressors. Differences in health parameters between captive and free-ranging orangutans need to be considered when planning conservation areas, translocation procedures, and rehabilitation protocols. Because survival of the orangutan is linked to animal and ecosystem health, results of this study will assist wildlife conservation programs by providing baseline health information.

1033: ***+.165***

This 589-page book is meant to be an introduction to the subject of mammal invasions and their threat to biodiversity and ecological integrity around the world. It represents a companion volume to Introduced Birds of the World. The book provides a brief description of 337 mammalian species

that have been introduced, reintroduced or translocated into new environments, with a summary of the history of introductions, habits, behaviour, distribution and influence for each. The history includes date of introduction, person or agency responsbile, location of release, and the fate of the introduction. The book is organized by Order, Family and Genus, with species placed alphabetically under each genus. The text is illustrated with distributional maps for several of the animals and with line drawings for a small number. A lengthy list of references is included at the end of the book, as well as an index to common names and an index to scientific names. The text is written in English. Users of the book will include students of ecological systems management and biological conservation and practioners in those fields.

1034: *+.318*

The development of new scientific techniques has led to significant advances in our understanding of biodiversity and the threats facing animal populations. Zoos have been at the forefront of the application of these techniques, ranging from cytogenetics to the analysis of small-population biology, with the aim of improving animal management and facilitating in situ conservation. Many of the key applications of genetic analysis are discussed; for example, assessing species diversity, utilizing studbook data, understanding genetic diseases and the related implications for captive-breeding and reintroduction, together with the latest technological developments. The increasing power of genetic analysis will offer fundamental insights into aspects of biology that are of direct concern to zoos.

1035: *+.345*

This paper reviews the occurrence and quality of reintroductions of plants and animals, and the use of reintroduction as a tool in conservation biology. The Re-introduction Specialist Group (RSG) of the IUCN/Species Survival Commission (SSC) was created in 1988 and the activities of the Group are described. Illustrations of innovative processes and practices, developed within improved frameworks of policies and legislation, including the Convention on Biological Diversity, show that reintroduction has become a more rigorous discipline with the potential to contribute to community-restoration programmes. The activities of the RSG have contributed to this achievement.

1037: *+.063*

The last lowland locality of Gentiana verna in the Czech Republic is a calcareous grassland near Rovna at Strakonice in South Bohemia. This locality was the subject of a recovery programme that included support of the remaining population by micropropagation. The plants were inoculated with arbuscular mycorrhizal fungi (AMF) after their transfer to ex vitro and the effect of AMF on their establishment and survival was studied. Although the conventional method of inoculation of G. verna using spores or colonized root segments as an inoculum source resulted in no or negligible root colonization, the transplantation of gentians to the locality Rovna was successful and the plants became colonized with AMF very rapidly in the field. Successful mycorrhization of gentians under experimental conditions occurred only via the extraradical mycelial network established by neighbouring mycorrhizal plant species (nurse plant effect). Different nurse plant species formed different morphological types of mycorrhiza when inoculated with the same fungal isolate. Gentians always had the Paris type of root colonization with intracellular hyphal loops and swellings. Intercellular hyphae, arbuscules and vesicles were not observed. No evidence for a positive growth response was found in G. verna.

1038: *-.037*

Previous work suggests that submergence of Lycaena dispar larvae during overwintering may play a significant role in this butterfly's population dynamics. Since potential re-introduction sites in eastern England are prone to regular seasonal flooding, we further studied the species' submergence tolerance with a view to formulating management protocols conducive to larval survivorship under periodic flood conditions. Simulated flooding regimes using captive-reared larvae showed that enforced submergence has a twofold effect: firstly, a direct increase in mortality after 28 days under water and, secondly, a longer term, post-diapause increase in mortality; manifest either as an inability of larvae to resume feeding, or a failure to complete development. Additionally, there was a marked difference in the response of "early" and "late" diapause larvae; the latter generally succumbing after shorter periods under water, and suffering higher total mortalities. Behavioural investigations suggest that, if afforded the opportunity, diapausing larvae can evade submergence by climbing onto the exposed sections of partially flooded host plants. Significantly, survival on partially flooded plants was found to be comparable to that on unflooded controls. Further re- introductions of L. dispar in the U.K. will probably necessitate a direct translocation of wild Dutch stock. As the flood tolerance of this source population remains largely undetermined, and given that re-introduction site hydrology will be generally unamenable to conservation-oriented manipulation, it is recommended. that restoration management be directed towards creating structural diversity in the vegetation of overwintering habitats, thereby providing potential "flood refugia" for hibernating larvae.

1039: *+.149*

This paper presents data obtained on the biology of the Marquesan Imperial-Pigeon, or Upe, a critically endangered species that is very poorly studied, during a reintroduction procedure of the species from Nuku Hiva to Ua Huka Island, which was initiated as a conservation priority to create a second population. Our data show that in the study area, the Upe preferentially used the plant species of the emergent tree stratum and some of the native tree species of the intermediate stratum. The species' diet includes fruit and grain but also flowers, leaves and insects. Data collected on nest sites, social interactions, courtship, vocalisations and measurements, including sex and age determination, are also presented.

1040: *+.056*

The leaf beetles Cryptocephalus coryli, C. decemmaculatus and C. nitidulus are of conservation concern and are included on the UK Biodiversity Action Plan. The distinctiveness of the disjunct remaining populations of these beetles was compared to that of more continuously distributed Cryptocephalus species. This was carried out with a view to defining evolutionary significant units (ESUs) in the rare species. A portion of the cytochrome b gene, an intergenic spacer and partial tRNA was analysed from 93 specimens of Cryptocephalus beetle (Coleoptera: Chrysomelidae). Considerable sequence divergence was apparent in all the species, even at an intersite scale when the distances between sampled localities were very small (< 1 km). Intrapopulation, intersite and interpopulation divergence observed in the rare species was reflected in the species that have a more continuous distribution, implying that dispersal ability in these species is poor and gene flow can be impeded by relatively trivial barriers to dispersal. The evidence suggests that the disjunct populations of the rare Cryptocephalus species can, tentatively, be considered as ESUs. This has important implications for management strategies and reintroductions.

1041: *+.184*

In a Zoo-FISH study chicken autosomal chromosome paints 1 to 9 (GGA1-GGA9) were hybridized to metaphase spreads of nine diverse birds belonging to primitive and modern orders. This comparative approach allows tracing of chromosomal rearrangements that occurred during bird evolution. Striking homologies in the chromosomes of the different species were noted, indicating a high degree of evolutionary conservation in avian karyotypes. In two species, the quail and the goose, all chicken paints specifically labeled their corresponding chromosomes. In three pheasant species as well as in the American rhea and blackbird, GGA4 hybridized to chromosome 4 and additionally to a single pair of microchroinosomes. Furthermore, in the pheasants fission of the ancestral galliform chromosome 2 could be documented. Hybridization of various chicken probes to two different chromosomes or to only the short or long chromosome arm of one chromosome pair in the species representing the orders Passeriformes, Strigiformes, and Columbiformes revealed translocations and chromosome fissions during species radiation. Thus comparative analysis with chicken chromosome-specific painting probes proves to be a rapid and comprehensive approach to elucidate the chromosomal relationships of the extant birds. Copyright (C) 2003 S. Karger AG, Basel

1042: *+.136*

The Delmarva fox squirrel, Sciurus niger cinereus, is a federally listed endangered subspecies whose range has been reduced by 90%. In an attempt to increase both population size and range, translocation sites were established beginning in the 1960's by moving squirrels from the natural range to sites outside the current range. Although translocations have served as the primary component of the DFS recovery program, there has been very little post-release examination of the genetics of the translocation sites. In this study, we developed ten microsatellite loci, screened the three polymorphic loci, and sequenced a 330 bp fragment of the mitochondrial control region in order to assess levels of genetic variation in natural and translocated regions of Delmarva fox squirrels and to compare them to Southeastern fox squirrels (S. n. niger). Although we found low levels of microsatellite polymorphism, there were no differences in heterozygosity between natural and translocated regions, or between Delmarva and Southeastern fox squirrels. We found high levels of polymorphism in the mitochondrial control region. Our patterns of haplotype diversity suggest incomplete lineage sorting of the two subspecies. In general, our data suggest that the current levels of genetic variation in the translocated sites are representative of those found in the natural population, and we encourage the continued use of translocations as a major component of Delmarva fox squirrel recovery.

1043: *-.009*

Silene hifacensis is a narrowly endemic plant, restricted to a few small populations on limestone cliffs in the Spanish province of Alicante and on the Balearic island of Ibiza. The species was collected to extinction in its original mainland location by the early 20th century. Attempts have been made to reintroduce S. hifacensis to this area but conservation efforts are limited by a lack of information on the geographic structure of genetic variation in the species. We used nuclear (allozyme) and chloroplast DNA (cpDNA) PCR/RFLP markers to investigate the structure of genetic variation in 2 mainland and 6 Ibizan populations. Levels of allozyme variation were low, with a mean of 2 alleles per polymorphic locus. Mean (over polymorphic loci) total allozyme diversity (H-tot) was 0.203 and mean within-population diversity (H-pop) was 0.085. Most diversity was explained by the between-population diversity component (G(pop). reg = 57%). Both mainland populations showed allozyme fixation. Three composite cpDNA haplotypes were identified. The first is unique to a mainland population that is also allozymically distinct from all the other populations. The second haplotype is found in the other mainland population and one

Ibizan population: these two populations are allozymically identical. The remaining Ibizan populations contain the third haplotype. The geographic distribution of allozymes and cpDNA haplotypes is discussed in terms of population history, dispersal and, speculatively, in terms of the possibility that there has been undocumented translocation of material between populations.

1044: *+.058*

Until recently, the dusky Canada goose (Branta canadensis occidentalis) was managed as one breeding population from the Copper River Delta (CRD), Alaska. Population numbers on the CRD have declined precipitously over the last three decades, due in part to changes in habitat. In 1981, a pair of Canada geese, presumably B. c. occidentalis, was reported nesting on Middleton Island (MID), in the Gulf of Alaska. Numbers of Canada geese on the island increased in the decade subsequent to a translocation of geese from CRD to MID, but it is unclear whether the increase is attributable to the translocation effort. We used genetic data derived from three classes of genetic markers to clarify relationships of Canada geese breeding in south-coastal Alaska. Geese were sampled from 5 populations: CRD, MID, Anchorage (ANC), Admiralty Island (ADM) in southeastern Alaska, and Green Island (GRN) in Prince William Sound (PWS). Mitochondrial DNA analyses demonstrate Canada geese from MID are nearly monomorphic for a unique haplotype fixed on GRN but not found in CRD or any other breeding population. Furthermore, nuclear markers consistently cluster MID with GRN to the exclusion of CRD. We suggest the current population on MID is not derived from birds translocated from CRD, but rather that MID was most likely colonised by birds inhabiting other island habitats within the PWS. Furthermore, since geese from the CRD share mtDNA haplotypes with geese from other breeding locales, they apparently share recent common ancestry and/or gene flow with populations representing other subspecies. Our genetic data raise questions about the validity of current management units of Canada geese.

1045: *+.119*

Dry, sandy scrub habitats of the Florida peninsula represent naturally fragmented remnants of xeric ecosystems that were widespread during the Pliocene and early Pleistocene. This habitat is characterized by high endemism, and distribution of genetic and evolutionary diversity among scrub " islands" is of compelling interest because Florida scrub is rapidly disappearing under human development. We compare range- wide diversity in mitochondrial cytochrome b sequences for three scrub- associated lizards with contrasting levels of habitat specificity. All species show strong geographic partitioning of genetic diversity, supporting the hypothesis that scrub fauna is highly restricted by vicariant separations. The mole skink (Eumeces egregius), the least habitat specific, has the lowest phylogeographic structure among the lizards (phi(st) = 0.631). The mtDNA geneology for E. egregius is not entirely concordant with the five recognized subspecies and supports a link between populations in central Florida (E. e. lividus) and the Florida Keys (E. e. egregius) rather than a previously proposed affiliation between northern and southern populations. The Florida scrub lizard (Sceloporus woodi) is the most habitat specific of the lizards and has the strongest phylogeographic structure (phi(st) = 0.876). The sand skink (Neoseps reynoldsi) falls between the mole skink and scrub lizard in terms of habitat specificity and phylogeographic structure (phi(st) = 0.667). For all three species, networks of mtDNA haplotypes coalesce on two central ridges that contain the oldest scrub. The geographic structure and deep evolutionary lineages observed in these species have strong implications for conservation, including strategies for translocation, reserve design, and management of landscape connectivity.

1046: *+.110*

Pleistocene fragmentation of the Great Bahama Bank resulted in one large and several small populations of rock iguanas (Cyclura cychlura). We explore patterns of genetic variation within and among these island populations using mitochondrial sequence data (partial ND4 to tRNA(Leu)) in combination with eight polymorphic microsatellite loci (2 to 10 alleles). Genetic data support two phylogeographically distinct groups, Andros Island and the Exuma cays. This result conflicts with current subspecific taxonomy in which three subspecies are described. Analyses of allelic data indicate that most island populations are currently demographically independent. Pairwise Fst values between eight island populations range from 0.18 to 0.63, and 6 of 135 individuals are misassigned in an assignment test. Population-genetic diversity is characterized using standard measures such as number of alleles and heterozygosity (H) in addition to a normalized Shannon-Weaver index of diversity (D). We find genetic diversity in the Andros Island population comparable to that in other non-piscine animals (avg. # of alleles = 5, avg. H = 0.56, avg. D = 0.66) while in the Exuma cays populations these measures are much lower (avg. # of alleles = 2.75-1.625, avg. H = 0.43-0.17, avg. D = 0.45-0.18). These data are used to discuss conservation management strategies, including prioritization and translocation.

1047: *+.251*

Underpinning the conservation management of Austropotamobius pallipes in the UK is the process of monitoring and reporting crayfish distribution. Should the current trend in the decline of A. pallipes continue, the species could be virtually extinct in mainland Britain within 30 years (SIBLEY, 2003). Conversely, if the increase in the distribution of non-indigenous crayfish species (NICS) continues at its current rate, the distribution (by 10 km squares) of these species could double within 15 years. These forward projections are based on a number of possibly unreliable assumptions; they illustrate however the magnitude of the challenge facing those concerned with the conservation of A. pallipes in the UK at this time. Recent work in crayfish conservation management in the UK has yielded guidance in several areas including monitoring, habitat enhancement and a re-introduction protocol for A. pallipes (KEMP and HILEY, 2003). Similarly, scientific research continues to inform our understanding of the movement and behaviour of NICS and explores new methods for the potential management of these species. In addition, the protection afforded to A. pallipes by current legislation is key to the long-term survival prospects of the species, albeit with a probable fragmented distribution, across the British Isles and continental Europe. Legal provisions in the UK derive in part from European instructions (e.g. EC Habitats and Species Directive) and also from national legislation (e.g. Salmon and Freshwater Fisheries Act (1975) and the Wildlife and Countryside Act (1981)). Also, a raft of "quasi-legislation" exists which requires responsible organisations in the UK to implement the white-clawed crayfish biodiversity action plan (BAP). Altogether these provisions constitute a considerable volume of legal protection for crayfish and provide the legal framework on which UK management policy and practice are based.

1048: *+.183*

When looking at the various national surveys performed in Metropolitan France since 1977 by the Conseil superieur de la peche, crayfish species distribution shows that nativespecies (Austropotamobius pallipes, Astacus astacus, Austropotamobius torrentium) are either rare, or in decline; while the introduced species (Orconectes limosus, Pacifastacus leniusculus, Procambarus clarkii, Astacus leptodactylus), are increasing. The ban of live introduced species transport, except for A. leptodactylus, didn't stop this process with a peculiar acceleration of P leniusculus propagation these last five years, certainly responsible for plague (Aphanomyces astaci) renewed outbreaks. The other measures in favor of native species, such as fishing limitations and

reintroduction plans, have not reversed the trend. So, it seems necessary to develop a conservation policy based on habitat preservation and on the respect of certain prophylactic rules which are to be determined. That can only be considered on watersheds of small area, easily controlled if local authorities are involved. The 55 French habitat decrees, and the 154 Natura 2000 zones with their management plans, seem to be a promising way to implement this type of actions in France. The case of Corsica, recently colonized by O. limosus, would require special measures to prevent the island from invasion by P leniusculus and P clarkii in the future.

1049: ***+.002***

Before 1994, the California condor (Gymnogyps californianus) captive program relied upon egg removal, double clutching, and hand rearing for the rapid increase of the captive population and subsequent reintroduction. Problems encountered with the first hand-reared released birds resulted in modification of the captive condor program to initiate parent rearing whenever possible. From 1994 through 1999, we examined incubation behavior in eight captive pairs of California condors at the San Diego Wild Animal Park using time-lapsed, dawn-to-dusk video recordings. Known pedigrees were the basis for pair formation rather than free choice. For males and females, we calculated the percent of time spent incubating, frequency of nest box entry with distinction between entries into a vacant or occupied nest box, egg manipulation, number of incubation bouts, nudge mate, egg pull, and aggressive interactions. There were no sex differences in the percent of time spent in incubation. Males entered occupied nest boxes significantly more often than females and such entries frequently resulted in aggression. Females had a higher mean rate of aggression than did males, but the difference was not significant. Because some pairs had higher combined rates of male/female aggression than others we categorized pairs as "compatible" or "incompatible" based on the results of paired sign tests. Incompatible pairs had a significantly greater proportion of entry into occupied nests, rates of nudge mate, and egg pull than the compatible pairs. Observed differences found within particular pairs in their daily time spent on nest and rates of aggression suggest that some of the forced pairs were less cooperative in their parental care duties. As more birds become available for captive propagation, there will be greater flexibility in establishing cooperative condor pairs and potentially increasing the number of parent-reared birds for release.

1050: ***+.175***

Stocks of anadromous American shad Alosa sapidissima, blueback herring A. aestivalis, and alewife A. pseudoharengus are being restored to the Susquehanna River in Maryland, Pennsylvania, and New York. This river once supported extensive American shad and herring fisheries that were lost with construction of four large hydroelectric dams in the lower 90 kin during 1904-1931. Fish reintroduction and project evaluation activities have been undertaken over a 3-decade period by numerous state, federal, and utility company partners. These included the culture and release of over 150 million marked larval and fingerling American shad and trap and transport of over 200,000 prespawning adults to suitable spawning waters above blockages. The American shad population returning to the lowermost dam on the Susquehanna grew from only a few hundred to over 150,000 fish in the past 16 years. Survival of out-migrating juvenile American shad through hydroelectric turbines has been evaluated and, where necessary, improved with project operational changes during peak movement periods. Settlement agreements reached with each of the utility companies led to construction of large-capacity fish elevators and ladders at the four lower river dams during 1991-2000. As a result, over 750 km of historic spawning habitat for anadromous fishes has been reopened for the first time in almost a century.

1051: ***+.160***

Among fishes of the family Clupeidae (which includes the shads), about 29 species are diadromous, most of them anadromous, though one is amphidromous and two are catadromous. The origin and evolution of diadromy in shads remains largely unexamined, and how often it has evolved is unknown. The implications of diadromy, nine of which follow, for the ecology, evolution, and biogeography of shads are diverse. (1) Diadromy suppresses evolutionary divergence and increase, in species diversity through facilitation of gene flow among populations that are otherwise isolated in separate river systems. (2) This gene flow is hindered, and so divergence and diversity are fostered, when diadromous shads are able to undertake accurate homing to natal habitats. (3) Diadromy facilitates dispersal and the acquisition of broad geographical ranges, because even where there is some homing, straying is likely. (4) Diadromy enables species to be among the first that invade newly available habitats (as with the retreat of glaciers) and also facilitates restoration of fish populations to habitats following short-term perturbation. (5) Diadromy appears, in general, to hinder successful human translocation of species to novel areas, probably as a result of highly evolved navigational powers that relate to solar or geomagnetic guidance mechanisms, though the only instance in which an anadromous shad translocation has been attempted was successful, and the species has continued to spread. (6) Diadromous migrations pose special problems for conservation, in relation to the need for fishes to have both upstream and downstream access during their life cycles. (7) There is latitudinal variation in the frequency of diadromy, with anadromous fishes tending to be more common at higher latitudes, but explanations of this variation that point to parallel gradients in marine productivity seem more elegant than real and are, at best, partial. (8) Diadromy has a significant and distinctive role in stream fish community assembly; gradients of upstream-downstream diversity in strongly diadromous fish communities that superficially appear to conform to the River Continuum Concept have an altogether different explanation: diversity is an outcome of differential upstream penetration by fish species migrating into rivers from the sea. (9) Diadromy has implications for the application of indices of biological integrity (IBI) to the extent that without the acquisition of extensive data sets of local origin and the elaboration of models of distribution, IBIs are largely inapplicable to fish communities that include a significant proportion of diadromous fishes.

1052: ***+.106***

Vulture Reintroduction Projects in the Alps. In the last century all four European vulture species nested in the Alps, albeit with different status, sometimes based upon unreliable historical data. Proposals for the reintroduction of the Bearded Vulture had already been put forward in the 1920's but the plan finally became operative with a program of reintroductions using the `hacking' technique starting in 1986 in the High Tauern in Austria. Following on from this, the reintroduction program was extended to four different Alpine sectors and the use of various release points. Altogether more than 100 individuals have been released with a current population of about 70 Bearded Vultures. The first successful breeding occurred in 1997 since when there has been a gradual increase in the number of pairs formed and young birds fledging. The Eastern Alps play regular host to Griffon Vultures coming from the Balkans. These over-summer particularly in the High Tauern range in Austria and the mountainous areas of Friuli and Slovenia. This phenomenon seems to be declining in recent decades in parallel with the decline in Griffon Vulture populations in south-eastern Europe and especially those of the Kvarner Archipelago (Croatia), which holds the northernmost colonies and from where, at least in recent years, most of the birds originate. Steps to help the species were proposed from the 1970's onwards. The main focus of efforts for this species have been interventions aimed at helping the species maintain and

consolidate its presence in the Alpine area, as well as contributing to the protection of the northern Adriatic colonies. To this end a project was undertaken in the Friulan Pre-Alps with the release of 60 birds between 1992 and 1998 and the establishment of a feeding site. The initiative has brought about the creation of nesting colonies in the area with about 60 Griffon Vultures present. It also acts as a focal point for Croatian birds which can number more than 50 birds during the summer months. The possibility to stop over at secure sites with a reliable food supply has reduced mortality rates for these birds (particularly the juveniles), allowing the expansion of the feeding range of the Croatian colonies and providing a fundamental contribution to the strategy for the conservation of the species in Eastern Central Europe. Griffon Vultures from other areas (France and Spain) have also been observed at the site and the colonies and feeding site acts as a magnet, drawing in other raptors including species which are rare in Italy. After the success of the project in the Cevennes, three further Griffon reintroduction projects have been launched in Provence (France) with the aim of bringing the species back to southern France and the French Alps, In these areas the reintroduction of the Black Vulture is also being planned.

1053: *+.356*

Conservation and reintroductions: poor planning, many projects. By examining the INFS (Istituto Nazionale per la Fauna Selvatica) mail archives, scientific literature, the main popular nature magazines, and bulletins from the most relevant nature conservation associations, it has been possible to assess the extent of raptor reintroduction projects proposed or in progress in Italy between 1995 and 2001. In total, 115 news events have been recorded, referring to 23 projects involving 8 different raptor species. These results suggest that there are many reintroduction programs, carried out by many different organizations and concerning a wide range of species, some of them characterizing a favourable conservation outcome. In Italy, reintroductions are therefore perceived as ordinary tools for raptor conservation and are often promoted at the local level, without consideration of the wider perspectives and critical evaluation parameters (i.e., actual effectiveness of the action, priority level, risks assessment of impacts on natural populations, etc.). Such a situation requires the development of a more formal reintroduction policy for raptors and other wildlife in Italy. In order to change the current approach to reintroductions, and for improving the quality level of the reintroduction efforts, it is suggested that only those projects that are explicitly supported by international and national action plans should be undertaken in the future; and moreover, each reintroduction program should be submitted for scientific and technical approval.

1054: *-.040*

In 2001 the California Department of Fish and Game requested that I conduct an independent review of the causes of mortality of California Condors and to specifically review the data on lead in the environment in California and the magnitude of the lead exposure hazard to condors. The scope of this study has included the compilation of data on mortality, movements of birds in the field, feeding, monitoring efforts, blood sampling, and the lead exposure data for other species within the condor range in California. This review also examines the potential sources of lead in the environment in California, and assesses the magnitude of the exposure risk of lead to California Condors. The California Condor Recovery Program has overcome many hurdles in the effort to increase the condor population and return birds to successful independent survival in the wild. Captive propagation to increase numbers has been accomplished at three facilities (San Diego Wild Animal Park, Los Angeles Zoo, and the Peregrine Fund World Center for Birds of Prey, Boise Idaho) with the hatching and rearing of more than 250 chicks over the past 20 years. Reintroduced California Condors now occupy most of the range within California that they used

during the period 1940-1987. Range expansion into Monterey County has occurred with reintroductions near Big Sur. The wild-hatched condors AC8 and AC9, which were trapped in 1986-7 and held in captivity for 14 years, were reintroduced in 2000 and 2001, and they use the traditional foraging areas of the Sierra foothills. Condors have suffered a high mortality rate in the wild. During the 1970s and 1980s approximately half of the known population disappeared from the wild, with many of the carcasses never found. During that period, at least 3 condors died of lethal exposure to lead. All remaining condors were trapped and kept in captivity until a reintroduction program was begun in the early 1990s. Since 1995, more than 140 condors have been released in California and Arizona, and 44 are free-flying in California at the current time. Four condors have died of lead poisoning since 1997 (1 in California, 3 in Arizona), and 26 condors have received emergency chelation treatment to reduce toxic lead levels (8 in California, and 18 in Arizona). AC8 nearly died in November 2002, when she ingested metallic lead near Hopper Mountain NWR. Expert veterinary care prevented her death, and several other potential lead mortalities since 2000. AC8 was released back to the wild in late December 2002, but she was shot and killed on the Tejon Ranch in early February 2003. Field biologists have systematically monitored lead exposure in condors since 1997. In California, every bird has had detectable lead in blood samples. 62% of the blood samples taken in Southern California have shown higher than background lead exposure (blood levels greater than 20 [mu]g/dI blood). 13 of 87 samples (15%) have exceeded 60[mu]g/dl, indicating the condors were clinically affected by lead poisoning, and 6 birds have had blood lead levels greater than 100 [mu]g/dl, indicating acute toxic exposure and requiring emergency veterinary intervention. The half-time for excretion of lead from condor blood is about 13 days, demonstrating that condors will quickly depurate lead if kept in a clean environment. The high number of condors with above background blood levels suggests that the birds are frequently exposed to lead in the wild. Metallic lead fragments have been positively identified in the digestive systems of two condors in California since 1984. Two additional condors (AC3 and 132) had metal fragments in their gut at death, but chemical analysis was not performed to confirm lead. Three other condors with high blood lead levels had X-ray identification of dense particles, also not confirmed by recovery and testing, making a total of seven condors in California suspected of having lead fragments in their digestive systems. In Arizona, seven condors have either had lead pellets recovered from their gizzards, or have been diagnosed by radiography. Six of these 14 birds died of lead poisoning, and the others were successfully treated for acute toxicity. Lead exposure and intoxication continue to be a very critical problem for both the California and Arizona populations. Seventeen of the 35 dead condors examined by pathologists have not been tested for lead exposure. High-resolution lead and copper analysis techniques should be conducted on feathers that were growing at the time of death of the archived condors to determine the extent of lead and copper exposure in all condors that have died and been recovered since 1982. Experimental studies with lead intoxication of other species of raptors indicate that condors are not more sensitive to lead intoxication than most species of birds. Turkey Vultures, however, are very resistant to the effects of lead, and are not a good surrogate species to compare with condors. Condors and Turkey Vultures also apparently have very different responses to ingested copper. Copper levels in the livers of condors analyzed at necropsy have all exceeded 20 ppm (21-181 ppm), although the significance of copper reside levels is unknown. Copper levels should be studied in feathers, to determine whether a relationship exists with lead exposure. Potential sources of lead within the condor range include residual atmospheric lead deposition from leaded gasoline exhaust, lead in the soil from natural deposits, from disposal of lead containing items, and lead ammunition on rifle ranges. Current industrial air emissions of lead in California are small, totally only 9301 pounds in 2001, and most emission sources are outside of the condor range and not in the prevailing wind pattern. Hunting within the condor range is extensive, and the number of game animals taken by hunters in 2001 was estimated at 106,049 for the eight counties within the condor range. This review estimates the

number of large animal carcasses and field-dressed visceral remains (gut-piles) left in the field at more than 30,000 annually. This includes the gut piles of 8,180 deer, 17,249 wild pigs, and the carcasses of 10,816 coyotes, plus unrecovered carcasses of deer and pigs crippled by hunters and depredation permit holders. The number of ground squirrels shot within the condor range is unknown, but almost certainly represents a significant source of carrion with the potential to contain lead fragments that could be ingested by both condors and other scavenging birds and mammals. The risk of lead exposure to condors is high, because of the amount of carrion left in the field. Direct observations of condors feeding on hunter-shot carrion are few, even with intense radio telemetry and visual surveillance, and every effort should be made to document and organize the field observation data to quantify the exposure hazard from hunter-shot carrion. Every effort should be made to educate hunters and depredation permitees of this potential problem, and to encourage or require the use of commercially available lead-free and "lead-safe" rifle and shotgun ammunition. Field and captive management efforts by the condor program have been highly professional and the overall program is very successful. The field program has concentrated on a feeding program to prevent exposure to lead, monitoring the birds to determine movements, capture of birds to keep radio transmitters functional and to collect blood samples to monitor for lead exposure, and emergency intervention to prevent the debilitation or death of birds exposed to lead. The program has become very labor intensive, but the efforts to prevent lead exposure have not been successful. If a reduction in condor lead exposure can be accomplished, the Condor Recovery Program shows every promise of successfully rebuilding a stable wild population.

1055: *+.128*

1. After re-introduction in 1991 of Eurasian otters Lutra lutra (L.) to the River Lee catchment (England), changes in range were assessed from spraint collections in the periods 1991-1994, 1995 1997 and 1998-2000. Diet, assessed from concerted spraint sampling within the first two periods, was compared between 1992-1994 and 1996-1997, with dietary preferences assessed for the initial phase using available fish stock data for that period.2. In 1991-1994, range encompassed much of the catchment, but reduced in 1995-1997 to a small area around the initial release site, when the otters were joined/replaced by natural immigrants presumed to come from a contiguous catchment. Range expanded in 1998-2000 to upper reaches of the catchment's major tributaries.3. Fish, mainly cyprinids, were the major prey taken in 1992-1994 and 1996-1997. In 1996-1997, fish abundant near the initial release site became more prominent, in particular chub Leuciscus cephalus, and (upstream) species characteristic of the initial range decreased in importance.4. Despite confirmed and unconfirmed sightings of females with cubs, it remains unclear whether the presence and apparent expansion of otters in the Lee catchment is the result of the reintroduction or of natural immigration/recolonization. Copyright (C) 2003 John Wiley Sons, Ltd.

1060: *+.278*

The aim of this study was to evaluate the recovery and survival of four species of unionid mussles [pimpleback, Quadrula pustulosa pustulosa (I. Lea, 1831); spike, Elliptio dilatata (Rafinesque, 1820); Higgins eye, Lampsilis higginsii (I. Lea, 1857); and pocketbook, Lampsilis cardium (Rafinesque, 1820)] that were experimentally relocated to in situ refugia in the St Croix River of Minnesota and Wisconsin, USA. In 1996, 150 mussels of each of the first three species (450 total) were relocated to three 5 x 5 m study grids (Site A), one near Lakeland, Minnesota, which served as a source-site control, and two in the experimental refuge 48 km upstream, near Franconia, Minnesota. In a second relocation in 1997, L. cardium was substituted for L. higginsii and 150 mussels of this and each of the other two species (450 total), were relocated to two study grids (Site B). The source site control was near Sunrise, Minnesota and the experimental refuge was 14

km downstream near Almelund, Minnesota. Mussel recovery, survival and substratum characteristics were evaluated annually at Site A for 2 years and for 3 years at Site B. Mean annual recovery of all three species ranged from 90 to 100% at Site A, and from 34 to 70% at site B. The mean annual survival of recaptured mussels ranged from 85 to 100% at Site A, and from 88 to 100% at Site B. The textural characteristics of the substratum differed significantly between the control and the two refuge locations at the beginning of the study, but did not differ from this initial status among subsequent years at Site A. At Site B, there was a significant shift in textural characteristics from large to smaller fractions over the four years. The relatively high survival of mussels during this study demonstrates the importance of proper handling and transport protocols when relocating mussels and the selection of suitable relocation habitat with stable substratum. When established correctly, in situ refugia may be a viable tool for preserving unionid mussels.

1061: ***+.146***

Kokako (Callaeas cinerea wilsoni) population recovery on the North Island of New Zealand depends primarily on control of key introduced mammal pests, especially ship rats (Rattus rattus) and brushtail possums (Trichosurus vulpecula). Recovery can still occur if pest control is pulsed (x years 'on'; y years 'off') because kokako sub-adults and adults are generally long-lived, although chick production is high only during 'on' years. Pulsing effort means that conservation resources can be extended to other sites or problems during 'off' years; that toxin input at any one site is reduced; and that project staff do not burn out by repeatedly working at a site. Mathematical modelling supports empirical evidence that pests need not be controlled every year in order to maintain or greatly increase kokako populations. It predicts that the total number of years during which there is pest control is the main factor determining population size. Three years of pest control in each 10 should be sufficient to at least maintain a population with 20 females when mean parameters apply, but pulsed control should still be effective with very pessimistic parameters. In the safest strategies, control should occur in minimum pulses of 2-3 years to avoid single poor years when few breeding attempts are made. Very small populations should first be increased to at least 20 females by translocation or continuous pest control. This will greatly reduce the probability of chance extinction, and increase the efficiency of subsequent pest control. The model will apply best to closed kokako populations below carrying capacity, in which pests are controlled over the entire block. Empirical data on the effects of habitat carrying capacity on kokako dispersal, and on the importance of stoats as predators of adult females are required to further strengthen the model. (C) 2002 Elsevier Science Ltd. All rights reserved.

1062: ***+.095***

The western barred bandicoot was reintroduced to the Australian mainland in 1995 after an absence of at least 60 years. The new population was derived from 14 animals, reintroduced to Heirisson Prong from Dorre Island in Shark Bay, Western Australia. Introduced predators (the European red fox and the feral cat) were controlled at the reintroduction site, but European rabbits were not. A large fenced area of natural vegetation within the reintroduction site was used as a secure refuge from mammalian predators. Bandicoots were released from this predator refuge to the 12 km 2 conservation site. Dispersal from the point of free release was minimal. The reintroduced population has persisted for 4 years and increased, with at least 175 bandicoots recruited to the population in this time. The recapture rate of marked bandicoots was low, suggesting that adult mortality was high. Reproductive output at Heirisson Prong appeared greater than that of the two surviving wild populations on Bernier and Dorre Islands. Litter size was similar, but there was an extended annual breeding season at the reintroduction site. Body condition of reintroduced and wild bandicoots were similar, although there was some indication

that reintroduced males may have been in poorer condition than their island counterparts. The litter size of bandicoots increased with a decrease in rabbit abundance, however, bandicoots were able to reproduce, maintain condition, and sustain recruitment to allow the population to increase despite the presence of rabbits. Two fox incursions occurred during the 4-year period of establishment, and feral cats were present on occasion in low numbers. Feral cats may be responsible for a lower rate of population increase than that observed on predator-free Dorre Island. Ongoing predator control is essential for any mainland reintroduction of bandicoots. (C) 2002 Elsevier Science Ltd. All rights reserved.

1063: ***+.170***

The behavioral development of reintroduced, captive-born animals and their wild-born offspring is understudied, limiting the scientific understanding and, therefore, utility of reintroduction as a conservation tool. Several reintroduction programs have shown that survival rates of captive-born animals are lower than those of their wild-born offspring. However, whether these differences are because of increased behavioral competency of wild-born animals or age-related factors is unknown. This study compared behavior of captive-born golden lion tamarins to that of their age-matched first- and second-generation descendents. Subjects included 134 golden lion tamarins living in and around the Poco das Antas Biological Reserve in Brazil. Overall, captive-born animals were deficient in locomotor and foraging skills as compared with their wild-born offspring, and some of these deficiencies persisted after two years in the wild. Locomotor and foraging differences were also observed between generations of wild-born animals, suggesting that behavioral change continued past the first generation. Recommendations for future reintroductions with this and other species include: (1) increased exposure to complex environments prior to release; (2) intensive post-release support; (3) introduction of naive animals with experienced conspecifics when possible; (4) comparisons of reintroduced and wild populations when possible; and (5) short-term management plans aimed at the survival of captive-born individuals combined with long-term plans focused on maximizing natural adaptive processes.

1064: ***+.134***

Maritime ringlet butterflies (Coenonympha tullia nipisiquit McDunnough) are rare, endangered salt marsh butterflies with larvae that survive periodic tidal submergence. Only six disjunct populations are known to exist. First-instar larvae were released and monitored in seven microhabitats within a salt marsh in Bathurst, New Brunswick, Canada. From resighting data, microhabitat-specific daily death rates were estimated with maximum likelihood. The survival of the larvae released in groups was compared by calculating minimum number of larvae known to be alive (MNKA). Death rates were low in microhabitats with the larval host plant, salt meadow cordgrass (Spartina patens (Aiton) Muhl.) and the major nectar source, sea lavender (Limonium carolinianum (Walter) Britton), a habitat with a moderate frequency of flooding. Because microhabitats have a large effect on the survival rate of young larvae, use of microhabitat profiles in salt marshes as habitat-quality indices is recommended in critical-habitat assessment and reintroduction efforts.

1065: ***+.479***

Declines in the number and range of prairie grouse (Tympanuchus spp.) in North America have prompted numerous translocation efforts to establish additional populations, but overall success of translocations has been low. Because success of a translocation is ultimately determined by the quantity and quality of habitat at the translocation site, evaluating habitat prior to translocation

should be a critical consideration. I used landscape characteristics surrounding 75 greater prairie chicken (T. cupido) leks and 75 unused points to develop a habitat model identifying suitability of landscapes for greater prairie chickens in Wisconsin. Presence of leks was positively associated with amount of grassland and wetland in the landscape and negatively associated with forest cover and distance from nearest known lek. The model correctly identified 94% of sample leks and unused points. I applied the model to digital landcover data of the entire state of Wisconsin to create a spatially explicit map predicting suitability of unoccupied landscapes for translocation of greater prairie chickens. Sites identified as suitable for greater prairie chickens agreed with results of other prairie chicken habitat models and landscapes identified as having high priority for conservation of grassland birds in Wisconsin. The most suitable landscapes had substantial public ownership but would likely require fine-grained management to meet all habitat requirements of greater prairie chickens. Landscape-level habitat models combined with accurate digital data provide an efficient means of objectively assessing habitat for prairie chicken translocation.

1066: ***+.064***

Recent studies illustrate the emerging field of restoration genetics, which is a synthesis of restoration ecology and population genetics. The translocation of organisms during the restoration of native ecosystems has provoked new questions concerning the consequences of sampling protocols and of intraspecific hybridization between locally adapted and transplanted genotypes. Studies are now underway to determine both the extent of local adaptation among focal populations and the potential risks of introducing foreign genotypes, including founder effects, genetic swamping and outbreeding depression. Data are needed to delineate 'seed transfer zones', or regions within which plants can be moved with little or no consequences for population fitness. Here, we address the revival of transplant and common garden studies, the use of novel molecular markers to predict population genetic consequences of translocation, and their combined power for determining appropriate seed transfer zones in restoration planning for native plant populations.

1067: ***-.165***

Desert bighorn sheep (Ovis canadensis) are known for their climbing skills in rugged and steep terrain. Occasionally sheep die from climbing accidents. Between 1979 and 1995, the Arizona Game and Fish Department reintroduced desert bighorn sheep to areas within their historic distribution: northwest, southwest, southeast, and central Arizona. Because at least half of reintroduced sheep (n = 412) were fitted with radiocollars and monitored monthly, we documented mortalities by climbing accidents. None of 54 mortalities in the southeastern region and 53 mortalities in the southwestern region was identified as climbing accidents. However, climbing accidents caused 5 of 42 sheep mortalities in the northwestern region and 4 out of 54 mortalities in the central region. Most climbing accidents (7 out of 9) happened <9 months after sheep were translocated. Our data suggest that translocated desert bighorn sheep can be vulnerable to climbing accidents due to lack of familiarity with local terrain. Differential vulnerability between regions might be related to differences in substrate, ruggedness of terrain, or both at release sites. Additionally, sheep translocated from less rugged terrain might be more likely to fall in new areas than sheep translocated from more rugged terrain.

1068: ***+.214***

We translocated five colonies of the highly social and co-operatively breeding Black-eared Miner Manorina melanotis, an endangered Australian honeyeater. Two colonies were released immediately (hard release) and two colonies were housed in aviaries for up to a week on-site and

then supplied with food for a further week following release (soft release). A fifth colony was released using a combination of methods. All four hard and soft released colonies contained dependent fledglings at the time of release. This appears to be the first translocation of a co-operative species where intact colonies containing multiple breeding females, each with a suite of helpers have been translocated successfully. Both hard and soft release treatments appeared equally successful during an initial monitoring period of up to two months. All four colonies maintained social cohesion, and displayed high levels of survival and site fidelity. Both hard release and one soft release colony attempted to breed within 600 m of their release site within eight weeks of release. The other soft release colony bred 12 months later. We believe the inclusion of dependent young in each translocated colony provided a focus for translocated colonies that promoted site faithfulness and colony cohesion. Results of long-term monitoring remain inconclusive and it is recommended that monitoring be repeated during several future breeding events. Given our findings, we recommend that when translocating highly social species every effort is made to translocate the entire group, hard release techniques be applied and stimuli that enhance group cohesion and site faithfulness (the presence of dependent young) be exploited.

1069: ***+.119***

The state of Washington and four Native American tribes on the east Olympic Peninsula cooperatively conducted single translocations of 17 Roosevelt elk into the Dosewallips drainage in 1995 and 24 elk into the Skokomish drainage in 1997, in attempts to augment the small resident herds. Three of the elk translocated into the Dosewallips and 12 of those translocated into the Skokomish were fitted with radio-collars and monitored for 3 yr post-translocation for survival and movement. Survival of elk translocated into the Dosewallips could not be calculated due to insufficient sample size, but survival of elk translocated into the Skokomish averaged 0.65 +/- 0.04 for the first 2 yr and was 0.80 the third year post-translocation. This compares with survival of 0.91 +/- 0.02 for 43 resident elk from six herds in the east Olympics from 1993-2000. Of the elk translocated into the Dosewallips, 41% remained with the resident herd for at least 1 yr, while only 4% of the translocated elk remained with the Skokomish herd for 1 yr. All radio-collared elk translocated into the Skokomish dispersed singly or in small groups an average of 20 kin in the general direction of the source herd. The population in the Dosewallips herd increased from 26 to 46 within 1 yr of the translocation, and has remained stable at the higher level. The Skokomish herd increased from 17 to 25 after the translocation, but subsequently declined to 15.

1070: ***-.070***

The reintroduction of wild boar from central Europe after World War II has contributed substantially to the range expansion of this species in Italy, where indiscriminate hunting in earlier times resulted in extreme demographic reduction. However, the genetic impact of such processes is not well-understood. In this study, 105 individuals from Italian and Hungarian wild boar populations were characterized for nine autosomal microsatellite loci. The Hungarian samples, and two central Italian samples from protected areas (parks) where reintroduction is not documented, were assumed to be representative of the genetic composition of the source and the target populations in the reintroduction process, respectively. Animals hunted in the wild in the Florence area of Tuscany (Italy) were then studied to identify the effects of reintroduction. The results we obtained can be summarized as follows: (i) none of the populations analysed shows genetic evidence of demographic decline; (ii) the three parental populations from Italy and Hungary are genetically distinct; however, the low level of divergence appears in conflict with the naming of the Italian and the European subspecies (Sus scrofa majori and Sus scrofa scrofa , respectively); in addition, the Italian groups appear to be as divergent from each other as they are from the

Hungarian population; (iii) most of the individuals hunted near Florence are genetically intermediate between the parental groups, suggesting that hybridization has occurred in this area, the average introgression of Hungarian genotypes is 13%, but approximate to 45% of the genetic pool of these individuals can not be directly attributed to any of the parental populations we analysed; (iv) analysis of microsatellite loci, though in a limited number, is an important tool for estimating the genetic effect of reintroduction in the wild boar, and therefore for the development of conservation and management strategies for this species.

1071: *+.022*

As part of a project to restore the American peregrine falcon (Falco peregrinus anatum) in Kentucky, cliff habitat, we initiated a series of pre-release evaluations designed to promote efficient allocation of project resources and to provide empirical support for decision-making. Pre-release evaluations included development of a demographic model examining the relative influence of first-year mortality, rate of philopatry, and founder cohort size on population persistence for 20 years. Using VORTEX, we simulated reintroductions in which 6-96 peregrine falcons were released annually for 3 consecutive years under conditions of first-year mortality and rate of philopatry that were 0.45-0.65 and 0.03-0.15, respectively. We used logistic regression to conduct sensitivity analysis. We compared standardized regression coefficients (bn/SEn) to quantify and rank the magnitude of effect on model outcomes at year 20 associated with changes in input parameter values. Founder cohort size accounted for the most variability, (b/SE=9.85) in the probability of persistence at year 20. First-year mortality and rate of philopatry affected the persistence of reintroduced peregrine falcon populations to a lesser extent (b/SE=4.86 and -0.11, respectively). High first-year mortality (0.65) precluded population reestablishment regardless of founder cohort size. Similarly, releasing <48 peregrine falcons annually for 3 consecutive years resulted in high extinction probability (gtoreq0.8) and low population size at year 20 (<0.6), regardless of variation in first-year mortality and rate of philopatry. To address the influence of high first-year mortality on population recovery, we recommend a formal assessment of risk of predation or other sources of mortality at potential release sites. However, program financial resources should be allocated largely to maximizing founder cohort size because, given identification and avoidance of potential release habitats where first-year mortality, likely would be high (i.e., gtoreq0.65), successful restoration of peregrine falcons in Kentucky cliff habitat will be predicated on maximizing the number of peregrine falcons released.

1072: *+.187*

This paper presents brief overview of the historical development of the Beaver (Castor fiber) population in Germany. Thanks to conservation legislation and undertaken protection measures the post-war population of subspecies C. f. albicus (Matsche, 1970) survived from extinction. That population has grown up afterwards to approximately 3,500 animals. Also reintroduction projects are described. The most successful one was carried out in Bavaria, southern Germany, with several subspecies involved (C. f. fiber, C. f. vistulanus, C. f. galliae). Currently the Beaver population in Bavaria is estimated at 2,500 to 2,800 animals. The total stock of Beavers (ca. 6,000) is the result of comprehensive management based on systematic research. Number of Beavers continues to grow up, but populations in suboptimal habitats cause now increasing conflicts with water supplies, agriculture and forestry. Ecological adaptations of Beavers admit them to live in areas developed and populated by humans.

1073: *+.392*

Captive breeding and release programs, widely used to supplement populations of declining species, minimize juvenile mortality to achieve rapid population growth. However, raising animals in benign environments may promote traits that are adaptive in captivity but maladaptive in nature. In chinook salmon, hatchery rearing relaxes natural selection favoring large eggs, allowing fecundity selection to drive exceptionally rapid evolution of small eggs. Trends toward small eggs are also evident in natural populations heavily supplemented by hatcheries, but not in minimally supplemented populations. Unintentional selection in captivity can lead to rapid changes in critical life-history traits that may reduce the success of supplementation or reintroduction programs.

1074: *+.051*

Exterminated at the beginning of the 19th century, the beaver was reintroduced along the upper Rhine with animals captured in the Rhone valley: 22 animals released on the river Muehlbach (1973) and the river Moder (1993-95) in Alsace, thus as 4 animals on the river Rench (1979) in Baden. Method and results of the monitoring realized at 1998-2002 on both riversides of the upper Rhine are presented. The presence of beavers is attested in 28 localities in Alsace and 5 in Baden. Current population size is estimated at 90-110 individuals divided in two subpopulations that are mostly localized near both release areas in Alsace. However, three decades after the first releases in Alsace, the species has not yet reoccupied many suitable habitats like drainage channels, residual parts of the old Rhine and most part of the Floodplains in Baden. Several limiting factors are supposed to be responsible for this situation as deaths caused by non-selective process of destroying against muskrat and coypou, or by road traffic. Propositions are made for improvement of the habitats just as strengthening and monitoring of the net of beaver subpopulations established in the French and the German part of the Floodplain of the upper Rhine.

1075: *+.080*

Predation is an important selective pressure in natural ecosystems. Among non-human primates, relatively little is known about how predators hunt primate prey and how primates acquire adaptive responses to counteract predation. In this study we took advantage of the recent reintroduction of radio-tagged harpy eagles (Harpia harpyja) to Barro Colorado Island (BCI), Panama to explore how mantled howler monkeys (Alouatta palliata), one of their primary prey, acquire anti-predator defences. Based on the observation that harpies follow their prey prior to attack, and often call during this pursuit period, we broadcast harpy eagle calls to howlers on BCI as well as to a nearby control population with no harpy predation. Although harpies have been extinct from this area for 50-100 years, results indicate that BCI howlers rapidly acquired an adaptive anti-predator response to harpy calls, while showing no response to other avian vocalizations; howlers maintained this response several months after the removal of the eagles. These results not only show that non-human primates can rapidly acquire an alarm response to a newly introduced predator, but that they can detect and identify predators on the basis of acoustic cues alone. These findings have significant implications both for the role of learning mechanisms in the evolution of prey defence and for conservation strategies, suggesting that the use of 'probing' approaches, such as auditory playbacks, may highly enhance an a priori assessment of the impact of species reintroduction.

1077: *+.133*

We studied habitat use and movements of a repatriated population of federally endangered Wyoming toads (Bufo baxteri) after the breeding season at Mortensen Lake, Albany County, Wyoming, USA. We followed 8 adult toads using telemetry (n = 68 relocations) during periods of

activity and observed 59 post-metamorphic juvenile toads (n = 59 locations). Adult toads used habitat with a greater mean vegetation canopy cover (mean = 52.6%) than juveniles (mean = 39.20%). We found adults farther from the shoreline (mean = 1.32 m) than juveniles (mean = 1.04 m). Substrates used by toads had a mean surface temperature of 20.31 degreesC for adults and 23.05 degreesC for juveniles. We found most adult and juvenile toads on saturated substrates. All adult toads sampled did not move outside of a 30 x 500 m area along the east-to-south shore where they were captured. Toads were active diurnally through the end of October. We found toads torpid at night. We compared our results to a similar study of the historic population and found that adult toads of the current population used denser vegetation than those of the historic population. Unlike many bufonids, terrestrial stages of the Wyoming toad appear to depend on saturated substrates. The best logistic regression predictors of adult and juvenile toad presence were surface temperature and distance to shore. Survey transects within the moist margin of the lake (! 10 m from water) and after substrates have reached temperatures greater than or equal to20 degreesC will likely yield more detections.

1078: *+.241*

1. The UK Biodiversity Action Plan (UKBAP) identifies invertebrate species in danger of national extinction. For many of these species, targets for recovery specify the number of populations that should exist by a specific future date but offer no procedure to plan strategically to achieve the target for any species.2. Here we describe techniques based upon geographic information systems (GIS) that produce conservation strategy maps (CSM) to assist with achieving recovery targets based on all available and relevant information.3. The heath fritillary Mellicta athalia is a UKBAP species used here to illustrate the use of CSM. A phase 1 habitat survey was used to identify habitat polygons across the county of Kent, UK. These were systematically filtered using relevant habitat, botanical and autecological data to identify seven types of polygon, including those with extant colonies or in the vicinity of extant colonies, areas managed for conservation but without colonies, and polygons that had the appropriate habitat structure and may therefore be suitable for reintroduction.4. Five clusters of polygons of interest were found across the study area. The CSM of two of them are illustrated here: the Blean Wood complex, which contains the existing colonies of heath fritillary in Kent, and the Orlestone Forest complex, which offers opportunities for reintroduction.5. Synthesis and applications. Although the CSM concept is illustrated here for the UK, we suggest that CSM could be part of species conservation programmes throughout the world. CSM are dynamic and should be stored in electronic format, preferably on the world-wide web, so that they can be easily viewed and updated. CSM can be used to illustrate opportunities and to develop strategies with scientists and non-scientists, enabling the engagement of all communities in a conservation programme. CSM for different years can be presented to illustrate the progress of a plan or to provide continuous feedback on how a field scenario develops.

1079: *+.019*

A total of 21 acidified Norwegian rivers are now being limed to re-establish or restore Atlantic salmon, Salmo salar L., stocks. Natural reproduction of Atlantic salmon was evident 1 year after the first year of liming in all rivers that had lost their native stocks (n=9) except for one river. The density of fry (age 0+) developed significantly more rapidly in rivers that supported remnant stocks than in rivers that had lost their stocks, based on data 5 years after treatment. Nine of the study rivers were supplied with hatchery-reared salmon, mainly unfed fry. Of the rivers with lost stocks, those which were supplied with fish had significantly higher densities than those that were not enhanced. On the other hand, rivers with remnant stocks that were supplied with fish had significantly lower densities of salmon fry than those that did not undergo such mitigation

measures. In 2001, all limed rivers yielded 41.9 t of salmon.

1080: ***+.008***

Mammalian carnivores are increasingly the focus of reintroduction attempts in areas from which they have been extirpated by historic persecution. We used static and dynamic spatial models to evaluate whether a proposed wolf reintroduction to the southern Rocky Mountain region (U.S.A) would advance recovery by increasing species distribution beyond what might be expected through natural range expansion. We used multiple logistic regression to develop a resource-selection function relating wolf distribution in the Greater Yellowstone region with regional-scale habitat variables. We also used a spatially explicit population model to predict wolf distribution and viability at several potential reintroduction sites within the region under current conditions and under two contrasting predictions of future landscape change. Areas of the southern Rocky Mountains with resource-selection-function values similar to those of currently inhabited areas in Yellowstone could potentially support >1000 wolves, 40% within protected areas and 47% on unprotected public lands. The dynamic model predicted similar distribution under current conditions but suggested that development trends over 25 years may result in the loss of one of four potential regional subpopulations and increased isolation of the remaining areas. The reduction in carrying capacity due to landscape change ranged from 49% to 66%, depending on assumptions about road development on public lands. Although much of the wolf population occurs outside core protected areas, these areas remain the key to the persistence of subpopulations. Although the dynamic model's sensitivity to dispersal parameters made it difficult to predict the probability of natural recolonization from distant sources, it suggested that an active reintroduction to two sites within the region may be necessary to ensure low extinction probability. Social carnivores such as the wolf, which often require larger territories than solitary species of similar size, may be more vulnerable to environmental stochasticity and landscape fragmentation than their vagility and fecundity would suggest.

1081: ***+.041***

During October-December 1989-1991, biologists from the Vermont Fish and Wildlife Department and the USDA Forest Service reintroduced 115 (88 male, 27 female) American Martens (Martes americana) into the southern half of the Green Mountain National Forest. During the years of release, brief radio-contact was made with 9 of the 13 (8 male, 5 female) radio-collared animals, and several of these may have established residency. Results of winter track count surveys suggested the presence of at least four Martens in 1990. During winter 1994-1995, Trailmaster(R) cameras and boxed camera systems detected Martens at two, and Fishers (Martes pennanti) at 11, of 20 sites. During winter 1997-1998, Fishers were detected at 37 of 47 boxed camera sites, but no Martens were detected. During summer 1997 and 1998, no Marten photos were recorded at 285 pressure-plate camera stations in a larger area that included all Marten release sites. Although post-release monitoring may have been insufficient to definitively confirm Marten presence, results from the 1997 and 1998 camera surveys indicate that a viable population of Martens was not established in southern Vermont, perhaps due to competition with Fishers.

1082: ***+.043***

With gray wolves restored to Yellowstone National Park, this ecosystem once again supports the full native array of large ungulates and their attendant large carnivores. We consider the possible ecological implications of wolf restoration in the context of another national park, Isle Royale, where wolves restored themselves a half-century ago. At Isle Royale, where resident mammals are

relatively few, wolves completely eliminated coyotes and went on to influence moose population dynamics, which had implications for forest growth and composition. At Yellowstone, we predict that wolf restoration will have similar effects to a degree, reducing elk-and coyote density. As at Isle Royale, Yellowstone plant communities will be affected, as will mesocarnivores, but to what degree is as yet undetermined. At Yellowstone, ecosystem response to the arrival of the wolf will take decades to unfold, and we argue that comprehensive ecological research and monitoring should be an essential long-term component of the management of Yellowstone National Park.

1083: ***-.030***

Wild dogs have been eradicated from most of South Africa. However, a large number of smaller isolated reserves offer the potential for metapopulation conservation management of this species through continued translocations among reserves. Wild dogs were released into the Pilanesberg National Park (500 km(2)), South Africa, in June 1999 from a combination of wild captured and captive-bred individuals. The reserve has lions but no spotted hyaenas. We document post-release spatial use, prey selection and breeding biology. Dogs used a very small area (13.4 km(2)) for their first denning period, and then ranged more widely, but avoided the central parts of the park. Movement patters and den site locations suggested that dogs avoided the presence of lions. Major prey species were kudu (50 %), impala (32 %) and waterbuck (14 %). Large prey, including adult male kudu (250 kg) and waterbuck (260 kg) were taken regularly through use of the boundary fence as an aid to capture. Wild dogs have bred three times since introduction, indicating that reserves as small as 200 km(2) may be suitable for introduction of wild dogs, and metapopulation management strategies may be a viable option as long as sympatric large predator populations are absent or managed appropriately.

1084: ***+.070***

The cooperation between international organisations for conservation of species and local wildlife rescue centers is not ideal at present. But the right to exist of the latter is undeniable and their position and contribution to conservation activities should be improved and intensified. Basic approaches were two workshops held by the Humane Society of the United States/International (HSUS/HSI) and the university of San Jose, Costa Rica, with together more than 200 participants (representatives from different governments and rescue centers, biologists and veterinarians) from North-, Central- and South America. Aims were to improve the rescue centers in animal husbandry, disease controlling, techniques of reintroduction to the wild and environmental education. Rescue centers which are specialized on certain taxa, like 'Profelidae', could contribute to projects of zoos and breeding centers by sharing their experiences. International trade with wild animals is directed and controlled by CITES. But what about national wild animal trade? Surveys in Costa Rica have shown, that in 24[degree] of the households, wild animals are (most illegally) kept. In the city of Santa Cruz de la Sierra (Bolivia), this percentage is almost doubled (47 [degree]). Due to bad terms of keeping, mortality, and, thus, sales are high. This shows once more, that an intact environment does not guarantee for intact animal populations. Education and ex situ conservation are important parts for all conservation projects and could be carried out by specialized wildlife rescue centers.

1085: ***+.248***

Historically, Kuwait is the home for 28 mammalian, over 300 bird, and 40 reptilian species. Expanding human population and technology are increasingly altering Kuwait's natural habitat. Such alterations have lead to the disappearance of many native wildlife species. Currently, eight

mammalian species have been eradicated locally but are available elsewhere in the Arabian Peninsula. I have developed a priority system for reintroduction efforts for these species or other wildlife species in the area. This system will help decision makers in efforts to conserve Kuwait's wildlife biodiversity. The application of this system requires the accumulation of basic information about species. The most important of such information is related to the historic range and major extinction factors for each of the local species. (C) 2003 Elsevier Science Ltd.

1086: ***+.078***

We analysed the mitochondrial DNA (mtDNA) from two historical samples of eastern North American wolves: the last wolf reported to have been killed in northern New York State (ca. 1890s) and a wolf killed in Maine in the 1880s. These wolves represent eastern wolves, presently classified as the gray wolf (Canis lupus) subspecies Canis lupus lycaon, which were present well before the expansion of western coyotes (Canis latrans) into these regions. We show the absence of gray wolf mtDNA in these wolves. They both contain New World mtDNA, supporting previous findings of a North American evolution of the eastern timber wolf (originally classified as Canis lycaon) and red wolf (Canis rufus) independently of the gray wolf, which originated in Eurasia. The presence of a second wolf species in North America has important implications for the conservation and management of wolves. In the upper Great Lakes region, wolves of both species may exist in sympatry or interbreed with each other, which impacts the accuracy of estimates of numbers of wolves of each species within this geographic region. Furthermore, the historical distribution of the eastern timber wolf (C. lycaon), as revealed by these skin samples, has important implications for the reintroduction of wolves into the northeastern U.S. states, such as New York and Maine.

1087: ***+.052***

We monitored the survival, dispersal, and home-range establishment of captive-bred, reintroduced puaiohi Myadestes palmeri, a critically endangered thrush endemic to the island of Kauai. Fourteen captive-bred, juvenile birds were released from hacktowers in January-February 1999 and monitored for 8-10 weeks using radiotelemetry. All 14 birds (100%) survived to 56 days post-release. Two birds (14.3%) dispersed greater than 3 kin from release site within 1 day of release. The remaining birds settled within 1 week and established either temporary home-ranges (mean area = 7.9 +/- 12.0 ha, range 0.4-31.9) or breeding home-ranges (mean area 1.2 +/- 0.34 ha, range 0.8-1.6). Temporary home ranges were abandoned by the beginning of the breeding season, and ultimately 6 of the 14 birds (43%) established breeding home ranges in the release area. The high survival rate bodes well for establishing additional populations through captive breeding and release; however, the 57% dispersal rate out of the target area means that several releases of birds may be necessary in order to repopulate a given drainage. Furthermore, observed dispersal and gene flow between the reintroduced and wild populations have important implications for management of the captive flock. Published by Elsevier Science Ltd.

1089: ***+.047***

British red squirrel populations have been subject to landscape management practices resulting in large-scale fragmentation and defragmentation of habitat, as well as numerous historical introductions of closely related subspecies. This unique population history allowed us to examine: (1) the morphological changes to a rare native species probably caused by hybridization with introduced subspecies; (2) the impact of landscape management, specifically reforestation, on the spread of these morphological changes across the north of England. British red squirrels represent

a peripheral population of Sciurus vulgaris, which is regarded as a separate subspecies (S. v. leucourus) to populations found on the European continent. British populations are in danger of extinction because of the introduction of the North American grey squirrel S. carolinensis. Repeated translocation of continental European S. vulgaris individuals to Britain over 150 years may be responsible for an alteration of the morphological characteristics of populations compared to the original subspecies description. The majority of examined populations possessed the coat colour characteristics associated with continental European subspecies. Only populations in the western region of Cumbria possessed coat colour characteristics similar to the described subspecies S. v. leucourus. Changes to landscape connectivity in Britain during the 1980s greatly altered dispersal patterns, resulting in an increase of gene flow from populations in the north-east of England and the Borders into Cumbria. The morphological characteristics of the Cumbrian population also changed over this period, from traits similar to the British subspecies before 1980, to traits similar to the continental European subspecies after 1980. This study demonstrates the threat landscape management practices and the introduction of closely related subspecies can present to rare peripheral populations.

1090: ***-.066***

1. Interactions between captive-reared and wild salmonids are frequent because hatcheries annually rear millions of fish for release in conservation programmes while many thousands of domesticated fish escape from fish farms. However, the outcome of competition between captive-reared and wild fish is not clear: wild fish may be smaller and less aggressive than hatchery fish, but they have more local experience and a prior residence advantage. Moreover, it is important to know whether any competitive differences are genetic (due to the process of domestication) or due to the rearing environment.2. We therefore examined the factors influencing competition for feeding territories in juvenile Atlantic salmon. We studied the effect of domestication by using three independent stocks of both domesticated and wild-origin fish, all of which were reared in a common hatchery environment. We also used fish from the same wild stocks that had been living in the wild. Territorial contests were staged in stream tank compartments between pairs of fish differing in origin or rearing environment; the relative importance of body size and prior residence was also assessed.3. All three stocks of domesticated fish were generally dominant over wild-origin fish when both had been raised in a common hatchery environment. If the wild-origin fish were given a 2-day period of prior residence on the territory this asymmetry in dominance was reversed. However, domesticated fish did not gain any additional advantage from being prior residents. The relative body size of the two contestants had a negligible effect on contest outcomes.4. Truly wild fish (i.e. those of wild origin that had also grown up in the wild) were generally dominant over domesticated or wild-origin fish that had been hatchery-reared. Differences in body size between contestants had no effect on the outcome.5. Synthesis and applications . These results show that, while juvenile farmed Atlantic salmon are inherently more aggressive than wild-origin fish, the hatchery environment reduces their ability to compete for territories with wild resident fish. Rearing salmon in conventional hatcheries for later release into the wild where natural populations already exist may not be a prudent conservation measure; it is preferable to plant eggs or first-feeding fry rather than attempt to 'help' the fish by rearing them through the early life stages.

1091: ***+.225***

Using microsatellite analysis, we investigated the reproductive success and genetic structure of a translocated colony of the Bahamian iguana (Cyclura cychlura inornata) 10-yr post introduction. We investigated the reproductive success of the founder males to determine if all were

contributing equally to the descendant gene pool. We compared the genetic diversity in the founder population with that in the incipient, translocated population to determine if the number of translocated individuals was sufficient to retain the genetic diversity derived from the source population. In 1999, the estimated population size for the translocated colony was between 75 and 90 individuals. Blood was taken from 35 iguanas (16 males, 18 females, and 1 juvenile) of mixed age, including the four original translocated males and three of the four original translocated females. Of the eight polymorphic microsatellite loci in C. cychlura, only five were polymorphic in C. c. inornata. With two exceptions, the low average allelic diversity and heterozygosity among these loci (2.4 and 0.45, respectively) hindered accurate determination of parentage among the founders. Nevertheless, the data indicated that the potential parental contributions appear equal for the founder males and that, at 10-yr Post introduction, the small amount of genetic diversity at the amplified loci in the founding population was retained. The low level of genetic variation appears to have no negative short-term effects on the translocated or other populations of C. cychlura inhabiting the Exuma Island chain, making them excellent candidates for translocation and repatriation programs if physiological and environmental requirements are met.

1092: *+.241*

Translocation is increasingly used to maintain evolutionary processes, particularly the genetic rescue of fragmented populations, but it is sometimes a costly and unsuccessful conservation tool. Succeeding at genetic rescue depends on the transfer of alleles between populations in the same way that successful parenthood consists in transferring alleles to the next generation. Moreover, there are large differences in the reproductive potential of males and females and growing evidence of sex-biased survivorship after translocation in sexually selected species. Under these circumstances the Trivers-Willard model (TWM), a founding and influential theory in behavioral ecology, makes predictions about how a parent should invest in the offspring of a different sex based on their ability to invest. Like parents, conservation managers also modify their investment in translocations to improve success. Thus, there are remarkable parallels between the predictions of modern evolutionary theory about parental investment in offspring of different sex and the investments of conservation managers in the translocation of alleles between populations via animals of different sex. The TWM for sex-biased parental investment could be a theoretical guide to the problem of maximizing the transfer of alleles for genetic rescue.

1093: *+.116*

Coyotes (Canis latrans) in Yellowstone National Park (YNP) have lived in the absence of wolves (Canis lupus) for over 60 years. I examined whether wolf reintroduction in 1995 and 1996 in YNP influenced coyote vigilance and foraging ecology. From December 1997 to July 2000, my co-workers and I collected 1708 h of coyote activity budgets. Once wolves became established in the Park, they once again provided a continuous source of carrion in the Lamar Valley and we found that coyotes began feeding on carcasses throughout the year. Although we documented that wolves killed coyotes, it also became clear that surviving coyotes quickly adjusted their behaviors when wolves were present. When coyotes were near wolves or in areas of high wolf use, they fed on carcasses much more; however, they increased the amount of time spent in vigilance activities and decreased rest. There appears to be a trade-off in which wolf kills provide a quick source of food that is energetically advantageous to coyotes; however, attendant costs included increased vigilance, decreased rest, and a higher risk of being killed. Changes in the behavior of coyotes in response to the reintroduction of this large carnivore may ultimately have wide-ranging cascading effects throughout the ecosystem.

1094: ***+.216***

This paper reviews and updates the distribution and status of two geographically distinct subspecies of New Zealand Saddleback Philesturnus carunculatus, a New Zealand forest passerine that is highly susceptible to predation by introduced mammals such as stoats and rats. The recovery of the North Island and South Island saddleback populations has been rapid since translocations to offshore islands free of exotic predators began in 1964, when both subspecies were on the brink of extinction. South Island saddlebacks have gone from a remnant population Of 36 birds on one island to over 1,200 birds spread among 15 island populations, with the present capacity to increase to a maximum Of 2,500 birds. We recommend that South Island saddleback be listed under the IUCN category of Near Threatened, although vigilance on islands for invading predators and their subsequent rapid eradication is still required. North Island saddlebacks have gone from a remnant population Of 500 birds on one island to over 6,000 on 12 islands with the capacity to increase to over 19,000 individuals. We recommend that this subspecies be downgraded to the IUCN category of Least Concern. The factors that limited the early recovery of saddlebacks are now of less significance with recent advances in predator eradication techniques allowing translocations to large islands that were formerly unsuitable. The only two predators that still cohabit some islands with saddleback are Pacific rats or kiore Rattus exulans and Weka Gallirallus australis, a flightless native rail. Although North Island saddlebacks coexist with kiore, South Island saddlebacks do less well in their presence, possibly because the relict population had no previous history with this species of rat. The impact of Weka as predators of saddlebacks is less clear, but population growth rates appear to be slowed in their presence. It is recommended that while current recovery strategies involving island habitat restoration and translocations be maintained, management effort should also be directed towards returning saddlebacks to selected, "mainland island" sites, where introduced pests are either excluded by predator-proof fences or controlled at very low levels by intensive pest management.

1095: ***+.075***

The introduction of threatened marsupials to islands affords a high degree of translocation success due to the lack of exotic species on islands, or the feasibility of eradicating them. The dibbler Parantechinus apicalis is a small marsupial endemic to the southwest of Australia. It is listed under international and national legislation as Endangered, and has been the focus of a successful conservation strategy to introduce captive-bred individuals to an island as a security measure, and as part of a formal Interim Recovery Plan. A total of 88 individuals were released in four groups on Escape Island from 1998 to 2000. The population was monitored using radiotelemetry and trapping techniques from 1998 to 2001. Breeding and dispersal of young occurred within the first year of release. Three years after the initial release, the third generation of wild-born dibblers had entered into the population. The total cost of this translocation exercise approximated $AUS 0.6 million. The conservation effort to give additional security to dibblers has been successful, at least in the short term, due to the collaboration between four organisations and a commitment to support a monitoring program of the released population over time. (C) 2002 Elsevier Science Ltd. All rights reserved.

1096: ***+.209***

The wolf population in Scandinavia has increased from functionally extinct to about 100 wolves since the 1970s. In 2001 we surveyed four groups of Swedes to analyze the relationship between experience, knowledge, and people's attitude toward wolves. Although all groups support the right of wolves to exist, Swedes who live in areas where wolves have been restored have more negative

attitudes than the general public. Attitudes toward wolves are not strong among the general public, thus changes are possible. Experience with wolf predation leads to more negative attitudes toward wolves. Hunters in areas with wolves have the most accurate knowledge about wolves but at the same time the most negative attitudes. But within all four groups as knowledge increases attitudes become more positive. Still, the most knowledgeable local hunters have less favorable attitudes than the least knowledgeable members of the general public. High proportions of the population do not care about wolves which makes it difficult to reach them with information, but does make them susceptible to rapid changes if wolves become a media topic. With the restoration of wolves, hunters, the strongest supporters of wolves in the 1970s, are now less supportive than the general public. (C) 2003 Elsevier Science Ltd. All rights reserved.

1097: ***+.131***

The single steps of the species recovery plan "green toad" are presented: It contains the clarification of the historical distribution and the mapping of the recent occurrences as basic research, furthermore investigations of all key populations as well as of the threats and causes of decline. A second step includes the conservation and management of all recent populations by means of legal protection under the nature conservation act and measures based on management plans. Step three plans for the partial recovery of the historical distribution and the connection of currently isolated populations. The measures involved a network of habitats, the consideration of habitat requirements in new mining licenses and other impact projects, as well as options for reintroductions with captive bred animals. The fourth step contains a long-term monitoring of all apparent populations. To spacely define steps two and three, a regional species recovery strategy were worked out for each centre of distribution in Lower Saxony. These species recovery strategies have the aim to stabilize and to connect the recent populations as well as establishing new populations as far as possible. In this paper the example chosen to illustrate the species recovery strategy is the situation in the Helmstedt district. Eleven recent sites with green toads still exist in the Helmstedt district, lying from one to several kilometres apart. Adult specimens and stages of reproduction can be found regularly in only a few sites, while in most sites toads can only be found irregularly, mostly as calling males. All colonised ponds are man-made and it concerns excavation sites, ponds for wastwater treatment, mud deposits and even ponds especially designed for the green toad. In terrestrial habitats only a few specimens have been observed, mostly within a distance from about 1 km maximum in the surrounding of their spawning sites in excavation sites or on field-paths. It can be assumed that migrations between certain sites are possible; an indication is the fast appearance of the species in recently created ponds. The main threats are the frequent drying up of their mostly shallow spawning waters and the continuous natural succession. As a conservation strategy proposals are ordered by priority for existing sites and survey areas suitable for habitat development are planned and presented.

1098: ***+.078***

The green toad is the most threatened species in Sweden. Today about 200 adults remain in three reproducing populations and a more diffuse occurrence of single or a few individuals mainly along the west and east cost of Scania. A conservation program is running since 2000. This includes captive breeding and reintroduction within the former range, restoration of old localities and the creation of many new ones. Our goal is to establish ten self sufficient populations within a six year period.

1099: ***+.135***

The spatial distributions of 20 female and 15 male Arabian oryx Oryx leucoryx, reintroduced into the fenced Mahazat as-Sayd protected area (2,244 km(2)) in western Saudi Arabia between 1990 and 1994, were examined from their release until the end of 1999. Over this period we observed a westward shift in home range location of most male and female founder oryx to include the Rangers' Camp within core areas of activity, despite rain falling in patches throughout most of the reserve. Sporadic and unplanned availability of water had occurred at the Camp during several years. The pre-release enclosure was also located at the Camp, and high quality shading areas could be found underneath portacabins. Oryx that maintained independence of the Camp tended to be older individuals and those released in the first years (1990-1992). Concentration of oryx in the western part of the protected area and around the Camp could potentially reduce the effective carrying capacity of the reserve, change the social structure of the population, facilitate the transmission of disease, modify habitat in the form of a piosphere (a zone of attenuating animal impact away from a watering point) around the Camp, and reduce potential genetic flow within the reintroduced population. Whereas wild-born oryx were observed at the Camp, founders were disproportionately represented, suggesting that potential problems associated with dependence on the Camp may diminish as the total population increases and ages.

1100: ***+.172***

Post-release monitoring, including abundance estimation, is an important part of reintroductions, providing a basis for management intervention designed to achieve long-term persistence. The Arabian oryx Oryx leucoryx became extinct in the wild in 1972, surviving as captive populations. Since 1982 reintroductions of Arabian oryx have taken place in Oman and Saudi Arabia. Modelling of oryx population dynamics has highlighted the importance of precise estimation of population size (N). Between 1990 and 2000 three methods of estimating N have been applied in Mahazat as-Sayd protected area in Saudi Arabia: derived population estimates (DPE) based on known births and deaths, distance sampling, and mark-resighting (MR). This study assesses the feasibility and precision of these methods. Inability to assess precision, interdependence of consecutive estimates, and the assumption that all gains and losses are recorded, make DPE of limited value. At current densities, distance sampling along 455 km of driven transects yields too few detections to derive precise estimates of N. To achieve a coefficient of variation of 20% it would be necessary to drive up to c. 2,900 km of transect; this amount of survey effort could be achieved through pooling of data across repeat surveys of established transects. MR estimates, based on re-sighting of collared oryx, have the potential to yield the most precise estimates of N when the proportion of marked animals reaches 30% of the total population. The most reliable MR estimates available indicate the Mahazat as-Sayd Arabian oryx population had grown to > 400 animals by 2000.

1101: ***-.061***

Museum specimens from the late 19th and early 20th centuries were surveyed for the single nucleotide polymorphism identified previously and used to diagnose populations of the federally threatened Northeastern Beach Tiger Beetle Cicindela d. dorsalis (Coleoptera: Carabidae). Widespread polymorphism was revealed throughout the historical range of this species, suggesting a relatively recent anthropogenic character fixation event associated with the extinction and fragmentation of populations. Implications for the phylogenetic species criterion and for the reintroduction of individuals to formerly occupied sites are discussed.

1102: ***+.244***

Nineteen of the 27 species of Colombian primates are under some type of threat due to habitat loss and illegal traffic of at least 13 species. There is a high number of individuals that are confiscated during official controls and, as a management strategy, their introduction or reintroduction it protected areas is proposed. A Saimiri sciureus population was introduced seven years ago in a 60 ha recreational park of dry forest, they were fed on artificial food in semicaptivity, engaging a high economic investment. The effect of progressive reduction of artificial food on the behavior and group composition was evaluated by comparing the values before and after the supply of artificial food reduction. The progressive reduction permitted the group to adapt and make use of the natural resources of the environment by increasing habitat exploration. We propose this strategy as a viable alternative for biological rehabilitation.

1103: *+.244*

Nineteen of the 27 species of Colombian primates are under some type of threat due to habitat loss and illegal traffic of at least 13 species. There is a high number of individuals that are confiscated during official controls and, as a management strategy, their introduction or reintroduction in protected areas is proposed. A Saimiri sciureus population was introduced seven years ago in a 60 ha recreational park of dry forest, they were fed on artificial food in semicaptivity, engaging a high economic investment. The effect of progressive reduction of artificial food on the behavior and group composition was evaluated by comparing the values before and after the supply of artificial food reduction. The progressive reduction permitted the group to adapt and make use of the natural resources of the environment by increasing habitat exploration. We propose this strategy as a viable alternative for biological rehabilitation.

1104: *+.028*

The Persian fallow deer (Dama mesopotamica)-among the rarest deer species in the world-has been gradually reintroduced, using individuals from a captive-bred population, in northern Israel since September 1996. As of October 2000, >80 animals were in the wild population. We studied seasonal and circadian attributes of deer home ranges to assess the success of the reintroduction in terms of behavioral adjustments to the wild. We used radiotelemetry to determine locations and analyzed home ranges with the adaptive kernel method. We defined 3 seasons: fawning (Mar-Jun), rut (Jul-Oct), and winter (Nov-Feb). For females (n = 16), rut home ranges were significantly larger than winter home ranges (449 +/- 45 ha [mean +/- SE] vs. 384 +/- 36 ha, P-1,P-15 = 0.013). During fawning, female home ranges were intermediate (424 +/- 51 ha). Males (n = 5) increased their home ranges in rut season (820 +/- 162 ha [mean +/- SE], P < 0.012) and shifted their locations toward the release point. In winter, males significantly decreased their home ranges (584 +/- 158 ha, P < 0.012), shifted their locations away from the release point, and almost no overlap of core areas was noticeable (1.8% overlap). In fawning (the time of antler casting and regrowth), males continued to shift away from the release point and decreased home ranges (358 +/- 66 ha, P = 0.049) with almost no overlapping of core areas (0.06% overlap). No statistically significant differences were found between day home ranges (males [n = 5]: 621 +/- 220 ha [mean +/- SD], females [n = 16]: 402 +/- 164) and night home ranges (males: 482 +/- 145, females: 389 +/- 183), although day core areas tended to be larger (in all males and 12 of 16 females). All documented aspects of seasonality in female and male home ranges are in accordance with the annual reproduction cycle, and are related to seasonal food availability. These results, combined with previous works, suggest that so far, the reintroduced Persian fallow deer have adjusted well to living in the wild and that the chances of achieving a self-sustaining wild population are good. However, further research for extended period should verify these conclusions.

1105: ***+.160***

We used radiotelemetry and field observations to Study survival and reproduction of 718 reintroduced elk (Cervus elaphus) in eastern Kentucky, USA, from 1997 to 2001. Capture-related injuries accounted for 49% of the transit and post-release mortality. Annual survival was high across all age and sex classes, and ranged from 0.90 (adult females) to 0.97 (yearling females). Calving rates increased from 66% in 1998 to 92% in 2000. A high nutritional plane may explain the relatively high reproduction among females bred as yearlings, consecutive-year pregnancies, and twinning. Such high survival and reproductive rates are characteristic of colonizing ungulate populations in areas devoid of predators and competitors. Future research should focus on Kentucky-born calves to more accurately determine the effects of meningeal worm (Parelaphostrongylus tenuis) and other factors on recruitment, colonization, and population establishment.

1106: ***-.038***

Meningeal worm (Parelaphostrongylus tenuis) has been implicated in the failure of several elk (Cervus elaphus) restoration attempts in the eastern United States. However, limited post-release monitoring and a paucity of published literature prevents a clear understanding of this parasite's role in past failures. During winters of 1997-2001, the Kentucky Department of Fish and Wildlife Resources translocated 1,044 elk from western states to eastern Kentucky (USA) in an effort to restore a free-ranging population. We monitored 521 radio-collared elk over 4 yr to determine the impact meningeal worm had on population establishment. Thirty (23%) of 129 non-capture related mortalities were attributed to meningeal worm. Twenty-two (73%) of these meningeal worm-caused mortalities were animals <3 yr old. if younger elk born in Kentucky suffer higher mortality rates than older translocated elk, the population growth observed during the initial years of restoration may be temporary. Additional research is necessary to determine the influence meningeal worm will have on elk population growth in Kentucky.

1107: ***+.051***

Hydrastis canadensis L. (goldenseal) is an endangered species of perennial wildflower native to eastern North America and is harvested for its root that contains the alkaloids berberine, hydrastine and canadine. Studies have shown this herb to have anti-inflammatory, antibiotic and anti-pyretic effects. Today, the number of wild goldenseal populations is on the decline, to the point of extirpation in some states, thought to be largely due to over-harvesting and exacerbated by the plant's reproductive strategy. Goldenseal reproduces by both vegetative and sexual means. Juvenile plants reproduce clonally and it takes two to three years for a plant to reach sexual maturity. At this stage the root is considered large enough to harvest. Thus, as wild harvesters collect mature plants, the probability of sexual reproduction is reduced. Consequently, it is thought that there is little genetic diversity within those wild populations that are subject to wild harvesting on a regular basis. Understanding germplasm diversity is critical for crop improvement and breeding programs for endangered species. This information also helps conservation efforts by offering data that can support preservation of habitat, collection monitoring and genetically sound reintroduction strategies. In this study we analyzed several populations of Hydrastis canadensis for genetic diversity within and among populations. The samples are from cultivated populations in Kentucky, Massachusetts, New York and Canada and wild populations from Kentucky, Ohio, West Virginia and New York. We used the Random Amplified Polymorphic DNA (RAPD) analysis technique to generate DNA profiles from our samples and to estimate genetic relatedness among and between populations. RAPD analysis is a relatively simple technique and offers the ability to

compare numerous sites within the genome with just a few primers. The results for the cultivated material show 72-86% similarity among the populations tested and the similarity for the wild populations of 20-67%. These numbers are in line with expected results when comparing a clonaly propagated group with the wild type.

1108: *+.275*

The box huckleberry (Gaylussacia brachycera (Michx.) Gray) is a slow-growing, dwarf evergreen woody groundcover that is native to both the mountains and coastal plains of Pennsylvania, Virginia, Kentucky, Tennessee, West Virginia, Delaware, and Maryland. It has glossy, dark green, fine-textured foliage, with new growth often red to maroon colored. The box huckleberry's global conservation status is listed as G3 and the state listing for Delaware, Maryland and Pennsylvania is S1 (critically imperiled). In the seven states in which it is native, there are less than 20 known populations of this species. Under permit, plants of box huckleberry have been collected from 14 native habitats in six states. Most of these plants have been established in a protected site at the U.S. National Arboretum in Washington, D.C. We hope to use these plants to achieve the following objectives: 1) In cooperation with the Maryland DNR, enhance the recovery of box huckleberry in the wild by reintroducing the plant back into its native habitat in Maryland, where only one plant remains; 2) determine molecular genetic distances among collected populations to guide decisions regarding conservation, preservation, and breeding; 3) determine optimum propagation and production methods so that this species may be evaluated by commercial nurseries as a slow-growing, native, evergreen landscape plant; and 4) perform controlled pollinations between accessions to create new genotypes.

1109: *+.056*

A role for molecular genetic approaches in conservation of endangered taxa is now commonly recognized. Because conservation genetic analyses provide essential insights on taxonomic status, recent evolutionary history and current health of endangered taxa, they are considered in nearly all conservation programs. Genetic analyses of the critically endangered Far Eastern, or Amur leopard, Panthera pardus orientalis, have been done recently to address all of these questions and develop strategies for survival of the leopard in the wild. The genetic status and implication for conservation management of the Far Eastern leopard subspecies are discussed. (C) 2003 Academie des sciences. Published by Editions scientifiques et medicales Elsevier SAS. All rights reserved.

1110: *+.162*

European water frogs are characterized by anthropic introductions and Rana ridibunda may be considered as an invasive species. As such translocations may result in introgression of exotic genes in native populations, i.e. genetic pollution, we studied genetic characteristics (on I I allozymic loci) of natural versus introduced water frogs. Our study contributed to (1) disclose 3 genetic markers allowing the identification of exotic frogs; (2) quantify the proportion of exotic frogs found in natural populations; and (3) suggest how genetic pollution may arise in these frogs. (C) 2003 Academie des sciences. Published by Editions scientifiques et medicales Elsevier SAS. All rights reserved.

1111: *+.149*

We focus on constraints faced by antelopes reintroductions in and environments, and propose keys to enhance their success, using the oryx project in Saudi Arabia as example:(1) Monitoring and

management of reintroduced populations appear more important than the number of released animals;(2) Because of the low accuracy of population size estimators, we recommend to implement a continuous monitoring and to use several estimators to assess the reintroduced population size;(3) Reintroduction schedule should take into account the unpredictability of food resources in and environments;(4) The re-establishment of desert antelopes depends as a priority on the enforcement of regulations to avoid poaching. (C) 2003 Academie des sciences. Published by Editions scientifiques et medicales Elsevier SAS. All rights reserved.

1112: *+.020*

The black-footed ferret (Mustela nigripes) of North America is critically endangered due in part to its extreme specialization on formerly stable and abundant prairie dogs (Cynomys). Its close relative, the Siberian polecat (M. eversmannii) seems to have been subjected to a varying environment that was not conducive to specialization. One source of environmental variation in Asian steppes was plague (caused by Yersina pestis), which was absent from North America. Introduction of plague to North America presents serious challenges to ferret recovery. Partial solutions to other biological and political problems have been found, resulting in improved production in captivity, increased survival post-release, and thriving populations in plague-free South Dakota. (C) 2003 Academie des sciences. Published by Editions scientifiques et medicales Elsevier SAS. All rights reserved.

1113: *+.273*

We studied survival and demography of black bears, Ursus americanus, in Banff National Park (BNP) from 1994 to 2000 to test the efficacy of National Park protection. We monitored 25 radiocollared bears an average of 1.9 years each for a total of 51.8 bear-years. Eighty-two percent of all mortality (n = 11) was human-caused, composed of highway mortality (36%), management mortality (27%) and management relocation (18%). Survival was influenced by season and management status. Once bears became a management problem, survival (0.66) was lower than several hunted populations. Adult (0.84) survival was comparable to other unprotected or partly protected populations. Cub (0.64) and yearling (0.67) survival, and reproductive rate (m(x) = 0.47 female cubs/inter-birth interval), was slightly lower than other populations in western North America. We combined survival and reproductive rates in a preliminary post-birth pulse age-class Leslie matrix model and estimated population growth rate as 0.95 (95% simulated C.I. 0.79-1.10). Sensitivity analyses showed X was most sensitive to changes in adult female survival. Responsible management agencies should reduce adult female highway mortality and the likelihood of becoming a management problem, while continuing monitoring to refine demographic analyses to adequately protect this population. (C) 2002 Elsevier Science Ltd. All rights reserved.

1114: *+.132*

Translocation of species for conservation purposes is a key element of many recovery programs. While the benefits of translocations seem obvious, potential negative effects have been described. For endangered beach mice (Peromyscus polionotus ssp.), repopulation of native, unoccupied habitat using translocated individuals has been extremely successful. Once populations are established, concerns over founder effects and isolation have led to proposals for continued secondary translocations. Unfortunately, little information is available to help formulate protocols for these actions. To test the effectiveness of translocation, the fates of 18 translocated Choctawhatchee beach mice (Peromyscus polionotus allophrys) were followed as they attempted to integrate into an established population. We found that translocated groups, repeated for two

seasons, tended to use larger homeranges and significantly more burrows than did resident mice. The monthly (31 day) survival rate of resident mice was 3:4 times greater than for translocated individuals. We hypothesize that differential predation pressure was directly linked to the failure of mice to integrate successfully into the existing population. Given our results, we must advise caution and that additional knowledge be obtained before translocations are used to supplement existing populations. (C) 2003 Elsevier Science Ltd. All rights reserved.

1115: ***+.147***

Monitoring is essential to evaluate the success of translocations, but is frequently neglected. One exception has been the reinforcement of the otter (Lutra lutra) population in the Derwent and Esk catchments in North East England, UK, between 1990 and 1993. Here, we use data on otter sprainting activity collected before, during and after translocations to identify relationships with vegetation, food resources and physical river characteristics. Sprainting activity increased significantly with trout density, stream order, and surrounding cover by woodland and semi-natural grassland vegetation, and decreased significantly with stream gradient. The form of these relationships was unimodal, sprainting activity peaking at intermediate levels of environmental variables. A logistic regression model including variables relating to fish density, the physical characteristics of the river and surrounding vegetation cover was able to predict the presence or absence of otter sprainting at different survey sites with an accuracy of 92%. Fish density and the physical characteristics of the river were the most important factors in the model. Models such as this are of practical use for assessing the likely success of future otter translocations, both in North East England and other regions of the UK and Europe. (C) 2002 Elsevier Science Ltd. All rights reserved.

1116: ***-.103***

The wild water buffalo is highly endangered, with the few remaining populations already affected or likely to be increasingly affected by hybridization with domestic buffalo. The work described here was done to evaluate a genetic method to discriminate wild from mixed ancestry (hybrid) and domestic animals, and to identify with high probability those most likely to be purebred wild. Samples from 45 animals (phenotypically classified into three groups - ten wild, 28 domestic and seven hybrid) were genotyped for ten microsatellite loci. Although genetic distances among the three groups were small, an assignment test identified two of the 'wild' and seven of the 'domestic' as hybrids. However, sample sizes also are small, indicating the need for a conservative approach in the first instance in using these results. As more animals are genotyped, assignments will become more accurate, and a translocation programme to establish a second Nepalese wild population in a protected area could be undertaken.

1117: ***+.279***

Conservation programmes increasingly involve the translocation of animals to reinforce failing populations or establish new ones. To help guide translocation programmes of swift foxes (Vulpes velox) or other imperilled species, we aimed to discern factors affecting translocation success among reintroduced swift foxes in Canada. Post-release movements characterized three stages. In the initial acclimation phase, foxes moved erratically and quickly distanced themselves from release sites. During the establishment phase, distances from the release site did not change significantly but daily movements were more wide-ranging than those of Concurrently tracked, resident swift foxes. In the final settlement phase, movements of translocated foxes reflected those of resident individuals. Radio-telemetry showed that survival and reproductive success were

highest for swift foxes with small dispersal distances, suggesting that measures C should be taken to acclimatize animals to release sites. Since females had lower survival rates than males, translocations should also use a greater proportion of females to establish balanced sex ratios in the population. Translocated juveniles dispersed less far but survived and reproduced as well as translocated adults, suggesting that juveniles can be used to establish translocated foxes in small, protected areas, while minimizing demographic effects on source populations. The fact that survival rates and litter sizes of translocated foxes were similar to those of resident animals indicates that translocation can be an effective reintroduction tool for this endangered species, and possibly other foxes.

1118: *+.058*

The helminth populations found in a group of wild boars collected in central Spain were compared to those in a group of animals imported from a French game farm that produces boars for restocking. Eleven helminth species, including ten nematodes and one acanthocephalan, were found. Gongylonema pulchrum and Macracanthorhynchus hirundinaceus were only detected in autochthonous wild boars, while Oesophagostomum dentatum, Ascaris suum, and Trichuris suis were detected in imported animals only. Autochthonous wild boars were more frequently and more intensely parasitised by Ascarops strongylina than the imported ones. No differences in prevalence nor intensity were found for the species Capillaria garfiai, Globocephalus urosubulatus, Metastrongylus sp., Physocephalus sexalatus and Simondsia paradoxa. To our knowledge, G. urosubulatus, G. pulchrum and S. paradoxa have not previously been described in wild boars in Spain. Our results highlight the risks of translocating wild animals, with regard to their helminth parasites. Until improved control measures are established, it would be wise to avoid long-distance translocations in order to prevent the potential introduction of foreign parasites. (C) 2003 Elsevier B.V. All rights reserved.

1119: *-.116*

The United States Fish and Wildlife Service has proposed restoring grizzly bears (Ursus arctos) to the Bitterroot ecosystem of central Idaho and western Montana, where grizzly bears were extirpated by 1932. We based estimates of future grizzly bear population size and distribution in the Bitterroot ecosystem on resource selection functions developed for the Yellowstone ecosystem and Swan Mountains in northwestern Montana. This method yielded an estimate of 321 grizzly bears in the Bitterroot recovery area. Our analysis suggests that habitat attributes, particularly the paucity of roads and human developments, will help to ensure a viable population of grizzly bears in the Bitterroot ecosystem.

1120: *+.057*

In recent years wildlife diseases (infectious and non-infectious) have played a relevant role in both wildlife conservation and public health. Global environmental changes have determined a bimodal evolution of wildlife. On one side a huge loss of biodiversity has been observed leading to the increasing of threatened or endangered species. In contrast few opportunistic taxa increased their aboundances and ranges. The above scenarios claim the intervention of wildlife veterinarians. In conservation the understanding of the ecological role of the host-parasite relationship and the perturbations on the host population dynamics have to be assessed and eventually modified. In public health the increased overlapping among wildlife, livestock, pets and human beings represents a risk for diseases spread (no matter in which directions). Serious limits are, still now, observed in the acceptance of this 'new world' by veterinary academics. As a consequence

curricula often fail in providing adequate skill at both undergraduate and graduate levels. An addressed approach towards wildlife diseases should be promoted as an essential component of environmental management.

1121: *-.060*

The population of the scallop Argopecten purpuratus in Mejillones Bay, Chile (23degreesS) was estimated using diving-quadrat techniques between July and August 1999. The Population, distributed over an area of 255 ha, was estimated at about 18 * 10(6) individuals, having a mean length of about 68 mm. Of this number, about 16 * 10(6) individuals were aggregated into a core area of 5 1 ha. A subsequent controlled harvest carried out in 33.5 ha of this area in November 2000 produced only 2 * 10(5) individuals, strongly suggesting that large scale clandestine (illegal) harvesting had occurred since the original population survey. Reseeding of collected scallops was carried out in an interior area of the bay which had low densities of scallops but highly similar physical and chemical seawater characteristics. Post-seeding mortality of the translocated scallops, determined two weeks after the transfer, was 12.5%, with a mean of 2.5 indiv./m(2) in an 8.2 ha area. The low initial mortality, compared with values in the literature, suggested that the methods of retrieval, transport, and restocking the scallops could serve as a viable repopulation strategy in other areas in the future.

1122: *+.025*

Allozyme electrophoresis was used to evaluate levels and distribution of genetic diversity in Iberian populations of the threatened coastal sand dune plant Stachys maritima (Lamiaceae). During the last century, this species was subjected to severe habitat fragmentation, mainly as a consequence of tourism activities and urban pressures, with a decreasing of area up to 99% in the Iberian Peninsula and a remaining total population size of ca. 250 individuals. Extremely low levels of genetic variation were detected (P = 14.0, A = 1.16 and H-e = 0.066). From the 20 interpretable loci found, only 4 were polymorphic and another one, the ldh-2 locus, showed a fixed heterozygous banding pattern, which may be attributed to gene duplication. Low values of diversity can be related to small population size and isolation of populations. The degree of threat has been increased from vulnerable (VU) to critically endangered (CR), according to the IUCN criteria. Conservation policies should be focused on maintaining population size and gene flow, in addition to preserving its habitat, although reintroductions and reinforcements appear to be necessary to ensure long-term viability of populations.

1123: *+.193*

Translocation is a commonly used tool in conservation management. However, because post-release monitoring has been infrequent in the past, reasons for the outcomes of translocations have often been unknown. Here, I review the reintroduction biology (including dispersal patterns, social organisation, survival, habitat use and foraging patterns) of a population of 26 South Island Saddlebacks (Philesturnus carunculatus carunculatus), on Motuara Island, New Zealand. After release on Motuara Island, South Island Saddlebacks dispersed widely through forest areas. During their first post-release breeding season, saddlebacks established territories of 1.9 ha-8.8 ha (X=4.21 ha, SD=2.42) in size, and territorial confrontations were very rare. Saddlebacks bearing both adult and subadult plumage held territories and attempted to breed, and successful breeding produced approximately 10 fledglings. Saddlebacks foraged on a variety of plant species, dead wood and the ground. Except for five-finger (Pseudopanax arboreus), a preferred foraging substrate, birds foraged in all plant material in proportion to its availability. Saddlebacks preferred

to forage in the lower levels of the forest. Although vegetation composition differed significantly between territories, all territories contained forest areas, and birds appeared to prefer foraging in larger sized trees. Large territory sizes, breeding attempts by young birds and rarity of territorial confrontations are most likely products of low population density. As density increases, birds are expected to occupy smaller territories, forage more efficiently within these smaller areas, start breeding at older ages, and possibly colonise scrub habitats. The translocated group sustained no more than 50% mortality at 8-10 months after release. In the past, translocations of 15-59 South Island Saddlebacks have been successful, suggesting that the relatively small founder group does not threaten the success of the transfer to Motuara Island. Saddlebacks are flexible in their habitat use, appear to readily adapt to 'new' environments and have high reproductive potential, increasing the likelihood of success of translocations of this species.

1124: *+.143*

This paper explores the relative effects of host plant dynamics and butterfly-related parameters on butterfly persistence. It considers an empty habitat network where a rare butterfly (Cupido minimus) became extinct in 1939 in part of its historical range in north Wales, UK. Surviving populations of the butterfly in southern Britain were visited to assess use of its host plant (Anthyllis vulneraria) in order to calibrate habitat suitability and carrying capacity in the empty network in north Wales. These data were used to deduce that only a portion (similar to 19%) of the host plant network from north Wales was likely to be highly suitable for oviposition. Nonetheless, roughly 65,460 eggs (3273 adult equivalents) could be expected to be laid in north Wales, were the empty network to be populated at the same levels as observed on comparable plants in surviving populations elsewhere. Simulated metapopulations of C. minimus in the empty network revealed that time to extinction and patch occupancy were significantly influenced by carrying capacity, butterfly mean dispersal distance and environmental stochasticity, although for most reasonable parameter values, the model system persisted. Simulation outputs differed greatly when host plant dynamics was incorporated into the modelled butterfly dynamics. Cupido minimus usually went extinct when host plant were at low densities. In these simulations host plant dynamics appeared to be the most important determinant of the butterfly's regional extirpation. Modelling the outcome of a reintroduction programme to C. minimus variation at high quality locations, revealed that 65% of systems survived at least 100 years. Given the current amount of resources of the north Wales landscape, the persistence of C. minimus under a realistic reintroduction programme has a good chance of being successful, if carried out in conjunction with a host plant management programme.

1125: *+.107*

We examined a remnant host plant (Primula veris L.) habitat network that was last inhabited by the rare butterfly Hamearis lucina L. in north Wales in 1943, to assess the relative contribution of several spatial parameters to its regional extinction. We first examined relationships between P. veris characteristics and H. lucina eggs in surviving H. lucina populations, and used these to predict the suitability and potential carrying capacity of the habitat network in north Wales. This resulted in an estimate of roughly 4500 eggs (ca 227 adults). We developed a discrete space, discrete time metapopulation model to evaluate the relative contribution of dispersal distance, habitat and environmental stochasticity as possible causes of extinction. We simulated the potential persistence of the butterfly in the current network as well as in three artificial (historical and present) habitat networks that differed in the quantity (current and X3) and fragmentation of the habitat (current and aggregated). We identified that reduced habitat quantity and increased isolation would have increased the probability of regional extinction, in conjunction with

environmental stochasticity and H. lucina's dispersal distance. This general trend did not change in a qualitative manner when we modified the ability of dispersing females to stay in, and find suitable habitats (by changing the size of the grid cells used in the model). Contrary to most metapopulation model predictions, system persistence declined with increasing migration rate, suggesting that the mortality of migrating individuals in fragmented landscapes may pose significant risks to system-wide persistence. Based on model predictions for the present landscape we argue that a major programme of habitat restoration would be required for a re-established metapopulation to persist for > 100 years.

1126: *+.070*

Neotropical parrots are among the most threatened groups of birds in the world, and many species are facing extinction in a near future. At the same time, the taxonomic position of many species remains unclear. Karyotype analysis has been used to elucidate the phylogenetic status of many bird groups, also providing important information for both in situ and ex situ conservation plans. The objective of the present study was to describe for the first time the karyotypes of the endangered Hyacinth Macaw, Anodorhynchus hyacinthinus, and of the Hawk-headed Parrot, Deroptyus accipitrinus. A diploid number of 2n = 70 and a karyotype similar to the main pattern previously found for the genera Ara, Cyanopsitta, Aratinga, Propyrrhura, Pionites, Pionopsitta, Nandayus, and Guaruba were found for both species. These karyotype descriptions can be a starting point for the genetic monitoring of these two declining species.

1127: *+.165*

The fringed darter (Etheostoma crossopterum) was introduced into two streams where neither the fringed darter nor any other species of the same subgenus occur but that otherwise have suitable habitat. Darters were collected from two streams in the Cache River basin. and a combination of darters from each source stream were moved to the receiving streams, which also were in the headwaters of the Cache River basin (20 males and 40 females to each). Darters were moved in late March 2001 at the beginning of their reproductive season, which continues for approximately 2 months. Fringed darter nests were present within 3 days at both introduction sites and nests were found thereafter until late May at which time searching for nests was terminated. Twenty-five nests were found in 2001 compared with 168 nests in 2002. A total of 8714 eggs were counted in 22 nests during 2001 with a mean of 413 eggs per nest (n = 19 nests). In September 2001 and 2002, surveys for juveniles and adults indicated successful recruitment in both streams. Mean size of juvenile darters differed significantly between the two introduction sites in 2001. The design of this study allowed for a rapid, initial assessment of the translocations, aspects of which appeared successful. Moving fishes at the beginning of their reproductive season could increase the chance of successful reproduction and eventual establishment. The protocol for translocation of fringed darters might be applicable to other similar and endangered darters such as Barrens (Etheostoma forbesi), relict (E. chienense), and duskytail darters (E. perenurum). Sampling was conducted in other areas of the Cache River basin in search of additional populations of fringed darters. An updated account is given of the distribution of the fringed darter in Illinois. (C) 2003 Elsevier Science Ltd. All rights reserved.

1128: *+.005*

In response to a policy of fire suppression since early in the 20th century, forest managers have recently initiated emergency programs of prescribed burning to reduce readily combustible fuel loads in many forests of the western United States. The effects of burning on woody plant

composition and structure are relatively well understood; however, little is known about the impact of burning on other taxa. I tested the response of butterflies to fire reintroduction in the Rogue River National Forest and Yosemite National Park. I established replicated transects on three different types of prescribed burn treatment (forest burns, fuel breaks, and riparian burns), as well as control sites, to monitor adult butterfly richness and diversity. Two to three times as many butterfly species occur in forest burns as controls, 13 times as many in fuel breaks as controls, and twice as many in riparian burns as controls. The results of this study suggest that the reintroduction of diverse fire management methods, especially riparian burning, will benefit butterfly diversity in coniferous forests. Further study is required to examine potential proscriptions against riparian burning, including erosion and invasive species encroachment. Both area and density of gaps in the forest canopy were found to explain large amounts of the variation in butterfly richness (R-2 =0.64 and R-2=0.80, respectively). This study demonstrates that using non-traditional taxa (e.g., butterflies instead of trees) to study ecosystem processes may help to provide valuable insights into alternative management strategies. (C) 2003 Elsevier Science Ltd. All rights reserved.

1129: *-.017*

We report on the social dynamics and behaviour of five lions reintroduced to the Welgevonden Private Game Reserve. Despite initially being together in a boma for three months, the lions did not stay together. Aggression was also noted after the release of a female, which had been placed in the boma earlier after sustaining an injury. The results showed different lions associating and breaking away from the group on various occasions, which illustrated the flexibility of lion social structure when prides are disturbed. The findings are similar to those observed in the Kruger National Park after a lion-cropping programme. The lions that were reintroduced on Welgevonden were variously related, and the way in which they finally associated in prides was based on their relatedness. This raised the question of whether association took place by chance, or whether there was some kind of kin-recognition in lions, resulting in them associating mostly with kin. It was not possible to prove kin-recognition in this study, but the association with kin could lead to genetic problems in the future due to inbreeding.

1130: *+.139*

The critically endangered Seychelles Black Paradise Flycatcher Terpsiphone corvina was once found on at least five of the inner (granitic) islands in the Seychelles archipelago, western Indian Ocean. Currently, it is only found on two islands, with c. 98% of the world population (c. 150-200 individuals) occurring on the 10-km(2) island of La Digue. Creation of additional island populations is therefore considered crucial in improving its conservation status. The presence of native broad-leaved plateau forest in proximity to wetland areas is proposed as an important selection criterion when considering the suitability of other islands for translocation, due to the presumed importance of insect prey (dependent on water) in the diet. We quantified habitat use, territory composition, the effect of water on invertebrate abundance, and foraging and breeding success to determine the importance of native broad-leaved woodland and wetland areas for Flycatchers. Flycatcher territories contained significantly more native broad-leaved woodland (88%) than its availability on the plateau (43%); Flycatchers used native tree species significantly more for both foraging (81%) and nesting (95%) than their availability within territories (71%); and territory size varied inversely with the density of native broad-leaved tree species. Native broad-leaved forest was associated with semipermanent and permanent water bodies and availability of native forest habitat was a good predictor of territory distribution. The number of aerial insects trapped was higher close to water, but there was no effect of the proximity to water

on the number of insects counted on foliage. The majority of identified prey species in adult diet were not dependent on water: Orthoptera and Lepidoptera were the most common prey groups, comprising 66% of identified prey items. There was no effect of proximity to water on foraging or breeding success (c. 35%): depredation was the major factor determining breeding success, and accounted for the majority of nest failures. The importance of wetland areas to Flycatchers therefore appears to have been over-emphasized. The implications for translocation and conservation of the Flycatcher are discussed.

1131: *+.244*

An approach to an overall management planning for the conservation of endangered species within protected natural areas is presented, based mainly on the development of methods for the diagnosis of the conservation status and the identification of critical life cycle stages (phenophases) of target species. This model includes other aspects inherent in the overall planning of a protected area so as to match its technical, human and economic resources in terms of research and conservation. We provide examples of some island endemics that are included in the "Recovery Plans for the Endangered Plants in the Canary Islands National Parks". The use of demography and genetics is particularly emphasised. We propose reintroductions as one of the most important tools for the management and conservation of endangered plants and the use of "system dynamics" tools as an alternative to traditional population matrix analysis. We provide examples of the application of system dynamics to population dynamics studies and to the design of management actions. (C) 2003 Elsevier Science Ltd. All rights reserved.

1132: *+.160*

The growth in standard length of endemic soft-muzzled trout Salmothymus obtusirostris, translocated to the Zrnovnica river about 25 years ago, was studied. Before the Prancevica dam was built at the other side of the karst mountain where the river springs from, the river Zrnovnica existed only temporarily and therefore did not provide a habitat for fish. The S. obtusirostris specimens caught ranged from 15.5 to 33.5 cm in standard length and from 80 to 425 g in weight. The growth in standard length of this population could be expressed by the following von Bertalanffy expression: L(t) = 48.68(1-e(-0.2379(t-0.1261))). These results indicate that S. obtusirostris adapted to the new environment. They also show translocation to be a useful tool for its conservation. Nevertheless, the protection of this endangered species should be of the utmost importance.

1133: *+.010*

Beavers were exterminated in Bavaria in 1867. The reintroduction, conducted by the "Bund Naturschutz in Bayern e.V." between 1966 and the late seventies, was very successful: The present beaver population in Bavaria is estimated at more than 6.000 individuals, and it is still growing and expanding into its former range. This success was paralleled by a growing number of conflicts between beavers and human land users in the densely settled and intensively used Bavarian landscape. Beavers fed on crops, beaver dams flooded forests, pastures and basements, beavers digged their burrows under roads, fields and high water dikes, and some of them felled apple trees in gardens. During the 1980s until the early 1990s, the beaver conflicts escalated, less because of the real world problems, but more because politicians and mass media abused this conflict between nature conservation and land owners for personal gain. Taking a closer look at beaver-human-conflicts, we found, that almost all conflicts root in the overuse of landscape by man: beaver conflicts happen mainly in a narrow strip along the river banks. Where man has left some

space to the rivers, there are only very few conflicts with beavers. Such unused areas are not only important for beavers and other plant and animal species, but more and mainly for man himself (e.g. for protection of water and prevention of floods).

1134: ***+.012***

In Austria the last beavers became extinct in the middle of the 19th century. More than 100 years later a successful reintroduction started with a small founder group (50 individuals, most of them from Poland) and was rarely realised by the people. Discovered beaver cuttings seemed to be small sensations and the species had a rather positive image. Unfortunately this changed rapidly when the beaver population started to grow and to disperse from protected areas to places intensively used by agriculture and forestry. Landowners complained about beaver damages and some questions like "Why do we need beavers? Will we be forced to live with that species? How many beavers are enough?" were asked more often.

1135: ***+.010***

Beavers were exterminated in Bavaria in 1867. The reintroduction, conducted by the "Bund Naturschutz in Bayern e.V." between 1966 and the late seventies, was very successful: The present beaver population in Bavaria is estimated at more than 6.000 individuals, and it is still growing and expanding into its former range. This success was paralleled by a growing number of conflicts between beavers and human land users in the densely settled and intensively used Bavarian landscape. Beavers fed on crops, beaver dams flooded forests, pastures and basements, beavers digged their burrows under roads, fields and high water dikes, and some of them felled apple trees in gardens. During the 1980s until the early 1990s, the beaver conflicts escalated, less because of the real world problems, but more because politicians and mass media abused this conflict between nature conservation and land owners for personal gain. Taking a closer look at beaver-human-conflicts, we found, that almost all conflicts root in the overuse of landscape by man: beaver conflicts happen mainly in a narrow strip along the river banks. Where man has left some space to the rivers, there are only very few conflicts with beavers. Such unused areas are not only important for beavers and other plant and animal species, but more and mainly for man himself (e.g. for protection of water and prevention of floods).

1136: ***+.012***

In Austria the last beavers became extinct in the middle of the 19th century. More than 100 years later a successful reintroduction started with a small founder group (50 individuals, most of them from Poland) and was rarely realised by the people. Discovered beaver cuttings seemed to be small sensations and the species had a rather positive image. Unfortunately this changed rapidly when the beaver population started to grow and to disperse from protected areas to places intensively used by agriculture and forestry. Landowners complained about beaver damages and some questions like "Why do we need beavers? Will we be forced to live with that species? How many beavers are enough?" were asked more often.

1137: ***+.016***

Once distributed along the Coastal Plain from South Carolina to Texas, the short-lipped ladies'-tresses, Spiranthes brevilabris Lindley (Orchidaceae), appeared in 1999 to be restricted to a single population in Levy County, Florida. That population consisted of 152 plants. Ongoing efforts to locate additional populations of this terrestrial orchid have been unsuccessful. We provide 1) a

technique to germinate seeds of this orchid in vitro using mycorrhizal fungi (symbiotic seed germination); 2) a technique to establish seedlings onto soil ex vitro; and 3) a description of the mycorrhizal fungi that prompted germination and establishment. Two fungal endophytes, both Epulorhiza spp. recovered from the roots of the epiphytic orchid Epidendrum magnoliae Muhl. (syn. E. conopseum) and S. brevilabris, were utilized in the inoculation of seed. Germination was rapid (<10 days), and a higher percentage of seedlings developed leaves in vitro when inoculated with the S. brevilabris-derived fungus as opposed to the E. magnoliae-derived fungus (25% versus 20%). Of 165+ laboratory-grown seedlings transplanted onto soil at six sites in Florida, 100% survived >1 month, and 17 initiated anthesis >6 months.

1138: ***+.152***

For the first time in decades the world population of the critically endangered golden-headed langur has increased. A total of nine langurs births (seven births in 2003), but only three animal losses have been recorded since October 2000, when the ZOOLOGICAL SOCIETY FOR THE CONSERVATION OF SPECIES AND POPULATIONS in partnership with Muenster Zoo initiated the Langur Conservation Project on Cat Ba Island, Vietnam. Increasing conservation awareness among the local indigenous people and improving habitat and wildlife protection constituted the main objectives of the conservation project during the past 2 1/2 years. Poaching could be brought under control through ranger work, a strictly protected langur sanctuary in Cat Ba National Park was established, and active involvement of lo-cal people in protection work takes place. Resulting from a major governmental development plan for Cat Ba Island, habitat destruction and fragmentation have increased and so has the pressure on Cat Ba National Park, that is currently in danger of loosing up to 30 % of its area. The langur project is working to alleviate threats to Cat Ba National Park by contributing to map the future park boundaries and by assisting to apply for UNESCO Biosphere Reserve nomination for the Cat Ba Archipelago. With about 60 individuals, scattered over several isolated subpopulations, the langur population still is extremely small and fragile, and new threats such as proposed military roads, this species still will need all the help it possibly can get. Even translocation of langurs seems to be unavoidable.

1139: ***+.218***

1. We studied a reintroduced population of the formerly critically endangered Mauritius kestrel Falco punctatus Temmink from its inception in 1987 until 2002, by which time the population had attained carrying capacity for the study area. Post-1994 the population received minimal management other than the provision of nestboxes.2. We analysed data collected on survival (1987-2002) using program MARK to explore the influence of density-dependent and independent processes on survival over the course of the population's development.3. We found evidence for non-linear, threshold density dependence in juvenile survival rates. Juvenile survival was also strongly influenced by climate, with the temporal distribution of rainfall during the cyclone season being the most influential climatic variable. Adult survival remained constant throughout.4. Our most parsimonious capture-mark-recapture statistical model, which was constrained by density and climate, explained 75.4% of the temporal variation exhibited in juvenile survival rates over the course of the population's development.5. This study is an example of how data collected as part of a threatened species recovery programme can be used to explore the role and functional form of natural population regulatory processes. With the improvements in conservation management techniques and the resulting success stories, formerly threatened species offer unique opportunities to further our understanding of the fundamental principles of population ecology.

1140: ***+.269***

Management plans for threatened or recovering large vertebrate species that are increasing in population size and range focus on the establishment of viable populations within set temporal limits. New Zealand (Hookers) sea lions (Phocarctos hookeri) were declared a threatened species in 1997, and New Zealand legislation requires that threatened species of marine mammals must be managed to reduce human-induced mortality and achieve a non-threatened status within 20 years. The present breeding distribution of P. hookeri is highly localised, with over 95% of total annual pup production located at Auckland Islands and almost all of the remainder at Campbell Island. Breeding elsewhere has been ephemeral or restricted to < 10 adult females. The only recorded sustainable breeding at a new location has been at Otago, South Island, New Zealand. This breeding population consisted of a total of four breeding females in 2002 and is derived from one immigrant female that gave birth to her first pup in the 1993/1994 breeding season. The New Zealand Department of Conservation management plan specifies that to achieve a non-threatened status P. hookeri (1) at Otago must increase in the number of breeding females to greater than or equal to 10, and (2) must establish greater than or equal to two new breeding locations within the 20-year time frame, each with greater than or equal to 10 breeding females. This study 1) projects the population growth trends at a new location (Otago) to see if it will achieve greater than or equal to 10 breeding females within the legislated time frame, and (2) examines the likelihood that other breeding locations will establish elsewhere given the demographic information available for this species. We present 20 deterministic and three stochastic Leslie matrix model scenarios for female population growth for the initial years following the start of breeding at a new location. Our results indicate that (1) a new breeding population derived from one immigrant female is unlikely to reach 10 breeding females in 20 years; this duration is more likely to be 23-41 years (deterministic models) or 23-26 years (stochastic model), (2) the likelihood of two new sites establishing within 20 years is unquantifiable, but the probability is low, and (3) if the legislated outcome and time limit are not revised in the population management plan, the feasibility and effectiveness of re-locating young females could be investigated. (C) 2003 Elsevier Ltd. All rights reserved.

1141: ***+.226***

The Egyptian vulture (Neophron percnopterus) breeds in Sicily and, in 22 years of monitoring, has shown a decline followed by a slight recovery. We used Generalised Linear Models to predict: (1) the distribution range, (2) the habitat selection within the range, (3) the quality (i.e., occupation rate, breeding success) of breeding sites. Some 60% of Sicily proved to be unsuitable, being either too densely forested and without cliffs for nesting, or too densely populated, along with intensive agriculture. The models converged, indicating that the pairs select a precise upland habitat where low cliffs, distant from urban areas, are surrounded by arboreal crops and Mediterranean vegetation. The variables predicting the quality of a site are also related to human disturbance, including the distance from a road or the presence of heavy traffic. Natural restocking, in the last 3 years, is occurring in quiet sites and in proximity to extensive grazing land rich in sheep and goat herds. These results focused on short-term programmes for local management of the species aimed at protracting the natural restocking: namely, the activation of (1) a stable system of artificial feeding stations, (2) nest site protection with joint activities for increasing public awareness, (3) a reintroduction project, by hacking, of juveniles born in captivity in Italian and European breeding centres.

1142: ***+.218***

Many species have strong habitat preferences that directly influence population viability. For successful reintroduction of threatened populations that rely on habitat structures, the correct

placement of artificial structures is also important to population persistence. In this paper, we present a hierarchical approach to the problem of translocating animals that rely on permanent habitat structures, in which we first use population dynamics data to identify areas of suitable habitat, and then identify optimal configuration for habitat structures. We use data collected from a non-endangered, conspecific population of the endangered riparian woodrat (Neotoma fuscipes riparia) to examine the degree to which the distribution of dens in translocation sites might influence the likelihood that animals persist in their new environment. We characterize the habitats in which dens occur, analyze their spatial clustering, and compare them to temporal changes in population status for sex and age classes. We compare the potential efficacy of translocation efforts using spatial analysis versus solely habitat-based approaches and identify the optimal spatial configuration of dens that should be considered in this translocation effort. We found that patterns of habitat use were positively correlated with overstorey cover, and animal weight was positively correlated with understorey cover. Woodrats appear to select den locations on the basis of understorey cover, but also benefit from dense overstorey cover and distance to nearest tree. Our results suggest that in translocation efforts, artificial dens should be placed in clusters within a radius of 15 m, as values above and below this value showed negative correlations with body mass. Translocations should occur after reproductive events, which occur in April and August for woodrats in southern California. Our analyses provide practical guidelines in determining appropriate timing and spacing for translocation events in the context of animal condition, minimizing disease transmission, and reproduction.

1143: *+.211*

Optimization and simulation modeling can be used to account for demographic and economic factors simultaneously in a comprehensive analysis of endangered-species population recovery. This is a powerful approach that is broadly applicable but under-utilized in conservation biology. We applied the approach to a population recovery analysis of threatened and endangered piping plovers (Charadrius melodus) in the Great Plains of North America. Predator exclusion increases the reproductive success of piping plovers, but the most cost-efficient strategy of applying predator exclusion and the number of protected breeding pairs necessary to prevent further population declines were unknown. We developed a linear programming model to define strategies that would either maximize fledging rates or minimize financial costs by allocating plover pairs to 1 of 6 types of protection. We evaluated the optimal strategies using a stochastic population simulation model. The minimum cost to achieve a 20% chance of stabilizing simulated populations was approximately $1-11 million over 50 years. Increasing reproductive success to 1.24 fledglings/pair at minimal cost in any given area required fencing 85% of pairs at managed sites but cost 23% less than the current approach. Maximum fledging rates resulted in >20% of simulated populations reaching recovery goals in 30-50 years at cumulative costs of <$16 million. Protecting plover pairs within 50 km of natural resource agency field offices was sufficient to increase simulated populations to established recovery goals. A range-wide management plan needs to be developed and implemented to foster the involvement and cooperation among managers that will be necessary for recovery efforts to be successful. We also discuss how our approach can be applied to a variety of wildlife management issues.

1144: *+.256*

Understanding the factors that underlie colonization success is crucial both for ecological theory and conservation practices. The most effective way to assess colonization ability is to introduce experimentally different sets of individuals in empty patches of suitable habitat and to monitor the outcome. We translocated mated female waterstriders, Aquarius najas, into 90 streams that were

not currently inhabited by the species. We manipulated sizes of propagules (from 2 to 16 mated females) and numbers of origin populations (one or two). Three origin populations were genetically different from each other, but they were less than 150 km from the streams of translocation. The results demonstrate clearly that both the larger propagule size and the high number of source populations have positive effects on the probability of colonizing a new stream. Thus, in addition to the stochastic factors related to the propagule size it may be essential to consider also the diversity of genetic origin for colonization success.

1145: *-.022*

Cook's petrel (Pterodroma cookii), a New Zealand endemic, now breeds on only three islands at the extremities of its former range. Holocene fossil bones have indicated sites of 11 extinct colonies on North and South Islands, showing that Cook's petrels preferred hills <1000 m high and 20-30 km inland. This is a forest-breeding, burrowing petrel. Atypically for a seasonally breeding gadfly petrel, some birds visit Hauturu (Little Barrier Island) nocturnally through the non-breeding season, but do not land. The breeding season extends from September to April at its northern-most colony on Hauturu but is a month later in the south at Whenua Hou (Codfish Island), where birds are heavier. The pre-laying exodus lasts about 28 days. Egg-laying extends over 38 days in the large northern colony, but barely 15 days in the smaller southern one. Incubation is mainly done in three 14-day spells. Chick-rearing takes about 87 days. Chicks attain almost twice the non-breeding adult weight. There is a desertion period of about 10 days before fledging. Pacific rats (Rattus exulans), feral cats (Fells catus) and weka (Gallirallus australis) have endangered these last colonies. Breeding success, most affected by rat predation of eggs and chicks, deteriorated seriously after extermination of feral cats from Hauturu, but improved significantly after eradication of rats from Whenua Hou. Rats are now a major threat on Hauturu. Cook's petrels should be considered for reintroduction to suitable mainland reserves, to enhance biodiversity and restore nutrient inflows.

1146: *+.069*

Trounson Kauri Park is a 445-ha fragment of kauri-podocarp forest located in western Northland, New Zealand, and is one of a series of "mainland island" ecosystem restoration projects managed by the Department of Conservation. One of the main objectives at Trounson is to reduce introduced mammalian pest numbers to a level that would allow recovery of those native species still present in the park and allow the reintroduction of local or regionally extinct species. \\Brushtail possums (Trichosurus vulpecula) and rodents (ship rats, Rattus rattus, Norway rats, R. norvegicus, and mice, Mus musculus) were targeted in poison baiting operations using 1080, various anticoagulants, cholecalciferol or cyanide pellets laid in bait stations within the park. These operations suppressed possum and rat numbers to low levels, but mouse numbers were reduced only for short periods. The reduction in possum and rat numbers allowed significant seasonal increases in kukupa (New Zealand wood pigeon, Hemiphaga novaeseelandiae) abundance. Feral cats (Felis catus) and mustelids (stoats, Mustela erminea, weasels, M. nivalis, and ferrets, M. furo) were trapped around the forest boundaries and at key locations within and around the park. The predator trapping consistently provided predation relief for North Island brown kiwi (Apteryx australis mantelli) chicks, which are extremely vulnerable to predation by stoats, such that enough survived in order to sustain and increase the size of the adult population. Attempts were made to re-establish breeding populations of North Island robins (Petroica australis longipes), North Island kokako (Callaeas cinerea wilsoni), and brown teal (Anas aucklandica chlorotis) (three species of locally extinct native birds) to Trounson, but these were only partially successful at best.

1147: ***+.019***

Although analyses of intraspecific variability are an important prerequisite for species identification assays, only a few studies have focused on population genetics and historical biogeography of sturgeon species. Here we present the first study on genetic variability of the last remaining Adriatic sturgeon, Acipenser naccarii, derived from mitochondrial and nuclear DNA. Our mitochondrial DNA analyses arranged individuals into three distinguished mitochondrial DNA haplogroups (Po1, Po2 and Buna). Two haplogroups (Po1 and Buna) were correlated to geographical distribution, whereas the third (Po2) was not. It was, however, very closely related to one lineage of its Ponto-Caspian sister species, A. gueldenstaedtii. The distribution of nuclear markers (microsatellites and amplified fragment length polymorphism) was strongly correlated to geographical distribution. An assignment test based on nuclear data placed no specimen of A. naccarii to A. gueldenstaedtii and vice versa. Therefore, the presence of gueldenstaedtii-like haplotypes within the Po population is either the result of a postglacial introgression or an ancestral polymorphism and does not indicate a hybrid population. The most valuable tool for forensic species identification purposes is one diagnostic deletion separating all A. naccarii from A. gueldenstaedtii. As both A. naccarii populations are genetically differentiated, stocking of sturgeon from the Po River in Italy into waters of the Buna River would jeopardize the genetic differences between both populations and should thus be avoided.

1148: ***+.219***

White-tailed deer (Odocoileus virginianus) were nearly extirpated from the southeastern USA during the late 19th and early 20th centuries. Recovery programmes, including protection of remnant native stocks and transplants from other parts of the species' range, were initiated in the early 1900s. The recovery programmes were highly successful and deer are presently numerous and continuously distributed throughout the southeastern USA. However, the impact of the recovery programmes on the present genetic structure of white-tailed deer remains to be thoroughly investigated. We used 17 microsatellite DNA loci to assess genetic differentiation and diversity for 543 white-tailed deer representing 16 populations in Mississippi and three extra-state reference populations. There was significant genetic differentiation among all populations and the majority of genetic variation (greater than or equal to 93%) was contained within populations. Patterns of genetic structure, genetic similarity and isolation by distance within Mississippi were not concordant with geographical proximity of populations or subspecies delineations. We detected evidence of past genetic bottlenecks in nine of the 19 populations examined. However, despite experiencing genetic bottlenecks or founder events, allelic diversity and heterozygosity were uniformly high in all populations. These exceeded reported values for other cervid species that experienced similar population declines within the past century. The recovery programme was successful in that deer were restored to their former range while maintaining high and uniform genetic variability. Our results seem to confirm the importance of rapid population expansion and habitat continuity in retaining genetic variation in restored populations. However, the use of diverse transplant stocks and the varied demographic histories of populations resulted in fine-scale genetic structuring.

1149: ***+.061***

A trial reintroduction of the European beaver (Castor fiber) to Scotland has been proposed and is awaiting Scottish Executive approval. Currently, no data have been published on the actual effects of beavers on the Scottish landscape, although many authors have predicted potential impacts. Such predictions have been based on the impacts of the beaver in other European countries. The

aim of this study is to provide a better predictive capability as to the potential effects on tree felling immediately following beaver reintroduction by using data of beavers in captivity. In 2002, four European beavers were released into two large, semi-natural enclosures - the Willow Carr Site and the Lake Site - in eastern Scotland. This paper represents data from the first year of a three-year monitoring programme to investigate the felling and feeding activities of these beavers. In absolute terms, willow (Salix spp.) were the favoured species at both sites, being felled in the greatest numbers, followed by alder (Abuts spp.) and birch (Betula spp.). In terms of relative abundance, only the selections against birch at both sites, and for willow at the Lake site, were found to be significant. No size-selectivity at the Willow Carr Site was evident, but significantly smaller than average trees of all three genera were felled at the Lake Site. Decreased felling activity was observed with increasing distance from the lodge at the Willow Carr Site, whilst most trees felled at the Lake Site were situated within the shallow margins of the lakes. Approximate felling rates were 0.5 and 0.8 trees per beaver per day, at the Willow Carr Site and Lake Site respectively.

1150: *+.045*

The European beaver (Castor fiber) returned to Denmark in 1999 when 18 beavers were released in Klosterheden State Forest District in the northwestern part of the country. A monitoring programme was initiated to trace the population and distribution of the beavers, beaver-human conflicts, and effects on flora and fauna. The status of flora and fauna in the reintroduction area was systematically investigated prior to the beaver reintroduction. By 2003, beavers inhabited the entire catchment basin in which they were released and had dispersed to a neighbouring river system 25-30 km away. Beaver kits were observed every year and the population was estimated at 51 individuals in 2003. The beavers mainly fed on willow scrubs during the winter season and non-woody plants in the summer. No damage was reported in forests or agricultural production areas but the beavers caused minor problems by flooding an arable field, gardens, meadows, and forest roads and by blocking inlets to a fish farm. Few significant alterations of water flow were recorded but the diversity of the wetland biotopes increased. The only negative effects appeared to be a restriction of spawning migration of sea trout (Salmo trutta) by beaver darns in brooks. Other fish species were thought to benefit from the beaver ponds. Ponds enhanced spawning potentials for amphibians and enabled new species of birds to breed in the area. Bats profited by more suitable hunting sites. Occurrence of otters (Lutra lutra) increased but no clear relationship with beaver distribution was demonstrated.

1151: *+.195*

The beaver (Castor fiber) was reintroduced to the Netherlands in 1988, in the Biesbosch National Park. In the following years beavers were also introduced in several newly developed nature areas, including the Gelderse Poort, a natural area 100 km upstream from the Biesbosch. Fifteen years later, we see a steadily growing beaver population, living in a still expanding ecological network of traditional nature reserves and newly developed nature areas, especially along the rivers Rhine, Meuse and IJssel. The number of beavers in the Netherlands is estimated at about 200-250 individuals.

1152: *+.012*

After a long period of absence, beavers (Castor fiber) reappeared in Flanders in the spring of 2000, first in the province of Vlaams-Brabant and in 2002 also in Limburg. The beavers originate from the unofficially reintroduced Walloon population. With a view to a future reintroduction project to

restock this not yet reproducing Flemish beaver population, a feasibility study was conducted at the request of AMINAL Nature Division (Ministry of the Flemish Government). This study concluded that the basins of the rivers Schelde and Dijle could carry a viable beaver population of at least 40 families that were all expected to stay in the same area and cause no problems in the wide vicinity. Even before the Flemish government had decided whether or not to proceed with the preparation of an official reintroduction, 20 Bavarian beavers of unknown age and sex were released on 11 April 2003 along the rivers Dijle and Laan. This happened unofficially, without any scientific follow-up and without preparing or informing the local population or other interest groups. Scarcely two months later at least two v beavers had already crossed the city of Leuven. Beavers are now permanently present along the rivers Dijle and Laan south of Leuven. Some traces have also been found on the Use. Complaints are coming in about damage to private as well as to public property and an adaptation of rat control methods is required. Since in the densely populated ulated Flanders many human-beaver conflicts can be expected, the pros and cons of beaver presence in Flanders should be weighed up carefully, taking all interest groups into account.

1153: *+.063*

The beaver (Castor fiber) returned to Wallonia mostly due to clandestine reintroductions of 100 Bavarian beavers between 1998 and 2001. Beaver settlements are now present in most of the river basins, sometimes in seemingly unsuitable areas or in highly urbanised zones. Beavers spread widely during the two years following the reintroductions. The Walloon authorities instituted proceedings against the perpetrators of these releases and, at the same time, instigated a survey and a structure for managing possible conflicts. Forest, fishing and muskrat trapping agents conduct the field survey work and transfer information to the Research Centre for Nature, Forests and Wood (CRNFB) and the Biernausaut Association. With this set-up, a new beaver site can be detected within one month. About 200-250 beavers are now present in Wallonia, at about 60 sites. Only small problems have occurred until now, but they are expected to increase as the beaver population grows and spreads. Thus, beaver management has to continue, to prevent conflicts with human activities but also in accordance with nature conservation objectives such as the implementation of the Natura 2000 programme. A future trans-border co-ordination for surveying beavers should be developed.

1154: *+.066*

During the 19th century the European beaver (Castor fiber) disappeared from most parts of France, except for the Rhone Valley. In the 1970s, beavers caught from the river Rhone were released in the Loire River, near the city of Blois. While this reintroduction was successful and led to the progressive recolonisation of the Loire River and its tributaries, the density of beaver colonies has remained low. Furthermore, habitat quality has decreased in the downstream part of the Loire, and beavers might not find enough suitable sites to settle. This study was designed to estimate the number of suitable places for beaver settlement in a downstream segment of the Loire River, which is still uncolonised, but already explored by pioneer individuals. For this purpose, we searched for relationships between signs of beaver presence (lodges, cut trunks, and remains of browsed plants) and vegetation features, bank characteristics, and human disturbance in a presently colonised region of the Loire River. Beavers selected sites dominated by 10-15 m tall trees to build lodges, irrespective of the tree species. Sites with cut trees were dominated by Salicaceae. Beavers fed on Salicaceae and numerous herbs. Based on these findings, vegetation features in the uncolonised stretch of the river are expected to be favourable to beaver settlement and feeding. However, beavers will have to face river bank protections and more intense levels of

human activity in most of the uncolonised area. These factors may dramatically limit the number of sites suitable for lodge building.

1155: *+.110*

European beavers (Castor fiber) have been reintroduced to many areas within their former range. The resulting populations are still in a phase of population growth and range expansion. From a management point of view it is of interest to understand the pattern of population development that these populations are, or will be, exhibiting. Based on data from two surveys of a province in southwestern Sweden, I have earlier proposed that reintroduced beaver populations may exhibit an irruptive pattern of development, possibly as a result of overutilization of resources and lack of predators. The aim of this study was to see if a repeated study, twelve years after the previous, would support or question the proposed pattern of population development. Data from three surveys of the same province were used. The overall population density for the total area had increased from 0.10 colonies/km2 in 1976, to 0.19 in 1987, and to 0.21 in 1999. However, when population density of local areas with time passed since colonisation was related, a peak in density (mean: 0,34 colonies/km2) after 25 years was revealed. Dividing the data into groups, areas colonised more or less than 25 years ago, and beaver population density decreased or increased since the previous survey (1987), showed that a negative change in population density was significantly more common in areas colonised more than 25 years ago. The results support the proposed pattern of population irruption in the studied beaver population.

1156: *+.138*

After being reduced to about 1,200 animals in eight isolated populations by the beginning of the 20th century, European beavers (Castor fiber) have powerfully recovered in both range and population, through relaxation of persecution, natural spread, and widespread reintroductions. Populations are now (2003) established in all countries within their former natural range in Europe except for Britain, Portugal, Italy, and the south Balkans (Greece, Albania, Bulgaria, Macedonia; status in Bosnia-Herzegovina is uncertain). In Asia, there are significant ficant populations in central Siberia, Kamchatka, and on the Amur; and small relict populations elsewhere in Siberia, and in Xinjiang (China)/western Mongolia. The current minimum population estimate is 639,000. Both populations and range are in rapid expansion. We present maps summarizing current knowledge of the world distribution of European beaver and the Eurasian distribution of the introduced American beaver (Castor canadensis), and tables of the most recent known population estimates for each country.

1157: *+.210*

The roles of behavioral studies of individuals in the conservation of wildlife, which should be important for the conservation of birds also, are briefly introduced and discussed in the light of two cases studies of the Asiatic Black Bear Ursus thibetanus and Sika Deer Cervus nippon in Japan. Some local populations of the bear, one of Japan's largest mammal species, are in danger of extinction. At Karuizawa town, Nagano Prefecture, central Japan, some bears have been visiting garbage disposal sites. Sixteen bears were captured, collared, and tracked using radio-telemetry, from July 1998 to September 2001. Three individuals were killed because they were staying within the town and judged as dangerous individuals. The other 16 bears were translocated. This individuals based management with radio-telemetry data can minimize risks of local population extinction of bears. On Kinkazan Island, Miyagi Prefecture, north-east Japan, most members of a 150-strong Sika Deer population were individually recognizable and have been tracked since

1989. Changes in body conditions, such as morphological and nutritional condition of individuals, and lifetime reproductive success were recorded. The data suggested that variances in lifetime reproductive successes among individuals were high, and the population dynamics can be strongly related to survival and reproductive success of the variation among individuals. This long-term study of the deer showed that reproductive strategies of individuals affected population dynamics. While, this study also showed that the population density, food availability, and stochastic variation in environment might seriously affect on survival and reproductive success of each individual.

1159: *+.068*

In the years 1999-2002, first studies were carried out on the metazoan parasites of Atlantic salmon (Salmo salar L.) recently reintroduced into the Elbe River drainage system, after more than 50 years after the complete extinction of the Elbe salmon population. A total of six helminth species were recorded from salmon smolts from three streams of the Elbe River basin (Kamenice River, Jestedsky and Libocansky Brooks) in North Bohemia, Czech Republic, where S. salar fingerlings have been released since 1998: Gyrodactylus truttae Glaser, 1974, Crepidostomum metoecus Braun, 1900, Diplostomum spathaceum (Rudolphi, 1819) metacercariae, Raphidascaris acus (Bloch, 1799) adults and encapsulated larvae, Cystidicoloides ephemeridarum (Linstow, 1872), and Neoechinorhynchus rutili (Moller, 1780). Except for D. spathaceum, all these freshwater parasites have been received from the helminth fauna of the co-habiting brown trout (Salmo trutta fario L.). Due to local ecological conditions, the parasite faunae of both salmon and brown trout exhibited distinct qualitative and quantitative differences in the three localities. The finding of G. truttae on S. salar represents a new host record. Three helminth parasites of marine origin, the cestodes Eubothrium crassum (Bloch, 1779) and Scolex pleuronectis Muller, 1788 plerocercoids, and the nematode Anisakis simplex (Rudolphi, 1809) larvae were found in the single examined adult S. salar from the Kamenice River. New data on the geographical distribution of some nematode parasites of Salino trutta fario L., Barbatula barbatula (L.) and Anguilla anguilla (L.) are presented.

1160: *+.243*

American Peregrine Falcons in California (Falco peregrinus anatum) have been managed in one of the largest reintroduction programs in the history of endangered species management. However, as for most other widespread natural populations, the spatial and temporal dynamics of California Peregrines have remained poorly understood. Longterm monitoring data on this highly managed species present a unique opportunity for retrospective analysis of the factors contributing to the successful recovery of Peregrine Falcons in California, as well as demographic differences between habitat types. We used a newly developed mark-recapture model (the Barker model), which allows the simultaneous use of recaptures, dead recoveries, and live resightings, to provide estimates of first-year, second-year, and adult survival of Peregrine Falcons in the mid- and south-coast regions of California. Annual survival rates for second-year and adult Peregrines were estimated at 86%. Through model fitting, we show positive effects of urban habitats on first-year survival. In our best-fit models, first-year birds fledged in urban areas had a survival rate of 65%, whereas rural (non-urban) first-year survival was only 28%. The introduction method also influenced first-year survival after dispersal; in rural areas, estimated survival of hacked young (after independence) was lower than estimated survival of wild-reared young. We also show that birds in urban habitats have significantly higher fecundity rates than birds in rural habitats, even though the fecundity of rural breeders has increased significantly over the last two decades. We argue that the strong habitat differences in first-year survival combined with lower fecundity rates

in rural habitats (due to slower improvement in eggshell thinning rates) facilitates spatial structuring of the California Peregrine population. Matrix population models constructed for both rural and urban habitats support this assessment. The temporally averaged population growth rate in urban habitats was estimated as lambda = 1.28, compared to lambda = 0.99 in rural habitats. Yearly analytical lambda values in rural habitat predict declining population growth (lambda < 1) throughout the 1980s and increasing population growth (lambda > 1) in the 1990s due to improved reproductive performance. These results indicate that the introduction effort was pivotal in recovering the rural, population in this portion of. its former habitat, because intrinsic growth rates alone would have been insufficient to yield the observed population recovery.

1161: *+.115*

Soil characteristics, disturbance histories, and species richness among distinct groups of plants and animals may be useful predictors of important conservation areas when data are limited. We used multivariate analysis of covariance to test the hypothesis that the species richness of plants, arthropods, herpetofauna, and breeding birds are correlated and increase with soil fertility (silt and clay content in sandy soils), soil variability, and hardwood midstory reduction in subxeric, nutrient-poor longleaf pine sandhills at Eglin Air Force Base in northwest Florida. During 1994-1995 and 1998-1999, we sampled 30 81-ha plots with varying fire-exclusion histories and at two spatial scales of resolution (10 x 40 m and 81 ha). The relationship between the number of plant species and percent silt and clay and its coefficient of variation was significant and positive at the plot level (81 ha) but not at the subplot level (10 x 40 m). Herpetofaunal species richness was the only faunal variable that increased with silt and clay content, but the coefficient of variation did not. Multivariate effects of hardwood reduction were significant in 1998-1999 but not in 1994-1995, which suggests that the reintroduction of fire increased species richness across taxa. Univariate effects of hardwood reduction were marginally significant for plant species richness at the subplot level and not significant for herpetofaunal species richness at either scale. Plant and arthropod species richness were not correlated. Herpetofaunal species richness was not correlated with that of other taxa (plant and animal). The species richness of plants and arthropods were each significantly positively correlated with the richness of breeding bird species at the subplot level during 1994-1995, whereas only arthropod species richness was positively correlated with bird species richness at the plot level in either year. Our results suggest that the restoration of fire regime may be the most effective tool with which to increase diversity in pyrogenic areas considered for conservation protection.

1162: *-.015*

In Alberta, Canada (1982-2001), and in Idaho, Montana, and Wyoming, United States (19872001), wolves (Canis lupus) killed various domestic animals, among which the major prey were sheep in the United States (68%, n = 494) and cattle in Canada (95%; n = 1633). Under recovery programs, the wolf population increased in the United States, and depredation events increased proportionately. In both countries, the number of domestic animals killed each year was correlated with the number of wolves killed by government authorities for depredation management. We tested the ability of antiwolf barriers made of flags hanging from ropes to impede wolf access to food and livestock. In 18 experiments, barriers prevented captive wolves (n = 9) from accessing food for up to 28 hours and allowed daily separation of wolves to administer contraceptive pills to a female wolf. Barriers prevented access by wild wolves to 100-m(2) baited sites during two 60-day tests. We also set barriers around three cattle pastures. In Alberta during two 60-day trials on 25-ha pastures, wolves approached barriers on 23 occasions but did not cross them, and no cattle were killed. Wolves killed cattle on neighboring ranches during the trials and before and after the

trials on the tested ranches. In Idaho four radio collared wolves crossed barriers and killed cattle in a 400-ha ranch after 61 days of barrier exposure. Our results suggest that antiwolf barriers are effective in deterring captive and wild wolves for >1 and 60 days, respectively, and that wild wolves switch to alternative livestock when excluded from one herd of livestock. Our depredation data indicate that protecting livestock from wolves reduces the necessity for killing wolves. Barriers could play a role among the limited set of preventive measures available and offer a cost-effective mitigation tool for the problem of livestock depredation on a local scale.

1163: *-.007*

As wolf (Canis lupus) populations recover in Wisconsin (U. S. A.), their depredations on livestock, pets, and hunting dogs have increased. We used a mail-back survey to assess the tolerance of 535 rural citizens of wolves and their preferences regarding the management of "problem" wolves. Specifically, we tested whether people who had lost domestic animals to wolves or other predators were less tolerant of wolves than neighboring residents who had not and whether compensation payments improved tolerance of wolves. We assessed tolerance via proxy measures related to an individual's preferred wolf population size for Wisconsin and the likelihood she or he would shoot a wolf. We also measured individuals' approval of lethal control and other wolf-management tactics under five conflict scenarios. Multivariate analysis revealed that the strongest predictor of tolerance was social group. Bear (Ursus americanus) hunters were concerned about losing valuable hounds to wolves and were more likely to approve of lethal control and reducing the wolf population than were livestock producers, who were more concerned than general residents. To a lesser degree, education level, experience of loss, and gender were also significant. Livestock producers and bear hunters who had been compensated for their losses to wolves were not more tolerant than their counterparts who alleged a loss but received no compensation. Yet all respondents approved of compensation payments as a management strategy. Our results indicate that deep-rooted social identity and occupation are more powerful predictors of tolerance of wolves than individual encounters with these large carnivores.

1164: *+.071*

The 10 km(2) island of La Digue, Republic of Seychelles, western Indian Ocean supports the last viable population of the Critically Endangered Seychelles Black Paradise-flycatcher Terpsiphone corvina. Small populations recorded on adjacent islands since the 1980s appear to be ephemeral and not self-sustaining. We document the results of the first island-wide survey of the flycatcher on La Digue using the playback of conspecific calls at random points. Previous surveys were based largely on counting the number of singing males. The survey was conducted between April and August 2001 and confirmed (i) the current world population is at least 200 individuals (109-145 territories, 218-290 individuals) in a c. 4.4 km(2) range, thus accurately quantifying the documented increase in flycatcher numbers since the late 1970s; (ii) territories were more widely distributed than previously recorded and not exclusively associated with coastal plateaux or freshwater bodies, contrary to previous descriptions; and (iii) distribution was determined largely by the presence of high canopy (native) broad-leaved tree species. The importance of canopy height to flycatchers was highlighted by the fact that localized loss of high canopy (native) forest (4%), in a 161 ha study area on the large western plateau, resulted in an equivalent reduction (4%) in the number of territories. In light of our findings we discuss the implications for conservation of the flycatcher on La Digue and its possible translocation.

1165: *+.203*

Translocations of threatened species play an increasingly important role in conservation management. However, few studies have examined what effects, if any, the translocation process itself (i.e. catching, handling, confining, transferring and releasing an animal into an unfamiliar environment) has on subsequent breeding success. Takahe Porphyrio hoclistetteri living on offshore "predator-free" islands in New Zealand are a model system for examining such effects because pre-breeding birds have been frequently translocated between established island populations before they pair up and breed at 2-3 years of age. We postulated that "translocated" breeders (i.e. breeders that had been raised on another island) would delay first breeding attempts and/or have lower reproductive success compared with "resident" birds (i.e. bred on the same island that they were raised). The results indicated that translocated birds did not delay breeding and had similar mean hatching and fledging success as resident pairs in their first breeding season and subsequent seasons combined. The results suggest that at least for large or long-lived birds such as Takahe, the effects of any stress from the translocation itself, or the release into an unfamiliar environment, might be either short-lived or not significant enough to hinder subsequent breeding success. We recommend that further research be carried out on other species to determine the baseline effects, if any, of translocations, so that they can be taken into account when considering other determinants of translocation success such as habitat suitability, number of individuals and timing of releases.

1166: ***+.292***

We studied the effect of food supplementation, female age and clutch order on productivity in a translocated species. Food supplementation increased clutch size from 3.9 to 4.4 (average) eggs per nesting attempt, and more than doubled fledging and recruitment success. Supplemented females started a second clutch 9 days sooner after fledging first clutch chicks than unfed females. During second clutches, supplemented females incubated the eggs for a shorter period of time (15.2 days vs. 16.8). Older females laid larger clutches (4.6 vs. 3.7 eggs) than yearlings and incubated second clutch eggs for a shorter period (15.4 vs. 16.6 days). Females laid more eggs in first clutches (4.2 vs. 3.8 eggs), and those eggs took approximately 30% longer to lay than eggs in second clutches. The successful maintenance of hihi populations on the available islands may be dependent on the permanent provision of supplemental food at nest sites. (C) 2003 Elsevier Ltd. All rights reserved.

1167: ***+.165***

The loggerhead shrike, Lanius ludovicianus, is a declining songbird that forms breeding aggregations. Despite such reports from several populations, only one statistical analysis of loggerhead shrike territory distribution has been published to date. I use a spatio-temporal simulation technique to test for deviations from randomness in the spatial distribution of point data that takes into account date of nest establishment. I apply this model to data on the distribution of shrike nests in Comanche County, Oklahoma, USA, collected over the 1998 and 1999 breeding seasons. When the data are considered without regard to date of nest-establishment, the results are equivocal; nests are significantly aggregated in 1999, but not 1998. However, when order of nest establishment is taken into account, later nests are significantly more likely to be closer than expected to conspecifics in both years. This is true even when the distribution of simple resources, such as suitable nest-trees, is included in the model. These results are consistent with the hypothesis that loggerhead shrikes 'prospect' for suitable habitat using the distribution of breeding conspecifics. This aspect of loggerhead shrike breeding ecology should be considered for both habitat conservation and captive breeding and reintroduction programs for this species. (C) 2003 Elsevier Ltd. All rights reserved.

1168: ***+.376***

In France, most civil engineering and excavation projects are at present accompanied by compensatory measures with the aim of preserving biodiversity. In order to avoid the destruction of a habitat of high conservation interest in NE France, harbouring two legally protected plant species, an experiment of soil translocation was conducted on an area of I ha. The donor site was an extensively managed mesophilic meadow and the receiving site was a neighbouring arable land. The vegetation of the translocated meadow was described 8 and 17 months after soil translocation, and compared (1) with vegetation resulting from more classical restoration techniques tested on the arable land (natural regeneration and seed mixture sowing) and (2) with the soil seed bank and vegetation previously present on the donor site. Results showed that the soil translocation technique permitted the development of many meadow species, including two legally protected species, and few ruderal species despite a large area of bare ground. This technique seems effective in terms of number and abundance of meadow species compared to natural regeneration and commercial seed sowing. In the case of the two classical methods, species richness was lower and only widespread species were present. Topsoil translocation provides a good compensatory method to avoid habitat and species destruction. However, the study should be continued, with the aim of assessing the longer term development and stabilization of the vegetation of the translocated meadow.

1169: ***+.321***

I analyzed the habitat selection of two Alpine marmots Marmota Marmota (Linnaeus, 1758) populations (A and B) re-introduced in the Friulian Dolomites Natural Park (Eastern Italian Alps) in 1977 and 1983 respectively. Population A showed a higher density of family units than the more recently introduced and still increasing population B. I mapped winter burrows and I conferred proportions of usage of habitat types with their availability by the Jacobs index. Population B positively selected fewer types of habitat than population A, and particularly selected those habitat types more strongly selected by A. Through stepwise discriminant function analysis and oneway ANOVA, I analyzed the characteristics of the winter burrow surroundings, by splitting up the two study areas into sample squares covering 1 ha each. The importance of the alpine meadows and pastures was subordinate to the presence of rocks, especially in the pastures. Comparing the results obtained separately for the two populations, the more recent and less dense population showed a more restrictive habitat and slope selection, in accordance with the ideal free distribution theory. In order to validate the habitat suitability model obtained by discriminant analysis I applied it to two other populations of Alpine marmots present in FDNP and in the Julian Prealps Natural Park.

1170: ***-.078***

The Spiny-tailed or Gidgee Skink, Egernia stokesii, has two western subspecies; one of which, Egernia stokesii badia, has declined in distribution and abundance because of habitat disturbance and is currently listed as threatened. This study examined all known locations of E. s. badia in the Western Australian Wheatbelt to determine its current status. Results show that it is still found over a large part of its previously known range in the eastern part of the northern Wheatbelt, but it has declined or disappeared from most localities in the central Wheatbelt. Reintroduction to previously occupied sites from populations occupying threatened localities (abandoned buildings, farm sheds, etc.) could be undertaken if suitable habitat (e.g. old hollow logs) is established or available at translocation sites and members of family groups are relocated together.

1172: ***+.071***

California condors are one of the most endangered species native to the mainland United States and are subject of intense effort regarding captive breeding and reintroduction. We analyzed 20 years of California condor egg records from the wild and from three captive propagation facilities for fertility, hatchability, and chick survivability, along with changes in egg size due to multiple clutching. Overall annual mean percent of fertile eggs was 80.2%, hatchability was 87.3%, and chick survivability to greater than or equal to 60 days was 95.5%. One egg-laying site had a significantly lower fertility rate (P less than or equal to 0.0001) than the other sites, which was probably due to pair incompatibility rather than any physiological factors. Egg volume of the first egg was significantly greater than both the second (t = 6.73, P = 0.0001) and third egg (t = 6.62, P less than or equal to 0.0001) of the season, while the second egg had a significantly greater volume (t = 3.20, P = 0.0084) than the third egg. Chicks from the second egg (t = 3.24, P = 0.029) and third egg (t = 7.94, P = 0.0014) of the season were significantly smaller than chicks from the first egg of the season. The decrease in egg measures and chick hatch weight due to multiple clutching did not affect hatchability or chick survivability. There were significant positive relationships (P < 0.0001) between fresh egg weight and chick hatch weight and between egg volume and chick hatch weight, as well as between fresh egg weight and egg volume. In spite of the decrease in fresh egg weight, egg volume and chick hatch weights, due to egg removal to stimulate double and sometimes triple clutching, the captive propagation program has been successful in producing birds for the restoration of this species. Zoo Biol 23: 489-500, 2004. (C) 2004 Wiley-Liss, Inc.

1173: *-.045*

A total of 85 brush-tailed bettongs (Bettongia penicillata) from Western Australia and two sites in South Australia were translocated to Yathong Nature Reserve (YNR) in western New South Wales in October 2001. Aerial baiting to control the introduced red fox (Vulpes vulpes) had been undertaken on YNR since 1996. Thirty-one bettongs were fitted with radio-transmitters at the time of release, and two subsequently. Trapping took place at irregular intervals after the translocation. In all, 73% of telemetered bettongs died within the first six months; all were dead within 13 months. Eight bettongs died within the first eight days immediately following their release, due to causes other than predation. These eight all originated from St Peter Island (SPI), South Australia. A low incidence of breeding on SPI supports the belief that this source population was in poor condition and unsuited for translocation. Overall, 19 of the 33 telemetered bettongs were killed by predators: 14 (74%) by feral house cats (Felis catus), two (11%) by birds, and three (16%) by predators, which, although they could not be fully identified, were not foxes. One month after release, surviving bettongs weighed less than they did at the time of their release (mean decrease in mass = 9.7%, range 2.6 - 22.4%, n = 11). Within two months of their release most had regained any lost mass (mean change in mass since release = - 0.3%, range - 5.9 to 10.5%). Food resources on YNR appeared sufficient to sustain adult brush-tailed bettongs, despite a period of severe drought. Small pouch young present at the time of release were subsequently lost. Females gave birth and carried small pouch young (up to 50 mm), but no young-at-foot were recorded. Bettongs did not disperse further than 10 km from their release site. Overall, 50% of aerial-tracking locations were no further than 3.2 km from the release site, and 92% no further than 7.0 km. This experimental translocation of brush-tailed bettongs failed due to predation by cats. It demonstrated that foxes were no longer a threat to wildlife on YNR and identified cats as the major impediment to the restoration of locally extinct fauna.

1174: *-.020*

Deer species (fallow, red, sambar, chital, rusa and hog deer) have formed wild populations in Australian habitats ranging from arid woodland to rainforest and are a growing management issue.

Data were obtained via an Australia-wide land-manager survey that collected information on the liberation, distribution, abundance and management of wild deer in Australia. It is estimated that there are 218 wild deer herds in Australia with 7% of these herds originating from acclimatisation society releases, 35% from deer farm escapes/releases and 58% from translocations (deliberate releases). On average, herds released by acclimatisation societies are estimated to be 107 years old, herds that have escaped from (or been released from) deer farms are 9 years old, and transplanted herds are 6 years old. It is estimated that Australia currently has 200000 wild deer, with 85% of these deer originally released by acclimatisation societies, 6% through escapes/releases from deer farms and 9% by translocation. Poor knowledge of the impacts of wild deer by land managers and the absence of consistent legislation governing the management of farmed and wild deer are factors that have exacerbated deliberate releases of deer and the escape of deer from farms. Management strategies for wild deer in Australia need to be developed by land managers to address the escape and release of deer from farms, the illegal translocation of deer into the wild and the management of existing wild deer herds.

1175: *+.042*

Although the theoretical effects of a severe reduction in effective population size (i.e. a bottleneck) are well known, relatively few empirical studies of bottlenecks have been based on extensive temporally spaced samples of a population both before and after a bottleneck. Here we describe the results of one such study, utilising the Jenolan Caves (JC) population of the brush-tailed rock-wallaby (Petrogale penicillata). When first sampled in 1985 (n = 20) the JC population comprised similar to 90 individuals. Subsequently the population crashed, and by 1992 only seven individuals remained. In 1996 the entire population (n = 10) was again sampled. Genetic diversity in the pre- and post-crash JC population was compared using 11 polymorphic microsatellite loci and PCR-SSCP analysis of the mitochondrial DNA control region. Only a single unique control region haplotype was detected in the pre- and post-crash JC population, although variant haplotypes were present in other P. penicillata populations. Of the 35 microsatellite alleles present in the pre- crash population, nine (26%) were lost during the bottleneck. The average number of rare alleles declined by 72%, allelic diversity was reduced by 30% and average heterozygosity declined by 10%. These observations are consistent with theoretical predictions. Additional analyses revealed that a P. penicillata female at Wombeyan Caves was the only survivor of a 1990/91 reintroduction attempt using animals from JC. Of the microsatellite alleles detected in this female, 21% (4/19) were no longer present in the post-crash JC population. Furthermore, the genetic profiles of animals from the recently discovered Taralga population indicate that they are not derived from JC stock, but represent a threatened remnant of a hitherto undetected natural P. penicillata population.

1176: *+.226*

Large blue (Maculinea anion) is probably the most famous butterfly species in Europe. It has been focusing attention of scientists, collectors, conservators since 18th century, especially in Great Britain. The mystery of the extraordinary life history was solved a hundred years ago by F. W. Frohawk and T. A. Chapman. Caterpillars are initially endophytic and live in Thymus flowerheads but at the beginning of the fourth (final) larval instar they leave the plants. If lucky larvae are adopted by Myrmica ants (Fig. 1) and they continue their development in nests preying upon host brood. Formerly large blue occurred locally on nearly 100 sites in Southern England. Fast decline was observed and until 1970 only 3 colonies survived (Fig. 2). Detailed ecological demands were not understood enough to undertake effective conservation measures. Key factors were discovered by J. A. Thomas in 1970s. Although M. anion larvae are transported to nests by workers of all

Myrmica species, the rate of survival differs significantly among species. A butterfly population is dependent on high number of M. sabuleti nests present near Thymus plants. The host ant is very sensitive to habitat quality and in English climate it is abundant only on heavily grazed southern slopes (Fig. 3). Changes in land management, abandonment as well as myxomatosis in 1950s, which almost completely eliminated rabbits - natural mowers, led to disappearance of M. anion habitat. After extinction, the reintroduction programme has started very soon, following proper habitat management. Large blue populations were restored successfully on some sites using Swedish stock; further reintroductions are planned. In continental Europe, M. anion is endangered in many countries, but habitat requirements vary in different climatic zones. In Poland, where declining is very fast, it is suspected that larvae at least at the NE part of the country develop in nests of different than M. sabuleti Myrmica species (Fig. 4). Conservation measures should respect this possible local specificity, but first of all further extensive ecological studies are urgently needed.

1177: ***+.062***

Current Species Status: Threatened (WA Wildlife Conservation Act 1950), Critically Endangered (ranking by WA Threatened Species Scientific Committee), Endangered (Commonwealth Environment Protection and Biodiversity Conservation Act 1999), Critical (Action Plan for Australian Reptiles, 1993), Critically Endangered under IUCN (2000) Red List Criteria A2c and D, listed as Critically Endangered in the IUCN 2000 Red List of threatened animals. Habitat requirements and Limiting Factors: Pseudemydura umbrina inhabits shallow, ephemeral, winter-wet swamps on clay or sand-over-clay soils with nearby suitable aestivating refuges. Clearing and drainage have destroyed most original habitat within its very small former range. Existing protected habitat marginal. Recovery Plan Objective: To decrease the chance of extinction of the Western Swamp Tortoise by creating at least three wild populations and increasing the total number of mature individuals in the wild to >50. Recovery Criteria: Criteria for successful achievement of the Objective are: 1. Complete extension of the Ellen Brook Nature Reserve to the west to include Western Swamp Tortoise habitat currently within private properties. 2. An increase in the number of adult, sub-adult and juvenile (> 2 years old) tortoises at Ellen Brook Nature Reserve to more than 50 by 2007. 3. Persistence of a population of more than 40 adult, sub-adult and juvenile (> 2 years old) tortoises at Twin Swamps Nature Reserve and reproduction (egg laying) of re-introduced tortoises demonstrated by 2007. 4. The creation of a population from captive-bred animals at Mogumber Nature Reserve of more than 35 adult, sub-adult and juvenile (> 2 years old) tortoises by 2007. 5. The maintenance of a captive population of at least 12 breeding adults producing at least 20 two-year-old animals each year. 6. The creation of a second captive colony at another accredited Zoo in Australia. 7. The creation of a semi-captive 'insurance' colony of at least 20 tortoises at the Harry Waring Reserve of UWA or some other site. 8. The selection by the Recovery Team and endorsement by relevant authorities of a third suitable translocation site. The criteria for failure to achieve the objective are: 1. A decline in numbers of the Western Swamp Tortoise in the wild. 2. Cessation or significant reduction (to less than 10 hatchlings per year) in captive breeding. 3. The maintenance of more than 50% of the non-hatchling world population of P. umbrina in a single captive colony. Recovery Actions: The Western Swamp Tortoise Recovery Team will coordinate implementation of the following actions. 1. Employment of Chief Investigator 2. Management of Ellen Brook, Twin Swamps and Mogumber Nature Reserves 3. Tortoise population monitoring 4. Captive breeding 5. Translocations 6. Education, publicity and sponsorship. Biodiversity benefits: The Western Swamp Tortoise represents the subfamily Pseudemydurinae monotypically and is little changed from fossils from the early Miocene at Riversliegh in Queensland. Ellen Brook and Twin Swamps Nature Reserves protect wetland ecological communities that are now threatened because of

clearing and drainage. Twin Swamps and Ellen Brook Nature Reserves protect threatened ecological communities and Mogumber Nature Reserve protects species of threatened plants.

1178: ***-.066***

The Hazel Grouse is mainly threatened by habitat loss as result of inadequate forest management (large clear-cuts, coniferous monocultures, ceasing of coppice forest use) and habitat fragmentation. The current distribution (map Fig. 1, c. 2000-4000 ind.) is confined to four isolated populations in the Rheinisches Schiefergebirge (continues in Belgium/Luxembourg), Black Forest, Bavarian and Bohemian Forests (continues to the Austrian Waldviertel) and Bavarian Alps (continues in Austria and Switzerland). In the Bavarian Alps and the Bavarian Forest, suitable habitats occur on large and less fragmented areas, so that the Hazel Grouse is not actually threatened there. The isolated and fragmented remaining populations in the Rheinisches Schiefergebirge and Black Forest are virtually endangered. However, conservation measures (e.g. forestry use in small mosaic patches acceptance of gaps, support of amentiferous pioneer tree species) could be performed relatively quickly and at rather low costs. The population of Capercaillie has declined drastically due to habitat destruction and fragmentation through e.g. large clear-cuts, short rotation periods, dense coniferous plantations, increase of forest roads, disturbances by tourism and high ungulate densities. The current occurrence (map Fig. 2, c. 1600 ind.) is restricted to a few isolated populations. The largest (meta-) population (800 ind.) occurs in the Bavarian Alps and is connected with Austrian and Swiss populations. The population in the other Bavarian mountains has dramatically declined (mainly c. 30 remaining birds in the Bavarian Forest, neighboured to c. 100 in the adjacent Bohemian Forest in Czech Republic). In the Fichtelgebirge there is a small, isolated population of about 40 ind. In the Black Forest (600 ind.), the decline was less pronounced due to intense conservation measures. In Thuringia, the population decline was severe (from 300 ind. in 1970 to 20-30 ind. in 1990, after translocation of wild-captured Russian birds now 30-40 ind.). The populations in Saxony and Hesse and the only lowland population in Lusatia (S-Brandenburg/N-Saxony) have become extinct. Reintroduction schemes in five mountain ranges have been stopped because of poor success. Viable meta-populations of Capercaillie will probably survive only,in the Alps and in the Black Forest, provided that forestry management fulfils the habitat needs of this grouse. The example of Fichtelgebirge demonstrates, however, that even small and isolated populations are able to survive for longer periods and are worthwhile to start conservation measures.

1180: ***+.101***

In the North Bilaspur Forest Division in Madhya Pradesh, India, the sloth bear(Melursus ursinus) population is ecologically isolated, and some bears have developed aggressive behavior. Available bear habitat is highly fragmented and degraded and is interspersed with human habitation. In this study we assessed habitat use patterns of sloth bears with the goal of establishing management guidelines to reduce human-bear conflicts. Goodness-of-fit comparisons showed that expected use of each habitat category differed from habitat availability. Bear sign was most frequent in sal forest followed by land near water, sal mixed forest, and mixed forest. However, bear use of terrain categories was not different from expected use. A large number of bear dens were near water and human settlements. Bears regularly used more than 50% of observed den sites. Because of nearness to human settlement and degraded habitat, bears largely depended on villages for food, resulting in frequent human-bear encounters, some ofwhich led to maulings and fatalities. One management priority is to protect highly preferred habitats of sloth bears. Bear population control and translocation of bears from isolated habitat patches to more suitable areas may be carried out simultaneously with education and awareness programs to conserve this species and to mitigate

human-bear conflicts on a long-term basis.

1181: *-.137*

For many years, the primary strategy for managing grizzly bears (Ursus arctos)that came into conflict with humans in the Greater Yellowstone Ecosystem (GYE) was to capture and translocate the offending bears away from conflict sites. Translocation usually only temporarily alleviated the problems and most oftendid not result in long-term solutions. Wildlife managers needed to be able topredict the causes, types, locations, and trends of conflicts to more efficiently allocate resources for pro-active rather than reactive management actions. To address this need, we recorded all grizzly bear-human conflicts reported in the GYE during 1992-2000. We analyzed trends in conflicts over time (increasing or decreasing), geographic location on macro- (inside or outside of the designated Yellowstone Grizzly Bear Recovery Zone [YGBRZ]) and micro- (geographic location) scales, land ownership (public or private), and relationship to the seasonal availability of bear foods. We recorded 995 grizzly bear-human conflicts in the GYE. Fifty-three percent of the conflicts occurred outside and 47% inside the YGBRZ boundary. Fifty-nine percent of the conflicts occurred on public and 41% on private land. Incidents of bears damaging property and obtaining anthropogenic foods were inversely correlated to the abundance of naturally occurring bear foods. Livestock depredations occurred independent of the availability of bear foods. To further aid in prioritizing management strategiesto reduce conflicts, we also analyzed conflicts in relation to subsequent human-caused grizzly bear mortality. There were 74 human-caused grizzly bear mortalities during the study, primarily from killing bears in defense of life and property (43%) and management removal of bears involved in bear-human conflicts (28%). Other sources of human-caused mortality included illegal kills, electrocution by downed power-lines, mistaken identification by American black bear(Ursus americanus) hunters, and vehicle strikes. This analysis will help provide wildlife managers the information necessary to develop strategies designedto prevent conflicts from occurring rather than reacting to conflicts after they occur.

1183: *+.108*

The NSW Threatened Species Conservation Act 1995 (TSC Act) is unique and effective in Australian jurisdictions in recognising threatened populations. Section II of the TSC Act provides for the listing of endangered populations. There were 28 endangered populations listed under the TSC Act (January 2003) for which there were two approved recovery plans, one publicly exhibited draft plan and four plans in preparation. Monitoring was being undertaken for only six populations. The TSC Act would be strengthened by: (i) adding an objective which states that recognising threatened entities is fundamental to the legislation; (ii) changing the definition of population to make it consistent with IUCN terminology; (iii) simplifying Section 11; (iv) better resourcing for faster preparation of plans and their implementation; and (v) including population monitoring in all recovery plans. The process of saving threatened species is occurring at the level of population; for example, through priority sites for threat abatement, translocation, replicates for research treatments and habitat protection. The objective of recognising and the process of listing threatened populations are fundamental to sound threatened species legislation.

1184: *+.167*

The history of extinction in the wild and present status of the European bison, Bison bonasus, is briefly summarised. Reasons for the extinction of this species include the overabundance of other large herbivores, cattle grazing in forests, war, and other political instabilities. Restitution (the recovery) of the species began with 54 animals and started with the reproduction of bison in zoos

and reserves, increasing population numbers and distribution as wide as possible, and resulting in release into the wild (1952). The European Bison Pedigree Book (EBPB) played a very important role in maintaining the purity of European bison herds. Present (year 2000) numbers and distribution of the species in enclosed (191) and free-roaming herds (31) is larger than shortly before its extinction in the wild; a situation that would seem to assure the bisons' better survival. At least 2,864 European bison have been registered by the EBPB (2001) globally, of which c. 60% were free- or semi-free ranging. The actual world population is a few hundred larger, because not all breeding centres reported data to EBPB. Many threats remain which could seriously influence the future development of European bison populations. Reintroductions were somewhat chaotic, not always respecting the separation of genetical lines and numbers increased slowly. Established free-ranging herds are often small and isolated, and tend to be subject to human influence, including use of traditional management methods, as well as supplemental feeding during winter. As yet, a viable population within a continuous range has not been established. Recent simulations have indicated that population of at least 100 individuals could be counted as demographically safe. There are only four such herds. Recent European bison gene pool consists of only 12 diploid sets of genes; those of the Lowland line originate from only seven founders. The world herd is highly inbred (F=20.2%), especially the Lowland line (F=32.4%), as found in the 1980s and those indices are still increasing. Inbreeding affects viability, the interval between calving and skeletal growth, to a higher degree in the Lowland-Caucasian line than in the Lowland line. Many indices suggest that the European bison has lost its genetic variability and this continues to occur. The genetic contribution of founders is uneven; remaining significantly unaltered over the last decades. Ecology of the species has been reviewed, based mostly on studies concerning the largest population in Bialowieza Forest. Natural mortality has considerably decreased due to a reduction in the natural forces of selection. However, diseases have recently appeared in several herds which may pose a serious threat to European bison populations. Balanoposthitis of unknown aetiology has appeared in a few herds, as well as tuberculosis in one herd. Both diseases may exert a deleterious effect on European bison herds. Future goals in conservation and management of European bison are recommended. Captive breeding is very important for preservation of the species and serves to maintain its genetic purity and reintroduction to natural ecosystems should continue. The establishment of the European bison Gene Resource Bank could be very important for the future of the species. In the wild, numbers must increase to attain the management goal of creating self-sustaining populations of both genetic lines. Regulated culling in local populations will be necessary for managing optimal bison habitat. Attention should however be given to the chosen scheme of elimination according to the sex and age of the groups. Further introductions to the wild are necessary to establish a 'continuous' range and ensure the long-term survival of viable populations. There are proposals to create such populations in the Eastern Carpathians, as well as in Russia. Lowland European bison are well distributed in Belarus, and together with Lithuanian and northern Polish populations they may create another region within the bison's contemporary range. These populations must be monitored and allowed to increase in size. Ecological corridors need to be established between them or a programme for assisted migration (translocations) developed. Pure Lowland and Lowland-Caucasian lines should be separated in the wild, as well as in enclosed breeding centres, up to the time of their likely natural contact. This isolation is of particular importance for preserving the genetic variability within Lowland-Caucasian line. Pure animals should be separated from European x North American bison hybrids within the Caucasus Mountains and elsewhere. Current genetic management requires a more balanced genetic contribution of animals representing founder genes, which are currently under-represented or not present in free-ranging herds, and must be identified and included in European bison populations. The aetiology and epidemiology of European bison diseases, especially of the male genital organs, should be determined and their effects on the risk of extinction assessed. The place of the European bison in ungulate communities and the species effect on the carrying capacity of

contemporary forest ecosystems in Europe need to be determined. A review of the European bison's status clearly indicates that its recent numbers and distribution are much better than before its extinction in the wild at the beginning of the 20th century. The European bison is classified as an Endangered (EN) species on the I UCN Red List of Threatened Species, mainly due to the many continued threats. While these threats exist, the European bison's full recovery and re-naturalisation in the wild cannot be guaranteed. It should therefore also be included in the Habitat Directive and in Appendix II of the Bern Convention.

1185: *-.072*

The new Canid Action Plan synthesises the current knowledge on the biology, ecology and status of all wild canid species, and outlines the conservation actions and projects needed to secure their long-term survival. Aiming at conservation biologists, ecologists, local conservation officials, administrators, educators, and all others dealing with canids in their jobs, the authors aspire to stimulate the conservation of all canids by highlighting problems, debating priorities and suggesting action. The 36 taxa of wild canids that comprise the family Canidae, ranging in size from the tiny fennec fox to the mighty grey wolf, and found in every continent except Antarctica, are special. They are special because they have, as perceived friend or foe, preoccupied the imaginations of mankind for millennia; because the breadth of their adaptations makes them enthralling to science; and because the contradictory facets of their relations with people perplex the conservationist. The increase in numbers of people, the spread of settlement, and the exploitation of natural resources of previously little-disturbed wild lands, together with persecution, are threatening some of these canids with extinction. The possibility that we are heedlessly, perhaps needlessly, mismanaging many of them is saddening; the probability that our negligence will force several more to extinction should fill us with bottomless dismay. It demands action, and that is why we have compiled this new Canid Action Plan. Following a short introduction and a chapter on phylogeny, classification, and evolutionary ecology of the Canidae (Part 1), Part 2 provides the latest information on the distribution, biology and conservation status of each species, organised by geographical region. The accounts also list current field projects, and their contact details are provided in an appendix. The Canid Specialist Group's members are active worldwide. Nine of the 36 taxa covered are threatened (3 Critically Endangered, 3 Endangered and 3 Vulnerable), and one is considered Near Threatened. Six species (7%) were listed as Data Deficient, and 20 (56%) species as Least Concern (Appendix 1). The threatened canids are: Darwin's fox (CR). Until recently, known only from the Island of Chiloe (Chile) until rediscovered 600km away in the coastal mountains, where domestic dogs threaten them with disease or direct attack. Red wolf (CR). Currently the subject of taxonomic debate, red wolves were declared Extinct in the Wild by 1980, but have been reintroduced into eastern North Carolina, where they are now locally common. Hybridisation with coyotes is the primary threat. Island fox (CR). Restricted to the six largest of the eight California Channel Islands, each island population is considered a separate subspecies, and four have declined precipitously. Threats include hyperpredation by golden eagles and the introduction of canine diseases. Ethiopian wolf (EN). Less than 500 individuals remain, confined to eight locations in the Ethiopian Highlands. Previously listed as Critically Endangered, continuous loss of habitat due to high-altitude subsistence agriculture remains the major threat, along with disease (particularly rabies). African wild dog (EN). Formerly distributed throughout sub-Saharan Africa, excluding rainforests, wild dogs have disappeared from 25 of the former 39 range states. More than half of the mortality recorded among adults is caused directly by human activity. Dhole (EN). Formerly distributed across Asia, dholes have undergone widespread decline and are threatened by depletion of their prey base, habitat loss, persecution, competition and disease. Dingo (VU). Austronesian people transported the dingo from Asia to Australia and other islands in between 1,000 and 5,000 years

ago. Pure dingoes occur only as remnant populations in central and northern Australia and in Thailand, and they are threatened by cross-breeding with domestic dogs. Bush dog (VU). Despite a supposedly widespread distribution in South American forests, this species is perceived as rare, and threatened by habitat conversion and human encroachment. Blanford's fox (VU). Present in arid mountainous regions of the Middle East and north-eastern Egypt eastwards to Afghanistan, where human development could pose a threat. In contrast to the threats faced by threatened canids, several species are thriving in human-dominated landscapes and incur the loathing of farmers and hunters alike. Red foxes are notoriously successful in urban settings, and coyotes, golden jackals, crab-eating and kit foxes seem able to thrive amidst human settlements. Management prompted by rabies control, fur harvest, and livestock predation leads to the slaughter of hundreds of thousands of canids annually. Part 3 of the Plan considers nine major issues in canid conservation, namely canid society, conservation genetics, assessing and managing diseases, management of canids near people, impact of exploitation and trade, survey and census techniques, captive conservation, reintroduction and meta-population management, and conservation education. Part 4 is arguably the most important section. It includes a chapter on the need for setting priorities and measuring success in canid conservation, and the detailed Action Plan for canid conservation into the 21st century. Although we have sought to refine and consolidate these entries, they represent the views of the many experts around the world who suggested them, who debated them in our workshops and in the forum of our international congress, hosted by the WiIdCRU in Oxford in September 2001. The list of proposed projects makes no claim to be comprehensive, but it is the result of extremely wide consultation. The plan itself, together with the databases concerning existing members and research projects, are all available on the web at http://www.canids.org. The Action Plan was prepared in parallel with our edited monograph entitled The Biology and Conservation of Wild Canids (Oxford University Press, 2004) which contains comprehensive reviews of the science underpinning this Action Plan, together with 14 case studies of wild canid biology.

1186: ***+.197***

Background. As part of SNH's species management decision-making framework there is a need to address the role of translocation or reintroduction of native species. This report uses international experience from six countries: USA, Sweden, Netherlands, Spain, Australia and New Zealand to assess how translocations have been used to further biodiversity policy, and its suitability as a species management tool. The report identifies the differing, but complementary, ways in which translocations have been used in longaltered environments, similar to Scotland, and those which are still undergoing rapid landscape and habitat changes. The results will contribute to the ongoing debate within Scotland. Main findings. In some countries, such as the USA and New Zealand, native species translocation is an established part of the biodiversity armoury, in others they are only recently beginning to be addressed. The CBD, EC conservation Directives and the Bern Convention have all emphasised the need for environmental and species sustainability. Translocation is identified as an important mechanism to achieve these objectives. There are few formal policy frameworks in place for the use of translocation as a tool for achieving biodiversity policy outcomes. The best developed identified translocation policy framework has been produced by the Department of Conservation in New Zealand. In most countries translocation is part of the toolkit of species recovery or action plans and accompanied by other policy elements Translocation is a long-term staged process. The ESA uses translocation according to need. It is applied equally to populations, races, sub species and species. The determinant is threat and need. In the USA the scientific case comes first. Biological criteria are paramount under the ESA. In Europe translocations are used as part of the suite of tools to help deliver individual biodiversity Plans, and protected areas were core to translocation programmes. In all countries national

landscape action plans are seen as essential prerequisites. There is no simple formula for deciding on the use or no use of translocation as a tool for biodiversity policy or outcomes. Review of international policy and practice for native species conservation translocations Commissioned Report No. 034 (ROAME No. F03NC04B) Contractor: EcoText COMMISSIONED REPORT Summary. Cost efficiency is rarely given overt consideration in choosing options within plans. Extinction is viewed as more expensive. Stakeholder engagement is key to delivery at every stage of recovery plans and in the success of translocation programmes. In all countries, cultural practices are important in determining the acceptability of translocation programmes. New Zealand has adopted a systematic, standardized procedure for evaluating the needs where translocation may be appropriate. There is a clear need for an unequivocal set of policies that identify the role and function of translocations in the Scottish context. Translocation cannot exist as a tool in isolation - it is part of a wider set of options used for delivering biodiversity policy and practice. A rigorously transparent approach to translocation will pay dividends, both in establishing the scientific case and in developing stakeholder support for individual projects.

1187: *+.143*

Scientists have been implementing a programme to reintroduce Bearded Vultures into the Alps during the last 25 years. This paper summarizes the results at the alpine scale. This effort has been facilitated through the implementation of the International Bearded Vulture Monitoring (IBM) Project of the National Park Hohe Tauern/EGS since 2001. A single database facilitates homogenous collection and collation of information over the entire study area. All alpine observations are analysed as soon as they reach the co-ordination office. After an evaluation process they are distributed to participants to give everybody access to the whole database. Data are transferred to Geographical Information Systems (GIS) for spatial analyses. Dispersal, pair formation and other relevant factors for population development are examined. The monitoring intensity throughout the Alps is regularly checked. With the help of external GIS tools, dispersal routes can be determined, resulting in a better understanding of the spatial distribution of the species by age and sex.

1188: *+.129*

Since 1984, when the population of Black Vultures in Europe was estimated at less than 300 pairs, a spectacular recovery has taken place in Spain where 1,334 breeding pairs were recorded in 2001. The main reasons for the recovery has been the reduction of various threats, including prohibition of the use of poison for predator control and protection of most breeding areas. The Black Vulture is now extinct in most of the other countries within its historical distribution range. Only one colony is left in the Balkans, in the Dadia Forest Reserve in Greece, and there are some generally declining colonies in the Caucasus. The total European population is now estimated at 1,456-1,489 pairs, of which 92% are in Spain. Conservation results achieved for the species in Spain show its capacity to respond to integral species and habitat protection. The conservation experience of Spain and western Europe in general is now being applied to the eastern part of the European population. The Black Vulture Conservation Foundation (BVCF) has been working since 1986 to re-establish a continuous population in the species' former European distribution area, by connecting the separate populations of Spain in the west and the Balkans in the east. Conservation of existing colonies is supported and re-stocking or reintroductions are carried out where the causal factors responsible for the population decline have been removed.

1189: *+.247*

In 2002, an "Action Plan for the Recovery and Conservation of Vultures on the Balkan Peninsula and Adjacent Countries" was drafted as a joint effort of national and international NGOs. Based on the Action Plan, Memoranda of Understanding have been negotiated and concluded between Albania, Macedonia and Bulgaria. The Action Plan is promoted by the Frankfurt Zoological Society (FZS), the Black Vulture Conservation Foundation (BVCF) and the Foundation for the Conservation of the Bearded Vulture (FCBV), with the support of LPO/FIR, BirdLife International, the Royal Society for the Protection of Birds (RSPB), IUCN-Europe, the Eastern Griffon Vulture Working Group (EGVWG), the Bulgarian Environmental Partnership Foundation (BEPF) and NGOs from the range countries. The Action Plan provides the most recent update on the status of the four European vulture species, describes the threats to the individual populations. and makes conclusions about the necessity for immediate conservation actions. Additionally it identifies the need for further research (status, threats, conservation situation), conservation actions (increasing natural food availability, establishment of feeding places, habitat conservation, activities against the use of poison, education, restocking and reintroduction) and coordination/co-operation events (workshops, seminars, public participation, Balkan Vulture web site) for the four vulture species in Albania, Macedonia, Bulgaria, Greece, Serbia, Montenegro and the Ukraine. It is to be extended to include additional countries. Projects are mainly financed by the FZS acid the first activities were implemented in 2002 and 2003, with the involvement of 28 different local NGOs and governmental institutions. First results have been encouraging, and this joint effort could lead to the recovery of vultures and their habitats in south-eastern Europe in the medium- and long-term.

1190: ***+.167***

The note presents 9 observations of adult female of Peregrine Falcon Falco peregrinus in the fields of Western Wielkopolska. It was a wintering individual which hunted city pigeons. Since 1990 a reintroduction program for Peregrine Falcon has been carried out. Following the release of over 200 individuals, breeding pairs were recorded only in towns. Due to the slow reconstruction of population, every observation of falcons, including the wintering ones, is important in determining new pairs.

1191: ***+.132***

Snake River sockeye salmon Oncorhynchus nerka were listed as endangered in 1991. Prior to listing, a captive broodstock program was initiated to prevent species extinction and to begin rebuilding the population. Reintroduction plans for captive broodstock progeny have followed a "spread-the-risk" philosophy incorporating multiple release strategies and lakes. Since 1993, more than 860,000 presmolts, 158,000 smolts, 325,000 eyed eggs and 880 adults have been reintroduced to the habitat. From this production, 312 anadromous sockeye salmon have returned to adult trapping facilities in the Sawtooth Valley of Idaho. Monitoring and evaluation efforts have focused on maximizing the use of limited hatchery-rearing space and on identifying and prioritizing the most successful reintroduction strategies. Comparisons of presmolt overwinter survival and out-migration success from nursery lakes have shown that (1) presmolts released directly to Redfish Lake in October have emigrated more successfully than presmolts reared in Redfish Lake net pens prior to release, (2) presmolts released directly to Alturas and Pettit lakes in October have emigrated more successfully than presmolts released directly to takes in July, and (3) presmolts reared at the Idaho Department of Fish and Game (IDFG) Sawtooth Fish Hatchery emigrated more successfully than presmolts reared at the IDFG Eagle Fish Hatchery following July releases to Alturas and Pettit lakes. Presmolts that spent one winter in Redfish Lake prior to out-migration were interrogated more successfully at lower Snake and Columbia River dams than

smolts released in the outlet of Redfish Lake. Smolt-to-adult return rates for anadromous adults produced by the captive broodstock program from 2000 to 2002 varied from 0.6% for unmarked fish returning in 2002 to 0.4% for a combined smolt and presmolt release group in 2000. Using captive broodstock techniques, the program has successfully prevented the extinction of Snake River sockeye salmon and increased Population abundance in the habitat. However, without Substantive improvements in smolt-to-adult survival, program efforts will likely be insufficient to rebuild the population to self-sustaining levels.

1192: *+.034*

The gene pool of Lowland European bison consists of only seven founders' genes and in consequence the high inbreeding within this line is observed. The four free roaming herds of Lowland European bison were analyzed. On the beginning the founding group for every herd was determined and the pedigree analysis was done. The founding group in Bialowieza Forest consists of 28 (9, 19) released animals and two calves, which fathers were not included in the group. The Borecka Forest herd started from 15 (7, 8) bison and 5 (2, 3) from Borki reserve, Knyszyn Forest with 6 (3, 3) and Pila forest district with 8 (4, 4) individuals. The contribution of the pair of founders (PLANTA and PLEBEJER) was highest in all groups but differed in size between herds. The comparison of founder equivalent (fe) showed that all analyzed Polish herds had more genetic variability in the moment of their creation than the captive part of the population. The highest distance (from 1.01 to 2.40%) was observed between free herds and the captive part of current population. The inbreeding coefficient for Bialowieska founding group was equal to 25.05% and lower than mean kinship for the group (32.15%). The difference between those two parameters was smaller for Borecka Forest (30.68% vs. 30.82%). In conclusion should be stated that free roaming Polish herds lost genetic variability during the process of creation and the process of heterozygosity decrease exists mainly because of herds' isolation.

1193: *+.100*

Problems of an active protection of dryas satyr, a species becoming extinct in Poland, are discussed. In total 30 localities of this species were known prior to1939. But during the period 1940 - 1978 only 5 localities were known. Thus 25 localities ceased to exists, including 18 situated in cool meadows and low peatbogs. During 1986 - 1995 only 2 localities remained, including one created by introduction in 1973. Up to 2004 the species was permanently established in four new localities in vicinity of Krakow after new introductions in 1997 and 1999. Its populations there amounts 30 to 400 specimens during a flight period. A success in introduction of dryas satyr is achieved by transfer of fertilized females to new sites. In this way an accidental transfer of diseases and parasitoids, possible during the transfer of caterpillars and pupae, was avoided. Females lay eggs in the most suitable ecological niches. In order to assure the survival of this species in the Polish lepidopterofauna, the number of new localities should be increased. This would prevent its extinction by accidental local disasters (fires, floods etc.).

1194: *+.058*

The activity pattern of the Sand Goanna Varanus gouldii in northern South Australia was estimated by radio telemetry during the summer months. Individuals were located within the Arid Recovery Reserve where introduced European Rabbits Oryctolagus cunniculus, Feral Cats Felis catus and European Foxes Vulpes vulpes were removed. Interaction between the re-introduced Greater Stick-nest Rat Leporillus conditor and the Sand Goanna, a potential significant predator in the absence of cats and foxes, was examined. Mean activity area for Sand Goannas was 5.9 ha (+/-

1.0, n = 9), and their summer activity was concentrated on sand dunes rather than the adjacent inter-dunal swales. Tracks of Sand Goannas were found more often around Greater Stick-nest Rat nests than control sites. However, Sand Goanna predation on re-introduced Greater Stick-nest Rats could not be confirmed and most deaths of radio-collared Greater Stick-nest Rats coincided with a period of exceptionally high maximum temperatures and were probably due to heat and dehydration. Sand Goannas scavenged Greater Sticknest Rat carcasses, and analysis of scats and stomach material revealed that although invertebrates were the most common prey item, mammal material (hair and bones) was present in 20% of scats.

1197: ***+.140***

Populations live in habitats whose quality varies spatially and temporally. Understanding how populations deal with these variable habitats can aid our understanding of theoretical issues, and practical issues of biological invasions and biodiversity conservation. I investigate these issues by superimposing simple models of population growth and dispersal on spatiotemporally fractal landscapes, and examining the properties of the landscapes, and of the populations inhabiting them. The properties of the simulated landscape sequences are comparable to those of real habitats. The simulated populations exhibit a range of dynamic behaviors; these behaviors are strongly influenced by the fractal parameters of the landscapes. The results may help explain several important phenomena seen in reintroductions of threatened and endangered species, introductions of biological control agents, and biological invasions. These phenomena include frequently observed lags between population introduction and initial population growth and spread, and the observed high frequency of failure of introductions.

1198: ***-.060***

In November 1993, unusual mortality occurred among endangered Laysan ducks on Laysan island, one of the remote refugia of the Northwestern Hawaiian Islands National Wildlife Refuge (USA). Ten live ducks were emaciated, and blood samples documented anemia, heterophilia, and eosinophilia. Pathology in 13 duck carcasses revealed emaciation, marked thickening of the proventricular wall, abundant mucus, and nodules in the gastrointestinal tract. Histology revealed granulemata associated with nematodes in the proventriculus, small intestines, and body walls of nine of 10 ducks examined on histology. We suspect that low rainfall and low food abundance that year contributed to enhanced pathogenicity of parasite infection, either through increased exposure or decreased host resistance. Because the Laysan duck is found only on Laysan island and is critically endangered, translocation of this species to other islands is being considered. Given that we have not seen pathology associated with Echinuria spp. in native waterfowl on other Hawaiian Islands and given the parasite's potential to cause significant lesions in Laysan ducks, it will be important to prevent the translocation of Echinuria spp.

1199: ***+.001***

The Vietnamese sika deer (Cervus nippon pseudaxis) is an endangered subspecies of economic and traditional value in Vietnam. Most living individuals are held in traditional farms in central Vietnam, others being found in zoos around the world. Here we study the neutral genetic diversity and population structure of this subspecies using nine microsatellite loci in order to evaluate the consequences of the limited number of individuals from which this population was initiated and of the breeding practices (i.e., possible inbreeding). Two hundred individuals were sampled from several villages. Our data show both evidence for limited local inbreeding and isolation by distance with a mean F(ST) value of 0.02 between villages. This suggests that exchange of

animals occurs at a local scale, at a rate such that highly inbred mating is avoided. However, the genetic diversity, with an expected heterozygosity (H(e)) of 0.60 and mean number of alleles (k) of 5.7, was not significantly larger than that estimated from zoo populations of much smaller census size (17 animals sampled; H(e)=0.65, k=4.11). Our results also suggest that the Vietnamese population might have experienced a slight bottleneck. However, this population is sufficiently variable to constitute a source of individuals for reintroduction in the wild in Vietnam.

1200: *+.119*

The current distribution of the Hermann's tortoise, Testudo hermanni (Gmelin, 1789), in Italy is very scattered. Most of populations occur along the Thyrrhenian coast, particularly southern Tuscany, and Latium, where the most high density was observed. Other populations, probably autochthonous, can be found in Campania and in Calabria. Along the Adriatic coast, the biggest populations can be found in Apulia, Abruzzo and Molise. The species also occurs in some sites of Sicily and Sardinia. In the northern Italy, the Hermann's tortoise has almost disappeared and can be found only in the Po River Delta, such as the Bosco della Mesola. Sites are mainly located in the coasts but, in some cases, also in the inland up to mountains slopes. Further sightings in other areas concerned isolated individuals probably released from captivity. In the present paper the results of a population study carried out in the Bosco della Mesola Reserve, are discussed. The population structure is dominated by adults over 20 years old. The home range size was larger for the females than for the males. Longer daily distances were covered in spring and summer, while movements were greatly reduced during post and pre-hibernation periods. Conservation strategies should be based on: (1) verifying the taxonomic status and the origin of the Italian population through the analysis of genetic distances; (2) monitoring all the major populations in order to assess their status and dynamics; (3) establishing new protected areas in Mediterranean coastal habitats, to protect autochthonous populations and in-crease population density in case of low numbers. In order to in-crease the reproductive success of the Hermann's tortoise within protected areas, enclosures should be set out where to raise pairs of breeders. Young turtles should be released in core areas of the reserves, without anthropic disturbance. Moreover, the sites where females laid eggs should be fenced by wire net, in order to prevent predation up to the hatching. The goal is to create a pool of specimens useful for reintroduction and restocking.

1201: *+.125*

Under the biological species concept, the intraspecific variability and true species richness of Palearctic mammals has often been overlooked, and therefore the need to conserve it. Recovery projects of endangered European mammals in Western Europe rely mainly upon translocation of conspecifics from viable populations in Central or Eastern Europe. From a wildlife management and restoration ecology point of view, many such recovery projects have been successful. However, from a biodiversity perspective it could be argued that they could have failed to protect the original European biodiversity. The increasing evidence of a complex phylogeographic pattern in many European mammals - especially in the Mediterranean region - has led to a reconsideration of the conservation unit and highlights the need for species-specific programmes for assuring the survival of threatened, distinctive populations. Such programs should also include captive breeding. It is therefore suggested that a two-level classification of captive breeding programmes is needed according to the degree of threat of concerned taxa, to maximise available resources without jeopardising in situ conservation. It is proposed to distinguish between a) level I captive breeding programmes, which are part of the conservation strategy for seriously threatened taxa and need to be financed by state or federal agencies, and b) "prophylactic" level II for vulnerable taxa or populations, and for which funds may be available mainly from the private sector. Available

evidence suggests that given adequate husbandry techniques and prerelease training, even captive-bred carnivores can be successfully reintroduced to the wild. However, a closer collaboration among zoological gardens, zoologists and agencies involved in wildlife conservation is needed to avoid ill-conceived, potentially dangerous captive-breeding and re-introduction projects.

1202: *+.147*

Ungulates in Italy have experienced in the last decade a further increase of their distribution and, possibly, consistence. This trend has been very obvious in the central and northern part of Italy, on the contrary, in south Italy the increase of ungulates populations is quite slow if any. A partial exception is Sardinia where Sardinian red deer (Cervus elaphus corsicanus) has been reintroduced into the four provinces of the island. In this frame the relevance of the increase of ungulates for the increase of wolves (Canis lupus) in Italy must be emphasized. Ungulates management has also experienced a general development in the last decade, even if deep differences between south and central-north Italy still persist. Well conducted reintroductions have allowed to fill many gaps in the species distribution. Ungulates hunting bags size, suggest an increased importance of ungulates in the hunting activities in Italy. Selective hunting with rifles on cervids and bovids was established in wide areas of north and central Italy, mainly as consequence of roe deer (Capreolus capreolus) widespread presence. Wild boar (Sus scrofa) hunting still represents a problem for ungulate management in Italy. Almost 50% of the provinces where wild boar is hunted does not collect hunting bag statistics, and only 35% of them try to roughly estimate the consistence of the populations. The traditional dogs hunting of wild boar has expanded to areas where wild boar was recently and illegally reintroduced, as south Italy and the Alps region, and this is a serious problem for the development of a more rational and correct hunting practices. New possibilities of hunting management of red deer in central Italy, and in the future of alpine ibex (Capra ibex), are to be considered. An increase of the presence of trained wildlife managers in public administrations is suggested, as a further step in the development of a proper management of ungulates and other wildlife. Scientific research on ungulates in Italy was, in the last decade, quite uneven both in the species and in the disciplines chosen. There is a clear opportunistic approach to subjects, that often has no link with the real necessities of wildlife management. The development of long term studies is suggested, together with a more efficient coordination among scientist in the choice of the research subjects. Last but not least a more efficient founding mechanisms is needed in order to avoid money waste and to promote serious research programmes.

1203: *+.099*

In 1968, an international team of experts, led by academician J. Kratochvil, made two reports on the historic and current occurrence of the European lynx Lynx lynx (Linnaeus, 1758) in Europe (KRATOCHVIL et al. 1968 a, b). Reports made for IUCN and WWF analysed the reasons for the extinction of the European lynx in many European countries and examined ways of its conservation in the countries where it was still found. At the beginning of 1960's, the Slovak population of the European lynx was estimated at 500 animals (HELL 1968). A number of the animals were captured in the six-ties for use in Slovak zoological gardens and some offspring were successfully reared (GRESSNER 1963, 1964). The first three lynx came to the Zoological Garden in Ostrava in December 1965; they were captured in the Slovak Beskydy (Beskids) Mountains. The first progenies were reared in 1967. KUNC (1967, 1969), STEHLIK (1971 b) and KUNC et STEHLIK (1982) encouraged regulation of the numbers of the European lynx by catching them into live traps. Thus, a total of 95 European lynx from the Slovak (Slovensky) Beskydy mountains, Slovensky Rudohorie, Slovensky Kras, Nizke and Zapadni Tatry (Low and Western Tatras), Velka Fatra and Vsetinske Vrchy came to Ostrava Zoo in the period from 1965 to

1992. Most of them came from permitted captures made in the above localities in Slovakia. In several instances there were young animals who came to the zoo after losing their mother (this was the case of lynx from the Nizke and Zapadni Tatry and Vsetinske Vrchy). Catching lynx in Slovakia was regulated - quotas for capture and the prices were determined by the Central Committee of the Slovak Hunters Union (Slovensky polovnicky zvaz) in Bratislava, who also inspected the activities. This co-operation was terminated in the Autumn 1984, when the Slovak Ministry of Culture and the Slovak Ministry of Agriculture and Food decided, and we were informed, that lynx in Slovakia would only be captured by the zoological gardens in Bratislava and Bojnice and, at a later stage, the Zoo in Kosice. For this reason, the Ostrava Zoo did not get its contract for the catching season of 1984/1985. This was the end of the participation of the Zoological Garden in Ostrava in the ongoing reintroduction of the European lynx in Vosges and in the Sumava Mountains (Bohemian Forest), and this proved to be a difficult matter to explain to our French partners. The relatively rich material in Ostrava Zoo was utilised for breeding, scientific work, education as well as promotion and for reintroduction programs (STEHLIK 1985). The research of the European lynx in Ostrava Zoo was based on suggestions made by SLADEK (1970) and was in harmony with the current requirements laid on zoological gardens. The acquired knowledge contributed to finding solutions to issues concerning the ecological function of beasts of pray in the wild and helped to determine an optimum closed season and an effective way of conserving the species. Based on this know-ledge gained from studying reproduction and post-natal development, STEHLIK (1979 a) called for total conservation of the European lynx in Slovakia, at least for a test period. From 1970 to 1992, Ostrava Zoo supplied 50 captured European lynx into several European reintroduction projects (Tab. 1). Its participation in the reintroduction of the European lynx was a pioneer step at the time, taking all the political measures into consideration that complicated the exports and imports of animals and made it practically impossible for people to travel abroad. We would receive a number of invitations to symposia (e.g. Murau, Spiegelau, Strasbourg in 1978) and frequent invitations to provide expert views in localities of reintroduction (e.g. Gran Paradiso), however, we had never received the permission to leave the country. We have to bear in mind that, in those days, there was only limited experience with the reintroduction of felines. NOVELL et JACKSON (1996) stated that there were attempts to reintroduce the wild cat (Fells silvestris) in Bavaria between the years 1984 and 1989, to reintroduce the Canadian lynx (Lynx canadensis) in the state of New York from 1988 to 1990 and that of the Bobcat (Lynx rufus) in the Cumberland Island, Georgia, in 1988 and 1989. It was not until 1988 that the Re-introduction Specialist Group (RSG) was formed within IUCN/SSC. Today, it has over 300 members all over the world, who are involved in overall reintroduction projects for animals and plants. In 1998, this group of experts prepared Guidelines for Re - introductions for IUCN. The guidelines declare that re-introduction is always a long, comprehensive and costly process, the main target of which is to found a viable population. It is an attempt to bring a species or sub-species back to the location of their historical occurrence where they had been made or had become extinct. Preparatory work for such projects includes evaluation of historical information, local ecological conditions, social behaviour of the species, the size of the home ranges, food availability, predators and diseases. The potential migration range is taken into consideration, socio-economic conditions are evaluated and an enlightenment campaign waged. Post-reintroduction activities include monitoring of individual animals, studying their adaptation, mortality evaluation and observation of the behaviour of reintroduced animals, demographic and ecological studies. An important role is played by publishing in mass-media and professional publications (IUCN 1998). Reintroduction of beasts of prey into their former localities is often difficult not only for biological but also for social and political reasons. Reintroduction of large beasts, especially, is a potential conservationist hazard, often inducing concern in the general public (CARBYN et FRITTS 1998). Typically, the problem is the minimum size of vial population and inbreeding (BALLOU 1998).

1205: ***+.240***

Burrow-nesting petrels have been extirpated from many traditional breeding sites by human-induced factors, especially the introduction of predatory mammals. Petrels have proven to be extraordinarily difficult to attract or restore to secure sites due to their strong philopatry, and low intrinsic rates of population growth. Translocation and/or attraction techniques are needed to establish additional populations of endangered petrels to restore species to part or all of their historic range and to restore the keystone role of petrels in terrestrial ecosystems. We attempted to restore a colony of Common Diving Petrels (Pelecanoides urinatrix) on Mana Island, New Zealand, by a combination of broadcasting vocalisations, and by transferring and hand-feeding nestlings until they fledged. Calls were broadcast at night almost continuously during 1993-2003, and 239 chicks were transferred during 1997-99. About half the chicks fledged, and 20 of these have returned to Mana Island, along with 51 unbanded birds. Fifteen of the returned chicks have bred on Mana Island, and at least 14 parent-reared chicks fledged in 2002.

1206: ***-.011***

Floristic and structural characteristics of vegetation inhabited by Southern Emu-wrens, Stipiturus malachurus, were examined for three geographically isolated subspecies in South Australia. Of the 47 plant species recorded in quadrats, 29 of these (61%) occurred in the habitat of a single subspecies. Structurally, there were no significant differences between habitats used by the three subspecies; however, some trends were apparent. All three subspecies used dense habitat; however, S. m. intermedius (Southern Mount Lofty Ranges) used habitat that, on average, was denser than habitats used by S. m. polionotum (Coorong) or S. m. halmaturinus (Kangaroo Island). Habitat used by all subspecies was most dense at ground level and decreased with height. S. m. intermedius used two distinct vegetation types (swamp and dry-heath) that were floristically, but not structurally, different. The habitat at Cox Scrub Conservation Park (CP), where S. m. intermedius existed prior to a wildfire in 1983, was neither floristically nor structurally different from that currently inhabited by S. m. intermedius. The continued absence of S. m. intermedius from Cox Scrub CP reflects its limited dispersal ability. Since the habitat is extensive, Cox Scrub CP would be a suitable translocation site for S. m. intermedius. Successful translocation would greatly reduce the risk of a single fire event eliminating a substantial proportion of the remaining population of S. m. intermedius.

1209: ***+.120***

The White-winged Guan (Penelope albipennis) is a cracid endemic to the Northwestern Peruvian dry forests that is critically endangered due to poaching pressure and habitat loss. In the year 2000, a species reintroduction program was established which consisted in the release of captive born and raised animals from the Barbara D'Achille captive breeding center. The guans were released in areas where the species had disappeared and that were safe for reintroduction and conservation. Between September 2001 and July 2003, 20 White-winged Guans were released at the Chaparrí Private Conservation Area, Lambayeque, Perú. The bird's adaptation to the area was measured through its dispersion, survival and reproduction. Based on the measured dispersion distance, it has been found that it is actually feasible to link the reintroduced population with the surrounding wild populations. Survival has been established at 55 %, measured two years after the animals were released. With respect to reproduction, 3 wild-born chicks were born from reintroduced parents.

1211: ***+.075***

To evaluate inter- and intra-populational divergence of the endangered Siberian crane a comparative analysis of mtDNA D-loop region (490 bp) sequences was performed for 17 captive cranes including 14 originating from the eastern and 3 from the central nesting area. Thirteen variable sites form 9 haplotypes, 6 belonging to the eastern nesting site individuals, 2 belonging to the central nesting site and one being common for both nesting areas. The haplotype network analysis did not show significant separation of these two groups. The data obtained could provide genetic grounds for the programs of restoration of the central nesting area population by captive breeding and reintroduction to the wild using the eastern nesting site population gene pool. These results could also be a basis for further evaluation of Siberian crane populations genetic divergence needed for its adequate protection.

1212: *+.275*

Most studies using demographic PVA models in a context of species restoration have concluded that rather than the rate of introduction, the total number of individuals released had the most important significant influence on the chance of success. In this article we use a genetic simulation model including deleterious and adaptive alleles to assess the impact of the method of release on the change in population mean fitness. We systematically compare a strategy that consists in releasing all individuals at the same time with a strategy that consists in staggering releases over a long period of time. Our results show that the former strategy is more beneficial for long-term fitness when considering advantageous genes only, while the latter is better when considering deleterious genes only. If deleterious and adaptive alleles are considered together, the best strategy depends then essentially on which of these types of alleles has the stronger influence on the change in total fitness. Although the relative contributions of the variance in total fitness due to adaptive and deleterious alleles may vary with the initial frequencies and the selective and dominance effects of these alleles, our results show that the optimal rate of release is mostly dependant on the expected long-term population size. Thus from a genetic view-point, the temporal release strategy of reintroduced populations should be considered with respect to their environment's carrying capacity.

1213: *-.000*

Although the Louisiana black bear (Ursus americanus luteolus) is currently listed as threatened under the Endangered Species Act, there have been no attempts to estimate range-wide abundance. This subspecies was thought to occupy a near contiguous range across southern Mississippi, Louisiana and east Texas but is now restricted to three isolated areas in Louisiana. In 1964, Louisiana initiated a restocking program in which black bears from Minnesota were introduced into two of these areas. It is not clear how the additions affected population structure or if substantial breeding occurred between native and introduced bears. Using baited sites to snare hair samples, and microsatellite DNA analysis to distinguish individuals, we estimated abundance of two geographically isolated bear populations in south central Louisiana: Inland and Coastal. Additionally, we examined genetic variation both within and between the two populations. Mark recapture analysis of the distribution of individual captures during two primary sampling periods resulted in population estimates of 77 +/- 9 for Coastal and 41 +/- 6 for Inland. Genetic analysis revealed significant population differentiation (F-ST = 0.206) between the two populations. The apparently smaller Inland population exhibited more diversity than the Coastal, which suggests that the genetic structure of the Inland population has been influenced by the reintroduction. Both of these populations are isolated and face considerable demographic and genetic threats, thus conservation measures to protect both are warranted. However, the Coastal population is more representative of Louisiana black bears prior to reintroduction and special consideration should be

given to insure its integrity.

1214: *+.144*

British S. vulgaris are classified as a separate subspecies, S. v leucourus, to mainland Europe. While S. vulgaris is not under threat across most of its Eurasian range, in Britain, Ireland and Italy populations are declining, mainly due to the introduction of the American grey squirrel (S. carolinensis). In this study, we conducted an extensive survey of mitochondrial DNA variation in British S. vulgaris populations and a preliminary survey of continental European populations. Our main aims were to determine the extent to which any populations of S. vulgaris in Britain are partially or wholly the product of artificial translocation of red squirrels from continental Europe, and whether continental population variation will provide information on post-glacial reafforestation patterns in Europe. We found that the majority of extant populations of British S. vulgaris are of continental ancestry, many with a very recent (last 40 years) Scandinavian ancestry. The Scandinavian haplotype has rapidly become the most dominant in northeastern Britain, despite not appearing in northern English populations until 1966. This suggests that these squirrels may have an adaptive advantage in the non-native spruce dominated conifer plantations of northern England. Our preliminary examination of continental populations demonstrated that they are sufficiently differentiated to allow a phylogeographic study of this species.

1215: *+.022*

The translocation of individuals from one population to another is a common technique in wildlife conservation. However, the outcome of translocation programs is not always properly evaluated and the relative contribution of released individuals to the resident population often remains unknown. We used mitochondrial DNA and autosomal genetic markers to evaluate the success of a translocation program of Eurasian otters (Lutra lutra) in Sweden. The program is regarded as successful because of subsequent population growths. Norwegian otters used for the restocking program could be genetically differentiated from Swedish otters. The releases took place at two sites. In an area south of the first site, where 47 otters were released, no genetic contribution of the introduced animals to the population could be observed and the genetic diversity was lower than before the releases. At the second site, the release of seven otters led to a change in genetic composition of the resident population. The results of this study suggest that the growth of the otter population after the restocking may not be as dependent on the releases as initially suspected. The genetic effects of the translocations appear to be restricted to areas in the immediate vicinity of the release sites.

1216: *+.213*

Genetic factors influence the population viability of rare species, yet the fitness consequences of inbred and outbred progeny are seldom tested empirically in reintroduction strategies designed for species recovery or habitat restoration. Rare and endangered plants of Silene (Caryophyllaceae) occur on four continents, including North America. In Oregon, inbred and outbred progeny were monitored for three years after experimental reintroduction of a narrow endemic, S. douglasii var. oraria, into formerly grazed habitat within its presumed historical range. Survival and reproduction were compared for progeny that were derived from the seeds of self-versus cross-pollinated flowers produced in situ at Cascade Head, a UNESCO Biosphere Reserve where the largest of three extant populations occurs. Progeny of cross-pollinated flowers had significantly greater field survival in all years than did offspring of selfed or open-pollinated flowers ($P<0.01$). Outbred progeny also significantly exceeded other treatment cohorts in canopy area, and produced more

reproductive stems and flowers than other progeny types of the same maternity. For plots varying in plant density, mortality was greatest in the high-density competitive regime but the survivors reached significantly larger sizes and reproductive capacities than in low density plots ($P<0.05$). In all, successful conservation plans involving reintroduction may require genetically diverse progeny to offset inbreeding depression as well as suitable planting densities and source populations.

1217: *+.079*

The Chihuahuan Desert Ecoregion (CDE), located in the central and northern regions of Mexico, and in southern Arizona and New Mexico and west Texas in the United States, comprises desert and semi-desert regions among the most biologically diverse in the world. CDE reptile and amphibian biodiversity alone consists of approximately 217 species. Many factors (e.g., habitat destruction) affect amphibian and reptile populations in the CDE. Though collection for the commercial trade may impact these populations, the magnitude of this impact has not been thoroughly investigated and is poorly understood. The objectives of this report are to: present information gathered on collection, trade, and regulation of the amphibians and reptiles of the CDE; assess the current and potential impacts of collection and trade; evaluate the effectiveness of existing regulations; and make recommendations regarding the steps needed to ensure that collection and trade are sustainable and do not pose a significant threat to CDE reptile and amphibian populations. Mexico Reptiles have long played an important role in the lives of native Mexican people, as a source of food, clothing, arts and crafts manufacture, traditional medicine, and other uses. The diversity of the Mexican CDE herpetofauna is among the highest in all desert ecoregions, with more than 130 reptile species known to exist in the Chihuahuan Desert. Mexico's legal framework includes laws, regulations, norms, international agreements,national plans, and governmental dispositions, all of which are used to promote the protection and sustainable use of Mexico's naturalresources, including its native reptiles. With limited exceptions - primarily involving noncommercial, scientific use -Mexico prohibits the export of native reptiles and amphibians, and all legal shipments must be accompanied by a permit. In addition to this legal regime, there are five protected areas in the CDE in Mexico, including the Cuatro Cienegas Area for the Protection of Flora and Fauna, the Canon de Santa Elena Area for the Protection of Flora and Fauna, the Mapim Biosphere Reserve, the Maderas del Carmen Area for the Protection of Flora and Fauna, and the Sierra Gorda Biosphere Reserve. These protected areas cover in excess of 1 million hectares. Though there is some targeted hunting of reptiles, especially for more commercially valuable species such as rattlesnakes and Bolson tortoises, collecting is frequently opportunistic. Domestic demand for pet reptiles has increased in the recent past, and there is an established trade in markets andsome pet stores within Mexico. Some of this trade is common in established markets, where other pet animals such as aquarium fishes orsongbirds are offered; however, the numerous street markets in major urban centers as well as at some busy crossroads and stretches of road also act as selling points. Most of this trade is targeted at nationals seeking personal pets or for resale in established businesses. At least 82 species, or approximately 63% of the approximately 130 reptile species found in the Mexico portion of the CDE, are subject tosome kind of trade. Most of this trade is targeted at demand for pets, followed by demands for meat, skins, and traditional medicine. Of the 82 species identified in domestic or international trade, 6 are listed in Appendix I or II of the Convention on International Trade in Endangered Species of Wild Fauna and Flora (CITES), 4 are classified as Threatened by IUCN-the World Conservation Union (2002), and 51 in the NOM 059 ECOL 2001. Seventeen of the 82 species are endemic to Mexico. Rattlesnakes, used for skins, rattles, meat, fat, venom, and as live animals, are the most commonly traded Chihuahuan Desert reptiles. Several reptile species, including Bolson tortoises Gopherus flavomarginatus, Coahuilan box turtle Terrapene coahuila and black spiny softshells

Apalone ater, may be particularly threateneddue to their population status. Five main distribution and trade centers for CDE reptile species were identified in three major areas, including: Plateros Magical/Religious Center, Zacatecas; Charco Cercado, San Luis Potos; three Mexico City Marketplaces (the Mercado de Sonora, the Mercado Nuevo San Lazaro, and the Mercado Emilio Carranza). Virtually all of the trade in reptiles and amphibians in these distribution and trade centers is illegal. There is also an international, illegal trade in Mexican reptiles and reptile products, primarily to the United States. Because Mexican law generally prohibits the export of native reptiles, virtually all export of live reptiles, reptile parts, and products from Mexico is illegal. However, the offer for sale of numerous CDE reptile species endemic to Mexico by reptile dealers outside of Mexico, especially in the United States, indicates that illegal exports are taking place. Although many of these species are now bred in captivity within the United States, the original founder stock was likely exported illegally from Mexico. Also, there are frequent seizures oflive reptiles, as well as parts and products, at the U.S./Mexico border. However, neither of these information sources gives a clear pictureof the extent of exports from Mexico. Demand for live rattlesnakes, skins, and parts for use in traditional medicine appears to have led to significant population reductions in some areas, such as Plateros, Zacatecas. However, basic biological information on most CDE reptile species is scarce, making it difficult to evaluate the impact of collection and trade on wild populations. This is compounded by the fact that these species face other threats in many areas, such as habitat loss by deforestation, agriculture, overgrazing, exotic species introduction, pollution, and watershed depletion. To ensure that collection and trade are not a threat to CDE reptile species in Mexico, TRAFFIC recommends the following: Enforcement: 1. Enforcement programs, which involve frequent inspections of important trade centers, should be enhanced to provide a more effective deterrent to illegal trade activities. 2. Wildlife inspector numbers should be increased and specific training provided (e.g., species identification, reptile handling, etc.) to ensure more effective regulation of the large-scale trade in reptiles and other wildlife. 3. Long-term investigations of reptile traffic networks, from their natural collecting areas to their final sale, are needed to fully understand the extent and impacts of this trade. 4. Protocols for disposing of confiscated wildlife, products, and byproducts, emphasizing the proper maintenance and handling of live specimens, including the creation of centers devoted to dealing with live confiscated wildlife, should be developed as a priority. Management: 5. The UMA (Unidad para la Conservacion, Manejo y Aprovechamiento Sustentable)system should be assessed to determine whether sufficient controls and monitoring mechanisms are in place. If so, the system should be promoted, especially with regard to captive production of reptiles. These efforts should focus on threatened species that are in significant demand for pets or other uses. Other UMA efforts might include exhibits, ecotourism, and reintroduction programs. 6. Expanded knowledge of the biology and size of the populations of these species is imperative, especially for threatened and heavily collected species.7. Standardized gathering and reporting of wildlife collection and trade information (investigations, seizures, repatriations, prosecutions, etc.) and greater facilitation of information exchange and consultation among government offices and other parties are greatly needed.8. Rehabilitation and release programs for seized animals, based on scientific data on the biology and distribution of each species, taking into account the potential for disease, genetic pollution, and other risks to wild populations, should be developed. Education: 9. Environmental education programs focusing on local communities should be developed, to increase awareness of Mexican laws andpromote the value of these communities' natural resources. United States of America Though dozens of reptile and amphibian species are exploited throughout the United States, little is known about the extent of these activities or their impacts on wild populations. This is particularly true for the large number of small lizards and snakes such as those found in the CDE. The collection and sale of reptiles andamphibians in the United States is generally regulated at the state level. For CDE species, the wildlife legislation and regulations of New Mexico, Arizona, and Texas are applicable

to activities involving reptiles and amphibians in each of those jurisdictions. Federal legislationand regulations generally apply to federally protected species, activities on federal lands, and interstate and international commerce. The domestic trade in amphibians and reptiles from the CDE is almost impossible to quantify, because take of most species is not regulated in New Mexico, trade from Arizona is illegal, and Texas has only recently begun gathering data from collectors. There is no reason to believe that large numbers of animals from Arizona are commercialized. Commercialization does occur in New Mexico, but there was no current information available at the time of this study. Information on collection and sale of nongame species was available from Texas for 1999. In Texas during 1999, 14 351 specimens of reptiles and amphibians were reported to be collected by 53 Resident or Nonresidentnongame permit holders. Of these, 4861 were amphibians and 9493 were reptiles. These numbers do not include rattlesnakes collectedfor rattlesnake roundups, and thus the overall figure is likely to be much higher. Ninety percent of all specimens collected in the CDE in Texas in 1999 originated in only five counties, with the top eight collectors accounting for 90.3% of the total harvest. Available data indicate that the number of snakes removed from the wild is not extraordinary. In 1999, the Texas Parks and Wildlife Department (TPWD) nongame database showed that 985 snakes were collected in the CDE. Of these, 325 were garter snakes Thamnophis spp. and 307 wererattlesnakes Crotalus spp. Seventy-three kingsnakes were collected (all species of Lampropeltis); 42 were gray-banded kingsnakes. Thirty-two ratsnakes Elaphe spp. and 48 Trans-Pecos ratsnakes Bogertophis subocularis were reported collected by nongame permit holders. Several other species were collected in smaller numbers. International trade in reptiles from the CDE in the United States is difficult to assess, due to a number of factors. Though some data are available from the U.S. Fish and Wildlife Service (USFWS), these data raise as many questions as they provide answers, largely because the data collected by the USFWS are often not species-specific, do not include any locality information, and frequently don't indicate the origin (wild-caught or captivebred) of the animals. Despite these limitations, USFWS data are of some value in an analysis of CDE species trade. A thorough review of U.S. export data showed that there are approximately 50 reptile species found at least in part in the CDE that appear to be involved in international trade. The export of turtle species found in the CDE likely involves few, if any, turtles actually collected from the CDE. For example, the export of red-eared sliders almost exclusively involves turtles produced on farms in Louisiana and elsewhere in the southeastern United States. The same is true for the export of most other turtle species found in trade. The export of live CDE snake and lizard species does not appear to be a significant concern. Most species are apparently being exported in small numbers, and at least some of these species are being produced in captivity, with little indication of increasing trade throughout the time period examined. A review of USFWS data showed that at least seven species found in the CDE are traded as parts or products. Though the export of native CDE species as parts or products involves far fewer species than are involved in the live trade, this practice may be having a far greater impact on individual species because the trade in parts and products is more likely to involve wildcaught adult animals. Of some concern is the exploitation of several venomous reptile species for meat, skins, and leather products. This is especially true of the Western diamond-backed rattlesnake, which is exported in a large array offorms. The most significant trade in this species appears to be meat, with well over 2000 kilograms of meat exported over the six-yearperiod examined. A significant volume of skins and skin products was also exported during this period, including 1616 skins, 712 shoes/boots, 1407 small leather products/skin pieces/leather trim, 34 handbags, 79 garments, 939 pieces of jewelry, and 753 unspecified items. Based on the lack of field studies and monitoring data on the trade in reptiles and amphibians, it is not possible to draw definiteconclusions about the threats posed by collection and trade. However, based on life history pattern, geographic extent of range, anddetectability, it is likely that the vast majority of reptile and amphibian species found in the CDE are widely enough distributed thatcollecting will not lead to regional or global species extinction. Almost all

amphibian and reptile collecting is done from roads or inaccessible canyons. Enormous areas are on private land or so remote that collecting for trade is inefficient, and there are extensive areas of suitable and occupied habitat that are never collected. Collecting and trade in the CDE probably should not be considered a threat to species that are relatively widely and continuously distributed, are small in size, and that possess a life history characterized by relatively short life span and high reproductive rate. The seven most collected species from the CDE in Texas in 1998-99 accounted for 79% of allspecies collected. It is impossible to evaluate quantitatively the impact of collecting on the populations that were exploited without detailed information on collecting effort, geographic extent of collecting, and demographic information on the exploited populations. However, the three amphibians-tiger salamander Ambystoma tigrinum, Couch's spadefoot Scaphiopus couchii, and green toad Bufo debilis-are common and widely distributed. The two lizards-common sideblotched lizard Uta stansburiana and marbled whiptail Cnemidophorus tigris-are alsowidespread and continuous in their distributions throughout much of the CDE. Gila monsters Heloderma suspectum, turtles in general, and the prairie rattlesnake Crotalus viridis are species that, based on natural history, may be locally vulnerable to overcollecting. Species with fragmented ranges, such as the massasauga Sistrurus catenatus or Western hog-nosed snake Heterodon nasicus, even if the geographical extent of the range is large, may also be susceptible to local population extirpation. Because of their vulnerability and the life history characteristics of long-lived organisms, turtle populations may be eliminated from specific sites from over-collecting. The yellow mud turtle Kinosternon flavescens and desert box turtle Terrapene ornata luteola were collected in large numbers in Texas. Though both species are common and widespread, yellow mud turtles are generally found when locally concentrated in ponds, stock tanks, and playas, and desert box turtles have a low reproductive rate. It is reasonable that these and similar species could be significantly impacted by intenstive collecting. The pattern of collecting in Texas indicates some generalities that are relevant to reptile and amphibian collecting throughout theregion. First, few collectors account for the great majority of specimens collected. Second, few species were collected in large numbers. The species that were collected in large numbers are widespread, small bodied, and, except for amphibians and turtles, are relatively short lived. A third generalization was that collecting was very patchy, with a small number of areas being intensively collected. In Texas, the same stretches of roads that have been popular with collectors for decades continue to produce specimens, and remain the principal areas used by collectors. There are enormous expanses of habitat throughout the entire ecoregion that are not visited by collectors. Thus, given available information, collection and trade do not appear to be a significant threat for the vast majority of CDE reptile and amphibian species in the United States. However, for a select number of species with particular vulnerabilities and/or life history characteristics, over-collecting may at least be impacting local populations and bear further attention. Based on this information, TRAFFIC recommends the following: Collection and Trade Monitoring: 1. To ensure that collection and trade of CDE amphibians and reptiles is sustainable,implementation of a legally binding monitoring system of take and trade in these groups is needed. Such a monitoring system must be based on the legal status of amphibians and reptiles and rules regulating their take. Such systems have been implemented in Texas and New Mexico and have provided valuable information on the trade of amphibians and reptiles in the state. Field Research: 2. It is not often feasible to census amphibian and reptile species directly because of the large geographic areas involved, the time and expense required to measure these populations, and the inherent difficulties in measuring amphibian and reptile populations with confidence. Instead, targeted research should be aimed at producing sufficient information about collecting amphibians and reptiles to guide management decisions. 3. Information is needed on the amount of suitable habitat available for species with relatively small distributions and that require very specific habitat types in relation to the amount of area where they are collected. 4. Research on source-sink phenomena in exploited amphibians and reptiles would be useful, and has yet to be

conducted for any exploited amphibian or reptile. Information on source-sink dynamics of selected species may clarify whether populations are selfsustaining, or to what extent habitat corridors and large uncollected areas are needed to sustain populations of exploited populations on a regional basis

1218: ***-.057***

In the article there are written the results of works with population of bitterling that are in danger of extinction. That populations used to live in reservoir that emerged as a result of the clay exploitation, than were moved to another reservoir that was the result of the clay and the sand exploitation. After four years there was affirmed a full success of introduction to the reservoir in Szejkowice (that emerged as a result of the clay exploitation) and to the reservoir in Rogoznik (emerged as a result of the sand exploitation). Owing to the acid water that was input to the reservoir near Rudy, there was determined the extinction of the populations of bitterling and bivalves. All works were led by consent of Department of Enviroment, financed and inspected by Provincial Nature Conservator.

1220: ***+.389***

The aim of this roundtable session was to discuss reintroduction of native crayfish and habitat restoration providing practical recommendations of value to environmental authorities, decision makers and local managers. A (re)introduction may be appropriate: a) to reintroduce a population recently lost, b) to extend the distribution of an ICS into historic range and c) to create new or isolated populations to conserve genetic diversity or the species. Before (re)introduction it is imperative to demonstrate the probability that the receptor locality is empty of crayfish, to know the reason for crayfish extinction, and that the reason is removed before restocking. Before restocking, the receptor locality should also be assessed for crayfish suitability, including: water type and chemistry, physical conditions (shelter), presence of crayfish plague, risk of NICS, predatory fish. In general, major constraints in a restocking project is money and availability of stocking material. General recommendations: a) if have abundant/readily available and acceptable donor population use adults and young/juveniles as available, b) if short of stock boost with hatchery rearing, and c) if want population quickly, stock as many as can afford and several times. Trade offs: time vs. cost. Genetic distinctiveness/possible spread of diseases must be considered. In general, the preferential ranking is donor from: 1) same watercourse, 2) same catchment, 3) adjacent catchment or one nearby in the same biogeographic region for crayfish, and 4) any other catchment. Habitat restoration can be a valuable action, and should be prioritised to: 1) give greatest overall benefit to the ecosystem, 2) benefit ICS and 3) be achievable and cost effective. Natural characteristics of waterbodies in local area should be considered. A key factor in the protection of ICS populations is the knowledge and attitude of local people. Objectives and actions should be agreed with local stakeholders to improve chances of success.

1221: ***+.044***

Conservation of the endangered noble crayfish (Astacus astacus) requires preservation of existing populations and reintroduction into suitable habitats, as currently underway in several regional projects in Germany and Poland. Therefore, knowledge about the genetic variation between populations is vital but, unfortunately, still restricted. By means of ISSR-PCR (Inter-Simple Sequence Repeats - Polymerase Chain Reaction) eight stocks in Germany and Poland were analysed. These semi-arbitrary multiloci markers had not previously been used in population studies of the noble crayfish. A total of 22 unambiguous and polymorphic markers were detected

to use in the subsequent statistical analysis. The number of polymorphic loci in one population ranged from 4 to 19. Therefore the ISSR markers proved suitable for assessing within- and between-population DNA variations and establishing significant separation of most of the stocks. Shannon's Index, a relative estimate of genetic diversity within populations, ranged from 0.06 to 0.51. UPGMA cluster analysis based on pairwise ROGERS' (1972) genetic distance revealed two main clusters corresponding to populations sampled in the western and eastern part of the study area. Results are relevant on the local, regional and supra-regional (landscape) level. The implications for conservational management and restocking programs are presented.

1222: ***+.216***

The Glomma and Halden watercourses in Norway were hit by crayfish plague in 1987 and 1989. Reintroduction of the noble crayfish started in 1989 in the Glomma and in 1995 in the Halden watercourse. Norway has especially good conditions for reintroduction of the native crayfish after crayfish plague, as there is no alien plague-carrying crayfish species in the country. In the Glomma watercourse, approx. 15 000 adult crayfish and 10 000 juveniles have been stocked while in the Halden watercourse the figures are 19 000 adults and 26 500 juveniles. All stocking sites were previously regarded as very good crayfish localities. Four years after stocking, natural recruitment was recorded at all adult crayfish stocking sites in the Glomma watercourse and at most sites in the Halden watercourse. Current crayfish density is, however, much lower than pre-plague densities even at the sites where population development has been in progress for more than 10 years. Extensive post-stocking movements were recorded among adult crayfish. Some sites seemed more suitable for settling, resulting in a great variation in CPUE between the different test-fishing sites. Juveniles seem more appropriate as stocking material if the goal is to re-establish a population in a particular area, due to their stationary behaviour, which seems to remain as they grow larger.

1223: ***+.163***

In Tyrolean like in other European freshwaters, crayfish populations decreased in numbers and qualities. They are today regarded as endangered animals. The Astacus astacus (Linnaeus, 1758) population of historical evidence in Tristacher See and its out flowing stream Tristacher Seebach (mentioned already by Emperor Maximilian I in 1504) became extinct in the late 1990s. After the restoration of the stream we started a species conservation programme with various specific protection measures, including breeding and restocking of young-of-the-year and adult A. astacus. Females, after having released their young in the hatchery, were stocked together with males in a 200-m-section of Tristacher Seebach, previously populated by A. astacus. In October, the young-of-the-year crayfish were released in another area of the same stream. To show the importance of habitat diversity and shelter, four sites for introduction were selected describing a gradient of habitat diversity. We monitored general characteristics of the population (sex, size, densities) and compared them to habitat conditions. Individual crayfish were tagged with gloss-paint pens to allow an observation of their movements between the different sections over the summer months. We found significant results when migration behaviour, population assemblage and habitat conditions were compared. Males frequently moved longer distances than females. Migration length corresponded to the gradient of available structures and shelter. Heterogeneous riprap was somewhat preferred to artificial shelter like bricks or plastic tubes. Sections without additional shelter showed almost no presence of crayfish. Sex and size distribution within assemblages appeared also to be affected by habitat conditions. Our results indicate the importance of monitoring in species reintroduction projects, as this research demonstrated the immediate effect and importance of habitat structure and affirmed the success of the protection measures. This may in fact help to avoid future failure.

1224: ***+.163***

In Tyrolean like in other European freshwaters, crayfish populations decreased in numbers and qualities. They are today regarded as endangered animals. The Astacus astacus (Linnaeus, 1758) population of historical evidence in Tristacher See and its out flowing stream Tristacher Seebach (mentioned already by Emperor Maximilian I in 1504) became extinct in the late 1990s. After the restoration of the stream we started a species conservation programme with various specific protection measures, including breeding and restocking of young-of-the-year and adult A. astacus. Females, after having released their young in the hatchery, were stocked together with males in a 200-m-section of Tristacher Seebach, previously populated by A. astacus. In October, the young-of-the-year crayfish were released in another area of the same stream. To show the importance of habitat diversity and shelter, four sites for introduction were selected describing a gradient of habitat diversity. We monitored general characteristics of the population (sex, size, densities) and compared them to habitat conditions. Individual crayfish were tagged with gloss-paint pens to allow an observation of their movements between the different sections over the summer months. We found significant results when migration behaviour, population assemblage and habitat conditions were compared. Males frequently moved longer distances than females. Migration length corresponded to the gradient of available structures and shelter. Heterogeneous riprap was somewhat preferred to artificial shelter like bricks or plastic tubes. Sections without additional shelter showed almost no presence of crayfish. Sex and size distribution within assemblages appeared also to be affected by habitat conditions. Our results indicate the importance of monitoring in species reintroduction projects, as this research demonstrated the immediate effect and importance of habitat structure and affirmed the success of the protection measures. This may in fact help to avoid future failure.

1225: ***+.090***

In December 1998, Alsace knows the first French reintroduction of Otter Lutra Lutra. Six individuals from the Breeding Centre of Hunawihr were released over one three years period. The territorial evolution of four otters is followed-up daily during an average of 269 days thanks to the radio-tracking. From September 2000, only signs of presence make it possible to monitor the population. Signs of breeding were probably discovered in 2000 and 2001. A study of the diet was engaged between April 2002 and March 2003. It shows the perfect opportunism of otters coming from captivity and consuming preys available which vary in quantity and quality among seasons. Fishes constitute 98% of the diet. The Cyprinidae are preferred in autumn and winter and then are replaced in spring and summer by the Cottidae, the Cobitidae and the Salmonidae. Today, not less than 130 km of rivers are occupied by offers. Nevertheless, since the end of the year 2001, the experiment is stopped because of genetic "incompatibility" between captive and wild populations of otters which penalizes the total success of the project and the stability of the species in Alsace.

1226: ***+.053***

Wolf management can be controversial, reflecting a wide range of public attitudes. We analyzed wolf management case histories representing a spectrum of approaches in Canada and the United States. During the early 20th century, wolves were considered undesirable. They were subject to persecution and were extirpated from large areas of their original range. With increased environmental awareness in the 1970s, attitudes toward wolves began to change. Wolf conservation became a focus of public interest, providing conditions that favored regional wolf recovery. However, in regions where livestock production or big-game hunting is valued, wolves have continued to be controlled by management authorities or through the actions of individual

citizens. With US wolf populations recovering in the conterminous states, a rule was approved to delist the species from endangered to threatened status under the Endangered Species Act. Notwithstanding the intent of legal instruments, history has demonstrated that societal values ultimately determine the survival of species such as the wolf.

1227: ***+.001***

Long-term maintenance of captive populations followed by release of captive animals into the wild is one of many approaches to endangered species conservation. To assess captivity's effects on behavior, a simulated predator was presented and response behaviors measured in oldfield mice, Peromyscus polionotus subgriseus. The animals tested were from four populations collected from Ocala National Forest, Florida, and held in captivity for varying numbers of generations: 35, 14, 2, and 0 (wild caught). Results show (1) that the more generations a population has been in captivity, the less likely an individual is to take cover after seeing a predator and (2) variance in predator-response behaviors increases with generations in captivity. These results point to two ways in which captivity can compromise animal behavior and, in turn, the success of reintroduction programs. First, because individuals from populations that have been in captivity for multiple generations seek refuge less often than their wild counterparts, they might experience increased mortality in the wild due to predation. Second, increased behavioral variance could translate into decreased survivorship upon reintroduction. Therefore, more individuals will need to be released to reach the targeted wild population size. (C) 2003 Elsevier Ltd. All rights reserved.

1228: ***+.007***

We present a spatially explicit population model for analysing the expansion of brown bears (Ursus arctos) after the reintroduction program in central Austria. The model is based on field investigations into brown bears in Austria and Slovenia and on current knowledge of brown bears. The landscape of the eastern Alps is represented by a GIS-derived raster map defining local habitat suitability and five major spatial barriers to dispersal. The population model follows the fate of individual bears and simulates reproduction, dispersal, home range establishment, and mortality in annual time steps. We indirectly adjust unknown or uncertain model parameters with 10-year data on the number of females with cubs in central Austria and determine key variables of population dynamics, such as population sizes and growth rates within different population nuclei, dispersal distances, or mortality rates, for model parameterisations that reproduce the data on females with cubs. We estimated a current (1996 - 2000) growth rate of the population in Austria and adjacent parts of Italy of some 14%; a high proportion of this growth was due to immigration from Slovenia. Consequently, the growth rate of the subpopulation in central Austria, which probably is isolated functionally (i.e., no exchange of females) from the nuclei along the Austrian - Slovenian border, yielded some 7%. This subpopulation may comprise seven residents, and we estimated for females a 33% risk of extinction during the 1992 - 2000 period. Validation and confirmation of our model results with data on bear densities that were not used for model construction and parameterisation supported our findings. The high female mortality rates, together with the vulnerability of the small population to chance events (i.e., demographic stochasticity), are the most pressing threat for the population in the eastern Alps. Our approach could be widely applied for analysing dynamics of rare and endangered species in which the paucity of data precludes an appraisal of the state of the population using standard methods.

1229: ***+.055***

It has been argued that spatially explicit population models (SEPMs) cannot provide reliable

guidance for conservation biology because of the difficulty of obtaining direct estimates for their demographic and dispersal parameters and because of error propagation. We argue that appropriate model calibration procedures can access additional sources of information, compensating the lack of direct parameter estimates. Our objective is to show how model calibration using population-level data can facilitate the construction of SEPMs that produce reliable predictions for conservation even when direct parameter estimates are inadequate. We constructed a spatially explicit and individual-based population model for the dynamics of brown bears (Ursus arctos) after a reintroduction program in Austria. To calibrate the model we developed a procedure that compared the simulated population dynamics with distinct features of the known population dynamics (= patterns). This procedure detected model parameterizations that did not reproduce the known dynamics. Global sensitivity analysis of the uncalibrated model revealed high uncertainty in most model predictions due to large parameter uncertainties (coefficients of variation CV approximate to 0.8). However, the calibrated model yielded predictions with considerably reduced uncertainty (CV approximate to 0.2). A pattern or a combination of various patterns that embed information on the entire model dynamics can reduce the uncertainty in model predictions, and the application of different patterns with high information content yields the same model predictions. In contrast, a pattern that does not embed information on the entire population dynamics (e.g., bear observations taken from sub-areas of the study area) does not reduce uncertainty in model predictions. Because population-level data for defining (multiple) patterns are often available, our approach could be applied widely.

1230: *+.059*

Myrica rivas-martinezii A. Santos and Sideritis discolor Bolle, two endangered endemic species of the laurel forest from the Canary Islands, were studied by using RAPD markers with the goal of assessing their within- and among-population apportionment of genetic variability. Ten and 11 oligonucleotide primers assayed in three populations of each of M. rivas-martinezii and S. discolor resulted in 32 molecular markers in the former species and 49 in the latter (90.6% and 100% polymorphic, respectively). The value of the coefficient of genetic differentiation among populations was very high for M. rivas-martinezii (F-ST=0.487) but only moderate for S. discolor (F-ST=0.149). These results suggest that gene flow among populations is extremely low in M. rivas-martinezii and quite substantial in S. discolor. The UPGMA cluster obtained from Dice's coefficient mixed individuals from different populations in S. discolor but not in M. rivas-martinezii, where they were grouped by their island of occurrence. On the basis of these results and given the vulnerability of these two species, we suggest protection of all natural populations. However, if habitat conservation is not possible, our results suggest that for S. discolor, the La Virgen population would make a good donor population because of geographical and genetic factors. The high genetic differentiation detected among populations of M. rivas-martinezii suggests that each island should be considered as a distinct management unit. However, considering the situation of this species on La Palma Island with only two isolated individuals, reintroductions of germoplasm from El Hierro may be a viable way to guarantee the existence of M. rivas-martinezii on that island.

1231: *+.117*

Viola pumila Chaix is known in Italy, only in small populations growing in the Po Valley, Emilia-Romagna Region (Italy). Due to its scarce distribution and to the scarcity of populations the species is considered rare and in danger of extinction in this territory. A project of integrated in situ/ex situ conservation has, therefore, been promoted. A few hundred plants were obtained in vitro from collected wild seeds and were subsequently transplanted in natural sites in the

Novellara e Reggiolo lowlands (Province of Reggio Emilia), and in the Botanical Garden of Modena with the aim to set up a conservation collection. This project was carried out thanks to co-operation between the Botanical Garden, local Administrations and the Association of Voluntary Ecological Wardens of Reggio Emilia.

1232: ***+.067***

The feasibility of translocation to establish a population of the eastern box turtle (Terrapene c. carolina) was studied at Floyd Bennett Field, Brooklyn, New York, USA. The 579 ha site, originally salt marsh, was filled during the 1920's to construct a now-abandoned airport. It consists primarily grasslands, native shrub thickets and woodlands, and mixed stands dominated by giant reed (Phragmites australis). These human-created uplands are managed by the U.S. National Park Service for recreation and ecological restoration. Prior to this work, the site did not support a population of this species, but it is historically native to adjacent uplands. T. c. carolina were collected from sites on Long Island, New York, that were undergoing development, and released after data on size, mass, age, and sex were recorded. From 1987 through 1990, 335 individuals were released auto developing woodlands. To provide data on dispersal, home range establishment, and initial survival, fifty-three of these were radio-tracked for up to seven years. Though individually variable, the T. carolina dispersed homeward. Of the 53 radio-tagged individuals, 13 (24.6%) left the site, 25 (47.2%) established home ranges, and 15 (28.3%) died before determination of home range establishment could be made. Most individuals established home ranges within a kilometer of the release point. However, some dispersed greater distances. Of the 25 individuals that established home ranges, 17 (68%) did so in the release year, two (8%) in outyear 1, three (12%) in outyear and three (12%) in outyear 3. Annual known survival over five years post-release was 71%. Though not statistically significant, annual survival was 64% over the first two years and 84% over the final three. Principal causes of "mortality" were dispersal from the site and pneumonia, both of which were greatest initially, plus winter kill, a random event. Patterns of growth, home age size, activity season, habitat use, annual reproductive output, and production of young were generally comparable to natural populations of T. carolina. These results suggest that translocation have potential for establishing new populations of T. carolina, though long term viability is still uncertain. However, any contemplated translocation would need to address the initially high loss dispersal and disease. Moreover, since there are few sites of adequate size and quality, at least 500 ha of predominantly woody habitat, lacking populations of this species, its appropriateness is limited.

1233: ***+.078***

Using large-scale data analysis to assess life history and behavioural traits: the case of the reintroduced White stork Ciconia ciconia population in the Netherlands.- The White stork Ciconia ciconia has been the object of several successful reintroduction programmes in the last decades. As a consequence, populations have been monitored over large spatial scales. Despite these intense efforts, very few reliable estimates of life history traits are available for this species. Such general knowledge however constitutes a prerequisite for investigating the consequences of conservation measures. Using the large-scale and long-term ringing and resighting data set of White storks in the Netherlands, we investigated the variation of survival and resighting rates with age, time and previous individual resighting history, and in a second step supplementary feeding, using capture-recapture models. Providing food did not seem to affect survival directly, but may have an indirect effect via the alteration of migratory behaviour. Large-scale population monitoring is important in obtaining precise and reliable estimates of life history traits and assessing the consequences of conservation measures on these traits, which will prove useful for

managers to take adequate measures in future conservation strategies.

1234: *+.269*

The behavior of reintroduced, captive-born animals is understudied, limiting the scientific understanding and utility of reintroduction as a conservation tool. This work describes changes in locomotor and foraging behaviors in captive-born golden lion tamarins over the first 18 months after their release into the wild. The subjects included 73 individuals living in and around the Poco das Antas Biological Reserve in Brazil between 1984 and 1996. The differences between animals that survived 6 months after release and those that did not indicate that initial deficiencies in locomotor and foraging abilities are related to survival. Behavioral changes in both juvenile and adult individuals during the first 6 and 18 months after release appear to be primarily related to locomotor abilities; however, the effect of provisioning on foraging abilities is unknown. Juvenile animals showed a larger number of changes relative to adults during the first 6 and 18 months, suggesting that placing tamarins into complex environments early in development may promote the expression of natural behaviors and increase survival opportunities after their release. However, when this is not possible, the best mechanism for reintroducing adult members of this species involves intensive post-release support rather than pre-release training, which confers few behavioral advantages. Recommendations for future reintroductions with this and other species include introducing animals to complex environments early in development, and collecting data systematically. (C) 2004 Wiley-Liss, Inc.

1235: *+.209*

This thesis deals with the nature conservation of reptiles and amphibians in the Netherlands, the present practices and what should be done to improve them. Most of the species of the herpetofauna, as these groups together are known, are in a state of continuing decline. Obviously something is wrong; apart from the role played in this decline by loss of habitat, measures in the field are not effective for the sustainable survival of these species. This thesis has been written to rectify this; based on current knowledge of the ecology of these protected animals and on own experiences with nature management, it is meant to provide a guide and practical tool when carrying out the appropriate measures in the field. The introduction describes work that has been done internationally, especially at European level, to protect the herpetofauna. Further, how this has influenced policy plans and legislation in the Netherlands. It also points out the shortcomings, both in the approach of the policymakers, as well as in the current training of nature managers, and how these are reflected by the measures taken in the field. This information provides a background for chapter 7, the last chapter of this thesis, where the practice of management is discussed. The remaining chapters include five articles on ecological research; they also show how much time is needed to improve our knowledge on the ecology of these animals. Also, not understanding it can unwittingly create marginal conditions for a species, as was the case for the Slow-worm and Sand Lizard with the forest management on the Utrechtse Heuvelrug in the 1980s; this is described in the first two articles. That long-term surveys of population dynamics are more than necessary, is shown by the great fluctuations in the presence and activity of the European tree frog over the six years it was studied in the southwest of the Netherlands. Methodology is also discussed in this third article, and a practical formula given for estimating population size. Pond characteristics form the subject of the next article, wherein statistical analysis illustrates how they can indicate the possible presence and therefore the suitability of a pond for amphibians. Practical indications are given, useful when constructing new ponds as part of the Pond Action Plans. The last article has implications for the reintroduction of species into new or former habitats. That there is phenotypic variation within species of amphibians occurring within 200 kilometres of each other, shows that,

to avoid failure, this should be taken into account in the selection both of the source population and the location of the habitat into which the animals are to be introduced. All these aspects are brought together in the final chapter where the practice of the conservation of both groups is discussed in turn. The habitats are the point of departure, and it is shown how conservation measures with plants or landscapes in mind can bring about deterioration or loss of habitat for a particular species of reptile or amphibian. The integration of management goals should be the approach for as far as is possible, but legal obligations may force us to take species specific measures into account for herpetofauna. Heathlands are of utmost importance for reptiles and the most relevant features of the management for this type of habitat are highlighted. For their part, amphibians find their major aquatic habitat in ponds. Details are given of the management for various amphibian species, both in ponds, and as far as we know it, in their habitat on land. The extensive literature on these subjects, found at the end of Chapters 1 and 7, as well as that of the articles, provides opportunity for going further into depth. The recommendations from Chapter 7, dealing with the structure of the vegetation to a considerable extent, should improve the situation of the herpetofauna in the Netherlands.

1236: ***+.260***

Limited data indicate that Shiras moose (Alces alces shirasi) occurred in low numbers in Idaho throughout the 1911, century. Harvest was allowed in Idaho during 1893-1898, after which seasons were closed. Shiras moose were fully protected in Idaho from 1899-1945. Moose populations increased during the 20(th) century and harvest seasons resumed in 1946. Harvest has focused on mature males, allowing continued population growth through the end of the 20(th) century. Rapid population growth during 1980-2000 resulted in moose dispersing westward from the Rocky Mountains and southward from the Panhandle region of Idaho. The management goal for moose in Idaho is to provide opportunities for recreational hunting and harvest of mature male moose. Although some managers assess moose populations directly by aerial survey, most managers rely on indirect measurements (e.g., hunter success rate and antler spread of bulls harvested) to assess the impact of harvest on moose populations. Other population indicators (e.g., dispersal into previously unoccupied areas, damage to private property) have been used as indicators of social tolerance for expanding moose populations. Where moose have approached the limit of social tolerance, attempts to stabilize or reduce populations by harvest of females and translocation of 'problem' moose have been utilized. Both a historic perspective of moose abundance and a revised statewide population estimate are provided.

1237: ***+.267***

This 344-page book is part of a series on Iguanas: Biology and Conservation. The book is divided into 3 parts, which are further divided into 20 individually authored chapters. The first part deals diverstiy, which further includes the evolution of iguans: an overview of relationships and a checklist of species, genetic contribution to Caribbean iguana conservation, genetic structure of the turks and caicos rock iguana and its implications for species conservation, and sodium and potassium secretion by iguana salt glands: acclimation or adaptation. The second part deals with behavior and ecology, which further includes behavior and ecology of rock iguanas, evidence for an appeasement display and populaiton differences. The thrid part deals with conservation, which further includes translocation strategies as a conservation tool for west Indian iguanas: evaluations and recommendations, and ecotourism and its potential impact on iguana conservation in a Caribbean. Remaining chapters include determinants of lek mating success in male galapagos marine iguanas: behavior, body size, condition, ornamentation, ectoparasite load, and female choice. The book highlights a list of contributors and their respective institutions. Each chapter

contains a list of references. The text is written in English. Users of this book will include zoologists, conservationists, and ecologists.

1239: *+.128*

Severe bottlenecks can reduce genetic diversity and increase inbreeding as individuals are forced to mate with close relatives, but it is unknown at what minimum population size the negative fitness consequences of bottlenecks are expressed. The New Zealand avifauna contains a large number of species that have gone through bottlenecks of varying severity, providing an exceptional opportunity to test this question by using the comparative method. Using decreased hatchability as a measure of fitness costs, we found that hatching failure was significantly greater among both native and introduced species that had passed through bottlenecks of <150 individuals. Comparisons between pre- and postbottleneck populations of introduced species suggest that hatching problems arise even in populations founded by <600 individuals. Our study confirms that hatching failure is widespread and persistent among birds passing through severe bottlenecks and that the population sizes at which this fitness cost is expressed are several times greater than the number of individuals currently used to found most new populations of endangered species. We recommend that conservation managers revise the protocols they use for reintroductions or they may unwittingly reduce the long-term viability of the species they are trying to save.

1240: *+.121*

Fishes listed under the U.S. Endangered Species Act receive intensive management, but evaluations of population characteristics are rare. We report population and habitat characteristics of federally threatened greenback cutthroat trout Oncorhynchus clarki stomias in 12 streams in north-central Colorado surveyed in 1998 and 1999. Our objectives were to assess population size, structure, and distribution, to evaluate a model developed by Harig and Fausch (2002) to assess translocation success, and to compare population characteristics to those reported in the recovery plan. Allopatric population sizes in 10 streams varied from 170 to 7,347 greenback cutthroat trout at least 75 mm long, whereas the 2 streams with brook trout throughout supported 0-142 greenback cutthroat trout. Populations displayed few upstream-downstream trends in fish abundance or mean length, and the coefficient of variation among sampled reaches within streams averaged 61%. As the density of fish at least 75 mm long increased, the percentage of juvenile (<75 mm) fish in a population increased, the percentage of adult (>125 mm) fish longer than 200 rum decreased, and the average weight of a 200-mm fish declined. The abundance of juvenile fish was positively correlated with summer water temperature. Population estimates were highly correlated with the translocation model index values, suggesting that the translocation model may apply to other cutthroat trout taxa in similar environments. Of the 12 streams for which our extensive field data are available, 4 satisfied the recovery criteria we studied and other criteria in the recovery plan; 8 did not. We conclude that many populations of greenback cutthroat trout, including some that meet recovery criteria, are still at risk of extinction and that a reliable monitoring protocol needs to be established.

1241: *+.109*

A single stocking of 611 wild flannelmouth suckers Catostomus latipinnis in 1976 represented the first successful reintroduction of a native fish in the lower Colorado River. Flannelmouth suckers ranging in age from young of the year to 24 years were captured during 19992001; their population was estimated as at least 2,286 (95% confidence interval, 1,847-2,998). Recruitment

appeared sporadic, consisting of consecutive years of low recruitment (<10%) supplemented by a stronger (31%) year-class. Historically, this native fish was rare and was believed extirpated from the lower river by 1975, but it now reproduces naturally in a reach dramatically altered by water development. This successful reintroduction indicates that one native fish can successfully tolerate environmental alterations whereas another, the razorback sucker Xyrauchen texanus, apparently cannot. Other opportunities may exist in altered rivers to benefit native fishes where they were absent or historically rare.

1242: ***+.034***

Bearded vulture populations in the Western Palearctic have experienced a severe decline during the last two centuries that has led to the near extinction of the species in Europe. In this study we analyse the sequence variation at the mitochondrial control region throughout the species range to infer its recent evolutionary history and to evaluate the current genetic status of the species. This study became possible through the extensive use of museum specimens to study populations now extinct. Phylogenetic analysis revealed the existence of two divergent mitochondrial lineages, lineage A occurring mainly in Western European populations and lineage B in African, Eastern European and Central Asian populations. The relative frequencies of haplotypes belonging to each lineage in the different populations show a steep East-West clinal distribution with maximal mixture of the two lineages in the Alps and Greece populations. A genealogical signature for population growth was found for lineage B, but not for lineage A; futhermore the Clade B haplotypes in western populations and clade A haplo-types in eastern populations are recently derived, as revealed by their peripheral location in median-joining haplotype networks. This phylogeographical pattern suggests allopatric differentiation of the two lineages in separate Mediterranean and African or Asian glacial refugia, followed by range expansion from the latter leading to two secondary contact suture zones in Central Europe and North Africa. High levels of among-population differentiation were observed, although these were not correlated with geographical distance. Due to the marked genetic structure, extinction of Central European populations in the last century re-sulted in the loss of a major portion of the genetic diversity of the species. We also found direct evidence for the effect of drift altering the genetic composition of the remnant Pyrenean population after the demographic bottleneck of the last century. Our results argue for the management of the species as a single population, given the apparent ecological exchangeability of extant stocks, and support the ongoing reintroduction of mixed ancestry birds in the Alps and planned reintroductions in Southern Spain.

1243: ***+.013***

Two new species of ricefishes or buntingi, Adrianichthys roseni and Oryzias nebulosus, are described from Lake Poso, Sulawesi Tengah, Indonesia, bringing to 12 the number of endemic ricefishes known from Sulawesi. Adrianichthys roseni and its sole congener, A. kruyti, are distinguished from other relatively large-bodied ricefishes, Xenopoecilus, in having orbits that project beyond the dorsal profile of the head; paired preethmoid cartilages (versus single or absent in Xenopoecilus); and 13-16 (versus 8-13) dorsal fin rays. Also, as in A. kruyti, the lower jaw of A. roseni is enclosed in the upper jaw when the mouth is closed; however, the upper jaw is not as large and broadly expanded as in A. kruyti. Adrianichthys roseni differs further from A. kruyti in having fewer scales in a lateral series (approximately 63-65 versus 70-75), attaining a smaller maximum recorded standard length (90 mm versus 109 mm), and having the lateral process of the pelvic bone in line with the fifth, rather than the eighth, pleural rib. Oryzias nebulosus shares with O. nigrimas, also from Lake Poso, a unique sexual dichromatism in preserved specimens: adult males are dark gray to black, whereas females are a lighter grayish-yellow to brown. Relative to

O. nigrimas, O. nebulosus is a small species (maximum recorded SL 33 mm versus 51 mm), with pelvic fins relatively anterior (lateral process of pelvic bone is in line with the third, fourth, or fifth, modally fourth, rather than the fourth or fifth, modally fifth, pleural rib), dorsal fin relatively anterior (origin above the 12-14th anal fin ray as opposed to the 15-17th anal fin ray), precaudal vertebrae 11-13, modally 12 (versus 13-14, modally 13), and relatively straight, narrow and slender (as opposed to curved, broad, and robust) ossified and cartilaginous portions of the gill arches. Endemic buntingi are threatened by introduction of exotic species, overfishing, and pollution. A conservation plan includes rearing native fishes for local use as ornamental fishes and possible reintroduction.

1244: ***+.072***

Comparative mapping of more than 100 region-specific clones from human chromosome 3 in Bornean and Sumatran orangutans, siamang gibbon, and Old and New World monkeys allowed us to reconstruct ancestral simian and hominoid chromosomes. A single paracentric inversion derives chromosome I of the Old World monkey Presbytis cristata from the simian ancestor. In the New World monkey Callithrix geoffroyi and siamang, the ancestor diverged on multiple chromosomes, through utilizing different breakpoints. One shared and two independent inversions derive Bornean orangutan 2 and human 3, implying that neither Bornean orangutans nor humans have conserved the ancestral chromosome form. The inversions, fissions, and translocations in the five species analyzed involve at least 14 different evolutionary breakpoints along the entire length of human 3; however, particular regions appear to be more susceptible to chromosome reshuffling. The ancestral pericentromeric region has promoted both large-scale and micro-rearrangements. Small segments homologous to human 3q11.2 and 3q21.2 were repositioned intrachromosomally independent of the surrounding markers in the orangutan lineage. Breakage and rearrangement of the human 3p12.3 region were associated with extensive intragenomic duplications at multiple orangutan and gibbon subtelomeric sites. We propose that new chromosomes and genomes arise through large-scale rearrangements of evolutionarily conserved genomic building blocks and additional duplication, amplification, and/or repositioning of inherently unstable smaller DNA segments contained within them. (C) 2003 Elsevier Inc. All rights reserved.

1245: ***-.228***

Between January and April 2000, the cyprinid fishes Zacco platypus and Squalidus chankaensis in Uji River, Kyoto Prefecture showed inactive swimming and hemorrhages on the fins, skin and eyes. No bacterial or viral agents seemed involved in the disease outbreak; however, numerous metacercariae of unidentified trematodes of the family Bucephalidae were found encysted in the fins, skin, musculature and eyes of diseased fish, suggesting these parasitic infections were the etiological agents. The metacercariae comprised two species, designated here as Metacercaria A and Metacercaria B. It is assumed that the infection started with an accidental introduction of infected first intermediate hosts, the freshwater mussel, Limnoperna fortunei, into Uji River, and that Z. platypus and S. chankaensis served as the second intermediate hosts, and the sheatfish Silurus biwaensis as the final host to complete their life cycles. This is the first case of bucephalid infections of freshwater fishes in Japan. The Yodo River system, including Uji River, is the only area in Japan where the bucephalid infections have so far been confirmed. Comments are made on the possible danger of translocation of even a single infected first, second or final host from Uji River to Lake Biwa or other water systems.

1246: ***+.160***

A stochastic population model has been developed for exploring the conservation management of the endangered fish trout cod (Maccullochella macquariensis) in the circumstances of incomplete understanding of the ecology of the species as well as the absence of appropriate data for the estimation of some vital rates. The model includes a stage-structured approach with environmental and demographic variation, and examines three types of density dependence: Beverton-Holt, Ricker and a biomass approach that we have developed to incorporate intraspecific competition beyond recruitment to 1-year olds. The stochastic model was used to explore the current and future status of the protected and last self-sustaining natural population of trout cod, restricted to a 200 km section of the Murray River in south-eastern Australia, under the different density-dependent mechanisms. Current practices for reintroducing trout cod were also evaluated. The analysis indicates that the protected natural population may be stable provided that it remains free from any significant disturbance. However, the analysis also indicates that trout cod may be very sensitive to any reduction in adult survival and remain potentially vulnerable to continued anthropogenic disturbance, in particular fishing. The analysis also indicates that the current practice of releasing fingerlings to establish a reintroduced population was more likely to fail than releasing on-grown 1-year-old fish at reintroduction sites. Furthermore, the traditional density-dependent mechanisms have less support than the applied biomass approach. The stochastic population model developed becomes a resource for guiding the conservation management and further research into the ecology of trout cod. Crown Copyright (C) 2003 Published by Elsevier B.V. All rights reserved.

1247: ***+.109***

This report covers the results of the western pond turtle head-starting and reintroduction project for the period of June 2002-September 2003. Wild hatchling western pond turtles from the Columbia River Gorge were reared at the Woodland Park and Oregon Zoos in 2002 and 2003 as part of the recovery effort for this Washington State endangered species. The objective of the program is to reduce losses to introduced predators like bullfrogs and largemouth bass by raising the hatchlings to a size where they are too large to be eaten by most of these predators. In 2002, 27 females from the two Columbia Gorge populations were equipped with transmitters and monitored until they nested. Four more females carrying old transmitters were also monitored; only one of these transmitters lasted through the nesting season. In 2003, 30 females were monitored. Twenty-three of the females monitored in 2002 nested and produced 84 hatchlings. The hatchlings were collected in fall 2002 and reared in captivity at the Woodland Park and Oregon zoos in the head-start program. Twenty-seven of the turtles monitored in 2003 nested. Six of the turtles nested twice, producing a total of 33 nests. The nests will be checked in September and October 2003 for hatchlings. Of 121 head-started juvenile western pond turtles collected in the Columbia Gorge during the 2001 nesting season, 119 were released at three sites in the Columbia Gorge in 2002, and 2 held over for additional growth. Of 86 turtles reared in the head-start program at the Woodland Park and Oregon Zoos fall 2002 through summer 2003, 67 were released at sites in the Columbia Gorge in summer of 2003, and 15 held over for more growth. Fifty-nine juveniles were released at Pierce National Wildlife Refuge in July 2002, and 51 released there in July 2003. Sixteen of those released in 2002 and 16 released in 2003 were instrumented with radio transmitters and monitored for varying amounts of time for survival and habitat use between the time of release and August 2003, together with juveniles from the 2001 release which were monitored from June 2001 through August 2003, and juveniles from the 2000 release which were monitored from August 2000 through August 2003. The number of functioning transmitters varied due to transmitter failures and detachments, and availability of replacement transmitters, as well as opportunities to recapture turtles. By August 15, 2003, a total of 39 turtles were being monitored: 6 from the 2000 release, 8 from the 2001 release, 10 from the 2002 release, and 15 from the 2003 release. During the 2002 field season trapping effort, 280 turtles were captured in the Columbia

Gorge, including 236 previously head-started turtles. During the 2003 trapping season, 349 turtles were captured in the Columbia Gorge; 304 of these had been head-started. These recaptures, together with confirmed nesting by head-start females and visual re-sightings, indicate the program is succeeding in boosting juvenile recruitment to increase the populations. Records were also collected on 160 individual painted turtles captured in 2002 and 189 painted turtles captured in 2003 during trapping efforts at Pierce NWR, to gather baseline information on this native population. Eight female painted turtles were monitored by telemetry during the 2002 nesting season; 4 nests were recorded for these animals, plus 35 nests located incidentally. Preferred habitat for nesting was identified based on the telemetry results, to be considered in anticipating future turtle habitat needs and in management planning at Pierce NWR. Bonneville Power Administration (BPA) funding supported activities in the Columbia River Gorge from June 2002 through September 2003.

1248: *+.108*

We compared the historic and current geographical ranges of 43 North American carnivores and ungulates to identify large-scale patterns in range contractions and expansions. Seventeen of the species had experienced range contractions over more than 20% of their historic range. In areas of higher human influence, species were more likely to contract and less likely to persist. Species richness had also declined considerably since historic times. The temperate grasslands and temperate broadleaf-mixed forest biomes lost the highest average number of species, while the boreal forest and tundra showed fewer numbers of species lost. Species contractions were a result of Euro-American settlement and postsettlement development in North America. These effects have been widespread and indicate a rapid collapse of species distributions over the course of only 1 to 2 centuries. The results of this study can be used to improve scientists' knowledge of historical reference conditions and to provide input for wildlife reintroductions and for the creation of wildlife reserves.

1249: *-.164*

During summer and autumn of 1994-1997, we determined the movements and mortality of 38 adult male Eastern grey squirrels (Sciurus carolinensis) that had been captured in urban-suburban backyards and translocated to a large forest. The squirrels did not fare well. Squirrels not found dead or classified as 'probable mortality' disappeared from the forest with a median time to disappearance of 11 days. Ninety-seven per cent (37 of 38) of the squirrels either died or disappeared from the release area within 88 days.

1250: *-.061*

As natural populations of endangered species dwindle to precarious levels, remaining members are sometimes brought into captivity, allowed to breed and their offspring returned to the natural habitat. One goal of such repatriation programmes is to retain as much of the genetic variation of the species as possible. A taxon of giant GalApagos tortoises on the island of Espahola has been the subject of a captive breeding-repatriation programme for 33 years. Core breeders, consisting of 12 females and three males, have produced more than 1200 offspring that have been released on Espanola where in situ reproduction has recently been observed. Using microsatellite DNA markers, we have determined the maternity and paternity of 132 repatriated offspring. Contributions of the breeders are highly skewed. This has led to a further loss of genetic variation that is detrimental to the long-term survival of the population. Modifications to the breeding programme could alleviate this problem.

1251: ***+.210***

In order to evaluate the success of a population of red-legged partridges Alectoris rufa reintroduced into a 33.7 km(2) study area, we estimated 15 demographic parameters from 1996 to 1999 by regularly monitoring the population through winter, spring and summer mapping censuses. During the study period, late winter population and spring dispersal decreased, pair number, brood number, brood production rate, chick survival and over winter losses were stable, whereas average brood size, juvenile number, age ratio, adult spring to summer survival, late summer population, and population growth rate from spring to late summer all increased. The population showed a moderate improvement in the overall productivity, mainly related to increased production of young and to a decrease in adult mortality. The improvement in breeding performance took place, in particular, after the second year of study with the conclusion of the releasing session of reared birds.

1252: ***+.211***

This paper explores my shifting understandings of interactions primarily between salmonid fish culture and fish conservation during the latter half of the 20th century. The idea that conspecific natural and cultured fish were largely interchangeable among phenotypically similar populations began to change with the advent of molecular genetic markers. With the gradual clarification of major geographic lineages beginning in the 1970s came awareness that translocations among anadromous lineages were generally destined for failure; in contrast, gene flow more readily occurred among non-anadromous lineages and sometimes, species. Within lineages, data concurrently were accumulating that showed adaptations to their respective environments distinguished cultured and wild populations. Reduced obstacles to gene flow at this level often resulted in homogenizations among wild and cultured fish in areas where widespread hatchery releases occurred; conversely, adaptive radiations in vacant habitats sometimes occurred over a few decades from single source hatchery releases. Current ideas relating to salmonid interbreeding, population substructure and culture evolved from these observations. Among lineages, resistance to gene flow is much greater between anadromous than purely freshwater populations or species. Within lineages, ease of gene flow in both anadromous and freshwater populations is problematical with regard to cultured and wild populations because large-scale supplementation programs erode local adaptations and fine-scale population substructures. At this level, a potential ability to regenerate natural substructure upon relaxation of supplementation is offset by uncertainties of time scales and intrinsic capabilities of homogenized populations. However, management that separates harvest and reproduction of wild and cultured subpopulations can minimize these losses. Some generality of this strategy to other fishes is supported by losses of local adaptations and outbreeding depression in black basses following population admixtures that parallel those observed in salmonids.

1253: ***+.125***

Many Pterodroma species are threatened, and translocations to predator-free islands are desirable for several species. As these birds are highly philopatric, only chicks that have not yet imprinted on their natal colony should be transferred but techniques to identify suitably aged Pterodroma nestlings are needed. We investigated feeding frequency, meal size and chick growth in Pycroft's petrel (P. pycrofti) on Red Mercury Island, New Zealand to provide this information. Mean daily probability of being fed decreased from 0.47 60-23 days before fledging to 0.004 in the last seven days before fledging. Mean meal size was 34 g and morphometric measurements at fledging were similar to mean adult measurements. The best indicator of chick age was wing length.

Transferable Pycroft's petrel chicks should have wings measuring 149 - 184 mm and weigh 218 - 250 g.

1254: *-.015*

The Chequered Skipper butterfly (Carterocephalus palaemon) is extinct in England but extant in Scotland and Continental Europe. The possibility of re-introducing the species is under active consideration by conservation bodies, but ecological differences between Scottish and English populations raise the question of which populations should donate individuals, Continental European, or Scottish? We used mitochondrial DNA sequences (CO I, CO II and Cyt b) from potential donor populations to test the hypothesis that ecological differences could have arisen as a result of differing routes of post-glacial colonisation from separate refugia and subsequent isolation of UK populations. Shared haplotypes between populations in Belgium, Norway, Scotland and England provides no evidence to support the hypothesis that populations in Scotland result from an alternative post-glacial colonisation route. As the genetic evidence remains equivocal we suggest that choice of donor stock for a re-introduction to England should be made primarily on ecological grounds.

1255: *+.151*

Barbary deer, Cervus elaphus barbarus, were reintroduced in the Mhebes reserve, Bizerte governorate (Tunisia). They came from the El Feidja reserve, situated in the Jendouba governorate (Tunisia). A comparison of the antler devellopment of stags of both reserves was made to get an indirect appreciation of the success of this reintroduction, and of habitat effect on the biology of the species. The following criteria, measured on the right-hand antlers collected between 1980 and 2000, were compared: beam, brow tine and tray tine lengths, coronet circumference and beauty. Four classes of probable age were distinguished after the number of tines on the right-hand antler: class 1 (1 tine, 1 year), class 2 (2 to 4 tines, 2 to 4 years), class 3 (5 tines, 5 to 7 years) and class 4 (6 tines, 8 to 10 years). As of class 2, the antler development in Barbary deer was lesser in Mhebes than in El Feidja. These results tended to show that the trophical conditions in the Mhebes reserve were not as good as in the El Feidja one, where salt licks have been distributed. The conclusion is that the management of the Mhebes reserve has become a priority.

1256: *+.038*

We used artificial social stimulation (decoys, vocalization playbacks, and artificial nests) to initiate group displays in six (two females, four males) Caribbean Flamingos Phoenicopterus ruber ruber that had not successfully bred since their introduction to Guana Island, British Virgin Islands, in 1992. During a control period prior to the introduction of stimuli, flamingos exhibited no social displays or nest building activities. All flamingos were observed approaching the decoy area as a flock within four hrs of the decoys being introduced, and Head-Flagging displays were exhibited by two birds within the first 24 hrs. In a 12-hr watch conducted two-weeks post decoy introduction, there were significantly more group display behaviours, as well as nest-building, as compared with the control period and immediately after the introduction (3.6% as compared with 0% and 0.35%). Two individuals performed the majority of group displays (although at least one social display posture was observed for each bird) and three birds exhibited nest-building behaviour. Overall, individuals spent most of their time feeding and resting/sleeping (> 95%) during all observation periods. We show for the first time that decoys and vocalization playbacks could have a positive impact on breeding success in the wild by inducing group displays and nesting behaviours in this group of introduced flamingos. We suggest that social attraction

techniques may be a useful tool to stimulate breeding in small captive and wild small populations of flamingos.

1257: ***+.265***

Since November 1997 the Madagascar Fauna Group has released 13 captive-bred black and white ruffed lemurs (Varecia variegata variegata) into the Betampona Reserve in eastern Madagascar. The release programme has three major aims: (1) to assess the ability of captive-bred V. v. variegata to adapt to life in their natural habitat; (2) to investigate the contribution that such a release programme can make to reinforcing the existing small wild population of V. v. variegata in Betampona; and (3) to contribute to the long-term protection and conservation of the reserve. Criteria for the selection of release candidates, veterinary screening and pre-release experience in naturalistic environments are described and discussed. Methods for post-release monitoring of health and behaviour are covered in detail. The importance of considering the social dynamics of the species involved is emphasised. The survival of five of the releasees, plus successful reproduction and integration with the wild population have led to the conclusion that the release was a success.

1258: ***+.006***

In South Africa efforts are currently being made to manage several sub-populations of African wild dogs (Lycaon pictus) occurring in isolated, fenced reserves, as a meta-population. This study represents an attempt to estimate the minimum reserve size for the reintroduction of a pack of wild dogs, as a sub-population. Minimum area requirements were based on the area required to support an adequate population of the most important prey species in the diet of a pack of wild dogs. A pack size of five is the threshold below which reproductive failure is likely, and the area requirements of five wild dogs are estimated to be 65 km(2) in northern, 72 km(2) in eastern and 147 km(2) in northeastern South Africa. The presence of perimeter fencing at release sites is a potentially complicating factor, however, as in some cases wild dogs learn to use fences as a hunting tool, permitting the capture of larger prey than is normal. In the event of this happening, larger areas may be required to prevent local population declines in preferred prey species. In general, the use of larger areas is advisable to allow for variation in prey population sizes and the prey profile of wild dogs post-release, and would also be necessary if wild dogs are to be reintroduced into an area with existing populations of lions and spotted hyaenas.

1259: ***+.123***

Understanding human attitudes often is vital to the success of restoration projects, especially those invoking controversial species such as carnivores. Support for restoration activities may differ depending oil residence location of the respondent; thus, understanding the spatial distribution of attitudes is important when selecting suitable restoration sites. We used black bear Ursus americanus) restoration as all example Of how to calculate and use a spatially explicit model of human attitudes based oil demographic variables to select restoration sites in Mississippi, USA. We sampled 2 populations (5,975 landowners adjacent to 10 Public land areas and a random sample of 490 Mississippians) to determine human attitudes toward black bear reintroduction in Mississippi. We chose a series of-reintroduction and demographic questions to develop 2 logistic regression models to predict support for reintroduction, and we validated the models. One model was developed oil all demographic variables (how many acres respondents owned in Mississippi and for how long, its well as their age, sex, education, income, community size, and race) to determine the relationship between support for reintroduction and demographic variables. The

second model used only, age, race, and sex demographic variables. Age, community size, race, sex, and number of years of landownership were significant predictors of support for hear restoration for the first model and age, race, and sex were significant predictors for the second model. Using the second model and the U.S. Census Bureau (1990) block group data, we predicted the proportion of each block group supporting restoration. Most of Mississippi halt block groups with >50% support for bear restoration. We were able to determine a statewide distribution of attitudes. Additionally, we documented that attitudes of landowners immediately, adjacent to public land may differ front those of residents of the area surrounding the public land (both adjacent and not). We suggest Using our approach for other restoration projects to better integrate human attitudes into the restoration program.

1260: *-.107*

1. Hen harriers Circus cyaneus prey on red grouse Lagopus l. scoticus and high breeding densities of harriers can limit the number of grouse available for shooting in the autumn. Ultimately, grouse hunting contributes to the maintenance of heather moorland, an ecologically important habitat for biodiversity in general and hen harriers in particular. Predation rates vary widely among harrier individuals. Understanding which factors influence this variation would be useful to target management to mitigate the effect of harriers on grouse, such as diversionary feeding.2. We used a simple habitat-based approach to test whether we could identify harrier nests to which most grouse were delivered. Using remote sensing habitat data, we tested whether delivery rates of dead grouse to the nest by hen harriers were higher for those pairs nesting in sites with more heather Calluna vulgaris. A relationship between heather cover and grouse delivery rates might have been expected as grouse densities were correlated with heather cover.3. After adjusting for annual variation in grouse abundance, the rate at which grouse were delivered to harrier nests was positively associated with the proportion of heather cover within 2 km of harrier nests. This was primarily due to the positive effect of heather cover on female delivery rates.4. This result allowed us to use habitat data to predict the harrier nests to which most grouse chicks would be delivered. Comparison of predictions of the model with observations of food delivery to nests indicated that, in terms of grouse chick delivery, the model correctly predicted the top 50% of harrier nests in five of six years.5. We undertook an experiment where carrion was provided to certain harriers at nest sites, in order to decrease their predation on grouse. Data from this experiment showed that when harriers were given diversionary food, the relationship between grouse predation rate and habitat was removed, with grouse predation reduced to negligible levels in most cases. This demonstrated the increased benefit of feeding birds with the highest proportion of heather cover within 2 km of their nest sites, rather than feeding birds at random within the conflict population.6. Synthesis and applications. The amount of heather cover around hen harrier nests can be used to predict which pairs will predate most grouse within a population. This information should facilitate targeted management practices, which may accrue greater benefit for grouse stocks and potentially reduce the conflict between grouse shooting and conservation of biodiversity.

1261: *+.242*

1. Few examples of habitat-modelling studies of rare and endangered species exist in the literature, although from a conservation perspective predicting their distribution would prove particularly useful. Paucity of data and lack of valid absences are the probable reasons for this shortcoming. Analytic solutions to accommodate the lack of absence include the ecological niche factor analysis (ENFA) and the use of generalized linear models (GLM) with simulated pseudo-absences.2. In this study we tested a new approach to generating pseudo-absences, based on a preliminary ENFA habitat suitability (HS) map, for the endangered species Eryngium alpinum. This method of

generating pseudo-absences was compared with two others: (i) use of a GLM with pseudo-absences generated totally at random, and (ii) use of an ENFA only.3. The influence of two different spatial resolutions (i.e. grain) was also assessed for tackling the dilemma of quality (grain) vs. quantity (number of occurrences). Each combination of the three above-mentioned methods with the two grains generated a distinct HS map.4. Four evaluation measures were used for comparing these HS maps: total deviance explained, best kappa, Gini coefficient and minimal predicted area (MPA). The last is a new evaluation criterion proposed in this study.5. Results showed that (i) GLM models using ENFA-weighted pseudo-absence provide better results, except for the MPA value, and that (ii) quality (spatial resolution and locational accuracy) of the data appears to be more important than quantity (number of occurrences). Furthermore, the proposed MPA value is suggested as a useful measure of model evaluation when used to complement classical statistical measures.6. Synthesis and applications. We suggest that the use of ENFA-weighted pseudo-absence is a possible way to enhance the quality of GLM-based potential distribution maps and that data quality (i.e. spatial resolution) prevails over quantity (i.e. number of data). Increased accuracy of potential distribution maps could help to define better suitable areas for species protection and reintroduction.

1262: *+.096*

Information on population size and distribution of the Komodo monitor (Varanus komodoensis) has so far been restricted to early reports or to surveys conducted on only part of the species' range. We carried out a study based on transects through sampling plots and single-catch trapping events to assess the extent to which the distribution of V. komodoensis has changed since the last comprehensive survey was conducted, in 1971. We also report on the status of the habitat and identify conservation priorities. Resident Komodo monitor populations are now found on only four islands in Komodo National Park and on the island of Flores in south-east Indonesia. Average population density estimates recorded on Flores were more than 60% lower than those reported for Komodo National Park. Habitat fragmentation and poaching of prey species currently represent the main threats to the Komodo monitor, and protection of monsoon forest in west and north Flores is crucial for the long-term conservation of the species.

1263: *+.252*

Queen conch (Strombus gigas) stocks in the Florida Keys once supported commercial and recreational fisheries, but overharvesting has decimated this once abundant snail. Despite a ban on harvesting this species since 1985, the local conch population has not recovered. In addition, previous work has reported that conch located in nearshore Keys waters are incapable of spawning because of poor gonadal condition, although reproduction does occur offshore. Queen conch in other areas undergo ontogenetic migrations from shallow, nearshore sites to offshore habitats, but conch in the Florida Keys are prevented from doing so by Hawk Channel. The present study was initiated to determine the potential of translocating nonspawning nearshore conch to offshore sites in order to augment the spawning stock. We translocated adult conch from two nearshore sites to two offshore sites. Histological examinations at the initiation of this study confirmed that nearshore conch were incapable of reproduction, whereas offshore conch had normal gonads and thus were able to reproduce. The gonads of nearshore females were in worse condition than those of nearshore males. However, the gonadal condition of the translocated nearshore conch improved, and these animals began spawning after three months offshore. This finding suggests that some component of the nearshore environment (e.g., pollutants, temperature extremes, poor food or habitat quality) disrupts reproduction in conch, but that removal of nearshore animals to suitable offshore habitat can restore reproductive viability. These results indicate that translocations are

preferable to releasing hatchery-reared juveniles because they are more cost-effective, result in a more rapid increase in reproductive output, and maintain the genetic integrity of the wild stock. Therefore, translocating nearshore conch to offshore spawning aggregations may be the key to expediting the recovery of queen conch stocks in the Florida Keys.

1264: *+.009*

During the twentieth century, the North Island kokako population in Taranaki, New Zealand, was reduced almost to extinction. Following initial habitat loss, predation by introduced brush-tailed possums and ship rats was identified as the main cause of decline. These predators can now be controlled to levels that will allow kokako populations to survive. This makes the translocation of kokako to Taranaki a possibility. This study examines the ecological requirements of kokako, predator control, translocation techniques, and evaluates the suitability of four Taranaki sites for translocations. The costs of predator control and benefits to other species are also considered. All four sites investigated proved ecologically suitable, but varied in terms of costs, practicality, and the benefits to other species. The two sites still holding remnant kokako-Waitaanga and Moki-Makino-are the most difficult sites to access and would be the most expensive to establish and operate. would be the cheapest site in which to establish predator control. Egmont is the second cheapest option and there are synergies with blue duck and North Island brown kiwi programmes. Waitaanga, Moki-Makino and Egmont are all large forest blocks (>10 000 ha) and predator control would be carried out in core areas (c. 4000 ha) with the risk that birds might disperse through the remainder of the forest. Whitecliffs is a more discreet block, and predator control could be carried out across its entire area.

1265: *+.198*

Brachycome muelleri Sonder (Corunna daisy) is an endangered annual herb, endemic to the upper Eyre Peninsula of South Australia. It is restricted to a single population occupying an area of approximately 3 ha on steep, south-facing cliff-foot slopes of the Baxter Hills. Its unique habitat, within an otherwise generally and region, optimises soil moisture retention through the provision of concentrated water catchment, shade, and low evaporation rates during the growing season. Weeds had the potential to significantly disrupt recruitment, growth and reproductive output, but grazing did not constitute a direct threat to the population. Fresh seeds were innately dormant, requiring a period of after-ripening before they would germinate. Dormancy was artificially broken by gibberellic acid (GA(3)) treatment. Germination was optimal at 20degreesC, and it was proposed that temperature plays an important role in regulating germination in situ. B. muelleri seeds did not respond to smoked water treatment, and GA(3) (1000 mg l(-1)) was recommended as a routine seed treatment to stimulate germination. Trial translocations using seed as founder propagules resulted in low establishment, growth and flowering rates. Established seedlings provided several advantages over seed as founder propagules, including higher success rates, more vigorous growth, and improved seed yields in the first season. Successful regeneration, proliferation and expansion of a new translocated population was observed and recorded over four consecutive years. We suggest that conservation management of this species should include the establishment of several new populations by translocation to suitable, isolated, weed-free sites. (C) 2003 Elsevier Ltd. All rights reserved.

1266: *+.092*

Thirty-two kokako (Callaeas cinerea wilsoni) were translocated to Kapiti I. between 1991 and 1997 to establish a viable 'insurance' population since the species is threatened on the New

Zealand mainland. Kokako were translocated from remnant mainland populations at Little Barrier I., Mapara Wildlife Reserve, and Mount Bruce National Wildlife Centre (a captive rearing facility). Population growth may at various times have been limited by an unrecognised male sex bias, by accidental poisoning, and by annual variation in food availability and mate choice behaviour of translocated kokako. The number of pairs (14) and presence of at least seven young birds in April 2003, balanced sex ratio, and the adequate amount of suitable habitat (at least 200 ha) indicate that the established kokako population on Kapiti I. appears likely to be self-sustaining. Monitoring provided valuable information about the lack of breeding females, to which managers responded by translocating young female kokako to Kapiti I. Lessons learnt from monitoring kokako on Kapiti I. are relevant to other translocations of kokako and perhaps other species.

1267: *+.071*

The last wild California Condor (Gymnogyps californianus) was brought into captivity in 1987. Captive breeding was successful and reintroduction efforts began in 1992. The current population is descended from 14 individuals belonging to three genetic "clans." This population bottleneck led to the loss of genetic variation and changes in allele frequencies, including a probable increase in the frequency of the putative allele for chondrodystrophy, a lethal form of dwarfism. We use studbook data to analyze the current genetic and demographic status of the population and explain how it is managed to meet specific goals. In August 2002 the population consisted of 206 individuals distributed among three captive-breeding facilities and three reintroduction sites. The population is managed to preserve genetic diversity using the concept of mean kinship. Growth of the total population has been between 10% and 15% per year since 1987, but the growth of the captive population has been only about 5% per year since 1992 due to the removal of chicks for reintroduction. Assuming that founding birds within clans were half-siblings, the birds used to found the captive population theoretically contained 92% of the heterozygosity present in the hypothetical wild base population. About 99.5% of this heterozygosity has been retained in the current population. Alleles from most founders are well represented across captive-breeding facilities and reintroduction sites. The genetic status of this population compares favorably with other species that have been rescued from extinction by captive breeding.

1268: *-.013*

Angiopteris chauliodonta, endemic to remote Pitcairn Island, was until recently thought to occur in only two small populations. Survey work carried out on the island in 1997 increased the number of populations to six, but as the total number of plants found was 774 (of which only 147 were mature adults) the species should be regarded as critically endangered. The species occurred in native fern-rich Homalium taypau and Metrosideros collina forest that was in many areas heavily invaded by Syzygium jambos and Lantana camara. RAPD analysis identified related populations but there was no correlation between genetic and geographical distance. The highest levels of genetic diversity was partitioned within populations (H-S = 0.154; DST 0.116), although the larger populations were not necessarily the most diverse. Threats are primarily due to forest clearance, invasive species and erosion. Conservation management for this species will be through reinforcement of existing populations to maximise their genetic diversity and translocation of new populations to suitable habitats. (C) 2003 Elsevier Ltd. All rights reserved.

1269: *+.148*

We modeled populations of lynx (Lynx canadensis) and snowshoe hares (Lepus americanus) to determine prey densities required for persistence of lynx translocated to the southern portion of the

species' range. The models suggested that a density of 1.1-1.8 hares/h is required for lynx persistence; these densities are higher than those reported for most hare populations across the USA. We found that lynx dispersal and density-independent mortality substantially increased the hare density required for lynx persistence. Reintroduction success was associated with number of release events, total number of animals released, and timing of release relative to the phase of the hare population cycle. However, no release protocol could override the negative effects of low prey density or high population losses. We conclude that successful lynx reintroduction requires high hare densities and minimal anthropogenic disturbance; few areas in the contiguous USA currently posses such qualities. (C) 2003 Elsevier Ltd. All rights reserved.

1270: *+.004*

The largest and most fecund population of the endangered Purshia subintegra is restricted to limestone mesas in Verde Valley, Arizona. USA, where habitat destruction is imminent. To examine factors limiting its distribution and potential for expansion, we compared recruitment and survival of seedlings growing in soils from occupied and unoccupied habitat in caged field experiments and compared survival of caged and wild seedling cohorts from 1998 to 2003. In field tests, seeds germinated in soils from occupied and unoccupied habitats. Seedling survival, however, was greatest in currently occupied habitat and dropped to zero in some unoccupied habitats with the onset of severe drought. Among 16 factors measured, soil moisture significantly explained between 62% and 71% of the variation in recruitment in both wild and caged plots. Shrubs conferred protection to wild seedlings, but decreased caged seedling survival. For 5 yr following germination, caged seedlings had greater survival than natural seedling cohorts indicating that reintroduction was comparatively more successful than natural recruitment. Expansion of P. subintegra into novel habitats is limited by soil moisture capacity, and this condition varied during the experimental time frame. Reintroductions to limestone mesas are possible and most promising if cages and supplemental watering are used.

1271: *+.091*

European otters declined dramatically from the 1950s, disappearing from many rivers. We report here on long-term monitoring (from 1977) in 3 catchments in western Britain that were recolonized naturally and in 2 catchments in eastern England that were reinforced by captive-bred otters. A minimum of 16-years data was collected on each river until 2002. At a series of sites in each study river, the percentages which were positive for otters and the number of spraints per sprainting Site were recorded and combined to produce an annual index of population. One western river, naturally recolonized, showed rapid early population growth for 5 years, followed by slower growth, while growth was steadier in 2 catchments which already held some otters at the beginning of the study. Colonization on the eastern rivers was slower, with greater fluctuations over time. Annual population growth rates were estimated at 1-7%, higher in the earlier years. A strategy for annual monitoring of otters is recommended.

1272: *+.101*

We assess the fate of 100 Leiopelma pakeka transferred in two batches from remnant forest on Maud Island to a new site at Boat Bay, 0.5 km away, in 1984-85. Seventy of the original 100 individual frogs were recaptured, plus 35 young recruits into the population. The 43 frogs released in 1984 settled closer to the release site than did the 57 released a year later, suggesting that many of the later arrivals avoided sites already occupied by frogs. Boat Bay frogs became heavier than frogs in the source population, presumably a reflection of lower population density and greater per

capita food supply. Numbers declined initially, but the frog population remained relatively stable after losses of founder individuals began to be offset by local recruitment. The mean annual survival rate after initial settlement was high (97%), indicating an average life expectancy of 33 years.

1273: ***+.131***

Gortyna borelii lunata is a rare species of moth with a widespread, but localised distribution in Europe. In Britain, the moth is restricted to coastal grassland habitats that support its larval foodplant (Peucedanum officinale), in the southeast of England. Threats to G. borelii lunata in Britain include inappropriate management and sea-level rise. This study investigates how to secure the future of the moth in Britain by managing existing colonies appropriately and determining whether translocations of the species, away from the dangers of flooding, would be feasible. A mowing experiment and a translocation trial were conducted. Results from the mowing experiment indicate that cutting annually in either August or November is detrimental to the abundance of the moth. It is recommended that where sites must be mown this be performed on rotation, leaving much of the site uncut each year. The translocation of the moth and its foodplant to a site away from the threats of flooding was very successful, demonstrating that the species establishes well and can survive on higher ground. It is concluded that to secure the future of the moth in Britain, a sustainable network of interconnected sites for the species should be created. Also, an appropriate management plan should be formulated for each site.

1274: ***+.100***

There is a scarcity of data on the seedling establishment success of plants inhabiting the sand coasts of the Mediterranean, despite on increasing interest in the conservation and restoration of dune habitats. A field experiment, in conjunction with a series of laboratory germination tests, was conducted to (1) determine the recruitment capacity of Pancratium maritimum, a perennial dune plant which is declining along the north-western Italian coasts, by natural seed dispersal and (2) test the ability of seeds to tolerate different environmental stress. We planted seeds at different burial depths and found that seeds from greater depths (6, 10 and 15 cm) showed higher emergence (35.0-46.6%) than seeds from a shallower depth (2 cm; 11.6%). On average, less than 18% of seeds remained dormant at the end of the germination period. None of the seedlings that emerged in autumn survived over winter, and 88% of those that emerged in spring died by the start of the second reproductive season as a result of desiccation. Therefore, less than 3% of the seeds resulted in established seedlings. Under laboratory conditions seeds germinated well (87.5-100%) in darkness as well as at 12 h alternating light/dark and showed little dormancy (27-35 days). Germinability of seeds remained up to 70% for at last one year of storage. Temperatures below 10degreesC or over 30degreesC inhibited germination; frost and heat were lethal for most seeds. Germination was totally inhibited by salinity and water stress. Recovery of germination occurred in seeds from solutions below 25% seawater or -0.6 MPa. These data provide useful information for conservation and reintroduction of this species.

1275: ***+.255***

We collected genetic and behavioural data on hihi (Notiomysts cincta, an endangered New Zealand bird) after reintroduction to Mokoia Island to assess the effect of extrapair copulation on effective population size (Ne), and investigate the potential for increasing Ne through behavioural management. DNA fingerprinting revealed that 46% of chicks (n = 188) resulted from extra-pair paternity, and 82% of broods (n = 56) had at least one extra-pair chick. Of the extra-pair young,

34% (n = 89) were from unpaired males, and the remainder were from paired males. Variance in reproductive success (VRS) among individuals changed between years, and the relative variance among males and females depended on the sex ratio. VRS increased when measured over longer time scales, the variance in recruits being three times higher than the variance in the number of hatchlings. Extra-pair copulation increased VRS by 150% in 1 year and decreased it by 30% in another year, but this only caused a 4% decrease and 8% increase, respectively, to N-e/N. Although there is potential to manage VRS in this species through behavioural management, a more important factor is adult lifespan, which is the main correlate of lifetime reproductive success as well as the determinant of generation time. The high annual mortality rate in Mokoia hihi (females = 64%, males = 52%) has prevented the population from growing, so the key factors limiting N and N-e/N are the same.

1276: *+.119*

Captive populations can exhibit more behavioral variation than their wild counterparts as a result of relaxed selective pressures in the captive environment. This increased variation can translate into decreased survivorship upon reintroduction to native habitats. Data show that captive populations of oldfield mice (Peromyscus polionotus subgriseus) exhibit such an increase in variation. Motivated by these results, we developed a series of calculations for a "release ratio" that can be used to determine the number of captive-bred animals needed to compensate for the increased variance. We present calculations of release ratios for behavioral and morphological variables with different distributions and illustrate the functional relationship between release numbers, increased variation, and change in average behavior and morphology. Our calculations indicated that the release of 130-150 captive-bred oldfield mice is equivalent to the release of 100 wildlike animals. Release ratios will vary among species, however, and perhaps among different populations of the same species and should be calculated separately for each situation. Development of the release ratio is the first rigorous effort to incorporate behavioral and morphological changes due to captivity into reintroduction planning. Release ratios will help conservation biologists ensure that the appropriate number of animals is released, thus increasing the success of reintroduction programs.

1277: *+.168*

In Europe at the beginning of 20th century, there were only four isolated areas where an estimated stock of about 2,000 beavers, Castor sp., lived. Many projects of beaver reintroduction and protection enabled the population to increase in size to grow up to approx. 400, 000 or more individuals today. Further planned and partially achieved reintroduction projects (in Scotland, Great Britain, Bulgaria, and Italy), which were carried out without any regard for the formerly autochthonous distribution of the beavers, and were neither following the IUCN (The World Conservation Union) guidelines for the reintroduction of animals, contributed to a mixed beaver population and therefore to a mixed genetic potential. There are no exact data on the survival of Castor fiber in mixed populations. The genetic pool of some original subspecies of Castor fiber is therefore in danger. Legal and illegal releases of North American beaver, Castor canadensis, took place in some European countries. The protection status of the beavers varies according to the country from total protection (endangered species status) to seasonal protection or protection in selected areas. This is why, in the states with legal beaver hunting, the rules and laws may vary and an international agreement is necessary. There are conflicts of interests between protectionists and "regulators" in central and western Europe where the countryside is intensively used and mostly cultivated. Renaturation of water systems and flowing-stream networks in river basins would be an important demonstration for an optimal share in ecological revalorization which,

supra-regionally, will be most important. The beaver as a symbolic animal of progressive nature protection is a subject for discussion. We need a new management and control-system for beavers in the future.

1278: ***-.069***

The presence of top predators can affect prey behaviour, morphology and life history, and thereby can produce indirect population consequences greater and further reaching than direct depredation would have alone. Raptor species in the Americas are recovering since restrictions on the use of dichlorodiphenyl-trichloroethane (DDT) and the implementation of conservation measures, in effect constituting a hemisphere-wide predator-reintroduction experiment, and profound effects on populations of their prey are to be expected. Here, we document changes in the behaviour of western sandpipers (Calidris mauri) at migratory stopover sites over two decades. Since 1985, migratory body mass and stopover durations of western sandpipers have fallen steadily at some stopovers in the Strait of Georgia, British Columbia. Comparisons between years, sites and seasons strongly implicate increasing danger from the recovery of peregrine falcons (Falco peregrinus) as a causal factor. A decade-long ongoing steep decline in sandpiper numbers censused on our study site is explained entirely by the shortening stopover duration, rather than fewer individuals using the site. Such behavioural changes are probably general among migratory shorebird species, and may be contributing to the widespread census declines reported in North America.

1279: ***+.080***

The Javan gibbon is one of the rarest species of gibbons, restricted as it is to the western half of the densely populated island of Java, Indonesia. Based on a study from 1994-2002 it was found that the Javan gibbon has a larger distribution range than previously assumed. It is not restricted to the forests of the province of West Java and significant populations occur in the central part of the island. To establish the presence of gibbons in an area focused research is needed and in the past certain populations were missed in rapid presence-absence surveys. Javan gibbons occur at population densities of c. 2.6 groups km(-2) (8-9 individuals km(-2)) in lowland and hill forest <1000 in asl and less than one group km(-2) (1.5 individuals km(-2)) in montane forest between 1000-1750 in asl. Based on the extent of remaining habitat in 15 of the largest populations of Javan gibbons, a conservative density estimate of one group km(-2), and exclusion of floaters (sub-adults that have not yet established a territory), it is estimated that some 4000-4500 Javan gibbons remain in the wild. This conservative estimate is considerable higher than assumed by conservation authorities. Given that large-scale deforestation on Java (the main threat to the survival of the species) dates back more than a century and has slowed down over the last decades, this suggests that the present IUCN status of Critically Endangered seems untenable. Following IUCN guidelines the species should therefore be down-listed to Endangered. As still considerable populations remain in unprotected areas of natural forest it is argued that, in order to effectively protect the species and in an attempt to increase its survival prospects, increased protection of these forest areas is the key to the survival of the species. It is recommended not to resort to expensive and intrusive captive-breeding programmes and reintroduction initiatives as this will inevitably divert the attention away from in-situ conservation. Any programme that costs a great deal of money over the years will inevitably seek to portray itself as necessary and relevant, and if caution is needed it is in the evaluation of current management options, based on the best possible information irrespective of previous investments.

1280: ***+.004***

The quali-quantitative inventoried data of apprehended species made by environmental authorities of Rio Grande do Sul State (RS) are presented in this work. We have assessed the wildlife avian apprehension protocols. One of those protocols have been registered by Batalhao de Policia Ambiental (BPA) from Brigada Militar do Estado do Rio Grande do Sul in the 1999-2000 term at Porto Alegre/RS metropolitan area. The other one has been registered by Instituto Brasileiro do Meio Ambiente e dos Recursos Naturais Renovaveis (IBAMA) in the 1998 to June 2000 term, in relation to RS. As result of birds apprehension analysis made by IBAMA and BPA, they had been accounted the total amount, from 1998 to 2000, of 3797 specimens distributed in 26 families, 66 genus and 93 species. The most apprehended species was Paroaria coronata (red-crested cardinal) with 1088 individuals representing 28.7% of the total. The most apprehended family was Emberezidae, in a total of 2756 specimens, representing 72.6% of the total. We can infer that the bird's translocation is sufficiently considerable, especially because a parcel of the trafficked species is not reached by the authorities and gets at its destination. From the apprehended parcel, at least 93 distinct species are part of the roll of birds that is attractive to the illegal commerce. Amongst these, the singing birds and the parrots are distinguished.

1281: ***+.039***

A multidisciplinary research focused on characterizing the genetic diversity and reintroduction of the micropropagated plants in a rare and endangered taxon; Syzygium travancoricicm Gamble was undertaken. Random Amplified Polymorphic DNAs (RAPDs) and Single Primer Amplification Reactions (SPARs) genetic markers revealed a high percentage of polymorphism (>81%) and similar mean genetic distances (0.39 to 0.28) among the selected populations. Clustering based on UPGMA and SHAN indices revealed minimum genetic differentiation between the populations. Micropropagated plants derived through axillary bud proliferation were hardened in the forest nurseries and reintroduced in the wild with the help of the forest department and the local communities with limited success. This study emphasizes that maintaining current levels of genetic diversity substituted with in vitro approaches for rapid multiplication, and involvement of local communities are effective means for conservation and reintroduction of many endangered species.

1282: ***+.208***

Extinction of tree snails of the genus Partula on Moorea, following introduction of the predatory snail Euglandina rosea, has challenged conservation biology during years of successive captive breeding of small rescued populations. An experimental release of three Partula species into a predator-proof patch of native forest on Moorea was designed to test: effectiveness of physical and chemical methods of predator exclusion and to evaluate behavior of animals bred for up to six generations in highly artificial environments. At the close of the experimental release, there had been multiple incursions of E. rosea, and too few Partula spp. remained to assess effects of captive breeding on ecological responses. However, results demonstrated the effectiveness of the exclosure under ideal maintenance and monitoring. Captive breeding methods were validated by reproduction and growth to sexual maturity in the wild as well as retention of genetic variability in the form of persistent color polymorphism in one species.

1283: ***+.070***

Introduced mammals are a major driver of extinction and ecosystem change, particularly on islands. Feral goats Capra hircus have been introduced to numerous islands worldwide and have had wholesale impacts on ecosystems. Techniques are now available, however, to eradicate goat

populations from islands, providing a powerful conservation tool. Goats were removed from Pinta Island, Galapagos, Ecuador after a 30-year eradication campaign, the largest removal of an insular goat population using ground-based methods. Over 41,000 goats were removed during the initial hunting effort (1971-82). In the following decade the island was twice wrongly declared free of goats. During this period, the island was visited irregularly but no monitoring programme was implemented. A revised campaign over 1999-2003, which included improved hunting techniques and monitoring, removed the final goats from the island. The use of Judas goats was critical in locating the remaining goats and as a tool to confirm eradication. A systematic monitoring programme is critical for confirming eradication and preventing future reintroductions. An earlier monitoring programme would probably have resulted in earlier eradication and significant financial savings. Given limited resources, island conservation programmes elsewhere should strive to increase eradication efficiency and learn from past campaigns.

1284: *+.156*

From 1998 to 2000 we used radio telemetry to study habitat use by greater rheas Rhea americana in rural areas. Captive-born greater rheas preferred pastures to grasslands and did not use crops. No differences in habitat use were found between wild and captive-born greater rheas. Wild individuals preferred pastures, showed less preference for grasslands, and did not use crops. Rheas used pastures and grasslands for nesting but they did not use crops. No differences in nesting success was detected between these habitats. Our results show that agroecosystems that include grasslands and pasture production would strongly contribute to the conservation of this species. However, poaching must be controlled to ensure long-term persistence of wild populations of greater rheas.

1285: *+.106*

The conservation status of 23 Hawaiian endemic palms, Pritchardia spp., is reviewed. Field survey reports, recovery plans, herbaria holdings and observations have been utilized to assess each taxon's current range and status. Eleven species are categorized as Critically Endangered, nine Endangered, two Vulnerable and one as Data Deficient when subject to the IUCN Red List criteria. Conservation management options are discussed. A large proportion of this genus is on the verge of extinction and will continue to decline in the wild without active conservation management. Recommendations involving long-term management include maintaining and protecting the existing wild populations, establishment of effective ex situ populations, reintroduction into the wild, and the establishment of procedures to deal with invasive plants and animals.

1286: *+.035*

Interspecific competition is one of several constraints that might prevent an individual from maximising its energy intake. When an interspecific competitor is introduced, an individual is often forced to shift its diet according to the intensity of the competitive pressure. In this paper, we explore whether the introduced American mink (Mustela vison Schreber) shifts its diet when the density of its potential competitor, the Eurasian otter (Lutra lutra L.), is increased. We compared the diets of otter and mink at the same location but at two moments in time when the relative densities of these two species were different while controlling for the abundance of aquatic prey. Mink and otters are semi-aquatic mammals belonging to the same guild of mustelids and otters are expected to be the dominant competitor because they are larger and better at hunting underwater. The diets of otters and mink overlap to a great extent but while otters specialise mainly on aquatic

prey, mink are able to exploit both aquatic and terrestrial prey. These observations prompted the hypothesis investigated in this work that at higher otter densities the diet of mink should change to include a higher proportion of terrestrial items. This hypothesis was supported by the data and at higher otter densities mink diet was observed to consist of a higher proportion of mammals and birds while fewer fish were present, although this pattern was present only in winter while no changes were observed in spring. Meanwhile the diet of otters remained basically unchanged. In the second part of the study, we investigated whether niche breadth and niche overlap between otter and mink changed at different otter densities. We found that niche overlap declined as the density of otters increased, in agreement with the prediction of habitat selection theory.

1287: *-.065*

Competition is considered to be a major evolutionary driving force within assemblages of related and morphologically similar species, but it is notoriously difficult to test and quantify especially amongst species that cannot be easily manipulated in a laboratory or in the field. By exploiting a re-introduction of Eurasian otters (Lutra lutra) in the Upper Thames catchment (UK) in 1999 we performed an experiment to test whether there was evidence of competition between otters and American mink (Mustela vison). Mink and otters are semi-aquatic mustelids belonging to the same guild. Otters are expected to be the dominant competitor because they are larger and better adapted at exploiting aquatic resources. Our hypothesis was that mink declines when the density of otters increases. We measured the effect of competition at the population level, by observing whether mink distribution and densities changed in an area of 1353 km(2) in association with the arrival of its putative competitor. We estimated distribution and densities by means of sign surveys and trapping. The results showed that otters were associated with a significant and rapid reduction in the densities of mink, while mink occupancy remained approximately the same in an area of 2464 km(2) that was used as a control. We observed that the spatial distribution of the mink population throughout its decline was influenced by the yearly cycle of mink activities, with areas being temporarily re-colonized during the dispersal season. Mink is an invasive species in the UK threatening the survival of some native species. Our findings suggest that the re-establishment of otter populations is likely to lead to a decline of mink that may, in turn, be beneficial to native species threatened by this invasive.

1288: *+.003*

1. Literature on the wolf Canis lupus, brown bear Ursus arctos and lynx Lynx lynx is reviewed to determine if sufficient semi-natural habitat exists in the UK for a viable population of any of these species and to assess the potential risks to human safety, livestock and economically valuable wildlife. Public attitudes to the recovery and reintroduction of some other mammals are also briefly reviewed.2. The large home range sizes and low population densities of large carnivores mean that the Scottish Highlands is the only UK region with the potential to support a viable population. Human population density is also lower in the Highlands and the density of wild ungulate prey higher than in many parts of Europe where large carnivores survive.3. Attacks on people have been recorded in Europe for healthy bears and for rabid bears and wolves but there are no reports of attacks by lynx. Bears are more carnivorous in the north of their range than in the south and although wild mammals seldom appear to be important prey serious predation of livestock can occur. Livestock predation is also reported for the wolf and the lynx but they appear to prefer wild prey if available. However, mass kills of up to 100 or more sheep are occasionally recorded for wolves.4. Attitudes to reintroductions and carnivores generally tend to be favourable amongst the general public, but negative amongst those most likely to be adversely affected. Fears for human safety and significant livestock predation with bears and wolves, respectively, suggest

that reintroduction of these species is unlikely to be acceptable in the foreseeable future. Reintroduction of the lynx may be feasible but habitat suitability and potential impact on vulnerable native wildlife need to be assessed. Socio-economic and legal issues also need to be addressed before such a reintroduction is considered.

1289: *+.174*

South Island Saddlebacks Philesturnus carunculatus carunculatus were once found throughout the South Island of New Zealand, but by the early 1960s were confined to the island of Big South Cape, in the extreme south of the country. All subsequent reintroduced populations of South Island Saddlebacks are derived from 36 surviving birds from this relict population. The aim of this study was to compare the breeding success of three recently reintroduced populations of Saddlebacks relative to their distance from, and habitat similarity to, the relict population. The three study islands show a latitudinal cline with Ulva, Breaksea and Motuara Islands located 60, 190 and 810 km north of Big South Cape, respectively. Saddlebacks on Ulva and Breaksea appeared to prefer to establish breeding territories in coastal scrub, the dominant habitat feature of Big South Cape. The area of coastal scrub habitat was much smaller on Motuara, where breeding territories were instead scattered through broadleaf forest habitat. Nesting success, calculated using Mayfield's method, was significantly greater on Ulva (73%) than on Breaksea (32%) or Motuara (19%) owing primarily to higher egg fertility and hatching success. Although egg failure rates were highest on Motuara, the island least similar to Big South Cape, they were also relatively high on Breaksea where the habitat was similar to Ulva and Big South Cape. Therefore, the results only partially support the hypothesis that nesting success should decrease with increasing habitat difference associated with increasing latitudinal distance from the source population. The data from this 1-year study lay the groundwork for examining further hypotheses on the effects of reintroducing endangered species outside their contemporary range, but within their historical range.

1290: *+.190*

Knowledge of scarlet macaw (Ara macao) biology during the post-fledging period is limited, yet information about this important life stage is critical to developing effective conservation strategies. We used radiotelemetry to study the post-fledging movements and behavior of scarlet macaws from an isolated and threatened population in Costa Rica. Our results indicate that monitoring and protection of young macaws during the initial 14 days post-fledging is a critical conservation measure. We identify coastal mangrove as an important habitat for the species during the post-fledging period. Young scarlet macaws gradually developed flight and feeding skills, learned behaviors and movement patterns from their parents, and were integrated into social groups during the post-fledging period. Our results underscore the challenges facing management programs based on captive rearing and stress the importance of in situ conservation focusing on nest cavity management and protection in cooperation with local communities. (C) 2003 Elsevier Ltd. All rights reserved.

1291: *+.103*

The Po'ouli (Melamprosops phaeosoma), a Hawaiian honeycreeper endemic to Maui, has a population of only three known individuals; no breeding pair currently exists, and their home ranges are too far apart for breeding to occur. Without timely intervention this monotypic genus will likely go extinct. Conservationists have faced a dilemma: facilitate breeding amongst the known individuals, manage their ecosystem to benefit uncounted Po'ouli, or a combination of both? Po'ouli biology is poorly known - but their remote home ranges are closely monitored. A

State and Federal Environmental Assessment in 1999 recommended that one Po'ouli be translocated into the home range of another in an attempt to facilitate breeding. This first manipulative recovery action was achieved in April 2002, and provided valuable new information for future captive management efforts, but upon release, radio telemetry confirmed that the translocated bird returned to its own home range after one day. We describe the recent progress that has been made to recover the Po'ouli, and critically evaluate the Po'ouli case study and the lessons learned from it that can help expedite recovery of other birds of extreme rarity. (C) 2003 Elsevier Ltd. All rights reserved.

1292: *+.150*

The decline of rabbit (Oryctolagus cuniculus) populations in Mediterranean Spain has been shown to be one of the main threats to rabbit-specialist predators such as the Iberian lynx (Lynx pardina) and the Spanish imperial eagle (Aquila adalberti), two species which are in serious danger of extinction. Consequently, corrective measures such as increasing rabbit populations by restocking have been carried out to help the recovery of predator populations. In order to determine the general applicability of rabbit restocking for predator conservation, we performed three experimental restockings and evaluated their success in relation to season, habitat quality and the number of animals introduced. Rabbits were released either in large or small numbers in all four seasons of the year, and in habitat types defined as 'poor' or 'rich' according to the food availability for rabbits. For each restocking we evaluated rabbit survival, degree of residency and expected population increase. As a rule, the highest restocking success occurred whenever smaller number of rabbits were released in the richest quality habitat. Restocking during the breeding season was less successful than in other seasons. Problems concerning intraspecific competition, reproductive failure and diseases are discussed. (C) 2003 Elsevier Ltd. All rights reserved.

1293: *-.039*

Collecting tortoises for the pet trade is one of the factors threatening species of Testudo in the Mediterranean area. The collection of Testudo graeca graeca for pets is described in southeast Spain, where the main European population of this subspecies coincides with an area where keeping tortoises in captivity is a long-established custom. This present paper, based on inquiries made to children, reveals that this practice continues to be a common activity, and estimates a captive population in the order of tens of thousand of tortoises. Tortoises are collected as a result of chance encounters with wild animals by local inhabitants, and without any commercial objectives. The captive breeding and the release of tortoises without any institutional control is also common. These activities could be an important threat for the species. Implications for conservation are discussed and a critical review of the conservation strategies developed in relation to this threat (trade control and re-introduction programmes) are presented. We suggest that environmental education programmes are necessary to reduce casual collection and to change the social perception of tortoises as pets. (C) 2003 Elsevier Ltd. All rights reserved.

1294: *-.003*

We analyse the spatial and temporal variation in non-natural mortality during a 40-year period of strong contraction of the geographic range of the Iberian lynx (Lynx pardinus), which shrank from 40,600 to 22,300 km(2). We recorded 1258 lynx deaths, an average of 31.5 losses per year over the study period. Given the reduced lynx population size, especially later in the period (around 1100 individuals), this level of non-natural mortality may have contributed significantly to the quick decline of the Iberian lynx. Non-natural mortality was not spatially correlated with, and

probably did not shape the pattern of, relative abundance of lynx across its core range, but may have reduced its absolute density. Lynx losses were caused mainly by traps set not only for predator control but also for rabbits (Oryctolagus cuniculus), the lynx's staple food. We did not find evidence that non-natural mortality was higher in small lynx populations through edge effects. The highest mortality levels were recorded in regions where small game was a valuable economic resource compared with other activities. Mortality decreased throughout the period because of changes in the prevailing game regimes rather than because of legal protection. The Iberian lynx is now critically endangered and effective protection should be urgently enforced, especially in small game estates, which are environmentally favourable for rabbits but risky for lynx due to predator control. Lynx reintroductions would be better attempted in traditional rabbit hunting areas. Some big game estates where small game is not exploited and predators are not controlled may be good candidates for lynx reintroduction too, provided that habitat is managed towards a suitable interspersion of woody cover and grassland. (C) 2003 Elsevier Ltd. All rights reserved.

1295: ***+.027***

A native fish strategy has been initiated to rehabilitate native fish populations in the Murray-Darling Basin, Australia. The reintroduction of large woody debris (LWD) into the Basin's large lowland rivers is one of the restoration activities in the strategy. The results from three separate studies undertaken on the Murray River between Yarrawonga and Tocumwal are presented on the relationship between carp (Cyprinus carpio), native species, and LWD to examine whether native species and carp compete for LWD habitat. The first study reports on the relative abundance of carp and native fish in a river reach. Since 1995 carp abundance has declined, whereas the abundance of native fish populations has remained relatively constant providing little support for the hypothesis that competition for LWD habitat is having effects at the population level effects. The second study reports on the relationship between LWD, river channel position, and its use as habitat by carp and native species. A statistically significant relationship was observed between native fish, LWD, the location within a meander, and curvature of the meander. There was no statistically significant relation between carp and any of these parameters indicating that carp utilise a variety of riverine habitats, whereas native species were strongly associated with LWD. The third study reports on an experiment that tested the response of carp to the placement of new LWD habitat. The response from carp was statistically inconclusive. The combination of these studies suggest that it is unlikely that carp and native species are directly competing for LWD habitat and it is unlikely that carp will inundate restored LWD habitats and preclude native species.

1296: ***+.103***

1. Although many reintroduction schemes for the Eurasian lynx Lynx lynx in Germany have been discussed, the implications of connectivity between suitable patches have not been assessed.2. We introduce an individual-based, spatially explicit dispersal model to assess the probability of a dispersing animal reaching another suitable patch in the complex heterogeneous German landscape, with its dense transport system. The dispersal model was calibrated using telemetric data from the Swiss Jura and based on a map of potential lynx dispersal habitat.3. Most suitable patches could be interconnected by movements of dispersing lynx within 10 years of reintroduction. However, when realistic levels of mortality risks on roads were applied, most patches become isolated except along the German-Czech border. Consequently, patch connectivity is limited not so much by the distribution of dispersal habitat but by the high mortality of dispersing lynx. Accordingly, rather than solely investing in habitat restoration, management efforts should try to reduce road mortality.4. Synthesis and applications. Our approach illustrates

how spatially explicit dispersal models can guide conservation efforts and reintroduction programmes even where data are scarce. Clear limits imposed by substantial road mortality will affect dispersing lynx as well as other large carnivores, unless offset by careful road-crossing management or by the careful selection of release points in reintroduction programmes.

1297: ***+.013***

The Japanese regional population of the Oriental white stork (Ciconia boyciana) became extinct in 1986. Mitochondrial DNA (mtDNA) D-loop region from 20 mounted specimens preserved at public facilities in Toyooka City, Hyogo Prefecture, Japan and its vicinity (n = 17), the area inhabited by the last of the Japanese population, and samples originating from China (n = 3) which were kept at a zoo was analyzed. After extracting DNA from small pieces of skin from mounted specimens, a 1210-bp region of the mtDNA D-loop region was analyzed. The haplotypes among 11 specimens of storks captured or found dead at Toyooka City just before the population became extinct were completely identical. Four haplotypes observed among the mounted specimens preserved in the vicinity of Toyooka City were differentiated from those of captive storks originating from China and Russia in a previous study. Therefore, the last Japanese population might have been a genetically unique group. However, phylogenetic analysis using the maximum likelihood method showed that haplotypes found in the Japanese regional population were closely related to the Chinese and Russian lineages (sequence difference = 2.1%). One mounted specimen collected in 1935 at Izushi village, in the vicinity of Toyooka City, showed the same haplotype as the captive storks from China, suggesting that genetic flow may have historically occurred between the populations of Japan and the continent. Recently, reintroduction for the Oriental white stork has been planned in Toyooka City. The planning for the recovery of extinct populations should not only involve translocation of species to the range from which it disappeared, but also reconstruction of regional populations while considering the genetic lineage between the extinct and introduced populations.

1298: ***+.009***

In the Sonoran Desert of North America, populations of the desert tortoise (Gopherus agassizii) occur in rocky foothills throughout southwestern Arizona and northwestern Mexico. Although tortoise populations appear to be isolated from each other by low desert valleys, individuals occasionally move long distances between populations. Increasingly, these movements are hindered by habitat fragmentation due to anthropogenic landscape changes. We used molecular techniques and radiotelemetry to examine movement patterns of desert tortoises in southern Arizona. We collected blood samples from 170 individuals in nine mountain ranges and analyzed variability in seven microsatellite loci to determine genetic differentiation among populations. Gene flow estimates between populations indicate that populations exchanged individuals historically at a rate greater than one migrant per generation, and positive correlation between genetic and geographic distance of population pairs suggests that the limiting factor for gene flow among populations is isolation by distance. Life history traits of the desert tortoise, a long-lived species with delayed sexual maturity, may severely constrain the ability of small populations to respond to disturbances that increase adult mortality. Historic gene flow estimates among populations suggests that recovery of declining populations may rely heavily on the immigration of new individuals from adjacent mountain ranges. Management strategies compatible with the evolutionary history of gene flow among disjunct populations will help ensure the long-term persistence of Sonoran desert tortoise populations.

1299: ***+.175***

We integrated genetics and demography into population modeling in the context of species restorations, in which both the origin of released individuals and the management strategy may influence the success of introduction. Through an explicit individual-based simulation approach, we investigated the effects of the age of released individuals by exploring the relative merits of releasing juveniles or adults to establish populations. We included the effect of genetic variability responsible for inbreeding depression and mutational meltdown. Our general analysis uncovered an interaction between the age of founders and the extent Of intrapopulation fitness variability, which substantially influenced the efficiency of selection in populations founded by juveniles and had subsequent positive consequences for long-term persistence compared with the case in which adults were released. We then applied the model to the case of the reintroduction of the Griffon Vulture (Gyps fulvus fulvus) to southern France, for which post-release data were available. The demographic aspects of this reintroduction were already analyzed and published, suggesting that it is more efficient to release adults than juveniles, despite an observed reduction of demographic parameters following the release of adults. In that context, the inclusion of genetic considerations qualitatively changes the conclusion, predicting reduced long-term extinction risk if juveniles rather than adults are released.

1300: *+.172*

We investigated how large carnivores, herbivores, and plants may be linked to the maintenance of native species biodiversity through trophic cascades. The extirpation of wolves (Canis lupus) from Yellowstone National Parkin the mid-1920s and their reintroduction in 1995 provided the opportunity to examine the cascading effects of carnivore-herbivore interactions on woody browse species, as well as ecological responses involving riparian functions, beaver (Castor canadensis) populations, and general food webs. Our results indicate that predation risk may have profound effects on the structure of ecosystems and is an important constituent of native biodiversity. Our conclusions are based on theory involving trophic cascades, predation risk, and optimal foraging, on the research literature; and on our own recent studies in Yellowstone National Park. Additional research is needed to understand how the lethal effects of predation interact with its nonlethal effects to structure ecosystems.

1301: *+.063*

Re-introduction is a technique widely used in the conservation of threatened bird species. With advances in aviculture the use of captive-produced individuals as the release stock is becoming more commonplace, and ideally, survival of captive-produced, released individuals should be no different from their wild-bred counterparts. During the late 1980s the Critically Endangered Mauritius kestrel (Falco punctatus) was successfully re-introduced into the Bambous mountain range, Mauritius, some 30 years after its local extinction. Between 1987 and 2001 the developing population was closely monitored enabling us to construct re-sighting histories for 88 released and 284 wild-bred kestrels. We used age-structured models in the survival analysis software program MARK to determine if an individual's origin influenced its subsequent survival. Our analysis indicated no compelling evidence for reduced survival among juvenile captive-reared and released individuals, relative to their wild-bred counterparts, across the majority of cohorts and only limited evidence of a cohort-specific effect. This study illustrates that despite the lack of a formal experimental approach it is still feasible to conduct an assessment of re-introduction outcomes and techniques. (C) 2003 Elsevier Ltd. All rights reserved.

1302: *+.180*

The objective of this project was to collect scientific data to assist in the development of guidelines for the humane relocation of threatened and endangered arboreal non-human primate species. A troop of 31 Lowland Sykes monkeys (Cercopithecus mitis albotorquatus) was habituated to fruit bait for capture in a village and relocation to a previously selected suitable site in a protected forest reserve approximately 30 km away. Sixty-five percent (n = 20) of the animals were captured and relocated. This subgroup comprised one adult male, eight adult females, two subadult females, three juvenile mates, four juvenile females and two infant males. Although the relocated group originated from one single group, post-translocation telemetry signals demonstrated that it split into two groups, which established themselves approximately 2-4 km apart in their new territory, the adult male eventually became solitary. The factors of importance for the successful capture and relocation of forest primates were found to include: proper understanding of troop home-range utilisation and of social bond organisation within the troop, method and period of habituation, method of release, suitability of the new habitat with respect to the ecological niche requirements of the species in question, and the period of post-relocation monitoring.

1303: ***+.071***

Reintroduction of captive-bred animals is a key approach in conservation attempts for many endangered species, however, post-release survival is often low. Rearing conditions may be unlike those encountered upon release and the animals may not have had experiences necessary for survival in the wild. Animals may also habituate in captivity to stimuli that may pose a danger after release and/or there may be selection for behavioural traits, in particular reduced fearfulness, that may not be suited for the wild. Here, variation in boldness was assessed in captive-bred swift fox (Vulpes velox) and tested for influence on survival after release. Radio-tracked individuals that died in the 6 months following release were those judged previously as bold. In the presence of novel stimuli in captivity, they had left their dens more quickly, approached more closely to the stimuli and shown more activities indicating low fear than did those that survived. These individuals were less suited for release. Future selection of release-candidates on the basis of behavioural variation should enhance the success of reintroduction programmes.

1304: ***+.179***

Adequate monitoring of amphibian populations will be increasingly important if global declines are to be understood and, where possible, reversed. The natterjack toad, Bufo calamita, is the rarest British amphibian and has been the subject of substantial conservation efforts. Monitoring strategies have aimed to quantify both the total numbers of populations and the status of individual populations. Between 1970-1999 the number of natterjack populations increased from 43 to 48 because successful translocations (11) outnumbered extinctions (6). Efforts to monitor individual populations increased over the period 1970-1999. Between 1990-1999 there were no detectable trends in B. calamita population size or breeding success in Britain, although power to detect any trends over 10 years was low. Calling activity and short-term trends in spawn counts were unreliable predictors of long-term population viability in the absence of extra information such as toadlet production. Long periods (> 10 years) of spawn counts are needed to demonstrate trends that are reflective of real population changes at the national level. Efforts to develop reliable methods for monitoring B. calamita in Britain could provide useful guidelines for work on some other declining species of amphibians.

1305: ***+.149***

Although population dynamics of giant pandas Ailuropoda melanoleuca remain poorly known,

basic principles can be safely transferred from studies of, and experience with, similar species. Without minimizing the importance of continued study of demographics, genetics, and behavior of pandas, I offer the following generalizations from our understanding of carnivore population biology in North America. First, elasticity analyses confirm that pandas have evolved life-histories that prioritize high survival of adult females. In comparison, reproductive rates are unimportant. Increases in survival of adult females will yield approximately 5 times the conservation benefit of proportional increases in reproductive output. Second, under circumstances likely to characterize most panda populations, survival rates of males (even adults) are also relatively unimportant. Thirdly, notwithstanding its well-deserved reputation as a slow breeder, giant panda populations are mathematically capable of growing surprisingly quickly, if habitats (and associated survival rates) allow. Finally, reintroductions of endangered species in western North America remind us of the critical role of maintaining large patches of unfragmented habitat. Wolves Canis lupus, extirpated from the western U.S. before the mid-20th century, have made a rapid and surprisingly painless recovery because wild areas and prey populations remained abundant. In contrast, black-footed ferrets Mustela nigripes, lost from the wild more recently, have encountered great difficulty in becoming reestablished, despite tremendous scientific efforts. It appears that ferrets may simply no longer have sufficient wild habitat (i.e., prey) to persist. The best science will not be capable of saving pandas if sufficient habitat is not available.

1306: ***+.040***

Present population structure and patterns of genetic diversity in forest trees have been influenced by past anthropogenic activities and the major aim of this review is to assess the scale and timing of this influence within Europe. Very little 'natural' forest (defined as areas that have not experienced a break in forest continuity because of cultural activities since conditions became suitable for tree growth) remains in Europe. Yet many contemporary European forests have developed by natural regeneration from local stock, so despite a massive anthropogenic impact, one can predict a survival of the 'natural' patterns of genetic diversity despite a general reduction in total diversity. Systematic interference with forests was closely associated with the spread of agriculture from south-east to north-west Europe during the last 10,000 years. It has been suggested that the spread of trees of early economic importance was linked to local agricultural development. However, closer analyses of spreading dynamics and comparisons with comparable spreading in the USA does not fully support this hypothesis. Indeed, modelling the present and past range limits (if forest trees has suggested that climate change is the major driving force for range changes, implying that genetic composition is primarily governed by natural processes. Anthropogenic activity did catalyse the spread of Fagus sylvatica through increasing the rates of forest disturbance. By the Roman period, there are documented cases of introduction of e.g. Aesculus hippocastanum and Castanea sativa to many parts of Europe. Countries with colonial histories have been importing undisputedly 'exotic' species for several centuries. Occasionally there is doubt over exotic status owing to lack of documentation or possible prehistoric introduction, e.g. Arbutus unedo in western Ireland. The translocation of native species with the aim of improving production potential is a relatively recent practice often documented in national histories of forestry. Thus despite considerable widespread and long-term intervention with European forest ecosystems, many of the observed patterns of genetic diversity observed in naturally regenerated forests primarily originate from natural processes. However, given the enormous recent increase in the long-distance translocation of seeds. this situation is rapidly changing in regions with intensive forest management. (C) 2004 Elsevier B.V. All rights reserved.

1307: ***+.102***

Brosimum alicastrum Sw. is a multi-purpose species and it is dominant in some American tropical forests. In Western Mexico, forests dominated by B. alicastrum, called mojoteras, have been dramatically altered. The present work aims to supply basic elements for the reintroduction of mojoteras in the Sierra de Manantlan Biosphere Reserve. The question we posed was: Are there nursing species for the reintroduction of B. alicastrum seedlings in secondary tropical dry forest? A hundred randomly chosen 2-year-old B. alicastrum seedlings were planted in five treatments: under the covering of 80 individuals belonging to four species (Tabebuia chrysantha (Jacq.) G. Nicolson, Thouinia serrata Radlk., Acacia macilenta Rose and Acalypha cincta Muell. Ara.) and 20 on open ground. After 1 year, the survival of Brosimum alicastrum seedlings was significantly different among the treatments ($G(2) = 12.11$; $P < 0.05$). The treatments with the highest significant B. alicastrum seedling survival rate were under the covering of Acalypha cincta and Thouinia serrata (55 and 40%, respectively), whereas under the covering of Acacia macilenta. Tabebuia chrysantha and on open ground, the survival rate was the lowest (<5%). (C) 2004 Published by Elsevier B.V.

1308: *+.140*

A total of 436 logs were used to create 20 engineered log jams (ELJs) in a 1.1 km reach of the Williams River, NSW, Australia, a gravel-bed river that has been desnagged and had most of its riparian vegetation removed over the last 200 years. The experiment was designed to test the effectiveness of reintroducing woody debris (WD) as a means of improving channel stability and recreating habitat diversity. The study assessed geomorphic and ecological responses to introducing woody habitat by comparing paired test and control reaches. Channel characteristics (e.g. bedforms, bars, texture) within test and control reaches were assessed before and after wood placement to quantify the morphological variability induced by the ELJs in the test reach. Since construction in September 2000, the ELJs have been subjected to five overtopping flows, three of which were larger than the mean annual flood. A high-resolution three-dimensional survey of both reaches was completed after major bed-mobilizing flows. Cumulative changes induced by consecutive floods were also assessed. After 12 months, the major geomorphologic changes in the test reach included an increase in pool and riffle area and pool depth; the addition of a pool-riffle sequence; an increase by 0.5-1 m in pool-riffle amplitude; a net gain of 40 m(3) of sediment storage per 1000 m(2) of channel area (while the control reach experienced a net loss of 15 m(3)/1000 m(2) over the same period); and a substantial increase in the spatial complexity of bed-material distribution. Fish assemblages in the test reach showed an increase in species richness and abundance, and reduced temporal variability compared to the reference reach, suggesting that the changes in physical habitat were beneficial to fish at the reach scale. Copyright (C) 2004 John Wiley Sons, Ltd.

1309: *+.256*

Plant species richness in rural landscapes of northern Europe has been positively influenced by traditional management for millennia. Owing to abandonment of these practices, the number of species-rich semi-natural grasslands has decreased, and remaining habitats suffer from deterioration, fragmentation, and plant species decline. To prevent further extinctions, restoration efforts have increased during the last decades, by reintroducing grazing in former semi-natural grasslands. To assess the ecological factors that might influence the outcome of such restorations, we made a survey of semi-natural grasslands in Sweden that have been restored during the last decade. We investigated how plant species richness, species density, species composition, and abundance of 10 species that are indicators of grazing are affected by (1) the size of the restored site, (2) the time between abandonment of grazing and restoration, (3) the time elapsed since

restoration, and (4) the abundance of trees and shrubs at the restored site. Only two factors, abundance of trees and shrubs and time since restoration, were positively associated with total species richness and species density per meter square at restored sites. Variation in species composition among restored sites was not related to any of the investigated factors. Species composition was relatively similar among sites, except in mesic/wet grasslands. The investigated factors had small effects on the abundance of the grazing-indicator species. Only Campanula rotundifolia responded to restoration with increasing abundance and may thus be a suitable indicator of improved habitat quality. In conclusion, positive effects on species richness may appear relatively soon after restoration, but rare, short-lived species are still absent. Therefore, remnant populations in surrounding areas may be important in fully recreating former species richness and composition.

1310: *-.125*

1. We used field and experimental data to test if white grub parasites (Diplostomatidae) are costly to White Sands pupfish (Cyprinodon tularosa), a threatened species restricted to four sites in the Chihuahuan desert, New Mexico.2. Of the four populations of C. tularosa, two are native and two are introduced. The two native populations (Malpais Spring and Salt Creek) are genetically distinct and have been isolated historically in dissimilar aquatic habitats (brackish spring and saline river, respectively). Two populations were established c. 1970 from translocation of Salt Creek fish to another saline river (Lost River) and another brackish spring (Mound Spring).3. Physid snails (Physidae) occur in the two brackish spring habitats but not the saline river habitats. These snails are first intermediate hosts for white grubs (Diplostomatidae). Therefore, the two freshwater populations are infected by diplostomatids. For the Mound Spring population, the ecological relationship of C. tularosa and diplostomatids has only recently occurred.4. In 1995, a population crash occurred for C. tularosa at Mound Spring, associated with a parasite outbreak. Diplostomatids were the presumptive cause of this crash, but this was inferred from observation of infection in collected fish.5. Two years of seasonal sampling of the two populations revealed that all collected fish were infected. Parasite intensities were significantly lower in winter compared to summer, suggesting that heavily infected fish were lost from the population on a seasonal basis.6. We conducted an artificial infection experiment to assess the costs of parasitism for previously uninfected C. tularosa females for various life-history traits. Under experimental conditions, diplostomatid infection caused increases in C. tularosa mortality and decreases in growth and fat storage. Individual-level costs of parasitism may translate to population-level patterns of parasitism for C. tularosa populations. Results from this study suggest that parasites may impact host overwinter survival, which is consistent with lower parasite intensities found during winters in wild populations.

1311: *+.076*

A comparison of four pollination treatments revealed that Antirrhinum valentinum is self-incompatible but shows low levels of spontaneous self-fertility. Seed sets, after hand cross-pollination and spontaneous self-fertilization, were significantly fewer than in control flowers, but fruit and seed mass were not significantly different. Pollen tube growth was slower after selfing than crossing, being arrested in the stigmata or in the upper part of the style. Multilocus outcrossing rates (t(m)) were high and t(m) - t(s) (t(s) = single-locus outcrossing rate) small, indicating low levels of biparental inbreeding. Despite that, the estimated neighborhood sizes were from six to seven individuals, which can be considered low because the number of seeds per fruit ranges between 150 and 200. This apparent incongruence is explained by the small size of populations together with the multiallelic gametophytic control of self-incompatibility, making

possible crossing between half-sibs. On the basis of these data, recommendations to develop reinforcement or reintroduction programs as well as for in vitro propagation of plants are given.

1312: *+.064*

Although the Mexican axolotl (Ambystoma mexicanum) is well known as a popular laboratory and aquarium animal, in the wild it is almost extinct and confined to the remnant canals of Lake Xochimilco on the edge of Mexico City. Loss of habitat, introduced fish, pollution and illegal collection for food and medicines have all played a role in its decline. Capitalizing on its high profile within local culture, a multidisciplinary conservation programme is being developed using the axolotl as a flagship species for the wider Xochimilco system. The programme is focusing on raising the profile of the axolotl and other species by promoting nature tourism and conservation education within the local community. Complementing these socio-economic initiatives is biological research on population ecology, survey methods and threat mitigation. The axolotl has been bred in captivity very successfully both in Mexico and elsewhere. However, reintroduction to Lake Xochimilco will not be a realistic option until the threats facing the species have been addressed. Equally, the disease and genetic risks posed by the release of captive bred stock need to be evaluated prior to any reintroduction.

1313: *+.189*

The hare, Lepus europaeus, production in 25 enclosures was monitored from 1999 to 2003. The average surface of the enclosures was 3.4 +/- 1.3 (SD) ha (range 1.1-6.6). Annually, 2 adult males and 3 adult females, preventively captured in protected areas, were randomly assigned to each enclosure in January. The hares produced in the enclosures were captured in October/December for restocking. The enclosures were cultivated with non-harvested crops. Every enclosure was surveyed with a GPS system. A total of 675 hares were produced in the enclosures with 165.4 produced hares for 100 introduced hares. A linear regression analysis showed that the % woodland, % bush-land and clay content of the soil negatively affected the hare production, while the % mixed-hay fields, % undersowed cereals, number of "small-fields" per ha, enclosure surface, Shannon index, number of cultivated-plant species and sand content of the soil seemed to have a positive affect A stepwise selection of the environmental characteristics reduced the effect-variables to "year", "surface", woodland-incidence (negative factor), clay-content (negative factor), and mixed-hay fields-incidence (positive factor). Semi-natural rearing seems to be a valuable system to produce hares for restocking and reintroduction, but the success of this technique seems to be linked to the environmental characteristics and crop management of the enclosures.

1314: *-.039*

Several species of wild animals are endangered with extinction in Cameroon. The ostrich, Struthio camelus, which is one of these animals, has been protected since 1994. In 2000, there were about fifty individuals left in the Waza National Park, in the far northern part of the country. The number of ostriches has steadily decreased in the park despite more than half a century of protection. This repressive management method has progressively evolved into a participative management method. The primary cause of this drop in number is poaching, which is carried out by the local populations searching for eggs whose shells are sold to tourists, and for adult animals killed for their meat, and even more so for their bone marrow which is widely used in traditional medicine. Commercial hunting is carried out by foreigners, the skin being used in the fabrication of hand-crafted objects. In addition to these problems, natural predation occurs, mostly by jackals, Can is

aureus, and hyenas, Hyaena hyaena, which targets the hatchlings often already weakened by the harsh climactic conditions. One of the alternatives for the conservation of this species is the introduction of its breeding in "game ranching" or "game farming" near the park and urban centers where there are consumers of the products taken from this animal. The products of such breeding farms or ranches could be used as animals for reintroductions into the wild, improving the visual tourism, and be valorized on the national meat- and craft markets.

1315: *+.205*

Community Based Natural Resource Management (CBNRM) has made its contribution to the Environment and Development Agenda of most Southern African countries, and perhaps even in other parts of the world. In Namibia, it has gained its merit and widespread support in the wildlife sector, through the internationally-acclaimed Conservancy Programme. This Programme was launched in 1998 and has demonstrated that wildlife stands a better chance of success and growth by giving the wildlife resource economic values to those who live and interact with the wildlife on a day-to-day basis. These economic values are realized through usufruct rights given to rural communities through a strong policy and legislative framework, which defines, clarifies and guarantees such rights. The result is a vibrant community-driven programme, encompassing over 60 communities mobilized into governance and wildlife management bodies, called Conservancies, involving over 200,000 people (in both registered and emerging Conservancies). This represents over 10% of the total population of Namibia, and close to 20% of the rural population. The 29 registered Conservancies alone involve 95,000 people and cover over 7 million hectares of land. This accounts for 22% of communal land and close to 9% of the total land area. Communities are not only empowered to manage the wildlife, but are also in a position to reap direct financial and other benefits as a result of their management effort. Financial and non-financial benefits accrued to Conservancies from wildlife and wildlife-based tourism, have nearly doubled annually since 1998. In 2003, Conservancies earned over US$ 2,430,000 from a diversity of wildlife and tourism ventures. The Ju/'hoansi San of Nyae Nyae in northeastern Namibia is one of the Namibia's most poverty-stricken and marginalized communities. This community created a 9,030-km(2) Conservancy in 1998. Largely as a result of the game reintroduction effort made since 1999, the game numbers increased, contributing significantly to the livelihoods of the members. The Conservancy provided 28% of all jobs (n=97) and about 44% of all incomes (US$ 341,390) during 2003. The CBNRM has the potential of broadening community rights and benefits beyond wildlife to also include other natural resources, but this will require careful consideration and planning when integrating wildlife with other forms of land use, and further advancing the CBNRM as mainstream strategy for rural development. Considering the current inequities in the agricultural sector between the elites and the rural poor, the major future stake of rural communities in the wildlife sector will be to safeguard the fundamental principles of the CBNRM.

1316: *+.080*

This report covers the results of the western pond turtle head-starting and reintroduction project for the period of October 2003 - September 2004. Wild hatchling western pond turtles from the Columbia River Gorge were reared at the Woodland Park and Oregon Zoos in 2003 and 2004 as part of the recovery effort for this Washington State endangered species. The objective of the program is to reduce losses to introduced predators like bullfrogs and largemouth bass by raising the hatchlings to a size where they are too large to be eaten by most of these predators. Sixty-nine turtles were over-wintered at the Woodland Park Zoo and 69 at the Oregon Zoo. Of these, 136 head-started juvenile turtles were released at three sites in the Columbia Gorge in 2004. Two were

held back to attain more growth in captivity. Thirty-four were released at the Klickitat ponds, 19 at the Klickitat lake, 21 at the Skamania site, and 62 at Pierce National Wildlife Refuge (NWR). This brought the total number of head-start turtles released since 1991 to 246 for the Klickitat ponds, 114 for the Klickitat lake, 167 for the Skamania pond complex, and 250 at Pierce NWR. In 2004, 32 females from the two Columbia Gorge populations were equipped with transmitters and monitored for nesting activity. Twenty-one of the females nested and produced 85 hatchlings. The hatchlings were collected in September and October and transported to the Woodland Park and Oregon zoos for rearing in the head-start program. Data collection for a four-year telemetry study of survival and habitat use by juvenile western pond turtles at Pierce NWR concluded in 2004. Radio transmitters on study animals were replaced as needed until all replacements were in service; afterward, the turtles were monitored until their transmitters failed. The corps of study turtles ranged from 39 in August 2003 to 2 turtles at the end of August 2004. These turtles showed the same seasonal pattern of movements between summer water and upland winter habitats observed in previous years. During the 2004 field season trapping effort, 345 western pond turtles were captured in the Columbia Gorge, including 297 previously head-started turtles. These recaptures, together with confirmed nesting by head-start females and visual resightings, indicate the program is succeeding in boosting juvenile recruitment to increase the populations. Records were also collected on 224 individual painted turtles captured in 2004 during trapping efforts at Pierce NWR, to gather baseline information on this native population. Bonneville Power Administration (BPA) funded approximately 60% of program activities in the Columbia River Gorge from October 2003 through September 2004.

1317: ***-.001***

Crested Ibis Nipponia nippon is an Endangered species that has experienced a severe but short-lived population bottleneck in recent years. Only one wild population and two captive populations exist in China today, all of which were probably initiated from two breeding pairs rediscovered in 1981. This paper represents the first study of the genetic diversity of Crested This by analysing sequence variation of a fragment spanning domains II and III of the mitochondrial DNA (mtDNA) control region of both wild and captive populations. Two haplotypes were identified in both wild and captive populations, and the captive population had a bias towards haplotype 1. The species as a whole demonstrated an extremely low level of genetic diversity, with a haplotype diversity and nucleotide diversity of 0.386 +/- 0.074 and 0.069% +/- 0.013%, respectively. Despite the rapid increase in numbers of birds under intensive management, the paucity of genetic diversity remains a real threat to the species. The mtDNA control region variations detected in the present study could provide significant information additional to the studbook data of Crested Ibis; therefore we suggest that special attention be paid to individuals with haplotype 2 when considering captive breeding management. Overall, great care should be taken in the selection of reintroduction sites for this rare bird, as species with low genetic diversity are thought to be more limited in their ability to tolerate a wide range of environmental extremes and diseases.

1318: ***-.072***

Historical data on the distribution and status of the purple gallinule (Porphyrio porphyrio) in Portugal were collected by bibliographic research, from museum catalogues and from questionnaires to wetland managers, ornithologists and birdwatchers. The population suffered severe decline and range contraction at the end of the 19th century and most of the 20th century, due mainly to habitat loss and degradation and excessive hunting. However, the population has begun to recover in the last decade mainly through direct protection of the species and of the remaining habitat, together with creation of artificial wetlands and a reintroduction programme,

and probably also due to immigration from Spain. The population size has increased about 10-fold and the range has expanded to most of its former area. The estimated Portuguese breeding population in 2002 was 49-67 pairs. Densities varied between 0.05 and 7 individuals/ha in recently colonized areas and stable (saturated) areas, respectively. Although the population is recovering, its small size, high fragmentation and several natural and anthropogenic threats lead us to consider it still a species of major concern in Portugal. (C) 2003 Elsevier Ltd. All rights reserved.

1319: *+.064*

Although the Mexican axolotl (Ambystoma mexicanum) is well known as a popular laboratory and aquarium animal, in the wild it is almost extinct and confined to the remnant canals of Lake Xochimilco on the edge of Mexico City. Loss of habitat, introduced fish, pollution and illegal collection for food and medicines have all played a role in its decline. Capitalizing on its high profile within local culture, a multidisciplinary conservation programme is being developed using the axolotl as a flagship species for the wider Xochimilco system. The programme is focusing on raising the profile of the axolotl and other species by promoting nature tourism and conservation education within the local community. Complementing these socio-economic initiatives is biological research on population ecology, survey methods and threat mitigation. The axolotl has been bred in captivity very successfully both in Mexico and elsewhere. However, reintroduction to Lake Xochimilco will not be a realistic option until the threats facing the species have been addressed. Equally, the disease and genetic risks posed by the release of captive bred stock need to be evaluated prior to any reintroduction.

1320: *+.101*

The reintroduction of gray wolves to Yellowstone National Park (YNP) provides a natural experiment regarding the effects of top predators on scavenger species. Fieldwork on the Northern Range of Yellowstone indicates that wolves facilitate carrion acquisition by scavengers, but it is unclear whether this represents a transient or permanent effect of wolf reintroduction. Here we present a wolf-elk model with human elk harvest and use it to investigate the long-term consequences of predator-prey dynamics and hunting on resource flow to scavengers. Our model shows that while wolves reduce the total amount of carrion, they stabilize carrion abundance by reducing temporal variation in the quantity of carrion and extending the period over which carrion is available. Specifically, the availability of carrion is shifted from reliance on winter severity and elk density to dependence on the strength of wolf predation. Though wolves reduce the overall abundance of carrion by lowering the elk population, this reduction is partially offset by increases in the productivity of an elk population invigorated by removal of the weakest individuals. The result of this is higher carrion production per elk in the presence of wolves. In addition, this yields an ecological explanation for the phenomena that predators increase the robustness of their prey: namely that by reducing the effect of density-dependent resource competition among elk, those that remain, even some of the older animals, are better fed and healthier as a result. Our model also suggests that human hunting has no effect on the distribution of carrion across the year but is crucial in determining the long-term abundance of carrion because of the effect of hunting on elk population levels. By reducing the proportion of cows in the annual hunt, which have historically been high in order to control the number of elk migrating north of the park, managers can allow an adequate supply of carrion without substantially reducing hunter take. The effects of a more tractable food resource is likely to benefit scavengers in Yellowstone and other areas of the world where wolves have been or are currently being considered for reintroduction. (C) 2004 Elsevier B.V. All rights reserved.

1321: ***+.166***

The white-winged guan Penelope albipennis (Aves, Cracidae) is a Critically Endangered bird endemic to northern Peru. We surveyed a protected area suitable for its reintroduction north of the species' current range. Following IUCN guidelines, four factors were evaluated: diversity and quantity of plant species that are part of the white-winged guan's diet, year-round water sources, resting and roosting cover, and extent of undisturbed forest. Several suitable small valleys or quebradas were inspected, but our evaluation focused on three. We identified all shrub and tree species to compare with habitat currently used by the guan. Cover for nesting places was assessed visually. We found species composition and microhabitat types to be similar to habitat presently used by wild guans. Thus, all habitat components are favourable for reintroduction. The high degree of protection of the area makes it particularly suitable as a reintroduction site. We recommend experimental extension of the species current range to mitigate the risk of extinction posed by catastrophic events.

1322: ***+.185***

Echinacea laevigata (Boynton and Beadle) Blake, Asteraceae, smooth coneflower, is a federally listed endangered species native to the piedmont of the southeastern United States. The majority of known populations occupy roadsides and utility rights-of-way, and the few undisturbed populations are declining in number and size as succession shades out the understories. The recovery plan for E. laevigata calls for reintroduction to the wild and safeguarding throughout the range of the species. In an experimental reintroduction, we tested several planting methods to determine the optimal method for establishing populations in the wild including transplanting seedlings bare-root versus rooted in potting soil, clustering seedlings versus planting them individually, and planting adult plants (1-3 year old) versus seedlings. Treatments were tested at two sites typical of E. laevigata habitat. The high survival rates and comparable growth among plants from all planting methods that we observed suggests that reintroduction is a promising conservation strategy for the species and that there are various options for introducing populations. Therefore, reintroduction practitioners may weigh logistical costs and benefits on a case-by-case basis when choosing reintroduction methods for E. laevigata. Based on theoretical predictions and our findings, we suggest that an optimal method for establishing populations is to plant adult plants (older than one year) in the spring, without soil amendment.

1323: ***+.013***

There are two recognized subspecies of impala in sub-Saharan Africa: the common impala (Aepyceros melampus melampus) - widespread in southern and east Africa - and the vulnerable black-faced impala (A. m. petersi) - found naturally in only a small enclave in southwest Africa. The Etosha National Park (NP) in Namibia harbours the largest and only protected-area population of black-faced impala, numbering some 1500 individuals. Due to translocations of the exotic common impala to commercial farms in Namibia during the past decades, the black-faced impala in Etosha is faced with the potentially serious threat of hybridization posed by secondary contact with the common impala inhabiting bordering farms. Using eight microsatellite DNA markers, we analysed 127 black-faced impala individuals from the five subpopulations in Etosha NP, to determine the degree, if any, of hybridization within the park. We found that (a) the black-faced impala were highly genetically differentiated from the common impala (pairwise theta-values ranged from 0.18 to 0.39 between subspecies; overall value = 0.27) and (b) black-faced samples showed high levels of genetic variability [average expected heterozygosity (H-E) = 0.61 +/- 0.01 SE], although not as high as that observed in the common impala (average H-E = 0.69 +/- 0.02

SE). (c) No hybridization between the subspecies in Etosha was suggested. A Bayesian Markov Chain Monte Carlo approach revealed clear distinction of individuals into groups according to their subspecies of origin, with a zero level of 'genetic admixture' among subspecies.

1324: *+.066*

1. In a population with Allee effects a positive relationship exists between fitness and population size or density. Allee effects may result in extinction thresholds and are therefore crucial in conservation and management. It has been shown theoretically that Allee effects can be driven by predation; however, there are few empirical data. Previous empirical work on Allee effects has emphasized that taxa with life-history characteristics such as co-operative breeding may be prone to such effects. Because predation is a general ecological mechanism, Allee effects may be more widespread than previously thought.2. We used a series of simple heuristic models to develop a theoretical framework for understanding predation-driven Allee effects as a function of predator functional and aggregative responses.2. Predators can create an Allee effect if they have a type I (linear) or type II (saturating) functional response without a type III (sigmoid) aggregative response, or vice versa. In addition, predation must be the main driver of prey dynamics, and prey must have little spatial or temporal refuge from predation.4. We highlighted several, mainly unrecognized, examples of predation-driven Allee effects from the literature, the majority of which came from systems that had been perturbed by exploitation or introduced predators.5. Synthesis and applications. Allee effects can arise from a general ecological process under a variety of different combinations of functional and aggregative responses. Allee effects may thus be present in a broad spectrum of different taxa with different types of life history, not only those taxa, such as broadcast spawners and co-operative breeders, on which empirical work has focused thus far. Conservation biologists and managers working with heavily exploited or threatened populations, or attempting reintroductions, should be aware of the possibility of a threshold population size or density below which extinction is likely. These thresholds can occur regardless of species life history, if predation is a major source of mortality and spatial and temporal predation refuges are limited.

1325: *+.315*

In the UK, research has improved our understanding of amphibian populations, their habitats, threats and the effectiveness of conservation measures. The greatest research effort has been directed to the protected and declining species, notably Triturus cristatus, Bufo calamita and Rana lessonae. However, several challenges arise when attempting to employ research findings as a tool to shape policy. Wild populations and threats to them are not often simple systems that invite straightforward investigation. Extrapolating from small studies to more comprehensive application can also generate problems, especially with widespread species. The standards of confidence commonly used in science may not be directly transferable to conservation policy, as in conservation it is often desirable to apply the precautionary principle. When constructing policies, it is important to be realistic about the constraints that may be imposed due to factors beyond the control of conservation agencies and researchers, notably those of a legislative or socioeconomic nature. There is a need for conservation practitioners to engage more closely with scientists, with a view to identifying the current knowledge gaps that hinder the achievement of conservation gains. The increasing success of B. calamita reintroductions provides an excellent illustration of such an application of scientific knowledge.

1326: *+.024*

The dramatic decline of amphibian populations worldwide is an urgent conservation issue. The root causes underlying many amphibian declines are unknown. One Michigan species of Special Concern, the Blanchard's cricket frog (Acris crepitans blanchardi), is declining at an alarming rate in the northern portions of its range. In May 2004, the National Amphibian Conservation Center (NACC) at the Detroit Zoo initiated a translocation project to reintroduce Blanchard's cricket frog to three manmade wetlands at the northern extent of its range. Calling surveys in south-east Michigan revealed several robust populations of cricket frogs including one site slated for development in the fall of 2004. Working with the developer and the Department of Environmental Quality, we successfully removed about 1,050 frogs and tadpoles from the threatened breeding ponds. The animals were released into two Michigan Department of Natural Resource restored lake plain prairie complexes with wetland features, and into a restored wetland near the NACC in fall of 2004. These translocated populations will be monitored in subsequent breeding seasons to assess the success of the translocation project.

1327: *+.036*

Zoos, by nature, are breeding grounds for high numbers of rodents. The use of poisons and traps has been the main tool for rodent control. By acquiring and reintroducing avian predators, specifically barn owls, into a zoo setting, the rodent population could be controlled naturally. Barn owls (Tyto alba) once flourished in Pennsylvania and surrounding areas, particularly in old wooden barns, but the development of new prefabricated barns has left the owls with fewer places to nest. This study focuses on installing several man-made nest boxes for three avian predators on the grounds of the Pittsburgh Zoo & PPG Aquarium and surrounding areas. In time, it is hoped that the owls will begin to breed and higher densities will be achieved, as well as a suppressed rodent population.

1328: *-.213*

An introduced pathogen, white pine blister rust (Cronartium ribicola), has caused declines in five-needled pines throughout North America. Simultaneously, fire exclusion has resulted in dense stands in many forest types, which may create additional stress for these generally shade-intolerant pines. Fire exclusion also allows fuels to accumulate, and it is unclear how affected populations will respond to the reintroduction of fire. Although white pine blister rust and fire exclusion are widely recognized threats, long-term demographic data that document the effects of these stressors are rare. We present population trends from 2168 individuals over 5-15 years for an affected species, sugar pine (Pinus lambertiana), at several burned and unburned sites in the Sierra Nevada of California. Size-based matrix models indicate that most unburned populations have negative growth rates (lambda range: 0.82-1.04). The growth rate of most populations was, however, indistinguishable from replacement levels (lambda = 1.0), implying that, if populations are indeed declining, the progression of any such decline is slow, and longer observations are needed to clearly determine population trends. We found significant differences among population growth rates, primarily due to variation in recruitment rates. Deaths associated with blister rust and stress (i.e., resource competition) were common, suggesting significant roles for both blister rust and fire exclusion in determining population trajectories. Data from 15 prescribed fires showed that the immediate effect of burning was the death of many small trees, with the frequency of mortality returning to pre-fire levels within five years. In spite of a poor prognosis for sugar pine, our results suggest that we have time to apply and refine management strategies to protect this species.

1329: *+.142*

Captive breeding of animals is widely used to manage endangered species, frequently with the ambition of future reintroduction into the wild. Because this conservation measure is very expensive, we need to optimize decisions, such as when to capture wild animals or release captive-bred individuals into the wild. It is unlikely that one particular strategy will always work best; instead, we expect the best decision to depend on the number of individuals in the wild and in captivity. We constructed a first-order Markov-chain population model for two populations, one captive and one wild, and we used stochastic dynamic programming to identify optimal state-dependent strategies. The model recommends unique sequences of optimal management actions over several years. A robust rule of thumb for species that can increase faster in captivity than in the wild is to capture the entire wild population whenever the wild population is below a threshold size of 20 females. This rule applies even if the wild population is growing and under a broad range of different parameter values. Once a captive population is established, it should be maintained as a safety net and animals should be released only if the captive population is close to its carrying capacity. We illustrate the utility of this model by applying it to the Arabian oryx (Oryx leucoryx). The threshold for capturing the entire Arabian oryx population in the wild is 36 females, and captive-bred individuals should not be released before the captive facilities are at least 85% full.

1330: *+.083*

We developed an interactive management model for wild and captive populations of the ploughshare tortoise or angonoka, Geochelone yniphora. Interactive management is based on the translocation of individuals between wild and captive populations to simulate a metapopulation. Demographic parameters of one captive and two wild populations of this rare tortoise were used to conduct a metapopulation viability analysis (MVA). The effectiveness of the conservation strategy proposed for this species was then evaluated by modifying the probability of extinction and growth of the metapopulation over a fixed period of time. Several alternative scenarios of interactive management were then tested and ranked in terms of their effect on the viability of the metapopulation. The model predicted that catastrophic events such as bush fires would likely have a negative effect on the future of remaining wild populations. However, the model also predicted that the use of captive-born offspring to establish additional wild populations would decrease the risk of extinction of the metapopulation as a whole. We believe that, when supported by sound knowledge of the demographic parameters of a species, the use of MVA as part of an interactive management program can be an effective conservation tool that allows assessment of the probable response of both captive and wild populations to different management alternatives. One of the most interesting aspects of this interactive management approach is the link between in situ and ex situ conservation. (C) 2004 Elsevier Ltd. All rights reserved.

1331: *+.297*

The maintenance of wild populations of Greater Rhea (Rhea americana) through effective management requires an understanding of their habitat requirements in terms of vegetation composition and field configuration. We studied the relative influence of some anthropogenic variables (presence of route, house and fences) and resource variables (presence of water source, composition and coverage of plant species, vegetation height and bare soil), on the habitat use by a population of rheas in a cattle ranch of Buenos Aires Province, Argentina. Habitat use was determined indirectly by documenting the number of faeces in summer, autumn-winter and spring 1999. The presence or absence of faeces was related to the measured variables through discriminant analysis that allowed the elaboration of predictive models of habitat use by this species. Contrary to what was expected, those variables related to human activity showed a low

predictive value on the habitat use by rheas when compared with resource variables. Rheas preferentially selected the stream area in all seasons and sites with great percent cover of Bupleurum sp., Phyla canescens, Sida leprosa, Plantago lanceolata, Trifolium repens, Lolium multiflorum, Stipa spp., and Stenotaphrum secundatum. Low vegetation height was another important component of rhea's habitat in summer and autumn-winter. The high accuracy level obtained by validation tests of this model supports its utility for the management of rhea populations in other cattle ranches of the region, and to analyze the suitability of other ranches for reintroduction programs. (C) 2004 Elsevier Ltd. All rights reserved.

1332: *+.274*

To assess the feasibility of restoring an extirpated species, it is crucial to identify the method of reintroduction that optimizes juvenile survival and growth so that adequate adult populations inay be established. Because Atlantic salmon Salmo salar fry are relatively expensive to rear, we compared the efficacies of two embryo-stocking methods and one fry-stocking method in producing age-0 Atlantic salmon parr in the Salmon River, New York. As measured by resulting part- densities, there were no differences in survival rates between embryos stocked with and without protective hatching boxes; the mean Survival of embryo-stocked fish to late summer was less than 0.01 %. Fry stocking produced significantly greater densities of late-summer parr than did embryo stocking, with site-specific Survival ranging Lip to 8%; the mean survival of fry was 2%. Considering the hatchery costs of rearing each life stage, the labor involved in stocking, and the resulting late-summer parr abundances, we recommend planting fry rather than embryos as a method of reintroducing Atlantic salmon. Additional studies are needed to evaluate the success of stocking older life stages of Atlantic salmon in the Salmon River, although factors other than juvenile survival are probably important in establishing and maintaining a spawning Population.

1333: *+.108*

Conservation efforts in the United Kingdom have increasingly sought to restore the populations and ranges of birds affected by changes to their habitat or by direct human persecution. One approach is reintroduction, although this is likely to be appropriate in only a limited set of circumstances, involving a small number of species. This paper reviews the use of reintroduction as a tool for conserving birds associated with lowland farmland, highlighting the benefits of this approach to conservation, as well as the potential dangers. Two case studies are presented to illustrate how different reintroduction techniques are required, depending on the species involved and the donor stock available. The internationally agreed, though not legally binding, IUCN guidelines for reintroduction projects are summarized, together with the requirements of The Wildlife & Countryside Act (1981) in relation to the release of birds into the wild in Britain. Potential changes to current legislation are suggested, to prevent inappropriate and potentially damaging reintroduction projects from being carried out in the future.

1334: *+.187*

To organize and prioritise species-specific conservation efforts, we delineate 'functional conservation units' for the threatened Alcon Blue butterfly Maculinea alcon in Belgium. We used detailed distribution data on the butterfly, its host plant and its habitat, present-day population sizes and its mobility and colonization capacity to determine functional conservation units (FCUs) on different spatial scales: FCU-1, i.e., the 12 presently occupied habitat patches plus the area within a range of 500 in surrounding them (the maximum local movement distance, based on mark-release-recapture data), FCU-2, i.e., the areas within a range of 2 km around the occupied habitat

patches (the maximum observed colonization capacity) and FCU-3, i.e., potential re-introduction sites (sites where M. alcon went extinct recently). We suggest different management and planning measures for each type of functional conservation unit and discuss translocation and re-introduction as 'intensive care' conservation measures for this threatened and sedentary species. (C) 2004 Elsevier Ltd. All rights reserved.

1335: *+.007*

The bearded vulture Gypaetus barbatus is a large, long-lived osteophagus vulture whose abundance and breeding range have drastically declined during the last century, making it one of the most endangered European bird species. We evaluated the extinction risk of the bearded vulture population in Corsica (a small, isolated breeding population of 8-10 pairs), one of the last extant populations in Western Europe, and estimated its probability of extinction to be 0.165 over the next 50 years. A sensitivity analysis to assess the influence of uncertain demographic rates showed that it is critical to estimate precisely the values of pre-adult survival. Neither the type nor the parameters of density dependence acting on fecundity and survival rates influenced much the extinction risk of the Corsican population. We evaluated the effect of four realistic conservation actions that could be implemented on the Corsican bearded vulture population and rank them in terms of their respective decrease of the current extinction risk faced by this population. We found that the release of two juveniles every other year for 12 years and the increase of fecundity due to selective food provisioning would reduce by more than one-half the current extinction risk of Corsican bearded vulture population. In contrast, even substantial increases in the carrying capacity through large supplemental feeding produced very modest decreases in the extinction risk, thus calling into question the efficacy of one of the main pan-European conservation strategies for this species. Reestablishing a population network within the Mediterranean could be a potentially better strategy, though its efficacy depends on natal dispersal among populations that is currently unknown. (C) 2004 Elsevier Ltd. All rights reserved.

1336: *+.255*

Knowledge of the causes of success or failure in introductions may provide information to help improve the chances that re-introductions succeed. However, the utility of this information will be influenced by the comparability of sets of introduced and re-introduced species. Here, we assess the extent of this similarity using recent historical data on introduced and re-introduced birds. Introductions are less often threatened than expected and have larger ranges and earlier ages at maturity than re-introductions. Introductions also generally have 'weedy' life-history characteristics relative to family-typical values, whereas those of re-introductions more closely conform to these typical values. However, introductions and re-introductions do not differ in migratory behaviour, mating system or dichromatism, nor do they show statistically consistent differences in body mass, incubation time or annual fecundity. The taxonomic composition of the lists of introductions and re-introductions is similar, with more species than expected on both lists. Overall, these results suggest that the characteristics of introductions and re-introductions are sufficiently similar that conclusions from studies of the former may reasonably be used to inform studies of the latter.

1337: *+.159*

Island populations are an interesting dichotomy in conservation biology. On the one hand, they can be a refuge for species where mainland populations have been decimated by loss of habitat and predation by exotic predators. On the other hand, island populations usually have reduced genetic

diversity and are more susceptible to extinction through genetic and demographic processes. Genetic variation and morphological characters were measured for island and mainland populations of Parantechinus apicalis, small dasyurid marsupials, restricted to southwest Australia. Genetic variation at seven microsatellite loci revealed low levels of heterozygosity (H-e = 0.20 - 0.44) and high levels of inbreeding (F-e = 0.40 - 0.72) in island populations compared with the mainland population (H-e = 0.73). A nested clade analysis revealed that allopatric fragmentation was probably responsible for the association between geographical location and control region haplotypes, which is consistent with the isolation of populations on islands and indicative of two main populations of P apicalis representing separate conservation units for management. While these results are typical of many island populations, they have important implications in terms of the conservation of threatened species in Australia and around the world, where island populations are a common source of founders for captive breeding and translocation to mainland sites.

1338: *+.179*

The endemic pink pigeon has recovered from less than 20 birds in the mid-1970s to 355 free-living individuals in 2003. A major concern for the species' recovery has been the potential genetic problem of inbreeding. Captive pink pigeons bred for reintroduction were managed to maximise founder representation and minimise inbreeding. In this paper, we quantify the effect of inbreeding on survival and reproductive parameters in captive and wild populations and quantify DNA sequence variation in the mitochondrial d-loop region for pink pigeon founders. Inbreeding affected egg fertility, squab, juvenile and adult survival, but effects were strongest in highly inbred birds (F greater than or equal to 0.25). Inbreeding depression was more apparent in free-living birds where even moderate levels of inbreeding affected survival, although highly inbred birds were equally compromised in both captive and wild populations. Mitochondrial DNA haplotypic diversity in pink pigeon founders is low, suggesting that background inbreeding is contributing to low fertility and depressed productivity in this species, as well as comparable survival of some groups of non-inbred and nominally inbred birds. Management of wild populations has boosted population growth and may be required long-term to offset the negative effects of inbreeding depression and enhance the species' survival.

1339: *+.023*

Large blue (Maculinea) butterflies are highly endangered throughout the Palaearctic region, and have been the focus of intense conservation research(1-3). In addition, their extraordinary parasitic lifestyles make them ideal for studies of life history evolution. Early instars consume flower buds of specific host plants, but later instars live in ant nests where they either devour the brood (predators), or are fed mouth-to-mouth by the adult ants (cuckoos). Here we present the phylogeny for the group, which shows that it is a monophyletic clade nested within Phengaris, a rare Oriental genus whose species have similar life histories(4,5). Cuckoo species are likely to have evolved from predatory ancestors. As early as five million years ago, two Maculinea clades diverged, leading to the different parasitic strategies seen in the genus today. Contrary to current belief, the two recognized cuckoo species show little genetic divergence and are probably a single ecologically differentiated species(6-10). On the other hand, some of the predatory morphospecies exhibit considerable genetic divergence and may contain cryptic species. These findings have important implications for conservation and reintroduction efforts.

1340: *+.063*

Published data, recent surveys and studies of museum specimens are combined to provide a list of

84 fishes for South Australia in five drainage divisions. The list includes 58 native species (44 restricted to freshwater) and 26 alien species. Seven endemics are recognised, namely Chlomydogobius eremius (Zeitz), Chlamydogobius gloveri Larson, Cralerocephalus dalhousiensis lvanstoff & Glover, Craterocelphalus eyresii (Steindachner), Craterocephalus gloveri Crowley & Ivanstoff, Mogurnda thermphila Allen & Jenkins and Neosilurus gloveri Allen & Feinberg. New records are reported for Cralerocephalus stercusmuscarum stercusmuscarum (Gunther), Galaxias trutaceus Valenciennes and Neochanna cleaveri (Scott), and a terapontid of uncertain status also is noted. Range extensions are reported for Nannoperca obscura (Klunzinger), Nannoperca australis Gunther and an undescribed species of Hypseleotris, and the presence of Galaxias olidus Gunther and Galaxias brevipinnis Gunther in particular regions is confirmed. Possible extirpations are reported for Ambaysis agassizii Steindachner, Gadopsis marmoratus Richardson, Galaxias rostratus Klunzinger, Maccullochella macquariensis (Cuvier), Macquaria australasica Cuvier, Mogurnda adspersa (Castelnau), Neochanna cleaveri and Prototroctes maraena Gunther. There is need for further evaluations of fish distributions, better systematic frameworks, clarifications of conservation status, reviews of the introduction and impacts of alien species and development of protective measures for fish species and communities and their ecosystems.

1341: *+.038*

The Tuamotu Sandpiper or Titi is the only surviving member of the Tribe Prosoboniini and is confined to eastern Polynesia. Formerly distributed throughout the Tuamotu Archipelago, it has been decimated by mammalian predators which now occur on nearly all atolls of the archipelago. Isolated sandpiper populations are currently known from only four uninhabited atolls in the Tuamotu. Only two of these are currently free of mammalian predators, such as cats and rats, and the risks of rat invasion on them are high. This paper outlines tasks necessary in the short term (within five years) to secure the species, together with longer term actions needed for its recovery. Short-term actions include increasing the security of existing populations, surveying for other potential populations, eradicating mammalian predators on key atolls, monitoring key populations, and preparing a recovery plan for the species. Longer term actions necessary for recovery include reintroductions, advocacy and research programmes.

1342: *-.092*

Line-transect surveys were conducted at a small remnant of semideciduous Atlantic Forest to estimate primate density and population size. The Mata Sao Jose has about 230 ha and it is located between Rio Claro and Araras (Sao Paulo State, southeastern Brazil). This forest fragment holds 23 non-volant mammal species distributed in seven orders e 13 families. From July 1999 to January 2001, a total of 125 km were walked and three primate species were recorded. The densities were estimated based on Distance software. Brown capuchin monkey Cebus nigritus (Goldfuss, 1809) (Cebidae) presented the highest population density (24.5 individuals/ km2, ranging between 19.6 and 32.6 individuals/ km2). Titi monkeys Callicebus nigrifrons (Spix,1823) (Pitheciidae) and tufted ear marmosets Callithrix aurita (E. Geoffroy, 1812) (Callitrichidae) presented 3.5 individuals/ km2 (ranging between 2.8 to 4.6 individuals/ km2). Total population size of brown capuchin monkeys was 56 individuals (ranging between 45 to 75 individuals) and the titi monkeys and tufted ear marmosets were eight individuals (ranging between six to 10 individuals). Titi monkeys and tufted ear marmosets are vulnerable to extinction and both persist at Mata Sao Jose, but at low population densities. These primate species are at local extinction risk, because populations at low densities are more suscetible to stochastic events, inbreeding depression and environmental variation. Translocation is the only plausible management action that could avoid local extinction of these primates.

1343: ***+.118***

Recent losses and fragmentation of tallgrass; prairie habitat to agriculture and urban development have led to corresponding declines in diversity and abundance of plants and birds associated with such habitat. Mowing and burning are alternative management strategies for restoring and rejuvenating prairies in fragmented landscapes, but their specific, comparative effects are the subjects of ongoing evaluation. We compared the responses of plant and bird communities on four sets of mowed, burned, and untreated sites of small (3-10 ha), fragmented tallgrass prairies at the DeSoto National Wildlife Refuge (DNWR), Iowa, U.S.A., during May-July in 1998 and 1999. Species richness and diversity of plants, resident grassland birds, and communities of birds associated with grassland edges (edge species) were independent of treatment. Although not affecting species richness and diversity in plant communities, mowed sites ranked lower in total plant coverage and total forb coverage than burned sites or untreated sites. In contrast, untreated sites had more coverage by shrubs, suggesting that mowing and burning did retard shrub encroachment. Overall, abundance and diversity of plants and birds were generally insensitive to management strategies. Small, fragmented sites of rare habitat may not respond in the short term to management treatments and may not be capable of supporting highly diverse communities, no matter how intensively manipulated. It is more probable that diversity of native prairie communities can be enhanced and restored only through long-term efforts, acquisition of large land units capable of supporting stable populations, and deliberate reintroduction of species of high conservation value.

1344: ***+.119***

In the past, the otter (Lutra lutra) was a predator in Dutch freshwater ecosystems. Due to hunting, fishing, destruction and fragmentation of its habitat, pollution, traffic mortality and disturbances due to recreation, the otter became extinct. Fourteen years after extinction, the otter has been reintroduced in the Weerribben, an area in the northwest of Overijssel province. There was much discussion about releasing otters in the Netherlands. This paper discusses the reintroduction project in the light of the IUCN (international Union for Conservation of Nature and Natural Resources) guidelines, which are internationally recognized and applied in many reintroduction projects. The IUCN guidelines stress the importance of biodiversity and public nature conservation awareness (important in a highly populated country as the Netherlands). The guidelines stipulate that the former causes of death should have been removed or minimised in the release area and that the chances of a spontaneous recolonisation by otters, sufficient to establish a viable population, should be small. Capturing otters for reintroduction should not have negative effects, the released otters should be genetically similar to the extinct otter population and the project has to be monitored. Assessment of the project to reintroduce otters into the Netherlands shows that that the IUCN guidelines were followed.

1345: ***+.177***

1. Species undergoing reintroduction offer a unique opportunity for clarifying their specific niche requirements because they are likely, if sufficiently mobile, to colonize the most suitable habitats first. Information drawn from the individuals released first might thus be essential for optimizing species' policy as reintroductions proceed.2. Bearded vultures were extirpated from the European Alps about a century ago. An international reintroduction programme using birds reared in captivity was launched in 1986; up to 2003, 121 individuals had been released at four different locations. Subsequent dispersion throughout the range has been far from homogeneous, resulting in a clumped occurrence of the first breeding pairs within three main zones that do not necessarily

coincide with release areas.3. In order to discern ecological requirements we performed a geographical information system (GIS) analysis of bearded vulture sightings collected in Valais (Swiss Alps) from 1987 to 2001. This area harbours no release site, is situated in the core of the Alpine range and has been visited by birds from all four release points.4. During the prospecting phase (1987-94, mostly immature birds), the most important variable explaining bearded vulture distribution was ibex biomass. During the settling phase (1995-2001), the presence of birds (mostly maturing subadults) correlated essentially with limestone substrates, while food abundance became secondary.5. The selection of craggy limestone zones by maturing bearded vultures might reflect nesting sites that are well protected against adverse weather, as egg laying takes place in the winter. Limestone landscapes, in contrast to silicate substrates, also provide essential finely structured screes that are used for bone breaking and temporary food storage, particularly during chick rearing. Finally, limestone substrates provide the best thermal conditions for soaring.6. Synthesis and applications. Extrapolated to the whole Alpine range, these findings might explain both the current distribution of the subadult/adult population and the absence of breeding records for bearded vultures around release sites in landscapes dominated by silicate substrates. As reintroduced bearded vultures tend to be philopatric, we suggest that population restoration would be more efficient if releases were concentrated within large limestone massifs. This case study of the bearded vulture illustrates the need for continual adaptive management in captive release programmes.

1346: *+.105*

The National Trust for the Cayman Islands has been implementing conservation efforts for Cyclura lewisi since 1990. Commencing with small-scale captive breeding, the program has expanded to include field research, large-scale captive breeding and head-starting, reintroduction and restocking, habitat protection and management, education, and awareness activities. A formal species recovery plan is in place. In the past 12 years, the size of the wild population appears to have declined from 100-200 to 10-25 animals and is functionally extinct. Principal causes for the decline are habitat loss, predation by introduced mammals, road kills, and continued exploitation by humans. Reproduction in the wild by animals released from the captive breeding population has been confirmed. However, in order to assure the greatest possible genetic diversity within the captive population, wild individuals with potentially distinct genetic constitutions must be captured and assessed before their genes become diluted as a consequence of interbreeding with released animals. Goals of 100 hatchlings and approximately 100 two-year-old iguanas for release annually are being approached. Options for restoring self-sustaining wild populations are limited to habitat islands, for which perimeter fencing will be essential to restrain iguanas and exclude predators. The recovery program is operating on institutional grants, program-generated income, and private donations, but we hope that this charismatic lizard will become able to support its own survival through carefully managed tourism activities and related commerce.

1347: *+.086*

The use of relocations, repatriations and translocations as amphibian and reptile conservation strategies has received much debate. In the case of endangered species, their use may outweigh the potential negative consequences. We performed an experimental repatriation of the eastern massasauga (Sistrurus catenatus catenatus), which has experienced range-wide population declines and extirpations. The experiment included measures to minimize negative conspecific effects to the donor populations as well as inter-species effects oil the release and donor sites. Snakes released during late July had lower mortality rates, larger home ranges, and gained more mass than snakes released in early September. The July release cohort also successfully reproduced, while no

breeding activity was observed with September release snakes. Results of this study suggest that repatriation may be a viable method of restoring eastern massasauga populations. We hope the methods and conservation measures used in this experiment will serve as a template for future repatriations.

1348: ***+.057***

The need for an individual-based approach in genetic management for game species has been identified and generally welcomed by a wide spectrum of stakeholders in the South African game industry. Different levels of "intensive breeding" exist in the game industry At the one extreme would be the closely controlled captive breeding programmes for carnivores such as white lions, Panthera leo, cheetah, Acinonyx jubatus, and wild dogs, Lycaon pictus. It is generally acknowledged that a system approaching a domestic stud book would be justified for these programmes, and such databases are already in place for some species. At the other end of the scale are the game-farm populations of moderately valuable antelope species, for example tsessebe, Damaliscus lunatus, and nyala, Tragelaphus angasi, where individual genetic information is probably neither necessary nor feasible. It is in the area between these two extremes where the concept of individual genetic information was investigated. Examples are the white rhino, Ceratotherium simum, African buffalo, Syncerus caffer, and the valuable populations of species from the Hippotraginae, roan antelope, Hippotragus equinus, and sable antelope, Hippotragus niger. An accurate National Genetic Database for game species based on individuals can contribute to issues such as the retention of genetic diversity, avoidance of hybridization, the conservation of evolutionary significant units (ESUs), regulation of translocations, avoidance of inbreeding and to parentage verification. The database contributes to the development and application of cross-species and species-specific microsatellite marker sets, the expansion of a national BIOSTORE and the application of these tools for the forensic analyses of poaching cases. It can also ultimately result in a reference sequence database for all game species at a national level.

1349: ***+.060***

Pre-Euro-American settlement forest structure and fire regimes for Jeffrey pine-white fir, red fir-western white pine, and lodgepole pine forests in the Lake Tahoe Basin (California and Nevada, USA) were identified using stand structural analysis and fire scars based on measurements of stumps of trees that were cut in the 19th century. Comparisons of the presettlement reference with contemporary conditions were then used to determine how and why contemporary conditions deviate from presettlement conditions and to guide ecological restoration. Contemporary forests varied in different ways compared to the presettlement reference. Contemporary Jeffrey pine-white fir forests have more and smaller trees, more basal area, less structural variability, and trees with a more clumped spatial distribution than presettlement forests. The mean presettlement fire-return interval for Jeffrey pine-white fir forests was 11.4 yr, and most (>90%) fires burned in the dormant season; no fire was recorded in the study area after 1871. Similar differences were identified in the structural characteristics of contemporary and presettlement red fir-western white pine and lodgepole pine forests. However, 19th-century logging changed the composition of red fir-western white pine forests, and these forests now have more lodgepole pine than red fir or western white pine. Comparison of contemporary forests with the presettlement reference suggests that restoration treatments in Jeffrey pine-white fir forests should include: (1) density and basal-area reduction, primarily of smaller diameter trees; (2) reintroduction of frequent fire as a key regulating disturbance process; and (3) increasing structural heterogeneity by shifting clumped tree distributions to a more random pattern. Restoration treatments in red fir-western white pine

forests should include: (1) a shift in species composition by a density and basal-area reduction of lodgepole pine; and (2) increasing structural heterogeneity by shifting tree distributions to a more random pattern. In lodgepole pine forests the restoration emphasis should be: (1) a density and basal-area reduction of small-diameter trees; and (2) an increase in structural heterogeneity that shifts tree spatial patterns from clumped to a more random distribution. Reintroduction of fire as a regulating process into high-elevation red fir-western white pine and lodgepole pine forests can be viewed as a longer-term restoration goal. The method for quantifying presettlement reference conditions from stumps complements, or is an alternative to, methods based on dendroecology or repeat photography.

1350: *+.121*

The White-winged Guan (Penelope albipennis) is a cracid endemic to the Northwestern Peruvian dry forests that is critically endangered due to poaching pressure and habitat loss. In the year 2000, a species reintroduction program was established which consisted in the release of captive born and raised animals from the Barbara D'Achille captive breeding center. The guans were released in areas where the species had disappeared and that were safe for reintroduction and conservation. Between September 2001 and July 2003, 20 White-winged Guans were released at the Chaparri Private Conservation Area, Lambayeque, Peru. The bird's adaptation to the area was measured through its dispersion, survival and reproduction. Based on the measured dispersion distance, it has been found that it is actually feasible to link the reintroduced population with the surrounding wild populations. Survival has been established at 55 %, measured two years after the animals were released. With respect to reproduction, 3 wild-born chicks were born from reintroduced parents.

1351: *+.149*

Case studies are presented for the western ringtail possum, Pseudocheirus occidentalis, and the quokka, Setonix brachyurus. The ringtail case. study summarises information from a recent review of the species' distribution and conservation status, collates information on ringtail populations within the areas covered by the Department of Conservation and Land Management's (CALM) fauna recovery program, Western Shield, and draws extensively on the recent findings from translocation programs. Critical to the case study is the finding that (i) monitoring of the response to fox control programs is poor, or non-existent, at all known sites, with the exception of research specific translocation sites. This may reflect the difficulty in censusing and monitoring this species; and (ii) translocation success has not been demonstrated at any translocation release site and the primary translocation site, Leschenault Peninsula Conservation Park, has suffered a significant population decline. Hypotheses are proposed to explain this decline and, in the absence of demonstrated translocation success, critical components for monitoring western ringtail possum translocations are recommended. Monitoring protocols are also recommended for other extant populations within the species' geographic range. The quokka case study also summarises information from a recent review of the species' distribution and conservation status and collates information on quokka populations within the areas covered by Western Shield. The case study also draws extensively on the findings from recent research from the northern jarrah forest. As for the ringtail case study, the quokka case study found monitoring is non-existent or poor, or. ad hoc at best, at all known sites, with the exception of research specific sites within the northern jarrah forest. Consistent with recently published research findings, intervention management is recommended at these northern jarrah forest sites. An active adaptive management framework, using fire as a tool to create the preferred structural habitat mosaic, is recommended. Both case studies highlight the need to review data collection, collation, analyses and reporting processes to

ensure data are collected in a manner to allow objective analyses and scrutiny. Under this proposed scenario, any conservation gains can be quantified and documented. Conversely, in circumstances where desired outcomes have not been met, the failure to meet set goals can be identified and mechanisms can be implemented to improve conservation management.

1356: ***+.050***

The effects of seasonal prescribed fire on the belowground ectomycorrhizal community and live fine root biomass were investigated before, 1 year after, and 2 years after prescribed underburning. Ectomycorrhizas were sampled from four replications of three treatments (fall underburning, spring underburning, and a nonburned control) in a randomized complete block design. Samples were separated in two subsamples representing the upper 5 cm and lower 5 cm of a soil core. Molecular tools were used to distinguish 140 restriction fragment length polymorphism (RFLP) species of fungi directly from the ectomycorrhizas. Prior to underburning, the number of RFLP species and amount of live root biomass were similar among treatment units and between upper and lower core samples. Fall underburning largely removed live root biomass to a depth of 10 cm and significantly reduced ectomycorrhizal species richness compared with spring underburning and the nonburned control for at least 2 years. RFLP species richness and live root biomass following spring underburning were generally similar to the nonburned treatment. The successful reintroduction of fire to the ecosystem to retain high species diversity of ectomycorrhizal fungi and achieve the desired future condition of large-tree ponderosa pine retention with low fuel loads may require more than underburning in a single season.

1357: ***+.038***

Translocation of European wild rabbit Oryctolagus cuniculus L. is one of the most frequent management tools to increase rabbit density in Spain, both as prey of several predators that are threatened with extinction and for sport hunting. Nevertheless the elevated short-term mortality by predation makes translocations unsuccessful and increases their biological cost. Information on the factors affecting the short-term survival and dispersal of translocated rabbits is required to improve release management and increase performance of translocated rabbits, and to avoid the use of non-selective lethal methods for predator control. In this study we tested electric fencing and night-shooting as alternative to traditional release protocols, and the effects of vegetation cover and warren fencing on short-term survival and dispersal of rabbits. Night shooting performed during the first nights after release increased significantly the survival of rabbits, by hindering the activity of carnivores in the release area. The use of an electric fence enclosure also increased the performance of rabbits, but was not efficient to constraint rabbit dispersal. Rabbits released in areas with low vegetation cover showed higher mortality and dispersal distances than rabbits released in high cover areas. Warren fencing decreased both the dispersal of rabbits and the adverse impact of predation in low cover areas, but had no effect in high cover areas. Selection of high cover areas or warren fencing in low cover areas seem to be the most advantageous release conditions to decrease the short-term predation impact, reducing the biological cost of rabbit translocations and the risks for endangered predators derived from the use of traditional predator control practices during translocations. (C) 2004 Elsevier Ltd. All rights reserved.

1358: ***+.073***

There is increasing evidence that culture is an important determinant of behavior in some non-human species including great apes and cetaceans (whales and dolphins). In some cases, there may be repercussions for population biology and conservation. Rapidly evolving "horizontal" cultures,

transmitted largely within generations, may help animals deal with anthropogenic change and even allow them to exploit it, sometimes with negative consequences for both the animals and humans. In contrast, stable "vertical" or "oblique" cultures, transmitted principally between generations, may impede adaptation to environmental change, and confound range recovery, reintroductions and translocations. Conformist stable cultures can lead to maladaptive behavior, which may be mistaken for the results of anthropogenic threats. They can also structure populations into sympatric sub-populations with distinctive cultural variants. Such structuring is common among cetaceans, among which sympatric sub-populations may face different anthropogenic threats or respond to the same threat in different ways. We suggest that non-human culture should be integrated into conservation biology when considering populations with such attributes, and also more generally by refining definitions of evolutionarily significant units and considering how cultural attributes may change our perspectives of non-humans. (C) 2004 Elsevier Ltd. All rights reserved.

1359: *+.111*

Pond-breeding amphibians require aquatic and terrestrial habitats to complete their lifecycles, and preservation of both habitats is necessary for maintaining local populations. Current wetland regulations focus primarily on aquatic habitats, and criteria to define critical upland habitats and regulations to protect them are often ambiguous or lacking. We examined the association between the presence of seven pond-breeding amphibian species and the landscape composition surrounding 54 wetlands located within the Till Plains and the Glaciated Plateau ecoregions of Ohio, USA. We quantified landscape composition within 200 m of the wetland ("core terrestrial zone") and the area extending from 200 m to 1 km from the wetland ("broader landscape context zone"). We constructed binary logistic regression models for each species, and evaluated them using Akaike Information Criterion. Presence of spotted salamanders (Ambystoma maculatum), Jefferson's salamander complex (A. jeffersonianum) and smallmouth salamanders (A. texanum) was positively associated with the amount of forest within the core zone. Presence of wood frogs (Rana sylvatica) was positively associated with the amount of forest within the core zone and the amount of forest within the broader landscape context zone. Presence of tiger salamanders (A. tigrinum tigrinum) was negatively associated with the cumulative length of paved roads within 1 km of the site, and presence of red-spotted newts (Notophthalmus v. viridescens) was negatively associated with the average linear distance to the five nearest wetlands. Overall salamander diversity was positively associated with the amount of forest within the core zone, and negatively associated with the presence of predatory fish and cumulative length of paved roads within 1 km of the site. Our results confirm the strong association between the structure of surrounding upland areas and amphibian diversity at breeding ponds, and stress the importance of preserving core terrestrial habitat around wetlands for maintaining amphibian diversity. (C) 2004 Elsevier Ltd. All rights reserved.

1360: *+.191*

In this paper we discuss the history of the koala and possum fur trade in Queensland, based mainly on material in Queensland Government archives, and review the development of legislation and wildlife management practices. The koala and possum harvests arose in response to a large increase in the abundance of koalas and possums in mid to late 19th century. A marked increase in skin prices in the early 20th century provided further stimulation to the harvests. Harsh rural conditions and economic downturns further raised interest in harvesting and the government regularly came under pressure to open trapping seasons for economic reasons. Regulated koala harvests operated intermittently from 1906 to 1927, whereas possum harvests were held more

frequently and over a longer period, from 1906 to 1936. Fluctuation in demand was a major influence on the industry, leading to the expansion of the industry after 1900, and the eventual demise of the possum industry in the 1930s. Strong demand over about three decades resulted from a range of factors influencing the international fur markets, associated in particular with warfare and economic conditions after World War I.Successive Queensland governments supported the harvest because of its economic value, despite having serious concerns about the viability of the koala harvest. The legislation to control the harvest was aimed at protecting possums and koalas from extinction and establishing a sustainable industry. The principal legislative devices used to manage the harvest were close seasons and establishment of sanctuaries, supported by various other measures. Animals were taken by baiting (cyanide), snaring and shooting. Control measures were ineffective in preventing breaches of the regulations, and widespread take occurred during close seasons. The Department of Agriculture and Stock attempted to manage the trade as a sustainable harvest, with annual assessment of populations, and determination of open seasons in response to population levels and market conditions. The harvest was supported by population conservation measures including the establishment of reserves and attempts to conserve populations with a restocking (translocation) program for both koalas and possums in areas thought to be over-harvested. Koalas and possums were translocated to a number of mainland sites and onto islands. The take of possums ranged from about 400,000 to 3,000,000 per annum and that of koalas from about 450,000 to nearly one million. In good seasons, top quality skins could bring at least 60 shillings per dozen. The poorest quality skins brought as little as 2(1)/(2) pence each. The industry was of considerable economic value, generating personal incomes substantially greater than the income from wages of many workers, and total returns comparable to those of the annual sales of gold for the State. The total number of trapping permits issued in a season varied from about 8,000 to over 9,000. Governments had serious reservations about the koala harvest, but supported the possum harvest much more strongly. Possum harvests were held more frequently, generated more income, and made an important contribution to the economy. Strong community opposition to koala harvests first arose in 1919, effectively signalling the end of the harvest for that species. By 1936, community opposition to the possum seasons had also become established. There was a market downturn at the same time and the possum harvest came to an end. Significant community opposition to harvests of both species arose in both urban and rural areas, the latter mainly because harvests were seen as interfering with grazier interests, causing death of stock from cyanide baits and disturbance to stock by shots and spotlights.

1361: *+.107*

Alagoas Curassow Mitu mitu has long been subjected to taxonomic discussions as to its validity as a full species or as a member of a polytypic assemblage which includes the Razor-billed Curassow Mitu tuberosum. In this paper we review the history of this highly threatened taxon (now extinct in the wild) endemic to north-eastern Brazil, including summarising and mapping known localities for the species. We analyse and discuss all previously published proposed diagnostic characters for M. mitu and confirm its validity as a full species based on diagnostic characters of a bicoloured bill, bare auricular patch, tawny-tipped tail and nearly all-black central rectrices (molecular evidence also support this conclusion). We detail surveys carried out in north-eastern Brazil in October-November 2001 which were unsuccessful in finding extant populations of M. mitu but encountered widespread hunting and some wood extraction in tile few remaining forest blocks. There is, however, landowner Support for protecting tile remaining forests in the majority of areas. The captive population history and management is given including information on current location and provenance. Before reintroduction of M. mitu to the wild is possible many conservation measures will be needed at the chosen forest location, including better protection, monitoring, and educational work with local people.

1362: ***+.127***

Small founder populations of whooping cranes are managed to maximize egg production for the purpose of reintroducing young to the wild. This results in an excessive number of hatched chicks that cannot be naturally reared by parents. Hand-rearing techniques have been developed to raise the additional hatches. However, hand rearing may affect the behavior of the birds and their chances of survival later in life. The objectives of this study were to determine the impact of rearing practices on the behavior of whooping crane chicks. The birds were reared under three commonly used rearing techniques: parent reared (PR), hand reared (HR), and hand reared with exercise (HRE). Fifty-six whooping crane chicks were observed by focal animal sampling from hatch to 20 weeks of age. During these observations, occurrences of comfort behavior, aggression, foraging, nonvigilance, sleep, vigilance, and other types of behavior were collected. Data were analyzed using mixed models repeated measures analysis of variance (ANOVA). Behavior was affected by rearing treatment, age, and time of day. PR birds spent more time being vigilant than HR and HRE birds. An inverse correlation was found between percentage of time foraging and vigilant ($r = -0.686$, $P < 0.0001$). However, there were no differences in the behavior of birds reared in HR or HRE programs. (C) 2004 Published by Elsevier B.V.

1363: ***+.059***

Aims This study was designed to compare levels of genetic variation and its partitioning in three related species of Antirrhinum. A. subbaeticum, A. pertegasii and A. pulveridentum, and to check the hypothesis that species with small total population size have lower levels of genetic variability than those with bigger ones. This information should contribute to the development of conservation strategies of rare endemic species of Antirrhinum. Methods One hundred and seventy-seven plants were screened for variability at 14 allozyme loci by means of horizontal starch gel. Parameters of genetic diversity, and its partitioning, were calculated. An indirect estimate of gene flow was based on the equation: Nut = (1 - G(ST))/4G(ST). Key Results Genetic variabilities in A. subbaeticum and A. pertegasii were found to be the lowest known for the genus, the within-population genetic diversity being correlated with population size in both species. The distribution of genetic diversity is strikingly different among species, with 85% of the total variation distributed among populations in A. subbaeticlun. 6% in A. pertegasii and 23% in A. pulverulentum. Estimated levels of gene flow were negligible for A. subbaeticum (0(.)04), high for A. pertegasii (3(.)92), and substantial for A. pulverulentum (0(.)83). Genetic and geographic distances were negatively correlated in A. pertegasii, whereas no significant correlation was found in the other two species. Conclusions Levels of total genetic diversity agree with the hypothesis that species with small total population size have lower levels of genetic variability than those with bigger ones. Strategies for the conservation of the species are recommended. such as preservation of natural populations and avoidance of possible causes of threat, as well as ex situ preservation of seeds, reinforcement of small populations of A. subbaeticum with plants or seeds from the same population. and avoidance of translocations among populations. (C) 2004 Annals of Botany Company.

1364: ***-.114***

We used radioimmunoassay to determine fecal corticoid concentrations and assess potential stress in 10 endangered whooping cranes (Grus americana) undergoing reintroduction to the wild. Fecal samples were collected shortly after hatching at a captive facility in Maryland, during field training in Wisconsin, and throughout a human-led migration to Florida. After a 14-day decline following hatching, fecal corticoid concentrations stabilized at baseline levels for the duration of

the captive period, despite exposure to potentially stressful stimuli. Shipment of the cranes to the field training site was correlated with an eight- to 34-fold increase in fecal corticoid concentrations, which returned to baseline levels within 1 week. Increases were positively correlated with age but not body weight at the time of shipping. Fecal corticoid concentrations during the training period increased slightly and exhibited greater variation than levels observed at the captive facility, but were well within expected norms based on previous studies. Fecal corticoid concentrations increased twofold following premigration physical examinations and placement of radiotransmitters, and persisted for up to 4 days before they returned to baseline levels. Though fecal corticoid concentrations and variation during the migration period were similar to training levels, there was an overall decline in fecal corticoid concentrations during the artificial migration. Acute stressors, such as capture, restraint, and severe storms, were associated with stress responses by the cranes that varied in accordance with lasting physical or psychological stimuli. The overall reintroduction process of costume-rearing, ultralight aircraft habituation, training, and artificial migration was not associated with elevations in fecal corticoid concentrations suggestive of chronic stress.

1365: *-.152*

Conserving genetic diversity of rare and endangered species and their evolutionary potential is one of the long-term goals of ex situ conservation. Some potential genetic risks in ex situ conservation in botanical gardens are presented. The preserved species may lack genetic representativity because of poor sampling. Inappropriate plantations. inadequate records and unclear kinships jeopardize endangered species to genetic confusion. inbreeding depression or outbreeding depression. Artificial selection and habitat conversion also potentially result endangered plants in adapting to ex situ conservation. which had been usually overlooked. All the genetic risks can decrease the success of reintroduction and recovery. Therefore. appropriate genetic management should be carried out in botanical gardens to decrease or avoid genetic risks in ex situ conservation.

1367: *+.115*

A team of biologists and field assistants conducted, between January 2002 and May 2003, a survey on the status of the Iberian lynx in Portugal. The survey was performed on previously identified lynx areas, during studies carried out in the 1970-s and 1990-s Intensive search for lynx scats, for DNA analysis, and camera trapping provided a basis for identifying potential lynx areas. Over 4200 km were covered during a global searching effort of 1975 man-hours. This effort resulted in the collection of 168 potential scats that were submitted to genetic validation with no positive lynx amplifications. Camera trapping was applied in a total effort of 5647 camera days, in three lynx potential areas. No positive detections were achieved. At the same time, a study on wild rabbit distribution reveals that most historical nuclei do not support lynx viable populations. Although we can not confirm extinction, the scenario is highly pessimistic. The Iberian lynx is presently in the verge of extinction. Intensive rabbit regression and massive habitat destruction are identified as the main causes of decline in recent decades. In the Portuguese lynx historical range, we could only identify significant areas suitable for lynx in the South-eastern part of the country, particularly in the Andalusian border, where we lack on recent evidence of lynx presence. Being aware of the considerable difficulties pointed above, the ICN developed a Conservation Action Plan for the Iberian lynx in order to provide a consistent and effective approach to conserve the species in Portuguese territory. This proposal describes guidance that retains future options, provides management consistent, offers necessary flexibility, in order to achieve the maximum goal of conserving the lynx in Portugal. Conservation measures have the goal of provide guiding

lines for conservations agents in order to conduct actions that can positively affect lynx and/or to help avoid negative impacts through thoughtful planning of activities. The proposal of Action Plan will be applied in all the areas located in the lynx historical distribution geographic area, that present suitable characteristics for the species presence or landscape features that can be optimize for lynx survival and that can be relevant for the species life-cycle, independently of their protection status. The goal of this plan is to apply pre-release strategic reintroduction activities to make possible, in a long-term, the reintroduction of Iberian lynx, in order to assure the viability of the species, as a fundamental element of Mediterranean ecosystems. For achieving this goal it will be necessary to establish a suitable connection between ex-situ and in-situ actions.

1368: *+.239*

In the article the state of international bison's total number has been demonstrated and the threat and actual problems of species preservation have been shown. The mane conception of National Belorussian program on conservation, resettlement and using of the European bison (Program 'Bison') have been expounded. The metapopulation model of free-living bison's subpopulations with different status and regimes including main fund (insured) - for bison's from nature reserves and national parks and reserve fund - for animals inhabiting territories of general economic usage, are recognized more optimal and realistic. The results of first stage of program fulfilment have been sum up. In 1994 -2000 78 bison were caught in National park "Byelaya Vezha forest" and became the founder of 5 new centers of free-living subpopulations. The bison's reintroduction was successful. In centers bison's number has been reached 226 individuals in 2004. There are 610 individuals in Belarus compared with 347 before the start of the Program "Bison".

1371: *+.165*

American black bears (Ursus americanus) have been extirpated from all but a few areas in southwestern Alabama, and the remaining habitat is being rapidly lost to development. Remnant bear populations exist near extensive (> 125,000 ha) bottomland hardwood forests in the Mobile-Tensaw Delta (MTD), but those bottomland areas are rarely used by bears. Reintroduction of black bears to the MTD may improve viability of the remaining bear populations in southwestern Alabama. To evaluate the suitability of this area for bears, we compared habitat conditions at the MTD with similar alluvial habitats at White River National Wildlife Refuge (White River NWR), where bears are numerous. We measured overstory, midstory, and understory vegetation in the MTD and on the North and South management units at White River NWR. We used principal components analysis and principal variable selection to identify 9 variables associated with 5 principal components (hard mast, soft mast, cavity tree availability, large tree availability, and total basal area) that best explained variation among study areas. Differences among the study areas were associated with hard mast, soft mast, and cavity tree availability ($P <= 0.001$). Hard and soft mast production in the MTD was lower than at White River NWR, but we believe it was adequate. However, suitable den trees, which may be a critical habitat component given the duration and severity of winter flooding, appeared to be lacking in the MTD.

1372: *+.195*

Few studies have documented intestinal parasites in populations of Neotropical primates, and many of those have been carried out on captive animals. This essay reviews the state of knowledge of intestinal parasites in primates focusing on Neotropical species. The factors that affect gastrointestinal infections in wild primates are presented, and the implications on the conservation of primates are discussed. The main factors that affect parasitic infections include primate density,

behaviour, age, reproductive condition and diet, as well as humidity and habitat fragmentation of the habitat. It is recommended that primate management and translocation programmes consider the age class structure of the groups and the availability of medicinal plant species as food. It is also important to determine which season is most appropriate for the translocation of primates. Taking these factors into account when designing management programs and making decisions about translocating primate groups will help deter the negative effects of intestinal parasitic infections and contribute to the successful conservation of primates.

1373: *+.057*

The grey partridge Perdix perdix species is divided into at least eight subspecies (Table 1), of which two exist in Finland. In this study, which is presented more detailed in English elsewhere (Liukkonen 2005), the mitochonclrial DNA (mtDNA) control region I (CR1) of 138 wild and 36 captive grey partridges (Table 2. Fig. 2) was sequenced. Two separate mtDNA haplotype lineages were found hereafter referred to as the eastern and the western lineage. The dominant Finnish haplotype differed from the dominant European haplotype by 15 nucleotidesubstitutions (3.7%, Fig. 1). Both lineages included onecore haplotype. The Finnish lineage dominated in the wild population (131/138 birds, 94.9%). whereas theEuropean lineage dominated in the captive stock (32/36 birds. 88.9%). The eastern core haplotype was observed in 53 wild individuals (38.4 % of all wild bird samples) and it dominated in the South-Ostrobothnia subpopulation 42/90 individuals). Four birds of this haplotype were also found in the subpopulations of North Ostrobothnia and South Finland and three birds in subpopulation of SW Finland. Five western haplotypes were found among the farmed birds, and three among the wild birds in this addition to the dominant western haplotype. The greatest differences between populations were found between the wild and captive subpopulations(Table 3). The highest nucleotide and haplotype diversities were found in South Finland and in North Ostrobothnia, where individuals representing both lineages were found. The lowest nucleotide and haplotype diversities were found in South Ostrobothnia (Table 3). The analysis of molecular variance showed that 79.8% of the total variance was explained by the variation of birds within subpopulations and 20.2% by the variation between the different subpopulations. When the captive stock was included in the analysis, 67.0% of the variation was explained by the variation between the different subpopulations and 33.0% by the variation of birds within the subpopulations. According to the significant pair wise [PHI],STS (Table 3) captive birds differed from all the wild populations. South Ostrobothnia was different from the North Ostrobothnia and Southern Finland subpopulations, and SW Finland was different from North Ostrobothnia. These results clearly show that DNA CR1 in the native stock in Finland differs from that in the farm stock. No clear geographical structuring could be found in the distribution of separate haplotypes into different subpopulations. Some common haplotypes were found (ip, i8. Li, i11, Fig. 1), but several unique haplotypes were also found (i3, iS, i15). The pattern of one core, some common and several unique haplotypes is similar to that found in the Finnish capercaillie Tetrao urogallus subpopulations. There were two common western Lp (19 inclividuals) and L6 (9 individuals) in the captive population. In addition to this, the captive stock also included tour single haplotypes (LI, L2, L4 and L.7). Four eastern birds were also found in the captive stock, two of which represented the core haplotype ip, one was i9-type and one, ipt1, was unique and not found in the wilt. The captive rearing and releasing of grey partridges in Finland has previously been carried out to increase the game bag. Recently, it has become more and more common to do this in order to strengthen natural populations and to reintroduce the species into areas where it has disappeared. However. the survival rate of released partridges has been poor. It is worth mentioning that despite the frequent releases of birds representing the European lineage into the subpopulation South Ostrobothnia only one bird (1, 90) of this lineage has found in this area. The lack of success in ntroductions may result from the maladaptation of

the European grey partridges to the climatic conditions of lie release areas in Finland. Although the survival of the eleased partridges seems to be poor, it is possible, that ome released birds survive in the wild and later breed. The tame birds breed better in captivity. As a result, because European grey partridges have been captive-bred in Finland for generations, it has become obvious that the farm stock has adapted to life in aviaries. These birds breed easily and produce a great amount of offspring. In contrast, if wild birds are taken into farms, they are generally unsuccessful because of the stress and fear they experience caused by human presence, and their numbers therefore decrease in farm stock. Genetic adaptation to captive conditions is recognised as a severe problem if captive populations are used for reintroductions. Avoiding genetic adaptation to captive conditions and preserving genetic variation in the farm stock are without any doubt the major challenges of captive rearing. Captive stock may to some extent be "improved" by introducing individuals from the wild, but this may not always be possible or expedient. It has become increasingly desirable to only captive-rear birds with native origin in Finland. Captive-rearing itself includes many pitfalls that have to be solved in order to produce "good-quality" birds for reintroductions. In this study I have clearly shown one pitfall in captive-rearing, i.e. the possibility of genetic differences between wild and the captive stocks. Introducing alien species or subspecies is not considered reasonable from the conservational point of view. This has already been taken into account in the management of the grey partridge at a national level. However, habitat protection and improvement of the environment, as well as hunting restrictions, will still be of high importance in theconservation of the Finnish grey partridge populations.

1374: ***+.053***

IMPACTS OF TSUNAMI IN 2004 (Updated to May 2005) The earthquake of December 26 2004 that struck 30km beneath the Indian Ocean off the Northwestern coast of Sumatra, Indonesia, affected 10 countries that share the waters of the Indian Ocean. It triggered massive tsunamis which caused extensive devastation on land and unprecedented loss of lives, and will go down as one of the worst human tragedies in history. Four out of the 10 Southeast Asian countries were affected by the December 26, 2004 earthquake and tsunami - Indonesia, Thailand, Malaysia and Myanmar, all with coastlines lying within the Indian Ocean. Indonesia The northern sections of the island of Sumatra suffered the most severe damage. Pre and post-tsunami satellite images of northern Sumatra show considerable changes to the coastline morphology, including changes to the bathymetry of the surrounding waters. The tsunamis infiltrated several kilometers in to mainland in many areas, destroying not just coastal areas but entire cities and villages. An early estimate of coral reef damage falls in the range of 30%. Thailand A total of six provinces were affected by the tsunami [long dash] Phuket, Ranong, Phang-Nga, Krabi, Trang and Satun, with Phang-Nga experiencing the greatest impact. A total of 174 sites covering the six affected provinces were assessed. 12% of the sites showed severe impact; with 47% of the sites showing low to moderate impact and 41% of the sites had no visible impact from the tsunami. Malaysia The tsunamis reached the northwestern coast of Malaysia 2-3 hours following the earthquake. Coral reefs along the northwestern region of Malaysia were assessed in March 2005, and no tsunami related damage was observed at all sites surveyed, including Pulau Langkawi, Pulau Paya and Pulau Perek. Myanmar Official government reports that followed stated that Myanmar was only mildly affected by the tsunami, and subsequent assessments by NGOs and unofficial reports by tour operators and tourists substantiated the government reports. An assessment of the coral reefs of western Mergui Archipelago by Reef Check Europe and WorldFish Center in March 2005 indicated that the reefs were literally untouched by the tsunami waves with little environmental damage. CURRENT STATUS OF CORAL REEFS Cambodia Coral reefs are distributed along the offshore islands and rocky coastlines of Cambodia, covering a total reef area of approximately 28.065 km2. Seven sites have been identified and established for long term monitoring. Diversity

studies have indicated that 111 species of hard coral, 17 species of soft corals and 9 species of seafans and seawhips are present in Cambodian waters. The average live coral cover at the seven monitoring sites range from 23% to 58%. Indonesia Indonesia is a large archipelagic nation with about 14% of the world's coral reef area. Results form coral monitoring activities between 1993 to 2003 showed an overall improvement in coral reefs in "poor" condition, while reefs in the good and excellent condition remained relatively stable and unchanged. However, progressive degradation of coral reefs within the last 10-15 years in several areas has been recognized. The best reefs were mostly restricted to remote areas or areas with some level of active management in place. Philippines Changes in the percent cover of different benthic lifeform categories on coral reefs in the Philippines that are in excellent condition indicate that the reefs may be experiencing a steady state of decline (from 5% to 3% to >1% of reefs in excellent condition). However, reefs in good condition are still present in the Celebes Sea, Southern Philippine Sea, Sulu Sea and the Visayas biogeographic regions. About 15% of the reefs surveyed in South China Sea and Sulu Sea sustained very high fish biomass. Singapore Singapore is a small island state with an estimated 54km2 of coral reefs. Almost the entire coastline of the main island, particularly the southern half have been altered by land reclamation and many of the southern offshore islands merged by reclamation to form larger islands. Coral reefs, once abundant along the southwest coastline of the main island have been lost to coastal reclamation and are now restricted to narrow fringing bands and patches among the southern offshore islands. Despite the limited reef area and high anthropogenic impacts, some reefs still support good coral cover (50% - 70% coral cover) and diversity (over 150 species of corals). Thailand The coastal areas of Thailand between latitudes 6[degree] and 13[degree]N offer suitable environmental conditions for coral reef development. Three types of coral distribution are recognized in Thailand - coral communities with no true reef structure; developing fringing reefs; and early formation of fringing reefs. The surveys of coral reefs in certain provinces during 2002-2004 showed obvious changes condition of some reefs within the Gulf of Thailand, while the condition of coral reefs in the Andaman Sea have remained relatively unchanged. Vietnam Coral reefs are distributed within the shallow waters of the main coastline and around the offshore islands. Coral reefs in better condition are found within the central coastal areas and around the southeastern islands, where temperatures are normally greater than 20[degree]C, and where the reefs experience some offshore influences. Recent coral survey data indicate a dominance of reefs with fair to poor condition (18% and 30% hard coral cover respectively at 50 monitoring sites). Declining abundance of key invertebrate and fish indicators also indicate a reduction of economically important reef organisms. Reefs in better condition were only recorded at some reefs in Ninh Thuan and Phu Quoc. China The lack of warm water along much of China's coast has inhibited coral reef growth. Typical fringing reefs occur mainly on part of coasts of Hainan Island and Taiwan Island. Before 1984, coral reefs in China were in good condition with more than 70% in coral cover. In the 1990s, coral reef conditions declined occurred in great pace due to the fast social-economic growth. Coral percent cover was 32% in Daya Bay in 1991 and 38% in Luhuitou, Sanya, in 1994. During the recent surveys in 2002 in China, coral percent cover was still low as 35% in Daya Bay and 19% in Luhuitou. However, some areas have higher coral percent cover such as 68% in Yongxing Island, Xisha Island. Hong Kong Coral Communities are mainly found in the eastern and northeastern coasts of Hong Kong. There are currently four marine parks and one marine reserve in Hong Kong and all the MPAs are now being monitored for various physical parameters and biodiversity and abundance of corals, fish, other invertebrates and algae. The corals within the marine parks in the northeast are so far, generally healthy and some increase in coral cover has been registered over the last four years. Taiwan Coral reefs are found in all the waters around Taiwan except in the sandy area on the west coast. No significant changes in coral cover among years were found between 1997 and 2004, because both increasing and decreasing trends were observed. However, more increasing trends at reef slope and more decreasing trends at reef flat were found, suggesting that the reef flat biota was more

frequently influenced by anthropogenic disturbances than that of the reef slope. Japan Corals widely distribute from Ryukyu Islands to northern islands of Japan including Kyushu, Shikoku and Honshu. Although most of the coral have been recovered gradually in 10 to 20 years after the outbreak of crown of thorns starfish (COTs), the number of COTs has been increasing again in 2000's. After the catastrophic bleaching event in 1998, relatively small scale bleaching occurred in 2001, 2002 and 2003. On the other hand, coral coverage is increasing in the northern islands because of elevated water temperature in winter relaxing the limits for coral growth in the area. Korea Korean waters do not have any typical coral reefs by reef building corals, because of the low water temperature. Only the area around Jeju Island affects the influence of warm current and has temperate, subtropical and tropical marine species. 134 of soft and hard coral species are found in Korea including 15 species of stony corals. Most of researches have been conducted on soft corals mainly in Jeju Island THREATS TO CORAL REEFS Cambodia There are many anthropogenic impacts to coral reefs including destructive fishing practices, over-fishing, coastal development, agricultural run-off, sedimentation and seaweed farming on reefs. Reef fish and invertebrates densities are low due to over-fishing. Indonesia Many coastlines are being rapidly degraded mainly through inappropriate land development. Rapid population growth, land-based industry, domestic waste and tourism have resulted in a wide range of environment and resource management problems. The marine resources are severally threatened by destructive fishing methods such as dynamite, cyanide, compressors, fish traps, reef gleaning and over fishing. Philippines Philippines has a large coastal population that is highly dependent on reef resources for their livelihoods. Over-fishing and destructive fishing practices are by far the greatest threats to the reefs, followed by coastal development and land-based activities. Singapore Ongoing land reclamation since the early 1970's has resulted in elevated sedimentation. Regular dredging of shipping lanes and the offshore dumping of dredged spoils contribute to the sediment load. This chronic impact has resulted in the decline in reef condition since 1986, with a marked reduction in the vertical growth zone along the reef slopes. Thailand The first tsunami disaster in the history of Thailand occurred on December 26, 2004. 13% of the reef monitored showed "high impact", with around 40% showing "no impact". 21%, 17% and 9% of the reefs showed "very low", "low" and "moderate" impact respectively. Vietnam There were serious outbreaks of the Crown-of-Thorns starfish in Van Phong and Nha Trang bays, while algal bloom killed almost all the coral reefs in Ca Na bay (Binh Thuan province), with the recovery occurring slowly after the event. Sedimentation continues to have a strong impact in Ha Long and Cat Ba islands. China Overfishing and destructive fishing practices have badly damaged coral communities around Hong Kong and Xisha islands, causing most high-value fish species to become locally extinct. Blast fishing has been widely practiced in Hainan Island. Cyanide fishing is carried out by large-scale commercial operators who take fish from remote areas such as Xisha Island and Nansha Island. Cyanides are also used for collecting aquarium fishes. Sedimentation, freshwater incursion, and sewage outflows have adversely impacted China's reefs, particularly near the mainland. Failure in proper managing the land resources have caused problems of siltation. Local governments has bombed out Mabian Zhou, Daya Bay for oil industry in late 1990s, although coral reef transplantation has been carried out to reduce the reef destruction, the damage to coral reefs is still great. Hong Kong Occasional bleaching, predation, human or storm disturbances have been observed in Hong Kong waters. High sedimentation due to dredging and land reclamation, high nutrients due to effluence of untreated sewage and high heavy metal contents due to industrial effluence are some of the main marine pollution problems in Hong Kong. Extensive mortality of corals was reported in South Ninepin attributable to heavy siltation brought about by dredging in Tung Long Wan Borrow area. Hong Kong waters are generally considered as being overfished. Taiwan Soil erosion and landslides frequently occur in coastal areas especially after storms, and these runoffs have carried large amounts of sediment and nutrients to reef areas. Sewage of most towns and villages in coastal areas are often ill-treated and discharged to reef areas. This induces serious nutrient

enrichment to reef ecosystems especially in northeastern and southern Taiwan, and Hsiaoliuchiu. Although blast and poison fishing methods have been officially banned, there are still sporadic reports on these illegal fishing methods. Anchor damages could be found sporadically on most reefs. Bottom-trawlers, with their heavy gears sweep over reef surface, have caused severe damage to coral reefs in Penghu Islands. Trampling and mechanical breakage of coral skeleton by divers and tourists have been a serious problem at diving hotspots in Lutao, Penghu, and southern Taiwan. Construction of fishing ports and coastal highway in northeastern and eastern Taiwan has caused damage to coral reefs. Extensive coral bleaching was observed in southern Taiwan, Penghu Is., and Hsiaoliuchiu reefs following heavy rainfalls associated with typhoons in June and July, 2004. Outbreak of a sea anemone, Condylactis nanwanensis, was noticed in Nanwan Bay, southern Taiwan and caused severe damage to coral reefs since it destroys coral tissue and weakens coral skeleton. Typhoon is another disturbance on corals in Taiwan. Coral communities at Chitou, Penghu Islands and Tiaoshi, Nanwan Bay were heavily damaged (up to 90% coral mortality) by Typhoon in 2001, and have not recovered since then. Japan The number of COTs has been increasing again in southern part of Japan since 2000's. After the catastrophic bleaching event in 1998, relatively small scale bleaching occurred in 2001, 2002 and 2003. Soil runoff by the coastal development is also one of the major threats on the reefs in Okinawa and mainland Japan. The corallivorous gastropod, Drupella is still major predator of corals in northern islands. Korea The increase of tourists for SCUBA diving and tourist submarine have become the cause of reef destruction. CORAL REEF MANAGEMENT Cambodia The Cambodian government has very limited human resources, infrastructures, and finances for scientific research and monitoring. There are no laws that relate to the protection of coral reefs, with the exception of a Fisheries law. A national action plan that integrates coral reef management and conservation, capacity building and maintenance, infrastructure and institutional development, improved legislations, better administrative framework and enforcement, public awareness, communication, education, and participation, biodiversity assessments and regular monitoring is urgently needed. Indonesia The new 1999 legislation (Autonomy laws No. 22/1999 and No. 25/1999) effectively integrated all previous legislations concerning marine management. The ministry of Forestry's Directorate General for Protection and Nature Conservation (PKA) is the agency responsible for nature conservation in Indonesia. The government has set a target to establish 10 million ha of marine conservation area; however, only some 5.8 million ha has been officially declared, which includes 37 marine conservation area. Coral reef ehabilitation and Management Program (COREMAP) have conducted public communication programs for the last two years. All target sites were positively affected by the campaign. Philippines The Department of Environment and Natural Resources (DENR) and the Department of Agriculture, Bureau of Fisheries and Aquatic Resources (DA-BFAR) are the two national government agencies that have the mandate to establish MPAs or fishery refuges. Under the 1998 Philippine Fisheries Code, at least 15% of the total coastal areas in each municipality should be identified as fish sanctuaries. Of over 600 MPAs that have been established in the Philippines, only around 10% of these MPAs are actually being managed effectively. A National Coral Reef Strategy is currently being prepared to provide a clear and integrated management framework for the protection, conservation and rehabilitation of the coral reefs in the country. Singapore There are no marine protected areas covering coral reefs, although proposals to protect identified reefs have been made. Currently, only two legislated nature areas have marine components, but they do not contain reefs. The establishment of the Biodiversity Center within the National Parks Board in 2004 has increased the focus on the marine environment. Artificial reefs and coral translocation activities have been initiated since the mid-1990's to address the issue of coral reef remediaion. Thailand There are numerous institutions involved in coral reef monitoring and management, coordinated through the Department of Marine and Coastal Resources and several universities. A National Coral Reef Strategy: Policies and Action Plan was adopted by the cabinet in 1992. All the major coral reefs in Thailand are assigned

to one of four management categories - general use zones, intensive tourism zones, ecotourism zones and ecosystem reserve zones. The majority of coral reefs are classified as ecotourism zones. There have been a few studies on community-based management of coral reefs in Thailand, such as Had Chaolao in Chantaburi Province where local people manage coral reefs for tourism. Vietnam National government and international agencies have supported the development of a network of marine protected areas in Vietnam. A national action plan for coral reef management is being developed under the framework of UNEP GEF/SCS project. China Coral reefs are protected and managed by the regulations, such as; the State Law of Marine Environment Protection issued in 1983 and new revised edition issued in 2000; the Hainan Province Regulation of Coral Reef Protection issued in 1998; and the State Law of Ocean Use Management issued in 2001 demand that all coastal development programs need accord with the Division of Marine Functional Zonation made by government. There are 3 Marine Coral Reefs Reserves in mainland of China and more MPAs are in the planning process. Marine Coral Reefs Reserves are "no-take" areas where only scientific research is allowed within the boundaries. A program named "Restoration of Coral Reef Ecosystem and Protection and Management of Its Biodiversity in South China Sea of China" is one of the priority programs of 21 Century Ocean Agenda of China. Hong Kong The most important legislature that has a direct bearing on the protection of coral communities is the Marine Parks Ordinance that enacted in 1995 and took effect in July 1996. This Ordinance provides for the designation, control and management of marine parks and marine reserves under the Country and Marine Parks Authority of the Agriculture and Fisheries Department (AFD). The Agriculture, Fisheries and Conservation Department (AFCD, former AFD) of the Hong Kong SAR Government is the lead agency overseeing the protection of Hong Kong marine parks. The Authority is advised by the Country and Marine Parks Board that is made up of representatives from different government departments, the academia, NGOs and the public. There are currently four Marine Parks and one Marine Reserve in Hong Kong. AFCD provides the funding for research teams from tertiary institutions to monitor the parks. Taiwan Most coral reefs in Taiwan are within national parks, national scenic areas or coastal conservation zones and these areas are protected by the National Park Law and the Coastal Environmental Protection Plan. However, the current laws are inadequate to protect coral reefs. The revision of laws and the planning of MPAs with effective management are currently processing by governmental administrations. Japan There are several management practices conducted in the local communities to control the disturbances, such as COTs and Drupella. The government has established the Law for the Promotion of Nature Restoration and focus on integrated coastal management involving different stakeholders for coral conservation. There are two programs of Nature Restoration Projects targeting coral conservation. Korea Southern coast of Jeju-do were designated as MPA in the name of Wetland Protection Areas by Ministry of Maritime Affaires and Fisheries (MOMAF) as well as Man and Biosphere (MAB) by UNESCO in 2002. There are some overlapping between designation of protected area and development plan in different level (agencies) and they made local stakeholders confused. Legislation in local/national level needs to be well coordinated in complementary integration

1375: ***-.094***

This project's goal was to restore populations of four rare fishes into Abrams Creek, Blount County, TN. These species, all on the US Endangered and Threatened Wildlife List, include two catfishes, the smoky madtom (Noturus bailey) and the yellowfin madtom (N.flavipinnis), the duskytail darter (Etheostoma percnurum) and the spotfin chub (Erimonax monachus). Captive propagation, reintroduction, and non-invasive monitoring techniques were used for this restoration effort, which began in 1986. By 2000, there was evidence of reproduction for all four species. As of 2003, the number of these species stocked in Abrams Creek was 3167 smoky madtoms, 1574 yellowfin madtoms, 3430 duskytail darters, and 11,367 spotfin chubs. Increasing population sizes

were indicated for three of the four fishes, and smoky madtom and duskytail darter abundances were nearly comparable to native populations in Citico Creek, Monroe County, TN.

1376: *+.080*

Diuris species exhibit a large amount of morphological variation. resulting in a lack of unique characters and hence difficulties in assessing phylogenetic relationships. This study used direct sequencing of the nuclear ITS and plastid trnT-F regions for assessment of the phylogenetic relationships among some closely related Diuris species from the state of Victoria. Australia. Yellow and purple-flowered Diuris species formed well-supported monophyletic sister clades. Relationships within the purple-flowered clade, including Diuris fragrantissima, were unresolved and purple-flowered species in the "punctata" group sampled for this study are genetically very closely related. This Study will have implications for the conservation of Diuris fragrantissima and the location of a suitable mycorrhizal symbiont for reintroduction of this critically endangered species.

1377: *+.144*

Laelia lobata (Lindl.) H.J. Veitch is among the 24 endangered orchid species of Rio de Janeiro city. This species, endemic to Rio de Janeiro state mountains, was known to occur out), at the top of the most-visited mountains in town, on Pao de Acucar rock and Pedra da Gavea rock. Today the Pao de Acucar rock population may well be extinct because of over-collecting practices. but some plants can still be found at Pedra da Gavea rock, in spite of past over-collecting at the Site. Currently the CORES (Coastal Orchid Restoration) Project has adopted a conservation strategy that covers both ex-situ and in-situ conservation of the species. Following the action plan for conservation, developed by the IUCN Orchid Specialist Group, the project has three objectives: (I) Determine the conservation Status of Laelia lobata, analyzing Population demography, floral biology, and population genetics of three samples (Pao de Acucar rock, Pedra da Gavea rock, and ex-situ propagates); (2) Micro-propagate this Species using symbiotic and asymbiotic germination of seeds; and (3) Educate climbers on the need to preserve the orchid's native habitat on the two hills. Reintroduction will be attempted only if necessary. By pursuing these objectives, we attempt to achieve the main purpose of the project, which is to remove Laelia lobata from the Red List of endangered orchids.

1378: *+.197*

Some 221 species of native orchids have been recorded in Singapore. Of these, however. ca. 170 orchid species are considered to be extinct. and only four are viewed as common. The orchid conservation program at the Singapore Botanic Gardens aims to monitor these species. to explore ways to conserve their germplasm, and to increase their number for subsequent reintroduction into appropriate habitats, including managed parks and roadsides. Thus far, we have successfully reintroduced Grammatophyllum speciosum, Bulbophyllum vaginatum, and Bulbophyllum membranaceum. Recently. we initiated the Orchid Cryo-Seed Bank Project, and have managed to successfully store seeds Of four native species. They are Dendrobium crumenatum, Spathoglottis plicata. Bulbophyllum vaginatum. and Dendrobium anosum.

1379: *+.037*

Ex situ conservation may be the immediate solution for endangered species, but prolongued captivity poses problems as populations adapt to man's environment. Here we present ail historical

and biological overview of such problems, and we end with a word for caution as to their efficacy when reintroduction is desired.

1380: *+.236*

Current Species Status: Blackburn's sphinx moth (Manduca blackburni) is federally listed as endangered (U.S. Fish and Wildlife Service 2000a). This taxon is currently known to occur on three of the seven major Hawaiian Islands. No known Blackburn's sphinx moth populations are entirely protected from the numerous factors threatening the species' recovery, and the moth is endangered throughout its range. Habitat Requirements and Limiting Factors: Blackburn's sphinx moth is currently found in association with topographically diverse landscapes that contain low to moderate levels of non-native vegetation. Vegetation types that support Blackburn's sphinx moth include dry to mesic shrub land and forest from sea level to mid-elevations. Soil and climatic conditions, as well as physical factors, affect the suitability of habitat within the species' range. Impacts to the moth's habitat from urban and agricultural development, invasion by non-native plant species, habitat fragmentation and degradation, increased wildfire frequency, ungulates, and direct impacts to the moth from non-native parasitoids and insect predators have significantly reduced the species' range (A. Medeiros, U.S. Geological Survey-Biological Resource Division, pers. comm., 2001; E. Van Gelder and S. Conant, in litt., 1998). Recovery Objectives: Needed conservation and recovery activities include protection, management, and restoration of habitat including out-planting of native Nothocestrum sp. (aiea) (confirmed and presumed) host plants (two of which are federally listed: N. breviflorum and N. peltatum); a Blackburn's sphinx moth captive breeding and/or translocation program; and research. This plan identifies 3 recovery units comprised of 13 management units which are geographic areas recently documented to contain Blackburn's sphinx moth populations and/or Blackburn's sphinx moth host plant populations and which shall be the focus of recovery actions. The three recovery units and their component management units contain habitat considered necessary for the longterm survival and recovery of Blackburn's sphinx moth (e.g., networks of suitable habitat patches and connecting lands). The overall objective of this recovery plan is to ensure the species' longtermconservation and to conduct research necessary to refine recovery criteria so that Blackburn's sphinx moth can be reclassified to threatened status, and eventually delisted. Goals include: (1) protect habitat known to support extant populations and high-quality habitat known to have supported moth populations in the past; (2) stabilize moth populations within their known distributions; and (3) conduct research necessary to refine recovery criteria to include threat information. Downlisting Criteria: One Blackburn's sphinx moth population on each island of Hawaii, Kahoolawe, and Maui must be well-distributed, naturally reproducing, and stable or increasing in size through one to two El Nino events or for at least 5 consecutive years of average rainfall conditions before downlisting may be considered. This criterion assumes future genetic studies (see recovery action 3.5 and 3.6) will confirm the species currently consists of multiple populations. If additional research reveals the species is actually comprised of one population, this criterion will need to be revised. Stable Blackburn's sphinx moth populations are defined in this recovery plan as those in which observed population declines are followed by a population increase to pre-decline levels. These criteria should provide for the maintenance of genetic variation that occurs in natural populations of Blackburn's sphinx moth by protecting all known, natural populations and the habitats upon which they rely. Furthermore, these criteria should provide some assurance that a single catastrophic event will not destroy all populations of this species. More specific downlisting criteria can be developed when completion of some of the recovery actions provides necessary information on the life history and ecology of this species and its host plants. Delisting Criteria: Before delisting of Blackburn's sphinx moth can be considered, all of the following four requirements must be met: (1) one moth population, within one management unit, must be naturally reproducing and stable or

increasing in size, through one to two El Nino events or a minimum of 5 consecutive years of average rainfall within the Kauai-Oahu Recovery Unit; (2) four moth populations, within four management units, must be naturally reproducing and stable or increasing in size, through one to two El Nino events or a minimum of 5 consecutive years of average rainfall on three different islands within the Maui Nui Recovery Unit (of those four, one within windward and one within leeward Maui Island); (3) two moth populations, within two management units, must be naturally reproducing and stable or increasing in size, through one to two El Nino events or a minimum of 5 consecutive years of average rainfall within the Big Island (Hawaii Island) Recovery Unit; and (4) a post-delisting monitoring plan and agreements to conduct post-delisting monitoring are in place and ready for implementation at the time of delisting. These criteria assume genetic studies (see recovery actions 3.5 and 3.6) will confirm the species currently consists of multiple populations. If additional research actually reveals the species is comprised of one population, these criteria will need to be revised. More specific delisting criteria can be developed when completion of some of the recovery actions provides necessary information on the life history and ecology of this species and its host plants. Specific Actions Needed: 1. Protect, manage, and restore habitat and control threats. 2. Expand existing wild Nothocestrum sp. host plant populations. 3. Conduct additional research essential to recovery of Blackburn's sphinx moth. 4. Develop and implement a detailed monitoring plan for Blackburn's sphinx moth. 5. Reestablish and augment, through captive propagation if necessary, wild Blackburn's sphinx moth populations within its historic range. 6. Develop and initiate a public information program for Blackburn's sphinx moth. 7. Validate recovery objectives. 8. Develop a detailed Post-Delisting Monitoring Plan for Blackburn's sphinx moth.

1381: *+.108*

We examined the public process used to develop the 1996-2001 Fortymile Caribou (Rangifer tarandus) Herd Management Plan adopted by state and federal management boards. The process differed from most government-supported planning processes because it was initiated by residents of Alaska and Yukon, and not by an agency. State, federal, and territorial agencies were asked to participate in and support development of a management plan that would include a broad range of interest groups. We describe the planning effort, issues addressed by the planning team that posed significant challenges during both the planning and implementation phases, and then identify unforeseen costs and benefits derived from the process. Critical decision points in plan development and implementation are discussed.

1382: *+.245*

Alstroemeria (Alstroemeriaceac), the Incas lily, an exclusively South American genus comprises near 90 species ranging from Venezuela (31 North) to Tierra del Fuego, Argentina (531 South). Alstroemeria species and hybrids have gained world-wide importance as cut flower crop due to its attractive flowers and the excellent keeping quality of them. Nevertheless, they are also suitable for growing in pots and for garden and landscape uses. The recent taxonomic revisions of the group for Brazil (Assis, 2001), Argentina (Sanso, 1996), and Chile (Bayer, 1987), the biosystematic research (Aagesen and Sanso, 2003; Sanso and Xifreda, 1998, 1999, 2001, 2003) and the chromosome studies (Sanso and Hunziker, 1998; Sanso, 2002) have facilitated the identification of the taxa and improved the basic knowledge of the genus. The nomenclature of the species, their geographical distribution, ecological characteristics and the interspecific relationships are now better understood. Many wild taxa, that are scarcely known so far, carry genetic traits that open possibilities for improvement and development of more attractive new varieties. For example, some of the Brazilian taxa have an important potential value in breeding

programmes because of their showy flowers and the rigid leaves, instead of the very tender foliage that possess most of the species; some Patagonian ones have rather short plant height, which is important for potted plants. Several species of the wild populations are nowadays threatened and with risk of extinction. Most of the species occupy reduced geographical areas. Species that inhabit plain regions are vulnerable because those fields are burned and cultivations are introduced. Patagonian species especially need to be protected, against the grazing of the animals. The cultivation of the native plants would help to the ex situ conservation. Reintroduction in some natural areas might also play an important role in order to maintain the landscape value.

1383: *+.352*

A population of Cicindela patruela patruela DeJean (Coleoptera: Carabidae: Cicindelini) existed until at least 1950 in a small area of eastern Washington, D.C., and adjacent Prince George's County, Maryland, USA. Suitable habitat for this population consisted of open sandy barrens with soils derived from Cretaceous sediments of the Potomac Group and vegetation characterized by oak and pine species, particularly Quercus marilandica Munchhausen and Pinus rigida Miller. This habitat was eliminated in the Washington area by extensive suburban housing construction, which was driven by rapid growth in the human population. Recommendations are provided for the reintroduction of C. patruela and for the restoration of suitable habitat at remnant natural areas.

1384: *+.128*

A micropropagation protocol was developed for the conservation of critically endangered Serbian perennial Nepeta rtanjensis (Lamiaceae). Rooted shoots were obtained from one-node stem segments and shoot tips on a half-strength Murashige and Skoog (MS) medium without growth regulators. The best pH of the medium for axillary buds induction and for rooting of shoots was found to be at 7 and/or 7.2 respectively. The addition of cytokinins to the culture medium did not significantly stimulated auxillary bud production as compared to the control. On the contrary, on media supplemented with high cytokinin concentrations, only dwarf shoots with rudimentary roots were obtained. All tested concentrations of 6-benzylaminopyrine (BAP) and kinetine (Kn) in combination with 0.1 mg 1(-1) indole-3-acetic acid (IAA) negatively affected the elongation and rooting of shoots. Plants micropropagated on hormone free medium and rooted in vitro were successfully acclimatized in greenhouse and in open field conditions. The result of successful acclimatization was the production of more than 7000 plantlets with normal sexually reproduction. They flowered, fruited and produced seeds which exhibited 47% germination. The survival rate of plants that were transferred to the open field for the acclimatization and exposed to the winter chill was 99%. The reintroduction of N. rtanjensis occurred in May 2004. One thousand plantlets were planted within the historic range of this plant species. The survival rate was also 99%.

1385: *+.116*

The great bustard is a globally-threatened species needing conservation action across Europe. This paper discusses key issues in the case for reintroducing the bird to Britain. Great bustards became extinct as a breeding species in Britain in 1832 probably as a result of hunting, agricultural change and inclement weather. The factors that caused the loss are no longer thought to operate. Suitable habitat exists in pockets across England and especially on Salisbury Plain where a large area is protected for military training and conservation purposes. The Plain combines short grass areas for lekking, long grassland for feeding and adjacent arable land for nesting. Pilot studies on arthropods in long grassland suggest that their density is sufficient for chick-rearing but the precautionary creation of additional food-rich areas among arable crops is recommended. Genetic

studies indicate that Britain's bustards probably belonged to the central European group and that restocking should not use birds from Iberia. Only Russia has sufficient birds to supply a reintroduction project and losses there through nest destruction are high. By rescuing eggs, artificially incubating them and transporting chicks to Britain, the project should have zero detriment to the donor population. Modelling indicates that 40 chicks will need to be brought to Britain for 5-10 years to build a founder population of 100 birds. Although focused on direct action in Britain, the project will promote grassland conservation across Europe and serve as a model for translocating bustards elsewhere.

1386: *-.136*

We radio-tagged and released 49 captive-reared Hispaniolan Parrots (Amazona ventralis) in Parque Nacional del Este (PNE), Dominican Republic, during 1997 and 1998. Our primary objective was to develop a restoration program centered on using aviary-reared birds to further the recovery of the critically endangered Puerto Rican Parrot (A. vittata). Hurricane Georges made landfall over the release area on 22 September 1998 with sustained winds of 224 km/h, providing us with a unique opportunity to quantify responses of parrots to such disturbances. Quantitative data on such responses by any avian species are scarce, particularly for Amazona species, many of which are in peril and occur in hurricane-prone areas throughout the Caribbean. Mean home ranges of 18 parrots monitored both before and after the hurricane increased ($P = 0.08$) from 864 ha (CI = 689-1039 ha) pre-hurricane to 1690 ha (CI = 10032377 ha) post-hurricane. The total area traversed by all parrots increased > 300%, from 4884 ha pre-hurricane to 15,490 ha post-hurricane. Before Hurricane Georges, parrot activity was concentrated in coastal scrub, tall broadleaf forest, and abandoned agriculture (conucos). After the hurricane, parrots concentrated their activities in areas of tall broadleaf forest and abandoned conucos. Topographic relief, primarily in the form of large sinkholes, resulted in "resource refugia" where parrots and other frugivores foraged after the hurricane. Habitat use and movement patterns exhibited by released birds highlight the importance of carefully considering effects of season, topography, and overall size of release areas when planning psittacine restorations in hurricane-prone areas.

1387: *+.269*

Determining whether animals select some habitats over others provides basic information about how animals meet their requirements for survival and reproduction. Habitat selection is therefore an important component of conservation research. Studies involving the release and establishment of threatened species on island refuges can be particularly insightful because breeding pairs should be able to select habitat of the highest quality within the range available. This study uses GIS technology to investigate the spatial distribution of breeding territories in relation to overall habitat availability of two threatened passerines, South Island saddlebacks (Philesturnus carunculatus carunculatus) and Stewart Island robins (Petroica australis rakiura)two years after their release onto predator-free Ulva Island. Both species established breeding territories around the periphery of the island in coastal forest fringe habitat and away from mature forest in the interior of the island. Compositional analysis suggested that both species prefer dense, fringe-type habitat with open ground cover and deep litter layers and avoid more mature forests, especially with moss cover. Thus habitat structure is likely to be more important for both species than plant-species composition. However, the possibility exists that the preference of coastal fringe habitat could represent an 'ecological trap', where habitat preference does not correspond to better quality habitat in terms of reproductive fitness. It will be useful to continue monitoring saddlebacks and robins to obtain data on survival and fecundity as the density of birds increases, and breeding pairs are forced to establish territories in what is presently perceived to be less preferred habitat in the

interior part of the island.

1388: ***+.025***

One of the greatest challenges for biologists today is to protect biodiversity against a backdrop of widespread habitat loss. Helenium virginicum Blake (Virginia sneezeweed) is a federally threatened herb associated with sinkhole margins and wet meadows in the Shenandoah Valley of Virginia and in the Ozark Highlands of Missouri. In this study we evaluated the influence of seed source (maternal line), planting site, water regime, and weed exclusion on survivorship, growth, and fecundity of H. virginicum with the objectives: (1) to establish two viable populations on public land in Missouri, and (2) to formulate reintroduction protocols for the species. We collected seed from the one known population in the state of Missouri. Seeds were germinated under controlled conditions and then transplanted to two sites on public land. Maternal line was found to have no significant effects on seed germination or seedling survivorship in the greenhouse for the first year. Maternal line, site, water regime, and weed exclusion cloth had no effects on the survivorship of H. virginicum at field sites. Maternal line, along with site, water regime, and weed exclusion cloth had significant effects on height at time of flowering and number of flowers produced by H. virginicum. The results of this study address some of the unknowns regarding the biology of H. virginicum and will be used to guide future reintroduction projects as well as the ongoing development of state and federal recovery plans.

1389: ***+.017***

Small mammal monitoring using live trapping was done along the upper section of river Drava in 2003-2004, using capture-mark-recapture. Our sample areas were located in a strictly protected alder gallery forest (Lankoci-erdo) and in a neighbouring plot under gradual reforestation which had been clear-cut in 2000. A total of 9 small mammal species were recorded in the two sites, each comprising a 1-hectare sampling grid. The small mammal community revealed here had three characteristic rodent species (Apodemus flavicollis, A. agrarius, Clethrionomys glareolus), and two frequent shrew species (Sorex araneus, Crocidura leucodon). With the populations assumed to be closed, population sizes of the three dominant rodents were estimated from daily capture data. The highest estimated values were obtained for the two Apodemus populations (A. agrarius and A. flavicollis). Population sizes of the bank vole could be estimated from the protected area only. A spatial analysis with the nearest neighbour method showed that individuals of the more frequent species aimed at an even distribution, both in 2003 when densities were lower, and in 2004 when densities were higher. The more detailed analysis of the spatial pattern in 2004 suggested that by the end of late autumn, the different species are already organised in a pattern of winter survival strategy. It was shown by spatial distribution data that in this period the area of the closed alder gallery forest was occupied by individuals of yellow-necked wood mouse and bank vole. In the inner, larger part of the 1 ha forest section, it was mostly yellow-necked wood mice that established their home ranges. Striped field mice occupied only the reforested areas. Using extensive areas, the reforested area was inhabited also by bank voles, which fact is a proof for population translocation between the two areas in this species.

1390: ***+.045***

The phylogeographic structure of the brown hare (Lepus europaeus) was studied by analysing mtDNA control region sequences of 98 individuals from continental and insular Greece, Bulgaria, Cyprus and northern Israel, together with 44 published sequences from Italy and central Europe. We found two distinct clades separated by an average nucleotide divergence of 6.6%, which may

correspond to a Balkan and to an Asia Minor refugium. The estimated time of separation of the two clades was dated back to 105,000490,000 years ago. These two clades coexist in the area of northeastern Greece and Bulgaria, most likely as a result of a post-glacial northward expansion. Within the southern Balkan refughium, network analyses showed geographical structuring, which supports the hypothesis of several isolated Late Pleistocene populations. The central European and Italian populations appear to have originated from a non-detected northern Balkan population that was genetically closely related to some northern Greek populations,as a result of postglacial expansion, translocations or a combination of both. Moreover, several cases of ancient and recent translocations by humans were detected, especially for some island populations, while the eastern Aegean islands off the Asia Minor coast were most likely colonized naturally through Late Pleistocene land bridge connection. The genetic analysis presented here provided a framework for designing proper conservation and management guidelines for this species. (C) 2004 Elsevier Inc. All rights reserved.

1391: *+.196*

Translocation-the deliberate, human-mediated movement of organisms-is a useful conservation tool most often employed in attempts to increase persistence of threatened or depleted species. Translocation projects involve difficult management decisions regarding the allocation of animals between sites. This research provides a rational scientific basis for these decisions. We use a stochastic population model and Stochastic Dynamic Programming to determine optimal translocation strategies for theoretical populations, and apply this framework to a case study on the Bridled Nailtail Wallaby (Onychogalea fraenata). The translocation problem is defined as follows: in each time step of the translocation program, a set number of individuals are sourced from a captive population and translocated to two sites. Management decisions involve the allocation of these individuals between the two sites, over time. We use a first-order Markov chain stochastic population model to simulate each translocated population. This model follows females only, and does not consider age structure. Translocated populations A and B are limited by carrying capacities K-A and K-B, with population dynamics governed by Markov chain transition matrices A(A) and A(B). In the time step of their translocation, translocated individuals are governed by a separate transition matrix A(T) with a higher mortality rate and no reproduction. The Stochastic Dynamic Program (SDP) has three states: the number of individuals in the first site (n(A) = 0,...,K-A), the number of individuals in the second site (n(B) = 0,...,K-B), and the time period of the translocation project (t = 0,...,T). Populations can change state by natural processes-as captured in the transition matrices-or by receiving translocated individuals. With X individuals available in each time step, Population A receives d individuals and Population B receives X-d individuals. The SDP steps backwards from the terminal time T where the final reward, given by an objective function, is received. For each time step all possible decisions-represented by the variable d-are evaluated for every combination of possible population sizes. The optimal decision is the one that produces the highest score, determined by the dynamic programming equation. We compare the results given for two different objective functions. The first is a long-term persistence objective function, which maximises the persistence of translocated populations e time steps after the end of the translocation program. The second is a total population size objective function, which maximises the number of animals present at the end of the translocation program. The SDP is applied to the Bridled Nailtail Wallaby by making each time step equal to four months, and by using realistic population parameters derived from relevant literature. The translocation program is assumed to run for four years (twelve time steps), with two individuals available in each time step. The carrying capacity of each site is set at 50. Population A has an approximate (per capita) growth rate of 1.305, while Population B has a growth rate of 0.87. When using the long-term persistence objective function (with epsilon = 128), the optimal decision for most states is to

translocate both available animals to Population B. These results satisfy the objective function, but are not sensible for a real-life translocation program, as population B has a declining growth rate. This indicates that the long-term persistence objective function considered here does not adequately express the goals of a translocation program. The results for the total population size objective function are more practically applicable. In these results the optimal decisions in each time step are dependent on the number of animals in Population A. The large difference in results between the two objective functions demonstrates the importance of careful consideration when specifying the goals of a project. This applies not only to translocation programs, but any project where clear decision-making is needed.

1392: *+.205*

Many populations have a negative impact on their habitat, or upon other species in the environment, if their numbers become too large. For this reason they are often managed using some form of control. The objective is to keep numbers at a sustainable level, while ensuring survival of the population. One such population is the koalas (Phascolarctos cinereus) of Kangaroo Island, South Australia. Between 1923 and 1925, 18 koalas were introduced to the island as a conservation measure to protect them (they were classified as a threatened species). Today, the Kangaroo Island koalas are considered to be a pest. Their overabundance has had a significant negative impact on the health of the Rough-barked Mannagum (Eucalyptus viminalis cygnetensis), along with other high-quality koala habitat. As a response to poor and insufficient habitat, numbers are predicted to decline sharply, and, because of the increased risk of extinction of the koalas and of other species, control and management programs have been proposed. Here we present models that allow population management programs to be assessed. Two common control regimes will be considered: reduction and suppression. Under the suppression regime the population is maintained close to a particular threshold through near continuous control, while under the reduction regime, control (for example culling or sterilisation) begins once the population reaches a certain threshold and continues until it falls below a lower pre-defined level. We discuss how to best choose the control parameters, and we provide tools that allow population managers to select reduction levels and control rates. Additional tools will be provided to assess the effect of different control regimes, in terms of population persistence and cost. In particular we consider the effects of each regime on the probability of extinction and the expected time to extinction, and compare the control methods in terms of the expected total cost of each regime over the life of the population. The usefulness of our results will be illustrated with reference to the control of the koala population. We select a suitable reduction level based on a specified probability of persistence, the genetic diversity of the population and the expected time between control phases. All are important in the management of native fauna. Firstly, while we are aiming to control the population, we wish to ensure the survival of the species without introducing risk additional to that faced prior to control. Next, genetic diversity, which is often overlooked when managing populations, is of utmost importance. The aim is to avoid inbreeding depression and to allow for evolutionary change. We must select a minimum reduction level that ensures a high probability of persistence, while maintaining an adequate level of genetic diversity. We find that a reduction level larger than that derived through these considerations will often be allowable in practice. To aid in selecting the reduction level, we also provide an explicit formula for the expected time between culling phases. Population managers can then select a reduction level so that the time between implementing successive controls is larger than some stipulated minimum (necessitated, for example, by resource constraints). The optimal rate of culling is then obtained by minimizing the cost of each culling phase, before finally selecting the optimal regime for control in terms of the expected cost of control over the life of the koala population. Our results can be easily extended to various control types (for example, sterilisation and translocation), and birth

and death rates other than the ones considered here (for example, we may employ logistic birth rates). Consequently, we anticipate that our approach will be useful in a variety of population management contexts.

1393: *+.113*

Attempts have been made to reintroduce elk (Cervus elaphus) in Ontario, Canada, since the early 1900's. These efforts are on-going and current plans are to establish viable elk populations in 6 pre-selected restoration sites in Ontario. Significant populations of white-tailed deer (Odocoileus virginianus) exist in close proximity to some of the proposed restoration sites. Therefore a decision was made to assess the potential impact of restoring elk in white-tailed deer wintering areas in Ontario, prior to the release of elk. The primary concerns regarding white-tailed deer/elk competition are transmission of the meningeal worm (Parelaphostrongylus tenuis) from white-tailed deer to elk and the winter carrying capacity of habitat where the restoration is to occur. A review of existing data revealed that significant winter concentrations of white-railed deer do exist in the vicinity of the Haliburton Highlands elk release site in Southern Ontario. However, that proposed release site is at least 10 km from white-tailed deer wintering areas and restrictive winters occur infrequently in that area. A review of the literature, as well as interviews with Provincial and State biologists and elk managers revealed that although there was anecdotal evidence of competition between elk and white-tailed deer in a few jurisdictions, there was neither quantitative nor qualitative data to support or refute those claims. However, as snow depths increase and elk switch to browse dominated diets, there is potential for white-tailed deer/elk competition for resources if sharing the same winter range, especially during severe winters. The chances that elk and white-tailed deer will share the same winter range will depend on where the released elk decide to settle for the winter, which may be influenced by availability of unused winter habitat and the quality of the available habitat. Therefore, elk will not be released directly into traditional white-tailed deer wintering areas in Ontario. A comprehensive research and monitoring program will be mandatory for future elk releases in Ontario.

1394: *+.207*

A review of the decline of, and conservation activities for, the adder or northern viper Vipera berus within Greater London, the 3,200 sq km London Natural History Society-recording area and with reference to its surrounding hinterland, totalling 6,400 sq km. One hundred and twenty-seven adder sites, approximating to populations, are mapped at the tetrad scale and date from 1800 to 2005. These records are found within the thirty-three London boroughs, and surrounding counties, including the old county of Middlesex, much of Surrey and parts of Essex, Kent, and Hertfordshire. Today in Greater London only five very small populations of adder remain, only two of which are possibly derived from original natural populations. Extinction here has been prevented by protective actions, restocking and introductions/reintroduction over the last twenty years. Outside Greater London and within the London Natural History Society survey area there are around a further eighteen remaining recorded populations most of which are small, many not fully protected and nearly all under threat. The past and present causes of adder decline are described and problems encountered in conserving adders in London over the last thirty years are discussed. Measures needed to conserve adders are described including habitat restoration and creation and management activities to avoid overgrazing by stock and minimize negative influences such as burning of fragmented habitats. Full listing of adder under Schedule 5 of the Wildlife & Countryside Act 1981 is considered appropriate, or protection via other existing or new legislation giving protection to the species and its habitat. New strategic approaches are needed to effectively coordinate suitable nature conservation activities within the adder's highly fragmented

current state.

1395: *+.207*

A review of the decline of, and conservation activities for, the adder or northern viper Vipera berus within Greater London, the 3,200 sq km London Natural History Society-recording area and with reference to its surrounding hinterland, totalling 6,400 sq km. One hundred and twenty-seven adder sites, approximating to populations, are mapped at the tetrad scale and date from 1800 to 2005. These records are found within the thirty-three London boroughs, and surrounding counties, including the old county of Middlesex, much of Surrey and parts of Essex, Kent, and Hertfordshire. Today in Greater London only five very small populations of adder remain, only two of which are possibly derived from original natural populations. Extinction here has been prevented by protective actions, restocking and introductions/reintroduction over the last twenty years. Outside Greater London and within the London Natural History Society survey area there are around a further eighteen remaining recorded populations most of which are small, many not fully protected and nearly all under threat. The past and present causes of adder decline are described and problems encountered in conserving adders in London over the last thirty years are discussed. Measures needed to conserve adders are described including habitat restoration and creation and management activities to avoid overgrazing by stock and minimize negative influences such as burning of fragmented habitats. Full listing of adder under Schedule 5 of the Wildlife & Countryside Act 1981 is considered appropriate, or protection via other existing or new legislation giving protection to the species and its habitat. New strategic approaches are needed to effectively coordinate suitable nature conservation activities within the adder's highly fragmented current state.

1396: *+.009*

Since 2002 hamsters are released in three hamster-friendly managed sites (Sibbe, Amby and Heer) in the most southern part of The Netherlands in the province of Limburg. As a result of these reintroductions, three small hamster populations are established. The management of the sites resulted too in an enormous amount of Field mice (Microtus arvalis), which is a favorite prey-species for a lot of predators. Unfortunately, the population of mice collapsed in spring due to agricultural activities. The predators (mainly foxes and marders) than switched to other prey and food, with a disastrous effect on the hamster population. Part of the hamster population escaped predation by moving to surroundings fields with a normal agricultural management. The long time survival of the hamster populations depends on the (im)possibility of creating good hamster-habitat patches within the reserves, in the surrounding of the reserves and between the reserves. In the coming years the management of the three sites must be intensified and crops must be cultivated on larger patches, instead of small stripes. Outside the reserves the main problem seems a shortage of cover and the total absence of food for winter-storage.

1397: *+.057*

In this chapter, I examined evidence for ungulate impacts on forest biodiversity in the absence of large carnivores. Wild populations from eight species that are distributed worldwide in temperate and boreal forests have been shown to have deleterious effects on biodiversity. Plant species are most often directly affected by ungulates, but additional trophic levels, composed of birds and mammals, are like-wise affected. The closer ungulate populations are to carrying capacity the broader their impact. Biodiversity in forests can be restored following ungulate reduction if conditions are favorable. The reintroduction of large carnivores is one component in a restoration

project that must take into account the potential of the site, invasive species, life history traits of target species, and disturbance regimes. Hunters are not a direct equivalent to large carnivores because they often affect numerical, but not functional, responses in the prey. Large carnivores should not be expected to regulate ungulate populations that are at or above carrying capacity. The culling of ungulate populations prior to carnivore introductions might enhance the ability of carnivores to act as biodiversity "managers." It is preferable to produce large spaces that are variable for both the density of ungulates and plant biodiversity, because microhabitats within the landscape will differ in both their potential for biodiversity and their ability to "shield" ungulates from predation. We have the opportunity to conduct large-scale natural experiments, and these should be used to test the ideas presented in this volume. Forest communities will exhibit a large diversity of responses when placed back under the influence of large carnivores. Whereas it is difficult to predict the response of any one site to reduced ungulate density, it is not hard to predict that the impact over the landscape scalewill be significant.

1398: *-.124*

Unlike many regions in the world where wild pigs (Sus scrofa) are threatened, in Australia they are a significant invasive species. As such, the molecular ecology of feral pigs was investigated to understand their social and population genetic structure. Samples from 269 adult animals were collected over their distribution ill southwestern Australia. Using 14 highly polymorphic microsatellite markers, we identified 7 inferred feral pig populations that had moderate heterozygosity (mean = 0.580) and displayed a high level of differentiation (mean R-ST = 0.180). In revealing the genetic structure of feral pigs, we detected anomalies in the pi native origin of some individuals. Samples from these animals were collected from 2 main areas: recently colonized regions that were previously uninfested, and established feral pig populations, where animals from geographically isolated areas had been introduced. In the latter, these corresponded to areas that were in close proximity to public road access and towns. Given the large distances immigrants were found from their population of origin (from 50 to > 400 km), the generally low levels of dispersal of southwest feral pigs, and the grouping and sex of these pigs, we suggest that these individuals have been deliberately and illegally translocated to supplement recreational hunting stocks. Additionally, we could not detect any genetic contribution in these feral pigs from domestic pig herds, suggesting that the deliberate release of domestic pigs to restock feral Populations is relatively uncommon. Our molecular data allowed some inferences regarding the Success or lack thereof of current, management practices, and offered considerable insights into the dynamics of the feral pig populations and identification of "new" approaches that may allow for better control of this highly destructive species.

1399: *-.279*

A total of 294 sera collected between 1999 and 2001 from eight captive and one free-ranging herds of Arabian oryx (Oryx leucoryx) distributed in Saudi Arabia (SA) and the United Arab Emirates (UAE) were assayed for antibodies against 13 selected viral agents. Arabian oryx have been exposed to bluetongue virus (BTV), epizootic hemorrhagic disease virus (EHDV), rinderpest virus (RPV), bovine respiratory syncytial virus (BRSV), bovine adenovirus 3 (BAV-3), cervid herpesvirus-1, foot-and-mouth disease virus, equine herpesvirus 9, and bovine viral diarrhea virus. The high seroprevalence to BTV and EHDV in the UAE and SA indicates that Arabian oryx are likely to be susceptible to infection by these viruses and therefore could act as a source of virus. to vectors during the infective stage of infection. Moreover, antibodies were detected against RPV and BRSV in sera from SA and against BAV-3 in sera from the UAE. No antibodies were found against bovine herpesvirus-1, caprine herpesvirus-1, enzootic bovine leucosis virus, and peste des

petits ruminants virus. On the basis of these results, caution should be applied when considering translocation of Arabian oryx, and only those proven to be free of infectious agents that might present a risk to other species should be moved.

1400: *+.072*

In the present study, we evaluated the genetic diversity of Panax notoginseng F H Chen, a domesticated species, and P stipuleanatus H T Tsai et K M Feng, an endangered wild species in southeastern Yunnan and adjacent areas in Vietnam, using sequences of the internal transcribed spacer (ITS) regions of nuclear ribosomal DNA and amplified fragment length polymorphism (AFLP) markers. Twenty-four accessions from three plantations of P. notoginseng and 51 samples from eight populations of P. stipuleanatus were assayed. A total of 694 bp of partial sequences of 18S, ITS1, 5.8S, ITS2, and partial sequences of 26S were obtained. No sequence variation was detected within P. notoginseng and nine sites (1.30%) were variable in P. stipuleanatus. Two-thirds of the variable sites were found between Langqiao and other populations. In P. notoginseng, four pairs of AFLP primer combinations generated 312 bands, of which 240 (76.9%) were polymorphic and 60.15% of the polymorphisms were harbored within plantations. Approximately 41.0% and 66.9% of bands were polymorphic in population D7 and 5589, respectively. In P. stipuleanatus, the same four primer combinations produced 346 bands, of which 334 (96.5%) were polymorphic and approximately 62.14% of polymorphisms were maintained within populations. Considerable variations were observed. The percentage of polymorphic bands ranged from 50.2% to 84.9% and the average over populations was 70.9%. Cluster analysis did not show correlation of genetic differentiation with the distinctive leaf morphology of P. stipuleanatus (i.e. one form with bipinnatifid leaflets and the other with undivided leaflets). Because over 40% of genetic variations were maintained among populations and because of the very restricted distribution of P. stipuleanatus, all natural populations of this species should be conserved in situ. Considering that there are variations in P. notoginseng within and among plantations, we suggest establishing a genetic resource conservation garden or reintroducing P. notoginseng into its native habitats in southwestern China. Such reintroduction should be carefully executed after large-scale screening of genetic variation within the species.

1401: *+.213*

1. Reintroductions provide a good opportunity to study density-dependent population growth, as populations can be studied at a range of densities and the change in density is not confounded with environmental conditions. An understanding of density dependence is also necessary to predict dynamics of reintroduced populations under different management regimens, and assess the extent to which they can be harvested for further reintroductions.2. We monitored a North Island saddleback (Philesturnus rufusater) population for 6 years after reintroduction to Mokoia, a 135 ha island in New Zealand that was made suitable for saddlebacks by eradicating introduced Norway rats (Rattus norvegicus). We modelled adult and juvenile survival using Program MARK, and modelled numbers of young fledged per pair using Proc Mixed in SAS with individual female as a random factor.3. Juvenile survival clearly declined as the population increased, and the decline was closely correlated with the number of breeding pairs. Reproduction also showed a clear decline that was explained by two factors: a difference in quality between territories occupied immediately after reintroduction and those occupied later, and an overall decline as the number of pairs increased. Reproduction was also strongly affected by age, and this needed to be accounted for when modelling density dependence.4. A stochastic simulation model incorporating these dynamics closely predicted the observed population growth. The equilibrium population size was insensitive to density dependence in reproduction, but highly sensitive to density dependence in

juvenile survival.5. The model is being used to plan management strategies for potential reintroductions of saddlebacks to mainland areas with predator control. The species is currently confined to predator-free islands and one fenced mainland sanctuary.

1402: *+.073*

In the first attempt to re-establish a population of the red-winged grasshopper, Oedipoda germanica (Latr.), threatened with extinction in Germany, 32 adults (19 females, 13 mates) were taken from a population in a limestone quarry, individually marked, and translocated to the "Leutratal" nature reserve near Jena, Thuringia, formerly inhabited by the species. A simulation model. with two scenarios was run to determine the probability of re-establishment of this small translocated population. One of the two scenarios indicated that populations of 20 females could survive with optimal life history parameters. In 1994, the year of translocation, during seven nights of observation between July and September, 25 adults were re-observed at least once. About 50% of the females and 70% of the mates were found in a 20m radius of the release point, whereas a maximum distance of about 70 m was recorded for a female. Using minimum female life-span and oviposition data from greenhouse experiments, 545 eggs were estimated to have been laid by the initial inoculum in 1994. In the subsequent years 1995 and 1996, only seven and three grasshoppers were observed within 30m of the release point in 1994. However, the translocated population obviously went extinct in 1997 because no O. germanica could be found in that or following years. By comparing source and release habitat, the possible causes of the failed re-establishment were elucidated. In the year of release a short mean adult life span of 26 days was observed, assumed to be the consequence of high predation, and resulting in a lower reproductive rate. In the following years, tow soil temperature of the release habitat probably delayed egg hatching for about 1 week, delaying phenology and thereby reducing the period over which eggs could be laid. A rapid population decline resulted in only three females found in the year before extinction. (c) 2005 Elsevier GmbH. All rights reserved.

1403: *+.186*

Himachal Pradesh, in India's North Western Himalayan State, is a global stronghold of two pheasant species; western tragopan Tragopan melanocephalus, regarded as the rarest of Himalayan pheasants and cheer pheasant Catreus wallichii. Declines in the wild populations of these species are a cause for great concern. Himalayan monal Lophophorus impeyanus, the state bird, is also threatened owing to excessive poaching for its colourful crest and meat. Furthermore, Himachal Pradesh is an important area for genetically pure commercially important red junglefowl Gallus gallus. Conservation of pheasants has hitherto been limited to captive breeding, resulting only in inbred stock not suitable for any reintroduction programme. The Wildlife Wing of the Himachal Pradesh State Forest Department has instigated the world's largest programme of conservation breeding of four species - western tragopan, cheer pheasant, Himalayan monal and red junglefowl. For ex situ conservation of these pheasants, existing pheasantries at Sarahan, Manali, Chail and Khariun are being redesigned at more appropriate sites. The in situ programme will cover intensive habitat status research in parallel with conservation breeding projects capable of providing 95 - 150 birds of these four pheasants each year. This paper reviews the in situ habitat and population status of all the pheasants found in Himachal Pradesh in relation to the protected area network, the ex situ populations in the pheasantries, and outlines plans for future strategies for the conservation of pheasants in the state.

1404: *-.096*

A literature review and surveys undertaken between October 2003 and March 2004 in Kaptai National Park, Chittagong Hill Tracts, Lawachara National Park and Satchari forest in Mawlavibazar district, Kurigram in Rajshahi and Sundarbans in Khulna provinces, established that 16 species of Galliformes occur in Bangladesh. Seven were recorded during the field surveys, with the remainder being reported in the literature. Of these species, Indian peafowl, Pavo cristatus and green peafowl P. muticus are currently extinct. Whitecheeked partridge Arborophila atrogularis, rufous-throated partridge A. rufigularis, rain quail Coturnix coromandelica, blue-breasted quail C. chinensis and grey francolin Francolinus pondicerianus are the least known species. Black francolin Francolinus francolinus, swamp francolin F. gularis and grey peacock-pheasant Polyplectron bicalcaratum are critically endangered and kalij pheasant Lophura leucomelana and Manipur bush quail Perdicula manipurensis are locally endangered. Of these, swamp francolin and Manipur bush quail are vulnerable globally. The rest of the species are data deficient. All galliform species are under great pressure from habitat loss, population fragmentation, hunting, environmental degradation and severe human disturbances. Some selected species including extinct ones are recommended for captive breeding and reintroduction. Research including long term monitoring and raising awareness are essential for conservation and the preparation of action plans for the galliform species of Bangladesh. International cooperation and technical support are urgently required.

1405: ***-.069***

The massasauga rattlesnake (Sistrurus catenatus) is locally threatened or endangered throughout most of its range. The status of the desert massasauga (S. c. edwardsii) is as follows: Arizona, protected; Colorado, Species of Special Concern; Kansas, unknown; New Mexico, no special status; Oklahoma, unknown; Texas, unknown; Mexico, unknown. The desert massasauga is listed as a Species of Special Concern by the Colorado Division of Wildlife primarily because of the limited distribution of well-documented, stable populations. It is listed as a sensitive species by the USDA Forest Service (USFS), Rocky Mountain Region (Region 2), where populations are stable but uncommon in southeastern Colorado and southwestern Kansas. Primary Threats Long-lived, low fecundity animals such as the massasauga are inherently vulnerable to population losses because of limited replacement potential. In Region 2, the primary threat to massasauga populations is habitat loss and degradation due to urbanization, farming, heavy livestock grazing, and water table drawdown due to diversion and well water use. Like other xeric habitats, shortgrass prairie is severely affected by soil disruption (e.g., tilling, overgrazing, urbanization), and the arid nature of this habitat makes recovery following release from disturbance very lengthy and perhaps incomplete, particularly given the growing threat of invasive weeds. Massasauga populations in Colorado have benefited passively from geographic isolation in terms of distance from urban centers, but pressures from Front Range human populations could eliminate this isolation. Like all rattlesnakes, the common result of human encounters is death, and the effects of direct persecution in remote areas are nearly impossible to evaluate. Highway/road mortality is another anthropogenic threat to massasauga population stability and persistence. Conservation easements and public lands (e.g., State Trust lands, National Forest System lands) provide some protection, but presently these do not include areas in the state with the largest desert massasauga populations Primary Conservation Elements, Management Implications and Considerations Protection and conservation of large, contiguous tracts of native shortgrass prairie habitat will be necessary for the long-term survival of the desert massasauga. Acquisition and management of lands by public agencies (e.g., USFS, Bureau of Land Management) and other groups will help to conserve this fragile habitat. However, much of Colorado is privately owned, often as large ranches (10,000 to 100,000 acres or more), so pursuit of conservation easement agreements with private property owners is likely a more productive means of providing broader protection. It is

further suggested that desert massasaugas rangewide receive protected, no-take status. Direct intervention (e.g., captive breeding, reintroduction) for preservation of Region 2 populations is not indicated or recommended at this time; however, these types of programs are being undertaken in other parts of the species' range. Populations of desert massasaugas in Colorado should be monitored at several levels. Sensitive and robust populations identified by the lead author of this assessment should be surveyed on a regular basis (i.e., 5 to 10 year interval, depending on funding availability) to ensure that new threats to the populations have not arisen. State and federal agencies should monitor land use changes, and if significant changes (e.g., urbanization, till farming, overgrazing) occur in areas occupied by massasaugas, impacts should be evaluated. Massasauga populations on National Forest System lands, in particular in the vicinity of the Baca County locality records, should be surveyed again in the near future with a concerted effort placed on the regions immediately adjacent to and within the Comanche National Grasslands. The Cimarron National Grasslands in southwestern Kansas should be surveyed extensively in late spring and early fall, when the likelihood of encounters is greatest, to determine unequivocally whether or not the desert massasauga occurs there (we expect it to occur there).

1406: *+.054*

Castilleja levisecta (Scrophulariaceae), the golden paintbrush, is an insect-pollinated herbaceous perennial found in the Pacific Northwest. Currently restricted to two island populations off British Columbia and nine populations (eight on islands) in Washington, C. levisecta is a rare species threatened with extinction. Allozymes were used to describe genetic diversity and structure in these eleven populations. Despite its threatened status and small geographic range, exceptionally high levels of genetic diversity are maintained within C. levisecta. All sixteen of the loci resolved were polymorphic within the species (P-s=100%), while the mean percentage of loci polymorphic within populations (P-p) was 65.7%. The mean number of alleles per polymorphic locus (AP(s)) was 2.94 within the species and averaged 2.38 within populations (AP(p)). Genetic diversity (H-es) was 0.285 for the species, whereas mean population genetic diversity (H-ep) was 0.213. Smaller populations had, on average, fewer observed alleles and less genetic diversity. A significant negative correlation (r = 0.72) was found between genetic identity and geographic distance, indicating reduced gene flow between distant populations. The most geographically isolated population was one of the larger populations, one of the most genetically diverse and the most genetically divergent. A wide range of pairwise population genetic identities (I = 0.771 - 0.992) was found, indicating considerable genetic divergence between some populations. Overall, 19% of the total genetic diversity was distributed among populations. Results of this survey indicate that genetic augmentation of existing populations is unnecessary. The high allelic diversity found for the species and within its populations holds promise for conservation and restoration efforts to save this rare and threatened plant species.

1407: *+.030*

Many populations of turtles (e.g. the European pond turtle Emys orbicularis in Poland) and tortoises are endangered. Thus, many protection programs of the animals, including highly manipulative ones (e.g. headstarting), are currently being carried out. Headstarting is a species protection technique that involves raising turtle hatchlings in captivity to an age of a few months up to several years, and then releasing them into a natural habitat. Usually the objective of this procedure is to grow hatchlings to a size at which they are less vulnerable to predators. However, recent analyses of headstarting programs suggest that they are fairly inefficient as tool to increase population size. Analysis suggests that headstarting programs can increase population size only if a large percentage of hatchlings is headstarted, so with large populations it is virtually impossible.

Additionally, if adult survivorship is decreased (even by only 1%), such method will be inefficient. Thus, if the reasons for population decline are not eliminated, a headstarting program cannot save populations from extinction. The real efficiency of such programs, however, also depends e.g. on the length of the headstarting program and the long-term survival rates of wild and headstarted individuals (factors that are currently not known). Headstarting programs should, at this point, still be considered experimental. Other possible to use techniques of protection of the European pond turtle are also ineffective (clutch protection), or data about ecology of the species are insufficient to use them (restitutions, building of artificial nesting places). Headstarting is useful only when used in tandem with a strategy that will reduce mortality of adult turtles. I recommend the initiation of headstarting program of the European pond turtle only when a population is in real danger of extinction, and only if it is impossible to reduce adult and subadult mortality. As morphological and genetic variations of the turtle in Europe are high, and only few information in the subjects from Poland area are available, translocations of individuals between populations should be present forbidden.

1408: *+.113*

Gibbons, like orangutans, are a group of threatened Asian apes, so that genetic monitoring of each species or subspecies is a pressing need for conservation programmes. We conducted a project to take, as far as possible, samples of known origin from wild-born animals from Sumatra and Borneo (Central Kalimantan) for genetic monitoring of agile gibbons. As a result, we found a whole arm translocation between chromosomes 8 and 9 (WAT8/9) specific to Sumatran agile gibbons. Furthermore, population surveys suggested that the form with the WAT8/9 seems to be incompatible with an ancestral form, suggesting that the former might have extinguished the latter from Sumatran populations by competition. In any case, this translocation is a useful chromosomal marker for identifying Sumatran agile gibbons. Population genetic analyses with DNA showed that the molecular genetic distance between Sumatran and Bornean agile gibbons is the smallest, although the chromosomal difference is the largest. Thus, it is postulated that WAT8/9 occurred and fixed in a small population of Sumatra after migration and geographical isolation at the last glacial period, and afterwards dispersed rapidly to other populations in Sumatra as a result of the bottleneck effect and a chromosomal isolating mechanism.

1410: *+.047*

With only two known wild populations, the Florida semaphore cactus [Consolea corallicola (Cactaceae)] is one of the rarest plant species endemic to South Florida. The species is known to occur in two populations in the Lower Florida Keys (currently seven individuals) and in the Upper Florida Keys at Biscayne National Park (approximately 570 plants). We used ISSR molecular markers to assess the levels of genetic variation of these two populations. We found no genetic diversity within these two populations and only four of the 64 ISSR loci showed differences between them. A single individual from an extirpated population of Big Pine Key is currently maintained in the ex situ collection of Fairchild Tropical Botanic Garden; this individual was also included in our ISSR study and it was genetically different from the ones in the wild, lacking six alleles found in the two wild populations. Therefore the species is currently comprised of three ISSR genotypes. These results are consistent with previous reproductive biology studies that suggested that C. corallicola does not propagate sexually and that asexual reproduction is the main life-history strategy of this species.

1411: *+.464*

The European network CRAYNET "European crayfish as keystone species-linking science, management and economics with sustainable environmental quality" emphasises knowledge-based management strategies. The CRAYNET meetings concentrate on certain topics: monitoring in conservation and management of natives; interaction between natives and aliens; control of aliens; habitat restoration; reintroduction and restocking; legislation; education. The Irish meeting (Kilkenny, 2003) "The endangered native crayfish Austropotamobius pallipes: bioindicator and heritage species" initiated debates about (1) what is a bioindicator, a flagship species or a heritage species?; (2) the problem of species complexes and the need to clarify and resolve taxonomy before management; and (3) what is meant by biodiversity in the context of European crayfish? The Norwegian meeting (Halden, 2003) "European native, crayfish with a special focus on Astacus astacus: linking socioeconomics and conservation " involved roundtables about (1) threats to indigenous population of crayfish at a landscape level; (2) exploitation, conservation, legislation; (3) reintroduction of indigenous crayfish, habitat restoration and monitoring; and (4) the urgent need for joint research about pathology. The present meeting (Innsbruck, 2004) "European native crayfish in relation to land-use and habitat deterioration with a special focus on Austropotamobius torrentim" aims to identify the species protection programs, to examine if legislation is working well in protecting vulnerable species, the state of advances about conservation genetics and how to make progress in education of the public. A permanent connection between the 12 core members provides opportunities to check the needed lines of research, to prepare recommendations, and to gather information for an atlas on the distribution of crayfish species in Europe as well as to prepare leaflets for the public.

1412: ***-.083***

The fish hosts of Margaritifera auricularia, the critically endangered Giant Pearlmussel, have been identified. Natural infestations were searched for in the lower Ebro river (Catalonia, Spain), where the last viable population of M. auricularia survives. No encysted glochidia were found on wild fish. In addition, artificial infections were performed on 8 native and 8 exotic fish species. Encystment and growth was observed on the gill filaments of three species: Acipenser baeri, an introduced fish (showing that our early hypothesis involving sturgeons cannot be rejected, although it is not sufficient nor necessary); Gambusia holbrooki, a widespread exotic that may be useful in captive breeding; and Salaria fluviatilis, which is highly susceptible to infection, was the only species where full development was observed, shares the bivalve's habitat, and is also endangered. The dramatic reduction of the Giant Pearlmussel's range can now be explained as due to a combination of habitat destruction and disruption of its life cycle. This knowledge can now be used in a recovery program centered on the rearing of juvenile pearlmussels in captivity for reintroduction to the wild.

1413: ***-.008***

Allozyme electrophoresis was used to determine the extent of clonality in four naturally occurring populations of Acanthocladium dockeri F. Muell. from the mid- north of South Australia. A total of 33 loci was scored for at least 16 plants from each population. The four known populations of A. dockeri represent four quite distinct genetic clones. Each natural population consists of a single genet, and no indication of any correlation between geographical proximity and genetic similarity was found. Seed set in. flowers was very low(0.6% of. florets), probably owing to low pollen germinability. Seedlings raised from A. dockeri seed collected at Hart displayed obvious genetic affinities to their parent, but were nevertheless genetically distinct. Differences were consistent with the derivation of this seed by selfing of the Hart genet. A. dockeri is considered at high risk of extinction because of its low genetic diversity, poor seed production, lack of seedling recruitment

and population growth by clonal reproduction alone. On the basis of the outcomes of this research, the following recommendations for conservation management of A. dockeri are made: (1) all four extant genets should be preserved in their respective habitats; (2) at least one ex situ collection of all four extant genets should be maintained at a secure site as an insurance against population loss; (3) further surveys are required to potentially locate new populations with superior fertility; and (4) mixing of clones or seed provenances into a single population should be avoided until appropriately designed experimental translocations have evaluated the possible consequences of such mixing on competitive, reproductive and genetic responses of populations.

1414: ***+.171***

We examined the role of the grass-tree Xanthorrhoea semiplana F. Muell. canopy in the survival of the nationally endangered orchid Caladenia (syn. Arachnorchis) behrii Schltdl. at three sites in the northern Adelaide region. We compared grazing and pollination of the flowering orchids within and away from the grass-tree canopy. Grass-trees generally provided significant protection from grazing, but orchids protected by grass-trees experienced reduced pollination for 2 out of 5 years. The net effect of these interactions, as measured by seed set, varied across years. When grazing pressure is high, orchids may benefit from grass-tree protection (facilitation), but under low grazing pressure, it is more probable that orchids set seeds away from grass-trees than under their canopy. Grazing pressure probably does not decrease in view of habitat fragmentation the importance of fragments as wildlife refuges. Therefore, factors affecting the survival of grass-trees, such as Phytophthora cinnamomi, may also affect orchid survival. Kangaroos which are often assumed to be responsible for most grazing in the Adelaide Hills are not the only predators of orchids, and a culling program has so far not resulted in a decrease of grazing pressure for the orchids. Translocation and fencing programs should examine the role of facilitative plants and grazers before spending precious conservation resources.

1415: ***+.066***

The study is part of the surveys carried out within the framework of the European Community Life Project "Econet - corridors for life", aiming, to create, in the Emilia-Romagna region (Northern Italy), an ecological network in the plain of the Modena and Bologna provinces. The regional status of two fish species of conservational interest (the panzarolo goby Knipowitschia punctatissima and the three-spined stickleback Gasterosteus aculeatus) has been investigated and the possibility of restoring some of their populations analysed. 29 sites in the regional territory were surveyed, in 16 of which the fish and in 13 the benthic macroinvertebrates communities were recorded. Both the fish species are confirmed as critically endangered oil a regional level and virtually extinct in the province of Modena, where a reintroduction could be tried in two water systems of the Tiepido stream alluvial fan (Castelnuovo Rangone and Modena districts).

1416: ***+.389***

After complete eradication, Eagle Owl Bubo bubo (L.) was re-introduced in the north-western hillside areas of Germany. Scientific research including diet-analyses were part of the re-introduction program to clarify, if food availability and prey choice limit reproduction and population development of the re-introduced Bubo bubo population. Here I compare prey choice of eagle owl and climatic- / habitat factors in the Eifel area and the population development of eagle owl and rabbit Oryctolagus cuniculus (L.), an important prey in many parts of Europe. Several and very different species, namely hedgehog, rabbit, hare, pigeons brown rat and common vole provide the basis of nutrition. This is presumably the reason for a stable and high

reproductive success and still increasing population of eagle owl in the Eifel, despite the dramatic decrease of rabbit due to the diseases RI-ID and Myxomatosis. Climatic conditions and suitable nest sites seem at present to be more important limiting factors for Bubo bubo in the Eifel area with it's relatively cool temperate climate than the abundance of single prey species. However, landscapes change fast and therefore abundance of prey species can reach critical values soon, therefore an adequate food supply is a key-factor for eagle owl. Thus, eagle owl protection always has to consider adequate hunting areas, especially in Special Protection Areas of the European Birds Directive.

1417: *+.113*

One of the conservation precautionary measures applicable to overexploited species is to implement protected population through translocation of exogenous individuals. The European lobster (Homarus gammmarus L. 1758) and the brown meagre (Sciaena umbra L. 1758) are species under strong fishing pressure. In summers 2001 and 2002 we released four translocated lobsters and two translocated brown meagres inside the Natural Marine Reserve of Miramare, where the species are naturally present. The spatial behaviour of the released animals was monitored using two different acoustic tracking systems (Track28 and VR1 receivers; Vemco Ldt). On the base both of bibliography and of our preliminary experimental outcomes, the lobsters were tagged by gluing the pinger to the dorsal surface of the cephalothorax and the brown meagres were tagged by implanting the pinger in the peritoneal cavity. All the animals, both lobsters and brown meagres, disappeared from the detection range of the hydrophones after a variable number of days (2-16 days of continuous monitoring). We suppose that they did not colonize the site, leaving the protected area. From the methodological point of view, VR1 mode proved to be a suitable tool to evaluate the colonisation of an area by transferred animals.

1418: *+.124*

The West Indian manatee (Trichechus manatus) is an exclusively herbivorous aquatic mammal. Recently, the stranding of live-orphaned calves has been the main threat to the species in northeastern Brazil. Since 1989, the Brazilian Manatee Project (PPB) has recovered 52 calf carcasses, of which 44 were alive. In 1994, "Lua" and "Astro" were the first manatees released from captivity in Brazil, and they have been tracked using radio telemetry methods. During daily tracking bouts, Lua's behavior and movement patterns were recorded, including reproductive behavior. On 17 December 2003, while she was in the Maracaipe Estuary, Lua gave birth to her first calf. Beginning on 18 December 2003, the mother/calf pair began repeated, tidally determined moves from the estuary used during high tides to the sandstone reefs used during low tides. Four days after the birth, an increase in motorized boat traffic in Maracaipe Estuary was observed. On 22 December, Lua and her calf moved to Serrambi Beach and remained outside the estuary in the reef area. On 25 December, the ninth day after the birth, Lua was sighted alone. On 26 December, the calf was found dead at Serrambi Beach. The place of birth supports the hypothesis that estuaries are birthing areas for manatees. The fact that Lua established her main fidelity site and gave birth in an area where manatees had previously been extirpated indicates a potential for reestablishing the species in its historical range via the rescue, rehabilitation, and reintroduction program developed by PPB. The calf's death confirms the fragility of the species' conservation. The last 44 live-orphaned calves seem to have been caused by habitat destruction and/or human disturbances within their habitat. The lack of effective coastal management programs, despite being mandated within the federal Environmental Protection Areas established in the region, are factors that severely impair conservation of the Antillean manatee in Brazil.

1419: ***+.195***

Leiopelma hamiltoni from Maud Island, Marlborough Sounds, New Zealand is confined to two populations totalling approximately 19,000 individuals. In May 1997, 300 L. hamiltoni from Maud Island were translocated to nearby Motuara Island in an effort to expand their distribution and lower the risk of extinction for the species. By August 2002, 155 of the translocated frogs had been recaptured and the population contained a range of young to old frogs. Population estimates indicated the population on Motuara Island had stabilised with losses of the translocated frogs offset by new recruits. The first juvenile frog was found in January 1998, only 10 months after the translocation and 42 recruits were captured by August 2002. Although initial survival was low for the translocated frogs, survival following the initial 2-month settling-in period was high (71-100%). New recruits produced on Motuara Island had survival rates of 29-88%. Capture-recapture analyses support the view that the survival estimates include a large dispersal component. The a priori criteria for a successful translocation were met; the appropriateness of the Motuara Island habitat for breeding and adult survival was demonstrated.

1420: ***+.192***

In the preservation of plant biodiversity, there are fundamental genetic and ecological similarities involved it]: (1) predicting the fate of small, isolated populations, (2) ensuring the successful reintroduction of endangered species back into natural habitats, and (3) understanding the establishment of species beyond their native ranges. In all three cases, populations small in size only experience high levels, of inbreeding and subsequently the expression of inbreeding depression. The ability of these populations to persist will depend, ill part on the magnitude of inbreeding depression and the ability of selection to remove (or purge) the genetic load that causes inbreeding depression. The loss of genetic variation caused by inbreeding or drift in these small populations Will restrict their evolutionary potential, placing a high premium on the ability of plants to respond plastically to environmental variation. When plants make appropriate phenotypic adjustments in truly novel environments while maintaining high fitness, we suggest the use of a new term, "opportunistic plasticity," for this attribute. Opportunistic plasticity may be particularly critical and advantageous in the case of plant introductions in ecological restoration where habitats resemble but are not identical to seed collection sites, or in the initial establishment phase of exotic species in non-native locations where many aspects of the environment differ from the native range. Overall phenotypic plasticity may also be all important weans of dealing with environmental heterogeneity in many small populations, but unfortunately we have limited data on the interaction between inbreeding and plasticity in plants. The science and practice of conservation biology has generally appreciated the importance of inbreeding, but we feel that it will benefit greatly by considering the potential interactions between inbreeding and phenotypic plasticity and their effects in the establishment and persistence of small populations.

1422: ***+.018***

The Red River of the North basin (RRNB) has an area of about 287,000 square kilometers of the upper Midwestern United States and south-central Canada. The river forms the North Dakota-Minnesota boundary and flows into Lake Winnipeg, Manitoba, and then, via the Nelson River, into Hudson Bay. While the Red River main stem remains a sinuous stream similar to early descriptions, the river's watershed has been altered dramatically by intensive agriculture, wetland drainage, channelization of tributary streams, and dam construction. Early land surveys described a landscape largely covered by prairie and wetlands. However, thousands of kilometers of ditches have been excavated to drain wetlands for agriculture in the United States in the late 1800s to the

1920s, and continuing, in Canada, to the present. Over 500 dams have blocked access to critical spawning habitat in the basin starting in the late 1800s. Also, during the mid-1900s, many of the tributaries were channelized, causing the loss of several thousand stream kilometers. While much of RRNBs fish assemblage remains similar to earliest historical records, the loss of the lake sturgeon Acipenser fulvescens is a notable change resulting from habitat loss and fragmentation, and overfishing. Additional localized extirpations of channel catfish Ictalurus punctatus, several redhorse Moxostoma species, sauger Sander canadensis, and other migratory fishes have occurred upstream of dams on several tributaries. Presently, efforts are underway to restore migratory pathways through dam removal, conversion of dams to rapids, and construction of nature-like fishways. Concurrently, lake sturgeon is being reintroduced in the hope that restored access to historic spawning areas will allow reestablishment of the species. Proposed construction of new flood control dams may undermine these efforts.

1423: ***+.240***

Three Pacific salmonid species Onchorynchus spp. have replaced the extirpated Atlantic salmon Salmo salar as the main migratory salmonid in the Lake Ontario drainage. One of those species, the nonnative rainbow trout O. mykiss, has become widely distributed within the historical Atlantic salmon habitat, occupying an ecological niche similar to that of juvenile Atlantic salmon. Consequently, both a tributary's carrying capacity for Atlantic salmon and competition from established normative species are important when considering the feasibility of Atlantic salmon restoration. Estimation of juvenile rainbow trout production will help evaluate the capacity of tributaries to produce salmonids that occupy similar niches. Geostatistical methods were applied to standardized and efficiency-corrected electrofishing data from three of New York's best salmonid-producing streams to precisely estimate juvenile rainbow trout populations. Results indicated that each study stream could produce 20,000-40,000 age-0 and 4,000-10,000 age-1 and older rainbow trout per year. Statistical interpolation indicated areas of significantly different production potential and points of significant changes in productivity. Closer examination of the niche similarity and competitive potential of these two species is needed to properly interpret these estimates with regard to Atlantic salmon restoration.

1424: ***+.185***

Stream temperature is an important factor influencing habitat suitability for cutthroat trout Oncorhynchus clarkii, but temperature data from headwater habitats are difficult to obtain. We tested the ability of easily obtained landscape and meteorological data to predict the mean daily temperature measured at 79 sites in tributaries to the Madison River, Montana. We also evaluated stream habitat suitability by using temperature predictions to estimate growth potential for age-0 westslope cutthroat trout O. c. lewisi. A model using mean daily air temperature, elevation, and channel gradient explained approximately 75% of the observed variation in mean daily stream temperatures. Classifications of habitat suitability based on predicted fish growth indicated that the majority (78%) of stream habitat in Madison River tributaries provides suitable or highly suitable habitat for westslope cutthroat trout. However, these higher-quality habitats occur primarily in lower-elevation reaches where westslope cutthroat trout have been displaced by normative salmonids. Linking potential fish growth to stream temperature predictions will help managers prioritize conservation efforts for this declining subspecies by predicting habitat suitability at potential reintroduction or population expansion sites.

1425: ***+.206***

Because most reintroduced species are rare, data on their dynamics are scarce. Consequently, reintroduction programs often rely on data from other species or captive populations to project the performance of the reintroduced population in the wild. We compared the reproductive success and survival of a Persian fallow deer (Dama mesopotamica) population reintroduced in Israel over the first 5 years of the project with the survival and reproduction parameters estimated while planning the reintroduction. In addition, we compared the actual growth of the wild population with the growth originally projected by a computer model in the original reintroduction program. We monitored 74 radio-collared individuals (57 females and 17 males) released semiannually 1996-2001. Survival during the first year after release was lower than later years (0.90 and 0.82 versus 0.95 and 0.88, for females and males, respectively). Such an impact was not anticipated in the original plan, but overall survival was higher than originally projected. As assumed in the reintroduction program, reproductive success improved significantly with time since release and overall, was higher than expected. The mean number of animals released annually was lower than planned. Overall, the growth of the reintroduced population was slower than projected, but the deviation was close to confidence limits and the pattern similar. After 5 years it appears that the original time frame of 8-10 years for project completion can be met or at worst will cause a 1-year delay. Over the short term of 5 years, projection models in reintroduction programs are useful tools for assessing the sustained use of the breeding core, depicting the dynamics of the population in the wild, providing a relatively accurate time frame for the successful completion of the project, and assessing project success.

1426: *+.155*

Reintroduction or re-enforcement programmes are major tools in species conservation, but there is a need for more studies that assess the influence of different husbandry and release methods on the survival of released animals. We investigated the survival of globally threatened Marbled Teal (Marmaronetta angustirostris) taken into captivity as ducklings when they became trapped in an irrigation channel, then released again after fledging. We used wing tags and mark-recapture models to estimate the survival of released teal. Ducklings rescued in 1996 (n = 53) were released soon after fledging in September and their survival was modelled for seven months until April 1997. Their apparent monthly survival rate (lower than true survival owing to loss of wing tags) was 0.85 +/- 0.12 (+/-s.e.). Ducklings rescued in 1997 (n = 44) were released together in February 1998 over five months after fledging, and their survival was modelled for six months from February until August. Their apparent monthly survival rate was 0.54 +/- 0.06. Ducklings rescued in 1998 (n = 159) were released in August-September soon after fledging and their survival was modelled for 10 months from August until June. Their apparent monthly survival rate was 0.83 +/- 0.07. Monthly survival was significantly higher for the 1996 and 1998 cohort, suggesting that retaining birds in captivity after fledging had a negative impact on post-release survival. When birds were released in February, a lower proportion survived until the breeding season three months later than when they were released five months earlier in September. (C) 2004 Elsevier Ltd. All rights reserved.

1427: *+.200*

This study reports on three scarlet macaw (Ara macao) reintroduction projects using hand-raised birds in Peru and Costa Rica. The habitats at the release sites ranged from pristine tropical forest to forest fragments in an agricultural matrix. The combined first-year survival was 74% and the annual post first-year survival was 96%. Survival rates were very high despite a wide range in predator communities. Number of birds released explained 70% of the variation in survival with birds from larger releases having higher survival rates. Behavioral evidence suggests that birds

established at the site facilitated survival of later releases. Breeding attempts were recorded at all three sites and hand-raised birds with wild mates successfully fledged young in Peru. Supplemental feeding post-release played an important role in keeping the birds near the release site and facilitating social interactions. This work shows that properly socialized hand-raised macaws can survive and breed in the wild but that ex-pets are not good release candidates. (C) 2004 Elsevier Ltd. All rights reserved.

1428: *+.272*

Insight regarding the genetic origin and composition of the studied population of the red-legged partridge (Alectoris rufa) is likely to provide general and critical information for the appropriate management and possible conservation of the species. The reintroduced population of red-legged partridges living in Pianosa Island (National Park Tuscany Archipelago) has proven to be sustainable: captive-bred individuals, morphologically assigned to the taxon A. rufa, were released to the island approximately 20 years ago, establishing an apparently well-adapted population. We have investigated this population by means of 10 microsatellite loci in order to shed light on its genetic structure. Considering that A. rufa is known to crossbreed with A. chukar, the Pianosa Island population was compared at the molecular level with a red-legged partridge breeding stock (Aulla, MS) as well as with a population of pure A. chukar. Our results indicate that the red-legged partridge population from Pianosa, morphologically identified as A. rufa, is actually partly introgressed with A. chukar, questioning its genetic purity and the possible use of this population as a starting stock for future reintroductions elsewhere.

1429: *+.090*

The little bustard (Tetrax tetrax) has declined rapidly across European farmland landscapes due to agricultural intensification. In France, the number of breeding males in agricultural habitats has been reduced by 92% over the last 20 years as a result of decreases in insect abundance and nest destruction during harvesting. An age-and sex-structured stochastic metapopulation model was formulated for the remaining little bustard population in south-west France and, using actual estimates of demographic rates obtained after 1997, its extinction risk over the next 30 years was estimated to be ca. 0.48. Limited population reinforcement has thus appeared as a potential conservation strategy for this species in agricultural habitats, while agro-environmental actions have begun to have an effect on habitat quality at the landscape level. Different strategies for the reinforcement of fledglings, including the number and frequency of releases and the number of release localities in relation to four criteria for choosing the release points, were evaluated in terms of their effect in reducing the extinction risk of local populations and of the metapopulation. The reinforcement of 100 fledglings per year for 5 years and choosing the actual release points using the current abundance were found to be the optimal choices for reducing the estimated extinction risk of the remaining little bustard population in south-west France.

1430: *+.095*

Relocation of endangered species can be an effective conservation tool if it does not mix populations that represent significant intraspecific variation. The threatened Italian agile frog, Rana latastei, has small populations with low genetic diversity: translocation has been proposed to improve the likelihood of survival of populations. Using a common environment experiment and field surveys, we investigated whether there were differences in larval growth and developmental rate between foothill and lowland R. latastei populations, to evaluate if they are evolutionarily significant units. In nature, the colder climate of the foothills causes delayed metamorphosis.

Conversely, in a common environment, larvae from foothill populations show faster growth and development. We did not find a significant egg-size related maternal effect or any differences in size at metamorphosis. We hypothesise that counter-gradient selection promoted fast growing phenotypes in a cold environment, where low temperatures slow down larval development. Foothill populations, despite being only a small geographical distance away from lowland populations, seem to be adapted to a colder climate and represent an evolutionarily significant unit. Different populations should, therefore, be managed independently, avoiding translocation. We suggest that evolutionary divergence between populations should be verified prior to planning relocation programmes, to prevent the risk of genetic homogenisation.

1431: ***+.225***

Translocation of primates is still a rare event. The translocation in 1984 of two research groups of wild baboons that had been studied for 12 years prior to translocation and observed for 18 years afterwards offers a comprehensive set of data with which to evaluate success. A comparison with indigenous baboon troops at the release site provides an independent control for assessing performance in the release area. Two success criteria are developed with the use of indicator measures that include birth rate, death rate, patterns of mortality and survivorship, body condition, intestinal parasites, and group size. The baboon translocation succeeded according to both criteria: the two troops were saved by the translocation, and they did as well or better than could be expected in their new home. Their performance matched or exceeded that of translocated groups of other primate species. (C) 2005 Wiley-Liss, Inc.

1432: ***+.211***

We used landmark-based geometric morphometric methods to describe patterns of body shape variation and shape covariation with size among populations of the threatened White Sands Pupfish (Cyprinodon tularosa), a species that occurs in dissimilar aquatic habitats. White Sands Puplish populations include two genetically distinct, native populations that have been historically isolated in Salt Creek, a saline river, and Malpais Spring, a brackish spring. In addition, two populations were established approximately 30 years before this study by translocation of fish from Salt Creek to Lost River (a saline river) and Mound Spring (a brackish spring). We found significant body shape variation among populations and between males and females. Body shapes were more slender for females than for males and more slender for saline river populations than brackish spring populations. Introductions of pupfish to new habitats resulted in significant departures in body shape and shape allometry from the native Salt Creek population. Shape divergence was more pronounced for the Mound Spring population, which is consistent with a greater change in abiotic conditions. Although Mound Spring pupfish, like Malpais Spring pupfish, were more deep-bodied than saline river pupfish, differences in body shape and the level of sexual dimorphism were significant between the two brackish spring populations, indicating that deep-bodied shapes may be achieved from different anatomical configurations. The significant shape divergence of introduced populations warrants consideration for the conservation of this rare species, as creation of refuge populations for native stocks is a current management strategy.

1433: ***+.081***

Perhaps no species elicits more polarized opinions in the United States than the gray wolf (Canis lupus). Both proponents and opponents of wolf recovery use symbolic language in an attempt to persuade others to change their attitudes and values. We used structured phone interviews with 1,300 registered voters to examine the attitudes of people living in Arizona, Colorado, and New

Mexico toward a proposed restoration of the gray wolf to the southern Rocky Mountains, and to examine the ability of persuasive arguments to change these attitudes. We found a high level of support for wolf restoration by residents of all 3 states; 64% of respondents favored reestablishing wolves in the southern Rockies, whereas 33% expressed opposition. Support was general across almost all demographic and other groups sampled, the exception being ranchers (44% in favor, 53% opposed). Persuasive arguments had little impact on respondents' attitudes toward wolves and their proposed restoration. Overall support for wolf reestablishment remained high and increased slightly after respondents heard persuasive arguments for and against wolf restoration. Yet most respondents (63.3%) did not change their level of support or opposition to the idea of reestablishing wolves after hearing persuasive arguments. Most people who did change their opinion increased the extremity of their responses, supporting attitudinal theory that predicts that people with strongly held attitudes will increase the extremity of their opinions after receiving more information. The attitudes people hold are critically important to the success of wolf restoration efforts. Although most of the public supports wolf restoration, polarization of the issue remains strong. This polarization poses a significant challenge to wildlife managers. If management agencies decide to pursue wolf restoration in the southern Rockies, efforts to mitigate strongly polarized positions should be given a high priority. Alternatively, if those agencies choose not to restore wolves, they likely will face significant controversy as unsatisfied wolf proponents make their feelings known.

1434: ***-.040***

The peninsular pronghorn (Antilocapra americana peninsularis) in the Baja California peninsula is an endangered subspecies. We constructed captive breeding facilities in the Vizcaino Desert within the current range of peninsular pronghorn to assist in population recovery. The captive breeding facilities included fences, shade, feeders, an irrigation system, observation towers, housing for caretakers, and a visitor center. We document the initial 6-year cycle of the captive management, 1 step of the recovery effort, from the first capture of 5 fawns in 1998 to 90 captive peninsular pronghorns by the end of 2003. We identified problems with the captive breeding facility (e.g., identification of animals and movements into, and accidents with, fences), but overall the facility has been a success. We projected the first potential translocation of animals into the wild in 2004. If the translocation of captive animals is successful, peninsular pronghorn may begin the recovery process in the wild.

1435: ***-.016***

Human-induced erosion regularly delivers massive quantities of fine sediments into streams and rivers forming large static bodies of sediment known as sand slugs, which smother in-stream habitat, alter community structure, and decrease biodiversity. Sand slugs are widespread in parts of southeastern Australia as well as in many other parts of the world, and there is now considerable interest in restoring such affected streams. The reintroduction of large timber is widely suggested as a strategy for restoring habitat complexity, but this has rarely been tested in sand slug-affected streams. We examined the response of fish populations to wood addition to two streams in southeastern Australia that have been impacted by sand slugs. Manipulated sites (three per treatment) had either one or four timber structures added, and these sites were compared with (three) unmanipulated (control) sites before and after the manipulation occurred. Despite a supraseasonal drought during the study, we observed short-term increases in the abundance of Mountain galaxias (Galaxias olidus) at the four-structure sites, while both the four-structure and the one-structure treatments appeared to buffer against drought-induced declines in two other species, River blackfish (Gadopsis marmoratus) and Southern pygmy perch (Nannoperca

australis), relative to controls. However, drought eventually caused the complete loss of surface water from these streams and the loss of fish from both manipulated and unmanipulated sites. Thus, although the study supports the use of timber structures as a means of increasing local fish abundances, these beneficial effects were, in these streams, contingent upon permanency of flow. Because sedimentation has depleted the number of permanent refuge pools in these creeks, recovery rates of the fauna (i.e., resilience) are likely to be slow. We therefore conclude that in streams subjected to frequent disturbance, restoring refugia may be as, if not more, important as restoring what we term residential habitat.

1436: ***-.020***

Salvaging from premining areas and translocation of succulent plants has been investigated for the revegetation of gypsiferous mine spoil in an arid region of South Africa. Given that facilitation effects are thought to outweigh competition effects in harsh environments, we hypothesized that the survival of translocated succulents would be higher when planted in multispecies clumps than alone and that the growth rate (measured as stem extension) and fruit-set would be greater for plants in clumps than for those planted alone. Two leaf-succulent species (Aridaria noctiflora ssp. noctiflora and Drosanthemum deciduum) and one stem-succulent species (Psilocaulon dinteri) were salvaged from the area destined for mining and translocated onto the mine spoil. These plants were planted either in a multispecies clump of the three species together or alone. One year after translocation, 67% of the plants survived. It was also found that the succulents used in these experiments survived in higher numbers when planted alone. Due to the similar root morphology of D. deciduum and P. dinteri, they competed for resources instead of facilitating each other's establishment. The results were variable for each of the species used, and neither growth nor seed-set was improved by clumping. These findings would also possibly vary from year to year with different abiotic conditions.

1437: ***+.105***

Breeding biology of the endangered Mauritius Olive White-eye Zosterops chloronothos, the least known extant species of the endemic Mauritian avifauna, was studied for three consecutive breeding seasons between 1998 and 2001. Fifteen territories were monitored each year. Six nests were found and closely monitored. Prior to this study, only two nesting episodes had been documented. Nest observations revealed that both male and female were involved in incubation, brooding and feeding of the young at both the nestling and fledgling stage. One nest was successful (this being the first observation of a successful nest for this species) with fledging and juvenile dependency periods of 14 and 61 days, respectively. The remaining five nests were depredated, with evidence suggesting predation by the introduced Ship Rat Rattus rattus. Five breeding pairs each produced a single fledgling during the three-year period and no pair produced more than one fledgling. Targetted poisoning and trapping of nest predators on the main island of Mauritius and translocation of Olive White-eyes to predator-free islands should be implemented as conservation measures. Further ecological studies of the Mauritius Olive White-eye are sorely needed to direct conservation management of this declining species.

1438: ***+.072***

The western barred bandicoot (Perameles bougainville) is an endangered species, free ranging on only two islands off the coast of Western Australia (Dorre and Bernier Islands). Conservation efforts are currently directed at reintroducing these marsupials into predator-proof enclosures and habitats in historical distribution ranges on the mainland in the southwest of Western Australia and

in South Australia. In September 2000, 19 western barred bandicoots were captured on Bernier Island for translocation, and 11 of these had evidence of at least one of the following eye conditions: corneal opacity, conjunctivitis, ocular discharge, and blepharitis. Five bandicoots were examined, and conjunetival and cloacal swabs were collected. Polymerase chain reaction for Chlamydiales was positive in four bandicoots. Four Chlamydiales types were identified by gene sequencing, including a strain of Chlamydia pecorum different from strains previously found in koalas and several new Chlamydiales genotypes. The bandicoots responded excellently to treatment with oxytetracyline weekly for 6 wk, and topical oxytetracycline and neomycin were administered topically to both eyes s.i.d. for 4 mo.

1439: *-.014*

Mbuna, the dominant fishes on the rocky shores of Lake Malawi, have become a major 'model system' for the study of rapid speciation and adaptive radiation. At least 295 putative species are known, of which more than 200 remain undescribed. There is no good evidence for monophyly in the mbuna, rather mitochondrial DNA phylogenies indicate that they are polyphlyetic with respect to benthic feeding cichlids of the genera Aulonocara, Alticorpus and some species of Lethrinops. Male mbuna hold territories for 18 months or more and breed year-round. All species are maternal mouthbrooders, but females do not guard free-swimming young. Mbuna are polygamous (both sexes). There is sexual dimorphism in size, colour and fin length, and many species show within-population colour polymorphism. Mbuna genera are largely differentiated on the basis of head, jaw and tooth morphology, but congeneric species are generally distinguished by male colour. Many morphologically specialized forms have broad diets and often feed on common easily obtainable resources. While it is likely that dietary and habitat niche partitioning contributes to species coexistence, this has never clearly been demonstrated under experimental conditions, Populations on spatially separated habitat patches are often genetically differentiated, probably because most species are specialized for life on rocky shores, and lack a dispersal phase in their life histories. Males seem to disperse more than females, but are able to home several kilometres back to their territories. Some closely related ecologically equivalent allopatric populations are differentiated in male colour. Those tested have been shown to mate at least partially assortatively. Sexual selection acting on male colour seems the most plausible mechanism for initial species divergence. The same colour forms seem to have arisen several times, suggesting frequent parallel evolution. The main conservation threat to mbuna at present seems to be translocation of species within the lake as a result of the aquarium trade. However, deleterious effects on indigenous populations are not documented. In the long run, sedimentation, pollution, introduction of alien species and the development of targeted food fisheries could be more serious threats.

1441: *+.172*

Dispersal is universally considered important for biodiversity conservation. However, the significance of long- as opposed to short-distance dispersal is insufficiently recognized in the conservation context. Long-distance dispersal (LDD) events, although typically rare, are crucial to population spread and to maintenance of genetic connectivity. The main threats to global biodiversity involve excessive LDD of elements alien to ecosystems and insufficient dispersal of native species, for example, because of habitat fragmentation. In this paper, we attempt to bridge the gap in the treatment of LDD by reviewing the conservation issues for which LDD is most important. We then demonstrate how taking LDD into consideration can improve conservation management decisions.

1442: *+.048*

The southern brown bandicoot (Isoodon obesulus) has undergone significant range contractions since European settlement, and it is now considered "Endangered" throughout south-eastern mainland Australia. This species currently has a highly fragmented distribution inhabiting a mosaic of habitats. This project uses mitochondrial DNA (mtDNA) and microsatellite data to determine levels of genetic diversity, population structure and evolutionary history, which can aid wildlife managers in setting priorities and determining management strategies. Analyses of genetic diversity revealed low levels of mtDNA variability (mean h = 50.42%, π = 0.76%) and divergence (mean d(A) = 0.29%) across all regions investigated, and was among the lowest recorded for marsupials. These data indicate a relatively small female effective population size, which is most likely a consequence of a large-scale population contraction and subsequent expansion occurring in pre-history (mismatch distribution analysis, SSD P-value = 0.12). Individuals from the Sydney region experienced significant reductions in microsatellite diversity (A = 3.8, H-E = 0.565), with the Garigal National Park (NP) population exhibiting "genetic reduction signatures" indicating a recent population bottleneck. Population differentiation analysis revealed significant genetic division amongst I. obesulus individuals from Sydney, East Gippsland and Mt Gambier regions (θ = 0.176 - 0.271), but could not separate the two Sydney populations (Ku-ring-gai NP and Garigal NP). Based on these data and habitat type, translocations could readily be made between the two Sydney populations, but not between the others. Phylogenetic comparisons between I. obesulus and I. auratus show little support for current Isoodon taxonomy, consistent with the findings of Pope et al. 2001. We therefore recommend the recognition of only three I. obesulus sub-species and suggest that these comprise a single morphologically diverse species that once was widespread across Australia.

1443: *-.047*

Lake minnow is a cyprinid fish species, which is critically endangered in Poland and protected by law, and also included in the Polish Red Data Book of Animals. This species is an inhabitant of small and shallow, isolated water bodies, vulnerable to the total destruction mainly due to swamp draining and amelioration. In recent decades, the rapid disappearance of Polish lake minnow populations has been observed, from about 80 described in literature ever to as little as 19 at present; most of them (13) known from Kaszuby region in northern Poland (Fig. 1). It is therefore considered necessary to urgently begin the active protection of this species, including either protection in situ or ex situ. The latter is now possible due to good knowledge of the techniques of lake minnow captive breeding and production of stocking material under controlled conditions. The strictly limited, local translocations of this species, based on the results of preliminary studies on genetic variability of Polish populations, are planned to be performed in the Mazowsze region (central Poland) in the nearest future, in order to increase there the number of existing populations from 2 to 4. Long-term monitoring will enable to evaluate efficiency of such activities as concerns the most endangered lake minnow populations in other regions in Poland.

1444: *+.156*

The aim of the work was to illustrate characteristics of the natural resources and the evaluation of the small mesotrophic Wiejki Lake situated in Grodecko-Michalowska Basin (Polnocnopodlaska Lowland, north-eastern Poland). The lake is one of only four such objects located in this part of the Polish Lowland. In this article, influence of lowering of groundwater levels and anthropogenic use on the lake vegetation in the period between 1953 and 2003 are also presented. Changes in natural environment surrounding the Wiejki Lake are described on the basis of aerial photographs and phytosociological records. In the past the lake occupied a considerably larger area, what is reflected in a mineral and organic gyttja layer a few metres thick occurring in the bottom of

organic deposits (Fig. 2). Low birch-willow thickets (Betula humilis-Salix rosmarinifolia community) and moss-sedge associations mostly determined the earlier vegetation of the lake. At present in the surroundings of the Wiejki Lake, seven plant communities are distinguished: Equisetetum fluviatilis, Caricetum rostratae, Caricetum diandrae, Salicetum pentandro-cinereae, Thelypterido-Betuletum, Ribeso nigri-Alnetum and dominant Festuca rubra communities. They create distinct zones around the water level mark (Fig. 4). Sites of shrubby birch Betula humilis listed in Polish Red Data Book of Plants and additionally three plant species strictly protected in Poland (Drosera rotundifolia, Dactyllorhiza maculata, Dactyllorhiza incarnata) were found in vicinity of the lake. In the natural environment of the Wiejki Lake, significant changes of vegetation were affirmed in the course of last 50 years. They are: forming of reed-mace Typha latifolia concentrations in littoral zone, narrowing of sedge and moss-sedge communities zones as well as encroaching of grey willow Salix cinerea to rush and sedge phytocoenoses (Fig. 3, 4). Birch-willow tickets were replaced by birch-alder forests Thelypterido-Betuletum and cultivated wet meadows. The overall water surface area has not changed in this period, however deepness of the lake has decreased to about 1,5 m. These changes are directly connected with thorough drainage made in the middle of the 1980s. The outcome of the work is to define a protection plan of the Wiejki Lake in which indispensable methods of nature conservation were specified. It provides for: rising of ground water level, elimination of drainage ditches, reintroduction of Lapland willow (Salix lapponum) and the removal of shrubs and trees in order to preserve sites of Betula humilis (Fig. 3). Finally, a change of the protection status of the object from ecological area to nature reserve is suggested.

1445: ***-.006***

Reintroduction projects may fail because captive-reared animals do not possess the behavioural skills required for survival in the wild. Rearing captive-bred animals in semi-natural enclosures prior to release has been used to improve the survival of reintroduced endangered species, but it is unclear how rearing environment influences the development of behaviour. This study examined the effect of rearing conditions on the behaviour of the goodeid Skiffia multipunctata, an endangered species of Mexican fish. Under standard laboratory conditions, the courtship, aggression, boldness and foraging behaviour of fish raised in aquaria was compared to that of fish reared in outdoor ponds. We present initial behavioural descriptions for this species and show that laboratory-reared fish displayed increased courtship, aggression and curiosity towards a novel predator in comparison to their pond-reared counterparts. Laboratory-reared fish also commenced foraging on a novel food item (Artemia) more rapidly than fish reared outdoors. These findings suggest that captive rearing environments promote the development of behavioural tendencies, such as boldness and aggression, which could be detrimental to the survival of reintroduced individuals. (C) 2004 Elsevier Ltd. All rights reserved.

1446: ***+.128***

The small Cape mountain zebra population in Gamka Mountain Nature Reserve represents a third of the entire gene pool of this endangered species and is thus vital for it's conservation. Presently, management of this population is largely hands off, with the belief that it will grow to levels which will allow it to form a source for the mixing of mountain zebra stocks in the future. The growth of this population however, has been slow and we investigated the influence of habitat and fire on this growth. Firstly, we used a diffusion model to perform a population viability analysis. This analysis indicated that the population had a low probability of attaining quasi-extinction in the next 50 years (G = 0.0032). However, our findings indicated that less than 30% of the reserve was suitable for mountain zebra and that the preferred habitat would have to be burnt at unnaturally short

intervals to sustain the present growth. We therefore argue that the risk of quasi-extinction to this population is greater than predicted and suggest that management options need to be implemented to reduce this risk. These options include; translocation to another protected area; acquisition of adjacent land; burning preferred habitat at unnaturally short intervals; forming a conservancy with adjacent landowners; leasing cultivated land for pasture. We suggest that only the latter two options are likely to stimulate mountain zebra population growth in the short term and that these should receive immediate attention. (C) 2004 Elsevier Ltd. All rights reserved.

1447: *+.058*

Little is known about the reproduction of the Iberian lynx (Lynx pardinus) even though it is the most endangered felid in the world. We studied during 9 years several reproductive parameters of the Iberian lynx in one of the subpopulations situated in Donana National Park (south-western Spain), by means of radio-tracking, direct observations and photo-trapping. The potential breeding subpopulation was usually composed of 3 adult females, which bred 83% of the total possible 29 female-reproductive year. The minimum total number of cubs born during the study was 64 (7.7 +/- 0.69 per year). There was no correlation between the number of cubs born or number of breeding females and population size of European rabbit (Oryctolagus cuniculus, the main prey of the lynx). All known births (n = 16) occurred in March except one in April and another in June. Mean litter size was 3.0 +/- 0.16 (n = 16, range = 2-4). Sex-ratio (n = 59 cubs) was 1.03:1.00 (females: males). Nevertheless, the commonest picture was a female with 2 cubs older than 3 months. Altogether, at age of 3 months, 75% of cubs survived. Number of lynx alive at 10 months old and before dispersing was 69% and 57%. respectively. Sex did not affect survival for any age. Normally, it was not possible to know the causes of the death of cubs younger than 3 months. Survival at this age was not related with mother, mother age, or rabbit abundance. All females that bred were older than 3 years. The age of last reproduction was 9 years. For 3 females that were tracked during almost their complete reproductive a life, the life time reproductive output was between 11 and 19 cubs. Iberian lynx reproductive parameters did not respond to wide changes in prey abundance during the study. Conservation plans considering the extraction of cubs with a low survival probability should be considered by managers, for instance, in translocation campaigns. (C) 2004 Elsevier Ltd. All rights reserved.

1448: *+.095*

Great crested newts (Triturus cristatus) are protected under European and UK legislation, but are frequently the subject of conflict between development and conservation in England. When this occurs, the developer is legally obliged to instigate a mitigation plan to reduce the impacts on the newts. This usually involves the translocation of newts coupled with habitat enhancement and creation. We reviewed mitigation projects carried out in England between 1990 and 2001 by (1) analysing licensing information collected by the governmental licensing authorities; and (2) a questionnaire survey of a sample of mitigation projects. Over half of the licensed projects on file contained no report of the work undertaken. There was an increase in the number of new translocation projects from less than 10 a year in the early 1990s to over 80 a year by 2000. This translates into about 1.5 million per year currently being spent on grew: crested newt mitigation projects. Most of these projects involved in situ translocations of newts to areas within or adjacent to the development site. The number of newts translocated per project declined over the same period, and was related to the total area of habitat destroyed and work effort. About 27% of great crested newt terrestrial habitat was destroyed during the developments along with about half of all ponds. Although the number of new ponds created compensated for the number of known great crested newt ponds lost, there was a net loss in terms of overall area of aquatic habitat. Where

follow-up monitoring of translocations was conducted, there was evidence of breeding at most sites one-year post-development, but it is unclear whether these populations were sustainable in the long-term. (C) 2004 Elsevier Ltd. All rights reserved.

1449: ***+.234***

Kaka (Nestor meridionalis), red-crowned parakeet (Cyanoramphus novaezelandiae), whitehead (Mohoua albicilla), tomtit (Petroica macrocephala), and bellbird (Anthornis melanura) have all recently been reintroduced to sites in or near Wellington city. Prior to or concurrent with these translocations, unmarked individuals of all five species were detected in forested reserves on Wellington peninsula. Based on the number of birds seen, and frequency of sightings, we suggest that red-crowned parakeets, whiteheads and bellbirds have established resident populations in some reserves independent of translocations. We attribute these successful re-establishments to the effective control of possums (Trichosurus vulpecula) and rats (Rattus sp.) undertaken by Greater Wellington Regional Council and the Department of Conservation.

1450: ***+.102***

A trial translocation to establish a new fluttering shearwater (Puffinus gavia) colony is reported. From 1991 to 1996, 334 fluttering shearwater chicks were transferred from Long Island to Maud Island, Marlborough Sounds, New Zealand. Chicks were artificially housed and hand-fed until fledging. Overall fledging success was 82%, 32 of the 273 chicks that fledged returned to Maud Island, and 30 have bred. Mean age of first breeding was 6.8 years (range 5-10 years). Returning chicks were heavier at fledging and spent longer on Maud Island than chicks that did not return. Transferred chicks showed typical post-fledging behaviour by dispersing to southeast Australian waters. The new colony has gradually increased, and 15 pairs bred in 2003/04. Methods developed have application to endangered species management.

1452: ***+.060***

The Przewalski's horse was extinct in the wild in the late 1960ties of the last century. But it survived in captivity, in zoos and animal pares. The successful breeding between 1950 and 1980 allowed the reintroduction to the wild after 1990. Different sites were checked in Mongolia, and the Gobi B-strictly protected area turned out to be the most suitable habitat. Between 1992 and 2004 89 Przewalski's horses were transported to the Gobi and 93 foals were born. 91 animals survived until now. Especially during winter 2000/2001 severe losses were registrated. By 2004 four harem-groups and one stallion-group were established in the wild. Since 1998 a substantial scientific program was established, which was helpful for the monitoring of the Przewalski-population and different habitat factors. Since 2002 the focus has changed; the Przewalski's horse is not the first goal of the project anymore, but the umbrella species for the development of the Gobi B-strictly protected area into a biosphere reserve.

1453: ***+.250***

Understanding the mechanisms by which climate and predation patterns by top predators co-vary to affect community structure accrues added importance as humans exert growing influence over both climate and regional predator assemblages. In Yellowstone National Park, winter conditions and reintroduced gray wolves (Canis lupus) together determine the availability of winter carrion on which numerous scavenger species depend for survival and reproduction. As climate changes in Yellowstone, therefore, scavenger species may experience a dramatic reshuffling of food

resources. As such, we analyzed 55 y of weather data from Yellowstone in order to determine trends in winter conditions. We found that winters are getting shorter, as measured by the number of days with snow on the ground, due to decreased snowfall and increased number of days with temperatures above freezing. To investigate synergistic effects of human and climatic alterations of species interactions, we used an empirically derived model to show that in the absence of wolves, early snow thaw leads to a substantial reduction in late-winter carrion, causing potential food bottlenecks for scavengers. In addition, by narrowing the window of time over which carrion is available and thereby creating a resource pulse, climate change likely favors scavengers that can quickly track food sources over great distances. Wolves, however, largely mitigate late-winter reduction in carrion due to earlier snow thaws. By buffering the effects of climate change on carrion availability, wolves allow scavengers to adapt to a changing environment over a longer time scale more commensurate with natural processes. This study illustrates the importance of restoring and maintaining intact food chains in the face of large-scale environmental perturbations such as climate change.

1454: *-.029*

Morangaya pensilis is an endemic moncitypic cactus genus from the southern part of the Baja California Peninsula, Mexico. Previously known only as scattered individuals occurring at a very low density in the highest areas of Sierra de La Laguna, the occurrence of a relatively more dense population (4-7 plants per 250 m(2)) is reported on Sierra Cacachilas, c. 40 km to the north. Disturbance to the species' habitat includes fragmentation, illegal collection and trade, and land use changes, especially to farming and cattle ranching. Seeds collected from Sierra Cacachilas had a relatively high viability (92%) but recruitment (of 2-month old seedlings) in the area was relatively low, despite above average rainfall in the year of measurement. M. pensilis is eligible for inclusion in Mexico's list of threatened species and fulfils the requirements for categorization as Vulnerable on the IUCN Red List. Management activities that are already taking place in this area include restrictions on cattle and goat grazing and consideration of areas suitable for translocation of the species.

1455: *+.149*

The distribution and abundance of the greater sage-grouse (Centrocercus urophasianus) have declined dramatically, and as a result the species has become the focus of conservation efforts. We conducted a range-wide genetic survey of the species which included 46 populations and over 1000 individuals using both mitochondrial sequence data and data from seven nuclear microsatellites. Nested clade and STRUCTURE analyses revealed that, in general, the greater sage-grouse populations follow an isolation-by-distance model of restricted gene flow. This suggests that movements of the greater sage-grouse are typically among neighbouring populations and not across the species, range. This may have important implications if management is considering translocations as they should involve neighbouring rather than distant populations to preserve any effects of local adaptation. We identified two populations in Washington with low levels of genetic variation that reflect severe habitat loss and dramatic population decline. Managers of these populations may consider augmentation from geographically close populations. One population (Lyon/Mono) on the southwestern edge of the species' range appears to have been isolated from all other greater sage-grouse populations. This population is sufficiently genetically distinct that it warrants protection and management as a separate unit. The genetic data presented here, in conjunction with large-scale demographic and habitat data, will provide an integrated approach to conservation efforts for the greater sage-grouse.

1456: ***+.032***

The newly described Gunnison sage-grouse (Centrocercus minimus) is a species of concern for management because of marked declines in distribution and abundance due to the loss and fragmentation of sagebrush habitat. This has caused remaining populations to be unusually small and isolated. We utilized mitochondrial DNA sequence data and data from 8 nuclear microsatellites to assess the extent of population subdivision among Gunnison sage-grouse populations in southwestern Colorado and southeastern Utah, USA. We found a high degree of population structure and low amounts of gene flow among all pairs of populations except the geographically adjacent Gunnison and Curecanti populations. Population structure for Gunnison sage-grouse was significantly higher than has been reported for greater sage-grouse (C. urophasianus). Further, we documented low levels of genetic diversity in some populations (particularly Dove Creek/Monticello and Pinon Mesa with an average of only 3.00 and 2.13 alleles per locus respectively) indicating that translocations from larger, more genetically diverse populations may be warranted. Bayesian analysis identified 3 potential migrants (involving San Miguel, Dove Creek/Monticello, Crawford, and Curecanti). Further, this analysis showed that 4 individuals from Cerro/Cimarron were more closely related to birds from San Miguel than to its geographically closer neighbors Gunnison and Curecanti. This suggests the Cerro/Cimarron area may act as a stepping stone for gene flow between San Miguel and Gunnison and that habitat restoration and protection in areas between these 2 basins should be a priority in an attempt to facilitate natural movement among these populations. Conservation plans should include monitoring and maintaining genetic diversity, preventing future habitat loss and fragmentation, enhancing existing habitat, and restoring converted sagebrush communities.

1457: ***+.106***

1. Large carnivores are currently recolonizing areas where they have been extinct for decades. This poses considerable challenges for wildlife managers, partly because the optimal harvesting strategies of prey populations may be affected. If the carnivores in such areas are under strict management control (as in Scandinavia), the predator will not show a numerical response to changes in prey density. Consequently, the density of prey is mainly determined by the vital rates of the prey population and the predation pressure. In this study we modelled the optimal harvesting strategy for a prey population in which there was no numerical response by the predator.2. Our model is an age-structured deterministic matrix model system. Optimal harvesting strategies are determined, measuring yield either as number of animals harvested or as mass of meat.3. First, using a moose population in Hedmark in south-eastern Norway as a case study, we demonstrate that continuing to harvest at the rates used prior to wolf recolonization will lead to a decline in the moose population.4. Secondly, harvesting quotas are specified by age and sex, usually with a high proportion of calves. Although wolves mainly kill juvenile moose (calves and yearlings), the relationship between harvest composition and yield is not affected by predation. Both in the presence and absence of predation, a high proportion of calves in the harvest gives the highest yield measured as the number of animals harvested, whereas a high proportion of adults maximizes the yield measured in terms of meat. Furthermore, a female-biased sex structure in the population gives a higher yield in both the presence and absence of the predator.5. Synthesis and applications. We have shown that managers facing the new challenges presented by recolonizing populations of large predators such as wolves should reduce the size of harvest quotas in order to avoid decreases in prey populations. However, the general relationship between the harvesting strategy and yield maximization is not affected by wolf predation. The harvest yield from cervid populations is often important to local economies, and moose is the single most important game species in Scandinavia. It is therefore important to implement optimal harvesting strategies under

these new conditions in order to prevent an unnecessary loss of yield, and success in this task may in turn affect local attitudes towards large carnivores.

1458: *-.070*

The European minnow, Phoxinus phoxinus (L.), constitutes a serious threat to natural brown trout, Salmo trutta L., stocks in Norway following its introduction and translocation. In the present study, the leaping capabilities of the European minnow (50-110 mm total length) and brown trout (64-255 mm total length) were investigated with the aim of constructing suitable waterfall barriers to prevent further unwanted dispersal of the minnow, but still allow passage of larger brown trout. No successful leap of minnows was recorded at 4.9-6.5 degreesC, even at height intervals as low as 3 cm. At 14.0-16.5 degreesC, minnows were able to force waterfall barriers up to 27 cm high. At 4.9-6.5 degreesC brown trout forced waterfall barriers up to 40 cm. Thus, building of such barriers in brooks and rivers can help prevent the dispersal of minnows, and still allow larger brown trout to pass during feeding and spawning migrations.

1459: *+.172*

To minimise the risks of unintentional translocation and further spread of the non-native cyprinid, Pseudorasbora parva (Temminck & Schlegel) in the UK, various morphological and meristic characters of this species were measured, with a view to isolating the characters which allow reliable identification of this species during the early stages of development. The combination of myomere numbers, the nature of the internal pigmentation associated with the notochord and the lack of a single row of external melanophores along the route of the lateral line organ reliably facilitate the ability to distinguish this species from the native fauna of the British Isles. Thus, for the first time, fishery managers and environmental agencies have a much needed tool to screen for the presence young-of-the-year of this species when authorising movements of other fish species.

1460: *+.049*

Here I present a detailed analysis of individual inbreeding coefficient effects on some reproductive parameters and longevity in three species of gazelles under different conservation status: vulnerable dorcas gazelle. endangered Cuvier's gazelle. and extinct in the wild mhorr gazelle. The novelty of this study stems from the inclusion of both males and females in analyses including a large database of information collected during two decades of periodical studbook inventories for these species. Translocations to different zoo locations of the extinct subspecies mhorr gazelle do not apparently affect reproductive performance (population sex ratio) or individual longevity. In agreement with previous works, the average inbreeding coefficients vary inter-specifically, being higher in Cuvier's, followed by mhorr and dorcas gazelles. This reflects the different population size of the founding individuals of each scepies captive population. Sexual maturity and age at first birth follow an allometric pattern, occurring at an earlier age in the smallest species (dorcas), followed by Cuvier's and then the mhorr gazelle. Twinning in Cuvier's gazelle depends on maternal experience. as it is less frequent in primiparous females. Inbreeding affects neither twinning nor sex ratio. Mhorr gazelles' studbook shows several causes of death and it emerges that a higher proportion of non-inbred females die due to pathologies than males. although both sexes show similar proportion of mortal pathologies when inbred. Multifactor ANOVA shows that longevity decreases with inbreeding level and that females live longer than males in the three species of gazelles studied. as expected in polygynous mamma Mhorr and dorcas non-inbred females show higher juvenile survival than males, whereas inbred individuals show a similar declining juvenile survival, particularly in mhorr and Cuvier's gazelles. Finally. it is discussed the

apparent inbreeding tolerance in Cuvier's species, and the great value keeping and studying long term data of well-monitored captive populations may prove to the conservation of threatened species. (C) 2004 Elsevier Ltd. All rights reserved.

1461: *+.043*

The escalating threats to ecosystems worldwide have lead to a need for efficient methods to breed animals in captivity and to prepare captive-born animals for release back to the wild. However, life in captivity may lead to modifications in the animals behaviour mainly by genetic changes, including behavioural adaptations such as reduced predator responses. Such modifications may seriously affect survival after a reintroduction. The present study was a first screening of behavioural and morphological variation between different captive populations in standardized test situations using red junglefowl as a model species. The birds were tested in three different test situations in order to measure anti-predatory behaviour. social behaviour and exploratory behaviour. The results of this study clearly show that there ate behavioural differences between the captive populations which potentially can be crucial for the animals in a reintroduction situation. However, the extent to which these differences are due to genetic changes caused by small breeding populations or adaptations to the different captive environments is not yet known. although morphological differences found suggest that genetic variation may cause some of the behavioural differences as well. The differences found imply that life in captivity can affect an animal's behaviour and even though the red junglefowl is merely used as a model here, this suggests that these aspects may be important to consider also in other species where reintroduction is a more central motive for keeping the animals in captivity. (C) 2004. Elsevier Ltd. All rights reserved.

1462: *+.012*

We assessed the effects of range disjunction, migratory habit, coloniality, and habitat structure on the genetic differentiation of North American Burrowing Owl (Athene cunicularia) populations. Burrowing Owls in North America comprise two forms or subspecies: A. c. floridana in Florida, separated by &SIM; 1,500 km from the western form, A. c. hypugaea, which ranges from Texas to California and north to southern Canada. Burrowing Owls tend to be loosely colonial, and both the Florida populations and southerly populations of A. c. hypugaea from California to Texas are nonmigratory. To assess genetic structure, we examined 201 individuals from nine western and six Florida populations at seven highly variable microsatellite DNA loci. Mean gene diversity (H-exp) was higher in the west than in Florida (0.539 and 0.341, respectively; $P < 0.05$). Populations within subspecies were essentially panmictic (A. c. floridana: $\theta = 0.038$, $\varrho = 0.014$; A. c. hypugaea: $\theta = 0.014$, $\varrho = 0.009$) and even genetic differentiation across subspecies was modest ($\theta = 0.051$, $\varrho = 0.014$). Nevertheless, the western and Florida forms were easily distinguished by any of several criteria, such as allelic absences in Florida, assignment tests, and well-supported branches on the inferred phylogenetic tree. Genetic differentiation was at least twice as great in resident Florida ($\theta = 0.038$) and California ($\theta = 0.021$) populations as in migratory western populations ($\theta = 0.012$), though 95% confidence intervals of theta estimates overlapped. We found no evidence of a genetic bottleneck that would result in evolutionary disequilibrium within subspecies. In the west, high observed heterozygosity values and evidence of gene flow suggest that population declines and patchy habitat, which currently imperil this species throughout much of its range, have not led to inbreeding or biologically meaningful genetic differentiation among the sampled populations.

1463: *+.146*

Experimental translocations of three endangered plants undertaken in South Australia confirmed the impact of specific factors thought to affect the survival and establishment of seedlings of each species. A trial involving Prostanthera eurybioides planted into several different microsites, found microsite to be a critical determinant of survival and growth. Herbivore grazing and weed competition adversely affected survival and growth of Acacia cretacea and Acacia whibleyana translocants, respectively. While these findings may not necessarily extrapolate to all species, common sense suggests that these three factors should be important considerations when planning other plant translocations. For example, attention needs to be given to the exact placement of individuals in relation to suitable edaphic, biotic and climatic factors around the receptor site. Where weeds threaten the population, they need to be controlled either before or at the time of planting. Furthermore, protection of new plantings from herbivores is likely to be crucial, particularly during the first few months after planting.

1464: *+.047*

Temperate grassy ecosystems are amongst Australia's most endangered ecosystems. Most remnants are small, fragmented and highly degraded. Practical methods for restoring native understorey species are urgently required. Dominant native grasses such as Kangaroo Grass (Themeda triandra Forssk.) and Tussock-grasses (Poa species) have been eliminated from many remnants by heavy grazing in the past. The reintroduction of these grasses is a critical step for understorey restoration. This paper W reviews the literature on Themeda seed biology and seedling establishment; 00 summarizes the lessons learnt from three major attempts to establish Themeda stands in south-east Australia; and (iii) identifies the research needed to enhance Themeda restoration. Considerable information is available on Themeda seed and establishment biology, and restoration exercises have shown that Themeda stands can be readily established by surface-spreading awned seeds in seed-bearing hay. However, many practical challenges remain, including the need to identify optimal sowing periods, create better seedbed conditions, develop practical mulching techniques, and improve weed control. The use of seed-bearing hay has constrained restoration to relatively small areas in the past. Future trials may benefit by using more concentrated seed products such as seed-bearing florets and pure seeds which permit larger areas to be restored at one time.

1465: *-.012*

The island fox (Urocyon littoralis) on Santa Catalina Island is among the most imperiled species on the Channel Islands due to a recent outbreak of canine distemper virus (CDV). The western subpopulation, which was not exposed to CDV, is a crucial element in the recovery of foxes by providing a source of animals for translocation and captive breeding. Using the program VORTEX, we developed a population viability analysis for the Santa Catalina Island fox to (1) address the likelihood of population persistence, (2) estimate the current susceptibility of the population to catastrophic events, and (3) evaluate the efficacy of current restoration strategies of releasing captive bred foxes and transplanting wild animals. Overall, we found the population to be susceptible to catastrophic events; a 50% increase in mortality every 20 years was sufficient to elevate the extinction risk above 5%. Current management activities entail the transplanting of 12 juvenile foxes annually, which may reduce the viability of the western subpopulation. A minimum population size of at least 150 foxes should be maintained in each subpopulation to reduce the risk of extinction due to demographic stochasticity. Releases of translocated and captive bred animals affect the speed of recovery on the eastern half of Catalina Island, but not the probability of extinction, which is near zero under current conditions. We conducted a sensitivity analysis for demographic parameters by incrementally varying survival, fecundity and density-dependence

parameters, while holding all other parameters constant. Sensitivity analyses identified mortality and mean litter size as the most sensitive parameters, while the implementation of density-dependence and environmental variation of model parameters did not seem to affect population performance. We conclude that the population of island foxes on Santa Catalina is currently at a critically low population level, but recovery of the species appears possible. (c) 2004 Elsevier B.V. All rights reserved.

1467: *+.175*

This report provides guidelines for the captive management of mudfish (Neochanna) species in New Zealand. This includes the requirements for captive rearing, breeding, stock maintenance, and the establishment of new Neochanna populations. The reproductive and developmental biology of Neochanna species has been an infrequently studied topic, with the exception of Canterbury mudfish (N. burrowsius). Further, spawning in captivity has been documented only once in the cases of black (N. diversus) and brown (N. apoda) mudfish, and never for the Chatham (N. rekohua) or Northland (N. heleios) mudfishes. As each species has differing characteristics that are likely to influence its requirements, and much is still to be learnt, the aim of this report is to collate information and provide preliminary guidelines. Improving the survival rate of eggs and fry through successful captive breeding and rearing may provide a pool of recruits for the establishment of further populations. Translocation is an important method of safeguarding against species extinction by spreading the risk among a greater number of populations. However, captive management and translocation are not without risk, and should be contemplated thoroughly. Neochanna species have proved a challenge to breed in captivity, and many translocation attempts have failed to establish populations. These disappointments illustrate that a greater understanding is required to direct future conservation efforts. Thus, this report provides practical guidance to encourage further research into the biology of Neochanna species based on observations from both successful and unsuccessful experiences of breeding Neochanna species in captivity.

1468: *+.113*

At least five species and six taxa of kiwi (Apteryx spp.) are recognised at present. All taxa are currently listed as threatened. Since the 1890s, translocation of kiwi populations to the offshore islands of New Zealand has been used in the conservation of the genus. This report identifies and reviews offshore (and lake-bound) islands where kiwi occur naturally, together with islands to which kiwi have been translocated. Brief, descriptive histories of populations and translocations are provided. At least 28 offshore islands (excluding Stewart Island) currently support populations of kiwi. A number of islands, nominated by DOC conservancies, which might be suitable for future translocations, are also listed. The criteria for this list of potential islands include: lack of predators; sufficient size (at least 100 ha); presence of suitable habitat; legal protection; and absence of conflicting conservation values. Before recommendations as to which species should be translocated (and to which islands) can be made, consultation with other interested parties and detailed investigations of the islands. suitability will be required.

1469: *+.201*

Throughout the latter half of the twentieth century, wildlife habitat destruction and species extinctions have greatly increased. As the number of endangered species grows, the use of reintroduction as a conservation tool against species extinction increases. This study focuses on a 16-month study on marsh deer reintroduced in the Jatai Ecological Station. The animals were radio tagged and tracked daily between December 1998 and April 2000. Displacement activity and

spatial preferences were monitored by triangulation. The animals wandered the floodplains inside the protected area and also a floodplain that is part of privately owned property on the western edge of the Jatai Ecological Station. During the study, most of the reintroduced marsh deer preferred the private floodplain area more than the floodplains inside the protected area. This preference revealed the ecological importance of the area and the necessity of its incorporation into the Jatai Ecological Station.

1470: *+.160*

In this work, in vitro clonal propagation of Drosophyllum lusitanicum (Dewy pine) was obtained from seedlings germinated in vitro. Seeds were collected in various populations identified in the Algarve region and germinated in vitro on MS medium supplemented with 0.5 mg l(-1) BA (6-benzyladenine) and 0.1 mg l(-1) GA(3) (gibberellic acid). The obtained shoots were used in several multiplication assays. The best results were observed in MS medium supplemented with 0.2 or 0.5 mg l(-1) zeatin. The highest rooting frequency (83%) was observed on 1/4MS medium supplemented with 0.2 mg l(-1) IBA (indole-3-butyric acid). Fifty percent of the plantlets were successfully acclimatized to ex vitro conditions, exhibiting normal development. Plans are underway to reintroduce the in vitro produced plants from this study in selected locations in their natural habitat.

1471: *+.254*

1. Najas flexilis (Willd.) Rostk. & Schmidt is a submerged annual macrophyte, rare in Europe, which is protected under the EC Habitats Directive.2. N. flexilis grows in deep, often coloured or turbid water in mesotrophic lakes. Because of this habitat preference it is difficult to locate and assess the ecological state of populations of the species for conservation monitoring purposes.3. A method is described based on plant community information that can be used to determine the baseline probability that conditions in a lake are suitable for supporting N.,flexilis growth. This can be applied to conservation management decisions, such as whether a detailed underwater survey is justified for monitoring the integrity of existing populations of N.flexilis, or whether the lake may be a suitable site for introduction, or reintroduction, of populations of the plant.4. Two methods of plant community description are compared: a quantitative micro-habitat scale approach and a whole-lake-scale qualitative approach. Plant community data collected using each method were grouped using TWINSPAN, and environmental descriptors of the sites comprising each plant community group were compared statistically.5. Micro-habitat-scale community groups differed significantly only in the light extinction coefficient, indicating the zone within the lake in which N.,flexilis occurred.6. The whole-lake-scale community groups differed in a number of environmental variables indicative of eutrophication and acidification, two major environmental threats to N. flexilis survival.7. This study suggests that a lake-scale qualitative plant community description would be a better indicator of site suitability for N.flexilis growth than a quantitative micro-habitat plant community description. This is because the whole-lake-scale approach could detect a difference ill the environmental factors that affect N.flexilis growth, which the micro-habitat scale approach could not. Copyright (c) 2005 John Wiley & Sons, Ltd.

1473: *-.060*

In Brazil, few efforts have been made to reintroduce the species seized from the illegal traffic of animals, so they are frequently released in habitats different from their own and without any type of identification. The introduction of such species in such habitats can cause great disturbance, at times irreparable. The present study addresses the breeding of a group reintroduction of Aratinga

aurucapilla specimens seized from the illegal traffic of sylvan animals and recuperated and medicated in a Selection Center. All the specimens were banded with adequate steel bands before being released. Soon after their reintroduction, the birds started nesting in artificial nests made of PVC tubes, having generated several offsprings. The birds and their offsprings are being monitored for the last seven years.

1474: *-.255*

The Przewalski's horse (Equus caballus przewalskii) was extinct in the wild by the mid 1960s. The species has survived because of captive breeding only. The Takhin Tal reintroduction project is run by the International Takhi Group; it is one of two projects reintroducing horses to the wild in Mongolia. In 1997 the first harem group was released. The first foals were successfully raised in the wild in 1999. Currently, 63 Przewalski's horses live in Takhin Tal. Little information exists on causes of mortality before the implementation of a disease-monitoring program in 1998. Since 1999, all dead horses recovered (n = 28) have been examined and samples collected and submitted for further investigation. Equine piroplasmosis, a tick-transmitted disease caused by Babesia caballi or Theileria equi, is endemic in Takhin Tal and was identified as the cause of death of four stallions and one stillborn foal. In December 2000, wolf predation was implicated in the loss of several Przewalski's horses. However, thorough clinical, pathologic, and bacteriologic investigations performed on dead and surviving horses of this group revealed lesions compatible with strangles. The extreme Mongolian winter of 2000-2001 is thought to have most probably weakened the horses, making them more susceptible to opportunistic infection and subsequent wolf predation. Other occasional causes of death since 1999 were trauma, exhaustion, wasting, urolithiasis, pneumonia, abortion, and stillbirth. The pathologic examination of the Przewalski's horses did not result in a definitive diagnosis in each case. Several disease factors were found to be important in the initial phase of the reintroduction, which could potentially jeopardize the establishment of a selfsustaining population.

1476: *+.182*

Conservation of mammal species often requires the application of predictive habitat models. While empirical models can indicate the potential suitability and distribution of recent habitat, they may fail to depict native habitat and distribution. Therefore, we advocate validating such models with archaeozoological data. To demonstrate the power of archaeozoological data in investigating native distribution patterns, we use the alpine chamois (Rupicapra rupicapra) as a model species. After experiencing a severe historical population bottleneck due to overexploitation, chamois populations recovered markedly during the last century. Fostered by humans and having profited from translocations, this alpine ungulate greatly expanded its range and began to invade forested areas both within and outside the Alps, where damage to vegetation was soon obvious. Consequently, a controversy arose concerning the natural distribution and habitat of chamois. To study the native habitat and distribution of alpine chamois in Switzerland, we focus on the Late Mesolithic and Neolithic period (6000-2200 BC). This period best Suits our purpose because pristine forests then dominated the landscape and human influence was as yet minimal. We describe two opposing habitat models: the alpine model assumes that chamois had survived only in alpine areas, whereas the forest model assumes that they also roamed in steep, entirely forested areas. We validate these models with archaeozoological data. Because the probability of chamois bone occurrence in prehistoric settlements is expected to decrease with increasing distance from chamois habitat, the models differ in their geographical predictions of chamois bone records. Applying logistic regression models, only settlement proximity to chamois forest habitat explains recoveries of fossil chamois bones. The resulting function of catchment

distances (i.e., the likelihood of hunting chamois depending on the distance between a settlement and the nearest chamois habitat) matches the spatial behavior of extant hunters within pristine forests. We conclude that Holocene chamois range in Switzerland naturally included steep and entirely forested regions, like the Jura Mountains. The recent invasion of these areas by chamois thus constitutes repatriation of native habitat. Accordingly, we propose a shift in perspective toward landscape integration of chamois.

1477: ***-.035***

Endemic to the islands of Guam and Rota in the Mariana islands, Mariana Crow Corvus kubaryi is the only corvid in Micronesia. Currently, it survives on Guam only because of translocation of individuals from Rota (1999-2003). island-wide surveys in 1982 and 1995 on Rota yielded population estimates Of 1,348 and 592 respectively, indicating a 56% decrease in only 13 years. A sharp decline in the only viable Mariana Crow Population has serious implications for conservation efforts on Rota and for efforts to re-establish the Guam population. However, the validity of the apparent decline has been debated among scientists and government management agencies. We augmented the 1982 and 1995 island-wide VCP surveys with (1) an additional island-wide survey conducted in 1998, and (2) roadside surveys conducted during 1991-1993 and again during 1999-2002. We also outline historical changes in Rota's limestone forest based on aerial photographs and historical information. Data from all surveys indicate a significant decline in the Mariana Crow population. Declines occurred especially along the north-central coast and in the area east of the airport known as As Dodo in the 1990s, but the data indicate an island-wide decline over the entire span of the surveys. Introduced predators, human persecution, and habitat loss and degradation by anthropogenic and natural causes have all contributed to the decline. Long-term preservation of this species will require effective brown treesnake Boiga irregularis control, habitat protection, continued monitoring and research, and increased public education and awareness of Rota's rare and endangered species.

1478: ***+.044***

We report the results of eight years of post-release monitoring of 37 wild-born, captive chimpanzees released into the Conkouati-Douli National Park, Republic of Congo. Overall survival was high, with 23 (62%) individuals remaining in the release zone, and only 5 (14%) confirmed dead. Released females regularly interacted with wild chimpanzees. Several females appeared to have integrated into wild groups for extended periods of time, and four released females gave birth to a total of five offspring. However, encounters with wild chimpanzees were a major cause of mortality in released males, and 40-50% of released males would have died without veterinary intervention. These sex differences are in accordance with knowledge of chimpanzee behavioural ecology. Our results demonstrate that wild-born, captive chimpanzees can be released into the wild successfully, under certain specific conditions. Most importantly, careful planning and preparation is critical at all stages; a suitable release area must be identified; potential risks to existing wild populations, including the possibility of disease transmission, must be minimised; and post-release monitoring is essential. Adolescent females are the most suitable candidates for release, as they appear to be able to integrate successfully into wild communities. However, males should not be released where wild chimpanzees occur, as they are likely to be attacked and killed. Release into the wild addresses the welfare of certain individual animals, although it clearly cannot address the fate of all captive, wild-born chimpanzees. Knowledge of how to successfully release chimpanzees into the wild also has both current and potential future benefits for the conservation of wild chimpanzee populations. (c) 2005 Elsevier Ltd. All rights reserved.

1479: ***+.240***

African wild dogs are endangered, and in South Africa as elsewhere, they inhabit a fraction of their former range. In this study, we assessed the potential for economic benefits derived from ecotourism to offset the costs of three wild dog conservation options using a contingent valuation study of the willingness of visitors to four protected areas to pay to see wild dogs at the den - within a viable population in a large protected area (Kruger National Park), through reintroduction into nature reserves, and through the conservation of wild dogs occurring on ranchland in situ. Results indicated that tourism revenue from wild dogs in large protected areas is more than sufficient to offset the costs and could potentially be used to subsidise wild dog reintroductions or the conservation of wild dogs in situ on ranchland. On ranchland and for reintroductions, tourism revenue was generally predicted to offset most of the costs of conserving wild dogs where predation costs are low, and to exceed the costs where willingness to pay is high, and/or where the costs of predation by wild dogs are zero. Conservation efforts should facilitate the derivation of eco-tourism-related benefits from wild dogs on ranchland and in private reserves to create incentives for wild dog conservation. Ecotourism should be part of a multifaceted approach to wild dog conservation which also includes education and awareness campaigns, and efforts to encourage landowners to cooperate to form conservancies. (C) 2004 Elsevier Ltd. All rights reserved.

1480: ***+.213***

Translocation is an important tool for the conservation of species that have suffered severe range reductions. The success of a translocation should be measured not only by the survival of released animals, but by the reproductive output of individuals and hence the establishment of a self-sustaining population. The bridled nailtail wallaby is an endangered Australian macropod that suffered an extensive range contraction to a single remaining wild population. A translocated population was established and subsequently monitored over a four year period. The aim of this study was to measure the reproductive success of released males using genetic tools and to determine the factors that predicted reproductive success. Captive-bred and wild-caught animals were released and we found significant variation in male reproductive success among release groups. Variation in reproductive success was best explained by individual male weight, survival and release location rather than origin. Only 26% of candidate males were observed to sire an offspring during the study. The bridled nailtail wallaby is a sexually dimorphic, polygynous macropod and reproductive success is skewed toward large males. Males over 5800 g were six times more likely to sire an offspring than males below this weight. This study highlights the importance of considering mating system when choosing animals for translocation. Translocation programs for polygynous species should release a greater proportion of females, and only release males of high breeding potential. By maximizing the reproductive output of released animals, conservation managers will reduce the costs of translocation and increase the chance of successfully establishing a self-sustaining population. (C) 2004 Elsevier Ltd. All rights reserved.

1481: ***-.028***

The wild grapevine, Vitis vinifera L. subsp. silvestris (Gmelin) Hegi, is considered to be an endangered taxon in Europe, mainly as a consequence of the introduction of pathogens from North America and of the destruction of its habitat. In the Rhine Valley, nearly all populations disappeared due to river management, the intensi. cation of forestry, and the introduction of phylloxera. After a growing awareness of the need to preserve endangered forest ecosystems, attempts to reintroduce wild grapevine in the Rhine Valley were performed, particularly in the

French nature reserves Erstein and Offendorf since 1992. However, regular surveys of the plants indicate the rapid decline of the populations. In 2002, we proposed to summarise the knowledge accumulated after 10 years of experiments. Results indicate that from the initial 91 individuals planted in 1992, only 14 survived in 2002 (2 in Erstein, 12 in Offendorf). The failure of the experiment may be explained by several factors: unsuitable sites (too shady, absence of support for the young plants), absence of monitoring, vandalism or predation. According to these results and recent knowledge of the ecology of the plant and of vines in general, new transplantation experiments are proposed in which the plants will be monitored during their establishment in the forests. The success of this second transplant (50 plants per reserve) will be enhanced by restoration projects of the Rhine River dynamics, with partial re-flooding. Floods should help to avoid, or at least to reduce, pest and disease expansion on future adult plants.

1482: *+.067*

Populations of harlequin frogs Atelopus are declining throughout their geographical range. In Peru six of the 11 nominally described species are Critically Endangered, and there are at least 17 undescribed additional forms (all representing apparently distinct species), the conservation status of which is unknown. Most Atelopus taxa in Peru have relatively small geographical ranges, some known only from single populations, and have only a limited chance of survival. Conservation will require: (1) improvement of the faunistic and taxonomic knowledge of the genus, (2) field surveys, including in remote areas, (3) assessment of all species and populations for chytridiomycosis and research on the agent's biology, (4) ex situ conservation measures to facilitate later reintroduction, and (5) involvement of national and international conservationists and local people.

1483: *+.028*

In species of great conservation concern, special attention must be paid to their phylogeography, in particular the origin of animals for captive breeding and reintroduction. The endangered European mink lives now in at least three well-separated populations in northeast, southeast and west Europe. Our aim is to assess the genetic structure of these populations to identify 'distinct population segments' (DPS) and advise captive breeding programmes. First, the mtDNA control region was completely sequenced in 176 minks and 10 polecats. The analysis revealed that the western population is characterized by a single mtDNA haplotype that is closely related to those in eastern regions but nevertheless, not found there to date. The northeast European animals are much more variable (pi = 0.012, h = 0.939), with the southeast samples intermediate (pi = 0.0012, h = 0.469). Second, 155 European mink were genotyped using six microsatellites. The latter display the same trends of genetic diversity among regions as mtDNA [gene diversity and allelic richness highest in northeast Europe (H-E = 0.539, R-S = 3.76), lowest in west Europe (H-E = 0.379, R-S = 2.12)], and provide evidences that the southeast and possibly the west populations have undergone a recent bottleneck. Our results indicate that the western population derives from a few animals which recently colonized this region, possibly after a human introduction. Microsatellite data also reveal that isolation by distance occurs in the western population, causing some inbreeding because related individuals mate. As genetic data indicate that the three populations have not undergone independent evolutionary histories for long (no phylogeographical structure), they should not be considered as distinct DPS. In conclusion, the captive breeding programme should use animals from different parts of the species' present distribution area.

1484: *+.318*

1. The genus Castor comprises two species: the Eurasian beaver Castor fiber, and the North American beaver Castor canadensis. Both species suffered from overexploitation, but have seen a revival since the 1920s due to increased protection and reintroduction programmes. Increases in the populations and distributions of species that are able to modify ecosystems have generated much scientific interest. Here we review the available literature concerning the possible ecological impact of beaver species in the Old and New World.2. Beavers, being ecosystem engineers, are among the few species besides humans that can significantly change the geomorphology, and consequently the hydrological characteristics and biotic properties of the landscape. In so doing, beavers increase heterogeneity, and habitat and species diversity at the landscape scale. Beaver foraging also has a considerable impact on the course of ecological succession, species composition and structure of plant communities, making them a good example of ecologically dominant species (e.g. keystone species).3. Nevertheless, the strength of beavers' impact varies from site to site, depending on the geographical location, relief and the impounded habitat type. Consequently, they may not be significant controlling agents of the ecosystem in all parts of their distribution, but have strong interactions only under certain circumstances. We suggest that beavers can create important management opportunities in the Holarctic, and this review will help land managers determine the likely outcome of beaver activity.

1485: ***+.018***

1. The effects of captivity on the behaviour of wild and domestic animals have been relatively well studied, but little has been published on morphological changes in wild animals in captivity. We review the evidence for changes in a wide variety of mammalian taxa, with non-mammalian examples where relevant.2. We consider the morphological effects of the process of domestication, and compare changes in both hard and soft tissues in captive and domestic animals with those in their wild counterparts. These include skull shape differences, brain size reduction, postcranial adaptations and digestive tract changes.3. We also summarize studies that have looked at morphological change in feral animals in comparison with their wild and domestic ancestors, and consider their use as an analogue for morphological change in captive-bred animals that have been released into the wild.4. We then discuss the importance of this work for the wider aims of conservation of endangered species and captive breeding over many generations, and emphasize the importance of studying these changes now, while for many species, the process is just beginning rather than many generations down the line, or immediately prior to release, where survival of captive-bred animals may be severely compromised.

1486: ***+.061***

In this study we determined the complete sequence of the mitochondrial DNA (mtDNA) control region of the Eurasian otter (Lutra lutra). We then compared these new sequences with orthologues of nine carnivores belonging to six families (Mustelidae, Mephitidae, Canidae, Hyaenidae, Ursidae, and Felidae). The comparative analyses identified all the conserved regions previously found in mammals. The Eurasian otter and seven other species have a single location with tandem repeats in the right domain, while the spotted hyena (Hyaenidae) and the tiger (Felidae) have repeated sequences in both the right and left domains. To assess the degree of genetic heterogeneity of the Eurasian otter in Italy we sequenced two fragments of the gene and analyzed length polymorphisms of repeated sequences and heteroplasmy in 32 specimens. The study includes 23 museum specimens collected in northern, central, and southern Italy; most of these specimens are from extinct populations, while the southern Italian samples belong to the sole extant Italian population of the Eurasian otter. The study also includes all the captive-reared animals living in the colony "Centro Lontra, Caramanico Terme" (Pescara, central Italy). The

colony is maintained for reintroduction of the species. We found a low level of genetic polymorphism; a single haplotype is dominant, but our data indicate the presence in central and southern Italy of two slightly divergent haplotypes. One haplotype belongs to an extinct population, the other is present in the single extant Italian population. Analyses of length polymorphisms and heteroplasmy indicate that the autochthonous Italian samples are characterized by a distinct array of repeated sequences from captive-reared animals.

1487: *+.162*

Aim We examine patterns of temporal turnover of common species in avian assemblages in North America to test the hypothesis that changes in avian diversity structure observed in these assemblages were associated with the colonization of common species. Location The contiguous United States and southern Canada. Methods We measured temporal turnover from 1968 to 2003 for 547 avian species at 1673 North American Breeding Bird Survey (BBS) routes. We used the Euclidian distance between expected and observed presence/absence vectors and randomization tests to place species into two categories, common and not-common, and into three categories for common species: (1) always common, (2) common and colonizing, and (3) common and extirpated. We used these categories to identify species experiencing extreme colonization and extirpation events and to examine changes in species composition at BBS routes. We also determined how these patterns were associated with changes in species richness and changes in similarity in species composition. Results Nine of the 547 species represented outliers, where the number of BBS routes colonized greatly exceeded the number extirpated; no species showed extreme values for extirpation. The nine species colonized BBS routes primarily in the upper Midwest and north-eastern United States. Presence of the nine species at BBS routes was correlated with increasing net gain in common species (difference between common colonized and common extirpated), higher levels of species richness and increasing species richness over time, more similar species compositions and increasing similarity over time, and a greater prevalence of common species over not-common species. The literature indicates that all nine species experienced some form of geographical range expansion during the time of the survey involving four elements: (1) introduction and invasion; (2) the ability to use human-altered environments, including habitats associated with agricultural, suburban, or urban areas; (3) intensive management activities, including habitat improvements and reintroductions and (4) the ability to use habitats formed through forest regeneration. These factors in combination point to anthropogenic activities and related land use histories as the primary drivers of change. One of the nine species colonized regions well outside its historic geographical range and the remaining eight species were native within the regions they colonized. Main conclusions Our results suggest that a combination of anthropogenic activities promoted, within certain regions of North America, the geographical expansion of a limited number of common species that were native to those regions. These colonization events were correlated with changes in diversity structure, implying that large-scale diversity patterns were being influenced by anthropogenic activities. These changes can be characterized primarily by gains in species richness, an increased prevalence of common species, and more similar species compositions. Thus, using simple large-scale measures of diversity could be problematic if recent biogeographical patterns of species diversity are not considered. Specifically, using species richness or an indicator species to assess diversity could bias assessments towards common species whose populations have recently benefited through anthropogenic activities.

1488: *+.092*

The progressive decline in red squirrel (Sciurus vulgaris) numbers in Wales has led to

conservation and reintroduction projects being established on the island of Anglesey. The recovery of the island's remnant wild population was initially successful, however concern remained over potential loss of genetic diversity resulting from an observed demographic bottleneck. We used mitochondrial DNA (mtDNA) control region sequences and six microsatellite loci to assess current levels of genetic variation in the population. Samples were monomorphic for control region sequences and a historic specimen from the same area carrying a different haplotype demonstrated a loss of mtDNA diversity during the last 20 years. Inclusion of other Welsh haplotypes indicated phylogeographic structure in the region, in contrast to previous UK studies. Genotyping results showed allelic diversity and heterozygosity to be less than 50% of that recorded in other UK populations, with strong evidence for a recent genetic bottleneck. A parallel reintroduction programme on Anglesey included genetic analysis of individuals during the selection of captive breeding pairs. We present analysis of sequence and microsatellite data, and subsequent management decisions taken to maximise diversity in the founder and F1 generations. Population and Habitat Viability Analysis applied to both populations modelled future levels of heterozygosity and allelic diversity. Supplementation of the remnant and reintroduced populations with translocated squirrels was simulated as a potential management tool; results support use of this strategy to reduce loss of diversity and increase survival. The limitations of applying conservation genetic theory within small-scale management projects are discussed.

1489: *+.085*

Populations of the endangered giant kangaroo rat, Dipodomys ingens (Heteromyidae), have suffered increasing fragmentation and isolation over the recent past, and the distribution of this unique rodent has become restricted to 3% of its historical range. Such changes in population structure can significantly affect effective population size and dispersal, and ultimately increase the risk of extinction for endangered species. To assess the fine-scale population structure, gene flow, and genetic diversity of remnant populations of Dipodomys ingens, we examined variation at six microsatellite DNA loci in 95 animals from six populations. Genetic subdivision was significant for both the northern and southern part of the kangaroo rat's range although there was considerable gene flow among southern populations. While regional gene diversity was relatively high for this endangered species, hierarchical F-statistics of northern populations in Fresno and San Benito counties suggested non-random mating and genetic drift within subpopulations. We conclude that effective dispersal, and therefore genetic distances between populations, is better predicted by ecological conditions and topography of the environment than linear geographic distance between populations. Our results are consistent with and complimentary to previous findings based on mtDNA variation of giant kangaroo rats. We suggest that management plans for this endangered rodent focus on protection of suitable habitat, maintenance of connectivity, and enhancement of effective dispersal between populations either through suitable dispersal corridors or translocations.

1491: *+.237*

The successful introduction of captive bred takhi or Przewalski's horse, Equus ferus przewalskii, into Mongolia in the 1990s is a good example of the benefits of ex situ conservation and one of the few examples of the recovery of an animal after it became extinct in the wild. This is also particularly interesting because virtually nothing was known about how takhi lived before they died out, and the introductions have enabled us to study how they have settled, and their ecology and behaviour within their former natural range. In this paper, we describe the movement, home range size and shape, and habitat use of takhi at one of the release areas, the 570 km(2) Hustai National Park in Mongolia. Harem home ranges varied between 129 and 2399 ha, with 80% core

areas of between 61 and 1196 ha. There was no relationship between range size and harem size, or length of time since release. Initially, harems stayed near their release enclosures, but over time they established home ranges further away. There was little overlap between home ranges of different harems, but neither was there evidence of exclusive range use. The more nutritious vegetation at lower elevations was preferentially selected. Thus the present situation looks good, but, as the population continues to grow, we anticipate that there will be potential problems related to intraspecific competition for water and vegetation resources, and the potential for hybridisation with domestic horses belonging to the local people. We consider the time it may take for takhi to reach carrying capacity within Hustai National Park and emphasise that continual monitoring of the population is essential because interventional management is likely to be required in the future. (c) 2005 Elsevier Ltd. All rights reserved.

1492: ***-.045***

We evaluated the effectiveness of supplemental stockings being proposed in the recovery of the federally endangered Key Largo woodrat (KLWR, Neototma floridana smalli) using a stage-based, stochastic model. Supplemental stockings were evaluated with a population model using current trapping and telemetry data along with published and unpublished KLWR data. Model simulations predicted the KLWR had > 70% probability of terminal extinction over the next 10 years even with the use of supplemental annual stockings. Supplemental stockings of KLWRs (<= 20 females) appear to delay the extinction of the species, but negative population trajectories accelerated after stockings cease. Model results illustrated the importance of determining limiting factors on the population prior to the use of supplemental stockings as a recovery option for this endangered woodrat population. (c) 2005 Elsevier Ltd. All rights reserved.

1493: ***+.271***

Management policies to save threatened species are not always successful, often due to the lack of a scientific basis and evaluation of the species response. We describe the ecological studies and the conservation actions taken between 1985 and 1992 on Cousin Island (29 ha, Seychelles) to safeguard the future of the highly threatened Seychelles warbler (Acrocephalus sechellensis), which until 1988 only occurred on this island. A detailed field study was designed to (1) identify the key processes influencing warbler demography, (2) identify appropriate management techniques to increase the warbler population, and (3), assess the influence of the resulting habitat management. Since 1980 the island has been saturated with c. 115 territories and c. 320 birds. The warbler is purely insectivorous. Morinda (Morinda citrifolia), the most insect rich tree, is preferred for foraging. The higher the insect abundance (and Morinda cover) in territories the higher the reproductive success and survival of warblers. Insect numbers were highest in the central part of Cousin and decreased towards the coast. Coastal territories protected by a salt tolerant hedge of Scaevola (Scaevola taccada) had more insects and higher reproductive success than unprotected territories. Between 1990 and 1992 Morinda trees were planted on the island and Scaevola along the coast. Although these habitat restoration measures have not resulted in higher numbers of adult warblers and territories due to habitat saturation, they have been successful in terms of improving the quality of existing breeding territories and with that the reproductive success of breeding birds (including the number of territories producing recruits), and the exchange of individuals (genetic material) between territories. We provide evidence that the high reproductive potential of this species is likely to improve the resilience of the species to catastrophic events. (c) 2005 Elsevier Ltd. All rights reserved.

1494: ***+.328***

The Australian freshwater crayfish, Cherax destructor is Cultured commercially and has been translocated throughout much of Australia. Previous investigation on C destructor using 16S rRNA sequences of samples collected from natural environments has revealed a significant phylogeographic structure in this species with three well supported geographically non-overlapping clades, namely 'northern' C. d. destructor, 'southern' C d. destructor and C. d. albidus. Movement of individuals beyond their natural range of distribution may have adverse effects on genetic integrity of the species. In the present Study, aspects of translocations of the species were genetically investigated. Sequences of the 16S rRNA gene region of the mitochondrial DNA (mtDNA) were obtained from samples collected in nine quasi-natural waterbodies, supplemented with sequences of samples obtained from 31 natural waterbodies examined in a previous study. Results of phylogeographic analysis provide evidence that certain haplotypes from major clades of C. destructor have been translocated. The findings of this Study have important implications for the conservation and management of genetic diversity within C. destructor.

1495: ***+.039***

The two key objectives of the recovery plan for the Federally threatened tiger beetle Cicindela dorsalis dorsalis Say are to protect populations within the Chesapeake Bay and to establish by reintroductions new populations in the U.S. northeast (New Jersey to Massachusetts). This article reports on the development and implementation of translocation work to establish a population of C. d. dorsalis at Gateway National Recreation Area, Sandy Hook, NJ, by using larvae from Chesapeake Bay populations. Previous experimental translocation trials in Virginia by using adults were unsuccessful because the adults dispersed from the translocation sites within 1-2 wk. Experimental translocations were conducted to test methods with larvae from several Virginia sites to Sandy Hook in September 1994 and 1995. The translocated larvae readily dug burrows, many survived the winter, and some emerged as adults the following summer. Additional translocations of > 475 larvae each year were conducted in early May 1997, 1999, and 2000. Peak numbers of emerging adults counted each year in July increased from 178 in 1997 to 749 in 2001. Adults exhibited normal behaviors in the field (foraging, thermoregulation, and mating) and recruited larvae each year. A population seemed to be successfully established, but adult numbers declined sharply after the 2001 peak to 142 in 2002, 43 in 2003, and six in 2004. We have little evidence for the cause of this sharp decline in adult numbers, but it may have resulted from predation by gulls, dispersal triggered by the high gull densities where beetles occurred, or perhaps from coastal storm impacts causing a progressive decline in survival and recruitment of the beetle population. The initial success of this translocation suggests that efforts using these methods should be continued, but closer monitoring at the translocation site is needed to determine the fate of the population. These methods also may be applicable to the recovery of other threatened or endangered tiger beetles.

1496: ***-.045***

Captive-reproduced stocks of some species of ungulates (Artiodactyla), and particularly the red deer (Cervus elaphus), fallow deer (Dama dama), roe deer (Capreolus capreolus) and the wildboar (Sus scrofa) are more or less extensively translocated in Italy, mainly for local reintroductions or restocking of exploited wild populations. However, captive breeding often involves the reproduction of non-indigenous individuals or the production of artificial hybrids. Consequently, translocations of captive-reproduced ungulates are of concern for the conservation of indigenous populations and gene pools. The impact of translocations should be evaluated within the background of the growing knowledge on population genetic and phylogeographic structure of ungulates. Molecular genetic markers are being used to map geographic genetic diversity, and

reconstruct the phylogeographic history of natural populations (i.e., in the roe deer). Molecular makers are also used to detect the consequences of domestication and identify hybrids between wild and domesticated populations (i.e., in the wildboar), or to detect inter-specific hybridisation (i.e., between the red deer and wapiti). Hybridisation of wild and domestic pigs, and diffusion of hybrids in nature is widespread in Italy. Admixture of indigenous and non-indigenous roe deer stocks is also widespread. Therefore, conservation and management of indigenous ungulates calls, for careful evaluation of captive-reproduced stocks.

1497: ***+.114***

Common wild rice (Oryza rufipogon Griff.), known as the ancestor of Asian cultivated rice (Oryza sativa L.), is the most important germplasm for rice improvement. The first male sterility gene was found in the wild rice, and introduced to the cultivated rice, which launched the fast development of the high-yielding hybrid rice. Other agronomically beneficial traits in the wild rice, such as rice tungro virus resistance, bacterial leaf blight (Xa21 gene) resistance and acid sulfate soil tolerance, have played important roles in rice breeding. China has the northernmost distribution area of wild rice possessing great genetic diversity. However, most of the populations of this species have disappeared in China over the last three decades, mainly caused by habitat loss, fragmentation and other human disturbances. Unfortunately, the decline of existing populations still continues. In the present study, we reviewed studies on genetic diversity and conservation of this wild rice in China, concentrating on population structure, pollen competition, pollen/gene flow from cultivated rice to wild rice, and ecological restoration in relation to in situ conservation. The relatively high genetic diversity of populations of O. rufipogon in China suggests that there is great value for conservation. Considerable gene flow from cultivated rice to wild rice may alter the genetic structure of natural populations of O. rufipogon and eventually lead to its genetic erosion. Pollen competition between wild and cultivated rice has caused a low rate of crop-to-wild gene flow, but it does not completely prevent gene flow from the crop. Effective isolation measures should be undertaken in the regions where in situ conservation of O. rufipogon is carried out. Reintroduction is an important alternative for the in situ conservation of wild rice species. As wild rice is an important genetic resource, both in situ and ex situ conservation strategies are needed.

1498: ***+.077***

1. The distribution of dispersal distances (the dispersal kernel) is a major determinant of spatial population dynamics, yet little is known about the shape of the dispersal kernel for most species. This is partly because of the relative difficulty of measuring dispersal, exacerbated by a lack of standardized protocols. We suggest that this problem can be addressed by using modelling approaches to aid the design of studies to quantify dispersal.2. In this study we present such an approach by optimizing seed trap sampling design using stochastic simulations. A number of alternative sampling designs (random placements, grid arrays, transects, sectors and annuli arrangements) for a point source were tested against a common kernel to assess the best methods for estimating the dispersal kernel.3. For a given source strength and total trap area, transects and sectors of traps usually provided better data for kernel estimation than random placement, grid arrays and annuli. Kernel estimation was improved by increasing the source strength (the number of dispersing propagules) and the trap area, as expected.4. When the 'true' kernel was unknown, transects were slightly better for identifying the thin-tailed exponential distribution, whereas sectors were better for detecting the fat-tailed half-Cauchy.5. In the case of anisotropic dispersal (here, dispersal biased in one direction), annuli and grid arrays performed better than transects and sectors when the anisotropy was unknown. However, when the anisotropy was anticipated, and the trap arrangements were adjusted accordingly, transects and sectors were better. This was true

regardless of source strength and total trap area.6. Synthesis and applications. This study presents a simulation approach to the design of dispersal experiments. While the general results of our simulations can be used by those designing field studies for plant point sources, the simulation approach itself can be modified for a wide range of organisms, dispersal mechanisms and dispersal measurement approaches. Thus, the approach presented here facilitates improvements of dispersal study designs, which in turn will increase the precision of dispersal kernel estimates and predictions of spatial population dynamics, including modelling of rates of spread or metapopulations. This is invaluable in a range of ecological applications, such as the management of rare or invasive species, predicting species' response to climate change, or planning species reintroductions.

1499: *+.146*

The effect of habitat destruction on the likelihood of species survival is often estimated based on the assumption that colonization and extinctions are in balance. This assumption is not sustainable in species where the dynamics of colonization and extinctions is slow in relation to landscape changes, such as in most plants. Here we use an alternative approach, a realistic, dynamic landscape-level model that does not rely on this assumption. It enables estimation of the effect of habitat destruction using field data on the biology of a species and on real landscape structure. Because our approach relies on direct comparisons of changes in population size and survival probability due to habitat changes, it can be easily extended to other conservation questions, such as assessing the effects of events causing the extinction of populations but allowing for recolonization, or identifying optimal reintroduction strategies. We applied this method to a perennial herb, Succisa pratensis, that is a typical grassland species. We combined detailed demographic data with information on the spatial distribution of suitable habitats to model species dynamics in the landscape under different scenarios. The results show that habitat destruction alone has little effect on regional survival. However, the effect of habitat destruction increases when combined with factors causing extinctions of, the existing populations that are expected to play a significant role in the study system. Our results further show that an optimal reintroduction strategy at the landscape level depends on the number of available seeds. The approach presented here was designed for studying systems where species colonization-extinction dynamics is slow compared with landscape changes. Such time lags and nonequilibrium dynamics have been suggested to be important features of many ecosystems and life forms, and this approach is thus likely to be useful for a wide range of future studies. The approach also allows the estimation of short-term effects of habitat destruction, i.e., situations that are nonequilibrium by definition. This is never possible with equilibrium models, giving the model a wide applicability for all types of organisms.

1500: *+.115*

Aside from Kruger National Park, no other suitable reserves of sufficient size exist in South Africa that will hold a viable population of wild dogs (Lycaon pictus). Consequently, conservation efforts have been focused on creating a metapopulation through a series of wild dog reintroductions into isolated fenced reserves. Additional potential exists for conserving wild dogs on private ranch land. Establishing the metapopulation was an expensive process, accounting for approximately 75% of the US$380,000 spent on wild dog conservation in South Africa during 1997-2001. The principal goal of the metapopulation project was to reduce the risk of catastrophic population decline. Now that this has been achieved, we developed a uniform cost-efficiency index to estimate the cost efficiency of current and potential future conservation strategies in South Africa. Conserving wild dogs in large protected areas was predicted to be the most cost-efficient

conservation strategy (449 packs/$100,000 expenditure). Establishing the metapopulation has been less cost efficient (23 packs/$100, 000), and expansion of the metapopulation was predicted to be even less cost efficient if predation by wild dogs results in additional costs, as is to be expected if private reserves are used for reintroductions (3-13 packs/$100,000). Because of low logistical costs, conserving wild dogs in situ on private ranch land was potentially more cost efficient than reintroducing wild dogs (14-27 packs/$100, 000). We recommend that donor funding be used to reintroduce wild dogs into transfrontier parks, when they are established, to maintain the existing metapopulation and to establish conservation programs involving wild dogs on private ranch land. Investing in the expansion of the metapopulation should be limited to state-owned nature reserves willing to carry predation costs without compensation.

1501: *+.071*

Conservation efforts involving introductions, reintroductions, and translocations of populations have an inherent and inescapable problem of small initial populations. Small founding populations are likely to have a small proportion of the genetic variability carried by the original population. This may manifest phenotypically through changes in individual morphology, such as decreased body size, increased degree of fluctuating asymmetry, or changing susceptibility to environmental stressors. I investigated the effects of population and landscape variables on the morphology and fluctuating asymmetry in individual bush-crickets by examining 584 individual Roesel's bush-crickets (Metrioptera roeseli) from 29 established populations from different propagule sizes that were introduced in areas previously unoccupied by the species. The introduction sites were in landscapes similar to where the species occurs naturally, but sites differed in connectivity and amount of surrounding suitable habitat. Individuals were caught up to 9 years after the initial introduction, and five different morphological traits were measured. All introduced individuals originated from the same population and individuals from this source population were also collected for comparison in the analyses. Male body weight and female body length were positively affected by initial population size and degree of connectivity of the introduction patch. Isolation also affected fluctuating asymmetry in male tibias, with a higher degree of asymmetry in males that came from more isolated populations. In a relatively short time period, 1 was able to detect the effect of isolation and small population sizes on morphology and asymmetry. Small propagule sizes and habitat isolation are both likely to have resulted in decreased genetic diversity, the latter by reducing population sizes through decreased survival. These results show the importance of both large propagule sizes and good connectivity of habitats when introducing populations. The differences between the sexes in response to the variables examined also indicate that studies on morphology and fluctuating asymmetry need to consider males and females separately to avoid inaccurate generalizations of the state of the population.

1502: *+.357*

We analysed the spatial distribution of home-ranges and the habitat use of re-introduced grey partridges Perdix perdix in relation to newly established ecologically enhanced areas, i.e. wild-flower strips and hedges, within an intensively cultivated area in Switzerland from which the species had become extinct. All birds settled within the ca. 30% of the study area where the proportion of enhanced areas was highest (5.8%). At the level of the individual family group, we found a significant over-proportional use of enhanced areas throughout the year. Furthermore, when the birds used the agricultural fields, the use densities strongly declined with distance from the nearest enhanced area. Thus, the availability and spatial distribution of ecologically enhanced areas were the main determinants of the partridges' range use. These results indicate that a framework of ecologically enhanced areas is a key habitat structure for grey partridges, and that

these enhancements, even if small in proportion, were the crucial first measure to restore the research area to an appropriate grey partridge habitat and, thus, the basis for successful re-introduction in this intensively cultivated area. © 2005 Elsevier Ltd. All rights reserved.

1503: *+.122*

We report natural abundance of (15)N content in a suite of plants and lichens from a sloping open peatland in coastal northern British Columbia, Canada. A wide range of delta(15)N values varying between -11 and +3 parts per thousand were observed in the studied species. Herbs, mosses, and shrubs generally grouped individually along the delta(15)N gradient from enriched to more depleted values. This grouping probably reflects similar nitrogen acquiring strategies within the group, but some species with different strategies showed deviated delta(15)N values from the cluster. Hummock-lawn gradient, which is illustrated as depth to water table, explains the variation in delta(15)N signature of cryptogams: the deeper the groundwater table, the more depleted is the (15)N. This trend might be explained by differences in nitrogen sources and/or differences in internal fractionation during nitrogen translocation processes. (c) 2005 Elsevier B.V. All rights reserved.

1504: *+.053*

For selected species, conservation breeding has become integrated into recovery plans, most often through the production of offspring for reintroduction into nature. As these programs increase in size and scope, it is imperative that conservation managers retain the biological integrity of the species. This study investigated the causes of morphological changes that are known to occur in black-footed ferrets (Mustela nigripes) maintained ex situ. In a previous study, ferrets maintained in captivity were 5-10% smaller in body size than pre-captive, in situ animals. In the present study, the authors compared nine morphological characters among ex situ animals and their in situ descendants. Within the ex situ population, cage types were compared to determine whether housing influenced morphometry. Black-footed ferrets born to reintroduced individuals quickly returned to their pre-captive size suggesting that a diminutive morphology ex situ did not have a genetic basis. Furthermore, cage type affected overall body size and shape; ulnas and tibias were as much as 9% shorter for ex situ animals. The authors hypothesise that small cage size and environmental homogeneity inhibit the mechanical stimuli necessary for long bone development. These findings have ramifications for ex situ managers who need to create artificial captive settings that promote natural physical development. In the absence of such an environment, 'unnatural' morphologies can result that may contribute to poor fitness or perhaps even domestication.

1506: *+.257*

The anthropogenic introduction of exotic species is one of the greatest modern threats to marine biodiversity. Yet exotic species introductions remain difficult to predict and are easily misunderstood because knowledge of natural dispersal patterns, species diversity, and biogeography is often insufficient to distinguish between a broadly dispersed natural population and an exotic one. Here we compare a global molecular phylogeny of a representative marine meroplanktonic taxon, the moon-jellyfish Aurelia, with natural dispersion patterns predicted by a global biophysical ocean model. Despite assumed high dispersal ability, the phylogeny reveals many cryptic species and predominantly regional structure with one notable exception: the globally distributed Aurelia sp.1, which, molecular data suggest, may occasionally traverse the Pacific unaided. This possibility is refuted by the ocean model, which shows much more limited

dispersion and patterns of distribution broadly consistent with modern biogeographic zones, thus identifying multiple introductions worldwide of this cryptogenic species. This approach also supports existing evidence that (i) the occurrence in Hawaii of Aurelia sp. 4 and other native Indo-West Pacific species with similar life histories is most likely due to anthropogenic translocation, and (ii) there may be a route for rare natural colonization of northeast North America by the European marine snail Littorina littorea, whose status as endemic or exotic is unclear.

1507: *+.253*

This chapter presents a vision of the involvement of zoos and aquariums, as centres of expertise insmall population management, in regional or global cooperative breeding programmes. To maximizevalue to conservation, ex situ populations need to be demographically stable, well-maintained and capable of self-sustaining reproduction. They should be distributed among several institutions and of sufficient size to maintain high levels of genetic diversity. However, many programmes have too few founders and participating institutions, depleted genetic diversity and/or poor breeding success. Strategies to enhance viability include: increasing breeding spaces, expanding from regional to global programmes, increasing the intensity of genetic management, improving husbandry practices through research, and importing founders from the wild or other regions. Population management includes demographic and genetic management, veterinary care and husbandry. The first involves monitoring numbers, and age, social and sex structure. Genetic management involves verifying taxonomic identity, and avoiding deleterious effects of inbreeding and loss of genetic diversity. Management decisions are developed through studbooks. Population data are held on a database system at the International Species Information System (ISIS), supported by registration and analysis software. Other management aspects discussed include confiscated and abandoned animals, ownership, and the impact of regulations on animal transfers. The chapter emphasizes that a primary goal of ex situ programmes is support (including demographic and genetic reservoirs) for in situ conservation. Metapopulation management involves managing a set of interacting populations under a common conservation goal. Its components may include ex situ populations, in-country breeding programmes, wild populations and genome banks. Transfer between populations may involve reintroduction. The chapter ends by stating that many wild populations are like ex situ ones - of small size with limited gene flow between them. The science of small population management developed primarily for managing ex situ populations is thus of direct relevance to field conservation. This expertise is a major contribution that zoos and aquariums can offer conservation.

1508: *+.135*

We assessed whether behavioral markers could be used to evaluate pair compatibility and predict pair bond success of captive-reared San Clemente loggerhead shrikes (Lanius ludovicianus mearnsi) released into the wild. Potential breeding pairs of shrikes were introduced at the Zoological Society of San Diego's captive-breeding facility and then moved to release cages located in suitable but unoccupied habitat. Courting pairs were affected negatively by the disturbance of translocation to a new location and generally needed a few days to reinitiate pair bonding in the release enclosures. We separated and returned pairs to captivity when intra-pair aggression or cessation of all courtship behavior occurred; all other pairs were released into the wild. The rate of nest approaches was the best marker to predict a successful release into the wild (i.e., pairs that remained near the release site and attempted to breed). Additionally, all experienced breeding pairs (i.e., pairs with males and females with prior captive breeding experience) exhibiting nesting behavior were successful, although previous experience alone does not ensure post-release breeding. Results from this study indicate the importance of assessing

behaviors of individuals paired for population augmentation. In using behavioral cues, identifying pairs with a low probability of success is possible, and replacing these pairs with individuals that have a higher likelihood of post-release success can occur. This strategy will be important to

1509: ***+.039***

We evaluated translocation as a method to promote recovery of black-tailed prairie dogs (Cynomys ludovicianus) after plague-induced population declines in colonies at the Charles M. Russell National Wildlife Refuge, Montana. We translocated prairie dogs in June of 1999 and evaluated the effects of translocation on colony area 1 year and 4 years later. We also assessed effects of release group size and estimated rates of population growth and survival. Initial size of experimental colonies was categorized as inactive (0 ha), small (0.1-2.0 ha), or large (2.0-6.6 ha); numbers of prairie dogs translocated to each colony size class were 0 (control), 60, and 120. Among inactive colonies, the control remained inactive and the colony receiving 60 prairie dogs grew to 1.5 ha by 2000 and after a second release in 2002 was 1.9 ha in 2003. The colony receiving 120 grew to 3.3 ha by 2000 and decreased to 2.6 ha by 2003. Patterns on small and large colonies after I year were less dramatic, but in each case the proportional increase in colony area was lowest on the control and highest on the colony receiving 120 prairie dogs. Patterns were more difficult to discern 4 years after translocations. It appears that as large colonies approach historic size, area growth decreases and is slower than on small colonies. Experimental colonies grew 24.6 ha (315%) by 2000 and 72.1 ha (924%) in 4 years compared to non-experimental colonies of similar size that grew 6.5 ha (23%) in 1 year and 26.5 ha (93%) by 2003. Monthly survival rates of prairie dogs during the first 3 months following translocation were higher on large colonies (0.79, 95% CI=0.75-0.82) than on small (0.67, 95% CI=0.62-0.72) or inactive colonies, (0.63, 95% CI=0.57-0.68). Monthly survival rates were relatively high (0.88-1.0) during subsequent intervals and did not vary among initial colony size classes. Recapture rates for all colonies were higher during the fall trapping session (0.83, 95% CI=0.76-0.90) than in the winter (0.59, 95% CI=0.49-0.69). Translocation was effective for restoration of prairie dog populations, particularly on inactive colonies.

1510: ***-.004***

Large mammals such as Asiatic elephant (Elephas maximus), rhino (Rhinoceros sp.), golden monkey (Rhinopithecus roxellana), gibbon (Hylobates sp.), giant panda (Ailuropoda melanoleuca), Pere David's deer (Elaphurus davidianus), sika deer (Cervus nippon), and brown bear (Ursus arctos) once lived in the Dongting Lake drainage area of the middle-lower reaches of Yangtze River. However, due to climate change change, geomorphological change and human disturbance, these large mammals were extirpated from the area. After humans settled in the Dongting Lake drainage area, human activity was the major cause of the extinction of local mammalian fauna, especially human hunting, habitat loss to human settlements and land reclamation. Rhino and Asiatic elephant were extirpated or emigrated from the area during the late period of the Northern Song Dynasty while Pere David's deer, golden monkey, gibbon, giant panda, sika deer and brown bear became extinct in the Dongting Lake Drainage Area in the late 19th century. According to our investigations during past years, such a process of species extinction is still escalating. We recorded 21 national key protected mammals in the 30 nature reserves or forest parks in the Dongting Lake drainage area. Among those species, five species are critically endangered, six species are endangered and ten species are vulnerable. Primary causes that threaten the survival of mammals are habitat loss, environmental pollution, hunting, and competition for food or habitat among wild animals. Impacts of human activities on survival of presently endangered mammalian species are much greater than ever before. These threats should

be considered when reintroducing Pere David's deer to the Dongting Lake region. A suitable habitat with enough foods and space and no human disturbance, a healthy founder population under close monitoring, and active conservation measures based on community co-management will be prerequisites for successful re-establishment of the extinct Pere David's deer in the Dongting Lake drainage area.

1511: *+.047*

Black rhinoceros (Diceros bicornis) are one of the most endangered mammal species in Africa, with a population decline of more than 96% by the end of the last century. Habitat destruction and encroachment has resulted in fragmentation of the remaining populations. To assist in conservation management, baseline information is provided here on relative genetic diversity and population differentiation among the four remaining recognized subspecies. Using microsatellite data from nine loci and 121 black rhinoceros individuals, and comparing the results with those of other African species affected in similar ways, Diceros bicornis michaeli retained the most genetic diversity (heterozygosity 0.675) compared with Diceros bicornis minor (0.459) and Diceros bicornis bicornis (0.505), suggesting that the duration of the known bottlenecks in these populations has only had a limited impact on diversity. Comparable and moderate degrees of population differentiation were found between D. b. minor, D. b. bicornis and D. b. michaeli. Results from the single sample available of the most endangered subspecies, Diceros bicornis longipes, showed the least diversity of all individuals examined. This information should assist conservation management decisions, especially those affecting population viability assessments and selection of individuals for translocations, and will also facilitate subspecies identification for ex situ individuals of uncertain origin.

1512: *+.069*

In 2000-2001 a national distribution survey of the badger (Meles meles) was undertaken. The survey was to contribute to the interim evaluation of the badger protection plan of the Ministry of Agriculture, Nature Management and Fisheries of the Netherlands. The execution of this protection plan started in 1984. The outcomes of this survey are presented in this article. The following aspects are described: (1) the historic development in the distribution, (2) the occupation rate of suitable badger habitat, (3) the development of settlement types, (4) the merger of subpopulations, (5) the continuity of occupation, (6) the output of translocations, and (7) the number of disappeared setts and the disturbances registered. In addition, a comparison is made with the results from the surveys of 1960, 1970, 1980, 1990 and 1995. The collected data originated mainly from fieldwork. For every 1-km square, the more than incidental use of badger setts was recorded. Earlier research (1995) on the habitat of the badger qualified 15% of the research area (about 25,000 km(2)) as suitable. In 2001, badgers occupy about 25% of the suitable habitats available to them. The northern and eastern parts of the study area had a relatively low occupation-rate, i.e. 4 to 5 times lower khan the rate for the middle and south, where the three largest populations are located. The increase of distribution in the north and east, however, was twice as much as in the middle and the south. In the Netherlands 4,400 badger setts have been recorded since 1960. During the survey more than 2,500 locations (57%) were visited. In the period 1995-2001, the distribution area of the badger increased with almost 30%. The distribution area of the nine splinter populations even increased by 48%. A decrease of 17% was recorded for the 25 dispersed populations. The average distance between the core populations existing from 1980 onwards decreased from 28 to 21 kilometres. For the period 1995-2001, in the regions of Gelderland-Veluwe and Zuid-Limburg the 18% increase in distribution area remained below the national average (29%). The large population in Gelderland-Veluwe even hardly grew (1%). The

much smaller population in the Reest valley, at the border of the provinces of Drenthe and of Overijssel, did not grow at all. In the region of Gelderland-Achterhoek, the growth was minimal if the contribution from translocations is excluded. The continuity in occupation increased. Since 1960, of the 1-km squares remained occupied in the following year of survey (n=2,536). 1,402 1-km squares were at least occupied once by badgers. Of these, 257 were new in 2001, whereas 253 1-km squares remained vacant after initial occupation. Continuous occupation since 1960 occurred in 115 1-km squares. The remaining 892 1-km squares were occupied more than once, but not continuously. The three main distribution areas, Gelderland-Veluwe, Maasvallei and Zuid-Limburg, showed an increase of occupied 1-km squares of 36%. This is well above the national average of 29%. These large populations cover 84% of the badger distribution in the Netherlands. The increase must be credited almost entirely to the growth of the Meuse valley population. In the period 1990-1995, the increase of 1-km squares occupied by the three largest populations was 4% below the national average, i.e. 12%. In this period, the growth is concentrated in Zuid-Limburg. In the period 1987-2001, the translocation of badgers contributed at least 4% (37 km(2)) to the total distribution of 2001. In this period, 210 badgers were translocated from enclosed release sites at 26 locations in seven provinces. At least 202 setts disappeared between 1995 and 2001, representing about 8% of the locations visited. One in five was occupied in 1995. The amount of destroyed setts decreased with almost 40% in comparison with the period of 1990-1995.

1513: *+.131*

This work presents new data from 48 maned sloths Bradypus torquatus captured between November 2002 and November 2003 in three regions of the Atlantic forest where the largest remnant populations of this species are found. Data from another long-term study, carried out from 1994 to 1996 and from 1999 to the present (n = 14), were also used, making a total sample of 62 sloths. Average adult body weight is 6594 +/- 236 g and average headbody length is 66.5 +/- 0.8 cm (n = 35), indicating that Bradypus torquatus is the heaviest of all four Bradypus species. Individuals from lower-montane forests (600-1000 in a.s.l.) are significantly larger (head-body length; t-test; P = 0.001) than individuals from the lowlands (< 350 ma.s.l.), suggesting altitudinal differentiation between populations. Sexual dimorphism, here reported for the first time, was found in body length (females are significantly larger than males) and in other external characters, such as mane size and form (darker and larger in males), and size and shape of the external genitalia. Most differences between sexes are, however, only discernible in reproductively active individuals. Breeding is slightly seasonal; minimum observed interbirth interval was 1 year and litter size was always one. Infants stayed with their mothers until 8-11 months old; started feeding on solid food as early as 2 weeks old but continued suckling until c. 4 months of age. Age of sexual maturity was estimated at 2-3 years and the oldest captured maned sloth was a healthy male who was at least 12 years of age. Overall, results indicated that maned sloths are similar in these aspects to congeneric species. Information presented here significantly improves the current knowledge on this endangered sloth species and is useful for the proper planning and implementation of in situ conservation strategies such as translocations and reintroductions.

1514: *+.096*

Rapid propagation of I. malabarica (Reichb.f.) J D Hook, an endemic and endangered orchid of the Western Ghats of Kerala, India through conversion of axillary buds to protocorm-like bodies (PLBs), and Subsequent plant regeneration was achieved. Growth regulators and sugar displayed significant influence in the induction of PLBs. In vitro derived shoots from field grown rhizomes of Ipsea cultured on Murashige and Skoog (MS) medium with 13.3 mu M N-6-benzyladenine (BA) containing 2% commercial grade sugar turned the axillary buds to PLBs within 25 days, and

developed a mean of 33.1 PLBs within 50 days. Kinetin (KIN) did not induce PLBs, but facilitated axillary bud proliferation. Transfer of PLBs on medium having same concentration of BA and sugar facilitated rapid multiplication, and developed a mean of 47.5 PLBs. No decline of PLB proliferation was observed up to 10(th) subculture. Half strength MS medium with 6.97 mu M KIN facilitated conversion of 98% PLBs to plantlets. On this media, a mean of 5.8 roots were also developed per shoot. Shoots developed bulbs during culture were grown to rhizomes. Increase of sugar to 6 or 8% hastened the development of bulbs/rhizomes. Reintroduction of PLB-derived plantlets in the natural habitat i.e. at Vellarimala (at 1300 m height) of the Western Ghats of Kerala was attempted as a means to assist in situ conservation. This is the first report of conversion of axillary buds to PLBs. The protocol enables to surmount the threat of extinction of this endemic and endangered orchid.

1515: *+.263*

In 1886 William T. Hornaday, the Chief Taxidermist of the US National Museum, led the 'Smithsonian Institution Buffalo Outfit' to Montana. The American bison was fast heading toward extinction and the mission of the expedition was to locate those of the elusive animals that remained and obtain specimens for the scientific collection at the museum. The successful expedition produced the most complete scientific series, and the finest artistic grouping of taxidermied specimens of the American bison anywhere in the world. Haunted by the scattered skeletal remains of the millions of slaughtered bison that peppered the Eastern Montana Plains, Hornaday fought to establish the National Zoological Park, which would provide sanctuary for bison and other endangered species, and a captive breeding program, which would result in the eventual reintroduction of the American bison to the wild.

1516: *+.002*

The Idaho ground squirrel, which consists of a northern (Spermophilus brunneus brunneus) and a southern subspecies (S. b. endemicus), has suffered from habitat loss and fragmentation, resulting in a reduction in both numbers and geographic range of the species. The northern Idaho ground squirrel (NIDGS) is listed as a threatened subspecies under the Endangered Species Act, and the southern Idaho ground squirrel (SIDGS) is a candidate. Because Idaho ground squirrel populations are small and often isolated, they are susceptible to inbreeding and loss of genetic diversity through drift. This research evaluates levels of genetic diversity and patterns of population divergence in both subspecies of Idaho ground squirrels. We hypothesized that NIDGS would exhibit lower genetic diversity and greater population divergence due to a longer period of population isolation relative to most SIDGS populations. Genetic diversity and divergence were quantified using 8 microsatellite loci. Contrary to expectations, SIDGS populations exhibited consistently lower levels of microsatellite diversity. Additionally, NIDGS exhibited only modest divergence among populations, while divergence levels among SIDGS populations were highly varied. Preliminary evaluations of mitochondrial DNA diversity and structure revealed lower diversity in NIDGS and some differences in gene flow that warrant further study. Based on our results, we suggest different management strategies for the two subspecies. Habitat restoration appears to be the most desirable conservation strategy for NIDGS populations. In contrast, low genetic diversity observed in SIDGS may warrant supplementation of isolated populations through translocations or captive breeding to mitigate further loss of genetic variability.

1517: *+.218*

Eurasian lynx are slowly recovering in Germany after an absence of about 100 years, and

additional reintroduction programs have been launched. However, suitable habitat is patchily distributed in Germany, and whether patches could host a viable population or contribute to the potential spread of lynx is uncertain. We combined demographic scenarios with a spatially explicit population simulation model to evaluate the viability and colonization success of lynx in the different patches, the aim being to conclude guidelines for reintroductions. The spatial basis of our model is a validated habitat model for the lynx in Germany. The dispersal module stems from a calibrated dispersal model, while the demographic module uses plausible published information on the lynx' life history. The results indicate that (1) a viable population is possible, but that (2) source patches are not interconnected except along the German-Czech border, and that (3) from a demographic viewpoint at least 10 females and 5 males are required for a start that will develop into a viable population with an extinction probability of less than 5% in 50 years. The survival rate of resident adults was the most sensitive parameter, and the best management strategy for the success of reintroduction would be to reduce the mortality of residents in the source patches. Nevertheless, the extremely low probability of connectivity between suitable patches makes most of the reintroduction plans isolated efforts, and they are therefore questionable in the long run. With such a model, the suitability of the single habitat patches can be assessed and the most appropriate management scheme applied. This study shows that simulation models are useful tools for establishing the comparative effectiveness of reintroduction plans aimed at increasing the viability of the species. (c) 2005 Elsevier Ltd. All rights reserved.

1518: *+.146*

In South Africa, wild dogs are limited to a single viable population in Kruger National Park. Current conservation efforts aim to develop a meta-population through the reintroduction of wild dogs into fenced reserves. However, significant potential also exists for conserving naturally occurring wild dogs in situ on ranchland. This study represents an assessment of the attitudes of southern African landowners towards wild dogs to determine the scope for conserving them on private land, and to identify the conditions under which conservation efforts might succeed. Over half of ranchers interviewed indicated that they would like to have wild dogs on their property. Younger ranchers were more positive than older ranchers, suggesting that traditional prejudices against wild dogs are fading. Attitudes were generally negative where ranches are game-fenced, and where cattle or consumptive wildlife utilisation dominate land use. Negative attitudes were typically related to economic costs associated with wild dogs, and conservation initiatives aimed at reducing costs or creating benefits from the species represent the most direct way to improve attitudes. Many ranchers recognised the potential ecotourism value of wild dogs, and attitudes were most positive where ranches belong to conservancies, and where ecotourism-based land uses predominate. Similar relationships were found between ranch/rancher characteristics and attitudes towards most large carnivores. Thus, our findings have general relevance for large carnivore conservation on private land in southern Africa. Encouraging the formation of conservancies should be a priority for carnivore conservation efforts on ranchland, to reduce conflict and promote coexistence between people and predators. (c) 2005 Elsevier Ltd. All rights reserved.

1519: *+.287*

For species whose decline preceded the modern era and whose distribution is in the developing world, it is difficult to map suitable habitat across its former range. Eld's deer (Cerrus eldi) is an endangered cervid whose range across Southeast Asia was reduced during the last century to disjoint populations in Myanmar and Cambodia. We used ecological data from the present populations to determine landscape and habitat parameters that would help us predict the occurrence of the species in forests not yet surveyed. The suitable-forest GIs model was created

using four readily available datasets for elevation, forest type, canopy closure, and human density. Comparison of the GIs model with 24 verified sightings of Eld's deer during recent large mammal surveys in Cambodil, found 22 sightings (92%) within predicted suitable forest. Use the suitable-forest GIs model to survey a province in southern Lao People's Democratic Republic, located a single. previously unreported population from 9 patches surveyed. In a separate analysis, a logistic regression model to predict Eld's deer habitat in Northern Cambodia found percent tree cover, presence of wetlands, and distance to villages as the best predictors of deer, similar to variables used in the GIs model, with the exception of the importance of wetlands. Using mean annual rainfall to rank suitable-forest patches identified in the GIs model indicated dry dipterocarp forests in Northeastern Cambodia and Northern Myanmar have the highest potential to conserve eld's deer. Examination of the suitable forest GIs map and current protected areas indicated only Cambodia, with 11% suitable forest protected. has placed sufficient dry dipterocarp forest under protected status. Other Southeast Asia countries have not recognized dry dipterocarp forest as a significant ecotype worthy of conservation status. (c) 2005 Elsevier Ltd. All rights reserved.

1520: *+.026*

Thirteen Cuvier's gazelles were relocated to a 6-ha acclimatization enclosure in Boukornine National Park (Boukornine NP) in Tunisia, where they are part of a reintroduction project. To determine the degree of adaptation and habitat use under the new conditions, the acclimatization enclosure was divided into 6 sections according to topography, plant cover and plant species in the area. Signs of gazelle activity were coded as feeding site, paths, passages, feces and resting places. Sampling was done in spring, summer and autumn from September 2000 to July 2001. Multivariate analysis using PATN analysis and chi(2) distribution tests were used to analyze the data. Multivariate analysis yielded 5 groups of biotopes according to the above variables. The chi(2) distribution test showed the significant effect of each variable on the presence of signs of gazelles. Cuvier's gazelles prefer areas with low and west to north facing slopes and scant plant cover; animals are attracted to the proximity of the fence as the limit of their territory and even though the presence of humans does not represent a disturbance, gazelles select areas far (> 50 m) from the supplementary feeding and water supply for their activities.

1521: *+.105*

The current distribution and status of five macaque species, Macaca fascicularis, M. mulatta, M. arctoides, M. assamensis and M. nemestrina, in Thailand were determined by questionnaire survey in the sub-district level and by field surveys. Among these 5 species of macaques, long-tailed macaques are the most successful species adapted to a wide range of ecological habitats in Thailand. We found this species at 68 localities during our survey, but in contrast at only 5- 10 localities for each of the other four species. In those troops where it was practically feasible, monkeys were temporarily caught, and data and samples were collected for morphological, physiological, and genetic analyses. Based on our analyses, long-tailed macaques have a wide geographical range in Thailand (from ca. 6[degree] N to 16[degree] N), and they can be lumped into either Indochinese or Sundaic groups separated by the boundary at the Isthmus of Kra. Two troops of M. fascicularis aurea were found on islands just below the Isthmus of Kra. The probable hybrid monkeys between long-tailed and rhesus macaques were found at the postulated hybrid zone (15-20[degree] N). The severe threats to Thai macaque populations are habitat deterioration and the artificial genetic pollution caused by pet release and monkey translocation.

1522: *+.151*

1. Climate change is recognized as a major threat to the survival of species and integrity of ecosystems world-wide. Although considerable research has focused on climate impacts, relatively little work to date has been conducted on the practical application of strategies for adapting to climate change. Adaptation strategies should aim to increase the flexibility in management of vulnerable ecosystems, enhance the inherent adaptability of species and ecosystem processes, and reduce trends in environmental and social pressures that increase vulnerability to climate variability.2. Knowledge of the specific attributes of climate change likely to impact on species or habitats is central to any adaptive management strategy. Temperature is not the only climate variable likely to change as a result of anthropogenic increases in greenhouse gases. In some regions changes in precipitation, relative humidity, radiation, wind speed and/or potential evapotranspiration may be more marked than for temperature.3. Uncertainty exists in the response of species and ecosystems to a given climate scenario. While climate will have a direct impact on the performance of many species, for others impacts will be indirect and result from changes in the spatiotemporal availability of natural resources. In addition, mutualistic and antagonistic interactions among species will mediate both the indirect and direct effects of climate change.4. Approaches to predict species' responses to climate change have tended to address either changes in abundance with time or in spatial distribution. While correlative models may provide a good indication of climate change impacts on abundance, greater understanding is generated by models incorporating aspects of life history, intra- and interspecific competition and predation. Models are especially sensitive to the uncertainty inherent in future climate predictions, the complexity of species' interactions and the difficulties in parameterizing dispersal functions. Model outputs that have not been appropriately validated with real data should be treated with caution.5. Synthesis and applications. While climate impacts may be severe, they are often exacerbated by current management practices, such as the construction of sea defences, flood management and fire exclusion. In many cases adaptation approaches geared to safeguard economic interests run contrary to options for biodiversity conservation. Increased environmental variability implies lower sustainable harvest rates and increased risks of population collapse. Climate change may significantly reduce habitat suitability and may threaten species with limited dispersal ability. In these cases, well-planned species translocations may prove a better option than management attempts to increase landscape connectivity. Mathematical models, long-term population studies, natural experiments and the exploitation of natural environmental gradients provide a sound basis for further understanding the consequences of climate change.

1523: ***+.326***

The realms of rare species conservation and metapopulation biology theory are often interrelated, and hence share several basic challenges. Two of the most important are the critical and frequently difficult tasks of distinguishing a priori between habitat and nonhabitat, and then delimiting suitable habitat patches in a study area. We combined classification tree analysis, a subset of classification and regression tree (CART) modeling, with digital data layers of environmental variables in a geographic information system (GIS) to predict suitable habitat and potential new population occurrences for turkeybeard (Xerophyllum asphodeloides), a rare liliaceous understory herb associated with southern Appalachian pine-oak (Pinus-Quercus) forests, in northwestern Virginia. Sample values from eight environmental data layers and population survey data were used in the modeling process to produce a cross-validated classification tree that predicted suitable habitat in the study area. Elevation, slope, forest type, and fire frequency were the four main explanatory variables in the model. Approximately 4% of the study area was classified into five suitable habitat classes, with a misclassification error rate of 4.74%. The final 13-leaf tree correctly classified 74% of the known presence areas and 90% of the known absence areas, and ground-truthing surveys resulted in the discovery of eight new occupied habitat patches. Results of this

study are important for conservation and management of X. asphodeloides, as well as for the applicability of the habitat modeling techniques to enhancing the study of metapopulations and disturbance regimes in Appalachian forests. In addition, they confirm the potential and value of CART and GIS-based modeling approaches to species distribution problems. Our model was successful at defining suitable habitat and discovering new populations of a rare species at the landscape scale. Similar application to other rare species could prove very useful for addressing these and other ecological and conservation issues, such as planning transplantation or reintroduction experiments, identifying metapopulation fragmentation thresholds, and formulating conservation strategies.

1524: *+.100*

Securing natural habitats into which reintroduced species are released and those into which they will radiate is essential for the long-term establishment and viability of reintroduced populations. Consequently, the ability to project future range expansion is critical in reintroduction programs. We evaluated whether numerical growth and spatial expansion of a reintroduced population can be projected based on short-term monitoring of a newly reintroduced population, by studying a Persian fallow deer (Dania mesopotamica) population reintroduced to Israel. Over a five-year period, five to six adult (>1 yr) females were released semi-annually, for a total of 53 over the research period. We developed an individual-based spatially explicit model that projected spatial expansion of the female population in the wild. Model parameters were derived from empirical studies on movement patterns, home range establishment, behavioral adjustments, and demographic dynamics of reintroduced females during the first three years of the reintroduction project. Significant factors that were found to affect home-range establishment and that were incorporated into the model were: occurrence of Mediterranean woodland; occurrence of moderate terrain; roads; built-up areas; and presence of conspecifics. We tested the model by comparing its projections with field observations at the end of the five-year period since the project's onset, i.e., two years beyond the time on which parameters were based. The model was able to predict the direction and rate of expansion of the population and the main activity areas in the wild during the last two years. By using the model for long-term projection, future potential activity centers and landscape connectivity, important for securing the lands required for the Persian fallow deer future radiation, can be identified.

1525: *+.090*

Successful nonlethal management of livestock predation is important for conserving rare or endangered carnivores. In the northwestern United States, wolves (Canis lupus) have been translocated away from livestock to mitigate conflicts while promoting wolf restoration. We assessed predation on livestock, pack establishment, survival, and homing behavior of 88 translocated wolves with radiotelemetry to determine the effectiveness of translocation in our region and consider how it may be improved. More than one-quarter of translocated wolves preyed on livestock after release. Most translocated wolves (67%) never established or joined a pack, although eight new packs resulted from translocations. Translocated wolves had lower annual survival (0.60) than other radio-collared wolves (0.73), with government removal the primary source of mortality. In northwestern Montana, where most wolves have settled in human-populated areas with livestock, survival of translocated wolves was lowest (0.41) and more wolves Proportionally failed to establish packs (83%) after release. Annual survival of translocated wolves was highest in central Idaho (0.71) and more wolves proportionally established packs (44%) there than in the other two recovery areas. Translocated wolves showed a strong homing tendency; most of those that failed to home still showed directional movement toward capture

sites. Wolves that successfully returned to capture sites were more likely to be adults, hard (immediately) rather than soft (temporarily held in enclosure) released, and translocated shorter distances than other wolves that did not return home. Success of translocations varied and was most affected by the area in which wolves were released. We suggest managers translocating wolves or other large carnivores consider soft releasing individuals (in family groups, if social) when feasible because this may decrease homing behavior and increase release-site fidelity.

1526: ***+.282***

Logistic regressions were used to study the relationship between habitat variables and the use of tropical watercourses by the West Indian manatee Trichechus manatus at Northeastern Costa Rica and Southern Nicaragua. Presence of manatees in watercourses was assessed through direct and reported sightings of individuals and feeding signs on aquatic vegetation. Indirect methods provided good approximations to the actual distribution that could not have been achieved through direct observations or aerial surveys. Best multivariate models showed that manatees were most present in watercourses that presented abundant aquatic vegetation, warm, and clear waters, high forest cover, and are wider than those where the species is absent. Although habitat variables that explain habitat use of manatees differed for the two sectors found within the study area, manatees preferred lagoons to other watercourses in both areas. These findings point to forest clearing on the shores as a threat for manatee conservation. Habitat variables are excellent predictors of manatee presence, and predictive models as those developed in this study can help assess potential distribution of manatees in areas where this information is lacking as well as to assist identify potential reintroduction areas. (c) 2005 Elsevier Ltd. All rights reserved.

1527: ***-.101***

This paper describes the effect of a disease outbreak on the success of a translocation for conservation management in a critically endangered species. Three juvenile kakapo from a group of 19 translocated birds died within 72 h of transport between New Zealand offshore islands. Clinical findings, gross necropsy changes, cytology, histopathology and bacterial culture confirmed systemic disease caused by Erysipelothrix rhusiopathiae. On the island from which the kakapo were sourced, positive cultures of E rhusiopathiae were obtained from the medulla of the ulna from 10 out of 15 seabird carcasses examined, suggesting that this could be the source of infection for the kakapo. Immediately after the diagnosis, all of the translocated birds were re-captured and treated with antibiotics. A vaccination programme has commenced using a commercial killed bacterin developed for turkeys. The disease outbreak has had costly implications for the population and conservation management of the species. This is the first report of erysipelas in wild parrots, and the first report of the management of erysipelas in a critically endangered wild population of birds.

1528: ***+.140***

The northern pademelon (Thylogale browni) is a small to medium-sized macropodid that is native to northern and central New Guinea, but is also found on some of the islands of the Bismarck Archipelago, such as New Britain, New Ireland and Lavongai, where it appears to have been introduced. In New Ireland, archaeological evidence indicates that it may have been introduced by prehistoric human agency c. 7,000 years ago. In the chain of islands that constitutes New Ireland Province, historical evidence indicates that the species also recently occurred in the Tabar, Lihir, Tanga and Feni island groups prior to undergoing a series of local extinctions and range contractions during the first half of the 20(th) century. Furthermore T browni also appears to have

declined on New Ireland and Lavongai, where it is now restricted to the remote mountainous interior. Much of the sudden range contraction coincided with the Pacific War (1942-1945), during which time blockaded Japanese troops confiscated local food produce. It is postulated that the privations of war led to an extended period of over-hunting which drove the species into local extinction in much of its former range. Furthermore, since the war, ongoing human pressures and a breakdown in the traditional ethnozoological translocation / re-stocking regimes which would normally have re-introduced this species to satellite islands, appears to have prevented T browni from regaining its former widespread distribution in the New Ireland Province Archipelago.

1529: ***+.123***

This paper stated briefly about the current situation of the Giant Panda (Ailuropoda melanoleuca) including its population, density, habitat and the adjacent communities. It also analyzed the main problems facing to the protection of the Giant Panda, including: (1) serious disturbance resulted in the isolated situation of the Giant Panda population; (2) the florescence of bamboos threatening the survival of wild population; (3) the conflict between the development of communities and the protection of the Giant Panda. Then, this paper put forward the essential policy and four strategic objects for the Giant Panda conservation as follows: (1) forming several continuous conservation regions by establishing a series of natural reserves; (2) allaying the obstruct to the Giant Panda made by perpetual project; (3) making overall action plan for the development of the community economy and implementing management together with community; (4) implementing the project of reintroduction of the captive Giant Panda. In the end, it pointed out specially ways and measures to actualize these policy and objectives.

1530: ***+.044***

We examined the physical habitat of nest sites chosen by hatchery Atlantic salmon, Salmo salar, in a recovery program for this extirpated species in Lake Ontario, Canada. We compared the sites used by these captive bred fish to a set of random locations in a wide range of available habitats. Compared to random locations in the stream, the nest sites chosen were lower in the relative abundance of sediment size classes that are detrimental to embryo and juvenile survival. In addition, the process of nest construction by these captive bred fish further reduced the proportions of these detrimental sediments. Although captive breeding may have changed some aspects of the nest site selection and construction behaviour, it has not caused a complete loss or major alteration of the trait and thus does not preclude hatchery fish from restoration or reintroduction programs.

1531: ***-.043***

In this study samples of ten species of fish were analyzed for concentrations of organochlorine pesticides, PCBs and heavy metals (Pb, Cd, and Cu). Fish were captured using electric fishing on ten sites along the Drome river (Rhone-Alpes region). Quantitative determination of the organochlorine and PCBs compounds was performed by gas chromatography-electron-capture detection (GC-ECD). The concentrations of heavy metals were determined by atomic absorption spectrophotometry. Samples contained detectable concentrations of lindane, PCBs, and heavy metals but at concentrations below the maximum residue limit (MRL). Non-parametric statistical analysis was performed to distinguish groups of sites with different levels of contamination. PCBs concentrations increased along the river with four groups of sites significantly different from each other. Cadmium concentrations were below the MRL. Lead contamination showed two groups significantly different and a repartition similar to PCBs. Copper contamination was correlated with the localization of vineyards. We assessed the potential effects of contamination the otter (Lutra

lutra). The concentrations of all pollutants analyzed in fish sampled in this study are lower than the threshold values described in literature. The Drome river is relatively unpolluted river, and the establishment of otter populations should not be affected by pollution. (c) 2005 Elsevier Ltd. All rights reserved.

1532: *+.122*

We reintroduced 33 peregrine falcons (Falco peregrinus) at two release areas with contrasting habitat configurations to assess landscape influences on dispersal. One site (Daniel Boone National Forest) had non-forested corridors within a forest matrix, whereas the other site (Tom Dorman State Nature Preserve) had forested corridors within an agricultural matrix. We used aerial telemetry and ground observation to assess differences in dispersal between sites and we used multivariate statistical analyses to detect combinations of response variables depicting landscape influences. Non-forested corridors at Daniel Boone affected post-fledging movements and initiation of dispersal, whereas no discernable pattern in dispersal initiation was observed at Dorman Preserve. Results included significant ($p < 0.05$) differences between peregrines at Daniel Boone and Dorman Preserve in (x) over bar +/- SD post-fledging area (PFA) size (2643.5 +/- 2599.0 and 931.7 +/- 732.1 ha, respectively), maximum movement distances (12.9 +/- 13.9 and 6.1 +/- 4.6 km, respectively), orientation of movements (second-order (x) over bar angle +/- 95% confidence interval (CI) = 280.8 +/- 58.6 (non-random orientation) and 358.8 +/- 98.8 (random orientation), respectively), time spent on the PFA (16.0 +/- 12.2 and 31.0 +/- 3.3 days, respectively) and selection of agricultural habitats (Euclidean distance vectors p = 0.66 and p = 2.50, respectively). Selection of agriculture by peregrines released on Daniel Boone reflected selection of corridors as part of pre-dispersal movement. These results, coupled with generally unorientated movement behaviour by Dorman Preserve peregrines, provided evidence for an influence of functional landscape connectivity on dispersal in peregrines.

1533: *+.209*

Efforts to evaluate the efficacy of translocation as a conservation tool have mostly been inadequate, particularly for reptiles and amphibians, leading many biologists to discount translocation as a viable management option. Nevertheless, with two-thirds of the world's tortoise and freshwater turtle species at risk, translocation may be one of the few remaining options for re-establishing extirpated populations and reconnecting fragmented ones. We translocated 106 gopher tortoises (Gopherus polyphemus) to a protected area within the historical range but with no resident tortoises and tested the effects of penning on site fidelity and activity area size. We assigned 38 adults and subadults to one of three penning treatments (9 months, 12 months and no penning) and radio-tracked them for 2 years. Penning significantly increased site fidelity and resulted in smaller activity areas. Our data suggest that translocation coupled with penning Will improve the likelihood of establishing self-sustaining tortoise populations.

1534: *+.249*

The successful eradication of introduced rodents from islets off the coast of Mauritius has led to local conservation bodies investigating the possibility of translocation as a measure of safeguarding endemic reptile populations. The present study was the first to determine the habitat and microhabitat requirements of Telfair's skinks (Leiolopisma telfairii) on Round Island, Mauritius, with a view to aiding future translocation projects to islands within their historic range. Contrasting preferences found for Telfair's skink at macro- and micro- habitat levels underline the importance of sampling at multiple ecological scales in such investigations. Significantly fewer

sightings of L. telfairii were recorded in bare rock habitats compared to more vegetated habitats. Conversely, at a microhabitat scale principal component analysis indicated structural characteristics were the primary determinant of microhabitat choice. The first dietary analysis of Telfair's skinks confirmed their status as omnivores. Cockroaches (Blattodea spp.) appeared to be a primary food source. Four exotic plant species were also present in faecal samples and the potential for L. telfairii to aid their dispersal is discussed. Implications for the long-term management and proposed translocation of Telfair's skinks are discussed. (c) 2005 Elsevier SAS. All rights reserved.

1535: *+.002*

We used radiotelemetry and population modeling techniques to examine factors related to population establishment of black bears (Ursus americanus) reintroduced to Felsenthal National Wildlife Refuge (NWR), Arkansas. Our objectives were to determine whether settling (i.e., establishment of a home range at or near the release site), survival, recruitment, and population viability were related to age class of reintroduced bears, presence of cubs, time since release, or number of translocated animals. We removed 23 adult female black bears with 56 cubs from their winter dens at White River NWR and transported them 160 km to man-made den structures at Felsenthal NWR during spring 2000-2002. Total movement and average circuity of adult females decreased from I month, 6 months, and 1 year post-emergence (F-2,F-14 =19.7, P < 0.001 and F-2,F-14 =5.76, P=0.015, respectively). Mean first-year post-release survival of adult female bears was 0.624 (SE=0.110, SEinterannual = 0.144), and the survival rate of their Cubs was 0.750 (SE=0.088, SEinterannual=0.109). The homing rate (i.e., the proportion of bears that returned to White River NWR) was 13%. Annual survival for female bears that remained at the release site and survived > 1-year post-release increased to 0.909 (SE=0.097, SEinterannual=0.067; Z=3.5, P < 0.001). Based on stochastic population growth simulations, the average annual growth rate (lambda) was 1.093 (SD=0.053) and the probability of extinction with no additional stockings ranged from 0.56-1.30%. The bear population at Felsenthal NWR is at or above the number after which extinction risk declines dramatically, although additional releases of bears could significantly decrease time to Population reestablishment. Poaching accounted for at least 3 of the 8 adult mortalities that we documented; illegal kills Could be a significant impediment to Population re-establishment at Felsenthal NWR should poaching rates escalate.

1536: *+.186*

Understanding how populations expand to recolonize former habitats is important to restoration efforts in wildlife management and conservation. Translocation of black bears (Ursus americanus) to Arkansas in the 1950s and 1960s has led to recolonization of former bear range in Oklahoma, with substantial increases in distribution and abundance of the species in Oklahoma over the last 15 years. We studied demographics of black bears in southeastern Oklahoma from May 2001 to November 2002 to provide insight into characteristics of recolonizing populations of large carnivores. We trapped 51 black bears (22 M, 29 F) 77 times and radiocollared 25 female bears. Sex ratios of adults and cubs were skewed toward females, and the age structure was younger than observed in other unharvested populations. Survival of adult females was estimated at 0.9 +/- 0.1, and fertility was estimated at 0.77 female young/female/year. Density on the study area was estimated at 0.21 bears/km(2) and the current finite growth rate (lambda) of the study population was estimated to be 1.11/year. Demographic characteristics of the Oklahoma population of black bears were similar to those of other recolonizing populations of large carnivores.

1537: *-.010*

Although numerous authors are investigating indirect effects of wolf recovery, the most fundamental ecological impact of the Greater Yellowstone Area wolf reintroduction, the effects of wolf predation on ungulate populations, remains unclear. We report on a 5-year comparative study of wolf (Canis lupus)-elk (Cervus elaphus) dynamics on an elk herd in the headwaters of the Madison River within Yellowstone National Park and the lower Madison elk herd that winters 40 km downriver outside the Park. A resident pack became established on the Madison headwaters area in 1997 and grew to multiple packs totaling 30-40 animals by 2002. During winter 1999 emigrates from Yellowstone established a pack on the lower Madison area. However, poor recruitment and low adult survival limited wolf population growth, with the area supporting a single pack, never exceeding 5 animals. Wolf kill rates on the lower Madison area were approximately double that documented for the Madison headwaters area. Moderate kill rates in the Madison headwaters, combined with high wolf densities and modest elk densities, resulted in an estimated 20% of the elk population being killed during winter and projections for a declining elk population. In contrast, high kill rates on the lower Madison area, combined with low wolf densities and high elk densities, resulted in winter predation estimates not exceeding 40% of the elk population. We suspect this level of mortality will be of little biological significance with respect to elk population trajectory. These results suggest that the effects of wolf predation on elk populations differ substantially over relatively small spatial scales, depending on a complex suite of interacting factors. Thus, we caution against generalizing the effects of wolf restoration on elk dynamics from any single study and encourage collaborations to develop comparative predator-prey studies that improve our understanding of wolf-ungulate interactions and enhance conservation.

1538: ***+.039***

Grasslands dominated by exotic annual grasses have replaced native perennial vegetation types in vast areas of California. Prescribed spring fires can cause a temporary replacement of exotic annual grasses by native and non-native forbs, but generally do not lead to recovery of native perennials, especially where these have been entirely displaced for many years. Successful reintroduction of perennial species after fire depends on establishment in the postfire environment. We studied the effects of vegetation changes after an April fire on competition for soil moisture, a key factor in exotic annual grass dominance. As an alternative to fire, solarization effectively kills seeds of most plant species but with a high labor investment per area. We compared the burn to solarization in a study of establishment and growth of seeds and transplants of the native perennial grass Purple needlegrass (Nassella pulchra) and coastal sage species California sagebrush (Artemisia californica). After the fire, initial seed bank and seedling densities and regular percent cover and soil moisture (0-20 cm) data were collected in burned and unburned areas. Burned areas had 96% fewer viable seeds of the dominant annual grass, Ripgut brome (Bromus diandrus), leading to replacement by forbs from the seed bank, especially non-native Black mustard (Brassica nigra). In the early growing season, B. diandrus dominating unburned areas consistently depleted soil moisture to a greater extent between rains than forbs in burned areas. However, B. diandrus senesced early, leaving more moisture available in unburned areas after late-season rains. Nassella pulchra and A. californica established better on plots treated with fire and/or solarization than on untreated plots. We conclude that both spring burns and solarization can produce conditions where native perennials can establish in annual grasslands. However, the relative contribution of these treatments to restoration appears to depend on the native species being reintroduced, and the long-term success of these initial restoration experiments remains to be determined.

1539: ***-.049***

This paper summarises published research on Przewalski's horse, Equus ferus przewalskii. Biology of the species is described, as well as its history in the wild and in captivity. Reintroduction efforts at Takhiin Tal and Hustai National Park are discussed, with current population levels given, as well as some survivorship data. Ecology of the Przewalski horse at the different reintroduction sites is described, showing similarities and differences. Finally future conservation of Przewalski's horse is discussed, with particular reference to the change in status from Extinct in the Wild to Endangered, based on the IUCN Categories and Criteria.

1540: *+.059*

The Eurasian beaver, Castor fiber L., suffered extreme demographic reduction through overhunting until the end of the 19th century. However, active protection measures have led to a powerful recovery in range and population numbers. The vast majority of beavers (83%) now occur in the former Soviet Union. The present study investigates the geographic distribution of genetic variation of C. fiber in this eastern part of the species range (former Soviet Union and Mongolia), with special emphasis on small isolated populations of the Asian subspecies C. fiber pohlei, C. fiber tuvinicus, and C. fiber birulai. The analysis yielded 12 different haplotypes, all of which were population specific. Results indicate that C. fiber displays great population structuration (F-ST = 0.985), coupled with an overall low level of genetic divergence (mean number of pairwise differences 7.262 +/- 3.435). In particular, the autochthonous populations in Mongolia or Siberia do not appear significantly different from samples from the European part of Russia, despite the great geographical distance. C. f birulai appears as the most divergent member, a fact that could result from its longer genetic isolation in an enclosed watershed. Examination of our data suggests a single recent origin of the present beaver population in eastern Europe and Asia.

1541: *+.057*

Introduced rats depredate every life stage of island nesting seabirds, but the extent of predation is rarely quantified. Introduced black rat (Rattus rattus) and native deer mouse (Peromyscus maniculatus anacapae) predation on Xantus's murrelet (Synthliboramphus hypoleucus scrippsi) nests was experimentally quantified using artificial nests before and after rat eradication on Anacapa Island (California). The staged rat eradication programme provided experimental treatments: in 2002 rats were eradicated on one island (East Anacapa Islet) and remained on two islands (Middle and West Anacapa Islets), providing a control comparison, and, in 2003, rats were eradicated from the remaining islands (Middle and West Anacapa Islets). In 2002, 96% of artificial nests were depreciated on control islands (rats present) with rats accounting for most predation. Nest predation on the treatment island (rats eradicated) in 2002 was significantly lower: 8% of artificial nests were depredated, mostly by endemic deer mice. In 2003, following rat eradication on the remaining islands (Middle and West Anacapa Islets), nest predation was reduced from 96% in 2002 to 3% of total nests in 2003. Predation of nests on East Anacapa Islet (rats eradicated in 2002) increased significantly due to reintroduction and recovery of native deer mouse populations, with 23% of artificial nests depredated. The inference is that rat predation on real Xantus's murrelet nests was responsible for the historically low nesting success and small population sizes of breeding murrelets on Anacapa Island. With rats removed, the hatching success of Xantus's murrelet chicks and the number of individuals nesting on Anacapa Island will increase dramatically. Artificial nest studies are particularly well suited to quantifying introduced rat impacts on hole and crevice nesting seabirds and can simultaneously serve as an effective monitoring tool to detect the presence of rats and the recovery of native nest predators.

1542: ***+.121***

Atlantic salmon (Salmo salar) was once native to Lake Ontario, however, its numbers rapidly declined following colonisation by Europeans and the species was extirpated by 1896. Government agencies surrounding Lake Ontario are currently undertaking a variety of studies to assess the feasibility of reintroducing Atlantic salmon. We released hatchery-reared adult Atlantic salmon into a Lake Ontario tributary to examine spawning interactions between this species and fall-spawning exotic salmonids found in the same stream. Chinook salmon, coho salmon and brown trout were observed interacting with spawning Atlantic salmon in nearly one-quarter of our observation bouts, with chinook salmon interacting most frequently. Whereas a previous investigation found that chinook salmon caused elevated agonistic behaviour and general activity by spawning Atlantic salmon, the present study found that interspecific courtship was the most common form of exotic interaction with spawning Atlantic salmon. In particular, we observed precocial male Chinook salmon courting female Atlantic salmon and defending the female against approach by male Atlantic salmon. We discuss the potential implications of these interactions on the Lake Ontario Atlantic salmon reintroduction programme.

1543: ***+.243***

How does society influence the management of an introduced native mammal that is destroying its own habitat-especially when that animal is considered threatened on parts of the mainland, is aesthetically pleasing, and is important to the tourism industry? If control through sterilization and translocation is part of the answer, what lessons can be learned from this program?

1544: ***+.080***

To counter losses of genetic diversity in reintroduced populations, species sometimes are reintroduced into networks of populations with the potential to exchange individuals. In reintroduced populations connected by gene flow, patterns of genetic structure initiated by the founding event may become obscured, and populations may eventually follow an isolation-by-distance model of genetic differentiation. Taking advantage of well-documented reintroduction histories of wild turkey populations in Indiana, we assessed the degree to which gene flow among reintroduced populations has obscured genetic signatures left by the founding events. Using a suite of nuclear microsatellite loci and sequence data from the mitochondrial control region, we characterized the level of genetic diversity and degree of genetic structure within and among: (1) reintroduced populations in isolated northern Indiana Fish and Wildlife Areas, (2) reintroduced populations in southern Indiana Fish and Wildlife Areas, where the distribution of populations is more continuous, and (3) source populations used for these reintroductions. We also utilized individual-based assignment tests to determine the relative contribution of source populations to the current distribution of alleles in reintroduced populations. Our results indicate that wild turkey reintroductions in Indiana have left distinct genetic signatures on populations that are detectable even after several decades. Although we found some case-specific evidence for gene flow, particularly in regions where populations are in close proximity, our data indicate on overall paucity of gene flow at a regional scale. Such post-reintroduction genetic monitoring has immediate implications for the design of optimal strategies to reintroduce wildlife for conservation and management.

1545: ***+.105***

Chinese alligator (Alligator sinensis) is a critically endangered species endemic to China. In this

study, the extent of genetic variation in the captive alligators of the Changxing Reserve Center was investigated using microsatellite markers derived from American alligators. Out of 22 loci employed, 21 were successfully amplified in the Chinese alligator. Sequence analysis showed loci in American alligators had a bigger average size than that of the Chinese alligators and the longest allele of an individual locus almost always existed in the species with longer stretch of repeat units. Eight of the 22 loci were found to be polymorphic with a total of 26 alleles present among 32 animals scored, yielding an average of 3.25 alleles per polymorphic locus. The expected heterozygosity (H (E)) ranged at a moderate level from 0.4385 to 0.7163 in this population. Compared to that in the American alligators, a lower level of microsatellite diversity existed in the Changxing population as revealed by about 46% fewer alleles per locus and smaller H (E) at the homologous loci. The average exclusion power and the ability to detect shared genotypes and multiple paternity were evaluated for those markers. Results suggested that when the polymorphic loci were combined, they could be sensitive markers in genetic diversity study and relatedness inference within the Chinese alligator populations. The level of genetic diversity present in the current Changxing population indicated an important resource to complement reintroductions based on the individuals from the other population. In addition, the microsatellite markers and their associated diversity characterized in this population could be utilized to further investigate the genetic status of this species.

1546: ***+.097***

Hill Mynah Gracula religiosa is one of the most popular bird pets worldwide due to its ability to mimic diverse sounds, especially human speech. However, Mynahs have rarely been bred in captivity, so nestlings from natural populations are in large demand, resulting in many populations being threatened with extinction. Both subspecies in Thailand, intermedia and religiosa, are costly and desired in the pet market. Captive breeding is one of the most practical strategies to solve a conservation problem of this nature and this report describes a success in breeding Hill Mynahs in captivity. Mated pairs were given free access to food, nest-cavities and nest materials. Reproductive behaviour in captivity was not different from that in the wild, with the exception that breeding occurred throughout the year, even during the non-breeding season for wild populations. Although there are doubts concerning the reintroduction of captive-bred birds and whether successful Hill Mynah breeding in captivity is an economically competitive alternative to poaching, it ensures species survival in captivity as the risk of extinction increases.

1547: ***+.075***

To facilitate recovery through captive breeding and foster-parenting programs of the endangered whooping crane, one of two eggs was removed from 62% of nests in Wood Buffalo National Park during 1967-1996. Egg removals were justified because cranes usually rear a single chick; the other dies to siblicide or predation. Concerns exist that the wild population might have recovered even faster if nests had not been disturbed. Here we show, contrary to expectation, that removing one of two eggs from a whooping crane nest actually increases the probability of nest success, and this effect is highly correlated with the dynamics of predators in the nesting area. These results beg the question: why do whooping cranes lay two eggs? We attribute two-egg clutches to occasional "good years" in which both chicks survive, compensating for higher. mortality in two-egg broods. Egg removal has benefited conservation allowing establishment of several captive flocks, supporting reintroduction of two new populations, and reducing the variance in reproductive. success of the wild flock thereby minimizing extinction risk. (c) 2005 Elsevier Ltd. All rights reserved.

1548: ***+.073***

We evaluated the genetic consequences and efficiency of conservation practices in Oryza rufipogon using microsatellite DNA markers. Spatial autocorrelation analysis from 12 microsatellite loci revealed that microsatellite alleles were exclusively distributed it? patches within the population, indicating that large populations were unlikely to be homogeneous. At? in situ conserved stand of O. rufipogon, which has been protected by a concrete wall from a large population, captured only 67.9% of the total genetic variation of the previous large population. The concrete wall was built to protect the wild rice. but it acted more as a physical barrier to gene exchanges between the two sides. An assignment test revealed only 11.1% putative seed exchanges across the wall. A reintroduced population was found to be genetically very diverse. About 76.3% of the total genetic variation detected in other populations was captured in this reintroduced population. and 24.8% of the total genetic variation in this population was not found in other populations. These results display two important findings for conservation of O. rufipogon. First, conserving one part of a large population of O. rufipogon will not preserve an adequate sample of the genetic variability, since populations are not homogeneous, and genotype distribution varies among localities. Second, a reintroduced population is not genetically depauperate, but it is too early to assess its long-term survival.

1549: ***+.157***

Background and Aims Isoetes sinensis (Isoeteaceae) is a critically endangered aquatic quillwort in eastern China. Rapid decline of extant population size and local population extinction have occurred in recent years and have raised great concerns among conservationists. Methods Amplified fragment length polymorphisms (AFLPs) were used to investigate the genetic variation and population structure of seven extant populations of the species. Key Results Eight primer combinations produced a total of 343 unambiguous bands of which 210 (61(.)2 %) were polymorphic. Isoetes sinensis exhibited a high level of intra-population genetic diversity (H(E) = 0(.)118; hs = 0(.)147; I = 0(.)192; P = 35(.)2 %). The genetic variation within each of the populations was not positively correlated with their size, suggesting recent population decline, which is well in accordance with field data of demographic surveys. Moreover, a high degree of genetic differentiation (F(ST) = 0(.)535; G(ST) = 0(.)608; theta(B) = 0(.)607) was detected among populations and no correlation was found between geographical and genetic distance, suggesting that populations were in disequilibrium of migration-drift. Genetic drift played a more important role than gene flow in the current population genetic structure of I. sinensis because migration of I. sinensis is predominantly water-mediated and habitat range was highly influenced by environment changes. Conclusions Genetic information obtained in the present study provides useful baseline data for formulating conservation strategies. Conservation management, including both reinforcement for in situ populations and ex situ conservation programmes should be carefully designed to avoid the potential risk of outbreeding depression by admixture of individuals from different regions. However, translocation within the same regional population should be considered as a measure of genetic enhancement to rehabilitate local populations. An ex situ conservation strategy for conserving all extant populations to maximize genomic representation of the species is also recommended.

1552: ***+.102***

In an effort to learn more about the potential for reintroduction of hare-wallabies to sites in Australia, 34 captive-bred hare-wallabies were released onto Peron Peninsula within the Shark Bay World Heritage Property in 2001 as part of an experimental reintroduction program. One

objective of this experiment was to characterise their behaviour and daytime refugia to identify suitable habitat for future releases. The mala (Lagorchestes hirsutus) and merrnine (Lagostrophus fasciatus) were fitted with radio-transmitters and tracked daily. Merrnine were more faithful to a previously occupied shelter than mala. Mala maintained a solitary daytime habit at all times. Within the study area, mala preferentially sought low-lying vegetation primarily comprising the species Lamarchea hakeifolia, which provided dense cover up to 1 m in height, under which they constructed scrapes. L. hakeifolia was preferred as shelter vegetation instead of Triodia (spinifex) hummocks, despite Triodia hummock habitat being preferred by mala in central Australia. Merrnine occupied taller vegetation with an open understorey to 1.5 m, although the extremity of the understorey remained dense at this height. Individual merrnine sometimes sheltered with conspecifics of the opposite sex. Since both species utilised floristically and structurally variable vegetation, we suggest that they have the ability to cope with vegetation that has been altered by changes in fire regimes and introduced herbivores. This is particularly important for future reintroduction exercises as the results suggest that vegetation characteristics required to support these species, particularly mala, may be variable rather than limited to perceived necessary habitat types in central Australia or on islands. These results should allow future reintroduction projects to consider a wider range of release-site options during their planning phase.

1553: ***+.241***

The authors present the problem of protection of Parnassius apollo, i.e. its distribution and number (along with their changes in Europe and Poland), its bionomy, dangers to the species, finally a forecast of changes in population and current methods of its protection. P. apollo has been observed since 1994 (11 generations) in the Kruczy Kamien nature reserve. Reintroduction of P. apollo in, for example, Lower Silesia, while reverting its range to that known from historical sources and complementing it with an additional net of stations, is accepted as an important goal of the programme of protecting this species in Po-land. 15-30 reintroduction centres are planned in order to acquire and maintain metapopulation; various locations and subsoils for caterpillar feeding plants need to be used. In the course of next 15-20 years, vital populations of P. apollo will be introduced onto the chosen locations (in the Sudety Mountains, the butterfly has been extinct already from the nineteenth century).

1555: ***+.103***

The galaxioid fishes are the dominant, most speciose group of freshwater fishes (with > 50 species) in the lands of the cool southern hemisphere, with representatives in western and eastern Australia, Tasmania, New Caledonia, Lord Howe Island, New Zealand, the Chatham, Auckland and Campbell Islands, Patagonian South America (Chile, Argentina), the Falkland Islands and South Africa. The group is most diverse in Australia and New Zealand. Lepido-galaxiidae is found only in Australia, Retropinnidae in Australia and New Zealand, and Galaxiidae across the entire range of the group. Many species are in serious conservation crisis for a diversity of reasons, including habitat deterioration and possibly fisheries exploitation, but there is enduring and pervasive information that shows that the group has been seriously impacted by the acclimatisation of salmonid fishes originating in the cool-temperate northern hemisphere, particularly brown and rainbow trout. With few exceptions, where these trout have been introduced there has been major decline in the galaxioids, especially Galaxiidae, as a result of a complexly interacting series of adverse impacts from these introduced fishes. In some places, centrarchids and cichlids may also have adverse impacts. In addition, there appear to have been adverse impacts from the translocation of galaxioids into communities where they do not naturally occur. In many instances it appears that displacement of the galaxioids has led to a situation where

galaxioids and salmonids no longer co-occur, owing either to displacement or predation, leading to fish communities in which there is no explicit evidence for displacement. These effects are resulting in the galaxioid fishes being amongst the most seriously threatened fishes known.

1556: *+.171*

Alter 40 years of intensive conservation work to stop persecution (shooting and poisoning), to improve habitat quality, to educate the public and to ensure an adequate food supply, the situation of the four species of vultures (Griffon Gyps fulvus, Black Aegypius monachus, Egyptian Neophron percnopterus, and Bearded Gypaetus barbatus), shows an overall positive trend in France. In 1981 a reintroduction programme for Griffon Vultures began in the south Massif Central (Grands Causses). To help re-establish this species to its former breeding areas, other similar programmes started in the 1990s in the Southern Alps. The success of these programmes encouraged us to go further and prepare the reintroduction of the Black Vulture, which became extinct from these regions at the end of the 19th century. Today, the populations of Griffon Vultures in France originating from reintroductions (about 210 pairs in 2005), represent about 25% of the total for the whole country (ca. 800 pairs in 2005, i.e. more than a ten-fold increase since 1960) and this proportion is continuously increasing. Started in 1992 in the Grands Causses, and in 2004 in the Southern Alps, the reintroduction of the Black Vulture is still in an initial stage. Nevertheless, in these young populations reproduction started 4 years after the first releases, with 28 young having fledged since 1996. To help the understanding of these ambitious programmes, the following topics are analysed: releasing techniques for both species, monitoring of the released birds, monitoring of the breeding populations, feeding strategy used to help the birds to become independent from the conservation structures and avoid causes of mortality. Also some data are given concerning the movements of these new populations and the interactions with the autochthonous ones (from Spain to Greece).

1557: *+.128*

In recent years there has been an increasing recognition of the role that reintroduction programmes can play in species conservation. Some of the best examples of successful bird reintroductions come from vulture projects. There may be fundamental features of the biology of these scavenging birds that make them particularly suitable for this management technique. IUCN has developed international recommendations on the procedures that should be followed before any species reintroduction programmes are planned. These will be reviewed in relation to their relevance for vulture projects. Fortunately there is considerable data from the reintroduction programmes for the California Condor in America and the European Griffon and Bearded Vulture in Europe, and these allow us to consider which are the most important factors to consider if reintroductions are likely to succeed. It is becoming apparent that the major challenge is probably in the selection of suitable release sites, and the management of birds once released. Vultures have extremely extensive foraging behaviour. This can expose them to a wide range of environmental hazards, and if these are not fully understood can lead to a low probability of self-supporting populations becoming established. The mortality agents acting on vulture populations are often quite unexpected, and extremely difficult to predict. Detailed monitoring of released birds is not only essential in order to identify the hazards that may face released birds, but also gives essential information for the conservation of wild populations. The role of supplementary feeding of released birds is also badly in need of more serious study. There are obvious advantages to this technique, but there may also be serious disadvantages if it prevents the development of natural normal foraging behaviour in released birds.

1558: ***+.095***

In order to assess the availability of areas for reintroducing the Bearded Vulture in Andalusia, Southern Spain, potential breeding sites were modelled. The GLM model selected four variables: altitude, topographic irregularity, distance to village and distance to the nearest neighbouring breeding pair. A GIS was used to obtain digital models for each variable. We validated the GLM model testing it with new nest sites in a different geographical context, without including distance to neighbouring pairs. According to the model, in Andalusia the areas with probability > 0.8 coincided with the main mountain ranges. Of these, we selected only protected areas within the historical range of the species. Thus, eight protected areas were considered and surveyed to evaluate their feasibility for reintroduction. We gathered information at two geographical scales:1-km and 15-km radius around high-probability cliffs. We summarised environmental information on habitat quality and threat level in a matrix that made it possible to compare the viability of reintroduction in each of these eight areas. Poisoning cases and the density of power lines were the most restrictive variables. Sierra Nevada National Park, which was expected to be one of the best areas, showed a high level of threat because of power lines, cable cars and outdoor activities. These threats, however, could be easily mitigated by implementing reintroduction actions. Two protected areas, the Cazorla, Segura y Las Villas Natural Park and the Alhama, Tejeda yAlmijara Natural Park, were the most suitable areas for a reintroduction.

1559: ***+.108***

Between 1996 and 2001 103 red kites were released in central Scotland as the second phase of red kite re-establishment in Scotland. This was the fourth site for red kite releases in the UK (the second in Scotland) and it was overseen by the employment of two project officers over the years of releases. This paper gives details of the release programme in central Scotland which used red kites imported mainly from Sachsen-Anhalt, Germany as donor stock. The paper outlines the minimum first year survival rates, and the breeding performance of the donor stock up until December 2002. The survival rate of birds in their first calendar year between 1998 and 2001 is estimated at a minimum of 57.6 %. Fifteen pairs of kites bred in central Scotland in 2002, with 24-25 young fledged. The population in central Scotland has been supplemented by birds from both Dumfries and Galloway and the north of Scotland, with 2 of the latter birds now breeding in central Scotland. The central Scotland red kite population is now considered self sustaining and at this stage that there will be no need for further importation or releases of birds. The central Scotland red kite reintroduction project is a good example of how international collaboration between conservationists can work to help threatened species. In this case Scottish and German statutory and non Governmental organisations have worked closely together to help conserve the red kite, a species whose world range is largely confined to Europe.

1560: ***-.017***

There is little published information on the pathogens present in New Zealand passerines. We report here on a preliminary survey of selected pathogens and haematology profiles for seven species in the Auckland region. Avian translocations are commonly used for the recovery of threatened species. Translocations may increase the risk of spreading disease to immunologically naive populations. It is therefore important to take every opportunity to gather baseline disease data and test hypotheses associated with disease. Blood, cloacal and faecal samples were collected from Fernbird Bowdleria punctata, Tui Prosthemadera novaeseelandiae, Bellbird Anthornis melanura, Tomtit Petroica macrocephala, New Zealand Robin Petroica australis, Whitehead Mohua albicilla and Starling Sturnus vulgaris during four translocations to and from Tiritiri

Matangi Island. Birds (n = 137) were also examined for external lesions typical of avian pox. Blood samples (n = 40) were screened by microscopy for Plasmodium spp., Atoxoplasma spp. and other blood parasites and a differential white blood cell count was made. Cloacal swabs (n = 38) were cultured for Yersinia spp., Salmonella spp. and Campylobacter spp. Faecal samples were screened for coccidia spp. (n = 28). An unidentified coccidian sp. and a Haemoproteus sp. were detected in one Fernbird and one robin respectively. No other organisms with the potential to cause disease were detected. Despite the effort required to complete disease screening, we argue that disease samples and baseline haematology normal values should be collected at all opportunities. We make recommendations for future disease screening, and discuss the importance and potential significance of disease to the conservation of New Zealand's biodiversity.

1561: *+.033*

The White-necked Crow (Corvus leucognaphalus) formerly inhabited St. Croix, Puerto Rico, and Hispaniola. It survives only in Hispaniola, where populations are declining for some of the same reasons primarily responsible for its extirpation from Puerto Rico: habitat fragmentation and loss, and shooting. Egg and chick depredation by the Pearly-eyed Thrasher (Margarops fuscatus) was also important in the extirpation of the Puerto Rico population. I studied the crow in the Dominican Republic intermittently from 1974 to 2004. The species occupies a wide range of habitats, including wet coastal and montane forest, pine forest, cactus forest, mangrove swamp, and palm savanna. Its vocal behavior is complex and diverse, more like that of ravens than of crows. White-necked Crows fed on a wide variety of plant and animal matter. Foraging crows selected plant materials in 76.4% of my observations, whereas animals made up 51.6% of the food items delivered to nestlings. Core activity areas of three breeding pairs averaged 9.8 +/- 3.7 ha, whereas their average territory size was 5.5 +/- 5.2 ha. Nest budding began in late January. Nests were bulky structures placed high in trees. Eggs were incubated 18-22 days and the nestling period was 35-44 days.

1562: *+.128*

Population models are useful tools to guide management as they allow us to project growth and persistence of wildlife populations under different scenarios. Nevertheless, good data are needed to produce reliable models, and this requirement is problematic in some situations. North Island saddlebacks (Philesturnus rufusater) were reintroduced to Boundary Stream Mainland Island in September 2004, and this was the first time this species had occurred in an unfenced mainland area since their extirpation in the 19th century. This situation creates a challenging scenario for population modelling, as this species has never been studied in the presence of mainland predators, and management of these predators will be the key factor determining whether the population survives. In this paper we present an approach for developing a "prior model" before a reintroduction takes place. We use data from the reintroduced saddleback population on Mokoia Island to develop a model of how saddleback populations are regulated in the absence of mammalian predators. We use this model to project growth of a reintroduced population when vital rates are reduced by predation and also to project responses of source populations to harvesting of birds for translocation. We then incorporate data from the reintroduced North Island robin (Petroica longipes) population at Paengaroa Mainland Island to model the relationship between population parameters and predator tracking rates. The combined model can be used to predict the level of predator control needed to ensure growth of the saddleback population, but the prediction is contingent on guessing the relative Vulnerability of robins and saddlebacks to predation. We envision using a Bayesian approach to update such prior models as site-specific data become available after reintroduction.

1563: ***+.195***

This 600-page book, entitled 'New Perspectives in the Study of Mesoamerican Primates: Distribution, Ecology, Behavior, and Conservation', is a comprehensive synthesis of recent advances in primate field research, ecology, and conservation biology in Mesoamerica. This volume is part of the series Developments in Primatology: Progress and Prospects. This volume highlights 4 major areas of research; evolutionary biology and biogeography, population demography and ecology, behavior, and conservation and management policies. The book is structured into 5 major parts and contains 23 individually-authored chapters. The text is in English and each chapter is individually referenced. The first chapter provides an overview of the Mesoamerican primate fauna, primate studies, and conservation concerns. Chapters 2 and 3 are found in part 1 of the book, which focuses on taxonomy and biogeography, and these 2 chapters individually discuss taxonomy and distribution of Mesoamerican primates and the biogeographic history of Mesoamerican primates. Population responses to disturbance are discussed in part 2 of the book and there are 5 chapters in this second part, chapters 4-8. Topics covered in these 5 chapters include, respectively: demographic features of Alouatta pigra (black howler) populations in extensive and fragmented forests; population structure of black howlers in Southern Belize and responses to Hurricane Iris; the effects of forest fragment age, isolation, size, habitat types, and water availability on monkey density in a tropical dry forest; forest fragmentation and its effects on the feeding ecology of black howlers from the Calakmul area in Mexico; and intestinal parasitic infections in black howlers in tropical rainforest in Lacandona, Chiapas, Mexico and the implications for behavioral ecology and conservation. Behavior and ecology is the theme of the part 3, which contains chapters 9-16, inclusive. More specific topics covered in these 8 chapters include, respectively: average body weight for Alouatta palliata palliata (mantled howler monkey); exploratory analysis of developmental plasticity in Costa Rican mantled howler monkeys; travel patterns and spatial mapping in Nicaraguan mantled howler monkeys; an experimental field study of the use of landmark cues to locate feeding sites in Cebus capucinus (wild white-faced capuchin monkeys); ontogenetic influences on positional behavior in Cebus and Alouatta; food choice by juvenile capuchin monkeys in a tropical dry forest; dominance and reproductive success in wild white-face capuchins; and post-conceptive mating in white-faced capuchins in terms of hormonal and sociosexual patterns of cycling, noncycling and pregnant females. Part 4 of the book focuses on conservation and management policies and this fourth part contains chapters 17-22. Specific topics covered in this fourth part include, respectively: growth of reintroduced Ateles geoffroyi (spider monkey) population on Barro Colorado Island, Panama; primates in agroecosystems and the conservation value of some agricultural practices in Mesoamerican landscapes; primate populations in the protected forests of Maya archeological sites in Southern Mexico and Guatemala; mapping primate populations in the Yucatan Peninsula, Mexico; a metapopulation approach to conserving the howler monkey in a highly fragmented landscape in Los Tuxtlas, Mexico; and quantifying fragmentation of black howler habitat after Hurricane Iris in 2001 in Southern Belize. The fifth and final part of the book provides a synopsis and perspectives, and this final part contains the final chapter, chapter 23, which discusses new perspectives in the study of Mesoamerican primates and includes concluding comments and conservation priorities. The book is indexed by species and by subject and contains 165 illustrations. The book also includes a list of the contributors and their respective institutions. This book will appeal to students and researchers in biology, anthropology, primatology, zoology, ecology, animal behavior, conservation biology, natural resource management, and to administrators responsible for conservation policies and government agencies.

1564: ***+.015***

The wildcat is one of the most endangered carnivore species in Europe. The population density declined and the distribution area became fragmented over the last century due to the hybridisation, loss of habitat, illegal hunting and road kills (Stahl and Artois 1994). However, a slight increase in the population density could be found in Belgium, France, Germany and Slovenia over the last few decades due to the recolonisation and reintroductions (Stahl and Artois 1994). Wildcats have been protected in Hungary since 1973, but the situation of this species was not been investigated until 1987. Mail questionnaire surveys were conducted between 1987 and 2001 to evaluate the changes of population density and distribution. Clear decrease of the wildcat population distribution range, constant and serious decrease in the population density could be found in Hungary between 1987 and 2001. The stable areas of the species' occurrence are the Transdanubian and the Northern Middle Altitude Mountains, the Drava plain, the Mecsek and the Villanyi Mountains and the forests of floodplains in the Great Plain. The protection of this species have to be intensified with a species protection plan, which contains the wildcat reserve areas with strict protective management measures.

1566: *+.076*

Morphologic variability and allozyme polymorphisms previously indicated that three different hare species coexist in the Iberian peninsula, Lepus europaeus, L. granatensis, and L. castroviejoi. Here, we show evidence of clear nuclear differentiation and distinctiveness among the three Iberian hare populations, applying 6 microsatellite loci to 100 hare specimens. This provides compelling information of a barrier to neutral gene flow between the three populations. In addition to previous results, our data confirm their status as distinct biological species. On the other hand, the genetic heterogeneity found between two natural L. europaeus populations, from the Iberian and Balkan peninsulas, provided useful information to assess the incidence of re-introduction programs in situations of isolated and locally adapted populations. These results suggest that re-stocking programs of L. europaeus in Spain using hares from non-Iberian populations should be avoided. (c) 2005 Deutsche Gesellschaft fur Saugetierkunde. Published by Elsevier GmbH. All rights reserved.

1567: *+.034*

The European mink is an endangered species in need of urgent conservation efforts, but whose decline and habitat requirements are only poorly known. In this study we developed a model for mink distribution in Biscay to catalogue variables influencing its presence/absence. Sites were described in terms of vegetation parameters, water quality, riverbank alteration, anthropogenic structures and American mink presence. Logistic regression analysis was used to identify ruling variables and synergies. The model extracted two variables of high significance: water quality and riverbank alteration. European mink was absent from polluted waters and canalised streams. The absence is explained by a decrease in prey availability. In the case of water pollution, bioaccumulation is considered to have a deleterious effect on the presence of the species. In the case of canalisation, the lack of adequate shelter areas and especially the depletion of food resources are also likely to play an important role explaining the absence of the species. Finally, the possible barrier effect of canalisation for European mink and its consequences are also discussed. For the remaining variables, only those describing habitat use by European mink within its home range seemed to have a little influence on the presence of the species. The presence of American mink was a poor predictor of the absence of its European counterpart. Based on the results, we suggest that improved riverbank management policies are needed to ensure the future of European mink in the area. Species introduction and reintroduction programs should consider our habitat model when searching for suitable areas for the species. Further research is also needed

on the effect of diverse types of water pollution on European mink.

1569: *+.152*

After the extinction of the Otter (Lutra lutra) in The Netherlands in 1988, measures were taken to restore otter habitat in lowland peat marshes in the north of the country. A spontaneous recolonisation was not expected, reintroduction started in 2002 with the release of 15 otters in the lowland peat marsh area of the Weerribben, followed by another 8 in 2004-2005 in the adjacent Wieden area. The otter population was monitored using radio-telemetry and genetic techniques based on DNA-fingerprinting of spraints. Until now of the 23 released otters, 14 settled in their release area, 2 otters died shortly after their release and 7 did not settle. At least 9 otters were born from 5 different females, indicating that habitat quality seems sufficient. At present in total 11 animals were found dead (8 adults and 3 newborns), of which 9 were killed by traffic collision. The population remains vulnerable for extinction, and the additional release of new otters to supplement the population is now of utmost importance together with further measures to improve habitat quality (i.e. corridors, fencing).

1570: *-.013*

The Eurasian lynx (Lynx lynx) used to occur throughout Europe, but its current distribution is restricted to the scattered large continuous forest regions. Core areas are the Carpathians, the Alps, and the Balkan. Some areas like the Harz, Pfalz, the Jura Mountains and Vosges, hold recently reintroduced or otherwise settled populations. Potential new habitat in western Germany and The Netherlands has a patchy distribution and is poorly connected with currently occupied core areas. An important obstacle is the dense infrastructural network of main roads, secondary roads, canals, railroads etc. Chances are being discussed whether the species would benefit from the realisation of a western European ecological network for large mammals.

1571: *+.008*

In many species genetic distances increase with geographic distances, resulting in the "isolation by distance" (IBD) pattern. However, more complex patterns usually are observed in heterogeneous habitats. Geographic obstacles limit gene flow in a discontinuous way and might confound a simple IBD relationship. Moreover, current population structure is not only determined by present-day evolutionary processes but also shaped by population history. Barriers to gene flow lead to differences in genepool composition among populations, so that molecular population genetics methods should allow these barriers to be detected. It is also possible to identify cryptic boundaries, which may represent secondary contacts among previously isolated populations. Landscape genetics approach that combines molecular population genetics and landscape ecology aimsto detect such genetic discontinuities and to correlate them with environmental features. In this paper, the effects of genetic and environmental factors that affect population genetic structure and population history, are explored with a focus on the followingexamples: (1) the common vole populations in heterogeneous habitats of the Biebrza valley in NE Poland; (2) red deer populations in France that experienced isolation and translocations; (3) different chromosome races of the common shrew in Poland forming hybrid zones and (4) two sympatric subspecies of the chequered skipper in the Bialowieza Primeval Forest, NE Poland. Implications of such approaches for evolutionary biology, ecology and conservation biology are discussed in the context of most recent achievements in the field.

1572: *+.086*

Extensive translocation of wildlife throughout North America has led to concerns regarding taxonomic integrity for a number of species. Often, multiple subspecies or variants were translocated into a common habitat or region, creating the opportunity for hybridization to occur. This issue is of particular concern to managers of wild turkeys (Meleagris gallopavo), a species in which considerable mixing of subspecies has occurred. We aim to quantify the subspecific status and degree of hybridization of individuals within an introduced population of Merriam's turkeys (M. g. merriami) in the Davis Mountains of Texas, USA, and within nearby Rio Grande turkey populations (M. g. intermedia). We used data from the Merriam's source population in New Mexico, USA, as a baseline reference for the genetic characteristics of the Merriam's subspecies. Nineteen years following the introduction event, microsatellite data indicate that the genetic integrity of the introduced population of Merriam's turkeys in the Davis Mountains Preserve has been eroded by both immigration from and hybridization with nearby Rio Grande populations. Data from the mitochondrial control region allow for further characterization of hybrid individuals and indicate that most hybrids were the result of immigrant Rio Grande males mating with resident Merriam's females. Our results attribute to the potential importance of hybridization in wildlife species and suggest that hybridization can be a rapid process capable of drastically altering the evolutionary integrity of animals in a region.

1573: ***+.018***

The presence of Eurasian lynx as a former native species in Britain during the Holocene is known from bones recovered from several sites. AMS radiocarbon dating of lynx bone recovered from two sites in the Craven area of northern England gave 1842 +/- 35 C-14 yr BP and 1550 24 14C yr BP, together representing the youngest dates for lynx from England, and in the case of the latter, the youngest for Britain as a whole. These dates support the view that the game animal whose occurrence in the nearby Lake District is described in the early 7th century Cumbric text Pais Dinogad, and whose translation to date has been problematic, is a lynx. The occurrence of lynx in early medieval Britain shows that earlier periods of climate change, previously blamed for the species' extinction in Britain, were not responsible. Instead, anthropogenic factors such as severe deforestation, declining deer Populations, and persecution, are likely to have caused the extirpation of lynx in Britain. Consequently, the lynx qualifies as a candidate for reintroduction. Large-scale reafforestation, the growth of deer populations, and more positive attitudes towards carnivores in modern society, could permit the restoration of lynx to Britain, particularly in Scotland. Copyright (c) 2005 John Wiley & Sons, Ltd.

1574: ***+.137***

Predictive models on breeding habitat preferences of Bonelli's eagle (Hieraaetus fasciatus; Aves: Accipitridae) have been performed at four different spatial scales in Castellon province, East of Iberian Peninsula. The scales considered were: (1) nest site scale (1x1 km(2) Universal Transverse Mercator (UTM) square containing the nest); (2) near nest environment (3x3 km(2) UTM square); (3) home range scale (5x5 km(2) UTM square); and (4) landscape level scale (9x9 km(2) UTM square containing the above mentioned ones). Topographic, disturbance, climatic and land use factors were measured on a geographic information system (GIS) at occupied and unoccupied UTM squares. Logistic regression was performed by means of a stepwise addition procedure. We tested whether inclusion of new subset of variables improved the models by increasing the area under the receiver operator characteristic plot. At nest site scale, only topographic factors were considered as the most parsimonious predictors. Probability of species occurrence increases with slope in craggy areas at lower altitudes. At the 3x3 km(2) scale, climate and disturbance variables were included. At home range and landscape level scales, models included climate, disturbance,

topographic and land use factors. Higher temperatures in January, template ones in July, higher rainfall in June, lower altitudes and higher slope in the sample unit increase probability of occurrence of Bonelli's eagle at broadest scales. The species seems to prefer disperse forests, scrubland and agricultural areas. From our results, we consider that there is a hierarchical framework on habitat selection procedure. We suggest that it is necessary to analyse what key factors are affecting Bonelli's eagle nest-site selection at every study area to take steps to ensure appropriate conservation measures. The combination of regression modelling and GIS will become a powerful tool for biodiversity and conservation studies, taking into account that application depends on sampling design and the model assumptions of the statistical methods employed. Finally, predictive models obtained could be used for the efficient monitoring of this scarce species, to predict range expansions or identify suitable locations for reintroductions, and also to design protected areas and to help on wildlife management.

1575: *+.118*

Population viability and metapopulation theory and models are heuristic tools that can be used to plan restorations and assess their success. Using examples from South Florida, USA, we review background information and ongoing reintroduction experiments with the federally endangered coastal perennial vine, beach jacquemontia, Jacquemontia reclinata (Convolvulaceae). All known wild populations are declining in isolated habitat fragments varying in size, occupied area, and degree of isolation. Eleven reintroduction sites ranging in size from 422 to 4800 m(2) within the extant species' range have been identified that have characteristics suitable for J. reclinata introductions and have land managers amenable to restoration efforts. Previous RAPD analysis indicated that genetic diversity of natural populations was positively correlated with population size; the two largest populations had the highest genetic diversity and the smallest populations had relatively low genetic diversity. Despite habitat fragmentation and large distances between some populations, migration rates were very high among populations (m = 4.05). Experimental crosses indicated the species has a mixed mating system. From 2001 to 2005, we have introduced 935 1 reclinata in seven experiments in five locations using plants propagated ex situ at Fairchild Tropical Botanic Garden. Reintroductions have dramatically increased the number of plants in the wild by 72%. Survival from the time of transplant to 2005 ranged from 2% to 97%, was not significantly correlated with metapopulation parameters, such as, founding population size, patch size, or connectivity to extant populations. Reintroduced plants are contributing seed and pollen to the wild populations, but no recruited seedlings have yet been observed. Although it may take decades before we can consider the reintroduced populations to be self-sustainable, we argue that planning restorations for rare species based on predictions from ecological theory is advisable to allow a higher probability of success. (c) 2006 Elsevier GmbH. All rights reserved.

1576: *-.101*

Animal management is the keystone of any modern programme for the prevention and control of rabies. Historically, "animal control" for local elimination of disease was largely equated with population reduction. However, with relatively few exceptions, culling alone has not led to effective control of rabies. In most documented examples of effective control of rabies in the 20th century; an integrated management approach was used that included public education, responsible stewardship of animal populations, manipulation of the population carrying capacity of the local habitat, and vaccination strategies.Globally, the greatest burden on human health that is attributable to this zoonosis is caused by uncontrolled rabies in dogs. Where political willingness, biomedical infrastructure, and economic stability permit the sustained use of control measures (e.g. stray animal removal and mandatory parenteral vaccination), canine rabies has been

significantly suppressed and even eliminated over large geographical areas. Examples include many island nations, most of North America, Europe, and increasingly in South America. Despite the effectiveness of such proven control techniques, however, their implementation in parts of Asia, Africa, and elsewhere has been limited, primarily because of a lack of dedicated resources and intersectoral cooperation, and also because of the burden of high-density populations of dogs. Implementation is often complicated by cultural and social factors, e.g. reluctance to cull apparently ownerless, nuisance animals that are suspected to have been exposed to rabies, partly on the basis of religious beliefs). Attempts to modify animal fertility (such as the encouragement of voluntary spay - neuter programmes or individual chemical contraception, and the extension of such actions to animals in the community) may provide ancillary support in line with other traditional methods of control of canine rabies. With the identification of complex situations in which wildlife rabies persists despite the elimination of canine rabies, e.g. in North America and Europe, cats can pose a significant public health risk requiring consideration of alternative approaches. In any model system, the threat of translocation of infected animals, unintentional or otherwise, provides a strong rationale for the creation of barriers to prevent reintroduction or exacerbation of the disease, and the maintenance of a minimum body of expertise related to surveillance, diagnosis, and the enactment of mitigating measures. While control activities have traditionally focused upon certain Carnivora species, bats represent another worldwide rabies reservoir. Indiscriminate killing of bats and destruction of roosts was once the norm, but such activities are not sanctioned by reputable organizations today. Even vampire bats, responsible for substantial effects on health and agricultural losses in the New World (Mexico to Argentina), should be targeted only by specific control applications, rather than by more widespread, unconventional, non-specific methodology. Bats should be excluded from human living quarters. Implementing measures to prevent bats from gaining access to homes should occur at an appropriate time when the bats are absent, especially to avoid sealing the non-flying young within a building. Although great progress has been made during the past four decades in the induction of herd immunity among free-ranging carnivores via oral vaccination against rabies, similar novel solutions have not been readily applied to bat populations. Given these challenges, new paradigm shifts are eagerly anticipated as additional biotechnological applications (including contraceptives and anticoagulants) are developed to deal with domestic animals and wildlife.

1577: ***+.071***

A review of mammal species and subspecies, which had enriched mammal fauna of Ukraine as a result of artificial introductions and natural invasions during few last centuries is presented. Totally, the list of fauna was supplemented by 36 species and subspecies mainly due to 3 groups: ungulates, rodents, and carnivores. A complete naturalization with formation of stable populations was established for just few species (raccoon dog, muskrat, American mink, etc.) and subspecies ("teleutka" form of red squirrel, ussurian form of wild pig, etc.). A success in forming of stable populations of introduced species depended of mastering of natural ecosystems by them. On the contrary, a success of invasive species occurs in urban systems (house mouse, Norway rat, Kuhl's pipistrelle, etc). Ecological effects of adventive fauna are analysed.

1578: ***+.085***

As one of the researchers involved in the project referenced in Ariana Rickard's article, Assessment of translocations of Blanchard's cricket frogs (Acris crepitans blanchardi) in southeast Michigan (this issue), I have a different interpretation of the results and accomplishments of the emergency translocation of cricket frogs from the Lakewood Farm construction site. Rickard's interpretation of this program as a failure may eventually prove to be accurate, but is, at best,

premature. Long-term monitoring is required before an accurate assessment of the success or failure of an established, self-sustaining population at the translocation sites can be completed. Though I agree with many basic principles presented in Rickard's article, translocation should not be eliminated as a possible conservation tool for this and other species until long term and more complete monitoring data are available. The inevitable destruction of habitat in this case required the translocation of parts of the endangered local population of Blanchard's cricket frogs to occur sooner than expected. Since a full feasibility study was not possible given the immediace of the situation, decisions were based on the best life history data available. Even if the population translocations that were attempted were complete failures (which remains to be determined), these failures will provide valuable information on which to base future plans for translocation when extensive planning can be done with more adequate time.

1579: ***+.110***

An entire population of Blanchard's cricket frogs (Acris crepitans blanchardi) threatened by construction, was translocated to three restored wetlands within the historic range of the species in the summer of 2004 and 2005. Working with the National Amphibian Conservation Center at the Detroit Zoo, state natural agencies and the developer, we moved about 1060 Blanchard's cricket frogs, a Michigan Species of Special Concern, from Lakewood Farms before housing construction began. This case study represents the first effort to track the effectiveness of translocations as a method of conserving cricket frogs in Michigan. I collected data on population structure and breeding success of the translocated populations of cricket frogs. I monitored nearby wild populations of cricket frogs to get baseline comparison data for the translocated frogs. Data on population size and breeding success were used to develop recommendations on amphibian translocation. Although initial breeding attempts were observed at all three release sites, and over 240 juvenile cricket frogs were seen at one of the translocation sites in August 2005, all translocated populations had declined rapidly by October 2005. The translocations apparently did not result in viable, self-sustaining populations of Blanchard's cricket frogs at any of the release sites. I recommend that no more cricket frog translocations occur until the causes of the decline is known and effectively removed at release sites. Conservation dollars and research should focus on habitat preservation and determining the causes of declining numbers of cricket frogs in the Midwest.

1580: ***-.009***

In a time of accelerated extinction rates and biodiversity loss, it may seem illogical to be concerned with methods limiting the reproduction of wild populations; however, there is an urgent need to inhibit the proliferation of a wide variety of species. The range of animals for which fertility control is desired makes the development of a single method impossible. The various reproductive strategies used by individual species, the desired outcome of contraceptive programs (reversible or irreversible, male or female directed) and our ignorance of the reproductive biology of many endangered species necessitate thorough species-specific investigations. As fertility control in humans is a reality and research into methods of fertility control is more advanced, humans serve as a model for developing contraceptive approaches for wild species. Population control by traditional methods (indirect or direct intervention by culling, poisoning, translocation, etc.) is increasingly unacceptable to the public, making human studies even more valuable for finding solutions to overabundant wild populations. This review compares and contrasts the range of contraceptive methods used in both wildlife and humans. (C) 2006 Elsevier Inc. All rights reserved.

1582: ***+.196***

Restoring connectivity is viewed as an important recovery option for fish species adversely affected by river fragmentation. This simulation study quantified the genetic and demographic effects of translocation on metapopulations of white sturgeon (Acipenser transmontanus) inhabiting a series of long (source) and short (sink) river segments. Genetic effects were predictable; upstream translocations increased introgression and downstream translocations had no effect. Demographic results suggest that indiscriminant efforts to reconnect populations may do more harm than good. Simulated river systems with high interspersion of long and short segments and a long segment far upstream tended to benefit most from translocation, but only when narrow screening or downstream passage was also provided below the river segment receiving fish. When combined with narrow screening, upstream translocation to a long segment subsidizing several downstream short segments produced the best results. Downstream passage outperformed narrow screening only when the translocation recipient was a short segment in a river system with low interspersion and no long, upstream river segment. This model-based evaluation of reconnection options has helped to refine ideas about restoring populations in fragmented rivers by predicting which options benefit riverine metapopulations as a whole.

1583: ***+.112***

Any initiative aimed at the management of a threatened species needs a good knowledge of its environmental requirements. Aims of this study were to individuate suitable areas for the reintroduction of crayfish species belonging to the Austropotamobius pallipes complex and to evaluate the causes of the local extinction in Tuscany (Italy) of some populations. Between May 2003 and September 2004, we collected ecological data from 19 streams of 4 catchments, 9 watercourses where crayfish were present (WI) and 10 where they were present in the recent past and absent now (WO), and we compared them. Multivariate analyses were performed using chemico-physical and biotic parameters to examine the relationships between their values and the presence of crayfish. The results did not allow us to find significant differences between the two categories of streams, suggesting their suitability for crayfish reintroduction.

1584: ***+.192***

Marmosets (Callithrix spp.) have been introduced widely in areas within Rio de Janeiro state assigned for the reintroduction of the endangered golden lion tamarin (Leontopithecus rosalia). The objetives of this study were to estimate the marmoset (CM) population in two fragments with reintroduced golden lion tamarin to quantify the association and characterize the interactions between species. The CM population density (0,09 ind/ha) was higher than that of the golden lion tamarin (0,06 ind/za). The mean association index between tamarins and marmosets varied among groups and seasons (winter=62% and summer=35%). During the winter, competition resulted in increases in territorial and foraging behavior when associated with marmosets. Evidence of benefits during the summer was reduced adult vigilance while associated to marmosets. Golden lion tamarins were also observed feeding on gums obtained from tree gouges made by the marmosets. Marmosets represented a threat for the conservation of golden lion tamarins.

1585: ***+.298***

We used a spatially explicit population model of wolves (Canis lupus) to propose a framework for defining rangewide recovery priorities and finer-scale strategies for regional reintroductions. The model predicts that Yellowstone and central Idaho, where wolves have recently been successfully

reintroduced, hold the most secure core areas for wolves in the western United States, implying that future reintroductions will face greater challenges. However, these currently occupied sites, along with dispersal or reintroduction to several unoccupied but suitable core areas, could facilitate recovery Of wolves to 49% of the area in the western United States that holds sufficient prey to support wolves. That percentage of the range with recovery potential could drop to 23% over the next few decades owing to landscape change, or increase to 66% owing to habitat restoration efforts such as the removal of some roads on public lands. Comprehensive habitat and viability assessments such as those presented here, by more rigorously defining the Endangered Species Act's concept of "significant portion of range," can clarify debate over goals for recovery of large carnivores that may conflict with human land uses.

1586: ***-.011***

The wattled crane (Grus carunculatus), a species highly dependent on wetlands, is the largest and rarest of the six African crane species. The once vast range of the wattled crane now consists of only three disjunct populations. The South African population has shown dramatic declines and supplementation of this population using eggs from south-central Africa has been proposed. The objectives of this study were to compare levels of genetic variation in South African and south-central African populations to determine if such supplementation is needed, and if so, whether the south-central African populations represent a genetically similar source for supplementation. We surveyed genetic variation in samples from South Africa, Zimbabwe, and Botswana using 12 microsatellite DNA loci and a 400-bp fragment of the mitochondrial D-loop. Samples from Zimbabwe and Botswana were deemed genetically similar and pooled to increase sample size. Subsequent analyses indicate that the pooled south-central and South African populations show differentiation in microsatellite DNA genotypes, as well as mitochondrial DNA. As the results from both genetic markers indicate genetic isolation, these populations should be managed as separate entities. As no indication was seen from either microsatellite or mtDNA data that significant loss of genetic diversity has occurred within South African wattled cranes, supplementation from outside populations may not be necessary at this time. (c) 2005 Elsevier Ltd. All rights reserved.

1587: ***+.159***

Knowledge of factors affecting the survival of individuals and their reproductive success is essential for threatened species management, but studies assessing these factors are lacking for many threatened rock-wallaby species. In this study we investigated the factors influencing the breeding performance of females and the survival of pouch young in a wild colony of the threatened brush-tailed rock-wallaby. Individuals were trapped between October 2000 and April 2004. More than 50% of the females in the colony were breeding below their full potential and giving birth to only one offspring per year. Most females within the colony bred in synchrony, with a substantial birth peak evident during autumn. Pouch young born in autumn left the pouch during spring and were weaned during summer and autumn when forage was most abundant. Pouch young born during the autumn birth peak or in winter had a substantially higher probability of surviving through to pouch emergence than those born during spring or summer. This study provides demographic parameters that may be used in population models and for comparison with other populations, particularly those that are small and declining. To optimise reproductive success in reintroduction programs, females in good condition and with small pouch young should be released at the end of the wettest season.

1588: ***+.193***

We evaluated the utility of a focussed, short-duration research program for investigation of two rare species that occur within an urban bushland remnant, with application for improving conservation management and translocation outcomes. Conospermum undulatum Lindl. is listed as Threatened and Macarthuria keigheryi Lepschi is listed as Vulnerable under the (Australian) Federal Environment Protection and Biodiversity Conservation Act 1999. The C. undulatum population lacked evidence of juvenile plants, indicating either lack of a suitable germination cue or failure of seedling establishment. The M. keigheryi population was browsed heavily by feral rabbits where exposed and thrived when sheltered within unpalatable vegetation from rabbits. Seeds of M. keigheryi were rapidly removed in an ant cafeteria-style experiment, although seeds of the related Macarthuria australis and an 'outgroup' species, Gompholobium tomentosum, were removed at equally high rates. Seed viability was high in M. keigheryi, but variable in C. undulatum. Germination rates, in untreated fresh seed, were 11% for C. undulatum and 0% for M. keigheryi and increased to 27 and 3%, respectively, when treated with gibberellic acid. Maximum strike rates for cuttings of 33 and similar to 75% were obtained for C. undulatum and M. keigheryi, respectively. Cutting-grown plants of M. keigheryi flowered and seeded profusely in the nursery within 7 months, providing a highly effective seed-orchard resource for research and conservation seed banking. Genetic fingerprinting (AFLP) indicated that the C. undulatum population at Perth Airport was not genetically distinct from nearby C. undulatum populations, despite its relatively high variability in leaf morphology. All C. undulatum populations contained moderate to high levels of genetic variation, with the percentage of AFLP markers polymorphic ranging from 48.6 to 64.9%, and heterozygosity from 0.167 to 0.202. Overall, the knowledge gained from this program of short duration will enable informed management and will underpin successful population enhancement through future translocations.

1589: ***+.131***

Prior to the late 1970's, the northern leopard frog (Rana pipiens) was a common and widely distributed amphibian throughout central and southern Alberta. The northern leopard frog has vanished from much of its former range in the province. The species was designated as Threatened under Alberta's Wildlife Act in 1996. Because the small number of remaining populations of leopard frogs are separated by large areas of unsuitable habitat, their re-establishment into previously occupied habitat may rely on transplanting individuals or egg masses from existing major breeding populations in southern Alberta. A reintroduction project was initiated in Magrath in spring 2002 with the primary objective of re-establishing a breeding population of northern leopard frogs in its formerly occupied native habitat in the area. After water quality testing and dissolved oxygen testing of potential receiving ponds, egg masses were collected from three different source populations in 2003, 2004 and 2005. The egg masses were transferred to the Pothole Creek area in Magrath, and reared in floating predator exclosures. Once the eggs had hatched and developed into mobile tadpoles they were released directly into suitable habitat. A total of 8502 tadpoles were released over the three years, in a pond known as Dudley's Pond, which had also served as the primary rearing site. Dispersal of frogs into surrounding habitat was observed and documented. Preliminary success of the reintroduction project was achieved in spring 2004 when yearling northern leopard frogs were observed after over-wintering successfully in Dudley's Pond. Another level of success was reached in spring 2005 with an additional year of successful over-wintering of both yearling and young-of-the-year frogs, and with breeding behaviour being observed. Dispersal of the frogs has also resulted in establishment of northern leopard frogs for several kilometres upstream and up to 10 kilometres downstream in the Pothole Creek drainage. The Magrath Northern Leopard Frog Reintroduction Project was established as a pilot project to test the effectiveness of local transplants using on-site rearing. It has successfully demonstrated that the technique is feasible, and provides a template for future northern leopard

frog reintroduction projects.

1590: *+.136*

The translocation of the marbled African lungfish Protopterus aethiopicus into Lake Baringo created a new fishery for the local community, who capture them primarily in a bottom-set long line fishery. Its introduction, development and current fishery status in the lake are documented. Annual catch data were obtained from the District Fisheries Office, while catch and effort data of the long line fishery were recorded at one active fish-landing site between February and October 2001. Lungfish comprise a significant component of commercial landings, sometimes exceeding catches of the Baringo tilapia Oreochromis niloticus baringoensis as the most landed species by weight. Daily catch rates and effort varied considerably, ranging from 5.51-15.18kg day(-1) and from 50-590 baited hooks day(-1), respectively. Daily catch per fisher ranged from 0-35.72kg, while the overall mean catch per unit effort (CPUE) was only 0.003kg per hook-hour. This study provides the only baseline CPUE data for a lungfish long line fishery that the authors are aware of, for comparison with future studies in this or other lakes where lungfish are similarly exploited. Recommendations for improved management and the maintenance of a viable lungfish long line fishery in Lake Baringo are presented.

1591: *-.036*

In forest restoration areas in northern Ethiopia, natural regeneration of Olea europaea ssp. cuspidata only occurs under pioneer shrubs. To assess the impact of erosion on the spatial distribution of Olea recruits, secondary dispersal of olive seeds by surface runoff was determined in and near the micro-environment of two important pioneers (Acacia etbaica and Euclea racemosa). We hypothesized that (1) Olea seeds deposited under the protective crowns of shrubs are less likely to be lost by surface wash than seeds in the bare inter-plant areas and (2) that seeds in the latter are more prone to be translocated by runoff to microsites under shrubs than to open areas. Runoff experiments were conducted in August- September 2003 in ten 3 x 3 m(2) plots laid out around isolated shrubs in two sites. Twenty-eight marked tracer seeds were dropped at the upper end of each plot at 0.10 in intervals. Ten seeds marked in another way were deposited under the crown in each plot simulating seed dispersal by birds. The seeds were collected after the rainy season and their positions recorded. In a third experiment, mulch under shrubs was removed and ten marked seeds were deposited under each crown. Rainfall simulation experiments were conducted with a maximum intensity of 120 mm h(-1) for a 10 min duration. For all experiments, seed movement was analyzed in relation to slope and shrub variables. Seed movement was limited in one site, where only 21% of the seeds were translocated. Movement was significantly higher in the other site (61%) although it had a lower slope gradient. The number of seeds moved by surface runoff was not significantly related to shrub species, slope gradient or slope roughness. There was a strong linear relationship between the distance parallel to the contour from the shrub center to the seed and the extent of the downhill translocation. No seeds moved to a position under a shrub crown and no shrub lost a seed deposited under its crown. Simulated rainfall after mulch removal resulted in a seed loss of nearly 33%.Micro-topographic structures under shrubs, especially under E. racemosa, cause some soil and sediment retention, but more importantly divert runoff water and concentrate flows to rills alongside shrubs. From an eco-geomorphological point of view, shrubs show poor seed trapping efficiency contradictory to expectation. Seeds in sediment flows are deposited in the bare inter-plant areas. However, seeds under shrubs are not likely to be lost by surface wash. Surface runoff has little effect on the general pattern of the primary seed rain. Since Olea recruits were exclusively found under shrubs and not in the spaces between shrubs in the studied field sites, it appears plausible that seeds deposited in the open field are almost without

doubt lost to post-dispersal seed predation, unsuccessful germination or seedling predation and mortality. Recovery of O. europaea in degraded shrubland communities therefore depends upon directed dispersal of seeds under shrubs by frugivorous birds. (C) 2005 Elsevier B.V. All rights reserved.

1592: ***+.114***

Whether communities respond smoothly or discontinuously to changing environemental conditions has important consequences for the preservation and restoration of ecosystems. Theory shows that communities may exhibit a variety of responses to environmental change, including abrupt transitions due to the existence of alternate states. However, there have been few opportunities to look for such transitions in nature. Here, we examine the impact of a two-orders-of-magnitude decrease and then increase in planktivore abundance in Wintergreen lake (Michigan, USA), caused by the extinction and reintroduction of two dominant fish species (largemouth bass, Micropterus salmoides, and bluegill, Lepomis macrochirus). Over a 16+ yr period of slow change from high planktivory to low planktivory back to high planktivory, the zooplankton community changed smoothly and predictably between states. In years of low planktivory, the zooplankton assemblage was dominated by a single, large, cladoceran species, Daphnia pulicaria, whereas in years of high planktivory, D. pulicaria disappeared and was replaced by a suite of small-bodied cladocerans. We quantified the multivariate change in zooplankton community dissimilarity and found that community state smoothly tracked changes in planktivore density in both a forward and backward direction. Thus, there was little evidence of discontinuity in this system where transitions are strongly driven by planktivory.

1593: ***+.040***

Low genetic variation is often considered to contribute to the extinction of species when they reach small population sizes. In this study we examined the mitochondrial control region from museum specimens of the Heath Hen (Tympanuchus cupido cupido), which went extinct in 1932. Today, the closest living relatives of the Heath Hen, the Greater (T. c. pinnatus), Attwater's (T. c. attwateri) and Lesser (T. pallidicinctus) Prairie-chicken, are declining throughout most of their range in Midwestern North America, and loss of genetic variation is a likely contributor to their decline. Here we show that 30 years prior to their extinction, Heath Hens had low levels of mitochondrial genetic variation when compared with contemporary populations of prairie-chickens. Furthermore, some current populations of Greater Prairie-chickens are isolated and losing genetic variation due to drift. We estimate that these populations will reach the low levels of genetic variation found in Heath Hens within the next 40 years. Genetic variation and fitness can be restored with translocation of individuals from other populations; however, we also show that choosing an appropriate source population for translocation can be difficult without knowledge of historic population bottlenecks and their effect on genetic structure.

1594: ***+.153***

To study the relative importance of inbreeding depression and the loss of adaptive diversity in determining the extinction risk of small populations, we carried out an experiment in which we crossed and self-fertilized founder plants from a single, large population of shore campion (Silene littorea Brot). We used the seeds these plants produced to colonize 18 new locations within the distribution area of the species. The reintroduced populations were of three kinds: inbred and genetically homogeneous, each made up of selfed seed from a single plant; inbred and mixed, made up of a mixture of selfed seeds from all founder plants; and outbred and mixed, made up of a

mixture of seeds obtained in outcrosses between the founders. We compared the inbred homogeneous populations with the inbred mixed to measure the effect of genetic diversity among individuals and the inbred mixed with the outbred mixed to measure the effect of inbreeding. Reintroduction success was seriously limited by inbreeding, whereas it was not affected by genetic diversity. This observation and the nonsignificant interaction between family and reintroduction location for individual plant characters suggest that the fixation of overall deleterious genes causing inbreeding depression posed a snore serious threat to the short-term survival of the populations than the loss of genes involved in genotype and environment interactions. Thus, reintroduction success was related to adaptive diversity. Preventing such fixation might be the most important consideration in the genetic management and conservation of shore campion populations.

1595: *+.313*

Because of widespread habitat fragmentation, maintenance of landscape connectivity has become a major focus of conservation planning, but empirical tests of animal movement in fragmented landscapes remain scarce. Wee conducted a translocation experiment to test the relative permeability of three landscape elements (open habitat, shrubby secondary vegetation, and wooded corridors) to movement by the Chucao Tapaculo (Scelorchilus rubecula), a forest understory bird endemic to South American temperate rainforest. Forty-one radio-tagged subjects were translocated (individually) to three landscape treatments consisting of small release patches that were either entirely surrounded by open habitat (pasture), entirely surrounded by dense shrubs, or linked to other patches by wooded corridors that were otherwise surrounded by open matrix. The number of days subjects remained in releasepatches before dispersal (a measure of habitat resistance) was significantly longer for patches surrounded by open habitat than for patches adjoining corridors or surrounded by dense shrubs. These results indicate that open habitat significantly constrains Chucao dispersal, in accord with expectation, but dispersal occurs equally well through wooded corridors and shrub-dominated matrix. Thus, corridor protection or restoration and management of vegetation in the matrix (to encourage animal movement) may be equally feasible alternatives for maintaining connectivity.

1596: *+.074*

Pen and field trials were used to test the effectiveness and cost-efficiency of wire netting and electric fence designs as barriers to feral cats, foxes and rabbits in northern South Australia. A 180 cm high wire netting fence with foot apron and a curved 'floppy' overhang effectively contained most rabbits, feral cats and foxes during pen trials and proved effective with intensively monitored paddock-scale exclosures. A reduced height fence of 115 cm did not reduce effectiveness of the fence during fence trials but paddock-scale trials are yet to be completed. Conventional 40 mm diameter hexagonal "rabbit netting" was not an effective barrier against young independent rabbits and it is recommended that 30 mm hexagonal netting should be used. A 60 cm wide external netting overhang, curved in an arc and supported by lengths of heavy gauge wire, effectively precluded more feral cats and foxes than a 30 cm wide overhang angled upwards. The 30 cm foot apron was augmented in erosion-prone dunes and watercourses by the addition of wider netting or rubber matting to prevent incursions. Posts, and particularly corners, were targeted by feral cats and foxes and the efficacy of the fence was improved by using steel, rather than timber posts. Electric wires offset from the netting at heights of 120 and 150 cm provided a shock to animals exploring the base of the overhang and further improved the fence efficacy. PVC conduit rollers on the top wire were not effective. Material costs ranged from AUD $8814 per km for the 115 cm high fence to AUD $12,432 per km for the 180 cm high fence with two electric wires. The non-

standard 30 mm hexagonal netting accounted for 57% of the material costs of the low netting fence. Increased demand for this netting may reduce the expense of rabbit exclusion. Expenses could also be reduced where existing stock fences are modified by the addition of netting. (c) 2005 Elsevier Ltd. All rights reserved.

1597: *+.183*

ISSR markers were used to analyze the genetic diversity and genetic structure of eight natural populations of Cupressus chengiana in China. ISSR analysis using 10 primers was carried out on 92 different samples. At the species level, 136 polymorphic loci were detected. The percentage of polymorphic bands (PPB) was 99%. Genetic diversity (He) was 0.3120, effective number of alleles (Ae) was 1.5236, and Shannon's information index (I) was 0.4740. At the population level, PPB = 48%, Ae = 1.2774, He = 0.1631, and I = 0.2452. Genetic differentiation (Gst) detected by Nei's genetic diversity analysis suggested 48% occurred among populations. The partitioning of molecular variance by AMOVA analysis indicated significant genetic differentiation within populations (54%) and among populations (46%; $P < 0.0003$). The average number of individuals exchanged between populations per generation (Nm) was 0.5436. Samples from the same population clustered in the same population-specific cluster, and two groups of Sichuan and Gansu populations were distinguishable. A significantly positive correlation between genetic and geographic distance was detected ($r = 0.6701$). Human impacts were considered one of the main factors to cause the rarity of C. chengiana, and conservation strategies are suggested based on the genetic characters and field investigation, e.g., protection of wild populations, reestablishment of germplasm bank, and reintroduction of more genetic diversity.

1598: *+.179*

ISSR markers were used to analyze the genetic diversity and genetic structure of eight natural populations of Cupressus chengiana in China. ISSR analysis using 10 primers was carried out on 92 different samples. At the species level, 136 polymorphic loci were detected. The percentage of polymorphic bands (PPB) was 99%. Genetic diversity (H (e)) was 0.3120, effective number of alleles (A (e)) was 1.5236, and Shannon's information index (I) was 0.4740. At the population level, PPB = 48%, A (e)=1.2774, H (e)=0.1631, and I=0.2452. Genetic differentiation (G (st)) detected by Nei's genetic diversity analysis suggested 48% occurred among populations. The partitioning of molecular variance by AMOVA analysis indicated significant genetic differentiation within populations (54%) and among populations (46%; $P < 0.0003$). The average number of individuals exchanged between populations per generation (N (m)) was 0.5436. Samples from the same population clustered in the same population-specific cluster, and two groups of Sichuan and Gansu populations were distinguishable. A significantly positive correlation between genetic and geographic distance was detected ($r=0.6701$). Human impacts were considered one of the main factors to cause the rarity of C. chengiana, and conservation strategies are suggested based on the genetic characters and field investigation, e.g., protection of wild populations, reestablishment of germplasm bank, and reintroduction of more genetic diversity.

1599: *+.174*

Censusing wild populations and detecting trends in population size over time is an important task in the conservation and management of wildlife. We compared two methods used to monitor numbers of chamois Rupicapra rupicapra and R. pyrenaica in two contrasting populations, and explored the relationship between the sampling effort and the repeatability of the results using resampiing methods. One population in the Alps had been stable at a high density for several

years, whereas the other population, located in the Pyrenees, was increasing exponentially, following a reintroduction. In both sites, a long-term monitoring programme based on individually marked chamois, allowed us to estimate population size using capture-mark-recapture methods (CMR). In addition, we calculated an index of population size as the mean number of animals observed on a foot transect surveyed repeatedly. We then compared whether trends estimated by each method were consistent. In the increasing population, both the index and the CMR estimates revealed an exponential increase in population size. In the stable population, neither the index nor the CMR estimate revealed any trend in size. Consistent results between the index and the CMR suggest that the index could be used to monitor trends in population size. Resampling techniques, however, pointed out that the index is only reliable when calculated over a sufficient number of surveys per year (10 in the Pyrenees, three in the Alps) and over a sufficient number of years of monitoring (about five years).

1600: *+.276*

Once an abundant and conspicuous presence in wetlands across much of north-central North America, Trumpeter Swan (Cygnus buccinator) poplulations were decimated in the mid- to late 1800s by a combination of market hunting, subsistence hunting, and habitat loss. Since then, restoration has focused primarily oil reintroduction efforts in which captive-reared birds are released and then monitored. From 1991 to 1993, 44 birds were released into Seney National Wildlife Refuge (Schoolcraft County, Michigan) in a multi-agency attempt to enhance the breeding population of this species in the Upper Great Lakes region. To provide information useful to swan restoration efforts elsewhere, this paper summarizes 14 years of Trumpeter Swan occupancy and productivity at Seney. In doing so, we document the first substantial inter-annual decline in swans on the Refuge and provide evidence that suggests birds may now be dispersing onto other lakes and wetlands in the eastern Upper Peninsula of Michigan. We also present information from which we infer processes regulating swan numbers and rates of productivity and discuss both the continued need for monitoring and the need for research to examine the effects swans might have on other components of aquatic ecosystems at the Refuge.

1601: *+.201*

Human activities have been diminishing greater rhea Rhea americana (Linnaeus, 1758) populations throughout their natural distribution. The reintroductions of captive-born greater rheas have been tried but without success; since the individuals reintroduced were killed by predators. Captive-born animals that have been isolated from predators for many generations can lose their predator recognition abilities. To enhance the survival rates of the reintroduced animals, researchers are now using antipredator training techniques. We studied the response of 15 zoo-borne greater rheas to antipredator training. The animals were divided into three groups: two test groups and one control group. We ran IS antipredator tests and four control testes with each group. Antipredator tests consisted of pairing a taxidermized predator model or a real predator (domestic dog) with a simulation of a capture procedure. Control tests consisted of presenting the predator model (jaguar) to the birds, after training but not associating it with an aversive event and recording behavioural responses. All tests were video-recorded and analysed a posteriori. Results showed that the trained rheas responded appropriately to the predators, becoming more vigilant and that there was considerable individual differences in response to antipredator training. The results demonstrated that antipredator training is effective and therefore an invaluable tool for reintroduction projects involving greater rheas. Furthermore, the methods employed in this research project should be applicable to other species of flightless birds.

1602: ***+.183***

In 1993, experiments on the restoration of calcareous grasslands on ex-arable fields were started in order to provide new habitats for species of a small nature reserve with ancient grasslands north of Munich (Germany). The effects of diaspore transfer by the application of seed-containing hay on vegetation establishment were studied on restoration fields with and without topsoil removal for 5 years. The aim of the study was to assess plant diversity for the evaluation of restoration success by different methods including determination of species with viable seeds in the hay by germination tests, phenological investigations on hay-transfer source sites at the time of harvest, and vegetation analyses on the restoration sites. Total seed content of the hay and the number and composition of plant species with viable seeds were affected by the time of harvesting and differed between a site which had been used as arable field until 1959 and ancient grassland sites. Nevertheless, the number of established hay-transfer species showed only few differences between restoration fields. The proportion of species transferred to restoration fields in relation to the number of species with viable seeds in the hay was between 69 and 89%. Five years after the hay transfer, the proportion of the established species was still between 58 and 76%. Up to now, topsoil removal had no significant effect on the number of established hay-transfer species. After triple hay application the absolute number of transferred grassland species was higher than on sites with single hay application, but restoration efficiency was lower because many of the species with viable seeds in the hay did not establish. In general, our results showed that the transfer of autochthonous hay is a successful method to overcome dispersal limitation in restoration projects.

1603: ***+.134***

Despite recent efforts to reforest cleared rainforest landscapes, in Australia and elsewhere, the value of reforested sites for rainforest-dependent reptiles is unknown. We surveyed the occurrence of reptiles in a range of reforestation types (monoculture and mixed-species timber plantations, diverse "ecological restoration" plantings and regrowth), as well as reference sites in pasture and rainforest, in tropical and subtropical Australia. We recorded 29 species of reptiles from 104 sites, including 15 rainforest-dependent species. Most rainforest reptiles were strongly associated with complex microhabitats (tree trunks, logs, rocks). The richness and abundance of rainforest-dependent reptiles varied between the different types of reforestation and between regions. In the tropics, rainforest reptiles were recorded in old timber plantations and ecological restoration plantings but not in young timber plantations or regrowth. Rainforest reptiles were recorded in few reforested sites in the subtropics. The occurrence of rainforest-dependent reptiles in reforested sites appears to be influenced by (1) habitat structure; (2) proximity to source populations in rainforest; and (3) biogeography and historical differences in the extent of rainforest. Restoration of cleared land for rainforest-dependent reptiles may require the development, or deliberate creation, of complex structural attributes and microhabitats in reforested sites. Where reforested sites are located away from rainforest, recolonization by rainforest reptiles may require the construction of corridors of suitable habitat between reforested sites and rainforest or the translocation of reptiles to reforested sites.

1604: ***+.083***

Despite their individual importance for population dynamics and conservation biology, the combined impacts of Allee effects and parasitism have received little attention. We built a mathematical model to compare the dynamics of populations with or without Allee effects when infected by microparasites. We show that the influence of an Allee effect takes the form of a tradeoff. The presence of an Allee effect in host populations may protect them, by reducing the

range of population sizes that allow parasite spread. Yet if infection spreads, the Allee effect weakens host populations by reducing their size and by widening the range of parasite species that lead them to extinction. These results have important implications for predicting the survival of threatened populations or the success of reintroductions, and may help define size ranges within which given populations should be maintained to prevent both epidemics and Allee effects driven extinctions.

1605: *+.157*

Although golden paintbrush historically inhabited the prairies of the Willamette Valley, Oregon, this Pacific Northwest prairie endemic is currently restricted to eleven sites in the Puget Trough of Washington and British Columbia. Recovery criteria call for the establishment of new populations throughout the species' historic range, including the Willamette Valley. We described vegetation and soil characteristics of representative golden paintbrush recovery sites in the Willamette Valley and compared them with those of remaining golden paintbrush populations in the Puget Trough. Potential golden paintbrush habitat in the Willamette Valley was ecologically distant from remaining populations. This disparity was likely related to regional differences in geology, climate, ocean proximity, and land-use history. Many of the species indicative of remaining populations in the Puget Trough were native perennials, while those of potential reintroduction sites in the Willamette Valley were introduced annuals. Soil characteristics of golden paintbrush sites were also distinct among the two ecoregions. Puget Trough sites were located on sandy soils with generally high levels of magnesium and sulfur, while Willamette Valley sites were found on silty-clay soils with high concentrations of potassium and phosphorous. Differences in soil texture, and magnesium and potassium concentrations were associated with plant community divergence among the two regions. We suggest using a plant functional group approach when comparing vegetation assemblages among Puget Trough and Willamette Valley sites, which allows comparison of taxonomically distinct communities that share ecological characteristics.

1607: *+.079*

Historical, anecdotal records of the state-endangered Miami blue butterfly, Cyclargus thomasi bethunebakeri (Comstock & Huntington) (Lepidoptera), have mentioned larval associations with the Florida carpenter ant, Camponotus sp. Recent population studies confirm that C. t. bethunebakeri larvae associate with Camponotus floridanus (Buckley) as well as another member of the genus, Camponotus planatus (Roger). Additionally, caterpillars have been observed tended by Crematogaster ashmeadi (Emery), Forelius pruinosus (Roger), and Tapinoma melanocephalum (Fab.). Field surveys of remaining Miami blue habitat and recent butterfly reintroduction sites reveal other potential ant associates, Paratrechina longicornis (Latreille) and Paratrechina bourbonica (Forel), and a host of possible predaceous ant species. The corresponding conservation implications are discussed. Detailed information is also presented about larval ant-associated organs and their mediation of this facultative symbiosis.

1608: *-.064*

The order of Acipenseriformes (sturgeon and paddlefishes) contains the economic most valuable species in world trade: the producers of black caviar. Mainly because of their high economic value, sturgeon and paddlefishes were and are the goal of many conservation programs worldwide. In this review, I present some of the main conclusions that can be drawn from previous conservation efforts. My review is divided into two parts. The first part deals with species identification methods, which are necessary for international trade control (Convention on

International Trade in Endangered Species of Wild Fauna and Flora). Considering the outcome of all previous forensic studies, I conclude, firstly, it is necessary to use large sample sizes to avoid misinterpretation of possible diagnostic substitutions, and, secondly, the combination of both DNA types (mitochondrial and nuclear markers) is recommended for correct species identification. The second part deals with the influence of restocking/release programs on native populations. The outcome of previous stocking efforts indicates that, in most cases, stocking with nonnative specimens is economically nonsense because most translocation efforts failed. In addition, in cases where such efforts were successful, they influenced negatively the genotypic structure (inbreeding and outbreeding depressions) of native populations. In fact, nonnative stocking does jeopardize adaptation and blurs the genetic differences used to discriminate populations. Furthermore, if it is necessary to release individuals to avoid extinction, released specimens should be as young as possible (homing fidelity).

1609: *+.039*

The closely related, endangered Braya longii and threatened Braya fernaldii (Brassicaceae) are endemic to the Limestone Barrens on the Northern Peninsula of Newfoundland, Canada. These species were not known to co-occur naturally, but anthropogenic disturbance has mediated contact. Future preservation strategies must consider population variability, breeding system and possible hybridization in the context of human use. Breeding system and hybridization were assessed using floral measurements and hand-pollination experiments under field conditions. Morphological analysis revealed the presence of two well-defined groups as well as population-level variation in floral characters. PCA confirmed intraspecific variation in both species, mediated by origin of disturbance. Natural populations of B. longii are differentiated due to very limited seed dispersal, whereas non-differentiated anthropogenically disturbed populations reflect the homogenising effect of aggregate movement among sites. Although both species are primarily autogamous, floral characters indicate B. longii has great potential for outcrossing compared with B. fernaldii. Outcrossed hand-pollinations resulted in significantly lower fruit set for B. fernaldii (< 18%) than for B. longii (64%). Hybridization potential was suggested by the presence of intermediate forms in wild populations as well as fruit set from hand-pollinations between the two species. These results indicate that conservation strategies should focus on preserving populations throughout the entire range of each species in order to capture genetic variation among populations. Human activities that promote the population homogenisation, such as movement of quarried gravels among sites, should be minimized. Reintroductions should be undertaken with caution to prevent possible hybridization. (c) 2005 Elsevier Ltd. All rights reserved.

1610: *+.002*

The pulmonate limpet Siphonaria compressa is South Africa's most endangered marine mollusc. It is endemic to just two localities: Langebaan Lagoon on the west coast, and Knysna Estuary on the south coast, and occurs only on the eelgrass Zostera capensis. In Langebaan Lagoon, eelgrass has fluctuated substantially over the last 34 years, and S. compressa has twice approached extinction. S. compressa is largely confined to the lower edge of the eelgrass beds there, being replaced higher up by another small gastropod, Assiminea globulus. We explored the physical and biological factors underlying the limpet's narrow habitat, using field observations, translocations, caging and transplant experiments. Abundance of S. compressa was positively correlated with Z. capensis cover and negatively correlated with shore height. When moved to the upper portions of the eelgrass bed, S. compressa had lower rates of persistence and survival than in the lowest zone. The lower limit of zonation for S. compressa was set indirectly by bioturbation by the sandprawn Callianassa kraussi, which excluded eelgrass from intertidal sandbanks. Transplants of eel-grass

into the sandbanks proliferated provided C kraussi was experimentally eliminated, and supported densities of S. compressa 20-fold greater than in control eelgrass beds, suggesting that high-shore eelgrass beds to which S. compressa is normally confined are suboptimal for the limpet. A. globulus showed patterns opposite to those of S. compressa: its persistence and survival were greatest in the tipper zone and it actively avoided the lower sections of these beds and never colonised eelgrass transplanted into sandflats lower on the shore. There was no evidence that competition between S. compressa and A. globulus influenced the zonation or abundance of either species. Rarity of S. compressa and its endangered status seem dictated by its extremely narrow and temporally changeable habitat-range, which is defined by physical stress in the high-shore and bioturbation by C kraussi in the low-shore. Fluctuations in eelgrass abundance and limitation of S. compressa to just two localities add substantially to the risks of extinction for this embattled stenotypic limpet. (C) 2006 Elsevier B.V. All rights reserved.

1611: *+.115*

Salmonid fishes represent a model system for addressing a wide range of biogeographic, evolutionary, and conservation questions. Studies on the genetic structure and phylogeographic pattern of Brachymystax lenok Pallas (Salmoninae, Salmonidae) populations are important for addressing the systematics, evolution, and effective conservation of the species. Partial sequences of the mitochondrial control region (835 bp) and cytochrome (cyt) b (1069 bp) were obtained by PCR amplification from 71 B. lenok individuals from 7 populations in the river systems of northern China. Analysis of molecular variance indicated that a high proportion of the total genetic variance was distributed among regions, supporting strong geographic structuring of mtDNA polymorphism. Phylogenetic analysis was conducted using Neighbor-joining (NJ), maximum likelihood (ML), and Bayesian approaches, based on the combined control region and cyt b sequences. Seventeen haplotypes were assigned to 3 clades that were related to geographic regions. No shared haplotypes were found among regions. The pattern of phylogenetic discontinuity, which is associated with spatial separation, is a result of both historical (long-term, zoogeographic barriers to gene flow) and contemporary (limited dispersal and gene flow capabilities) components. Based on our results, together with geological age data, we inferred that after entering the Amur River drainage, lenoks spread southwardly along inland drainages and hypothesize the dispersal route of the species in the water systems of eastern China. We further propose that each of the 3 evolutionarily distinct groups of lenok populations should be protected in order to avoid the loss of biodiversity. It is highly recommended that management efforts focus on riverine conservation, and avoid translocations among populations of different regions.

1612: *-.044*

1. Although captive breeding and reintroduction is a high-profile management tool for many threatened species, it is unclear how long-term captive breeding can influence fitness attributes such as natural defences to predators.2. Induced defences that have evolved in the Mallorcan midwife toad Alytes muletensis in response to introduced predators were compared in natural and reintroduced populations that had a common ancestry, and in short-term and long-term captive populations that differed in ancestry.3. Defences against predators were maintained in a reintroduced population derived from stock that had passed through three to eight generations of captive breeding prior to release into a predator-free area. Heterozygosity did not differ between natural and reintroduced populations, but the reintroduced population displayed lower allelic richness.4. A comparison between populations maintained for different lengths of time in captivity revealed a significant reduction in one defensive trait in stock maintained for more than eight generations. Neutral genetic variation (i.e. heterozygosity and allelic richness) did not differ

between the short-term captive population and a natural population, but there was a significant loss of genetic variation in the long-term captive population.5. Synthesis and applications. The results suggest that relatively high levels of heterozygosity and important fitness attributes can be maintained for a few generations in breeding programmes for threatened species despite small numbers of founders and the absence of natural selection. Nevertheless, both fitness and heterozygosity may eventually start to deteriorate in the long term, and this may have implications for reintroduction strategies.

1613: *+.140*

A stage-structured population model was developed to predict which of nine hypothetical translocation scenarios was likely to produce the best outcome for the rare Hamilton's frog (Leiopelma hamiltoni McCulloch). Model outcome was measured in terms of population growth rate and probability of extinction. Only females were modelled. The model predicted that moving at least 20 female adult frogs was the best strategy, and moving subadult frogs alone, or no frogs at all was the worst in terms of mean growth rate of both populations combined. When the new population was considered separately, introducing subadults alone was the worst strategy in terms of mean growth rate and extinction probability. Extinction of the donor population was most likely when 40 adult females were removed, and the extinction risk was reduced when only 20 were removed. We consider the most reasonable management strategy confirmed by the modelling and supporting qualitative data- is the translocation of 20 adult and 20 subadult female frogs (with the concurrent translocation of 40 males). This scenario provides a balance between risk of extinction in the donor population and probability of success in the translocated population.

1614: *+.116*

The duck-billed platypus is an extraordinary mammal. Its chromosome complement is no less extraordinary, for it includes a system in which ten sex chromosomes form an extensive meiotic chain in males. Such meiotic multiples are unprecedented in vertebrates but occur sporadically in plant and invertebrate species. In this paper, we review the evolution and formation of meiotic multiples in plants and invertebrates to try to gain insights into the origin of the platypus meiotic multiple. We describe the meiotic hurdles that translocated mammalian chromosomes face, which make longer chains disadvantageous in mammals, and we discuss how sex chromosomes and dosage compensation might have affected the evolution of sex-linked meiotic multiples. We conclude that the evolutionary conservation of the chain in monotremes, the structural properties of the translocated chromosomes and the highly accurate segregation at meiosis make the platypus system remarkably different from meiotic multiples in other species. We discuss alternative evolutionary models, which fall broadly into two categories: either the chain is the result of a sequence of translocation events from an ancestral pair of sex chromosomes (Model I) or the entire chain came into being at once by hybridization of two populations with different chromosomal rearrangements sharing monobrachial homology (Model II).

1615: *+.068*

The recent rediscovery of the polecat (Mustela putorius) in Scotland has raised the question of whether the species has been present at low abundance all along or was covertly reintroduced. We assess the exceptionality of the recent sighting. The analysis suggests that the polecat was reintroduced. (c) 2005 Elsevier Ltd. All rights reserved.

1616: *+.130*

Gould's petrel (Pterodroma leucoptera leucoptera) was restricted, essentially, to a single breeding locality - Cabbage Tree Island, Australia. As a safeguard against extinction, an additional breeding colony was established on nearby Boondelbah Island, where artificial nesting habitat was created by installing 100 plastic nest boxes. Over two years, a total of 200 nestling Gould's petrels were translocated from Cabbage Tree island to these boxes. Colonies on both islands were then monitored for a further four years. Selection of nestlings for transfer was based on prior knowledge of growth, plumage development and emergence from the burrow, and aimed to select only birds that were 11-28 days from fledging (DBF) in 1999 and 11-22 DBF in 2000. of the first 100 nestlings translocated to Boondelbah Island (in March 1999), 95 fledged successfully 8-27 days after transfer (mean = 17.3 days). Of the second 100 nestlings translocated (in March 2000) all successfully fledged 9-22 days after transfer (mean = 15.1 days). The removal of young had no discernible effect on the subsequent breeding productivity of the donor pairs. In all, 41 Gould's petrels have been recorded at the new colony on Boondelbah Island, where at least 27 nest boxes have been visited. Ten translocated fledglings (nine male, one female) have returned to the translocation site, taking up nest boxes that were, on average, 5.5 m from the box from which they fledged. An additional 27 non-translocated birds, of unknown origin, have also nested in nest boxes on Boondelbah, along with four birds previously known from Cabbage Tree Island. TWO nestlings transferred to Boondelbah island have returned to Cabbage Tree Island. Within five years of the first translocation, the newly established colony on Boondelbah island has produced a total of 24 eggs and 14 fledglings. The translocation technique developed for Gould's petrel has broad applicability, being readily adaptable for other burrow-nesting seabirds. (c) 2005 Elsevier Ltd. All rights reserved.

1617: *-.095*

Loss of genetic variation in populations may have other effects than inbreeding depression and loss of adaptive potential. In the case of the numerous gynodioecious plant species having cytonuclear systems of sex determination with dominant nuclear restorers of the male function, reduced genetic variation and increased inbreeding would increase the proportion of non-restorer recessive genes found in homozygosity, and therefore that of female plants producing pistillate flowers. This could have opposite effects on the extinction risk of the population. Female plants produce no pollen and may cause pollen limitation, but at the same time they may reduce average homozygosity and inbreeding depression because they are forced outcrossers. We observed that inbreeding indeed increased the frequency of female plants and pistillate flowers in the population, and that this effect was accompanied by reductions in population recruitment independent of inbreeding depression and likely due to pollen limitation. These results suggest that dominant nuclear restorers of pollen production speed up the extinction of small populations of Silene littorea, and that sex determination mechanisms might be an important factor to consider in the conservation of many plant species. (c) 2005 Elsevier Ltd. All rights reserved.

1618: *+.141*

Under the rationale that behavioral studies may contribute to the conservation of threatened species, we analyzed mating behavior of the crayfish Austropotamobius italicus in the laboratory. Our objectives were to investigate whether nonrandom mating by size occurs in this species and to explore the role exerted by mate choice and male-male competition in inducing mating. We observed 17 "quartets" (one female and three differently sized males), divided into three groups based on the female size. To understand the influence of male-male competition on mating, we compared six quartets with six "duets" (one female and one male). Our results clearly show that mating in A. italicus is nonrandom caused by the clear mating advantage of larger males. In fact,

as compared to smaller size categories, large males paired more often and for a longer time, and interrupted the final phase of mating less frequently. Male size-advantage mostly derives from an active female choice for large partners, combined with dominance of big males in intrasexual competition. However, a restricted mating period and the low number of receptive females may make males relatively indiscriminate in their overt behavior towards females. Female selection of big males and male-male competition over mates, also in the form of sperm competition, should result in a skewed reproductive success among males. As a consequence, populations with few big males would be characterized by a low heterozigosity and therefore by little genetic variation upon which selection acts. Our results should warn managers to pay more attention to the size structure of crayfish populations that are used for reintroductions.

1619: *+.284*

Background: Assessing the suitability of a habitat prior to the release of animals is vital. Proper assessment of the flora will allow reintroduction programmes to determine whether the area will be capable of supporting the released animals in the long-term. Here data are presented from an island in Central Kalimantan, Indonesia which has been used as a release site for agile gibbons (Hylobates agilis albibarbis) since January 2003.Results: Methods and results regarding fruit abundance, fruit productivity, tree density and diversity are presented. This information is then analysed in the context of the island's suitability to sustain released gibbons and without impact on the resident fauna. Based on the above ecological characteristics, the final carrying capacity of the island is estimated to be between 3 and 19 gibbons.Conclusion: These data highlight the need to survey areas being considered for release of gibbon prior to the release taking place. For reintroductions to be successful, long-term habitat assessment is vital, both pre- and post-release.

1620: *+.132*

The French Broad River originates in North Carolina, flows west into Tennessee and at its confluence with the Holston River forms the Tennessee River. Douglas Dam, located on the French Broad River 52 km above its mouth, is operated primarily for peaking hydroelectric power and flood control. Prior to completion of the dam in 1943, the lower French Broad River contained about 53 species of freshwater mussels and 100 species of fish. By 1977, the fauna in the 52-km-long tailwater was reduced to 12 species of mussels and 42 native species of fish. Improvements in tailwater conditions occurred following initiation of minimum flows in 1987, and consistent reaeration of discharge in 1993. From 1988 to 2002, we sampled three sites (4, 28, and 39 km downstream of the dam) to monitor the fish assemblage. Each year since 1988, we have collected one or more additional species, indicating continued immigration. We collected 82 native and 9 exotic species of fish overall, but the maximum of 67 species in I year suggests that some species reside in the tailwater at low densities or all immigrants may not successfully colonize the tailwater. There is limited potential for most extirpated species of mussels to naturally recolonize the tailwater because source populations are isolated. Consequently, 19 754 adult mussels of 19 species were introduced between 1997 and 2000. Survival of translocated mussels has been high, and successful reproduction of at least one translocated species has occurred. Additionally, four mussel species are naturally colonizing the tailwater. Colonization and recruitment of additional mussel species is expected as populations of their host fishes increase. We believe that the improved conditions of the tailwater may allow for the re-establishment of sustaining populations of 30 mussel species of historic occurrence, but the continued operation of Douglas Dam as a peaking hydroelectric project will reduce the probability of successfully reintroducing some species. Published in 2006 by John Wiley & Sons, Ltd.

1621: ***+.022***

Eco-tourism depending on wildlife is becoming increasingly profitable and landowners are beginning to favor game farming and ecotourism. In these areas, large-scale translocation of wildlife involves a diversity of species and large populations. The African buffalo (Syncerus caffer) is one of the major tourist attractions in Zambia. It accounts for 8.7% and 12.4% of the total animal species hunted in the Game Management Areas and the total hunting revenue earned in Zambia, respectively. It is ecologically an important animal species essential for the purpose of habitat control and facilitating the provision of suitable grazing pastures. However, the rearing of the African buffalo on game ranches has been hampered by its carrier state of the Southern Africa Terroritory (SAT) serotypes of foot and mouth disease virus (FMD). The African buffalo is also known to be a carrier of Theileria parva lawrencei, the causative agent of corridor disease (CD) that continues to have devastating effects on the livestock industry in Zambia. In addition, the importation of buffaloes from countries with populations endemic to bovine tuberculosis is highly restricted. Veterinary regulations in Zambia, strongly advocate against the translocation of buffaloes from protected areas to private ranches for disease control purposes thereby mounting a considerable constraint on the economic and ecological viability of the industry. It is hoped that this review will motivate the relevant government authorities in exploiting ways in which this animal species play a central role in eco-tourism.

1622: ***+.183***

A set of Chinese muntjac (Muntiacus reevesi) chromosome-specific paints has been hybridized onto the metaphases of sika deer (Cervus nippon, CNI, 2n = 66), red deer (Cervus elaphus, CEL, 2n = 62) and tufted deer (Elaphodus cephalophus, ECE, 2n = 47). Thirty-three homologous autosomal segments were detected in genomes of sika deer and red deer, while 31 autosomal homologous segments were delineated in genome of tufted deer. The Chinese muntjac chromosome X probe painted to the whole X chromosome, and the chromosome Y probe gave signals on the Y chromosome as well as distal region of the X chromosome of each species. Our results confirmed that exclusive Robertsonian translocations have contributed to the karyotypic evolution of sika deer and red deer. In addition to Robertsonian translocation, tandem fusions have played a more important role in the karyotypic evolution of tufted deer. Different types of chromosomal rearrangements have led to great differences in the genome organization between cervinae and muntiacinae species. Our analysis testified that six chromosomal fissions in the proposed 2n = 58 ancestral pecoran karyotype led to the formation of 2n = 70 ancestral cervid karyotype and the deer karyotypes is more derived compare with those of bovid species. Combining previous cytogenetic and molecular systematic studies, we analyzed the genome phylogeny for 11 cervid species.

1623: ***-.178***

The introduction of chronic, infectious diseases by colonizing populations (invasive or reintroduced) is a serious hazard in conservation biology, threatening the original host and other spillover species. Most research on spatial invasion of diseases has pertained to established host populations, either at steady, state or fluctuating through time. Within a colonizing population, however, the spread of disease may be influenced by the expansion process of the Population itself. Here we explore the simultaneous expansion of a colonizing population and a chronic, nonlethal disease introduced with it, describing basic patterns in homogeneous and structured landscapes and discussing implications for disease management. We describe expected outcomes of such introductions for three qualitatively distinct cases, depending on the relative velocities at

which the population and epidemic expand. (1) If transmissibility is low the disease cannot be sustained, although it may first expand its range somewhat around the point of introduction. (2) If transmissibility is moderate but the wave-front velocity for the population; v(p,) is higher than that for the disease, v(d), the disease wave front lags behind that of the Population. (3) A highly transmissible disease, with v(d) > v(p), will invade sufficiently rapidly to track the spread of the host. To test these elementary theoretical predictions, we Simulated disease outbreaks in a spatially structured host population occupying a real landscape. We used a spatially explicit, individual-based model of Persian fallow deer (Dama mesopotamica) reintroduced in northern Israel, considering a hypothetical introduction of bovine tuberculosis. Basic patterns of disease expansion in this realistic setting were similar to our conceptual predictions for homogeneous landscapes. Landscape heterogeneity, however, induced the establishment of population activity centers and disease foci within them, leading to jagged wave fronts and causing local variation in the relative velocities at which the population and epidemic expanded. Based on predictions from simple theory and simulations of managed outbreaks, we suggest that the relative velocities at which the population and epidemic expand have important implications for the impact of different management strategies. Recognizing which of our three general cases best describes a particular outbreak will aid in planning an efficient strategy to contain the disease.

1624: ***+.008***

On 10 September 2004, 36 North Island saddleback (Philesturnus carunculatus rufusater) were transferred to Boundary Stream Mainland Island (BSMI), Hawkes Bay, from Cuvier Island, New Zealand. This translocation, consisting of 11 adult males, 18 adult females, 6 juvenile males, and 5 juvenile females, was the first attempt to transfer a species which was previously extinct on the mainland to a mainland habitat with low predator abundance. The two key risks of this transfer were loss of individuals through predation, and dispersal. This study investigated the rate of initial mortality and the role of dispersal on the success of the reintroduction. A survey conducted 6 weeks after release resulted in an estimated survival rate of 57%. Ten saddlebacks had tail-mounted transmitters attached prior to release. These saddlebacks were located once per week. The mean daily dispersal distance of transmittered birds was 30 m (n = 10), with adults dispersing 42 m (n = 6) and juveniles dispersing 16 m (n = 4) per day. The largest total distance dispersed away from the release site was 1952 m. The risk of dispersal after release may be minimised by transferring juveniles.

1625: ***+.112***

Species interactions research and monitoring was initiated in 1989 to investigate ecological interactions among fish in response to proposed supplementation of salmon and steelhead in the upper Yakima River basin. This is the fourteenth of a series of progress reports that address species interactions research and supplementation monitoring of fishes in the Yakima River basin associated with the Yakima/Klickitat Fisheries Project. Data have been collected before and during supplementation to characterize the ecology and demographics of non-target taxa (NTT) and target taxon, and to monitor interactions and supplementation success. Major topics of this report are associated with implementing NTT monitoring prescriptions for detecting potential impacts of hatchery supplementation, and monitoring fish predation indices. This report is organized into seven chapters, with a general introduction preceding the first chapter. This annual report summarizes data collected primarily by the Washington Department of Fish and Wildlife (WDFW) between January 1, 2005 and December 31, 2005 in the Yakima River basin, however these data were compared to data from previous years to identify preliminary trends and patterns. Summaries of each of the chapters included in this report are described below. Chapter 1. Release of large

numbers of hatchery origin salmon has the potential to negatively impact other taxa (non-target taxa, NTT). To determine changes in NTT status that could be related to hatchery smolt releases, we compared the abundance, size structure, and distribution of 14 non-target taxa before and seven years after annual spring releases of about 1 million yearling smolts (coho and Chinook) in the Yakima River, Washington. We compared any observed changes in status to predetermined containment objectives that were judged to reflect acceptable levels of impact. We utilized detection strategies that would balance our ability to detect changes and the chances of falsely associating a change with supplementation. With the exception of steelhead size, all of the changes we observed were within the containment objectives established for the project. The main stem Yakima River steelhead size index has decreased through the post-supplementation period although the decrease was not significant (-1%, P>0.05). Our analysis suggests that the depressed size of the steelhead index was not related to supplementation activities. For instance, we could not detect any differences in the sizes of rainbow trout between areas of high and low spring Chinook abundance. Results from status monitoring of 14 NTT after seven years of hatchery releases suggest that risk containment actions are not necessary at this time. Chapter 2. Salmon supplementation and reintroduction programs have the potential to negatively impact other valued fish taxa, which are not the target of enhancement (non-target taxa). We evaluated the impacts of spring Chinook salmon supplementation and coho salmon reintroduction (hereafter supplementation) to rainbow, steelhead, cutthroat, and bull trout after five years of stocking approximately one million yearling smolts in the upper Yakima Basin between 1999 and 2003. Field methods included backpack electrofishing and snorkeling in tributaries, and drift-boat electrofishing in the main stem. We used three sequential steps in our evaluation: First, we determined if spatial overlap occurred between supplementation fish and non-target taxa. Second, if overlap occurred, we determined if a change in abundance, size, or biomass occurred during supplementation. Lastly, if a change occurred we determined if the change could be reasonably attributed to supplementation. Salmon rarely overlapped cutthroat and bull trout in tributaries, but some overlap of cutthroat occurred in relatively high elevations of the main stem, and considerable overlap with rainbow trout occurred in tributaries and the main stem. With the exception of steelhead, the lower 90% confidence limit of abundance, size, and biomass was above the containment objective for non-target taxa that overlapped significantly with salmon. We used rainbow trout as an analog for steelhead. The lower 90% confidence limit of rainbow trout abundance and size in tributaries, and abundance, size, and biomass in the main stem was below the containment objective for steelhead trout. However, comparisons of rainbow trout abundance, size, and biomass between tributaries and main stem sections with relatively high and low salmon abundance revealed that the change was likely not the result of supplementation (BACIP P>0.05). Our data indicate that early stages of salmon supplementation have not impacted valued trout species in the upper Yakima Basin beyond predetermined containment objectives. Chapter 3. Electrofishing is one of the most widely used methods to sample salmonid fishes because it is relatively easy to do, is relatively inexpensive, can be done in a variety of conditions, and can be done with relatively low impacts to fish and other animals. The utility of electrofishing as a means to sample fish is evident by the abundance of literature on the theory and practice of electrofishing, the application to abundance estimation, species richness or community structure sampling, and estimating the size structure of fish populations. Despite its popularity, several studies have revealed that historical electrofishing practices and commonly made assumptions should be reconsidered and in some cases abandoned. This chapter provides practical assistance to fisheries biologists for optimizing the utility of the data produced by applying appropriate sampling designs, good planning, and optimal electrofishing techniques. Chapter 4. We compared the backpack electrofishing capture efficiencies and Petersen-type mark-recapture abundance estimates of resident rainbow trout Oncorhynchus mykiss that had recovered for 24 h versus 3 h after electrofishing, handling, marking, and release in 13, 100 m sites in four Yakima Basin

tributary streams in central Washington State. Our results indicatethat the catchability of rainbow trout was not significantly different between the two recovery periods examined (P = 0.27). Similarly, Petersen-type mark-recapture abundance estimates were not significantly different between the two recovery periods (P = 0.20). Despite vigilant effort at installing and maintaining block nets during the 24 h period, we detected fish movement out of 75% of our sites. In addition, our block nets collapsed or were compromised in 36% of our sites when we used a 24 h recovery period and therefore, valid estimates could not be calculated. In contrast, no movement or net failure was detected during a 3 h recovery period. Some of the advantages we observed using a 3 h recovery period between mark and recapture backpack electrofishing include: (1) Increased probability of generating a population estimate with a minimum threat of block net failure; (2) Lower probability of violating the movement assumption associated with the Petersen-type mark-recapture estimator; and (3) Field sampling can be completed with a single site visit in a single day. We believe that these advantages should be considered when designing sampling protocols for enumerating stream fish. Chapter 5. Long-term trend monitoring of resident rainbow trout Oncorhynchus mykiss in the Yakima River Basin is a vital component of the Yakima Klickitat Fisheries Project non-target taxa of concern (NTTOC) monitoring and evaluation program. Concerns that supplementation may negatively affect the resident rainbow trout population in the upper Yakima River contributed to the development and implementation of the program. Information on the abundance, size, and distribution of rainbow trout in the Yakima River are collected annually in 5 sections of the Yakima River between Roza Dam and the Cle Elum River confluence. The information collected in these sections is presented under the risk containment monitoring framework and can be difficult to interpret outside of that context. The purpose of this report chapter is to provide managers, anglers, special interest groups, and all interested readers a summarized version of Yakima River rainbow trout data collected under the YKFP's NTTOC monitoring program in the Yakima Basin. Chapter 6. The size of predators that consume the most fish and the size of prey fish that are the most vulnerable to predation are important factors when assessing the predation risks to valued prey fish such as Chinook salmon Oncorhynchus tshawytscha in the Pacific Northwest. We found that the risk of predation by nonnative smallmouth bass Micropterus dolomieu to native salmonids in the lower Yakima River generally decreased with increasing predator and prey size. Smallmouth bass 150-199 mm FL consumed 42.9% of all of the salmonids consumed by smallmouth bass. Overall, most of the salmonids were consumed by smallmouth bass <250 mm (69.6%), and the vast majority by bass <300 mm (83.6%). Smaller smallmouth bass were much more abundant than large bass and the proportion of smallmouth bass that contained salmonid prey items in the gut decreased with increasing bass size. Salmonids >100 mm FL were rarely consumed by smallmouth bass. We found that the maximum percent length of salmonids consumed by smallmouth bass was 56.6%. In addition, the percent length of salmonids consumed decreased with increasing smallmouth bass size. Smallmouth bass generally ate salmonids at lengths that were <50% of their capacity and at an average of 25% of their length. The introduction of smallmouth bass to the Yakima River appears to have changed the size-based predation risk dynamics in the lower Yakima River that were historically represented by northern pikeminnow Ptychocheilus oregonensis. Chapter 7. One of the strategies that managers can use to reduce predation impacts to valued fish species is by swamping predators with more prey than they can eat. We examined whether this approach was viable by calculating the maximum bioenergetic consumption of non-native smallmouth bass Micropterus dolomieu on ocean-type Chinook salmon Oncorhynchus tshawytscha juveniles in the Yakima River throughout the spring between 1998 and 2002. In addition, we examined the functional response of smallmouth bass to different abundances of Chinook by comparing annual consumption estimates and mortality to an index of Chinook salmon abundance. We found that the smallmouth bass population consumed ocean-type Chinook salmon well below their bioenergetic potential. However, individual smallmouth bass that were piscivorous were eating other food items at a level

near satiation. Furthermore, the maximum consumption potential was relatively low prior to mid-April, and then increased substantially to a peak in May. Among years, predation by smallmouth bass increased as oceantype Chinook salmon density increased but there was no asymptote that would indicate a swamping threshold. We did not detect that percent mortality was decreasing with increasing consumption, thereby exhibiting a type III functional response on ocean-type Chinook salmon prey. Predation mortality to hatchery ocean-type Chinook salmon could be reduced within a year by releasing ocean-type Chinook salmon that will emigrate quickly prior to mid-April, when predation potential is still very low. However, attempting to swamp predators with hatchery Chinook salmon to benefit naturally produced Chinook salmon poses uncertain benefits. Considerable swamping is occurring by other naturally produced fish species in the Yakima River. Therefore, it is important for managers to consider impacts to other species because they could have indirect impacts on other species that are highly valued by managers. All findings in this report should be considered preliminary and subject to further revision unless they have been published in a peer-reviewed technical journal (i.e., see General Introduction).

1626: *-.132*

The captive southern white rhinoceros (Ceratotherium simum simum) population is not self-sustaining. Many founders reproduced, but reproduction among captive-born (F-1) females has been extremely sluggish. Thus the conservation breeding program for this species faces a looming crisis. Using behavioral observations of a large captive population and a questionnaire survey circulated to facilities worldwide, several hypotheses for F-1 female reproductive failure were evaluated. Counter to predictions regarding behavioral deficiency in sociosexual behaviors, F-1 females were at least as proficient as F-0 females for all behavioral measures. Males also showed no sociosexual preferences for F-0 over F-1 females. Results indicate that most reproductive failure occurs post-copulation. The reigning root-cause hypothesis for F-1 female reproductive failure postulates that F-0 females are behaviorally dominant and suppress reproduction in F-1 females. However, no evidence for behavioral dominance was found and F-1 females housed with F-0 females were more likely to reproduce than those housed without F-0 females. Such social facilitation of reproduction is beneficial to F-1 female reproduction, but does not explain differential reproduction between F-1 and F-0 females. Because the design controlled for current conditions, these results point to development in captivity as the root cause of postcopulatory reproductive failure in F-1 females. (c) 2005 Elsevier Ltd. All rights reserved.

1627: *+.335*

We used a probabilistic decision tree to help select among alternative recovery strategies for the Po'ouli, a critically endangered forest bird endemic to the Hawaiian island of Maui. The Po'ouli is one of the rarest birds in the world, with only three individuals known, and no breeding pairs. The most urgent conservation need for the species is to create a breeding pair and obtain eggs for captive propagation. Seven recovery strategies had been proposed, and there was disagreement among conservation workers about which strategy to pursue. In addition to lengthy discussions, a decision tree was introduced to provide an objective way of quantifying the chance of success under each alternative. All available information was used to attempt evaluation of each node in the decision tree. The overall chance of obtaining eggs was calculated by multiplying estimates at each node under each alternative. All options produced low estimates of potential success, but two options, removal of all birds to captivity and placement of the birds in a field aviary in an accessible location, produced similar estimates of success. The US Fish and Wildlife Service and the Hawaii Division of Forestry and Wildlife eventually agreed that removal to captivity was the preferred alternative because it could be implemented quickly, which was important considering

the advanced age of the birds, and would make it easier to provide veterinary care and ensure their safety from predators, severe weather, and vandals. Decision trees and other structured models should not be relied on exclusively, but they can provide an objective method of helping to make difficult conservation decisions and provide a record of complex thought processes used in reaching a determination. The case of the Po'ouli may serve as a template for navigation towards a decision to recover other species of extreme rarity. (c) 2005 Elsevier Ltd. All rights reserved.

1628: *+.196*

A population viability analysis (PVA) using the computer package VORTEX was conducted to assess the minimum viable population (MVP) of the Atlantic Forest endemic marsupial Micoureus paraguayanus. The objectives were: to estimate demographic and genetic MVPs that could be used as quasi-extinction thresholds for future modeling, to estimate the minimum area of suitable habitat (MASH), and to use these results to apply IUCN red list criteria so as to suggest its proper status classification. The model predicted that populations of 100 and 2000 individuals were necessary to achieve demographic and genetic stability, respectively, within a time frame of 100 years. The model was sensitive to changes in inbreeding depression, mortality and reproduction. MASH estimated to contain genetically viable populations reached 1300 ha. Fortunately, there still are quite a number of forest remnants equal to or larger than this. Isolation is suggested as the principal threat facing M. paraguayanus. Therefore, promoting conditions for dispersal together with efforts dealing with translocation, should prove to be the most appropriate management strategies for M. paraguayanus at this stage. A landscape pattern composed of large patches holding MVPs and sets of smaller patches harboring viable metapopulations that maximize probability of dispersal can provide a viable scenario for the conservation of M. paraguayanus.

1629: *+.160*

Carotenoids are essential dietary components utilized not only in pigmentation but also as immuno-stimulants and antioxidants. Reduced availability can have consequences on individual health and survival, thus making carotenoids a good indicator of environmental stress. We compared carotenoid profiles and plumage colour characteristics of an endangered passerine species in New Zealand, between its remnant island source population and two reintroduced island populations. Circulating carotenoids were predominantly lutein (mean of 82.2%) and zeaxanthin (mean of 14.8%), and these were the major carotenoids present as yellow pigments in the males' plumage. There were clear differences in total carotenoid concentrations and plumage colour among the three populations. Circulating carotenoid concentration was significantly higher in one of the reintroduced populations, and the yellow plumage of males was significantly higher in both reintroduced populations in comparison with the remnant population (reflected as a significant increase in hue). Understanding how these differences arise may be of importance to this species given the health benefits carotenoids impart and our ability to select plant species containing these compounds or artificially supplement them.

1630: *-.178*

A primary threat to amphibians in North America is the loss of wetland areas used for reproduction, especially small, temporary, and isolated wetlands. The Illinois chorus frog (Pseudacris streckeri illinoensis) is particularly vulnerable and exists today in a highly fragmented distribution limited to a few isolated populations in Arkansas, Illinois, and Missouri. Precision land-leveling combined with seasonal drought conditions has resulted in a significant population decline and range contraction for this species in Arkansas. Distributional surveys conducted from

1987 through 2004 indicate a 61% range contraction from a maximum of 59 km(2) to a current range of approximately 23 km(2). Additionally, there has been a lack of recruitment the past 2 years for a species that typically possesses a 2-3-year lifespan. Because the Clean Water Act will only protect isolated vernal pools if the continued existence of a threatened or endangered species is jeopardized, the future of this subspecies of chorus frog in Arkansas is both tenuous and problematic. In the absence of immediate protection and habitat modification through the reintroduction of depressions, we argue extirpation of this species in Arkansas may be imminent. The increasing use of precision land-leveling may have implications for other amphibian species worldwide.

1631: *+.276*

In conservation biology there is a basic need to determine habitat suitability and availability. Astroblepus ubidiai (Siluriforms), the only native fish in the highlands of Imbabura province in the Ecuadorian Andes, was abundant in the past in the Imbakucha watershed and adjacent drainages, but currently it is restricted to a few isolated refuges. Conservation actions are needed if this unique fish is to persist. A Habitat Suitability Index (HSI) for the species has been developed in order to aid management decisions. In this HSI model biomass density (B) was selected as a better indicator of habitat quality than either abundance or density. A population well-being index (PI) was constructed with the combination of B and an indicator of fish health (proportion of fish in the population with parasites and deformities). Based in other models of benthic fish the habitat variables current velocity, flow, depth, width, cover, invertebrate composition, vegetation type, terrestrial vegetation, land use, substrate, temperature, pH, TDS, oxygen, altitude, and slope were included in the analysis. An anthropogenic perturbation index (H) and a fragment isolation index (FII) were developed and included as habitat variables as well. The HSI model was applied to refuges and a sample of 15 aquatic bodies without fish populations within the study region. From the sampled sites without A. ubidiai 26.6% presented low quality, and the remaining 73.3% had medium quality according to the HSI estimated. Good quality habitat for dispersal, escape or translocations is rare in the region. The low HSIs estimated in some of the refuges suggests that current populations are not settled in the most favorable habitat but in the habitat least favorable to the agents of decline.

1632: *-.086*

Spotted suslik Spermophilus suslicus is one of the most endangered mammal species in Poland. Over the last 50 years, it lost more than 90% of its populations and about 70% of its individuals. In order to establish a conservation and reintroduction program, the knowledge of population structure of the species is crucial. We have developed nine polymorphic microsatellite loci to analyse the population structure. Six of the primer sets also amplify polymorphic markers in the European suslik Spermophilus citellus.

1633: *+.288*

Hand-raised Scarlet Macaws (Ara macao) are being reintroduced in two forested sites in Costa Rica: in the seasonally dry Northwest, and the wet Southwest of the country. Overall Mayfield first year survivorship estimates were 92% (n=13) and 100% (n=25), respectively. All individuals are individually identifiable and the older macaws have home-ranges of between 8 and 72 km2 in each site. The macaws show a propensity for dietary exploration and have been observed feeding on 30 and 39 species of plants (or their fruits) in each site, respectively. Their high survival rate, dietary range, and propensity to remain in the release populations are attributed to: good vigor on release,

intensive pre-release exposure to a wide variety of locally-available fruits, supplemental feeding post-release, and the daily provision of a nutritionally-insignificant amount of sunflower seeds (creating foci for socialization). The reintroduction of psittacines can be successful when the factors that have caused their extinction are controlled. Reintroduction efforts are also appealing to the media and can provide excellent forums for general conservation education.

1634: *+.039*

We tested two methods of releasing endangered, captive-bred Burrowing Owls (Athene cunicularia) in Saskatchewan, Canada. The first technique involved releasing pairs of captive-reared, adult owls. Twelve of 26 pairs remained together using this technique, while another six individuals paired with wild owls. Pairing/nesting success was poor when enclosures were left in place for only 3 d prior to release; success improved when enclosures remained for 5 d or until clutch initiation. At least 19% of released adults died during the breeding season, compared to only 3.7% for wild owls. At least five released adults failed to migrate. None of the captive-release adults returned to the study area in subsequent years, whereas 19% of banded wild owls returned during the same period. One of 62 offspring from released pairs returned to breed in a subsequent year; this recruitment rate was not different than that of offspring produced by wild adults. The second release technique involved fostering captive-hatched owlets into wild nests. We fostered 54 owlets at three different ages. Fostered chicks were accepted by wild owls; their growth, survival, and behaviors did not differ from their wild siblings'. Our results suggest that adults raised in captivity can breed successfully in the wild, but there are questions about their ability to migrate successfully. Fostering captive chicks into wild nests showed some success, but also had some limitations.

1635: *+.147*

As wild primate populations decline, numbers of orphaned primates, sanctuaries, and attempts to release primates back to the natural environment increase. Release projects frequently are poorly documented despite IUCN guidelines recommending post-release monitoring and systematic data collection as central to the process. Since 1996, Habitat Ecologique et Liberte des Primates (HELP) has been releasing wild-born orphaned chimpanzees into natural habitat in the Conkouati-Douli National Park, Republic of Congo. HELP developed a post-release monitoring system as an integral component. We present activity budgets and diet of released chimpanzees, and compared them to those of wild chimpanzee, as primary indicators of successful release. Feeding, moving, and resting dominated activity budgets, reflecting the overall patterns in wild populations. Diet was diverse and dominated by fruit, and the released chimpanzees showed specialization on a smaller number of species, as in many wild communities. The high survival rates of the chimpanzees and overall success of the release program are attributed to careful planning and post-release support facilitated via the monitoring process. Systematic post-release data collection monitoring has confirmed that wild-born chimpanzees can adjust behaviorally and nutritionally to the wild. Survival statistics of the reintroduced chimpanzees-confirmed 56%, possible 88%- reflect the behavioral adaptability.

1636: *+.104*

Banksia goodii (rare and endangered) and B. gardneri var. gardneri (widespread) are closely-related rhizomatous evergreen sub-shrubs of southwestern Australian scrub-heath and woodland. They have 17 and 177 known populations, respectively, mostly small remnants due to landscape fragmentation from agricultural activities. Bioclimatic profiles developed using BIOCLIM indicate

that B. gardneri tolerates a wider range of climatic conditions than B. goodii, which has a very narrow predicted range. These results do not match what one would predict from their comparative biology. Specifically, their post-fire survival and resprouting vigour, rates of seedling growth and soil penetration, and susceptibility to seedling predators are similar. A field trial established along a steep climatic gradient of growing season length, showed that both species could extend their ranges beyond the distributions predicted by BIOCLIM, especially B. goodii. Seedlings of both species survived for at least 8 years at sites with two (but not three) months shorter and one month longer growing season than experienced by natural B. goodii populations. The rarity of B. goodii is a result of its recent origin, dispersal limitation, possibly habitat specialization (dense woodland), and the impacts of habitat degradation and fragmentation within its current range. Under these circumstances, it is highly improbable that any sort of bioclimatic modelling could predict its potential climatic envelope. A stage-based model of B. goodii shows that under natural conditions the species is stable because of extremely low natural adult mortality (indeed, undetected), but plant losses due to human interference cannot be compensated due to its low levels of sexual reproduction. Population growth of B. goodii, and its potential for recovery, depends on population size and survival of seedlings and juveniles. Reproductive output per adult increases with population size, with populations of < 8 plants being sterile. Conservation management should target these factors as well as prevent the destruction of existing adults through additional land clearing or other threatening processes. Thus, habitat degradation, fragmentation and loss are likely to have a greater influence than any predicted global climate change on species survival. Translocation experiments appear to have far more scope in predicting the outcomes of climate change than bioclimatic modelling. As a consequence of poor dispersal ability and low reproductive rates, as well as habitat fragmentation, these banksias cannot migrate as the climate changes, but they already have a wide climatic tolerance and seem likely to withstand climate change in situ.

1637: *+.134*

Seasonal changes in paddy field and river foraging habitats used by herons and egrets were examined to determine the relative value of the two foraging habitats at the reintroduction site of the oriental white stork in Toyooka basin, Hyogo Prefecture, Japan. Herons and egrets gathered at the site to breed. Population size fluctuations were driven by changes in the paddy field: three times as many herons and egrets occurred in the paddy field than at the river. Therefore, it would seem that the herons and egrets depended on the paddy field for food during the breeding season, when the largest amount of food is required. During the non-breeding season, however, the majority of the herons and egrets did not forage in the paddy field, and particularly not in the rice ground. This suggests that the quality of the paddy field as a foraging habitat decreased during the non-breeding season, probably because the area had become dry. In contrast, the river provided a constant supply of food to the herons and egrets throughout the year. The river habitat may have been saturated with foraging herons and egrets, because their numbers did not increase during the non-breeding season, when they did not forage in the paddy field. It is necessary to increase the aquatic fauna of the paddy field during the non-breeding season for the success of this reintroduction.

1638: *+.197*

A total of 350-600 huemul (Hippocamelus bisulcus) remain as fragmented groups along 1,850 km of Argentine Andes. Their conservation depends on accurate knowledge of the species' requirements and the factors preventing their recovery. The Regional Delegation for Patagonian National Parks (RDP) erroneously alleged that huemul status is satisfactory, and current in situ

efforts are sufficient to guarantee recovery. Therefore, conservation centers are regarded unnecessary and the associated risks too high, especially because previous attempts with manipulations have failed. No data support these claims, instead many subpopulations have disappeared recently even in national parks (NP) which hold < 0.01 huemul/km(2). Causes preventing recovery or recolonization are unknown. Current pressures on huemul subpopulations include increased economic activities and alien species. Normal ranges for many biological parameters or population performance of huemul are unknown. Focus is on habitat studies using presence as surrogate for what should be studied on survival and reproduction. Factors important to small-sized populations or preventing recovery remain unstudied. RDPs insistence on indirect methodology prevents implementation of other potentially more promising research approaches. The lack of consensus regarding the necessity and feasibility of a conservation center prevented its establishment and related census flights in unprotected sites. RDP currently forecloses aerial census and capturing and thus prospects for a huemul conservation center, and the proposition of establishing such a center was neither discussed nor incorporated into the national recovery plan. Helicopter captures have been used successfully on deer in huemul habitat. Captures and translocation of huemul occurred since 1830; several zoos kept them successfully up to 10 years, and natural tameness facilitated husbandry. Recently, Chile successfully caught and transported huemul by helicopter to stock a private center. Unknowns can be addressed easily on semicaptive deer; other questions can be studied through reintroductions, employing adaptive management. RDP places faith in NP providing viable subpopulations. However, it remains doubtful whether some 220 huemul living in > 22,000 km(2) of parks can guarantee species survival. For Argentine cervids, absence of studies and management plans due to lack of funds is typical. Considering the actual situation and future perspectives, it appears doubtful that recovery will be achieved based on strategies similar to those employed in the past.

1639: *+.073*

Logistic regression is a statistical tool widely used for predicting species' potential distributions starting from presence/absence data and a set of independent variables. However, logistic regression equations compute probability values based not only on the values of the predictor variables but also on the relative proportion of presences and absences in the dataset, which does not adequately describe the environmental favourability for or against species presence. A few strategies have been used to circumvent this, but they usually imply an alteration of the original data or the discarding of potentially valuable information. We propose a way to obtain from logistic regression an environmental favourability function whose results are not affected by an uneven proportion of presences and absences. We tested the method on the distribution of virtual species in an imaginary territory. The favourability models yielded similar values regardless of the variation in the presence/absence ratio. We also illustrate with the example of the Pyrenean desman's (Galemys pyrenaicus) distribution in Spain. The favourability model yielded more realistic potential distribution maps than the logistic regression model. Favourability values can be regarded as the degree of membership of the fuzzy set of sites whose environmental conditions are favourable to the species, which enables applying the rules of fuzzy logic to distribution modelling. They also allow for direct comparisons between models for species with different presence/absence ratios in the study area. This makes them more useful to estimate the conservation value of areas, to design ecological corridors, or to select appropriate areas for species reintroductions.

1640: *-.024*

The reintroduction of large mammals is often considered a priority conservation action in highly

industrialized countries in which many of these species have been depleted. However, species reintroduction after decades of absence may involve important risks for human activities and ecological communities, such as favoring the spread of diseases. An example of a potentially troublesome reintroduction is the wild boar, which may act as a reservoir of diseases, e.g., classical swine fever, and cause high economic losses, and has become a species of concern in several European countries for both ecological and recreational reasons. Failure to prevent the disease consequences of species restoration can negate its conservation benefits. Here we evaluated the probability of both successfully reintroducing wild boar into Denmark and limiting their contact with domestic pig farms to which they might spread disease. For this purpose, we developed a spatially explicit, individual-based population model that incorporates information on boar habitat and demography information from Central European populations. We then compared model predictions with the spatial distribution of farms to achieve a spatial assessment of the contact risk. The most restrictive model scenario predicted that nearly 6% of Denmark provides habitat conditions that would allow wild boar to reproduce. The best habitats for reintroduction were aggregated in seven different areas throughout the country in which the extinction probability was < 5%. However, the expected population expansion was very limited in most of these areas. Both the number of suitable areas and the potential for population expansion greatly increased when we relaxed our habitat assumptions about boar forest requirements; this provided a more conservative scenario for a cautious risk analysis. We additionally found that part of the risk of contact with piggeries was associated with the magnitude of the expansion, although the nonrandom spatial pattern of farm distribution also had a strong influence. The partitioning of risks into those related to population expansion and those related to farm distribution allowed us to identify trade-offs between restoring boar populations and minimizing risks in different potential areas and under different risk scenarios; as a result, we rejected some of the particularly high-risk areas for potential reintroduction of the species. Our approach illustrates how the joint quantification of anticipated reintroduction success and associated risks can guide efforts aimed at reconciling species recovery and the affected health and economic interests.

1641: *-.024*

The reintroduction of large mammals is often considered a priority conservation action in highly industrialized countries in which many of these species have been depleted. However, species reintroduction after decades of absence may involve important risks for human activities and ecological communities, such as favoring the spread of diseases. An example of a potentially troublesome reintroduction is the wild boar, which may act as a reservoir of diseases, e.g., classical swine fever, and cause high economic losses, and has become a species of concern in several European countries for both ecological and recreational reasons. Failure to prevent the disease consequences of species restoration can negate its conservation benefits. Here we evaluated the probability of both successfully reintroducing wild boar into Denmark and limiting their contact with domestic pig farms to which they might spread disease. For this purpose, we developed a spatially explicit, individual-based population model that incorporates information on boar habitat and demography information from Central European populations. We then compared model predictions with the spatial distribution of farms to achieve a spatial assessment of the contact risk. The most restrictive model scenario predicted that nearly 6% of Denmark provides habitat conditions that would allow wild boar to reproduce. The best habitats for reintroduction were aggregated in seven different areas throughout the country in which the extinction probability was < 5%. However, the expected population expansion was very limited in most of these areas. Both the number of suitable areas and the potential for population expansion greatly increased when we relaxed our habitat assumptions about boar forest requirements; this provided a more conservative scenario for a cautious risk analysis. We additionally found that part of the risk

of contact with piggeries was associated with the magnitude of the expansion, although the nonrandom spatial pattern of farm distribution also had a strong influence. The partitioning of risks into those related to population expansion and those related to farm distribution allowed us to identify trade-offs between restoring boar populations and minimizing risks in different potential areas and under different risk scenarios; as a result, we rejected some of the particularly high-risk areas for potential reintroduction of the species. Our approach illustrates how the joint quantification of anticipated reintroduction success and associated risks can guide efforts aimed at reconciling species recovery and the affected health and economic interests.

1642: *+.139*

Information about geographic distributions is required for species conservation and management. Ultimately, this information is derived from records of occurrence. However, the reliability and availability of occurrence records are variable. A conceptual framework for evaluating the reliability of occurrence records is provided. Only records associated with physical evidence, especially a museum voucher specimen, are considered verified. However, errors in species identification or location are possible even for verified records. In addition, biases exist in occurrence records because they generally are collected haphazardly. Other sources of bias include sampling error associated with small areas or range limits and aspects of the species' biology that make it unlikely to be documented. A practical method is provided for interpreting a species' distribution in a particular area given a paucity of reliable occurrence records. Factors that must be considered for including such areas of interest within the range of a species include: (1) plausible reason for the paucity of records; (2) continuous suitable habitat between the area of interest and localities of reliable occurrence; and (3) absence of biogeographic breaks in the distribution of other organisms with similar evolutionary histories. The possible distribution of wolverine (Gulo gulo) and Canada lynx (Lynx canadensis) in New Mexico provides a case study of this approach. It is concluded that the mountains of north-central New Mexico should be considered within the natural range of wolverine and Canada lynx. (c) 2005 Elsevier Ltd. All rights reserved.

1643: *+.057*

Soapweed (Yucca glauca Nuttall) was approved for provincial listing as an Endangered species in 2003 by the Minister of Alberta Sustainable Resource Development. This vascular plant species has an obligate mutualistic relationship with its specialized moth pollinator, the yucca moth (Tegeticula yuccasella Riley), which is also considered Endangered both provincially and federally. There are two known natural populations; one population is on the Agriculture and Agri-food Canada Research Substation in Onefour and the other is on the Pinhorn Grazing Reserve along half a kilometre of southwest-facing coulee slope of the Milk River drainage. Little is known about the Pinhorn population of soapweed, except that there is little evidence of sexual reproduction at the site. In keeping with the province's commitment to the Accord for the Protection of Species at Risk, the National Framework for the Conservation of Species at Risk, combined with requirements in Alberta's Wildlife Act and SARA, a recovery plan was completed for the soapweed and yucca moth in 2006. To inform the development of recovery actions, a comprehensive inventory of the Pinhorn population of yucca was carried out in 2004. During the survey, clones (collections of soapweed rosettes separated by less than 15 cm of open soil) were counted. Evidence of previous flowering, number of inflorescences (flowering stalks), number of dead rosettes, number of live rosettes, number of fruit per clone and, number of emergence holes in fruit was also recorded. A much larger population of yucca was found at the Pinhorn site than previously reported, although this may be due to varying methods. Evidence of fruiting and larval emergence confirms that both sexual reproduction of soapweed and reproduction of yucca moth

are occurring at the Pinhorn site, but at a very low rate, despite a substantial amount of flowering among clones. The limited number of yucca moths at the site could have major implications for the long-term health of the soapweed population, as well as the yucca moth population. Herbivory was observed on a number of inflorescences at the site, reducing the number of flowers and fruit produced. Translocation of moths to the Pinhorn site and ungulate-proof fencing should be considered to address these limiting factors.

1644: *+.064*

The Northern Bald Ibis Geronticus eremita has undergone a long history of decline over at least four centuries, having been distributed over much of north and northeast Africa and the Middle East. Two distinct populations have been identified which are genetically distinct. The main western population occurs in Morocco and now numbers around 100 pairs. A relict population of two pairs persists in Syria, providing a precarious opportunity to keep the eastern population going in a truly wild state. Turkish birds are now only semi-wild, but are still a very important genetic resource for a time when reintroduction methodology has been developed further. It is thought that birds used to winter in Sudan, Eritrea, Saudi Arabia and Yemen. Post-1989 records in Saudi Arabia and Eritrea suggested that an undiscovered breeding colony remained in the Middle East. The Northern Bald Ibis is still classified as "Critically Endangered" because of its small range and population. The improvement of the population in Morocco is very recent and is mainly due to conservation and management actions. Where this is missing, the decline of a population appears dramatically, like in Syria over the last 20 years. The main threats to the species over the centuries have been a combination of direct persecution but also the loss of steppe and non-intensive agricultural areas. The chief threats the species now faces differ among the countries where it still occurs. In Morocco, preventing the loss of feeding areas and disturbance to breeding sites are the most important priorities. Illegal buildings and disturbance close to the breeding cliffs and changes in farming on the feeding grounds are the threats that may have the most severe impact on the population. In Syria, there are even greater challenges. Hunting is the main threat to the tiny population, and there is the need to control land-use pressures and other local and regional awareness issues. Knowledge of where the birds overwinter is urgently required to reduce potential threats there. Although Turkey has only a semi-wild population, it has to be managed well to build up the genetic stock. The Northern Bald Ibis is susceptible to pesticides and contaminated water sources, and particular attention to this is needed in all areas where the birds forage in all three countries. The key priority for conservation is to ensure the protection of the Moroccan population, which has two sites where it occurs. The Souss-Massa National Park was designated specifically to protect nesting and feeding areas. The main targets recognized increase the number of Northern Bald Ibis colonies in Morocco as well in Syria and Turkey were: * to maintain agriculture and grazing regimes in order to achieve sustainable exploitation of rangelands and halt the advance of desertification processes; * to promote alternative sustainable grazing regimes and energy use, coupled with the promotion of socio-economic development of local communities; * to control firewood collection to prevent destruction or degradation of feeding areas; * to stop hunting; * to control the construction of illegal buildings on or near to breeding and feeding sites; * to reduce the risk of intoxication. Considerable progress has been made over recent years with methodology that should help with potential reintroduction attempts in future. Establishing a resident population is now a real possibility following work carried out in Austria. But there are still important challenges to getting a migratory population established, an objective that could well prove to be possible in future. Further work in this area will be useful, but much more detailed information on ecological requirements and previously occupied sites will be necessary. However, this should not in any way distract from the top priorities in Morocco and in Syria tomaintain areas of breeding and feeding habitat for these remaining known wild breeding

populations.

1645: *+.034*

Uncertainties in species definitions can have important consequences for biodiversity conservation because taxonomic rank is used as a criterion to assess the conservation priorities of threatened organisms. The Vulnerable St Lucia whiptail lizard Cnemidophorus vanzoi, considered a single species, is the sole representative of its genus in the Caribbean region, found on Maria Major and Maria Minor islands off the coast of St Lucia. However, a recent study revealed significant morphological and phylogenetic differences between the two populations and recommended they should be managed as two separate entities. We surveyed the two populations and estimated them to comprise 1,985 and 29 individuals on Maria Major and Minor, respectively. The Maria Minor population is currently at a critically low level and consequently highly susceptible to demographic and genetic stochasticity and catastrophic events, in particular the colonization of invasive mammalian predators. If our goal is to conserve biodiversity and evolutionary potential we face a dilemma in formulating the optimum strategy for the management of these two threatened populations on the species boundary. We discuss some potential management options but also raise this issue for discussion in the conservation biology community.

1646: *+.323*

The ibex Capra pyrenaica has recently recolonized its former Portuguese range from a contiguous Spanish protected area. The first observations of ibex in Portugal were in Peneda-Geres National Park in 1998. In 2001 we began a survey to confirm ibex presence in Portuguese territory, and to determine the current status of the species there. There are three ibex nuclei in the general area of the international border, and they are expanding their geographical range with two of the nuclei almost restricted to Portuguese territory. In 2003 the ibex population consisted of a minimum of 75 individuals. Different founder histories have led to distinct age structures in each nucleus, but in general they exhibit the population dynamics typical of a recently reintroduced population with high reproductive potential. Priority conservation actions for this newly established population need to include increased monitoring, and reinforcement of the population with individuals from elsewhere. Effective conservation will require collaboration between staff of both Portuguese and Spanish protected areas and an integrated Spanish-Portuguese conservation plan.

1647: *-.087*

We censused wild buffalo Bubalus arnee in Koshi Tappu Wildlife Reserve, Nepal, in March 2004 using methods employed in earlier surveys, and estimated a population of 159 animals. Since the last census in 2000 the management situation has deteriorated. Guard posts have been evacuated due to the Maoist insurgency. The entire Army Battalion usually posted in the Reserve was at Headquarters at the time of this study and therefore there were no patrols over most of the Reserve, and much human encroachment. Mortality from flooding and road deaths, and possibly poaching for meat, were evident, and males suffered more mortality overall than females. Despite these threats the population had increased since 2000, albeit at a lower rate than previously. Active management, including interventions within the Reserve and a translocation of some individuals to Chitwan National Park, are recommended.

1648: *+.146*

1. One important goal in conservation biology is to characterise evolutionary lineages within

endangered species before management decisions are taken. Here, we assess population differentiation in the freshwater crayfish Austropotamobius pallipes, an endangered species endemic to western Europe and provide valuable information for the conservation of French populations.2. Analysis of five microsatellite loci in 44 populations revealed very different within population levels of genetic diversity (0.000 < H-0 < 0.564). Two groups, corresponding to northern and southern French populations, showed a high degree of genetic differentiation in both allele frequencies and allele sizes. Comparison of these results with previous studies of A. pallipes strongly suggests that the divergence between northern and southern populations could have occurred during the last glaciation period of the Pleistocene from one Atlantic and one Mediterranean refuge.3. Evidence for genetic admixture between these two lineages was revealed by correspondence analyses in southern populations, probably as the result of artificial translocations.4. French populations appeared significantly differentiated among the different river drainages and were highly structured within rivers. The impact of population size, population bottlenecks and founder events on the population genetic differentiation are discussed.5. Based on these results, we propose the designation of two evolutionarily significant units for A. pallipes in France. Our data also support the maintenance of separate demographic management strategies for crayfish inhabiting different river systems. However, genetic analyses will have to be combined with demographic and ecological data for sustainable conservation programmes.

1650: *+.042*

Translocation is frequently used to return rehabilitated animals to the wild, and is an important tool for the population management of endangered species. Whilst experimental field manipulations are important in determining optimal rehabilitation and translocation strategies, they are rarely implemented in practice. We used an experimental approach to examine the effects of translocation on post-release survival and behaviour, and the impact of introductions on the recipient wild population, using the European hedgehog (Erinaceus europaeus), the most common mammal admitted to British wildlife hospitals. The post-release survival and behaviour of five groups were compared: three different translocation treatments, one wild population at the release sites and one control wild population away from the release sites. individuals that were held in captivity prior to translocation had a better survival rate on release than individuals that were translocated with a minimum time spent in captivity. We suggest that temporary captivity improves chances of survival by allowing the build up of fat reserves and reducing manipulation stress suffered on release. No evidence was found for intra-specific competition between introduced individuals and the recipient wild hedgehog population. (c) 2006 Elsevier Ltd. All rights reserved.

1651: *+.144*

The ultimate success of reintroduction programs for endangered species depends on the ability of reintroduced animals to breed in the wild. We studied the nesting success and breeding biology of a reintroduced population of Puaiohi (Myadestes palmeri) on the island of Kaua'i, Hawaii. Thirty-four captive-bred Puaiohi were released into the Alaka'i Swamp in 1999-2001 and monitored using radiotelemetry. Ten females and two males paired with wild and other released birds, including one polygynous trio. From March to September, 31 nests were built. Mean clutch size was 2.0 eggs, daily nest survival was 0.97 +/- 0.01 (mean +/- SE) and overall nest success was 0.40 +/- 0.02. We confirmed predation, most probably by rats (Rattus spp.), as the greatest cause of nest failure, occurring at 38% of active nests with known fates, and causing the death of two nesting adult females. Ground-based rodent control proved ineffective at protecting nest attempts. Successful nests fledged an average of 1.4 young each (n = 10), and 85% of fledglings survived at

least two weeks. Importantly, breeding behavior and success were comparable to those of wild Puaiohi. This is the first record of breeding in the wild from captive-bred endangered Hawaiian passerines. The ability of captive-bred Puaiohi to survive and breed successfully in the wild bodes well for future releases of this and other endangered passerines, but high predation rates on nests and nesting females highlights the importance of maintaining and restoring safe habitat for recovery.

1652: *+.020*

From 1999 to 2003, 83 captive-reared Capercaillies were fitted with transmitters, released in the Harz Mountains region, and subsequently tracked by radio telemetry. The purpose was to achieve an effective record of survival chances, spacing, habitat use and feeding habits of the released birds, in order to evaluate the success of the project and to improve measures for future reintroduction projects. Some 23% (n = 18) of the radio-marked birds survived until either the transmitter or contact was lost. The median survival duration was 13 days, whereby hens (18 days) survived longer than cocks (12 days). The highest losses (79%, n = 48) occurred within the first four weeks after release. Of 61 deaths recorded, 62% were victims of foxes, 10% were killed by Goshawks, and 7% by Lynxes. In addition to mortalities where cause of death was unclear, individual birds were killed by traffic, dogs or died of under-nourishment. The main causes of the high mortality rate can be sought in the physiological and ethological deficits, which result from rearing in captivity. These include in particular a lack of predator-experience, and a probable inability to adjust adequately to food sources in the wild. Additionally, 5% of the losses were related to predators attracted to the area of the acclimatization pen. The released birds had activity ranges ("search areas") from less than 1 ha to up to 17.000 ha, whereby the majority of activity ranges (77%) comprised up to 1.000 ha. The recorded size of the activity range, as well as the maximum distance from the release site, increased with the length of the location period. The wide-ranging dispersal of the released birds in some cases was not only due to the generally marked dispersion of the juvenile Capercaillies, but possibly also to the unsuitability of the habitat at the release sites. Within the activity ranges birds established smaller centres of activity which they frequented until at least death, or loss of the transmitter. The size of the activity centres (n = 17) varied between 0.5 and 327 ha. In 70% of all cases, the birds covered a daily distance of not more than 500 m. Some 67% of all locations fell within a radius of 3 km from the release site. The greatest distances recorded were up to a maximum of 22 km (female). Arising from project experience, release direct from transport boxes is recommended. This helps to avoid additional losses at the acclimatization pen, and the release site can be selected more flexibly. Other improvement measures in the rearing and release methods are essential (e.g. early intensified diet provision appropriate to the species, training programmes, adequate disease prophylaxis), without which further releases of captive-reared grouse into the wild can no longer be justified.

1653: *+.202*

The rapid loss of native orchid habitat throughout ecologically important areas (e.g., Florida) has prompted researchers to develop appropriate plans for the propagation and reintroduction of many native orchid species. Ideally, symbiotic orchid seed germination methods are utilized in the production of orchid seedlings to be used in plant reintroduction programs. In the current study we (1) describe an efficient symbiotic seed germination protocol to germinate seeds of the rare sub-tropical terrestrial orchid Habenaria macroceratitis; (2) discuss the in vitro fungal specificity demonstrated by this species; and (3) describe the effects of three photoperiods(0/24 h, 16/8 h, 24/0 h L/D) on in vitro symbiotic seed germination of H. macroceratitis. Six fungal mycobionts were isolated from both vegetative and flowering plants of H. macroceratitis from two

geographically distinct sites. Symbiotic seed germination percent was highest (65.7%) and protocorm development was most advanced (Stage 2) when seeds were cultured with fungal mycobiont Hmac-310. Seeds of H. macroceratitis demonstrated a degree of specificity toward fungal mycobionts isolated from plants originating from the same site where seed was collected. Continual darkness (0/24 h L/D) inhibited initial seed germination (Stage 1; 17.1%), but stimulated subsequent protocorm development (Stage 2; 53.5%). These findings will aid in developing an efficient symbiotic seed germination protocol for the conservation of this rare Florida terrestrial orchid, and may prove useful in the conservation of other sub-tropical terrestrial orchid species.

1654: *+.222*

The introduction of rats and other mammalian predators has caused many New Zealand species to decline. Predator control is now being used to reverse these declines in selected mainland areas, and a footprint-tracking index is used to assess effectiveness of control. To assess the meaning of this index for native populations, it I,,, necessary to model the functional relationships between predator-tracking rates and vital rates of native populations. We monitored North Island robins (Petroica longipes) for 5 years after reintroduction to Paengaroa Mainland Island, and rat levels changed dramatically over this period due to changes in management policy. We used the resulting data to model how vital rates varied with rat tracking, using Akaike's Information Criterion to compare alternative models for each vital rate. We fitted survival models to mark-resighting data obtained in tri-annual surveys of the reserve, and we fitted fecundity models to data on numbers of independent young produced by individual females. The best model for annual adult survival was = 0.64P(0.24F) where p is the complement of the tracking rate (i.e.,,] where 0 = 100% tracking) and F is sex (0 = male, 1 = female). The best model for annual fecundity per female was 2.26(0.46(U)) [1 - e-0.46(/1-p)(p) U is the female's pairing status (0 = paired, 1 = unpaired). For juvenile survival (from independence to adulthood), it was ambiguous whether survival was constant (= 0.39) or changed with rat levels (s = 0.49p(0.58)). We used the delta method to obtain a 95% confidence interval for each vital rate at any rat tracking rate. The models allow the growth of the population to be projected at any tracking rate, and they provide a starting point for projecting growth of any population in relation to a predator-control index.

1655: *-.003*

Wildlife managers often have a good understanding of the threats faced by populations, but they need to know the intensity of management required for populations to survive. Managers therefore need quantitative projections for populations under different management regimes rather than just qualitative comparisons. However, quantitative projections are subject to tremendous uncertainty, particularly for small populations monitored for short time spans. We assess the level of predator control needed for a reintroduced population of North Island robins (Petroica longipes) to grow, accounting for uncertainty associated with parameter estimation, model structure, and demographic stochasticity. The robin population grew when exotic rats were reduced to low levels (< 10% of footprint tunnels tracked in 24 hr) by regular maintenance of poison bait stations. However, the population declined after baiting was stopped 3 years after the reintroduction (March 2002), and it had fallen to 4 pairs by September 2004. We created a simulation model incorporating relationships between vital rates (survival and fecundity) of the robin population and rat tracking rate estimated from 5 years of data. We ran the model 10, 000 times at each rat tracking rate, with vital rates sampled from distributions (defined by estimates and standard errors) at the start of each run. Output from a deterministic model suggested that, (finite rate of increase) would be > 1 if rat tracking were < 20%, and up to 1.2 with rat tracking at 1%. However, 95%

confidence intervals for X extended < 1 at any tracking rate. With demographic stochasticity added, there was > 20% probability of further decline in 5 years even when the expected X was 1.2. With all forms of uncertainty included, 41% of simulations projected a further decline over 5 years if the rat tracking rate were 10%. This proportion was reduced to 30% if initial population size was increased to 20 pairs. Our analysis therefore showed it was most likely that the robin population would grow if intensive rat control were reinstated, particularly if the population was supplemented, but there was substantial risk the population would continue to decline under such management.

1656: *+.105*

During 1997 and 1998, we implemented a pilot investigation to compare survival, site fidelity, and reproductive characteristics ofrelocated wild northern bobwhites (Colinus virginianus) with that ofresident birds. We captured wild bobwhites (n = 74) on managed lands in southern Georgia and relocated them (> 1.6 km from capture sites) to sites nearby where density estimates revealed that population density was low compared to surrounding sites. We equipped translocated birds with radiotransmitters and released them in groups of 8-12. We also captured resident birds (n = 166) and simultaneously monitored them via radiotelemetry. We found no difference in survival (P = 0.82), nest production (P = 0. 19), or nest survival (P = 0.85) between relocated and resident bobwhites. This suggests that relocating wild bobwhites does not negatively impact their survival or reproductive output. Based on the results of the pilot study, we implemented a large-scale relocation to determine whether relocation can increase native bobwhite populations. Following the pilot study, during 2000-2002, we relocated wild bobwhites (n = 202) within property boundaries to 3 different sites where population densities were low. Although only 2 sites experienced a significant population increase, hunting records suggested a positive population response for all sites where relocation occurred. Hence, relocation of wild bobwhites prior to breeding season may augment low-density populations, isolated populations, or voids within populations. The utility of translocation may facilitate preservation and conservation of the northern bobwhite by augmenting restoration efforts focused on habitat management, affording species preservation in isolated habitats, and increasing population dynamics and demographics via genetic enrichment.

1657: *-.120*

Wild boar (Sus scrofa L.) were introduced in the island of Cyprus in 1990, when five animals were imported from Greece for game farming. In 1994, wild boars were illegally released in Lemesos (Limassol) Forest and in 1996 in the Troodos National Forest Park. Soon the population increased and dispersed throughout the park. In 1997, the government of Cyprus decided to eradicate wild boar because of the danger of transmitting diseases to livestock and to prevent possible environmental destruction. To control the wild boar, hunting was permitted and game wardens were instructed to eliminate the free-ranging animals. In 2004, no animals were observed in localities where they had been seen before. Surveys in September 2004 (Troodos National Park) and January/February 2005 (Troodos Forest, Pafos Forest, and Lemesos Forest) revealed no signs of recent wild boar presence. The reasons for the possible failure of wild boar to establish in Cyprus are discussed.

1658: *+.042*

The European wild rabbit (Oryctolagus cuniculus) is a staple prey species in Mediterranean ecosystems. The arrival and subsequent spread of rabbit hemorrhagic disease throughout

southwestern Europe, however, has caused a decline in rabbit numbers, leading to considerable efforts to enhance wild rabbit populations, especially through habitat management. Because rabbit population dynamics depend on habitat suitability and changes in habitat structure and composition subsequent to habitat management, I evaluated the effects of population dynamics on the long-term impact of rabbit hemorrhagic disease on rabbit populations. I used an age-structured model with varying degrees of population productivity and turnover and different habitat carrying capacities, and I assumed the existence of a unique, highly pathogenic virus. My results suggest that disease impact may be highly dependent on habitat carrying capacity and rabbit population dynamics, and the model provided some insight into the current abundance of wild rabbits in different locations in southwestern Europe. The highest disease impact was estimated for populations located in habitats with low to medium carrying capacity. In contrast, disease impact was lower in high-density populations in habitats with high carrying capacity, corresponding to a lower mean age of rabbit infection and a resulting lower mortality from rabbit hemorrhagic disease. The outcomes of the model suggest that management strategies to help rabbit populations recover should be based on improving habitats to their maximum carrying capacity and increasing rabbit population productivity. In contrast, the use of strategies based on temporary increases in rabbit density, including vaccination campaigns, translocations, and temporal habitat improvements at medium carrying capacities, may increase disease impact, resulting in short-term decreases in rabbit population density.

1659: *+.104*

Introducing rare plants to new sites for conservation to offset effects of habitat destruction requires detailed knowledge of habitat requirements, plant demography, and management needs. We conducted a factorial experiment replicated at three coastal prairie sites to test the effects of clipping frequency and litter accumulation on seed germination, seedling survival, reproduction, and seedling recruitment of introduced populations of the endangered, tall-stature, annual forb, Holocarpha macradenia (DC.) E Greene.. Clipping favored H. macradenia, primarily by enhancing seed germination and flower production. Litter accumulation had no effect on seed germination, even after 5 years of treatments. Seedling recruitment was highly site specific with large numbers of recruits recorded at only one of three sites. Although recruitment of seedlings was higher in clipped plots for 2-3 years, by 4-5 years after introduction very few seedlings survived to reproduction in any treatment. We attribute this result to a combination of poor habitat quality, small population size, and lack of a seed bank. We were unsuccessful in introducing this relatively well-studied species of concern to apparently suitable habitat at multiple sites in multiple years, which suggests that translocating rare plant populations to mitigate for habitat destruction is an expensive and highly uncertain endeavor.

1660: *+.112*

As a widely adopted conservation tool, translocation refers to the movement of living organisms from one area into another with free release, commonly including introduction, reintroduction and supplement. Many factors influence the success of reintroduction projects, mainly from biological traits, natural environment, sociobiological conditions and reintroduction manner. Meanwhile, great opportunities and challenges are also brought to basic ecological research accompanied by the conduction of introduction or reintroduction of wildlife. As a flagship species, the giant panda is endemic to China and distributed in separated Qinling, Minshan, Qionglaishan, Daxiangling, Xiaoxiangling and Liangshan Mountains. Due to severe fragmentation of habitats and random factors from environment and small isolated population, pure in-site conservation perhaps cannot assure long-term persistence of these populations in the wild. With increase of numbers of captive

giant pandas and improvement of habitat quality, reintroduction of captive giant pandas into small wild isolated populations is presumed to be an effective conservation measure and conditions before reintroduction is basically met. Issues urgent to be solved before reintroduction of captive giant pandas are also discussed in the end.

1661: *-.131*

Northeast Sika Deer (Cervus nippon hortulorum), the main subspecies of domestic Sika Deer in China, is currently facing the threatening of extinction in the wild, however, the breeding centers of northeast Sika Deer distributed in all over the country. In order to design effective reintroduction strategies for this subspecies, we have investigated the genetic diversity and genetic structure of the domestic Chinese Sika Deer populations by analyzing part of the sequence of mitochondrial DNA control region in 45 individuals sampled from 9 domestic populations. The domestic populations of Chinese Sika Deer exhibited evident genetic diversity relative to the other subspecies of Sika Deer (C. nippon), but no strong genetic structure difference was found among different populations. These results indicate that the domestic population of northeast Sika Deer in northeast China can be used as the founders of reintroduction project, and that the founders of reintroduction project should come from several domestic populations of northeast Sika Deer from northeast China.

1662: *+.130*

Restoration practices are often based on trial and error or anecdotal information because data from controlled experiments are not available. In wet meadow restorations of the upper Midwest United States, Reed canary grass (Phalaris arundinacea L.) is controlled with spring burning and spring glyphosate herbicide applications, but the relative effectiveness of either treatment with respect to P. arundinacea growth and life history has not been assessed. We designed a multiyear field experiment to evaluate effects of burning and herbicide application timings on P. arundinacea populations. Burning did not reduce P. arundinacea biomass but reduced the P. arundinacea seed bank, potentially limiting recolonization of P. arundinacea. Glyphosate applications in late August and late September were more effective than in mid-May (due to enhanced glyphosate translocation to rhizomes), such that two mid-May applications reduced P. arundinacea biomass to a level equivalent to that achieved by one late-season application. Phalaris. arundinacea recolonized rapidly from the seed bank and, in plots that received suboptimally timed (mid-May) herbicide, from rhizomes. Establishment of native species was very low, likely due to competition with recolonizing P. arundinacea. Unplanted species (from the seed bank and refugial populations) accounted for the majority of non-P. arundinacea biomass. Recolonization of other species was strongly limited by a threshold level of P. arundinacea biomass. Adequate site preparation (over multiple growing seasons) and aftercare (selective removal of P. arundinacea) will be the key to facilitating subsequent wet meadow vegetation establishment. This research provides an example of the importance of experimental evidence as the basis to improve the efficiency of restoration practices.

1663: *+.027*

When monitoring rare insect species, or when surveying faunas within nature reserves, it is desirable not to use indiscriminate lethal sampling techniques. In this investigation we assessed the usefulness of simple tree-mounted wooden shelters to monitor endemic weta (Orthoptera) in nature reserves in Canterbury, New Zealand. Fifty shelters were placed out at six sites and examined at three-monthly intervals for a year. A wide variety of invertebrates were found

utilizing the shelters, with Arachnida, Blattodea. and Collembola being the most common occupants. After three months over 80% of the shelters exhibited signs of use by invertebrates, increasing to 96% after 12 months. Only seven tree weta (Anostostomatidae) and one (dead) ground weta (Hemiandrus sp.) were observed in the shelters over the full 12 month period. There were 52 observations of cave weta (Rhaphidophoridae) in the shelters, 36 of which occurred at one site, Orton Bradley Park. Occupation of the shelters by cave weta was not affected by soil conditions, light intensity or aspect of the shelter. However, cave weta exhibited a preference for shelters less than 50 cm. above the ground and for shelters attached to kanuka and vines. Although weta were found in only a small proportion (9%) of the shelters, this method proved useful in confirming the presence of weta without risk of harming vulnerable populations. These shelters are inexpensive and easy to manufacture and have potential for long-term non-lethal monitoring of weta and as a collection/carriage device for live specimens used in conservation translocations.

1664: *+.224*

The use of headstarting and related techniques as management tools for threatened and endangered species remains controversial, due in part to a lack of empirical data on their effectiveness. Here, we present data on pre-natal mortality, growth, and survival during headstarting as well as growth, survival, and reproduction following release for an unprotected population of plains gartersnakes. We combine these data with data on growth and survival of comparably sized wild-caught snakes to provide an overall evaluation of the potential effectiveness of headstarting. Depending on rearing conditions, proportions of live births varied from 0.79 ('worst-case') to 0.94 ('best-case'), survival during headstarting ranged from 0.74 ('worstcase') to 0.88 ('best-case'), and second-year survival following release ranged from 0.11 ('worst-case') to 0.40 ('best-case'). In comparison, survival of free-ranging snakes was 0.16 in their first year and 0.40 in their second year. Assuming the proportion of live births in nature is close to 1, expected survival to reproductive maturity among free-ranging snakes is 0.06, whereas that for headstarted snakes ranges from 0.07 ('worst-case') to 0.33 ('best-case'). The growth rate of headstarted snakes following release was similar to that of similar-sized free-ranging snakes, and headstarted snakes were successful at reproduction. Together, these results suggest that, if carefully implemented, headstarting may be in effective management tool for endangered plains gartersnake populations in Ohio and for other natricine snakes with similar demographic characteristics.

1665: *+.015*

The Cape Fear shiner (Notropis mekistocholas) is a recently described cyprinid species endemic to the Cape Fear River Basin of North Carolina, USA. Only five populations of the fish remain; thus, it is listed as endangered by the U.S. Government. Determining habitat requirements of the Cape Fear shiner, including water quality and physical habitat, is critical to the survival and future restoration of the species. To assess water quality in the best remaining and in the historical habitats, we conducted a 28-d in situ bioassay with captively propagated Cape Fear shiners. Fish were deployed at 10 sites in three rivers, with three cages per site and 20 fish per cage. Water and sediment samples were collected and analyzed for selected metals and organic contaminants. Passive sampling devices also were deployed at each site and analyzed for organic contaminants at test termination. Fish survival, growth (as measured by an increase in total length), and contaminant accumulation were measured on completion of the bioassay. Survival of caged fish averaged 76% (range, 53-100%) and varied significantly among sites and rivers. Caged fish accumulated quantities of cadmium, mercury, polychlorinated biphenyls, and other persistent contaminants over the test duration and grew significantly at only four sites. No apparent relations were observed between exposure to or accumulation of a specific contaminant and reduced growth

or survival of fish among all the sites. However, a generalized hazard assessment showed that certain sites exhibited trends in cumulative contaminant presence with reduced fish survival and growth, thereby enabling the identification of the existing riverine habitat most suitable for reintroduction or population augmentation of this endangered fish.

1666: *+.056*

The three French species of Parnassius are linked to mountain habitats. P. mnemosyne scattered in the Alps and the Prealps, in the Vivarais - Aigoual and Puy de Dome - Aubrac ranges in the Massif central, and the Pyrenees. It feeds on Corydalis (Fumariaceae) species and is found in mountain and subalpine forests. It has only slightly regressed in marginal localities of its habitat. P. sacerdos is only found in the Alps, in alpine and subalpine zones, along the shores of the torrents. Its usual food plant is Saxifraga aizoides, but the taxon gazeli, from the Mercantour, is linked to Sedum roseum. Its distribution is stable. P. apollo was to be found in all the French mountain ranges, from the mountain zone (with "abyssal" localities down to 300 m) to the alpine zone, always in open landscapes. It feeds on Crassulaceae, with local specializations. It has strongly regressed and has become extinct in the Vosges and the north and the lower plateaux of the Jura; in the Massif-Central, it has vanished from mont Pilat, Puy, de Dome, Forez massif, southern Causses (Larzac and Causse Noir). Its populations have strongly diminished in Vivarais, Cevennes, Cantal, Puy de Sancy and the Prealps. It is still abundant in the Alps and the Pyrenees. Habitat choice is very precise in the three species. Growth cycle regulation, characterized by embryonic diapause, seems to play an important role in the extent of the elevation range. A rise of the lower limit of its habitat has been observed in recent years. The confinement of individuals within a given habitat seems to involve a social component. Population density is fairly constant and variation in size is accompanied with shrinking of the colonized area. Occupation of new habitats occurs during demographic expansive phases. Population size is strongly variable. Demographic depressions correspond to unfavourable climatic phases. An especially grave crisis, consecutive to 1989 and 1990 warm winters, struck southern French colonies and caused the extinction of some of them, previously thriving. Usually, during these crises, the colonies retract in the most favourable "hardcore" localities. The individuals from large populations display an active dispersal behaviour, while those from small populations are much more sedentary. Many subspecies have been described by "classical" taxonomists. Their informative value about the actual population genetic structure is rather poor. Combining wing pattern morphometry and electrophoresis study of enzyme polymorphism discloses a geographical variation of genetic structure. Both types of markers also show a marked decrease of gene diversity correlated with geographical isolation. This decrease seems to be accompanied by a decrease of phenotype "buffering" and a "closure" of population structure. This phenomenon is obvious in P. mnemosyne at Sainte-Baume, P. apollo in the Massif Central and P. sacerdos gazeli in the Mercantour. P. mnemosyne displays the most clear-cut geographical differentiation, which could correspond to a somewhat ancient history and a stable settlement of populations in the Quaternary, with some recent extensions. The genetic groups observed correspond to units defined ecogeographically and historically. In P. sacerdos, the most prominent feature is the isolation of the taxon gazeli, suggestive of a quasi-species status. The other populations from the southern Alps are differentiated along an E-W gradient. In P. apollo, variation is complex and confused. It may be the sign of an eventful recent history, with numerous episodes of directional selection which wiped out many polymorphisms. However, three large geographical entities can be disclosed : the Alps and the Jura, the Pyrenees and the Massif Central. Ecological and genetic characteristics of Parnassius populations suggest a metapopulation structure. Spatial and numerical variations appear to take place through retraction-inflation cycles around constant "hard cores". In the species concerned, the role of local stochasticity in population turnover seems to be overwhelmed at present times by large-scale climatic events, with extinction

prevailing upon colonization. There are two major causes of Parnassius regression : the closure of open spaces in mountain zones under oceanic influence, and (global) warming in southern regions. The first factor is linked to the abandonment of traditional land use, especially sheep grazing and coppicing. Some Vivarais populations were destroyed by deliberate afforestation. The present legislation for species protection is inefficient. National parks are well located for conservation ; the Cevennes national Park, where the problem is severe, may play an important role. Conservation actions must imply numerous partners : state, local communities, the Forest Service (Eaux et Forets), organizations of amateur entomologists. Small scale actions are precarious and large scale ones out of reach. Reintroductions are conceivable, but will only be successful if seriously designed. A husbandry policy in mountain zones should have a very positive indirect effect, especially if the ecological requirements of Parnassius are taken into account.

1667: *+.047*

The translocation of species to a new site plays an important role in the conservation of many threatened birds; however, the problems and processes involved in planning and implementing such translocations are rarely reported. In order to establish a second secure 'insurance' population of the endangered Rarotonga Monarch Pomarea dimidiata, or Kakerori, 30 young birds were moved from the Takitumu Conservation Area on Rarotonga to the 2,700 ha island of Atiu between 2001 and 2003. The translocation of this single-island endemic was to a site outside the historical range of the species, because the small, but rapidly growing, population on Rarotonga was considered to be highly vulnerable to a catastrophe, such as a cyclone, or the arrival of a new bird disease or predator. The translocation followed consultation with local communities, an assessment of the suitability of islands in the southern Cook Islands, and an assessment of the disease risk posed by the translocation. The translocation appears to have been successful because Rarotonga Monarchs have bred well in a variety of forest habitats on Atiu.

1668: *-.030*

The identification and recovery of endangered species is difficult because of their rarity, the continuing threats to their survival, and inadequate funding for research and conservation. There have been some success stories, but also a number of failures. Have biologists learned from our failures, or are we repeating the same mistakes? While habitat availability and cost are important limitations to species recovery, other, more easily addressed issues also hamper recovery programs. The Wyoming toad (Bufo baxteri) is an endangered species whose recovery has been stalled by problems that are common to species recovery efforts, especially for animals without significant "charisma." I summarize the research undertaken on the Wyoming toad since its listing, highlight the difficulties in building a scientifically based recovery program, and identify some of the unmet challenges impeding recovery. Although specific to the Wyoming toad, these recommendations are relevant to recovery programs facing similar issues.

1669: *+.231*

Saxifraga hirculus is a postglacial relict in Central Europe, whose populations suffered a dramatic decrease in the 19th and 20th centuries. However, few researchers have been interested in its ecological requirements in Central Europe. This article synthesizes previous knowledge supplemented by original data from the last large population (Switzerland).S. hirculus is a weak competitor which needs precise ecological conditions. It grows on bryophyte carpets in neutral to slightly acid wetlands, with stable water table close to the soil surface (optimum between 8 and 14 cm) but does not stand long flooding. However, it requires a good oxygen supply, with roots 2-3

cm under the soil surface, generally not reached by water, with running, cold water through loose, fibric peat. Its optimal conditions are in spring fens, but it grows in other types of wetlands as well. However, overgrowing by shrubs, sedges or Sphagnum in natural successions may threaten the species with extinction, as did drainage and peat extraction previously. Now, its survival in Central Europe depends on an adequate management of the ecosystems. Moderate grazing (cattle or sheep) or mowing help to limit competition with taller Carex species. Reintroduction of disappeared populations or creation of new ones from cultivation in botanical garden is possible, but appropriate sites are rare. in some cases, substrate management could improve the conditions in somewhat inadequate situations. This management in four directions can be flexibly applied in different situations to progress to optimal conditions for the conservation of this valuable species. (c) 2006 Elsevier Ltd. All rights reserved.

1670: *-.131*

The Poouli (Melamprosops phaeosoma) is a highly endangered Hawaiian honeycreeper endemic to Maui, and is currently one of the World's rarest birds. Only two wild individuals of this species are now known to exist, and they are restricted to the windward slopes of Haleakala volcano on east Maui. Studies of the more common honeycreepers on the Hawaiian islands describe a diverse array of spatial use and movement patterns, which vary according to specific ecological needs. In contrast, spatial use and movement of Poouli are very poorly known, despite continuous field monitoring of all three individuals since 1997. We analyzed annual data by breeding season between 1995 and 2001 for three individuals, and eight days of telemetry data derived from the radio-tracking of one individual in 2002, using GIS and conventional methods, to estimate home range size, and to interpret these data alongside those for other honeycreepers. We estimated mean home range sizes of 8.43 hectares (ha) from annual re-sights using kernel analysis and 2.14 ha using minimum convex polygons, and 8.44 and 3.51 ha respectively from telemetry data. Our estimates conform to those derived for other insectivorous honeycreepers, but indicate that Poouli may forage widely to support their diet of forest snails. Our home range size estimates are compatible with estimates of population density for Poouli that were derived from field transects between 1975 and 1985, suggesting that such field methods may be a reliable density estimator for rare forest birds.

1671: *+.211*

A study was conducted to approach the techniques and strategies for urban wildlife management from three aspects including species, habitat and landscape based on the status of Harbin. It is suggested that some important habitats should be set up as urban reserves to maintain wildlife populations, and population rising curve could be used to control some over-producing populations by separation and contraception. Disturbance is also an important concern in management, which helps to increase the community biodiversity by imposing some necessary disturbance. The management strategy based on disturbance gradients could settle the contradiction between the development of city and the maintenance of biodiversity. The selection of surrogate species is a shortcut for community management, and a group of indicator species could assure to obtain a more accurate monitoring result. A permit mechanism for the feeding and trading of alien species should be established, and the species reintroduction could be made with the improvement of urban environment and the melioration of habitat quality, and the introduction of more third class carnivores could fulfill niche vacancy of urban ecosystem. The management of habitat should take into consideration the characteristics of urban wildlife so as to meet the need of wildlife in different periods and to provide various cover resources. Corridors between patches should be built to minimize the influence of landscape fragment and heighten the landscape

connectivity of urban greenbelts, which would be helpful to support more wildlife in the limited habitat area.

1672: ***+.179***

Species reintroduction programmes, in prioritizing areas for reintroductions, have traditionally used tools that include measures of habitat suitability and evaluations of area requirements for viable populations. Here we add two tools to this approach: evaluation of ecological requirements of species and evaluation of future suitability for species facing changing climates. We demonstrate this approach with two species for which reintroduction programmes are in the planning stages in Mexico: California condor Gymnogyps californianns and Mexican wolf Canis lupus baileyi. For the condor, we identify three areas clustered in the Sierra San Pedro Martir, Baja California; for the wolf, we identify a string of suitable sites along the Sierra Madre Occidental of western Mexico. We discuss the limitations of this approach, identifying ways in which the models illustrated could be made more realistic and directly useful to reintroduction programmes.

1673: ***-.000***

Molecular forensic methods are being increasingly used to help enforce wildlife conservation laws. Using multilocus genotyping, illegal translocation of an animal can be demonstrated by excluding all potential source populations as an individual's population of origin. Here, we illustrate how this approach can be applied to a large continuous population by defining the population genetic structure and excluding suspect animals from each identified cluster. We aimed to test the hypothesis that recreational hunters had illegally introduced a group of red deer into a hunting area in Luxembourg. Reference samples were collected over a large area in order to test the possibility that the suspect individuals might be recent immigrants. Due to isolation-by-distance relationships in the data set, inferring the number of genetic clusters using Bayesian methods was not straightforward. Biologically meaningful clusters were only obtained by simultaneously analysing spatial and genetic information using the program (BAPS) 4.1. We inferred the presence of three genetic clusters in the study region. Using partial Mantel tests, we detected barriers to gene flow other than distance, probably created by a combination of urban areas, motorways and a river valley used for viticulture. The four focal animals could be excluded with a high certainty from the three genetic subpopulations and it was therefore likely that they had been released illegally..

1674: ***-.005***

SMITH, M. (Department of Biological Sciences, Southern Illinois University, Edwardsville, IL 62026) AND E. HUNSLEY (Edwardsville High School, Edwardsville, IL 62025). Effects of achene pattern, achene density and competition on germination in Boltonia decurrens, a threatened plant of the Illinois River Valley. J. Torrey Bot. Soc. 133: 528-534. 2006-Boltonia decurrens is a plant that occurs only on floodplains of the Illinois River and in the area where the Illinois, Missouri and Mississippi Rivers merge. The number of naturally occurring populations has declined over the past 100 years. Boltonia decurrens is on the federal list of threatened species, and is listed as threatened in Illinois and as a species of concern in Missouri. Decline of the species is generally attributed to the loss of suitable habitat due to the alteration of the hydrology of the Illinois River. Without regular flooding, which provides appropriate habitat for the species, a B. decurrens population is replaced within three to five years by a variety of herbaceous species; its survival is dependant upon seedling establishment to renew extant populations and to colonize new sites. In this study, we assessed the effects of achene pattern (aggregated or random) and

density (high, medium and low) on the germination of B. decurrens' achenes in the presence of three competitors: Polygonum pensylvanicum, Aster pilosus and A. lateriflorus. Percent germination of B. decurrens' achenes was significantly higher in random compared to aggregate treatments, indicating that there was no positive effect of conspecific aggregation of achenes on germination nor a negative effect of nearby competitor achenes. Boltonia decurrens' germination was highest at low density in the random treatment, compared to the same density in aggregated achenes, thus suggesting the possibility of an autoallelopathic effect. Although B. decurrens' germination was not affected by the presence of competitors, there were significant differences in germination among species: Aster lateriflorus had higher percent germination than P. pensylvanicum, A. pilosus or B. decurrens, and germination of B. decurrens was significantly higher than P. pensylvanicum or A. pilosus. Data from this study will provide insights into the role of competition during germination in floodplain communities and be useful to conservation personnel when they evaluate potential sites for reintroduction or plan control measures to target the species that pose the greatest threat to B. decurrens at a population site.

1675: *+.037*

The endangered California Condor (Gymnogyps californianus) was reduced to a total population of 22 birds by the end of 1982. Their captive-bred descendants are now being released back into the wild in California, Arizona, and Baja California, where monitoring indicates they may accumulate lead to toxic levels. Fragments of ammunition in the carcasses of game animals such as deer, elk, and feral pigs not retrieved by hunters or in gut piles left in the field have been considered a plausible source of the lead, though little direct evidence is available to support this hypothesis. Here, we measured lead concentrations and isotope ratios in blood from 18 condors living in the wild in central California, in 8 pre-release birds, and in diet and ammunition samples to determine the importance of ammunition as a source of exposure. Blood lead levels in pre-release condors were low (average 27.7 ng/mL, SD 4.9 ng/mL) and isotopically similar to dietary and background environmental lead in California. In contrast, blood lead levels in free-flying condors were substantially higher (average 246 ng/mL, SD 229 ng/mL) with lead isotopic compositions that approached or matched those of the lead ammunition. A two-endmember mixing model defined by the background Pb-207/Pb-206 ratio of representative condor diet samples (0.8346) and the upper Pb-207/Pb-206 ratio of the ammunition samples (0.8184) was able to account for the blood lead isotopic compositions in 20 out of the 26 live condors sampled in this study (i.e., 77%). Finally, lead in tissues and in a serially sampled growing feather recovered postmortem from a lead-poisoned condor in Arizona evidence acute exposure from an isotopically distinct lead source. Together, these data indicate that incidental ingestion of ammunition in carcasses of animals killed by hunters is the principal source of elevated lead exposure that threatens the recovery in the wild of this endangered species.

1676: *+.259*

Translocation, the intentional release of captive-propagated and/or wild-caught animals into the wild in an attempt to establish, reestablish, or augment a population, is a commonly used approach to species conservation. Despite the frequent mention of translocation as an aid in threatened or endangered species recovery plans, translocations have resulted in the establishment of few sustainable populations. To improve the effectiveness of translocation efforts, it is essential to identify and adopt features that contribute to successful translocations. This study analyzed 148 translocations of the endangered Gila topminnow (Poeciliopsis occidentalis) to identify various factors that have significantly influenced translocation success. We quantified success as the "persistence time" of translocated populations and used survival analysis to interpret the role of

several factors. The following factors affected persistence times of translocated populations: season in which the fish were translocated, habitat type of the translocation site, and genetic origin of the fish stocked. In general, factors associated with stocking, the population stocked, and the site of translocation can significantly affect the persistence of translocated populations and thus increase the probability of translocation success. For Gila topminnow, future translocations should be undertaken in late summer or fall (not early summer), should occur into ponds (not streams, wells, or tanks), and should generally utilize individuals from genetic lineages other than Monkey Spring. For other species, a key lesson emerging from this work is that life history attributes for each translocated species need to be considered carefully.

1677: *+.184*

Restoration of habitat for endangered species often involves translocation of seeds or individuals from source populations to an area targeted for revegetation. Long-term persistence of a species is dependent on the maintenance of sufficient genetic variation within and among populations. Thus, knowledge and maintenance of genetic variability within rare or endangered species is essential for developing effective conservation and restoration strategies. Genetic monitoring of both natural and restored populations can provide an assessment of restoration protocol success in establishing populations that maintain levels of genetic diversity similar to those in natural populations. California's vernal pools are home to many endangered plants, thus conservation and restoration are large components of their management. Lasthenia conjugens (Asteraceae) is a federally endangered self-incompatible vernal pool annual with gravity-dispersed seeds. Using the molecular technique of intersimple sequence repeats (ISSRs), this study assessed levels and patterns of genetic variability present within natural and restored populations of L. conjugens. At Travis Air Force Base near Fairfield, California, a vernal pool restoration project is underway. Genetic success of the ecologically based seeding protocol was examined through genetic monitoring of natural and restored populations over a three-year period. Genetic diversity remained constant across the three sampled generations. Diversity was also widely distributed across all populations. We conclude that the protocol used to establish restored populations was successful in capturing similar levels and patterns of genetic diversity to those seen within natural pools. This study also demonstrates how genetic markers can be used to inform conservation and restoration decisions.

1678: *+.324*

Strategies are needed to recover the ocelot Leopardus pardalis from the endangered species list. Recently, a population viability analysis (PVA) was developed which concluded that combinations of different recovery strategies were needed to effectively reduce ocelot extinction probability in the United States (US), with habitat protection and restoration identified as the most effective recovery scenario. We expanded this PVA model by incorporating landscape data to develop a more realistic habitat-based PVA for ocelots in southern Texas. We used RAMAS/(GIS) software to conduct a habitat-based PVA by linking landscape data with a demographic metapopulation model. The primary goal of this study was to provide a model for evaluating ocelot recovery strategies in the US. Each model scenario was simulated 1000 times over 50 years and we defined extinction as one individual remaining. Using the RAMAS/(GIS) program we identified 11 possible ocelot habitat patches (i.e., subpopulations) occurring in southern Texas. In addition, based on the habitat-based PVA model we found that combinations of different recovery strategies were needed to effectively reduce ocelot extinction probability in the US, with reducing road mortality the single most effective strategy. Short-term recovery strategies should include reducing ocelot road mortality, and translocation of ocelots into the US from northern Mexico. Long-term

recovery strategies should include the restoration of habitat between and around existing ocelot habitat patches and the establishment of a dispersal corridor between ocelot breeding populations. (c) 2006 Elsevier Ltd. All rights reserved.

1679: *+.083*

Allen Cays Rock Iguanas (Cyclura cychlura inornata) are native to two small islets (Leaf and U Cay) in the north-central Bahamas. These populations were nearly extirpated in the early 1900s because of heavy hunting pressure (for food), but increased to a total of ca. 150 lizards in 1970, and now number over 500 (not including juveniles). Over the past several decades poaching has declined, but tourist visitation (including nearly daily supplemental feeding of iguanas) has increased. To examine human impacts on the demography of these iguanas, survival, population growth rates, and population sizes for subadult and adult (> 25 cm snout-vent length) males and females on the two cays were estimated based on mark-recapture data collected over a 25-year period (1980-2004). As predicted, annual survival probability was higher on U Cay (with less human visitation) than on Leaf Cay, was higher in females than in males (which are bolder), and exhibited a declining trend. Both populations more than doubled during this study, but population growth rates declined to near zero in recent years. These data reflect the importance of human impacts, but also suggest that the populations may be nearing carrying capacity. The rapid population growth observed on these cays, and that seen for several other translocated iguana populations, suggest that if unnatural causes of mortality are reduced or eliminated, island populations of iguanas are capable of rapid recovery. The inexpensive establishment of assurance colonies on undisturbed "islands" should be considered for any comprehensive management plan for endangered species of iguanas. (c) 2006 Elsevier Ltd. All rights reserved.

1680: *-.061*

We documented the fate of 29 cohorts of propagated Barrens topminnows Fundulus julisia stocked as juveniles and adults (n(total) = 2770 fish) into 17 springheads and small ponds in middle Tennessee in 2003 and 2004. Annual mortality rates were calculated after estimating the number of individuals of each cohort remaining 1-18 mos after fish were stocked. Lighted larval fish traps were deployed at seven reintroduction sites and the Type Locale to determine whether topminnows could reproduce in the presence of the introduced-transplanted Western mosquitofish Gambusia affinis. At stocking sites harboring mosquitofish (n = 12), their density ranged from 0.4 to 66.3 mosquitofish per m(2). Annual mortality of stocked Barrens topminnows ranged from 45 to 100% and 24 cohorts experienced annual mortality greater than 95%. Mortality was not related to mosquitofish density or the mean size at stocking. The robustness of Barrens topminnows did not differ in the presence or absence of mosquitofish, suggesting that interspecific competition for food was not occurring. Larval Barrens topminnows were collected at two reintroduction sites and the Type Locale, but juvenile recruits were produced only at sites lacking mosquitofish. The findings of this study, and concurrent laboratory studies, support the hypothesis that mosquitofish predation on larval Barrens topminnows was the primary mechanism in failed reintroductions and is the greatest threat to wild and reintroduced populations of this imperiled species.

1681: *-.069*

The Chinese alligator (Alligator sinensis) is a critically endangered species in China. Wild Populations of Chinese alligator are on the edge of extinction. Through a release program, some captive-bred alligators will be selected and released into the wild to supplement and renew natural Populations. The purpose of this Study was to investigate the genetic variation of captive-bred

Chinese alligators by AFLP markers and to select individuals with maximally different genetic backgrounds for release. Forty-three captive-bred alligators of the second filial generation from the Anhui Research Center for Chinese Alligator Reproduction (ARCCAR) were surveyed Using four primer combinations, yielding 117 AFLP markers. According to AFLP fingerprints, six samples had distinctly different band patterns compared to other samples. When the six samples were removed from the analysis, there were 19 monomorphic loci and 98 polymorphic loci yielding 84% polymorphic loci. Moreover, the genetic similarity (GS) among 37 samples varied from 0.13-0.97, and the average was 0.7503 +/- 0.0064 standard error (SE). When the six samples were included, the GS value among the 43 samples declined and varied from 0.06-0.97, and the average was 0.6523 +/- 0.0079 SE. Based on a cluster analysis using UPGMA, a dendrogram of the 43 alligators was constructed. According to the cluster analysis and gender of the 43 samples, eight Chinese alligators with very different genetic backgrounds were selected and suggested for release with two groups in the future.

1682: *+.003*

The Asian Vigna group of grain legumes consists of six domesticated species, among them black gram is widely grown in South Asia and to a lesser extent in Southeast Asia. We report the first genetic linkage map of black gram [Vigna mungo (L.) Hepper], constructed using a BC1F1 population consisting of 180 individuals. The BC1F1 population was analyzed in 61 SSR primer pairs, 56 RFLP probes, 27 AFLP loci and 1 morphological marker. About 148 marker loci could be assigned to the 11 linkage groups, which correspond to the haploid chromosome number of black gram. The linkage groups cover a total of 783 cM of the black gram genome. The number of markers per linkage group ranges from 6 to 23. The average distance between adjacent markers varied from 3.5 to 9.3 cM. The results of comparative genome mapping between black gram and azuki bean show that the linkage order of markers is highly conserved. However, inversions, insertions, deletions/duplications and a translocation were detected between the black gram and azuki bean linkage maps. The marker order on parts of linkage groups 1, 2 and 5 is reversed between the two species. One region on black gram linkage group 10 appears to correspond to part of azuki bean linkage group 1. The present study suggests that the azuki bean SSR markers can be widely used for Asian Vigna species and the black gram genetic linkage map will assist in improvement of this crop.

1683: *+.049*

The cycad Ceratozamia mirandae is endemic to Chiapas, Mexico. Demographic studies were made in two of its populations in the Sepultura Biosphere Reserve under different conservation conditions; in the nucleus zone "Tres Picos" (conserved) and buffer zone "La Sombra" (disturbed and under management). Spatial distribution of C. mirandae was aggregated, showed a clumped local distribution on shallow soils on steep slopes and male and female cones appear to be synchronous in both populations. The population structure was of type I (Bongers) for both sites. Individuals between the sites showed differences in growth pattern. The oldest plants (80-90 cm tall) were estimated to be about 490 years at "La Sombra". The finite growth rate (lambda) in the buffer zone population showed a tendency for decrease whilst in the nucleus zone this estimate remained stable. The highest elasticity values lied in the transition of the first three classes of the "La Sombra" population, in "Tres Picos" this corresponded to adult plants between 20 and 30 cm tall. Given the above, it is proposed that in the nucleus zone, reproductive adults should be of highest conservation priority, whereas in the buffer zone seedling reintroduction should be carried out regularly until the population increases. We recommend an IUCN Red List category of Vulnerable (VU C, 2a), largely due to difficult-to-control destructive annual forest fires that occur

in this Reserve.

1684: ***+.210***

In South Africa, a plan was launched to manage separate sub-populations of endangered African wild dogs Lycaon pictus in several small, geographically isolated conservation areas as a single meta-population. This intensive management approach involves the re-introduction of wild dogs into suitable conservation areas and periodic translocations among them. Here, we sought to evaluate the relevance of taking the formation of new reproductive units into consideration in promoting such translocation attempts. For this purpose, we analysed the behavioural process of integrating translocated wild dogs into new packs in pre-release holding facilities in Hluhluwe-iMfolozi Park, one of the meta-population conservation areas. In addition, we reviewed findings from other wild dog translocation attempts in regard to the outcome of using variously composed groups and captive-bred animals for the artificial creation of new packs for translocation purposes. We show the importance of social integration before release for wild dog re-introductions and translocations to be successful. We also present a set of proximate factors, including management manipulation of social relationships, which promote pack formation in pre-release holding facilities. This demonstrably enhances the efficiency of costly wild dog re-introductions and translocations, thereby illustrating the implications of sociality for endangered species recovery.

1685: ***+.299***

Information front more than hundred published sources, augmented by previously unpublished expert knowledge, is compiled to build tip a species account for the Western Palaearctic planorbid gastropod Anisus vorticulus (Troschel, 1834), threatened at European level and recently listed in the EU Habitats Directive. The account summarizes the available data about the species, including identification, environmental requirements, life cycle, dispersal, food, geographic distribution and threats, together with recommendations for site management and survey procedures. The species occurs in both natural habitats (lake littoral, streams, river floodplains) and man-made habitats (drainage ditches, excavations). It is mostly associated with calcareous, moderately well vegetated habitats, especially with abundant floating plant coverage. The species has normally an annual life-cycle but the few available data suggest a high spatial and inter-annual variability in its phenology. To date, conservation measures for the species included vegetation removal and translocation. A dearth of quantitative and general information is obvious for almost all aspects of the biology and ecology of the species and prevents informed conservation recommendations being made.

1686: ***+.202***

Alvars are habitats characterised by thin soils on limestone bedrock. The largest alvar area in the world is the Stora Alvaret, Oland (25,500 hectares), which is characterised by the presence of several plant communities of high conservation value. Emigration at the end of the nineteenth century reduced land-use intensity resulting in scrub encroachment, mainly by Juniperus communis L. This later accelerated, leading to abandonment because grazing in the low-productivity pastures was uneconomic. Monitoring in permanent plots over a twenty-year period (1971-91) showed an increase in juniper cover over time and a clear correlation with a decline of other vascular plant species. This resulted in a gradual loss of both a valuable mosaic structure and a high level of bio-diversity in the landscape. An EU funded LIFE project (1996-99), which examined the effects of grazing and scrub clearance, is outlined. Various management techniques were tested and evaluated, providing valuable information for the present large-scale restoration

and management programme supported by the European Union agri-enviromuent schemes. In 1994 less than 60% of the Stora Alvaret was grazed. By 2005 this had increased to 98 %. The reintroduction of grazing has resulted in a significant increase in the abundance of twelve out of 72 vascular species in permanent plots (1997-2001). Sheep and cattle numbers increased in Oland during the last decade, whereas they decreased in Sweden as a whole over the same period. Sixteen nature reserves have been established in Stora Alvaret, and a functioning alvar pasture landscape has been re-established. Almost all alvars in Oland have been designated as Natura 2000 areas.

1687: *+.090*

The Andalusian hemipode (Turnix sylvatica sylvatica) (Order: Turniciformes, Family: Turnicidae), formerly distributed in several Mediterranean countries, is a critically endangered bird, if not already extinct. Subspecies of the T. sylvatica complex, in turn composed by nine subspecies are widely distributed in Africa and southern Asia. The last free-ranging Andalusian hemipodes were shot by hunters near Donana National Park (Spain) in 1981. Therefore, this species could be the last bird species getting extinct in Iberia and Europe in the XXth century. This investigation deals with the phylogenetic relationships of the Andalusian hemipode with the supposedly congeneric T. varia, T. tanki, T. suscitator and T. pyrrhothorax, and with the supposedly conspecific T. sylvatica lepurana, which is the geographically nearest buttonquail population (occurring in central and southern Africa). A 606 bp long fragment of the cytochrome b gene (approx. 1140 bp) of the mitochondrial DNA was sequenced, using both museum skins (the only available source for T. s. sylvatica) or blood/tissue samples from contemporary individuals (remaining species and subspecies). Seven haplotypes were found: two each for T. varia and T. s. lepurana, and one each for T. tanki, T. suscitator, T. pyrrhothorax, and T. s. sylvatica. Sequence divergence values obtained from pairwise distances between the T. sylvatica group haplotypes and the other species, ranged from 19.4 to 25.9%. The low genetic divergence between T. s. sylvatica and T. s. lepurana (0.00-0.01%) confirmed that the current classification based on morphological characters is correct, and that these two taxa may should be considered as subspecies. This close relationship would permit an introduction T. sylvatica where the species was last seen in Spain (i.e., Donana National Park). This area is now strictly protected and human persecution is no longer a problem.

1688: *+.065*

Understanding the factors that drive the dynamics of remnant populations of long-lived species presents a unique challenge for conservation management. The long-lived Brothers Island tuatara Sphenodon guntheri is represented by one natural, self-sustaining population on 4-ha North Brother Island, New Zealand, and two small, translocated populations. The North Brother Island population was almost driven to extinction by extreme habitat modification and collecting in the late 19th century. Analysis of a long-term (1957-2001) dataset, following the population's recovery, reveals a significant decline in tuatara body condition over time, which is more pronounced in females. Declining body condition, coupled with very low reproductive output, may be symptomatic of a density-dependent response to elevated population size exacerbated by resource limitation. Sex-specific effects that disadvantage females could compromise this small population, particularly as it exhibits a male-biased sex ratio. We recommend removal of infrequently used structures and habitat restoration to alleviate intense resource competition. Population-level manipulation should be considered if future monitoring indicates an increasingly male-biased sex ratio and continued decline of female body condition.

1689: ***+.218***

As an alternative to kin selection, group augmentation theory provides a framework for evolutionary mechanisms maintaining cooperative breeding when individual fitness is positively related to group size. It is expected that a cooperator group would accept or adopt unrelated foreigners when it is below a critical threshold size and group members could thus benefit from recruiting additional helpers. In re-introduction attempts, this would allow for a group to be augmented artificially before release, which would enhance its chance to establish itself successfully in the release area. This possibility was tested using endangered African wild dogs Lycaon pictus studied in Hluhluwe-iMfolozi Park, South Africa. Here, we report on the first successful artificial integration of an unrelated adult female with her three male pups into an existing pack. In addition, post-release monitoring data are presented, including how a yearling male displaced the dominant male that adopted him as a pup, adding to the controversy over the evolutionary stability of group augmentation as a route to cooperative breeding. This study thus demonstrates how theory from evolutionary ecology can be applied to practical wildlife management, and vice versa.

1690: ***-.010***

Background: In Europe the mountain hare (Lepus timidus) exists in Great Britain, Norway, Sweden, Finland, parts of the Alps and in Eastern Europe, but not in Denmark. Interspecific hybridization has been demonstrated between native Swedish mountain hares and introduced brown hares (Lepus europaeus). During the data collection in a study concerning Danish brown hares we identified 16 hares with a single very divergent haplotype. Results: Phylogenetic analysis shows that the divergent Danish haplotype is most closely related to the Swedish mountain hare. The frequency of Lepus timidus mtDNA haplotype in the Eastern Danish hare populations is estimated to 6%. Conclusion: In contrast to what is known, the Danish hare populations are not pure L. europaeus populations but include introgressed brown hares with Swedish L. timidus mtDNA. The most probable explanation of this is natural migration or translocation of introgressed brown hares from Sweden. The impurity of hare populations has implications for conservation and population genetics.

1691: ***+.023***

Background: In Europe the mountain hare (Lepus timidus) exists in Great Britain, Norway, Sweden, Finland, parts of the Alps and in Eastern Europe, but not in Denmark. Interspecific hybridization has been demonstrated between native Swedish mountain hares and introduced brown hares (Lepus europaeus). During the data collection in a study concerning Danish brown hares we identified 16 hares with a single very divergent haplotype.Results: Phylogenetic analysis shows that the divergent Danish haplotype is most closely related to the Swedish mountain hare. The frequency of Lepus timidus mtDNA haplotype in the Eastern Danish hare populations is estimated to 6%.Conclusion: In contrast to what is known, the Danish hare populations are not pure L. europaeus populations but include introgressed brown hares with Swedish L. timidus mtDNA. The most probable explanation of this is natural migration or translocation of introgressed brown hares from Sweden. The impurity of hare populations has implications for conservation and population genetics.

1692: ***+.021***

Targeted sampling for gobiid fishes in the Port River estuarine system adjacent to Adelaide, South

Australia, identified four previously unrecorded species. Significant range extensions along the east-west coastline of southern Australia are reported for the Australian endemic flatback mangrove goby Mugilogobius platynotus (Gunther, 1861), largemouth goby Redigobius macrostoma (Gunther, 1861) and Krefft's frill goby Bathygobius kreffti (Steindachner, 1866) plus the alien Trident goby Tridentiger trigonocephalus (Gill, 1859). Moreover, M platynotus, R. macrostoma and T. trigonocephalus are new records to the fish fauna of the state of South Australia. While it is clear that T trigonocephalus has invaded another southern Australian port, there is difficulty in determining the status of the three Australian endemics as being either native to the area or recent introductions (e.g. through ship mediated translocation) due to a previous paucity of sampling and the cryptic nature of goby behaviour that may have prevented historic detection. The long-term existence of suitable habitat on the one hand suggests that these populations are naturally occurring in the Port River. However, a drastically altered estuarine environment, the high incidence of other translocated marine organisms in the system and goby biological traits suiting transportation in ship ballasts or hull fouling conversely casts doubts over their origin. Contrasting management scenarios of conservation versus potential eradication for these newly discovered species highlights a dilemma for biodiversity conservation in an altered environment.

1693: *-.036*

Prairie grouse populations are difficult to reestablish after extirpation. Following translocation, distances individuals move from the release site appear to affect restoration success. Previous authors have suggested assessing lek, nest-brood, and winter habitat when selecting release sites. We examined movement of 131 (66 M and 65 F) radiomarked Columbian sharp-tailed grouse (Tympanuchus phasianellus columbianus) translocated during 1999-2002 as part of management effort to restore populations to historical ranges in northeastern Nevada, USA, an area where sharp-tailed grouse have not been observed in the wild since the 1950s. We released grouse at 2 sites. We chose the initial site based on its physiographic and vegetation similarities to capture sites in Idaho, USA, particularly shrub-steppe at lower elevations and mountain shrub at higher elevations, and used it during 1999 and 2000 (34 M, 18 F in 1999; 42 M, 26 F in 2000). Females released at this site moved greater distances than males through time, with no differences between years. We changed the release site based on nest locations of previously translocated females. The second site was 10 km south of site 1 and we used it in 2001 and 2002 (36 M, 22 F in 200 1; 14 M, 5 F in 2002). Grouse released at this site moved substantially shorter distances than did the grouse initially released, and movement distances did not differ by gender or year. During 2004 we observed 23 grouse displaying on a lek near site 2 and observed no grouse near site 1. Our results support the hypothesis that nest-site availability is an important component to release-site selection insofar as sharp-tailed grouse in our study moved less when released into habitat that had been selected for nesting by previously released grouse.

1694: *+.020*

It is presented a bird survey of a forest fragment at the upper Rio Doce valley carried out from April 2002 to November 2004. The region is within one of the most important Brazilian hot spot for biological conservation: the Atlantic Forest. The fragment is within 'Estacao de Desenvolvimento Ambiental de Peti' (EPDA-Peti) under the premises of a hydroelectric power station run by the Minas Gerais Energetic Company. It holds 605 ha of a mosaic of secondary growth forest patches from different ages, characterised mainly by semi deciduous forest. Bird census were carried out through transect, mist-net captures, point counts and recording bird vocalizations. The present survey was compared to an unpublished survey performed in 1989 to

find out possible extinctions and colonization. It was recorded 231 species, belonging to 57 families. This represents 33% of all 682 bird species recorded for the Atlantic forest blome of east Brazil. Thirty-three species are considered endemic to the Atlantic Forest and one is considered endemic to the Cerrado biome of central Brazil. Five species are threatened in the state of Minas Gerais and one species is globally threatened to extinction, the red-billed curassow Crax blumenbachii Spix, 1825 (Cracidae). It was found 35 species not reported before for the area. Also, 52 species went locally extinct from the fragment. The EPDA-Peti holds a significant number of the Atlantic Forest bird species, and long-term bird monitoring on such fragments will reveal important aspects for the understanding of colonization and extinction in the biome.

1695: ***+.132***

Although species reintroduction is useful as both an integral part and a performance indicator of habitat restoration, it is not a risk-free process. Evaluation of potential reintroduction sites is often crucially important in reducing chances of failure. Despite its importance, there is no standard way to do so. This study applied a systematic scheme to evaluate five potential reintroduction sites for the endangered Karner Blue butterfly (Lycaeides melissa samuelis Nabokov) in Ontario, Canada, by looking at both biotic and abiotic aspects. Field data were collected in 2003 from these sites, and three potential founder butterfly sites in the United States. We used data collected from the U.S. sites to determine the minimum standards for ecological requirements of the Karner Blue. Data from the Ontario sites were then compared against these standards. The results show that all five potential reintroduction sites are of lower quality, at least in certain aspects, compared with the three potential founder butterfly sites. This implies that success is not guaranteed if the Karner Blue is reintroduced into these sites under current habitat conditions. Further site restoration is required and should focus on the shortcomings identified for individual sites in this study. The lessons from this study are useful for potential reintroduction site assessment in other restoration projects because they reveal whether the sites are ready for species reintroduction and, if not, how they need to be improved.

1696: ***+.106***

The Bohemian Forest straddles the upland border between Bavaria, the Czech Republic and Upper Austria. The Ural Owl is believed to have been extinct in the region since 1926, when the last bird was shot. With the foundation in 1970 of the Bavarian, Forest National Park within the Bohemian Forest belt, the possibility of re-establishing the original faunal diversity was discussed, e.g. by re-introduction of locally extinct species. The occurrence of Strix uralensis at this western fringe of the species' range represented a relict of post-glacial times, when forest spread over Central Europe. A reintroduction project relied on the release of young, captive-bred owls. The captive breeding stock consisted of owls from Sweden (4 male), Romania (2 female), Slovenia (2 male), Croatia, Finland, European Russia (1 male each) and Slovakia (1-2 female). From this foundation stock, between 5 and 11 breeding pairs were assembled in the National Park between 1972 and 2005, with a further 2 - 7 pairs elsewhere (zoo parks, rehabilitation centre, private keeper) during the same period. Since the first breeding success in 1973, 204 young Ural Owls have been reared (2 by hand, 202 by parent birds). Average date of first egg, March 21(st) (March 4(th) - April 16(th); n = 48); of hatching about April 22(nd) (n = 50); of leaving the nest-hole about May 24(th) (n = 38). Average clutch size (n = 93) was 2.4 eggs (max. 5-6), with average 3 eggs in replacement clutches. Ural Owl eggs are rather small in proportion to the body size of the female owl and measured 51.4 x 42.3 mm on average, smaller eggs overlapping in size those of the Tawny Owl Strix aluco. From this breeding programme, 212 Ural Owls (189 young birds and 23 older than 1 year) were released into the Bavarian Forest National Park between 1975 and 2005. Experiments

with the adoption of 10 day old-nestlings by free-living Tawny Owls and four week old owlets by free-living Ural Owls were successful, but the use of Tawny Owls as foster parents exposed the risk of hybridisation in these sibling species. To aid establishment, food was presented near the release pen (to compensate for the lack of prey offered by parent birds) and about 60 large nest boxes were erected to bridge the lack of natural breeding sites in younger forest stands. Of 37 owls recovered (33 found dead and 4 weakened birds recaptured) most had died by electrocution or by collision with cars or forest fences (27%); a few individuals had been shot. Owls released in their first year dispersed 11.4 km on average (max. 52 km; n = 29). Above this age migratory behaviour disappears and older released birds moved only 2.7 km on average (n = 4). Based on 330 field observations and the monitoring of 29 radio-tagged owls, the preferred habitats can be defined as old mixed mountainous forest or old growth stands rich in beech trees, in climatically favourable settings, so long as these are interrupted by meadows, wind-blow glades or gaps resulting from insect infestation. The first wild breeding attempt occurred in 1985, the first successful breeding in 1989 (4 young). Between 1981 and 2005 a total of 49 broods were recorded, 31 of these being successful with at least 59 young reared (1.3 young per brood initiated or 1.9 young per successful brood). To date > 6 pairs of Ural Owls have become established within the Bavarian Forest National Park, whose 240 km(2) have a theoretical carrying capacity of 10 breeding pairs. In order to reach a minimum viable population of 30 breeding pairs in the wider Bohemian Forest, cross-border cooperation is essential. Since 1991 a reintroduction programme has been running in the Sumava National Park (720 km(2)), where 87 Ural Owls were released between 1995 and 2006. Some 2-3 breeding pairs are now confirmed here. In Muhlviertel, Upper Austria, two owls were released in 2001 but this pilot project has not been continued although breeding has since been suspected. The rising proportion of broad-leaved trees and the development of old growth forest without timber extraction should lead to improvement in habitat quality, at least in the two national parks. An increase in Ural Owls across the wider region would be fundamental to the success of further conservation measures. Further support of the still young population in the Bohemian Forest appears indispensable, with cross-border participation by the national parks in Bavaria and Bohemia, and the nature conservation authorities and NGOs in Upper Austria.

1697: ***+.053***

The Great Philippine Eagle Pithecophai,a jcffelyi is regarded as one of the most endangered eagles in the world. Habitat loss and human persecution continue to impact the species adversely. These problems are complicated further by a number of human-induced factors. Yet despite these obstacles, sustained conservation initiatives over the years have began to yield positive results. The current population status of the species and threats are outlined in this report. In situ and ex situ actions associated with the conservation of the species show how these threats are being addressed. Advances made to further our understanding of the species' biology and ecology are also discussed. With continuing success in the captive propagation of the Philippine Eagle, the program is now in the process of initiating reintroductions. This is anticipated to take Philippine wildlife conservation into its next level. Recent developments and plans to strengthen recovery goals for the species are also presented in this paper.

1698: ***-.001***

Hedgehogs (Erinaceus europaeus) are being killed on the Uists, in the Outer Hebrides, Scotland, in an attempt to improve the breeding success of ground nesting birds. Translocation of hedgehogs was considered as an option, but dismissed on welfare grounds. The principal concern was that translocated animals would starve. The present study set out to test this hypothesis. Twenty hedgehogs from the Uists were released on the Scottish mainland at Eglinton Country Park, Irvine

and radio tracked for a month to ascertain whether or not the animals were going to starve in their new environment. Seven of the twenty radio tracked hedgehogs showed significant weight gains, five maintained their weight, and three lost weight. Two of the hedgehogs that lost weight died during the study. There were three early deaths from predation and drowning, one from a pre-existing tumour. If these deaths and the individual that vanished on the first night are removed from the analysis the results indicate an 80% survival rate one month after translocation. If all early deaths by predation and drowning are attributed to the unfamiliarity of the translocated hedgehogs with the terrain, the survival rate is 67% one month after translocation. The data also indicate that there is an advantage for females to weigh at least 550 g on release. Although conclusions should be drawn with care due to the limited sample size, study period and research approach our study suggests that concerns over the welfare of translocated hedgehogs are not well-founded, thus questioning the principal objection to such an undertaking.

1699: *-.063*

As a charismatic carnivore that is vulnerable to extinction, many studies have been conducted on predation by the cheetah Acinonyx jubatus. Cheetah are generally considered to capture medium-sized prey; however, which species are actually preferred and why has yet to be addressed. We used data from 21 published and two unpublished studies from six countries throughout the distribution of the cheetah to determine which prey species were preferred and which were avoided using Jacobs' index. The mean Jacobs' index value for each prey species was used as the dependent variable in multiple regression, with prey abundance and prey body mass as predictive variables. Cheetah prefer to kill and actually kill the most available prey present at a site within a body mass range of 23-56 kg with a peak (mode) at 36 kg. Blesbok, impala, Thomson's and Grant's gazelles, and springbok are significantly preferred, whereas prey outside this range are generally avoided. The morphological adaptations of the cheetah appear to have evolved to capture medium-sized prey that can be subdued with minimal risk of injury. Coincidentally, these species can be consumed rapidly before kleptoparasites arrive. These results are discussed through the premise of optimality theory whereby decisions made by the predator maximize the net energetic benefits of foraging. Information is also presented that allows conservation managers to determine which prey species should be in adequate numbers at cheetah reintroduction sites to support a cheetah population. Conversely, these results will illustrate which potential prey species of local conservation concern should be monitored for impact from cheetahs as several species are likely to be preyed upon more frequently than others.

1700: *-.207*

The Columbia basin subpopulation of pygmy rabbit Brachylagus idahoensis was listed as endangered by the United States Fish and Wildlife Service in November 2001, and no pygmy rabbits have been seen in the wild since spring 2002. Captive propagation efforts have attempted to increase population size in preparation for reintroduction of animals into central Washington. Disseminated mycobacteriosis due to Mycobacterium avium has been the most common cause of death of adult captive pygmy rabbits. Between June 2002 and September 2004, mycobacteriosis was diagnosed in 28 captive adult pygmy rabbits (representing 29% of the captive population), in contrast to 18 adult pygmy rabbits dying of all other causes in the same time period. Antemortem and postmortem medical records were evaluated retrospectively to describe the clinical course of mycobacteriosis in pygmy rabbits, physical examination findings, and diagnostic test results in the diagnosis of mycobacteriosis in pygmy rabbits. Various treatment protocols, possible risk factors for mortality. and recommendations for prevention of mycobacteriosis were evaluated also. Compromised cell-mediated immunity appears to be the best explanation at this time for the

observed high morbidity and mortality from mycobacterial infections in pygmy rabbits.

1701: *+.036*

The lack of adequate documentation of wildlife translocations, particularly details regarding the source stock used, have potentially serious implications for wildlife management. Poor documentation of translocations may lead to unintentional mixing of distinct types, potentially causing problems for future management, design of harvest programs, and evolutionary stability. In Kansas, we employed molecular tools and assignment methods to uncover the cryptic distribution of wild turkey subspecies resulting from decades of poorly documented translocations. Pure forms of Eastern (Meleagris gallopavo silvestris) and Rio Grande turkeys (Meleagris gallopavo intermedia) remain in many portions of the state, and future translocation programs now have the option to keep these distinct by prohibiting translocations between regions containing different subspecies. In addition, we documented 3 zones of hybridization: 1) at the interface between Eastern and Rio Grande subspecies, 2) in southwest Kansas where immigrant Merriam's turkeys (Meleagris gallopavo merriami) are mixing with translocated Rio Grande turkeys, and 3) surrounding an undocumented translocation of Eastern turkeys within a region characterized primarily by Rio Grande turkeys. The DNA-based techniques employed in this study were extremely informative tools for characterizing the distribution of wild turkeys in Kansas and suggest that such tools could be applied in a multitude of similar situations in other wildlife species.

1703: *+.086*

For the past several years, there has been growing interest in understanding the dynamics of parasites in ecosystems, as well as the diversity of ways in which they interfere with conservation and health preoccupations. Although it is widely recognized that many conservation practices (e.g., wildlife translocations, species removal, food supplementation) may be associated with parasite-related problems, less attention has been devoted to exploring the parasitological consequences of the overcrowding of animals in protected wildlife areas. Here, we discuss this important ecological/epidemiological problem, presenting at the same time an over-view of the main questions and challenges in this field. Using empirical and theoretical examples chosen from the literature, we focus particularly on the interactions between the overcrowding of free living species and parasite population dynamics, the evolution of parasite virulence, the indirect effects on the structure of invertebrate communities, as well as the nutritional value of prey species. We argue that conservation policies should be aware more than ever of this problem, especially given the serious health risks currently posed by the spread of virulent viruses (e.g., avian influenza).

1704: *-.240*

The current status of most West African birds is little known and may change quickly with increasing human population pressure and agriculture, road, tourism, hunting and mining developments. Following documented declines of raptors in Sudan and the Southern Sahel zones, I compared the number of birds counted along the same eight extensive transect counts in 1971-1973 (3,703 km) and 2004 (3,688 kin) in and steppes, acacia woodlands and desert mountains of northern Mali and Niger (Adrar des Iforhas, Air, Tenere). The once widespread Ostrich Struthio camelus is now extinct west of Chad. No Arabian Ardeotis arabs and Nubian Bustards Neotis nuba were seen in 2004 (216 in 1970s) nor any Ruppell's Griffon Gyps rueppellii and Lappet-faced Vultures Torgos tracheliotus (114 and 96 respectively recorded in the 1970s). From Adrar to Tenere, just one Egyptian Vulture Neophron perenopterus was recorded in 2004 (vs 75 in 1970s),

but it was still common in the oases of Kawar (27 vs 38). These data are exploratory and the current status of the species involved should be further documented. Nevertheless, they are a serious warning about the future of several taxa. Overhunting, aggravated by overgrazing and degradation of acacia woodlands ire obvious causes of the collapse of Ostrich and bustards. The near-extinction of wild ungulates, intensified use of cattle, increased disturbance and poisoning of predators may have been critical in the dramatic decline of vultures. An effective bunting ban, updates on the status of threatened species, reintroduction of Ostrich, enforcement of existing nature reserves and design of a new one in northern Mali are among the most urgent steps to take if the large birds of the vast subdesert areas of West Africa are to be conserved.

1705: *+.042*

The last remaining population of the Crested Ibis Nipponia nippon in the wild was studied in the Qinling Mountains, Yangxian County, Shaanxi Province, central China, over 24 breeding seasons with particular attention being paid to the nesting behaviour and breeding success. The average clutch size varied significantly among years (2.84 +/- 0.77; 11 = 271), suggesting that food abundance, which was distinct in different areas, was indeed a limiting factor. The mean hatching success was 80.2%, and ranged from 35.7% to 100%. Egg losses were due to three reasons: infertility or the eggs being addled, predation, and human disturbance. Three reasons accounting for chick death were highlighted: shortage of food, predation, and disturbance from local inhabitants. However, the overall breeding success of the Crested Ibis, which averaged 65.6%, was much higher than that of many nidicolous birds and was clearly dependent not only on the stability of pair maintenance but also on human conservation and protection measures. The relatively higher proportion of unsuccessful nests at altitudes between 500-700 m and 701-900 m was linked with the more frequent human activities, predation and lower stability of nesttrees in such areas. The fact that there was no significant variation in the number of successful nests or breeding success across different altitudinal zones demonstrated that, to a large degree, habitats used by the Crested Ibis were now suitable for breeding. The rapid increase in the species' numbers in recent years has been achieved through effective protection measures, including legislation, management of population and habitat, and regular surveys and monitoring. A reintroduction programme has been put into effect, protecting the population from a chance catastrophe such as communicable diseases within its limited range.

1706: *+.210*

The isolation of animal populations due to urban activities provides a useful framework for studying the consequences of landscape fragmentation. We studied a population of natterjack toads (Bufo calamita) in an urban park near Paris, France. In 2001 and 2002 we used radio-tracking to estimate the terrestrial movements of adults around their breeding sites. Twenty-four toads were equipped with internal transmitters in 2001 to record movements during and after the breeding period. In 2002, 19 males were released at 300 and 380 meters from their breeding ponds. Natterjack toad movements around and outside their breeding ponds were reduced compared to previous observations on this species. The only exchanges that were observed occurred between closely neighbouring breeding sites. During a translocation experiment in 2002, 58% of the displaced males returned to their site of capture and this happened mainly during the breeding period. The remaining 42% stayed close to the release site. There was no exchange of males between distant breeding sites. Natterjack toad conservation needs to take into account the high fidelity to a breeding site and the reduced breeding dispersal and homing ability of these animals. Conservation biology in urban landscapes constitutes a specific urban ecology with specific concepts such as 'population area'. Information from this study can assist land managers in

establishing protected areas of high habitat quality around breeding ponds in urban areas, and managing parks for the protection of amphibian populations, particularly by facilitating exchanges between available areas.

1707: ***-.294***

Although less than other animal groups, amphibians are sometimes concerned by the problems related to the introduction of alien specimens into natural populations. They may be victims of such introductions (especially of amphibians, fishes and other aquatic predators), or cause problems to other species through introduction outside their range. The problems posed by introductions, reintroductions and population reinforcements are discussed in a more general way. Introductions of alien species outside their range (faunistic pollution), or of alien specimens into other populations of the same species or of another interfertile species (genetic pollution), beside creating ecological problems, hinder or impede subsequent study of the history and evolution of these populations. For evolutionary biologists, they amount to a destruction of their object of study. Furthermore, such operations carry an optimistic but misleading message to the public, according to which destructions of the environment caused by human activities would be reversible. It is urgent that the main concepts of genetics and taxonomy be given more weight in decisions regarding reintroductions of animals into threatened populations or habitats.

1708: ***+.397***

Background: We conducted Geographic Information System (GIS) habitat analyses for lesser prairie-chicken (LPCH, Tympanuchus pallidicinctus) conservation planning. The 876,799 ha study area included most of the occupied habitat for the LPCH in New Mexico. The objectives were to identify and quantify: 1. suitable LPCH habitat in New Mexico, 2. conversion of native habitats, 3. potential for habitat restoration, and 4. unsuitable habitat available for oil and gas activities. Results: We found 16% of suitable habitat (6% of the study area) distributed in 13 patches of at least 3,200 ha and 11% of suitable habitat (4% of the study area) distributed in four patches over 7,238 ha. The area converted from native vegetation types comprised 17% of the study area. Ninety-five percent of agricultural conversion occurred on private lands in the northeastern corner of the study area. Most known herbicide-related conversions (82%) occurred in rangelands in the western part of the study area, on lands managed primarily by the US Bureau of Land Management (BLM). We identified 88,190 ha (10% of the study area) of habitats with reasonable restoration potential. Sixty-two percent of the primary population area (PPA) contained occupied, suitable, or potentially suitable habitat, leaving 38% that could be considered for oil and gas development. Conclusion: Although suitable LPCH habitat appears at first glance to be abundant in southeastern New Mexico, only a fraction of apparently suitable vegetation types constitute quality habitat. However, we identified habitat patches that could be restored through mesquite control or shinoak reintroduction. The analysis also identified areas of unsuitable habitat with low restoration potential that could be targeted for oil and gas exploration, in lieu of occupied, high-quality habitats. Used in combination with GIS analysis and current LPCH population data, the habitat map represents a powerful conservation and management tool.

1709: ***+.411***

Background: We conducted Geographic Information System (GIS) habitat analyses for lesser prairie-chicken (LPCH, Tympanuchus pallidicinctus) conservation planning. The 876,799 ha study area included most of the occupied habitat for the LPCH in New Mexico. The objectives were to identify and quantify: 1. suitable LPCH habitat in New Mexico, 2. conversion of native habitats, 3.

potential for habitat restoration, and 4. unsuitable habitat available for oil and gas activities.Results: We found 16% of suitable habitat (6% of the study area) distributed in 13 patches of at least 3,200 ha and 11% of suitable habitat (4% of the study area) distributed in four patches over 7,238 ha. The area converted from native vegetation types comprised 17% of the study area. Ninety-five percent of agricultural conversion occurred on private lands in the northeastern corner of the study area. Most known herbicide-related conversions (82%) occurred in rangelands in the western part of the study area, on lands managed primarily by the US Bureau of Land Management (BLM). We identified 88,190 ha (10% of the study area) of habitats with reasonable restoration potential. Sixty-two percent of the primary population area (PPA) contained occupied, suitable, or potentially suitable habitat, leaving 38% that could be considered for oil and gas development.Conclusion: Although suitable LPCH habitat appears at first glance to be abundant in southeastern New Mexico, only a fraction of apparently suitable vegetation types constitute quality habitat. However, we identified habitat patches that could be restored through mesquite control or shin-oak reintroduction. The analysis also identified areas of unsuitable habitat with low restoration potential that could be targeted for oil and gas exploration, in lieu of occupied, high-quality habitats. Used in combination with GIS analysis and current LPCH population data, the habitat map represents a powerful conservation and management tool.

1710: ***+.153***

The mammal fauna of the semi-arid Mallee region of Victoria differs markedly from that in temperate southern parts of the state, and includes numerous species that also occur in arid environments of central Australia. A total of 48 species of native mammals are recorded from the region, including at least 10 species known only from the Blandowski expedition of 1856-57. Marked changes have occurred to the fauna over the last 150 years, including the regional loss of at least 13 species (with five taxa globally extinct), and a further 10 species have been introduced to the area and established wild populations. Species loss has been greatest among the bandicoots. rodents and dasyurids, despite historical reports that some of these species were widespread and common. Loss has been greatest among species of medium body-size (0.05 - 0.5 kg); 78% (7/9) of species in this range have disappeared compared with 30% in other size-classes. In contrast, there is no evidence of loss among the four families of bats present, which together comprise one-third of the known native fauna. We describe the main components of the present mammal fauna, together with their relative abundance, distribution and conservation status. No species of native mammal is endemic to the Mallee region. Those most closely associated with mallee vegetation in Victoria arc the Mallee Ningaui, Little Pygmy Possum and Mitchell's Flopping Mouse. The future of the mammal fauna of the Mallee region over the next century, particularly of species regarded as 'threatened' or 'near threatened', depends on the choices made concerning human land-use in this region. Geographic areas particularly important to long-term conservation are the extensive vegetation blocks of the dunefields (Sunset Country, Big Desert), the Murray River corridor, and the plains of the far north-west where native vegetation links the dunefields with Contiguous mallee ecosystems in South Australia and New South Wales. Reintroduction of mammal species to the Mallee region also offers potential benefits for conservation, ecosystem restoration and a greater community appreciation of mallee ecosystems. However, for effective conservation the knowledge base for the mammal fauna of the Malice region must extend beyond inventory and survey to a greater understanding of the ecology of species and assemblages, and the processes that determine their distribution and abundance.

1711: ***+.153***

The mammal fauna of the semi-arid Mallee region of Victoria differs markedly from that in

temperate southern parts of the state, and includes numerous species that also occur in arid environments of central Australia. A total of 48 species of native mammals are recorded from the region, including at least 10 species known only from the Blandowski expedition of 1856-57. Marked changes have occurred to the fauna over the last 150 years, including the regional loss of at least 13 species (with five taxa globally extinct), and a further 10 species have been introduced to the area and established wild populations. Species loss has been greatest among the bandicoots. rodents and dasyurids, despite historical reports that some of these species were widespread and common. Loss has been greatest among species of medium body-size (0.05 - 0.5 kg); 78% (7/9) of species in this range have disappeared compared with <30% in other size-classes. In contrast, there is no evidence of loss among the four families of bats present, which together comprise one-third of the known native fauna. We describe the main components of the present mammal fauna, together with their relative abundance, distribution and conservation status. No species of native mammal is endemic to the Mallee region. Those most closely associated with mallee vegetation in Victoria arc the Mallee Ningaui, Little Pygmy Possum and Mitchell's Flopping Mouse. The future of the mammal fauna of the Mallee region over the next century, particularly of species regarded as 'threatened' or 'near threatened', depends on the choices made concerning human land-use in this region. Geographic areas particularly important to long-term conservation are the extensive vegetation blocks of the dunefields (Sunset Country, Big Desert), the Murray River corridor, and the plains of the far north-west where native vegetation links the dunefields with Contiguous mallee ecosystems in South Australia and New South Wales. Reintroduction of mammal species to the Mallee region also offers potential benefits for conservation, ecosystem restoration and a greater community appreciation of mallee ecosystems. However, for effective conservation the knowledge base for the mammal fauna of the Malice region must extend beyond inventory and survey to a greater understanding of the ecology of species and assemblages, and the processes that determine their distribution and abundance.

1712: *+.085*

This paper reports the movement patterns of two hard released, repatriated Egyptian Tortoises, Testudo kleinmanni, into Omayed Protectorate, Egypt. Upon release, both tortoises immediately dispersed away from the release point. The maximum dispersal distances from the release site were 1455 m and 113 1 m. These tortoises had exceedingly large activity ranges (mean 72.2 ha) which were roughly 10 times the size recorded for other T kleinmanni populations. There was also some spatial overlap and potential competition with livestock for vegetation, as 35 % of tortoise relocations were found within 2 m of livestock tracks. We suggest that any future tortoise repatriations into Omayed Protectorate should consider methods such as soft releases that could potentially reduce the initial long distance dispersals and the exceptionally large activity ranges

1713: *+.071*

Techniques to breed the Genji firefly, Luciola cruciata, a lampyrid (firefly) native to Japan, we developed at Tama Zoo, Tokyo. In order to determine captive husbandry procedures, the staff made close observations on the life cycle of this species in nature. It took more than a decade to establish the breathing program in an outdoor water channel at the Tama Zoo. Patterned after a freshwater ecosystem, the channel system enables the insect to undergo the simulated "food-chains" cycle for completing the metamorphic process. Captive-reared larvae, also known as "glowworms", and knowledge on husbandry have been utilized to reintroduce the species in its former natural ranges.

1714: *+.150*

Detailed studies of howendangered species use their environments at varied habitat scales are crucial if they are to be conserved and managed effectively. In this study, we used spool-and-line tracking to investigate the microhabitats used by the brush-tailed bettong (Bettongia penicillata) and the burrowing bettong (B. lesueur), two species with geographical ranges that have been dramatically reduced since European settlement in Australia. The study was carried out at Scotia Sanctuary, in semiarid western New South Wales, where both species have been recently reintroduced. The nocturnal movements and foraging of bettongs were associated with sites containing more canopy cover (mean 10-25%) than was available on average (0-10%). Models generated to predict the probability of bettong movements or activity points showed positive correlations with ground vegetation cover and ground vegetation height. Other microhabitat components of varying importance, including sand cover, litter cover, litter depth, crust cover, and distance to shrub/tree, were incorporated into these models. Species comparisons indicated that, although slight differences occurred in the way each species moved through the habitat, both species foraged in areas with similar microhabitat characteristics. While the models should have broad utility for the selection of favourable habitat for future release sites for B. penicillata and B. lesueur, further studies of diet and food availability are recommended to re. ne them further.

1716: *+.044*

The Laysan Teal Anas laysanensis is endemic to the Hawaiian Islands, where it has been restricted to Laysan Island over the last 150 years. Individuals of this endangered species have recently been translocated to the two largest islands that comprise Midway Atoll National Wildlife Refuge, to reduce the risk of the Lavsan Teal becoming extinct. Post-release monitoring with the aid of radio-telemetry was conducted to determine the success of the reintroduction attempt during October 2004-2007. The population was found to have increased after three breeding seasons, from forty-two founders sourced directly from Laysan, to a population of >= 192 post-fledglings juveniles and adults.

1717: *+.209*

Management of mustelid species such as fishers and martens requires an understanding of the history of local populations. This is particularly true in areas where populations were extirpated and restored through reintroduction efforts. During the late 19th and 20th centuries fishers (Martes pennanti) and American martens (Martes americana) were extirpated from much of their southern range, including Michigan and Wisconsin. Both species have been restored to varying degrees in these states following multiple reintroductions and translocations. We describe the status of the original populations and changes in their status over time, and include source locations, release sites, release and reintroduction dates, and demographic characteristics of released animals. This synthesis is crucial for evaluating the relative success of reintroductions in Michigan and Wisconsin, and, combined with knowledge of the current condition of these populations, can provide valuable guidance on the future management of these species. We also assess the reintroduction of fishers and martens in Michigan and Wisconsin and discuss strategies for successful reintroductions.

1718: *+.086*

Crayfish are consumed as a luxury food item in many parts of the World. The high economic importance of crayfish caused the translocation of some species (Procambarus clarkii, Pacifastacus leniusculus, Cherax destructor, C. quadricarinatus, Orconectes limosus, O. rusticus ve Astacus leptodactylus) from their natural habitats into different water resources. On the other hand, in

general, the translocation of crayfish species gives rise to negative environmental effects and they are accepted as a nuisance creature in their new habitat. The main reason of crayfish introduction at the start is that economical benefits, but at the end, crayfish are distributed commonly as uncontrolled populations in water resources. The negative effects of crayfish introductions are disappearance of native species, distribution of diseases into new habitats, consumption of fish eggs, reduction of fish stocks, excess consumption of water plants and direct or indirect impacts on water invertebrates. For example, the introduction of the native species from North America into Europe had negative impacts on the native species populations. Similarly, approximately 7,000 ton/year A. leptodactylus was exported from Turkey to Europe before the crayfish plague was arrived to Turkey. Crayfish export was stopped between 1986 and 1990, and it was only 320 ton in 1991. Although there has been an increase in the harvest of A. leptodactylus in Turkey in recent years (2317 ton in 2004), there is a danger to import non-native crayfish species into Turkey in order to expand crayfish production. In conclusion, it is thought that non-native crayfish species introductions into Turkey must be certainly forbidden because of the fact that the observed negative impacts of non-native crayfish species introductions carried out in many parts of the world. However, in comparison to the other native crayfish species of Europe, A. leptodactylus is accepted as a better species for reproduction and rearing. It has also an economic importance in European markets. For these reasons, to increase crayfish production of Turkey, before thinking the introduction of any non-native crayfish species it is necessary to increase studies scientifically on the biology, ecology, reproduction, feeding, juvenile rearing, conservation of populations, and diseases of A. leptodactylus.

1720: *+.186*

Status and trends in the abundance of populations of federally endangered Oregon chub Oregonichthys crameri, small floodplain minnows endemic to the Willamette Valley of western Oregon, were investigated by estimating fish abundance and from extensive fish surveys of 650 off-channel habitats from 1991 through 2004. The recent discovery of previously unknown populations of Oregon chub, some occurring in subbasins where they were presumed extinct, combined with successful reintroductions into suitable habitats have resulted in the improved status of this species. In 1991, eight populations of Oregon chub were known to exist. In 2004, we identified 33 populations of Oregon chub in the Willamette River basin. Ten of these populations, including the two most abundant populations, were introduced. The status of Oregon chub is approaching the recovery plan goal for downlisting the species to threatened. Nonnative fishes, which were found to be widespread in off -channel habitats preferred by Oregon chub, are the largest threat to full recovery and delisting of this species.

1722: *+.130*

Many of the world's cat species face growing threats to their continued survival in nature. For some species, managed captive populations may provide a reservoir for future reintroduction or genetic augmentation. Because most zoo populations are derived from small founder sizes and are subject to loss of genetic variation over time, periodic infusion of founder alleles is necessary to avoid the dire consequences of inbreeding. Collection and freezing of semen from free-living nondomestic felids offers a viable option for introducing founder genes into captive populations without removal of animals from the wild. The effective application of this strategy requires established protocols for safely capturing and anaesthetising wild cats coupled with suitable methods of semen recovery, processing and cryopreservation under field conditions. In small-sized non-domestic felids, the general feasibility of this approach is being explored in two studies of black-footed cats and Pallas' cats. Two factors - relatively low sperm numbers per ejaculate and

compromised status of frozen-thawed cat spermatozoa - suggest that in vitro fertilisation (IVF) and embryo transfer present the most efficient use of this limiting resource in small-sized cats. Our studies with captive felids have explored alternative methods of sperm cryopreservation that are adaptable to field situations and shown that frozen-thawed spermatozoa from Pallas' cats, ocelots, and fishing cats exhibit adequate function to fertilise heterologous and/or homologous oocytes in vitro. Most recently, we investigated the fertilising capacity of frozen-thawed spermatozoa obtained from wild Pallas' cats in Mongolia. Combined with improved methods for embryo culture and transfer in small cat species, sperm banking in situ will facilitate introduction of new founders into captive populations without causing further depletion of their wild counterparts. As one component of holistic conservation programs, including ongoing support of field ecology studies in range countries, this reproductive strategy serves to further strengthen linkages among imperiled ex situ and in situ cat populations.

1723: ***+.191***

The DPR n. 357, of 12th March 1997, (as modified and integrated by DPR 120/2003) has introduced the requirement of an authorisation by the competent regional authority for any reintroduction or augmentation of species included in annex D of the Habitat directive, or in annex I of the Bird Directive 79/409/CEE. The authorisation must be based on a detailed feasibility study, that the regional authority shall communicate to the Ministry of Environment, also providing an independent evaluation of the study carried on by the INFS (Italian Wildlife Institute), the ICRAM (Central Institute of Research Applied to the Sea) or by other relevant national scientific institutions. Objectives of the present volume are: to provide general principles for the reintroduction and augmentation of species of community interest; to define contents of the feasibility study required for the authorisation of reintroductions or augmentations; to identify criteria for the evaluation of the feasibility study. Despite recovery of species should primarily be based on in site interventions, it is acknowledged that in some cases reintroductions are a powerful conservation tool. However, considering the potential undesired effects that translocations may cause and the limiter. proportion of successful reintroductions, it is critical that the feasibility study assesses - among other aspects - aims of the reintroduction, risks for the source populations, risks of undesired effects and chances of success. The DPR 120/2003 also introduced a ban of introduction of alien species into the wild, with the aim of preventing impacts to species, habitat and areas of community interest. In this regard, the present volume defines the principles for enforcing the general ban of intentional introduction of alien species. In particular, considering the impacts caused by biological invasions, for any intentional introduction of alien species a prior authorisation by the Ministry of Environment is required, based on a comprehensive risk assessment. Such study shall in particular analyse the impacts caused by the species in other geographical contexts, the probability of establishment in the wild, the potentiality of spread, and the available techniques to control the population in the future.

1724: ***+.246***

Some techniques to account and calculate the abundance of Marmota bobak via interval estimation of the number of its families at certain sites of its habitat are described. They were applied for quantity estimation of the species in some Cis-Volga districts of the Saratov region in 2000. By our account data, the said territory (above 4 million ha) contained from 27 to 40 thousand individuals of M. bobak in 2000. The trend towards increasing the abundance of Al. bobak noted in European and West-Asian steppes of the CIS countries in the last decades takes place in the Saratov region as well. The appearance of M. bobak In a few districts is most likely due to natural processes and reintroduction.

1725: ***+.046***

Approximately 300 Asiatic lions Panthera leo persica are confined to the 1,883 km(2) Gir forests in Gujarat, western India. To establish a second home for the Asiatic lion in its former range, Kuno Wildlife Division (1,280 km(2), with a core 345 km(2) Sanctuary) has been identified in Madhya Pradesh. To assess whether the Sanctuary has sufficient wild ungulates to support a population of lions 17 transects totaling 461 km were surveyed over an area of 280 km(2) in early 2005. The density of potential ungulate prey was 13 animals km(-2). There are also c. 2,500 feral cattle, left behind by translocated villagers; the cattle are considered to be buffer prey in case droughts adversely affect the populations of wild ungulates. Control of poaching, moving of two villages, grassland management and building a rubble wall around the Division to keep out livestock would lead to a substantial rise in the population of ungulates (to c. 20 animals km(-2)) by the end of 2007. This density would support the first group of five lions (three females and two males) due to be reintroduced in the beginning of 2008. Even if all the three females raise cubs there will be sufficient wild prey by the end of 2009 to support the males, females and cubs.

1726: ***+.296***

The expansion of conservation estate in South Africa has seen large predators increasingly reintroduced in order to restore ecological integrity, conserve threatened species and maximise tourism. Reintroductions occurred at fenced, ecotourism sites in South Africa's Eastern Cape Province. Lion Panthera leo reintroduction began in 2000 and has been highly successful with a population of 56 currently extant in the region arising from 35 reintroduced individuals. The African wild dog Lycaon pictus population has increased to 24 from a founder population of 11. Reintroduction of spotted hyaenas Crocuta crocuta also appears successful, although reintroductions of leopards Panthera pardus and cheetahs Acinonyx jubatus have been less successful. Here we review the successes and failures of the reintroductions that have occurred in the region and describe recommendations to assist future translocations. Ecological attributes of each species affected the success with which they were reintroduced. Soft-release techniques, adequate fencing, appropriate socioeconomic environment, the order of predator reintroduction with subordinate species released prior to dominant ones, adequate prey base and adequate monitoring all improved the success of reintroductions. Carrying capacity for large predators is unknown and continued monitoring and, we fear, intensive management will be necessary in virtually all modern day conservation areas.

1727: ***-.102***

The least chub lotichthys phlegethontis, a small cyprinid endemic to Bonneville Basin, Utah, is at risk of becoming a threatened species in Utah. Captive propagation has become necessary to preserve genetically distinct populations and to produce specimens for reintroduction. We conducted two spawning studies to develop intensive aquaculture techniques for the least chub. In the two studies, 162 adults (78 females) produced a total of 12,492 fry. Adult broodstock density (0.03-0.20 fish/L), male: female sex ratio (1: 1, 1:2, and 2: 1), and spawning substrate transfer frequency (one versus two times per week) did not significantly affect production. In the second study, female mortality was significantly different between treatments, as more female mortalities occurred in tanks with one spawning substrate transfer per week than in tanks with two such transfers. Single females paired with single males produced multiple clutches at 2- to 3-week intervals. The relationship between fry production and female body size (g) appeared to be positive and linear between 1.0 and 2.2 g but was unclear for larger fish.

1728: *-.032*

AIM: To assess the prevalence of faecal excretion of Salmonella serovars by wild tuatara (Sphenodon punctatus) on Stephens Island, New Zealand.METHODS: One hundred cloacal swabs obtained as part of health-screening for the translocation of adult tuatara from Stephens Island were subjected to general aerobic culture and enrichment, and cultured specifically for Salmonella spp.RESULTS: No Salmonella spp were cultured from any of the cloacal samples, which suggests that, at the 95% confidence interval, the maximum prevalence of tuatara in the island population that were shedding Salmonella spp not detected by our sample size was 1.5%. Mixed bacteria were grown from the 70 cloacal swabs cultured aerobically. A predominant organism was evident in 30 cultures, and these were identified as Hafnia alvei type 1 (n=16) and type 2 (n=7), Corynebacterium spp (n= 4), Klebsiella oxytoca (n= 2), and Moraxella spp (n= 1).CONCLUSIONS: The absence of intestinal carriage of Salmonella spp by the tuatara sampled in this study may indicate either lack of exposure, or an innate resistance to intestinal colonisation in tuatara. The significance of the other bacteria cultured as potential pathogens to the tuatara and as zoonotic risks is also uncertain. Wildlife managers should screen translocated reptiles for Salmonella spp, and thereby avoid exposing wild and managed populations to infection.

1729: *+.291*

In the last century black rhinoceros numbers declined significantly in Africa. To ensure protection of the species and to also maintain a sustainable population, a national conservation strategy has been established to encourage population growth within South Africa. Part of this strategy involves translocation of black rhino from a high density source reserve, Hluhluwe-Umfolozi, to several, suitable, low density translocation reserves. This strategy encourages not only growth in the high density source reserve by lowering the population and hence reducing the effects of density but also encourages growth in new reserves. This has the additional benefits of encouraging tourism and economic growth in new areas of the country and also lowers the risk of extinction of the species from disease and poor environmental conditions. Current population models have supported the idea that the global population of the endangered black rhinoceros will increase faster if animals are translocated from high-density reserves to new suitable reserves of low density. However previous analyses of this problem may have not modelled the new populations appropriately and this may lead to sub-optimal implementation of government translocation policies. To gain a better understanding of this, and the dynamics of small, translocated populations, an IBM of a small rhino population has been developed and tested. This model has then been linked to existing models of the source reserve to get an overall indication of global population dynamics. The main purpose of this model is to simulate the population growth of the endangered black rhino in South Africa in order to predict the outcomes of different translocation strategies. Firstly the effects of translocating different life stages and sexes in optimising translocation reserve numbers are compared. Secondly, by varying the percentage removed from the source the relative effects on the source, translocated and global populations can be observed. The study investigates strategies that will satisfy two conditions:i. Populations at the source reserves must be maintained at a reasonable level.ii. The global population of Black Rhino must be at a maximum, provided the first condition is satisfied. From the model, different strategies that satisfy the opposing opinions of the park managers, who seek low translocation to preserve income, and the conservation specialists, who seek high translocation rates to many different reserves are developed. Specifically, it is hoped that the outcomes of these simulations enable both park managers and animal conservationist to make balanced, well informed decisions. The joint IBM - source reserve model gives a good indication of the overall global population dynamics and in particular the relationship between source and translocation reserves. From the

simulations it is suggested that translocation of approximately 10 to 15 percent of the source reserve population with a focus on translocating a higher percentage of adults, produce a maximum global population.

1730: *+.186*

1. Changes in otter Lutra lutra distribution in Italy were examined by analysing geographical, historical and survey data.2. As in other European countries, otters declined sharply in Italy during the late 1970s and throughout the 1980s. Between 1985 and 2004 the species became extinct, except for some reintroductions, in northern and most of central Italy while the species appears to have substantially recovered in its southern range. This recovery of the otter population is apparently not due to increased research effort, but reflects a real expansion of range.3. Differences in the degree of range reduction during the second half of the 20th century and in the availability of suitable habitats are probably the major factors that have determined the two opposite trends shown by otter populations in the last 20 years.4. Better knowledge of otter status and distribution is needed for effective conservation management. We suggest that a national survey of the Italian otter population that employs genetic approaches should represent a first step of the national action plan for otter conservation.

1731: *-.019*

Re-introductions of captive-bred animals are increasingly common in wildlife conservation and it is important that they fulfil their potential. To foster this goal we examined variations in stress levels in a captive-bred population of water voles Arvicola terrestris in response to housing conditions and radio-collaring, using weight loss and leukocyte coping capacity (LCC) as measures of relative stress, to investigate the impacts of housing conditions, handling and radio-collaring on this species. Thirty-eight water voles (22 males and 16 females) were used in the investigation, 25 housed in outdoor enclosures and 13 in laboratory cages. During the 6-week study, LCC, body weight and urine refractive index (URI, an indicator of hydration levels) were recorded once a week for each individual in weeks 1, 2, 4 and 6. After the first sample, radio-collars were attached to 20 individuals (10 males and 10 females) taken from both housing types. Throughout the experiment laboratory-cage housed voles weighed less, had lower LCC scores - indicating a reduced ability to combat infection - and had higher URIs than outdoor-enclosure voles. This suggests that the laboratory-cage voles were more stressed and dehydrated than the outdoor-enclosure voles. Weights and LCC scores of both housing groups decreased as the study progressed, suggesting that elements of the study, such as repeated handling, may have caused stress to both groups. Evidence suggested a short-term effect of radio-collaring on immuno-competence. We conclude that captive housing conditions, repeated manipulation and radio-collaring had demonstrable physiological effects on the water voles studied. We recommend that the effects of husbandry and tagging practices upon captive-bred mammals be closely studied as part of the quest to improve the success of the re-introductions to which they contribute.

1732: *+.109*

Brachypelma vagans is an endangered tarantula included in Appendix II of CITES because of its popularity as a pet. However, little is known about its ecology, in particular microhabitat choices. In this work we analyse the structure of the vegetation surrounding burrows of this tarantula, as well as soil preference for the burrow at different sites in relation to the density of the species. We also offer the first description of the burrow of this spider. Brachypelma vagans mostly uses open sites with low vegetation for establishing its burrow, rather than primary or secondary forest. The

burrows are essentially built in sites with deep clay soils, whereas sandy soils or soils with a high density of roots and stones are avoided. The soil characteristics may be the key factor determining the presence of B. vagans. The burrow of B. vagans is deep and complex, exhibiting various chambers, even if only one seems to be used by the occupant. The structure of the burrow and soil characteristics are obviously related. These results could help determine the real distribution of B. vagans within its geographic range, and might be useful for a better management focused on protection and reintroduction.

1733: *+.176*

Spiders have been advocated as valuable bio-indicators of forest ecosystem "health." However, the numbers and types of spiders that are recorded at a site will usually be highly dependent on the sampling method employed. The use of lethal, indiscriminate invertebrate sampling techniques is undesirable when investigating rare species, or sampling within areas of high conservation status. Therefore we used non-lethal artificial tree-mounted shelters to monitor arboreal spiders in nature reserves near Christchurch, New Zealand. After three months, over 60% of the shelters had been used by spiders, increasing to 91% after twelve months. There were significant differences in the numbers of spiders found in the shelters at the different sites. However, factors such as the species of tree the shelter was attached to, ground vegetation, and levels of incident light did not affect the likelihood of a shelter being occupied. The species composition of the spider faunas in those sites regarded as high quality forest remnants was dissimilar to the faunas found in the low quality reserves. However, although spiders were more abundant in the high quality sites compared with the poorest stands of woodland, they were not more species rich. The shelters are inexpensive and easy to manufacture and are useful for long-term non-lethal monitoring of spider communities. They also have good potential as a tool for studying spider phenology, population dynamics, behavior, and as a collection/carriage device for live specimens used in conservation translocations.

1734: *+.036*

Habitat fragmentation is one of the more important contributors to species endangerment, but one form of fragmentation, here called dispersal fragmentation, can often go unobserved for many years after it has occurred. Many species live in naturally fragmented habitats, but the local populations are interconnected genetically and demographically by dispersal through the environmental matrix in which the habitats are embedded. Because of dispersal, the local populations are not truly fragmented evolutionarily or ecologically. However, when human activities alter the environmental matrix such that dispersal is no longer possible, the population does indeed become fragmented even though they initially are present in the same habitats. An example of dispersal fragmentation via an altered environmental matrix is provided by the eastern collared lizard (Crotaphytus collaris collaris). This lizard lives on open, rocky habitats, called glades, that are embedded in the forests of the Ozarks, a highland region located primarily in Missouri and Arkansas in the USA. Forest fire suppression has reduced this habitat, resulting in severe habitat fragmentation, disruption of gene flow, loss of genetic variation within glade populations, and local extinction without recolonization. Beginning in 1982, glade habitats were restored by clearing and burning in the Peck Ranch area of the Missouri Ozarks, a region where the lizards had gone extinct. Starting in 1984, lizard populations were translocated from other Missouri glades onto restored glades at the Peck Ranch. Although these translocated populations survived well on the restored glades, no movement was detected between glades, some just 50 m apart, and no colonization of nearby restored glades, some just 60 m away, Occurred between 1984 and 1993. Fragmentation, lack of colonization, no gene flow, and loss of genetic variation

still persisted despite translocation reversing some of the local extinction. Fire scar data from trees and tree stumps indicated that forest fires were common in this area prior to European settlement, so in 1994 a new management policy of prescribed burning of both the glades and their forest matrix was initiated. Once the forest had been burned, the lizards could disperse kilometers through the forest, thereby reestablishing the processes of dispersal, gene flow, colonization, and local extinction followed by recolonization. This resulted in a dramatic increase in population size and inhabited area. By incorporating a landscape perspective into the management strategy, the eastern collared lizard has been successfully reestablished in a region of historic extirpation.

1735: ***+.500***

Conservation resources and interest are disproportionately directed towards large charismatic mammals, both in zoos and in situ. The order Rodentia represents c. 40% of known mammalian species, and rodents (and other small mammals) could be viewed by zoos as tools to demonstrate their commitment to conservation through local captive-breeding and reintroduction programmes. However, it is necessary to identify the true degree of threat to species before investing the limited resources available to conservation efforts. Population declines at the local or regional level do not necessarily indicate a threat to species survival at the global level, and interest in small mammals and the location of the majority of zoos do not appear to overlap with hotspots of endemicity for rodents. Education, especially if coupled with habitat-improvement activities, is essential to increasing public participation and support for biodiversity conservation. In this article we present the general principles that should be considered when establishing conservation priorities for small mammals, specifically in Europe but which apply equally across the globe.

1736: ***-.348***

Dramatic declines and extinctions of amphibian species have occurred worldwide over the last three decades owing to the introduction of chytridiomycosis. This emerging infectious disease is caused by the chytrid fungus Batrachochytrium dendrobatidis, a virulent water-borne pathogen of many amphibian species. It has caused epidemic waves of high mortality as it spread through susceptible wild populations in Australia, North, Central and South America, and New Zealand, and is now endemic in surviving populations in these continents and in Europe and Africa. The prevalence of chytridiomycosis in the international amphibian trade is high and import of infected frogs into zoos has caused disease epidemics in established amphibian collections. Management of disease spread requires effective national and international quarantine and control strategies. Although B. dendrobatidis is susceptible to a range of commonly used disinfectants, there is no universally effective treatment regime for infected amphibians. Zoological institutions can play a key role in preventing pathogen spread between captive facilities, and in disease surveillance, captive-breeding and reintroduction programmes, to limit the impact of this formidable disease on wild amphibian populations.

1737: ***+.016***

Zoo and wildlife veterinarians play an evolving role in regional management plans. Veterinarians serve as advisors, collecting and collating information regarding the medical issues of a species or taxa and making recommendations. Veterinary involvement increases with participation in captive propagation programmes, reintroduction programmes and in situ conservation efforts. With the increasing threat of emerging diseases, veterinarians have increasing responsibilities to these programmes. Many of the factors responsible for the emergence of infectious diseases also threaten the environment and wildlife populations. Understanding the potential impact a disease

could have on a wildlife population begins by knowing the current health status of that population, identifying critical health factors and recognizing possible threats. Baseline health information is not always available for many wildlife populations. Veterinarians working with captive populations can build this missing database. This is a review of the ways in which veterinarians have contributed to the collection and collation of information that is essential when developing regional management plans. It includes current veterinary guidelines available to zoo veterinarians in the United States and Europe. Veterinarians can improve their contributions to conservation programmes by expanding their knowledge of epidemiology, wildlife biology, ecology and environmental science and by partnering with those who have this expertise.

1738: *+.263*

The urgency to recover the remnant wild population of the Californian Condor, almost extinct in the 1980s, started a captivity reproduction program that was developed in Los Angeles and San Diego Zoos, in order to reintroduce Californian Condors in protected areas of California and Arizona since 1995, and in Sierra de San Pedro Martir (SSPM) National Park in Baja California, Mexico, since 2002. By 2007, 28 birds (4 or 5 birds per year) have been transferred to SSPM. Through the installment of a management and monitoring program, 24 California Condors survive very well in SSPM. The first breeding couple initiated their reproduction process this year. In this article the recovery process of the Californian Condor is described and an important question is set forth: Is the habitat of the XXI century adequate for the Californian Condor's surviving? While we resolve the habitat's dilemma we have to continue with the process of Californian Condor recovery. In Mexico, the recovery program is the first reintroduction experience with a species that had been previously extinct in the country, and now can fly free in Baja California's skies. We have still a long road to walk before we can say that the Californian Condor has returned to the nature.

1739: *+.157*

On the request from the Swedish Government, the needs for and prerequisites of action plans were elicited for marine fishes and shellfishes where fishing could be considered a threat and where national international legislations entail protection. On another request from the Swedish Government, the needs for and prerequisites of action plans were elicited for local fish stocks for which general management actions would not suffice. Fish populations are not only affected by fishing; several other factors in the environment exist, and in many cases the general or traditional managements are ineffective. During the last century larger lakes, running waters and coastal waters have been subjected to increased load of nutrients. Airborne transports (some of them from far away) of substances have cause acidification of water bodies. The acid rain lead to decline pH, sometime to such low values that fish reproduction are impeded, toxic substances injurious to the environment are released from sediments, or even that fish are killed. The constructions of hydro-electrical power plants have changed the environment in rivers and streams, the water current change, the natural variation of the water level are disturbed, species composition of both plants and animals are changed. In the future the global warming might cause new changes that effect the fish populations, both the sizes of the populations, species composition, reproduction and growth. For example higher temperatures, changes in precipitation, salinity, and changes of winds and water currents might be important. Thus, a number of factors affect the living conditions of the fish species. Since national legislation is subordinated to the Common Fishery Policy, protection of most of the threatened marine species can on by effectuated by the EU commission and the ministry of council. The presented action proposals are to complement the ongoing work and the information gathered will be highly relevant to the environmental quality objective "A balanced

marine environment, flourishing coastal areas and archipelagos" and "Flourishing lakes and watercourses", but also two other environmental quality objectives "Nothing but natural acidification" and "No eutrophication'. Action plans have been developed for those species which according to the Swedish 2005 Red list are considered as threatened and where fishing make up a significant part of this threat. Suggested action plans Common actions to protect cartilagenous fish In order to bring down the bycatches of sharks, skates and rays in the shrimp fishery, it is recommended to the EU commision that sorting grids attached to the trawl should be mandatory in Skagerrak and the North Sea. Furthermore, fishermen (both commercial and recreational) should be informed about the need to protect cartilagenous fish and be incited, if possible, to release them unharmed to the sea. Species specific actions Spiny dogfish Squalus acanthias A zero TAC should be implemented as ICES recommends 0 catch. Considering waters under Swedish jurisdiction the fishery on dogfish can be constrained by introducing compulsory fishery licence until a zero TAC has been implemented on the community level. Tope shark Galerhinus galeus Advice on a suitable TAC is needed fron ICES. There is also a need for an inventory of Swedish shark catches. Thornback ray and Blue skate Raja clavata; Dipturus batis These two species are already prohibited to fish and to land, however as usually only the wings of rays are landed it is hard to separate these species from other non-threatened ray species. It is therefore recommended that rays should only be allowed to be landed as uncutted. Wels catfish Silurus glanis Spawning sites and primary feeding are for the juveniles have to be surveyed any documented. Peled Coregonus peled In order to draw up a management plan for the species it is necessary to collect more information concerning the biology of the species. It is also important to lay down taxonomic status of the species. Spring-spawning vendace Coregoni, trybomi It is necessary to develop an appropriate test-fishing method for the species. The taxonomic status of the species has to be confirmed by use of modern DNA-technique. After this has been done it might be relevant to reintroduce the species and to restore the environmental conditions. European eel Anguilla anguilla A national management plan is under construction with the goals to increase the number of returning eels to the Sargass Sea by reducing the fishing mortality rates. An inventory of migratory impediments has been undertaken and stockings of elvers and juvenile eel have been a common practice during a long period of time. Noble crayfish Astacus astacus There is a need for developing methods eradicating the introduced signal crayfish Pacifastacus leniusculus). Thereafter the noble crayfish can be reintroduced. Atlantic salmon (the landlocked population of River Gullspangsalven) Salmo salar No additional actions are needed. Sea lamprey Petromyzon marinus The spawning success has to be continuously surveyed. The natural condition for the species should be recreated, thereafter reintroduction might be considered. Halibut Hippoglossus hippoglossus Important nursery areas should be identified and protected. A minimum legal size could be introduced to reduce the number of juveniles killed. Since the species move over large areas and Sweden only stands for a small part of the fishery it is important to protect this species at both the community and international level. Pollack Pollachius pollachius A TAC should be implemented also in the North Sea, Skagerrak and Kattegat. Spawning areas should be identified and protected from fishing. Because the species is very stationary the introduction of marine protected areas within Sweden could be a plausible measure. Artic charr, southern populations Salvelinus alpinus (Salvelinus umbla) The problems for the charr populations are acute in Lake Vattern. In this lake scientific test-fishing data are needed in order to reveal the situation and status of the charr in the lake. The mortality due to angling has to be quantified. The interactions between the charr and the introduced Atlantic salmon have to be analysed in detail. Cod Gadhus morhua A prerequisite for the recovery of the cod is that ICES advices on management are followed and that the discards and unreported catches are effectively reduced. Cod are caught in mixed fishery, so there is a need for coordinated management actions with other species. A national management plan is also of utmost importance and a reduction of both recreational and commercial fishing in inshore areas. Asp Aspius aspius The natural condition for the species should be recreated, thereafter reintroduction

might be considered Ling Molva molva The knowledge of biology of the species should be increased and tools for investigated species at hard bottoms should be developed. Roundnose grenadier Cotyphaenoide rupestris Introduction of sorting grids in shrimp trawls should be implemented in order to reduce bycatches of all deep-sea fishes such as roundnose grenadier. Atlantic sturgeon Acipenser oxyrinchus The species is extirpated in the Baltic region and the possibilities for spontaneous reintroduction are very low, therefore hatchery rearing and stocking is needed. However, before this action is taken it is necessary to investigate the reasons for the extinction and to survey the natural condition for reintroduction. European river lamprey Lampetra fIuviatilis Complementary management regulations ought to be introduced. Reintroduction might be necessary if it becomes apparent that other actions are ineffective. Belica Leucaspius delineatus A survey of the existing populations and the distribution are needed. Reintroduction might be necessary if it becomes apparent that other actions are ineffective. Haddock Melanogrammus aeglefinus A prerequisite for recovery is an effective] reduced fishery in the Kattegat. A national management plan is also needed. Spawning areas should be identified and marine protected areas implemented. Sweden should incite cooperation with Denmark to investigate how the original population structure may have looked like, and facilitate recolonisation in the waters along the Swedish west coast. Norway redfish Sebastes viviparus Introduction of sorting grids in shrimp trawls should be implemented in order to reduce bycatches of all deep-sea fishes such as Norway redfish. Turbot Psetta maxima Due to the general decline of turbot in the Baltic Sea, there is a need for coordination with other countries. However, as it is a stationary species there are also good opportunities for local management. Possible actions could be area or time specific protections of spawning and nursery areas or a limit in maximal mesh size to protect the biggest females. In addition important spawning and nursery areas should be protected from exploitation. Eelpout Zoarces viviparus The reasons for the decrease are unknown but the development of the stock should be followed and surveys in the Bothnian Sea should be undertaken. Investigations of the extent of bycatches in the eel fishery and the impact of cormorant predation are recommendable. Vimba Abramis vimba More information and knowledge on the distribution and conservation status of the vimba are needed; and that from all habitats: brackish water, lakes and running waters. Grayling-coastal populationsThymallus thymallus The reasons for the decrease are unknown. It is necessary to increase knowledge on life-history and ecology to suggest meaningful action plans

1741: ***+.063***

Asiatic wild asses and Przewalski horses initially inhabited steppe, semi-desert and desert areas, but Przewalski horses became extinct in the wild, and kulans are under threat of disappearance. The Bukhara Breeding centre (Uzbekistan) was created in 1976 for conservation and reintroduction of wild ungulate species. In 1977-1978, 5 kulans (2 males and 3 females) from Barsa-Kelmes Island on the Aral lake were introduced to the reserve. The group increased to 25-30 animals in 1995-1998, when 5 Przewalski horses from Moscow and St. Petersburg zoos were introduced to the same territory. We analyzed the home ranges, preferred habitats and social interactions of these closely related species during 1995-1999 by season and group composition. Horses and kulans each formed a reproductive group and a secondary bachelor group. The home range of the secondary group in both species was larger than that of the reproductive group and seemed to be less dependent on watering places. Kulans and Przewalski horses demonstrated different strategies of habitat use. They can share one area without serious conflicts avoiding competition by the temporal differentiation in the usage of key sites.

1742: ***-.018***

The proactive strategy for restoring Florida's depleted queen conch Strombus gigas population includes increasing the spawning stock by translocating reproductively deficient individuals into existing spawning aggregations where our previous research has shown that the translocated conch will develop normally. However, there may be unintended and potentially negative ecological ramifications if the translocated conch displace the native conch. To examine this issue, we translocated non-reproductive adult conch into 2 offshore spawning aggregations and used acoustic telemetry to track them relative to similarly tagged native conch. At one aggregation, the home-range sizes of native conch were significantly smaller than those of the translocated conch, which led to a significant reduction in the static interaction (i.e. home-range overlap) between the 2 groups of conch. However, the dynamic interaction (i.e. sociality coefficient) among the translocated conch was significantly higher. We hypothesize that these results were driven by reproduction: the translocations took place in July during the breeding season, and because the translocated conch were not yet in reproductive condition they moved into forage habitats, while the natives occupied the breeding habitats. At the other aggregation, there were no significant differences in the movements or interactions between native and translocated conch, a result probably due to the mosaic of habitats at this site, where breeding and forage areas are in close proximity. Our results indicate that displacement of natives did not occur at either site, and that translocating conch into spawning aggregations does not have adverse consequences relative to the interactions among conspecifics.

1743: ***+.226***

Florida ziziphus Ziziphus celata is a self-incompatible clonal shrub comprising fewer than a dozen remnant, uniclonal populations in south-central Florida, USA. We model the population viability of this highly endangered species based on 9 yr of data from 2 populations. The matrix projection model is based on hypothetically independent 'plants' defined as clumps of ramets within a 25 cm radius. Seedling recruitment is unknown in the uniclonal study populations and is not modeled. The populations modeled here have stable demographies characterized by high survival and stasis, but variable levels of new plant production (clonal recruitment). Population growth rates suggest protracted long-term declines in population size, but predicted extinction rates over 50 yr are as high as 20 % in one population. Changes in new plant production and survival had greater effects on population growth rates than did changes in growth rates of individual plants. Augmenting clonal plant production and protecting survival of new 'plants' are short-term management goals. The establishment of sexually reproducing populations through the translocation of cross-compatible genotypes is a long-term necessity for the persistence of Florida ziziphus populations.

1744: ***-.072***

At the time of European settlement in Australia in 1788, Magpie Geese (Anseranas semipalmata) were abundant, as they remain today, across northern Australia but were also common on swamps and on coastal and inland river floodplains in south-eastern Australia. However, by the early 1900s Magpie Geese had suffered a serious contraction of range from the south-east. In this study, we review all available records of the Magpie Goose and compile a list of processes potentially causing their decline. Historical changes in distribution are then compared with time frames of threatening processes to identify processes most likely to have driven the observed changes. The results suggest that the decline was primarily from loss of wetland habitat and hunting, although lesser threats such as poisoning, predation by Red Foxes (Vulpes vulpes) and severe drought may have increased the rate of decline. Since protection of the species from hunting in the 1930s in eastern and southern states and successful reintroductions in Victoria, New South Wales and South Australia, the species has returned to parts of its former range. However, populations are unlikely

to return to their former numbers in the south-east as management of water resources has greatly reduced areas of breeding habitat and drought refuges. We suggest a program of management for southern populations of the Magpie Goose that includes long-term monitoring, engagement of landholders, identification and protection of existing wetlands, creation of new habitat, and reduction of disturbance from humans, introduced pests and livestock.

1745: ***+.089***

Many factors, both biotic and abiotic, impede the success of reintroductions. Among these is the difficulty that small populations have in surviving and thriving. For many species of animals and plants, populations at low density or of small size suffer from a weak or even a negative growth rate, either of which increases their extinction risk; this is called the Allee effect. As reintroductions are almost invariably characterized by small propagule sizes, the importance of the Allee effect must be considered in order to assess its potential impact on the probability of success in reintroductions. In this paper, we highlight the ubiquity of the Allee effect, the importance of this process as predicted by theoretical models, and its potential role in the failure of previous reintroduction attempts. We emphasize that although previous reintroduction programs have shown that release size is paramount to the success of reintroductions, no link has yet been formally demonstrated between Allee effects and reintroduction success. We encourage researchers in ecology to further investigate the role of the Allee effect on the probability of reintroduction success, using available data on comparable processes such as invasive species and the propagation of biological control agents where possible. We also urge biodiversity managers to consider this potential impediment to the survival, increase, and spread of small groups of reintroduced individuals in conservation programs.

1746: ***+.080***

The translocation of animal species, that is the transfer of individuals from a source population to a release area to restock, is a widespread practice in conservation biology and wildlife management. However, translocation is likely a severe test for the translocated individuals as they are subjected to stressful and inevitable disturbances: physical handling, transport and release into an unknown environment. Moreover, other disturbances may be induced by translocation, for example in case of social disorganization among translocated individuals or of a large difference in habitat quality between origin and release areas. These disturbances induced by the translocation process may have a negative impact upon the translocated individuals, through reduced survival or breeding success, or abnormal behaviour. Consequently, founding a viable population by the mean of a translocation is not easy. Then, it is necessary to determine which factors are involved in the failure or the success of a translocation. Numerous studies carried out in many animal species throw some light on the determinism of translocation success, which nevertheless remains rather complex due to the various factors concurrently involved, and which often differs among species. However, some general principles may stand for the determinism of translocation success. Thus, wildlife managers may promote translocation success by intervening at several levels, in particular by choosing which individuals to release, from which population or environment to remove them and in which release area, or by conducting both adequate habitat management and translocation protocol.

1747: ***+.080***

The successful restoration of plant species in the wild depends on knowledge of the species' habitat requirements and genetic, demographic, and ecological traits that may increase

vulnerability to stochastic extinction processes. A few studies have reported experimental projects that attempted to re-establish populations of endangered plants; however, their experimental designs often led to ambiguous results. This paper reviews the common practices in plant restoration and re-establishment programs and reports on an experiment that we performed on an endangered plant (Arenaria grandiflora) in the Parisian region. Our aim is to provide advice on how a restoration experiment should be conducted in order to maximize its success.

1748: ***+.247***

It is well recognized that reintroductions have previously suffered from insufficient monitoring, and as a result reintroduction protocols and guidelines now have stringent monitoring requirements. It is important, however, that monitoring is done strategically. To be cost efficient, monitoring must be designed to address key questions relevant to reintroduction success and must be allocated to reintroductions where monitoring will have the greatest value. While the traditional aim of reintroductions was to recover endangered species, there is now a much greater emphasis on reintroduction as part of ecological restoration programmes. This often means that whole suites of species are re-introduced, including common as well as endangered species. We recommend the following approach for strategically monitoring reintroductions in restoration programmes. First, monitoring should be allocated not just to the rarest species, but to focal species chosen based on restoration objectives. Second, monitoring should primarily aim to assess the suitability of the habitat for supporting the species in the long-term, rather than the short-term effects of different release strategies (e.g., soft versus hard release). Third, an experimental approach should be used where possible to test the extent to which habitat factors such as food and predators are limiting population viability. We provide examples from our research on 2 species of New Zealand forest birds being reintroduced as part of ecological restoration programmes.

1749: ***+.199***

Parrots (order: Psittaciformes) are the most common captive birds and have attracted human fascination since ancient times because of their remarkable intelligence and ability to imitate human speech. However, their genome organization, evolution and genomic relation with other birds are poorly understood. Chromosome painting with DNA probes derived from the flow-sorted macrochromosomes (1-10) of chicken (Gallus gallus, GGA) has been used to identify and distinguish the homoeologous chromosomal segments in three species of parrots, i.e., Agapornis roseicollis (peach-faced lovebird); Nymphicus hollandicus (cockatiel) and Melopsittacus undulatus (budgerigar). The ten GGA macrochromosome paints unequivocally recognize 14 to 16 hybridizing regions delineating the conserved chromosomal segments for the respective chicken macrochromosomes in these representative parrot species. The cross-species chromosome painting results show that, unlike in many other avian karyotypes with high homology to chicken chromosomes, dramatic rearrangements of the macrochromosomes have occurred in parrot lineages. Among the larger GGA macrochromosomes (1-5), chromosomes 1 and 4 are conserved on two chromosomes in all three species. However, the hybridization pattern for GGA 4 in A. roseicollis and M. undulatus is in sharp contrast to the most common pattern known from hybridization of chicken macrochromosome 4 in other avian karyotypes. With the exception of A. roseicollis, chicken chromosomes 2, 3 and 5 hybridized either completely or partially to a single chromosome. In contrast, the smaller GGA macrochromosomes 6, 7 and 8 displayed a complex hybridization pattern: two or three of these macrochromosomes were found to be contiguously arranged on a single chromosome in all three parrot species. Overall, the study shows that translocations and fusions in conjunction with intragenomic rearrangements have played a major role in the karyotype evolution of parrots. Our inter-species chromosome painting results

unequivocally illustrate the dynamic reshuffling of ancestral chromosomes among the karyotypes of Psittaciformes. Copyright (c) 2007 S. Karger AG, Basel

1750: ***+.075***

We evaluated reproductive isolation of Chinook salmon (Oncorhynchus tshawytscha) life history types that have been reintroduced to northern Idaho, USA. Analysis of 1003 samples at six microsatellite loci revealed strong reproductive isolation between ocean- and stream-type Chinook salmon (fall and summer spawn timing, respectively) within the Clearwater River sub-basin (F-ST = 0.148, P < 0.00001). Very little evidence for gene flow among the two life history types was observed as assignment tests correctly assigned 99.6% of individuals in reference collections to either ocean- and steam-type Chinook salmon. Assignment of naturally reared juveniles indicated that both life history types were present with 24.1% stream-type and 75.9% ocean-type. Previous studies suggest high levels of divergence among the two life history types in natural populations, and our study verifies the persistence of reproductive isolation among types following colonization of habitat. Successful colonization of new habitat by (re)introduced species is likely influenced by diversity in life history types and this strategy has lead to naturally spawning populations in a variety of available habitats in the Clearwater River.. As many populations of O. tshawytscha are listed as threatened or endangered under the U.S. Endangered Species Act, hope for recovery lies not only in effective management and habitat improvement, but adaptability of this species.

1751: ***+.113***

Since 1985, China has established three breeding herds of Pere David's deer: the Beijing Pere David's Deer Park (39 degrees 07'N, 116 degrees 03'E), the Dafeng Pere David's Deer Nature Reserve (33 degrees 05'N, 120 degrees 49'E) and Shishou (Tianezhou) Pere David's Deer Nature Reserve (29 degrees 49'N, 112 degrees 33'E), through reintroductions of about 30-40 founders. Since establishment, all three populations have grown steadily. However, genetic backgrounds in those populations are still unknown. We studied the genetic diversity in Pere David's deer and genetic consequences of population relocations in China. We revealed that genetic diversity was extremely low in Pere David's deer populations in China. Only a single mtDNA D-loop haplotype was found in the deer, furthermore, only five polymorphic microsatellite loci were screened out from 84 pairs of species-transferred primers. Genetic makeup in the three Pere David's deer populations were significantly different (P < 0.01). H-E and allelic richness in the Tianezhou population Were the highest (0.54, 2.60, n = 31), Beijing population (0.52, 2.4, n = 125) showed the second highest measures, while the Dafeng population (0.46, 2.39, n = 39) measured lowest. Our results suggest that effective management of a species of low genetic diversity like the Pere David's deer should consider the genetic background of each founder to make sure genetic variations are preserved in both source population and relocated population. Now, the Tianezhou population is the most appropriate source population in China when establishing new Pere David deer populations in the wild.

1752: ***-.003***

The historic rarity of the critically endangered Grand Cayman blue iguana, Cyclura lewisi, has prevented detailed research on this species prior to this decade. This study primarily used focal animal observations, transect sweeps, and radio-telemetry to document the behavioral ecology of a captive-bred, reintroduced population of adult C. lewisi in a botanic park on Grand Cayman. Activity budgets and foraging budgets are presented for each sex in each season, and for all data combined. Distributions of basking time and active time differed between the two seasons.

Iguanas both emerged earlier and retreated later in the summer than in the fall. Of the small percentage of time spent active, iguanas mostly foraged and engaged in locomotion. Iguanas spent little time in trees or bushes or inside of retreats during the day. Observations of extensive tongue-touching of retreats, substrates, and feces suggest an importance of chemosensory ability in C. lewisi. Iguanas primarily consumed plant matter, of which the majority was non-cultivated plants in the park and surrounding area. Limited geophagy, coprophagy, and consumption of invertebrates were observed. Supplemental feeding contributed little to the overall diet of iguanas, but appeared to make iguanas more aggressive towards humans. Iguanas were heavily habituated to human and vehicular disturbances, which were common occurrences. Aspects of the behavioral ecology of C. lewisi reported here will assist in the conservation of this and other endangered iguanas.

1754: *+.216*

Conservation management options for southern African elephants range from local to regional scales. Here we review these options and argue in favour of actions that will deal with the causes rather than symptoms of elephant numbers that are locally high. Metapopulation theory ensures population persistence, while our approach extends this in order to stabilise elephant numbers regionally. By allowing for the development and maintenance of regional sinks, we may also limit numbers in sources. This application of the metapopulation metaphor is a powerful ecological platform from which to manage elephant numbers and impact through southern Africa. Our approach engages the causes of the apparently high abundance of elephants in parts of southern Africa. It moves away from the practice of dealing only with numbers (symptoms) when managing the impact of elephants on other species. While providing an ecological basis for the development of elephant management options, this needs to be melded with social, political and economic realities through southern Africa. In this regard we are encouraged by the ongoing development of several Transfrontier Conservation programmes and Peace Parks across the region. (c) 2006 Elsevier Ltd. All rights reserved.

1755: *+.262*

The 1991-1994 reintroductions of Florida endangered Pseudophoenix sargentii to 13 Florida Keys sites represent a rare example of a successful multi-agency long-term effort to conserve a long-lived palm. To assess reintroduction success, we compared population demographics with and without reintroduced plants and conducted population viability analyses. Since 1991, the wild population has increased 6.4-fold. Survival from 2000-2004 was 94%, growth was positive (lambda = 1.013), and there was no predicted extinction risk. Recent wild population growth is attributed to good seedling recruitment and removing the greatest threats. After 14 years, reintroductions had 43% survival, increased total plants in the wild by 27%, and expanded the species' distribution. Reintroduced plants had faster maturation rates, improved population age structure, and enhanced population growth (lambda = 1.032). Success varied with transplant year, location, microsite, and original transplant size. Failures in 1991 and at some historic sites emphasize the need for a multi-year, multisite approach to reintroductions to buffer against stochastic losses. Rockland hammocks and the tops of coastal berms had greatest plant growth and survival. Large transplants had the greatest survival. Because no reintroduced plants are reproductive, transitions between stages are extremely slow, and plants may require > 30 years to mature, continued institutional dedication to long-term monitoring will be required to assess whether the populations are self-sustaining. Horticultural expertise and ex situ collections complimented support of land managing agencies for the species' preservation. These first rare plant reintroductions to Florida State Parks opened avenues for more plant conservation efforts

and public interpretation. (c) 2006 Elsevier Ltd. All rights reserved.

1756: ***+.068***

During the last decades, various primate species around the world have been translocated from their threatened native habitats into new protected areas. Lemur populations living in the littoral forests of southeastern Madagascar are isolated in small forest fragments and are threatened by human pressures on this habitat. To avoid extinction of a remnant population of Eulemur collaris living in a forest fragment destroyed by charcoal makers, 28 individuals were transferred into a new protected area of approximately 230 ha within the Mandena Forest (M15/M16) in 2000 and 2001. Three groups of E. collaris were followed systematically for four years after their release at the new site. The size and composition of the translocated groups were monitored each year. After an initial phase of instability and death of several animals, the population increased to 36 individuals. Birth rates were similar to those of non-translocated groups living in largely undisturbed habitat. During the four years, animals gained weight. After translocation, the original groups split up in subunits consisting of one adult female and one or two adult males. Subsequently, several Cryptoprocta ferox, the largest living Carnivora on Madagascar, moved into the area, where they were previously unknown. They predated heavily on the translocated E. collaris and threaten the persistence of this population. The results show that E. collaris can be translocated successfully, however, translocation activities need continuous monitoring and possibly additional management.

1757: ***+.085***

The Fijian crested iguana (Brachylophus vitiensis) is restricted to tropical dry forest habitat and has been extirpated from over 80% of its original range primarily because of habitat destruction. A large population on Yadua Taba island has been proposed as a source for iguana translocations. This study aimed to determine the dietary and habitat requirements of the herbivorous B. vitiensis on Yadua Taba to identify essential tree species. Between September 2005 and June 2006 we examined the diet of B. vitiensis using faecal analyses, while perch preferences were examined using field surveys. Faecal analyses identified 26 plant species in the diet of B. vitiensis, while field surveys recorded iguanas in 33 tree species. The most common diet species largely overlapped with the most common perch species. There were no major seasonal shifts, sex differences, or age-class variations in diet or perch preferences. These results suggest that while B. vitiensis occurs in and consumes several plant species on Yadua Taba, it is primarily dependent on only a few species. To improve translocation success, future translocations of B. vitiensis need to ensure that these important tree species are present at new sites.

1758: ***+.042***

Ecosystem fragmentation results in major changes in several environmental and biotic parameters that affect the ability of plant populations to persist. All stages of the plant life cycle may be influenced in either negative or positive ways by the changed biophysical settings caused by fragmentation and associated changes in the surrounding landscape. This may result in plant populations being lost or significantly reduced from patches of native vegetation, leading to the need for active management intervention. This intervention may include management of threatening processes, reversal of ecosystem degradation, or the reintroduction of plants of species that have been lost from an area. These management actions range from preventative management through to active restoration. In the present paper I explore the question of whether there is a limit to the degree of intervention that is desirable in conservation terms, beyond which we are no

longer conserving but rather cultivating and gardening, i.e. creating an artificial and potentially unsustainable system. I discuss this question in relation to management of remnant vegetation in urban and agricultural settings and suggest that a careful mix of species-based and process-based management is required for us to succeed in the goal of biodiversity conservation in fragmented landscapes.

1759: *+.367*

Ex situ seed conservation aims to support species survival in the wild. This can be achieved by contributing genetic material for reintroduction. The goals of reintroduction are to increase both plant and population numbers, create self-sustaining populations and ultimately remove a species from its threatened listing. Quality seed collections with a broad genetic base are required to achieve this goal. Storage conditions that minimise deterioration of seeds will maximise the quality of seeds available for future use. Additionally, ex situ seed conservation provides long-term insurance against species or genotype loss until actual or potential threats can be removed. As threats to biodiversity escalate the most judicious conservation strategies will be ones that combine available resources to provide the highest possible degree of protection. Banked seeds are available irrespective of season and periods of low fecundity. Forward planning of reintroduction projects can be achieved with knowledge of the quantity and quality of banked seed. This paper discusses the challenges facing ex situ seed conservation while highlighting the benefits of integrating ex situ seed storage and plant reintroduction to help provide for better conservation outcomes.

1760: *+.140*

This review summarises scientific knowledge concerning the mycorrhizal associations, pollination, demographics, genetics and evolution of Australian terrestrial orchids relevant to conservation. The orchid family is highly diverse in Western Australia (WA), with over 400 recognised taxa of which 76 are Declared Rare or Priority Flora. Major threats to rare orchids in WA include habitat loss, salinity, feral animals and drought. These threats require science-based recovery actions resulting from collaborations between universities, government agencies and community groups. Fungal identification by DNA-based methods in combination with compatibility testing by germination assays has revealed a complex picture of orchid-fungus diversity and specificity. The majority of rare and common WA orchids studied have highly specific mycorrhizal associations with fungi in the Rhizoctonia alliance, but some associate with a wider diversity of fungi. These fungi may be a key factor influencing the distribution of orchids and their presence can be tested by orchid seed bait bioassays. These bioassays show that mycorrhizal fungi are concentrated in coarse organic matter that may be depleted in some habitats (e.g. by frequent fire). Mycorrhizal fungi also allow efficient propagation of terrestrial orchids for reintroduction into natural habitats and for bioassays to test habitat quality. Four categories of WA orchids are defined by the following pollination strategies: (i) nectar-producing flowers with diverse pollinators, (ii) non-rewarding flowers that mimic other plants, (iii) winter-flowering orchids that attract fungus-feeding insects and (iv) sexually deceptive orchids with relatively specific pollinators. An exceptionally high proportion of WA orchids have specific insect pollinators. Bioassays testing orchid-pollinator specificity can define habitats and separate closely related species. Other research has revealed the chemical basis for insect attraction to orchids and the ecological consequences of deceptive pollination. Genetic studies have revealed that the structure of orchid populations is influenced by pollination, seed dispersal, reproductive isolation and hybridisation. Long-term demographic studies determine the viability of orchid populations, estimate rates of transition between seedling, flowering, non-flowering and dormant states and

reveal factors, such as grazing and competition, that result in declining populations. It is difficult to define potential new habitats for rare orchids because of their specific relationships with fungi and insects. An understanding of all three dimensions of orchid habitat requirements can be provided by bioassays with seed baits for fungi, flowers for insects and transplanted seedlings for orchid demography. The majority of both rare and common WA orchids have highly specific associations with pollinating insects and mycorrhizal fungi, suggesting that evolution has favoured increasing specificity in these relationships in the ancient landscapes of WA.

1761: *-.066*

Ex situ conservation of threatened species may lead to behavioural adaptation, which can affect success of reintroduction attempts. In previous studies, we investigated the effects of captivity on the behaviour of red jungle fowl (Gallus gallus) and found that captive populations differed behaviourally as well as genetically. The aim of the present study was to compare the behaviour of two of the previously studied populations, raised under identical conditions. Eggs were collected from birds at Copenhagen zoo (Cop) and Gotala research station (Got) and were incubated and hatched together. Twenty-eight birds (16 Got and 12 Cop) were reared together and tested in eight different behavioural tests, measuring different aspects of fear-related behaviours as well as exploratory and social behaviours. The study revealed several differences in fear-related behaviours between the populations but none in exploratory or social behaviours. In general, one of the populations (Cop) showed more intense fear behaviours than the other (Got), which instead were less fearful in their behaviours. This indicates that breeding animals in captivity may lead to behavioural modifications, which can affect the outcome of reintroductions. The results further suggest that fear-related behaviours are dependent on the genetic background of the animals while social behaviours may be more influenced by the social environment. Since fear-related behaviours, such as predator avoidance and fear of humans, are essential for a life in the wild, these aspects are crucial for the breeding of animals in captivity for conservation purposes. (c) 2006 Elsevier B.V. All rights reserved.

1762: *+.085*

It is widely known that the adverse effects of stress must be considered in animal conservation programmes. However, a full consideration of how and where stress occurs in animal conservation programmes has not been undertaken, especially in translocation and reintroduction programmes. The literature concerning these types of programmes shows high levels of mortality, despite researchers' consideration of the effects of stress. However, an analysis of the literature shows that many conservation biologists have only a superficial knowledge about stress. For example, most do not understand the importance of subclinical stress or the fact that the effect of successive stressors can be additive or accumulative. While most conservation biologists know that stress is bad for animal health, few have considered its adverse effects on cognitive abilities, which an animal needs to survive in the wild (e.g. memory). In this paper we conclude with suggestions for improving the efficiency of animal conservation programmes in terms of the number of animals surviving after reintroduction or translocation. The most important conclusion from this review of the literature is that there needs to be a greater interchange of information between animal welfare and animal conservation scientists. (c) 2006 The Association for the Study ofAnimal Behaviour. Published by Elsevier Ltd. All rights reserved.

1763: *+.216*

With a total area of 8900 km(2), Puerto Rico is the smallest of the Greater Antilles. It is divided in

three physiographic regions or areas of relief: the mountainous interior, the karst region, and the coastal plains and valleys. The island comprises six ecological life zones: subtropical dry forest, subtropical moist forest, subtropical wet forest, subtropical rain forest, lower montane wet forest and lower montane rain forest. The herpetofauna of Puerto Rico consists of 25 species of amphibians (19 native, six introduced) and 56 species of reptiles (52 native, four introduced). The goal of this paper is to describe some of the present studies directed towards the conservation of Puerto Rican herpetofauna. Eleutherodactylus karischmidti, E. jasperi and E. eneidae have not been seen or heard since 1976, 1981 and 1990, respectively, and are probably extinct. Since 2000, the potential causes of amphibian declines in Puerto Rico have been studied, and a synergistic interaction between climate change (increased dry periods) and disease (chytridiomycosis) have been proposed as an explanation for the patterns observed. Recovery efforts for Peltophryne lemur include a captive-breeding program, reintroductions island-wide educational outreach, protection and restoration of existing habitat, and the creation of new breeding ponds. Among reptiles, the first conservation efforts to protect Epicrates inornatus were limited to trying to halt collection and hunting. However, current strategies to preserve the boa include gathering basic biological information, habitat conservation, and educational outreach. Recent efforts for the conservation of Trachemys s. stejnegeri combine three research approaches to clarify the status of local populations: a mark-recapture-release study, field monitoring of reproductive activity (i.e., nocturnal patrolling to identify nesting activity), and field assessment of the potential impact of introduced species, particularly identification of predatory species and exotic turtles. Recovery initiatives for Cyclura stejnegeri include management of invasive mammals, a headstart program for hatchling iguanas, and the assessment of the etiology of a condition causing blindness in adult iguanas. A reforestation project aimed at recovering a local herpetofaunal assemblage after disturbances in a limestone valley in northern Puerto Rico is discussed. As population sizes of common colonizers such as Eleutherodactylus and Anolis increased, larger forest-interior and predatory species like Epicrates inornatus, Alsophis portoricensis and Anolis cuvieri followed. Finally, the Mona Island marine turtle monitoring program is discussed and compared to other similar programs in Puerto Rico. As these and other similar conservation efforts provide scientifically based management recommendations, we hope to succeed in conserving the diverse herpetofauna that characterizes Puerto Rico.

1764: ***+.148***

Salmon supplementation and reintroduction programs have the potential to negatively impact other valued fish taxa that are not the targets of enhancement (nontarget taxa [NTT]). Impacts of the supplementation of spring Chinook salmon Oncorhynchus tshawytscha and the reintroduction of coho salmon O. kisutch (hereafter supplementation) on populations of rainbow trout O. mykiss, steelhead (anadromous rainbow trout), cutthroat trout O. clarkii, and bull trout Salvelinus confluentus were evaluated after 5 years of stocking approximately I million yearling smolts in the upper Yakima River basin between 1999 and 2003. Field methods included backpack electrofishing and snorkeling in tributaries and drift-boat electrofishing in the main stem. We used three sequential steps in our evaluation: (1) we determined whether spatial overlap occurred between supplemented fish and NTT; (2) if overlap occurred, we determined whether a change in abundance, size, or biomass occurred during supplementation; and (3) if a change occurred, we determined whether the change could be reasonably attributed to supplementation. Salmon rarely overlapped cutthroat trout or bull trout in tributaries, but some overlap with cutthroat trout occurred in relatively high elevations of the main stem and considerable overlap with rainbow trout occurred in tributaries and the main stem. Except in steelhead, the lower 90% confidence limit (CL) of abundance, size, and biomass was above the containment objective for NTT that overlapped significantly with salmon. We used rainbow trout as an analog for steelhead. The lower

90% CL of rainbow trout abundance and size in tributaries and the main stem and biomass in the main stein was below the containment objective for steethead. However, comparisons of rainbow trout abundance, size, and biomass between tributaries and main-stem sections with relatively high and low salmon abundances revealed that the change was probably not the result of supplementation (before-after control-impact paired site analysis: $P > 0.05$). Our data indicate that early stages of salmon supplementation have not impacted trout species in the upper Yakima River basin beyond predetermined containment objectives.

1765: *+.141*

Translocation is a common tool for restoring wildlife populations; however, potential genetic consequences include reduced levels of diversity within and increased divergence among populations. Elk (Cervus elaphus) were extirpated across much of North America by the early 20th century, but subsequent translocation programs restored the species to much of its historic range. The effects of these reintroductions on current patterns of genetic diversity in the western United States are largely unknown. We predicted that populations initiated with few founders and those experiencing slow postreintroduction growth would exhibit lower levels of diversity than other reintroduced populations. We used 12 microsatellite markers to examine patterns of genetic variability across 5 reintroduced populations of elk and 2 source herds from the Greater Yellowstone Ecosystem. The northern and southern Yellowstone source herds, which migrate to wintering areas separated by more than 260 km, exhibited similar levels of genetic diversity and high levels of gene flow, identified through both direct (i.e., assignment tests) and indirect measures. Levels of genetic diversity also were relatively high in all populations (unbiased heterozygosity, H-E = 0.51-0.60; allelic richness based on a sample size of 21, AR(21) = 3.3-4.0) and did not differ significantly between source and reintroduced populations or among reintroduced populations. We observed low to moderate levels of differentiation (Weir and Cockerham's F-ST statistic, theta = 0.01-0.08) and small genetic distances (Nei's standard genetic distance, D-S = 0.02-0.11) between populations. The relatively high levels of genetic diversity and low differentiation observed among our sampled populations are in stark contrast to observations of low diversity and high differentiation among isolated reintroduced populations of elk in the eastern United States. These results suggest that gene flow that includes other elk populations in the western United States may aid in preserving genetic diversity and limiting genetic divergence.

1766: *-.122*

Many captive stocks of economically or otherwise valuable species were established before the decline of the wild population. These stocks are potentially valuable sources of genetic variability, but their taxonomic identity and actual value is often uncertain. We studied the genetics of captive stocks of the threatened lesser white-fronted goose Anser erythropus maintained in Sweden and elsewhere in Europe. Analyses of mtDNA and nuclear microsatellite markers revealed that 36% of the individuals had a hybrid ancestry. Because the parental species are closely related it is unlikely that our analyses detected all hybrid individuals in the material. Because no ancestral polymorphism or introgression was observed in samples of wild populations, it is likely that the observed hybridisation has occurred in captivity. As a consequence of founder effect, drift and hybridisation, captive stocks were genetically differentiated from the wild populations of the lesser white-fronted goose. The high level of genetic diversity in the captive stocks is explained at least partially by hybridisation. The present captive stocks of the lesser white-fronted goose are considered unsuitable for further reintroduction, or supplementation: hybridisation has involved three species, the number of hybrids is high, and all the investigated captive stocks are similarly affected. The results highlight the potential shortcomings of using captive-bred individuals in

supplementation and reintroduction projects, when the captive stocks have not been pedigreed and bred according to conservation principles.

1767: *+.076*

We present a phylogenetic and morphological study of the grassland earless dragon, Tympanocryptis pinguicolla, an endangered habitat specialist that occurs in a few isolated populations in eastern Australia. Tympanocryptis pinguicolla occurred historically in Victoria in south-eastern Australia, but has not been seen since 1990, and current populations are known in New South Wales and Canberra in south-eastern Australia. Recently, new populations identified as T. pinguicolla were discovered on the Darling Downs, Queensland. Translocation of individuals between these populations has been suggested as a conservation management strategy to maintain genetic diversity. To address this issue, we undertook a phylogenetic study of all major populations of T. pinguicolla using a 1838 bp region of mitochondrial DNA, incorporating ND1, ND2, COI and eight tRNA genes. We incorporated specialized degraded-DNA techniques to amplify DNA from historical museum specimens, as no extant tissue was available for Victorian populations. Our results, which include morphological analysis, provide convincing evidence that populations currently identified as T. pinguicolla do not comprise a monophyletic species, as the populations from the Darling Downs are more closely related to T. tetraporophora than to T. pinguicolla. In addition, we find that there is a significant level of haplotype divergence between populations of T. pinguicolla, indicating that these lineages separated at least 1.5 mya. Our results suggest translocation may not be an appropriate management strategy and our findings that Darling Downs populations are not T. pinguicolla will significantly influence the conservation management of this species in Queensland.

1768: *+.069*

Populations of a threatened aquatic plant, Nymphoides peltata, have rapidly degenerated under the influence of recent artificial changes in Lake Kasumigaura of Japan. To estimate the potential of soil seed banks for genetic restoration of the species, we used 10 microsatellite markers to analyze the genetic variation in adults and in seedlings that emerged from soil seed banks. About 187 leaf samples from the cultured stocks that were collected in 17 adult subpopulations in 1995 and 2000 and from three subpopulations that were newly discovered in 2002 were analyzed. As a result, only 18 genets could be identified, suggesting that clonal diversity of the adult population had already become extremely low. Genetic tests were performed on 430 seedlings from seed banks at six locations of natural lakeshores and three of the restoration sites that were artificially constructed in an attempt to assign them to the remnant adult population; many of the seedlings showed genetic variation different from the adults. Furthermore, the seedlings preserved seven alleles that had been lost from remnant adults. However, they had lower average numbers of alleles and heterozygosity levels (NA = 1.5-3.1, H (E) = 0.146-0.487) than the remnant adults (NA = 3.5, H (E) = 0.539) and showed high inbreeding coefficients, suggesting that the seed banks were produced by inbreeding. Thus, although the seed banks had a certain potential to restore genetic diversity, the fitness reduction in seed banks caused by inbreeding could affect the success of restoration based on seed banks.

1769: *-.072*

The green and golden bell frog (Litoria aurea) has a widespread distribution along the south-east coast of Australia. The species range, however, is highly fragmented and remaining populations are predominately isolated and restricted to the coastline. Previously, the range extended further

inland and the species was considered common. Here we report a study designed to identify the phylogeographic and conservation genetic parameters of L. aurea. Mitochondrial DNA sequences were examined from 263 individuals sampled from 26 locations using both phylogenetic and population analyses. Despite a general consensus that amphibians are highly structured we found no phylogeographic divisions within the species, however, there was significant structure amongst extant populations (F (ST)=0.385). Patterns of haplotype relatedness, high haplotypic diversity (mean h=0.547) relative to low nucleotide diversity (mean pi=0.003) and mismatch distribution analysis supported a Pleistocene expansion hypothesis with continued restricted dispersal and gene flow. We conclude that the genetic structure of the species may permit 'well managed' intervention to mediate gene flow amongst isolated populations and provide some guidelines for the implementation of such conservation strategies.

1770: *+.170*

Reintroductions are conducted frequently throughout the world, and some source populations are harvested repeatedly to provide animals for translocation. The responses of these source populations to harvest should be monitored, and the resulting data used to refine population models will guide management After North Island Robins (Petroica longipes) were reintroduced to Tiritiri Matangi, New Zealand, in 1992, the population became a source for robins for additional reintroductions in the region. We constructed an initial model for the population on the basis of the data collected from 1992 to 1998 and used it to predict the population's response to the first translocation of robins from the island in the autumn (March) of 1999. We then analyzed postharvest data on survival (with mark-recapture analysis) and fecundity (with generalized linear-mixed modeling) to reassess and update the model. In the initial model, juvenile survival was assumed to be limited by the island's fixed carrying capacity, with excess juveniles dying over winter, hence, the autumn harvest was expected to cause an immediate increase in juvenile survival. In postharvest analysis, however most juvenile mortality occurred before autumn, and the best predictor of juvenile survival was the number of breeding pairs present the previous spring (start of the breeding season). Consequently, the updated population model predicted sustainable harvest levels about half those given by the initial model, and this model has been used to guide the number of individuals removed for two subsequent translocations. The ongoing development of the model has been invaluable for assuring conservation authorities that the population is not being unsustainably harvested, which has allowed surplus animals to be used to establish new populations. Our case study illustrates the value of an adaptive approach to harvesting source populations for reintroduction and illustrates the value of such studies for understanding the density-dependent mechanisms regulating populations.

1771: *+.189*

From 1998 to 2002, the Blackfeet Tribe and Defenders of Wildlife reintroduced 123 captive-reared swift foxes (Vulpes velox (Say, 1823)) to the Blackfeet Reservation in northern Montana. Because yearling swift foxes are colonizers of vacant habitats and are at the periphery of range expansion, we estimated 1st-year survival and explored aspects of dispersal to ascertain the contribution of wild-born juveniles to the reintroduction effort. First-year survival (post den emergence) of swift foxes averaged 0.38 and 0.36 for 2003-2004 and 2004-2005, respectively. Half of the females that survived to 1 June of their 1st year reproduced. Most dispersal (77%) and mortality (85%) occurred in autumn, with 80% of mortalities attributable to predation. Dispersal distance was not different among sexes and averaged 10.4 km. Understanding the contribution of yearling swift foxes - the colonizers - to reestablishing populations is important because several entities are currently reintroducing swift foxes and some states have expanding populations.

1772: ***+.144***

Although conservation and restoration practitioners have focused on maximizing above-ground population size and seed set of rare plants, a clear understanding of seed bank dynamics is crucial to managing these species. Santa Cruz tarplant (Holocarpha macradenia) is a threatened annual forb restricted to coastal prairie habitats in central California. Holocarpha produces disk achenes germinating within a year of production and ray achenes forming a persistent seed bank. We constructed both deterministic and stochastic demographic models for a restored Holocarpha population, using demographic rates measured separately for unmanipulated plants and plants growing in plots where vegetation was clipped. The deterministic models indicated that regardless of germination from the seed bank, the population would decline without clipping or similar treatments that enhance survival and reproductive output. Deterministic models showed only a slight positive effect of increased ray seed germination rates on population growth, which would need to be balanced against a potential loss of buffering against environmental variation as the dormant seed bank was reduced. Our stochastic simulations suggested that extinction risk for Holocarpha populations would be minimized by intermediate levels of ray seed germination. Thus, managers should focus on improving the performance of aboveground plants before considering actions to stimulate germination, since the former will yield a greater increase in deterministic population growth and not sacrifice any buffering effect of the seed bank. This case study emphasizes the importance of considering dormant seeds and seed banks in designing successful restoration and management strategies for plant species at risk of extinction. (c) 2006 Elsevier Ltd. All rights reserved.

1773: ***+.155***

The pygmy bluetongue lizard, Tiliqua adelaidensis, occupies spider burrows as home sites. It is an endangered species, known from only 19 small natural grassland sites in the midnorth of South Australia, all on privately owned land. Habitat requirements of the pygmy bluetongue lizard were investigated at four sites. Both within and between sites, lizards were more likely to be found in areas with a greater number of deep spider burrows. Areas where lizards were not found tended to lack these burrows. Strong site similarities were found for a range of habitat parameters examined. Within these grasslands there was no specific vegetation community associated with areas occupied by pygmy bluetongue lizards. However there was a distinct vegetation community associated with an absence of lizards. Generally there was no difference in the abundance and diversity of ground dwelling invertebrates between areas with and without lizards. As the only protected area of natural grassland within the known distribution, Mokota Conservation Park was assessed as a potential reintroduction site. It was found to be unsuitable due to a low number of deep spider burrows and a vegetation community similar to that found in uninhabited areas of known lizard inhabited sites. Unless other conservation areas can be established, preservation of this lizard will rely on habitat management by private land holders. Community goodwill and informed advice to the land holders will be essential in this process. (c) 2006 Elsevier Ltd. All rights reserved.

1774: ***+.168***

Re-introduction programs for endangered animals operate under the hope that protected habitats can sustain viable populations that rely little on humans. The goal of these programs is to supply animals with the resources and skills they need to succeed in the modem wild. However, predicting the set of skills necessary to respond to unpredictable selection events is difficult and efforts sometimes fail as animals respond inappropriately to environmental variation because they

lack behavioral flexibility. Population resilience to environmental change may be enhanced if all members of a population do not exhibit the same response when selection pressures change. In many species individual animals express behavioral types that exhibit alternative responses to the same stimuli. Yet when animals are prepared for release to the wild, there is rarely consideration of consistent behavioral variation between individuals. Since experience influences both behavioral and physiological responses to varied stimuli and can shape the future behavioral type of captive animals, pre-release environmental enrichment may be successful in facilitating the expression of varied behavioral types in populations slated for release. This approach to environmental enrichment requires a departure from a 'one size fits all' strategy and may also involve exposure to increased challenge and competition. In addition, there is a need for empirical evidence to better understand the role of environmental enrichment and behavioral types on post-release success. The zoo environment provides an excellent arena for examining the development and expression of behavioral types and for taking a novel functional approach to environmental enrichment research that may prove to be very important to re-introduction efforts. (c) 2006 Elsevier B.V. All rights reserved.

1775: *+.027*

Reintroductions of threatened species are increasingly common in conservation. The translocation of a small subset of individuals from a genetically diverse source population could potentially lead to substantial inbreeding depression due to the high genetic load of the parent population. We analysed 12 years of data from the reintroduced population of North Island robins Petroica longipes on Tiritiri Matangi Island, New Zealand, to determine the frequency of inbreeding and magnitude of inbreeding depression. The initial breeding population consisted of 12 females and 21 males, which came from a large mainland population of robins. The frequency of mating between relatives ($f > 0$; 39%, n=82 pairs) and close relatives ($f=0.25$; 6.1%) and the average level of inbreeding ($f=0.027$) were within the range reported for other small island populations of birds. The average level of inbreeding fluctuated from year to year depending on the frequency of close inbreeding (e.g. sib-sib pairs). We found evidence for inbreeding depression in juvenile survival, with survival probability estimated to decline from 31% among non-inbred birds ($f=0$) to 11% in highly inbred juveniles ($f=0.25$). The estimated number of lethal equivalents based on this relationship (4.14) was moderate compared with values reported for other island populations of passerines. Given that significant loss of fitness was only evident in highly inbred individuals, and such individuals were relatively rare once the population expanded above 30 pairs, we conclude that inbreeding depression should have little influence on this robin population. Although the future fitness consequences of any loss of genetic variation due to inbreeding are uncertain, the immediate impact of inbreeding depression is likely to be low in any reintroduced population that expands relatively quickly after establishment.

1776: *+.006*

Exotic species that invade remote islands, usually following human settlement, have had catastrophic effects on native biota. However, on islands it is increasingly feasible to eradicate key exotic species allowing extant native species to recover in situ or to return naturally. The practice of marooning threatened species on islands where the threat is absent, irrespective of whether the threatened species once occurred on the island, is well established. However, less focus has been given to the 'island' as the management unit on which to return extirpated species or related surrogates for extinct species. We use the example of Lord Howe Island as a case study to explore options for island restoration should the remaining critical exotic pests (rodents and perhaps owls in this case) be eradicated as planned. Lord Howe Island, in the south-west Pacific Ocean, is

remote, biologically diverse, has a high degree of endemism, and was only discovered by humans in 1778. Consequently, the original and exotic biota and their interactions are all better known than for many islands with a more ancient human history. Two species of plants, nine terrestrial birds, one bat and at least four invertebrates have been lost from the island since 1778. One plant and two invertebrates could be returned as conspecifics. One plant and all the terrestrial birds that are extinct could be replaced by closely related species from elsewhere in the Pacific Ocean. Decisions on replacing extinct species with surrogates should be based on the taxonomic relatedness of the candidates for reintroduction: the same species before subspecies before genera, with functional replacement being a further filter on candidates that are not the same species. In our opinion, taxa with functional equivalence but without taxonomic relatedness would not be acceptable candidates for reintroduction.

1777: *+.351*

We used isozymes (16 loci in 11 enzymatic systems) from Laelia speciosa, an endemic and endangered epiphytic orchid of Mexico, to assess the genetic diversity and population genetic structure in nine populations distributed along its geographic range, as well as to detect those populations that are genetically unique and therefore deserve high-priority protection. On average, the genetic diversity was high (percentage of polymorphic loci, P-p = 76%, mean number of alleles per locus, A = 3.34, the average observed heterozygosity H-O = 0.302, the average expected heterozygosity H-E = 0.382). Moderate levels of inbreeding (f = 0.216, 95% confidence interval = 0.029-0.381) were found. Low levels of genetic differentiation were observed among populations (theta(p) = 0.040); however, there was a significant correlation between geographic and genetic distances among the populations (Mantel test: r(2) = 0.43, P < 0.05). Populations located within the same mountain range were genetically more similar. Private alleles were found, so proper management requires protection and maintenance of genetic diversity throughout its range. In case of reintroduction, we suggest using individuals propagated from seeds from as many capsules as possible, from close populations. An ex situ conservation strategy also is proposed.

1778: *+.278*

Translocation is a useful management option for conservation of threatened animal species. It can be used to increase the range of a species, augment the numbers in a critical population, or establish new populations and hence spread the risk of extinction through local catastrophes. As it is an important and expensive conservation tool, translocation management decisions must be carefully considered, with the objective of the translocation project in mind. By analysing the translocation problem within a decision-theory framework, we find optimal management decisions that are rational and transparent. We illustrate our approach using a case study of the bridled nailtail wallaby (Onychoyalea fraenata). Our particular translocation question is: if we have a set number of wallabies to translocate in each time period and two translocation sites, how many wallabies should we put at each site given the state of each population to maximise the benefit to the species? We model the translocated populations with first-order Markov chain stochastic population models, and use stochastic dynamic programming to determine the optimal management decisions. We look at two sites with different growth rates - one increasing and one decreasing - and compare the optimal strategies for two different objective functions. The first is a long-term persistence objective function, which maximises the persistence of translocated populations a large number of time steps after the end of the translocation program. The second maximises total population size at the end of the translocation program. Although these objective functions are similar, they generate surprisingly different optimal translocation strategies. When maximising the long-term persistence of the translocated populations, translocation decisions are

not important as long as an increasing population is established. This indicates that site quality - rather than the number and timing of translocations - primarily determines the long-term persistence of populations. When maximising total population size, the optimal strategy is to add to the increasing population unless it is above a size where it is likely to reach its carrying capacity over the planning timeframe. As translocation decisions are important in fulfilling the objective, this objective function is more useful in creating practical advice for translocation managers. The discrepancy between the optimal strategies given by the two objectives demonstrates the importance of careful consideration when specifying the goals of a project. This observation applies not only to translocation programs, but any project where clear decision-making is needed. (c) 2006 Elsevier B.V. All rights reserved.

1779: ***+.291***

Sex- and species-specific singing is an integral part of gibbon behaviour. Young gibbons learn to sing by copying their parents. Analysis of the recorded great calls of wild-born, captive-raised agile gibbons (Hylobates albibarbis) from the Kalaweit Gibbon Rehabilitation Project, Central Kalimantan, Indonesia and wild-raised agile female songs in two locations around Kalimantan are presented herein. We found high variance of song characteristics both within and between populations. This result shows that captive-raised gibbon females can learn flexibility in the song pattern in the great call phase. We also demonstrate variation in song patterns between distinct populations, indicating a genetic component to the levels of flexibility in the great call. There is a clear genetic component, as gibbons raised alone produce the species-specific song and there is a clear level of individuality to the great call component of the song. This distinct individuality supports the hypothesis that the song of gibbons is a mechanism of intra- and inter-specific communication about the status of individual gibbons and pairs e.g. social and physical status. In addition evidence is presented suggesting that separate populations of the same species may have distinct dialects. Implications for rehabilitation and reintroduction of gibbons are discussed.

1780: ***+.084***

In this article we present the results of a study, from 1980 to 1991, which involves the census of nests and breeding pairs of Gypaetus barbatus, Gyps fulvus and Neophron percnopterus in eastern Herzegovina. These three vulture species has since disappeared from their breeding habitats. The presented data may help the planning and development of a strategy for the successful reintroduction conservation of vultures in Bosnia and Herzegovina.

1781: ***-.016***

The remaining populations of ocelot (Leopardus pardalis albescens) in the United. States are reduced to 2 isolated populations in southern Texas, with the next closest populations occurring in central Tamaulipas, Mexico. The species is listed as endangered, and recovery of populations in Texas eventually might require translocations from larger source populations. We sequenced the mitochondrial DNA control region from individuals from Texas and northern Mexico and compared these data to existing sequences derived from ocelots in other parts of its range in southern Mexico, Central America, and South America. Nucleotide diversity was lower in Texas than in Mexico, suggesting a loss of genetic variation as a consequence of fragmentation and increased genetic drift. Phylogenetic analyses showed a close relationship between populations in Texas and northern Mexico that encompass the range of the subspecies L. pardalis albescens. Based on these data, the best source population for a recover, plan involving ocelot translocations would be northern Mexico, because this region seems to form a discrete management unit (both

ecologically and phylogenetically) that includes Texas.

1782: *+.113*

In 1997, a plan to restore Elk (Cervus elaphus) to Ontario was approved by the provincial government. The objective of the Ontario elk restoration program, a multipartnered collaboration, was to restore a species that had been extirpated from the province during the 1800s. During 1998-2001, 460 elk were acquired from Elk Island National Park, Alberta, for release in four areas of Ontario. As greater than 90% of the elk were radio collared, monitoring provided detailed information on the dynamics of the four populations. Comprehensive research projects using graduate students were implemented to determine the environmental impact of releasing elk in Ontario. Those studies are in progress or have been completed and include the effect of wolf predation on restored elk, white-tailed deer and elk resource overlap, the development of genetic profiles for elk, and solutions for elk/human conflicts. Mortality of the released elk averaged 41% (190/460) during 1998-2004 with annual mortality generally declining over time in each release area. The primary causes of elk mortality included wolf predation (25% of mortalities), illegal shooting (13%), stress-related emaciation (13%) (partially due to the stress of relocation), bacterial infections (7%), and collisions with vehicles (6%). Productivity has been high in one of the release areas with 24-65% of the cows being observed with calves during late winter surveys. However, productivity has been low in two of the northern release areas due to a variety of factors including wolf predation. In some areas, dispersion of elk appeared to be related to the length of time animals were kept in pens prior to release. The precalving population estimate for Ontario in March 2004 was 375-440 elk. A comprehensive program review was conducted in 2003/2004 that included recommendations relating to the future management of elk in Ontario.

1783: *+.059*

Recent molecular data on the maternally inherited mitochondrial (mt) DNA have challenged the traditional view that the now extinct Baltic sturgeon population belonged to the European sturgeon Acipenser sturio. Instead, there is evidence that American sea sturgeon Acipenser oxyrinchus historically immigrated into the Baltic Sea. In this study, we test the hypothesis that A. oxyrinchus introgressed into, rather than replaced, the A. sturio population in the Baltic. We established four single nucleotide polymorphisms (SNPs) in the nuclear MHC II antigen gene with a species-specific SNP pattern. Using an ancient DNA approach and two independent lines of molecular evidence (sequencing of allele-specific clones, SNaPshot), we detected both A. sturio and A. oxyrinchus alleles in the available museum material of the now extinct Baltic sturgeon population. The hybrid nature of the Baltic population was further confirmed by very high levels of heterozygosity. It had been previously postulated that the immigration of the cold-adapted A. oxyrinchus into the Baltic occurred during the Medieval Little Ice Age, when temperature likely dropped below the degree inducing spawning in A. sturio. Under this scenario, our new findings suggest that the genetic mosaic pattern in the Baltic sturgeon population (oxyrinchus mtDNA, sturio and oxyrinchus MHC alleles) is possibly caused by sex-biased introgression where spawning was largely restricted to immigrating American females, while fertilization was predominantly achieved by abundant local European males. The hybrid nature of the former Baltic sturgeon population should be taken into account in the current reintroduction measures.

1788: *-.185*

Use of the subspecies as the basic unit in the conservation of endangered caribou (Rangifer tarandus) would produce a "melting pot" end-product that would mask important genotypic,

phenotypic, ecological, and behavioral variations found below the level of the subspecies. Therefore, we examined options for establishing the basic conservation unit for an endangered caribou population: use Of Subspecies based on taxonomy, subspecies based solely on mtDNA, Evolutionarily Significant Units, and the geographic population. We reject the first three and conclude that the only feasible basic unit for biologically and ecologically sound conservation of endangered caribou in North America is the geographic population. Conservation of endangered caribou at the level of the geographic population is necessary to identify and maintain current biodiversity. As deliberations about endangered caribou conservation often involve consideration of population augmentation, we also discuss the appropriate augmentation protocol for conserving biodiversity. Management of a critically endangered caribou population by augmentation should only be initiated after adequate study and evaluation of the genotype, phenotype, ecology, and behavior for both the endangered caribou and the potential 'donor' caribou to prevent the possible 'contamination' of the endangered caribou. Translocation of caribou into an endangered population will have failed, even if the restocking efforts succeed, if the donor animals functionally alter the population's gene pool or phenotype, or alter the ecological and behavioral adaptations of individuals in the endangered population. Most importantly, a seriously flawed restocking would risk irreversibly altering those functional characteristics of caribou in an endangered population that make them distinct and possibly unique. It might even result in the loss of the endangered population, thus eliminating a uniquely evolved line from among the caribou species.

1789: ***+.081***

This study represents the first attempt to determine post-release survival of a captive-bred owl in Africa. We released six captive-bred African Grass-Owls Tyto capensis into suitable habitat and, using radio telemetry, determined their daytime roost sites. One bird left the study area soon after release and did not yield data. Of the remaining five owls, three survived for at least 30 days in the wild, and all weaned off supplementary food onto a natural diet of wild rodents. Birds had no sign of supplementary food in their pellets after four, 28 or 33 days. Our results show similar success rates to those of both wild and captive-bred owls studied elsewhere in the world. Roost range area varied from 0.08-0.66 km(2). We conclude that captive breeding and release programmes may be a viable conservation option for African Grass-owls, if required in the future.

1790: ***+.223***

Recently, conservation estate in South Africa's Eastern Cape Province has increased 10-fold resulting in large predators being increasingly reintroduced to restore ecological integrity and maximize tourism. We describe the reintroductions of large carnivores (>10 kg) that have occurred in the Eastern Cape and use various criteria to assess their success. Lion Panthera leo reintroduction has been highly successful with a population of 56 currently extant in the region and problems of overpopulation arising. The African wild dog Lycaon pictus population has increased to 24 from a founder population of 11. Preliminary results for spotted hyaenas Crocuta crocuta also indicate success. Wild populations of leopards Panthera pardus exist on several reserves and have been supplemented by translocated individuals, although deaths of known individuals have occurred and no estimate of reproduction is available. Cheetah Acinonyx jubatus reintroduction has also been less successful with 36 individuals reintroduced and 23 cubs being born but only 41 individuals surviving in 2005. Criteria for assessing the success of reintroductions of species that naturally occur in low densities, such as top predators, generally have limited value. Carrying capacity for large predators is unknown and continued monitoring and intensive management will be necessary in enclosed, and possibly all, conservation areas in the Eastern Cape to ensure conservation success.

1791: ***+.133***

The success of efforts to re-establish mammalian carnivores within their former range is dependent on three key factors: methodological considerations, the biological requirements of the target species, and the involvement of local human communities for whom large carnivores pose a threat. We consider the role of these factors in the first 13 years of an effort to re-establish wild lions in northern KwaZulu-Natal Province, South Africa. We employed soft-release methods to mitigate the characteristic problems associated with restoration of large carnivores. A pre-release captivity period facilitated acclimatization of reintroduced lions and promoted long-term bonding of unfamiliar individuals into cohesive groups. All individuals remained in the release area and established enduring, stable home ranges. Reintroduced lions successfully reproduced and raised 78% of their cubs to independence. Human activity was the cause of all post-release mortality. Despite rapid population growth and the re-establishment of the species at Phinda Private Game Reserve, the population is small and isolated with little prospect for re-colonizing additional areas where the species has been extirpated, or for connecting with other isolated lion populations in the region. Accordingly, although we essentially overcame the short-term technical and biological challenges facing lion reintroduction, the long-term value of the Phinda population for addressing the conservation issues facing the species remains equivocal.

1792: ***+.092***

Quaking aspen (Populus tremuloides Michx.), a common species in North America, is a minor species in the Sierra Nevada of California. However, the limited coverage of aspen in this area appears to carry a disproportionate biodiversity load: numerous species are dependent on the unique components of aspen forests for habitat. Land managers in the region believe the species is declining due to fire suppression policies of the past century. Recent research from other regions shows mixed results when assessing the extent of decline. This review focuses on the crossroads between human and natural history to describe a broader picture of aspen ecology in the Sierra Nevada. The method used here combines a review of the ecological literature with historical synthesis. A central conclusion is that the current "decline" in aspen must be placed in the context of an unusual regeneration pulse brought on by intensive Euro-American resource extraction activities of the late 19th century. We address unique features of the Sierra aspen population, the interface of climate change and human-caused disturbance, and conservation strategies for restoration of an aspen community more closely aligned with contemporary climate-disturbance cycles. Conservation recommendations include reintroduction of mixed-severity natural fires and complimentary wildlife, such as top predators, where practical, plus allowance for local flexibility where deviations are appropriate based on ecology and social concerns.

1793: ***+.230***

Precise and unbiased estimates of demographic parameters are necessary for effective population monitoring and to parameterize population models (e.g., population viability analyses). This is especially important for endangered species, where recovery planning and managers' decisions can influence species persistence. In this study, we used mark-recapture methods to estimate survival of fledged juveniles (hatch-yr [HY]) and adult (after-hatch-yr [AHY]) Laysan ducks (Anas laysanensis), an endangered anatid restricted to Laysan Island in the northwestern Hawaiian Islands. To better understand population dynamics, we examined how survival varied as a function of Laysan duck density during 1998-2004. Using random effects models, we also quantified process variation in survival, thereby quantifying the appropriate source of variation for future population models. The dataset supported variation in survival that was time (yr), age (AHY vs.

HY), and sex specific. Due to small sample sizes, we did not examine time specificity in the survival of HY ducks. Survival of HY ducks was 0.832 (SE = 0.087) for females (n = 21) and 0.999 (SE < 0.001) for males (n = 15) during 1998-2001. Trends in time and density lacked support as sources of variation in the survival of AHY ducks during 1998-2004. After-hatch-year survival ranged from 0.792 (SE = 0.033) to 0.999 (SE < 0.001). Where we modeled survival as a random effect, annual survival for AHY females was 0.981 (SE = 0.017) and process variation (sigma(S)) was 0.034. For AHY males, annual survival (mu(S)) was 0.906 (SE = 0.019) and process variation (sigma(S)) was 0.040. This information will improve existing population viability analysis models for Laysan ducks. We believe that monitoring the source and translocation populations will be paramount for increasing our understanding of Laysan duck dynamics, recovery planning, and population management.

1794: *-.057*

In the past decade, many Indian states have reported an increase in Leopard (Panthera pardus fusca) populations outside forests, in certain areas, accompanied by a large number of attacks on people. This high density was attributed to declining natural habitats and prey species, and the increased survival of Leopards in croplands where they preyed on tended, as well as feral domestic animals. That Leopard cubs were frequently found in agricultural fields was thought to also indicate rising Leopard populations. We use data from our human-leopard conflict study in Junnar, Maharashtra, along with information from three other conflict sites in India, to propose that the reason for this increase in. Leopard population and conflict is related to the sustained translocation of 'problem' Leopards into nearby forests. That sustained releases could lead to population increases was never considered before, even though translocation is known to be a procedure for increasing populations of species at or close to the site of release. Although scientists do not recommend translocation as a management strategy for 'problem' carnivores, it is currently the legally recommended method of dealing with 'problem' large cats in India. Such faulty policies will only further hamper the conservation of this species, which is hunted in large numbers for illegal wildlife trade.

1795: *+.294*

Despite many successful reintroductions of large mammalian herbivores throughout the world, remarkably little attention has focused on how these actions affect native and exotic vegetation at reintroduction sites. One such herbivore is tale elk (Cervus elaphus nannods), which was on the brink of extinction in the mid 1800s, but now has numerous stable populations due to intensive reintroduction efforts. Here, we summarize results from a 5-year exclosure experiment that explored the effects of tule elk on a coastal grassland in northern California. Elk significantly altered the species composition of this community; the response of annual species (dominated heavily by exotic taxa) was dramatically different from perennial species. Elk herbivory increased the abundance and aboveground biomass of native and exotic annuals, whereas it either had no effect on or caused significant decreases in perennials. Elk also decreased the cover of native shrubs, suggesting that these herbivores play an important role in maintaining open grasslands. In addition, elk significantly reduced the abundance and biomass of a highly invasive exotic grass, Holcus lanatus, which is a major problem in mesic perennial grasslands. Our results demonstrate that the successful reintroduction of a charismatic and long-extirpated mammal had extremely complex effects on the plant community, giving rise to both desirable and undesirable outcomes from a management perspective. We suspect that these kinds of opposing effects are not unique to tule elk and that land managers will frequently encounter them when dealing with reintroduced mammals.

1796: ***+.256***

with recent increases in the numbers of species reintroduction projects and reintroduction-related publications, there is now a recognizable field of reintroduction biology. Nevertheless, research thus far has been fragmented and ad hoc, rather than an organized attempt to gain reliable knowledge to improve reintroduction success. We reviewed 454 recent (1990-2005) peer-reviewed papers dealing with wildlife reintroductions from 101 journals. Most research has been retrospective, either opportunistic evaluations of techniques or general project summaries, and most inference is gained from post hoc interpretation of monitoring results on a species-by-species basis. Documentation of reintroduction outcomes has improved, however, and the derivation of more general principles via meta-analyses is expected to increase. The fragmentation of the reintroduction literature remains an obstacle. There is scope to improve reintroduction biology by greater application of the hypothetico-deductive method, particularly through the use of modeling approaches and well-designed experiments. Examples of fruitful approaches in reintroduction research include experimental studies to improve outcomes from the release of captive-bred animals, use of simulation modeling to identify factors affecting the viability of reintroduced populations, and the application of spatially explicit models to plan for and evaluate reintroductions. We recommend that researchers contemplating future reintroductions carefully determine a priori the specific goals, overall ecological purpose, and inherent technical and biological limitations of a given reintroduction and that evaluation processes incorporate both experimental and modeling approaches. We suggest that the best progress will be made when multidisciplinary teams of resource managers and scientists work in close collaboration and when results from comparative analyses, experiments, and modeling are combined within and among studies.

1797: ***+.110***

During. 1997-1999 32 Woodland Caribou (Rangifer tarandus caribou) were translocated from) the Sustut Herd to the Telkwa Mountains in westeentral British Columbia to augment recovery of the Telkwa Caribou Herd. The animal were fitted with radiocollars and located during 1997-2000 to determine selection of habitat features and terrain variables. Six Caribou calves were also collared to determine Causes and timing, of calf mortality during summer 1999. Defining available habitat for newly translocated animals is often arbitrary and subjective. :in(] we based the analyses on ranks for habitat use and availability as this is less sensitive to the inclusion or exclusion of a questionable resource. This method represents some loss of information l but provides indications of the relative importance of various habitat types without classifying any as avoided. High elevation habitat (> 1700 masl) on moderate slopes (16 - 45 degrees) received the highest ranks. as did "warm" (136-315 degrees) aspects and forests > 250 years old. Three calves died shortly after birth. One calf appeared to have been killed by predation. likely by a Golden Eagle (Aquila chrysaetos), and one calf was abandoned by the cow. Cause of death for the third Calf is Unknown, To assess habitat use associated with calving we compared summer locations with data obtained throughout rest of 1999 for eight cows with calves and eight without Calves. We found signigicant difference in use of elevation during calving . I, e when cows with calves remained at high elevations and barren cows generally descended to lower elevation habitat. Surveys conducted in 2005. five years after the completion of the initial study. procuced a count of approximately 90 Caribou. This suggests that in the short term. the was successful in re-establishing a self-sustaining Caribou population in the Telkwa Mountains.

1798: ***+.104***

Translocation could be used as a tool in conservation of the threatened Mojave Desert Tortoise (Gopherus agassizii) by moving individuals from harm's way and into areas where they could contribute to conservation of the species. Numerous factors may affect the success of translocations, including the conditions experienced by tortoises in holding facilities while awaiting translocation. The tortoises available for our translocation study had been provided supplemental water during their years spent in a captive holding facility, potentially inducing carelessness in water conservation. In addition to generally investigating the efficacy of translocation, we compared the effects of continuing with the effects of ceasing the holding facility's water supplementation regimen. After exposure to one of the two water regimens, all tortoises were given the opportunity to hydrate immediately prior to release. We examined behavior, body mass, carapace length, movement, and mortality of tortoises for two activity seasons following release to the wild. Water supplementation was correlated with high rates of carapace growth and distant movements by males after release. Lengthy movements following translocation may be problematic for conservation planning, but this should be evaluated in light of the goals and circumstances of each translocation project. Although the mortality rate was 21.4% in 1997, data suggest that drought conditions at the site rather than the translocation itself negatively affected the tortoises. None of the tortoises died during their second season at the site. Our results indicate that translocation should be considered a useful tool in conservation of the Desert Tortoise. (C) 2006 Elsevier Ltd. All rights reserved.

1799: *-.051*

Reintroductions are important tools for the conservation of individual species, but recently more attention has been paid to the restoration of ecosystem function, and to the importance of carrying out a full risk assessment prior to any reintroduction programme. In much of the Highlands of Scotland, wolves (Canis lupus) were eradicated by 1769, but there are currently proposals for them to be reintroduced. Their main wild prey if reintroduced would be red deer (Cervus elaphus). Red deer are themselves a contentious component of the Scottish landscape. They support a trophy hunting industry but are thought to be close to carrying capacity, and are believed to have a considerable economic and ecological impact. High deer densities hamper attempts to reforest, reduce bird densities and compete with livestock for grazing. Here, we examine the probable consequences for the red deer population of reintroducing wolves into the Scottish Highlands using a structured Markov predator - prey model. Our simulations suggest that reintroducing wolves is likely to generate conservation benefits by lowering deer densities. It would also free deer estates from the financial burden of costly hind culls, which are required in order to achieve the Deer Commission for Scotland's target deer densities. However, a reintroduced wolf population would also carry costs, particularly through increased livestock mortality. We investigated perceptions of the costs and benefits of wolf reintroductions among rural and urban communities in Scotland and found that the public are generally positive to the idea. Farmers hold more negative attitudes, but far less negative than the organizations that represent them.

1800: *+.109*

The Endangered Primate Rescue Center is both a part of and the operational base of the "Vietnam Primate Conservation Programme" of the Frankfurt Zoological Society. Since its establishment in 1993, the Endangered Primate Rescue (EPRC) has published regularly the "EPRC-Newsletter." The Vietnamese Journal of Primatology creates a platform to make all the newsletter information accessible to a broader circle of readers. Reports from the EPRC should be regularly published in the journal. The number of primates at the EPRC increased continuously from the end of 1993 (8

individuals) to the end of 2006 (145 individuals). The number of taxa has also increased from two to fifteen. By the end of 2006, 109 individuals from nine taxa had been born at the center. Among those born were four species born for the first time ever in captivity: the Delacour's langur, Hatinh langur, Cat Ba langur, and grey-shanked douc langur. The center has 43 cages for langurs and gibbons with a total surface area of 2,750 m2, 6 indoor enclosures comprising 110 m2, and 13 cages for lorises with a total surface area of 100 m2. The quarantine station has four outdoor enclosures (total 60 m2), two indoor enclosures (total 50 m2), a surgery room and a preparation room. Of particular significance are the two electrically-fenced semi-wild enclosures with primary forest (roughly 2 and 5 ha). The staff at the center has grown to currently 20 Vietnamese workers, five Vietnamese biologists, one Vietnamese project assistant, two foreign animal keepers, and one foreign project leader. One foreign veterinarian has been working at the center from 1997 to autumn 2006. In cooperation with Vietnamese and foreign institutions and universities, the EPRC has contributed immensely to knowledge of the systematics, molecular genetics, locomotion, nutrition and feeding ecology of Indochinese primates. Van Long Nature Reserve is an important contribution to the protection of the largest and probably only viable population of the "Critically Endangered" Delacour's langur. In 2005, preparations began for a long-term reintroduction program in Phong Nha - Ke Bang National Park. The first step is the construction of a 20 ha electrically-fenced semi-wild area. The first species selected for the reintroduction is the Hatinh langur. The EPRC contributes in several ways to raising awareness about wildlife conservation, especially primate protection and education. Numerous publications and TV reports called attention to the highly endangered and endemic Vietnamese primates. Besides a basic financial contribution to the EPRC provided by the Frankfurt Zoological Society, finances originate from numerous conservation organizations, zoos and private people who contribute to the "Vietnam Primate Conservation Programme". Without their ongoing support the EPRC would not be able to continue its work for primate conservation in Vietnam.

1801: ***+.193***

Over the past 20 years, there has been a growing awareness of the impact of genetic factors on the success of reintroduction programs for fish and other species. One primary genetic criterion to be considered in the design and implementation of reintroduction programs is the maximization of genetic diversity within reintroduced populations. Reintroduction has become an important management tool for the imperiled lake sturgeon Acipenser fulvescens. However, little published work has evaluated current lake sturgeon reintroduction programs in terms of their ability to transfer genetic diversity from source populations to reintroduced populations. We evaluated the success of an ongoing lake sturgeon reintroduction program based upon its ability to adequately transmit the genetic diversity of the Lake Winnebago source population into reintroduced populations in the Mississippi and Missouri rivers. Additionally, a nonreintroduced single year-class from a hatchery population established from the Lake Winnebago stock was included in this study to determine how much of a source population's genetic diversity could be captured in a single stocking event. Reintroduced populations exhibited levels of genetic diversity similar to that of their source population, and estimates of genetic differentiation revealed very little divergence between source and reintroduced population pairs. Significant levels of genetic differentiation between the Lake Winnebago and nonreintroduced hatchery fish, as well as evidence of a bottleneck within the hatchery fish, indicated that the small number of parents used in a single-year stocking event may not adequately exploit a source's available genetic diversity. Therefore, a multiple-year stocking strategy may be most appropriate for lake sturgeon reintroduction programs.

1802: ***+.150***

Relocations of gopher tortoises (Gopberus polyphemus) in Florida, USA, are frequently employed as mitigation tools when tortoises occupy land desired for development. Here we present information about retention and health of a relocated population of gopher tortoises 17 years after relocation. We combine our 17-year postrelocation data with earlier surveys 1 year and 2 years postrelocation to examine whether retention rates change over time. We also evaluate whether retention rates vary by age and gender. Of 74 gopher tortoises relocated in 1985, 31 were present in 2002. We found a 1-year retention rate of 42%, with retention rates of 100% each year thereafter, when we used the percentage of relocated individuals captured at each survey. We found a I-year retention rate of 73%, a retention rate of 92% from year 1 to year 2, and an overall retention rate of 98.5% from year 2 through year 17, based on the assumption that all individuals present in later surveys were present in earlier surveys. We found no significant difference in retention rate over the 17-year period for adults and juveniles and for adult males and females. Relocated gopher tortoises showed natural growth patterns, indicating good health, but 35% of these gopher tortoises had >= 1 symptom of upper respiratory tract disease, a disease associated elsewhere with population declines of tortoises. Thus, retention rates of relocated gopher tortoises change over time, with relatively low retention during the first year postrelocation but nearly 100% retention in subsequent years. In general, our study shows that relocations can successfully lead to long-term retention of gopher tortoises, but we predict that without management (e.g., fire management and predator control) this relocated population is not viable.

1803: *+.142*

Many cave species exhibit k-selected life history characteristics, i.e., long life span, slow growth, and low reproductive effort, which can reduce their ability to survive rapid changes in environmental conditions and their potential for recovery from events that reduce their densities. In this study, we examined aspects of the life history, growth rate, and reproductive timing of the U.S. federally listed Illinois cave amphipod, Gammarus acherondytes, to assess its potential for recovery. Sampling between October 2003 to March 2005 with nonlethal techniques in Reverse Stream Cave, Illinois, U.S.A. showed that pre-juveniles and juveniles generally constituted > 50% of the population and thus strongly influenced fluctuations in population density. Except in September 2004, ovigerous females were collected every month, indicating that reproduction occurred year-round. Distinct peaks of pre-juveniles were observed from February to May and August to October. The seasonal recruitment of young appeared to be correlated with incubation time and flood events. Because it was difficult to track individual cohorts. the exact time to maturity or the life span could not be determined empirically. However, the minimum time to maturity was estimated at 14 to 16 months using a modeled growth curve. Our results suggest that G. acherondytes should recovery rapidly from perturbations if left undisturbed. A management strategy to aid recovery could be to reduce cave visitation during the major phases of recruitment. Additionally, our results indicate that establishing a laboratory population for use in future studies or to propagate individuals for reintroduction should require only one-and-a-half to two years. Our study shows how basic life history information obtained via non-lethal sampling can provide managers with vital information to develop conservation strategies for endangered species.

1804: *+.306*

Here we critically review the scale and extent of adaptive genetic variation in Atlantic salmon (Salmo salar L.), an important model system in evolutionary and conservation biology that provides fundamental insights into population persistence, adaptive response and the effects of anthropogenic change. We consider the process of adaptation as the end product of natural selection, one that can best be viewed as the degree of matching between phenotype and

environment. We recognise three potential sources of adaptive variation: heritable variation in phenotypic traits related to fitness, variation at the molecular level in genes influenced by selection, and variation in the way genes interact with the environment to produce phenotypes of varying plasticity. Of all phenotypic traits examined, variation in body size (or in correlated characters such as growth rates, age of seaward migration or age at sexual maturity) generally shows the highest heritability, as well as a strong effect on fitness. Thus, body size in Atlantic salmon tends to be positively correlated with freshwater and marine survival, as well as with fecundity, egg size, reproductive success, and offspring survival. By contrast, the fitness implications of variation in behavioural traits such as aggression, sheltering behaviour, or timing of migration are largely unkown. The adaptive significance of molecular variation in salmonids is also scant and largely circumstantial, despite extensive molecular screening on these species. Adaptive variation can result in local adaptations (LA) when, among other necessary conditions, populations live in patchy environments, exchange few or no migrants, and are subjected to differential selective pressures. Evidence for LA in Atlantic salmon is indirect and comes mostly from ecological correlates in fitness-related traits, the failure of many translocations, the poor performance of domesticated stocks, results of a few common-garden experiments (where different populations were raised in a common environment in an attempt to dissociate heritable from environmentally induced phenotypic variation), and the pattern of inherited resistance to some parasites and diseases. Genotype x environment interactions occurr for many fitness traits, suggesting that LA might be important. However, the scale and extent of adaptive variation remains poorly understood and probably varies, depending on habitat heterogeneity, environmental stability and the relative roles of selection and drift. As maladaptation often results from phenotype-environment mismatch, we argue that acting as if populations are not locally adapted carries a much greater risk of mismanagement than acting under the assumption for local adaptations when there are none. As such, an evolutionary approach to salmon conservation is required, aimed at maintaining the conditions necessary for natural selection to operate most efficiently and unhindered. This may require minimising alterations to native genotypes and habitats to which populations have likely become adapted, but also allowing for population size to reach or extend beyond carrying capacity to encourage competition and other sources of natural mortality.

1805: ***+.097***

Using a multi-dimensional ecological design, this study aimed first to analyse whether local environmental conditions can account for the spatial segregation of two Italian native decapods, the crayfish Austropotamobius italicus and the river crab Potamonfluviatile, in Central Italy freshwater ecosystems. Second, we aimed to analyse which environmental variables were more closely associated with the presence/absence of the two decapods in specific sites within their distribution area. Following a factorial design, a total of 32 sites were selected in two neighbouring geographic areas, one occupied by crayfish and one by crabs. Within each distribution area, eight streams where the decapod was present and eight where it was not present were selected. At each site, macro-invertebrate community composition and 16 abiotic variables were recorded and analysed with multi-variate methods. Variations in physical (minimum and maximum temperatures), chemical (calcium, oxygen, nitrate and nitrite) and geomorphological (substrate composition) parameters explained spatial segregation of P. fluviatile and A. italicus in the study area. The occurrence of crayfish reflected variations of chemistry (such as pH, calcium, nitrate and nitrite concentrations), temperature, water depth and substrate composition. On the contrary, the presence of the river crab, within its occurrence zone, was not associated to any biotic and abiotic parameters and was probably affected by anthropogenic pressure and uncontrolled harvesting. These findings provide fundamental ecological data for the maintenance

of the two decapod natural populations as well as for the selection of areas and streams adequate for their reintroduction. (C) 2007 Elsevier Ltd. All rights reserved.

1806: ***+.287***

Swift foxes (Vulpes velox) were once common prairie inhabitants throughout western North America and were integral components within ecosystems and some Native American tribal cultures. In response to extirpation from tribal lands, the Blackfeet Tribe and Defenders of Wildlife reintroduced 123 captive-raised swift foxes from 1998 to 2002 to the Blackfeet Indian Reservation, Montana, USA. We used two success criteria, a population growth rate >= 1.0 and an index count >= 100 foxes, to determine if the reintroduction was a short-term success. We radiocollared and monitored swift foxes from 2003 to 2005 to estimate survival and fecundity. The swift fox population grew at a rate of 16% during 2003/2004 and 14% in 2004/2005. In addition, field crews observed 93 foxes in the summer of 2005. The swift fox population reached one, and very nearly both, of our short-term success criteria. In light of swift fox sign in areas where we were unable to observe foxes despite being aware of their presence, we believe there were >= 100 foxes present in 2005. Based on our success criteria and the discovery of swift foxes 110 km from the release site, we consider this reintroduction a short-term success with promise for long-term success. The Blackfeet Tribe and Defenders of Wildlife have attained their goal of restoring a culturally important species to Tribal lands and have initiated a comeback of swift foxes beyond the Reservation border to the Rocky mountain Front in Montana. Collaborative projects between tribes and non-governmental groups can play a vital role in our effort to conserve biologically and culturally significant species. (C) 2006 Elsevier Ltd. All rights reserved.

1807: ***-.042***

Small or decreasing populations call for emergency actions like, for example, captive breeding programs. Such programs aim at rapidly increasing population sizes in order to reduce the loss of genetic variability and to avoid possible Allee effects. The Lesser Kestrel Falco naumanni is one of the species that is currently supported in several captive breeding programs at various locations. Here, we model the demographic and genetic consequences of potential management strategies that are based on offspring sex ratio manipulation. Increased population growth could be achieved by manipulating female conditions and/or male attractiveness in the captive breeders and consequently shifting the offspring sex ratio towards more female offspring, which are then used for reintroduction. Fragmenting populations into wild-breeding and captive-breeding demes and manipulating population sex ratio both immediately increase the inbreeding coefficient in the next generation (i.e. decrease N-e) but may, in the long term, reduce the loss of genetic variability if population growth is restricted by the number of females. We use the Lesser Kestrel and the wealth of information that is available on this species to predict the long-term consequences of various kinds of sex-ratio manipulation. We find that, in our example and possibly in many other cases, a sex-ratio manipulation that seems realistic could have a beneficial effect on the captive breeding program. However, the possible long-term costs and benefits of such measures need to be carefully optimized.

1808: ***+.045***

We investigated the potential for released captive-reared grey partridges Perdix perdix to restock regions from which the species has disappeared. Birds were released at two sites in Scotland (not concurrently) from 1997 to 2003 and monitored via spring and autumn counts, night-time surveys and radio-telemetry. Some wild female partridges were caught and radio-tagged each spring for

comparison with reared females. The survival rate of 520 captive-reared birds released in autumn until the following spring was poor (around 10% overall) and was not significantly different for radio-tagged and visibly-marked birds. Carrying a radiotag did not alter the body condition of the birds. The breeding-season survival rate of released hens averaged 30% and was not significantly different to that of 44% for wild hens. However, the power to detect significant differences was low due to the small number of survivors. The major cause of mortality throughout was predation (82 and 55% of losses at the two sites), with red foxes Vulpes vulpes and raptors being the most significant predators. Reared partridges at the study site with legal predator control had higher survival rates than those at the other site, but this was not true for wild hens as raptor predation compensated for declining mammalian predation rates (19% of deaths were due to raptors at the site without predator control, 56% at the site with predator control). We suggest that the vulnerability to predation of reared birds was most likely due to inappropriate antipredator behaviour or an increased risk of predation near to release pens. Of the partridges that survived long enough to breed, three times more wild hens reached incubation than reared hens. No reared hens raised chicks in their first breeding season whilst the only hen that survived long enough to breed in her second year raised 14 young. Captive-reared grey partridges could not be used to increase the species' range unless 1) the number surviving their first year increased, 2) the higher breeding rate of two-year-old females suggested here was ubiquitous, and unless 3) appropriate management was in place first. Methods for improving the success of releases are discussed.

1809: ***-.057***

Scimitar-horned oryx, now considered extinct in the wild, persists in large numbers in captivity. In this first molecular genetic study on this species, we explore the patterns of genetic diversity across European, North American, and a few other captive groups using microsatellite markers and mitochondrial control region sequencing. Strong population structure was not evident from microsatellite data but we discovered deep divergence within the mitochondrial DNA haplotypes from a network analysis where three disconnected networks were obtained, with estimated divergence times of c. 2.1-2.7 million years. Mismatch distribution analyses suggest population expansions c. 1.2 and 0.5 million years ago. We discuss our findings in the context of historical climatic changes in North Africa and use information obtained on current patterns of genetic diversity within captive groups to make recommendations for future captive management and reintroduction strategies.

1810: ***+.192***

Seven years into this new millennium, species and habitat loss continue at an accelerated rate. While there have been individual examples of conservation success, the trend towards catastrophic loss of biological diversity persists. If we are to be successful in saving even a handful of critically endangered species, it is clear that they will need to be intensively managed using a variety of in situ and ex situ approaches. The highest profile ex situ conservation strategy is captive breeding. Although its relative role in an overall conservation management plan varies, captive breeding may present the only viable option for propagating the future of a species once rendered extinct in the wild. The study of Iyengar et al. in this issue of Molecular Ecology on one such species, the scimitar-horned oryx (Oryx dammah), represents an important contribution to ex situ conservation, demonstrating how critical insights into demographic history and population genetic structure obtained using molecular approaches may significantly contribute to captive breeding and reintroduction strategies.

1811: ***+.149***

Translocations are used increasingly to conserve populations of rare freshwater mussels. Recovery of translocated mussels is essential to accurate assessment of translocation success. We designed an experiment to evaluate the use of passive integrated transponder (PIT) tags to mark and track individual freshwater mussels. We used eastern lampmussels (Lampsilis radiata radiata) as a surrogate for 2 rare mussel species. We assessed internal and external PIT-tag retention in the laboratory and field. Internal tag retention was high (75-100%), and tag rejection occurred primarily during the first 3 wk after tagging. A thin layer of nacre coated internal tags 3 to 4 mo after insertion, suggesting that long-term retention is likely. We released mussels with external PIT tags at 3 field study sites and recaptured them with a PIT pack (mobile interrogation unit) 8 to 10 mo and 21 to 23 mo after release. Numbers of recaptured mussels differed among study sites; however, we found more tagged mussels with the PIT-pack searches with visual confirmation (72-80%) than with visual searches alone (30-47%) at all sites. PIT tags offer improved recapture of translocated mussels and increased accuracy of posttranslocation monitoring.

1812: *+.036*

Bothriocephalus acheilognathi is an introduced tapeworm in North America often reported as a serious ecological threat to native fishes. In this paper, we report the first record of B. acheilognathi in the Big Bend region of the Rio Grande in Texas (known as the Rio Bravo del Norte in Mexico). Identification of B. acheilognathi was confirmed by morphologic and genetic techniques (sequences of ITS2 and V4-18S rRNA genes). Its prevalence was 27% and its intensity ranged from I to 5 individuals in a January 2006 collection of 115 red shiners Cyprinella lutrensis. In addition, it was found in the Tamaulipas shiner Notropis braytoni, a Rio Grande endemic and a new host record. The occurrence of B. acheilognathi might have negative ecological impacts on endemic fishes in the Rio Grande. Several of the fishes that could serve as definitive hosts are of conservation concern. Its occurrence also might affect the success of reintroducing the Rio Grande silvery minnow Hybognathus amarus, which is federally listed as endangered, in this portion of the Rio Grande.

1813: *-.044*

The relative importance of genetic and non-genetic factors in extinction liability has been extensively debated. Here, we examine the levels of genetic variability at 13 (seven informative) loci in wild and captive populations of two endangered species of Mexican Goodeid fish, Ameca splendens and Zoogoneticus tequila. Allelic diversity was higher in the wild populations, and F-IS lower. Values of theta (= 4Ne mu) were estimated using a coalescent approach. These implied that the effective population size of all captive populations of A. splendens were smaller than that of the wild population; qualitatively similar results were obtained using an analytical method based on within-population gene identity disequilibrium. However, the wild population of Z. tequila did not show a significantly greater estimate of theta. We used the Beaumont approach to infer population declines, and found that both species showed clear evidence of a decline in effective population size, although this was stronger and probably occurred over a longer period of time in Z. tequila than in A. splendens. The decline in Z. tequila probably occurred before captive populations were established. We discuss implications for the conservation of critically endangered populations.

1814: *+.345*

Muskellunge, Esox masquinongy, were an important component of the Green Bay ecosystem prior to mid 1900s, but were extirpated by over-fishing, pollution, habitat degradation, and the

introduction of exotic species. The Green Bay ecosystem improved after the passage of the Clean Water Act, and the Wisconsin Department of Natural Resources (WI DNR) started a muskellunge reintroduction program in 1989. Monitoring the results of reintroduction efforts is necessary to achieve the program goal of establishing a self-sustaining population. We used available data to provide a 2005 spawner abundance estimate for a Green Bay tributary, estimates of contributions to that spawning stock from fall fingerling and yearling stocking, a weight-length relationship, a growth analysis, and a description of size and age at maturity. Our results indicate that stocking efforts have been successful in producing an adult population, with yearlings contributing to the spawning stock at a higher proportion than fingerlings (14.69:1). Our weight-length and growth analyses suggest that Green Bay muskellunge are unlikely to reach record length, but that it is possible for females to achieve record weight. The rapid growth of Green Bay muskellunge results in their maturing at larger sizes than other stocks, but the relationship between age and maturity is not well understood. Reintroduction efforts in Green Bay have created stocked populations capable of supporting trophy fisheries, but evidence of successful natural reproduction has not been observed. Future research should focus on the reproductive requirements of muskellunge reintroduced into altered habitats.

1815: *+.230*

For conservation purposes islands are considered safe refuges for many species, particularly in regions where introduced predators form a major threat to the native fauna, but island populations are also known to possess low levels of genetic diversity. The New Zealand archipelago provides an ideal system to compare genetic diversity of large mainland populations where introduced predators are common, to that of smaller offshore islands, which serve as predator-free refuges. We assessed microsatellite variation in South Island robins (Petroica australis australis), and compared large mainland, small mainland, natural island and translocated island populations. Large mainland populations exhibited more polymorphic loci and higher number of alleles than small mainland and natural island populations. Genetic variation did not differ between natural and translocated island populations, even though one of the translocated populations was established with five individuals. Hatching failure was recorded in a subset of the populations and found to be significantly higher in translocated populations than in a large mainland population. Significant population differentiation was largely based on heterogeneity in allele frequencies (including fixation of alleles), as few unique alleles were observed. This study shows that large mainland populations retain higher levels of genetic diversity than natural and translocated island populations. It highlights the importance of protecting these mainland populations and using them as a source for new translocations. In the future, these populations may become extremely valuable for species conservation if existing island populations become adversely affected by low levels of genetic variation and do not persist.

1816: *+.109*

Reintroduction of fire and grazing, alone or in combination, has increasingly been recognized as central to the restoration of North American mixed-grass and tallgrass prairies. Although ecological studies of these systems are abundant, they have generally been observational, or if experimental, have focused on plant species diversity. Species diversity measures alone are not sufficient to inform management, which often has goals associated with life form groups and individual species. We examined the effects of prescribed fire, light cattle grazing, and a combination of fire and grazing on three vegetation components: species diversity, groups of species categorized by life form, and individual species. We evaluated how successful these three treatments were in achieving specific management goals for prairies in the Iowa Loess Hills

(U.S.A.). The grazing treatment promoted the greatest overall species richness, whereas grazing and burning and grazing treatments resulted in the lowest cover by woody species. Burning alone best achieved the management goals of increasing the cover and diversity of native species and reducing exotic forb and (predominantly exotic) cool-season grass cover. Species-specific responses to treatments appeared idiosyncratic (i.e., within each treatment there existed a set of species attaining their highest frequency) and nearly half of uncommon species were present in only one treatment. Because all management goals were not achieved by any one treatment, we conclude that management in this region may need refining. We suggest that a mosaic of burning and grazing (alone and in combination) may provide the greatest landscape-level species richness; however, this strategy would also likely promote the persistence of exotic species. Our results support the need to consider multiple measures, including species-specific responses, when planning and evaluating management.

1817: *+.160*

In previous centuries human activities had a profound effect on the distribution of Eurasian lynx (Lynx lynx) in Europe. The species has since been reintroduced to several areas but still much former range remains unoccupied. A relatively poor coloniser, it is likely that further reintroductions will be required to restore the species to potentially suitable areas. However, in the human-modified landscapes of Europe, where extensive wooded habitats are often fragmented, it is important to assess the potential lynx population size that could be supported in the available habitat. A previous study identified two networks of potential lynx habitat in Scotland. The present study further explores the feasibility of reintroducing lynx to Scotland by estimating the potential population size for the identified habitat by considering the availability of prey. An examination of lynx and wild ungulate densities from four areas in Europe, revealed a highly significant relationship between lynx density and the density of ungulate biomass. Based on the biomass represented by the densities of roe, red, sika and fallow deer occurring in two potential lynx habitat networks in Scotland, it was predicted that habitat in the Highlands could support 2.63 lynx 100 km(-2), while the Southern Uplands could support 0.83 lynx 100 km(-2). Applied to the amount of identified habitat, it is estimated that a Highlands habitat network could support around 400 lynx, and a Southern Uplands network, around 50. Scotland could support a large population of Eurasian lynx, which at current estimates, would be the fourth largest in Europe. (c) 2007 Elsevier Ltd. All rights reserved.

1818: *-.056*

The deleterious effects of inbreeding have been documented in species both in wild and in captivity. A large proportion of inbreeding depression is caused by deleterious recessive alleles. Inbreeding is expected to affect many aspects of fitness. Main objectives for genetic management of conservation breeding programs are the maintenance of genetic variability and the avoidance of inbreeding. The risk of depression (lower viability or fertility) as a result of mating between two distantly related individuals need to be considered. The spread of single deleterious allele may affect the overall aim of conservation breeding programs. Allozyme studies and DNA assays have provided valuable information for conservation programs. Quantitative genetic estimation gives better outlook for endangered species management than single locus estimates. Reintroduction programmes (present and past) of different endangered species have been listed.

1819: *+.093*

Karyotypes of chicken (Gallus gallus domesticus; 2n = 78) and mallard duck (Anas platyrhynchos;

2n = 80) share the typical organization of avian karyotypes including a few macrochromosome pairs, numerous indistinguishable microchromosomes, and Z and W sex chromosomes. Previous banding studies revealed great similarities between chickens and ducks, but it was not possible to use comparative banding for the microchromosomes. In order to establish precise chromosome correspondences between these two species, particularly for microchromosomes, we hybridized 57 BAC clones previously assigned to the chicken genome to duck metaphase spreads. Although most of the clones showed similar localizations, we found a few intrachromosomal rearrangements of the macrochromosomes and an additional microchromosome pair in ducks. BAC clones specific for chicken microchromosomes were localized to separate duck microchromosomes and clones mapping to the same chicken microchromosome hybridized to the same duck microchromosome, demonstrating a high conservation of synteny. These results demonstrate that the evolution of karyotypes in avian species is the result of fusion and/or fission processes and not translocations.

1820: *-.006*

Africa's large predator guild competes for a limited food resource base. To minimize the degree of competition, we hypothesized that the two largest members of this guild and its fiercest competitors, the lion and the spotted hyaena, would partition their activity patterns to avoid interacting. We used 96-h continuous follows of focal animal(s) to determine when the six radio-collared lions and eight radio-collared spotted hyaenas, reintroduced into Addo Elephant National Park in 2003/2004, were active using a binomial measure of activity which was defined as movements > 100 m during each hourly period. Contrary to our predictions, lions and hyaenas did not partition their activity times, probably because of their current low population densities. Both species exhibited a crepuscular activity pattern although hyaenas were far less active during daylight. A sub-adult lioness minimized competitive interactions by becoming diurnal. This is likely to be a common strategy for lions that have been expelled from their natal pride to become nomadic, as it allows them to minimize kleptoparasitic and agonistic interactions from competitively dominant conspecifics and competitors. The increase in testosterone that occurs in males upon reaching sexual maturity, darkens their pelage and causes them to be more directly impacted by the heat, and thereby affords females an opportunity to escape from males during hot temperatures. Similarly, the longer pelage of young hyaenas restricts their activity to the cooler night-time.

1821: *+.239*

The founder effect leads to the great genetic differentiation between the translocated populations and the source population, and the genetic diversity of translocated population is usually lower than the source population. The Hainan Eld's Deer (Cervus eldi hainanus) is a world threatened species and ranked in the first class of national key protected species. Since 1976, population restoration programs, including in situ and ex situ conservation, have successfully brought this deer back from the brink of extinction. We employed 10 microsatellite DNA loci to index genetic variation in the one source (Datian) and five introduced populations (Bangxi, Ganshiling, Fengmu, Jinniuling and Wenchang). The genetic variability was low (H-e approximate to 0.3) for each of the six populations. Five translocated populations harboured 1, 3 or 5 monomorphic loci respectively, while no monomorphic locus in the source population. Significant differentiation existed between Bangxi and Datian populations, and no significant differentiation was detected between Ganshiling and Datian populations. Our results suggest that founder effect have played a role in the genetic consequence, and the influence on different translocated populations is different. We thought the possible reasons that might lead to the different influence were founder size, founding history and population structure of founder. The study provided suggestions for

establishing new translocated populations in Hainan in future and provided scientific guide for ex situ conservation of other endangered species.

1822: ***+.232***

The first part 1993-2003 of the migratory fish program on the Rhone-Mediteffanean-Corsica basin demanded equipping and engineering, research and statutory measures to improve the circulation of the fish in the Rhone-MediterraneanCorsica basin. Studies permitted to improve the knowledge of taxonomy, biology and ecology of the twaite shad in the Rhone and showed the evidence of an endemic subspecies (Alosa fallax rhodanensis), which re-enforces the interest of a stock restoration program to conserve the biodiversity. Studies have also allowed to pinpoint the optimal shad migration period and different factors influencing the reproduction ascent of the adults, and were particularly useful in a qualitative analysis of the extension of the zone colonized by shads, which has grown these past years. This first part of the migratory fish program ended in 2003, and its aim, "The return of the shad in the Bas-Rhone below the river Ardeche and its right side tributaries (Gardon, Ceze, Ardeche)", was successful since shads often migrate and reproduce in the river Ardeche. The second part 2004-2008 of the migratory fish program on the Rhone-Mediterranean-Corsica basin deals both with a greater number of species and a larger geographical zone. For shad, the program wants to assess results obtained previously in the Rhone basin and to extend its migration to the left side tributaries of the Rhone and other French Mediterranean basins. For eel, the program wants to develop the stock for reproduction and fishing, to open growth areas by clearing obstacles downstream. For lampreys and migratory salmonids, it is recommended to get further information about their status, their geographical distribution and their populations. For the sturgeon, since its disappearance from the Rhone in the 70's, a feasibility study for its reintroduction will deal with both specific determination and river analysis. All studies will finally allow to get scientific, technical and financial information for the definition of a new geographical strategy 2009-2013.

1823: ***+.229***

Myricaria laxiflora is an endangered plant that grows in the flood zone along the Yangtze River in the Three Gorges area from 70 m to 155 m above sea level. To understand the spatial distribution patterns of the species and to provide information for developing conservation strategies, we used field surveys to study its seed reproduction and dispersion, and used growth chambers to study seed germination. Results showed that M. laxiflora produced many flowering branches, inflorescences and seeds. Seeds were very small and output was high although biomass allocation to reproduction was low (similar to 4%). Reproductive allocation was strongly correlated with the biomass of stems and leaves. Seeds were dispersed either by the wind or the river current. Wind-dispersed seeds usually settled within 25 m from parent plants leading to a clumped distribution of individuals in populations. Water-dispersed seeds often landed and established on strands of firth where the fine sediment and gentle sloping were available. Seedlings that emerged from water-dispersed seeds were distributed along the water flood line. The life-span of M. laxiflora seeds was about 7 days. Seeds could germinate within 24 h when they absorbed adequate amounts of water. Soil water content was a key factor limiting the establishment ability of M. laxiflora. Experiments showed that the minimum soil water content for germination to occur was 10% on sand or 17% on sandy soil substrates, and the optimal conditions were on saturated soils. The water content of sandy soils on the riverbank was lower than 10% in autumn, the dry season, and seeds were able to germinate only on sandy beaches that were intermittently inundated by the fluctuating river current. These characteristics of seed dispersal and germination limit the ability for M. laxiflora to expand its distribution. These results provide information essential for the conservation and

reintroduction of this endangered species.

1824: *+.040*

The Iberian lynx (Lynx pardinus) is the most endangered felid in the world. Only about 160 individuals remain in 2 separate metapopulations in Southern Spain (Sierra Morena and Do (n) over tilde a na). We obtained blood samples of 20 lynxes captured from 2004 to 2006, and determined the Prevalence of infection and genetic diversity of Cytauxzoon spp. using 18S rRNA PCR and sequence analysis. Prevalence of infection was 15% (3 of 20). Cytauxzoon sp. was only detected in Sierra Morena. For phylogenetic analysis, we used the sequences reported in the present study and those characterized in different domestic and wild felids and ticks from North and South America, Asia and Europe. Three different Cytauxzoon sp. sequences were obtained. They were closely related to that obtained from a Spanish cat, but diverged in LIP to 1 center dot 0% with respect to the only previously reported sequence from an Iberian lynx. Conversely, the latter sequence Clustered together with C. manul sequences obtained front Pallas cats (Otocolobus manul) in Mongolia. Our analysis yields a separate cluster of C. felts sequences from cats, wild felids and ticks in the United States and Brazil. These results suggest that at least 2 different Cytauxzoon spp. may be present in Iberian lynx. The apparent absence in one of the areas, together with the possibility of fatal cytauxzoonosis in lynxes makes necessary disease risks to be taken into account in management conservation strategies, such as translocations and re-introductions.

1826: *+.197*

Rifleman, or fitipounamu Acanthisitta chloris, is New Zealand's smallest endemic passerine. The species has a fragmented distribution and is threatened in the Rakiura region in the south of the South Island. The only known population of South Island rifleman A. c. chloris in the Rakiura region persisted on Codfish Island/Whenua Hou. To create a second population of rifleman in Rakiura, 30 caught from Codfish Island were reintroduced onto nearby Ulva Island in February 2003, the first translocation of rifleman. Survival and dispersal were monitored for 1 month post-release, and subsequently during the first and second breeding seasons. Mortality was greatest during holding and transfer, with low to moderate post-release mortality. All founding pairs bred in the first breeding season, and both founders and offspring bred in the second season. Dispersal across the island was greater for offspring. A simple deterministic matrix model indicated positive annual population growth (lambda = 1.33), and low risk of shortterm extinction. Holding/transfer techniques should be improved for future reintroductions, and longer-term monitoring should be undertaken for a more accurate assessment of vital rates. Based on the survival of founding birds, reproduction by the release generation and their offspring, and high probability of population persistence, the rifleman reintroduction was considered to be successful and a good model for future reintroductions of small passerine birds.

1827: *+.173*

Apex predators are often threatened with extinction, and reintroduction is one method conservation managers are using to secure their persistence. Yet the ability to predict what these predators will eat upon reintroduction is lacking. Here we test predictions of the diet of the lion (Panthera leo), derived from dietary electivity index and optimality theory, using independent data collected from reintroduced and resident populations. We solved the Jacobs' index preference equation for each prey species of the lion using values calculated by Hayward and Kerley (2005) and prey abundance data from 4 reintroduction sites and one resident lion population over several

years. We then compared these estimates with actual kill data gathered from each site and time period, using the log-likelihood ratio and linear regression. The model precisely predicted the observed number of kills in 9 of the 13 tests. There was a highly significant linear relationship between the number of lion kills predicted to occur at a site and the number observed for all but one site ((x) over bar r(2) = 0.612; beta = 1.03). Predicting predator diet will allow conservation managers to stop responding and start planning in advance for reintroductions and environmental variation. Furthermore, ensuring that sufficient food resources are available is likely to increase the success of reintroduction projects. In addition, managers responsible for threatened prey species will be able to predict the vulnerability of these species to predation in the event of predator reintroductions or changes in abundance. These methods are applicable to virtually all large predators that have been sufficiently studied.

1828: *-.009*

A 31-km-long reach of the Wolf River, from near the town of Pall Mall, Fentress County, to the backwaters of Dale Hollow Lake, Pickett County, Tennessee was surveyed by snorkeling and wading using visual and tactile methods. A total of 45 sites were sampled and live mussels were observed at 24. We collected nine species alive (seven are considered to be species of concern) and shells of three additional species. Although mussel densities were low ((X) over bar = 5.4 individuals/person-h), recruitment of most species was occurring. The Wolf River contains the last known populations of the fluted kidneyshell (Ptychobranchus subtentum) and the purple lilliput (Toxolasma lividus) and some of the last known populations of the pheasantshell (Actinonaias pectorosa), the Cumberland moccasinshell (Medionidus conradicus), and the Tennessee clubshell (Pleurobema oviforme) in the Cumberland River drainage within Tennessee. Populations of these five species in the Wolf River could provide a parental source for propagation and subsequent reintroductions into other Cumberland River tributaries.

1829: *+.220*

Understanding what determines a species' range is a central objective in ecology and evolutionary biology. It has important applications for predicting species distributions and how they might respond to environmental perturbations. This paper describes a mechanistic approach to predict past and present distribution of the Japanese serow (Capricornis crispus) on Honshu, Japan. We applied state-of-the-art microclimate and animal biophysical/ behavioral models coupled with climate and vegetation data to estimate the distribution of potential range expansion under protection. We tested the model results against detailed empirical distribution data from the Ministry of the Environment for a five-prefecture area in central Honshu. We also applied the models to time-series land use/ cover maps to investigate the historical transitions in habitat suitability during 1947-1999 in the Arai-Keinan region. This is the first time to our knowledge that mechanistic models have successfully predicted the landscape scale distribution of a mammal species in the absence of other animal species interactions, such as predators. In this case, animal energetics/ behavior-plant interactions seem to be critical. Forest cover appears to be important in summer and winter for suitable serow habitats. The energetics model results indicate that the serow can overheat in some open environments in midday hours in summer. In winter, simulation results suggested that forest cover provides effective refuge to avoid increased metabolic demands of cold temperatures and strong winds. The model simulations suggested that land use/ cover changes documented during 1947-1999 resulted in increased suitable serow habitat due to expanding forest cover from agricultural marginalization and ecological succession. The models provide a unique tool for estimating species' range expansion under protection or for selecting suitable reintroduction sites.

1830: ***+.189***

The Black-flanked Rock-Wallaby (Petrogale lateralis lateralis) was once widespread throughout Western Australia but due to a combination of factors its range has declined significantly and its present distribution is limited to a few widely scattered isolated Populations. It is gazetted as vulnerable tinder Western Australian legislation and requires active management to ensure its survival. Translocating species to areas of Suitable habitat, when Coupled with predator control, is an effective method of expanding species distribution as well as increasing Population numbers. This Study investigated the requirernents for both effective translocation and site assessment in relation to the development of tourism based on black-flanked rock-wallabies. A species-specific habitat ranking system was devised to identify Suitable areas for translocated Populations. This was followed by an assessment of the tourism potential of the identified sites. When both sets of results were added together potential sites were identified that Could satisfy both habitat and tourism rcquirements. Avon Valley National Park and Billyacatting Nature Reserve were found to be the most suitable sites for translocating rock-wallabies on the basis of suitable habitat and the potential for Subsequent development of wildlife tourism. Viable breeding Population size, feral predator control, competing introduced herbivore control and fire management are all identified as aspects that require management action. Tourism management requires stakeholder liaison, possible zoning and separation strategies and appropriate waste disposal at tourism sites. Public contact with translocated animals requires an educative approach that avoids any feeding of wildlife. Relatively close contact between visitors and wildlife may be achievcd through a process of habituation. Such strategies should however be subject to review.

1831: ***-.035***

The last remaining population of Megalagrion xanthomelas (Selys-Longchamps) resides in a 100 meter reach of stream located on the grounds of Tripler Army Medical Center, Oahu. Because actions may be taken that might jeopardize this only known Oahu population, it has been considered imperative to establish a second population to prevent M. xanthomelas from going extinct on Oahu. An attempt to establish this species at a stream in the Dillingham area of Oahu was made in 1998, but unfortunately was unsuccessful. Because the Tripler population is so small and restricted in distribution, a second effort at translocation was attempted at a new location. We estimated the population size of M. xanthomelas at the Tripler site in 1997 and again in 2003 by mark-recapture and concluded that the Tripler population was stable and could withstand the removal and translocation of a small number of adults and larvae. A stream site located in Makiki Valley was selected for its lack of alien predators such as crayfish, prawns, and mosquito fish, and a number of adults and immatures were translocated to the Makiki site in August 2004. Monitoring of the Tripler and the Makiki sites is ongoing and an additional translocation of M. xanthomelas to Makiki is planned. Future conservation plans should also include the assistance of from the general public through avenues such as stocking of backyard ponds with M. xanthomelas.

1832: ***+.144***

Changes in forest vegetation and the population size of forest plants were studied in two deciduous woodlands after the reintroduction of the 'coppice with standards' management system. This type of forest management was practised in southern Limburg for centuries but ceased in the mid-twentieth century. This resulted in increased shading of the understory of the forests, as a result of which many populations of herbs and shrubs declined and several species became nearly or completely extinct. After the reintroduction of this management system in two highly diverse nature reserves in the mid-1970s, most of the old coppice stools died and a dense vegetation

dominated by Clematis vitalba and Rubus species developed. However, continuation of the management led to much better results during recent years. Our observations show that new stools have developed since 1997,and many critical plant species have recovered from their initial decline, while the dominance of other, highly competitive plant species was reduced. Apparently, coppicing is a prerequisite for the restoration and conservation of the high species diversity in these woodlands.

1833: ***+.338***

Understanding the link between habitat use and components of fitness can yield useful insight into the environmental conditions necessary for population maintenance and can help promote effective habitat management. This information is especially important for species that are in decline or otherwise of conservation concern. Populations of brown-headed nuthatches (Sitta pusilla), an obligate cavity nester, have declined throughout their range, primarily due to extensive habitat loss and degradation. To help guide habitat management for this species, we identified habitat features associated with variation in the number of offspring fledged within 2 populations in southern Florida, USA. The most important predictor of productivity was the date on which a nest attempt began, with earlier nests producing more fledglings. The number of large pine (Pinus elliottii var. densa) snags and, to a lesser extent, the number of small pine trees surrounding a nest site were positively associated with productivity. We recommend that land managers in southern Florida focus on providing abundant large pine snags because doing so will increase productivity and also may increase nest-site availability and the percentage of individuals that breed each year. Prescribed burning may be an effective way to increase the abundance of large pine snags; however, land managers should exercise caution when doing so because of the trade-off between snag recruitment and snag consumption that accompanies the use of fire. We lack the data required to predict the fire-return interval that optimizes this trade-off, but until these data are available we recommend increasing the spatial heterogeneity in fire-return interval and lengthening the fire-return interval in some areas to 5-6 years.

1834: ***-.011***

Although interspecific killing among carnivores can drive populations toward extinction, it is generally unknown how these intraguild interactions vary among populations, and whether the threat for vulnerable species can be mitigated. We studied imperiled populations of swift foxes (Vulpes velox) in Canada and kit foxes (Vulpes macrotis) in Mexico to determine potential differences in survival or predator-avoidance strategies. Survival rates were significantly lower in Canada than in Mexico because of mortality caused by coyotes (Canis latrans) and golden eagles (Aquila chrysaetos), and the potential for population recovery is likely higher for the Mexican fox population. Differences in body size between coyotes and foxes, diet, group sizes, intraspecific home-range overlap, home-range sizes of coyotes, and movements of coyotes relative to foxes were similar among study areas. However, Canadian foxes had home ranges that were approximately 3 times larger than those in Mexico, and Canadian foxes were most frequently killed on their home-range peripheries. Home ranges of kit foxes decreased in size as the availability of black-tailed prairie dog (Cynomys ludovicianus) colonies increased and associated refuge holes, which foxes could use to escape predation, were significantly more abundant in Mexico than in Canada. Small home ranges of foxes probably reduced encounters with coyotes in Mexico, and a high availability of refuges likely allowed foxes to elude predators when such encounters did occur. Differences in survival of foxes relative to mortality caused by coyotes demonstrate that interactions between carnivores can vary greatly between populations and that, in some situations, vulnerable species may be able to coexist with dominant carnivores despite a lack

of large-scale habitat partitioning.

1835: *+.050*

The radiated tortoise (Geochelone radiata) is an endangered species endemic to Madagascar. It inhabits the semiarid spiny forest of the southern part of the island, an ecosystem heavily affected by habitat destruction. Furthermore, illegal harvesting greatly threatens this species. The main objective of our study was to acquire better knowledge of its genetic structure, in order to take appropriate management decisions concerning, for instance, the reintroduction of confiscated individuals. Our hypothesis was that rivers represent effective barriers to tortoise dispersal despite the fact that they are dry most of the year. We used 13 polymorphic microsatellite markers to compare samples from six populations across the range of the species. All analyses (Fisher's exact tests, F (ST) values, AMOVA) indicated that the radiated tortoise exhibits moderate levels of genetic structure throughout its range. In addition, we used a multiple regression approach that revealed the importance of rivers to explain the observed structure. This analysis supported the role of the Menarandra and Manambovo Rivers as major barriers to the dispersal of most radiated tortoises, but Markov chain Monte Carlo simulations revealed that low levels of recurrent gene flow may explain why F (ST) values were not higher. We identified three distinct conservation units with relatively high assignments rates (87%), which should be valuable for the management of the species. This is the first study to report the genetic structure of a species sampled throughout the Malagasy spiny forest.

1836: *+.017*

The continuing global decline of large carnivores has catalyzed great interest in reintroduction to restore populations and to reestablish ecologically functional relationships. I used variation in the distribution of four Holarctic prey species and their behavior as proxies to investigate the pace and intensity by which responses are lost or reinvigorated by carnivore repatriation. By simulating the presence of wolves (Canis lupus), tigers (Panthera tigris), and brown bears (Ursus arctos) at 19 transcontinental sites, I assayed three metrics of prey performance in areas with no large terrestrial carnivores (the polar islands of Greenland and Svalbard), extant native carnivores (Eastern Siberian Shield, boreal Canada, and Alaska); and repatriated carnivores (the Yellowstone region and Rocky Mountains). The loss and reestablishment of large carnivores changed the ecological effectiveness of systems by (1) dampening immediate group benefits, diminishing awareness, and diminishing flight reaction in caribou (Rangifer tarandus) where predation was eliminated and (2) reinstituting sensitivity to carnivores by elk (Cervus elaphus) and moose (Alces alces) in the Yellowstone region to levels observed in Asian elk when sympatric with Siberian tigers and wolves or in Alaskan moose sympatric with wolves. Behavioral compensation to reintroduced carnivores occurred within a single generation, but only the vigilance reaction of bison (Bison bison) in Yellowstone exceeded that of their wolf-exposed conspecifics from boreal Canada. Beyond these overt responses by prey, snow depth and distance to suitably vegetated habitat was related to heightened vigilance in moose and elk, respectively, but only at sites with carnivores. These findings are insufficient to determine whether similar patterns might apply to other species or in areas with alien predators, and they suggest that the presumed excessive vulnerability of naive prey to repatriated carnivores may be ill-founded. Although behavior offers a proxy to evaluate ecological effectiveness, a continuing challenge will be to understand bow naive prey respond to novel or introduced predators.

1837: *+.160*

The importance of strongly interactive predators has been demonstrated in many ecosystems, and the maintenance or restoration of species interactions is a major priority in the global conservation of biodiversity. By limiting populations of prey and/or competitors, apex predators can increase the diversity of systems, often exerting influences that cascade through several trophic levels. In Australia, emerging evidence points increasingly towards the dingo (Canis lupus dingo) as a strongly interactive species that has profound effects on ecosystem function. Through predatory and competitive effects, dingoes can alter the abundance and function of mesopredators including the introduced red fox (Vulpes vulpes) and feral cat (Felis catus), and herbivores including the European rabbit (Oryctolagus cuniculus). These effects often benefit populations of native prey, and diversity and biomass of vegetation, but may not occur under all circumstances. For example, the social structure of dingoes is of great importance; a pack subject to minimal human interference regulates its own numbers, and such packs appear to have fewer undesirable impacts such as predation on livestock. Despite abundant observational evidence that the dingo is a strong interactor, there have been few attempts to test its ecological role experimentally. Given the well-recognized importance of species interactions to ecosystem function, it is imperative that such experiments be carried out. To do this, we propose three broad questions: (i) do dingoes limit the abundance of other predators or prey? (ii) do dingoes affect the ecological relationships of other predators or prey (e.g. by altering their spatial or temporal activity patterns)? and (iii) does the removal or reintroduction of dingoes entrain ecological cascades? Finally, we discuss the design of appropriate experiments, using principles that may also be applied to investigate species interactions on other continents. Research might seek to clarify not only the impacts of dingoes at all trophic levels, but also the mechanisms by which these impacts occur.

1838: ***+.204***

Although reintroduction programmes are often implemented for recovering animal populations, projects seldom monitor the long-term survival of released animals. In addition, although many releases may occur in the same area, little is known about how the survival of successive release batches is affected by the presence of conspecifics and density dependence. Here, we use multi-state capture-recapture modelling (combining information from recaptures and recoveries) to analyse the survival of two batches of Hermann's tortoise Testudo hermanni hermanni released in a 10-year interval and monitored for 18 years at the Ebro Delta (western Mediterranean). We also tested whether the released animals experienced lower survival (i.e. a release cost) before becoming familiar with the new environment. Although we used a hard-release method, neither group experienced a short-term release cost. Annual survival of both groups differed and was not negatively affected by density-dependent factors. Annual survivorship of the first group of released tortoises was constant and very high (0.945, SE=0.011), and similar to that estimated from several natural populations. The presence of a terrestrial predator in 1 year (before the release of the second group) significantly decreased the survival of tortoises (0.819, SE=0.073). Strikingly, survival of the second batch was significantly lower than that of the first group after the first years of release (0.775, SE=0.049). Although survivorship for the first group suggested that habitat quality was high, the second group seemed not to acclimate well to the new environment, possibly due to the presence of resident tortoises. From a management perspective, reintroduction programmes of the Hermann's tortoise are a successful strategy for its recovery. Nevertheless, it seems advisable to avoid: (1) the release of tortoises at the core of well-established populations and (2) areas with a high density of predators, which can jeopardize the reintroduction success, especially when the number of released individuals is small.

1839: ***+.213***

Matrix models and population viability analysis (PVA) have become useful tools to understand population attributes and dynamics. Demography analysis gives valuable information for the management of threatened species, and can be used to create action plans for their conservation. PVA is particularly useful in those species with small population sizes difficult to sample. By calculating the individual fate of each member of the population, PVA simulates temporal population changes and estimates extinction risk over a time period. Here we use these models to analyse the population of axolotl Ambystoma mexicanum, which has decreased more than six times within only 5 years. Its natural environment (the Xochimilco aquatic system within Mexico City) has deteriorated significantly in the last decades. The matrix analysis showed large oscillations in the axolotl population growth rate (damping behaviour), which could explain the fast density reduction in only few generations. Younger ages (eggs and larvae) showed the highest sensitivity and elasticity values, suggesting that the lack of food sources such as zooplankton or the increased predation by exotic carp and tilapia are capable to reduce axolotl density. PVA shows low extinction probabilities using laboratory data for younger ages. However, a small reduction in egg or larvae survival rate is capable to increase extinction probabilities to 100% in 20 years. Based on these results, we found that the best strategy to restore the axolotl population is to increase the survival rate of eggs and larvae by restoring the habitat, eradicating introduced fish and improving water quality, rather than implementing a reintroduction programme.

1843: *+.026*

Translocation is a key strategy for conserving native subspecies of cutthroat trout Oncorhynchus clarkii that have declined markedly throughout their native range. Previous research showed that successful translocations of cutthroat trout in high-elevation southern Rocky Mountain streams were more likely in streams with warm summer water temperature and led to the hypothesis that cold summer temperatures govern translocation success by limiting recruitment. We tested this by measuring the density and size of age-0 cutthroat trout (greenback cutthroat trout O. c. stomias and Colorado River cutthroat trout O. c. pleuriticus) in six headwater streams in north-central Colorado that varied in thermal characteristics. Surveys were conducted at peak emergence during 3 years in two widely spaced study reaches in each stream. Fry density increased with Celsius degree-days accumulated during the growing season but did not vary significantly among years. We used laboratory data on the growth and survivorship of age-0 cutthroat trout in three temperature regimes that were similar to those studied in the field to determine expected survivorship and size of fry at the start of winter, which occurred several weeks after peak emergence in our warmer reaches and coincided with emergence in colder reaches. Our results support the hypothesis that recruitment of native cutthroat trout in Colorado is limited by cold water temperatures that reduce growth and recruitment. High-elevation streams, like those studied, that accumulate 900-1,200 Celsius degree-days during the growing season afford the best opportunity for cutthroat trout recruitment and translocation success. Streams that provide 800-900 degree-days probably sustain recruitment in some years, and those with less than 800 degree-days are generally unsuitable for translocations because of a greater risk of recruitment failure and the smaller sizes attained by fry by the onset of winter.

1844: *+.089*

In restoring species, reasons for introducing limited numbers of individuals at different locations include costs of introduction and maintenance, limited founder supply, and risk "bet hedging." However, populations initiated from few founders may experience increased genetic drift, inbreeding, and diversity loss. We examined the genetic diversity of an isolated stand of more than 5,000 American chestnut trees relative to that of the 9 surviving stand founders (out of 10 total)

planted in the 1880s. We used minisatellite DNA probes to reveal 84 genetic markers (circa 24 loci) among the nine founders, and their genetic diversity was compared with three separate plots of descendant trees, as well as with two natural stands. The descendants were circa 7.3% more heterozygous than the founders (mean estimated H = 0.556 vs. 0.518, respectively; $p < 0.0001$). Genetic differentiation was not pronounced ($FST < 0.031$), and no markers, including those at low frequency among the founders, were lost in the descendants. The founders and natural transects were not significantly different in H or similarity (mean proportion of bands shared). Special planting or mating protocols for establishment of a vigorous American chestnut population from a low number of founders may not be required to avoid strong effects of genetic drift and inbreeding. These results demonstrate that loss of genetic diversity following reintroduction of a limited number of founders is not always inevitable, such as this case where the species is highly outcrossing, expression of heterozygous advantage may occur, the original founders remain as gene contributors over generations, and the establishing population expands constantly and rapidly.

1845: ***-.055***

Homing behaviour in the New Zealand long-tailed bat (Chalinolobus tuberculatus), a temperate insectivorous species, was investigated at Grand Canyon Cave, central North Island. A pilot study of nine adult male bats was conducted to determine whether use of the cave was regular enough for a homing study. Eight bats returned to the cave over the 3-week monitoring period, six on the night following release. Nine additional bats carrying radio transmitters were then released at three sites (three at each site) c. 5, 10 and 20 km due east of the cave; sites estimated to be located within, on the border of, and outside the population's known familiar area respectively. All but one of these nine bats were subsequently detected at the cave. Results suggest that adult long-tailed bats are able to return home following displacement both inside and outside their familiar area. Implications of these findings for translocations of bats and the possession of a potential long-distance navigation system by this species are discussed.

1846: ***+.102***

Lesser short-tailed bats (Mystacina tuberculata) have recently been translocated to Kapiti Island in an attempt to form a new population of this threatened species. However, the island's vegetation is regenerating, and there was doubt that the forests provided enough large trees with cavities for bats to roost in. This study measured the availability of tree-trunk cavities of the right size for potential roost sites on Kapiti Island, and assessed if habitat restoration would be required to increase the translocation's chance of success. First, trees with cavities accessible to us were sampled in six of Kapiti Island's forest types. Size variables known to affect roost site selection by lesser short-tailed bats at the tree and cavity level were measured. Trees were classified as containing cavities that could potentially provide suitable roosts if their values for all variables measured fell within the range of roosts used by lesser short-tailed bats in natural populations. Roosts were classified as suitably sized for solitary bats or for colonies, using measurements from both types of roosts in natural populations. Second, the density of these potential roost cavities was calculated. Cavities of a size potentially suitable for colonies were found in four of the six forest types at densities ranging from 3.2 +/- 3.2 SE to 52.4 +/- 14.0 trees per ha. Density of potential solitary roosts was much higher. Not all potential cavities will be suitable because they may be damp, poorly insulated, or have an unsuitable microclimate. Nevertheless, our estimates indicated that the two most extensive forest types each contained thousands of potential cavities of a size suitable for colonies of lesser short-tailed bats. In addition, there were tens of thousands of cavities large enough to shelter solitary bats. Roost habitat restoration appears unnecessary to

assist translocated Mystacina tuberculata on Kapiti Island.

1847: ***+.031***

Invasion of globally threatened ecosystems dominated by arbuscular mycorrhizal plants, such as the alkaline prairies and serpentine barrens of eastern North America, by species of ectomycorrhizal (ECM) pine (Pinus) seriously threatens the persistence, conservation, and ongoing restoration of these rare plant communities. Using Maryland serpentine barrens and an Ohio alkaline prairie complex as model systems, we tested the hypothesis that the invasiveness of Virginia pine (Pinus virginiana L.) into such communities is regulated by the spatial pattern of ECM fungal inoculum in the soil. ECM colonization of pine seedlings can occur by (1) hyphae growing from the roots of mature ECM pines colonizing nearby seedlings (contagion model), (2) pine seedlings being infected after germinating in open areas where spores are concentrated in feces of animals that have consumed sporocarps (centers of infection model), and (3) colonization from spores that are wind-dispersed across the landscape (background model). To test these models of dispersal of ECM fungal inoculum into these barrens, we used autocorrelation and spatially explicit mapping techniques (semivariance analysis and kriging) to characterize the distribution and abundance of ECM inoculum in soil. Our results strongly suggest that ECM fungi most often disperse into open barrens by contagion, thereby facilitating rapid pine colonization in an advancing front from mature pine forests bordering the barrens. Spatial patterns consistent with the centers of infection model were present but less common. Thus, current management techniques that rely on cutting and fire to reverse pine invasion may be ineffective because they do not kill or disrupt hyphal mats attached to mature roots of neighboring pines. Management alternatives are discussed.

1848: ***-.184***

Habitat destruction and illegal wildlife trade are the main reasons for psittacids' population decay. The Scarlet macaw (Ara macao cyanoptera) is listed as an endangered species in Mesoamerica by the IUCN. Only scattered populations of this species remain from its original historical distribution. Its population size has been estimated to 4000 individuals dispersed in small isolated areas in Mexico, Belize, Guatemala, Honduras, Nicaragua, and Costa Rica. Environmental authorities in Latin America confiscate dozens of these birds every year. Nonetheless, because their origin is unknown, most of them die in confinement areas. In the best scenario, surviving animals are donated to zoos. This project's goal is to identify wild population markers by means of DNA sequences that will allow us to recognize the geographic source of confiscated macaws. By pinpointing the origin of these birds, reintroduction measures can be undertaken. The data gathered from wild populations will let us infer their genetic structure and gene flow patterns. Finally, genetic surveys of captive individuals will allow us to implement a breeding program that might reduce the inbreeding risk or incompatible mix-up of genetic lineages.

1849: ***+.265***

The Mediterranean tortoise (Eurotestudo hermanni) (Gmelin, 1789) has been present in the Garraf Natural Park since 1992 as part of a reintroduction project of the species coordinated by the Catalonian Department of Environmental Affairs (Department de Medi Ambient i Habitatge de la Generalitat de Catalunya). Up to August 2006, a total amount of 1 120 specimens, out of the 1997 pre-selected, have been placed in the accommodation enclosure and afterwards released. The sex ratio is unbalanced, males outnumbering females 1,41 male : 1 female (figures from April 2006). Factors which negatively influence the introduced colonies are revealed and measures for effective

habitat management to favour the implantation of the species, proposed.

1850: *-.092*

Infectious diseases have been responsible for large-scale declines in many endangered animals. Disease outbreaks in small populations have probably led to the eventual extinction of such endangered animals in the wild. The endangered Asiatic lion (Panthera lea persica) population may also face such threats. This was evident from this study on captive Asiatic lions from western India, which were sampled from December 1998 to March 1999. Fifty-six Asiatic lions, including 17 hybrid lions (Afro-Asian crosses) from six captive centers in western India, were tested for antibodies against canine distemper virus (CDV), feline parvovirus (FPV), feline immunodeficiency virus (FIV), and feline leukemia viral (FeLV) antigen. Agar gel immunodiffusion test and dot immunobinding assay were employed for CDV and FPV antibody detection. Commercial enzyme-linked immunosorbent assay kits were used for FIV antibody and FeLV antigen detection. Forty-nine of 56 lions (87.5%) were positive for CDV. All 56 (100%) lions were positive for FPV antibodies. There were no detectable levels of FIN antibodies and FeLV antigens. It was observed that CDV and FPV, two viruses known to cause high mortality in captive carnivores, were widely prevalent in these captive Asiatic lions. It is suggested that these seropositive animals will have the potential to pose a risk of infection to other seronegative animals. Hence, it is imperative to carefully consider any movement, translocation, or reintroduction of these animals to new regions. It is also recommended that these animals be required to undergo standard quarantine and disease screening protocols. The lack of exposure to pathogens, such as FIV and FeLV, would also be a risk, and, hence, identification of reservoirs and screening of in-contact animals is highly recommended. Vaccinations must be considered, using killed or other suitable viral vaccines, which have been proved to be safe, effective, and efficacious in endangered felids.

1851: *-.074*

Delineating a species' geographic range using the spatial distribution of museum specimens or even contemporary detection-non-detection data can be difficult. This is particularly true at the periphery of a species range where species' distributions are often disjunct. Wolverines (Gulo gulo) are wide-ranging mammals with discontinuous and potentially isolated populations at the periphery of their range. One potentially disjunct population occurred in the Sierra Nevada Mountains, California, USA, and appears to have been extirpated by the 1930s. Many early 20th century naturalists believed that this population was connected to other populations occurring in the Cascade Range of northern California, Oregon, and Washington, USA, but a recent analysis of historical records suggests that California wolverines were isolated from other populations in North America. We used DNA extracted from museum specimens to examine whether California wolverines were isolated. Both nuclear and mitochondrial DNA data indicate that California wolverines were genetically distinct from extant populations, suggesting long-term isolation. We identified 2 new control region (mitochondrial DNA) haplotypes located only within California. We used these data and referenced sequences from the Rocky Mountains, USA, to make inferences regarding potential wolverine translocations into California. In addition, we used these genetic data to make inferences about wolverine conservation throughout western North America.

1852: *-.123*

The scientific evidence that California condors (Gymnogyps californianus) are frequently sickened and killed by lead poisoning from spent ammunition supports the conclusion that current levels of

lead exposure are too high to allow reintroduced condors to develop self-sustaining populations in the wild in Arizona and, by inference, in California. The evidence for lead poisoning and its source comes from the following sorts of data: 1) 18 clinical necropsies revealing high levels of lead in body tissues and (or) presence of lead shotgun pellets and bullet fragments in digestive tracts; 2) moribund condors showing crop paralysis and impending starvation with toxic levels of lead in their blood; 3) widespread lead exposure among free-flying condors, many with clinically exposed or acute levels; 4) temporal and spatial correlations between big game hunting seasons and elevated lead levels in condors; and 5) lead isotope ratios from exposed condors showing close similarity to isotope ratios of ammunition lead but isotope ratios in less exposed condors being similar to environmental background sources, which are different from ammunition lead. Simple population models reveal harmful demographic impacts of unnatural mortality from lead on population trajectories of reintroduced condors. Recent innovations in the manufacture of nonlead shotgun pellets and bullets with superior ballistics now provide for a simple solution to the problem of lead ingestion by condors, many other species of wildlife, and human beings: substitute nontoxic forms of ammunition for traditional lead-based ammunition. The substitution of nontoxic ammunition would be highly efficacious for hunting, economically feasible, and the right thing to do.

1853: *-.113*

The grey partridge became extinct in the province of Siena (central Italy) in the late seventies, whereas the red-legged partridge had already disappeared by the beginning of the twentieth century. Some reintroduction attempts of both species carried out in the 1980s gave encouraging but not definitive results, and failed after an initial success. This was probably due to the low number of birds released, the small size of the re-introduction areas, their isolation, the farm-bred origin of the partridges, and hunting. In the province of Siena, for the first time in Italy, a large-scale reintroduction program of grey and red-legged partridges was experimented. The project started up in 1995 with seven reintroduction areas for grey and four for red-legged partridge, and was extended to 19 areas (22,562 ha) for grey and 7 (6858 ha) for red-legged partridge in 2002. Population viability analyses for both species showed that if reintroduced populations were isolated they would be extinct in a few years. Therefore, a metapopulation approach was adopted (contemporary releases in reintroduction areas close to each other). In each area, 100-1000 partridges per year were released for a minimum of 3 years, from different farms in order to achieve the maximum initial genetic diversity. Releases were effected in late summer (August-September) in acclimatization pens containing 10-20 aviaries. The reintroduced population showed marked variability of some demographic parameters, such as pair density and brood production rate; instead, average brood size was relatively constant across the study areas, but with annual variations. Reintroduction success was limited to a few areas only, mainly depending on the habitat characteristics of the areas, their surface area and isolation, and on the degree of care for the birds during the acclimatization period.

1854: *+.257*

Reintroduction of wolves (Cams lupus) to Yellowstone National Park in 19951996 has been argued to promote a trophic cascade by altering elk (Cervus elaphus) density, habitat-selection patterns, and behavior that, in turn, could lead to changes within the plant communities used by elk. We sampled two species of willow (Salix boothii and S. geveriana) on the northern winter range to determine whether (1) there was quantitative evidence of increased willow growth following wolf reintroduction, (2) browsing by elk affected willow growth, and (3) any increase in growth observed was greater than that expected by climatic and hydrological factors alone,

thereby indicating a trophic cascade caused by wolves. Using stem sectioning techniques to quantify historical growth patterns we found an approximately twofold increase in stem growth-ring area following wolf reintroduction for both species of willow. This increase could not be explained by climate and hydrological factors alone; the presence of wolves on the landscape was a significant predictor of stem growth above and beyond these abiotic factors. Growth-ring area was positively correlated with the previous year's ring area and negatively correlated with the percentage of twigs browsed from the stem during the winter preceding growth, indicating that elk browse impeded stem growth. Our results are consistent with the hypothesis of a behaviorally mediated trophic cascade on Yellowstone's northern winter range following wolf reintroduction. We suggest that the community-altering effects of wolf restoration are an endorsement of ecological-process management in Yellowstone National Park.

1855: *-.289*

An emerging infectious facial cancer threatens Tasmanian devils with extinction. The disease is likely to occur across the range of the devil within 5 years. This urgent time frame requires management options that can be implemented immediately: the establishment of insurance populations, in captivity, wild-living on islands, and aiming for eradication in areas that can be isolated. The long-term options of the spontaneous or assisted evolution of resistance or development of a field-deliverable vaccine are unlikely to be available in time. The disease's characteristic allograft transmission through intimate contact simplifies isolation of insurance populations and breaking transmission in suppression trials. Better knowledge of contact matrices in wild devils will help focus timing and demographic targets of removals. A metapopulation approach is needed that integrates captive and wild-living island and peninsula (disease suppression) populations to minimize the loss of genetic diversity over 50 years until either extinction and reintroduction can occur, resistance evolves or a field-deliverable vaccine is developed. Given the importance of the insurance populations and the low genetic diversity of devils, a conservative target for retention of 95% genetic diversity is recommended. Encouraging preliminary results of the first disease-suppression trial on a large peninsula show fewer late stage tumors and no apparent population decline. Limiting geographic spread or suppressing the disease on a broadscale are both unlikely to be feasible. Since the synergy of devil decline and impending fox establishment could have devastating consequences for Tasmanian wildlife, it is crucial to manage the dynamics of new and old predator species together.

1856: *+.110*

Substantial range declines of the greater bilby (Macrotis lagotis) and the burrowing bettong (Bettongia lesueur) are thought to have had dramatic effects on ecosystem processes in the Australian arid zone because of their impacts on surface soils. The reintroduction of bilbies and bettongs into a reserve in central Australia provided an opportunity to compare their ecosystem impacts with those of two prevalent fossorial animals; the exotic European rabbit (Oryctolagus cuniculus) and the native sand goanna (Varanus gouldii). Bilbies and bettongs dug deeper and wider pits, excavating significantly more soil than rabbits or goannas. Pit coverage was four-times greater, and significantly more soil was excavated in the reserve where bilbies, bettongs and goannas were enclosed together compared with a site outside the reserve where rabbits and goannas co-occurred, or within the reserve where goannas occurred alone. Goannas dug fewer holes outside the reserve than in either of the reserve paddocks. Litter and viable seed were restricted almost exclusively to the pits, and soil from pits had higher levels of labile carbon than non-pit surface soils. Compared with surface soils, bilby, bettong and goanna pits contained relatively more labile carbon than rabbit pits. The significantly greater soil excavation by the

bilbies and bettongs, and higher concentrations of carbon in their pits, relative to rabbit and goanna pits, demonstrate that these reintroduced fossorial mammals play important roles in the creation of fertile patches in arid landscapes. The results suggest that the extirpation of Australia's mammal fauna has been accompanied by a loss of key ecosystem processes. Crown Copyright (C) 2007 Published by Elsevier Ltd. All rights reserved.

1857: ***+.223***

Successful conservation initiatives often lead to rapid increases in large carnivore densities to the extent that overpopulation occurs. Yet conservation managers have no way of knowing the carrying capacity of their reserves. Here we derived relationships between the preferred prey (species and weight range) of Africa's large predator guild and their population densities to predict their carrying capacity in ten South African conservation areas. Conservation managers intervened at several of these sites because of evidence of predator overpopulation and these provided independent tests of our predictions. Highly significant linear relationships were found between the biomass of the preferred prey species of lion, leopard, spotted hyaena and African wild dog, and the biomass of prey in the preferred weight range of cheetah. These relationships are more robust than previous work for lion, cheetah and leopard, and novel for spotted hyaena and African wild dog. These relationships predicted that several predators exceeded carrying capacity at four sites, two where managers expressed concerns about overpopulation due to a decline in wildlife abundance and two where carnivores were actively removed. The ability to predict the carrying capacity of large predators is fundamental to their conservation, particularly in small enclosed reserves. Every predator that preys on large, readily surveyed wildlife can have its carrying capacity predicted in this manner based on the abundance of its preferred prey. This will be beneficial for reintroduction attempts, threatened species management, overpopulation estimation, detecting poaching and in investigating intra-guild competition. (c) 2007 Elsevier Ltd. All rights reserved.

1858: ***-.049***

Captive rearing is increasingly used as an interim strategy to maintain at-risk butterfly populations while long-term recovery techniques are developed. However, it is seldom feasible to measure effects of captive rearing on small, fragile, and highly mobile organisms, such as butterflies, in wild populations. We use a series of general population viability models to assess the demographic effects of a number of captive rearing strategies, defined by the source of butterflies brought into captivity (the at-risk "small" population vs. a different, stable "large" population) and the number brought into captivity. In general, captive rearing increases population viability, although the benefits are small for rapidly declining or highly stochastic populations. Taking butterflies from the large population is consistently better than taking butterflies from the small population, as long as the large population is not also at imminent risk of extinction. We then modify these models to include two possible risks of captive rearing: captive-reared individuals might not perform as well as wild individuals, and captive-reared individuals might decrease the population growth rate of the wild population, e.g., by introducing deleterious alleles or diseases. Reductions in individual performance do not change optimal captive rearing strategies. Short-term extinction risk is also robust to small (less than or similar to 5%) reductions in population growth rate. Although studying performance of captive-reared individuals in the wild is often not feasible, it is often possible to monitor performance while in captivity. Captive rearing is a robust way to maintain severely at-risk populations in the short-term, though it cannot replace long-term solutions. (c) 2007 Elsevier Ltd. All rights reserved.

1859: ***+.088***

The removal of sludge and domestic waste from an old disposal site south of the city of Salzburg was required in order to redevelop the area into a business park together with a slip road connecting the latter to the motorway nearby. A pre-study in 2001 showed high amphibian and reptile diversity in this area. Since all amphibian and reptile species are strictly protected by federal nature conservation laws, a strategy for translocation and compensation measures had to be worked out as one part of an environmental impact assessment (EIA). At the beginning of 2002, ten spawning ponds were created in three receptor areas of similar or larger size than the donor site. Additionally an area of 2000 m2 in a shrub encroachment dominated by willows (Salix spp.) at receptor site 02 was cleared and restructured with gravel heaps and egg deposition sites. The business park area itself was surrounded by a total of 5000 m of concrete barriers to shut out amphibians, reptiles and other small terrestrial animals. Further 29 ponds and puddles and habitat structures for reptiles were created as compensation measures after the completion of the translocation. Altogether 8125 specimens (7349 amphibians, 776 reptiles) of seven amphibian (Rana temporaria, Bufo bufo, Hyla arborea, Triturus alpestris, Triturus vulgaris, Triturus cristatus, Salamandra salamandra) and five reptile species (Anguis fragilis, Lacerta agilis, Zamenis longissimus, Natrix natrix, Coronella austriaca) were captured and transferred to the new habitats. Leaving aside all amphibian larvae, a total number of 2208 individuals were rescued, which is more than 7.5-times the number of pre-study recordings. Compared to the pre-study two further species were recorded in small numbers. During three years of monitoring (2003-2005) a total number of 181 observations of amphibians or reptiles in 69 localities were documented. In 33 of 39 (85%) newly created ponds the presence of 2-7 amphibian species was documented. Successful reproduction of 1-6 species was observed in 10 ponds. Reproduction was also documented for four reptile species at receptor site 02. Only the smooth snake was observed just once without indication of reproduction. Different methods of capturing herpetofauna are discussed and general implications are proposed.

1860: ***+.039***

In small populations of monogamous species sex ratio bias and sex-skewed demographic traits could lead to higher extinction probabilities than in other mating systems. Therefore a knowledge of bias in sex ratio, mortality and movement would be useful to determine the optimal strategy for sampling founders prior to reintroduction. We used molecular sexing to sex wild-hatched cohorts of two colonies (one native and one reintroduced) and four released groups of griffon vultures Gyps fulvus in France. In wild-hatched cohorts the sex ratio was not different from equilibrium whatever the year. Similarly no bias was detected in the sex ratio of founding stocks. Recoveries, recaptures, movements and philopatry were not skewed according to sex in wild-hatched and released groups. Our study revealed that no sex bias occurred during the griffon vulture life cycle (i.e. birth, death and movement). Consequently, random sampling may be appropriate to constitute founding stock in reintroduction programmes for monomorphic vultures.

1861: ***+.038***

The Endangered Corsican red deer Cervus elaphus corsicanus was extirpated from Corsica in the early 1970s, at which time the Sardinian population fell to < 250 individuals. The Sardinian authorities agreed to protect this subspecies and to secure its reintroduction in Corsica, a natural choice, considering ethological and historical descriptions. Since the beginning of 1985, when the first deer destined for captive breeding and eventual reintroduction arrived in Corsica, the population increased from 13 Sardinian founders to 106 captive animals under constant

monitoring in three enclosures (Quenza, Casabianda and Ania di Fium'Orbu). The sites of Quenza, Chisa and Santo Pietro di Venaco were selected by the Regional Nature Park of Corsica for the reintroduction into the wild that began in 1998. Currently the size of the whole Corsican population is c. 250 individuals. These deer are still closely monitored and studied, both in enclosures and in the wild, to secure the long-term conservation of this subspecies. The Corsican and Sardinian populations together now total slightly > 1,000, and the subspecies could therefore be downgraded to Near Threatened on the IUCN Red List.

1862: *+.079*

1The mammal fauna of the British Isles has seen major perturbations since the end of the last ice age, some natural and some anthropogenic. Today, 61 species of terrestrial mammals breed in the British Isles, but only 39 of them are native species, the rest have been introduced. Furthermore, 19 native species have disappeared from the fauna including all the large predators, lynx Lynx lynx, wolf Canis lupus and brown bear Ursus arctos.2Inevitably, these changes in species composition have been accompanied by major changes in community function including changes in patterns of energy flow through the mammalian community. For example, a high percentage of all the energy now flows through the introduced rabbit Oryctolagus cuniculus population and red deer Cervus elaphus, which, in the absence of natural predators, are living at extremely high densities.3Could the reintroduction of species help to reverse such changes in community structure and function? The successful return to the wild of species such as the Arabian oryx Oryx leucoryx and beaver Castor fiber has shown that reintroductions are certainly possible. However, the impact on community function of returned species is more difficult to evaluate.4This question is addressed in relation to the consequences for deer populations of any possible reintroduction of the wolf to Scotland. Based on what we know of wolves elsewhere, predictions are made about the likely demography and patterns of killing behaviour of introduced wolves. These values are then used to parameterize a Leslie matrix simulation of the impact of wolves on contemporary populations of red deer in Scotland. The simulations suggest very strongly that wolves are very unlikely to have any significant impact on the high-density populations of deer now living in the Scottish landscape.

1863: *+.130*

Translocations are widely used to reintroduce threatened species to areas where they have disappeared. A continuum multi-species model framework describing dispersal and settling of translocated animals is developed. A variety of different dispersal and settling mechanisms, which may depend on local population density and/or a pheromone produced by the population, are considered. Steady state solutions are obtained using numerical techniques for each combination of dispersal and settling mechanism and for both single and double translocations at the same location. Each combination results in a different steady state population distribution and the distinguishing features are identified. In addition, for the case of a single translocation, a relationship between the radius of the settled region and the population size is determined, in some cases analytically. Finally, the model is applied to a case study of a double translocation of the Maud Island frog, Leiopelma pakeka. The models suggest that settling occurs at a constant rate, with repulsion evidently playing a significant role. Mathematical modelling of translocations is useful in suggesting design and monitoring strategies for future translocations, and as an aid in understanding observed behaviour.

1864: *+.010*

Molecular genetic techniques have been widely used to evaluate management actions, including the success of species reintroductions. However, conclusions drawn from genetic characterizations must be interpreted in the context of the sampling design and degree of uncertainty underlying genetic parameter estimation and assumptions of analyses performed. For example, failure to correctly identify and sample appropriate groups of individuals for comparative analyses will bias estimates of summary measures of genetic diversity, intersample variance in gene frequency, and derivations of effective population size or degree of reduction or bottlenecks in numerical abundance. We critically evaluate the foundational assumptions underlying the sampling design and analytical methods employed by Swanson and colleagues. Inaccuracies in reporting the founding population history of American marten (Martes americana) in Michigan and high levels of uncertainty underlying estimates of effective population size, bottleneck history, and demographic sustainability suggest that the authors' genetic data are misrepresented.

1865: *+.320*

Reintroduction efforts require knowledge of how many animals are needed for successful establishment. Population viability analysis can be used to predict trajectories of introduced populations and tree squirrels provide an ideal model system to investigate this challenge. Conservation action is needed because more than 80% of species of tree squirrels are of precarious conservation status in some portion of their range. We combined data from closely related species of tree squirrels and used VORTEX to determine how many squirrels are needed to successfully establish populations of 6 species (Sciurus aberti, S. carolinensis, S. niger, S. granatensis, S. vulgaris, and Tamiasciurus hudsonicus). We ran multiple simulations to account for betweenpatch differences in breeding success (resource availability) and variation between years in different habitats. In the best-case scenarios, populations could be successfully established with fewer than 35 individuals for all species and as few as 15 for a subset of species. Empirical evidence from introductions of tree squirrels supports our simulation results, with 93% of populations of greater than 10 squirrels surviving more than 50 years. With relatively few individuals needed for establishing new squirrel populations, reintroductions are feasible and useful as a buffer for imperiled species.

1866: *+.257*

1. Adaptive management involves the development of predictive models, strategic manipulation of management actions to gain information, and subsequent updating of the models and management. The paradigm has several characteristics that make it an effective approach for determining requirements of re-introduced populations.2. Adaptive management was applied to the re-introduction of hihi Notiomystis cincta, a New Zealand forest bird that had been reduced to a single island population. Following three previous failed re-introductions, we initiated an 8-year series of management manipulations when hihi were re-introduced to Mokoia Island in 1994.3. We developed a population model for projecting outcomes under potential management scenarios, and updated it on an annual basis. The population model combined submodels for survival and reproduction that were selected from sets of candidate models using an information-theoretic approach. All projections incorporated demographic stochasticity, and later projections incorporated uncertainty associated with model selection and parameter estimation.4. The programme showed that some actions (e.g. the provision of sugar water during breeding season and mite control) substantially increased the population's growth rate, but that persistence was uncertain under any management scenario. The population growth rate was shown to be constrained by a low adult survival rate that was unaffected by supplementary feeding, and was associated with a feature of the island (high density of Aspergillus fumigatus spores) that could

not be remedied by management. Hihi were therefore removed from Mokoia. However, the management actions shown to be effective on Mokoia have now been used to produce sustained growth in three other re-introduced hihi populations.6. Synthesis and applications. The results illustrate how adaptive management can facilitate successful species recovery. Without manipulation of management treatments, the Mokoia hihi re-introduction would have just been another failure that provided no useful information. Instead, our manipulations allowed us to identify effective management actions that were successfully applied to other re-introduced populations, and allowed us to identify a limiting factor that had not been previously considered. We have illustrated how other characteristics of the adaptive management approach (flexible treatments, ongoing monitoring, early model development, quantitative projections and incorporation of uncertainty) were essential to the programme.

1867: *+.048*

The Nilgiri tahr, Hemitragus hylocrius Ogilby, is an endangered mountain goat distributed in southern Western Ghats of Peninsular India. Monitoring tahr habitats and selection of sites for management interventions and reintroduction of the tahr in their natural environment require an understanding of habitat suitability and hence this study was undertaken. The estimated tahr population in Kerala was around 1,000 individuals on 11 sites. The Eravikulam National Park (ENP) was chosen for developing the Habitat Suitability Index (HSI) model since it has the sole viable population of about 700 individuals. Based on the habitat utilization, the ENP was divided into different blocks. The relational trends between block-wise tahr density and the critical habitat variables viz., altitude, extent of cliff and availability of principal food species were examined through correlation analysis for evolving habitat suitability criteria. This formed the basis for deriving suitability indices, ranging from 0 to 1, which were subsequently used for developing the HSI models. In order to find out how well the HSI models captured variation in tahr density, they were subjected to regression analysis. The analyses indicate that the HSI models were satisfactory considering the limited number of factors involved. However, the models should be evaluated in other habitat conditions especially in. fragmented habitats considering their degradation factors such as human disturbance, grazing pressure.

1868: *+.420*

Increasing our ability to synthesize and compare fisheries assessment data among species and across geographic boundaries should facilitate a better understanding of the broad-scale condition of fish resources and necessary management strategies. We describe the Conservation Success Index (CSI), a new tool to analyze the status of native salmonids and facilitate protection, restoration, reintroduction, and monitoring efforts. The CSI provides a framework to evaluate indicators of population integrity, habitat integrity, and future security within native salmonids across all subwatersheds within their historic range. To date, the CSI has been completed for seven native trout and char species with summary status maps, data sheets, spatial analyses of management needs, and analysis of climate change and energy development impacts available at http://tucsi.spatialdynamics.com. Additional species are added annually. Case studies using Bonneville cutthroat trout and brook trout illustrate how the CSI provides a multi-scaled description of management priorities that can be combined with site-specific data to design needed restoration. Although specifically developed by Trout Unlimited to prioritize the organization's conservation work and to assist our members in understanding broad-scale conservation needs, the CSI may be useful to other organizations as a fisheries management or environmental education tool.

1869: *-.078*

The Indian rhinoceros (Rhinoceros unicornis) is a highly endangered species that inhabits only three political states. Recently, Zschokke and Baur [Zschokke, S., Baur, B., 2002. Inbreeding, outbreeding, infant growth, and size dimorphism in captive Indian rhinoceros (Rhinoceros unicornis). Canadian journal of Zoology 80, 2014-2023] found that the offspring of matings between captive Indian rhinoceros individuals from the Kaziranga and Chitwan populations had high mortality rates. These authors suggested that these two populations are partially genetically incompatible and, thus, they proposed that these would be separated into two subspecies. In this study we compiled data from a captive population with data from Dudhwa National Park (India), where rhinoceroses were successfully reintroduced in 1984. In Dudhwa, the breeding male came from the Kaziranga population and four out of the five breeding females came from the Chitwan population. In spite of these different origins, the Dudhwa population has bred very well. We analyzed, the factors influencing infant mortality of 22 Dudhwa and 181 captive calves. outbreeding (matings between animals from Kaziranga and Chitwan) did not play any role in infant mortality. From our data, we can conclude that parity, not outbreeding, is responsible for infant mortality in the Indian rhinoceros. Thus, we are unable to agree with Zschokke and Baur's suggestion that the Chitwan and Kaziranga populations belong to separate subspecies. Rather, we propose that the hybridization of captive animals from Chitwan and Kaziranga, as well as those in the wild, should help save the genetic diversity of this highly endangered species. (C) 2007 Elsevier Ltd. All rights reserved.

1870: *-.077*

illegal hunting of rhinoceros in East Africa was widespread in the late 1970s. Today, rhinoceros numbers remain perilously low. The Eastern black rhinoceros (Diceros bicornis michaeli) is restricted to protected areas within Kenya and Tanzania and the few protected areas in Kenya where rhinoceroses are found are reaching carrying capacity. The Serengeti-Mara Ecosystem represents the best potential site for population growth of the Eastern black rhinoceros. Populations within the Serengeti-Mara Ecosystem remain low and augmentation of the current population has been proposed. Using historical census data collected prior to the illegal hunting that occurring in the 1970s, we determined the historical distribution and population of rhinoceros in the Masai Mara National Reserve and Serengeti National Park. The population was approximately 460 animals. We developed a habitat suitability model for the black rhinoceros using the spatial location of historical count data matched with contemporary vegetation and landscape variables. illegal hunting still remains a significant threat to the rhinoceros. Therefore, we determined areas where the likelihood of a rhinoceros being discovered and then targeted by illegal hunters was highest. This information can be used by managers as a starting point for an assessment for reintroduction if other factors are taken into account. This case study exemplifies the importance and potentially unforeseen applications of long term ecological datasets for species conservation. (C) 2007 Elsevier Ltd. All rights reserved.

1871: *+.120*

Transferring plants between populations of rare species has often been proposed to increase population size and replenish genetic variation. While this approach has many advantages, it may also disrupt local adaptation. However, the scale over which plants adapt to local conditions is hard to predict. To detect local adaptation, we conducted reciprocal transplant experiments in the field with six populations of the rare perennial herb, Aster amellus. We sowed seeds in 2003 and 2004 (called 'Experiment 2003' and 'Experiment 2004') and transplanted adult plants in 2004. We

evaluated genetic differences between populations and ecological differences between habitats and tested which differences explain the degree of local adaptation. The number of juveniles from the local populations was 68% and 42% higher than the number of juveniles from the foreign populations in 'Experiment 2003' and 'Experiment 2004', respectively, indicating local adaptation. However, not all populations of A. amellus adapted to their local conditions. Differences in local climate and in vegetation composition particularly affected local adaptation. In contrast to transplanted seeds, transplanted adult plants from local populations did not overall perform better than plants from foreign populations. We conclude that transfer of seeds is a more appropriate technique than transfer of adult plants in conservation practice because it more likely prevents non-adapted genotypes from establishing. Material for the transfers should come, not necessarily from the closest, but rather from ecologically similar habitats. (C) 2007 Elsevier Ltd. All rights reserved.

1872: *+.286*

Animals that undergo a habitat shift face a number of challenges as they move between habitats; for example, they may encounter new predator species and may be vulnerable as they adapt to their new surroundings. An ability to adapt quickly to the new environment is likely to influence post-transition survival, and an understanding of the development of this ability is important in species that we rear for conservation and reintroduction programmes. Juvenile cod, Gadus morhua, undergo a habitat shift during their development, and they are also a species where reintroduction work has been tried. Here, we describe an experiment that investigated the effects that rearing environment has on cod shoaling behaviour. Cod were tested just after they had undergone the transition from a pelagic to a more benthic existence. We found that cod reared in either an enriched or in a plain, standard hatchery environment differed in terms of their shoaling responses; the shoaling tendency of fish reared in enriched tanks varied between testing environments, but fish reared in plain environments responded in the same way across the testing conditions. We discuss the influence of early experience on the development of appropriate behavioural responses and the importance of this for captive-reared species that are released into the wild.

1873: *+.196*

The Eurasian beaver Castor fiber, formerly occurred across the Palaearctic, but was nearly eradicated in the 19th century. Due to reintroductions in the 20th century, beaver populations are increasing and now extend into highland areas. Natural still waters are scarce in highlands of Central Europe. Therefore the question arises, "Are beaver ponds essential habitats for amphibians?", especially since fishes, predators of amphibian larval stages, also inhabit beaver ponds. We investigated the amphibian fauna of one typical valley in the Eifel, that was colonized by beavers in 1981, and compared areas with and without beaver ponds. All anuran species of the region occupied beaver ponds, including species that were absent (Alytes obstetricans, Bufo bufo and Rana kl. esculenta) or rare (Rana temporaria) in natural waters. Alytes obstetricans obviously benefited from pond construction and the removal of trees by beavers which leads to sunny plots along the slopes of the valley, crucial habitat for this species. The urodelans Salamandra salamandra, Triturus alpestris and Triturus helveticus were widely distributed in beaver ponds. Our results show clearly, that beaver altered landscapes offer high quality habitats for amphibians in our study area. Due to a considerable increase of habitat heterogeneity in impounded streams, the predator Salmo trutta was not able to extirpate the amphibian fauna. We conclude that the historic effects of beavers need to be considered for a proper understanding of patterns of amphibian distribution and habitat requirements in Central European Highlands. Furthermore, beaver-created

landscapes will be of future relevance for conservation of endangered species, like Alytes obstetricans.

1874: *-.026*

This paper reviews an ongoing, large-scale multidisciplinary experiment designed to study the possibilities of rehabilitating forest structure and species composition through logging, dead wood creation and fire in managed Norway spruce (Picea abies) forests in southern Finland. These forests have been utilized for several centuries with intensive management and clear-cut harvesting, which has been the dominant practice in Finland since World War II. During this era, the forest structure has become relatively even-aged, and the amount of dead wood has been reduced considerably. Simultaneously, due to an effective fire suppression policy, the role of fire in Finnish nature has been almost completely eliminated. One of the key species in biodiversity, aspen (Populus tremula), has also been actively removed from the forests in the past. Forest restoration activities, such as the creation of dead wood and the reintroduction of fire to forest management, have been suggested in conservation and restoration programmes. So far we have studied the immediate effects of restorative actions on forest structure, regeneration, soil nutrient status, understorey and epixylic vegetation, lichens and beetles. In the larger Evo research area we have also studied the population structure of aspen in both protected and managed forests. Our early results show that it is possible, through active forest restoration, i.e. the creation of dead wood and prescribed burning, to rehabilitate boreal forest diversity, even when a significant part of the wood volume is harvested for commercial use. Despite the fact that the immediate effects of fire on many species groups were negative, the long-term effects are expected to be predominantly positive. There is currently a decline in aspen populations in Finnish forests. The absence of large aspens in managed forests and the absence of younger trees/cohorts in conservation areas, combined with high mortality, is a significant threat to aspen-dwelling species. We conclude that studies on active restoration treatments, together with long-term inventories of several species groups, are necessary in order to assess the impacts of varying restoration practices for cost-efficient large-scale applications. (C) 2007 Published by Elsevier B.V.

1875: *+.343*

The Tianmu Mountain National Nature Reserve (TMNNR) preserves the only primeval forest of China cedar (Cryptomeria fortunei) in the world. In order to assist in planning the protection of China cedar habitat and propose a scientific zoning project for TMNNR, we established a nature reserve geographic information system (NRGIS) of TMNNR using a geographic information system (GIS) and remote sensing (RS). In support of NRGIS, we produced a 3-dimensional simulation of TMNNR and obtained a predictive model through a combination of logistic regression modeling and multivariate analysis. The results demonstrate that slope direction, soil type, and annual precipitation are the main factors affecting China cedar habitat. The existence probability of China cedar increases with (converted) slope direction and annual precipitation. Yellow soil (FAO classification) is more suitable for the growth of China cedar in comparison to red soil (FAO classification). Finally, we predicted suitable habitats for China cedar using a model and function zoning for TMNNR. We suggest that it is necessary to analyze key factors affecting China cedar habitat in order to ensure appropriate conservation measures. NRGIS has been found indispensable for studying, protecting, and managing China cedar forest and TMNNR. NRGIS provides not only modeling information but also the means for monitoring this rare species, identifying suitable locations for reintroduction, and carrying out visible and dynamic management of the whole reserve.

1876: *+.160*

Knowing the distribution of endangered species is of substantial importance for conservation. We considered a useful approach for modeling species distribution when managing information from atlases and museums but when absence data is not available. By modeling the distribution for Graellsia isabelae, a threatened moth species, we assessed its current conservation status and identified its most relevant distribution explanatory variables using Geographic Information System and Generalized Linear Models. The distribution model was built from 136 occurrence records and 25 digitized explanatory variables at a 10 X 10 km resolution. Model predictions from logistic-regressed pseudo-absences, obtained from a presence-only method (Ecological-Niche Factor Analysis), explained 96.23% of the total deviance. We found that the best predictor variables were summer precipitation, aridity, and mean elevation. With respect to host plants, the presence of G. isabelae associated mainly with Scots pine (Pinus sylvestris) and Austrian pine (P. nigra). The finding of 8 areas, exclusively in the eastern Iberian territory, and a larger unoccupied habitat in the western Iberian Peninsula indicates that this species is probably not in equilibrium with its environment by historical factors. Sites of Community Importance under protection do not seem sufficient to maintain current populations, necessitating the protection of suitable neighboring habitats. Our methodology is useful to manage the conservation status of species for which reliable absence data is not available. It is possible to determine those variables that most affect the distribution of species as well as the potential suitable areas with the purpose of evaluating protected areas, connectivity among populations, and possible reintroductions.

1877: *+.048*

1. Interference competition with wolves Canis lupus is hypothesized to limit the distribution and abundance of coyotes Canis latrans, and the extirpation of wolves is often invoked to explain the expansion in coyote range throughout much of North America.2. We used spatial, seasonal and temporal heterogeneity in wolf distribution and abundance to test the hypothesis that interference competition with wolves limits the distribution and abundance of coyotes. From August 2001 to August 2004, we gathered data on cause-specific mortality and survival rates of coyotes captured at wolf-free and wolf-abundant sites in Grand Teton National Park (GTNP), Wyoming, USA, to determine whether mortality due to wolves is sufficient to reduce coyote densities. We examined whether spatial segregation limits the local distribution of coyotes by evaluating home-range overlap between resident coyotes and wolves, and by contrasting dispersal rates of transient coyotes captured in wolf-free and wolf-abundant areas. Finally, we analysed data on population densities of both species at three study areas across the Greater Yellowstone Ecosystem (GYE) to determine whether an inverse relationship exists between coyote and wolf densities.3. Although coyotes were the numerically dominant predator, across the GYE, densities varied spatially and temporally in accordance with wolf abundance. Mean coyote densities were 33% lower at wolf-abundant sites in GTNP, and densities declined 39% in Yellowstone National Park following wolf reintroduction.4. A strong negative relationship between coyote and wolf densities ($\beta = -3.988$, $P < 0.005$, $r(2) = 0.54$, $n = 16$), both within and across study sites, supports the hypothesis that competition with wolves limits coyote populations.5. Overall mortality of coyotes resulting from wolf predation was low, but wolves were responsible for 56% of transient coyote deaths ($n = 5$). In addition, dispersal rates of transient coyotes captured at wolf-abundant sites were 117% higher than for transients captured in wolf-free areas.6. Our results support the hypothesis that coyote abundance is limited by competition with wolves, and suggest that differential effects on survival and dispersal rates of transient coyotes are important mechanisms by which wolves reduce coyote densities.

1878: ***+.116***

Wildlife diseases are in fashion. This is creating an explosion of related knowledge. Despite this, the dynamics of both wildlife and diseases and the changes in livestock and wildlife management make it increasingly difficult to overview the current situation of wildlife diseases in Europe. This paper aims to discuss the available management possibilities and to highlight current research priorities. One area that causes severe concern to authorities is diseases largely under control in domestic populations but still existing as a reservoir in wildlife. Multihost situations are also of concern for wildlife management and conservation, as diseases can affect the productivity and density of wildlife populations with an economic or recreational value. Concern about emerging diseases is rising in recent years, and these may well occur at the fertile livestock-wildlife interface. Wildlife-related zoonoses are a diverse and complex issue that requires a close collaboration between wildlife ecologists, veterinarians and public health professionals. A few risk factors can be identified in most of the relevant wildlife diseases. Among them are (1) the introduction of diseases through movements or translocations of wild or domestic animals, (2) the consequences of wildlife overabundance, (3) the risks of open air livestock breeding, (4) vector expansion and (5) the expansion or introduction of hosts. Wildlife disease control requires the integration of veterinary, ecology and wildlife management expertise. In addition to surveillance, attempts to control wildlife diseases or to avoid disease transmission between wildlife and livestock have been based on setting up barriers, culling, hygienic measures, habitat management, vector control, treatments and vaccination. Surveillance and descriptive studies are still valuable in regions, species or diseases that have received less attention or are (at least apparently) emerging. Nonetheless, limiting the research effort to the mere reporting of wildlife disease outbreaks is of limited value if management recommendations are not given at the same time. Thus, more experimental approaches are needed to produce substantial knowledge that enables authorities to make targeted management recommendations. This requires policy makers to be more aware of the value of science and to provide extra-funding for the establishment of multidisciplinary scientific teams.

1879: ***+.112***

The jewelled gecko (Naultinus gemmeus) may be close to extinction in Central Otago due to habitat removal and modification by post-settlement fire and pastoralism. This study aimed to characterise the habitat of sites in dry parts of Central Otago where jewelled geckos still exist and assess how habitat has changed at sites where the species is no longer present. This species is likely to have once been widespread across Central Otago, when native woody vegetation was present at most mid- to low-altitude sites. However, only two widely separated populations are now known, both at the edges of the Central Otago area. In this study, sites in dry parts of Central Otago where jewelled geckos have previously been reported were surveyed and habitat features were recorded during summer 2006-07. It was found that the distribution of jewelled geckos in Central Otago appears to be determined by the presence of refugia from historic fires and extreme low temperatures in rocks or vegetation; persistence of diverse, woody, native vegetation; and site aspect and altitude. No jewelled geckos were observed during this study. Although this was probably largely due to the cryptic nature of this species and the short survey duration, some sites were extremely degraded and suitable habitat has been removed or severely modified over most of the area. Determining where jewelled geckos have persisted in dry parts of Otago, and the characterisation of the habitat both at these sites and at sites where the species is no longer found will help to guide drylands restoration, the prioritisation of sites for protection and the reintroduction of native species.

1880: +.116

The Millerbird (Acrocephalus familiaris) is an endemic Northwestern Hawaiian Islands reed warbler that existed until about 1923 on Laysan Island (A.f. familiaris) and currently occurs in a small population on Nihoa Island (A. f. kingi). The two populations are described as separate subspecies or species on the basis of size and plumage differences. We assessed genetic variation in blood samples from 15 individuals in the modern Nihoa population using approximately 3000 base pairs (bp) of mitochondrial DNA (mtDNA) sequence and 14 microsatellite loci. We also obtained up to 1028 bp of mtDNA sequence from the fragmented DNA of museum specimens of three birds collected on Nihoa in 1923 and five birds collected on Laysan in 1902 and 1911 (ancient samples). Genetic variation in both marker types was extremely low in the modern Nihoa population (nucleotide diversity [pi] = 0.00005 for mtDNA sequences; observed heterozygosity was 7.2% for the microsatellite loci). In contrast, we found three mtDNA haplotypes among the five Laysan individuals (pi = 0.0023), indicating substantially greater genetic variation. The Nihoa and Laysan taxa differed by 1.7% uncorrected mtDNA sequence divergence, a magnitude that would support designation at the subspecies, and perhaps species, level relative to other closely related Acrocephalus species pairs. However, in light of strong ecological similarity between the two taxa, and a need to have additional populations to prevent extinction from stochastic effects and catastrophes, we believe these genetic differences should not deter a potential translocation of individuals from Nihoa to Laysan.

1881: +.082

Habitat destruction and introductions of invasive species have been primary causes of endemic species loss on oceanic islands. This paper addresses the question of whether a reintroduced population of a critically endangered island endemic, the Mauritius fody (Foudia rubra) is competing or co-existing with an exotic congener, the Madagascar fody (F. madagascariensis). We investigated habitat use and feeding ecology of the Mauritius fody during two early phases of its reintroduction onto a restored islet, and compared these parameters with the sympatric population of Madagascar fodies. The number of Mauritius fody territories increased from 4 to 20 in a seven month period following release, and overall mean territory area (c. 1 ha) was significantly larger than that of the more abundant Madagascar fody (c. 0.07 ha). The initial four territories were located in mature coastal forest, whilst those of the Madagascar fody were characterised by open canopy, smaller trees and grass-dominated ground cover. Mauritius fodies foraged mainly in dead leaves for insects, whilst the Madagascar fody fed primarily on the seeds of a native grass species. Mauritius fody territories later expanded to include a range of habitats. Differing ecological requirements indicate that divergence between these congeners may be sufficient for them to coexist and exploit contrasting resources on restored islets. We discuss the implications of these findings for both the long-term restoration of the Mauritius fody and other avian recovery programmes and highlight the critical importance of long-term post-release monitoring to assess the success of reintroduction to offshore islets as a long-term conservation strategy. (C) 2007 Elsevier Ltd. All rights reserved.

1882: +.233

Relocations of species have become a tool widely used in nature conservation, but insects have rarely been considered as targets. Here, we present a translocation project of the field cricket (Gryllus campestris L. 1758), which is a threatened species at the northern edge of its range. Only ten populations were left in Lower Saxony (Germany), illustrating the need for urgent conservation measures. After 10 years of monitoring and management of an isolated population,

213 nymphs were captured and released at another nature reserve in summer 2001. The size of the new population increased significantly from 27 singing males in spring 2002 to 335 singing males in spring 2005. The occupied area increased from 5.66 ha to 33.14 ha. Altogether, the translocation project was evaluated as successful, but the inland dune proved to be not as suitable for the species as initially expected. Our results indicate that translocations of highly reproductive insect species are promising, as long as the release locality contains sufficiently large areas of suitable habitat and a high number of wild juveniles from a closely located and large source population are released in a climatically favorable period. Management and restoration of habitats, as well as continuous monitoring are of crucial importance for the success of the translocation project. Moreover, the importance of a high quality of cooperation between conservationists, authorities, foresters, farmers, financiers and scientists cannot be overstated.

1883: *+.034*

The Przewalski's horse (Equus ferus przewalskii) became extinct in the wild in the 1960s, but survived as a species due to captive breeding. There have been several initiatives to re-introduce the species in central Asia, but until now only two projects in Mongolia establish free-ranging populations. Data on basic ecology and behavior of the species prior to extinction are largely lacking and thus a good documentation of the reintroduction process is essential. Between 13 May and 2 September 2003 we documented the time budget-, behavioral synchrony- and body score development of a newly released Przewalski's horse group in the Gobi area of SW Mongolia. Contrary to our expectations, the newly released Przewalski's horses did not show the expected succession of an exploration-, acclimatization-, and established phase. Grazing activity was very high after the release, decreased to a minimum in July and increased again towards the end of the study in September. Resting activity followed the opposite trend, whereas moving activity was more or less constant over the entire observation period. Behavioral synchronization of the group was high throughout the study period and immigration or emigration of members did not result in a de-synchronization of the group. The body score index never dropped, but rather increased for all group members. Our data suggests that captive bred Przewalski's horses experience little behavioral and nutritional stress when being released into the desert steppe of the Gobi regions after 1 year in an adaptation enclosure. (C) 2006 Elsevier B.V. All rights reserved.

1884: *+.127*

Asian turtles face an extinction crisis, and so it is imperative that systematists accurately determine species diversity in order to guide conservation strategies effectively. We surveyed mitochondrial and nuclear DNA (mtDNA and nuDNA) variation of the heavily exploited Mauremys mutica complex, a clade of Asian turtles that contains the endangered M. mutica from Japan, Taiwan, China and Vietnam, and the critically endangered Mauremys annamensis from central Vietnam. We discovered extensive mtDNA and nuDNA variation among samples that did not correspond to the currently recognized taxonomy. Both nuDNA and mtDNA data suggest that M. mutica is paraphyletic with respect to M. annamensis. Surprisingly, M. annamensis exhibits a previously unknown mtDNA structure in the form of two clades that are paraphyletic to M. mutica. These data reveal that the currently recognized taxonomy of the mutica complex does not reflect the genetic diversity of our samples. Unfortunately, many conservation-oriented captive-breeding efforts for turtles are also based on trade samples such as the ones studied here. These efforts include plans to breed trade-rescued individuals and release their progeny into the wild. Because our genetic survey reveals that the taxonomic identity of these samples does not reflect genetic diversity, we raise serious questions about the efficacy of these programs. In order to address conservation issues and provide more accurate estimates of evolutionary lineages within

Mauremys, we recommend continued surveys for wild populations of the mutica complex to provide new genetic material and additional distributional data, attempts to extract DNA from historic museum specimens and a shift in conservation focus to in situ preservation of wild populations and associated habitat.

1885: *+.035*

Following a dramatic population decline in 1999, captive-breeding and translocation programs were initiated to recover the Santa Catalina Island fox Urocyon littoralis. Neonatal losses during the first year of captive breeding raised concerns, but little information was available on which to base reproductive expectations in captivity, and pregnancy rates and pup mortality had not been assessed in free-ranging foxes. In 2002 and 2003 we evaluated the relative contributions of captive breeding and translocation to population recovery by comparing pregnancy rates and perinatal mortality in free-ranging and captive Santa Catalina Island foxes, and by comparing pregnancy rates and perinatal mortality were also compared between resident free-ranging foxes and foxes that had been captive bred and released, or translocated as juveniles. Pregnancy rates and fetal number were determined using ultrasound. Free-ranging pregnant foxes were followed via radiotelemetry, and a combination of camera-traps, observation and targeted trapping was used to determine how many pups survived to weaning. Video cameras and observations were used to determine the weaning success for captive foxes. The adult pregnancy rate for free-ranging foxes (95.0%) was higher than for adult captive foxes (47.6%; P=0.003). Perinatal mortality for pups born to free-ranging mothers (43.2%) was higher than for pups born to captive mothers (15.0%, P=0.055). Adult pregnancy rates and perinatal mortality were 100 and 25.0% for translocated and captive-bred foxes combined, and 92.3 and 53.6% for resident wild foxes. The average weaned litter size (+/- standard deviation) for free-ranging foxes (1.8 +/- 0.6) was similar to that for captive foxes (2.1 +/- 0.4). Successful pup production by translocated and captive-bred foxes supports the utility of these strategies to recover island foxes. Our approach, integrating veterinary and field biology techniques to assess the contributions of different management strategies to population recovery, can be utilized for other endangered species.

1886: *+.201*

Translocation of individuals between, isolated populations is practised, or envisaged, for a variety of reasons but requires careful consideration. Linklater [Linklater, WL., 2003. A novel application of the Trivers-Willard model to the problem of genetic rescue. Conserv. Biol. 17, 906-909] proposed that the number of individuals of each sex translocated into a target population for the purposes of maintaining genetic diversity could be chosen using parental investment theory when the manager is allocating limited resources between the sexes as a parent invests in offspring of different sex. In this paper, by adapting basic ideas in the parental investment literature, we formulate a model to capture Linklater's proposal and provide a thorough mathematical analysis of the model. Granted the necessary species-specific biological information and translocation protocols to determine the model parameters in any instance in which the model is applied, the analysis indicates that a practical algorithm can be constructed to generate predictions of the optimal number, sex ratio, and division of resources for translocation with the goal of genetic rescue. This work is in line with recent calls for a more theoretical approach to translocation by providing quantitative models which can be subjected to scientific scrutiny and provide protocols for translocation planning. In particular, our model is intended to promote the design of translocations which study means of investment and the measurement of response to that investment. (C) 2007 Elsevier B.V. All rights reserved.

1888: ***+.103***

Lynx were reintroduced to the Jura Mountains in the mid-1970s. A first retrospective update of the situation in France and Switzerland was undertaken 10 years later. Since then, real-time collection of occasional observations has been going on in both countries. The monitoring methods have been standardised since the beginning of the 1990s using, among other methods, a network of observers. During 1972-2001, 862 observations were collected in Switzerland and in this paper, these are used to describe the colonisation of the Swiss Jura Mountains and the present distribution of lynx. A comparison with the results of French researchers shows that during the first years of recolonisation, most of the observations were collected in the Swiss part of the Jura Mountains. Today, the French territory has become the core area of the population and includes about 2/3 of the population. The population has recovered after a period of reduced presence at the beginning of the 1990s. The range occupied perma nently by lynx in the Swiss Jura Mountains is estimated to be 2,100 km, representing a potential population of 17-23 resident individuals. The range occupied permanently by lynx in the Jura Mountains on both sides of the border is estimated to be 7,100 km(2). Depending on the degree of saturation in the population, this represents a potential population of 56-78 resident individuals. Compared to existing habitat suitability models, most of the suitable habitat has already been occupied. In the future more importance will be given to the exchange of information across the international border and the participation of local people in the survey. I recommend that active monitoring using camera-traps be carried out periodically as a supplement to the ongoing passive surveillance system.

1889: ***+.057***

Lynx Lynx lynx returned to a semi-natural, human dominated landscape in the Jura Mountains in France and Switzerland after reintroductions in the early 1970s. Controversy has resulted from lynx attacking sheep and preying on game species such as roe deer Capreolus capreolus and chamois Rupicapra rupicapra. We review the history of the lynx, the transition of the landscape and fauna in the Jura Mountains, and recent findings from long-term field studies on the species. Possible threats to the survival of the population are assessed. The ecological conditions for the existence of the lynx in the Jura Mountains have improved since the species was eradicated in the 19th century. Both habitat and prey base are suitable for maintaining the population. Immediate threats include traffic accidents and illegal killings. Long-term threats include small population size and genetic impoverishment as a result of the post-reintroduction bottleneck. We recommend conservation and management that involve local people and cooperation at national and international scales. Fragmentation of the habitat and the management system should be avoided, and landscape linkages from the Jura Mountains to adjacent mountain ranges should be established to promote a lynx metapopulation.

1890: ***+.096***

Beneficial soil microorganisms are integral to nutrient availability and uptake for plants in restoration. They include mycorrhizal fungi and nitrogen-fixing bacteria, together with the soil microbial populations which contribute to nutrient availability. Around 70% of jarrah forest plant species form arbuscular mycorrhizas, and approximately a quarter also form ectomycorrttizas. Many are also legumes. In addition, around 70 orchid species depend on mycorrhizal symbioses. Therefore, symbiotic soil microorganisms are important in the ecosystem. Arbuscular mycorrhizal fungi recover to pre-mining levels in bauxite restoration in five years. Ectomycorrhizal fungi are poorly adapted to disturbance; however, they are able to reinvade through wind-blown spores. The density of ectomycorrhizal fungi has been found to be equivalent in seven-year-old restoration and

adjacent forest, but both abundance and diversity are correlated with development of a litter layer. Fortunately, rhizobia are known to be tolerant of soil disturbance, and failure of N-fixation by legumes has not been reported in restoration. Other N-fixing symbioses, such as between Allocasuarina and Frankia or Macrozamia and Nostoc, have not been investigated in restored mines. Soil microbial biomass C achieves near equivalence after about eight years and appears to be driven by vegetation productivity and related inputs of C into the soil. There is little field evidence that the absence, or very low levels, of soil microbial symbionts will have a substantial impact on plant growth in restoration. Therefore, deliberate reintroduction of these microorganisms does not appear justified. However, soil management to enhance the survival of soil biological components is recommended.

1891: *+.029*

The Crest-tailed Mulgara (Dasycercus cristicauda), a species listed as vulnerable under the Commonwealth Environment Protection and Biodiversity Conservation Act (1999) and as a Schedule I species under the Western Australian Conservation Act (1950), was once found throughout and central Australia and Western Australia, but its geographic range has been significantly reduced. The spatial distribution and shape of Mulgara burrows is described for an area that was subsequently cleared in the Pilbara of Western Australia. The area contained a substantial cover (>> 50% cover) of spinifex (Triodia sp.) tussocks to about 600 mm high and scattered shrubs when first searched in June 2006 but had been burnt (November 2006) by the time Mulgara were to be translocated before the vegetation was cleared in January 2007. Burrows contained between two and nine entrances, tunnels were mostly on a single level and to a depth of about 300 mm. The lumen for a burrow entrance was typically an arch over a flat bottom with a height of 70-80 mm, and a width of 80-100 mm at the base. Internal tunnels were mostly 50-70 mm wide. Burrows entrances in the burnt landscape were mostly in the open. There was one burrow per 2.5 ha in the area searched, but this was probably a 'hot-spot' for Mulgara for the region. Four Mulgara were caught in 750 Elliott trap-nights and five by digging out 65 recently active burrows in an area of about 22 ha. From this we concluded that a substantial trapping effort was required to trap all the Mulgara in an area. If Mulgara are being translocated from an area, then we would recommend an intensive trapping program combined with searching for and digging out all recently active burrows in the area. Strategies to enhance the capture of Mulgara are discussed.

1892: *+.282*

Many factors important to reproduction are difficult to quantify for wild mammals, yet an understanding of them is often critical to species' recovery programs, particularly those involving captive breeding and reintroduction. We examined management variables employed by the Vancouver Island man-not captive breeding program during 1998-2005 to determine how such variables influenced production of young and litter sizes. We then tested the ability of factors that were identified as important to predict production of young in the breeding program in 2006. For previously paired animals, production of young was significantly greater when they had been paired with the same mate for >= 1 year before the breeding season. When these females had been with a mate for < 1 year, production of young was more than 2 times greater among 2-4 year olds compared to older age classes. Among previously paired females, 5-7 year olds and those that had produced young previously tended to produce young more often than other categories. Litter size was positively influenced by visual contact between a female and other pairs. For previously paired females, age, the amount of time paired with their mate, and previous production of young accurately predicted 75% of production of young in 2006. We propose that results from out study

be combined with genetic considerations to plan future pairings in the breeding program and to assist in the selection of individuals for reintroduction to the wild. Methods reported herein may be applicable to recovery programs for other imperiled mammals.

1893: ***+.237***

The current strategy for conserving white-clawed crayfish, Austrapotamobius pallipes, contained in the United Kingdom Biodiversity Action Plan includes reintroduction to isolated water, free of signal crayfish, Pacifastacus leniusculus. The purpose of this project was to improve the chances of successful reintroduction of white-clawed crayfish into the River Lathkill in the Peak District National Park and to demonstrate how the method might be applicable to other reintroduction and captive breeding schemes. The method focussed on the habitat requirements of white-clawed crayfish, in particular the need of younger crayfish to conceal themselves from predators. Cages were built which provided juvenile crayfish with hides. The cages were tested in the river and juvenile growth and survival were recorded. Adult crayfish survival was only 50% annually whilst survival of juveniles was much less. Despite this, the habitat within the cages protected crayfish during moult and is thought to have increased their survival rate. The project has provided evidence for development of a successful reintroduction and captive breeding programme for white-clawed crayfish that will enable the conservation strategy to be undertaken more effectively.

1894: ***+.155***

We used in microsatellite loci to examine rangewide population structure and interpopulation gene flow in the federally threatened Coachella Valley fringe-toed lizard (Uma inornata). Our results indicate low population differentiation consistent with high gene flow, recent colonization and range expansion, and/ or frequent local extirpation/recolonization events. Given high historical gene flow among populations and current isolation of remaining populations, conservation planning for this species should include monitoring of potential deleterious effects that may result from reduction in gene flow, such as inbreeding and loss of genetic variation, to ensure maintenance of ecological and evolutionary population processes adequate for long-term survival of the species.

1895: ***+.039***

Translocation of individuals among extant populations is an important tool in species conservation that allows managers to supplement dwindling populations and potentially alleviate the deleterious effects of inbreeding. Ideal translocation strategy should consider historical relationships among existing populations to avoid potential disruption of population subdivision and local adaptation. Here, we examine mitochondrial sequence variation in the endangered blue duck Hymenolaimus malacorhynchos, a New Zealand endemic riverine specialist, to facilitate informed decision making in future translocations. Behavioural observations suggest that blue duck dispersal is limited and may result in genetic structure within and between regional populations. We analysed 894 base pairs of mitochondrial control region in 78 adult blue ducks sampled from 11 river catchments across the species' range (representing four regions in the North Island and three regions in the South Island) and found strong and significant genetic structure both within and among islands. These results, combined with a 2.0% sequence divergence between islands, indicates that North Island and South Island blue ducks should be treated as separate management units. The relationship between genetic differentiation and geographic distance for blue ducks on the South Island conformed to an "isolation by distance" pattern. Overall, we recommend that translocations of blue ducks should not be made between the North and the South Islands and

those within each island should be restricted to neighbouring catchments.

1896: ***+.134***

Approximately 40% of the living tortoises and freshwater turtles of the world are considered threatened species and several are critically endangered due to a variety of anthropogenic causes. Captive breeding and the creation of assurance colonies, with subsequent translocation, is an important conservation strategy for some of these species and will likely become more important for others. At present there is a diversity of programs, including those done in-situ (within the natural range of threatened species), or ex-situ (out of natural range). Captive breeding occurs in large commercial farms and small intensive non-commercial ventures. In order to help achieve success, these programs need to include genetic management of their captive populations and understand the genetic implications of their actions to eventual translocated wild populations. Concerns include the loss of genetic diversity within small populations, including inbreeding depression and inappropriate mixing of turtles belonging to different genetic lineages and loss of cryptic lineage diversity. Although many captive breeding programs do not include genetic management of their populations at present, those that do will have greater conservation value in the future. As wild populations of species disappear, captive populations may provide stock for reintroductions. Comprehensive and conscientious record keeping and data management over the long time that populations are held captive are of utmost importance. This paper reviews the various captive breeding and translocation activities that can be put to use in the conservation of freshwater turtles and tortoises.

1897: ***-.005***

Many of the world's turtles and tortoises are currently threatened with population extinction. Some species are so threatened in their native habitats that the only way to ensure their survival may be through captive propagation, an endeavor in which many private conservation organizations are currently engaged. In this review we outline the genetic issues that need to be considered when establishing captive breeding colonies of tortoises and turtles for eventual reintroduction or population supplementation. The first section of this review stresses the importance of creating breeding units that are based on the population structure of a species in its native range. We discuss how molecular methods and the concepts of evolutionarily significant units (ESUs) can be used to define breeding units for captive breeding colonies and determine their geographic origin for reintroduction. In the second section we discuss the need to maintain the genetic variability of the colony members and the techniques that are available to achieve this goal.

1898: ***+.099***

Cochlearia polonica, a narrow endemic of southern Poland, is one of the rarest and most endangered species of the European flora. All natural populations are extinct and the species has survived in only one transplanted population derived from 14 original individuals. Using AFLPs, the genetic variation and spatial structure of this population were analysed approximately 30 years after transplantation. The incidence of polymorphic AFLP bands (30.46%) is low compared with data from a natural population of another Cochlearia species, C. tatrae. Principal co-ordinates and spatial autocorrelation analyses demonstrated the presence of significant genetic structure. It is recommended that conservation efforts on C. polonica should preserve the complete population area, because local extinctions may lead to a loss of genetic information. The presence of genetic structure should also be taken into account during the sampling of material (plants or seeds) for ex situ conservation measures. (c) 2007 The Linnean Society of London, Botanical Journal of the

Linnean Society, 2007, 155, 527-532.

1899: ***+.020***

Population life-history traits such as the propensity to move across inhospitable landscapes should be shaped by exposure to landscape structure over evolutionary time. Thus, birds that recently evolved in landscapes fragmented by natural disturbances such as fire would be expected to show greater behavioral and morphological vagility relative to conspecifics that evolved under less patchy landscapes shaped by fewer and finer-scaled disturbances, i.e., the resilience hypothesis. These predictions are not new, but they remain largely untested, even for well-studied taxa such as neotropical migrant birds. We combined two experimental translocation, i.e., homing, studies to test whether Ovenbird, Seiurus aurocapilla, from the historically dynamic boreal mixedwood forest of north-central Alberta (n = 55) is more vagile than Ovenbird from historically less dynamic deciduous forest of southern Quebec (n = 89). We found no regional difference in either wing loading or the response of homing Ovenbird to landscape structure. Nevertheless, this study presents a heuristic framework that can advance the understanding of boreal landscape dynamics as an evolutionary force.

1900: ***+.133***

In order to establish a wild population, Hubei Shishou Milu Natinal Nature Reserve introduced 30 (8 male, 22 female) and 34 (10 male, 24 female) Milu (Pere David ' s deer) from the Beijing Milu Park to establish a breeding herd in 1993 and 1994, respectively. Since June 2000, we have monitored the population trends of reintroduced P re David' s deer in Shishou Milu National Nature Reserve. Until the end of the calving season of 2006, there were 522 P re David' s deer in the reserve. The growth of Milu population fits the equation: N-t = 84e(0.226t). The sex ratio in the Milu population was 1 : 1.22 (male : female) before the calving season of 2006. Owing to heavy floods in the Yangtze River during the summer of 1998, some Milu escaped from the fenced area in the reserve to the south of the Yangtze River. According to the fitted population growth model, the population growth rate of Milu inside the reserve was 0.226 which was lower than that (0.267) of the Milu that live outside the reserve. The mean annual population growth rate of the Milu that live outside the reserve was also higher than the population inside the fenced reserve. Compared with those of the Dafeng Milu National Nature Reserve, the mean annual birth rate (26.8%, P = 0.010) and the mean annual population growth rate (21.7%, P = 0.038) of the Milu population in Shishou Milu National Nature Reserve were significantly higher than that of the Dafeng population. However, there was no significant difference between the mean annual mortality rates between the Shishou and Dafeng populations. Due to habitat deterioration and human interference in the Shishou nature reserve, some conservative measures should be taken urgently to improve the living conditions of Milu because the growth of the population has started to show a density-dependent pattern [Acta Zoologica Sinica 53. (6) : 947 - 952, 2007].

1901: ***-.079***

As the Arabian Ostrich Struthio camelus syriacus, a distinct subspecies, became extinct in the wild during the mid-20(th) century, the most closely related subspecies, S. c. camelus occuring in north-eastern Africa, has been chosen for reintroduction into Saudi Arabia. A few individuals of this Red-necked Ostrich were obtained from Sudan in 1988-89 from a private collection, and in 1994 a few birds were translocated to Mahazat as-Sayd Protected Area into a 25 ha fenced enclosure. So far a total of 96 Red-necked Ostriches has been released into the fenced Mahazat as-Sayd and the estimated population is between 125 and 150 individuals. Since captive flocks of Ostriches were

translocated to Mahazat, their survival rate increased by > 41% up to the end of 2000. On an average 22-30 chicks are hatched annually. A total of 137 Ostriches was recorded dead over the period of 13 years during the drought period. Both captive-bred and wild-born adults and young died of starvation and thirst, despite being provisioned with alfalfa and water during several years.

1902: ***+.207***

Aims Isoetes sinensis (Isoeteaceae) is a critically endangered aquatic fern ally in China. Understanding the genetic differentiation of quantitative traits and local adaptation of the remnant populations should provide insight into the adaptation of different populations to their local environment and useful information for formulating appropriate conservation strategy. Methods The quantitative traits of nine isozyme multilocus genotypes from three remnant populations of I. sinensis were investigated using nested analysis based on random block design in common-garden experiments. Population differentiation of allozyme (F-ST) and quantitative traits (Q(ST)) were estimated using the Bayesian approach. Important findings Of 14 quantitative traits examined, ten were found to be significantly different among populations and three were significantly different within populations. Multiple comparison by Turkey tests indicated that the mean values of five traits were the highest for the Songyang population and lowest for the Xiuning population, whereas the mean values of the macrosporangium characteristics and plant height were highest for the Jiande population and lowest for the Xiuning population. This result probably reflected different scenarios of founder effect, habitat competition ability and trade-off between vegetative and reproductive growth. Local adaptation was evaluated as a difference of QST vs. FST value, and eight important reproductive fitness traits were found to be significant (Q(ST) > F-ST, p <= 0.05) under the selfing assumption, suggesting local adaptability in the remnant populations. It is more likely that there is a potential risk of outbreeding depression if genetic enhancement is implemented by translocation of individuals from different populations. Therefore, translocation among these remnant populations is not recommended. Instead, we recommend increasing gene flow within each population and improving habitat management by reducing competition with companion species in in Situ conservation for Xiuning and Songyang populations. In the case of Jiande population, minimizing human disturbance should be considered as a top priority and an in situ conservation plot should be designated for the population.

1903: ***-.100***

Many translocation methods have been tested in southern Europe in recent decades to increase the translocation success of the European wild rabbit (Oryctolagus cuniculus L.) for both conservation and game purposes. The main problem experienced during such translocations is the high short-term (7 days) mortality attributed to predation during the days immediately following rabbit release. In this study, we test the effect of the exclusion of terrestrial predators on the survival of translocated rabbits for recovery purposes. Four translocation plots (4 ha with 18 artificial warrens each) were constructed, two of them with a fence to exclude terrestrial predators. In all, 724 rabbits were released to the translocation plots in five batches and forced to remain inside warrens for 7 days. Following liberation, exclusion of predators did not increase rabbit survival in the short term. Contrary to expectations, three months after release, survival of rabbits in the unfenced plot was slightly, but not significantly, higher than in the fenced plot (0.57 and 0.40 respectively). Although predator control is a frequent management practice associated with rabbit translocations, our results suggest that it may not favour rabbit survival rate as much as the adaptation of rabbits to the release site.

1904: ***+.111***

Overabundant populations of kangaroos pose substantial management problems in small parks on the fringe of urban areas in Australia. Translocation is impractical and culling is often not publicly acceptable, but fertility control offers an acceptable alternative. One potential contraceptive is levonorgestrel, which provides effective long-term contraception in women, and prevents births in some marsupials for up to five years. We evaluated the long-term efficacy of levonorgestrel in free-ranging eastern grey kangaroos (M. giganteus) at two sites in Victoria, Australia. We trapped 25 adult females at one site (Portland Aluminium), treating 18 with two subcutaneous 70-mg levonorgestrel implants and seven with control (inert) implants. We darted 25 adult females at the other site (Woodlands Historic Park), treating all with two 70-mg levonorgestrel implants. We monitored the reproductive status of the kangaroos, as indicated by the obvious presence of a pouch young, in spring each year for up to seven years. In the first three years at Portland, 81-86% of levonorgestrel-treated females were infertile, compared with 12 - 29% in the control group, but the effectiveness of fertility control declined over time. At this site, the proportions of treated females breeding in the fourth, fifth, sixth and seventh years of the trial were 36%, 50%, 67% and 100% respectively. Fecundity at Woodlands was similar. Although this protocol achieved fertility control for several years, it was likely that more than one treatment or a higher dose rate would be required for effective fertility control in this long-lived species.

1905: ***+.181***

It has been previously suggested that the characteristics that are driving the taxonomic homogenisation of the global avifauna, through the extinction of native bird species and the establishment of exotic bird species, are opposite sides of the same coin. One of the most important tools that conservation biologists and wildlife managers have to ameliorate the extinction of a species is to reintroduce populations to stronghold areas from which they have been extirpated or were not previously common. In this paper, we address the question of what the study of exotic bird introductions can tell us to inform the translocation of native species. We review the relative importance of the five factors that have been suggested significantly to influence the successful establishment of non-native species: introduction effort, environmental matching, species' interactions, species' life histories, and phylogenetic relatedness. Current evidence suggests that introduction effort will be an important determinant of release, but how many individuals need to be released, and in how many separate release events, is contingent on characteristics of species and environment. The importance of climate matching for introduction success suggests that the success of translocations will depend greatly on the study and amelioration of the problem that caused the initial population decline. This is most problematic in situations where the decline is associated with human-induced climate change. Migratory and sexually selected species may be harder to re-establish, but related species may differ substantially in their likelihood of success. We suggest that further insights into the reintroduction process may be gained particularly by studying species that are experiencing a threat in their native range but which are also being widely released as exotics outside of this range.

1906: ***+.074***

This review takes a broad perspective on mammalian invasions and considers genetic aspects of both natural colonisation and conservation-related translocations as a backdrop to the genetics of introductions of wildlife-management concern. Genetics can help characterise invading populations in useful ways and can reveal, with greater or lesser precision, the geographical sources of invasions, their timing and how many individuals were involved. Invading mammals may affect the genetics of natives indirectly or directly, and it is important to be able to document this. There is a need to consider both 'organism invasion' and 'gene invasion'. Genetics often

provides an unexpected perspective on invasion biology. Examples illustrating all these points are provided through the article.

1907: ***-.162***

Understanding the potential threats of predation can play a crucial role in conservation management of threatened species. We investigated the frequency of sublethal injuries to live individuals of the endangered pygmy bluetongue lizard (Tiliqua adelaidensis). We found that there was no significant difference in the frequency of injury between males and females. However, there was a significant difference in injury frequency between adults and subadult animals and also between two close, but isolated, populations of pygmy bluetongue lizards. These data can be used, with caution, to understand the predation risks in natural populations of this species. They also suggest that predation would be a significant hazard that must be considered in any translocation program that is considered for this species.

1908: ***+.203***

Reintroductions are attempts to return species to parts of their historical ranges where they were extirpated, and might involve release of either captive-bred or wild-caught individuals. The poor success rate of reintroductions worldwide has led to frequent calls for greater monitoring, and since 1990 there has been an exponential increase in the number of peer-reviewed publications related to reintroduction. However, these publications have largely been descriptive accounts or have addressed questions retrospectively based on the available data. Here, we advocate a more strategic approach where research and monitoring targets questions that are identified a priori. We propose ten key questions for reintroduction biology, with different questions focusing at the population, metapopulation and ecosystem level. We explain the conceptual framework behind each question, provide suggestions for the best methods to address them, and identify links with the related disciplines of restoration ecology and invasion biology. We conclude by showing how the framework of questions can be used to encourage a more integrated approach to reintroduction biology.

1909: ***+.056***

Lakeside daisy (Tetraneuris herbacea Greene, Asteraceae) was re-introduced into dry gravel prairie at three locations within Manito Prairie Nature Preserve, Tazewell County, Illinois in 1988 with additional plantings at four other preserve locations in 1994 and 1995. Monitoring documented the disappearance of all but one population within five years following reintroduction. This population has persisted for nineteen years, but declined to one flowering and 63 non-flowering individuals in 2003. Long-term survival of this taxon at Manito Prairie Nature Preserve appears unlikely.

1910: ***+.211***

The management history of the Koala Phascolarctos cinereus in Victoria is unique and spectacular. Management of Koala populations began in Victoria in about 1910, at which time the species was undergoing a severe decline in population number and distribution. The fortuitous transfer of small numbers of Koalas to two coastal islands in the late 19(th) Century allowed intensive conservation management to begin in 1923, and it has continued almost unabated for the subsequent 84 years. Initially, Koalas were marooned for conservation purposes on four other large coastal islands, and several smaller ones, including two in the Murray River. These island populations were then used

to re-introduce the species to remaining habitat across the former natural range of the species in Victoria and south-east South Australia. In the process intractable over-browsing problems were inadvertently created at ten sites. Since about 1985, the sole reason for translocation has been to protect natural values from the impacts of Koala over-browsing. Since 1995, considerable research effort has been directed at finding suitable in-situ population control mechanisms. During the 84 year program more than 24 000 Koalas were translocated to about 250 release sites and Koala populations have been successfully re-established in most areas of suitable habitat in Victoria. The genetic costs of using inbred populations as the source of animals for re-introduction are perhaps yet to be fully realised.

1911: *-.000*

Attacks by Australian Magpies Gymnorhina tibicen on humans are among the most common forms of human-wildlife conflict in Australia, especially in suburban environments. Despite the familiarity of these interactions, remarkably little is known about the phenomenon, significantly undermining attempts to develop a sound basis for management. To redress this, a series of studies spanning most of a decade were carried out in Brisbane, designed to explore all dimensions of the conflict. This paper describes several relevant aspects of the phenomenon including temporal patterns of reproduction and attacks, and summarises key findings of investigations into community attitudes towards managing what is a favourite Australian species. In particular, it was established that lethal control was opposed by a clear majority of survey respondents while translocation as an option for management was supported. As a result, comprehensive studies into this approach were carried out, establishing that the technique reduced specific conflicts. However, we were unable to account for the fate of most released birds.

1912: *+.236*

In tribute to the 30(th) anniversary of the successful reintroduction of chamois in northern Velebit, the first part of the article presents the monograph "Chamois", written by the forester Milan Knezevic and published in Sarajevo 70 years ago (1938). The book, based on the author own study of the Balkan chamois subspecies and imbued with love of the true hunter for the game, has not lost any of its significance and interest. The second part of the article is dedicated to the life and work of Milan Knezevic (Bihac, 1879 - Zavidovici, 1944), an exceptionally talented game writer. He graduated from the Forestry Department of Technical High School in Sarajevo in 1898. Shortly after passing the state exam in 1911, he became a forest administration manager and worked in a number of forest offices across Bosnia and Herzegovina. From 1925 to 1935 he was in charge of the hunting and fishing department within the Forestry Directorate in Sarajevo. This period of his life, marked by his passionate love for hunting, nature and wild life, proved to be the most fruitful. Despite his rough life, frequent dismissals from the service and the inability to affirm himself as an intellectual, he was a prolific hunting writer In addition to the monograph "Chamois" and a number of articles published in Zagreb-based "Hunting-Fishing Journal", he succeeded in completing, in cooperation with his son Ratko Knezevic, his most valuable work "The Wolf - Life, Harmfulness and Extermination", which was only published in 1956 by the Institute of Forestry and Wood Industry of Bosnia and Herzegovina. The last part of the article deals with the successful reintroduction of chamois in northern Velebit. Organized by the Republic Institute for Nature Protection in Zagreb and Forest Administration in Senj, the reintroduction was conducted on two occasions: the first time in the autumn of 1974 with 9 introduced chamois from Prenj (Bosnia and Herzegovina) and the second time four years later, on 10 October 1978, with 5 adult animals from the Kamnik Alps (Slovenia). The newly established population inhabiting the National Park of Northern Velebit and the hunting grounds "Sveti Juraj" and "Jablanac" is

estimated at about 400 animals and is subjected to game management (hunting).However, despite these encouraging numbers, we cannot be satisfied with the current status of chamois in Croatia from the game hunting aspect. In the Croatian part of the Dinaric mountain range, from Gorski Kotar to the hinterland of Dubrovnik, there are vast areas that are very suitable for successful breeding of chamois; yet, these areas are completely under-utilized in this respect. According to some calculations made by competent experts who are thoroughly acquainted with the conditions in the terrain, about 70, 000 ha would suffice for successful chamois breeding programes on Mt. Velebit. The major part of this area is chamois habitat of the first site class. In terms of animal number this area could support about two thousand chamois, with an annual income of 480 animals and harvest of 250 animals (Car 1972). The same number of chamois that were released in northern Velebit should also be released in central and southern Velebit for this purpose (Mikulic, 1982).

1913: ***+.403***

The scientific literature contains numerous descriptions and predictions of the effects of climate change on wildlife populations and ecosystems. Recently, resource managers and planners have proposed "adaptation strategies" to help wildlife and ecosystems adjust to the effects of a changing climate. In this report, we review the scientific literature on climate change adaptation as it relates to biodiversity conservation and wildlife management. We also review a series of actual climate change adaptation plans that have been developed in the U.S.A., Canada, England, Mexico, and South Africa. From these reviews, we identify eighteen general strategies that could be used to manage the effects of climate change on wildlife and biodiversity. We recommend that any strategy for managing the effects of climate change on wildlife and ecosystems be deployed within an adaptive management framework, in order to enable managers to learn from previous management activities, and to respond quickly and creatively to the challenges posed by climate change. For each of the eighteen strategies, we provide a brief summary and discussion of its advantages and disadvantages (including availability of tools or techniques for implementation, as well as relative costs). We present a decision tree to help natural resource managers select the most appropriate set of strategies for use in particular management situations. Strategies related to land protection and management include: increasing the amount of protected areas; improving representation and redundancy within natu- ral area networks; improving the management of existing natural areas to maximize resilience; designing new natural areas and restoration sites to maximize resilience in the face of climate change; protecting predicted movement corridors, "stepping stones," and refugia; focusing restoration and management efforts on the maintenance of ecosystem function rather than specific assemblages and components; increasing overall landscape permeability to species movements; and reducing non- climate stressors on natural areas and ecosystems. Strategies related to direct species management include: focusing conservation resources on species most likely to become extinct; translocation of select species; captive breeding of select species; and the reduction of non-climate stressors affect- ing individual species. Strategies related to monitoring and planning include: reviewing existing monitoring programs to insure that the information needed for the adaptive manage- ment of climate change effects is being collected; incorporating information on potential climate change impacts into species and land management plans; developing dynamic landscape conservation plans; and insuring that wildlife and biodi- versity are included in broader adaptation plans developed by local, regional, or national governments. Strategies in the legislative and regulatory arena include: reviewing existing laws, regulations, and policies regarding wildlife and natural resource management, to insure that these instruments provide managers with the flexibility needed to address effects of climate change; and proposing new legislation and regulations as needed to give managers additional tools and approaches to facilitate responses to climate change.

1914: *+.056*

Noturns munitus (Frecklebelly Madtom), is a diminutive catfish restricted to large rivers in the Mobile Basin and Pearl River drainages in the Southeastern United States. We conducted surveys of 13 major tributaries of the Alabama, Cahaba, and Tombigbee river systems in the Mobile Basin to determine use of tributaries by N. munitus. Our surveys found only one specimen in Oakmulgee Creek, a large tributary to the Cahaba River and one of the few rivers in which stable Populations of N. munitus remain. We combine results from Our recent Survey with a review of the literature and museum records for N. munitus throughout its range to present a consolidated status report. Our review indicates that N. munitus is Currently greatly reduced from its former range, and is in decline in most of the drainages it still inhabits. We recommend federal protection for the species under the Endangered Species Act. We also provide suggestions for future research and management actions for the species.

1915: *+.084*

The European pond turtle (Emys orbicularis) is endangered in many parts of its distribution area. Reintroduction programs are one of possibilities to protect of the species. However, survivorship of adult turtles is very high. Thus populations of such species are very sensitive on increase in mortality of adults (transferring adult individuals to other populations is tantamount to increase of mortality in parent population). Another chance is to use headstarted animals. I used life tables to model such possibility. Results of the studies indicate some essential problems with reintroductions using headstarted animals, e.g. results will be visible after long time, males will mature earlier, and large number of animals is needed. The real efficiency of such programs, however, also depends on behavior and fecundity of headstarted animals, factors that are currently not known. Reintroduction using headstarted turtles should still be considered experimental, and I am not in a position to recommend initiation of such programs.

1916: *+.114*

We conducted an experimental release of European pond turtles (Emys orbicularis) in an area of natural habitat from which the species had apparently disappeared around the early 20th century. Three groups of turtles were released in April 2000 (nine females and four males), May 2001 (five females and eigth males), and April 2002 (six females and three males), and were monitored until September 2002 using telemetry (over 10 000 locations). Movements based on telemetry are summarised for each individual. The release is considered a success. All 35 turtles survived in the wild. They established their home ranges near the release point, with no tendency to move in a particular direction. Maximum distance travelled was 5120 m from the release point, but the majority did not disperse. Clutches were observed on the artificial nesting site and hatchlings emerged in 2002. This three years program shows that turtles were able to survive in their new environment and to settle, forage and reproduce at the release site. Translocation can be considered a viable conservation strategy for this locally endangered species. However, as a sustainable supply must be used for the next releasing steps, a farming project has to be implemented to supply several new cores along the Rhone river.

1917: *+.056*

Native fish assemblages in large rivers in the United States have changed dramatically in the past century We use regional examples of 10 rivers in continental North America to commence to evaluate these changes in fish assemblages. Four of the 10 rivers are the 1st, 4th, 10th, and 16th

longest in the United States. To a large extent, translocations and introductions of normative and alien species, flow alteration, and habitat loss have been the primary causative factors of decline of native fish assemblages. Mainstream dams, irrigation diversions, channel alteration and simplification, groundwater mining, agriculture, and pollution have been the primary drivers of the negative impact on historical aquatic habitats. Introduction and translocation of fishes into river systems within the United States over the past century have been largely directed at increasing sport fishing opportunity. However, accidental introductions and those for bait, the aquarium trade, and biological control in combination also have impacted native assemblages through habitat competition, hybridization, and direct predation. As a result, many of the native species are either listed or becoming increasingly rare. A critical question relative to the theme of this Fourth World Fisheries Congress is, "Can we reconcile the current situation and conserve and sustain native fish assemblages in many of these rivers in the United States?" Conservation activities including hatchery and refuge propagation, management by river basin or watersheds, barrier construction, and safe harbor agreements are being used to sustain native fish assemblages. Notwithstanding, the current plight of native fishes in many river basins in North America can be described as a "battle against extinction."

1918: *+.035*

The Lesio-Louna and south-west Leini Reserves in the Bateke Plateau region of the Republic of Congo are not generally included as part of the geographic range of de Brazza's monkey (Cercopithecus neglectus). I present here observations made between 2002 and 2007 showing the species to be widely distributed within the gallery forests of the two reserves. Most sight records were of one or two individuals, although groups of up to six were also observed. De Brazza's monkeys could be heard calling on approximately 50% of days at project camps, and it was the most frequently detected large mammal during MacKinnon List surveys. Detectability was signiicantly lower during surveys that began after 07:00, a inding that may explain why the species has been largely overlooked during structured wildlife surveys. A review of grey literature suggests that the reserve management activities linked to a gorilla reintroduction program have led to recovery of the species following years of heavy hunting. However, the connectivity of the gallery forests of the Leini watershed to the major forest areas to the east is threatened by human activity. The maintenance of forest corridors to avoid isolation of the gallery forests may be an important consideration for the long-term sustainable management of the region.

1919: *+.093*

Genetic diversity within and among three populations of Tectona philippinensis Benth. & Hook.f. (Verbenaceae) is analyzed based on trnL intron sequence variations. Leaf samples were collected from Luzon Mainland (Lobo and San Juan, Batangas) and Iling Island (Occidental Mindoro). DNA extraction, PCR amplification, DNA sequencing, sequence alignment, genetic variation and statistical analysis were consequently conducted. The trnL intron sequence of T philippinensis has about 505-520 bps with a G/C content of 34%. Cluster analysis shows that the sequences of the T philippinensis are unique from its close generic relative in the family Labiatae. It also shows that the three populations of T philippinensis are homogenous, with unique sequences expressed in some Iling Island individuals. Except for the homogenous composition in Lobo, the calculated haplotype diversity for the populations is 0.257 and nucleotide diversity of 0.00077-0.00227. The lower nucleotide diversity within the San Juan individuals (0.00077) and within Lobo individuals (0.0) indicate that these mainland (sub)populations are relatively derived compared to the Iling group (0.00227). F-ST values of 0.023-0.047 indicate negligible genetic differentiation. Sequence polymorphism analyses using neutrality tests show that there is only one population for the species

with a tendency to develop two subpopulations, i.e., Iling subpopulation and San Juan-Lobo subpopulation. In-situ and ex-situ conservation efforts should take into account both the Iling Island and Luzon Island (San Juan-Lobo) populations.

1920: *+.165*

At least three currently named subspecies of the rock dwelling skink Egernia stokesii from Western Australia (E s. stokesii, E. s. aethiops and E s. badia) have restricted distributions or are considered endangered. We tested preferences of the more common eastern form of E stokesii (E s. zellingi) for various artificial refuges made from paving bricks by offering alternative structures in enclosures and recording times spent on each. We found that lizards preferred refuges with more crevices. They did not discriminate between structures where the crevices were located higher or lower or between structures that did or did not have adjacent small bushes. However, lizard basking position was influenced by crevice height or by proximity of a bush. Our results are relevant where translocations of animals are a component of conservation management, and where artificial refuges are added to induce relocated individuals to remain or to increase resources for existing populations.

1921: *+.027*

Once widespread throughout Victoria, the Eastern Barred Bandicoot Perameles gunnii has declined to near extinction on the Australian mainland due to habitat loss and predation by exotic predators. The last remaining wild population occurs in Hamilton, western Victoria. Founders for a captive breeding program were taken from this population in 1988, which has persisted without predator control or supplementation from captive-bred animals. The species was reintroduced to eight sites from 1989: Woodlands Historic Park, Hamilton Community Parklands, Mooramong, Floating Islands Nature Reserve, Lake Goldsmith, Lanark, Cobra Killuc, and Mount Rothwell. Although all reintroduced populations initially increased, declines were observed during the mid- to late 1990s, from which they have never recovered. A combination of drought and predation by Red Foxes Vulpes vulpes and Cats Felis catus is thought to be responsible. Currently, management techniques focus on intensive control of Red Foxes by poison baiting and shooting, and by construction and maintenance of predator barrier-fences at some sites. Understanding which characteristics lead to the success or failure of a reintroduction site is paramount to the success of the recovery programme for this species. This paper reviews the status of all reintroduction sites, and examines characteristics that could account for the performance of their populations.

1922: *+.167*

Optimising breeding performance in a seriously endangered species such as the black rhinoceros (Diceros bicornis) is essential. Following an estimation of the population demography of the black rhinos in Solio Game Reserve, Kenya and a habitat evaluation, a model of the Ecological Carrying Capacity showed there was a serious overstocking. Data analysis of the first year of rhino monitoring indicated a poor breeding performance of 3.8% and a poor inter-calving interval in excess of 36 months. Biological management was required to improve the performance of the population by the removal of a significant number of individuals [long dash] 30 out of the 87. The criteria for selection were: to take no young animals i.e. around 3.5 years of age, to take no breeding females i.e. those with calves, to take care to maintain some breeding males in Solio, to attempt to ensure some breeding males are part of the 'new' population, to take care to leave a balanced population, to take care to create a balanced population in the 'new' population, to move those individuals that were hard to identify and to keep individuals which were easy for visitors to

see. The need for careful candidate selection, the comparison of the population demography pre- and post-translocation, and the effect of the translocation activity on the remaining Solio population are discussed. While it will take many more months, even years, to fully ascertain the effect, beneficial or otherwise, of the biological management of the herd, preliminary results were encouraging.

1923: *+.165*

We report the results of the first release project of 36 captive-bred mandrills into the Lekedi Park, Gabon. A mortality rate of 33% was recorded in the first year post-release, with dependent infants the most affected age class, as a result of environmental stress and malnutrition. A programme of provisioning ensured that individuals remained in a good physical condition. During the second year the death rate decreased to 4%, and 6-month survival rate of infants was 100%. Over time the mandrills extended their spatial use of the park, although their exploration remained limited. Our results demonstrate that provisioning and the lack of knowledge of ecological characteristics of the new, complex seasonal habitat were the likely causes of this situation. After 2 years, reproduction and survival appear stable. While habitat preservation and in situ species protection are the best conservation options, release projects may constitute a viable short-term solution for particular species. In this context, this first release of captive-bred mandrills could play a role in the future conservation of this Vulnerable species.

1924: *+.113*

The research was conducted in the Endangered Wild Life Reproduction Centre (CRAVE), with 6 reproduction groups of Great Curassow (Crax rubra), composed of pairs or two females and one male. The eggs were put in incubators. After 28-30 days, new born chicks were transferred to a grow place. At 110 days of age, the curassows were transferred to a pre release protected area in Cerro Azul Wild Life Research Centre, located in Pilas de Canjel, Peninsula de Nicoya, an area from where the species had disappeared 50 years ago. Some 60 to 90 days later, curassows were released in groups in order to follow them more easily, count them and observe their behavior. Following this procedure, from 2000 through 2004, 128 Great Curassows were born in captivity, from which 104 were released. The proportion female-male born and released was of 2:1. In 2002, we observed the first chick born in the wild, for a total of more than 60 up to 2006. A large number of them are still alive and their behavior is very similar to that of other natural populations. After 7 years, the objective of establishing a viable population has been reached.

1925: *+.212*

The White-winged Guan (Penelobe albipennis) is a Peruvian endemic cracid classified as critically endangered due to hunting pressure and habitat loss. It inhabits a narrow strip in the Peruvian northern dry forests, one of the worlds most threatened ecosystems. It was thought to be extinct until its rediscovery in 1977. In 1978 the "White-winged Guan Conservation Project" began. The wild population has been previously evaluated in 1978, 1987 and 1990 and is estimated at 300 individuals. Although the species is protected by national laws, other conservation measures have been taken to insure its long term survival, including public awareness, education in local schools, research and other measures. One of these measures is a captive breeding program which has been in operation since 1980 to supply selected birds for two reintroduction programs, one of them running since 2001. Here I present preliminary results of a new population survey made from 2001-2005, and compare the results to the three previous evaluations, establishing a population trend and making recommendations for further surveys. I also analyze how the reintroduced

populations and the protected areas system are supporting conservation of the wild populations. I also assess the main threats to the species, ranking the most serious and how they could be mitigated. 1 provide a new approach for assessing the White-winged Guans area of distribution based, on recent ,research, with new data on the species habitat composition. Finally, I make recommendations for the species long term conservation.

1926: *+.094*

The Red-browed Parrot (Amazona rhodocorytha), an endemic species of the Tropical Atlantic Coastal forest of southeastern Brazil, is globally threatened and listed as endangered by BirdLife International, the World Conservation Union, and the Ministry of Environment of Brazil. The available information on this species is from old papers and regional bird inventories. Our research goals were to establish distribution; relative abundance and habitat conditions of the species in the state of Espirito Santo. We produced maps with 331 records of the species from our own field work and another 869 records from information provided by local people. Our maps also show the vegetation type, climate, elevation, remaining forest areas and forest patches with conservation priority The data suggest that the species is associated with high forests in regions with altitudes below 300 m. We found the species in habitats ranging fro m well preserved areas, such as "Reserva Biologica de Sooretama", to disturbed areas, such as papaya (Carica papaya, Caricaceae) plantations and fields with sparse trees. The number of Red-browed Parrots encountered during this study was 2295 individuals. The major threat to the species in the study area is apparently illegal trade. As many individuals were seen as pets among local people, as well as in the Reintroduction Center of Wild Animals (CEREIAS).

1927: *+.123*

The Blue-and-yellow Macaw (Ara ararauna), once native to the island of Trinidad, was extirpated in the early 1960's, primarily due to nest poaching for the pet trade. Between 1999 and 2004, the Cincinnati Zoo and Botanical Garden, Trinidad's Ministry of Environment and the Centre for the Rescue of Endangered Species of Trinidad and Tobago (CRESTT) translocated wild-caught birds from Guyana to Trinidad. During acclimation in a pre-release flight cage, the flight-readiness of the first 14 birds was monitored as the main criterion for release. Nine of the 14 birds released (64%) survived and produced 12 chicks in three nesting seasons. Three years later 20 additional wild-caught birds were imported and the criterion for their release was expanded. Trained villagers spent time each day carefully detailing the behavior of the macaws using an ethogram. In addition to flight-readiness, they noted which birds were aggressive or formed bonded pairs, which other birds stayed together and what native foods they ate. There was 100% survival of the first 12 flight-ready birds released from the second flock. Bonded pairs and social groups that were released stayed together, and exhibited behaviors indicating healthy social structure. When five additional birds were released, they integrated with members of the original flock, and also had 100% survival. Fourteen additional chicks were produced in three more nesting seasons. This study suggests that releasing birds in pairs and socially compatible groups might enhance their chances of survival in the wild.

1928: *+.350*

Understanding resource selection by animals is important when considering habitat suitability at proposed release sites within threatened species recovery programmes. Multi-scale investigatory approaches are increasingly encouraged, as the patchy distribution of suitable habitats in fragmented landscapes often determines species presence and survival. Habitat models applied to

a threatened New Zealand forest passerine, the South Island saddleback (Philesturnus carunculatus carunculatus), reintroduced to Ulva Island (Stewart Island) found that at landscape scale breeding pairs' preferences for sites near the coast were driven by micro-scale vegetation structure. We tested these results by examining models of breeding site selection by a reintroduced saddleback population on Motuara Island (Marlborough Sounds) at two scales: (1) micro-scale, for habitat characteristics that may drive breeding site selection, and (2) landscape scale, for variations in micro-scale habitat characteristics that may influence site colonisation in breeding pairs. Results indicated that birds on Motuara Island responded similarly to those on Ulva Island, i.e. birds primarily settled at the margins of coastal scrub and forest and later cohorts moved into larger stands of coastal forest where they established breeding territories. Plant species composition was also important in providing breeding saddleback pairs with adequate food supply and nesting support. However, Motuara Island birds differed in their partitioning of habitat use: preferred habitats were used for nesting while birds were foraging outside territorial boundaries or in shared sites. These differences may be explained because Motuara has a more homogeneous distribution of microscale habitats throughout the landscape and a highly bird-populated environment. These results show that resource distribution and abundance across the landscape needs to be accounted for in the modelling of density-bird-habitat relationships. In the search for future release sites, food (invertebrates and fruiting tree species) should be abundant close to available nesting sites, or evenly spread and available throughout the landscape.

1929: *+.158*

A population of the strictly protected sand lizard had to be temporarily relocated to conduct landscaping measures for the Rhineland-Palatine Garden Show 2008 in Bingen. Therefore an adjoining area has been restructured appropriately to keep the animals during construction of the fair area. After completion the animals will repopulate their former habitats. Overall, attention was paid to a high level of micro-structures in the cultivation area. After removing parts of the existing shrub vegetation piles of rock, sand and brushwood have been set up as hideouts and places for hibernation and egg deposition, and flowering plants were brought in to increase biotope capacity. The trapping mainly took place after hibernation from mid-March to egg deposition in mid-May. Thus it could be avoided that a large portion of the younger animals difficult to trap fell victim to the construction works. Besides, the lower night temperatures in spring time made it easier to catch the hematocryal animals. Sufficient lair options for hibernation, thermo regulation, protection from predators or competition within the species, and a high density of arthropods as food source are of outmost importance for the husbandry of the animals with a high density of individuals. In order to foster reproduction piles of dark and humid sand were heaped up. According to present stage the measure was successful since an accompanying monitoring programme proved successful reproduction without identifying significant population losses. Intensive publicity created a huge acceptance amongst the local people for this protection measure.

1930: *+.070*

The Amphibian Conservation Action Plan has proposed captive breeding as a potential tool to address amphibian declines. However this potential can only be realised if sufficient skills and experience exists within captive breeding establishments to be able to respond. With at least 25% of Madagascar's amphibians categorised as Vulnerable. Endangered or Critically Endangered captive breeding may well have a role to play in the conservation of amphibians on the island. An analysis of world zoos using ISIS databases, a questionnaire to European Zoos and interviews with private breeders holding Malagasy amphibians were used to determine the level of knowledge and expertise gained over the last ten years. Of the 226 Malagasy amphibians, 27 species are currently

kept in zoos. Only a few institutions have historical records of breeding for several generations or keeping large numbers of Malagasy frogs. Private breeders are keeping approximately the same number of species as zoos. Intercommunication and collaboration between zoos has been highlighted in this paper through a case study of captive colonies of Scaphiophryne gottlebei. Communication between private breeders occurs through the Internet and regular trade meetings. There remains a need to draw up husbandry protocols for captive breeding populations and ensure effective record-keeping for a complete understanding of the success of these captive breeding programmes. Furthermore further collaboration between private breeders and zoos should be encouraged. Finally the reintroduction potential of particular species should be assessed. The results demonstrate that current expertise in the captive management of Malagasy amphibians is limited. There is a need to develop this capacity through increasing our repertoire of species kept in captivity and developing husbandry guidelines. More rigorous record-keeping would allow for monitoring of future breeding success. Finally we suggest that in order to harness the combined expertise in managing threatened amphibians in captivity collaboration between private breeders and zoos should be encouraged.

1931: *+.166*

Land snails of the genus Placostylus Beck, 1837 are found only in the Western Pacific, from the Melanesian Plateau to New Zealand. While the ecology of the New Zealand Placostylus species is generally well known, having been studied for more than 20 years, and there is some published information on the one species of Placostylus from Lord Howe Island, there is very little published information on the six New Caledonian species. These New Caledonian taxa are all endangered, but their conservation is hampered by a lack of information about the life-history, biology and ecology especially relating to Placostylus bondeensis, P. eddystonensis, P. scarabus and P. caledonicus. The data that is available comes mostly from studies on the two remaining species - P. fibratus and P. porphyrostomus. Here we review what is known of the activity patterns, growth rates, life-histories, habitat use, nutrition, movement patterns and causes of population decline in these two species and collate the available information from the limited and mostly unpublished literature. The two species are threatened in different ways Placostylus fibratus is favoured as food, and as a result, has been over-collected from the wild, while P. porphyrostomus is threatened by habitat modification and destruction, and by predation by introduced rodents. We make several recommendations for the conservation of New Caledonian Placostylus which ultimately may depend on the work we describe here on captive breeding and management. Translocations to predator-free sanctuaries coupled with augmentation of existing populations may be necessary to maintain populations of these species in the wild.

1932: *-.106*

Repeated population bottlenecks can lead to loss of genetic variation and normally should be avoided in threatened species to preserve evolutionary potential. We examined the effect of repeated bottlenecks, in the form of sequential translocations, on loss of genetic variation in a threatened passerine, the saddleback (Philesturnus carunculatus carunculatus), a species that has recovered from a remnant population with historically low levels of genetic variation. Although a slight but nonsignificant loss of alleles may have occurred between the first-order translocation and the extirpated source population, first-, second-, and third-order translocated populations had very similar levels of genetic variation to each other. The most obvious difference among the seven island populations appeared to lie in allele frequencies with little or no loss of alleles among extant populations. Although sequential translocations are known to cause loss of variation in genetically diverse species, our study indicates that genetically depauperate species may be less

sensitive to loss of genetic variation through founder events presumably because the few remaining alleles are well represented in founding individuals. These results show that ancient bottlenecks may have a long-term effect on genetic variation, to the extent that contemporary population bottlenecks may leave no appreciable genetic signature. Our results suggest that subjecting genetically depauperate endangered species to sequential translocations could be used to rapidly establish new populations without further eroding genetic variation.

1933: ***+.702***

Although trait evolution over contemporary timescales is well documented, its influence on ecological dynamics in the wild has received much less attention particularly compared to traditional ecological and environmental factors. For example, evolution over ecologically relevant timescales is expected in populations that colonize new habitats, where it should theoretically enhance fitness, associated vital rates of survival and reproduction, and population growth potential. Nonetheless, success of exotic species is much more commonly attributed to ecological aspects of habitat quality and 'escape from enemies' in the invaded range. Here, we consider contemporary evolution of vital rates in introduced Chinook salmon (Oncorhynchus tshawytscha) that quickly colonized New Zealand and diverged over c. 26 generations. By using experimental translocations, we partitioned the roles of evolution and habitat quality in modifying geographical patterns of vital rates. Variation in habitat quality within the new range had the greatest influence on broad geographical patterns of vital rates, but locally adapted salmon still exhibited more than double the vital rate performance, and hence fitness, of nonlocal counterparts. The scope of this fitness evolution far exceeds the scale of divergence in trait values for these populations, or even the expected fitness effects of particular traits. These results suggest that contemporary evolution can be an important part of the eco-evolutionary dynamics of invasions and highlight the need for studies of the emergent fitness and ecological consequences of such evolution, rather than just changes in trait values.

1934: ***+.027***

Mexican and red wolves were both faced with extinction in the wild until captive populations were established more than two decades ago. These captive populations have been successfully managed genetically to minimize mean kinship and retain genetic variation. Descendants of these animals were subsequently used to start reintroduced populations, which now number about 40-50 Mexican wolves in Arizona and New Mexico and about 100 red wolves in North Carolina. The original captive Mexican wolf population was descended from three founders. Merging this lineage with two other captive lineages, each with two founders, has been successfully carried out in the captive population and is in progress in the reintroduced population. This effort has resulted in increased fitness of cross-lineage wolves, or genetic rescue, in both the captive and reintroduced populations. A number of coyote-red wolf hybrid litters were observed in the late 1990s in the reintroduced red wolf population. Intensive identification and management efforts appear to have resulted in the elimination of this threat. However, population reintroductions of both Mexican and red wolves appear to have reached numbers well below the generally recommended number for recovery and there is no current effort to re-establish other populations.

1935: ***-.071***

The widespread occurrence of free-ranging domestic or feral carnivores (dogs, cats) or ungulates (pigs, goats), and massive releases of captive-reproduced game stocks (galliforms, waterfowl) is raising fear that introgressive hybridization with wild populations might disrupt local adaptations,

leading to population decline and loss of biodiversity. Detecting introgression through hybridization is problematic if the parental populations cannot be sampled (unlike in classical stable hybrid zones), or if hybridization is sporadic. However, the use of hypervariable DNA markers (microsatellites) and new statistical methods (Bayesian models), have dramatically improved the assessment of cryptic population structure, admixture analyses and individual assignment testing. In this paper, I summarize results of projects aimed to identify occurrence and extent of introgressive hybridization in European populations of wolves (Canis lupus), wildcats (Felis silvestris), rock partridges and red-legged partridges (Alectoris graeca and Alectoris rufa), using genetic methods. Results indicate that introgressive hybridization can be locally pervasive, and that conservation plans should be implemented to preserve the integrity of the gene pools of wild populations. Population genetic methods can be fruitfully used to identify introgressed individuals and hybridizing populations, providing data which allow evaluating risks of outbreeding depression. The diffusion in the wild of invasive feral animals, and massive restocking with captive-reproduced game species, should be carefully controlled to avoid loss of genetic diversity and disruption of local adaptations.

1936: *+.005*

The recovery of the grey wolf in Yellowstone National Park is an outstanding example of a successful reintroduction. A general question concerning reintroduction is the degree to which genetic variation has been preserved and the specific behavioural mechanisms that enhance the preservation of genetic diversity and reduce inbreeding. We have analysed 200 Yellowstone wolves, including all 31 founders, for variation in 26 microsatellite loci over the 10-year reintroduction period (1995-2004). The population maintained high levels of variation (1995 H(0) = 0.69; 2004 H(0) = 0.73) with low levels of inbreeding (1995 F(IS) = -0.063; 2004 F(IS) = -0.051) and throughout, the population expanded rapidly (N(1995) = 21; N(2004) = 169). Pedigree-based effective population size ratios did not vary appreciably over the duration of population expansion (1995 N(e)/N(g) = 0.29; 2000 N(e)/N(g) = 0.26; 2004 N(e)/N(g) = 0.33). We estimated kinship and found only two of 30 natural breeding pairs showed evidence of being related (average r = -0.026, SE = 0.03). We reconstructed the genealogy of 200 wolves based on genetic and field data and discovered that they avoid inbreeding through a wide variety of behavioural mechanisms including absolute avoidance of breeding with related pack members, male-biased dispersal to packs where they breed with nonrelatives, and female-biased subordinate breeding. We documented a greater diversity of such population assembly patterns in Yellowstone than previously observed in any other natural wolf population. Inbreeding avoidance is nearly absolute despite the high probability of within-pack inbreeding opportunities and extensive interpack kinship ties between adjacent packs. Simulations showed that the Yellowstone population has levels of genetic variation similar to that of a population managed for high variation and low inbreeding, and greater than that expected for random breeding within packs or across the entire breeding pool. Although short-term losses in variation seem minimal, future projections of the population at carrying capacity suggest significant inbreeding depression will occur without connectivity and migratory exchange with other populations.

1937: *-.015*

A considerable fraction of the world's biodiversity is of recent evolutionary origin and has evolved as a by-product of, and is maintained by, divergent adaptation in heterogeneous environments. Conservationists have paid attention to genetic homogenization caused by human-induced translocations (e.g. biological invasions and stocking), and to the importance of environmental heterogeneity for the ecological coexistence of species. However, far less attention has been paid

to the consequences of loss of environmental heterogeneity to the genetic coexistence of sympatric species. Our review of empirical observations and our theoretical considerations on the causes and consequences of interspecific hybridization suggest that a loss of environmental heterogeneity causes a loss of biodiversity through increased genetic admixture, effectively reversing speciation. Loss of heterogeneity relaxes divergent selection and removes ecological barriers to gene flow between divergently adapted species, promoting interspecific introgressive hybridization. Since heterogeneity of natural environments is rapidly deteriorating in most biomes, the evolutionary ecology of speciation reversal ought to be fully integrated into conservation biology.

1938: ***+.430***

I conducted a wild turkey (Meleagris gallopavo) research needs survey to determine informational needs of natural resources professionals in Minnesota. The most common information or research need for habitat management included identification of turkey habitat requirements and improved understanding of turkey responses to habitat manipulations. The most common turkey ecology information needs were related to turkeys occurring on the northern edge of their range and included factors such as winter sources of food, mortality factors, depredation, and competition between turkeys and other species. Information needs for harvest management focused primarily on the population/permit setting process. Finally, respondents wanted information on urban turkey issues, and strongly advocated ending the turkey translocation program.

1939: ***+.208***

This meeting, which focuses on Lynx, contains 21 abstracts, all written in English and Serbian, on a variety of topics. Topics include molecular neurobiology, population monitoring of the European ground squirrel, microsatellite variability of European ground squirrel, management of European ground squirrel habitats in lower Austria, burrow entrance angle and grass height influence reintroduction success, the importance of nest material selection in nest insulation quality, and effect of habitat fragmentation in the European ground squirrel. Other topics include range wide phylogeography of European ground squirrel, female-kin structure in a colony of the yellow ground squirrel, and structure and variability of alarm calls.

1940: ***+.249***

The first transfer of European ground squirrels (Spermophilus citellus) in Slovakia was carried out in 1992-1993. Until now, restitution of ground squirrel populations has been successful at two localities (Kuchyna and Biele Vody). The following prerequisites have to be met for Successful restitution: release of a high number of individuals (about 500) during several consecutive years (on average 5 years) in an adequate habitat and continuous monitoring and protection of the ground squirrel population. The individuals used for restitution were captured at airports (M. R. Stefanik Airport in Bratislava and the Kosice Airport). In case of future extinction of the colonies at the airports, captive breeding of ground squirrels is very important for next restitution projects. Therefore captive breeding of the species was established in the Bojnice zoo in 2007. Altogether 15 individuals were brought to the zoo and the first successful reproduction was observed in spring 2008. During several years of captures and observation we found 3 species of Eulipotyphla, 5 species of Rodentia, I species of Lagomorpha and 5 species of Carnivora on a grassy area at the M. R. Stefanik airport in Bratislava. The occurrence of the solitarily living common hamster (Cricetus cricetus) with colonial ground squirrel is remarkable. The coexistence of two similar species of predators (Mustela eversmanni and M. putorius) is also interesting.

1942: ***+.144***

African Protected Area Conservation and Science during the past several years, non-invasive monitoring of steroid metabolites in faeces of elephants has become an increasingly popular technique to generate more information about the causal relationship between hormones and behaviour in both living elephant species. this is important knowledge which can be used to optimise local conservation and wildlife management by finding new strategies for better elephant population management and control. in this context, however, information about an actual involvement of the hypothalamic-pituitary-adrenal axis during assumable stressful events is still limited, especially for wildlife populations. One difficulty in discovering such information is often the lack of reliable data for hormone baseline levels. therefore, the aim of this study was to determine baseline concentrations of faecal glucocorticoid metabolites that could be expected within age classes and between seasons in African elephants (Loxodonta africana) in the kruger national park (KNP). a total of 374 faecal samples were collected from randomly located family herds in the southern KNP between may 2002 and august 2005. the samples were analysed for immunoreactive concentrations of faecal glucocorticoid metabolites using a validated enzyme immunoassay for 3 alpha,11oxo-cortisol metabolites (3 alpha,11oxo-CM). all samples were grouped according to the estimated age class of the subject using a field method based on bolus diameter, and regarding the ecological season collected. No significant differences in faecal 3 alpha,11oxo-CM concentrations were found across age classes (H-3 = 7.54; p = 0.057), but the mean 3 alpha,11oxo-CM concentration of samples collected in the dry season (n = 196) was significantly higher than in the wet season (n = 178) (u = 15206.50; p = 0.032), which indicates a possible physiological stress situation due to a decline in food quantity and quality. the information generated in this study represents a reliable data set for baseline concentrations of faecal glucocorticoid metabolites for elephants within the KNP and can be used to measure the stress-related effects of translocations, management actions and the impact of chosen land use activities.

1943: ***+.041***

Southern British Columbia, Canada, is the northwestern range limit of the American badger (Taxidea taxus) and supports a nationally endangered subspecies. We initially investigated space-use, diet, and demography in southeastern British Columbia to characterize range-limit ecology. Resident badgers in the northern part (NP) of our study area were extirpated or nearly so during our study (lambda = 0.7), whereas the southern (SP) badger population remained viable (lambda = 1.2). This apparent difference in viability between NP and SP may have been confounded by timing because research occurred later in SP; litter size, number of Columbian ground squirrels (Spermophilus columbianus) consumed, and home range size were each correlated nearly equally to latitude and date of research, and survivorship was greater later in the study for both the NP and SP. Collectively, these factors indicated temporal, not just spatial, variability. Therefore, we translocated badgers into the NP to 1) determine whether the NP had lost its capacity to support badgers or had merely experienced the variability expected at a range limit and, if the latter, 2) initiate recovery. Translocated animals and their offspring had kit production equivalent to that of SP residents, adult survivorship intermediate between the NP and SP residents, and no confirmed kit mortality, with population growth projected (lambda = 1.3). Diet of translocated individuals was similar to that of residents. Home ranges of translocated females were intermediate between the 2 resident groups, and home ranges of translocated males were not different than either resident group. juvenile dispersal dates and distances were similar to those of residents for each sex. Our results were consistent with the extirpation of the NP being driven by temporally variable conditions or the effect of random events expected at range limits. The extirpation of NP did not

appear to have been primarily due to any permanent loss of the NP's capacity to support badgers. At 3.5 years after starting translocations, badgers remained in the NP within an apparently growing population. We found translocation to be a useful diagnostic and conservation tool for badgers at their northern limit. Its utility may extend to countering the fluctuations typical of other rare, range-limit species.

1944: *+.004*

Disease can threaten the restoration of endangered species directly by substantially decreasing host survival or indirectly via incremental decreases in survival and reproduction. During a biomedical survey of reintroduced populations of the highly endangered black-footed ferret from 2002 to 2005, microfilariae discovered in the blood were putatively identified as Dirofilaria immitis, and widespread screening was initiated using a commercially available antigen-based ELISA test. A subset of animals (n = 16) was screened for D. immitis using a highly sensitive PCR-based assay. Microfilariae were also molecularly and morphologically characterized. Of 198 animals at six reintroduction sites, 12% bad positive results using the ELISA test. No antigen-positive animals which were screened via PCR (n=11) had positive PCR results, and all antigen-positive animals (n=24) were asymptomatic. No significant differences were found in body mass of antigen-positive (male: 1223 +/- 82 g [mean +/- SD], female: 726 +/- 75 g) vs. antigen-negative (male: 1,198 +/- 19 g, female: 710 +/- 53 g) individuals (P=0.4). Antigen prevalence was lower in juveniles (3%) than adults (12%; P=0.03), and higher in in situ, captive-reared individuals (33%) than wild-born individuals (10%; P=0.005). Morphologic analysis of microfilariae revealed they were neither D. immitis nor any other previously characterized North American species. PC R amplification of die 5S spacer region of rDNA revealed that the filarial sequence shared only 76% identity with D. immitis. This previously unidentified filarial sequence was present in all antigen positive animals (11 of 11 tested). It appears that black-footed ferrets were infected with a previously undescribed species of filaria whose antigen cross-reacted with the ELISA assay, although further analysis is needed to make a conclusive statement. Nonetheless, this previously undescribed filaria does not appear to threaten recovery for this highly endangered mammal.

1945: *+.297*

Complex interactions between an individual's genotype and its environment determine characteristics such as body size. However, gene-environment interactions should not be seen as being restricted to individual ontogeny: the diversity of the local gene pool can be greatly influenced by habitat variables and population history (e.g., landscape connectivity and propagule size). In this paper I use a model species, the bushcricket Metrioptera roeseli, and data from long-term experimental population introductions to examine individual body size as an indicator of the constraints placed on the gene pool by ecological variables following colonization of new environments. These broad-scale population-environment interactions are useful in understanding species ecology, species invasions and in managing successful reintroductions in conservation biology.

1946: *+.128*

Habitat modification, pollution, overfishing, poaching, competition from non-indigenous species, and diseases have led to the extinction in Europe of many populations of indigenous crayfish. Under the rationale that any programme of reintroduction should be preceded by a thorough understanding of habitat requirements of the species of concern, we studied the microhabitat use of an Austropotamobius pallipes population in Tuscany, central Italy. Microhabitat use was

assessed for water depth, current velocity, substrate, percentages of boulders, underwater tree roots, and in-stream vegetation cover. Results show that A. pallipes' habitat use is size-partitioned. Smaller individuals mostly occupy stream edges in shallow waters with submerged roots, whereas larger individuals use deeper waters, often associated with boulders. Crayfish spatial distribution is restricted mostly to the microhabitats characterised by extensive cover and slow current velocity. The study highlights the importance of habitat heterogeneity and cover elements for the protection of this indigenous species.

1947: ***+.184***

Prior to 2004, some biologists claimed river otters (Lontra canadensis) were extirpated in New Mexico, USA. In November 2004, the first physical evidence of the river otter in New Mexico in over 50 years was found between Grassy and Albino Canyons on the Los Pinos branch of Navajo Reservoir in San Juan County. With the observation of river otters and their scats, tracks, and nesting site or den (nestled in a rocky escarpment and overtaken from beavers) in La Jara Canyon in the summer of 2007, the known range of this species in New Mexico is extended to a second county (Rio Arriba) and a second river (San Juan) in the state. We also provide evidence for a persistent population of the species occurring in multiple localities and dates in the early 21st century. With previous sightings occurring prior to translocations with other river otter subspecies, we raise the question if the recent river otter observations belong to those of the exotic translocated subspecies, the native L. c. sonora, or a hybrid between the two. Other commensal wetland and animals and plants living on or among rocks are listed. Management recommendations are provided to protect this population from non-target otter trapping focused on beaver removal.

1948: ***+.090***

The mollusc Patella ferruginea, endemic to the Mediterranean, is the most endangered marine species of the list of the European Council Directive 92/43/EEC and it is presently under serious risk of extinction. Survival, growth rates and life-history of this species were studied for the first time in this species. A total of 570 specimens (420 introduced in a new habitat and 150 as control) were marked and monitored over a three-year period. Growth rates observed were mainly related to the availability of microalgal food. The mortality rate of transplanted specimens was high (50% mortality immediately after transplant). Seasonality in growth rates was observed in both control and transplanted specimens, with greater growth rates detected in spring-summer (warm season) than in autumn-winter (cold season). Smaller specimens of P. ferruginea had the greatest growth rates in comparison with the bigger specimens, therefore the potential ability to adapt in a new habitat was higher for small specimens immediately after removal. An elevated growth rate (appearing as a light-ring in the border of the shell) was detected immediately after translocation, following which growth rate progressively stabilized over time. Using differential equations and the von Bertalanffy model, the longevity of P. ferruginea was estimated to range between 8.89 and 35.72 years depending on the environment. Transplantation should not be considered as a conservation measure given the elevated mortality rate.

1949: ***+.055***

Populations of at-risk butterfly species are declining at an alarming rate. Conservation strategies emphasize a mix of restoration of butterfly habitat, captive propagation, and reintroduction of butterflies to repopulate sites at which populations have gone extinct and to augment declining populations. We review the use of these strategies to conserve butterflies for 25 British species

with Species Action Plans and 25 American species listed as Endangered, Threatened, or Candidate under the US Endangered Species Act and found in the continental US. Based on a broad review of published and unpublished literature and 47 interviews with agency staff, we find that the majority of species require active restoration (n = 47 of 50) and that most species receive restoration enhancements (n = 45), but only for a few species are ecological responses to this management monitored (n = 15). In addition, we find that most conservation strategies recommend reintroduction (n = 34) and it has been attempted for 21 British species but for only 5 American ones. Captive propagation is recommended for 12 of 25 American species and has been attempted for 8. Documentation of both reintroduction and captive propagation is limited, with the number of founders known forjust over half of the species. We conclude that advancing butterfly conservation will require systematic recording and communication of activities in readily accessible venues, improved experimental design and monitoring, enhanced use of ecological modeling, and improved knowledge of species-specific biology. Project designs that connect on-the-ground efforts to ecological responses of at-risk butterfly species would have tremendous impacts on our ability to use scarce resources to recover these species.

1950: ***+.341***

The Iberian Lynx Conservation Breeding Program follows a multidisciplinary approach, integrated within the National Strategy for the Conservation of the Iberian lynx, which is carried out in cooperation with national regional and international institutions. The main goals of the ex situ conservation programme are to: (1) maintain a genetically and demographically managed captive population; (2) create new Iberian lynx Lynx pardinus free-ranging populations through re-introduction. To achieve the first goal, the Conservation Breeding Program aims to maintain 85% of the genetic diversity presently found in the wild for the next 30 years. This requires developing and maintaining 60-70 Iberian lynx as breeding stock. Growth projections indicate that the ex situ programme should achieve such a population target by the year 2010. Once this goal is reached, re-introduction efforts could begin. Thus, current ex situ efforts focus on producing psychologically and physically sound captive-born individuals. To achieve this goal, we use management and research techniques that rely on multidisciplinary input and knowledge generated on species' life history, behaviour, nutrition, veterinary and health aspects, genetics, reproductive physiology, endocrinology and ecology. Particularly important is adapting our husbandry schemes based on research data to promote natural behaviours in captivity (hunting, territoriality, social interactions) and a stress-free environment that is conducive to natural reproduction.

1951: ***+.092***

Perth Zoo released a sub-adult female Sumatran orang-utan Pongo abelii in November 2006 into the protected Bukit Tigapuluh National Park in Jambi, Sumatra, where the Sumatran Orang-utan Conservation Programme (SOCP) is trying to re-establish an orang-utan population. This was the first release of a captive-born orang-utan into the wild. Temara is being closely tracked and monitored by Perth Zoo and SOCP staff. The aim is that she will be followed for at least 2 years. This paper describes: the pre-release preparation, the release process, the benefits of the programme, the monitoring process and the post-release results.

1952: ***+.259***

The effectiveness of flagship species as a conservation tool is controversial, and amphibians are not usually regarded as meeting the strategic criteria that flagships demand. Capitalizing on the historical, cultural and economic importance of the Axolotl Ambystoma mexicanum at Lake

Xochimilco, Mexico, a conservation programme for this species and its habitat was developed using the Axolotl as a flagship. The threats to the lake are complex and stem from the unsustainable use of its resources. The needs and livelihoods of local stakeholders must therefore be taken into account before attempting to address the threats. The programme therefore focused on developing nature tourism by training local boatmen (remeros) in environmental interpretation. Surveys showed that the boatmen increased their incomes and job satisfaction after training, and that the environmental interpretation programme improved relevant knowledge and awareness of visitors. Although ongoing threats mean that reintroduction of captive-bred Axolotls is not appropriate, zoos with captive populations of Axolotls supported the programme regionally and internationally by providing publicity, funds, staff expertise, training support and themed educational activities. By raising both funds and awareness for the wider conservation of Lake Xochimilco, the Axolotl is probably the first amphibian flagship to be launched successfully.

1953: *+.084*

This 366-page book is part of a series on ecology of saprotrophic basidiomycetes. The book is organized into three sections, which are further divided into seventeen individually authored chapters. The first chapter deals with mycelial networks-structure and dynamics. The second chapter deals with enzymes of saprotrophic basidiomycetes. The third chapter deals with mycelial networks-nutrient uptake, translocation and role in ecosystems. The remaining chapters deals with fruit bodies-their production and development in relation to environment, population biology of forest decomposer basidiomycetes, interactions between saprotrophic basidiomycetes and bacteria, interactions between basidiomycota and invertebrates, distribution and function of litter basidiomycetes in coniferous forests, distribution and role of mat- forming saprobic basidiomycetes in a tropical forest, basidiomycete community development in temperate angiosperm wood, distribution patterns of wood-decay basidiomycetes at the landscape to global scale, saprotrophic basidiomycetes in grasslands-distribution and function, ecology of marine and freshwater basidiomycetes, and conservation-selection criteria and approaches. The book highlights a list of contributors and their respective institutions. Each chapter contains a list of references. The text is written in English and indexed by subject with tables, and figures. Users of this book will include mycologists, biochemists, and ecologists.

1954: *+.176*

CURRENT SPECIES STATUS The Mojave population of the desert tortoise (Gopherus agassizii) (all tortoises north and west of the Colorado River in Arizona, Utah, Nevada, and California) was listed as Threatened on April 2, 1990. A recovery plan was published in June 1994 together with a supplement identifying proposed Desert Wildlife Management Areas. Critical habitat was also designated in 1994 in all four states supporting the species. Based on information in this recovery plan, the recovery priority number is classified as 6C and is predicated upon a) a high degree of threat, which has increased since 1994; b) a low potential for recovery, based on current uncertainties about various threats and our ability to manage them; c) listed population below the species level; and d) potential conflict with development or other forms of economic activity (USFWS 1983). We anticipate that implementation of this revised recovery plan will resolve key uncertainties about threats and management, thereby improving recovery potential. HABITAT REQUIREMENTS AND LIMITING FACTORS Desert tortoises occupy a variety of habitats from flats and slopes dominated by Larrea tridentata (creosote bush) scrub at lower elevations to rocky slopes in Coleogyne ramosissima (blackbrush) and Juniperus spp. (juniper) woodland ecotones at higher elevations. Desert tortoises occur from below sea level to an elevation of 2,225 meters (7,300 feet). Throughout most of the Mojave Desert, tortoises occur most commonly on gently

sloping terrain with sandy-gravel soils and where there is sparse cover of low-growing shrubs, which allows establishment of herbaceous plants. Soils must be friable enough for digging of burrows, but firm enough so that burrows do not collapse. Typical habitat for the desert tortoise in the Mojave Desert has been characterized as Larrea tridentata scrub where precipitation ranges from 5 to 20 centimeters (2 to 8 inches), the diversity of perennial plants is relatively high, and production of ephemerals is high. The vast majority of threats to the desert tortoise or its habitat are associated with human land uses. The threats identified in the 1994 Recovery Plan, and that formed the basis for listing the tortoise as a threatened species, continue to affect the species. However, despite clear demonstration that these threats impact individual tortoises, there are few data available to evaluate or quantify the effects of threats on desert tortoise populations. While current research results can lead to predictions about how local tortoise abundance should be affected by the presence of threats, quantitative estimates of the magnitude of these threats, or of their relative importance, have not yet been developed. Thus, a particular threat or subset of threats with discernable solutions that could be targeted to the exclusion of other threats has not been identified for the desert tortoise. In this revised recovery plan, we underscore the need to build on our understanding of individual threats, yet place new emphasis on understanding their multiple and synergistic effects due to the failure of simple threat models to inform us about tortoise abundance. The desert tortoise requires 13-20 years to reach sexual maturity, has low reproductive rates during a long period of reproductive potential, and individuals experience relatively high mortality early in life. These factors make recovery of the species difficult. Even moderate downward fluctuations in adult survival rates can result in rapid population declines. Thus, high survivorship of adult desert tortoises is critical to the species' persistence, and the slow growth rate of populations can leave them susceptible to extirpation events in areas where adult survivorship has been reduced. Another factor integral to desert tortoise recovery is maintaining the genetic and ecological variability within and among populations to allow tortoises to adapt to changes in the environment over time. Because desert tortoises occupy large home ranges, the long-term persistence of extensive, unfragmented habitats is essential for the survival of the species. The loss or degradation of these habitats to urbanization, habitat conversion from frequent wildfire, or other landscape-modifying activities place the desert tortoise at increased risk of extirpation. RECOVERY STRATEGY The 1994 Recovery Plan described a strategy for recovering the desert tortoise, which included the identification of six recovery units, recommendations for a system of Desert Wildlife Management Areas within the recovery units, and development and implementation of specific recovery actions. Maintaining high survivorship of adult desert tortoise was identified as the key factor in recovery. We recognize that the most significant challenge in the implementation of the 1994 Recovery Plan was not the number or types of actions implemented, but rather the coordination, description, documentation and evaluation of implementation of the actions. As a result, the revised strategy emphasizes partnerships to direct and maintain focus on implementing recovery actions and a system to track implementation and effectiveness of recovery actions. Strategic elements within a multi-faceted approach designed to improve the 1994 Recovery Plan are: 1. Develop, support, and build partnerships to facilitate recovery. 2. Protect existing populations and habitat, instituting habitat restoration where necessary. 3. Augment depleted populations in a strategic manner. 4. Monitor progress toward recovery. 5. Conduct applied research and modeling in support of recovery efforts within a strategic framework. 6. Implement a formal adaptive management program. The Desert Tortoise Management Oversight Group will be the partnership (Element 1) responsible for providing "executive-level" support and direction for recovery implementation, thus tying the entire program together. Regional Recovery Implementation Teams will include a member of the Desert Tortoise Recovery Office to provide guidance and coordination to land/wildlife managers and stakeholders on the teams, which will be responsible for developing step-down recovery-action plans and implementing those actions on the ground. The adaptive management program (Element 6) provides a formal framework with

which the partnerships can make better, more informed, and more explicit decisions. Through the partnership and adaptive management elements, habitat management (Element 2) and population augmentation (Element 3) actions will be prioritized, implemented, and reported. Aggressive management needs to be applied within existing tortoise conservation areas, as defined herein, or other important areas identified by Recovery Implementation Teams. Monitoring (Element 4) effects of these specific actions, as well as progress toward overall recovery, will again feed into the adaptive management system and inform managers on recovery progress. Finally, applied research and modeling (Element 5) will help us better understand desert tortoise ecology and better define our expectations of management actions. RECOVERY GOALS, OBJECTIVES, AND CRITERIA The goals of the recovery plan are recovery and delisting of the desert tortoise. The recovery criteria represent our best assessment of the conditions that would most likely result in a determination that delisting of the desert tortoise is warranted. Recovery criteria should ideally include the management or elimination of threats, addressing the five statutory (de-)listing factors. However, even though a wide range of threats affect desert tortoises and their habitat, very little is known about their demographic impacts on tortoise populations or the relative contributions each threat makes to tortoise mortality. Therefore, specific and meaningful threats-based recovery criteria cannot be identified at this time. In the meantime, we assume that threat mitigation will have been successful if the current recovery criteria have been met (taking into consideration any head-starting or translocation efforts). Specific recovery actions, including research, must be implemented to identify sets of threats that contribute to a greater number of mortality mechanisms or affect size structure or fecundity. As quantitative information on threats and tortoise mortality is obtained, more specific threats-based recovery criteria may be defined during future recovery plan review and revision. Recovery Objective 1 (Demography). Maintain self-sustaining populations of desert tortoises within each recovery unit into the future. Recovery Criterion 1. Rates of population change ([lambda]) for desert tortoises are increasing (i.e., [lambda]>1) over 25 years (a single tortoise generation), as measured a) by extensive, range-wide monitoring across tortoise conservation areas within each recovery unit, and b) by direct monitoring and estimation of vital rates (recruitment, survival) from demographic study areas within each recovery unit. Recovery Objective 2 (Distribution). Maintain well-distributed populations of desert tortoises throughout each recovery unit. Recovery Criterion 2. Distribution of desert tortoises throughout each tortoise conservation area is increasing over 25 years (i.e.,[psi] > 0). Recovery Objective 3 (Habitat). Ensure that habitat within each recovery unit is protected and managed to support long-term viability of desert tortoise populations. Recovery Criterion 3. The quantity of desert tortoise habitat within all desert tortoise conservation areas is maintained with no net loss until tortoise population viability is ensured. When parameters relating habitat quality to tortoise populations are defined and a mechanism to track these parameters established, the condition of desert tortoise habitat should also be demonstrably improving. RECOVERY ACTIONS The recovery actions for each strategic element are as follows: 1. Develop, Support, and Build Partnerships to Facilitate Recovery 1.1. Establish regional, inter-organizational Recovery Implementation Teams to prioritize and coordinate implementation of recovery actions. 2. Protect Existing Populations and Habitat 2.1. Protect intact desert tortoise habitat. 2.2. Reduce factors contributing to disease (particularly upper respiratory tract disease). 2.3. Establish/continue environmental education programs. 2.4. Increase law enforcement. 2.5. Restrict, designate, close, and fence roads. 2.6. Restore desert tortoise habitat. 2.7. Install and maintain urban or other barriers. 2.8. Sign and fence boundaries of sensitive or impacted areas. 2.9. Secure lands/habitat for conservation. 2.10. Restrict off-highway vehicle events within desert tortoise habitat. 2.11. Connect functional habitat. 2.12. Limit mining and minimize its effects. 2.13. Limit landfills and their effects. 2.14. Reduce excessive predation on tortoises. 2.15. Minimize impacts from horses and burros. 2.16. Minimize livestock grazing. 3. Augment Depleted Populations through a Strategic Program 3.1. Develop protocols and guidelines for the population augmentation

program, including those specific to head-starting and translocation. 3.2. Identify sites at which to implement population augmentation efforts. 3.3. Secure facilities and obtain tortoises for use in augmentation efforts. 3.4. Implement translocations in target areas to augment populations using a scientifically rigorous, research-based approach. 4. Monitor Progress toward Recovery 4.1. Monitor desert tortoise population growth. 4.2. Monitor the extent of tortoise distribution in each recovery unit. 4.3. Track changes in the quantity and quality of desert tortoise habitat. 4.4. Quantify the presence and intensity of threats to the desert tortoise across the landscape. 5. Conduct applied research and modeling in support of recovery efforts within a strategic framework 5.1. Characterize stable age distributions of stable or increasing populations. 5.2. Determine factors that influence the distribution of desert tortoises. 5.3. Conduct research on the restoration of desert tortoise habitat. 5.4. Improve models of threats, threat mitigation, and desert tortoise demographics. 5.5. Conduct research on desert tortoise diseases and their effects on tortoise populations. 5.6. Resolve population structure of the desert tortoise across its range. 6. Implement an Adaptive Management Program 6.1. Revise and continue development of a recovery decision support system. 6.2. Develop/revise recovery action plans. 6.3. Amend land use plans, habitat management plans, and other plans as needed to implement recovery actions. 6.4. Incorporate scientific advice for recovery through the Science Advisory Committee. TOTAL ESTIMATED COST OF RECOVERY $159,000,000 plus additional costs that cannot be estimated at this time. DATE OF RECOVERY If recovery actions are implemented promptly and are effective, including continued implementation of the current monitoring program, recovery criteria could be met by approximately 2025.

1955: *+.162*

Global biogeography and phylogeography have gained importance as research topics in zoology, as attested by the steady increase in the number of journals devoted to this topic and the number of papers published. Yet, in a globalising world, with species reintroductions, invasions of alien species, and large-scale extinctions, unravelling the true biogeographic relationships between areas and species may become increasingly difficult. We present an introduction to the symposium 'Biogeography: explaining and predicting species distributions in space and time' held in Amsterdam in 2007, and the resulting papers as published in this special issue, including papers on crustaceans, birds and mammals.

1956: *+.248*

This 354-page book, which contains 17 chapters divided into 4 major sections, belongs to the series Developments in Primatology: Progress and Prospects, and discusses conservation in the 21st century taking gorillas as a case study. Section 1 of the book contains 1 chapter that emphasises on current status of wild gorilla populations and strategies for their conservation. Section 2 contains 6 chapters that elucidate about conservation medicine for gorilla conservation, sanctuaries and reintroduction, responsible tourism, chimpanzee conservation and theatre, the value of long-term research taking mountain gorilla as a case study, and the art and zen of camera trapping. Section 3 contains 5 chapters that discuss an experiment in managing the human animal, approaches to corridor planning, linking the Community Options Analysis and Investment Toolkit (COAIT), an integrates goematics research programme for mountain gorilla behavior and conservation, and biomaterials in gorilla research. Section 4 contains 5 chapters that emphasise on transboundary conservation in the Virunga-Bwindi Region, the Great Ape World Heritage Species Project, conservation through scientific collaboration, zoos and conservation, and the Bushmeat Crisis Task Force. The book highlights a list of contributors and their respective institutions. The text of the book is written in English. Users of the book will include conservationists and

terrestrial ecologists.

1957: ***+.134***

The European pond turtle (Emys orbicularis) is threatened and in decline in several regions of its natural range, due to habitat loss combined with population fragmentation. In this work, we have focused our efforts on studying the genetic diversity and structure of Iberian populations with a fine-scale sampling (254 turtles in 10 populations) and a representation from North Africa and Balearic island populations. Using both nuclear and mitochondrial markers (seven microsatellites, similar to 1048 bp nDNA and similar to 1500 bp mtDNA) we have carried out phylogenetic and demographic analyses. Our results show low values of genetic diversity at the mitochondrial level although our microsatellite dataset revealed relatively high levels of genetic variability with a latitudinal genetic trend decreasing from southern to northern populations. A moderate degree of genetic differentiation was estimated for Iberian populations (genetic distances, F-ST values and clusters in the Bayesian analysis). The results in this study combining mtDNA and nDNA, provide the most comprehensive population genetic data for E. orbicularis in the Iberian Peninsula. Our results suggest that Iberian populations within the Iberian-Moroccan lineage should be considered as a single subspecies with five management units, and emphasize the importance of habitat management rather than population reinforcement (i.e. captive breeding and reintroduction) in this long-lived species.

1958: ***+.054***

The Azorean bat (Nyctalus azoreum) is endemic to the Azores archipelago and is listed as endangered due to its reduced and fragmented distribution range. We assessed genetic diversity at eight microsatellite loci in 280 individuals from 14 locations throughout six islands. Overall, we found that the Azorean bat populations are not genetically impoverished. Indeed, the number of alleles per locus ranged from 8 to 10 and the observed heterozygosity ranged from 0.77 in Terceira to 0.83 in Faial. The highest genetic diversity and level of private alleles was observed in S. Miguel, the largest island, and the closest to the mainland. Private alleles occurred at all islands except in Graciosa. Global and pairwise F-ST among islands were all statistically significant, suggesting restricted gene flow. These results, together with those of factorial correspondence analysis, Bayesian clustering method, and individual assignment tests, corroborate the conclusions of a previous mtDNA based study, providing strong support for the existence of two major subpopulations: one includes all islands of the Central Group and the other corresponds to S. Miguel. Gene flow between them is very limited, suggesting that management plans should avoid translocations between these subpopulations.

1959: ***-.013***

Population augmentation with translocated individuals has been shown to alleviate the effects of bottlenecks and drift. The first step to determine whether restoration for genetic considerations is warranted is to genetically monitor reintroduced populations and compare results to those from the source. To assess the need for genetic restoration, we evaluated genetic diversity and structure of reintroduced (n = 3) and captive populations of the endangered black-footed ferret (Mustela nigripes). We measured genotypic changes among populations using seven microsatellite markers and compared phenotypic changes with eight morphometric characters. Results indicated that for the population which rapidly grew post-reintroduction, genetic diversity was equivalent to the captive, source population. When growth languished, only the population that was augmented yearly maintained diversity. Without augmentation, allelic diversity declined precipitously and

phenotypic changes were apparent. Ferrets from the genetically depaupertate population had smaller limbs and smaller overall body size than ferrets from the two populations with greater diversity. Population divergence (F(ST) = 0.10 +/- 0.01) was surprisingly high given the common source of populations. Thus, it appears that 5-10 years of isolation resulted in both genotypic divergence and phenotypic changes to populations. We recommend translocation of 30-40 captive individuals per annum to reintroduction sites which have not become established quickly. This approach will maximize the retention of genetic diversity, yet maintain the beneficial effects of local adaptation without being swamped by immigration.

1960: *+.083*

It is generally considered that limiting the loss of genetic diversity in reintroduced populations is essential to optimize the chances of success of population restoration. Indeed, to counter founder effect in a reintroduced population we should maximize the genetic variability within the founding group but also take into account networks of natural populations in the choice of the reintroduction area. However, assessment of relevant reintroduction strategies requires long-term post-release genetic monitoring. In this study, we analyzed genetic data from a network of native and reintroduced Griffon vulture (Gypsfulvus) populations successfully restored in Southern Europe. Using microsatellite markers, we characterized the level of genetic diversity and degree of genetic structure within and among three native colonies, four captive founding groups and one long-term monitored reintroduced population. We also used Bayesian assignment analysis to examine recent genetic connections between the reintroduced population and the other populations. We aimed to assess the level of fragmentation among native populations, the effectiveness of random choice of founders to retain genetic variability of the species, the loss of genetic diversity in the reintroduced population and the effect of gene flow on this founder effect. Our results indicate that genetic diversity was similar in all populations but we detected signs of recent isolation for one native population. The reintroduced population showed a high immigration rate that limited loss of genetic diversity. Genetic investigations performed in native populations and post-released genetic monitoring have direct implications for founder choice and release design.

1961: *+.009*

The originally diverse ciscoe fish fauna of the Laurentian Great Lakes has suffered many extinctions and local extirpations. Bloaters (Coregonus hoyi) are presumed extirpated from Lake Ontario and the reintroduction of this deepwater fish is under consideration. Given the demographic fluctuations of this species in the other Great Lakes and its recent intralacustrine origin, we sought to identify a genetically diverse and similar source of C hoyi via an analysis of genetic diversity and population structure using 10 microsatellite loci. Despite well-documented demographic declines, we found no genetic evidence of bottlenecks in 12 C hoyi samples from the four potential donor lakes (Huron, Michigan, Superior and Nipigon). By contrast, evidence of bottlenecks in historical samples of C. artedi from Lake Ontario suggested that standard genetic methods frequently used to identify population bottlenecks can only detect very severe and long-lasting demographic declines in naturally large populations. Patterns of genetic differentiation and assignment tests indicated that C hoyi from Lake Huron and Lake Michigan, which are not differentiated, are genetically most similar to Lake Ontario ciscoes. The small available sample of deepwater ciscoes recently caught in Lake Ontario did not allow determining if these represent a small undetected C hoyi population or a recent invasion of the deep section by C artedi. On the basis of genetic criteria, we conclude that C hoyi from any location within Lake Huron or Lake Michigan would be judicious sources of breeders for reintroducing C. hoyi in Lake Ontario.

1962: *+.067*

Between 2002 and 2004, populations of the Common Hamster (Cricetus cricetus) were translocated on three different sites in Lower Saxony (Germany). To date, little is known about the success of translocation measures. Hamster mobility has to be considered in choosing the location of compensation areas. In this study, the capture-mark-recapture method yielded information on the remigration capability of Common Hamsters. In total, 72 adult hamsters (3 1 males and 41 females) were captured, individually marked and transported to their new habitats, the compensation areas. These sites were directly connected to the origin habitats without any barriers. The rate of remigrated animals was 25 % and the frequency was up to 4 times per individual. Male hamsters remigrated a maximum of 460 m, females 260 m. The high tendency towards remigration can be avoided by locating the compensation area far away from the hamsters' familiar landmarks. A univariate regression model predicted that the probability of remigration approximates 0 after 700 in. To minimise the mortality of translocated hamsters, disruptive influences (e.g. roads) should be also located beyond this range. To support population preservation, compensation areas have to be managed in a hamster-friendly manner and must be suitable for the Common Hamster.

1963: *+.157*

The Common Hamster (Cricetus cricetus) population in Alsace, France is severely threatened and declining. The protection of these populations, living in the extreme western part of their distribution range, has become an issue of prime importance for the conservation of the species in Europe. In France, the species acquired the status of a protected species in 1993, and as such became the subject of a first conservation program in 2000-2004. It was implemented by the Office national de la chasse et de la faune sauvage at the request of the Ministry of Ecology and Sustainable Development. The success of the actions that had been implemented was assessed in 2005. The monitoring studies showed that the conservation plan was unable to stop the population decline. At that time, the species was found in only 62 of the 387 Alsatian villages in which it still was present in the early 20(th) century (WENCEL et al. 2003). Based on these results, it was decided to extend conservation program to the period between 2007 and 2011.Here, the authors present Common Hamster conservation programs in France and describe experiments conducted during the first national conservation program. Proposals for actions in the future are presented, and all partners concerned are encouraged to contribute to their successful implementation.

1964: *+.041*

The Seychelles subspecies of yellow-bellied mud turtle, Pelusios castanoides intergularis (Family Pelomedusidae), is restricted to six islands of the Seychelles group. Four breeding populations remain and the extant wild population was estimated at only 120 adults in 2005. Populations continue to decline due to ongoing marsh drainage and river canalization. Legal protection of wetland habitats is urgently required, and ongoing reintroductions to protected areas need to continue to secure the future of this species.

1965: *+.020*

The Seychelles subspecies of black mud turtle, Pelusios subniger parietalis (Family Pelomedusidae), is restricted to six islands of the Seychelles group. Five breeding populations exist and the wild population was estimated to be about 660 adults in 2005. Populations continue to decline due to ongoing marsh drainage. Legal protection of wetland habitats is urgently required

and ongoing reintroduction to protected areas need to continue to secure the future of this species.

1967: ***+.232***

There has been increasing interest in recent years in reintroducing birds lost from Britain (or parts of it) as a result of human activities. In many respects, reintroduction is an extension of other 'hands-on' approaches to conserving birds, including creation and management of suitable habitats, protection of nests and the provision of food or artificial nest-sites. Carefully considered reintroduction projects can be expensive and take many years to plan, implement and monitor but, in addition to restoring the species in question, may have substantial benefits for a wide range of wildlife. In contrast, poorly planned or inappropriate projects can be detrimental to wildlife. Current legislation is, we believe, in need of revision to ensure that future reintroductions achieve their objectives and are of a high standard in their implementation. Case studies are used that illustrate the achievements of well-organised bird reintroductions in Britain and to highlight: some of the problems that can arise. Finally, we look ahead to the role that reintroductions could play in the future conservation of British birds.

1968: ***+.108***

Knowledge of the habitat requirements of threatened species at both local and landscape scales is crucial for maintaining viable populations and for making conservation and management decisions. Here, we use live trapping and radio-tracking to investigate habitat use by four species of threatened marsupials - burrowing bettongs (Bettongia lesueur), brush-tailed bettongs (B. penicillata), greater bilbies (Macrotis lagotis), and bridled nailtail wallabies (Onychogalea fraenata). The study populations had been re-introduced to Scotia Sanctuary in western New South Wales, Australia, within a predator-proof area. All showed preferences for particular macrohabitats while resting by day, with M. lagotis and B. penicillata selecting Eucalyptus woodland with Triodia understorey and B. lesueur and O. fraenato selecting Eucalyptus woodland with shrubs. However, they showed no such partiality at night. Bettongia penicillata used areas with Triodia and litter but few herbs for shelter, while burrows of M. lagotis avoided shrubs. Habitat components that influenced trap captures were: crust cover and herb layer cover (negative) for B. penicillata, trees <5 in in height and number of shrubs (both negative) for B. lesueur, crust cover for M. lagotis, and crust cover and trees <5 in high for O. fraenata (both negative). There was also a negative association at this scale between B. penicillata and both B. lesueur and M. lagotis, suggesting the possibility of competition. our results support the idea that studies at multiple spatial scales are crucial to understand the habitat use and requirements of threatened fauna, and should therefore be incorporated into future re-introduction programs. (C) 2007 Elsevier Ltd. All rights reserved.

1969: ***+.165***

Populations of the European wildcat (Felis silvestris) are only slowly recovering in Central Europe after a severe decline in the last centuries and require specific conservation plans in many areas. However, detailed information on wildcat occurrence and habitat requirements is still scarce and controversial. We present a fine-scale habitat selection model for wildcats based on detailed species and land use information and evaluate its accuracy to predict habitat distribution in new areas. We analysed habitat use within home ranges using single locations of 12 radio-tracked individuals from south western Germany. Several competing models were fitted and compared using generalised linear mixed models (GLMM) and information-theoretic approaches. Radio-tracking data of 9 and 10 wildcats from two distant areas were used to evaluate the models. The

selected model predicted habitat associated to close distance to forest, watercourses and meadows and a critical distance to villages, single houses and roads. To predict area suitable for home ranges we superimposed rules derived from home range attributes at a higher level of selection. Predictions from the combination of the fine-scale habitat model and home range rules matched well with more than 2000 wildcat observations of southwestern Germany. We discuss the application of the model in wildcat conservation for finding potential reintroduction sites, identifying small isolated populations and aiding in the evaluation of the needs of mitigation and compensation within the scope of the European Habitats Directive. (C) 2007 Elsevier Ltd. All rights reserved.

1970: ***+.230***

The federally threatened Florida Scrub-Jay (Aphelocoma coerulescens) has suffered dramatic losses in recent years primarily because of overdevelopment of its unique scrub habitat. Ensuring the survival of this species will require not only measures to reduce habitat loss, but also efforts to increase the numbers of birds in small scattered populations to a point at which they are resilient to extinction from stochastic population fluctuations. Here we evaluate the utility of providing supplementary food during the pre-breeding season as a means of increasing the reproductive output of Florida Scrub-jays. Data collected from 2000 to 2007 at Archbold Biological Station in south-central Florida indicate a marked effect of supplemental feeding on reproductive output. This increased output appeared to result primarily from larger clutch sizes produced by supplemented female breeders as a result of their advanced laying. Supplementation also increased offspring survival probabilities and the early laying permitted more renesting attempts. Furthermore, annual variation in several reproductive measures was dampened in supplemented birds, suggesting that supplemental feeding may reduce environmental or stochastic effects on reproductive output. Although a number of potential problems may be associated with supplemental feeding (e.g., predator entrainment and increased opportunity for disease transmission), it can serve as a valuable tool for management agencies that wish to rapidly increase local carrying capacity. In addition, food supplementation might benefit translocation efforts both by increasing the number of potential translocation candidates from a donor population and then by supplementing the newly established population to promote rapid growth. (c) 2007 Elsevier Ltd. All rights reserved.

1971: ***+.006***

Reintroduction of extirpated populations creates a unique context that can exacerbate the effects of interactions among species. Thus, reintroduced populations may be particularly vulnerable to predators and competitors, including native species with which they historically coexisted. in this study, we evaluated the effect of native fishes on Survival of reintroduced Atlantic salmon (Salmo salar) in the Connecticut River basin, where the native salmon population is extinct. juvenile salmon are stocked annually in many Connecticut River tributaries. We sampled salmon reintroduction sites across tributaries with different fish communities to determine whether native fish reduce the success of salmon reintroductions (N=19 site-years). Increased density of slimy sculpin (Cottus cognatus), a native generalist predator, was associated with reduced recruitment of reintroduced salmon. Salmon first-summer survival declined with increased sculpin density across sites, and low first-summer survival led to reduced densities of overyearling salmon the subsequent year. Hierarchical partitioning analysis showed that the negative relationship between sculpin and salmon was independent of potentially confounding variation in other fish community or habitat characteristics. Negative effects of native, historically-sympatric species, particularly generalist predators, can impede restoration of extirpated populations. (c) 2007 Elsevier Ltd. All

rights reserved.

1972: *-.045*

In South Africa, a plan was launched to manage separate sub-populations of endangered African wild dogs (Lycaon pictus) in several small, geographically isolated, conservation areas as a single meta-population. This intensive management approach involves the re-introduction of wild dogs into suitable conservation areas and periodic translocations among them. To assess the attitudes towards re-introduced wild dogs, we conducted a questionnaire survey of multiple stakeholders-local community members, private landowners and tourists-in and around Hluhluwe-iMfolozi Park (HiP), one of the meta-population conservation areas. Here, we document conflicting human interests over the re-introduced wild dogs. Tourists in HiP, on the one hand, expressed overwhelmingly positive opinions about wild dogs across personal details of the respondents, but especially after having seen free-ranging wild dogs. On the other hand, we found misconceptions and perceptions that were more negative among the rural population around HiP, again largely independent of personal details of the participants, although educated respondents voiced more favourable views of wild dogs. These negative attitudes were in particular due to perceived and real threats of livestock losses. In a follow-up questionnaire survey, we also discovered apparent shortcomings of a previous short-lived conservation education programme among the local communities adjacent to HiP. Consequently, the mitigation of the conflict between wild dogs and rural people requires an understanding of the conditions under which livestock predation occurs, the encouragement of practices that prevent such predation, and increasing local tolerance of co-existence with wild dogs through both economic and non-monetary incentive schemes as well as continued conservation education.

1973: *+.155*

The beaver population has fortunately increased thanks of protective measures as well as legal protection and through reintroduction projects (partly backed up by research, partly by actionism only). It is questionable whether the currently stock numbers introduced from different areas in Europe - of 15,000 to 20,000 beavers are correct, because different data inventory methods for beaver population give rise to doubt. Today, we can hardly still speak of the symbolic animal of nature conservation" or a "key species for restoration of flood plains". The beaver - on the contrary - develops more and more often to a "political" rodent that will be fewer and fewer tolerated by the community. Different legislations in the individual federal states, law enforcement regulations and different compensatory hamper dealing with the EU law-protected beavers. Beaver habitats disappear continuously and beavers come more often into conflicts with the peoples usage interests, although spacious areas are still available for a natural resettlement in Germany [long dash] a spreading trend is still existent. It is about time to embark on new ways towards beaver management.

1974: *-.058*

In Lake Martin (16,000 ha), Alabama, black bass Micropterus spp. tournaments Occur nearly every weekend at a single site (Wind Creek State Park [WCSP]), which could cause fish to accumulate at this release site and potentially cause negative impacts to the Population. Over a 7-month period, nearly 6,600 tournament-caught largermouth bass M. salmoides and spotted bass H. punctulatus were injected with a coded wire tag at different body locations before release at WCSP After release, black bass were collected,with electrofishing up to 1.5 years following release at: 0-10 km front the release site and scanned for a tag. Although variable, a substantial proportion (10-

70%) of tournament-caught black bass comprised the black bass population within 3 kin of WCSP up to 3 months after release. After 3 months, proportions of tagged black bass within 10 km of WCSP decreased dramatically and by an order of magnitude after 1 year, which strongly suggested that these fish dispersed from WCSP. Over a 2- to 70-d period after release from a tournament, relative weights of tournament-released black bass were typically less than fish not released in tournaments. In addition, we collected and aged black bass throughout Lake Martin; relative weights of both black bass species were less and spotted bass growth was lower within 10 km of WCSP compared to other regions of Lake Martin. Even though black bass dispersed front the WCSP release site, these negative Population effects were attributed to the constant translocation and accumulation of tournament-caught fish in this region of Lake Martin. In water bodies Such as Lake Martin where mass translocation of black bass Occurs annually at a single site, the use of live-release boats to transport tournament-caught black bass and the promotion and use of alternative release sites should be encouraged to reduce possible localized negative population effects.

1975: ***-.023***

The Growling Grass Frog Litoria raniformis has undergone population declines throughout its range over the past 20 years and is listed as a threatened species both in Victoria and nationally. The species was last recorded in 1979 in the area now occupied by the Portland Aluminium Smelter in south-western Victoria, but the reasons for its disappearance from the area are unclear. Approximately 50 wetlands remain in the buffer zone of the smelter and we investigated the suitability of 20 of these wetlands for the reintroduction of the species. We measured a set of habitat parameters at the smelter (Smelter sites) and at five wetlands in south-western Victoria that had recent records of Growling Grass Frogs (Extant sites). Discriminant function analysis identified a number of variables that discriminated between Extant and Smelter sites, particularly the extent of algae and the percentage of shoreline covered by bare ground, by short terrestrial vegetation and by other cover types. We recommend the planting of emergent aquatic vegetation and the encouragement of floating and submerged vegetation to increase habitat suitability for Growling Grass Frogs in the smelter wetlands.

1976: ***-.014***

Reintroduction programs are used widely in conservation to reduce a species' risk of extinction and amphibians are considered suitable candidates for such programs because of their behavioural simplicity and high reproductive output. The Green and Golden Bell Frog Litoria aurea is an endangered species that has been reintroduced into several areas within its natural range, but the outcome of these programs remain unknown. This paper presents the results from the first release of the bell frog in the Hunter Region of New South Wales. This reintroduction released 850 tadpoles into a closed system of three ponds and rehabilitated habitat. Tadpole survival was high but following metamorphosis a decline in numbers began that continued for 13 months and resulted in the disappearance of all released bell frogs. The cause of this decline was investigated and eventually attributed to infection by the Amphibian Chytrid Fungus Batrachochytrium dendrobatidis. These results emphasize the importance of including regular chytrid testing in the monitoring of both natural populations and reintroduction programs, particularly as few sick and dead animals were found to indicate its presence.

1977: ***+.167***

The Green and Golden Bell Frog Litoria aurea is threatened with extinction, but generally occurs

in disturbed sites and has successfully colonized some sites that are essentially artificial. It should therefore be possible to promote recovery of the species by increasing the availability of suitable habitat through habitat modification or creation and, where necessary, translocating individuals into these habitat areas. Apparently suitable habitat for this species had been created at Long Reef Golf Course in the northern Sydney suburb of Collaroy. We translocated approximately 9,000 captive-bred tadpoles from Taronga Zoo into these habitat areas over 7 years. This program has not led to the establishment of a self-sustaining population of the Green and Golden Bell Frog at Long Reef Golf Course and must therefore be considered unsuccessful. It has had partial success as some released tadpoles metamorphosed into frogs, some of these developed into adults, and a few males were recorded calling, However, breeding by these introduced animals has not been recorded, and, in the absence of continuing tadpole releases, the number of bell frogs has declined to zero. It has, however, provided a number of guidelines for future similar programs. Success with this program has been limited by fish, time of tadpole release and water temperature, and hence we recommend that future translocations of Green and Golden Bell Frog tadpoles should be carried out during spring or summer and should target ponds that are warm and fish-free. Program success was also limited by the numbers of tadpoles available for release. The lack of tadpoles for spring/summer release since the 2003/2004 breeding season has prevented evaluation of new, relatively warm ponds. Any captive-breeding program for this frog species must therefore be successful, in its own right, if it is to provide tadpoles for release. Disease is unlikely to have influenced the outcomes of this program, but should always be considered a potentially-important factor.

1978: ***+.126***

Captive bred Green and Golden Bell Frog Litoria aurea tadpoles were introduced to a coastal wetland near Pambula on the far south coast of New South Wales. The reintroduction involved the release of approximately 5000 captive-bred tadpoles and subsequent monitoring. Before the reintroduction could take place several requirements had to be satisfied. A re-introduction proposal was prepared for the Department of Environment and Conservation (DEC) and independently reviewed by two referees, pre-release surveys of frogs were undertaken to determine if L aurea existed on the site and to ascertain if the pathogen Bactrachochytrium dendrobatidis (chytrid) was present within the endemic frog population. The captive bred tadpoles were tested for the presence of chytrid prior to release. The water quality was tested at the proposed release site and potential predators were removed. The project required maintenance and breeding of the captive population, and post release surveys. The project took 4.5 years from submission of the proposal to the first release of tadpoles and to date has cost approximately $190K, of which only $37K was funded. It is anticipated that ongoing costs will be in the order of $25K per year for the next four years (2008-12). Details of the project costs and chronology of actions are given in order to assist others who wish to undertake similar projects. So far two adults have been detected 13 months post-release.

1979: ***-.036***

Translocations involving the endangered Green and Golden Bell Frog Litoria aurea have been under way in the Greater Sydney area since 1993. Case studies for four of these translocations are presented; the translocation sites being at Botany, Marrickville, Long Reef and Arncliffe. Bell frogs have persisted at only one of these sites since their introduction (Arncliffe). The success or failure of each translocation has provided insights into the habitat requirements and management of bell frogs. In unsuccessful translocations, the reasons for the inability to establish a permanent population became more apparent with monitoring; at Botany, young bell frogs failed to survive

the winter because of inadequate or inappropriate over-winter habitat being available; at Long Reef, foraging and breeding habitat were inadequate; at Marrickville, urban predators and disease eliminated frogs. Other factors also appear to have a significant effect on the likely outcome of the translocation. These include: the proximity of a source population, the presence or absence of predatory fish, pond water temperature and the timing of the release of tadpoles at translocation sites. Despite the difficulties and uncertainties associated with habitat creation and the establishment of translocated frogs, translocations remain as a last resort strategy for the conservation of frog populations that may otherwise be lost.

1980: *+.130*

At least four populations of the red-crowned parakeet (Cyanoramphus novaezelandiae) have been established via translocation within New Zealand over the last 40 years, but reproductive parameters of these populations have not been documented. We quantified differences in clutch parameters and reproductive success for a translocated population of this species on Tiritiri Matangi Island over two breeding seasons. Overall clutch parameters and estimates of reproductive success were consistent with reported values from natural populations. However, we found previously unreported differences in clutch size, hatching success and brood size between breeding seasons. The number of fledglings produced per breeding pair increased significantly from 1.4 to 3.4 fledglings during our two-year study. In contrast, egg volume and fertility per clutch did not vary during the same period. Overall, 7 eggs were laid per breeding pair but only 2.22 nestlings fledged, representing a 63.8% loss of initial reproductive potential. Losses during the incubation stage were caused by partial and total hatching failure, whereas starvation of nestlings caused all losses during the brood-rearing stage. Hatching success during our study was lower than that reported for wild populations of this and other parrot species, and remained lower even during the most productive breeding season. We found no cases of predation on eggs or nestlings during our study despite the presence of native and exotic avian predators on Tiritiri Matangi Island. We show that clutch size, brood size and changes in loss between breeding seasons are determinants of reproductive output in translocated red-crowned parakeet and also that reproductive output can vary greatly between breeding seasons. Finally, if reduced hatching success is the result of small founder size, management of parakeets should consider the movement of larger and more genetically diverse flocks.

1981: *+.100*

The two South American ratites (Lesser Rhea, Pterocnemia pennata and Greater Rhea, Rhea americana) are categorised as 'Near Threatened' in the Red List of Threatened Species of the International Union for Conservation of Nature and Natural Resources, and are included in Appendices 1 and II of Convention on International Trade in Endangered Species of Wild Fauna and Flora. The wild populations of rheas are affected by human activities, such as illegal hunting and egg harvesting, and conversion of natural habitats into croplands. On the other hand, commercial farming of rheas has been expanding in the last two decades. Studies on farms have led to improved production, not only for commercialisation, but also for the release of individuals to the wild. Effectiveness of this latter strategy was confirmed by fieldwork and radiotelemetry. Molecular studies revealed that genetic variability of captive populations of Greater Rhea were similar to those of wild ones, and they do not show signs of inbreeding depression or differences in allele frequencies. Consequently, as natural populations of rheas continue to decline, farms will arguably serve as genetic reservoirs and provide a source of individuals for repopulation. Therefore captive breeding should be regarded as having high value as a conservation tool for native ratites.

1982: ***+.130***

Rhizanthella gardneri R. S. Rogers is an entirely subterranean mycoheterotrophic orchid known only from two isolated populations within south-western Western Australia (WA). This rare species appears restricted to habitats dominated by species of the Melaleuca uncinata complex. R. gardneri purportedly forms a tripartite relationship with Melaleuca(1), via a connecting mycorrhizal fungus, for the purpose of carbohydrate and nutrient acquisition. Here, we quantify key climate, soil and vegetation characteristics of known R. gardneri habitats to provide baseline data for monitoring of known R. gardneri populations, to better understand how R. gardneri interacts with its habitat and to identify possible new sites for R. gardneri introduction. We found that the habitats of the two known R. gardneri populations show considerable differences in soil chemistry, Melaleuca structure and Melaleuca productivity. Multivariate analyses showed that both multidimensional scaling (MDS) and principal components analysis (PCA) ordinations of soil chemical characteristics were very similar. Individual sites within populations were relatively similar in all attributes measured, whereas overall northern and southern habitats were distinct from each other. These results suggest that R. gardneri can tolerate a range of conditions and may be more widespread than previously thought, given that there are extensive areas of Melaleuca thickets with similar habitat characteristics across south-western WA. Variability within the habitats of known R. gardneri populations suggests translocation of this species into sites with similar vegetation may be a viable option for the survival of this species.

1983: ***+.139***

Arachnorchis concolor and A. pilotensis are two rare orchid species with contrasting spatial distributions found in south-eastern Australia. A. concolor is known from similar to 220 plants, with the largest population found in southern central Victoria and the remaining smaller populations similar to 100 km north. Some taxonomic uncertainty surrounds the affiliations of these disjunct populations. A. pilotensis is known from similar to 100 plants in a single location near the Beechworth region of north-eastern Victoria. Small populations such as these can show extreme demographic and/or genetic constraints and careful management is required to ensure their long-term persistence. The present study used amplified fragment length polymorphism (AFLP) markers to describe the levels of relatedness among plants from both species and to determine levels of genetic diversity for each species as well as levels of differentiation among A. concolor populations, to assist with species management. Species-level genetic diversity was lower in A. pilotensis (PLP 44%, Hj 0.182) than A. concolor (PLP 58.2%, Hj 0.202). Genetic diversity also varied among A. concolor populations but this does not appear to relate to population size. High levels of inbreeding were evident in A. concolor (f, 0.828) in contrast to moderate levels observed in A. pilotensis (f, 0.466). Genetic relatedness maps, generated by principal coordinates analyses, indicated significant differentiation among A. concolor populations with some substructuring also apparent within A. pilotensis. Management implications for the two species, with respect to sourcing of material for translocation and augmentation of pollination events within populations, are discussed in light of these findings.

1984: ***+.143***

We developed a spatially explicit population-viability-analysis (PVA) model to evaluate different management options for the endangered Florida Grasshopper Sparrow (Ammodramus savannarum floridanus). We also conducted a sensitivity analysis to determine which parameters had the greatest effect on population viability. The model was most sensitive to environmental variation and least sensitive to carrying capacity, initial abundance, and level of correlation in demographic

rates between subpopulations. The model that represented present conditions predicted a 22% chance that the population will fall below the extinction threshold of 60 male Florida Grasshopper Sparrows within 50 years. Reintroduction to large prairie sites and prairie restoration reduced the chance of falling below the extinction threshold and increased the number of subpopulations occupied. Loss of dry prairie located on private land adjacent to existing Florida Grasshopper Sparrow subpopulations increased the probability of falling below the extinction threshold to 66%. We caution that the strength of PVAs is not in predicting absolute values of viability or number of individuals but, rather, in evaluating the relative effects of different management options. We found that increasing the amount of prairie habitat, especially core habitat, had a strong positive effect on viability. If the Florida Grasshopper Sparrow reaches recovery goals developed by the U.S. Fish and Wildlife Service (10 subpopulations with >50 males per site), we estimate that the metapopulation has a 99% probability of remaining above the extinction threshold. Our analyses indicate that the Florida Grasshopper Sparrow is currently vulnerable to extinction, but various managernent practices can increase population viability.

1985: ***+.291***

Western subspecies of the Australian skink Egernia stokesii are considered endangered and translocation to unoccupied areas of suitable habitat has been proposed as a conservation strategy. We investigated the internal structure of artificial refuges that might induce translocated lizards to remain at the site of release. In a laboratory environment, individual lizards were offered choices of alternative structures as refuges. They preferred deeper and narrower refuge structures, with a single entrance rather than two entrances. They showed a slight tendency to avoid PVC structures when plywood or brick paving alternatives were available. Soft sand or bard brick substrate were equally accepted. The results suggest that the use of brick pavers may be a practical management strategy to provide extra refuges for the lizards, but further trials are needed with a greater range of temperatures that are representative of field conditions.

1986: ***+.155***

Gopher tortoises on lands to be developed may be translocated as a conservation measure, sometimes to areas already occupied by the species. We assessed the success of this type of translocation by monitoring the movements, spatial positioning, health, and reproductive activity of translocated and resident individuals at a site in central Florida from 2001 to 2004. By several criteria, the translocation was a success. Most translocated individuals remained on-site for at least one year, home ranges of resident individuals were not significantly different before and after translocation, home ranges of translocated individuals fit within the range of estimates reported in the literature, and neither body condition nor reproduction of either group of individuals could be shown to be affected by the translocation. On the other hand, several resident individuals altered their habitat use after translocation and the spatial positioning of resident individuals was different than that of individuals throughout; so, some potential exists for future off-site movements. The study illustrates two practical problems in assessing translocation success: lack of adequate pre-translocation data for both resident and translocated individuals, which interferes with documentation of translocation effects, and the necessarily small sample sizes, which reduces statistical power.

1987: ***+.068***

Castor fiber Linnaeus, 1758 is the only indigenous species of the genus Castor in Europe and Asia. Due to extensive hunting until the beginning of the 20th century, the distribution of the formerly

widespread Eurasian beaver was dramatically reduced. Only a few populations remained and these were in isolated locations, such as the region of the German Elbe River. The loss of genetic diversity in small or captive populations through genetic drift and inbreeding is a severe conservation problem. However, the reintroduction of beaver populations from several regions in Europe has shown high viability and populations today are growing fast. In the present study we analysed the population genetic structure of a natural and two reintroduced beaver populations in Germany and Austria. Furthermore, we studied the genetic differentiation between two beaver species, C. fiber and the American beaver (C. canadensis), using RAPID (Random Amplified Polymorphic DNA) as a genetic marker. The reintroduced beaver populations of different origins and the autochthonous population of the Elbe River showed a similar low genetic heterogeneity. There was an overall high genetic similarity in the species C. fiber, and no evidence was found for a clear subspecific structure in the populations studied.

1988: ***-.092***

This paper discusses the problems raised by the phenomena of biotic pollution, which result from introductions, reintroductions and population reinforcements in animals and plants. The negative ecological consequences of the translocation of specimens outside the distribution range of their species (taxonomic, i.e., faunistic or floristic pollution), especially in the cases of "invasive species", have long been known. In contrast, it is less usually recognized that so-called "population reinforcements", often aiming at increasing the population size in order to reduce inbreeding, also pose problems. These operations consist in the introduction in a population of specimens originating from another population of the same species or of another interfertile species. This results in a modification of the genetic structure of the receptor population (genetic pollution), and also in some cases, in species where behaviour is in part transmitted by imitation or learning, of some behavioural traits of the autochtonous animals (cultural pollution). The struggle against genetic pollution is not motivated, as construed by some, by a racist ideology, or by the idea that the introduced specimens would always bring alleles responsible for a lower adaptation to the local conditions than those of the autochtonous population, thus reducing its fitness (although this sometimes occurs). It is justified by the fact that this genetic (and sometimes behavioural) mixture will later make difficult impossible the study of the history, adaptations and evolution of the receptor population, as allowed for example nowadays by phylogeographic studies. The latter take advantage of the existence of genetic polymorphism and of its variation in space and time to reconstruct the phylogenetic trajectory of natural populations. Species are not black boxes containing identical individuals and the fact that they bear the same scientific name does not mean that different specimens can be mixed or replaced by each other without consequences. For evolutionary biologists, biotic pollutions amount to a destruction of their object of study. Besides, such operations send an optimistic but misleading message to the public, according to which destructions of the environment caused by human activities would be reversible at little cost - so that there would be no urgent need to modify the relationship between our societies and their environment. The basic concepts, methods and aims of genetics, phylogeography and taxonomy should be afforded more weight in the decisions regarding the reintroduction of organisms into reduced populations or threatened habitats.

1989: ***+.064***

The Arabian Oryx (Oryx leucoryx) is listed as critically endangered by the International Union for the Conservation of Nature and a reintroduction effort currently is underway involving three release sites in the Negev desert of southern Israel. We describe a simulation model developed to project future population trends in view of uncertainties concerning establishment of migration

corridors among release sites and post-reintroduction increases in natality rates. We first evaluate ability of the model to simulate observed trends in population growth following reintroductions of Arabian Oryx in Oman, and trends observed to date in the Israeli Negev. We then use the model to project future scenarios in the Israeli Negev under all combinations of three assumptions regarding establishment of migration corridors and two assumptions regarding post-reintroduction increases in natality rates. Model projections suggest (1) no individual site supports a viable population (minimum viable population size has been estimated at 100 individuals) under any of the scenarios; (2) if there are no post-reintroduction increases in natality rates, the area supports a viable metapopulation (101 individuals) only with establishment of migration corridors among all sites; (3) if there are post-reintroduction increases in natality rates, the area supports a viable metapopulation (247 individuals) with establishment of migration corridors among all sites and also with establishment of migration corridors only between Sites A and C (172 individuals). Model projections also suggest that uncertainty regarding natality and migration, which are the rates most critical to management decisions, might be reduced substantially by continued field monitoring of Sites A and C over the next 5 or 6 years; management of Site B (mountainous terrain) remains critical either until increases in natality rates and the establishment of a migration corridor between at least Sites A and C (open, flat terrain) have been confirmed, or until the decision is made to abandon Site B in favor of the establishment of an alternative release site. (C) 2007 Elsevier B.V. All rights reserved.

1990: *+.243*

In this study, we present the first data about putative source populations of the vagrant Subantarctic fur seal, Arctocephalus tropicalis, found on the Brazilian coast, through the comparison of their mitochondrial DNA control sequences to exclusive haplotypes from the main breeding colonies of the species. The results indicated that, despite the majority of the vagrant individuals are from Gough Island (the closest breeding site to the Brazilian coast), they also come from other reproductive colonies, such as Crozet Island, a distance around 16,500 km from the Brazilian coast. Furthermore, the molecular data identified three possible management units: (1) Gough, (2) Amsterdam, and (3) Marion, Macquarie and Crozet. This significant genetic subdivision must be taken into account in any future management plan for the species conservation, including rehabilitation and even reintroduction of vagrant fur seals.

1991: *-.059*

1. In the high Mountains of Cordoba (Central Argentina) large native herbivores were replaced completely by domestic livestock early in the 20th century. Recently, livestock were excluded in a large portion of the mountains to reduce alarming soil erosion rates, leading to an unnatural situation as the area has a long evolutionary history of large herbivore grazing. Many of the birds living in this area are endemic subspecies. Lack of large herbivore grazing can reduce plant diversity, but the response of birds is unknown.2. We surveyed birds in 46 1.8-ha transects distributed across eight vegetation units under (a) traditional livestock rearing and (b) 4 years of livestock exclusion. We described bird communities per transect using density and richness. The effects of grazing situation and vegetation units on these parameters were analysed by two-way analysis of variance (ANOVA). Additionally, we analysed bird composition through detrended correspondence analysis (DCA).3. Livestock exclusion caused, in all vegetation units, significant reductions in observed bird density and richness when all species were included in the analysis, and when the 12 endemic subspecies were considered separately. An analysis for each guild showed a similar pattern but differences were significant only for richness of insectivorous and granivorous birds. Vegetation units always showed significant differences in bird density and

richness, with no significant interactions between vegetation and grazing situations. Community composition described through DCA was different between vegetation units but not between grazing situations. Endemic birds were associated mainly with rocky areas.4. Synthesis and applications. In areas where large native herbivores are locally extinct, extensive exclusion of domestic livestock is not recommended where bird conservation is also a priority. Where possible, we suggest reintroducing native large herbivores. Where reintroductions are not feasible, livestock must be excluded only from those sites where it is most necessary, and maintained elsewhere at reduced stocking rates. To mimic past natural grazing regimes more effectively, livestock grazing regimes should incorporate temporal fluctuations at seasonal, yearly and decade scales.

1992: *+.296*

1. We evaluated one of the most extensive efforts to date to re-introduce an endangered species: attempts to establish an actively managed meta-population of African wild dogs Lycaon pictus in South Africa.2. Using an information-theoretic approach, known-fate modelling in program MARK was employed to estimate the survival of re-introduced wild dogs and their offspring, and to model covariate effects relative to survival. Multiple a priori hypotheses on correlates of re-introduction success were tested (collated from extensive individual experiences) using different re-introduction attempts as natural quasi experiments.3. Survival analyses revealed that the determinants of re-introduction success can be reduced to two factors relevant for management, suggesting that wild dog re-introductions should be attempted with socially integrated animals that are released into securely fenced areas, unless measures are implemented to mitigate human-related mortalities outside protected areas.4. Synthesis and application. This study illustrates that monitoring and evaluation of conservation efforts, complimented with expert knowledge, forms the foundation of informed decision-making to underpin management recommendations with scientific evidence, particularly if the proposed actions are controversial.

1993: *+.072*

In order to evaluate the population projection for the huemul of the,south (Hippocamelus bisulcus) in Central Chile (Nevados de Chillan), a stochastic simulation model was applied. This population has continually decreased in size over the last de cade and empirical estimates suggest that in a short time the current relicts of this population would extinguish locally. The simulation model was applied using life history and environmental data. Survival rate, mean fecundity and carrying capacity were modeled over a time span of 100 years. The population age structure, sex ratio and local density average were determined on the,basis of historical (1975-2003) records. The results indicate that this is a high risk population with a mean extinction time between 27 and 42 years. The population decline could, be explained by effects generated mainly by sustained anthropic perturbation occurring in a large proportion of primary habitats and by stochastic environmental perturbations. The relevance of several protection measures is discussed, including increment for the area of available primary habitat, conservation of corridors among fragments, and translocation of individuals from the existing southern populations in XI Region of Chile.

1994: *+.120*

1. Edge effects are a highly cited issue in conservation biology with a wealth of empirically described impacts on invertebrates, but the mechanisms underlying the responses of individual species to habitat edges have been seldom tested. One often-raised hypothesis is that individual species vary in their ability to survive along a gradient of changing microclimatic conditions across habitat edges.2. I used a phylogenetically controlled, translocation experiment to investigate

edge-driven variation in adult mortality rate for two closely related species that have contrasting abundance patterns with respect to forest-grassland edges; the habitat generalist Mecodema fulgidum Broun (Coleoptera: Carabidae) and the forest-dwelling Mecodema rugiceps Sharp.3. Adult individuals of both species were live-caught and translocated into containers at varying distances along an edge gradient extending from 1024 m inside a large forest fragment and 64 m into the surrounding grassland matrix. Experimental containers were connected to the above- and below-ground environments via mesh-covered panels, and beetle mortality over a 4-month period in the 2006/2007 austral summer was recorded.4. Overall, mortality was higher for the habitat specialist M. rugiceps than for the generalist M. fulgidum. Neither species exhibited an edge-related trend in mortality, nor were mortality rates correlated with microclimatic gradients across the edge.5. Weather records for the summer indicated that M. rugiceps was more sensitive than M. fulgidum to a soil moisture deficit that became more pronounced as summer progressed. Analysis of a 10-year climate record shows that soil moisture deficits occur annually, suggesting that seasonal variation in soil moisture may contribute to the variation in the distribution and abundance of the two species.

1995: *+.111*

Reintroduction programs aim at reinstalling a self-sustained population into the wild via a period of supplementation with captive-bred individuals. This procedure can rapidly generate inbreeding among offspring because of the mating scheme and this inbreeding might be further enhanced by the reintroduction scenario. First, we used simulations to assess the consequences of breeding designs on mean inbreeding index F among offspring when the genetic diversity of breeders, the number and sex ratios of breeders, and the proportion of successful crosses vary. A high number of breeders, a balanced sex ratio, a high proportion of effective crosses and a genetically diverse source population generally contribute to lower F values. However, moderately high (>= 20) numbers of breeders combined with all but the most biased sex ratios produced mean F values near minimal values. The variability in F was negligible in all parameter combinations except for a very small number of breeders (5) and very biased sex ratios (<= 1M : 19F). We also simulated the long-term inbreeding dynamics in the introduced population under various demographic scenarios. Our main finding was that the annual number of introduced offspring is a decisive factor in establishing long-term F values in the supplemented population. Low supplementation levels (10(2)) quickly generated an almost completely inbred population whereas high levels (>= 10(4)) produced stable F values close to that of the introduced offspring. Simulations were run based on the life history and specific demographics of the bloater (Coregonus hoyi), whose reintroduction in Lake Ontario is being considered.

1996: *+.362*

The rate of recovery of invertebrate communities following habitat restoration has received little attention, despite the importance of invertebrates in ecosystem dynamics. in experimental trials in a mined peat bog in New Zealand, we compared the short-term rate of beetle community re-assembly at sites restored using management techniques varying in cost and effort to implement, and subsequently examined the long-term rate of beetle community convergence towards the 'target' community structure of an undisturbed peat bog. There was a direct relationship between the rate of beetle community re-assembly and the cost and effort applied to plant community restoration treatments (processed peat with no seed, processed peat with seed, and direct habitat translocation). To test the longer term rate of beetle community convergence following habitat restoration, we monitored beetle assemblages on a chronosequence of experimental habitat islands in which restoration was initiated 1, 13, 24, 25, 42 and 72 months previously With increasing age,

the plant community became more diverse and structurally complex, and beetle community composition converged rapidly on the target community structure of an undisturbed peat bog. Using a simple linear regression analysis on ordination axis scores, we obtained the quantitative prediction that the beetle community on restored islands would converge on the average community composition of an undisturbed peat bog within just 7-8.5 years. Our results clearly show that rapid invertebrate community re-assembly can occur in direct response to management techniques focused on establishing vegetation structure. Even the use of a low-cost restoration technique (processed peat with seed added) was effective in initiating remarkably rapid invertebrate community re-assembly in cutover peat bogs within 10 years. (C) 2008 Elsevier Ltd. All rights reserved.

1997: ***+.100***

Populations with small effective sizes are at risk for inbreeding depression and loss of adaptive potential. Variance in reproductive success is one of several factors reducing effective population size (N-e) below the actual population size (N). Here, we investigate the effects of polygynous (skewed) mating and variation in female breeding success on the effective size of a small population of the Gunnison sage-grouse (Centrocercus minimus), a ground nesting bird with a lek mating system. During a two-year field study, we recorded attendance of marked birds at leks, male mating success, the reproductive success of radio-tagged females, and annual survival. We developed simulations to estimate the distribution of male reproductive success. Using these data, we estimated population size ((N) over cap) and effective population size N-e for the study population. We also simulated the effects of population size, skewed vs. random mating, and female breeding failure on N-e. In our study population, the standardized variance in seasonal reproductive success was almost as high in females as in males, primarily due to a high rate of nest failure (73%). Estimated N-e (42) was 19% of (N) over cap in our population, below the level at which inbreeding depression is observed in captive breeding studies. A high hatching failure rate (28%) was also consistent with ongoing inbreeding depression. In the simulations, N-e was reduced by skewed male mating success, especially at larger population sizes, and by female breeding failure. Extrapolation of our results suggests that six of the seven extant populations of this species may have effective sizes low enough to induce inbreeding depression and hence that translocations may be needed to supplement genetic diversity. (C) 2007 Elsevier Ltd. All rights reserved.

1998: ***-.094***

This review focuses on the success and survivorship of captive-born versus wild-caught carnivores used in reintroductions. Previous reviews have suggested that reintroduction projects using captive-born animals are less likely to be successful than projects translocating wild-caught animals. The purpose of this paper is to examine this statistically and investigate how captivity may affect the survival of reintroduced carnivores. We examined results published in previous reviews, and found evidence to support that reintroduction projects using wild-caught animals are significantly more likely to succeed than projects using captive-born animals. We further compiled our own review of 45 case studies in carnivore reintroduction projects (in 17 species across S families) to investigate survival rates rather than overall project 'success'. We found that (1) wild-caught carnivores are significantly more likely to survive than captive-born carnivores in reintroductions; (2) that humans were the direct cause of death in over 50% of all fatalities and (3) that reintroduced captive-born carnivores are particularly susceptible to starvation, unsuccessful predator/ competitor avoidance and disease. (C) 2007 Elsevier Ltd. All rights reserved.

1999: ***+.074***

Despite the apparent risks of the introduction of non-indigenous ungulates to biodiversity, relatively little is known globally about the pathways of introduction, propagule pressure and realized impacts of ungulate introductions. These issues were examined here by investigating ungulate introductions to South Africa within a global context. Across countries globally, introduced ungulate richness is not related to indigenous ungulate richness, and several countries are clear outliers. South Africa is second only to the USA in the number of ungulate species introduced to date. Zoos have traded more ungulate species and individuals to non-zoo recipients than to other zoos, highlighting the tensions that exist between in situ and ex situ conservation goals. Introductions to, and extralimital introductions within South Africa have increased through time, with propagule pressure being highest in areas with high human population density. The long distances ungulates have been translocated raise concerns for genetic homogenization. Translocations of indigenous ungulate species extralimitally have significantly altered range sizes, typically to a greater extent than is expected from range shifts associated with global climate change. Although ungulate introductions and translocations are likely to have impacts on biodiversity, evidence for such impacts in South Africa, and elsewhere, is limited. Whilst arguments may be made for a precautionary approach to ungulate introductions, an evidence-based one is much more likely to deliver efficient and convincing conservation decision-making. [copyright] 2008 Elsevier Ltd. All rights reserved.

2000: ***+.063***

The works on the restoration of European beaver in Europe (Sweden) be-an in the 1920s, in Russia - in the 1930s. In Finland, Canadian beaver was introduced in the 1930s. At the present time, both species dwell in the most part of Eastern Fennoscandia. A problem of competition and displacement of one species by another one has arisen. In southern Karelia, European beavers occupy the areas, where Canadian beavers were introduced, i.e. the new species is substituted for the indigenous one. However, Canadian beavers being settled over the north-eastern Karelian territories, penetrated to Arkhangel'sk region, into the habitat of European beaver. Specific features of ecology of both species based on the material obtained in nature, under similar environmental conditions are analyzed. The pathways and some consequences of the species expansion and some aspects of their interrelationships are discussed.

2001: ***+.034***

We evaluated the role that endangered species reintroduction efforts can play in the larger context of ecosystem restoration. To do so, we examined interactions between endangered giant tortoises (Geochelone nigra hoodensis), currently being reintroduced to Isla Espanola, Galapagos, and an arboreal cactus (Opuntia megasperma var. megasperma), which is itself endangered and a keystone resource for many animals on the island. We collected information on spatial patterns of occurrence of cacti, tortoises, and woody vegetation and compared recruitment of juvenile cacti in areas occupied versus unoccupied by tortoises. Reintroduced tortoises appeared to suppress cactus recruitment near the few remaining adult cacti at the study site, but facilitate it at longer distances, with tortoise-cactus interactions mediated by the presence of woody vegetation, which likely alters tortoise movements and thereby patterns of cactus seed dispersal. The net effect of tortoises on cacti appeared to be positive insofar as tortoise presence was associated with greater recruitment of juveniles into cactus populations. Our study provides support for reintroducing endangered reptiles and other animals to aid ecosystem restoration in areas where they might once have played an important role in grazing upon and dispersing plants.

2002: *-.056*

habitats dominated by herbaceous plants on thin, rocky soils occur within the forests of eastern North America. Although these habitats vary in origin, structure, geology, and species composition, all contribute greatly to regional biodiversity by harboring endemic and/or rare plants. Little is known about how disturbances affect plant populations in these ecosystems. Fire once was a frequent natural disturbance in the Ketona dolomite glades of Alabama, an ecosystem harboring eight endemic taxa and numerous other species of conservation concern. We designed an experiment to determine how the reintroduction of fire into the glades and surrounding long leaf pine forests affects populations of rare glade plant species. Experimental and control plots were established within the glades. Experimental plots were burned in April 2004, and all plots were surveyed during two subsequent growing seasons (2004 and 2005). Populations of three of 14 species of conservation concern declined significantly after the initial fire but recovered the next year. Among other herbaceous species, only five and two differed in population size in 2004 and 2005, respectively. In 2004, more species were more abundant in control than burned plots, but this difference was not detected in 2005. Multivariate community-level analyses of species presence-absence suggested that the effects of fire were negligible by the 2005 survey. Populations of young trees that had invaded the glades declined dramatically as a result of treatment fires. These results suggest that the reintroduction of fire will not harm glade species and may help prevent encroachment of the surrounding forest.

2003: *+.102*

Agri-environment schemes are the most widely adopted political measure to maintain and restore farmland biodiversity in Europe. However, abiotic and biotic factors often limit the success of ecological restoration. Among the biotic factors, the size of the local and regional species pool is a major constraint. This is only well documented for plants. We therefore wanted to know if a small regional species pool can also limit restoration efforts of invertebrates. Specifically, we tested if by relocating grasshoppers from further away, we could overcome regional species pool limitations on hay meadows under the Swiss agri-environment scheme, so-called Ecological Compensation Area meadows (ECA meadows). All meadows had been under restoration for 6 years and were formerly intensively used hay meadows. Two grasshopper species, Euthystira brachyptera and Mecostethus parapleurus, were selected; E. brachyptera was not found in the regional species pool and M. parapleurus had nearly disappeared. In 2004, 1,400 grasshopper individuals of each species were taken from the nearest large source populations and distributed equally on seven ECA meadows and seven control meadows. In 2005, we evaluated whether the species had successfully established. Only one individual of M. parapleurus was found. We conclude that a small regional species pool is not the only constraint for the reestablishment of grasshoppers on restoration meadows. Also, other factors such as habitat quality appear to constrain the reestablishment of grasshoppers on restoration meadows. Additional restoration efforts specifically targeted at grasshopper restoration are needed, and innovative techniques have to be developed to overcome the relocation constraints.

2004: *+.295*

Pancratium maritimum L. (Sea Daffodil) is a bulbous perennial plant inhabiting sandy dunes along Bulgarian Black Sea coast. Its large scented white flowers are highly attractive and often collected by tourists. Loss of natural populations and habitats led to protection measures: the species is included in the Red Data Book of Bulgaria and is under the regulations of the Biodiversity Act. Mature seeds of R maritimum collected from the wild were used as initial material for in vitro

culture initiation. The seeds were surface sterilized using two-step procedure (Panayotova et al. (in press)). Fast germination on water agar was followed by relatively slow shoot development on solidified MS medium supplemented with BAP and NAA. Growth of the obtained cultures was improved by modification of the auxin and cytokinin levels in the media. Rapid growth and leaf elongation was observed by cultivation of bulblets in liquid MS medium supplemented with 2 mg 1(-1) BAP and 0.15 mg 1(-1) NAA. Subsequent subcultivation on solidified medium resulted in shoot formation between the scales of the bulblet sectors within 2 weeks. Such promising results give opportunities to develop a rapid propagation method for conservation and reintroduction purposes.

2005: ***+.094***

Most Central European Capercaillie populations have been declining during the last century. In the Jura Mountains, at the border between Switzerland and France, remaining Capercaillie populations are now isolated and endangered. In this study, land-use and Capercaillie presence data were used to identify key landscape parameters by logistic regression modelling. We found that Capercaillie prefers areas at the highest altitude in the Jura Mountains that are characterised by continuous forests and stands with intermediate canopy cover. At the local scale, winter habitat selection revealed a preference for open forests with a sparse canopy cover dominated by spruce and fir. Capercaillie avoided dense undercanopy and understorey, especially when dominated by beech. Population viability and sensitivity analyses underlined the crucial importance of adult female survival, chick survival and breeding success for populations maintenance. Legal bases, scientific knowledge and technical measures are now available to conserve the flagship species Capercaillie within the Jura Mountains. Capercaillie-adapted forestry requires a mosaic distribution of habitat types, with a matrix of open forests where fir is favoured, and understorey kept sparse. Preliminary essays indicate that grouse-adapted forestry costs are similar or even lower than present costs. To increase survival and breeding success, one option is to diminish human disturbance by limiting access to Capercaillie breeding and wintering areas. An action plan for the species should avoid more costly and intensive approaches such as the reintroduction of birds from other populations. Capercaillie conservation represents a major challenge rising from various and contradictory leisure, tourist and rural development activities. Collaboration with different stakeholders and state agencies for forest and wildlife conservation should complete the positive effects of grouse forestry with an effective protection from human disturbance.

2006: ***-.017***

1. Zingel asper is one of the most endangered freshwater fish of Western Europe. Because of critical habitat loss and fragmentation, stocking or reintroduction protocols are sometimes considered even though few data are available about the behaviour and biology of the species.2. The present study is the first attempt to explore the population genetic structure of juveniles (young of the year, YOY) of this endangered species using genetic markers (microsatellite loci). In the Beaume River (ardeche, France), Z. asper exhibits very low densities (10-80 fish per hectare), and it was expected that annual recruitment would comprise only few clutches (full- or half-sibs groups).3. However, sibship reconstruction in one YOY cohort showed that more than 60% of the adult population reproduced. Dispersal rate in early YOY was high: relatedness estimates showed that only few months after hatching, YOY sibs were mixed among study sites. A large number of breeders together with high dispersal rates explain the high genetic variability observed in small populations of Z. asper.4. Conservation strategies for the species are discussed based on these results, emphasizing the importance of conserving population genetic variability and preserving habitat connectivity for juveniles.

2007: *+.203*

It is safe to say that there have not been more Eurasian lynx (Lynx lynx) in Europe for several centuries. The last 50 years have seen the natural recovery of the Scandinavian, Baltic and Carpathian populations. The tiny Balkan population has persisted. A total of 15 reintroduction attempts have been made. The sum total of all this is that we are no longer talking about a crisis action of rescuing a species from the edge of extinction (although the Balkan population is of great conservation concern). Instead we are trying to reintegrate the large carnivore species (Table 1) into our landscapes and moving towards a long term, robust and flexible management system.

2009: *-.215*

We analyse the causes of mortality for the Bearded Vulture in Europe. Shooting (31%), intentional poisoning (26%), collision (18%) and unintentional poisoning (12%) were the most important causes of mortality. No differences were found between sexes or age classes (nonadults and adults) for any of the causes of death. When the four main categories of mortality were grouped in periods of 3 years from 1986 (coinciding with the species' reintroduction to the Alps) to 2oo6, mortality showed significant temporal variation. The results suggest that while the number of collision/electrocution deaths has remained stable or increased slightly, the number of cases of shooting has declined during the last 6 years, while at the same time intentional and unintentional poisonings have increased. We found substantial differences between causes of mortality recorded for birds located by chance (75% related to shootings and collisions with powerlines) and radio-tagged birds (86% related to intentional and unintentional poisoning), suggesting biases in methodology for monitoring mortality. The results suggest that human persecution continues to be the main factor contributing to unnatural mortality for European Bearded Vultures. Future management actions should concentrate on the creation of protocols for the collection of carcasses and detailed analyses to determine and mitigate anthropogenic sources of mortality.

2010: *+.057*

Red deer (n = 149) from eight geographical locations, including the endangered endemic populations from the Tyrrhenian islands (Sardinia and Corsica), were analysed at eight polymorphic microsatellite loci. Two questions were addressed: (1) Is there a founder effect in the Corsican population, which was reintroduced to the island using Sardinian deer after the species' extinction on Corsica? (2) What is the origin of the Tyrrhenian or Corsican red deer (Cervus elaphus corsicanus)? Our results showed signs of a founder effect for the red deer on Corsica in that these deer showed differentiation from the Sardinian population as measured by F-ST values, assignment tests (with and without a priori definition of populations) and individual-based dendrograms. Genetic variability, however, did not differ significantly between the two populations. With respect to the phylogeography of C. e. corsicanus we found that both deer from North-Africa and Mesola on the Italian mainland were genetically close to the Corsican red deer, but phylogenetic trees based on genetic distances were only poorly supported statistically. Among all populations studied the Mesola red deer showed the lowest distance values from Corsican red deer and yielded allele frequencies that were more similar to those of C. e. corsicanus than were those of North-African red deer. These results are in line with recent palaeontological and archaeozoological findings which suggest that the Corsican red deer is derived from small Italian red deer introduced from the mainland to Sardinia and Corsica during the Late Neolithic and just before the beginning of Classical Antiquity, respectively. They also suggest a possible recent introduction of Tyrrhenian red deer to North-Africa (rather than the other way around), thus accounting for the close genetic relationship (especially based on mitochondrial DNA) that has

repeatedly been found between C. e. corsicanus and C. e. barbarus.

2011: ***+.119***

Spatially explicit decision support systems are assuming an increasing role in natural resource and conservation management. In order for these systems to be successful, however, they must address real-world management problems with input from both the scientific and management communities. The National Training Center at Fort Irwin, California, has expanded its training area, encroaching U.S. Fish and Wildlife Service critical habitat set aside for the Mojave desert tortoise (Gopherus agassizii), a federally threatened species. Of all the mitigation measures proposed to offset expansion, the most challenging to implement was the selection of areas most feasible for tortoise translocation. We developed an objective, open, scientifically defensible spatially explicit decision support system to evaluate translocation potential within the Western Mojave Recovery Unit for tortoise populations under imminent threat from military expansion. Using up to a total of 10 biological, anthropogenic, and/or logistical criteria, seven alternative translocation scenarios were developed. The final translocation model was a consensus model between the seven scenarios. Within the final model, six potential translocation areas were identified.

2012: ***+.181***

1. Spanish populations of the white-clawed crayfish have declined sharply over the last three decades. Although Austropotamobius pallipes was once widely distributed and very abundant in most of the limestone basins of the country, outbreaks of crayfish plague since 1978 have reduced its populations, and now only some 500-600 small populations are left.2. Consequently, the species now enjoys protection under national legislation. Management decisions regarding the conservation of a threatened species require an understanding of the genetic structure of its populations.3. Using random amplified polymorphic DNA (RAPD) fingerprinting the genetic variability of 11 populations of A. pallipes was assessed over the species' range in Spain, and their phylogenetic relationships determined.4. Substantial genetic differentiation was detected among the populations tested; no clear relationship was found between patterns of genetic variability and hydrological basin. The RAPD markers showed the degree of genetic variability of these populations to be similar to, and in some cases slightly higher than, that reported in previous studies on other Spanish and European populations of A. pallipes.5. The results offer hope for the recovery of this species in Spain, and provide information that might be useful in the management of crayfish reintroduction programmes. Copyright (c) 2007 John Wiley & Sons, Ltd.

2013: ***+.042***

The endangered Mariana Swiftlet, Aerodramus bartshi (Mearns, 1909), occurs in its native habitat on only three islands worldwide-Guam, Saipan, and Aguiguan. It is locally extinct on the islands of Rota and Tinian, and numbers have declined on Guam. On Saipan and Aguiguan, the bird remains common. We present previously unpublished data from reports lodged with the Commonwealth of the Northern Mariana Islands Division of Fish and Wildlife combined with an analysis of arrival count data from surveys conducted regularly on Saipan (1985-2005) and opportunistically on Aguiguan (19852002). Direct counts of swiftlets arriving at nesting caves did not permit islandwide population estimates but provided an index useful for assessing relative abundance. On Aguiguan, swiftlets occurred in only a few of the available caves; the population was small, more densely concentrated than on the other islands, and relatively stable. On Saipan, swiftlet numbers declined for the first part of the monitoring period (1985-1992), then increased

significantly (1998-2005), and now stand at their highest level (>5,000 birds) since 1985. Large between-year fluctuations, high variation in colony attendance patterns, and occasional abandonment and recolonization of some caves were evident during the 20-yr monitoring period. Of the potential constraints to the population, pesticide use, typhoons and supertyphoons, habitat alteration by feral animals, human disturbance in the nesting caves, and predation remain areas of concern. Conservation measures may have lessened some disturbance events and nest damage by cockroaches, while other measures, such as translocation, may improve the species' chances of persistence.

2014: *+.080*

Population units that merit separate management and are of conservation concern have been called evolutionary significant units. Two divergent lineages of the European rabbit Oryctolagus cuniculus occur naturally in Spain, with a well-marked geographical distribution. We analysed the frequency and importance of rabbit translocations in central-southern Spain and whether this practice, carried out by hunters and conservationists, could cause the mixture of two clearly different evolutionary significant units. We carried out interviews in 1993 and 2002 at 60 locations to determine the presence and intensity of translocations during both decades. The distribution of the lineages was obtained using mtDNA analysis of hunted rabbits in 2003-2005. We demonstrate that rabbit translocation was used frequently in the 1980s and increased in the 1990s. Up to 43% of the studied areas translocated rabbits in the latter decade, whereas only 25% did so in the 1980s. Our results show that neither the origin of the introduced rabbits nor their genetic lineage were taken into account in most of the translocations. We found rabbits of lineage A in several localities within the distribution area of lineage B, and vice versa, probably as a consequence of translocations. The distribution of both lineages is likely to have been altered by human activity and this could represent the loss of the results of 2 million years of genetic differentiation with possible attendent ecological consequences. Consequently, authorities should more closely regulate rabbit translocations and convey to both hunters and conservationists the importance of not mixing the lineages by translocations.

2015: *+.129*

Over the last 30 years bearded vultures (Gypaetus barbatus) have been reintroduced to their former habitats in the Alpine region, after having been exterminated about 200 years ago by direct persecution. Before starting active reintroduction an intensive captive breeding programme was launched in the year 1978. From the year 1986, in which the first four young vultures have been released, year by year on average eight young birds were set free by the so called "hacking" method. National parks in Austria, Italy, Switzerland and France provided best conditions for the successful reintroduction of bearded vultures. Until the year 2007 exactly 150 birds have been released, with a proven presence of 123. The first pair bred successfully in France (Haute Savoy) in the year 1997. Since that time the number of breeding pairs resp. trios has reached the number of eight to ten. Their reproduction rate now corresponds with the number of offspring bred in captivity in the breeding network. A cost analysis of the entire reintroduction programme revealed that each of the young vultures reared in the captive breeding programme produced costs of an average of 65 000 Euro up to its release. Additional costs linked with the monitoring and the active releasing and safeguarding of the young birds cannot be estimated. The database for the monitoring and its management created additional costs of about 25 000 Euro per year. These facts are leading to the conclusion that it is more cost-efficient to conserve species in their natural habitats. Every intervention causing the loss of species will cause severe financial burdens to future generations for the re-establishment of functioning ecosystems.

2016: *-.045*

The European wild boar is an important game species, subjected to local extinctions and translocations in the past, and currently enormously and worryingly expanding in some areas where management is urgently required. Understanding the relative roles of ancient and recent events in shaping the genetic structure of this species is therefore not only an interesting scientific issue, but it represents also the basis for addressing future management strategies. In addition, several pig breeds descend from the European wild boar, but the geographical location of the domestication area(s) and the possible introgression of pig genomes into wild populations are still open questions. Here, we analysed the genetic variation in different wild boar populations in Europe. Ten polymorphic microsatellites were typed in 252 wild boars and the mtDNA control region was sequenced in a subset of 145 individuals. Some samples from different pig breeds were also analysed. Our results, which were obtained considering also 612 published mtDNA sequences, suggest that (i) most populations are similarly differentiated, but the major discontinuity is found along the Alps; (ii) except for the Italian populations, European wild boars show the signature of a postglacial demographic expansion; (iii) Italian populations seem to preserve a high proportion of preglaciation diversity; (iv) the demographic decline which occurred in some areas in the last few centuries did not produce a noticeable reduction of genetic variation; (v) signs of human-mediated gene flow among populations are weak, although in some regions the effects of translocations are detectable and a low degree of pig introgression can be identified; (vi) the hypothesis of an independent domestication centre in Italy is not supported by our data, which in turn confirm that Central European wild boar might have represented an important source for domestic breeds. We can therefore conclude that recent human activities had a limited effect on the wild boar genetic structure. It follows that areas with high variation and differentiation represent natural reservoirs of genetic diversity to be protected avoiding translocations. In this context controlling some populations by hunting is not expected to affect significantly genetic variation in this species.

2017: *-.013*

Rare. parasitic plants pose an interesting challenge to restoration practitioners. In addition to the problems associated with small population size, rare parasites may also be limited by their host requirements. We examined how the performance of a rare Pacific Northwest hemiparasite, Castilleja levisecta, was affected by the availability of different host combinations in the greenhouse and in the field. Castilleja levisecta individuals were grown with two individuals of the grass Festuca roemeri, two individuals of the aster Eriophyllum lanatum, one individual of each of these species (a "mixed" treatment). or without any host. We did not find Support for the complimentary diet hypothesis, which predicts that parasites grown with multiple host species perform better than individuals grown alone or with a single host. In the greenhouse, C levisecta individuals grown in the mixed treatment had greater stem growth than those planted with F. roemeri, but did not differ front E. lanatum or no-host treatments. In the field, vole activity had indirect effects oil C. levisecta survival mediated through host species: vole tunneling and C levisecta mortality were strongly associated with [lost treatments including E. lanatum. Vole tunneling and C levisecta mortality were strongly associated with [lost treatments including E. lanatum. Field survival of no-host and F roemeri treatments were significantly higher than those grown with E. lanatum. Our results emphasize the importance of basing conservation decisions oil experimental research conducted under conditions similar to those of the intended application. as greenhouse results were it poor predictor of field performance. For restoration of endangered hemiparasitic plants, we recommend planting with hosts that are not attractive to herbivores.

2018: +.170

To examine effects of relocation on eastern box turtles (Terrapene carolina), we compared home ranges and movement patterns of 10 resident and 10 relocated box turtles in Davidson, North Carolina, USA. Home ranges of relocated turtles were approximately 3 times larger than those of resident turtles when measured by minimum convex polygons, 6 times larger than resident turtles when measured with 95% kernels and 7.5 times larger than resident turtles when measured by 50% kernels. Relocated turtles also moved a greater average distance per day than resident turtles. Additionally, 5 relocated turtles experienced mortality or disappearance compared to no mortality or disappearance of resident turtles. Our results raise questions about the success of relocation as a management strategy for eastern box turtles.

2019: -.022

Hoolock Gibbon (Hylobates hoolock Harlan 1834) is the only ape that occurs in the Indian subcontinent. The species is classified as endangered and their numbers have declined throughout their geographic range primarily due to habitat destruction. We determined the current status of the Hoolock Gibbons in Bangladesh. A total of 35 sites (10 in the north-eastern region and 25 in the south-eastern region) were surveyed for Hoolock Gibbons from 2002 to 2005. A total of 282 Hoolock Gibbons in 96 groups was estimated to live in 24 of the 35 sites visited. Several sites have lost all their Hoolock Gibbons within the last 15 years. Karnafuli (part of Kaptai National Park) in the south-east and Lawachara National Park in the north-east were recognised as two major strongholds of Hoolock Gibbons. Conservation of the remaining Hoolock Gibbons of Bangladesh rests on the future management of their patchy forest habitats. We recommend the cessation of illegal deforestation, habitat restoration and translocation of individuals to maintain these last Hoolock Gibbon populations in Bangladesh.

2020: +.054

The Lesser Rhea (Pterocnemia-Rhea-pennata pennata) has a complex reproductive system that combines polygyny with sequential polyandry, in which males build the nest, fully incubate the eggs and care for the young. As occurs with the Greater Rhea (Rhea americana), Lesser Rhea females sporadically lay eggs outside the nest ('orphan' eggs), which are not incubated and thus fail to hatch. We have examined the orphan eggs of Lesser Rhea over two separate breeding seasons to determine their abundance and fertility status. During 2004 and 2005, weekly ground searches for orphan eggs were conducted in a wild population of Lesser Rhea in northwestern Patagonia, Argentina. During these searches the total number of nests, eggs in each nest and orphan eggs laid outside the nests was recorded. Orphan eggs represented approximately 7% of the total eggs laid in a breeding season. Six fresh orphan eggs were artificially incubated, four of them being fertile. Orphan eggs seemed to have two origins: some were laid near deserted nests in the early to mid-reproductive season; others were probably laid by first-time breeders and were found later in the reproductive season. Given the near-threatened status of the Lesser Rhea, harvesting and artificial incubation of orphan eggs, which otherwise would be unproductive, may contribute significantly to the conservation of this species, i.e., 'recovered' birds could be used for reintroduction or reinforcement of wild Lesser Rhea populations.

2021: -.137

Raccoons occur on a number of islands in the Bahamas and the Lesser Antilles in the West Indies. Zooarcheological studies have long suggested that these animals are not native to the West Indies.

Originally, Caribbean populations were described as endemic insular species Procyon maynardi (Bahamas), P. minor (Guadeloupe), and P. gloveralleni (Barbados), a classification that was recognized throughout much of the 20th century. More recently, studies of qualitative morphology and a review of historical publications and documents have been used to bolster arguments that these populations of raccoons are not unique species worthy of special conservation attention, but invasive populations of the North American raccoon (P. lotor) introduced in recent centuries. Raccoons in the Bahamas and the French Antilles appear to be spreading onto other islands with human assistance, but the population on Barbados is now apparently extinct. We present evidence from the mitochondrial control region, including sequence data from the extinct population on Barbados generated using ancient DNA protocols, indicating that all 3 major insular populations of West Indian raccoons are conspecific with P. lotor and probably originated via recent translocations from eastern North America. Like normative populations of raccoons that have been established elsewhere (e.g., in Alaska, Japan, and Europe), the raccoons of the West Indies deserve no special taxonomic recognition or conservation status. They may be destructive to native wildlife on West Indian islands where they have been introduced, particularly if their spread to and across other islands continues.

2022: *+.305*

In this study we examined long-term variation in the selection of nesting cliffs for the recovering population of the Bearded Vulture Gypaetus barbatus inhabiting the Spanish Pyrenees. We focussed on variables indicating a high probability of cliff occupancy as determined by a previously published model. Although the breeding population increased from 53 to 93 territories between 1991 and 2002, the breeding range expanded only slightly. New and old nesting cliffs had similar habitat features in relation to topography, altitude and degree of human influence, but the distance between occupied cliffs was reduced (from 11.1 to 8.9 km). Thus the probabilities of occupation predicted by the model were lower for newly colonized locales. Our study shows that territory compression may occur without serious modification of nesting habitat quality. These results may arise from the lack of strong territorial behaviour by Bearded Vultures and the availability of high-quality cliffs. The relatively low quality of sites in adjacent mountains may prevent the expansion of the breeding range, but conspecific attraction may also play a role. Our study confirms that monitoring changes in key variables important to habitat selection is useful in determining long-term trends in settlement patterns in heterogeneous environments. The results also suggest that the available nest-site selection model may accurately predict cliff occupancy by Bearded Vultures in those areas where the distance to the nearest neighbour is not a limiting factor. In particular, the model may be useful in establishing priority areas for reintroduction.

2023: *-.006*

Management of invasive species involves choosing between different management strategy options, but often the best strategy for a particular scenario is not obvious. We illustrate the use of optimization methods to determine the most effcient management strategy using one of the most devastating invasive forest pests in North America, the gypsy moth (Lymantria dispar), as a case study. The optimization approach involves the application of stochastic dynamic programming (SDP) to a metapopulation framework with different infestation patch sizes, with the goal of minimizing infestation spread. We use a novel "moving window" approach as a way to address a spatially explicit problem without being explicitly spatial. We examine results for two cases in order to develop general rules of thumb for management. We explore a model with limited parameter information and then assess how strategies change with specific parameterization for the gypsy moth. The model results in a complex but stable, state-dependent management strategy for a

multiyear management program that is robust even under situations of uncertainty. The general rule of thumb for the basic model consists of three strategies: eradicating medium-density infestations, reducing large-density infestations, and reducing the colonization rate from the main infestation, depending on the state of the system. With specific gypsy moth parameterization, reducing colonization decreases in importance relative to the other two strategies. The application of this model to gypsy moth management emphasizes the importance of managing based on the state of the system, and if applied to a specific geographic area, has the potential to substantially improve the efficiency and cost-effectiveness of current gypsy moth eradication programs, helping to slow the spread of this pest. Additionally, the approach used for this particular invasive species can be extended to the optimization of management programs for the spread of other invasive and problem species exhibiting metapopulation dynamics.

2024: *+.030*

Many populations have recovered from severe bottlenecks either naturally or through intensive conservation management. In the past, however, few conservation programs have monitored the genetic health of recovering populations. We conducted a conservation genetic assessment of a small, reintroduced population of Mauritius Kestrel (Falco punctatus) to determine whether genetic deterioration has occurred since its reintroduction. We used pedigree analysis that partially accounted for individuals of unknown origin to document that (1) inbreeding occurred frequently (2.6% increase per generation; N-el = 18.9), (2) 25% of breeding pairs were composed of either closely or moderately related individuals, (3) genetic diversity has been lost from the population (1,6% loss per generation; N-ev = 32.1) less rapidly than the corresponding increase in inbreeding, and (4) ignoring the contribution of unknown individuals to a pedigree will bias the metrics derived from that pedigree, ultimately obscuring the prevailing genetic dynamics. The rates of inbreeding and loss of genetic variation in the subpopulation of Mauritius Kestrel we examined were extreme and among the highest yet documented in a wild vertebrate population. Thus, genetic deterioration may affect this population's long-term viability. Remedial conservation strategies are needed to reduce the impact of inbreeding and loss of genetic variation in this species, We suggest that schemes to monitor genetic variation after reintroduction should be an integral component of endangered species recovery programs,

2025: *+.051*

For more than 10 years, ecologists have been discussing the concept of ecosystem engineering (i.e., nontrophic interactions of an organism that alters the physical state of its environment and affects other species). In conservation biology, the functional role of species is of interest because persistence of some species may be necessary for maintaining an entire assemblage with many threatened species. The great capricorn (Cerambyx cerdo), an endangered beetle listed in the European Union's Habitats Directive, has suffered a dramatic decline in the number of populations and in population sizes in Central Europe over the last century. The damage caused by C. cerdo larvae on sound oak trees has considerable effects on the physiological characteristics of these trees. We investigated the impacts of these effects on the species richness and heterogeneity of the saproxylic beetle assemblage on oaks. We compared the catches made with flight interception traps on 10 oaks colonized and 10 oaks uncolonized by C. cerdo in a study area in Lower Saxony (Germany). Our results revealed a significantly more species-rich assemblage on the trees colonized by C. cerdo. Colonized trees also harbored more red-listed beetle species. Our results suggest that an endangered beetle species can alter its own habitat to create favorable habitat conditions for other threatened beetle species. Efforts to preserve C. cerdo therefore have a positive effect on an entire assemblage of insects, including other highly endangered species. On

the basis of the impact C. cerdo seems to have on the saproxylic beetle assemblage, reintroductions might be considered in regions where the species has become extinct.

2026: ***+.014***

Markhors (Capra falconeri) are among the most endangered mammal species, and several conservation measures, including ex situ breeding, are implemented to prevent their extinction. We studied sequence diversity and differentiation of the first hypervariable segment of the mitochondrial DNA control region among C. f. heptneri and C. f. megaceros kept in four zoos in relationship to lineages of other wild and domestic goats, to assess for the first time the level of molecular distinctness and variability among those subspecies, and to check for possible introgression by related Capra taxa, such as domestic goats. Levels of differentiation between some Capra falconeri lineages and modern domestic goats were similar to levels between other wild goat species (i.e., Capra aegagrus, Capra ibex) and domestic goats. Among pure markhor lineages, paraphyly was observed for C. f. heptneri, suggesting occurrence of shared ancestral polymorphism among markhor subspecies and/or ancient or recent gene exchange between subspecies. Interestingly, 35.7% of all studied markhors from three zoos are introgressed by the domestic goat. Furthermore, despite relatively small breeding group sizes, markhors have maintained a relatively high proportion of mtDNA variation within zoo groups. In any case, the existence of markhors introgressed with domestic goat DNA in zoos should be considered when selecting markhors for ex situ breeding programs with the aim of building up a stock for later reintroduction into the wild.

2027: ***+.108***

In an attempt to reduce the high extinction risk inherent to small island populations, we translocated wild Laysan teal Anas laysanensis to a portion of its presumed prehistoric range. Most avian translocations lack the strategic post-release monitoring needed to assess early population establishment or failure. Therefore, we monitored the survival and reproduction of all founders, and their first-generation offspring using radio telemetry for 2 years after the first release. Forty-two Laysan teal were sourced directly from the only extant population on Laysan Island and transported 2 days by ship to Midway Atoll. All birds survived the translocation with nutritional and veterinary support, and spent between 4 and 14 days in captivity. Post-release survival of 42 founders was 0.857 (95% CI 0.86-0.99) during 2004-2006 or annualized 0.92 (95% CI 0.83-0.98). Seventeen of 18 founding hens attempted nesting in the first two breeding seasons. Fledgling success was 0.57 (95% CI 0.55-0.60) in 2005 and 0.63 (95% CI 0.62-0.64) in 2006. The effective founding female population (N-e) was 13. We applied these initial demographic rates to model population growth. The nascent population size increased to > 100 after only 2 years post-release (lambda=1.73). If this growth rate continues, the size of the Midway population could surpass the source population before 2010.

2028: ***+.074***

The endangered St. Francis Satyr (Neonympha mitchellii francisci) is a small sedentary butterfly and one of the rarest in North America. Our study examined various quantitative aspects of this butterfly's biology, including the distributional range, habitat associations, population size and trends, demographic parameters and spatial aspects of population structure. The range of N.m. francisci distribution is restricted to Dol) lands at Ft. Bragg, North Carolina, where the butterfly utilizes wetland habitats, predominantly those that have previously been impounded by beaver. In situ habitat associations and captive rearing experiments indicate that multiple sedges in the genus

Carex, particularly C. mitchelliana, may be important larval food plants. Subpopulation estimates range between 49-739 individuals at any one site, while cumulative population estimates range between 700-1400 individuals for all accessible areas on Ft. Bragg. Habitats occupied by N.m. ftancisci are frequently subject to burning or flooding and thus butterfly subpopulations are extremely dynamic, fluctuating in response to these disturbances. This regular disturbance regime dictates that dispersal is necessary for population persistence. Several inter-colony movements were measured during capture-recapture studies and we observed both subpopulation extinctions and colonization of new habitat through the period of our studies. Conservation of N.m. ftancisci depends on accommodating unique aspects of its populations, including its dependence on beaver and its multi-tiered metapopulation structure.

2029: *+.059*

More than 70 years of fire suppression has influenced forest dynamics and led to the accumulation of fuels in many forests of the United States. To address these changes, forest managers increasingly seek to restore historical ecosystem structure and function through the reintroduction of fire and disturbance processes that mimic fire such as silvicultural thinning. In oak forests of eastern North America, prescribed fire and thinning are important tools used to facilitate oak (Quercus spp.) regeneration and recruitment. The global scientific community is increasingly raising concerns about the accumulation of atmospheric CO,, and its potential to impact global climate; therefore, activities such as prescribed fire and thinning that can influence the carbon balance of terrestrial ecosystems should be evaluated. We used field measurements and modeling with the PnET-II carbon balance model in oak forests of southern Ohio, USA, to (1) assess the efficacy of prescribed fire and silvicultural thinning in facilitating oak recruitment and regeneration, and (2) quantify the impacts of these treatments on aboveground carbon stocks and net primary production. Silvicultural thinning increased recruitment of maples, but oak recruitment was minimal. Prescribed burning caused an increase in the mortality rate of oaks' major competitor (Acer rubrum L.) in the overstory (stems >= 10 cm DBH), but oak mortality also increased following the bum treatments. Our measurements of stem growth suggest that the timing of the prescribed fires coincided with the initiation of growth in oaks, which may have created vulnerability in these species that are generally considered fire-resistant. The pre-treatment aboveground biomass of overstory trees was approximately 233 Mg/ha (Mg = 1 X 10(6) g). Prescribed burning had significant impacts on the mortality of stems; however, it had no significant effects on the aboveground net primary production (ANPP). Thinning removed approximately 30% of the aboveground biomass and resulted in significant but transient (1 year) reduction of ANPP (386 and 560 g C m(-2) year(-1) for thinned and non-thinned stands, respectively). In sum, thinning created recruitment opportunities in our study area, but these opportunities were captured by maples, and oak recruitment was minimal. Prescribed fire caused mortality in oaks and maples, and the oak mortality may have been related to the coincidence of the burn treatment and the initiation of oak stem growth. Finally, our data suggest that there is a transient impact of thinning on ANPP, but that there is no long-term effect of thinning and/or burning treatments on the aboveground carbon uptake in oak forests. (C) 2007 Elsevier B.V. All rights reserved.

2030: *+.209*

Frankfurt Zoological Society (FZS) started the "Vietnam Primate Conservation Program" in 1991. Included in this program are the management of the Endangered Primate Rescue Center, primate protection, primate habitat protection, primate research, and education. At the end of 2007 the EPRC housed 141 individuals of 17 taxa. Nine individuals of 5 taxa were born during the year and

16 individuals died; nine of those that died were pygmy lorises. Eight captive bred Hatinh langurs were transferred in September to Phong Nha-Ke Bang National Park for the reintroduction project. Sixteen confiscated animals arrived at the center (1 slow loris, 11 pygmy lorises and 4 douc langurs). A big loss for the breeding program at the EPRC has been the disappearance of two female white-cheeked gibbons, most probably shot on the semi-wild area, close to the EPRC and reception area of the national park. The care of all the EPRC animals has been continuously managed by a staff of 20 Vietnamese workers under the supervision of an experienced animal keeper from Zoos in Berlin and Leipzig, Germany. Five biologists and one project assistant complete the staff of the EPRC. Scientific research was carried out in cooperation with several Universities from the USA, the Zoological Museum Dresden, Germany, and with the German Primate Center. Students from German Universities have been working for several months to continue the work on the Primate Data Base. One survey was carried out on Hon Heo Peninsula, Khanh Hoa Province, to assess the status of a black-shanked douc langur population which was discovered in 2006. The support of protection activities at Van Long Nature Reserve continued. Salaries, equipment, and uniforms were provided for 20 guards. A PhD student from Duke University, USA started an ecological study of the Delacour's langurs with support from FZS. A three-week primate training course was organized by Conservation International, Hanoi National University, and FZS for students and staff of protected areas and Forest Protection Departments. A one-week primate training course was organized by Da Nang University and FZS, and carried out for students at Da Nang University. The project assistant of the "Vietnam Primate Conservation Program", and one of the foreign head keepers of the center participated in a three-week training course "Conservation of Endangered Species" at the Durrell International Training Conservation Center at Jersey Zoo, UK. Several TV reports from German and Vietnamese TV stations were made and broadcasted. The guidebook "Protected Animals of Vietnam" was completed. The guidebook should be a tool for the rangers of the Forest Protection Departments and other law enforcement authorities. Posters, postcards, and T-shirts were printed as information and education material.

2031: ***-.042***

In the middle of the last century, the Europe-wide highly endangered "Violet Copper" Lycaena helle, was still widespread in the lowlands of North-East Germany. Today, this species can only be found in the valley of the lower River Uecker. All known historic habitats of the species in Brandenburg were compiled and compared with the habitats of the western Central European population of L. helle. A distribution map will be provided. Finally, the paper discusses conditions and possibilities for the reintroduction of the species in former habitats of North-East Germany.

2032: ***+.083***

In 1999 Canada lynx (Lynx canadensis) were reintroduced to the southern Rocky Mountains and in 2000 the species was listed as threatened under the Endangered Species Act in the contiguous United States (Colorado Division of Wildlife 2000, U.S. Fish and Wildlife Service 2000). To better evaluate the progress of this reintroduction, we conducted field studies to estimate population densities of snowshoe hares (Lepus americanus), the primary prey of lynx in Colorado, USA. We conducted our field studies in southwestern Colorado in winters 2002 and 2003. We estimated population densities in forested stands of mature Engelmann spruce (Picea engelmannii)-subalpine fir (Abies lasiocarpa) and mature lodgepole pine (Pinus contorta) using mark-recapture data and 3 methods for estimating effective area trapped: half trap interval, mean maximum distance moved (MMDM), and half MMDM. In Engelmann spruce-subalpine fir, we found density estimates ranged from 0.08 +/- 0.03 (SE) hares/ha to 1.32 +/- 0.15 hares/ha and in

lodgepole pine, density estimates ranged from 0.06 +/- 0.01 hares/ha to 0.34 +/- 0.06 hares/ha, depending on year and method used for estimating effective area trapped. Our density estimates are similar to those reported at the low phase of the hare cycle in populations to the north (<0.1-1.1 hares/ha), where Canada lynx persist (Hodges 2000a). Although density estimates are a useful comparative tool, they depend upon methods used to estimate effective area trapped. Therefore, we urge caution in comparing our density estimates with those from other areas, which may have used dissimilar methods. We also examined effects of temperature and moon phase on capture success of snowshoe hares; extremely low temperatures affected capture success but moon phase did not. Capture success can be improved by trapping snowshoe hares at temperatures above their lower critical temperature (T-1c). If abundance estimates are derived from mark-recapture studies then effects of temperature should be included when modeling capture probabilities.

2033: ***+.150***

Under the Endangered Species Act, documenting recovery and federally mandated population levels of wolves (Canis lupus) in the Northern Rocky Mountains (NRM) requires monitoring wolf packs that successfully recruit young. United States Fish and Wildlife Service regulations define successful breeding pairs as packs estimated to contain an adult male and female, accompanied by >= 2 pups on 31 December of a given year. Monitoring successful breeding pairs will become more difficult following proposed delisting of NRM wolves; alternatives to historically intensive methods, appropriate to the different ecological and regulatory context following delisting, are required. Because pack size is easier to monitor than pack composition, we estimated probability a pack would contain a successful breeding pair based on its size for wolf populations inhabiting 6 areas in the NRM. We also evaluated the extent to which differences in demography of wolves and levels of human-caused mortality among the areas influenced the probability of packs of different sizes would contain successful breeding pairs. Probability curves differed among analysis areas, depending primarily on levels of human-caused mortality, secondarily on annual population growth rate, and little on annual population density. Probabilities that packs contained successful breeding pairs were more uniformly distributed across pack sizes in areas with low levels of human mortality and stable populations. Large packs in areas with high levels of human-caused mortality and high annual growth rates had relatively high probabilities of containing breeding pairs whereas those for small packs were relatively low. Our approach can be used by managers to estimate number of successful breeding pairs in a population where number of packs and their sizes are known. Following delisting of NRM wolves, human-caused mortality is likely to increase, resulting in more small packs with low probabilities of containing breeding pairs. Differing contributions of packs to wolf population growth based on their size suggests monitoring successful breeding pairs will provide more accurate insights into population dynamics of wolves than will monitoring number of packs or individuals only.

2034: ***-.109***

The Chatham Island taiko Pterodroma magentae (tchaik) is one of the World's most endangered seabirds with a population size of between 120-150 individuals that includes only 8-15 breeding pairs. Molecular techniques were used to identify the sex of taiko, which is difficult to assign morphologically. Blood samples were obtained from almost the entire known living population and from some birds now thought to be dead. We report an approximately even sex ratio in taiko chicks and adults associated with breeding burrows, but a large male-biased ratio in non-breeding adult birds caught on the ground. This finding suggests that unpaired males may be having difficulty in attracting females to burrows and that this situation may be an example of the Allee effect, that reduced density of potential mates acts to decrease population productivity.

Identification of the sex of taiko using a molecular technique has important implications for the conservation management of this critically endangered species, including the future transfer of taiko chicks to a predator-excluded breeding site.

2035: *+.234*

While it is widely acknowledged that nurse-plants facilitate the establishment of seedlings in drylands, there are several anecdotal reports on associations between cacti and rocks. We assessed the preference for rocks or shrubs from the spatial distribution of eight cactus species (Mammillaria spp.). We experimentally assessed if distribution results from facilitation evaluating the survival of Mammillaria pectinifera seedlings growing next to rocks or under nylon sunblockers that simulated shrub shading. We found that more than half of the species avoided shrubs, while 50% of them were positively associated with rocks. Longevity and growth were highest for seedlings placed next to stones, so it may be concluded that the observed association is due to differential survival and not to other processes such as non-random seed dispersal. It is broadly accepted that the association between cacti and shrubs is mainly due to shading, however it may also represent a cost for the seedling in terms of photosynthetically active radiation. Rocks may provide a fresh and moist environment without reducing sunlight. Our results are relevant for conservation and reintroduction programs. It is important to continue the research on stress amelioration in drylands and to consider other kinds of nurse objects besides plant canopies. (c) 2007 Elsevier Ltd. All rights reserved.

2036: *+.195*

1. Understanding the structure of threatened populations, particularly those that exist in degraded or fragmented habitats is crucial for their effective management and conservation. Recently developed methods of individual-based analysis of genetic data provide an unprecedented opportunity to understand the relationships amongst fragmented populations.2. In the present study, population structure of an important cyprinid species (Tor douronensis), which is indigenous to Sarawak, Malaysia, is investigated as part of an ongoing conservation effort to restore threatened wild populations of the species. The population structure inferred using data from seven autosomal microsatellite loci was generally consistent with geography and habitat fragmentation.3. The results indicate that there are two well-defined clusters of T. douronensis in Sarawak, namely the 'northeastern' and the 'southwestern' clusters. In addition, a further subdivision was observed in each of the clusters distributed between river systems. Low levels of gene flow were also observed and migrants between habitat fragments were identified, possibly resulting from human-mediated translocations.4. Implications of the findings for management and conservation of T. douronensis are discussed.

2037: *+.273*

A fuzzy logic knowledge based decision support system, Wildlife Introduction Advisor, was developed using NetWeaver to standardise the decision making process of selecting communal conservancies for wildlife translocation in Namibia. Wildlife translocations are a valuable conservation tool with benefits for both local communities and wildlife populations, but are often undertaken in an ad hoc fashion. In Namibia, communal conservancies present a conservation opportunity for the expansion of wildlife ranges but the economic opportunities associated with wildlife create difficult situations of having to decide who gets animals and who does not. The Wildlife Introduction Advisor is the result of experts' agreement on structuring and prioritising the main factors that need to be considered. Both the validity of the system outputs and their

sensitivity to input changes were evaluated through a series of tests, which show that it is possible to use a fuzzy logic decision support system to aid the selection of translocation sites for wildlife species. A knowledge based decision support tool such as Wildlife Introduction Advisor is an effective tool for a wildlife agency because it standardises the decision making process and thus enhances transparency and management accountability. (c)therefore 2007 Elsevier Ltd. All rights reserved.

2038: ***+.172***

Repatriations and translocations are among the most frequent actions deployed for conservation of amphibians all over the world. However, very few studies have analysed the effectiveness of these actions. The aims of this study were i) to measure the success of repatriations of Rana latastei carried out within a conservation project in Lombardy (Northern Italy) from 1999 to 2001, and ii) to analyse how the environmental factors affected repatriation success. Eighteen ponds were surveyed from February to April 2006, for an overall of 45 field surveys. Each pond was carefully checked for frogs and egg masses. We classified each pond as newly excavated or pre-existing according to habitat management actions and we measured 16 variables concerning micro- and macro-habitat characteristics, human disturbance and presence of predators. Rana latastei was found in 33.3% of monitored sites and the repatriation outcome of tadpole release did not differ between newly excavated and non managed ponds. Habitat variables influenced the success of repatriations, which increased in sites with higher percentage of woodlands. Finally, repatriation outcome was also negatively influenced by human disturbance and predator occurrence.

2039: ***+.190***

Several conservation efforts are being made to recover European rabbit populations (oryctolagus cuniculus) on the Iberian Peninsula. Some of them focus on bur-row management; others involve building different types of warren. A few studies have examined site selection for warren building, and these studies have considered only warren placement within sites and not the broader area surrounding these locations. The objective of this study was to evaluate how landscape pattern determines habitat selection by rabbits for warren building at different spatial scales. Landscape, home range scale, and microhabitat were the spatial scales used in this study. Warrens were not uniformly distributed over the study area but, rather were concentrated in areas with a high abundance and cover of Retama monosperma and high vegetation cover. Rabbits preferred digging warrens in areas with low fragmentation and where patches are few, large, and contiguous. Based on our results, we suggest that a study of landscape structure should be carried out before design habitat management, recovery or translocation programs. Such studies will need to take into account the physiognomy and size, shape, and continuity of patches in fragmented landscapes. Rabbit conservation programs must address areas that provide not only the maximum potential rate of intake, but also good soil and vegetation cover conditions for warren building and suitable surrounding areas. (c) 2008 Elsevier Masson SAS. All rights reserved.

2040: ***+.119***

Simple correlated random walk (CRW) models are rarely sufficient to describe movement of animals over more than the shortest time scales. However, CRW approaches can be used to model more complex animal movement trajectories by assuming individuals move in one of several different behavioural or movement states, each characterized by a different CRW. The spatial and social context an individual experiences may influence the proportion of time spent in different movement states, with subsequent effects on its spatial distribution, survival and fecundity. While

methods to study habitat influences on animal movement have been previously developed, social influences have been largely neglected. Here, we fit a 'socially informed' movement model to data from a population of over 100 elk (Cervus canadensis) reintroduced into a new environment, radio-collared and subsequently tracked over a 4-year period. The analysis shows how elk move further when they are solitary than when they are grouped and incur a higher rate of mortality the further they move away from the release area. We use the model to show how the spatial distribution and growth rate of the population depend on the balance of fission and fusion processes governing the group structure of the population. The results are briefly discussed with respect to the design of species reintroduction programmes.

2041: *+.103*

Interspecific hybridization has increasingly become regarded as a serious threat to the genetic integrity and persistence of rare plants. Oenothera glazioviana (Onagraceae) is a horticultural species that has escaped cultivation and now threatens the narrow Pacific coastal endemic O. wolfii with hybridization. Reports of morphologically intermediate and ecologically aggressive forms prompted this investigation into the extent of hybridization over the range of O. wolfii. In particular, this study identifies populations of pure and hybrid origins. We used multivariate methods to characterize the morphological variation of Oregon and northern California coastal Oenothera populations. Putatively pure O. wolfii and O. glazioviana individuals do not overlap in many floral characteristics. We found morphologies ranging between the 2 species in northern California populations, however, supporting the inference of hybridization. Remote O. wolfii populations in Oregon were smaller in almost all characters, and discriminant analysis was able to distinguish the rare species from both hybrids and O. glazioviana. However, 5 of 10 O. wolfii populations overlapped significantly with hybrid populations for individual traits and composite morphology, and trait values outside the range of O. wolfii were discovered in 2 populations previously considered to be pure. We also discuss the morphological evidence in light of these species' chromosomal complexes and environmental factors.

2042: *+.375*

The World Conservation Union (1987) defines a translocation as a release of animals with the intention of establishing, reestablishing, or augmenting an existing population. Despite frequent use as a tool for the management of threatened and endangered wildlife, the full benefits of translocations often go unrealized. In this article, I demonstrate how translocations can achieve outputs for conservation management, conservation science, and the wider human community, using North Island (NI) Saddleback or Tieke (Philesturnus rufusater) as an illustrative example. From a conservation management perspective, NI Saddleback have been salvaged from a relic population of less than 500 birds on 484-ha Hen Island to a metapopulation of approximately 6,000 birds on 13 offshore islands and at two mainland New Zealand sites. These translocations have reduced the risk of global extinction for this species and helped restore the ecosystems involved. All these translocations have occurred in the past 42 years from known source populations and with known numbers of birds released. The resulting replicated serial population bottlenecks provide numerous scientific opportunities for conservation and biological research. Although the first Saddleback translocations were to reserves closed to the public, subsequent translocations have been to open reserves, providing the wider human community with an opportunity to see and be actively involved in the management of a threatened endemic species. This has raised the profile of both NI Saddleback and other species and has provided wider community conservation benefits. These three outputs illustrate the value of translocations for resource management and conservation science and for increasing community interest,

participation, and investment in biological conservation.

2044: *-.098*

During the last two centuries, lynx populations have undergone severe declines and extinctions in Europe. The Alpine lynx, once distributed across the whole Alpine arc, became extinct due to direct human prosecution and deprivation of its main prey in the 1930s. Similar to the Iberian lynx Lynx pardinus, its taxonomy has been subject to several controversies. Moreover, knowing the taxonomic status of the Alpine lynx will help to define conservation units of extant lynx populations in Europe. In this study, we investigated two mitochondrial DNA regions in museum specimens (n=15) representing the autochthonous Alpine population and in samples from extant Eurasian lynx Lynx lynx populations in Europe and Asia (n=17). Phylogenetic analysis (cytochrome b, 345 bp) placed the Alpine lynx within the Eurasian lynx lineage. Among all individuals examined, seven different haplotypes (control region, 300 bp) were observed but no unique Alpine haplotype was discovered. Haplotypes of the extinct Alpine population were identical to previously described haplotypes in Scandinavian lynx signifying a recent genetic ancestry with current European populations. Moreover, our genetic data suggest two distinct glacial refugia for the Carpathian and Balkan population. Overall this study demonstrates that historical DNA from extinct populations can help to disentangle the phylogenetic relationships and historical biogeography of taxa with only a limited number of extant populations remaining.

2045: *+.233*

Freshwater mussel (Superfamily Unionoidea) communities are important components of food webs, and they link and influence multiple trophic levels. Mussels filter food from both the water column and sediment with ciliated gills. Differences in cilia structure and arrangement might allow mussel species to partition food resources. Mussels are omnivores that feed across trophic levels on bacteria, algae, detritus, zooplankton, and perhaps, dissolved organic matter. Living mussels and their spent shells provide or improve habitat for other organisms by providing physical structure, stabilizing and bioturbating sediments, and influencing food availability directly and indirectly through biodeposition of organic matter and nutrient excretion. Effects of mussel communities on nutrient translocation and cycling depend on mussel abundance, species composition, and environmental conditions. Nutrient-related mussel effects influence multiple trophic levels. Healthy mussel communities occur as multispecies assemblages in which species interactions are probably very important. Food limitation and competition among species have been documented, but so have positive species interactions, and rare species have been shown to benefit energetically from living in species-rich communities. Effects of mussel species on ecosystem services and food webs vary across spatial and temporal scales, and the relative importance of competition and facilitation might change at different scales.

2046: *+.163*

Metapopulation dynamics are influenced by spatial parameters including the amount and arrangement of suitable habitat, yet these parameters may be uncertain when deciding how to manage species or their habitats. Sensitivity analyses of population viability analysis (PVA) models can help measure relative parameter influences on predictions, identify research priorities for reducing uncertainty, and evaluate management strategies. Few spatial PVAs, however, include sensitivity analyses of both spatial and nonspatial parameters, perhaps because computationally efficient tools for such analyses are lacking or inaccessible. We developed GRIP, a program to facilitate sensitivity analysis of spatial and nonspatial input parameters for PVAs created in

RAMAS Metapop, a widely applied software program. GRIP creates random sets of input files by varying parameters specified in the PVA model including vital rates and their correlations among populations, the number and configuration of populations, dispersal rates, dispersal survival, initial population abundances, carrying capacities, and the probability, intensity, and spatial extent of catastrophes, while drawing on specified parameter distributions. We evaluated GRIP's performance as a tool for sensitivity analysis of spatial PVAs and explored the consequences of varying spatial input parameters for predictions of a published PVA model of the sand lizard (Lacerta agilis). We used GRIP output to generate standardized regression coefficients (SRCs) and nonparametric correlation coefficients as indices of the relative sensitivity of predicted conservation status to input parameters. GRIP performed well; with a single analysis we were able to rank the relative influence of input parameters identified as influential by the PVA's original author, S. A. Berglind, who used three separate forms of sensitivity analysis. Our analysis, however, also underscored the value of exploring the relative influence of spatial parameters on PVA predictions; both SRCs and correlation coefficients indicated that the most influential parameters in the sand lizard model were spatial in nature. We provide annotated code so that GRIP may be modified to reflect particular species biology, customized for more complex spatial PVA models, upgraded to incorporate features added in newer versions of RAMAS Metapop, used as a template to develop similar programs, or used as it is for computationally efficient sensitivity analyses in support of conservation planning.

2047: ***+.156***

Pedigree analysis has clear benefits for the genetic management of threatened populations through the evaluation of inbreeding, population structure and genetic diversity. The use of pedigrees is usually restricted to captive populations and few examples exist of their exclusive use in managing free-ranging populations. One such example is the management of the takahe (Porphyrio hochstetteri), a highly endangered, flightless New Zealand rail at risk from introduced mammalian predators and habitat loss. During the 1980's and 90's, as part of the takahe recovery programme, birds were translocated from the sole remnant population in Fiordland to four offshore islands from which introduced predators had been eradicated. The subsequent "island" population, now numbering 83 and thought to be at carrying capacity, has been closely monitored since founding. Detailed breeding records allow us to analyse the island pedigree, which is up to 7 generations deep. Gene-drop analysis indicated that 7.5% of genetic diversity has been lost over the relatively short timeframe since founding (2.1 generations on average; total genetic founders = 31) due to both a failure to equalise founder representation early on and subsequent disproportionate breeding success (founder equivalents = 12.5; founder genome equivalents = 6.6). A high prevalence of close inbreeding will have also impacted on genetic diversity. Predictions from pedigree modelling suggest that 90% genetic diversity will be maintained for only 12 years, but by introducing a low level of immigration from the Fiordland population and permitting the population to grow, 90% GD could be maintained over the next 100 years. More generally, the results demonstrate the value of maintaining pedigrees for wild populations, especially in the years immediately after a translocation event.

2048: ***-.076***

Natural populations of the endangered western barred bandicoot (Perameles bougainville) now exist on only two islands in Shark Bay, Western Australia. Our aim was to investigate genetic diversity in natural, reintroduced, and captive populations of the bandicoots and to assess the extent of divergence between the populations. The contemporary isolation of the natural populations has resulted in heterogeneity of allele frequency between the islands, which has acted

to maintain a higher combined diversity than would be expected from either population on its own. These findings highlight how remnant island populations can act as genetic reservoirs to maximize diversity for reintroductions into a species former range. Although diversity is high between island populations, diversity within populations, based on six microsatellite loci, are amongst the lowest ever recorded for populations of marsupials. The mtDNA sequence data indicate that the two remaining natural populations show only minor divergence from each other, with the five haplotypes separated by just single base pairs. The reintroduced population and captive colonies show evidence for the loss of diversity related to genetic drift operating on small isolated populations.

2049: ***+.230***

Hainan Eld's deer (Cervus eldi hainanus) experienced a dramatic decline in the late 1960s through early 1970s and by 1976 only 26 deer remained in Datian of Hainan Island, China. Since then, conservation efforts have successfully rescued this deer from extinction. We employed 10 microsatellite DNA loci to index genetic variation in the one source (Datian) and two introduced populations (Bangxi and Ganshiling) and suggest implications for the conservation of the species. A total of 40 alleles at 10 loci were examined from 198 deer blood samples. The source population harbored all 40 alleles, while the Bangxi and Ganshiling translocated populations contained 24 and 26 alleles, respectively. The genetic variability was low (H(e) approximate to 0.33) for each of the three populations. No significant difference in genetic variability between the three populations was detected ($P > 0.05$); yet significant differentiation was found among the three populations. Our results suggest that founder effects and genetic drift have affected the two translocated populations. For conservation we recommend the three populations be managed as a meta-population. When establishing future reintroductions, the founder population should have a size larger than the original 26 founders in Datian population or be composed of a cohort of over 20 same-age individuals with 1:1 sex ratio. Genetic monitoring for both the source and translocated populations should be continuously conducted in order to assess the effectiveness of deer conservation in the future.

2050: ***-.026***

The reintroduction of endangered species is a potentially useful conservation strategy, which in the case of freshwater unionid mussels, must be preceded by the successful laboratory rearing of juvenile mussels on their host fishes. However, an understanding of the genetic and environmental implications of reintroductions of artificially propagated mussels is required. Unfortunately, there is a dearth of information on these issues with respect to freshwater mussels. In general, regarding the genetic effects of reintroductions, small founder populations may lead to low heterozygosity (reduced genetic variability) in the reintroduced populations, which can make them more susceptible to extinction. Captive breeding programs may also alter the genetic composition of species through artificial selection, whether intentional or unintentional. Captive breeding may also affect an individual's interactions with conspecifics or predators by altering behaviour. Genetic problems in reintroduced populations also have the potential to affect wild populations, particularly by reducing variability among populations of the same species and eliminating local adaptation. There is also the possibility that diseases, parasites, or exotic species may be spread when populations are relocated or augmented. Recommendations related to the minimization of these impacts are presented for freshwater mussels, with the recognition that many of the issues will require additional study.

2051: ***+.205***

Some 2,000 orphaned chicks of African Penguins Spheniscus demersus were hand-reared and released back into the wild on Robben and Dassen Islands following the Treasure oil spill in June 2000. Of these chicks, 1,787 were flipper banded. This paper reports on the subsequent survival rate and breeding success of those individuals seen on Robben Island from 2001-2006. Survival to breeding age and their subsequent breeding success of hand-reared chicks was no different from that of naturally-reared chicks. Over a four-year period, pairs where at least one partner was a hand-reared chick produced an average of more than 1.6 chicks per year. Combining the data on survival with that on breeding success indicates that 1,000 hand-reared chicks will produce around 1,220 chicks themselves over their lifetimes, making this a worthwhile conservation intervention.

2052: ***+.249***

The coastal population of the black-headed dwarf chameleon Bradypodion melanocephalum is threatened by rapid urban expansion in and around Durban which lies in the centre of the chameleon population's distribution, Translocations of threatened species from urban development sites is a mitigation method that is used in various parts of the world. The translocation of B. melanocephalum largely by volunteers from a proposed light industrial business park to two neighbouring areas with partially restored habitats was eventually successful in one recipient area and unsuccessful in the other. The results suggest that translocations of B. melanocephalum from development construction sites to recipient areas will only be successful if adequate time and resources are available for: a) finding and securing suitable. areas for habitat restoration; b) sufficient restoration of the indigenous vegetation in the recipient areas before the capture and translocation of B. melanocephalum from the proposed development sites; c) searching for, and capture of, all B. melanocephalum on the development sites, and; d) long-term management of the restored habitat in a manner that does not result in significantly elevated mortality or dispersal of B. melanocephalum. Monitoring of B. melanocephalum numbers in the recipient sites, by at least two people per transect, is important to gauge the success or otherwise of the translocation. Monitoring can give insights into the controllable factors that have an impact on the numbers of chameleons, and provide suggestions as how to improve the management of the habitat.

2053: ***+.098***

De Hoop Nature Reserve and a neighbouring conservancy contain the most genetically diverse subpopulation of the Endangered (IUCN) Cape mountain zebra (Equus zebra zebra Linnaeus 1758). Although vital for the long-term stability of the meta-population, the population had received limited monitoring post-1999. We summarize data obtained during a population monitoring programme established in 2005. Ninety-nine individuals were identified indicating a decline in annual population growth from 6.6% (1995-1999) to 4.5% (1999-2005). The population was male biased and the deficit of females is likely to have prevented additional breeding herd formation resulting in excess nonbreeding males. These animals are currently of limited reproductive value to the meta-population and may be contributing to the decline in reproductive potential at De Hoop by competing for limited resources. One solution may be to translocate 'excess' males to reinforce existing small populations or establish new populations with females from elsewhere provided that a minimum of 78 animals is maintained at De Hoop to limit genetic loss. Population monitoring and effective management strategies for the De Hoop population and the meta-population are vital to ensure the long-term survival of Cape mountain zebra and for the success of other species recovery programmes.

2054: ***+.233***

Interspecific competition can have a substantial impact on sympatric carnivore populations and may threaten reintroduction attempts of threatened or endangered species. Coyotes (Canis latrans) are the primary threat to recovery of red wolves (C. rufus) in the wild, through hybridization and loss of the red wolf genotype and habitat occupancy that reduces space available for wolf occupation. We built a stochastic simulation model (using data collected from a recovering red wolf population in northeastern North Carolina as well as from the literature) to examine spatial dynamics of sympatric red wolves and coyotes (independent of habitat influences) and to elucidate the potential role of coyotes on wolf recovery and reintroduction success. Survival of juvenile and adult wolves had the greatest impact on wolf population size and likelihood of extinction. Introducing coyotes to the model had a substantial negative impact on wolf numbers, and the model was highly sensitive to the estimates of the competitive impact of coyotes on red wolves, through declines in wolf productivity. We simulated coyote management from either removal (lower coyote survival) or surgical sterilization (lower coyote reproductive rates) and found that both management strategies increased viability of red wolf populations, especially during initial colonization. Our results suggest that coyotes can inhibit red wolf reintroduction success through competitive interactions, but that management of coyote populations can improve the probability of successful wolf recovery. Additional information on spatial dynamics and dietary overlap between coyotes and wolves in the recovery area is needed to further elucidate the current and potential competitive impact of coyotes on red wolf populations. (c) 2008 Elsevier B.V. All rights reserved.

2055: ***-.015***

In-situ captive rearing of endangered passerines for reintroduction has rarely been used as a conservation tool. Nests of Mauritius fodies threatened with predation by introduced mammalian predators were harvested from the wild, and chicks were reared to independence for release onto an offshore, predator-free island. The daily probability of the survival was higher in captivity than in the wild, and 69 chicks were reared to fledging of which 47 would have been expected to fledge in the wild. Harvesting of nests probably had little impact on the wild population. Captive breeding trials on Mauritius. fodies showed that large numbers of individuals could be produced for a release program from a small number of pairs if enough space was provided. Artificial incubation of passerine eggs and rearing of chicks can be used to increase the productivity of endangered taxa. Zoos can play an important role in in-situ conservation programs through provision of avicultural expertise and training of local staff.

2056: ***-.339***

Butternut (Juglans cinerea L.) is a native, cold-tolerant, hard-mast species formerly valued for its nuts and wood, which is now endangered. The most immediate threat to butternut restoration is the spread of butternut canker disease, caused by the exotic fungus Sirococcus clavigignenti-juglandacearum Nair, Kostichka & Kuntz. Other threats include the hybridization of butternut with the exotic Japanese walnut (Juglans ailantifolia Carr.) and poor regeneration. The hybrids, known as buartnuts, are vegetatively vigorous, highly fecund, more resistant than butternut to butternut canker disease and difficult to identify. We review the vegetative and reproductive morphological traits that distinguish butternut from hybrids and identify those that can be used by field biologists to separate the taxa. No single trait was sufficient to separate butternut from hybrids, but pith color, lenticel size, shape and abundance, and the presence or absence of a notch in the upper margin of leaf scars, can be used in combination with other traits to identify butternuts and exclude most hybrids. In at least one butternut population, reduced symptoms of butternut canker disease were significantly associated with a dark barked phenotype. We also describe two

randomly amplified polymorphic DNA (RAPD) markers that differentiate butternuts from hybrids based on DNA polymorphism. Together, these results should assist in the identification and testing of non-hybrid butternut for breeding and reintroduction of the species to its former habitats.

2057: ***+.232***

Reintroduction or rehabilitation plans for fish populations in many systems (e.g., lakes) are complicated by limited data on ecological and genetic characteristics before human disturbances occurred. While no two lakes have identical physical and biological characteristics, a growing body of empirical evidence nevertheless indicates that parallel patterns of population structuring may evolve within northern temperate fish species. Examining the population structuring in undisturbed lakes of similar physical and biological characteristics may thus provide insight into the probable historical extent and causes of both population structuring and connectivity in human-impacted lakes. Here, we review research on the population structuring and evolution of migratory brook trout Salvelinus fontinalis in a relatively undisturbed, postglacial lake (Mistassini Lake, Quebec). We provide information on lake habitat use, the morphology and life history characteristics of populations, diets, lakewide genetic population structure, seasonal migration characteristics between spawning and feeding areas, population evolutionary histories, and the prevalence of lake spawning. The biology of Mistassini Lake brook trout has a compelling number of similarities with what is known about that of the "coaster" form in Lake Superior and lake-migratory brook trout elsewhere. Our review also has several implications for the rehabilitation of coaster populations with respect to (1) clarifying the degree of natural connectivity between populations; (2) predicting the likelihood of recolonization of vacant habitats; (3) choosing candidate source populations for translocations; and more broadly, (4) understanding the spatial scale of probable local adaptation. Mistassini Lake therefore provides a useful case study that applies to lake-migratory trout elsewhere. We hope that our research will stimulate managers and biologists working on similar systems with pronounced human disturbances to consider the interplay between ecology and evolution in future conservation efforts.

2058: ***+.203***

Twelve years after six (2[male][male]4[female]) pygmy hogs were captured for conservation breeding purposes from the last surviving wild population in Manas National Park, 16 (7[male][male]9[female][female]) captive bred hogs were re- leased in Sonai Rupai Wildlife Sanctuary in north-west Assam between 4th and 9th May 2008. This event not only constituted the first such reintroduction attempt, but the first of a proposed series of releases in this sanctuary; hopefully to be followed by similar series of releases into 'vacant' habitats in two other protected areas within the known or presumed former range of this species in this region. This project is being conducted by Pygmy Hog Conservation Programme (PHCP), a collaborative ven- ture between PPHSG, Durrell Wildlife Conservation Trust and local government authorities. This agreement, the first of its kind in India, was first signed in 1995, and was intended to facilitate implementation of a wide-ranging conservation action for this species originally drafted and approved by both the Union Ministry and Assam State Forest Department in 1981! Both the PPHSG and Durrell have played instru- mental roles in the development of the Action Plan and in subsequent evolution of this Programme, for which purposes Durrell also kindly provided or sourced the majority of funding support and provided di- verse other assistance for the past 30 or more years. These animals were bred at the PHCP research and breeding centre at Basistha, near Guwahati, before being transferred to a specially constructed 'pre-release' facility in Potasali, on the outskirts of Nameri National Park, east of Sonai Rupai Wildlife Sactuary; both of which areas had been identified as likely future reintroduction sites. Whilst at Potasali, every effort was made to 'pre-condition' the

animals for eventual release by maintaining them in integrated social groups, in simulated natural habitats intended to encourage natural foraging, nest-building and other behaviours; whilst also minimising human contact to mitigate tameness and other behavioural characteristics consequent upon their captive management. Whilst in Potasali radio-harnesses designed for post-release monitoring studies were field-tested during trial attachments to two individuals in each group, thereby unexpectedly exposing serious problems in the use of these harnesses, which the animals were either able to escape from or which caused unacceptable injuries. Possible alternative means of monitoring were devised and initiated, including training the ani- mals to visit random bait sites and screening for field 'sign'. After 5 months tenure in the 'pre-release' enclosures at Potasali, these hogs were transferred in early May to temporary 'soft-release' enclosures constructed for this purposes in a relatively secluded, but easily accessible area of natural habitat in the far interior of the Sonai-Rupai Sanctuary. These enclosures were also rigged with two lines of electric fencing and kept under continual surveillance as a precaution against potential predators and to deter incursion by wild elephants. The animals were maintained for a further two to three weeks in these enclosures before being released, by the simple expedient of removing sections of fence in each of the three enclosures and allowing the animals to find their own way out.

2059: *+.209*

Primula denticulata ssp. sinodenticulata (Primulaceae) is an herbaceous perennial in southwest China. Many gardeners recommend it for alpine garden construction or greenhouse cultivation. Due to habitat destruction, however, this plant is rapidly decreasing in number and size of populations. To conserve this plant for our future reintroductions and breeding, 10 natural populations of Primula denticulata ssp. sinodenticulata were analyzed using morphological markers. Morphological diversity among and within populations were analyzed based on 8 phenotypic traits of 30 plants from each population. Phenotypic variance analysis showed that significant variation existed among and within populations. Coefficient of variations (CVs) ranged between 4.73% and 9.90%, and phenotypic differentiation coefficients (Vst) ranged from 15.41% to 40.69%, the mean value was 28.54% and within-population variation comprised a majority of total phenotypic variation. UPGMA Cluster analysis based on morphological traits showed that the 10 populations could be divided into three groups. According to our results, conservation of this species' genetic resources should focus on protecting populations with higher morphological variation, and in situ conservation should be carried out in their original habitats.

2060: *+.127*

Small-bodied, riverine minnows that historically characterized fish assemblages of Great Plains river, in North America have declined because of river fragmentation, dewatering, river channel degradation, river salinization and normative species introductions. The Pecos bluntnose shiner Notropis simus pecosensis, a member of this guild, persists in one segment of the Pecos River, New Mexico, USA. We characterized habitat associations for the species at two spatial scales. In general, N. s. pecosensis associated with fluvial habitats, but velocity association depended on body size, with larger individuals using swifter habitats. All N. s. pecosensis associated with relatively low depths (3-51 cm), which were most abundant in sites with relatively wide river channels (>25 m), especially when discharge was between 0.5 and 4.0 m(3) s(-1). The Pecos River sub-segment that is occupied by the core population of N. s. pecosensis (V-ii) had a unique combination of being buffered from direct dam effects by intervening segments and sub-segments, high sub-segment length, substantial sediment inputs from numerous uncontrolled tributaries, substantial base flow provided by irrigation return flows and groundwater inflows, high channel width in relation to discharge and low salinity. Although no unoccupied Pecos River segment

appears to be suitable for N. s. pecosensis, habitat restoration opportunities exist within all occupied sub-segments (V-i, V-ii and V-iii) via base flow enhancement and river channel restoration. Restoration that offsets chronic effects of dams may be necessary to conserve the species. Restoration would also benefit other rare riverine minnows that coexist with N. s. pecosensis. Copyright (C) 2008 John Wiley & Sons, Ltd.

2061: *-.105*

We examined the levels and apportionment of genetic variation of the 11 known subpopulations of Atractylis preauxiana at 95 RAPD loci to help streamline a conservation strategy for this Canarian endemic taxon, which is in a critical situation because of the constant exposure of plants to intensive, uncontrolled anthropic action in the last few decades. Our results revealed low genetic variation levels that match with the general picture of demographic and habitat degradation that this taxon is undergoing. Although geographic isolation between Tenerife and Gran Canaria is an effective barrier to gene flow, genetic heterogeneity within islands is also substantial, plausibly due to the negative impact of fragmentation on genetic variation. Our genetic results, together with declining population sizes, poor seedling survival, and recent population extinctions, compellingly indicate that A. preauxiana is undergoing an extinction ratchet, whereby every further local extinction will add up to the probability of total species' extinction. Our genetic results suggest that mitigating the deleterious consequences of this effect entails urgent mixed reinforcements of all sub-populations with sub-populations from the same island and urgent translocation of the two sub-populations from Tenerife that are doomed to extinction to ecologically suitable areas, together with seed collection and preservation in a convenient ex situ banking facility.

2062: *-.117*

The recently-described Arunachal macaque Macaca munzala is so far known only from western Arunachal Pradesh, north-east India. Here we present the first conservation status assessment for the species. Our surveys enumerated a total of 569 individuals in the Tawang and West Kameng districts of the State. The species seems to be tolerant of anthropogenic habitat change but is vulnerable to hunting. A low infant to adult female ratio suggests that not all adult females reproduce at any given time, and females do not give birth every year. The macaques are persecuted largely in response to crop damage, with the practice of keeping them as pets providing an added incentive to hunting. The species is, however, able to attain remarkably high densities in the absence of hunting. Crop damage by the macaque is widespread; patterns of crop damage are similar across altitudinal zones and do not seem to be correlated with macaque density. The species will need to be protected in human-modified landscapes, and the issues of crop damage and retaliatory persecution need to be addressed urgently.

2063: *+.252*

Establishing new populations by transferring founder individuals from source populations has been effective for managing the recovery of many threatened species including some weta (Orthoptera: Anostostomatidae) in New Zealand. These large-bodied flightless insects are 'flagship species' for insect conservation in New Zealand and many are rare or threatened. The declining abundance of most weta species, particularly giant weta, can be attributed to the introduction of mammalian predators, habitat destruction, and habitat modification by introduced mammalian browsers. New populations of some weta have been established in locations, particularly on islands, where these threats have been eliminated or severely reduced in order to reduce the risk of extinction. Some populations were established to provide food for endemic vertebrates, ecosystem

restoration and ready access for the general public. We illustrate how methods for both transferring weta and monitoring them have become more sophisticated by using a series of case studies. Other transfers of weta not included in the case studies are also summarised. We conclude by re-iterating the importance of documenting the transfer and post-release monitoring for all insect transfers, both for biogeographical reasons and to provide information to improve future transfers.

2064: *+.180*

The second half of the 20(th) century was characterised by intense processes of urbanisation, industrialisation and agricultural mechanisation, leading to a fragmentation of the agricultural and forest landscape. This, in turn, reduced the bio-permeability of the territory and affected the dispersion of many wild species. Brown hare (Lepus europeus) dispersion is dramatically affected by habitat fragmentation, presence of predators, intense tillage and elevated hunting pressure. Consequently, the only stable populations of hare are often in no-hunting areas where wildlife management is efficient. It is necessary, therefore, to identify not only additional areas suitable for reproduction, but also the most suitable dispersion pathways for hares, in order to optimise management. In the present study, by means of a Geographic Information System (GIS), a deterministic hare suitability model was developed on the basis of a multicriterial approach and fuzzy logic. Subsequently, a friction surface was derived from the suitability map in order to describe the land bio-permeability. Finally, on the basis of species potential, the spread of hares from stable population areas (source areas) to the remaining territory was simulated. The area of study was the province of Viterbo (central Italy). The suitability map showed good discrimination ability (ROC=0.705). The hare dispersion simulation map allowed the potential spreading of this species throughout the provincial territory to be analysed. Isolated or less connected zones were highlighted, allowing the distribution of habitat enhancements, and/or the institution of new no-hunting areas devoted to the reproduction and consequent spread of hares throughout the territory, to be localised. The presented flexible and reiterable methodology could prove useful for wildlife management and hunting planning over a wide area. It would thus provide an important contribution to reducing the importance of animal translocation and favouring an increase in native resources spontaneously spreading throughout a territory. In a more general sense, this study is in accordance with the sustainable land management perspective, meeting the requirements of environmental protection, without compromising the anthropic development of non-urban areas.

2065: *+.179*

Guidelines designed to aid in the restoration of rare species have been previously proposed using two primary strategies to select individuals for augmentation and reintroduction: mixing progeny from different populations or separating individuals from different populations. Understanding the genetic structure and diversity of an endangered species can offer insights into conservation management strategies. We used random amplified polymorphic DNA markers to assess the genetic structure and diversity of Jacquemontia reclinata, a federally endangered species endemic to Southeastern Florida. We sampled 20 percent of total number of individuals from eight of the ten known wild populations. Across individuals high levels of polymorphic loci (94.7%) were found and larger populations had greater genetic diversity. Cluster and ordination analyses found that one population was genetically differentiated from all the others; this population grows in a unique habitat. Most genetic variation (77.5%) was found within populations, and genetic distances between populations were not explained by their geographic distances. We recommend the use of two management units in restoration programs for J. reclinata, one consisting of the genetically differentiated population and the second consisting of the other seven populations

sampled.

2066: *+.167*

Estimating historic distributions of species is a critical step in evaluating current levels of habitat loss, evaluating sites for potential restoration and reintroductions, and for conservation planning at a landscape scale. However historic distributions can be difficult to estimate objectively because substantial habitat changes may have occurred prior to comprehensive surveys. As a means to address this question, we evaluated a novel approach by creating spatial niche models for two species of psammophilic lizards. Using a partitioned Mahalanobis D-2 analysis and abiotic variables that were independent of anthropogenic change, we created niche models for the federally threatened Coachella Valley fringe-toed lizard (Uma inornata) and for the flat-tailed horned lizard (Phrynosoma mcallii). The niche models estimated that within the Coachella Valley there were originally 32,164 ha of potential habitat for the fringe-toed lizard and 33,502 ha of potential habitat for the horned lizard. After screening these estimates of historic habitat for current conditions that would render that potential habitat unsuitable, we calculated a 91-95% loss of potential habitat for the fringe-toed lizard and an 83-92% loss for the horned lizard. Unlike the fringe-toed lizard, the horned lizard also occurs outside the Coachella Valley. Conducting a similar analysis throughout its range would provide an objective estimate of the total habitat loss experienced by this species. This information could be used to address whether granting it federal or state protection is warranted. For species whose distributions can be modeled with abiotic variables such as soils, elevation, topography, and climate, this approach may have broad applications for resolving questions regarding their current levels of habitat loss and regional conservation planning. (c) 2008 Elsevier Ltd. All rights reserved.

2084: *+.100*

1. Populations of white-clawed crayfish (Austropotamobius pallipes) have undergone substantial declines across Europe. Remaining populations tend to be fragmented and in many catchments they are restricted to upland streams. Information is needed concerning their spatial ecology to assist with conservation and rehabilitation of existing fragmented populations, as well as possible reintroductions.2. A novel method for the long-term tagging of white-clawed crayfish was used to study the spatial ecology of a white-clawed crayfish population fragment in a small, moderate-gradient upland stream. Internal passive integrated transponder (PIT) tags enabled adult crayfish (carapace length > 27 mm) to be permanently tagged, each with a unique identification code and resulted in a high number of recaptures. Of 501 crayfish tagged 413 were subsequently relocated at least once.3. Crayfish did not make extensive movements, the median annual distance moved was 84.8 m yr(-1), equivalent to annual net movement of 0.233 m day(-1), substantially less than reported in previous studies. The lower levels of movement may reflect the study encompassing all seasons, including winter, when crayfish are relatively inactive.4. Significantly more crayfish moved downstream compared with upstream and distances moved downstream were significantly greater than those in an upstream direction. This may be linked to the relatively high gradient of the stream and a reduced passability of the abundant riffles to upstream movements of crayfish.5. A small weir acted as a barrier within the stream, preventing upstream movements of crayfish. Even small barriers may limit the movements of white-clawed crayfish and have the potential to limit connectivity within populations and prevent expansion or recolonization.6. The results presented suggest that fragmented populations of white-clawed crayfish in upland streams are unlikely to expand rapidly and reconnect to other population fragments, even where habitat is suitable. In establishing stream populations care should be taken to ensure that even small barriers to movement are removed unless these are intended to exclude non-native biota, especially

crayfish. Copyright (c) 2007 John Wiley & Sons, Ltd.

2085: ***+.289***

The European mink (Mustela lutreola) is a small mammal, which belongs to the Mustelidae family (Carnivora). Earlier, the range of distribution of this species encompassed much of the European continent. During the 20th century, the numbers of European mink declined and the range of its distribution became reduced to three fragmented populations; today this species faces extinction. The urgent necessity for effective conservation efforts to protect the European mink is accepted by the governmental organizations as well as scientific communities of most European countries. In this paper, the reasons for the disappearance of European mink are reviewed and results of past conservation efforts based on captive breeding and reintroduction programmes are critically evaluated in the broad context of modern concepts of conservation genetics and reproductive biology. The data recently obtained on the reproduction and pre-implantation development of European mink and the prospects of incorporation of modern reproductive technologies into the conservation programme of this species are discussed.

2086: ***-.060***

We use the information provided by 36 RAPD loci and 15 morphological traits to describe and construe the population differentiation in A. arbuscula, a Canarian endemic Asteraceae threatened with extinction that exhibits a disjunct distribution in the islands of Gran Canaria (var. schizogynophylla) and Lanzarote (var. arbuscula). Our evaluation of morphological characters and the extent of RAPD differentiation found sets the stage for a taxonomic restructuring to hoist both var. arbuscula and var. schizogynophylla to subspecies category. Our genetic results suggest that fragmentation and generally low population sizes are jeopardising the survival of this species through a predominance of stochastic processes in microevolutionary dynamics, especially in Gran Canaria, where subpopulations exhibit the lowest levels of genetic variation and gene flow. If, as most Asteraceae, these plants have a sporophytic self-incompatibility system, the scarce reproductive turnover observed in the much smaller subpopulations from Gran Canaria (ssp. schizogynophylla) is possibly more influenced by a deficiency of S-alleles (that would be provoking the unavailability of compatible mating types, and a cascade of deleterious effects associated with the Allee effect) than by a reduced pollinator visitation frequency. Based on the depauperated values of genetic parameters for this subspecies, urgent mixed subpopulation reinforcements and reintroductions (with specimens belonging to the same subspecies) seem advisable on the grounds that they might allow the isolated habitat remnants to retrieve from inbreeding through the introduction of a new stock of S-alleles and the subsequent production of genotypes that may have a higher selective value.

2087: ***-.046***

The global amphibian crisis has resulted in renewed interest in captive breeding as a conservation tool for amphibians. Although captive breeding and reintroduction are controversial management actions, amphibians possess a number of attributes that make them potentially good models for such programs. We reviewed the extent and effectiveness of captive breeding and reintroduction programs for amphibians through an analysis of data from the Global Amphibian Assessment and other sources. Most captive breeding and reintroduction programs for amphibians have focused on threatened species from industrialized countries with relatively low amphibian diversity. Out of 110 species in such programs, 52 were in programs with no plans for reintroduction that had conservation research or conservation education as their main purpose. A further 39 species were

in programs that entailed captive breeding and reintroduction or combined captive breeding with relocations of wild animals. Nineteen species were in programs with relocations of wild animals only. Eighteen out of 58 reintroduced species have subsequently bred successfully in the wild, and 13 of these species have established self-sustaining populations. As with threatened amphibians generally, amphibians in captive breeding or reintroduction programs face multiple threats, with habitat loss being the most important. Nevertheless, only 18 out of 58 reintroduced species faced threats that are all potentially reversible. When selecting species for captive programs, dilemmas may emerge between choosing species that have a good chance of surviving after reintroduction because their threats are reversible and those that are doomed to extinction in the wild as a result of irreversible threats. Captive breeding and reintroduction programs for amphibians require long-term commitments to ensure success, and different management strategies may be needed for species earmarked for reintroduction and species used for conservation research and education.

2088: ***+.272***

Refuge populations of Cyprinodon macularius and Cyprinodon eremus, the extant members of the endangered desert pupfish complex, have been maintained for up to 33 years in semi-natural refuges. We examined the success of the refuge program in maintaining diversity at four microsatellite DNA loci in 24 refuge populations of C. macularius and six of C. eremus that include, respectively, seven and four lineages representing original translocations from the wild. These lineages have been maintained with essentially no inoculations of genetic material from the wild and, except for one refuge, no intermixing of lineages. Comparison with wild-source populations showed marked declines in diversity within local refuges and within lineages, but relatively minor declines for the composite of all refuge populations for each species. In genetic makeup, the refuge populations generally clustered by lineage, indicating significant genetic drift early in lineage history. The results indicate that, with relatively minor adjustments in management, the refuge program can successfully preserve a large portion of the wild genetic diversity in the desert pupfish complex.

2089: ***-.018***

The Iberian lynx Lynx pardinus has suffered a dramatic reduction in its range throughout the Iberian Peninsula and at present is one of the most endangered mammals in the world. The latest studies report that, out of the 48 breeding areas that existed in 1990, only two populations are left in southern Spain. As a consequence, some of the formerly largest populations, such as Montes de Toledo (central Spain), are to all intents and purposes regarded as extinct. To determine the current distribution of Iberian lynx outside the two recognized populations, we surveyed five different areas where the species is considered extinct and collected 581 faeces for the genetic identification of the species. We identified 18 samples as belonging to Iberian lynx in four out of the five areas studied, providing clear evidence for the presence of lynx in central Spain. In some areas the species was detected repeatedly at different localities and on different dates, indicating a regular occurrence of an unknown number of individuals. The conservation implications of these results are discussed in terms of the genetic importance of the individuals found and future reintroductions of the species from an ongoing captive-breeding programme.

2090: ***-.122***

Fecal glucocorticoid metabolite (FGM) analysis provides a non-invasive method for studying the physiological response of wildlife to a variety of stressors and is a ground-breaking monitoring technique in wildlife management and conservation. The conservation benefits of successful

wildlife translocation restocking efforts are significant but understandably stressful for the animals being captured, removed from familiar habitat, held in captivity in many cases and subsequently released into an unfamiliar environment. It is imperative that we identify non-invasive methods for evaluating stress in translocated animals, especially in endangered species. Twenty Grevy's zebra Equus grevyi were translocated to Meru National Park as part of a Kenya Wildlife Service re-population initiative. FGMs were monitored from the time of capture, during captivity and post-release as an indicator of the stress of translocation and acclimation to the new environment. FGMs from representative non-translocated zebra were used as a further control. When held in pens at Meru Park 3-4 and 5-6 weeks after capture, the zebra had higher FGMs (25.1 +/- 1.2 and 23.4 +/- 1.3 ng g(-1)) than either at the time of capture (14.6 +/- 2.1 ng g(-1)) or non-translocated controls (16.2 +/- 1.2 ng g(-1)). This suggests that the stress of captivity elevated FGMs. FGM concentrations returned to pre-capture concentrations c. 11-18 weeks after the zebra were released into Meru Park. The return of FGM concentrations to baseline suggests successful acclimation to the new environment. This study supports the use of FGM analysis as an assessment technique in wildlife management projects involving the movement of endangered large mammals with application for monitoring stress in a wide array of conservation projects involving translocation, reintroduction and rehabilitation.

2091: ***+.021***

We report on the recent growth of upland aspen (Populus tremuloides Michx.) thickets in northwestern Yellowstone National Park, USA following wolf (Canis lupus L.) reintroduction in 1995. We compared aspen growth patterns in an area burned by the 1988 fires to aspen growth patterns in an adjacent unburned area. Elk (Cervus elaphus L.) are the principal ungulates that use this area to meet foraging needs. Within a 2 m x 6 m belt transect established in each aspen thicket, we measured aspen densities and recorded annual browsing and height information on the three tallest post-1988 aspen stems. We found greater densities ($p < 0.01$) in the burned area relative to the unburned area. A decline in the percentage of stems browsed in the burned area began in 1997, with no measured browsing occurring since 2001. In contrast, the percentage of stems browsed in the unburned area began declining in 2002, with 41% of stems still being browsed in 2004. We hypothesize that the combined effect of fire and a subsequent decrease in herbivory following wolf reintroduction facilitated aspen growth. We further propose that, in addition to any changes in elk density in recent years, a recoupling of fire with increased predation risk from wolves may create a positive feedback loop that improves aspen recruitment. (c) 2008 Elsevier B.V. All rights reserved.

2092: ***+.294***

We collected Chinese cobras (Naja atra) from one island (Dinghai) and four mainland (Huangshan, Lishui, Quanzhou, and Baise) populations in southeastern China, and used sequence data derived from the ND2 (1032 bp) and cytochrome b (1117 bp) genes and molecular variance estimates to investigate the population genetic structure of the species. Our sequence data show that: (1) the three eastern (Dinghai, Huangshan, and Lishui) populations are genetically segregated from the two southern (Quanzhou and Baise) populations; (2) the Quanzhou and Baise populations consist of two well-defined subclades, suggesting that the two populations have been well differentiated; (3) N. atra from the Huangshan population do not differ from those from the Lishui population, and lineage sorting in the northeastern part of the cobra's distributional range has not yet been completed because of the young age of Zhoushan Islands. The three eastern populations, the Quanzhou population, and the Baise population should be regarded as different management units (MUs). For these MUs, we suggest that in-situ protection measures should be taken because

of their genetic uniqueness. Re-introductions or translocations are required to protect or re-establish natural populations of N. atra, but great care should be taken to enhance or retain local genetic variation.

2093: ***+.322***

Through dam-building activity, beavers (Castor canadensis) play an important role in creating pond and wetland habitat for bird communities. Their impact may be intensified in semiarid landscapes and may increase with increasing dam density on it stream. Out objective was to examine relationships between dam density, riparian area characteristics, and the riparian bird community in it semiarid landscape. In 2002 and 2003 we surveyed riparian birds, riparian area characteristics, and the number of dams along 1.2-km sections of 11 streams in sagebrush steppe regions of Wyoming. We categorized the riparian bird community into 2 assemblages based on their affiliation with terrestrial or aquatic riparian habitats. Average width of the woody riparian zone, average riparian shrub height, and percent cover of emergent vegetation all had significant positive relationships with dam density, but percent cover of ponded water did not. Species richness an(] abundance of all riparian birds, and of the terrestrial assemblage, increased significantly with increasing woody riparian zone width. In contrast, richness and abundance of the aquatic assemblage were significantly positively influenced by cover of emergent vegetation and ponded water. When we accounted for riparian area characteristics, we found that total species richness, total abundance, and aquatic assemblage abundance were each positively correlated with dam density, suggesting that dam density is related to other riparian characteristics selected by birds. Our results suggest increasing dam-building activity may be important in creating favorable riparian conditions for it rich and abundant bird community in semiarid regions.

2094: ***-.071***

Historically, Gopher Tortoises (Gophents polyphemus) occurred in fire-maintained habitats; however, many of these areas have been fire-suppressed. Herein, we examine the spatial distribution of Gopher Tortoises 17-18 years after fire reintroduction to a fire-suppressed area. Our study took place at Archbold Biological Station (ABS) in south-central Florida. Fires were suppressed at ABS from 1927 until 1985, at which time fire was reintroduced to this area. During 2002 and 2003, we surveyed Gopher Tortoise burrows in 17 burn units of sandhill and scrubby flatwoods with varied fire histories. Using density of active burrows as an indicator of Gopher Tortoise density, Gopher Tortoise burrow densities were highest in recently and frequently burned areas. Because fire influences habitat structure, we also gathered information on the amount of canopy cover and bare ground at each study site. Density of active Gopher Tortoise burrows increased with bare ground and decreased (but not significantly so) with canopy cover. Backward stepwise ridge regression analyses showed that number of fires was a more important predictor of active Gopher Tortoise burrow density than habitat structure (amount of bare ground). In sum, our study demonstrates that reintroducing fire to fire-suppressed areas is beneficial to Gopher Tortoises, and this response is not solely caused by changes in habitat structure.

2095: ***-.137***

The Saker Falcon (Falco cherrug) was formerly abundant and widespread in Bulgaria. In the 20th Century numbers declined markedly following World War II as a result of changes in agricultural practices that dramatically altered the landscape. In addition government sponsored campaigns to eradicate predatory birds and rodents directly affected the Saker Falcon population and its favoured prey species, the European Souslik (Spermophilus citellus). By the end of the 1980's the

Saker Falcon population had diminished to an estimated 30-50 breeding pairs. Subsequently, the change of government in 1989 saw an increase in nest robbery and illegal poaching, and now the Saker is believed to be extinct as a breeding species in the country. With the accession of Bulgaria to the European Union and the establishment of a network of Natura 2000 protected sites there is an opportunity to restore the Saker Falcon through a programme of reintroduction. This article describes the feasibility study currently being undertaken to examine the potential and logistics of such a project.

2096: ***-.169***

Vulture Populations across the Indian Subcontinent collapsed in the 1990s and continue to decline. Repeated population surveys showed that the rate of decline was so rapid that elevated mortality of adult birds must be a key demographic mechanism. Post mortem examination showed that the majority of dead vultures had visceral gout, due to kidney damage. The realisation that diclofenac, a non-steroidal anti-inflammatory drug potentially nephrotoxic to birds, had become a widely used veterinary medicine led to the identification of diclofenac poisoning as the cause of the decline. Surveys of diclofenac contamination of domestic ungulate carcasses, combined with vulture population modelling, show that the level of contamination is sufficient for it to be the sole cause of the decline. Testing on vultures of meloxicam, an alternative NSAID for livestock treatment, showed that it did not harm them at concentrations likely to be encountered by wild birds and would be a safe replacement for diclofenac. The manufacture of diclofenac for veterinary use has been banned, but its sale has not. Consequently, it may be some years before diclofenac is removed from the vultures' food supply. In the meantime, captive populations of three vulture species have been established to provide sources of birds for future reintroduction programmes.

2097: ***+.184***

Belding's Yellowthroat (Geothlypis beldingi) inhabits freshwater marshes the length of the state of Baja California Sur. A gap in occurrence from approximately 25 degrees 50'N to 24 degrees 50'N separates the Subspecies goldmani to the north from beldingi to the South. According to BirdLife International's (2000, 2007) population estimates, the most important sites for the species are San Ignacio (537-648 birds) and La Purisima (203-450) in the north and San Jose del Cabo (219-480) and Punta San Pedro (70) in the south. Half of 12 presumed breeding sites in the north and five of 14 in the south were discovered within the last ten years. The species apparently no longer occurs at one historical site in the south. Since 2000, two records from Guerrero Negro and one from Bahia Tortugas approximately 140 km and > 200 km respectively northwest of the known breeding range demonstrate the species' dispersal ability. Belding's Yellowthroat is of utmost conservation concern, but the most recent conservation summaries exaggerated the species' plight, in particular by under-appreciating the bird's capacity for long-range dispersal and the ability of marsh habitat to regenerate quickly. Formal Studies of the species should be undertaken and marsh creation should be incorporated in the state's development plans. Previous calls for reintroduction efforts should not be heeded, at least for the time being.

2098: ***+.236***

Social behaviour is extremely widespread in the animal kingdom and has a heritable component in many species. However, the degree to which social behaviour is phenotypically plastic and influenced by conditions individuals experience during early ontogeny is less well understood. Using the guppy as a model species, we examined the importance of early social environment upon the development of a number of social behaviours. We reared guppies at relatively low and

high conspecific densities to investigate how early experience impacted shoaling behaviour and social learning ability in this species. Guppies reared at low densities had a significantly higher shoaling tendency than guppies reared at higher densities. Furthermore, individuals reared at low densities located a food resource more often and quicker than individuals reared at high densities in a foraging maze trial with trained demonstrators. After 8 consecutive days of maze trials we removed the demonstrators to investigate social learning skills. Guppies reared at low densities located food faster alone than guppies reared at high densities, implying that they were more adept at socially learning foraging information. This intriguing relationship between early social environment and the development of shoaling behaviour and social learning skills may have considerable implications for captive breeding programs in conservation and aquaculture. (C) 2008 The Association for the Study of Animal Behaviour. Published by Elsevier Ltd. All rights reserved.

2100: *+.409*

Parental care of unrelated young In species able to recognize their own offspring Is called adoption. Here, we establish adoption as a novel conservation tool for endangered African wild dogs (Lycaon pictus) to augment the size of existing packs In situ and to enhance gene flow among isolated populations. Three cases of facilitated adoption of orphaned pups by unrelated free-ranging wild dogs are reported. All pups were successfully integrated Into an adoptive pack. Besides Individual fitness benefits accruing from larger pack size, we propose that wild dogs may also adopt pups due to benefits relating to the future reproductive value of unrelated individuals. This study will hopefully stimulate further research on the adaptive value and conservation Implications of adoption in other endangered species.

2101: *+.213*

Once common at Isle Royale National Park (IRNP), the American Marten Mantes americana may have been extirpated during the early 20th century. We compiled historical and recent records to assist in evaluating its status there. Ten records were reported between 1873 and 1929 representing a minimum of 20 American Marten individuals. No observations were recorded between 1930 and 1990. From 1991 to 2006, 28 reports were received, including tracks, sightings, faeces, and photographs. We assessed the plausibility of a remnant population persisting undetected for 60 years by extending the historical record forward for American Marten by about 20 years. We further assessed the potential for recolonisation considering American Marten ecology and scientific investigations that have occurred at IRNP. Whether an undetected remnant population has experienced a bottleneck brought about by over-trapping or a recent immigration event has occurred, the importance of this isolated population increases with regards to scientific inquiry and conservation considering the mission of the National Park Service (NPS). NPS policy states the following with respect to animal population management: "pre-serve and restore the natural abundances, diversities, dynamics, distributions, habitats, and behaviors of native animal populations and the communities and ecosystems in which they occur; restore native animal populations in parks when they have been extirpated by past human-caused actions; and minimize human impacts on native animals, populations, communities, and ecosystems, and the processes that sustain them". Understanding historical prevalence of American Marten at IRNP, as we have outlined here, will inform and guide future management and research of this species within the park.

2102: *+.113*

1. This review highlights the status of the European wild rabbit Oryctolagus cuniculus, which is threatened within its native range and yet is a highly successful colonizer across its worldwide, introduced range.2. The European wild rabbit is a keystone species in Iberia, and the survival of a range of threatened predator species, including the Iberian lynx Lynx pardinus and Spanish imperial eagle Aquila aldabertii, is dependent upon the restoration of rabbit populations. Although not native to the UK, the rabbit also performs significant ecosystem services for nationally rare UK species, by maintaining short sward heights in heathland and grassland ecosystems, and serving as a prey item for populations of predators.3. We identify the European wild rabbit as an excellent model to demonstrate the wide range of complex effects that an introduced mammalian species may exert on ecosystems to which it has been introduced. These effects include habitat degradation following overgrazing, competition with native mammals and facilitating meso-predator release and hyperpredation.4. We also show that rabbit eradication from some sites may generate more problems than are solved because of the impacts of trophic cascades stemming from dependence on rabbits by native predator assemblages.

2103: *+.135*

1. The severe and early destruction and fragmentation of woodland habitats due to human activities is thought to have been a leading factor in the extirpation from Britain of several large, forest-dependent mammal species, such as the Eurasian lynx Lynx lynx. However, during the 20th century, Scotland in particular has experienced rapid, large-scale reafforestation. In order to assess if this reafforestation has been sufficient to permit the potential restoration of extirpated forest mammal species with large spatial requirements, a Geographical Information System (GIS) analysis of potential habitat of one species, the Eurasian lynx, was performed for the Scottish mainland.2. A rule-based analysis, incorporating data and expert opinion from Switzerland, an environmentally similar area where lynx now occur, was used to identify patches of suitable lynx habitat in Scotland. A connectivity analysis was used to investigate whether and how these patches are connected to form larger interconnected networks of potential lynx habitat that would allow lynx to sufficiently interact with one another to form a single interbreeding population.3. Scotland has over 20 000 km(2) of suitable lynx habitat split into two main networks of interconnected patches: the Highlands (c. 15 000 km(2)) and the Southern Uplands (c. 5000 km(2)). A further 800 km(2) of potential habitat, contiguous with the Southern Uplands lynx habitat network, lies across the border in England. Although connectivity between the Highlands and Southern Uplands networks is currently weak, the implementation of measures to mitigate the barrier effects of busy roads in central Scotland could facilitate the movement of lynx between the two areas.4. Based on the availability of prey resources, Scotland could support around 400 adult and subadult lynx in the Highlands and around 50 in the Southern Uplands. A Scottish population of this size would be the fourth largest lynx population in Europe considering current population estimates.

2104: *+.345*

1. In a context of increasing human impact on ecosystems and species distributions, population restoration (introductions, reintroductions, reinforcements) is an essential management tool, especially for plant species with limited colonization ability. However, detailed demographic surveys following restoration and comparisons of demographic rates between restored and natural populations, although essential for identifying the key factors of restoration success, are lacking.2. We compared the demography over 10 years of six natural and two experimentally introduced populations of the narrowly endemic, cliff-dwelling, self-incompatible plant species Centaurea corymbosa. We analysed the fate of two cohorts of individuals that emerged simultaneously from seed introduction and natural germination. We then built a matrix model of population dynamics

(using 6 years of data) and compared the demographic rates and asymptotic growth rate between the natural and introduced populations.3. Overall, survival rates were higher in the introduced than in the natural populations, either due to better habitat conditions at the cliff scale or to better conditions in microsites selected for seed introduction compared to those reached by chance following natural seed dispersal.4. In contrast, introduced populations exhibited lower fecundity than natural populations, probably due to the introduction protocol which led, in combination with self-incompatibility, to severely reduced mate availability.5. Despite clear differences in population dynamics between introduced and natural populations, no significant difference in the asymptotic growth rates could be detected, because the higher survival compensated for the lower fecundity in introduced populations.6. Synthesis and applications. Creating new populations of C corymbosa in suitable unoccupied sites seems straightforward, provided that the introduction protocol allows sufficiently high fecundity. This key parameter for restoration success can be optimized by sowing seeds from several sources at high density and in several consecutive years, which should increase mate availability for self-incompatible flowering individuals. We suggest that population introduction might be successful for many (endemic) plant species whose geographical range is mainly limited by low colonization ability, especially in Mediterranean landscapes. We show that the simultaneous monitoring of restored and natural populations enables identification of the key parameters to be targeted for management optimization of restored populations.

2105: *+.245*

1. Under the effects of rapid environmental change, such as climate change and land degradation, assessment of plant species potential distribution is becoming increasingly important for conservation purposes. Moreover, land administrators need reliable predictions of species suitability for planning a wide range of management activities.2. In this study, we used the recent Maxent algorithm for modelling the niche of Arnica montana within a Site of Community Importance in the Alps, with the ultimate aim of providing a rigorous evidence base for management of this locally threatened species. We built a final suitability map taking into account (i) the minimization of spatial autocorrelation through the use of a constrained random split of sampled data; (ii) the use of a stepwise selection of predictors in order to obtain a reduced model containing only meaningful variables; (iii) the comparison of the predictive power of three sets of environmental predictors; (iv) the identification of the most suitable areas by overlaying predictions of three competing models; (v) the use of divergence maps as a complement to conventional performance comparison assessments.3. Maxent improved accuracy both on training and test data sets. Elevation, geomorphology and hosting habitats performed as effective primary predictors. A reduced model based on the outcomes of a preliminary stepwise selection analysis of predictors gave the best accuracy score on test data. Two parts of the study area have been selected for management as a result of areas of agreement between the three competing models.4. Synthesis and applications. There remain important methodological issues that need to be improved in order to increase confidence in niche modelling and ensure that reintroduction and management activities for threatened or rare plant species are based on reliable distribution models. Modellers can improve predictions of plant distribution by addressing methodological topics that are often overlooked, as demonstrated for A. montana in this study.

2106: *-.122*

The White-headed Duck is a globally threatened species historically recorded from Spain in the west to China in the east. It has suffered major population declines, local extinctions and range fragmentation. Several projects have attempted to reintroduce captive-bred birds into parts of the

former range in Europe, but with little success. Two captive stocks currently exist, one originating from Pakistan in 1968 and the other originating from Spain in 1982. This study compares the suitability of these captive stocks for specific reintroduction projects by using 11 microsatellite markers and mtDNA control region sequences to assess genetic differences between captive populations and wild birds from Spain and Greece. No significant population structure was found and all microsatellite alleles recorded in captive birds originating from Pakistan were also observed in the wild Spanish population. A higher diversity of alleles was observed in wild birds from Greece than from Spain, probably due to the effects of a strong bottleneck experienced in Spain in the 1970s. Compared with wild populations, both captive stocks have suffered a significant loss of diversity in microsatellites and mitochondrial DNA owing to founder effects and/or genetic drift, and therefore may not be well suited for release programmes. We recommend the development of a more diverse captive breeding programme based on birds taken from different areas of the range, in particular by supplementing the Spanish population with birds from North Africa. Our study shows the value of molecular tools in developing conservation programmes for threatened bird species and has implications for the design of recovery programmes.

2107: ***+.085***

This second state-of-the-lake report for Lake Michigan identifies progress made during 2000-2004 in meeting the fish-community objectives established for the lake in 1995. An oral public report, providing more- extensive data than given here, was made in 2005. During 2000-2004, a geographical information system for the lake and its basin was expanded considerably and is expected to provide a means for establishing priorities for habitat remediation. This system was used in the development of a report, under review, identifying environmental objectives for the lake. Recently introduced invertebrates, particularly dreissenid mussels (Dreissena spp.), continued to impact the lake's food web. Their proliferation coincided closely with severe reductions in populations of the amphipod Diporeia spp. Round goby (Neogobius melanostomus), first reported in the lake in 1995, continued to expand its range and apparently nearly eliminated two inshore species, the johnny darter (Etheostoma nigrum) and mottled sculpin (Cottus bairdi), but the impact of the goby on other fishes was unresolved. Northern snakehead (Channa argus), an aquarium fish, was found in a Chicago harbor, although this specimen did not appear to have been part of an established population. The yield of lake whitefish (Coregonus clupeaformis), the most-important commercial fish in the lake, reached a modern low in 2004, falling just below the lower bound (1.8 million kg) of the fish-community objective. Although the abrupt decline in its favorite food, Diporeia, had caused a drop in condition of lake whitefish, the recent lower yield of lake whitefish was attributed to reduced fishing effort, not reduced fish abundance. At the time of the first state-of- the-lake report, which covered events through 1999, yield of yellow perch (Perca flavescens) was low because of an early life-stage bottleneck and imposition of more-restrictive fishing policies aimed at preserving spawning stocks. Spawning-stock biomass improved during this reporting period due to recruitment of a strong 1998 year-class and better survival of adults. Another strong year-class, produced in 2003, is expected to aid in the recovery of this species. Nonetheless, reductions in the biomass of zooplankton, caused by the filtering activities of dreissenids, make attainment of the objective for yellow perch unlikely. Because the lake sturgeon (Acipenser fulvescens) population remained listed as threatened, increased efforts were directed at describing its ecological status and genetic characteristics, improving rearing and stocking methods, and reassessing the need for new regulations. Spawning populations have been confirmed in eight rivers, and reintroductions have been initiated in four rivers. An alarmingly high number of adult sturgeon were found washed up on beaches. Although the causes of these mortalities have not been confirmed, type E botulism is suspected. The biomass of planktivores

(pelagic prey fishes) during this reporting period remained below the target range, similar to what their biomass was at the end of 1999. The energy density of alewife (Alosa pseudoharengus), a key planktivore, was 23% less during 2002-2004 than in 1979-1981, making them less nutritious as prey for top predators. This decline in energy density is likely related to the deterioration of Diporeia populations. The weight-at-age of Chinook salmon (Oncorhynchus tshawytscha), the main consumer of planktivores, reached a recent low in 2003, and poor growth continued on into 2004. During this reporting period, Renibacterium salmoninarum, the cause of bacterial kidney disease, continued to be detected in Chinook salmon, and nearly half of the lake trout (Salvelinus namaycush) eggs tested expressed early mortality syndrome, which remains a serious threat. The number of naturally reproduced Chinook salmon smolts increased from about 2.5 million in the years just before 2000 to an average of more than 4.0 million during this reporting period. The yield of Chinook salmon was below the objective of 3.1 million kg during 2001-2003 but rose to 3.9 million kg in 2004. The harvest was not considered sustainable, even though stocking was cut in 1999, because growth, ration, and prey abundance were declining. Little progress was made in rehabilitating lake trout during this reporting period[long dash]abundance of adults declined as a result of an increased population of sea lampreys (Petromyzon marinus). Natural reproduction of lake trout remained inconsequential and is not expected to change until the sea lamprey population is reduced and stocking is increased to recommended levels. Our major recommendations to the Lake Michigan Committee are: (1) develop a process for addressing recommendations in this and previous state-of-the-lake reports, (2) embrace an ecosystem approach to fishery management, (3) oversee completion of the draft environmental objectives for the lake, and (4) revise the lake's fish- community objectives to account for the changes in the lake that occurred after 1995.

2108: ***+.208***

Understanding the dynamics of populations at low density and the role of Allee effects is a priority due to concern about the decline of rare species and interest in colonization/invasion dynamics. Despite well-developed theory and observational support, experimental examinations of the Allee effect in natural systems are rare, partly because of logistical difficulties associated with experiments at low population density. We took advantage of fish introduction and removal in alpine lakes to experimentally test for the Allee effect at the whole-ecosystem scale. The large copepod Hesperodiaptomus shoshone is often extirpated from the water column by fish and sometimes fails to recover following fish disappearance, despite the presence of a long-lived egg bank. Population growth rate of this dioecious species may be limited by mate encounter rate, such that below some critical density a colonizing population will fail to establish. We conducted a multi-lake experiment in which H. shoshone was stocked at densities that bracketed our hypothesized critical density of 0.5-5 copoepods/m(3). Successful recovery by the copepod was observed only in the lake with the highest initial density (3 copepods/m(3)). Copepods stocked into small cages at 3000 copepods/m(3) survived and reproduced at rates comparable to natural populations, confirming that the lakes were suitable habitat for this species. In support of mate limitation as the mechanism underlying recovery failure, we found a significant positive relationship between mating success and density across experimental and natural H. shoshone populations. Furthermore, a mesocosm experiment provided evidence of increased per capita population growth rate with increasing population density in another diaptomid species, Skistodiaptomus pallidus. Together, these lines of evidence support the importance of the Allee effect to population recovery of H. shoshone in the Sierra Nevada, and to diaptomid copepods in general.

2109: ***+.248***

Habitat manipulation to favour prey species is a potential alternative management technique to conventional predator control methods. New Zealand lizards are suitable taxa for evaluating the effectiveness of habitat manipulation, but to do so we need good baseline information about the study population as well as reliable sampling techniques. To this end, the three main aims of this research were to conduct a baseline survey of lizard distribution and species composition along the entire length of Kaitorete Spit, Canterbury; to develop a new sampling method (artificial retreats) for Canterbury geckos (Hoplodactilus 'Canterbury'); and to test the relative effects of habitat and predator manipulation on survival of McCann's skinks (Oligosoma maccanni). Pitfall trapping identified differences in species distribution across duneland, farmland and shrubland habitats, and a decline in capture rates of spotted skinks (O. lineoocellatum 'Central Canterbury') over a 3-year period. Canterbury geckos preferred artificial retreats made from Onduline over those made from corrugated iron and concrete, whereas skinks showed no preferences. It was also found that Onduline retreats could be used to detect Canterbury geckos following translocation and to estimate population size. Annual survival probability of McCann's skinks increased at sites with predator exclosures, but stayed constant at control sites and sites with artificial retreats. Therefore, predator control, but not the addition of artificial retreats, is predicted to benefit McCann's skinks. Our recommendations include predator control for spotted skinks near Birdlings Flat, Kaitorete Spit, and the development of standard guidelines for using artificial retreats.

2110: *+.123*

The Lesser White-fronted Goose Anser erythropus is globally threatened, being recognised as Vulnerable by IUCN and ranked by BirdLife International as 'SPEC 1' within Europe, denoting a European species of global conservation concern. It is listed on Annex 1 of the European Council Directive on the conservation of Wild Birds (79/409/EEC, 2 April 1979), in Column A of the Action Plan under the African-Eurasian Migratory Waterbird Agreement (AEWA) and in Annex II 'Strictly protected species' of the Bern Convention. Lesser White-fronted Geese are long-distance Palearctic migrants, currently breeding discontinuously in the sub-arctic zone from northern Fennoscandia to eastern Siberia. The wintering/staging areas and migration routes are only partially known. Population and range decline The global population of Lesser White-fronted Goose has declined rapidly since the middle of the 20th century. The decrease in numbers has been accompanied by fragmentation of the breeding range and is continuing to affect all populations, giving rise to fears that the species may go extinct. Overhunting and habitat loss are considered to be the main threats. BirdLife International estimates a decrease in numbers in the range of 30% to 49% during the period 1998[long dash]2008. Four subpopulations can be recognised, three of which are surviving components of the species' formerly more extensive breeding range: * Fennoscandian population (breeding in the Nordic countries and the Kola Peninsula of north-westernmost Russia); * Western main population (nesting in northern Russia to the west of the Taimyr Peninsula); and * Eastern main population (nesting from the Taimyr Peninsula eastwards and wintering in China). The fourth subpopulation has been created by the release of captive-bred birds within the former range of the Fennoscandian population in Sweden and by the establishment of a human-modified flyway. The Fennoscandian and Western main populations underwent significant declines during the 20th century and continue to decrease, due primarily to hunting pressure and habitat loss along migration routes and in the wintering areas. The supplemented/reintroduced population appears to be increasing slowly, but views differ markedly on the ethical and scientific merits of the conservation measures applied to this species and their potential implications (e.g. hybridisation risk with other species). Scope of this Action Plan This Action Plan deals with conservation of two of the three wild populations [long dash] namely the Fennoscandian population and Western main population [long dash] given that the Eastern main population does not occur within the AEWA Agreement Area or the territory of

Member States of the European Union. The Eastern main population is therefore only mentioned when a global context or comparison is required. The Action Plan also takes into account the population derived from captive-bred birds and used for restocking in Swedish Lapland, migrating to winter in the Netherlands. According to previous agreements between the Fennoscandian Range States and in line with AEWA's mission, the main focus of this plan is the conservation of the wild populations. Principal Range States Lesser White-fronted Geese occur regularly in at least 22 States within the European Union and/or AEWA Agreement Area. These are referred to as 'Principal Range States' in the Action Plan and have the major responsibility for its implementation. These states are listed below. The letters in brackets denote the relevant populations of Lesser White-fronted Goose (F = Fennoscandian; WM = Western main; R = supplemented/reintroduced): EU Principal Range States Bulgaria (F, WM) Estonia (F) Finland (F) Germany (F, WM) Greece (F) Hungary (F) Lithuania (F) Netherlands (R) Poland (F,WM) Romania (WM) Sweden (F,R) Non-EU Principal Range States Azerbaijan (WM) Iraq (WM) Islamic Republic of Iran (WM) Kazakhstan (F,WM) Norway (F) Russian Federation (F,WM) Syrian Arab Republic (WM) Turkey (F,WM) Turkmenistan (WM) Ukraine (F,WM) Uzbekistan (WM) Threats There is strong evidence that the most important factors driving the continued decline in numbers and fragmentation of the range of the Lesser White-fronted Goose (both the Fennoscandian and Western main subpopulations) are those that cause high mortality among fully grown birds. These factors operate primarily on the staging and wintering grounds, given that studies in the breeding range have failed to detect any adverse impacts that are of significant magnitude to explain the population crash. Although the species is legally protected, on paper at least, across virtually its entire range, hunting is considered to be the primary cause of mortality and the single most important threat that this Action Plan has to tackle. The loss and degradation of suitable habitat is currently considered to be an important but secondary threat to survival of full-grown birds. However, its significance as a likely driver for the historical declines and range changes during the 20th century should not be underestimated. Focus and content of the Action Plan (see Chapter 5) Action Plan Goal To restore the Lesser White-fronted Goose to a favourable conservation status within the AEWA Agreement Area. Action Plan Purpose To stop and reverse the current population decline and range contraction. Results required for delivering the Purpose and Goal Result 1: Mortality rates are reduced Result 2: Further habitat loss and degradation are prevented Result 3: Reproductive success is maximised Result 4: No introgression of DNA from other goose species into the wild population occurs as a result of further releases and DNA introgression from already released birds from captive breeding programmes is minimised Result 5: Key knowledge gaps filled Result 6: International cooperation maximised For each Result, Objectively Verifiable Indicators, Means of Verification, Priority and Timescale are identified, in addition to the specific activities needed to achieve the desired Result (see Chapter 6). Principles of Implementation 1. An International Lesser White-fronted Goose Working Group shall be established, consisting of governmental representatives of all Range States. The governmental representatives shall be free to bring in their own experts and to call on their support as required. The Working Group shall be chaired by the AEWA Secretariat (subject to additional, dedicated human and financial resources being made available to the Secretariat) and will operate in accordance with Terms of Reference to be developed by the AEWA Secretariat, approved by the Range States and endorsed by the AEWA Technical Committee. 2. The main priority for the conservation of the Lesser White-fronted Goose is the maintenance of the wild populations breeding in Fennoscandia and Russia. 3. The efficiency of conservation measures is to be assessed by the International Lesser White-fronted Goose Working Group. 4. Implementation and future modification of this International Single Species Action Plan [long dash] and all related decisions [long dash] shall be undertaken with transparency and accountability so that progress can be subject to scientific scrutiny at any time. 5. Each Range State shall consider support for 'on-the-ground' conservation measures, particularly along the Lesser White-fronted Goose flyway(s) that

traverse(s) its territory. 6. Particular attention shall be paid to mortality due to hunting and urgent targeted measures shall be implemented to reduce the magnitude of this threat, the success of which shall be promptly and regularly reviewed and evaluated. 7. Supplementing wild populations with captive-bred birds shall be considered if other conservation measures are not as quickly efficient as needed and should populations continue to decline. As with any other captive breeding, reintroduction or supplementation initiatives this project will be subject to consideration and practical advice by the Committee for captive breeding, reintroduction and supplementation of Lesser White-fronted Geese in Fennoscandia (see below). 8. The SSAP should be regularly adapted and updated every 5 years.

2111: *+.113*

How will climate change affect species' reproduction and subsequent survival? In many egg-laying reptiles, the sex of offspring is determined by the temperature experienced during a critical period of embryonic development (temperature-dependent sex determination, TSD). Increasing air temperatures are likely to skew offspring sex ratios in the absence of evolutionary or plastic adaptation, hence we urgently require means for predicting the future distributions of species with TSD. Here we develop a mechanistic model that demonstrates how climate, soil and topography interact with physiology and nesting behaviour to determine sex ratios of tuatara, cold-climate reptiles from New Zealand with an unusual developmental biology. Under extreme regional climate change, all-male clutches would hatch at 100% of current nest sites of the rarest species, Sphenodon guntheri, by the mid-2080s. We show that tuatara could behaviourally compensate for the male-biasing effects of warmer air temperatures by nesting later in the season or selecting shaded nest sites. Later nesting is, however, an unlikely response to global warming, as many oviparous species are nesting earlier as the climate warms. Our approach allows the assessment of the thermal suitability of current reserves and future translocation sites for tuatara, and can be readily modified to predict climatic impacts on any species with TSD.

2112: *+.165*

New populations of threatened species tire often established as a conservation measure. However, if only a few individuals contribute to subsequent generations, these populations may have limited genetic diversity. Such genetic bottlenecks can result in inbreeding depression. reduced fitness. and even extirpation of populations. Eight isolated populations of Sacramento perch Archoplites interruptus established through anthropogenic translocations were examined for evidence of genetic bottlenecks. Sacramento perch are endemic to two regions of California but have been entirely extirpated from their native range; the remaining populations are essential for conservation of the species. Using 12 microsatellite DNA loci, we determined that genetic bottlenecks occurred in six of the populations. Allelic richness, richness of private alleles, and effective Population size differed significantly among populations. Strong differentiation among the extant populations probably resulted from differences in the Sources used to establish the populations and from genetic drift due to the small population sizes. These results indicate that genetic bottlenecks are frequent when new, isolated populations of a species are established. Although these extant populations have persisted despite bottlenecks, future Sacramento perch populations Should be established by drawing from the most diverse of the current populations and should be monitored with genetic markers to evaluate diversity and the possible need for further stocking. We combine three measures of genetic diversity (allelic richness, private allelic richness, and effective population size) to recommend potential source populations.

2113: *+.123*

We analysed 25 years (1980-2004) of demographic data on a small re-introduced population of endangered African wild dogs (Lycaon pictus) in Hluhluwe-iMfolozi Park (HiP), South Africa, to describe population and pack dynamics. As small populations of cooperative breeders may be particularly prone to Allee effects, this extensive data set was used to test the prediction that, if Allee effects occur, aspects of reproductive success, individual survival and population growth should increase with pack and population size. The results suggest that behavioural aspects of wild dogs rather than ecological factors (i.e. competitors, prey and rainfall) primarily have been limiting the HiP wild dog population, particularly a low probability of finding suitable mates upon dispersal at low pack number (i.e. a mate-finding Allee effect). Wild dogs in HiP were not subject to component Allee effects at the pack level, most likely due to low interspecific competition and high prey availability. This suggests that aspects of the environment can mediate the strength of Allee effects. There was also no demographic Allee effect in the HiP wild dog population, as the population growth rate was significantly negatively related to population size, despite no apparent ecological resource limitation. Such negative density dependence at low numbers indicates that behavioural studies of the causal mechanisms potentially generating Allee effects in small populations can provide a key to understanding their dynamics. This study demonstrates how aspects of a species' social behaviour can influence the vulnerability of small populations to extinction and illustrates the profound implications of sociality for endangered species' recovery.

2114: *+.024*

The Common spadefoot (Pelobatus fuscus) occurred mainly in mesotrophic waters on sandy soils. Near these reproduction waters, they need bare soil were they can easily dig in. This type of habitat is not rare in the east of The Netherlands. In spite of this, the Dutch population decreased since 1990 with ca 60% and is at the moment endangered. There are 35 small populations left and most of them (70%) depend on only one reproductionwater! The last 15 years a large number of protective measures for both water- and land habitats have been taken, but so far the effect seems very low. The authors suggest that in most small populations the lack of recovery when habitat quality is restored is due to the loss of genetic variation that prevents populations to grow. Therefore they advise to start with the addition of new genetic material (genetic resque) to small populations and reintroductions at short notice.

2115: *+.150*

Aim The aim of this paper is to examine taxonomic homogenization in ungulates globally and at the local scale in South Africa. Specifically, we aim to examine the roles of distance, scale, time, extinctions vs. introductions, and extralimital vs. extraregional introductions in the homogenization of ungulate biotas, and to determine pathways of introduction of ungulate species globally and the proximate explanatory variables of ungulate introductions in South Africa. Location Forty-one countries globally and three spatial resolutions in South Africa. Methods Indigenous, extirpated and established introduced ungulate species data were obtained for countries globally, and at a quarter-degree grid-cell resolution in South Africa. Homogenization was calculated using Jaccard's index of similarity (JI) for countries globally and for three spatial resolutions in South Africa. Zoo holdings and transfer data from the International Species Information System database were used to investigate the relationship between non-indigenous ungulate species introductions and the number of non-indigenous ungulate species in zoos. Relationships between JI and species richness, and between numbers of introductions and several environmental and social factors were examined using generalized linear models. Results Homogenization in ungulates was 2% for countries globally and 8% at the coarsest resolution in South Africa. Homogenization increased with increasing resolution and with time, but it decreased

with increasing percentage change in species richness. Globally, introductions contributed more to homogenization than did extinctions. Within South Africa, extralimital introductions contributed more to the homogenization of ungulate assemblages than did extraregional ones, and ungulates were typically introduced to high-income areas with high human population and livestock densities. The same was not true in the past, when ungulates were introduced to ungulate species-poor areas. The number of non-indigenous ungulate species established in a country is significantly related to the number of non-indigenous ungulate species in zoos in the country, possibly owing to sales of surplus animals from zoos. Main conclusions Ungulate faunas are homogenized at both the global scale and in South Africa, with extralimital introductions being of considerable significance regionally. In consequence, increasing attention will have to be given to the conservation consequences of ungulate translocations, both within particular geopolitical regions and across the globe.

2116: *+.061*

Burrows created by other organisms provide important refugia for many vertebrates and invertebrates. With severe range declines of most of Australia's burrowing mammals, the introduced European rabbit may have replaced the role of native burrowers. The vertebrate and invertebrate fauna using the warrens of the native burrowing bettong and greater bilby were compared with fauna using warrens of the introduced European rabbit and control sites without active warrens in and South Australia. Echidnas, broad-banded sandswimmers, hemipterans and coleopterans, particularly Brises caraboides, revealed a preference for warrens over sites without warrens. Iridomryrmex sp. E (rufoniger gp.) was recorded at significantly higher abundance at bettong rather than rabbit warrens and Melophorus ants apparently benefited from the bare ground around warrens of both native burrowers and rabbits. Identification of small mammal preferences for different warren types was confounded by low predation rates inside the Arid Recovery Reserve, where cats and foxes had been exterminated to allow reintroduction of native burrowers. Due to the widespread use of the below ground and surface features of warrens by commensal native vertebrates and invertebrates, consideration should be given to reintroducing native burrowers following the removal of rabbits in and Australia. (c) 2008 Elsevier Ltd. All rights reserved.

2117: *+.203*

Captive breeding programs are increasingly being initiated to prevent the imminent extinction of endangered species and/or populations. But how well can they conserve genetic diversity and fitness, or re-establish self-sustaining populations in the wild? A review of these complex questions and related issues in salmonid fishes reveals several insights and uncertainties. Most programs can maintain genetic diversity within populations over several generations, but available research suggests the loss of fitness in captivity can be rapid, its magnitude probably increasing with the duration in captivity. Over the long-term, there is likely tremendous variation between (i) programs in their capacity to maintain genetic diversity and fitness, and (ii) species or even intraspecific life-history types in both the severity and manner of fitness-costs accrued. Encouragingly, many new theoretical and methodological approaches now exist for current and future programs to potentially reduce these effects. Nevertheless, an unavoidable trade-off exists between conserving genetic diversity and fitness in certain instances, such as when captive-bred individuals are temporarily released into the wild. Owing to several confounding factors, there is also currently little evidence that captive-bred lines of salmonids can or cannot be reintroduced as self-sustaining populations. Most notably, the root causes of salmonid declines have not been mitigated where captive breeding programs exist. Little research has also addressed under what

conditions an increase in population abundance due to captive-rearing might offset fitness reductions induced in captivity. Finally, more empirical investigation is needed to evaluate the genetic/fitness benefits and risks associated with (i) maintaining captive broodstocks as either single or multiple populations within one or more facilities, (ii) utilizing cryopreservation or surrogate broodstock technologies, and (iii) adopting other alternatives to captive-rearing such as translocations to new habitats. Management recommendations surrounding these issues are proposed, with the aim of facilitating meta-analyses and more general principles or guidelines for captive-breeding. These include the need for the following: (i) captive monitoring to involve, a priori, greater application of hypothesis testing through the use of well-designed experiments and (ii) improved documentation of procedures adopted by specific programs for reducing the loss of genetic diversity and fitness.

2118: ***+.017***

Populations of New Zealand's large indigenous land snails have been reduced to small scattered remnants by habitat destruction and predation. It is possible to rear some of these snail species in captivity, but it is important that they can then be released back into the wild to establish new populations. Eleven Placostylus hongii snails released from captivity onto Limestone Island, Whangarei Harbour, New Zealand on 5 August 2002 were monitored after release. All eleven snails died within < 0.25[long dash]1.63 years. The snails were equipped with harmonic radar transponders to facilitate finding them each time the island was visited. Six juveniles developed into adults, five after 0.25[long dash]0.47 years and one after 1.02[long dash]1.26 years on the island. A single newly hatched juvenile found on 13 August 2003 showed some breeding had occurred. The snails remained within 3 m of the small grove of trees where they were released. They moved 0.3[long dash]11.1 m between recaptures and were followed for total distances of 0.4[long dash]19.7 m with most displacements being uphill. Failure to establish was possibly due to a long dry period with high temperatures during the summer of 2003/04, together with soil that dried hard, thus preventing the snails from burrowing. Further research is needed to determine the causes of mortality in translocated snails and how these can be mitigated before further captive-rearing followed by translocation is considered.

2119: ***-.109***

The populations of many species are declining worldwide, and conservation efforts struggle to keep pace with extinction rates. Conservation biologists commonly employ strategies such as translocation and reintroduction, which move individuals of endangered species from one part of their range to another. Because individuals from endangered populations are nonexpendable, identifying any potential barriers to the establishment of viable populations prior to release of individuals should be a priority. This study evaluates the potential for learned communication signals to constrain conservation strategies such as reintroduction in an endangered species, the Thick-billed Parrot (Rhynchopsitta pachyrhyncha). We conducted vocal surveys at three geographically distinct breeding populations in the Sierra Mad e Occidental of Chihuahua, Mexico. Acoustic analyses utilizing both spectrogram cross-correlations and parameter measurements from spectrograms revealed no significant differences among the three sites in two common call types. Calls did vary among individuals within a site. The apparent lack of significant geographic variation across sampled sites suggests that differences in learned communication signals are unlikely to pose a barrier to the integration of translocated individuals from different populations into newly established populations.

2120: ***+.081***

Quantitative and spatial data for orchid pollination are scarce and may be important tools for reintroduction and conservation; however, conclusions cannot be drawn on the basis of the typically infrequent and unpredictable pollination events. We carried out a novel, retrospective, spatial analysis of the pollination of the entire population of two miniature orchids, Erycina crista-galli and Notylia barkeri, on coffee bushes in plantations at 900 m in Soconusco, south-eastern Mexico. The numbers of mature flowering plants of both species in the experimental site were similar. Notylia barkeri produced nearly four times as many flowers, but a similar proportion of the total number of flowers produced was pollinated (1.23% and 1.48% for N. barkeri and E. crista-galli, respectively). An estimated 29 919 977 (+/- 4 983 995) seeds were produced by N. barkeri, nearly 12 times more than E. crista-galli at 1 009 414 (+/- 147 000). The pollinators of N. barkeri chose flower clusters at random and pollinated various flowers within a patch, whereas the pollinators of E. crista-galli chose patches of flowers slightly more systematically, with less dependence on flower density, and appeared to dedicate less attention to each patch. For both species, pollinators slightly favoured larger clusters of flowers and left many individual and groups of flowers unvisited. To restore populations of these orchids in coffee plantations as a replacement habitat, N. barkeri should be planted in small, separate groups and E. crista-galli in larger groups of individuals, dispersed regularly throughout the selected site to maximize the possibility that the flowers will be discovered by pollinators. (C) 2008 The Linnean Society of London, Botanical Journal of the Linnean Society, 2008, 158, 448-459.

2121: *+.179*

Most turtle species require high adult survivorship to maintain stable populations. Translocations are often implemented to conserve turtle populations but may cause demographic disturbance as a result of increased mortality or dispersal of released animals. The gopher tortoise (Gopherus polyphemus) is one of the most frequently translocated turtle species. Short-term monitoring indicates that dispersal by released tortoises is common, but few long-term data are available to determine if losses of translocated animals continue for multiple years. We used 12 years of mark-recapture data to investigate long-term apparent survival of two groups of gopher tortoises translocated during separate periods to St. Catherines Island, Georgia, USA. We analyzed capture histories in program MARK to compare apparent survival of newly released tortoises and previously established translocated tortoises and also to determine whether apparent survival varied with sex or maturity. Apparent annual survival did not vary between adult males and females (0.98 +/- 0.01), but was lower in sexually immature tortoises (0.84 +/- 0.05). We documented a temporary reduction in apparent survival of newly released adult (0.75 +/- 0.06) and immature tortoises (0.45 +/- 0.26) during the first year after release that may be attributed in part to permanent dispersal. However, for both maturity classes, apparent survival of newly released tortoises was consistently high and matched that of previously established animals during the remainder of the study. Additional long-term studies of both translocated and naturally-occurring populations are needed to improve management of remaining tortoise populations. (c) 2008 Elsevier Ltd. All rights reserved.

2122: *+.313*

1. Conservation biologists need tools that can utilize existing data to identify areas with the appropriate habitat for species of conservation concern. Regression models that predict suitable habitat from geospatial data are such a tool. Multiple logistic regression models developed from existing geospatial data were used to identify large-scale stream characteristics associated with the occurrence of mountain suckers (Catostomus platyrhynchus), a species of conservation concern, in the Black Hills National Forest, South Dakota and Wyoming, USA.2. Stream permanence, stream

slope, stream order, and elevation interacted in complex ways to influence the occurrence of mountain suckers. Mountain suckers were more likely to be present in perennial streams, and in larger, higher gradient streams at higher elevations but in smaller, lower gradient streams at lower elevations.3. Applying the logistic regression model to all streams provided a way to identify streams in the Black Hills National Forest most likely to have mountain suckers present. These types of models and predictions can be used to prioritize areas that should be surveyed to locate additional populations, identify stream segments within catchments for population monitoring, aid managers in assessing whether proposed forest management will potentially have impacts on fish populations, and identify streams most suitable for stream rehabilitation and conservation or translocation efforts.4. When the effect of large brown trout (Salmo trutta) was added to the best model of abiotic factors, it had a negative effect on the occurrence of mountain suckers. Negative effects of brown trout on the Mountain sucker Suggest that management of recreational trout fisheries needs to be balanced with mountain sucker conservation in the Black Hills. However, more spatially explicit information on brown trout abundance would allow managers to understand where the two species interact and where recreational fisheries need to be balanced with fish conservation. Copyright (c) 2008 John Wiley & Sons, Ltd.

2123: *+.036*

Release of Galliformes species is common management practice in Europe and North America. Here, we attempt to synthesise available information on the release of wild-caught and captive-reared galliforms, and discuss flow rearing and release techniques affect release success. Galliforms are released into the wild to increase hunted populations with after-hunting-season releases, to establish new populations, to augment threatened populations, and to be used for 'put and take' shooting. We conclude that the release of artificially reared galliforms after the hunting season is not suitable practice to increase an already viable population. Release for 'put and take' shooting is ail alienated form of hunting, and it raises ethical questions. 'Put and take' should be strictly examined in the context of wildlife conservation, hunting culture, philosophy and economy. In the case Of Population establishment and augmentation, translocation of wild-caught birds is by far the best choice. The alternative choice is release of naturally or semi-naturally reared birds and anti-predator trained birds. Galliform release should not be a stereotyped process with many failures, but rather a practice in which correct techniques are used for the pursued aim.

2124: *+.141*

The present population size of the Ural Owl Strix uralensis in Europe is estimated at 43,000 and 121,000 (mean 82,000) breeding pairs. But only about 6,000 pairs live in central and south-eastern Europe. The attempts at re-introducing the Ural Owl in the Bavarian/Bohemian Forest are promising. The same positive developments can be seen in the populations in the Polish Carpathians, in the Czech Beskides, in the Slovak Tatra Mts., in the western Ukraine, in northeastern Hungary and in Slovenia. From the last-mentioned country the species immigrates to the province of Udine (northeastern Italy) and to Carinthia (southern Austria). Open areas in forests are very favourable for the Ural Owl, because a high food supply in form of voles and yellow-necked mice can develop. In the same way the growing number of gaps in forests caused by hurricanes and/or beetle-attacks may lead to an extension of the breeding area of this big owl in central Europe.

2125: *+.069*

Note about the occurrence and observations of the Pearly Parakeet (birds, Psittacidae) in the state

of Pernambuco, NE Brazil. A group of twelve individuals of Pearly Parakeet was found inhabiting an Atlantic Forest fragment in Recife, NE Brazil. The individuals seemed to be well adapted to the place. In non systematic visits to the area between 2004-2006 we observed some items of the diet, defense behavior against predators and population increase. The origin of the individuals is probably of improper release or escape from captivity, since this species doesn't inhabit the Atlantic Forest. Since it is a threatened species, this work also aims to contribute to conservation efforts, like reintroduction programs in areas of original distribution where the species is extinct.

2126: ***+.234***

An important goal of native plant restorations was to reconstitute populations that are genetically similar to native ones, thereby increasing the probably of successful establishment and persistence. We examined the extent to which this goal has been accomplished in Great Lakes restorations of Ammophila breviligulata Fern., a beachgrass species that is widely used for habitat restoration and is considered threatened in the study areas. In parallel studies on Lake Michigan and Lake Superior, we used polymorphic Intersimple Sequence Repeat markers to assess genetic similarity between well-established and new native populations, restored populations, and restoration propagules obtained from two commercial suppliers. Native populations were generally more diverse than expected for a clonal species, whereas the commercially cultivated releases were monotypic. One of the commercial releases used in Minnesota was exclusively found in restored populations and did not occur in any other native population at this site. The propagules used in the newly planted restoration in Illinois were derived from a release that commercial suppliers maintain was derived from a native Michigan population, as opposed to a selected release. Diversity in this restoration was equivalent to that native Illinois' populations; however, many of the genotypes were not of local origin. Overall, study underscores the importance of obtaining baseline genetic surveys of remnant native populations and restoration propagules before restoration efforts are initiated, especially when the populations are threatened or endangered.

2127: ***+.139***

Though translocations of rare populations should be considered only as the last resort for species' conservation, when habitat destruction is imminent, it may be the only means to preserve a species. With over half the known, wild federally endangered Crenulate leadplant (Amorpha herbacea var. crenulata), Fabaceae, growing on unprotected land slated for development, preserving this unprotected population was critical. We rescued whole plants, cuttings, and seeds for an experimental translocation. Into a restored pine rockland, once dominated by the invasive exotic tree Brazilian pepper (Schinus terebinthifolius), we transplanted plants from different sources and of different sizes. Plants used were rescued from an unprotected site, seedlings, and 1-, 2-, and 7-year-old plants from Fairchild Tropical Botanic Garden's ex situ collection, creating a novel population in a new habitat. We also evaluated which propagule type and source had the best survival, growth, and reproduction. After 40 months, overall transplant survival was 71%. Large whole plants, rescued and nursery grown, had the best survival rates (86 and 78%), whereas cuttings had 67% survival and seedlings had only 26% survival. The restored site, once nearly a monoculture of S. terebinthifolius, is now dominated by 104 native plant species, including 17 naturally recruited state listed, plus the one translocated federally endangered plant species. In addition, one federally threatened snake species was observed on the site. These studies demonstrate that botanic garden collections not only play a vital role in the conservation of species' genetic diversity but also can be used as source material for habitat restoration.

2128: ***+.185***

After their extinction in the first half of the 20th century, mainly by hunting, Ospreys returned to Bavaria as a breeding species in 1992 when one pair nested. Protection of the birds by foresters and provision of artificial nests helped the population grow to 4 pairs in 2008. Three of the females were hatched in Brandenburg and Saxony. Nesting pairs are monitored annually. In total 41 young have fledged to date. The slow population growth seems typical for small, remote subpopulations. Habitat requirements and means for the restoration of the natural breeding range are discussed, such as the provision of artificial nests and translocation of young to potential nesting areas.

2129: *+.196*

The Galapagos land iguanas (genus Conolophus) have faced significant anthropogenic disturbances since the 17th century, leading to severe reduction of some populations and the extinction of others. Conservation activities, including the repatriation of captive-bred animals to depleted areas, have been ongoing since the late 1970s, but genetic information has not been extensively incorporated. Here we use nine species-specific microsatellite loci of 703 land iguanas from the six islands where the species occur today to characterize the genetic diversity within, and the levels of genetic differentiation among, current populations as well as test previous hypotheses about accidental translocations associated with early conservation efforts. Our analyses indicate that (i) five populations of iguanas represent distinct conservation units (one of them being the recently discovered rosada form) and could warrant species status, (ii) some individuals from North Seymour previously assumed to be from the natural Baltra population appear related to both Isabela and Santa Cruz populations, and (iii) the five different management units exhibit considerably different levels of intrapopulation genetic diversity, with the Plaza Sur and Santa Fe populations particularly low. Although the initial captive breeding programmes, coupled with intensive efforts to eradicate introduced species, saved several land iguana populations from extinction, our molecular results provide objective data for improving continuing in situ species survival plans and population management for this spectacular and emblematic reptile.

2130: *-.213*

Emerging infectious diseases pose a considerable threat to wildlife globally. One such disease that has apparently emerged in recent years in New Zealand is avian malaria, with Plasmodium infections being detected in numerous species for the first time. Although the overall significance of this apparent emergence is not yet known, infection by Plasmodium has been diagnosed as a cause of mortality in several native species in captivity. Here we investigate the epidemiology of the most recently confirmed case, with our results having potentially important implications for native bird management. Avian malaria caused the death of five mohua or yellowheads (Mohoua ochrocephala) at Orana Wildlife Park in Canterbury during 2003-05, after their translocation from the Blue Mountains (Otago) in 2003. A lack of detectable Plasmodium infection in wild mohua in both the Blue Mountains and the nearby Catlins region, in contrast to an unusually high prevalence in wild bird Populations at Orana Park at the time of the outbreak, indicates that infection was most likely acquired by the birds after translocation, This evidence, although not conclusive, strongly argues for assessment of the risk of greater (and potentially deleterious) exposure to malarial parasites to be undertaken prior to native bird translocation. A mosquito investigation carried out at Orana Wildlife Park identified the ubiquitous indigenous mosquito Culex pervigilans as the likely disease vector. Hence, management of this mosquito species (in addition to the exotic Cx. quinquefasciatus, a known vector of avian malaria in other countries) is a potentially useful preventative measure against disease outbreaks in native bird populations of conservation value in New Zealand.

2132: ***+.405***

Rich in biological diversity, South Africa's natural habitats are internationally recognized as a conservation priority. Biodiversity loss continues, however, and limited scope to enlarge the state-protected areas, combined with funding shortages for public parks, means that conservationists are increasingly turning to private landowners for solutions. The recent boom in privately owned wildlife ranches in South Africa has the potential to contribute to conservation in South Africa. This paper explores the benefits, limitations, and challenges of private wildlife ranching as a tool for conservation in South Africa through interviews with key stakeholders working within conservation and wildlife ranching, and through case studies of threatened species programs. Respondents suggested that wildlife ranches contribute to conservation positively by maintaining natural areas of habitat and by providing resources to support reintroduction programs for threatened species. However, they reported a number of limitations centered on three themes that generally arise due to the commercial nature of wildlife ranching: (1) tourist preferences drive the industry, (2) predators are persecuted to protect valuable game, and (3) inadequate resources are made available for professional conservation management and planning on ranches. In addition to challenges of combining economic gain with conservation objectives, ranchers face a number of challenges that arise because of the small, enclosed character of many ranches in South Africa, including the need to intensively manage wildlife populations. In order to enhance the role of wildlife ranching within conservation, clear guidance and support for ranchers is likely to be required to boost endorsement and minimize economic loss to ranchers.

2133: ***+.405***

Rich in biological diversity, South Africa's natural habitats are internationally recognized as a conservation priority. Biodiversity loss continues, however, and limited scope to enlarge the state-protected areas, combined with funding shortages for public parks, means that conservationists are increasingly turning to private landowners for solutions. The recent boom in privately owned wildlife ranches in South Africa has the potential to contribute to conservation in South Africa. This paper explores the benefits, limitations, and challenges of private wildlife ranching as a tool for conservation in South Africa through interviews with key stakeholders working within conservation and wildlife ranching, and through case studies of threatened species programs. Respondents suggested that wildlife ranches contribute to conservation positively by maintaining natural areas of habitat and by providing resources to support reintroduction programs for threatened species. However, they reported a number of limitations centered on three themes that generally arise due to the commercial nature of wildlife ranching: (1) tourist preferences drive the industry, (2) predators are persecuted to protect valuable game, and (3) inadequate resources are made available for professional conservation management and planning on ranches. In addition to challenges of combining economic gain with conservation objectives, ranchers face a number of challenges that arise because of the small, enclosed character of many ranches in South Africa, including the need to intensively manage wildlife populations. In order to enhance the role of wildlife ranching within conservation, clear guidance and support for ranchers is likely to be required to boost endorsement and minimize economic loss to ranchers.

2134: ***-.062***

Fragmentation of natural populations can have negative effects at the genetic level, thus threatening their evolutionary potential. Many of the negative genetic impacts of population fragmentation can be ameliorated by gene flow and it has been suggested that in wind-pollinated tree species, high or even increased levels of gene flow are a feature of fragmented populations,

although several studies have disputed this. We have used a combination of nuclear microsatellites and allele-specific PCR (AS-PCR) analysis of chloroplast single nucleotide polymorphisms (SNPs) to examine the levels and patterns of genetic diversity and population differentiation in fragmented populations of juniper (Juniperus communis) in Ireland and inform conservation programs for the species. Significant population differentiation was found for both chloroplast and nuclear markers, indicating restricted gene flow, particularly over larger geographic scales. For conservation purposes, the existence of genetically distinct clusters and geographically localised chloroplast haplotypes suggests that the concept of provenance should be taken into account when formulating augmentation or reintroduction strategies. Furthermore, the potential lack of seed dispersal and seedling establishment means that ex-situ approaches to seed and seedling management may have to be considered.

2135: ***-.020***

The effectiveness of rare plant conservation will increase when life history, demographic, and genetic data are considered simultaneously. Inbreeding depression is a widely recognized genetic concern in rare plant conservation, and the mixing of genetically diverse populations in restoration efforts is a common remedy. Nevertheless, if populations with unrecognized intraspecific chromosome variation are crossed, progeny fitness losses will range from partial to complete sterility, and reintroductions and population augmentation of rare plants may fail. To assess the current state of cytological knowledge of threatened and endangered plants in the continental United States, we searched available resources for chromosome counts. We also reviewed recovery plans to discern whether recovery criteria potentially place listed species at risk by requiring reintroductions or population augmentation in the absence of cytological information. Over half the plants lacked a chromosome count, and when a taxon did have a count it generally originated from a sampling intensity too limited to detect intraspecific chromosome variation. Despite limited past cytological sampling, we found 11 plants with documented intraspecific cytological variation, while 8 others were ambiguous for intraspecific chromosome variation. Nevertheless, only one recovery plan addressed the chromosome differences. Inadequate within-species cytological characterization, incomplete sampling among listed taxa, and the prevalence of interspecific and intraspecific chromosome variation in listed genera, suggests that other rare plants are likely to have intraspecific chromosome variation. Nearly 90% of all recovery plans called for reintroductions or population augmentation as part of recovery criteria despite the dearth of cytological knowledge. We recommend screening rare plants for intraspecific chromosome variation before reintroductions or population augmentation projects are undertaken to safeguard against inadvertent mixtures of incompatible cytotypes.

2136: ***+.184***

Animal relocations have become a common tool in nature conservation, but the genetic consequences of such projects have rarely been studied in insects. As both natural and artificial formation of new populations may lead to genetic drift (founder effect), decreased genetic diversity and increased rates of inbreeding, genetic analyses can provide valuable information to evaluate the success of a relocation project. The field cricket (Gryllus campestris) has been subjected to reintroduction and translocation projects in England and northern Germany. Here, we present a microsatellite study on the population genetics of one recently established population of this species in comparison with several older populations and some recently colonized sites. our results show that the translocation did not result in a significant loss of genetic diversity, when compared to source and other natural populations suggesting that translocation. of a high number of nymphs from different subpopulations may be a suitable method to decrease the loss of genetic

diversity and reduce the risk of inbreeding. Furthermore, the translocation had no negative effect on the source population, which reached a new maximum population size in 2006. An assignment test showed that individuals from the translocated population (F4 generation) were still assigned to the source populations, whereas two young subpopulations that originated by natural colonization from the central population about ten years ago already formed separate genetic clusters. As the strong fragmentation of G. campestris populations in northern Germany hampers natural colonization of newly created potential habitats, translocation projects seem to be an appropriate method to preserve this species. (c) 2008 Elsevier Ltd. All rights reserved.

2137: *+.074*

The diversity and genetic structure of the cycad Dioon sonorense (De Luca, Sabato & Vazq. Torres) Chemnick, T.J. Greg. & S. Salas-Mor. were evaluated from the four known populations in tropical dry forests of Sonora, Mexico. The estimates of genetic diversity in the 19 loci analysed were: mean number of alleles per locus (A = 2.0), percentage of polymorphic loci (P = 81.6) and expected heterozygosity (H(E) = 0.314). The subpopulations were found to be in Hardy-Weinberg equilibrium at the local level (F(is)), even though we found a global excess of homozygotes (F(it) = 0.13). Genetic variation attributed to differences among populations was 15%. Our results indicate that the geographical isolation caused by the historical effects of the Pleistocene among populations has probably generated clinal variation of the allelic frequencies at two loci, in relation to their latitudinal distribution. We compare our results with the long-lived southern hemisphere trees Fitzroya and Nothofagus in relationship to the Pleistocene glaciations. We also infer that the populations have become fragmented recently due to increasing pressure of habitat conversion, disturbance and illegal extraction of plants and seeds for the international horticultural trade. We therefore recommend three aspects: (i) propagation of this species for reintroduction, (ii) declare the localities of its northern distribution as a protected area, and (iii) an amendment of the current International Union for Conservation of Nature Red List categorisation for this species to 'Critically Endangered'. (C) 2008 Elsevier Ltd. All rights reserved.

2138: *+.013*

The lesser kestrel (Falco naumanni) is one of the most endangered bird species in Europe, and a captive breeding and reintroduction project was established. A breeding project is vulnerable to pathogens, e.g., mycoplasmas, reducing the reproductive success and carrying the risk to release pathogens with the birds to the wild. Therefore, 18 infertile eggs and 43 dead in shell embryos of the breeding project, as well as 27 nestlings and 34 adult birds of the captive and three different free-ranging populations were investigated for the occurrence of mycoplasmas by culture and a Mycoplasma genus-specific polymerase chain reaction. All eggs, embryos, and hand-reared nestlings from the captive group were negative. In contrast, all parent-reared nestlings and 88% of the adults were positive. Mycoplasma falconis and unidentifiable mycoplasmas were detected in all groups. Mycoplasma buteonis was found in the captive and only in two of the three free-ranging populations. Sequencing the 16S rRNA gene of six randomly selected unidentified isolates showed that five isolates were similar and most likely had been found previously in a falcon from Germany. The remaining isolate demonstrated a very high homology to unidentified Mycoplasma isolates obtained previously from semen samples of raptors. The results suggest that these isolates might represent two new species. Mycoplasmas seem not to play a major role as pathogens in the breeding project, and there is no evidence that releasing birds poses a risk to the free-ranging population with regard to mycoplasmas. The study seems to be the first to describe the occurrence and role of mycoplasmas in the lesser kestrel.

2139: ***+.066***

Anthropogenic hybridization in wildlife has been identified as one of the main causes of genetic homogenization, highlighting the need for identification and evaluation of populations at risk. Relocation of wildlife for game management purposes is a widespread practice that may promote the admixing of genetically different populations, subspecies or species. We undertook a large-scale study on the Iberian Peninsula to assess the extent of hybridization in red-legged partridge Alectoris rufa populations, which have been subject to extensive restocking of farm-reared individuals. Using a polymerase chain reaction-restriction fragment length polymorphism technique to assess the prevalence of individuals with mtDNA from other species, we examined samples of A. rufa from museum specimens (229), extant wild populations (955) and game farms (530). We found widespread occurrence of chukar partridge Alectoris chukar mtDNA lineages in samples obtained from game farm partridges (63% of game farms) and from wild partridges (45% of populations), but no allochthonous mtDNA lineages were found in museum partridges. We also found that the probability of occurrence and the number of partridges with allochthonous lineages was higher in localities where recent restocking had occurred. In addition, investigation of trends in bag records and the numbers of game farms over the past 30 years suggests that the general decline of wild populations has been accompanied by an increase in game farm facilities. These results suggest that supplemental stocking practices are threatening the integrity of the wild population gene pool. We recommend that rural development policies and associated wildlife management programs focused on maintaining high stock densities for hunting also need to consider the impact of game management on the genetic integrity of game populations.

2140: ***+.097***

Captive breeding has become globally important in endangered species recovery, yet it is fraught with problems such as maintenance of genetic diversity, and adaptation to captivity. We studied survival and population dynamics of a captive population of endangered Columbia Basin pygmy rabbits Brachylagus idahoensis, from 2003 to 2007, to evaluate its potential for supporting reintroduction and recovery of wild extirpated populations in shrub-steppe ecosystems of eastern Washington, USA. We developed stochastic population viability analysis models in Vortex and RAMAS to assess performance and surplus production of the captive population. This pygmy rabbit population has low adult survival beyond 1 year and dependency on high juvenile recruitment for population growth. Low juvenile survival and high variability in stochastic growth rates result in high variability in annual productivity. Our analysis showed that the captive population at n=75 cannot sustain a steady annual harvest of >= 30 rabbits for reintroduction and supplementation without increasing the risk of quasi-extinction (n=30) to 59%. We conducted sensitivity analysis on maternity, carrying capacity and survival rates to identify critical values for model parameters that would lower extinction risk to the captive population when used as a source of rabbits for reintroduction. Increasing juvenile survival and recruitment into the first breeding class is the most effective method for enhancing the breeding program. Our population models suggest that captive breeding and recovery programs for short-lived lagomorphs present significant conservation challenges because of the need to rapidly grow such populations to overcome demographic and genetic challenges.

2141: ***+.262***

Rare butterfly conservation requires understanding of butterfly interactions with the resource conditions that influence population growth. Exotic plant invasions can reduce butterfly population size and growth by displacing key resources, degrading habitat conditions and directly impacting

fitness, but exotic plants may also be incorporated into native butterfly diets, rendering practicing conservation biologists a choice between exotic plant control and butterfly population persistence. Euphydryas editha taylori (Nymphalidae), a candidate endangered butterfly species in the Pacific Northwest of North America, switched from an unknown native larval host plant to become entirely dependent on an exotic larval host Plantago lanceolata in extant western Oregon populations. Furthermore, the last Oregon butterfly populations are surrounded by two exotic grasses, Brachypodium sylvaticum and Festuca arundinacea, both of which can dominate remnant native prairies and degrade native grassland structural conditions. When given a choice of habitat conditions, E. e. taylori oviposited on larval host plants surrounded by increasing abundance of short-statured native bunchgrasses and adult nectar resources, indicating that females select egg-laying sites based on habitat conditions rather than just host plant presence. Both larval and adult resources substantially diminished with increasing cover of exotic grasses and were nearly absent when B. sylvaticum and F. arundinacea dominated. Butterfly resource loss with increasing exotic grass cover, the documentation of contemporary subpopulation extinction, current historic site conditions and grass invasion history in the Willamette Valley suggest that mass E. e. taylori extinction in western Oregon by the late 1970s was due to B. sylvaticum and F. arundinacea invasion. To prevent the extinction of E. e. taylori, land managers must accept that the anthropogenically mediated host switch to P. lanceolata must be conserved and that reintroductions/augmentation with a potential native host plant species is unlikely to be effective because larval development is timed to P. lanceolata growth.

2142: ***+.067***

The removal of individuals from a population may occur for several reasons and responses of populations will vary depending on the magnitude and nature of the removal and the life history of the species. An understanding of the effects of loss of individuals on these populations, and the mechanism of replacement, will be important to conservation. This maybe particularly important where wild individuals are used for the increasingly popular conservation strategy of translocation. During the recent translocation of the endangered eastern bristlebird (Dasyornis brachypterus), two monitoring sites were established in the wild source population, one where removals were to take place and another as a control to assess the impact of the removals on the population. The removal of 44 eastern bristlebirds across 3 years from a single area in the source population had no significant detectable impact in the numbers of individuals surveyed. Individuals that were removed appeared to have been replaced within 6 months of their removal, although to a lesser extent in the later part of the study. The origin of the replacement eastern bristlebirds was unknown and the quick recovery was suggested to be a result of juvenile dispersal, perhaps combined with territory uptake by previously non-territorial and non-calling (thus undetectable) individuals within the population. Such a surplus may be a result of insufficient suitable habitat for population expansion, and will also have implications for monitoring populations of rare and cryptic species. It is also suggested that some territorial species may have several mechanisms that can replace losses of individuals from a population.

2143: ***+.241***

Translocations have been used for decades to restore or augment wildlife populations, yet more often than not, little to no data and/or arbitrary means are used for determining translocation success. The objectives of our study were to describe nesting habitat utilised by the greater sage grouse translocated into an extant population and to identify factors related to nest success, thereby demonstrating the adaptability of the birds to their new environment and producing one measure of long-term translocation success. We trapped female grouse individuals during the

spring on and near leks of source populations, fitted them with radio-transmitters, and released them in the morning onto an active lek in an extant population in Strawberry Valley, Utah. We monitored translocated females for nesting activity and documented nesting attempts, nest success, clutch size and embryo viability. Data were recorded on habitat variables associated with nest sites and paired-random sites, including factors known to be important for resident females that nested successfully. We used logistic regression and an a priori information-theoretic approach for modelling nest v. paired-random sites and successful v. unsuccessful nest sites. Our analyses suggested that crown area of the nest shrub and percentage grass cover were the two variables that discriminated between nest and paired-random sites. Females that nested successfully selected sites with more total shrub canopy cover, intermediate size-shrub crown area, aspects other than NW and SE, and steeper slopes than for unsuccessful nests. After being translocated from distant sites with differing habitat characteristics, these birds were able to initiate a nest, nest successfully, and select micro-habitat features similar to those selected by resident sage grouse across the species range. Our results demonstrate the adaptability of the translocated female sage grouse individuals and produce one tangible measure of long-term translocation success.

2144: *+.235*

Habitat fragmentation can have several adverse genetic impacts on populations. Assessing the extent of these threatening processes is essential in conservation management. In the present study, we investigated the genetic population structure of the endangered pygmy bluetongue lizard, Tiliqua adelaidensis, which is now restricted to a few small fragments of its previously more extensive grassland habitat. The aim of our study was to investigate genetic diversity and gene flow both among and within sample sites. The information will assist in making recommendations for habitat conservation and translocation programs. We collected DNA from 229 individuals from six isolated sample sites and genotyped them for 16 polymorphic microsatellite loci. Across all six sample sites, observed heterozygosity ranged from 0.75 to 0.82. There was no evidence of population bottlenecks and little evidence of inbreeding due to consanguineous mating. Genetic differentiation was low to moderate although significant for all pairs of sample sites (F-ST = 0.021-0.091). Results from Bayesian clustering analyses revealed distinct clusters in the overall sample and suggested restricted gene flow between sample sites separated by distances ranging from 1.7 to 71.6 km. By using spatial autocorrelation, we also found a significant genetic structure within sample sites at distances up to 30 m, suggesting restricted gene flow even in small patches of continuous habitat. It will be important to preserve this finely clustered population structure in captive breeding and translocation programs. Increasing opportunities for gene flow through habitat corridors or population augmentation may help maintain genetic diversity and prevent an increase in differentiation. Although endangered species do not always present model systems for studying fragmentation, our approach shows how important genetic information can be acquired to aid conservation in highly fragmented ecosystems.

2145: *+.099*

Control of introduced predators is critical to both protection and successful reintroduction of threatened prey species. Efficiency of control is improved if it takes into account habitat use, home range and the activity patterns of the predator. These characteristics were studied in feral cats (Felis catus) and red foxes (Vulpes vulpes) in arid South Australia, and results are used to suggest improvements in control methods. In addition, mortality and movement patterns of cats before and after a poison-baiting event were compared. Thirteen cats and four foxes were successfully fitted with GPS data-logger radio-collars and tracked 4-hourly for several months. High intra-specific variation in cat home-range size was recorded, with 95% minimum convex polygon (MCP) home

ranges varying from 0.5 km(2) to 132 km(2). Cat home-range size was not significantly different from that of foxes, nor was there a significant difference related to sex or age. Cats preferred habitat types that support thicker vegetation cover, including creeklines and sand dunes, whereas foxes preferred sand dunes. Cats used temporary focal points (areas used intensively over short time periods and then vacated) for periods of up to 2 weeks and continually moved throughout their home range. Aerial baiting at a density of 10 baits per km(2) was ineffective for cats because similar high mortality rates were recorded for cats in both baited and unbaited areas. Mortality was highest in young male cats. Long-range movements of up to 45 km in 2 days were recorded in male feral cats and movement into the baited zone occurred within 2 days of baiting. Movement patterns of radio-collared animals and inferred bait detection distances were used to suggest optimum baiting densities of similar to 30 baits per km(2) for feral cats and 5 per km(2) for foxes. Feral cats exhibited much higher intra-specific variation in activity patterns and home-range size than did foxes, rendering them a potentially difficult species to control by a single method. Control of cats and foxes in arid Australia should target habitats with thick vegetation cover and aerial baiting should ideally occur over areas of several thousand square kilometres because of large home ranges and long-range movements increasing the chance of fast reinvasion. The use of temporary focal points suggested that it may take several days or even weeks for a cat to encounter a fixed trap site within their home range, whereas foxes should encounter them more quickly as they move further each day although they have a similar home-range size. Because of high intra-specific variability in activity patterns and home-range size, control of feral cats in inland Australia may be best achieved through a combination of control techniques.

2146: ***+.047***

During the year 2008, the census and monitoring of the breeding population of Osprey (Pandion haliaetus) in Spain has been undertaken under a variety of schemes that are ongoing in the Spanish regions within the species'range. In the Balearic Islands, such schemes are the Osprey Conservation Plan of the Balearic Government, in coordination with the Cabrera Maritime-Terrestrial National Park administration. In the Canaries, the work was undertaken in the frame of the Endangered Species Monitoring Programme of the Government of the Canary Islands, whilst the relevant tasks in the Chafarinas Isles have been carried out by the Spanish National Parks Authority. In Andalusia, the responsi- bility was within the team in charge of the reintroduction programme for the species in the 'Donana'Ecology Research Unit (Estacion Biologica de Donana). Overall, 31 pairs have been recorded, of which 15 in the Balearic Islands, 14 in the Canary Islands, 1 in the Chafarinas Isles and 1 in Andalusia. All insular stocks belong to a distinct group showing unique features such as the nesting on sea cliff cornices or rocky outcrops just off shore, and sedentary adults with local juvenile recruitment. However, the only pair breeding in peninsular Spain belongs to the group showing continental features, which is attached to inland waters and nests usually on trees, although these can be replaced by posts or power line pylons. The only breeding pair in the Chafarinas Isles belongs to the Northern Moroccan breeding nucleus. The 31 breeding pairs in current records entail an 18% decrease as compared to population numbers during the period 2000-2002, when maximum estimates amounted to 38 pairs. Nonetheless, after analysing these data into detail, the conclusion seems to be that maximum ? gures were overestimated at that time, when the minimum population level estimated was 31 pairs. Taking into account the latter ?gure, the population of Osprey in Spain would have remained stable since 2002. This suggests that the Spanish population of the species has not improved during the last 10 years, despite the stock of suitable habitat still avai- lable within the species' traditional territories, which would allow for some population growth. Therefore, the recovery of the species recorded during the 1980s and 1990s has been halted or might have even reversed during the last decade, falling well below the 72-97 pairs estimated back in 1950. In fact, amongst the endangered raptors

living in Spain, the Osprey is the one having the smallest population. The Osprey's current range in Spain is virtually the same as in 1991; none of the territories or isles where the species once bred has been re-colonised. As com- pared to the records for the years 2000-2002, only the populations in the Isle of Cabrera (Balearic Islands) and in Andalusia have increased, although this might have occurred also in La Gomera Island (Canary Islands). Nonetheless, a decre- ase has been recorded for the same period in the Islands of Mallorca and Menorca (Balearics), Tenerife, Alegranza and Montana Clara (Canaries). The global productivity in 2008 has been 1.17 [plus or minus] 1.05 SD (n = 24; Balearics: 1.55; Canaries: 1.1), with breeding success being 1.40 [plus or minus] 0.99 (n = 20; Balearics: 1.70; Canaries: 1.1). These values are low as compared to their counterpart in conti- nental Europe breeding sites, although they are similar to records obtained within the Mediterranean breeding nucleus of the Island of Corsica. The Osprey populations in Spain are currently in poor condition; there is no connection between the two main breeding populations, which host 93.5% of the total stock, and the recruitment of individuals for these sites out of continen- tal locations is unlikely. Consequently, the conservation status of Osprey in Spain has been classi? ed as 'Critically Endangered'.

2147: *+.147*

The monotypic family Bretschneideraceae comprises only Bretschneidera sinensis Hemsl with a sparse distribution ranging from south western China to northern Vietnam as a Tertiary relict tree. B. sinensis is also included in the China Plant Red Data Book as a rare plant. Seed germination. seedling emergence and translocation were investigated in this paper to review some of the germination and reintroduction requirements for effective conservation of B. sinensis. Results showed that aril removal and surface sterilization of seeds were necessary for the control of decay fungi pre-chilling treatment (4-5 degrees C) or imbedding, in wet sand (10-15% MC) could partly released shallow seed dormancy: the optimal temperature for seed germination was 25 degrees C: light played a positive role in seed germination. Seeds sowed at the depth of 2 cm in sandy soil gave maximum seedling emergence, and deeper planting significantly decreased germination. The germination percentage could also be highly enhanced by in vitro germination.

2148: *+.314*

Trends in adaptation to new habitats of orphaned bear cubs in the period of rearing and after release into the wild have been revealed. The pattern of their distribution in a biotope is contingent on its exploration in search for food, with the intensity and scale of this process increasing as the animals grow. The results indicate that the methods of rearing used in the Ussuri Nature Reserve are effective and promising.

2149: *+.132*

The roach (Rutilus rutilus) is a widely distributed European fish. An inhabitant of many rivers and still water bodies of the United Kingdom, it is most abundant in low velocity lotic environments that are typical of the middle and lower courses of river systems. The roach is an adaptable, mobile species, undergoing seasonal and daily migrations to locate preferred feeding grounds, migrations to overwintering areas and annual migrations to spawn once sexual maturity is attained (3-4 years). A single female can produce in excess of 15,000 eggs, which contributes to the roach's vagility (success in the struggle for existence). An important species in the ecology of freshwaters, the roach eats a very wide range of food and adapts its diet to the food available. It is also eaten by a wide range of predators. These factors, of migratory behaviour and the potential to produce many offspring, lead to a hypothesis that roach populations will show little spatial genetic

differentiation within interconnected environments such as rivers. In addition to its ecological value, the roach is an important part of the coarse fisheries of England and Wales. It is a staple species in the interests of anglers. The coarse fisheries are worth close to one billion pounds annually, and, consequently, the interests of anglers are an important factor in the continued maintenance of high fish numbers in rivers. Little is known about the population genetics of most coarse fish, as interest has often focussed on the commercially more valuable salmonids. To date, management considerations for coarse fish have not included determination of 'genetic management units'. The main purpose of this study was to use a combination of mitochondrial (mt)DNA and nuclear (microsatellite) DNA markers to determine the levels of genetic diversity within, and connectivity among, contemporary roach populations, and to report this information in a way that could be of use in the future management of this species in the UK. Like many northern European freshwater fish species, the roach shows little mtDNA genetic variation. This is consistent with a hypothesis that the UK population originated by postglacial demographic expansion and colonisation from a European refuge(s). The patterns of mutations that link mtDNA haplotypes suggest at least two phylogroups colonised and now inhabit the UK. Roach samples from all 15 rivers examined were genetically distinct. This was principally due to frequency differences of two common haplotypes (A and B), but also because of the presence and/or elevated frequency of rare haplotypes, particularly in smaller and/or coastal rivers. The most genetically distinct rivers were the Severn, Kentish Stour, Bristol Avon, Medway and Sussex Ouse. In broad terms three geographical areas could be defined, based on mtDNA genetic composition: the Thames drainage and all rivers to the south of it; the Severn drainage and all rivers west and southwest of it; English rivers north of the Thames and east of the Severn. The significant genetic differentiation among drainages confirms the intuitive prediction that fish populations of these drainages are reproductively isolated, and that a moderately diverse natural population of UK roach has not been genetically homogenised by anthropogenic influences (fish translocation, modification of water courses, linking drainages via canals). Genetic diversity was studied in roach populations in the main river courses of two rivers, the Thames and the Suffolk Stour. These roach populations exhibit moderately high levels of genetic diversity (for a northern European freshwater fish) at nuclear (microsatellite) DNA loci. Statistically significant differences in gene frequencies, between most sampled sites, indicate that roach populations in these rivers consist of genetically differentiated local sub-populations. Significant differentiation indicates that there has been little large-scale interbreeding and limited gene flow among sub- populations. An isolation-by-distance pattern of genetic differentiation and signal of genetic relatedness was higher than expected within short distances (7.7 to 12.7km). This all suggests a typical meta-population structure for UK roach populations within drainages. The emerging picture is that roach populations do not exchange large numbers of effective migrants (i.e. ones that interbreed) on short timescales, so sub- populations within areas of ~10km may be demographically uncoupled from neighbouring sub-populations. However, in the longer term, levels of gene flow (migration) are high enough to maintain genetic cohesion across the total population of a river/drainage (i.e. no sub-population is reproductively isolated). There are differences between the roach populations in the Thames and the Stour: the Thames has a stronger pattern of isolation-by-distance, whereas the Stour shows lower rates of migration, overall higher levels of genetic structuring and less association of river distance with genetic differentiation. The greater population sizes in the Thames, coupled with more river traffic, mean that the opportunities for migration are probably greater in the Thames than the Stour. Gene flow in the Thames is primarily downstream, whereas bi-directional gene flow is more common in the Stour. The preponderance of downstream gene flow, at least in the Thames, is consistent with the observation that levels of genetic diversity are slightly higher downstream (although not significantly so), with the exception of tidal sites. The lower levels of genetic variation in the tidal populations, coupled with increased genetic differentiation of these sites from others upstream, may indicate a biologically significant (i.e.

selectively important) difference between tidal and fully freshwater sub-populations. An analysis of genetic variation in the roach can inform, and has implications for, monitoring, stocking and other management practices. Populations in UK river drainage basins are sufficiently different from one another, genetically, to warrant attention as individual management units. Within-drainage management should also utilise genetic information. Large and heterogeneous catchments, such as the Thames, may hold genetically differentiated local sub-populations, indicating the relative isolation of some tributaries from the rest of the catchment, especially if the confluence of a tributary with the main channel lies below the tidal halocline. Levels of genetic differentiation among sites in both the Thames and the Stour (and probably in many UK rivers) are significant. However, they are so low that, in terms of conservation of genetic biodiversity, both rivers can be seen as comprising single genetic populations. In the short term, population genetic processes act over limited geographical scales (tens of km at most) and so demographic management and monitoring should be considered at such scales. When restocking local reaches, we recommend that introduced fish are sourced from as near as possible to the introduction site, but use of fish from any source site within the same drainage should be acceptable, and not cause significant changes to the genetic character of the overall population. However, there may be exceptions to this general recommendation, especially where the habitat is notably different from that of the rest of the river, for example at the tidal halocline, where it may be preferable to encourage rapid natural recolonisation.

2153: *+.086*

Background: Successful conservation of endangered species such as Apium repens requires sound information about their ecology and habitat demands. Currently, knowledge about this species is very limited.Aims: Our objectives were to analyse the ecology and habitat demands of A. repens to achieve a better understanding of the factors relevant for its conservation.Methods: We set up a transplantation trial and conducted experiments on competitive ability and inundation tolerance of A. repens; these were complemented by an analysis of 85 vegetation releves and land use and soil analyses of 24 north German populations.Results: Both intra- and interspecific competition had negative impacts on the growth of A. repens. Disturbance alleviated the impact of competition. Apium repens tolerated fresh water inundation of up to 56 days; salt water inundation proved to be fatal. Transplantation showed that seedlings are suitable founder propagules for reintroduction schemes and that disturbance is vital for their prospering.Conclusions: Apium repens is a light-demanding weak competitor that is dependent on disturbances and well-adapted to ground-water fluctuations and inundation. We recommend that disturbance regimes and hydrological conditions of extant populations should be maintained and suggest seedling-based reintroductions as a valuable tool for conserving the species.

2154: *+.125*

A reintroduction experiment of Bidens cernua L., a species included in the Red List of Italian Flora, was carried out at Lake Posta Fibreno (Lazio, central Italy). There were no significant differences in the length of the phenological phases between the reestablished population (P(r)) and the natural one (P(n)). The length of the phenological cycle, from seedling emergence to the end of the fruiting phase, was 207 +/- 3 days. The relative growth rate in height (RGR(H)) and relative growth rate in mass (RGR(m)) were significantly ($p < 0.05$) higher for P(r) (0.047 +/- 0.001 cm cm(-1) d(-1) and 0.057 +/- 0.001 g g(-1) d(-1), respectively) than for P(n) (0.045 +/- 0.001 cm cm(-1) d(-1) and 0.055 +/- 0.001 g g(-1) d(-1), respectively). There were significant ($p < 0.05$) differences for plant height (H) and total leaf area per plant (TLA) between P(r) and P(n), the latter having the lower values. Total plant dry mass (PDM) was significantly higher for P(r),

and the above-ground/below-ground dry mass ratio was 6.7 +/- 0.4 and 4.7 +/- 0.2 for P(r) and P(n), respectively. On the whole, results demonstrate that the P(r) of B. cernua has the potential to develop in a manner that is demographically similar to the P(n) present in the Posta Fibreno Lake protected area.

2155: *+.091*

The extinction of species across the globe is accelerating, directly or indirectly due to human activities. Biological impoverishment, habitat fragmentation, climate change, increasing toxification, and the rapid global movement of people and other living organisms have worked synergistically to diminish ecosystem function. This has resulted in unprecedented levels of disease emergence, driven by human-induced environmental degradation, which poses a threat to the survival and health of biodiversity. The emerging discipline of conservation medicine addresses these concerns through the following entities: humans; global climate; habitat destruction and alteration; biodiversity, including wildlife populations; domestic animals; and pathogens, parasites and pollutants. Furthermore, conservation medicine focuses on explicit linkages between these entities. As a crisis discipline, the usefulness of conservation medicine ultimately will depend on its applicability to solving problems. The perspectives and scientific findings of conservation medicine provide input into biomedical education; and policy and management of ecosystems, habitats and imperiled species. A sentinel species is one that has presented itself, or has been selected, to provide insight into the state (health) of an ecosystem, based on user-defined (e.g., researchers, conservationists or policymakers) objectives (e.g., disease, parasites, toxics, climate change, habitat destruction), coupled with the utility and vulnerability of this species to the perceived stress. The scientific information generated by the sentinel species should empower stakeholders and decision-makers to take mitigative action or support predictive capabilities; the "utility" of the species selected should consider its value and relevance to conservationists and to society at large (e.g., education and outreach; social sciences). Wild canids may serve as excellent sentinel species of emerging canine vector-borne diseases. Several canine vector-borne diseases or antibodies to these pathogens have been identified in wild canids including visceral leishmaniosis, Lyme disease, heartworm, hepatozoonosis and anaplasmosis to name a few. These reports are relatively recent as they relate to wildlife-domestic animal interactions, globalisation, translocations, habitat fragmentation and climate change. These pathogens and their relationship to wild canids are described herein. Further research needs to be performed to elucidate the role of the 36 extant species of wild canids in the epidemiology of canine vector-borne diseases.

2156: *+.149*

Paddlefish Polyodon spathula historically occurred in the Ohio and Allegheny River systems, extending into the headwater sections of the Allegheny River in New York and Pennsylvania. At the turn of the 19th century, paddlefish were reported in the Allegheny River near the cities of Salamanca and Olean, New York. The last published historical account of paddlefish in Pennsylvania occurred in 1919 at the mouth of the Kiskiminetas River, a major Allegheny River tributary. The demise of paddlefish in Pennsylvania and New York has been attributed to channelization, dams, gravel dredging, and water quality degradation. The construction of Kinzua Dam in 1968 prohibited paddlefish to the upper Allegheny River system. As late as 1986, paddlefish in Pennsylvania were listed as extirpated, and they continue to be classified as such in New York. In order to re-establish self-sustaining populations, Pennsylvania initiated a stocking program in 1991 in the upper Ohio and lower Allegheny rivers. In 1998, New York initiated a complimentary stocking program approximately 160 km upstream in the Allegheny Reservoir

(above Kinzua Darn). A second stocking location, Conewango Creek, was added in 2006 in a relatively unaltered section of the historic range. Free ranging adult paddlefish were captured by gill nets and "reliable source" reports were documented in Pennsylvania and New York. No evidence of natural reproduction or year-class structure has been documented in either state. Pennsylvania plans to increase the size of stocked fish and New York plans to increase stocking densities as hatchery space permits. Both states will continue to monitor and assess the reintroduction of paddlefish to the upper Ohio River basin.

2157: *+.173*

Dispersal is fundamental to the persistence of wild populations. Historically, swift foxes Vulpes velox of the northern Great Plains of North America have been thought to be poor dispersers. Short-grass prairie is optimal habitat for swift foxes but can be fragmented in the northern Great Plains. We wanted to assess whether wild-born, juvenile swift foxes from two proximate but distinct reintroduced populations had potential to move from one population to the other. We found five animals exhibiting long bouts of dispersal, much further than averages previously reported. One female fox traversed the long distance between the two populations and survived for at least three breeding seasons in the wild. We believe our findings are significant for conservation because they show that swift foxes are not poor dispersers and that patches of short-grass prairie previously thought to be too isolated (> 25 km) for natural movement may be recolonized or be suitable for reintroductions of swift foxes.

2158: *+.385*

Urban streams globally are characterised by degraded habitat conditions and low aquatic biodiversity, but are increasingly becoming the focus of restoration activities. We investigated habitat quality, ecological function, and fish and macroinvertebrate community composition of gully streams in Hamilton City, New Zealand, and compared these with a selection of periurban sites surrounded by rural land. A similar complement of fish species was found at urban and periurban sites, including two threatened species, with only one introduced fish widespread (Gambusia affinis). Stream macro invertebrate community metrics indicated low ecological condition at most urban and periurban sites, but highlighted the presence of one high value urban site with a fauna dominated by sensitive taxa. Light-trapping around seepages in city gullies revealed the presence of several caddisfly species normally associated with native forest, suggesting that seepage habitats can provide important refugia for some aquatic insects in urban environments. Qualitative measures of stream habitat were not significantly different between urban and periurban sites, but urban streams had significantly lower hydraulic function and higher biogeochemical function than periurban streams. These functional differences are thought to reflect, respectively, (1) the combined effects of channel modification and stormwater hydrology, and (2) the influence of riparian vegetation providing shade and enhancing habitat in streams. Significant relationships between some macroinvertebrate community metrics and riparian vegetation buffering and bank protection suggest that riparian enhancement may have beneficial ecological outcomes in some urban streams. Other actions that may contribute to urban stream restoration goals include an integrated catchment approach to resolving fish passage issues, active reintroduction of wood to streams to enhance cover and habitat heterogeneity, and seeding of depauperate streams with native migratory fish to help initiate natural recolonisation.

2159: *+.157*

In 2006 the department of veterinary. hunting and fishery of the canton Basel-Landschaft started a

project to restock the white-clawed crayfish (Austropotamobius pallipes, Red list of Swiss species 2). Between 18 and 141 individuals from three donor sites were resettled in nine different receptor sites. Three donor sites and nine receptor sites were visited by nightly excursions to determine the density and the distribution of the white-clawed crayfish between the beginning of July and the beginning of September 2008 at a total distance of 9.75 km. By reviewing the literature a data entry form containing 20 parameters was created to assess the habitat quality in each donor and receptor site. An estimation of the stocks of the white-clawed crayfish in the donor sites revealed 2000 individuals for the Bennwilerbach (111 individuals/100 m shore length). 500 and 370 individuals for the sections in the Lutzel at Neumuhle at id Neuhuus (68 individuals/100 In shore length) and 83 individuals for the Bosenbach (13 individuals/100 m shore length). During two excursions between 1.6 % and 21.9 % of the stocked crayfish were discovered at each receptor site, but no juveniles could be found. In five other receptor sites no crayfish was observed. The best correlation between the abundances of the white-clawed crayfish and the recorded habitat data of the donor sites wits generated by live parameters: wood debris. fine roots in the stream bed, natural Steep Shores, Shelters Under trees and roots as well as a substrate consisting of stones and rubbles. Using, these parameters it method for assessing watercourse Sections Was created. so that white-clawed crayfish call be reintroduced ill the sections with the greatest habitat potential to enhance the success.

2160: *+.249*

Ranching, stock enhancement and restocking are management approaches involving the release of wild or hatchery-bred organisms to enhance, conserve or restore fisheries. The present study, conducted from April 2002 to November 2005, evaluated the effectiveness of releasing wild and hatchery-reared (HR) mud crabs in the mangroves of Ibajay, Aklan, Philippines where preliminary studies demonstrated declining fishery yields, abundance and size of crabs. Comparison of survival and growth of wild-released and HR Scylla olivacea and HR Scylla serrata demonstrated the effect of nursery conditioning, size-at-release and species differences. Overall yield and catch per unit effort (CPUE) increased by 46% after stock enhancement trials. Recapture rates of released crabs were highest in wild-released S. olivacea and in crabs measuring 65.0-69.9mm carapace width (CW) and lowest in non-conditioned HR S. serrata. Growth rates were highest for conditioned HR S. olivacea and lowest for conditioned HR S. serrata (11.7 and 3.7mm month(-1) respectively). Fishing mortality was highest for S. olivacea, whereas natural mortality was greater for S. serrata. Conditioning hatchery-bred animals before release is also important in obtaining higher survival. S. olivacea was the more appropriate of the two species for release in mangrove habitats inundated with low-salinity water. However, there is a need for site-specific studies to evaluate the effectiveness of releases.

2161: *-.067*

Species reintroduction is a management strategy used to conserve endemic fish biodiversity. The present study investigated stocking on-grown endangered trout cod (Maccullochella macquariensis) in the Murrumbidgee River, Australia. The hypothesis that post-juvenile dispersal underpins the long-term scarcity of adults recorded at fingerling stocking locations was also tested. Radio-trackingwas used to quantify dispersal of stocked sub-adults (2-year old hatchery fish, n=27) compared with fish originally stocked as fingerlings (unknown-age wild fish, n=31), but we encountered poor survivorship of the former group (survivorship=9% and 95%, respectively, at 13 months post release). The hatchery group exhibited both limited dispersal and large-scale dispersal (up to 55 km) downstream from the release site. Wild fish exhibited limited net dispersal, occupying home-ranges within a 13-km reach and occasionally undertook large-scale excursions

(10-70 km). It is concluded that (1) re-establishment of cod populations based on release of on-grown fish is not straightforward, and (2) adults of this species have an ability to disperse away from stocking sites. The study demonstrates the benefit of using radio-tracking to monitor the movement and survivorship of stocked threatened fish and indicates a need to consider the effects of hatchery rearing when conducting fish reintroductions.

2162: +.075

This study investigated the levels of genetic diversity and variation exhibited by red and sika deer in Ireland, along with the extent and regional location of hybridisation between these two species. Bi-parental (microsatellites) and maternally-inherited (mitochondrial DNA) genetic markers were utilised that allowed comparisons between 85 red deer from six localities and 47 sika deer from 3 localities in Ireland. Population genetic structure was assessed using Bayesian analysis, indicating the existence of two genetic clusters in sika deer and three clusters in red deer. Levels of genetic diversity were low in both red and sika deer. These genetic data presented herein indicate a recent introduction of sika deer and subsequent translocations in agreement with historical data. The origins of the current red deer populations found in Ireland, based on genetic data presented in this study, still remain obscure. All hybrid deer (red/sika) found in this Study were found in Wicklow, Galway and Mayo where the 'red-like' deer exhibited sika deer alleles/haplotypes, and vice versa in the case of Wicklow. Molecular methods proved invaluable in the identification of the hybrid deer because identification of hybrids based on phenotypic external appearances (pelage and body proportions) can be misleading. Areas where red and sika deer are sympatric need to be assessed for the level and extent of hybridisation occurring and thus need to be managed in order to protect the genetic integrity of 'pure' red deer populations. (C) 2009 Deutsche Gesellsehaft fur Saugetierkunde. Published by Elsevier GmbH. All rights reserved.

2163: +.062

The red deer (Cervus elaphus) is one of the most widely distributed species of deer in Europe. Due to its economic value as game species or its negative impacts on forestry, agriculture and conservation areas, most populations are currently managed, with strategies and intensity of the management varying between countries. In Britain, and less certainly in Ireland, red deer have been continuously present since the end of the last glaciation and constitute the largest population of red deer in Europe. Although they thrived in the past when forests were abundant, the current distribution of red deer in the British Isles is uneven, with the largest numbers being found in Scotland and few and more localised populations in England, Wales and the Republic of Ireland. In the British Isles, Lis in many other parts of Europe, there is a long history of man interacting with deer populations including local extinctions, multiple translocations and introductions of exotic species of deer. Among introduced exotic species of deer, the Japanese sika (Cervus nippon) is the one of most concern. After introduction of small numbers at multiple locations in Britain and Ireland from 1860 onwards, sika have increased in population number and range in areas with good forest cover, and where they overlap with red deer there is a risk of hybridisation. Due to recent increases in numbers and range of red and sika deer, both species pose a range of management challenges which are not easy to solve. In this review we summarise the history and status of these two species in Great Britain and Ireland, describe current management and discuss management options for the future. (C) 2009 Deutsche Gesellschaft fur Saugetierkunde. Published by Elsevier GmbH. All rights reserved.

2164: +.131

Population viability analysis of a Gold-spotted pond frog (Rana chosenica) populationat Cheongwon-gun, Chungbuk, in South Korea was conducted and we proposed several suggestionsfor effective conservation and re-introduction of the species. Simulating a developed modelover 1,000 times predicted that the population will exist over 30 years with a relatively low growthrate of 0.113, but with a high probability of extinction as 81.1%. Population growth and extinctionprobability were the most greatly depended on the rate of successful metamorphosis. In the caseof outbreak of amphibian diseases such as Chytridiomycosis and Ranavirus, the population willbe easily extinct within 4 years with 100% probability. In a habitat of which carrying capacity is200, to successfully re-introduce an extinct population, it is initially needed to put 100 individualsof which 83% is males and its age structure is normal-distributed. If we additionally conducts artificialsupplementation of 10% individuals every 2 years from 4 years to 10 years after initial reintroduction,the population will become a stable with 0.297 growth rate and 0.290 extinction rate. Our results are the first case of amphibian population viability analysis in Korea and could be usedto develop effective conservation and re-introduction plans for endangered Gold-spotted pond frog.

2165: *+.335*

Estimation of abundance is important for assessing population responses to management actions. Accurate abundance estimates are particularly critical for monitoring temporal variation following reintroductions when the management goal is to attain population sizes capable of sustaining harvest. Numerous reintroductions have taken place in the Great Lakes region of North America, including efforts to restore extirpated fishers (Martes pennanti) and American martens (M. americana). We used a DNA-based noninvasive hair-snaring method based on one trap design and trapping -grid configuration, and evaluated capture-mark-recapture (CMR) analytical approaches to simultaneously estimate population size for co-distributed fishers and American martens in a 671-km(2) area of the Ottawa National Forest in the western Upper Peninsula of Michigan, USA. We included harvest as a final recapture period to increase probability of recapture and to evaluate potential violations of geographic closure assumptions. We used microsatellite markers to identify target species, eliminate congener species, and provide individual identity for estimation of abundance. Population estimates for fishers and martens on the study area ranged from 35 to 60 and 8 to 28, respectively. Estimators incorporating harvest data resulted in up to a 40% increase in abundance estimates relative to estimators without harvest. We considered population estimates not including harvest data the most appropriate for the study due to timing of sampling and environmental factors, but inclusion of harvested individuals was shown to be useful as a means to detect violations of the assumption of geographic closure. We suggest improvements on future CMR sampling designs for larger landscape scales of relevance to management through incorporation of habitat or historical harvest data. Noninvasive genetic methods that simultaneously estimate the numerical abundance of co-distributed species can greatly decrease assessment costs relative to traditional methods, and increase resulting demographic and ecological information. (JOURNAL OF WILDLIFE MANAGEMENT 73(1): 26-34; 2009)

2166: *-.197*

Reintroduction of endangered Whooping Granes (Grus americana) in eastern North America has successfully established a migratory population between Wisconsin and Florida. Eighty birds (47 males, 33 females) were released between 2001 and 2006, and all birds were tracked following release with satellite and/or VHF monitoring devices. By the end of 2006, 1.7 deaths (12 males, five females) were recorded from this population. Postmoretem findings and field data were evaluated for each bird to determine the cause of death. Causes included predation (n=8, 47%),

trauma (n=2, 12%), and degenerative disease (n = 1, 6%); the cause or death was undetermined for 35% (n=6) of the birds. Based oil physical evidence, the primary predator of the birds was the bobcat (Lynx rufus). Limited roosting habitat availability or bird behavior were likely prime factors in the occurrence of predation. Traumatic injuries and mortality, were caused by gunshot, electrical utility lines, and an unknown source. The lone case of degenerative disease was due to chronic exertional myopathy associated with translocation. Available postmortem testing did not indicate the presence of infectious disease in this limited sample.

2167: *-.063*

Enot Zukim, an oasis on the shore of the Dead Sea, was originally prized for its diversity of herbaceous cover and meadow-like landscapes. It has been monitored for about 20 years during changes induced by removal of Bedouin grazing, accumulation of combustible biomass, subsequent wildfires, decline in water quality and quantity, invasion by Phragmites australis, reintroduction of grazing by donkeys to reduce the P. australis, and expansion of Tamarix sp. coverage. Monitored permanent plots failed to document the reduction in P. australis but did show the change in composition and decline of species diversity with Tamarix sp. expansion. Conservation management faces a dilemma at Enot Zukim: when grazing reduces the invasive P. australis coverage, it seems that Tamarix sp. increases in its place. We do not yet know an effective strategy to both reduce wildfires and control all major invasive plant species.

2168: *-.044*

Planned conservation efforts for tree snails of the endangered genus Achatinella, endemic to the island of O'ahu, Hawai'i, will include translocations among the remaining wild and captive-bred populations. In order to establish optimal levels of artificial migration among neighboring groups of snails within fragmented populations, efforts to determine natural dispersal rates through direct observation were initiated. Capture-mark-recapture (CMR) efforts have proved inadequate for obtaining the requisite dispersal estimates, due to low recapture probabilities. In addition, snail dispersal beyond the boundaries of a finite CMR study site was indistinguishable from mortality. In the preliminary study reported here, both the low recapture probability and dispersal detection problems of past CMR efforts were addressed by using harmonic radar tracking. This approach yielded rough dispersal estimates that were unattainable using CMR alone by providing 100% recapture rates even beyond the normal survey area boundaries. Extensive snail movements within clusters of connected trees were frequently observed after tracking for merely a few hours, although movements between unconnected trees were rare and recorded only after monthly survey intervals. Just 11 out of 40 tracked snails made between-tree movements (average distance of 4.94 +/- 1.52 m) during the entire 7-month study, and provided the only data utilizable for inferring gene flow in and out of subpopulations. Meteorological data loggers were deployed when tracking began to look for an association between such snail movement and weather fluctuations. The resultant data indicate that increases in both wind gusts and humidity facilitate dispersal (R-2=0.77, p-value < 0.001), and that passive wind dispersal alone may be responsible for many snail movements (R-2=0.59, p-value=0.0014). Despite having provided coarse estimates of short-term dispersal and corresponding wind influences, the limitations of the radar method can be substantial.

2169: *+.030*

Habitat loss and degradation continue to be the leading cause in species extinctions and expatriations. With the global human population increasing at exponential rates, the environmental

pressures currently facing Galliformes will continue to intensify. Reintroductions and translocations are two methods that have been used to offset some of these environmental pressures in the past, however in the event that no suitable habitat exists, other options, such as conservation introductions, should be considered. Conservation introductions have recently played a crucial role in conservation initiatives for several species around the globe, but have gone relatively unnoticed by galliform conservationists. This paper reviews the use of conservation introductions as a tool for galliform conservation by looking at the use, feasibility, implications and ethics of introducing Galliformes into non-native areas.

2170: *+.053*

Once thought to be extinct, a small population of black-footed ferrets (Mustela nigripes; ferret) was discovered in 1981 near the town of Meeteetse, western Wyoming. By 1987, only 18 individual ferrets remained, all of which were taken into captivity in an effort to avert extinction and initiate a captive breeding programme. The ferret depends on three prairie dog species (Cynomys spp.) for survival, and use prairie dogs as prey and prairie dog burrows for shelter and dens. It was the conversion of native grasslands to agriculture and decades of intensive prairie dog poisoning in the 19005 that originally brought the ferret to the brink of extinction. Since 1987, an international ferret recovery programme has overcome significant challenges to experience many remarkable successes. From 1987 to 2007, about 6000 ferrets have been produced in captivity and over 2400 have been reintroduced at 17 separate project sites in the western U.S. and Mexico. Still, reintroduction success varied widely and some efforts were compromised by prey/ habitat impacts from an introduced disease (sylvatic plague), drought and politics (with attendant land management failures). The most recent population estimate indicates that wild ferret populations are only about 20% of a long-standing "downlisting" objective (population improvements that would change species status from "critically endangered" to "threatened"). Although the Endangered Species Act and other environmental law in the U.S. provides mandates and guidance for recovery of endangered species, and associated management of federal public lands, perhaps the biggest obstacle to recent recovery progress stemmed from political interference and neglect toward environmental law and policy (as displayed by the U.S. 2000-2008 Administration). The technical expertise, capabilities and raw habitat exist to fully recover the ferret in the wild. However, complete recovery can only achieved with strong recommitments by state and federal agencies to principles set forth in the Endangered Species Act and action to improve habitat conditions over the ferret's historical range. A specific example of how political interference impacted ferret recovery is addressed in this opinion paper, as well as a recommendations perhaps applicable to recovery of the Iberian lynx and other imperiled species.

2171: *+.040*

The Iberian lynx (Lynx pardinus) is the most highly endangered fetid species in the world. One of the best known surviving lynx populations lives in and around Donana National Park, within the most highly protected natural setting left of the lynx's historic distribution. The population has remained stable at around 40-50 individuals for the past 20-25 years, the majority of which presently live outside Park boundaries. This makes the Donana lynx population particularly vulnerable to extinction. If one more lynx territory disappears inside the National Park, model results indicate that extinction of the Donana lynx population could happen within the next 15 years. Recovery of the reproductive source areas inside the national park, coupled with the translocation of a few lynxes from the Sierra Morena population, and supported by the restoration and increased carrying capacity of the source areas inside the park, would reduce the probability of extinction of the entire Donana lynx metapopulation to below 5% in the next too years. In this

chapter we discuss specific considerations for the selection of areas for Iberian lynx translocation inside the national park, together with the biological characteristics of translocation candidates (lynx age and status) and best timing for the actual translocation.

2172: *+.115*

We translocated 32 bobcats (Lynx rufus; 3.1 bobcats/lo km2) to a coastal barrier island, Cumberland Island, Georgia, USA, during 1988-1989 to restore a native predator. Annual survival of adults was 93% (SE=2.6%) for the first three years and recaptured bobcats exhibited an average weight gain of o.8 kg (12% increase), and we documented reproduction. Marsh rabbits (Sylvilagus palustris), white-tailed deer (Odocoileus virginianus) and hispid cotton rats (Sigmodon hispidus) were the principal prey species. By 1997-1998, prey use changed, in which white-tailed deer and marsh rabbits occurred less frequently in scats and all other species occurred more frequently. No bobcats retained areas of exclusive use from conspecifics of the same sex. Estimates and indices of deer abundance indicated that following reintroduction of bobcats the deer population declined and remained low but body weights of deer averaged 11.0 kg greater in 1997 compared to 1989. On nine plots containing 87 oak trees, where oak regeneration at each tree was measured in 1990, the number of trees with seedlings or root sprouts increased from 52 to 86 and the average number of seedlings per plot increased by 153.5. On plots that contained seedlings and sprouts in both 1990 and 1997, average height increased 4.6 cm (95% Cl=4.0-5.2). Our observations of bobcat use of deer as a primary prey species, a decline in deer abundance, and an increase in oak regeneration indicated that bobcats caused a trophic cascade effect on the island. Research prior to the restoration of bobcats indicated deer were abundant and deer browsing suppressed tree regeneration, and apparently deer were suitable prey for bobcats because of their abundance and small size. Post-release monitoring of a reintroduced species provides information to understand why a reintroduction project succeeds or fails. Moreover, restoration projects of predator populations should consider monitoring trophic level characteristics of ecosystems because the role of predators in ecosystems in poorly understood, especially vertebrate predators. If monitoring programmes were developed to test theories of community population dynamics, there would be potential to better understand food webs of terrestrial ecosystems and trophic level inter-relationships.

2173: *+.275*

The Canada lynx (Lynx canadensis) occurs throughout the boreal forests of northern North America. Colorado represents the southern-most historical distribution of lynx, where the species occupied the higher elevation, montane forests in the state. Lynx were extirpated or reduced to a few animals in the state by the late 1970's. Given the isolation of Colorado to the nearest northern populations, the Colorado Division of Wildlife considered reintroduction as the only option to attempt to reestablish the species in the state. The goal of the Colorado lynx reintroduction programme is to establish a viable population of lynx in this state. Evaluation of incremental achievements is an interim method of assessing if the reintroduction effort is progressing towards success. There are seven critical criteria for achieving a viable population: i) development of release protocols that lead to a high initial post-release survival of reintroduced animals; 2) long-term survival of lynx in Colorado; 3) development of site fidelity by the lynx to areas supporting good habitat in densities sufficient to breed; 4) reintroduced lynx must breed; 5) breeding must lead to reproduction of surviving kittens; 6) lynx born in Colorado must reach breeding age and reproduce successfully, and 7) recruitment must be equal to or greater than mortality. The first lynx were released in Colorado in February 1999. Site fidelity and survival were documented through intensive monitoring of individuals through telemetry. Reproduction was first documented

during the 2003 reproduction season. Successful breeding seasons were documented in 2004, 2005 and 2006. A female lynx born in Colorado in 2004 was the mother of one of these litters which documented the first recruitment of Colorado-born lynx into the Colorado breeding population. Results to date have demonstrated that the Colorado Division of Wildlife has developed release protocols that ensure high initial post-release survival followed by high long-term survival, site fidelity, reproduction and recruitment of Colorado-born lynx into the Colorado breeding population. What is yet to be demonstrated is whether Colorado can support sufficient recruitment to offset annual mortality for a viable lynx population over time. Monitoring continues in an effort to document such viability.

2174: *+.131*

Knowledge about the reproductive physiology of lynx could help improve conservation practices. The goal of this study was to validate fecal hormone metabolite assays for Canada lynx and develop a basic understanding of their reproductive physiology. Fecal androgen assays were validated for males, and analysis revealed a clear seasonal increase in androgen expression immediately prior to and during the breeding season. The validation of fecal estrogen and progestagen assays for females was not entirely convincing, but nevertheless informative. A significant rise in fecal estrogens was observed during the breeding season for females. Assay validation may be partially confounded by the unusually long persistence of corpora lutea in Lynx species, which could produce different hormone profiles compared to other female mammals. Lastly, a fecal glucocorticoid metabolite assay was validated for both males and females. We found preliminary evidence that females may have a more pronounced physiological stress response than males, although this does not necessarily imply that females are more sensitive to stress. Chronic stress (e.g. translocation and holding) does appear to suppress androgen expression in males. Further research is needed to gain a clearer understanding of how environmental stressors may impact Canada lynx reproduction, and thereby affect their population size.

2175: *+.050*

The Eurasian lynx studbook (ESB) was established in 2002 after recommendations from the EAZA Fetid TAG (Taxon Advisory Group). The reasons to establish this studbook were manifold. Almost 50% of the lynxes in captivity were of unknown origin, many reintroductions had been taking place, many lynxes were bred and many different studies were being conducted with the captive specimens. In an attempt to address these problems in an organized manner, all zoos were asked to send their historical registration on lynxes, and this data was compared with the information received from ISIS (International Species Information System). Through careful investigation of the studbook many discrepancies and problems were identified. Specifically, it was found out that the level of inbreeding in the captive population was very high. Secondly, the number of lynxes of unknown origin was also high. In addition, there were a large number of identified lynxes of mixed genetic origin. Finally, many lynxes were kept in suboptimal captive conditions. Recommendations were sent out to zoos urging cooperation to avoid further inbreeding of the population, to improve enclosure design and husbandry procedures for this species and to convince involved institutions to participate in the genetic studies aimed at determination of the various subspecies. The subspecies problem is an important one for the programme, since it is very difficult to manage the captive population if it is unclear how many subpopulations it consists of. Furthermore, as there are still zoos involved in reintroduction projects, it is imperative to find out the origin of lynxes that are targeted for reintroduction. A Swiss research group is currently involved in genetic sampling of lynxes and has already set up a databank for some subspecies. Zoos keeping lynxes are encouraged to cooperate as much as

possible by providing samples of known subspecies. In this way, the ESB serves as a catalyst for the EAZA institutions that provide genetic samples from their lynxes.

2176: *+.147*

One of the main roles of conservation genetics is to support conservation practice by providing specific recommendations and procedures for the management of ex situ programmes and other conservation actions. However, these recommendations must be tailored to the particularities of the life, demographic and evolutionary histories of the species and populations we deal with. In the establishment of the Iberian Lynx Ex situ Conservation Programme the first decision that had to be taken was whether to mix the two wild populations (Sierra Morena and Donana) or to treat them as two different units. Although differentiation for neutral molecular markers was large between groups, this might be explained by the predominant action of genetic drift in recent times. Moreover, no differences on potentially adaptive features have been reported between both populations and these are unlikely given the known demograhpic history of the species and its dispersal capacity. Consequently, they were treated as a single unit, and translocations of animals from Sierra Morena to Donana were advised. Next step was determining the proportion of individuals to be captured from each of the wild populations to maximize levels of starting genetic diversity. Based on the genetic variation within and between both populations, the optimal contribution of each to the captive stock was estimated in 64% of individuals from Sierra Morena and 36% from Donana. By mid-2008 the captive population included four founders from Donana and 24 from Sierra Morena. Empirical analysis of the captive population revealed an expected heterozygosity a little below the potential maximum, mostly due to insufficient representation of Donana population. Management of the captive populations has been driven by two main criteria: using all the available founders as breeders (to allow the maintenance of their genetic information) and the application of a minimum coancestry mating scheme. According to the latter principle, matings between individuals coming from different wild populations were favoured, as one can be sure that they are less related than couples formed within populations. The Ex situ Programme has been so successful that initial growth projections have been surpassed, and the original carrying capacity of the Programme has been reached two years in advance of original predictions. Therefore, continuing the expansion of the Programme to new centers is urgently needed to cope with population increase. In parallel, captive individuals for reintroduction to the wild might become available already by 2009. Once all the planned centers are running at carrying capacity, both wild and captive populations should be managed jointly to keep the highest levels of global genetic diversity, while optimising the exchange of individuals between them.

2177: *+.086*

This paper aims to summarise the general principles of the genetic management of conservation breeding programmes with the aim of reintroduction. One of the most important aims for such programmes is to retain as much gene diversity as possible. Gene diversity represents the evolutionary potential captured within the population and is correlated with population fitness. Populations in captivity are often small, lack gene flow between subpopulations without human intervention, and live under unnatural conditions. This makes captive populations vulnerable to genetic changes that may affect reintroduction success, such as loss of genetic variation through genetic drift and inbreeding, inbreeding depression, and genetic adaptation to captivity. Gene diversity can best be maintained in captive populations by i) maximising the number of founders (without compromising the wild source population); 2) maximising the growth rate in the growth stage of the population (implying good knowledge of natural history and captive husbandry); 3) maximising carrying capacity, and 4) basing the pairings of individuals, especially during the

capacity stage of the Programme, on their mean kinship (mk) values, while 5) minimising inbreeding. The mk value of an individual is a measure for the relatedness of this individual to the entire population. Animals with low mk values have few relatives in the population and vice versa. If an individual with few relatives dies, chances are high that unique genetic variation is lost forever. In contrast, most of the genetic material of an individual with many family members (i.e., high mk) is likely also present in its relatives. By giving breeding priority to low mk individuals, combining individuals with similar mk values for mating, and minimising inbreeding, the amount of gene diversity retained can be maximised. A degree of compromise will be necessary to take into account the biological and social characteristics of the species, non-genetic peculiarities of the individuals involved, as well as practical circumstances. Finally, the captive born individuals best chosen for reintroduction are those that benefit the gene diversity of the wild population, but are genetically well represented in the captive population. The above principles are explained while paying particular attention to the specific case of the Iberian lynx.

2178: *+.213*

The conservation status of the Iberian lynx was found to be extremely critical by the end of the zoth Century, when only 150 individuals remained in the wild secluded into two isolated populations, Donana and eastern Sierra Morena, both located in Andalusia. The Andalusian Government, together with a number of partners, adopted different conservation measures that received support from the European Union in the form of two LIFE - Nature conservation projects. These projects have proved essential to avoid the potential extinction of the Iberian lynx in the wild and to stabilize both free - ranging populations. Presently, in situ conservation efforts are focused on the following objectives: 1) Maintaining and expanding the two existing populations; 2) Recovering extinct population nuclei - following IUCN reintroduction criteria; 3) Maximizing genetic diversity by "genetically connecting" the two existing populations; 4) Continuing to promote local, national and international support to ensure the recovery of this highly endangered species. Nowadays, the Sierra Morena population continues to grow - both numerically and in surface area - at an annual basis. In fact, the number of lynxes has increased from 38 adults (individuals of more than one-year-of-age) registered in 2001 to 95 in zoo8. The Donana population remains stable and a translocation programme, with the ultimate goal of genetic and demographic reinforcement, is currently taking place. Also, in situ efforts are presently focused on the recovery of historical population nuclei through reintroduction programmes. Preparation works are being carried out since 2005 and the first releases are scheduled to begin in 2009.

2179: *+.252*

From the first half of the 20th century onwards, the Portuguese Iberian lynx population was distributed in three major nuclei: Sado Valley, Malcata and Contenda-Barrancos. In the following decades these areas were subjected to a process that culminated in the specie's considerable regression, probably as consequence of a major allocation of potential habitat to forestry and of prey scarcity as a result of viral diseases. The most recent survey, conducted from 2002 till 2004, revealed that the species is presently on the verge of extinction. Aware of the critical situation of the Iberian lynx in Portugal, the Institute of Nature Conservation and Biodiversity developed a Conservation Action Plan for the Iberian lynx in order to provide a consistent and effective approach to conserve the species in Portuguese territory. The on-going Action Plan is being applied in all Natura 2000 Sites, located in the lynx historical distribution that present suitable characteristics for the species potential presence or landscape features that can be optimised for lynx survival and that can be relevant for the species life-cycle. The goal of this Plan is to apply pre-release strategic reintroduction activities in order to make it possible, in the long-term, the

reintroduction of Iberian lynx. Integrated in the plan, there are several ongoing conservation projects, which include habitat and prey restoration and the construction of a breeding centre that will be integrated within the overall Iberian Lynx Ex situ Conservation Programme.

2180: *+.230*

A new Strategy for the Conservation of the Iberian Lynx (Lynx pardinus) has recently been approved by the Spain's maximum authorities in Environmental Policy at the Sectorial Conference for the Environment. The new Strategy has been developed in a different working framework from the one that led to the first Strategy for the Conservation of the Iberian Lynx in 1999. The demographic situation of the Iberian lynx has never been worse. However, there have never been so many human and financial resources available, and the species has never been the focus of so much public attention and concern. The ultimate goal of the Strategy is to ensure that the Iberian lynx becomes a functional part of the Mediterranean scrubland habitat again. To this end, the recovery of the species involves both successfully managing the remaining populations and choosing and restoring areas to carry out reintroduction projects that will lead to the establishment of new wild populations. The new Strategy has set a roadmap for the conservation and recovery of the Iberian lynx, as well as specific numerical targets that must be met in a given period of time. These targets include: 1) Stabilize the populations by combating the causes of threat to the species; 2) Increase the number of individuals in the wild populations so that the Iberian lynx can be downlisted from Critically Endangered (CR) to Endangered (EN) by 2011; and 3) Increase the number of wild populations, so that the species can be downlisted from Endangered (EN) to Vulnerable (VU) by 2020. According to the Spanish system, the first target should be achieved through Regional Recovery Plans, which must adopt the guidelines established in the National Strategy and develop them fully and efficiently. Achieving the second goal requires increasing the number of individuals in the lynx populations until at least one of them has more than 50 mature individuals, which must not amount to more than 90% of all the wild mature individuals. If necessary, "Restocking" and "Population Exchange Projects" are recommended to help increase the abundance of lynxes in the existing populations. To achieve the third target, the combined wild populations must comprise at least 250 mature individuals and not show signs of decline. This could only be attained through "Habitat Restoration and Reintroduction Projects" carried out in all the Autonomous Communities of Spain where the Iberian lynx occurs or occurred until recent times.

2181: *+.045*

The European wild rabbit (Oryctolagus cuniculus L.) is a keystone species in Mediterranean ecosystems of the Iberian Peninsula. During the last decades wild rabbit populations have suffered a dramatic decline because of habitat loss and especially due to the incidence of myxomatosis and Rabbit Haemorrhagic Disease (RHD). Currently, enhancement of rabbit populations is a primary concern in conservation; however, the impact of RHD in rabbit population dynamics seems to be one of the major challenges to rabbit recovery. Under the theoretical insights obtained in a previous RH D epidemiology modelling approach, we evaluated the possible outcomes of several rabbit management strategies. Following model assumptions, habitat improvement was the best way - alone or in combination with other management strategies - to permanently increase rabbit densities in populations at equilibrium with RHD. The enhancement of rabbit populations in areas that had not yet reached equilibrium with RHD seemed to be more complex, likely due to possible interactions of disease with other factors like predation. The misuse of translocations arose as an added obstacle to rabbit enhancement because of underlying mechanisms, such as apparent disease-mediated competition, that could yield harmful effects on native populations. More

research is needed in order to evaluate the implications of RHD on rabbit biology and to provide novel approaches to rabbit management.

2182: ***+.024***

On the River Ticino (NW Italy), the release, in 1997, of a pair of otters Lutra lutra, possibly reinforced by the escape of a further pair, allowed the establishment of a small breeding population. In summer 2008, a survey was carried out using the 'standard method' to determine Current otter distribution. Data on otter diet were also collected through the analysis of 36 spraints. Otter presence was recorded at 3 of 10 sampling stations, along a 2.6 km long section of the river. Considering only this stretch, the mean number of sprainting sites per 100 in was 0.10, while the mean number of spraints per 100 m was 0.19. Fish - mainly Salmo trutta, Perca fluviatilis, Phoxinus phoxinus and Chondrostoma genei -, formed the bulk of the otters' diet. The prosecution of any reintroduction project requires an updated feasibility study which provides for management actions aimed to favour the expansion of the species in northern Italy.

2183: ***+.060***

The wild rabbit (Oryctolagus cuniculus) and the Iberian lynx (Lynx pardinus) are endemic species of the Iberian Peninsula. The Iberian lynx is a specialist predator, listed as critically endangered, and rabbit represents the bulk of its diet as well as of many other Iberian predators. The decline of this emblematic predator has been recurrently explained by means of rabbit decline in Spain. A recent study has shown that wild rabbit populations have declined 55% in three decades in the country. Habitat destruction and fragmentation by infrastructures, the abandonment of marginal crops, the intensification of farmland ecosystems, and the direct and continuous persecution by farmers to preserve their crops have been claimed as the main factors that account for this dramatic decline. In parallel, the impact of both myxoma virus and rabbit haemorrhagic disease (RHD) implied a relevant increase in rabbit mortality rates, while the species coped with a strong hunting pressure and an increase of competition with other wild herbivores. At present, all of these threats have not yet disappeared and conservation efforts have been very scarce. However, previous to the rabbit decline, the area of distribution of the Iberian lynx had already suffered a reduction higher than 50%. This fact suggests that the extinction forces operating on Iberian lynx populations were other than rabbit population decline. The most probable hypothesis is that the main factors that accounted for this reduction were the direct persecution of the species and the massive non-selective predator control. Indeed, rabbit is abundant enough to maintain stable populations of Iberian lynx in many places, but the authorized predator control is ongoing and intensive, preventing lynx establishment. In fact, Iberian lynx populations are now located in areas where predator control is not applied and rabbits are really scarce. The Conservation National Plan for the Iberian Lynx has focused on introducing rabbits into these marginal areas, favoring the increase of Iberian lynx productivity. It is time to add as one of the main objectives of the lynx recovery plans the removal of the causes of non-natural mortality to guarantee the survival of dispersing individuals and the success of future reintroductions. Despite the minor relevance of rabbit recovery to Iberian lynx conservation plans, we advocate for a National Plan for rabbit recovery to coordinate the actions that guarantee the conservation of wild rabbit populations in Spain, and thus to preserve also this endemic key species of the Mediterranean ecosystems.

2184: ***-.144***

The dormouse Muscardinus avellanarius has disappeared from a large part of its range in England, probably because of habitat fragmentation and deterioration. Reintroductions, mainly using

captive-bred animals, have been generally successful at re-establishing populations within this lost part of the species' range. with only 2, perhaps 3, out of 15 known to have failed. However, the establishment of new isolated populations is not seen as an end point to the project and attention is now focused on improving habitat connectivity around the reintroduction sites, to enable the founder populations to spread to nearby woods.

2185: ***+.014***

In this paper we present new approach to the problem of conservation the small and endangered populations of black grouse. We elaborated and checked in the field non-invasive methods enabling to obtain the genetic material without removing birds from natal populations. The main idea is to mate the tame, especially accustomed hen from the aviary with wild cock. After mating the hen lays the eggs in the aviary and we obtain the first generation which has a half of the gene pool coming from the population of its father. Consecutive, repeated crossing the females from such broods with next wild males leads in few years to bring the genes from original population to the aviary in non-invasive manner. Another method is to collect semen from wild males using a stuffed female. We describe some technical methods of such manipulations with the birds and present some preliminary results. These methods enable obtaining birds bred in aviary for future program of reintroduction.

2187: ***+.029***

The Australian lungfish, Neoceratodus forsteri, exists as remnant natural populations in two rivers of south-east Queensland, Australia, and several translocated populations. Lungfish habitats have been impacted by agriculture and forestry, alien plants and fish and by river impoundment and regulation of flows. The species has been listed as vulnerable under Australian Commonwealth legislation. A proposal to construct Traveston Crossing Dam on the free-flowing main channel of the upper Mary River could seriously threaten the lungfish. The dam can be stopped by Commonwealth legislation if important populations of lungfish in the Mary River are likely to be significantly impacted by the new dam. This paper assembles evidence that impoundment of the Mary River and regulation of river flows are likely to decrease and fragment important lungfish populations, disrupt the breeding cycle, reduce juvenile recruitment, and isolate and decrease habitat availability/quality to such an extent that the species is likely to decline. Proposed mitigation strategies include fish transfer facilities, provision of flow releases from the dam (environmental flows) to sustain lungfish habitat and breeding downstream, and translocation of hatchery-reared juvenile lungfish into suitable natural habitats. These mitigation efforts may not be sufficient to secure the genetic diversity and long-term viability of lungfish populations in the Mary River.

2188: ***+.107***

Relocation has become an important tool in conservation biology. So far little is known about the suitability of translocation to restore bat populations. We evaluated the conditions for successful translocations amongst 2 bat species: the greater horseshoe bat Rhinolophus ferrumequinum and the lesser horseshoe bat R. hipposideros. Both species underwent a dramatic decline in Western and Central Europe in the second half of the 20th century, but some populations have recently started to recover. Due to their sedentary habits, natural recolonisation of their formerly vast inhabited range advances only slowly. Translocation could solve this problem. In 2006, we conducted translocation experiments with 11 greater and 7 lesser horseshoe bats within Switzerland. Bats were captured at large colonies, released into relict colonies, radiotracked for up

to 10 d and checked for presence during the following years. Ten out of the 13 individuals released at distances <20 km from the donor roost homed. None of the 5 bats released at distances >40 km expressed homing tendencies. Within 3 d of release, 1 greater and 3 lesser horseshoe bats died (2 of shock). Lesser horseshoe bats seem to react very sensitively to translocation. Long-distance translocation of 2 greater horseshoe bats led to short-term settlement in the release area; since its translocation, the sole female released at long distance has been regularly observed in the receiver colony. Apparent species-specific differences in tolerance to translocation underline the necessity of studying the focal species instead of surrogate species in translocation projects.

2189: *-.065*

Translocation is a powerful tool that has been used in the conservation of a wide range of taxa. However, few translocations of bats have been attempted and we know of no successes. The few translocations which have been attempted have either failed due to dispersal from the release site or have not been monitored sufficiently to determine the cause of failure. We assessed the short-term success of a translocation of lesser short-tailed bats Mystacina tuberculata by the New Zealand Department of Conservation, where 3 release methods were used to minimise dispersal or mortality: bats were juveniles, were maintained in captivity at the release site, and were provided with supplementary food and roosts following release. Success was assessed by determining if founders remained at the release site and maintained condition (weight), Recapture showed that at least 9 of the 20 bats remained at the release site 232 d after release. There was weak evidence that bats lost weight, although final weights were comparable to those of bats from a natural population. However, all bats captured 8 mo after release had damaged, infected ears and some were balding. The problem was treated but recurred, and bats were returned to captivity. Our results are the first to demonstrate that translocated bats can remain at their release site and survive. However, disease may be an issue in future translocations.

2190: *+.200*

Translocation is an increasingly popular conservation tool from which a wide range of taxa have benefited. However, to our knowledge, bats have not been translocated successfully. Bats differ behaviourally, morphologically and physiologically from the taxa for which translocation theory has been developed, so existing guidelines may not be directly transferable. We review previous translocations of bats and discuss characteristics of bats that may require special consideration during translocation. Their vagility and homing ability, coloniality, roost requirements, potential ability to transmit diseases, susceptibility to anthropomorphic impacts, and cryptic nature have implications for establishing populations, effects of these populations on the release site, and ability to monitor translocation success following release. We hope that our discussion of potential problems will be able to supplement the existing, more generic guidelines to provide a starting point for the planning of bat translocations.

2191: *-.394*

Psittacine beak and feather disease (PBFD) is a highly infectious and potentially fatal viral disease of parrots and their allies caused by the beak and feather disease virus (BFDV). Abnormal feather morphology and loss of feathers are common clinical symptoms of the disease. PBFD also damages the lymphoid tissue and affected birds may die as a result of secondary bacterial or fungal infections. The disease is therefore of concern for conservation biologists and wildlife managers, as it is immunosuppressive and can become an additional threatening factor among critically endangered psittacines. We conducted a PCR-based screening for BFDV in a wild

population of the Red-fronted Parakeet (Cyanoramphus novaezelandiae) on Little Barrier Island, New Zealand, during a translocation of this species. Fifty-four parakeets were captured and feather samples collected for molecular screening. We detected BFDV DNA from 15 individuals, but only two showed external signs attributable to PBFD, namely abnormal feather morphology or colouration, loss of feathers and haemorrhagic feathers. Our survey represents the first positive identification of BFDV in wild New Zealand endemic psittacines and confirms the risk of spread of the virus between wild populations within this global hotspot of endemic psittacine diversity.

2192: *-.022*

The three species of emu-wrens (Maluridae : Stipiturus) are small passerines found in arid to mesic habitats across southern Australia. The geographical distribution of nucleotide sequence diversity of mitochondrial DNA (mtDNA) in emu-wrens was investigated to assess the systematic status of Stipiturus (Aims 1 and 2), particularly in eastern Australia, and to examine closely the population structure of Southern Emu-wren (S. malachurus) in the Mount Lofty Ranges of South Australia (Aim 3). Aim 1: Phylogenetic relationships among the three species of Stipiturus, based previously on plumage and allozyme data, are confirmed by our mtDNA data, which support recognition of Mallee Emu-wren (S. mallee) as a distinct (evolutionary) species from, and sister lineage to, Rufous-crowned Emu-wren (S. ruficeps) rather than Southern Emu-wren. These relationships indicate that any interaction between Mallee and Southern Emu-wrens in south-eastern South Australia is a secondary contact. Aim 2: Within the Southern Emu-wren, which currently comprises eight subspecies, phylogenetic relationships among haplotypes from eastern Australia are not concordant globally with subspecies boundaries, but correspond in part to the most recent classification. A quantitative statistical evaluation of the taxonomic implications of phenotypic variation in these birds is warranted. Eastern haplotypes of Southern Emu-wren show strong phylogeographic structure indicative of allopatric divergence in refugia, with subsequent expansion without widespread introgression. Aim 3: Within the endangered populations of the Mount Lofty Ranges, historical isolation and demographic independence of central and southern populations is supported, corresponding with distribution data, but confirmation using nuclear markers is required. Despite separate subspecific designation of emu-wrens of the Mount Lofty Ranges (which are endangered) and south-eastern South Australia, they share mtDNA haplotypes without strong differentiation and emu-wrens of south-eastern South Australia should be preferred over Kangaroo Island birds for translocations into the MLR, although translocation among populations of the Mount Lofty Ranges is probably a wiser initial strategy to minimise the impact of ecological differences and risk of introduction of disease.

2193: *+.069*

A new checklist of 36 metazoan parasites recorded in European eels Anguilla anguilla in Ireland is presented and reviewed. Some of these parasite taxa are eel specialists but most utilize a range of fish hosts. Many were accidentally brought to Ireland during fish introductions. Changing distributions of preferred intermediate hosts have affected some parasite species. Commercial transport of eels has been implicated in the introduction and spread of several potentially pathogenic parasites, including the Asian nematode Anguillicola crassus. The current status of this and two Pseudodactylogyrus species, similarly introduced to Ireland, is discussed. Analysis of parasite assemblages of Irish eel populations indicates that individual host characteristics, such as size and diet, are important at the infra-community level. Likewise, variation in biotic and abiotic features of ecosystems is reflected in composition and structure of eel parasite component communities. Environmental changes, Such as eutrophication and species introductions, were found to affect eel parasite assemblages. Better regulation of fish introductions and translocations

is needed to protect the ecological integrity of Ireland's freshwater systems and to avoid economic damage by nonindigenous parasites. Restrictions on live eel transport and on eel stocking programs may be necessary to protect recreational fisheries and the Irish aquaculture industry.

2194: *+.195*

Forty thousand American eel Anguilla rostrata elvers were released in a 400-ha lake in an eel-free watershed in eastern Quebec in 1999. Subsequent sampling enabled the measurement of poststocking movements, growth, and sex ratio. Populations showed limited movements, and occupancy extended less than 3 km into inflowing tributaries by 2003. Annual growth increments in the lake (118 mm/year) were the highest reported for the species, but growth increments in rivers (40 mm/year) were typical of those found elsewhere. Four of seven eels whose sex could be determined were females, in contrast to other sites in the St. Lawrence watershed where females are more than 99% of the population. American eel translocation to growth areas that have been blocked by artificial barriers may be a useful means to increase production of silver eels.

2195: *+.041*

After decades of absence, large carnivores are being restored or recolonizing substantial portions of their historical ranges worldwide. The reintroduction of wolves (Canis lupus) to Yellowstone National Park in 1995 is one such example of this larger trend. While the Greater Yellowstone Area was rich in prey abundance and diversity, how wolves would respond to the varying prey assemblages within the park was an open question. Wolves were expected to readily adapt to the elk-rich (Cervus elaphus) area of northern Yellowstone, but restoration success and subsequent wolf population ecology was less certain in the central portion of the park, where formidable bison outnumber elk and prey density was more patchy and seasonal due to severe winters. We describe wolf reintroduction efforts in the central Yellowstone area and the population ecology of wolf packs in this system as it transitioned from no wolves to an established population. We focused on pack dynamics in this relatively small area that supports dense ungulate prey in winter, but dispersed prey in summer.

2196: *+.004*

A key goal in road ecology is to determine which species are most vulnerable to the negative effects of roads on population persistence. Theory suggests that species that avoid roads are less likely to be negatively affected by roads than those that do not avoid roads. The goal of this study was to take a step toward testing this prediction by evaluating the behavioral response to roads and traffic of a species whose populations are known to be negatively affected by roads and traffic, the northern leopard frog (Rana pipiens). We studied the movement patterns of northern leopard frogs during their spring migration from overwintering sites in a river to various breeding ponds that were disconnected from the river by roads. We performed short-distance translocations of migrating frogs, followed them visually, and documented their movement coordinates following each hop, both near the roads and in non-roaded areas. We found that frogs took longer to move near roads with more traffic and that their movement was quickest in areas without roads nearby. Frogs tended to deviate more from a straight-line course when they were released near roads than compared with control areas, but this response was independent of traffic volume. All frogs released near roads attempted to cross the road. On very low traffic roads (10.86 mean vehicles per hour), 94% of frogs crossed the road successfully, whereas at higher traffic roads (58.29 mean vehicles per hour) 72% were successful. Our results suggest that frog's inability to avoid going onto roads and their slow movement combine to make them particularly vulnerable to road

mortality, which likely explains the strong negative effects of roads on frog population abundance. Conservation efforts should focus on preventing frogs from accessing the road surface through the use of drift fencing and culverts.

2197: ***+.074***

The paper summarizes development of the White-tailed Eagle (Haliaeetus albicilla) population in the Czech Republic (CR). The White,tailed Eagle ceased breeding in the CR at the end of the 1911, century. The last known breeding occurred in South Bohemia in 1882. After about a hundred years' break, in the mid-80s of the 20,h century, the species started to breed in two core areas in the CR (South Bohemia and South Moravia). The breeding Population in South Bohemia was backed up by a reintroduction project (a total of 9 individuals released 1978-1985). The White-tailed Eagle Subsequently colonized almost the whole Country. In 2000, 25 territories were occupied, in 2005 44 (76% increase) and in 2007 Lip to 55 breeding territories were recorded (120% increase in 7 years). The bulk of the population breeds in lowland areas with a high representation of ponds or wetlands. The highest altitude at which breeding has been confirmed is 700-750 in a.s.l. The most important wintering places are South Moravia and the Trebonsko region in South Bohemia. Birds from Poland (6), Finland (2), Estonia (1) and Germany (1) have been recorded in the CR. The diet of the White-tailed Eagle in the CR consists mainly of fish and waterfowl. Locally, a higher representation of mammals can be recorded (mainly carcasses or grallochs of hoofed game, or Common Vole Microtus arvalis). To protect the eagles, 3 SPAs within the NATURA 2000 project were declared (Trebonsko, Palava, Nove Mlyny middle reservoir). A significant conservation problem of the last years is poisoning by carbofuran. During 2003-2008, 16 individuals were proved or supposed to be poisoned in the CR. In the same period, two cases of shot eagles were reported and two nests were de stroyed by man. A complete bibliography on the White-tailed Eagle in the CR is included.

2198: ***+.259***

In 2006, the Ballinderry River Enhancement Association secured two years funding to establish the "Ballinderry White-clawed Crayfish Austropotamobius pallipes Breeding and Reintroduction Programme". The project has established Ireland's first ark site for A. pallipes in a natural lake. The lake is used as the last in a series of settling ponds in the Acheson & Glover Group's Pomeroy Sand Pit, near the headwaters of the Ballinderry River, Co. Tyrone. An artificial habitat bay was created and 150 Ballinderry A. pallipes introduced. Since the introduction of A. pallipes to the ark site, trapping has shown that the population is healthy, breeding and occupying available habitat in the lake. It is hoped that in the future, A. pallipes can be re-introduced to parts of the river where the species is currently absent but previously known to have occurred.

2199: ***-.152***

Porcelain disease caused by the microsporidian parasite Thelohania contejeani is common in noble and white-clawed crayfish populations throughout Europe. In the UK, prevalence of the disease is normally very low, 5% or less, in healthy white-clawed crayfish populations. However, there are several aspects of the disease that are of concern to conservationists. Signal crayfish have recently been confirmed as harbouring multiple microsporidian species, including T. contejeani, while remaining asymptomatic. Certain white-clawed crayfish populations are experiencing very high rates of porcelain disease for as yet unknown reasons. Ark sites are being increasingly made for indigenous crayfish populations at risk, and considerations must be made regarding porcelain disease rates in donor populations. Finally, the potential import of two alien Thelohania species in

infected redclaw Cherax quadricarinatus or other Australian crayfish species is a concern, as it is not known if white-clawed crayfish are susceptible to porcelain disease caused by these microsporidians. Here we provide a basic introduction to porcelain disease and its impacts on crayfish, and address the above conservation concerns.

2200: ***+.099***

The Kermadec red-crowned parakeet Cyanoramphus novaezelandiae was driven to extinction on Raoul Island over 150 years ago by introduced cats Felis catus and rats (Rattus norvegicus and R. exulans). These predators were eradicated from the island (2,938 ha) between 2002-04 during the world's largest multi- species eradication project. In 2008 we documented a unique recolonisation event when parakeets were observed to have returned to Raoul, presumably from a nearby island group, The Herald Islets (51 ha). We captured and aged 100 parakeets, of which 44% were born in 2008, and breeding was observed on Raoul Island. This represents the first evidence of nesting of this species on Raoul Island since 1836. Our findings highlight the global conservation potential for island avifaunas by prioritising eradication areas through consideration of proximity of remnant populations to target management locations, instead of the classical translocation approach alone. The natural recolonization of parakeets on Raoul Island from a satellite source population is to our knowledge, a first for parrot conservation and the first documented population expansion and island recolonization of a parrot species after removal of invasive predators.

2201: ***+.219***

The present report is an attempt of a comprehensive look at the present state of the occurrence in Poland, threats and protection of the endangered cyprinid fish species, Eupallasella percnurus. The extensive field survey, carried out in 2002-2009, resulted in unexpectedly numerous discoveries of sites entirely unknown to the knowledge. The total number of E. percnurus sites, presently existing in the territory of Poland, can be assumed at 157. Vast majority of them (90) are situated in the Pomorskie Voivodeship, whereas 44 exist in the Lubelskie Voivodeship and 15 in the Mazowieckie Voivodeship. Among the latter, 6 are those established recently by means of fish translocations - both juveniles originating from the controlled conditions (5 sites) or wild individuals taken from a local population (one site). The translocations as well as the inventory field survey and other activities were performed within the long-term (15 years) project for the protection of E. percnurus populations in the Mazowieckie Voivodeship. The project is the only one of this kind in the whole country. The existing E. percnurus sites are situated within the historical range of occurrence of this species in Poland. Approximately 43% of the sites are single water bodies, all the remaining ones form their larger complexes. Most of the sites (62%) are those of the anthropogenic origin (mainly former peat excavations). As little as 25% of the Polish sites are likely to survive safely for a long time, another 25% are those remaining under strong threat, resulting most commonly from both low water depth and water deficit. Nearly 45% of the existing sites will presumably be protected within the European Ecological Network Natura 2000 in the Special Areas of Conservation (SACs). Good knowledge of the present E. percnurus occurrence and the promising results of the project currently carried out in the Mazowieckie Voivodeship (including successful translocations) can form a good scientific and practical basis for more comprehensive project of its protection everywhere in Poland, where populations of this species turned out to occur. Establishing of such a project is considered to be an urgent need.

2202: ***+.261***

For extirpated Atlantic Salmon (Salmo salar) in Ontario, fluvial habitat for spawning and early

growth would be required to re-introduce the Lake Ontario population. Based on a review of habitat suitability, the Ontario Ministry of Natural Resources identified three tributaries for restoration, Credit River, Duffins Creek and Cobourg Creek, all of which supported salmon historically. Based on a drainage area of 87,100 ha, the conservation targets for the Credit River were estimated to be 8.61 X 106 eggs and 3,631 female spawning salmon (1 lake winter; average 1.8 kg), assuming a conservation egg deposition target of 2.4 eggs/m2. Conservation targets (number of female spawners) for the other two tributaries would be less. All accessible rearing habitat in the rivers could be essential for survival for three reasons: 1) allowable harm assessment indicated that population viability is sensitive to juvenile mortality; 2) only 3 of the original 27 rivers inhabited by salmon are targeted for restoration; and 3) identification of the whole river as important habitat is consistent with the proposed approach for inner Bay of Fundy salmon. Methods are proposed for identifying the amount of fluvial habitat needed if an adult population recovery target is identified on a lake basis rather than a tributary basis. Identification of important habitat in Lake Ontario proper could be deferred until more is known about migration routes and feeding areas. Residences (as defined in SARA) may apply to Atlantic Salmon for redds (egg incubation), but a Fisheries and Oceans Canada policy on residences is still pending. Based on known reproducing populations of other Salmoninae in Lake Ontario, suitable habitat for Atlantic Salmon is likely available, and re-introduction of this extirpated species is ecologically feasible.

2203: ***+.189***

In the past, crayfish were present in most of the open European waters and they were caught regularly. Nowadays, crayfish are threatened by environment degradation and by spreading of non-native crayfish which carry the crayfish plague. Introduction and repatriation could be one of the possibilities to improve this situation. The model study of crayfish stocking, presented in this paper, suggests that crayfish introduction to well selected locality could be successful. Although, native crayfish are listed in Habitat Directive, Annex II, the repatriation and commercial use are handled differently in each country. More strict rules focused on repatriation, manipulation and breeding of crayfish are applied in the Czech Republic, in comparison with other European countries. At the same time, a suggestion that commercial use of native crayfish for repatriation and introduction purposes, but also for consumption, need not be in contrast with their conservation and vice versa, has been frequently presented by some Czech and European specialists. Such actions could also have a positive reflection in socioeconomic and educational points of view. The impartial and pragmatic discussion between specialists, not only on international but also on national levels, is desirable and very important.

2204: ***+.123***

The distribution, population's number, conservation status and bioecological peculiarities of Persian wild goat (Capra aegagrus Erxleben) have been analised. Possibilities for re-introduction and the most suitable habitats of the species in Bulgaria have been also discussed.

2205: ***+.133***

Between 1999 and 2007, more than 2,600 adult Pacific lampreys Entosphenus tridentatus (formerly Lampetra tridentata) were reintroduced to the Umatilla River, where they had been extirpated by poisoning, from nearby locations in the Columbia River consistent with the International Union for Conservation of Nature and Natural Resources guidelines. Reintroduced adult Pacific lampreys were able to find suitable spawning habitat, construct nests, and deposit viable eggs (81-93% mean egg viability per nest). Their larvae were able to feed and grow.

Median lengths for age 0+, 1+, and 2+ larvae were 19, 63, and 109 mm, respectively. Mean density of larvae in survey plots increased over time from 0.08 to 6.56 larvae/m(2). Geographical distribution of larvae in the river increased downstream, but larvae failed to become established in the lower Umatilla River where water flows were regulated for irrigation. Annual abundances of trapped, recently metamorphosed, out-migrating larvae increased during the study from nearly zero to 180,000, but not in all years, which suggests that many might not be surviving migration to the Columbia River, possibly because of irrigation withdrawals. Abundances of trapped, returning adult lamprey also increased from 2003 to 2006, which corresponded with the period when adult lampreys that were the progeny of reintroduced lampreys were expected to return, but long-term monitoring is necessary to confirm that increases were the result of the reintroduction. Our results also demonstrated that even if presumptive causes of extirpation were known and removed before reintroduction, success is not guaranteed. Reintroduction not only assists in redistributing animals to parts of their historical range, but in conjunction with monitoring, it may be essential to identify additional limiting factors that were unknown at reintroduction.

2206: ***+.240***

Sturgeons are representatives of the most ancient vertebrates, the ancestors of which appeared on the Earth several hundred million years ago. Some species of the Acipenseriformes order, including the Adriatic sturgeon (Acipenser naccarii) are threatened with extinction. However, progress in science and technology, inter alia, in ichthyology and aquaculture, may enable us to conserve and restore these endangered species. We give here a biological overview of the principal characteristics of sturgeons and their habitat. Sturgeons are mostly anadromous migrants and reveal a large capacity to grow in seawaters with extremely rich feeding stocks. In some coastal areas of the Caspian Sea the benthos biomass totals 50-500 g/m(2) and sometimes exceeds 1000 g/m(2). As to the sturgeon restocking of the Mediterranean Sea, it is very likely that this basin could provide adequate feeding stocks for the Adriatic sturgeon, though specific issues need additional studies. All sturgeons reproduce in fresh waters. Generally, sturgeons prefer spawning grounds located at some distance from the estuary with gravel substrate and a rapid flow of water (more than 1 m/s). There are strong doubts about the adequacy of the current hydrological regime in the Guadalquivir River for the successful natural reproduction of the Adriatic sturgeon. Therefore we would suggest that sturgeon hatcheries should be developed for restoration of the Adriatic sturgeon population in its historical area. In this respect the experience of artificial reproduction of sturgeon in Russia and other states could be quite helpful.

2207: ***+.181***

Reintroductions are an important tool in conservation biology but frequently fail. Factors influencing reintroduction 'success' are rarely tested experimentally. We examined the relationship between habitat quality and reintroduction success in an experimental reintroduction of populations of water voles (Aruicola terrestris) in the UK.We released cohorts of 44 water voles into 12 replicate 800 m stretches of river, each supporting a different habitat abundance. Water voles initially established at nine sites, failing to establish at three sites due to predation from American mink (two sites) and atypically severe flooding post-release (one site). For sites where voles established, at those with higher vegetation abundance more of the release cohort survived (initial survival rates range 0.43-0.61), and post-establishment survival rates (range 0.45-0.80) and population densities (range 2.1-5.4 voles per 100 m of habitat) were higher. A further two populations were lost to American mink predation post-establishment. Reintroductions are commonly designated as either a 'success' or a 'failure'. The principal cause of a failed release in our study was insufficient mink control. However, whilst seven of our 12 reintroductions were

'successful', our results indicated substantial variation in the population densities and survival rates that the replicate habitats could support. This highlights the need to ensure that any habitat selected for a reintroduction is the best obtainable. (C) 2008 Elsevier Ltd. All rights reserved.

2208: *+.300*

Future climate change constitutes a major threat to Earth's biodiversity. If anthropogenic greenhouse gas emissions continue unabated, 21(st) century climate change is likely to exceed the natural adaptive capacity of many natural ecosystems and a large proportion of species may risk extinction. A recurrent finding is that the degree of negative impact depends strongly on the dispersal potential of the species. However, there is a growing realization that many, if not most species would be unlikely to disperse as fast and far as required. As a consequence, it has been proposed that species at risk should be actively translocated into unoccupied, but environmentally suitable areas that are likely to stay suitable over the next 100 or more years (assisted colonization or assisted migration). This solution is controversial, though, reflecting negative experiences with introduced exotics and probably also the traditional emphasis in conservation management on preserving a certain local, often historical situation with a static species composition, and a tendency among ecologists to think of biological communities as generally saturated with species. Using the European flora as a case study, we here estimate the main environmental controls of plant species richness, assess how the maximum observed species richness depends on these environmental controls, and based here on estimate how many species could at least be added to an area before further species additions would perhaps inevitably lead to corresponding losses locally. Our results suggest that there is substantial room for additional plant species across most areas of Europe, indicating that there is considerable scope for implementing assisted colonization as a proactive conservation strategy under global warming without necessarily implicating negative effects on the native flora in the areas targeted for establishment of translocated populations. Notably, our results suggest that 50% of the cells in Northern Europe, the likely target area for many translocations, could harbor at least 1/3 as many additional species as they have native species. However, we also emphasize that other, more traditional conservation strategies should also be strengthened, notably providing more space for nature and reducing nitrogen deposition to increase population resilience and facilitate unassisted colonization. Furthermore, any implementation of assisted colonization should be done cautiously, with a careful analysis on a species-by-species case.

2209: *+.108*

The Yabby Cherax destructor has a natural distribution across inland river systems in central and south-eastern Australia. Within that range it supports important recreational and commercial fisheries, and is also widely used for aquaculture and as a pet in the aquarium trade. Recently, the species has become established in parts of Australia outside its natural range. This paper documents sites of translocation in the coastal drainages of eastern New South Wales, Australia. Potential implications of these translocations are discussed with regard to the native aquatic fauna of the region. We draw particular attention to: (i) the native species of amphibian that are threatened by predation by introduced yabbies, and (ii) the native crayfish fauna threatened by competition with this species. The Fitzroy Falls Crayfish Eucistacus dharawalus has a distribution restricted to one small catchment and C. destructor has been recently translocated into this creek. On the basis of these data, we propose to nominate C. destructor as a key threatening process under the Threatened Species Conservation Act 1995 and Euastacus dharawalus as a threatened species, also under that Act.

2210: ***+.198***

The greater bilby Mocrotis logotis once occupied about 70% of the Australian mainland but is now restricted in occurrence to just 20% of its former range. It is classified as Extinct in New South Wales. We assessed the major components of the diet of a recently re-introduced population of bilbies at Scotia Sanctuary, in western New South Wales, from faecal material collected over a period of 13 months. Animals consumed more invertebrates than green plant material and seeds throughout the study, although there was temporal variation in the presence of these food categories. Five orders of invertebrates were identified, with Coleoptera (beetles) occurring in more than 80% of faecal samples and Isoptera. (termites) and Formicidae (ants) in 48% and 40%, respectively. These results identify key components of the diet of the first population of bilbies in New South Wales since the early 201 century and, combined with detailed studies of habitat requirements and prey abundance, should assist with selection of additional sites to expand the distribution of this iconic species further in future.

2211: ***+.118***

We studied a population of the critically endangered Malherbe's parakeet (Cyanoramphus malherbi), following the release of 62 captive-bred individuals on Maud Island, New Zealand, to identify and characterise nesting sites in a novel island environment. Previous work on Malherbe's parakeets consisted of limited observations on remnant mainland populations. The age of breeding pairs on Maud Island was 7.2 +/- 4.7 months and included both captive-bred individuals of the first release flock and individuals hatched on Maud Island within a year of the first release. Nests were found in hollows of mamaku (Cyathea medullaris), vacant nests of sacred kingfisher (Todiramphus sanctus), a hole in the ground and a hollow in a kohekohe (Disoxylum spectabile). Active nests were found in the austral spring, summer and autumn. Clutch size was 5 eggs. The fledging of three Malherbe's parakeets was confirmed for one nest 43 days after hatching. Observations of newly fledged individuals around the island indicate that at least seven successful nesting attempts occurred. Consistent with other studies in Cyanoramphus parakeets, our results suggest that availability of nesting sites on small islands may not be a limiting factor for the establishment of additional populations of Malherbe's parakeets via captive breeding and translocation. The formation of breeding pairs at an early age, the use of diverse nesting sites in regenerating vegetation, and the evidence of successful breeding shortly after release on an island represent encouraging prospects for the conservation of New Zealand's rarest parakeet.

2212: ***+.186***

The greater bilby, Macrotis lagotis, is a species of conservation significance in the and and semiarid zones of Australia. A species recovery program has been underway since the mid-1990s but the incorporation of molecular genetic data within the program has been difficult due to the problems of obtaining regular, population-wide samples of this trap-shy and sparsely distributed species. In this study, we demonstrate that faecal pellets collected from around burrows in the dry, arid habitat of western Queensland provide a viable source for DNA extraction and analysis. Faecal DNA was used to generate population-level estimates of microsatellite and mtDNA diversity for comparison with previous estimates for the natural population derived from tissue samples. Data were used to assess both the reliability of faecal-derived genotypes and the extent of any diversity loss since the previous study. Microsatellite diversity recorded from eight polymorphic markers for the natural population (A = 4.31 +/- 0.30, H(E) = 0.76 +/- 0.03) was comparable with the previous study, indicating little change in genetic diversity for the natural population in the 10-year interim. Faecal genotypes generated for the recently reintroduced

population matched the known number of founders as well as a known genotype, providing support for the reliability of the faecal DNA approach. The captive and reintroduced populations had significantly lower diversity levels than the natural population (A = 3.59 +/- 0.28, H(E) = 0.68 +/- 0.03; A = 3.57 +/- 0.20, H(E) = 0.65 +/- 0.03 respectively). Mitochondrial control region analysis, incorporating nested clade phylogeographic analysis (NCPA), agrees with earlier findings that populations of bilbies across the arid zone in Australia have only recently become fragmented, but the case for Queensland bilbies being strongly differentiated from other regions is diminished. Implications from this study include the need to further supplement the captive and reintroduced populations with additional out-bred individuals and that faecal DNA can be used effectively for ongoing monitoring and management of this species.

2213: *+.037*

Acacia attenuata Maiden and Blakely, is a vulnerable shrub, endemic to south-east Queensland, Australia. The population ecology and genetics of the species were examined throughout its range to assist with conservation and recovery of the species. South-east Queensland is experiencing massive population expansion and the associated housing and infrastructure development is having an impact on the remnant vegetation in the region. Population sizes differed significantly ($P < 0.05$) and were smaller in the southern urbanised parts of the species distribution. Genetic diversity of A. attenuata was high in comparison to other Acacia species. Genetic diversity was not significantly correlated with population size or isolation. There was a high degree of genetic similarity among populations (F(ST) = 0.101). Populations were effectively inbred (F = 0.482); however, inbreeding was not correlated with population size, density, isolation or reproductive activity. Uniform high levels of genetic diversity and low population differentiation suggest that A. attenuata once had a more continuous distribution. A population that was due to be translocated because of a development decision was also assessed as part of the research. The population at the development site (AA14 - Bundilla) was the largest and one of the most genetically variable sites, thus the genetic diversity of the population needs to be conserved within the translocation. The translocation process is reported here and occurred based on the information on genetics and ecology provided by this study. Population density and the proportion of seedlings and juveniles were significantly negatively correlated with time since fire. Fire regimes of 5-10 years are optimal for A. attenuata population regeneration and persistence, thus active fire management will be required for both the translocated population and for other populations within the urban and peri-urban areas, where competing demands make fire management controversial and difficult.

2214: *+.125*

Understanding the causes of rarity and ways of managing populations of rare species is essential for their successful conservation. The present study applies the conceptual model of a hierarchy of causes to Lasiopetalum pterocarpum E. M. Benn. & K. Shep. (a critically endangered species) to understand better its reproductive and ecological attributes, possible reasons for its rarity and to determine whether this model assists in developing management strategies. L. pterocarpum subpopulations from Serpentine National Park were censused to record abundance, plant health, phenology, flower and fruit production and the presence of any seed bank. These characteristics were matched to criteria in the hierarchies of cause model. There was no evidence of recent seedling recruitment at any subpopulation. Hand-pollination produced a flower-to-fruit conversion proportion similar to that found in the field and self- and cross-pollinations produced virtually the same fruit set. Seed store in soil from beneath the native subpopulations and at a translocation site showed seed was patchily distributed and infrequent. L. pterocarpum is an obligate seeder, killed by. re and dependent on disturbance to break seed dormancy. However, smoke has no effect on

germination. Seed production does not constrain population growth, because seedling regeneration after. re in 1999 was prolific at sites where plants had been growing. In the hierarchies of cause framework, the main causes of rarity for this species are taxon ecology, life-history strategy and stochasticity. Thus, concentrating active management on factors related to life history such as mosaic patch burning, fencing after. re to exclude vertebrate grazers, weed control and establishment of translocated populations will aid the preservation of this species in the wild.

2215: *+.325*

Many Caladenia species have been reduced to extremely small and/or fragmented populations, and reintroduction/translocation into natural or rehabilitated habitats, by using ex situ propagated plants or via direct seeding, represents an important adjunct in conservation planning. However, Caladenia species are some of the most difficult terrestrial orchid taxa to propagate, in part because of the specificity of the mycorrhizal associations and the need to provide growing conditions that suit both the mycorrhizal fungi and Caladenia plants. The present paper reviews recent advances in Caladenia propagation and reintroduction methods, including in vitro seed germination, transferral from in vitro to nursery environments, ex vitro symbiotic germination (germination in inoculated nursery media), nursery cultivation, the use of nurse plants and reintroduction of Caladenia into natural habitats by using seed, dormant tubers or growing plants. Techniques discussed in the present paper increase the options for future Caladenia conservation programs, especially for those species currently on the brink of extinction.

2216: *+.093*

By conducting reintroductions of the endangered terrestrial orchid Diuris fragrantissima D. L. Jones & M. A. Clem. we compared planting at the following three stages of the natural perennial growth cycle: as actively growing symbiotic plants in spring and autumn and as dormant tubers in summer. Plants reintroduced in spring and autumn were incorporated into randomised treatments involving soil aeration and addition of a mycorrhizal fungus. The addition of a mycorrhizal fungus and soil aeration together significantly increased survival and flowering of plants reintroduced in spring, whereas they had no significant effect on plants reintroduced in autumn. Addition of a fungus without soil aeration did not improve plant survival or flowering. Reintroducing actively growing plants was more successful than reintroducing dormant tubers, with 32.5% and 29.1% plants (reintroduced in spring and autumn, respectively) and 11.0% of tubers persisting after 4 years. Although survival of reintroduced plants declined at a rate of 16.9% per year for 4 years following reintroduction, survival of remnant plants remained relatively constant, ranging from 80.0 to 93.0%. Tuber size was positively correlated with survival and flowering of reintroduced plants for 2 years following reintroduction. A general trend was observed towards the increased likelihood of re-emergence and flowering of plants that flowered in previous seasons.

2217: *+.115*

Attempts were made to reintroduce water insects (five stonefly species and one mayfly species) into several third order streams in Rhineland-Palatine and Hessen, Germany. All these streams had been strongly affected by waste water and rubbish and had lost most of their macroinvertebrates. As a consequence of the installation of several purification plants in the past three decades, water quality has improved. Since no stoneflies returned, 700-1300 eggs of three different species (Isoperla goertzi, I. oxylepis, and I. grammatica) were exposed in the Selz brook (1997). In February 1998 a few larvae and in 1999 about 10,000 eggs of Perla marginata were added in the Walluf brook, and in 1998, 500 larvae of Oligioneuriella rhenana each in the Ruwer, Alf and Elz

brooks. After 10 years, I found only one single larva of Perla marginata in the Walluf brook. In the laboratory, the total number of eggs produced per female could be determined by rearing. On that basis coupled with the assumption of about 3% survival chances for the embryonic and larval development and of about 50% survival chances for the adult females, it was calculated that at least two females of I. goertzi, eight females of I. exylepsis and seven females of I. grammatica were required to found a new and sustainable population.

2218: *+.007*

1. A proposed trial reintroduction of the Eurasian beaver (Castor fiber L.) to Scotland has recently been approved (May 2008). A previous proposal Was turned down by the licensing authority, partly over the perceived risks to woodland within a Special Area of Conservation.2. This paper presents data oil two years of willow (Salix spp.) and aspen (Populus tremula L.) regrowth following tree Felling by captive beavers within two large semi-natural enclosures ill eastern Scotland. Both willow and aspen are highly preferred by beavers.3. Regrowth of Willow sterns was quantified from Cut Stumps. felled logs and partially severed ('incompletely felled') trees, and compared With growth of steins in the canopies of untouched willows.4. Maximum annual regrowth was observed in incompletely felled trees, followed by cut stumps and then unfelled control trees. Mean regrowth rates were ca 18 m regrowth per metre or measured tree section per year, ill incompletely Felled trees. This was approximately 12 times more annual regrowth than that observed ill unfelled trees. despite apparently selective browsing of the regrowth of incompletely felled trees by roe deer.5. Although incompletely felled willows represent a relatively small component of felling activity (9% of all felled willow), they contribute disproportionately to the biomass of beaver-engineered riparian woodlands. creating a more multi-layered habitat structure.6. Willow regrowth ill inundated areas was avoided by deer, and harvesting of regrowth by beavers was rare.7. Significant regrowth from 11 stumps of aspen in 2 years post-felling Occurred by suckering. with 85 Stickers of mean height 2.1 m being recorded, all of which were subsequently avoided by browsing deer and beavers. S. These data Suggest that rapid regeneration of willow and aspen Will Occur in riparian Woodlands in the event of major felling activity by Eurasian beaver. even in the presence of low to moderate levels of roe deer browsing and that the conservation status of both these trees or the wider habitats that they form Would not be threatened by a Well planned and managed reintroduction of beavers to Scotland.
Copyright (C) 2008 John Wiley & Sons, Ltd.

2219: *+.107*

1. In 2006, two periods of hypoxia resulted in the death of approximately 35 tonnes of black bream (Acanthopagrus butcheri) in Lake Indoon, a small inland lake in Western Australia.2. Acanthopagrus butcheri was the first fish species to be recorded in this lake, along with the mosquitofish (Gambusia holbrooki) which was also observed during sampling in 2006. Acanthopagrus butcheri appears to have been introduced to Lake Indoon between 1998 and 2003 and formed a self-sustaining population. It is believed to have been deliberately introduced for the purpose of creating a recreational fishery, despite the existence of substantial penalties for illegal translocation of fish in Western Australia.3. Recent human-induced environmental changes, including rising groundwater and salinization, have probably aided the establishment of both species in Lake Indoon. The importance of salinity to recruitment success by A. butcheri was indicated by the presence of only two age classes in 2006, with estimated recruitment dates coinciding with the years of highest recorded salinity in the lake.4. The 'fish kills' provided an opportunity to examine aspects of A. butcheri biology in a relatively low salinity environment which is atypical for this estuarine species. In particular, the recruitment period in Lake Indoon

was delayed until autumn/winter, rather than spring/summer as seen in other populations. Biological responses in Lake Indoon have implications for natural populations living in estuarites with modified salinity regimes.5. The ecological, social and economic impacts potentially arising from the introduction of fish to Lake Indoon, which is an important migratory bird habitat and a recreational amenity for local residents and tourists, illustrate the complexities of fish translocation and the need for rigorous assessment before stocking to identify potential costs and benefits. Copyright (C) 2008 John Wiley & Sons, Ltd.

2220: *+.195*

Forty thousand American eel Anguilla rostrata elvers were released in a 400-ha lake in an eel-free watershed in eastern Quebec in 1999. Subsequent sampling enabled the measurement of poststocking movements, growth, and sex ratio. Populations showed limited movements, and occupancy extended less than 3 km into inflowing tributaries by 2003. Annual growth increments in the lake (118 mm/year) were the highest reported for the species, but growth increments in rivers (40 mm/year) were typical of those found elsewhere. Four of seven eels whose sex could be determined were females, in contrast to other sites in the St. Lawrence watershed where females are more than 99% of the population. American eel translocation to growth areas that have been blocked by artificial barriers may be a useful means to increase production of silver eels.

2223: *+.009*

Reintroductions of muskoxen (Ovibus moschatus), European bison (Bison bonasus), and moose (Alces alces) have occurred recently in Russia. Although the process of capturing and moving muskoxen was problematic in remote areas, the reintroduction of animals from Canada and the USA successfully restored this extirpated species, and the current population in northern Russia serves as a source for further transplants. European bison populations were stagnant and suffered from inbreeding in Russia prior to reintroduction of captive animals from throughout Europe. The population in Orlovskoye Polesie National Park has experienced population growth with improved genetic potential. Of concern is that reintroductions in other areas of Russia were unsuccessful and the global population of European bison is not improving. Moose from the Penzhina River area in Russia were successfully reintroduced to the Kamchatka Peninsula where they were absent for >400 years. The population is growing and dispersing across the peninsula from the transplant sites, and is among the largest physically in Eurasia.

2226: *+.041*

BackgroundThe harpy eagle (Harpia harpyja) is the largest Neotropical bird of prey and is threatened by human persecution and habitat loss and fragmentation. Current conservation strategies include local education, captive rearing and reintroduction, and protection or creation of trans-national habitat blocks and corridors. Baseline genetic data prior to reintroduction of captive-bred stock is essential for guiding such efforts but has not been gathered previously.Methodology/FindingsWe assessed levels of genetic diversity, population structure and demographic history for harpy eagles using samples collected throughout a large portion of their geographic distribution in Central America (n=32) and South America (n=31). Based on 417 bp of mitochondrial control region sequence data, relatively high levels of haplotype and nucleotide diversity were estimated for both Central and South America, although haplotype diversity was significantly higher for South America. Historical restriction of gene flow across the Andes (i.e. between our Central and South American subgroups) is supported by coalescent analyses, the haplotype network and significant FST values, however reciprocally monophyletic lineages do not

correspond to geographical locations in maximum likelihood analyses. A sudden population expansion for South America is indicated by a mismatch distribution analysis, and further supported by significant ($p<0.05$) negative values of Fu and Li's DF and F, and Fu's FS. This expansion, estimated at approximately 60 000 years BP (99 000–36 000 years BP 95% CI), encompasses a transition from a warm and dry time period prior to 50 000 years BP to an interval of maximum precipitation (50 000–36 000 years BP). Notably, this time period precedes the climatic and habitat changes associated with the last glacial maximum. In contrast, a multimodal distribution of haplotypes was observed for Central America suggesting either population equilibrium or a recent decline. SignificanceHigh levels of mitochondrial genetic diversity in combination with genetic differentiation among subgroups within regions and between regions highlight the importance of local population conservation in order to preserve maximal levels of genetic diversity in this species. Evidence of historically restricted female-mediated gene flow is an important consideration for captive-breeding programs.

2227: *-.122*

Sixteen percent of the Israeli native freshwater fishes have become extinct and 19% are critically endangered The situation in other and and semi-and countries is similar Are all the endangered species doomed to become extinct) The case of the restoration of the wild populations of the Yarqon bleak clearly shows that there is an alternative The Yawn bleak, a cyprinid fish endemic to the Mediterranean rivers in Israel, almost became extinct following the drought of 1998-1999 Several clays prior to the drying of the streams. ca. 150 fish from two basins were brought to the Ichthyological Laboratory at Tel Aviv University They were carefully treated and housed in a breeding centre Within five years, we had managed to produce more than 14,000 fish First attempts to reintroduce the fish to the Yawn River, made in 2002 and 2003, failed, in that the adults did not breed Following an experiment which showed that the fish need suitable spawning sues and shelter sites for juveniles, several aquatic sites were engineered according to knowledge acquired during the research and were stocked with laboratory-horn fish in 2006 During 2007 and 2008, juvenile Yarqon bleak of various sizes were found in eight out of 11 monitored sues The success to save the Yargon bleak shows that endangered fish species can be rescued This requires action to be taken along two fronts 1) raising public awareness and 2) a professional approach that includes relevant research and implementation of the results

2229: *+.184*

Large-scale losses of seagrass beds have been reported for decades and lead to numerous restoration programs. From worldwide scientific literature and 20 years of seagrass restoration research in the Wadden Sea, we review and evaluate the traditional guidelines and propose new guidelines for seagrass restoration. Habitat and donor selection are crucial: large differences in survival were found among habitats and among donor populations. The need to preferably transplant in historically confirmed seagrass habitats, and to collect donor material from comparable habitats, were underlined by our results. The importance of sufficient genetic variation of donor material and prevention of genetic isolation by distance was reviewed. The spreading of risks among transplantation sites, which differed in habitat characteristics (or among replicate sites), was positively evaluated. The importance of ecosystem engineering was shown in two ways: seagrass self-facilitation and facilitation by shellfish reefs. Seagrass self-facilitative properties may require a large transplantation scale or additional measures. (C) 2008 Elsevier Ltd. All rights reserved.

2230: *+.248*

Legislative changes during recent decades resulted in a massive shift away from livestock towards game ranching in southern Africa, resulting in significant increases in the abundance and distribution of many wildlife species. However, there are problems associated with game ranching from a conservation perspective, including persecution of predators, overstocking, introductions of exotic species and genetic manipulation of 'huntable' species. We suggest here that most of these problems could be overcome through promoting the formation of conservancies, where adjacent ranches remove internal fencing to form larger collaborative wildlife areas. Larger areas permit the reintroduction of the full range of indigenous mammals, tending to result in a land-use shift from high-offtake, low-value consumptive utilization towards higher value forms of hunting and ecotourism. Under these land-use conditions, ranchers tend to be more tolerant of predators and often actively reintroduce them. Freedom of movement for wildlife populations increases resilience to environmental shocks. The collaborative management agreements typical of conservancies tend to align more closely with conservation objectives than on single ranches. Fortuitously, there are financial advantages associated with conservancies: land-use options in conservancies are more profitable and there are economies of scale associated with cooperative management. Land within conservancies is likely to appreciate in value and attract external investment. In addition, conservancies are more conducive to developing partnerships with indigenous communities and investors and may thus increase the political and social sustainability of game ranching. However, ranchers are fiercely independent and may be resistant to removing fences due to the perception that they may relinquish control over their land and wildlife. Strategies are required to overcome such reluctance and promote the formation of conservancies to enhance the conservation value of game ranch land.

2231: ***+.147***

Reintroductions are commonly used for re-establishing self-sustainable populations in formerly inhabited areas. Reintroductions are expensive, and thus, it is worth performing a thorough demographic analysis of current and likely future population trajectories to guide strategic decisions on release policy. Bearded vultures Gypaetus barbatus were exterminated from the Alps in the late 19th century, mainly due to human persecution. To re-establish them, captive-bred young have been released annually since 1986. Since the first successful breeding in the wild in 1997, the population has increased to 9 pairs in 2006. It is not known, however, for how long releases should be continued to obtain a self-sustaining, viable population. We estimated age-specific survival probabilities with a mark-resighting model and quantified fecundity rates of released individuals. Using the resulting demographic estimates, we built a stochastic population model to estimate population growth rates, and explored the value of continuing to release birds for varying periods into the future. Annual survival probabilities were high (first year of life, 0.88; later years, 0.96); average annual fecundity was 0.6 fledglings per breeding pair. Using the estimated survival probabilities, projected population growth rates would increase with additional years of releases. Yet, the population would grow, even if releases had stopped after 2006. Only if mortality increased by >= 50% would the population start to decline. Synthesis and applications. Our population dynamics model provides essential information to optimize decision-making within a major reintroduction programme. From a demographic viewpoint, releases of captive-raised bearded vultures can be ceased in the Alps. The resources freed could be redirected towards a close demographic surveillance of the free-ranging population, with periodic evaluation of its viability and the option to release birds if deemed necessary. Birds available from the captive stock could be used for reintroductions in other areas where the bearded vulture is extinct.

2232: ***-.070***

Captive reintroductions often suffer high mortality, with predation as one source. Many species learn about predators; thus training captive-born animals to recognize predators may increase survivorship. We adapted variants of methods developed for birds to attempt to condition monkeys to mob a predator. Captive-reared cotton-top tamarins (Saguinus oedipus) did not differentiate between a snake and a rat, confirming previous research that na < ve cotton-top tamarins do not fear snakes. Tamarins then viewed a living snake during playback of mobbing calls. In posttests, tamarins did not mob the snake. We then eliminated the pretest to avoid potential habituation and placed a stillborn tamarin with the snake in an attempt to heighten arousal. In posttests, tamarins again did not mob the snake, although they did increase their rates of calls, indicating mild arousal. Overall, none of 8 groups of cotton-top tamarins learned to mob a predator. Potential reasons for failure include conditioning multiple subjects at once and the lack of an experienced demonstrator. Observing a demonstrator was not necessary for birds to acquire mobbing, but may be necessary for tamarins.

2233: ***+.114***

Sturgeon disappeared from the Rhone River in the mid 70's without certitude about which species it was and about the existence of a sympatry between European sturgeon, Acipenser sturio, and Adriatic sturgeon, A. naccarii, in this watershed. In order to reach a reliable specific determination of this extinct sturgeon population, archaeo-zoological remains of the Jardin d'Hiver in Arles city, on the Rhone river banks, were genetically analysed, following strict criteria of authentication for the ancient DNA work. The rich collection of Arles sturgeon bone remains stems from human activities between the 6th and the 2nd Century BC. Sequences of 86 bp of the cytochrome b gene were obtained on four bones, from different anatomical parts of the fish and from different archaeological layers. All gave A. sturio diagnostic sequences. This preliminary analysis is an essential first step in the project of sturgeon reintroduction in the Rhone River. Thus, further analyses on a larger sample are necessary to comfort this result and to solve the question of sympatry with A. naccarii.

2234: ***+.200***

Translocations are important tools in the field of conservation. Despite increased use over the last few decades, the appropriateness of translocations for amphibians and reptiles has been debated widely over the past 20 years. To provide a comprehensive evaluation of the suitability of amphibians and reptiles for translocation, we reviewed the results of amphibian and reptile translocation projects published between 1991 and 2006. The success rate of amphibian and reptile translocations reported over this period was twice that reported in an earlier review in 1991. Success and failure rates were independent of the taxonomic class (Amphibia or Reptilia) released. Reptile translocations driven by human-wildlife conflict mitigation had a higher failure rate than those motivated by conservation, and more recent projects of reptile translocations had unknown outcomes. The outcomes of amphibian translocations were significantly related to the number of animals released, with projects releasing over 1000 individuals being most successful. The most common reported causes of translocation failure were homing and migration of introduced individuals out of release sites and poor habitat. The increased success of amphibian and reptile translocations reviewed in this study compared with the 1991 review is encouraging for future conservation projects. Nevertheless, more preparation, monitoring, reporting of results, and experimental testing of techniques and reintroduction questions need to occur to improve translocations of amphibians and reptiles as a whole.

2235: ***+.212***

The appropriateness of using carrying capacity (CC) estimates to indicate habitat utilisation for a particular species, and thus as a tool for conservation population planning, has been questioned. We argue individual fitness is driven by resource availability, and we therefore assume individuals select habitats with a higher quality, abundance, and availability of key resources. In the past such selection has been related to the CC of a habitat. We tested whether we can use CC estimates to indicate habitat selection by individuals using a selective forager, black rhinoceros Diceros bicornis, for which CC approaches underpin species conservation plans. We tested for correlation of individuals' habitat selection with predicted CC values at three spatial scales of selection. Individual selection was not related to the value of the habitat according to our CC estimates for any of the three scales we tested at. We discuss how density-dependence, environmental variables, scale of selection, individual variation and intra- and inter-specific dynamics may have influenced these results. Following this, we question the use of a priori calculations of potential resource quality and abundance of habitats (CC estimates), which do not take into account the various factors that influence an animal's selection of a habitat, as an indicator of species habitat selection. We raise caution regarding the use of such CC models to determine optimal population numbers for an area. (C) 2008 Elsevier Ltd. All rights reserved.

2236: *+.074*

Despite the apparent risks of the introduction of non-indigenous ungulates to biodiversity, relatively little is known globally about the pathways of introduction, propagule pressure and realized impacts of ungulate introductions. These issues were examined here by investigating ungulate introductions to South Africa within a global context. Across countries globally, introduced ungulate richness is not related to indigenous ungulate richness, and several countries are clear outliers. South Africa is second only to the USA in the number of ungulate species introduced to date. Zoos have traded more ungulate species and individuals to non-zoo recipients than to other zoos, highlighting the tensions that exist between in situ and ex situ conservation goals. Introductions to, and extralimital introductions within South Africa have increased through time, with propagule pressure being highest in areas with high human population density. The long distances ungulates have been translocated raise concerns for genetic homogenization. Translocations of indigenous ungulate species extralimitally have significantly altered range sizes, typically to a greater extent than is expected from range shifts associated with global climate change. Although ungulate introductions and translocations are likely to have impacts on biodiversity, evidence for such impacts in South Africa, and elsewhere, is limited. Whilst arguments may be made for a precautionary approach to ungulate introductions, an evidence-based one is much more likely to deliver efficient and convincing conservation decision-making.
(C) 2008 Elsevier Ltd. All rights reserved.

2237: *+.082*

A comparative analysis of the habitats of Baldellia ranunculoides (subsp. ranunculoides and subsp. repens) and Baldellia alpestris (Alismataceae) was carried out across central and western Europe. Soil samples from 43 populations were analyzed and the composition of accompanying vegetation was analyzed by canonical correspondence and indicator species analysis. Significant differences in the habitat conditions and the accompanying vegetation were found between the three taxa. B. ranunculoides subsp. repens is growing in lowland water bodies on acidic substrates (pH 5-6.3) which are markedly richer in organic matter and poorer in cations, especially in Ca^{2+}. B. ranunculoides s. str. is also a lowland taxon, but grows on more mineral and basic substrates (pH 7-9), usually rich in Ca^{2+}. The endemic B. alpestris grows in the most distinct habitats, on substrates with low pH and with very low Ca^{2+} concentrations, at much higher altitudes than the

other taxa and in very specific plant communities that have almost no affinities with those of the two lowland Baldellia-taxa. The Baldellia-taxa are threatened by eutrophication of their habitats. Moreover, many of the extant populations, in particular those of B. ranunculoides s. str., are very small and will be threatened by environmental stochasticity. Reintroduction and the creation of new populations might reduce the risk of extinction. Information given on the detailed habitat preferences of the Baldellia-taxa could help conservation efforts for these endangered taxa and identify suitable sites for (re-) introductions. (C) 2008 Elsevier B.V. All rights reserved.

2238: *+.399*

The loss of wetlands and semi-natural grasslands throughout much of Europe has led to a historic decline of species associated with these habitats. The reinstatement of these habitats, however, requires spatially explicit predictions of the most suitable sites for restoration, to maximize the ecological benefit per unit effort. One species that demonstrates such declines is the white stork Ciconia ciconia, and the restoration of habitat for this flagship species is likely to benefit a suite of other wetland and grassland biota. Storks are also being reintroduced into southern Sweden and elsewhere, and the a priori identification of suitable sites for reintroduction will greatly improve the success of such programmes. Here a simple predictive habitat-use model was developed, where only a small but reliable presence-only dataset was available. The model is based on the extent and relative soil moisture of semi-natural pastures, the extent of wetlands and the extent of hayfields in southern Sweden. Here the model was used to predict the current extent of stork habitat that is suitable for successful breeding, and the extent of habitat that would become suitable with moderate habitat restoration. The habitat model identifies all 10 occupied nesting sites where breeding is currently successful. It also identifies similar to 300 km(2) of habitat that is predicted to be suitable stork habitat, but that is presently unused; these sites were identified as potential areas for stork reintroduction. The model also identifies over 100 areas where moderate habitat restoration is predicted to have a disproportionate effect (relative to the restoration effort) on the area of suitable habitat for storks; these sites were identified as priorities for habitat restoration. By identifying areas for reintroduction and restoration, such habitat suitability models have the potential to maximize the effectiveness of such conservation programmes.

2239: *+.079*

Game species exploitation usually causes an increase in their distribution, further from their own dispersal potential, but we know too little about the ecological effects that these human-induced expansions may produce. The realized niche of the red deer was analyzed by means of habitat suitability modelling in the present study. Two populations inhabiting different geographic areas have been distinguished according to their origin, one is native to the study area and the other was translocated as a result of human hunting interests. Translocated red deer showed strong distributional overlap with the native Iberian ibex. However, a comparative analysis of the potential distribution of native red deer and Iberian ibex populations did not yield a significant niche overlap. Thus, we conclude that the observed niche overlap between the Iberian ibex and the red deer in the study area would not have taken place without human intervention. We discuss these results in the light of biological invasions and native species translocation programmes, and conclude that human-induced range expansion of native species should be regarded as a specific case of invasive species.

2240: *+.139*

During the last 150 years, nine-banded armadillos (Dasypus novemcinctus) have increased their

range and abundance in the southeastern United States. When foraging, armadillos cause damage to agricultural crops, as well as structural damage to driveways and foundations. Homeowners frequently use translocation to reduce local armadillo abundance. Despite its popularity with the general public, however, the appropriateness of nuisance wildlife translocation presents concerns for biologists. Our objective was to address some of these concerns by examining survival and movements of translocated armadillos. We translocated 12 armadillos (9 male, 3 female) equipped with radio-transmitters and compared their survival and movements to that of 29 (11 male, 18 female) resident armadillos. Most (92%) of the translocated animals dispersed from their release site within the first few days after release. Resident armadillos generally maintained stable home ranges. We found evidence that translocated animals were able to return to their original capture sites. Therefore, we recommend against translocating nuisance armadillos.

2241: ***-.068***

The U.S. Environmental Protection Agency has proposed permitting ballast water discharges-a benefit of which would be to reduce the economic damages associated with the introduction and spread of aquatic invasive species. Research on ship-borne aquatic invasive species has been conducted in earnest for decades, but determining the economic damages they cause remains troublesome. Furthermore, with the exception of harmful algal blooms, the economic consequences of microscopic invaders have not been studied, despite their potentially great negative effects. In this paper, we show how to estimate the economic benefits of preventing the introduction and spread of harmful bacteria, microalgae, and viruses delivered in U.S. waters. Our calculations of net social welfare show the damages from a localized incident, cholera-causing bacteria found in shellfish in the Gulf of Mexico, to be approximately $706,000 (2006$). On a larger scale, harmful algal species have the potential to be transported in ships' ballast tanks, and their effects in the United States have been to reduce commercial fisheries landings and impair water quality. We examine the economic repercussions of one bloom-forming species. Finally, we consider the possible translocation within the Great Lakes of a virus that has the potential to harm commercial and recreational fisheries. These calculations illustrate an approach to quantifying the benefits of preventing invasive aquatic microorganisms from controls on ballast water discharges.

2242: ***-.056***

The topmouth gudgeon, Pseudorasbora parva, has been described as Europe's most invasive fish. To control their UK invasion, some lentic populations at risk of causing fluvial dispersal have been eradicated. The first of these operations was from a lake in north-west UK in March 2005 using rotenone application; prior to eradication, their mean density was estimated as 6.1 m(-2) whereas since eradication, no P. parva have been recorded. Prior to rotenone application, the majority of native fishes were removed, held off-site and reintroduced following degradation of rotenone to safe levels. In the three growth seasons since their reintroduction and P. parva eradication, the abundance, somatic growth rate and production of roach Rutilus rutilus and common bream Abramis brama have increased significantly; production is now driven by a lower number of comparatively larger, faster growing individuals. These data suggest that the eradication of this P. parva population has been highly beneficial for the growth, recruitment and production of these native species.

2243: ***+.216***

Temporal variation in survival, fecundity, and dispersal rates is associated with density-dependent and density-independent processes. Stable natural populations are expected to be regulated by

density-dependent factors. However, detecting this by investigating natural variation in density is difficult because density-dependent and independent factors affecting population dynamics may covary. Therefore, experiments are needed to assess the density dependence of demographic rates. In this study, we investigate the effect of density on demographic rates of the Seychelles Warbler (Acrocephalus sechellensis). This species, endemic to a few islands in the Indian Ocean, went through a severe population bottleneck in the middle of the last century, with only; 30 individuals left on one small island, but has since recovered. Our monitoring shows that since reaching the island's carrying capacity, population density has remained stable. However, we detected neither density-dependent reproduction nor survival on the basis of natural density variation during this stable period. For conservation reasons, new populations have been established by transferring birds to nearby suitable islands. Using the change of numbers during the process of saturation as a natural experiment, we investigated whether we can detect regulation of numbers via density-dependent survival and reproduction within these new populations. We found that populations were mainly regulated by density-dependent reproduction, and not survival. Variation in density between islands can be explained by food abundance, measured as insect density. Islands with the highest insect densities also had the highest bird densities and the largest breeding groups. Consequently, we suggest that the density-dependent effect on reproduction is caused by competition for food.

2244: *-.042*

As on many other islands, most of the seabird species breeding in Portugal's Azores archipelago are now restricted to tiny remnants of their former nesting range, owing in large part to the introduction of mammals by humans. Praia Islet, 0.12 km2 in size, is home to five seabird species, four of which fall under the European listing of "conservation concern." Introduced rabbits (Oryctolagus cuniculus) on Praia had accelerated soil erosion through overgrazing, destroyed seabird nests, and competed for burrows with petrels. Restoration of seabird habitat on the islet began in 1995 and involved rabbit eradication, control of soil erosion, native plant reintroduction, and installation of artificial nests for common terns (Sterna hirundo), roseate terns (Sterna dougallii), and Madeiran storm-petrels (Oceanodroma castro). Rabbits were eradicated in 1997 using broadcast pellets containing poison. Since then, soil erosion has decreased, many of the reintroduced native plants have started to spread, and tern and Madeiran storm-petrel breeding numbers on the islet have increased by 1,548% and 43%, respectively. However, Praia Islet seabirds remain vulnerable owing to human recreational activities. This case study highlights the need to consider local conditions carefully when assessing restoration options to effect rapid mammal eradication. It also confirms the value of combining measures aimed at restoring native vegetation and improving seabird habitat quality with alien herbivore eradication.

2245: *+.316*

Active adaptive management (AAM) is an approach to wildlife management that acknowledges our imperfect understanding of natural systems and allows for some resolution of our uncertainty. Such learning may be characterized by risky strategies in the short term. Experimentation is only considered acceptable if it is expected to be repaid by increased returns in the long term, generated by an improved understanding of the system. By setting AAM problems within a decision theory framework, we can find this optimal balance between achieving our objectives in the short term and learning for the long term. We apply this approach to managing the translocation of the bridled nailtail wallaby (Onychogalea fraenata), an endangered species from Queensland, Australia. Our task is to allocate captive-bred animals, between two sites or populations to maximize abundance at the end of the translocation project. One population, at the original site of

occupancy, has a known growth rate. A population potentially could be established at a second site of suitable habitat, but we can only learn the growth rate of this new population by monitoring translocated animals. We use a mathematical programming technique called stochastic dynamic programming, which determines optimal management decisions for every possible management trajectory. We find optimal strategies under active and passive adaptive management, which enables us to examine the balance between learning and managing directly. Learning is more often optimal when we have less prior information about the uncertain population growth rate at the new site, when the growth rate at the original site is low, and when there is substantial time remaining in the translocation project. Few studies outside the area of optimal harvesting have framed AAM within a decision theory context. This is the first application to threatened species translocation.

2246: ***+.095***

Data on geographical ranges are essential when defining the conservation status of a species, and in evaluating levels of human disturbance. Where locality data are deficient, presence-only ecological niche modelling (ENM) can provide insights into a species' potential distribution, and can aid in conservation planning. Presence-only ENM is especially important for rare, cryptic and nocturnal species, where absence is difficult to define. Here we applied ENM to carry out an anthropogenic risk assessment and set conservation priorities for three threatened species of Asian slow loris (Primates: Nycticebus).Borneo, Java and Sumatra, Southeast Asia. Distribution models were built using maximum entropy (MaxEnt) ENM. We input 20 environmental variables comprising temperature, precipitation and altitude, along with species locality data. We clipped predicted distributions to forest cover and altitudinal data to generate remnant distributions. These were then applied to protected area (PA) and human land-use data, using specific criteria to define low-, medium- or high-risk areas. These data were analysed to pinpoint priority study sites, suitable reintroduction zones and protected area extensions. A jackknife validation method indicated highly significant models for all three species with small sample sizes (n = 10 to 23 occurrences). The distribution models represented high habitat suitability within each species' geographical range. High-risk areas were most prevalent for the Javan slow loris (Nycticebus javanicus) on Java, with the highest proportion of low-risk areas for the Bornean slow loris (N. menagensis) on Borneo. Eighteen PA extensions and 23 priority survey sites were identified across the study region. Discriminating areas of high habitat suitability lays the foundations for planning field studies and conservation initiatives. This study highlights potential reintroduction zones that will minimize anthropogenic threats to animals that are released. These data reiterate the conclusion of previous research, showing MaxEnt is a viable technique for modelling species distributions with small sample sizes.

2247: ***+.056***

To describe and analyse phylogeographical patterns in the endangered endemic lizard Podarcis lilfordi from across its remaining range and thereby establish baseline information on genetic diversity that will help determine conservation priorities and assist future reintroduction programs. Balearic Islands, Spain. We analysed mitochondrial DNA (2382 bp sequence from eight genes) from 118 individuals and characterized the relationships among haplotypes using parsimony networks, as well as phylogenetic inference. Analyses of historical gene flow and population growth were used to provide further insights into population histories. Four unconnected parsimony networks were obtained that mirrored the main clades in the phylogenetic tree: (I) all Menorcan populations, (II) Dragonera, Malgrats and Toro islands (Western Mallorca) (III and IV) and the remaining populations from Cabrera and Mallorca. Two major haplotype groups were detected in Menorca (I) and these provided signatures of a demographic expansion and

asymmetrical historical gene flow, respectively, concordant with the expected direction of colonization from south to north of the island. Populations from western Mallorca (II) showed evidence of historical allopatric fragmentation events following isolation around the start of the Pleistocene. In networks III and IV, Cabreran populations appear to have become isolated from north and south Mallorca quite recently, with asymmetric gene flow indicating a northwards dispersal direction.P. lilfordi is a genetically diverse species that shows substantial mtDNA structuring both between regions and, at a finer scale, between some islet populations within regions. The precarious state of some islet populations shown here to be quite divergent (e.g. Toro island in western Mallorca) means that conservation of this intraspecific biodiversity requires urgent action.

2248: *+.092*

The freshwater pearl mussel is a typical flag species, which attracts public interest in nature conservation. Its flag role is related to ability to create pearls. We have reviewed historical information on the pearl mussel exploitation and its potential influence on the persistence of the species. The Sudety Mts with the upper sections of the B6br, Nysa Luiycka and Kwisa rivers abounding in pearl mussels had become the well known area of pearl fishing since the 17th century. The first evidence of overfishing, recorded in the first half of the 18th century, was a decree issued in 1729 by August II Mocny (Friedrich August I), king of Poland and Saxon elector, forbidding pearl fishing due to the low population of the pearl mussel. Kacper Ludwig Trebluth, the last person granted by king of Poland and Saxon elector, August III Sas (Friedrich August II) a licence for pearl fishing (in the Sudety Mts. in the period of 1753-1769), could not earn his livelihood from this activity. The last records of pearl mussels shoals were, provided by local naturalists from the upper Kwisa river at the end of the 19th century . It is difficult to identify factors which caused the species decline. It is obvious that similarly as in other countries of Europe, the species decline is associated with the intensive development of agriculture and industry, which caused major changes in the landscape. One factor seems to be more important than previously believed: intense river and streams regulation (artificial stone-lined channels). The development of watermills and local industry, and the intense development of human settlements forced the regulation of any stream; in addition, water quality decreased due to agriculture (deforestation) and industrial sewage. It is rather improbable that muskrat Ondatra zibethicus could influence the pearl mussel population because that species was purposefully released from captivity in Czech (1905) after the pearl mussel had become extinct. In Poland no intensive search of freshwater pearl mussels was carried out. The species has been regarded as extinct since 1960. The inventory of water courses in the Sudety Mts., done in 2006-2007, did not show the species presence, except for a few well preserved shells found in Koci Potok (Fig. 2). It may indicate that a relic population can still live in this stream or that some subfossil remnants have been excavated by the stream. The inventory has also shown that the number of water courses which could meet the habitat requirements of pearl mussels is very low. On the other hand, land abandonment has caused spontaneous restoration of some streams in Sudety Mts. The general improvement of environmental conditions in the whole region, especially in terms of water quality, as well as the plans of indigenous salmonid fish reintroduction create habitat conditions appropriate for the freshwater pearl mussel. A chance of restoring or reintroducing the species has been assessed, taking into account the interest of local communities.

2249: *+.155*

Distribution models are commonly used to generalise across species distributions, to project future potential range changes, and to identify potential areas for species reintroductions and recovery

plans. Building several models that incorporate different potential causal factors is a useful way of formalising alternative hypotheses. We developed a series of models to test hypotheses about the factors influencing the distribution of a species of conservation importance - the hen harrier Circus cyaneus. A climate-based model using continental distribution data was consistent with the continental distribution and observational studies in Britain. According to the climate-model the parts of Britain occupied by the hen harrier are the least climatically suitable.Habitat-based models using detailed distribution data from seven Scottish areas explained the recent British distribution well, with birds largely confined to heather dominated areas. These patterns were inconsistent with historical data on the species' distribution, its habitat use in other parts of its range and with the climate-based model. Our burn intensity index of gamekeeper activity was highly correlated with climatic suitability within the best 25% of 10 km squares by modelled habitat suitability, negatively associated with the productivity data and associated with a decrease in abundances between 1998 and 2004. Gamekeeper activity may be keeping hen harriers out of the most climatically suitable areas with habitat similar to that which they currently occupy within Britain and or keeping the population numbers too low and isolated for the natural re-expansion of the species into parts of the range where it was historically extirpated. (C) 2008 Elsevier Ltd. All rights reserved.

2250: *+.180*

Distribution models are commonly used to generalise across species distributions, to project future potential range changes, and to identify potential areas for species reintroductions and recovery plans. Building several models that incorporate different potential causal factors is a useful way of formalising alternative hypotheses. We developed a series of models to test hypotheses about the factors influencing the distribution of a species of conservation importance [long dash] the hen harrier Circus cyaneus. A climate-based model using continental distribution data was consistent with the continental distribution and observational studies in Britain. According to the climate-model the parts of Britain occupied by the hen harrier are the least climatically suitable. Habitat-based models using detailed distribution data from seven Scottish areas explained the recent British distribution well, with birds largely confined to heather dominated areas. These patterns were inconsistent with historical data on the species' distribution, its habitat use in other parts of its range and with the climate-based model. Our burn intensity index of gamekeeper activity was highly correlated with climatic suitability within the best 25% of 10 km squares by modelled habitat suitability, negatively associated with the productivity data and associated with a decrease in abundances between 1998 and 2004. Gamekeeper activity may be keeping hen harriers out of the most climatically suitable areas with habitat similar to that which they currently occupy within Britain and or keeping the population numbers too low and isolated for the natural re-expansion of the species into parts of the range where it was historically extirpated.

2251: *+.201*

1. Robust redhorse Moxostoma robustum is an imperiled, potadromous fish in the south-eastern USA. Initial recovery efforts have focused on supplementing existing populations and establishing refugial populations through extensive stocking programmes. However, assessment of the Success of these programmes has not vet been conducted, and there are few reports evaluating the effectiveness of such programmes with other potadromous species.2. Radio telemetry was employed to assess the effectiveness of a stocking programme aimed at addressing whether stocked individuals Would remain in an area free of introduced predators and ascertaining the ability of stocked fish to integrate into a resident population.3. Hatchery-reared robust redhorse were captured from refugial populations established in other river systems and were transferred to

the Ocmulgee River, Georgia where a population of hatchery-reared individuals and an unknown number of wild fish reside.4. These transferred robust redhorse exhibited an exploratory phase for the first 3 months before adopting behaviour patterns, including spawning migrations, that were consistent with those reported for wild fish in other systems. However, some individuals seemed unable to locate suitable spawning habitat.5. Approximately half of the radio-tagged fish remained within the area free of introduced predators.6. At least some radio-tagged robust redhorse fully integrated into the resident population as evidenced by their presence in spawning aggregations with resident individuals.7. The effectiveness of a stocking programme is dependent upon the ability of stocked individuals to integrate into an existing population or replicate the behaviour and functionality of a resident Population. Evaluations of stocking programmes should incorporate assessments of behaviour in addition to surveys to estimate abundance and survivorship and genetic assessments of augmentation of effective Population sizes. Published in 2008 by John Wiley & Sons, Ltd.

2252: *+.109*

Landscape corridors connecting habitat patches may help overcome the genetic and demographic problems of small and isolated populations. An elegant field experiment shows that some Costa Rican forest birds will use 'riparian' (river margin) corridors to get back home, but they can be picky a out corridor quality.

2255: *-.018*

Oribi (Ourebia ourebi) are listed as endangered and one of the options to increase their numbers and populations is to breed them in captivity and subsequently reintroduce them into suitable habitat. A captive breeding facility for oribi in KwaZulu-Natal, South Africa, had been used to reintroduce captive-bred oribi on to the neighbouring agricultural/game farm for the previous 10 years. However, the success of these reintroductions was unknown. Consequently the aim of this study was to monitor the success of the reintroduction of captive-bred oribi. As in previous years, 10 captive-bred oribi were released by the owners of the facility onto the neighbouring farm. Using radio-telemetry and mortality sensors, survivorship of the reintroduced oribi was monitored, and the factors involved in a successful or failed reintroduction attempt identified. Within two months of release, seven of the 10 oribi were dead. The causes of mortality varied, but predation by natural predators and humans was a major factor. Consequently no further reintroductions on to this property were advised. Several factors of concern were raised and need to be addressed in future captive breeding, reintroduction and management of oribi.

2256: *+.088*

This report summarises findings from a 5-year research project (2003-200-) investigating the extent of loss of genetic diversity and inbreeding across various New Zealand threatened birds. Introduced predators and habitat loss are impacting on many New Zealand native species, but many species also have exceptionally low genetic diversity as a consequence of persisting in small and isolated populations. Research indicated that temporary bottlenecks associated with founder events during translocations do not contribute as much to loss of genetic variation as the small, finite population sizes of island sites. The build-up of inbreeding within closed island populations can result in further reductions in individual fitness. There is evidence of moderate inbreeding depression in a reintroduced population of North Island robins (Petroica australis longipes) on Tiritiri Matangi, and weak inbreeding depression in takahe (Porphyrio mantel/1) translocated to offshore islands. To what extent reduced individual fitness translates to reduced population growth

rates depends on the frequency of close inbreeding, the magnitude of inbreeding depression and which life history traits (i.e. fecundity versus survival) are most affected. Genetic management of New Zealand threatened species should not take priority over other management concerns such as controlling predators or improving habitat quality, but it does need more attention than it currently receives. Recommendations for genetic management emulating from this research should not be viewed in isolation, but considered alongside other contributing factors to help inform management decisions. Moreover, the maintenance of genetic diversity should become a fundamental component in long-term management strategies for threatened species in New Zealand.

2257: *+.191*

Population density affects dispersal success because residents can hinder or facilitate immigration into a new site, via a "social fence effect" or "social attraction" (or "conspecific attraction"), respectively. These mechanisms can affect the dynamics of fragmented populations and the success of translocations. However, information on the settlement behaviour of dispersers is rare. We conducted a manipulative field experiment using wild water voles, which exist in metapopulations along waterways in Scotland. We translocated 17 young of dispersal age into either an occupied site or a vacant site containing good habitat, which had recently become extinct due to a feral predator (American mink) moving through. We monitored the movements of translocated voles using radio telemetry. Translocated voles were less likely to settle in occupied sites with higher densities of residents, suggesting a possible social fence effect at high density. There was evidence of a social attraction mechanism, because voles never remained at new sites unless another individual arrived soon after translocation, and they were more likely to settle in occupied or colonised sites than vacant ones. Voles remained in the transient phase of dispersal for many days, and often followed a "stepping stone" trajectory, stopping for several days at successive sites. We suggest that trajectories followed by dispersing water voles, the time scale and long dispersal distances found in this species are conducive to locating conspecifics at low density and colonising vacant habitat. These results are encouraging for prospects of metapopulation persistence and future translocation success.

2258: *+.104*

Tropical dry forests are a unique and threatened ecosystem in the Pacific and globally. In Fiji, the endangered Fijian crested iguana (Brachylophus vitiensis) is endemic to tropical dry forests. Yadua Taba Island contains one of the best remaining stands of tropical dry forest in the Pacific along with the largest (and only secure) population of R. vitiensis in Fiji and has been proposed as a translocation source for iguana conservation. In this Study we determined the major vegetation types on Yadua Taba and identified forest habitat preferences of B. vitiensis to (1) characterize the island's habitats for tropical dry forest regeneration monitoring and (2) understand which forest types are preferred by iguanas for future translocation projects. Vegetation data were collected using reconnaissance, entitation, line transects, and aerial photos. Iguana abundance data were collected by nocturnal surveys of permanent transects. Six major vegetation types were identified of which tropical dry forest was the largest (46% of the island), followed by a combination of rocky cliff-shrubland/grassland vegetation (26%). Our conservative estimate of B. vitiensis population size on Yadua Taba is 12,000 Iguanas, the majority of which occur in tropical dry forest. Superabundance of the dry forest understory tree Vavaea amicorum, the favorite fruit species of iguanas, may help account for the high density of Iguanas observed. These results highlight the ecological link between tropical dry forest and B. vitiensis and emphasize the importance of rehabilitation or conservation of tropical dry forest habitat in potential Iguana

translocation sites as part of the management plan for B. vitiensis throughout the Fiji Islands.

2259: ***+.002***

Short-distance translocation (SDT) is commonly used to mitigate snake-human interactions, yet little is known about its effectiveness or its effects on behavior and welfare of snakes. Between April 2004 and October 2005, we evaluated SDT as a conservation and management tool by investigating how 500-m SDT affected spatial ecology, body condition, and behavior of western rattlesnakes (Crotalus oreganus) surgically implanted with radiotransmitters in a field study near Osoyoos, British Columbia, Canada. Of 14 rattlesnakes subjected to SDT, 12 (85.7%) returned on >= 1 occasion (range 1-7 times) to the general area they were removed from. Rattlesnakes that underwent SDT showed an increase in total distance moved over an active season compared to non-translocated snakes, but there was no evidence to suggest SDT had an effect on activity range size. There was no evidence to suggest SDT affected body condition, behavior, or mortality rates. Short-distance translocation to high-quality undisturbed habitats was unsuccessful as a long-term solution to snake-human conflict because most translocated snakes returned to conflict areas within a short time ((x) over bar 19.9 +/- 68.7 days). However, SDT may be an effective short-term tool to manage snake-human conflict in areas where human presence is seasonal or short-lived if careful attention is paid to species-specific biological needs, habitat quality at the release site, and the location of the release site in relation to conflict areas. (JOURNAL OF WILDLIFE MANAGEMENT 73(3):419-425;2009)

2260: ***+.271***

White-tailed Eagles Haliaeetus albicilla became extinct in Britain in 1918 following prolonged persecution. Intensive conservation efforts since the 1970s have included the re-introduction of the species to Britain through two phases of release of Norwegian fledglings in western Scotland in 1975-85 and 1993-98. Population growth and breeding success have been monitored closely to the present day, aided by the use of patagial tags to individually mark most released birds as well as a high proportion of wild-bred nestlings. This study reviews the growth and demography of this re-introduced population, and makes comparisons with other European populations. For the first time, we compare the demographic rates of released and wild-bred birds in the Scottish population. Breeding success in the Scottish population has increased over time as the average age and experience of individuals in the population have increased, and success tends to be higher where one or both adults are wild-bred. Current levels of breeding success remain low compared with some other populations in Europe, but similar to those in Norway where weather conditions and food availability are likely to be most similar. Survival rates in Scotland are similar to those recorded elsewhere, but survival rates of released birds are lower than those of wild-bred birds, especially during the first 3 years of life. Despite the effect of lower survival rates of released birds in limiting overall population growth rate, the recent rate of growth of the Scottish population remains high relative to other recovering populations across Europe. Differences in demographic rates of wild-bred and released birds suggest that in future re-introduction programmes, steps to maximize the success and output of the earliest breeding attempts would help ensure the most rapid shift to a population composed largely of wild-bred birds, which should then have a higher rate of increase.

2261: ***+.004***

Upper respiratory tract disease (URTD) caused by Mycoplasma agassizii has been hypothesized to contribute to the decline of some wild populations of gopher tortoises (Gopherus polyphemus).

However, the force of infection (FOI) and the effect of URTD on survival in free-ranging tortoise populations remain unknown. Using four years (2003-2006) of mark-recapture and epidemiological data collected from 10 populations of gopher tortoises in central Florida, USA, we estimated the FOI (probability per year of a susceptible tortoise becoming infected) and the effect of URTD (i.e., seropositivity to M. agassizii) on apparent survival rates. Sites with high (>= 25%) seroprevalence had substantially higher FOI (0.22 +/- 0.03; mean +/- SE) than low (<25%) seroprevalence sites (0.04 +/- 0.01). Our results provide the first quantitative evidence that the rate of transmission of M. agassizii is directly related to the seroprevalence of the population. Seropositive tortoises had higher apparent survival (0.99 +/- 0.0001) than seronegatives (0.88 +/- 0.03), possibly because seropositive tortoises represent individuals that survived the initial infection, developed chronic disease, and experienced lower mortality during the four-year span of our study. However, two lines of evidence suggested possible effects of mycoplasmal URTD on tortoise survival. First, one plausible model suggested that susceptible (seronegative) tortoises in high seroprevalence sites had lower apparent survival rates than did susceptible tortoises in low seroprevalence sites, indicating a possible acute effect of infection. Second, the number of dead tortoise remains detected during annual site surveys increased significantly with increasing site seroprevalence, from; 1 to; 5 shell remains per 100 individuals. If (as our results suggest) URTD in fact reduces adult survival, it could adversely influence the population dynamics and persistence of this late-maturing, long-lived species.

2262: *+.214*

In order to confirm the persistence of a Fisher (Manes perinatal) population reintroduced in 1990 in Cooking Lake-Blackfoot Provincial Recreation Area. central Alberta, we inventoried trails in the winters of 2006 and 2007. We recorded the presence of Fishers in 16 locations, and we confirmed the presence of at least two animals. We believe that the presence of Fishers 17 years after their release in the recreation area indicates that there is a self-sustaining population.

2263: *+.068*

Invasive species are an important driver of global biodiversity loss. Under international legislation, the UK has an obligation to eradicate or to control the alien, invasive American mink. Using a large-scale field experiment, we tested the effectiveness of a specified mink removal strategy, identified through earlier modelling work, in reducing the relative abundance of mink. We found that mink removal could be effective in reducing mink populations with four months or less of trapping per year, over only 2-3 years, but that for small sites (c. 20 km) a flexible, reactive approach, coupled with continual monitoring for mink presence is necessary. Survival of reintroduced water voles at four sub-sites within our mink removal sites suggest that the reactive mink removal strategy adopted in this study was sufficient for water vole protection. We discuss the use of an adaptive management approach in local mink management, and consider the wider implications of our results for invasive species control on mainlands. (C) 2008 Elsevier Ltd. All rights reserved.

2264: *+.131*

Conservation managers are in the unenviable position of trying to conserve and restore biodiversity, without having a definitive timeframe to restore it to. Currently, managers around the world focus on various timeframes from recent to historical, but without a definitive target, countless conservation problems arise. Managers need to determine what constitutes a native species, which species to reintroduce, whether selective breeding should be implemented to

resurrect supposedly extinct organisms, targets on population levels, whether assisted migration should be employed when climate change alters the environmental envelope of a species surrounded by human-altered landscapes, and how to manage for stochasticity and evolutionary processes. Without having definitive goals to target, these issues are difficult/impossible to address. It is only by discussing these important issues that some consensus will be attained that allow us to stop responding to crises and start predicting the future of biodiversity and plan and respond accordingly.

2265: *+.095*

Expected consequences of global warming include habitat reduction in many cool climate species. Rock ptarmigan is a Holarctic grouse that inhabits arctic and alpine tundra. In Europe, the Pyrenean ptarmigan inhabits the southern edge of the species' range and since the last glacial maximum its habitat has been severely fragmented and is restricted to high-alpine zones or 'sky islands'. A recent study of rock ptarmigan population genetic in Europe found that the Pyrenean ptarmigan had very low genetic diversity compared with that found in the Alps and Scandinavia. Habitat fragmentation and reduced genetic diversity raises concerns about the viability of ptarmigan populations in the Pyrenees. However, information on population structuring and gene flow across the Pyrenees, which is essential for designing a sound management plan, is absent. In this study, we use seven microsatellites and mitochondrial control region sequences to investigate genetic variation and differentiation among five localities across the Pyrenees. Our analyses reveal the presence of genetic differentiation among all five localities and a significant isolation-by-distance effect that is likely the result of short dispersal distances and high natal and breeding philopatry of Pyrenean ptarmigan coupled with severe habitat fragmentation. Furthermore, analysis of molecular variance, principal component analysis and Bayesian analysis of genetic structuring identified the greatest amount of differentiation between the eastern and main parts of the Pyrenean chain separated by the Segre Valley. Our data also show that the Canigou massif may host an isolated population and requires special conservation attention. We propose a management plan which includes the translocation of birds. If a sky island structure affects genetic divergence in rock ptarmigan, it may also affect the genetic structure of other sky island species having low dispersal abilities.

2266: *+.002*

Knowledge about the phylogenetic history, genetic variation and ecological requirements of a species is important for its conservation and management. Unfortunately, for many species this information is lacking. Here we use multiple approaches (phylogenetics, population genetics and ecological modelling) to evaluate the evolutionary history and conservation status of Capra walie, an endangered flagship species of wild goat endemic to Ethiopia. The analysis of mitochondrial cytochrome b and Y-chromosome DNA sequences suggests that C. walie forms a monophyletic clade with Capra nubiana, but potentially has been isolated for up to 0.8 million years from this closely related species. Microsatellite DNA analyses show that C. walie has very low genetic variation (mean heterozygosity=0.35) compared with other endangered mammals. This reduced variation likely derives from a prolonged demographic decline and small effective population size. Ecological niche modelling using the bioclimatic features of habitats occupied by C. walie, suggests ecological differences between C. walie and C. nubiana, and identifies the areas most suitable for future reintroductions of C. walie. The genetic and bioclimatic data suggest that C. walie is distinct and requires immediate conservation actions including genetic monitoring and reintroductions to establish independent populations. This study illustrates how combining noninvasive sampling along with genetic and ecological (bioclimatic) approaches can help assess

conservation status of poorly known species.

2267: ***+.150***

This study presents an assessment of the establishment success of smallmouth yellowfish, Labeobarbus aeneus (Burchell 1822), in the Great Fish River (GFRI), where they were introduced by the Orange-Fish River interbasin water transfer scheme and in the Glen Melville Reservoir (GMR), where the species was subsequently introduced from the GFRI. Edge Analysis of asteriscii validated an annual deposition rate of one growth increment per year and sampled fish were aged at between 0 and 10 years old. Combined sex length-at-age (L) was described by L(t) = 650(l - e(-0.066(t + 4.22))) mrn FL for GMR and L(t) = 498 (l - e(-0.23(t + 0.373))) mm FL for GFRI. Natural mortality rate was estimated at 0.56/yr in GFRI and 0.96/yr in GMR. Condition factor was significantly higher in the GFRI than in GMR. In comparison with other populations, the GFRI population had reproductive and growth traits that were similar to those in its natural riverine range. Reproductive assessment showed ripe male and female fish in October to December in the GFRI, while in the GMR, ripe male fish but few ripe female fish were recorded. This was attributed to the lack of spawning cues in the reservoir. As a result, L. aeneus were considered established in the GFRI, but were not established and likely dependent on introductions in the GMR.

2268: ***-.016***

The Bali mynah Species Survival Plan (SSP (R)), an Association of Zoos and Aquariums program, strives to maintain the genetic and demographic health of its Population, avoid unplanned changes in size, and minimize the risk of Population extinction. The SSP Population meets current demographic and genetic objectives with a Population size of 209 birds at 61 institutions and 96%,, genetic diversity (GD) retained from the Source Population. However. participating institution have expressed concerns regarding space allocation, target Population size (TPS). breeding restrictions, inbreeding depression. and harvest in relation to future Population availability and viability. Based oil these factors. we assess five questions with a quantitative risk assessment, specifically a population viability analysis (PVA) using ZooRisk software. Using In individual-based stochastic model, we project potential population changes under different conditions (e.g. changes in TPS and genetic management) to identify the most effective management actions. Our projections indicate that Under current management conditions, population decline and extinction are Unlikely and that although GD will decline over 100 years the projected loss does not exceed levels acceptable to Population managers (less than 90% GD retained). Model simulations indicate that the combination of two genetic management strategies (i.e. priority breeding based oil mean kinship and inbreeding avoidance) benefits the retention of GD and reduces the accumulation of inbreeding. The Current TPS (250) is greater than necessary to minimize the risk of extinction for the SSP Population but any reduction in TPS must be accompanied by continued application of genetic management. If carefully planned, birds can be harvested for transfer to Bali for a reintroduction program without jeopardizing the SSP population . Zoo Biol 28:230-252, 2009. (C) 2009 Wiley-Liss. Inc.

2269: ***+.214***

White-tailed deer abundance has been determined at local scales, reducing its uses for management and conservation at the landscape-level. Our objective was to determine if normalized difference vegetation index (NDVI) is a predictor of white-tailed deer abundance. Field data was generated for two areas, one temperate and one tropical, the first one in Sierra de

San Luis, municipality of Agua Prieta, Sonora and the second in Sierra Los Pavos, municipality of Sahuaripa, Sonora. Density of deer was estimated using pellet count surveys. We found a significant relationship between NDVI and density in the temperate site, but a non-significant one at the tropical area. This difference appears to be attributed to anthropogenic differences and not biotic relationships. This index appears useful to determine reintroduction sites in temperate areas of northeastern Mexico.

2270: *+.141*

Migration barriers are a major reason for species loss and population decline of freshwater organisms. Significant efforts have been made to remove or provide passage around these barriers; however, our understanding of the ecological effects of these efforts is minimal. Installation of a fish passage facility at the Landsburg Dam, WA, USA provided migratory fish access to habitat from which they had been excluded for over 100 years. Relying on voluntary recruitment, we examined the effectiveness of this facility in restoring coho (Oncorhynchus kisutch) salmon populations above the diversion, and whether reintroduction of native anadromous species affected the distribution and abundance of resident trout (O. mykiss and O. clarki). Before the ladder, late summer total salmonid (trout only) density increased with distance from the dam. This pattern was reversed after the ladder was opened, as total salmonid density (salmon + trout) approximately doubled in the three reaches closest to the dam. These changes were primarily due to the addition of coho, but small trout density also increased in lower reaches and decreased in upper reaches. A nearby source population, dispersal by adults and juveniles, low density of resident trout and high quality habitat above the barrier likely promoted rapid colonization of targeted species. Our results suggest that barrier removal creates an opportunity for migratory species to re-establish populations leading to range expansion and potentially to increased population size. Copyright (C) 2008 John Wiley & Sons, Ltd.

2271: *+.289*

Animal movement and habitat selection behavior are important considerations in ecology, and remain a major issue for successful animal reintroductions. However, simple rules are often used to model movement or focus only on intrinsic environmental cues, neglecting recent insights in behavioral ecology on habitat selection processes. In particular, social information has been proposed as a widespread source of information for habitat evaluation. We investigated the role of explicit breeding habitat selection strategies on the establishment pattern of reintroduced populations and their persistence. We considered local movement at the scale of a single population. We constructed a spatially-implicit demographic model that considered five breeding habitat selection rules: 1) random, 2) intrinsic habitat quality, 3) avoidance of conspecifics, 4) presence of conspecifics and 5) reproductive success of conspecifics. The impact of breeding habitat selection was examined for different release methods under various levels of environmental heterogeneity levels, for both long and short-lived monogamous species. When heterogeneity between intrinsic habitat patch qualities is high, the persistence of reintroduced populations strongly depends on habitat selection strategies. Strategies based on intrinsic quality and conspecific reproductive success lead to a lower reintroduction failure risk than random, conspecific presence or avoidance-based strategies. Conspecific presence or avoidance-based strategies may aggregate individuals in suboptimal habitats. The release of adults seems to be more efficient independent of habitat selection strategy. We emphasize the crucial role of oriented habitat selection behavior and non ideal habitat selection in movement modeling, particularly for reintroduction.

2272: ***+.209***

Surprisingly few faunal taxa worldwide have experienced an improvement in conservation status through direct conservation action. One of the few is Gould's Petrel (Pterodroma leucoptera leucoptera) - a threatened species that breeds only in New South Wales. In the early 1990s, the breeding population of this subspecies was small (<250 pairs) and declining. Each year, adult mortality at the breeding grounds exceeded the number of young produced. A recovery programme, focused on reducing adult mortality, commenced in 1993. As a result of the recovery actions undertaken, the Gould's Petrel is now increasing in numbers. Also, the rainforest where this seabird breeds is now regenerating after being degraded for almost a century by the introduced European Rabbit (Oryctolagus cuniculus). The creation of artificial nesting habitat and the development of effective translocation procedures have led to the establishment of a second breeding colony of Gould's Petrel, further reducing the risk of extinction. In this paper, we explore the key elements we believe to be responsible for the success of this particular recovery programme: (i) a strong underpinning of robust ecological research; (ii) adaptive management; (iii) monitoring and reporting; (iv) a multidisciplinary approach; and (v) a willingness to accept risk. We conclude with some suggestions to improve the current recovery planning process.

2273: ***+.122***

The cryptic Eastern Bristlebird (Dasyomis brachypterus) is an endangered endemic of south-eastern Australia. Its distribution is highly fragmented with only two populations exceeding 500 individuals. Consequently, recovery planning includes translocation to increase the number of viable populations. The Eastern Bristlebird is typically found in low, dense vegetation. The species occurs in 26 different plant communities throughout its range, which suggests that it might be considered a habitat generalist. However, two studies based on aural surveys have demonstrated that it was conspicuous at heath-wood ecotones. Radiotracking was used to overcome reliance on aural surveys and to investigate the habitat of 12 Eastern Bristlebirds at 50-m wide heath-wood ecotones in two sites at Jervis Bay. Although individual birds appeared either to prefer or avoid the heath, ecotone or wood, there was no consistent pattern of habitat selection and there was no attraction to, or avoidance of, the heath-wood edge at species level. The present study provides further evidence that although heath-wood ecotones may provide suitable habitat for some individual Eastern Bristlebirds, the species is neither dependent on, nor confined to, heath-wood ecotones. This knowledge was an important consideration in the selection of two host sites for recently conducted reintroductions of the species.

2274: ***+.031***

To relate the recent Iberian lynx decline to changes in the distribution of the European rabbit after the haemorrhagic disease outbreak of 1989. As Iberian rabbits evolved in two geographically separated lineages, being the recent lynx range practically restricted to the southwestern lineage, we also test if differential range dynamics exists for these lineages, with the consequent implications for lynx conservation and reintroduction planning. The Iberian Peninsula. We modelled environmental favourability for the lynx based on its distribution before 1989, and for the rabbit using distribution data collected primarily after 1989, and validated them using independent abundance data. We compared both models and combined them in a lynx occurrence forecast. We correlated the prevalence of southwestern rabbit lineage with the environmental favourability for the rabbit. The environmental lynx model correlated with past lynx abundance data, but did not reflect its recent strong range contraction. The rabbit model correlated with recent rabbit abundance, but was negatively correlated with the environmental model for the lynx. The

combination of both models forecasted lynx occurrence in a few separated nuclei, which encompass all recent lynx records. The prevalence of rabbit's southwestern lineage correlated negatively with favourability for the rabbit. The region to which the lynx became confined before 1989 is currently less favourable for rabbits, whereas more favourable areas remain outside lynx reach. This differential favourability correlates with rabbit phylogeographical structure, suggesting that the southwestern lineage is facing more unfavourable conditions or is less resilient to recent diseases. The loss of concordance between lynx distribution and the whole rabbit phylogeographical structure has prevented lynx persistence in northeastern rabbit lineage areas, which should be considered in lynx reintroduction planning. Similar conservation problems could affect other ecologically interacting species whose distributions' overlapping has sharply diminished.

2275: *+.180*

Alligator snapping turtle (Macrochelys temminckii) populations have declined across much of the southeastern United States in recent decades, due at least in part to overcollection. Recently, however, legal protection from large-scale harvesting has been granted to the species in all states where it is native, thereby drastically reducing one of the greatest threats to its survival. There is growing interest in captive propagation of alligator snapping turtles for reintroduction where populations have been decimated. In conjunction with one such effort, we analyzed the physiological effects of temperature on embryonic and posthatching development. Results indicate that extreme high and low incubation temperatures negatively affected embryo survival, and high incubation temperatures corresponded with shorter incubation time but also produced smaller hatchlings. The effects of temperature on gonadal differentiation indicated that the upper pivotal temperature was approximately 27.5 degrees C. Posthatching growth was faster at warmer water temperatures, and there was little to no acclimation of metabolic rate to exposure to either incubation or water temperature. We conclude that intermediate (27.5 degrees-28.5 degrees C) incubation temperatures produce a female-biased mixed sex ratio and maximize hatching success and hatchling size while increasing incubation duration only slightly over that at the higher temperatures. In addition, posthatching growth was positively influenced by hatchling body temperature; therefore, warmer water temperatures (similar to 30 degrees C) decreased the time required to rear turtles to a size suitable for reintroduction.

2276: *+.045*

This study evaluates the potential impacts of the release of the giant tortoise, Dipsochelys arnoldi, to vacant habitat within the species' presumed historic range. Five individuals (3 males and 2 females) were released in December 2006 in the isolated Grande Barbe area of Silhouette Island, Seychelles. A comprehensive vegetation survey of all plant species within feeding height of the tortoises in Grande Barbe was conducted. These data, combined with daily feeding observations, were used to calculate feeding rates and diet preferences. Tortoises were observed to have a mean diurnal active time of 257 minutes per day exhibiting a mean feeding rate of 3.86 g of plant material consumed per minute of active time. Individuals were recorded feeding on 18 of 26 recorded plant species, 9 of which were selectively foraged. Species composition of plant species ingested differed between sexes. Males selectively foraged on 6 plant species and females selectively foraged on 7 species. Only 2 plant species were selectively foraged by both sexes.

2277: *+.175*

The use of surrogate species in conservation planning has been applied with disappointing results

on relatively large sets of species. It could still prove useful for optimizing conservation efforts when considering a small set of species with similar ecological requirements, however few field tests of this nature have been carried out. The aim of this research is to compare the response of three arboreal rodent species-the fat dormouse (Glis glis), the hazel dormouse (Muscardinus avellanarius) and the red squirrel (Sciurus vulgaris)-to habitat loss and fragmentation, with the aim of identifying priorities for conservation and evaluating possible optimization of conservation efforts under different scenarios: habitat restoration and selection of focal patches. We studied the distribution of the three species in a sample of patches in a highly fragmented landscape in central Italy, using a patch-landscape scale approach. The distribution was studied by using hair tubes, nestboxes and nocturnal surveys. The three species showed analogous responses to increasing isolation and decreasing size of habitat patches; what differed however, was the magnitude of responses. Our results show possible application of surrogacy within this restricted group of species, however several caveats arise depending on the conservation strategy and available funding. If habitat restoration is the objective, then the fat dormouse should be the target species for guiding size and isolation of patches. On the other hand, the magnitude of the differences and patch requirements for this species, question the feasibility of these conservation actions. If selection of focal patches for conservation is the objective then selecting the fat dormouse as a focal/umbrella species would overlook areas suitable for the other two species. Feasible optimisation of conservation efforts may be possible only between the red squirrel and the hazel dormouse.

2278: ***+.021***

Using a nearly range-wide sampling, we investigated phylogeographic differentiation and mitochondrial diversity of Testudo horsfieldii, the only tortoise species confined to Central Asia. We identified three major haplotype clades with mainly parapatric distribution that do not correspond well to the currently recognized three subspecies. One clade is restricted to the Fergana Valley and seems to represent a previously overlooked evolutionarily significant unit. Another clade, consisting of several largely parapatrically distributed haplotypes, occurs in the north and the central southern part of the species' range. The third clade, likewise comprising several largely parapatrically distributed haplotypes, was identified from the south-eastern corner of the Caspian Sea in the west, from Afghanistan and Pakistan in the east and from two more northerly sites in western and south-eastern Uzbekistan. It is possible that this clade also occurs in eastern Turkmenistan and adjacent Afghanistan, regions not sampled for the present study. The generally parapatric distribution of individual haplotypes, even within each of the three major clades, suggests advanced lineage sorting, either due to limited dispersal abilities, glacial isolation in distinct local microrefuges or both acting in accord. The localized distribution of endemic haplotypes in the northern and central plains as well as in the mountainous eastern and southern parts of the distribution range supports the existence of multiple microrefuges there. Records of haplotypes of distinct clades in sympatry or close geographic proximity are likely the result of Holocene range expansions. In recent years, thousands of confiscated steppe tortoises were released into the wild. The detected mitochondrial differentiation offers a powerful tool for nature conservation, as a means of determining the geographic origin of confiscated tortoises and selecting suitable reintroduction regions.

2279: ***-.243***

Coelomactra antiquata is a commercially important bivalve species, but has been suffering from severe population decline due to over-exploitation and the deterioration of environmental conditions. Previous genetic survey of C. antiquata conducted with allozymes combined with

morphology revealed high levels of genetic differentiation between northern and southern populations which suggests a cryptic species might exist in C. antiquata. To test this hypothesis, amplified fragment length polymorphisms (AFLPs) and 16S rRNA gene sequence were used to re-evaluate the spatial genetic structure of six populations of C. antiquata along the coast of China. Both genetic markers display a sharp genetic break between the four northern populations (northern lineage) and two southern population (southern lineage). Large numbers of private alleles (AFLP) were found within the northern or southern populations and a deep divergence of about 6.5% in 16S rRNA gene sequence between the northern and southern lineages suggests the occurrence of potential cryptic or sibling species of C. antiquata. Applying previously published rates of mutation, divergence between the two lineages is estimated to have occurred approximately 3 million years ago and may be due to allopatric isolation during the middle Pliocene times. While no genetic differentiation was found within the northern or southern populations in both AFLP and 16S mtDNA markers, the results indicate that the northern and southern lineage should be managed separately and any translocation between the two areas should be avoided.

2280: ***+.246***

The release program of European Roe Deer Capreolus capreolus was launched in 1989 when the Royal Society for the Conservation of Nature (RSCN) received a donation of four individuals (two males and two females) from Turkish-Bulgarian border. The aim of the program was to establish a viable population in the former habitat of the species. The program was ongoing for almost 18 years at Ajloun Forest Reserve. On 19 January 2006, 26 individuals (11 males and 15 females) were released in the reserve. After collecting information relating to the breeding and release program, a rapid assessment survey was conducted during August and September 2007. Based on visual census, spoor routes, opportunistic observations and interviews method, a distribution map was produced and it was found that the released population had dispersed inside the reserve. Occasionally, they were found to have wandered outside the reserve, where they used surrounding vineyards for feeding and resting; a total of four individuals of two males and two females were counted. The RSCN has succeeded in establishing a free viable population, in its natural habitat, using effective long-term on-site management. It would be recommendable to establish a socio-economic program, and ongoing outreach programs in order to raise the awareness of the importance of protecting endangered species, and secure continuous funding. a further recommendation would be to provide training in deer management for the ecologist and further staff on the reserve and to implement a monitoring program for the released herd in order to insure the success of the reintroduction program.

2281: ***-.062***

The use of captive rearing to promote recovery of endangered butterflies has substantially increased over the last decade. These programs have the potential to play a significant role in butterfly population recovery, but the effects of captive conditions are poorly understood and rarely are traits of captive individuals assessed relative to traits in their founding populations. To develop rearing protocols and investigate possible effects of captive conditions, we reared Puget blue butterflies (Icaricia icarioides blackmorei), a subspecies closely related to the endangered Fender's blue (I. i. fenderi) which is limited to Oregon, USA. We reared individuals from two wild populations in Washington, USA to investigate two approaches for egg collection (collect eggs in the wild vs. collect eggs from adult females which were brought to a greenhouse for oviposition) and three diapause environments (in indoor facilities at two independent locations vs. outdoors in enclosures). Survival from egg to adult was similar across all captive groups which survived past

diapause and was less than 10%. Captive reared individuals were lighter and had smaller wings and shorter body lengths than their founding populations for both sites. Based on our findings, we recommend that rearing programs compare characteristics of reared individuals to individuals from the founding population to quantify possible effects of captive conditions, diapause individuals in natural environments, and for programs with survival rates similar to rates in the wild, consider alternatives to augment declining populations and reintroduce historic ones.

2282: ***+.203***

There have been numerous transfers of the large-bodied orthopteran, the Mahoenui giant weta (Family Anostostomatidae: Deinacrida mahoenui), over the past 19 years but there has been limited follow-up monitoring to ascertain establishment and breeding of transferred populations. Recent surveys carried out at all the locations where this weta were transferred, found weta at four of the seven transferred sites. The most important factor determining the success of past transfers is the absence of introduced mammalian predators, particularly rats, at a site. At two sites, Mahurangi Island Scenic Reserve and Warrenheip, weta appeared to be flourishing and have successfully established new populations in the absence of rats. If mammals are present at a site, the occurrence of dense prickly gorse to protect giant weta from predation is another important factor in their survival. Weta were found at very low densities (only single specimens were captured) at Mangaokewa Scenic Reserve and Tikikaru (private land) and it is likely their populations are not viable in the long term. Further efforts to establish Mahoenui giant weta populations should be in mammal-free sanctuaries containing native forest. More intensive post-transfer monitoring using radio-transmitters would enable better understanding of their behaviour after transfer. In addition, long-term regular monitoring of transferred populations is required, particularly those where the likelihood of rat re-invasion is high.

2283: ***+.001***

Since 1996, The Peregrine Fund has released California condors (Gymnogyps californianus) in the Grand Canyon region of northern Arizona with the goal of establishing a self-sustaining population, disjunct from other released populations in California and Baja California. A free-ranging population of more than 60 individuals now ranges within northern Arizona and southern Utah and has produced 9 wild young. The most frequent cause of death is lead poisoning from the ingestion of lead bullet fragments and shotgun pellets in the remains of gun-killed animals. In response, the Arizona Game and Fish Department has effectively reduced lead occurrence within the foraging range of the condors through hunter education and the promotion of nonlead ammunition. Most hunters have participated in the program. Throughout the course of the reintroduction effort, veterinary science and application have played essential roles in diagnosing fatalities and treating lead-exposed condors, a species with such a low natural reproductive rate that every adult is significant to the population.

2284: ***+.009***

While freshwater systems provide important goods and services for society, they are threatened by human activity. Fragmentation is one of the most serious ecological concerns in the riverine environment. Historical and cultural values may conflict with nature restoration. Here we use the Zwalm sub-basin (Scheldt basin, Belgium) as a case study for reconciling the restoration of the native fish fauna with the preservation of historical water mills (320-1000 years old). We assessed the genetic structure of a barrier-sensitive species, the three-spined stickleback Gasterosteus aculeatus, to estimate the impact of fragmentation on a local to catchment scale. We show how

population genetic approaches may be used to generate guidelines for restoration and management, and advance the science of river restoration. Dispersal was lower in above- than in below-mill populations, and water mills provoked an average loss of almost 4% of the genetic variation. This loss accumulated to 40% over the entire system (similar to 23 km, 13 barriers). The impact of individual mills strongly increased with upstream distance and water mill height. One mill provoked significant genetic differentiation, despite the presence of a fish passage. This detailed picture of the genetic connectivity in stickleback is indicative for the basin's depauperate fauna. Many species share the same migratory pathways and barriers to dispersal. The physical properties of the water mills are likely to have similar effects on species with a similar genetic structure to stickleback. Synthesis and applications. Population genetic studies may be particularly useful during the planning of river restoration and associated ecological studies. In the case of the Zwalm sub-basin, we propose a number of management actions, such as building new fish passages and translocating individuals to above-mill populations. These will counter the negative impact of the water mills on the genetic variation in aquatic fauna, whilst retaining their cultural-economical value and limiting the restoration costs. Simulations suggest that reassessment of stickleback genetic structure after a decade should reveal whether or not restoration actions have been effective.

2285: ***+.285***

Burbot Lota lota are of global conservation concern particularly near the southern extent of the species range. Populations in small streams lack quantitative descriptions of resource selection. Microhabitat and reach-scale habitat were surveyed at occupied and unoccupied locations. Data were analyzed with logistic regression to develop resource selection function models which predicted the probability of occurrence and abundance categories for microhabitat and reach-scale habitat, respectively. Information-theoretic model selection methods were used in an exploratory context, and four competing models were multi-model averaged to create a microhabitat resource selection function. The sum of Akaike's Information Criterion weights across the four competing models indicated that substrate type, substrate embeddedness, and depth were equally the most important microhabitat explanatory variables. At the reach scale, high correlation among explanatory variables precluded multivariate model development. Stream-reach gradient was the most supported univariate model. Three additional models competed as important; mean summer temperature, sinuosity, and dominant substrate type. The created resource selection functions can be used in ranking potential translocation sites, used in instream flow assessments, and provide habitat selection information that can provide context for habitat management decisions.

2286: ***+.069***

Forest canopy and subcanopy data were collected from and compared among five disjunct bottomland hardwood forests in the Lower Mississippi Alluvial Valley, each with known occurrence of a population of the federally endangered shrub Lindera melissifolia. All study sites are cut-over forests, underlain by hydric soils, and have a seasonal high water table. Canopy and subcanopy species are similar among sites, but species differ in relative importance, and flood tolerant tree species exceed that of flood intolerant species. Distribution of L. melissifolia colonies within each study site was not associated with mean tree density or d.b.h. Forest composition and structure at each study site reflect hydrologic regime, topography, historical disturbance, and an absence of recent disturbance. Results of this study provide a quantitative description of bottomland forests that currently sustain L. melissifolia populations. This information may be utilized for development of forest management plans aimed at ensuring continued sustainability of existing L. melissifolia populations and assessing other bottomland hardwood forests for potential

reintroduction of this endangered species.

2287: ***-.253***

Translocation and reintroduction have become major conservation actions in attempts to create self-sustaining wild populations of threatened species. However, avian translocations have a high failure rate and causes for failure are poorly understood. While 'stress' is often cited as an important factor in translocation failure, empirical evidence of physiological stress is lacking. Here we show that experimental translocation leads to changes in the physiological stress response in chukar partridge, Alectoris chukar. We found that capture alone significantly decreased the acute glucocorticoid (corticosterone, CORT) response, but adding exposure to captivity and transport further altered the stress response axis (the hypothalamic-pituitary-adrenal axis) as evident from a decreased sensitivity of the negative feedback system. Animals that were exposed to the entire translocation procedure, in addition to the reduced acute stress response and disrupted negative feedback, had significantly lower baseline CORT concentrations and significantly reduced body weight. These data indicate that translocation alters stress physiology and that chronic stress is potentially a major factor in translocation failure. Under current practices, the restoration of threatened species through translocation may unwittingly depend on the success of chronically stressed individuals. This conclusion emphasizes the need for understanding and alleviating translocation-induced chronic stress in order to use most effectively this important conservation tool.

2288: ***+.352***

Knowledge about reproduction is critical for predicting the viability of wildlife populations in nature and for managing breeding programmes in captivity. Intensive species-based studies are the priority, because reproductive mechanisms are extraordinarily diverse, even within the same taxonomic family. Carnivores deserve more attention as such species are highly vulnerable to environmental change and human persecution. The present review provides contemporary illustrations of how reproductive science is contributing to understand unique reproductive mechanisms that are both of fundamental and applied interest. In the case of the endangered African wild dog (Lycaon pictus) free-living in South Africa, non-invasive faecal corticosteroid assessments have yielded new insights about the impact of animal relocation and reintroduction on adaptive responses, reproductive fitness and survival. For the maned wolf (Chrysocyon brachyurus), advances have been made in characterizing and comparing reproductive traits in free-ranging vs captive individuals. For the cheetah (Acinonyx jubatus), recent studies have focused on the cryosensitivity of sperm and the ability to develop a field-friendly sperm cryo-method. The by-product has been a large-scale frozen repository of sperm from wild-caught cheetahs useful for infusing new genes into ex situ populations. Finally, rigorous, multi-disciplinary and cross-institutional reproductive studies of the black-footed ferret (Mustela nigripes), including the use of artificial insemination, have contributed to the remarkable recovery and restoration of this species, once on the brink of extinction. In summary, advances in reproductive science are not necessarily related to 'assisted breeding'. However, understanding the unique ways of carnivore reproduction greatly contributes to species management and conservation.

2289: ***+.164***

Relocating populations of philopatric turtle species is a relatively rare method used for species conservation that has had equivocal results. The purpose of this study is to report On the Status of a population of ornate box turtles, Terrapene ornata ornata Agassiz, that Was introduced into a

restored tallgrass prairie habitat 160 km from its native location. Front 1998-2003. a total of 56 individual turtles and one nest were collected from their natal habitat and were released into either a 5-ha or 1.75-ha enclosure. Most of the turtles were scute marked prior to release to facilitate recognition of specific individuals. In 2006-07. we resampled the population to determine if any turtles Survived the relocation, if any local reproduction could he detected, and if specific microhabitats were being selected. We captured 14 T. ornata ornata, 11 of which were definitively Pall Of the Original turtle introduction. Three juvenile turtles were found and aged to be 5 years, suggesting local reproduction is Occurring oil site. Finally. although some turtles move through the prairie habitat throughout the fall Season. Only a small sandy microhabitat was used for hibernating. Our results suggest that relocating ornate box turtle populations to avoid mortality can be used as a Successful conservation practice provided that the individuals are first released into a confinement to restrict their philopatric instincts.

2290: *+.053*

Estimating abundance of carnivore populations is problematic because individuals typically are elusive, nocturnal, and dispersed across the landscape. Rare or endangered carnivore populations are even more difficult to estimate because of small sample sizes. Considering behavioral ecology of the target species can drastically improve survey efficiency and effectiveness. Previously, abundance of the black-footed ferret (Mustela nigripes) was monitored by spotlighting and generating indices of relative abundance because reintroduced populations were slow to establish. Indices, however, lack variance estimates and are costly to generate for the black-footed ferret. We therefore used spotlight surveys and live-trapping in conjunction with a robust mark-recapture estimator to improve abundance monitoring for the black-footed ferret, one of North America's most endangered carnivores. We estimated abundance of the black-footed ferret at Shirley Basin, Wyoming, USA, using correlated density estimates and Program MARK. We compared our results to 2 indices of relative abundance, minimum number alive and predicted number of ferrets from litter counts. The correlated density estimate for the black-footed ferret ((N) over cap (R) 229; 95% CI = 161-298) was similar to minimum number alive ((N) over cap 192) and predicted number of ferrets from litter counts ((N) over cap 235). The efficiency and effectiveness of survey methods we used for the black-footed ferret were high by carnivoran standards. Our results suggest that the sampling approach we utilized can be implemented for a fraction of the cost and effort required to generate 2 indices of relative abundance for the black-footed ferret. Although we recommend managers implement a similar survey approach to monitor abundance of reintroduced populations of the black-footed ferret, analysis with sparse data sets will be problematic. Until the black-footed ferret becomes widespread and abundant at a reintroduction site, spotlighting will remain preferable as a means to generate indices of distribution and relative abundance for the black-footed ferret. (JOURNAL OF WILDLIFE MANAGEMENT 73(5): 669-676; 2009)

2291: *+.270*

Using data from 396 breeding attempts over an 8-year period, we investigated age- and stage-specific survival rates and their modifying factors in a closed island population of the New Zealand stitchbird (or hihi, Notiomystis cincta Du Bus). Survival probability generally increased over time; however, at each life-history transition, survival in the new stage started lower than at the end of the previous stage, creating a 'saw-tooth' function of age-related survival. The probability of an egg hatching was low (0.73 +/- 0.01): most likely a consequence of genetic bottlenecks previously endured by this population. There was strong support for a positive relationship between hatching rate and the subsequent survival of the female parent, and hatching success declining for females > 4 years old. Nestling survival probability increased as a function

of brood size and days since hatching, and decreased relative to daily maximum ambient temperature and hatching date. Support for models including ambient temperature was greater than for other covariates, with the majority of this temperature-mediated survival effect being restricted to the early nestling stage. Fledglings had low survival rates in the first two weeks after leaving the nest, with post-fledging survival related to the fledgling's mass. Two months after fledging, juvenile survival probability plateaued and remained relatively constant for the following autumn, winter and spring/summer breeding season. There was no effect of sex or season on adult survival probability. However, there was strong support for age-specific variation in adult survival, with survival likelihood increasing during the first four years before showing evidence of a senescence decline.Within-stage survival increases were likely related to stage-specific selection pressures initially weeding out individuals of poorer phenotypes for the environment specific to each life-history stage. Such a mechanism explains the initial high mortality at life-history transitions; a well-adapted phenotype for one stage may not necessarily be so well adapted for subsequent stages. These patterns are not only valuable for examining life-history theory, but also for understanding the regulation of vital rates in an endangered species and providing a basis from which better population management models and harvesting regimes can be derived.

2292: ***+.261***

Within the monitoring plan of guanaco (Lama guanicoe) reintroduction in "Quebrada del Condorito" National Park (Cordoba, Argentina), we registered the behaviour of the translocated animals in March 2007. These were obtained from the search, detection and scan-sampling of the first group translocated. From the fourteen detected behaviours during the daytime activity, the most important was the feeding (60%). During autumn and winter, the invested time in this behaviour was more important than in spring and summer. During spring, the guanacos spent less time foraging, and increased the time they spent laid down on the ground. The invested time in behaviour by this monitored group was as expected for this species. Between the main monitored behaviours in presence of human beings, the watching and escape were the most important ones. This happened mostly with people riding horses (between 100 and 500 meters from the animals).

2293: ***+.125***

The crested ibis (Nipponia nippon) had declined severely from a common species to only two pairs in last century. To analyze the declining process, we established a GIS database with historical occurrences of the crested ibis based on published literatures, and layers of environmental factors such as elevation, wetland, and human activities. We compared the environmental factors at the occurrence sites in different periods to quantify the changes of habitat use across time. To address the spatial deviation of the occurrences and check the effect of measuring scale on habitat use, we calculated the environmental factors in a serial measuring scales from 1 to 161 x 161 km(2). Our results indicated that the crested ibis traditionally lives in habitat with higher wetland density and higher human impact, then it gradually moved to areas with higher elevation and lower human impact in last century. In 1980-2000 the crested ibis stayed at a very high elevation, lower wetland density and lower human impact as possible consequences of human activities such as using fertilizer and pesticide, drying the over wintering rice paddies, and direct hunting. Our quantitative analysis of the habitat use matched well the previous published statements (which have no numerical evidences) on the declination of the crested ibis. We suggest to reestablish the habitat with traditional farming practice that the bird has adapted as a major solution for applications such as conservation planning and reintroduction.

2294: ***+.293***

Over the last two decades wild boar Sus scrofa Linnaeus, 1758 became the most intensively managed game species in Bulgaria. In order to delineate the population genetic structure, which is essential for sustainable wildlife management, we screened 10 porcine microsatellites across 289 wild boar samples originating from all relevant bioregions of the country. Results based on F(ST) values, Bayesian clustering methods and a multi-dimensional scaling analysis can be summarised as follows: (1) two main genetic groups were revealed for the Bulgarian data set: the first one included individuals collected from the Balkan Range Mountain and the northern part of the country and the second one comprised individuals from the Rhodope, Osogovo, Iskur Range and Rila mountains in southern and south-western Bulgaria; (2) all Bulgarian wild boar populations showed a higher level of genetic diversity compared to four populations from Germany which were included for out-group comparison, and (3) wild boar sampled from a game enclosure were found to be genetically divergent from the other Bulgarian populations, indicating human impact on population genetic structure most likely resulted from fencing and former translocation actions. The evolutionary background leading to the two defined management units as well as conservation and management strategies are discussed.

2295: *-.021*

The yellow carnation Dianthus guliae Janka is a rare endemic of the Italian peninsula. As numerous extinctions have occurred in the past, very few populations are still present. Two years of field surveys revealed high mortality and an absence of recruitment in the southernmost populations of this species. Work in a botanical garden, under semi-natural conditions, revealed the occurrence of proterandrous hermaphroditism and self-compatibility. The durable flower lifespan, the competitive effects among flowers and the different outcomes from spontaneous and hand-performed pollinations (with both self- and cross-pollen) suggested low pollination rates in the experimental stand. Pollinator exclusion experiments revealed a low frequency of delayed autonomous selfing, suggesting that self-fertilization in D. guliae relies mainly on facilitated selfing and geitonogamy. Studies of inbreeding depression during the early life-history stages revealed significant differences between selfed and crossed progenies in terms of seed mass, germination rates, developmental vigour and mortality rates. Therefore, when pollen delivery is scarce, the plant may fail reproductive assurance via autonomous selfing. The influence of inbreeding depression contributes to a further reduction in recruitment chances in very small D. guliae populations. Experimental reintroductions are urgent to avoid local extinction at the southern periphery of its range.

2296: *+.172*

Species reintroductions are used commonly as a tool for conservation, but rigorous, quantitative assessments of their outcome rarely occur. Such assessments are critical for determining success of the reintroduction and for identifying management actions needed to ensure persistence of reintroduced populations. We collected 9 years of demographic data on populations of brown-headed nuthatches (Sitta pusilla) and Eastern bluebirds (Sialia sialis) reintroduced via translocation into Long Pine Key, Everglades National Park, Florida, USA. Realized population growth of brown-headed nuthatches was positive in the first 3 years after cessation of translocations (lambda(2002) = 1.15, SE = 0.13; lambda(2003) = 1.28, SE = 0.12; lambda(2005) = 1.32, SE = 0.20) but became negative thereafter (lambda(2006) = 0.67, SE = 0.10; lambda(2007) = 0.77, SE = 0.13). Realized growth rate for the Eastern bluebird population did not vary among years and indicated either a stable or a slowly declining population (lambda = 0.92, SE = 0.04). Reintroductions were a qualified success; they resulted in the re-establishment of populations of both species, but neither population grew to the extent expected and both remained at risk of

extinction. (JOURNAL OF WILDLIFE MANAGEMENT 73(6): 955-964; 2009)

2297: ***+.234***

One of the goals of the Florida panther (Puma concolor coryi) recovery plan is to expand panther range north of the Caloosahatchee River in central Florida. Our objective was to evaluate the potential of that region to Support panthers. We used a geographic information system and the Mahalanobis distance statistic to develop a habitat model based on landscape characteristics associated with panther home ranges. We used cross-validation and an independent telemetry data set to test the habitat model. We also conducted a least-cost path analysis to identify potential habitat linkages and to provide a relative measure of connectivity among habitat patches. Variables in our model were paved road density, major highways, human population density, percentage of the area permanently or semipermanently flooded, and percentage of the area in natural land cover. Our model clearly identified habitat typical of that found within panther home ranges based on model testing with recent telemetry data. We identified 4 potential translocation sites that may support a total of approximately 36 panthers. Although we identified potential habitat linkages, out least-cost path analyses highlighted the extreme isolation of panther habitat in portions of the study area. Human intervention will likely be required if the goal is to establish female panthers north of the Caloosahatchee in the near term.

2298: ***+.144***

The aim of this study was to assess potential post-bottleneck temporal genetic differentiation following the reintroduction of the species into the Bialowieza Forest. Variability of 12 polymorphic microsatellite markers was analysed for 178 individuals born between 1955 and 2005, divided by birth year into five temporal groups. Low overall allelic richness (AR) per locus (AR = 2.0) and a low overall expected heterozygosity (H-E = 0.28) were observed. The overall F-IS was not significantly different from zero. The mean F-IS values were, however, significantly different from zero for individuals born between 1955 and 1965 (F-IS = 0.19). A Bayesian computation was used to estimate effective population size (N-e) for each temporal group. We observed relatively small values of N-e ranging from 7.9 to 28.4. The low N-e values confirm that, despite a rapid growth of the bison population following the founder event, N-e increased only slowly. (C) 2009 The Linnean Society of London, Biological Journal of the Linnean Society, 2009, 97, 801-809.

2299: ***-.111***

Populations of Partulina redfieldi, an achatinelline tree snail studied in four isolated trees, grew 100-900% between 1983 and 1995. Beginning in 1995, populations declined by 85%, and shells of rat-killed snails accumulated beneath the trees. While rat-marked shells were always present in the study area, numbers increased significantly. Despite a rat-abatement program begun in 1995, the snails continued to disappear, which we conclude was due to continued rat migration into the study area, despite baiting, and a switch in rat-food preference toward the snails. In neighboring forest where tree canopies are more continuous, snail density is lower and rat predation is not apparent. Captive-bred snails were successfully introduced to a small unoccupied tree in the same area in 1989, and this population suffered the same fate as the natural snail populations. Since 2000, P. redfieldi populations have remained low and rat predation continues.

2300: ***+.072***

The peregrine falcon (Falco peregrinus anatum) has not yet recolonized natural cliff sites in Illinois and much of the lower Midwestern US, and remains restricted to urban areas. We constructed a landscape-linked population viability analysis using RAMAS/GIS software to compare possible reintroduction strategies for the species. Habitat-specific (i.e. cliff and urban) demographic parameters such as survival, fecundity, and dispersal rates were derived from the Midwest Peregrine Society Database for peregrines in the central Mississippi River region during 1982-2006. We simulated a base scenario of no reintroduction and 18 models of reintroduction with varying cohort sizes, supplementation schedules, and number of reintroduction sites, and used the Lake Superior population to test our model. Our analysis indicated that even without reintroductions in Illinois, the peregrine population in the lower Midwestern region is slowly increasing and is not likely to go extinct. Recolonization of cliff sites in southern Illinois likely will occur via dispersal from urban populations, however further research on dispersal rates between urban areas and cliffs is needed. Analysis indicated that the most cost-effective reintroduction strategy would be priced at approximately $280,000 and would result in only two additional breeding pairs compared to the no-action scenario. Thus, funds would be more effectively used in other management efforts such as habitat preservation. This study provides an example of how post-release monitoring can be used to inform future reintroduction plans. Published by Elsevier Ltd.

2301: ***+.213***

Background: Habitat management for reproductively challenged rare species is a problem when there is insufficient knowledge of their autecology. This study investigated reproductive failure in the rare grass Calamagrostis porteri ssp. insperata (Swallen) C. Greene (Reed bentgrass). Does the management recommendation of high light stimulate clonal growth, flowering, and seed production?Location: Shawnee National Forest, IL, USA, and in a greenhouse and an experimental garden at Southern Illinois University, Carbondale, IL, USA.Methods: Clones obtained from the three known Illinois populations were grown in a glasshouse under experimental light and soil moisture treatments. After 3 years, plants from the high light treatment were planted outside in an experimental garden where the light treatments were maintained for two more years. In the field, vegetative and flowering tiller density, canopy cover, and associated biotic and abiotic variables including abundance of cooccurring plant species were monitored for 5 years. The overhead tree canopy was cleared over a portion of one population.Results: In the glasshouse, plants increased in size under high light and moist soil, and there were size differences among populations. Sixty-six per cent (20 of 30) of the genets flowered when planted outdoors under full sunlight but did not produce seed. In the field, flowering only occurred in Calamagrostis growing in the cleared area, but no seed were produced. The plants in the flowering population were smaller than plants in the other two populations. The herbaceous community associated with Calamagrostis in the open diverged from the communities remaining under the shade.Conclusions: This study highlights the difficulty of managing reproductively challenged rare species. Calamagrostis populations can be managed to enhance clonal growth, but establishment of new populations would require translocation of vegetative material as it is highly unlikely that seed can be obtained.

2302: ***+.077***

Once extinct in the wild, great progress has been made over the past 18 years at reintroducing Black-footed Ferrets Mustela nigripes within historical habitats of North America. Since 1987, more than 6,000 Ferrets have been produced in captive breeding centres, facilitating the release of 3,094 captive-born Ferrets at 18 reintroduction sites across the western United States and northern

Mexico. In addition, 147 wild Ferrets have been translocated from existing reintroduction sites to start or supplement other reintroduced Ferret populations. Allocations of Ferrets to reintroduction sites are determined by the U.S. Fish and Wildlife Service in consultation with the Black-footed Ferret Recovery Implementation Team, an advisory group comprised of 72 members representing 48 government agencies, Indian tribes, universities and conservation organisations. Allocation decisions are made using a ranking matrix consisting of biological, primary, factors (e.g. habitat and survival) and non-biological, secondary and tertiary, factors (e.g. planning, funding, and other project capabilities). Currently, there is an estimated minimum wild population of at least 824 individuals. However, only approximately 300 of those individuals are adult animals and contribute to the 'down-listing' goal of 1,500 breeding adults. In addition, only four sites have documented the ability to maintain at least 30 breeding adults over multiple years and thus contribute to the second 'down-listing' goal of maintaining at least 10 separate populations. Despite considerable progress, the programme faces obstacles such as disease and public acceptance of the Ferret's principal prey, prairie dogs Cynomys. Full recovery of the species will require continued vigilance of many involved partners, and greater support by the public, state and federal agencies, tribes, and non-governmental organisations to maintain and increase habitat for prairie dogs and Ferrets across the former ranges of these species.

2303: *+.036*

Liolaemus lutzae Mertens, 1938 is a critically endangered lizard endemic to the restinga habitat of the state of Rio de Janeiro. We surveyed 25 restinga habitats in order to locate remaining populations, evaluate the status of the species, and determine the nature of local habitat degradation. We found remnant populations of L. lutzae in 18 restinga habitats of six municipalities. The conservation status of each population varied between areas: the population of Grumari, in Rio de Janeiro municipality, is the most preserved and the population of Praia do Forte, in Cabo Frio, is the most disturbed. No L. lutzae were found in Niteroi municipality. The most destructive type of habitat degradation identified was the removal of beach vegetation associated with the construction of coastal roads and/or sidewalks, destruction of the vegetation due to trampling, vehicle traffic and garbage dumping. Our data revealed that generally, beach habitats under a larger number of impact sources were those with smaller population sizes of L. lutzae. We consider that the most effective conservation measure for L. lutzae is the strict protection of its habitat, with restoration of the original beach vegetation. Finally, we recommend vegetation recovery to be followed by a program of reintroduction of the species in localities where it has been eradicated.

2306: *+.079*

The present paper reviews the use and exchange of genetic resources of the migratory freshwater fish Pangasianodon hypophthalmus (Sauvage 1878) (the striped or sutchi catfish). This species is naturally distributed in the Mekong River and Chao Phraya River basins, and is cultured in several countries, but current production occurs predominantly in the Mekong Delta, Vietnam. Catfish aquaculture in Vietnam has evolved from extensive systems using wild-caught seed to an intensified farming system that is entirely dependent on hatchery-produced seed. Genetic improvement programmes on catfish have started in Vietnam, but are still in their infancy. Genetic studies have revealed several subpopulations of the species. Apart from selective breeding and the production of hybrids with closely related species, no other technologies have been applied to improve the performance of catfish. The use and exchange of P. hypophthalmus genetic resources have brought benefits to rural communities. Aquaculture development of catfish has evolved from being seen as an exploitation of natural resources to an activity that can reduce pressure on wild

fish populations. Management of aquaculture stocks need to be rationalised to minimise the potential impacts it might cause to wild populations.

2307: *+.202*

There are 58 species of Clarias recognized in FishBase (as of January 2009), 33 in Africa and 25 in Asia. Aquaculture of clariids is important with 30 countries reporting a total production of over 300 000 t worth nearly US$400 million in 2006. Most production involves the African Clarias gariepinus (Burchell, 1822) and three Asian species, Clarias batrachus (Linnaeus, 1758), Clarias macrocephalus (Gunther, 1864) and Clarias fuscus (Lacep'de, 1803). In much of Asia, hybrids of introduced C. gariepinus with native species dominate aquaculture and may pose threats to the purity and viability of wild populations. Many local strains have evolved within farms, some of which have been described and included in genetic management programs. Genetic variation among species and populations is significant, but to date little work on selective breeding of the group has been reported. Conservation efforts have so far focused on ex situ methods, primarily for farmed stocks, but these are few and expensive and farmed stocks are often of lower genetic diversity than wild stocks. In situ conservation of genetic material, both for aquaculture and for the maintenance of fitness in wild populations in light of changes occurring in the watershed, needs to be considered as a more viable long-term strategy. The preservation of ecosystem functional integrity is thus a prerequisite for the long-term conservation of Clarias genetic resources for food and aquaculture.

2308: *+.232*

This review describes the global use and exchange of salmonid genetic resources for human food from fisheries and aquaculture. Trends in population abundance, variations in the harvest of wild stocks, historic transfers and worldwide translocations of stocks for fisheries and aquaculture are briefly described for seven species of Pacific salmon (Oncorhynchus), for Atlantic salmon (Salmo salar) and for Arctic charr (Salvelinus alpinus). Also considered are the tools currently used to assist in the conservation of endangered wild populations (e.g. captive breeding programmes and gene banks) and the major techniques developed to enhance the production of cultured stocks (selective breeding, hybridization, sex control, chromosome set manipulation and gene transfer). The review briefly discusses the significance of salmon production to the economy of selected countries and the complexity of allocating economic value to long-range migratory fisheries resources that also hold direct and indirect value for aboriginal/native communities and recreational users.

2309: *+.069*

Most ecologists believe that climate change poses a significant threat to the persistence of native species. However, in some areas climate change may reduce or eliminate non-native invasive species, creating opportunities for restoration. If invasive species are no longer suited to novel climate conditions, the native communities that they replaced may not be viable either. If neither invasive nor native species are climatically viable, a type of "transformative" restoration will be required, involving the translocation of novel species that can survive and reproduce under new climate conditions. Here, we illustrate one approach for restoration planning by using bioclimatic envelope modeling to identify restoration opportunities in the western United States, where the invasive plant cheatgrass (Bromus tectorum) is no longer climatically viable under 2100 conditions projected by the Geophysical Fluid Dynamics Laboratory (GFDL2.1) coupled atmosphere-ocean general circulation model. We then select one example of a restoration target

area and identify novel plant species that could become viable at the site in the wake of climate change. We do so by identifying the closest sites that currently have climate conditions similar to those projected at the restoration target area in 2100. This approach is a first step toward identifying appropriate species for transformative restoration.

2310: *+.163*

Species native to ecotones are often overlooked in restoration efforts despite the increasing rarity of ecotone habitat. In fragmented, fire-suppressed landscapes, true ecotone may no longer exist. Restoration biologists interested in reintroducing ecotone species must decide whether to plant them in historic ecotones maintained by manual thinning or whether to opt for discrete restoration areas that are easier to maintain. We investigated these two alternatives with Lantana canescens, a rare tropical shrub native to the ecotone between pine and hardwood forests of Miami-Dade County, Florida, U.S.A. Our short-term findings show that after 15 and 18 months, survival of transplants was 69% in a restored site and 65% and 84% in two historic ecotone sites. The restored site had significantly higher photosynthetically active radiation (PAR) (75%) than the historic ecotones (25-39%). Correspondingly, 267 seedlings have recruited at the restored site, whereas only 8 have emerged at both historic ecotone sites. Seedling establishment was associated with higher PAR at the restored site. We found that overall population sustainability was higher at the restored site where there is the additional benefit of less maintenance. Our work suggests that, by reducing succession, a discrete restoration area can approach the historic conditions of hardwood/pine forest ecotone more closely than degraded historic ecotones themselves. We present a viable solution for conserving rare ecotone species when their natural habitat and the processes that maintained it no longer exist.

2311: *+.001*

Hydrologic restoration of Hatches Harbor, a tide-restricted marsh on Cape Cod (Massachusetts), has resulted in significant plant community changes 7 years following the reintroduction of seawater. Since 1999, incremental increases in flow through a tide-restricting dike have facilitated the rapid decline of salt-intolerant vegetation, while encouraging the expansion of native salt marsh taxa. These changes show strong spatial gradients and are correlated with marsh surface elevation, distance from the point of seawater entry, and porewater salinity. Common reed (Phragmites australis) has not decreased in abundance but has migrated a considerable distance upslope. In the wake of this retreat native halophytes have proliferated. Now that maximum flow through the existing dike structure has been reached, continued recovery may be limited less by changing physicochemical conditions and more by rates of growth, seed dispersal, and seed germination of salt marsh taxa.

2312: *-.069*

Thirty-one North Island weka (Gallirallus australis greyi) were released on Pakatoa Island (26 ha), Hauraki Gulf, New Zealand in Aug 1996. The population then fluctuated between c.19 and 182 individuals, including c.6-55 pairs. The last of the translocated weka died between Jan and Jun 1998, during a drought and after the rodenticide Talon (R) was laid to kill Norway rats (Rattus norvegicus), and only weka <1 year old survived. Most young raised in Dec 2001-Jan 2002 died during a drought in Feb - Mar 2002. The weka population increased during a period of higher rainfall from mid-1998 to Dec 2001. The higher population resulted in smaller home ranges, higher frequencies of diurnal spacing calls, more aggressive behaviour, and a higher incidence of plumage damage. The large fluctuations in population size on Pakatoa I suggests that future

translocations of weka should select islands with wetter and less variable rainfall patterns

2313: *+.067*

This paper reports a study of two anteater (Tt1 and Tt2) female adults, which were translocated and monitored by radio-tracking in the Brazilian Cerrado biome. The displacement of both animals was approximately 6 km in six days; and they both were established in open savanna physiognomies. Tt1 came to death 21 days after its release, and it was not possible to determine its home range. Tt2, on the other hand, survived for four months and established a home range of 1.06 km(2) (MCP 95%). There is a need for further studies to assess the feasibility of translocation and, therefore, the predictions proposed by the International Union for Conservation of Nature should be considered.

2314: *+.220*

Reduced genetic diversity can result in short-term decreases in fitness and reduced adaptive potential, which may lead to an increased extinction risk. Therefore, maintaining genetic variation is important for the short- and long-term success of reintroduced populations. Here, we evaluate how founder group size and variance in male reproductive success influence the long-term maintenance of genetic diversity after reintroduction. We used microsatellite data to quantify the loss of heterozygosity and allelic diversity in the founder groups from three reintroductions of tuatara (Sphenodon), the sole living representatives of the reptilian order Rhynchocephalia. We then estimated the maintenance of genetic diversity over 400 years (similar to 10 generations) using population viability analyses. Reproduction of tuatara is highly skewed, with as few as 30% of males mating across years. Predicted losses of heterozygosity over 10 generations were low (1-14%), and populations founded with more animals retained a greater proportion of the heterozygosity and allelic diversity of their source populations and founder groups. Greater male reproductive skew led to greater predicted losses of genetic diversity over 10 generations, but only accelerated the loss of genetic diversity at small population size (< 250 animals). A reduction in reproductive skew at low density may facilitate the maintenance of genetic diversity in small reintroduced populations. If reproductive skew is high and density-independent, larger founder groups could be released to achieve genetic goals for management.

2315: *+.230*

Founder populations in reintroduction programmes can experience a genetic bottleneck simply because of their small size. The influence of reproductive skew brought on by polygynous or polyandrous mating systems in these populations can exacerbate already difficult conservation genetic problems, such as inbreeding depression and loss of adaptive potential. Without an understanding of reproductive skew in a target species, and the effect it can have on genetic diversity retained over generations, long-term conservation goals will be compromised. In this issue of Molecular Ecology, Miller et al. (2009a) test how founder group size and variance in male reproductive success influence the maintenance of genetic diversity following reintroduction on a long-term scale. They evaluated genetic diversity in two wild populations of the iconic New Zealand tuatara (Fig. 1), which differ greatly in population size and genetic diversity, and compared this to genetic diversity in multiple founder populations sourced from both populations. Population viability analysis on the maintenance of genetic diversity over 400 years (10 generations) demonstrated that while the loss of heterozygosity was low when compared with both source populations (1-14%), the greater the male reproductive skew, the greater the predicted losses of genetic diversity. Importantly however, the loss of genetic diversity was ameliorated after

population size exceeded 250 animals, regardless of the level of reproductive skew. This study demonstrates that highly informed conservation decisions could be made when you build on a solid foundation of demographic, natural history and behavioural ecology data. These data, when informed by modern population and genetic analysis, mean that fundamental applied conservation questions (how many animals should make up a founder population?) can be answered accurately and with an eye to the long-term consequences of management decisions.

2316: *+.055*

This paper aimed to investigate human-tiger conflict during the year 2000-2004 and has reviewed a number of potential measures for resolving the conflicts as part of conserving the Sumatran tigers which can be applied in the study area. These include technical measures which deal mainly with the individual 'problem' animal that comes into conflict and human dimension measures which focus on raising human tolerance toward tigers and reducing the negative effects of co-existence between human and tigers. Some of the measures namely, disruptive stimuli modification, raising tolerance through education and cultural perspective approaches had been tried for a short period. The success or otherwise of these measures in the field have also contributed to the conclusions drawn and the recommendations made as a result. The data are supplemented with more information, including the insight on the effect of conflicts between inhabitant and wildlife, the perspectives of villagers regarding human-tiger conflicts and the causal factors of individual conflict incidents. The reluctance of local community to report incidents prevents conservation agencies for delivering an immediate and effective response and it stimulates the killing of Sumatran tigers that benefits both hunters and livestock owners through the sale of tiger derivatives. This study also proposed a procedure for handling human-tiger conflict that hopefully may be tested in any other area.

2318: *+.083*

Although several phylogenetic studies of Gyps species have been conducted, few studies have addressed the genetic diversity of these species out a finer scale such as microsatellite analyses. We collected samples of migratory adults and nestlings from four species of vultures in six different localities. We analyzed the samples using microsatellites in order to determine the genetic distance as well as the amount of variation within and among Gyps species populations. Low genetic diversity in Long-billed Vultures (G. indicus) was probably indicative of a single population with no immigration mid low gene flow. As this species is critically endangered, future conservation programs should consider genetically suitable stock for a breeding and reintroduction program. High genetic diversity in African White-backed Vultures (G. africanus) was likely indicative of a number of populations, with immigration and gene flow. We confirmed previous findings of low genetic differentiation among Griffon Vulture (C. fulvus) populations, which indicated high mobility and gene flow among these populations.

2319: *+.100*

DiTommaso, A., Clements, D. R., Darbyshire, S. J. and Dauer, J. T. 2009. The Biology of Canadian Weeds. 143. Apocynum cannabinum L. Can. J. Plant Sci. 89: 977-992. Hemp dogbane, Apocynum cannabinum (Apocynaceae), is a perennial herb with white to greenish flowers in terminal clusters that produces pencil-like pods 12-20 cm long. A highly variable plant, A. cannabinum may be distinguished from spreading dogbane (Apocynum androsaemifolium) by its shorter corolla (2-6 nun compared with 5-10 mm), erect greenish-white petals (compared with recurved or spreading pinkish petals), seeds more than 3 mm long (compared with seeds less than

3 mm), and more erect leaves (compared with spreading or drooping leaves), although frequent hybridization between the two species obscures the identity of some individuals. Hemp dogbane is native to the United States and southern Canada, but most abundant in the upper Mississippi River Valley and east to the Atlantic coast. It has been increasing in other areas, and becoming more of a problem where conservation tillage is adopted. It infests crops such as corn (Zea mays), soybeans (Glycine max), wheat (Triticum aestivum), sorghum (Sorghum bicolor) and forages, and may cause livestock poisoning due to cardiac glycosides within its milky sap (but livestock generally avoid it). Potential medicinal uses of these compounds have been investigated, and the roots are a source of fibre. Control of A. cannabinum with various herbicides is difficult due to a thick cuticle, and one solution may be to target susceptible stages, such as seedlings or early spring growth. Cultivation may also control A. cannabinum, but care must be taken not to promote the proliferation of the plant through regrowth from fragmented roots and rhizomes. Rotation with alfalfa also reduces populations of A. cannabinum.

2320: *-.017*

The decimation of aquatic wildlife through overexploitation is usually perceived as a marine phenomenon, yet it has also been common in freshwater ecosystems. Fish and other aquatic animals were superabundant when Europeans first arrived in North America and Australia, and were intensively exploited soon after. Contemporaneously, the construction of barriers in rivers increasingly prevented many species from migrating. Populations usually crashed as a result. Natural resource managers have not fully considered the ecological impacts of the devastation of these species to the environmental degradation that we see today, yet these impacts are likely to be pervasive. Nor have resource managers embedded the role of these species in river restoration. We argue that the functions of these depleted stocks need to be considered and perhaps reestablished if river restoration efforts are to be successful. The establishment of freshwater protected areas may be the most effective way to do this.

2321: *+.039*

In South Africa, the oribi, Ourebia ourebi is an endangered small antelope that requires conservation management and intervention to prevent their extinction. Use of translocation for the conservation of subpopulations facing local extinction in the wild has been proposed. In this study, fifteen oribi from threatened populations were captured and released (November 2004) on a private game reserve in KwaZulu-Natal, South Africa. Radio telemetry was used to monitor the dispersal and survival of translocated oribi. Only one death occurred during that period. All other released oribi survived, and most remained in close proximity of the release site for more than a year postrelease. Reproduction also occurred. This suggests that, translocation could be a viable option for conserving wild populations of oribi, assuming that other factors, such as availability of suitable grassland habitat and that poaching has been curtailed, are met.

2322: *+.218*

Knowing which environmental characteristics constrain forest understory species' distribution and reproduction can inform conservation decisions about habitat management and locations for reintroduction efforts. Differential plant performance is common in varying environmental conditions, such as levels of canopy closure. Actaea elata (tall bugbane) is a rare, perennial, forest understory herb found from Oregon to British Columbia. Populations throughout this species' range commonly occur in managed forests. We assessed the importance of environmental variation on the distribution and flowering of A. elato at both microhabitat and site-level scales. At four

sites encompassing a variety of forest types, we measured several biotic and abiotic variables at vegetative and reproductive plants, as well as at random points. We measured these same variables at three sites where A. elata has been extirpated. Within occupied sites, reproductive plants were more likely to be located in canopy gaps than were vegetative plants. Both vegetative and reproductive plants were more likely to be found in areas of high herb cover compared to random points. These results indicate that gaps in the canopy may be critical for this species' ability to reproduce, but survival may be possible in a broader range of forest understory conditions (similar to other co-occurring herb species). Random points at extirpated and occupied sites both tended to be located in areas of lower canopy openness and moisture availability than points in occupied sites where A. elata was present. Extirpated sites were either densely forested or clearcut, while occupied sites contained stands of intermediate density. Circaea alpina, Geranium robertianum, and Lactuca muralis were indicator species for A. elato and normally indicate moist soils, with Circaea alpina being a strong indicator of the presence of flowering A. elata. Taken together, our results suggest that maintenance of canopy gaps is important to promote flowering, and understory species that indicate moist sites, particularly Circaea alpina, can be used as aids to locate new populations of A. elata. (C) 2009 Elsevier B.V. All rights reserved.

2323: *+.032*

Alien predators can have catastrophic effects on ecosystems and are thought to be much more harmful to biodiversity than their native counterparts. However, trophic cascade theory and the mesopredator release hypothesis predict that the removal of top predators will result in the reorganization of trophic webs and loss of biodiversity. Using field data collected throughout arid Australia, we provide evidence that removal of an alien top-predator, the dingo, has cascading effects through lower trophic levels. Dingo removal was linked to increased activity of herbivores and an invasive mesopredator, the red fox (Vulpes vulpes), and to the loss of grass cover and native species of small mammals. Using species distribution data, we predict that reintroducing or maintaining dingo populations would produce a net benefit for the conservation of threatened native mammals across greater than 2.42 x 10(6) km(2) of Australia. Our study provides evidence that an alien top predator can assume a keystone role and be beneficial for biodiversity conservation, and also that mammalian carnivores more generally can generate strong trophic cascades in terrestrial ecosystems.

2324: *-.184*

Rock hyraxes (Procavia capensis) are categorized as 'Least Concern' in the 2008 IUCN Red List of Threatened Species. In South Africa they were once listed as vermin in the old Cape Province due to their high population numbers and impact on grazing. However, about 10 years ago, populations in the KwaZulu-Natal province became locally extinct. This resulted in the recent reintroductions of rock hyraxes, purchased at annual wildlife auctions in the province. Success of these reintroductions was unknown as there had been no post-release monitoring. This study determined the success of reintroducing rock hyraxes, using two source populations, namely rock hyraxes that had been in captivity for 16 months (n = 17) and those from the wild (n = 9). Captive rock hyraxes did not have site fidelity after release and after three months could not be found. All wild rock hyraxes, except one whose fate is unknown, were found dead within 18 days of release. One had an accidental death while the rest were preyed upon. In conclusion, the reintroduction of captive and wild rock hyraxes likely failed due to predation. This may have been a consequence of group disintegration, probably as a result of incorrect group composition, captive stress, and type of release. Suggestions to improve the success of future rock hyrax reintroductions are provided.

2325: ***+.219***

Wood abundance in aquatic systems has been dramatically reduced compared to historical levels due to anthropogenic activities that led to wood removal and stream simplification. As a result, reintroduction of wood to aquatic systems is now a widely used and relatively well-studied restoration technique for increasing habitat complexity. Although stream periphyton (biofilm) and invertebrates serve as food sources for a variety of predators including fish, birds and bats, data on how lower trophic levels respond to wood placement are relatively scarce. The purpose of this study was to test the hypothesis that periphyton biomass and aquatic invertebrate density were higher on Engineered Log Jams (ELJs) than on inorganic substrates in two large Pacific Northwest river systems. Among years and rivers, periphyton biomass and invertebrate densities were significantly higher on ELJs than on cobbles within the same reach. Invertebrate communities on ELJs were dominated by meiofauna (<500 mu m), whereas cobbles were dominated by larger chironomids. We attribute these trophic level differences to substrate type, as we did not detect taxonomic differences between cobbles in reaches with and without ELJs. We show that adding wood to reaches with little or no naturally occurring wood increased overall habitat surface area and thereby the potential for increased productivity relative to reaches with low levels of wood. Finally, wood supports a unique community of invertebrates that are often overlooked in lotic system studies but may be contributing substantially to overall biological diversity. Published in 2009 by John Wiley & Sons, Ltd.

2326: ***+.002***

Translocation outcomes for mobile species can be affected by post-release movement of individuals, yet few population reintroduction and supplementation projects consider propensity to move as a selection criterion when selecting individuals to release or sites for release. We investigate the influence of release age (juvenile or subadult), the size of the release group and the size of the wild population at the release site on movements of Critically Endangered kaki (black stilt) Himantopus novaezelandiae. Over 460 subadult and juvenile kaki have been released during 12 years at nine sites in the Waitaki Basin, New Zealand, with the aim of supplementing specific sub-populations. Among the survivors that reached breeding age, 32% of released kaki ended up away from their release sites, i.e. away from the subpopulations they were intended to augment and 15% of these birds were in unmanageable areas where monitoring cannot take place. Kaki released as juveniles (2-3 months) made more long moves and moved further from the release site during 2 months post-release. The presence of conspecifics affected behaviour after release: released birds were more likely to remain closer to the release site when the size of the wild population at the release site was large, and kaki released in larger groups were more likely to make more longer moves. Despite initial differences in mobility, long-term monitoring revealed equal proportions of 2-year old survivors that had been released as juveniles and subadults returning and being observed consistently at release sites, emphasizing the value of long-term monitoring in informing release strategies for population restorations.

2327: ***+.055***

The Near Threatened white rhinoceros Ceratotherium simum went extinct in Botswana during the 20th century because of poaching. Several attempts have been made to reintroduce the species. From 2001 to 2003 four batches (a total of 32 individuals) of white rhinos were released in the Moremi Game Reserve. All were fitted with transmitters, ear notched and monitored on a regular basis. Rhinos released in the last batch moved significantly further from the release site compared to early batches. Six female rhinos from the last batch dispersed out of the Reserve. Activity area

(95% minimum convex polygon; MCP) sizes decreased with years after release and increasing density of rhinos but only density had an effect on the core area (50% MCP) sizes. We conclude that the number of rhinos present in the area of release should be carefully considered before further individuals are released. When released in an area with rhinos that have established territories, the newly reintroduced individuals may be forced to disperse. If other areas of suitable habitat are available elsewhere in the same protected area, animals should be released at different sites to avoid unwanted long-term dispersal and to use the inverse density-dependent activity area sizes to maximize the rhino population in an area.

2328: ***+.052***

Worldwide, the family of the sturgeons is near extinction. Reasons are different human factors like energy production in rivers with the interruption of the river continuum, the pollution of water bodies and overfishing because of the valuable caviar. The Danube has five native sturgeon species, two landlocked species and three migrating species from the Black Sea which migrated partially up to the Bavarian Danube for spawning. In the Austrian part of the Danube only small quantities of the small sterlet can be found, which are still threatened. It is imminent to undertake steps for protection and support of the remaining population. Beside the sterlet there are efforts to reintroduce the land locked ship sturgeon into Austrian water bodies. The main problem is that the species is very rare and only occasionally caught in the middle Danube. A reintroduction of this species would be an attractive and ecological welcome addition to the fish fauna of the Austrian Danube.

2329: ***+.068***

Restoration and reintroduction efforts for the sicklefin redhorse Moxostoma sp. have been initiated by state. tribal. and federal agencies owing to the limited geographic distribution of this species and threats associated with the physical alteration of its habitat. A critical component of a Successful reintroduction is that the source and recipient populations have similar genetic resources and life history patterns. We used 10 microsatellite loci to estimate and compare indices of genetic diversity between the Little Tennessee River population of wild adults and the hatchery broodstock being used for initial reintroduction efforts. We also compared relatedness values for the broodstock used for restoration efforts. There were no significant difference,.; between hatchery broodstock and wild adults with respect to average gene diversity. but the average number of alleles for each brood year was., significantly less than thin for wild Adults. While this trend persisted when the 2007 and 2008 brood years (combined) were compared with wild adults, the reduction was not significant. Finally, all hatchery crosses were among unrelated individuals. Our results highlight the importance Of using genetic information to assist restoration and reintroduction efforts.

2330: ***+.040***

We evaluated the potential for reintroducing Lahontan cutthroat trout Oncorhynchus clarkii henshawi. a species listed under the Endangered Species Act, into a lacustrine system where the biotic community has changed dramatically since the species' extirpation there. Since 2002. 76,547 Lahontan cutthroat trout have been reintroduced into Fallen Leaf Lake, California; we used creel surveys. diet data, mark-recapture methods. bioenergetics modeling, and netting data across seasons to evaluate the habitat use, growth, and relative abundance of Lahontan cutthroat trout and the abundance. diet. habitat use, and predation by nonnative species. Sampling totals (n = 2) and survey observations (n = 3) indicate low survival and abundance of reintroduced fish and together

with creel data indicate the importance of epilimnetic habitats across size-classes. Despite substantial growth. Lahontan cutthroat trout exhibited low condition factor values (average = 0.69). We found substantial predation pressure front a large population of lake trout Salvelinus namaycush ((N) over cap = 8.799 fish: 95% confidence interval [CI] 4,990-16,530 fish): analysis of lake trout diets showed an increase in piscivory and in the percentage of stomachs containing Lahontan cutthroat trout anchor tags with increasing predator size. Overall. we estimated that lake trout consumed over 38% of reintroduced Lahontan cutthroat trout (mean number consumed = 7,736 Fish: 95% CI = 4,388-14,534 fish). With bioenergetics modeling, however. we estimated that lake trout consumed considerable amounts of salmonid biomass during this period (mean biomass = 3,137 kg: 95% Cl = 1 779 5,993 kg), which greatly exceeded the biomass of Lahontan cutthroat trout reintroduced in 2006. During the stratification period, there was little overlap in habitat use between lake trout and Lahontan cutthroat trout, but overlap was high during the spring and autumn. We found moderate-sized populations of nonnative brown trout Salmo trutta, kokanee O. nerka (lacustrine sockeye salmon), and rainbow trout O. mykiss. Together, our results suggest that Lahontan Cutthroat trout have few refugia front direct and indirect negative interactions with normative species and that alternative approaches are needed.

2331: *+.013*

The endangered Mohave tui chub Gila bicolor mohavensis, which occurs only in three populations in southern California, has not been previously spawned in captivity. Captive breeding of imperiled fishes can be important to conserve biodiversity and aid in native fish conservation efforts by reducing the collection of wild fish for translocations, providing individuals for experimental studies, and ensuring species survival. To spawn Mohave tui chub, we lowered water temperature to 10 degrees C, held this temperature constant for 30 d, and then warmed the tank at a rate of 1 degrees C per day to reach ambient air temperature (20-22 degrees C). We used a photoperiod of 10 h light : 14 h dark and adjusted the photoperiod to 14 h light : 10 h dark when the tank reached 15 degrees C. These photoperiod and water temperature regimes were designed to simulate Mojave Desert springtime conditions. Artificial plants were provided as spawning substrate. Three spawning events produced over 1,700 larval fish. Fish not subjected to this photoperiod and water temperature regime did not spawn. Simulation of a spring photoperiod and temperature regime was associated with successful spawning of Mohave tui chub in captivity and may be effective for spawning other endangered cyprinids with similar requirements.

2332: *+.077*

Branchiobdellid annelids are usually found as commensal symbionts associated with crayfish populations, but knowledge of their dispersion and ecology in Europe is generally scarce. We hypothesized that their geographic extension of species and populations may mirror the distribution history of their hosts. We analysed potential host specificities and the geographic distribution of species from the Italian and Austrian Tyrol and Carinthia by characterizing the morphological and genetic features. On the three indigenous crayfish species Astacus astacus, Austropotamobius pallipes and Austropotamobius torrentium, we identified four branchiobdellid species based on morphological characteristics: Branchiobdella hexodonta, Branchiobdella pentodonta, Branchiobdella balcanica and Branchiobdella parasita. In contrast to the morphological classification, phylogenetic analysis using mitochondrial cytochrome c oxidase I (CO-I) sequences identified five main lineages: B. balcanica, B. hexodonta, Branchiobdella italica, B. parasita and B. pentodonta. The arrangement of branchiobdellid species corresponded generally to the geographical distribution of their crayfish hosts' locations but also confirmed previous assumptions of crayfish translocations. Our study provides the first application of ideas on the

association of freshwater crayfish and their ectosymbionts to be used for discussing the biogeography of crayfish populations. The phenotypical and genotypical analysis also demonstrated so far ignored effects of human activities at both macro-ecological and micro-ecological levels.

2333: ***+.132***

Conservation management requires knowledge of how a target species interacts with other species. Some relatively common species can modify the environment to the advantage of rarer, endangered species. Thus, local enhancement of those common species can positively influence remaining populations of the rarer species. The endangered pygmy bluetongue lizard Tiliqua adelaidensis inhabits burrows that are constructed by lycosid and mygalomorph spiders. We recorded 490 burrows in a 1 ha plot at the end of one season, and then observed at regular intervals the formation and loss of burrows, and the changes in occupancy status of each burrow over the next season. We found spiders in 94% of all newly constructed burrows and deduced that they had built the burrows. We found no evidence that lizards dug new burrows or deepened existing burrows. The numbers of both lizards and spiders in the burrows declined over the spring and summer, with lizards moving from their burrows more often early in the season than later. However, there was no strong trend for lizards to replace spiders in burrows. In fact, lizards tended to occupy deeper burrows than spiders, suggesting little negative impact of lizards on spiders. However, spiders had a positive impact on lizards by providing the refuge burrows central for lizard survival. Although lizards readily accept artificial burrows, long-term conservation for the lizards must include viable spider populations to maintain a supply of suitable burrow refuges.

2334: ***+.114***

The American black bear (Ursits americanus) experienced a significant range contraction during the 19th and 20th centuries due to a variety of anthropogenic factors. Although previous molecular studies of black bears provided insight into historic and contemporary forces shaping phylogeographic patterns, none included black bears from the central part of the species distribution. Understanding the historical aspects of the connectivity and genetic differentiation of black bears in this region is important for proper management and conservation programs, but this understanding is confounded by poorly documented translocation efforts and population expansion. To address these issues, we generated mitochondrial DNA sequence data for 409 black bears from 15 populations in North America. Two sampling localities (Manitoba, Canada, and Minnesota) were source populations for translocation into western Arkansas and Louisiana. Major conclusions from our study include: black bears in western Arkansas were affected genetically by the translocation program; eastern Oklahoma has been repopulated by westward expansion of bears from Arkansas with a mixture of translocated bears and remnant individuals, black bears in Louisiana were not affected genetically by the translocation program; black bears in western Texas and northern Mexico dispersed there from the Southeastern United States; and bears in White River National Wildlife Refuge (eastern Arkansas) share closer genetic affinities with U. a. luteolus than they do with the widespread U. a. americanus.

2335: ***+.111***

The contemporary genetic structure of animal populations is sculpted by past events, including demographic bottlenecks and expansions and movement of animals by humans. In an analysis of microsatellite DNA of black bears (Ursus americanus; n = 540) across California, we discovered distinct population structure and genetic evidence of 2 historic colonization events. First,

genotypes of bears sampled in southern California are most related to those from the Yosemite National Park region and not with spatially intervening populations. Historical records recount the translocation of 28 black bears from the Yosemite National Park area of the central Sierra Nevada to the San Bernardino Mountains in southern California in the 1930s. Second, before colonization of California by Europeans, the Central Coast region was inhabited by the now extinct California grizzly bear (Ursus arctos californiensis), but not black bears. Following an apparent competitive release and range expansion during the past century, black bears now inhabit the Central Coast region of California. Black bears in California's Central Coast display lower genetic diversity (founder effect) and a genetic signature most closely allied with black bears from the southern Sierra Nevada. In both these cases, molecular genetic techniques allowed historical reconstruction of anthropogenic events leading to changes in animal distributions.

2336: *-.233*

1. Birds of prey and driven-grouse shooting are at the centre of a long-standing human-wildlife conflict. Hen harrier predation can reduce grouse shooting bags, limit grouse populations and cause economic losses. Despite legal protection, hen harrier numbers are severely depleted on driven-grouse moors.2. In limited trials, provision of supplementary food to hen harriers greatly reduced their predatory impact on young grouse, but did not result in higher grouse densities for shooting. Consequently, grouse moor managers have failed to adopt the technique.3. A recent Forum paper has called for a trial 'population ceiling scheme' for hen harriers, arguing that this represents the best way to increase hen harrier numbers on driven-grouse moors. Once densities exceed the agreed ceiling, the excess would be translocated to other suitable habitat.4. Whilst a 'ceiling' scheme might work, it would be difficult to implement and we believe that other approaches to population recovery should be tested first.5. While driven-grouse shooting makes an important economic contribution to some rural communities, some grouse moor owners receive considerable sums of public money. Despite this, many moors are in poor condition, the ecosystem services they supply may be at risk from both climate change and current management practices, and grouse numbers are in decline. The socio-economic and environmental implications of alternative models of grouse management need urgent examination.6. Synthesis and applications. If driven-grouse shooting is only viable when birds of prey are routinely disturbed and killed, then we question the legitimacy of driven-grouse shooting as a sustainable land use. Moorland owners need to consider more broadly sustainable shooting practices for the 21st century.

2337: *+.207*

The Rio Grande drainage is an important and imperiled wetland of the US/Mexican border arid lands. There is a desire to restore otter populations in this river by interested parties. In order to follow IUCN guidelines for restoration, biologists need learn more fully the situation prior to implementation of restoration management. A prerequisite for proper restoration conservation is to know the organism's taxonomy (i.e., what taxa or species and subspecies one is dealing with), distribution, and relative abundance. The historic and current distribution of the Nearctic otter (Lontra canadensis) and Neotropical otter (L. longicaudis) in the borderlands of US and Mexico are reviewed in this paper. The evidence indicates that otters were native to the Rio Grande valley and has been recorded in the languages and customs of Native Americans such as the Pueblo people prior to European settlement of the area. The first Spanish documents we were able to find whereby otters were recorded, date to the middle 16th century. Otters during historical times were probably more numerous than previously thought and one of the first wildlife laws in the borderlands revolved around a moratorium on trapping the otter and beaver. Presently, populations of otters occur in 1) the Rio San Pedro of Chihuahua, a tributary of the Rio Conchos entering the

Rio Grande from the southeast, 2) the upper Rio Grande near the Colorado/New Mexico border, and 3) the middle Pecos River in southeastern New Mexico entering the Rio Grande from the west. These observations are corroborated by multiple observations by competent observers and in the case of the first population, otter photos and sign. These populations are centered on areas with macro-habitats characterized by a river flowing through 1) deep canyons, or 2) ancillary wetlands. Considerable more detailed survey work is needed to determine the full extent of the distribution of otters in the Rio Grande drainage. A genetic study is critically needed to determine the true taxonomic affiliation of these recently discovered populations. A moratorium on translocations should be put in place for the Rio Grande to conserve the native populations already existing.

2338: *-.034*

The Pyrenees are becoming an environmental reservoir. The acute human depopulation experienced during the twentieth century and the progressive appropriation of large parts of the mountainous territory by the state in order to implement conservation policies have resulted in the return, via reintroduction or natural regeneration, of bears, wolves, beavers, river otters, marmots, mouflon, feral goats, and deer, among other species. This development, however, has not occurred without social and scientific controversy and leads to questions about territorialization and governmentality. Herders perceive wild animals as unregulated public property subsidized by the work of the local populace. Agriculturalists see their fields trespassed on a daily basis by animals they cannot kill because of their protected status. Ranchers, under extremely strict sanitation regulations, see their animals coming into contact with these unchecked wild populations. The work and living space of the mountain communities has fallen under the jurisdiction of external institutions and constituencies that value conservation and ecotourism above local subsistence.

2339: *-.124*

Acute and chronic lead (Pb) poisoning have been recognized as some of the most important causes of mortality for raptors worldwide. We simultaneously examined the recent, medium-term and long-term lead exposure of the endangered bearded vulture (Gypaetus barbatus) from the Pyrenees (northern Spain and southern France). One hundred and one blood samples from 87 captured individuals and tissue samples (liver and bone) from a further 43 dead individuals were analyzed for lead residues. The majority of individuals examined had very low lead concentrations in blood, liver and bone. However, two individuals showed elevated blood Pb levels, two individuals showed liver lead concentrations indicative of excessive lead exposure and one individual showed bone lead levels indicative of chronic lead poisoning, suggesting that the Pyrenean population is not free from the risk of poisoning. We found that Pb exposure was significantly higher in adult individuals as well as in the northern (France) and eastern (Catalonia) range of their distribution. These differences could be related to different feeding habits between age classes (pre-adults are more linked to supplementary feeding sites) and differences in hunting practices between regions (in some regions, carcasses and offal of game animals are not retrieved). Blood, liver and bone lead levels found were slightly higher during the hunting season than outside of the hunting season. Lead presents an unnecessary threat to adult birds and the only way to remove this risk is to ban all hunting with lead within the range of distribution of the endangered bearded vulture. Acute and chronic lead poisoning should be considered in differential diagnosis in any diseased or injured wild bearded vulture, especially subadult and adult individuals, and the potential risk of Pb poisoning should be considered in future reintroduction programs. (C) 2009 Elsevier Inc. All rights reserved.

2340: *+.360*

The potential distribution of critically rare or endangered species is necessary to assess species conservation status and guide recovery plans. Habitat models based on remotely sensed geospatial data are increasingly used to predict the suitability of sites for rare and endangered species, but in rapidly changing landscapes, habitat evaluations must reflect temporal as well as spatial variation of environmental suitability in order to properly inform management. We used field measurements of species occurrence, a 22-yr time series of satellite images, and the Maximum Entropy modeling approach (Maxent) to monitor spatio-temporal variation in habitat suitability of an endangered butterfly that uses riparian wetlands modified by beaver activity. We modeled the niche of the St. Francis' satyr Neonympha mitchellii francisci in an environment of remotely sensed metrics and projected the niche model over space and time to evaluate habitat dynamics and target sites for reintroduction efforts. Suitable habitat for the subspecies is currently distributed across the study area; however, most of the suitable area is unoccupied, and patches of the most suitable habitat have shifted over time in response to beaver activity and subsequent wetland succession, suggesting a negative interaction between dispersal limitation and landscape dynamics. Landcover changes complicate the recovery of critically threatened species such as N. m. francisci, but habitat monitoring over time can improve recovery plans, offer adaptive management strategies, and provide more exact criteria for species status assignment. Spatio-temporal extensions of the niche/habitat concept are made possible by long-term archives of remotely sensed data, and will likely prove most useful in rapidly changing landscapes.

2341: *+.129*

The population density of the white-tailed sea eagle Haliaeetus albicilla is very low in many countries. In last twenty years, the sea eagle population in South Bohemia was restored by strict protection subsidized by reintroduction. The active help consisted of feeding during winter and building of artificial nests. A new sea eagle breeding population arose in the Trebon basin area in the early 1980's. Until this time sea eagles had used former breeding places only for wintering, probably coming from the Baltic. The South Bohemian sea eagle population is very unique: it exists in a densely man-occupied landscape, mainly in areas with very intensive carp breeding in artificial fishponds and was partly artficially (help to wintering birds and reintroduction of some individuals) restored. The experience from South Bohemia may have importance for populations of the sea eagle in other areas of its occurence, primarily in the continental conditions [Current Zoology 55 (5) 315-318, 2009]

2342: *+.055*

The small population of brown bears in central Austria originated from a single migrant bear that had settled in the area in 1972 and three bears that were released in the years 1989-1993. Subsequently, the population has been monitored by radio-tracking and collecting data on bear signs and observations. In 2000 we started a genetic monitoring program of the population with the aim to obtain data on population size, sex ratio, relationships as well as movements of individuals. We present results from six years of genetic monitoring, which were combined with field observations. During this time 1,005 hair and faecal samples were gathered in an area of[3,000 km(2), most of them in the core area of <1,000 km(2). Furthermore we analysed blood samples from captured individuals. Eight microsatellite and two sex determination loci were employed for DNA profiling. The number of detected individuals is surprisingly low, ranging from 5-8 per year. Concerning relationships the analysis reveals that all genotyped individuals are descendants of the founder individuals indicating that no immigration took place. Only one male and three females (mother and 2 daughters) took part in reproduction. Considering the fact that 28 bears were born in this region since 1991 the question arises where the bears disappear to. Our

results suggest that subadult bears migrate from the core area. However, indices of bear occurrence outside the core area are rare and migration could be proved only for two young males. Other explanations, such as increased natural mortality and illegal hunting are discussed.

2343: *+.089*

The Orchidaceae is characterised by a diverse range of life histories, reproductive strategies and geographic distribution, reflected in a variety of patterns in the population genetic structure of different species. In this study, the genetic diversity and structure was assessed within and among remnant populations of the critically endangered sexually deceptive orchid, Caladenia huegelii. This species has experienced severe recent habitat loss in a landscape marked by ancient patterns of population fragmentation within the Southwest Australian Floristic Region, a global biodiversity hotspot. Using seven polymorphic microsatellite loci, high levels of within-population diversity (mean alleles/locus = 6.73; mean H(E) = 0.690), weak genetic structuring among 13 remnant populations (F(ST) = 0.047) and a consistent deficit of heterozygotes from Hardy-Weinberg expectation were found across all populations (mean F(IS) = 0.22). Positive inbreeding coefficients are most likely due to Wahlund effects and/or inbreeding effects from highly correlated paternity and typically low fruit set. Indirect estimates of gene flow (Nm = 5.09 using F(ST); Nm = 3.12 using the private alleles method) among populations reflects a historical capacity for gene flow through long distance pollen dispersal by sexually deceived wasp pollinators and/or long range dispersal of dust-like orchid seed. However, current levels of gene flow may be impacted by habitat destruction, fragmentation and reduced population size. A genetically divergent population was identified, which should be a high priority for conservation managers. Very weak genetic differentiation indicates that the movement and mixing of seeds from different populations for reintroduction

2344: *+.043*

The absence of an organism from a landscape for a long time can be a major barrier to the restoration of that species due to factors such as environmental conditions changing since extinction. This can make it difficult to assess the feasibility of reintroduction when an extirpated species cannot, by definition, be observed in the landscape of interest. In such situations, two important options for conservation scientists include: (1) to draw on insights from analogous ecosystems where the organism is extant, or where it has been successfully reintroduced and (2) to undertake research into the reintroduction in the location of interest under controlled experimental conditions. The idea of reintroducing wolves (Canis lupus) to the Scottish Highlands provides an excellent case study of such a situation. A key argument for reintroduction has been that native red deer (Cervus elaphus) numbers, considered by many to be ecologically unsustainable, would be reduced through wolf predation. To date, research into the ecological value of reintroduction has focused on this important issue. However, new research, emerging from wolf reintroduction projects in North America, suggests that nonlethal 'behaviourally-mediated' effects of wolves also have a profound effect on deer behaviour and consequently on the ecosystems in which they live. In short, deer avoid places or browse less where there is a high risk of wolf predation, which allows previously inhibited tree regeneration. The implications for wolf reintroduction in Scotland are that changes in deer behaviour could be as important as lethal effects, and that fewer wolves may be needed than indicated by predator-prey modelling to have significant positive impacts on ecosystems in the Scottish Highlands. Understanding the relative likely contributions of both lethal and nonlethal effects in the Scottish context will be challenging because nonlethal impacts result from an interaction between deer behaviour in response to wolf predation and particular landscapes and ecosystem features. While a full reintroduction may be far off, research must begin

in the near term. There would be considerable scientific merit in establishing a large, controlled experiment (for example on an island or in a fenced area) in the Scottish Highlands to examine the relative lethal and nonlethal effects of wolves on deer and ecosystem restoration. In this paper, a long-term pathway for scientific research to provide sound ecological evidence to inform future decision-makers is proposed. (C) 2009 Elsevier Ltd. All rights reserved.

2345: *+.067*

Recovery of the endangered Vancouver Island marmot (Marmota vancouverensis) is contingent upon releases of captive-born marmots into natural habitats. Success of such re-introduction programs largely depends on the ability of released animals to survive in the wild. However, whether and to what extent survival and cause-specific mortality rates of captive-born marmots differ from those of their wild-born counterparts remains unknown. We used radio-telemetry (1992-2007) and mark-resighting (1987-2007) data to estimate seasonal and annual survival rates of the Vancouver Island marmot, to compare survival and cause-specific mortality rates of captive-born marmots that have been released into the natural habitat with those of wild-born marmots, and to test for the effect of age-at-release on survival of the released marmots. Analysis of radio-telemetry data suggested no difference in survival of males versus females. However, annual survival of captive-born marmots released into the wild was low ($S = 0.605$; 95% CI = 0.507-0.696) compared to wild-born marmots ($S = 0.854$; 95% CI = 0.760-0.915). Marmots released as 2-year-old or older survived more successfully than those released as yearlings. Additional forensic evidence reinforced the idea that predation was the most important cause of mortality. Causes of death differed significantly between captive-born and wild-born marmots. Predation by golden eagles (Aquila chrysaetus) was the most important cause of mortality for captive-born marmots, whereas predation by wolves (Canis lupus) and cougars (Fells concolor) was more important for wild-born marmots. Age-specific apparent annual survival rate, estimated using the combined mark-resighting and radio-telemetry data, was lowest for pups ($S = 0.500$; 95% CI = 0.375-0.616) and highest for yearlings and adults ($S = 0.656$; 95% CI = 0.604-0.705); and apparent survival of 2-year-old was similar to that of yearlings and adults ($S = 0.649$; 95% CI = 0.527-0.754). Our results, based on the analysis of radio-telemetry data, suggest that delaying release of captive-born marmots until 2 years of age may enhance their probability of survival in the wild, and will likely improve the success of the release program. (C) 2009 Elsevier Ltd. All rights reserved.

2346: *+.167*

Climate change is causing many organisms to migrate to track climatically-suitable habitat. In many cases, this will happen naturally, but in others, human intervention may be necessary in the form of 'assisted colonisation'. Species re-establishments in suitable parts of their historic ranges provide an opportunity to conserve some species and to test ideas about assisted colonisation. Here, bioclimatic models of the distributions of two extinct British butterflies, Aporia crataegi and Polyommatus semiargus, were used to investigate the potential for re-establishment in Britain. Generalised additive models and generalised linear models were created to describe the species' European distributions for the period 1961-1990. All models projected the British climate during this period to be suitable for both species. Thirty-year climate projections for the periods 1991-2020 and 2021-2050, and for three climate change scenarios, were then put into the models to generate projections of climatic suitability throughout the 21st century. British climate was projected to remain highly suitable for A. crataegi, but to decline somewhat for P. semiargus. Southern and eastern Britain were found to be the areas most likely to support suitable climate. This difference between the species appeared to be due in part to decreasing summer rainfall in

climate change projections, as this should only benefit A. crataegi. It is concluded that, with further study of habitat requirements, both species could be reintroduced to Britain as part of a long-term European conservation strategy. (C) 2009 Elsevier Ltd. All rights reserved.

2347: *+.021*

The ultimate goal of most translocation efforts is to create a self-sustaining wild population of a species deliberately moved from one part of their range to another. As follow-up of a translocation attempt is often difficult, causes for failure are relatively unknown. Dispersal away from the release site is one potential source of failure because it decreases the likelihood of the released population establishing itself post-translocation. In this study, we used chukar Alectoris chukar as a surrogate for translocated game birds in order to conduct a large-scale experimental study. We observed that these desert-adapted birds demonstrate a strong fidelity for specific water sources. We also report the propensity for the translocated individuals to either disperse and return to their original water source site or remain at the release site. During two field seasons, we observed opposing behaviors such that the proportion of individuals returning to the capture site, versus those remaining at the release site, shifted between years. We analyzed this change between the years as well as within the years to assess the potential underlying causes such as translocated distance, differences in rainfall between seasons and water source type. We concluded that homing behavior was strong in this non-migratory bird species and that strength of this homing behavior varied, potentially due to conditions surrounding the limiting resource, water availability. The large-scale, original data presented here may help to explain why some releases result in a successfully established population while other releases result in widely dispersed individuals.

2348: *+.213*

We evaluated translocation as a restoration technique for southern fox squirrels (Sciurus niger niger) by capturing squirrels from sic donor populations in coastal South Carolina and releasing them into unoccupied habitats on St. Phillips (n = 24) and Hall Islands (n = 28), South Carolina, during Jan. 1999-Jan. 2000. We monitored survival, home range size and habitat use during <= 90-d post release, 91-180-d post release and >365-d post release. Annual survival on St. Phillips Island (71%) was similar to reports for other populations in the Southeast, but survival on Hall Island (34%) was lower than reported for those populations. Home range sizes of males and females on St. Phillips Island and females on Hall Island decreased from <= 90-d to 91-180-d post release, and were similar to those of reported populations by >365-d post release. Home range size for males on Hall Island remained large throughout our study. During <= 90-d post release, males and females on St. Phillips Island established home ranges containing a disproportionate amount of tall grass marsh causing that habitat to rank higher than mature pine habitat. Tidal flats were represented less in home ranges than expected. By 91-180-d post release, squirrels on St. Phillips Island used habitats within their home ranges randomly excerpt for males at <= 90-d who used food plots more than mature pines and all habitats ranked above fallow fields and young planted pines. Our data suggested translocation is a useful tool in the management southern fox squirrels.

2349: *+.121*

Numerous animal species have been introduced to areas from which they were previously absent, and many of these have become invasive, with substantial impacts. However, in other cases, impacts are assumed from theory. Empirical demonstrations are uncommon, making evidence-based conservation policy difficult to achieve. Here we review the broader ecological and conservation lessons from recent work on non-indigenous species in two southern systems, the

policy implications thereof, and the subsequent changes to policy as a result of this work. First, we discuss invasions in the Antarctic region. Strong relationships exist between numbers of animal invasions and numbers of human visitors to Southern Ocean Islands, abiotic factors are often limiting for introduced species, homogenization across islands differs among taxonomic groups, and control actions can rapidly result in unintended consequences. This knowledge has influenced national policy and decisions within the Antarctic Treaty System. Second, we discuss ungulate introductions and translocations, both in South Africa and elsewhere. We show that substantial homogenization has resulted from both processes. However, firm evidence for impacts of ungulate introductions and translocations is sometimes difficult to find, despite the theoretical likelihood thereof. Such a lack of information may have profound consequences for the effective implementation of policy.

2350: *+.078*

Background: The harpy eagle (Harpia harpyja) is the largest Neotropical bird of prey and is threatened by human persecution and habitat loss and fragmentation. Current conservation strategies include local education, captive rearing and reintroduction, and protection or creation of trans-national habitat blocks and corridors. Baseline genetic data prior to reintroduction of captive-bred stock is essential for guiding such efforts but has not been gathered previously.Methodology/Findings: We assessed levels of genetic diversity, population structure and demographic history for harpy eagles using samples collected throughout a large portion of their geographic distribution in Central America (n = 32) and South America (n = 31). Based on 417 bp of mitochondrial control region sequence data, relatively high levels of haplotype and nucleotide diversity were estimated for both Central and South America, although haplotype diversity was significantly higher for South America. Historical restriction of gene flow across the Andes (i.e. between our Central and South American subgroups) is supported by coalescent analyses, the haplotype network and significant F(ST) values, however reciprocally monophyletic lineages do not correspond to geographical locations in maximum likelihood analyses. A sudden population expansion for South America is indicated by a mismatch distribution analysis, and further supported by significant ($p<0.05$) negative values of Fu and Li's D(F) and F, and Fu's F(S). This expansion, estimated at approximately 60 000 years BP (99 000-36 000 years BP 95% CI), encompasses a transition from a warm and dry time period prior to 50 000 years BP to an interval of maximum precipitation (50 000-36 000 years BP). Notably, this time period precedes the climatic and habitat changes associated with the last glacial maximum. In contrast, a multimodal distribution of haplotypes was observed for Central America suggesting either population equilibrium or a recent decline.Significance: High levels of mitochondrial genetic diversity in combination with genetic differentiation among subgroups within regions and between regions highlight the importance of local population conservation in order to preserve maximal levels of genetic diversity in this species. Evidence of historically restricted female-mediated gene flow is an important consideration for captive-breeding programs.

2351: *+.200*

A 312 bp segment of the mitochondrial cytochrome b gene was sequenced from 132 sea bass Lates calcarifer individuals from nine populations across Peninsular Malaysia. Phylogenetic analysis and analysis of molecular variance within and among populations showed no significant geographical structuring. Several populations formed discrete units while others were of mixed populations. The former group suggests a low gene flow among some populations while the latter suggests that widespread translocations have impacted the other wild and cultured local populations. The data from this study have important implications for fishery management, conservation of sea bass

stocks and translocation policy for aquaculture and stock enhancement in Peninsular Malaysia.

2352: ***+.306***

Background: The usual paradigm for translocations is that they should not take place in declining populations until the causes(s) of the decline has been reversed. This approach sounds intuitive, but may not apply in cases where population decline is caused by behavioral or demographic mechanisms that could only be reversed by translocation itself.Methodology/Principal Findings: We analyzed a decade of field data for Pyrenean brown bears (Ursus arctos) from two small populations: the growing Central population - created from a previous translocation and the endemic Western population - believed to be declining because of excessive human-caused mortality. We found that adult survival rates for both populations were as high as those observed for most other protected brown bear populations. However, the Western population had much lower reproductive success than the Central population. Adult breeding sex ratio was male-biased in the Western population and female-biased in the Central population. Our results exclude high anthropogenic mortality as a cause for population decline in the West but support low reproductive success, which could result from sexually selected infanticide induced by a male-biased adult sex ratio or inbreeding depression. Using a stochastic demographic model to compute how many bears should be released to ensure viability, we show that the Western population could recover provided adequate numbers of new females are translocated.Conclusions/Significance: We suggest that a translocation could take place, even if the decline has not yet been reversed, if the translocation itself removes the biological mechanisms behind the decline. In our case, the ultimate cause of low reproductive success remained unknown (infanticide or inbreeding), but our proposed translocation strategies should eliminate the proximate cause (low reproductive success) of the decline and ensure population recovery and viability.

2353: ***+.015***

In 2005, Katala Foundation started planning and building the Katala Institute for Ecology and Biodiversity Centre (KIEBC). It is meant as a centre for environmental education of the local human population, youth, plus national and international visitors. Breeding programmes and, if possible, reintroduction schemes for endangered endemic species, such as Philippine cockatoo (Cacatua haematuropygia) and Philippine pond turtle (Siebenrokkiella leytensis) are being developed. The centre aims to become a research facility for ecology and biodiversity conservation, especially management of endangered species, preservation and habitat restoration. A number of the required buildings and facilities have been completed recently.

2354: ***+.278***

Tropical botanical gardens (TBGs) should have a leading role in in situ conservation by directly promoting several initiatives, including the reintroduction of important or valuable native species, focused habitat restoration, 'assisted migration' of species that are vulnerable to climate change, and creative local collaboration with governments, NGOs and indigenous peoples. Compared with temperate gardens, TBGs face heightened challenges for ex situ conservation, including greater absolute amounts of biodiversity, need for resource mobilization, risk of introducing invasive species and potential genetic introgression within living collections. Meanwhile, the ecosystems surrounding TBGs have undergone widespread and rapid conversion. Here, we provide several illustrations of the effectiveness of TBGs in achieving their mission of preserving tropical biodiversity at the frontier of in situ ecosystem management.

2355: ***+.129***

The Cape Fear shiner Notropis mekistocholas is an endangered minnow endemic to the Cape Fear River basin of North Carolina; only five populations remain, all of which are declining. Determining the population densities and habitat requirements of the species is critical to its survival and restoration planning. We conducted population surveys (four sites) and instream microhabitat suitability analyses (six sites) on the Rocky and Deep rivers to (1) estimate the population density of Cape Fear shiners, (2) quantify the use, availability, and suitability of microhabitats, and (3) determine whether physical habitat alterations were a likely cause of local extirpations and whether instream habitat limits the occurrence and density of this species. Density ranged from 795 fish/ha to 1,393 fish/ha (4,768-7,392 fish/km) at three of the sites surveyed and was too low to be estimated at the fourth site. The fish most frequently occupied riffles and velocity breaks at moderate depths over gravel substrates. It occupied microhabitats nonrandomly with respect to availability; the microhabitats occupied were similar between spawning and postspawning seasons but shallower during spawning. Comparisons of suitable habitat among sites where the fish is extant, rare, or extirpated suggest that suitable substrate (gravel) is lacking where the fish is rare and that suitable microhabitat combinations, especially with respect to water velocity, are rare at all sites. Potential reintroduction sites where the species is rare or extirpated were shallower than extant sites, and one site where the fish is extirpated contained suitable physical habitat but lacked adequate water quality. Another site where the species is rare would require substrate alteration to improve conditions. The survival and recovery of the Cape Fear shiner is dependent on the protection of remaining suitable physical habitat with approaches that consider instream habitat, water quality, and biotic interactions as well as human uses and alterations of the river, riparian zone, and watershed.

2356: ***+.184***

The chamois is a useful species with which to investigate the combined genetic impact of habitat fragmentation, over hunting, and translocations. Genetic variation within and between chamois (genus Rupicapra) populations was analyzed in 259 individuals from 16 sampling sites located in Italy, Spain, Slovakia, and the Czech Republic. Two mitochondrial DNA markers (control region and cytochrome b) and 11 nuclear microsatellites were typed. The principal results of this study can be summarized as follows: 1) high and significant differentiation between almost all chamois populations is observed even on a microgeographical scale, probably caused by the patchy distribution of this species, sharp geographical barriers to gene flow, and drift effects related to recent bottlenecks; 2) historical translocation events have left a clear genetic signature, including interspecific hybridization in some Alpine localities; 3) the Apennine subspecies of chamois, Rupicapra pyrenaica ornata, shows a high and similar level of divergence (about 1.5 My) from the Pyrenean (Rupicapra pyrenaica pyrenaica) and the Alpine (Rupicapra rupicapra) chamois; therefore, the specific status of these taxa should be revised. These results confirm the potential of population genetic analyses to dissect and interpret complex patterns of diversity in order to define factors important to conservation and management.

2357: ***-.295***

Population structure and genetic diversity were examined using partial mitochondrial cytochrome b gene sequences of four wild, one reintroduced, and five captive populations of the endangered cyprinid Hemigrammocypris rasborella from three river systems in the easternmost region of the species' range in Shizuoka Prefecture, central Honshu, Japan. We detected loss of genetic diversity from portions of the wild and captive populations, as well as suspected nonindigenous haplotypes

in some captive, reintroduced, and even wild populations. Given the population structure revealed, we suggest that the populations should be managed with consideration for both the endemism and viability (avoidance of inbreeding depression) of the local populations.

2358: *+.188*

Large-scale restoration of quality habitats is often considered essential for the recovery of threatened pond-breeding amphibians but only a few successful cases are documented, so far. We describe a landscape-scale restoration project targeted at two declining species-the crested newt (Triturus cristatus Laur.) and the common spadefoot toad (Pelobates fuscus Wagler)-in six protected areas in southern and southeastern Estonia. The ponds were restored or created in clusters to (i) increase the density and number of breeding sites at local and landscape levels; (ii) provide adjacent ponds with differing depths, hydroperiods and littoral zones and (iii) restore an array of wetlands connected to appropriate terrestrial habitat. In only 3 years, where 22 of the 405 existing ponds (5%) were restored and 208 new ponds (51%) created, the number of ponds occupied by the common spadefoot toad increased 6.5 times. Concerning the crested newt and the moor frog (Rana arvalis Nilsson), the increase was 2.3 and 2.5 times, respectively. The target species had breeding attempts in most of the colonised ponds-even more frequently than common species. Also, the amphibian species richness was higher in the restored than in the untreated ponds. The crested newt preferably colonised ponds that had some submerged vegetation and were surrounded by forest or a mosaic of forest and open habitats. The common spadefoot toad favoured ponds having clear and transparent water. Our study reveals that habitat restoration for threatened pond-breeding amphibians can rapidly increase their numbers if the restoration is implemented at the landscape scale, taking into account the habitat requirements of target species and the ecological connectivity of populations. When the remnant populations are strong enough, translocation of individuals may not be necessary.

2359: *-.032*

Allium roylei Stearn of family Alliaceae is a potential gene reservoir harbouring genes imparting resistance against downy mildew and leaf blight. Except for one, all other populations of this species worked out, till date, are complex translocation heterozygotes. This bottleneck of sterility coupled with extrinsic pressures is threatening the very survival of this important species. This necessitates immediate enforcement of various conservation strategies to protect and proliferate this taxon. The present communication is a step towards these efforts.

2360: *+.248*

Reestablishment of locally extinct populations and augmentation of declining populations are management activities used with increasing frequency in the conservation of imperiled fishes in the United States. Unfortunately, these options were not always carefully or appropriately used in past cases, partly owing to a lack Of guidelines that address scientifically-based protocols for propagation, translocation, reintroduction, and augmentation (PTRA). PTRA programs are,in important management tool for the recovery of imperiled fishes when undertaken with careful planning, including everything from determining that PTRA is necessary to incorporating knowledge of life history and genetics into the PTRA plan. In addition, PTRA programs must also assemble advisory groups, obtain funding and permitting, construct and maintain propagation facilities, and raise community awareness of the program. Because such diverse skills are needed, Successful PTRA programs should prepare for long-term partnerships to achieve the goal of recovery.

2361: ***+.067***

In South Africa, a plan was launched to manage separate sub-populations of endangered African wild dogs (Lycaon pictus) in several small, geographically isolated conservation areas as a single meta-population. This intensive management approach involves the re-introduction of wild dogs into suitable conservation areas and periodic translocations among them. Despite the initial failures and high costs associated with wild dog re-introductions and translocations, there is no predictive framework available to quantify which management protocol is the most efficient. We therefore developed an individual-based model of wild dog population and pack dynamics, which accounts for the wild dogs' social complexity. The model appeared to capture the essential characteristics of a real wild dog population from Hluhluwe-iMfolozi Park, South Africa and to be relatively robust to parameter uncertainty, suggesting that the model is valid enough for addressing management problems. The model enabled us to quantify a critical initial number of packs (two) and individuals per pack (six) necessary for a re-introduced wild dog population to establish itself in the release area. We also found a practically feasible intervention regime at which a re-introduced wild dog population had the best chance of persistence: intermittently adding packs (at least every 6 years) and harvesting disperser groups (as often as every 4 years) for translocation to other release sites, without threatening the small source population. This study demonstrates that individual-based models can be a powerful decision-support tool in re-introduction planning and provides insight into how populations made up of social groups have dynamics, and ultimately persistence, determined by individual behaviour. (C) 2009 Elsevier Ltd. All rights reserved.

2362: ***+.074***

Sensitivity analyses of population growth in desert tortoise (Gopherus agassizii) have shown no consensus on the limiting vital rate. More importantly, the most sensitive vital rate might not be the most readily manipulated by management, so it begs the question of what actions would be most effective. We compared 13 management alternatives using a vital rate sensitivity analysis that is valid regardless of age structure, and is sensitive to initial population size and time frame, to determine the efforts required for equivalent population growth. We evaluated three time frames, each with five initial population sizes and three initial age distributions. To achieve equivalent population growth, mortality of older females needed to be reduced less than did mortality of other age classes. Similarly, fewer adults needed to be introduced to a population to have the same effect as releasing juveniles, but differences among adult age classes were trivial. A single release (headstarting) required fewer total individuals than did annual releases to achieve the same population growth. Also, the same population growth was more easily achieved when the initial age structure was deficient of young animals. Interestingly, because small tortoises are difficult to survey, some management alternatives could result in increased population size but decreased numbers of countable individuals over short to intermediate (25 years) time frames. Our paper demonstrates an approach to determine what constitutes equivalent management actions for population growth, thus allowing managers to more directly compare expected gains toward population recovery achieved by their resource-allocation decisions. (C) 2009 Elsevier Ltd. All rights reserved.

2363: ***+.108***

Captive breeding for species of conservation concern is the act of bringing rare or endangered animals into captivity with the hope of rearing sustained captive populations for eventual reintroduction into the wild. Within captivity, genetic changes can occur that may reduce a species' ability to persist once a population is reintroduced back into its natural habitat. We sought to

determine the efficacy of recommendations made to minimize genetic adaptation to captivity by addressing the following questions: (i) Are these recommendations already being carried out in captive programs? (ii) How practical is each recommendation? and (iii) Which recommendations call for future investigation? We performed an extensive search of the published literature for studies of non-domestic, non-model, captive animals in which the investigators used and reported a strategy that can minimize genetic adaptation to the captive environment. We found different forms of each recommendation already being executed in captive programs to varying degrees. in all, we reviewed 90 articles covering four broad categories of strategies. We conclude that the best approach to minimize genetic adaptation is to reduce the number of generations that a species spends in captivity. If this is not possible, then we suggest attempting to minimize generations first by delaying reproduction and then by cryopreservation of germplasm. Other strategies are effective to varying degrees depending on the species' natural history. A large gap in the current literature is the interactive effects of multiple strategies being implemented simultaneously, future research should focus on this issue. (C) 2009 Elsevier Ltd. All rights reserved.

2364: ***+.256***

Red squirrels have undergone a 30% contraction of their range in the last 10 years in Ireland, a decline attributed to the introduced grey squirrel. Large regions in the west of Ireland are free of both species of squirrel, due to the isolation of forests there and their relatively recent planting. The potential of these forests as translocation sites for red squirrels was investigated to ascertain the possibility of increasing the range of red squirrels in Ireland. Nineteen red squirrels were moved into Derryclare wood, Co. Galway using a soft release into enclosures, and their subsequent survival and settlement in the wood was monitored, using trapping and radio-tracking. The successful release of 94.7% of squirrels from the enclosure, and 68.4% survival to the start of the following year's breeding season were in excess of the target survival rates of 75 and 50%, respectively. Five of the females were found to be lactating in May 2006, and seven offspring captured in August 2006. A squirrel was observed in a follow-up visit in October 2007. Radio-tracked red squirrels tended to remain in the general vicinity of release, incorporating supplementary feeders as part of their home ranges. Squirrels also fed on the natural food available in the wood, with feeding signs readily observed in the wider woodland. The techniques used proved very successful in survival, retention and future reproduction of the translocated population. Timing of release may be an important consideration for future translocations with survival better in summer-released animals.

2365: ***+.069***

Note about the occurrence and observations of the Pearly Parakeet (birds, Psittacidae) in the state of Pernambuco, NE Brazil. A group of twelve individuals of Pearly Parakeet was found inhabiting an Atlantic Forest fragment in Recife, NE Brazil. The individuals seemed to be well adapted to the place. In non systematic visits to the area between 2004-2006 we observed some items of the diet, defense behavior against predators and population increase. The origin of the individuals is probably of improper release or escape from captivity, since this species doesn't inhabit the Atlantic Forest. Since it is a threatened species, this work also aims to contribute to conservation efforts, like reintroduction programs in areas of original distribution where the species is extinct.

2366: ***+.109***

We conducted opportunistic observations on the diet of translocated orange-fronted parakeets (Cyanoramphus malherbi) on Maud Island to provide a first account of the diversity of food types

ingested in the wild by this critically endangered species. Orange-fronted parakeets consumed fruits and leaves of 14 plant species as well as non-dietary items such as bark sticks and grit. Of dietary items, 96% were on plant species and 4% invertebrates. Of the plant species ingested 10% were non-natives. A major dietary component consisted of fruits and leaves of mahoe (Melicytus ramiflorus). In contrast to the only other published account of the diet of orange-fronted parakeets, invertebrates constituted a minor part of identified ingested items. This may be related to the different composition of vegetation at the study sites, the low parakeet population density during the time of our study and methodological restrictions during our survey. Our observations on undocumented food items add information about the biology of New Zealands' rarest parakeet species and indicate dietary flexibility of the species highlighting the potential of other regenerating islands as release sites to expand the geographic distribution of orange-fronted parakeets.

2367: ***+.079***

Screening for pathogenic micro-organisms is an essential component of translocation-based conservation management. While there are some data on pathogens in New Zealand passerines, little is known about the distribution and prevalence of pathogens infecting New Zealand Psittaciformes. We conducted a survey for pathogens of the vulnerable New Zealand endemic red-crowned parakeet Cyanoramphus novaezelandiae in two wild populations (Little Barrier Island and Raoul Island), and in a translocated population (Tiritiri Matangi Island). A total of 101 cloacal samples were tested for Salmonella and Yersinia. Of these, 82 samples were also tested for Campylobacter. None of these microorganisms were detected. Although our sampling effort was insufficient to detect a low prevalence of Campylobacter, modelling of minimum detectable prevalence of Salmonella and Yersinia indicates that these micro-organisms would have been detected if present as common or chronic conditions of red-crowned parakeets at these sites.

2368: ***-.118***

This paper discusses human-elephant conflict (HEC) in Koundinya Wildlife Sanctuary (KWS), one of the two sites in Andhra Pradesh colonized by elephants during the 1980s after dispersing from sites in Tamil Nadu and Karnataka states. The nature and extent of the past and present HEC, causes for the conflict, mitigation measures adopted, and their effectiveness are discussed based on a one year study (January-December 2005). The findings reveal that the primary reason for the decline in HEC is due to the decline in elephant numbers, especially adult bulls in the case of man slaughter, and that the crop damage mitigation measures adopted by the Forest Department have not been a success on the whole. As for tackling HEC, we suggest translocation of the animals to other elephant habitats as the existing small population (12 individuals) is theoretically speaking not viable to survive into the future and due to the problems facing the Sanctuary, unless the Forest Department is keen on conserving the species in KWS for which management measures are recommended.

2369: ***+.028***

The Kashmir Deer or Hangul Cervus elaphus hanglu, a critically endangered deer, is one of the four easternmost subspecies of Red Deer found in Asia and is endemic to the mountains of Kashmir in the north-western Himalayan region of India. At present, the only viable Hangul population is confined to the 141 sq. km Dachigam National Park (NP), with a few isolated Hangul herds in its adjoining protected areas. Here, we present our recent (2001-2008) assessment of the Hangul's status and conservation in the Kashmir region based on intensive monitoring in

Dachigam NP and extensive surveys carried out all over the Hangul's erstwhile stronghold and range. Our range-wise surveys indicate that at present the last surviving and genetically viable Hangul population of 140-170 individuals is restricted to Dachigam NP. A few isolated Hangul populations are also present in the adjoining conservation reserve areas of Bren-Nishat (11 Hangul), including Cheshmashahi Forest Reserve, south-west of Dachigam NP, where a direct sighting of two Hangul females was made in autumn; Khrew (2-6 Hangul); Khanagund (1-2 Hangul); Shikargah (7-12 Hangul) and Overa Wildlife Sanctuary (6 Hangul). Besides, Hangul use the Surfrao and Akhal blocks of Sindh Forest Division, north-east of Dachigam NP, during spring and summer. A group of about 12 Hangul was sighted north of the holy Amarnath cave, which falls just outside the demarcated boundaries of the Overa-Aru and Baltal-Thajwas wildlife sanctuaries, east of Dachigam NP. The current population trends indicate that the species could go extinct if the necessary serious interventions are not made immediately. This study attributes the decline in Hangul population to low breeding, female biased sex ratio, the problem of survival of the young, inadequate recruitment of fawns to adulthood due to factors such as considerable predation by the Leopard Panthera pardus and Asiatic Black Bear Ursus thibetanus, poaching and continued degradation of Hangul summer habitats in Upper Dachigam, along with biotic interference in winter habitats, and the movements of Hangul in summer to unprotected areas in Sindh Forest Division outside Dachigam NP and the excessive biotic interferences therein. Significant parasitic infestations have also been found in faecal samples of Hangul in Dachigam NP. The Hangul population in Dachigam NP and its adjoining areas thus need; immediate attention. An intensive population monitoring programme, studies of the reproductive ecology and movement patterns of the Hangul and monitoring its health to understand better the factors affecting the population growth and biology and other aspects of Hangul ecology are required for effective management and long term conservation. Population studies indicate a decrease in genetic heterozygosity over time and thus there is a need for urgent measures to arrest the loss in heterozygosis and declining trend of the Hangul population. There is an urgent need for a Hang 11 recovery plan to be developed that includes field surveys to identify corridors to help dispersion and reintroduction of Hangul to its former distribution range and habitat protection in Upper Dachigam and other potential Hangul habitats outside Dachigam. A captive breeding plan for the Hangul is important to repopulate existing good habitats in the Hangul range, beginning with the Shikargah-Overa ranges in Lidder Valley.

2370: *.000*

The mitochondrial cytochrome b gene was sequenced for six individuals of the pygmy madtom, Noturus stanauli, a globally imperilled catfish, from both known localities in Tennessee (U.S.A.) separated by over 1055 river km. Phylogenetic and population genetic analyses revealed little divergence between these populations for this locus. (C) 2009 The Authors Journal compilation (C) 2009 The Fisheries Society of the British Isles

2371: *+.178*

There are few effective or efficient methods for monitoring arboreal forest lizards, especially in areas with low lizard densities. This is problematic for their conservation and research. I developed a novel technique for capturing lizards using closed-cell foam covers as artificial retreats placed on tree trunks. I tested the method at three sites in New Zealand by comparing lizard occupancy rates with the outcomes from conventional methods (lizard houses, g-minnow traps, pitfall traps, Onduline artificial retreats, and spotlighting). At the site where Duvaucel's Geckos (Hoplodactylus duvaucelii) were abundant, most methods detected their presence, but the foam cover technique detected geckos much more effectively (39 geckos per 100 observations).

Also, I was able to sample juvenile lizards better using foam covers than any other technique, and both sexes used foam covers equally. On a per unit effort basis, night spotlighting resulted in 0.6-1.6 geckos per hour, whereas covers returned 3.1 geckos per hour. At the other two sites where either Pacific Geckos (H. pacificus) or Forest Geckos (H. granulatus) were at low densities, only foam covers detected them. Lizard houses typically caught either zero to one gecko per 100 observations; whereas, at these sites, foam covers returned four geckos per 100 observations, despite low gecko abundance. This is a significant improvement in detection. Foam covers offer improved population sampling of arboreal forest lizards, improved efficiency, and lower costs compared to other sampling techniques. They may also reduce some potential biases experienced when using other sampling techniques. This study suggests that foam covers are an effective method for inventorying and monitoring arboreal lizard communities.

2372: *+.092*

Alpine marmots were introduced in the French Pyrenees between 1948 and 1988. The exact number of re-introduced individuals is unknown, but it oscillated around 400. The likely preference of marmots for the southern sunny slopes rapidly facilitated their expansion to the southern Pyrenees, where the lack of both natural predators and of important interspecific competitors also likely facilitated an important expansion of this species. There is only one attempt to broadly calculate the population of marmots in the southern Pyrennes, estimating a population of around 10,000 individuals. However, there exist no reliable data to calculate the potential distribution of this new colonizing species in the southern Pyrenees, and a map of. the potential distribution of the species is necessary to see whether alpine marmots can potentially establish in sites where it might be necessary to manage its populations for various reasons. We developed a map of potential distribution based on census carried out in summer of 2007 in an area of more than 600 km. We censused more around 300 colonies together with around 300 random points to characterized habitat selection variables. A map with a pixel size of 15 x 15 m has been obtained based on preferred habitats and also on distances to other habitats for the whole southeastern Pyrenees.

2373: *+.004*

A key goal in road ecology is to determine which species are most vulnerable to the negative effects of roads on population persistence. Theory suggests that species that avoid roads are less likely to be negatively affected by roads than those that do not avoid roads. The goal of this study was to take a step toward testing this prediction by evaluating the behavioral response to roads and traffic of a species whose populations are known to be negatively affected by roads and traffic, the northern leopard frog (Rana pipiens). We studied the movement patterns of northern leopard frogs during their spring migration from overwintering sites in a river to various breeding ponds that were disconnected from the river by roads. We performed short-distance translocations of migrating frogs, followed them visually, and documented their movement coordinates following each hop, both near the roads and in non-roaded areas. We found that frogs took longer to move near roads with more traffic and that their movement was quickest in areas without roads nearby. Frogs tended to deviate more from a straight-line course when they were released near roads than compared with control areas, but this response was independent of traffic volume. All frogs released near roads attempted to cross the road. On very low traffic roads (10.86 mean vehicles per hour), 94% of frogs crossed the road successfully, whereas at higher traffic roads (58.29 mean vehicles per hour) 72% were successful. Our results suggest that frog's inability to avoid going onto roads and their slow movement combine to make them particularly vulnerable to road mortality, which likely explains the strong negative effects of roads on frog population abundance.

Conservation efforts should focus on preventing frogs from accessing the road surface through the use of drift fencing and culverts.

2374: *+.218*

Reintroduction of endangered or extinct species is becoming major restration activities for conservation of biodiversity and ecosystems. The crested ibis (Nipponia nippon), once extinct in Japan, was released into natural environment on Sado Island, Japan, in September 2008. Since orthopteran insects are thought to be one of the major prey for the crested ibis, it is important to clarify the factors influencing the distribution and abundance of orthopterans. We investigated abundance of five orthopterans (Oxya yezoensis, Atractromorpha lata, Conocephalus maculatus, Ruspolia lineosa and Teleogryllus emma) on Sado Island in September 2008. Local-scale environmental factors including grass-height and land-use type (arable or fallow) were recorded in the field. Landscape-scale factors such as proportion of paddy fields and total length of forest edge in the buffer circle surrounding the paddy fields were extracted using GIS. Various sizes of buffer circles were generated around each paddy to identify appropriate spatial scales. Model selection was performed with a generalized linear model using the AIC to find the best model for accounting for the abundance or the probability of occurrence of these species. Landscape factors were important for all species except A. lata. The proportion of paddy fields had a significant positive effect on the abundance of O.yezoensis which was conspicuously dominant. The interaction between the proportion of paddy fields and the length of forest edge had a significant effect on C. maculatuas and T. emma. At the local scale, intermediate grass-height enhanced the abundance of O. yezoensis and C. maculatus and the occurrence of R. lineosa, and fallow fields were the suitable habitats for T. emma. Our results suggest that maintaining lowland paddy fields containing fallows and reducing the frequency of mowing surrounding paddy fields are effective for increasing the orthopteran abundance and diversity in the paddy-dominated ecosystems.

2375: *+.207*

Translocations are increasingly important tools for endangered species conservation, but their success is often uncertain. We analyzed 125 time series of grazing mammal translocations in South African protected areas. Some 94% of translocations succeeded (66% unambiguously) even though most populations began with <15 individuals and most of the species involved are of conservation concern. Adding new individuals to existing small populations increases per capita growth rates and seems to prevent translocations from failing. Growth of the translocated populations is both greater and less variable than wild mammal populations and appears less affected by the typically important ecological factors (e.g., initial propagule size, precipitation, reserve size, or presence within historical range). One-third of the populations showed robust signs of density dependence but we detect few examples of Allee effects. Our results, from empirical time series of small populations, offer new insights into achieving success for translocation programs limited to releasing few individuals.

2376: *+.064*

We isolated and characterized eight polymorphic microsatellite loci for redside dace (Clinostomus elongatus), a colorful North American cyprinid that is threatened or endangered throughout most of its range. The number of alleles per locus ranged from three to eighteen, with observed heterozygosity ranging from 0.31-0.92. Cross-amplification revealed that these markers will also be useful for examining closely related and more distantly related species, including the rosyside dace (Clinostomus funduloides), Lahontan redside shiner (Richardsonius egregius), hornyhead

chub (Nocomis biguttatus), and central stoneroller (Campostoma anomalum). These microsatellite loci will provide a valuable set of tools for examining fine and coarse scale population structure, exploring reproductive success, and testing outcomes of proposed conservation initiatives (e.g. captive breeding and translocation of wild individuals) for redside dace.

2377: *-.044*

Translocation is a strategy commonly used to maximize the persistence of threatened species, but it may sometimes lead to undesirable genetic consequences. The northern quoll (Dasyurus hallucatus) is a carnivorous marsupial that is critically endangered in Australia's Northern Territory due to rapid population declines in areas recently colonized by the exotic cane toad Chaunus [Bufo] marinus. In 2003, 64 quolls were translocated to two offshore islands to establish insurance populations and reduce the species' risk of extinction. In this study, we assessed genetic diversity at five microsatellite loci in the translocated populations, two endemic islands and three mainland populations. In the short-term (three generations), the translocated populations showed a slight but non-significant reduction in genetic diversity (A = 4.1-4.2; H (e) = 0.56-0.59) compared to the mainland source populations (A = 5.0-8.4; H (e) = 0.56-0.71). In comparison, high genetic erosion was observed in the endemic island populations (A = 1.5-2.9; H (e) = 0.11-0.34). Genetic bottlenecks were detected on both endemic islands and in one mainland population, indicating recent reductions in population size. Our results are consistent with previous studies describing greater losses of genetic diversity on islands compared to mainland populations. Divergence from ancestral allele frequencies in the translocated populations also suggests effects due to founder events. This study, although short-term, highlights the importance of continued monitoring for detecting changes in genetic diversity over time and makes a significant contribution to our understanding of the effects of founder events on island populations.

2378: *+.078*

Babcock's leopard tortoises (Stigmochelys pardalis babcocki) are taken to rehabilitation centers in KwaZulu-Natal province, South Africa, because they are either escaped, unwanted, or confiscated pets, or else are confiscated from persons who acquire them illegally from the wild. South African rehabilitation centers are reluctant to euthanize tortoises, and there are few tortoise sanctuaries. Consequently, the local conservation authority, Ezemvelo KwaZulu-Natal Wildlife, developed a release protocol based on International Union for Conservation of Nature and Natural Resources guidelines, to facilitate the release of rehabilitated S. p. babcocki into the wild. The present study was done to determine whether rehabilitated animals could be successfully released into the wild, judged by whether individuals were able to survive in the wild. Seventeen apparently healthy individuals, with longer than 100-mm carapace length, that had been in captivity for more than 2 months in a large rehabilitation center were released into the wild. These rehabilitated animals, with attached radiotelemeters, were hard-released at 2 different sites within the historical range of the species and were monitored over a year. One of the tortoises was returned to captivity because of disease, 3 were killed intentionally or accidentally by humans, one died probably due to being turned over by another animal, 3 others died from a combination of disease, starvation and/or dehydration, and the fate of 6 was unknown. Because only 2 animals survived 13 months after release at one of the sites and only one was known to have survived 25 months after release at the other site, rehabilitated S. p. babcocki were not successfully released into the wild. However, recommendations to improve the probability of success of future releases of rehabilitated S. p. babcocki into the wild are provided.

2379: *+.076*

Currently orangutans are found in widely fragmented and isolated populations. Sumatran orangutan is primarily found in northern Sumatra, and the Bornean orangutans is distributed in Central, West, and East Kalimantan, Sarawak and Sabah. The determination of intra- and inter-species variation between Bornean and Sumatran orangutans is been stated to be essential for both the management of orangutan reintroduction projects and the planning of conservation strategies to preserve the remaining wild populations. This study aimed to identify two species of Orangutans (Pongo sp.) by means of RFLP (Restriction Fragment Length Polymorphisms) analyses of mitochondrial DNA (mtDNA). An approximately 540 bp single fragment of the ND5 gene near the 5'-region was PCR amplified for all samples tested. Digestion pattern for both AluI and MseI were different between two groups of ND5 fragments in this study. Present result showed a rapid protocol to identify these two species by means of RFLP (Restriction Fragment Length Polymorphism) analyses of mtDNA (mitochondrial DNA). This technique can be applied easily to rehabilitation centres and zoos to resolve species discrimination problem.

2380: *+.108*

Without seeking independent scientific review, Interior Secretary Ken Salazar recently approved a 14 January 2009 Bush administration rule to remove endangered species protection from the northern Rocky Mountain (NRM) Distinct Population Segment (DPS) of gray wolves less than 14 years after their reintroduction to Idaho and Wyoming. The "delisting" rule does not adequately address lack of genetic connectivity between Yellowstone wolf packs and other NRM populations, for which reason a federal court overturned tire 2008 predecessor of the rule. The US Fish and Wildlife Service defies its own policies by delisting the Idaho and Montana portions of the DPS while Wyoming wolves remain endangered. Criteria for this delisting are inconsistent with prior delistings of recovered birds and mammals. New scientific understanding of species recovery argues for a higher delisting threshold for the NRM gray wolf metapopulation. Finally, we argue that ecosystem recovery should be a recovery criterion for this unique keystone predator.

2381: *+.231*

Translocations have become one of the most commonly used tools for biodiversity restoration worldwide, however one out of three re-introduction plans fails to create a viable population or to successfully reinforce the existing one. We used results from the analysis of individual-based information on the reintroduction of a threatened waterfowl species, the crested coots Fulica cristata, to provide guidelines to maximise re-introduction success. We found that about a third of the post-release mortality took place within the first month after release. This immediate 'cost of release' in terms of local survival or 'release risk factor' seems to be a common feature of re-introduction projects, and it is likely due to the inexperience of captive-born individuals to face the new environment. This hypothesis was supported by the positive association between survival and time spent in the wild Results suggested that coots released between February and May have a slightly higher survival. A joint measure of survival and breeding probabilities indicated that birds released in late winter (February-March) had a higher chance to survive and reproduce compared to birds released later in the year. From an applied perspective our results can be used within an adaptive management framework to determine the optimum period of release, providing substantial support for future decision-making in the management of waterfowl, and other long-term projects of re-introduction of threatened vertebrate species. (C) 2009 Elsevier Ltd. All rights reserved.

2382: *+.091*

Ex situ conservation of animal populations may benefit from captive-breeding programmes, but these are criticised because they are assumed to be difficult, time-consuming and expensive, while they do not guarantee success. However, such assumptions remain untested in most organisms; for example, introductions could be very useful for recovering populations of small-sized species with short generation time, no learned behaviours, and ease to rear in captivity. Here, we document an easy, cheap and successful reintroduction programme of the lacertid lizard Psammodromus algirus. Two captive-bred cohorts (178 juveniles in 2001 and 187 in 2002) were released in four woodland fragments (0.9-5.2 ha) at two localities (B and V): B housed a stable lizard population whereas V apparently lacked a viable population of lizards. We monitored introduced and native lizards during 2002 and 2003, and carried out a corroborative searching in 2006 which confirmed the existence of a lizard population at site V. Introduced lizards had higher activity and dispersed more frequently among woodland fragments than native ones. Survivorship and growth rates were similar for both groups, but introduced juveniles were about 25% larger than native ones, due to both early hatching and better rearing conditions. The whole procedure was easily implemented in our Faculty facilities (mean hatching and hatchling survival rates of 0.90 and 0.87), and cost less than 20,000 E (excluding salaries). Therefore, similar programmes may be of wide application in small animals and of practical importance for species with a meta-population structure living in fragmented landscapes. (C) 2009 Elsevier Ltd. All rights reserved.

2383: ***-.105***

The costs of wildlife conservation distribute unequally across society. Compensation can potentially redress inequities and raise local tolerance for endangered wildlife that damage property. However, the rules for payments generate controversy, particularly as costs mount and species recover. In Wisconsin (USA), gray wolf damage payments grew notably over 28 years and eventually undermined budgets for conserving other endangered species. We measured attitudes to compensation among 1,364 state residents, including those who voluntarily contributed funds and those likely to receive compensation, and we interviewed elected officials about the politics of payment rules. Most respondents endorsed compensation for wolf damages to livestock-even when wolves are no longer endangered-but opposed payments for wolf damage to hunting dogs on public land. Most donors opposed killing wolves and over one-fourth unconditionally rejected a wolf hunt. We predict the latter donors would stop contributing funds for compensation if the state were to implement a proposed wolf hunt. Controversy over payment rules reveals clashing values regarding wildlife between those receiving and those paying for compensation. Moreover, the costs of compensation ratchet up as endangered species recover and claims of entitlement expand. Hence we recommend conservationists use sunset clauses and an adaptive management of compensation programs.

2384: ***+.062***

Managers are sometimes faced with a situation where one endangered species increases the vulnerability of another one. According to our late-winter helicopter survey of Finland's two small populations of wild-forest reindeer (Rangifer tarandus fennicus), the eastern one decreased by half during the last 7 years. This is probably due to the return of the wolf (Canis lupus) to the area. Our data show that the annual recruitment rate of reindeer is strongly correlated with wolf density. Calf mortality was high and stable during the first months after birth. The wolf is classified as an endangered species in Finland. Thereby, our study area provides an example of a triggered situation in which a locally abundant, endangered predator increases the vulnerability of a threatened prey. There are basically two policy options for avoiding extinction: (1) to directly control the predation risk or (2) to reach further out into the ecosystem to control those factors that

have led to high abundance of predators. One potential direct management action is increased wolf control in the primary summer home ranges of female reindeer. In the long term, wolf predation on wild-forest reindeer would decline if the abundance of moose (Alces alces) could be lowered, because high moose density supports high abundance of wolves. Another noteworthy option is the reintroduction of reindeer into regions where the wolf still exists at low densities.

2385: *+.097*

The presence of the Mediterranean pond turtle Mauremys leprosa in the Ebre Delta (Catalonia, north-east Spain) is well documented after the late 1970s, when the first reptile distribution lists were published. Owing to the fact that the species was considered scarce, a reintroduction programme based on the release of individuals at sites with potential habitat suitability was launched. From 1999 to 2001, 234 turtles of different ages were released and subsequently monitored until 2007, in order to make a conservation diagnosis using five assessment criteria. These criteria were body condition, individual growth, reproduction, survival and population growth rate. Despite the relatively large number of turtles released, no viable population resulted from the programme. Assessment criteria suggested that: (1) released individuals showed good physical condition and satisfactory growth (the only positive results); (2) reproduction was almost absent; (3) local survival was reduced compared with that of Spanish wild populations of the species; (4) temporary emigration was high; (5) the growth rate of the population was negative. In conclusion, the results suggested that the habitat at the Ebre Delta marshes may not be favourable to the species, and that the scarce historical data record may indicate a relatively low-quality habitat; the few records may correspond to individuals dispersing from the river, a more suitable habitat. Thus, we conclude that historically scarce records may be the result of natural patchiness and heterogeneous distributions, and they are not necessarily a good indicator of relict, decimated populations. Good assessment criteria, as those proposed and used here, are necessary tools to assess results in reintroduction projects to recover endangered chelonian populations.

2386: *-.071*

Twenty-five years ago, the snow leopard Uncia uncia, an endangered large cat, was eliminated from what is now Sagarmatha National Park (SNP). Heavy hunting pressure depleted that area of most medium-large mammals, before it became a park. After three decades of protection, the cessation of hunting and the recovery of wild ungulate populations, snow leopards have recently returned (four individuals). We have documented the effects of the return of the snow leopard on the population of its main wild prey, the Himalayan tahr Hemitragus jemlahicus, a 'near-threatened' caprin. Signs of snow leopard presence were recorded and scats were collected along a fixed trail (130 km) to assess the presence and food habits of the snow leopard in the Park, from 2004 to 2006. Himalayan tahr, the staple of the diet, had a relative occurrence of 48% in summer and 37% in autumn, compared with the next most frequent prey, musk deer Moschus chrysogaster (summer: 20%; autumn: 15%) and cattle (summer: 15%; autumn: 27%). In early summer, the birth rate of tahr (young-to-female ratio: 0.8-0.9) was high. The decrease of this ratio to 0.1-0.2 in autumn implied that summer predation concentrated on young tahr, eventually altering the population by removing the kid cohort. Small populations of wild Caprinae, for example the Himalayan tahr population in SNP, are sensitive to stochastic predation events and may be led to almost local extinction. If predation on livestock keeps growing, together with the decrease of Himalayan tahr, retaliatory killing of snow leopards by local people may be expected, and the snow leopard could again be at risk of local extinction. Restoration of biodiversity through the return of a large predator has to be monitored carefully, especially in areas affected by humans, where the lack of important environmental components, for example key prey species, may make

the return of a predator a challenging event.

2387: ***+.295***

Population growth and spread of recently reintroduced species is crucial for the success of their reintroduction. We analysed what limits the spread of two congeneric butterfly species Maculinea teleius and Maculinea nausithous, over 10 years following their reintroduction. During this time, their distributions appeared to be limited to a few sites although it was thought that more suitable habitats were available. Thus, we question, does the quality or the spatial arrangement of their habitat limit their spread? Although adult individuals of both species can select high-quality plots, we show that selection of suitable plots in the area of reintroduction is spatially constrained. A low colonization probability of unoccupied distant plots of high quality was found for both species. The abandonment of occupied plots in Ma. teleius was also found to be dependent on the distance to occupied plots. We conclude that the spatial distribution of the two species during the 10 years following reintroduction was limited by the spatial arrangement of their habitat, rather than by the availability of high-quality plots. The spatial constraints in movement can explain observed source-sink structures when female butterflies deposit their eggs on low-quality plots. We conclude that although these species have very similar life histories, they require different approaches to their conservation due to subtle differences in adult habitat use and movement. Conservation of Ma. teleius should concentrate on improving local habitat quality, whereas conservation of Ma. nausithous is predicted to be more effective by creating a spatial network of suitable habitat plots, such as along road verges.

2388: ***+.158***

The coastal population of the black-headed dwarf chameleon Bradypodion melanocephalum in KwaZulu-Natal appears to be centred on the eThekwini Municipal Area, which is experiencing rapid urban development. This population occurs in few statutory protected areas, all of which are small. In order to conserve successfully the coastal population in KwaZulu-Natal, knowledge of its geographic range and location of potentially suitable habitat is required. Bradypodion melanocephalum was until recently known mainly from Durban, with isolated records from areas to the north and south. A cartographic model was used to estimate the distribution of the coastal population in KwaZulu-Natal. The model predictions were ground-truthed, and the model was found to have a positive predictive power of 0.57 and a negative predictive power of 0.84. The relatively low positive predictive power of the model appeared related to habitat management that is not appropriate for the conservation of B. melanocephalum. The geographic range was then estimated using a Maxent model to predict climate suitability while at the same time taking cognizance of a dispersal barrier, the output of which was further refined in a geographic information system to exclude areas of unsuitable landcover. The variables included in the final model were mean daily minimum temperature of the coldest month and mean daily maximum relative humidity of the coldest month. Both these variables relate to dew formation in the dry winter period, and dew appears to be a critical water source for B. melanocephalum during winter. The Maxent model estimates greater probabilities Of Occurrence towards the coast, where rapid urbanisation is occurring. This urbanisation is one of the threats to the survival of the coastal population of Bradypodion melanocephalum. The utility of translocation of B. melanocephalum to areas that will not be developed within a development site in comparison to translocation off-site as mitigation to ensure the conservation of the coastal population in rapidly urbanizing areas needs to be investigated further.

2389: ***-.099***

In order to conserve the last autochthonous population of the European Fallow Deer, Dana dama dama (Linnaeus, 1758), the Turkish Government began a breeding programme at Duzlercami near Antalya in 1966. The programme began with 7 animals and the numbers continuously increased until the mid-1980s, when they reached over 500 animals. However, the population then collapsed until the year 2000 and did not recover. Today it comprises less than 130 individuals. The reasons for the population collapse are not fully understood but are thought to be a combination of several factors related to increasing human pressure such as urbanisation, recreational activities, and poaching. The population is much below the carrying capacity of the area. Attempts to re-introduce Fallow Deer into other areas of Turkey have not been successful but should be further considered as an option to minimise the risk of extinction, as at present the entire gene pool of the Turkish autochthonous population is concentrated at Duzlercami.

2390: *+.101*

History of Thai zoos started in 1934 with the foundation of Dusit Zoo in Bangkok. In 1954 this zoo was transferred from Bangkok municipality to the newly founded Zoological Park Organization (ZPO). During the following decades four further zoos were established within the ZPO, namely Chiang Mai, Khao Kheow Open Zoo in Chonburi, Korat Zoo in Nakhon Ratchasima and Songkhla. Due to rapid wildlife decline in Southeast Asia the necessity of ex-situ conservation was recognized and a captive breeding management system, called "Species Champions", was established. For each managed species one zoo is designated to become its breeding centre and staff of this zoo is responsible for its management. The ZPO zoos are home to some exceptional and unique stocks of rare and endangered species, e.g. the currently biggest and most successfully reproducing group of Red-shanked douc langurs at Dusit Zoo, the biggest captive stock of Clouded leopards and the first and only breeding Asian black-necked storks at Khao Kheow Open Zoo and almost half of the world's known captive population of Red-headed vultures at Korat Zoo. The long-term goal of many breeding programmes is a reintroduction of species locally or even totally extinct in Thailand. This has already been practiced with several species, e.g. Painted stork and Eld's deer.

2391: *+.083*

Context. Translocation as a non-lethal management tool to eliminate individual 'problem' animals is commonly used but rarely evaluated.Aim. We sought to evaluate whether translocating stock-raiding leopards (Panthera pardus) into a protected area with resident conspecifics in Botswana is effective in dealing with 'problem' carnivores.Methods. We assessed release-site fidelity, ranging behaviour, individual survival and reduction of conflict after translocation of four translocated leopards in relation to four resident conspecifics, which were tracked from 24 days up to 4 years. Key results. None of the translocated leopards showed release-site fidelity; they either returned to the trap site or showed extensive roaming behaviour after release. Three of the four translocated leopards reportedly resumed stock-raiding and were shot when ranging outside of protected areas, which apparently was a consequence of being released into an area already occupied by territorial conspecifics. On the basis of satellite telemetry, the linear movement distance of one translocated leopard (1249 km) and the range size of three resident female leopards (513 +/- 124 km(2)) are the largest on record.Conclusions. From comparing the survival and ranging behaviour of translocated leopards with that of resident conspecifics, we conclude that translocation was not an effective management tool for dealing with stock-raiding leopards in our study.Implications. Rather than translocating 'problem' carnivores, efforts should focus on reducing the potential for problems to develop, most importantly on improving livestock-husbandry practices.

2392: ***+.157***

Abundant populations of elk (Cervus elaphus) are cherished game in many regions of the world and also cause considerable human-wildlife conflicts through depredation on agriculture and speciality crops, lack of regeneration to native ecosystems, collisions with vehicles and transmission of disease between free-ranging and farmed hoofstock. Management of elk varies, depending on current and historical agency objectives, configuration of the landscapes elk occupy, public perception, population density and behaviour of elk. Selection of the method to manage elk often requires knowledge of timing of impacts, duration relief from elk damage is desired, cost-effectiveness of management activities, tolerance of impacts, public perception of management strategies and motivation or habituation of elk to determine the likelihood of success for a proposed management action. We reviewed methods that are available to control abundant populations of elk that include lethal (e.g. hunting, sharpshooting) and non-lethal (e.g. fertility control, frightening) options. We promote an integrated approach that incorporates the timely use of a variety of cost-effective methods to reduce impacts to tolerable levels. Lethal options that include regulated hunting, sharpshooting and aerial gunning vary by likelihood of success, duration needed for population reduction, cost to implement reduction and public perceptions. Several non-lethal options are available to affect population dynamics directly (e.g. fertility control, translocation), protect resources from damage (e.g. fences, repellents) or influence space use of elk on a regular basis (e.g. harassment, frightening, herding dogs, humans). Public perception should be considered by agencies that are looking for feasible methods to control populations of elk. Disturbance to residents or visitors of public property may influence methods of management employed. Future research should explore the duration of harassment needed to avert elk from sensitive areas and costs to implement such programs. Several methods in our review were implemented on deer and additional research on elk and other cervids in conflict with human interests would provide a much needed component to our understanding of management methods available for ungulate species.

2393: ***+.154***

Context. The deliberate accumulation of faeces or scats in one location is a common behaviour used for social signalling. The endangered pygmy bluetongue lizard, Tiliqua adelaidensis, is a solitary-living species that appears to use scats as social signals. Previous studies have found that these lizards approach burrows more cautiously if there is a conspecific scat present and behave differently to scats from male and female conspecifics, indicating that these lizards can recognise scat-based conspecific cues. For endangered species, understanding how an animal uses and responds to chemical cues has the potential to become a powerful tool in conservation management.Aims. To investigate whether these solitary lizards might be using scats as social signals by testing whether scats were being deposited in a non-random pattern designed to maximise their exposure to the nearest neighbour conspecifics.Method. In the present study, we investigated the spatial pattern of scatting behaviour by pygmy bluetongue lizards in the field.Results. Scats were consistently deposited in one direction that was significantly aligned with the nearest occupied burrow of a neighbouring lizard. The same deposition sites were used when previous scats were either destroyed by rain, or experimentally moved to a different location. Male lizards deposited scats significantly closer to their own burrows when neighbours were closer. Key conclusions. The results were consistent with the lizards using scats to advertise their presence and status. The scat-deposition patterns observed in the present study suggest that scats are used as an olfactory signal associated with social organisation of pygmy bluetongue lizards.Implications. Current plans for the conservation management of this endangered species include translocations. By understanding how these lizards respond to, and use chemical cues, we may be able to use this

knowledge to aid in translocation programs, e.g. by pre-establishing territories for individuals before they are introduced into the population.

2394: *+.214*

Context. Giant pandas (Ailuropoda melanoleuca) are restricted to six mountain ranges at the edge of the Tibetan Plateau. One of these ranges, the Qinling Mountains, contains the highest density of giant pandas and is home to similar to 20% of those remaining in the wild. Commercial logging and other developments have resulted in habitat fragmentation, and an efficient and powerful conservation network is now needed for the species in this area.Aims. This study sought to assess giant panda habitat and estimate the carrying capacity of this reserve network. Our goal was to improve the function and carrying capacity of the reserve network and facilitate population growth and gene flow among subpopulations of giant pandas.Methods. We use habitat suitability models to assess the efficacy of conservation networks. With estimation of carrying capacity by home range, we can reveal issues facing reserves and populations of endangered species they contain. Here, we define key habitat, linkages, corridors and overall connectivity and then use habitat network modelling and spatial analyses to design a conservation landscape for giant pandas across their Qinling Mountains stronghold. Key results. We found that 91% of giant panda sightings were in suitable or marginally suitable habitat. The total area of giant panda habitat present in the Qinling Mountains is similar to 1600 km(2) fragmented across four key habitat blocks by national roads or other human activity. The current nature reserve network encompasses 71% of available suitable habitat and 62% of available marginal habitat, meaning a significant proportion of panda habitat remains outside the current conservation network. We found that giant panda reserves across this region are not equal in their carrying capacity; some reserves contain an overabundance of giant pandas and the wellbeing of these populations are in doubt.Conclusions. Our results highlight the potential risk of high densities and bamboo flowering events to the safety of giant pandas. With poor population size and heavy isolation, small populations will not persist without translocation.Implication. Redrawing the reserve network to correct localised problems may improve the function of the giant panda protection system, build capacity in the reserve network, and decrease human-wildlife conflict. We propose a new reserve and adjustment of the borders and region for three reserves.

2395: *-.273*

Context. Aerial application of poison bait pellets is an established and widely used method for removing invasive rodents and restoring insular ecological processes. However, the non-target effects of saturation poisoning require very careful consideration and precautionary risk-avoidance strategies.Aims. We assessed the risk of primary and secondary poisoning by rodenticides to terrestrially foraging lesser sheathbills (Chionis minor marionensis), Gough moorhens (Gallinula comeri) and Gough buntings (Rowettia goughensis) at Marion and Gough Islands.Methods. Birds taken into temporary captivity were offered non-toxic bait pellets dyed different colours and the carcasses of house mice (Mus musculus). In addition, dead mice were offered to these three species in the field, as well as to sub-Antarctic skuas (Catharacta antarctica) at both islands. Response to captivity was assessed by daily weighings. Key results. Individual birds either gained or lost mass overall during their 4-7 days in captivity. Whereas all captive birds pecked at the pellets, minimal amounts were consumed. However, Gough moorhens offered pellets in the field did consume them. Sheathbills (in captivity and in the field) and moorhens (in the field) consumed mouse carcasses, whereas buntings in captivity ate little from them. Sub-Antarctic skuas offered mouse carcasses in the field at both islands readily consumed them. At Gough Island some, but not all, skuas consumed bait in the field.Conclusions. Although the levels of assessed risk to primary

and secondary poisoning differed among the three main species studied, it is recommended that populations for subsequent reintroduction be taken into temporary captivity before and during a poison-bait exercise as a precautionary measure. It is not deemed necessary to take sub-Antarctic skuas into captivity because they will be largely absent during a poisoning exercise in winter (the most likely period).Implications. Captive studies to assess susceptibility to primary and secondary poisoning are useful for determining positive risk; however, cage effects can cause false negatives by altering behaviours, and should be conducted with complimentary field trials. Where endemic species show any degree of risk (e.g. are vulnerable to the poison, regardless of how it might be ingested), precaution dictates that the risk be mitigated.

2396: *+.035*

Context. Nest boxes are a useful tool in the reintroduction, conservation and monitoring of many hollow-using species.Aims. All forms of nest-box monitoring involve some form of invasion, often upsetting their continued use by occupants. We conducted a pilot study to investigate and validate the innovative use of temperature dataloggers (iButtons(R)) to remotely monitor nest-box use, leaving the nest-box occupants untouched.Methods. In captivity, iButton recordings revealed the duration and time of day when each of the three nest-box designs was occupied by Pseudocheirus occidentalis (western ringtail possums); the accuracy of occupancy data was validated by unobtrusive infrared video recording. In the field, where translocated P. occidentalis and naturally occurring Trichosurus vulpecula (common brushtail possum) populations are present, hair sampling at the nest-box entrances (in addition to iButton recording) was used to identify the mammal species present. Key results. Nest-box use by captive P. occidentalis validated iButtons as a useful remote-monitoring tool, with <5-6% error for two nest-box designs. Although there was limited use of nest boxes at the field site, our results confirmed that iButtons are useful for remote-monitoring of nest-box use in the field; iButton data revealed both short (<2 h) and long (>10 h) periods of continuous occupancy (T. vulpecula only). In addition to the convenience (to researcher and animal) of continuous (24-h) monitoring with minimal disturbance, a major advantage from using iButtons is that occupancy can be matched with environmental temperature or rainfall records, as well as other events (e.g. storms or frost).Conclusions. iButtons are a useful remote-monitoring tool of nest boxes, and it is possible that their use in this manner may be extended to tree-hollow occupation. Most importantly, this approach can inform us as to the conditions under which the nest boxes are used by fauna, as well as preferences for different nest-box designs.Implications. It is important to note that the criteria used for determining the presence or absence in the nest box (i.e. temperature difference, T(in)-T(out), of 2 degrees C) in the present study will not be relevant for all nest-box designs and before using these methods, the thermal properties of the nest box or tree hollow will require investigation.

2397: *+.187*

Context. There are few cases where a species has been removed from a list of threatened species as a result of conservation efforts. One such example is the woylie (also known as the brush-tailed bettong), Bettongia penicillata ogilbyi, which was removed from state (Western Australian), national (Australian) and international lists in 1996, following the successful implementation of the species' recovery plan. Since downgrading of its conservation status, the woylie has been considered conservation dependent. Conservation efforts continued in the form of toxic baiting to control the species' principal predator, monitoring to identify trends in distribution and abundance, and translocation to help restore ecosystem function and further secure the conservation status of the species. Recent observations of a decline in abundance of the species have prompted a review of its conservation status.Aims. To assess the conservation status of the woylie in 2006 against

IUCN criteria and to investigate the value of continued conservation efforts following the delisting of the species.Methods. Monitoring data were collated and parameters required to assess the conservation status of the woylie against IUCN criteria were investigated. The various processes associated with conserving the species, such as translocation and monitoring, were also assessed. Key results. The species underwent a rapid decline between 2001 and 2006, reducing the population by similar to 75% to an estimated 10 000 individuals. The decline has not been consistent across occurrences and of particular concern are the declines observed at Perup/Lake Muir, Dryandra and Batalling, which were previously considered amongst the largest and most stable occurrences. In 2006, the species qualified for listing as Endangered using IUCN criteria. The resources allocated to translocation and monitoring the delisted woylie have ultimately resulted in managers being in a much better position to understand and act when an unforeseeable population decline occurred.Conclusion. Conservation efforts and population monitoring of delisted species must continue at a level where changes in distribution or abundance, which are significant enough to support relisting, can be detected.Implications. Threatened species lists should not be the primary consideration in allocation of resources to conservation efforts.

2398: *+.136*

The Iberian lynx, the most endangered cat in the world, is presently found only in two isolated populations in southern Spain. Natural expansion from these populations is limited which turns Iberian lynx reintroduction programs into the only alternative to save the species from extinction. Prey availability is one of the top considerations for predator reintroductions. In this paper, we review the state of the art regarding wild rabbit (lynx's main prey) biology, status and management in the Iberian Peninsula, and future perspectives for Iberian lynx conservation. Historically, wild rabbits have sharply declined in the Iberian Peninsula, mainly as a consequence of habitat loss and the arrival of viral diseases. Most Iberian rabbit populations are still declining so different management techniques are employed to revert this scenario. Population monitoring, adjusting hunting pressure, predator control, habitat management, restocking and rabbit vaccination are the most frequently employed management tools. Surprisingly, strong empirical evidence is still lacking to support the usefulness and impact of most of these management techniques. Hence, for the success of future Iberian lynx reintroductions, efforts need to be made to suppress knowledge gaps of rabbit ecology and management at several levels, namely: the study of basic biological parameters from natural free populations, the implementation of an Iberian rabbit monitoring framework based on standardised rabbit monitoring protocols (that produces systematic and periodic comparable results), the study of the impact of predator control, the assessment of both the costs vs. benefts of vaccinating wild rabbits against viral diseases and the effectiveness of habitat management. Finally, the creation of a working platform congregating researchers, hunters and game managers, conservationists and further sectors involved in wild rabbit management is essential for the defnition of a global strategy that defends collective interests and serves the ultimate goal of conserving this lagomorph.

2399: *+.060*

Although on a local scale Iberian lynx distribution is determined by the availability of prey rabbits, recent modelling analyses have uncovered broad-scale disagreements between these two species' distribution trends. These analyses showed also that the lynx had become restricted to only a fraction of the rabbit's genetic diversity, and that this could be jeopardising its survival in the face of environmental hazards and uncertainty. In the present paper, a follow-up was carried out through the building of lynx and rabbit distribution models based on the most recent Spanish mammal atlas. Environmental favourability values for lynx and rabbit were positively correlated

within the lynx's current distribution area, but they were negatively correlated within the total Spanish area where lynx used to occur in the 1980's. Environmental favourability for rabbits was signifcantly higher where lynx maintains reproductive populations than where it recently disappeared, indicating that rabbit favourability plays an important role and can be a good predictor of lynx persistence. The lynx and rabbit models were extrapolated to predict favourable areas for both species in Spain as well as in Portugal, on the original scale of the distribution data (10x10 km) and on a 100 times finer spatial resolution (1x1 km). The lynx and rabbit models were also combined through fuzzy logic to forecast the potential for lynx occurrence incorporating information on favourable areas for its main prey. Several areas are proposed as favourable for lynx expansion or re-introduction, encompassing both countries and both genetic lineages of the rabbit.

2400: *+.107*

The Laysan Teal Anas laysanensis is a non-migratory duck that survives as a single relict population on a small remote Pacific atoll, Laysan island (4 km(2)). The species suffered range contraction and isolation after mammalian predator introductions to the Hawaiian archipelago. An understanding of spatio-temporal behaviour on Laysan island may help guide conservation priorities such as habitat restoration and reintroductions on additional islands. This study therefore analysed factors influencing spatio-temporal variation in Laysan Teal's habitat use. Diurnal, nocturnal and crepuscular (i.e. twilight) behaviour and home range utilisation (95% and 50% fixed kernels) of nesting and non-breeding adults on Laysan Island were determined using radio telemetry. Total home range (mean +/- s.e.) was 17.69 +/- 4.28 ha with core area use of 2.57 +/- 0.54 ha (n = 27). There was little overlap between core diurnal and nocturnal activity centres. Total home range of non-breeders was larger than that of nesters, and crepuscular movements were larger than diurnal and nocturnal movements. Time of day influenced the Laysan Teal's use of vegetation type and behaviours. Differences in behaviour, prey abundance, and rainfall were observed between years of this study, and Laysan Teal were detected spending more time foraging at night in 2004 during drier environmental conditions compared to 2005, a wet year. Since Laysan Teal do not migrate or disperse from Laysan Island, significant inter-annual differences in rainfall and food abundance are likely to strongly influence resource use and behaviour within their very limited geographic range. These results emphasise that habitat management for threatened species, especially those with restricted mobility and small ranges, should accommodate their circadian use of resources, and inter-annual environmental variability.

2401: *+.357*

On the analysis of nature-resources' potential condition, development of nature reserve fund territories and expansion of European bison in Bukovinian Carpathians is realized the grounding of organization of scientific complex for breeding, conservation and restoration of a rare species (Bison bonasus L.) on basis of zoological reserve "Zubrovytsa" with an area of 27 thousand ha.

2402: *+.111*

The article summarizes the occurrence of bison in the last 40,000 years is known evidence. The species were a characteristic animal of the Ice age, at that time a significant numbers were living in the Carpathian Basin. The cavemen who live here regularly hunted on them. The conquering of our ancestors may have met a large number of animals, which is hunting for a higher rank. The last specimen died out from Hungary at the end of the 1500s. However the bison remained in Transylvania by the end of the 19th century. The Carpathian Basin would have been a suitable for

bison, but the habitat loss and intensive hunting extinctted it. In recent years the possibility of reintroduction of the species raised. The densely populated country is not located in an area where free herds could live freely. Free stocks to develop, unfortunately there is little chance, but any larger gamefarm is justified.

2403: *-.021*

Home range and food habits of tigers (Panthera tigris tigris) were studied in Sariska Tiger Reserve from July 2008 to June 2009. Three tigers (one male and two females) were radio-collared and reintroduced in Sariska Tiger Reserve from Ranthambhore Tiger Reserve, Western India during 2008-2009. The reintroduced tigers were monitored periodically through ground tracking using "triangulation and homing in techniques." The estimated annual home ranges were 168.6 km(2) and 181.4 km(2) for tiger and tigress-1 respectively. The estimated summer home range of tigress-2 was 223.4 km(2). In total, 115 kills and 103 scats of tigers were collected to study the food habits. The line transect method was used to estimate the prey availability. The density of peafowl (Pavo cristatus) was found to be highest (125.2 +/- 15.3/km(2)) in Sariska followed by livestock (Bubalis bubalis and Bos indicus) (59.9 +/- 22.3/km(2)), chital (Axis axis) (46.7 +/- 9.5/km(2)), sambar (Rusa unicolor) (26.2 +/- 4.9/km(2)), common langur (Semnopithecus entellus) (22.8 +/- 6.5/km(2)), nilgai (Boselaphus tragocamelus) (19.5 +/- 3.3/km(2)) and wild pig (Sus scrofa) (15.4 +/- 4.4/km(2)). Tigers fed on seven prey species as shown by kill data. Tigers' scat analysis revealed the presence of five prey species. Prey selection by tigers based on scat analysis was in the following order: sambar> chital> nilgai> livestock> common langur. It is proposed to restock the tiger population initially with five tigers in Sariska and subsequent supplementation of two tigers every three years for a period of six years, which will allow the population to achieve demographic viability. Removal of anthropogenic pressure from the national park will be crucial for the long term survival of tigers in Sariska.

2404: *+.049*

Within Illinois. the state-endangered snuffbox mussel Epioblasma triquetra (Rafinesque) is currently found only in a small stretch of the Embarras River in Douglas and Coles counties and is considered one of the rarest freshwater mussels in the state. To assess the current population status of E. triquetra in Illinois, I estimated density, length frequency, and sex ratio of the Embarras River population. I also examined the status of the snuffbox mussel's host fish, the logperch Percina caprodes (Rafinesque), to determine if the fish was present in this area Seven sites in the Embarras River were sampled for freshwater mussels and fishes during the summers of 2007 and 2008 using common sampling protocols. Only five adult (>55 mm) E. triquetra males were collected from two sites, suggesting this species is functionally extirpated in Illinois. Although P. caprodes was collected at five sites including the two that housed E. triquetra, it occurred at low densities. It seems unlikely that E triquetra can recover naturally in Illinois, and carefully planned translocation or augmentation methods might be required to restore the species.

2405: *+.342*

Genetic population analysis using molecular markers is probably the most important issue in conservation genetics and today it is a very useful tool for the study of species subjected to sustainable use. Caiman latirostris (broad-snouted caiman) is one of the two crocodilian species cited for Argentina. Their wild populations were drastically reduced in the 1950s and 1960s due to commercial hunting and intense alteration of their habitat, and C. latirostris was included in the Appendix I of CITES. Since 1990, management plans that use ranching system (harvest of wild

eggs, captive rearing and reintroduction to nature) began in Argentina. Through these management activities, Argentine caiman populations were numerically increased and transferred to the Appendix II of CITES that allows the regulated trade of their products. Genetic population studies are being developed together with these sustainable use plans because genetic monitoring is considered essential in management program execution. This chapter includes genetic population studies about broad-snouted caiman in Santa Fe province, Argentina. Analysis related to variability, differentiation and genetic structure were carried out through isozyme electrophoresis, RAPD markers, and quantitative traits. Furthermore, paternity studies were conducted using microsatellite markers. The obtained results indicate that the broad-snouted caiman populations analyzed have low to intermediate genetic variability values, a significant population differentiation, and a high phenotypic variability for some of the morphometric traits studied. In addition, we found indications that C. latirostris mating system could include multiple paternity behavior, since we found more than one paternal progenitor in at least one of the families analyzed. Although the utility and broad applicability of molecular studies are widely accepted, this approach should be complemented by population analyses conducted by means of traditional methods such as morphometry, cytogenetics, ecology, and ethology to get a deeper biological knowledge of the species. To increase the efficiency in the use of natural resources the development of suitable legal guidelines as well as their effective implementation becomes very important to protect wildlife.

2406: *+.302*

Written historical records are widely used to estimate the previous distributions of the larger mammals in southern Africa. However, such records have some limitations and the use of those older than 100 years has been questioned. Written historical records, from the broader Eastern Cape, South Africa, were investigated to examine this contention critically. They were classified according to record quality (acceptability of identification and precision of locality) and analysed according to two levels: 'all' species and 'noticeable' versus 'non-noticeable' species. Records that comprise acceptable identification and precise locality information are the most suitable for mapping historical distributions; they form 33% of the records for the 27 mammal species analysed. A further 49% of the records have acceptable identification but imprecise locality information; they can fulfil a useful function when supported by records where both parameters are of good quality. Thus, the majority (82%) of written historical records from the study area are useful for compiling historical distribution maps and the quality of these records is consistent back to 1750 for this data set. The number and quality of written historical records varies between species. Historical distribution data should be evaluated for reliability (quality) and degree of usefulness, rather than simply discarded a priori.

2407: *+.186*

Background Scottish Natural Heritage (SNH), in collaboration with specialists based across Europe and the UK and with full national consultation, has conducted an extensive assessment of the feasibility and desirability of reintroducing the European beaver (Castor fiber) to Scotland. This accords with UK Government obligations to consider the desirability of reintroducing certain extinct species under Article 22 of the European Council Directive 92/43/EEC on the Conservation of Natural Habitats and of Wild Flora and Fauna (the 'Habitats Directive'). The European beaver became extinct in Scotland as a result of over-hunting around the 16th Century. Further, the European beaver is one of 32 species included in Scotland's "Species Action Framework" as a focus of new management action for five years from 2007 for SNH and a range of partners. The Species Action Framework, launched in 2007 by Ministers, sets out a strategic

approach to species management in Scotland. As a result of the findings of the feasibility and desirability studies, a trial reintroduction of up to four families of European beaver to Knapdale Forest, Argyll, by the Scottish Wildlife Trust (SWT) and the Royal Zoological Society of Scotland (RZSS) on behalf of the 'Scottish Beaver Trial' partnership was sanctioned by the Minister of the Environment in 2008. The trial aims to conduct a five year robustly monitored reintroduction which will facilitate decision makers tasked with considering the feasibility and desirability of reintroducing European beaver to the whole of Scotland. Once the trial has been completed, SNH will provide a final report to the Scottish Government. The aim of this report is to provide an independent critical review, based on scientific literature and expert opinion, of the impacts of beaver on fish and fish stocks and to collate new, updated information which can be used in the wider consideration of beaver reintroduction to Scotland. Main findings * The results of meta-analysis highlight a bias towards studies of beaver/ fish interactions in North America (90) compared to Europe (8). * The meta-analysis indicated the main positive impacts of beaver activity on fish cited were increased habitat heterogeneity, greater area for rearing and overwintering, higher invertebrate production, and the provision of refuge from both high and low flows. * The main negative impacts of beaver activity cited were barriers to fish movement due to the construction of dams, loss of spawning habitat due to siltation, and reductions in oxygen levels in beaver ponds leading to fish kills. * Overall, positive impacts (157) were cited more frequently than negative impacts (102). * The impact of beaver on fish populations is spatially and temporally variable, and differs inter- and intraspecifically. * The results of an Expert Opinion Survey (EOS) that involved 45 North American and European experts (70% return) revealed that the majority of fisheries scientists and managers tended to suggest that the overall impact of beavers on fish populations was positive (58% of items). * The impact of beavers on the abundance and productivity of migratory salmonids was considered positive. * The impact of beaver dams on the movement of aquatic organisms in tributary streams, including upstream and downstream migrating salmonids, and on the availability of suitable salmonid spawning habitat was generally considered negative. * A high level of agreement (50%) was achieved for half of all items responded to.

2408: *+.252*

Monitoring of over a two decade period (1987-2007) of mugger (Crocodylus palustris) population of River Vishwamitra (Gujarat State. India) indicates the present status of the species in and around Vadodara City to be the most noticeable and unique The population found in Vishwamitri-Dhadhar River System represents a unique case study of relationship between a crocodilian species and humans The population of muggers is growing with at the late of 7 77 animals per year and has leached over 100 Also, mugger conflict is increasing, a total of 292 muggers were rescued from human settlements and translocated, including 38% small (under 1 m). 48% large (1 - 2 m) and 14% huge sized (over 2 in) muugers But few of them returned to the same location in the Vishwamitri Rivet A total of 14 crocodile attacks were recorded, including six that were fatal The present study provides recommendations and an action plan lot the long-term mugger conservation in the area

2409: *+.096*

We argue that the introduction of non-native extant tortoises as ecological replacements for extinct giant tortoises is a realistic restoration management scheme, which is easy to implement. We discuss how the recent extinctions of endemic giant Cylindraspis tortoises on the Mascarene Islands have left a legacy of ecosystem dysfunction threatening the remnants of native biota, focusing on the island of Mauritius because this is where most has been inferred about plant-

tortoise interactions. There is a pressing need to restore and preserve several Mauritian habitats and plant communities that suffer from ecosystem dysfunction. We discuss ongoing restoration efforts on the Mauritian offshore Round Island, which provide a case study highlighting how tortoise substitutes are being used in an experimental and hypothesis-driven conservation and restoration project. The immediate conservation concern was to prevent the extinction and further degradation of Round Island's threatened flora and fauna. In the long term, the introduction of tortoises to Round Island will lead to valuable management and restoration insights for subsequent larger-scale mainland restoration projects. This case study further highlights the feasibility, versatility and low-risk nature of using tortoises in restoration programs, with particular reference to their introduction to island ecosystems. Overall, the use of extant tortoises as replacements for extinct ones is a good example of how conservation and restoration biology concepts applied at a smaller scale can be microcosms for more grandiose schemes and addresses more immediate conservation priorities than large-scale ecosystem rewilding projects.

2410: *+.020*

We assessed the distribution and conservation status of bonnet macaques (Macaca radiata), rhesus macaques (Macaca mulatta) and Hanuman langurs (Semnopithecus entellus) in the state of Karnataka, India. Karnataka is situated in southwest India with an area of 191,791 km(2). A total of 9697 km of vehicular survey was made from November 2001 to July 2004. We also visited 107 temples/tourist spots to determine the presence of primates. Bonnet macaques and Hanuman langurs were widely distributed, whereas rhesus macaques were not found in the state. However, bonnet macaques were absent in a few districts in the northern plains and Hanuman langurs were absent in some districts of the southern plains. A total of 205 groups of bonnet macaques and 139 groups of Hanuman langurs were sighted. The relative encounter rate of both species differed across biogeographic zones. Bonnet macaques were largely encountered in the Western Ghats and the Southern Plateau whereas Hanuman langurs were abundant in the Western Ghats and Northern Plains. We found that bonnet macaques have been eliminated from about 48% temples/tourist spots where they occurred in the recent past. The Hanuman langur population of Dharwar-Haliyal Road was assessed during April 2003, and we found that the present population size was about 38% of a previous survey in 1961. Habitat change, hunting/trapping and translocation were the major factors causing a decline in the langur population.

2413: *-.038*

Removing invasive rats from islands is a powerful conservation tool, and practitioners are now targeting larger islands for rat eradication. As they do so, they face the challenge of mitigating for potential non-target impacts on native biodiversity that may be susceptible to rodenticides. We report on the eradication of black rats Rattus rattus from Anacapa Island, California, in 2001-2002, which was the first-ever invasive rodent eradication from an entire island where an endemic rodent was present and the first aerial application of a rodenticide in North America. As a mitigation strategy we staggered the rodenticide application over 2 years and held a representative sample of the Anacapa deer mouse Peromyscus maniculatus anacapae in captivity. We also mitigated for bird species potentially susceptible to brodifacoum poisoning and monitored aspects of the terrestrial and marine environments. The free-ranging native rodent Population severely declined following rodenticide applications but reintroduction and translocation efforts were successful, and the population quickly recovered to pre-eradication levels. Non-target impacts also included mortality of raptors, gulls and passerines, including high mortality of rufous-crowned sparrows Aimophila ruficeps obscura despite planned mitigation. All observed non-target impacts are expected to be ephemeral; however, further monitoring should reveal details on the dynamics of those impacts.

Brodifacoum was not detected in the marine environment or in significant amounts in terrestrial soil, plants and arthropods. Seabird benefits from the rat eradication were quickly realized.

2414: *+.140*

The Alagoas Curassow Mitu mitu is considered extinct in the wild. Since 1979, two females and a male caught in the wild have bred successfully in captivity, and, in 1990, hybridizations between M. mitu and Razor-billed Mitu M. tuberosum were performed. By June 2008, there were around 130 living birds in two different aviaries. We sequenced two regions of the mitochondrial DNA of both captive stocks of Alagoas Curassows. We unequivocally identified hybrids that have haplotype typical of M. tuberosum. However, unless the original studbook can be recovered there is no confident way to discriminate "pure" M. mitu birds for breeding and reintroduction purposes. Allied with morphological data gathered in an independent study, we suggest that conservation actions need to focus on specimens with diagnostic phenotypic characters of M. mitu, and avoid birds with mitochondria, genetic contribution of M. tuberosum. Although we have detected low levels of genetic variability among captive birds, the steady increase of the captive population suggests that inbreeding depression and hybridization are not a reproductive hindrance. Reintroduction of some of these potential hybrid birds in the original area of occurrence of the Alagoas Curassow may be the only hope to fill in the ecological niche left vacant. An educational program involving local communities to conserve future reintroduction of curassows and their restored habitat is highly recommended. Accepted 12 November 2009.

2415: *+.059*

We have investigated historical records of observations of Lesser White-fronted Goose Anser erythropus of the original Fennoscandian population in the area in Sweden where captive-reared goslings of the same species were released during 1981-1999. The release project has earlier been regarded as a re-introduction project. The data in this review include reports from many sources and we can present earlier unpublished observations from the area. The large number of observations of birds from the Fennoscandian population proves that the release of goslings in the actual area was a supplementation of a small but extant population and not a re-introduction.

2416: *+.109*

1. The freshwater pearl mussel Margaritifera margaritifera is endangered throughout Europe. 2. Historically, mussels were described on the basis of shell characteristics. In more recent years with the advent of molecular techniques many 'species' of molluscs have been found to be ecophenotypes. 3. The pearl mussel is found in numerous rivers throughout Ireland and the UK with varying degrees of superficial differences. It is has been thought that the most divergent form is found in the Nore River, Ireland, M. margaritifera durrovensis. 4. The current investigation considers shell shape differences (using elliptic fourier descriptors) in mussels from a variety of rivers in Ireland in relation to river pH. 5. Results suggest that M. margaritifera has a fairly plastic phenotype, with a gradient of shape change in relation to water pH. 6. M. margaritifera durrovensis does not appear to be morphologically unique from other populations studied, instead occurring at one end of the shell shape gradient. 7. Findings also suggest that shell shape may be characteristic to individual rivers. The existence of phenotypically distinct groups of Margaritifera margaritifera has particularly important implications for the future conservation of the species. 8. Ex-situ conservation and reintroduction efforts will need to consider both the genotypic and phenotypic suitability of mussels if translocation is to be used as a viable conservation tool in the future.

2418: ***+.243***

Reintroduction programmes need to be monitored as a way of gauging potential causes of their success or failure. This, in turn, can be used to improve the likelihood of future translocation success. Since the 1990s, stitchbird (or hihi: Notiomystis cincta) translocations have been intensively monitored, with comparisons between two of these projects (Tiritiri Matangi Island - a successful introduction, and Mokoia Island an unsuccessful introduction) often compared and contrasted as a means of identifying factors important in translocation success for this species. A consistently low adult survival rate on Mokoia Island in conjunction with a study showing a high prevalence of aspergillosis (a fungal disease of the respiratory tract caused by Aspergillus fumigatus) in adult stitchbirds led to this disease being commonly discussed as a major factor responsible for the difference in translocation outcomes. However, A. fumigatus infection rates have never been compared between the two stitchbird populations; thus, population differences in adult survival may have resulted from other factors. One possibility is that survival differences between populations were influenced by differing predation pressures from morepork (or ruru: Ninox novaeseelandiae). Evidence of stitchbird predation by moreporks and the fact that morepork density on Mokoia Island was markedly higher than on Tiritiri Matangi Island provides sonic support for this hypothesis. It is important that all plausible hypotheses for differences in survival be considered so that we can better evaluate future conservation strategies that target the recovery of this species.

2419: ***+.122***

The translocation of animals between populations is becoming a common conservation technique, which, in addition to the target species, unfortunately inevitably involves any parasitic organisms they host. Many of these organisms can become pathogenic in a new environment. We assessed the prevalence of intestinal carriage of Salmonella, a common disease organism in wildlife worldwide, in New Zealand native lizards on eight islands off the coast of New Zealand. Cloacal swabs were obtained from 703 lizards and cultured for Salmonella, using four aerobic enrichment and culture methods at two incubation temperatures. We recorded six environmental and physical variables that may affect Salmonella carriage. Logistic regression revealed that two variables were significantly correlated to the presence/absence of Salmonella. In general, Salmonella was found predominantly but not exclusively in six species of lizard and in beach habitats. Salmonella prevalence on the islands was not correlated to previous lizard translocations to the island. More research is required to determine the pathogenicity to New Zealand wildlife of the Salmonella serovars identified in this study. Pending further research, translocations of the six species highlighted in this study or to beach habitats should include disease screening in order to prevent the spread of this potential pathogen.

2420: ***+.357***

An understanding of the mechanisms influencing habitat selection in reintroduced bird populations is fundamental for successful translocation programmes. Plant species composition, abundance, structure and food availability are likely to influence animal movement and habitat choice, but few studies have evaluated their combined effect on habitat selection of translocated birds. Stewart Island robins (Petroica australis rakiura) and South Island saddlebacks (Philesturnus carunculatus carunculatus) are two threatened New Zealand bird species that have been reintroduced to Ulva Island (Stewart Island). We hypothesised that their initial settlement patterns were driven by habitat quality. We tested this hypothesis by comparing habitat components between occupied and unoccupied habitats as the population grew after initial tanslocation. We also modelled

probabilities of site selection as a function of the composition and structure of vegetation, availability of food (invertebrate composition) and nesting resources (cavity type). Founding pairs of both species first established territories in coastal habitat in the western part of the island, which is characterised by structurally complex broad leaved vegetation. Birds also selected sites with a greater abundance and diversity of food resources. Thus in the early stages of population establishment robins and saddlebacks appear to select high quality habitat that offers enhanced cover and foraging opportunities.

2421: *+.232*

New Zealand's offshore and outlying islands have long been a focus of conservation biology as sites of local endemism and as last refuges for many species. During the c. 730 years since New Zealand has been settled by people, mammalian predators have invaded many islands and caused local and global extinctions. New Zealand has led international efforts in island restoration. By the late 1980s, translocations of threatened birds to predator-free islands were well under way to safeguard against extinction. Non-native herbivores and predators, such as goats and cats, had been eradicated from some islands. A significant development in island restoration in the mid-1980s was the eradication of rats from small forested islands. This eradication technology has been refined and currently at least 65 islands, including large and remote Campbell (11216 ha) and Raoul (2938 ha) Islands, have been successfully cleared of rats. Many of New Zealand's offshore islands, especially those without predatory mammals, are home to large numbers of breeding seabirds. Seabirds influence ecosystem processes on islands by enhancing soil fertility and through soil disturbance by burrowing. Predators, especially rats, alter ecosystem processes and cause population reductions or extinctions of native animals and plants. Islands have been promoted as touchstones of a primaeval New Zealand, but we are now increasingly aware that most islands have been substantially modified since human settlement of New Zealand. Archaeological and palaeoecological investigations, together with the acknowledgement that many islands have been important mahinga kai (sources of food) for Maori, have all led to a better understanding of how people have modified these islands. Restoration technology may have vaulted ahead of our ability to predict the ecosystem consequences of its application on islands. However, research is now being directed to help make better decisions about restoration and management of islands, decisions that take account of island history and key drivers of island ecosystem functioning.

2422: *+.109*

After the extinction of the Eurasian Otter in North Rhine Westphalia during the first half of the 20th century, the species has been recorded only sporadically. In 2009, two roadkills were reported in Munsterland region and afterwards a small population was found nearby. The animals have been genetically investigated using their faeces. At least six individuals could be discriminated genetically going with Otter populations in the region of Liineburger Heide in Lower Saxony. During winter 2009/20I0 successful reproduction could be observed in Munsterland region. In addition, a small population was detected in the border area of the Netherlands and Germany. There. at least two females, stemming from the Dutch reintroduction project, and one male of probably German origin share territories. Additionally. single records of the Eurasian Otter in North Rhine Westphalia after the extinction are listed. In conclusion, a resettlement of North Rhine Westphalia by the Eurasian Otter seems to he ongoing. We suggest a countrywide monitoring program.

2423: *+.118*

Reintroductions and translocations are increasingly used to repatriate or increase probabilities of persistence for animal and plant species. Genetic and demographic characteristics of founding individuals and suitability of habitat at release sites are commonly believed to affect the success of these conservation programs. Genetic divergence among multiple source populations of American martens (Martes americana) and well documented introduction histories permitted analyses of post-introduction dispersion from release sites and development of genetic clusters in the Upper Peninsula (UP) of Michigan < 50 years following release. Location and size of spatial genetic clusters and measures of individual-based autocorrelation were inferred using 11 microsatellite loci. We identified three genetic clusters in geographic proximity to original release locations. Estimated distances of effective gene flow based on spatial autocorrelation varied greatly among genetic clusters (30-90 km). Spatial contiguity of genetic clusters has been largely maintained with evidence for admixture primarily in localized regions, suggesting recent contact or locally retarded rates of gene flow. Data provide guidance for future studies of the effects of permeabilities of different land-cover and land-use features to dispersal and of other biotic and environmental factors that may contribute to the colonization process and development of spatial genetic associations.

2424: *+.167*

Recent technological advances have resulted in a dramatic renaissance of population genetics and its application to species ecology and conservation. This review summarizes the progress made in applying these new techniques, notably hypervariable genetic markers (microsatellite loci and mitochondrial control region), to the study of marsupials. Since 1990, population genetic studies have overwhelmingly been of marsupial species from eastern and southern Australia, largely focusing on threatened species or those with restricted distributions. To date, over 500 polymorphic microsatellite loci have been isolated from 38 marsupial species (six American, 32 Australian), including representatives from 13 of the 18 extant marsupial families. Levels of microsatellite diversity identified within the 209 marsupial populations (43 species) so far examined have varied greatly, although the range is similar to those reported from eutherians and other vertebrates. Marsupial populations with high levels of genetic diversity tend to be those from relatively abundant or widespread species, while those with lower levels are typically species with restricted distributions, that are threatened, found on islands or have been established via translocation. Although data for most families are still limited, bandicoots, koalas and wombats appear less and phalangerids more diverse than the marsupial average. The application of these hypervariable genetic markers to investigate marsupial species ecology has substantially improved our understanding of population biology, behaviour and reproduction in many species, as well as informing conservation initiatives and management plans for many threatened marsupial taxa. It is hoped that in the future, marsupial population genetic studies can be expanded to include a larger number of South American and New Guinean species, as well as a better representation of arid, tropical, widespread and abundant Australian species.

2425: *-.057*

The melanocortin-1 receptor (MC1R) regulates melanogenesis in mammals within the mammalian melanocyte and the hair follicle. Common variations (polymorph isms) in the MC1R gene are associated with normal differences in skill and hair colour. So far, a unique MC1R allele (E+) has been identified in European wild boar (Sus scrofa scrofa), associated with the wild-type coat colour (variable shades of brown) that is not found in any of the domestic breeds. In addition, a series of alleles found in pigs, some of which observed only in particular breeds, have been proposed as markers in breed traceability systems. The current study is an attempt to detect

possible hybrids between wild boars and domestic pig breeds as well as to identify races of pig that are not purebred. For this purpose, wild boars were analysed against Large White pigs, applying the PCR-restriction fragment length polymorphism (RFLP) method. A high percentage (16.7%) of hybrids was detected within a breeding station compared with the percentage of hybrids within the populations of free-ranging wild boar (5.0%). These results should be taken into consideration for future restocking operations to avoid the chance of outbreeding depression, which is more intense when local Populations are introgressed by gene pools from domesticated, usually inbred, animals. (C) 2008 Deutsche Gesellschaft fur Saugetierkunde. Published by Elsevier GmbH. All rights reserved.

2426: *+.015*

Discontinuous wildlife reserves can lead to inbreeding depression for fragmented populations of threatened species. To offset such effects, conservation managers frequently turn to translocation strategies, such as the one-migrant-per-generation rule (OMPG), which relies on many unrealistic assumptions of Wright's (1931) island model. We therefore propose an alternate translocation approach based on the natural dispersal rates of focal species, using two endangered Hawaiian tree snails species, Achatinella sowerbyana and A. mustelina, as practical examples. The rate at which tree snails historically dispersed across reserve boundaries can be used to guide contemporary translocation across those dispersal barriers. Snail movements were monitored for three years using capture-mark-recapture (CMR) methods, and analyzed with a multi-strata model in program MARK to obtain survival and dispersal rates. We tested and ranked models, including age, time, weather, and location effects on survival, dispersal, and capture probabilities. Annual mortality ranged from over 50% to less than 20%, by site, mirroring expectations from anecdotal observations of predator abundances. Monthly dispersal rates between isolated tree clusters were recorded between 3% and 24% of a population, depending on the population's exposure to severe weather rather than its species designation. Simulations based on dispersal-distance distributions were then applied to estimate emigration rates beyond the finite study sites. Emigration rates ranged among sites from 0.7% to 6.7% of the population per month, translating to between 6 and > 100 emigrants per year, depending on the density of snails at each site and the site's dimensions. The site boundaries are directly analogous to current and future reserve designs, and we show how such emigration rates can be used to guide two-way translocation rates across such artificial barriers.

2427: *+.152*

Translocation of common macropodids has recently emerged as a management option for addressing impacts of destruction of their habitat in urban areas. However, little is known of the likely success of such translocations. Eastern grey kangaroos (Macropus giganteus) were translocated from a residential development site at the Gold Coast (Queensland) to a nearby 288 ha council conservation area. Objective criteria were developed as a basis for site selection. Ten adults were radio-tracked for up to a year after release or until death (if earlier). The majority of the animals soon moved outside the partially fenced conservation area into adjacent vegetated areas, to a distance of up to 6 km. Animals moved in a somewhat random fashion through suitable habitat then, after a variable period of time, generally settled into fairly stable home ranges. Out of the translocated animals, an estimated 80% were still alive four months after release and an estimated 60% were still alive after one year. All surviving animals appeared to be in good condition and health. While the translocation was reasonably successful in the short term, the site is considered far from ideal due to threats associated with urban development in adjacent areas. However, it was very difficult to find a site meeting all the necessary criteria. Capture, release and

monitoring were expensive in time and money, especially considering the small number of animals involved. It is concluded that translocation is not likely to be a widely applicable option for dealing effectively with destruction of eastern grey kangaroo habitat, associated with development.

2428: *+.106*

The marsupial family Potoroidae (bettongs and potoroos) has been severely affected in Australia by European settlement. Of the 11 known contemporary species, three have become extinct and six are listed on the IUCN Red List 2008 with a range of status assignations from 'conservation dependent' to 'critically endangered'. Four of the species in this family are still in decline. Possible causes for the decline of potoroids include predation by the introduced European red fox (Vulpes vulpes) and cat (Felis catus), competition from introduced herbivores and loss of habitat. Translocation has become an extremely important and popular tool for the management of wildlife, particularly to increase the ranges of endangered species and return them to parts of their former distribution. In this chapter, we document and analyse the outcomes of all translocation attempts for species within the Potoroidae. We located a total of 50 translocation attempts in the literature for three potoroid species. Sixty percent of translocations occurred in Western Australia. Only 33.3% of all translocations have been successful, with limiting factors associated mainly with predation. Success rates have been higher on islands or when the source population was derived from captive individuals. This review highlights the need to publish the results of all translocation attempts to increase their likelihood of success, and recognises the benefits associated with translocations of members of this family in the future.

2429: *+.184*

Recent advances have transformed the field of population genetics and led to a dramatic renaissance in the application of these techniques to the study of ecology and conservation. We review the progress made in applying these tools, notably hypervariable genetic markers (microsatellite loci and mitochondrial control region) and new statistical methods, to the study of wild macropodoid populations. Population genetic studies have been conducted on 20 macropodoid species, more species than any other marsupial group, although most studies have focused on threatened species or those with restricted distributions. A substantial number of microsatellite loci (n = 79) have been isolated from seven macropodoid species, and have been used to successfully amplify polymorphic loci in representatives of all three extant macropodoid families and most genera. Levels of microsatellite diversity identified within the 61 macropodoid populations so far examined have varied enormously, although the range of values is similar to those reported from eutherians and other vertebrates. Macropodoid populations with high levels of genetic diversity tended to be those from relatively abundant or widespread species, while those with lower levels were typically from species with restricted distributions, that are threatened or found on islands or have been established via translocation. The level of genetic structuring among macropodoid populations was variable, but consistent with known species biology. Limited structure has been documented in widespread vagile species such as kangaroos, while moderate or high amounts of genetic structure were found in habitat specialists, such as rock-wallabies. Studies to date suggest that dispersal for macropodoids is male-biased, which is broadly consistent with behavioural observations. Although the power to detect fine-scale genetic substructuring within a population has only recently become available, two studies have independently detected a pattern of differential spatial structuring between male and female rock-wallabies within single colonies. This suggests strong female philopatry and that within-colony dispersal is also male-biased. Data on the genetic mating systems of macropodoids in the wild are available for only four species.

These studies have largely confirmed behavioural-based mating system hypotheses but have also shed light on alternative mating strategies. The potential of hypervariable genetic markers to investigate ecological patterns has been powerfully demonstrated within wild macropodoid populations. These studies have improved our understanding of macropodoid population biology, behaviour and reproduction, as well as informing conservation initiatives and management plans for many threatened macropodoid taxa. It is hoped that, in the future, population genetic studies can be expanded to include a larger number of widespread and abundant macropodoid species, especially those in northern Australia that have been less severely affected by European settlement.

2430: *+.318*

We had surveyed about two species of Falconiformes Falconidae from February 2004 to July 2007. And available for surrogate mother of Common Kestrel Falco tinnunculus used method of clutch size control purposed to increase of individual number of Peregrine Falcon Falco peregrinus. 2) We think after collected the eggs Peregrine Falcon, to induce the additional egg-laying(clutch size control) egg gathering is very important, after the last egg-laying should be done at least within 15days. 3) Advantages of Common Kestrel as a surrogate mother of Peregrine Falcon. ① Peregrine Falcon and the same time, mating behavior and laying. ② Egg's shape and color are similar. ③ Compare to other birds of prey, Common Kestrel has a high population, and strong propagation power. ④ During raising the young falcon, it must not be tamed to people but keep its wild type. 4) Reintroduction of Peregrine Falcon has a significant meaning in maintaining ecological balance and biodiversity. moreover, stable supply of Peregrine Falcon can give a contribution in development of hawking which is our traditional culture and come into the spotlight as a high a high quality leisure sports in today. 5) According to this study,the study can be adapted to other birds of prey, and can be used as a basic material in restoration or conservation of birds of prey which is endangered. Moreover, this can provide an opportunity of observation and learning about Peregrine Falcon to people who want to know the environment.

2431: *+.170*

Mountain gazelle Gazella gazella in Saudi Arabia are listed as 'vulnerable' by the IUCN. At present, the species' survival is secured by extensive captive-breeding programmes and reintroductions into protected areas. Two reintroduction attempts (Ibex Reserve and Uruq Bani Ma'Arid protected areas) in Saudi Arabia have been undertaken in the past two decades. Post-monitoring of released individuals is essential for the success of such reintroduction programmes; however, cryptic species like mountain gazelles are extremely difficult to observe directly. As radio-tracking is a cost-intensive and invasive post-monitoring technique, we asked: how can reintroduced or remnant pockets of natural gazelle populations be monitored indirectly? Here, we propose the use of latrine mapping as an effective, cost-efficient and non-invasive tool to survey the social organization of reintroduced mountain gazelles as ail indicator for repatriation success. In this study, we used released radio-collared animals to characterize the spatial distribution of latrines within female group home ranges. Distance to the next latrine, latrine size, as well as numbers of fresh faecal pellet groups per latrine or presence of urination marks were used as dependent variables for step-wise backward multiple regressions and were correlated with various ecological factors. Most dependent variables were correlated with distance or direction from the nearest tree, but not indicative of home-range cores. Only latrine densities were distinctly higher in core areas of female group home ranges.. and no pattern of peripheral marking was detected. Hence, latrine density is a good indicator of home-range use in female group home ranges. Mapping latrines and determining latrine densities are therefore the methods of choice to survey

mountain gazelle populations.

2432: ***+.059***

Predatory interactions involving large carnivores and their ungulate prey are increasingly recognized as important in structuring terrestrial communities, but such interactions have seldom been studied in the temperate Neotropics. Here, the large carnivore guild is limited to a single species, the puma Puma concolor, native prey populations have been drastically reduced and lagomorphs and ungulates have been introduced. We examined puma dietary patterns under varying abundances of native camelid prey - guanacos and vicunas - in protected areas of northwestern Argentina. We collected puma feces from seven protected areas, and sampled each area for the relative abundance of camelids using on-foot strip and vehicle transects. In one area, where longitudinal studies have been conducted, we examined the remains of vicunas and guanacos for evidence of puma predation in 2004-2006. We compared our results with a study conducted in 1978-1983, and contrasted the frequency of carcasses showing signs of puma predation with estimates of camelid abundance. Across sites, we observed a positive and significant relationship between camelid Consumption by pumas and camelid abundance, with pumas about nine times more likely to consume camelids where the latter were most abundant. The temporal variation in predation rates on camelids differed by species. Guanacos, which did not change in abundance between periods, showed a slight decrease (1.5 times) in the relative frequencies of individuals killed by pumas. Conversely, vicunas increased in abundance by a factor of similar to 7 between periods, coinciding with an c. 3.4 times increase in individuals showing evidence of puma predation. Some protected areas of northwestern Argentina are conserving the trophic interaction between pumas and native camelid prey. This interaction may be the basis of the far-reaching community effects described for analogous systems oil other continents. It also has implications for the possible recovery of or reintroduction of camelids to areas with high puma densities, where predation losses can be expected to be high, and possibly prohibitive.

2433: ***+.329***

Currently, biodiversity conservation is regarded as one of the most important environmental issues in the 21st century. However, The educational approaches on biodiversity conservation is not actively developed. This study was conducted to develop the educational program on biodiversity conservation related with endangered species conservation for improving elementary and middle school girl students' knowledge on biodiversity, inspiring the value and importance of biodiversity, and improving the attitude toward the conservation of biodiversity. Because the biodiversity loss of wetlands such as rice-fields, rivers, marshes has made the breeding population of Oriental White Stork(Ciconia boyciana) endangered in Korea, this subject was very effective for students to learn the cause and effect of biodiversity loss realistically. The education program was conducted to 68 local elementary and middle girl students from 2007 to 2009 and consists of three main activities such as feeding on storks in captivity, sampling living organisms in rice-fields, and designing the future stork living village. The results didn't show a positive effect on improving the knowledge on the biodiversity, but a positive effect on improving the attitude toward the conservation of biodiversity. And the number of taxa that students noticed in rice-fields has increased significantly. In conclusion, this educational program promoted students to concern about and take up a positive attitude on the biodiversity conservation issue.

2434: ***+.207***

A reintroduction programme is a way to restore a species that has become extirpated from a part or all of its range. The Swift fox Vulpes velox has been the focus of a number of reintroductions over areas of Canada and the northern United States from which it had previously been extirpated. A common problem of reintroduction programmes is the lack of published information about the technical aspects of actually capturing, handling, transporting and releasing animals. This information is useful for finding ways to mitigate stress and thus improve the post-release survival of individuals. This review gathers together the available literature along with unpublished reports on previous Swift fox reintroductions and recommends improvements to release protocols from initial capture and/or handling and post-release strategies so as to lessen stress and potentially increase survival of reintroduced Swift fox.

2435: +.255

The importance of captive breeding has evolved as zoos themselves have evolved. Beyond allowing captive populations to be self-sustaining, zoos can contribute to species recovery and reintroduction by improving reproductive rates and developing monitoring techniques that provide data critical to understanding reproductive processes. Several threatened canid species have recently been saved through captive-breeding efforts in partnership with reintroduction programmes. Two of those, the Mexican grey wolf Canis lupus baileyi and the Island fox Urocyon littoralis, provide case studies. For both species, data generated during monitoring revealed important basic features of their biology and also provided critical information to managers that could be used to enhance reproductive rates. Both species are also part of reintroduction programmes that exemplify successful partnerships between the United States Fish and Wildlife Service and North American zoos.

2436: +.174

Bears need to learn appropriate survival and behavioural skills in the first 1 or 2 years of life. They can acquire those skills fully only if raised by their mothers in the natural habitat. Releasing captive-born and/or hand-reared cubs threatens their life expectancy because individuals will have problems finding food and shelter, and experience intra- and inter-specific predation. Additionally, bears reared in captivity may cause behavioural and genetic pollution of the indigenous free-living population. The release of bears cannot be called 'reintroduction'. The surplus of bears currently in captivity should be resolved by control of reproduction and investment in efforts to prevent situations whereby wild-born bears become orphaned and captive. The existing captive population should be given the best possible care and be used as ambassadors to raise public awareness about situation of free-living conspecifics. The above statements are corroborated by experiences with European brown bears Ursus arctos.

2437: +.119

We present data on the reproductive ecology of the Orinoco crocodile (Crocodylus intermedins) in a newly established population at the El Frio Biological Station, Venezuela, from 2003 to 2007. Nesting occurs during the dry season, and hatching of young takes place at the beginning of the rainy season. Elliptical hole-like nests are constructed in artificial sand beaches with a median nest depth of 42.6 cm. Nest depth is positively correlated with female total size, enabling us to predict the size of the female based on nest characteristics. Temperature in the egg chamber was on average 31.9 degrees C. The thermal amplitude of the nest was positively correlated with nest depth, and less than 1.3 degrees C when the nest was deeper than 30 cm. The average clutch size was 41.2 eggs, the average clutch mass was 4256.2 g, and egg viability was 75.4%. The average

length, width and weight of eggs was 7.61 cm, 4.73 cm and 111.07 g, respectively. As part of the conservation programme, we also artificially incubated eggs from the species. Hatching rate in the incubator was 84.3%. Total length and mass at hatching were 28.6 cm and 66.9 g, respectively. Our data demonstrate that head-starting our population through egg incubation is a suitable conservation strategy for this endangered species.

2438: *+.318*

Although the stone crayfish, Austropotamobius torrentium, is one of the most threatened freshwater invertebrates in Europe, only scattered knowledge about its population characteristics and freshwater habitat exists. Thus, recently discovered populations in Carinthia (Austria) and their habitat conditions were characterized in small forest brooks near Millstatter Lake. We investigated population size and structure, together with abiotic and biotic features in three brooks: Weirerbach, Tschernbachl and St. Jakober Bachl. Sex, carapace length, weight and injuries were noted for every individual and its preferred habitat was determined. Based on the analysis of abiotic (morphology of the watercourses, physico-chemical parameters) and biotic parameters (macroinvertebrate fauna) our investigations suggest that the presence of A. torrentium is linked to intact natural water courses with a high heterogeneity of width, depth, current velocity and substrate. A high density of A. torrentium was correlated with high substrate variability and a well developed riparian vegetation cover. Correspondingly, a diverse fauna of larval Diptera, Ephemeroptera, Plecoptera and Trichoptera indicated good habitat quality. As indigenous crayfish are increasingly threatened by various reasons and conservation measures will become necessary, our results provide essential information for reintroductions and other activities to improve the situation for A. torrentium.

2439: *+.207*

In South Africa, the most common primate in rehabilitation centres is the vervet monkey (Chlorocebus aethiops). Here we evaluated the efficacy of releasing two vervet monkey troops into the wild, using the standard methods employed by an established rehabilitation centre. Two troops were assembled over 2-3 years. Coloured ear tags identified adults, subadults and juveniles. Radiocollars were placed on all the adults and subadults of both troops (41% of all individuals). Each troop was released at a suitable site after 2 nights in a holding cage, and supplementary food was provided for 2 months after release. For 10 months, locations of both troops were recorded, as well as the presence/absence of individuals and their general behaviour. The smaller troop survived better than the larger troop, with only 6 of 35 individuals (17%) confirmed alive in the large troop compared with 12 of 24 (50%) in the small troop. Large numbers of missing monkeys make it difficult to determine whether the release was a success. However, results suggest that rehabilitated vervet monkeys could be successfully released in the future. Recommendations are provided for consideration in future releases. Copyright (C) 2010 S. Karger AG, Basel

2440: *+.154*

The distribution of marron in the southwest of Australia has seen many changes since European settlement. Reconstructions of their range from historical records suggested that marron inhabited the waters between the Harvey River and Denmark River. Due to translocation, their range has expanded as far north as the Hutt River and as far east as Esperance. Although at present marron still exist in all the original rivers within the southwest, their distribution within these rivers has contracted. Poor water quality, salinity, low rainfall and environmental degradation in the upper and lower reaches have restricted marron populations. Historically, management decisions in the

Recreational Marron Fishery have been based on fishery-dependent CPUE data collected using a logbook survey and phone survey. A critical assumption has been that the fisheries-dependent CPUE values were proportional to abundance. However, raw or nominal fisheries-dependent CPUE effort data are seldom proportional to abundance and relative abundances indices based on nominal and even standardised CPUE data are notoriously problematic and often provide little useful guidance for management. Although, the fishery-dependent programs provide high quality data on changes in the fishery, in isolation, these data provided limited information on the effects of fishing and the impact of fishery regulations on marron abundance. Standardising the fishery-dependent CPUE data for just one (introduction of snare-only areas during the 1990s) of the numerous management changes illustrated the signifcant bias in raw, nominal CPUE data. The use of biased fishery-dependent data as measures for Recreational Marron Fishery productivity was probably one of the contributing factors limiting the success of developing predictive models using non- fishery variables (e.g. rainfall, river flow). After a thorough review of (historical) sampling methods, a new fishery-independent annual research program using inexpensive box traps was implemented in 2006. Trapping allowed technical staff to sample several sites (2-4) simultaneously instead of just one site per night. More importantly, traps were set late afternoon and retrieved the following morning, removing the serious occupational health and safety issues associated with the historical late night (18:00- 1:00) sampling trips using drop nets and scoop nets. Furthermore, trapping removed the high level of subjectivity (e.g. operator skill level) associated with the traditional methods, especially scoop netting. Trap data appeared to be the most suitable as an index of relative abundance of marron. Interestingly, comparing trap catches with density data obtained through visual surveys using scuba revealed that at least over soft substrate in dams, trap catches can be used as both a measure of relative and absolute (#/m2) abundance. There were large differences in the fshing mortality and rates of exploitation of the two key indicator stocks, Wellington Dam and the Warren River. Fishing mortality and subsequent rates of exploitation were much greater in the habitat poor dam stock relative to the relatively pristine river stock. This was attributable to the differences in habitat and food availability between these systems. Marron are also susceptible to teleost fish predation during the juvenile part of their life history. For example, small (<30 mm Orbital-Carapace Length, OCL), juvenile marron were preyed upon by feral/introduced fish like redfin perch and trout and native predators such as, freshwater catfish, aquatic birds, longneck turtles and water rats. However, the current study did not find native predators to have a significant impact on marron recruitment to the fishery. Among the feral/introduced predatory fish, redfin perch (>20 cm Standard Length, SL) consumed by far the most marron throughout the year. Small trout feed predominantly on insects with larger individuals (>30cm SL) shifting towards fish and crayfish, including marron. The highest marron densities were found in water bodies with complex hide habitat despite large numbers of introduced predatory fish like redfin perch, rainbow and brown trout; highlighting the importance of complex habitat in reducing intra and interspecifc predation and maintaining recruitment to the fshery. As such, no evidence was found to support the hypothesis that marron stocks are signifcantly (recruitment) limited due to predation in systems with complex habitat. The Recreational Marron Fishery has targeted (adult) marron since the introduction of a minimum legal size of 57 mm OCL in 1952. The reduction of the fishing season from 135 days ([approximately]500,000 marron) in the 1970s and 1980s, to 16 days ([approximately]50,000 marron) in the early 2000s, has significantly reduced the impact of fshing mortality. Marron densities and population structure were strongly infuenced by habitat type. Both trapping and visual surveys using scuba, clearly demonstrated that complex habitat sustained higher densities of marron over a wide size range, while fewer, but mainly larger marron, occurred over soft, fat substrate. The key indicator populations studied in the Warren River and Wellington Dam had the highest densities of marron of all the surveyed water bodies but the population dynamics in each dam were strikingly different. In both water bodies, juveniles initially grow at roughly the same

rate but the population in Wellington Dam is stunted, very few animals larger than 60 mm OCL. While in the Warren River animals between 60-90 mm OCL were common and continued to grow, tagging data showed that in Wellington Dam adult marron would moult but not increase in size. The study demonstrated that the Wellington Dam stock was effectively fully exploited and had very low productivity. By contrast, the Warren River stock had relatively low fishing exploitation and a high productivity driven by higher growth rates of trappable individuals coupled with higher densities. The differences in densities and growth of larger individuals were attributed to differences in habitat quality and food resources. The differences in exploitation rates were attributed to the greater fsher accessibility in the dam relative to the river. These differences are likely to be similar in other dam and river stocks (with the exception of Harvey Dam) and thus have direct implications for the management of the two sectors of the fishery. A key finding of the study is that water quality (salinity) and the quality and availability of complex habitat (intact riparian vegetation providing woody debris in rivers and a lack of complex habitat in dams) are the critical bottlenecks restricting marron distribution and abundance in the southwest of WA. Little temporal and spatial variation was observed in both ovarian (potential) and pleopodal (effective) fecundity among a dozen marron populations. Size-at-maturity showed little temporal but considerable spatial variation ranging from 30-70 mm OCL. Preliminary results indicated that at present the minimum legal size in the Margaret, Blackwood and Moore Rivers might be insuffcient to protect the female breeding stock. Furthermore, it appeared that in some populations (e.g. Shannon River), females were not reproductively active every year and may trade-off growth for reproduction. Alternating between growth and reproduction has been demonstrated for Tasmanian and Victorian freshwater crayfsh populations where low water temperatures restrict the growing/breeding season. Limited information is available on the early phases of juvenile marron in the feld. Visual surveys demonstrated that in dams juvenile abundance is highly correlated with the presence of complex, hard substrate. Several designs of 'habitat traps' were successfully tested in Wellington Dam and Warren River to capture juvenile marron. However, the design of the 'habitat trap' will need some further adjustment and testing, before this method can be used as a reliable recruitment abundance index. Tagging juvenile marron with small (<1mm), internal coded micro wire tags was highly successful and proved to be a potentially powerful tool to study juvenile biology in the feld. Laboratory experiments showed that tagging had no effect on mortality or growth of small juvenile marron and tag retention was high. The degradation (salinisation, reduced surface and groundwater infow) of the rivers in the southwest is a threat to both the recreational marron fsheries and the marron populations upon which it is based and has resulted in reduction of the inland range of the species. The exclusion of recreational fshing from public dams (e.g. Stirling Dam and possible Logue Brook Dam and Wellington Dam in the near future) reduces the size of the marron fshery but simultaneously creates unoffcial protected areas for marron and other native aquatic fauna. The major threat to the ongoing sustainability of the fshery is the decline in the health of the freshwater systems in which it currently occurs. As such, a key recommendation is to promote inter-agency cooperation in addressing the decline of river health; particularly with regard to water quality and riparian vegetation. The future of the recreational marron fshery lies in also promoting it as a skilful, exciting, inexpensive and fun outdoor activity that can be enjoyed by the whole family. As such, creation of a snare-only, 'Trophy' marron fshery with conservative bag and possession limits throughout the whole fshery would greatly simplify research, compliance and management. More importantly, such an approach would allow for a considerable increase in season length that will allow more people to participate or organise multiple trips, enhancing visitation to the southwest region and further contributing to regional economies.

2441: ***+.193***

In Sweden, pikeperch (Sander lucioperca) is found in many lakes and along the brackish Baltic Sea coast. The species is subject to extensive commercial and recreational ish- ing, and each year large numbers of young pikeperch from mainly one source (Lake Hjalmaren) are released to support existing fisheries and to introduce the species into new areas. Surprisingly little is known about the population structure of pikeperch, and so far no evaluations of the large scale supplemental releases have been carried out [long dash] neither with respect to effects on genetic characteristics of recipient populations, nor to the assumed beneicial effects on fisheries. This study, carried out at the Swedish Board of Fisheries, had two main aims: (1) to use genetic data (nine microsatellites) for a detailed assessment of genetic structure in Lakes Hjalmaren and Malaren, and adjacent coastal areas, and (2) to evaluate both short and long term effects of supplemental stocking programs that have been going on for decades in two coastal areas and in Rivers Dalalven and Ljusnan. Genetic analysis of a total of 1 147 individuals from 21 sampling sites revealed that the pikeperch appears genetically homogenous in Hjalmaren and in the central basins of Malaren, respectively, whereas distinct subpopulations were identified in less accessible parts of Malaren (and in the more remote Rivers Dalalven and Ljusnan). Along the adjacent coast, the species is subdivided into several genetically distinct local populations, despite decades of large scale stocking. The level of genetic connectivity seems to be more pronounced within large lakes than in coastal areas, possibly because suitable spawning areas along the coast are less common and thereby the populations become more fragmented and isolated. In the coastal areas with supplemental stocking only minor genetic effects from ntroduced Lake Hjalmaren ish could be seen, with rather low accumulated admixture rates (1[long dash]4 %). In contrast, the studied resh water localities in Rivers Dalalven and Ljusnan were found to be significantly more influenced by stocking. In particuar, the pikeperch in Hedesundafjardarna River Dalalven) has been heavily intro-gressed; the current population represents a hybrid swarm where as much as about 50 % of the genetic material seems to be of stocked origin (i.e. from Lake Hjalmaren). The corresponding admixture rate in Orsjon River Ljusnan) is 11 %. Possible negative itness effects on natural populations due o introgression from stocked pikeperch remains to be studied, however. Despite the signiicant genetic change detected in River Dalalven, it is doubtful whether the stocking program has resulted in increasing fishing opportunities and catches, which has been the prime motive for carrying out these activities. A genetically based evaluation of a sample of individual pikeperch caught in the recreational fishery in 2008 showed that a majority of the analyzed ish were in fact wild born (hybrids in various generations). The ow proportion of stocked fish in the catch 0-8 %) agrees fairly well with the gene flow from introduced fish over the years estimated to about 10 % per generation. An almost complete dominance of wild born pikeperch was also observed in samples from the other localities with stocking. The difference in levels of genetic admixture between study areas seems mainly to be due to different relationships between the amount of stocking and the level of natural production. Still, there are indications that the introduced freshwater ish may have had somewhat lower survival and/or reproductive success (i.e. itness) when stocked at the coast. Additional studies will be needed, though, such as e.g. breeding experiments in various salinities, to evaluate in further detail whether or not local adaptations to fresh and brackish environments do exist in pikeperch. Although one should not generalize too much from a single study, several of these results are of relevance for the practical management of pikeperch. To ensure a sustainable ishery, the local populations (stocks) which have been identiied should be managed separately, e.g. by regulating the fishery in relation to the local productivity. Our results also show that stocking of pikeperch aimed at enhancing an existing population should not be carried out until an assessment of the natural productivity has been performed - otherwise there is an obvious risk that the number of stocked pikeperch remains low in relation to the wild born ones, resulting in minor positive effects on the ishery. However, even when stocking is not enhancing the ishery to any major extent, the long-term effects through gene low and introgression may still be significant when recurrently releasing genetically different fish from other areas. Thus,

stocking of foreign pikeperch should not be carried out except for reintroduction purposes. When planning to introduce pikeperch into areas where the species does not occur, other aspects (than genetics) need to be taken into account, such as effects on other fish species and the local ecosystem as a whole.

2442: ***-.108***

MONITORING OF RARE AND ENDANGERED SPECIES OF MEDICINAL PLANTS IN THE PERM TERRITORYA.A. Ustinova, V.D. Belonogova, PhD; G.I. Oleshko, PhDPerm State Pharmaceutical AcademyRare and endangered species of medicinal plants in the Perm Territory were monitored. The most typical habitats were identified for 6 species; the rarity status was established; limiting factors were revealed. A reintroduction program and a pion of measures for their recreation were complied; a living collection was made; the geoinformotion system "Rare and endangered medicinal plants of the Red Book of the Perm Territory" was developed.

2443: ***+.185***

Despite the current expansion of community-based marine conservation initiatives in the Pacific, few studies have specifically addressed their ecological efficiency to restore or enhance reef invertebrate resources. This paper investigated the effects of two very small (< 0.05 km(2)) recent village-based marine reserves (tabu areas) located along the shallow fringing reef of Emau island, Vanuatu. Surveys focused on heavily harvested species (namely trochus, giant clams and green snails) and involved both experienced scientists and local villagers. Abundance, density and individual size data were collected by snorkelling along random transect belts located inside and outside the tabu areas, using simple PVC measuring tools specifically developed for participative monitoring. Habitat was assessed using a photographic method to quantitatively describe varied reef substrata. Resource recovery varied between the areas as a result of species-specific responses to contrasted reserve characteristics and local management practices. Fast-growing mobile Trochus niloticus exhibited strong positive abundance and size responses only within the older larger tabu area through the combined effects of protection from harvesting and translocation actions by local fishers. Similar trends were observed to a lesser extent for sessile slow-growing giant clams (Tridacna spp.), but these were not significant after four years of closure. Despite historical evidence of their presence in the area, surveys emphasized the severe population collapse of the heavily targeted green snail (Turbo marmoratus). Under certain conditions, very small-scale reserves, such as those implemented by village-based conservation initiatives, can rapidly and efficiently enhance local reef invertebrate resources. It is still unclear whether the changes are sufficient to restore critical levels of spawning biomass at larger scale and reverse the severe depletion of invertebrate resources occurring in Vanuatu.

2444: ***+.158***

As an attempt to help conserve the endangered European mink Mustela lutreola (Linnaeus, 1761), a reintroduction program was started in a nature reserve in Saarland, Germany; the present study is part of this ongoing reintroduction project. Within the first 2 yr period, 48 (16 male, 32 female) founder animals were reintroduced, out of a total of about 75 animals intended for release in the course of the 3 yr project. Prior to reintroduction, the animals were acclimatized using soft-release pens where they were preconditioned with natural prey. A number of individuals (n = 18) were set free in groups of mating partners (female female male, female male) and in mother-offspring groups. Thirty-three European mink (14 male, 19 female) were fitted with intraperitonial transmitters and radio-tracked and surveyed via live-trapping. Animals were monitored from April

2006 to May 2008 to determine the distribution, size and temporal changes in home range. Released animals showed linear home ranges spreading along rivers and brooks. Home range sizes were 7.2 km (88 ha) for 1 adult male, 0.2-5.9 km (1-505 ha) for adult females, around 1.7-5.6 km (17-132 ha) for juvenile males and 9.2 km (778 ha) for 1 juvenile female, respectively. The released adults showed inter-, but only few intra-, sexual home range overlaps. In contrast, juveniles did not show any home range overlaps. In winter, home range size decreased to a minimum of about 1 km. Further, home range size for females decreased around parturition but increased successively at time of weaning. The activity pattern of released animals was highest at night (especially around dusk and dawn).

2445: ***+.193***

To ensure the success of reintroduction programs, it is important to monitor the post-release behavior and survival of released animals. In this study, the post-release movement and behavior of 5 wild and 5 head-started hawksbill turtles Eretmochelys imbricata were monitored using ultrasonic telemetry. Their dispersal directions and recaptures may indicate that wild turtles perform homing migrations. However, the head-started turtles showed non-uniform patterns in dispersal movements. Four head-started turtles moved out of the monitoring area in various directions, whereas one turtle stayed within the monitoring area for approx. 10 mo. These results might indicate that head-started turtles wander aimlessly in their new surroundings. Signal reception patterns indicated that wild turtles were active in the daytime and rested under the coral at night. Although the head-started turtles also rest at night, their resting places did not seem to be sheltered from hazardous sea conditions or adequate for efficient resting. Therefore, head-started hawksbill turtles appear to need pre-release training such as exposure to structures or ledges in the rearing tank so they can utilize similar structures in the wild for shelter during rest periods and maximize their dive duration by employing these as a roof to counteract the positive buoyant effect of inhaled air. Prey analysis of a head-started turtle captured incidentally demonstrates that these turtles can make feeding adaptations to adjust to the natural environment. These findings provide constructive information for the implementation and improvement of head-start programs.

2446: ***+.110***

Genetic management of fragmented populations poses logistical and theoretical challenges to conservation managers. Simulating changes in genetic diversity and differentiation within and among fragmented population units under different management scenarios has until now rarely used molecular marker data collected from present-day populations. Here we examine the genetic implications of management options for the highly fragmented yet globally significant orang-utan population in the Lower Kinabatangan Wildlife Sanctuary, Sabah, Malaysia. We simulated the effects of non-intervention, translocation, corridor establishment and a mixture of the latter 2 approaches on future genetic diversity in this population using the stochastic simulation software VORTEX and a well-described molecular dataset for 200 individuals from within the Sanctuary. We found that nonintervention resulted in high extinction risks for a number of subpopulations over short demographic timescales (<5 generations). Furthermore, the exclusive use of either translocation or corridor establishment as a management tool was insufficient to prevent substantial levels of inbreeding using demographically and logistically feasible translocation rates and was insufficient to prevent inbreeding and extinction in the most isolated subpopulations using conservative corridor establishment rates. Instead, a combination of modest translocation rates (1 ind. every 20 yr) and corridor establishment enabled even the most isolated subpopulations to retain demographic stability and constrain localised inbreeding to levels below a threshold of 0.1. Our simulations suggest that this mixed management approach is both a pragmatic and potentially

successful course of action and that this combination may be useful in other species and fragmented populations in the future. The use of presentday molecular data in stochastic simulations requires further development, but here we show that it can aid predictive modelling.

2447: *-.100*

Understanding predator-prey relationships can be pivotal in the conservation of species. For 2 decades, desert tortoise Gopherus agassizii populations have declined, yet quantitative evidence regarding the causes of declines is scarce. In 2005, Ft. Irwin National Training Center, California, USA, implemented a translocation project including 2 yr of baseline monitoring of desert tortoises. Unusually high predation on tortoises was observed after translocation occurred. We conducted a retrospective analysis of predation and found that translocation did not affect the probability of predation: translocated, resident, and control tortoises all had similar levels of predation. However, predation rates were higher near human population concentrations, at lower elevation sites, and for smaller tortoises and females. Furthermore, high mortality rates were not limited to the National Training Center. In 2008, elevated mortality (as high as 43%) occurred throughout the listed range of the desert tortoise. Although no temporal prey base data are available for analysis from any of the study sites, we hypothesize that low population levels of typical coyote Canis latrans prey (i.e. jackrabbits Lepus californicus and other small animals) due to drought conditions influenced high predation rates in previous years. Predation may have been exacerbated in areas with high levels of subsidized predators. Many historical reports of increased predation, and our observation of a range-wide pattern, may indicate that high predation rates are more common than generally considered and may impact recovery of the desert tortoise throughout its range.

2448: *+.233*

Considering the drastic changes in primate habitats, we must search for management strategies to maintain primate populations in the wild. In the present study, a group of howler monkeys Alouatta palliata was followed after being translocated to a 90 ha tropical forest used for under-story palm Chamaedorea elegans cultivation. The group's behavior was recorded in the new environment. There was no evidence of behavioral alterations due to the different habitat conditions, the translocation process or the palm cultivation activities at the release site. These results indicate that reintroduction, even to fragments used for cultivation purposes, is a viable conservation approach for howler monkey conservation in the Los Tuxtlas region of Mexico.

2449: *-.089*

The translocation of wild birds, commonly conducted as part of management of threatened and endangered populations, is a potentially stressful procedure that may have an impact on their subsequent survival. Corticosterone is the main avian stress hormone, and we examined the relationship between the corticosterone response to initial capture and handling, change in mass during a short period of captivity between capture and release, and subsequent survival after release during a translocation of North Island Saddlebacks (Philesturnus rufusater), an endemic New Zealand bird. In common with other birds, Saddlebacks had marked corticosterone responses to capture and handling. Saddlebacks confined in aviaries for 1-2 days between initial capture and release lost mass, but those confined for 3 days gained mass. The change in mass of birds after one night of confinement was not correlated with the initial corticosterone response. Survival after release to one year was high (70%) relative to four other monitored North Island Saddleback translocations (mean 53%, range 41-71%) but was not related to corticosterone response. The

absence of a relationship between corticosterone response and survival may reflect benign conditions at the translocation site as suggested by this high rate of survival. The relationship between this acute corticosterone response and chronic stress and whether these physiological responses may potentially predict how individuals cope with these protocols warrants further investigation.

2450: *+.354*

In Flanders, several fish species are endangered due to water pollution and habitat deterioration. However, water quality is improving and efforts are being made to restore habitat quality, allowing natural recovery of several fish species. For other species, an integrated approach for protection and recovery of their populations is required. Fish stocks are built up with respect to genetic origin. Mathematical models were developed and used to evaluate habitat suitability. Reintroduction is considered when both water quality and habitat are suitable. Appropriate measures in relation to habitat demands of fish are being taken with relevant partners involved in integrated water management; stopping biodiversity loss and the EU Habitats Directive are the policy frame for these measures. The angling sector also finances scientific research as part of the fish permits proceeds.

2452: *-.088*

Biotic responses to climate change will vary among taxa and across latitudes, elevational gradients, and degrees of insularity. However. clue to factors such as phenotypic plasticity, ecotypic variation, and evolved tolerance to thermal stress. it remains poorly Understood whether losses should be greatest in populations experiencing the greatest climatic change or living in places where the prevailing climate is closest to the edge of the species' bioclimatic envelope (e.g., at the hottest, driest sites). Research on American pikas (Ochotona princeps) in montane areas of the Great Basin during 1994-1999 suggested that 20th-century population extirpations were predicted by a combination of biogeographic, anthropogenic. and especially climatic factors. Surveys during 2005-2007 documented additional extirpations and within-site shifts of pika distributions at remaining sites. To evaluate the evidence in support of alternative hypotheses involving effects of thermal stress oil pikas, we placed temperature sensors at 156 locations within pika habitats in the vicinity of 25 sites with historical records of pikas in the Basin. We related these time series of sensor data to data on ambient temperature from weather stations within the Historical Climate Network. We then Used these highly correlated relationships, combined with long-term data front the same weather stations. to hindcast temperatures within pika habitats from 1945 through 2006. To explain patterns of loss, we posited three alternative classes of direct thermal stress: (1) ICMC cold stress (number of clays below a threshold temperature) (2) acute heat stress (number of days above a threshold temperature) and (3) chronic heat stress (average summer. temperature). Climate change was defined as change in our thermal metrics between two 31-yr periods: 1945-1975 and 1976-2006. We found that patterns of persistence were well predicted by metrics of climate. Our best models suggest some effects of Climate change; however recent and long-term metrics of chronic heat stress and acute cold stress, neither previously recognized as sources of stress for pikas, were sonic of the best predictors of pika persistence. Results illustrate that extremely rapid distributional shifts can be explained by climatic influences and have implications for conservation topics such as reintroductions and early-warning indicators.

2453: *+.124*

Ten years of demographic and genetic monitoring of Stachys maritima in Catalonia (2001-2010). Implications for a recovery plan.- Stachys maritima is a species typical of the coastal dunes, with a wide distribution within the Mediterranean Basin. In spite of this, the species shows a clear regression. In Catalonia, it has been observed an important reduction of its populations since early 20th century, where it has disappeared from several localities in which it was relatively common (Tarragones, Barcelones). Herein we present the results of the demographic monitoring of the species during the last 10 years (2001-2010) in the known localities in Catalonia. Besides corroborating the disappearance (northern Sant Marti d'Empuries), the re-discovering (Llobregat Delta beach) and the detection of new populations (inner dunes of the Montgri), a large year-to-year fluctuation of the monitored populations is stated; the possible reasons are discussed. In addition, the present work also includes the results of the allozyme diversity analysis of the new detected populations as well as the rediscoveries of the period 2004-2008, which were not included in a former study of genetic diversity carried out in 2002-2003. It is necessary to emphasize that the contribution of the new populations to the genetic diversity of Stachys maritima is very small, which can be attributed to their limited population size and/or to founder effects. Despite that the species is included in the Annex 2 ("En Perill d'Extincio") within the Cataleg de Flora Amenacada de Catalunya (Catalogue of Endangered Flora of Catalonia), and some "soft" conservation measures have been applied at local level (signposting of the beach accesses, environmental education, etc.) coupled with other more significant measures (e.g. translocation of individuals discovered in an artificial sandbank), it would be necessary the coordinated action and the scientific support of any initiative of conservation that could be carried out. The general frame to initiate actions of conservation should be the recovery plan of Stachys maritima, whose draft and application is mandatory in accordance to the Cataleg.

2455: ***+.179***

The Yangtze River dolphin or baiji, a freshwater cetacean found in the mid-lower Yangtze River and neighboring lake and river systems, experienced a precipitous population decline throughout the late twentieth century driven by unsustainable by-catch in local fisheries and habitat degradation. An intensive survey in 2006 failed to find any evidence that the baiji still survives, and the species is now highly likely to be extinct. Although considerable protective legislation was put in place from the late 1970s onwards in China, notably laws banning harmful fishing practices and the establishment of a series of reserve sections in the main Yangtze channel, regulations were difficult or impossible to enforce and in situ reserves proved unable to provide adequate protection for baiji. More intensive species-specific recovery strategies also received considerable national and international attention, with extensive deliberation for over twenty years about an ex situ recovery program that aimed to establish a translocated breeding population of baiji under semi-natural conditions. However, minimal financial or logistical support for this active baiji conservation strategy was ever provided by the international conservation community. A more dynamic international response is required if other threatened river dolphin species are to be conserved in the future.

2456: ***+.136***

Globalization and increasing human impact on natural aquatic systems have facilitated the movement of species and the establishment of nonindigenous species enhancing hybridisation opportunities between naturally allopatric species. In this review, we focus on a special case of natural hybrid speciation and the consequences of recent anthropogenic hybridisation in the water frog complex (Pelophylax esculentus complex), which consists of two parental species, Pelophylax lessonae and Pelophylax ridibundus and a hybrid taxon. The hybrid water frogs

reproduce hybridogenetically and eliminate the genome of the syntopic water frog species. Although the actual cause triggering chromosome exclusion remains elusive, it has been proposed that chromosome elimination takes place prior to meiosis and may involve enzymatic degradation of the discarded genome. Translocations of water frogs in Western Europe have become frequent the last decade leading to rapid expansion of the range of the marsh frog P. ridibundus. Subsequent hybridisation of the exotic P. ridibundus may dramatically affect the viability and maintenance of hybrid water frog populations throughout Europe. Interestingly, the impact of this introduced species may differ depending on their geographic origin, which defines the ability to induce genome elimination. This may result in fertile or sterile hybrids, making global conservation guidelines challenging. We predict a severe genetic and ecological impact of nonindigenous P. ridibundus prompting for strict conservation measures to reduce species translocations and for studies on the geographic origin of exotic frog species.

2457: *-.044*

In Australia, numerous small mammal species have suffered extinction or severe declines in distribution and abundance following European settlement. The extent of these declines from forested areas of southeastern Australia, however, remains poorly understood. In this paper we use sub-fossil deposits of the sooty owl (Tyto tenebricosa tenebricosa) as a tool for understanding the diversity of the small mammal palaeocommunity. These results are compared to the contemporary sooty owl diet from the same geographical region to investigate the degree of small mammal decline following European settlement. Of 28 mammal species detected in sub-fossil deposits and considered prey items of the sooty owl at the time of European settlement, only 10 species were detected in the contemporary sooty owl diet. Numerous small mammal species have not only recently suffered severe declines in distribution and abundance but have also recently undergone niche contraction, as they occupied a greater diversity of regions and habitats at the time of European settlement. For some species our understanding of their true ecological niche and ecological potential is therefore limited. The species that underwent the greatest declines occupied open habitat types or were terrestrial. The severity of decline is also likely to have resulted in severe disruption of ecosystem functions, with wide scale ecosystem consequences. There is an urgent need to improve small mammal conservation, to maintain crucial ecosystem functions performed by small mammals. It is recommended that broad-scale exotic predator control programs are conducted which may also provide suitable conditions for the re-introduction of locally extinct species. (C) 2009 Elsevier Ltd. All rights reserved.

2458: *-.074*

Reintroduction of captive-bred individuals for supplementation or re-establishment of wild populations has become increasingly important to prevent the extinction of many endangered species. Despite the importance of reintroductions for conservation biology, few studies have empirically evaluated the potential impacts of reintroductions of naive organisms on disease outbreaks in native wild populations. Here, we use a model organism, the guppy (Poecilia reticulata) and its ubiquitous parasite (Gyrodactylus turnbulli) to evaluate: (i) the most effective release procedure (i.e. en masse vs. gradual release) that minimises host mortality and parasite load, (ii) the effect of pre-exposure to the parasite on host susceptibility, and (iii) the potential advantage of removing the most susceptible individuals before release. We could not detect a statistically significant difference in host survival rates between release protocols and pre-exposure regime. However, the parasite population went extinct significantly more often when pre-exposed fish were released. Furthermore, the parasite load of individual guppies by the end of the reintroduction was significantly lower in treatments where fish had been pre-exposed to parasites

than in treatments with naive guppies. Additionally, pre-exposure of hosts provided important information about their level of resistance when reintroduced. These findings suggest that pre-exposure to native parasites could be beneficial for the survival rate of captive-bred animals when reintroduced in the wild. We discuss the practicalities of these potential release strategies. (C) 2009 Published by Elsevier Ltd.

2459: *+.125*

Native plant species are routinely planted or sown in ecological restoration projects, but successful establishment and survival depend on where and how seeds are collected. Research suggests that it is important to use locally adapted seeds. Local populations often show a home-site advantage and non-local genotypes may be maladapted to local environmental conditions. Furthermore, intraspecific hybridisation of local and non-local genotypes may have a negative impact on the genetic structure of local populations via mechanisms such as outbreeding depression. Many species show a strong small-scale genetic differentiation between different habitats so that matching habitats of the restoration and donor site can be more important than minimizing geographical separation. It is a challenge to identify appropriate seed sources because strong small-scale population differentiation makes it difficult to delineate geographically defined seed zones to which seed exchange should be limited. Moreover, it is important to consider the genetic diversity of introduced material because it may be crucial to avoid genetic bottlenecks, inbreeding depression and poor establishment of plant populations. Repeated propagation in stock, which is often required to obtain a sufficient amount of seeds, can further reduce genetic diversity and may select for particular genotypes. Negative impacts of improper seed choice for nursery planting stock may become detectable only after many years, especially in long-lived and slow growing plants. Although scientific information on many species remains limited, the increasing demand for translocation of seed means that mandatory regulations are necessary. Guidelines should prescribe a specification of seed provenance, a record of genetic diversity of wild collections and rules for subsequent processing such as direct transfer and propagation of stock or seed orchards. We use a literature review to evaluate current legislation and to develop recommendations for herbaceous and woody species.

2460: *+.108*

The in situ management of many orchid species is problematic because individual species' ecology and habitat requirements are poorly understood. Here, the requirements of nine Pterostylis species are investigated for the first time. Individual species' abundances were recorded from 35 sites and correlated with known environmental and climatic variables using canonical correspondence analysis to determine which variables may explain species distribution. Altitude, aspect, drainage, precipitation, radiation, temperature and moisture index were identified as important variables that influence distribution patterns. The positioning of several members of the Pterostylis longifolia species complex in ordination space was poorly resolved, as was the relationship between Pterostylis pedoglossa and Pterostylis parviflora. Distinct ecological partitioning was evident among the remaining three species. This study has identified important environmental variables that can be assessed in the field and assist in the detection of suitable habitat for orchid translocations.

2461: *+.076*

This study investigated the diversity and specificity of mycorrhizal fungi associated with five Diuris (Orchidaceae) taxa in south-eastern Australia, as part of a reintroduction program for the

endangered species Diuris fragrantissima. We compared fungi isolated from D. fragrantissima occurring naturally in the only remaining population with those from artificially cultivated plants and reintroduced plants 18 months after planting in a new field site west of Melbourne. Genetic similarity of nuclear internal transcribed spacer and nuclear large subunit DNA sequences showed that Diuris taxa associate with a narrow taxonomic range of fungi within the cosmopolitan family Tulasnellaceae in the Rhizoctonia alliance. All fungal isolates induced host seed germination and hence were considered mycorrhizal. Fungal isolates from naturally occurring D. fragrantissima plants showed a higher level of genetic similarity than fungi isolated from cultivated plants. This observation suggests that, historically, the species may have associated with a more genetically variable range of Tulasnella fungi. Artificially cultivated D. fragrantissima were propagated aseptically from seed and spontaneously formed mycorrhizal associations within 6 months of transfer to potting media. Wild collected D. fragrantissima plants maintained in cultivation for over 30 years were found to contain mycorrhizal fungi similar to those isolated from naturally occurring plants in 2004-2006. Mycorrhizal associations in artificially cultivated D. fragrantissima were present in 18 randomly sampled plants 18 months after reintroduction. Further, associations formed between several reintroduced plants and a fungus concurrently inoculated into site soil. We propose that future orchid reintroductions may benefit from the concurrent addition of suitable mycorrhizal fungi to site soil. Maintenance of orchid mycorrhizal relationships after reintroduction is essential to improve long-term viability of reintroduced populations.

2462: *+.244*

The Growling Grass Frog (Litoria raniformis) is a large, semi-aquatic tree-frog that is distributed widely across southern Australia, including eastern South Australia, Victoria, Tasmania, southern New South Wales and (formerly) the Australian Capital Territory. Despite being once widespread and abundant, L. raniformis is today recognised as threatened in all states in which it occurs, and considered nationally vulnerable to extinction. While historical perturbations have been important factors in this decline (e.g., droughts and the introduction of exotic disease), habitat loss, fragmentation and degradation continue to be significant issues for the conservation of L. raniformis. Of particular concern in this regard are the many remnant populations of the species that occur in urbanising landscapes, such as on the fringe of Melbourne in southern Victoria. It is now clear that the ongoing decline of L. raniformis in these landscapes is driven by the outward spread of urban development, and that this decline will continue unless sound conservation programs are enacted. These guidelines are designed to facilitate the development of such conservation programs. Based on nearly a decade of research on the population dynamics and habitat requirements of L. raniformis on the urban fringe of Melbourne, they describe wetland-level and landscape-level objectives for habitat management, and outline protocols for surveys aimed at determining wetland occupancy by the frog. The former is crucial for the planning of conservation reserves, because it can inform decisions about the prioritisation of wetlands to be protected, enable identification of enhancement opportunities at existing wetlands, and guide the design of dedicated artificial wetlands for the frog. Survey protocols are also of vital importance, because accurate assessments of wetland occupancy are needed to plan habitat management and monitor the success of such initiatives. In addition to these core objectives, the guidelines also broach some secondary issues for the management of L. raniformis in urbanising landscapes. Techniques other than occupancy-based surveys are reviewed as a means of prioritising and monitoring habitat management actions, including surveys aimed at establishing the reproductive success of remnant populations, and mark-recapture and radio- telemetry for examining population size, survival and dispersal rates. Two experimental approaches to management are also considered: underpasses under roads to facilitate dispersal, and translocation of populations from wetlands ear-marked for destruction. The potential effectiveness of these approaches is reviewed,

and advice provided on the context in which they should be applied. These guidelines are aimed at the broad range of individuals and organisations involved in conservation of L. raniformis in urbanising landscapes, including both public land managers and conservation agency staff, individuals and organisations involved in urban development (developers, consultants, etc.), and private land holders who may wish to implement conservation initiatives for the species on their properties. In doing so, the guidelines aim to promote a scientifically validated and consistent approach to the species' management in these landscapes.

2464: *+.186*

We examined the suitability of using translocations as a method to create a new population of Egyptian tortoises Testudo kleinmanni in an area where the species historically occurred. We released 109 tortoises, comprising 57 males, 48 females and 4 juveniles. Dispersal from the release site influenced survival and retention rate, i.e. the proportion of individual tortoises found after the original release. The number of times a tortoise was recaptured decreased as the minimum distance at which it was found from the release site increased. In addition, live tortoises were significantly more likely to be found at shorter minimum distances from the release site than were dead tortoises. The sex ratio of pre-released tortoises tended to be different from the sex ratio of tortoises found during later surveys, with females proportionally more likely to be found than males. Pre-release mass was not a significant predictor of an individual tortoise being recaptured. Retention rates of future reintroductions may be improved by allowing tortoises to acclimatize and develop fidelity to the release site before they are translocated.

2465: *+.113*

Freshwater pearl mussels (Unionidae: Bivalvia) rank among the most endangered aquatic invertebrates, and this has recently prompted a number of initiatives designed to propagate the species through captive breeding. Yet there are few guidelines to aid in freshwater mussel culture for conservation, and few or no results on the fate of released juveniles. We reviewed various ex situ strategies for freshwater mussel conservation with emphasis on the freshwater pearl mussel Margaritifera margaritifera (L.), one of the most critically endangered unionids. Captive breeding could help safeguard critically endangered populations, but current rearing methods need to be optimised. Areas in particular need of research include the collection and storage of viable glochidia, the development of efficient rearing systems, and the formulation of algal diets. Likewise, the degree of host specificity warrants further investigation, as this will largely dictate the success of reintroduction programmes. Finally, we note that more information is needed on the degree of genetic structuring and post-release survival before translocation programmes can be recommended. As with other conservation projects, captive breeding of the freshwater pearl mussel cannot compensate for loss of critical habitats and is likely to be most efficient in combination with in situ conservation, not in isolation.

2466: *+.175*

Sturgeon were among the first fish species that revealed a population decline in response to the increased anthropogenic pressures upon the river systems in the 19th century. Safeguarding and remediation attempts were taken only after the species was almost extinct, relying on potential populations in the historic range. Through support of the German Federal Agency for Nature Conservation, a first pilot project was initiated by the in the mid 1990s attempting to identify potential donor populations for ex - situ measures. At the same time forty juvenile Adpenser sturio originating of a controlled reproduction of wild spawners from the Gironde River were transferred

from the French Cemagref to the Leibniz - Institute for Freshwater Ecology and Inland Fisheries in Berlin for experimental purposes and broodstock development, providing the nucleus of the German ex-situ stock. Habitat assessments were carried out to identify suitable river systems for remediation of the species. Genetically the French; A. slurio have been proven suitable for the reintroduction in the German North Sea tributaries. Since 2007 offspring of the first ex-situ reproductions in France were used to expand the future broodstocks both in France and Germany as well as to restock the Gironde system. These transfers also were utilized to carry out the first experimental release in the Elbe River system since 2008. First insights into the migration behaviour and habitat utilization were obtained through a telemetry study between the release site at Lenzen (river - km 485) to Hamburg Harbour (river - km 614). Furthermore, an outlook on the future requirements with regard to adaptation of rearing techniques to allow the adaptation of fish designated for release as well as options for stakeholder involvement are discussed.

2467: *+.101*

Loss of biodiversity and future of french bird populations. The overall situation of the conservation status, past changes and current trends of bird populations in France are different between habitats and species groups, but general declines are more prominent than a few increases, due to the consequences of economic and human population growth. In agricultural areas (>50% of the territory), most species are in marked decline, whereas many of them were considered common until recently. Only the Carrion Crow is increasing. Bird species in forest (>25% of the territory) are more stable, even though modern forestry and disturbance have significant impacts. Wetlands and coastal habitats have the highest number of decreasing species and some of the strongest declines. Among species groups, storks, cranes, herons and allied, as well as some raptors, have recently increased. Many seabirds, waders, Galliformes, rails and breeding ducks are decreasing. Among passerines, open country and several mediterranean species, as well as long distance migrants, are also decreasing. The most important cause of losses comes from agriculture, both habitat changes, declines of food resources and pollution. Increasing urbanization and associated infrastructures (>10% of the territory) are both a net loss of habitats and a source of significant mortality (cats, collisions, powerlines, ...) and habitat fragmentation. Even though hunting pressure is still high, it may have severe impacts on a few species (waterbirds, Woodcock, thrushes). Other factors such as climate changes, tourism, pollutions, overfishing, etc ... have still little documented overall impacts on bird populations. Traditional conservation measures (protected areas, hunting restrictions, public information) are still among the most successful. New tools, such as intensive management and restauration of species and habitats, as well as reintroductions, are developped through national and european legislation and funding, with more long-term results.

2468: *+.060*

Siberian Grouse (Falcipennis Falcipennis, Hartlaub, 1855) is an endemic species of Russia, a rare species included in the Red Data Book of Russia and Red List of IUCN. Catastrophic decrease in its population is observed everywhere. As a means to protect its population the authors suggest developing technology of breeding these birds in open-air cages, forming an open-air population in a new area. The study of Siberian Grouse was carried out at Karasuk permanent base of Institute of Systematics and Ecology of Animals of SB RAS of the jointly with the Novosibirsk Zoo for many years. Methods of keeping, feeding and breeding this species were developed. Two ways of growing fledglings were used: under a female and in brooders. Both technological (mixed fodder, grain mixture and forage additives) and natural feed (larch and spruce needles, berries, greenery and, sometimes, live arthropods) were fed. Natural feed in the ration was a must as a part of the

population was intended to be reintroduced into the wild. Taiga forests of Maslyaninsky region of Novosibirskaya oblast were chosen as a new area. Larch and spruce growing there, as well as berries, were a staple diet for the birds. All birds were ringed before release. The birds were taken to the place of reintroduction in a truck at night time and were released in the morning. The area chosen was 12km away from Maslyanino settlement. The total number of the reintroduced birds was 170 at the age from 3 months to 3 years old. The main part of them was reintroduced in April before the beginning of breeding and the rest of them were released in the beginning of September. Monitoring of their state was done by hunting inspectors, ecological inspectors and local volunteers. The birds were met mostly in autumn both soon after the introductions (several days to several weeks), and several months after them as a rule those were single individuals or couples met on taiga earth roads or on sides of asphalt roads not farther than 30km away from the place of reintroduction, and only once at a distance of 90km. A hunter came across a female bird with its brood of 6 fledglings in June 2008 at a distance of 20km away from the place of reintroduction. The results of forming a reserve population of Siberian Grouse demonstrated that birds grown in open cages survived in new situations during one life cycle. An encounter of a Siberian Grouse brood provided evidence that the process of forming a new population seemed to proceed to the next stage [long dash] reproduction of reintroduced individuals.

2469: ***+.367***

This 363-page book titled "Molecular Approaches in Natural Resource Conservation and Management" is written in English. This book contains 13 individually authored chapters. The topics covered include importance of biodiversity discovery in conservation, gene flow, genetic modification in crops, ecosystem genetics, conservation potential through vertebrate sex-determining genes, adaptive differentiation dynamics in European oaks, population genomics in conservation, counterintuitive evolutionary trends, hybridization in endangered animal taxa, pollen-seed movement in disturbed tropical landscape, allelic recharge from bottleneck events recovery, landscape alteration, genetic diversity conservation, dune restoration in threatened local population, evolutionary consideration in recovery planning, molecular methods in genetic management improvement, wildlife reintroduction and evolutionary toxicology. Each chapter is extensively referenced and also includes a list of contributors to this edition and their respective institutions. The book will be of interest to all those working or studying in the field of wildlife management and conservation.

2470: ***+.041***

We collected primary data on 13 European ground squirrel reintroduction projects carried out in the Czech Republic, Slovakia and Poland since 1989. During these projects more than 3,200 ground squirrels were reintroduced at 15 sites or used for reinforcement of 5 populations. Reintroductions can be considered successful at 7 sites where settlement and reproduction of the released individuals were observed. At other 7 sites reintroductions failed and the result of reintroduction is still unknown at one site. Proportion of long existing reintroduced colonies is even much lower. Results of reinforcements are unclear at all 5 sites. The main problems of reintroductions were the low number of released individuals, unsuitable methodology of releasing and inappropriate site management. For future reintroduction attempts we recommend to release a sufficient number of individuals and to use artificial burrows as well as temporary fencing of the site of release. Long-term management of the site and regular monitoring of the newly established population are also necessary.

2471: ***-.185***

Objective-To characterize infection patterns and identify factors associated with avian mycobacteriosis among zoo birds that were housed with infected enclosure mates.Design-Matched case-control study.Animals-79 birds with avian mycobacteriosis (cases) and 316 nondiseased birds (controls) of similar age and taxonomic group that were present in the bird collection of the Zoological Society of San Diego from 1991 through 2005.Procedures-Inventory and necropsy records from all eligible, exposed birds (n = 2,413) were examined to determine disease incidence and prevalence in the exposed cohort. Cases were matched in a 1:4 ratio to randomly selected controls of similar age and taxonomic grouping. Risk factors for mycobacteriosis (demographic, temporal, enclosure, and exposure characteristics as well as translocation history) were evaluated with univariate and multivariable conditional logistic regression analyses.Results-Disease prevalence and incidence were estimated at 3.5% and 8 cases/1,000 bird-years at risk, respectively. In the multivariable model, cases were more likely to have been imported into the collection, exposed to mycobacteriosis at a young age, exposed to the same bird species, and exposed in small enclosures than were controls. Odds for disease increased with an increasing amount of time spent with other disease-positive birds. Conclusions and Clinical Relevance-The low incidence of mycobacteriosis and the risk factors identified suggested that mycobacteria may not be easily transmitted through direct contact with infected enclosure mates. Identification of risk factors for avian mycobacteriosis will help guide future management of this disease in zoo bird populations, (J Am Vet Med Assoc 2010;236:211-218)

2472: ***+.176***

Background: While wild chimpanzees are experiencing drastic population declines, their numbers at African rescue and rehabilitation projects are growing rapidly. Chimpanzees follow complex routes to these refuges; and their geographic origins are often unclear. Identifying areas where hunting occurs can help law enforcement authorities focus scarce resources for wildlife protection planning. Efficiently focusing these resources is particularly important in Cameroon because this country is a key transportation waypoint for international wildlife crime syndicates. Furthermore, Cameroon is home to two chimpanzee subspecies, which makes ascertaining the origins of these chimpanzees important for reintroduction planning and for scientific investigations involving these chimpanzees. Results: We estimated geographic origins of 46 chimpanzees from the Limbe Wildlife Centre (LWC) in Cameroon. Using Bayesian approximation methods, we determined their origins using mtDNA sequences and microsatellite (STRP) genotypes compared to a spatial map of georeferenced chimpanzee samples from 10 locations spanning Cameroon and Nigeria. The LWC chimpanzees come from multiple regions of Cameroon or forested areas straddling the Cameroon-Nigeria border. The LWC chimpanzees were partitioned further as originating from one of three biogeographically important zones occurring in Cameroon, but we were unable to refine these origin estimates to more specific areas within these three zones. Conclusions: Our findings suggest that chimpanzee hunting is widespread across Cameroon. Live animal smuggling appears to occur locally within Cameroon, despite the existence of local wildlife cartels that operate internationally. This pattern varies from the illegal wildlife trade patterns observed in other commercially valuable species, such as elephants, where specific populations are targeted for exploitation. A broader sample of rescued chimpanzees compared against a more comprehensive grid of georeferenced samples may reveal 'hotspots' of chimpanzee hunting and live animal transport routes in Cameroon. These results illustrate also that clarifying the origins of refuge chimpanzees is an important tool for designing reintroduction programs. Finally, chimpanzees at refuges are frequently used in scientific investigations, such as studies investigating the history of zoonotic diseases. Our results provide important new information for interpreting these studies within a precise geographical framework.

2473: ***+.200***

Background: While wild chimpanzees are experiencing drastic population declines, their numbers at African rescue and rehabilitation projects are growing rapidly. Chimpanzees follow complex routes to these refuges; and their geographic origins are often unclear. Identifying areas where hunting occurs can help law enforcement authorities focus scarce resources for wildlife protection planning. Efficiently focusing these resources is particularly important in Cameroon because this country is a key transportation waypoint for international wildlife crime syndicates. Furthermore, Cameroon is home to two chimpanzee subspecies, which makes ascertaining the origins of these chimpanzees important for reintroduction planning and for scientific investigations involving these chimpanzees.Results: We estimated geographic origins of 46 chimpanzees from the Limbe Wildlife Centre (LWC) in Cameroon. Using Bayesian approximation methods, we determined their origins using mtDNA sequences and microsatellite (STRP) genotypes compared to a spatial map of georeferenced chimpanzee samples from 10 locations spanning Cameroon and Nigeria. The LWC chimpanzees come from multiple regions of Cameroon or forested areas straddling the Cameroon-Nigeria border. The LWC chimpanzees were partitioned further as originating from one of three biogeographically important zones occurring in Cameroon, but we were unable to refine these origin estimates to more specific areas within these three zones.Conclusions: Our findings suggest that chimpanzee hunting is widespread across Cameroon. Live animal smuggling appears to occur locally within Cameroon, despite the existence of local wildlife cartels that operate internationally. This pattern varies from the illegal wildlife trade patterns observed in other commercially valuable species, such as elephants, where specific populations are targeted for exploitation. A broader sample of rescued chimpanzees compared against a more comprehensive grid of georeferenced samples may reveal 'hotspots' of chimpanzee hunting and live animal transport routes in Cameroon. These results illustrate also that clarifying the origins of refuge chimpanzees is an important tool for designing reintroduction programs. Finally, chimpanzees at refuges are frequently used in scientific investigations, such as studies investigating the history of zoonotic diseases. Our results provide important new information for interpreting these studies within a precise geographical framework.

2474: ***+.089***

The distribution pattern, age structure and species composition of concomitant community of Bretschneidera sinensis Hemsl. were investigated in Nankun Mountain Nature Reserve, Guangdong province. Results showed that the distribution area of this population was narrow and concentrated; the age structure was incomplete and decline, especially II, III saplings were scarce; The concomitant community belonged to typical sub-tropical zone; whereas the species composition was unstable and the dominant species was not significant. It was advised that in situ conservation of the saplings should be emphasized by getting rid of the fast-growing species such as Acer fabri, Rhododendron cavaleriei and Cinnamomum parthenoxylon to create better circumstance with sunshine, water and fertilizer; and for the adult tree elder than IV, the number and density of Alniphyllum fortunei and Machilus chinensis which have the similar spatial niches to B. sinensis should be monitored and thinned out timely to help the adult tree get better canopy space. The reintroduction of seedlings at uneven age of B. sinensis should be carried out actively to promote the recovery and development of the population.

2475: ***+.117***

A large number of die-off of globally threatened Arabian Oryx (Oryx leucoryx), and Arabian Sand Gazelle (Gazella subgutturosa marica) were recorded from 1999 to 2008 in fenced Mahazat as-

Sayd Protected Area (PA) in western-central Saudi Arabia. Mortalities of animals have been recorded during summer months when the rainfall is negligible or insignificant. Deaths were due to starvation because of reduced availability, accessibility and quality of food plants in the area. In total, 560 oryx and 2815 sand gazelle deaths were recorded since the reintroduction projects began till the end of 2008. Mortalities of animals were higher in 1999-2001, 2006, 2007 and 2008. Grazing of oryx habitat depends on rainfall and animals move over great distances in response to rain. The fence around Mahazat as-Sayd PA prevents natural movements of animals, and artificially concentrates the ungulate populations into possibly unfavourable habitat. The sand gazelle is a highly gregarious and migratory species, moving long distances in search of good quality pastures. Populations of sand gazelle in Central Asia are also known to migrate over large distances, covering several hundred kilometers. It is therefore likely that by preventing natural movements of sand gazelles and oryx, fencing may have reinforced the effects of stressful conditions such as drought. To reduce the catastrophic effects, a Strategy and Action Plan was developed in August 2008 to manage oryx and gazelle within the reserve and with provision for food and water at the five camps in the reserve as emergency plan to minimize mortalities.

2476: *+.269*

1. Non-invasive genetic sampling (NGS) of hair and faeces has become an important tool for monitoring wildlife populations, but many managers question the feasibility and cost-effectiveness of these methods for long-term monitoring. To address this question, more studies are needed that simultaneously evaluate the effectiveness and efficiency of multiple NGS designs in the same study area.2. In 2003-2004, we carried out an experimental study of NGS for a small brown bear Ursus arctos population established by translocation in the Italian Alps. We evaluated and compared the effectiveness and efficiency of three NGS approaches including two systematic designs, baited hair traps and transect sampling of hair and faeces, and opportunistic collection of faecal and hair samples. Effectiveness was evaluated in terms of the number of samples collected, bears identified, genotyping success and error rate, detection frequencies, individual movement and spatial distribution of the species. We also evaluated the suitability of the data collected for population size estimation using single- and multi-session approaches. Efficiency was assessed by calculating total cost/genotyped sample, cost/unique bear identified and cost/bear sample.3. During 2 years of sampling, 1164 samples and 15 unique genotypes were obtained. From these genotypes, we documented reproduction, an increase in the minimum population size of bears in the study area and important information on specific bears causing damages to property.4. The optimal sampling strategy combined systematic hair trapping and opportunistic sampling, as the pooled data set efficiently provided large sample quantities, the highest number of identified bears, multiple individual detections, information on bear distribution and suitable data for population size estimation.5. We provide an example of how the efficiency of NGS monitoring can be improved by integrating sampling into routine duties of existing field personnel.6. Synthesis and applications. This study provides baseline data for monitoring brown bears in the Italian Alps and has important implications for NGS of other small populations in human-dominated landscapes. Conservation of small populations in such habitats will benefit from multiple strategies that obtain critical demographic, spatial and genetic information in a cost-effective manner.

2477: *+.037*

Assisted colonization could help prevent the extinction of threatened and endangered species by intentionally moving a species to a region where it has not occurred in the recent past, but should survive under future climate scenarios. Where species are naturally localized and confined to patchy habitats, assisted colonization might be the only means for population dispersal across

human landscape barriers such as urban and agriculture areas. The major risk associated with assisted colonization is introducing ecologically harmful species. Previous policy papers have described management options for deciding when to move a species to mitigate for climate change. We build on this previous work by examining management options and policy solutions for assisted colonization under the U.S. Endangered Species Act (ESA). On its surface, the ESA statutory language appears to provide the legal framework for allowing assisted colonization, as the U.S. Congress gave the U.S. Fish and Wildlife Service (USFWS) broad discretion to manage populations of endangered species. However, current USFWS regulations are an impediment to assisted colonization for many endangered animal species, whereas regulations do not necessarily restrict assisted colonization of endangered plants. Because of this discrepancy, we recommend a review of the regulatory language governing movements of endangered species.

2478: *+.031*

We used microsatellites to assess ongoing captive breeding and reintroduction programs of the lesser kestrel. The extent of genetic variation within the captive populations analysed did not differ significantly from that reported in wild populations. Thus, the application of widely recommended management practices, such as the registration of crosses between individuals in proper stud books and the introduction of new individuals into the genetic pools, has proven satisfactory to maintain high levels of genetic variation. The high rates of hatching failure occasionally documented in captivity can therefore not be attributed to depressed genetic variation. Even though genetic diversity in reintroduced populations did not differ significantly when compared to wild populations either, average observed heterozygosities and inbreeding coefficients were significantly lower and higher, respectively, when compared to the captive demes where released birds came. Monitoring of reproductive parameters during single-pairing breeding and paternity inference within colonial facilities revealed large variations in breeding success between reproductive adults. The relative number of breeding pairs that contributed to a large part of captive-born birds (50-56%) was similar in both cases (28.6 and 26.9%, respectively). Thus, the chances for polygyny (rarely in this study), extra- pair paternity (not found in this study) and/or social stimulation of breeding parameters do not seem to greatly affect the genetically effective population size. Independently of breeding strategies, the release of unrelated fledglings into the same area and the promotion of immigration should be mandatory to counteract founder effects and avoid inbreeding in reintroduced populations of lesser kestrels.

2479: *-.013*

The ability to distinguish between native and translocated populations is important in conservation biology, but can be problematic, especially near range edges. We describe here the use of variable genetic markers as an approach to resolving this issue. Natterjack toads Bufo calamita have a very restricted distribution in south-west Ireland. Populations in the main distribution area are well documented, but there are also some recently discovered ones at a widely separated site. We used genetic evidence to investigate whether the recently discovered populations are newly established or of ancient origin, using three criteria: genetic diversity, genetic structure and estimated time since divergence. The results highlighted some difficulties of using genetic data from range edge populations, where such issues are most likely to arise but where diversity (and thus statistical power) is often low. On balance the genetic data weakly supported the hypothesis that the newly discovered populations are not recent translocations. It will always be important in such investigations to use genetic inferences in combination with other independent lines of evidence (such as fossils) wherever possible.

2480: ***+.147***

The population of elk (Cervus elaphus roosevelti) inhabiting Afognak Island, Alaska, USA arose from an introduction of 8 individuals from an established population in Washington, USA in 1929, and recently peaked at approximately 1,400 individuals. We examined indices of diversity for 15 microsatellite loci in the Afognak population and compared them to levels in the parent population to determine effects of translocation and demography on genetic variation. The Afognak population differed significantly ($P < 0.0001$) from the source population in both allele and genotype frequencies. Allelic richness, number of private alleles and multilocus heterozygosity, but not percent loci polymorphic, were significantly lower in Afognak elk. Mean inbreeding coefficients within Afognak ($f = 0.019$) and source ($f = -0.006$) populations did not differ significantly from zero. Despite the demographic bottleneck, no evidence of a genetic bottleneck in the Afognak population was detected using a test for heterozygosity excess or mode shift of allele frequencies. Simulations indicated that rapid population growth after the translocation resulted in heterozygosity excess for only 8 years. Conversely, a statistic testing for a bottleneck signature in the ratio of allele number to allele size range (M-ratio) was significant for both the Afognak and source populations, suggesting that the Afognak population had effectively undergone serial bottlenecks. Nonetheless, Afognak failed to show a smaller M-ratio than the parent population, suggesting a failure of that statistic to detect the bottleneck associated with introduction. We show that a severe bottleneck followed by rapid population growth may be undetectable using available tests.

2481: ***+.115***

The Puerto Rican crested toad (Peltophryne lemur) is currently composed of a single wild population on the south coast of Puerto Rico and two captive populations founded by animals from the northern and southern coasts. The main factors contributing to its decline are habitat loss, inundation of breeding ponds during storms, and impacts of invasive species. Recovery efforts have been extensive, involving captive breeding and reintroductions, habitat restoration, construction of breeding ponds, and public education. To guide future conservation efforts, genetic variation and differentiation were assessed for the two captive colonies and the remaining wild population using the mitochondrial control region and six novel microsatellite loci. Only two moderately divergent mitochondrial haplotypes were found, with one fixed in each of the southern and northern lineages. Moderate genetic variation exists for microsatellite loci in all three groups. The captive southern population has not diverged substantially from the wild population at microsatellite loci ($F(ST) = 0.03$), whereas there is little allelic overlap between the northern and southern lineages at five of six loci ($F(ST) > 0.3$). Despite this differentiation, they are no more divergent than many populations of other amphibian species. As the northern breeding colony may not remain viable due to its small size and inbred nature, it is recommended that a third breeding colony be established in which northern and southern individuals are combined. This will preserve any northern adaptive traits that may exist, and provide animals for release in the event that the pure northern lineage becomes extirpated.

2482: ***+.025***

For endangered plants interspecific hybridization occurring in ex situ collections may lead to failure of reintroduction actions. We used Sinojackia xylocarpa, a well documented Chinese endemic species that is extinct in the wild, as a model case to address this concern. We used paternity analyses to assess the spontaneous hybridization and patterns of pollen flow between S. xylocarpa and its congener species, S. rehderiana, in conserved populations in Wuhan Botanic

Garden. Interspecific hybridization events were detected in seven out of eight maternal trees of S. xylocarpa, and an average of 32.7% seeds collected from maternal trees of S. xylocarpa were hybrids. The paternity of 93 out of 249 seedlings from S. xylocarpa assigned to S. rehderiana provided convincing evidence that spontaneous interspecific hybridization occurred extensively in the living garden collection we studied. Different patterns of pollen dispersal (predominantly short-distance vs. long-distance pollination) were observed between intra-and interspecific hybridization events in the garden. Pollen dispersal within the ex situ populations was not restricted by distance, as evidenced by a lack of significant correlations between the average effective pollen dispersal distance (delta) and the geographic distances (d1 and d2) between maternal and paternal trees. The interspecific pollen-dispersal distance ranged from 10 to 620.1 m (mean 294.4 m). Such extensive hybridization in ex situ collections could jeopardize the genetic integrity of endangered species and irrevocably contaminate the gene pool if such hybrids are used for reintroduction and restoration. We recommend strongly that measures be taken to minimize the genetic risks of this kind of hybridization, including establishing buffer zones in ex situ collections, manipulating flowering phenology, testing seed lots before use in reintroduction programs, and controlling pollination for seed purity.

2483: *+.256*

Barriers are used to achieve diverse objectives in conservation and biosecurity. In conservation management, fences are often erected to exclude introduced predators and to contain diseased animals or invasive species. Planning an efficient conservation fence involves a number of decisions, including the size and design of the enclosure. We formulated the first general framework for building a fence that minimizes long-term management costs by balancing the expense of constructing a more secure barrier against the costs of coping with more frequent failures. The approach systematically considers the range of potential solutions to a well-defined fencing problem and results in a solution that maximizes conservation return on investment. We illustrated this method by designing efficient fences to address two different conservation goals: exclusion of invasive predators from populations of threatened eastern barred bandicoots (Perameles gunnii) and maintenance of isolated populations of healthy Tasmanian devils (Sarcophilus harrisii). A systematic approach to conservation fencing allows the best fence design to be chosen quantitatively and defensibly. It also facilitates conservation decisions at a strategic level by allowing fencing to be compared transparently with alternative conservation management actions.

2484: *+.337*

The absence of top-level predators in many natural areas in North America has resulted in overabundant ungulate populations, cascading negative impacts on plant communities, and the loss of biodiversity and ecosystem processes. Meanwhile, distinct population segments of the gray wolf (Canis lupus) have been removed from the list of endangered and threatened species, implying an end to wolf recovery and reintroductions. Me propose another paradigm for wolf conservation, one that emphasizes ecosystem recovery instead of wolf recovery. Improvements in technology an enhanced understanding of the ecological role of wolves, lessons from other countries, and changing public attitudes provide a new context and opportunity for wolf conservation and ecosystem restoration. Under this new paradigm, small populations of wolves, even single packs, could be restored to relatively small natural areas for purposes of ecosystem restoration and stewardship. We acknowledge the complications and challenges involved in such an effort, but assert that the benefits could be substantial.

2485: *-.008*

Tropical island species and ecosystems are threatened worldwide as a result of increasing human pressure. Yet some of these islands also lend themselves to restoration, as they are physically defined units that can be given focused attention, as long as resources are available and clear conservation targets are set. Cousine Island, Seychelles, is a tropical island that has received such intensive restoration. From a highly degraded island in the 1960s, the island has now been restored to what is believed to be a semblance of the natural state. All alien vertebrates have been eradicated, as have 25 invasive alien plants. Cultivated plants are now confined to one small section of the island. Poaching of nesting marine turtles has been stopped, leading to an increase in turtle breeding numbers. The shearwater population has increased in size with poaching activities under control. The Sooty tern has also returned to the island to breed. The coastal plain has been restored with over 2,500 indigenous shrubs and trees, which have now grown into a forest carpet. There are strict quarantine procedures on the island, keeping it free of rats, mice, various alien invertebrates and potentially invasive alien plants. Three threatened Seychelles endemic land birds (Seychelles warbler, Seychelles magpie robin and Seychelles white-eye) have been introduced and are thriving, with these introductions contributing to both the magpie robin and the white-eye being downgraded from CR to EN (the warbler remains at VU). Ecotourism, and nature conservation for the local inhabitants, have been introduced in a way that does not reduce the improved compositional, structural and functional biodiversity of the island. The result of the restoration effort appears to be sustainable in the long term, although challenges still remain, especially with regards to adequate clean water and a non-polluting power supply on the island. Cousine is thus paving the way in the art and science of tropical island restoration as a legacy for future generations.

2486: *+.105*

Relocation is one of the strategies used by conservationists to deal with problem cheetahs in southern Africa. The success of a relocation event and the factors that influence it within the broader context of long-term viability of wild cheetah metapopulations was the focus of a Bayesian Network (BN) modelling workshop in South Africa. Using a new heuristics, Iterative Bayesian Network Development Cycle (IBNDC), described in this paper, several networks were formulated to distinguish between the unique relocation experiences and conditions in Botswana and South Africa. There were many common underlying factors, despite the disparate relocation strategies and sites in the two countries. The benefit of relocation BNs goes beyond the identification and quantification of the factors influencing the success of relocations and population viability. They equip conservationists with a powerful communication toot in their negotiations with land and livestock owners, which is key to the long-term survival of cheetahs in southern Africa. Importantly, the IBNDC provides the ecological modeller with a methodological process that combines several BN design frameworks to facilitate the development of a BN in a multi-expert and multi-field domain. (C) 2009 Elsevier B.V. All rights reserved.

2487: *+.209*

We report results of a 4-year translocation effort to reestablish a breeding population of Evermann's Rock Ptarmigan (Lagopus muta evermanni) in the Near Islands group of the western Aleutian Archipelago. Habitat restoration was completed by eradication of introduced foxes from Agattu Island by 1979. We captured and moved 75 ptarmigan from Attu Island to Agattu Island during 2003-2006, and monitored 29 radio-marked females in the last 2 years of the study. We compared the demography of newly translocated birds (n = 13) with resident birds established

from translocations in previous years (n = 16). Mortality risk was increased by translocation and 15% of females died within 2 weeks of release at Agattu Island. All surviving females attempted to nest but initiated clutches 8 days later in the breeding season and laid 1.5 fewer eggs per clutch than resident females. Probability of nest survival ((x) over bar +/- SE) was good for both translocated (0.72 +/- 0.17) and resident females (0.50 +/- 0.16), and renests were rare. Probability of brood survival was higher among translocated (0.85 +/- 0.14) than resident females (0.25 +/- 0.12), partly as a result of inclement weather in 2006. Fecundity, estimated as female fledglings per breeding female, was relatively low for both translocated (0.9 +/- 0.3) and resident females (0.3 +/- 0.2). No mortalities occurred among radio-marked female ptarmigan during the 10-week breeding season, and the probability of annual survival for females in 2005-2006 was between 0.38 and 0.75. Translocations were successful because females survived, successfully nested, and recruited offspring during the establishment stage. Post-release monitoring provided useful demographic data in this study and should be a key component of translocation programs for wildlife restoration. Future population surveys and additional translocations may be required to ensure long-term viability of the reintroduced population of ptarmigan at Agattu Island. Received 31 July 2008. Accepted 16 July 2009.

2488: ***+.107***

Effective conservation planning for endangered species depends on an understanding of space use patterns. Black-footed ferrets Mustela nigripes depend on prairie dogs Cynomys sp. as prey and use their burrow systems for shelter. The availability of areas with high densities of active prairie dog burrows is the major factor thought to affect their selection of sites and resources. However, we have little knowledge about how the spatial distribution of active prairie dog burrows might influence the spatial organization and home-range size of ferrets. We monitored the movements of black-footed ferrets on a black-tailed prairie dog C. ludovicianus colony in South Dakota to document ferret space use patterns. Home ranges of female ferrets were 22.9 - 95.6 ha in size ((x) over bar =56.3 ha, SE=19.7, N=6), while male ferret home ranges were on average more than twice as large as those of females ((x) over bar =128.3 ha, SE=68.5, N=3). The home-range size of female ferrets was correlated with mean active prairie dog burrow utilization distribution (UD) value within ferret home ranges, where home-range size decreased as active prairie dog burrow UD value increased (r(2)=0.974, P < 0.001, N=6). Ferret space use overlapped more extensively than previously reported, with up to 43% UD overlap between a ferret and the nearest adjacent ferret of the same sex. Areas of overlap tended to have higher active prairie dog burrow UD values, suggesting that the spatial distribution of active prairie dog burrows influenced both home-range size and the amount of space use overlap between ferrets. These findings emphasize the potential influence of resource distribution on carnivore sociobiology and the importance of considering that distribution in assessing habitat for the reintroduction of specialized species.

2489: ***+.036***

The western pond turtle (Actinemys marmorata) has lost most of its habitat ill the Central Valley of California to agricultural activities, flood Control, and urbanization. Although a few areas Still support this turtle, most habitats are now altered by humans. Aquatic habitats near population centers also may become release sites For a variety of introduced turtles, which Could Compete with the native A. marmorata. In 1999, 2002, and 2007, I trapped at the Fresno and Hanford wastewater-treatment facilities to determine presence and numbers of A. marmorata at settling ponds in these facilities. I caught 213 A. marmorata at. Fresno and 106 at Hanford. No other species of till-ties was Caught. Turtles at both sites grew rapidly and had a mean size Of clutch of 8.2 (Fresno) and 8.5 eggs (Hanford), which are the highest mean size of clutch reported for this

species. Although not esthetically appealing to people, both sewage-treatment facilities provide habitat for A, marmorata and these could Stock For future reintroductions of this species to more natural, rehabilitated aquatic habitats in nearby areas.

2490: *+.164*

We studied 2 years of postrelease telemetry data of elk (Cervus elaphus) translocated to their historic range limit in Ontario, Canada and sought to determine if postrelease movements were related to behavior, demography of released animals, or site-specific attributes such as length of holding period. During 1998-2004 we radio-tracked 341 elk in 10 release groups via ground and aerial telemetry and monitored movement patterns relative to gender, age, and pre-release holding period (4-112 days). We found that elk that were held for short periods prior to release (4-11 days) moved longer distances than those subject to extended conditioning (17-112 days), suggesting that an extended conditioning period is beneficial from the standpoint of promoting philopatry. When all elk were pooled by sex and age class, male calves remained in closer proximity (8.0 +/- 13.2 km) to release sites than adult females (19.1 +/- 20.6 km), adult males (19.7 +/- 15.1 km), and female calves (14.4 +/- 20.4 km). Most calves dispersed in a southeasterly direction whereas adults tended to travel southwest. Our results reveal that elk movement characteristics are influenced by factors such as release protocol and group demographics; these findings provide further insight regarding appropriate release methods for restoring natural populations near their historical range limit.

2491: *+.177*

In this study we systematically examined species-specific exploration behaviour and behavioural acclimatisation of a black rhino Diceros bicornis founder group post-translocation. The study was carried out in a fenced region, approximately 350 km(2) in size and directly adjacent to Etosha National Park in Namibia. Rhinos were radio-tracked and movement data were complemented by detailed habitat descriptions at the centres of rhino activity with vegetation plot sampling. Rhinos used both geological formations of the study area: Otavi dolomite and Etosha calcrete substrate. The latter was dominated by Acacia spp. due to former land use for livestock farming. The size of total and seasonal home ranges and core areas, as well as home range establishment patterns and habitat use, are highly variable among individual rhinos. Home ranges of most individuals from our study are among the largest recorded for the species. We found age class specific patterns of home range establishment, typical mating and social behaviour, seasonal changes of home range and core area size, and clear shifts in spatial behaviour over time. The results provide our best estimate to date for the natural exploration behaviour and behavioural acclimatisation of black rhinos in a semi-arid savannah ecosystem.

2492: *+.011*

Species that pass repeatedly through narrow population bottlenecks (< 100 individuals) are likely to have lost a large proportion of their genetic variation. Having genotyped 92 Raso larks Alauda razae, a Critically Endangered single-island endemic whose world population in the Cape Verdes over the last 100 years has fluctuated between about 15 and 130 pairs, we found variation at 7 of 21 microsatellite loci that successfully amplified, the remaining loci being monomorphic. At 6 of the polymorphic loci variation was sex-linked, despite the fact that these microsatellites were not sex-linked in the other passerine birds where they were developed. Comparative analysis strongly suggests that material from several different autosomes has been recently transferred to the sex chromosomes in larks. Sex-linkage might plausibly allow some level of heterozygosity to be

maintained, even in the face of persistently small population sizes.

2493: *+.298*

Managers of species that exist as metapopulations are faced with many decisions. In this paper we use a decision-theory framework to examine a fundamental management question: Should we focus on decreasing the local extinction probability of subpopulations by increasing the size of their patch, or should metapopulation viability be improved by constructing more patches? Using a spatially implicit stochastic metapopulation model and stochastic dynamic programming (SDP), we found the optimal solution to this problem for both the finite- and infinite-time horizon cases. We showed that the SDP solutions outperform a range of heuristic management strategies. The optimal strategy for a given parameter set depends heavily on metapopulation parameters, and it is difficult to make generalizations about the optimal restoration strategy a priori. Although heuristic strategies perform well in some cases, it is not possible to judge their performance until the SDP solution has been computed, and for this reason we advocate the use of SDP as a management tool in restoration. We demonstrate the use of SDP by deriving an optimal management strategy for a population of the Mount Lofty Ranges Southern Emu-wren (Stipituras malachurus intermedius).

2494: *+.133*

Assessing the potential for threatened species to persist and spread within fragmented landscapes requires the identification or core areas that can sustain resident populations and dispersal corridors that can link these core areas with isolated patches of remnant habitat. We developed a set of GIS tools, simulation methods, and network analysis procedures to assess potential landscape connectivity for the Delmarva fox squirrel (DFS; Sciurus niger cinereus), an endangered species inhabiting forested areas on the Delmarva Peninsula, USA. Information on the DFS's life history and dispersal characteristics, together with data on the composition and configuration of land cover on the peninsula, were used as input data for an individual-based model to simulate dispersal patterns of millions of squirrels. Simulation results were then assessed using methods from graph theory, which quantifies habitat attributes associated with local and global connectivity. Several bottlenecks to dispersal were identified that were not apparent from simple distance-based metrics, highlighting specific locations for landscape conservation, restoration, and/or squirrel translocations. Our approach links simulation models, network analysis, and available field data in an efficient and general manner, making these methods useful and appropriate for assessing the movement dynamics of threatened species within landscapes being altered by human and natural disturbances.

2495: *-.186*

The flightless dung beetle Circellum bacchus (Fabricius, 1781) is a unique, ectothermic dung beetle that is of conservation concern due to a massive decline in its distribution. Very little is known about its conservation ecology and the upgrade of roads in one of its last strongholds, South Africa's Addo Elephant National Park, led to concerns that road kill was threatening the population because drivers could not see the beetles due to their similar colour as the upgraded roads. We tested whether the upgraded, black, tar roads led to more road kills than the original sandy-red, gravel roads using counts of live and dead beetles along transects through similar habitats of the park. There was no significant difference between the number of live and dead dung beetles on the tar or gravel roads illustrating that the infrastructure improvements themselves are not threatening the persistence of the species. The high levels of vehicle-derived mortality along roads, however, suggest that road kills may be a threatening process with potentially 100,000 C.

bacchus killed on roads annually (although 45,000 is a more conservative estimate). Further research is needed to ascertain whether this off-take is sustainable and to formulate mitigation measures.

2496: *-.181*

We studied the effect of a recolonizing wolf (Canis lupus L., 1758) population on a resident lynx (Lynx lynx (L., 1758)) population in south-central Sweden. Wolf and lynx share the same prey species, western roe deer (Capreolus capreolus (L., 1758)), and the size difference between the two species suggests a strong potential for interference competition. The spatial distributions of lynx family groups (n = 378) over four winters were not significantly affected by the increase in size and range of the wolf population. Survival of lynx kittens until 9 months of age did not differ significantly inside (54%; n = 37) and outside (62%; n = 42) wolf territories, and female lynx (a = 3) selected natal den sites (n = 19) in the same local area before and after wolf establishment. Furthermore, lynx home-range size (n = 42) did not increase as a result of presence of wolves and space use by female lynx (n = 3) was not affected by wolf establishment. We found no evidence of cleptoparasitism by wolves on roe deer killed by lynx. We conclude that the intensity of interference and exploitation competition between wolves and lynx was low.

2497: *+.343*

From 2001 to 2005, 71 costume-reared juvenile Whooping Cranes Grits americana were led by ultralight aircraft from Wisconsin to a winter release site on the west-central Gulf Coast of Florida. A strategy was developed and implemented to maximize first winter survival while preventing exposure to non-costumed humans and tame Sandhill Cranes, maximize social bonding between males and females, promote safe roosting and wild behaviour, and minimize harassment by Whooping Cranes from earlier releases. Methods were improved each year; these modifications included enlargement of an open-topped release pen, creation of an artificial roosting substrate, addition of a top-netted pen, and holding birds at a distant pen site until older birds had cleared the release area. These techniques resulted in high survival and successful adaptation to the wild after migration from the winter release site.

2498: *+.170*

This meeting, which contains 167 English abstracts, focusses on bat research news. Topics include, bat migration in Western Baltic Sea, microhabitat structure and prey abundance in foraging site selection of Myotis capaccinii, movements of marked Myotis myods between hibernacula and summer nursery roosts and among nursery roosts, phylogeography of mouse-eared bats, Myotis myotis and Myods blythii present in Anatolia and Turkish Thrace, the distribution patterns of Pipistreilus kuhlii and Hypsugo savii in Hungary, activity and foraging habitats of Myotis capaccinii in Southern France and implications for species conservation and impact of climate change on European bats according to their biogeographical patterns.

2499: *+.099*

The Ord's kangaroo rat is a rare, nocturnally-active rodent distributed through the Middle Sand Hills and surrounding sandy regions of southeast Alberta. Abundance is highest in areas of loose sandy soils, particularly those associated with active sand dunes. In recent decades, much of the available sand dune habitat has become stabilized by vegetation. Stabilized sand dunes are rarely occupied by kangaroo rats in Alberta. Thus, sand dune stabilization represents rapid habitat loss

and fragmentation for the species and has led to a number of disjunct and isolated sub-populations within the province. Recent research has identified the location of habitats for the Ord's kangaroo rat in Alberta, including a number of isolated patches of natural habitat. Population projections based on computer simulation models have indicated that the two most isolated sub-populations in Alberta (Empress Sand Dunes sites and Dune Point sites) are at the highest risk of extirpation due to their extreme isolation, despite their high habitat quality. Other small, isolated sub-populations may also be susceptible to extirpation because of their increased vulnerability to disease and stochastic effects, fewer mating opportunities which can lead to lower animal recruitment, increased inbreeding, and low rates of immigration and rescue. Translocations of individuals from productive source sites in the population to small and isolated or declining sites has been identified as a management tool to mitigate local extirpations and strengthen regional population persistence. Various translocation experiments have been performed on kangaroo rats and other heteromyid rodents in the United States. Most have met with limited success, a sign that the feasibility of using kangaroo rat translocations as a conservation tool is not well understood. This report provides recommended methods for translocating Ord's kangaroo rats in Alberta based on the successes and limitations observed in previous studies, with particular consideration of the unique properties of the Alberta population (e.g., bot fly parasitism). This report also describes knowledge gaps and recommended research that should be undertaken to determine whether Ord's kangaroo rat translocations can be used in Alberta to mitigate population decline and local extirpation of isolated habitats. Although the feasibility of using translocations as a management tool is not well understood, it is hoped that future research in Alberta can identify best practices for translocations to provide a valuable conservation tool for managers who wish to ensure the future persistence of this endangered species.

2500: *+.063*

The reintroduction of African elephants into fenced game reserves throughout South Africa has presented managers with several challenges. Although elephants are a natural part of southern African ecosystems, their confinement to fenced protected areas in South Africa has exacerbated their potential to impact their habitats negatively. However, many studies investigating the impact of elephants have failed to control for the effects of other browsers on the vegetative community. In this study, we used location data on an elephant herd to delineate high-use and low-use areas. This paired design allowed us to minimize confounding factors that could explain differences in the structure, diversity and utilization of woody species. We found little evidence to suggest elephant-mediated change in, or selection for, the structure or diversity of woody species; however, our results suggest that elephants may be altering the composition of species by preferentially using areas with higher canopy diversity and by enhancing sapling recruitment. Although stripping of bark was higher in high-use areas, there was no evidence of differential mortality of tree species. Therefore, in our study area, and over the current time scale, elephants are having a negligible impact on the vegetative community.

2501: *-.001*

Background: Animals captive bred for reintroduction are often housed under conditions which are not representative of their preferred social structure for at least part of the reintroduction process. Specifically, this is most likely to occur during the final stages of the release programme, whilst being housed during transportation to the release site. The degree of social stress experienced by individuals during this time may negatively impact upon their immunocompetence.Methodology/Principal Findings: We examined two measure of stress-body weight and Leukocyte Coping Capacity (LCC) - to investigate the effects of group size upon

captive-bred water voles destined for release within a reintroduction program. Water voles were housed in laboratory cages containing between one and eight individuals. LCC scores were negatively correlated with group size, suggesting that individuals in larger groups experienced a larger degree of immunosuppression than did individuals housed in smaller groups or individually. During the course of the study mean body weights increased, in contrast to expectations from a previous study. This was attributed to the individuals sampled being sub-adults and thus growing in length and weight during the course of the investigation.Conclusions/Significance: The reintroduction process will inevitably cause some stress to the release cohort. However, for water voles we conclude that the stress experienced may be reduced by decreasing group size within captive colony and/or transportation housing practises. These findings are of significance to other species' reintroductions, in highlighting the need to consider life-history strategies when choosing housing systems for animals being maintained in captivity prior to release to the wild. A reduction in stress experienced at the pre-release stage may improve immunocompetence and thus animal welfare and initial survival post-release.

2502: ***+.128***

The white-tailed deer (Odocoileus virginianus) is the most widely distributed and studied ungulate in the American continent. This species is found throughout Mexico, except on the peninsula of Baja California and some areas of northern Chihuahua and Sonora. In this study we compared three geographic distribution models (Kellogg 1956; Hall 1981; Villarreal 1999) of white-tailed deer subspecies on a national scale, by state and by principal vegetation types. We found that neither the number of subspecies (13 or 14 of the 38 recognised subspecies), nor the geographical limits between subspecies coincided completely between models. Furthermore, for several subspecies, marked differences in distribution area were found depending on the distribution model used. Using multivariate analyses, we found that the 14 subspecies can be separated into three groups associated with different vegetation types: the northern subspecies associated with shrub land, the Pacific subspecies associated with temperate forest and tropical dry forest, and the south-eastern subspecies associated with tropical evergreen forest, cloud forest and tropical semi-deciduous forest. We suggest the classification of the 14 subspecies into three ecoregions. The data analyzed here is relevant to the management and conservation of the white-tailed deer subspecies and/or geographical variations in Mexico; it is also important to avoid the translocation of individuals into inappropriate areas with respect to their evolution and adaptation to different ecoregions.

2503: ***+.058***

Live-harvests from source populations for translocation are key to rapid recovery for many species. Contrary to common assumption, however, reduced density might not immediately improve vital rates because animal recolonization is slow - creating management uncertainty about harvest adequacy or sustainability. Reports measuring animal recolonization are rare. We measured the response of the 19 same- or opposite-sex neighbours of 11 live-harvested black rhinoceros (Diceros bicornis minor) in Hluhluwe-iMfolozi Park, South Africa, by comparing the size and location of their activity areas for an average two years before and after harvest. The only significant change was a decolonization response by opposite-sex neighbours, especially males after the harvest of a female neighbour. Recolonization of habitat after harvest, at least by neighbours, might not just be slow but also further delayed by the disruption of long-standing breeding relationships with important implications for the spatial pattern and frequency of harvest.

2504: ***+.457***

We use the case of the Eurasian lynx Lynx lynx in the Alps to discuss how to implement existing directives and recommendations, as well as how to integrate biological concepts, into practical conservation and wildlife management. Since 1995 the occurrence of lynx in the Alpine countries has been monitored and reported by the Status and Conservation of the Alpine Lynx Population expert group Both the area of occupancy and the estimated number of individuals increased from 1995-1999 to 2000-2004 The estimated number of lynx is 120-150 across the Alps and the area of occupancy 27,800 km(2), in six distinct sub-areas. In the highly fragmented Alpine habitat lynx populations expand slowly, even in situations of high local density and when suitable habitat is available Thus, almost 40 years after the first reintroduction, < 20% of the Alps have been recolonized by lynx. In addition to biological and ecological factors, the persistent disagreements about the return of the lynx between conservationists and other land-users, including livestock breeders and hunters, and the political fragmentation of the Alps (with different regional priorities and large carnivore policies), has prevented the creation of a consensus regarding pan-Alpine conservation goals for the lynx and the implementation of conservation measures such as translocations and reintroductions We discuss possible approaches in the light of new guidelines for population level management plans for large carnivores recently developed on behalf of the European Commission.

2505: ***+.109***

The term 'field propagation and release' refers to the breeding of captive adults in large field enclosures, allowing them to raise their young, and then releasing those young from that location. This technique is currently being implemented in Canada as one of several recovery tools for the endangered eastern loggerhead shrike Lanius ludovicianus migrans During 2001-2007 a total of 360 shrike fledglings were produced in field propagation enclosures and 301 were released from these enclosures Annual return rates of birds released since 2004 are 2-6.6%. Seventeen released birds have been re-sighted, including 10 birds that have returned to the breeding grounds the following season to produce young with wild mates. The high annual return rate of release birds and the successful integration of these birds into the wild breeding population represent important milestones for the recovery of this population The management technique we describe here has the potential to be applicable to other species that require natural habitat for breeding and/or are reliant on a suite of parent-learned behaviours that cannot be accommodated for or adequately replicated within intensive close captive-breeding or hand-rearing conditions

2506: ***-.204***

Laysan Ducks are endemic to the Hawaiian archipelago and are one of the world's most endangered waterfowl. For 150 yr, Laysan Ducks were restricted to an estimated 4 km(2) of land on Laysan Island in the northwestern Hawaiian Islands. In 2004 and 2005, 42 Laysan Ducks were translocated to Midway Atoll, and the population increased to approximately 200 by 2007. In August 2008, mortality due to botulism type C was identified, and 181 adult, fledgling, and duckling carcasses were collected from August to October. Diseased birds were (build on two islands within Midway Atoll at multiple wetlands; however, one wetland contributed most carcasses. The epidemic was discovered approximately 14-21 days after the mortality started and lasted for 50 additional days. The details of this epidemic highlight the disease risk to birds restricted to small island populations and the challenges associated with managing newly translocated endangered species. Frequent population monitoring for early disease detection and comprehensive wetland monitoring and management will be needed to manage avian botulism in endangered Laysan Ducks. Vaccination may also be beneficial to reduce mortality in this small, geographically closed population.

2507: ***+.172***

The distribution of the oriental weatherloach, Misgurnus anguillicaudatus, together with related environmental factors were surveyed at 185 paddy field locations on Sado Island in Japan. This was associated with the reintroduction of the Japanese crested ibis, Nipponia nippon, which prefers to feed on the loach. Loach were found to be present at 90 locations (49%). Analysis with GIS, GLM and AIC revealed that positive factors for the loach distribution included the presence of an earth ditch, the connections at outlets and the proportion of paddy field area within an 800 m radius. Conversely, pumping-up water irrigation and flow irrigation affected loach distribution negatively. In an interview survey that was conducted concurrently, older farmers in the area recalled that the loach had once been distributed over almost the entire island (89/96, 93%) about half a century ago. The farmers also suggested that the impact of agricultural chemicals was one of the main reasons for loach reduction or local extinction. A map of the current potential loach distribution was made using the best fit model from GIS variables. The loach was expected to be distributed in the Kuninaka region with high probability (60-100%) and in patches in the Osado and Kosado regions with low-medium probability (10-50%). As a feasible scenario for the conservation of the loach, another predictive map of the loach distributions was made using a model fit based on the GIS variables and outlet connection, in which the all of the outlets were presumed to be connected to the ditches without gaps. In this case, the loach were expected to be distributed almost throughout the paddy fields with medium-high probability (40-100%). In another scenario that presumed the complete land consolidation of all the paddy fields, the probability of loach occurrence was low (0-30%) throughout the island with the exception of the island's center in the Kuninaka region.

2508: ***-.001***

Populations of the Little Bustard Tetrax tetrax in the farmlands of Europe have declined greatly over the last century. In Western Europe, France now holds the only remaining migratory population, which currently numbers fewer than 300 displaying males. However, the movements of these birds are virtually unknown, in spite of the important implications of this information for the conservation of this species. We identified migratory movements and overwintering areas of French migratory populations, using wild individuals fitted with satellite or radio-transmitters. Little Bustards completed their migration journey over a relatively short time period (2-5 days), with nocturnal migration flights of 400-600 km per night. All birds overwintered in Iberia. In addition, we tested the consequences of captive rearing on migratory movements. French wild adults and French captive-bred juveniles behaved similarly with regard to migration, suggesting that hand-raising does not alter migratory movements. However, birds originating from eggs collected in Spain and reared in western France did not migrate, suggesting a genetic component to migratory behaviour. These results therefore suggest that a conservation strategy involving the release in France of birds hatched from eggs collected in Spain may imperil the expression of migratory movements of the French population. More generally, to maintain the integrity of native populations, the introduced individuals should mimic their migratory movements and behaviour.

2509: ***+.173***

Chromosomal rearrangements and copy number variants (CNVs) play key roles in genome evolution and genetic disease; however, the molecular mechanisms underlying these types of structural genomic variation are not fully understood. The availability of complete genome sequences for two bird species, the chicken and the zebra finch, provides, for the first time, an ideal opportunity to analyze the relationship between structural genomic variation (chromosomal

and CNV) and recombination on a genome-wide level. The aims of this study were therefore threefold: (1) to combine bioinformatics, physical mapping to produce comprehensive comparative maps of the genomes of chicken and zebra finch. In so doing, this allowed the identification of evolutionary chromosomal rearrangements distinguishing them. The previously reported interchromosomal conservation of synteny was confirmed, but a larger than expected number of intrachromosomal rearrangements were reported; (2) to hybridize zebra finch genomic DNA to a chicken tiling path microarray and identify CNVs in the zebra finch genome relative to chicken; 32 interspecific CNVs were identified; and (3) to test the hypothesis that there is an association between CNV, chromosomal rearrangements, and recombination by correlating data from (1) and (2) with recombination rate data from a high-resolution genetic linkage map of the zebra finch. We found a highly significant association of both chromosomal rearrangements and CNVs with elevated recombination rates. The results thus provide support for the notion of recombination-based processes playing a major role in avian genome evolution.

2510: *+.247*

The resurrection of Asiatic lions (Panthera leo persica) from the brink of extinction is a remarkable conservation success story. Yet, occurrence of lions as a single population makes them vulnerable to extinction from genetic and environmental factors. Asiatic lions exist as a single free ranging population of 360 individuals in Gir Protected Area (PA; about 290 lions) and surrounding satellite areas (68 lions), namely Girnar Wildlife Sanctuary (WLS), coastal areas, hill ranges extending from Mitiyala-Savarkundla-Palitana-Shihor spreading across Junagadh, Amreli and Bhavnagar districts of Gujarat State, India. This paper traces the conservation history, current conservation pressures, and critically evaluates current conservation planning based on lion ecology and existing anthropogenic pressures. Conservation proposals for translocation of lions to alternate habitats in Kuno WLS and Barda WLS are awaiting final implementation. An alternate initiative is aimed at developing satellite lion habitats, improving corridor connectivity, and facilitating natural dispersal and expansion of lion habitats. The paper evaluates human population living within 2 km boundary of lion habitats and between Gir PA and satellite habitats to show that dispersing lions have to cross heavily populated habitations taking refuge in agriculture fields and scattered forest patches. Satellite habitats vary in size from 18 km(2) (Mitiyala WLS) to 250 km(2) (scattered forests Hipavadli-Savarkundla-Palitana zone along Shetrunji river) and are inadequate to maintain natural ranging and movement requirements of territorial lions. These habitats are varied in vegetation, terrain, human pressure, and distance to source population. Though satellite lion habitats are important suboptimal habitats for dispersing lions, long-term conservation planning require planned restoration of mosaic habitats for growing populations.

2511: *+.398*

Money is often a limiting factor in conservation, and attempting to conserve endangered species can be costly. Consequently, a framework for optimizing fiscally constrained conservation decisions for a single species is needed. In this paper we find the optimal budget allocation among isolated subpopulations of a threatened species to minimize local extinction probability. We solve the problem using stochastic dynamic programming, derive a useful and simple alternative guideline for allocating funds, and test its performance using forward simulation. The model considers subpopulations that persist in habitat patches of differing quality, which in our model is reflected in different relationships between money invested and extinction risk. We discover that, in most eases, subpopulations that are less efficient to manage should receive more money than those that are more efficient to manage, due to higher investment needed to reduce extinction risk. Our simple investment guideline performs almost as well as the exact optimal strategy. We

illustrate our approach with a case study of the management of the Sumatran tiger, Panthera tigris sumatrae, in Kerinei Seblat National Park (KSNP), Indonesia. We find that different budgets should be allocated to the separate tiger subpopulations in KSNP. The subpopulation that is not at risk of extinction does not require any management investment. Based on the combination of risks of extinction and habitat quality, the optimal allocation for these particular tiger subpopulations is an unusual case: subpopulations that occur in higher-quality habitat (more efficient to manage) should receive more funds than the remaining subpopulation that is in lower-quality habitat. Because the yearly budget allocated to the KSNP for tiger conservation is small, to guarantee the persistence of all the subpopulations that are currently under threat we need to prioritize those that are easier to save. When allocating resources among subpopulations of a threatened species, the combined effects of differences in habitat quality, cost of action, and current subpopulation probability of extinction need to be integrated. We provide a useful guideline for allocating resources among isolated subpopulations of any threatened species.

2512: *+.079*

The last recorded presence of the Eurasian otter (Lutra lutra) in the Netherlands dates from 1989 and concerned a dead individual. In 2002 a reintroduction programme was started, and between June 2002 and April 2008 a total of 30 individuals (10 males and 20 females) were released into a lowland peat marsh in the north of the Netherlands. Noninvasive genetic monitoring based on the genetic profiles obtained from DNA extracted from otter faeces (spraints) was chosen for the post-release monitoring of the population. To this end, the founding individuals were genotyped before release and spraints were collected in the release area each winter from 2002 to 2008. From June 2002 to April 2008 we analysed the genetic profile of 1,265 spraints on the basis of 7-15 microsatellite loci, 582 of which (46%) were successfully assigned to either released or newly identified genotypes. We identified 54 offspring (23 females and 31 males): the females started to reproduce after 2 years and the males after 4 years. The mating and reproductive success among males was strongly skewed, with a few dominant males fathering two-thirds of the offspring, but the females had a more even distribution. The effective population size (Ne) was only about 30% of the observed density (N), mainly because of the large variance in reproductive success among males. Most juvenile males dispersed to surrounding areas on maturity, whereas juvenile females stayed inside the area next to the mother's territory. The main cause of mortality was traffic accidents. Males had a higher mortality rate (22 out of 41 males (54%) vs. 9 out of 43 females (21%)). During winter 2007/08 we identified 47 individuals, 41 of which originated from mating within the release area. This study demonstrates that noninvasive molecular methods can be used efficiently in post-release monitoring studies of elusive species to reveal a comprehensive picture of the state of the population.

2513: *+.037*

Eurasian otter populations strongly declined and partially disappeared due to global and local causes (habitat destruction, water pollution, human persecution) in parts of their continental range. Conservation strategies, based on reintroduction projects or restoration of dispersal corridors, should rely on sound knowledge of the historical or recent consequences of population genetic structuring. Here we present the results of a survey performed on 616 samples, collected from 19 European countries, genotyped at the mtDNA control-region and 11 autosomal microsatellites. The mtDNA variability was low (nucleotide diversity = 0.0014; average number of pairwise differences = 2.25), suggesting that extant otter mtDNA lineages originated recently. A star-shaped mtDNA network did not allow outlining any phylogeographic inference. Microsatellites were only moderately variable (H (o) = 0.50; H (e) = 0.58, on average across populations), the average allele

number was low (observed A (o) = 4.9, range 2.5-6.8; effective A (e) = 2.8; range 1.6-3.7), suggesting small historical effective population size. Extant otters likely originated from the expansion of a single refugial population. Bayesian clustering and landscape genetic analyses however indicate that local populations are genetically differentiated, perhaps as consequence of post-glacial demographic fluctuations and recent isolation. These results delineate a framework that should be used for implementing conservation programs in Europe, particularly if they are based on the reintroduction of wild or captive-reproduced otters.

2514: *+.106*

We provide mitochondrial sequence variation of the invasive fish Gambusia holbrooki from 24 European populations, from Portugal to Greece. Phylogeographic structure in Europe was compared with genetic data from native samples (USA) and historical records were reviewed to identify introduction routes. Overall, data agree with records of historical introductions and translocations, and indicate that the most abundant haplotype throughout Europe originated from North Carolina and corresponded to the first introduction in 1921 to Spain, being transferred to Italy in 1922 and to many countries afterwards. Our results also show that at least another independent introduction occurred first in France and subsequently from France to Greece. Haplotypes of G. affinis were not detected in our European sampling effort but historical records and other data suggest that this species was introduced to Italy in 1927 and it might be present. At the continental scale, there is less diversity in Europe than in North America, in agreement with the low number of introduced fish. At the local scale, some European populations gained diversity from multiple introductions and from "de novo" mutations.

2515: *+.211*

The riparian brush rabbit (Sylvilagus bachmani riparius) is an endangered species found in dense, brushy habitat in the California's Central Valley. We implemented a reintroduction program to bolster populations at a Federal Wildlife Refuge and to assess factors influencing mortality and subsequent survival of released individuals. Between July 2002 and July 2005, we reintroduced 325 captive-bred Individuals to unoccupied habitat within their historic range using a soft-release strategy and monitored their subsequent survival with radiotelemetry Longer time in soft-release pens resulted in increased monthly survival Rabbits were most susceptible to post-release mortality during the first 4 weeks following reintroduction and both body mass and length of time in the soft-release enclosure influenced this relationship. When we controlled for release mortality during this acclimation period, subsequent monthly survival probabilities were most strongly influenced by release year (year 1 vs. years 2 and 3) and by a catastrophic flooding event; length of time in the soft-release enclosure remained an important variable in longer-term survival. Cause of mortality was unknown for the majority of deaths (61.9%), but predation (including presumptive predation) was the greatest known cause of death in translocated rabbits (26 4%) Reintroduction programs should employ an adaptive management approach with ongoing monitoring of target animals and concurrent analysis to allow managers to adjust methods as conditions dictate. (C) 2010 Elsevier Ltd. All rights reserved.

2516: *+.169*

European Bison (Bison bonasus) barely escaped extinction in the early 20th century and now only occur in small isolated herds scattered across Central and Eastern Europe. The species survival in the wild depends on identifying suitable habitat for establishing bison metapopulations via reintroductions of new herds We assessed European Bison habitat across the Carpathian

Mountains, a stronghold of European Bison and one of the only places where a viable bison metapopulation may be possible. We used maximum entropy models to analyze herd range maps and habitat use data from radio-collared bison to identify key habitat variables and map European Bison habitat across the entire Carpathian ecoregion (210.000 km(2)) Forest cover (primarily core and perforated forests) and variables linked to human disturbance best predict bison habitat suitability. Bison show no clear preference for particular forest types but prefer managed grasslands over fallow and abandoned fields Several large, suitable, but currently unoccupied habitat patches exist, particularly in the eastern Carpathians. This available suitable habitat suggests that European Bison have an opportunity to establish a viable Carpathian metapopulauon, especially if recent trends of declining human pressure and reforestation of abandoned farmland continue. Our results also confirm the suitability of a proposed Romanian reintroduction site Establishing the first European Bison metapopulation would be a milestone in efforts to conserve this species in the wild and demonstrate a significant and hopeful step towards conserving large grazers and their ecological roles in human-dominated landscapes across the globe. (C) 2009 Elsevier Ltd All rights reserved.

2517: *+.123*

Three of Malaysia's endangered large mammal species are experiencing contrasting futures. Populations of the Sumatran rhino (Dicerorhinus sumatrensis) have dwindled to critically low numbers in Peninsular Malaysia (current estimates need to be revised) and the state of Sabah (less than 40 individuals estimated). In the latter region, a bold intervention involving the translocation of isolated rhinos is being developed to concentrate them into a protected area to improve reproduction success rates. For the Asian elephant (Elephas maximus), recently established baselines for Peninsular Malaysia (0.09 elephants/km(2) estimated from one site) and Sabah (between 0.56 and 2.15 elephants/km(2) estimated from four sites) seem to indicate globally significant populations based on dung count surveys. Similar surveys are required to monitor elephant population trends at these sites and to determine baselines elsewhere. The population status of the Malayan tiger (Panthera tigris jacksoni) in Peninsular Malaysia, however, remains uncertain as only a couple of scientifically defensible camera-trapping surveys (1.66 and 2.59 tigers/100 km(2) estimated from two sites) have been conducted to date. As conservation resources are limited, it may be prudent to focus tiger monitoring and protection efforts in priority areas identified by the National Tiger Action Plan for Malaysia. Apart from reviewing the conservation status of rhinos, elephants and tigers and threats facing them, we highlight existing and novel conservation initiatives, policies and frameworks that can help secure the long-term future of these iconic species in Malaysia.

2518: *+.116*

Sinojackia xylocarpa is a Chinese endemic species that is extinct in the wild but extant in botanical gardens. Microsatellites were used to investigate the genetic diversity and mating system of this species for future use in a reintroduction program. Ex situ conserved populations of S. xylocarpa maintain intermediate levels of genetic diversity (H(E) = 0.570-0.640). However, a general and significant heterozygote excess was found, with a mean F(IS) of -0.103. S. xylocarpa was determined to be predominantly outcrossing (t(m) = 0.992; t(s) = 1.092). Population size and genetic diversity were found to be positively correlated (r = 0.991; P = 0.084). Principal coordinate analysis (PCA) suggests that all extant individuals are derived from two source populations. Reintroduction strategies of S. xylocarpa were proposed on the basis of these results. (C) 2009 Elsevier Ltd. All rights reserved.

2519: *-.012*

The endangered minnow Iberochondrostoma almacai is an endemic Iberian cyprinid with a restricted and fragmented distribution. Here, we describe the genetic structure of the species and infer its demographic history from six nuclear-encoded microsatellite loci and mitochondrial cytochrome b sequences. Genetic diversity was low (microsatellite H-e < 0.45; mtDNA pi < 0.0015), and both markers resolved two groups: one from the northern Mira drainage and one from the Arade and Bensafrim drainages. The relatively low differentiation between these groups (0.09 < F-ST < 0.29; 0.31 < (ST) < 0.57) suggests past headwater captures and/or that populations were large until recently. The genetic diversity and differentiation estimates were compared with those for other three endangered cyprinids inhabiting similar intermittent rivers. Microsatellite data indicate a population decrease in the last 100-2400 years, probably as a result of anthropogenic disturbance. Human activities together with an intermittent flow of these rivers apparently led to local extinctions with consequent fragmentation and contraction in range. We recognize two management units corresponding to the two genetic groups identified. To maintain/increase genetic diversity, we recommend habitat restoration actions and measures to increase gene flow within and/or between the two units, under controlled reproductive programmes. Ecological experiments should be performed to ensure the success of supplementation among the two units. Moreover, the reintroductions in unoccupied drainages are suggested if further data confirm the presence of I. almacai in the recent past.

2520: *+.266*

An experimental elk herd of 25 animals was released into the Chequamegon National Forest near Clam Lake, Wisconsin in 1995. This herd thrived and increased to nearly 100 animals within 10 years. The project's experimental phase ended and responsibility for the herd transferred from the University of Wisconsin-Stevens Point to the Wisconsin Department of Natural Resources. In June 2000, the Wisconsin Natural Resources Board approved a management plan for the Clam Lake elk herd. This plan established core and buffer areas, described habitat and population management techniques, and set population objectives. The success of the Clam Lake introduction and the attractiveness of elk has spurred several other localities around Wisconsin to voice an interest in establishing local elk herds. When adopting the Clam Lake management plan, the Natural Resources Board set forth guidelines for further reintroduction efforts. These guidelines require, among other things, the evaluation of the biological and ecological suitability of other areas for elk and indicate that factors such as the quality and quantity of available habitat, the amount and proximity of agriculture, and public attitudes regarding elk should be included. Geographic information systems (GIS) have been valuable tools for the analysis of habitat suitability. Most past efforts, however, have considered the biological aspects of suitability, with little effort focused on social information or on identifying potential conficts with other resources or land uses. In addition, past efforts to model habitat have covered either a very large area (e.g., a state or larger) or a more specifc area (e.g., a county or other smaller area). We developed a GIS-based habitat suitability model using ArcInfo[registered trademark] (ESRI, Redlands, CA) for the state of Wisconsin. Data inputs to the model included both biological elements (mostly land cover information derived from satellite imagery) and social elements (agriculture, road density, and land ownership). The intent of this model was to conduct a statewide evaluation of areas potentially suitable for elk reintroduction. We identifed 15 areas of "suitable" elk habitat in Wisconsin. Most areas were located in the northern forest region of the state, with two patches in the central forest. Suitable elk patches generally had suffcient forest cover (both deciduous and coniferous) with little to no agriculture, low road density, and large amounts of public land. Suitable patches ranged in size from 192 km2 (79 mi 2) to over 6,000 km2 (2,450 mi2). Winter

cover was the least suitable of the biological elements. We also identifed potential conficts with captive elk herds and rare plant species for each suitable patch. Some caution must be exercised when interpreting our statewide results. We used only generalized land cover information, which could result in areas identifed as being suitable actually having very low habitat quality. Thus, it will be important to evaluate these areas in more detail with more specifc forestry and social information.

2521: *-.095*

The larval biology of Patella,ferruginea is studied for the first time. Development in the species first showed two complete and equal cleavages whereas the third cleavage was unequal, resulting in an embryo with 4 micromeres and 4 macromeres. Early trochophores were detected 19 hours post-fertilization and pretorsional veligers appeared 27 hours post-fertilization. Early stages of development are very similar to those shown by other related limpet species, with higher developmental times than those recorded for Patella caerulea and similar to those obtained in Patella vulgata. However, in vitro fertilization and the obtainment of spats in a massive amount could be the solution for replenishing threatened or extinct populations of this extremely endangered Mediterranean species. The results of the present study represent a first approach in order to produce great amounts of spats in laboratory conditions for further reintroduction projects aiming for conservation of the species.

2522: *+.166*

The distribution of mockingbird species among the Galapagos Islands prompted Charles Darwin to question, for the first time in writing, the 'stability of species'. Some 50 years after Darwin's visit, however, the endemic Floreana mockingbird (Mimus trifasciatus) had become extinct on Floreana Island and, today, only two small populations survive on two satellite islets. As Darwin noted, rarity often precedes extinction. To avert extinction, plans are being developed to reintroduce M. trifasciatus to Floreana. Here, we integrate evolutionary thinking and conservation practice using coalescent analyses and genetic data from contemporary and museum samples, including two collected by Darwin and Robert Fitzroy on Floreana in 1835. Our microsatellite results show substantial differentiation between the two extant populations, but our coalescence-based modelling does not indicate long, independent evolutionary histories. One of the populations is highly inbred, but both harbour unique alleles present on Floreana in 1835, suggesting that birds from both islets should be used to establish a single, mixed population on Floreana. Thus, Darwin's mockingbird specimens not only revealed to him a level of variation that suggested speciation following geographical isolation but also, more than 170 years later, return important information to their place of origin for the conservation of their conspecifics.

2523: *+.227*

Gray wolves (Canis lupus) were extirpated from Yellowstone National Park in the 1920s. The ensuing seven decades marked a period when wild ungulates, principally Elk (Cervus elaphus), extensively used woody browse species in the upper Gallatin and northern winter ranges, thus limiting the capability of establishing plants to grow more than 100 cm in height. Following the reintroduction of wolves in the mid-1990s, we evaluated patterns of woody browse species recovery within riparian areas of these winter ranges. Measurements indicated that cottonwood (Populus spp.) recruitment (growth of seedlings/sprouts into tall saplings and trees) was occurring for the first time in several decades. A spatially patchy increase in the heights of young willow (Salix sp.) and cottonwood in the upper Gallatin and northern winter ranges, respectively, was also

found within riparian transects comprising nearly 20 km in total length. Within some transects, heights of woody species have begun to exceed 200 cm (the approximate upper browse level of Elk). Results are consistent with the reestablishment of a tri-level trophic cascade involving wolves, ungulates, and riparian vegetation. We additionally present conceptual models of vegetation recovery, illustrating differences in plant height responses to behaviorally and density-mediated trophic cascades. Northern Yellowstone's "experiment in time," whereby wolves were removed and then reintroduced, provides new insights regarding how top predators can influence the structure and biodiversity of terrestrial ecosystems. Restoration ecologists and policymakers should consider the potential benefits of large predators as an option for helping restore degraded ecosystems.

2524: *+.149*

The increased translocation of plant species for biodiversity restoration and habitat creation has provoked a debate on provenance and genotypic diversity of the used plant material. Nonlocal provenances are often not adapted to the local environmental conditions, and low population genotypic diversity may result in genetic bottlenecks hampering successful establishment. We tested provenance differentiation of four plant species used in agri-environment schemes to increase biodiversity of agricultural landscapes (wildflower strips). Provenances were collected close to the experimental field and at four further sites of different distances ranging from 120 to 900 km. In two of these provenances, different levels of genotypic diversity were simulated by sowing seed from a high and low number of mother plants. We found a large provenance differentiation in fitness-related traits, particularly in seedling emergence. There was no evidence for a general superiority of the local population. The productivity was greater in populations of high genotypic diversity than in those of low diversity, but the effect was only significant in one species. Productivity was also more constant among populations of high diversity, reducing the risk of establishment failure. Our results indicate that the choice of an appropriate provenance and a sufficient genotypic diversity are important issues in ecological restoration. The use of local provenances does not always guarantee the best performance, but a spread of superior alien genotypes can be avoided. A sufficient genotypic diversity of the sown plants might be a biological insurance against fluctuations in ecosystem processes increasing the reliability of restoration measures.

2525: *+.038*

Er-Situ Cultivation and In-Situ Management as Contribution to the Protection of Species The examples of Cypripedium calceolus, Gladiolus palustris, Gentianella bohemica and Gentianella lutescens The research study investigated the following issues: 1) To what extent can ex-situ cultures be established of species listed in the appendices of the Habitats Directive of the EU? 2) Which simple in-situ measures work to enhance seedling-recruitment? 3) Which are the economic framework conditions of these measures regarding ecological compensation? First of all, germination experiments for G. palustris, G. lutescens and G. bohemica were carried out in a climate chamber both in a laboratory and in outdoor pot-cultures. G. palustris and G. lutescens exhibited the highest germination rates when subjected to cold-stratification schemes. Fitness of juvenile plants was positively affected by moderate substrate moisture during summer months, moderate fertilization, good aeration of the substrate, and application of VA-Mycorrhiza-substrates. Multiple regression analysis revealed, however, that the number of flowers in Gentianella plants was significantly correlated with the pot depth.In-situ measures showed that in sowing experiments significantly more seedlings of G. palustris could be recorded in plots where vegetation and litter layer were removed. Similarly, juvenile plants of C. calceolus could only be

recorded in plots where the litter and topsoil layer had been removed three years before. If topsoil and litter layer were removed in a patch around Cypripedium plants, these plants showed significantly more flowers after three years than untreated individuals. The calculation of costs carried out by a nursery showed that in-situ management and ex-situ cultivation of a Cypripedium population with the aim to yield material for re-introduction would cost 15,180 over a period of eight years.Finally, the study discusses the in-situ measures investigated in this study as well as the establishment of ex-situ cultivation of highly endangered species and species listed in the Appendices of the Habitats Directive in the light of necessary ecological compensation measures.

2526: ***+.095***

After roughly a 60-year absence, wolves (Canis lupus) immigrated (1979) and were reintroduced (1995-1996) into the northern Rocky Mountains (NRM), USA, where wolves are protected under the Endangered Species Act. The wolf recovery goal is to restore an equitably distributed metapopulation of >= 30 breeding pairs and 300 wolves in Montana, Idaho, and Wyoming, while minimizing damage to livestock; ultimately, the objective is to establish state-managed conservation programs for wolf populations in NRM. Previously, wolves were eradicated from the NRM because of excessive human killing. We used Andersen-Gill hazard models to assess biological, habitat, and anthropogenic factors contributing to current wolf mortality risk and whether federal protection was adequate to provide acceptably low hazards. We radiocollared 711 wolves in Idaho, Montana, and Wyoming (e.g., NRM region of the United States) from 1982 to 2004 and recorded 363 mortalities. Overall, annual survival rate of wolves in the recovery areas was 0.750 (95% CI = 0.728-0.772), which is generally considered adequate for wolf population sustainability and thereby allowed the NRM wolf population to increase. Contrary to our prediction, wolf mortality risk was higher in the northwest Montana (NWMT) recovery area, likely due to less abundant public land being secure wolf habitat compared to other recovery areas. In contrast, lower hazards in the Greater Yellowstone Area (GYA) and central Idaho (CID) likely were due to larger core areas that offered stronger wolf protection. We also found that wolves collared for damage management purposes (targeted sample) had substantially lower survival than those collared for monitoring purposes (representative sample) because most mortality was due to human factors (e.g., illegal take, control). This difference in survival underscores the importance of human-caused mortality in this recovering NRM population. Other factors contributing to increased mortality risk were pup and yearling age class, or dispersing status, which was related to younger age cohorts. When we included habitat variables in our analysis, we found that wolves having abundant agricultural and private land as well as livestock in their territory had higher mortality risk. Wolf survival was higher in areas with increased wolf density, implying that secure core habitat, particularly in GYA and CID, is important for wolf protection. We failed to detect changes in wolf hazards according to either gender or season. Maintaining wolves in NWMT will require greater attention to human harvest, conflict resolution, and illegal mortality than in either CID or GYA; however, if human access increases in the future in either of the latter 2 areas hazards to wolves also may increase. Indeed, because overall suitable habitat is more fragmented and the NRM has higher human access than many places where wolves roam freely and are subject to harvest (e.g., Canada and AK), monitoring of wolf vital rates, along with concomitant conservation and management strategies directed at wolves, their habitat, and humans, will be important for ensuring long-term viability of wolves in the region.

2527: ***+.288***

We use a state dependent life history model to predict the life history strategies of female steelhead trout (Oncorhynchus mykiss) in altered environments. As a case study of a broadly applicable

approach, we applied this model to the American and Mokelumne Rivers in central California, where steelhead are listed as threatened. Both rivers have been drastically altered, with highly regulated flows and translocations that may have diluted local adaptation. Nevertheless, evolutionary optimization models could successfully predict the life history displayed by fish on the American River (all anadromous, with young smolts) and on the Mokelumne River (a mix of anadromy and residency). The similar fitness of the two strategies for the Mokelumne suggested that a mixed strategy could be favored in a variable environment. We advance the management utility of this framework by explicitly modeling growth as a function of environmental conditions and using sensitivity analyses to predict likely evolutionary endpoints under changed environments. We conclude that the greatest management concern with respect to preserving anadromy is reduced survival of emigrating smolts, although large changes in freshwater survival or growth rates are potentially also important. We also demonstrate the importance of considering asymptotic size along with maximum growth rate.

2528: *+.016*

In Poland, the European Pond Turtle attains the northern border of its geographical range. It was common in our country until 19th Century but now breeds only in a few isolated places, although among them the Lgczna-Wlodawa Lake District in Eastern Poland, housing the biggest population in Central Europe. Occasional records of single individuals have also been noted from almost all of the country. Its present numbers have been estimated at 700-800 individuals. A decline of its population is mostly due to human activity: drainage of water and marshy areas, regulation of rivers, elimination of local ponds and ox-bow lakes, which results in habitat loss, and destruction of breeding grounds by overgrowing and afforestation (Jablonski 2001, Najbar 2001a, Rybacki 2003). The first mention about the existance of European Pond Turtle in the South Wielkopolska (SW Poland), in the vicinity of Chocz in Pleszew district, was given by Rzaczynski (1742). The breeding was confirmed only once, in 1938, by the sighting of five young turtles near Zerkow in Jarocin district. Since the 1930s till 1960s single specimens were caught or observed near Kalisz, Baszkow and Przedborow (Berger 1957, Iwanowski et al. 1966, Aniola, Kuiniak 1968) and around 1980 one turtle was caught on Przygodzice Fishponds (Ostrow Wielkopolski district). In 1998 observations of several turtles was published from Witaszyce (Bajda 1998), but with no evidence that these animals belonged to this species, and not others, imported from abroad (e.g. popular in Poland Trachemys scripta elegans). On 4th November 2005 an adult female of native subspecies E. o. orbicularis (Fig. 2) was observed and photographed on the Rybin Fishponds (Kobyla Gora commune, Ostrzeszow district) in the Barycz river valley. In the summer 2007, one adult turtle was caught in a drained canal ca 700 meters ago, but without photos it is impossible to say if it was the same specimen. According to the interviews with local people, turtles were observed on these fishponds during 1950s and 1960s. Maybe a small population found refuge here, but it may have been the last representative of this long-life species. Rybin Fishponds are situated 16 km east from the forest ponds near Goszcz (Olesnica district) where 40 and 20 2-years old European Pond Turtles, origin from the Lgczna-Wlodawa Lake District, were reintroduced in 1998 and 2001 respectively.

2529: *-.007*

Till the end of the 1950s the huchen occurred in Poland in autochthonous localities in the Czarna Orawa River system and in the Czadeczka Stream (Silesian Beskid Mts). It then became extinct in both these river systems as a result of poaching and water pollution. During our studies in May 2007 deleterious environmental changes have been noted in the Czadeczka and its tributaries (poverty and scarcity of the ichthyofauna, considerable water pollution, hydrotechnical

constructions) which precludes successful restitution of the species which is endemic to the Danube River catchment area. In the Czarna Orawa with its tributaries the original environmental conditions have been preserved which, with restitution attempts launched in 2000, makes the return of the huchen likely.

2530: *+.133*

Among the especially interesting and rare plants on the Balkan Peninsula are tertiary relics and endemics belonging to the tropical family Gesneriaceae: Haberlea rhodopensis Friv (Rhodope silivryak, Orpheus' flower) and representatives of the genus Ramonda. Haberlea rhodopensis Friv. is included in the European list of rare, in danger of extinction and endemic plants and in the Bulgarian list of endangered plants. In recent years, our team has extended in a new directions the traditions of many years' investigations of this interesting species at the Dept. of Plant physiology and Molecular biology. We started a thorough exploration of the various habitats and ecological characteristics of Haberlea rhodopensis Friv. Simultaneously, at the Plant Biotechnology laboratory at the University of Plovdiv we started the establishment of a live collection of in vitro Haberlea rhodopensis Friv. from various populations and habitats in Bulgaria. Such in vitro live collection with the aim of conservation and investigation of the natural population is created for a first time in Bulgaria. This is accomplished through an in vitro system for regeneration and propagation, modified by our research group. The live collection of in vitro Haberlea rhodopensis Friv. plants will be a donor for conservation and reintroduction of adapted in vitro plants in their natural endangered habitats and also for physiological studies of drough tolerance, and multidisciplinary comparative analyses.

2531: *-.068*

The re-introduction of extirpated species is a valuable conservation tool. Red kites Milvus milvus are declining over much of their European range and have been re-introduced to England and Scotland, following their extinction due to widespread human persecution during the 19th century. Considerable regional variation in population growth exists. This study aims to identify the proximate demographic and ultimate environmental constraints on red kites in north Scotland, a region with low population growth. Productivity in north Scotland was high compared to other Scottish and Welsh populations and equal to English populations with high population growth rates. In north Scotland, annual survival of wild-fledged birds was low for first-year birds compared to other Scottish populations and second-year survival declined over time. In north Scotland, 40% of 103 red kites found dead were killed illegally, mainly by direct poisoning. In the absence of illegal killing, we estimate that annual survival rates in wild red kites might increase from 0.37 to 0.54, 0.72 to 0.78 and 0.87 to 0.92 for first, second-year and adult birds respectively. Demographic rates from this study produce population trends that recapitulate observed trends for the north Scotland population (leading to a population of c40 pairs by 2006). Models in which the additive illegal killing mortality is excluded, predict a population trajectory and size (c300 pairs by 2006) very similar to that found in the Chilterns, a rapidly growing population (320 pairs in 2006) in south-east England re-introduced at the same time, but where rates of illegal killing are much lower. We conclude that illegal killing of red kites is the cause of poor population growth in north Scotland and the key challenge facing government is to find a way to eliminate this killing. (C) 2010 Elsevier Ltd. All rights reserved.

2532: *+.250*

1. The establishment of refuge populations has become a common management tool for threatened

fish species in recent years, yet the effects of translocation are not fully understood in a conservation context.2. This paper examines the hypothesis that phenotypic changes have occurred during the formation of two refuge populations of the nationally rare powan (a freshwater fish species) which were established in Loch Sloy and Carron Valley Reservoir in Scotland.3. Significant differences in head morphology, size and growth between the founder and refuge populations and between the two refuge populations were demonstrated. These changes are probably due to a combination of founder effects, intense selection and phenotypic plasticity. These changes can undermine the rationale behind the establishment of refuge populations.4. The results call into question the usefulness of translocation as a conservation measure; however, there are times when this is the only viable management option available. The future of translocation and the validity of establishing refuge populations for powan conservation are discussed.
Copyright (C) 2010 John Wiley & Sons, Ltd.

2533: *+.397*

Since reintroduction programmes involve moving animals from captive or wild environments and releasing them into novel environments, there are sure to be a number of challenges to the welfare of the individuals involved. Behavioural theory can help us develop reintroductions that are better for both the welfare of the individual and the conservation of populations. In addition to modifying captive environments to prepare animals for release to the wild, it is possible to modify the animals' experience in the post-release environment. For releases to be more successful, they need to better accommodate the ecological and psychological needs of individuals. A better understanding of sensory ecology how animals acquire and respond to information in their environment is needed to develop new, more successful management strategies for reintroductions. Sensory ecology integrates ecological and psychological processes, calling for better synergy among researchers with divergent backgrounds in conservation and animal welfare science. This integrative approach leads to new topics of investigation in reintroduction biology, including more careful consideration of post-release stress and the role of social support. Reintroductions are essentially exercises in 'forced' dispersal; thus, an especially promising avenue of research is the role of proximate mechanisms governing dispersal and habitat selection decisions. Reintroduction biologists have much to gain from the study of mechanism because mechanisms, unlike function or adaptive value, can be manipulated to enhance conservation and welfare goals.

2534: *+.253*

Background: Decision analysis and game theory [1,2] have proved useful tools in various biodiversity conservation planning and modeling contexts [3-5]. This paper shows how game theory may be used to inform group decisions in biodiversity conservation scenarios by modeling conflicts between stakeholders to identify Pareto-inefficient Nash equilibria. These are cases in which each agent pursuing individual self-interest leads to a worse outcome for all, relative to other feasible outcomes. Three case studies from biodiversity conservation contexts showing this feature are modeled to demonstrate how game-theoretical representation can inform group decision-making. Methodology and Principal Findings: The mathematical theory of games is used to model three biodiversity conservation scenarios with Pareto-inefficient Nash equilibria: (i) a two-agent case involving wild dogs in South Africa; (ii) a three-agent raptor and grouse conservation scenario from the United Kingdom; and (iii) an n-agent fish and coral conservation scenario from the Philippines. In each case there is reason to believe that traditional mechanism-design solutions that appeal to material incentives may be inadequate, and the game-theoretical analysis recommends a resumption of further deliberation between agents and the initiation of

trust-and confidence-building measures. Conclusions and Significance: Game theory can and should be used as a normative tool in biodiversity conservation contexts: identifying scenarios with Pareto-inefficient Nash equilibria enables constructive action in order to achieve (closer to) optimal conservation outcomes, whether by policy solutions based on mechanism design or otherwise. However, there is mounting evidence [6] that formal mechanism-design solutions may backfire in certain cases. Such scenarios demand a return to group deliberation and the creation of reciprocal relationships of trust.

2535: *+.312*

The Red-cockaded Woodpecker (Prcordes borealis) is an endangered species endemic to pine (Pones spp) forests of the southeastern United States I examined Red-cockaded Woodpecker foraging behavior to learn if there were male/female differences at the Savannah River Site. South Carolina The study was conducted in largely young forest stands (<50 years of age) in contrast to earlier foraging behavior studies that focused on more mature forest The Red-cockaded Woodpecker at the Savannah River site is intensively managed Including monitoring, translocation, and installation of artificial cavity inserts for roosting and nesting Over a 3-year period. 6,407 foraging observations covering seven woodpecker family groups were recorded during all seasons of the year and all tunes of day The most striking differences occurred in foraging method (males usually scaled 145% of observations] and females mostly probed [47%]). substrate used (females had a stronger preference [93%] for the trunk than males [79%]), and foraging height from the ground (mean SE foraging height was higher for males [111 +/- 05 m] than females [9 8 +/- 0 5 m] Niche overlap between males and females was lowest for substrate (85 6%) and foraging height (87 8%), and highest for tree species (99 0%) tree condition (98 3%), and tree height (96 4%) Both males and females preferred to forage in older, large pine trees The habitat available at the Savannah River Site was considerably younger than at most other locations. but the pattern of male/female habitat partitioning observed was similar to that documented elsewhere within the range attesting to the species' ability to adjust behaviorally Received 24 March 2009 Accepted 9 November 2009

2536: *+.070*

Natal dispersal is common among many bird species with females usually dispersing farther than males We examined postfledging and natal dispersal ecology of Crested Ibis (Nippoma nippon) in the Qinling Mountains. China by marking nestlings with either color bands or radio transmitters Family groups. consisting of juveniles alter fledging and their parents. dispersed progressively farther from nest sites after fledging in a southerly direction with a mean (+/- SD) direction of 189 +/- 46 5 degrees and a mean distance of 20 3 +/- 7 0 km Mean natal dispersal distance of females (9 6 kin) was significantly greater than for males (5 9 km) (t = 2 14. P = 0 03) Most individuals moved southeasterly from natal areas and dispersal movements of individual ibis were concentrated at the mean direction(mu = 185 6 degrees. (sic) = 86 1 degrees) First reproduction by females (3 64 +/- 1 36 years) was not significantly earlier than for males (4 17 +/- 1 47 years) The results of this study will be useful for reintroduction programs for critically endangered species Received 20 January 2009 Accepted 20 November 2009

2537: *+.123*

Creeks in the Pinaleno Mountains of Arizona were renovated in the 1960s to remove nonnative salmonids, and were stocked with native Apache trout (Oncorhynchus apache). Cursory observations of distribution of fish following attempts to transplant have been reported, but

following the Clarks Peak Fire of 1996 more thorough investigations of the aquatic communities were needed. In 1997, we sampled communities of fish with a backpack electrofisher and we collected aquatic invertebrates with a dip-net. Lengths of fish were recorded and scales were removed for assessment of age. We collected 398 putative Apache trout 60-257 mm in length and 0-3 years of age from four creeks, while four others contained no fish. Densities of invertebrates were variable and did not appear to be correlated with presence of fish or drainage. Translocation of Apache trout to Grant and Ash creeks has resulted in reproducing populations of trout with adequate densities and rates of growth to serve as potential future sources of fish for stocking. Marijilda Canyon and Big creeks also supported reproducing populations, but at a much lower density and may not, be suitable as sources of fish for stocking. However, genetic information from these populations is needed to determine their suitability for service as replicate stocks.

2538: ***-.170***

Aggressive interactions, differences in chela size, and the effect of chela size on outcomes of aggressive interactions were studied in a laboratory setting using the federally protected (endangered) Orconectes shoupi (Nashville Crayfish), and two sympatric species, O. placidus (Bigclaw Crayfish) and O. durelli (Saddle Crayfish). Orconectes placidus and O. durelli are potential threats to O. shoupi through competitive or aggressive interactions. Understanding such interactions could help explain species distributions, provide insight on additional threats, and guide management decisions regarding Nashville Crayfish translocations. Aggressive interactions were examined with 30-min videotaped trials between body-size-matched hetero- and conspecific pairs. The predicted influence of chela size on outcomes of aggressive interactions was also analyzed. Our results demonstrated that O. shoupi males and females were significantly more aggressive than O. placidus. However, O. durelli females won more encounters and were slightly more aggressive than O. shoupi females. Significant differences in chela size were detected in some body-size-matched species and gender pairings: O. shoupi males had narrower chelae than O. durelli males; and O. shoupi females had longer and wider chelae than O. placidus females, and longer but narrower chelae than O. durelli females. Although chela size appeared to play a role in dominance, it was not the only factor influencing outcomes of aggressive interactions. Our laboratory results did not identify displacement threats to O. shoupi from O. placidus, and therefore do not preclude introduction of O. shoupi into habitat occupied by O. placidus to meet recovery plan objectives. However, interspecific aggression in the presence of a vital resource (e.g., food or shelter) was not tested here and should be investigated to provide a more comprehensive evaluation of possible threats to O. shoupi.

2539: ***+.098***

A reappraisal of the conservation status of the New Zealand frog fauna is presented using the 2008 version of the New Zealand Threat Classification System. Of New Zealand's four extant endemic species, three are judged to be 'Threatened' (Leiopelma hamiltoni being 'Nationally Critical', and L. pakeka and L. archeyi being 'Nationally Vulnerable') and one 'At Risk' (L. hochstetteri 'Declining'). Three Leiopelma species are listed as extinct-they are known from bone deposits in caves throughout the country until some time in the last 1000 years. Three introduced and naturalised Litoria species are abundant in New Zealand although two (L. aurea and L. raniformis) are threatened in their country of origin (Australia). An additional unidentified frog taxon from northern Great Barrier Island is listed as 'Data Deficient'.

2540: ***+.043***

P>1. Landscape connectivity, the ability of species to move between different elements of a landscape, has been evaluated mainly by expert opinion, proxy data or homing experiments, all of which have major limitations. Cost distance modelling can overcome these limitations, but the resistance values of different landscape elements are difficult to estimate.2. Here, we present a novel method combining step selection functions with cost distance modelling to assess functional landscape connectivity. Instead of relying on movement metrics, the method uses a case-control design to assess whether the chosen steps differ from a random sample of alternatives of similar lengths. Alternative models of landscape connectivity and dispersal behaviour are represented as maps of resistance values, and compared using an information-theoretic approach to select those hypotheses that maximize the discrepancy between chosen steps and random alternatives.3. We applied this method to daily locations recorded along the dispersal paths of 38 juvenile North Island robins Petroica longipes in a fragmented pastoral landscape in New Zealand. We compared models with different resistance values for four recognized vegetation types in the landscape and assessed gap-crossing behaviour by changing the resistance value of pasture as a function of distance to the closest woody vegetation.4. Model comparison showed that juvenile robins move in decreasing order of preference through native forest, plantations and shrubland, and showed a marked reluctance for flying over pasture. Under the best model, the largest gap crossed was 110 m.5. Synthesis and applications. In combination with data on the total cost distances travelled by dispersers, cost distance models of landscape connectivity can be used to predict distributions of dispersal distances in any landscape with similar vegetation types. They can therefore predict responses of species to landscape management or predict spatial dynamics of populations following reintroduction. Our method is potentially applicable to any dispersal data, even with a relatively small number of locations recorded in complex landscapes, meaning models can be fitted to data that cannot be analysed using previous method. Tools are freely available for download to allow researchers and wildlife managers to apply our methods to their own data.

2541: ***-.031***

P>1. Even when we cannot eradicate an invasive species, we may be able to reduce its ecological impact. In Australia, a critically endangered predator, the northern quoll Dasyurus hallucatus is threatened by the invasion of the highly toxic cane toad Bufo marinus. Following toad invasion, quoll populations have become extinct across Northern Australia. Toads are continuing to spread, and will soon invade the Kimberley, one of the quoll's last strongholds. To prevent future local extinctions, we need a new approach for mitigating the impact of cane toads on this iconic predator.2. We investigated whether conditioned taste aversion (CTA) could be used to modify quoll predatory behaviour and mitigate toad impacts. We successfully induced an aversion to live toads in juvenile northern quolls by feeding them a dead toad containing a nausea-inducing chemical (thiabendazole).3. To investigate whether CTA enhanced quoll survival, we fitted radiocollars to 31 toad-smart and 31 toad-naive quolls, and monitored their survival after reintroduction to the wild. We analysed telemetry data using the program MARK to investigate whether survival was influenced by sex or experimental treatment (toad-smart vs. toad-naive).4. Five of 17 (29%) toad-naive male quolls died shortly after release, as soon as they encountered and attacked large cane toads. In toad-naive quolls, apparent survival rates were higher for females (0 center dot 84) than for males (0 center dot 58), reflecting a sex difference in the propensity to attack toads. In both sexes, toad-smart quolls had higher apparent survival rates than did toad-naive conspecifics (mean daily apparent survival rates for females, 0 center dot 94 vs. 0 center dot 84 respectively; for males, 0 center dot 88 vs. 0 center dot 58).5. Synthesis and applications. Wildlife managers could aerially deploy taste aversion baits in the field, ahead of the toad invasion front, to teach toad-naive quolls to avoid live cane toads before the toads invade such areas. This approach has wider applications, and could also be used to mitigate the impacts of invasive

vertebrate predators on threatened fauna. When invasive predators cannot be eradicated, CTA may provide a feasible way to maintain populations of endangered fauna in the presence of the invader.

2542: *+.024*

The fact that human-large carnivore relationships tend to be full of material and social conflicts raises applied questions concerning the origin of human perceptions linked to these animals and more theoretical questions concerning the link between identification and relational processes. This study, based on ethno-ethological surveys in the Republic of Macedonia (SE Europe), aims to show that the widely contrasting species specific behavioural characteristics of brown bears, wolves and Eurasian lynx influence local perceptions of these species through the nature and frequency of their interactions with humans. It appears that a high frequency of interactions allows the relational processes to dominate, leading people to modify their actions in response to the behaviour and ecology of the species. However, the fact that the virtual absence of interactions with lynx has not prevented the construction of a particular image of the species also highlights the complexity of the relationship between the level of interactions and people's perception about animals.

2543: *+.111*

A rapid shoot multiplication protocol was established for the endangered cactus Mammillaria mathildae to reintroduce it to its natural habitat. In vitro-germinated seedlings were used as the source of explants. Three explant sources (apical, lateral, and basal excised from in vitro-germinated seedlings) were tested. Shoot multiplication was induced in Murashige and Skoog (MS) medium supplemented with different 6-benzylaminopurine/indole-3-acetic acid (BA/IAA) combinations (0, 22.19, 44.39 and 0, 1.43, 2.85, 5.71, respectively). Explants developed abundant callus in the presence of any BA/IAA concentration, whereas hormone-free media produced 0.59 +/- 0.11 new shoots (with a 41% callus development) from basal explants. Apical and lateral explants produced 1.14 +/- 0.07 and 4.09 +/- 0.13 new shoots, respectively, without callus generation. Plantlets originating from lateral explants developed a vigorous rooting system after 2 months growing on MS medium supplemented with 30 g.L(-1) sucrose. Under greenhouse conditions, 98% of micropropagated M. mathildae survived. Plantlets were reintroduced in an experimental plot near to Juriquilla's wild population of M. mathildae; over 52% of the outplanted M. mathildae lot declined after 5 months. Water availability was associated with the decline of outplanted populations during the first month (43%).

2544: *-.194*

The European wild rabbit (Oryctolagus cuniculus) is a keystone species in the Iberian Mediterranean ecosystems being the staple prey of protected and endangered predators. Wild rabbits were once widespread, but the introduction of two viral diseases, myxomatosis in the 1950s and the rabbit hemorrhagic disease in 1989, resulted into a dramatic decline of its populations. Sarcoptic mange is a highly contagious parasitic infection caused by Sarcoptes scabiei. The first cases of sarcoptic mange in a wild rabbit population are recorded from a hunting area in Majorca (Balearic Islands, Spain). Five of 32 inspected rabbits (14.7%) were affected, with similar prevalences in summer and autumn. Sarcoptes scabiei were obtained from the edge of the lesions of two of the rabbits. The most frequently observed lesions were small areas of alopecia and crusts in the limbs. Affected limbs presented also a marked hypertrichosis and an apparent abnormal growth of the nails. One rabbit also presented lesions around mouth and nostrils. Parasitized rabbits were in significant lower body condition than healthy ones. According to

previous reports of mange epizooties in other na < ve wild species that caused marked short-term effects in their populations, this disease may pose a risk for the conservation of wild rabbit and their predators. Whether mange is endemic in rabbits from Majorca or it has been introduced is unknown. Game managers are encouraged to be aware of introducing sarcoptic mange during rabbit translocations.

2545: *+.099*

Rabbit restocking is widely used in Spain for conservation and/or hunting purposes; however, the success of rabbit restocking is generally low. Thus, many studies have assessed ways to reduce this problem, one of which is the use of a "soft-release" procedure whereby rabbits are acclimated to their release site for a variable time period prior to release. This study assessed the short-term effects of two soft-release confinement periods on the survival of rabbits during an experimental restocking program carried out in southwest Spain. The post-release survival rate of rabbits confined at the release site for six nights was significantly higher than that of rabbits confined for three nights. The longer acclimation period after rabbit translocation minimized mortality while rabbits adapted to their new environment.

2546: *+.185*

There is global interest in recovering locally extirpated carnivore species. Successful efforts to recover Louisiana black bear in Louisiana have prompted interest in recovery throughout the species' historical range. We evaluated support for three potential black bear recovery strategies prior to public release of a black bear conservation and management plan for eastern Texas, United States. Data were collected from 1,006 residents living in proximity to potential recovery locations, particularly Big Thicket National Preserve. In addition to traditional logistic regression analysis, we used conditional probability analysis to statistically and visually evaluate probabilities of public support for potential black bear recovery strategies based on socioeconomic characteristics. Allowing black bears to repopulate the region on their own (i.e., without active reintroduction) was the recovery strategy with the greatest probability of acceptance. Recovery strategy acceptance was influenced by many socioeconomic factors. Older and long-time local residents were most likely to want to exclude black bears from the area. Concern about the problems that black bears may cause was the only variable significantly related to support or non-support across all strategies. Lack of personal knowledge about black bears was the most frequent reason for uncertainty about preferred strategy. In order to reduce local uncertainty about possible recovery strategies, we suggest that wildlife managers focus outreach efforts on providing local residents with general information about black bears, as well as information pertinent to minimizing the potential for human-black bear conflict.

2547: *+.139*

Habitat fragmentation, degradation, and loss have taxed early-successional species including the Northern Bobwhite (Colinus virginianus) and numerous grassland obligate birds. Translocation is often applied to counteract the consequences of habitat fragmentation through the creation, reestablishment, or augmentation of wild populations for the purposes of conservation, biodiversity maintenance. However, the implementation of these techniques is often conducted without valid experimental designs and therefore lacks robust, empirical data needed to evaluate and advance the knowledge and application of translocation. Despite the increasing amount of habitat management applied to patches among fragmented landscapes, a paucity of source populations often limits natural (re) colonization. As such, translocation may serve as a surrogate

to natural dispersal, but its efficacy among fragmented landscapes is uncertain. Few studies exist that have assessed site fidelity, movement, and survival of individuals following translocation among fragmented landscapes. Thus, we experimentally evaluated the efficacy of translocation using known-fate and multi-strata models to evaluate hypotheses of temporal, biological, and group effects on survival and movement of translocated and resident bobwhites. We did not detect differences in survival or movement between translocated and resident bobwhites, suggesting that movement of individuals to a fragmented habitat does not negatively influence these demographic attributes. Based on these data, we suggest that two site-specific criteria should be met prior to instituting translocation: habitat management should be conducted to ensure that quality habitat exists and the patch size should be a minimum of 600 ha of quality habitat (poorer sites may warrant even larger patches). Translocation is a viable conservation method for increasing abundance in patches when habitat quality is high but source populations are limited.

2548: ***+.053***

All known populations of the nationally critical tree daisy, Olearia gardneri, were studied using AFLP markers. With fewer than 160 individual plants, Olearia gardneri is the third-rarest tree in New Zealand and a good model with which to study evolutionary process in fragmented endangered plants. Genetic variation was at similar levels to other long-lived tree species in New Zealand and also as in other studies to date had poor correspondence between genetic and geographic distance. Genetic factors such as inbreeding depression and the loss of genetic diversity might lower fitness and have substantial consequences for evolution and survival of rare threatened plants. Due to the decline of this species in recent times it is imperative that conservation measures are undertaken, including revegetation. Despite considerable emphasis on "eco-sourcing" in plant recovery programmes there is strong evidence that this may not be the best strategy for O. gardneri due to breeding system and population size considerations.

2549: ***+.071***

The major decline of Houbara Bustards-sensu lato-throughout their range has led to their classification as 'vulnerable' on the IUCN Red List. Within this context, a captive breeding program was established in Morocco to restock North African wild populations of Houbara Bustard (Chlamydotis undulata undulata). At present, founders originate from locations that are currently being reinforced through the addition of captive bred individuals. However, it is planned to restock others areas of the species' range. Before this could be considered, an accurate knowledge of the genetic structure of the wild recipient population is essential to delineate potential conservation units. We therefore sampled populations throughout most of the remaining range, and used several methods to evaluate their genetic differentiation by means of both mtDNA and microsatellite markers. Very little genetic differentiation was found for both genetic markers (I broken vertical bar(st) ranged from -0.181 to 0.118 and F (st) ranged from -0.004 to 0.019). This suggests that C. u. undulata populations could be managed as a single Conservation Unit.

2550: ***+.087***

Resolving the taxonomy and historic ranges of species are essential to recovery plans for species at risk and conservation programs that aim to restore extirpated populations. In eastern North America, planning for wolf population restoration is complicated by the disputed historic distributions of two wolf species: the Old World-evolved gray wolf (Canis lupus) and the New World-evolved eastern wolf (C. lycaon). We used genetic and morphometric data from 4- to 500-year-old Canis samples excavated in London, Ontario, Canada to help clarify the historic range of

these two wolf species in the eastern temperate forests of North America. We isolated DNA and sequenced the mitochondrial control region and found that none of the samples were of gray wolf origin. Two of the DNA sequences corresponded to those found in present day coyotes (C. latrans), but morphometric comparisons show an eastern wolf, not coyote, origin. The remaining two sequences matched ancient domestic dog haplotypes. These results suggest that the New World-evolved eastern wolf, not the gray wolf, occupied this region prior to the arrival of European settlers, although eastern-gray wolf hybrids cannot be ruled out. Furthermore, our data support the idea of a shared common ancestry between eastern wolves and western coyotes, and that the distribution of gray wolves at this time probably did not include the eastern temperate forests of North America.

2551: ***+.096***

The short-haired bumblebee (Bombus subterraneus) is the rarest of four Bombus species introduced to New Zealand (NZ) from the United Kingdom (UK) in the nineteenth century, and is now extinct in the UK. The NZ population has been proposed as a source for re-introduction of this species to the UK. However, very little is known about the biology of this species and research is hampered by the difficulty of exact identification as it co-occurs with two morphologically similar species (B. hortorum and B. ruderatus). In this study we have developed simple PCR-based molecular identification tools to rapidly, cheaply, and reliably identify not only B. subterraneus, but also two other species: B. hortorum and B. terrestris. We have combined a species-specific internal primer with two non-specific external primers that amplify 426 bp of the Bombus Cytochrome b gene, to produce a presence/absence PCR test that is combined with a positive internal control. The result is a set of molecular tools that will allow us to separate three cryptic species and facilitate basic research on the biology of New Zealand's rarest bumblebee.

2552: ***+.060***

Choosing the provenance of seed used in ecological restoration could entail its success. An alternative approach to examine local adaptation in seed sourcing is the assessment of genetic structure and diversity based on molecular markers. These types of analyses focus on the genetics of the target plant itself and eliminate the genetic influence of associated organisms, such as EpichloA < By impacting the fitness of their host, such symbionts may influence population genetic structure and diversity. Therefore, seed sourcing for grasses must consider the influence of their endophytes to increase seed translocation success and minimize the risks associated with this practice. To delineate seed zones for restoration of the alpine fescue Festuca eskia Ramond ex. DC. (Poaceae), we assessed population genetic differentiation and diversity patterns in the species including endophyte occurrence along altitudinal and longitudinal gradients in the Pyrenees Mountains. Twenty-three populations were analysed for endophyte status, and three STS and one SSR marker were used to examine genetic differentiation and diversity patterns. Results showed that F. eskia hosts an asexual form of EpichloA << and infection frequency within populations decreased from East to West (100 vs. 8-25%). Molecular markers separated F. eskia into two East and West groups, and endophyte infection and genetic patterns were congruent with molecular data. Little evidence for genetic differentiation or difference in endophyte occurrence associated with altitude was detected. Little variation was found in within population diversity, regardless of provenance altitude and site, and/or endophyte infection frequency. The results of this study suggested the establishment of two distinct management units for F. eskia seed sourcing restoration.

2553: ***+.191***

Predation pressure on vulnerable bird species has made predator control an important issue for international nature conservation. Predator removal by culling or translocation is controversial, expensive, and time-consuming, and results are often temporary. Thus, it is important to assess its effectiveness from all available evidence. We used explicit systematic review methodology to determine the impact of predator removal on four measurable responses in birds: breeding performance (hatching success and fledging success) and population size (breeding and postbreeding). We used meta-analysis to summarize results from 83 predator removal studies from six continents. We also investigated whether characteristics of the prey, predator species, location, and study methodology explained heterogeneity in effect sizes. Removing predators increased hatching success, fledging success, and breeding populations. Removing all predator species achieved a significantly larger increase in breeding population than removing only a subset. Postbreeding population size was not improved on islands, or overall, but did increase on mainlands. Heterogeneity in effect sizes for the four population parameters was not explained by whether predators were native or introduced; prey were declining, migratory, or game species; or by the study methodology. Effect sizes for fledging success were smaller for ground-nesting birds than those that nest elsewhere, but the difference was not significant. We conclude that current evidence indicates that predator removal is an effective strategy for the conservation of vulnerable bird populations. Nevertheless, the ethical and practical problems associated with predator removal may lead managers to favor alternative, nonlethal solutions. Research is needed to provide and synthesize data to determine whether these are effective management practices for future policies on bird conservation.

2554: ***-.003***

The Texas tortoise (Gopherus berlandieri) is a state-protected, threatened species in Texas. The expansion of agricultural practices and urban development are major causes of habitat degradation for the species. To provide genetic data that can inform conservation planning for this species, genetic variation, population structure, and the process that maintains the observed structure were examined in the Texas populations of G. berlandieri. Microsatellite genetic variation of 8 polymorphic loci was examined for a total of 138 individuals collected from 10 sampling areas in southern Texas. Assignment tests, F statistics, and analysis of molecular variance indicated that G. berlandieri forms weak population differentiation into northern and southern groups, with a boundary at southern Duval County. A test of isolation by distance and indirect estimation of the number of migrants (Nm) suggest recent gene flow between the 2 groups. Estimation of the extent of contemporary migration appears to be complicated by human translocation of tortoises, and an asymmetrical direction of migration needs further examination. Gopherus berlandieri is weakly differentiated because of ongoing migration as evidenced by a pattern of isolation by distance. Given the limited population structure and continuous habitat degradation, designation of 2 management units may not be warranted. Conservation efforts, rather, should emphasize connectivity between the groups to maintain genetic variation in both groups.

2555: ***+.120***

The Arrau turtle, an endangered species, is management-dependant for the recovery of its populations. The objective of this research, carried out in the reproductive season of 2003 (January - April), was to determine through in situ tests, transplant treatments that allow to maximize the hatching success, reducing the deformed young's rate and increase the efficiency of this management technique. 27 nests events were selected and three were let in natural conditions. The 24 clutches to transplant were divided in two groups of 12. Four clutches from the first group were buried at a 50 cm deep, while other four were buried at 60 cm and the rest at 70 cm. The twelve

clutches from the second group were divided on halves to obtain 24 half clutches from them, eight half clutches were buried at 50 cm, eight half clutches at 60 cm and the other eight were buried at 70 cm. A two-way ANOVA was applied to determine differences between the two nesting sizes, between the three different depths and between the six treatments (depth + nesting size). In addition, a one-way ANOVA permitted to evaluate differences between transplanted treatments and control group. Half clutches at 70 cm deep offered the best conditions of incubation (high hatching success, the least percentages of deformed young and the most efficient treatment). The half clutches at 60 cm treatment was not adequate as a translocation technique. Half clutches obtained better results than complete clutches.

2556: *+.007*

Leiopelma hochstetteri is an endangered New Zealand frog now confined to isolated populations scattered across the North Island. A better understanding of its past, current and predicted future environmental suitability will contribute to its conservation which is in jeopardy due to human activities, feral predators, disease and climate change. Here we use ecological niche modelling with all known occurrence data (N = 1708) and six determinant environmental variables to elucidate current, pre-human and future environmental suitability of this species. Comparison among independent runs, subfossil records and a clamping method allow validation of models. Many areas identified as currently suitable do not host any known populations. This apparent discrepancy could be explained by several non exclusive hypotheses: the areas have not been adequately surveyed and undiscovered populations still remain, the model is over simplistic; the species' sensitivity to fragmentation and small population size; biotic interactions; historical events. An additional outcome is that apparently suitable, but frog-less areas could be targeted for future translocations. Surprisingly, pre-human conditions do not differ markedly highlighting the possibility that the range of the species was broadly fragmented before human arrival. Nevertheless, some populations, particularly on the west of the North Island may have disappeared as a result of human mediated habitat modification. Future conditions are marked with higher temperatures, which are predicted to be favourable to the species. However, such virtual gain in suitable range will probably not benefit the species given the highly fragmented nature of existing habitat and the low dispersal ability of this species. (C) 2010 Elsevier Ltd. All rights reserved.

2557: *+.181*

Climate change and habitat destruction are widely recognized as major threats to species' survival. As a result of these anthropogenic impacts, species are often forced into novel landscapes where their persistence is difficult to predict. Knowledge of how individuals move or disperse through the landscape, choose habitat in which to settle, and produce offspring which survive to repeat the process can greatly improve our ability to predict species' persistence. The field of behavioral landscape ecology uses a strong theoretical base to explore, often experimentally, how the behavior of a particular species is affected by heterogeneous and rapidly changing landscapes and can offer valuable insight for managing species in the face of human-induced environmental changes. When interpreted by modelers, results of landscape-level behavioral experiments can be quantified for use in predictive models. To this end, we summarize the methods and results of research using direct experimental manipulation techniques broken into the following categories: translocations, playback experiments, food resource manipulations, manipulations of reproductive success, direct manipulations of the landscape, and manipulations of predation risk. We review and place in a theoretical framework the results from this emerging body of research regarding how organisms move in and respond to different types of landscapes, both natural and human-altered. We go onto highlight the potential of each experimental method to quantify different

processes, which may be useful when interpreted by modelers attempting to parameterize predictive models. Finally, we suggest future directions for experimental research that will allow for greater integration of behavioral landscape ecology and predictive modeling. (C) 2010 Elsevier Ltd. All rights reserved.

2558: ***-.149***

As the number and diversity of animal species meriting conservation intervention increases, improving the success of translocation efforts is vital and understanding the role of physiological stress in translocation failure is essential. Though the short-term acute stress response is critical for survival in the wild, chronic stress results in pathology and occurs when the physiological stress response system is pushed beyond the normal capacity and becomes dysregulated. As this review discusses, translocation may inevitably lead to a state of chronic stress; however, this translocation-induced stress does not doom an animal to post-release failure or preclude translocation as a conservation tool. Rather, because chronic stress in translocated animals may actually be the norm rather than the exception, "stress" can be considered a predictable factor to incorporate into translocation planning. In terms of translocation failure, we argue that the role of stress is an indirect one. Translocation-induced chronic stress increases the overall vulnerability of the individuals and, as a result, decreases the probability that the population will become self-sustaining. In this review, we further propose techniques to potentially decrease the total number and magnitude of stressor exposures throughout the translocation procedure in order to diminish the incidence of chronic stress-related pathology. Aspects of the translocation activities, such as capture techniques and care procedures during captivity, can be adjusted to accomplish this. Though stress may be an inevitable component of translocation, reducing the impact and time course of chronic stress on the physiology and behavior of translocated animals will increase the likelihood of translocation success as measured by the formation of a new, self-sustaining population (C) 2010 Elsevier Ltd. All rights reserved.

2559: ***+.249***

The Mediterranean climate regions of Western Australia and South Africa are recognized as global hot spots of diversity. Both are threatened by climate changes that are projected to have significant impacts on the quantity and variability of rainfall and affect key ecosystem drivers (e.g. fire regimes). This poses significant challenges to monitoring programs designed to detect these impacts. Effective monitoring of the impact of climate change on biodiversity (rather than individual species) requires a cross-disciplinary, coordinated, focused and integrated approach. Ideally, this should involve a multidisciplinary team of specialists working to a common plan on the same set of plots. The contributions of 'citizen scientists' are potentially useful if well managed. Biodiversity per se (across all kingdoms of life, and including the levels of the gene, population and community) should be monitored, especially key species interactions and processes. Forestcheck is an example of such a program which has been applied in forests in south-west Western Australia since 2001. In concert with measuring the direct impact of climate change on biodiversity and the indirect impact of factors that affect biodiversity (such as disease, invasive species, fire regime and habitat removal), there is a need for a proactive focus on creating, maintaining and monitoring resilience to climate change impacts in ecosystems. It is also necessary to monitor the effectiveness of management actions such as vegetation thinning, changes in fire regimes, species translocations and revegetation of farmland to link isolated protected areas in agricultural landscapes, remnant native vegetation in rangelands and extensive protected areas. A pluralist approach is recommended. This should include natural experiments, matched photographs where available, passive adaptive management, active adaptive management

and traditional reductionist scientific investigation. The resultant synthesis of information from this range of sources is likely to be a predictive, robust and credible record of historical change.

2560: *+.140*

In 2003 a reintroduction program of osprey started in the region of Andalusia, SW Spain, in order to recover the former breeding population in mainland Spain and to improve the situation of the species in the Mediterranean basin. From 2003 to 2009, 129 young ospreys were released by means of hacking. In 2009, the first breeding pair reared successfully three chicks in the Odiel Marshes for the first time in mainland Spain since 1981, when the species become extinct. The first breeding pair constitutes a significant indicator for the evaluation of the project and the beginning of a future population.

2561: *+.027*

1. Species reintroductions are an increasingly fashionable tool among conservation practitioners for restoring locally and nationally extinct populations. For a reintroduction programme to be successful, an understanding of the causes of the decline of a species is required. This, however, proves challenging when baseline data on the distribution and abundance of the species are limited.2. This paper uses evidence from historical literature to map the former distribution of the burbot (Lota lota) within UK rivers before its extinction in the early 1970s. A scoring system was developed to model anecdotal descriptions of burbot abundance from the collected literature.3. The former distribution was divided into four geographical areas based loosely on catchment boundaries. The literature identifies 42 rivers in eastern England in which the burbot was likely to have existed. The status of the species in the Thames catchment is still unclear as information from written sources is contradicted by evidence from the archaeological record.4. The findings indicate that the year of source data was a significant predictor of burbot abundance across the former population as a whole and for three of the four geographical areas (the Trent catchment, the Fenland rivers and the Yorkshire rivers). The timing of the burbot's decline showed differences between the geographical regions, with the Trent catchment exhibiting an earlier decline than the Fenland and Yorkshire areas. Copyright (C) 2010 John Wiley & Sons, Ltd.

2562: *-.021*

Flies constitute a cost to animals by feeding from them, passing on disease or by preventing them feeding effectively. These costs could be more severe to animals suffering from some kind of stress such as reintroduction as part of a conservation programme. This study focused on the effects of fly disturbance on Przewalski horses (Equus ferus przewalskii) reintroduced into Hustai National Park in Mongolia. As the reintroduced horses could not be handled, we took three approaches: direct fly sampling, sampling flies on a ridden domestic horse (Equus ferus caballus) and monitoring their behaviour, and observations of Przewalski horse behaviour. Flies were sampled in traps and on a domestic horse in grazing and resting places used by the Przewalski horses: more flies were found in valley grazing places than ridge resting places. The number of flies was related to temperature, with most found between 15 degrees C and 30 degrees C, peaking at 22 degrees C, however taken together no environmental or seasonal variable satisfactorily explained the number of flies. Comfort behaviour, the behavioural response of horses to the presence of flies, increased with temperature and decreased with wind speed in both domestic and Przewalski horses, and increased with elevation for Przewalski horses. During warm months Przewalski horses had a daily pattern of moving up to high, bare places where there was no forage during the warmer hours of the day. It is likely that they moved to escape flies, but were followed

by them. The cost of flies to horses and their potential impact on the reintroduced population is discussed. (C) 2010 Elsevier B.V. All rights reserved.

2563: *-.007*

In recent years populations of howler monkeys (Alouatta palliata) in southeastern Mexico have decreased substantially due to the transformation and loss of natural habitats. This is especially evident in the Santa Marta mountain range, Veracruz, Mexico where several studies have evaluated the impact of fragmentation on howler monkey populations in order to propose management programs for their conservation. The conditions generated by fragmentation likely change the rates of parasitic infection and could decrease howler survival. In this study, gastrointestinal parasite species richness, prevalence, and egg density of infection were determined in howler groups inhabiting five forest fragments at the Santa Marta mountain range. Two hundred and seventy-eight fresh fecal samples were collected between October 2002 and April 2003. Three parasite species were found during the dry and the wet season in all forest fragments sampled: one unidentified species of Eimeriidae; Topanoxyuris minutus (Oxyuridae); and Controrchis biliophilus (Dicrocoeliidae). Both the prevalence of T. minutus and infection density for all parasites differed between seasons and fragments (the largest fragment consistently differed from other fragments). Host density, distance to the nearest town, fragment size, fragment shape, and total basal area of food trees explained parasite prevalence, but each species had a different pattern. Although parasite richness was lower, prevalence and density were higher than values reported for howlers in conserved forests. These results suggest that the establishment of biological corridors and animal translocation programs must take into account the parasite ecology of each fragment to avoid higher infection rates and preclude potential consequent mortality. Am. J. Primatol. 72:539-548, 2010. (C) 2010 Wiley-Liss, Inc.

2564: *+.161*

Traditional methods of monitoring gray wolves (Canis lupus) are expensive and invasive and require extensive efforts to capture individual animals. Noninvasive genetic sampling (NGS) is an alternative method that can provide data to answer management questions and complement already-existing methods. In a 2-year study, we tested this approach for Idaho gray wolves in areas of known high and low wolf density. To focus sampling efforts across a large study area and increase our chances of detecting reproductive packs, we visited 964 areas with landscape characteristics similar to known wolf rendezvous sites. We collected scat or hair samples from 20% of sites and identified 122 wolves, using 8-9 microsatellite loci. We used the minimum count of wolves to accurately detect known differences in wolf density. Maximum likelihood and Bayesian single-session population estimators performed similarly and accurately estimated the population size, compared with a radiotelemetry population estimate, in both years, and an average of 1.7 captures per individual were necessary for achieving accurate population estimates. Subsampling scenarios revealed that both scat and hair samples were important for achieving accurate population estimates, but visiting 75% and 50% of the sites still gave reasonable estimates and reduced costs. Our research provides managers with an efficient and accurate method for monitoring high-density and low-density wolf populations in remote areas.

2565: *+.275*

Harvest of furbearers through trapping has been challenged by anti-trapping organizations for centuries, with organizational goals often including prohibition of all forms of trapping. Challenges to trapping may also include dissention among state wildlife agencies, pro-hunting

organizations, and pro-trapping organizations. Despite recent efforts by anti-trapping organizations and occasional dissention among consumptive-use groups, national trends in snaring regulations included less restrictive regulations through time. This positive trend may offer opportunities for state wildlife agencies and pro-trapping organizations to enhance the public image of trapping, increase recruitment of trappers, and reverse the increasing trend of wildlife damage and associated costs. We offer support and suggestions to state wildlife agencies and pro-trapping organizations to help achieve these goals, with their partnership likely having a synergistic effect. Although we attempt to illuminate approaches for increasing support for trapping within the constraints of the cultural norms of the United States, we hope our approaches are useful to and promote dialogue in other jurisdictions experiencing similar problems.

2566: ***+.034***

Wildlife managers often need to assess the current health status of wildlife communities before implementation of management actions involving surveillance, reintroductions. or translocations We estimated the sensitivity and specificity of a commercially available domestic canine rapid diagnostic antigen test for canine parvovirus and a rapid enzyme-linked immunosorbent assay for the detection of antibodies toward Anaplasma phagocytophilum on populations of fishers (Marles pennanti) and sympatric gray foxes (Urocyon cinereoargenteus) Eighty-two fecal samples from 66 fishers and 16 gray foxes were tested with both SNAP (R) PARVO rapid diagnostic test (RDT) and a nested polymerase chain reaction (PCR) Whole blood samples from 23 fishers and 53 gray foxes were tested both SNAP 4Dx (R) RDT and immunofluorescence assays (IFAs) The SNAP PARVO RDT detected no parvovirus, whereas PCR detected the virus in 17 samples Eleven samples were positive using the SNAP 4Dx RDT, whereas 46 samples tested by IFA were positive for A phagocytophilum Both RDTs had low sensitivity and poor test agreement These findings clearly, demonstrate the importance of validating It RDTs developed for domesticated an bel ire using them for wildlife populations

2567: ***-.069***

Captive-reared Whooping Cranes (Grus americana) released into Florida for the resident reintroduction project experienced unusually high mortality and morbidity during the 1997-98 and 2001-02 release seasons Exposure to infectious bursal disease virus (IBDV) serotype 2 as evidenced by seroconversion was suspected to be the factor that precipitated these mortality events Very little is known about the incidence of I BID in wild bird populations Before this study, natural exposure had not been documented in wild birds of North America having no contact with captive-reared cranes, and the prevalence and transmission mechanisms of the virus in wild birds were unknown Sentinel chickens (Gallus gallus) monitored on two Whooping Crane release sites in central Florida, USA, during the 2003-04 and 2004-05 release seasons seroconverted, demonstrating natural exposure to IBDV serotype 2 Blood samples collected from Wild Turkeys (Meleagris gallopavo) and Sandhill Cranes (Grits canadensis) in eight of 21 counties in Florida, USA, and one of two counties in southern Georgia. USA, were antibody-positive for IBDV serotype 2, indicating that exposure from wild birds sharing habitat with Whooping Cranes is possible The presence of this virus in wild birds in these areas is a concern for the resident flock of Whooping Cranes because they nest and raise their chicks in Florida, USA However, passively transferred antibodies may protect them at this otherwise vulnerable period in their lives.

2568: ***+.006***

Wild animal populations lace threats from pathogens from both intentionally released captive

animals and domestic animals that accompany human settlements From December 2004 through August 2005. we studied free living macaws and parrots in the Tambopata National Reserve in the Peruvian Amazon and semicaptive domestic fowl in human settlements adjacent to the reserve In 1992-1993, large macaws (Aras spp) that were serologically positive for Salmonella Pullorum were released into this reserve, which hosts dense populations of free-hying parrots and macaws We collected cloacal swabs from 64 birds and cultured for Salmonella spp via standard laboratory methods All 35 psittacines tested were culture negative for Salmonella spp, while 31% of 29 domestic kiwi were culture positive Our findings suggest that the domestic. fowl that accompany human settlement in this region carry and shed Salmonella spp that could threaten wild bird populations in and around the reserve

2569: ***+.467***

Adaptive management has a long history in the natural resource management literature, but despite this, few practitioners have developed adaptive strategies to conserve threatened species. Active adaptive management provides a framework for valuing learning by measuring the degree to which it improves long-run management outcomes. The challenge of an active adaptive approach is to find the correct balance between gaining knowledge to improve management in the future and achieving the best short-term outcome based on current knowledge. We develop and analyze a framework for active adaptive management of a threatened species. Our case study concerns a novel facial tumor disease affecting the Australian threatened species Sarcophilus harrisii: the Tasmanian devil. We use stochastic dynamic programming with Bayesian updating to identify the management strategy that maximizes the Tasmanian devil population growth rate, taking into account improvements to management through learning to better understand disease latency and the relative effectiveness of three competing management options. Exactly which management action we choose each year is driven by the credibility of competing hypotheses about disease latency and by the population growth rate predicted by each hypothesis under the competing management actions. We discover that the optimal combination of management actions depends on the number of sites available and the time remaining to implement management. Our approach to active adaptive management provides a framework to identify the optimal amount of effort to invest in learning to achieve long-run conservation objectives.

2570: ***+.027***

Invasive species are widespread and can have devastating effects on biota, especially insular biota. Invasive species eradications are increasingly employed to promote island recovery to preinvasion states. However, it remains unclear if additional restoration actions may be required on islands that were once heavily reliant on seabird guano for ecosystem functions. Active seabird augmentation has been suggested as necessary to exact ecosystem recovery on contemporary timescales in some cases. I use two experiments on offshore islands in Cook Strait, New Zealand, to test the hypothesis that seabird restoration will restore island ecosystem functioning following invasive rodent removal. The first is a small-scale single-island fertilization experiment that simulates seabird recovery. This experiment tested the recovery potential of offshore islands and was used to infer the density of seabirds needed to elicit ecosystem recovery. The second is a large-scale natural experiment that takes advantage of eight islands with differing rodent eradication and seabird restoration histories. I compared ecosystem functioning variables (delta(15)N, C:N ratios in soil, plants, and spiders, as well as arthropod abundance and diversity) on two islands that had rodents eradicated and two islands undergoing seabird augmentation with two control islands (never invaded by rodents) and two positive control islands (currently invaded by rodents). The results suggest that islands do have the potential for recovery given nutrient amendments, but that

islands with rodents eradicated and islands undergoing seabird augmentation have not recovered most of their ecosystem function. Finer, intra-island analysis showed that seabird restoration projects have the potential to speed the recovery process, but that the projects on the studied seabird restoration islands were not advanced enough to produce island-wide recovery. The results suggest that high seabird densities (5-10 burrows/m(2)) are needed to promote recovery to never-invaded control levels. Seabird augmentation, through chick translocation and/or social facilitation with decoys, vocalization playbacks, and/or mirrors can supplement passive seabird recovery on islands where seabirds have been extirpated or extremely reduced by invasive predators. Such restoration efforts may be necessary to promote ecosystem recovery on contemporary timescales.

2572: *+.111*

1. The freshwater pearl mussel Margaritifera margaritifera is endangered throughout Europe.2. Historically, mussels were described on the basis of shell characteristics. In more recent years with the advent of molecular techniques many 'species' of molluscs have been found to be ecophenotypes.3. The pearl mussel is found in numerous rivers throughout Ireland and the UK with varying degrees of superficial differences. It is has been thought that the most divergent form is found in the Nore River, Ireland, Margaritifera m. durrovensis.4. The current investigation considers shell shape differences (using morphometrics - elliptic Fourier descriptors) in mussels from a variety of rivers in Ireland in relation to river pH.5. Results suggest that M. margaritifera has a fairly plastic phenotype, with a gradient of shape change in relation to water pH.6. Margaritifera m. durrovensis does not appear to be morphologically unique from other populations studied, instead occurring at one end of the shell shape gradient.7. Findings also suggest that shell shape may be characteristic to individual rivers. The existence of phenotypically distinct groups of Margaritifera margaritifera has particularly important implications for the future conservation of the species.8. Ex situ conservation and reintroduction efforts will need to consider both the genotypic and phenotypic suitability of mussels if translocation is to be used as a viable conservation tool in the future. Copyright (C) 2010 John Wiley & Sons, Ltd.

2573: *-.039*

This technical memorandum is divided into three sections. Section 1 summarizes the proceedings of the second workshop on Shark Predation on Hawaiian Monk Seals sponsored by the Hawaiian Monk Seal Research Program (HMSRP) of the Pacific Island Fisheries Science Center (PIFSC) and also the Pacific Islands Regional Office (PIRO) of the National Marine Fisheries Service (NMFS). Section II reviews knowledge to date about shark predation on preweaned and newly weaned monk seal (Monachus schauinslandi) pups and NMFS' mitigation attempts at French Frigate Shoals (FFS) and elsewhere in the Northwestern Hawaiian Islands (NWHI). This section also provides a more comprehensive picture of the issues than time permitted at the workshop. Section III summarizes HMSRP's premises about the nature of shark predation based on peer-reviewed science, inferences, expert opinions, and field experience. HMSRP's positions on controversial aspects of the issue are stated, and a number of appendices are included that detail plans to be executed in 2009 and mitigation ideas for the future. Section I. Workshop II Report Workshop II was held on November 5-6, 2008 in Honolulu, Hawaii. Participants included representatives from PIFSC, PIRO, Papahanaumokuakea Marine National Monument (the Monument), U.S. Fish and Wildlife Service (USFWS), State of Hawaii Department of Land and Natural Resources (DLNR), Marine Mammal Commission (MCC), and the Hawaiian Monk Seal Recovery Team. The primary goal of this workshop was to exchange ideas and opinions from different management and scientific perspectives about the predation problem and suggest a logical course of action. Presentations describing the endangered status of the Hawaiian monk

seal, the shark predation problem at FFS, and the first workshop on the issue all set the stage for the second workshop's discussions. Hawaiian Institute of Marine Biology (HIMB) scientists reviewed previous shark research in FFS, reported the results of their 2008 research efforts, and presented their 2009 research plan which was aimed at gathering fine-scale movement data on sharks. HMSRP described 2008 mitigation activities and mitigation strategies for the future. The 2008 mitigation strategy focused solely on the application of a suite of deterrents and devices around Trig Island and the translocation of weaned pups to "safe" islets, although the lethal removal of select sharks had also received support at Workshop I. Outcomes of Workshop II included an evaluation of previous research efforts, development of definitive statements about the predation problem which was agreed on by all Workshop participants, identification of knowledge gaps, and a prioritized list of suggested actions for upcoming field seasons. Workshop participants advocated improved deterrent design, improved and informed removal of sharks displaying predatory behavior, and a need for analyses on previous data and the collection of additional data on seal and shark behavior. Ideas, such as the use of barriers to keep sharks away from nearshore areas and sonic-tagging pups, were discussed and their implementation recommended. Section II. Knowledge to Date about the Shark Predation at FFS and its Mitigation The genus Monachus is in crisis; with just two extant representative species, the Hawaiian monk seal offers the best chance of its persistence. However, the Hawaiian monk seal population itself is heading toward extinction. Numerous threats afflict the species across its range. Shark predation on preweaned and newly weaned pups contributes to a unique and extreme situation at FFS that peaked in 1997[long dash]1999 and stands out from the trends observed at other sites in the NWHI. Since then, predation has declined to 6-11 pups a year, an unsustainable rate as a result of falling birth rates. Galapagos sharks (Carcharhinus galapagensis) and tiger sharks (Galeocerdo cuvier) both potentially feed on marine mammals; however, HMSRP has only observed Galapagos sharks attacking and killing pups in nearshore water. Mitigation activities by HMSRP conducted over the last decade include harassment of sharks, intensive observation, translocation of weaned pups, deployment of devices to deter predation, and shark removal. Section III. HMSRP Premises, Positions and Post-Workshop Developments HMSRP has developed premises about the identity and number of sharks likely involved, shark wariness to human activity, and opinions about shark culling based on peer- reviewed science, inference, expert opinion and ample experience with the situation at FFS. Post-workshop, HMSRP systematically compared all mitigation actions proposed, detailing the potential benefits and drawbacks based on its premises, positions, workshop recommendations and stakeholders' perspectives. A 2009 field plan was created that includes: (1) logistical and financial support for HIMB shark scientists to conduct shark tagging studies at FFS, (2) the systematic application and comparison of 3 treatments (human presence, deterrents and a control) at 2 pupping sites, (3) the design and installation of a custom-made remote surveillance camera system on 1 pupping site, and (4) additional behavioral monitoring of sharks and seals.

2574: ***+.018***

Translocation is defined as the human-managed movement of living organisms from one area for free release in another. Throughout the world, increasing numbers of animals are translocated every year. Most of these movements involve native mammals, birds and fish, and are made by private and national wildlife agencies to augment existing populations, usually for sporting purposes. The translocation of endangered species, often to reintroduce them into a part of the historical range from which they have been extirpated, has also become an important conservation technique. The main growth in reintroduction projects over the last decade has involved smaller animals, including amphibians, insects and reptiles. The success of potentially expensive, high-profile wildlife translocation projects depends to a large extent on the care with which wildlife

biologists and their veterinary advisers evaluate the suitability of the animals and chosen release site, and on the ability of the translocated animals to colonise the area. The veterinary aspects of reintroduction projects are of extreme importance. There are instances of inadequate disease risk assessment resulting in expensive failures and, worse still, the introduction of destructive pathogens into naive resident wildlife populations. In this paper, some of the disease risks attending wildlife translocation are described. Risk assessment, involving the examination of founder and recipient populations and their habitats, is now a pre-requisite of managed movements of animals.

2575: ***+.061***

Two examples of the introduction of non-indigenous invasive species are reviewed: the grey squirrel in Europe (United Kingdom, Ireland and Italy) and the brushtail possum in New Zealand. Both have become very successful in their respective non-native habitats since their introductions in the mid-to-late 19th Century. Both species impact extensively on native biodiversity, environmental sustainability, forestry, and agriculture through a range of direct and indirect mechanisms. Management is currently mainly by lethal control, namely poisoning, trapping and shooting. Such methods of control are, however, increasingly contentious for both species, and alternative, non-lethal methods of population control, e.g. fertility control, are being developed. The case studies highlight many of the issues in invasive animal control; for example, prevention being better than control, lack of good understanding of impacts and the success of control measures on reducing impacts, interactive impacts on native biodiversity and ecosystems, the telling influence of public opinion on management options and, lastly, the need to better inform and educate the public.

2576: ***-.063***

After habitat destruction, invasive alien species are the second leading cause of biodiversity loss, particularly in freshwater ecosystems. They also alter the structure and functioning of ecosystems, lead to biotic homogenisation, and eventually threaten human economies and health. This review aims to synthesise some of the existing information about the world distribution, vectors of spread, and impacts of two important components of freshwater ecosystems, crayfish and fishes. Analysis of the available literature shows that crayfish and fish species, once moved outside their native range, are likely to establish self-reproducing populations, spread from the point of introduction and become invasive. Efforts to manage these populations are difficult and expensive, which warrants the provision of effective preventative measures. Unfortunately, the state of our knowledge of the mechanisms in play in crayfish and fish invasions is still limited, which suggests that much greater attention and investment should be directed to studies in this field.

2577: ***+.045***

Mycoplasmas are commensals and pathogens of various avian species, and are also regularly found in birds of prey, although their significance to birds' health remains unclear. Here we describe two novel Mycoplasma isolated from the upper respiratory tract of four Eurasian griffon vultures (Gyps fulvus) housed in a wildlife recovery centre in Sardinia (Italy). By sequencing the 165 rRNA gene and the entire 16S/23S intergenic spacer region, the new strains were classified within the Mycoplasma taxonomy at the group and cluster levels, showing that the two isolates fall into the Mycoplasma synoviae and Mycoplasma hominis clusters of the hominis group, respectively. We combined molecular tools and immunoblotting methods in order to further characterize these isolates, and antigenic analyses overall confirmed the molecular findings.

Different levels of pathogenicity and prevalence of these strains might have different implications for the conservation and reintroduction of vultures. (C) 2010 Elsevier Ltd. All rights reserved.

2578: ***-.017***

The terrestrial orchid genus Caladenia contains many species which are threatened with extinction. They have highly specific associations with Sebacina vermifera and closely related fungi, and conservation of these terrestrial orchids, in part, relies on symbiotic propagation to produce plants for reintroduction and ex situ conservation collections. However, little is known of the diversity of mycorrhizal fungi associating with natural populations. Here, restriction fragment polymorphism analysis, internal transcribed spacer and nuclear large subunit sequencing and symbiotic seed germination were used to investigate the taxonomic and functional diversity of fungal isolates from single populations of six endangered Caladenia species and one common species across the same biogeographic range. Fifty-nine fungal isolates were collected for investigation including ten isolates from the six endangered species Caladenia audasii, Caladenia amoena, Caladenia sp. aff. fragrantissima (Central Victoria), Caladenia sp. aff. patersonii, Caladenia rosella and Caladenia orientalis and 49 isolates from six populations of the common species Caladenia tentaculata. While the common species associated with three distinct S. vermifera-like taxa, the six endangered species were restricted to one of these fungal taxa. No direct relationship between the taxonomic identity of the fungi and their ability to stimulate seed germination was observed; however, the majority of the fungi isolated from the Caladenia species were capable of germinating seed in vitro, indicating their mycorrhizal status and potential for symbiotic propagation in conservation programmes.

2579: ***+.374***

Socially learned behavior can be a crucial factor in how animals interact with their environment and, thus, in conservation and management. For species in which social learning and culture are important determinants of behavior, several factors complicate conservation and management. These include the rapid spread of novel behavior through social learning, the inhibition of adaptive behavior because of cultural conformism, the evolution of maladaptive behavior, and the development of culturally isolated but sometimes sympatric groups. These factors can affect habitat suitability, movements, how animals react to anthropogenic effects, and genetic structures. Social learning and culture may be important factors in translocation success, and should sometimes be considered when delineating population units for conservation and management. We should aim to protect cultural as well as genetic diversity. Unfortunately, clear data on social learning and culture in the wild are scarce. Hence, the ideas and methods outlined in this special issue have great potential.

2580: ***+.062***

Conservation managers of oak woodlands have been reintroducing fire both as an ecological process per se and to assist in restoring native plant communities. To increase our understanding of the impacts of reintroduced fire on ground-dwelling invertebrates we examined the response of ants and spiders to a late season (autumn) prescribed fire conducted in a blue oak (Quercus douglasii) woodland ecosystem in northern California. Twelve 100 m x 100 m plots were established, six plots received a burn treatment and the remaining six plots were unburned controls. Ants and spiders were sampled using pitfall traps left open continuously and collected approximately every 32 days. Sampling was conducted over a year, consisting of four pre-burn and nine post-burn collections. Abundance was analyzed using a repeated measures ANOVA,

which showed seed-harvester ants decreased significantly in the two months following the fire. Total spider abundance also showed a significant decrease in two months, although this did not occur immediately after the burn. One spider hunting guild, the 'diurnal ambush' group (Thomisidae) remained suppressed for up to nine months. Correspondence analysis measures of ant species abundance with environmental and vegetation variables (percent rock, bare ground, plant species richness and height of herbaceous vegetation) were higher than expected by chance, which assists in explaining some of the responses. Findings from this study revealed that the reintroduction of autumn burns has modest and short-term effect on the invertebrates sampled, suggesting that late season fires are compatible with other conservation goals for oak woodland ecosystems.

2581: *+.309*

Our ability to evaluate the effectiveness of conservation interventions is primarily reliant on and often limited by the available evidence. As claimed conservation success (or failure) might merely be an artefact of the quantitative approach used for evaluation, both in terms of locating and analysing data, cross-validation of results is recommended. By cross-validation we mean using two (or more) different methods for evaluation and comparing the results. An initial assessment of the effectiveness of African wild dog (Lycaon pictus) reintroductions in South Africa was re-evaluated using a systematic review approach. This cross-validation differed from the previous evaluation in two important aspects, which define the systematic review process: comprehensive data searching; and meta-analysis. The original dataset was confirmed to be complete by an exhaustive search to locate additional data. Both the initial assessment and the meta-analysis suggested that wild dog reintroductions are successful in the short-term, with high survival rates of the released animals and their offspring. The meta-analysis corroborated the importance of pre-release socialisation in promoting post-release survival at the pack level. In contrast, the initial assessment found that additional covariates affect the survival of reintroduced wild dogs at the individual level. This study emphasises the importance of cross-validating management recommendations in endangered species recovery programmes using an evidence-based approach to assess and communicate the reliability of results. (C) 2009 Elsevier GmbH. All rights reserved.

2582: *+.235*

Reintroduction of Sumatran orangutans can be an important tool to conserve the species in the face of the current decline of wild populations. Monitoring ex-captive orangutans during the reintroduction process provides insight into their adaptation to forest life and is important for evaluating the success of such programs. We investigated activity patterns, food choice, height use, and nest-building abilities of 8 immature orangutans at a reintroduction station in Sumatra. All focal individuals spent most of their time in the forest feeding, but at a lower proportion than wild conspecifics. Among the focal individuals, the behavior of orangutans that avoided human contact differed in several aspects from those that were human bonded. Their diet was composed mainly of fruits, similar to the food choice of wild orangutans, and their ground-avoidance and superior nest-building abilities indicated more effective predation-avoidance behavior vs. that of human-bonded animals. Finally, these immatures showed regular social contact with more experienced conspecifics, which ultimately may facilitate the social learning of local feral expertise. Human-bonded individuals, in contrast, preferentially stayed on the ground and at low heights and rarely built nests, thereby increasing predation risk. Their choice of leaves over fruits as foods indicated a less effective foraging strategy. Nevertheless, this group exhibited individual learning trends toward an adult-like foraging pattern and height use. We conclude that social interactions with conspecifics and humans, especially during an early developmental period, can

affect adaptation to forest life and probably also influence the success of orangutan reintroduction processes.

2583: ***+.236***

The divergence of conspecific recognition signals (CRS) among isolated populations facilitates the evolution of behavioral barriers to gene flow. The influence of CRS evolution on signal effectiveness in isolated populations can be assessed by testing the salience of changes in CRS from surviving ancestral populations but founder events are rarely detected. The population history of the North Island (NI) saddleback Philesturnus rufusater is absolutely known following conservation translocations which increased the number of populations from 1 to 15. With one exception there is no gene flow between these populations. The translocations have generated interisland divergence of male rhythmical song (MRS), a culturally transmitted CRS. We conducted an experimental test of behavioral discrimination in NI saddlebacks exposed to familiar and unfamiliar MRS and found that responses were significantly stronger for familiar MRS, consistent with a model of contemporary cultural evolution leading to discrimination between geographic song variants. Significantly, this result demonstrates the rapid tempo with which discrimination of CRS might evolve within isolated populations and supports both bottleneck and cultural mutation hypotheses in CRS evolution. The evolutionary implications of contemporary cultural evolution in the production and perception of CRS merit debate on the time frames over which conservation management is evaluated.

2584: ***+.018***

Endangered species recovery plans are frustrated by small, spatially structured populations where understanding the influence of birth, death, and dispersal is difficult. Here we use a spatially explicit, long-term study to describe dispersal in the Cape Sable seaside sparrow (Ammodramus maritimus mirabilis). Since 1990, this species declined > 50%. It occurs as several geographically isolated subpopulations in the Florida Everglades. We characterize dispersal, recognizing that our sampling, as well as the species' distribution, is spatially heterogeneous. The annual movements of juveniles and adults are statistically heavy-tailed. That is, while most individuals are recaptured locally, a significant portion exhibit long-distance dispersal. Individuals move between subpopulations to distances > 30 km. Not accounting for the spatial heterogeneity of sampling or the species range itself underestimates dispersal and can lead to ineffective management decisions. Recovery focused on translocation will be less successful than strategies that protect habitat and increase breeding.

2585: ***+.190***

It would be much easier to assess the effectiveness of different reintroduction methods, and so improve the success of reintroductions, if there was greater standardization in documentation of the methods and outcomes. We suggest a series of standards for documenting and monitoring the methods and outcomes associated with reintroduction projects for birds. Key suggestions are: documenting the planned release before it occurs, specifying the information required on each release, postrelease monitoring occurring at standard intervals of 1 and 5 years (and 10 for long-lived species), carrying out a population estimate unless impractical, distinguishing restocked and existing individuals when supplementing populations, and documenting the results. We suggest these principles would apply, largely unchanged, to other vertebrate classes. Similar methods could be adopted for invertebrates and plants with appropriate modification. We suggest that organizations publically state whether they will adopt these approaches when undertaking

reintroductions. Similar standardization would be beneficial for a wide range of topics in environmental monitoring, ecological studies, and practical conservation.

2586: *+.449*

Plant conservation urgently needs a concept that would unify different aspects of population viability as parts of conservation methodology. Such unification is especially lacking for ex situ conservation. We introduce a novel conservation approach in which ex situ collections maintained in natural or semi-natural environment and preserving both neutral and adaptive genetic diversity are a part of a complementary ex situ-in situ conservation strategy. Our approach is the first that explicitly takes into account ecologically significant (i.e. adaptive) variation of plants in both ex situ and in situ conservation actions. Using this approach we provide detailed guidelines for (1) representative sampling of the populations; (2) collection maintenance; and (3) utilization for in situ actions.

2587: *+.148*

In South Africa, more than 30 small, enclosed game reserves have reintroduced lions over the last two decades, which now house more than 500 individuals. There is a high risk of inbreeding in these fragmented, fenced and isolated populations, which may be compounded by a lack of management guidelines. A population of 11 founder lions Panthera leo was reintroduced to Madikwe Game Reserve in 1995, and this population has in turn become a source for reestablishing other populations. Only four lineages were reintroduced, founder males were related to founder females, and since 1997, only one male lineage maintained tenure for > 9 years, resulting in breeding with direct relatives. Interventionist management to limit lion population growth and inbreeding in Madikwe has taken the form of translocating, trophy hunting and culling of mainly sub adult lions. Despite this management, inbreeding started 5 years after reintroduction. Reproductive performance and thus population growth in Madikwe were dependent on the overall lion population density. When lion density was low, females first gave birth at a significantly younger age and produced larger litters, resulting in a high population growth rate, which decreased significantly when lion density in the park reached carrying capacity, that is, 61 lions. This might have profound consequences for future reestablishment of lion populations when restocking new reserves: our study illustrates the need for founder populations of reintroduced endangered predator species to be as large and genetically diverse as possible, and thereafter new genetic material should be supplemented. The development of such management guidelines is becoming very important as large predator populations become increasingly fragmented and managed as metapopulations.

2588: *+.211*

Headstarting has become a popular tool employed by wildlife managers to help animal species, specifically those lacking or providing minimal parental care-offset extinction. However, many researchers challenge the conservation value of headstarting and urge proponents to monitor headstarted individuals following release into the wild to evaluate the success of headstart programs. As part of an experimental headstarting program managed by the Iguana Verde Foundation in Costa Rica, we conducted a 1.5-month radiotelemetry study of 11 headstarted 2 year old green iguanas (Iguana iguana) following their release into the wild at the Gandoca-Manzanillo Wildlife Refuge. Headstarted iguanas were compared to their wild counterparts (two radiotelemetered and 18 opportunistically-encountered) with respect to changes in growth, arboreal microhabitat use, social aggregation, activity ranges and movements. Male and female

headstarted iguanas exhibited similar behaviours and headstarted iguanas were similar to wild iguanas for most variables measured. Thus, the headstarted green iguanas were clearly capable of short-term (1.5-month) survival in the wild and their apparently normal behaviours reflected the suitable conditions under which they were raised. The results provide insight into the ecology of green iguanas and will help guide headstarting and reintroduction programs for iguanas at this location and endangered iguanas elsewhere.

2589: ***+.208***

Habitat selectivity by European beaver (Castor fiber L., 1758) was studied in 226 km of river channels during their colonization of the Morava River basin (the Czech Republic), which had not been occupied by beavers for hundreds of years. The colonization started after initial reintroductions in 1991 and 1992. Annual increases in colonization of the river system from 1995 to 2007 were 15.5 +/- 9.4 SD km year(-1) and varied greatly between these years (min 0 km, max 33 km). Beavers appeared to be following a dispersal pattern, in which distant sites are often colonized before close-by sites. The selection of habitat variables during the colonization process varied. In the early phase of colonization, there were many areas with optimal habitat that were not occupied by beavers, and habitat selection appeared chaotic. After this early phase, the most significant habitat variable related to beaver occupation was shown to be the presence of willow (Salix spp.) species. In the later phases, the beaver population expanded into suboptimal habitat. Settlement distance from roads, railways, and urbanized areas became less. When comparing the generalized linear models, a model from the winter of 2003/2004 had the best overall accuracy and showed excellent agreement among observed and fitted values (Cohen's kappa = 0.75). The model suggests that beavers established their home ranges at first in optimal habitat, which had not been occupied before and then subsequently in suboptimal/marginal areas.

2590: ***-.070***

In this study, we investigated what problems urban marmosets (Callithrix penicillata) face in a city environment through the analysis of responses to callouts (N = 348) made by the environmental police of Belo Horizonte, Minas Gerais, Brazil, in the period from 2002 to 2007. Our objective was to characterise the problems faced by the marmosets and human city dwellers. The environmental police responded to two types of callouts: (1) solicitation whereby a person called them to report a problem (N = 218); and (2) the report of a hurt or injured animal (N = 127). On average, one callout per week was made in relation to urban marmosets. We found no time of year effects in relation to callouts, or any effect of gender or age of the person making the callout (P > 0.05). Furthermore, we found no environmental (e.g. percentage of "green area") or socioeconomical variables (e.g. salary levels) of the city's administrative regions associated with callouts (P > 0.05). The majority of callouts resulted in the attempt to capture marmosets (N = 345), and usually, only one animal was captured (N = 309). Many of these animals were released into city forest fragments (N = 146). Some sick animals were sent to veterinary clinics (N = 25) whereas others or confiscated animals were sent to the government's wildlife processing centre (N = 143). From this data, we were able to make a series of recommendation about how the management of urban marmosets could be improved.

2591: ***+.249***

Background: The knowledge of both potential distribution and habitat suitability is fundamental in spreading species to inform in advance management and conservation planning. After a severe decline in the past decades, the griffon vulture (Gyps fulvus) is now spreading its breeding range

towards the northwest in Spain and Europe. Because of its key ecological function, anticipated spatial knowledge is required to inform appropriately both vulture and ecosystem management.Methodology/Findings: Here we used maximum entropy (Maxent) models to determine the habitat suitability of potential and current breeding distribution of the griffon vulture using presence-only data (N = 124 colonies) in north-western Spain. The most relevant ecological factors shaping this habitat suitability were also identified. The resulting model had a high predictive performance and was able to predict species' historical distribution. 7.5% (similar to 1,850 km(2)) of the study area resulted to be suitable breeding habitat, most of which (similar to 70%) is already occupied by the species. Cliff availability and livestock density, especially of sheep and goats, around 10 km of the colonies were the fundamental factors determining breeding habitat suitability for this species.Conclusions/Significance: Griffon vultures could still spread 50-60 km towards the west, increasing their breeding range in 1,782 km(2). According to our results, 7.22% of the area suitable for griffon vulture will be affected by wind farms, so our results could help to better plan wind farm locations. The approach here developed could be useful to inform management of reintroductions and recovery programmes currently being implemented for both the griffon vulture and other threatened vulture species.

2592: *+.094*

Genetic methods for the reintroduction of primates Saguinus, Aotus and Cebus (Primates: Cebidae) seized in Bogota, Colombia. Primates are one of more confiscated taxa by the environmental authorities in Bogota, Colombia. During 2008, 133 monkeys were confiscated; samples from 115 of them were sequenced by the mitochondrial cythocrome oxidase II gene (mtCOII) and 112 sequences obtained were of high quality. These sequences were compared with those obtained by our research group from individuals directly sampled in the field, with precise geographic origin. So, a more specific geographic area of the Colombian territory could be considered for a correct rehabilitation treatment during the reintroduction of these confiscated animals. The main results with five primate species were: 1- For all the specimens analyzed of Saguinus copus, they could be liberated in any geographical area of its distribution range, since only one gene pool was found. 2- For the 14 Aorta sp. individuals sequenced from the SDA (Environmental District Secretariat), one of them (A. vociferans) was coming from the Amazon, seven exemplars belonged to A. griseimembra from the Magdalena Valley and the Colombian Caribbean coasts, four individuals represented to A. brumbacki from the Colombian Eastern Llanos, and two were associated to A. azarae azarae from Northern Argentina and Paraguay (which means that illegal traffic of animals is arriving to Colombia from other South-American countries). 3-Out 14 Cebus albifrons sequenced, two belonged to the geographical area of C. a. versicolor, one to C. a. pleei, 10 to C. a. leucocephalus and one could be not assigned because its sequence yielded a great genetic divergence with respect to the other specimens sequenced of this species. 4- The two Cebus capucinus sequenced showed to be associated to a gene pool found in the Northern of Choco, Sucre and Cordoba Departments. 5- Out 11 Cebus apella sequenced, 10 showed to belong to the gene pool presented in the Colombian Eastern Llanos and highly related (but differentiable) to Cebus apella open from the French Guyana. It could be named C. a. fatuellus sensu Groves (2001). One exemplar sequenced could be not related with the other C. apella analyzed, nor the related taxa to the aforementioned species =C. a. paraguayanus C. cay; C. xanthosternos; C. nigritus). Rev. Biol. Trop. 58(3): 1049-1067. Epub 2010 September 01.

2593: *+.243*

The iconic plains bison (Bison bison) have been reintroduced to many places in their former range, but there are few scientific data evaluating the success of these reintroductions or guiding the

continued management of these populations. Relying on mark-recapture data, we used a multistate model to estimate bison survival and breeding transition probabilities while controlling for the recapture process. We tested hypotheses in these demographic parameters associated with age, sex, reproductive state, and environmental variables. We also estimated biological process variation in survival and breeding transition probabilities by factoring out sampling variation. The recapture rate of females and calves was high (0.78 +/- 0.15 [SE]) and much lower for males (0.41 +/- 0.23), especially older males (0.17 +/- 0.15). We found that overall bison survival was high (>0.8) and that males (0.80 +/- 0.13) survived at lower rates than females (0.94 +/- 0.04), but as females aged survival declined (0.89 +/- 0.05 for F >= 15 yr old). Lactating and non-lactating females survived at similar rates. We found that females can conceive early (approx. 1.5 yr of age) and had a high probability (approx. 0.8) of breeding in consecutive years, until age 13.5 years, when females that were non-lactating tended to stay in that state. Our results suggest senescence in reproduction and survival for females. We found little support for the effect of climatic covariates on demographic rates, perhaps because the park's current population management goals were predicated from drought-year conditions. This reintroduction has been successful, but continued culling actions will need to be employed and an adaptive management approach is warranted. Our demographic approach can be applied to other heavily managed large-ungulate systems with few or no natural predators.

2594: *+.050*

We live-trapped American black bears (Ursus americanus) and sampled DNA from hair at White River National Wildlife Refuge, Arkansas, USA, to estimate annual population size (N), growth (lambda), and density. We estimated N and lambda with open population models, based on live-trapping data collected from 1998 through 2006, and robust design models for genotyped hair samples collected from 2004 through 2007. Population growth was weakly negative (i.e., 95% CI included 1.0) for males (0.901, 95% CI = 0.645-1.156) and strongly negative (i.e., 95% CI excluded 1.0) for females (0.846, 95% CI = 0.711-0.981), based on live-trapping data, with N from 1999 to 2006 ranging from 94.1 (95% CI = 70.3-137.1) to 45.2 (95% CI = 27.1-109.3), respectively, for males and from 151.4 (95% CI = 127.6-185.8) to 47.1 (95% CI = 24.4-140.4), respectively, for females. Likewise, mean annual l based on hair-sampling data was weakly negative for males (0.742, 95% CI = 0.043-1.441) and strongly negative for females (0.782, 95% CI = 0.661-0.903), with abundance estimates from 2004 to 2007 ranging from 29.1 (95% CI = 21.2-65.8) to 11.9 (95% CI = 11.0-26.9), respectively, for males and from 54.4 (95% CI = 44.3-77.1) to 27.4 (95% CI = 24.9-36.6), respectively, for females. We attribute the decline in the number of females in this isolated population to a decrease in survival caused by a past translocation program and by hunting adjacent to the refuge. We suggest that managers restructure the quota-based harvest limits until these growth rates recover.

2595: *+.088*

When 2 populations are mixed for whatever reason, the outcome can be difficult to predict. In 1993, 2 populations of an atyid shrimp (Paratya australiensis:Atyidae) from different subcatchments of the Brisbane River, Queensland, Australia, were mixed as a result of a translocation. At the time, they were thought to represent slightly divergent populations of a single species. However, subsequent molecular analysis showed that they were significantly divergent. An analysis of patterns at one of the sites in 2001 suggested that the translocated lineage might be sending the resident population extinct in one of the streams. Analysis of mitochondrial deoxyribonucleic acid (DNA) and 3 allozyme loci indicated that the explanation for the outcome might be related to asymmetrical hybridization and nonviability of hybrids between resident

females and translocated males. We present data for 2002 and an analysis of samples that had been collected prior to the 2001 study, but not analyzed. We tested 3 hypotheses: 1) the translocated genotypes would continue to increase in relative frequency at sites above the translocation site because their site of origin was at higher altitudes, to which they were expected to be better adapted; 2) at intermediate sites, the relative frequency of translocated alleles in juveniles would increase relative to in their parents; and 3) resident genotypes would survive better than translocated genotypes within a generation, especially at lower and intermediate elevations. Translocated genotypes increased extremely rapidly at the most upstream site. Consistent evidence was present for higher relative frequency of translocated alleles in juveniles relative to their parents, both in the generation following the 2001 study and in the pre-2001 samples. With the exception of in the upstream site, translocated genotypes had lower fitness within a generation than resident genotypes. Thus, the translocated genotypes had a higher reproductive success but were less well adapted to their local environment than residents, a situation that decreased population fitness overall. Such an outcome demonstrates a rarely reported effect of translocations and mixing of divergent lineages.

2596: ***-.003***

The population of giant Canada geese (Branta canadensis maxima) breeding in eastern South Dakota has increased dramatically since reintroduction efforts began in the 1960s. May breeding population levels of giant Canada geese exceeded population management goals set by the South Dakota Department of Game, Fish and Parks (SDGFP) by the mid-1990s, and the population has continued to increase into the 2000s. This population increase was accompanied by an increase in goose-related conflicts such as crop depredation. In 1996, a September hunting season was implemented in select counties in eastern South Dakota in an effort to reduce the giant Canada goose population. After its implementation, some hunters and biologists were concerned that the early September season was causing Canada geese to disperse from areas open to hunting due to hunting pressure. Herein, we describe post-molt movements by geese, particularly in relation to the September hunting season. We caught Canada geese in 7 counties in eastern South Dakota during the summer molting period, 2000 to 2003. We attached VHF (n = 153) and satellite transmitters (n = 43) on adult female geese with broods. We monitored movements of marked geese weekly from July through the fall freezing period. For this study, we considered major movements any post-molt movement >= 40 km from the wetland in which the goose was banded prior to October 15. Forty-six percent of marked geese made major movements from July to September, and 43% moved during the first week of the September season, indicating that the season may have triggered their post-molt movement. Major movements were primarily in a northerly direction, and the longest documented post-molt movement was 474 km north. It appears that the onset of the September hunting season may have caused geese to move immediately before or during the first 10 days of the season. Post-molt movements prior to the September hunting season may simply have been a function of established, learned traditions, but the punctuated movement of geese during the opening weekend of the hunting season may have resulted from geese responding to the hunting season itself.

2597: ***+.122***

The population of giant Canada geese (Branta canadensis maxima) in eastern South Dakota has increased substantially since reintroduction efforts began in the 1960s. Breeding population estimates of Canada geese exceeded the population management objective of the South Dakota Game, Fish and Parks by the mid-1990s and has continued to increase at an estimated rate of 3 to 5% per year. Goose-related crop damage complaints have also increased. In 1996, a September

hunting season (September 1 to 15) was implemented in 10 counties in eastern South Dakota and was expanded in 2000 to include most of eastern South Dakota. We initiated this study during 2000 to 2004 to estimate survival, harvest, and recovery rates of giant Canada geese. We captured and leg-banded Canada geese in 7 counties in eastern South Dakota during the summers of 2000 to 2003. Of the total leg-banded sample (n = 3,839), we recovered 648 bands during the same year that they were placed on geese (i.e., direct harvest rate), and we recovered 645 banded geese in later years (i.e., indirect recovery rate). Estimates of annual survival rate (95% Cl) for adults and immatures were 0.52 (0.46 to 0.59) and 0.68 (0.57 to 0.79), respectively. Estimates of annual recovery rates (95% Cl) for adult and immature geese were 0.16 (0.13 to 0.19) and 0.18 (0.14 to 0.21), respectively. Of the total recoveries, 77 and 69% of direct and indirect band recoveries, respectively, occurred in South Dakota. The composite harvest rate estimate during the period studied was 0.22 (0.20 to 0.24). Forty-nine percent of adult recoveries and 44% of immature recoveries (direct and indirect pooled for both age classes) occurred during the September season. In comparison to a previous band-recovery study of resident giant Canada geese in eastern South Dakota, survival rates for both adult and immature geese have declined, while recovery and harvest rates have increased. Survival estimates for this study were some of the lowest documented for giant Canada geese. However, it appears that even with a September hunting season targeting the local breeding population, declines in adult survival documented during this study are not reducing the population. Alternative management strategies may be necessary to reduce the population to achieve the management objective.

2598: *+.015*

Food composition of otter, Lutra lutra, was studied by the analysis of 349 spraints found during one year period (2003-2004) at the River Kamenice (Czech Republic), where Atlantic salmon, Salmo salar, fry have been stocked regularly since 1998 in a reintroduction programme for the species. Brown trout, Salmo trutta m. fario, dominated otter diet and formed 29% of all prey items and 62% of biomass of all fish eaten. The second most abundant prey (27%) was common sculpin, Cottus gobio, followed by Atlantic salmon, and grayling, Thymallus thymallus. The proportion of salmon in the diet of otters amounted to 14.5% in numerical abundance of all prey items taken and 2% in biomass of fish component of the diet. The majority (71.5%) of fish eaten by otters had a total length between 61 to 200 mm.

2599: *+.174*

In Central Europe several plant species of dry grasslands are particularly rare. Here I investigate whether habitat requirements, reproduction, and dispersal potential can contribute to the rarity of Astragalus exscapus (Fabaceae) growing in dry grassland habitats in dry regions of Europe. In addition, I question whether historic events might have contributed to the present-day rarity of A. exscapus. To assess habitat requirements of A. exscapus, vegetation composition and soil characteristics were studied in 37 populations in central Germany. Production and dispersal potential of seeds were investigated in 10 populations, and germination and recruitment were assessed in experimental plots in three populations. Vegetation of the habitats included most dry grassland community types occurring in the central German dry region indicating a broad ecological niche of the species within dry subcontinental grasslands. Soil characteristics of the habitats also spanned a wide range. Seed production was moderate. 98% of the seeds sown in the laboratory germinated whereas under natural conditions 20% of the seeds developed seedlings. Half of these seedlings survived for one year but only 4.5% for two years. 90% of the seeds were dispersed less than 50 cm distance indicating a low dispersal potential. I conclude that A. exscapus is mainly limited in dispersal but recruitment limitation might also be important in explaining its

rarity. Furthermore, former climate change and postglacial reforestation of the area very likely contribute to the rarity of A. exscapus.

2600: *+.104*

Dams without fish passage facilities block access to much of the historic spawning habitat of spring Chinook salmon (Oncorhynchus tshawytscha) in Oregon's Willamette River basin. Adult salmon are routinely outplanted above the dams to supplement natural production, but many die before spawning despite extensive suitable habitat. In 2004-2007, we examined prespawn mortality patterns using live detection and carcass recovery data for 242 radio-tagged outplants. Total prespawn mortality was 48%, but variability was high, ranging from 0% to 93% for individual release groups. Prespawn mortality was strongly condition dependent, consistently higher for females than males and higher for early release groups. Across years, warm water temperature in the migration corridor and at the collection site was associated with sharply higher mortality. Results highlight a need for better evaluations of the effects of adult mortality on population reintroduction and recovery and relationships among prespawn mortality, dam-related temperature change and salmon life history and behaviour.

2601: *+.019*

The Eastern Bristlebird, Dasyornis brachypterus, is an endangered species occurring in a limited number of locations in eastern Australia. Over its distribution, D. brachypterus is confronted by continuing loss and fragmentation of its habitat and catastrophic fire events. To aid the development of a long-term plan for the protection and management of D. brachypterus, we developed ten polymorphic microsatellite markers to characterise levels of genetic diversity and the degree of population subdivision of remnant populations. Genotyping a small number of birds (n = 25) revealed between 6 and 11 alleles per locus, high expected heterozygosity (range = 0.72-0.89), and a fit to Hardy-Weinberg equilibrium for nine of ten loci. This set of microsatellite markers provides the capacity for genetic research to be incorporated in a recovery plan for D. brachypterus, including informing managers of the scale over which translocation or supplementation should be conducted.

2603: *+.204*

Maintaining animal movement in fragmented landscapes depends upon the levels of connectivity among habitat patches, which in turn may depend upon the landscape matrix. Little is known about how the matrix affects dispersal abilities, in part because few experimental tests have been conducted. We experimentally translocated 142 migratory American Redstarts (Setophaga ruticilla) and resident Jamaican Todies (Todus todus) 0.6-4 km from their territories across landscapes fragmented by pen-urban development and bauxite mining and continuous forest. Redstarts returned more rapidly and with greater success than todies across all landscapes, with 95% of redstarts returning in an average of 2.5 days versus 60% of todies in >20 days. Return success was best predicted by translocation distance for redstarts and by sex for todies, with a trend of fewer birds returning when released in bauxite landscapes. Return time was strongly affected by matrix type, with both species returning more rapidly in a forested relative to a bauxite matrix and intermediately in a pen-urban matrix. These findings provide strong experimental evidence that land cover surrounding forested habitat influences species mobility. (C) 2010 Elsevier Ltd. All rights reserved.

2604: *+.216*

Eurasian lynx in Scandinavia are subject to regular harvest and lethal control to reduce depredation on domestic livestock and semi-domestic reindeer. Here we introduce the use of total reproductive value to model the effects of current harvest on population dynamics and to propose sustainable harvest strategies for lynx. Demographic stochasticity strongly influences lynx population dynamics. Analyses of the number of lynx shot in relation to the number of family groups registered in annual censuses showed proportional harvest in large parts of Norway because the quotas were higher at larger population sizes. In other areas of Norway the number of lynx shot was independent of population size. The analyses of the model showed that a pure proportional harvest strategy may lead to rapid extinction of lynx populations. In contrast, applying a threshold or proportional threshold harvest strategy in which no harvest occurs below a given threshold can result in the maintenance of viable populations. Thus, this study shows that harvest without any lower threshold for stopping harvest will result in rapid extinction of lynx populations. Accordingly, lynx harvest is not likely to be sustainable if the illegal killing of animals is not controlled because poaching can result in a de facto proportional harvest even at very small population sizes. Under the influence of the large demographic stochasticity in lynx populations this harvest would result in short expected times to extinction. This gives an empirical demonstration that a correct choice of harvest strategy is essential for maintenance of viable populations of harvested species. Our analyses illustrate that parameters determining the viability of small populations can be estimated from individual-based demographic data from a sample of individuals without using time series of fluctuations in population size, which facilitates quantitative analyses of how harvest or removal of individuals, e.g. for captive breeding or translocations, affect the expected lifetime of populations. (C) 2010 Elsevier Ltd. All rights reserved.

2605: *+.215*

The range-wide habitat status of many endangered species is unclear. We evaluated the status and spatial distribution of the habitat of the endangered giant panda (Ailuropoda melanoleuca) across its entire geographic range (i.e., six mountain regions located in Sichuan, Shaanxi and Gansu provinces, China) by integrating field and remotely sensed data to develop a habitat distribution model. Results suggest that current suitable habitat corresponds to ca. 1/4 of the habitat baseline (i.e., maximum amount of habitat possible). The highest proportion of suitable habitat relative to the baseline is in the Qinling mountain region. Overall, around 40% of the suitable habitat is inside nature reserves, but the proportion of habitat inside them varied among different mountain regions, ranging from ca. 17% (Lesser Xiangling) to ca. 66% (Qinling). The habitat model also predicted the occurrence of potentially suitable habitat outside the currently accepted geographic range of the species, which should be further evaluated as potential panda reintroduction sites. Our approach is valuable for assessing the conservation status of the entire habitat of the species, for identifying areas with significant ecological roles (e.g., corridors), for identifying areas suitable for panda reintroductions, and for establishing specific conservation strategies in different parts of the giant panda geographic range. It might also prove useful for range-wide habitat analyses of many other endangered species around the world. (C) 2010 Elsevier Ltd. All rights reserved.

2606: *+.336*

I discuss future challenges and opportunities in genetic approaches to biodiversity conservation. Resolving taxonomy uncertainties and identifying diverged evolutionary units within species are both bedevilled by a plethora of definitions: the challenge for the conservation community is to come to an agreed definition of species and for a unit within species for conservation purposes. For genetic management in the wild, the main challenge is to apply well-established genetic

principles to management, especially of fragmented populations. Fears about outbreeding depression are preventing rational use of gene flow for genetic rescue: predicting the risk of outbreeding depression is the most important unmet scientific challenge in the field. The major challenge in genetic management of captive populations of threatened animal species is to institute explicit management to minimize genetic adaptation to captivity, so that reintroduction success is maximized. The development of low cost genome sequencing offers many research opportunities and challenges. For example, there are opportunities to identify genes involved in speciation and a major challenge is to devise molecular tests to predict reproductive isolation between populations. Genomics offers opportunities to provide higher precision estimate for many parameters of importance to conservation. A major challenge is to devise means to assess, on a genome-wide basis, genetic diversity that is important to adaptive evolution. There is a challenge to develop simple inexpensive means to monitor genetic diversity of species on a global scale. Many of the most important practical challenges concern application of current genetic knowledge to the management of threatened species. (C) 2010 Elsevier Ltd. All rights reserved.

2607: *+.098*

Pacifastacus leniusculus (an invasive species in European water bodies) was detected for the first time in the Andalusia Region (S. Spain) in the year 2000. Since 2005, a continuous control management programme has been carried out by the Environmental Regional Government. Management efforts aimed to reduce the population size, to contain the dispersal and reduce the probability of deliberate translocation into other rivers caused by illegal captures. A combination of techniques was used, including crayfish traps, manual removal from artificial refuges and electrofishing. In the 2005-2009 period, 31 374 specimens were captured. The mean catch rate per worker and day declined from 30.4 +/- 3.2 specimens in the first year to 9.8 +/- 1.7 in the fourth year, therefore suggesting a sharp decrease in population size. Summer was the period of mating and maximum yields, whereas minimum yields were obtained in Winter, coinciding with egg incubation in burrows. The results obtained and the experience gained will provide essential baseline information for the future management of non-native crayfish in the region.

2608: *+.133*

Density-dependent feedback mechanisms provide insights into the population dynamics and interactions of large herbivores with their ecosystem. Sex ratio also has particularly important implications for growth rates of many large mammal populations through its influence on reproductive potential. Therefore, the interrelationships between density-dependent factors, comprising density, sex ratio and underlying growth rates (r) were examined for the Eastern black rhino (Diceros bicornis michaeli) living in three rhino sanctuaries in Kenya using four population models. The exponential and logistic models gave similar results and the former were accepted because they better portrayed the actual situation on the ground. Sex ratios in all sanctuary populations were positively correlated with r but interpreted with realization of other factors also affecting r. We caution that the results of population models should be interpreted alongside ground-truthed observations. We recommend that future translocation strategies should take into account sex and age structures of the donor population, while future studies of density dependence should take into account both biotic and abiotic factors. ResumeDes mecanismes de feedback de densite-dependance chez les grands herbivores donnent un apercu de la dynamique des populations et de l'interaction avec l'ecosysteme. Les sex-ratios ont aussi d'importantes implications pour la dynamique des populations de nombreux grands mammiferes, specialement par leur influence sur le potentiel reproducteur. On a etudie ces relations croisees entre les facteurs densite-dependants du rhino noir de l'Est Diceros bicornis michaeli, le sex-ratio et le taux de

croissance (r) sous-jacent dans trois sanctuaires de rhinos du Kenya en utilisant quatre modeles de population. Les modeles exponentiel et logistique donnaient des resultats similaires, les resultats du premier etant acceptes parce qu'ils representaient la situation actuelle sur le terrain. Les sex-ratios de toutes les populations etaient positivement lies ar mais interpretes en realisant que d'autres facteurs affectent aussi r. Nous attirons l'attention sur le fait que les resultats de la modelisation des populations doivent etre interpretes tout en les confirmant par des observations sur le terrain; nous recommandons des strategies de translocations qui prelevent des individus dans les diverses structures de sexe et d'age de la population d'origine; et nous suggerons que de futures etudes de densite-dependance tiennent compte de facteurs biotiques et abiotiques.

2609: *+.205*

Background: Parrots are one of the most frequently kept and bred bird orders in captivity. This increases poaching and thus the potential importance of captive populations for rescue programmes managed by zoos and related institutions. Both captive breeding and poaching are selective and may be influenced by the attractiveness of particular species to humans. In this paper, we tested the hypothesis that the size of zoo populations is not only determined by conservation needs, but also by the perceived beauty of individual parrot species assessed by human observers.Methodology/Principal Findings: For the purpose of data collection, we defined four sets of species (40 parrots, 367 parrots, 34 amazons, 17 macaws). Then, we asked 776 human respondents to evaluate parrot pictures of the selected species according to perceived beauty and we analyzed its association with color and morphological characters. Irrespective of the species set, we found a good agreement among the respondents. The preferred species tended to be large, colorful, and long-tailed.Conclusions/Significance: We repeatedly confirmed significant, positive association between the perceived beauty and the size of worldwide zoo population. Moreover, the range size and body size appeared to be significant predictors of zoo population size. In contrast, the effects of other explanatory variables, including the IUCN (International Union for Conservation of Nature) listing, appeared insignificant. Our results may suggest that zoos preferentially keep beautiful parrots and pay less attention to conservation needs.

2610: *+.044*

Little is known about the ecology of the Chinese Giant Salamander (Andrias davidianus), a critically endangered species. Such information is needed to make informed decisions concerning the conservation and management of this species. Four A. davidianus raised in a pool were released into their native habitat on 04 May 2005 and were subsequently radio-tracked for approximately 155-168 days. Following their release, the giant salamanders traveled upstream in search of suitable micro-habitats, and settled after 10 days. Later, a devastating summer flash flood destroyed the salamanders' dens, triggering another bout of habitat searching by the animals. Eventually, the salamanders settled in different sections of the stream where they remained until the end of the study. On average, each habitat searching endeavor took 7.5 days, during which a giant salamander explored a 310 m stretch of stream with a surface area of about 1157 m(2) and occupied 3.5 temporary dwellings. Each giant salamander spent an average of 144.5 days in semi-permanent micro-habitats, and occupied territories that had a mean size of 34.75 m(2). Our results indicate that the Chinese giant salamander responds to habitat disturbance by seeking new habitats upstream, both water temperature and water level affect the salamander's habitat searching activity, and the size of the salamander's semi-permanent territory is influenced by the size of the pool containing the animal's den.

2611: *+.248*

The successful re-introduction of grey wolves to the western United States is an impressive accomplishment for conservation science. However, the degree to which subpopulations are genetically structured and connected, along with the preservation of genetic variation, is an important concern for the continued viability of the metapopulation. We analysed DNA samples from 555 Northern Rocky Mountain wolves from the three recovery areas (Greater Yellowstone Area, Montana, and Idaho), including all 66 re-introduced founders, for variation in 26 microsatellite loci over the initial 10-year recovery period (1995-2004). The population maintained high levels of variation (H-O = 0.64-0.72; allelic diversity k = 7.0-10.3) with low levels of inbreeding (F-IS < 0.03) and throughout this period, the population expanded rapidly (n(1995) = 101; n(2004) = 846). Individual-based Bayesian analyses revealed significant population genetic structure and identified three subpopulations coinciding with designated recovery areas. Population assignment and migrant detection were difficult because of the presence of related founders among different recovery areas and required a novel approach to determine genetically effective migration and admixture. However, by combining assignment tests, private alleles, sibship reconstruction, and field observations, we detected genetically effective dispersal among the three recovery areas. Successful conservation of Northern Rocky Mountain wolves will rely on management decisions that promote natural dispersal dynamics and minimize anthropogenic factors that reduce genetic connectivity.

2612: *-.071*

Probably no conservation genetics issue is currently more controversial than the question of whether grey wolves (Canis lupus) in the Northern Rockies have recovered to genetically effective levels. Following the dispersal-based recolonization of Northwestern Montana from Canada, and reintroductions to Yellowstone and Central Idaho, wolves have vastly exceeded population recovery goals of 300 wolves distributed in at least 10 breeding pairs in each of Wyoming, Idaho and Montana. With > 1700 wolves currently, efforts to delist wolves from endangered status have become mired in legal battles over the distinct population segment (DPS) clause of the Endangered Species Act (ESA), and whether subpopulations within the DPS were genetically isolated. An earlier study by vonHoldt et al. (2008) suggested Yellowstone National Park wolves were indeed isolated and was used against delisting in 2008. Since then, wolves were temporarily delisted, and a first controversial hunting season occurred in fall of 2009. Yet, concerns over the genetic recovery of wolves in the Northern Rockies remain, and upcoming District court rulings in the summer of 2010 will probably include consideration of gene flow between subpopulations. In this issue of Molecular Ecology, vonHoldt et al. (2010) conduct the largest analysis of gene flow and population structure of the Northern Rockies wolves to date. Using an impressive sampling design and novel analytic methods, vonHoldt et al. (2010) show substantial levels of gene flow between three identified subpopulations of wolves within the Northern Rockies, clarifying previous analyses and convincingly showing genetic recovery.

2613: *+.091*

Chemical communication in mammals includes an array of specific behaviours that are often ignored in terms of their potential relevance to conservation. Often used during territorial or social interactions between animals, chemical communication can also be used as a tool in reintroduction programmes. Reintroductions still exhibit high failure rates and methods to improve success should be investigated. The Eurasian beaver Castor fiber has been widely reintroduced across Europe after its near extinction in the 19th century. Using olfactory studies in the beaver, we aim to demonstrate how scent transfers a range of information about the sender which can be used to monitor social and territorial behaviour along with general well-being. Scent manipulation can be

used to reduce human-beaver conflicts, and aid reintroduction success through reducing stress and territorial conflicts, and by influencing dispersal and settlement. Two species of beavers, the Eurasian beaver and the North American beaver Castor canadensis, occupy freshwater habitats throughout North America and in parts of South America, most of Europe and parts of Asia. Most of the reviewed literature concerns the wild Eurasian beaver, its chemical communication and conservation; however, captive studies and those addressing North American beavers are also included. Chemical communication is advanced and has been well documented in this highly territorial species. However, few studies directly link olfaction with conservation practices. Olfactory studies in beavers can provide non-invasive methods to monitor translocated animals and indicators of health. We conclude that chemical analysis, olfactory studies and behavioural manipulations involving semiochemicals have important impacts on conservation and can generate practical solutions to conservation problems including aiding animal capture, captive stress reduction, breeding pair formation and release site fidelity.

2614: *+.184*

The goal of the Endangered Species Act is to improve the chances of listed species' survival by increasing population levels (US Fish and Wildlife Service in American burying beetle (Nicrophorus americanus) recovery plan. Newton Corner, MA, p 80, 1991). If successful, a species will be delisted, but in order to achieve the goal of species recovery the demography, habitat preferences, reproductive biology, and cause of the species decline must be understood. Like many rare invertebrates, information about the endangered American burying beetle (Nicrophorus americanus) prior to listing consisted of the taxonomic description and morphological characterization. Surveys for N. americanus provide data that can be integrated into spatial models to help predict suitable habitat. Our objective was to model the potential distribution of N. americanus and to evaluate these models ability to generate maps of potential habitat, thus focusing recovering efforts. We chose six modelling algorithms that utilized both presence and absence data from beetle surveys conducted throughout eastern Oklahoma. Using area under the curve (AUC) as our evaluation statistic, we found that ten of the twelve models performed within the AUC index category of "potentially useful" (AUC 0.7-0.9). Models utilizing presence only data performed well compared to models built with presence/absence data. This may indicate the weakness of using absence data to indicate unsuitable habitat. Lack of integration into the model of biotic interactions may also be affecting model performance. To improve model performance, the causes of N. americanus's endangered status and its population shrinkage should be considered. Although the best models were not highly accurate, the map of suitable habitat can help to inform conservation biologists of areas with a likelihood of N. americanus presence. Overgenerous models can mislead conservation planners in thinking that more areas are highly suitable. If resources are limited for planning preserves and areas of reintroduction, it may be better to be conservative and to limit consideration to the most suitable habitat.

2615: *-.101*

Translocation programs releasing animals into the wild need to assess the potential risks associated with the exchange of parasites and other pathogens between native and translocated species. We assessed the composition of the parasite communities in sympatric native and introduced primates. Over a 3-yr period we monitored the gastrointestinal parasites of 3 primate species living in the isolated ecosystem of Rubondo Island National Park, Tanzania: translocated chimpanzees (Pan troglodytes) and guerezas (Colobus guereza) and the indigenous vervets (Chlorocebus aethiops pygerythrus). We detected Troglodytella abrassarti and Enterobius cf. anthropopitheci only in chimpanzees and Chilomastix mesnili in chimpanzees and guerezas. In vervets, we recorded

Anatrichosoma sp. and Subulura sp., previously reported in Rubondo chimpanzees. We found Blastocystis sp., Giardia sp., Iodamoeba buetschlii, Entamoeba coli, Entamoeba spp., Trichuris sp., Strongyloides spp., spirurids (cf. Protospirura muricola), and undetermined strongylids in all 3 primate species. Considering the absence of Protospirura muricola in other wild populations of chimpanzees and guerezas, it has probably been acquired from the native vervets, as have Anatrichosoma sp. and Subulura sp. Lower parasite load in Rubondo chimpanzees, in comparison with wild populations at other study sites of this species, might be due to their stay in captivity in Europe before being released on the island. Despite a lack of any apparent health problems from infections in introduced Rubondo primates, parasite monitoring during reintroduction/introduction projects is necessary to decrease potential risks resulting from the exchange of parasites between translocated and native species.

2616: *+.237*

P>1. Anthropogenic environmental changes have the capacity to disrupt natural population dynamics. For amphibians with complex life cycles, it is important to understand how environmental perturbations interact with variation in larval period duration to affect the timing of dispersal and the abiotic conditions under which terrestrial movements occur.2. Widespread hybridization between California tiger salamanders and introduced tiger salamanders has created a situation in which length of larval development and metamorphic timing varies because of extrinsic variation in abiotic factors such as temperature, and also as a result of individual-specific variation in genomic admixture.3. We examined how line-cross type (e.g. F1, F2, backcrosses, etc.), morphology and temperature interact to affect the performance of emigrating immature salamanders. We performed endurance trials on a mechanized treadmill to simulate dispersal following metamorphosis. Our study provides insights into the interaction of environmental change and genomic composition on locomotor performance and the spread of the hybrid swarm.4. We found that temperature had a significant positive impact on endurance, potentially making it an important factor driving dispersal distances in the wild. Line-cross type also affected performance, with F1 hybrids demonstrating the greatest movement capacity. However, we did not observe an interaction between line-cross type and temperature.5. These results demonstrate that an increase in ambient temperature may enhance the dispersal ability of hybrid individuals and accelerate the spread of the hybrid swarm. An increase in the rate of introgression of non-native tiger salamander alleles into formerly pure native habitats places enhanced importance on the proper management of populations near the boundary of the hybrid swarm and emphasizes the need for management actions that prevent the intentional or accidental translocation of non-native species.

2617: *+.196*

Predators are predominantly valued for their ability to control prey, as indicators of high levels of biodiversity and as tourism attractions. This view, however, is incomplete because it does not acknowledge that predators may play a significant role in the delivery of critical life-support services such as ecosystem nutrient cycling. New research is beginning to show that predator effects on nutrient cycling are ubiquitous. These effects emerge from direct nutrient excretion, egestion or translocation within and across ecosystem boundaries after prey consumption, and from indirect effects mediated by predator interactions with prey. Depending on their behavioural ecology, predators can create heterogeneous or homogeneous nutrient distributions across natural landscapes. Because predator species are disproportionately vulnerable to elimination from ecosystems, we stand to lose much more from their disappearance than their simple charismatic attractiveness.

2618: ***+.001***

Scats from marine otters were collected from the entire Peruvian distribution range along the Pacific coast. Partial mtDNA control region sequences (265 bp) were successfully amplified and analysed in 37 out of 87 samples. Based on spatial distribution and home range information of marine otters we assumed our final data set to represent at least 24 different individuals, yielding surprisingly high genetic variability (11 haplotypes, h = 0.86, pi = 0.0117). No unequivocal evidence of genetic sub-structuring, a bottleneck or isolation by distance could be detected. This study presents the first genetic data in this endangered species and highlights the significance of the Peruvian gene pool for the establishment of reserves, potential future expansion, recolonisation or translocations.

2619: ***+.050***

For several centuries, game management has involved translocations of non-native individuals of many species to reinforce local native populations. However, there are few quantitative studies of potentially negative effects on population viability as expected when taxa with different local adaptations hybridise. The European red deer has been subject to particularly many translocations. Around 1900, a total of 17 red deer of Hungarian (Cervus elaphus hippelaphus) and German (C. e. germanicus) origin were introduced onto the island of Otteroya in Norway where few native red deer (C. e. atlanticus) remained n similar to 13). To assess interbreeding, the present stock on Otteroya and the indigenous Norwegian and Hungarian populations were characterised in 14 microsatellite loci and in the control region of mtDNA. An intermediate level of genetic variation in the Otteroya population and the presence of population specific alleles from both the indigenous Norwegian and the Hungarian population demonstrate that the introduced red deer interbred with the native. Even distributions of one indigenous and one non-indigenous mtDNA haplotype in the Otteroya population and two point estimates of admixture indicate similar genetic contributions from the two parental populations into the hybrid stock. Low numbers of migrants identified with Bayesian assignment tests demonstrate low recent gene flow from Otteroya into the Norwegian mainland population. The Otteroya hybrid stock has grown vastly in numbers during recent decades, suggesting a high population viability. We observed that the body mass of red deer on Otteroya was similar or greater than in adjacent indigenous Norwegian stocks, indicating that population performance has not been reduced in the hybrid stock and that gene flow probably has not had any negative effects.

2620: ***+.131***

In conservation biology, understanding the causes of endangerment is a key step to devising effective conservation strategies. We used molecular evidence (coalescent simulations of population changes from microsatellite data) and historical information (habitat and human population changes) to investigate how the most-isolated populations of giant pandas (Ailuropoda melanoleuca) in the Xiaoxiangling Mountains became highly endangered. These populations experienced a strong, recent demographic reduction (60-fold), starting approximately 250 years BP. Explosion of the human population and use of non-native crop species at the peak of the Qing Empire resulted in land-use changes, deforestation, and habitat fragmentation, which are likely to have led to the drastic reduction of the most-isolated populations of giant pandas. We predict that demographic, genetic, and environmental factors will lead to extinction of giant pandas in the Xiaoxiangling Mountains in the future if the population remains isolated. Therefore, a targeted conservation action-translocation-has been proposed and is being implemented by the Chinese goverment.

2621: *+.269*

We applied the quasi in situ conservation strategy, described in an accompanying paper, to a critically endangered plant species, Iris atrofusca from the Northern Negev, Israel. As the first steps of this strategy implementation we performed habitat and demographic observations; creation of two living collections outside the natural populations, but within the same ecological conditions; and relocation experiments. Plants in the living collections got established and showed high reproductive potential. In the relocation experiments, 3 years after introduction of rhizomes, no firm conclusions could be made about factors limiting species distribution at either large or small scale, but microhabitat was important for relocation success. We conclude that complex conservation approach that includes quasi in situ strategy should be useful for an endangered species that is distributed over variable ecological conditions.

2623: *+.217*

Along with other amphibian populations in Europe and elsewhere, natterjack toad Bufo calamita populations in Britain have declined since at least 1960. Conservation management since the 1970s has aimed to halt the decline and maintain viable populations at key sites throughout the species' recent historical range. Here, we assess population trends from 1985 to 2006 at 20 British B. calamita sites and evaluate the role of active management in maintaining good conservation status. We investigated the effects of 25 climatic, site-characteristic and conservation management variables on population trends using general linear models. In single-variable analyses, rainfall variables showed negative relationships with population trends. Among the site characteristics, being located at the very edge of the species' range (northern Irish Sea coast) and occurrence of common toad (B. bufo) were negatively related to B. calamita population trends. Management history (populations established via translocation as opposed to native populations) had a significant positive effect; as had sites that received greater translocation releases, undergone Species Recovery Programme management, and where common toad was absent. In multiple-variable analyses, the combined effects of management history and average pre-breeding season rainfall accounted for inter-site variation in population trends. The rainfall effects in single- and multiple-variable analyses were strongly influenced by three sites with very high rainfall whilst no clear effect was apparent for the remaining sites. This study highlights the role of climatic factors in population decline, and the importance of conservation management in stabilizing population trends. Climate change over the next 50-100 years is predicted to have limited impacts on most B. calamita populations in Britain, but strongly positive impacts on the most threatened populations located at the very edge of species' range that will benefit from reduced precipitation. A need for active conservation management will remain for the foreseeable future.

2624: *-.094*

A study was undertaken between 1 January 2006 and 16 February 2007 to identify haemoparasites and gastrointestinal parasites infecting African wild dogs (Lycaon pictus) in KwaZulu-Natal (KZN) province, South Africa. Blood and faecal samples were opportunistically collected from wild dogs immobilized for collaring or translocation purposes (n = 24). Three common domestic canine gastrointestinal parasites, Toxocara canis, Dipylidium caninum and Ancylostoma spp., and two genera of canid protozoan GI parasites, Sarcocystis and Isospora, were identified in 12 fresh faecal samples. The seroprevalence of Ehrlichia canis from 24 individual serum samples analysed was 83%. However, only 21% of the 14 whole-blood smears evaluated for the presence of E. canis morulae within monocytes were positive. Twelve whole-blood smears were evaluated for the presence of Babesia canistrophozoites within erythrocytes and revealed 0% prevalence. Although

there is currently no evidence of direct parasite-related mortality in the KZN population, the presence of internal parasites may be more detrimental to the overall health status of African wild dogs with immunosuppression as a result of other disease conditions, translocation stress, or inbreeding depression.

2625: ***-.007***

The historical and prehistorical occurrences of the Nile crocodile (Crocodylus niloticus) in the Eastern Cape and Western Cape provinces, South Africa, are poorly known. This can lead to confusion in the conservation policy and management of protected areas because it is uncertain where it might be appropriate to reintroduce or conserve the species. The problem is confounded because the recorded information is summarized in two sources that are hard to access. This information is examined and discussed. It shows that, historically, living crocodiles were seen or killed on the coast as far south as 32 degrees 18'S; 28 degrees 50'E, in the Dwesa State Forest, Eastern Cape, in the late 19th and early 20th centuries. However, there are museum specimens of skulls and teeth collected from localities farther south, between the mouths of the Keiskamma and the Keurbooms Rivers. These are probably of widely different ages, but older than the historical era beginning in the 16th century. There is no information on their geological or archaeological provenance. This suggests that the species' range limits expanded and contracted at least once through approximately 2 of latitude before the historical period. The former government of Transkei reintroduced the species to the Dwesa-Cwebe Nature Reserve in c. 1980. There is no evidence to suggest that it would be appropriate to reintroduce crocodiles south or westward of this population.

2627: ***+.185***

Astragalus gines-lopezii is a perennial herb, endemic to the south of the Iberian Peninsula. It has been classified as 'Endangered' by the Extremadura Regional Catalogue and placed in the equivalent category (EN D) of the National Red List because only one population in the world is known. Conservation of A. gines-lopezii requires a greater understanding of its population size, seed ecology and germination conditions. The latter issues are particularly important when establishing a reintroduction programme. This species was observed in nine colonies on the same mountainside, the largest of which consists of 60 plants. In this study, we compare the ability of the seeds to germinate under various conditions. Unlike most other members of the Fabaceae, the seeds of A. gines-lopezii do not need physical or chemical pre-treatment in order to germinate; instead they require a photoperiod for optimal germination. This study will facilitate a more in-depth understanding of this rare and poorly known species.

2628: ***+.218***

Translocation, the intentional movement of living organisms from one area to another is increasingly being used as a conservation tool to overcome barriers to dispersal. A dichotomy exists for conservation-oriented translocations: on one hand, there are those that release plants or animals into known historic ranges and on the other hand, there are releases outside historic distributions. Misuse of or attempts to redefine established terms and a proliferation of variants of new terms such as assisted colonization, confuse and hamper communication. The aim of this opinion article is to describe and define a conservation translocation spectrum, from species reintroductions to assisted colonization, and beyond, and in so doing provide a standard framework and terminology for discussing translocation options. I suggest that we are moving along this spectrum, away from the dictates of historical species distribution records, toward the

inclusion of more risky interventions that will be required to respond to habitat shifts due to anthropogenic impacts. To some extent rapid climate change changes everything, including how we should view introductions versus reintroductions. We need to seriously consider adding other approaches to our conservation toolbox. Assisted colonization will start us along this path, acknowledging as it does the accelerated rate of habitat change and the problems of attempting to preserve dynamic systems. The next step along the conservation translocation spectrum may be for reintroduction biology and restoration ecology to more comprehensively join forces on carefully selected projects to use species introductions to create novel ecosystems through active ecological community construction.

2629: *+.197*

The creation of new populations in conservation reserves plays an increasingly important role in reducing the extinction risk of endangered plants. We experimentally introduced seedlings of the federally endangered forb Astragalus bibullatus into protected limestone cedar glades in central Tennessee and tested how source population, transplant season, and glade site affected the demographic performance of transplants over 7 years. Plants derived from seed from three source populations survived at similar rates at introduced sites, but exhibited different germination rates in greenhouse cultivation trials. Even though glade sites were seemingly similar in abiotic conditions and in close proximity (< 400 m), survival rates varied widely among sites. Plants introduced in the spring never produced seed and were nearly four times more likely to die than plants introduced in the fall. Within 3 years of outplanting, 20% of plants introduced in the fall produced seed, yet sexual reproduction ceased after the third year and only one seedling recruit was observed after 7 years of monitoring. This resulted in small subpopulations that were not self-sustaining probably due to demographic stochasticity. In addition to highlighting the need for long-term monitoring of introduced populations to draw appropriate conclusions, our study emphasizes the importance of properly timing outplantings with the appropriate seasons and having multiple introduction sites as a hedge against environmental variability. However, overcoming seed limitation during the early stages of population growth and determining "safe sites" for seedling recruitment remains a central challenge in introducing endemic plants into seemingly suitable but unoccupied habitat.

2630: *+.098*

An understanding of recent evolutionary processes is essential for the successful conservation and management of contemporary populations, especially where they concern the introduction or invasion of species outside their natural range. However, the potentially negative implications of intraspecific introductions and invasions have attracted less attention, although they also represent a potential threat to biodiversity, and are commonly facilitated through human activities. The European bitterling (Rhodeus amarus) is a small cyprinid fish that decreased greatly in its distribution during the 1970s and 1980s and was subsequently included on many European conservation lists. This decline appears to have reversed, and the extent of its distribution now exceeds its former range. We used a combination of 12 microsatellite markers and cytochrome b sequences on a large data set (693 individuals) across the current range of the European bitterling to investigate possible scenarios for its colonization of Europe. We show that the inferred history of colonization of Europe was largely congruent between mitochondrial and nuclear markers. The most divergent mtDNA lineages occur in the Aegean region but probably are not reproductively isolated as the Aegean populations also displayed mtDNA haplotypes from other lineages and nuclear data indicated their close relationship to Danubian populations. Much of Europe is currently populated by descendants of two main lineages that came to natural secondary contact in

western Europe. An approximate Bayesian computation analysis indicates different dates for admixture events among western and central European populations ranging from the last deglaciation (natural) to the last few centuries (human-assisted translocations).

2631: *+.270*

The Oriental White Stork (Ciconia boyciana) and the Crested Ibis (Nipponia nippon) are wetland species listed as "Endangered" on the IUCN Red List of Threatened Species The two species were once common on the Korean peninsula, but have experienced a severe population reduction in the past decades Currently, they are officially extinct in Korea At present, reintroduction programs to release the birds to the wild are in progress in Korea as well as in Japan In this study, we surveyed the historic breeding sites of the two species using the literature and face-to-face interviews with local people as a step toward determining appropriate breeding habitats for reintroduction We found 26 historic breeding sites for the Oriental White Stork in Chungcheong-do and Gyeonggi-do, but did not find any breeding sites for the Crested Ibis These findings suggest that the Oriental White Stork was resident while the Crested Ibis was a winter visitor to Korea Based on these results, we discuss the possibilities for successful reintroduction of the two species in Korea

2632: *+.129*

The dwarf bulrush (Typha minima Funck ex Hoppe) is an endangered pioneer plant species of riparian flood plains. In Switzerland, only 3 natural populations remain, but reintroductions are planned. To identify suitable source populations for reintroductions, we developed 17 polymorphic microsatellite markers with perfect repeats using the 454 pyrosequencing technique and tested them on 20 individuals with low-cost M13 labeling. We detected 2 to 7 alleles per locus and found expected and observed heterozygosities of 0.05-0.76 and 0.07-1, respectively. The whole process was finished in less than 6 weeks and cost approximately USD 5000. Due to low costs and reduced expenditure of time, the use of next-generation sequencing techniques for microsatellite development represent a powerful tool for population genetic studies in nonmodel species, as we show in this first application of the approach to a plant species of conservation importance.

2633: *+.306*

Mountain gazelle (Gazella gazella) in Saudi Arabia is listed as 'vulnerable' by the IUCN. At present the species' survival is secured by extensive captive-breeding programs and re-introductions into protected areas. Post-monitoring of released individuals is essential for evaluating the success of such re-introductions but managers have difficulties in deciding whether food, water and other resources are sufficient to sustain a stable population. This study reports on data collected during standardized road transect counts in two wadis of the Ibex Reserve into which gazelles were previously released, and aims to compare the success of both re-introduction attempts with resource availability and home range size. Results from step-wise backward multiple regressions identified food availability and population density as significant predictors for home range size. The low amount of available food may have increased the competition between non-related females and therefore led to an increased overlap between non-group members resulting in increased dispersal rates. This information will allow interpretation of habitat suitability and provides possible reasons for the population decrease at both study sites. The data will enable conservation mangers to identify future re-introduction sites and will help improving the success of future re-introductions in Saudi Arabia or other parts of the species range. (C) 2010 Elsevier Ltd. All rights reserved.

2634: ***+.138***

AimWe searched for evidence of floristic homogenization in widespread oceanic archipelagos. LocationTwelve oceanic archipelagos in the Atlantic and Pacific oceans: Ascension, Azores, Canary, Cape Verde, Desventuradas, Easter, Galapagos, Hawaii, Juan Fernandez, Madeira, Puerto Rico and Savage. MethodsBy using Jaccard's index, we established the floristic similarity between pairs of archipelagos at two stages: original (pre-European; J(o)) and current flora (J(c)). Then, we calculated Delta J = J(c)-J(o), where positive differences imply that similarity has increased floristic homogenization. ResultsWe found that floristic similarity increased fourfold on average, from 1.6% to 6.3% for original and current floras, respectively. In fact, we recorded 64 cases in which floristic similarity increased and only two in which it decreased. The importance of invasions exceeds that of extirpations as a driver of biotic change by more than an order of magnitude (2679 versus 142 species, respectively). Main conclusionsThe vascular floras of these 12 insular oceanic systems have increased in compositional similarity, a phenomenon consistent with the trend towards biotic homogenization. It can be characterized as a teleconnected process that operates across vast geographical distances, driven by the unprecedented capacity of humans for translocation. Trends in biotic homogenization differ depending on the geographical location (i.e. Pacific < Atlantic archipelagos) and phylogenetic groups (island vascular plants > island birds reported in a previous study), emphasizing the complexity of biotic change.

2635: ***+.239***

As species extinction rates continue to rise, zoos have adopted a more active role in the conservation of endangered species. A central concern is to preserve genetic diversity of zoological populations. Accordingly, when selecting individuals to transfer to new or existing populations, zoo managers must consider the genetic effects on all populations involved. We propose a quadratic integer programming (IP) model to identify a group of individuals to transfer that maximizes genetic diversity within two subpopulations. We then reduce this model to a linear IP formulation and apply it to the California condor (Gymnogyps californianus) studbook. After simplifying the linear IP model, optimality is achieved within a reasonable time limit when a limited number of individuals are relocated. We also develop a local improvement algorithm (LIA) to efficiently provide near-optimal solutions when we increase the number of transferred individuals. The LIA quickly obtains optimal solutions when few individuals are transferred and in most cases, the LIA outperforms MetaMK, an existing program used to select animals for transfer. (C) 2010 Elsevier Ltd. All rights reserved.

2636: ***-.115***

Avian captive breeding programs pose a particular challenge with migratory birds due to natal dispersal and high mortality during migration. In Canada a captive breeding program for the eastern loggerhead shrike (Lanius ludovicianus migrans) has released over 400 juveniles since 2001, but the fate of almost all these birds is unknown. In 2007 and 2008, we used radio-telemetry of captive-reared juveniles to determine pre-migration survival and dispersal movements away from the release site. Overall, 76% (29 of 38) of shrikes survived from release to the initiation of migration and the daily survival rate was 0.987. Most deaths (78%) occurred in females, suggesting a possible sex-biased pre-migration mortality. Shrikes typically dispersed independently, there was little overlap between dispersal sites, and the average pre-migration dispersal distance from the release site was 4.2 km (SD 2.4). Release date was negatively correlated with time spent at the release site prior to dispersal, but did not have a significant effect on survival, time spent at dispersal sites or distance of dispersal. Migration initiation date ranged

over 6 weeks and early-hatched juveniles were the first to begin migration. Using aerial telemetry, we located five of 29 birds that had begun migration at distances up to 180 km from the release site; no birds were found dead after initiating migration despite the extensive search area. High juvenile survival to migration for captive-reared juveniles suggests that the captive breeding and release program has high potential to augment wild populations of the eastern loggerhead shrike. (C) 2010 Elsevier Ltd. All rights reserved.

2637: ***+.140***

Sex-biased natal dispersal is prevalent in many avian species, with females typically dispersing further than males. We examined natal dispersal patterns in an growing reintroduced population of hihi (stitchbird. Notiomystis cincta) on Tiritiri Matangi island in New Zealand. Hihi nest almost exclusively in the nest boxes provided throughout the island, and breeding locations have been recorded since the 1997-1998 season. The population grew from 16 to 170 birds in the 10 years since establishment in 1995, and also experienced substantial changes in adult sex ratio over this period. We calculated distances from natal to breeding locations to examine the dynamics of natal dispersal with sex, population density and sex ratio, and maternal and environmental effects. Overall, females dispersed significantly further than males, with permutation tests indicating that mean female dispersal distance was significantly higher than expected if they selected boxes at random, and male mean dispersal distance was significantly less than expected if they selected boxes at random. General linear mixed modelling also revealed sex-specific changes in dispersal behaviour over time that were correlated with changes in population density and sex ratio, and a strong effect of the maternal females' identity. The mean dispersal distance of males decreased with density, with the number of the juveniles in the cohort as the best correlate. We suggest that males are able to disperse further to gain the highest quality open territories at lower population densities, whereas dispersal distances become increasingly constrained by territory availability, and possibly variation in mating strategy, at higher densities. In contrast, female dispersal showed a weak positive density-dependent relationship, but was more strongly influenced by sex ratio, with dispersal distances decreasing with an increasing male bias in the population. We also found a strong effect of the maternal female that could not be explained solely by nest box location. Overall, these results imply a critical role of social context, including population size, density, and sex ratio in influencing natal dispersal dynamics in a closed, insular population of hihi, and have important conservation management applications for future translocation design and implementation for this endangered species.

2638: ***+.082***

Changes in land-use have resulted in the decline of many formerly common plants of nutrient-poor grasslands in Europe. Recently, extensification schemes have been applied at sites in order to restore former habitat conditions. However, the establishment of rare and endangered plants is often severely limited by the lack of propagules both in the seed bank and in the surrounding landscape. For such species deliberate introductions may be necessary to overcome these limitations. In a 7-year study, we assessed the importance of gaps created by sod cutting, of plant stage, and of plant origin for the restoration of populations of Scorzonera humilis, a threatened long-lived plant of nutrient-poor, wet grasslands. The effect of gaps on seedling emergence and survival varied strongly among the 12 sites. Gaps increased survival at nutrient-rich, but reduced it at nutrient-poor sites. Remarkably, young plants grown for only 5 weeks in the laboratory and transplanted into the same sites had much higher survival than seedlings from seeds sown and there were no differences in survival between nutrient-rich and nutrient-poor sites. The field performance of the plants from the various populations of origin varied depending on the site into

which they were transplanted, indicating genotype by environment interactions and genetic differentiation among populations, but there was no home-site advantage. While sowing only succeeded in producing adult plants in five sites, transplanting succeeded at 10 sites. Our results suggest that transplanting young plants could be a much more effective and faster way to establish new populations than sowing seeds.

2639: *+.027*

We evaluated landscape-scale forest restoration treatment implementation and effectiveness in meeting objectives in a ponderosa pine forest at Mt. Trumbull, Arizona, U.S.A. The goal of the project was to alter forest structure by thinning and burning to more closely resemble forest conditions prior to Euro-American settlement in 1870. We measured 117 permanent plots before (1996/1997) and after (2003) treatments. The plots were evenly distributed across the landscape (approximately 1,200 ha), about half of which was an untreated control. We evaluated treatment implementation and effectiveness based on 1870 structure and/or goals outlined by managers. The success of treatment implementation varied: about 94% of the area originally planned for restoration was treated in some manner by 2003, but only 70% received the full planned treatment (thin and burn). Although density of ponderosa pines > 2.5 cm was reduced significantly by 66% from approximately 429 pines/ha to approximately 146 pines/ha in the treated area, the targeted residual density was exceeded by 111-256% (all plots) or 10-85% (thinned and burned plots). Thirteen percent of the pre-settlement pines died in the treated area by 2003, but 9% percent also died in the control, indicating that pre-settlement pines in untreated areas were nearly as vulnerable as those exposed to restoration treatments. Large snags increased 45%, and 65% of logs > 50 cm were retained, achieving implementation goals. Although restoration treatments were not implemented totally to specifications, they were effective in attaining the overall project goal of restoring more open forest structure while preserving more than 75% of the pre-settlement pines. Canopy fuel loads were substantially reduced, allowing for the reintroduction of surface fires.

2640: *+.040*

Recently, a conservation strategy developed to restore populations of black-tailed prairie dog (Cynomys ludovicianus) suggested reintroducing animals into the Chihuahuan Desert grasslands of the southwestern United States. Rainfall in desert habitats is lower and more variable compared to rainfall near the center of the prairie dog's range. Additionally, peak rainfall comes months after prairie dogs reproduce in these desert systems. Thus, southwestern populations may be less prolific and fluctuate more than those found in northerly climes. Using mark-recapture and mark-resight techniques, we estimated reproduction and monthly survival from 577 individuals inhabiting 6 reintroduced colonies from 2003 to 2005 in the northern Chihuahuan Desert. During 2003 precipitation was 64% of the long-term average, whereas both 2004 and 2005 had near-average precipitation. Probability that a female became pregnant, number of juvenile prairie dogs emerging from maternity burrows, and date of emergence were all correlated to adult female body mass. Adult monthly survival decreased from >0.95 during spring to 0.70 in summer 2003, following a rapid loss in adult body mass that coincided with low precipitation. In 2003 monthly juvenile survival was near zero on 2 of the 3 largest colonies and growth rates of juveniles were half that of subsequent years. Estimated population size declined by 68% (range = 18-91%) from 2003 to 2004, and 5 of 6 populations declined an average of 75% from their original introduction size. Prairie dog populations in desert environs may have a high risk of extirpation caused by weather patterns indicative of desert climates. Our results are important for those managers involved in the conservation of prairie dogs and we suggest that regional differences should be carefully considered prior to any reintroduction effort.

2641: ***+.220***

1. Despite conservation programmes (India 1975, Nepal 1978) gharial populations (Gavialis gangeticus) have declined over their entire distribution range. Information about the current status and main threats is needed to implement effective conservation measures.2. This study presents a survey (2003/2004) of the largest Nepalese gharial population in the Chitwan National Park that has benefited from regular re-introduction of young gharials since 1981.3. Population size estimates fluctuate between 34 (2003) and 38 (2004). The reintroduction programme, although of limited success, has helped to maintain the gharial population.4. Gharials bask preferentially in large sand banks, and these sites must be protected.5. The main threats are from a dam that causes fish depletion and flushes gharials from the protected area, sand mining and grazing that destroy basking sites, fishing that causes food shortage, drift nets that kill gharial, and water pollution.6. Improvement in the survival of reintroduced gharials is needed. Strict protection of preferred basking sites and prohibition of fishing in the main settling zones are the principal conservation measures while in the long term, education and participatory management by local people are also necessary. Copyright (C) 2010 John Wiley & Sons, Ltd.

2642: ***+.061***

The giant pearl mussel Margaritifera auricularia, previously widespread in Western Europe, still survives in a few aging core populations, which have not been recruiting for several decades. The extirpation of its main host-fish, the European Sturgeon Acipenser sturio, is believed to be the main cause of its rarefaction. A few decades seem to be left to save the giant pearl mussel from extinction. A European Sturgeon restoration plan is ongoing, but given the late sexual maturity of this species, we cannot expect a return of sexually mature individuals in rivers in France before ca. 15 years. In order to restore ecosystems' functionality and save the Giant Pearl Mussel from extinction, we suggest the reintroduction of a similar species: the Atlantic Sturgeon A. oxyrinchus. This species is the least threatened of the Acipenseridae and was present in France at least between 3 000 BC and 200 AD. The interspecific competition risk with the European Sturgeon, as well as the introgression risk, can be eliminated as both species have coexisted in the past. The possibility of reintroducing the Atlantic Sturgeon gives new insights for the recovery of Giant Pearl Mussel populations.

2643: ***+.074***

Elephant's infant is considered extremely difficult to re-introduce into the wild. On 21st of November 2009, an eight day old elephant's infant was found strayed from its group at Shyampur forest of the Haridwar forest division. For the first time in the history of Uttarakhand, attempts had been made by forest officials to re-introduce the orphaned baby elephant into the wild. It is noteworthy that during the introduction attempts, group of seven elephants had taken the baby within group, but left her behind after a while. They had responded from all directions to the cries of the baby elephant but the attempts were in vain. Radha [long dash] the domesticated elephant at Chilla forest of the Rajaji National Park nurtured the infant for 10 days before infant's death and Radha's behaviour always illuminated something new about elephant's life. It was the first attempt to re-introduce the infant to wild in north-west India in which some lessons came forward and could be helpful in management of elephants and in documentation of conservation-oriented action plan. Additionally, studies on the behaviour of wild elephants are highly required and recommended so that we can ensure the future survival of this endangered species.

2644: ***+.138***

Background: Assisted migration or translocation of species to ameliorate effects of habitat loss or changing environment is currently under scrutiny as a conservation tool. A large scale experiment of assisted migration over hundreds of kilometres was tested on a morph from a commercial fishery of southern rock lobster Jasus edwardsii, to enhance depleted populations, improve the yield and sustainability of the fishery, and test resilience to a changing climate. Methodology and Principal Findings: Approximately 10,000 lower-valued, pale-coloured lobsters were moved from deep water to inshore sites (2 in Tasmania [TAS] and 2 in South Australia [SA]) where the high-value, red morph occurs. In TAS this was a northwards movement of 1 degrees latitude. Growth was measured only in TAS lobsters, and reproductive status was recorded in lobsters from all locations. Pale females (TAS) grew 4 times faster than resident pale lobsters from the original site and twice as fast as red lobsters at their new location. Approximately 30% of translocated pale lobsters deferred reproduction for one year after release (SA and TAS), and grew around 1 mm yr(-1) less compared to translocated pale lobsters that did not defer reproduction. In spite of this stress response to translocation, females that deferred reproduction still grew 2-6 mm yr(-1) more than lobsters at the source site. Lobsters have isometric growth whereby volume increases as a cube of length. Consequently despite the one-year hiatus in reproduction, increased growth increases fecundity of translocated lobsters, as the increase in size provided a larger volume for producing and incubating eggs in future years. Conclusions and Significance: Assisted migration improved egg production and growth, despite a temporary stress response, and offers a tool to improve the production, sustainability and resilience of the fishery.

2645: ***+.363***

The whio/blue duck (Hymenolaimus malacorhynchos) is an endangered species that is listed as Nationally Vulnerable under the New Zealand Threat Classification System (2008) due to a significant decline in abundance and distribution. Active management is needed to ensure the species' long-term survival, as whio are dependent on in situ management in suitable large-scale river systems, and require protection from the key threat of stoat (Mustela erminea) predation. This is the second national recovery plan for whio and follows on from the previous (1997[long dash]2007) recovery plan. This 10-year plan is a guide for the Department of Conservation (DOC) and interest groups involved in conserving whio. Its first priority is to secure populations to a minimum of 400 pairs at eight 'Security Sites' throughout New Zealand by 2014. To achieve this, the whio recovery programme will control introduced predators, and target research towards gaining a further understanding of whio biology, threats and management requirements. The second priority is to recover or re-establish populations throughout their former range ('Recovery Sites'). The recovery programme will continue to investigate translocation/re-establishment techniques and improved predator control management. Public awareness, education and community involvement are important tools that will be used to assist the recovery of whio.

2646: ***+.151***

Translocation is often considered a viable conservation strategy, despite the absence of species-specific post-translocation data. Macrochelys temminckii (Alligator Snapping Turtle) populations have declined across their range and they may be considered candidates for translocation, but few studies have examined the response of individuals to movement events. I monitored M. temminckii with radiotelemetry in northwest Louisiana to provide baseline data regarding the species' response to translocation. I calculated average distances moved per day, measured water depths, and recorded growth of translocated and resident turtles. There was no observed mortality during the study, and translocated turtles gained mass and increased shell dimensions, indicating they effectively located resources after translocation. Resident individual shell dimensions

increased, but some residents lost mass, possibly due to early recapture and reweighing dates. Movement distances were within the ranges reported by previous researchers. These data contribute baseline information concerning M. temminckii conservation biology.

2647: *+.052*

Isoetes hypsophila Hand.-Mazz. (lsoetaceae) is an endangered quillwort endemic to the Qinghai-Tibetan Plateau. Genetic variation and population structure of 12 I. hypsophila populations were examined by using amplified fragment length polymorphism (AFLP) markers. Eight primer pairs produced a total of 472 unambiguous bands, of which 229 (48.5%) were polymorphic. Intra-population genetic variation of Isoetes hypsophila was low (P(p) = 14.9%, H(E) = 0.039, hs = 0.084 and I = 0.061). The F statistics calculated by different approaches consistently revealed high genetic differentiation among populations, contributing approximately 50% of total gene diversity. Four genetic groups corresponding to four geographic regions were detected, indicating significant geographic structure. Our results suggest that both ongoing evolutionary forces (e.g., inbreeding system, bottlenecks resulting from fluctuation of water level) and historical events (e.g., orogenic movements which reduce gene flow among populations, repeated population retreat, and recolonization during ice ages), may have contributed to the genetic structure of I. hypsophila. In conservation, attention should be paid to preserving every population, and intra-region translocations can currently be recommended.

2648: *+.148*

Pacific lion-paw scallops were collected from natural aggregations in Laguna Ojo de Liebre (Pacific Ocean), the Gulf of California, and from aquaculture facilities for genetic diversity analyses. Mitochondrial DNA sequencing uncovered two highly supported clades separated by 2.5% divergence. Data from ten microsatellite markers suggest individuals from these mitogroups are introgressed, raising questions about the mitotype origin. Some evidence suggests gene flow between La Paz and Ojo de Liebre; otherwise the Gulf of California and Ojo de Liebre are acting as two distinct populations. It is unclear whether translocations between sites have influenced the observed genetic structure or whether gene flow has been facilitated by past geologic events. Finally, scallops spawned for aquaculture are unique from the wild and have significantly less diversity. These results warrant the attention of managers and producers who should work to monitor and conserve genetic diversity in both wild and aquaculture populations.

2649: *+.140*

Propagation of the cycad Dioon edule aimed at sustainable management in the state of Veracruz, Mexico has been ongoing since 1990 under the supervision of staff at Francisco Javier Botanic Garden, with the principal objectives of addressing illegal trafficking and habitat destruction. Plant sales have been limited. Nevertheless these, along with the sale of other managed forest products, have given the cycad producers and other villagers enough incentive to conserve 80 hectares of cycad habitat and to discourage illegal collecting. This model was taken up by four similar nurseries in the buffer zones of two biosphere reserves in Chiapas for the propagation of four additional cycad species and two endangered Chamaedorea palms. A further biosphere reserve in Puebla hosts a similar nursery for the critically endangered D. caputoi. Here the producers are paid through the Reserve authority for cultivation and reintroduction of the cycad. All species were studied at the population level prior to and during nursery establishment. Cultivation knowledge has been passed on to the farmers as well as limited help in marketing. Seedling reintroduction experiments have been carried out but further demographic studies of D. edule and C. mirandae

have given reason to re-think reintroduction strategies. There is great potential for these nurseries to act as shelter for rescued plants during civil engineering projects. The marketing problem is still an issue and has been approached by the involvement of conservation authorities in Chiapas to assist the producers with permit paperwork and to seek markets. This experience is an important example of botanic garden extension to rural communities in Mexico that covers several articles of the Convention on Biological Diversity.

2650: *-.022*

The Asian fish tapeworm Bothriocephalus acheilognathi (Cestoda: Bothriocephalidea) is an introduced fish parasite in the southwestern United States and is often considered a serious threat to native desert fishes. Determining the geographic distribution of nonnative fish parasites is important for recovery efforts of native fishes. We examined 1,140 individuals belonging to nine fish species from southwestern U. S. streams and springs between January 2005 and April 2007. The Asian fish tapeworm was present in the Gila River, Salt River, Verde River, San Pedro River, Aravaipa Creek, and Fossil Creek, Arizona, and in Lake Tuendae at Zzyzx Springs and Afton Canyon of the Mojave River, California. Overall prevalence of the Asian fish tapeworm in Arizona fish populations was 19% (range = 0-100%) and varied by location, time, and fish species. In California, the prevalence, abundance, and intensity of the Asian fish tapeworm in Mohave tui chub Gila bicolor mohavensis were higher during warmer months than during cooler months. Three new definitive host species-Yaqui chub G. purpurea, headwater chub G. nigra, and longfin dace Agosia chrysogaster-were identified. Widespread occurrence of the Asian fish tapeworm in southwestern U. S. waters suggests that the lack of detection in other systems where nonnative fishes occur is due to a lack of effort as opposed to true absence of the parasite. To limit further spread of diseases to small, isolated systems, we recommend treatment for both endo- and exoparasites when management actions include translocation of fishes.

2651: *+.090*

1. Wildlife reintroductions can help to restore populations and save species from extinction. However, success rates of reintroductions are low due to inherent problems, such as behavioural deficiencies resulting in high post-release predation rates among captive-bred animals. In particular, the released animals may use habitats maladaptively, leading to an ecological trap, i.e. preference for low-quality habitats leading to reduced survival and/or breeding success. Ecological traps in reintroductions can be identified only through intensive studies of habitat preferences and survival of known individuals, but such studies are lacking. 2. We investigated habitat preferences and their relationship with survival by radio-tracking reintroduced, captive-bred grey partridges Perdix perdix, a widely reintroduced commercial game species and native farmland bird of conservation concern in the UK. The low success rate of grey partridge releases could be due to maladaptive habitat use and a possible ecological trap. 3. Grey partridges released as pairs in spring showed preferences for crop and field margin habitat. The use of crops had a positive, and use of field margins a negative, association with survival, suggesting field margins could serve as ecological traps for released grey partridges. Predation rates were high, and field margins probably hosted concentrated predator activity. 4. Grey partridges released as family groups (coveys) in autumn exhibited a preference for game covers, i.e. areas of tall vegetation specifically planted to provide cover for game birds, and mortality rates were lower than in spring. Habitat use did not affect survival in autumn. 5. Synthesis and applications. We have shown that newly released grey partridge pairs behave maladaptively by preferring a habitat which has a negative association with survival. In grey partridge reintroductions, we recommend releasing grey partridge family groups in autumn rather than releasing pairs of birds in spring, and providing game covers that could

induce the groups to settle onto the release areas. In reintroductions in general, the habitat preferences of the released animals should be documented together with their fates, to enable detection of possible ecological traps that could threaten the success of these conservation efforts.

2652: ***+.014***

The African wild dog (Lycaon pictus) is one of Africa's most endangered species and therefore classified as endangered by IUCN. Earlier distributions included most of Africa but currently the African wild dog only has populations larger than 300 individuals in three countries (Botswana, Tanzania and South Africa). In 1998, a plan was launched in South Africa to manage sub-populations of the African wild dog in several small, geographically isolated, conservation areas. This management program involved the reintroduction of wild dogs into suitable conservation areas and periodic translocations among them. We used the stochastic population simulation model VORTEX to evaluate the Limpopo Valley Conservancy in the north of South Africa, as a possible reintroduction site for African wild dogs. The simulations showed that the size of the initial population released only had a small effect on the population dynamics. However, when individuals were supplemented and harvested over a longer period the probability of persistence increased. Number of females breeding, male mortality, and carrying capacity were key factors in the population dynamics, but according to VORTEX the severity of natural catastrophes had the greatest influence on the extinction risk and inbreeding. We suggest that the reintroduction program may be successful, if areas are properly secured, the dogs are held in a boma before release, wild animals or at least a mix of wild and captive animals are used for the release and the animals are vaccinated against rabies. It is, however, essential to continue monitoring followed by modelling efforts to re-evaluate the success of the reintroduction program. (C) 2009 Elsevier GmbH. All rights reserved.

2653: ***+.092***

A century ago, tigers (Panthera tigris Linnaeus, 1758) were so common in parts of Southeast Asia as to be considered pests, and governments sponsored their killing. Habitat loss and fragmentation, market-driven poaching and loss of prey have since led to the disappearance of Indochinese tigers from most their former range. Despite 15 years of dedicated tiger conservation funding, national estimates of Indochinese tiger subpopulations can at best only be roughly approximated. The future for the subspecies appears grim unless very focused efforts can be applied to stabilize and recover subpopulations. On a regional scale, the 2 proposed subspecies Panthera tigris corbetti and P. tigris jacksoni are effectively managed as separate conservation units. Evaluating where to place conservation efforts should consider the vulnerability (likelihood of extinction) and irreplaceability (likelihood that an area contributes uniquely to regional conservation) of tiger subpopulations. Only 1 site in Thailand supporting < 200 individuals (Huai Kha Khaeng-Thung Yai) is considered low vulnerability, and is irreplaceable. Five sites in Lao, Thailand and Peninsular Malaysia are medium vulnerability and irreplaceable. Priorities at these 6 sites are to double tiger numbers within 10 years through protection and monitoring. Seven sites in Lao, Thailand and Myanmar are high vulnerability and irreplaceable, and might be recovered if government commitment to tigers, staff capacity and legal frameworks for tiger protection are established. Tigers are extremely vulnerable or even extinct in Cambodia's Eastern Plains and the site is irreplaceable for tigers because it represents the only large (> 10 000 km2) block of dry forest habitat available in the region. A reintroduction program is the only option to recover tigers there.

2654: ***+.125***

Over recent decades, investigators have studied many aspects of the natural history of the threatened and evolutionarily distinct Leiopelma frogs of New Zealand, effectively integrating natural history with conservation. To exemplify this, seven aspects of natural history (systematics, senses and defenses, threats, distribution and habitat, reproduction, demography, pathology) are related to 13 conservation needs, and the main linkages identified. This provides both a review of the frogs' natural history and an illustration of their conservation needs. Leiopelmatids have declined markedly and lost species, with three larger species (L. auroraensis, L. markhami, L. waitomoensis) now extinct, and four extant species (L. archeyi, L. hamiltoni, L. hochstetteri, L. pakeka) all threatened and on the amphibian EDGE list. Leiopelma archeyi tops that list. Potential threats include invasive mammalian predators and emerging diseases, particularly Amphibian Chytrid Fungus (Batrachochytrium dendrobatidis). Distribution surveys have clarified the frogs' current status, extending known ranges of some (i.e., L. archeyi, L. hochstetteri), and confirming restricted ranges of others (i.e., L. hamiltoni, L. pakeka). Observations on captive Leiopelma clarified patterns of reproduction and development, allowed assessment of evolutionary relationships, and are relevant to captive management of threatened populations. Long-term demographic studies represent some of the most lengthy population research on wild anurans, providing conservation-relevant data, e.g., revealing a decline in L. archeyi in the late 1990s, coinciding with finding chytridiomycosis in the species. While Leiopelma taxonomy needs more resolution, our knowledge of the natural history of these frogs has substantially informed conservation management, embracing programs dealing with habitat restoration, translocation, adaptive management, captive breeding, and disease prevention.

2655: *+.184*

P>Consideration of key sociological factors, including the views and attitudes of the local population, is critical to species reintroduction project success. This study examined the opinions of anglers, conservationists and the general public to a potential reintroduction of burbot, Lota lota L., to UK rivers. An online survey targeted the key stakeholder groups and a questionnaire was used to obtain a snapshot of public attitudes. In both studies, more than 90% of respondents supported reintroduction. Anglers, participants with prior knowledge of the species, males and respondents who were not a member of a conservation organisation, exhibited the most positive attitudes. Anglers and participants with prior knowledge of the species cited burbot as an angling opportunity, suggesting these groups view burbot as an exploitable resource. Overall, the surveys indicated that reintroduction of the burbot, should it be deemed feasible on ecological and biological grounds, would not be met by major public opposition.

2656: *+.051*

Reintroductions are a common approach for preserving intraspecific biodiversity in fragmented landscapes. However, they may exacerbate the reduction in genetic diversity initially caused by population fragmentation because the effective population size of reintroduced populations is often smaller and reintroduced populations also tend to be more geographically isolated than native populations. Mixing genetically divergent sources for reintroduction purposes is a practice intended to increase genetic diversity. We documented the outcome of reintroductions from three mixed sources on the ancestral composition and genetic variation of a North American fish, the slimy sculpin (Cottus cognatus). We used microsatellite markers to evaluate allelic richness and heterozygosity in the reintroduced populations relative to computer simulated expectations. Sculpins in reintroduced populations exhibited higher levels of heterozygosity and allelic richness than any single source, but only slightly higher than the single most genetically diverse source population. Simulations intended to mimic an ideal scenario for maximizing genetic variation in

the reintroduced populations also predicted increases, but they were only moderately greater than the most variable source population. We found that a single source contributed more than the other two sources at most reintroduction sites. We urge caution when choosing whether to mix source populations in reintroduction programs. Genetic characteristics of candidate source populations should be evaluated prior to reintroduction if feasible. When combined with knowledge of the degree of genetic distinction among sources, simulations may allow the genetic diversity benefits of mixing populations to be weighed against the risks of outbreeding depression in reintroduced and nearby populations.

2657: ***+.167***

The Tokyo bitterling Tanakia tanago (Cyprinidae) was once found throughout the Kanto Plain, central Japan, but most of their habitats have been lost due to human activities such as urbanization and improvement of paddy fields. Subsequently, conservation efforts, including captive breeding and reintroduction, have been ongoing. However, the genetic relationships among populations of this species including captive and remnant wild populations have been uncertain and thus management units for this species have been unidentified. We examined the population differentiation among 12 populations, including four wild and eight captive populations, and their relative genetic diversities to assist in conservation management decisions. Phylogeographic analyses based on partial mitochondrial cytochrome b gene sequences and microsatellite polymorphisms revealed four geographically associated genetic groups in the populations. Northern Tochigi populations have diverged from other populations (0.77% of d (A)), likely stemming from allopatric fragmentation following a change in the route of the Naka River, which occurred during the middle of the Pleistocene epoch. Microsatellite analysis has revealed that the genetic diversity of each population is generally low, and that most of the populations have experienced genetic bottlenecks. For future in- and ex-situ conservation programs to succeed, the population structure and genetic variability of remnant populations need to be taken into consideration.

2658: ***+.634***

The current shortfall in effectiveness within conservation biology is illustrated by increasing interest in "evidence-based conservation," whose proponents have identified the need to benchmark conservation initiatives against actions that lead to proven positive effects. The effectiveness of conservation policies, approaches, and evaluation is under increasing scrutiny, and in these areas models of excellence used in business could prove valuable. Typically, conservation programs require years of effort and involve rigorous long-term implementation processes. Successful balance of long-term efforts alongside the achievement of short-term goals is often compromised by management or budgetary constraints, a situation also common in commercial businesses. "Business excellence" is an approach many companies have used over the past 20 years to ensure continued success. Various business excellence evaluations have been promoted that include concepts that could be adapted and applied in conservation programs. We describe a conservation excellence model that shows how scientific processes and results can be aligned with financial and organizational measures of success. We applied the model to two well-documented species conservation programs. In the first, the Po'ouli program, several aspects of improvement were identified, such as more authority for decision making in the field and better integration of habitat management and population recovery processes. The second example, the black-footed ferret program, could have benefited from leadership effort to reduce bureaucracy and to encourage use of best-practice species recovery approaches. The conservation excellence model enables greater clarity in goal setting, more-effective identification of job roles within programs,

better links between technical approaches and measures of biological success, and more-effective use of resources. The model could improve evaluation of a conservation program's effectiveness and may be used to compare different programs, for example during reviews of project performance by sponsoring organizations.

2659: *+.110*

Asian turtles have declined substantially in recent decades because of the large-scale collection of wild individuals for the food, pet, and medicine trades. This has hampered studies into the ecology and natural history of turtles in their natural habitats because many species have become so rare that they are simply unavailable for study. One way to re-establish or supplement these turtle populations is through translocation (either of wild-caught individuals or those raised in farms). However, successful translocation is partly dependent upon released individuals maintaining site fidelity. We translocated 16 big-headed turtles, Platysternon megacephalum, in southern China to study their movements and microhabitat use in the wild and use this information to evaluate the feasibility of translocation as a conservation technique for this species. Translocated turtles generally moved short distances (daily range: 0-89.6 m), with males moving further than females and both sexes moving further at night than during the day. Additionally, big-headed turtles rarely used terrestrial habitats (the maximum distance an individual was located from the stream was 5.8 m) and remained hidden in refugia most of the time. Some sex differences in microhabitat use were also apparent; females were visible less often, spent more time on land (which coincided with the nesting season), remained closer to the stream bank, used shallower water, and used different types of refugia than males. The very short distances that bigheaded turtles moved, combined with their consistent fidelity to the stream and cryptic behavior, all suggest that this species would be a good candidate for larger-scale translocation experiments. To our knowledge, this is the first published study of movement patterns and habitat use of translocated semiaquatic or aquatic turtles outside of Europe and the first published radiotelemetry study of turtles from mainland China.

2660: *+.083*

Efforts to protect endangered species sometimes involve removing animals from sites to be developed and translocating them to protected sites. This method has rarely been successful. Protected kangaroo rats in the San Joaquin Valley of California continue to lose habitat to development. In 2001, I monitored four Tipton kangaroo rats(Dipodomys nitratoides nitratoides) and seven Heermann's kangaroo rats (D. heermanni ssp.) fitted with radio transmitters that were translocated away from development at an electrical substation to protected native land of the San Joaquin Valley, Kern County, California. I released translocated kangaroo rats into individual artificial burrows that were spaced 10-15 m apart and that were provisioned with bird seed. Only 1 individual survived the 45 days of the study. All four Tipton kangaroo rats were dead within 5 days of release, and all appear to have been eaten by predators. Two Heermann's kangaroo rats appeared to have been killed by conspecifics, three were killed by predators, and the fate of one was undetermined. If translocation is to be considered a useful conservation measure, kangaroo rats need to be released into habitat that is unoccupied, or nearly so, by conspecifics and the use of additional methods of protecting translocated individuals may be necessary.

2661: *+.387*

Conservation scientists increasingly recognize the need to incorporate the social sciences into policy decisions In practice, however, considerable challenges to integrating the social and natural

sciences remain In this article we review the US Fish and Wildlife Services (FWS) 2009 decision to remove the northern Rocky Mountain population of gray wolves from the federal list of endangered species We examine the FWS s arguments concerning the threat posed by humans attitudes toward wolves in light of the existing social science literature Our analysis found support for only one of four arguments underlying the FWS s assessment of public attitudes as a potential threat to wolves Although we found an extensive literature on attitudes toward wolves, the FWS cited just one empirical research article We conclude that when listing decisions rest on assumptions about society, these assumptions should be evaluated using the best available natural and social science research

2662: *-.036*

The Eurasian wild boar, Sus scrofa, is an important game species and is widely distributed in Eurasia. The recent demographic expansions allied to man mediated translocations and reintroductions, reshaped the genetic variability of wild boar populations, and gave rise to management problems. Genetic variability and the population structure of the European populations are not well-known, in particular in the Iberian Peninsula. In the present study, we assessed the phylogenetic relationship among 17 Iberian wild boar populations and several Eurasian wild and domestic pigs by sequencing 660 bp of the mitochondrial (mt)DNA control region. This analysis was extended to the two autochthonous Portuguese pig breeds, Bisaro and Alentejano, and their relationship with Eurasian pigs was also established. High levels of gene diversity were found in Iberian wild boar and Portuguese domestic pigs, as well as a significant genetic difference among and within populations. Iberian wild boar and Portuguese pig breeds belong to the main European clade but diverge with animals from Central Europe, showing a high proportion of private (Iberian) haplotypes. These results suggest the existence of glacial refugia for Sus scrofa in the Iberian Peninsula during the Last Glacial Maximum. No signs of Asian mtDNA introgression from Asian populations were found in the present survey. (C) 2010 The Linnean Society of London, Biological Journal of the Linnean Society, 2010, 101, 797-822.

2663: *+.138*

Sponges belonging to the genus Cliona are common inhabitants of many coral reefs, and as bioeroders, they play an important role in the carbonate cycle of the reef. Several Cliona species maintain intracellular populations of dinoflagellate zooxanthellae (i.e., Symbiodinium spp.), which also form symbioses with a variety of other invertebrates and protists (e.g., corals, molluscs, forminifera). Unlike the case of coral symbioses, however, almost nothing is known of the metabolic interaction between sponges and their zooxanthella symbionts. To assess this interaction, we performed a tracer experiment to follow C and N in the system, performed a reciprocal transplant experiment, and measured the stable carbon isotope ratio of Cliona spp. with and without zooxanthellae to study the influence of environment on the interaction. We found strong evidence of a transfer of C from zooxanthellae to their sponge hosts but no evidence of a transfer of N from sponge to zooxanthellae. We also saw significant influences of the environment on the metabolism of the sponges. Finally, we observed significant differences in carbon metabolism of sponge species with and without symbionts. These data strongly support hypotheses of metabolic integration between zooxanthellae and their sponge host and extend our understanding of basic aspects of benthic-pelagic coupling in shallow-water marine environments.

2664: *+.107*

Ceratopteris pteridoides (Hook.) Hieron. is an endangered aquatic homosporous fern in China.

Genetic diversity and structure of eight populations collected from the mid-lower reaches of Yangtze River were investigated using amplified fragment length polymorphisms (AFLPs). A low level of gene diversity was found at the population level (P-P = 17.4%, H-E = 0.039 and I = 0.063), which possibly resulted from its high degree of inbreeding, clonal growth and short life history of this species. C. pteridoides contained high clonal diversity (PD = 0.757, D = 0.992). High population differentiation was revealed by partitioning of genetic diversity (G(ST) = 0.707), and the AMOVA analysis consistently showed that 72.3% of the total genetic diversity was attributable to among-population diversity. Based on the genetic information from UPGMA cluster and principal coordinate analysis, two management units have been identified, and translocation within each management unit is recommended. (C) 2010 Elsevier Ltd. All rights reserved.

2665: *+.113*

The European wild rabbit Oryctolagus cuniculus has been recently reclassified as Near Threatened in its natural range in the Red List of Endangered Species by the IUCN, and a huge conservation effort is being undertaken in Spain for this keystone species. Restocking is a frequent measure for wild rabbit population reinforcements and it is also part of predator conservation programmes. However, it can have a negative influence on the resident rabbit population when it is not carried out carefully. In our work, using a model selection procedure based on a theoretic information approach, we analyze which factors favor the presence of sarcoptic mange in some wild rabbit populations in Catalonia (north-eastern Spain), as well as the trend of rabbit abundance in affected and non-affected hunting estates. Presence of mange depends on animal abundance and on restocking numbers (22.09%). From the mean rabbit abundance (30 rabbits hunted km-2) and the mean restocking rate (18 rabbits km-2), the probability of being affected increased in nearly 5% when the restocking rate increased in one unit. Rabbit abundance (2001-2007) depends on the presence of mange and on the effect of the year (23.86%), and clearly declined in the hunting estates with mange, whereas abundance is maintained in mange-free zones. Our results indicate that a sanitary control is necessary when restocking wild rabbit populations. Furthermore, restocking is shown to be a potential way of introducing pathogens to resident populations, especially under conditions of high density.

2666: *-.001*

Males and females of most dimorphic ruminants segregate outside the mating season, which may necessitate that conservation efforts focus on differential resources used by the sexes. Dimorphic bighorn sheep Ovis canadensis are one of the rarest ungulates in North America with some populations listed as endangered. Water sources are important for the persistence of populations of bighorns, especially in a changing climate. Understanding whether the sexes use different water sources could influence the conservation of this species and the habitats they occupy; however, little research exists regarding this important topic. We tested hypotheses relating to use of water sources by reintroduced male and female bighorns in Utah, USA. We investigated whether use of this resource differed across seasons by sex, and if sexes used water more during drought compared with non-drought conditions. Bighorns used small, adjacent core areas during segregation, and males and females used different sources of water during that time. Males visited water sources used by females more during aggregation. Males and females used water sources more in summer, and males visited water sources more during rut than did females. Males and females did not use water sources more during drought compared with non-drought conditions; however, sexes visited water sources more during the season following drought than following non-drought conditions, indicating a time-lag in use of this resource. Our results highlight the importance of water sources used by sexes of bighorns, and indicate that the existing criterion for

distance of bighorn reintroductions from water may be inadequate for successful establishment of populations. We recommend conservationists assess availability of water sources near habitat used by male and female ungulates before conserving and manipulating habitat, siting artificial sources of water and reintroducing animals.

2667: *+.095*

Invasive species threaten biodiversity; hence, predicting where they may establish is vital for conservation. Our aim is to provide a robust predictive model for an invasive species suitable for managers acting at both global and regional scales. Specifically, we investigate one of the world's worst invasive species [the red-eared slider turtle (RES) Trachemys scripta elegans] and one of the world's biodiversity hotspots (New Zealand) as our representative systems. We used climate data and location records to define a bioclimatic envelope for the species. Multimodel inference was used to predict areas suitable for RES establishment, weighting in favour of models with low false-negative and high true-positive rates in predictive cross-validation tests. Our performance criterion was the partial area under the curve of a receiver operating characteristic plot where sensitivity exceeded 0.95. We generated both conservative (best-case scenario) and liberal (worst-case scenario) predictions, based on different levels of information about breeding potential. All predictions were expressed on a standard scale of suitability relative to existing known distribution. Globally, the best climate matches for RES outside of their native range in North America include south-east Asia, and parts of Europe, areas where RES have already established. The best available site in New Zealand is considered climatically more suitable than 16% of global sites where RES have bred successfully. While RES can survive in several areas throughout New Zealand, the potential to establish a self-sustaining (i.e. breeding) population appears restricted to the upper areas of the north island where the mean daily temperatures in the hottest month exceed 18 degrees C. The methods developed here were designed to reduce false-negative predictions as that represents a precautionary approach for species that pose a biosecurity risk. They could readily be adapted, however, to reduce false-positives when predicting areas suitable for translocation of rare and endangered species.

2668: *+.150*

A series of field studies examined the effectiveness of using a grass blade/stem to extract tiger beetle larvae (Coleoptera Carabidae Cicindelinae) from their burrows without damaging the larva or the burrow - a procedure often called fishing We found that larvae of many species can be efficiently sampled at rates equal to or exceeding other methods Extracted larvae have a low percentage of injury and can be assessed for parasitism and condition They can be returned to either their own burrow or an altenative burrow where they can be re sampled The results of these tudies indicate that fish ing for tiger beetle larvae can be an effective tool for monitoring populations where larvae can be returned to existing burrows In addition the fishing technique allows for relocation of organisms while eliminating the impacts associated with excavating threatened species or larvae from rare habitats

2669: *+.142*

A survey of breeding Cirl Buntings Emberiza cirlus in the United Kingdom in 2009 recorded an estimated 862 territories (95% confidence limits 785-975), in 136 occupied tetrads. These were confined largely to south Devon, but a small population now exists in Cornwall as a result of an ongoing reintroduction project. From the lows of the 1980s, the UK Cirl Bunting population continues to recover, in terms of both abundance and distribution. Between 2003 and 2009, the

population estimate increased by 24% and the number of occupied tetrads by 15%. However, there is little evidence to suggest that the species is naturally recolonising areas beyond its core range in south Devon. Further work will evaluate recent trends in relation to agri-environment scheme delivery.

2670: *+.167*

Artificial incubation is a conservation technique used to provide founders for new or to augment existing reptile populations with minimal impact on the original population. It relies on the premise that hatching success of eggs in artificial conditions is high relative to natural nests. Our goal was to assist with the rescue of populations on the brink of extinction by incubating eggs produced by Tuatara from small islands inhabited by introduced rats. We incubated eggs produced over an 18 year period by Tuatara originating from Little Barrier, Cuvier, Stanley, and Red Mercury Islands, New Zealand, while they were in captivity awaiting return after rat eradications. The most successful results came from the Little Barrier stock where the population numbers increased dramatically, with eggs produced by all four mothers and high hatching success. Stanley Island stock produced the least successful outcome. Adults from Stanley Island suffered high mortality in captivity, and surviving females produced few eggs, with comparatively low hatching success. On balance, rescuing the genetic stock of remnant populations through captive incubation gives conservation programs time to deal with causes of decline and to plan for future success. However, supplementation in the future from other wild populations is likely to be necessary to ensure long term genetic variability and therefore viability of these populations, in particular Stanley Island.

2671: *+.058*

A hypothesis of phylogenetic relationships is proposed for the subgenus Pancallia Rivalier 1961 of the genus Cicindela Linnaeus 1758 (Coleoptera Cicindelidae). Sixteen adult morphological characters were identified and scored for the six taxa currently recognized in this subgenus: Cicindela (Pancallia) angulicollis Horn 1900, C. (P.) aurofasciata Dejean 1831, C. (P.) goryi Chaudoir 1852 (new status), C. (P.) princeps ducalis Horn 1897, C. (P.) princeps princeps Vigors 1825, and C. (P.) shivah Parry 1848. Cladistic parsimony analysis of the resulting taxon-character matrix using the computer program NONA identified a single most parsimonious tree for these six taxa. Cicindela (Pancallia) angulicollis is the sister-species of the rare C. (P.) shivah, a species of current conservation interest in India. Cicindela (Pancallia) princeps is the sister-species of a monophyletic clade containing C. (P.) aurofasciata and C. (P.) gotyi. Cicindela (Pancallia) goryi is restored to full species status based on differences in morphology, behavior, and ecological associations which separate this taxon from C. (P.) aurofasciata.

2672: *+.014*

Attempts to conserve threatened species by establishing new populations via reintroduction are controversial. Theory predicts that genetic bottlenecks result in increased mating between relatives and inbreeding depression. However, few studies of wild sourced reintroductions have carefully examined these genetic consequences. Our study assesses inbreeding and inbreeding depression in a free-living reintroduced population of an endangered New Zealand bird, the hihi (Notiomystis cincta). Using molecular sexing and marker-based inbreeding coefficients estimated from 19 autosomal microsatellite loci, we show that (i) inbreeding depresses offspring survival, (ii) male embryos are more inbred on average than female embryos, (iii) the effect of inbreeding depression is male-biased and (iv) this population has a substantial genetic load. Male susceptibility to

inbreeding during embryo and nestling development may be due to size dimorphism, resulting in faster growth rates and more stressful development for male embryos and nestlings compared with females. This work highlights the effects of inbreeding at early life-history stages and the repercussions for the long-term population viability of threatened species.

2673: ***-.066***

Ceropegia fantastica Sedgw. (Asclepidaceae) is a critically endangered, endemic species in Western Ghats of India. The fruit and seed setting are very low and in vitro propagation is the only viable alternative for its sapling raising and restoration of this plant's population in the wild natural environment through reintroduction. Attempts have been made here for regeneration of this species through in vitro technique using nodal segments as explants and up to 13 multiple shoots were initiated on Murashige and Skoog's (MS) basal medium supplemented with 6-benzyl aminopurine (1.5 mg l(-1)). Shoots were multiplied by routine periodic subcultures. The shoots of 3-4 cm length were isolated and rooted on MS basal medium (without CaCl(2)) containing indole-3-buteric acid (1 mg l(-1)). The rooted plantlets were hardened and successfully established in pots. More than 250 hardened plantlets in two successive years were transferred to their natural habitats of Western Ghats.

2674: ***+.148***

Context. The value of animal reintroduction as a conservation tool is debated. This is largely because the limited quantity of research that has been conducted on animal reintroductions has shown varying degrees of success in establishing new populations. The reasons why some reintroductions are successful, whereas others are not, are often not clear.Aims. The present research aims to determine whether reptile reintroduction into restored mine pits is a potential management technique for managing and conserving reptile populations within a mined landscape.Methods. Twelve Napoleon's skinks were trapped then fitted with 0.9-g transmitters. Half were reintroduced into 5-year-old restored mine pits and the other half into unmined forest. Bodyweights, movement patterns and macro-habitat selection were recorded weekly during November and then monthly until March. Key results. Skinks reintroduced into restored sites quickly moved into unmined forest. Both groups of skinks moved large distances, but those reintroduced into restored sites travelled further than did control skinks and took longer to reduce their distances travelled, showing possible stress as a result of release into unsuitable habitat. Eventually, almost all skinks found suitable habitat in unmined forest and settled into these areas while continuing to gain weight.Conclusions. Reintroduction was an ineffective technique for facilitating colonisation of restored minesites by Napoleon's skink. Lack of suitable micro-habitats within restoration areas, such as ground logs and coarse, woody debris piles, is likely limiting the use of these areas by Napoleon's skinks and is likely to be the cause of their failure to remain or settle in restored sites after reintroduction.Implications. Determining the habitat requirements of skinks and replicating this in restoration sites would seem the more appropriate management option than is reintroduction, and this may be the case for other reptiles and habitat specialists.

2675: ***+.011***

Aleutian Cackling Geese Branta butchinsii leucopareia were feared extinct until a remnant population was discovered on Buldir island by Robert "Sea Otter" Jones in 1962. Population declines, primarily due to predation by Arctic Foxes Alopex lagopus introduced to the breeding islands, resulted in the listing of Aleutian Cackling Geese as endangered in 1967. Fox removal, translocation of captive birds and captive breeding programmes boosted the remarkable recovery

of this sub-species from 790 individuals in 1967 to > 30,000 in 2001, when it was removed from the United States' Endangered Species List. Population estimates currently exceed 100,000 birds. However, the population recovery has brought complex management issues, including the harvest of a once-endangered sub-species and conflict with agricultural interests. This review comes 50 years following rediscovery of the remnant population, 20 years after initial reclassification from endangered to threatened, and 10 years after formal delisting from the United States' Endangered Species Act. This review describes the events leading up to the bird's recovery, details management actions taken on behalf of the sub-species, and recommends strategies for ensuring that this conservation success story continues into the future.

2676: *+.102*

Although Greater Sage-Grouse (Centrocercus urophasianus) face a suite of predators in sagebrush (Artemisia spp.) communities across the species' range, none of these predators specializes on sage-grouse. Greater Sage-Grouse are susceptible to predation from egg to adult, leading to the hypothesis that predator control would be an effective conservation tool for sage-grouse populations. Therefore, I reviewed the literature pertaining to predator communities across the range of Greater Sage-Grouse and assessed the effects of predation on sage-grouse life history. I then provided a framework for evaluating when predator management may be warranted. Generally, nest-success rates and adult survival are high, suggesting that on average predation is not limiting. However, in fragmented landscapes or in areas with subsidized predator populations, predation may limit population growth. Few studies linked habitat quality to mortality rates, and fewer still linked these rates to predation. Predator management studies have not provided sufficient evidence to support implementation over broad geographic or temporal scales, but limited information suggests predator management may provide short-term relief for a population sink. Evaluating the need for predator management will require linking reduced demographic rates to habitat quality (fragmentation or degradation) or predator populations out of the natural range of variability (exotic species of subsidized populations). Alternatively, managers might consider predator management in translocation efforts to buffer recently released individuals from potentially elevated predation rates. Future work should quantify predator and alternate prey communities in habitats used by Greater Sage-Grouse.

2677: *+.117*

Due to the critically low number, poor genetic status and much reduced possibility of natural re-colonisation (isolated population), the Lower Silesian Capercaillie population was nearing extinction. So, in 2009 a program of re-introduction of this species was initiated in the area of Ruszow Forest Inspectorate. During two years 24 young Capercaillies were released into the wild. The birds were obtained from the breeding facility for forest gallinaceous birds run at the Wild Animals Park in Kadzidlowo, and from the State Forests capercaillie breeding facility operated at the Wisla Forest Inspectorate. The birds coming from Kadzid3owo (12 individuals) were reared using the "born to be free" method developed by Dr. A. Krzywinski. The method was elaborated with an idea to use it for the species posing particular difficulties in terms of reintroduction. In autumn, 60% of young Capercaillies were fitted with radio-transmitters containing activity and mortality sensors. The survival rate of young birds is high and the main cause of mortality was predation. The Ruszow Forest Inspectorate applies a number of various practices aimed at improving the quality of the environment, considered in view of the habitat requirements of Capercaillies. Among the most important are: reducing the number of terrestrial predators, improving the shelter conditions by planting spruce in forest undergrowth, controlling the amount of undergrowth and herbaceous layer, lessening the crown density in tree stands, improving

feeding conditions by cutting old unproductive patches of bilberries, increasing moisture levels in the ground via the construction of small-scale retention projects, and eliminating foreign invasive plant species. Furthermore, educational campaigns are carried out, mainly for young people.

2679: ***+.170***

The Common hamster (Cricetus cricetus) has declined dramatically throughout Europe, especially in the most western part of Europe (the Netherlands, Belgium, West-Germany and northeast of France). The decline has led to strict protection of the species within Europe. At this time conservation projects are in progress to protect the remaining populations. In the Netherlands and Belgium the hamster population has drastically declined in number and range. In the Netherlands, for example, the hamster population declined to only 14 individuals in 1999. This has led to rapping of the last remaining individuals. A breeding program with the last remaining individuals and Belgian and German hamsters has been established. Since 2002 hamster populations are created with the offspring of these individuals. This resulted in three genetically different breeding lines: Dutch; Dutch-Belgian and Dutch-German. These breeding lines have been released in different areas. This study analyses five Dutch hamster populations (Amby, Heer, Puth, Sibbe and Sittard) and two Belgian populations (Berthem and Widooie). Four populations have been restocked with captive-bred hamsters with "new" alleles. This study analysed three questions regarding the genetic diversity in reintroduced and restocked populations of the Common hamster: 1) Is the genetic diversity of the reintroduced populations affected as a result of a founder-effect ? 2) Is the genetic composition changed after some years ? 3) Did the restocking result in an increase in genetic diversity ? The genetic diversity of reintroduced hamster populations is not affected as the result of any founder effects. The genetic variation of the reintroduced population (t = 0) is compared with the genetic variation of the first measurement period (t = 1). Genetically, not much has changed. No new alleles were detected, and no considerable change in heterozygosity has been found. Just a small increase in Allelic Richness (Rs) in the Amby and Sibbe area has been found. The genetic composition in the Puth area hasn't changed in four years. In Sittard the situation is different. The population starts to differ from this source, regarding its increasing Fst value. Also the heterozygosity slightly increases during five years. The increase in genetic diversity might be due to immigration of individuals from a neighbouring population. Introduction of individuals with new alleles (restocking) resulted in an increase of genetic diversity in the Heer, Berthem and Widooie areas. Restocking led to an increase in the number of alleles, Allelic Richness and heterozygosity. Further research is needed to examine the effect of this increase in genetic diversity and the population growth. Conservation of the Common hamster still remains the most important goal, but research contributes to knowledge for better protection of the species.

2680: ***+.202***

Populations of the Common hamster (Cricetus cricetus) are declining in Western Europe. Habitat fragmentation and changes in agricultural land use are the main reasons for the decline. In the Netherlands, a species protection plan was adopted in 2000, consisting of a reintroduction program and arrangements to create an interlinked hamster habitat of 500 ha. Now, it is eight years ago since the first hamsters were reintroduced in hamster reserve Sibbe (46 ha). The purpose of this research was to evaluate crop management in this reserve, by analysing the vegetation cover. Results show that on average, 45% of the Sibbe reserve provided sufficient cover for hamsters over the years. The best cover providing crop was alfalfa, as it offered sufficient cover during the longest time span, and provided most hectares with at least 80% cover. The crop that offered sufficient cover earliest in spring was winter oats, so it is recommended to cultivate this crop more frequently. In the Sibbe reserve, harvest was timed early in the season, as the average cover of

harvested crops started already to decline from early August. This might negatively affect the development of the second litter. To reach a growing hamster population, at least two litters are required. Consequently, it is recommended to postpone harvest until mid September. More direct research focused on the Dutch hamsters themselves is needed to improve preservation of the Common hamster

2681: *+.148*

The Common hamster (Cricetus cricetus, L. 1758) was once widespread and numerous throughout Europe between the latitudes 44[degree]- 59[degree]N and the longitudes 5[degree]- 95[degree]E (NIETHAMMER 1982). Its distribution is generally limited by climatic conditions and the existence of cohesional, well drained soil. Hamsters hibernate and store food in their burrows as supply for times of activity between torpor bouts. In the last four decades a continuous population decline has been observed especially at the westernmost distribution range in Belgium, Germany, France and the Netherlands, but in Poland also a decline of at least 80 % has been documented recently. In eight out of 18 European countries Cricetus cricetus is currently classified from "susceptible" up to "critically endangered". In seven countries no suitable data was available to classify the conservation status of the species and only two countries estimate it to he still common. Conservation measures are carried out in four countries only. In six countries (Belarus, Hungary, Moldova, Serbia, Russia and Ukraine) the hamster is not protected and may still be pest controlled and/or trapped for its fur. Together with Slovakia these are the same countries which possess no profound data to determine the conservation status. These are alanning statistics as those countries comprise the main part of the overall European distribution range of the Common hamster! Furthermore in at least ten countries data deficiency is the main reason for the lack of knowledge on the future population trend. The main threats for the Common hamster are habitat loss and fragmentation and modern, monocultural agriculture. Therefore the overall conservation strategy should be: Updating the knowledge on the distribution, population density and vitality especially in the eastern part of its range within the next two to five years. Intensifying and support research on population ecology, dynamics and genetics as well as 'the efficiency of conservation measures. Devising long-term conservation plans on the basis of above results for every country. Undertaking efforts to educate and inform the public to increase the acceptance of the species Increasing the co-operation between countries through regular meetings to exchange information and experiences. Wild populations should be protected by means of habitat conservation. Especially in those countries (Czech Republic, Hungary, Romania, Ukraine, Russia) where Cricetus cricetus is still abundant these measures should be taken in advance preferably in areas which are not liable to future habitat loss and fragmentation due to building activities. To maintain the Common hamster in the western part of its range an intensification of the efforts in terms of habitat protection, restoration and management, reduction of future habitat loss, conservation breeding and reintroduction is necessary.

2682: *+.032*

For centuries humans have reduced and transformed Mediterranean-climate oak woodland and savanna ecosystems, making it difficult to establish credible baselines for ecosystem structure and composition that can guide ecological restoration efforts. We combined historical data sources, with particular attention to mid-1800s General Land Office witness tree records and maps and twentieth century air photos, to reconstruct 150 years of decline in extent and stand density of Valley oak (Quercus lobata Nee) woodlands and savannas in the Santa Clara Valley of central coastal California. Nineteenth century Valley oak woodlands here were far more extensive and densely stocked than early twentieth century air photos would suggest, although reconstructed

basal areas (7.5 m2/ha) and densities (48.9 trees/ha) were not outside the modern range reported for this ecosystem type. Tree densities and size distribution varied across the landscape in relation to soil and topography, and trees in open savannas were systematically larger than those in denser woodlands. For the largest woodland stand, we estimated a 99% decline in population from the mid-1800s to the 1930s. Although most of the study area is now intensely developed, Valley oaks could be reintroduced in urban and residential areas as well as in surrounding rangelands at densities comparable to the native oak woodlands and savannas, thereby restoring aspects of ecologically and culturally significant ecosystems, including wildlife habitat and genetic connectivity within the landscape.

2683: ***+.151***

This 497-page book in English titled "Red Panda-Biology and Conservation of the First Panda" is a volume in its series. This book contains 26 individually-authored chapters. Each chapter is extensively referenced. Topics covered include red panda's role in economy and culture, evolution of the family Ailuridae, phenotypic and geographic diversity of the lesser panda Parailurus, taxonomy and phylogeny of Ailurus, reproduction and placentation of the red panda, management, husbandry and veterinary medicine of red pandas, and status and distribution of red panda, Ailurus fulgens fulgens. Also discussed are project Punde Kundo, conservation initiatives in China, records and reports of red pandas, Ailurus fulgens from areas with warm climates and release and reintroduction of captive-breed red pandas into Singalila National Park in India. This book includes a list of the contributors and their respective institutions. This book will be of interest to all those working or studying in the fields of wildlife management and ecology.

2686: ***+.052***

Resolution Island (20,800 ha) in Fiordland, New Zealand, has long held great potential as a sanctuary for the protection and reintroduction of highly threatened bird species. In 2008, the New Zealand Department of Conservation initiated a programme to eradicate wild stoats (Mustela erminea) from Resolution Island. Following the establishment of a trapping network, but prior to the traps being set, hair-snagging devices were deployed on approximately one quarter of the island, in order to obtain an independent estimate of population density. Stoat hair samples were collected from devices approximately daily over a 10-day period. DNA was extracted from 117 hair samples, and resulting genotypes were analysed using the spatially explicit mark-recapture software DENSITY, which provided a population density estimate for the study area of 0.48 stoats km-2 (95% CL 0.31 [long dash] 0.74; CV 23%). Hair tubes underestimated the 'minimum number alive' population density calculated from the number of stoats subsequently captured in kill-traps (an estimate of 1.4 stoats km-2) but provided precise information on detection parameters. They also gave an independent measure of initial trapping success with 21 out of 22 stoats detected in tubes being subsequently caught in traps. The above data in a Lincoln-Peterson index, with hair samples as the mark and trap samples as the recapture, gave a population estimate slightly above the actual number trapped. In a preliminary analysis, we modelled trap-capture data in a Bayesian framework and estimated that the probability of stoats persisting would be <1% after 10 consecutive checks with no captures. These models also yield a population slightly higher than the number of animals actually caught. We conclude that DOC150 traps were efficient at detecting stoats, but trapping stoats to extinction on Resolution Island will not be achieved in the near future and that initial trap spacing may have contributed to this.

2687: ***+.083***

The Lepidoptera (moths and butterflies) of Quail Island located in Lyttelton Harbour, Banks Ecological Region, Canterbury, New Zealand were surveyed between 2007 and 2009. Four other Banks Peninsula sites were also sampled. In total, 146 species of Lepidoptera were found on Quail Island, which is currently undergoing ecological restoration. This is relatively rich for a small island with limited habitat diversity, but not as rich as nearby Banks Peninsula sites. None of the known Banks Peninsula endemic moths were recorded on the island. Recommendations are made for additional plantings to encourage the establishment of greater numbers of moth species and particular moth species are suggested for reintroduction to the island.

2688: *+.135*

The maintenance of genetic diversity is thought to be fundamental for the conservation of threatened species. It is therefore important to understand how genetic diversity is affected by the re-introduction of threatened species. We use establishment history and genetic data from the remnant and re-introduced populations of a New Zealand endemic bird, the hihi Notiomystis cincta, to understand genetic diversity loss and quantify the genetic effects of re-introduction. Our data do not support any recent bottleneck events in the remnant population. Furthermore, all genetic diversity measures indicate the remnant hihi population has retained high levels of genetic diversity relative to other New Zealand avifauna with similar histories of decline. Genetic diversity (N-A, alleles per locus, allelic richness, F-IS and H-S) did not significantly decrease in new hihi populations founded through re-introduction when compared to their source populations, except in the Kapiti Island population (allelic richness and H-S) which had very slow post-re-introduction population growth. The N-e/N-c ratio in the remnant population was high, but decreased in first-level re-introductions, which together with significant genetic differentiation between populations (F-ST & Fisher's exact tests) suggest that extant populations are diverging as a result of founder effects and drift. Importantly, simulations of future allele loss predict that the number of alleles lost will be higher in populations with a slow population growth, fewer founding individuals and with nonrandom mating. Interestingly, this species has very high levels of extra-pair paternity which may reduce reproductive variance by allowing social and floater males to reproduce a life history trait that together with a large remnant population size may help maintain higher levels of genetic diversity than expected.

2689: *+.071*

Invertebrate survey conducted to establish species knowledge to assess habitat and diversity of ground dwelling invertebrates in proposed Houbara bustard Chlamydotis undulata macqueenii release sites of the Western Region of Abu Dhabi Emirate. The specific invertebrate species diet of Houbara Bustard in this region is poorly known and this study helps to understand those needs for attempts restore Houbara Bustard populations in the wild. Pitfall traps were used to study the diversity and seasonal abundance of invertebrates, especially ground dwelling darkling beetles (Tenebrionidae) at five desert sites in the Western Region of Abu Dhabi Emirate from March 2009 to February 2010. Traps were checked monthly and analysis was done based on the total catch of Tenebrionids from all study sites. The most abundant were Tenebrionid specimens. From all sites a total of 5720 Tenebrionid beetles were trapped, representing 15 species. The most dominant were Adesmia stoeckleini rasalkhaymana, Prionotheca coronata, Erodius octacostatus, Blap skollari kollari, Pimelia arabica, Pimelia arabica emiri, Apentanodesarabica and Paraplatyopepopovi. The species P. coronata, P. arabica, Mesostena puncticollis, A. arabica and P. popovi were found in all sites while species A. stoeckleini rasalkhaymana was confined to Site I. Number of Tenebrionids trapped in winter and midsummer were low, they reached peak in the spring when the weather was moderate and plant diversity highest. The appearance and disappearance of the beetles were

strongly linked with seasonal changes.

2690: *+.111*

Freshwater environments and their fishes are particularly vulnerable to climate change because the persistence and quality of aquatic habitat depend heavily on climatic and hydrologic regimes. In Australia, projections indicate that the rate and magnitude of climate change will vary across the continent. We review the likely effects of these changes on Australian freshwater fishes across geographic regions encompassing a diversity of habitats and climatic variability. Commonalities in the predicted implications of climate change on fish included habitat loss and fragmentation, surpassing of physiological tolerances and spread of alien species. Existing anthropogenic stressors in more developed regions are likely to compound these impacts because of the already reduced resilience of fish assemblages. Many Australian freshwater fish species are adapted to variable or unpredictable flow conditions and, in some cases, this evolutionary history may confer resistance or resilience to the impacts of climate change. However, the rate and magnitude of projected change will outpace the adaptive capacities of many species. Climate change therefore seriously threatens the persistence of many of Australia's freshwater fish species, especially of those with limited ranges or specific habitat requirements, or of those that are already occurring close to physiological tolerance limits. Human responses to climate change should be proactive and focus on maintaining population resilience through the protection of habitat, mitigation of current anthropogenic stressors, adequate planning and provisioning of environmental flows and the consideration of more interventionist options such as managed translocations.

2691: *+.026*

Brown bears have lost most of their range on the European continent. The remaining western populations are small, isolated and highly endangered. The Dinaric-Pindos brown bear population is the western-most stable population and the fourth largest in Europe. It has been recognized as a potential source for recolonization of populations whose survival is at risk. Indeed, several translocations of Dinaric bears to Italy, Austria and France have recently been made. Despite the importance of the Dinaric bear population, its genetic status remains poorly understood. Using tissue samples from 156 hunted or accidentally killed Dinaric bears in Croatia, this study analysed genetic diversity at 12 microsatellite loci, as well as population structure and past reductions in size. In addition, a subset of 59 samples was used to assess diversity of the mitochondrial DNA control region. The results indicate that Dinaric bears have high nuclear genetic diversity, as compared to other extant brown bear populations, despite genetic evidence of a bottleneck caused by past persecutions. However, haplotype diversity was low, probably as a result of male-biased dispersal and female philopatry. Not surprisingly, no evidence of population sub-structure was found using nuclear markers, as the bear habitat has remained continuous and the highway network has been built only recently. Management should focus on maintaining habitat connectivity and keeping the effective population size as large as possible. In addition, when removing individuals, care should be taken not to further deplete the population of rare haplotypes. A coordinated transboundary management of the entire Dinaric-Pindos brown bear population should be a priority for its long-term conservation. (C) 2010 Deutsche Gesellschaft fur Saugetierkunde. Published by Elsevier GmbH. All rights reserved.

2692: *+.176*

Eurasian beavers Castor fiber, formerly threatened with extinction, have been widely reintroduced since the 1920s. Reintroductions and studies of possible reintroductions are continuing. The

International Union for Conservation of Nature (IUCN) guidelines for reintroductions state that 'the source population should ideally be closely related genetically to the original native stock'. Palaeoecological studies suggest that the species survived the last Ice Age in two refugia: in the west in Iberia and Southern France and in the east in the Black Sea region. The post-Ice Age population of Western Europe, including Great Britain, recolonized from the western refugium. Recent mitochondrial deoxyribonucleic acid studies strongly support this view, and extant beaver populations are clearly divided into eastern and western evolutionarily significant units (ESUs). The western ESU is composed of three stocks which survived the 19th and early 20th century as very small, isolated populations. They are very closely related to each other. Each is genetically depauperate, apparently as a result of genetic drift at low population levels. There is evidence of inbreeding depression and of phenotypic abnormalities in beaver populations descended from unmixed stocks. The evidence suggests three coherent management options for sourcing reintroduction stock for Great Britain and for unoccupied areas of western continental Europe. These are (i) use animals from a single western ESU stock; (ii) intentionally mix animals from two or all three of the surviving western ESU stocks; (iii) make an informed exception to the IUCN guidelines and reintroduce animals of mixed eastern and western ESU provenance. These options are discussed with regard to IUCN guidelines, conservation biology and animal welfare considerations. It would be advantageous if a common policy on the origin of reintroduction stock were agreed by the national agencies responsible.

2693: *+.195*

Major global changes (e.g., human impact or climatic cycles) have a severe impact on the distribution and diversity of species such as the vulnerable European noble crayfish Astacus astacus. This is the first large-scale study regarding haplotype diversity of A. astacus in central and southeastern Europe. We analyzed a partial sequence of the mitochondrial gene cytochrome oxidase subunit I from 416 specimens of 92 crayfish stocks of three European river basins (Black Sea, North Sea and Baltic Sea). Twenty-two haplotypes were identified, and one common haplotype was found across the whole study area. We detected differences in the genetic diversity between major river catchments (Phi(ST): 0.03481 to 0.20387). The high haplotype diversity (H(D) = 0.794 +/- 0.024) and high number of private haplotypes suggests a glacial refuge in the Balkan area. The very low haplotype diversity in central Europe (H(D) = 0.299 +/- 0.038 and H(D) = 0.163 +/- 0.058) could be a result of human translocation and/or founder effects due to postglacial re-colonization. Nevertheless, the high frequency of private haplotypes in all major catchment areas indicates a differentiation of noble crayfish populations throughout Europe despite the extensive human translocation of crayfish. The results of this study support the establishment of conservation management plans for this vulnerable species.

2694: *+.008*

The Floreana Mockingbird (Mimus trifasciatus) is one of the rarest bird species in the world, with an estimated 550 individuals remaining on two rocky islets off the coast of Floreana, Galapagos, Ecuador, from which the main population was extirpated more than 100 yr ago. Because they have been listed in critical danger of extinction, a plan to reintroduce this species to Floreana has been initiated. Determining the health status of the source mockingbird populations is a top priority within the reintroduction plan. We report the health status, over the course of 4 yr, of 75 Floreana Mockingbirds on Champion Island and 160 Floreana Mockingbirds on Gardner-by-Floreana, based on physical examinations, hematology, hemolysis hemagglutination assay, exposure to selected infectious disease agents, and ecto- and endoparasite counts. Birds on Gardner-by-Floreana had higher body condition index scores, packed cell volumes, total solids, and

lymphocyte counts. Additionally, Gardner-by-Floreana birds had lower heterophil counts, eosinophil counts, and heterophil:lymphocyte ratios. No Chlamydophila psittaci DNA or antibodies to paramyxovirus-I, adenovirus-II, or Mycoplasma gallisepticum were found in any of the mockingbirds tested. Ectoparasites were present on birds from both islands, although species varied between islands. A coccidian species was found in eight of the 45 fecal samples from birds on Gardner-by-Floreana, but none of 33 birds examined from Champion. Birds on Gardner-by-Floreana were classified as healthier than those on Champion based on clinical and laboratory findings. These health data will be analyzed in conjunction with genetics, population structure, and disease presence on Floreana for developing recommendations for the Floreana Mockingbird reintroduction plan.

2695: ***+.144***

The population of the Gyps vultures has declined in South Asian region to 80-95% in recent years. The primary cause of this rapid decline is attributed to the widespread use of a NSAID, Diclofenac in the livestock, however, other causes of vulture decline are habitat destruction, pesticides poisoning etc. In order to restore the population of Gyps vultures conservation centres have been established in South Asia. The primary aim of these centres is to hold safely a population of the species affected. Once the environment is safe for vultures, they can help in reintroductions or supplementations to the wild. In Pakistan such a centre has been established at Changa Manga for Gyps bengalensis. This centre is managed by WWF - Pakistan in partnership with the Punjab Wildlife and Parks Department, whilst technical and financial support of The Hawk Conservancy Trust, UK and keystone funding comes from the Environment Agency Abu Dhabi, UAE. The Birds of Prey have many success stories of population restoration through captive breeding and reintroduction, some examples are California Condor Gymnogyps californianus, Mauritius Kestrel Falco punctatus, Peregrine Falcon Falco peregrinus and Griffon vulture Gyps fulvus. Techniques such as double clutching and hacking have been successful in increasing the breeding rate and improving reintroduction success. In Changa Manga a large communal aviary holds fifteen Gyps bengalensis. Sex determination has been done genetically and birds have been inserted microchipped and ringed for individual recognition. Within the aviary, potential breeding birds have access to artificial nest sites (shallow woven baskets, wooden baskets) and nests retrieved from the wild. A roosting/nesting ledge has also been developed for potential breeding pairs. Ungulates are kept alive for at least five days before they are slaughtered to be fed to the birds, this is done to ensure that had there been any Diclofenac injected is excreted out the system. Furthermore, breeding facility for rabbits has been developed and goats are also bred at the facility to maintain a variety in the diet and sustainability. The aviary at the Conservation Centre at Changa Manga has a capacity of approximately 30 vultures and four separate breeding aviaries have been developed already. These are arranged so that all the pairs are able to see each other that would help in synchronising breeding. The project faces the challenges of implementation of Diclofenac ban in veterinary practice, increase awareness to stop the use of human formulations in the veterinary practice and to increase the founder population of G. bengalensis in the conservation centre. Successful captive breeding and release programme has been internationally agreed as the most appropriate conservation intervention of the conservation of Asian Gyps vultures.

2696: ***+.091***

Captive breeding is one of the best ways to ensures future existence of any plant or animal. Through captive breeding practices efforts made to breed the animal species which are endangered or threatened, so that they could be reintroduced in their natural habitats. In Pakistan, all Wildlife

and Forest Departments are working at provincial level. In captive breeding centers, Gazella bennettii, Axis porcinus, Boselaphus tragocamelus, Antilope cervicapra, Crocodylus palustris, Chelonia mydas japonica, Lepidochelys olivacea olivacea, Capra aegagrus hircus, Cranes, Peafowls and Pheasants kept for breeding. Successful breeding and reintroduction of Antilope cervicapra in Cholistan, Gazella bennettii and Pavo cristatus in Salt Range, Axis porcinus in Daphar Irrigated Plantation has encouraged the Wildlife and Forest Departments for further work. Captive breeding ensures the continuity of the progeny to ultimate survival rate of the species and helps to maintain healthy population for education, awareness and ecological studies.

2697: ***+.370***

Nordens Ark is a non-profit foundation aiming to provide self-sustainable viable populations of threatened species through conservation breeding and reintroduction programmes. In 2001, an important decision was made to be part of a scientific society with close contacts to universities, and to appoint a professor in conservation biology as the scientific leader. Together with the universities, new programmes were developed where traditional academic knowledge could be combined with the experience and practical hands-on knowledge of the zoo world. Components of these programmes are held at Nordens Ark on a contracted basis. Nordens Ark has developed international research schools together with Gothenburg University, and conservation aspects of threatened wild animal species and domestic breeds provide the overarching theme. Nordens Ark has successfully taken part in breeding and reintroduction programmes for, among others, the White-backed woodpecker Dendrocopos leucotos, the Peregrine falcon Falco peregrinus, the Lesser white-fronted goose Anser erythropus, the Green toad Bufo viridis and the Fire-bellied toad Bombina bombina. Nordens Ark has also been contracted by national and international conservation authorities or organizations to ensure that the best possible conservation measures are taken when conflicts arise between infrastructure projects and the conservation of threatened species.

2698: ***-.032***

The white-winged guan Penelope albipennis (Cracidae) is endemic to the dry forests of northwestern Peru and considered Critically Endangered due to hunting and habitat destruction. Efforts to conserve this species included captive breeding and reintroduction. Before release, birds were screened for avian diseases to ensure the reintroduction of healthy guans. This work describes the efforts carried out during the years 2000 - 2007, to determine which diseases occur in captive guans and in backyard fowl surrounding the breeding centre, and to establish a pre-release health protocol. A health study was carried out in 95% of the captive population and sick backyard fowl, looking for Galliformes diseases: Avian Influenza (AI), Newcastle Disease Virus (NDV), Infectious Bursal Disease (IBD), Infectious Bronchitis Virus (IBV), Avian Leucosis Virus (ALV), Reticuloendotheliosis Virus (REV), Mycoplasma gallisepticum (MG), Mycoplasma sinoviae (MS), Salmonella Enteritidis (SE) and Salmonella Pullorum/ Gallinarum (SP/SG). We report for the first time avian pathogens in white-winged guans. Released guans have successfully bred, indicating viability of the reintroduction programme. The species showed positive titers to Infectious Bursal Disease, Infectious Bronchitis, Mycoplasma synoviae, Salmonella Enteritidis and S.Pullorum/Gallinarum. Backyard fowl were additionally positive to Newcastle Disease and Mycoplasma gallisepticum. Future reintroductions should follow the pre-release health protocol. At the breeding centre, periodical health screenings would allow the detection of new pathogens and would monitor the incidence of the known ones. The extension of the screenings in the released and wild guans would compare the prevalence and presentation of avian pathogens. Future conservation of the white-winged guan depends partially on the captive population and

should incorporate the health component.

2699: ***+.302***

In 2008 the National University of Mexico (UNAM) launched the Program for Water Management, Use and Re-use (PUMAGUA) with three main objectives: (1) to reduce water consumption by 50%; (2) to improve the quality of drinking and treated wastewater according to the most strict water quality norms; and (3) to promote participation of the entire population of the university in the efficient use of water. The first stage of PUMAGUA is taking place at the main campus of Mexico City, the University City. The campus covers an area of approximately 3 km(2), includes around 100 buildings, and hosts a population of about 150,000. Before the university was built, the area was covered by xerophytic brushwood, and more than 350 species of plants and around 150 species of birds were registered. However the vegetation has been replaced by buildings, parking lots and more than 150 hectares have been planted with grass and exotic plants. It is estimated that these areas are irrigated with an average of more than 1.5 millions liter of drinking water per day. The original vegetation is nowadays restricted to the Ecological Reserve of El Pedregal de San Angel (REPSA), which covers 270 hectares. Therefore, one of the actions suggested by PUMAGUA is to substitute some of the 150 hectares planted with grass and exotic plants and restore it with the original brushwood vegetation. The consequence would be a considerable reduction in water resource use for irrigation and the promotion of biodiversity conservation. PUMAGUA encourages active participation of the university's community in this matter. Consequently gardeners have been invited to workshops where the proposal of substituting vegetation has been presented, and also over twenty biology students have carried out considerable research related to this subject. Specific research activities have been the description of irrigation methods in different areas of the campus, the survey of the community's acceptance to change gardens and permit the re-growth of endemic vegetation in large areas as well as gardens of some departments. In the Department of Social and Political Sciences (adjacent to REPSA, and thus with an important function as a buffer zone) a zonification was carried out in order to determine the areas where reintroduction of native vegetation is feasible. The zonification was guided by physical and environmental characteristics, as well as the recreational use of the area. An outstanding achievement in this sense is the creation of a "show garden" inside de Botanical Garden of UNAM. It has an educational purpose, demonstrating visitors how native plants are integrated into the landscape of the university, where esthetical and ecological elements are combined and intergrated. Biology students have prepared a plant guide for this garden, where their ecological value is pointed out in terms of preserving biodiversity and reducing water consumption.

2700: ***+.110***

This study advances black bear management in East Texas by understanding residents' attitudes toward the return of black bear to the region and potential reintroduction efforts. Six counties in the study have the potential to be impacted by a returning population. The objective was to assess public knowledge, opinions, and attitudes of stakeholders related to black bears, management of particular species, and their habitats. Surveys were mailed to 2,000 households in the six counties. More than half of the respondents indicated they favored bears dispersing on their own but did not support agency intervention to reintroduce them. Respondents worried about problems that may arise from dispersal. Managers may use the results to identify areas of potential conflict and employ public information and education campaigns to encourage a public more receptive of black bears and management efforts.

2701: ***+.128***

We developed 12 microsatellite loci for the endangered minnow species, Tanichthys albonubes, using PCR-based isolation of microsatellite arrays. These new markers were tested in 26 individuals from a wild population collected from Guangzhou in China and 26 individuals from a cultured strain. The number of alleles ranged from two to nine and the expected heterozygosity from 0.177 to 0.853. The wild population had significantly higher allelic richness than the cultured strain, with a mean allelic richness of 5.52 (range = 3.69-8.64) and 3.13 (range = 1.99-5.73) for the wild population and the cultured strain, respectively. No evidence of a recent bottleneck was detected in the wild population, but it was found in the cultured strain based on the BOTTLENECK test. These primers can be used to understand the demography and to examine genetic differences between the cultured T. albonubes strains and wild populations to help determine conservation and reintroduction strategies.

2702: ***+.211***

We examined genetic variation in blood cockles in an effort to obtain information useful for the sustainability, management, and the stability of this species as a major commodity in the fisheries sector. Ten populations of cockles were sampled from the north to the south of the west coast of peninsular Malaysia. The cockles were collected in collaboration with the Fisheries Research Institute, Penang. The population genetic analysis of the cockles were studied via RAPD-PCR and mtDNA sequencing. Three hundred individuals were analyzed with RAPD-PCR experiments. High gene diversity over all loci was observed (Shannon index = 0.549 +/- 0.056 and Nei's gene diversity = 0.4852 +/- 0.0430 among 35 loci). The second method, mtDNA sequencing, was employed to complement the information obtained from RAPD-PCR. The gene selected for mtDNA sequencing was cytochrome c oxidase I (COI). One hundred and fifty individuals were sequenced, yielding a partial gene of 585 bp. Statistical analysis showed homogeneity in general but did reveal some degree of variability between the populations in Johor and the rest of the populations. The Mantel test showed a positive but nonsignificant correlation between geographic and genetic distances (r = 0.2710, P = 0.622), as in the RAPD analysis. We propose that the homogeneity between distant populations is caused by two factors: 1) the translocation of the spats; 2) larvae are carried by current movement from the north of the peninsula to the south. The different genetic composition found in Johor could be due to pollution, mutagenic substances or physical factors such as the depth of the water column. This population genetic study is the first for this species in peninsular Malaysia. The data from this study have important implications for fishery management, conservation of blood cockles and translocation policies for aquaculture and stock enhancement programs.

2703: ***-.145***

The wild boar (S. scrofa L.) is a generalist feeder that occasionally predates on semi-aquatic and terrestrial molluscs. We document for the first time the massive predation of wild boar on an aquatic epibenthic prey, the endangered freshwater mussel M. margaritifera. During the severe drought of 2009 an important part of the Negro mussel population was found predated by wild boar in a stretch subject to an episodic low level of water. Severe drought and boar predation caused the death of around 40% of the individuals in this metapopulation in the lower reaches of the river, which is the largest population near the southern limit of its global distribution. We propose a survey of the mussel colonies during periods of drought and the translocation of the mussels to deeper areas of the river in order to avoid this endangered mussel being accessible during the summer to the scavenging of wild boar.

2704: ***+.068***

Many sea turtle nesting colonies are in decline worldwide, and a common conservation practice maximizes hatchling production by translocating eggs from threatened nests to protective beach hatcheries. Typically, translocated eggs are 'doomed', or at risk of death due to tidal inundation, predation, or poaching. Sea turtles exhibit temperature-dependent sex determination. We determined how primary sex ratios, estimated from incubation temperatures, were affected by egg clutch translocation to a beach hatchery. We monitored incubation temperatures of eastern Pacific leatherback turtles Dermochelys coriacea in hatchery and in situ clutches at Playa Grande, Costa Rica, throughout each nesting season from 1998 to 2007. In situ clutches were estimated to be 90% female, whereas hatchery clutches (9% of clutches) were estimated to be 64% female. Taking into account differences in hatching success of in situ and hatchery nests, the overall sex ratio was 83% female. The Playa Grande hatchery abiotic environment (sand temperatures, water inputs) was similar to that in situ. However, metabolic heating was significantly reduced in hatchery clutches. The most likely explanation is that temperatures in hatchery clutches were cooler (less female-biased) due to decreases in the number of metabolizing embryos since hatchling success was lower in hatchery clutches than in situ clutches. Alteration of both primary sex ratios and hatching success is the tradeoff for reducing the risk of death to egg clutches by translocation to a hatchery. This tradeoff is not unique to Playa Grande leatherback turtles, and it is a strong indication that hatchery translocation should be used cautiously.

2706: ***+.216***

With a total area of 8900 km(2), Puerto Rico is the smallest of the Greater Antilles. It is divided in three physiographic regions or areas of relief: the mountainous interior, the karst region, and the coastal plains and valleys. The island comprises six ecological life zones: subtropical dry forest, subtropical moist forest, subtropical wet forest, subtropical rain forest, lower montane wet forest and lower montane rain forest. The herpetofauna of Puerto Rico consists of 25 species of amphibians (19 native, six introduced) and 56 species of reptiles (52 native, four introduced). The goal of this paper is to describe some of the present studies directed towards the conservation of Puerto Rican herpetofauna. Eleutherodactylus karlschmidti, E. jasperi and E. eneidae have not been seen or heard since 1976, 1981 and 1990, respectively, and are probably extinct. Since 2000, the potential causes of amphibian declines in Puerto Rico have been studied, and a synergistic interaction between climate change (increased dry periods) and disease (chytridiomycosis) have been proposed as an explanation for the patterns observed. Recovery efforts for Peltophryne lemur include a captive-breeding program, reintroductions island-wide educational outreach, protection and restoration of existing habitat, and the creation of new breeding ponds. Among reptiles, the first conservation efforts to protect Epicrates inornatus were limited to trying to halt collection and hunting. However, current strategies to preserve the boa include gathering basic biological information, habitat conservation, and educational outreach. Recent efforts for the conservation of Trachemys s. stejnegeri combine three research approaches to clarify the status of local populations: a mark-recapture-release study, field monitoring of reproductive activity (i.e., nocturnal patrolling to identify nesting activity), and field assessment of the potential impact of introduced species, particularly identification of predatory species and exotic turtles. Recovery initiatives for Cyclura stejnegeri include management of invasive mammals, a headstart program for hatchling iguanas, and the assessment of the etiology of a condition causing blindness in adult iguanas. A reforestation project aimed at recovering a local herpetofaunal assemblage after disturbances in a limestone valley in northern Puerto Rico is discussed. As population sizes of common colonizers such as Eleutherodactylus and Anolis increased, larger forest-interior and predatory species like Epicrates inornatus, Alsophis portoricensis and Anolis cuvieri followed.

Finally, the Mona Island marine turtle monitoring program is discussed and compared to other similar programs in Puerto Rico. As these and other similar conservation efforts provide scientifically based management recommendations, we hope to succeed in conserving the diverse herpetofauna that characterizes Puerto Rico.

2707: ***+.023***

I present an up-to-date annotated list of the herpetofauna of Martinique, and try to explain the causes responsible for the eradication of species such as Leptodactylus fallax, Boa sp. and Leiocephalus herminieri. Mabuya mabouya and Liophis cursor have not been seen for decades and may have been extirpated. It cannot be established that the mongoose was responsible; Didelphis marsupialis, of recent introduction, may have played an important role. Introduced and invasive species are numerous in Martinique: Chaunus marinus, Scinax ruber, Eleutherodactylus johnstonei, Gymnophthalmus underwoodi, Iguana iguana, Gekko gecko, Hemidactylus mabouia, without considering escaped pets and the dubious case of Allobates chalcopis as an endemic species. I also present the restoration plan for Iguana delicatissima in the French West Indies and the conservation work for this species in Martinique; increase of nesting areas, translocation, creation of numerous protected areas, and control of I. iguana. Of a total of 13 endemic and indigenous species from Martinique, three are definitely and a further two are probably eradicated. Including Guadeloupe, the French West Indies have the highest loss of herpetological biodiversity among all the islands in the West Indies.

2708: ***-.037***

The Burmese Star Tortoise, Geochelone platynota (Family Testudinidae) is a medium-sized tortoise (carapace length to ca. 30 cm) endemic to the dry zone of central Myanmar. Little is known concerning the ecology of G. platynota in the wild. The species occurs in xerophytic habitats typical of the dry zone, as well as grazed pastures, hedgerows, and agricultural fields. Its diet consists largely of grass and other vegetation, but fruit, mushrooms, snails, and insect remains have been recovered in feces. Mating occurs from June to September, followed by egg-laying from October through February. The mean size of 27 clutches was 4.4 eggs, and a positive relationship was noted between female size and clutch size. Based on a limited sample, the home range of males is somewhat larger than that of females. During cool and dry periods, activity declined and tortoises sheltered in bamboo thickets, undercut banks, and rock crevices. Geochelone platynota occurs both macro- and microsympatrically with Indotestudo elongata in the dry zone, but the ecological relationship between the two species remains poorly understood. The few available data suggest that G. platynota is ecologically extinct in the wild, as a result of historic long-term subsistence harvesting and more recent over-collecting to supply illegal international food and pet markets. The last known wild populations in Shwe Settaw and Minzontaung Wildlife Sanctuaries, and Myaleik Taung are now apparently reduced to non-viable levels. Future conservation efforts hinge on developing and implementing successful captive breeding and reintroduction programs in Myanmar. Currently, offspring are being produced at several rearing facilities in Myanmar, but persistent rampant poaching precludes the reintroduction of tortoises into protected areas.

2709: ***+.011***

Mauremys reevesii, Reeves' Turtle (or Chinese Three-keeled Pond Turtle) (Family Geoemydidae), is a moderate-sized aquatic species (carapace length to 300 mm) widely distributed in East Asia throughout central and eastern continental China, exclusive of the most southern, western, and

northern regions, and including Taiwan, southern Japan, and part of the Korean peninsula. However, the native distribution has been extended by human-aided translocations. The turtle lives in freshwater habitats in lowland areas with still or slowly moving water. Although no concrete data are available regarding the status of most populations, it is apparent that most have experienced major declines as a result of habitat destruction and/or commercial over-exploitation. Intentional and accidental releases of specimens from continental Asia may be altering the genetic stock of Japanese populations. Effective conservation of the species will require habitat protection and regulations to control the collection and transportation of wild animals. Additional studies are needed on the status, life history, and demography of the species.

2710: *+.100*

Hermann's Tortoise, Testudo hermanni (Family Testudinidae), is a medium-sized terrestrial species (average carapace length ca. 130 to 180 mm), widespread in the European Mediterranean region. Currently two subspecies are distinguished: T. h. hermanni in Western Europe and T. h. boettgeri in Eastern Europe, the border between them being the Po Valley in northeastern Italy. The species inhabits most Mediterranean vegetation habitats, but typically semi-open formations of stony, sun-drenched hills with low and sparse vegetation and grass. Some East European populations are found at up to 1300 m of elevation, but most populations are below 500 m. Sexual dimorphism is moderate, with females on average 12% larger than males. A morphological distinction from most other tortoise species is the presence of a horny claw-like scale at the tip of the tail that is more developed in males than in females. The maximum number of clutches laid annually is 3, but most frequent are 1 to 2 clutches per year. Maximum clutch size is 7 eggs for T. h. hermanni and 9 eggs for T. h. boettgeri, with a mean clutch size of 3.3 and 4.3 eggs, respectively. Annual survival of adults is estimated to range from 85 to 97%. Most western populations of the species are in strong decline and have very restricted distributions. Eastern populations appear to be more stable, though some populations also show a strong decline. Primary threats are destruction and alteration of habitats (mostly by forest fires, expansion of human settlements and infrastructure, and changes to traditional use of forest, pastoral practices, and agriculture), harvesting for the pet trade, and increases in the population size of native predators (mainly mammal carnivores and wild boars). Proposed conservation measures include habitat restoration and improvement, creation of reserves to protect the species, and environmental education. Captive breeding and reintroduction programs are necessary only for the most threatened populations.

2711: *+.175*

Species lists are essential to understand both temporal and distributional patterns of taxa. Based on data compiled by CEO (Centro de Estudos Ornitologicos), Willis and Oniki (2003), and from a search of more than 50 theses, dissertations, monographs and technical works, we listed all bird species recorded in the State of Sao Paulo. These records are composed of skins and other evidence collected and deposited in collections, and on photographs and voice samples. A total of 793 species were registered, distributed in 25 orders and 85 families, and corresponding to 45% of the Brazilian avifauna. Reasons for this high diversity are related to the environmental diversity found in the state, influenced by altitudinal and geographical ranges, different phytophysiognomies, presence of a coastal region, and areas of contact between forest ecosystems and Cerrado. Results of the Biota project contributed to a better understanding of how birds respond to anthropogenic alterations of the environment, such as habitat fragmentation. The main ornithological research groups are still based in universities and museums. Deficiencies of knowledge concerning bird studies in Sao Paulo are related to the lack of standardization of survey methodologies; paucity in the monitoring of threatened species in the long term; restricted

knowledge about species capacity to use matrix; and lack of refinement in the delimitation of evolutionary units and their distribution, which is essential for species reintroduction in regions where they have gone extinct.

2712: *+.092*

To support the sturgeon reestablishment program in Poland, DNA analyses of the extinct sturgeon populations of Odra and Vistula rivers were performed. According to the data obtained, the dominant sturgeon species since the third century BC was Acipenser oxyrinchus, rather than A. sturio as previously stated. Two ancient populations of A. oxyrinchus from Poland, one dated for the third century BC and one for the ninth to thirteenth centuries AD, were genetically characterized based upon mtDNA region (used here) and microsatellite analysis. The two populations are genetically close to A. oxyrinchus populations from the St. John River population, NB, Canada. Starting from 2004, fertilized eggs obtained from sturgeons in the St. John River were transferred to Poland. In 2006/2007, the first experimental stocking was performed. The fish maintained as a brood stock, as well as those used for stocking, were genetically characterized to monitor the level of genetic polymorphism.

2713: *+.109*

The European sturgeon, Acipenser sturio L., was present in rivers of the north-western Adriatic Coast together with the Adriatic sturgeon, Acipenser naccarii Bonaparte 1836, and the great sturgeon, Huso huso L., while it was the only species on the Tyrrhenian Coast. It was the most common sturgeon species in the Po River, where the first signal of decline, at the end of the nineteenth century, became a progressive trend in the first half of the following century and accelerated in the 1950s, when industrial development took place. In a different way, the most important Tyrrhenian population in the Tiber River underwent a rapid decline and disappearance in the 1920s, due to increasing fishing and pollution. In Italy, sturgeon fishing was declared illegal in the 1980s, but in a few years the species disappeared. Nowadays, a reintroduction plan could take advantage of the experience gained with the recovery plan carried out for A. naccarii.

2714: *+.199*

In response to ongoing local extinction of species and the current biodiversity crisis, the number of reintroduction programs aiming to establish new populations of rare species in the wild has increased. However, only a small proportion of these programs has been planned and monitored scientifically and comparative multi-species studies are missing in this context. Therefore, the relative importance of factors involved in reintroduction success is poorly known. In 2007, we assessed population growth since introduction as a measure of establishment success of 25 wetland species (rare or extinct in the wild nationwide) and a total of 50 populations in Switzerland that had been introduced at seven restored sites with apparently adequate environmental conditions between 1997 and 2005. We related establishment success to 32 life-history traits of these species obtained from the BiolFlor database, to initial number of introduced plants (propagule pressure with 1-130 individuals introduced per population), and to the ecological distance between source sites and restored sites based on vegetation records. Our results clearly showed the importance of close ecological similarity between source and introduction sites for successful establishment of wetland species into restored pond habitats. In contrast, neither life-history traits nor propagule pressure were related to establishment success in our study. Based on our results, we strongly recommend enforcing ecological studies prior to reintroduction to accurately assess the suitability of restored sites. To unambiguously assess the key determinants of

successful establishment, future reintroduction programs should be set-up according to experimental designs. (C) 2010 Elsevier Ltd. All rights reserved.

2715: *+.200*

Ex situ collections in botanic gardens have great potential in contributing to the conservation of rare plants. However, little is known about the effects of cultivation on the genetic diversity and fitness of garden populations, about genetic changes due to unconscious selection and potential adaptation to the artificial conditions. We compared the genetic variability and fitness of the rare, short-lived perennial Cynoglossum officinale from 12 botanic gardens and five natural populations in Germany. Genetic variability was assessed with eight nuclear microsatellites. Plants were grown in a common garden and performance was measured over 2 years. Mean genetic diversity was very similar in botanic garden and natural populations. However, four of the garden populations exhibited no genetic variability at all. Moreover, the genetic diversity of garden populations decreased with increasing duration of cultivation, indicating genetic drift. Plant performance from natural and garden populations in terms of growth, flowering and seed production was similar and in garden populations only seed mass was strongly related to genetic diversity. Several lines of evidence indicated genetic changes in garden populations in response to cultivation. Seed dormancy was strongly reduced in garden populations, and in response to nutrient addition garden plants increased the size of their main inflorescence, while wild plants increased the number of inflorescences. These changes could be maladaptive in nature and reduce the suitability of garden populations as a source for reintroductions. We suggest that botanic gardens should pay more attention to the problem of potential genetic changes in their plant collections. (C) 2010 Elsevier Ltd. All rights reserved.

2716: *-.098*

The European mink, an endemic semiaquatic small mustelid formerly widespread on this and continent, is nowadays critically endangered. An European Endangered Species Conservatio des Program EEP was initialized to conserve this species for future reintroductions. The association EuroNerz e.V. is member of this EEP. During the mating season captive European mink behave in a highly aggressive and individually manner. Due to this, mating takes place centralized in a breeding station. Subsequently the ER- pregnant females are lent to wildlife parks zoos for rearing the cubs. These institutions their visitors act as important multipliers for the aim of "public relations". With its captive population, EuroNerz e.V. supports reintroduction projects, of which one is described here as a case study. The chances for the conservatio m.e of the European mink are discussed.

2717: *+.327*

Research and translocations of brush-tailed rock-wallabies (Petrogale penicillata) in New South Wales have, in conjunction with studies in Victoria and Queensland, provided extensive insights yet also document the high variability in the species' response to management. Nonetheless, experts are being asked to quantify predicted response for cost benefit prioritisation models that will rank threatened species and populations worthy of future funding, with little consideration of the basic principles behind adaptive management. The weaknesses of these prioritisation models must be evaluated carefully by experts in order that appropriate advice is provided which genuinely assists decision-making. I explore the questions facing rock-wallaby ecologists as a case study of how much more we need to know and learn within adaptive approaches to conservation before our predictions are robust.

2718: *+.011*

A morphologically distinct subspecies of black-footed rock-wallaby (Petrogale lateralis pearsoni), is naturally found only on North Pearson Island (similar to 160 ha) in the Investigator Group, SA, where it was isolated by rising sea levels similar to 10 500 years ago. Subsequent translocations have seen additional populations established on Middle-South Pearson Island (similar to 53 ha) in 1960 and Wedge Island (96 ha) in 1975. We have used 10 hypervariable microsatellitc loci to examine the levels of genetic diversity in the endemic (n = 38) and translocated (n = 45-77) P. l. pearsoni populations compared with mainland P. lateralis populations (n = 19-52). Results show that all sampled P. l. pearsoni populations have very low levels of genetic diversity (A = 1.5-1.9; H-E = 0.02-0.13) compared with mainland populations (A = 3.5-12.7; H-E = 0.54-0.87). Intriguingly, more diversity was detected in the translocated Middle-South Pearson population than in its source population from North Pearson Island. In contrast, the Wedge Island population was almost monomorphic. Overall, the severe loss of genetic diversity (up to 98%) in P. l. pearsoni populations appears to result from random genetic drift on a small isolated population, exacerbated by some subsequent one-off translocation events. Although additional supplementary translocations are recommended to enhance genetic diversity, populations of P. l. pearsoni are likely to remain inherently vulnerable to extinction and therefore of special conservation concern.

2719: *+.240*

In 2008, after 9 years of presumed local extinction, brush-tailed rock-wallabies (Petrogale penicillata) were reintroduced at Moora Moora Creek in the Grampians National Park, western Victoria. Since little is known about this species in Victoria, the reintroduction presented an important opportunity to gain information on the species' ecology. Radio-tracking was undertaken and home range determined for three individuals released 11 months before this study and a further five individuals that were released at the commencement of the study in October 2009. Home-range size showed little variation amongst individuals, with a mean overall home range of 26 ha (+/- 1.69, s.e.) and a mean core home range of 2.5 ha (+/- 0.24, s.e.). Newly reintroduced individuals showed higher levels of association with wallabies from the same release and greater site fidelity when known conspecifics were close. Within 5 months of release, newly reintroduced animals showed home ranges similar in both size and distribution to those of animals released 11 months prior.

2720: *-.050*

We investigated the ecological relationships, reproductive biology and demography of four shrub taxa restricted to ironstone ranges in south-western Australia, to assess the feasibility of post-mining reintroductions. We found that three taxa were restricted to narrow fissures in massive ironstone and the fourth was restricted to fissures and skeletal soils over ironstone. In all taxa, adult plants were the most abundant life stage in populations and produced seeds annually. Newly emerged seedlings were observed in low numbers each winter of three census years, with the highest rates occurring when winter rainfall was above average in the semiarid Mediterranean climate. Mortality was highest and most variable for <1-year-old seedlings (50-93%), 1-year-old seedlings (17-67%), juveniles (21-54%) and vegetative adults (6-50%), and was lowest and least variable for the reproductive adults (2-7%). The restriction of three of our study taxa to narrow fissures excludes the option of using seedlings in reintroductions. Using seeds, although possible, will be both an inefficient and a high-risk strategy for at least three of the four taxa studied. This is because of the low frequency of years when winter rainfall is sufficient to stimulate high rates of seed germination, coupled with the consistently high rates of seedling mortality in most years, and

no easy method for determining which rock fissures will be suitable for plant establishment. The more widespread taxa showed reproductive and demographic characteristics similar to those of the taxa restricted to the narrow fissures, indicating that establishment of many species will be difficult in this environment.

2721: *+.056*

The ornamental trade is one of the major vectors of freshwater animal species translocation worldwide. The Australian redclaw crayfish Cherax quadricarinatus was brought into Singapore through the trade and culture of aquarium specimens during the late 1980s. From 2000 to the present, redclaw crayfish have been observed in at least 3 of Singapore's 13 reservoirs, including 2 inland reservoirs in the Central Catchment Nature Reserve (CCNR). The current paper presents the first in situ population data of feral C. quadricarinatus populations originating from ornamental trade and includes updated distribution records in Singapore based on recent surveys and museum samples. We report a C. quadricarinatus range expansion in 2 additional reservoirs (1 coastal and 1 inland), and the presence of multiple size cohorts confirms the establishment of reproducing populations in 2 coastal reservoirs and 1 CCNR reservoir. This is alarming as the current distribution borders Singapore's oldest and arguably most important nature reserve (Bukit Timah Nature Reserve: BTNR) and encircles the single remaining substantial freshwater swamp forest in the country (Nee Soon). The Nee Soon swamp forest and BTNR have extremely high conservation value as both areas together harbour the richest native freshwater fauna and flora of the island. Therefore, any range expansion of C. quadricarinatus in Singapore may have severe impacts on its native freshwater fauna and demands immediate attention. As C. quadricarinatus is one of the many invasive freshwater species occurring from the aquarium trade in Singapore, we argue that trade control might prevent further establishment of ornamentals.

2722: *+.246*

The Persian fallow deer (Dama mesopotamica) reintroduction project of the Israel Nature and Parks Authority is based on a permanent breeding core (Hai-Bar Carmel) established in Israel in 1976 from 2 males and 5 females, before the formulation of the guidelines for reinstruction by the IUCN, with no strategic long-term planning, and little consideration of conservation principles and monetary consequences. By the mid 1990s the breeding core had nearly 50 adult females and it became evident that a reintroduction program should be prepared. The existence of a permanent breeding core offered flexibility in protocol and the possibility of a long-term approach based on multiple releases. Using a maximum sustained yield approach, IUCN criteria, and simulations of population performance we formulated a release strategy and a time frame for the project, based on repeated releases carried out sequentially in three reserves in northern Israel with good corridors connecting them. The project began with releases in the Kziv reserve with continuous post-release monitoring and an adaptive management approach. Reproductive success was dampened during the initial years after release, but increased to expected levels thereafter. Survival was higher than expected. Animals from later releases used formerly released animals as cue and established a home range faster. Annual home-range dynamics and social structure were comparable to other similar deer species. The deer transported viable seeds of many species by ingestion (endozoochory) and thinned the forest canopy allowing for better understory growth. Simulations based on empirical data indicated that pre-project demographic simulation offered reliable projections. A growth model incorporating the empirical data on dynamics, habitat preferences, and social structure during the first 2.5 years enabled the construction of a spatially realistic individual-based population model that reliably projected the numerical and spatial growth of the population over a 5-year period. This model was then used to assess future risks due

to human sprawl. Due to agricultural damage, the project was forced in 2003 to select a new less favourable site in the Judean hills (central Israel) with no habitat linkage to the former location. Release in this area was based on individuals from the Hai Bar Carmel and from a second breeding core established in the Jerusalem Biblical Zoo. The less favourable site and behavioural problems of the zoo animals hampered the success of the reintroduction. In 2010 the northern region of the Israel Nature and Parks Authority approved a second release site in the Galilee. Using the spatially realistic model described above we reassessed the multiple-site approach, considering options of releases in 1-10 sites carried out in parallel or sequentially. These simulations indicated that the best results, in terms of numerical growth and spatial expansion, would be obtained by repeated releases in two sites carried out sequentially. Computer simulations combined with a permanent breeding core enabled robust planning and an adaptive management approach. Post-release monitoring provided important data for assessing reintroduction procedures and for future management of the species. This reintroduction has greatly enhanced the survival prospects of the Persian fallow deer, and their reintroduction has reestablished important ecosystem processes.

2723: *+.294*

The diet of the critically endangered Western giant eland (Tragelaphus derbianus derbianus Gray, 1847) has never been studied. We studied the food preferences of a Western giant eland group translocated from its native Sudanian habitat to a wildlife reserve in a Sahelian area in relation to the abundance of food in the new habitat. The study generated the first qualitative and quantitative data on this eland subspecies' diet. The translocated group fed only on 33 different plant species available in its new habitat. Isoberlinia doka, and Julbernadia sp., two species eaten by the other eland subspecies did not occur here. The food preference for most ligneous species did correspond to their high availability in the translocation area (like Acacia seyal, 32.56% versus 28.26% abundance in the new habitat, Acacia ataxacantha, 19.7% versus 17.03%, and Azadirachta indica, 7.11% versus 4.71%; only Grewia bicolor, 7.48% versus 10.14% did not). In contrast, the food preference for many herbaceous species did not, as some were preferred (like Merremia pentaphylla, 35.41% versus 11.01%, and Peristrophe bicalyculata, 2.58% versus 0.00%), and others avoided (like Corchorus tridens, 1.72% versus 2.20%, Cucumis melo, 0.43% versus 5.73%, and Abutilon ramosum, 0.21% versus 7.05%). The most eaten Cassia tora (42.7%) was also the most abundant (36.56%). Our data will provide conservation managers with information on how to protect the species' natural environment and to make appropriate management decisions with respect to the proclamation of protected areas or the identification of core zones, mainly for future (re) introduction of satellite populations. (C) Koninklijke Brill NV, Leiden, 2011.

2724: *+.221*

In the present work we analyzed the genetic structure of the populations of the terrestrial tortoise Testudo graeca graeca in southeastern Spain, identified as a recent range expansion from North Africa. The study and interpretation of the species' genetic spatial pattern could provide clues to the processes related to the species' arrival and, because of its endangered status, is especially useful in implementing appropriate management measures. We used microsatellite markers to analyze 17 populations located in the coastal region of the species' range in southeastern Spain, and an external group of Algerian tortoises. Three genetic units with a high level of spatial coherence and moderate levels of admixture resulted from a cluster analysis, and an isolation-by-distance pattern covering the entire study area was detected. These results suggest that southeastern Spanish populations show a complex spatial genetic pattern resulting from their isolation from North African populations and their natural dispersal in this region. Finally, our

work shows that conservation actions such as captive breeding, introductions or translocations, may have played a relevant role in the modification of the genetic structure of some populations in southeastern Spain. Therefore, these types of conservation measures should be carried out with more caution.

2725: *+.419*

Anthropogenic climate change has significant consequences for the sustainability and productivity of agroforestry ecosystems upon which millions of smallholders in the tropics depend and that provide valuable global services. We here consider the current state of knowledge of the impacts of climate change on tree genetic resources and implications for action in a smallholder setting. Required measures to respond to change include: (1) the facilitated translocation of environmentally-matched germplasm across appropriate geographic scales, (2) the elevation of effective population sizes of tree stands through the promotion of pollinators and other farm management interventions; and (3) the use of a wider range of 'plastic' species and populations for planting. Key bottlenecks to response that are discussed here include limitations in the international exchange of tree seed and seedlings, and the absence of well-functioning delivery systems to provide smallholders with better-adapted planting material. Greater research on population-level environmental responses in indigenous tree species is important, and more studies of animal pollinators in farm landscapes are required. The development of well-functioning markets for new products that farmers can grow in order to mitigate and adapt to anthropogenic climate change must also consider genetic resource issues, as we describe.

2726: *+.076*

European red deer are known to show a conspicuous phylogeographic pattern with three distinct mtDNA lineages (western, eastern and North-African/Sardinian). The western lineage, believed to be indicative of a southwestern glacial refuge in Iberia and southern France, nowadays covers large areas of the continent including the British Isles, Scandinavia and parts of central Europe, while the eastern lineage is primarily found in southeast-central Europe, the Carpathians and the Balkans. However, large parts of central Europe and the whole northeast of the continent were not covered by previous analyses. To close this gap, we produced mtDNA control region sequences from more than 500 red deer from Denmark, Germany, Poland, Lithuania, Belarus, Ukraine and western Russia and combined our data with sequences available from earlier studies to an overall sample size of almost 1,100. Our results show that the western lineage extends far into the European east and is prominent in all eastern countries except for the Polish Carpathians, Ukraine and Russia where only eastern haplotypes occurred. While the latter may actually reflect the natural northward expansion of the eastern lineage after the last ice age, the present distribution of the western lineage in eastern Europe may in large parts be artificial and a result of translocations and reintroduction of red deer into areas where the species became extinct in historical times.

2727: *+.110*

We evaluated the status of lynx in the Swiss Alps for the period 2005-2009. Even though the number of lynx presence signs remained almost stable between the present (2,068 signs) and previous pentad (2,091), there was a 7.6% increase in the area occupied by the 5-km circular buffers around the confirmed lynx signs of presence over the five years period (12,637 km(2)). The north-western Swiss Alps (VI) remained the compartment with the highest number of chance observations. It was followed by compartments central Switzerland west (III) and north-eastern Switzerland (II). These sub-populations acted as source in the current pentad, as signs of

reproduction were reported almost every year. The translocation to north-eastern Switzerland is still the only significant contribution to the spatial increase of the lynx range in the last 10 years in the Swiss Alps. The small and vulnerable north-eastern Swiss lynx sub-population plays an important role for the Alpine population. There is hope that in the future this sub-population could act as stepping stone to the eastern Alps and together with individuals dispersing from the central Switzerland west (III) sub-population would enable to found a new sub-population in central Switzerland east (IV). The status of the sub-population in the Valais (VII) is less clear. As only few signs of reproduction and mortalities were reported over the pentad, it acted more as sink than a source population. From the few signs of lynx presence reported in the remaining compartments (Grisons V, central Switzerland east IV and Ticino VIII) we concluded that only a few single lynx that did not yet establish the typical social organisation occur there. An occupancy-based population estimate from a parallel study resulted in about 111 (SE = 10) independent lynx for the period 2005-2009. This is higher than the 60-90 individuals estimated for the previous pentad.

2728: *+.100*

Large-scale declines of grey partridges (Perdix perdix) since the 1980s have led to local extinctions in the species range. As part of a UK recovery programme, we aimed to identify the best methods of re-establishing grey partridges through releasing in areas of extinction where a suitable environment has been restored. In East Anglia and southern England we followed the fates and breeding success of radio-tagged (one site per region) and colour-ringed birds (12 sites per region) of individuals released using five different techniques. The average resighting rate after the first 6 months post-release was 20% for bantam-reared and artificially-reared fostered young, 7% for unfostered young, 10% for full-grown birds in autumn-released coveys and 9% for spring-released adults. For birds that survived the first 6 months, the percentage resighted after a second 6-month period averaged 35%. Across both regions, 65% of grey partridge losses were due to predation of which 58% were killed by mammalian predators and 37% by raptors. Of birds still alive during the breeding season, 88% established their breeding territory within 1.5 km of the release location. There were no detectable differences in breeding success between release methods, but the proportion of females with broods among released birds was a third lower than among wild birds. We recommend re-establishing grey partridges by first releasing autumn coveys, followed by fostering. However, where wild birds are still present, the conservation focus should be on habitat improvements and predation control. (C) 2010 Elsevier Ltd. All rights reserved.

2729: *+.305*

Population manipulations such as translocation are becoming increasingly important tools in the management of rare and declining species. Evaluating the effectiveness of such manipulations requires comprehensive monitoring of population processes, including dispersal, survivorship, and reproduction. We investigated the mating system of a translocated population of gopher tortoises (Gopherus polyphemus) established through multiple releases, which occurred primarily during 1987-1994. During 2006-2007, we sampled and genotyped 27 candidate males (candidate sires), 34 candidate females (candidate dams), and 121 offspring from 19 clutches at five polymorphic microsatellite loci to determine the relative frequency of multiple paternity and to estimate individual reproductive success. Multiple paternity was detected in 57% of clutches genotyped, and females of single-sire clutches and females of multiple-sire clutches were of similar size. Reproductive success varied among male tortoises, and successful sires were significantly larger than males to which no offspring were attributed. Among successful sires, previously established males sired a disproportionate number of the offspring sampled, despite being significantly smaller

than subsequently released males. The high variance in individual reproductive success and the apparent reproductive advantage associated with prior residence observed in this gopher tortoise population has important implications for the design of future translocation projects. (C) 2010 Elsevier Ltd. All rights reserved.

2730: ***-.050***

We conducted an intensive survey of the Vulnerable eastern hoolock gibbon Hoolock leuconedys along the west bank of the Salween River in southern China, covering all known hoolock gibbon populations in China. We found 40-43 groups, with a mean group size of 3.9, and five solitary individuals. We estimated the total population to be < 200. In the nine groups for which we recorded composition, seven comprised one adult pair and 0-3 offspring and the other two groups both comprised one adult male and two adult females. The population is severely fragmented, in 17 locations, with the largest subpopulation containing only five family groups. Compared with the population in 1985 and 1994 five subpopulations have declined and gibbons have been extirpated from nine localities, although we discovered two previously unknown subpopulations. Commercial logging, illegal hunting, agricultural encroachment and population fragmentation pose serious threats to the future of H. leuconedys in China. An integrated conservation plan, including nature reserve establishment/expansion, enforcement of existing laws, conservation education, translocation and conservation-oriented research are needed to ensure the survival of H. leuconedys in China.

2731: ***+.121***

Dominance of warm-season grasses modulates tallgrass prairie ecosystem structure and function. Reintroduction of these grasses is a widespread practice to conserve soil and restore prairie ecosystems degraded from human land use changes. Seed sources for reintroduction of dominant prairie grass species include local (non-cultivar) and selected (cultivar) populations. The primary objective of this study was to quantify whether intraspecific variation in developing root systems exists between population sources (non-cultivar and cultivar) of two dominant grasses (Sorghastrum nutans and Schizachyrium scoparium) widely used in restoration. Non-cultivar and cultivar grass seedlings of both species were isolated in an experimental prairie restoration at the Konza Prairie Biological Station. We measured above- and belowground net primary production (ANPP and BNPP, respectively), root architecture, and root tissue quality, as well as soil moisture and plant available inorganic nitrogen (N) in soil associated with each species and source at the end of the first growing season. Cultivars had greater root length, surface area, and volume than non-cultivars. Available inorganic N and soil moisture were present in lower amounts in soil proximal to roots of cultivars than non-cultivars. Additionally, soil NO(3)-N was negatively correlated with root volume in S. nutans cultivars. While cultivars had greater BNPP than non-cultivars, this was not reflected aboveground root structure, as ANPP was similar between cultivars and non-cultivars. Intraspecific variation in belowground root structure and function exists between cultivar and non-cultivar sources of the dominant prairie grasses during initial reestablishment of tallgrass prairie. Population source selection should be considered in setting restoration goals and objectives.

2732: ***+.138***

Translocations to recover native fishes have resulted in mixed success. One reason for the failure of these actions is inadequate assessments of their feasibility prior to implementation. Here, we provide a framework developed to assess the feasibility of one type of translocation-

reintroduction. The framework was founded on two simple components of feasibility: the potential for recipient habitats to support a reintroduction and the potential of available donor populations to support a reintroduction. Within each component, we developed a series of key questions. The final assessment was based on a scoring system that incorporated consideration of uncertainty in available information. The result was a simple yet transparent system for assessing reintroduction feasibility that can be rapidly applied in practice. We applied this assessment framework to the potential reintroduction of threatened bull trout Salvelinus confluentus into the Clackamas River, Oregon. In this case, the assessment suggested that the degree of feasibility for reintroduction was high based on the potential of recipient habitats and available donor populations. The assessment did not provide a comprehensive treatment of all possible factors that would drive an actual decision to implement a reintroduction, but it did provide a fundamental level of feasibility assessment that is often lacking in practice.

2733: *+.174*

Context. Animal translocations are an important conservation tool; however, post-release dispersal can hinder successful population establishment. Playback of conspecific song attracts dispersing individuals in some species, although its application following animal translocation has yet to be rigorously investigated.Aims. To determine whether conspecific song can be used as an 'acoustic anchor', we adopted an experimental approach during the translocation of 60 North Island robins (Petroica longipes).Methods. At one of two release locations, we broadcast song at natural rates from four speakers (4 h per morning), for 9 days following release; we set the second release location as a control where identical conditions were established but no playback occurred. To assess the impact of playback, we monitored speaker and control locations, surveyed tracks around the release areas, and radio-tracked robins over nine playback days and an additional 9 days. Key results. Most robins left both immediate release areas; however, our results showed that (1) more robins (6 birds on 14 of the 18 days), in particular females (3 birds), approached the playback location than the 'flagged' control location (3 male birds on 5 of the 18 days), (2) individual robins returned to the playback location repeatedly, unlike those at the control site, and (3) robins also visited the playback location longer after playback than they did silent control locations. In contrast, radio-telemetry data from five robins suggested that general dispersal was not influenced by playback. Two radio-tracked females moved over long distances (some to >3 km from their release location), whereas two radio-tracked males remained relatively close to the release sites.Conclusions. We demonstrated a short-term attraction effect of playback over a period of several weeks for some birds, particularly females. In contrast, we detected fewer birds over a shorter period at the silent control release site, where no females were detected. However, long-term monitoring at both sites suggested that the effect of playback on reducing post-release dispersal was transitory.Implications. The lack of a clear and lasting effect of acoustic anchoring on dispersal in the present study has provided information on the limited utility of song playback as a conservation management tool for this species. Consideration of the species' ecology and suitability for 'acoustic anchoring' must be made before playback is employed as a conservation measure to reduce excess post-translocation dispersal.

2734: *-.029*

Context. The poor survivorship of animals released into the wild for translocation, reintroduction or rehabilitation may be cited as a reason not to release experimental animals, but there is only limited information available on the fate of ex-research animals returned to the wild.Aims. This study tested the hypothesis that there is no difference in the recapture of bandicoots used for physiological experiments and control bandicoots.Methods. Six adult male bandicoots were

trapped and maintained in captivity for three weeks for physiological experiments, then released at the capture site. Sixteen other bandicoots were captured and released immediately. Seven weeks after the release of the bandicoots used for physiological studies, follow-up trapping was carried out, and the survival, body mass and distance moved of recaptured bandicoots was recorded. Key results. Survivorship did not differ statistically between bandicoots used for physiological experiments and control bandicoots, with five of six experimental bandicoots (83%) and 11 of 16 control bandicoots (69%) recaptured. Bandicoots used for physiological experiments lost a significantly greater proportion of body mass than control animals, but this occurred in captivity, not after release. The distance between recaptures for both groups (0-224 m) was consistent with previously published observations.Conclusions. My results suggest that bandicoots maintained in captivity for non-invasive physiological experiments can be successfully released, with survivorship at least as high as that of control animals.Implications. This study provides researchers, wildlife managers, and animal ethics committees with information to assist with making judgements concerning the fate of ex-research animals.

2735: ***+.244***

Captive breeding is one of a myriad of tools at the disposal of conservationists. It can fulfil specific tasks that should be an integral part of the overall conservation action plan for a species. Captive breeding and other types of intensive management of individuals and populations often become necessary when human caused threats (habitat destruction, exploitation etc.) have caused the population of a species to become so small and fragmented that even if the human caused threats could be magically reversed, the species would still have a high probability of extinction purely due to random demographic and genetic events, environmental variation and catastrophes; or when the continuing, unchecked decline in population size indicates that this will soon become the case. Provided sufficient knowledge on the biology and husbandry of the species exists, breeding individuals in the relative safety of captivity, under expert care and sound management may provide an insurance against extinction, and/or a stock for reintroduction or reinforcement efforts, and/or opportunities for education, raising of awareness, scientific and husbandry research and other contributions to conservation. Important challenges include recognising when "the time is right", identifying the precise role of the captive breeding efforts within the overall conservation action plan, setting realistic targets in terms of required time spans, population sizes, founder numbers, resources etc., ensuring sound management and cooperation and developing much needed new technical methods and tools. The above is illustrated with examples from the Arabian Peninsula.

2736: ***+.080***

Captive breeding has the potential to play a pivotal role in conserving threatened species, among others by providing a healthy "safety net" population with which to buffer dwindling numbers in the wild. The Arabian Leopard Panthera pardus nimr is Critically Endangered on the IUCN Red List. Captive breeding is an essential component of conservation for this species. Many experts are of the opinion that the chances for survival of the Arabian Leopard in the wild are much reduced without the potential for reintroduction of animals. The captive breeding programme has been operating on a regional level since 1999, although the First Arabian Leopards registered in the studbook were caught in 1985. The current living population consists of 42 males, 32 females, and three unsexed leopards; nineteen are wild caught (of which 3 are siblings) and a substantial number of these do not actively participate in the breeding programme. The program focuses on ensuring a genetically sound population that closely resembles the wild population. Current and predicted trends within the population are compared with recommended trends and graphically

illustrated using dedicated population management software, PM2000.

2737: ***+.156***

In Saudi Arabia, a conservation and restoration programme for Oryx Oryx leucoryx, Pallas, 1766 was started in 1989 by the Saudi Wildlife Commission (formerly the National Commission for Wildlife Conmservation and Development). Concurrent conservation programmes were launched for the protection of large areas within the former range of the Arabian Oryx, and captive breeding at the National Wildlife Research Center (NWRC). Together, these have enabled the restoration of wild self-sustaining populations of Arabian Oryx in Saudi Arabia using animals from the 'World Herd' to improve their genetic variability. The success of the oryx conservation programme is described here together with the constraints faced in the arid environments and the consequent lessons learnt. As rainfall has a strong influence on the presence of annual plants, it is the single most important factor in the production of grazing. Poor rainfall had a major detrimental impact on forage in the re-introduction sites from 1999 to 2008 and mortality of oryx was higher during this period. As oryx historically moved over great distances in response to rain, the fence around one site, the Mahazat as-Sayd Protected Area, prevents natural movements of animals and artificially concentrates ungulate populations into seasonally unfavourable habitat. We propose some management strategies to minimize mortalities in the wild, and assesses post-release monitoring and adoption of various estimation techniques to assess oryx populations in both the fenced and free-ranging areas. As poaching is still a problem, strict law enforcement and a public-awareness programme to inform citizens of the biological and historical significance of the Arabian Oryx is recommended.

2739: ***+.271***

Marine biodiversity is increasingly threatened by multiple processes, and management strategies therefore must explicitly address the synergistic effects of multiple threats to marine species. The effects of harvesting and habitat degradation may be magnified for many coastal marine fishery species that rely on structurally complex nursery habitats to enhance survival and growth of postlarval and juvenile life history stages. Fishery management strategies that do not account for processes reducing juvenile survival and growth may overestimate the amount of biomass that can be taken; similarly, conservation and restoration strategies for nursery habitats that do not account for variable recruitment may fail. We used the blue crab Callinectes sapidus as a case study to investigate the population-level effects of harvest and seagrass habitat loss and fragmentation. We used available data to parameterize a stochastic stage-based model to test combinations of management strategies, namely reduced harvest rates and introductions of juvenile crabs to nursery habitat. Under a no-harvest scenario, large continuous areas of seagrass supported the largest blue crab populations. However, when harvest rates exceeded 20%, median population abundance was maximized in seascapes composed of smaller, fragmented seagrass patches. Populations in isolated patches of seagrass benefitted more from the introduction of crabs rather than harvest reduction, but the opposite was true for crab populations inhabiting highly connected seagrass seascapes. Management of species that use seagrass beds as nursery habitat must consider the spatial context of multiple threats and their potential synergies to maintain population persistence.

2740: ***-.011***

Huemul (Hippocamelus bisulcus) is a native deer of Patagonia whose endangered status has raised concerns for several decades, and yet conservation efforts to reverse this situation have not

succeeded for most populations. Captive breeding projects attempted in the past were short-lived; animals were often lost due to poor methodology or unsanitary conditions during capture, transport stresses and rudimentary husbandry, and reintroductions could not be realised. Despite inappropriate capture and transport techniques of the past, a few individuals did make it to captive centres where they managed to survive for several years, with a minimum of eight births recorded. Regardless of the successes, it is the past failures that impinge upon today's conservation efforts. In Argentina, a recent financially backed proposal - establishing a huemul breeding centre and including an in situ reintroduction program - was prevented by the prevailing opinion that captive breeding was neither feasible nor a necessary conservation tool for huemul. In Chile, the Huilo Huilo Foundation was able to obtain government consent and to establish the only captive breeding project in the last two decades with the main objective of reintroducing individuals in the future. Here we present some of the historical accounts to demonstrate the suitability of the species to captivity. We then describe the Chilean semi-captive breeding program (begun in 2005) including capture, transport, site selection, construction design and maintenance procedures of the two centres. The first centre has grown from an initial two adults to nine individuals. The second centre, which initially served for rehabilitation of an injured male, is awaiting arrival of some females. The success of the current program demonstrates that huemul can do well in captivity, and wherever considered beneficial, could serve as a significant conservation tool for the recovery of the species, inclusive of a research program and reintroductions to qualified sites.

2741: *-.096*

A key method of species conservation is captive breeding and release into the wild. This is usually the option of last resort when the organism of concern is either locally or functionally extinct. The endangered freshwater pearl mussel M. margaritifera has suffered huge declines across its biogeographic range, and as a result there is much interest in captive breeding and release as a conservation tool to halt its decline. This paper outlines an experimental protocol, which attempts to adhere to IUCN guidelines for reintroductions as far as is possible. The objective is to augment a declining M. margaritifera population in the Ballinderry River, Northern Ireland using juvenile mussels bred from captive stock from the natal river. Four hundred and ninety five mussels grown in captivity since 1998 have been tagged using Passive Integrated Transponder (PIT) tags. Fifteen tagged mussels were used in a pilot study, five of which were a control group to monitor stress during transport. Nine of the remaining ten have been relocated using the PIT tag receiver, and five of these have been visually confirmed as alive; none of the mussels in the control group suffered any ill effects as a result of transport. From the remaining tagged mussels, approximately 350 individuals in two size classes will be released across three sites within the river. Two periods of release were carried out; February and September 2009. Mussels were randomly allocated across three sites and taken to each site to be released, along with a control group to test for the effect of stress during transport on mussel mortality. Growth, survival and dispersal of translocated mussels will be monitored at intervals of six months. In this study, we provide an example of a scientifically informed protocol for introductions of captive-bred Margaritifera margaritifera (L.) into the wild to supplement declining populations or re-establish the species where it has become extinct.

2742: *+.114*

Macadamia jansenii is endemic to south-east Queensland, Australia, and is currently known from a single population 180 km north of the nearest wild population of its congener, the edible Macadamia integrifolia. A recently developed Macadamia recovery plan identified that this population was under significant threat and recommended a reintroduction program to safeguard

against chance extinction of the single wild population. This study undertook demographic population census surveys, genetic analysis, habitat analysis and niche modelling to determine the potential long-term viability for the species, and to guide search and reintroduction programs. We expanded the known population size of the species to similar to 60 individuals (1 m and taller) spread over a 900-m distance along a single creek clumped into three subpopulations. There was moderate genetic diversity in the species and the subpopulations showed little genetic differentiation. We developed a potential habitat model combining abiotic variables and vegetation associations, and mapped areas of potentially suitable habitat for M. jansenii within its local area. These maps are being used to target searches for other populations and to guide the location of introduction populations.

2743: ***+.182***

American shad Alosa sapidissima are in decline throughout much of their native range as a result of overfishing, pollution, and habitat alteration in coastal rivers where they spawn. One approach to restoration in regulated rivers is to provide access to historical spawning habitat above dams through a trap-and-transport program. We examined the initial survival, movement patterns, spawning, and downstream passage of sonic-tagged adult American shad transported to reservoir and riverine habitats upstream of hydroelectric dams on the Roanoke River, North Carolina and Virginia, during 2007-2009. Average survival to release in 2007-2008 was 85%, but survival decreased with increasing water temperature. Some tagged fish released in reservoirs migrated upstream to rivers; however, most meandered back and forth within the reservoir. A higher percentage of fish migrated through a smaller (8,215-ha) than a larger (20,234-ha) reservoir, suggesting that the population-level effects of transport may depend on upper basin characteristics. Transported American shad spent little time in upper basin rivers but were there when temperatures were appropriate for spawning. No American shad eggs were collected during weekly plankton sampling in upper basin rivers. The estimated initial survival of sonic-tagged American shad after downstream passage through each dam was 71-100%; however, only 1% of the detected fish migrated downstream through all three dams and many were relocated just upstream of a dam late in the season. Although adult American shad were successfully transported to upstream habitats in the Roanoke River basin, under present conditions transported individuals may have reduced effective fecundity and postspawning survival compared with nontransported fish that spawn in the lower Roanoke River.

2744: ***+.227***

BACKGROUND A series of efforts to establish a refuge population of vendace (Coregonus albula) originating from the threatened population of Bassenthwaite Lake in north-west England was made at Loch Skeen (or Loch Skene), Dumfries and Galloway, in south-west Scotland during the late 1990s. Subsequent sampling at Loch Skeen by gill netting in 2003 revealed that these efforts had been successful and that a reproducing population has been established, although a full population study including an assessment of abundance was not undertaken. Given a continuing deterioration in the status of the vendace in Bassenthwaite Lake, where no biological specimens have been recorded since 2001, the refuge population of Loch Skeen will prove invaluable in any future reintroduction attempt. The objective of this project was to apply a Site Condition Monitoring assessment protocol for whitefish to the vendace refuge population of Loch Skeen. This involved quantitative hydroacoustics and survey gill netting followed by assessment of results against a series of criteria covering abundance, population demographic structure, and maintenance of habitat quality. MAIN FINDINGS Geometric mean abundance of all sizes of all fish was 386.2 fish ha-1, with lower and upper 95% confidence limits of 106.6 and 1399.3 fish ha-

1, respectively. A total of 91 fish of three species, i.e. 42 brown trout (Salmo trutta) (length range 120 to 299 mm, weight range 22 to 336 g), 10 minnow (Phoxinus phoxinus) (length range 40 to 73 mm, weight range 1 to 4 g) and 39 vendace (length range 122 to 215 mm, weight range 16 to 111 g) was recorded from inshore, offshore bottom and offshore surface habitats. The vendace were aged from 1 to 6 years with all encompassed age classes being recorded. The overall male : female sex ratio was 0.5:1 (13 males : 24 females), with two immature individuals of age 1 year being of indeterminable sex. Both sexes generally reached maturity at 2 years. The above overall fish abundance converted to a geometric mean abundance of vendace of 231.7 fish ha-1, with lower and upper 95% confidence limits of 64.0 and 839.6 fish ha-1, respectively. The mean percentage contribution by small individuals to the combined offshore populations (identification to species was not possible) was 81%, with lower and upper 95% confidence limits of 58% and 105%, respectively. On the basis of the above and other features, the overall condition of the vendace refuge population of Loch Skeen was considered to be favourable.

2745: ***+.155***

BACKGROUND This report details the use of stable isotope analysis to provide information on the ecology of populations of the threatened vendace (Coregonus albula) translocated to two Scottish lochs (Lochs Earn and Skeen). Samples were collected during 2007 from vendace, other fishes, and putative food resources in the two lakes. These data were used to examine the ecology of vendace in these translocated populations with particular reference to trophic ecology, habitat use and interactions between vendace and other species. MAIN FINDINGS Analysis of carbon ([delta]13C) and nitrogen ([delta]15N) stable isotope ratios from the two translocation sites provided a wealth of ecological information at individual, species and ecosystem levels. Unfortunately only a single vendace was available from Loch Earn. However, comparisons of stable isotope values from different members of the fish community revealed apparent trophic and habitat segregation at the species level, i.e. between vendace, brown trout, Arctic charr, rainbow trout and three-spined sticklebacks. Comparison of stable isotope values from putative prey (benthic macroinvertebrates, zooplankton) and primary producers (epilithic algae and phytoplankton) revealed strong isotopic differences between lake habitats. Clustering in [delta]15N values indicated that the Loch Earn Arctic charr may include two sub-populations. Loch Earn brown trout were characterised by extreme isotopic variation indicating the presence of individual specialisation within the population. There was no evidence that the Loch Earn Arctic charr population was subject to negative trophic interactions with brown or rainbow trout. Vendace, brown trout and minnows were analysed from Loch Skeen, along with prey items removed from fish stomachs. Analysis of stable isotope ratios revealed that the different species were largely isotopically distinct. Vendace showed little overlap with brown trout and no overlap with minnow. Brown trout again displayed considerable variation at the individual level, with isotopic differences between individuals captured from offshore and inshore habitats. Brown trout displayed an increase in trophic level ([delta]15N) as they grew, but there was no isotopic evidence that brown trout were consuming vendace in Loch Skeen.

2746: ***+.148***

BACKGROUND The vendace Coregonus albula is a lake dwelling fish that is currently the rarest indigenous freshwater fish species in Great Britain. Of four populations that existed here at the start of the 20th Century, Derwentwater in Cumbria is believed to be the last remaining lake which continues to support a viable natural vendace population. The vendace is protected in Great Britain by the Wildlife and Countryside Act (1981). Several conservation projects for vendace have been undertaken in the last decade on behalf of Scottish Natural Heritage, the Environment Agency and

Natural England. These include vendace introductions to Loch Skeen and Daer Reservoir in southwest Scotland, and to Sprinkling Tarn in Cumbria. Vendace have also been placed in the Black Loch in Galloway as a holding stock for future introductions. The main objective of this project was to create new safeguard stocks in Black Esk Reservoir, Loch Valley and/or Loch Trool in southwest Scotland. MAIN FINDINGS Throughout an extended field programme in December 2007 nets of appropriate mesh sizes were set in Derwentwater to catch spawning vendace. Eggs collected there were fertilised on site and taken to a hatchery set up for this purpose at Barony College, Dumfries. This work was carried out with considerable staff and equipment contributions from the Environment Agency. The catches of vendace (13) were much lower than anticipated as were the number of eggs collected (<10,000). Of two batches of eggs delivered to the hatchery, the first (and largest) failed, leaving only some 3,600 fertile eggs for distribution later. At a meeting of the UK BAP (Vendace) group in January it was decided that because of the low number of eggs collected and the small number of parents contributing, the most appropriate course of action was to supplement earlier vendace introductions to Daer Reservoir and leave the Black Esk Reservoir and Lochs Valley and Trool for future use. Vendace egg incubation is lengthy and during the later stages of incubation, in March, the eggs were transported to Daer Reservoir.

2747: *+.223*

European mink is a small semiaquatic mustelid. Current Spanish population is lesser than 500 minks, along 2,300 kilometres of rivers. There are only three populations in Europe: the western one (Spain and France), the Romanian one (Danube Delta) and the Russian one (several subpopulations). The isolate and the small size of population, the lost of habitat, la presence of pollutants in water, the higher mortality by human causes (road casualties), the presence and ecological competition with American mink and minks infected by Aleutian mink disease, are the main threats on this species in Spain, and in other European countries. Since 1992, several researches are carried out in Spain. Some involved monitoring of Spanish population using appropriate methodology, based on prospecting and trapping animals alive. Other studies are ecological and ethological research (activity patterns, home ranges, habitat use, spatial distribution), population dynamic (sex-ratio, age-ratio, relative and absolute density, life expectancy), genetic characterization of population. The actions focusing the direct conservation are reduction of human mortality, conservation of genetic of species, prevention and control of diseases, elimination and control of American mink populations, definition and develop of "ex-situ" reproductive program and study of future viability of a reintroduction and population reinforcement program. The actions focusing to habitat are conservation of present habitat, recuperation of missing or deteriorated habitat, try connect different reproductive populations and Special Interest Areas (very good habitat to feed, shelter and reproduce), and reduction of level of pollutants in the water.

2748: *-.007*

Monitoring the distribution and movements of a species following reintroduction can aid resource managers in assessing release-site fidelity, rates of spread, initial project success, and feasibility of (or need for) future releases. We used radio-telemetry to monitor an entire founding population of 70 elk (Cervus elaphus) during 16 months following their reintroduction to eastern Ontario, Canada. At the end of the study, elk were widely scattered over a 27,000 km2 area. Dispersal distances ranged from 2 to 142 km; 50% of animals moved > 40 km from the release site. Dispersal distances differed by time periods and age but not sex. Calves dispersed significantly shorter distances than adults and many mature elk were isolated during the rut. In contrast to a random distribution model, movements had a strongly southwestern directional bias, perhaps

owing to prevailing winds from the same direction. Mortality during the study period was 27%; the primary causes of known mortalities were emaciation, collision with automobiles, and illegal shooting. During the first 11 / 2 years, lack of release-site fidelity and high dispersal coupled with animal-human conflicts and mortalities likely contributed to an initial lag in population growth. Resource managers planning animal reintroductions should consider using methodologies that enhance site fidelity following release.

2750: *-.034*

Genetic variation in two translocated populations of North Island saddleback (Philesturnus rufusater) on Kapiti Island and at Zealandia was investigated using five microsatellite loci and compared with the source populations in the Hauraki Gulf. Although the absolute number of alleles in the two populations was low (3 alleles per locus), both populations carried all the alleles found in their immediate source populations, but lacked one rare allele found in only one individual from the original remnant population on Hen Island. Overall heterozygosity was high and inbreeding coefficients were low. Population viability analyses showed that these populations will likely reach carrying capacity by the middle of this decade, and genetic simulations predicted that they should retain between 90% (Kapiti) and 95% (Zealandia) of the heterozygosity of their sources. The difference between the two populations is most likely due to the prolonged post-translocation bottleneck on Kapiti when rats were still present on the island. While our results suggest that additional top-up translocations would be unnecessary and unwarranted at this time, further work on potentially selected loci or inbreeding depression could justify this decision to be revisited.

2751: *+.079*

Context. Objective assessments of habitat requirements for endangered species are often lacking when planning management strategies, and inappropriate landscape manipulation can sometimes turn an endangered species into a pest. Recent expansive population growth of the rock hyrax Procavia capensis in northern Israel has been attributed largely to the proliferation of man-made boulder piles on the fringes of new residential developments.Aims. The hyrax is a protected species, but when in proximity to residential areas it can be a garden pest and is medically important as a reservoir of cutaneous leishmaniasis. Management should thus consider preservation of hyrax populations in combination with minimising pest potential. We examined the hypothesis that hyraxes prefer artificial boulder piles to natural outcrop crevices as den sites.Methods. We surveyed all 57 potential den sites in a 1 x 1 km area around a village in northern Israel, and conducted logistic regression to examine the correlation of hyrax presence with site type (pile or crevice), size, distance from the village, distance from other den sites and network centrality within the den site network. We used the Aikaike information criterion (AIC) to compare logistic models. Key results. Occupancy was well predicted by site type, site size, and distance from other sites, explaining 59% of the variation in the logistic regression. These three predictors were selected both by considering the combination of predictors that gave the lowest AIC value, and also by the stepwise logistic algorithm. Conclusions and implications. Hyrax den site preference, and in particular preference for boulder piles over natural crevices, should be integrated into managing this species simultaneously for conservation and pest control in the face of continuing residential encroachment on natural areas.

2752: *+.007*

The orchid Dactylorhiza incarnata (L) Soo is a highly polymorphic species listed as endangered in

many regional red lists of Central Europe and Scandinavia. The dramatic decline of its populations during recent decades has been caused by the loss and degradation of their natural and semi-natural habitats (fens and wet meadows, respectively) as a result of secondary succession following intensification or abandonment of traditional land use. In this study, we analysed the effects of abandonment and re-introduction of mowing on the long-term (28 years) population dynamics of Dactylorhiza incarnata at Lake Barsbek in northern Germany. In this area, to preserve a remaining population of D. incarnata, an annual mowing regime was re-established in 1981 on site M-1 of the investigated plots after a period of abandonment. Annual mowing was introduced on a second site, M-2, in 1987. Two- to three-year mowing was introduced on a third site, M-3. Site A, abandoned since 1970, was used as a reference. On each of these sites, flowering individuals were counted once a year. Population structure and accompanying vegetation were recorded simultaneously. In 2006, light measurements were carried out in the mowed areas. The D. incarnata population at M-1 increased exponentially during the first 10 years after reintroduction of mowing. Pronounced decreases in the number of flowering individuals were recorded in 1997 and 2003. Population dynamics at M-2 generally resembled the temporal development at M-1. D. incarnata disappeared on site A during the investigation period, while vegetation height and litter layer increased by 60 and 100%, respectively. D. incarnata was able to withstand reduced light availability to a certain extent by increasing its vertical growth (shade avoidance). It is concluded that the maintenance of D. incarnata populations in Central Europe requires continuation or re-establishment of wet meadow management. On previously abandoned sites, an initially higher mowing frequency is recommended. Management intensity can be reduced after phytomass production of the vegetation has declined. (C) 2011 Elsevier GmbH. All rights reserved.

2753: ***+.098***

The fish fauna of a 2.9 km long semi-natural main drain ("Mithlenhauser Fleet") within the upper river Weser estuary marshland near Bremen was removed and transferred to a new built water course due to motor-way construction. The drain lies within a Special Area of Conservation (SAC) regarding Habitats Directive, and retained a high population of Spined loath (Cobitis taenia), a species appearing in Annex II of the Habitats Directive. The fishing was done by means of quantitative electrofishing and intensive hand search following water-surface draw-down and dividing the watercourse into 150 m long sections. A total of 20728 individuals from 22 species and in addition 10000 cvprinid larvae were translocated. In comparison with other north western German marshland water bodies there was a higher percentage of phytophilic species. The Spined loach (Cobitis taenia) showed with 2018 individuals/ha remarkably high abundances in comparison with literature data. A value of > 2000 individuals/ha within the abundance criteria to classify a population to be in a favourable conservation status concerning Habitats Directive is supposed to be too high. Likewise populations showing a favourable conservation status are to be found in water bodies with a predominant organic substrate. The Crucian carp (Carassius carassius) occurred in a notable population size also. Conservation measurements should be enforced especially for this typical and representative floodplain species in the future because of a large-scale decline of populations, which is leading to an increasing degree of threat in many European countries. On the other hand the fish fauna showed deficiencies pertaining to diadromous species like European three-spined stickleback (Gasterosteus aculeatus) and eel (Anguilla anguiila).

2754: ***+.021***

Release of captive-bred red-legged (Alectoris rufa) partridges is used to reinforce hunting areas

where wild populations have decreased in southern European countries. However, breeders have often used other species to improve acclimation to captivity (e.g. Alectoris chukar), producing different degrees of hybridized individuals. In this study, three hunting reserve partitions, characterized by the different likelihood of contact with captive-reared partridges, were sampled and genotyped with 22 microsatellite markers to check for the existence of A. rufa x A. chukar hybridization and to compare the genetic composition of restocked and non-restocked red-legged partridge populations. Our results reveal the efficiency of the marker set used to differentiate among closely related A. rufa partridge populations, and the different genetic composition between captive-reared individuals and wild ones, but also the hybridization with A. chukar partridges on cynegetic farms. These facts must be taken into account and genetic controls of farm breeding stocks should be performed before restocking, both to avoid introgression in wild populations and to guarantee the reintroduction of partridges of known genetic origin in each area.

2755: *+.013*

Context. Feral cats and foxes pose a significant threat to native wildlife in the Australian arid zone and their broadscale control is required for the protection of threatened species.Aims. The aim of this research was to trial aerial poison baiting as a means of controlling feral cats and foxes in northern South Australia.Methods. Eradicat baits or dried meat baits containing 1080 poison were distributed by air over areas of 650 to 1800 km(2) in trials from 2002 to 2006. Different baiting density, frequency, bait type and area were trialled to determine the optimum baiting strategy. Baiting success was determined through mortality of radio-collared animals and differences in the track activity of cats and foxes in baited and unbaited areas. Key results. Quarterly aerial baiting at a density of 10 baits per square km successfully controlled foxes over a 12-month period, while annual baiting led to reinvasion within four months. Despite the majority of radio-collared cats dying after baiting, a significant decline in cat activity was only recorded during one of the eight baiting events. This event coincided with extremely dry conditions and low rabbit abundance. Rabbit activity increased significantly in baited areas over the study period in comparison with control areas.Conclusions. Despite trialling different baiting density, frequency and area over a five-year period, a successful long-term baiting strategy for feral cats could not be developed using Eradicat baits or dried meat baits.Implications. Broadscale control of feral cats in the arid zone remains a significant challenge and may require a combination of control methods with flexible delivery times dependent on local conditions. However, it is doubtful that current methods, even used in combination, will enable cat numbers to be reduced to levels where successful reintroductions of many threatened wildlife species can occur.

2756: *-.115*

Myrmecobius fasciatus Waterhouse, 1836, is a small to medium-sized dasyuromorph marsupial known as the numbat. M. fasciatus is unusual among marsupials in that it is diurnal and feeds exclusively on termites, and it has a number of characteristic adaptations associated with this specialized niche. M. fasciatus has at least 8 postcanine teeth in the lower jaw; the dentition is variable between individuals and even between the 2 sides of the jaw of the same individual. Although widespread throughout southern Australia at the time of European settlement, M. fasciatus is currently restricted to 2 naturally occurring populations in the southwestern portion of Western Australia, and some additional populations within its historic range resulting from successful reintroductions. It is currently listed as "Endangered."

2757: *+.202*

Observed high pup and juvenile mortality, as a result of starvation conditions that were prevalent in the Hawaiian monk seal (Monachus schauinslandi) population in the Northwestern Hawaiian Islands (NWHI), led to the development of a rehabilitation and reintroduction program for underweight monk seals. During 1984-1995, the program collected 98 young female monk seals whose mortality appeared certain (underweight and in some cases ill) from French Frigate Shoals (FFS) and attempted to nourish and rehabilitate these animals to enable their release as healthy individuals. Six additional young females of normal size were relocated from FFS (5) and Oahu (1) to Kure Atoll where their survival rate was expected to be higher than at their birth islands. The goal of the program was salvaging the reproductive potential of young female monk seals to aid in the recovery of this endangered species. Of the 104 animals collected, 17 died in captivity, 13 were converted to permanent captivity (for health or behavioral reasons), and the remaining 74 were released at Kure Atoll or Midway Islands within 14 mo of collection. Survival during the first year post-release was compromised, compared to native seals (born at the release site) but was similar to survival of natives in the second year. The released monk seals migrated among the three westernmost atolls at a higher rate than native seals. Monk seal monitoring continued through 2005 when 32 animals were known to be alive; they, with their offspring, constituted at least 12 to 14% of the animals in the three western NWHI populations. Captive-care management strategies were developed in a rapid response and varied greatly as did the success. These results are critical to the development of future captive-care initiatives that may be necessary to mitigate the continuing high loss of young monk seals in the NWHI.

2758: *+.057*

Monk seals (genus Monachus) are among the most endangered mammals in the world, with all species sharing a similar history of anthropogenic overexploitation. Reductions in population abundance have been accompanied by the loss of genetic diversity in Mediterranean (M. monachus) and Hawaiian (M. schauinslandi) monk seals. Both species are characterized by extremely low variability at all genetic markers tested to date, including microsatellite loci, the mitochondrial control region, and major histocompatibility complex class I genes. Genetic variation is partitioned differently in the two species. The Hawaiian monk seal does not exhibit spatial population structure throughout its range in the Hawaiian Archipelago (F-ST = 0.00); therefore, it is unlikely that the translocation of monk seals will result in genetic incompatibilities. In contrast, Eastern Mediterranean and Western Saharan M. monachus populations are reproductively isolated (F-ST = 0.56), though the distribution of alleles likely signifies a once contiguous range sundered by the extirpation of geographically intermediate subpopulations. Given the magnitude of genetic differentiation, moving Mediterranean monk seals between eastern and western populations could result in reduced overall fitness; additional data is required to assess the risk of moving monk seals within a population (e.g., the Eastern Mediterranean). Recent advances in genomics will facilitate future investigation into the reproductive success of both species and guide managers in their quest to recover Mediterranean and Hawaiian monk seal populations.

2759: *+.122*

In conservation, difficult decisions concerning prioritization of different geographic areas must be made. Large-scale prioritization methods such as defining biodiversity hotspots and crisis ecoregions have paved the way; however, these efforts then need to be localized. Various approaches have been taken, such as defining evolutionarily significant units (ESUs), management units, and core habitat areas within the range of a taxonomic species. These units can then be ranked as candidates for conservation, with the goal of preserving a given species by protecting

only a subset of populations. Here we used a combination of amplified fragment length polymorphisms (AFLPs) and DNA sequence data to elucidate the relationships within and among populations of the spinytailed iguana Ctenosaura melanosterna throughout its range in Honduras. Our findings indicate that there are 2 ESUs corresponding to geographically and ecologically distinct island and mainland habitats. Management strategies consisting of translocation or captive breeding and release should not consider the island and mainland populations exchangeable. In addition, the narrow geographic range of each group suggests that no region or subpopulation is likely expendable. This study demonstrates a situation, most likely increasingly common in conservation, in which it seems that only the high priority areas remain.

2760: ***-.067***

Crayfish plague infection, caused by the oomycete Aphanomyces astaci, is believed to lead to a total mortality of the populations of highly susceptible freshwater crayfish species like the noble crayfish Astacus astacus. It has therefore been customary in Finland to restock affected water bodies relatively soon after the population has been wiped out in an acute episode of crayfish plague. In many cases, these re-introductions have failed without any explanation. During a follow-up study of a small lake, no restocking was undertaken after an outbreak had struck the population in 2001. The crayfish plague agent was isolated three years after the outbreak and again a year later in 2005. This capability of A. astaci to survive supported by a weak noble crayfish population may explain the often recorded failures of repopulation attempts, and recurrent episodes of acute crayfish plague in certain lakes.

2761: ***+.102***

Although Greater Sage-Grouse (Centrocercus urophasianus) face a suite of predators in sagebrush (Artemisia spp.) communities across the species' range, none of these predators specializes on sage-grouse. Greater Sage-Grouse are susceptible to predation from egg to adult, leading to the hypothesis that predator control would be an effective conservation tool for sage-grouse populations. Therefore, I reviewed the literature pertaining to predator communities across the range of Greater Sage-Grouse and assessed the effects of predation on sage-grouse life history. I then provided a framework for evaluating when predator management may be warranted. Generally, nest-success rates and adult survival are high, suggesting that on average predation is not limiting. However, in fragmented landscapes or in areas with subsidized predator populations, predation may limit population growth. Few studies linked habitat quality to mortality rates, and fewer still linked these rates to predation. Predator management studies have not provided sufficient evidence to support implementation over broad geographic or temporal scales, but limited information suggests predator management may provide short-term relief for a population sink. Evaluating the need for predator management will require linking reduced demographic rates to habitat quality (fragmentation or degradation) or predator populations out of the natural range of variability (exotic species of subsidized populations). Alternatively, managers might consider predator management in translocation efforts to buffer recently released individuals from potentially elevated predation rates. Future work should quantify predator and alternate prey communities in habitats used by Greater Sage-Grouse.

2762: ***-.015***

Moll's (1988) report to the Illinois Endangered Species Protection Board recommended attempts to locate viable populations of alligator snapping turtles (Macrochelys temminckii) before considering management actions. We surveyed 18 sites in southern Illinois and captured 2,671

turtles, none of which were M. temminckii. No verifiable records were obtained from our solicitation of observations in two regulatory publications of the Illinois Department of Natural Resources. Our findings are supported by other chelonian studies and a lack of documented records of M. temminckii in Illinois during the past quarter century. We conclude recovery of this species is unlikely in Illinois without direct management such as translocation or release of captive-raised individuals.

2763: *+.107*

A population of slow-worms were monitored throughout August and September 2010 at a roadside location near Dobwalls, Cornwall. The population was released onto the site in 2009 and one of the aims of the study was to determine if the population was surviving in their new habitat. Follow up work post-translocation is rarely done in the UK. The vegetation was predominantly saplings, young shrubs and rough grassland and black roof felt was used as refugia to attract the slow-worms. The site was visited two to three times a week and the temperature, weather, invertebrates present, sex or age and location of the slow-worms were recorded. The results found that the site was abundant in invertebrate species that slow-worms could feed on and it was unlikely their distribution was determined by the food source alone. It was also apparent that the site contained smaller microhabitats of damp, sunny and overgrown areas that influenced the slow-worms. location. It is possible that some individuals had migrated up to 267m from the release point in the one year of being on site. It is a complex combination of many environmental factors that determine where the slow-worms are on site. The weather conditions including the temperature are very important factors in observing the slow-worms under the refuges. The optimum conditions were 15iAEC with at least 40% cloud cover and a moderate breeze. When it was too cold or hot very few were recorded. The distribution of the population in terms of gender and age was heavily weighted towards females and juveniles. This could be due to the fact that they tend to bask and utilise the refuges more or because there were more females in the population but probably due to both. A number of recommendations have been made including an extended monitoring area and a more detailed food source survey.

2764: *-.031*

Reintroduction of captive-bred animals into suitable habitats is an important technique for the long-term conservation and recovery of populations of endangered species in fragmented landscapes. Inbreeding depression is an inherent risk when using captive populations for reintroduction programs and needs to be carefully assessed prior to reintroduction. In this study, we evaluated inbreeding levels within a captive breeding program and one remnant wild population of Cabot's Tragopan (Tragopan caboti), an endangered pheasant species endemic to China, for which reintroduction is now an essential conservation strategy for long-term population persistence. Fifteen highly polymorphic microsatellite loci were developed to genotype individuals. Inbreeding coeficients (FIS) reveal that there is no evidence of inbreeding within the Tragopan Breeding Center of Beijing Normal University (TBCBNU) captive population and the remnant population from the Wuyi-Yandang Mountains. Diversity of origin, large founder population size and a rational breeding strategy are the most critical factors preventing inbreeding depression within the TBCBNU captive population. We suggest that the TBCBNU population is a suitable candidate stock for T. caboti reintroduction programs and that there is an urgent need to better coordinate and strengthen reproduction management of captive T. caboti populations to sustain the long-term ex situ conservation of the species.

2765: *+.138*

Eradications of invasive mammals have become increasingly complex and expensive. Increased public exposure and involvement in decisions about island eradications mean that conservation scientists must be prepared to justify the benefits of proposed eradications and defend the science used to measure cause and effect of agents of decline. Here I assess the biological, scientific and political outcomes of eradications on those islands in New Zealand from which all introduced mammal populations have been removed. By 2010, 147 populations of 13 species of vertebrates had been removed from a least 95 islands with a total area of 32,000 ha. Identified benefits to biodiversity were through in situ recovery, translocations or metapopulation management on the islands. These include improved prospects for 16 species of invertebrates, two species of frogs, three taxa of tuatara (Sphenodon spp.), 23 species of lizards, 32 taxa of terrestrial birds and 16 taxa of seabirds. The eradications can also be used to test hypotheses about the impacts of invasive species on native ecosystems. Considerable effort has been applied to understanding the effects of Pacific rats (Rattus exulans). There are now published accounts of the effects of these rats on plants, lizards, tuatara and seabirds, often using well designed field experiments. However, the effects of most other invasive vertebrates are poorly documented. Furthermore, impressive accounts of biodiversity achievements obscure potential problems. These include the genetic effects of small relict populations or small founders from translocations. Nonetheless, there has been acceptance of the value of these eradications at the highest political levels, government support for assistance in developing countries, and global export of technologies developed. A deeper understanding of the effects of invasive species, good reporting systems, and frequent communication and defence of benefits will be needed to gain public acceptance of increasingly ambitious projects.

2766: *-.006*

In fisheries, recovery actions for species of concern sometimes involve reintroductions of multiple species. As an example, desert pupfish Cyprinodon macularius and Gila topminnow Poeciliopsis occidentalis historically had sympatric distributions and inhabited similar environments, and they are often reintroduced into the same waters. Success of these stockings has varied greatly, and a hypothesis developed among conservationists that desert pupfish populations were more likely to establish and persist if they were stocked first and allowed to establish before Gila topminnow were stocked. We examined historical records of desert pupfish reintroductions and conducted laboratory experiments to evaluate this hypothesis. For the reintroductions, we evaluated 29 ponds where four general stocking strategies (treatments) were implemented: (1) only desert pupfish were stocked, (2) desert pupfish were stocked first and allowed to establish and then Gila topminnow were stocked, (3) both desert pupfish and Gila topminnow were stocked simultaneously, and (4) Gila topminnow were stocked first and allowed to establish before desert pupfish were stocked. Desert pupfish established populations and persisted in 100% of desert pupfish-only and desert pupfish-first ponds and 57% of simultaneous-stocking and Gila topminnow-first ponds. In laboratory experiments utilizing 2.4-m-diameter tanks, the same four treatments plus one additional treatment (in which only Gila topminnow were stocked) were implemented; each treatment was replicated four times. Desert pupfish abundance increased in 75% of the desert pupfish-only trials and 50% of the desert pupfish-first trials but decreased in 75% of simultaneous-stocking trials and 100% of Gila topminnow-first trials. Gila topminnow increased in abundance in all treatments and trials but increased the least when desert pupfish were stocked first. Our results support the original hypothesis and emphasize the need to evaluate species interactions in multiple-species reintroductions. To increase success, desert pupfish should be stocked in isolation or stocked first and allowed to establish before Gila topminnow are stocked.

2767: ***+.081***

The endangered Przewalski's horse is the closest relative of the domestic horse and is the only true wild horse species surviving today. The question of whether Przewalski's horse is the direct progenitor of domestic horse has been hotly debated. Studies of DNA diversity within Przewalski's horses have been sparse but are urgently needed to ensure their successful reintroduction to the wild. In an attempt to resolve the controversy surrounding the phylogenetic position and genetic diversity of Przewalski's horses, we used massively parallel sequencing technology to decipher the complete mitochondrial and partial nuclear genomes for all four surviving maternal lineages of Przewalski's horses. Unlike single-nucleotide polymorphism (SNP) typing usually affected by ascertainment bias, the present method is expected to be largely unbiased. Three mitochondrial haplotypes were discovered-two similar ones, haplotypes I/II, and one substantially divergent from the other two, haplotype III. Haplotypes I/II versus III did not cluster together on a phylogenetic tree, rejecting the monophyly of Przewalski's horse maternal lineages, and were estimated to split 0.117-0.186 Ma, significantly preceding horse domestication. In the phylogeny based on autosomal sequences, Przewalski's horses formed a monophyletic clade, separate from the Thoroughbred domestic horse lineage. Our results suggest that Przewalski's horses have ancient origins and are not the direct progenitors of domestic horses. The analysis of the vast amount of sequence data presented here suggests that Przewalski's and domestic horse lineages diverged at least 0.117 Ma but since then have retained ancestral genetic polymorphism and/or experienced gene flow.

2768: ***+.035***

Captive breeding and release is a tool used by conservation biologists to re-establish populations of endangered or locally extinct species. Reintroduced animals that have been bred in captivity must learn to meet the challenges posed by free living, and to adjust to local environmental conditions, food and water sources. How well reintroduced animals might meet these challenges is uncertain as few longitudinal studies have investigated the physiology of reintroduced animals or the implications of this for successful establishment of new populations. Here we have evaluated long-term, seasonal energy and water use by reintroduced yellow-footed rock-wallabies (Petrogale xanthopus celeris), an endangered medium-sized marsupial that inhabits rocky outcrops across Australia's arid and semiarid rangelands. Captive-bred rock-wallabies were reintroduced to an area within the known boundaries of their former range, in south-western Queensland, Australia. Post-release water turnover rates (WTR) and field metabolic rates (FMR) were measured during their first wet summer and dry winter, by means of the doubly labelled water method. Total body water (73.1%), FMR (1650.0 kJ day(-1)), female fecundity (100%), and male and female body masses and survival were consistent between seasons, but rates of water turnover were significantly lower for all animals during the dry winter (174.3 mL day(-1)) than during the wet summer (615.0 mL day(-1)). There were no significant differences in WTR or FMR between males and lactating females (in either season).

2769: ***+.216***

We studied the conservation status of some populations of spadefoot toad, Pelobates fuscus, in Piedmont, by using predictive models, with the aim of foreseeing their extinction risks. The applied model allowed to test the sensitivity of the species using several parameters that affect the demographic trend, to formulate hypotheses, case by case, on the observed population decline. We also measured the contribution given in terms of survivor- ship from the systems organized in metapopulations and testing the opportunity provided by reintroduction actions. The need to deine

the necessary parameters for the construction of the model, put in evidence the main deiciencies in the knowledge of the species biology, upon which addressing future ield research.

2770: ***+.213***

Rewilding is a strategy for the conservation of complete, self-sustaining ecosystems, primarily involving the protection and, where necessary, reintroduction, of populations of keystone species in large, connected reserve networks. A potential method of preserving ecosystem functions and biodiversity, it is now receiving a great deal of practical and political attention, particularly in North America. In Scotland, where many native species have been extirpated in the relatively recent past, rewilding has clear relevance and may provide an overarching set of objectives for current programmes of native woodland restoration and species reintroductions. Nevertheless, rewilding is not widely used as a term or strategy in Scottish conservation. This review considers the development of the concept and its possible application in Scotland, and identifies substantial scope for rewilding, in terms of the restoration and protection of large areas of wild land, and of the reintroduction of native species which have been driven to extinction by human activity. As the environmental, social and economic benefits which are likely to result from a programme of rewilding in Scotland outweigh the potential drawbacks, the adoption of rewilding is recommended as one aim of environmental policy.

2771: ***-.010***

Many species of gazelles (Gazella spp.) are nowadays threatened by hunting, poaching, habitat loss and habitat deterioration. Conservation efforts for this group not only face the problem of maintaining remnant populations, but often natural populations have been extirpated from the wild. In some cases though, captive breeding programs exist that might provide a valuable source for future reintroductions. A major problem in this context is that phylogeographic relationships among different (potentially locally adapted) populations, and even basic phylogenetic relationships between species, are only poorly understood, thus hampering the assignment of management units, breeding groups, or stocks for reintroduction projects. Our present study focused on dorcas gazelles (G. dorcas and 'G. saudiya') from the species' entire distribution range, with samples originating from western Saharan Africa into Saudi Arabia. In stark contrast to previous studies reporting on pronounced genetic structure in taxa like mountain gazelles (G. gazella), we detected only low genetic diversity and no evidence for major phylogenetic splits when analyzing two mitochondrial genetic markers. Using a coalescent approach we inferred a steep population decline that started app. 25,000 years before present and is still ongoing, which coincides with pre-historic human activities in Saharan Africa. Our phylogenetic analyses, statistical parsimony network analysis and inferred colonization patterns shed doubt on the validity of various described subspecies of G. dorcas.

2772: ***+.319***

Many species of lemurs can be found in European Zoos. Some French zoos, which are very implicated in the conservation of the lemurs, are the founders of several captive populations of lemurs. If the examples of reintroductions of captive-born lemurs are rare, the captive populations are nevertheless a potential reservoir of individuals for wild populations, particularly in conservation projects including a metapopulation management of isolated populations due to fragmentation of their habitat. The captive populations of lemurs are powerful "ambassadors" of wild populations that allow to alert the public on their disappearance and to the situation of Madagascar and to promote fundraising in favour of in-situ conservation of these species. Indeed

today, the presence of these lemurs in captivity is the only source of funding for some conservation projects. Moreover, the captive populations contribute to research and allow, like wild populations and in a complementary way, a better understanding of species for implementing appropriate conservation strategies.

2774: *+.072*

BackgroundThe reduction in the amount of food available for European avian scavengers as a consequence of restrictive public health policies is a concern for managers and conservationists. Since 2002, the application of several sanitary regulations has limited the availability of feeding resources provided by domestic carcasses, but theoretical studies assessing whether the availability of food resources provided by wild ungulates are enough to cover energetic requirements are lacking. Methodology/FindingsWe assessed food provided by a wild ungulate population in two areas of NE Spain inhabited by three vulture species and developed a P System computational model to assess the effects of the carrion resources provided on their population dynamics. We compared the real population trend with to a hypothetical scenario in which only food provided by wild ungulates was available. Simulation testing of the model suggests that wild ungulates constitute an important food resource in the Pyrenees and the vulture population inhabiting this area could grow if only the food provided by wild ungulates would be available. On the contrary, in the Pre-Pyrenees there is insufficient food to cover the energy requirements of avian scavenger guilds, declining sharply if biomass from domestic animals would not be available. Conclusions/SignificanceOur results suggest that public health legislation can modify scavenger population trends if a large number of domestic ungulate carcasses disappear from the mountains. In this case, food provided by wild ungulates could be not enough and supplementary feeding could be necessary if other alternative food resources are not available (i.e. the reintroduction of wild ungulates), preferably in European Mediterranean scenarios sharing similar and socio-economic conditions where there are low densities of wild ungulates. Managers should anticipate the conservation actions required by assessing food availability and the possible scenarios in order to make the most suitable decisions.

2775: *+.218*

Translocations of Baltic whitefish (Coregonus sp.) into Austrian Alpine lakes have created 'artificial hybrid zones', threatening the genetic integrity of native lineages. We evaluate the genetic structure of Coregonus in Austrian lakes and characterize hybridization and introgression between native and introduced lineages. Fifteen populations (N = 747) were assessed for allelic variation at eight microsatellite loci and a reduced set (N = 253) for variation across two mtDNA genes (cyt b and NADH-3). Bayesian approaches were used to estimate individual admixture proportions (q-values) and classify genotypes as native, introduced or hybrids. q-value distributions varied among populations highlighting differential hybridization and introgression histories. Many lakes revealed a clear distinction between native and introduced genotypes despite hybridization, whereas some locations revealed hybrid swarms. Genetic structure among lakes was congruent with morphological divergence and novelty raising speculation of multiple taxa, including a population south of the Alps, outside the putative native range of Coregonus. Although statistically congruent with inferences based on nuclear markers, mitochondrial haplotype data was not diagnostic with respect to native and non-native lineages, supporting that the Alpine region was colonized post-glacially by an admixture of mtDNA lineages, which coalesce > 1 Ma. Mechanisms promoting or eroding lineage isolation are discussed, as well as a high potential to conserve native Alpine lineages despite the extensive historical use of introduced Baltic stocks.

2776: ***+.131***

AimThe role of human activities in species biogeography can be difficult to identify, but in some cases molecular techniques can be used to test hypotheses of human-mediated dispersal. A currently accepted hypothesis states that humans mediated the divergence of two species of spiny-tailed iguanas in the Ctenosaura hemilopha species complex, namely C. conspicuosa and C. nolascensis, which occupy islands in the Sea of Cortes between the peninsula of Baja California and mainland Mexico. We test an alternative hypothesis that follows the traditional knowledge of the Seri Indians and states that the divergence of these species was not mediated by humans.LocationMexico, including Baja California, Sonoran and Sinaloan coastal regions, and Isla San Esteban and Isla San Pedro Nolasco in the Sea of Cortes. MethodsWe analysed mitochondrial (cytochrome b and cytochrome c oxidase subunit III) DNA sequences from four species in the C. hemilopha species complex. Maximum parsimony and Bayesian inference were used to infer matriarchal genealogical relationships between the species and several outgroup taxa. Bayesian methods were used to estimate divergence times for the major nodes on the trees based on previously published, fossil-calibrated priors. ResultsOur analysis indicated that lineages within the C. hemilopha species complex diverged long before human colonization of the Americas. The divergence of C. nolascensis and C. conspicuosa could not be attributed to Seri translocations. The matriarchal genealogy of the species complex currently defies a simple biogeographical interpretation. Main conclusionsWe conclude that humans did not mediate the divergence of C. nolascensis and C. conspicuosa. This conclusion is consistent with the traditional knowledge of the Seri people. These results demonstrate the utility of molecular techniques in investigating potential cases of human-mediated dispersal of plants and animals, and reinforce the importance of considering traditional knowledge in the formation of scientific hypotheses and the interpretation of results.

2777: ***+.238***

P>1. Despite being expensive, complicated and less successful than the conservation of primary habitat, translocation is rapidly gaining importance as a conservation approach due to accelerated loss of natural environment. Finding the optimal abiotic and biotic conditions needed for successful translocation of plants can be difficult for species with limited information on prior distribution. Unfortunately, this is often the case with endangered plant species, including those urgently needing action.2. We present a method of evaluating the relative importance of multiple environmental parameters in translocation success. This method is based on the application of variation partitioning in canonical ordination and it allows usage of not only multiple independent biotic and abiotic variables, but also multiple dependent variables for fitness estimates.3. In this study, six soil parameters together with the abundance of 61 plant species and their total biomass were used to explain the variation in translocation success of Iris atrofusca plants among 22 microsites. The relative importance of each of the three factors was estimated using ordination techniques.4. Soil characteristics and total biomass of other plants did not significantly affect the performance of translocated irises, but the species composition of the surrounding vegetation did have a significant effect. The abundance of relatively rare species was closely correlated with iris performance. It is likely that these species do not affect the irises directly but instead represent environmental conditions not measured in this study, which are necessary for the survival of irises.5. Synthesis and applications. Variation partitioning appears to be a highly promising method for planning the translocation of plants and evaluating success due to its ability to estimate the unique contribution of each of two or more sets of environmental factors. It can be used to monitor success, and to identify the key contributory factors, in experimental translocations preceding actual introduction of plants in conservation programmes.

2778: ***+.179***

South Africa has a rich flora which exhibits among the highest species density in the world, distributed across nine biomes that support an impressive diversity of animal life. However, a variety of human actions, invasion by alien species, natural disturbances and climate change collectively impact negatively on the great diversity of both plant and animal species. In situ conservation has long been practised, primarily in nature reserves, complemented by ex situ conservation in national botanic gardens, but in vitro plant conservation is not common. In the context of animal biodiversity conservation, the Wildlife Biological Resource Centre of the National Zoological Gardens utilises cryo-banking as one of its major focuses and is now poised to expand as the repository for the cryoconservation of plant germplasm, particularly for indigenous recalcitrant-seeded and poor-seeding species. However, there are particular problems associated with successful germplasm cryostorage of such tropical and subtropical plants. As we see the science and application of cryobiology and cryoconservation as cross-cutting and transdisciplinary, we have entrained formal networking among scientists offering a range of specialisations aimed at a deeper understanding of common problems and practical outcomes to facilitate both plant and animal biobanking. The endeavours are aimed at elucidating the basis of both successes and failures in our efforts to attain optimal outcomes. With focus on best practices, standard operating procedures, validation and risk management for cryopreserved and cold-stored plant and animal material, our ultimate aim is to facilitate restoration by the safe reintroduction of indigenous species.

2779: ***+.118***

This special issue is dedicated to the in vitro tools and methods used to conserve the genetic diversity of rare and threatened plant species from around the world. Species that are on the brink of extinction because of the rapid loss of genetic diversity and habitat come mainly from resource-poor areas of the world and from global biodiversity hotspots and island countries. These species are unique because they are endemic, and only a few small populations or sometimes only a few individuals remain in the wild. Therefore, the challenges to support conservation by in vitro measures are many and varied. The editors of this invited issue solicited papers from experts from Asia, Africa, Europe, Australia, and North and South America. This compilation of articles describes the efforts in these diverse regions toward saving plants from extinction, and details the direct application of in vitro and cryopreservation methods. In addition, these contributions provide guidance on propagation of rare plants, including techniques for large-scale propagation, storage, and reintroduction. The in vitro techniques for conserving plant biodiversity include shoot apical or axillary-meristem-based micropropagation, somatic embryogenesis, cell culture technologies and embryo rescue techniques, as well as a range of in vitro cold storage and cryopreservation protocols, and they are discussed in depth in this issue.

2780: ***+.116***

Arabian oryx (Oryx leucoryx) had faced extinction in the wild more than three decades ago and was saved by the prudent efforts of captive breeding programs. A clear understanding of the molecular diversity of contemporary Arabian oryx population is important for the long term success of captive breeding and reintroduction of this potentially endangered species. We have sequenced the segments of mitochondrial DNA including 12S rRNA, 16S rRNA, cytochrome b (Cyt-b) and control region (CR) genes of 24 captive-bred and reintroduced animals. Although the sequences of 12S rRNA, 16S rRNA and Cyt-b were found to be identical for all the samples, typical sequence variations in the CR gene were observed in the form of 7 haplotypes. One of

these haplotypes has been reported earlier while the remaining 6 haplotypes are novel and represent different lineages from the founders. The haplotype and nucleotide diversities were found to be 0.789 and 0.009 respectively. The genetic distances among the 7 mtDNA haplotypes varied from 0.001 to 0.017. These findings are of potential relevance to the management of captive breeding programs for the conservation of Arabian oryx.

2781: *+.092*

We examined the efficacy of a translocation program in which large numbers of leopards (Panthera pardus fusca) were trapped in human-dominated landscapes where livestock attacks were common and human attacks rare and released into adjoining forested areas in an attempt to reduce leopard presence and mitigate conflicts at the capture site. In the year starting in February of 2001, 29 leopards were captured in the human-dominated rural landscape of the Junnar region (4275 km2, 185 people/km2), Maharashtra, India, and released an average of 39.5 km away in adjoining forests. Eleven leopards were also relocated to the same forests from other districts. Prior to the large-scale translocation program, an average of four leopard attacks on humans occurred each year between 1993 and 2001. After the translocation program was initiated, the average increased substantially to 17 attacks. Linear and logistic models showed that attack frequency increased in Junnar following nearby releases of leopards and decreased when leopards were removed for releases far away; that attacks became more lethal when the number of leopards introduced from other districts increased; and that attacks were most likely to occur in the regions where the largest number of leopards had been introduced from other areas. These results suggest that leopards did not stay at the release sites and that translocation induced attacks on people. Potential explanations for these results include increased aggression induced by stress of the translocation process, movement through unfamiliar human-dominated landscapes following release, and loss of fear of humans due to familiarity with humans acquired during captivity. Our results show that reactive solutions to attacks on humans by leopards, such as translocation, could in fact increase human-leopard conflict. Measures to reduce human-carnivore conflicts may include more effective compensation procedures to pay livestock owners for the loss of animals to predation by carnivores, providing better methods of protection for livestock, and encouraging greater social acceptance of the presence of carnivores in human-dominated landscapes.

2782: *-.158*

The Hawaiian monk seal (Monachus schauinslandi) is one of the most critically endangered marine mammals. Less than 1200 individuals remain, and the species is declining at a rate of approximately 4% per year as a result of juvenile starvation, shark predation, and entanglement in marine debris. Some of these problems may be alleviated by translocation; however, if island breeding aggregates are effectively isolated subpopulations, moving individuals may disrupt local adaptations. In these circumstances, managers must balance the pragmatic need of increasing survival with theoretical concerns about genetic viability. To assess range-wide population structure of the Hawaiian monk seal, we examined an unprecedented, near-complete genetic inventory of the species (n = 1897 seals, sampled over 14 years) at 18 microsatellite loci. Genetic variation was not spatially partitioned ((theta) over cap (w) = -0.03, p = 1.0), and a Bayesian clustering method provided evidence of one panmictic population (K = 1). Pairwise F-ST comparisons (among 7 island aggregates over 14 annual cohorts) did not reveal temporally stable, spatial reproductive isolation. Our results coupled with long-term tag-resight data confirm seal movement and gene flow throughout the Hawaiian Archipelago. Thus, human-mediated translocation of seals among locations is not likely to result in genetic incompatibilities.

2783: ***+.047***

The number of individuals translocated and released as part of a reintroduction is often small, as is the final established population, because the reintroduction site is typically small. Small founder and small resulting populations can result in population bottlenecks, which are associated with increased rates of inbreeding and loss of genetic diversity, both of which can affect the long-term viability of reintroduced populations. I used information derived from pedigrees of four monogamous bird species reintroduced onto two different islands (220 and 259 ha) in New Zealand to compare the pattern of inbreeding and loss of genetic diversity among the reintroduced populations. Although reintroduced populations founded with few individuals had higher levels of inbreeding, as predicted, other factors, including biased sex ratio and skewed breeding success, contributed to high levels of inbreeding and loss of genetic diversity. Of the 10-58 individuals released, 4-25 genetic founders contributed at least one living descendent and yielded approximately 3-11 founder-genome equivalents (number of genetic founders assuming an equal contribution of offspring and no random loss of alleles across generations) after seven breeding seasons. This range is much lower than the 20 founder-genome equivalents recommended for captive-bred populations. Although the level of inbreeding in one reintroduced population initially reached three times that of a closely related species, the long-term estimated rate of inbreeding of this one population was approximately one-third that of the other species due to differences in carrying capacities of the respective reintroduction sites. The increasing number of reintroductions to suitable areas that are smaller than those I examined here suggests that it might be useful to develop long-term strategies and guidelines for reintroduction programs, which would minimize inbreeding and maintain genetic diversity.

2784: ***+.130***

The rapidity of climate change is predicted to exceed the ability of many species to adapt or to disperse to more climatically favorable surroundings. Conservation of these species may require managed relocation (also called assisted migration or assisted colonization) of individuals to locations where the probability of their future persistence may be higher. The history of non-native species throughout the world suggests managed relocation may not be applicable universally. Given the constrained existence of freshwater organisms within highly dendritic networks containing isolated ponds, lakes, and rivers, managed relocation may represent a useful conservation strategy. Yet, these same distinctive properties of freshwater ecosystems may increase the probability of unintended ecological consequences. We explored whether managed relocation is an ecologically sound conservation strategy for freshwater systems and provided guidelines for identifying candidates and localities for managed relocation. A comparison of ecological and life-history traits of freshwater animals associated with high probabilities of extirpation and invasion suggests that it is possible to select species for managed relocation to minimize the likelihood of unintended effects to recipient ecosystems. We recommend that translocations occur within the species' historical range and optimally within the same major river basin and that lacustrine and riverine species be translocated to physically isolated seepage lakes and upstream of natural or artificial barriers, respectively, to lower the risk of secondary spread across the landscape. We provide five core recommendations to enhance the scientific basis of guidelines for managed relocation in freshwater environments: adopt the term managed translocation to reflect the fact that individuals will not always be reintroduced within their historical native range; examine the trade-off between facilitation of individual movement and the probability of range expansion of non-native species; determine which species and locations might be immediately considered for managed translocation; adopt a hypothetico-deductive framework by conducting experimental trials to introduce species of conservation concern into new areas

within their historical range; build on previous research associated with species reintroductions through communication and synthesis of case studies.

2785: *+.165*

Conservation diagnosis of ex situ techniques (e.g. releasing chicks by hacking) cannot be evaluated without quantitative assessment of the fate of individuals, mainly their survival and recruitment. Here we use a long-term monitoring at a large spatial scale of a hacking programme (i.e. chick translocations) for Audouin's gulls in an uninhabited site so as to establish a new breeding patch and reinforce the meta-population. The success of the programme relied on the assumption that birds tend to recruit to the site where they fledge (i.e. they were philopatric). Multi-state capture-recapture models applied to a large dataset (more than 43,500 resights at 30 colonies during 1999-2010) were used to evaluate the survival of released chicks and the probability of being philopatric. Adult survival was high, but juvenile survival was lower than that of wild gulls, suggesting that there was a cost associated with hacking only during their first year of life. As expected, most released birds returned to the hacking site, but very few (including immigrants from abroad) attempted to breed here. Dispersal was inversely correlated with distance from the hacking site and positively associated with the population size of the patch (i.e. conspecific attraction). Even though most hacking procedures met the ecological conditions to succeed, results clearly showed that the program failed to establish a new breeding site. The ability of prospecting and the attraction made by conspecifics at established sites may be a constraint for the success of hacking programs, particularly for social and nomadic species. (C) 2010 Elsevier Ltd. All rights reserved.

2786: *+.199*

Reintroduction of native species has become increasingly important in conservation worldwide for recovery of rare species and restoration purposes. However, few studies have reported the outcome of reintroduction efforts in plant species. Using data from the literature combined with a questionnaire survey, this paper analyses 249 plant species reintroductions worldwide by assessing the methods used and the results obtained from these reintroduction experiments. The objectives were: (1) to examine how successful plant species reintroductions have been so far in establishing or significantly augmenting viable, self-sustaining populations in nature; (2) to determine the conditions under which we might expect plant species reintroductions to be most successful; (3) to make the results of this survey available for future plant reintroduction trials. Results indicate that survival, flowering and fruiting rates of reintroduced plants are generally quite low (on average 52%, 19% and 16%, respectively). Furthermore, our results show a success rate decline in individual experiments with time. Survival rates reported in the literature are also much higher (78% on average) than those mentioned by survey participants (33% on average). We identified various parameters that positively influence plant reintroduction outcomes, e.g., working in protected sites, using seedlings, increasing the number of reintroduced individuals, mixing material from diverse populations, using transplants from stable source populations, site preparation or management effort and knowledge of the genetic variation of the target species. This study also revealed shortcomings of common experimental designs that greatly limit the interpretation of plant reintroduction studies: (1) insufficient monitoring following reintroduction (usually ceasing after 4 years); (2) inadequate documentation, which is especially acute for reintroductions that are regarded as failures; (3) lack of understanding of the underlying reasons for decline in existing plant populations; (4) overly optimistic evaluation of success based on short-term results; and (5) poorly defined success criteria for reintroduction projects. We therefore conclude that the value of plant reintroductions as a conservation tool could be improved by: (1)

an increased focus on species biology; (2) using a higher number of transplants (preferring seedlings rather than seeds); (3) taking better account of seed production and recruitment when assessing the success of reintroductions; (4) a consistent long-term monitoring after reintroduction. (C) 2010 Elsevier Ltd. All rights reserved.

2787: ***+.286***

The physiology of an animal determines the range of environmental conditions under which it can survive. Surprisingly, relatively few conservation studies have used physiology to make predictions about the performances of translocated individuals in their new environment. Tuatara Sphenodon punctatus are of international significance as the last rhynchocephalian reptile. Natural populations are now restricted to similar to 30 offshore islands in northern New Zealand, where survival of at least one population is threatened by climate change. Translocation to cooler regions (further south, but within the past latitudinal range of the genus) is an important option for future management. However, the genetic suitability of available source populations for environments that are currently cooler is unclear. To help predict the success of translocation, we examined the effects of cool temperature on juveniles sourced from one of the most southerly natural populations and compared these with three lizard species that inhabit southern New Zealand. We found that tuatara possess the same range of preferred body temperature, feeding responses to cool temperatures and critical thermal minimum as these three lizard species. However, tuatara did not digest at temperatures below 12 degrees C, suggesting that new reintroduction sites must offer enough basking opportunities to allow digestion of prey in winter. As tuatara showed responses to cold temperatures similar to lizards from southern New Zealand, we predict that tuatara will survive if translocated south of their current range, but whether they will be able to produce self-sustaining populations still needs investigation. Physiological predictions from the current study should now be combined with measurements of population performance in an experimental translocation.

2788: ***+.033***

As human populations expand and nonhuman animals decline, understanding the interactions between people and wildlife is essential. For endangered species, appreciating the effect of human disturbance can be important for their conservation. However, a human disturbance angle is often absent from ecological research, despite growing evidence of the negative impact of non-fatal human interference. Here, we monitored Hainan Eld's deer living within a reserve and translocated animals living amongst villagers. We show that translocated deer deviated from a crepuscular activity pattern and became increasingly nocturnal, and most active when villagers were not. It appears that translocated deer adapted over time to human disturbance and this pattern is similar to that of other species during periods of hunting. People do not pose an actual threat to Eld's deer, but their presence triggered a response akin to predator avoidance and may be interfering with broader aspects of their biology and conservation.

2789: ***+.112***

The endangered annual plant Limnanthes floccosa ssp. californica Arroyo is restricted to vernal pools in Butte County, California. To identify populations with unique genetic resources, guide reintroduction efforts, and design seed collection scenarios for long-term ex situ seed storage we determined extant genetic diversity and structure by surveying 457 individuals from 21 distinct populations using nine polymorphic microsatellite markers. We found low within population genetic diversity: low allelic diversity (1.9 [0.06 SE] alleles/locus); low heterozygosity (H (obs) =

0.10 +/- A 0.018, H (exp) = 0.19 +/- A 0.015), and a high fixation index (0.556 +/- A 0.044). The number of polymorphic loci ranged between 11 and 89%. Bayesian ordination determined 20 distinct populations and we found high genetic structure among these (F (st) = 0.65, P < 0.0001). We identified notable gene flow barriers across populations, confirming regional structuring between three previously defined population density centers and two outlying populations (F (st) = 0.21, P < 0.0001). Population size estimates ranged between similar to 50 and > 5,000 extant plants per site. Our study confirms previous isozyme-based results and suggests that the loss of any population would represent a significant loss in the species' genetic diversity. Recovery requires active restoration of existing populations and permanent habitat protection. We recommend close comparison of microhabitats of declining populations with genetically similar populations, to determine the potential for human assisted gene flow via seed movement to recover declining populations.

2790: *+.075*

Translocation and reintroduction are important tools for the conservation or recovery of species threatened with extinction in the wild. However, an understanding of the potential genetic consequences of mixing populations requires an understanding of the genetic variation within, and similarities among, donor and recipient populations. Genetic diversity was measured using two independent marker systems (microsatellites and AFLPs) for one island and four small remnant mainland populations of Setonix brachyurus, a threatened medium sized macropod restricted to fragmented habitat remnants and two off-shore islands in southwest Australia. Microsatellite diversity in the island population (R (s) = 3.2, H (e) = 71%) was similar to, or greater than, all mainland populations (R (s) = 2.1-3.9, H (e) = 34-71%). In contrast, AFLP diversity was significantly lower in the island population (PPL = 20.5; H (j) = 0.118) compared to all mainland populations (mean PPL = 79.5-89.7; mean H (j) = 0.23-0.29). Microsatellites differentiated all (mainland and island) populations from each other. However, AFLP only differentiated the island population from the mainland populations-all mainland populations were not significantly differentiated from each other for this marker. Given a known time since isolation of the island population from the mainland (6,000 years ago), and an overall more conservative rate of evolution of AFLP markers, our results are consistent with mainland populations fragmenting thousands of years ago (but < 6,000 years), probably as a consequence of reduced rainfall and the constriction of the preferred mesic habitat of quokkas. Our results also support a recent history of severe population bottlenecks in mainland populations, and a long history of bottlenecks of the island population, but reflect a recent explosion in numbers since European occupation of the island. Our results indicate that translocation of island populations to supplement mainland populations would introduce genetically markedly differentiated, and possibly maladapted, individuals.

2791: *+.109*

Relocation programs are often initiated to restore threatened species to previously occupied portions of their range. A primary challenge of restoration efforts is to translocate individuals in a way that prevents loss of genetic diversity and decreases differentiation relative to source populations-a challenge that becomes increasingly difficult when remnant populations of the species are already genetically depauperate. Trumpeter swans were previously extirpated in the entire eastern half of their range. Physical translocations of birds over the last 70 years have restored the species to portions of its historical range. Despite the long history of management, there has been little monitoring of the genetic outcomes of these restoration attempts. We assessed the consequences of this reintroduction program by comparing patterns of genetic variation at 17

microsatellite loci across four restoration flocks (three wild-released, one captive) and their source populations. We found that a wild-released population established from a single source displayed a trend toward reduced genetic diversity relative to and significant genetic differentiation from its source population, though small founder population effects may also explain this pattern. Wild-released flocks restored from multiple populations maintained source levels of genetic variation and lacked significant differentiation from at least one of their sources. Further, the flock originating from a single source revealed significantly lower levels of genetic variation than those established from multiple sources. The distribution of genetic variation in the captive flock was similar to its source. While the case of trumpeter swans provides evidence that restorations from multiple versus single source populations may better preserve natural levels of genetic diversity, more studies are needed to understand the general applicability of this management strategy.

2792: *+.180*

The genetic variability among Italian populations of the white-clawed crayfish (Austropotamobius pallipes) was examined to determine their phylogeography and to assess their conservation status as a management unit. A fragment of the mitochondrial DNA COI gene of 107 specimens from ten populations was sequenced, and the phylogenetic relationships were established. Two out of three haplotypes sampled in two French populations from the Rhne basin were shared with Italian populations. Despite a moderate level of genetic variability within the Italian populations of A. pallipes, no genetic structure was revealed. It has been suggested that there have been translocation events throughout the Alpine barrier between the North-Western Italian basins and the Rhne basin. Genetic exchangeability of the French and Italian populations was demonstrated in this study, and a shift of conservation efforts towards the native, congeneric Austropotamobius italicus is recommended.

2793: *+.050*

Georgia plume (Elliottia racemosa, Ericaceae) is a threatened, woody plant endemic to Georgia's Coastal Plain region in the southeastern United States. Populations of the plant have a fragmented distribution within a restricted range and are characterized by low genetic diversity and a lack of sexual recruitment. Georgia plume cannot be effectively propagated using conventional methods. We have developed an in vitro shoot regeneration system that is effective with explants obtained from mature plants in the wild. The objective of this study was to determine the efficacy of using this in vitro protocol to regenerate proliferating shoot cultures from 34 georgia plume genotypes obtained from divergent populations. Young expanding leaves were cultured on Gamborg's media supplemented with 10 mu M thidiazuron and 5 mu M indole-3-acetic acid. After 8 weeks, tissues were transferred to a shoot elongation medium with 25 mu M 2-isopentenyl adenine. Of the 34 genotypes tested, 91% formed shoot primordia and 85% regenerated shoots within 6 months of inoculation. This study verifies that tissue culture can be used to produce adventitious shoots from a wide range of georgia plume genotypes. Within a coordinated conservation program, tissue culture is a feasible system to use for safeguarding and reintroduction of genetically diverse plant material, which may be critical to the survival of this rare species.

2794: *+.184*

Woodsia ilvensis has become extinct from its last known natural localities in Estonia and has not been rediscovered since 1977. This fern grew in northern and north-western Estonia in areas of suitable habitat. Considering that habitat conditions may have changed in previous localities, an experimental project was started to test if it would be possible to reintroduce W. ilvensis into new

localities where suitable habitat conditions exist. Two experiments were performed, one on an old stone wall, constructed of stones collected from the surrounding fields, and another on two granite boulders in two localities, one where the surrounding soil was acidic, and the other where the soil was basic. The plants were grown from spores of wild provenance received from Finland via the seed and spore exchange system of botanical gardens. Results confirmed that individual plants can establish and persist for at least 10 years on stone walls without maintenance. The experiment on boulders failed, as plants did not establish there. Young, 2-year old mature individuals proved to be the best stage for planting out onto the stone walls in this case study. The best indicators for selecting suitable habitat were characteristic plant species of the natural community. Here I discuss the experimental methods used and first results of the experiment.

2795: *-.189*

Examples from a variety of taxa demonstrate that under certain circumstances, the exclusion or translocation of 'problem' animals is ineffective in resolving human-wildlife conflicts and may even elicit new problems elsewhere. Damage caused by badger setts (burrows) is an important source of human-wildlife conflict in the UK and is commonly managed by excluding badgers from all or part of problem setts. We used records of licences issued for the management of such problems and a novel statistical approach to assess spatiotemporal associations between problem cases in England from 2002 to 2005. We predicted that management at urban badgers' setts, and particularly exclusion of badgers from urban main setts, would give rise to subsequent problems at focal setts and in neighbouring areas. Frequencies of problems occurring at individual setts were similar in urban and rural areas. In areas neighbouring setts subjected to management action, the background frequency of problems was higher in urban than in rural areas, reflecting the occurrence of problems at a higher proportion of urban setts. The frequency of new cases arising at or in the vicinity of managed setts within a critical time period after management action was not significantly different from the background frequency of problems for any combination of land use, sett type and management approach. This finding suggests that the measures currently employed for managing problem setts do not importantly increase the likelihood of problems reoccurring in the same location or emerging nearby.

2796: *+.199*

Background: A key challenge for conservation biologists is to determine the most appropriate demographic and genetic management strategies for wildlife populations threatened by disease. We explored this topic by examining whether genetic background and previous pathogen exposure influenced survival of translocated animals when captive-bred and free-ranging bighorn sheep (Ovis canadensis) were used to re-establish a population that had been extirpated in the San Andres Mountains in New Mexico, USA.Results: Although the free-ranging source population had significantly higher multi-locus heterozygosity at 30 microsatellite loci than the captive bred animals, neither source population nor genetic background significantly influenced survival or cause of death. The presence of antibodies to a respiratory virus known to cause pneumonia was associated with increased survival, but there was no correlation between genetic heterozygosity and the presence of antibodies to this virus.Conclusions: Although genetic theory predicts otherwise, increased heterozygosity was not associated with increased fitness (survival) among translocated animals. While heterosis or genetic rescue effects may occur in F1 and later generations as the two source populations interbreed, we conclude that previous pathogen exposure was a more important marker than genetic heterozygosity for predicting survival of translocated animals. Every wildlife translocation is an experiment, and whenever possible, translocations should be designed and evaluated to test hypotheses that will further improve our

understanding of how pathogen exposure and genetic variability influence fitness.

2797: ***+.213***

The development and implementation of a population supplementation and restoration plan for any endangered species should involve an understanding of the species' habitat requirements prior to the release of any captive bred individuals. The freshwater pearl mussel, Margaritifera margaritifera, has undergone dramatic declines over the last century and is now globally endangered. In Northern Ireland, the release of captive bred individuals is being used to support wild populations and repatriate the species in areas where it once existed. We employed a combination of maximum entropy modelling (MAXENT) and Generalized Linear Mixed Models (GLMM) to identify ecological parameters necessary to support wild populations using GIS-based landscape scale and ground-truthed habitat scale environmental parameters. The GIS-based landscape scale model suggested that mussel occurrence was associated with altitude and soil characteristics including the carbon, clay, sand, and silt content. Notably, mussels were associated with a relatively narrow band of variance indicating that M. margaritifera has a highly specific landscape niche. The ground-truthed habitat scale model suggested that mussel occurrence was associated with stable consolidated substrates, the extent of bankside trees, presence of indicative macrophyte species and fast flowing water. We propose a three phase conservation strategy for M. margaritifera identifying suitable areas within rivers that (i) have a high conservation value yet needing habitat restoration at a local level, (ii) sites for population supplementation of existing populations and (iii) sites for species reintroduction to rivers where the mussel historically occurred but is now locally extinct. A combined analytical approach including GIS-based landscape scale and ground-truthed habitat scale models provides a robust method by which suitable release sites can be identified for the population supplementation and restoration of an endangered species. Our results will be highly influential in the future management of M. margaritifera in Northern Ireland. (C) 2010 Elsevier Ltd. All rights reserved.

2798: ***+.082***

Diseases in wildlife have been recognised as having the potential to affect human health, livestock health and species conservation. In order to assess and respond to these potential risks in an effective and a proportionate way, the UK Government initiated development of the Wildlife Health Strategy to provide a framework for decision making. The England Wildlife Health Strategy (EWHS) has been developed through extensive consultation. Discussions and negotiations with government departments, agencies, non-governmental public bodies and wildlife organisations were held to obtain advice and input on specific and specialised aspects of wildlife health. A series of workshops to investigate the application of innovative science to wildlife health policy contributed further. A formal public consultation was held that proposed a range of actions to implement the strategy. A summary of responses to this consultation was published in October 2007. The EWHS was published in June 2009 and provides a framework for a generic four-stage approach to wildlife health that can be adopted by decision makers both within and outside government.

2799: ***+.195***

Assisted Colonization (AC) has been proposed as one method of aiding species to adapt to the impacts of climate change. AC is a form of translocation and translocation protocols for threatened species, mostly for reintroduction, are well established in Australia. We evaluate the information available from implementation of translocations to understand how existing policies and

guidelines should be varied to plan, review and regulate AC. While the risks associated with AC are potentially greater than those of reintroductions, AC is likely to be the only available method, other than germplasm storage and establishment of captive populations, of conserving many taxa under future climate change. AC may also be necessary to maintain ecosystem services, particularly where keystone species are affected. Current policies and procedures for the preparation of Translocation Proposals will require modification and expansion to deal with Assisted Colonization, particularly in relation to risk management, genetic management, success criteria, moving associated species and community consultation. Further development of risk assessment processes, particularly for invasiveness, and guidelines for genetic management to maintain evolutionary potential are particularly important in the context of changing climate. Success criteria will need to respond to population establishment in the context of new and evolving ecosystems, and to reflect requirements for any co-establishment of interdependent species. Translocation Proposals should always be subjected to independent peer review before being considered by regulators. We conclude that consistent approaches by regulators and multilateral agreements between jurisdictions are required to minimize duplication, to ensure the risk of AC is adequately assessed and to ensure the potential benefits of AC are realized.

2800: *+.155*

Evolution occurs rapidly and is an ongoing process in our environments. Evolutionary principles need to be built into conservation efforts, particularly given the stressful conditions organisms are increasingly likely to experience because of climate change and ongoing habitat fragmentation. The concept of evolutionary resilience is a way of emphasizing evolutionary processes in conservation and landscape planning. From an evolutionary perspective, landscapes need to allow in situ selection and capture high levels of genetic variation essential for responding to the direct and indirect effects of climate change. We summarize ideas that need to be considered in planning for evolutionary resilience and suggest how they might be incorporated into policy and management to ensure that resilience is maintained in the face of environmental degradation.

2801: *+.183*

Altitude influences forest structure and food abundance and distribution, which in turn affect primate feeding and ranging patterns. Javan gibbons (Hylobates moloch) are endemic to forests spanning a broad range of altitudes on Java, Indonesia. Most information about Javan gibbon behavior comes from studies in lowland forests, while the vast majority of wild gibbons remaining inhabit hill and lower montane forests. We studied the diets, activity patterns, and ranging behavior of three gibbon groups in hill/lower montane (950-1,100m asl) forest in the Gunung Halimun-Salak National Park (GHSNP) from April 2008 to March 2009. The mean home range size was 37 ha and the mean daily path length was 1,180 m. The study groups spent 36% of time feeding, 41% resting, 15% traveling, 6% engaging in social behavior, and 2% in aggressive interactions. Fruit was the most important food (63% of feeding time) followed by leaves (24%), and flowers (12%). Our results suggest that Javan gibbons in higher elevation habitats have substantially larger home ranges than lowland populations, despite broad similarity in their activity budgets and diets. Conservation managers should consider the effects of altitude and habitat quality on gibbon ranging behavior when developing habitat corridors, selecting sites for translocation or reintroduction projects, and designating and managing protected areas. Am. J. Primatol. 73:270-280, 2011. (C) 2010 Wiley-Liss, Inc.

2802: *-.056*

Micropropagation is a useful technique for ex situ multiplication and restoration of critically endangered plant species, but the sexual reproductive behaviour of micropropagated plants is seldom evaluated prior to reintroduction. We examined the critically endangered species Rulingia sp. 'Trigwell Bridge', with only three remaining plants known in the wild, as a model case to examine this issue. Abnormalities in micropropagated plants of this species related to four floral traits (lengths of sepals, petals and anthers and width of anthers). The number of pollen grains per flower of abnormal individuals was lower than in plants with apparently normal flowers (wild types), but not significantly so (P = 0.068). Pollen viability for the abnormal plant (0.87 +/- 0.26%) was significantly lower than for the plants exhibiting wild-type floral morphology (45.42 +/- 4.47%). Experimental manipulations were used to examine the mating behaviour of normal and abnormal plants. The results showed that both male and female reproductive failure was linked to individuals exhibiting abnormal flowering attributes. Such aberrant reproductive performance in a micropropagated rare species predicates caution when using micropropagated plants in reintroduction programmes, highlighting the importance of screening for reproductive normality prior to release of micropropagated plants (especially for critically endangered species where reliance on in vitro propagation methods is often a necessity). (c) 2011 The Linnean Society of London, Botanical Journal of the Linnean Society, 2011, 165, 278-284.

2803: ***+.397***

A suite of ecological and genetic factors are likely to contribute to reintroduction performance. Potential factors include the ecological similarity between seed source and introduction site, population size and genetic diversity of seed sources, and the habitat quality of the introduction site. We conducted common garden experiments with golden paintbrush (Castilleja levisecta), an endangered species from the Pacific Northwest, U.S.A., in order to test hypotheses about reintroduction performance and to provide management recommendations. Ten common gardens, each composed of C. levisecta individuals grown from seed from six of the remaining populations, were planted into field conditions and monitored during two growing seasons. Plant community characteristics were important predictors of observed variation in C. levisecta performance. Exotic species-cover at common garden sites was associated with a reduction in performance of first-year C. levisecta transplants, while survival to the second growing season increased with increasing similarity in plant functional groups between source and common garden sites. Although measures of genetic diversity, population size, and geographic distance are often used to make conservation decisions during species recovery, here they were poor predictors of C. levisecta performance and establishment. We recommend choosing material for reintroduction from ecologically similar habitats, rather than those most proximate geographically, and selecting recovery sites with low exotic species abundance.

2804: ***-.071***

The Asiatic black bear is one of the most endangered mammals in South Korea owing to population declines resulting from human exploitation and habitat fragmentation. To restore the black bear population in South Korea, 27 bear cubs from North Korea and Russian Far East (Primorsky Krai) were imported and released into Jirisan National Park, a reservoir of the largest wild population in South Korea, in 2004. To monitor the success of this reintroduction, the genetic diversity and population structure of the reintroduced black bears were measured using both mitochondrial and nuclear DNA markers. Mitochondrial D-loop region DNA sequences (615 bp) of 43 Japanese black bears from previous study and 14 Southeast Asian black bears in this study were employed to obtain phylogenetic inference of the reintroduced black bears. The mitochondrial phylogeny indicated Asiatic black bear populations from Russian Far East and

North Korea form a single evolutionary unit distinct from populations from Japan and Southeast Asia. Mean expected heterozygosity (H-E) across 16 microsatellite loci was 0.648 for Russian and 0.676 for North Korean populations. There was a moderate but significant level of microsatellite differentiation (F-ST = 0.063) between black bears from the 2 source areas. In addition, genetic evidences revealed that 2 populations are represented as diverging groups, with lingering genetic admixture among individuals of 2 source populations. Relatedness analysis based on genetic markers indicated several discrepancies with the pedigree records. Implication of the phylogenetic and genetic evidences on long-term management of Asiatic black bears in South Korea is discussed.

2805: ***+.004***

P>1. Male-biased adult sex ratios are frequently observed in free-ranging populations and are known to cause changes in mating behaviours including increased male harassment of females, which can cause injury to females and/or alter female behaviour during breeding.2. Although we can explain why such behaviours may evolve and have studied their impacts on individuals when it does, we know very little about the demographic consequences of harassment caused by changes in adult sex ratio.3. Using a 12-year longitudinal data set of a free-living and endangered New Zealand passerine, the hihi (Notiomystis cincta), we show that a changing adult sex ratio has little or no effect on adult female survival or the number of fledglings produced per female. This is despite clear evidence of male harassment of breeding females when the sex ratio was male biased (up to three males per female).4. The length of the study and major fluctuations in sex ratio observed made it possible to obtain narrow confidence or credible intervals for effect sizes, showing that any effect of sex ratio on demographic rates were small.5. Our results provide rare empirical evidence for the demographic consequences of biased adult sex ratios in the wild and particularly in a conservation context.

2806: ***+.118***

Following the reintroduction of the Bearded Vulture (Gypaetus barbatus) in the western Alps in 1987, the species reappeared in the Gran Paradiso National Park, Italy, where it had not been recorded since 1930. We analysed 1157 sighting records collected inside the park borders from 1989 to 2007. The number of sightings per year was not correlated with the number of captive-bred individuals released during the same year in the western Alps but was positively correlated with the number released one year prior and two years prior. Bearded Vultures were recorded mainly at higher altitudes during warmer months, and at lower altitudes when the terrain was mostly covered by snow, as were the two most abundant ungulates of the park, the alpine ibex (Capra ibex) and the alpine chamois (Rupicapra rupicapra), whose carcasses were primary food sources for vultures. Three habitats were used with a frequency significantly higher than expected based on availability: vegetated cliffs and screes, forest-scrub mosaic, and agriculture. Bare rocks and deciduous forests were used less frequently than expected, and other habitats were used in the same proportion as expected, including alpine grassland, coniferous forests, and alpine heaths and scrubs.

2807: ***+.086***

Genetic endemism of island organisms and the threat to such organisms provided by artificially introduced genes are aspects of major interest in evolutionary and conservation studies of fishes. In this paper the genetic population structure of the oriental weatherloach, Misgurnus anguillicaudatus, in Sado Island of Japan was elucidated by phylogeographic analysis based on

partial mitochondrial control region sequences. The specimens were sampled at 62 sites in Sado Island and 14 sites on the mainland close to the island. We found various haplotypes of different origins, most of which had already been reported from the mainland and other places of Japan. This suggests that the loach has been historically introduced to the island from various regions of Japan. Of the 62 sites on the island, cultured/nonnative individuals were confirmed to have been stocked at eight specific sites for feeding of re-introduced Japanese crested ibis (Nipponia nippon). By a Mantel test, geographical and genetic distances were not significantly correlated among the local populations in Sado Island. However a significant correlation was found when the eight stocked local populations were excluded from the analysis. This implied that the genetic distribution pattern of the loach on the island has been disturbed by the stocking. In addition, the nucleotide diversity values of stocked local populations were significantly higher than those of other local populations, also a likely outcome of the stocking. In conclusion, the loach on the island likely had their origins in multiple historical introductions and colonizations, where more recent stocking for the ibis has caused further genetic disturbance to their local populations.

2808: ***-.059***

Feral hogs (Sus scrofa) are among the most widely-distributed mammals in the world and have the highest reproductive output compared with other ungulates. Worldwide, feral hogs are increasing in range and numbers. Human feral hog conflicts include impact on abundance and richness of plant and animal species, crop damage, predation on livestock, vehicle collisions, and disease transmission. We reviewed methods employed to mitigate the impact of feral hogs on human activities and discussed these methods in terms of effectiveness, feasibility, costs, and social acceptance. Traditional methods of control include trapping, angering, shooting, poisoning, and Judas hogs. Nonlethal methods of control include fertility control, fencing, repellents, diversionary feeding, and translocation. The review indicated that successful eradications of feral hogs from islands have been achieved by combining different control methods and by establishing post-eradication monitoring to ascertain that the eradication had been completed. Conversely, on the mainland and in countries where feral hogs have long been established, management of human feral hog conflicts often relies on population size reduction through hunting and poisoning the animals or on exclusion fencing and diversionary feeding. In the majority of instances, population control is not based on previous knowledge of local densities or on predicted impact of control on population size. Based on these results, we propose a framework of criteria to guide decisions regarding the suitability of different options to manage human feral hog conflicts in different contexts.

2809: ***+.060***

The lecture and discussion in the symposium was organized on conservation strategies for preventing the extinction of Tsushima leopard cat (Prionailurus bengalensis euptilurus) that is listed as Critically endangered species (I A) in the Red Data Book edited by the Ministry of Environment of Japan. For the conservation of Tsushima leopard cats, continuous efforts in related regions of the projects should be made including 1) comprehensive approaches in terms of the administration, local government and organizations, and the attitude of local people towards the animals in the wild habitat, 2) a peripheral support by veterinarian organization for preventing the transmission of infectious diseases mediated by domestic cats, which is a part of cause of population decrease, and 3) captive breeding in ex-situ conservation in zoos for the reintroduction. There has been limited progress on the reintroduction program of endangered mammals in Japan. The present project will be expected to play a key role for the future revolution of wildlife conservation.

2810: +.040

Nest-site and nesting material used by the Chinese alligator (Alligator sinensis) was studied at the Gaojingmiao Breeding Farm, Langxi County, Anhui, China from May to September 2009. In this study, artificial nesting materials were placed in 43 potential nesting sites before the nesting season, 11 of which were used. Additionally, eight nests were built at natural sites without artificial nesting materials provided. Seven environmental variables were measured at each nest site: distance from water, height from water surface, sunlight duration, nearest bank slope, nest site slope, vegetation coverage and concealment. Statistical analyses indicated that concealment was significantly different between used and unused nest sites, with concealment being significantly correlated to the use of materials-placed sites. In comparing the nests at artificial vs. natural sites, only the nearest bank slope differed significantly. Further, principal component analysis of natural nests indicated that the duration of nest exposure to sunlight and vegetation coverage were more influential than the other factors studied.

2811: +.222

Environmental conditions under which species reproduce have major consequences on breeding success and subsequent fitness. Therefore breeding habitat choice is ultimately important. Studies rarely address the potential fitness pay-offs of alternative natural breeding habitats by experimental translocation. Here we present a new tool to study fitness consequences of free living birds in different habitats. We translocated a migratory passerine, the pied flycatcher (Ficedula hypoleuca), to a novel site, where pairs were subjected to a short stay (2-4 days) in a nest box-equipped aviary before being released. We show that it is technically possible to retain birds in the new area for breeding, allowing the study of reproductive consequences of dispersal under natural conditions. The translocation resulted in an extension of the interval between arrival and egg laying of four days, highlighting the importance of having an adequate control group. Clutch size and nestling parameters did not differ significantly between translocated and unmanipulated females, which suggests that the procedure did not affect birds in their reproductive performance later on. This method could be applied broadly in evolutionary and ecological research, e.g., to study the potential fitness benefits and costs for dispersing to more northern latitudes as a way of adapting to climate change.

2812: +.049

For a successful re-introduction program, we need artificial nests that are accepted by the birds. The designs of the artificial nest box used in the Singapore Hornbill Project are described. Up to 62.5% of the nest boxes erected were utilised by the Oriental Pied Hornbill in Pulau Ubin. All occupied nests yielded successful occupation by the females, with eggs laid and at least one chick fledging; except for one nest where the chicks drowned as a result of a heavy rainstorm. A pair of captive Oriental Pied Hornbill from the Jurong Bird Park was introduced into the area of the southern Bukit Timah forest on 12 December 2008. The pair made frequent visits to the artificial nest box utilised in the previous cycle within a temporary aviary, and the female sealed herself inside the nest on 26 December 2008, just two weeks after relocation. For the first clutch, 4 eggs were laid at an average of 4.2 +/- 0.4 days apart. After 30 days and 29.1 days of incubation respectively, calculated on the assumption that the first and second eggs were the fertile eggs, the first and second chick hatched. The female was confined in the nest box for 99.3 days from 26 December 2008 to 4 April 2009, with successful fledglings at 63.5 days old (chick #1) and 60 days old (chick #2). The female was again sealed inside the nest box on 30 May 2009, 56 days after she emerged from the nest with her two fledglings from the first clutch. This resulted in one successful

fledgling at 57.5 days old, one instance of cannibalism (chick #2 at 3d 15hr old), and two instances of infanticide-cannibalism (chick # 3 and # 4, at 3d 7hr and 1h old respectively). Only one chick fledged from the second clutch. This paper describes the first record and data of two successful clutches within one calendar year, first observation of a male committing cannibalism, and the first instance of a chick leaving the nest box earlier than the female, in our population of Oriental Pied Hornbills. This paper was presented at the 5(th) International Hornbill Conference jointly organised by the National Parks Board (Singapore) and the Hornbill Research Foundation (Thailand), in Singapore on 22(nd)-25(th) March 2009.

2813: ***+.172***

Obligate myrmecophilic butterfly species, such as Phengaris (Maculinea) teleius and P. nausithous, have narrow habitat requirements. Living as a caterpillar in the nests of the ant species Myrmica scabrinodis and M. rubra, respectively, they can only survive on sites with both host ants and the host plant Great Burnet Sanguisorba officinalis. After having been reintroduced into a nature reserve in the Netherlands in 1990, both butterfly species expanded their distribution to linear landscape elements such as road verges and ditch edges outside this reserve. As additional habitat of both butterfly species, vegetation management of these landscape elements became important. Our results show that a management beneficial for Phengaris butterflies should aim to increase the nest density of Myrmica species, at the same time reducing the density of nests of the competitor Lasius niger or at least keeping them at a low density. Unfavourable grassland management under which L. niger thrives, includes not mowing or flail-cutting the grass, or depositing dredgings along the side of the ditch. Management favourable for the two Myrmica species differs, demanding some flexibility if both species are to benefit. M. scabrinodis is best supported with early mowing of the road verge vegetation or late mowing in the nature reserve, both of which result in an open vegetation and warm microclimate. In contrast, the nest sites of M. rubra should be left undisturbed during the summer, and mown in late autumn. Mowing of butterfly habitat should be avoided between mid-June and mid-September as this would remove the flowerheads of the Sanguisorba plants, on which the butterflies lay their eggs.

2814: ***+.213***

The Marsh Fritillary butterfly, Euphydryas aurinia (Rott.), is regarded as the classic species that requires landscape-scale management in order to maintain viable populations. This paper describes the background to an extinction of this species in Northern England, United Kingdom, and the action taken to reintroduce and monitor the progress of sub-populations between 2007 and 2009. A captive breeding stock was secured using the last 155 known wild caterpillars in 2004 and supplemented by 95 caterpillars collected under licence from the nearest extant populations in western Scotland. Through captive breeding the source population for subsequent reintroduction was increased dramatically and 42,400 caterpillars were used in 2007 to reintroduce the butterfly to four locations in North and West Cumbria. Each of the release sites is within known historical network areas where the butterfly had thrived in past decades. The intention was to restore multiple habitat patches across each network area and eventually establish viable metapopulations across these landscapes. Adults and larval webs have been monitored since 2007 and show that introduction has been successful at three out of the four locations. The failure at the fourth site appears to be unrelated to habitat condition and the causes are currently being investigated. Work is continuing to secure appropriate management of suitable habitat within each landscape area and the number of sub-populations in one area will be increased in the 2010 season.

2815: ***+.126***

P>1. Most hypotheses for translocation success are elaborate, hierarchical, and untested combinations of socio-ecological predictors. Empirical support for those tested is vulnerable to spurious single-predictor relationships and does not account for the hierarchy amongst predictors and non-independence amongst individuals or cohorts. Testing hypotheses as a priori multi-level models promotes stronger inference.2. We apply a 25-year (1981-2005) data base including 89 reintroduction and 102 restocking events that released 682 black rhinoceros Diceros bicornis into 81 reserves to test 24 hypotheses for translocation success, defined as survival to 1 year post-release. We made information-theoretic comparisons of hypotheses represented as hierarchical models incorporating random effects for reserve and release cohort predictors of death.3. Mortality rates after restocking were higher than for reintroductions (13 center dot 4 cf. 7 center dot 9%, respectively) due largely to intraspecific fighting. No predictors strongly influenced reintroduction success, although cohorts consisting entirely of adult males were 8 center dot 2% of individuals but contributed 21 center dot 9% of deaths, and reserves with lowest carrying capacities (i.e. < 0 center dot 1 rhino km-2) had a 16 center dot 3% mortality rate. Most models for restocking success were not supported. Only those including age class received substantial support. Age was the only predictor to strongly influence death rates. Predictors previously thought influential, like population density, reserve area and quality, and cohort size, were not supported.4. Synthesis and applications. Simple rules succeeded where complex ecological and demographic hypotheses failed to predict survival after translocation of critically endangered black rhinoceros. Results support bold attempts by managers at translocations towards species recovery in most ways that they have historically occurred. Groups of rhinoceros of different size and composition can be successfully moved over large distances between different ecological contexts. Also, the release of cohorts into reserves that are relatively small, poorer habitat or already stocked need not be avoided so long as calves and all-male cohorts are not reintroduced, and only adults used for restocking. Our analysis demonstrates the importance of information-theoretic comparisons of a priori hierarchical models to test hypotheses for conservation management. We caution against interpreting simple correlations or regression amongst a large number of nested ecological and demographic variables.

2816: *+.088*

Pet and food trade are among the major threats for many chelonians species worldwide. We investigate the impact of the present and past trade in European pond turtles (Emys orbicularis, E. trinacris) on their extant distribution pattern. Using a comprehensive mtDNA dataset of cytochrome b haplotypes derived from more than 1,550 individuals, we assigned wild-caught allochthonous and captive turtles from six European countries to their native regions across the entire distribution range. We found allochthonous haplotypes in all countries surveyed, providing evidence of long-distance translocations owing to past and present trade, illegal poaching or collection by tourists. In summary, we identify source regions of allochthonous turtles and past and present trade routes. Moreover, we point out future directions which would contribute to conservation and management of these threatened species.

2817: *+.042*

Reintroduced populations of threatened species are often founded by a small number of individuals, but maximising genetic diversity is often a criterion for founder selection. Reintroduction of pregnant females has been proposed as a means of maximising productivity and genetic diversity, but it is unclear whether the release of pregnant females increases the effective number of founders. Ten male and 20 gravid female egg-laying skinks (Oligosoma suteri) were reintroduced to Korapuki Island from Green Island, New Zealand in 1992. We sampled the

populations on both Green and Korapuki Islands to examine the effect of reintroduction on the genetic structure and fitness of egg-laying skinks following release. The population on Korapuki Island showed multiple genetic signatures of a bottleneck that were not detected in the population on Green Island. At the individual level, juveniles on Korapuki Island were more homozygous than adults on Korapuki and Green Islands. However, we did not find evidence of inbreeding depression using two performance-based surrogates of fitness. Further, the population on Korapuki Island had a significantly larger effective population size than would have been expected by reintroduction of 30 skinks, based on 10,000 simulated populations. The reintroduction of gravid females aided in increasing the effective number of founders, and may be a viable option for maximizing genetic diversity in reintroduced populations, particularly for long-lived species. However, the continued loss of genetic variation in reintroduced populations may have more insidious long-term consequences, such as the loss of adaptive potential, which cannot be assessed in the short-term.

2818: *-.039*

It is crucial to understand the genetic health and implications of inbreeding in wildlife populations, especially of vulnerable species. Using extensive demographic and genetic data, we investigated the relationships among pedigree inbreeding coefficients, metrics of molecular heterozygosity and fitness for a large population of endangered African wild dogs (Lycaon pictus) in South Africa. Molecular metrics based on 19 microsatellite loci were significantly, but modestly correlated to inbreeding coefficients in this population. Inbred wild dogs with inbreeding coefficients of a parts per thousand yen0.25 and subordinate individuals had shorter lifespans than outbred and dominant contemporaries, suggesting some deleterious effects of inbreeding. However, this trend was confounded by pack-specific effects as many inbred individuals originated from a single large pack. Despite wild dogs being endangered and existing in small populations, findings within our sample population indicated that molecular metrics were not robust predictors in models of fitness based on breeding pack formation, dominance, reproductive success or lifespan of individuals. Nonetheless, our approach has generated a vital database for future comparative studies to examine these relationships over longer periods of time. Such detailed assessments are essential given knowledge that wild canids can be highly vulnerable to inbreeding effects over a few short generations.

2819: *+.258*

From the time since the decline of the wild rabbit Oryctolagus cuniculus in southern Europe, various techniques and methods have been explored with a view to restoring wild rabbit populations or increasing rabbit resilience, for both conservation and game purposes. Rabbit restocking and habitat management are among the measures most often applied. Some efforts have been made to increase refuges for wild rabbits, mainly through the construction of artificial warrens. The present study evaluates the response of a wild rabbit population introduced to artificial warrens of varying sizes. This involves comparisons of the density of rabbits in the warrens, rabbit density change between seasons of low and high rabbit population density and the productivity index for large and small warrens in rabbit populations living under semi-natural conditions. Our results show that large warrens had higher rabbit abundance than had small warrens, but significantly lower rabbit density. No differences in density increase or productivity index were found with respect to warren size. The results suggest that it is preferable to build many small warrens for conservation of wild rabbit populations, but, in the event that only a few warrens are built, it is advisable that they be large.

2820: *+.104*

"Laguna de las Ilusiones" is an isolated urban lake in the city of Villahermosa, Tabasco in Mexico that retains a population of Morelet's crocodile (Crocogyhts moreletii). During the years 2007 and 2008 we studied 22 natural nests of this species. Most of the nests (53%) were built with vegetal material (leaf-litter, stems and thin branches). Also, we recorded human waste as part of the materials for nest construction. Mean clutch size was 30.4 +/- 10.8 eggs by nest (nests n = 19, eggs n = 578). Mean egg volume is significantly different among nests, an indication of size differences between the nesting females. Physical characteristics of the nests in 2007-2008 were compared with nests found at the same area during 1991-1994. We found that nest diameter, shore distance and clutch size were significantly higher in 2007-2008. Hatching in "Laguna de las Ilusiones" was low during 2007-2008 (33.9% +/- 31.21) compared with other crocodilians. All new born crocodiles in 2007 and 2008 were males. We discuss how the city may affect the nesting of Morelet's crocodiles, influencing different aspects such as, the use of new materials for nest construction, increasing the size of the basal area of the nests, or promoting the search for a nesting site away from the shore. We recommend a management of the crocodiles with female reintroduction and artificial incubation in the "Laguna de las Ilusiones" to facilitate conservation of this population.

2821: *-.005*

Despite it being the most abundant mountain dwelling ungulate of Europe and the Near East, the taxonomy, systematics and biology of the chamois are still imperfectly known. Although neither species of chamois is at risk, several subspecies are threatened (Rupicapra rupicapra cartusiana, Rupicapra rupicapra tatrica and Rupicapra rupicapra balcanica; Rupicapra pyrenaica ornata. Rupicapra rupicapra asiatica is data-deficient but probably threatened). A life history with apparently contradictory relationships between survival, sexual dimorphism and mating system suggests a unique survival strategy not yet fully understood. Over the last century, morphologic, biometric, behavioural and genetic features have been studied to shed light on the phylogeography and monophyly or polyphyly of the chamois as well as on the number of existing species and subspecies of the genus Rupicapra. The dispersal hypothesis, according to which R. rupicapra migrated westward from eastern Europe in the Quaternary, confining R. pyrenaica to the southernmost regions of Europe, has been recently called into question by some molecular analyses that yielded contradictory results. In spite of subtleties relevant to each method of analysis, an overall evaluation of differences between the R. rupicapra and the R. pyrenaica groups strongly supports the functional separation of the taxa into two species. Further studies on the ecology of chamois, as well as on the epidemiology of severe diseases, e.g. sarcoptic mange, are needed to improve the management of viable populations. Before translocations and reintroductions are carried out, the risk of hybridization leading to genetic extinction should be evaluated.

2822: *+.185*

During the Last Glacial Maximum, European red deer Cervus elaphus occurred in refugia in Iberia/southern France, Italy, the Balkans and the Carpathians. Most of Europe, including large parts of the east and north-east, is now inhabited by red deer from the western lineage. The eastern lineage is largely confined to south-eastern Europe; a third lineage comprises Sardo-Corsican and Barbary red deer.Sardo-Corsican, Barbary and Mesola red deer are genetically unique units. They exhibit low levels of genetic diversity and deserve particular protection, since conservation strategies should target genetic information. Hybridization between sika Cervus nippon and red

deer occurs rarely, but may lead to extensive introgression, particularly in parts of the British Isles. Further expansion of both species may lead to increased hybridization in continental Europe. Although hunting has an impact on red deer gene pools, the main threat today is habitat fragmentation in human-dominated landscapes. The resulting increase in genetic drift and inbreeding reduces variability in isolated populations and may lead to inbreeding depression. To support vital meta-populations, migration corridors should be established.

2823: ***+.005***

The critically endangered Mangrove Finch (Camarhynchus heliobates) is a habitat specialist restricted to mangroves, with a global population size of about 100 individuals. Due to its extremely limited geographic range and low dispersal capabilities, translocations of individuals to different mangrove areas within its historic range have been considered. We studied foraging behaviour, food abundance and nest site choice to support decisions for the choice of sites for translocation. Mangrove Finches principally searched for their food in dead wood, litter and the apical buds of red mangrove (Rhizophora mangle), where they mainly fed on spiders, caterpillars and beetles (adults and larvae). The intense use of litter confirmed that separation from a direct flow of seawater in the mangroves was an important component of habitat suitability. Even though red mangrove is an important feeding substrate, Mangrove Finches cannot safely build their nests in the branch structure of this tree species: they always placed nests in the outermost branches of the lower canopy of black (Avicennia germinans) and white (Laguncularia racemosa) mangrove trees and showed a pronounced preference for black mangrove where available. Nests were located in tall trees within patches of high mangroves that were often flooded during high tide. Such mangroves are rare on the Galapagos,which thus may contribute to the natural rareness of the finch.

2824: ***+.015***

The Griffon Vulture (Gyps fulvus) is considered to be socially monogamous. However, extra-pair fertilizations are suspected due to observations of extra-pair copulations in some populations. We performed parentage studies based on ten polymorphic microsatellite markers in two reintroduced colonies of Griffon Vulture. Out of 40 genotyped chicks, we found eight chicks whose genotypes mismatched those of their observed parents. Two could be explained by the occurrence of a null allele at one locus. The six remaining mismatches detected relied on mismatches at one locus, and they were not detected when we increased the potential genotyping error rate. We thus conclude that the Griffon Vulture is genetically monogamous, at least in low-density populations.

2825: ***+.151***

Maintenance of viable populations of many endangered species will require conservation action in perpetuity. Efforts to conserve these species are more likely to be successful if their reliance on conservation actions is assessed at the population level. Woodland caribou (Rangifer tarandus caribou) were extirpated recently from Banff National Park, Canada, and translocations of caribou to Banff and neighboring Jasper National Park are being considered. We used population viability analysis to assess the relative need for and benefits from translocation of individuals among caribou populations. We measured stochastic growth rates and the probability of quasi extinction of four populations of woodland caribou with and without translocation. We used two vital rates in our analysis: mean adult female survival and mean number of calves per breeding-age female as estimates of mean fecundity. We isolated process variance for each vital rate. Our results suggested the Tonquin caribou population in Jasper is likely to remain viable without

translocation, but that translocation is probably insufficient to prevent eventual extirpation of the two other populations in Jasper. Simulated reintroductions of caribou into Banff resulted in a 53-98% probability of > 8 females remaining after 20 years, which suggests translocation may be an effective recovery tool for some caribou populations.

2826: ***+.243***

During 2003 and 2004, 25 mature Kootenai River white sturgeon Acipenser transmontanus were dual tagged with sonic and radio transmitters, and translocated upstream approximately 50 km from their current spawning site which is comprised of silt and sand and with low egg survival. The release site was higher gradient and cobble and gravel substrate (canyon reach). The purpose of this study was to join mature white sturgeon with a more suitable incubation and rearing habitat and determine if translocation had legitimacy as a management measure. Residence times in the study varied among years and release groups and movement patterns were difficult to discern. In 2003, all males moved downstream out of the canyon reach within 2 days of release. Of the three females released in 2003, one remained in the canyon reach for 13 days while the other two moved downstream within 4 days. However, five eggs were collected on one substrate mat in the canyon reach in 2003 near river kilometer 261.6, although the eggs were too early in their developmental stage to verify fertilization. In 2004, adults were released in two different time frames, an early and late period. Only one female was part of the early release group in 2004, but three of the remaining six males from this group stayed in the canyon reach for over 3 weeks, the longest of any group. Combining years, males stayed in the canyon reach longer than females; white sturgeon released early in the spawning season stayed in the canyon reach longer. The release of white sturgeon in groups or in areas where other white sturgeon were residing may increase the residency in the study reach. It is feasible to move mature white sturgeon into the more suitable spawning habitats, however, the population level benefits are difficult to determine from this research, and should be weighed against other management and recovery actions.

2827: ***-.037***

The objective of this study was to evaluate the effects of acclimation to flow and site-specific physicochemical water conditions on poststocking dispersal and physiological condition of age-1 hatchery-reared pallid sturgeon. Fish from three acclimation treatments were radio-tagged, released at two locations (Missouri River and Marias River), and monitored using passive telemetry stations. Marias treatment was acclimated to flow and site-specific physicochemical conditions, Bozeman treatment was acclimated to flow only, and controls had no acclimation (reared under traditional conservation propagation protocol). During both years, fish released in the Missouri River dispersed less than fish released in the Marias River. In 2005, Marias treatment dispersed less and nearly twice as many fish remained in the Missouri River reach as compared to control fish. In 2006, pallid sturgeon dispersed similarly among treatments and the number of fish remaining in the Missouri River reach was similar among all treatments. Differences in poststocking dispersal between years were related to fin curl which was present in all fish in 2005 and only 26% in 2006. Pallid sturgeon from all treatments in both years had a greater affinity for the lower reaches of the Missouri River than the upper reaches. Thus, release site influenced poststocking dispersal more than acclimation treatment. No difference was observed in relative growth rate among treatments. However, acclimation to flow (i.e., exercise conditioning) prevented fat accumulation from rupturing hepatocytes. Acclimation conditions used in this study did not benefit pallid sturgeon unless physiological maladies were present. Overriding all treatment effects was stocking location; thus, natural resource agencies need to consider stocking location carefully to reduce poststocking dispersal.

2828: ***+.321***

P>1. Where populations are confined to fragmented, human-dominated landscapes, preventing declines and extirpations will often rely on metapopulation management. Spatially-explicit population viability analyses provide tools to evaluate how well the local management efforts can be combined to conserve metapopulations across large areas. Yet, metapopulation models have rarely been combined with tools to assess the cost-effectiveness of different conservation strategies.2. European bison Bison bonasus only occur in small, fragmented populations, making their long-term survival dependent on establishing a metapopulation across eastern Europe. We parameterized a European bison metapopulation model based on time-series of bison demography and a habitat suitability map to assess the viability of bison populations in the Carpathians and the relative cost-effectiveness of (i) reintroductions, (ii) wildlife overpasses and (iii) anti-poaching measures in establishing a viable bison metapopulation.3. Our results suggest that the Carpathians could support a viable metapopulation of European bison provided that active efforts are taken to safeguard bison and connect isolated herds. With such steps, our model forecasts that bison numbers could increase substantially over the next 100 years as local populations increase and bison recolonize parts of the Carpathians.4. Reintroductions appear to be the most cost-effective approach for establishing a viable bison metapopulation among our scenarios, especially when coupled with wildlife overpasses to improve connectivity among herds. The most promising region for a bison metapopulation in the Carpathians was south-eastern Poland, Ukraine and northern Romania. We identified several candidate regions for reintroductions and wildlife overpasses, especially in the border region of Romania and Ukraine. Site-specific assessments of both habitat suitability, and the costs and benefits of a large bison population, should target those regions.5. Synthesis and applications. Our results highlight how careful conservation planning can identify solutions to preserve large mammals in human-dominated landscapes. Choosing the most effective option from a range of management strategies is a central challenge for wildlife managers. We have shown that incorporating cost-effectiveness analyses into metapopulation models can elucidate the relative value (gain per unit cost) of different conservation management options, allowing decision makers to choose cost-effective options to preserve large mammals. Our model projections also provide hope for establishing a viable free-ranging European bison population in the Carpathians, one of the last relatively wild areas in Europe.

2829: ***+.029***

P>1. When managing endangered species the consequences of making a poor decision can be extinction. To make a good decision, we must account for the stochastic dynamic of the population over time. To this end stochastic dynamic programming (SDP) has become the most widely used tool to calculate the optimal policy to manage a population over time and under uncertainty.2. However, as a result of its prohibitive computational complexity, SDP has been limited to solving small dimension problems, which results in SDP models that are either oversimplified or approximated using greedy heuristics that only consider the immediate rewards of an action.3. We present a heuristic sampling (HS) method that approximates the optimal policy for any starting state. The method is attractive for problems with large state spaces as the running time is independent of the size of the problem state space and improves with time.4. We demonstrate that the HS method out-performs a commonly used greedy heuristic and can quickly solve a problem with 33 million states. This is roughly 3 orders of magnitude larger than the largest problems that can currently be solved with SDP methods.5. We found that HS out-performs greedy heuristics and can give near-optimal policies in shorter timeframes than SDP. HS can solve problems with state spaces that are too large to optimize with SDP. Where the state space size precludes SDP, we argue that HS is the best technique.

2830: -.305

An infectious disease caused by Squirrelpox virus has contributed to the decline of red squirrels, Sciurus vulgaris, in the British Isles. Because of the heightened disease surveillance activity in red squirrels, adenovirus infection with associated mortality has been detected. Adenoviral disease is described in other rodent species usually associated with stressors. Here we 1) describe the pathologic findings in red squirrels found dead with adenoviral infection and gastrointestinal disease, and 2) investigate the epizootiology of the disease through pathologic investigation, scanning surveillance, and virologic studies. Ten red squirrels involved in conservation studies were diagnosed with adenoviral infection by electron microscopy or PCR. All squirrels exhibited diarrhea and small intestinal inflammation or hemorrhage was evident in seven cases. Lesions indicative of splenic lymphocytolysis were observed in one squirrel and leukocytic hepatitis in another. No adenovirus was detected in grey squirrels, Sciurus carolinensis, inhabiting the same forest area, but previous serologic studies showed that grey squirrels cannot be discounted as a reservoir of the virus. Scanning surveillance showed that 12% of 493 red squirrels had diarrheal disease and two of 13 free-living red squirrels with diarrheal disease had adenovirus infection. Adenoviral disease in declining free-living wild red squirrel populations in the British Isles occurs at a detectable frequency and its impact on the conservation of this species deserves further attention.

2831: +.104

Wildlife translocations, the deliberate movement of animals from one part of their distribution to another, are increasingly used as a conservation method for the reestablishment of rare and endangered species. The objective of this study was to examine the movement patterns and macro- and microhabitat use of translocated and resident spur-thighed tortoises. This translocation was considered a soft-release as the tortoises were forced to be relatively inactive due to their being released at the beginning of the aestivation season. Our results suggest that forced aestivation soft-releases may succeed in reducing dispersal by forcing spur-thighed tortoises to spend time at the release site as the majority of translocated tortoises had similar activity range sizes and movement path tortuosity as resident tortoises. Spur-thighed tortoise conservation will require protecting habitat at multiple scales, with the remaining native forests in the country of Jordan being important to the spur-thighed tortoise during the activity and aestivation/hibernation seasons, as this macrohabitat was used significantly more than the human-modified habitats. Microhabitat structures such as leaf litter and availability of large stones may also be especially important in human-modified landscapes, as these microhabitats may help reduce the effects of habitat degradation.

2832: +.086

We documented date and duration of each breeding phase, breeding rate, nursing behavior, parental care, and leveret survival of the Tehuantepec jackrabbit (Lepus flavigularis), a critically endangered lagomorph. Between June 2006 and May 2008, we observed 60 adult radio-collared jackrabbits in Oaxaca, Mexico. Tehuantepec jackrabbits exhibit breeding behaviors 250 clays out of the year, with a high-intensity period during the rainy season (May October). Females give birth to 2 leverets 32 days after copulation. Directly after birth, leverets are put into "beds" or "nests," which are depressions in the ground covered by prairie grass (Jouvea pilosa). Females return to nurse and groom the leverets once per day until the leverets are weaned (12 days after birth). The breeding season and parental care behaviors of Tehuantepec jackrabbits are similar to those of other jackrabbits. Females produced an average of 2 litters per breeding season. The breeding rate

for the Tehuantepec jackrabbit (4 leverets per breeding female per breeding season) is lower than the average of other species in the genus Lepus. The survival rate of Tehuantepec jackrabbit leverets (50% at day 19) is higher than that of other leporids. Predation by domestic dogs (Canis familiar-is) is the main cause of mortality Understanding reproductive behavior is critical for captive breeding, reintroduction, and conservation efforts for endangered leporids such as the Tehuantepec jackrabbit.

2833: *+.151*

Black-tailed prairie dogs, Cynomys ludovicianus, now inhabit a small fraction of their original range in the Great Plains. We monitored a population of black-tailed prairie dogs at Scotts Bluff National Monument, Nebraska, from colonization in 1981 until 2009 (28 years). Colony boundaries were mapped by delineating clip lines and active burrows; population densities were estimated via visual counts. Estimates of total population size revealed 4 distinct periods of changing dynamics: (1) a linear increase, (2) a decline and prolonged depression, (3) an exponential increase, and (4) a period of high variability. Area occupied revealed similar, although less-defined trends, whereas densities fluctuated greatly (8-80 individuals . ha(-1)). Even after almost 30 years, this population remains relatively small. Decreases in the population may have been due, in part, to predation by badgers, although sylvatic plague cannot be ruled out. Black-tailed prairie dogs are recognized as keystone grassland species, and attempts are underway to reintroduce them to parts of their historic range. Our data suggest that black-tailed prairie dogs possess high potential for rapid population growth and decline, regardless of colony size. Therefore, either human-assisted or natural dispersal events may be important in establishing colonies in suitable habitat.

2834: *+.180*

The Critically Endangered black-faced lion tamarin Leontopithecus caissara has a restricted distribution consisting of small mainland and island populations each with distinct habitats in coastal south-eastern Brazil. The conservation action plan for the species recommends translocation to increase population size and promote genetic exchange among threatened isolated populations, measures that require an understanding of habitat and resource requirements. We investigated habitat selection by the black-faced lion tamarin, comparing habitats and examining frequency of use by two insular groups and two mainland groups. Using this understanding of habitat preferences we were able to refine current estimates of the carrying capacity (K) for this species. Habitats preferred by mainland groups were swamps and inundated areas and secondary forest. Submontane forest was avoided, as were hydromorphic lowland forest and urbanized areas. Island groups used mainly tall lowland forest and arboreal restinga (forest on sandy soil). The finding that L. caissara avoids montane forest sheds light on its restricted distribution to low elevation coastal plains. An estimate of K indicated c. 700 individuals, which could be increased to c. 1,500 with appropriate conservation management measures. Findings agree with the supposition that lion tamarins are typical of mature Atlantic forest, with little altitudinal variation but flexible in their use of successional stages. Future conservation management measures should account for differences in habitat use, selection and differences between island and mainland habitats.

2835: *+.145*

In 1994 Alan Rabinowitz decried what he regarded as lackadaisical attempts by governments, NGOs and international funding agencies to conserve the Sumatran rhinoceros Dicerorhinus

sumatrensis. Sixteen years on it is timely to evaluate whether his warnings were heeded. We review the current conservation status of D. sumatrensis throughout its range and the latest threats and challenges complicating efforts to conserve this species. Recent data from governments, NGOs and researchers indicate that the global population could be as low as 216, a decline from c. 320 estimated in 1995. Based on lessons learnt and expert opinions we call on decision makers to focus on two core strategies for conservation of D. sumatrensis: (1) the translocation of wild individuals from existing small, isolated or threatened forest patches into semi-in situ captive breeding programmes, and (2) a concomitant enhancement of protection and monitoring capacities in priority areas that have established these breeding facilities or have recorded relatively high population estimates and track encounter rates. At least USD 1.2 million is required to implement these strategies annually in four priority areas: Bukit Barisan Selatan and Way Kambas National Parks on Sumatra, and Danum Valley Conservation Area and Tabin Wildlife Reserve on Sabah. Given that conservation funds are rarely secure and D. sumatrensis is still in decline we call on potential donors to help secure and augment existing capacities of organizations in these four priority areas before committing resources to elucidate the status of the species in other areas such as Gunung Leuser and Taman Negara National Parks.

2836: ***+.177***

Terrestrial wildlife migrations, once common, are now rare because of ecosystem fragmentation and uncontrolled hunting. Botswana historically contained migratory populations of many species but habitat fragmentation, especially by fences, has decreased the number and size of many of these populations. During a study investigating herbivore movement patterns in north-west Botswana we recorded a long-distance zebra Equus burchelli antiquorum migration between the Okavango Delta and Makgadikgadi grasslands, a round-trip distance of 588 km; 55% of 11 animals collared in the south-eastern peripheral delta made this journey. This was unexpected as, between 1968 and 2004, the migration could not have followed its present course because of the bisection of the route by a veterinary cordon fence. As little evidence exists to suggest that large-scale movements by medium-sized herbivores can be restored, it is of significant interest that this migration was established to the present highly directed route within 4 years of the fence being removed. The success of wildlife corridors, currently being advocated as the best way to re-establish ecosystem connectivity, relies on animals utilizing novel areas by moving between the connected areas. Our findings suggest that medium-sized herbivores may be able to re-establish migrations relatively quickly once physical barriers have been removed and that the success of future system linkages could be increased by utilizing past migratory routes.

2837: ***+.098***

Premise of the study: Microsatellite primers were developed for the native perennial cycad Cycas taitungensis to evaluate the genetic variation of this endangered insular species. Methods and Results: Using a magnetic bead enrichment method and EST data, 16 primer sets were developed and identified for the native Taiwan cycad C. taitungensis. The primers amplified dinucleotide, trinucleotide, and complex repeats with 1-9 alleles per locus. Most primers also amplified DNA from C. revoluta and C. debaoensis.Conclusions: These results indicate the utility of primers for future studies of the genetic structure of C. taitungensis. In addition, the primers are useful for further phylogeographic studies between C. taitungensis and C. revoluta, which is a closely related species.

2838: ***+.259***

Anthropogenic activities have significantly altered freshwater fish communities. Extirpations of deepwater coregonines (Coregonus spp.), a diverse group of fish species, have left vast areas of the Laurentian Great Lakes devoid of a deepwater fish community. Currently, fisheries managers are considering restoring populations by reintroducing deepwater coregonines from Lake Superior and Lake Nipigon. However, little is known about the historical ecology of deepwater coregonines, and species characterization has proved difficult. We used stable isotope analysis of museum-preserved and contemporary specimens to investigate if (1) coregonine species historically occupied distinct niches and (2) the pattern of trophic niche partitioning has changed over the last century. Across all lakes, individual species occupied distinct trophic niches, confirming that these species were ecologically distinct. Understanding trophic niche partitioning helps resolve uncertainty about distinctness of species within and across lakes and may provide a better ecological basis for rehabilitation of Great Lakes food webs and ecosystems.

2839: ***+.065***

Research on the allocation of resources to manage threatened species typically assumes that the state of the system is completely observable; for example whether a species is present or not. The majority of this research has converged on modeling problems as Markov decision processes (MDP), which give an optimal strategy driven by the current state of the system being managed. However, the presence of threatened species in an area can be uncertain. Typically, resource allocation among multiple conservation areas has been based on the biggest expected benefit (return on investment) but fails to incorporate the risk of imperfect detection. We provide the first decision-making framework for confronting the trade-off between information and return on investment, and we illustrate the approach for populations of the Sumatran tiger (Panthera tigris sumatrae) in Kerinci Seblat National Park. The problem is posed as a partially observable Markov decision process (POMDP), which extends MDP to incorporate incomplete detection and allows decisions based on our confidence in particular states. POMDP has previously been used for making optimal management decisions for a single population of a threatened species. We extend this work by investigating two populations, enabling us to explore the importance of variation in expected return on investment between populations on how we should act. We compare the performance of optimal strategies derived assuming complete (MDP) and incomplete (POMDP) observability. We find that uncertainty about the presence of a species affects how we should act. Further, we show that assuming full knowledge of a species presence will deliver poorer strategic outcomes than if uncertainty about a species status is explicitly considered. MDP solutions perform up to 90% worse than the POMDP for highly cryptic species, and they only converge in performance when we are certain of observing the species during management: an unlikely scenario for many threatened species. This study illustrates an approach to allocating limited resources to threatened species where the conservation status of the species in different areas is uncertain. The results highlight the importance of including partial observability in future models of optimal species management when the species of concern is cryptic in nature.

2840: ***+.348***

Habitat loss threatens large mammals worldwide, and their survival will depend on habitat in human-dominated landscapes. Conservation planners thus face the challenge to identify areas of least conflict with land use, yet broadscale species distribution models rarely incorporate real landscape patterns nor do they identify potential conservation conflicts. An excellent example of such conservation challenges is provided by European bison (Bison bonasus). Almost extinct by the early 20th century, bison can only survive in the wild if large metapopulations are established, but it is unclear where new herds can be reintroduced. Using European bison as an example we

conducted a continental-scale habitat assessment based on real landscape patterns. Our specific aims here were to (1) map European bison habitat throughout the species' former range, (2) examine whether broadscale habitat suitability factors differ from previously reported fine-scale factors, and (3) assess where suitable habitat occurs in areas with low potential for conflict with land use. We assessed habitat suitability using herd range maps for all 36 free-ranging European bison herds as habitat use data. Habitat suitability maps were compared with maps of land cover, livestock density, agricultural constraints, and protected areas to assess potential conservation conflicts. Our models had high goodness of fit (AUC 0.941), and we found abundant potential bison habitat. European bison prefer mosaic-type landscapes, with a preference for broad-leaved and mixed forests. European bison metapopulations do not appear to be limited by habitat availability. However, most potential habitat occurred outside protected areas and has substantial potential for conservation conflicts. The most promising areas for establishing large bison metapopulations all occur in Eastern Europe (i.e., the Carpathians, the Belarus-Ukraine borderlands, and several regions in European Russia). The future of European bison and that of other large mammals in the wild thus clearly lies in Eastern Europe, because habitat there is most abundant and least fragmented, and because the potential for conflict with land use is lower. More generally we suggest that broadscale habitat assessments that incorporate land use can be powerful tools for conservation planning and will be key if large herbivore and carnivore conservation is to succeed in a human-dominated world.

2841: ***+.037***

The Siamese crocodile (Crocodylus siamensis), listed in CITES Appendix I, is considered one of the most critically endangered crocodilians in the world, and the reintroductions of it have been tried. Investigation of genetic variation for Siamese crocodile can help to conserve and improve this endangered species. Fourteen microsatellite loci were developed and twelve polymorphic loci were used to investigate the genetic variation and genetic bottleneck hypothesis on 48 captive individuals sampled in Guangdong Provincial Wildlife Rescue Center in Guangzhou. The allele number of polymorphic markers ranged from 2 to 10 per locus, with the average of 4.357. Observed heterozygosity ranged from 0.063 to 0.649 and expected values ranged from 0.259 to 0.844. The Shannon information index and Polymorphic Information Content showed that most of the loci were highly informative with an overall mean of 0.941 and 0.440, respectively. The bottleneck analysis provided evidence of a significant genetic signature of population decline.

2842: ***+.035***

The dogma that gray wolf (Canis lupus) population densities in naturally occurring systems are limited almost solely by available ungulate biomass is based upon studies that fit straight line linear regressions (Type 1 numerical response) to data collected at 32 sites across North America. We fit Type 1, 2, and 3 response functions to the data using linear and nonlinear regression as appropriate and found that the evidence supported wolf population regulation by density-dependence as much as limitation by prey availability. When we excluded 4 of 32 points from the original data set because those points represented exploited or expanding wolf populations the data suggested that wolf populations are self regulated rather than limited by prey biomass by at least a 3:1 margin. In establishing goals for sustainable wolf population levels, managers of wolf reintroductions and species recovery efforts should account for the possibility that some regulatory mechanism plays an important role in wolf population dynamics. (C) 2011 The Wildlife Society.

2843: ***+.148***

Reintroductions are a common strategy to restore ecosystem integrity, especially when top predators are involved. Reintroductions are often time consuming, expensive, and controversial, and thus understanding what aspects are important for a successful program is needed. Focusing on the example of the reintroduction of Canada lynx (Lynx canadensis) to Colorado, we investigated how different release protocols (RP) affected mortality within the first year post-release. We found that average monthly mortality in the study area during the first year decreased with time in captivity from 0.205 (95% CI = 0.069, 0.475) for lynx having spent up to 7 days in captivity to 0.028 (95% CI = 0.012, 0.064) for lynx spending > 45 days in captivity before release. Our results also suggest that keeping lynx in captivity beyond 5-6 weeks accrued little benefit in terms of monthly survival. We found that, on a monthly average basis, lynx were as likely to move out (P = 0.196, SE = 0.032) as well as back onto (P = 0.143, SE = 0.034) the reintroduction area during the first year after release. Mortality was 1.6 times greater outside of the study area, suggesting that permanent emigration and differential mortality rates on and off reintroduction areas should be factored into sample size calculations for an effective reintroduction effort. A post-release monitoring plan is critical to providing information to assess aspects of RP and to improve survival of individuals. Future lynx and other carnivore reintroductions may use our results to help design reintroduction programs including both the release and post-release monitoring protocols. (C) 2011 The Wildlife Society.

2844: *+.187*

The effects of anthropogenic habitat modification are evident on tropical Pacific islands where forests have been extensively converted to plantations of Coconut Palm (Cocos nucifera). We evaluated resource selection in the critically endangered Tuamotu Kingfisher (Todiramphus gambieri gertrudae), which is confined to a single population on the low-elevation island of Niau Atoll in French Polynesia. Our analyses indicate that resources associated with foraging underlie space use and habitat selection of Tuamotu Kingfishers at multiple spatial scales. At the island scale, the occurrence of the species was best predicted by habitat features associated with foraging opportunities, including agricultural coconut forest with open understory, hunting perches, and exposed ground. Conversely, the species' distribution was negatively associated with undisturbed vegetation, including primary feo forest and fallow coconut plantation. At the home-range scale, utilization distributions of radiomarked Tuamotu Kingfishers also indicated that birds selected agricultural coconut forest and least preferred native feo forest. Observations further indicated that foraging birds selected coconut plantations managed with prescribed burning for hunting. The Tuamotu Kingfisher is a rare example of a threatened tropical species that likely benefits from agricultural management, and our findings provide support for conservation strategies based on establishing rescue populations on other islands with coconut plantations. We suggest that incorporating agricultural coconut forests into conservation planning could help prevent the extinction of several kingfisher species that historically relied on the natural broadleaf habitats that are now almost entirely absent from insular Pacific Oceania. Received 13 August 2010, accepted 22 February 2011.

2845: *+.086*

Many species endemic to isolated islands are of conservation interest because of concerns over the potentially devastating effects of environmental stochasticity and the pending threat of anthropogenic influences and invasive species. The effective size (N-e) of these species is a key parameter in their conservation because it predicts the detrimental effects of inbreeding or genetic drift and can be used to inform management plans. We used microsatellite allele frequencies (4 loci) and mtDNA (control region; cytochrome b) sequences to assess the genetic diversity and the

effective number of breeders (N-b) in the Nihoa Millerbird (Acrocephalus familiaris kingi), a critically endangered passerine endemic to the northwest Hawaiian island of Nihoa. Using samples collected in 2007 and 2009, our results reveal extremely low levels of genetic diversity at both microsatellites and mtDNA, and both approximate Bayesian computation (ABC) and sibship methods indicate that the effective number of breeders (N-b) for this species is between 5 and 13 individuals. Our analysis highlights the utility of ABC and sibship methods for estimating N-e in species with low genetic polymorphism or few loci. We compare our results to a recent genetic study of this species and document the loss of alleles at two microsatellite loci and one unique mtDNA haplotype. We discuss our findings in the context of the planned translocation of Nihoa Millerbirds from Nihoa to Laysan Island. Received 16 June 2010, accepted 17 December 2010.

2846: ***+.122***

Background: Clostridium botulinum strains can be divided into four physiological groups that are sufficiently diverged to be considered as separate species. Here we present the first complete genome of a C. botulinum strain from physiological group III, causing animal botulism. We also compare the sequence to three new draft genomes from the same physiological group.Results: The 2.77 Mb chromosome was highly conserved between the isolates and also closely related to that of C. novyi. However, the sequence was very different from the human C. botulinum group genomes. Replication-directed translocations were rare and conservation of synteny was high. The largest difference between C. botulinum group III isolates occurred within their surprisingly large plasmidomes and in the pattern of mobile elements insertions. Five plasmids, constituting 13.5% of the total genetic material, were present in the completed genome. Interestingly, the set of plasmids differed compared to other isolates. The largest plasmid, the botulinum-neurotoxin carrying prophage, was conserved at a level similar to that of the chromosome while the medium-sized plasmids seemed to be undergoing faster genetic drift. These plasmids also contained more mobile elements than other replicons. Several toxins and resistance genes were identified, many of which were located on the plasmids.Conclusions: The completion of the genome of C. botulinum group III has revealed it to be a genome with dual identity. It belongs to the pathogenic species C. botulinum, but as a genotypic species it should also include C. novyi and C. haemolyticum. The genotypic species share a conserved chromosomal core that can be transformed into various pathogenic variants by modulation of the highly plastic plasmidome.

2847: ***+.030***

How Much is a Bluethroat? The economic value of wild birds - an exemplary appraisementThe economic value of animal species living in the wild which are neither hunted nor used is very difficult to be determined. The fact that wild animals have a real value can be derived e.g. from the costs of reintroduction programmes - unfortunately there are hardly any cost analyses. Costs of measures necessary for the preservation of affected species can be described in the context of compensation duties depending on legal species protection. These costs provide the base for the economic valuation of wild species in the paper presented. In order to determine their economic values the spatial requirements of the animals have been combined with the production costs for their essential biotopes. This approach has been tested using the 109 bird species of Annex I of the Birds Directive as an example.

2848: ***+.201***

In 2001, the sudden collapse of the Columbia Basin population of pygmy rabbits prompted the initiation of a captive breeding program to facilitate reintroduction, but reproductive success in

captivity has not met expectations. Therefore, the objective of this study was to characterize the reproductive and adrenal endocrinology of this endangered rabbit species so that appropriate management strategies could be developed to monitor animal welfare and increase reproduction. Fecal samples were collected from 27 female pygmy rabbits over three breeding and non-breeding seasons. HPLC analyses verified the presence of progesterone in the excreta of pygmy rabbits, but the majority of progestagen metabolites were unidentified polar compounds. By contrast, > 70% of glucocorticoid immunoactivity was associated with cortisol. Longitudinal fecal hormone profiles during pregnancy were characterized by a large spike in progestagens shortly after mating, a gradual increase in progestagen and glucocorticoid concentrations throughout gestation and a return of hormones to baseline soon after birth (Day 24). The spike in progestagens 1 day after mating was a significant discovery for this species and appears to provide a reliable means of determining if a successful mating has occurred. Seasonal analyses of hormone excretion found that progestagen baselines did not vary between the breeding and non-breeding seasons, but, as expected, were highest during the breeding season in association with pregnancy. Across seasons, the lowest concentrations of glucocorticoids were associated with the highest rates of offspring production and survival, suggesting a possible link between heightened adrenal activity and lowered reproductive fitness in pygmy rabbits. Published by Elsevier Inc.

2849: ***-.086***

Many of the species at greatest risk of extinction from anthropogenic climate change are narrow endemics that face insurmountable dispersal barriers. In this review, I argue that the only viable option to maintain populations of these species in the wild is to translocate them to other locations where the climate is suitable. Risks of extinction to native species in destination areas are small, provided that translocations take place within the same broad geographic region and that the destinations lack local endemics. Biological communities in these areas are in the process of receiving many hundreds of other immigrant species as a result of climate change; ensuring that some of the 'new' inhabitants are climate-endangered species could reduce the net rate of extinction.

2850: ***+.120***

The Key Largo woodrat is an endangered rodent endemic to the island of Key Largo in the Florida Keys. After several reports documented a steep decline in the population, the US Fish and Wildlife Service developed a recovery plan, including captive breeding and reintroduction. Captive breeding efforts were to be focused on providing animals for future reintroduction to protected areas on Key Largo. However, little was known about the husbandry needs or reproductive behavior of this elusive nocturnal species. In 2005, Disney's Animal Kingdom (R) received 11 animals and began to systematically investigate methods of breeding Key Largo woodrats. Since the program's inception, 30 pups have been born and successfully parent reared. In this report, we describe some of the husbandry techniques that have contributed to the success of the Key Largo woodrat captive breeding program at Disney's Animal Kingdom (R). The results obtained may be of use to other facilities maintaining woodrats and other rodent species. Zoo Biol 30:318-327, 2011. (C) 2010 Wiley-Liss, Inc.

2851: ***+.124***

Landscape composition and configuration, often termed as habitat loss and fragmentation, are predicted to reduce species population viability, partly due to the restriction of movement in the landscape. Unfortunately, measuring the effects of habitat loss and fragmentation on functional

connectivity is challenging because these variables are confounded, and often the motivation for movement by target species is unknown. Our objective was to determine the independent effects of landscape connectivity from the perspective of a mature forest specialist-the northern flying squirrel (Glaucomys sabrinus). To standardize movement motivation, we translocated 119 squirrels, at varying distances (0.18-3.8 km) from their home range across landscapes representing gradients in both habitat loss and fragmentation. We measured the physical connectedness of mature forest using an index of connectivity (landscape coincidence probability). Patches were considered connected if they were within the mean gliding distance of a flying squirrel. Homing success increased in landscapes with a higher connectivity index. However, homing time was not strongly predicted by habitat amount, connectivity index, or mean nearest neighbour and was best explained as a simple function of sex and distance translocated. Our study shows support for the independent effects of landscape configuration on animal movement at a spatial scale that encompasses several home ranges. We conclude that connectivity of mature forest should be considered for the conservation of some mature forest specialists, even in forest mosaics where the distinction between habitat and movement corridors are less distinct.

2852: ***+.074***

In Canada, habitat loss has pushed many more species to the brink of extinction than expected in a region with extensive wilderness. However, species richness gradients depend strongly on climate, so species are concentrated in southern regions, where agricultural and urban land uses are both intensive and extensive. Agricultural pesticide use is associated with increasing rates of species endangerment in the south, but long-range transport of persistent organic pollutants is an emerging issue in remote northern regions. Because their distributions reflect climate so strongly, climate change threatens species throughout Canada. Evidence indicates that species' distributions, phenologies, and interactions with pests and diseases are changing more rapidly in response to climate change than global mean values. Nevertheless, climate change is expected to impose dispersal requirements that surpass species' maximum rates. Habitat losses may interact with climate change to impair species' dispersal still further, creating the potential for widespread disruption of biological systems in the most diverse areas of Canada. New research is urgently needed to address questions, and the ethics, around species translocation, ecosystem engineering to anticipate future environmental conditions, and strategies to facilitate the persistence of rare species in landscapes dominated by human activities.

2853: ***+.141***

Objective evaluations of wildlife reintroductions are vital for increasing the success of future efforts to re-establish endangered species. Attempts to reintroduce one of the most endangered mammals in North America, the black-footed ferret (Mustela nigripes), have been ongoing for 18 years with no quantitative assessment of factors related to reintroduction success. We examined relationships between ferret reintroduction success and factors associated with disease outbreaks, release strategies, and the distribution and abundance of their primary prey, prairie dogs (Cynomys sp.), at 11 reintroduction sites. The most important factor related to ferret reintroduction success was a cumulative metric incorporating both size of the area occupied by prairie dogs and density of prairie dog burrows within that area. Each of the four successful sites had prairie dog populations that occupied an area of at least 4300 ha. No sites with <4300 ha of prairie dogs were successful in maintaining >= 30 adult individual ferrets over multiple years without augmentation even if they had a high prairie dog burrow density. The overarching importance of the availability of high-quality habitat suggests managers should prioritize actions that maintain and enhance the availability of large areas with high prairie dog burrow density, which are becoming increasingly

rare due to anthropogenic impacts and disease outbreaks. (C) 2011 Elsevier Ltd. All rights reserved.

2854: +.081

A remaining challenge for animal reintroductions is how best to aid individuals transitioning from captivity into the wild. We tested two techniques - 'soft' vs. 'hard' release - in a 7-year study on endangered Western burrowing owls (Athene cunicularia hypugaea) in British Columbia, Canada. Traditionally, captive-reared yearling adults were released into the wild, as pairs, directly from artificial burrows (hard-release). Only 46% of 201 owls released in this way (2001-2004) stayed at release sites. To test for improved success, soft-releases were performed in the same manner as concurrent hard-releases (2005-2007), except above-ground enclosures confined each soft-release pair to the vicinity of their burrow for 2 weeks before complete release. Of 140 soft-released owls, 86% stayed at release sites: whereas, 66% of 100 hard-released owls stayed. Breeding-season survival was 70% for soft-released owls vs. 50% for hard-released owls. On average, soft-released owls produced 50% more fledglings than concurrently hard-released owls. Post-fledging survival was 69% and first-year return rate was 7.0% for fledglings from soft-released parents, compared to 50% and 4.0% for fledglings from hard-release parents. Ultimately, 0.17 offspring were recruited into the local wild breeding population per soft-released pair, compared to 0.05 local recruits per hard-released pair. We recommend that enclosure-based soft-releases be used for reintroduction of burrowing owls in British Columbia and elsewhere in North America. We encourage other tests for improved release success with soft-releases, particularly for species with a high tendency for dispersal or those likely to experience significant predation pressure. (C) 2011 Elsevier Ltd. All rights reserved.

2855: +.081

The western hoolock gibbon is an endangered primate, restricted to South-East Asia. The combination of being strictly arboreal and having a frugivorous diet makes the species very vulnerable to any negative changes to their habitat. In northeast India, habitat loss and fragmentation combined with hunting and the species' relatively low abundance are the major threats. For long-term conservation of the species, a comprehensive plan of action is required. A Strategic Planning workshop has been or- ganized with concerned stakeholders to develop guidelines for the conservation of the western hoolock gibbon in Assam, India. Ten landscapes in Assam have been identified for long-term conser- vation of the species based on two criteria, habitat integrity and biological importance. A Gibbon Con- servation Committee is being proposed to review overall gibbon research and conservation activities in the state and link them to better management of the habitats. Regular gibbon censuses, monitoring selected populations to identify trends, and a central gibbon database are the major recommenda- tions, along with habitat improvement and improved connectivity between small fragments. Identifica- tion of isolated groups, characterization of fragmented forests, and development of a translocation program for meta-population management are additional parts of the plan. The development of sepa- rate education and outreach programs to address different sections of society were also emphasized in the strategic plan.

2856: +.045

The loss and degradation of prairie-oak habitats has resulted in significant changes in bird species breeding distributions and populations. Among the 49 species highly associated with prairie-oak habitats, 21 have experienced extirpations, range contractions, and/or regional population declines. Three species have been regionally extirpated as breeding species since the 1940s, including

Lewis's woodpecker, which historically occurred throughout the region. Eleven species have experienced local or ecoregional extirpations and/or range contractions. The predominant pattern of range contraction starts at the northern edge of a species range and moves southward. Nine species have relatively small regional populations, six with limited distribution in the Klamath Mountains ecoregion, and three with small and patchily distributed breeding populations throughout the region. There are nine species with significantly declining regional population trends with a high degree of confidence based on Breeding Bird Survey data, and five with similar declines using Christmas Bird Count data. Several other species may be declining based on a lower degree of confidence in the data or anecdotal observations. These include both endemic subspecies, streaked horned lark and Oregon vesper sparrow, which have regional population estimates of <2,000 and <3,000 birds, respectively. Six species have expanded their range in prairie-oak habitats in the last 50 years. The predominant pattern of range expansion starts at the northern edge of a species range and moves northward. Recommended actions to support prairie-oak bird conservation include range-wide and local inventories and monitoring to determine status, and evaluations and implementation of reintroductions or federal listings as appropriate.

2857: ***-.125***

The overwhelming majority of animal conservation projects are focused on vertebrates, despite most of the species on Earth being invertebrates. Estimates state that about half of all named species of invertebrates are parasitic in at least one stage of their development. The dilemma of viewing parasites as biodiversity or pest has been discussed by several authors. However, ticks were omitted. The latest taxonomic synopses of non-fossil Ixodidae consider valid 700 species. Though, how many of them are still extant is almost impossible to tell, as many of them are known only from type specimens in museums and were never collected since their original description. Moreover, many hosts are endangered and as part of conservation efforts of threatened vertebrates, a common practice is the removal of, and treatment for external parasites, with devastating impact on tick populations. There are several known cases when the host became extinct with subsequent coextinction of their ectoparasites. For our synoptic approach we have used the IUCN status of the host in order to evaluate the status of specifically associated hard-ticks. As a result, we propose a number of 63 coendangered and one extinct hard-tick species. On the other side of the coin, the most important issue regarding tick-host associations is vectorial transmission of microbial pathogens (i.e. viruses, bacteria, protozoans). Tick-borne diseases of threatened vertebrates are sometimes fatal to their hosts. Mortality associated with pathogens acquired from ticks has been documented in several cases, mostly after translocations. Are ticks a real threat to their coendangered host and should they be eliminated? Up to date, there are no reliable proofs that ticks listed by us as coendangered are competent vectors for pathogens of endangered animals.

2858: ***-.069***

Contamination of their carrion food supply with the non-steroidal anti-inflammatory drug diclofenac has caused rapid population declines across the Indian subcontinent of three species of Gyps vultures endemic to South Asia. The governments of India, Pakistan and Nepal took action in 2006 to prevent the veterinary use of diclofenac on domesticated livestock, the route by which contamination occurs. We analyse data from three surveys of the prevalence and concentration of diclofenac residues in carcasses of domesticated ungulates in India, carried out before and after the implementation of a ban on veterinary use. There was little change in the prevalence and concentration of diclofenac between a survey before the ban and one conducted soon after its implementation, with the percentage of carcasses containing diclofenac in these surveys estimated

at 10.8 and 10.7%, respectively. However, both the prevalence and concentration of diclofenac had fallen markedly 7-31 months after the implementation of the ban, with the true prevalence in this third survey estimated at 6.5%. Modelling of the impact of this reduction in diclofenac on the expected rate of decline of the oriental white-backed vulture (Gyps bengalensis) in India indicates that the decline rate has decreased to 40% of the rate before the ban, but is still likely to be rapid (about 18% year(-1)). Hence, further efforts to remove diclofenac from vulture food are still needed if the future recovery or successful reintroduction of vultures is to be feasible.

2859: *+.146*

Background: The reduction in the amount of food available for European avian scavengers as a consequence of restrictive public health policies is a concern for managers and conservationists. Since 2002, the application of several sanitary regulations has limited the availability of feeding resources provided by domestic carcasses, but theoretical studies assessing whether the availability of food resources provided by wild ungulates are enough to cover energetic requirements are lacking.Methodology/Findings: We assessed food provided by a wild ungulate population in two areas of NE Spain inhabited by three vulture species and developed a P System computational model to assess the effects of the carrion resources provided on their population dynamics. We compared the real population trend with to a hypothetical scenario in which only food provided by wild ungulates was available. Simulation testing of the model suggests that wild ungulates constitute an important food resource in the Pyrenees and the vulture population inhabiting this area could grow if only the food provided by wild ungulates would be available. On the contrary, in the Pre-Pyrenees there is insufficient food to cover the energy requirements of avian scavenger guilds, declining sharply if biomass from domestic animals would not be available.Conclusions/Significance: Our results suggest that public health legislation can modify scavenger population trends if a large number of domestic ungulate carcasses disappear from the mountains. In this case, food provided by wild ungulates could be not enough and supplementary feeding could be necessary if other alternative food resources are not available (i.e. the reintroduction of wild ungulates), preferably in European Mediterranean scenarios sharing similar and socioeconomic conditions where there are low densities of wild ungulates. Managers should anticipate the conservation actions required by assessing food availability and the possible scenarios in order to make the most suitable decisions.

2860: *+.142*

Background: A third of all known freshwater mollusk extinctions worldwide have occurred within a single medium-sized American drainage. The Mobile River Basin (MRB) of Alabama, a global hotspot of temperate freshwater biodiversity, was intensively industrialized during the 20(th) century, driving 47 of its 139 endemic mollusk species to extinction. These include the ancylinid limpet Rhodacmea filosa, currently classified as extinct (IUCN Red List), a member of a critically endangered southeastern North American genus reduced to a single known extant population (of R. elatior) in the MRB.Methodology/Principal Findings: We document here the tripling of known extant populations of this North American limpet genus with the rediscovery of enduring Rhodacmea filosa in a MRB tributary and of R. elatior in its type locality: the Green River, Kentucky, an Ohio River Basin (ORB) tributary. Rhodacmea species are diagnosed using untested conchological traits and we reassessed their systematic and conservation status across both basins using morphometric and genetic characters. Our data corroborated the taxonomic validity of Rhodacmea filosa and we inferred a within-MRB cladogenic origin from a common ancestor bearing the R. elatior shell phenotype. The geographically-isolated MRB and ORB R. elatior populations formed a cryptic species complex: although overlapping morphometrically, they

exhibited a pronounced phylogenetic disjunction that greatly exceeded that of within-MRB R. elatior and R. filosa sister species.Conclusions/Significance: Rhodacmea filosa, the type species of the genus, is not extinct. It persists in a Coosa River tributary and morphometric and phylogenetic analyses confirm its taxonomic validity. All three surviving populations of the genus Rhodacmea merit specific status. They collectively contain all known survivors of a phylogenetically highly distinctive North American endemic genus and therefore represent a concentrated fraction of continental freshwater gastropod biodiversity. We recommend the establishment of a proactive targeted conservation program that may include their captive propagation and reintroduction.

2861: *+.173*

Appropriate monitoring tools are essential for assessing the effectiveness of management for all threatened insect taxa. In New Zealand the large-bodied flightless orthopterans in the genus Deinacrida have mostly been monitored by searching through habitat during the day or spotlighting at night but this is time consuming and the results depend on the skill of the searcher. Recently, footprint tracking tunnels, similar to those used for monitoring small mammals in New Zealand, were found to be effective for detecting adults of various giant weta species. In this study, we compared the abundance of Cook Strait giant weta (CSGW) in the vicinity of the tunnels, estimated by mark-recapture, with the number of tracking tunnels tracked by weta. We found strong indications that both baited and unbaited tracking tunnels can be used to estimate the number of adult weta present but that this probably depends on their responses to meteorological conditions which are not yet understood. Our results also show that footprint tracking tunnels are more effective for detecting adult CSGW than searching for these insects at night and that baiting tracking tunnels with peanut butter increases their effectiveness for detecting adult CSGW. We confirmed how far Cook Strait giant weta moved each night on Matiu-Somes Island by attaching transmitters to them and found that day roosts of three adult males were on average 8.6 m apart each day and those of adult females were on average 21.3 m apart. Both the low recapture rates of marked adult CSGW and the nightly displacements of those with transmitters suggest that adult CSGW show no site fidelity and are clearly capable of moving large distances each night. However, an individual weta is unlikely to track more than one tunnel per night if tunnels are 30 m apart. Tracking tunnels have the potential to be used with some other insects, provided their footprints are diagnostic. An advantage of using tracking tunnels is that they are non-lethal and would therefore be particularly suitable for monitoring other large threatened insect taxa.

2862: *+.022*

Goitred gazelle (Gazella subgutturosa) rank among the most endangered mammals on the Arabian Peninsula and the Asian steppes. Past conservation efforts have been plagued by confusion about the phylogenetic relationship among various-phenotypically discernable-populations, and even the question of species boundaries was far from being certain. This lack of knowledge had a direct impact on conservation measures, especially ex situ breeding programmes, hampering the assignment of captive stocks to potential conservation units. Here, we provide a phylogenetic framework, based on the analysis of mtDNA sequences of a number of individuals collected from the wild and captivity throughout the species' natural range. Our analyses revealed a polyphyly within the presumed species of G. subgutturosa resulting in two distinct clades: one on the Arabian Peninsula, Iraq, Jordan, Syria and Turkey (Gazella marica; sand gazelle) and one genetically diverse larger clade from the rest of its Asian range (G. subgutturosa; goitred gazelle). Additionally, we provide a quick method (PCR-RFLP) to analyse the taxonomic affiliation of captive gazelles that will be used for re-introductions into the wild.

2863: ***+.078***

Survival and recruitment of the whistling thorn (Acacia drepanolobium Sj circle divide stedt) seedlings was studied in Olpejeta Conservancy, Kenya, between 1999 and 2009. Seedlings were monitored for damage by drought and herbivores as well as survival and recruitment into adult trees. We determined variability in seedling damage, survival and recruitment before and after two management interventions; the elephant translocation in 2001 and the expansion of the reserve in 2007. Seedling survival over 8 years was low, with mortalities being initially high between 2000 and 2001. Reduction in elephant numbers was followed by a gradual reduction in seedling mortality. We recorded a 47% survival over 8 years; 63% of the mortality having been caused by browsers, drought or other factors. Only 8 (0.75%) of the seedlings had grown into trees. Seedlings experienced greater damage at higher densities and exhibited a storage effect underneath grass cover. Survival and recruitment of A. drepanolobium seedlings is very low and of concern. The expansion of conservation area and the opening of wildlife migration corridors have, however, resulted in a steady recovery for seedlings. We recommend enclosing sections of the conservancy to exclude grazers and browsers such as elephants and to allow seedling survival and recruitment. ResumeLa survie et le recrutement des jeunes plants d'Acacia drepanolobium Sj circle divide stedt ont eteetudies au Ol Pejeta Conservancy, au Kenya, entre 1999 et 2009. Les jeunes plants furent suivis tant au point de vue des dommages causes par la secheresse et par les herbivores qu'a celui de la survie et du recrutement jusqu'au stade adulte. Nous avons determine la variabilite des dommages causes aux plants, de leur survie et de leur recrutement avant et apres deux interventions de gestion: la translocation d'elephants en 2001 et l'extension de la reserve en 2007. La survie des jeunes plants fut faible sur ces huit annees, la mortaliteetant particulierement forte entre 2000 et 2001. La reduction du nombre d'elephants fut suivie d'une reduction progressive de la mortalite des plants. Nous avons enregistre un taux de survie de 47% sur huit ans, 63% de la mortalite ayant ete causee par les herbivores, la secheresse ou d'autres facteurs. Seuls huit (0.75%) jeunes plants sont devenus des arbres. Les jeunes plants subissaient plus de degats lorsque leur densiteetait plus forte et presentaient un effet de stockage sous la couverture herbacee. La survie et le recrutement des jeunes plants d'Acacia drepanolobium sont tres faibles, voire inquietants. L'extension de la superficie de la Conservancy et l'ouverture de corridors de migration pour la faune sauvage ont cependant abouti a une restauration prononcee des jeunes plants. Nous recommandons de cloturer certaines sections de la reserve pour en exclure les herbivores, comme les elephants, afin de permettre la survie et le recrutement des jeunes plants.

2864: ***+.056***

Anopheles nili is one of the major malaria vectors in Africa with a wide geographic distribution. However, the taxonomic and population genetic studies on this species are scarce. New research tools are urgently needed to genetically characterize this important malaria vector. In this study, a high-resolution cytogenetic map was developed for An. nili polytene chromosomes. Chromosomes were straightened and subdivided into 46 numbered divisions according to the banding pattern. Population analysis of An. nili females collected in Burkina Faso revealed the presence of two highly polymorphic inversions on the 2R chromosomal arm. A statistically significant departure from Hardy-Weinberg equilibrium due to a deficit in heterozygotes was detected for inversion 2Rb. To determine chromosome homologies and gene order conservation between An. nili and other major malaria vectors, PCR probes based on the An. gambiae coding sequences were mapped to An. nili chromosomes. Comparative mapping demonstrated that An. nili chromosomes have an An. stephensi-like arm association and that whole-arm translocations and paracentric inversions were the major types of rearrangement in evolution of these mosquitoes. The minimum number of fixed inversions among An. nili, An. gambiae, and An.

stephensi was calculated using the Multiple Genome Rearrangements (MGR), Genome Rearrangements In Man and Mouse (GRIMM), and Sorting Permutation by Reversals and block-INterchanGes (SPRING) programs. The data suggest that the An. nili is, at least, as diverged from An. gambiae as An. stephensi. We provide evidence that 2La/a arrangement of An. gambiae is present in outgroup species An. nili and An. stephensi confirming the ancestral status of the 2La inversion in the An. gambiae complex. Availability of the new polytene chromosome map, polymorphic inversions, and physically mapped DNA markers for An. nili will further stimulate population genetic, taxonomic, and genomic studies of this neglected malaria vector. (C) 2010 Elsevier B.V. All rights reserved.

2865: *+.126*

P>1. Urbanization represents a major threat to biodiversity world-wide because it causes permanent degradation and fragmentation of biologically rich natural communities. This is particularly acute in coastal plains and river valleys, where cities are typically located. Although movement is essential to the persistence of populations in fragmented landscapes, little is known about how development and transportation corridors affect the movements of wildlife in the urban context.2. We conducted a series of translocation experiments within the urban landscape of Calgary, Alberta, Canada, to assess the permeability of selected landscape elements for two species of forest songbirds with contrasting adaptabilities to urban development and migratory behaviours: the black-capped chickadee Poecile atricapillus, an urban-adaptable year-round resident, and the yellow warbler Dendroica petechia, an urban-sensitive Neotropical migrant.3. Birds were caught in riparian habitats and translocated either within the riparian corridor of origin or across the urban matrix. Riparian treatments included continuous forest, one or several transportation bridges and a major river. In the urban matrix, birds were translocated across a single major road, well- or poorly-treed developed areas, or multiple gaps.4. Using Cox regression we found that the presence of gaps in forest cover explained more variation in return time than the amount of forest cover for both species. Multiple gaps, in particular, resulted in significantly longer return times compared with continuous forest. Chickadees exhibited longer return times when translocated across linear gaps associated with bridges or roads. In contrast, yellow warbler movements appeared to be more constrained by urban development.5. Synthesis and applications. Our results suggest that improving the permeability of urban landscapes for songbirds can be achieved by preserving connectivity along riparian corridors and other major swaths of natural vegetation while minimizing gaps in vegetation throughout the urban matrix. Our study also demonstrated a cumulative effect of multiple barriers, species-specific response thresholds to canopy cover and gap width, and an important effect of distance-to-territory on movement behaviour. Finally, we demonstrated the utility of 'most forested route' as a new, animal-based approach for quantifying the permeability of heterogeneous landscapes.

2866: *+.079*

Game species are often manipulated by human beings, whose activities can deeply affect their genetic make-up and population structure. We focused on a geographically isolated wild boar population (Sardinia, Italy), which is classified, together with the Corsican population, as a separate subspecies (Sus scrofa meridionalis). Two hundred and ten wild boars collected across Sardinia were analysed with a set of 10 microsatellites and compared with 296 reference genotypes from continental wild populations and to a sample of domestic pigs. The Sardinian population showed remarkable diversity and a high proportion of private alleles, and strongly deviated from the equilibrium. A Bayesian cluster analysis of only the Sardinian sample revealed a partition into five subpopulations. However, two different Bayesian approaches to the assignment

of individuals, accounting for different possible source populations, produced consistent results and proved the admixed nature of the Sardinian population. Indeed, introgressive hybridization with boars from multiple sources (Italian peninsula, central Europe, domestic stocks) was detected, although poor evidence of crossbreeding with free-ranging domestic pigs was unexpectedly found. After excluding individuals who carried exotic genes, the population re-entered Hardy-Weinberg proportions and a clear population structure with three subpopulations emerged. Therefore, the inclusion of introgressed animals in the Bayesian analysis implied an overestimation of the number of clusters. Nonetheless, two of them were consistent between analyses and corresponded to highly pure stocks, located, respectively, in northwest and southwest Sardinia. This work shows the critical importance of including adequate reference samples when studying the genetic structure of managed wild populations. Heredity (2011) 106, 1012-1020; doi:10.1038/hdy.2010.155; published online 22 December 2010

2867: *+.086*

Noninvasive genetic techniques have become indispensible tools in wildlife conservation and management. Here, we report the development of the first set of microsatellite markers for the Eurasian beaver (Castor fiber). All 15 loci show considerable variation within the sampled region in southwestern Germany, with number of alleles ranging from two to six alleles per locus. A comparison between tissue and hair samples revealed that amplification success was only slightly lower for hair samples, making their use in noninvasive monitoring feasible. Despite some evidence for false alleles and allelic dropout, 77% of all loci were genotyped successfully among all hair samples and loci tested. The developed markers will be used for subspecies differentiation and reconstruction of dispersal routes, following reintroductions in Central Europe.

2868: *+.134*

The population structuring and low genetic diversity of the Manchurian zokor Myospalax psilurus Milne-Edwards, 1874, an East Asian endemic included in the Red List of Russia, were demonstrated. Two separate geographical groups differing in the level of their genetic diversity were found on the territory of the Primorskii krai. The subpopulation located closest to the main area of this species was determined as ancestral. A subspecies differentiation of the Primorskii krai and Transbaikal M. psilurus populations was shown, as was the monophyletic origin of M. psilurus and its high divergence from M. aspalax. The animals from northern localities are recommended for reintroduction in nature under species recovery programs in Primorskii krai.

2869: *+.087*

The Lesser Kestrel Falco naumanni is one of the most endangered birds in Europe. Spanish populations have suffered large declines and disappeared from large areas of former distribution, thus leading to many reintroduction programmes. One of the main factors that could affect the population growth of reintroduced populations is the availability of suitable habitat for breeding and foraging. We investigated whether nest site availability is a limiting factor for a newly established population of Lesser Kestrels in eastern Spain. We developed univariate models to understand the relationship between building characteristics (area, height, roof condition, etc.) and occupancy and abundance of Lesser Kestrels, and multivariate models to predict the availability of nest-sites. Our results showed that the species selected medium-sized buildings with extensive land use in the surrounding area and an absence of trees. In addition, Lesser Kestrel abundance was explained by roof condition and distance to the nearest building. Multivariate models predicted that most of the buildings were not suitable for nesting by Lesser Kestrels and thus

population growth may be limited due to lack of nest sites. Lack of suitable nest sites, conspecific attraction and Allee effects are all processes that may be limiting, resulting in the low population and colony sizes that were found and predicted. Based on this, we recommend the provision of nest boxes, the use of special tiles and the construction of breeding towers to improve building quality and therefore increase colony size. We also critically question the use of reintroduction projects to restore locally extinct populations for two reasons. First in this case the reintroduced population is near some larger colonies and there is a strong tendency for Lesser Kestrels to move from small to large colonies. Secondly, in a general strategy for the conservation of Lesser Kestrels, conservation of the main colonies is the priority rather than dedicating human and economic resources on manipulative actions for the establishment of new colonies.

2870: *+.147*

Genetic diversity and population structure of ex-situ conservation populations from two remnant Isoetes sinensis populations and one remnant I. orientalis population were investigated using microsatellite markers. A total of 56 alleles were identified in 720 individuals across the seven microsatellites, with a mean value of 8. 43 alleles per locus. High genetic diversity was found in subpopulations with the mean polymorphism information content (PIC) of 0. 707. Low genetic differentiation was revealed among conservation subpopulations (G(ST) = 0. 070), which may be due to higher gene flow among ex-situ subpopulations (N-m = 3. 59). Gene flow between subpopulation pairs was significantly affected by the relative position and water flow state among subpopulations under ex-situ conservation. One-way ANOVA indicated that the dispersal ability of Iseotes spores or sporophytes along the main flow direction was stronger than that along the weak flow direction, which suggested water flow had a great impact on the gene flow of Isoetes. This was in accordance with UPGMA analysis, which revealed that the subpopulations tended to cluster with the neighbor subpopulations connected by water flow. The results indicated that genetic mixing or genetic assimilation may have occurred among ex-situ conservation populations from different remnant populations of I. sinensis and I. orientalis, suggesting a possible risk of out-breeding depression if genetic enhancement was implemented by translocation of individuals from different remnant populations. Therefore, translocation among these remnant populations with local adaptation or significant evolution is not recommended to construct ex-situ conservation or reintroduction populations.

2871: *+.208*

Reintroductions of wildlife populations to their former range in California are often undertaken without systematic, spatially-explicit habitat analyses as part of feasibility studies. This has been true for the tule elk (Cervus elaphus nannodes), a California endemic subspecies brought to the brink of extinction a century ago. We evaluated the Grasslands Ecological Area of Merced County as potential habitat for a future free-ranging herd. The study area was modeled using three variables: cover/forage, habitat diversity, and human impacts. Within 11,650 ha of likely usable habitat, we found two large areas of very high quality habitat (totaling 4,638 ha). These areas contained forage and cover in close proximity, low levels of human disturbance, and a variety of habitats for use by elk. Carrying capacity of these areas was estimated at 180-320 individuals. We suggest that this type of systematic evaluation should be a component of future reintroduction efforts for tule elk and other native species of California wildlife.

2872: *+.069*

From March 2002 to May 2011 about a hundred water bodies in the Mazowieckie Voivodeship

(Poland) were investigated with the goal of discovering new sites inhabited by the endangered cyprinid lake minnow, Eupallasella percnurus (Pall.). Ten sites were discovered that had been previously unknown. During the same period, six new sites were created by translocation of cultured juvenile individuals into suitable water bodies not inhabited by this species. These measures were undertaken as part of a long-term (2002-2015), voivodeship-wide program for the active protection of this species, which is the only one of its kind in Poland. Of the sixteen sites currently in existence, ten are suffering from advanced water basin shallowing and/or vegetation overgrowth. However, good water conditions throughout 2010 and in the first half of 2011 have meant that only six sites are presently considered as critically threatened, whereas seven appear to be of least vulnerability. At present, five sites (31.3% of all sites in the voivodeship) are protected within the European Ecological Natura 2000 Network.

2873: ***-.024***

In July and August 2010, the Lincoln-Petersen capture-recapture method was used to assess the size and basic structure of a newly established population of the endangered in Poland cyprinid fish lake minnow, Eupallasella percnurus (Pall). The population that inhabits a small (0.08 ha) peat excavation site in the village of Kowalicha near Radzymin (Mazowieckie Voivodeship, Poland) was initiated in 2004-2006 using the translocations of a total of 1530 cultivated individuals, mostly juveniles aged 0+. The total size of the E. percnurus population was estimated to be 600 individuals excluding aged 0+. The population size is not considered to be large in comparison with other populations inhabiting water bodies of similar surface areas. Males proved to be predominated by females [long dash] phenomenon typical of populations of this species [long dash] but at relatively high ratio of approximately 1:4. Most of females were aged 2+, whereas most of males were older (3+). These data indicate that the population remains in the early stages of development. The Lincoln-Petersen method proved useful in the studies of the size and structure of E. percnurus populations. Keywords: lake

2874: ***+.290***

The objective of the study was to characterize the physical and chemical water parameters of Eupallasella percnurus (Pall.) habitats, to identify the habitat preferences of this species, and, in particular, to determine which parameters would be useful in selecting water bodies for translocation to establish viable new populations as part of active protection programs for this species. Results obtained from seven water bodies in central Poland over a minimum of a four-year period are analyzed. Water temperature, oxygen saturation, conductivity, pH, and concentrations of mineral forms of nitrogen and phosphorus were determined. The key parameter for assessing habitat quality was identified as water pH ranging from 4.9 to 9.1. Low pH values appear to cause disturbances in natural fish recruitment. Populations from water bodies with water pH not lower than 5.0 did not exhibit any effects of unfavorable environmental conditions despite periodic water temperatures that significantly exceeded optimal values for this species (> 25[degree]C) and dangerously low levels of water oxygen saturation (< 14%).

2875: ***+.168***

Two populations of lake minnow, Eupallasella percnurus (Pall.), from Bledzewo and Kowalicha were investigated. The Bledzewo population has existed for many decades, while Kowalicha was established between 2004 and 2006 by translocations of cultured juveniles. The genetic variation and genetic distance between these populations was evaluated. The size of the genetic bottleneck/founder effect that affected genetic variation was also investigated. In most of the

examined microsatellite loci, only 2-3 alleles/population were detected. Heterozygosity across the investigated loci was low with ranges of Ho: 0.24-0.45 and He: 0.24-0.50. Both populations remained at Hardy-Weinberg equilibrium. The M value was low, and suggested a reduction in the genetic variation because of the founder/bottleneck effect. The genetic distance between populations was high (FST 0.23, ai2 2.96). High genetic differences existing between the investigated E. percnurus populations and possibly other ones suggest that the inter-population translocations of this species should be preceded by an evaluation of the genetic differences existing between source and destination populations.

2877: *+.083*

The Grizzled Giant Squirrel is endemic to southern India and Sri Lanka. In India it is distributed in isolated populations with less than 500 mature individuals, restricted mostly to patchy riverine habitats. We have recorded the presence of this species (minimum 14 individuals) across eight locations in Hosur forest division along the Cauvery riverine forest, north of earlier reported locations in the Eastern Ghats. The documentation of this species in the study area adds to our understanding of its distribution. These squirrels are canopy dwellers, hence discontinuous forest restricts their movement and dispersal. Measures including stop auctioning Tamarindus indica fruits on large scale for commercial purpose by Forest Department, restoration of habitat, maintenance of canopy continuity, reducing anthropogenic pressure and translocating squirrels from larger populations are suggested to enhance the long-term survival of this habitat specialist, which is on the brink of local extinction in the study area. We also suggest a comprehensive population assessment of the species to reevaluate its global status.

2878: *+.102*

Duvaucel geckos (Hoplodactylus duvaucelii) are large endemic lizards that have been extirpated from the New Zealand mainland due to introduced mammalian predators. This species has subsequently become the subject of species translocation conservation management in an endeavour to increase the number of populations and as a part of island ecological restoration. Blood sampling of a captive adult male after tail autotomy led to the discovery of a Rickettsia-like organism within this gecko's erythrocytes. We conclude that this infection was acquired at the gecko's source location, a remote island closed to the general public and lacking potential invasive parasite reservoir reptiles. The likely vector is the native mite Geckobia naultina. This finding represents valuable new baseline information about the health parameters of this threatened species. Particularly in the light of the paucity of reported blood parasitism in New Zealand reptiles, the conservation management of this species through relocation and captive-breeding, and the on-going concerns regarding the introduction of novel parasites to New Zealand. (C) 2011 Elsevier B.V. All rights reserved.

2879: *+.087*

Since threatened species are generally incapable of surviving in their natural environments, ex situ conservation programs are required to preserve them from total extinction. Captive breeding provides the tool to rear sustained populations. Knowledge about biology of such species is critical for managing such breeding programmes. In this paper, we look at the sex variation in calves surviving to 30 days in captive populations of two endangered polygynous gazelles, Gazella cuvieri and Gazella dama mhorr. We examined which of the following mother traits may affect sex allocation: her age; experience; and inbreeding coefficient. Because twins are quite common in Cuvier's gazelle, we also analyse whether offspring sex is mediated by litter size in this gazelle. In

both species the overall sex ratio did not differ from unity at the age of 30 days. These results support the suggestion that for a given population to be in equilibrium, a 1: 1 calf sex ratio is to be expected, with females in good condition producing sons and females in poor condition producing daughters. We also found that offspring sex is not significantly related to the same mother traits in the two gazelles studied. Cuvier's gazelle findings showed that only maternal inbreeding influenced offspring sex, the less inbred the mother the higher the probability of producing sons. In contrast, none of the maternal traits studied were related to offspring sex in Mohor gazelle. Two nonexclusive explanations are given to justify differences observed between these two species; one relates to a probable differential cost of producing either sex; the other refers to the actual homozygosis level in captive populations of each species. For Cuvier's gazelle litter size has no effect on offspring sex when the sex of the littermate is taken into account. This result fits predictions derived from Trivers-Willard model: mother in good conditions (less inbred) produce the sex with higher fitness returns (i.e., male), no matter if born at single or twins brood. Many variables are likely to affect progeny sex ratio. We have presently examined only a few. But our results might be of interest in management decisions and conservation programs such as reintroductions. (C) 2010 Elsevier GmbH. All rights reserved.

2881: *+.040*

Males are homogametic (ZZ) and females are heterogametic (WZ) with respect to the sex chromosomes in many species of butterflies and moths (insect order Lepidoptera). Genes on the Z chromosome influence traits involved in larval development, environmental adaptation, and reproductive isolation. To facilitate the investigation of these traits across Lepidoptera, we developed 43 degenerate primer pairs to PCR amplify orthologs of 43 Bombyx mori Z chromosome-linked genes. Of the 34 orthologs that amplified by PCR in Ostrinia nubilalis, 6 co-segregated with the Z chromosome anchor markers kettin (ket) and lactate dehydrogenase (ldh), and produced a consensus genetic linkage map of similar to 89 cM in combination with 5 AFLP markers. The O. nubilalis and B. mori Z chromosomes are comparatively co-linear, although potential gene inversions alter terminal gene orders and a translocation event disrupted synteny at one chromosome end. Compared to B. mori orthologs, O. nubilalis Z chromosome-linked genes showed conservation of tissue-specific and growth-stage-specific expression, although some genes exhibited species-specific expression across developmental stages or tissues. The O. nubilalis Z chromosome linkage map provides new tools for isolating quantitative trait loci (QTL) involved in sex-linked traits that drive speciation and it exposes genome rearrangements as a possible mechanism for differential gene regulation in Lepidoptera.

2882: *+.205*

Background and Aims Plants show patterns of spatial genetic differentiation reflecting gene flow mediated by pollen and seed dispersal and genotype x environment interactions. If patterns of genetic structure are determined largely by gene flow then they may be useful in predicting the likelihood of inbreeding or outbreeding depression but should be less useful if there is strong site-specific selection. For many Australian plants little is known about either their population genetics or the effects on mating systems of variation in pollen transfer distances. Experimental pollinations were used to compare the reproductive success of bird-adapted Grevillea mucronulata plants mated with individuals from a range of spatial scales. A hierarchical survey of microsatellite DNA variation was also conducted to describe the scale of population differentiation for neutral markers. Methods The effects of four pollen treatments on reproductive performance were compared. These treatments were characterized by transfer of pollen from (a) neighbouring adults; (b) an adjacent cluster of adults (30-50 m distant); (c) a distant cluster (>5 km distant); and (d)

open pollination. Sets of 17.9 +/- 3.3 leaves from each of 15 clusters of plants were genotyped and spatial autocorrelation and F statistics were used to describe patterns of genetic structure. Key Results Grevillea mucronulata displayed evidence of both inbreeding and outbreeding depression, with 'intermediate' pollen producing consistently superior outcomes for most aspects of fitness including seed set, seed size, germination and seedling growth. Significant genotypic structuring was detected within clusters (spatial autocorrelation) and among adjacent clusters and clusters separated by >5 km distance (F(ST) = 0.07 and 0.10). Conclusions The superior outcome of intermediate pollen transfer and genetic differentiation of adjacent clusters suggests that G. mucronulata selection disfavours matings among closely and distantly related neighbours. Moreover, the performance of open-pollinated seedlings was poor, implying that current mating patterns are suboptimal.

2883: *-.002*

Low-elevation islands face threats from sea level rise (SLR) and increased storm intensity. Evidence of endangered species' population declines and shifts in vegetation communities are already underway in the Florida Keys. SLR predictions indicate large areas of these habitats may be eliminated in the next century. Using the Florida Keys as a model system, we present a process for evaluating conservation options for rare and endemic taxa. Considering species characteristics and habitat, we assess central issues that influence conservation options. We contrast traditional and controversial options for two animal and two plant species giving special emphasis to perceptions of ecological risk and safety from SLR and suggest courses of action. Multiple strategies will be required to spread extinction risk and will be effective for different time periods. Global climate change presents an uncertain, perhaps no-analog future that will challenge land managers and practitioners to re-evaluate equilibrium-state-conceived laws and policies not only for these taxa, but for many facing similar threats. To embrace conservation in a changing world will require a new dialogue that includes controversial ideas, a review of existing laws and policies, and preparation for the oncoming change.

2884: *+.139*

Modelling post-release survival probabilities of reintroduced birds can help inform 'soft-release' strategies for avian reintroductions that use captive-bred individuals. We used post-release radiotelemetry data to estimate the survival probabilities of reintroduced captive-bred Red-billed Curassow Crax blumenbachii, a globally threatened Cracid endemic to the Brazilian Atlantic Rainforest. Between August 2006 and December 2008, 46 radiotagged Curassows from the Crax Brazil breeding centre were reintroduced to the Guapiacu Ecological Reserve (REGUA), Rio de Janeiro state, Brazil, in seven different cohorts. Reintroduced birds were most vulnerable during the first 12 months post-release from natural predation, domestic dogs and hunting. Annual post-release survival probability was high (75%) compared with published estimates for other Galliform species. However, when considering survival in all birds transported to REGUA (some birds died before release or were retained in captivity) and not only post-release survival, phi in this study was closer to estimates for other species (60%). The duration of the pre-release acclimatization period within the soft-release enclosure and the size of the released cohorts both positively influenced post-release survival of reintroduced Curassows. Our results are relevant to future Cracid reintroductions and highlight the importance of utilizing post-release monitoring data for evidence-based improvements to soft-release strategies that can significantly enhance the post-release survival of captive-bred birds.

2885: *-.043*

Severe population declines led to the listing of southern California Rana muscosa (Ranidae) as endangered in 2002. Nine small populations inhabit watersheds in three isolated mountain ranges, the San Gabriel, San Bernardino and San Jacinto. One population from the Dark Canyon tributary in the San Jacinto Mountains has been used to establish a captive breeding population at the San Diego Zoo Institute for Conservation Research. Because these populations may still be declining, it is critical to gather information on how genetic variation is structured in these populations and what historical inter-population connectivity existed between populations. Additionally, it is not clear whether these populations are rapidly losing genetic diversity due to population bottlenecks. Using mitochondrial and microsatellite data, we examine patterns of genetic variation in southern California and one of the last remaining populations of R. muscosa in the southern Sierra Nevada. We find low levels of genetic variation within each population and evidence of genetic bottlenecks. Additionally, substantial population structure is evident, suggesting a high degree of historical isolation within and between mountain ranges. Based on estimates from a multi-population isolation with migration analysis, these populations diversified during glacial episodes of the Pleistocene, with little gene flow during population divergence. Our data demonstrate that unique evolutionary lineages of R. muscosa occupy each mountain range in southern California and should be managed separately. The captive breeding program at Dark Canyon is promising, although mitigating the loss of neutral genetic diversity relative to the natural population might require additional breeding frogs. (C) 2011 Elsevier Ltd. All rights reserved.

2886: *+.215*

Hatcheries often produce bold fish that are maladapted to survive in the wild, as absence of predators and selection for fast growth tend to favour risk-taking behaviors. Not surprisingly, losses of hatchery fish through predation can be high immediately after release and this may account for the failure of many ex-situ fish conservation programmes. For supportive-breeding to be useful, it is essential that released fish are able to display natural behaviors. We compared the performance of juvenile Atlantic salmon reared in environmentally-enriched tanks receiving natural prey and subjected to simulated predator attacks with fish reared under standard hatchery conditions while keeping densities constant. No differences were detected between controls and environmentally enriched fish in survival, final size or nutritional status. Yet, changes in rearing conditions had rapid and marked effects on risk-taking behavior. Environmentally enriched fish were 2.1 times less willing, and took significantly longer to leave shelter, than controls within two weeks of enrichment. Thus our study indicates that it is possible through environmental enrichment to modify at least one component of fishes' behavior known to have clear adaptive implications, i.e. the propensity of hatchery-reared fish to take excessive risks. Ex-situ conservation could therefore benefit from rearing fish in naturalized, structurally complex environments with natural prey to promote the development of more natural behaviors. (C) 2011 Elsevier Ltd. All rights reserved.

2887: *+.189*

The relevance of chemical communication to mammalian conservation is not often the focus of scientific investigation. Our review identifies and discusses ten key areas in which the study of chemical communication aids conservation behaviour. Articles (n = 140) were revealed, most were concerned with population monitoring (22.50%), reducing human-wildlife conflicts (18.93), influencing habitat selection (18.57%). increasing welfare of captive animals (12.86%), encouraging captive breeding (12.86%), reducing predation (5.71%), and increasing the success of release programmes (5.00%). Few articles (<4%) were found relating olfactory studies to health status of wild populations, reducing hybridization or as indication of pollution. A growing number

of articles are addressing how olfactory studies may aid conservation, but more rigorous experimental testing and manipulations are required. The vast majority of studies linking olfaction with conservation involved the population monitoring of wild carnivores. We suggest that animal behavioural studies and manipulations of chemical communication can have significant impacts on conservation in these areas, which should be further developed to generate practical applications. Areas of future study include chemical communication of aquatic mammalian species, the transfer of olfactory cues under water, and the identification of genetic markers that may link 'personality' with olfactory responses. Linking olfactory studies to fitness, either on an individual or population scale, particularly in a wider ecological context is more likely to increase conservation value. Animal translocations and reintroduction programmes may offer a means to do this and could be an important area to direct future studies. (C) 2011 Elsevier Ltd. All rights reserved.

2888: *+.173*

The North American river otter (Lontra canadensis) is recovering from near extirpation throughout much of its range. Although reintroductions, trapping regulations and habitat improvements have led to the reestablishment of river otters in the Midwest, little is known about how their distribution is influenced by local- and landscape-scale habitat. We conducted river otter sign surveys from Jan. to Apr. in 2008 and 2009 in eastern Kansas to assess how local- and landscape-scale habitat factors affect river otter occupancy. We surveyed three to nine 400-m stretches of stream and reservoir shorelines for 110 sites and measured local-scale variables (e.g., stream order, land cover types) within a 100 m buffer of the survey site and landscape-scale variables (e.g., road density, land cover types) for Hydrological Unit Code 14 watersheds. We then used occupancy models that account for the probability of detection to estimate occupancy as a function of these covariates using Program PRESENCE. The best-fitting model indicated river otter occupancy increased with the proportion of woodland cover and decreased with the proportion of cropland and grassland cover at the local scale. Occupancy also increased with decreased shoreline diversity, waterbody density and stream density at the landscape scale. Occupancy was not affected by land cover or human disturbance at the landscape scale. Understanding the factors and scale important to river otter occurrence will be useful in identifying areas for management and continued restoration.

2889: *+.066*

Novel populations pose unusual challenges for wildlife managers because knowledge regarding the source of these populations is essential to develop sound management approaches. One example that illustrates the complexity of this issue is the small population of red squirrels (Tamiasciurus hudsonicus) identified in northeastern Illinois in the 1970s. To elucidate the source of the red squirrel population in Illinois, we examined both contemporary and less recent patterns of genetic structure using nuclear microsatellite loci and mitochondrial DNA. Analyses revealed the Illinois subpopulation was primarily comprised of descendents of immigrants from Indiana, but there was also evidence of a translocation of squirrels from Minnesota. We recommend continued protection for the red squirrel in Illinois due to its restricted geographic range, small population size, and status as a native population. (C) 2011 The Wildlife Society.

2890: *+.150*

Previous research from 2001 to 2006 on an experimentally released elk (Cervus elaphus) population at Great Smoky Mountains National Park (GSMNP or Park) indicated that calf recruitment (i.e., calves reaching 1 yr of age per adult female elk) was low (0.306, total SE =

0.090) resulting in low or negative population growth (lambda = 0.996, 95% CI = 0.945-1.047). Black bear (Ursus americanus) predation was the primary calf mortality factor. From 2006 to 2008, we trapped and relocated 49 bears (30 of which were radiocollared) from the primary calving areas in the Park and radiomonitored 67 (28 M: 39 F) adult elk and 42 calves to compare vital rates and population growth with the earlier study. A model with annual calf recruitment rate correlating with the number of bears relocated each year was supported (Delta AIC(c) = 0.000; beta = 0.070, 95% CI = 0.028-0.112) and a model with annual calf recruitment differing from before to during bear relocation revealed an increase to 0.544 (total SE = 0.098; beta = -1.092, 95% CI = -1.180 to -0.375). Using vital rates and estimates of process standard errors observed during our study, 25-yr simulations maintained a mean positive growth rate in 100% of the stochastic trials with lambda averaging 1.118 (95% CI = 1.096-1.140), an increase compared with rates before bear relocation. A life table response experiment revealed that increases in population growth were mostly (67.1%) due to changes in calf recruitment. We speculate that behavioral adaptation of the elk since release also contributed to the observed increases in recruitment and population growth. Our results suggest that managers interested in elk reintroduction within bear range should consider bear relocation as a temporary means of increasing calf recruitment. (C) 2011 The Wildlife Society.

2891: *-.023*

Disease can dramatically influence the dynamics of endangered wildlife populations, especially when they are small and isolated, with increased risk of inbreeding. In Hluhluwe-iMfolozi Park (HiP), a small, enclosed reserve in South Africa, a large lion (Panthera leo) population arose from a small founder group in the 1960s and started showing conspicuous signs of inbreeding. To restore the health status of the HiP lion population, outbred lions were translocated into the existing population. In this study, we determined the susceptibility to bovine tuberculosis (bTB), and the prevalence of antibody to feline viruses of native lions, and compared the findings with those from translocated outbred lions and their offspring. Antibodies to feline herpesvirus, feline calicivirus, feline parvovirus, and feline coronavirus were present in the lion population, but there was no significant difference in antibody prevalence between native and translocated lions and their offspring, and these feline viruses did not appear to have an effect on the clinical health of HiP lions. However, feline immunodeficiency virus (Fly), which was previously absent from HiP, appears to have been introduced into the lion population through translocation. Within 7 yr, the prevalence of antibody to FIV increased up to 42%. Bovine tuberculosis posed a major threat to the inbred native lion population, but not to translocated lions and their offspring. More than 30% of the native lion population died from bTB or malnutrition compared with < 2% of the translocated lions and their offspring. We have demonstrated that management of population genetics through supplementation can successfully combat a disease that threatens population persistence. However, great care must be taken not to introduce new diseases into populations through translocation.

2892: *+.049*

One subadult male specimen of a reintroduced mountain lion was monitored using the radio telemetry technique. The monitoring lasted 110 days, being randomly distributed. Data of the monitoring through radiotelemetry suggested that the home range of this specimen was of approximately 26km(2). This sets as the smallest home range described for this species. However, the importance of this basic monitoring should be emphasized, since information of the behavior of a large feline reintroduced in its natural habitat after a period in captivity is incipient.

2893: ***+.199***

The black-footed ferret (Mustela nigripes), once extinct in the wild, remains one of the most critically endangered mammals in North America despite 18 years of reintroduction attempts. Because black-footed ferrets are specialized predators of prairie dogs (Cynomys sp.), a better understanding of how black-footed ferrets select resources might provide insight into how best to identify and manage reintroduction sites. We monitored ferret resource selection at two reintroduction sites with different densities of prairie dog populations one that contained a high density of prairie dogs (Conata Basin, South Dakota) and one that was lower (UL Bend, Montana). We evaluated support for hypotheses about ferret resource selection as related to the distribution of active burrows used by black-tailed prairie dogs (Cynomys ludovicianus), interactions between ferrets, and habitat edge effects. We found support for all three factors within both populations; however, they affected ferret resource selection differently at each site. Ferrets at Conata Basin tended to select areas with high prairie dog burrow density, closer to the colony edge, and that overlapped other ferret ranges. In contrast, ferrets at UL Bend tended not to select areas of high active prairie dog burrow density, avoided areas close to edge habitat, and females avoided areas occupied by other ferrets. The differences observed between the two sites might be best explained by prairie dog densities, which were higher at Conata Basin (119.3 active burrows per ha) than at UL Bend (44.4 active burrows per ha). Given the positive growth of ferret populations at Conata Basin, management that increases the density of prairie dogs might enhance ferret success within natural areas. To achieve long-term recovery of ferrets in the wild, conservationists should increasingly work across and outside natural area boundaries to increase prairie dog populations.

2894: ***+.194***

Allee effects, the reduction of vital rates at low population densities, can occur through several mechanisms, all of which potentially apply to reintroduced populations. Reintroduced populations are initially at low densities, hence Allee effects can potentially lead to reintroduction failure despite habitat quality being sufficient to allow long-term persistence if the population survived the establishment phase. The probability of such failures can potentially be reduced by releasing large numbers of organisms, by reducing post-release dispersal or mortality through management, or by directly managing the Allee effects, e.g., by implementing predator control or food supplementation until population size increases. However, such measures incur costs, as large releases have a greater impact on source populations, and management actions require financial and other resources. It is therefore essential to compare the costs and benefits of attempting to reduce Allee effects in reintroduction programs. Here we advocate the use of structured decision-making frameworks whereby alternative strategies are nominated, probability distributions of outcomes obtained under different strategies, and utilities assigned to different outcomes. We illustrate the potential application of such decision frameworks using projections from a stochastic population model including Allee effects. As there will seldom be estimates of Allee effects available from the species or system involved, it will be necessary to predict these effects based on the biology of the species and data from other systems. In doing so, it is important to identify mechanisms for proposed Allee effects, and to avoid misleading inferences from correlations subject to confounds. In particular, naive interpretations of correlations between numbers released and reintroduction success may exaggerate the benefits of releasing large numbers.

2895: ***-.027***

Poaching was the primary cause of extermination of black rhinos in Zambia. From an estimated population of 12,000 individuals in the 1960s, the population declined to extinction by 1993.

Reported incidences of spoors and unverified sightings of individuals continued to be reported to the Zambia Wildlife Authority but none of these reports was verified and the species was assumed to be locally exterminated by 1995. In 2003 a National Rhino Conservation Plan was formulated and in the same year, with the technical guidance of the Rhino Specialist Group and financial support of the Frankfurt Zoological Society a rhino reintroduction programme begun. To date there are 27 black rhinos with six births and four mortalities recorded. None of the mortalities were due to poaching. There are plans to continue the reintroduction programme in other national parks where the rhino existed by 1972 when most of the national parks were established. As the black rhino population was being established, the out-of-range white rhino was also introduced in Mosi oa Tunya National Park, where the population size was recorded to be seven and another pair was introduced in the newly established Lusaka National Park No. 20 in March 2011.

2896: *+.053*

Intraperitoneal implantation of radio-transmitters is a useful method of monitoring free-ranging aquatic and semi-aquatic mammals; however, some researchers are concerned about the physiological effects of such implants. Few studies have investigated the long-term consequences of intraperitoneal implants on survival or reproductive performance. An adult female North American River Otter (Loon-a canadensis) surgically equipped with an intraperitoneal radio-transmitter and released in northwestern Pennsylvania in June 1990 as part of a reintroduction project was killed in March 1999. The North American River Otter was estimated lobe 10 years old and was pregnant with two fetuses at the time of her death. Our observation suggests that wild North American River Otters surgically equipped with intraperitoneal radio-transmitters can live long after implantation of the radio-transmitter and continue to reproduce successfully.

2897: *+.175*

The transplantation is an important method for the restoration of degraded ecosystem. However, it is unclear how the choice of species and transplantation mode affects the community dynamics during recovery from a disaster, particularly for long-lived organisms such as corals. To address this issue, we study a population dynamic model of multiple species in multiple habitats connected by larval dispersal. We first consider two species showing the trade-off relationship between growth rate and mortality and examine three restoration goals to evaluate the effectiveness of transplantation: (1) total coverage; (2) species diversity; (3) spatial heterogeneity of species composition. To promote the rapid development of total coverage, the transplantation of fast-growing species should be adopted. To maintain a high level of regional species diversity, the transplantation of slow-growing species or short-dispersal species is effective. Next, we suppose four genera of corals - Acropora, Pocillopora, Porites, and Favites - as an example of coral community in Okinawa where Pocillopora is facing to local extinction. In addition to three indexes; (4) recovery of locally endangered species is evaluated as a restoration goal. Results show that to promote the recovery of Pocillopora, the transplantation of the same species is clearly the most effective choice. In contrast, the transplantations of Acropora and Porites led to undesirable results. In summary, these results indicate that both the restoration goal and the transplanted species must be carefully selected before conducting transplantation operations. (C) 2011 Elsevier Ltd. All rights reserved.

2898: *+.304*

Population viability analysis (PVA) is a frequently used conservation tool for the assessment of long-term survival of populations. Based on demographic information from published literature, a

PVA model using VORTEX was constructed for the burbot Lota lota (L.), a freshwater fish species believed extinct in the United Kingdom since the 1970s. The model was constructed to evaluate the persistence of a single burbot population over a 100-year period as part of an assessment of the feasibility of reintroducing the species to rivers of its former distribution in England. Population persistence was highly variable and dependent on juvenile survival and spawning regularity. Under high juvenile survival scenarios, all populations persisted and were maintained at the carrying capacity. At low juvenile survival, population persistence was highly influenced by spawning regularity. Sensitivity analysis indicated that females must successfully spawn approximately every second year to guarantee 95% population survival. The analysis suggests that the long-term viability of any potential reintroduced burbot population will be dependent on the environmental conditions related to spawning success.

2899: ***+.189***

Reintroduction projects represent viable options for animal conservation. They allow the establishment of new local populations and may contribute to recreating functional networks within a metapopulation. In the latter case, landscape connectivity may be a major determinant of the phase of spread of the reintroduced populations. Here, we deal with an example of a red deer (Cervus elaphus) translocation planned to enable the connection among existing isolated populations of the species in the Italian Alps. Our aim was to assess whether the analysis of landscape suitability and the simulation of dispersal of released individuals could shed light on the actual process of population spread. For these purposes, we adopted a modelling approach using radiotracking data to develop a habitat suitability map. On the basis of this map, we simulated the dispersal of the animals after release and we then compared the simulation results with the outcome of null models and with the observed population redistribution. The results suggest that the spread of the subpopulation was easier north-westward than southward. Taking into account landscape suitability, our simulations produced a reliable estimate of the ease of colonization of the valleys neighbouring the release-site and they allowed the identification and validation of a potential pathway for animal dispersal. The suitability model based on the monitoring of individuals in the earliest phase of establishment shed light on the spread of the population and on its potential connections with other deer subpopulations.

2900: ***+.093***

Aim Members of the tropical tree snail family Partulidae are endemic to Pacific high oceanic islands and typically have single-island ranges. Two nominal Papua New Guinean species, Partula carteriensis and Partula similaris, deviate from familial norms by having extensive multi-island ranges that include low islands. We hypothesized that undocumented anthropogenic introductions may underlie this regional biogeographical anomaly and evaluated this hypothesis with novel field distributional and genotypic data. Location Papua New Guinean archipelagos between 1.4 and 11.4 degrees S and 146.5 and 154.2 degrees E.Methods Ethanol-preserved museum lots of P. carteriensis (from New Britain, Bismarck Archipelago) and P. similaris (from Woodlark, Boiaboiawaga and Goodenough islands) were genotyped for a standard mitochondrial marker, cytochrome c oxidase subunit I (COI), and the resulting haplotypes were subjected to phylogeographical analyses. Results All four genotyped populations showed very little genetic or conchological differentiation, irrespective of nominal taxonomic status, the archipelago sampled or whether the island was low, high, oceanic or continental. Partula carteriensis and P. similaris exhibit atypical distributions on larger high islands, being restricted to coastal villages and absent from native forest. Main conclusions Our results strongly indicate that P. carteriensis and P. similaris are conspecific, although a formal taxonomic revision is beyond the scope of this present

study. They collectively exhibit the most heterogeneous geographical range known among partulids and their explicitly synanthropic association with high island coastal villages strongly implicates human introduction as the regional dispersal mechanism. We currently lack insights into the timeframe (apart from regional prehistory) and cultural context of these translocations. We also lack a convincing source population, and it may be necessary to survey the partulid fauna of the neighbouring Solomon Islands to identify one. Partulids are critically endangered throughout much of their range and the discovery of populations that apparently thrive in human-altered landscapes is noteworthy. Their study may provide clues of broad relevance to partulid conservation.

2901: *-.002*

For species that are habitat specialists or sedentary, population fragmentation may lead to genetic divergence between populations and reduced genetic diversity within populations, with frequent inbreeding. Hundreds of kilometres separate three geographical regions in which small populations of the endangered Eastern Bristlebird, Dasyornis brachypterus, a small, ground-dwelling passerine that occurs in fire-prone bushland in eastern Australia, are currently found. Here, we use mitochondrial and microsatellite DNA markers to: (i) assess the sub-specific taxonomy designated to northern range-edge, and central and southern range-edge D. brachypterus, respectively, and (ii) assess levels of standing genetic variation and the degree of genetic subdivision of remnant populations. The phylogenetic relationship among mtDNA haplotypes and their spatial distribution did not support the recognised subspecies boundaries. Populations in different regions were highly genetically differentiated, but in addition, the two largest, neighboring populations (located within the central region and separated by similar to 50 km) were moderately differentiated, and thus are likely closed to migration (microsatellites, F (ST) = 0.06; mtDNA, F (ST) = 0.12, I similar to (ST) = 0.08). Birds within these two populations were genotypically diverse and apparently randomly mating. A long-term plan for the conservation of D. brachypterus's genetic diversity should consider individual populations as separate management units. Moreover, managers should avoid actively mixing birds from different populations or regions, to conserve the genetic integrity of local populations and avoid outbreeding depression, should further translocations be used as a recovery tool for this species.

2902: *+.150*

Reintroduction of captive-reared animals has become increasingly popular in recent decades as a conservation technique, but little is known of how demographic factors affect the success of reintroductions. We believe whether the increase in population persistence associated with reintroduction is sufficient to warrant the cost of rearing and relocating individuals should be considered as well. We examined the trade-off between population persistence and financial cost of a reintroduction program for Crested Coots (Fulica cristata). This species was nearly extirpated from southern Europe due to unsustainable levels of hunting and reduction in amount and quality of habitat. We used a stochastic, stage-based, single-sex, metapopulation model with site-specific parameters to examine the demographic effects of releasing juveniles or adults in each population for a range of durations. We parameterized the model with data from an unsuccessful reintroduction program in which juvenile captive-bred Crested Coots were released between 2000 and 2009. Using economic data from the captive-breeding program, we also determined whether the strategy that maximized abundance coincided with the least expensive strategy. Releasing adults resulted in slightly larger final abundance than the release of nonreproductive juveniles. Both strategies were equally poor in achieving a viable metapopulation, but releasing adults was 2-4 times more expensive than releasing juveniles. To obtain a metapopulation that would be

viable for 30 years, fecundity in the wild would need to increase to the values observed in captivity and juvenile survival would need to increase to almost unity. We suggest that the most likely way to increase these vital rates is by increasing habitat quality at release sites.

2903: *+.059*

Reintroduction guidelines recommend that 'adequate' numbers of individuals be released to minimize loss of genetic diversity, but these numbers are rarely quantified. We present a framework for assessing the number of individuals required for an island reintroduction that takes account of allele loss both during the founding event and in the following establishment phase with a low population size. This is the first attempt to model release numbers for reintroductions in order to preserve alleles with a specified initial frequency, while taking post-release mortality rates, population growth rates and site carrying capacity into account. Probability of allele retention was sensitive to both release number and post-release demography. The rate of allele loss was strongly influenced by both environmental stochasticity and delayed population growth but was little affected by increasing the annual turnover rate of the breeding population. We illustrate the model's application using parameter estimates from a threatened New Zealand passerine, the mohua Mohoua ochrocephala, for which reintroduction is a common management tool. Our modelling indicates that when population growth is moderate (lambda = 1.3), c. 60 individuals would need to be released to achieve at least 95% certainty that alleles at an initial frequency of 0.05 would be retained after 20 years (five overlapping generations), which is double the number typically released in translocations of mohua and other threatened forest passerines in New Zealand.

2904: *+.153*

The numbers of black rhino Diceros bicornis in Africa declined dramatically during the last century due primarily to poaching and latterly habitat transformation and fragmentation and as such, significant concerns exist with regard to the long-term population viability and the management of these fragmented populations. A considerable proportion of the remaining black rhino (ssp. minor) are found within South Africa where they largely fall under the protection and management of Ezemvelo KwaZulu-Natal (KZN) Wildlife. Here we provide information on the genetic diversity, population differentiation and level of inbreeding among 77 Diceros bicornis minor individuals sampled in seven protected areas within the KZN Province of South Africa and a single population from Zimbabwe founded from the KZN population. For reference purposes with the cluster analyses, we included four individuals from ssp. bicornis and four individuals from ssp. michaeli. We found low levels of differentiation among ssp. minor populations across the KZN Province; this result is not unexpected given the history of establishments and translocations between reserves. In fact, we argue that the translocations conducted by Ezemvelo KZN Wildlife have contributed to the acceptable levels of heterozygosity and minimal inbreeding which characterize the majority of protected areas in the province. Although the overall genetic diversity in D. b. minor is lower than that present in both Diceros bicornis bicornis and Diceros bicornis michaeli, we do not feel that it is any cause for concern at this stage as it still falls within the range reported for other large mammals across Africa. The information presented here forms the basis of an ongoing monitoring programme aimed at providing vital information which, when taken with ecological and other data, will direct the future management decisions regarding translocations between reserves in South Africa and the exchange of individuals with other countries.

2905: *+.124*

Captive breeding has become an important tool in species conservation programmes. Current management strategies for ex situ populations are based on theoretical models, which have mainly been tested in model species or assessed using studbook data. During recent years an increasing number of molecular genetic studies have been published on captive populations of several endangered species. However, a comprehensive analysis of these studies is still outstanding. Here, we present a review of the published literature on ex situ conservation genetics with a focus on molecular studies. We analysed 188 publications which either presented empirical studies using molecular markers (105), studbook analyses (26), theoretical work (38), or tested the genetic effects of management strategies using model species (19). The results show that inbreeding can be minimized by a thorough management of captive populations. There seems to be a minimum number of founders (15) and a minimum size of a captive population (100) necessary in order to minimize a loss of genetic diversity. Optimally, the founders should be unrelated and new founders should be integrated into the captive population successively. We recommend that genetic analyses should generally precede and accompany ex situ conservation projects in order to avoid inbreeding and outbreeding depression. Furthermore, many of the published studies do not provide all the relevant parameters (founder size, captive population size, H-o, H-e, inbreeding coefficients). We, therefore, propose that a general standard for the presentation of genetic studies should be established, which would allow integration of the data into a global database.

2906: *+.058*

The Chinese alligator (Alligator sinensis) is considered the most critically endangered crocodilian as a result of the near total loss of its habitat and its extremely small and fragmented wild populations. Plans for population recovery lie mostly with wetland restoration and the reintroduction of captive-reared animals. We carried out a first-trial release of 3 adult Chinese alligators (1a (TM),, 2a (TM) Euro) into a pond at the Hongxing conservation site, Xuancheng, southern Anhui Province; the animals were radio-tracked from May to October in 2003. We hypothesized that after a period of adaptation, the alligators would establish definable home ranges. Two (1a (TM),, 1a (TM) Euro) of the 3 alligators were monitored for the whole of the tracking period. The male had an annual home-range size of 7.61 hm(2), and the female 4.00 hm(2). Water temperature and pond water level were two important factors influencing the alligators' distributions, and daily movements. The radio-tracked alligators had overlapping home ranges, which notably included the one substantial island in the pond; that island is the only known nesting site of the local native wild alligators. Aggressive interactions between the released alligators and native wild alligators were observed during the breeding season around this island. All the three reintroduced alligators survived the winter of 2003 and were alive in the same pond in 2008. We concluded that the Hongxing conservation site provided a suitable habitat for the reintroduced alligators. However, the low water level in the pond resulting from farmland irrigation in August and September can be a substantial threat to the alligators' survival. Therefore, regulations on irrigation in summer and autumn are needed to balance the water needs of the alligators and agriculture.

2907: *+.122*

Captive populations provide a precious genetic resource for endangered animals and a source of individuals for reintroduction to depleted habitats. Therefore, accuracy in determining paternity is of vital importance for managing captive populations and in selecting representative individuals of known genetic characteristics for release. In this study, we established a fast and effective method to conduct paternity testing for captive giant pandas in the Wolong population. This technique uses two highly polymorphic microsatellites initially, subsequent use of five less polymorphic markers

and then paternity exclusion testing carried out using the giant panda paternity exclusion program we have developed. Our results revealed that (1) both sets of markers successfully identified the real fathers in 25 cases of paternity testing and (2) the success rate of paternity exclusion varied with the degree of polymorphism of the markers used. Subsequently, we conducted correlation analysis between the success rates of paternity identification with these markers, parameters of genetic diversity and tests of neutrality. We found that the paternity exclusion power of microsatellites was significantly correlated with the number of alleles (Na), expected heterozygosity (H (E)) and observed homozygosity statistic (F (O)) (all $P < 0.05$). From this, we developed a new variable, NaxH (E)/F (O), showing a highly significant positive correlation with the resolution power of microsatellites ($P = 0.001$). Moreover, the first two highly polymorphic loci gave a 100% success rate of excluding non-paternal males because they yielded higher values of NaxH (E)/F (O) than the other five less polymorphic markers. Thus, the NaxH (E)/F (O) parameter appears suitable to serve as a criterion for selecting microsatellite markers, which could be used for high-resolution molecular techniques of paternity determination among a range of captive animals besides giant pandas.

2908: *+.214*

We studied survival and causes of mortality of radiocollared cougars (Puma concolor) on the Greater Yellowstone Northern Range (GYNR) prior to (1987-1994) and after wolf (Canis lupus) reintroduction (1998-2005) and evaluated temporal, spatial, and environmental factors that explain variation in adult, subadult, and kitten survival. Using Program MARK and multimodel inference, we modeled cougar survival based on demographic status, season, and landscape attributes. Our best models for adult and independent subadults indicated that females survived better than males and survival increased with age until cougars reached older ages. Lower elevations and increasing density of roads, particularly in areas open to cougar hunting north of Yellowstone National Park (YNP), increased mortality risks for cougars on the GYNR. Indices of ungulate biomass, cougar and wolf population size, winter severity, rainfall, and individual characteristics such as the presence of dependent young, age class, and use of Park or Wilderness were not important predictors of survival. Kitten survival increased with age, was lower during winter, increased with increasing minimum estimates of elk calf biomass, and increased with increasing density of adult male cougars. Using our best model, we mapped adult cougar survival on the GYNR landscape. Results of receiver operating characteristic (ROC) analysis indicated a good model fit for both female (area under the curve [AUC] = 0.81, 95%CI = 0.70-0.92, n = 35 locations) and male cougars (AUC = 0.84, 95%CI = 0.74-0.94, n 49 locations) relative to hunter harvest locations in our study area. Using minimum estimates of survival necessary to sustain the study population, we developed a source-sink surface and we identify several measures that resource management agencies can take to enhance cougar population management based on a source-sink strategy. (c) 2011 The Wildlife Society.

2909: *+.228*

Understanding survival and cause-specific mortality of native and translocated animals can help biologists design more effective recovery programs. We estimated survival rates for 181 native mountain quail (Oreortyx pictus) in west-central Idaho from 1992 to 1996 and for 199 translocated mountain quail in western Idaho and eastern Washington in 2005 and 2006. Spring-summer survival of native birds over 4 yr ranged from 0.210 (SE = 0.116) to 0.799 (SE = 0.103) and fall-winter survival in 2 yr was 0.523 (SE = 0.089) and 0.244 (SE = 0.084). Annual survival rates were 0.418 (SE = 0.088) and 0.174 (SE = 0.065). Spring-summer survival rate of translocated birds was 0.215 (SE = 0.044) in 2005 and 0.059 (SE = 0.021) in 2006. We modeled biweekly survival as a

function of sex, age, movement rate, native versus translocated status, and linear time trend, and then we added year and 3 weather covariates (mean biweekly precipitation and maximum and minimum temperatures). Year and climate variables improved the a priori top model which included movement rate and native versus translocated status. Higher mortality rates due to predation coincided with movements to breeding habitat in late winter, periods of higher temperatures in the spring and summer, and periods of higher precipitation and colder temperatures during the fall-winter seasons. High movement rates of native birds in winter to avoid snow and by translocated birds when dispersing may have led to greater exposure to predators and consequently lower survival rates. Mountain quail can experience low and variable survival, stressing the potential need for multiple years of releases in restoration efforts in the eastern portion of their range. More attention is needed to identify optimal habitat (including nest sites) for restoring mountain quail populations to reduce movements, lower mortality risks, and provide conditions for withstanding periods of unfavorable weather. (c) 2011 The Wildlife Society.

2910: *+.151*

Black-footed ferrets (Mustela nigripes) apparently were extirpated from all native habitats by 1987, and their repatriation requires a combination of captive breeding, reintroductions, and translocations among sites. Improvements in survival rates of released ferrets have resulted from experience in quasi-natural environments during their rearing. Reestablishment of a self-sustaining wild population by 1999 provided the 1st opportunity to initiate new populations by translocating wild-born individuals. Using radiotelemetry, we compared behaviors and survival of 18 translocated wild-born ferrets and 18 pen-experienced captive-born ferrets after their release into a prairie dog colony not occupied previously by ferrets. Translocated wild-born ferrets moved significantly less and had significantly higher short-term survival rates than their captive-born counterparts. Using mark recapture methods, we also assessed potential impacts to the established donor population of removing 37% of its estimated annual production of kits. Annual survival rates for 30 ferret kits remaining at the donor subcomplex were higher than rates for 54 ferret kits at the control subcomplex (unmanipulated) for males (+82%) and females (+32%). Minimum survival of translocated kits did not differ significantly from survival of those at the control subcomplex. Direct translocation of young, wild-born ferrets from site to site appears to be an efficient method to establish new populations.

2911: *-.120*

Predation can be a critical factor influencing recovery of endangered species. In most recovery efforts lethal and nonlethal influences of predators are not sufficiently understood to allow prediction of predation risk, despite its importance. We investigated whether landscape features could be used to model predation risk from coyotes (Canis latrans) and great horned owls (Bubo virginianus) on the endangered black-footed ferret (Mustela nigripes). We used location data of reintroduced ferrets from 3 sites in South Dakota to determine whether exposure to landscape features typically associated with predators affected survival of ferrets, and whether ferrets considered predation risk when choosing habitat near perches potentially used by owls or near linear features predicted to be used by coyotes. Exposure to areas near likely owl perches reduced ferret survival, but landscape features potentially associated with coyote movements had no appreciable effect on survival. Ferrets were located within 90 m of perches more than expected in 2 study sites that also had higher ferret mortality due to owl predation. Densities of potential coyote travel routes near ferret locations were no different than expected in all 3 sites. Repatriated ferrets might have selected resources based on factors other than predator avoidance. Considering an easily quantified landscape feature (i.e., owl perches) can enhance success of reintroduction

efforts for ferrets. Nonetheless, development of predictive models of predation risk and management strategies to mitigate that risk is not necessarily straightforward for more generalist predators such as coyotes.

2912: *-.078*

Black-footed ferrets (Mustela nigripes) likely were extirpated from the wild in 1985-1986, and their repatriation depends on captive breeding and reintroduction. Postrelease survival of animals can be affected by behavioral changes induced by captivity. We released neutered Siberian polecats (M. eversmanii), close relatives of ferrets, in 1989-1990 on black-tailed prairie dog (Cynomys ludovicianus) colonies in Colorado and Wyoming initially to test rearing and reintroduction techniques. Captive-born polecats were reared in cages or cages plus outdoor pens, released from elevated cages or into burrows, and supplementally fed or not fed. We also translocated wild-born polecats from China in 1990 and released captive-born, cage-reared black-footed ferrets in 1991, the 1st such reintroduction of black-footed ferrets. We documented mortality for 55 of 92 radiotagged animals in these studies, mostly due to predation (46 cases). Coyotes (Canis latrans) killed 31 ferrets and polecats. Supplementally fed polecats survived longer than nonprovisioned polecats. With a model based on deaths per distance moved, survival was highest for wild-born polecats, followed by pen-experienced, then cage-reared groups. Indexes of abundance (from spotlight surveys) for several predators were correlated with mortality rates of polecats and ferrets due to those predators. Released black-footed ferrets had lower survival rates than their ancestral population in Wyoming, and lower survival than wild-born and translocated polecats, emphasizing the influence of captivity. Captive-born polecats lost body mass more rapidly postrelease than did captive-born ferrets. Differences in hunting efficiency and prey selection provide further evidence that these polecats and ferrets are not ecological equivalents in the strict sense.

2913: *+.016*

Habitat complexity has been recognised to exert a significant influence on the abundance and diversity of benthic invertebrates. This issue is especially important for the management of endangered species. The recruitment of limpet species was monitored monthly for one year on natural and artificial surfaces. Control plots showed the highest mean number of species and individuals settled per plot, followed by rough then smooth plots. Control plots presented the highest mean diversity values followed by rough and smooth plots. Recruits of the endangered limpet Patella ferruginea were mainly observed during the spring, from April to June. Recruitment seemed to be influenced by both the heterogeneity and nature of the substratum. P. ferruginea repopulation programmes involving the translocation of recruits on experimental plates should be conducted using similar materials to the natural substratum, such as granite or limestone, rather than plastic, avoiding surfaces with low levels of heterogeneity, and taking into account that translocation of adults is not feasible due to the high mortality observed. (C) 2011 Elsevier Ltd. All rights reserved.

2914: *-.144*

Seed removal by water erosion may explain the sparse vegetation cover in systems like the Chinese Loess Plateau, which is characterized by severe soil erosion. The seeds from 16 species found on the plateau were examined in relation to the likelihood of their removal by erosion, as tested by rainfall simulation experiments. The experiments were performed over 1-m(2) plots with slopes of 10 degrees, 15 degrees, 20 degrees and 25 degrees for 60 min at intensities of 50 mm

h(-1), 100 mm h(-1) and 150 mm h(-1), respectively. Seed loss occurred at simulated rainfall intensities of 100 mm h(-1) and 150 mm h(-1), with total seed loss rates of 26-33% and 59-67%, respectively. Most seeds were displaced, even at 50 mm h(-1). The degrees of seed loss and displacement varied among species. These data, in combination with data from our former research on propagule, seedling and population development in these species, indicate that the species with high seed loss rates either compensate by having a soil seed bank that produces seedlings during the growing season or reproduce by vegetative propagation; the species with no seed loss are still sparsely distributed. Seed germination and seedling survival seem to be more important than seed loss in determining establishment in these regions of the Loess Plateau. Seed translocation by water erosion, however, contributes to the observed distribution of vegetation in this geographic region. (C) 2011 Elsevier B.V. All rights reserved.

2915: *+.121*

The efficiency of three strategies carried out by fisheries managers to restore native Mediterranean brown trout populations threatened by non-native Atlantic populations were assessed. The strategies tested were (i) genetic refuge area where stocking is banned, (ii) stocking with Mediterranean fry and (iii) translocation of wild Mediterranean spawners. Using two discriminatory microsatellite loci between Atlantic and Mediterranean alleles, we compared the genetic composition of samples before and after the changes of practices. The three strategies had several detectable effects in the standing populations causing strong temporal changes in departures from Hardy-Weinberg equilibrium and linkage equilibrium between loci and the apparition of new Mediterranean alleles. The significant reductions in the proportions of Atlantic alleles observed over time can mostly be explained by the disappearing of the pure non-native Atlantic trout after the stopping of hatchery releases. The results, however, also suggest that the active strategies carried out by managers led to intraspecific introgression between both non-native Atlantic and native Mediterranean strains.

2916: *+.181*

1. Dams, ubiquitous features in many lotic ecosystems, are believed to have many broad-ranging and predominantly negative effects on stream biota. Whereas the impacts of larger dams are well studied, few studies have quantified effects of small dams on streams.2. Recent surveys found numerous locations where mussels were abundant and larger in reaches immediately downstream from small dams. We examined mussel shell growth and resource conditions in Sandy Creek, a small (third-order) tributary of the Tallapoosa River in east-central Alabama (U. S. A.), to determine whether larger populations and individuals result from more rapid growth or longer lifespans of mussels downstream from the dam.3. Growth rates for populations occurring immediately downstream from the dam (mill reach), c. 5 km downstream from the dam (downstream reach) and upstream from the impoundment (upstream reach) were compared with environmental conditions (seasonal measures of nutrient concentrations and water chemistry) and food availability [total suspended solids (TSS)]. Water temperature was continuously monitored using data loggers.4. Analysis of length-at-age data using multiple growth models found that mill reach mussels grew faster than both up-and downstream populations. This dam appears to substantially increase water temperatures and may extend the shell growth period in the mill reach. TSS quantity varied seasonally between sites but was generally highest in the impoundment and mill reach during spring and autumn. TSS quality was highest in the upstream reach from spring through autumn but was highest in the impoundment and mill reach during winter.5. Our data suggest that some small impoundments enhance conditions for freshwater mussel growth in downstream reaches. However, we do not know how far downstream this subsidy extends or how

different species respond to mill dam augmentation. Regardless, mounting evidence suggests that this phenomenon is geographically and taxonomically widespread in eastern North America. Heretofore, undocumented positive effects of small dams suggest that some older dams may warrant protection or restoration if downstream reaches support imperilled mussel populations. Further, some small dams may prove useful conservation tools for natural resource managers attempting to identify sites for mussel culture facilities or translocation refugia.

2917: ***-.057***

The study on the population status and human impacts on the endemic and endangered Swayne's hartebeest (Alcelaphus buselaphus swaynei) was carried out in Nechisar Plains, Nechisar National Park, between 2009 and 2010. Direct and total count methods were applied in the population census. Data collected since 1967 were also examined to indicate trends of Swayne's hartebeest populations and their translocation status. In eight illegal Guji Oromo settlements, a total of 134 household samples were identified for group discussion and interview. A trend on livestock population was carried out. The total count of Swayne's hartebeest was 12, 11 and 12 during the 1st, 2nd and 3rd counts, respectively. The population has been decreasing and nowadays only 12 individuals remain. Most respondents had a negative attitude towards conservation areas. The increase in livestock population from 1985 to 1996 was 47.8%. It also increased by 49.9% and 56.5% during 2006 and 2010, respectively. There were 12,531 heads of livestock that depended on the grass plains illegally. Overstocking rate of livestock, illegal resource exploitation and loss of wildlife habitat were the major problems encountered. Active measures have to be implemented to control the human impact and safeguard the future of Swayne's hartebeest.

2918: ***-.004***

The Anacapa deer mouse is an endemic subspecies that inhabits Anacapa Island, part of Channel Islands National Park, California. We used mitochondrial DNA cytochrome c oxidase subunit II gene (COII) and 10 microsatellite loci to evaluate the levels of genetic differentiation and variation in similar to 1400 Anacapa deer mice sampled before and for 4 years after a black rat (Rattus rattus) eradication campaign that included trapping, captive holding and reintroduction of deer mice. Both mitochondrial and microsatellite analyses indicated significant differentiation between Anacapa deer mice and mainland mice, and genetic variability of mainland mice was significantly higher than Anacapa mice even prior to reintroduction. Bayesian cluster analysis and Principal Coordinates Analysis indicated that East, Middle and West Anacapa mice were genetically differentiated from each other, but translocation of mice among islands resulted in the East population becoming less distinct as a result of management. Levels of heterozygosity were similar before and after management. However, numerous private alleles in the founder populations were not observed after reintroduction and shifts in allele frequencies occurred, indicating that the reintroduced populations experienced substantial genetic drift. Surprisingly, two mitochondrial haplotypes observed in an earlier study of Anacapa deer mice were lost in the 20 years prior to the rat eradication program, leaving only a single haplotype in Anacapa deer mice. This study demonstrates how genetic monitoring can help to understand the re-establishment of endemic species after the eradication of invasive species and to evaluate the effectiveness of the management strategies employed.

2919: ***+.026***

The red-legged partridge, Alectoris rufa (Phasianidae), is a game bird hunted throughout its range (Italy, France with Corsica Island, Iberian Peninsula). The release into the wild of farmed birds of

unknown origin coupled to the hybridization with the exotic chukar Alectoris chukar (East Mediterranean to East Asia) has led to the reduction of the spatial component of genetic variability and to the pollution of the genome of A. rufa, respectively. On the mainland, A. chukar genes occur according a decreasing gradient from Italy to the Iberian Peninsula. Corsica hosts a number of A. rufa x A. chukar hybrids, but at a much lower incidence than nearby Italy. We sampled 97 red-legged partridges in different habitats of Corsica [lower-Mediterranean: Desertu di l'Agriate; rural: Nessa-Felicetu; mountainous: Vivariu-Venacu and Fium'Orbu-Taravu (FT)]. We investigated kinship between Corsican and continental A. rufa populations by sequencing the mitochondrial DNA (mtDNA) Cytochrome-b gene in a subset (n = 60) of island specimens as well as in 105 partridges sampled on mainland Europe. All 97 Corsican partridges were genotyped at eight microsatellite DNA loci in order to estimate intraspecific relationships at a finer scale. We also used microsatellite data from previous studies to compare the genotypes of A. rufa reared in the only island farm with those of wild conspecifics. Corsican partridges grouped in the only statistically reliable and diverging mtDNA clade. Microsatellites provided evidence for the genetic isolation of the FT mountain population, whose low level of hybridization with A. chukar had been unveiled in a former paper. Both mtDNA and microsatellite markers revealed that released captive partridges did not enter the wild breeding populations to any great extent. We suggested banning A. rufa translocation from Corsica to the continent to comply with the disclosed genetic kinship, and vice versa to contain the spreading of A. chukar genes in to the A. rufa population.

2920: ***+.308***

There is an increasing awareness that adaptive differences among local populations may affect the success of translocation programmes. A mismatch in habitat quality of the target localities and in the local adaptations of the translocated individuals may reduce the success rate of the translocation programme. The green toad Bufo viridis is the most threatened amphibian in Sweden and has been the focus of an extensive translocation programme of eggs, tadpoles and juvenile toads to several localities with apparently favourable conditions for green toads. However, the success of these measures has been poor. In this study, we investigated the extent of local adaptation in the green toad by examining population divergence and the effect of thermal and saline conditions on larval performance in four Scandinavian populations. In a common garden experiment, we measured larval survival and development as well as the occurrence of spinal deformations. In addition, we quantified pond temperature and water salinity, two important environmental variables for larval performance in anurans in the breeding ponds as well as in seven additional localities included in the conservation programme. We found significant variation among the localities in water temperature and salinity, and significant among-population divergence in larval life history traits and spinal deformations, including both trait means and plastic responses to salinity and temperature. The available evidence suggests that at least part of this divergence is adaptive. We did not find direct support for local adaptation affecting the success of the translocations, however, we argue that the population origin and the impact of rearing conditions on the fitness-related larval traits should be taken into account in the introduction measures of the Swedish green toad conservation programme as well as in translocation programmes in general.

2921: ***+.166***

Inter-simple sequence repeat (ISSR) markers were used to determine the genetic variation and genetic differentiation of cultured and wild populations of Trachidermus fasciatus, an endangered catadromous fish species in China. Six selected primers were used to amplify DNA samples from 85 individuals, and 353 loci were detected. Relatively low genetic diversity was detected in the

cultured population (the percentage of polymorphic loci PPL = 73.80%, Nei's gene diversity h = 0.178 2, Shannon information index I = 0.276 9). However, the genetic diversity at the species level was relatively high (PPL = 91.78%; h = 0.258 3, I = 0.398 6). The UPGMA tree grouped together the genotypes almost according to their cultured and wild origin, showing distinct differences in genetic structure between wild and cultured populations. The pairwise F (st) values confirmed significant genetic differentiation between wild and cultured samples. The cultivated population seems to be low in genetic diversity as a result of detrimental genetic effects in the captive population. The results suggest that ISSR markers are effective for rapid assessment of the degree of diversity of a population, thus giving important topical information relevant to preserving endangered species.

2922: *+.041*

Across much of North America, river otter (Lontra canadensis) populations were extirpated or greatly reduced by the early 20th century. More recently, reintroductions have resulted in restored populations and the recommencement of managed trapping. Perhaps the best example of these river otter reintroductions occurred in Missouri, regarded as one of the most successful carnivore recovery programs in history. However, abundance estimates for river otter populations are difficult to obtain and often contentious when used to underpin management activities. We assessed the value of latrine site monitoring as a mechanism for quantifying river otter abundance. Analyses of fecal DNA to identify individual animals may result in an improved population estimate and have been used for a variety of mammal species. We optimized laboratory protocols, redesigned existing microsatellite primers, and calculated genotyping error rates to enhance genotyping success for a large quantity of river otter scat samples. We also developed a method for molecular sexing. We then extracted DNA from 1,421 scat samples and anal sac secretions (anal jelly) collected during latrine site counts along 22-34-km stretches representing 8-77% of 8 rivers in southern Missouri in 2009. Error rates were low for the redesigned microsatellites. We obtained genotypes at 7-10 microsatellite loci for 24% of samples, observing highest success for anal jelly samples (71%) and lowest for fresh samples (collected within 1 day of defecation). We identified 63 otters (41 M, 22 F) in the 8 rivers, ranging from 2 to 14 otters per river. Analyses using program CAPWIRE resulted in population estimates similar to the minimum genotyping estimate. Density estimates averaged 0.24 otters/km. We used linear regression to develop and contrast models predicting population size based on latrine site and scat count indices, which are easily collected in the field. Population size was best predicted by a combination of scats per latrine and latrines per kilometer. Our results provide methodological approaches to guide wildlife managers seeking to initiate similar river otter fecal genotyping studies, as well as to estimate and monitor river otter population sizes. (C) 2011 The Wildlife Society.

2923: *+.014*

The red-cockaded woodpecker (Picoides borealis; hereafter RCW) was listed as federally endangered in 1973 after a population decline due primarily to habitat loss. Habitat fragmentation produces isolated populations of RCWs and managers often translocate subadult birds to augment existing populations. Although several studies have examined success of translocating subadults, detailed studies examining translocations of adults have been limited. We evaluated the feasibility and success of using adult RCWs for translocation and augmentation of existing populations in Morehouse Parish, Louisiana, 2006-2009. We translocated 41 primarily adult RCWs, consisting of 12 potential breeding groups (PBGs) and 5 single bird groups to suitable habitat at the Morehouse Parish Conservation Area (MPCA). Fifty-nine percent of translocated RCWs remained on the MPCA and 45% became breeders. Thirty-four percent of translocated RCWs were breeding after

being on the MPCA for 2 breeding seasons, suggesting that translocated adult RCWs can augment the breeding population within 2 years of translocation. Fledglings contributed by translocated RCWs ranged from 11% to 30% of the total fledglings on the MPCA. There were 5 PBGs established on the MPCA from translocated RCWs and 20% of the fledglings had at least 1 translocated parent. Success rates for translocation of adults in our study were lower than previous studies where subadults were used. However, previous research suggested that demographically isolated groups have a high risk of abandonment and extirpation, and thus do not contribute to the recovery of the species. The success rates we observed suggest that translocating adult groups may be a useful tool in RCW recovery, and hence should be considered by managers when demographically isolated groups occur. (C) 2011 The Wildlife Society.

2924: *+.069*

The wildcat (Felis silvestris ssp.) is a conservation concern largely due to introgressive hybridization with its congener F. s. rates, the common domestic cat. Because of a recent divergence and entirely overlapping ranges, hybridization is common and pervasive between these taxa threatening the genetic integrity of remaining wildcat populations. Identifying pure wildcats for inclusion in conservation programs using current morphological discriminants is difficult because of gross similarity between them and the domestic, critically hampering conservation efforts. Here, we present a vetted panel of microsatellite loci and mitochondrial polymorphisms informative for each of the 5 naturally evolved wildcat subspecies and the derived domestic cat. We also present reference genotypes for each assignment class. Together, these marker sets and corresponding reference genotypes allow for the development of a genetic rational for defining "units of conservation" within a phylogenetically based taxonomy of the entire F. silvestris species complex. We anticipate this marker panel will allow conservators to assess genetic integrity and quantify admixture in managed wildcat populations and to be a starting point for more in-depth analysis of hybridization.

2925: *+.331*

The aims of this work were to introduce Lippia junelliana into cultivation, to compare the essential oil accumulation between cultivated and wild plants, and to reintroduce micropropagated plants in the location of the original population. The leaves and inflorescences of cultivated plants accumulated, on a dry weight basis, higher amounts of essential oil than their wild counterparts. Thus, total essential oil accumulation of cultivated plant parts was also significantly higher than that of wild counterparts. The cultivated plants showed the same essential oil profile than the wild plants. This work demonstrates that cultivation can be a more efficient vehicle to both preserve and exploit L junelliana, than collection from the wild, because higher yields of biomass and oil accumulation can be achieved, while essential oil composition is less affected by the different treatments. The reintroduction of new plants into the species' original location has proved to be a viable alternative for their in situ preservation or enrichment planting. This model of introduction of aromatic plants into cultivation through micropropagation could be a useful technique to recover valuable chemotypes from the wild in the search for new alternatives in the agriculture and for the preservation of natural resources for future generations. (C) 2010 Elsevier B.V. All rights reserved.

2926: *+.090*

A key first step in any reintroduction programme is to identify the former native range of the species in question. Such information provides the geographical framework for the project, within

which potential reintroduction sites can be assessed. This study provides a historical review of the former distribution and status of the burbot, Lota lota, a freshwater gadoid extirpated from England. The review examines records of burbot presence and abundance from a range of historical and anecdotal sources and provides a qualitative evaluation of the species' decline in English rivers. Burbot were found to have inhabited 42 rivers in eastern England ranging from the River Skerne in County Durham to the River Blyth in Suffolk. The literature suggests the species persisted in greater numbers in a few isolated areas such as the Yorkshire Derwent and the Great Ouse. The review also indicates that rather than be confined to slow-flowing lowland rivers, burbot occupied a range of aquatic environments including small upland headwaters as well as some lake systems.

2927: ***+.125***

Aim Australian acacias (1012 recognized species native to Australia, which were previously grouped in Acacia subgenus Phyllodineae) have been moved extensively around the world by humans over the past 250 years. This has created the opportunity to explore how evolutionary, ecological, historical and sociological factors interact to affect the distribution, usage, invasiveness and perceptions of a globally important group of plants. This editorial provides the background for the 20 papers in this special issue of Diversity and Distributions that focusses on the global cross-disciplinary experiment of introduced Australian acacias. Location Australia and global. Methods The papers of the special issue are discussed in the context of a unified framework for biological invasions. Distributions of species were mapped across Australia, their representation in bioclimatic zones examined and the potential global distribution of the group modelled. By collating a variety of different lists, we determined which Australian acacias have reached different stages in the introduction-naturalization-invasion continuum in different parts of the world. Paradigms and key research questions relating to barriers to invasion, stages of invasion and management perceptions are sketched. Results According to our global database of Australian acacia records, 386 species have been moved outside Australia by human agency, 71 species are naturalized or weedy, and 23 are unequivocally invasive. Climatic models suggest that about a third of the world's land surface is climatically suitable for Australian acacias. Many species are commercially important crops or are useful for other purposes and have been extensively planted, and many different human perceptions of Australian acacias exist in different parts of the world. The papers in the special issue cover all the barriers, stages and processes that define biological invasions and touch on many aspects: history and the human dimension; aspects of the species pool; species traits; biotic interactions; climate and niche; and management. Main conclusions Australian acacias are an excellent model group for examining interactions between evolutionary, ecological and socio-economic drivers of species introductions. New insights have emerged on the biological, ecological and evolutionary correlates of naturalization and invasion, but human usage factors permeate all explanatory models. Understanding and managing introduced Australian acacias requires a fundamental and integrative appreciation of both intrinsic (e.g. species traits) and extrinsic (e.g. human usage and perceptions) aspects.

2928: ***+.157***

The critically endangered Western Derby eland Taurotragus derbianus derbianus, representing <200 wild individuals, undoubtedly needs a coordinated conservation programme. To promote the survival of this subspecies, a single worldwide semi-captive population was established in Senegal in 2000, with one male and five female founders transferred from the Niokolo Koba National Park. To determine a lone-term conservation strategy, we used demographic and pedigree data based on continuous monitoring of reproduction during 2000 - 2009 in breeding enclosures in the Bandia

and Fathala Reserves, in conjunction with modelling software. In 2009, the semi-captive population consisted of 54 living individuals (26 males and 28 females), managed using the minimal kinship strategy. The female breeding probability was 84%, annual calf and adult mortality rates were 5.09% and 3.27%, respectively, and the annual population growth rate was 1.36. As the population grew, the animals were progressively separated into five herds within two reserves. A pedigree analysis revealed an effective population size of 6.72 and an N(e)/N ratio of 0.13. The population retained 77% of the gene diversity (GD). The founder genome equivalent (FGE = 2.21) was relatively low due to the overrepresentation of one founder male. Although the mean level of inbreeding (F) reached 0.119, a significant potential GD (92%) was still retained. In this article, we predict GD development in this population in the next 100 years with the inclusion of new founders. If the whole wild population were included, we could maintain 90% of GD. As this option is not practically feasible, we present three options with the goal of maintaining 75% GD. We highly recommend capturing new founders from the remaining wild population to ensure the survival of the subspecies at least in semi-captivity, which could allow possible reinforcement of the wild population or reintroduction in the future. The semi-captive population, if appropriately constituted and genetically managed, could play a considerable role in Western Derby eland conservation.

2929: *+.103*

We report the salinity tolerance of snails to evaluate how parasite communities with complex life cycles have been altered by translocations of the White Sands pupfish Cyprinodon tularosa to habitats with altered salinity levels. Native and introduced pupfish populations co-occur with the gastropods Physa acuta and associated white grub parasite, Posthodiplostomum minimum, at two brackish springs (Malpais and Mound), but physids are absent from the two saline habitats occupied by White Sands pupfish. We conducted a salinity challenge experiment to test the hypothesis that environmental salinity limits the distribution of physid snails. A 22-d survival experiment with the Malpais Spring physid population indicated that exposure to elevated salinities significantly reduced survival. We also saw sequential declines in survival and reproduction that were proportional to salinity exposure. Salinity of 7% was the apparent threshold for these effects. These results have implications for the use of translocation as a conservation tool in the management of fish populations.

2930: *-.046*

Many species of gazelles (Gazella spp.) are nowadays threatened by hunting, poaching, habitat loss and habitat deterioration. Conservation efforts for this group not only face the problem of maintaining remnant populations, but often natural populations have been extirpated from the wild. In some cases, though, captive breeding programs exist that might provide a valuable source for future reintroductions. A major problem in this context is that phylogeographic relationships among different (potentially locally adapted) populations, and even basic phylogenetic relationships between species, are poorly understood, thus hampering the assignment of management units, breeding groups or stocks for reintroduction projects. Our present study focused on Dorcas gazelles (G. dorcas and G. saudiya) from the species' entire distribution range, with samples originating from western Saharan Africa into Saudi Arabia. In stark contrast to previous studies reporting on pronounced genetic structure in taxa such as Mountain gazelles (G. gazella), we detected low genetic diversity and no evidence for major phylogenetic lineages when analyzing two mitochondrial genetic markers. Using a coalescent approach we infer a steep population decline that started approximately 25,000 years before present and is still ongoing, which coincides with human activities in Saharan Africa. Our phylogenetic analyses, statistical

parsimony network analysis and inferred colonization patterns shed doubt on the validity of various described subspecies of G. dorcas.

2931: *-.098*

Avian malaria is recognised as a potential threatening factor for endangered New Zealand birds; nevertheless, analyses of its prevalence are few and often retrospective, following outbreaks in managed species. We conducted an opportunistic polymerase chain reaction (PCR)-based survey for Plasmodium on a remnant population of red-fronted parakeet (Cyanoramphus novaezelandiae) on Little Barrier Island alongside an analysis of haematology profiles as a first assessment of the effects of this parasite on parakeets. We sampled 22 parakeets and detected Plasmodium relictum DNA in nine samples (prevalence 40.9%; 95% CI = 20.49-61.51%). One successfully amplified sequence corresponded to P. relictum haplotype GRW4. Lymphocyte and heterophil to lymphocyte counts did not differ between PCR-positive and PCR-negative parakeets. However, it is unclear which state of the infection cycle the parakeets experienced during our sampling scheme. From a management perspective, our results indicate that translocation of parakeets from Little Barrier Island to sites where introduced reservoirs of P. relictum occur is a sound management option given the current exposure to this microorganism at the source site.

2932: *-.020*

The hihi is an endangered New Zealand species and its survival depends on the health of translocated populations. Low nestling survival was detected at Zealandia-Karori Sanctuary (KS) during the 2008-09 breeding season with 34% of eggs surviving to fledge. Samples obtained from live and dead hihi nestlings showed that several disease syndromes contributed to nestling deaths. Cause of death was diagnosed by necropsy and histopathology for 25 nestlings. Mortality was highest (60%) in nestlings <= 7 days old and was associated with seasonally low minimum daily temperatures (<11 degrees C). Deaths in this age group were most commonly attributed to poor pulmonary aeration and starvation. Ventriculitis was associated with mortality in eight 6-19-day-old nestlings with no indication that weather was implicated. The remaining deaths were from a variety of unrelated events or causes. Candida albicans, Aspergillus sp. and an unidentified haemoparasite were found among the nestling population. There was no evidence of coccidia or other intestinal parasite infections. In contrast to translocated populations at other sites, nesting material recovered from nest boxes contained low densities of tropical fowl mites (Ornithonyssus bursa). To improve survival for nestlings up to 7 days old, extra food provisioning and nest box weather protection during inclement weather is suggested.

2933: *+.098*

The hihi (or stitchbird, Notiomystis cincta) is a New Zealand endemic nectivorous forest bird now restricted to one pristine island. Relocation to establish viable hihi populations on other islands has been the main conservation action since the early 1980s. To date, hihi reintroductions to young growth islands have had poor success despite the absence of mammalian predators. It was thought that past failures were due to food limitation, but research suggests that food limitation alone cannot account for their poor survivorship. Post-mortems of dead hihi has shown that aspergillosis caused by Aspergillus fumigatus is a major mortality factor and there is current concern regarding their susceptibility to this fungal disease. In this paper we develop and assess the hypothesis that A. fumigatus limits hihi population viability on modified islands, and suggest that A. fumigatus is a potential indicator species for habitat disturbance. We report that the prevalence of A. fumigatus spores in the soil is much higher in young growth forests and forest edge habitats. Results suggest

that hihi mortality rates between islands are potentially due to differential exposure to A. fumigatus spores. We assess relationships between habitat disturbance, A. fumigatus contamination and hihi mortality rates by testing the following predictions: (1) that densities of A. fumigatus spores will be higher on modified islands, (2) that densities of A. fumigatus spores on islands will be correlated with hihi mortality rates and (3) that densities of A. fumigatus spores will be higher at the forest edge than in the interior. We test each of these predictions using soil samples, air samples and samples of nectar from plant species fed on by hihi.

2934: ***+.225***

Sustained reproduction is critical for successful restoration of extirpated populations. Evaluating success of restoration efforts requires an understanding of reproductive capacity in such populations. We examined age-specific reproductive capacity of a Lontra canadensis (River Otter) population in southern Missouri that was the focus of intense restoration efforts in the 1980s and early 1990s. We collected carcasses of 387 legally harvested female Otters from 1996-1999. Otters ranged in age from 1-11 years, with juveniles (<1 yr) the dominant (41.1%) age class. Mean annual corpora lutea counts were high (<(x)over bar> = 2.48 +/- 0.12) and present in 72.8% of adult (>1 yr) Otters. Our results indicate that the southern Missouri River Otter population has one of the greatest recorded potential reproductive capacities for that species.

2935: ***-.233***

The discovery of an isolated population of the dice snake near Meilen by F.A. THIEL in 1883 and the entering of this faunistic novelty into the zoological literature are described. The particular geographical and climatic situation of the dice snake habitat at Meilen is portrayed and the connection of this isolate with the occurrence of the species in the river system of the Elbe and its tributaries Eger (Ohfe), Vltava and Berounka, respectively, in the Czech Republic is discussed. This is followed by a description of the fate of the Meilen population that was marked by continuous anthropogenic impairment up to the near-complete destruction of its habitat through the construction of roads and ended with the extinction of the population around 1936/38. In 1999/2000, a reintroduction project that covered the whole of Germany saw the attempt of re-establishing the dice snake along the River Elbe at Meif3en. A total of 152 individuals were released in Meilen, consisting for a smaller part of wild-caught and for a larger portion of captive-bred specimens of dice snakes from the rivers Eger (Ohfe) and Berounka in the Czech Republic. The reintroduced population subsequently suffered severe losses through high traffic volumes on the roads, shrinking habitats, and the extreme floods of the Elbe in 2002. Estimated at presently comprising a mere 25 specimens at maxi-mum, it is at its existential limits. As negative influences continue to be exerted, no reliable prognosis for a more positive future can be suggested.

2936: ***+.233***

A recent complete assessment of the conservation status of 5487 mammal species demonstrated that at least one-fifth are at risk of extinction in the wild. We retrospectively identified genuine changes in extinction risk for mammals between 1996 and 2008 to calculate changes in the International Union for Conservation of Nature (IUCN) Red List Index (RLI). Species-level trends in the conservation status of mammalian diversity reveal that extinction risk in large-bodied species is increasing, and that the rate of deterioration has been most accelerated in the Indomalayan and Australasian realms. Expanding agriculture and hunting have been the main drivers of increased extinction risk in mammals. Site-based protection and management, legislation, and captive-breeding and reintroduction programmes have led to improvements in 24

species. We contextualize these changes, and explain why both deteriorations and improvements may be under-reported. Although this study highlights where conservation actions are leading to improvements, it fails to account for instances where conservation has prevented further deteriorations in the status of the world's mammals. The continued utility of the RLI is dependent on sustained investment to ensure repeated assessments of mammals over time and to facilitate future calculations of the RLI and measurement against global targets.

2938: *+.095*

Beaver (Castor canadensis) are currently the focus of many wetland restoration projects as a keystone species and an ecosystem engineer. This species forms multi-season pair bonds, making sex ratio an especially important consideration for restoration projects. However, beavers lack external sexual dimorphism (with the exception of lactation) and current methods of sex identification have error rates a parts per thousand yen10%. We improved on previous methods of genetic sex identification for beaver by developing a test that uses a short nuclear fragment in addition to an established Y chromosome-specific sex-determining region (SRY) marker. This method had 100% accuracy with 28 known-sex samples and 95% PCR success with no inconsistencies for 42 hair samples from beavers of unknown sex. We used this test to inform a beaver restoration study and increase the probability of population establishment from translocated animals.

2939: *+.219*

In reintroduction projects, ethology studies play a significant role in evaluating the behaviour of the individuals in habitats where they are reintroduced. We studied foraging and vigilance time allocation of a guanaco (Lama guanicoe) population reintroduced in Quebrada del Condorito National Park (QCNP), in the central mountains of Crdoba, Argentina. On average, individuals showed a higher proportion of time invested in vigilance and a lower proportion of time invested in foraging than a previously studied guanaco population belonging to the same ecological region as the source population, suggesting that at the time of this study, the reintroduced population was not fully adapted to the new habitat or suffered from an increased predation pressure. On the other hand, as expected for the species, throughout the study period, males allocated more time to vigilance than females, both sexes increasing vigilance during the reproductive period, and females increasing foraging time allocation during the post-reproductive period. Taking into account that further reinforcement to the existing population is planned, the present results may contribute to the elaboration of management strategies aimed at the successful establishment of guanaco in QCNP.

2940: *-.028*

1. Throughout Europe, the range of many deer species is expanding. We provide current distribution maps for red deer Cervus elaphus, sika Cervus nippon, fallow deer Dama dama and muntjac deer Muntiacus sp. in Ireland, and estimates of range expansion rates for red deer, sika and fallow deer.2. There was a considerable expansion in the ranges of red deer, sika and fallow deer between 1978 and 2008. The compound annual rate of expansion was 7% for red deer, 5% for sika and 3% for fallow deer. The total range increase was 565% for red deer, 353% for sika and 174% for fallow deer. The potential implications of these expansions are discussed.3. There are unknown numbers of red-sika hybrid deer in some parts of Ireland. Range expansion is likely to lead to further hybridizations with implications for the genetic integrity of deer stocks.4. Sightings of free-roaming muntjac deer were first recorded in 2007. The distribution of confirmed sightings

of single and multiple animals in the eastern region of Ireland suggests multiple releases.5. Deer are already impacting on both the economic and biodiversity values of habitats in Ireland, where, at present, no sustainable deer management policy exists.

2941: *+.266*

Animals can select breeding sites using non-social information (habitat characteristics) and social information (conspecific presence or abundance). The availability of both types of information is expected to vary over time during the colonization of a new area, conditioning their use by colonizers. However, if and how both types of information are exploited during the colonization process remains unclear. We hypothesized that non-social information should be predominant at the beginning of a colonization episode (when conspecific presence is low) and that social information should gain in importance as the colonization progresses. We tested this hypothesis by studying habitat selection by the Griffon Vulture Gyps fulvus, a long-lived colonial raptor, during a natural colonization process spanning 40 years. In NW Spain, the population showed a sharp increase from 15 breeding pairs in three colonies in the 1970s to 586 breeding pairs in 120 colonies in 2008, expanding its range from 90 km(2) in the 1970s to 6403 km(2) in 2008, with directions of expansion following areas rich in nesting cliffs. The main determinants of habitat selection varied over time. Livestock density and the characteristics of nesting cliffs were the main predictors of settlement at the onset of colonization. Breeding density of conspecifics increased its importance over time, having the greatest relative weight in habitat selection later in the colonization process. Our results indicated a prevalent use of non-social information during the early stages of the colonization and an increasing role of social information as the expansion progressed.

2942: *+.021*

Suggestions that the extinct Vegas Valley leopard frog (Rana fisheri = Lithobates fisheri) may have been synonymous with one of several declining species have complicated recovery planning for imperiled leopard frogs in southwestern United States. To address this concern, we reconstructed the phylogenetic position of R. fisheri from mitochondrial and nuclear sequence data obtained from century-old museum specimens. Analyses incorporating representative North American Rana species placed archival specimens within the clade comprising federally Threatened Chiricahua leopard frogs (Rana chiricahuensis = Lithobates chiricahuensis). Further analysis of Chiricahua leopard frogs recovered two diagnosable lineages. One lineage is composed of R. fisheri specimens and R. chiricahuensis near the Mogollon Rim in central Arizona, while the other encompasses R. chiricahuensis populations to the south and east. These findings ascribe R. chiricahuensis populations from the northwestern most portion of its range to a resurrected R. fisheri, demonstrating how phylogenetic placement of archival specimens can inform recovery and conservation plans, especially those that call for translocation, re-introduction, or population augmentation of imperiled species.

2943: *+.009*

Human-mediated movement of wildlife is a common practice in North America. Some translocations have occurred where local populations were thought to be extinct or simply not present. In Alaska's Alexander Archipelago, mountain goats (Oreamnos americanus) were not considered indigenous and were introduced to Baranof Island in 1923. However, a range-wide survey using microsatellites and mitochondrial DNA revealed a distinct genetic subpopulation endemic to the island. In this study, we attempted to clarify the evolutionary history of the

mountain goats on Baranof Island by examining sequence variation in the Y chromosome. We first screened five regions of the Y chromosome in a subset of mountain goats from across their native range. We detected a single polymorphic site in the SRY promoter, and subsequently sequenced this gene in 100 mountain goats. A unique Y chromosome polymorphism was restricted to Baranof Island and an area near Haines, Alaska, and not detected in the presumed source population. An island-to-mainland dispersal scenario from a cryptic refugial population during the retreat of the Cordilleran ice-sheet would account for this distribution. Overall, these data support the hypothesis that a glacial relict population of mountain goats was present on the island prior to introduction. Based on a combination of mitochondrial, microsatellite, and Y chromosome data, we recommend recognizing Baranof Island mountain goats as an evolutionary significant unit.

2944: ***-.012***

For some highly endangered species there are too few reproductively capable animals to maintain adequate genetic diversity, and extraordinary measures are necessary to prevent extinction. We report generation of induced pluripotent stem cells (iPSCs) from two endangered species: a primate, the drill, Mandrillus leucophaeus and the nearly extinct northern white rhinoceros, Ceratotherium simum cottoni. iPSCs may eventually facilitate reintroduction of genetic material into breeding populations.

2945: ***+.028***

Hybridization between introduced and endemic ungulates, resulting from anthropogenic actions, has been reported for several species. Several studies of such events contain the common themes of extralimital movements, problematic phenotypic and genetic detection, and imperfect management. In southern Africa, the endemic black wildebeest (Connochaetes gnou) currently faces a serious threat of hybridization and introgression. This species survived near extinction and consequent genetic bottlenecks in the late 1800s and in the 1930s. Initiatives by private fanners followed by conservation authorities led to a dramatic recovery in numbers of this species. However, in an ironic twist, the very same advances in conservation and commercial utilisation which led to the recovery of numbers are now themselves threatening the species. Injudicious translocation has brought the species into contact with its congener, the blue wildebeest (Connochaetes taurinus), and in recent times, hybridization between the species has occurred at numerous localities in South Africa. Consequently, a significant proportion of the national black wildebeest population potentially carries a proportion of introgressed blue wildebeest genetic material. We discuss completed and ongoing attempts to find molecular markers to detect hybrids and highlight the difficulty of detecting advanced backcrosses. Additional avenues of research, such as work on morphology (cranial and postcranial elements), estimating of the probability of introgression and modelling of diffusion rates are also introduced. In addition to the difficulty in detecting hybrid animals or herds, the lack of consensus on the fate of hybrid herds is discussed. Finally, in an environment of imperfect information, we caution against implementation of management responses that will potentially induce a new genetic bottleneck in C. gnou.

2946: ***+.153***

The Far Eastern Leopard (Panthera pardus orientalis; Schlegel, 1857) is perhaps the world's most endangered large felid subspecies occurring in a single population of,30 adults, and faces immediate risk of extinction unless additional populations can be established within its historical range in the Russian Far East. We used locations of leopard tracks (and their ungulate prey) collected from snow track surveys from 1997 to 2007 to develop resource selection functions

(RSF) to identify potential habitat for reintroduction. We compared models that include prey versus those based on landscape covariates. and also included covariates related to human-induced mortality. To estimate potential population size, we used a habitat-based population estimate based on the ratio of population size and RSF value of occupied range. Far Eastern leopards selected for areas with high ungulate density, lower-elevation Korean pine forests on southwest facing slopes, and in areas far from human activity. Using this RSF model, we identified a total of 10,648 km(2) in eight patches > 500 km(2) of potential Far Eastern leopard habitat that could harbor a potential population of 105.3 (57.9-147.2) adults. In combination with the existing population, successful reintroductions could result in a total of 139.2 (76.5-194.6) adult leopards, a 3-4-fold increase in population size. Our habitat models assist the reintroduction planning process by identifying factors that predict presence and potential suitable habitat. Identifying the highest quality, most connected patches, in combination with appropriate selection and training of released animals, is recommended for successfully reintroducing Far Eastern leopards, and potentially other endangered carnivores into the wild. (C) 2011 Elsevier Ltd. All rights reserved.

2947: *+.058*

Expansion of finfish aquaculture will see increased incentive for translocation of stocks across geopolitical boundaries. The potential for genetic contamination of stocks arising from translocation and subsequent release or escape of translocated and/or genetically mixed stocks may be a significant risk to wild populations. Assessment of risk requires knowledge of the local population structure for the species across the range that translocation might occur and it is in this context we undertook a population genetic survey of stocks of Yellowtail Kingfish across temperate Australia and New Zealand. Seven polymorphic microsatellite loci were used to analyse a total of 272 individuals sampled from New Zealand, New South Wales, Victoria, South Australia and Western Australia. A subset of individuals from each sample locality was also assessed for variation at the mitochondrial gene ND4. Pairwise analysis of sample location and Bayesian analysis showed that Western Australia S. lalandi were genetically distinct from S. lalandi sampled from the other localities. No difference was found between New Zealand S. lalandi and eastern (New South Wales) or central (South Australia and Victoria) Australian fish. The mitochondrial analysis supported the microsatellite data with western samples possessing unique haplotypes compared with all other sites which shared haplotypes. With the expansion of the Yellowtail Kingfish aquaculture industry and likely translocation of stocks, there is a need for a review of translocation policies that consider genetic diversity as a factor in the development of Yellowtail Kingfish aquaculture in the region. (C) 2011 Published by Elsevier B.V.

2948: *+.109*

1. After a major decline, the UK otter Lutra lutra population is now recovering in its known strongholds (northern England, Wales and Borders and southwest England) and also in central England where the population had become small, fragmented and was reinforced with captive bred individuals. Bayesian clustering and GIS are used here to identify the genetic structure of the UK otter population and to assess expansion from strongholds and the contribution of introduced otters. Large carnivores have recently started to recolonize landscapes where they were formerly absent, especially in developed countries and understanding the expansion of these populations is essential for informing conservation management, linking fragmented populations and re-establishing gene flow.2. Three Bayesian clustering techniques were used (STRUCTURE, GENELAND SPATIAL and BAPS4 SPATIAL) to estimate the number of otter populations (K). A novel progressive partitioning approach was tested to identify genetic substructuring at various hierarchical levels using successive partitions at K = 2.3. Four regional populations were identified

that reflect known population history. Isolated populations in southwest England and in Wales and its borders showed the lowest levels of genetic diversity. Higher diversity and private alleles in northern and central England reflect the proximity to genetically diverse Scottish populations and the positive effect of reintroductions.4. Progressive partitioning was used to produce a more detailed analysis, by allowing comparison and combination of clusters identified by different techniques and by avoiding the subjective estimation and choice of K.5. Synthesis and applications. Although the otter population is increasing, our data show little sign of population expansion from the stronghold regions into central England, instead reflecting the success of population reinforcement in this area. Our progressive partitioning approach allows the identification of fine-scale substructure (11 groups) that enables the prioritization of management effort including identifying barriers to dispersal within and between populations and monitoring of introduced individuals.

2949: *+.084*

Reintroductions are commonly employed to preserve intraspecific biodiversity in fragmented landscapes. However, reintroduced populations are frequently smaller and more geographically isolated than native populations. Mixing genetically, divergent sources are often proposed to attenuate potentially low genetic diversity in reintroduced populations that may result from small effective population sizes. However, a possible negative tradeoff for mixing sources is outbreeding depression in hybrid offspring. We examined the consequences of mixed-source reintroductions on several fitness surrogates at nine slimy sculpin (Cottus cognatus) reintroduction sites in south-east Minnesota. We inferred the relative fitness of each crosstype in the reintroduced populations by comparing their growth rate, length, weight, body condition and persistence in reintroduced populations. Pure strain descendents from a single source population persisted in a greater proportion than expected in the reintroduced populations, whereas all other crosstypes occurred in a lesser proportion. Length, weight and growth rate were lower for second-generation intra-population hybrid descendents than for pure strain and first-generation hybrids. In the predominant pure strain, young-of the-year size was significantly greater than any other crosstype. Our results suggested that differences in fitness surrogates among crosstypes were consistent with disrupted co-adapted gene complexes associated with beneficial adaptations in these reintroduced populations. Future reintroductions may be improved by evaluating the potential for local adaptation in source populations or by avoiding the use of mixed sources by default when information on local adaptations or other genetic characteristics is lacking.

2950: *+.112*

The Iberian lynx (Lynx pardinus) is the most endangered wild feline species and the only feline listed as critically endangered by the International Union for the Conservation of Nature. Successful conservation actions rely on accurate knowledge of the species' distribution and decline. Anecdotal unverified reports have overestimated the distribution of the Iberian lynx and misrepresented the severity of its decline. We reconstructed the Iberian lynx range from 1940 to 2000 using only records verified with indisputable physical evidence. We collected data from the 2 major scientific vertebrate collections in Spain, trophies registered by hunting authorities, and miscellaneous private collections. Of 320 lynxes collected during 1940-2007, 261 contained adequate date and location information for this study. The overall species range in 1940 included 15 subpopulations occupying 65 verified 10- x 10-km grid cells. Three large subpopulations (Montes de Toledo, eastern Sierra Morena, and Donana) accounted for 86.6% of records. The species had a steady decline from 1940 until the 1990s, when lynxes remained in only 2 isolated subpopulations. Our reconstruction of verified lynx distributions since 1940 illustrates how most

local extinctions occurred before disease outbreaks among prey, previously assumed to be the principal cause of lynx declines. Rabbit diseases alone cannot account for observed lynx declines, and we suggest that human-caused mortality from direct hunting and indiscriminate predator control programs likely played a larger role in the species' decline. Our verified maps provide a more accurate history of the Iberian lynx distribution in Spain than was available previously. Ideally, this information can help managers outline priority areas for conservation and reintroduction programs to reinforce and restore important subpopulations.

2951: *-.069*

The conservation and management of Saddlebacks (Philesturnus carunculatus) and other New Zealand birds, currently relies on the translocation of individuals to predator-free sites. Avian malaria has been identified as one of the diseases to be tested for prior to translocations in New Zealand, with the aim of translocating disease-free individuals. We describe avian malaria lineages and their seasonal prevalence in 2007-2008 in Saddlebacks from Mokoia Island, a source of birds for translocations, and investigate their pathogenicity. Three lineages of avian malaria were found at low prevalence (<= 10.6%) and parasitemia (all but one infection were below 1/10,000 erythrocytes), typical of chronic infections. Two lineages clustered with previously identified lineages of Plasmodium relictum and one with a lineage of Plasmodium (Huffia) elongation. Prevalence of malaria infection was higher in the spring with no significant difference in prevalence between juvenile and adult birds. We found no effect of stress on infections or any indication of pathogenicity.

2952: *+.241*

1. Improving the understanding, prediction and management of range expansions is a key challenge for ecology. Over recent years, there has been a rapid increase in modelling effort focussed on range expansions and a shift from predominantly theoretical developments towards application. This is especially the case in the field of invasion biology and also in relation to reintroductions and species responses to climate change.2. While earlier models were exclusively analytical, individual-based models (IBMs) are now increasingly widely used. We argue that instead of being viewed as competing methodologies, analytical and individual-based methods can valuably be used in conjunction.3. We use a mechanistic wind dispersal model to generate age-specific dispersal kernels for the invasive shrub, Rhododendron ponticum. To demonstrate the utility of employing both modelling approaches, this information along with demographic parameters is incorporated into an IBM and an analytical, integrodifference model. From both models, the equilibrium rate of spread is calculated.4. Estimates of wavespeeds were similar for the two models, although slower rates of spread were consistently projected by the IBM. Further, our results demonstrate the wavespeed to be sensitive to the characterisation of age structure in the model; when few age classes are used, much higher rates of spread are projected.5. The analytical model is extremely efficient at providing elasticity analysis of the wavespeed, which can provide helpful information for management. We gain qualitatively similar results using the IBM but obtaining the results is time-consuming and, because the model is stochastic, they are noisy and harder to interpret. We argue that analytically derived transient elasticity analyses are needed for the many cases where success of control is measured on a relatively short time horizon.6. To demonstrate the flexibility of the IBM approach, we run it on a real landscape comprising different habitat types. The comparison of two different control scenarios is an example of the utility of this approach for more tactical applications.7. As a general conclusion of the study, we emphasise that analytical and individual-based approaches offer different, but complementary, advantages and suggest how their joint use can facilitate the improvement in biodiversity management at a range

of spatial scales.

2953: ***+.011***

In South Africa there are efforts to manage reintroduced subpopulations of the Vulnerable cheetah Acinonyx jubatus in small reserves (10-1,000 km(2)) as a managed metapopulation. We estimated areas required to support cheetahs given varying prey densities, prey profiles and presence/absence of competing predators. A recent population and habitat viability assessment indicated that 20 subpopulations of 10 cheetahs or 10 subpopulations of 15 cheetahs are required to retain 90% of the heterozygosity of free-ranging cheetahs and to overcome stochastic events in the absence or presence of lions Panthera leo, respectively. We estimate that 203 +/- SE 42 km(2) (range 48-466 km(2)) is required to support to cheetahs in the absence of lions, whereas 703 +/- SE 311 km(2) (166-2,806 km(2)) is required to support 15 cheetahs given equal numbers of lions, and 2,424 +/- SE 890 km(2) (727-3,739 km(2)) given equal numbers of leopards Panthera pardus, spotted hyaenas Crocuta crocuta, wild dogs Lycaon pictus and lions. Existing subpopulations of cheetahs generally occur at densities higher than our mean predicted densities but usually within the range of predicted densities. The large area requirements of cheetahs have implications for the development of the managed metapopulation. Sourcing reintroduction sites of the sizes required to support recommended subpopulation sizes will be difficult. Consequently, innovative measures to increase the carrying capacity of reserves for cheetahs and/or to enlarge reserves will be required. Managers may be forced to stock cheetahs close to or beyond the carrying capacity of their reserves. Consequently, careful management of reintroduced subpopulations will be required to prevent declines in prey populations.

2954: ***-.161***

Case studies of well-documented snake reintroductions are limited, despite their potential value for conservation and ecosystem recovery. The Endangered woma Aspidites ramsayi is a large boid snake that has declined considerably and is now threatened throughout much of central Australia. We describe a trial release of captive-bred womas into the feral predator-free Arid Recovery Reserve in northern South Australia. All of the reintroduced womas were killed within 4 months, with predation by the mulga snake Pseudechis australis confirmed or implied in all cases. Lessons learned for the conditioning of captive-bred snakes for wild release and the role of the mulga snake in structuring Australian arid-zone snake assemblages are discussed.

2955: ***+.108***

The release of tortoises after confinement and treatment in rehabilitation centres includes the risk that released individuals may infect wild conspecifics with foreign parasites and other pathogens. The recent monitoring of the release of rehabilitated leopard tortoises (Stigmochelys pardalis) onto private land in KwaZulu-Natal province, South Africa, revealed that the health-checking protocol at the wildlife rehabilitation centre was inadequate. As a result the provincial conservation authority placed a moratorium on further leopard tortoise releases until a comprehensive health-checking protocol was in place. This protocol is detailed here to ensure the success of the release of confiscated and rehabilitated leopard tortoises, and that this does not come at cost to the wild leopard tortoise populations.

2956: ***-.075***

Two orphaned tigers cubs aged 8-10 months of either sex were monitored over a period of one and

half years between October 2008-March 2010 in the fragmented landscape of Ranthambhore Tiger Reserve (RTR), India. Both animals dispersed out into fragmented landscapes, however the male cub accidently ventured into human habitation and resulted in conflict. The cub was rescued and rehabilitated in an adjoining forest. The female cub dispersed naturally outside the national park area into Chambal ravines. Both the animals were monitored using camera trap and pugmark based tracking method during the study period. Both tiger cubs adapted well in the human dominated (fragmented) landscape, caused minimal conflict, and survived their first winter and summer till they attained the age of >= 24 months. This paper presents the survival of orphaned cubs in fragmented landscape and highlights the importance of translocation as a tool for alleviating conflict.

2957: ***+.138***

The deteriorating demographic status of the endangered Hawaiian monk seal has motivated renewed and expanded proposals for conservation action, including translocation of seals to improve survival. Over the past three decades, numerous monk seal translocations have been conducted with a variety of objectives, including mitigating shark predation and conspecific male aggression, reducing human-seal interactions, and taking advantage of favorable foraging habitats to improve survival. Here, we analyze our cumulative experience with translocation of Hawaiian monk seals. We found a strong correlation between the time seals remained in the vicinity of the release site and their age. Recently weaned pups (with little or no at-sea foraging experience) exhibited high fidelity to release sites commensurate with that shown by untranslocated pups to their birth location. In contrast, juvenile and adult seals tended to stray from their release locations farther and sooner. Nevertheless, when 21 adult male seals were moved more than 1000 km from Laysan Island in the Northwestern Hawaiian Islands (NWHI), to the main Hawaiian Islands (MHI), they subsequently dispersed among the MHI; however, only one was observed to return to the NWHI. Translocated seals appeared to survive at rates comparable to seals native to the release site. Outcomes suggest that in most cases the intended objectives of translocations were achieved. Except for one notable case, translocations within the MHI to arrest human-seal interactions were mostly unsuccessful. These findings will be essential for informing successful large-scale translocation plans in the future. Published by Elsevier Ltd.

2958: ***-.010***

Identifying the genetic processes derived from habitat fragmentation is critical for the conservation of endangered species. We conducted an integrated analysis of genetic patterns in the endangered Dupont's lark (Chersophilus duponti), a circum-Mediterranean songbird threatened by the loss and fragmentation of natural steppes in recent decades. After sampling all the remaining Spanish populations and the two closest North African ones, we found that the Mediterranean Sea acts as a major barrier against gene flow and that recent habitat fragmentation is isolating Spanish populations at different spatial scales. While we found a historical signal of gene flow among Spanish regions, a coalescent model supported that the ancestral panmictic population is evolving into several different units in the absence of current gene flow, genetic drift being more intense in the smaller and more isolated populations. Moreover, small-scale spatial autocorrelation analyses showed that genetic differentiation is also acting within populations. The spatial genetic structure, significant levels of inbreeding and high relatedness within patches raise concerns on the viability of most of the extant populations. We highlight the urgency for steppe patches to be protected, expanded and reconnected, considering the genetic clusters identified here rather than the previously considered eco-geographic regions occupied by the species. Meanwhile, translocations could be considered as a complementary, faster management action to attenuate the crowding and

genetic effects of population fragmentation and the extinction risk of small populations without compromising the current local adaptations, culture diversity and genetic clusters already known for the species. (C) 2011 Published by Elsevier Ltd.

2961: *-.099*

There are numerous examples demonstrating that selection has greatly influenced phenotypes in wild-harvested species. Here, a significant reduction in horn size in trophy desert bighorn sheep rams over 30 years in a reintroduced population in Aravaipa Canyon, Arizona is documented. After examining the potential effects of a detrimental change in the environment, inbreeding depression, and hunter-caused evolutionary change, it appears that environmental deterioration, apparently from the effects of drought, may be a major cause of the decline in horn size. In particular, the reduction in ram horn size is positively associated with reduced winter lifetime rainfall over the 3 decades. Over the same period, the demographic indicator lamb-to-ewe ratio has also declined in the Aravaipa population. On the other hand, lamb-to-ewe ratio has not declined statewide in Arizona, and the population size in Aravaipa appears to be increasing, suggesting local- and trait-specific effects. Using a theoretical context, neither inbreeding depression nor hunter selection by themselves appear to the sole causes of the lower horn size. However, some combination of environmental factors, inbreeding depression, and hunter selection may have caused the decrease in observed horn size. It is not clear what management actions might be successful in countering the environmental effects on horn size, but supplemental feeding and cattle removal are suggested while translocation is suggested to counter the effects of inbreeding depression and reduced hunting and translocation are suggested to counter the effects of hunter selection.

2962: *+.113*

The amplified fragment length polymorphism (AFLP) technique was used to examine genetic variation among old and newly emerged individuals of Hyophorbe lagenicaulis (the Round Island bottle palm) on Round Island to assess surviving levels of diversity in the wild population and to evaluate the suitability of old cultivated stocks on Mauritius as a source of seed for reintroduction. The analysis of AFLP data for 48 individuals of H. lagenicaulis (individuals from Round Island and elsewhere), two H. verschaffeltii, two H. vaughanii, one H. amaricaulis and one H. indica yielded 81 variable and six monomorphic bands. Analysis of molecular variance (AMOVA) and Shannon's indices showed a high level of genetic variation within the wild population on Round Island and a smaller amount of genetic variation among cultivated individuals. A neighbor joining analysis resulted in an unrooted network of genetic distances in which the five Hyophorbe spp. were separated and much variation within H. lagenicaulis was recovered. The Round Island populations of H. lagenicaulis contain representatives of the genetic variation found within the species as a whole. However, a few individuals, both wild and cultivated, represent apparently rare AFLP profiles, and thus, if a more representative distribution of genotypes is wanted for the wild population, cultivated sources could be introduced to Round Island from Mauritian gardens and plantings. (C) 2011 The Linnean Society of London, Botanical Journal of the Linnean Society, 2011, 167, 301-310.

2963: *-.066*

Forecasts of species endangerment under climate change usually ignore the processes by which species ranges shift. By analysing the 'climate paths' that range shifts might follow, and two key range-shift processes dispersal and population persistence - we show that short-term climatic and

population characteristics have dramatic effects on range-shift forecasts. By employing this approach with 15 amphibian species in the western USA, we make unexpected predictions. First, inter-decadal variability in climate change can prevent range shifts by causing gaps in climate paths, even in the absence of geographic barriers. Second, the hitherto unappreciated trait of persistence during unfavourable climatic conditions is critical to species range shifts. Third, climatic fluctuations and low persistence could lead to endangerment even if the future potential range size is large. These considerations may render habitat corridors ineffectual for some species, and conservationists may need to consider managed relocation and augmentation of in situ populations.

2964: *+.276*

Although large-scale biodiversity declines are ongoing, certain conservation actions have made a positive difference. Rates of extinction and endangerment of vertebrate species, for instance, have probably been reduced via conservation interventions. Such conservation actions operate at different spatial scales. Habitat preservation and endangered species recovery are examples of conservation successes at microscales. Mesoscale conservation includes regional cooperation among neighboring countries that has arrested population declines of endangered species, such as mountain gorillas. At macroscales, public pressure on multinational corporations has sometimes resulted in their abandoning environmentally damaging practices or suppliers with poor environmental records. Overall, conservation projects such as these need more long-term funding and greater political and popular support, and must also include provisions to evaluate and document their outcomes. As we discuss here, a focus on conservation successes achieved at different scales can help to promote these aims and guide future conservation victories.

2965: *-.002*

Sierra Nevada bighorn sheep (Ovis canadensis sierrae) experienced a severe population decline after European settlement from which they have never recovered; this subspecies was listed as endangered under the United States Endangered Species Act (ESA) in 1999. Recovery of a listed species is accomplished via federally mandated recovery plans with specific population goals. Our main objective was to evaluate the potential impact of disease on the probability of meeting specific population size and persistence goals, as outlined in the Sierra Nevada bighorn sheep recovery plan. We also sought to heuristically evaluate the efficacy of management strategies aimed at reducing disease risk to or impact on modeled bighorn populations. To do this, we constructed a stochastic population projection model incorporating disease dynamics for 3 populations (Langley, Mono, Wheeler) based on data collected from 1980 to 2007. We modeled the dynamics of female bighorns in 4 age classes (lamb, yearling, adult, senescent) under 2 disease scenarios: 5% lower survival across the latter 3 age classes and persistent 65% lower lamb survival (i.e., mild) or 65% reduced survival across all age classes followed by persistent 65% lower lamb survival (i.e., severe). We simulated management strategies designed to mitigate disease risk: reducing the probability of a disease outbreak (to represent a strategy like domestic sheep grazing management) and reducing mortality rate (to represent a strategy that improved survival in the face of introduced disease). Results from our projection model indicated that management strategies need to be population specific. The population with the highest growth rate ((lambda) over cap; Langley; (lambda) over cap = 1.13) was more robust to the effects of disease. By contrast, the population with the lowest growth rate (Mono; (lambda) over cap = 1.00) would require management intervention beyond disease management alone, and the population with a moderate growth rate (Wheeler; (lambda) over cap = 1.07) would require management sufficient to prevent severe disease outbreaks. Because severe outbreaks increased adult mortality, disease

can directly reduce the probability of meeting recovery plan goals. Although mild disease outbreaks had minimal direct effects on the populations, they reduced recruitment and the number of individuals available for translocation to other populations, which can indirectly reduce the probability of meeting overall, range-wide minimum population size goals. Based on simulation results, we recommend reducing the probability of outbreak by continuing efforts to manage high-risk (i.e., spatially close) allotments through restricted grazing regimes and stray management to ensure recovery for Wheeler and Mono. Managing bighorn and domestic sheep for geographic separation until Sierra Nevada bighorn sheep achieve recovery objectives would enhance the likelihood of population recovery. (C) 2011 The Wildlife Society.

2966: ***+.108***

We used an individual-based population model to perform a viability analysis to simulate population growth (lambda) of 167 elk (Cervus elaphus manitobensis; 71 male and 96 female) released in the Cumberland Mountains, Tennessee, to estimate sustainability (i.e., lambda > 1.0) and identify the most appropriate options for managing elk restoration. We transported elk from Elk Island National Park, Alberta, Canada, and from Land Between the Lakes, Kentucky, and reintroduced them beginning in December 2000 and ending in February 2003. We estimated annual survival rates for 156 radio-collared elk from December 2000 until November 2004. We used data from a nearby elk herd in Great Smoky Mountains National Park to simulate pessimistic and optimistic recruitment and performed population viability analyses to evaluate sustainability over a 25-year period. Annual survival averaged 0.799 (Total SE = 0.023). The primary identifiable sources of mortality were poaching, disease from meningeal worm (Parelaphostrongylus tenuis), and accidents (environmental causes and unintentional harvest). Population growth given pessimistic recruitment rates averaged 0.895 over 25 years (0.955 in year 1 to 0.880 in year 25); population growth was not sustainable in 100% of the runs. With the most optimistic estimates of recruitment, mean lambda increased to 0.967 (1.038 in year 1 to 0.956 in year 25) with 99.6% of the runs failing to be sustainable. We suggest that further translocation efforts to increase herd size will be ineffective unless survival rates are increased in the Cumberland Mountains. (C) 2011 The Wildlife Society.

2967: ***+.312***

Translocations are being increasingly proposed as a way of conserving biodiversity, particularly in the management of threatened and keystone species, with the aims of maintaining biodiversity and ecosystem function under the combined pressures of habitat fragmentation and climate change. Evolutionary genetic considerations should be an important part of translocation strategies, but there is often confusion about concepts and goals. Here, we provide a classification of translocations based on specific genetic goals for both threatened species and ecological restoration, separating targets based on genetic rescue of current population fitness from those focused on maintaining adaptive potential. We then provide a framework for assessing the genetic benefits and risks associated with translocations and provide guidelines for managers focused on conserving biodiversity and evolutionary processes. Case studies are developed to illustrate the framework.

2968: ***-.066***

Genetic structure and diversity of nine Japanese kokanee (landlocked) Oncorhynchus nerka stocks and anadromous O. nerka from the North Pacific and the Canadian Lake Cultus population were examined using microsatellite and mitochondrial DNA. Sequence analyses of the cytochrome b

region of mtDNA for Japanese kokanee O. nerka stocks on Honshu and Hokkaido islands revealed that most Japanese stocks were monomorphic of one major haplotype, which was also dominant in the Lake Cultus population and anadromous O. nerka in the North Pacific. Assignment tests using microsatellite DNA revealed that there was no clear-cut population structure in Japanese kokanee O. nerka stocks. (C) 2011 The Authors Journal of Fish Biology (C) 2011 The Fisheries Society of the British Isles

2969: ***+.245***

Soil seed bank composition and dynamics are crucial elements for the understanding of plant population and community ecology. Earthworms are increasingly recognized as important dispersers and predators of seeds. Through direct and indirect effects they influence either positively or negatively the establishment and survival of seeds and seedlings. Seedling establishment is affected by a variety of earthworm-mediated mechanisms, such as selective seed ingestion and digestion, acceleration or deceleration of germination, and seed transport. Earthworm casts deposited on the soil surface and the entrance of earthworm burrows often contain viable seeds and constitute important regeneration niches for plant seedlings and therefore likely favour specific seed traits. However, the role of earthworms as seed dispersers, mediators of seed bank dynamics and seed predators has not been considered in concert. The overall effect of earthworms on plant communities remains little understood. Most knowledge is based on laboratory studies on temperate species and future work has to explore the biological significance of earthworm-seed interactions under more natural conditions. In this review we summarize the current knowledge on earthworm-seed interactions and discuss factors determining these interactions. We highlight that this interaction may be an underappreciated, yet major driving force for the dynamics of soil seed banks and plant communities which most likely have experienced co-evolutionary processes. Despite the experimental bias, we hypothesize that the knowledge gathered in the present review is of crucial relevance for restoration and conservation ecology. For instance, as earthworms emerge as successful and ubiquitous invaders in various ecosystems, the summarized information might serve as a basis for realistic estimations and modelling of consequences on native plant communities. We depict promising directions of future research and point to the need to consider above- and belowground interactions in order to mechanistically understand the driving forces of plant community assembly. (C) 2011 Elsevier Masson SAS. All rights reserved.

2970: ***+.103***

Pathogens are a significant driver of biodiversity loss. We examine two wildlife disease management strategies that have seen growing use, sometimes in combination: (i) trapping-and-culling infectious animals (disease control), and (ii) trapping-and-translocating healthy animals to a reserve, with possible future reintroduction. A reserve can improve conservation when there is no disease. But, when infection exists, we show investing in the reserve may counteract disease control. We find jointly pursuing both strategies is sub-optimal when the reserve is costly to maintain. Numerically, we examine management of Devil Facial Tumor Disease, which has generated extinction risks for Tasmanian Devils. Disease control (though not eradication) is generally part of an optimal strategy, although a reserve is also optimal if it can be maintained costlessly. This implies preserving the original population by addressing in situ conservation risks, rather than translocating animals to a reserve and giving up on the original population, is generally the first-best. (C) 2011 Elsevier Inc. All rights reserved.

2971: ***+.094***

Characterizing the effects of landscape features on genetic variation is essential for understanding how landscapes shape patterns of gene flow and spatial genetic structure of populations. Most landscape genetics studies have focused on patterns of gene flow at a regional scale. However, the genetic structure of populations at a local scale may be influenced by a unique suite of landscape variables that have little bearing on connectivity patterns observed at broader spatial scales. We investigated fine-scale spatial patterns of genetic variation and gene flow in relation to features of the landscape in desert tortoise (Gopherus agassizii), using 859 tortoises genotyped at 16 microsatellite loci with associated data on geographic location, sex, elevation, slope, and soil type, and spatial relationship to putative barriers (power lines, roads). We used spatially explicit and non-explicit Bayesian clustering algorithms to partition the sample into discrete clusters, and characterize the relationships between genetic distance and ecological variables to identify factors with the greatest influence on gene flow at a local scale. Desert tortoises exhibit weak genetic structure at a local scale, and we identified two subpopulations across the study area. Although genetic differentiation between the subpopulations was low, our landscape genetic analysis identified both natural (slope) and anthropogenic (roads) landscape variables that have significantly influenced gene flow within this local population. We show that desert tortoise movements at a local scale are influenced by features of the landscape, and that these features are different than those that influence gene flow at larger scales. Our findings are important for desert tortoise conservation and management, particularly in light of recent translocation efforts in the region. More generally, our results indicate that recent landscape changes can affect gene flow at a local scale and that their effects can be detected almost immediately.

2972: ***+.098***

After release to the wild, captive reared salmon have shown lower foraging rates on natural prey and impaired ability to avoid natural predators and thus lower survival compared with wild-born conspecifics. Here, we examine whether captive breeding influences learning of foraging on natural prey and how enriched rearing methods may improve foraging on natural prey by Atlantic salmon (Salmo salar) parr. We reared offspring of hatchery or wild salmon of the same population in either a standard or enriched environment at production-scale densities. The enriched environment featured submerged overhead shelter, varying water current, depth and direction and consequently alterations in food dispersion. Parr reared in the enriched environment expressed higher feeding rates, and parr of wild origin started to forage earlier on natural prey. The enriched method promoted foraging of hatchery reared parr on natural prey and is easily applicable to commercial production of salmonids for reintroduction or stock enhancement purposes.

2973: ***+.130***

This meeting, which focuses on multiplying human impacts bordering open space-challenges for wildlife habitat and connectivity protection, contains approximately 67 oral abstracts in English. Topics include evaluation of wildlife guards at access roads, using cameras effectively to monitor wildlife, current status of trumpeter swan reintroduction at the flathead indian reservation, and evaluating the genetic distinctiveness of the salmon river drainage bighorn sheep and their connectivity to neighboring populations. Other topics are winter ecology of the shiras moose on the mount haggin wildlife management area, Montana electronic precipitation map, and keeping common species common-inventory and monitoring for a diversity of wildlife species.

2974: ***+.212***

The Azorean endemic gymnosperm Juniperus brevifolia (Seub.) Antoine is a top priority species

for conservation in Macaronesia, based on its ecological significance in natural plant communities. To evaluate genetic variability and differentiation among J. brevifolia populations from the Azorean archipelago, we studied 15 ISSR and 15 RAPD markers in 178 individuals from 18 populations. The average number of polymorphic bands per population was 65 for both ISSR and RAPD. The majority of genetic variability was found within populations and among populations within islands, and this partitioning of variability was confirmed by AMOVA. The large majority of population pairwise F (ST) values were above 0.3 and below 0.6. The degree of population genetic differentiation in J. brevifolia was relatively high compared with other species, including Juniperus spp. The genetic differentiation among populations suggests that provenance should be considered when formulating augmentation or reintroduction strategies.

2975: ***-.105***

Plant reintroductions include labor-intensive, costly, and time-consuming work and often cannot guarantee a successful outcome. In order to maximize the chances of success, it is therefore of utmost importance to appropriately select target species, release site, cultivation and reintroduction methodology, and management technique of the out-planting site. Case studies, best practice and experiences of plant reintroductions are however not sufficiently disseminated to the plant conservation community, most often remaining in unpublished internal reports to which access is difficult. We suggest that this is a major problem for conservationists and it requires the establishment of a framework for rapid and effective broadcasting of information on plant reintroduction programs. We propose a set of variables for a centralized web-based interface which could provide the necessary information in a standardized and accessible form.

2976: ***-.017***

The Turks Island Boa (Epicrates c. chrysogaster) is endemic to the Turks and Caicos Islands and is currently known from only 11 islands. The subspecies has likely been extirpated from several islands in its historic range, and all remaining populations are threatened with extirpation owing to habitat loss, introduced feral predators, malicious killing, and vehicle strikes. To assist conservation efforts, we undertook a genetic analysis of 53 individual E. c. chrysogaster, representing five island populations, with the goal of identifying existing population structure and genetic diversity. For each snake sampled, we sequenced one mitochondrial and two nuclear genes, resulting in 1591 bp of sequence, and screened nine microsatellite loci. All individuals were found to be monomorphic at the four microsatellite loci that amplified, and only three individuals were found to vary (by a single nucleotide polymorphism) in either nuclear gene. Nine mitochondrial haplotypes were found, with a maximum sequence divergence of,1%. Taken together, these data indicate shallow genetic divergence in this subspecies, possibly owing to a lack of historical population structure and small population size when the Turks and Caicos Banks were each single islands during the last glacial maximum. Epicrates c. chrysogaster appears to represent a single evolutionarily significant unit, a significant finding suggesting that conservation strategies focusing on ecologically intact populations might be more appropriate than alternate strategies involving near-impossible reversal of declining populations on heavily disturbed islands. In addition, reintroduction programs would likely not disrupt any significant historical population structure.

2977: ***+.159***

Biodiversity conservation in an era of global change and scarce funding benefits from approaches that simultaneously solve multiple problems. Here, we discuss conservation management of the

island scrub-jay (Aphelocoma insularis), the only island-endemic passerine species in the continental United States, which is currently restricted to 250-square-kilometer Santa Cruz Island, California. Although the species is not listed as threatened by state or federal agencies, its viability is nonetheless threatened on multiple fronts. We discuss management actions that could reduce extinction risk, including vaccination, captive propagation, biosecurity measures, and establishing a second free-living population on a neighboring island. Establishing a second population on Santa Rosa Island may have the added benefit of accelerating the restoration and enhancing the resilience of that island's currently highly degraded ecosystem. The proactive management framework for island scrub-jays presented here illustrates how strategies for species protection, ecosystem restoration, and adaptation to and mitigation of climate change can converge into an integrated solution.

2978: *-.015*

The extent and causes of crucian carp Carassius carassius decline were assessed during an initial study of c. 25 ponds in north Norfolk, eastern England, U.K., which was then replicated (a validation study) on another c. 25 ponds in an adjacent area. Of these ponds, c. 40 are known to have contained C. carassius during the 1970s1980s. In the initial and validation studies, C. carassius were found in only 11 of these ponds, yielding declines of 76% (five of 21 ponds) and 68% (six of 19 ponds), respectively (72% decline overall). Non-native cyprinids, including goldfish Carassius auratus and common carp Cyprinus carpio and their hybrids with C. carassius, were observed in 20% of the ponds. Causes of C. carassius local extinction from 21 ponds were confidently determined as desiccation due to drought, terrestrialization and habitat deterioration, hybridization and competition with non-native cyprinids, agricultural land reclamation and predation (after the introduction of pike Esox lucius). This study led to C. carassius being designated as a Biodiversity Action Plan (BAP) species in the county of Norfolk, the first formal conservation designation for the species in the U.K. The C. carassius BAP plan aims to halt the decline of this much overlooked species through reintroductions and selective stocking of suitable ponds within the native range of the species.

2979: *+.055*

Polar bears were found as summer residents on the St. Matthew Islands in the northern Bering Sea from the time of their discovery in the mid-18th century until the late 19th century, when the last bears were presumably shot by crews from Canadian and American sealers and a U.S. revenue cutter. Historical documents suggest that the killing of the last summer-resident polar bears on the St. Matthew Islands was an indirect consequence of the controversy between the United States and Great Britain over management of the fur seal harvest and the associated pelagic hunting of these seals. Although polar bears have continued to be present near the St. Matthew Islands in winter, when sea ice is present, a metapopulation of summer-resident bears has not-reestablished on these islands. In 1972, the State of Alaska considered a proposal to reestablish a summer-resident polar bear population on the St. Matthew Islands, and since 2008, when the United States listed the polar bear as a threatened species, such reestablishment has been suggested as a conservation strategy. However, given the observed changes in local Bering Sea ice conditions in recent decades, the lack of detailed information on the population ecology and habitat dependencies of the historical St. Matthew bears, and the unavailability of an analogous extant metapopulation of polar bears for comparison, it is highly unlikely that reestablishment of summer-resident polar bears on the St. Matthew Islands could be realized.

2980: *-.054*

Muskrats (Ondatra zibethicus) prey on freshwater mussels, many species of which are threatened or endangered. Muskrat populations have been reduced in some streams where North American river otters (Lontra canadensis) were reintroduced, and it has been hypothesized that otter reintroduction could be used as a tool for conservation of mussels. We used occupancy estimation methods to evaluate the ecological relationship between muskrats and otters by collecting presence-absence data based on field sign found at bridge crossings in eastern and central Kentucky. Mean detection probabilities (ps) and occupancy probabilities (psi s) for muskrats were 0.692 (SE = 0.045) and 0.723 (SE = 0.071) and for otters were 0.623 (SE = 0.036) and 0.662 (SE = 0.069), respectively. Otter occupancy was related negatively to distance from release sites, which suggests that the otter population is still expanding its range. A 2-species interaction model indicated that the occupancy by muskrats and river otters was independent, and we conclude that river otter reintroduction would not be an effective strategy for conserving mussels.

2981: *+.314*

Until 2003 Cottus perifretum was believed to be extinct from the Demer River basin in Flanders, Belgium. However, that year a relict population of this bullhead species was found in the Dorpbronbeek. The Research Institute for Nature and Forest (INBO) and the Agency for Nature and Forests (ANB) launched a conservation project to preserve this population by re-introducing cultured progeny to the Zevenbronnenbeek. This headwater stream was carefully selected from seven evaluated potential locations within the Demer River basin based on water and habitat quality and food availability. In October 2008, 1220 cultured age 0+ bullhead were released. To enlarge the chance for success, ceramic roof tiles were added to the stream as artificial spawning substrates. In 2009, the success of the re-introduction was assessed. The recaptured fish had grown since their release and were in visual good condition, but most important, natural reproduction occurred.

2982: *+.099*

Atlantic Salmon (Salmo salar) once was a common fish in German rivers. In the course of the industrial revolution the water quality decreased, the rivers were straightened and gradually fragmented by weirs and dams. As a consequence the salmon populations drastically decreased and finally became extinct by the middle of the last century. About 20 years later, in 1978, the first salmon was released in an attempt to re-establish this valuable species. Today re-introduction programmes are running at all former important salmon rivers, namely at the rivers Rhine, Ems, Weser, Elbe as well as some smaller rivers. This contribution provides a short overview on the status of these re-introductions, the success stories as well as the drawbacks of salmon re-introduction in German rivers.

2983: *+.068*

The reintroduction of Atlantic salmon in to tributaries of the Rhine River in the German Federal States of Rhineland-Palatinate and Hesse is implemented by the fisheries authorities of the two States (Lander). Stocking is conducted in Rhineland-Palatinate since 1994 and in Hesse since 1995. In phase I (19942003) various strains were tested in parallel. In phase II (since 2003/2004) only the Swedish Atran strain was imported. It was hypothesized that the strain Atran would perform particularly well because of a similar spawning time to the original stock of River Sieg and a comparable Multiple-Sea-Winter ratio to many original stocks in the Rhine River. Returning salmon were monitored annually since 1996 in Rhineland-Palatinate and since 2002 in Hesse. Between 1996 and 2008 a total of 533 returners were recorded in the two programmes, with 52.9%

being males and 38.4% females (sex unknown: 8.7%). 2007 and 2008 yielded the highest ratio of Multiple-Sea-Winter (MSW) salmon so far. In 2008 74% of the individuals with known length had spent two or more winter in the sea, thus raising the average length to 80.3 cm. Overall trends show an increase in the total number of recordings and MSW salmon ratio, together with an increase of mean length correlated with the latter. The percentage of female MSW returners increased by 1077% compared to 2007. Until 2008 natural reproduction was recorded in five river systems (total: 11 streams). In 2008 reproduction was demonstrated in 11 tributaries, an increase of three compared to 2007. The density of juvenile 0+ salmon found in immediate vicinity of several spawning sites in the rivers Saynbach, Wisper, Nister and Sieg exceeded 100 individuals per 100 m2. In 2008 approximately 85% of the suitable and accessible habitat in rivers Nister and Saynbach (Rhineland-Palatinate) were inhabited by wild 0+ salmon, followed by River Wisper (Hesse) with 80%. As a consequence of an increasing shortage of available stocking material a brood-stock programme was established in the Atlantic Salmon Centre Hasper Talsperre. The rearing-programme started in 2006 and aims to become independent from further imports by the year 2011. The brood-stock consists (almost) exclusively of the strain Atran (Sweden). Wild juveniles make up around 30% of the brood-stock. The wild juveniles were sampled from several spawning areas in nine streams with documented natural reproduction. A genetic examination of the brood-stock generation 2004 and 2005 and of the offspring of Atran returners to River Sieg indicated no loss of genetic diversity and revealed a similar genetic structure of the brood-stock and the offspring of Atran returners to River Sieg. It is concluded that the high reproductive success of the non-native strain Atran observed here corroborates the hypothesis of timing of spawning and Multiple-Sea-Winter ratio being key factors in the selection of a successful donor strain for reintroduction.

2984: ***+.142***

Post-release monitoring data of reintroduced captive-bred birds can be utilized to help optimize future avian reintroduction programs. We present a case study of broad interest to reintroduction and conservation biologists interested in investigating movements and habitat use by reintroduced captive-bred birds. We used radio telemetry to monitor reintroduced captive-bred red-billed curassow Crax blumenbachii at a private reserve, Rio de Janeiro state, Brazil. During August 2006 and October 2008, 25 radio-tagged individuals (15 females and 10 males, all <30 months old) were monitored over a 25-month period. Evaluation of home-range size and habitat use revealed that captive-bred curassows should be released only into forest areas with adequate riverine habitat that are larger than the minimum home-range movements of the proposed population. Curassows also utilized pastureland, cultivated areas and secondary forests, suggesting that the proximity of release sites to such habitats may not be entirely detrimental for future reintroductions. Site fidelity for reintroduced birds was low, and there was a tendency for resident curassows to move away when new cohorts were released into the area. Determining how habitat characteristics, displacement by newly released cohorts, adjustments to their new surroundings or cohort social interactions influence post-release movements of resident birds at release sites over prolonged time frames would improve our knowledge on the impacts of releasing further captive-bred individuals into habitats with extant populations. Critically, the movement patterns of reintroduced curassows identified in this study demonstrate that avian post-release monitoring must be considered over an appropriate time frame and we highlight how different conclusions may be generated depending on the duration of post-release monitoring. It may take more than 2 years for reintroduced captive-bred sub-adults to become established following release and that post-release monitoring of similar duration may not be adequate for large avian species such as Cracids.

2985: ***-.101***

The ocelot Leopardus pardalis has become a conservation priority in the US as a result of severe population decline and loss of habitat during the 20th century. Only two small populations remain in this country. Their short-term viability is threatened by the disappearance of dense thornshrub communities, human-caused mortality and demographic stochasticity. The influence these factors have on ocelot persistence must be considered to develop effective conservation initiatives. We therefore examined neutral genetic diversity and connectivity among ocelots in the US and northeastern Mexico using 25 autosomal microsatellites and a 395-bp segment of the mitochondrial control region. Genetic variation was lowest in the population occurring on Laguna Atascosa National Wildlife Refuge, Texas (autosomal microsatellite HE=0.399 and mtDNA-haplotype diversity=0) and highest in northeastern Mexico (0.637 and 0.73, respectively), while intermediate on private lands in Willacy County, Texas (0.553 and 0.252, respectively). Significant genetic differentiation between the two Texas populations was observed, despite their close proximity (similar to 30 km). Both populations were also significantly divergent from northeastern Mexico. The absence of any detectable gene flow implies that the human modified landscape of the Lower Rio Grande Valley in southern Texas acts as a strong barrier to ocelot movement, disrupting metapopulation dynamics and contributing to loss of diversity. As a consequence, continued genetic erosion among the Texas populations is expected. The lack of movement through the fragmented landscape also suggests it is unlikely ocelots will recolonize unoccupied habitat patches along the Lower Rio Grande and the delta interior where agriculture and urban land uses predominate. The continued rapid development will exacerbate this problem. These factors threaten the persistence of the Texas populations and limit their recovery. Translocations are necessary to link ocelot populations in the US.

2986: ***-.053***

In England Pulsatilla vulgaris is a threatened herb that declined from 130 to 33 sites between 1750 and the 1960s due to ploughing-up of calcareous grassland. We examined the subsequent fate of these populations using documentary evidence and field survey. Demographic trends were related to changes in grassland composition, structure and management and responses to increased above-ground competition (caused by reduced grazing) were simulated in a 10-year shading experiment. Since 1968 P. vulgaris has been lost from 16 sites and gradually declined on four others. However, the total population size increased by 258% due to the reintroduction of winter grazing on three sites. This produced a significantly shorter, more herb-rich sward, with a lower cover of Bromopsis erecta (c. 10%), than on sites where populations remained stable or declined. Experimental shading had a significant negative effect on plant survival and flowering performance. These results confirmed that reduced grazing is now one of the major threats to species dependent on short swards, especially on isolated sites where livestock farming is no longer economically viable. That many of these declines took place on nature reserves highlights the difficulties of managing isolated grasslands, and the urgent need to re-instate grazing on reserves supporting populations of threatened species in otherwise intensively managed lowland landscapes. (C) 2011 Elsevier Ltd. All rights reserved.

2987: ***+.006***

Ten reintroduction attempts were conducted in and around the Arid Recovery Reserve in northern South Australia between 1998 and 2008. Five locally-extinct mammal species and one reptile species were reintroduced into a fenced Reserve where cats, foxes and rabbits were excluded. Reintroductions of the nationally threatened greater stick-nest rat, burrowing bettong, greater bilby and western barred bandicoot were all considered successful based on short and medium-term success criteria. These criteria included continued survival after 8 years, increased distribution

across the large Reserve and, most importantly, recovery after a drought event. The trial reintroductions of the numbat and woma python into the Reserve were unsuccessful due to predation by native avian and reptilian predators respectively. Outside the Reserve, where cats and foxes were present but controlled through poison baiting, reintroduction attempts of the greater bilby and burrowing bettong were unsuccessful. High mortality was attributed to cat and fox predation with dingoes also contributing to post-release mortality in bettongs. However, a reintroduction of burrowing bettongs into a fenced area with low rabbit and cat abundance has, to-date, met short-term and medium-term success criteria. Results suggest that the absence or severe restriction of exotic mammalian predators was the critical factor responsible for the success of the mammal reintroductions. Determining thresholds of predator activity below which successful reintroduction of threatened species can occur, are needed to improve the science of reintroduction biology in Australia. (C) 2011 Elsevier Ltd. All rights reserved.

2988: ***+.081***

The great-tailed grackle Quiscalus mexicanus was introduced by Aztec Emperor Auitzotl beyond its native range in 16th century Mexico. Haemig (2011) provides valuable details on the transport, introduction, establishment, and spread of this species. However, here I argue that while this is an interesting account of a historical bird translocation, this is not the reason why this species has undergone one of the greatest range expansions of any native North American species during the 20th century. Fossil records indicate that the species was already present in areas further north before this historical translocation. Moreover, it is a very plastic species and human commensal. Therefore, the northward expansion of this tropical species has been aided by anthropogenic habitat changes rather than a consequence of the active translocation by the Aztec Emperor Auitzotl.

2989: ***+.429***

The introduction of great-tailed grackles Quiscalus mexicanus by Aztecs in pre-Columbian times reported by Haemig (Ardeola, 58: 387-397) probably constitutes the most detailed information on an ancient exotic bird introduction. There is however sparse evidence, compiled here, showing that several domesticated and non-domesticated bird species were translocated even thousands of years ago by different civilizations, often ending on exotic introductions which last until current times. Given that different ancient cultures showed a great fascination for exotic birds and intensively traded them, we should expect a number of still unraveled old-time introductions worldwide. The combination of information from old-written documents, archeological and fossil findings, stable isotope analyses, art museums, and the most advanced molecular techniques should add exotic introduction surprises in the coming years.

2990: ***+.164***

A project to reintroduce the Spanish imperial eagle in the province of Cadiz (Andalusia, Spain) began in 2002. The aim was to restore the former breeding nucleus, to encourage subpopulation interconnection and to ensure the long-term persistence of the southern metapopulation of this endemic Iberian species. A population reinforcement programme also began in 2005 at a location in the Donana area (Andalusia, Spain) to improve the viability of this endangered subpopulation. Between 2002 and 2010, a total of 73 young Spanish imperial eagles were released at these four locations by means of hacking. As a result, in 2010 a released male bred successfully with a non-reintroduced female and two young were reared. This was the first successful breeding event recorded in Cadiz province since 1960. An additional territorial pair also settled in the release area

in 2010 and four more translocated individuals have made breeding attempts within nearby breeding subpopulations since 2006. The settlement and breeding of reintroduced individuals is the starting point of a future population and constitutes a relevant indicator for the evaluation of the project.

2991: ***+.223***

The Newell's shearwater (Puffinus auricularis newelli), an IUCN and ESA listed species, faces terrestrial threats from predation, fallout (attraction to artificial lights) and collision with powerlines. Various indices suggest the population has declined by ~75% in the past two decades. Population modeling is required for Habitat Conservation Plan (HCP) and Recovery Planning to consider the benefits of existing and proposed management actions to the Kauai population. Population scenarios modeled here included a) stable, realistic and optimal growth; b) threats of predation, fallout and powerline collision; and c) management actions of minimizing fallout and powerline mortality, the Save Our Shearwater rescue program, predator control, predator eradication and chick translocation. The growth rate (lambda) produced in our worst case threat scenario for all threats (0.906) fell within the range of annual change suggested by ornithological radar data from 1993- 2010 using only Newell's shearwater traffic (0.899), and Save Our Shearwater data of Newell's shearwater fledglings from 1988-2009 (0.905). When considered independently, fallout and powerline minimization have the potential to increase growth rate by up to 0.5% and 0.3%, respectively, but would prove more effective in areas where concurrent colony management is planned. The Save Our Shearwater program could theoretically increase growth rate by up to 0.8% if fledglings recovered and released experience the same survival as fledglings' not experiencing fallout. While experiencing powerline strike and fallout threats, predator control with 90% effectiveness produced population growth rates from 0.976 to 0.991 and predator eradication produced growth rates from 0.986 to 1.00, suggesting additional growth of 0.2% - 2.4% would be required to stop a decline in these areas. We allowed translocated populations to grow at 1.012 because chicks would likely only be moved to areas free of terrestrial threats. Combined management scenarios with 1) 10% population subjected to predator control, 5% to predator eradication, and 100 chicks translocated over five years, and 2) 20% population subjected to predator control, 10% to predator eradication, and 400 chicks translocated over 10 years, would provide a benefit of 2,000 and 4,000 birds over 25 years, respectively, compared to no management undertaken. The benefits from these actions have clear potential to offset incidental take proposed in HCP planning. A recovered (i.e. stable, self-sustaining) Newell's shearwater population in 25 years will likely be much reduced in size from what exists today. However, it is certainly realistic that subsets of the population can achieve positive growth rates when predation, fallout and powerline mortality are removed. Combined management actions likely to provide the greatest potential benefit should be targeted to prevent this species from becoming extinct, including predator control of Northwest colonies, predator eradication projects in combination with aggressive powerline and fallout minimization in the same region, and chick translocation to threat-free environments.

2992: ***+.065***

Kenya's black rhinoceros population declined by more than 98% from 20,000 individuals in the 1970s to around 400 individuals in 1990 due to the effects of poaching, at which time the surviving individuals were isolated in a series of demographically inviable subpopulations. An initial management exercise translocated the survivors into four high security sanctuaries to control poaching and enhance breeding, and this measure successfully arrested the decline. Subsequently, new sanctuaries were established and the metapopulation size reached 650 animals

by 2008. However, translocations and the current management strategy that partitions the metapopulation into 'montane' and 'lowland' rhinoceros may have substantial consequences at the population level and their impact on population genetic diversity has not been investigated. In this study, 12 of the 16 extant subpopulations were analysed using 408 bp of mitochondrial control region sequence (n = 170) and nine microsatellite loci (n = 145). Both markers detected moderate to high genetic diversity (h = 0.78 +/- 0.027, n = 170; H-O = 0.70 +/- 0.087, n = 145) consistent with previous studies on Diceros bicornis michaeli. However, mtDNA and nDNA diversity varied substantially between subpopulations. The results suggest that the Masai Mara is more differentiated, inbred and isolated than other subpopulations. It also suggests that there are neither distinct montane and lowland groups nor other detectable historical barriers to gene flow. Instead the large majority of genetic diversity was partitioned at the level of individuals; highlighting the need to conserve as many individuals as possible. Future translocations should consider the genetic profile of individuals and the demographic history of both the donor and recipient subpopulations.

2993: *+.204*

Animal translocations are human-induced colonizations that can represent opportunities to contribute to the knowledge on the behavioral and demographic processes involved in the establishment of animal populations. Habitat selection behaviors, such as social cueing, have strong implications on dispersal and affect the establishment success of translocations. Using modeling simulations with a two-population network model (a translocated population and a remnant population), we investigated the consequences of four habitat selection strategies on post-translocation establishment probabilities in short-and long-lived species. Two dispersal strategies using social cues (conspecific attraction and habitat copying) were compared to random and quality-based strategies. We measured the sensitivity of local extinctions to dispersal strategies, life cycles, release frequencies, remnant population and release group sizes, the proportion of breeders and the connectivity between populations. Our results indicate that social behaviors can compromise establishment as a result of post-release dispersal, particularly in long-lived species. This behavioral mechanism, the "vacuum effect", arises from increased emigration in populations that are small relative to neighboring populations, reducing their rate of population growth. The vacuum effect can drive small remnant populations to extinction when a translocated group is large. In addition, the magnitude of the vacuum effect varies non-linearly with connectivity. The vacuum effect represents a novel form of the behaviorally mediated Allee effect that can cause unexpected establishment failures or population extinctions in response to social cueing. Accounting for establishment probabilities as a conditional step to the persistence of populations would improve the accuracy of predicting the fates of translocated or natural (meta) populations.

2994: *-.026*

White-nose syndrome(WNS) has caused recent catastrophic declines among multiple species of bats in eastern North America(1,2). The disease's name derives from a visually apparent white growth of the newly discovered fungus Geomyces destructans on the skin (including the muzzle) of hibernating bats(1,3). Colonization of skin by this fungus is associated with characteristic cutaneous lesions that are the only consistent pathological finding related to WNS4. However, the role of G. destructans in WNS remains controversial because evidence to implicate the fungus as the primary cause of this disease is lacking. The debate is fuelled, in part, by the assumption that fungal infections in mammals are most commonly associated with immune system dysfunction(5-7). Additionally, the recent discovery that G. destructans commonly colonizes the skin of bats of Europe, where no unusual bat mortality events have been reported(8-10), has generated further

speculation that the fungus is an opportunistic pathogen and that other unidentified factors are the primary cause of WNS11,12. Here we demonstrate that exposure of healthy little brown bats (Myotis lucifugus) to pure cultures of G. destructans causes WNS. Live G. destructans was subsequently cultured from diseased bats, successfully fulfilling established criteria for the determination of G. destructans as a primary pathogen(13). We also confirmed that WNS can be transmitted from infected bats to healthy bats through direct contact. Our results provide the first direct evidence that G. destructans is the causal agent of WNS and that the recent emergence of WNS in North America may represent translocation of the fungus to a region with a naive population of animals(8). Demonstration of causality is an instrumental step in elucidating the pathogenesis(14) and epidemiology(15) of WNS and in guiding management actions to preserve bat populations against the novel threat posed by this devastating infectious disease.

2995: *-.075*

From July 2010 to January 2011,scan sampling and behavioral sampling methods were used to study the behavior of Crested Ibis (Nipponia nippon) in Yangxian Crested Ibis Acclimation Park (n(YX) = 30) and Huayang Crested Ibis Acclimation Base (n(HY) =22). The acclimation conditions of the two training cages were also compared. The results indicated that the time budget of foraging behavior were significantly different in the two training populations. In autumn,the Yangxian birds showed more paddling,explore pecked,biting throw and washing food behaviors (P < 0.01),while the searching and filter pecked behaviors were significantly less than what of the Huayang birds (P < 0.01). There was no tearing food behavior in Huayang. In winter, the foraging behavior differences were similar to what in autumn, however, there was more hollow-out behavior in Yangxian birds(P < 0.05) and there was no paddling, tearing and biting-throw food behavior in Huayang birds. In Huayang training cage, the Crested Ibis frighten-flying time (96.5 +/- 84.9 s) was significantly longer than what of the birds in Yangxian cage (40.6 +/- 51.3 s), and the landing site tendency was also different. From July to mid December 2010, some of the birds in Huayang showed abnormal behavior of roosting on the ground at night. The shape of the food and the foraging site quality in the training cage were main factors caused the foraging behavior difference. The frighten-flying time and landing site tendency of the ibis maybe related to the shelter abundance and openness of the training cage. The shortage of perch in Huayang cage caused some birds' abnormal behavior of roosting on the ground.

2996: *-.018*

The translocation of snakes has been viewed as a useful tool to augment declining populations and to mitigate human-wildlife conflicts, even though released snakes often exhibit relatively high mortality. We radio-tracked 13 Amur Ratsnakes (Elaphe schrenckii) in the Woraksan National Park in South Korea from July 2008 to May 2009. Two of these snakes were residents, and 11 had been illegally captured in areas remote from the study site and were donated by the park office. During the study period, six of the translocated snakes were lost: two were killed by predators, one died of unknown causes, and the radio signals of three of the snakes were lost. In the field, the ratsnakes laid eggs in early August, moved into hibernacula in late November, and moved away from the hibernacula in mid-April. Compared to the resident snakes, five of the translocated snakes traveled approximately 1.3 times farther per week, and the home ranges of the translocated snakes were three to six times larger than those of the resident snakes. In addition, the translocated snakes were found underground more frequently than the resident snakes. The management recommendations resulting from this study will guide biologists and land use managers in making appropriate decisions regarding release sites and the use of gravid females in the translocation of this endangered ratsnake.

2997: *-.155*

Large mammals re-introduced into harsh and unpredictable environments are vulnerable to stochastic effects, particularly in times of global climate change. The Mongolian Gobi is home to several rare large ungulates such as re-introduced Przewalski's horses (Equus ferus przewalskii) and Asiatic wild asses (Equus hemionus), but also to a millennium-old seminomadic livestock herding culture. The Gobi is prone to large inter-annual environmental fluctuations, but the winter 2009/ 2010 was particularly severe. Millions of livestock died and the Przewalski's horse population in the Gobi crashed. We used spatially explicit livestock loss statistics, ranger survey data and GPS telemetry to provide insight into the effect of a catastrophic climate event on the two sympatric wild equid species and the livestock population in light of their different space use strategies. Herders in and around the Great Gobi B Strictly Protected Area lost on average 67% of their livestock. Snow depth varied locally, resulting in livestock losses following an east-west gradient. Herders had few possibilities for evasion, as competition for available winter camps was high. Przewalski's horses used three different winter ranges, two in the east and one in the west. Losses averaged 60%, but differed hugely between east and west. Space use of Przewalski's horses was extremely conservative, as groups did not attempt to venture beyond their known home ranges. Asiatic wild asses seemed to have suffered few losses by shifting their range westwards. The catastrophic winter 2009/ 2010 provided a textbook example for how vulnerable small and spatially confined populations are in an environment prone to environmental fluctuations and catastrophes. This highlights the need for disaster planning by local herders, multiple reintroduction sites with spatially dispersed populations for re-introduced Przewalski's horses, and a landscape-level approach beyond protected area boundaries to allow for migratory or nomadic movements in Asiatic wild asses.

2998: *-.016*

It has been recognized the need for studies to determine the long-term viability of populations of endangered species, such as the Tehuantepec jackrabbit (Lepus flavigularis) populations. Currently the total population is estimated at less than 1000 individuals. We developed a viable population analysis to determine the extinction risk of the Tehuantepec jackrabbit population of Santa Maria del Mar, Oaxaca. A total of 31 scenarios were modeled in order to test independent and combined effect of flooding, predation by domestic dogs and poaching; also we tested the effect of inbreeding depression and a hypothetical reintroduction program. The results show that the Tehuantepec jackrabbit population is at high risk of extinction. The population survived over the 500 years of simulation only in the model which involved the hunt, besides the base model. The effects of the three catastrophic scenarios in combination, as well as the inbreeding, increased the risk of extinction up to 100% and an average of 41.60 ± 25.88 years. Based on our results, we propose that conservation and management strategies should include the elimination of threats that affect the Tehuantepec jackrabbit, as well as improvement of habitat quality. Also, assess the relevance of a translocation program with individuals from other populations.

2999: *+.110*

Towards the end of the Middle Ages large carnivores like the lynx (Lynx lynx) and the wolf (Canis lupus) were extinct in most regions of Central Europe due to loss of habitat and direct persecution. Changing attitudes towards nature conservation led to legal protection and to several reintroduction projects of lynx in Central Europe in the second half of the 20th century (e.g. Swiss Alps, Swiss Jura Mountains, Vosges, Bohemian Forest, the Harz Mountains and Slovenia). For reintroducing lynx in Black Forest, South-West Germany, an NGO ("Luchsinitiative e.V.") was

founded in 1986. However, the idea of reintroduction also led to severe controversies between hunters and lynx advocates, mostly nature conservationists and foresters. In 1997 an application to reintroduce lynx, posed by the "Luchsinitiative", was refused by the responsible Ministery of food, rural area and consumerism in Baden-Wuerttemberg (MLR), but the controversy was still going on. In 2004, the Ministery launched the "Arbeitsgruppe Luchs" (AG Luchs), a facilitated round-table working group including all stakeholders who would be affected by a potential reintroduction. The objectives of the "AG Luchs" were to improve mutual understanding and to coordinate research and actions for conservation of lynx. In 2008, the Forest Reseach Institute of Baden-Wuerttemberg and the University of Freiburg were charged to conduct a transdiciplinary research project with the following objectives: - investigating habitat suitability, - quantifying the chances of natural immigration of lynx from Switzerland, including road kill and poaching mortalities, - assessing how the attitudes of the affected groups concerning the lynx are constituted and legitimated. Using a regression model we found that there is enough suitable habitat for about 70 resident lynx in the Black Forest. Based on a population model the probability of establishing a population by natural immigration was estimated to only a few percent. Due to the conservative dispersal behaviour of the species, combined with habitat fragmentation and poaching, lynx can not be expected to recolonize suitable habitat in the Black Forest, as it was previously expected. The social scientific analysis of causal connections for poaching of lynx by single hunters was based on an explanatory model originating from the sociology of law. This model predicts the willingness to illegal behavior due to different social variables. The model was applied on the act of poaching lynx and tested for plausibility considering the rational choice theory. Further variables could be added to the model which improved its predictive capability. On the basis of an analysis derived from scientific literature, press articles and dialogues with affected or concerned actors, such as hunters, farmers and conservationists, a complex of interacting factors could be identified that influence the willingness to poach lynx. The group of so-called 'progress variables' showed to be most important for the reduction of illegal mortality of lynx, namely the degree of normative deviance of the hunter's peer group, the density of lynx, the valuation of carnivores by the hunter, the perceived competence of the legislative authority, the. freedom of action for the hunters, the valuation of hunting by the society, the quality of group interactions between hunters and conservationists, and the ecological knowledge of both, hunters and conservationists. The results of the second socio-economic investigation showed that the conflict between the stakeholders does not primarily centre on the lynx and its behavior but rather on the interaction between the concerned stakeholder groups. This interaction influences the groups' attitudes towards the return of the lynx and creates a symbolic meaning: the presence or absence of the lynx is perceived as a symbol/sign of a group's ability to implement/enforce its interests and value orientations at the expense of others. The conflict surrounding the lynx is indeed part of a greater group conflict about the definition of societal values. Inherent in this situation is the perception of actors that their specific group characteristics and values are threatened by the values of other groups who strive to impose these upon others. In the case of the affected hunters and farmers this leads to a psychological response towards the interests and actions of the lynx-advocates as well as towards in-group bias and out-group discrimination. During the investigation the members of the "AG Luchs" were kept up to date and took part in discussing the results. As a consequence of the research project the "AG Luchs" launched the so called "Transfer- and Communication Project handling large carnivores in Baden-Wuerttemberg". Objectives of this project are - informing the stakeholders about the biology of large carnivores and their impact on hunting and farming - rearranging the identified conflict-dynamics between the stakeholders using interactive mediation. Modules of the project are the development and the support of an interactive internet forum (www.forum-grossraubtierebw.de) as well as improving the mediation skills of people involved in the monitoring of large carnivores. The centrepiece of the project is the installation of so called "Lokale Foren Grossraubtiere" in four chosen regions: Round tables with up to 15 participants of

the different stakeholder-groups will be informed about the background of the conflict dynamics. Based on these new insights, and with the assistance of facilitators, the participants are then encouraged to develop strategic plans to handle potential scenarios like livestock depredation. By doing this in a proper facilitated way the mutual confidence of the participants is expected to increase and the conflicts to be mitigated.

3000: *+.053*

Forty male bridled nailtail wallabies Onychogalea fraenata were translocated from an on-site captive breeding compound to two release areas beyond the 8000 ha conservation fences at Scotia Sanctuary (far western New South Wales) in late July 2010. We tested the hypothesis that site fidelity (facilitated by spreading soil laden with female bridled nailtail wallaby odour at the release site) would increase survivorship by restricting animals to Scotia where intensive pest animal control has occurred. Two groups of fifteen animals were fitted with radio collars and released at the two areas (odour-added and odour-free) and monitored intensively for three months. Seven of the bridled nailtail wallabies survived this period, 19 died and four remain unaccounted for. Of the 19 that died, three were killed by introduced red foxes Vulpes vulpes, two by wedge-tailed eagles Aquila audax and one by a dingo/dog Canis lupus dingo. Two bridled nailtail wallabies died from pneumonia. The causes of death for the remaining 11 individuals are unknown. Following their release, 13 bridled nailtail wallabies remained on Scotia whilst the other 13 left the sanctuary (excluding the four that were censored). Those individuals that stayed on Scotia had much higher survival (46%) than the dispersers (8%). This result demonstrates the importance of encouraging the released animals to remain within the area that is subject to intensive predator control. The bridled nailtail wallabies were released at two sites: in an attempt to encourage site-philopatry we added soil laden with bridled nailtail wallaby urine and faeces at one of these sites. Males released here tended to travel less far, and had higher survival, than the males released at the 'odour-free' site. We believe the wandering males were searching for mating opportunities. Philopatry may be encouraged and survival increased if females are released with males in future phases of the project. We note that the bridled nailtail wallaby population in Scotia's 8000 ha feral free area, and also in Scotia's captive breeding colony, continued to increase during the initial three months of the translocation.

3001: *-.024*

In Australia, populations of mammals within the critical weight range (CWR) of 35 to 5500 g have been severely affected by European settlement, with twenty-two species having become extinct over the past 200 years. Many highly threatened CWR mammals, such as bilbies, bandicoots and numbats, are insectivorous or omnivorous, and invertebrates comprise a significant portion of their diet. Additionally, such mammals cause significant disturbance to arthropod habitats through burrowing and engage in a range of other interactions with arthropods, including mutualisms, parasitism and competition. The loss of this trophic level is thus likely to have had considerable impacts on arthropods. Here, I consider the potential effects of the dramatic decline of native omnivores on the abundance, diversity, composition, morphology and functional roles performed by arthropods. I also discuss reintroductions such as that at Scotia Sanctuary in western NSW and other conservation sites as a model for understanding the pre-European state of arthropod-CWR mammal interactions.

3002: *-.034*

Australian native mammal species within the 35 g-5500 g critical weight range (CWR) have been

declining rapidly over the last two centuries, with eighteen species becoming extinct. Inhabitants of arid and semi-arid zones are among those most at risk of extinction. Mammal declines threaten the efficiency of invertebrate-driven ecosystem processes such as nutrient recycling by artificially increasing the realised niche overlap for dung resources used by invertebrates involved with dung decomposition. Native dung beetles are one of the main taxa involved in dung decomposition, an ecosystem function necessary for nutrient recycling. Many native dung beetle species strongly prefer marsupial dung, due to their co-evolutionary history. Threatened populations of CWR species can be protected through species reintroductions. However, the long term absence of mainland CWR mammals may have compromised the effectiveness of dung decomposition as an ecosystem function by reducing dung availability. The compatibility of current coprophage assemblages with 'novel' inputs from reintroduced CWR species should therefore be questioned. Assessing the potential for persistence and/or relocation of coprophages in mainland habitats associated with CWR species will be an important part of restoring and monitoring habitats used for species recovery.

3003: *+.129*

In June 2011, Prague Zoo organised the first independent transport of Przewalski's horses to Mongolia, and took 4 horses to the Khomiin Tal reserve in the west of the country. The main target of the project'Return of the Wild Horses'was to enhance the genetic diversity and increase the populations already existing in that locality (Bobek et al, 2011). Adaptation of the new arrivals continues successfully and in 2012 even first offspring were born (Us, pers. comm). Not long after the first successful transport we started thinking about carrying on with the project in the following year. The second transport was managed in mid July 2012, but contrary to 2011 the final destination was a strictly protected area (SPA) of Gobi B.This piece of land, which under Mongolian laws has a higher degree of protection than a national park, is located in the south-west corner of Mongolia, very close to the Chinese border. From the point of life of the Przewalski's horse, it is a vital territory. The area of Gobi B was the last known refuge of the species. It was just there the very last Przewalski's horses in the wild were spotted by the end of the 1960's (Ryder 1993). When the populations of the Przewalski's horse in captivity started reaching reasonable levels, first ideas were expressed about the possibilities of reintroducing the horse into the wild (Kus, 2000). The Gobi B area was selected as a site for repatriating Przewalski's horses by the German foundation of Christian Oswald Stiftung (Oswald, 1995). By the late 1990's projects leadership and management was overhauled by International Takhi Group (ITG) established in 1999 as a nongovernmental organisation in Switzerland and Mongolia to continue and extend the project. New research and scientific data started firmly integrated into the decision making process in Przewalski's horses repatriation efforts in Gobi B area.(Pfisterer et all, 2012)

3004: *+.251*

Unprecedented rates of extinction have lead to the development of fields, such as conservation genetics, in an attempt to better understand biodiversity and consequently devise conservation programs to maintain the genetic integrity of species. We discuss the utility and application of conservation genetics, using examples from Australian freshwater crayfish, with a view to applying this methodology to the sole native crayfish species found in Japan, Cambaroides japonicus (De Haan). This species is threatened by the non-indigenous crayfish Pacifastacus leniusculus (Dana) through displacement, competition pressures, and susceptibility to Aphanomyces astaci Schikora (the crayfish plague, carried by the invasive species). Examining the genetic diversity within C. japonicus could allow populations of high conservation priority to be identified (i.e., genetically distinct populations) or, in contrast, show that this species is genetically

homogenous. Such genetic continuity may suggest that other conservation methods (e.g., translocations and restocking populations) may be suitable for this species of crayfish. Identifying genetic variability, or lack thereof, is a key step in dictating the future direction of any conservation measures for C. japonicus.

3005: *+.005*

We present the first population estimate for the little known and critically endangered Malherbe's parakeet Cyanoramphus malherbi inhabiting Maud Island, New Zealand. From March 2007 to May 2009 we conducted surveys for the species at this site to document the status of this translocated population and to determine the relative value of Maud Island for the conservation of this species. Using a modified version of the mark-resighting method, we estimated that the Maud Island population of Malherbe's parakeets has gone from an initial founder group of 11 captive-bred parakeets released on site, to a maximum of 97 during our survey period (assuming a 72% survival rate between trimesters). Out of a total of 221 sightings, 22% corresponded to un-marked individuals hatched on site. Our estimate of population size, coupled with the high reproductive potential of the species, suggests that translocation of captive-bred individuals to sanctuaries free of invasive predators is an effective management method for increasing the global population size of the species and eventually downgrade its IUCN threat category.

3006: *+.221*

The in situ management of the critically endangered grand skink Oligosoma grande currently hinges on the on-going health of a single large sub-population at Macraes Flat, Otago. Given its vulnerability, it was considered desirable to establish additional sub-populations to ensure the long-term survival of the species. A spatial meta-population simulation of grand skinks at Macraes Flat suggested that this could be facilitated by the translocation of grand skinks into areas of predator protected habitat. Areas identified by modelling as suitable translocation sites were ground-truthed by an experienced survey team in 2008. In October 2009 we began a translocation trial. We moved nineteen grand skinks from three locations to the translocation site. The founder population was made up of ten juveniles and nine sexually mature grand skinks. Seasonal estimation of persistence and abundance using a photographic re-sight methodology allowed the short- and medium-term performance of the translocation to be assessed. High initial persistence rates suggested immediate homing was not a factor of concern. After one year, all translocated juveniles had persisted, but only four of the original nine adults remained at the release site. While the loss of adults was to some extent offset by the birth of the young-of-the-year (total skinks start: n = 19, finish: n = 20) there was a moderate loss of [approximately] 10% in terms of the population's expected reproductive value. Overall, we viewed the outcome as favourable and on that basis undertook a follow up translocation.

3008: *+.002*

Although the rough-scaled python Morelia carinata of north western Australia is one of the most morphologically distinctive python species worldwide, it remains virtually unstudied. Our fieldtrips have increased the number of animals seen in the wild from 2 to 12, and the 5 animals brought into captivity produced 7 clutches totalling 71 viable offspring over a period of 6 years. In this paper we provide quantitative information on reproduction and growth of these captive animals, as well as qualitative information about behaviour and dietary habits. Reflecting their close phylogenetic relationship with green tree pythons Morelia viridis from northeastern Australia, captive rough-scaled pythons are sedentary animals that spend most of their time tightly

coiled in arboreal or saxicolous ambush sites. Females produce relatively small clutches of 10 to 14 eggs, which hatch as large, slender-bodied offspring averaging 406 mm snout-vent length (SVL) and 16.9 g in weight. Growth is rapid, with captive males attaining sexual maturity at around 1000 mm SVL and approximately 18 months of age; females mature at 1400 mm and 30 months. Many hatchlings were reluctant to accept mammalian prey unless anuran or avian scent had been added beforehand, suggesting that frogs may be an important dietary component of juvenile snakes in the wild. In turn, frog-eating may render these snakes vulnerable to the imminent invasion of cane toads Rhinella marina into the northwestern Kimberley. Continued maintenance of captive stocks can provide for potential future reintroduction to the wild if toads (or some other threatening process) imperil existing wild populations of rough-scaled pythons.

3009: *+.073*

The Saker Falcon (Falco cherrug) is a rare species with a very controversial present breeding status in Bulgaria. In 2009 a team of Bulgarian and foreign organizations came up with a feasibility study, discussing the need for reintroducing Saker Falcons and the means of completing the task in the country. That study formulated a number of criteria for Saker Falcon site suitability and identified the area of the Central Balkan Mountains as most suitable to support and maintain a reintroduced population of the species. This article presents a set of preparatory pilot measures aiming at guaranteeing optimal conditions for the Saker Falcon reintroduction in terms of food supply and electrocution risk mitigation. A set of activities were carried out to support a colony of European Susliks (an important prey for Saker Falcons) through mowing and clearing tall grass and shrub vegetation, preparing temporary holes for the animal. The management measures did not lead to a visible effect on the Suslik population during the first project year. Habitat management and the monitoring should continue 2-3 more years to see if these measures would increase Suslik numbers and enlarge their occupied area. In addition to that, a section of 20 kV electricity distribution network in the proposed reintroduction area was studied in order to assess the potential electrocution risk for the birds. The study located a total of 488 pylons of 6 different types and evaluated their potential threat. As a result a region with a relatively low electrocution risk was identified to assist the selection of initial Saker Falcon releases by hacking.

3010: *+.128*

This brief report aims at describing the sightings of 6 individuals of Rock ptarmigan (Lagopus muta helvetica), which took place on Mount Altissimo in Nago (Baldo mountain range) and in surrounding areas between January and March 2011. This species underwent local extinction in the period 1972-1975 and was afterwards addressed with a reintroduction attempt in 2002- 2003. This report provides further information concerning wandering individuals [long dash] quite rarely [long dash] spotted on Mount Baldo during autumn-winter time between 1988-2010, as well as it relates recent sightings on Alpine foothills in Western Trentino. Lastly, prospects of potential Rock ptarmigan recolonization are shortly discussed.

3012: *+.121*

The reintroduction of trumpeter swans to the north central United States appears to be a conservation success story. For the most part, population management goals have been met or exceeded. The population cannot be considered self-sustaining, however, because 90% of the swans migrate short distances to wintering sites where supplemental feeding occurs. The remaining 10% migrate longer distances to areas where adequate open water and forage occur naturally. To determine how these 2 different wintering habits might affect mortality, we used

mark-resight data gathered between 2000 and 2008 to estimate and compare annual survival rates for long- and short-distance migrant swans marked in Wisconsin. Apparent annual survival rates were similar for long- (0.81, SE = 0.019) and short- (0.81, SE = 0.022) distant adult migrants but were higher for long-distance sub-adult (0.86, SE = 0.036) migrants than for short-distance sub-adult migrants (0.7, SE = 0.046). We also estimated seasonal survival of long-distance migrants to determine if the migratory periods are a time of high mortality. We found little evidence for seasonal variation in survival and estimates for both migratory and non-migratory seasons were very high (>0.97). Overall, the results suggest that little mortality occurs during migration and long-distance migrants are able to survive at rates at least equal to, but probably higher than, short-distance migrants. (C) 2011 The Wildlife Society.

3013: *+.110*

We carried out an experimental study to determine the serological response against myxoma virus (MV) and rabbit hemorrhagic disease virus (RHDV) in wild rabbits using commercial vaccines. Seroconversion against MV ranged between 72.7% and 97.2% in animals vaccinated by subcutaneous and intradermal route, respectively, whereas between 75.0% and 77.8% of the animals presented antibodies against RHDV after inoculation with subcutaneous and intradermal vaccines, respectively. Regardless of the inoculation route, vaccination against MV resulted in a significant increase of seropositivity 5 days post-vaccination (dpv), which did not occur in animals vaccinated against RHDV. Furthermore, seroconversion against MV was significantly higher and faster in intradermally vaccinated rabbits as compared to those inoculated subcutaneously due to either the route of application and/or the type of vaccine used. The results indicated that vaccination significantly increased the prevalence of antibodies against MV and RHDV and suggested that the vaccines currently available induce a safe and effective immune response against both diseases in wild rabbits. Vaccination may be a useful management tool to control both viral diseases in field conditions, particularly in wild rabbits captured for translocations and restocking purposes in which a large number of animals are handled. (C) 2011 The Wildlife Society.

3014: *+.091*

Translocating prairie dogs from areas in or near human developments to wildlands can reduce conflicts with humans or supplement wild populations, but translocation methods differ in cost and fate of translocated individuals is often difficult to assess. We translocated 74 Gunnison's prairie dogs from 1 source colony in downtown Flagstaff, Arizona (urban) and 75 from 1 source colony in lower density housing outside the city (suburban) to 2 abandoned, recipient colonies on open grasslands 50 km north of the city (wildland). We released animals into uncaged, pre-existing burrow entrances (hard release) or into temporary cages over pre-existing burrow entrances (soft release). We captured 15 (10%) marked animals 1 year post-translocation at the 2 recipient colonies, 7 from soft release treatments and 8 from hard release treatments but visual surveys indicated a minimum of 57 adult prairie dogs and 76 pups present. Adult prairie dogs in all photographs taken by automated cameras placed at burrow entrances at each recipient colony had ear tags, suggesting that most animals at these colonies were survivors from translocation and that survival was likely higher than 10%. By 1 year post-release, recipient colonies occupied an area roughly 918 times that of source colonies. Urban Gunnison's prairie dogs can be successfully translocated to abandoned wildland colonies without using soft release methods, but animals may disperse widely. Given the cost and effort translocation requires, and the fact that all 6 confirmed mortalities were from human shooting, we recommend temporary restrictions on shooting at recipient colonies until populations have met management goals. (C) 2011 The Wildlife Society.

3015: *+.165*

Within the past several decades, seabird populations have been actively restored in locales where they were reduced or extirpated. Chick translocation, acoustic vocalization playbacks, and decoys are now used widely to lure breeding seabirds to restoration sites. In this first worldwide review of seabird restoration projects we evaluate the factors affecting project success or failure and recommend future directions for management. We identified 128 active restoration projects that were implemented to protect 47 seabird species in 100 locales spanning 14 countries since active restoration methods were pioneered in 1973. Active seabird restoration can achieve conservation goals for threatened and endangered species, and for species affected by anthropogenic impacts (e.g., oil spills, invasive species, fisheries). It also can be used to relocate populations from undesired breeding locales to more favorable locations, and to establish multiple breeding locations to reduce risks posed by catastrophic events. Active restoration can help to restore ecological processes, as large seabird colonies function to cycle marine nutrients to terrestrial ecosystems and create habitats for commensal species. Active restoration is especially appropriate where the original causes of decline are no longer working to suppress colony establishment and growth. Successful restoration efforts require careful planning and long-term commitments. We introduce the different forms of active seabird restoration techniques, review their utility for different seabird species, and use case studies to suggest how to optimize this technique to restore seabird species globally. Wildlife managers can use this review to guide their seabird restoration projects in the planning, implementation, and monitoring stages; tailor their restoration to seabird-specific life histories; and identify areas for further research to improve restoration utility in the future. (C) 2011 The Wildlife Society.

3016: *-.016*

We document causes of death in free-ranging California Condors (Gymnogyps californianus) from the inception of the reintroduction program in 1992 through December 2009 to identify current and historic mortality factors that might interfere with establishment of self-sustaining populations in the wild. A total of 135 deaths occurred from October 1992 (the first post-release death) through December 2009, from a maximum population-at-risk of 352 birds, for a cumulative crude mortality rate of 38%. A definitive cause of death was determined for 76 of the 98 submitted cases, 70% (53/76) of which were attributed to anthropogenic causes. Trash ingestion was the most important mortality factor in nestlings (proportional mortality rate [PMR] 73%; 8/11.), while lead toxicosis was the most important factor in juveniles (PMR 26%; 13/50) and adults (PM R 67%; 10/15). These results demonstrate that the leading causes of death at all California Condor release sites are anthropogenic. The mortality factors thought to be important in the decline of the historic California Condor population, particularly lead poisoning, remain the most important documented mortality factors today. Without effective mitigation, these factors can be expected to have the same effects on the sustainability of the wild populations as they have in the past.

3017: *+.213*

Conservation strategy for maintaining and protecting tuatara (Sphenodon punctatus), a rare endemic reptile from New Zealand, includes the reinstatement of populations through the past historical range. A proposal exists to translocate tuatara from Stephens Island in Cook Strait to the Orokonui Ecosanctuary (Te Korowai o Mihiwaka), a coastal site in southern New Zealand. The proposed site is within the former latitudinal range of the genus, but lies outside the current distribution of tuatara, where the climate is warmer. In this study, we examined whether cool incubation temperature is a limiting factor for the proposed reintroduction of tuatara to Orokonui.

The tuatara is a species with temperature-dependent sex determination, with only females being produced at low incubation temperatures. Thus, cool southern temperatures may produce only females, even if incubation temperatures are high enough to support successful development. We experimentally translocated tuatara eggs to the ecosanctuary and found that nest temperatures were consistently below those of nests in their current natural distribution and would produce only female hatchlings if successful incubation occurred. An addition of sand to soil did not raise temperatures sufficiently to produce both sexes. However, additional assessments of soil temperatures in a third year indicated that some new sites were warm enough for males to be produced. Given that other aspects of site suitability appear favourable, and that global temperatures are predicted to rise in the near future, which should produce a more viable incubation environment, translocation of this long-lived reptile to the southern ecosanctuary is worth further exploration. However, monitoring of female nesting behaviour, including nest locations, depths and resulting temperatures, will be essential. Our study demonstrates an experimental approach for assessing site suitability for translocation that may be relevant to other egg-laying reptiles.

3018: ***+.143***

The Black-footed Rock-wallaby (Petrogale lateralis MacDonnell Ranges Race), or warru, as it is known by Anangu, the traditional owners of the region, formerly inhabited the rocky hills of the Anangu Pitjantjatjara Yankunytjatjara (APY) Lands in northwest South Australia. However, introduced carnivores and inappropriate fire regimes have decimated the population, and there are now only 150200 animals remaining in the wild. This prompted the formation of the Warru Recovery Team (WRT), a collaboration between Traditional Owners, Anangu communities and scientists, who are working together to recover warru populations across the APY Lands. The team are working on the Warru Reintroduction Project, which is combining modern science and the traditional ecological knowledge of Anangu to reintroduce warru back into the APY Lands. Between 2007 and 2009, 22 iti-warru (warru-joeys) were taken to Monarto Zoo (Monarto, South Australia) to initiate the captive population. These zoo-warru have successfully bred in captivity, and in 2011, six founder animals and five captive bred warru were returned to the APY Lands. They are being held in a 97-ha predator-proof warru enclosure that will allow zoo-warru to adjust to the local environment and to learn the survival skills of their ancestors, prior to being released into the wild. Lessons learnt from the release of warru into warru pintji will inform future release situations, as well as management of the in situ warru population, which remains the priority of the WRT.

3019: ***+.017***

The European ground squirrel (Spermophilus citellus) is endangered and in decline. Populations are increasingly fragmented, and only a coordinated conservation effort at the European level may guarantee its long-term survival. To obtain a general population genetic picture on a larger geographic scale, we screened 117 individuals from seven local populations in Hungary, Romania, and Austria for allelic variation at eleven microsatellite loci. We found a high (23.4%) proportion of private alleles, and a moderate to somewhat elevated level (15.27%) of partitioning of genetic diversity among populations, compared to that found in many other terrestrial mammals. Genetic variability was significantly higher than in earlier studied Czech populations that are considered genetically depleted, but significantly lower than in undisturbed populations of S. suslicus and S. brunneus, that are similar to the European ground squirrel in their ecological requirements, reproductive biology, and social organization. Genetic diversity was also lower than in most presumably "undisturbed" populations of other Sciurid species. This, together with the observed

level and pattern of genetic differentiation among populations, such as no significant increase of genetic differentiation with geographic distance and similar variance of genetic differentiation between populations independent of geographic distance, indicated the prevalence of relatively strong drift effects for all populations. A Bayesian STRUCTURE analysis and a factorial correspondence analysis concordantly revealed a fairly complex genetic composition of local populations, but no major geographic trend in the pattern of the genetic composition. Overall, the results suggest disintegration of local colonies that might earlier have been more connected genetically. The STRUCTURE analysis also suggested anthropogenic translocations among single Hungarian populations. Our data on genetic diversity and its distribution do not object to such conservation measures. Translocation of individuals particularly from nearby populations may increase the chances of survival of small and isolated populations and counteract inbreeding at low densities. (C) 2011 Deutsche Gesellschaft fur Saugetierkunde. Published by Elsevier GmbH. All rights reserved.

3020: ***+.182***

The choice of suitable sites for establishment of a new population of a species is the first step in a translocation programme. However, evaluation of a large number of sites can be demanding of time and money; practitioners need time- and cost-effective evaluation tools. A predictive model for the preliminary evaluation of potential reintroduction sites was developed using the endangered quillwort Isoetes malinverniana as a case study. The reliability of three water habitat variables (pH, conductivity, and clarity) as predictors of the presence/absence of I. malinverniana was tested. Three sets of logistic models were produced on the basis of the mean values of pH and conductivity measured over 3, 4 and 6months to understand whether frequency of measurement affected the reliability of the models. Models of each set were ranked according to AIC and their reliability was then tested using data from the literature. The conductivity of water and pH were the most effective predictors of the suitability of sites for the reintroduction of I. malinverniana. In particular there was a negative relationship between the presence of I. malinverniana and these variables. Because of seasonal fluctuations in the variables considered, at least 4months monitoring are required to obtain reliable results. Despite this, the method is still advantageous in terms of costs, compared with repeated measurements of a wide range of chemical characteristics at a large number of sites. The main goal of this method is to limit expensive chemical analysis to a few sites chosen after the exclusion of unsuitable sites, through application of the model. Considering the similar ecophysiological features of quillworts from oligotrophic waters, the same model or conceptual framework could be applied to other quillworts and isoetid species growing in degraded areas and needing active conservation. Copyright (C) 2012 John Wiley & Sons, Ltd.

3021: ***-.165***

Context. The reintroduction of dingoes into sheep-grazing areas south-east of the dingo barrier fence has been suggested as a mechanism to suppress fox and feral-cat impacts. Using the Western Division of New South Wales as a case study, Dickman et al. (2009) recently assessed the risk of fox and cat predation to extant threatened species and concluded that reintroducing dingoes into the area would have positive effects for most of the threatened vertebrates there, aiding their recovery through trophic cascade effects. However, they did not formally assess the risk of dingo predation to the same threatened species.Aims. To assess the risk of dingo predation to the extant and locally extinct threatened vertebrates of western New South Wales using methods amenable to comparison with Dickman et al. (2009).Methods. The predation-risk assessment method used in Dickman et al. (2009) for foxes and cats was applied here to dingoes, with minor modification to accommodate the dietary differences of dingoes. This method is based on six independent

biological attributes, primarily reflective of potential vulnerability characteristics of the prey. Individual-attribute scores were used to derive an overall risk score. Key results. Up to 75 (94%) of the 80 extant species were predicted to be at risk of dingo predation (71% at high risk) regardless of any effect dingoes might have on foxes or cats. Upto 17 of the 21 (81%) locally extinct species were predicted to be at high risk of dingo predation using this approach. The re-establishment of even low-density dingo populations may have negative effects on at least 22% of extant threatened vertebrates.Conclusions. The generic risk-assessment method was insensitive, and experienced difficulty in describing the true nature of canid predation risk. Despite this weakness, however, it is clear that several threatened vertebrates are susceptible to dingo predation. Prior to the re-establishment of dingoes, we recommend that dingo predation risks to all vertebrates (threatened or otherwise) be assessed using more sensitive and descriptive techniques, and we strongly caution against the positive management of dingoes under current ecological conditions.Implications. The results of this study imply that dingoes present similar levels of direct risk to threatened species as foxes and feral cats, and dingo predation of threatened species should be formally considered in any proposal encouraging dingo populations in western New South Wales.

3022: *-.048*

In 2008 Przewalski's gazelle Procapra przewalskii, endemic to the Qinghai-Tibetan Plateau, China, was recategorized from Critically Endangered to Endangered on the IUCN Red List. The species is still, however, threatened, and the human population and accompanying domestic livestock are increasing around Qinghai Lake. Here we provide up-to-date information on the distribution and population size of the species, evaluate its current conservation status and discuss the difficulties of protecting this gazelle species. We used both distance sampling and total counts to survey 16 sites where the species has been reported and found it at 13 of these, occupying a total area of c. 250 km(2). Population size estimated from distance sampling (1,635) and total counts (1,544) was similar. About 20% of the gazelles located were in newly discovered areas for the species. The results indicate an overall growth in the population of Przewalski's gazelle since 2003, although some subpopulations have declined or been extirpated. In spite of conservation efforts Przewalski's gazelle is still threatened by habitat degradation and loss, habitat fragmentation, fencing, intensified competition with domestic livestock and predation. Further growth of this gazelle population is constrained by limited habitat availability and human-gazelle conflict. We recommend that management and conservation strategies need to incorporate comprehensive knowledge of the gazelle, long-term monitoring, and captive breeding of injured and orphaned gazelles to form a potential pool of individuals for future reintroductions to the historical range of the species outside the Qinghai Lake basin.

3023: *+.187*

The great bustard Otis tarda became extinct in the UK during the 19th century due to a combination of factors, including hunting, egg collection and changes in agriculture. In 2003 a 10-year licence was granted to begin a trial to reintroduce the species back to the UK. Here we report on the first 5 years of the trial and assess the progress made towards establishing a founder population. From April 2004 to September 2009 a total of 102 great bustard chicks were imported from Russia and 86 released on Salisbury Plain. Monitoring showed that post-release survival was 18% in the first year following release, and that mortality of released bustards was mainly attributable to predation and collisions. Estimated adult survival was 74%, although the sample size was small. All known surviving great bustards are faithful to the surroundings of the release site, returning throughout the year. A lek has been established where males have been observed

displaying to females. The first nesting attempt was in 2007, and in 2009 two females aged 3 and 4 years successfully nested, fledging one chick each. Models incorporating the new demographic estimates suggest that at the end of the 10-year trial period the project can expect to have 8-26 adults as a founder population.

3024: *+.132*

In order to re-establish a population of Mountain Gazelles (Gazella gazello(Pallas, 1776)) in the Tuwayq Mountains, central Saudi Arabia, a re-introduction program was initiated in 1990, with 11 releases between 1990 and 2007. The number of gazelles in the Ibex Reserve initially increased during the 5 years after the First release but then diminished dramatically. After about 10 years the decrease levelled out and the population remained more or less stable at about 0.9-1.9 gazelles / km(2) (60-80 gazelles) in the two wadi systems into which gazelles were released. This population is likely to remain small due to limited food as a result of droughts and also to competition with livestock, both of which apparently lead to increased dispersal to sites outside the Reserve. This, in turn, results in increasing conflict with humans. Food and shelter availability and the effects on home range size, dispersal behaviour, and population density of the already existent population on further repatriation success, and population establishment are reviewed in more detail. Further releases of Mountain Gazelles into the Ibex Reserve need to be carefully considered. Improved protection of the naturally occurring Mountain Gazelle population is imperative for the survival of this species in Saudi Arabia.

3025: *+.102*

The "translocation of species into habitable locations outside of their current ranges, termed assisted migration, has been proposed as a means of saving vulnerable species from extinction as a result of climate change. We explore the use of this controversial technique using a threatened keystone species in western North America, whitebark pine (Pinus albicaulis), as a case study. Species distribution models predict that whitebark pine will be extirpated from most of its current range as temperatures rise over the next 70 years. However, the same models indicate that a large area within northwestern British Columbia, Canada, is climatically suitable for the species under current conditions and will remain so throughout the 21st century. To test the capacity of whitebark pine to establish relative to Climatic and habitat features within its predicted climatic range, we planted seeds from seven populations in eight locations spanning from 600 km southeast to 800 km northwest of the northern boundary of the current species range. During the first three growing seasons, germination occurred in all locations. Nearly three times as many treated (induced maturation and broken dormancy) than untreated seeds germinated, and most treated seeds germinated a year earlier than the untreated seeds. Germination, survival, and growth were primarily influenced by seed mass, site climate conditions related to the duration of snow cover, and provenance temperature. Our experiment provides a preliminary test of models predicting the existence of climatically suitable whitebark pine habitat north of the current species ranges. More broadly, our techniques and results inform the development of scientific guidelines for assisting the migration of other species that are highly threatened by climate change. Applied case studies of this kind are critical for assessing the utility of species distribution models as conservation planning tools.

3026: *+.145*

The tiger population that once inhabited the Korean peninsula was initially considered a unique subspecies (Panthera tigris coreensis), distinct from the Amur tiger of the Russian Far East (P. t.

altaica). However, in the following decades, the population of P. t. coreensis was classified as P. t. altaica and hence forth the two populations have been considered the same subspecies. From an ecological point of view, the classification of the Korean tiger population as P. t. altaica is a plausible conclusion. Historically, there were no major dispersal barriers between the Korean peninsula and the habitat of Amur tigers in Far Eastern Russia and northeastern China that might prevent gene flow, especially for a large carnivore with long-distance dispersal abilities. However, there has yet to be a genetic study to confirm the subspecific status of the Korean tiger. Bone samples from four tigers originally caught in the Korean peninsula were collected from two museums in Japan and the United States. Eight mitochondrial gene fragments were sequenced and compared to previously published tiger subspecies' mtDNA sequences to assess the phylogenetic relationship of the Korean tiger. Three individuals shared an identical haplotype with the Amur tigers. One specimen grouped with Malayan tigers, perhaps due to misidentification or mislabeling of the sample. Our results support the conclusion that the Korean tiger should be classified as P. t. altaica, which has important implications for the conservation and reintroduction of Korean tigers.

3027: *-.024*

Boreal caribou were extirpated from the Charlevoix region (Quebec) in the 1920s because of hunting and poaching. In 1965, the Quebec government initiated a caribou reintroduction program in Charlevoix. During the winters of 1966 and 1967, a total of 48 boreal caribou were captured, translocated by plane, and released within enclosures; only their offspring (82 individuals) were released in the wild. Between 1967 and 1980, a wolf control program was applied to support caribou population growth. The caribou population, however, remained relatively stable at 45-55 individuals during this period. During the 1980s, the population grew slowly at a rate of approximately 5% each year to reach a peak of 126 individuals in 1992. At that time, Bergerud & Mercer (1989) reported that the Charlevoix experiment was the only successful attempt at caribou reintroduction in the presence of predators (in North America). Afterwards, the population declined and since then it has been relatively stable at about 80 individuals. Here we reviewed the literature regarding the ecology and population dynamics of the Charlevoix caribou herd since its reintroduction, in an attempt to critically assess the value of reintroduction as a conservation tool for this species. Indeed, the Charlevoix caribou herd is now considered at very high risk of extinction mostly because of its small size, its isolation from other caribou populations, and low recruitment. The Charlevoix region has been heavily impacted by forestry activities since the early 1980s. Recent studies have indicated that these habitat modifications may have benefited populations of wolves and black bears-two predators of caribou-and that caribou range fidelity may have exposed caribou to higher predation risk via maladaptive habitat selection. As females are ageing, and females and calves suffer high predation pressure from wolves and bears respectively, we suggest that the future of this reintroduced herd is in question and that they are facing a high probability of extinction in the near future if further action is not taken.

3031: *+.140*

The 1995/1996 reintroduction of gray wolves (Canis lupus) into Yellowstone National Park after a 70 year absence has allowed for studies of tri-trophic cascades involving wolves, elk (Cervus elaphus), and plant species such as aspen (Populus tremuloides), cottonwoods (Populus spp.), and willows (Salix spp.). To investigate the status of this cascade, in September of 2010 we repeated an earlier survey of aspen and measured browsing and heights of young aspen in 97 stands along four streams in the Lamar River catchment of the park's northern winter range. We found that browsing on the five tallest young aspen in each stand decreased from 100% of all measured leaders in 1998 to means of <25% in the uplands and <20% in riparian areas by 2010.

Correspondingly, aspen recruitment (i.e., growth of seedlings/sprouts above the browse level of ungulates) increased as browsing decreased over time in these same stands. We repeated earlier inventories of cottonwoods and found that recruitment had also increased in recent years. We also synthesized studies on trophic cascades published during the first 15 years after wolf reintroduction. Synthesis results generally indicate that the reintroduction of wolves restored a trophic cascade with woody browse species growing taller and canopy cover increasing in some, but not all places. After wolf reintroduction, elk populations decreased, but both beaver (Caster canadensis) and bison (Bison bison) numbers increased, possibly due to the increase in available woody plants and herbaceous forage resulting from less competition with elk. Trophic cascades research during the first 15 years after wolf reintroduction indicated substantial initial effects on both plants and animals, but northern Yellowstone still appears to be in the early stages of ecosystem recovery. In ecosystems where wolves have been displaced or locally extirpated, their reintroduction may represent a particularly effective approach for passive restoration. (C) 2011 Elsevier Ltd. All rights reserved.

3032: *-.138*

The massive destruction and deterioration of the habitat of Oryx leucoryx and illegal hunting have decimated Oryx populations significantly, and now these animals are almost extinct in the wild. Molecular analyses can significantly contribute to captive breeding and reintroduction strategies for the conservation of this endangered animal. A representative 32 identical sequences used for species identification through BOLD and GenBank/NCBI showed maximum homology 96.06% with O. dammah, which is a species of Oryx from Northern Africa, the next closest species 94.33% was O. gazella, the African antelope. DNA barcode sequences of the mitochondrial cytochrome C oxidase (COI) gene were determined for O. leucoryx; identification through BOLD could only recognize the genus correctly, whereas the species could not be identified. This was due to a lack of sequence data for O. leucoryx on BOLD. Similarly, BLAST analysis of the NCBI data base also revealed no COI sequence data for the genus Oryx.

3033: *+.346*

Challenges for the conservation of threatened plants and natural habitats in French overseas territories.-The French overseas territories represent a broad range of climatic and biogeographical conditions, from sub-arctic to equatorial, resulting in a richness, diversity and uniqueness of their floras that mirrors that of the global flora. These territories range widely in area (from small oceanic islands to large continental regions) and human population densities, leading to more or less pronounced anthropogenic impacts on natural ecosystems. Threats to the French overseas flora are generally the same (habitat destruction, biological invasions, species overexploitation, climate change) but with varying importance depending on the territory. Conservation measures, including the establishment of lists of protected species or habitats and new protected areas, as well as habitat restoration, species reintroduction or population reinforcement of threatened species, also vary depending on the territory, in relation with their legal status, and the concern of local populations and authorities toward the conservation of their natural heritage. We underline the necessity and urgency to better know and conserve this flora (more than 10 000 vascular plants, including 3480 endemics, and with 685 protected species) of not only national but also regional and global importance.

3034: *+.097*

Parasites are essential and dynamic components of global biodiversity. They are drivers for genetic

diversity and play an important role in host population dynamics, in inter- and intraspecific interactions, and in wildlife conservation. At high intensities or in naive hosts, they can cause disease and have population-level impacts. Some parasites of wildlife, zoonotic parasites, are also known to infect people and have negative health and socioeconomic consequences. Parasites reflect environmental integrity and provide insight into community composition, interaction, and food web dynamics. Current host[long dash]parasite assemblages reflect the complexity of historical and contemporary processes. Shifting environmental conditions, domestic animal encroachment, and range expansions of wildlife or vectors can lead to parasite invasions with consequences for the health of endemic hosts and the biodiversity of parasites. Parasites are influenced by climate, both incremental (long-term) and more ephemeral (extreme) events. They can exploit favorable weather conditions to greatly augment their transmission potential. Interactions between climate and parasites can lead to explosive outbreaks of disease or parasite range expansions, with subsequent cascading effects on host population dynamics and ecosystems. Importantly, parasites add new dimensions to protected area planning and animal introductions and translocations. In polar and alpine regions, life history patterns of parasites are strongly adapted for transmission and dissemination in highly seasonal, extreme environments characterized by low host species diversity. Parasites are important components of biodiversity in these regions, linking hosts to the ecosystem through numerous interactions. In this chapter I use muskoxen and their parasites as an example to illustrate the importance of understanding existing parasite biodiversity, ecology, and host[long dash]parasite interactions in the Arctic and as a foundation to address modern conservation issues.

3035: ***+.158***

Different varieties of Asiatic wild ass (Equus hemionus) inhabit the Middle East. In Iran distribution of E. hemionus var onager is limited to two small and isolated populations in two protected area, Bahram-e-gour protected area and Touran protected area. In 2010 onager was re-introduced to Kalmand-Bahadoran protected area in Yazd province. We have used Vortex 9.73 software to calculate the extinction risk of the re-introduced population using four scenarios based on different supplementation. In the first scenario, initial re-introduced population was simulated without supplementation. In the second scenario, 3 female adults were added to population in the second year. In the third scenario, 3 female adults and 1 male added to population in the second year. Finally 5 female adults added to population in the second year and simulation was carried out in four different conditions. Vortex is an individual-based simulation model that uses for population viability analysis. The re-introduced population was investigated during a year (from July 2010 until July 2011) and used data of this species in Bahram-e-Goor protected area and gathered other required information from captive breeding to run the Vortex software. Across all simulations scenario 1 (Initial re-introduced population without supplementation) had population declines with the highest probability of extinction (0.83) and the greatest loss of diversity. Results indicated that the re-introduced onager population in Kalmand-Bahadoran protected area needs at least a few more transfers from the wild or captive breeding center. The analysis demonstrated that an increase in effective population will lead to an increase in genetic variation and survival rate.

3036: ***+.200***

The addax (Addax nasomaculatus) is a critically endangered antelope that is currently maintained in zoos through regional, conservation breeding programs. As for many captive species, incomplete pedigree data currently impedes the ability of addax breeding programs to confidently manage the genetics of captive populations and to select appropriate animals for reintroduction. Molecular markers are often used to improve pedigree resolution, thereby improving the long-term

effectiveness of genetic management. When developing a suite of molecular markers, it is important to consider the source of DNA, as the utility of markers may vary across DNA sources. In this study, we optimized a suite of microsatellite markers for use in genotyping captive addax blood samples collected on FTA cards. We amplified 66 microsatellite loci previously described in other Artiodactyls. Sixteen markers amplified a single product in addax, but only 5 of these were found to be polymorphic in a sample of 37 addax sampled from a captive herd at Fossil Rim Wildlife Center in the US. The suite of microsatellite markers developed in this study provides a new tool for the genetic management of captive addax, and demonstrates that FTA cards can be a useful means of sample storage, provided appropriate loci are used in downstream analyses. Zoo Biol 31:98-106, 2012. (C) 2011 Wiley Periodicals, Inc.

3037: ***+.028***

Present genetic patterns of marine organisms not only result from historical and contemporary ecological factors, but also from anthropogenic activities. Disentangling the relative effects of these factors can provide valuable insights into management and protection of exploitable species. The commercially important marine clam Tegillarca granosa is representative of species that are translocated within East Asia for coastal aquaculture purposes. We conducted a nucleotide sequence analysis of mitochondrial cytochrome c oxidase subunit I and nuclear internal transcribed spacer 1 markers in T. granosa to investigate its genetic diversity and distribution in 2 marginal seas (the East and South China Seas) of the northwestern Pacific. Based on phylogenetic inferences, we identified 2 evolutionarily significant units (ESUs) with high genetic distance between them for both markers. The high genetic distance may be associated with the historical isolation of the marginal seas during low sea level periods. One ESU was widely distributed in both seas, whereas the other was restricted to 2 disjunct localities in the South China Sea. Based on the isolation by distance analysis ($p = 0.068$) and comparison of patterns of co-occurring species, this pattern appears to be mostly attributable to the human-mediated translocations among coastal waters rather than natural range expansion. Furthermore, from a conservation viewpoint, the southern ESU is now facing high extinction risk because of mitochondrial introgression and smaller, fragmented populations; consequently, immediate proper management is required to protect the endangered populations representing this lineage.

3038: ***+.280***

Effective solutions for integrating development of agriculture and conservation of biodiversity at a landscape scale remain to be identified. This article presents a case study on an intensively farmed French cereal plain, where the reintroduction of grasslands has been proposed to protect the Little Bustard, a threatened European bird species. Although this solution may seem trivial at first glance, we analyze the design reasoning from which it resulted in order to highlight the innovative paths it opened. We apply C-K theory, a design theory that distinguishes concepts (i.e., unknown proposals) from knowledge. Our analysis reveals the links between the production of scientific knowledge and the generation of various solutions. It also highlights that specifying the ecological functions of grasslands facilitates their management. In the cereal plain, some of these functions give grasslands the status of common goods. This consideration opens new possibilities for managing agricultural landscapes in a way that reconcile agriculture and conservation.

3039: ***+.212***

In 1960, the Golden lion tamarin Leontopithecus rosalia was almost extinct in the wild and the captive population, with poor reproduction and survival, was not well established. In the 1970s,

after many improvements, the captive population began to grow and the Poco das Antas Biological Reserve was created to protect the species. In the 1980s, long-term research was begun on the demography and socio-ecology of the Golden lion tamarins, along with community environmental education and a reintroduction programme of captive-born animals (initially in the reserve, later in neighbouring private forests). About 30 zoos contributed the 146 captive-born reintroduced tamarins, and provided information on social behaviour, nutrition and health that was critical to developing reintroduction strategies. In 1994, threatened groups isolated in small fragments were rescued and translocated to a protected forest. Both programmes have been successful as measured by survival and reproduction after release, and both techniques have established growing populations. Although new threats (introduction of exotic primates) continue to challenge our efforts to preserve the species, there is no doubt of the success of almost 30 years of the Golden Lion Tamarin Conservation Programme.

3040: *+.002*

Attempts to maintain refuge populations of Devils Hole pupfish Cyprinodon diabolis in artificial tanks have achieved limited success. Previous studies have documented changes in morphological, behavioral, and genetic characteristics of refuge populations, which suggest that environmental conditions (and thus selective pressures) differ from those found in Devils Hole (DH). Physical, chemical, and biological characteristics were compared among the three Devils Hole pupfish refuges (Hoover Dam, School Springs, and Point of Rocks) and between each refuge and DH. In contrast to the thermally constant environment in DH (similar to 33 degrees C), mean refuge temperatures were cooler and fluctuated on a weekly and seasonal basis. On two occasions, extreme temperature fluctuations lasting several weeks (due to water supply malfunctions) were recorded at the Hoover Dam (6 degrees C decrease) and School Springs (22 degrees C decrease) refuges. The physical design of the refuges precludes surface runoff from entering them; thick layers of anoxic sediment accumulated, particularly in School Springs (mean depth = 19.1 cm; range = 1-30 cm) and Point of Rocks (mean depth = 8.9 cm; range = 5-39 cm), thus burying the rocky substrate designed to replicate the upper shelf in DH. All three refuges had higher mean oxygen concentrations and lower coefficients of variation (CVs; range = 12.2-16.9%) than the DH upper shelf (CV = 52.5%), which receives limited direct sunlight. Attached algal standing crops differed several-fold among refuges (range = 31.0-231.7 g of ash-free drymass/m(2)) but were not significantly different between seasons. In contrast, benthic biomass values reported from DH were smaller and varied seasonally. Aquatic invertebrate taxa that were abundant in DH were rare or absent in the refuges. These results demonstrate how artificial refuge environments deviate from natural conditions in DH and further illustrate the challenges faced when attempting to establish and maintain long-term refuge populations as a conservation strategy to preserve threatened and endangered fishes.

3041: *+.051*

ALBRECHT, M. A. AND J. C. PENAGOS Z. (Center for Conservation and Sustainable Development, Missouri Botanical Garden, PO Box 299, St. Louis, MO 63166). Seed germination ecology of three imperiled plants of rock outcrops in the southeastern United States. J. Torrey Bot. Soc. 139: 86-95.-2012. The success of rare plant conservation programs depends upon understanding the ecological factors that regulate seed dormancy and germination. In this study, we characterize the germination niche with respect to temperature and light of three imperiled perennials that are endemic to rock outcrops in the southeastern United States: Astragaltes bibullatus, Claytonia ozarkensis, and Conradina verticillata. Our results show that a majority of seeds for each species are unable to germinate at habitat temperatures prevailing after dispersal in

early summer. Seeds of the rockface endemic Claytonia ozarkensis germinated to high rates in darkness at 5 C, suggesting germination is confined to winter and that seeds are unable to persist beyond one germination season (transient seed bank). For Conradina verticillata, 44% of seeds germinated without cold stratification, while the remainder required cold stratification and light to overcome physiological dormancy. Following cold stratification, Conradina verticillata seeds germinated in light at cool (15/5 degrees C) but not at warm (30/15 degrees C) temperatures, although overall seed viability was low (26%). Seeds of Astragahts bibullatus germinated to low rates (< 10%) in seasonal temperature sequences, confirming that this species forms a persistent seed bank. Seeds of Astragalus bibullatus lost viability following heat shock for 30 min at 125 degrees C, but germinated to > 50% following mechanical scarification and incubation at 30/15 degrees C. Results from this study can be used to maximize germination for ex situ and reintroduction programs, and provide insight into managing wild populations.

3042: *-.004*

Although formerly an abundant species, the Eurasian Griffon (Gyps fulvus Hablizl, 1783) has undergone a dramatic decline in Herzegovina. Such an unfavorable trend may be associated with frequent poisoning incidents (consumption of poisoned baits), shortage of food and hunting. This species disappeared from its breeding habitats in Herzegovina during the last decade of the 20th century. The extinction was probably caused by military activities during the civil war. Using data that were collected over a period of long-term (1980-1991) monitoring of the breeding population, we discovered optimal environmental conditions for the nesting of the Eurasian Griffon Vulture in Herzegovina. Information on nest-site preference is valuable for conservation programs and the possible reintroduction of the Eurasian Griffon, not only in Herzegovina, but also to a much wider region. During the study period, we observed 61 nests and 252 nesting cases in four colonies of Eurasian Griffon Vulture. Most nests were located on limestone and dolomite rocks. The average altitude of nests was 378 m a.s.l.; most of nests (85%) were located below 500 m a.s.l. Also, the majority of nests were located on west-exposed sites.

3043: *+.087*

Invasive species are a global problem, which costs the world economy billions of dollars and world ecosystems millions of tons of herbicides, pesticides and other cides. Anthropogenic translocation of freshwater copepods associated with early shipping activities was postulated for some time, but was never tested with molecular tools. Here, we examine global molecular diversity of one cyclopoid species, test if the current cosmopolitan distribution is a result of anthropogenic translocation or natural dispersal, and investigate a possibility of cryptic speciation. We use patterns of haplotype frequency of DNA and RNA sequences of four genes (12S, 16S, 18S and cytB) and 11 populations (from England, Scotland, France, Germany, USA, New Zealand and Australia) to test inter-and intrapopulation variability, and three different methods (neighbour joining (NJ), maximum likelihood (ML) and maximum parsimony (MP)) for reconstructing their phylogenetic relationships. They were then tested against two competing hypotheses, and complemented by comparative morphology of microcharacters. Reconstructed phylogenies present strong evidence for anthropogenic translocation, with the same haplotype found in highly disjunct populations in Western Australia, Germany and the USA. Four different clades were revealed with the 12S, 16S and cytB genes, probably representing four cryptic species. Morphological examination of females of two clades contributed a set of microcharacters that can be used in the future taxonomic revision of this species complex. We prove for the first time that cuticular pores and sensilla are homologous structures. This research provides evidence for both homogenization of world freshwater fauna and our inadequate methods of identifying some of its

most common species.

3044: *-.116*

The bull trout Salvelinus confluentus is an apex predator in native fish communities in the western USA and is listed as threatened under the U. S. Endangered Species Act (ESA). Restoration of this species has raised concerns over its potential predatory impacts on native fish fauna. We held a five-person expert panel to help determine potential impacts of reintroducing bull trout into the Clackamas River, northwest Oregon, on the viability of four anadromous salmonid populations that are listed as threatened under the ESA: spring and fall Chinook salmon Oncorhynchus tshawytscha, coho salmon O. kisutch, and winter steelhead O. mykiss. The panel session was rigorously structured and used a modified Delphi process with structured expert elicitation, disclosure, discussion, and brainstorming. Each panelist distributed 100 score points among seven categories of potential bull trout impact (from no impact to very high impact) on extinction probabilities for the anadromous salmonids. Results were provided by individual panelists rather than as a group consensus and were summarized as means and variations in scores to express the panelists' individual uncertainty, variability among the panelists, and expected differences among the affected salmonids. Score results suggested that panelists viewed the potential impact of bull trout as very low or moderately low for spring Chinook salmon, coho salmon, and winter steelhead and mostly none to very low for fall Chinook salmon. Panelists also provided 19 possible monitoring activities and 21 possible management actions for assessing potential impacts and taking remedial action if bull trout are found to have unacceptable adverse effects. Results of the panel were used by the U. S. Fish and Wildlife Service to help craft and execute a plan to reintroduce bull trout into the Clackamas River system under the ESA. This rigorous expert panel process can be used for a wide range of evaluations in situations where empirical data are sparse or ecological interactions are too complex for explicit analytic solution.

3045: *+.125*

Defining areas of potential distribution for large carnivores is a critical step for generating conservation strategies. Ecological niche modelling is an important tool for identifying potential areas for conservation of carnivores, such as American black bears (Ursus americanus) in the Sierra Madre Occidental (SMOcc) and the Sky Islands (SI) region of Northwest Mexico. Our objective was to define areas and environmental factors that influence bear distribution and understand the causes of their absence. We used GARP (genetic algorithm for rule-set prediction) to define the potential area of distribution using historical and current records of black bear (n = 582) and 23 bioclimatic and physical variables. We obtained a consensus model with a high probability of occurrence and power prediction representing 80% of the SMOcc (221,078 km(2)), including the SI region (Sonora and Chihuahua deserts). The ecological dimensions of the model include temperate dry and mixed forest, low rainfall, low temperatures, and elevation above 1,500 m, with considerable slope variation. Information provided by residents of Aguascalientes, Chihuahua, Durango, and Zacatecas indicate that the species was extirpated in central and southwest Durango and Zacatecas about 50 years ago, coinciding with the use of 1080 poison (sodium fluoroacetate) to eradicate livestock predators, combined with habitat loss, fragmentation, and excessive hunting in the region. These factors precipitated the regional extirpation of the species. Areas such as those we have identified may be important sites for the reintroduction of black bears.

3046: *+.066*

The Miami blue butterfly, Cyclargus thomasi bethunebakeri, is a state-endangered taxon in Florida and a candidate for federal listing. This once common butterfly saw a dramatic decline in population number and abundance in the 1970s and 1980s, but significant collections of individuals prior to this decline are deposited in natural history museums. Using museum specimens, we quantified the genetic diversity in a historical population present in Key Largo, Florida in 1940, 1960, and 1980. Genetic diversity was consistently high within this historical population, but diversity was observed to decrease over the decades sampled. A comparison of historical diversity from the Key Largo population with the extant populations on Bahia Honda State Park (BHSP) and Key West National Wildlife Refuge (KWNWR) revealed differences in allelic frequencies, but only minor differences in the overall number of alleles. The historical distribution of butterflies throughout the Florida Keys further suggests a metapopulation structure. This structure involved partially-isolated populations of C. t. bethunebakeri that were loosely connected via gene flow and that underwent localized extinction and colonization events along the chain of suitable habitat in the Florida Keys. It appears that a "mini-metapopulation" currently exists on BHSP and KWNWR; structures that are similar to the historical metapopulation structure and distribution of populations on a larger scale. Knowledge of historical distribution helps to plan future reintroduction events with captive-bred butterflies. Additional populations of butterflies may represent undiscovered genetic diversity that, if appropriate, may be further incorporated into captive-breeding efforts. (C) Koninklijke Brill NV, Leiden, 2012.

3047: ***+.279***

Southern Africa's Cape Floristic Region (CFR) is a global priority for conservation action, with 41 native large mammal species considered in ongoing conservation schemes. This study reviews historic and paleozoological evidence suggesting that an additional species [long dash] the roan antelope (Hippotragus equinus) [long dash] is native to the region and warrants consideration in conservation efforts. A single observation in 1778 suggests that a population of roan antelope formerly inhabited the CFR in the vicinity of Plettenberg Bay (Western Cape, South Africa). The fossil record is consistent with this observation, showing that roan antelope inhabited the southern coast of the CFR for the last ~20,000 years. Roan antelope were likely extirpated from the CFR during the late 1700s, broadly corresponding to the extinction of the blue antelope (H. leucophaeus) and the near-extinction of the bontebok (Damaliscus pygargus pygargus). If the goal of conservation efforts is to establish viable populations of extant species that are native to the region, then roan antelope is a prime candidate for conservation action and reintroduction to the CFR.

3048: ***+.143***

Understanding phylogenetic diversity over large temporal scales as afforded by the fossil record allows for the identification of the history of taxonomic diversity in extant taxa. Identification of such long-term trends in lineage 'health' is a critical, but commonly underutilized method for helping to prioritize species for conservation. The modern Australian koala (Phascolarctos cinereus) is a case in point. It is widely debated whether the modern koala should be an immediate candidate for conservation. Although modern populations have seen recent declines in some regions, in other areas koalas are overabundant, with translocation, contraceptive, and evening culling programs suggested as population control measures. The view from the fossil record is that koalas (family Phascolarctidae) have suffered a dramatic, progressive long-term decline in diversity (e.g., four genera and eight species in the late Oligocene, compared to only one genus and species at present). At no time in the known history of the Phascolarctidae has phylogenetic diversity been as low as today. Climate change, leading to enhanced variability in seasonality,

increased aridification, and habitat change has had a negative impact on phascolarctid diversity through time, and has been a determining factor in the geographic range of the modern koala. Do such observations warrant adding the modern koala to the list of threatened species? Although the answer to this question remains outside the scope of this chapter, it should be remembered that extinction of the extant koala would mark the loss of not only of a single species, but also of an entire family of endemic Australian marsupial.

3049: ***+.087***

Internationally, re-introductions of endangered species into their former ranges have largely failed. Here we assess a successful reintroduction program of the endangered trout cod (Maccullochella macquariensis) and examine factors contributing to this success. Stocking of marked fish (all stocked fish were marked) occurred between 1997 and 2006 in the Ovens River, south-eastern Australia, where trout cod were historically abundant but locally extinct by the 1980s. We found no natural recruits (i.e. from spawnings of stocked fish in the wild) over the age of six, indicating that natural recruitment started at most five years after stocking began. Of the 83 fish we examined for sexual maturity, 12 were immature, 20 were male, and 51 were female. The body length at which 50% of the population can be considered mature was 325 and 250 mm for females and males, respectively. The length at which 90% of the population was mature was 394 and 318 mm for females and males, respectively. The smallest mature female was 245 mm. Average relative fertility was 9 eggs g(-1) fish weight. The results we obtained provide valuable insights into the aspects contributing to the success of reintroduction programs for endangered freshwater species.

3050: ***+.170***

Habitat destruction, invasive species, climate change and other threats to plant diversity are requiring increased conservation efforts. Priority is appropriately given to in situ conservation, but the important contribution that ex situ conservation can make is increasingly being recognised. In Finland, extensive seemingly intact natural areas have so far led authorities to consider ex situ conservation activities largely unnecessary. Botanic gardens, with estimated living collections of 80 000 plant species worldwide, cultivate valuable plant material that can be used in recovery and reintroduction programmes. Recently, gardens all over the world have started to survey their collections. The results to date have revealed some very valuable collections but also highlighted inadequate databasing, narrow genetic representation and various genetic problems. As a part of an EU Life+ funded initiative, 4 Finnish botanic garden collections were investigated in order to find nationally threatened vascular plant species of known wild origin. Accessions were assessed for their potential use in future reintroduction programmes by ranking the quality of origin data and genetic intactness. Seventy-seven accessions from 56 vascular plant target taxa were found cultivated as living plants, representing 18% of Finland's nationally threatened taxa. The findings of this Finnish survey are similar to studies completed for botanic gardens elsewhere showing that there are deficiencies in intraspecific and within-population diversity. However, the accuracy of origin data and genetic intactness of the accessions were comparatively high. This survey forms the basis of the current development of a national plant ex situ action plan.

3051: ***+.032***

Seed germination (effects of light, temperature. NaCl and KNO3) of the coastal endangered species Crucianella maritima was investigated by testing seeds from three different populations. Data were analyzed by means of Generalized Linear Mixed Model (GLMM). The principal results showed that germination of C maritima seeds was characterized by photoinhibition, absence of

primary dormancy and salt-induced secondary dormancy, with no need for high nutrient availability (KNO3). Intraspecific differences in germination pattern emerged, apparently due to a different seed mass. These results show important germination traits of C. maritima which should be taken into account in possible reintroduction attempts aimed at restoring threatened populations of this species. (C) 2011 Academie des sciences. Published by Elsevier Masson SAS. All rights reserved.

3052: *+.146*

Australian dingoes have recently been suggested as a tool to aid biodiversity conservation through the reversal or prevention of trophic cascades and mesopredator release. However, at least seven ecological and sociological considerations must be addressed before dog populations are positively managed.1. Domestication and feralisation of dingoes have resulted in behavioural changes that continue to expose a broad range of native and introduced fauna to predation.2. Dingoes and other dogs are classic mesopredators, while humans are the apex predator and primary ecosystem engineers in Australia.3. Anthropogenic landscape changes could prevent modem dingoes from fulfilling their pre-European roles.4. Dingoes are known to exploit many of the same species they are often presumed to 'protect', predisposing them to present direct risks to many threatened species.5. The assertion that contemporary dog control facilitates the release of mesopredators disregards the realities of effective dog control, which simultaneously reduces fox and dog abundance and is unlikely to enable increases in fox abundance.6. The processes affecting threatened fauna are likely a combination of both top-down and bottom-up effects, which will not be solved or reversed by concentrating efforts on managing only predator effects.7. Most importantly, human social and economic niches are highly variable across the ecosystems where dingoes are present or proposed. Human perceptions will ultimately determine acceptance of positive dingo management. Outside of an adaptive management framework, positively managing dingoes while ignoring these seven considerations is unlikely to succeed in conserving native faunal biodiversity but is likely to have negative effects on ecological, social and economic values.

3053: *+.032*

Population genetics can reveal otherwise hidden information involving a species' history in a given region. Koalas were thought to have been virtually exterminated from the Australian state of Victoria during the koala fur trade of the late 1800s. Koalas in the South Gippsland region of Victoria were examined using microsatellite markers to infer population structure and gene flow and to locate a possible remnant gene pool. The results indicate that the South Gippsland koala population had higher genetic diversity (A = 5.97, H-o = 0.564) than other published Victorian populations, and was genetically distinct from other koala populations examined. South Gippsland koalas, therefore, may have survived the population reductions of the koala fur trade and now represent a remnant Victorian gene pool that has been largely lost from the remainder of Victoria. This paper illustrates that historic anthropogenic impacts have had little effect on reducing the genetic diversity of a population in the South Gippsland region. However, the South Gippsland population is now subject to threats such as logging and loss of habitat from housing and agriculture expansion. Our results suggest that the South Gippsland koalas require an alternative conservation management program.

3054: *+.098*

Nonnative species have been shown to negatively impact the native community in which they are introduced. In the Great Lakes, competition with nonnative salmonids may be hindering the

restoration efforts of Atlantic salmon Salmo salar, a once-native top predator in Lake Ontario. We examined the effects of brown trout S. trutta and rainbow trout Oncorhynchus mykiss, two nonnative fishes in Lake Ontario, on the social dominance and growth rate of juvenile Atlantic salmon from three strains being used for reintroduction efforts in Lake Ontario. Using seminatural stream channels, we found that the presence of either rainbow trout or brown trout reduced aggression, dominance, and food consumption of the Atlantic salmon. Brown trout had the strongest effect, increasing aggression levels in the channels by a factor of two and sharply reducing the dominance of Atlantic salmon. When both nonnatives were present, initiated aggression by Atlantic salmon decreased by a factor of three and food consumption halved as compared with when the salmon were alone. Consequently, over a 7-d time period, standard growth rate of the Atlantic salmon dropped from no change in mass when alone to a value of -0.3% per day when with the nonnative species. Of the three strains tested, one strain was least affected by the nonnative trouts, implicating genetic differences among the strains and suggesting that one strain may have greater poststocking success in Lake Ontario tributaries with naturalized populations of brown trout and rainbow trout.

3055: ***+.286***

Like many fishes native to western Great Plains streams, the Arkansas darter Etheostoma cragini has declined, apparently in response to changes in flow regimes and habitat fragmentation. We investigated the effectiveness of translocation as a management strategy to conserve this threatened species in the Arkansas River basin of southeastern Colorado. We used a multiscale design to sample the darter and several attributes of its habitat at the local 10-m site scale, the 3.25-km translocation segment scale, and the 10-km riverscape scale in all 19 streams where it had been previously translocated. The darter was captured in 11 of the 19 streams, although 5 were completely dry when visited. Arkansas darters had reproduced in 10 of the 11 streams (one criterion in the state recovery plan), and 6 streams also met a second criterion for abundance (>500 individuals). However, the populations in only two streams unequivocally met a third criterion of being self-sustaining, because the other four streams had been stocked annually with hatchery-reared Arkansas darters. We used multistate occupancy estimation, based on two consecutive dipnetting surveys, to determine the habitat characteristics correlated with site occupancy and the detectability of Arkansas darters at the local site scale. The probability of detection for the darter at occupied sites was high for both age-groups, 92% for age-0 darters and 75% for age-1 darters, and was a function of day of year (age 0) and habitat depth (age 1). Cool stream temperature (a site-scale variable) and sufficient length of available habitat (a riverscape-scale variable) were the strongest predictors of site occupancy for both age-groups. The models are useful in identifying new sites for translocations and factors that may place current populations at risk. Effective conservation will require land management practices that conserve, protect, and restore contiguous stream segments with cool temperatures that can sustain Arkansas darters.

3056: ***-.035***

Two Florida state agencies (Florida Fish and Wildlife Conservation Commission [FWC] and Florida Department of Agriculture and Consumer Services) have regulatory and management programs to reduce the potential for escape or release of nonnative fishes from aquaculture facilities. The barramundi perch Lates calcarifer, a large, predatory euryhaline fish native to the Indo-Pacific, is classified as a conditional species by the FWC. Aquaculturists may possess this species only under strict biosecurity requirements. In 2006, a barramundi perch aquaculturist in central Florida initiated fee fishing by the public in outdoor culture ponds. The FWC conducted a risk analysis to assess the probability and potential consequences of barramundi perch population

establishment in Florida, specifically considering the increased risk of illegal barramundi perch introduction into state waters to create a sport fishery. Using a generic risk analysis review process, a stakeholder panel determined that the overall risk from barramundi perch aquaculture was medium or high; a subsequent assessment by FWC biologists found the overall risk to be high. Nonregulatory risk management options to reduce the chance of escape and to prevent reproduction were considered impractical or ineffective, and a regulatory solution was recommended to mitigate the risk. The FWC enacted regulations that prohibited outdoor culture and fee fishing for facilities that acquire barramundi perch in the future, while allowing the existing facility to continue operations only under current ownership. The generic risk analysis provides a useful and flexible framework for conducting risk analyses involving assessors with varied technical backgrounds. We strongly recommend face-to-face meetings to increase the opportunities to reach consensus on risk estimates and uncertainty. Providing specific factors for risk assessors to consider (e.g., translocation of barramundi perch by wildlife, likelihood of successful spawning, and impacts to sportfishing) facilitates development of focused risk management options.

3057: *+.222*

Translocation to island reserves is a common strategy in New Zealand and elsewhere for safeguarding species against introduced predators. When successful, however, the closed nature and relatively small size of many island sanctuaries can result in populations quickly reaching their carrying capacity, which in itself can present further challenges such as reduced productivity and population growth rates associated with density-dependent effects as well as increased rates of inbreeding. As part of its management strategy, small numbers of the highly endangered takahe (Porphyrio hochstetteri) were translocated during the 1980s and 90s from the last remaining natural population on the mainland of New Zealand to four offshore islands where introduced predators had been eradicated. We used logistic regression and generalised linear models to assess trends in population growth and recruitment and to evaluate whether the island metapopulation displays density-dependent effects on productivity. Our results indicate that the island metapopulation appears to have reached its carrying capacity, as reflected in an increasing ratio of non-breeding to breeding adults, and recent declines in juvenile production. These density-dependent effects are likely to constrain management strategies aimed at maintaining genetic diversity and minimising inbreeding. A recommendation to increase the immigration rate of takahe onto islands via translocations of birds from the source population on the mainland may be ineffective unless surplus birds are removed.

3058: *+.045*

Mortality and/or dispersal immediately after release can cause translocated populations to fail over both the short and long term, particularly at mainland sanctuaries. However, post-release mortality and dispersal can be limited by releasing individuals with an increased probability of survival and site attachment. We monitored a South Island saddleback (tieke; Philesturnus carunculatus carunculatus) population, translocated to a mainland sanctuary, for one year after release to understand the combined influence of post-release mortality and dispersal on initial establishment. We related settlement propensity to three individual characteristics (sex, age class, and previous pairing history) that are known to play a role in the success of reintroductions of other species. Observations of 38 individually marked birds within the sanctuary declined sharply immediately after release, similar to a pattern of post-release mortality observed after a saddleback translocation onto an offshore island. Thereafter, observations declined more gradually until after the start of the first breeding season (5 months after release), likely due to dispersal out of the

sanctuary. By the middle of the first breeding season, significantly more subadults than adults survived and remained at the release site, which we attribute to differences in territorial status prior to translocation. Although only 18% of the released birds survived and settled inside the sanctuary at one year after release, our results suggest that, for saddlebacks, releasing a greater proportion of subadults than adults could have a positive effect on reintroduction outcomes at mainland sites where dispersal is of concern.

3059: ***+.124***

A craniometrical variability of four samples of raccoon dog Nyctereutes procyonoides (total 205 skulls) from introduced in Tver and Vologda regions populations was investigated on different stages of its expansion using multivariate analysis. Alongside with evident sexual dimorphism in general skull sizes, variability of both sexes is similar, but extent of morphological divergence is differ. Morphological specificity of wide settled from initial places of introduction animals is less appreciable, and samples form continual subsets are connected by transitional morphological forms. The group from border with Vologda region forms a separate cluster with rather high morphological distance to all the rest samples. This divergence most probably is connected with reproductive barrier of Rybinsk water-storage basin and can be considered as founder effect.

3060: ***+.282***

In Tuscany, the red-legged partridge (Alectoris rufa) became extinct at the beginning of the 20th century. Recently, some attempts have been made to re-establish wild populations in Tuscany using farm reared birds, but in most cases the released populations have shown difficulties in reaching sufficient viability, even in areas where the habitat can be considered suitable. Modern technologies for rearing game-birds may be not suitable for reintroduction purposes. For this reason we carried out preliminary research to evaluate the survival of red legged partridges reared under natural condition (Natural) compared to those farm reared (Artificial). Natural rearing occurred in a large pen where birds were allowed to mate freely and to nest. Natural reared birds reached a larger size than artificially-reared birds. Partridges were released in a 7.56 sq km protected area located in the province of Leghorn (western Tuscany, Italy). We followed the fate of 22 young radio-tagged red-legged female partridges (half Natural and half Artificial) and 56 young red-legged partridges (25 Artificial and 31 Natural) marked with different coloured ponchos. The survival of Natural radio-tagged red-legged partridges was double that of the Artificial red-legged partridges. After six months, the re-sighting rate of the Natural poncho-marked birds reached 22.6% whereas none of the Artificial stock was re-sighted. The preliminary results of this research suggest that Natural rearing may be an important tool to improve the success of partridge reintroduction.

3061: ***+.384***

Habitat connectivity is important for the survival of species that occupy habitat patches too small to sustain an isolated population. A prominent example of such a species is the European bison (Bison bonasus), occurring only in small, isolated herds, and whose survival will depend on establishing larger, well-connected populations. Our goal here was to assess habitat connectivity of European bison in the Carpathians. We used an existing bison habitat suitability map and data on dispersal barriers to derive cost surfaces, representing the ability of bison to move across the landscape, and to delineate potential connections (as least-cost paths) between currently occupied and potential habitat patches. Graph theory tools were then employed to evaluate the connectivity of all potential habitat patches and their relative importance in the network. Our analysis showed

that existing bison herds in Ukraine are isolated. However, we identified several groups of well-connected habitat patches in the Carpathians which could host a large population of European bison. Our analysis also located important dispersal corridors connecting existing herds, and several promising locations for future reintroductions (especially in the Eastern Carpathians) that should have a high priority for conservation efforts. In general, our approach indicates the most important elements within a landscape mosaic for providing and maintaining the overall connectivity of different habitat networks and thus offers a robust and powerful tool for conservation planning. CC-BY Creative Commons Attribution 3.0 Unported

3062: *+.081*

To assess the status of lynx we analysed lynx signs of presence within the range of the Italian Alps from 2005 to 2009. A total of 268 signs have been collected, compared to 411 signs during the previous pentad. The distribution of the confirmed signs of lynx presence is confined to three concise areas: the North-eastern Alps of Friuli VG, the Trentino province and the Ossola valley in the Piedmont region. Occupancy modelling revealed a decrease of the lynx range by one third: The estimated number of occupied 100 km(2) cells decreased from 34 (pentad: 2000-2004) to 21 (pentad: 2005-2009). Less than 10% of the Italian Alps are colonized. We estimated the number of lynx present in all the Italian Alps at less than 15 individuals. Therefore, the persistence of lynx in the Italian Alps highly depends on immigration from neighbouring countries.

3063: *+.089*

Context. Timing (mean birthdate) and synchrony (variance around that date) of births can influence survival of young and growth in ungulate populations. Some restored populations of ungulates may not adjust these life-history characteristics to environments of release sites until several years after release, which may influence success of reintroductions.Aims. We quantified timing and synchrony of births from 2005 to 2007 in four populations of reintroduced bighorn sheep (Ovis canadensis) occupying two ecoregions (Central Basin and Range and Wasatch and Uinta Mountains) in Utah, USA, to investigate whether bighorns would adjust these life-history characteristics to environmental conditions of the two ecoregions. We also compared timing and synchrony of births for bighorns in their source herd (Antelope Island) with bighorns in an ecologically similar release site (Stansbury Mountains) during 2006 and 2007.Methods. We relocated female bighorns to record birthdates of young, and observed groups of collared bighorns to quantify use of elevation by those ungulates. We also calculated the initiation, rate and timing of peak green-up by ecoregion, using the normalised difference vegetation index. Key results. We quantified 274 birthdates, and although only separated by 57 km, bighorn populations occupying the Central Basin and Range Mountains gave birth an average of 29 days earlier than did those on the Wasatch and Uinta Mountains, which corresponded with the initiation of vegetation green-up. Additionally, bighorn sheep on the Stansbury Mountains (ecologically similar release site) gave birth at similar times as did bighorns on Antelope Island (source area).Conclusions. Populations of bighorn sheep that were reintroduced into adjacent ecoregions adjusted timing of births to environments and green-up of vegetation in restoration areas. Timing and synchrony of births for reintroduced bighorn sheep in an ecologically similar release site were the same as those of their source area.Implications. Consideration should be given to the adjustment of timing and synchrony of births when reintroducing bighorns, especially when animals are released into different ecoregions. Also, biologists should select release sites that are ecologically similar to source areas, thereby reducing potential negative effects of animals adjusting timing and synchrony of births to environmental conditions of restoration areas.

3064: ***+.164***

Habitat fragmentation, hybridization, and competition with nonnative salmonids are viewed as major threats to Lahontan cutthroat trout Oncorhynchus clarkii henshawi. Understanding Lahontan cutthroat trout behavior and survival is a necessary step in the reintroduction and establishment of naturally reproducing populations of Lahontan cutthroat trout. We used weekly radiotelemetry monitoring to examine movement patterns, habitat use, and apparent survival of 42 hatchery-reared Lahontan cutthroat trout in a 16.5-km stretch of the Truckee River, Nevada, across three reaches separated by barriers to upstream movement. We found differences in total movement distances and home range sizes of fish in different reaches within our study area. Fish used pool habitats more than fast water habitats in all reaches. Time of year, stream temperature, and fish standard length covariates had the strongest relationship with apparent survival. Monthly apparent survival was lowest in January, which coincided with the lowest flows and temperatures during the study period. Our results verify the mobility of Lahontan cutthroat trout and indicate that conditions during winter may limit the survival and reintroduction success in the portions of the Truckee River evaluated in this study.

3065: ***+.174***

We investigate optimal timing of augmentation of an endangered/threatened species population in a target region by moving individuals from a reserve or captive population. This is formulated as a discrete-time optimal control problem in which augmentation occurs once per time period over a fixed number of time periods. The population model assumes the Allee effect growth functions in both target and reserve populations and the control objective is to maximize the target and reserve population sizes over the time horizon while accounting for costs of augmentation. Two possible orders of events are considered for different life histories of the species relative to augmentation time: move individuals either before or after population growth occurs. The control variable is the proportion of the reserve population to be moved to the target population. We develop solutions and illustrate numerical results which indicate circumstances for which optimal augmentation strategies depend upon the order of events.

3066: ***+.089***

After being reduced to about 1,200 animals in eight isolated populations by the beginning of the 20th century, Eurasian beavers (Castor fiber) have powerfully recovered in range and population, through widespread reintroductions, relaxation of persecution, and natural spread. Populations are now (2010) established in all countries within their former natural range in Europe except for Portugal, Italy, and the south Balkans (Greece, Albania, Bulgaria, Macedonia, Kosovo, Montenegro). In Asia, there are significant populations in West and Central Siberia; and small relict populations elsewhere in west and south Siberia, and in Xinjiang (China)/western Mongolia. The current minimum world population estimate for Eurasian beavers is 1.04 million, though this figure includes some populations of introduced North American beavers (C. canadensis) in the NW and Far Eastern Russian Federation, where the ranges of the two species occur in close proximity and to some extent overlap. Both populations and range are in rapid expansion, and in 2008 the species was reclassified by IUCN as Least Concern, though some subspecies remain threatened. We present maps summarizing current knowledge of the world distribution of Eurasian beaver and the Eurasian distribution of the introduced North American beaver, and tables of the most recent known population estimates for each country, broken down by region for the Russian Federation.

3067: *+.208*

Translocation is among several tools available to conservation managers, either to augment existing populations, or to establish populations in previously occupied habitat, or in habitat identified as suitable for the future persistence of the species. Translocated reptiles do not always become established at the release site. We simulated a translocation site for an Endangered Australian skink, the pygmy bluetongue lizard Tiliqua adelaidensis, to investigate whether adding food would encourage released individuals to disperse less. We provided artificial burrows in a central release area within circular cages and found that lizards were more likely to remain in a burrow, spent less time exposed on the ground surface and were less likely to move out of the central area when food was provided. These modified behaviours are likely to encourage translocation success if lizards with added food expose themselves less frequently to predators, and if fewer of those lizards disperse away from the translocation site in the early days after release. We suggest that the provision of supplementary food will be an important component of any trans location programme for this lizard.

3068: *+.029*

Reintroductions are often used to re-establish a self-sustaining population of a species as a conservation method. Despite their prevalence, few reintroductions have followed recent international recommendations to publish details such as appropriate site-selection, criteria for success and experimental analyses of the reintroduction. Here we report on the first experimental reintroduction of the Brown Treecreeper (Climacteris picumnus), a ground-foraging Australian woodland passerine. Seven social groups (43 individuals) were released into two nature reserves in south-eastern Australia. Using a robust comparison of habitat-restoration treatments, we evaluate the influence of these treatments and demographic parameters of the Brown Treecreepers on measures of success of the reintroduction. Although individual Brown Treecreepers lost an average of 5.82% of their bodyweight during translocation, survival during the first 24 h and the first 3 days after reintroduction was high and was not significantly influenced by habitat treatments at the release site. There was, however, evidence of high levels of mortality in the first 2 months after release, but there was no influence of sex or age on apparent survival. These apparent losses may be attributable to longer-term effects of translocation stress, lack of familiarity with habitat or insufficient effectiveness of restoration treatments. Although this reintroduction appears to have failed, we present details on all aspects of the reintroduction to provide vital information and lessons learned regarding procedures and outcomes.

3069: *-.004*

The subtropical coastal heathlands of southeast Queensland contain many rare and threatened species and have undergone considerable habitat loss in recent years due to high levels of urbanisation. We used morphological and microsatellite markers to investigate the reproductive ecology, in relation to fire, and population genetic structure of the endangered shrub Allocasuarina emuina. Highly significant differences ($P < 0.001$) were detected among populations for all cone- and seed-related morphological traits, which revealed distinct northern and southern groups of populations, a pattern repeated in the genetic component of the study. Results also indicated that the reproductive viability of A. emuina is more related to fire interval than population size and confirmed that seed viability declines with time since fire. The highest level of genetic diversity for the species was observed in the population on Mt Emu (AE4A; H-E = 0.688). Contrary to the expectations of population genetic theory, no relationship was found between the level of genetic variation and population size, density or degree of isolation, hinting that genetic diversity is being

conserved as a result of polyploidy and apomixis. However, genetic signatures in the northern populations indicated that unidirectional dispersal of genetic material from the putatively refugial population on Mt Emu to the surrounding coastal plain has been occurring with recession of sea levels following the last interglacial. The findings of the study will aid in both the conservation of natural populations and translocations of A. emuina and have significant implications relating to the biogeographical history of a considerable number of co-occurring heathland taxa.

3070: *+.044*

Quebrada del Condorito National Park is located in the upper belt of the mountains of central Argentina and preserves a heterogeneous rangeland area. After the creation of the National Park, in 1996, domestic livestock were gradually removed to avoid soil loss and degradation due to overgrazing in this fragile ecosystem. Lack of large-scale herbivory allowed the expansion of tussock grasslands over grazing lawns. In 2007 a guanaco (Lama guanicoe) population was reintroduced; this large native herbivore, that had become extinct in the region was selected, because it is a low-impact grazer. Habitat selection by the guanaco population reintroduced to the National Park was studied. Seven habitat types previously defined for the region were considered, each one exhibiting a particular dominant plant growth form and different percent cover of plant species. Guanacos made a positive selection of moist and dry grazing lawns, and avoided tussock grasslands and forests. The reintroduced guanacos selected landscapes with short plants and a high percentage of perennial graminoids and forbs, which are guanacos' preferred food items. The results indicate that availability of forage of a nutritive value and dominant plant growth form largely explain habitat selection by guanaco in the National Park; this information can be useful for both the ongoing guanaco reintroduction project and the design of management strategies aimed at ecological restoration of this important rangeland region of central Argentina.

3071: *+.015*

Xerothermic species are rare and threatened in central and eastern Europe. In light of the continuing loss of steppe-like habitats due to anthropogenic fragmentation and degradation, the evaluation of genetic variation in populations inhabiting them is of immediate importance if appropriate conservation measures are to be undertaken. Here we report on the genetic diversity of the rare leaf beetle Crioceris quatuordecimpunctata, whose populations in central and eastern Europe inhabit highly geographically isolated areas. All of the studied populations (in Poland, Ukraine, and Slovakia) were differentiated at the mitochondrial marker COI. However, with respect to the nuclear marker ITS1, Polish populations were monomorphic, but distinct from all other populations. The distinctiveness of the studied populations was confirmed by Wolbachia screening, which showed that all populations carried different strains (one or two), which were probably transferred independently from other insects. On the other hand, no diversity was found in any marker within particular populations, which could be caused (at least for mtDNA) by a Wolbachia selective sweep. Crioceris quatuordecimpunctata probably consists of isolated populations, which went through narrow bottlenecks leading to a drastic reduction in their genetic diversity. As these populations are reciprocally monophyletic for mtDNA haplotypes and show a significant divergence of allele frequencies at nuclear loci, they could be classified as evolutionarily significant units (ESUs). In addition, DNA barcodes were used to identify Asparagus officinalis as the host plant for members of all studied populations. These data should be valuable in efforts to conserve populations of C. quatuordecimpunctata (e.g., for guiding reintroductions).

3072: *+.028*

Context. Translocation has become an increasingly common tool in the conservation of species. Understanding the movement patterns of some species can be important to minimise loss of individuals from the translocation release site.Aims. To describe seasonal and sex-biased movements within populations of an endangered Australian lizard.Methods. We monitored seasonal movement in the endangered pygmy bluetongue lizard (Tiliqua adelaidensis) by using pitfall trapping, with a total of 49 440 trap-nights from three sites over 2 years. Other studies have shown that individual pygmy bluetongue lizards normally remained closely associated with their spider burrow refuges, with very little movement. Thus, we interpreted any captures detected through pitfall trapping as out of burrow movements. We investigated whether there was any seasonal, age or sex bias in moving individuals. Key results. We found that male pygmy bluetongue lizards were more likely to move than were females. After adults, neonates were the second-most captured age class. Spring was the peak movement time for adults, whereas movement of neonates occurred in autumn. Key conclusions. The majority of movement can be attributed to males in the breeding season, whereas females move very little.Implications. The present study provides some baseline data that would allow more informed decisions about the most appropriate individuals in a population to choose for a translocation program and the times to conduct translocations to allow the maximum chance for establishment.

3073: ***+.021***

The northern bettong (Bettongia tropica) (Potoroidae), is an endangered macropod with a restricted distribution. We combined radio-tracking and trapping data with microsatellite genotypes to infer the mating system and local dispersal patterns of this species, and discuss their relevance to translocations. We defined the mating system as 'overlap promiscuity' (sensu Wittenberger 1979), though we cannot rule out serial monogamy. We found significant effects of proximity (average distance between parents = 190 m) and male weight, but not size, on the likelihood of paternity, suggesting that closer, heavier males have greater mating success. The average distance between putative pairs of relatives suggested that most dispersal occurred over short distances, with the distance between 'related' females significantly lower than that between related males (0.9 km versus 1.3 km). A spatial autocorrelation analysis showed high female relatedness across distances of up to 435 m, equivalent to half an average home range width. Conversely, male pairs had low relatedness across 0 to 870 m. These results suggested that female young often settle next to their mother, while males avoid nesting within their father's home range. Both limited natal and 'mating' dispersal may have contributed to the strong genetic structure previously reported for this species.

3074: ***+.300***

Although also formerly present in two lochs in South West Scotland, populations of vendace (Coregonus albula) were until recently believed to persist in only two U.K. lakes, i.e., Bassenthwaite Lake and Derwent Water in North West England. However, although Derwent Water has retained its natural mesotrophic conditions and the status of its vendace population is acceptable, monitoring at Bassenthwaite Lake has failed to record any specimens since 2000 and the species has recently been declared locally extinct. Bassenthwaite Lake has experienced increasing problems from eutrophication, sedimentation and the introduction of fish species including roach (Rutilus run/us) and ruffe (Gymnocephalus cernuus), both of which have more recently also been introduced to Derwent Water and give concern over possible food competition and egg predation. In addition to monitoring, considerable efforts have been made in recent years to conserve vendace through the protection and improvement of their habitats and the establishment of refuge populations. The latter has resulted in the establishment of a population

originating from Bassenthwaite Lake in Loch Skeen of South West Scotland, with further attempts still in progress at two other sites. Public awareness of the conservation of this species, which is the U.K.'s rarest freshwater fish, has also been actively promoted.

3075: *+.151*

Every partridge counts, successful techniques used in the captive conservation breeding programme for wild grey partridge in Ireland.-Between 1998 and 2001 the last remaining wild grey partridge (Perdix perdix) population in Ireland faced imminent extinction with an estimated spring population of 4-6 pairs, and an autumn population of 22-24 birds. A captive breeding programme began in 2002 with two pairs of grey partridge. In the most successful year in 2010, 39 pairs produced a total of 510 chicks. Average chick survival rate was 65.13%. At 88.9 the highest chick survival rate was achieved in 2011. Chick survival of parent reared birds in captivity is defined by the number of juveniles surviving at age six weeks: similar to estimations used for wild populations of grey partridge. Family coveys were released in late summer to early autumn. In most instances the entire family cohort was released as one unit. However, in coveys of twenty or above, an average of five parent reared poults were held back as breeding stock for the following year. In early spring of the following year, birds held back were paired with single males or females trapped from the wild. The techniques we used were traditional and labour intensive but highly effective. We recommend that other grey partridge recovery projects should consider captive breeding using the methods employed in this programme to compliment other game management methods used.

3076: *+.049*

Spatial behaviour and survival of translocated wild brown hares. - The fragility of many populations of brown hares in Western Europe is a concern for managers, hunters and naturalists. We took advantage of a locally high density population to use wild individuals to restock areas where the species had disappeared or was close to disappearing. The aim of the project was to assess the evolution of the spatial behaviour after release using radio tracking. Over 150 wild brown hares were translocated, one third of which were fitted with radio collars. In addition, fifteen individuals were radio tagged and released back into the source population as a control. Most individuals settled in less than two months and their seasonal home range, once settled, was similar to that observed in the source population. Mean duration of tracking was not significantly different between the two groups. Moreover, two years after the last translocation, tagged individuals can still be observed, but most hares present are not tagged, which indicates natural reproduction of the released individuals. The translocation of wild individuals thus appears to give encouraging results.

3077: *+.003*

The Gartcosh Industrial Site, North Lanarkshire is home to the largest known population of great crested newts (Triturus cristatus,) in Scotland. Economic development of the site required the translocation of the great crested newt and four other amphibian species from existing ponds to a purpose built reserve around the periphery. Monitoring the effectiveness of translocation as a mitigation method has shown that in this case, the breeding adult population is being maintained at levels comparable with the previous site although there are indications of possible declines with other life stages. Longer term monitoring is required at a level more in-depth than currently planned. The aquatic and terrestrial habitat created appears sufficient to support the population although there are problems with fragmentation, both within the site and connections to external

locations. There is still pressure for further development in an area that could affect the newt population.

3079: -.032

Both Ireland and Britain have lost many large mammal species owing to habitat loss, conflict with humans and hunting for sport, fur or some other commodity. Reintroductions offer an effective way of restoring former indigenous species but, until recently, this has largely involved birds of prey in Ireland and Britain. Re-wilding has been proposed as a way of re-establishing whole mammalian communities in order to restore ecosystems and prevent further extinctions of other species dependent on one or a few keystone species. Pleistocene re-wilding is rejected in this paper as unrealistic but Mesolithic re-wilding is regarded as feasible because virtually all former indigenous species still survive globally or can be replaced by domestic descendants or close relatives. A form of re-wilding, naturalistic grazing, is practiced in some areas in Europe in the mistaken belief that predators have little or no impact on herbivore populations. However, evidence from the reintroduction of wolves to Yellowstone National Park indicates that they have direct impacts through predation and indirect impacts by creating 'landscapes of fear' that exclude herbivores, leading to vegetational and other species' recovery. The terrestrial mammal faunas of Ireland and Britain have been continually changing since late glacial times, partly in response to dramatically changing climatic and environmental conditions, but also owing to the direct and indirect impact of human activities. However, rising sea levels and differing proximities to continental Europe had a profound impact on the composition of the post-glacial mammal faunas of Ireland and Britain which has subsequently affected their natural and anthropogenic development during the Holocene. Our knowledge of faunal change has come primarily from recent advances in the analysis of the fossil and archaeological records as well as phylogeographical studies of contemporary mammal populations although there remain many gaps. Despite these possible deficiencies it seems likely that much of Ireland's terrestrial mammal fauna is introduced but the few natural post-glacial colonizers often represent morphological and genetically distinct forms indicating Ireland is a refugium or centre of endemism in Europe. Curiously, Ireland's post-glacial mammal fauna had a diverse array of large predators and few herbivores - there is no modern comparator of this unique mammalian community so that we can only speculate as to its ecological functioning and stability. With so much of the expected mammalian crew missing from Ireland and the inevitable loss of important mammalian biodiversity that would result from attempts to restore the 'natural' post-glacial mammal fauna what is the future of Ireland's mammals?

3080: -.006

Reintroduction is a widespread method for saving populations of endangered species from extinction. In spite of recent reviews, it is difficult to reach general conclusions about its value as a conservation tool, as authors are reluctant to publish unsuccessful results. The Mohor gazelle is a North African gazelle, extinct in the wild. Eight individuals were reintroduced in Senegal in 1984. The population grew progressively, albeit slowly, during the first 20 years after release, but then declined dramatically, until the population in 2009 was estimated at no more than 13-15 individuals. This study attempts to determine the likelihood of gazelle-habitat relationships to explain why the size of the gazelle population has diminished. Our results show that the Mohor gazelle in Guembeul is found in open habitats with less developed canopy where the grass is shorter, suggesting the possibility that changes in habitat structure have taken place during the time the gazelles have been in the Reserve, reducing the amount of suitable habitat. Reintroduction design usually concentrates on short-term factors that may affect survival of the released animals

and their descendants (short-term achievement), while the key factors for assessing its success may be those that affect the long-term evolution of the population.

3081: *+.215*

The project Status and Conservation of the Alpine Lynx Population (SCALP) is an ongoing program aiming to co-ordinate the lynx monitoring and propose conservation activities in the Alps. The SCALP project was initiated from several active lynx researchers as an informal group in the early 1990s twenty - years after the reintroductions in Switzerland, Italy, Slovenia, and Austria. To propose adequate management measures, a sound monitoring of the Alpine lynx population needs to be in place. In the early 1990s the first efforts were made to put all available data on lynx presence together. The least common denominator of data collection in the Alps was - and still is - the compilation of direct and indirect signs of lynx presence. To standardise the interpretation of the data collected, SCALP experts agreed on a categorisation of occurrence records, where each record is evaluated retrospectively whether it can be verified for correct species identification and whether it has been verified for correct species identification. Therefore, for the monitoring of the lynx throughout the Alps in the frame of the SCALP surveys, the collected data are classified in three categories according to the following SCALP criteria: Category 1 (C1): "Hard facts", verified and unchallenged observations; Category 2 (C2): Observations controlled and confirmed by a lynx expert (e.g. trained member of the network); Category 3 (C3): Unconfirmed category 2 observations and all observations such as sightings and calls which, if not additionally documented, by their nature cannot be verified. The SCALP criteria allow to both combine and distinguish reliable and only partly reliable data for a better interpretation of the actual distribution.

3082: *+.157*

E.P. Kruchenkova. Foreign conservation programs: structure and rating of success. There are two ways to save rare species: 1. in-situ management of endangered populations in the wild which may last for a very long period, and 2. ex-situ formation of stable captive groups to provide animals for reintroduction to the nature. This last stage, in fact, determines the success of the whole project. However, ecologists and ethologists from one side and "conservationists" from the other used to estimate the success of the same conservation programs in different ways. To clear up the situation, data on 134 cases of reintroduction described in two IUCN issues of Global Re-introduction Perspectives (2008 and 2010) have been analyzed. The similar structure of all programs made the task easier. Only 33 projects were devoted to mammals, 22 of them concerned ungulates and Primates. It was found out that most of conservation programs implied just translocation of wild animals and could not be discussed as real reintroduction. Only 12% of all projects led to forming of stable self-sustaining populations in the wild and could be estimated as highly successful. Authors of all projects came to a conclusion that long-term captive keeping and breeding of animals resulted in reduction of probability of their reintroduction success.

3083: *+.175*

Background: The endemic Italian roe deer (Capreolus c. italicus) is threatened by introgressive hybridization with the introduced and expanding European subspecies Capreolus c. capreolus. Population genetic surveys show that some populations in central Italy are not yet admixed with the introduced subspecies.Question: Is it possible to identify and map the distributions of native and admixed roe deer populations?Methods: We obtained and analysed diagnostic mitochondrial DNA control-region sequences and individual genotypes at 11 autosomal microsatellite loci in

1051 roe deer samples collected from the entire distribution of Italian roe deer and from reference populations of European roe deer. We used classical and Bayesian statistical approaches to describe the genetic substructure of roe deer populations in Italy. We used admixture analyses and landscape genetic tools to map the fine-scale distributions of Italian roe deer populations and locate their admixture zones.Results: A very few fragmented patches of the Italian roe deer do survive in central Italy. Although these populations are seriously threatened by hybridization with expanding European roe deer, they can be genetically identified and, by means of translocations, saved from genetic extinction.Discussion: Italian roe deer populations exist and are still viable, but their survival is threatened by the expansion of reintroduced European roe deer. The rapid identification of suitable and pristine areas to which pure individuals from remaining patches could be translocated appears the best way to preserve the Italian roe deer genetic pool.

3084: ***+.283***

BackgroundThe conservation of species structured in metapopulations involves an important dilemma of resource allocation: should investments be directed at restoring/enlarging habitat patches or increasing connectivity. This is still an open question for Maculinea species despite they are among the best studied and emblematic butterfly species, because none of the population dynamics models developed so far included dispersal. Methodology/Principal FindingsWe developed the first spatially and financially explicit Population Viability Analysis model for Maculinea alcon, using field data from The Netherlands. Implemented using the RAMAS/GIS platform, the model incorporated both local (contest density dependence, environmental and demographic stochasticities), and regional population dynamics (dispersal rates between habitat patches). We selected four habitat patch networks, contrasting in several basic features (number of habitat patches, their quality, connectivity, and occupancy rate) to test how these features are affecting the ability to enhance population viability of four basic management options, designed to incur the same costs: habitat enlargement, habitat quality improvement, creation of new stepping stone habitat patches, and reintroduction of captive-reared butterflies. The PVA model was validated by the close match between its predictions and independent field observations on the patch occupancy pattern. The four patch networks differed in their sensitivity to model parameters, as well as in the ranking of management options. Overall, the best cost-effective option was enlargement of existing habitat patches, followed by either habitat quality improvement or creation of stepping stones depending on the network features. Reintroduction was predicted to generally be inefficient, except in one specific patch network. Conclusions/SignificanceOur results underline the importance of spatial and regional aspects (dispersal and connectivity) in determining the impact of conservation actions, even for a species previously considered as sedentary. They also illustrate that failure to account for the cost of management scenarios can lead to very different conclusions.

3085: ***+.049***

Fungal populations that reproduce sexually are likely to be genetically more diverse and have a higher adaptive potential than asexually reproducing populations. Mating systems of fungal species can be self-incompatible, requiring the presence of isolates of different mating-type genes for sexual reproduction to occur, or self-compatible, requiring only one. Understanding the distribution of mating-type genes in populations can help to assess the potential of self-incompatible species to reproduce sexually. In the locally threatened epiphytic lichen-forming fungus Lobaria pulmonaria (L.) Hoffm., low frequency of sexual reproduction is likely to limit the potential of populations to adapt to changing environmental conditions. Our study provides direct evidence of self-incompatibility (heterothallism) in L. pulmonaria. It can thus be hypothesized that

sexual reproduction in small populations might be limited by an unbalanced distribution of mating-type genes. We therefore assessed neutral genetic diversity (using microsatellites) and mating-type ratio in 27 lichen populations (933 individuals). We found significant differences in the frequency of the two mating types in 13 populations, indicating a lower likelihood of sexual reproduction in these populations. This suggests that conservation translocation activities aiming at maximizing genetic heterogeneity in threatened and declining populations should take into account not only presence of fruiting bodies in transplanted individuals, but also the identity and balanced representation of mating-type genes.

3086: ***+.033***

Ireland holds a low diversity of breeding raptors as a result of its location on the western edge of Europe but also due to historical persecution leading to the loss of at least seven species. Recolonisation by Buzzards Buteo buteo and the recent reintroduction of three species, Golden Eagle Aquila chrysaetos, White-tailed Eagle Haliaeetus albicilla and Red Kite Milvus milvus, has helped redress such losses. Monitoring for raptors is carried out by the statutory agencies, NGOs and two university research groups. Decadal and semi-decadal surveys are undertaken nationally in the Republic of Ireland and Northern Ireland for Peregrine Falcon Falco peregrinus and Hen Harriers Circus cyaneus, respectively. Long term monitoring projects have been established for some key species such as Barn Owl Tyto alba. However, some species receive little monitoring effort (e.g. Sparrowhawk Accipiter nisus, Merlin F. columbarius). A dedicated Raptor Monitoring Scheme to determine long-term population trends across a range of species is lacking and remains an urgent priority. Development of an Action Plan for raptors and/or single key species would further help identify priorities and raise awareness of the need of monitoring for raptors.

3087: ***+.049***

Fungal populations that reproduce sexually are likely to be genetically more diverse and have a higher adaptive potential than asexually reproducing populations. Mating systems of fungal species can be self-incompatible, requiring the presence of isolates of different mating-type genes for sexual reproduction to occur, or self-compatible, requiring only one. Understanding the distribution of mating-type genes in populations can help to assess the potential of self-incompatible species to reproduce sexually. In the locally threatened epiphytic lichen-forming fungus Lobaria pulmonaria (L.) Hoffm., low frequency of sexual reproduction is likely to limit the potential of populations to adapt to changing environmental conditions. Our study provides direct evidence of self-incompatibility (heterothallism) in L. pulmonaria. It can thus be hypothesized that sexual reproduction in small populations might be limited by an unbalanced distribution of mating-type genes. We therefore assessed neutral genetic diversity (using microsatellites) and mating-type ratio in 27 lichen populations (933 individuals). We found significant differences in the frequency of the two mating types in 13 populations, indicating a lower likelihood of sexual reproduction in these populations. This suggests that conservation translocation activities aiming at maximizing genetic heterogeneity in threatened and declining populations should take into account not only presence of fruiting bodies in transplanted individuals, but also the identity and balanced representation of mating-type genes.

3088: ***+.049***

Fungal populations that reproduce sexually are likely to be genetically more diverse and have a higher adaptive potential than asexually reproducing populations. Mating systems of fungal species can be self-incompatible, requiring the presence of isolates of different mating-type genes

for sexual reproduction to occur, or self-compatible, requiring only one. Understanding the distribution of mating-type genes in populations can help to assess the potential of self-incompatible species to reproduce sexually. In the locally threatened epiphytic lichen-forming fungus Lobaria pulmonaria (L.) Hoffm., low frequency of sexual reproduction is likely to limit the potential of populations to adapt to changing environmental conditions. Our study provides direct evidence of self-incompatibility (heterothallism) in L. pulmonaria. It can thus be hypothesized that sexual reproduction in small populations might be limited by an unbalanced distribution of mating-type genes. We therefore assessed neutral genetic diversity (using microsatellites) and mating-type ratio in 27 lichen populations (933 individuals). We found significant differences in the frequency of the two mating types in 13 populations, indicating a lower likelihood of sexual reproduction in these populations. This suggests that conservation translocation activities aiming at maximizing genetic heterogeneity in threatened and declining populations should take into account not only presence of fruiting bodies in transplanted individuals, but also the identity and balanced representation of mating-type genes.

3089: *+.049*

Fungal populations that reproduce sexually are likely to be genetically more diverse and have a higher adaptive potential than asexually reproducing populations. Mating systems of fungal species can be self-incompatible, requiring the presence of isolates of different mating-type genes for sexual reproduction to occur, or self-compatible, requiring only one. Understanding the distribution of mating-type genes in populations can help to assess the potential of self-incompatible species to reproduce sexually. In the locally threatened epiphytic lichen-forming fungus Lobaria pulmonaria (L.) Hoffm., low frequency of sexual reproduction is likely to limit the potential of populations to adapt to changing environmental conditions. Our study provides direct evidence of self-incompatibility (heterothallism) in L. pulmonaria. It can thus be hypothesized that sexual reproduction in small populations might be limited by an unbalanced distribution of mating-type genes. We therefore assessed neutral genetic diversity (using microsatellites) and mating-type ratio in 27 lichen populations (933 individuals). We found significant differences in the frequency of the two mating types in 13 populations, indicating a lower likelihood of sexual reproduction in these populations. This suggests that conservation translocation activities aiming at maximizing genetic heterogeneity in threatened and declining populations should take into account not only presence of fruiting bodies in transplanted individuals, but also the identity and balanced representation of mating-type genes.

3090: *+.246*

1. The freshwater pearl mussel, Margaritifera margaritifera (L.), is Northern Ireland's most imperilled freshwater species and is known to survive in six rivers in Counties Tyrone and Fermanagh. 2. Ex-situ conservation, specifically captive breeding for the purposes of population supplementation and reintroduction has been identified as an important component in current and future conservation strategies for the species through collaboration between Government, Ballinderry Fish Hatchery Ltd. and Quercus, Queen's University Belfast. 3. This protocol specifically adapts IUCN Guidelines for Reintroductions of the freshwater pearl mussel in Northern Ireland informed by over a decade of ex-situ conservation experience and research. 4. The protocol is based on a review of the current status of the species in Northern Ireland, the aims and objectives of ex-situ conservation, current and proposed captive breeding, selection of brood stock, genetic provenance, biosecurity, population supplementation and reintroductions including aspects of handling and transport, choosing a release site, soft verses hard release strategies, post-release monitoring and other conservation strategies such as in-situ conservation. It also considers

socioeconomic and legal requirements including financial and political support, policy and licensing. 5. To maximise the likelihood of long-term effective conservation of M. margaritifera in Northern Ireland a total of 38 Actions are recommended.

3091: *+.044*

Britain's only native crayfish species, the white-clawed crayfish Austropotamobius pallipes are threaten through rapid population declines caused by non-indigenous species, habitat degradation and pollution. This study focuses one of the last remaining small A. pallipes population in the Creedy Yeo River, Devon. This study aims to decipher whether the use of artificial refuge traps (ARTs) or baited traps are the most effective method of catching this species, to aid translocation efforts to conserve the remaining population under threat from the American signal crayfish Pacifastacus leniusculus. The capture rate of each type of trap was studied at four sites along the river, along with the size and gender of crayfish caught and a survey of the habitat conditions. The ARTs were already in place along the river and baited traps were installed the day before a translocation, using fresh sardines as bait. The results showed that ARTs caught more crayfish of a lower size in comparison to the baited traps which caught large adult crayfish. There was no significant difference in the capture rate of either trap type and no relationship was found between habitat variables of shade cover, siltation level and main refuge types in the riverbank or channel. To conclude, this study suggests that there are a wide multitude of factors determining condition that can influence the capture rate of crayfish. The most consistent capture method was using the ARTs, as baited traps were unreliable in low density populations. This can be applied to aid conservation methods in other low density river populations.

3092: *-.060*

Captive breeding programs are widely used for the conservation and restoration of threatened and endangered species. Nevertheless, captive-born individuals frequently have reduced fitness when reintroduced into the wild. The mechanism for these fitness declines has remained elusive, but hypotheses include environmental effects of captive rearing, inbreeding among close relatives, relaxed natural selection, and unintentional domestication selection (adaptation to captivity). We used a multigenerational pedigree analysis to demonstrate that domestication selection can explain the precipitous decline in fitness observed in hatchery steelhead released into the Hood River in Oregon. After returning from the ocean, wild-born and first-generation hatchery fish were used as broodstock in the hatchery, and their offspring were released into the wild as smolts. First-generation hatchery fish had nearly double the lifetime reproductive success (measured as the number of returning adult offspring) when spawned in captivity compared with wild fish spawned under identical conditions, which is a clear demonstration of adaptation to captivity. We also documented a tradeoff among the wild-born broodstock: Those with the greatest fitness in a captive environment produced offspring that performed the worst in the wild. Specifically, captive-born individuals with five (the median) or more returning siblings (i.e., offspring of successful broodstock) averaged 0.62 returning offspring in the wild, whereas captive-born individuals with less than five siblings averaged 2.05 returning offspring in the wild. These results demonstrate that a single generation in captivity can result in a substantial response to selection on traits that are beneficial in captivity but severely maladaptive in the wild.

3093: *+.144*

Given the increasing expansion of human dominated landscapes it often becomes necessary to relocate endangered and at-risk species from existing habitat areas. Further, there is growing

research stating that climatic and atmospheric changes attributed to climate change are already affecting species distributions and geographic ranges, requiring endangered species to be relocated. Existing reserve design models mostly focus on choosing the optimal land area given existing species distributions and have not incorporated species relocation as a criterion. This paper introduces linear integer programming formulations for the relocation of multiple populations of a species at risk to clustered conservation areas. We present a basic clustered relocation model and extend the model to minimize the distances of relocation. We apply the models to a dataset related to Gopher Tortoise (GT), a keystone species currently considered 'at risk', at Ft. Benning Georgia where expanding military training needs require the relocation of GTs. We present the results and discuss the trade-off between compactness and relocation considerations using an efficiency frontier. (C) 2011 Published by Elsevier B.V.

3094: ***+.243***

Species translocations are remarkable experiments in evolutionary ecology, and increasingly critical to biodiversity conservation. Elaborate socio-ecological hypotheses for translocation success, based on theoretical fitness relationships, are untested and lead to complex uncertainty rather than parsimonious solutions. We used an extraordinary 89 reintroduction and 102 restocking events releasing 682 black rhinoceros (Diceros bicornis) to 81 reserves in southern Africa (1981-2005) to test the influence of interacting socio-ecological and individual characters on post-release survival. We predicted that the socio-ecological context should feature more prominently after restocking than reintroduction because released rhinoceros interact with resident conspecifics. Instead, an interaction between release cohort size and habitat quality explained reintroduction success but only individuals' ages explained restocking outcomes. Achieving translocation success for many species may not be as complicated as theory suggests. Black rhino, and similarly asocial generalist herbivores without substantial predators, are likely to be resilient to ecological challenges and robust candidates for crisis management in a changing world.

3095: ***+.078***

We analysed 131 common impala (Aepyceros melampus melampus) samples from two provinces in South Africa (Limpopo and KwaZulu-Natal) that are separated by the Drakensberg Mountain Range using sequences of the mitochondrial control region and seven polymorphic nuclear microsatellite loci. In line with earlier studies on bovid species, we found very high values of genetic diversity, particularly at the mtDNA locus with an overall nucleotide diversity of 3.6% and an overall haplotype diversity of 0.98. All statistical approaches confirmed a significant population differentiation between Limpopo and KwaZulu-Natal, suggesting that areas of unsuitable habitat caused by the presence of the Drakensberg Range and the Indian Ocean coastal belt act as a barrier to gene flow. Only few individuals with signs of admixed origin were indicative of translocations or rare migration between the two provinces. Combination of our mtDNA data set with those of previous studies on impala from south-western, southern and eastern Africa revealed the highest diversity in South Africa. This is in line with the hypothesis of a southern glacial refuge from which various African ungulate species spread northeast during the Holocene, although in the case of impala further analyses based on larger data sets will be necessary to definitively settle this question.

3096: ***+.068***

Declining populations of less than 250 mature individuals are symptomatic of many Critically Endangered cycads, which, globally, comprise the most threatened group of organisms as a result

of collecting and habitat loss. Survival plans focus on law enforcement, reintroduction, and augmentation programmes using plants from the wild and botanical gardens. Augmentation is one of the few remaining options for cycad populations, although the assumed benefits remain untested and there is a possibility that augmentation from different sources could compromise the genetic integrity of existing populations, especially when garden plants have no provenance data. We studied Encephalartos latifrons, a South African endemic, which is a typical Critically Endangered cycad. We studied the extent and structure of genetic diversity in wild and ex situ populations to assess the potential benefits and risks associated with augmentation programmes. We examined 86 plants using amplified fragment length polymorphisms (AFLPs). The 417 AFLP markers thus generated yielded a unique DNA fingerprint for each plant. Wild populations retain high levels of genetic diversity and this is reflected among the ex situ holdings at the Kirstenbosch Botanical Garden. No population differentiation is evident, indicating a single panmictic population, consistent with moderately high levels of gene flow between subpopulations and a sexual mode of reproduction. Bayesian clustering identified four genotype groups in the wild, as well as a genotype group only found in ex situ collections. Our results indicate that E. latifrons would benefit from augmentation programmes, including the use of undocumented collections, and careful management of breeding plants would increase the heterogeneity of propagules. (C) 2011 The Linnean Society of London, Biological Journal of the Linnean Society, 2012, 105, 293308.

3097: *+.044*

Questions: Does stand age influence the direction and rate of post-fire successional dynamics in coastal Calluna heaths and can old degraded heath vegetation be restored through reintroduction of fire?Location: Coastal heaths in the Tarva archipelago, central Norway.Methods: We investigated revegetation dynamics after experimental fires set in young (8 years since last fire) and old (> 50 years since last fire) grazed heath stands. A repeated measures design was used, with floristic data recorded in permanent plots in the post-fire successions (n = 12) over a 7-year period. The data were analysed using multivariate ordination techniques (PCA, RDA and PRC) and mixed effects models.Results: The age of Calluna stands strongly influenced post-fire succession, different trends due to age explained 10.4% of variation in floristic data. Young heath showed faster succession towards pre-fire community composition than old heath, and this could partially be explained by succession-related factors: young heath had lower cover of mosses and lichens in the pre-burned vegetation, and lower cover of litter early in succession. Young heath had a less pronounced overall community response to fire than old heath. Vegetative regeneration of C. vulgaris was absent in both old and young heath, but Calluna still re-established as the dominant species within 5-7 years in both young and old stands. Regeneration dynamics were also affected by habitat conditions, different trends due to habitat explained 6% of variation.Conclusions: Our study demonstrates that old stands do develop characteristic heathland vegetation and structure after fire, and while potential invasives into the system such as trees and rhizomatous species are present, they do not impair Calluna regeneration or vegetation development towards the target heathland community composition and structure. Further, as our young stands are only in their second fire rotation after restoration, we suggest that characteristic dynamics of managed heathlands can re-establish relatively rapidly, even in severely degenerated sites (450 years since last fire). Site-specific factors also need to be considered. We conclude that there is restoration potential in old heaths, despite slow dynamics in the first rotation.

3098: *+.009*

Complex Triticeae genomes pose a challenge to genome sequencing efforts due to their size and repetitive nature. Genome sequencing can reveal details of conservation and rearrangements

between related genomes. We have applied Illumina second generation sequencing technology to sequence and assemble the low copy and unique regions of Triticum aestivum chromosome arm 7BS, followed by the construction of a syntenic build based on gene order in Brachypodium. We have delimited the position of a previously reported translocation between 7BS and 4AL with a resolution of one or a few genes and report approximately 13% genes from 7BS having been translocated to 4AL. An additional 13 genes are found on 7BS which appear to have originated from 4AL. The gene content of the 7DS and 7BS syntenic builds indicate a total of similar to 77,000 genes in wheat. Within wheat syntenic regions, 7BS and 7DS share 740 genes and a common gene conservation rate of similar to 39% of the genes from the corresponding regions in Brachypodium, as well as a common rate of colinearity with Brachypodium of similar to 60%. Comparison of wheat homoeologues revealed similar to 84% of genes previously identified in 7DS have a homoeologue on 7BS or 4AL. The conservation rates we have identified among wheat homoeologues and with Brachypodium provide a benchmark of homoeologous gene conservation levels for future comparative genomic analysis. The syntenic build of 7BS is publicly available at http://www.wheatgenome.info.,

3099: ***+.097***

Populations of large carnivores are declining globally, and analysis of public discourse about carnivores is useful for understanding public opinion and influences on management and policy. Portrayal of carnivores in the media affects public perceptions and support for their conservation. We conducted a content analysis of 513 articles about Florida panthers (Puma concolor coryi) published from 2003 to 2006 in newspapers with local circulation in core panther habitat in southwest Florida and papers with statewide circulation to compare the differences in the amount of coverage and portrayals of panther risks to people and property on the basis of proximity of human communities to panthers. Local papers published significantly more news articles and significantly longer news articles primarily about panthers. Articles in local and statewide papers used both episodic frames, which focus on specific occurrences (e.g., a panther sighting or predation) and thematic frames, which focus on general trends (e.g., abundance of panthers over time). Local articles more often emphasized risks that panthers might harm people, pets, or livestock than statewide papers. Our results are consistent with theory that proximity to humancarnivore conflict influences perceptions and salience of risks posed by large carnivores. Most articles mentioned panthers as a secondary topic, which we believe was a result of the relevance an endangered carnivore has in discussions of public land management, development, and regulations in Florida. Claims made by sources quoted in each article had a neutral to positive depiction of panthers, and most quotations were from federal and state agency scientists. We suggest continued use by the media of agency sources provides the opportunity for clear, concordant messages about panther management. Content analysis provides a way to monitor media portrayal of carnivores for consistency with agency outreach goals.

3100: ***+.117***

Translocation is used to reestablish wild populations of animals, but translocation projects often do not meet their objectives because postrelease mortality of animals is high. One reason for translocation failure is that the behavioral or ecological requirements of released animals are unmet. Maintaining founder-group social relationships during release can affect reestablishment of social species. Solitary territorial species with stable neighbors (restricted dispersal and lifetime occupation of a home range) of the same species may also benefit from the maintenance of these social relationships during translocation. We translocated Stephens kangaroo rats (Dipodomys stephensi), a solitary species listed as endangered under the U.S. Endangered Species Act, with

and without neighboring kangaroo rats. We compared the settlement (establishment of a stable home range) decisions and fitness of kangaroo rats between the 2 treatments. Kangaroo rats translocated with neighbors traveled shorter distances before establishing territories, had higher survival rates, and had significantly higher reproductive success than kangaroo rats translocated without neighbors. Number of offspring was 24-fold higher for kangaroo rats translocated with neighbors than those translocated without neighbors. Differences in behavior following release may partially explain differences in survival between the 2 groups. Immediately following release, animals translocated with neighbors fought less and spent significantly more time foraging and digging burrows than animals translocated without neighbors. Our results indicate that even for solitary species, maintaining relationships among members of a translocated group of animals can influence translocation success. This study is the first empirical demonstration of the fitness consequences of disrupting social relationships among territorial neighbors.

3101: *+.144*

We devised a novel approach to model reintroduced populations whereby demographic data collected from multiple sites are integrated into a Bayesian hierarchical model. Integrating data from multiple reintroductions allows more precise population-growth projections to be made, especially for populations for which data are sparse, and allows projections that account for random site-to-site variation to be made before new reintroductions are attempted. We used data from reintroductions of the North Island Robin (Petroica longipes), an endemic New Zealand passerine, to 10 sites where non-native mammalian predators are controlled. A comparison of candidate models that we based on deviance information criterion showed that rat-tracking rate (an index of rat density) was a useful predictor of robin fecundity and adult female survival, that landscape connectivity and a binary measure of whether sites were on a peninsula were useful predictors of apparent juvenile survival (probably due to differential dispersal away from reintroduction sites), and that there was unexplained random variation among sites in all demographic rates. We used the two best supported models to estimate the finite rate of increase (?) for populations at each of the 10 sites, and for a proposed reintroduction site, under different levels of rat control. Only three of the reintroduction sites had ? distributions completely >1 for either model. At two sites, ? was expected to be >1 if rat-tracking rates were <5%. At the other five reintroduction sites, ? was predicted to be close to 1, and it was unclear whether growth was expected. Predictions of ? for the proposed reintroduction site were less precise than for other sites because distributions incorporated the full range of site-to-site random variation in vital rates. Our methods can be applied to any species for which postrelease data on demographic rates are available and potentially can be extended to model multiple species simultaneously.

3102: *-.123*

Reintroductions are increasingly utilized for the conservation of endangered avian species. To avert disease-related failures, studies to determine disease risks should be performed prior to the implementation of any avian reintroduction program. The presence, and prevalence, of disease-causing agents in both the source population and in birds at the site of reintroduction may help better direct reintroduction programs. In this study, we determined the prevalence of parasitic and pathogenic agents in chickens and wild birds on Floreana Island prior to the reintroduction of the critically endangered Floreana mockingbird Mimus trifasciatus. We investigated avian diseases on Floreana in 175 chickens and 274 wild birds. In addition to a number of clinical abnormalities, chickens tested positive for antibodies to paramyxovirus-1 (30%), adenovirus (11.3%) and seven other pathogens of concern for both domestic and wild birds. Wild birds on Floreana had antibodies to paramyxovirus-1 (3.0%) and adenovirus (2.4%). This is the first report of possible

spillover of disease from domestic to wild birds in the archipelago. Based on these findings, and the lack of disease exposure documented in the source mockingbird population, we recommend improved poultry biosecurity measures on Floreana, and that mockingbirds only be reintroduced in areas on the island far from poultry and human presence and following further prerelease analyses. This study provides valuable data for the reintroduction of this iconic bird species and serves as a template for other avian reintroduction programs.

3103: ***-.016***

Members of the order Psittaciformes (parrots and cockatoos) are among the most long-lived and endangered avian species. Comprehensive data on lifespan and breeding are critical to setting conservation priorities, parameterizing population viability models, and managing captive and wild populations. To meet these needs, we analyzed 83 212 life-history records of captive birds from the International Species Information System (ISIS) and calculated lifespan and breeding parameters for 260 species of parrots (71% of extant species). Species varied widely in lifespan, with larger species generally living longer than smaller ones. The highest maximum lifespan recorded was 92 years in Cacatua moluccensis, but only 11 other species had a maximum lifespan over 50 years. Our data indicate that while some captive individuals are capable of reaching extraordinary ages, median lifespans are generally shorter than widely assumed, albeit with some increase seen in birds presently held in zoos. Species that lived longer and bred later in life tended to be more threatened according to IUCN classifications. We documented several individuals of multiple species that were able to breed for more than two decades, but the majority of clades examined had much shorter active reproduction periods. Post-breeding periods were surprisingly long and in many cases surpassed the duration of active breeding. Our results demonstrate the value of the ISIS database to estimate life-history data for an at-risk taxon that is difficult to study in the wild, and provide life-history data that is crucial for predictive modeling of future species endangerment and proactively management of captive populations of parrots.

3104: ***+.082***

Populations of Houbara Bustards have dramatically declined in recent years. Captive breeding and reintroduction programs have had limited success in reviving population numbers and thus new technological solutions involving molecular methods are essential for the long term survival of this species. In this study, we sequenced the 694 bp segment of COI gene of the four specimens of Asian Houbara Bustard (Chlamydotis undulata macqueenii). We also compared these sequences with earlier published barcodes of 11 individuals comprising different families of the orders Gruiformes, Ciconiiformes, Podicipediformes and Crocodylia (out group). The pair-wise sequence comparison showed a total of 254 variable sites across all the 15 sequences from different taxa. Three of the four specimens of Houbara Bustard had an identical sequence of COI gene and one individual showed a single nucleotide difference (G > A transition at position 83). Within the bustard family (Otididae), comparison among the three species (Asian Houbara Bustard, Great Bustard (Otis tarda) and the Little Bustard (Tetrax tetrax)), representing three different genera, showed 116 variable sites. For another family (Rallidae), the intra-family variable sites among the individuals of four different genera were found to be 146. The COI genetic distances among the 15 individuals varied from 0.000 to 0.431. Phylogenetic analysis using 619 bp nucleotide segment of COI clearly discriminated all the species representing different genera, families and orders. All the four specimens of Houbara Bustard formed a single clade and are clearly separated from other two individuals of the same family (Otis tarda and Tetrax tetrax). The nucleotide sequence of partial segment of COI gene effectively discriminated the closely related species. This is the first study reporting the barcodes of Houbara Bustard and would be helpful in future molecular studies,

particularly for the conservation of this threatened bird in Saudi Arabia.

3105: ***+.062***

Maintaining aquatic biodiversity in urban or suburban areas can be problematic because urban landscapes can be nearly devoid of aquatic habitats other than engineered basins for storm water management. These areas are usually of questionable value for fish, but we examined a case study in which five regionally imperiled fish species were reintroduced into an artificial storm water detention pond and subsequently thrived. Although not a formal experiment, postintroduction survey data suggested that three of the five species maintained high population densities for 10 years after initial stocking, and two persisted in lower numbers. Success was likely due to a combination of unique design features and prior habitat preparation that resulted in clear water conditions that supported dense vegetation. Stocked fish persisted despite occasional bouts of low dissolved oxygen and increased chloride levels resulting from road salt application within the watershed. Transplanted fish served as a source population for both research and further reintroduction experiments. We suggest that, for some fish species, habitat preservation has a middle ground between natural habitats and completely artificial environments that require constant husbandry and that storm water systems could be used to create engineered sanctuaries within the human landscape that have many potential benefits for both humans and fish.

3106: ***+.163***

Postrelease monitoring is an important aspect of reintroduction projects, one outcome of which is to allow an assessment of the initial success of the reintroduction, often measured by quantifying survival and reproduction rates. In long-lived species, accurate estimations of demographic parameters are difficult to obtain, and therefore assessment of reintroduction success in such species is challenging. To assess the initial success of a reintroduction program for the long-lived, slow-reproducing, and critically endangered western lowland gorilla Gorilla gorilla gorilla, we analyzed postrelease monitoring data from 2 reintroduced populations, in the Bateke Plateau region of the Republics of Congo and Gabon, to quantify several demographic parameters, and compared our results with published data on wild gorilla populations. Annual survival rate of the 51 released gorillas was 97.4%, 9 females gave birth to 11 infants at an annual birth rate of 0.196 births per adult female, and first-year survival of the infants was 81.8%. Annual birth rate within the reintroduced populations is not significantly different from that given for wild western gorilla populations, and other demographic parameters fall within the range of published data for wild gorilla populations. Our analysis illustrates that the reintroduction program has been successful in terms of the initial measures of postrelease survival and reproduction, and our quantitative data should facilitate the development of a population model that can predict the probability of population persistence and therefore provide an indication of longer-term reintroduction success.

3108: ***+.352***

Habitat connectivity is important for the survival of species that occupy habitat patches too small to sustain an isolated population. A prominent example of such a species is the European bison (Bison bonasus), occurring only in small, isolated herds, and whose survival will depend on establishing larger, well-connected populations. Our goal here was to assess habitat connectivity of European bison in the Carpathians. We used an existing bison habitat suitability map and data on dispersal barriers to derive cost surfaces, representing the ability of bison to move across the landscape, and to delineate potential connections (as least-cost paths) between currently occupied and potential habitat patches. Graph theory tools were then employed to evaluate the connectivity

of all potential habitat patches and their relative importance in the network. Our analysis showed that existing bison herds in Ukraine are isolated. However, we identified several groups of well-connected habitat patches in the Carpathians which could host a large population of European bison. Our analysis also located important dispersal corridors connecting existing herds, and several promising locations for future reintroductions (especially in the Eastern Carpathians) that should have a high priority for conservation efforts. In general, our approach indicates the most important elements within a landscape mosaic for providing and maintaining the overall connectivity of different habitat networks and thus offers a robust and powerful tool for conservation planning. (C) 2011 Elsevier Ltd. All rights reserved.

3109: ***+.071***

We provide the first genetic analysis of the Bruneau Hot Springsnail (Pyrgulopsis bruneauensis), a federally listed (endangered) hydrobiid gastropod that is distributed in spring-fed habitats along a short reach of the Bruneau River in southwestern Idaho and threatened with extinction by groundwater withdrawal. Partial sequences of mitochondrial cytochrome c oxidase subunit I (COI) and NADH dehydrogenase subunit I (NDI) were obtained from 51 specimens from six sites spanning the narrow geographic range of P. bruneauensis. A Bayesian analysis of the combined dataset resolved this species as a well supported clade which differed from other regional congeners by 4.66-10.62% sequence divergence (COI). The 11 observed COI haplotypes in P. bruneauensis formed two divergent (1.42 +/- 0.7%) subgroups that co-occurred at five of the six collecting sites. COI haplotype diversity was substantial (ranging up to 0.9111) in all but one sample, while nucleotide diversity was low (<0.01). AMOVA detected small but significant variation among sites, although only one sample was significantly differentiated by pairwise comparisons. Haplotype composition varied widely among the collecting localities and no obvious geographic pattern was detected. These findings suggest that translocation of snails, which was considered as a possible measure in the P. bruneauensis recovery plan, should be preceded by assays to ensure selection of appropriately genetically diverse source populations.

3110: ***+.010***

Assessing patterns of genetic structure and diversity of threatened species has become an essential tool for determining conservation status and designing management strategies. We examine the genetic structure of the Sierra Madre sparrow (Xenospiza baileyi), a species restricted to fragmented patches of subalpine bunchgrass in three small isolated areas of northwestern and central Mexico. Coding and non-coding regions of mtDNA (1,878 bp) from individuals of the only three known populations revealed the existence of a single major lineage, with closely related haplotypes being shared between populations across the range. The sharing of haplotypes between the distant northwest and central populations (similar to 800 km) suggests a recent fragmentation of a formerly contiguous population. Despite a lack of large-scale phylogeographic structure, haplotype frequencies at local scales revealed significant genetic differentiation and high F-ST values between all three remaining populations, even between localities separated by less than 12 km. These results suggest restricted gene flow and limited dispersal, likely due to the species' inability to cross areas of unsuitable habitat. On the basis of genetic interchange and ecological equivalence criteria, we recommend that the species be managed as a single unit, permitting the strengthening of the small population in the northwest with individuals from central Mexico, and/or the translocation of individuals to new areas of suitable habitat.

3111: ***+.099***

Since being declared extinct in the wild in 1972, the Arabian oryx has been the subject of intense and sustained effort to maintain a healthy captive population and to reintroduce the species to its ancestral range. Previous reintroductions and associated genetic assessments focused on the release of closely managed zoo animals into Oman and included observations of inbreeding and out-breeding depression. Here we describe the use of multiple unmanaged herds as source populations for a new reintroduction project in the United Arab Emirates, allowing a comparison between studbook management and uncontrolled semi-captive breeding approaches to the conservation of genetic diversity. Results of mitochondrial control region sequencing and 13-locus microsatellite profiling highlight a severe lack of diversity within individual source populations, but a level of differentiation among populations that supports the formation of a mixed founder herd. The combined release group contained a similar level of diversity to each of the intensively managed captive populations. The research includes the first genetic data for animals held on Sir Bani Yas Island, a former private reserve which until recently held over 50% of the world's Arabian and scimitar-horned oryx and is recognized as having huge potential for re-establishing endangered antelope species in the wild. The genetic assessment provides the first stage of an ongoing genetic monitoring programme to support future supplemental releases, translocations and genetic management of reintroduced populations.

3112: ***+.044***

The release of animals to reestablish an extirpated population is a decision problem that is often attended by considerable uncertainty about the probability of success. Annual releases of captive-reared juvenile Whooping Cranes (Grus americana) were begun in 1993 in central Florida, USA, to establish a breeding, non-migratory population. Over a 12-year period, 286 birds were released, but by 2004, the introduced flock had produced only four wild-fledged birds. Consequently, releases were halted over managers' concerns about the performance of the released flock and uncertainty about the efficacy of further releases. We used data on marked, released birds to develop predictive models for addressing whether releases should be resumed, and if so, under what schedule. To examine the outcome of different release scenarios, we simulated the survival and productivity of individual female birds under a baseline model that recognized age and breeding-class structure and which incorporated empirically estimated stochastic elements. As data on wild-fledged birds from captive-reared parents were sparse, a key uncertainty that confronts release decision-making is whether captive-reared birds and their offspring share the same vital rates. Therefore, we used data on the only population of wild Whooping Cranes in existence to construct two alternatives to the baseline model. The probability of population persistence was highly sensitive to the choice of these three models. Under the baseline model, extirpation of the population was nearly certain under any scenario of resumed releases. In contrast, the model based on estimates from wild birds projected a high probability of persistence under any release scenario, including cessation of releases. Therefore, belief in either of these models suggests that further releases are an ineffective use of resources. In the third model, which simulated a population Allee effect, population persistence was sensitive to the release decision: high persistence probability was achieved only through the release of more birds, whereas extirpation was highly probable with cessation of releases. Despite substantial investment of time and effort in the release program, evidence collected to date does not favor one model over another; therefore, any decision about further releases must be made under considerable biological uncertainty. However, given an assignment of credibility weight to each model, a best, informed decision about releases can be made under uncertainty. Furthermore, if managers can periodically revisit the release decision and collect monitoring data to further inform the models, then managers have a basis for confronting uncertainty and adaptively managing releases through time.

3113: ***+.120***

Inbreeding depression is frequently a concern of managers interested in restoring endangered species. Decisions to reduce the potential for inbreeding depression by balancing genotypic contributions to reintroduced populations may exact a cost on long-term demographic performance of the population if those decisions result in reduced numbers of animals released and/or restriction of particularly successful genotypes (i.e., heritable traits of particular family lines). As part of an effort to restore a migratory flock of Whooping Cranes (Grus americana) to eastern North America using the offspring of captive breeders, we obtained a unique dataset which includes post-release mark-recapture data, as well as the pedigree of each released individual. We developed a Bayesian formulation of a multi-state model to analyze radio-telemetry, band-resight, and dead recovery data on reintroduced individuals, in order to track survival and breeding state transitions. We used studbook-based individual covariates to examine the comparative evidence for and degree of effects of inbreeding, genotype, and genotype quality on post-release survival of reintroduced individuals. We demonstrate implementation of the Bayesian multi-state model, which allows for the integration of imperfect detection, multiple data types, random effects, and individual- and time-dependent covariates. Our results provide only weak evidence for an effect of the quality of an individual's genotype in captivity on post-release survival as well as for an effect of inbreeding on post-release survival. We plan to integrate our results into a decision-analytic modeling framework that can explicitly examine tradeoffs between the effects of inbreeding and the effects of genotype and demographic stochasticity on population establishment.

3114: ***+.074***

Positive demographic responses have been reported in several species where the immigration or supplementation of genetically distinct individuals into wild populations has resulted in a genetic rescue effect. However, rarely have researchers incorporated what could be considerable risk of outbreeding depression into planning for genetic management programs. We assess the genetic effects of an experiment in genetic management involving replicate populations of California bighorn sheep (Ovis canadensis californiana) in Oregon, USA, which previously experienced poor productivity and numerical declines. In the experiment, two declining populations were supplemented with ewes from a more genetically diverse population of California bighorn sheep in Nevada. We incorporated analysis of genetic samples representing both experimental populations prior to supplementation, samples from the supplemented individuals, and samples collected from both experimental populations approximately one generation after supplementation. We used genetic analyses to assess the integration of supplemented and resident populations by identifying interpopulation hybrids. Further, we incorporated demographic simulations to assess the risk of outbreeding depression as a result of the experimental augmentation. Finally, we used data from microsatellites and mitochondrial sequences to determine if genetic management increased genetic diversity in the experimental populations. Our analyses demonstrated the success of genetic management by documenting interpopulation hybrids, identifying no evidence for outbreeding depression as a result of contact between the genetically distinct supplemented and resident populations, and by identifying increased population-level metrics of genetic diversity in postsupplementation populations compared with presupplementation levels.

3115: ***-.081***

Sarcoptic mange was recently described in the wild European rabbit (Oryctolagus cuniculus) in north-eastern Mediterranean Spain, the first such infection reported in this species anywhere in the world. This finding has created concern in conservationists and game managers given that an

outbreak of mange after a translocation would have catastrophic consequences for naive rabbit populations in other parts of Spain. A retrospective sero-survey using an 'in house' ELISA test based on the use of a recombinant antigen aimed at determining the rates of contact with Sarcoptes scabiei was carried out on sera from 966 rabbits collected between 1993 and 2010 in Spain. Antibodies were found in 13% of wild rabbits in 60% of the 53 areas surveyed, as well as in 16 of the 17 Spanish provinces and islands studied. Seropositive rabbits were found amongst the oldest samples analyzed and in all studied years. Antibodies were also detected in 36% of rabbits from the protected island of Dragonera, where rabbits have probably not been released since the 1970s. On Mallorca, where 89 rabbits were inspected for both lesions and antibodies, the prevalence of lesions (5.6%) was much lower than the seroprevalence (22.5%), indicating that rabbits often survive infection or that ELISA detects infected rabbits before they develop visible lesions. Seroprevalence was higher in areas with medium levels of rabbit abundance, no restocking and high rainfall. The results show that mange is widespread in rabbits and that the mite is not a recent introduction. Thus, sarcoptic mange could be considered as an enzootic disease in the wild rabbit and so prophylactic measures implemented during rabbit translocations are to be encouraged to avoid local outbreaks in naive populations. (C) 2011 Elsevier B.V. All rights reserved.

3116: *+.208*

From observations conducted in a suburban property in Perth, Western Australia, over 22 years, it appears that a single pet cat may have exterminated a population (est. 40-50 animals) of the lizard Ctenotus fallens over two years, but with the greatest impact in just the first few months. C. fallens did not begin to recolonise the site until six years after the cat had moved away. The observations support the hypothesis that extinctions of wildlife in suburbia following the introduction of cats can be swift. They also suggest that C. fallens is a suitable species for reintroduction experiments into suburban Perth, comparing the success of reintroductions at sites where cats are confined with those where cats roam freely.

3117: *+.374*

1. Availability of suitable habitat is a prerequisite for species reintroduction success, and to ensure population persistence, investigations of a species habitat utilisation throughout its life history should be conducted as part of a feasibility study.2. Habitat utilisation models for burbot, Lota lota, developed using data from field studies conducted in France and Germany and information from the literature were used to assess the feasibility of reintroducing burbot into rivers of its former native range in eastern England.3. Per cent tree roots, aquatic vegetation and flow types were important predictors of adult burbot abundance. Furthermore, the habitat utilisation models were supplemented with information from the literature, which suggested that off-channel habitat such as wetlands and backwaters is important for spawning and nursery stages.4. An assessment of the habitat availability in the rivers of the burbot's former native range using variables related to spawning and nursery and adult life stages showed that although adult habitat was widely distributed, the availability of spawning and nursery habitat was less abundant, potentially limiting successful reestablishment.5. Potential suitable habitat was concentrated in the central and southern areas of the species' former English distribution. Overall, rivers of the burbot's former range potentially afford suitable habitat to sustain a reintroduced population. However, sites should be preferentially selected on the basis of having appropriate spawning and nursery areas.

3118: *-.033*

Non-local population stocking can have adverse genetic effects on wild populations through loss

of genetic diversity and introgressive hybridization. The grey partridge (Perdix perdix) has been an important European game species for centuries, widely subject to translocation and stocking. After c. 80 years of releasing reared grey partridges in Denmark, this study investigated whether an indigenous Danish grey partridge still existed. If so, they would (1) belong to the western European clade (W1) and (2) be more closely related to the historical, indigenous grey partridges than to farm-bred partridges. These predictions were tested by analysing the variation in both the mitochondrial control region (CR1) and microsatellite markers in museum samples representing the ancestral indigenous Danish grey partridge, contemporary wild grey partridges and farmed grey partridges from the five largest farms in Denmark. Phylogeography and population structure analyses showed traces of the indigenous Danish grey partridges amongst recent wild partridges in certain areas and significant genetic differences between farmed partridges and historical and recent partridges. The results also showed that the indigenous Danish grey partridges belonged to the western European clade (W1 haplotype). A foreign stocking effect was detected on the remote island of Bornholm, where the current population originated from introduced Danish and Bohemian grey partridges. The loss of haplotype diversity over time in certain geographical areas probably results from serious declines in wild Danish grey partridge numbers in recent decades. This, combined with the observation that hybridization between released stocked and wild partridges can occur, may complicate recovery of partridge populations. (C) 2012 The Linnean Society of London, Biological Journal of the Linnean Society, 2012, 105, 694710.

3119: ***+.116***

Interplay between the complex geography, hydrogeomorphological history, past climatic changes, and anthropogenic pressures is likely responsible for the current diversity and species' distribution of freshwater fishes in the Iberian Peninsula. To further disentangle the evolutionary processes promoting the diversification of endemic Iberian Cyprinids through time and space, we explored the patterns of genetic diversity of the Iberian arched-mouth nase, Iberochondrostoma lemmingii (Steindachner, 1866), using molecular markers rendering at different timescales: the mitochondrial gene cytochrome b and seven microsatellite loci. Both markers showed significant differentiation of populations though the relative genetic distances among populations were different between markers. Mitochondrial DNA results indicate the isolation of hydrographic basins as the main driver of population differentiation, with Tejo as the centre of diversification. The results also support connections between Tejo, Guadiana, and Guadalquivir, with levels of divergence suggesting an earlier severance of Guadalquivir, whereas Guadiana and Tejo maintained connections until a more recent past. Establishment of more peripherial populations in small southern basins (Quarteira and Almargem) could have been ruled by founder events. However, the analysis of present-day genetic configuration suggested by microsatellite data implies, for the first time, the involvement of other factors in the evolution of arched-mouth Iberian nase populations. Relative low genetic distances between inter-basin populations (Tejo and Guadiana) and the lack of concordance between differentiation and geography suggest a possible influence of human-mediated translocations in the population genetic patterns of I. lemmingii. High intra-basin differentiation levels were found within Tejo and Guadiana and may be associated with factors intrinsic to the species (e.g. low dispersal capability) or natural and/or artificial barriers to gene flow. The low vagility of the species appears to be an important factor influencing the evolutionary processes shaping the phylogeographical patterns of I. lemmingii, which could be relevant for the conservation of this threatened species. (C) 2012 The Linnean Society of London, Biological Journal of the Linnean Society, 2012, 105, 559572.

3120: ***+.030***

Deliberate taxon substitution is a much discussed but rarely enactioned concept in restoration ecology. We describe the successful establishment of a translocated population of Snares Island snipe (Coenocorypha huegeli) on Putauhinu I, which lies alongside Taukihepa (Big South Cape I), the last stronghold of the extinct South Island snipe (C. iredalei). Thirty Snares Island snipe were captured on North East I, Snares Is in Apr 2005 and released 3-5 days later on Putauhinu I. A survey on Putauhinu I in Mar 2011 resulted in the capture of 54 descendants of the released birds and a population estimate of at least 320 birds. This is one of few documented translocations of an organism with the specific objective of replacing a closely related extinct taxon. As a result, the Snares Island snipe is probably more abundant than at any time in its evolutionary history.Miskelly, C.M.; Charteris, M.R; Fraser, J.R. 2012. Successful translocation of Snares Island snipe (Coenocorypha huegeli) to replace the extinct South Island snipe (C. iredalei). Notornis 59 (1&2): 32-38.

3121: *+.233*

In the next century, safeguarding plant species against extinction from complete land conversion may require introducing species to novel locations. Although regulatory agencies caution against translocation outside of known historic ranges, when most wild populations and their habitats have been severely altered few viable options may be available for conserving rare plants. We introduced 345 endangered Amorpha herbacea var. crenulata along a pine rockland/transverse glade gradient with similar attributes to historically known occurrences for south Florida, USA, and monitored their survival and growth for five years. The experimental phase addressed: (1) Is the recipient site suitable for colonisation of this species despite hydrological manipulation in the region? (2) Can translocated plants grow equally well in four microhabitats along a gradient within the recipient site? We characterised soil water content, soil nutrient, and vegetation cover to assess the microhabitats at the recipient site. From 2006 to 2008 plants survived in all four microhabitats, but had highest survival in pineland. Translocated plants grew best in microhabitats with less grass cover and higher P content - the pineland and the restoration glade. Through 2008 we observed consistently higher soil water content with less total vegetation cover in pineland and significantly higher P content in the restoration glade. Using 2006-2008 data, we implemented the adaptive management phase, moving 20 plants from the lowest survival microhabitat to the highest survival microhabitat. This tactic improved the survival of plants by 2011, though growth rates of moved plants did not improve. Short-distance translocation, assessing environmental attributes related to plant survival and growth, quantifying similarity of soil, temperature, precipitation, and community as in this study are recommended to evaluate prospective introduction sites for translocations within or outside of range. (c) 2011 Elsevier GmbH. All rights reserved.

3122: *+.197*

Only ca. 3000 individuals of Hex khasiana Purk. are surviving today. The tree species is endemic to Khasi hills of northeast India, and is critically endangered. For improving the conservation status of the species, potential area and habitat for reintroduction were predicted using Maximum Entropy (MaxEnt) distribution modelling algorithm. The model was developed using 16 locality data in the native range of Khasi hills, and 16 environmental parameters including enhanced vegetation index (EVI) and digital elevation data. The model predicted that the suitable habitats of I. khasiana was restricted to an area of approximate to 500 km(2) in the Khasi hills of Meghalaya. The distribution of potential habitats was strongly influenced by elevation and the EVI layers for the period April-May, which corresponds to the flowering phase of the species, thus indicating the importance of flowering stage in determining the species distribution. Population status was positively correlated with higher model thresholds in the undisturbed habitats confirming the

usefulness of the habitat model in population monitoring, particularly in predicting the successful establishment of the species. The study delineated the potential habitats in the higher elevations of Khasi hills within the current home range where the species can be reintroduced. (C) 2011 Elsevier B.V. All rights reserved.

3123: ***+.062***

Many breeding colonies of Procellariiformes have been threatened with extinction. Chick translocation has been shown to be an effective method for establishing new "safer" colonies of burrow-nesting species, but techniques for surface-nesting species have not been fully developed. The entire breeding population of the threatened Short-tailed Albatross Phoebastoria albatrus is restricted to two sites, Torishima Island and the Senkaku Islands, and neither site is secure due to volcanic activity or political instability. The Short-tailed Albatross Recovery Team has recommended facilitating the recovery of this species by establishing at least one additional colony through the translocation and hand-rearing of chicks at a safe historical breeding site. To evaluate the feasibility of this approach, we hand-reared 10 post-guard phase chicks of two related species in 2006-2007: Laysan Albatross P. immutabilis translocated from Midway Atoll to Kaua'i Island, Hawai'i and Black-footed Albatross P. nigripes translocated from a nearby islet in the Ogasawara (Bonin) Islands to Mukojima Island, Japan. In these pilot studies, 40% of Laysan Albatross chicks and 90% of Black-footed Albatross chicks fledged successfully. Following this groundwork, 40 post-guard phase Short-tailed Albatross chicks were translocated from Torishima Island to Mukojima Island in February 2008-2010 and hand-reared to fledging. Their fledging success has been 100% in all three years. Fledging body sizes were similar or greater in hand-reared chicks at the release site than parent-reared chicks on Torishima Island. There were significant differences in levels of some blood chemistry parameters between pre-fledging hand-reared and parent-reared chicks. The techniques developed in our studies have broad-reaching implications for the future conservation of threatened populations of other surface-nesting seabirds.

3124: ***+.379***

Until recently, Bermuda Petrel Pterodroma cahow (IUCN Category: 'Endangered') bred only in sub-optimal habitat on four small islets in north-east Bermuda. Although intensive management of the population since 1962 has led to a substantial increase in population size (now approaching 100 pairs), the nesting habitat on these four islets is being increasingly inundated, eroded and destroyed by high seas associated with hurricanes and storms. To ensure the long-term conservation of the species a decision was made to establish a new colony at a more secure site on nearby Nonsuch Island, where they once bred in large numbers. Between 2004 and 2008, 104 near-fledged nestlings were translocated to artificial burrows on Nonsuch Island, where they were hand-fed meals of fish and squid. All but three translocated birds fledged successfully, with the first returning to Nonsuch Island in February 2008. The first Bermuda Petrel egg on Nonsuch Island in more than 300 years was laid in January 2009, and the resultant fledgling departed in June of the same year. By the end of the 2009/10 breeding season, a total of 18 Bermuda Petrels have been recorded on Nonsuch Island, 17 were translocated as near-fledged nestlings, and one bird came from the existing colonies. A total of five eggs have been produced, resulting in two fledglings. The establishment of this new colony, at a site that is much more secure than the existing nesting sites, greatly enhances the conservation prospects of the species and demonstrates the importance of translocation as a tool for the conservation of threatened seabirds.

3125: ***+.143***

Tigers Panthera tigris continue to decline despite the best efforts of the worldwide scientific and conservation communities. Prey depletion has been linked to this decline, but a clear definition of what constitutes preferred prey and preferred prey weight range does not exist. This is critical information if we are to assess tiger reintroduction potential, monitor unforeseen poaching of predators and prey, and successfully conserve the species. Here we reviewed the available literature on tiger diet and prey availability and calculated Jacobs's electivity index scores from 3187 kills or scats of 32 prey species. We found that wild boar and sambar deer are significantly preferred by tigers, with red deer and barasingha likely to be significantly preferred also with a larger sample size. Prey body mass was the only variable that related to tiger prey preference with species weighing between 60 and 250? kg preferred by tigers yielding a ratio of predator to preferred prey of 1:1, which is similar to other solitary felids. This information can be used to predict tiger diet, carrying capacity and movement patterns, as it has been for Africa's large predator guild, and has important implications for tiger conservation throughout its distribution.

3126: *+.147*

Dispersal is a key parameter for understanding demography and is consequently critical to conservation management. Peregrine Falcons (Falco peregrinus) in eastern North America exemplify a population that collapsed to extirpation and, subsequent to reintroduction, experienced a rapid, dispersal-based expansion back into its former range. To understand how dispersal and its correlates may be impacting falcon demography and encounter rates, we looked for (a) patterns of sex- and site-based differences in the dispersal movements of Peregrine Falcons from Pennsylvania, U.S.A.; (b) trends in density dependence in dispersal distances; (c) linkages between natal- and breeding-site characteristics; and (d) causes of death of Peregrine Falcons. Female peregrines showed significantly greater natal dispersal distances (337.7 +/- 164.5 km; n = 24) than did males (161.4 +/- 177.7 km; n = 21), but there were no statistically significant differences in dispersal distances of all birds or in breeding-site selection among birds originally hatched at bridges and buildings. Causes of death appeared typical for a large raptor in a human-dominated environment and the population showed no effects of density dependence on natal dispersal behavior. These results suggest that the peregrine population in Pennsylvania will continue to grow on its current trajectory, at least for some time. This finding has important implications for interpreting when this formerly endangered species may reach a carrying capacity in Pennsylvania.

3127: *+.160*

Translocating European ground squirrels Spermophilus citellus has become a popular conservation tool. However, few release techniques have been carefully evaluated. To contribute to an evidence-based ground squirrel translocation guide for wildlife managers, we evaluated conditions of habitat manipulation (grass height and artificial burrow entrance angle) which we expected to affect. settlement of translocated ground squirrels during the critical period after release. In a field experiment, we translocated 173 individuals in southeastern Hungary in 2007. We released the animals into angled or vertical artificial burrows and manipulated grass height. We found that animals preferred angled (similar to 30 degrees) artificial burrows, which facilitate digging, and medium-height grass (18 cm +/- 1.5). Moreover, although ground squirrels generally are associated with short grass habitats, overhead protection by grasses is valuable after a translocation. This result implies that in order to accomplish a translocation, it is not sufficient to only know the habitat preference of a species in undisturbed situations, but also how and to what extent habitat characteristics should be manipulated to increase the chances of success.

3128: ***+.022***

Ecological restoration typically focuses on promoting vegetation recovery in degraded habitat or reintroducing endangered animals to enhance their regional or global persistence. Here, we argue that attention should also be devoted to vertebrate reintroductions in overhunted but floristically intact tropical forests in order to prevent insidious regime shifts in these systems. Growing evidence suggests that tropical forests deprived of seed-dispersing animals exhibit replacement of fleshy fruiting trees by species with abiotic seed dispersal. Left unchecked, this process could eventually render the forest uninhabitable by frugivores through reduced density and diversity of their food plants. In tropical areas where hunting can be controlled, we contend that frugivore reintroduction, regulation of wild fruit harvest by humans, and outplanting of native fruiting trees should be deployed as management tools long before the systems are in need of traditional habitat restoration.

3129: ***+.148***

Relocation of threatened populations is a common method employed in conservation. However, environmental differences in the new habitat may reduce the survival of relocated populations, while phenotypic plasticity may enhance the likelihood of establishment of relocated populations in their novel environment. Conservation of the British white-clawed crayfish (Austropotamobius pallipes; IUCN Red Data List Endangered) often involves relocation of threatened populations into isolated ponds (Ark Sites), where risk of competition with invasive crayfish is minimized. In this study, the morphology (using 12 morphometric variables) of A. pallipes in wild populations was investigated with respect to eco-geographic variables. A field cage experiment was carried out to compare the relative survival, growth, and change in morphology of crayfish from lotic (stream) and lentic (pond) donor habitats following relocation to a lentic recipient habitat. In the wild, lentic crayfish were broader than their lotic counterparts, which may reflect an increase in branchial (gill) volume in adaptation to an oxygen-poor benthic environment. In the relocation experiment there was no difference in the length, growth, or survival of animals from relocated lentic, lotic, or control populations. However, crayfish derived from a lotic population showed an increase in carapace width and areola width over the 4-month growing season following relocation. This evidence for phenotypic plasticity suggests that crayfish are resilient to relocation, and that they can adapt morphologically to novel environmental conditions. Relocation of threatened populations of A. pallipes may therefore prove a useful technique in the conservation of white-clawed crayfish populations within the UK. Copyright (c) 2012 John Wiley & Sons, Ltd.

3131: ***+.157***

Captive breeding of animals is often cited as an important tool in conservation, especially for fishes, but there are few reports of long-term (<50 years) success of captive breeding programs, even in salmonid fishes. Here we describe the captive breeding program for Eagle Lake rainbow trout, Oncorhynchus mykiss aquilarum, which is endemic to the Eagle Lake watershed of northeastern California. The population in Eagle Lake has been dependent on captive breeding for more than 60 years and supports a trophy fishery in the lake. Nevertheless, the basic life history, ecological, and genetic traits of the subspecies still seem to be mostly intact. Although management has apparently minimized negative effects of hatchery rearing, reestablishing a wild population would ensure maintenance of its distinctive life history and its value for future use as a hatchery fish. An important factor that makes reestablishment possible is that the habitat in Eagle Lake is still intact and that Pine Creek, its major spawning stream, is recovering as habitat. With the exception of an abundant alien brook trout (Salvelinus fontinalis) population in Pine Creek,

the habitat factors that led to the presumed near-extinction of Eagle Lake rainbow trout in the early twentieth century have been ameliorated, although the final stages of reestablishment (eradication of brook trout, unequivocal demonstration of successful spawning migration) have still not been completed. The Eagle Lake rainbow trout story shows that long-term captive breeding of migratory salmonid fishes does not necessarily prevent reestablishment of wild populations, provided effort is made to counter the effects of hatchery selection and that natural habitats are restored for reintroduction. Long-term success, however, ultimately depends upon eliminating hatchery influences on wild-spawning populations. Extinction of Eagle Lake rainbow trout as a wild species becomes increasingly likely if we fail to act boldly to protect it and the Eagle Lake watershed.

3132: ***+.212***

Reintroductions and other conservation translocations have become increasingly important conservation tools, albeit with variable success. Genetic variation is one factor, which may influence reintroduction success. Genetic variation in reintroduced populations can be augmented by increasing the number of founders or by admixing animals from different source populations. At present there is no clear understanding of the relative importance of the two. Here we address this question by combining detailed demographic information about the reintroduction history of 40 Alpine ibex populations with genetic data from neutral markers, including coalescent-based estimates of the number of genetic founders. Number of genetic founders was a better predictor of present-day genetic variation than number of released founders, indicating that differential survival of founders can substantially affect the genetic variation of reintroduced populations. The degree of admixture in the founder group had about twice as much impact on genetic variation than the number of founders. Thus, to maintain genetic variation in reintroduced populations, releasing animals from different sources might be more important than releasing many animals from a single source. This even applies to cases such as the Alpine ibex where all individuals descended from a single ancestral population, and where the admixture was only between sub-populations created by the reintroduction program and thus between populations with relatively little genetic differentiation. (C) 2012 Elsevier Ltd. All rights reserved.

3133: ***-.019***

Animal translocation success rate is generally low, with the causes of failure poorly understood without comprehensive and protracted monitoring. Here we examine the outcome of a translocation of endangered North Island kokako (Callaeas wilsoni) from two adjacent song neighborhoods in New Zealand, each with individual vocal traditions (c. 75% of phrases unshared) to a single release site. We conducted detailed radio-telemetry to monitor post-release dispersal over 50 days during four serial releases of 20 birds while we broadcast neighborhood-specific song around the release site. The birds moved substantial distances after release, however overall short-term release site dispersal was not as great as predicted by a random walk model, suggesting an attraction to playback and/or a reluctance to explore areas away from the release site. This apparent attraction was not specific to a given song neighborhood, however. Although the post-release mortality rate (22% over 31 days) was relatively high in this translocation, we did not detect an effect of sex, age, source origin, or duration of captivity on mortality. We show that habitat use during this acclimation period was disproportionate to availability - the birds' preferred habitat was similar to that at the capture site. At least four pairs formed, with two and three confirmed breeding in the first and second seasons post-release respectively. Mate choice was non-assortative with respect to song neighborhood, revealing that reduced phrase sharing rates found in adjoining neighborhoods are not a barrier to pair formation. We compare this example with other

kokako translocations and make recommendations for future translocations. (C) 2011 Elsevier Ltd. All rights reserved.

3135: ***+.314***

The North American beaver (Castor canadensis) has not been considered native to the mid- or high-elevations of the western Sierra Nevada or along its eastern slope, although this mountain range is adjacent to the mammal's historical range in the Pit, Sacramento and San Joaquin rivers and their tributaries. Current California and Nevada beaver management policies appear to rest on assertions that date from the first half of the twentieth century. This review challenges those long-held assumptions. Novel physical evidence of ancient beaver dams in the north central Sierra (James and Lanman 2012) is here supported by a contemporary and expanded re-evaluation of historical records of occurrence by additional reliable observers, as well as new sources of indirect evidence including newspaper accounts, geographical place names, Native American ethnographic information, and assessments of habitat suitability. Understanding that beaver are native to the Sierra Nevada is important to contemporary management of rapidly expanding beaver populations. These populations were established by translocation, and have been shown to have beneficial effects on fish abundance and diversity in the Sierra Nevada, to stabilize stream incision in montane meadows, and to reduce discharge of nitrogen, phosphorus and sediment loads into fragile water bodies such as Lake Tahoe.

3136: ***-.055***

Many species of Australian robins (Petroicidae) are declining in wooded landscapes across southern Australia, perhaps because they are unable or unwilling to disperse across the matrix between remnants, or because they experience high mortality while doing so. Eastern Yellow Robins Eopsaltria australis have declined in parts of southeastern Australia, and have gone extinct in some woodland remnants. We translocated adult Yellow Robins to remnants from which they had disappeared, and followed their survival and behaviour. Nine Yellow Robins were translocated to a 100 ha remnant in 2001 (seven birds) and 2002 (two birds). One bird disappeared immediately and a second after 2 weeks, but the rest stayed for at least 2 months, with two birds surviving to at least 4 and 5 years. One reintroduced pair bred each year from 2001 to 2005, producing six fledglings. Two colour-banded Yellow Robins also arrived unaided from another remnant 7 km away. Fifteen Yellow Robins were translocated to nine small remnants (<60 ha) in 2005-06. Seven birds disappeared within 3 days of their release, apparently killed by predators. The remainder survived for up to 7 weeks, but none was found breeding. Translocated Robins moved up to 3 km from their release sites through natural or planted corridors, and up to 1 km through a matrix of scattered trees. Small remnants, which surviving Robins ultimately vacated, had fewer shrubs than sites where they bred. The results suggest that Eastern Yellow Robins can disperse through a landscape with corridors or scattered trees. However, they may suffer predation and reject sites that are too small or of poor habitat quality. Management to retain Eastern Yellow Robins in a fragmented landscape should enlarge remnants, protect scattered trees, plant or regenerate strategic clumps of trees and shrubs in gaps between remnants, and allow shrubs to regenerate within remnants.

3138: ***+.034***

Freshwater biodiversity has declined dramatically in Europe in recent decades. Because of massive habitat pollution and morphological degradation of water bodies, many once widespread species persist in small fractions of their original range. These range contractions are generally

believed to be accompanied by loss of intraspecific genetic diversity, due to the reduction of effective population sizes and the extinction of regional genetic lineages. We aimed to assess the loss of genetic diversity and its significance for future potential reintroduction of the long-tailed mayfly Palingenia longicauda (Olivier), which experienced approximately 98% range loss during the past century. Analysis of 936 bp of mitochondrial DNA of 245 extant specimens across the current range revealed a surprisingly large number of haplotypes (87), and a high level of haplotype diversity (Hd = 0.875). In contrast, historic specimens (6) from the lost range (Rhine catchment) were not differentiated from the extant Raba population (F-ST = 0.02, p = 0.61), despite considerable geographic distance separating the two rivers. These observations can be explained by an overlap of the current with the historic (Pleistocene) refugia of the species. Most likely, the massive recent range loss mainly affected the range which was occupied by rapid post-glacial dispersal. We conclude that massive range losses do not necessarily coincide with genetic impoverishment and that a species' history must be considered when estimating loss of genetic diversity. The assessment of spatial genetic structures and prior phylogeographic information seems essential to conserve once widespread species.

3139: ***+.228***

The application of scientific-based conservation measures requires that sampling methodologies in studies modelling similar ecological aspects produce comparable results making easier their interpretation. We aimed to show how the choice of different methodological and ecological approaches can affect conclusions in nest-site selection studies along different Palearctic meta-populations of an indicator species. First, a multivariate analysis of the variables affecting nest-site selection in a breeding colony of cinereous vulture (Aegypius monachus) in central Spain was performed. Then, a meta-analysis was applied to establish how methodological and habitat-type factors determine differences and similarities in the results obtained by previous studies that have modelled the forest breeding habitat of the species. Our results revealed patterns in nesting-habitat modelling by the cinereous vulture throughout its whole range: steep and south-facing slopes, great cover of large trees and distance to human activities were generally selected. The ratio and situation of the studied plots (nests/random), the use of plots vs. polygons as sampling units and the number of years of data set determined the variability explained by the model. Moreover, a greater size of the breeding colony implied that ecological and geomorphological variables at landscape level were more influential. Additionally, human activities affected in greater proportion to colonies situated in Mediterranean forests. For the first time, a meta-analysis regarding the factors determining nest-site selection heterogeneity for a single species at broad scale was achieved. It is essential to homogenize and coordinate experimental design in modelling the selection of species' ecological requirements in order to avoid that differences in results among studies would be due to methodological heterogeneity. This would optimize best conservation and management practices for habitats and species in a global context.

3140: ***-.078***

A wild male Oriental White Stork Ciconia boyciana was found dead on 27 February 2007. He appeared in August 2002, and was named "Hachi-goro" by residents of Toyo-oka City, among whom he was popular and where a reintroduction project of this species has been underway since 2005. Here we present ecological and veterinary evidence, and suggest that he. was weakened greatly during a violent interaction and defeat to a released male, and that this resulted in his death soon after the interaction.

3141: ***+.204***

Translocations are frequently used to restore extirpated carnivore populations. Understanding the factors that influence translocation success is important because carnivore translocations can be time consuming, expensive, and controversial. Using population viability software, we modeled reintroductions of the fisher, a candidate for endangered or threatened status in the Pacific states of the US. Our model predicts that the most important factor influencing successful reestablishment of a fisher population is the number of adult females reintroduced (provided some males are also released). Data from 38 translocations of fishers in North America, including 30 reintroductions, 5 augmentations and 3 introductions, show that the number of females released was, indeed, a good predictor of success but that the number of males released, geographic region and proximity of the source population to the release site were also important predictors. The contradiction between model and data regarding males may relate to the assumption in the model that all males are equally good breeders. We hypothesize that many males may need to be released to insure a sufficient number of good breeders are included, probably large males. Seventy-seven percent of reintroductions with known outcomes (success or failure) succeeded; all 5 augmentations succeeded; but none of the 3 introductions succeeded. Reintroductions were instrumental in reestablishing fisher populations within their historical range and expanding the range from its most-contracted state (43% of the historical range) to its current state (68% of the historical range). To increase the likelihood of translocation success, we recommend that managers: 1) release as many fishers as possible, 2) release more females than males (55-60% females) when possible, 3) release as many adults as possible, especially large males, 4) release fishers from a nearby source population, 5) conduct a formal feasibility assessment, and 6) develop a comprehensive implementation plan that includes an active monitoring program.

3142: ***+.226***

We used radiotelemetry and recapture to monitor survival and body condition of 36 captive-reared Ozark Hellbenders (Cryptobranchus alleganiensis bishop!) released at two sites on the North Fork of the White River, Missouri, from May 2008 to August 2009. At the end of our study 16 salamanders were alive, 13 had died, and the fate of seven could not be determined. Captive-reared hellbenders released at a site with densely arranged boulders exhibited approximately 1.5-fold higher annual survival (0.7467; daily survival = 0.9992 +/- 0.0004 95% Cl) than hellbenders released at a site where boulders were patchily distributed (0.4816; daily survival = 0.9980 +/- 0.0007 95% Cl). When compared to log-transformed length-mass relationships developed for wild hellbenders from the same river in the 1970s, mean body condition of hellbenders at the patchy boulder site was about average at the end of the study (mean residual distance = -0.0273 +/- -0.0234 SE, n = 7; range = -0.1375-0.0486), while mean body condition of hellbenders at the dense boulder site was above average (mean residual distance = 0.0423 +/- 0.0402 SE; n = 8; range = -0.0374-0.1088). In addition to lower survivorship and body condition, a greater proportion of hellbenders at the patchy site accrued physical abnormalities (6 of 13 vs. 2 of 14), carried leech parasites (9 of 16 vs. 4 of 14), and carried the fungus Batrachochytrium dendrobatidis (3 of 11 vs. 1 of 13). A 'site only' model of survival was most supported, though additional supported models suggested increased mass at release may have increased daily survivorship. While more work is needed to determine the impact of translocation on long-term population dynamics of Ozark Hellbenders, our study demonstrated that about half of a translocated population of captive-reared hellbenders can survive while maintaining or increasing in body condition during their first year post-release, given release sites are well selected.

3143: ***+.203***

The purpose of this work is to estimate the density and the population size of four primate species

[Alouatta clamitans Cabrera, 1940; Callicebus nigrifrons (Spix, 1823); Callithrix aurita (E. Geoffroy, 1812); Cebus nigritus (Goldfuss, 1809)] which occur in a fragment of Atlantic forest of approximately 350 hectares located in Pouso Alegre, state of Minas Gerais, as well as to give subsidies for the conservation of those species in the area. The population surveying was carried out through the distance sampling method in linear transects (Distance Sampling). Data were collected between April and August 2008 from four transects deployed in the study area. The density and population size were calculated using the software Distance 5.0 and were estimated in 23,83 +/- 9,78 ind./km(2) for Callicebus nigrifrons, 14,76 +/- 5,92 ind./km(2) for Callithrix aurita and 7,71 +/- 2,13 ind./km(2) for Cebus nigritus. The population size was estimated in 83,0 +/- 34,0 individuals for C. nigrifrons, 52,0 +/- 20,8 individuals for Callithrix aurita and 27,0 +/- 7,4 individuals for Cebus nigritus. With regard to the howler monkey (A. clamitans), it was stated out that just a group with six individuals survive in the area. In conclusion, the chances for these isolated populations to survive are slim due to the risk of stochastic events. The creation of ecological corridors connecting the study area to the other fragments, besides the translocation of individuals from other areas of the Atlantic forest to this region, could provide alternatives to ensure the viability of these populations in a long-term. Therefore, it is necessary to consolidate public policies in Pouso Alegre that lead to the creation, enlargement and management of conservation units and incentives for the adoption of productive practices based on sustainability in these areas of ecological interest.

3144: *+.160*

Question: To what extent is restoration of vegetation in coastal grasslands delayed by accumulation of nutrients after abandonment of traditional management and subsequent reed encroachment? How does nutrient flow in the plant-soil system react to reintroduction of grazing? Location: Coast of Baltic Sea, western Estonia.Methods: Abandoned, continuously managed and restored coastal meadows were selected in four different study regions and their vegetation composition sampled. Nitrogen, P, K, Na, Ca and Mg concentrations and C/N ratios were determined in both vegetation and soil. Differences between management groups were evaluated.Results: Comparison among different management groups revealed several differences in both relative and total amount of nutrients in soil and vegetation. Most soil properties of restored sites were similar to those in abandoned sites. Carbon stock in the soil profile doubled after abandonment, total N concentration in the top soil layer increased while plant available P concentration decreased. The phytomass and chemical composition of phytomass rapidly changed back to a 'normal' level after restoration. Species composition remained different, but species typical of coastal grasslands were present in restored sites. There was a strong site specificity in the results.Conclusions: Re-establishment of grazing had a rapid impact on plant biomass of coastal grasslands. Species composition responded more slowly, but target species returned relatively quickly. Slow recovery of soil properties, however, means that the results of restoration may be fragile and return of tall-growth vegetation is very probable if managent intensity declines. Long restoration periods should be planned to reach pre-abandonment environmental conditions when using non-destructive restoration methods.

3145: *+.169*

Reintroductions are considered an important part of the action plans and recovery strategies of endangered ground squirrel species, but so far little is known about their proper methodology. We collected primary data on 12 European ground squirrel reintroduction projects carried out at 14 localities in the Czech Republic, Slovakia and Poland since 1989. We focused on seven methodological aspects of each reintroduction: selection of release site, method of releasing, date

of releasing, origin of released animals, total number of released animals, mean number of released animals per season and reintroduction site management. The method of releasing was found to be the key factor in determining the settlement of animals at the target locality. Only soft releasing methods, i.e. the use of enclosures and/or artificial burrows, ensure that animals remain at the target locality. The other factors significantly determining reintroduction success are the number of released animals per season (at least 23 animals required) and the total number of released animals (a minimum of 60 individuals). Long-term management of the site and regular monitoring of the newly established population are necessary. Our recommendations, based on experience with the successes and failures of previous reintroductions, could largely improve the efficiency of future reintroductions of highly endangered species.

3146: *+.137*

Species relocation programmes are increasingly performed with the intention of establishing a self-sustaining population of threatened or declining native species. However, the use of experimental quantitative approaches in species relocation programmes is still relatively uncommon, despite a number of international studies recommending clear guidelines and standards. This paper evaluates species relocation programmes conducted within Australia to assess how programmes performed in relation to such standards. The search techniques identified 54 species relocation programmes, the majority of which were reintroductions (52%) and supplementations (30%). Only 25 (46%) of the species relocation programmes claimed success, with a lack of effective predator control recognized as contributing to the failure of 14 programmes. There was considerable variation in the quality of species relocation programmes in relation to key features such as whether the programme integrated experimental approaches with testable hypotheses, whether there were explicit statements of criteria for success, whether suitable habitat was identified for the release site and whether long-term monitoring was conducted. We propose guidelines to improve scientific rigour and success rates of species relocation programmes.

3147: *+.117*

1 The Eurasian beaver Castor fiber suffered a drastic reduction in both geographical range and population size, due to human persecution, until the end of the 19th century. After the adoption of protection measures, natural expansion and reintroductions led to the recovery of this species over much of its European range. 2 We review historical events that led to the recovery of beavers in France, and summarize the status of beavers in various river systems. Beaver establishment in France is a story of overall success: several major river systems are presently occupied, so that the species is no longer at risk in France. 3 However, beaver recolonization took place in parallel with increasing human impacts on the environment. In addition to natural limiting factors, anthropogenic factors impeded beaver settlement in many areas. Today, beavers often occupy suboptimal habitats and, as a consequence, come into conflict with human activities. Effective solutions for preventing beaver damage include the restoration of riparian habitats to discourage crop damage and the provision of physical barriers to protect crops. 4 Beaver populations reintroduced into France all originate from the relict Rhone population. However, in recent years, beavers from populations in neighbouring countries have been expanding into north-eastern France. Therefore, our review of beaver origin and distribution in these countries may contribute to the development of appropriate national management strategies and towards important decisions, e.g. the decision to try to keep Rhone beavers genetically isolated, or to allow populations to mix. 5 The recently discovered presence of North American beavers Castor canadensis in three countries surrounding France has raised an important issue. This species may

out-compete C. fiber in places where the species come into contact. A programme based on field-trapping sessions and genetic analyses has recently been initiated in some western countries in order to eradicate this non-native species.

3148: *+.142*

Literature data are reviewed on the origin, distribution, economic benefits and impacts of common carp Cyprinus carpio L. in the Mediterranean region. Despite the ubiquity of domesticated and feral forms, wild populations of the genetically pure ancestor are still found in confined areas of Thrace and Northern Anatolia, and possibly in eastern parts of Greece. Introductions and translocations throughout the region from at least Roman times have been driven by a combination of historical, economic and cultural motives, which have contributed to the spread of the species into many freshwater systems. Although impacts have been either documented or suspected in most areas of distribution, and intervention by biomanipulation successfully implemented in some water bodies, there is a compelling need for more focused research in more vulnerable areas characterised by drier/warmer and overall more unpredictable climate conditions, which appear to favour successful common carp population dynamics. In contrast to large-scale (i.e. country-wide) control measures, likely to prove unfeasible because of the intrinsically high costs associated and/or loss of revenue from sport fishing and fisheries activities, localised (integrated) management actions, followed by post-intervention monitoring, are likely to benefit targeted water bodies for increased amenity and restoration value.

3149: *+.075*

Translocation, introduction, reintroduction, and assisted migrations are species conservation strategies that are attracting increasing attention, especially in the face of climate change. However, preventing the extinction of the suite of dependent species whose host species are threatened is seldom considered, and the effects on dependent species of moving threatened hosts are unclear. There is no published guidance on how to decide whether to move species, given this uncertainty. We examined the dependenthost system of 4 disparate taxonomic groups: insects on the feather-leaf banksia (Banksia brownii), montane banksia (B. montana), and Stirling Range beard heath (Leucopogon gnaphalioides); parasites of wild cats; mites and ticks on Duvaucel's gecko (Hoplodactylus duvaucelii) and tuatara (Sphenodon punctatus); and internal coccidian parasites of Cirl Bunting (Emberiza cirlus) and Hihi (Notiomystis cincta). We used these case studies to demonstrate a simple process for use in species- and community-level assessments of efforts to conserve dependents with their hosts. The insects dependent on Stirling Range beard heath and parasites on tigers (Panthera tigris) appeared to represent assemblages that would not be conserved by ex situ host conservation. In contrast, for the cases of dependent species we examined involving a single dependent species (internal parasites of birds and the mite Geckobia naultina on Duvaucel's gecko), ex situ conservation of the host species would also conserve the dependent species. However, moving dependent species with their hosts may be insufficient to maintain viable populations of the dependent species, and additional conservation strategies such as supplementing populations may be needed.

3150: *+.258*

This study investigated the reproductive parameters of free-ranging rehabilitant female orangutans. We aimed to assess the factors that influence these parameters and provide information that could assist with the management of orangutan reintroduction programs. We analyzed the birth records of free-ranging female rehabilitants at Bukit Lawang, Bukit Tigapuluh,

Sepilok, Camp Leakey, Kaja Island, Sungai Wain, and Meratus and compared them with reproductive parameters reported in wild and zoo populations. Females' ages at first birth were 10.6-14.7 years, significantly earlier than those of wild and zoo orangutans. Computed inter-birth intervals (IBIs) calculated by the Kaplan-Meier method were 65.1-90.1 months; the values for Camp Leakey and Bukit Lawang rehabilitants were significantly shorter than those reported for wild Sumatran orangutans. Infant mortality rates were 18-61%; the values for Bukit Lawang and Sepilok were significantly higher than those reported for wild Sumatran and zoo orangutans. In rehabilitants, young ages at first birth and shorter IBIs may result from the high energy intake enabled by provisioning, although the possibility exists that they reflect underestimations of age on arrival at rehabilitation centers. The observed high infant mortality rate may reflect poor mothering skills due to human rearing and/or increased disease transmission. This study demonstrates that accelerated reproductive rates (younger age at first birth and shorter IBI) are common in provisioned rehabilitant females on both Sumatra and Borneo.

3151: *+.027*

In South Africa, vervet monkeys (Chlorocebus aethiops) are frequently persecuted, resulting in large numbers of injured and/or orphaned animals. Rehabilitation centres aim to care for these monkeys and ultimately return them to the wild whenever possible. However, it is unknown whether rehabilitation is successful in its goal of creating wild-living, independent, self-sustaining troops due to limited published research in this area. This study describes the release and subsequent fate of a troop of rehabilitated vervet monkeys over a 6-month period. A troop of 16 monkeys was released into the Ntendeka Wilderness Area, a protected part of Ngome Forest, by the WATCH (Wild Animal Trauma Centre and Haven) rehabilitation centre in KwaZulu-Natal, South Africa. Monitoring data were evaluated with regard to survival, mortality, suitability of the release site, breeding, condition, troop composition, behaviour, group dynamics, ranging patterns and the effectiveness of monitoring tools. The release was considered to be a partial success in that the troop exhibited behaviour, group dynamics and ranging patterns similar to wild conspecifics. However, the survival rate was low and the troop was judged to be non-self-sustaining. The main problems identified were the limited lifetimes of radio collars, which resulted in missing animals and caused monitoring to be cut short, illegal hunting activities, predation and a small troop size with few adults. The authors recommend improvements that may increase success, such as retaining troops in release enclosures for longer periods, releasing a larger troop with more adults that more closely matches wild troop composition, selecting a release site at least 3 km from the nearest human settlement and the use of GPS collars to allow for a longer monitoring period encompassing all seasonal conditions. Furthermore, all primates for release should be medically screened so as to avoid potential negative impacts on wild populations.

3152: *+.109*

1. Turfs transplanted from native vegetation can be used to restore diverse plant communities on disturbed sites. There is, however, limited understanding of optimal turf size and the tolerance of different plant communities and species to transplanting.2. The effects of turf size in restoration of alpine plant communities were studied in SW-Iceland. Treatments tested in 2-m 2 plots were as follows: planting of sixteen 5 x 5 cm turfs, four 10 x 10 cm turfs, one 20 x 20 cm turf or one 30 x 30 cm turf; a 20 x 20 cm turf shredded and spread over the plot and controls without turfs. The 10-cm thick turfs were extracted from nearby heath and grassland vegetation and planted in mineral soil and road verges at 260-410 melevation. Species composition, cover and colonization were monitored for three growing seasons.3. Grassland vegetation tolerated division into small turfs better than heath vegetation, but responses varied by functional groups. Cover of dwarf-shrubs

decreased with decreasing turf size; grass cover was highest in plots with 5 x 5 cm turfs and lowest in plots with shredded turfs, while moss cover increased most rapidly in plots with shredded turfs.4. Synthesis and applications. Optimum turf size for the restoration of native species varied among functional groups of plants and decreased as follows: evergreen dwarf-shrubs > deciduous dwarfshrubs > sedges > grasses > mosses. Turfs that are at least 20-30 cm in diameter may be needed for the transplantation of dwarf-shrubs, while turfs as small as 5 cm in diameter can be used to establish many grass species. Even smaller units can be used to facilitate moss colonization. Turfs that are salvaged from development projects can be a valuable source of native species for use in restoration schemes. Turf size for transplanting should be selected with regard to donor vegetation, growth form and abundance of the target species.

3153: *+.287*

With 28% of the 350 species of parrots considered threatened, numerous conservation efforts have been initiated for these species. Among these, the restoration or establishment of new populations has increasingly relied on reintroductions as a conservation strategy, often with mixed or uncertain results. We reviewed the results and methodologies of 47 distinct releases and reintroductions of psittacines in nine different countries worldwide over the past 25 years to identify common denominators of successful efforts. To do so, we established a uniform and objective definition of reintroduction success (first-year survival >0.50 and released birds breeding with conspecifics, either captive-reared or wild), and applied generalized linear models and information-theoretic model selection to multiple datasets to identify important predictor variables. We identified several likely predictors of successful psittacine reintroductions, relating to predation mitigation, habitat quality, and post-release supplementation that may provide guidance for future efforts. We also advocate SWOT analysis for objectively evaluating the suitability of potential reintroduction sites. Published by Elsevier Ltd.

3154: *+.216*

Island populations and populations established by reintroductions are prone to extinction, in part because they are vulnerable to deterministic and stochastic phenomena associated with geographic isolation and small population size. As population size declines, reduced genetic diversity can result in decreased fitness and reduced adaptive potential, which may hinder short- or long-term population viability. We used 32 microsatellite markers to investigate the conservation genetics of a newly established population of Evermann's Rock Ptarmigan (Lagopus muta evermanni) at Agattu Island, in the western Aleutian Archipelago, Alaska. We found low genetic diversity (observed heterozygosity = 0.41, allelic richness = 2.2) and a small effective population size (N-e = 28.6), but a relatively large N-e/N ratio = 0.55, which was attributed to multiple paternity in 80% of the broods and low reproductive skew among males (lambda = 0.29). Moreover, successful breeding pairs were less related to each other than random male-female pairs. For conservation efforts based on reintroductions, a mating system with high rates of multiple paternity may facilitate retention of genetic diversity, thereby reducing the potential for inbreeding in small or isolated populations. Our results underscore the importance of quantifying genetic diversity and understanding the breeding behavior of translocated populations.

3155: *+.058*

The Common hamster (Cricetus cricetus) has declined by more than 99% in the westernmost part of its range in Belgium, the Netherlands and the adjacent German federal state of North Rhine-Westphalia (BNN region) during recent decades. Various conservation schemes are ongoing to

support the remaining populations, including restoration of the habitat, captive breeding and reintroductions. One of the factors determining the success of conservation actions is the genetic constitution of the remaining populations. We therefore measured the genetic variation in current BNN hamster populations and compared the outcome with the genetic variation in museum samples from the historical, non-fragmented, population. Most of the current populations have lost the majority of their rare alleles and individual animals have become nearly homozygous. Since different alleles became fixed in different populations, this has resulted in strong genetic differentiation between current populations and reflects the strength of drift and inbreeding processes in small and isolated populations. Despite this differentiation, the total gene diversity of these small populations combined is not much less than that of the historical population. Hence, the main genetic difference between historical and present is not in terms of total genetic variation or number of alleles in the BNN region, but in the distribution of this variation over the populations.

3156: *-.211*

This article reviews the biology, ecological effects, and management of the domestic cat (Felis catus) in the Pacific basin. The cat is one of the most controversial invasive species in the Pacific region because of its complex relations with humans. At one extreme, well-fed domestic house pets are allowed outdoors where they may hunt native animals; at the other, unsocialized feral cats have replaced native predators as apex predators or occupy a new niche on oceanic islands, where they have devastated native faunas. In the middle are stray cats that are still socialized around humans. Feral and stray cats can be reservoirs of diseases that infect free-roaming domestic cats, humans, and wildlife. Given these problems, the best response would be to keep domestic cats indoors, restrict cat breeding, and remove feral populations. However, most Pacific basin societies have failed to reach a consensus on the cat problem, so solutions are ad hoc, often lacking in any scientific basis, and reflect our conflicting views. Compromise management might best fall into three broad classes: (1) eradication of cats should be confined to islands and other areas of high native biodiversity where reintroduction can be prevented; (2) in a landscape of low or moderate biological value, efforts should be made to educate the public to reduce the impact of their cats on remaining wildlife, while excluding cats from "islands" of elevated biodiversity values or human sensitivity; (3) in drastically simplified urban ecosystems, management perhaps should occur only in response to local complaints.

3157: *+.171*

Translocation and reintroduction are used to reduce extinction risk associated with a small population and range size in threatened mammal species. We evaluated the outcome of a reintroduction of the bridled nailtail wallaby Onychogalea fraenata to Avocet Nature Refuge, a private refuge in central Queensland, Australia. This macropod was also reintroduced to Idalia National Park in western Queensland in 1996 and occurs in one natural population in central Queensland. We estimated population growth, adult and juvenile survival, and distribution changes since the last release of O. fraenata to Avocet in 2005, and evaluated female reproductive success and health. Although animals were in good condition, population size was a tenth of that of the 1996 Idalia reintroduction reported after 3 years and, unlike at Idalia, juvenile survival at Avocet was low. The likely causes are consistent with predictors of translocation and reintroduction failures in mammals. These are predation, the small number of individuals in each release, the likely suboptimal health status of reintroduced individuals, drought, and possibly lack of dispersal from the small area of preferred habitat. The lessons of this reintroduction are that future attempts are likely to have the best chance of success if they occur in non-drought years, at

sites with large, non-fragmented areas of brigalow forest, involve the release of large groups of animals together, and are accompanied by intensive, long-term baiting to control introduced predators.

3158: *+.056*

Slater's skink, Liopholis slateri, is an endangered, burrow dwelling scincid, confined to the desert river floodplains of central Australia. This species has undergone a significant population decline over the past 40 years probably due to a loss of suitable habitat for burrow construction caused by changes in land use, the invasion of exotic weeds and altered fire regimes. In this paper we describe the characteristics of natural burrows and their physical association with other environmental features. Lizards were found to construct relatively complex, multi-entranced (up to 10 entrances) burrow systems in mounds of soil, ranging from 4.5-33 cm in height and 3.12-10.36 m basal circumference, that had formed under shrubs ranging from 0.42 3.22 m in height. We also found that the temperature inside one burrow was substantially lower during the hottest part of the day, and showed substantially less daily temperature variation than experienced outside of the burrow. We found no evidence that lizards had a preferred compass direction for orientating their burrow openings. This study provides baseline data to enable the development of artificial burrow systems for use in future habitat restoration projects, possible translocations and captive breeding programmes.

3159: *+.242*

Species occurrence and abundance models are important tools that can be used in biodiversity conservation, and can be applied to predict or plan actions needed to mitigate the environmental impacts of hydropower dams. In this study our objectives were: (i) to model the occurrence and abundance of threatened plant species, (ii) to verify the relationship between predicted occurrence and true abundance, and (iii) to assess whether models based on abundance are more effective in predicting species occurrence than those based on presence-absence data. Individual representatives of nine species were counted within 388 randomly georeferenced plots (10 m x 50 m) around the Barra Grande hydropower dam reservoir in southern Brazil. We modelled their relationship with 15 environmental variables using both occurrence (Generalised Linear Models) and abundance data (Hurdle and Zero-Inflated models). Overall, occurrence models were more accurate than abundance models. For all species, observed abundance was significantly, although not strongly, correlated with the probability of occurrence. This correlation lost significance when zero-abundance (absence) sites were excluded from analysis, but only when this entailed a substantial drop in sample size. The same occurred when analysing relationships between abundance and probability of occurrence from previously published studies on a range of different species, suggesting that future studies could potentially use probability of occurrence as an approximate indicator of abundance when the latter is not possible to obtain. This possibility might, however, depend on life history traits of the species in question, with some traits favouring a relationship between occurrence and abundance. Reconstructing species abundance patterns from occurrence could be an important tool for conservation planning and the management of threatened species, allowing scientists to indicate the best areas for collection and reintroduction of plant germplasm or choose conservation areas most likely to maintain viable populations. (C) 2012 Elsevier B.V. All rights reserved.

3160: *+.105*

Management and care of the nondomestic ruminant neonate are similar in principle to domestic

animal practice. Housing of the dam, conditions for birth, preparation for intervention, and plans for treatment and hand-rearing of sick neonates must all be considered carefully before undertaking nondomestic ruminant breeding. Unfortunately, neonatal losses tend to be much higher in nondomestic calves before weaning than in domestic cattle, sheep, and goat herds.1 With continued habitat and population declines in wild species, successful captive breeding of nondomestic herds becomes more important to species sustainability and potential reintroduction programs. The primary challenges contributing to neonatal losses in nondomestic ruminants are often animal temperament and adaptation to captivity. Only through experience can some of these challenges be overcome. However, by understanding some species-specific behavioral tendencies and the fractious nature of nondomestic ruminants in general, we can improve our success in managing and maintaining healthy populations of nondomestic ruminants in captivity.

3161: *+.020*

1. Global climate change is predicted to raise water temperatures and alter flow regimes in northern river systems. Climate-related factors might have profound impacts on survival, reproduction and distribution of freshwater species such as red-listed noble crayfish (Astacus astacus) in its northern limit of distribution.2. In this study, noble crayfish capture data over 27 years from the River Ljungan, Sweden, were examined. Time series of catch per unit effort (CPUE) were analysed in relation to the North Atlantic Oscillation (NAO) index, regional weather factors and water flow. CPUE was assumed to reflect differences in population size. Two models were constructed to explore the relative impact of different climate factors and density dependence on variability of catch sizes.3. The most parsimonious model for CPUE time series, explaining 72% of the variance in CPUE, included density-dependent population dynamics of the crayfish and climate or weather factors. The specific effect from density dependence in the model was 37%, while climate/weather factors contributed with 35% of the variation. The most important climate/weather factors are variations in NAO index and water flow. Temperature did not improve the model fit to capture data.4. The best model was evaluated using independent data sets that gave correlations between model predictions and data ranging from 0.44 to 0.53. The density dependence shows a time lag of 1 year, while climate variables show time lags from 2 to 6 years in relation to CPUE, indicating effects on different cohorts of the crayfish population.5. Both density dependence and climatic factors play a significant role in population fluctuations of noble crayfish. A 6-year time lag for NAO index is puzzling but indicates that some as yet unidentified factors related to NAO might act on the juvenile stages of the population. Water flow shows a 2-year lag to the CPUE, and high flow in the river may affect adult survival. The reasons for fluctuation of crayfish catches in response to climate need to be identified, and fishing quotas should consider the different cohort sizes because of variation in environment. Reintroduction programmes for crayfish need to consider effects of climate change when designing management strategies.

3162: *+.113*

Establishment of persistent plant populations may be restricted by limitations on the numbers of seeds, emergence of seedlings, or survival to reproductive maturity. The relative importance of these phases in establishment of new populations, particularly restorations, is poorly understood. In an experiment to quantify seedling emergence and juvenile survival of Echinacea angustifolia during its reintroduction to previously agricultural sites, we evaluated effects of two types of vegetation and prescribed burning at four times relative to sowing. We collected achenes from prairie remnants in western Minnesota, United States, and, each October 20002002, overseeded them into nearby study plots either in recently planted stands of native grasses or in oldfields abandoned 40 years earlier. For each cohort, we determined germinability of achenes in the

laboratory and, in the field, monitored seedling emergence the following spring and subsequent survival in annual censuses through summer 2009. Germinability ranged from 20 to 37%, varying significantly among collection years. Seedlings emerged in every treatment combination, but emergence rarely exceeded 8% of achenes sown. Burns during the spring prior to sowing tended to enhance emergence, but to differing degrees depending on the year and vegetation. Burning in the spring after sowing reduced emergence. Burning enhanced juvenile survival in restored plots but not in oldfields. Strategies to reintroduce this species should include burning in the spring before sowing, sowing large quantities of seed, avoiding burning in the spring following sowing, and burning at least once within the first 6 years.

3163: *+.107*

The creation of new populations of rare and endangered plant species has become well-established as a standard technique in conservation and restoration ecology. However, much remains unknown about the actual rates of success or failure of such reintroductions. Recent research suggests that in part this reflects under-reporting of failures. In 2000, the authors published a paper reporting rates of success in reintroducing eight perennial plant species into two reserves near Boston, MA, in 19941995. In 2010, the authors conducted a recensus of the experimental sites 15 years after reintroduction; almost all the populations reported in 2000 had disappeared. The implications for reintroduction methodology, with respect to establishing and reporting both successful and unsuccessful experiments are discussed.

3164: *+.060*

Assisted colonization (AC), or the intentional translocation of populations to compensate for risks related to climate change, is receiving increasing attention. It has been recently suggested by that rather than relocating endangered species, a focus should be placed on local adaptations of foundation or keystone species, and that these local ecotypes should be moved within their own range. Hence, this type of relocation could be applied with minimal risk in many restoration efforts. We think that caution is needed when considering the translocation of these foundation species, even within their range. Many recent studies have shown that foundation species can influence community structure and ecosystem processes through heritable traits, which suggests a genetic basis for ecosystem services. Thus, the translocation of different genotypes of foundation species might lead to unexpected results of colonization and might not be as predictable as Kreyling et al. have argued. Here, in our response, we stress how AC of foundation species can have important evolutionary consequences that might be impossible to reverse. We propose, whenever possible, (1) to favor population mixes of the foundation species to minimize the potential negative effects of specific genotypes; and (2) to collect from adjacent populations along ecological clines of the foundation species to mimic natural processes of migration under climate change.

3165: *+.155*

Recent advances highlight the potential for predators to restore ecosystems and confer resilience against globally threatening processes, including climate change and biological invasions. However, releasing the ecological benefits of predators entails significant challenges. Here, we discuss the economic, environmental and social considerations affecting predator-driven ecological restoration programmes, and suggest approaches for reducing the undesirable impacts of predators. Because the roles of predators are context dependent, we argue for increased emphasis on predator functionality in ecosystems and less on the identities and origins of species

and genotypes. We emphasise that insufficient attention is currently given to the importance of variation in the social structures and behaviours of predators in influencing the dynamics of trophic interactions. Lastly, we outline experiments specifically designed to clarify the ecological roles of predators and their potential utility in ecosystem restoration.

3166: *-.113*

Branchiobdellidans or crayfish worms are clitellate annelids and ectosymbionts of freshwater crayfish. An investigation of branchiobdellidan infestation was undertaken in a population of endangered white-clawed crayfish (Austropotamobius pallipes) in the river Aire, UK. Thirty two percent of animals were infested either by the adult parasite or their cocoons (n=107). Parasite burden increased with host size, but did not differ with sex. Observations of crayfish gill tissue revealed a strong positive relationship between melanization of filaments and parasite prevalence and burden. Taxonomic identification revealed that 1 species of branchiobdellidan was present, Branchiobdella astaci. The first sequences were generated for this species and phylogenetically analysed alongside published sequences for 5 other branchiobdellidan species in Europe. The position of B. astaci within the genus Branchiobdella was confirmed, and it was found to cluster as a sister group to B. parasita.

3167: *+.060*

Georgia plume, Elliottia racemosa (Ericaceae), is a small tree endemic only to the state of Georgia, where it is listed as a threatened species. Information about genetic relatedness is critical for establishing approaches for safeguarding, reintroduction, and conservation of this rare species. The genetic relationships among and within selected georgia plume populations were evaluated using random amplified polymorphic DNA (RAPD) in conjunction with site visits at which time a census and GPS survey were conducted. Populations ranged from those containing eight to over 1000 individuals with most populations containing few plants (less than 50 individuals). With one exception, small populations with less than 50 individuals had more genetic similarity than populations with greater numbers of plants. Two protected populations containing large numbers of individuals were sampled extensively. Genetic similarity of individuals was not associated with plant proximity within a population. The small number of individuals and geographic isolation characteristic of many populations were associated with high within-population genetic similarity. Conservation priorities should be given to preserving as many different populations as possible to retain the genetic diversity of the species. Whether the narrow genetic variation found in some populations may be contributing to lack of sexual reproduction in the wild is an area for further study.

3168: *+.035*

The Eastern Bristlebird (Dasyornis brachypterus) is an endangered endemic passerine of south-eastern Australia. The re-establishment of extirpated populations through translocation was identified as a key action in New South Wales to address the threats to this species associated with habitat fragmentation and widespread and frequent fire. At Jervis Bay during 20032005, 50 birds were translocated from Bherwerre Peninsula to Beecroft Peninsula. In the Illawarra in 2008, 50 birds were translocated from Barren Grounds Nature Reserve to Cataract. At Jervis Bay, monitoring indicated that after 7 years, (i) there was no detectable impact on the source population from the removal of birds and (ii) the count at Beecroft Peninsula was 94 birds, with dispersal up to 6.3 km from the release point. In the Illawarra, (i) the source population was recovering 3 years post-removal and (ii) the maximum count at Cataract was 15 birds after 3.5 years, including

evidence of breeding, and after 3 years, the maximum dispersal was 7 km from the release point. Both translocations adhered to five key principles as follows. (i) Feasibility analysis prior to each project was favourable. (ii) For 17 pre-stated criteria for success, 14 and 10, respectively, were met for Jervis Bay and Illawarra. (iii) Financial accountability was achieved with detailed statements showing budgets of $201k and $92k, respectively, for Jervis Bay and Illawarra. (iv) Ecological research was incorporated into both projects. (v) The results of each project are progressively being published. The re-introduction at Jervis Bay has succeeded, and we are optimistic about the Illawarra re-introduction.

3169: ***+.140***

Translocating birds to a new area of habitat to restore or supplement depleted populations may pose a significant threat to the translocated individuals. While for many species, translocated individuals appear to move larger distances than resident animals, species with poor dispersal capacity may be restricted in movements and translocation methods may need to accommodate differences in movements to ensure success. In this study, designed to provide insights to inform our broader programme of translocations in New South Wales, Australia, we investigated post-release movements in the endangered, semi-flightless Eastern Bristlebird (Dasyornis brachypterus). We predicted that movements would be minimal, with few differences between males and females, similar to published information for a resident un-manipulated population. Following the release of 45 birds at a host location at Jervis Bay, NSW, over a 3-year programme, we followed individuals for up to 2 weeks using radio-tracking. The translocated birds had larger maximum movements and moved through much larger home ranges than non-translocated individuals from the resident population. Translocated birds moved 300 m further after release when conspecifics were present. Males moved further than females and tended to have larger home ranges, although average daily displacement did not differ. We concluded that the semi-flightlessness of the species does not result in minimal movements. Release at a small number of locations in the new habitat was considered appropriate for the species, as animals seem to move enough to find new unoccupied areas in a relatively short period. This work provided us with increasing confidence to continue with further translocations.

3170: ***+.001***

The Vancouver Island marmot (Marmota vancouverensis; VIM) is one of North America's most endangered species with fewer than 150 individuals remaining in the wild. A captive breeding program was established across four facilities in Canada as an insurance population and source of animals for reintroduction to the wild. The purpose of this study was to gather information about the basic reproductive biology and behavior of this species, which is essential to improve captive breeding programs. Regular fecal samples were obtained from adult female (n = 14) and male (n = 10) marmots, 2 years of age and older, over 13 breeding seasons (23 months duration posthibernation) for steroid hormone analysis. Enzyme immunoassays were validated for quantifying fecal testosterone metabolite concentrations for males, and fecal estrogen and progesterone metabolite concentrations for females. Results indicated that fecal progesterone metabolite concentrations can be used to monitor ovulation and pregnancy. Behavioral monitoring through infrared video surveillance was conducted in four breeding pairs over a 2-year period (n = 7 behavioral profiles). Breeding behaviors correlated strongly with changes in reproductive endocrine profiles. A high frequency of play behavior or wrestling was observed in conjunction with breeding activity before an elevation in progesterone metabolite concentrations. Impending parturition was associated with increased aggression and exclusion of the male from the maternal nestbox as well as an increase in nesting activity. Observational data combined with hormonal

analysis suggest that female VIMs are induced ovulators and that multiple breeding attempts may be required for ovulation and conception. Gestation appears to be approximately 34 days from peak breeding activity (32 days from estimated ovulation). Fecal testosterone concentrations suggest that testicular activity is seasonal with the reproductive activity occurring immediately posthibernation. Monitoring breeding behavior is a useful means of indicating estrus, conception and pregnancy, which can also be supported by the hormonal analysis of daily fecal samples of individual animals. Zoo Biol 31:275290, 2012. (c) 2011 Wiley Periodicals, Inc.

3171: ***-.084***

Background: To conserve critically endangered predators, we also need to conserve the prey species upon which they depend. Velvet geckos (Oedura lesueurii) are a primary prey for the endangered broad-headed snake (Hoplocephalus bungaroides), which is restricted to sandstone habitats in southeastern Australia. We sequenced the ND2 gene from 179 velvet geckos, to clarify the lizards' phylogeographic history and landscape genetics. We also analysed 260 records from a longterm (3-year) capture-mark-recapture program at three sites, to evaluate dispersal rates of geckos as a function of locality, sex and body size. Results: The genetic analyses revealed three ancient lineages in the north, south and centre of the species' current range. Estimates of gene flow suggest low dispersal rates, constrained by the availability of contiguous rocky habitat. Mark-recapture records confirm that these lizards are highly sedentary, with most animals moving < 30 m from their original capture site even over multi-year periods. Conclusion: The low vagility of these lizards suggests that they will be slow to colonise vacant habitat patches; and hence, efforts to restore degraded habitats for broad-headed snakes may need to include translocation of lizards.

3172: ***-.093***

Background: To conserve critically endangered predators, we also need to conserve the prey species upon which they depend. Velvet geckos (Oedura lesueurii) are a primary prey for the endangered broad-headed snake (Hoplocephalus bungaroides), which is restricted to sandstone habitats in southeastern Australia. We sequenced the ND2 gene from 179 velvet geckos, to clarify the lizards' phylogeographic history and landscape genetics. We also analysed 260 records from a longterm (3-year) capture-mark-recapture program at three sites, to evaluate dispersal rates of geckos as a function of locality, sex and body size.Results: The genetic analyses revealed three ancient lineages in the north, south and centre of the species' current range. Estimates of gene flow suggest low dispersal rates, constrained by the availability of contiguous rocky habitat. Mark-recapture records confirm that these lizards are highly sedentary, with most animals moving < 30 m from their original capture site even over multi-year periods.Conclusion: The low vagility of these lizards suggests that they will be slow to colonise vacant habitat patches; and hence, efforts to restore degraded habitats for broad-headed snakes may need to include translocation of lizards.

3173: ***+.068***

Translocation success of Frithia humilis Burgoyne, an endangered succulent plant: preliminary results. A Frithia humilis population, growing on a licensed mining plot near Witbank was saved from obliteration in 2009. It was translocated to three habitats, which have been monitored since 2010. Thus far, flowering has increased despite decreasing population numbers; the populations seem stable.

3174: ***-.040***

The ex situ monitoring of Frithia humilis Burgoyne as a control study of a translocation project. Frithia humilis is an endangered succulent window plant. Ex situ had more flowers than translocated. The natural and the ex situ population was the highest at the 3-6 age class and the translocation population at the <3 age class. Age class 3-6 had the most plants in 2011.

3175: *+.210*

Mantisia spathulata Schult. and M. wengeri Fischer, two critically-endangered, endemic and rare species of the genus Mantisia (Zingiberaceae), have been rediscovered from Lunglei province of Mizoram, India, after two decades. For sustainable conservation and utilization of the Mantisia species, in vitro seed and clonal propagation methods have been developed earlier by our research group and plantlets have been reintroduced to their natural habitat for species recovery. To comprehend the plausible reasons for endemism and endangeredness of both the species at DNA level, they were analyzed to assess natural genetic variation using three different polymerase chain reaction (PCR) based DNA markers viz. random amplified polymorphic DNA (RAPD), inter simple sequence repeat (ISSR) and directed amplification of minisatellite DNA regions (DAMD), both individually and cumulatively, which are popularly regarded as single primer amplification reaction (SPAR) methods. A total of 107 primers belonging to three SPARs are used which collectively endow low genetic variation (15 and 20 %, respectively) in both M. spathulata and M. wengeri. The use and efficacy of SPAR methods to reveal the natural genetic variation in Mantisia species at intra-specific level has been recorded for the first time. To impede the extinction risk of these two species of genus Mantisia, large scale conservation strategies including in situ and ex situ conservation are recommended.

3176: *+.309*

Reintroduction of beaver (Castor spp) may facilitate rehabilitation of freshwater habitats providing a cost-effective sustainable means of improving ecological conditions. Despite extensive research, debate and consultation, a general consensus on the impact of beaver on fishes has proven elusive because of variability in biological response. This paper provides a systematic review of the impacts of beaver dams on fishes and fish habitat based on a meta-analysis of the literature and expert opinion. Research is regionally biased to North America (88%). The most frequently cited benefits of beaver dams were increased habitat heterogeneity, rearing and overwintering habitat and flow refuge, and invertebrate production. Impeded fish movement because of dams, siltation of spawning habitat and low oxygen levels in ponds were the most often cited negative impacts. Benefits (184) were cited more frequently than costs (119). Impacts were spatially and temporally variable and differed with species. The majority of 49 North American and European experts considered beaver to have an overall positive impact on fish populations, through their influence on abundance and productivity. Perceived negative effects related to the movement of aquatic organisms in tributary streams, including upstream and downstream migrating salmonids, and the availability of suitable spawning habitat.

3177: *+.139*

In this study, we report the genetic population structure of the Fire-bellied toad Bombina bombina in Brandenburg (East Germany) in the context of conservation. We analysed 298 samples originating from 11 populations in Brandenburg using mitochondrial control region sequences and six polymorphic microsatellite loci. For comparison, we included one population each from Poland and Ukraine into our analysis. Within Brandenburg, we detected a moderate variability in the mitochondrial control region (19 different haplotypes) and at microsatellite loci (9-12 alleles

per locus). These polymorphisms revealed a clear population structure among toads in Brandenburg, despite a relatively high overall population density and the moderate size of single populations (100-2000 individuals). The overall genetic population structure is consistent with a postglacial colonization from South East-Europe and a subsequent population expansion. Based on genetic connectivity, we infer Management Units (MUs) as targets for conservation. Our genetic survey identified MUs, within which human infrastructure is currently preventing any genetic exchange. We also detect an unintentional translocation from South East to North West Brandenburg, presumably in the course of fish stocking activities. Provided suitable conservation measures are taken, Brandenburg should continue to harbor large populations of this critically endangered species.

3178: *-.067*

For conservation managers tasked with recovering threatened species, genetic structure can exacerbate the rate of loss of genetic diversity because alleles unique to a sub-population are more likely to be lost by the effects of random genetic drift than if a population is panmictic. Given that intensive management techniques commonly used to recover threatened species frequently involve movement of individuals within and between populations, managers need to be aware not only of pre-existing levels of genetic structure but also of the potential effects that intensive management might have on these patterns. The Mauritius parakeet (Psittacula echo) has been the subject of an intensive conservation programme, involving translocation and reintroduction that has recovered the population from less than 20 individuals in 1987 to approximately 500 in 2010. Analysis of genotype data derived from 18 microsatellite markers developed for this species reveals a clear signal of structure in the population before intensive management began, but which subsequently disappears following management intervention. This study illustrates the impacts that conservation management can have on the genetic structure of an island endemic population and demonstrates how translocations or reintroductions can benefit populations of endangered species by reducing the risk of loss of genetic diversity.

3179: *+.168*

The Saint Croix ground lizard (Ameiva polops) is a Critically Endangered species endemic to Saint Croix, U.S. Virgin Islands. Although it is completely extirpated from Saint Croix Island (last seen in 1968), two small natural satellite populations survive on two islets off St. Croix: one on Protestant Cay (estimated at similar to 30 individuals in 2002); and one on Green Cay (estimated at similar to 180 individuals in 2002). Two additional small populations exist that were founded with individuals translocated from the two surviving natural populations. One is on Ruth Island, a man-made islet off St. Croix, founded in 1990 with 10 individuals from Protestant Cay. The other is on Buck Island, similar to 2.5 km from Saint Croix, founded in 2008 with 57 individuals from Green Cay. All populations are vulnerable to catastrophic events such as hurricanes, sea level rise, introduction of exotic species, and landscape transformation. Herein, we used mitochondrial and nuclear-microsatellite markers to examine levels of genetic diversity within extant populations of A. polops and the degree of genetic differentiation among them. We also conducted analyses to search for signatures of recent bottlenecks in these populations and to estimate their effective population size (N (e)). We found low genetic variability within populations of this lizard, comparable to that observed in other threatened vertebrates. We also found significant genetic differentiation among the three populations examined, as well as signatures of recent bottlenecks and critically low N (e) values in all populations. Based on our results, we suggest two different conservation units for A. polops: (1) Green Cay and its replicate population at Buck Island; and (2) Protestant Cay and its replicate population at Ruth Island. We discuss the implications of our

findings on the conservation and management of A. polops.

3180: *+.147*

Translocation programs are a common strategy to increase the number of viable populations of threatened freshwater fishes. Yet, only in a minority of cases the success or failure of translocations has been assessed through a quantitative analysis of demographic traits, compensatory responses, life-histories and population dynamics of the threatened species. A paradigmatic case a translocation program combining both management- and research-oriented activities is represented by the Marble Trout Conservation Program, which started in 1993 in the upper reaches of the Soca, Idirjca and Baca river basins (Slovenia) for the conservation of stream-dwelling marble trout . In order to enhance the viability of the species, two new populations were created in 1996 by stocking 500 marble trout aged 1+ in previously fishless streams (Gorska and Zakojska) within the core habitat of the species. The new populations have been systematically monitored for 15 years by individually tagging and sampling marble trout. Our analyses show that deterministic extinction of marble trout populations are unlikely and that high-magnitude environmental stochasticity (i.e., severe floods) is the only main cause of local population extinction, despite the high resilience to flood-induced massive mortalities exhibited by marble trout through compensatory mechanisms (e.g., relaxation of density-dependent body growth and survival at low densities). Fishless headwaters, probably characterized by a history of recurrent severe floods, should not be considered as candidate sites for the creation of new populations. Fewer individuals than originally reintroduced (i.e., 500 fish aged 1+ in each stream) might be sufficient to establish viable populations, since compensatory mechanisms are likely to regulate population size around stream carrying capacity in a few years. Besides enhancing the species viability, translocation programs can provide an excellent framework for the estimation of ecological traits (e.g., life-histories, demography, population dynamics etc.), identify potential vulnerabilities and thus guide well-formed management actions for the threatened species.

3181: *+.191*

A new set of 18 microsatellite loci was developed for the threatened Australian freshwater fish southern purple-spotted gudgeon Mogurnda adspersa (Eleotridae) using a next generation sequencing approach. A total of 84 fish from two populations (including one rescued into captivity) were successfully genotyped at all markers using a multiplex approach. As expected for threatened species, we observed relatively low genetic variation across most loci (average allelic diversity = 5.4; average heterozygosity = 0.380). No evidence for linkage disequilibrium was detected and all loci were in Hardy-Weinberg equilibrium. This new set of microsatellite markers will benefit substantially the ongoing conservation program of a critically endangered population of M. adspersa that involves captive breeding, relatedness and paternity analyses, reintroduction, and landscape genetics.

3182: *+.680*

The role of ex situ activities for the conservation of biodiversity, and of zoos and aquaria in particular, is open to continuing debate. The present note highlights the conservation breeding potential of zoological gardens and aquaria in the European union, but it also recognises the lack of a convincing scientific and legal framework that encourages ex situ activities for 'exotic' species. If ex situ programmes are considered essential for global biodiversity conservation, the EU should not limit itself to regulating zoos through the zoo directive, but should actively promote and support their ex situ conservation activities.

3183: ***+.249***

Population viability analysis (PVA) is a tool to evaluate the risk of extinction for endangered species and aid conservation decision-making. The quality of PVA output is dependent on parameters related to population dynamics and life-history; however, it has been difficult to collect this information for the giant panda (Aliuropoda melanoleuca), a rare and endangered mammal native to China, confined to some 30 fragmented habitat patches. Since giant pandas are long-lived, mature late, have lower reproductive rates, and show little sexual dimorphism, obtaining data to perform adequate PVA has been difficult. Here, we develop a parameter sensitivity index by modeling the dynamics of six giant panda populations in the Minshan Mountains, in order to determine the parameters most influential to giant panda populations. Our data shows that the giant panda populations are most sensitive to changes in four female parameters: initial breeding age, reproductive rate, mortality rate between age 0 and 1, and mortality rate of adults. The parameter sensitivity index strongly correlated with initial population size, as smaller populations were more sensitive to changes in these four variables. This model suggests that demographic parameters of females have more influence on the results of PVA, indicating that females may play a more important role in giant panda population dynamics than males. Consequently, reintroduction of female individuals to a small giant panda population should be a high priority for conservation efforts. Our findings form a technical basis for the coming program of giant panda reintroduction, and inform which parameters are crucial to successfully and feasibly monitoring wild giant panda populations.

3184: ***+.249***

Population viability analysis (PVA) is a tool to evaluate the risk of extinction for endangered species and aid conservation decision-making. The quality of PVA output is dependent on parameters related to population dynamics and life-history; however, it has been difficult to collect this information for the giant panda (Aliuropoda melanoleuca), a rare and endangered mammal native to China, confined to some 30 fragmented habitat patches. Since giant pandas are long-lived, mature late, have lower reproductive rates, and show little sexual dimorphism, obtaining data to perform adequate PVA has been difficult. Here, we develop a parameter sensitivity index by modeling the dynamics of six giant panda populations in the Minshan Mountains, in order to determine the parameters most influential to giant panda populations. Our data shows that the giant panda populations are most sensitive to changes in four female parameters: initial breeding age, reproductive rate, mortality rate between age 0 and 1, and mortality rate of adults. The parameter sensitivity index strongly correlated with initial population size, as smaller populations were more sensitive to changes in these four variables. This model suggests that demographic parameters of females have more influence on the results of PVA, indicating that females may play a more important role in giant panda population dynamics than males. Consequently, reintroduction of female individuals to a small giant panda population should be a high priority for conservation efforts. Our findings form a technical basis for the coming program of giant panda reintroduction, and inform which parameters are crucial to successfully and feasibly monitoring wild giant panda populations.

3185: ***+.082***

Worldwide, approximately 168 bird species are captive-bred for reintroduction into the wild. Programs tend to be initiated for species with a high level of endangerment. Depressed hatching success can be a problem for such programs and has been linked to artificial incubation. The need for artificial incubation is driven by the practice of multiclutching to increase egg production or by

uncertainty over the incubation abilities of captive birds. There has been little attempt to determine how artificial incubation differs from bird-contact incubation. We describe a novel archive (data-logger) egg and use it to compare temperature, humidity, and egg-turning in 5 whooping crane (Grus americana) nests, 4 sandhill crane (G. canadensis) nests, and 3 models of artificial incubator; each of which are used to incubate eggs in whooping crane captive-breeding programs. Mean incubation temperature was 31.7 degrees C for whooping cranes and 32.83 degrees C for sandhill cranes. This is well below that of the artificial incubators (which were set based on a protocol of 37.6 degrees C). Humidity in crane nests varied considerably, but median humidity in all 3 artificial incubators was substantially different from that in the crane nests. Two artificial incubators failed to turn the eggs in a way that mimicked crane egg-turning. Archive eggs are an effective tool for guiding the management of avian conservation breeding programs, and can be custom-made for other species. They also have potential to be applied to research on wild populations. (C) 2012 The Wildlife Society.

3186: ***+.249***

Population viability analysis (PVA) is a tool to evaluate the risk of extinction for endangered species and aid conservation decision-making. The quality of PVA output is dependent on parameters related to population dynamics and life-history; however, it has been difficult to collect this information for the giant panda (Aliuropoda melanoleuca), a rare and endangered mammal native to China, confined to some 30 fragmented habitat patches. Since giant pandas are long-lived, mature late, have lower reproductive rates, and show little sexual dimorphism, obtaining data to perform adequate PVA has been difficult. Here, we develop a parameter sensitivity index by modeling the dynamics of six giant panda populations in the Minshan Mountains, in order to determine the parameters most influential to giant panda populations. Our data shows that the giant panda populations are most sensitive to changes in four female parameters: initial breeding age, reproductive rate, mortality rate between age 0 and 1, and mortality rate of adults. The parameter sensitivity index strongly correlated with initial population size, as smaller populations were more sensitive to changes in these four variables. This model suggests that demographic parameters of females have more influence on the results of PVA, indicating that females may play a more important role in giant panda population dynamics than males. Consequently, reintroduction of female individuals to a small giant panda population should be a high priority for conservation efforts. Our findings form a technical basis for the coming program of giant panda reintroduction, and inform which parameters are crucial to successfully and feasibly monitoring wild giant panda populations.

3187: ***+.005***

Translocation of threatened species is a tool used increasingly to conserve biodiversity, but the suite of co-dependent species that use the threatened taxa as hosts can be overlooked. We investigate the preliminary impact of translocating three threatened plant species on insect species and the integrity of insect assemblages that depend on these plants as their hosts. We compare the insect assemblages between natural populations of the threatened species, related non-threatened plant species growing wild near the threatened plants, and threatened plants translocated to another site approximately 40 km away. We used host breadth models and a coextinction risk protocol to determine which insect species are potentially host-specific on the threatened plants, and then assessed these insects' potential presence at the translocation site. We found that insect assemblages on naturally-occurring threatened plants had more individuals, higher species density and higher species richness than assemblages on translocated plants. For one plant species, , species composition differed significantly between wild and translocated populations (< 0.001).

Furthermore, four insect species that were host-specific to and were not detected on the translocated plants. Instead, translocated plants supported insect assemblages more similar to those of related plant species from the surrounding area. We conclude that threatened plant translocations that involve seed collection and propagation may have limited benefit for individual dependent species or the supported insect assemblage. Additional conservation actions will be required to maintain the diversity of insect assemblages and host-dependent relationships.

3188: *-.314*

Translocations of species are expected to be used increasingly to counter the undesirable effects of anthropogenic changes to ecosystems, including loss of species. Methods to assess the risk of disease associated with translocations have been compiled in a comprehensive manual of disease-risk analysis for movement of domestic animals. We used this manual to devise a qualitative method for assessing the probability of the occurrence of disease in wild animals associated with translocations. We adapted the method such that we considered a parasite (any agent of infectious or noninfectious disease) a hazard if it or the host had crossed an ecological or geographical barrier and was novel to the host. We included in our analyses hazards present throughout the translocation pathway derived from the interactions between host immunity and the parasite, the effect of parasites on populations, the effect of noninfectious disease agents, and the effect of stressors on hostparasite interactions. We used the reintroduction of Eurasian Cranes (Grus grus) to England to demonstrate our method. Of the 24 hazards identified, 1 was classified as high risk (coccidia) and 5 were medium risk (highly pathogenic avian influenza virus, Mycobacterium avium, Aspergillus fumigatus, tracheal worms [Syngamus sp. and Cyathostoma sp.], and Tetrameres spp.). Seventeen other hazards were considered low or very low risk. In the absence of better information on the number, identity, distribution, and pathogenicity of parasites of wild animals, there is uncertainty in the risk of disease to translocated animals and recipient populations. Surveys of parasites in source and destination populations and detailed health monitoring after release will improve the information available for future analyses of disease risk. We believe our method can be adapted to assess the risks of disease in other translocated populations.

3189: *+.035*

The return of the Eurasian Lynx to Central Europe has led to a number of conflicts. A primary subject of discussion involves its predation on other wildlife species. Here, we investigated the influence of lynx on its main prey, Roe Deer, in the Bavarian Forest National Park in south-eastern Germany. We compared the survival rates of deer before and after reintroduction of lynx. The analysis is based on data from 1984 to 1988 and 2005 to 2008 of 88 and 99 radio-collared Roe Deer, respectively. During the first period, 35 deer deaths were documented; during the second period, 41 deaths were documented. The causes of death in the second period were lynx 44%, road kill 15%, hunting 12%, and other causes 29%. We used the Cox model to determine the influence of covariables on the hazard rate, which made it possible to consider interactions between the variables. The resulting model includes the four main effects sex, age, presence of lynx, and severity of first winter, and the three interactions-presence of lynx:sex, age:severity of first winter, and sex:severity of first winter, which had a statistically significant influence on Roe Deer survival.

3190: *+.069*

In Mediterranean ecosystems, rabbits are a key prey species for many predators, such as the

Iberian lynx, which is threatened with extinction and has gone extinct locally in several regions of its historical distribution range. One of these regions is Serra da Malcata Nature Reserve, Portugal, which is also currently proposed as a potential site for reintroduction. We intended to investigate annual variation, potential time trends and the effects of management practices on the rabbit population in Serra da Malcata as a model for future potential reintroduction areas. The rabbit population was monitored over 12 years (from 1997 to 2009) by counting latrines along linear transects. These data were used to estimate rabbit occupancy, colonization and extinction patterns using a likelihood-based method including habitat, population and topographic covariate effects. Our results suggest that initial occupancy, when management practices were absent, was driven by the presence of Erica spp. and Cistus ladanifer shrubs and by distance to summits. Site colonization was positively influenced by the presence of edges between shrubs and pastureland and by patterns of rabbit distribution in the previous sampling season. On the other hand, local extinction was negatively influenced by edges. We conclude that the increase in rabbit occupancy and local colonization patterns was clearly associated with management actions (particularly, the creation of pasturelands), although the recovery of the species was noticeably limited by previous patterns of spatial distribution.

3191: ***+.067***

Translocation of endangered species to habitats where exotic predators have been removed is now a common conservation practice around the world. Many of these translocated populations have thrived, and they are often used as sources for the harvesting of individuals for translocations to sites where exotic predators still exist, albeit at reduced densities. This study investigates how isolation from exotic predators affects the ability of individuals to recognize such predators using the North Island robin (Petroica longipes) as a model. The study was carried out in three robin populations in the North Island, New Zealand: a translocated population on Tiritiri Matangi Island, where exotic mammalian predators are absent; a population reintroduced from Tiritiri Matangi Island to Wenderholm Regional Park, a mainland site where these mammals are controlled to low densities; and a mainland population at Benneydale where exotic predatory mammals are common. The response intensity of robins to a model stoat was high at Benneydale and low at Tiritiri Matangi and Wenderholm. This result indicates that isolation from mammalian predators on Tiritiri Matangi has suppressed the ability of North Island robins to recognize these predators. It is possible that the low predatory mammal densities at Wenderholm have reduced robin contact with stoats, therefore reduced the opportunity for robins to learn to recognize stoats. Thus, translocation of individuals from populations without predators to places where key predators still exist could be unsuccessful if translocated individuals fail to perform appropriate anti-predator behaviours.

3192: ***+.170***

In this paper, an economic model was constructed to determine the optimal wolf population and distribution across the Northern Rocky Mountains. Both ecological and economic concepts were incorporated in an implicitly spatial social welfare maximization problem. This interdisciplinary model relies on multiple data sources, including current wolf population and distribution information, opportunity cost to local landowners, and contingent valuation studies to determine willingness-to-pay for wolves. Economic models tend to externalize ecological concerns and ecological models often omit the complex human dimensions of conservation policy. Accordingly, this model can serve as a guide for integrating best practices from both fields. The model presented here is sufficiently general to apply to wolves in other ecosystems and to other highly interacting species such as beavers and bison. The Northern Rocky Mountain wolf was used as an

example of how this economic model works, but this model can be applied far more broadly.

3193: ***+.118***

The concept of refugee species provides a theoretical framework towards increasing the predictive power of the declining population paradigm through identifying species which are expected to suffer from a declining population syndrome. Using a simple habitat model as a framework, refugee species are defined as those that can no longer access optimal habitat, but are confined to suboptimal habitats, with consequences of decreased fitness and density, and attendant conservation risks. Refugee species may be difficult to detect in the absence of information on prior habitat use and fitness and their observed ecology will be constrained by the habitat limits forced on them. Identification of refugee species, characterisation of pre-refugee ecology and the restoration of such species to optimal habitat is critical to their successful conservation. The concept is showcased by addressing the conundrum of a large grazing bovid, the European bison Bison bonasus, being managed as a forest specialist, despite its evolutionary background, dental morphology, neonatal behaviour, diet and microhabitat selection being characteristic of a grazing species inhabiting open, grass-rich habitats. It is hypothesized that a combination of increasing replacement of open steppe by forest cover after the last postglacial period and increasing human pressure forced bison into forests as a refuge habitat. This process was then reinforced through active management of bison in forests as managers committed themselves to the bison as forest species paradigm. A research agenda to test this hypothesis using an experimental approach in the conservation management of European bison by introducing populations into diverse habitat types is suggested.

3194: ***-.019***

Translocation has been widely studied as a tool for conservation management to restore or enhance degraded populations. On the contrary, few studies have been conducted on translocation for commercial purposes. In this study, we evaluate the genetic consequences of translocation of wild individuals of Pinctada margaritifera on farmed and adjacent wild populations. We tested the hypotheses that translocations would induce high genetic heterogeneity in farmed populations and this heterogeneity would then leak into the adjacent wild populations. In fact, farmed samples exhibit high levels of heterogeneity and low pairwise relatedness compared to wild populations, highlighting the pooling of genetically divergent populations into farms. We also demonstrate that this heterogeneity is transmitted to adjacent wild populations as a result of interbreeding. Adjacent wild populations tend to have higher genetic diversity values and greater pairwise relatedness coefficient with farmed populations than wild populations. Overall, pearl culture in French Polynesia promotes the mixing of unrelated individuals in farmed locations and reduces genetic divergence among geographically distant populations as well as among farmed and wild populations of a same lagoon. We also studied for the first time a farmed population originating from spat collected in a lagoon where release of hatchery-produced larvae occurred 10 years ago and we were able to identify four distinct genetic groups. These groups contribute highly to reproduction and caused considerable genetic drift in the lagoon, suggesting that hatchery-produced larvae are neither sustainable method for pearl culture nor for conserving the diversity of P. margaritifera in French Polynesia.

3195: ***+.018***

Bald Eagles (Haliaeetus leucocephalus) were one of the upper-trophic-level avian predators on the Channel Islands, California, prior to their extirpation by 1960 caused in part by large amounts of

DDT discharged into the Southern California Bight. From 2002 to 2006, 61 Bald Eagles were reintroduced onto the northern Channel Islands, as part of a 5-yr feasibility study conducted under the auspices of the Montrose Settlement Restoration Program. In December 2005, a yearling Bald Eagle female was found on Santa Rosa Island with a broken wing and elevated lead levels in her blood of 52.2 ug/dl (0.522 ppm). This incident raised concerns that lead poisoning could be a potential threat to the restoration effort and prompted further investigation. Femurs from five female and two male Bald Eagles reintroduced to the northern Channel Islands were collected postmortem for analyses of lead and other metals. Lead levels detected in femurs of these birds ranged from 0.2 to 55.0 ppm (dry weight). Lead levels in liver were also determined for two of the seven Bald Eagles. Analysis of Bald Eagle movement data from satellite telemetry transmitters suggested that eagles that spent the most time on Santa Rosa Island had the highest lead levels. The results of this study suggested that spent ammunition containing lead found in carrion (offal and entire carcasses) from deer and elk hunting on Santa Rosa Island may have been a primary source of contamination. The on-island hunt program converted to nontoxic bullets in 2007 and ended in late 2011.

3196: ***+.113***

Release methods used in species restoration can affect the success of establishment and survival of released animals. We evaluated the effect of age at release and sex on the length of the dependence period of hacked captive-bred juvenile Harpy Eagles (Harpia harpyja). Between 2002 and 2007, we released 34 (19 males and 15 females) young eagles in Panama and Belize. To test the effect of age, these eagles were divided into two age classes: younger age class 1 (5-7 mo old) and older age class 2 (18-22 mo old). Survival (hacking success) was lower for the younger release age (70%) compared to the older release age (100%; Z = -2.05, P = 0.040). This difference in hacking success was attributed to the extended period of dependence on provisioned food by the younger (18.9 +/- 1.3 mo [SE]) compared to older eagles (1.5 +/- 0.8 mo). Between-sex comparisons showed that the average length of the dependence period was longer for males of age class 1 (males = 21.8 mo vs. females = 14.3 mo) and for females of age class 2 (females = 2.7 mo vs. males = 0 mo). Cox regression models indicated that the interaction of age at release and sex had a significant effect on the dependence period, and that age at release was the most influential variable. Eagles released at 18 mo or older showed increased survival and shorter dependence periods. Hacking can be used to successfully release captive-bred Harpy Eagles into the wild, but this technique was more efficient when delayed from fledging age (when falconers traditionally hack falcons) to nearer the Harpy Eagle's age of independence.

3197: ***+.037***

The reintroduction of large predators provides a framework to investigate responses by prey species to predators. Considerable research has been directed at the impact that reintroduced wolves (Canis lupus) have on cervids, and to a lesser degree, bovids, in northern temperate regions. Generally, these impacts alter feeding, activity, and ranging behavior, or combinations of these. However, there are few studies on the response of African bovids to reintroduced predators, and thus, there is limited data to compare responses by tropical and temperate ungulates to predator reintroductions. Using the reintroduction of lion (Panthera leo) into the Addo Elephant National Park (AENP) Main Camp Section, South Africa, we show that Cape buffalo (Syncerus caffer) responses differ from northern temperate ungulates. Following lion reintroduction, buffalo herds amalgamated into larger, more defendable units; this corresponded with an increase in the survival of juvenile buffalo. Current habitat preference of buffalo breeding herds is for open habitats, especially during the night and morning, when lion are active. The increase in group size

and habitat preference countered initial high levels of predation on juvenile buffalo, resulting in a return in the proportion of juveniles in breeding herds to pre-lion levels. Our results show that buffalo responses to reintroduced large predators in southern Africa differ to those of northern temperate bovids or cervids in the face of wolf predation. We predict that the nature of the prey response to predator reintroduction is likely to reflect the trade-off between the predator selection and hunting strategy of predators against the life history and foraging strategies of each prey species.

3198: *+.320*

Translocation is an important tool for wildlife conservation and biodiversity restoration, but an inefficient one because of the unpredictability of success. Predictors of success such as habitat quality of the release site and number of individuals released have been identified, but the dynamics of successful translocations remain poorly understood. In particular, little is known about the relationship of individual post-release movements to population establishment. In 2004, Laysan teal Anas laysanensis were reintroduced by translocating 20 wild birds from Laysan Island to Midway Atoll. Twenty-two additional wild founders were brought the next year. We monitored the survival, reproductive success and movements of the 42 translocated individuals and their offspring for 4 years. Additionally, we monitored population size from 2004 to 2010. Unlike most translocations, we did not observe elevated post-release mortality despite flight-feather trimming to prevent immediate dispersal off-island: first year survival was >?90% and survival rates until 2009 were 0.65 +/- 0.08 for founding adults. Laysan teal flew between the two main islands of Midway Atoll, and offspring had significantly larger maximum movement distances than founders. We monitored 84 nests and observed a significant, negative relationship of home range size to productivity for founding females. Flightless founders did not show fidelity to their release sites, but had strong fidelity to annual home ranges after attaining flight. Although we observed a component Allee effect on mate-finding, this did not translate into a demographic Allee effect, and generally, the high fitness of founders contributed substantially to successful population establishment. Laysan teal abundance increased linearly until 2009, but showed evidence of population regulation afterwards. The population estimate was 473 (95% confidence interval 439-508) in 2010. On the much larger main Hawaiian Islands, we expect greater post-release movement, a stronger component Allee effect, lower survival and lower reproductive rates because of predation to preclude successful reintroductions of this species to sites without predator management.

3199: *+.088*

Previous work has shown that captive environments can relax selective pressures on various traits, resulting in significantly more trait variance in captive-bred versus wild populations. This increased variance could be one cause of the high mortality rates observed when captive-bred populations are released into the wild, because a significant proportion of the released population exhibits traits that are no longer adapted to the wild. Here we use evolutionary simulation to examine the effects of relaxed selection on trait variance and population persistence when populations are reintroduced into static and changing environments. Our simulations show that when wild environments remained stable and selective pressures on given traits were quite strong (1) relaxation of selective pressures led to significant increases in trait variance; (2) trait variance rapidly decreased when pressures were restored; (3) the smaller the population size, the lower the probability of persistence; and (4) there was often a significant lag time between reintroduction and eventual extinction, reinforcing the importance of long-term monitoring after release. When captive-bred populations were reintroduced into changing environments, strength of selection in

the release habitat was the factor that most influenced survivorship. Amount of change between the source and release habitat and the population's carrying capacity also influenced survivorship, but to a lesser extent.

3200: *+.001*

Harperella (Harperella nodosum) is the only federally listed endangered plant species in the Chesapeake and Ohio Canal National Historical Park (C&O Canal NHP) in the National Park Service in Maryland and one of four federally listed endangered plant species in West Virginia. This paper contrasts unsuccessful and successful efforts to reintroduce harperella seedlings in the main stem of the flood-prone Potomac River and on opposite sides of the river in damp sites in western Maryland and in streams in northeastern West Virginia. The reintroduction efforts in Maryland unsuccessfully transplanted seedlings on cobble bars on the Maryland side of the Potomac River and in artificial sites in the prism of the C&O Canal where harperella had never been found; the reintroduction efforts in West Virginia successfully transplanted seedlings on cobble bars in two tributaries of the Potomac River in West Virginia where harperella populations once flourished but in the last decade or so had been decimated by floods and ice damage. This investigation has contributed to defining harperella's life-cycle characteristics and ecological requirements. The data and observations presented can be used as a guide for reintroducing harperella seedlings and, by extension, other semiaquatic plant species into appropriate sites.

3201: *+.153*

The Columbia Basin pygmy rabbit (Brachylagus idahoensis) is critically endangered and the focus of a captive-breeding program. However, reproductive success in captivity to date has not been sufficient to sustain reintroduction efforts. The goal of this study was to investigate patterns of fecal progestagen and glucocorticoid excretion in females during mating, gestation, and lactation and identify hormonal relationships to reproductive success. Fresh fecal samples were collected from 48 adult, female rabbits over 3 breeding seasons at a frequency of 4-7 samples per week. Results showed that a large (17-fold) increase in progestagen concentrations 1 day after mating provides a reliable means of determining if a successful mating occurred. In general, higher glucocorticoid concentrations during the breeding season, specifically during mating and gestation, were associated with lower reproductive success. Females that failed to conceive during the breeding season had higher glucocorticoid and lower progestagen baseline concentrations than females that did conceive. Glucocorticoid excretion during late gestation, but not lactation, was negatively associated with litter success, suggesting it affects offspring survival more during the prenatal than the postnatal period. Progestagen and glucocorticoid concentrations at the end of gestation were positively related to litter size, which may be an important factor in juvenile survival. In summary, higher concentrations of fecal glucocorticoids during the breeding season were associated with reduced conception rates and survival of subsequent litters. Ultimately, identifying what factors cause elevated glucocorticoids in pygmy rabbits could provide opportunities to alleviate negative stressors and increase the reproductive output of the captive population.

3202: *-.012*

The threatened Bluetail Mole Skink (Plestiodon egregius lividus) is limited to the xeric habitats of the southern Lake Wales Ridge in central Florida. To generate important data for conserving this species, we characterized genetic variation at the mitochondrial cytochrome-b gene and seven microsatellite loci among multiple populations. We compared the Bluetail Mole Skink's pattern of

genetic diversity and differentiation to those of two other lizards with similar geographic and habitat distributions the Florida Sand Skink (Plestiodon reynoldsi) and the Florida Scrub Lizard (Sceloporus woodi). The Bluetail Mole Skink was highly variable at the genetic markers, and significant genetic differentiation occurred among scrub patches. Patches can be divided into central and southern Lake Wales Ridge groups. Our results also suggest that each sampled habitat patch should be treated as a population and reintroductions should minimize the distance between recipient and source locations to limit altering the potential long-term pattern of genetic differentiation among Bluetail Mole Skinks on the Lake Wales Ridge. The Bluetail Mole Skink and the Florida Sand Skink had similar genetic diversity, and all three lizards had comparable patterns of genetic differentiation. The concordance of genetic differentiation among these species is further evidence suggesting that similar conservation issues face the three species, namely, preserving the remaining xeric habitat. It is likely, therefore, that conservation efforts directed at the more common Florida Sand Skink and Florida Scrub Lizard would benefit the Bluetail Mole Skink.

3203: ***-.129***

In recent decades, dozens of studies have involved attempts to introduce native and desirable nonnative plant species into grasslands dominated by invasive weeds. The newly introduced plants have proved capable of establishing, but because they are rarely monitored for more than four years, it is unknown if they have a high likelihood of persisting and suppressing invaders for the long term. Beyond invaded grasslands, this lack of long-term monitoring is a general problem plaguing efforts to reintroduce a range of taxa into a range of ecosystems. We introduced species from seed and then periodically measured plant abundances for nine years at one site and 15 years at a second site. To our knowledge, our 15-year data are the longest to date from a seeding experiment in invaded, never-cultivated grassland. At one site, three seeded grasses maintained high densities for three or more years, but then all or nearly all individuals died. At the second site, one grass performed similarly, but two other grasses proliferated and at least one greatly suppressed the dominant invader (Centaurea maculosa). In one study, our point estimate suggests that the seeded grass Thinopyrum intermedium reduced C. maculosa biomass by 93% 15 years after seeding. In some cases, data from three and fewer years after seeding falsely suggested that seeded species were capable of persisting within the invaded grassland. In other cases, data from as late as nine years after seeding falsely suggested seeded populations would not become large enough to suppress the invader. These results show that seeded species sometimes persist and suppress invaders for long periods, but short-term data cannot predict if, when, or where this will occur. Because short-term data are not predictive of long-term seeded species performances, additional long-term data are needed to identify effective practices, traits, and species for revegetating invaded grasslands.

3204: ***+.022***

Historically the wolf (Canis lupus) was hated and extirpated from most of the contiguous United States. The federal Endangered Species Act fostered wolf protection and reintroduction which improved the species' image. Wolf populations reached biological recovery in the Northern Rocky Mountains and upper Midwest, and the animal has been delisted from the Endangered Species List in those areas. Numerous studies in National Parks suggest that wolves, through trophic cascades, have caused ecosystems to change in ways many people consider positive. Several studies have been conducted in Yellowstone National Park where wolf interactions with their prey, primarily elk (Cervus elaphus), are thought to have caused reduction of numbers or changes in movements and behavior. Some workers consider the latter changes to have led to a behaviorally-mediated

trophic cascade. Either the elk reduction or the behavioral changes are hypothesized to have fostered growth in browse, primarily willows (Salix spp.) and aspen (Populus spp.), and that growth has resulted in increased beavers (Castor Canadensis), songbirds, and hydrologic changes. The wolfs image thus has gained an iconic cachet. However, later research challenges several earlier studies' findings such that earlier conclusions are now controversial, especially those related to causes of browse regrowth. In any case, any such cascading effects of wolves found in National Parks would have little relevance to most of the wolf range because of overriding anthropogenic influences there on wolves, prey, vegetation, and other parts of the food web. The wolf is neither a saint nor a sinner except to those who want to make it so. Published by Elsevier Ltd.

3205: *-.046*

Assisted colonization of endangered species to locations outside their native ranges in response to projected climate change has emerged as a potential, but highly controversial conservation tool. The debate has been largely philosophical and hypothetical as little biological data exist in the literature. In 2006, nearly 1000 endangered wild orchid plants belonging to 29 species were translocated to higher elevations in subtropical southwestern China in response to inundation threats from a hydropower project. We took advantage of this upward translocation to address one of the main biological concerns associated with assisted colonization, i.e. whether the target endangered species can survive in the novel environment that is projected to be suitable for them, sometime in the future. We assessed the impacts of two extreme weather events, translocation shock and herbivory, on survival of 20 of these species and 462 individuals that were translocated beyond their current range vs. within that range. A cold spell in 2008 on average caused 10% mortality, less than the mortality rate from herbivores. However, the cold spell was the only force that extirpated an out-of-range population. No mortality resulted from a drought event in 2010. The 5-year survival percentages were not different between low and wide elevation species (69.3% mean +/- 36.3% standard deviation vs. 67.3% +/- 30.9%). Orchids represent 10% of flowering plant diversity and are among the most endangered group of organisms due to a combination of their often specialized ecological requirements, habitat destruction, and overexploitation. The demonstrated ability to survive extreme environmental challenges indicates that assisted colonization may be a viable conservation tool for the many endangered orchids worldwide due to climate change and/or other reasons. (C) 2012 Elsevier Ltd. All rights reserved.

3206: *+.106*

We used data on alleles of seven polymorphic microsatellite loci in 142 individuals of the star cactus Astrophytum asterias from five subpopulations to estimate genetic parameters. Four of the subpopulations had high levels of heterozygosity and allelic diversity compatible with efficient outcrossing, and low F-ST-values that suggested high rates of gametic gene flow effected by winged-insect pollinators. The fifth subpopulation that was small and occupied the smallest area had low levels of heterozygosity and allelic diversity, which can be caused by small populations, inbreeding, geographic isolation, and founder effect. Our research indicated that all subpopulations, except one, were genetically suitable sources of propagules for reintroduction or for augmentation of other populations.

3207: *+.354*

This investigation presents an annotated checklist of the living mammals reported to occur in Tennessee as well as recently extirpated species and reintroduced taxa. Data were obtained from field collections, literature records, and selected mammal collections in North America. Species

included in the checklist represent 8 orders, 23 families, and 86 species. The work provides information relating to distribution, habitat, and conservation status of each species and should be useful in planning management and conservation programs in Tennessee.

3208: ***+.082***

We examined overwintering behavior in gopher tortoises (Gopherus polyphemus) translocated to the northern periphery of their geographic range by using miniature temperature data loggers during 2 winters. All adult and juvenile tortoises monitored with temperature loggers survived overwintering; however, during the course of the study 2 translocated juvenile tortoises without temperature loggers died during winter months. Onset and termination of overwintering were not different between the 2 yrs and were not correlated with mean above-ground air temperature. Mean overwinter duration was 127 +/- 9 d SD and 128 +/- 13 d SD during 2002-2003 and 2004-2005, respectively. Tortoises experienced temperatures as low as 7 degrees C and as high as 31 degrees C while overwintering; however, most (12 of 15) tortoises experienced very little (< 1 degrees C) mean daily temperature fluctuation despite air temperatures regularly dropping below 0 degrees C and exceeding 20 degrees C. The overall mean temperature of overwintering tortoises was 12.4 degrees +/- 0.8 degrees C (2002-2003) and 12.6 degrees +/- 1.2 degrees C (2004-2005). Large fluctuations in temperature occurred when tortoises actively basked, and half of the monitored tortoises did, particularly juveniles, which accounted for 42% of winter basking events. Our results suggest that, given timely access to suitable refugia at recipient sites, overwinter mortality of translocated adult individuals may be minimal.

3209: ***+.295***

The destruction of prairies has led to the decline of the ornate box turtle (Terrapene ornate) across much of its range. Land management agencies are considering translocation programs to restore populations to areas from which they have been extirpated. For these conservation efforts to be successful, long-term posttranslocation monitoring is necessary to ensure that translocated individuals behave and use habitat similarly to unmanipulated individuals. We conducted a 3-yr radiotelemetry study of a potential source population of ornate box turtles to provide baseline data on home range size and site fidelity pretranslocation. Adult males and females did not differ in minimum convex polygon home range size (mean 4.0 ha), 95% fixed kernel home ranges (mean 2.6 ha), or 50% fixed kernel home ranges (mean 0.4 ha). Both sexes showed high site fidelity to annual home ranges and to previously used overwintering sites, although distance between subsequent overwintering sites was less for females than for males. At our study site, ornate box turtles have relatively small home ranges and exhibit strong site fidelity. Translocation programs for this species should closely monitor movements of translocated individuals to assess whether they are successfully establishing new home ranges or attempting to return to their site of origin. Moreover, the high site fidelity exhibited by this species suggests that newly translocated individuals may be at increased mortality risk because they are unfamiliar with suitable overwintering and/or nesting sites in their new location. The results of our study will be used to ensure that sites to which animals are translocated are comparable to the site of origin in terms of home range size requirements and important habitat features. In addition, our data serve as a critical baseline to which the habitat use and movement patterns of monitored animals posttranslocation can be directly compared to assess the success of the translocation.

3210: ***+.184***

A revised list of the butterflies of the La Trobe Wildlife Sanctuary and adjacent nature

conservation reserves near Melbourne is presented based on intermittent observations and collections between 1989 and 2011. A total of 31 species is recorded, of which 22 (71%) are considered to be resident. Of the resident species, nine (41%) specialise as larvae on Poaceae. Populations of three resident species that were previously absent have become established via different pathways: Trapezites symmomus was translocated deliberately in 1988, Trapezites phigalioides appears to have been introduced unintentionally in the late 1990s, while Toxidia doubledayi invaded the area naturally during a period of range expansion during the 1990s. The introduction of the hesperiid Trapezites symmomus involved several key elements, namely community education in relation to the principles of butterfly conservation, involvement of local science students and park rangers, increasing the extent and integrity of the butterflies' habitat through collection and propagation of the larval food plant, translocation of the larval stage of the butterfly, and long-term monitoring of the species over 20+ years. Ecological restoration and conservation management of the reserves estate over the past two decades appears to have benefited the grass- and Lomandra-feeding specialists and other butterflies associated with the understorey ground layer. The success of the T symmomus introduction suggests that other more threatened species in the Melbourne area associated with monocot food plants could potentially be established in conservation reserves through such translocation programs. (The Victorian Naturalist 129 (3), 2012, 86-97)

3211: *+.057*

Space-use strategies are essential behavioural skills during the fledging-to-dispersal period, when physical capabilities and the ability to explore the external world are developed. Here we describe the space-use strategies of 13 radio-tagged ospreys Pandion haliaetus, released as part of a reintroduction project in Central Italy, during their post-fledging dependence period. The ospreys remained within a radius of about 1 km of the hacking tower for about twenty days after release. Later they began to explore more of their surroundings. The occurrence and frequency of explorations further than 1 kin from the release point generally increased with time, even though juveniles continued to frequent the vicinity of the release pens. The translocated ospreys showed marked aggregation perhaps because the period spent at the hacking tower could have reinforced the feeling of belonging to the same brood, promoting a consequent strong association among the young. Also, the absence of parents may have led juveniles to aggregate as a compensation behaviour for the lack of parental care. These explanations, which are not mutually exclusive, may have resulted in more time being spent in intra-specific interactions, accounting for the long pre-dispersal phase that we observed in the present study.

3212: *+.340*

Background: The conservation of species structured in metapopulations involves an important dilemma of resource allocation: should investments be directed at restoring/enlarging habitat patches or increasing connectivity. This is still an open question for Maculinea species despite they are among the best studied and emblematic butterfly species, because none of the population dynamics models developed so far included dispersal.Methodology/Principal Findings: We developed the first spatially and financially explicit Population Viability Analysis model for Maculinea alcon, using field data from The Netherlands. Implemented using the RAMAS/GIS platform, the model incorporated both local (contest density dependence, environmental and demographic stochasticities), and regional population dynamics (dispersal rates between habitat patches). We selected four habitat patch networks, contrasting in several basic features (number of habitat patches, their quality, connectivity, and occupancy rate) to test how these features are affecting the ability to enhance population viability of four basic management options, designed to

incur the same costs: habitat enlargement, habitat quality improvement, creation of new stepping stone habitat patches, and reintroduction of captive-reared butterflies. The PVA model was validated by the close match between its predictions and independent field observations on the patch occupancy pattern. The four patch networks differed in their sensitivity to model parameters, as well as in the ranking of management options. Overall, the best cost-effective option was enlargement of existing habitat patches, followed by either habitat quality improvement or creation of stepping stones depending on the network features. Reintroduction was predicted to generally be inefficient, except in one specific patch network.Conclusions/Significance: Our results underline the importance of spatial and regional aspects (dispersal and connectivity) in determining the impact of conservation actions, even for a species previously considered as sedentary. They also illustrate that failure to account for the cost of management scenarios can lead to very different conclusions.

3213: *+.241*

Background: Ecological replacement involves the introduction of non-native species to habitats beyond their historical range, a factor identified as increasing the risk of failure for translocations. Yet the effectiveness and success of ecological replacement rely in part on the ability of translocatees to adapt, survive and potentially reproduce in a novel environment. We discuss the welfare aspects of translocating captive-reared non-native tortoises, Aldabrachelys gigantea and Astrochelys radiata, to two offshore Mauritian islands, and the costs and success of the projects to date.Methodology/Principal Findings: Because tortoises are long-lived, late-maturing reptiles, we assessed the progress of the translocation by monitoring the survival, health, growth, and breeding by the founders. Between 2000 and 2011, a total of 26 A. gigantea were introduced to Ile aux Aigrettes, and in 2007 twelve sexually immature A. gigantea and twelve male A. radiata were introduced to Round Island, Mauritius. Annual mortality rates were low, with most animals either maintaining or gaining weight. A minimum of 529 hatchlings were produced on Ile aux Aigrettes in 11 years; there was no potential for breeding on Round Island. Project costs were low. We attribute the success of these introductions to the tortoises' generalist diet, habitat requirements, and innate behaviour.Conclusions/Significance: Feasibility analyses for ecological replacement and assisted colonisation projects should consider the candidate species' welfare during translocation and in its recipient environment. Our study provides a useful model for how this should be done. In addition to serving as ecological replacements for extinct Mauritian tortoises, we found that releasing small numbers of captive-reared A. gigantea and A. radiata is cost-effective and successful in the short term. The ability to release small numbers of animals is a particularly important attribute for ecological replacement projects since it reduces the potential risk and controversy associated with introducing non-native species.

3214: *-.046*

Background: Establishment success in newly founded populations relies on reaching the established phase, which is defined by characteristic fluctuations of the population's state variables. Stochastic population models can be used to quantify the establishment probability of newly founded populations; however, so far no simple but robust method for doing so existed. To determine a critical initial number of individuals that need to be released to reach the established phase, we used a novel application of the "Wissel plot", where ln(1 P0(t)) is plotted against time t. This plot is based on the equation P0(t) = 1 - c1e-[omega]1t, which relates the probability of extinction by time t, P0(t), to two constants: c1 describes the probability of a newly founded population to reach the established phase, whereas [omega]1 describes the population's probability of extinction per short time interval once established. Results: For illustration, we applied the

method to a previously developed stochastic population model of the endangered African wild dog (Lycaon pictus). A newly founded population reaches the established phase if the intercept of the (extrapolated) linear parts of the "Wissel plot" with the y-axis, which is ln(c1), is negative. For wild dogs in our model, this is the case if a critical initial number of four packs, consisting of eight individuals each, are released. Conclusions: The method we present to quantify the establishment probability of newly founded populations is generic and inferences thus are transferable to other systems across the field of conservation biology. In contrast to other methods, our approach disaggregates the components of a population's viability by distinguishing establishment from persistence.

3215: *+.100*

The activity patterns of the Badgers (Meles meles) reintroduced from Shandong to Shanghai was observed during November 2010 to February 2011. The results showed that in winter, these badgers mainly fed on meat and acted once every 2 - 4 days. The duration of the activity was short. Activity of reintroduced badgers depended on temperature in seminatural area. The reintroduced badgers can adapt to artificial condition and overwinter smoothly.

3216: *+.128*

Recent genetic analysis has shown that the extinct Caspian Tiger (P. t. virgata) and the living Amur Tigers (P. t. altaica) of the Russian Far East are actually taxonomically synonymous and that Caspian and Amur groups historically formed a single population, only becoming separated within the last 200 years by human agency. A major conservation implication of this finding is that tigers of Amur stock might be reintroduced, not only back into the Koreas and China as is now proposed, but also through vast areas of Central Asia where the Caspian tiger once lived. However, under the current tiger conservation framework the 12 "Caspian Tiger States" are not fully involved in conservation planning. Equal recognition as "Tiger Range States" should be given to the countries where the Caspian tiger once lived and their involvement in tiger conservation planning encouraged. Today, preliminary ecological surveys show that some sparsely populated areas of Central Asia preserve natural habitat suitable for tigers. In depth assessments should be completed in these and other areas of the Caspian range to evaluate the possibility of tiger reintroductions. Because tigers are a charismatic umbrella species, both ecologically and politically, reintroduction to these landscapes would provide an effective conservation framework for the protection of many species in addition to tigers. And for today's Amur Tigers this added range will provide a buffer against further loss of genetic diversity, one which will maintain that diversity in the face of selective pressures that can only be experienced in the wild.

3217: *+.001*

As habitat fragmentation and climate change degrade the suitability of natural habitats, many species are likely to become threatened with local or global extinction. In the future, conservation of species and management of degraded areas may need to employ radical solutions, such as assisted dispersal and colonisation. Here, we report the findings of a small scale, two-fold investigation into potential management actions for reintroducing functionally important species (dung beetles) in a fragmented system. We tested two a priori hypotheses: 1) dung supplementation will increase dung beetle population sizes in the short term; and 2) introducing dung beetles into a previously uninhabited area (assisted dispersal) will establish viable populations. Although we expected population sizes to increase following dung supplementation, there was no significant effect on beetle population sizes on eight small islands when compared to

eight control islands. However, introducing 827 individuals of the common species Paragymnopleurus maurus to two previously uninhabited islands had mixed results. On one island, the newly founded population persisted beyond 51 days but rapidly declined on the other. Follow-up investigations suggested that invasive, predatory yellow crazy ants (Anoplolepis gracilipes) may have prevented population establishment on the island where this failed. Assisted dispersal, and colonisation, may provide mechanisms to combat some effects of fragmentation and climate change if isolation is the primary cause of the population decline, but success depends on extensive prior suitability assessment. Although suggestive, our results should be interpreted with caution because of experimental limitations.

3218: *-.048*

Seabirds are highly vagile and can disperse up to thousands of kilometers, making it difficult to identify the factors that promote isolation between populations. The endemic Hawaiian petrel (Pterodroma sandwichensis) is one such species. Today it is endangered, and known to breed only on the islands of Hawaii, Maui, Lanai and Kauai. Historical records indicate that a large population formerly bred on Molokai as well, but this population has recently been extirpated. Given the great dispersal potential of these petrels, it remains unclear if populations are genetically distinct and which factors may contribute to isolation between them. We sampled petrels from across their range, including individuals from the presumably extirpated Molokai population. We sequenced 524 bp of mitochondrial DNA, 741 bp from three nuclear introns, and genotyped 18 microsatellite loci in order to examine the patterns of divergence in this species and to investigate the potential underlying mechanisms. Both mitochondrial and nuclear data sets indicated significant genetic differentiation among all modern populations, but no differentiation was found between historic samples from Molokai and modern birds from Lanai. Population-specific nonbreeding distribution and strong natal philopatry may reduce gene flow between populations. However, the lack of population structure between extirpated Molokai birds and modern birds on Lanai indicates that there was substantial gene flow between these populations and that petrels may be able to overcome barriers to dispersal prior to complete extirpation. Hawaiian petrel populations could be considered distinct management units, however, the dwindling population on Hawaii may require translocation to prevent extirpation in the near future. Heredity (2012) 109, 19-28; doi:10.1038/hdy.2012.7; published online 21 March 2012

3219: *+.099*

Dispersal is crucial for gene flow and often determines the long-term stability of meta-populations, particularly in rare species with specialized life cycles. Such species are often foci of conservation efforts because they suffer disproportionally from degradation and fragmentation of their habitat. However, detailed knowledge of effective gene flow through dispersal is often missing, so that conservation strategies have to be based on markrecapture observations that are suspected to be poor predictors of long-distance dispersal. These constraints have been especially severe in the study of butterfly populations, where microsatellite markers have been difficult to develop. We used eight microsatellite markers to analyse genetic population structure of the Large Blue butterfly Maculinea arion in Sweden. During recent decades, this species has become an icon of insect conservation after massive decline throughout Europe and extinction in Britain followed by reintroduction of a seed population from the Swedish island of Oland. We find that populations are highly structured genetically, but that gene flow occurs over distances 15 times longer than the maximum distance recorded from markrecapture studies, which can only be explained by maximum dispersal distances at least twice as large as previously accepted. However, we also find evidence that gaps between sites with suitable habitat exceeding similar to 20 km induce genetic

erosion that can be detected from bottleneck analyses. Although further work is needed, our results suggest that M. arion can maintain fully functional metapopulations when they consist of optimal habitat patches that are no further apart than similar to 10 km.

3220: *+.006*

Aspen (Populus tremuloides) on the northern Yellowstone winter range has declined over the last half-century. Beaver (Castor canadensis) were reintroduced in Eagle Creek in 1991 in an attempt to reverse this trend. In 2005, we assessed the efficacy of this project by quantifying the long-term effects of beaver on aspen stands and the riparian area in this drainage. Between 1990 and 2005, the canopy cover of mature aspen decreased more than 62%, whereas immature aspen cover more than tripled, resulting in a total aspen canopy cover decrease ($p < 0.05$) from 43 to 25% (a loss of 7.25 ha). Willow canopy cover increased from 10 to 14% during the same period. The impacts of beaver on aspen stands were estimated by comparing vegetative changes among control sites with less than 10% beaver use ($n = 5$), active beaver sites ($n = 6$), sites inactive for 13 years ($n = 7$), sites inactive for 46 years ($n = 4$), and sites inactive for 711 years ($n = 5$). Aspen sprout and sapling densities were greater ($p = 0.01$) on sites which were active and inactive for 13 years compared to the other sites. Aspen ramets were not able to grow taller than 2 m on sites without beaver activity for 411 years due to ungulate herbivory. Although beaver stimulated the growth of aspen sprouts and saplings, ungulate herbivory prevented successful aspen recovery in the Eagle Creek drainage of the northern Yellowstone winter range 14 years after beaver reintroduction.

3221: *+.115*

Differences between continental and island species in their ability to recognize mammalian predators are well documented, but how quickly acquired predator recognition behaviour declines or is lost when animals are translocated to sites without mammals has not been fully investigated. We compared predator recognition in a 'mainland' population of Stewart Island robins, Petroica australis rakiura, where rats have been present since the 1600s, and in a reintroduced island population where rats were recently eradicated and the resident robins have experienced a rat-free environment for about one generation. We found that the rat-free island population showed little fear or recognition of a model rat and were less agitated and more likely to approach and consume food in front of the model rat relative to robins on the mainland. The results of our study suggest that endemic species that acquire the ability to recognize mammalian predators lose this ability relatively quickly when translocated to islands where mammals are absent. These results raise questions about the costs of maintaining mammalian recognition behaviour in mammal-free environments, even when avian predators are present. They also raise concerns about whether island sanctuaries are appropriate sources for harvesting for reintroductions back to the rat-infested mainland. (C) 2012 The Association for the Study of Animal Behaviour. Published by Elsevier Ltd. All rights reserved.

3222: *+.061*

Newly established populations are susceptible to founder events that reduce genetic variation. This may be counterbalanced by gene flow after populations become established or founders coming from genetically different populations. However, initial gains in genetic diversity may be short-lived if there is limited mixing between lineages and subsequent inbreeding, or if one lineage sweeps to fixation through selection or genetic drift. Here, we report on the genetic changes taking place within two newly established populations of intertidal snail over a 15-year period (similar to 10 generations). Each translocation was set up using multiple, genetically distinct source

populations. Our data show that higher levels of variation in the translocated populations compared to the source populations were maintained over time for both nuclear (microsatellite) and mitochondrial genes. Small changes in allele and haplotype frequencies were observed in the source populations and in one of the translocated populations, but marked changes were evident in the other, where there was a dramatic shift towards the genetic make-up of one of the source populations. These genetic changes occurred despite relatively large numbers of founders (200374 adults) and no evidence of the population experiencing a severe reduction in effective population size. Our study shows that the genetic composition of newly established populations can vary greatly over time and that genetic outcomes can be highly variable, and significantly different from initial expectations, even when they are established using high numbers of individuals and involve source populations from the same geographic regions.

3223: ***+.304***

Large assemblages of animal bones and/or shells from archaeological sites can provide data valuable for modern conservation efforts, e.g., by providing accurate historical baselines for species reintroductions or habitat restoration. Such data are underused by natural scientists, partly due to assumptions that archaeological materials are too biased by prehistoric human actions (the so-called "cultural filter") to accurately reflect past biotic communities. In order to address many paleobiological, archaeological, or applied research questions, data on past species, communities, and populations must first be demonstrated to be representative at the appropriate level. We discuss different ways in which one kind of cultural bias, human transport of specimens, can be tested at different scales, using freshwater mussel shells from prehistoric sites in the Tombigbee River basin of Mississippi and Alabama to show how representativeness of samples can be assessed.

3224: ***-.054***

Large predators have been reintroduced to an increasing number of protected areas in South Africa. However, the conditions allowing both prey and predator populations to be sustained in enclosed areas are still unclear as there is a lack of understanding of the consequences of such reintroductions for ungulate population dynamics. Variation in lion numbers, two decades after their first release, offered a special opportunity to test the effects of predation pressure on the population dynamics of seven ungulate species in the 960 km(2) Hluhluwe-iMfolozi Park (HiP), South Africa. We used two different approaches to examine predator prey relationships: the population response of ungulates to predation pressure after accounting for possible confounding factors, and the pattern of ungulate adult mortality observed from carcass records. Rainfall patterns affected observed mortalities of several ungulate species in HiP. Although lion predation accounted for most ungulate mortality, it still had no detectable influence on ungulate population trends and mortality patterns, with one possible exception. This evidence suggests that the lion population had not yet attained the maximum abundance potentially supported by their ungulate prey; but following recent increases in lion numbers it will probably occur soon. It remains uncertain whether a quasi-stable balance will be reached between prey and predator populations, or whether favoured prey species will be depressed towards levels potentially generating oscillatory dynamics in this complex large mammal assemblage. We specifically recommend a continuous monitoring of predator and prey populations in HiP since lions are likely to show more impacts on their prey species in the next years. (c) 2012 Elsevier Masson SAS. All rights reserved.

3225: ***+.176***

Bioclimatic envelope models use associations between aspects of climate and species' occurrences to estimate the conditions that are suitable to maintain viable populations. Once bioclimatic envelopes are characterized, they can be applied to a variety of questions in ecology, evolution, and conservation. However, some have questioned the usefulness of these models, because they may be based on implausible assumptions or may be contradicted by empirical evidence. We review these areas of contention, and suggest that criticism has often been misplaced, resulting from confusion between what the models actually deliver and what users wish that they would express. Although improvements in data and methods will have some effect, the usefulness of these models is contingent on their appropriate use, and they will improve mainly via better awareness of their conceptual basis, strengths, and limitations.

3226: ***-.058***

Awareness of parasite risks in translocations has prompted the development of parasite management protocols, including parasite risk assessment, parasite screening and treatments. However, although the importance of such measures seems obvious it is difficult to know whether the measures taken are effective, especially when working with wild populations. We review current methods in one extensively researched case study, the endemic New Zealand passerine bird, the hihi Notiomystis cincta. Our review is structured around four of the 10 questions proposed by Armstrong & Seddon (Trends in Ecology & Evolution, 2008: 23, 20-25) for reintroduction biology. These four questions can be related directly to parasites and parasite management and we recommend using this framework to help select and justify parasite management. Our retrospective study of recent disease and health screening in hihi reveals only partial overlap with these questions. Current practice does not focus on, or aim to reduce, the uncertainty in most steps of the risk assessment process or on evaluating whether the measures are effective. We encourage targeted parasite management that builds more clearly on available disease risk assessment methodologies and integrates these tools within a complete reintroduction plan.

3227: ***+.301***

Scrophularia takesimensis Nakai is a Critically Endangered plant species endemic to Ulleung Island, Republic of Korea. We provide updated information on the distribution and conservation status of this species. We located 39 subpopulations and counted a total of 443 individuals, including some reintroduced. Observations of dried and broken branches, with fruits, of S. takesimensis along the coast may indicate dispersal by sea. The construction of coastal roads is the main threat to the species. To conserve this species more effectively we recommend that: (1) the two habitats identified as a priority for conservation should be afforded special protection, (2) habitats to the seaward side of coastal roads are more suitable than habitat on the landward side for in situ conservation, and (3) the presently known subpopulations require continuous protection and monitoring.

3228: ***+.114***

Tigridiopalma magnifica, a perennial herb and the only species in the genus Tigridiopalma (Family Melastomataceae) is rare and endemic to China where it is categorized as Critically Endangered on the national Red List. Twelve locations with populations of T. magnifica have been identified (1 extinct, 11 extant). T. magnifica only grows in the surface soil on stone walls or rocks under the canopy of secondary forests and plantations and has no specific associated plant species. Canopy closure, soil water content and the distance to the closest stream are the main factors influencing

the distribution of T. magnifica. We reintroduced T. magnifica plantlets produced by tissue culture into three locations: one within the species' original range, and 11.5 and 400 km from the species' original range. After 11 months survival rate was 40-58% but survival was higher and plantlet crowns were larger at the location within the species' original range than at the other two sites. The combination of advanced propagation techniques and ecological restoration could facilitate reintroduction and conservation of T. magnifica and other rare and threatened plants. This example of the successful reintroduction of a rare, threatened herb has implications for human-assisted migration and colonization of rare plant species under future climate change scenarios.

3229: ***-.097***

The gastrointestinal and external parasites and their level of infestation in the Arabian oryx (Oryx leucoryx) were studied at King Khalid Wildlife Research Center (KKWRC) in Thumamah, Riyadh Province, Saudi Arabia. Fecal samples were collected from 36 oryx while they were being handled for routine management at KKWRC; the samples were examined for gastrointestinal parasites. Protozoan oocysts detected in the present study were Eimeria saudiensis (prevalence 63.9%) and Cuptosporidium sp. Strongyle-type nematode eggs were present in 75% of the fecal samples. Nematodirus spathiger eggs (prevalence 55.6%) and Trichuris sp. eggs (prevalence 13.9%) were also recovered from the fecal samples. Additionally, 3 of 44 (6.8%) serum samples tested positive for toxoplasmosis antibodies. Carcasses of 6 oryx that died at the center were necropsied and all were infected with Camelstrongylus mentulatus, Trichostrongylus probolurus, and Nematodirus spathiger, whereas 3 animals were infected with Trichuris cervicaprae at relatively low intensities. Cysticerci of Taenia hydatigena were recovered from 3 of 6 necropsied oryx.

3230: ***+.034***

The Antillean manatee (Trichechus manatus manatus), a subspecies of the West Indian manatee, is a large-bodied marine mammal found in fresh, brackish, and marine habitats throughout the Caribbean Islands and Central and South America. Antillean manatees in Brazil are classified as critically endangered, with a census size of approximately 500 individuals. The population in the Northeast region of Brazil is suspected to have approximately 300 manatees and is threatened by habitat alteration and incidental entanglement in fishing gear. A high incidence of dependent calf strandings have been identified near areas of altered critical manatee habitat. The majority of the calves are neonates, discovered alive, with no potential mothers nearby. These calves typically require human intervention to survive. Since 1989 the calves have been rescued (N=67), rehabilitated, and released (N=25) to supplement the small wild manatee population. The rescued calves, and those born in captivity, are typically, not released to their rescue location, mainly for logistical reasons. Therefore, phylogeographic analyses can help to identify related populations and appropriate release sites. Here, mitochondrial DNA analyses identified low haplotype (h=0.08) and nucleotide (p=0.0026) genetic diversity in three closely related haplotypes. All three haplotypes (M01, M03, and a previously unidentified haplotype, M04) were found in the northern portion of the region, while only a single haplotype (M01) was represented in the south. This suggests the presence of two genetic groups with a central mixing zone. Release of rehabilitated calves to unrelated populations may result in genetic swamping of locally adapted alleles or genotypes, limiting the evolutionary potential of the population. The small population size coupled with low genetic diversity indicates that the Northeast Brazil manatee population is susceptible to inbreeding depression and possible local extinction. Further conservation measures incorporating genetic information could be beneficial to the critically endangered Brazilian manatee population. Published 2012. This article is a U.S. Government work and is in the public domain in the USA.

3231: ***-.045***

Although the spur-thighed tortoise, Testudo graeca, is one of the most widely distributed species of tortoises, its natural populations are threatened through its whole range. Particularly at south-eastern Spain, the species is mainly threatened by habitat destruction and over-collection, given that this chelonian has been traditionally considered an appreciate pet. As south-eastern Spanish wildlife recovery centers shelter hundreds of captive animals mainly coming from illegal trade or captive-bred, there is a strong debate about what to do with these animals: maintaining them in captivity all along their lives or reintroducing them to wildlife. It is well known that the reintroduction of captive animals supposes a risk for the wild population due to the uncertainty of their genetic origin and to the possible spread of infectious diseases. However, despite the increasing evidence that infectious agents are a potential health hazard for wildlife, little is known about the risk that introduced parasites could suppose for the wild populations of spur-thighed tortoise. The present study investigates for the first time the presence of helminth eggs and worms in faeces from 107 wild and captive individuals collected from mid-March to mid-June 2010, and relates the findings to different environmental and host variables. Sixteen oxyurid species and the ascarid Angusticaecum holopterum were identified. This last nematode and the oxyurid species Tachygonetria palearticus and T. seurati had not been reported in Spanish wild T. graeca previously. The prevalence of oxyurid eggs and worms were 94% and 70%, respectively; while, ascarid eggs and worms were found in 26% and 5% of tortoises, respectively. Ascarid infections affected mostly captive animals and were associated to caparace deformities and symptoms of upper respiratory tract disease ($p < 0.05$). Oxyurid infections were not associated to negative health traits and prevalence increased with age. In free-living tortoises, the distribution of pharingodonid genera also varied according to habitat; moreover, T. longicollis, T. pusilla, conica, T. robusta and Mehdiella stylosa where significantly more frequent in wild compared to captive tortoises ($p < 0.05$). Study results highlight important differences in the nematode fauna of captive and free-living tortoises and questions one more time if the reintroductions of captive animals suppose a risk for the wild population since the former ones can harbor and distribute among free populations pathogens like ascarid nematodes. (C) 2012 Elsevier B.V. All rights reserved.

3232: ***-.064***

Reintroductions are an increasingly common conservation restoration tool; however, little attention has hitherto been given to different methods for monitoring the stress encountered by reintroduced individuals. We compared ten potential measures of stress within four different categories (neuroendocrine, cell function, body condition and immune system function) as proxies for animal welfare in water voles being reintroduced to the Upper Thames region, Oxfordshire, UK. Captive-bred voles were assessed pre-release, and each month post-release for up to five months. Wild-born voles were captured in the field and assessed from two months post-release. Plasma corticosteroid, hydration and body condition of captive-bred voles differed between their pre-release measures and both their first ("short-term") recapture, and their final recapture ("long-term" release), however only body condition and immunocompetence measured using the Nitroblue Tetrazolium (NBT) test were significantly different post-release between the first and last recaptures. Captive-bred animals had lower fat reserves, higher weight/length ratios and better immunocompetence (NBT) than did wild-born voles. Captive-bred males had higher ectoparasite burdens compared to wild-born males and, as reintroduction site quality decreased, became less hydrated. These observations indicate that some methods can identify changes in the stress response in individuals, highlighting areas of risk in a reintroduction programme. In addition, a single measure may not provide a full picture of the stress experienced; instead, a combination of measures of different physiological systems may give a more complete indication of stress during

the reintroduction process. We highlight the need to monitor stress in reintroductions using measures from different physiological systems to inform on possible animal welfare improvements and thus the overall success rate of reintroductions.

3233: *+.296*

Environmental changes currently pose severe threats to biodiversity, and reintroductions and translocations are increasingly used to protect declining populations and species from extinction. Theory predicts that establishment success should be higher for more variable groups of dissimilar individuals. To test this 'diversity promotes establishment' hypothesis, we introduced colour polymorphic pygmy grasshoppers (Tetrix subulata) to different sites in the wild. The number of descendants found at the release sites the subsequent year increased with increasing number of colour morphs in the founder group, and variation in founder groups also positively affected colour morph diversity in the established populations. Since colour morphs differ in morphology, physiology, behaviour, reproductive life history and types of niche used, these findings demonstrate that variation among individuals in functionally important traits promotes establishment success under natural conditions, and further indicate that founder diversity may contribute to evolutionary rescue and increased population persistence.

3234: *+.067*

Effective treatment methods to eliminate infection with Batrachochytrium dendrobatidis (Bd) are required for development of sustainable captive survival assurance populations of amphibians and to reduce the risk of introducing Bd to new locations as part of amphibian trade or reintroduction programs. Treatment with itraconazole baths at 100 mg l(-1) is commonly used in captive amphibians, but side effects are observed in some amphibian species and life stages. Naturally occurring outbreaks of chytridiomycosis in Wyoming toads Anaxyrus baxteri and White's tree frogs Litoria caerulea were treated with lower-dose itraconazole baths (e.g. 50 mg l(-1) for White's tree frogs) and followed post-treatment with serial Taqman PCR testing to confirm elimination of Bd infection. Post-treatment PCR tests were consistently negative for the presence of Bd and treatment was deemed successful. Although this was not a controlled clinical trial, results suggest that lower doses of itraconazole may be effective for treatment of chytridiomycosis with resulting cost savings to amphibian conservation programs and a potential for a reduction in dose-related side effects from itraconazole treatment. Prospective clinical trials of alternative itraconazole treatment protocols are encouraged.

3235: *+.267*

Understanding the divergence of behavioural signals in isolated populations is critical to knowing how certain barriers to gene flow can develop. For many bird species, songs are essential for conspecific recognition and mate choice. Measuring the rate of song divergence in natural populations is difficult, but translocations of endangered birds to isolated islands for conservation purposes can yield insights, as the age and source of founder populations are completely known. We found significant and rapid evolution in the structure and diversity of bird song in North Island saddlebacks, Philesturnus rufusater, in New Zealand, with two distinct lineages evolving in < 50 years. The strong environmental filters of serial translocations resulted in cultural bottlenecks that generated drift and reduced song variability within islands. This rapid divergence coupled with loss of song diversity has important implications for the behavioural evolution of this species, demonstrating previously unrecognised biological consequences of conservation management.

3236: ***+.310***

1. The conservation of salmonid inter- and intra-specific diversity is a well-known challenge, and general management guidelines and conservation processes are available. However, research demonstrating the outcomes of practical conservation actions is largely lacking. 2. We monitored the spatiotemporal genetic and demographic evolution of a native Mediterranean brown trout population in a river in the French Alps to assess the efficacy and early effects of genetic refuge (i.e. cessation of stocking) and wild trout translocation strategies. We also studied the use of angling as a tool to limit the introgression of the wild standing population. 3. We found that the rate of non-native alleles in wild populations was age dependent, underpinning the importance of using age profiles in the design of genetic conservation studies. 4. Genetic refuge and direct translocation of wild trout resulted in a rapid and significant decrease in the percentages of non-native alleles. Moreover, the genetic refuge strategy resulted in a significant reduction in the number of pure non-native individuals, without changing trout densities, whilst direct translocations resulted in the establishment of dense, self-sustaining native trout populations. Direct translocations changed the distribution of genotype categories and increased densities up to 55-fold in 3 years. Our results also showed that angling resulted in a selective pressure on non-native trout introduced at fry stage, whereas non-native trout issued from natural recruitment were not affected. 5. Our study provides insights for improving the efficacy of practical conservation policies and can be used in other native freshwater fish conservation plans. Proactive measures such as direct translocation need to be implemented together with passive approaches such as genetic refuge policies. Before implementing such actions, accurate genetic and demographic studies at small geographical scales are essential to ensure that no self-sustaining population of non-native fish is present. To obtain rapid colonisation, we recommend introducing fish along whole river sections rather than concentrating on a few river stretches. Angling pressure can be used as an additional tool to improve restoration.

3237: ***+.226***

Protecting populations in their natural habitat allows for the maintenance of naturally evolved adaptations and ecological relationships. However, the conservation of genetic resources often requires complementary practices like gene banks, translocations or reintroductions. In order to minimize inbreeding depression and maximize the adaptive potential of future populations, populations chosen for ex situ conservation should be selected according to criteria that will result in a reduction of global coancestry in the population. Generally, large populations should reveal lower coancestry and higher genetic variation than small populations. If detailed knowledge about coancestry is lacking, census population number (N (c)) can be used as a proxy for required characteristics. However, a simple measure of N (c) may be misleading in particular cases as genetic processes rely on effective population size (N (e)) rather than N (c) and these two measures may differ substantially due to demographic processes. We used an example of English yew to address whether N (c) can be a good predictor of genetic parameters when used in conservation programs. Using microsatellite markers, we estimated allelic richness, inbreeding and coancestry coefficients of six relatively large yew populations in Poland. Each population was characterized by N (e) using the linkage disequilibrium method. Our results showed that populations of English yew were subject to substantial divergence and genetic drift, with both being inversely proportional to the effective subpopulation size (N (e)). Additionally, allelic richness appeared proportional to N (e) but not to N (c) . However, the N (e) /N ratio differed greatly among populations, which was possibly due to different population histories. From the results we concluded that choosing source populations based only on their census size can be fairly misleading. Implications for conservation are briefly discussed.

3238: +.192

We review the indexed scientific literature (233 papers) dealing with 'restocking' of vertebrates, i.e. reinforcement of wild populations by release of individuals of the same species. We found evidence that restocking may have desired beneficial effects such as: increased genetic diversity and mitigation of Allee effects in small populations; increased size or even salvation of threatened populations; increased harvest opportunities; and, redirection of harvest pressure from wild to captive-bred individuals. However, restocking may also have negative effects like changes in behaviour, morphology, and demography in recipient populations, as well as enhancement of pathogen spread. Negative genetic effects on recipient populations include homogenisation, introduction of non-native genes, and loss of local adaptation. Research thus far is strongly biased towards birds and mammals, and geographically to Europe and North America. Restocking for conservation purposes has been studied more than that for harvest management, while the latter may be of far greater importance in terms of number of released individuals. Demographic and genetic effects have been studied more than effects on behaviour, which in turn have received more attention than effects on morphology and pathogen spread. There is a general tendency for research on restocking to be fragmented taxonomically and by biological sub-disciplines. Our review demonstrates that restocking practices may and do cause significant disruptions of natural patterns in wild recipient populations. It also highlights the diversity, frequency and extent of these activities, leading us to argue that restocking is an emerging conservation and ecosystem resilience issue of global significance. Based on this review we outline monitoring and research needs for the future. We also provide guidelines to practitioners in conservation, game management, fisheries, epidemiology and other fields involved in restocking of vertebrates, who are likely to benefit from merging their perspectives and adopting a more cross-taxonomical and interdisciplinary attitude when laying out future agendas for evaluation and policy-making in this field. (c) 2012 Elsevier GmbH. All rights reserved.

3239: +.256

In light of limited conservation funding, global conservation initiatives are increasingly focused on regions of the planet that have been identified as valuable on the basis of their species diversity, the vulnerability of resident species to extinction, or the perceived pristine nature of their ecosystems. Regions that have been resilient to high rates of extinction have not yet been systematically considered in conservation efforts. We used published range maps for 392 vertebrate species to compare historical and current species ranges. We used the results of the comparison to identify regions of the globe in which no known vertebrate species has been extirpated in the past 200 years. In 17 regions, no detectable vertebrate extinctions occurred in the past 200 years. In 6 other regions, reintroductions of species restored the full historic complement of vertebrate species. The effects of humans on a landscape, as measured by the human-footprint index, although useful, was not a singularly good predictor of faunal intactness because more than 20% of intact land area was in heavily affected areas (50% of Earth's land area), and several regions where humans have had very little effect did not have intact faunas. Only 22% of intact land area was within protected-area networks. High-latitude areas were particularly underrepresented; they made up 3 of the 4 least-protected areas in our analyses. Our results indicate that although protected areas are in some cases associated with the prevention of extinctions, there are many regions in which human activity coexists with intact vertebrate assemblages. In addition, our new approach for assessing the value of global regions for conservation identifies several regions that are not represented in other prioritization metrics.

3240: -.006

Given the conflict with human interests that in many cases results in the extirpation of large carnivores, acceptance of their reintroduction is a considerable challenge. By the 1980s Mexican wolves (Canis lupus) were extinct in the wild. In 1998 a population was reintroduced in the Blue Range Mountains of New Mexico (U.S.A.). Efforts to reintroduce the species in Mexico have been ongoing since the late 1980s. Four teams working independently identified 6 areas in northern Mexico in the historic range of Mexican wolves, where reintroductions could potentially be successful. Each team used different methods and criteria to identify the areas, which makes it difficult to prioritize among these areas. Therefore, members of the different teams worked together to devise criteria for use in identifying priority areas. They identified areas with high, intermediate, and low potential levels of conflict between wolves and humans. Areas with low potential conflict had larger buffers (i.e., distance from human settlement to areas suitable for wolves) around human settlements than high- and intermediate-conflict areas and thus were thought most appropriate for the first reintroduction. High-conflict areas contained habitat associated with wolf presence, but were closer to human activity. The first reintroduction of Mexican wolves to Mexico occurred in October 2011 in one of the identified low-conflict areas. The identification of suitable areas for reintroduction represents a crucial step in the process toward the restoration of large carnivores. Choice of the first reintroduction area can determine whether the reintroduction is successful or fails. A failure may preclude future reintroduction efforts in a region or country.

3241: *+.049*

Some species have insufficient defenses against climate change, emerging infectious diseases, and non-native species because they have not been exposed to these factors over their evolutionary history, and this can decrease their likelihood of persistence. Captive breeding programs are sometimes used to reintroduce individuals back into the wild; however, successful captive breeding and reintroduction can be difficult because species or populations often cannot coexist with non-native pathogens and herbivores without artificial selection. In captive breeding programs, breeders can select for host defenses that prevent or reduce pathogen or herbivore burden (i.e., resistance) or traits that limit the effects of parasitism or herbivory on host fitness (i.e., tolerance). We propose that selection for host tolerance may enhance the success of reintroduction or translocation because tolerant hosts generally have neutral effects on introduced pathogens and herbivores. The release of resistant hosts would have detrimental effects on their natural enemies, promoting rapid evolution to circumvent the host resistance that may reduce the long-term probability of persistence of the reintroduced or translocated species. We examined 2 case studies, one on the pathogenic amphibian chytrid fungus (Batrachochytrium dendrobatidis [Bd]) and the other on the herbivorous cactus moth (Cactoblastis cactorum) in the United States, where it is not native. In each case study, we provide recommendations for how captive breeders and managers could go about selecting for host tolerance. Selecting for tolerance may offer a promising tool to rescue hosts species from invasive natural enemies as well as new natural enemies associated with climate change-induced range shifts.

3242: *+.287*

Translocations are commonly used conservation actions that aim at establishing new, self-sustaining populations of threatened species. However, many translocated populations are not self-sustaining but managed through supplemental feeding from the onset. Often, the decision to start managing is ad hoc, but managers will eventually have to make decisions for the future, for example, stop intervening, continue as it is or change the quantity of food provided. Such a decision requires managers to quantify the importance of supplemental feeding in determining the

performance and population dynamics of translocated populations, information that is rarely available in the published literature. Using the hihi as a case study, we examined the importance of supplemental feeding for the viability of a translocated population in New Zealand. We found that supplemental feeding positively affected the survival and abundance of translocated adult hihi but also found evidence of negative density dependence on recruitment. We present two stochastic population models that project the hihi population under different management scenarios, quantitatively assessing the impact supplemental feeding has had on the population. Our results illustrate how important long-term targeted monitoring is for robust decision making about adaptive management.

3243: *+.175*

The Pacific lamprey (Entosphenus tridentatus) is in decline in the Columbia River Basin, and translocating adult lamprey to bypass difficult migration corridors has been implemented since 2000. We describe and report results from two current translocation programs, provide context for use of translocation, and discuss potential benefits, risks, and uncertainties. Both translocation programs appear to have increased the number of spawning adults and the presence of larvae and juveniles; however, any subsequent increase in naturally spawning adults will require at least one, and likely more, generations to be realized. It was seen that the number of adults entering the Umatilla River increased beginning four years after the first translocations. Potential benefits of translocation programs are increased pheromone production by ammocoetes to attract adults, increased lamprey distribution and abundance in target areas, increased marine-derived nutrients, and promotion of tribal culture. Potential risks include disruption of population structure and associated genetic adaptations, disease transmission, and depletion of donor stocks.

3244: *+.037*

Managed relocation is defined as the movement of species, populations, or genotypes to places outside the areas of their historical distributions to maintain biological diversity or ecosystem functioning with changing climate. It has been claimed that a major extinction event is under way and that climate change is increasing its severity Projections indicating that climate change may drive substantial losses of biodiversity have compelled some scientists to suggest that traditional management strategies are insufficient. The managed relocation of species is a controversial management response to climate change. The published literature has emphasized biological concerns over difficult ethical, legal, and policy issues. Furthermore, ongoing managed relocation actions lack scientific and societal engagement. Our interdisciplinary team considered ethics, law, policy, ecology, and natural resources management in order to identify the key issues of managed relocation relevant for developing sound policies that support decisions for resource management. We recommend that government agencies develop and adopt best practices for managed relocation.

3245: *+.120*

We studied the genetic structure of a red deer (Cervus elaphus) population in 8 woodlands of northeastern Poland and 1 in western Belarus and compared it with the documented history of the population in the region. Red deer nearly went extinct in the region in the 18th and 19th centuries. In the mid-19th century, reintroductions began and continued until the mid-1960s. Animals were translocated from various sites in Poland and other European countries. We genotyped 303 individuals using 14 microsatellite loci and sequenced 253 individuals for a fragment of the control region (mitochondrial DNA [mtDNA]). The microsatellite analyses demonstrate that 3

genetically separate subpopulations exist, but 4 according to mtDNA. All haplotypes found in northeastern Poland are closely related to haplotypes from northern and northwestern Europe. The only individuals that could have originated from autochthonous red deer populations, rather than introductions, were found in Napiwoda Forest. The present regional genetic structure of the species is consistent with the known history of red deer translocations. Current patterns of genetic diversity in these populations are determined by the interaction of past human management and contemporary natural migrations. (c) 2012 The Wildlife Society.

3246: *-.100*

Even large fragments of tropical forest lose bird species over time. The speed of disappearance and permanence of these losses depend on a variety of factors, but the pattern of species loss appears more universal. We still do not understand the degree to which different mechanisms lead to population declines and local extinction in fragmented tropical landscapes. We review data currently available to evaluate three of the leading hypotheses, which include altered community dynamics producing mesopredator release and indirect effects of elevated herbivore populations; sensitivity of forest birds to altered microclimatic conditions in fragments, and the constraint of poor dispersal ability that reduces connectivity in fragmented landscapes. We find that all three mechanisms require additional study and suggest steps to be taken in future studies.

3248: *+.100*

All offshore oil and gas platforms have finite economic life spans. One of the decommissioning options for these platforms is complete removal, requiring the use of explosives to dislodge the support structure from below the seafloor. Off California, this decommissioning option would kill large numbers of platform-associated and commercially important groundfishes that inhabit the bases of the platforms, and may potentially affect regional fish populations. Capturing and translocating fishes before removing a platform might mitigate the effects of platform removal. In this study, we acoustically tagged 79 rockfishes and lingcod from three oil platforms in the east Santa Barbara Channel and translocated them to a natural reef inside a state marine reserve at Anacapa Island to determine whether individuals would home back to their platforms of capture, or take up residency at their new location. Movements between natural and platform habitats were monitored over a two-year period. Twenty-five percent of all tagged fishes translocated to a natural reef returned to their home platforms relatively quickly, traveling distances from 11 km to >= 18 km, in 10.5 h to 17 d. Those that did not home took up residency at Anacapa Island, moved to Santa Cruz Island or out of the range of detection. Although a small proportion of fish (25%) homed back to the platforms, a higher proportion (75%) remained at their platforms of release. Those that homed back to their platform of capture did so relatively quickly (avg 15 +/- 31 d). Lingcod had the highest probability of homing back to their platform of capture, typically doing so in < 1 day. These results suggest that fish translocation may be a successful, but costly mitigation strategy for platforms that require full decommissioning and that some species may be more successfully translocated than others.

3249: *-.111*

To determine the presence of viral pathogens in natural areas a survey was conducted on an opportunistic sample of fifty eight wild (Felis silvestris silvestris) and feral cats (F. s. caws). The biological materials included serum, lung tissue extract and stool. Feline leukemia virus p27 antigen was detected in 13/50 serum/lung tissue extract samples (26%), canine distemper virus antibodies were detected in 2/26 serum/lung tissue extract samples (7.7%), feline coronavirus

RNA was present in 6/29 stool samples (20.7%) and feline parvovirus DNA in 2/29 stool samples (6.9%). Canine distemper virus RNA was not detected. Feline immunodeficiency virus and feline coronavirus antibodies were not detected. Evidence of exposure to feline leukemia virus, canine distemper virus, feline coronavirus and feline parvovirus was found in wild and feral cats raising the importance of performing a comprehensive survey to correctly evaluate the potential threat of infectious diseases to endangered species, namely to the wildcat and to the Iberian lynx, which is meant to be reintroduced after 2012 in Portugal. (C) 2012 Elsevier B.V. All rights reserved.

3250: *-.021*

The banded leaf monkey (Presbytis femoralis femoralis) is critically endangered in Singapore and affected by widespread deforestation in southern Peninsular Malaysia. The Singapore population has recovered from a low of 15-20 to more than 40 individuals, but prior to our study it was unclear how severely the past bottleneck had depleted the genetic diversity of the population. Here, we provide the first analysis of the genetic variability based on seven samples (ca. 20% of population) collected over two years of fieldwork. We find only two haplotypes that differ only in one variable site for the hypervariable region I (HV-I) of the mitochondrial d-loop. Compared to available population-level data for other colobines (proboscis monkey, Yunnan snub-nosed monkey, Sichuan snub-nosed monkey, Angolan black and white colobus), the banded leaf monkey population in Singapore has the lowest number and the most similar haplotypes. This low genetic variability is the next challenge for the conservation of the population. Protected habitats in prospering urban environment may become important sanctuaries for endangered species, but reintroductions may have to be considered in order to restore genetic variability that was lost during past bottlenecks.

3251: *+.098*

The hihi (or stitchbird, Notiomystis cincta) is a New Zealand endemic nectivorous forest bird now restricted to one pristine island. Relocation to establish viable hihi populations on other islands has been the main conservation action since the early 1980s. To date, hihi reintroductions to young growth islands have had poor success despite the absence of mammalian predators. It was thought that past failures were due to food limitation, but research suggests that food limitation alone cannot account for their poor survivorship. Post-mortems of dead hihi has shown that aspergillosis caused by Aspergillus fumigatus is a major mortality factor and there is current concern regarding their susceptibility to this fungal disease. In this paper we develop and assess the hypothesis that A. fumigatus limits hihi population viability on modified islands, and suggest that A. fumigatus is a potential indicator species for habitat disturbance. We report that the prevalence of A. fumigatus spores in the soil is much higher in young growth forests and forest edge habitats. Results suggest that hihi mortality rates between islands are potentially due to differential exposure to A. fumigatus spores. We assess relationships between habitat disturbance, A. fumigatus contamination and hihi mortality rates by testing the following predictions: (1) that densities of A. fumigatus spores will be higher on modified islands, (2) that densities of A. fumigatus spores on islands will be correlated with hihi mortality rates and (3) that densities of A. fumigatus spores will be higher at the forest edge than in the interior. We test each of these predictions using soil samples, air samples and samples of nectar from plant species fed on by hihi.

3252: *+.325*

Understanding the effects of land-use on threatened ecosystems is of special relevance for nature conservation. The aim of our study was to use Orthoptera as ecological indicators for succession in

Central European steppe grasslands. Orthoptera showed a clear response to succession. Each successional stage harboured a unique assemblage. Species richness of habitat specialists was highest in the earliest seral stages. In contrast, density of all species peaked at the intermediate successional stage. Early successional stages are mostly likely to be preferred by specialized Orthoptera because they provide warm suitable oviposition sites (bare ground) and microclimatic conditions. The density peak in the mid-successional stage probably reflects a trade-off between favourable ambient temperatures for optimal development, sufficient food and shelter against predators. Although all successional stages of steppe grassland are relevant for conservation, early and mid-successional stages are the most important. Consequently, conservation management should aim at reintroduction of a traditional, low-intensive land use for abandoned steppe grasslands. As an optimal land use, we recommend traditional rough grazing with sheep and goats, which creates a heterogeneous habitat structure with bare ground, and avoids the accumulation of litter, favouring Orthoptera. (C) 2012 Elsevier Ltd. All rights reserved.

3253: ***+.144***

Phylogeographic information on European grayling, Thymallus thymallus, is still fragmentary for the Northern Adriatic basin. In this article, we provide complete mitochondrial DNA control region sequence data of 456 grayling specimens from 21 sampling sites across distinct river drainages. Thirty-seven haplotypes were resolved and clustered into Danubian, Atlantic and Adriatic lineages. The latter clade, composed of 16 new haplotypes, was identified in 12 out of 17 Adriatic sampling sites and reached frequencies of 0.97 within single water courses of the Adige and the Po drainages. However, native Adriatic haplotypes were accompanied by Danubian and/or Atlantic variants in all cases. A positive correlation between hatchery haplotype frequency and annual stocking input pointed to a direct effect of stocking intensity on the genetic architecture of wild populations, although natural trans-basin colonisation may have additionally complicated the situation. However, both the extent and patterns of introgression between native and foreign strains, as well as microgeographic population structure within the Adriatic lineage will be clarified by future molecular surveys, based on nuclear genetic markers. Until then, conservation management must include an immediate cessation of stocking of commercial grayling stocks, as well as the prohibition of grayling translocation, even at the intra-drainage level.

3254: ***-.149***

Protection of humans and livestock from disease has been used to justify many aggressive and costly wildlife control programs. Recent regulatory changes on livestock carcass disposal aimed at controlling the spread of bovine spongiform encephalopathy in Canada have led to substantial increases in exposed livestock carcass dumps. Such "boneyards" are known to attract grizzly bears (Ursus arctos), which leads to human-bear conflict. We compiled data on human-grizzly bear interactions in an agricultural landscape in southwestern Alberta over a 12-year time period (1999-2010) overlapping regulatory changes. Boneyards increased markedly after regulations were enacted and grizzly bear incidents increased correspondingly, particularly those related to dead livestock. The high rate of conflict results in frequent management captures, relocations, and translocations that create a likely population sink. Although work is underway to reduce human-bear interactions, revisions are needed to recent regulatory changes, such that they take wildlife into account. When combined with programs aimed at ensuring proper storage of attractants, we believe that such policy reforms will make it possible for humans to coexist with grizzly bears in southwestern Alberta. (C) 2012 The Wildlife Society.

3255: ***+.036***

Orange-fronted parakeets (Cyanoramphus malherbi) are New Zealand's rarest parakeet species with a global population of less than 500 individuals on remnant mainland populations and reintroduced populations on offshore islands. Since there is limited information about habitat preferences by this species on offshore islands I characterised habitat use on Maud Island, where captive-bred parakeets were introduced in 2007. I compared the vegetation characteristics of 29 plots (each 25 m(2)) where parakeets were encountered and 23 plots randomly selected. Parakeets were observed foraging in 96.6% of the plots. Plots used by parakeets showed significantly higher density of stems under 20 cm dbh and a higher canopy than random plots. Used plots also tended to have greater canopy cover and lower understory and ground vegetation covers. These results indicate that orange-fronted parakeets use ecotones of broadleaf coastal forest-manuka scrub, and pine plantations-manuka scrub for foraging highlighting the potential value of islands with mixed patches of these vegetation types as future refuges for this critically endangered species. Ortiz-Catedral, L. 2012. Habitat use by the critically endangered orange-fronted parakeet (Cyanoramphus malherbi) on Maud Island: its relevance for future translocations. Notornis 59 (3&4): 148-152.

3256: ***-.158***

The Houbara bustard captive-breeding programme was started in 1986 in an attempt to restore the population of this native species. Two sites were selecte re-introduction, Mahazat as-Sayd and Saja Umm Ar-Rimth protected areas in Saudi Arabia. All re-introductions were done in accordance with IUCN Guidelines. A total of 1005 Houbara were moved to Mahazat and 970 (493 males and 477 females) released between 1991 and 2010. Of those 970 birds were released, 100-150 could still be located at the middle of May 2010 while the rest were missing, possibly due to failed transmitters. The estimated current population of Houbara in Mahazat lies between 250-300. In Saja, the re-introduction was started in 2003 and a total of 256 Houbara bustard were released up to 2010 of which 134 were females and 122 males. By mid-May 2010, around 35 houbara were still alive from the 2003 to 2009 releases. Mortality after release is a key issue and due mainly to predation by mammals (foxes and cats), uncontrolled hunting, some cases of starvation and poaching.

3257: ***+.064***

The Southern Corroboree Frog Pseudophryne corroboree is a small myobatrachid frog from south-eastern Australia that has rapidly declined in recent decades largely due to disease, caused by infection with the amphibian chytrid fungus Batrachochytrium dendrobatidis. As a key recovery effort to prevent the imminent extinction of this species, an ex situ captive breeding program has been established in a collaborative partnership between Australian zoological institutions and a state wildlife department. Despite initial difficulties, successful captive breeding protocols have been established. Key factors in achieving breeding in this species include providing an adequate pre-breeding cooling period for adult frogs, separation of sexes during the non-breeding period, allowing female mate-choice via the provision of numerous males per enclosure and permitting the females to attain significant mass prior to breeding. Difficulties were experienced with egg and larval mortality in early years, though these issues have since been largely resolved. To date, the success of captive breeding from 2010-2012 has permitted the reintroduction of 1,060 captive-produced eggs and an increasing captive population. size that will support conservation research and provide insurance against further declines.

3258: ***+.079***

Bay scallop (Argopecten irradians) populations existed in Chesapeake Bay until 1933, when they declined dramatically due to a loss of seagrass habitat. Since then, there have been no documented populations within the Bay. However, some anecdotal observations of live bay scallops within the lower Bay suggest that restoration of the bay scallop is feasible. We therefore tested whether translocated adults of the southern bay scallop, Argopecten irradians concentricus, could survive during the reproductive season in vegetated and unvegetated habitats of the Lynnhaven River sub-estuary of lower Chesapeake Bay in the absence of predation. Manipulative field experiments evaluated survival of translocated, caged adult scallops in eelgrass Zostera marina, macroalgae Gracilaria spp., oyster shell, and rubble plots at three locations. After a 3-week experimental period, scallop survival was high in vegetated habitats, ranging from 98% in their preferred habitat, Z. marina, to 90% in Gracilaria spp. Survival in Z. marina was significantly higher than that in rubble (76%) and oyster shell (78%). These findings indicate that reproductive individuals can survive in vegetated habitats of lower Chesapeake Bay when protected from predators and that establishment of bay scallop populations within Chesapeake Bay may be viable.

3259: *+.276*

One of the challenges of understanding habitat requirements of endangered species is that the remaining populations may not be in optimal habitat, requiring experimentation to determine optimal habitat and to guide management. A better knowledge of its habitat requirements is important for the conservation of Streptanthus bracteatus, a rare annual of central Texas woodlands. The habitat requirements of a rare, declining species like S. bracteatus can also provide insights into anthropogenic habitat degradation and into previous disturbance regimes. We conducted a garden experiment and a transplant experiment to determine the effect of different light environments on the growth and reproduction of S. bracteatus. Higher levels of light improved S. bracteatus performance, especially fecundity. The optimum level of combined canopy and understory cover at the height of a S. bracteatus plant (a parts per thousand currency sign0.5 m above ground) was less than 50 %. The remaining populations of S. bracteatus are in sub-optimal habitat because it is not open enough. The results are consistent with the hypothesis that this species was a "fire-follower." The results also support the hypotheses that central Texas woodlands were once more open and that fire played an ecological role in these woodlands, an example of the ecological requirements of a rare species revealing past community structure and dynamics.

3260: *+.286*

Sport hunting may help in controlling cervid populations over large areas. As with natural predators, several environmental factors can influence sport harvest. A better understanding of the environmental variables that limit the efficiency of sport hunting could provide guidelines for more efficient wildlife management using hunting. We studied white-tailed deer (Odocoileus virginianus) hunting on a high deer density island where hunting was the sole form of predation. Our objective was to study the behavior of sport hunters and determine the habitat characteristics (e.g., abundance of deer forage, visibility of the deer from the hunter's point of view, and accessibility of the territory to hunters) that are associated with a successful harvest. We collected movements and harvest site location data from 477 hunters equipped with handheld Global Positioning System (GPS) units. Harvest sites were visited and characterized, along with a paired random site, to determine the environmental conditions associated with a successful hunt. We also developed a model to predict the daily number of deer seen by hunters considering weather conditions, hunter characteristics (e.g., age, experience), and date of hunting. We used the mean number of deer seen per hunter per day as a relative index of local density in each hunted territory. At both the site and landscape scales, the combination of visibility and access had a positive effect

on the distribution of harvested deer. Habitat types with less visual obstruction from vegetation enabled hunters to see more deer in a given day. At the site scale, harvested deer were located in areas with a lower density of access routes compared to areas where hunters travelled throughout the day. Using an innovative approach of studying hunter behavior with GPS technology, digital maps, and questionnaires, we highlighted the factors associated with hunter success. Our study suggests that habitat characteristics could be modified to increase harvest by improving accessibility and visibility near roads. Creating openings in mature and regenerating forest near access roads could make sport hunting a more efficient management tool, but the potential impact of increased forage availability in forest openings should not be overlooked. (c) 2012 The Wildlife Society.

3261: *+.068*

We translocated 120 Agassiz's desert tortoises to 5 sites in Nevada and Utah to evaluate the effects of translocation on tortoise survivorship, reproduction, and habitat use. Translocation sites included several elevations, and extended to sites with vegetation assemblages not typically associated with desert tortoises in order to explore the possibility of moving animals to upper elevation areas. We measured survivorship, reproduction, and movements of translocated and resident animals at each site. Survivorship was not significantly different between translocated and resident animals within and among sites, and survivorship was greater overall during non-drought years. The number of eggs produced by tortoises was similar for translocated and resident females, but differed among sites. Animals translocated to atypical habitat generally moved until they reached vegetation communities more typical of desert tortoise habitat. Even within typical tortoise habitat, tortoises tended to move greater distances in the first year after translocation than did residents, but their movements in the second or third year after translocation were indistinguishable from those of resident tortoises. Our data show that tortoises translocated into typical Mojave desert scrub habitats perform well; however, the large first-year movements of translocated tortoises have important management implications. Projects that employ translocations must consider how much area will be needed to contain translocated tortoises and whether roads need fencing to prevent the loss of animals. (c) 2012 The Wildlife Society.

3262: *+.166*

The distributional ranges of many species are contracting with habitat conversion and climate change. For vertebrates, informed strategies for translocations are an essential option for decisions about their conservation management. The pygmy bluetongue lizard, Tiliqua adelaidensis, is an endangered reptile with a highly restricted distribution, known from only a small number of natural grassland fragments in South Australia. Land-use changes over the last century have converted perennial native grasslands into croplands, pastures and urban areas, causing substantial contraction of the species' range due to loss of essential habitat. Indeed, the species was thought to be extinct until its rediscovery in 1992. We develop coupled-models that link habitat suitability with stochastic demographic processes to estimate extinction risk and to explore the efficacy of potential climate adaptation options. These coupled-models offer improvements over simple bioclimatic envelope models for estimating the impacts of climate change on persistence probability. Applying this coupled-model approach to T. adelaidensis, we show that: (i) climate-driven changes will adversely impact the expected minimum abundance of populations and could cause extinction without management intervention, (ii) adding artificial burrows might enhance local population density, however, without targeted translocations this measure has a limited effect on extinction risk, (iii) managed relocations are critical for safeguarding lizard population persistence, as a sole or joint action and (iv) where to source and where to relocate animals in a

program of translocations depends on the velocity, extent and nonlinearities in rates of climate-induced habitat change. These results underscore the need to consider managed relocations as part of any multifaceted plan to compensate the effects of habitat loss or shifting environmental conditions on species with low dispersal capacity. More broadly, we provide the first step towards a more comprehensive framework for integrating extinction risk, managed relocations and climate change information into range-wide conservation management.

3263: *+.144*

Oreochromis mossambicus (Peters 1852) are native to the eastward flowing rivers of central and southern Africa but from the early 1930s they have been widely distributed around the world for aquaculture and for biological control of weeds and insects. While O. mossambicus are now not commonly used as an aquaculture species, the biological traits that made them a popular culture species including tolerance to wide ranging ecological conditions, generalist dietary requirements and rapid reproduction with maternal care have also made them a 'model' invader. Self-sustaining populations now exist in almost every region to which they have been imported. In Australia, since their introduction in the 1970s, O. mossambicus have become established in catchments along the east and west coasts and have the potential to colonise other adjacent drainages. It is thought that intentional translocations are likely to be the most significant factor in their spread in Australia. The ecological and physical tolerances and preferences, reproductive behaviour, hybridization and the high degree of plasticity in the life history traits of O. mossambicus are reviewed. Impacts of O. mossambicus on natural ecosystems including competitive displacement of native species, habitat alteration, predation and as a vector in the spread of diseases are discussed. Potential methods for eradicating or controlling invasive populations of O. mossambicus including physical removal, piscicides, screens, environmental management and genetic technologies are outlined.

3264: *+.257*

European bisons were eradicated in the wild at the beginning of the 20th century. Intensive efforts of conservation breeding have meanwhile led to the re-establishment of a worldwide population of more than 4,400 animals. From the 50s onwards bisons have been reintroduced to the wild, which means that about two third of the world population are roaming freely. In 2003 an initiative has been established to also allow the European bisons in Western Europe living in the wild in a private forest area in the south-west of North Rhine-Westphalia. This requires a management adapted to the special framework conditions of the region. To start with, a feasibility study investigated the general suitability of the habitat, the acceptance in the region and the financial conditions. From October 2009 onwards a project promoted by the Federal Agency for Nature Conservation and the State Ministry of the Environment North Rhine-Westphalia prepared a small group of bisons for their release. The results of the accompanying scientific investigations will be the base for the subsequent decision of the state ministry on the release of the bisons in winter 2012/2013.Finally, the paper discusses the particular importance of small, intensively managed populations of the European bison in the wild, even in Western Europe.

3265: *+.004*

The Siamese Crocodile (Crocodylus siamensis) is one of the most endangered crocodilians in the world, and wild populations throughout Southeast Asia have precipitously declined over the last 50 years. Although initially feared extinct in Thailand, surveys in 2001 located a remnant population of C. siamensis in Kaeng Krachan National Park (KKNP), an extensive (2,915 km(2))

protected area along the Thai-Myanmar border. Our objectives were to assess the conservation status of C. siamensis populations within the park, determine if reproduction is occurring, and develop conservation recommendations based on these findings. We used a combination of nocturnal spotlight counts, track and sign surveys, and village interviews to census crocodile populations in KKNP from 2009-2011. Interview data suggest crocodiles occasionally enter Kaeng Krachan Reservoir, although we observed none during spotlight counts. No evidence of crocodiles was found on the Mae Pradone River. We recorded 10 detections of crocodile sign (tracks and scat) along the Petchburi River, although overall detection rates were low (<0.30/km). We found three nests along the Petchburi River from 2009-2011. Differences in mean egg width among clutches suggest one to three females nested. Clutches were considerably larger than those reported from other wild populations, but contained only non-viable eggs, possibly due to an insufficient number (or complete absence) of males in the Petchburi River. Collectively our survey data suggest at least four, and perhaps as many as six non-hatchling C. siamensis inhabit KKNP. The viability of this small population is doubtful and without direct conservation action, extinction appears inevitable. To avoid this fate, the existing population should be augmented using crocodiles obtained from commercial farms in Thailand.

3266: *+.151*

Background: The spread of wildlife pathogens into new geographical ranges or populations is a conservation concern for endangered species. Cystodiscus australis and Cystodiscus axonis are two species of myxosporean parasites infecting Australian frogs and tadpoles that have been recently recognised as important disease agents impacting amphibian conservation. Yet despite their importance to wildlife health, the mechanism of emergence for these parasites is unknown. We hypothesise that these parasites are capable of being accidentally translocated with their amphibian hosts in fresh produce (agricultural, horticultural and industrial) shipments into naive environments and host populations.Methods: We surveyed 33 Australian "Banana box" frogs from Sydney fruit markets during 2011 using faecal smears and multiplex species specific PCR on DNA isolated from frog faeces or using histopathology to demonstrate the presence of both C. australis and C. axonis.Results: One of the "Banana box" frogs, the Dainty green tree frog (Litoria gracilenta) was positive for C. australis and C. axonis in its faeces and continuously shed the parasites for eight months.Conclusions: We present a possible mechanism for the emergence of Cystodiscus parasites and a non-invasive screening method to be used as a diagnostic test. In the future, vigilance and communication between wildlife managers/researchers and veterinarians will provide valuable information about these parasites, their host range and true distribution. This will aid risk management assessments for threatened populations within the range of Cystodiscus parasites and ultimately enhance conservation efforts.

3267: *+.092*

In 1986 the return of the largest bird species in the European Alps started with the release of 4 zoo-born Bearded Vultures. The methods of enforced breeding and rearing has been developed by Alpenzoo Innsbruck, where the continuous breeding results since 1973 gave the initiative for the International Bearded Vulture Reintroduction Program. Nearly 40 Zoos and 5 Breeding Centres are organised by the EEP under the chair of Dr. Hans Frey, Vet. med. Univ. of Vienna, Austria. Under the umbrella of WVVF, Frankfurt Zoological Society and some other conservation bodies, the releasing and monitoring program is in progress to establish a stable population of Bearded Vultures in the Alps. Within 20 years from 1986 up to 2006 146 fledglings have been released on 12 sites across the Alps. In 2007 in addition 8 zoo-born specimens were at disposal. In 1997 the first successful breeding in the field occurred. Within 10 years 43 offspring have been raised by

their parents in the wild.

3268: *-.099*

Captive breeding programs have been used to save several endangered birds from extinction. The existence of a considerable number of successful releases of captive-born birds into the wild demonstrated that it is possible to establish a species in an area of its historical range (reintroduction) and to reinforce an existing wild population that was in decline (restocking). Most reintroduction programs are complicated and expensive, involving a multi-disciplinary approach to problem solving. One of the major problems of many current ex-situ conservation programs is the low fertility rate of the endangered species in captivity. In the current review, some research programs on assisted bird reproduction are introduced, and new diagnostic and therapeutic methods for avian infertility are described.

3269: *-.110*

While governments normally take action to eradicate or control feral populations of introduced species, management becomes problematic in the rare event of an inadvertent reintroduction of a locally extinct species to its former range. Free-living wild boars became extinct in Britain around 700 years ago, but animals have recently escaped from farms and recolonised parts of England. There has been much debate about the potential ecological and economic impacts of the presence of feral populations of wild boar in England. Predicted negative impacts include disease transmission to domestic pigs, crop damage and problems caused by the species' rooting behaviour. Perhaps the strongest argument in favour of the wild boars in England is the restoration of a formerly native species. However, for the re-established populations to have an intrinsic value as an addition to English biodiversity, it would be preferable if the animals were genetically pure wild boars. We used four genetic marker systems to assess the genetic status of a wild boar population in the Forest of Dean, western England. We found high frequencies of alleles of domestic origin at the mitochondrial control region and a nuclear pseudo-gene. Microsatellite-based analyses also suggested that English wild boars had a mixed wild boar/domestic pig ancestry. Therefore, it is debatable whether the wild boar in the Forest of Dean can be regarded as a restored native species.

3270: *+.065*

Conservation of species threatened by habitat fragmentation is a major global challenge, and determining the genetic and demographic processes associated with isolation and reductions in population size will be critical for an increasing number of species. We conducted controlled crosses and field germination experiments to quantify the effects of inbreeding and outbreeding in the declining orchid Gymnadenia conopsea in two Norwegian populations that differ in size. We further compared our results with published estimates of inbreeding depression in orchids. There was severe inbreeding depression for seed production (delta = 0.41-0.67) and germination (delta = 0.46-0.66) in both populations, with stronger inbreeding depression in the large population. Compared to outcrossing, selfing reduced female fitness (number of seeds per fruit x proportion of seeds germinating) by 76 and 54 % in the large and small population, respectively. The magnitude of inbreeding depression for seed production was higher than the average reported for orchids, while for germination it was similar to earlier estimates. The large population also experienced considerable outbreeding depression for seed production (delta = 0.23-0.27), germination (delta = 0.33) and female fitness (delta = 0.47) following crosses with a population 1.6 km away. The strong inbreeding depression indicates that both populations harbour a substantial genetic load,

and suggests that fragmentation may reinforce population decline in G. conopsea via increased inbreeding. Moreover, the local outbreeding depression indicates substantial genetic differentiation at a moderate spatial scale. This has important implications for the use of crosses between populations or plant translocations as conservation approaches.

3271: *+.151*

Genetic diversity is one of several factors affecting extinction risk in vulnerable populations. In addition to informing conservation management strategies, data on genetic variability can also shed light on the recency and magnitude of historic bottlenecks. The pine hoverfly Blera fallax is one of the rarest invertebrates in the UK, known from just two sites in Scotland. It belongs to an often overlooked, species-rich community that is fundamental to forest function, the saproxylics (that depend on dead wood). To assist current conservation management for B. fallax, including captive breeding and translocations, it is important to know whether genetic factors will limit the success of recovery. Using 12 microsatellite loci, we compared the genetic variation in Scottish and Swedish specimens (Swedish populations are thought to represent a more outbred B. fallax population). As expected, the Scottish population showed significantly lower levels of polymorphism, expected heterozygosity and allelic richness than the Swedish population. Furthermore, significant genetic differentiation was found between the two B. fallax populations (F-ST = 0.134). We then used an allele frequency-based approach and a Bayesian coalescent-based method to assess genealogical history and detect recent changes in population size. Unexpectedly, data from not only the Scottish but also the Swedish population indicated a strong and relatively recent decline that was more pronounced in Scotland. We discuss the implications of our findings for future conservation management planning, the first undertaking of its kind for saproxylic species in Britain.

3272: *+.100*

We investigated the origins and persistence of European pine marten (Martes martes) populations across the British Isles by identifying mitochondrial DNA (mtDNA) sequences from contemporary populations (sampled since 1981) and comparing these with those of older 'historical' museum specimens (pre-1981) originally collected from the same geographic areas. Excluding Scotland, where the haplotype composition of populations appears to be unchanged, haplotypes found in contemporary and historical marten populations elsewhere differed both temporally and geographically. While these data suggest that the contemporary Irish population is descended from a relict population that passed through an early to mid 1900s bottleneck, the historical and contemporary English and Welsh populations differ in their abundance of specific mtDNA control region haplotypes. These data appear to suggest that particular haplotypes may have been lost from England and Wales at some point in the early to mid 1900s, but further nuclear DNA work is required to determine whether this shift has occurred by rapid genetic drift in the mtDNA control region or whether relict populations have been replaced by pine martens from elsewhere. If the reported shifts in mtDNA haplotypes reflect population extirpation events, historical pine marten populations of England and Wales would appear to have become extinct in the twentieth century (in Wales after 1950 and in England after 1924). Additionally, the recent occurrence of haplotypes originating from continental Europe, and of M. americana, suggest that relict populations of England and Wales have been replaced by, or hybridised with, occasional released, escaped and/or translocated animals. The implications of these results for pine marten conservation, and particularly reintroduction, are discussed.

3273: *+.084*

Currently world crane populations are decreasing. Many crane species are endangered (EN) or vulnerable (VU) as classified by IUCN, including Sarus Crane (Grus sp.). For Thailand, Grus antigone shapii was listed as extinct in the wild (EW). Cranes are wetland species. Loss of wetland areas due to threats and human activities were major causes of the disappearance of crane populations in Thailand. Reintroduction science is widely accepted for the conservation of rare and endangers species including Grus antiogone shapii. In 1997, the Zoological Park Organization of Thailand succeeded in reproducing Sarus cranes in captivity that were ready to be reintroduced to natural wetlands. However, there were many factors, and not only the number of populations, to be considered before reintroducing captive wildlife back into a wild habitat, which are mostly influenced by human. Therefore, the first phase of reintroduction for the Sarus crane project was to screen and select the most suitable wetland habitats. Criteria and indicators were developed and applied. Eight criteria, four indicators and forty-three verifiers that corresponded to reintroduction science and sustainability of natural resources management, such as landscape conservation, habitat integrity, threats and disturbance and awareness of community were considered. However, the key factor for the successful and sustainable reintroduction of the Sarus crane in Thailand was not only the integrity of the habitat but also the participation of all stakeholders.

3274: *+.271*

Giant weta (Orthoptera: Anostostomatidae) are large flightless New Zealand insects vulnerable to predation from introduced mammals. Some species have been transferred to islands or mammal-free mainland sanctuaries to establish additional populations. Radiotelemetry was used to investigate behaviour, movements and survival of adult Cook Strait giant weta (Deinacrida rugosa) immediately after translocation into Karori Sanctuary, New Zealand, to describe their initial movements, and to assess the importance of this establishment phase in relation to the long-term viability of the population. The average distance moved between consecutive daytime refuges for translocated male D. rugosa within Karori Sanctuary was significantly further than for resident weta on Matiu-Somes Island. In contrast, translocated female weta moved significantly smaller distances between consecutive daytime refuges within Karori Sanctuary than those on Matiu-Somes Island. Translocated D. rugosa travelled significantly further between consecutive daytime refuges between 19 and 45 days after release than during the first 19 days and more than 45 days of radiotracking. Deinacrida rugosa survived well following translocation and there was only limited evidence of predation despite an increased abundance of indigenous avian and reptilian predators being present, and the presence of low numbers of mice. The establishment potential of this population was not adversely affected by movements and survival of the weta immediately after translocation. It still remains to be seen if a self-sustaining population of D. rugosa develops in Karori Sanctuary but the indications are that the species is present because progeny of the translocated weta are regularly seen within Karori Sanctuary. Radiotelemetry provided valuable insights into the behaviour of adult D. rugosa and it could be appropriate for monitoring other large bodied invertebrates.

3275: *+.059*

Captive rearing and translocation are often used concurrently for species conservation, yet the effects of these practices can interact and lead to unintended outcomes that may undermine species recovery efforts. Controls in translocation or artificial-propagation programs are uncommon; thus, there have been few studies on the interacting effects of these actions and environmental conditions on survival. The Columbia River basin, which drains 668,000 km2 of the western United States and Canada, has an extensive network of hydroelectric and other dams, which impede and slow migration of anadromous Pacific salmon (Oncorhynchus spp.) and can increase

mortality rates. To mitigate for hydrosystem-induced mortality during juvenile downriver migration, tens of millions of hatchery fish are released each year and a subset of wild- and hatchery-origin juveniles are translocated downstream beyond the hydropower system. We considered how the results of these practices interact with marine environmental conditions to affect the marine survival of Chinook salmon (O. tshawytscha). We analyzed data from more than 1 million individually tagged fish from 1998 through 2006 to evaluate the probability of an individual fish returning as an adult relative to its rearing (hatchery vs. wild) and translocation histories (translocated vs. in-river migrating fish that traveled downriver through the hydropower system) and a suite of environmental variables. Except during select periods of very low river flow, marine survival of wild translocated fish was approximately two-thirds less than survival of wild in-river migrating fish. For hatchery fish, however, survival was roughly two times higher for translocated fish than for in-river migrants. Competition and predator aggregation negatively affected marine survival, and the magnitude of survival depended on rearing and translocation histories and biological and physical conditions encountered during their first few weeks of residence in the ocean. Our results highlight the importance of considering the interacting effects of translocation, artificial propagation, and environmental variables on the long-term viability of species.

3276: ***+.144***

Climate change poses a particular threat to species with fragmented distributions and little or no capacity to migrate. Assisted colonization, moving species into regions where they have not previously occurred, aims to establish populations where they are expected to survive as climatic envelopes shift. However, adaptation to the source environment may affect whether species successfully establish in new regions. Assisted colonization has spurred debate among conservation biologists and ecologists over whether the potential benefits to the threatened species outweigh the potential disruption to recipient communities. In our opinion, the debate has been distracted by controversial examples, rather than cases where assisted colonization may be a viable strategy. We present a strategic plan for the assisted migration of tuatara (Sphenodon punctatus), an endemic New Zealand reptile. The plan includes use of extant populations as reference points for comparisons with assisted-colonization populations with respect to demography, phenotypic plasticity, and phenology; optimization of genetic variation; research to fill knowledge gaps; consideration of host and recipient communities; and inclusion of stakeholders in the planning stage. When strategically planned and monitored, assisted colonization could meet conservation and research goals and ultimately result in the establishment of long-term sustainable populations capable of persisting during rapid changes in climate.

3277: ***+.190***

Halley described three options for sourcing Eurasian beavers Castor fiber for reintroduction in Britain and Western Europe: (i) use animals from populations within a single western evolutionarily significant unit (ESU); (ii) mix animals from two or three western ESUs; and (iii) mix animals from eastern and western ESUs. Option three contravenes International Union for Conservation of Nature guidelines, so debate should focus on options one and two. We believe there is a need for further genetic analysis, before a final decision can be made. This decision should not be heavily influenced by cost or be determined by the genotypes of existing captive or escaped populations.

3278: ***+.012***

The High Mountains of Crdoba, Argentina have a long evolutionary history of grazing by large herbivores. However, about 400 years ago, European livestock were introduced and gradually replaced native herbivores. Since the 1920s, domestic herbivores have been the only large herbivores present in the area, causing severe soil erosion and a threat to the system diversity. The endemic fauna of the region includes four amphibian species. We evaluated the effect of livestock rearing on amphibian diversity and water bodies in woodlands and grasslands of the High Mountains of Crdoba. The work was conducted on stream stretches and ponds in two contrasting grazing situations: an area with livestock presence and another area where livestock was excluded 14 years ago. The following variables were recorded at each sampling site: amphibian richness and abundance, percentage of emergent, submerged and peripheral vegetation in areas surrounding the water bodies, water pH, and water dissolved O-2. No significant differences were detected in amphibian diversity between streams of both grazing situations, whereas a greater diversity ($p < 0.01$) was observed in ponds in grazed grasslands. Our results suggest that livestock rearing, qualitatively measured as grazing and 14 years of livestock exclusion, in the study area would not have negative effects on amphibian diversity. This finding might be due to the long evolutionary grazing history of the area, large-scale livestock exclusion exhibiting a novel scenario. The ongoing reintroduction of native grazers may provide the benefits of grazing without the consequent soil erosion and habitat degradation associated with domestic livestock.

3279: ***+.098***

Active conservation has often been successful in reversing or arresting population declines of endangered species. However, examples of Critically Endangered species recovering in the absence of human intervention are extremely rare. We censused the Raso lark, a single-island endemic of the Cape Verdes archipelago, annually from 2001 to 2010. Between 2004 and 2010, the world population grew from 65 to 470 individuals. This remarkable increase occurred without conservation intervention, but correlated strongly and positively with rainfall. Because of this population increase, the mean age of birds reduced and the population shifted from male skew, a consequence of higher male survival, towards one where the sexes were more equally represented. This study illustrates the dramatic effect that natural changes in climatic conditions may have on the recovery of endangered species. However, the current favourable situation may not persist, and we suggest a translocation to another Cape Verdean island be urgently considered. We conclude that temporal trends in the population dynamics of endangered populations need to be considered when planning and implementing species recovery plans.

3280: ***-.003***

It has recently been suggested that goitered gazelles (Gazella subgutturosa and Gazella marica) have paraphyletic maternal origin, and that the mitochondrial cytochrome b gene fragment can be used for species identification prior to reintroduction of the gazelles. Although there is a large geographic area where the gazelles have intermediate morphology, previous researchers have not inferred any signs of mitochondrial haplotype introgression, and it is thought that the introgression, if it exists, is male-biased. We studied mitochondrial haplotypes of morphologically typical G. subgutturosa from two geographic locations. Goitered gazelles from eastern Turkey, morphologically identical to G. subgutturosa, had haplotypes identical to G. marica. This finding confirms ongoing maternal gene introgression from G. marica to G. subgutturosa. Our suggestion is that there is a natural gene flow between these two nominal species, and morphological characters together with recombinant genetic markers rather than mitochondrial DNA should be used to differentiate among individuals from areas close to the contact zone.

3281: ***+.082***

Conservationists previously described the need for research into using translocation to rescue threatened populations. We conducted an experimental translocation of Tuamotu kingfishers (Todiramphus gambieri gertrudae) to provide foundational information about movement ecology and inform conservation planning for the critically endangered bird. We captured and radio-marked mated pairs of kingfishers on Niau Atoll, French Polynesia, where approximately 125 individuals compose the last remaining population of Tuamotu kingfishers. One bird from each pair was translocated to a reintroduction site on the opposite side of the island, and the other pair member was released back onto the home territory. Home-ranging pair members remained on territories and used habitats disproportional to availability, as determined by Brownian bridge analysis. Translocated individuals made multiple forays from the release site to explore the surrounding landscape, during which habitats were used in proportions similar to availability. The exploratory behavior resembled that of dispersing juvenile birds, which suggested that adults retain the ability to explore, and that dispersal theory may be a useful basis for planning translocations. Vacancies on donor territories were filled within 1-10 days. Our results indicated that translocation was not effective for range expansion on Niau because birds readily returned to donor areas; however, translocation to other islands remains a potential conservation strategy for Tuamotu kingfishers.

3282: ***+.045***

In 2009 and 2010 16 Norwegian Eurasian beavers (Castor fiber) were reintroduced to Knapdale, Scotland as part of a 5-yr reintroduction trial (Scottish Beaver Trial). Despite numerous reintroduction programs throughout Europe there is no published information concerning recommended health surveillance during beaver reintroduction and only one publication describing causes of mortality. We describe the establishment of a health surveillance program based on International Union of Conservation of Nature (IUCN) and governmental guidelines, and report preliminary results based on the fecal and blood samples following the completion of the first stage of reintroduction. Animals underwent at least one general anesthetic to allow collection of fecal and blood samples and a thorough clinical examination. No bacterial enteric pathogens such as Salmonella spp., Campylobacter spp., or Yersinia pseudotuberculosis were isolated, nor were Giardia spp. or Cryptosporidium spp. However, numerous helminths including Travassosius rufus and Stichorchis subtriquetrus were detected. Five animals were positive for Leptospira antibodies. This included Leptospira saxkoebing, Leptospira canicola, Leptospira copenhageni, Leptospira icterohaemorrhagiae, Leptospira autumnalis, and Leptospira javanica. The highest loss of animals (20%) was during the statutory 6-mo rabies quarantine period. No common cause of death was determined. The rabies quarantine conditions were waived for four remaining animals, three of which were introduced to the wild successfully. The authors recommend the shortest possible quarantine period when introducing beavers, but allowing for the minimum recommended IUCN 35 days to allow for implementation of the initial stage of the health surveillance program, examination of animals, sample collection, and processing.

3283: ***+.283***

Translocation has become increasingly important for conserving island species. Limited tools are available for guiding the selection of translocation sites, however, particularly when establishing rescue populations outside of historic ranges. We developed a Bayesian network model framework for translocation site selection for island birds. The model consisted of four primary components including ecological requirements for survival, anthropogenic threats at the population

establishment site, effects the translocated species may have on native species, and operational support associated with the translocation process and ongoing management. We then used the model to identify potential sites for the establishment of a wild population of Guam Micronesian kingfishers (Todiramphus cinnamominus cinnamominus) on an island outside the bird's historic range. Conditional probabilities that guided model evaluations were allocated using information from the literature, expert opinions, and a training set of islands outside the region under consideration for releases. The model was used to evaluate 239 islands where a translocation population of Micronesian kingfishers could be established. Five islands, all in the Federated States of Micronesia, were identified as being suitable for assisted colonization, including Kosrae, Yap, Faichuk, Weno and Fefan. Sensitivity analysis showed a correspondence between model variables and island characteristics indicated by the literature as being the most important for successful translocation. We found the Bayesian network model to be a useful tool for translocation site selection despite limited information on the natural history of the Guam Micronesian kingfisher and the factors that impact the success of translocations. (C) 2012 Elsevier Ltd. All rights reserved.

3284: *+.107*

Examination of age structures and sex ratios is useful in the management of northern river otters (Lontra canadensis) and other forbearers. Reintroductions and subsequent recolonizations of river otters have been well documented, but changes in demographics between expanding and established populations have not been observed. As a result of reintroduction efforts, immigration from Arkansas and northeastern Texas, and other efforts, river otters have become partially reestablished throughout eastern and central Oklahoma. Our objective was to examine age structures of river otters in Oklahoma and identify trends that relate to space (watersheds, county) and time (USDA Animal and Plant Health Inspection Service county trapping records). We predicted that river otters in western areas of the state were younger than river otters occurring farther east. From 2005-2007, we obtained salvaged river otter carcasses from federal and state agencies, and we live-captured other river otters using leg hold traps. Seventy-two river otters were sampled. Overall, sex ratios were skewed toward females (1F:0.8M), but they did not differ among spatiotemporal scales examined. Teeth were removed from salvaged and live-captured river otters (n = 63) for aging. One-year old river otters represented the largest age class (30.2%). Proportion of juveniles (<1 y old) in Oklahoma (19.0%) was less than other states. Mean age of river otters decreased from east-to-west in the Arkansas River and its tributaries. Mean age of river otters differed between the Canadian River Watershed (0.8 y) and the Arkansas River Watershed (2.9 y) and the Canadian River Watershed and the Red River Watershed (2.4 y). Proportion of juveniles did not differ among spatiotemporal scales examined. Similar to age structure variations in other mammalian carnivores, colonizing or growing western populations of river otters in Oklahoma contained younger ages than more established eastern populations.

3285: *-.049*

Despite an increasing appreciation of the disease risks associated with wild-life translocations, the effects which captive breeding programs exert on parasite communities remain understudied. This may be attributed, in part, to the current lack of rapid and cost-effective techniques for comparing parasite assemblages between host populations. Terminal restriction fragment length polymorphism (T-RFLP) analysis of the rDNA region encompassing the internal transcribed spacers (ITS-1 and ITS-2) and 5.85 rRNA gene was used to characterise bursate nematode communities (suborder Strongylida) across two captive and two non-captive colonies of the threatened brush-tailed rock-wallaby. Petrogale penicillata. A clone library was constructed and a

restriction enzyme selected to differentiate the predominant operational taxonomic units (OTUs) by the unique peak profiles they generated. The prevalence, intensity of infection and comparative structure of strongylid assemblages was evaluated for each of the host colonies. Compared to wild conspecifics, captive wallabies exhibited a reduced prevalence of infection and significantly lower faecal egg counts. T-RFLP revealed that a high proportion of the OTUs co-occurred across three of the four study locations. Despite this, the composition of strongylid assemblages was significantly different between the colonies, even when host translocation events had occurred. These results suggest that captive breeding programs may exert a profound impact on parasitic helminth assemblages. Developing efficient techniques for characterising community dynamics in potentially pathogenic organisms is critical to the long term success of species recovery efforts worldwide. (c) 2012 Elsevier Inc. All rights reserved.

3286: ***-.042***

Based on sources from the literature and several archives, the historical distribution and economic relevance of huchen in the province of Salzburg was analysed. This historical situation was compared with current information about occurrence, angler's catches, and stocking activities. Up to the beginning of the 20th century, huchen was an abundant and widespread species in the lower course of the Salzach river catchment (upstream as far as Golling), and was of high economic relevance in local commercial fishery. In the upstream reaches, low densities were recorded up to the area of Mittersill. In the river Enns, single specimens occurred up to Radstadt. The status of huchen as an autochthonous species of the River Mur upstream a cataract at Murau remains unclear. Today, except of occasional observations of introduced huchen in the River Mur downstream Tamsweg, the distribution is restricted to the lower reaches of the river Salzach. There, re-introduction projects were started in the upstream reaches (Tennengau) already three decades ago. Nevertheless, due to serious human pressures (e.g. regulations, hydro-electrical power plants), very low densities are documented, and stocks are almost exclusively conserved by stocking activities. As a consequence, annual angler's catches are limited to single specimens. Detailed suggestions for future management strategies are provided.

3287: ***-.460***

Wildlife reintroductions select or treat individuals for good health with the expectation that these individuals will fare better than infected animals. However, these individuals, new to their environment, may also be particularly susceptible to circulating infections and this may result in high morbidity and mortality, potentially jeopardizing the goals of recovery. Here, using the reintroduction of the grey wolf (Canis lupus) into Yellowstone National Park as a case study, we address the question of how parasites invade a reintroduced population and consider the impact of these invasions on population performance. We find that several viral parasites rapidly invaded the population inside the park, likely via spillover from resident canid species, and we contrast these with the slower invasion of sarcoptic mange, caused by the mite Sarcoptes scabiei. The spatio-temporal patterns of mange invasion were largely consistent with patterns of host connectivity and density, and we demonstrate that the area of highest resource quality, supporting the greatest density of wolves, is also the region that appears most susceptible to repeated disease invasion and parasite-induced declines. The success of wolf reintroduction appears not to have been jeopardized by infectious disease, but now shows signs of regulation or limitation modulated by parasites.

3288: ***-.090***

Background: Small, isolated populations often experience loss of genetic variation due to random

genetic drift. Unlike neutral or nearly neutral markers (such as mitochondrial genes or microsatellites), major histocompatibility complex (MHC) genes in these populations may retain high levels of polymorphism due to balancing selection. The relative roles of balancing selection and genetic drift in either small isolated or bottlenecked populations remain controversial. In this study, we examined the mechanisms maintaining polymorphisms of MHC genes in small isolated populations of the endangered golden snub-nosed monkey (Rhinopithecus roxellana) by comparing genetic variation found in MHC and microsatellite loci. There are few studies of this kind conducted on highly endangered primate species.Results: Two MHC genes were sequenced and sixteen microsatellite loci were genotyped from samples representing three isolated populations. We isolated nine DQA1 alleles and sixteen DQB1 alleles and validated expression of the alleles. Lowest genetic variation for both MHC and microsatellites was found in the Shennongjia (SNJ) population. Historical balancing selection was revealed at both the DQA1 and DQB1 loci, as revealed by excess non-synonymous substitutions at antigen binding sites (ABS) and maximum-likelihood-based random-site models. Patterns of microsatellite variation revealed population structure. F-ST outlier analysis showed that population differentiation at the two MHC loci was similar to the microsatellite loci.Conclusions: MHC genes and microsatellite loci showed the same allelic richness pattern with the lowest genetic variation occurring in SNJ, suggesting that genetic drift played a prominent role in these isolated populations. As MHC genes are subject to selective pressures, the maintenance of genetic variation is of particular interest in small, long-isolated populations. The results of this study may contribute to captive breeding and translocation programs for endangered species.

3289: ***+.118***

Dams constructed along waterways interrupt the dispersion and migration of aquatic organisms, affecting mainly the abundance of migratory fish species. Translocation mechanisms have been constructed at dams aiming to minimize their impact on fish species migration behavior. There is little information available about the effect of the construction of dams on the genetic structure of the Neotropical migratory fish fauna. Therefore, RAPD molecular markers and microsatellites were utilized to evaluate the diversity and genetic structure of the migratory species Leporinus elongatus (piapara) in the Canoas Complex-Paranapanema River-Brazil. Ten groups were sampled in the fish ladders of the hydroelectric dam Canoas I and Canoas II during the reproductive period in three consecutive years. Both markers showed a high level of genetic diversity within these groups. The microsatellite markers demonstrated a loss of heterozygosity and a considerable level of inbreeding in the species. The genetic differentiation found among the groups with both markers utilized is within a range from low to moderate. The data obtained with the parameter of genetic diversity among the groups led to the conclusion that the groups of L. elongatus of the Canoas Complex are structured as a single population composed of sub-populations with low genetic diversity among them. The data on genetic diversity and population structure of L. elongatus are of great importance for the development of the species management and conservation programs in the Canoas Complex, which can also be utilized in aquaculture programs.

3290: ***+.014***

In August 2011 the first Delacour's langurs (Trachypithecus delacouri) were introduced into Van Long Nature Reserve. This has been the first planned and monitored release of any leaf-eating langur. Prior to reintroduction several studies were carried out on the ecology, behavior, nutrition of the species, population genetics and on habitat conditions and carrying capacity of the area. Van Long Nature Reserve is divided into four more or less isolated parts. One smaller part of the nature

reserve harbors the world's largest population of this species. This population increased through the elimination of poaching and human activities and is currently the only population with long-term viability. Several isolated populations outside Van Long Nature Reserve have been exterminated during the last decade or decreased dramatically due to poaching. To connect the largest Delacour's langur population in the smaller part of the nature reserve with a relic population in the largest part of the nature reserve is the goal of the reintroduction. A larger population in this part would increase the possibility of an exchange of individuals supporting the panmixia and the genetic stability of the nature reserve's whole population.

3291: *-.074*

Congo Cay, U.S. Virgin Islands, has high value for breeding seabirds and is a potential reintroduction site for the endangered Virgin Islands tree boa (Epicrates monensis granti). However, introduced ship rats (Rattus rattus) undermine its conservation value. Three unsuccessful eradication attempts have been conducted since 1990, with the latest in 2006; rats were trapped 1.5 years later. We examined microsatellite DNA and mitochondrial DNA sequences of ship rats from Congo Cay and three other nearby cays to determine if rats found after the most recent eradication effort were surviving individuals or reinvaders from neighboring cays; we had no pre-eradication samples. Only one mitochondrial haplotype was present, implying that historically there was a single invasion or if multiple invasions, rats came from a single source with limited haplotype diversity. Low genetic variation on Congo Cay suggested either a population bottleneck resulting from survivors or a founder event resulting from invaders. F (ST) estimates, cluster distances, migrant detections, and factorial correspondence analysis indicated low but meaningful levels of gene flow between Congo and Lovango Cays and between Mingo and Grass Cays. Except for two alleles, all other alleles found on Congo were also present on Lovango. Without pre-eradication samples we could not eliminate the possibility of survivors from a failed eradication. However, our data suggest reinvasion from Lovango Cay was likely and that future eradication efforts should consider both pairs of cays as eradication units. Cay juxtaposition and orientation along with ocean currents may explain rat movement, or lack thereof, among these cays.

3292: *+.363*

Preserving genetic health is an important aspect of species conservation. Allelic diversity is particularly important to conserve, as it provides capacity for adaptation and thus enables long-term population viability. Allele retention is difficult to predict beyond one generation for real populations with complex demography and life-history traits, so we developed a computer model to simulate allele retention in small populations. AlleleRetain is an individual-based model implemented in R and can be applied to assess management options for conserving allelic diversity in small populations of animals with overlapping generations. AlleleRetain remedies the limitations of similar existing software, and its source code is freely available for further modification. AlleleRetain and its supporting materials can be downloaded from https://sites.google.com/site/alleleretain/ or CRAN (http://cran.r-project.org).

3293: *+.250*

Noninvasive genetic sampling has increasingly been used in ecological and conservation studies during the last decade. A major part of the noninvasive genetic literature is dedicated to the search for optimal protocols, by comparing different methods of collection, preservation and extraction of DNA from noninvasive materials. However, the lack of quantitative comparisons among these

studies and the possibility that different methods are optimal for different systems make it difficult to decide which protocol to use. Moreover, most studies that have compared different methods focused on a single factor collection, preservation or extraction while there could be interactions between these factors. We designed a factorial experiment, as a pilot study, aimed at exploring the effect of several collection, preservation and extraction methods, and the interactions between them, on the quality and amplification success of DNA obtained from Asiatic wild ass (Equus hemionus) faeces in Israel. The amplification success rates of one mitochondrial DNA and four microsatellite markers differed substantially as a function of collection, preservation and extraction methods and their interactions. The most efficient combination for our system integrated the use of swabs as a collection method with preservation at -20 degrees C and with the Qiagen DNA Stool Kit with modifications as the DNA extraction method. The significant interaction found between the collection, preservation methods and the extraction methods reinforces the importance of conducting a factorial design experiment, rather than examining each factor separately, as a pilot study before initiating a full-scale noninvasive research project.

3294: *+.098*

Effective management of overabundant animal populations is a difficult challenge for wildlife managers around the globe. Translocation is often considered a viable management tool, whereby individual animals are removed from areas of high population density and released in areas where densities are lower. Typically, the success of a translocation program is measured at the population source, with little attention given to the fate of translocated individuals. Here we use a koala (Phascolarctos cinereus) translocation program from southeastern Australia as a case study to investigate the effects of translocation on individual animals. The koala is an iconic species that occurs at high densities in some parts of its southern range, leading to numerous conservation and animal welfare issues. Between 1997 and 2007 over 3,000 koalas from a high-density island population were captured, surgically sterilized, and translocated to the mainland. Annual post-translocation surveys at release sites revealed densities of =0.4?koalas/ha, despite release densities of 1.0?koala/ha. Radiotracking studies indicate that low densities were because of both mortality and high dispersal of translocated individuals. We observed a mortality rate of 37.5% for translocated koalas in the first 12 months post-release. No deaths occurred among animals that were not sterilized and translocated. Translocated koalas moved greater distances than non-translocated animals. Monitoring of translocated individuals should be performed routinely during translocation programs for overabundant species. Due consideration must be given to what is an acceptable level of mortality for translocated individuals. Although often considered an ethically acceptable management technique (especially for iconic and charismatic species), translocation may not always be the best option from an animal welfare perspective. (c) 2012 The Wildlife Society.

3295: *+.411*

Translocation success of Greater Sage-Grouse (Centrocercus urophasianus) is generally measured by documenting whether translocated individuals survive and reproduce at the release site. However. demographic parameters, such as annual survival of translocated individuals, provide a more accurate measure of translocation success. We translocated 60 female sage-grouse from Oregon and Nevada to Clear Lake National Wildlife Refuge, California, during 2005-2010 to augment a small population of resident grouse. We radio-marked each translocated female and a sample of resident female sage-grouse, recorded their locations, and monitored their survival at monthly intervals over the study period. We observed most (55/60) translocated birds near (<100 m from) the only lek in the study area within one week of their release, To examine factors

influencing survival, we developed a set of a priori models that included the effects of translocation status, season (breeding vs. nonbreeding), year, and age on annual survival and compared the models AlCc values using program MARK. The null model was the best supported model and received 335 of the model weight. Models that included survival during the first year posttranslocation, age, and season, however, were competitive (<2 Delta AlCc) with the top model. However, the 1 coefficient distinguishing breeding from nonbreeding season survival was the only coefficient whose 95% confidence interval did not overlap zero; monthly survival during the breeding season (0.952 +/- 0.014) was lower than during the nonbreeding season (0.960 +/- 0.008). The model average estimate of annual survival for female sage-grouse in our study area was 59.6% (95% CI 47.9-70.1). Our analyses provide little support for a difference in survival between translocated and resident sage-grouse, :mud our annual survival estimates were comparable to annual survival estimates of resident sage-grouse in other locations. Our results suggest that when current recommendations for translocation protocols are followed, translocated female sage-grouse survive just as well as resident individuals and quickly integrate into the local population.

3296: ***+.099***

. 1. The western forests of Mexico are rich in species of stingless bee, possibly a consequence of the diverse habitats found across different altitudes of the Trans-Mexican Volcanic Belt (TMVB) and the Pacific coast (PAC). 2. Scaptotrigona hellwegeri is an example of a stingless bee species found across the TMVB and PAC. It is currently considered a single species. Its TMVB populations have become rare and rapidly impacted by human-modified habitats. Translocation of S. hellwegeri colonies from the PAC tropical lowlands to TMVB subtropical highlands has been considered, as colonies at PAC are more abundant and even used in stingless beekeeping. 3. Morphometric analyses of meristic characters plus genetic analysis at microsatellite loci and sequencing of the cox1 region of mtDNA were used to evaluate phenotypic and genetic differentiation in S. hellwegeri from TMVB and PAC. 4. Significant morphometric differences were detected between S. hellwegeri from TMVB and PAC. Corrected nuclear multilocus F'ST was 0.592 (P < 0.01), supporting the view that TMVB and PAC populations are markedly genetically differentiated. A 573 bp sequence of the cox1 region of mtDNA showed six sites of divergence, with sequence divergence between PAC and TMVB populations of 1.1%. 5. Our morphometric and genetic results make evident that S. hellwegeri from TMVB and PAC are significantly differentiated and represent two genetic lineages. An immediate recommendation is to restrict the movement of colonies from the lowland PAC regions to TMVB, where colonies are currently scarce.

3297: ***+.107***

Wild Secale species, Secale africanum Stapf., serve as a valuable source for increasing the diversity of cultivated rye (Secale cereale L.) and provide novel genes for wheat improvement. New wheat - S. africanum chromosome 1R(afr) addition, 1R(afr)(1D) substitution, 1BL.1R(afr)S and 1DS.1R(afr)L translocation, and 1R(afr)L monotelocentric addition lines were identified by chromosome banding and in situ hybridization. Disease resistance screening revealed that chromosome 1R(afr)S carries resistance gene(s) to new stripe rust races. Twenty-nine molecular markers were localized on S. africanum chromosome 1R(afr) by the wheat - S. africanum introgression lines. Twenty markers can also identically amplify other reported wheat - S. cereale chromosome 1R derivative lines, indicating that there is high conservation between the wild and cultivated Secale chromosome 1R. Nine markers displayed polymorphic amplification between S. africanum and S. cereale chromosome 1Rafr derivatives. The comparison of the nucleotide

sequences of these polymorphic markers suggested that gene duplication and sequence divergence may have occurred among Secale species during its evolution and domestication.

3298: ***+.314***

Since the 1970s, Paradise Park Wildlife Sanctuary in Cornwall, United Kingdom, has built up a captive flock of red-billed choughs Pyrrhocorax pyrrhocorax and over 30 years has developed successful methods of keeping, breeding, and appropriately socializing them in captivity. A total of 77 nests reached the egg stage with 27 nests producing at least one young and 48 young fledging in total. Several components are important in achieving successful breeding and socialization. Provision of live food, especially ant's eggs and small mealworms and crickets, in the first days after hatching is essential, improving the condition of adults and survival of nestlings. Situating aviaries in quiet areas, away from public view, is important. Socialization in family groups during the winter months and allowing choughs to choose partners induces better compatibility. Introduction of nest cameras greatly improved young survival through early identification of health problems enabling treatment of young between hatching and 10-days old, when mortality is otherwise highest, and enabling precautionary medication shortly after hatching. We show that clutch size increases significantly with female age and that direct intervention such as artificial egg incubation and hand rearing can be successful and worthwhile, but its requirement is reduced by closer monitoring. Red-billed choughs provide a good model species to further develop captive management and release techniques that can then be applied to critically endangered species that show similar social and long-learning behaviors. Captive breeding programs can play an important role in such work through provision of suitable birds and supporting avicultural expertise. Zoo Biol. 31:725-735, 2012. (c) 2012 Wiley Periodicals, Inc.

3299: ***+.035***

This article discusses two related issues of the captive breeding and reintroduction of the Oriental pied hornbill (Anthracoceros albirostris) in order to increase its population in the natural habitats. Oriental pied hornbills were bred in Khao Kheow Open Zoo. Three pairs were separated and kept in breeding cages. Females occupied artificial nests between February and April 20052007. Eggs were laid and incubated between February and March each year from 2005 to 2007. Nestlings hatched in late March and left the nest in late April 20052007. Each breeding pair was fed with approximately 400? g of food each day. All three pairs reproduced resulting in mature offspring of seven in 2005, six in 2006, and five in 2007. Four of sixteen 3-year-old birds were randomly selected and equipped with a GPS receiver on their backs. Activities of the birds attached and unattached with GPS were not significantly different. The first two birds (one female, one male with GPS) were reintroduced on August 5, 2006, and another nine birds (four birds with GPS) were reintroduced on December 26, 2006. The average home range of these reintroduced birds was 0.13?km2. Their foods consisted of wild plants and animals in the home range. The first reintroduced pair was able to breed naturally by laying and hatching eggs in an artificial nest. Two juveniles left the nest in April 2008. These results indicate that both captive breeding and reintroduction are potentially important ways to increase the population of the Oriental pied hornbill in natural habitats. Zoo Biol 31:683-693, 2012. (c) 2011 Wiley Periodicals, Inc.

3300: ***+.120***

Changes in the structure of woodlands and forests, caused by shifts in management, stand maturity, and composition, have been implicated in the population decline of some bird species in Europe and North America. One such species is the Marsh Tit (Poecile palustris). We investigated

relationships between Marsh Tit occupation (derived from territory mapping) and vegetation structure, tree species composition, and proximity to woodland edge in a British woodland, using a combination of 5 years of occupation data and high-resolution (0.5 and 1 m), large-scale (155 ha) habitat models derived from remote sensing. The results demonstrated that Marsh Tit occupation was linked to vegetation characteristics through the woodland's full vertical profile and related significantly and positively with overstory height, tree canopy closure, and the coverage of understory vegetation below the overstory. Marsh Tit occupation was lower within 50 m of the woodland perimeter, where habitat structure was less favorable than in the woodland interior. No preference was shown for areas rich in any particular prevalent tree species. Our results suggest that widespread changes in woodland structure resulting from abandonment by managers are unlikely to be responsible for the decline of the Marsh Tit in Britain and that reintroduction of active management that prevents woodland maturation could be detrimental to remaining populations. The study demonstrates a novel approach to integrating territory maps and remote-sensing data to permit highly detailed analyses of bird-habitat interactions and may have wider implications for woodland management and related bird species.

3301: *+.088*

We tested the effectiveness of observers on horseback for searching for signs of activity of bilbies reintroduced to a former pastoral lease in central Western Australia. As a means of transport, horses were able to traverse country not readily accessible to motorised transport including all-terrain vehicles, were productive in terms of area searched per unit time, were an elevated platform from which to observe signs of activity, and had a low impact on soil and vegetation. In this trial, a distance of 27 km was traversed with observers visually inspecting 121.5 ha for signs of bilby activity. Ten fresh (active) bilby burrows were detected, equating to an active burrow every 12.15 ha. Further research will focus on estimating bilby population from the number of active burrows.

3302: *+.016*

Avian malaria is an important cause of the decline of endemic Hawaiian honeycreepers. Because of the complexity of this disease system we used a computer model of avian malaria in forest birds to evaluate how two proposed conservation strategies: 1) reduction of habitat for mosquito larvae and 2) establishment of a low-elevation, malaria-tolerant honeycreeper (Hawaii Amakihi) to mid-elevation forests would affect native Hawaiian honeycreeper populations. We evaluated these approaches in mid-elevation forests, where malaria transmission is seasonal and control strategies are more likely to work. Our model suggests the potential benefit of larval habitat reduction depends on the level of malaria transmission, abundance of larval cavities, and the ability to substantially reduce these cavities. Permanent reduction in larval habitat of >80% may be needed to control abundance of infectious mosquitoes and benefit bird populations. Establishment of malaria-tolerant Amakihi in mid-elevation forests increases Amakihi abundance, creates a larger disease reservoir, and increases the abundance of infectious mosquitoes which may negatively impact other honeycreepers. For mid-elevation sites where bird populations are severely affected by avian malaria, malaria-tolerant Amakihi had little impact on other honeycreepers. Both management strategies may benefit native Hawaiian honeycreepers, but benefits depend on specific forest characteristics, the amount of reduction in larval habitat that can be achieved, and how malaria transmission is affected by temperature.

3303: *+.054*

Predators must ignore unhelpful background "noise" within information-rich environments and focus on useful cues of prey activity to forage efficiently. Learning to disregard unrewarding cues should happen quickly, weakening future interest in the cue. Prey odor, which is rapidly investigated by predators, may be particularly appropriate for testing whether consistently unrewarded cues are ignored, and whether such behavior can be exploited to benefit prey. Using wild free-ranging populations of black rats, Rattus rattus, an alien predator of global concern, we tested whether the application of bird-nesting odors before the introduction of artificial nests (odor preexposure), enhanced the survival of birds eggs (prey) compared with areas where prey and nesting odors were introduced concurrently. In areas where predators had encountered prey odor before prey being available, the subsequently introduced eggs showed 62% greater survival than in areas where prey and odor were introduced together. We suggest that black rats preexposed to prey odor learned to ignore the unrewarding cue, leading to a significant improvement in prey survival that held for the 7-d monitoring period. Exploiting rapid learning that underpins foraging decisions by manipulating sensory contexts offers a nonlethal, but effective approach to reducing undesirable predatory impacts. Techniques based on olfactory preexposure may provide prey with protection during critical periods of vulnerability, such as immediately following a prey reintroduction. These results also highlight the potential benefits to species conservation to be gained from a greater understanding of the cognitive mechanisms driving alien predator behavior within ecological contexts.

3304: *-.032*

The Philippine crocodile (Crocodylus mindorensis) is considered one of the most endangered of the crocodilian species. Rumors or anecdotal concerns have existed for some time as to the possibility of hybrid individuals existing in a captive collection under consideration for providing reintroduction candidates; however, visual observations failed to identify suspected hybrids. Samples were collected from 619 Philippine crocodiles from several captive facilities and two free-ranging populations. Mitochondrial DNA D-loop (601 bp) fragments were sequenced for each crocodile and compared to 28 individuals representing ten crocodile species. Among Philippine crocodiles, 48 variable sites (47 parsimony informative sites) were identified, which defined six C. mindorensis haplotypes and one C. porosus-derived haplotype. Data were also generated for a 965 bp fragment of the ND4 subunit gene fragment for two samples of each D-loop haplotype. Among them, 91 variable sites (90 parsimony informative site) were identified, which defined three C. mindorensis haplotypes and one C. porosus-derived haplotype. From the nuclear genome, the C-mos gene was successfully amplified for the 388 bp partial fragment for all Philippine crocodile samples. Only two variable sites were identified. These sequences were compared to GenBank sequences for C. porosus. Of the 619 Philippine crocodile samples, 57 samples were found to harbor D-loop haplotypes identified as C. porosus and 31 of those harbored C-mos mutational sites diagnostic for C. porosus introgression. All individuals indicating C. mindorensis x C. porosus hybridization were sampled from the Palawan Wildlife Rescue and Conservation Center.

3305: *+.281*

To assist with conservation of Endangered Patagonian Huemul Deer (Hippocamelus bisulcus), the Huemul Task Force (HTF) reassessed information on appendicular morphology, paleobiogeography, and historical distribution as potential factors in recovery efforts. Traditional claims of being a mountain specialist of the Andes were refuted by empirical evidence showing huemul morphology to coincide with other cervids rather than the commonly implied homology to rock-climbing ungulates. It thus supports historical evidence of huemul in treeless habitat and reaching the Atlantic coast, which cannot be dismissed as past erroneous observations. Instead,

pre- and post-Columbian anthropogenic impacts resulted in huemul displacement from productive sites and in survival mainly in remote and marginal refuge areas. The process of range contraction was facilitated by easy hunting of huemul, energetic incentives from seasonal fat cycles and huemul concentrations, the change from hunting-gathering to a mobile equestrian economy, and colonization with livestock. However, areas used presently by huemul, as supposed mountain specialists, are also used by wild and domestic ungulates that clearly are not considered mountain specialists, whereas the only other Hippocamelus successfully uses areas homologous to tree-less Patagonia. Rigid application of modern habitat usage to infer past habitat use and ignoring historic extra-Andean accounts is unwarranted; these conclusions reached by the HTF indicate new opportunities for recovery efforts by considering morphological and historical evidence. For instance, reintroductions to other portions of the landscape used formerly by huemul, which tend to be more productive sites than those currently occupied by many huemul groups, would present a promising avenue.

3306: *+.124*

Habitat fragmentation is hypothesized to influence movements of animals between isolated habitat fragments and to affect survival of animals moving between fragments. Translocation experiments can provide quantitative information on movements and survival. We assessed potential barriers to dispersal and survival of pygmy rabbits (Brachylagus idahoensis), a species of conservation concern that is hypothesized to be sensitive, after translocation, to fragmentation of its sagebrush habitats. We measured homing tendency and estimated survival of pygmy rabbits after short-distance (1-2 km) experimental translocations at sites in southeastern Oregon. We captured, radio-tagged, and translocated 59 pygmy rabbits across 3 landscape categories of habitat fragmentation. We used logistic regression to compare among landscapes the odds of homing, after accounting for sex and displacement distance of individuals. We used known-fate models in program MARK to estimate survival rates of rabbits after translocation. Fifteen percent of translocated pygmy rabbits successfully homed to within 150 m of their original capture locations. Individuals translocated across fragmented landscapes with patchy cover of big sagebrush were the most likely to home, whereas rabbits translocated across relatively continuous big sagebrush cover bisected by a road were least likely to home. We also found that pygmy rabbits that homed had higher survival rates than those that did not return to their home areas, and rabbits that settled near roads had lower survival rates than those that did not settle near roads. The proximity of the largest patch of big sagebrush also had a positive influence on the survival of rabbits after translocation. Our results indicate that fragmentation does not necessarily impede movements nor does it necessarily reduce survival.

3307: *+.188*

Refugee species have been confined to suboptimal habitat through historic anthropogenic factors. If this is unknown, management might actively conserve these species in suboptimal habitat assuming it represents optimal habitat. Similarly, species distribution modelling (SDM) might misguide conservation management of refugee species by only using presence data from suboptimal habitats. We illustrate this by commenting on a recent SDM for European bison that reconstructed the historic distribution of the species. We challenge the interpretation of this model by suggesting an alternative historic biogeography based on the refugee species concept. We argue that, in the case of refugee species, historic reconstructions using SDM cannot be used as a template for conservation management. Rather, experimental re-introduction programmes should provide us with population performance and life history data from a range of suboptimal to optimal habitats. Such data could be used in mechanistic niche modelling to predict potential

distribution of refugee species.

3308: ***+.041***

Among the loss of genetic diversity due to population declines, population fragmentation and habitat loss, hybridization also stands as a threat to Morelet's crocodile (Crocodylus moreletii) populations. Genetic surveys in Belize and the Yucatan Peninsula have detected evidence of hybridization with the American crocodile (C. acutus). Admixture between these two species is most likely driven by human-mediated translocations. Along the central gulf coast of Mexico, C. moreletii populations are presumed to be purebred. To test this, we use nine microsatellite loci and sequence data from the mitochondrial control region to detect if C. acutus alleles have introgressed into populations of C. moreletii from central Veracruz. In 2010, C. moreletii was transferred from Appendix I to II of CITES based on a whole species demographic analysis, which indicated that populations had recovered across its range. Our study shows that populations in central Veracruz are purebred, although they exhibit low levels of genetic diversity most likely caused by inbreeding. Our data also suggest there is fragmentation among populations of C. moreletii, which may lead to further loss of genetic variation. Due the purity and low genetic diversity of C. moreletii populations from central Veracruz, we recommend increased protection and active management practices that take genetic data into account.

3309: ***+.031***

The taxonomy of African black rhinoceros (Diceros bicornis) remains unresolved. Maintaining levels of genetic diversity and species rescue by reintroduction and restocking requires its resolution. We compared the sequences of the mitochondrial DNA (mtDNA) control region for a total of 101 D.bicornis from three subspecies: D.b.minor, D.b.michaeli and D.b.bicornis. A single unique haplotype was found within the 65 D.b.minor samples from KwaZulu-Natal (KZN) Province, South Africa, 55 of which came from Hluhluwe-iMfolozi Game Park (HiP) and Mkuzi Game Reserve (MGR) source populations. However, six different haplotypes were represented in eleven D.b.minor samples from Zimbabwe. Similarly, published autosomal microsatellite data indicate low levels of diversity within the KZN D.b.minor populations. The low levels of mtDNA diversity within the KZN metapopulation point to the possible need for genetic supplementation. However, there is a need to determine whether the low levels of genetic variation within KZN D.b.minor are a result of the recent bottleneck or whether KZN historically always had low diversity.

3310: ***+.207***

Most of the European grassland butterfly species are dependent on species rich grasslands shaped by low intensity farming. Conservation of these specialist species in agricultural landscapes relies on knowledge of their essential resources and the spatial distribution of these resources. In The Netherlands, the dusky large blue Phengaris (Maculinea) nausithous butterflies were extinct until their reintroduction in 1990. In addition, a spontaneous recolonization of road verges in an agricultural landscape occurred in 2001 in the southern part of The Netherlands. We analyzed the use of the essential resources, both host plants and host ants, of the latter population during the summers of 2003 and 2005. First we tested whether the distribution of the butterflies during several years could be explained by both the presence of host plants as well as host ants, as we expected that the resource that limits the distribution of this species can differ between locations and over time. We found that oviposition site selection was related to the most abundant resource. While in 2003, site selection was best explained by the presence of the host ant Myrmica

scabrinodis, in 2005 it was more strongly related to flowerhead availability of the host plant. We secondly compared the net displacement of individuals between the road verge population and the reintroduced population in the Moerputten meadows, since we expected that movement of individuals depends on the structure of their habitat. On the road verges, butterflies moved significantly shorter distances than on meadows, which limits the butterflies in finding their essential resources. Finally we analyzed the availability of the two essential resources in the surroundings of the road verge population. Given the short net displacement distances and the adverse landscape features for long-distance dispersal, this landscape analysis suggests that the Phengaris population at the Posterholt site is trapped on the recently recolonized road verges. These results highlight the importance of assessing the availability of essential resources across different years and locations relative to the movement of the butterflies, and the necessity to careful manage these resources for the conservation of specialist species in agricultural landscapes, such as this butterfly species.

3312: *+.104*

This commentary discusses the findings and conclusions of the paper Drug resistant human Staphylococcus aureus findings in sanctuary apes and its threat to wild ape populations. This paper confirms the zoonotic transfer of Staphylococcus aureus in a sanctuary setting. The assertion that this in itself is enough to reconsider the conservation potential of ape reintroduction provides an opportunity to discuss risk analysis of pathogen transmission, following IUCN guidelines, using S. aureus as an example. It is concluded that ape reintroduction projects must have disease risk mitigation strategies that include effective biosecurity protocols and pathogen surveillance. These strategies will assist with creating a well planned and executed reintroduction. This provides one way to enforce habitat protection, to minimise human encroachment and the risks from the illegal wildlife trade. Thus reintroduction must remain a useful tool in the conservation toolbox. Am. J. Primatol. 74:1076-1083, 2012. (c) 2012 Wiley Periodicals, Inc.

3313: *-.101*

Reintroduction of sanctuary apes to natural habitat is considered an important tool for conservation; however, reintroduction has the potential to endanger resident wild apes through the introduction of human pathogens. We found a high prevalence of drug-resistant, human-associated lineages of Staphylococcus aureus in sanctuary chimpanzees (Pan troglodytes) from Zambia and Uganda. This pathogen is associated with skin and soft tissue diseases and severe invasive infections (i.e. pneumonia and septicemia). Colonization by this bacterium is difficult to clear due to frequent recolonization. In addition to its pathogenic potential, human-related S. aureus can serve as an indicator organism for the transmission of other potential pathogens like pneumococci or mycobacteria. Plans to reintroduce sanctuary apes should be reevaluated in light of the high risk of introducing human-adapted S. aureus into wild ape populations where treatment is impossible. Am. J. Primatol. 74:1071-1075, 2012. (c) 2012 Wiley Periodicals, Inc.

3314: *-.076*

An important question that arises is what to do when an invasive exotic is a species threatened with extinction within its original distribution and there are few cases in the world illustrating this situation. These species potentially compete with local species for resources and may displace native species or, may in some cases, weaken the gene pool of the native species. The simple eradication of the invasive population could reduce the species' gene pool, and the eradication process might affect local sympatric species. We recommend a program including identification of

areas within the natural range where the species is extinct, removal of the causes of extinction in those areas, then gradual removal of the species from its introduced range and release in the relocation areas following proper guidelines for reintroduction of species.

3315: ***+.337***

Most sequential decision-making problems in conservation can be viewed conceptually and modelled as a Markov decision process. The goal in this context is to construct a policy that associates each state of the system with a particular action. This policy should offer optimal performance in the sense of maximizing or minimizing a specified conservation objective Dynamic programming algorithms rely on explicit enumeration to derive the optimal policy. This is problematic from a computational perspective as the size of the state space grows exponentially with the number of state variables. We present a state aggregation method where the idea is to capture the most important aspects of the original Markov decision process, find an optimal policy over this reduced space and use this as an approximate solution to the original problem. Applying the aggregation method to a species reintroduction problem, we demonstrate how we were able to reduce the number of states by 75% and reduce the size of the transition matrices by almost 94% (324 vs. 5184), and the abstract action matched the optimal action more than 86% of the time. We conclude that the aggregation method is not a panacea for the curse of dimensionality, but it does advance our ability to construct approximately optimal policies in systems with large state spaces.

3316: ***+.166***

At Booderee National Park, south-eastern Australia, the intensive control of the introduced red fox (Vulpes vulpes) resulted in a major increase in the abundance of a browsing macropod, the swamp wallaby (Wallabia bicolor). This has led to a major decrease in the abundance and biomass of a range of palatable plant species. Fox control has also started a trophic cascade that has resulted in a decline in the abundance of the greater glider (Petauroides volans) a folivorous arboreal marsupial, mediated either through increased predation by owls or increased competition with common brushtail possums (Trichosurus vulpecula). We identified five potential scenarios for managing the effects of over-abundant swamp wallabies on the ecosystem as a whole. These were (1) the present scenario of continued intensive fox control and four possible scenarios to redress the problem: (2) ceasing fox control; (3) intensive fox control and intensive wallaby control; (4) introducing dingoes and ceasing fox control; and (5) introducing dingoes and maintaining fox control. We used an ecosystem modelling approach based on a fuzzy cognitive map (FCM) to predict relative estimates of abundance for each scenario for a wide range of taxa in the Booderee National Park ecosystem likely to be affected by each scenario. We addressed uncertainty in our knowledge of the interactions between species by creating alternative models of the system by removing one or more of the uncertain links between species and varying the strength of the remaining interactions in the FCM and aggregated predictions from 100,000 models to estimate the effect of uncertainty on the predictions from our FCM model. In comparison with the current scenario of intensive fox control, scenario 3 had the greatest likelihood of improving the status of palatable plants. Scenarios 2 and 4 reduced the abundance of a range of medium-sized mammals but improved the status of greater gliders, whereas the predicted effects of scenario 5 were uncertain. The FCM modelling approach developed here provided a valuable tool for managers to learn about the potential ecosystem wide effects of management actions while incorporating the likely effects of uncertain knowledge on system outcomes.

3317: ***-.106***

Translocation is a common conservation tool and often involves founders that are reared in captivity. Why some translocations succeed and others fail is not well understood, but may be related to phenotypic changes brought about by captivity. We found that variation in speed and body condition index among a small group of captive-reared Otago skinks (Oligosoma otagense) did not influence their survival after release to the wild. In the first 12 months, 75% of skinks persisted, and this reduced to 58% by 18 months. After combining our results with data for other Oligosoma species, we found that captive-reared skinks pre-release have a higher body condition index and are about 50% slower than wild lizards; however, slower speeds are not consistently associated with higher body condition indices. We suggest that reduced speeds of captive lizards are a function of physiological and behavioural changes associated with captivity, but not necessarily obesity.

3318: *+.129*

Persistence of forest-dependent species in fragmented landscapes strongly relies on sufficient dispersal between patches, making it important to understand how animal movements are affected by the intervening matrix. Movements can be influenced through selection or avoidance of land cover based on their perceived suitability for foraging or providing cover. The composition and configuration of the matrix will, therefore, most likely be an important factor to consider when estimating connectivity between patches. To address this, we performed translocation experiments to understand how forest birds used different land cover types in a fine-grained matrix of a fragmented Afromontane biodiversity hot spot (Taita Hills, Kenya). Our results revealed that use of land cover types for both the forest specialist Cabanis's greenbul Phyllastrephus cabanisi and for the forest generalist white-starred robin Pogonocichla stellata was disproportional to their availability. However, this effect was influenced by matrix configuration; in patchy matrices, land cover selection was more pronounced compared with more uniform matrices, especially for the forest specialist. At the scale of movement steps, risk avoidance seemed to be a strong factor in the route decisions for both species. Observed steps contained on average lower proportions of open land cover and did less frequently intersect built-up areas than expected. P.? stellata did not differentiate between the alternative land cover types, whereas P.? cabanisi preferred steps that contained more indigenous forest. The observed negative relationship between degree of forest dependency and matrix permeability implies that for members of the Taita bird community, which are even more dependent on intact forest habitat (i.e. the critically endangered Taita thrush Turdus helleri), current permeability of the matrix may be even lower. Matrix restoration to improve connectivity may, therefore, be a crucial instrument for the long-term survival of forest-dependent species in these fragmented Afromontane landscapes.

3319: *+.040*

Large terrestrial carnivores are particularly prone to factors constraining levels of population genetic diversity because of their low densities and high spatial requirements. We studied the pattern of Eurasian lynx Lynx lynx population genetic variability in the westernmost part of its natural range from Scandinavia to the Carpathian Mountains (north-central Europe) based on 190 samples using 613 base pair-long sequences from the mitochondrial DNA control region (mtDNA-cr). We examined whether the population history or contemporary habitat constraints of this large and mobile carnivore could have significantly affected its genetic structure. We recorded nine mtDNA haplotypes, including five not previously reported. Lynx from Latvia and Estonia had the highest variability with haplotype and nucleotide diversities of 0.810.88% and 0.440.47%, respectively. In contrast, there was no polymorphism present in peripheral populations from Norway and the Carpathian Mountains. Lynx populations were strongly differentiated [analysis of

molecular variance (AMOVA): F ST?=?0.570, P? Spatial Analysis of Molecular Variance identified four separate groups of populations: (1) Norway, Finland and Estonia; (2) Latvia and North Eastern Poland; (3) the Bialowieza Primeval Forest; (4) the Carpathians. The patterns of genetic diversity and differentiation suggested a number of discrete populations that are poorly connected by contemporary gene flow and could therefore be considered demographically independent. The peripheral location of these populations, habitat fragmentation and the strict territorial structure of lynx populations are factors likely contributing to the observed patterns. The study provides suggestions for active conservation/management decisions including translocations or reintroductions of lynx.

3320: *+.047*

Wildlife translocation is increasingly used to mitigate disturbances to animals or habitat due to human activities, yet little is known about the extent to which translocating animals causes stress. To understand the relationship between physiological stress and translocation, we conducted a multiyear study (20072009) using a population of desert tortoises (Gopherus agassizii) near Fort Irwin, California. Blood samples were collected from adult tortoises in three treatment groups (resident, translocated and control) for 1 year prior to and 2 years after translocation. Samples were analyzed by radioimmunoassay for plasma total corticosterone (CORT), a glucocorticoid hormone commonly associated with stress responses in reptiles. CORT values were analyzed in relation to potential covariates (animal sex, date, behavior, treatment, handling time, air temperature, home-range size, precipitation and annual plant production) among seasons and years. CORT values in males were higher than in females, and values for both varied monthly throughout the activity season and among years. Year and sex were strong predictors of CORT, and translocation explained little in terms of CORT. Based on these results, we conclude that translocation does not elicit a physiological stress response in desert tortoises.

3321: *+.353*

Microsite availability is crucial for recruitment success in natural populations as well as populations being established for restoration projects. Understanding the specific microsite requirements of a particular species targeted for restoration will increase the probability of success of any restoration project. Surface mining for coal represents one of the largest anthropogenic disturbances to the forests of the eastern United States. The original natural range of the American chestnut (Castanea dentata (Marsh.) Borkh.) overlaps the extent of the Appalachian Coal Basin. With American chestnut being readied for reintroduction trials, we sought here to determine some of the effects of microsite conditions on the establishment success of American chestnut on mine sites reclaimed using new, compaction-reducing techniques (i.e., "end-dump" reclamation) that create a series of loosely dumped mounds roughly 8-m diameter and 3-m tall to serve as a planting substrate. Specifically, we examined the effects of distance from existing forest edge, amount of existing cover of vegetation, small-scale topographic position, and a small set of soil variables on the growth and survival over three seasons of American chestnut seedlings planted on a reclaimed mine site in east-central Ohio. We found decreased tree survival adjacent to existing forest edges and greater annual growth rates at distances of 20 and 50 m from the existing forest edge. Microtopographic position had a significant effect on seedling growth and survival seedlings planted higher on mounds had increased mortality and lower growth than those on the side slopes of those mounds. The amount of existing vegetative cover also affected survival and growth; trees growing in plots with higher vegetative cover values showed increased growth and survival. The compaction-reducing reclamation approach used here is relatively new and novel. Promising results that have been observed to date using American chestnut and this method seem to indicate

that the combination may be very effective at restoring functional forests on lands degraded by surface mining.

3322: *+.198*

The ongoing global amphibian decline calls for an increase of habitat and population management efforts. Pond restoration and construction is more and more accompanied by breeding and translocation programs. However, the appropriateness of translocations as a tool for conservation has been widely debated, as it can cause biodiversity loss through genetic homogenization and can disrupt local adaptation, eventually leading to outbreeding depression. In this study, we investigated the genetic structure of two translocated populations of the critically endangered fire-bellied toad Bombina bombina at its north western distribution edge using supposedly neutral genetic markers (variation in the mitochondrial control region and microsatellites) as well as a marker under selection (major histocompatibility complex (MHC) genes). While one of the newly established populations showed the typical genetic composition of surrounding populations, the other was extremely diverged without clear affinity to its putative source. In this population we detected a profound impact of allochthonous individuals: 100% of the analyzed individuals exhibited a highly divergent mitochondrial haplotype which was otherwise found in Austria. 83% of them were also assigned to Austria by the analysis of microsatellites. Interestingly, for the adaptive marker (MHC) local alleles were predominant in this population, while only very few alleles were shared with the Austrian population. Probably Mendelian inheritance has reshuffled genotypes such that adaptive local alleles are maintained (here, MHC), while presumably neutral allochthonous alleles dominate at other loci. The release of allochthonous individuals generally increased the genetic variability of the affected population without wiping out locally adaptive genotypes. Thus, outbreeding depression might be less apparent than sometimes thought and natural selection appears strong enough to maintain locally adaptive alleles, at least in functionally important immune system genes. (C) 2012 Elsevier GmbH. All rights reserved.

3323: *-.058*

The first occurrence of Cobitis paludica (de Buen, 1930) in the Segura River Basin (SE Iberian Peninsula) The aim of the present report is to describe the establishment of viable populations of Cobitis paludica (de Buen, 1930) in the Segura River Basin. We found two isolated populations: one located in the upper part of the Segura River and the mouth of its tributary, the Zumeta River, and another in the Mundo River, between the Talave and Camarillas reservoirs. We hypothesised that the introduction of this species may be attributable to the deliberate or accidental introduction by anglers or fish translocation from the Tajo-Segura interbasin water transfer system. C. paludica is a threatened endemic fish species from the Iberian Peninsula, and it exhibits sharply declining populations. Therefore, further investigation is needed to assess the genetic origin of the populations reported in this report and to monitor the population trends to determine the population status and the appropriate management plan in the Segura River Basin.

3324: *-.143*

Aspen in the Greater Yellowstone Ecosystem are hypothesized to be recovering from decades of heavy browsing by elk due to a behaviorally mediated trophic cascade (BMTC). Several authors have suggested that wolves interact with certain terrain features, creating places of high predation risk at fine spatial scales, and that elk avoid these places, which creates refugia for plants. This hypothesized BMTC could release aspen from elk browsing pressure, leading to a patchy recovery in places of high risk. I tested whether four specific, hypothesized fine-scale risk factors are

correlated with changes in current elk browsing pressure on aspen, or with aspen recruitment since wolf reintroduction, in the Daly Creek drainage in Yellowstone National Park, and near two aspen enclosures outside of the park boundary. Aspen were not responding to hypothesized fine-scale risk factors in ways consistent with the current BMTC hypothesis.

3325: ***-.061***

Recolonising native mammals have the potential to cause environmental and agricultural damage. However, if their future distribution can be predicted, effective control measures can be scheduled beforehand to prevent the onset of damage. In this study, we predicted the future range expansion of recolonising wild boar Sus scrofa populations in the Chiba Prefecture, Japan, using simulations. Wild boars were extinct in the Chiba Prefecture until the 1970s, but since then, a new naturalised population has spread, probably due to release for hunting. Recently a small, isolated, naturalised population was found in the northern part of the prefecture, which was considered to be a new release. We divided the Chiba Prefecture (5,156 km(2)) into 3-km grids and, based on nuisance control records, we examined the 'presence' of wild boar populations from 2002 to 2007 and in 2010. We simultaneously estimated habitat suitability and dispersal probability of the source population via range-expasion modelling. We predicted the future distribution by the use of stochastic simulations for 20 years after 2010. According to the simulations, the wild boar populations will expand into the southern and northern regions of the Chiba Prefecture at a rate of 2,153 km/year, and crop damage should be expected in these areas in the future. Range expansion into the northern region of the prefecture will be completed by around 2025. If the northern isolated population is removed, it will be possible to delay the range expansion for about five years. The eradication of a small isolated population in the northern Chiba Prefecture may have significant economic benefits because the crop production in this area is relatively large.

3326: ***-.076***

Seven North American beavers Castor canadensis (Cc) were introduced into Finland in 1937 to supplement an ongoing reintroduction of the nearly extinct Eurasian beaver C. fiber (Cf). At that time, many zoologists recognised only one species. However, in 1973, chromosome counts (Cf = 48, Cc = 40) acknowledged two species, and Cc became an invasive alien. Recently, expanding populations of both species have converged on two fronts in Finland and northwestern Russia. According to Gause's competitive exclusion principle, two species with identical niches cannot coexist indefinitely. The imminent question is whether coexistence or competitive exclusion will ultimately result, with the possible regional extirpation or eventual extinction of Cf. We reviewed published cases of interspecies contact and compared their life history, ecology and behaviour. The few published incidences of contact were inconclusive with respect to competitive advantage. Body size is similar, but Cc litter size is slightly greater. Only minor differences in life history, ecology and behaviour were found to exist, suggesting nearly complete niche overlap. Though competitive exclusion resulting in the extinction of a native mammal by an alien congener at the continental landscape scale has been rare, the process may be difficult to detect due to potential time lags of centuries. Thus, there is a distinct risk that Cc may eventually competitively exclude Cf at all landscape scales. As no country in Eurasia obviously wants an invasion of Cc, and as most national conservation laws and international treaties forbid the spread of alien species, we advocate that the precautionary principle be adhered to and an attempt to eradicate Cc from Eurasia be seriously considered. Successful eradication is still possible if the will to do so exists. Here, we outline an eradication strategy.

3327: ***+.183***

The modeling of potential and current distribution of species has become a very active research area. Generally, modeling is based on the concept of ecological niche, and is supported by the use of computer programs. The main objective of this project was to develop a potential distribution model of Pinus martinezii in the Cuitzeo Lake basin using data of environmental variables, and presence of the target species. To this purpose, the environmental factors that determine the distribution of P. martinezii were identified, and a bioclimatic profile of the species was made. The modeling was based on a spatial multicriteria analyisis. The attributes were grouped into 3 criteria: geopedologic, morphometric and climatic conditions. The resulting potential distribution map showed that there are 2 main areas of potential distribution of P. martinezii, and some isolated areas where populations have not been found. The spatial model is an important tool for planning conservation and reforestation efforts, as well as to plan additional field surveys and identifying sites where the specie can be reintroduced.

3328: *+.017*

Reduced genetic variation is a severe threat for long-term persistence of endangered animals. Immigration or translocation of new individuals may result in genetic rescue and increase the population viability of the endangered population or species. Unfortunately, studying genetic rescue in wild populations is very difficult, but breeding programs of endangered species can contribute to our knowledge of the diverse effects of genetic rescue. A recovery breeding program of common hamsters in the Netherlands enabled the study of genetic rescue in an endangered rodent as a few wild hamsters from two nearby and also highly threatened populations were added to the breeding stock. Litter size increased over the years, but no relation between inbreeding levels and litter size was found. Average litter size benefited from the genetic variation introduced by a hamster from Germany, but hamsters from Belgium had no effect on litter size. Rather than alleviating inbreeding depression the genetic rescue effect observed in this population seems to originate from the introduction of beneficial alleles by the German male. Breeding programs using several populations may increase the success of reintroductions and long-term persistence of these populations.

3329: *+.049*

Fungal populations that reproduce sexually are likely to be genetically more diverse and have a higher adaptive potential than asexually reproducing populations. Mating systems of fungal species can be self-incompatible, requiring the presence of isolates of different mating-type genes for sexual reproduction to occur, or self-compatible, requiring only one. Understanding the distribution of mating-type genes in populations can help to assess the potential of self-incompatible species to reproduce sexually. In the locally threatened epiphytic lichen-forming fungus Lobaria pulmonaria (L.) Hoffm., low frequency of sexual reproduction is likely to limit the potential of populations to adapt to changing environmental conditions. Our study provides direct evidence of self-incompatibility (heterothallism) in L. pulmonaria. It can thus be hypothesized that sexual reproduction in small populations might be limited by an unbalanced distribution of mating-type genes. We therefore assessed neutral genetic diversity (using microsatellites) and mating-type ratio in 27 lichen populations (933 individuals). We found significant differences in the frequency of the two mating types in 13 populations, indicating a lower likelihood of sexual reproduction in these populations. This suggests that conservation translocation activities aiming at maximizing genetic heterogeneity in threatened and declining populations should take into account not only presence of fruiting bodies in transplanted individuals, but also the identity and balanced representation of mating-type genes.

3330: *-.026*

Human-elephant conflict (HEC) threatens the survival of endangered Asian elephants (Elephas maximus). Translocating "problem-elephants" is an important HEC mitigation and elephant conservation strategy across elephant range, with hundreds translocated annually. In the first comprehensive assessment of elephant translocation, we monitored 16 translocations in Sri Lanka with GPS collars. All translocated elephants were released into national parks. Two were killed within the parks where they were released, while all the others left those parks. Translocated elephants showed variable responses: "homers" returned to the capture site, "wanderers" ranged widely, and "settlers" established home ranges in new areas soon after release. Translocation caused wider propagation and intensification of HEC, and increased elephant mortality. We conclude that translocation defeats both HEC mitigation and elephant conservation goals.

3332: *+.248*

Plantations of fast-growing tree species may be of use in conservation by accelerating the restoration of forest habitat on abandoned farmland and increasing connectivity in fragmented landscapes. The objective of this study was to determine if hybrid poplar plantations can be suitable habitats for the reintroduction of native forest plant species and, if so, which abiotic factors predict successful reintroduction. Four species of forest herb species (Trillium grandiflorum, Sanguinaria canadensis, Maianthemum racemosum, Asarum canadense), of which three have legal conservation status, were transplanted into experimental plantations of two hybrid poplar clones and nearby second-growth woodlots at six sites in southern Quebec, Canada. The transplanted individuals were protected from deer browsing with exclusion cages. After two years, the plant responses of all four species were stable or increased over two years in both types of hybrid poplar plantations. Sanguinaria showed a better response in the plantations than in the woodlots, preferring the rich post-agricultural soils of the plantations with low C:N ratios. Asarum and Maianthemum showed no significant difference between stand types, while Trillium grew better in the woodlots than in the plantations. Much of the variability in the response of the latter three species was unexplained by the measured environmental variables. These results suggest that certain forest herb species can be reintroduced as juvenile plants into plantations, knowing that their spontaneous recolonization is often limited by dispersal and/or seedling establishment. Plantations could also contribute to the conservation of biodiversity by providing an environment for the cultivation of forest herb species as an alternative to their destructive harvest from natural populations.

3333: *-.013*

Herein we clarify the taxonomy of Fluminicola coloradensis Morrison (1940), which was described for populations in the Green River and Bonneville basins but has also been treated as restricted to the former watershed and conspecific with (currently unassigned) Snake River basin populations of Fluminicola hindsi sensu Taylor (1966). Bayesian analyses of DNA sequences from 2 mitochondrial genes congruently resolved F. coloradensis and Snake River basin populations of F. hindsi sensu Taylor as a strongly supported, shallowly structured clade. Haplotypes were extensively shared by Bonneville, Snake River and Green River populations; AMOVA did not detect significant variation among basins for either gene. Morphological variation was minor. Based on these results, we assign the Snake River basin populations to F. coloradensis. We attribute the limited differentiation of widely ranging F. coloradensis to its well-integrated habitats and to dispersal mediated by geologically recent drainage transfers. The broadly disjunct population in the Owyhee River drainage may be a product of translocation, as evidenced by

detection of only the most common haplotypes in these snails. Our finding that F. coloradensis is more widely distributed than previously thought suggests that it may not require conservation attention rangewide, although some geographic subunits may be at risk.

3334: *+.192*

The Blue-and-yellow Macaw, once native to the island of Trinidad, was extirpated in the early 1960s primarily due to nest poaching of chicks for the pet trade. Between 1999 and 2004, the Cincinnati Zoo and Botanical Garden, Trinidad and Tobago's Ministry of Environment and the Centre for the Rescue of Endangered Species of Trinidad and Tobago (CRESTT) reintroduced wild-caught birds from Guyana to the Nariva Swamp in Trinidad. After quarantines, testing and physical examinations, the birds were acclimated in a pre-release flight cage and the flight readiness of the first 14 birds was monitored as the main criterion for release. Nine of the 14 birds released (64%) survived and produced 12 chicks in three nesting seasons. Three years later 20 additional wild-caught birds were imported from Guyana and the criterion for their release was expanded to include social behaviors such as pair bonding and compatible groups. There was 100% survival of the 17 flight-ready birds released from the second flock. Bonded pairs and compatible groups that were released stayed together and exhibited behaviors indicating healthy social structure. Fourteen additional chicks were produced in three more nesting seasons. Twenty-six of the 31 birds released (84%) survived. Nesting success continued with the surviving population now estimated at 86 birds. This represents a 230% increase over 12 nesting seasons. Several factors have contributed to the survival and reproductive success of the reintroduced birds. This status report identifies some of these factors and suggests that Blue-and-yellow Macaws can be successfully reintroduced to a habitat from which they were extirpated when conditions are favorable.

3335: *+.059*

Post - released monitoring, of hog deer, Eld's deer and sambar deer in Salakphra grassland, Salakphra Wildlife Sanctuary, Kanchanaburi Province was procedure during August 16th, 2011 and April 2013. The objectives were to monitor the released hog deer, Eld's deer and sambar deer. Radio tracking, camera traping and direted observing were used to investigate the adaptability of the animal. In the case of habitat preparing, grassland improvement covered an area of 10 ha approximately by prescribe burning was done. Ten artificial salt lick sites and 21 water sources were also done in the released area. Thirty-one hog deer, 19 males and 12 females, were translocated in to the area, 4 males and I female were tagged with radio collar. Six Eld's deer, 3 males and 3 females were translocated. Four of them, 2 males and 2 females were tagged with radio collar. Ten sambar deer, 4 males and 6 females were translocated. Two of them, 1 male and 1 female were tagged with radio collar. The monitoring results from radio telemetry all year round found that the male hog deer had 124.44 ha avearge home range size. The size of the female was 79.16 ha. The average home range size of male Eld's deer was 114.69 ha. Whereas those of the female was 92.55 ha. The overall home range of the female sambar deer was 153.10 ha. The result from camera trapping during 2011 and 2012 found that 19 wildlife spececies inhabited in the area. The occurance of sambar deer had positive coreration with other 4 wildlife species significantly. Whereas the occurance of hog deer and Eld's deer had positive correlation with other I wildlife species significantly. Dhole and guar that had never been occured in the area were also photographical recorded in 2012. The result from fecal pellet group count method showed ecological density of sambar deer around the area was 0.13 individuals/ha whereas those of the barking deer was 0.03 individuals/ha. The released hog deer gave 10 newborns during the study period. Nevertheless 11 of the released individuals were died, 7 of them due to translocation and

ear tag

3336: ***+.249***

Comprising 50%-75% of the world's fauna, insects are a prominent part of biodiversity in communities and ecosystems globally. Biodiversity across all levels of biological classifications is fundamentally based on genetic diversity. However, the integration of genomics and phylogenetics into conservation management may not be as rapid as climate change. The genetics of hybrid introgression as a source of novel variation for ecological divergence and evolutionary speciation (and resilience) may generate adaptive potential and diversity fast enough to respond to locally-altered environmental conditions. Major plant and herbivore hybrid zones with associated communities deserve conservation consideration. This review addresses functional genetics across multi-trophic-level interactions including "invasive species" in various ecosystems as they may become disrupted in different ways by rapid climate change. "Invasive genes" (into new species and populations) need to be recognized for their positive creative potential and addressed in conservation programs. "Genetic rescue" via hybrid translocations may provide needed adaptive flexibility for rapid adaptation to environmental change. While concerns persist for some conservationists, this review emphasizes the positive aspects of hybrids and hybridization. Specific implications of natural genetic introgression are addressed with a few examples from butterflies, including transgressive phenotypes and climate-driven homoploid recombinant hybrid speciation. Some specific examples illustrate these points using the swallowtail butterflies (Papilionidae) with their long-term historical data base (phylogeographical diversity changes) and recent (3-decade) climate-driven temporal and genetic divergence in recombinant homoploid hybrids and relatively recent hybrid speciation of Papilio appalachiensis in North America. Climate-induced "reshuffling" (recombinations) of species composition, genotypes, and genomes may become increasingly ecologically and evolutionarily predictable, but future conservation management programs are more likely to remain constrained by human behavior than by lack of academic knowledge.

3337: ***+.057***

Like other European native crayfish species, the stone crayfish, Austropotamobius torrentium, has disappeared from many freshwater systems for various, mostly anthropogenic reasons during the last 150 years. In the Austrian Federal State Vorarlberg, this species is protected through the EU Habitats Directive and national legislation and must be maintained at, or restored to, the so called "favourable conservation status". According to the legal requirements, and based on relevant field study results, we assessed the conservation status of A. torrentium on this regional scale for the first time. We show that the populations suffered significantly from insufficient habitat size and availability, as well as pollution risks due to land-use and degraded or lacking riparian vegetation. The observed isolation of all existing populations will have an impact over the long term. Based on these findings, we state the urgent need for conservation measures, not only because of nature conservation considerations, but also from a legal point of view. Therefore, we define the crucial aspects needed for a future management plan. In this context, we propose reintroductions of native crayfish to be a valuable management tool, but also show the difficulty linked to this measure due to a scarcity of target habitats in this densely populated region of the Alps. Thus, conservation efforts in the field should be linked with the establishment of protected areas, thereby setting local priorities for the benefit of the threatened stone crayfish.

3338: ***+.099***

Conservation of endangered British crayfish (Austropotamobius pallipes) involves translocation to isolated waterbodies (Ark Sites) and reintroductions from hatchery stock. The species is restricted in the wild to relatively alkaline waterbodies. The optimum pH conditions that maximise growth and survival are unknown. This information would benefit the selection of Ark Sites and hatchery water chemistry. Here we measure growth and survival of juvenile A. pallipes in the laboratory using three pH levels from within the species' natural range. Survival at pH 8.6 was high (94%), whereas that at lower pH levels was low (pH 6.5, 25%; pH 7.1, 34%). Growth (moult increment and frequency) was also higher at pH 8.6. Survival was also lower for Thelohania contejeani-infected animals, with a trend towards higher mortality in infected individuals at lower pH. Electron microscopy revealed a lower cuticle thickness at pH 6.5 than higher pH which was associated with an apparent increase in the number of endocuticle layers. We therefore recommend that high pH (ca. 8.6) be used as a target for Ark Site selection and hatchery water chemistry in order to maximise survival and growth.

3339: *+.073*

The European medicinal leech was for centuries important component of therapy and it has direct application also in modern medicine. Many protease inhibitors were purified from leech saliva, and Hirudo medicinalis Linnaeus, 1758 has been approved as a prescription medical device in some countries. Due to its potential application, medicinal leaches are permanently captured from nature, however the main threat is associated with the destruction of habitats, changes of water regime, climatic changes and dessication of marshes. The comeback of use of leeches in modern medicine (microsurgeries, such as plastic and reconstructive surgeries) and reclamation of wetlands emphasize the need of effective monitoring and protection. While there are only few sites where medicinal leech has been recorded or mentioned during last decade in Slovakia (all of the in southern lowland regions), an affective inventory and monitoring programme is necessary for species conservation with following priorities: (1) Confirming the current distribution of the medicinal leech in Slovakia; (2) Provision of appropriate advice to maintain the remaining populations. Attempts to restore the fragmented populations could include (a) establishing the satellite populations close to potentially vulnerable sites where the medicinal leech currently occurs; (b) reintroduction at other suitable localities, to secure the continuing presence of the medicinal leech in Slovakia.

3340: *+.246*

A potential means of mitigating the impact that development has on animals is to move affected individuals to new areas where development will not occur or to release individuals back to a site after activities have ceased. In the case of translocation, the desired outcome is that the majority of the translocated individuals will survive and reproduce in a new, uninhabited and protected site, such that there is no net loss of endangered animals. Because of project displacement, we moved 144 Tipton kangaroo rats Dipodomys nitratoides nitratoides, a state and federally listed endangered species, to a preserve north of Bakersfield, California, USA, in December 2006 that had no Tipton kangaroo rats, but was known habitat. To help determine short-term survivorship, we radio-tracked 22 individuals for 30 d post-release. We placed 14 radio-collared kangaroo rats in cages with artificial burrows (soft release) on site and 8 radio-collared individuals in artificial burrows without cages (hard release). We placed all other kangaroo rats in artificial burrows with (n = 86) or without (n = 36) cages. The percentage survivorship of radio-tagged kangaroo rats that were soft-released (58.3%) was greater than that of radio-tagged animals hard-released (37.5%), but the differences were not significant. Trapping over 3 yr showed a small, but persistent, population of Tipton kangaroo rats. We caught 38 new Tipton kangaroo rats in these 3 yr. Based

on an AMOVA of genotypic data from 2 microsatellite loci of Tipton kangaroo rats translocated to the site, unmarked young caught on site are consistent with being offspring of the translocated animals. Future research should address the value of caging kangaroo rats on sites prior to release, the relative merit of short-term removal of competing species, and appropriate habitat management strategies.

3341: *-.179*

Because of the precarious condition of small cetacean species and subpopulations listed as Endangered or Critically Endangered by the IUCN, use of captive breeding for conservation has been suggested for some of them, and will likely be suggested for others. A successful captive breeding program for a new species cannot be implemented until reliable capture and husbandry techniques have been developed. Techniques for assisted reproduction and reintroduction may also be needed. We review attempts to capture, maintain, and breed poorly known small cetaceans and discuss assisted reproductive technologies (ART) that have been used to enhance captive breeding efforts for other small cetaceans. We conclude that the techniques required for successful captive breeding of most Endangered or Critically Endangered small cetacean species have not been sufficiently developed. Development of these techniques should begin before a species or population is Critically Endangered. In particular, ARTs tend to be species specific, necessitating considerable time, money, and research to develop for each species of concern. Critically Endangered populations cannot afford to lose the individuals needed for technique development. The fairly large captive population sizes necessary (to avoid loss of genetic diversity, inbreeding, and genetic adaptation to captivity), limited space available in aquariums, and high costs of captive breeding and reintroduction programs make it unlikely that captive breeding will play a major role in the conservation of most small cetaceans. The substantive conservation measures needed to prevent extinction of Critically Endangered small cetaceans is reduction or elimination of their primary threats, which are usually by-catch and habitat loss.

3342: *+.288*

We propose a two-stage translocation strategy to conserve metapopulations of endangered species. The concept takes advantage of variation in vital rates among subpopulations to increase individual fitness, improve species status, and maintain metapopulation structure for long-term resiliency. We simulate two-stage translocation scenarios for conserving the Hawaiian monk seal Monachus schauinslandi, a critically endangered species which exhibits highly variable juvenile, but consistently favorable adult, survival rates. Moving young seals from areas of lower to higher juvenile survival and subsequently returning them to their source site once they have reached an appropriate age improves population reproductive value. We present a decision framework for implementing two-stage translocation in a manner that minimizes risks while increasing the likelihood of desired outcomes. Two-stage translocation may be effective for metapopulations of other rare species which exhibit variation in vital rates among subpopulations and a life-stage bottleneck due to factors that are not amenable to in situ mitigation.

3343: *+.258*

Free-ranging African elephants live in a fission-fusion society, at the centre of which is the matriarch. Matriarchs are generally older females that guide their families to resources and co-ordinate group defense. While much is known about elephant society, knowledge is generally lacking about how age affects the physiology of wild elephants. Investigation of the ovarian activity of free-ranging elephants could provide insight into the reproductive ageing process, with

implications for population management. Faecal samples were collected from 46 individuals ranging in age from 14 to 60 years for a 2-year period, and progestagen metabolite analyses were used to examine relationships between social status, age, season, and ovarian activity in female elephants in Addo Elephant National Park, South Africa. Social status was the strongest predictor of faecal progestagen metabolite concentrations in non-pregnant elephants, with grand matriarchs (n = 6) having the lowest values compared with matriarchs (n = 21) and non-matriarch females (n = 19). Likewise, social status and age were the strongest predictors of faecal progestagen metabolite concentrations in pregnant elephants (n = 27). The number of years since a nonpregnant female gave birth to her last calf (post-partum duration) was longer for older females with a higher social status, as well as during the dry season. Our results indicate that social standing and age of elephants are related to reproductive function, and that older females exhibit reductions in ovarian capacity. These results expand our understanding of reproduction and fertility throughout an elephant's lifespan, and the factors that impact gonadal function in free-ranging females. Given that possible over-abundance of elephants in areas such as Addo Elephant National Park is fuelling the debate over how best to manage these populations, knowledge about the reproductive potential of high-ranking females can provide managers with biological data to identify the best candidates for controlling growth through translocation or contraception.

3344: *+.008*

The global range of the Aldabra white-throated rail Dryolimnas cuvieri aldabranus, the last surviving flightless bird in the Indian Ocean, was restricted to only three islands of Aldabra Atoll in 1998. It was extirpated on the islands of Grand Terre (before the late 1800s) and Picard (soon after 1910), mainly due to the introduction of feral cats by early settlers. In 1999, following the eradication of cats from Picard, 18 Aldabra rails were successfully reintroduced. After the reintroduction, population growth of the Aldabra rail on Picard was predicted to continue to approximately 1,000 pairs by 2010. In this paper, we report on the long-term effectiveness of the reintroduction by updating the Aldabra rail population estimate on Picard 12 years after the translocation and one year after the predicted maximum was expected to be reached. We confirm the predicted carrying capacity on Picard has been reached and probably exceeded; report a reliable survey method for the Aldabra rail, which can be applied to other terrestrial bird species; and recommend subsequent monitoring and conservation management strategies for the Aldabra rail and potentially other species of rail.

3345: *+.150*

Numerous Virgin Islands plants have become rare or extinct over the last 200 years due to cultivation, development, and introduction of exotic species. St. John endemic Solanum conocarpum (Solanaceae) has fewer than 200 known individuals currently extant in the wild. DNA fingerprinting with RAPD PCR was used to assess the population genetics of S. conocarpum and congener S. polygamum. In addition, controlled crosses were performed to determine whether the plant was self-incompatible. Finally, seedlings were outplanted and monitored to (1) restore small populations and (2) compare performance of individuals from the largest and smallest populations of the species. Population genetic analysis revealed that species level diversity in S. conocarpum remains high, but that virtually all diversity is confined to a single population. The crosses performed indicated that the species is completely self-incompatible. While seedlings from the smallest population performed poorly in the greenhouse by comparison with plants from the large population with higher genetic diversity, both groups performed equally well in the field over a three-year period. The species' high level of overall diversity and ability to survive even when diversity is reduced suggest that prospects for conservation are good. In order to ensure the

survival of the species, all populations should be brought up to sizes large enough to allow outcrossing, and ex-situ conservation should be employed.

3346: *+.101*

Coenopopulations of *Craniospermum subvillosum* Lehm., the endemic species of Baikal region, were studied. Intrapopulation and interpopulation variabilitiy of morphological features of the plants, their fruits and seeds were investigated. The criteria were identified to estimate the state of the coenopopulations. *C. subvillosum* is a diplochorous plant, this strategy determining the size of its populations in different habitats. The protection measures for *C. subvillosum* include monitoring of its coenopopulations and reintroduction.

3348: *+.048*

Wild chimpanzee populations are still declining due to logging, disease transmission and hunting. The bushmeat trade frequently leads to an increase in the number of orphaned primates. HELP Congo was the first project to successfully release wild-born orphan chimpanzees into an existing chimpanzee habitat. A collection of post monitoring data over 16 years now offers the unique opportunity to investigate possible behavioural adaptations in these chimpanzees. We investigated the feeding and activity patterns in eight individuals via focal observation techniques from 1997-1999 and 2001-2005. Our results revealed a decline in the number of fruit and insect species in the diet of released chimpanzees over the years, whereas within the same period of time, the number of consumed seed species increased. Furthermore, we found a decline in time spent travelling, but an increase in time spent on social activities, such as grooming, as individuals matured. In conclusion, the observed changes in feeding and activity patterns seem to reflect important long-term behavioural and ecological adaptations in wild-born orphan released chimpanzees, demonstrating that the release of chimpanzees can be successful, even if it takes time for full adaptation.

3349: *+.014*

Ecological restoration of mined peatlands in North America involves active reintroduction of bog plant species. Animals are not actively reintroduced, thus the re-establishment of peatland fauna must occur either by inoculation along with introduced plant material or by dispersal. We examined the extent to which insects are reintroduced to restored sites with plant material by rearing insects from shredded vegetation collected in three donor sites. We assessed differences in abundance, diversity, and composition of taxonomic and trophic groups among seasons and sites. Abundance and species richness did not differ by season, but species assemblages did. The three sites were significantly different in abundance, but not in species richness and assemblages. Few insects emerged from the vegetation, suggesting that shredded plant material may not be the primary source of insect colonists. Insects likely recolonize by active or passive dispersal from the surrounding area. The species pool was similar among donor sites; consequently a mined site could be inoculated with vegetation from another peatland in the same region and this would not affect the insect assemblages at the initial stage of establishment. Diapause may be a major factor for emergence success among seasons of collection. Knowledge of how restoration techniques influence establishment of insect communities will help predict longer-term outcomes of restoration on biotic communities in peatlands.

3350: *+.093*

The successful establishment of trees in pastures may be related to species, seed size, and weed control treatments. This study experimentally evaluated the emergence, establishment after 2 years, and growth of individuals of 7 tree species with different seed sizes sowed under three weeding methods (no weeding, grasses clipped, and grasses removed). The experiment was carried out in a tropical abandoned pasture in Midwestern Brazil, dominated by the exotic invasive grass Urochloa brizantha. The effect of seed size on seedling emergence was significant and was more intense in the no weeding and weeding treatment plots than in grasses clipped. Furthermore, an increase in seed size resulted in an increase in the probability of establishment of tree species, although this pattern also differed among weeding treatments. The increase in seed size reflected negatively on tree growth in the absence of grasses, whereas in the presence of grasses there was an inverse relationship. The experimental results suggested that the use of seeds of different sizes for direct seeding in pastures is a possible strategy of Fabaceae tree species reintroduction in pastures within the Cerrado. Fabaceae species with seeds larger than 100 mg can establish in areas with no weeding, whereas species with seeds smaller than 50 mg can establish preferentially in areas with weeding, growing faster than larger ones. Thus, weeding strategies can determine the success of direct seeding of different species in restoration projects of tropical pastures.

3351: *+.226*

Natural resources managers are being asked to follow practices that accommodate for the impact of climate change on the ecosystems they manage, while global-ecosystems modelers aim to forecast future responses under different climate scenarios. However, the lack of scientific knowledge about short-term ecosystem responses to climate change has made it difficult to define set conservation practices or to realistically inform ecosystem models. Until recently, the main goal for ecologists was to study the composition and structure of communities and their implications for ecosystem function, but due to the probable magnitude and irreversibility of climate-change effects (species extinctions and loss of ecosystem function), a shorter term focus on responses of ecosystems to climate change is needed. We highlight several underutilized approaches for studying the ecological consequences of climate change that capitalize on the natural variability of the climate system at different temporal and spatial scales. For example, studying organismal responses to extreme climatic events can inform about the resilience of populations to global warming and contribute to the assessment of local extinctions. Translocation experiments and gene expression are particular useful to quantify a species' acclimation potential to global warming. And studies along environmental gradients can guide habitat restoration and protection programs by identifying vulnerable species and sites. These approaches identify the processes and mechanisms underlying species acclimation to changing conditions, combine different analytical approaches, and can be used to improve forecasts of the short-term impacts of climate change and thus inform conservation practices and ecosystem models in a meaningful way.

3352: *+.229*

Human influence typically impacts on natural populations of conservation interest. These interactions are varied and sometimes complex, and may be negative and unintended or associated with conservation and management strategy. Understanding the details of how these interactions influence and are influenced by natural evolutionary processes is essential to the development of effective conservation strategies. In this study, we investigate a species in Britain that has experienced both negative impact through overhunting in historical times and management efforts through culls and translocations. At the same time, there are regional populations that have been less affected by human influence. We use mtDNA and nuclear microsatellite DNA markers to

investigate patterns of connectivity and diversity and find multiple insular populations in Britain that probably evolved within the Holocene (when the habitat was free of ice). We identify three concurrent processes. First, surviving indigenous populations show highly provincial patterns of philopatry, maintaining and generating population structure on a small geographic scale. Second, founder populations into habitat extirpated of native populations have expanded, but remained largely insular. Third, introductions into established populations generate some admixture. We discuss the implications for the evolution of diversity of the integration of natural processes with anthropogenic influences on population size and distribution.

3353: *-.007*

Analysis of global positioning system (GPS) location clusters (GLCs) is becoming increasingly popular in studies of carnivore ecology. While promising, this application of GPS technology is still poorly developed for most species. We applied this method to study predation and maternal behavior of the Eurasian lynx (Lynx lynx) in the Dinaric Mountains. Low population densities, rugged terrain, dense vegetation, and administrative borders make studies of this endangered population using traditional methods and a limited budget very challenging. We used the geographic information system and linear mixed-effects models to understand the movement of lynx during the consumption process and denning period and estimate lynx kill rates. A total of 99 % of kills were found at GLCs longer than 30 h and with minimum two locations within 300 m. We confirmed 86 % of potential kills and all potential dens that were searched for in the field. High success in predicting kill and den sites showed that the Eurasian lynx is a suitable species for application of the GLC analysis methods. Comparison of field-confirmed kills with model predictions showed the possibility for remote estimation of approximate kill rates in Eurasian lynx. Movements of the lynx were primarily affected by daytime period, time since the last kill/den translocation, lynx category, and their interactions. Based on the empirical data, we programmed simulations of lynx movements and elaborated recommendations for more efficient field procedures and study designs (GPS schedules) for future studies. We believe that our findings and approach will also benefit studies of other species with similar behavior.

3354: *+.193*

A method is described and tested for identifying and prioritizing actions to facilitate recovery (restoration) and/or conservation (maintenance) of populations of threatened marine species. The exercise was based on established approaches for terrestrial species and on assessing each species according to degree of threat and recovery/conservation potential. Assessment of both degree of threat and recovery/conservation potential was informed by researching the relevant life-history traits of each species and existing knowledge of natural fluctuations in abundance. Rarity was a key consideration in assessing degree of threat but rarity measures for cetaceans and pelagic fishes were not available and a new methodology was therefore developed. Likely actions for maintenance or recovery of a population of a species were specified under the headings: Site Management, Translocation, Enforcement, Research, Monitoring and Wider Environment. The recovery/conservation goal for each species was identified according to SMART (Specific, Measurable, Attainable, Relevant and Time-bound) criteria. The terrestrial approach transferred well to marine species but with some adaptation as the marine environment is different to terrestrial ecosystems in the pressures and activities that are likely to adversely affect species, to our knowledge of decline in species and to the ecological processes that are likely to aid recovery. The species researched are prioritized for action according to degree of threat and recovery/conservation potential. Recovery/conservation goals are specified and the reasons for proposed actions are explained. Identifying measures for recovery or conservation was often

difficult because the cause of decline or the threats to species were unknown or unclear. Better collation of relevant information would create a stronger evidence base, assist the provision of better advice, and therefore support better decision-making by managers. Application of the methodology to other marine species of conservation concern in a particular biogeographical or administrative area needs more meaningful lists than are currently used of species that are rare, scarce, in decline or threatened with decline. Copyright (C) 2012 John Wiley & Sons, Ltd.

3355: ***-.038***

The interaction among several parasites in European rabbits (Oryctolagus cuniculus) is crucial to host fitness and to the epidemiology of myxomatosis and rabbit hemorrhagic disease. These diseases have caused significant reductions in rabbit populations on the Iberian Peninsula. Most studies have focused on the epidemiology and pathogenesis of these viruses individually, and little is known about interactions between these viruses and other parasites. Taking advantage of an experimental restocking program in Spain, the effects of coccidian and nematode infections on the probability of having detectable antibody to myxoma and rabbit hemorrhagic disease viruses were tested in European wild rabbits. For 14 mo, we monitored rabbit abundance and parasite loads (coccidia and nematodes) in three reintroduced rabbit populations. While coccidian and nematode loads explained seasonal antibody prevalences to myxoma virus, the pattern was less clear for rabbit hemorrhagic disease. Contrary to expectations, prevalence of antibody to myxoma virus was inversely proportional to coccidian load, while nematode load seemed to play a minor role. These results have implications for viral disease epidemiology and for disease management intended to increase rabbit populations in areas where they are important for ecosystem conservation.

3356: ***-.149***

The ecological restoration of fire-suppressed habitats may require a multifaceted approach. Removal of hardwood trees together with reintroduction of fire has been suggested as a method of restoring fire-suppressed longleaf pine (Pinus palustris) forests; however, this strategy, although widespread, has not been evaluated on large spatial and temporal scales. We used a landscape-scale experimental design to examine how bird assemblages in fire-suppressed longleaf pine sandhills responded to fire alone or fire following mechanical removal or herbicide application to reduce hardwood levels. Individual treatments were compared to fire-suppressed controls and reference sites. After initial treatment, all sites were managed with prescribed fire, on an approximately two-to three-year interval, for over a decade. Nonmetric multidimensional scaling ordinations suggested that avian assemblages on sites that experienced any form of hardwood removal differed from assemblages on both fire-suppressed sites and reference sites 3-4 years after treatment (i.e., early posttreatment). After >10 years of prescribed burning on all sites (i.e., late posttreatment), only assemblages at sites treated with herbicide were indistinguishable from assemblages at reference sites. By the end of the study, individual species that were once indicators of reference sites no longer contributed to making reference sites unique. Occupancy modeling of these indicator species also demonstrated increasing similarity across treatments over time. Overall, although we documented long-term and variable assemblage-level change, our results indicate occupancy for birds considered longleaf pine specialists was similar at treatment and reference sites after over a decade of prescribed burning, regardless of initial method of hardwood removal. In other words, based on the response of species highly associated with the habitat, we found no justification for the added cost and effort of fire surrogates; fire alone was sufficient to restore these species.

3357: ***+.144***

The population dynamics of threatened invertebrates have important implications for their conservation and restoration. The Seychelles giant millipede (SGM), Sechelleptus seychellarum, is a threatened and functionally important macro-detritivore endemic to the Seychelles granitic islands. Here, we studied the population dynamics of the SGM from 1998 to 2009 on Cousine Island, Seychelles, to make practical restoration recommendations. Large fluctuations in millipede densities were found between 1998 and 2009. In 2002, 2003, 2005, and 2007 millipede densities were low, while densities were high in 1998 and 2009. Although the SGM is active all year round, millipede surface activity was positively correlated with rainfall, with millipede density high during the wet NW monsoon period (i.e., October to April) and low during the SE trade wind period (i.e., May to September). Female:male:juvenile ratios were approximate to 3:1:1. The implications of these results for restoration are that translocations should preferably be done in years of high millipede densities and during the wet season. Furthermore, chemical control of the invasive ant Pheidole megacephala, which is currently being carried out on Cousine Island and in future could be conducted on other Seychelles islands, should preferably be done during the low rainfall months, as the SGM readily consumes the hydramethylnon-based bait.

3358: ***+.142***

Supplemental feeding is widely used after the translocation of animals and is presumed to increase post-release survival or reproductive output. However, the results of empirical studies on supplemental feeding are equivocal and research is needed to determine the mechanisms by which supplemental feeding affects health and behaviour. Here, we studied the effect of supplemental feeding on the Brown Teal, or Pateke (Anas chlorotis), an endangered duck endemic to New Zealand, following four translocations of captive-bred individuals. Radio-telemetric monitoring showed no significant effect of supplemental feeding on post-release survival. Male birds dispersed further than females, and supplemental feeding decreased post-release dispersal. To reduce heterospecific competition at Brown Teal feeders, we also tested an exclusion device designed to prevent the main heterospecific competition, the Purple Swamphen (Pukeko in New Zealand, Porphyrio porphyrio melanotus), from accessing supplemental food. Although this device decreased the presence of Purple Swamphens at feeders, it also decreased use of feeders by Brown Teal. Ultimately, we concluded that supplemental feeding has value as a conservation tool for Brown Teal, particularly during releases in managed areas. Further studies on feeder design, as well as spatial and temporal patterns of use of feeders, are needed to maximise the positive effect of supplemental feeding on success of translocations.

3359: ***+.125***

North Island Saddlebacks (Philesturnus rufusater) have been the subject of a well-documented sequence of translocations to isolated islands around New Zealand. We measured corticosterone stress responses in North Island Saddlebacks derived from the ancestral population and from a series of three sequential translocations. Each translocation might impose a selective pressure on the founder populations of North Island Saddleback. This selective filter might favour a particular corticosterone response linked through an associated coping ability. Evidence of selection would be reflected in a progressive shift in the mean corticosterone response related to the number of sequential translocations. Mean corticosterone responses to a standardised stressor were highest in birds descended from a population subject to two sequential translocations. However, responses measured at localities representing the original source population and populations derived from one and three sequential translocations were not significantly different from each other. Accordingly, our data suggest that translocations do not necessarily cause directional selection on the stress responses of these vulnerable populations of birds.

3360: *-.032*

Four species of Diuris temperate terrestrial orchids from wild and captive populations were tested for the presence of polyadenylated RNA viruses. The genomes of three exotic viruses were determined: two potyviruses, Bean yellow mosaic virus and Ornithogalum mosaic virus, and the polerovirus Turnip yellows virus. The genomes of five indigenous viruses were detected, including four novel species. They were the potyvirus Blue squill virus A, another potyvirus, two proposed capilloviruses, and a partitivirus. Partitivirus infection is of interest as this group of viruses is also associated with endophytic fungi (mycorrhizae) that are necessary for the germination, growth, development of many terrestrial orchids. Sequence divergence data indicate post-European, pre-European, and endemic origins for these viruses via inoculum from introduced and native plants. The implications of the findings of this study for orchid conservation, and particularly reintroduction programs where viruses may be spread inadvertently to wild populations from infected propagation sources, are discussed. (C) 2012 Elsevier B.V. All rights reserved.

3361: *-.020*

Ancient DNA is becoming increasingly recognised as a tool in conservation biology to audit past biodiversity. The widespread loss of Australian biodiversity, especially endemic mammal populations, is of critical concern. An extreme example occurred on Depuch Island, situated off the north-west coast of Western Australia, where an unidentified species of rock-wallaby (Petrogale sp.) became extinct as a result of predation by red foxes. Two potential candidate species, Petrogale lateralis and P. rothschildi, both have ranges adjacent to Depuch Island, making identification based on geography difficult. A museum bone (one of the only surviving Depuch Island specimens) was subjected to standard ancient DNA analyses and procedures. Mitochondrial DNA cytochrome b and hypervariable control region were targeted for species identification. Ancient DNA was successfully recovered from the bone: 200 base pairs (bp) of control region and 975 bp of the cytochrome b gene. Bayesian phylogenetic analyses were employed to model the Depuch Island rock-wallaby DNA sequences together with sequences of other rock-wallaby taxa from GenBank. Evidence suggests that of the two Petrogale lateralis subspecies proposed to have inhabited Depuch Island, Petrogale lateralis lateralis was identified as the most likely. The identification of the Depuch Island rock-wallaby population may assist in the reintroduction of an insurance population of Petrogale lateralis lateralis, which is becoming increasingly threatened on mainland Australia.

3362: *+.159*

The aoudad (Ammotragus lervia) is a wild ruminant considered the living ancestor of domestic sheep and goats. The original distribution of aoudads includes several countries in the North of Africa, but it has been introduced, for trophy-hunting purposes, into other countries (USA, Mexico and Spain). The species was declared vulnerable in the 2012 IUCN's Red List and is also included in the CITES II Appendix. Surprisingly, little is known about its conservation status or the reproductive biology of the natural populations. There are some reports of the application of basic assisted-reproduction techniques in captive aoudads. In this preliminary study, we explored the feasibility of implementing assisted reproduction procedures in captive aoudad females using non-traditional techniques for in vivo embryo production. This approach was used to obtain the best results using the minimum possible number of animals. Three aoudad females were synchronized using a domestic sheep protocol and subcutaneous osmotic pumps for the delivery of follicle-stimulating hormone. A mini-surgical approach combined with laparoscopy was performed to obtain in vivo-produced embryos. All females had an ovulatory response of more than three

corporea lutea, but only five good quality morulae were obtained from one female. Those were cryopreserved by vitrification using a Cryotop (R). In conclusion, our approach was successful in obtaining in vivo embryos using a limited number of females. Further studies are necessary to optimize the fertilization rate and clarify the effect of our protocol on embryo implantation and the production of offspring.

3363: *+.151*

Gaur, which became locally extinct before 1995 in Bandhavgarh Tiger Reserve (BTR), Central India, is an endangered animal per Schedule - I of the Indian Wildlife Protection Act (1972). A re-introduction program was therefore created to rebuild the gaur population in BTR, both to enhance the long-term survival of the species and to restore natural biodiversity. After re-introduction, the home range, habitat use and food habits of gaur (Bos gaurus gaurus) were studied in BTR, India, from January 2011 to January 2012. Nineteen gaurs (five males - three radio-collared and 14 females - nine radio-collared) were re-introduced from Kanha Tiger Reserve to Bandhavgarh Tiger Reserve in January 2011. The reintroduced gaurs were monitored periodically through ground tracking and satellite GPS fixes. The mean annual group size of gaur was estimated at 7.3 +/- 0.76 (SE). The overall estimated summer, monsoon and winter home ranges of gaur were 290 km(2), 137 km(2) and 155 km(2) (Minimum Convex Polygon) respectively. The overall individual male home ranges varied from 135 to 142 km(2), and overall individual female home ranges varied from 32 to 169 km(2). Radio collared locations were plotted on a classified (LISS III) habitat map of Bandhavgarh Tiger Reserve to evaluate the habitat use and availability in each season. Habitat preference was computed using Bonferroni confidence interval method, compositional analysis and Ivlev's index. In summer, gaur largely preferred grassland (P<0.0001), whereas in monsoon and winter, gaur preferred bamboo mixed forest (P<0.0001). Gaur avoided open mixed forest (P<0.0001) and agricultural land in all three seasons. Data on food habits were collected through opportunistic sightings. In total, gaur fed on 68 plant species. The present study has reported first-time information on ranging patterns of reintroduced gaur and their degree of preferences for different vegetation and terrain types across seasons, which will be very useful to the park administration for future conservation of this endangered species and for habitat intervention.

3364: *+.026*

Deforestation and a changing climate threaten the health and survival of lemurs in Madagascar. An important component of lemur health, parasite infection can reduce fitness and survival outcomes. Future lemur parasite richness, abundance and distribution may be highly influenced by climate change. Current knowledge of lemur parasites is narrow in geographic and temporal scope, with sampling at a limited number of sites, and thus far, there have been no attempts to assess the effects of climate change on lemur parasite distributions. We used geospatial tools to predict the distributions of six lemur parasites of high frequency and pathogenic potential. We then assessed how anticipated climate shifts in Madagascar may alter the distributions of these lemur parasites in the future. Under current climate conditions, we found that the focal parasites exhibited widespread potential distributions across Madagascar, covering 12-26% of surface land area and 40-86% of forested area. Our analyses also showed that parasites responded differently to projected climate changes, with shifts ranging from a contraction of current distributions by 7% to an expansion of 60%. A predicted net expansion in parasite distribution may expose naive lemur hosts to new parasites, which could have a profound effect on lemur health. Those parasites with the greatest potential for harmful effects are predicted to experience the largest expansion in range. Predicting these changing distributions will be critical for assessing population health, improving protected area design, preparing for reintroduction efforts and addressing potential parasite risk in

lemurs, humans and domestic animals. (C) 2012 Elsevier Ltd. All rights reserved.

3365: *+.211*

The mycorrhizal associations of Borya mirabilis Churchill,T.B.Muir & Sinkora are described for the first time, based on seasonal observations over 12 months. Roots of vascular plants associated with B. mirabilis in the field were also examined for mycorrhizal associations; Callitris rhomboidea R.Br. ex Rich. & A.Rich. was found to be most morphologically similar. The usefulness of associated plants to inoculate ex-situ populations and as indicators of potential translocation sites for B. mirabilis is discussed.

3366: *+.090*

1. Herbivores and granivores represent one of the most influential drivers of plant abundance and population dynamics. Their effect can be, in turn, modulated by biotic or abiotic factors such as community composition, habitat characteristics or space heterogeneity.2. Recent approaches to the study of herbivore and granivore impacts on plants have considered the combined action of multiple herbivore species, the effect of herbivores on several plant life stages or the effect of environmental gradients on these interactions. However, studies addressing the effect of multiple herbivore species on different plant life stages are still lacking.3. We estimated the combined effect of multiple exotic herbivores (European rabbits, Oryctolagus cuniculus; black rats, Rattus rattus; and house mouse, Mus musculus) on four different life stages of an endangered plant species (Medicago citrina, Fabaceae). Mortality for seed, seedling and sapling was estimated at three types of plots (open, rat exclusion and rat + rabbit exclusion) replicated at four sites (N = 3 per site and treatment) within Cabrera Island (Balearic Islands, western Mediterranean). Browsing of reproductive adults was simulated under common-garden conditions (Soller Botanic Garden, Mallorca Island) and its effect on reproductive effort and success measured.4. European rabbits and black rats had complementary impacts on the different life stages of M. citrina. These included independent effects on different life stages (seed predation by rats, seedling predation by rabbits), which resulted in multiplicative increases in plant mortality, and concurrent effects on the same life stage (sapling predation). In addition, the simulated-herbivory experiment showed that a low rate of canopy removal (25% of initial biomass) already causes a strong decrease in fruit set (from 54% to 30%), but increasing rates of canopy removal do not increase this effect.5. Synthesis. Our results stress the importance of considering the combined effects of different herbivores on several life stages of the plant's life cycle and their consecutive effects on population dynamics. From an applied point of view, future reintroduction attempts of M. citrina in Cabrera Island should consider measures to either control the populations of both exotic herbivores or mitigate their impacts on the earlier recruitment stages of the plant (seeds, seedlings and saplings).

3367: *+.168*

Context. Knowledge of the habitat selection of reintroduced species is crucial to successful re-establishment of viable populations and effective conservation decision-making.Aims. The aim of our research was to examine habitat selection by reintroduced Eld's deer (Cervus eldi) in a human-dominated landscape.Methods. The study was conducted during the period from July 2005 to November 2007 in the Chihao region, a human-dominated area located in western Hainan Island, China. Radio-telemetry was used to monitor 15 collared deer to gain their location information. Resource selection functions were used to quantify habitat selection of the study population at the landscape and home-range scales in both wet and dry seasons. Key results. At the landscape scale, Eld's deer showed selection for habitats with scrubland, high elevation, gentle slope, close to water

sources and roads. At the home-range scale, Eld's deer showed selection for habitats with dense forest, scrubland, grassland, low elevation and far away from roads, but they randomly used habitats without special consideration to the distance to water sources. At both landscape and home-range scales, Eld's deer showed strong avoidance of villages. In addition, Eld's deer showed increased selection of sparse forests and decreased use of grasslands in the dry season, as compared with the wet season at both spatial scales. Sexual differences in habitat selection existed in reintroduced Eld's deer. Males showed stronger avoidance to human disturbance, whereas females selected vegetation with higher forage availability but poor hiding cover, especially during the antler-growing period (i.e. wet season).Conclusions. The habitat selection of reintroduced Eld's deer was scale-dependent. As a non-fatal anthropogenic factor, human disturbance had a strong influence on habitat selection of Eld's deer. They more strongly selected slope habitats at relatively high elevations. However, our results also indicated that the reintroduced Eld's deer had certain adaptive ability and tolerance to the disturbed environment.Implications. This work provides insight into the habitat selection of reintroduced Eld's deer in a human-dominated landscape. If the essential food resources are available, the regions at a relatively high elevation with low human disturbance can be considered as potential sites of future Eld's deer reintroduction.

3368: *+.074*

The cougar Puma concolor was part of the pre-European fauna of the north-eastern USA. It was extirpated in the late 1800s and since the late 1900s there have been discussions concerning its reintroduction to the region. One site considered is Adirondack State Park in northern New York. In 1981 an assessment of the feasibility of returning cougars concluded that the Park had adequate prey and forest cover to support a small population of cougars but that conflicts with humans would cause the demise of this population within 10 years. Thus reintroduction at that time was not advised. Since then knowledge of cougar ecology and how cougars interact with humans has increased substantially. Based on information compiled since the 1980s I conducted a landscape-scale analysis to assess whether cougars could live in the Park. The results indicate that cougars could occupy 15,300-17,000 km(2) (61-69%) of the Park, with minimal contact with human habitation. Based on reported cougar densities the Park could support a population of 150-350 cougars. These cougars would consume < 10% of the adult deer population annually and fawn production would be sufficient to replace these losses. Human and road densities in the Park are similar to those of the Black Hills, South Dakota and southern Florida, both of which have viable populations of cougars. I concluded that Adirondack State Park could support a population of cougars. What is now required is the will to bring them back.

3369: *+.106*

Legislative changes during the 1960s-1970s granted user rights over wildlife to landowners in southern Africa, resulting in a shift from livestock farming to wildlife-based land uses. Few comprehensive assessments of such land uses on private land in southern Africa have been conducted and the associated benefits are not always acknowledged by politicians. Nonetheless, wildlife-based land uses are growing in prevalence on private land. In Namibia wildlife-based land use occurs over c. 287,000 km(2). Employment is positively related to income from ecotourism and negatively related to income from livestock. While 87% of meat from livestock is exported >= 95% of venison from wildlife-based land uses remains within the country, contributing to food security. Wildlife populations are increasing with expansion of wildlife-based land uses, and private farms contain 21-33 times more wildlife than in protected areas. Because of the popularity of wildlife-based land uses among younger farmers, increasing tourist arrivals and projected impacts of climate change on livestock production, the economic output of wildlife-based land

uses will probably soon exceed that of livestock. However, existing policies favour livestock production and are prejudiced against wildlife-based land uses by prohibiting reintroductions of buffalo Syncerus caffer, a key species for tourism and safari hunting, and through subsidies that artificially inflate the profitability of livestock production. Returns from wildlife-based land uses are also limited by the failure to reintroduce other charismatic species, failure to develop fully-integrated conservancies and to integrate black farmers sufficiently.

3370: *+.455*

The effects of management on ecosystem diversity, structure and function must be understood for the sustainable integration of conservation and development. A potential source of experimentation and learning in ecosystem management is the array of private protected areas worldwide. Autonomous management systems can be seen as natural experiments, presenting an opportunity to explore the consequences of manipulating ecosystem properties. By quantifying management diversity and developing an index of management intensity we assessed the ecological correlates of private protected area management within the savannah biome in South Africa. Management intensity is positively correlated with herbivore density, predator density and ecotourism lodge density and negatively with herbivore community heterogeneity, reintroduction success and primary productivity at the local protected area scale. However, these trade-offs are tantamount to functional diversity as different management systems play unique roles in the regional socio-ecological and socio-economic systems, which range from animal production centres high in commercial value to low density areas that may sustain landscape processes. Furthermore, fenced private protected areas are necessary to safeguard rare species that cannot sustain viable populations in altered ecosystems. Thus, when considered at the regional scale, a private protected area network that constitutes a patchwork of management systems will create a coincident conservation and production landscape. We suggest that maintaining management heterogeneity will provide net benefits to biodiversity and potentially galvanize locally sustainable, wildlife-based economies.

3371: *+.012*

Despite formidable challenges and few successes in reintroducing large cats from captivity to the wild, the release of captives has widespread support from the general public and local governments, and continues to occur ad hoc. Commercial so-called lion Panthera leo encounter operations in Africa exemplify the issue, in which the captive breeding of the lion is linked to claims of reintroduction and broader conservation outcomes. In this article we assess the capacity of such programmes to contribute to in situ lion conservation. By highlighting the availability of wild founders, the unsuitability of captive lions for release and the evidence-based success of wild-wild lion translocations, we show that captive-origin lions have no role in species restoration. We also argue that approaches to reintroduction exemplified by the lion encounter industry do not address the reasons for the decline of lions in situ, nor do they represent a model that can be widely applied to restoration of threatened felids elsewhere.

3372: *+.175*

Carapa procera DC. (Meliaceae) is a widespread species occurring in the woodlands of Africa and Latin America. It is a medicinal plant overexploited by the rural populations of southern Mali. Leaves, fruits and seeds, the bark of the trunk and the roots are used in the preparation of medicine for human and animal health. Such uses result in enormous pressures on natural stands of the species, which have great difficulty in regenerating naturally. Cultivation of C. procera was

investigated through studies of its seed physiology and seedling production techniques for reintroduction into its natural habitat. Results showed that fresh seeds of C. procera had a moisture content of over 50% (based on fresh weight) and >= 90% germination. When seeds were dried in a ventilated room condition, their moisture content dropped below 20% after 2 weeks, resulting in a total loss of their germination capacity. Carapa procera seeds can therefore be described as recalcitrant seeds. Stored in kegs containing wood chips, 50% of (small size) seeds germinated after one month and 33% after three months, maintaining a 23.5% moisture content. This study recommends that seed moisture content should be kept at above 35% in order to maintain seed viability in storage. We found that using a substrate of silty clay in the nursery allowed a good growth of seedlings (>= 40 cm), which could be planted out in the field within three months. In order to sustainably use and conserve C. procera in the savannah areas of West Africa, it is recommended that the species be cultivated through the establishment of plantations in its habitat by rural communities.

3373: *+.129*

Blue sheep (Pseudois nayaur) are the key prey of the endangered snow leopard (Panthera uncia) in the Himalayan region of Nepal. However, the snow leopard population has recently expanded back into the Sagarmatha (Mt. Everest) National Park where the blue sheep are currently absent, and the evidence of snow leopard depredation of livestock can be found. A solution to this prospective human-wildlife conflict is the translocation of blue sheep back into this area. The aims of this study are (1) to characterize the spatial and environmental factors related to current blue sheep distributions in three areas: Annapurna Conservation Area, Shey Phoksundo National Park and Kanchanjunga Conservation Area, and (2) to use these characteristics to assess the suitability of two areas: Sagarmatha (Mt. Everest) National Park and Langtang National Park for blue sheep translocation. Blue sheep were found to occur in 14,603 km(2) of Nepal, with 7343 km(2) (49%) inside protected areas. Blue sheep preferred the alpine meadow, pasture and grassland with the mean annual precipitation between 200 and 1000 mm and most frequently occurred at elevations between 3300 and 5100 m with soil combinations of Haplumbrepts, Dystrochrepts and Cryumbrepts.Overall, the Sagarmatha (Mt. Everest) National Park was significantly similar to the Kanchanjunga Conservation Area (Bray-Curtis similarity coefficient S > 72%) in terms of vegetation, elevation, precipitation, soil, aspect and slope. Therefore, the translocation of blue sheep from the Kanchanjunga Conservation Area to the Sagarmatha (Mt. Everest) National Park is recommended in accordance with the main conservation aim of supporting the population of snow leopards and other predators in the park. However, before translocation can proceed, a further detailed study is required on human disturbance, grass biomass and climate change at specific release sites as well as decisions on the number of blue sheep to be relocated.

3374: *+.232*

Translocations are an important conservation strategy for many species. However simply observing demographic growth of a translocated population is not sufficient to infer species recovery. Adequate genetic representation of the source population(s) and their long-term viability should also be considered. The woylie Bettongia penicillata ogilbyi has been subject to more formal translocations for conservation than any other marsupial that, up until recently, has resulted in one of the most successful species recoveries in Australia. We used mitochondrial and nuclear DNA markers to assess the genetic outcomes of translocated woylie populations. These populations have lost genetic variability, differentiated from their source population and the supplementation program on two island populations appears to have failed. We discuss the conservation implications that our results have for managing threatened species, outline some

general recommendations for the management of present and future translocations and discuss the appropriate sampling design for the establishment of new populations or captive breeding programs that may mitigate the genetic 'erosion' seen in our study species. This research provides some practical outcomes and a pragmatic understanding of translocation biology. The findings are directly applicable to other translocation programs.

3375: *+.062*

Plant mating systems can vary significantly in both space and time, influencing a range of demographic and genetic processes critical for the persistence of plant populations. Spatial and temporal mating-system variations were investigated in Banksia cuneata (A. S. George), a rare bird-pollinated shrub occurring in a highly fragmented landscape. Substantial variation in the mating system was observed, with the magnitude of temporal variation within a population often as high as the level of spatial variation among populations within a season. A significant reduction in outcrossing rates and increased temporal variation in both outcrossing rates and correlated paternity were observed in disturbed populations. Doubling of the paternal neighbourhood and a trend to increased outcrossing rate was found after augmentation of a disturbed population where population size was increased from 57 to 214 adult plants. A large increase in the fixation index for seeds compared with adults was observed in all populations, with the magnitude of the difference showing temporal and spatial uniformity. We suggest that temporal mating-system variation warrants increased consideration, not only in assessing the effects of habitat fragmentation on plant populations, but also in the design and establishment of successful translocation, augmentation and restoration programs.

3376: *+.198*

Densities of abalone in southern California have been dramatically reduced by over-fishing and disease, leading to the collapse of some populations, and low fertilization rates may be hindering population recovery. This is a pattern typical of abalone species globally. However, movement may produce clustered distributions that promote fertilization success in broadcast spawners, such as abalone, even at low regional densities. We translocated wild, adult pink abalone Haliotis corrugata to an existing pink abalone patch to create a high-density aggregation, and then used acoustic telemetry to characterize abalone movement and monitor aggregation maintenance for a period of 14 mo in the Point Loma kelp forest near San Diego, California. Abalone showed a minimal flight response to handling that did not differ between the resident or translocated groups. Most individuals exhibited small home ranges (median area 183 m(2)) and homing behavior consisting of regular back-and-forth movement to a single point. Nomadic movement was also observed in several individuals. Though site fidelity may help maintain aggregations, abalone density decreased, and nearest neighbor distances increased at our site to near-initial levels after 18 mo via a combination of mortality, large movements of a few individuals, and small, incremental movements of most individuals. No coordinated movements that would suggest spawning behavior were observed. Translocation of wild abalone to produce aggregations may not result in high rates of fertilization success that promote population recovery. However, the homing behavior observed may provide more opportunities for mating than expectations based on static measures of density and aggregation state.

3377: *+.050*

High extinction rates and loss of biodiversity is a critical conservation matter. Twenty-two Australian mammal species have become extinct in the last 200 years. Of the 95 mammal species

under the EPBC Act, 51 have recovery plans and 15 of these have captive components. Zoo-based programs as part of a recovery plan can 'buy' time for critically endangered species. In Australia, programs are established as a result of government recovery plans, though more consultation with the zoo industry in initial development phase is needed. Often by the time the decision is taken to remove individuals from the wild for captive breeding, the source populations are fragmented and small. As zoo-based programs become more successful, issues with space limitations arise. This may be rectified with regular release of individuals or avoidance of maintaining post-reproductive and/or non-reproductive animals long-term. Those involved in recovery efforts should make few assumptions on the captive productivity of species, unless the species has been held before. Success of the captive component of a recovery program can be measured by the number of animals bred for release and the level of genetic diversity retained by the program. Although recovery teams are responsible for strategy development, good communication between parties from an early stage is essential. Where a captive component is needed, a more unified strategy, developed early, should provide our native fauna with a realistic chance of recovery. Zoo-based breeding programs are useful in assisting with the preservation of some Australian fauna, whilst for others they will have limited relevance.

3378: ***+.125***

BackgroundUnderstanding the mechanisms by which natural populations cope with environmental stress is paramount to predict their persistence in the face of escalating anthropogenic impacts. Reef-building corals are increasingly exposed to local and global stressors that alter nutritional status causing reduced fitness and mortality, however, these responses can vary considerably across species and populations.Methodology/Principal FindingsWe compare the expression of 22 coral host genes in individuals from an inshore and an offshore reef location using quantitative Reverse Transcription-PCR (qRT-PCR) over the course of 26 days following translocation into a shaded, filtered seawater environment. Declines in lipid content and PSII activity of the algal endosymbionts (Symbiodinium ITS-1 type C2) over the course of the experiment indicated that heterotrophic uptake and photosynthesis were limited, creating nutritional deprivation conditions. Regulation of coral host genes involved in metabolism, CO2 transport and oxidative stress could be detected already after five days, whereas PSII activity took twice as long to respond. Opposing expression trajectories of Tgl, which releases fatty acids from the triacylglycerol storage, and Dgat1, which catalyses the formation of triglycerides, indicate that the decline in lipid content can be attributed, at least in part, by mobilisation of triacylglycerol stores. Corals from the inshore location had initially higher lipid content and showed consistently elevated expression levels of two genes involved in metabolism (aldehyde dehydrogenase) and calcification (carbonic anhydrase).Conclusions/SignificanceCoral host gene expression adjusts rapidly upon change in nutritional conditions, and therefore can serve as an early signature of imminent coral stress. Consistent gene expression differences between populations indicate that corals acclimatize and/or adapt to local environments. Our results set the stage for analysis of these processes in natural coral populations, to better understand the responses of coral communities to global climate change and to develop more efficient management strategies.

3379: ***+.297***

Natural refuges are sometimes supplemented with artificial refuges to enhance populations of endangered species, or to improve the success of translocation and relocation programs. The design and structure of these artificial structures should incorporate key features of natural refuges. We aimed to improve the design of artificial burrows currently used in the conservation of the pygmy bluetongue lizard, Tiliqua adelaidensis, by comparing burrows with or without a basal

chamber. We found that lizards chose burrows with chambers significantly more often, but that neither the size of the chamber, nor the substrate lining the chamber influenced the choice. Incorporating a basal chamber into the design of artificial burrows should provide more favourable artificial refuges for these lizards and should be incorporated into future conservation management programs.

3380: *+.008*

Translocation is a powerful tool in conservation management, but one of the major problems of this tool is dispersal after release. Translocated animals might disperse from prime habitat and face unsuitable habitat and possible increased exposure to predators. This might lead to decline of a translocated population and could compromise the success of translocation. We assessed whether short-term confinement within enclosures at the translocation site can significantly decrease post release movement, if confinement allowed animals to become familiar with the new habitat, and to overcome handling related stress. We simulated the translocation of an Australian lizard, the endangered pygmy bluetongue lizard Tiliqua adelaidensis, into the centre of a large enclosure and compared the behaviour between individuals confined to the central region for one or five days before release. We found that lizards confined for five days spent less time basking, and were more likely to disperse than lizards confined for just one day. We suggest that short-term confinement of lizards induces additional stress and that extra days of short-term confinement will not necessarily improve the success of a translocation. Future research could determine the effectiveness of different durations of confinement for releasing animals at translocation sites.

3381: *+.141*

The Pedder galaxias (Galaxias pedderensis) from Lake Pedder, Tasmania, Australia, is one of the world's most threatened freshwater fish. The flooding of Lake Pedder in 1972 for hydroelectric power generation caused a major change to the ecosystem that initiated an irreversible decline in the Pedder galaxias within its natural range. The flooding inundated another headwater catchment and native and introduced fish from this catchment colonised the impoundment. Numbers of the Pedder galaxias declined markedly as the impoundment matured and as colonising fish proliferated. Surveys in the 1980s confirmed the parlous state of the population, highlighting the need for conservation intervention. Several urgent conservation actions were undertaken to save the species from extinction. Translocation was considered the most important recovery action, given the critically low numbers in the wild. The species is now extinct from its natural range and is known from only two translocated populations. The conservation program, and specifically the translocation recovery action, saved the Pedder galaxias from extinction. The conservation management was extremely challenging since rapidly declining fish numbers needed timely and critical decisions to underpin the future of the fish. Recommendations are provided arising from this case study to guide conservation of freshwater fish in similar circumstances.

3382: *+.162*

Translocation is an increasingly popular conservation management activity worldwide, but the success of translocation is often not measured or reported. A population of the endangered Macquarie perch was imperilled by the damming in 1977 of the Queanbeyan River, near Canberra in south-eastern Australia. In November 1980, 66 adult Macquarie perch (309-389-mm total length) individuals were collected from the newlyformed reservoir, and translocated approximately 4 km upstream into the Queanbeyan River past a waterfall (which prevented access to spawning habitat). Five years of post-translocation monitoring at the release sites resulted in the

capture of only a single individual in late 1981. Consequently, monitoring ceased because the translocation was assumed to have failed. However, subsequent angler reports and a preliminary survey in 1991 confirmed that some translocated fish had survived, and a small recruiting population had established. More intensive follow-up surveys and subsequent monitoring from 1996 to 2006 demonstrated an established population with consistent recruitment until 2001. However, after 2001, there was no evidence of recruitment and the population is now undetectable, with the prolonged 'millennium drought' (1997-2010) being the most plausible cause. The present study demonstrates the potentially ephemeral nature of assessments of success and failure, and the importance of targeted long-term monitoring programs.

3383: *+.096*

The lower reaches of the expansive Murray-Darling Basin, Australia, are a hotspot for freshwater biodiversity. The regional ecosystem, however, has been significantly altered by river regulation, including local and catchment-wide water abstraction. Freshwater fishes have suffered from the resultant altered flow regime, together with other threats including habitat degradation and alien species. Impacts reached a critical point (imminent species extinction) during a prolonged drought (1997-2010) that lead to broad-scale habitat loss and drying of refuges during 2007-2010, and urgent conservation measures were subsequently instigated for five threatened small-bodied fish species. A critical response phase included ad hoc interventions that were later incorporated within a broader, coordinated multi-agency program (i.e. the Drought Action Plan and Critical Fish Habitat projects). On-ground actions included local translocation, alien species control, in situ habitat maintenance (e.g. earthworks, environmental water delivery), fish rescues, artificial refuge establishment and captive breeding. Improved river flows signalled an initial phase of recovery in 2011-2012 that included reintroductions. The present paper aims to document the actions undertaken in the Lower Murray, and review successes and lessons from practical examples that will help guide and inform management responses to conserve fish in modified systems subjected to severe water decline.

3384: *+.077*

Approximately 40% of Australian freshwater fish species are of conservation concern, largely because of the impacts of river regulation, habitat fragmentation and alien fishes. Murray hardyhead is a threatened fish endemic to the southern Murray-Darling Basin in Australia, which has declined significantly in range and abundance since European settlement. Conservation of the species has relied largely on environmental watering of off-channel wetlands where isolated populations persist. This became problematic during recent drought (1997-2010) because of competing demands for limited water, and resentment towards environmental watering programs from communities that themselves were subject to reduced water entitlements. In response, emergency conservation measures prioritised the delivery of environmental water to minimise applied volumes. Captive maintenance programs were established for fish rescued from four genetically distinct conservation units, with varying levels of breeding success. Several translocations of wild and captive-bred fish to surrogate refuge sites were also conducted. Future recovery of the species should secure existing natural and stocked populations and translocate fish to additional appropriate sites to spread risk and reinstate natural pathways for dispersal. The approach to the conservation of Murray hardyhead during extreme environmental conditions provides insights to inform the management of fishes in other drought-prone regions of the world.

3385: *+.113*

Freshwater fish are a highly threatened group and recovery of these threatened species is an increasingly difficult ecological and social challenge. There are many different on-ground recovery actions available to managers, but no synthesis of what, how or why these recovery actions have been deployed. The present paper reviews 428 reported on-ground recovery actions from a survey of practitioners of threatened freshwater-fish recovery in Australia. Recovery actions were grouped into 12 categories, with the most commonly utilised recovery categories being harvest control, translocation, habitat enhancement and stock enhancement. Major drivers of recovery actions were general conservation concern, recovery plans and emergency responses. The number of recovery actions grew significantly in the decade beginning 2000 as the impacts of prolonged drought in south-eastern Australia intensified. In all, 58% of recovery actions occurred in the Murray-Darling Basin, although this region holds only 27% of the 74 listed threatened freshwater fish in Australia. Few or no recovery actions were reported for many species, and few actions occurred in northern or western parts of the country. More than 80% of recovery actions reportedly had some form of monitoring. The diversity of management interventions is reviewed, and patterns and issues are identified to guide future recovery efforts.

3388: ***+.136***

Apium bermejoi is a stoloniferous plant endemic to the island of Menorca (Balearic Islands). It is found only at one locality, and it is listed as Critically Endangered (according to the IUCN criteria). We describe the main results of population restoration actions undertaken under the Recovery Plan for this species, including the following: 1) introduction at two new localities (2008), 2) reinforcement of the original wild and the introduced populations, and 3) a programme for monitoring population dynamics (including both wild and introduced populations) spanning four years (2006-2010). The plant material for the introduction and reinforcement projects was generated from seeds gathered in the wild. We carried out a monthly census of all of the individuals/patches and emerged seedlings, from which we assessed their survival at 3-4months. The survival rates of the planted individuals in the two new localities after three months were found to be 59.0% and 56.3%, and more than 80% of the surviving plants produced fruits. A seasonal pattern was observed based on the minimum cover values recorded in the censuses taken at the end of summer, with an increase detected during autumn, and maximal cover values recorded during May/June. The A. bermejoi populations showed large inter-annual fluctuations in both the number of patches and area of occupancy. The number of seedlings varied across the study years, and their survival was linked to specific meteorological events, such as severe storms and dry and hot spells during autumn. The initial phase of introduction for this species has been overall successful, but a final evaluation can only be made on a long-term basis.

3389: ***+.182***

Conservation translocation of species varies from restoring historic populations to managing the relocation of imperiled species to new locations. We review the literature in three areas-translocation, managed relocation, and conservation decision making-to inform conservation translocation under changing climates. First, climate change increases the potential for conflict over both the efficacy and the acceptability of conservation translocation. The emerging literature on managed relocation highlights this discourse. Second, conservation translocation works in concert with other strategies. The emerging literature in structured decision making provides a framework for prioritizing conservation actions-considering many possible alternatives that are evaluated based on expected benefit, risk, and social-political feasibility. Finally, the translocation literature has historically been primarily concerned with risks associated with the target species. In contrast, the managed relocation literature raises concerns about the ecological risk to the recipient

ecosystem. Engaging in a structured decision process that explicitly focuses on stakeholder engagement, problem definition and specification of goals from the outset will allow creative solutions to be developed and evaluated based on their expected effectiveness.

3390: *-.205*

Many endemic species on islands are vulnerable to predation and local extinction by introduced rats (Rattus spp.). As a result, the reintroduction of species to predator-free sanctuaries is a successful conservation strategy, especially in New Zealand. Nevertheless, reintroduced populations, even those that reach high densities, are still vulnerable to predation in the event of a rat reinvasion, and may also be susceptible to non-target poisoning during a subsequent eradication operation. We quantify for the first time the changes in population size and survival rate of a well-established, reintroduced species (Stewart Island robins, Petroica australis rakiura) following the reinvasion and eradication of Norway rats (Rattus norvegicus) on Ulva Island, New Zealand, in 2011. The robin population declined by nearly one-third (31.5%; 432 to 296 adults) in the breeding season following the rat reinvasion and eradication. The survival rate of robins prior to the poison operation was only slightly lower than expected, which suggests the growing population of Norway rats may have had a relatively minor negative effect on robin survival. In contrast, the majority of the decline occurred immediately following the poison operation. This suggests the robins were susceptible to non-target poisoning from the brodifacoum poison bait, although the robin population would have likely declined even further if Norway rats had not been eradicated. Our results indicate the importance of developing permanent surveillance systems on island sanctuaries to detect and kill rats upon arrival in order to avoid the potentially high rates of non-target poisoning associated with post-invasion, large-scale eradication operations.

3391: *+.059*

Capercaillie historically occurred in the lowland of East Germany once in the so-called pine heathland belt (Kiefernheidegurtel). In the middle of the 19th century the total population of Capercaillies ranged from 550 to 600 animals in the area called Lusatia, which nowadays belongs to the federal state of Brandenburg. All major populations in the south of Brandenburg disappeared before 1950. The remaining population was circularly concentrated around the city of Finsterwalde. The last free ranging Capercaillie in the region of Lower Lusatia was observed in October 1998. The main causes for the population decline in S Brandenburg were the intensification of forestry, habitat deterioration caused by open coal mining and the military use of forests. Within the framework of an official species protection programme, in November 2011 a pilot study for the reintroduction of Capercaillies in the region of Lower Lusatia was initiated. It was planned to release 30 wild Capercaillies captured in Sweden in an area called "Liebenwerdaer Heide" in 2012 and another 30 birds in the area of "Rochauer Heide" in 2013. Within the context of scientific documentation, the progress of the relocation will be analysed over the project period of three years. Some aspects which are to be examined are e.g, analysis of the dispersal of the released birds, habitat use and reproduction activities. In May and October 2012, 28 hens from Sweden were already released in the "Liebenwerdaer Heide". After relocation to the new area, most of the hens went through an orientation phase of 4-6 weeks before they settled for a longer time in a specific area. Some hens moved more than 30 km away from their release points and even changed between neighbouring forestal areas. Here, some hens moved more than 10 km across open habitat. It is confirmed that until March 2013, 12 of the 28 released hens have died. Six hens were killed by predators (four by Northern Goshawk, two by Red Fox or marten), two hens collided with a radio pole and a power line, respectively, and one was killed by a domestic dog in a garden. The causes of death of three hens could not be established. If the current project

study yields results which justify a reintroduction of Capercaillies in the region of Lower Lusatia, translocation should be continued until the re-establishment of a self-sustaining population in Lower Lusatia is achieved.

3392: ***+.095***

In the Kadzidlowo Wildlife Park a new method of release named "Born to,be free" was developed. Using this method a grouse female is put in an aviary located in a natural environment. The aviary has got small openings which enable the young birds to go outside from the very beginning of their live. The first experiments involved Lynx Lynx lynx, and in 2004 this method was used for Black Grouse Tetrao tetrix. The especially accustomed females together with the newly hatched nestlings were carried to heathland and put in small open-work aviaries, from which the chicks could go outside. During the following years the females were put in aviaries on heathland earlier, and so they built their nests there. It is very important for the mother hen to keep vocal contact with nestlings all the time. It was found that the chicks were very active looking for food, and came back to the mother hen for brooding and for the night. They reacted to her warning voice when a flying predator appeared. In 2008 attempts to rear Capercaillies Tetrao urogallus with the "born to be free" method were started using especially accustomed Black Grouse females. The young Capercaillies were kept in the forest until October. They were then caught and used to start a basic herd. It has previously been observed in Black Grouse that it is very important to cultivate a proper behaviour into nestlings, particularly against predators. In 2009, the first young (about two-and-half-month old) Capercallies reared with this method, together with an adult female, were transported to Lowland Silesian forests and were kept according to the scheme developed in Kadzidlowo until the natural release dispersion. The first five young birds (three males and two females) were introduced in such a way, and the mother, after the young had become independent, was carried back to Kadzidlowo. During the following three years release was continued, and 22 of 31 birds were equipped with radio transmitters. Almost all birds (30 out of 31 released) survived the first three months after release. This is a high success rate in comparison to the great losses that usually occur during the initial period when birds are released using traditional methods (captive-rearing). In summary, it seems that the presence of the mother is very important not only during the early period of nestlings' lives, but also later when the birds become independent as they do in nature. In 2012, for the first time seven young Black Grouse were introduced in the Mazurian Lake District by using the same method. All of them were equipped with telemetry GPS/GSM. Six birds survived the first three months.

3393: ***+.121***

Between 1999 and 2003, 145 Western Capercaillies caught in the wild in Russia were translocated to the Schiefergebirge area of Thuringia (Germany). The birds came from the Yaroslavl' and Kostroma areas, c. 260 and 320 km NE Of Moscow. Control of the project's success was carried out by radio telemetry. Between 1999 and 2002 a total of 25 Capercaillies were equipped with transmitters. The establishment of home range sizes was carried out using the Minimum Convex Polygon (MCP) and Kernel methods. Information on 16 birds (seven males and nine females) was used in the radio telemetry data evaluation. Each bird was independently localized at least 30 times and had survived at least two months. With a median home range (MCP) of 436 ha (mean: 844 ha), males had significantly smaller home ranges than females, with a median of 857 ha (mean: 1,008 ha). The median of the maximum distance from the release site was 3,157 m for all birds (mean: 4,407 m). The difference between the sexes was not statistically significant. The survival period was established for 19 males and 14 females. The median survival period of all Capercaillies caught in Russia whose transmitter data could be evaluated (n = 25), combined with

additional ring recoveries (n = 8), was 100 days (mean: 286 days). A comparison of the survival period of these 33 birds with 33 Capercaillies released into the wild from a breeding project in Thuringia showed a highly significant difference. The median survival period of the latter birds was 17 days (mean: 25 days). This comparison proved that birds caught in the wild are far more suitable for supporting a population and in reintroduction projects. From 1999 to 2007, the loss of 21 males and 16 females was registered. 81% of male (17 birds) and 56 % of female losses (nine birds) were caused by Red Fox Vulpes vulpes or Mantes spec. A further 14% of males (three birds) and 31 % of females (five birds) were taken by Goshawks Accipiter gentilis, while the remainder collided with forestry fences (one male and two females). By employing radio telemetry and direct observations, the use by the Russian Capercaillies of traditional structures (leks, wintering centres) that had always been used by the autochthonous population was confirmed. Two new leks were established at traditional sites, where a maximum of three displaying males was observed in the course of eight years. On at least eight different sites territorial behaviour by males was observed during the study period. Alongside radio telemetry, population monitoring was also carried out in selected centres of occurrence, employing both sight records and indirect indications (droppings, feathers, tracks, and sand-bathing places). The maximum total population in the Thuringia Schiefergebirge was estimated to be 7-9 birds in 2012. Successful breeding could be confirmed in only seven cases during the period 2000-2008.

3394: *+.041*

Since 1950, more than 4,800 Capercaillies have been released in the course of 11 release projects in different parts of Germany. Eight projects have been finished, only three ate still in progress (Thuringia, Higher Sauerland and Lower Lusatia). In 2012 a new pilot study (translocation of wild birds) was started in Lower Lusatia, which is continued only after a critical assessment. Five out of ten release projects definitely failed (Harz, Ebbegebirge, Odenwald, Rhon, central Black Forest). In Thuringia and Higher Sauerland the currently very small populations are maintained because of continued releases, hi northern Black Forest the presence of individuals of the autochthonous population masked any effect of the releases. A positve population trend was observed in the Bohemian/Bavarian Forest, but that increase can not without doubt be attributed to the release experiment. The release of birds reared in captivity failed to be a successful method. Those birds suffered from physiological and ethological deficiencies such as insufficient predator detection, insufficient development of organs and muscles, reduced digestive ability (non-typical characteristics of digestive tract and microbial composition within caecum) and abnormal behaviour (e.g. aggressive males and tame birds), leading to very high mortality. Despite enormous efforts the rearing of Capercaillies qualifying for living in the wild proved to be challenging. Unless succeeding in developing appropriate rearing and training methods, which enable to get viable birds regarding digestion and behaviour, further releases of captive-reared Capercaillies appear to be useless and should not be started. Translocation of capercaillie caught in the wild gave much better results. Nevertheless, success can be expected only in large areas of adequate habitat quality supporting a population of at least 100 individuals. Supporting a metapopulation system instead of a single isolated remnant population is recommended.

3395: *+.084*

The whitefish (Coregonus lavaretus (L.)) exists as only one native population in Wales, U.K., where it occurs in Llyn Tegid and is known as the gwyniad. This population is of national conservation importance but faces a number of environmental threats. Although eutrophication has not directly impacted on gwyniad habitat availability, associated low oxygen levels in the hypolimnion periodically approach its tolerance limit of 2 mg L-1. Anthropogenic water level

fluctuations and a 1980 introduction of ruffe (Gymnocephalus cernuus (L.)), a predator of gwyniad eggs, also pose potential threats. Despite these concerns, gill-netting surveys in the 1960s, 1991, 2003 and 2008 have shown no marked changes in gwyniad population biology in terms of length, weight and condition factor. Furthermore, hydroacoustic surveys from 2003 to 2009 have shown an increasing trend in total fish abundance. On the basis of gill-netting data, this increase is shared by gwyniad which in July 2009 reached a population density of 2,302.2 fish ha(-1) (lower and upper 95% confidence limits of 1,795.8 and 2,951.4 fish ha(-1), respectively). Nevertheless, a precautionary approach has been adopted to the conservation of this unique population and from 2005 to 2007 a translocation programme transferred 81,300 eggs from 366 male and 50 female gwyniad to the nearby water body of Llyn Arenig Fawr. Monitored hatching rates were generally high and in 2009 a hydroacoustic and gill-netting survey revealed that although the fish community of this site was sparse (1.7 fish ha(-1), with lower and upper 95% confidence limits of 0.8 and 3.8 fish ha(-1)), it now included adult gwyniad. Although it does not yet confirm successful translocation, this observation does demonstrate that the gwyniad is able to reach sexual maturity in the recipient site.

3396: ***+.226***

For the majority of plant species of conservation concern, seed banking and traditional propagation methods are the most efficient ways of meeting the ex situ and recovery conservation goals of Global Strategy for Plant Conservation (GSPC) Target 8. However, there are estimated to be 5000 or more endangered species for which these methods will not be adequate conservation tools. These "exceptional" species are those with recalcitrant seeds or those that produce few or no seeds. In vitro methods can provide alternative procedures for propagating and preserving germplasm in the long term for these species. Research at the Center for Conservation and Research of Endangered Wildlife (CREW) with several U.S. endangered species has shown the potential of these methods. In vitro propagation can provide plants for reintroduction and research when traditional propagation methods are not adequate. Phytotissue banking can be used for long-term ex situ conservation when seed or embryo banking is not possible. In vitro methods are also needed for recovery when embryo banking of recalcitrant seeds is possible. The full implementation of in vitro methods is constrained by information, scientific, and economic challenges, but the need for its use in meeting the needs of exceptional species should provide impetus for overcoming these challenges and making these methods an integral part of an overall ex situ conservation strategy.

3397: ***+.074***

Freshwater mussels (Unionidae) are among the most endangered groups of organisms in the world. North America, and, particularly, the southeastern United States, are both hotspots of freshwater mussel diversity and areas of special concern due to habitat degradation by changes in land uses (stream alteration and impoundment), impact of exotic species, and loss of host fish necessary for completion of mussel life cycles. The conservation and recovery of freshwater mussel species is now a priority throughout North America, with particular focus in the southeast U.S. Efforts aimed at captive propagation and reintroduction are hindered, however, by significant gaps in our understanding of mussel assemblages, spatial arrangements of populations, naturally occurring sex ratios and male spawning contributions, and genetic variability within wild populations. To begin to fill these gaps, here we utilized an RNA-seq-based approach to develop molecular resources in Villosa lienosa, the little spectaclecase. Sequencing of barcoded, pooled tissue samples within a single lane of Illumina HiSeq followed by assembly using Trans-ABySS generated 778,234 contigs with average length of 707.5 bp from 162 million filtered reads. Data

analysis allowed the identification of 23,742unigene hits against the NCBI nr database, and 36,582 microsatellites with sufficient flanking sequence for primer design. Microsatellite validation indicated a 36% polymorphic rate (16/44 tested markers) in the tested population (26 individuals) with an average of five alleles per marker. Analysis of differentially expressed genes between heat-stressed and untreated controls allowed identification of 604 genes involved in the stress response pathways. Real-time RT-PCR validation of gene expression results utilizing individual samples generally confirmed RNA-seq patterns (correlation coefficient 0.847, p

3398: *+.160*

The paper presents soil conditions of Pulsatilla vernalis in the Polish lowland. There were determined species preferences in relation to physical and chemical properties, grain size composition and the type of soil. It has been found that P. vernalis prefers very poor sandy, acid, dry and permeable soils, with small water capacity. The range of habitat parameters at the lowland sites differs, however, from the conditions at the mountain sites. Due to specific habitats of the species, new ecological indicator values were proposed for the lowland sites of P. vernalis. The results of soil analysis presented in this paper, as well as other data related to biology of the species, should be accounted for in the active protection plan, including the processes of reintroduction and introduction of the species.

3399: *+.265*

The negative effects of introduced nest predators on the breeding success of endemic New Zealand parrots are well documented, as is their role in the general decline of these species. In contrast, little is known about the intrinsic intra-brood dynamics responsible for modulating fledging success in parrots breeding at sites free of introduced nest predators. We studied red-crowned parakeets over two breeding seasons on Tiritiri Matangi, an offshore island free of introduced mammalian predators. We analysed the patterns of hatching and survival, and sex-specific growth of nestlings in relation to clutch initiation date and natural levels of hatching asynchrony. We also explored the relationships of nestling sex and hatching rank on survival. Earlier laid clutches resulted in larger broods which in turn produced more nestlings in better body condition, independent of the sex of the nestlings. Similar to many other bird species with extensive hatching asynchrony, last hatched red-crowned parakeets suffered higher mortality than other hatch ranks. Primary sex ratios and sex ratios at hatching and fledging did not deviate significantly from parity. Our results indicate that in the absence of nest predation by exotic predators, the timing of clutch initiation and brood reduction due to starvation of last hatched nestlings are the most important determinants of nestling survival and growth in red-crowned parakeets. Consequently, from a conservation management perspective, close monitoring of target populations during the breeding season is recommended to estimate nestling survival and nest productivity when planning the timing of capture of wild birds for translocations or harvesting potential breeding stock for captive breeding programmes.

3400: *+.221*

Tiritiri Matangi Island is a Scientific Reserve located in the Hauraki Gulf, New Zealand. In 1986, two years after the start of a ten-year planting programme on the island, members of the Ornithological Society of New Zealand, Auckland, began a monitoring programme of the bird populations. A biannual survey scheme commenced in April 1987, counting birds on predetermined transects and at listening posts. This paper focuses on the spring dataset (November) to provide an overview of changes in relative abundance of birds from 1987 to 2010.

Over this time, a revegetation programme, the successful translocation of 11 native bird species to the island and eradication of kiore (Pacific rat Rattus exulans) have altered the dynamics of the environment. Overall, an increase in indigenous avian biodiversity and abundance was recorded, although the increase was dominated by two species, the tui (Prosthemadera novaeseelandiae) and bellbird (Anthornis melanura). Substantial increases in population abundance were observed in the translocated species recorded in the counts. Exotic species and common forest passerines (fantail Rhipidura fuliginosa, grey warbler Gerygone igata, silvereye Zosterops lateralis) declined. Some of the possible reasons for these changes are discussed.

3401: *+.117*

Tiritiri Matangi Island ('Tiri') in the Hauraki Gulf of the northern North Island of New Zealand was deforested, pastorally farmed, and then farming was abandoned in 1972. This history is typical of many northern New Zealand islands. The island's modern history is less typical; since 1984 it has been the focus of a major restoration project involving thousands of volunteers. No original forest remains, but grazed secondary forest in a few valley bottoms covered about 20% of the island when farming was abandoned. Tin's wild vascular flora was recorded in the 1900s and again in the 1970s. From 2006-2010 we collated all past records, herbarium vouchers, and surveyed the island to produce an updated wild flora. Our results increase the known pre-1978 flora by 31% (adding 121 species, varieties and hybrids). A further six species are listed as known only from the seed rain; 32 species as planted only; and one previous wild record is rejected. These last three decades have seen major changes on the island: the eradication of the exotic seed predator Pacific rat, Rattus exulans, in 1993; the planting of about 280,000 native trees and shrubs during 1984-94 as part of a major restoration project along with a massive increase in human visitation; and the successful translocation of 11 native bird species and three native reptile species. More than two-thirds of the additions to the flora are exotic species, and over half of these are being controlled because of their weedy nature. The 32,000 humans who visit Tiri each year are suspected to be the main vectors of the new exotic plant species added to the flora. The recent planted forest, which covers 64% of the island, has transformed most of the former pasture and bracken fern cover; many of the exotic herbs of open areas are surviving in anthropogenic habitats (mown tracks and lawns); however, 75 species recorded during 1905-1977 appear to have become extinct. We recommend adoption of tighter quarantine requirements, control of more weed species, removal of hybrid ngaio and certain native species, more regular plant surveys, specific rare herb management, and promotion of the Hauraki Gulf threatened flora. We conclude by predicting that over the next 20 years there will be an increase in bird-dispersed seed, increase in seabird guano habitat, few new native tree species, and a continued increase in the proportion of shade-tolerant trees.

3402: *+.144*

One of the quandaries faced by ecological researchers is whether they should continue to invest in ongoing projects or whether they should shift their attention to new species or systems that may have received less attention. While research on Tiritiri Matangi has touched on a wide range of species and topics, the long-term projects on the reintroduced robin population (20 years) and hihi population (17 years) have accounted for the bulk of the published research, with 57 papers featuring these populations published to the end of 2009. This literature has made contributions to several disciplines (wildlife management, population ecology, behavioural ecology, conservation genetics, ornithology and wildlife disease) at both the local and international level. However, most of these published papers use less than 5 years of data, so most of the results made possible from the long-term data sets have yet to be published. We illustrate how long-term monitoring has

allowed us to continually improve our understanding of the dynamics of these populations, and how this has allowed us to interpret and predict the effects of management. This management includes the 1993 poison drop, follow-up translocations to both populations, food provisioning and mite control for hihi, and most importantly, the ongoing harvesting of both populations for reintroduction to other locations. We are now able to detect complex and subtle processes that required many years of data, and present information on the number of years required to obtain various results. These results are now allowing us to develop long-term models integrating factors such as density-dependent regulation, demographic and environmental stochasticity. These models are not only relevant to the long-term viability and management of Tiritiri Matangi populations, but to small populations worldwide.

3403: *+.477*

Translocation has played a key role in modem New Zealand conservation. This is particularly evident on Tiritiri Matangi where 12 species of bird have been translocated between 1974 and 2013. Eleven of these species have successfully established on the island, six as large self-sustaining populations, one large managed population, two small managed populations and two small establishing populations. Several of these populations are sufficiently fecund to sustain harvest for translocation to other sites, with eight species being translocated in >33 translocation events since 1983. Tiritiri Matangi provides a useful case study for the evolution of modem New Zealand conservation. There have also been substantial benefits associated with these translocations for resource managers, scientists and particularly community-based conservation efforts.

3404: *+.297*

Tiritiri Matangi Island is one of the oldest community-driven island restoration projects in New Zealand. While great effort has been directed towards recovery of vegetation and avian communities since the 1980s, restoration of the island's reptile fauna has not been initiated until early 2000s. Tiritiri Matangi supports only three remnant reptile species, which is considerably low given the island's size and geographic location. In recognition of this and the importance of reptiles in ecosystem function, translocations of several reptile species have been undertaken. The translocations presented opportunities for integrating in-depth scientific studies in regard to applied conservation management of native reptiles with experimental approaches. This review summarises research efforts on Tiritiri Matangi to date, including post-graduate studies that have contributed to: (1) baseline information on resident species (Oligosoma moco, O. aeneum, Woodworthia maculata, O. smithi & Naultinus elegans); (2) understanding the importance of seabird co-habitation for Sphenodon punctatus; (3) post-release behaviours (dispersal and habitat selection) of Hoplodaczylus duvaucelii; (4) body colour adaptation of O. smithi following translocation; (5) quantifying avian predation on lizard populations; and (6) measuring the short-term success of all translocations. Numerous research opportunities remain, either on existing populations or future translocations to the island. Emphasis has been placed on the involvement of public and local community volunteers in all reptile research. These groups are key stakeholders in the restoration of Tiritiri Matangi. Measurement of translocation success for New Zealand reptiles is dependent on long-term monitoring (> 10 years) and research, since these endemic reptiles exhibit distinctive characteristics such as slow maturity, low reproductive rates, and very high longevity. The process of restoration of a fully functioning New Zealand ecosystem is similarly slow, therefore, long-term study or monitoring will also enable assessment of the island's restoration outcome over time.

3405: *+.181*

The Southern Ground-Hornbill Bucorvus leadbeateri (SGH) is regarded as Vulnerable globally and Endangered in South Africa as a result of losing close to 70% of its range and 50% of its historic population in the country. One of the conservation tools being used to address this issue and restore the population to its historic range is reintroductions. The aim of this study was to identify and prioritise probable reintroduction sites in the Mopane Bioregion and the Limpopo and Mpumalanga sections of the Lowveld Bioregion of South Africa (all in the Savanna Biome), by using a niche-based modelling technique (Maxent) combined with GIS analyses. Suitable SGH habitat was determined for farms in the study area and evaluated for the absence of mappable threats. Three priority areas were identified for reintroductions. These areas are in a near-natural state, offer sufficient habitat, are free of mappable threats and are close to formally protected areas. Field surveys of these three priority areas are needed next to validate their suitability for reintroduction purposes. This is the first spatially explicit reintroduction plan for the SGH that has been developed and will contribute to conservationists' efforts to conserve the SGH.

3406: *-.038*

Translocation of fishes within and between drainage basins is widely recognised as a threatening process to Australian native fishes. While many translocations are deliberate, for example for fisheries enhancement, it is possible that translocation can occur naturally. In the Wet Tropic region of Australia, the widespread eastern rainbowfish, Melanotaenia splendida, has begun to colonise the Atherton tablelands. This is of particular concern because the area is home to several endangered endemic species such as the Lake Eacham rainbowfish, M. eachamensis, and its allies. It is likely that some of the translocations have occurred through the use of this species as bait, but the recent invasion of Lake Eacham may have occurred naturally via the movement of eggs between nearby streams running into Lake Tinaroo. Here we determine whether rainbowfish eggs could be transported over land by examining their desiccation tolerance. In the first experiment we plucked eggs from spawning media and exposed them to air for varying amounts of time. The results show almost 100% mortality after just 15min. Rainbowfish eggs have a web-like projection that enables them to adhere to aquatic vegetation. In the second experiment, therefore, eggs were exposed to air attached to simulated weed (wet acrylic wool). Around 20% of eggs on the simulated weed where still viable after an hour. The results show that rainbowfish eggs could readily be transported between catchments by aquatic birds or human leisure activities such as kayaking or swimming. The implications for conservation management of Australian freshwater species is discussed.

3407: *-.013*

Wildlife management personnel often transport human food-conditioned (FC) bears (Ursidae) from developed areas (areas with high human-use) to undeveloped areas to reduce the number of bear incidents and property damage in developed areas. Our goal was to determine if American black bears (Ursus americanus) return to developed areas after being transported to undeveloped areas in Yosemite National Park. Using capture records (1992-2011) for 29 bears transported in 2006-08, we determined if FC (n = 20) and not human food-conditioned (NFC; n = 9) bears were equally likely to return to developed areas following transport. We also reported the fate of these transported bears through 2011. We found that FC bears were more likely to return to developed areas than NFC bears. Of the 16 returning bears, 15 were FC (9 juveniles, 6 adults) and one was NFC. The other 8 NFC bears were never reported as entering developed areas, and no NFC bears were reported as killed. By 2011, 65% of FC bears (13 of 20) were euthanized by wildlife

management personnel (n = 10) or harvested near developed areas (n = 3). We recommend that Yosemite National Park discontinue the transport of FC bears and consider removing problem bears from the population.

3408: *+.057*

In the Nordic countries, the wood-living lucanid beetle Ceruchus chrysomelinus is a rare species connected to natural forests. In Sweden, it is red-listed and classified as endangered (EN). In an action plan for its preservation done by the Swedish Environmental Protection Agency, it was suggested that the result of a translocation of C. chusomelinus made in 1995 should be evaluated. The translocation was done by moving some logs containing high numbers of the beetle into an area devoid of the species but with expected suitable habitat. Our aim was thus to study this translocation, and specifically we asked if the establishment has been successful, how much the species have dispersed, which wood qualities the species use and how large supply of suitable wood there is on the site. The study was performed during 2012 in Pansaruddens nature reserve, a spruce dominated forest close to Uppsala in Sweden. Circular study plots were established with a radius of 75 m around the translocated logs in both sites. All lying dead wood trunks >= 10 cm in breast height within the study plots were investigated. Generalized linear models showed that the presence of C. cluysomelinus could be explained by high degree of decay, absence of white rot and by a large size of the log. The species had dispersed, but only very short distances were detected (<10 m from the translocated logs). On average the total amount of lying dead wood for the two study plots were 52 respectively 35 m(3)/hectare. Only 4% of the total dead wood supply was suitable for C. chrysomelinus, when considering the wood qualities found important for the species in this study.

3409: *+.207*

The endangered perennial plant Annamocarya sinensis (Dode) Leroy is a tertiary relict tree restricted to southeastern China and northern Vietnam. To explore endangerment mechanisms, develop protection strategies, and guide reintroduction efforts for this species, we investigated genetic diversity and population structure by surveying 70 individuals from three distinct populations using 12 polymorphic microsatellite markers. We found high genetic diversity for A. sinensis as indicated by high allelic diversity (allelic number = 4.667 +/- 0.436, effective number of alleles = 2.913 +/- 0.249), excess heterozygosity (observed heterozygosity = 0.586 +/- 0.039, expected heterozygosity = 0.582 +/- 0.029), and low fixation index (-0.028 +/- 0.057). Our research revealed low genetic differentiation (F-ST = 0.066 +/- 0.011) and no correlation between genetic distance and geographic distance. Analysis of molecular variance attributed 87% of the variance to differences within the population, whereas 13% was distributed among populations. The protection strategy should aim to protect as many populations as possible. Promoting sexual reproduction among various genotypes and establishing an outcrossing program are advisable for A. sinensis.

3410: *+.286*

European rabbit restocking is one of the most frequent actions in hunting estates and conservation projects in Spain, France and Portugal where rabbit is a keystone species. The aim of this work was to review current knowledge regarding rabbit restocking in accordance with the IUCN (1998) guidelines for re introduction in order to identify gaps in knowledge and highlight the techniques that improve the overall success rate. Eight of 17 items selected from these guidelines were identified as partly studied or unknown, including important items such as the management and

release of captive reared wild rabbits, the development of transport and monitoring programs, the application of vaccine programs, and post release long term studies. Researchers should therefore concentrate their efforts on bridging these knowledge gaps, and wildlife managers should consider all the factors reviewed herein so as to establish accurate management guidelines for subsequent rabbit restocking programs.

3411: *+.150*

The analysis of genetic diversity is routinely used to identify divergent intraspecific units and contribute to the knowledge base of biodiversity. In this study we used mitochondrial genetic diversity to propose three management units (MUs) for the Davy's naked-backed bat (Pteronotus davyi), an insectivorous forest-dwelling species that is distributed in tropical and subtropical areas of America. We analyzed a 555 bp segment of the mitochondrial DNA (mtDNA) control region in 144 individuals from 18 localities spread across the species distribution range in Mexico. Our results demonstrated that the mitochondrial genetic diversity of R davyi is distributed in three MUs, namely Gulf North, Pacific-Veracruz and Southeastern, with conservation priority, due to either the high mitochondrial genetic diversity or the high proportion of unique haplotypes, for the following populations: Playa de Oro, Arroyo del Bellaco and Catemaco in the Pacific-Veracruz region, and Agua Blanca, Sardina, Calakmul, Calcehtok and Kantemo from the Southeastern region. The Gulf North unit shows signs of the recent loss of genetic variability. These proposed conservation units could be considered a generalized model of conservation for other species of cave-dwelling bats that share the same habitats.

3412: *-.066*

Context. Criticisms of wildlife restocking operations typically focus on concerns that translocations can lead to the introduction of pathogens, and risk the integrity of locally adapted genetic diversity. Restocking programs aiming to stabilise population declines of European brown hares using captive-bred individuals have been carried out in several European countries, including Greece.Aims. To assess the potential for imported hares to introduce novel strains of European brown hare syndrome virus (EBHSV) during restocking operations, by (1) inferring the origin of wild Greek hares on the basis of their mitochondrial DNA (mtDNA) haplotype, (2) screening the hares to detect and characterise EBHSV, and (3) determining whether certain hare origin-EBHSV combinations occur in the wild.Methods. RNA extraction, polymerase chain reaction (PCR) amplification, and sequence and phylogenetic analyses of EBHSV were performed on 53 hares. Diagnostic RFLP markers of the mtDNA were used to infer the origins of sampled hares. Key results. Thirty-three hares had 'typical' native Greek haplotypes and 20 had mtDNA haplotypes matching those found in imported and released hares. Twelve of the latter and none of the former were positive for EBHSV. Phylogenetic analysis showed that nine virus isolates formed a single genetic lineage distinct from northern-central European ones. Three virus sequences from three imported reared-and-released hares, from Chalkidiki, were closely related to the northern-central European EBHS viruses.Conclusions. Alien strains of EBHSV are co-introduced with released captive-bred animals, possibly resulting in negative impacts on populations of Greek hares that have not evolved resistance to these novel virus strains.Implications. The identification of these allopatric EBHSV strains has led the authorities to ban captures and transportations of local brown hares for any restocking operation. We consider it imperative to reinforce microbiological and genetic controls before further releases of captive-bred game species in the wild in Greece.

3413: *+.079*

SEVERNS, P. M. (Department of Botany and Plant Pathology, Oregon State University, Corvallis, OR 97331). Precautionary hand pollination suggests outbreeding depression between potential seed donor populations for a rare wetland plant. J. Torrey Bot. Soc. 140: 20-25. 2013.-Pyrrocoma racemosa var. racemosa, is a rare, endemic species found in six remnant Willamette Valley wetland prairie sites in western Oregon, USA. Due to its rarity and association with high-quality wetland habitat, P. r. var. racemosa is a focal species for reintroduction and genetic rescue. However, no genetic studies for this species have been undertaken. I conducted a pollen addition experiment between the two largest P. racemosa populations to determine the effects of selfing, intrapopulation crosses and interpopulation crosses on plant fitness. An ANOVA indicated that the percentage of filled achenes from intrapopulation crosses was significantly greater (approximate to 30% more filled achenes) than both interpopulation crossing and selfing, which did not statistically differ from each other. Pollen viability, inferred through flourescein diacetate staining, did not differ statistically over the time span that it took to gather and perform the pollen addition treatments, suggesting that the diminished seed set from the interpopulation crosses was due to genetic incompatibility. Although the two P. r. var. racemosa populations are only about 6 km apart, the established seed transfer zones for other locally rare plants are considerably larger and if applied would not likely provide an effective genetic safeguard.

3414: *+.436*

Measures to conserve threatened populations of three species of fish in Scotland, Arctic charr Salvelinus alpinus (L.), Powan Coregonus lavaretus (L.) and Vendace Coregonus albula (L.), are described. The methods employed include in situ research, management and legislation, and successful ex situ translocation to new refuge sites.

3415: *-.047*

Management of freshwater fishes in zoos and aquariums for conservation breeding is a key area where much can be achieved with limited facilities and a comparatively modest financial outlay. Some species now survive only in captivity following in situ threats such as habitat loss. Fortunately, short generation length and high fecundity mean that populations of freshwater fishes can be readily maintained in anticipation of ecosystem reinstatement and a reintroduction programme, where conditions are appropriate. However, diseases that occur in captive populations may constrain any species recovery programme. Conversely, any disease not previously found in the wild population should not be present in captive fishes when the animals are reintroduced to the wild. Mycobacteriosis has regularly been identified in episodes of morbidity and mortality in groups of Extinct in the Wild and other threatened freshwater fishes maintained for conservation breeding. It is, therefore, a common impediment to breeding and reintroduction programmes. For any programme to succeed, the issue of disease management needs to be addressed and solutions found to the challenges. An overview of the elements that must be considered when mycobacteriosis is detected in captive populations of threatened freshwater fishes and ways in which these can be managed in aquariums are discussed here.

3417: *+.201*

The ideal conservation planning approach would enable decision-makers to use population viability analysis to assess the effects of management strategies and threats on all species at the landscape level. However, the lack of high-quality data derived from long-term studies, and uncertainty in model parameters and/or structure, often limit the use of population models to only a few species of conservation concern. We used spatially explicit metapopulation models in

conjunction with multi-criteria decision analysis to assess how species-specific threats and management interventions would affect the persistence of African wild dog, black rhino, cheetah, elephant, leopard and lion, under six reserve scenarios, thereby providing the basis for deciding on a best course of conservation action in the South African province of KwaZulu-Natal, which forms the central component of the Maputaland-Pondoland-Albany biodiversity hotspot. Overall, the results suggest that current strategies of managing populations within individual, small, fenced reserves are unlikely to enhance metapopulation persistence should catastrophic events affect populations in the future. Creating larger and better-connected protected areas would ensure that threats can be better mitigated in the future for both African wild dog and leopard, which can disperse naturally, and black rhino, cheetah, elephant, and lion, which are constrained by electric fences but can be managed using translocation. The importance of both size and connectivity should inform endangered megafauna conservation and management, especially in the context of restoration efforts in increasingly human-dominated landscapes.

3418: *+.122*

Gibson PG, Olden JD, O'Neill MO. In review. Beaver dams shift desert fish assemblages toward dominance by non-native species (Verde River, Arizona, USA). Ecology of Freshwater Fishes. PLEASE email J. Olden (olden@uw.edu) if you use this data in your research. AbstractThe reintroduction of beaver (Castor canadensis) into arid and semi-arid rivers is receiving increasing management and conservation attention in recent years, yet very little is known about native vs. non-native fish occupancy in beaver pond habitats. Streams of the American Southwest support a highly endemic, highly endangered native fish fauna and abundant non-native fishes, and here we investigated the hypothesis that beaver ponds in this region may lead to fish assemblages dominated by non-native species. We sampled fish assemblages within beaver ponds and within unimpounded stream reaches in the mainstem and in tributaries of the free-flowing upper Verde River, central Arizona. Non-native fishes consistently outnumbered native species, and this dominance was greater in pond than in stream assemblages. Few native species were recorded within ponds. Multivariate analysis indicated that fish assemblages in beaver ponds were distinct from those in stream reaches, in both mainstem and tributary locations. Individual species driving this distinction included abundant non-native green sunfish (Lepomis cyanellus) and western mosquitofish (Gambusia affinis) in pond sites, and native desert sucker (Catostomus clarkii) in streams. Overall, this study provides the first evidence that, relative to unimpounded stream habitat, beaver ponds in the Verde River basin support abundant small-bodied non-native fishes, which could have negative impacts on co-occurring native fish populations. Copyright: CC BY

3419: *+.038*

After the quasi-extinction of much of the European vertebrate megafauna during the last few centuries, many reintroduction projects seek to restore decimated populations. However, the future of numerous species depends on the management scenarios of metapopulations where the flow of individuals can be critical to ensure their viability. This is the case of the bearded vulture Gypaetus barbatus, an Old World, large body-sized and long-lived scavenger living in mountain ranges. Although persecution in Western Europe restrained it to the Pyrenees, the species is nowadays present in other mountains thanks to reintroduction projects. We examined the movement patterns of pre-adult non-breeding individuals born in the wild population of the Pyrenees (n=9) and in the reintroduced populations of the Alps (n=24) and Andalusia (n=13). Most birds were equipped with GPS-GSM radio transmitters, which allowed accurate determination of individual dispersal patterns. Two estimators were considered: i) step length (i.e., the distance travelled per day by each individual, calculated considering only successive days); and ii) total dispersal distance (i.e.,

the distance travelled between each mean daily location and the point of release). Both dispersal estimators showed a positive relationship with age but were also highly dependent on the source population, birds in Andalusia and Alps moving farther than in Pyrenees. Future research should confirm if differences in dispersal distances are the rule, in which case the dynamics of future populations would be strongly influenced. In summary, our findings highlight that inter-population differences can affect the flow of individuals among patches (a key aspect to ensure the viability of the European metapopulation of the endangered bearded vulture), and thus should be taken into account when planning reintroduction programs. This result also raises questions about whether similar scenarios may occur in other restoration projects of European megafauna.

3420: *+.136*

Reintroduction or reinforcement (RorR) of wild populations is a common conservation strategy. Many conservation projects involve the release of individuals of poorly studied species. This may lead to inefficient results or negative impacts on the conservation efforts. Here, we provide new insights into the conservation implications and potential consequences of a skew in the sex ratio of released birds and of the number of birds supplemented for the demography of a long-lived dimorphic bird species, the Andean condor ( ). We demonstrate that a RorR conservation program may be less effective in conserving a species if the sex ratios of the releases and the recipient populations are not considered. We also show that releases can reduce population declines but only if carried out over long periods (i.e., several decades). This can mean high costs for release programs and the added challenge of maintaining programs over time. If RorR programs are to be implemented, bearing in mind the importance of properly assessing their effectiveness, we urge conservation researchers and managers to consider the implications of sex ratio biases for wild populations, and particularly for dimorphic species with sexually despotic behaviour.Vulturgryphus

3421: *+.074*

Freshwater mussels (Unionidae) are among the most endangered groups of organisms in the world. North America, and, particularly, the southeastern United States, are both hotspots of freshwater mussel diversity and areas of special concern due to habitat degradation by changes in land uses (stream alteration and impoundment), impact of exotic species, and loss of host fish necessary for completion of mussel life cycles. The conservation and recovery of freshwater mussel species is now a priority throughout North America, with particular focus in the southeast U.S. Efforts aimed at captive propagation and reintroduction are hindered, however, by significant gaps in our understanding of mussel assemblages, spatial arrangements of populations, naturally occurring sex ratios and male spawning contributions, and genetic variability within wild populations. To begin to fill these gaps, here we utilized an RNA-seq-based approach to develop molecular resources in Villosa lienosa, the little spectaclecase. Sequencing of barcoded, pooled tissue samples within a single lane of Illumina HiSeq followed by assembly using Trans-ABySS generated 778,234 contigs with average length of 707.5 bp from 162 million filtered reads. Data analysis allowed the identification of 23,742unigene hits against the NCBI nr database, and 36,582 microsatellites with sufficient flanking sequence for primer design. Microsatellite validation indicated a 36% polymorphic rate (16/44 tested markers) in the tested population (26 individuals) with an average of five alleles per marker. Analysis of differentially expressed genes between heat-stressed and untreated controls allowed identification of 604 genes involved in the stress response pathways. Real-time RT-PCR validation of gene expression results utilizing individual samples generally confirmed RNA-seq patterns (correlation coefficient 0.847, p

3422: *+.122*

Habitat selection is an important behavioural process widely studied for its population-level effects. Models of habitat selection are, however, often fit without a mechanistic consideration. Here, we investigated whether patterns in habitat selection result from instinct or learning for a population of grizzly bears (Ursus arctos) in Alberta, Canada. We found that habitat selection and relatedness were positively correlated in female bears during the fall season, with a trend in the spring, but not during any season for males. This suggests that habitat selection is a learned behaviour because males do not participate in parental care: a genetically predetermined behaviour (instinct) would have resulted in habitat selection and relatedness correlations for both sexes. Geographic distance and home range overlap among animals did not alter correlations indicating that dispersal and spatial autocorrelation had little effect on the observed trends. These results suggest that habitat selection in grizzly bears are partly learned from their mothers, which could have implications for the translocation of wildlife to novel environments.

3423: *+.285*

Habitat restoration can play an important role in recovering functioning ecosystems and improving biodiversity. Restoration may be particularly important in improving habitat prior to species reintroductions. We reintroduced seven brown treecreeper (Climacteris picumnus) social groups into two nature reserves in the Australian Capital Territory in south-eastern Australia. This study provided a unique opportunity to understand the interactions between restoration ecology, behavioural ecology and habitat ecology. We examined how experimental restoration treatments (addition of coarse woody debris, variations in ground vegetation cover and nest box installation) influenced the behaviour and microhabitat use of radio-tracked individuals to evaluate the success of restoration treatments. The addition of coarse woody debris benefited the brown treecreeper through increasing the probability of foraging on a log or on the ground. This demonstrated the value of using behaviour as a bio-indicator for restoration success. Based on previous research, we predicted that variations in levels of ground vegetation cover would influence behaviour and substrate use, particularly that brown treecreepers would choose sites with sparse ground cover because this allows better access to food and better vigilance for predators. However, there was little effect of this treatment, which was likely influenced by the limited overall use of the ground layer. There was also little effect of nest boxes on behaviour or substrate use. These results somewhat confound our understanding of the species based on research from extant populations. Our results also have a significant impact regarding using existing knowledge on a species to inform how it will respond to reintroduction and habitat restoration. This study also places great emphasis on the value of applying an experimental framework to ecological restoration, particularly when reintroductions produce unexpected outcomes.

3424: *+.109*

Populations of the Large-flowered Sandwort (Arenaria grandiflora L.) in the Fontainebleau forest (France) have declined rapidly during the last century. Despite the initiation of a protection program in 1991, less than twenty individuals remained by the late 1990s. The low fitness of these last plants, which is likely associated with genetic disorders and inbreeding depression, highlighted the need for the introduction of non-local genetic material to increase genetic diversity and thus restore Fontainebleau populations. Consequently, A. grandiflora was introduced at three distant sites in the Fontainebleau forest in 1999. Each of these populations was composed of an identical mix of individuals of both local and non-local origin that were obtained through in vitro multiplication. After establishment, the population status (number of individuals, diameter of the

plants, and number of flowers) of the introduced populations was monitored. At present, two populations (one of which is much larger than the other) persist, while the third one became extinct in 2004. Analyses of the ecological parameters of the introduction sites indicated that differences in soil pH and moisture might have contributed to the differences in population dynamics. This introduction plan and its outcome attracted interest of local community, with those who supported the plan and regarded its 10-year result as a biological success (i.e., persistent populations were created), but also those who expressed reservations or disapproval of the plan and its outcome. To understand this controversy, a sociological study involving 27 semi-structured interviews was carried out. From these interviews emerged three areas of controversy: alteration of the identity of the plant, alteration of the identity of its territory, and the biological and ethical consequences of the techniques used for the experimental conservation.

3425: *-.002*

The giant panda is a characteristic endangered species in our country. Owing to the influence of human activities, the wild giant panda (Ailuropoda melanoleuca) habitats were severely fragmented resulting in restricted gene interflow between the isolated small populations, which perhaps cannot be reinforced or long-term conserved. For protecting the endangered species such as the giant panda, reintroduction captive-bred is one of the effective measures which applied as guidance for reinforcing and establishing sustaining wild population. Such researches have been reported successfully in the past, which indicated that the restricted gene interflow lowered the survive ability in such small isolated populations owing to the effects of inbreeding and genetic drift. Therefore we should consider the genetic background of the current populations first, for the captive giant panda reintroduction aims to improve gene pool of the wild population. The microsatellite and mitochondrial molecular marker analysis show that the existing wild and captive panda populations are still moderate or moderate above genetic level. Populations have a larger evolution potential which provides genetics support for reintroduction. In addition, a relative lower gene diversity was observed in Daxiangling and Xiaoxiangling population. So it is the key to release individual in these populations primarily. Furthermore, Qinling population should be particularly managed and protected in the future. The rational choice concerning on coefficient of kinship, higher preference with genetic diversity, and adverse selection of deleterious gene were emphasized by the genetic matters of reintroduction. This genetic background plays a significant role in reintroduction decision-making of captive-bred. This paper discusses mainly on the genetic diversity and genetic structure within giant panda population in order to provide reference for the success of eventual reintroduction of giant panda into the wild.

3427: *+.072*

As anthropogenic activity makes deeper incursions into forests, fragmenting habitat, wildlife is forced into closer proximity to humans leading to increased incidences of humanwildlife conflict and wildlife displacement. These same incursions facilitate poaching for the commercial trade in dead and live animals. As a direct result, the number of sanctuaries and internally displaced animals (IDAs) in need of sanctuary placement and rehabilitation are increasing. We focus on internally displaced primates given the prevalence of primate-focused facilities and anthropomorphic considerations surrounding this taxonomic group. Surveys were distributed globally to map the extent and range of native primate sanctuaries and species. Over 70 facilities care for more than 6,000 native primates comprising 64 species, with almost half listed as endangered or critically endangered. As not all sanctuaries were identified at the time of the survey distribution, we estimate that the actual number of facilities is closer to double this number with a captive population in excess of 10,000 individual primates. Native primate sanctuaries hold

significant numbers of primates in long-term captive care, with less than half (37%) identified as candidates for release. The surveyed sanctuary population accounts for 35% of the world's captive primates, as compared to ISIS-registered (where ISIS is International Species Information System) zoological facilities, although we estimate that the actual population is closer to 58%. For some species, the sanctuary population represents the only population in captivity. We discuss the prevalence of range-state sanctuaries and their primate populations, and issues surrounding their future development and management. Am. J. Primatol. 75:116-134, 2013. (c) 2012 Wiley Periodicals, Inc.

3428: *-.027*

Disanthus cercidifolius var. longipes is an endangered shrub endemic to China. Ten natural populations were investigated over the whole distribution area in order to explore its population and resource status, and two ex situ conservation populations were also surveyed in 2009 and 2010. Habitat status of each population and its community characteristics were described in detail based on field investigation. The reasons why this species is endangered were also explored. Results demonstrated that geographic distribution ranges were limited to several parochial areas, resources were very indigent, and populations were highly isolated from each other. Special life history traits of the species (i.e. reproductive biology problems) may impact self-reproduction. Seeds spreading only by elasticity from crazing capsules, together with poor mutual exchange among populations due to geographic isolation, may restrict population self expansion. These difficulties impact its endangered status. External factors such as plant diseases, insect pests, natural disaster and artificial disturbance cannot be ignored. In-situ and ex-situ conservation combined with reintroduction are effective measures for the protection and scientific management of D. cercidifolius var. longipes.

3429: *+.279*

It is essential to find the combination of factors associated with ecosystem invasibility, as this forms part of basic knowledge on biological invasions and provides important information to guide management and conservation decisions. We surveyed 325 sampling sites in Catalonia to investigate relationships between crayfish presence and a series of biotic and abiotic factors, including fish abundance and species richness, geographical features, and water mineralization and eutrophication. Abiotic data provided by 29 environmental variables were studied by principal-components analysis. We then used a combination of three statistical approaches (comparison of average scores, general linear mixed models, and hierarchical partitioning analysis) to determine the potential relationship between crayfish occurrence and predictors. Our findings seem to indicate that the presence of crayfish was associated with geographical features, water mineralization and eutrophication, and the introduction of non-indigenous fish species to Catalonia. Our results also suggest that re-establishment of the natural hydrology of Mediterranean streams could hinder the spread of Procambarus clarkii. This, combined with preservation of headwater streams and attempts at local extirpation of P. clarkii, would favour native species and, potentially, enable the successful reintroduction of the native white-clawed crayfish (Austropotamobius pallipes species complex).

3430: *+.073*

Steppe-like habitats in Europe are seriously threatened as a result of fragmentation and anthropogenic degradation, at least in western and central parts. Considering the dramatic loss of steppe-like habitats, the evaluation of genetic variation in populations of steppe species is of

immediate importance if appropriate conservation measures are to be undertaken. In this paper, we examine the genetic diversity of the highly endangered populations of the leaf-beetle Cheilotoma musciformis, which inhabits only a limited area in south-central Poland, which is geographically isolated from the continuous range of this species. Both mitochondrial and nuclear markers show that the Polish populations are distinct from Slovakian and Ukrainian ones. These regional populations should be considered independent conservation units. On the other hand, very little (mtDNA) or no (nuclear DNA) diversity has been found among the Polish subpopulations. This leads to the conclusion that this species has gone through a strong bottleneck leading to a drastic reduction in its genetic diversity prior to the establishment of present-day populations. Host plants have been identified for this species using barcodes, and the only hosts for the Polish and Ukrainian samples are sainfoins Onobrychis spp. while for the Slovakian sample it is either Dorycnium pentaphyllum or Lotus spp. (all Fabaceae). All of these data can be very valuable for the conservation of C. musciformis populations (e.g. for reintroductions).

3431: *+.063*

Rates of introgression from non-indigenous into native populations are increasing worldwide, often as a result of anthropogenic translocation events. In ungulates translocations have been common, especially among deer. European red deer consists of two distinct lineages, one western and one eastern. These probably originate from different glacial refuges, but it is unknown to what extent they hold different adaptations. Here we address dispersal and introgression into the Norwegian mainland population from an introduced island stock consisting of an admixture of both European lineages. The last decade this stock has grown considerably in number and dispersal could be expected to have increased. We therefore used samples separated by a 5 year interval from Otteroya, adjacent mainland areas and a more distant sub-population. Bayesian assignment analysis verified the genetic structure and identified dispersal between the Otteroya stock and the adjacent mainland coastal areas. Three individuals (two newly sampled) with second or third generation non-indigenous origin were found among the adjacent mainland samples (5 and 3 %, respectively). Two individuals with first and second generation mainland-origin were found on Otteroya (old samples). This suggests some non-indigenous introgression from Otteroya into the mainland Norwegian population.

3432: *+.132*

Swift fox (Vulpes velox) were historically distributed in southwestern South Dakota including the region surrounding Badlands National Park (BNP). The species declined during the mid-1800s, largely due to habitat loss and poisoning targeted at wolves (Canis lupus) and coyotes (Canis latrans). Only a small population of swift foxes near Ardmore, which is located in Fall River County, South Dakota, persisted. In 2003, a reintroduction program was initiated at BNP with swift foxes translocated from Colorado and Wyoming. Foxes released in the years 2003, 2004 and 2005 were translocated from Colorado (BNP-Colorado) whereas in 2006, released foxes were translocated from Wyoming (BNP-Wyoming). Our objective was to evaluate genetic diversity and structure of the restored swift fox population in the area surrounding BNP compared to source fox populations in an area of Colorado and Wyoming, as well as the local swift fox population neighboring BNP near Ardmore in Fall River County, South Dakota. A total of 400 swift foxes (28 released in 2003, 28 released in 2004, 26 released in 2005, 26 released in 2006, 252 wild-born foxes, 40 individual foxes from the Ardmore area of South Dakota) was genotyped using twelve microsatellite loci. We report mean gene diversity values of 0.778 (SD = 0.156) for the BNP-Colorado population, 0.753 (SD = 0.165) for the BNP-Wyoming population, 0.751 (SD = 0.171) for the BNP population, and 0.730 (SD = 0.166) for the Fall River population. We also obtained F-

st values ranging from 0.014 to 0.029 for pair-wise comparisons of fox populations (BNP, Fall River, BNP-Wyoming, BNP-Colorado). We conclude that the reintroduced fox population around BNP has high genetic diversity comparable to its source populations in Colorado and Wyoming. Although genetic diversity indicates that the reintroduction was successful, additional time is necessary to fully evaluate long-term genetic maintenance and interconnectivity among these populations.

3433: *+.059*

The Mohave tui chub (Siphateles bicolor mohavensis) is the only fish native to the Mojave River, California. The fish were displaced by introduced arroyo chubs (Gila orcutti) throughout most of their range, starting in the 1930s. Two potentially relictual populations and two transplanted populations were genetically characterized using 12 microsatellite DNA loci, along with contemporary cyprinid populations in the Mojave River. We found only un-hybridized Mohave tui chubs in the refuge populations, and only un-hybridized arroyo chubs in the Mojave River. The two largest Mohave tui chub populations (Lake Tuendae and China Lake) exhibit similar, comparatively high genetic variation. Another large population (Camp Cady) with low genetic diversity shows the effect of a bottleneck of ten individuals during the historic founding event. The fourth population (MC Spring) has the fewest alleles, lowest heterozygosity, and is the most divergent, suggesting that genetic drift from a persistently low effective population size has reduced genetic diversity since its apparent isolation in 1934. We recommend instituting artificial gene flow to rebuild genetic variation in Camp Cady from both Lake Tuendae and China Lake, and the establishment of new populations with founders from both Lake Tuendae and China Lake. Additionally, we comment on the infeasibility of restoring populations of Mohave tui chub in their historic habitats.

3434: *+.066*

Although the genetic and ecological effects of population declines in endangered species have been well studied, little is known of the social consequences. Changes in signaling behavior may result in disrupted communication and affect both reproductive and conflict-resolution activities. The North Island Kokako (Callaeas wilsoni) is an endangered, duetting (i.e., alternating, coordinated singing by mated pairs) songbird endemic to New Zealand temperate rain forests. Scattered populations (approximately 1500 individuals in 13 surviving and 11 translocated populations) in isolated conservation areas of different sizes have been rescued from extirpation and are currently recovering. We examined key song attributes of the Kokako to assess whether population size or growth rate are related to song complexity, the reduction of which may compromise effective communication. We analyzed song repertoire size and phrase-type sharing (i.e., Jaccard index of similarity), vocal performance (singing rates, song switching rates, and diversity of phrase types), and song syntactical characteristics (i.e., unpredictability in sequences of phrase types) in surviving and translocated populations (populations of approximately 19250 territorial individuals). Population size was positively correlated with a population's song repertoire, song diversity, and switching of song phrase types and negatively correlated with shared phrase types and variation in syntactical structure of songs. Population growth rate correlated positively with pair repertoire size, population repertoire size, and singing rates during song bouts. As for solo-singing species in fragmented landscapes, songs in the fragmented populations of Kokako appear to be undergoing microevolution as occurs in island colonization events. Our results suggest that vocal changes in small populations could affect population establishment and growth, particularly in multiple-source translocations. We believe measurement of vocal behavior could be used as a supplement to periodic population censuses to allow more

frequent monitoring of population size.

3435: *+.135*

Overabundant populations of cervids have induced drastic negative effects on plant communities in several regions worldwide. Antlerless deer harvest by sport hunters has been proposed as a potential solution to overabundance because the philopatric behavior of female deer is expected to limit recolonization of hunted zones. The efficiency of this method, however, has rarely been tested in the wild. Using a large-scale experimental design, we reduced white-tailed deer (Odocoileus virginianus) density within 5 20-km2 areas on Anticosti Island (Quebec, Canada). Our objective was to harvest 50% of antlerless deer in each site during the first year of the study in 2002, and 30% from 2003 to 2006. We monitored deer density, vegetation abundance and growth as well as deer life-history traits during 6 years in these experimental sites and in 5 control sites where harvest rate was 57%. Overall, we achieved 93% of harvest objectives. Contrary to our expectations, however, deer density, vegetation abundance and growth, and deer life-history traits did not vary differently in experimental and control sites during the study period. They rather varied stochastically but synchronously. We discuss several alternative hypotheses that may explain these results, including 1) compensatory mechanisms, 2) biases in density estimates, 3) limited access to territory for hunters, 4) large target areas for localized management, 5) low hunter density, 6) recolonization by surrounding deer, 7) slow plant response under canopy cover, and 8) bottom-up mechanisms. Given the large efforts invested in this study, we conclude that the local control of abundant cervid populations through sport hunting may be difficult to achieve in many natural environments. (C) 2012 The Wildlife Society.

3436: *+.235*

Agricultural intensification has caused a decline in structural elements in European farmland, where natural habitats are increasingly fragmented. The loss of habitat structures has a detrimental effect on biodiversity and affects bat species that depend on vegetation structures for foraging and commuting. We investigated the impact of connectivity and configuration of structural landscape elements on flight activity, species richness and diversity of insectivorous bats and distinguished three bat guilds according to species-specific bioacoustic characteristics. We tested whether bats with shorter-range echolocation were more sensitive to habitat fragmentation than bats with longer-range echolocation. We expected to find different connectivity thresholds for the three guilds and hypothesized that bats prefer linear over patchy landscape elements. Bat activity was quantified using repeated acoustic monitoring in 225 locations at 15 study plots distributed across the Swiss Central Plateau, where connectivity and the shape of landscape elements were determined by spatial analysis (GIS). Spectrograms of bat calls were assigned to species with the software batit by means of image recognition and statistical classification algorithms. Bat activity was significantly higher around landscape elements compared to open control areas. Short- and long-range echolocating bats were more active in well-connected landscapes, but optimal connectivity levels differed between the guilds. Species richness increased significantly with connectivity, while species diversity did not (Shannon's diversity index). Total bat activity was unaffected by the shape of landscape elements. Synthesis and applications. This study highlights the importance of connectivity in farmland landscapes for bats, with shorter-range echolocating bats being particularly sensitive to habitat fragmentation. More structurally diverse landscape elements are likely to reduce population declines of bats and could improve conditions for other declining species, including birds. Activity was highest around optimal values of connectivity, which must be evaluated for the different guilds and spatially targeted for a region's habitat configuration. In a multi-species approach, we recommend the reintroduction of structural

elements to increase habitat heterogeneity should become part of agri-environment schemes.

3437: *-.042*

Growing deterministic and stochastic threats to many wild populations of large vertebrates have focused attention on the significance of captive breeding for conservation. Nanger dama mhorr and Gazella cuvieri, two Sahelo-Saharan species, have declined dramatically since the 1950s, apparently due to excessive hunting and habitat degradation. Today, the earlier is extinct in the wild and the latter survives only in small numbers in a few isolated parts of their range, and captive breeding programmes currently provide an important tool for rearing sustained populations. In natural and captive populations, the largest percentage of mortality is among juveniles. This is most relevant, from an evolutionary perspective, as it has a profound influence on population dynamics and demography, and is pivotal to the conservation and management of captive endangered populations. This study explored the juvenile mortality curve during the first 6 months of life of the species mentioned earlier in captivity. Then, we looked at the causes of this mortality by examining potential mother-dependent as well as offspring-dependent sources of juvenile mortality. The resulting curves show that the critical period of mortality is the first 14 days of life. We also found that parity and longevity of the mother affected juvenile mortality in Mohor gazelles. Calves born to primiparous as well as those born to short-lived mothers were more prone to die than those born to either multiparous or long-lived mothers. In Cuvier's gazelles, juvenile mortality was explained by the interaction between parity and mother's age and litter composition. Neither mother nor offspring inbreeding had any effect on juvenile mortality in either population. Precise knowledge of the biological factors affecting juvenile mortality is increasingly important for the conservation of large mammals, whether they are captive, managed, reintroduced or simply fragmented, as the neonates are the population's potential recruits.

3438: *+.171*

To investigate the extent to which European silver eels Anguilla anguilla, originating from stocking programmes in the Baltic Sea tributaries, effectively contribute to the spawning stock, two hundred and seventy-four formerly stocked A. anguilla. emigrating from the Schwentine River near Kiel, Germany, were tagged with T-Bar anchor tags. A total of 29 Anguilla spp. were recaptured (c.11%) up to 14months after release. Stocking history of recaptured A. anguilla. was confirmed by otolith microchemistry. Recapture locations were concentrated around the outlet of the Baltic Sea (Danish Belt Sea) with 62% of all recaptures reported here or in the Kattegat. Recaptured Anguilla spp. showed a reduction in both LT and mass (mean +/- s.d.=1 center dot 5 +/- 0 center dot 9cm and 125 center dot 3 +/- 50 center dot 1g) while average total fat content remained in the order of values previously reported as high enough to provide energy resources to allow successful completion of the spawning migration (mean +/- s.d.=28 center dot 4 +/- 4 center dot 4%). The documented mean rate of travel (0 center dot 8kmday1), however, indicated a delay in the target-oriented migration that might be interpreted as a delayed initial migration phase of orientation towards the exit of the Baltic Sea.

3439: *+.217*

The butterfly Boloria aquilonaris is a specialist of oligotrophic ecosystems. Population viability analysis predicted the species to be stable in Belgium and to collapse in the Netherlands with reduced host plant quality expected to drive species decline in the latter. We tested this hypothesis by rearing B.aquilonaris caterpillars from Belgian and Dutch sites on host plants (the cranberry, Vaccinium oxycoccos). Dutch plant quality was lower than Belgian one conferring lower

caterpillar growth rate and survival. Reintroduction and/or supplementation may be necessary to ensure the viability of the species in the Netherlands, but some traits may have been selected solely in Dutch caterpillars to cope with gradual changes in host plant quality. To test this hypothesis, the performance of Belgian and Dutch caterpillars fed with plants from both countries were compared. Dutch caterpillars performed well on both plant qualities, whereas Belgian caterpillars could not switch to lower quality plants. This can be considered as an environmentally induced plastic response of caterpillars and/or a local adaptation to plant quality, which precludes the use of Belgian individuals as a unique solution for strengthening Dutch populations. More generally, these results stress that the relevance of local adaptation in selecting source populations for relocation may be as important as restoring habitat quality.

3440: ***+.199***

Many demographic parameters of imperiled fishers (Manes pennanti) in the Pacific Northwest remain poorly understood but are necessary to develop conservation strategies; herein we report on fisher reproduction, recruitment, and dispersal on the Hoopa Valley Indian Reservation, California, to help fill key knowledge gaps. Forty radiocollared, breeding-age females exhibited denning behavior on 80 (87%) of 92 opportunities between 2005 and 2011. Twenty-eight female fishers weaned offspring in 55 (65%) of 85 adequately monitored denning opportunities. Two-year-old female fishers were less likely than older females to den and wean kits. We counted 52, and extracted and marked 51, kits comprising 28 litters of 19 females between 2005 and 2008. Average litter size was 1.9 kits (27 females, 24 males, and 1 unknown) 4-12 weeks postbirth. Mean distances between natal dens and centroids of newly established ranges for 7 juvenile females was 4.0 km (range = 0.8-18.0 km); this distance for 1 male was 1.3 km. The recruitment rate of juveniles that successfully established a home range per adult female was 0.19 (0.16 for females and 0.02 for males). Our results suggest that managers should work toward increasing female survival rates and consider translocations to increase and expand existing fisher populations.

3441: ***+.154***

Translocation of plants and animal populations between environments is one of the major forms of anthropogenic perturbation experienced by pristine populations, and consequently, human-mediated hybridization by stocking practices between wild and exogenous conspecifics is of increasing concern. In this study, we compared the expression of seven candidate genes involved in multifactorial traits and regulatory pathways for growth as a function of level of introgressive hybridization between wild and domestic brook charr to test the null hypothesis of no effect of introgression on wild fish. Our analyses revealed that the expression of two of the genes tested, cytochrome c oxidase VIIa and the growth hormone receptor isoform I, was positively correlated with the level of introgression. We also observed a positive relationship between the extent of introgression and physiological status quantified by the Fulton's condition index. The expression of other genes was influenced by other variables, including year of sampling (reflecting different thermal conditions), sampling method and lake of origin. This is the first demonstration in nature that introgression from stocked populations has an impact on the expression of genes playing a role in important biological functions that may be related with fitness in wild introgressed populations.

3442: ***-.008***

A species of Galapagos tortoise endemic to Espanola Island was reduced to just 12 females and

three males that have been bred in captivity since 1971 and have produced over 1700 offspring now repatriated to the island. Our molecular genetic analyses of juveniles repatriated to and surviving on the island indicate that none of the tortoises sampled in 1994 had hatched on the island versus 3% in 2004 and 24% in 2007, which demonstrates substantial and increasing reproduction in situ once again. This recovery occurred despite the parental population having an estimated effective population size <8 due to a combination of unequal reproductive success of the breeders and nonrandom mating in captivity. These results provide guidelines for adapting breeding regimes in the parental captive population and decreasing inbreeding in the repatriated population. Using simple morphological data scored on the sampled animals, we also show that a strongly heterogeneous distribution of tortoise sizes on Espanola Island observed today is due to a large variance in the number of animals included in yearly repatriation events performed in the last 40years. Our study reveals that, at least in the short run, some endangered species can recover dramatically despite a lack of genetic variation and irregular repatriation efforts.

3443: ***+.195***

Captively reared animals can provide an immediate demographic boost in reintroduction programs, but may also reduce the fitness of colonizing populations. Construction of a fish passage facility at Landsburg Diversion Dam on the Cedar River, WA, USA, provided a unique opportunity to explore this trade-off. We thoroughly sampled adult Chinook salmon (Oncorhynchus tshawytscha) at the onset of colonization (20032009), constructed a pedigree from genotypes at 10 microsatellite loci, and calculated reproductive success (RS) as the total number of returning adult offspring. Hatchery males were consistently but not significantly less productive than naturally spawned males (range in relative RS: 0.700.90), but the pattern for females varied between years. The sex ratio was heavily biased toward males; therefore, inclusion of the hatchery males increased the risk of a genetic fitness cost with little demographic benefit. Measurements of natural selection indicated that larger salmon had higher RS than smaller fish. Fish that arrived early to the spawning grounds tended to be more productive than later fish, although in some years, RS was maximized at intermediate dates. Our results underscore the importance of natural and sexual selection in promoting adaptation during reintroductions.

3444: ***-.110***

As part of a conservation initiative, we released captive-bred individuals of European mink (Mustela lutreola) onto a Baltic island 'sanctuary', Hiiumaa (Estonia), and investigated the development of their diet in the wild. Fifty-four animals out of the 172 released were equipped with radio collars and tracked in 2000-2003 intensively after release. Based on the analysis of the contents of 564 collected scats, we monitored how the diet of released individuals changed after release and how this was affected by habitat and by season. Diet changed as they adapted to the wild: some prey consumed immediately after release were later substituted with prey more typical of wild European mink elsewhere. The mink's tendency to take typical prey increased (crayfish, 3; fish, 1.5; and small mammals, 2 times), while the proportion of atypical prey decreased more than five times in 60 days after release. Once established in the wild, the composition of the diet and its variation between seasons, habitats or weather conditions were similar to that reported elsewhere for wild European mink. There is no indication therefore that the components of the diet provided in captivity persisted in the wild after the adaptation period. We suggest that the adaptation of released carnivores to natural prey merits more attention in reintroduction projects.

3445: ***+.190***

The population of the medicinal plant, Malabar nut (Justicia adhatoda L) is shrinking in Dun valley due to habitat fragmentation, invasion by Lantana camara, over-exploitation, and an ever-increasing human population - the most important being the increasing demand on land for agriculture, industries and the urbanization. Predicting potential geographic distribution of the species is important from species and habitat restoration point of view. This paper reports the results of a study carried out in the Lesser Himalayan foothills in India (Dun valley) on potential distribution modeling for Malabar nut using Maxent model. The Worldclim bioclimatic variables, slope, aspect, elevation, and the land use/land cover (based on IRS LISS-III) data and 46 spatially well-dispersed species occurrence points were used to predict the potential distribution off. adhatoda in ca. 1877 km(2) study area. Jackknife test was used to evaluate the importance of the environmental variables for predictive modeling. Maxent model was highly accurate with a statistically significant AUC value of 92.3. The approach could be promising in predicting the potential distribution of medicinal plant species and thus, can be an effective tool in species restoration and conservation planning. (C) 2012 Elsevier B.V. All rights reserved.

3446: *+.064*

Koalas were introduced into Framlingham Forest, south-west Victoria, in 1971 and the population grew rapidly. By the 1990s the forest was suffering severe defoliation and many trees preferred by Koalas had been over-browsed. In 1998/99 around 1100 Koalas were captured, the males sterilised and animals translocated to other suitable habitats in western Victoria. Some habitat restoration was subsequently undertaken. In 2007 a deliberately lit fire destroyed most eucalypt foliage and many Koalas were killed or burned and removed by wildlife carers and DSE staff. A survey in 2011 found only two Koalas in the area. A Koala management plan for Framlingham Forest has been prepared. (The Victorian Naturalist 130 (1) 2013, 37-40)

3447: *+.213*

Animal restocking is a widely used conservation tool to restore relict populations of endangered species. However, success of such operations is often low due to poor short-term survival and experimental evidence is required to improve restocking results. We tested the impact of different release conditions on survival and reproduction of captive bred common hamsters (Cricetus cricetus L 1758), a highly endangered species in Western Europe. As predation plays a determinant role for released hamsters, especially during the first days after restocking, we performed two release experiments intending to reduce mortality: we tested (1) the efficiency of terrestrial predator proof electric fences and (2) the impact of improved shelter availability. We assessed both survival rate and reproductive success by radiotracking 70 hamsters between release date and the end of their aboveground active period. Reducing contact between released animals and predators thanks to electric fences had a strong positive impact on hamsters' survival and allowed them to have enough time to reproduce. It also appeared that release of hamsters was more efficient in wheat crop than in alfalfa. As expected, wheat harvest, inducing a sudden lack of shelter, negatively impacted restocking success. Finally, lifetime after release affected the number of litters per female and varied with individual characteristics: it decreased with burrow change frequency and was slightly lower for males. We conclude that electric fences associated with permanent well-developed vegetative cover like unharvested wheat seem to be suitable for releasing hamsters. (C) 2012 Elsevier Ltd. All rights reserved.

3448: *+.113*

It is widely accepted that the red fox subspecies of southeastern North America (Vulpes vulpes

fulva) is descended from red foxes imported from Europe to the American colonies for sport hunting. Thus, V. v. fulva is considered an exotic invasive organism that has apparently expanded its range to occupy much of the contiguous United States, with exception of the range of relict native populations in the western mountains and Sacramento Valley. I examined the evidence for importations and found that all claims of introduction stemmed to just two literature sources that were based on vague second-hand information. Together with results of morphological and genetic studies, this information indicates that V. v. fulva is native to North America. Thus, historical shifts in distribution and abundance of V. v. fulva and current management approaches should be re-evaluated in light of native status. Evaluating original sources of information can curtail the proliferation of inaccurate information and conclusions that influence conservation decisions. (c) 2012 Elsevier Ltd. All rights reserved.

3449: ***-.034***

Populations forced through bottlenecks typically lose genetic variation and exhibit inbreeding depression. 'Genetic rescue' techniques that introduce individuals from outbred populations can be highly effective in reversing the deleterious effects of inbreeding, but have limited application for the majority of endangered species, which survive only in a few bottlenecked populations. We tested the effectiveness of using highly inbred populations as donors to rescue two isolated and bottlenecked populations of the South Island robin (Petroica australis). Reciprocal translocations significantly increased heterozygosity and allelic diversity. Increased genetic diversity was accompanied by increased juvenile survival and recruitment, sperm quality, and immunocompetence of hybrid individuals (crosses between the two populations) compared with inbred control individuals (crosses within each population). Our results confirm that the implementation of 'genetic rescue' using bottlenecked populations as donors provides a way of preserving endangered species and restoring their viability when outbred donor populations no longer exist.

3450: ***+.020***

Many amphibians have declined globally due to introduction of the pathogenic fungus Batrachochytrium dendrobatidis (Bd). Hundreds of species, many in well-protected habitats, remain as small populations at risk of extinction. Currently the only proven conservation strategy is to maintain species in captivity to be reintroduced at a later date. However, methods to abate the disease in the wild are urgently needed so that reintroduced and wild animals can survive in the presence of Bd. Vaccination has been widely suggested as a potential strategy to improve survival. We used captive-bred offspring of critically endangered booroolong frogs (Litoria booroolongensis) to test if vaccination in the form of prior infection improves survival following re exposure. We infected frogs with a local Bd isolate, cleared infection after 30 days (d) using itraconazole just prior to the onset of clinical signs, and then re-exposed animals to Bd at 110 d. We found prior exposure had no effect on survival or infection intensities, clearly showing that real infections do not stimulate a protective adaptive immune response in this species. This result supports recent studies suggesting Bd may evade or suppress host immune functions. Our results suggest vaccination is unlikely to be useful in mitigating chytridiomycosis. However, survival of some individuals from all experimental groups indicates existence of protective innate immunity. Understanding and promoting this innate resistance holds potential for enabling species recovery.

3451: ***+.116***

Habitat fragmentation is considered a contributing factor to declining populations of northern

bobwhite (Colinus virginianus). Some population strongholds exist within large expanses of habitat; however, many regions of the species' range have become fragmented and populations therein have become nearly extirpated. Our objectives were to determine whether combined habitat management and bobwhite translocation could restore bobwhite populations in habitat patches within a fragmented landscape. We translocated 550 bobwhites to 2 sites (>= 660 ha; Caldwell and Fayette counties) in the Post Oak Savannah ecoregion of Texas, USA, during 2004-2006. We compared survival, home-range size, and reproduction between translocated bobwhites in a fragmented landscape and resident bobwhites in contiguous habitat (Brooks County). Pooled over the 3-year study, translocated bobwhites had lower survival (6 Apr-15 Aug, 2004-2006; (S) over cap = 0.35; n = 165 bobwhites) than did resident bobwhites ((S) over cap = 0.56; n = 224 bobwhites; P < 0.001). Translocated bobwhites also had larger home ranges (<(x)over bar> = 398.1 ha; n = 55 bobwhites) than resident bobwhites ((x) over bar = 10.9 ha; n = 28 bobwhites; P = 0.003). Moreover, percent of hens nesting (95% CI = 36 +/- 16.4%) and nesting rate (95% CI = 1.1 +/- 0.2 nests/hen) were lower for translocated bobwhites than for resident bobwhites (79 +/- 12.4% and 1.6 +/- 0.3 nests/hen, respectively). Our restoration efforts were unsuccessful; relative abundance of bobwhites remained low (<= 1.0 covey heard/point) on translocation sites despite intensive translocation efforts. Restoring bobwhite populations in areas with few remaining bobwhites may be beyond the realm of practical management in this fragmented ecoregion. 2012 The Wildlife Society

3452: ***+.150***

The goal of this study was to monitor the recovery of the populations of Bukhara deer (BD) within its historical distribution area in Central Asia. Restoration activities were based onscientific knowledge gained from a 30 year study of its ecology, behavior and communication. The research methods included: mapping acoustic communication - recording and acoustic analyses of the vocalizations; visual recording of animals in the field; long-term visual observations of individually recognized animals; recording and mapping of traces. In1989 there were around 900 BD in all groups, with potential for population growth up to 4000 - 5000 animals. The Bukhara deer restoration activities (WWF project) included technical support for the nature reserves still inhabited by the species, anti-poaching activities, reintroduction in suitable sites at the limits of the historical distribution, ecological education, local communities involvement, etc. All restoration activities were accompanied by species monitoring. As a result of this scientifically based approach to species restoration the total Bukhara deer number increased from 350 in 1999 to 1900 in 2011; the efficiency and cost effectiveness of the suggested methods were proven by the results obtained.

3453: ***+.031***

Assisted colonization-the deliberate translocation of species from unsuitable to suitable regions-is a controversial management tool that aims to prevent the extinction of populations that are unable to migrate in response to climate change or to survive in situ. The identification of suitable translocation sites is therefore a pressing issue. Correlative species distribution models, which are based on occurrence data, are of limited use for site selection for species with historically restricted distributions. In contrast, mechanistic species distribution models hold considerable promise in selecting translocation sites. Here we integrate ecoenergetic and hydrological models to assess the longer-term suitability of the current habitat of one of the world's rarest chelonians, the Critically Endangered Western Swamp Tortoise (Psuedemydura umbrina). Our coupled model allows us to understand the interaction between thermal and hydric constraints on the foraging window of tortoises, based on hydrological projections of its current habitat. The process can then

be repeated across a range of future climates to identify regions that would fall within the tortoise's thermodynamic niche. The predictions indicate that climate change will result in reduced hydroperiods for the tortoises. However, under some climate change scenarios, habitat suitability may remain stable or even improve due to increases in the heat budget. We discuss how our predictions can be integrated with energy budget models that can capture the consequences of these biophysical constraints on growth, reproduction and body condition.

3454: *+.257*

Predictive models are frequently used to define the most suitable areas for species protection or reintroduction. Land-cover variables can be used in different ways for distribution modelling. The surface area of a set of land-cover classes is often used, each land-cover presence/absence or the distance to them from any point of the study area can be preferred; multiple types of landcover variables may be combined to produce a single model. This paper assesses whether different approaches to using land-cover variables may lead to different ecological conclusions when interpreted for conservation by focusing on the distribution of the salamader Salamandra salamandra longirostris, an endangered amphibian subspecies in the south of the Iberian Peninsula. Twenty-eight land-cover classes and another 42 environmental variables were used to construct four different models. Three models used a unique type of land-cover variable: either the presence of each class, the surface area of each class or the distance to each class, with all three variable types jointly entered in a fourth model. All models attained acceptable scores according to some criteria (discrimination, descriptive and predictive capacities, classification accuracy and parsimony); however most of the assessment parameters computed indicated a better performance of the models using either the surface area of land classes or the distance to them from every sampled square, compared to the model using class presences. The best scores were obtained with the fourth model, which combined different types of land-cover variables. This model suggested that oak forest fragmentation in favour of herbaceous crops and pastures may have negative effects on the distribution of S. s. longirostris. This was only partially suggested by the first three models, which considered a single type of land-cover variable, demonstrating the importance of considering a multi-variable analysis for conservation planning.

3455: *-.254*

Small populations may suffer more severe pollen limitation and result in Allee effects. Sex ratio may also affect pollination and reproduction success in dioecious species, which is always overlooked when performing conservation and reintroduction tasks. In this study, we investigated whether and how population size and sex ratio affected pollen limitation and reproduction in the endangered Ottelia acuminata, a dioecious submerged species. We established experimental plots with increasing population size and male sex ratio. We observed insect visitation, estimated pollen limitation by hand-pollinations and counted fruit set and seed production per fruit. Fruit set and seed production decreased significantly in small populations due to pollinator scarcity and thus suffered more severe pollen limitation. Although frequently visited, female-biased larger populations also suffered severe pollen limitation due to few effective visits and insufficient pollen availability. Rising male ratio enhanced pollination service and hence reproduction. Unexpectedly, pollinator preferences did not cause reduced reproduction in male-biased populations because of high pollen availability. However, reproductive outputs showed more variability in severe male-biased populations. Our results revealed two component Allee effects in fruit set and seed production, mediated by pollen limitation in O.acuminata. Moreover, reproduction decreased significantly in larger female-biased populations, increasing the risk of an Allee effect.

3456: *-.211*

Both Impatiens glandulifera and Fallopia japonica are highly invasive plant species that have detrimental impacts on native biodiversity in areas where they invade and form dense monocultures. Both species are weakly dependent on arbuscular mycorrhizal fungi (AMF) for their growth and, therefore, under monotypic stands, the AMF network can become depauperate. We evaluated the impact of I. glandulifera and F. japonica on the performance (expressed as shoot biomass) of three UK native species (Plantago lanceolata, Lotus corniculatus and Trifolium pratense) grown in soil collected from under stands of both invasive plants and compared to plants grown in soil from under stands of the corresponding native vegetation. All native species had a higher percentage colonisation of AMF when grown in uninvaded soil compared to the corresponding invaded soil. P. lanceolata and L. corniculatus had a higher biomass when grown in uninvaded soil compared to corresponding invaded soil indicating an indirect impact from the non-native species. However, for T. pratense there was no difference in biomass between soil types related to I. glandulifera, suggesting that the species is more reliant on rhizobial bacteria. We conclude that simply managing invasive populations of non-native species that are weakly, or non-dependent, on AMF is inadequate for habitat restoration as native plant colonisation and establishment may be hindered by the depleted levels of AMF in the soil below invaded monocultures. We suggest that the reintroduction of native plants to promote AMF proliferation should be incorporated into future management plans for habitats degraded by non-native plant species.

3457: *+.177*

Selection of sites for successful restoration of impacted shellfish populations depends on understanding the dispersion capability and habitat requirements of the species involved. In Strangford Lough, Northern Ireland, the horse mussel (Modiolus modiolus) biogenic reefs cover only a fraction of their historical range with the remaining reefs badly damaged and requiring restoration. Previous experimental trials suggest that translocation of horse mussels accelerates reef recovery and has therefore been proposed as a suitable restoration technique. We used a series of coupled hydrodynamic and particle dispersal models to assess larval dispersion from remnant and translocated populations to identify suitable areas for adult live M. modiolus translocation in Strangford Lough, Northern Ireland. A maximum entropy model (MAXENT) was used to identify if dispersing larvae could reach habitat suitable for adult M. modiolus. From these we predicted if translocated mussels will reseed themselves or be able to act as larval sources for nearby reefs. The dispersal models showed that the remnant M. modiolus populations are largely self-recruiting with little connectivity between them. The majority of larvae settled near the sources and movement was largely dependent on the tides and not influenced by wind or waves. Higher reef elevation resulted in larvae being able to disperse further away from the release point. However, larval numbers away from the source population are likely to be too low for successful recruitment There was also little connectivity between the Irish Sea and Strangford Lough as any larvae entering the Lough remained predominantly in the Strangford Narrows. The areas covered by these self-seeding populations are suitable for M. modiolus translocation according to the MAXENT model. As a result of this work and in conjunction with other field work we propose a combination of total protection of all remaining larval sources and small scale translocations onto suitable substrata in each of the identified self-recruiting areas. (C) 2013 Elsevier B.V. All rights reserved.

3458: *+.187*

According to JOrgensen, the definition of reintroductions is crucial to their proper implementation and she highlights a number of ambiguities in existing definitions, particularly associated with the concept of historic range. We could not agree more and have incorporated her suggested term of indigenous range rather than historic range into the current revision of the InternationalUnion for the Conservation of Nature (IUCN) Guidelines for Reintroductions and other Conservation Translocations (in preparation by IUCN Species Survival Commission Reintroduction and Invasive Species Specialist Groups). We also agree with JOrgensen's interpretation that reintroductions are not always necessitated by humans causing the extirpation of species. However, we disagree with other aspects of JOrgensen's argument such as the critique of Seddon, the interpretation of previous IUCN guidance documents, and the recommendation that the conservation community rethink the basic definition of reintroduction rather than moving toward other translocation-based interventions. With regard to the latter point, we emphasize that reintroductions are part of a spectrum of translocations and to focus on reintroductions alone would overlook the fact that introductions beyond a species' indigenous range are being attempted. The new revision of the IUCN guidelines incorporates the whole conservation translocation spectrum and aims to avoid the ambiguities of previous definitions highlighted by JOrgensen.

3459: *+.098*

Wildlife populations have been introduced to new areas by people for centuries, but this human-mediated movement can disrupt natural patterns of genetic structure by altering patterns of gene flow. Insular populations are particularly prone to these influences due to limited opportunities for natural dispersal onto islands. Consequently, understanding how genetic patterns develop in island populations is important, particularly given that islands are frequently havens for protected wildlife. We examined the evolutionary origins and extent of genetic structure within the introduced island population of red squirrels (Sciurus vulgaris) on the Channel Island of Jersey using mitochondrial DNA (mtDNA) control region sequence and nuclear microsatellite genotypes. Our findings reveal two different genetic origins and a genetic architecture reflective of the introductions 120 years ago. Genetic structure is marked within the maternally inherited mtDNA, indicating slow dispersal of female squirrels. However, nuclear markers detected only weak genetic structure, indicating substantially greater male dispersal. Data from both mitochondrial and nuclear markers support historic records that squirrels from England were introduced to the west of the island and those from mainland Europe to the east. Although some level of dispersal and introgression across the island between the two introductions is evident, there has not yet been sufficient gene flow to erase this historic genetic footprint. We also investigated if inbreeding has contributed to high observed levels of disease, but found no association. Genetic footprints of introductions can persist for considerable periods of time and beyond traditional timeframes of wildlife management.

3460: *+.071*

The diet of great apes consists of several hundred plant species. The factors determining diet differences have been examined between populations but not within a population, probably due to the confounding effect of seasonal fluctuations on fruit availability. In Sumatran orangutans (Pongo abelii), fruit availability appears to be sufficiently high year round to have little influence on diet composition, which in turn allows for addressing this question. We examined the diet of eight adult female orangutans at Ketambe, Sumatra, and investigated whether fig and non-fig fruit availability, association time, and/or home range measures influenced dietary overlap between female dyads. Between most pairs, females' diets were different: 16 out of 23 pairs had a significantly low diet species overlap. Only fig diet overlap was influenced (negatively) by the

availability of non-fig fruit. Association time only influenced (positively) fig diet overlap. Hence, orangutans gathered in fig trees when non-fig fruit availability was low. Home range measures did not influence overall diet overlap. To our knowledge, this is the first study showing that, while controlling for confounding factors, individuals with similar energetic requirements, from the same population and sharing the same area, make different dietary choices relatively to their preferred (non-fig) fruit constituting the majority of their diet. Social transmission, with putative matrilineal diet traditions, suitably explains these results. We discuss the implications of the findings for orangutan conservation, namely on reintroduction and the felling of fig trees.

3461: *+.021*

Captive rearing of endangered species for later release is a method used to augment critically small populations, although studies have shown lower survival and fitness for individuals raised in captivity. Since 1992, recovery efforts for the endangered Great Lakes piping plover population have included captive rearing, but released young have lower survival than wild plover chicks. We tested the hypothesis that captive-reared chicks lack the ability to recognize predators by using visual and auditory stimuli. Vocalizations of predators and non-predators were combined with parental alarm calls, predator silhouettes, and a control to test for vigilant responses in captive-reared chicks prior to release. We tested for increases in average percent time spent on vigilant behaviors associated with exposure to predator and non-predator stimuli using linear mixed models. Chicks exhibited the greatest increase in vigilance when a treatment was combined with a parental alarm, regardless of whether the treatment was a predator or non-predator. Compared to white noise, average vigilance increased from 1.7% to 21.9% during non-predator treatments (95% CI: 8.0-34.2%) and to 78.3% during predator treatments (95% CI: 65.7-91.1%), indicating that chicks innately recognize avian predators as threatening. Average vigilance increased by 56.4% during predator treatments compared to non-predator treatments (95% CI: 49.8-64.8%), but individuals did not appear to differentiate between specific predators or non-predators. Our results provide strong evidence that captive-reared chicks innately recognize avian predators, which suggests that decreased post-release survival in captive-reared piping plovers is most likely caused by some other captivity-induced difference or by the lack of a parentally-taught behavior. (c) 2013 Elsevier B.V. All rights reserved.

3462: *-.033*

To better understand the future. spread of chronic wasting disease, we conducted a genetic assessment of mule deer Odocoileus hemionus population structure across the state of Montana, USA. Individual based analyses were used to test for population structure in the absence of a priori designations of population membership across the sampling area. Samples from the states of Wyoming, Colorado and Utah were also included in the analysis to provide a geographic context to the levels of population structure observed within Montana. Results showed that mule deer across our entire study region were characterized by weak isolation by distance and a lack of spatial autocorrelation at distances > 10 km. We found evidence for contemporary male bias in dispersal, with female mule deer exhibiting higher mean individual pairwise genetic distance than males. We tested for potential homogenizing effects of past translocations within Montana, but were unable to detect a genetic signature of these events. Our results indicate high levels of connectivity among mule deer populations in Montana and suggest few, if any, detectable barriers to mule deer gene flow or chronic wasting disease transmission.

3463: *+.238*

Within the context of a limited number of Brown Pelican (Pelecanus occidentalis) breeding sites, promoting new colonies can mitigate localized threats to regional populations. To assess the efficacy of short-distance (similar to 5 km) translocations and use of decoys to establish new colonies, and thereby increase statewide population viability, research was conducted within the Isles Dernieres archipelago, Louisiana. Translocadons of 323 Brown Pelican chicks to an un-colonized island were performed from 2007 to 2009, and from 2008 to 2010, 108 Brown Pelican decoys were deployed on a separate island void of nesting. From 2008 to 2010 band re-sighting surveys detected only one transplanted Brown Pelican chick that returned to the release site. Further, < 1% of translocated individuals were observed throughout the archipelago, compared to 5% and 9% of banded individuals encountered that fledged from nearby islands. Low detection of translocated Brown Pelicans may be due to translocation stress that can result in disorientation and social disorganization, which may promote increased roaming. At sites with decoys, no loafing or nesting Brown Pelicans were observed. Further, behavioral surveys suggest there was no difference in interest of passing Brown Pelicans to decoys compared to paired control survey areas without decoys. Despite past successes of translocations and decoys for establishing new colonies of Brown Pelicans and other waterbird species, Brown Pelican conservation may be best promoted via restoration and protection of current colony sites. Received 6 April 2012, accepted 17 October 2012.

3464: *+.110*

Social discrimination based on geographic variation in territorial signals is taxonomically widespread-most studies have found stronger reactions to local than to foreign signals. In birds with male-only song, this discrimination is thought to result in social exclusion and has been suggested as a behavioral barrier to interpopulation genetic exchange. However, little information exists on duetting species in this context, and nothing is known of how "mixed-dialect" pairs are perceived, despite their confirmed occurrence. We addressed these deficiencies using a duetting, endangered Passerine, the kokako (Callaeas wilsoni). We used reciprocal stereo playback experiments between 2 fragmented populations to present duets from local and foreign dialects (Experiment 1). Additionally, we assessed responses to mixed-dialect pairs by synthesizing duets, a novel technique, to determine which sex contributes the most salient duet components (Experiment 2). Territorial pairs vocally responded to local duets with less delay, and produced more song phrases with a lower diversity, than they did in response to playback of foreign duets. Pairs responded to mixed-dialect duets with equal overall strength regardless of the sex of the local component. Responses to mixed and pure local duets were qualitatively similar. From an evolutionary perspective, this suggests that mixed-dialect pairs can successfully defend territories without social penalties, improving the likelihood of cross-dialect gene flow. These findings have particular importance for conservation efforts like translocation, which often involve individuals from multiple, culturally distinct populations.

3465: *+.068*

The extinctions of keystone megafauna during the Pleistocene and Holocene continue to affect extant species and ecosystems. This is particularly acute in southern and western Madagascar where two now extinct species of giant tortoises were once amongst the most dominant herbivores. The extinct giant tortoises are likely to have influenced ecosystem processes by being effective dispersers of large seeds, keeping the understorey open, cycling nutrients, and indirectly regulating fire regimes. As a result of their extinction, ecological interactions and ecosystems have been altered. Given that there is evidence that the extant Aldabran giant tortoise, Aldabrachelys gigantea, is closely related to at least one of these Madagascan giant tortoises, Aldabrachelys

abrupta, we propose using captive Aldabran giant tortoises to restore missing ecological functions. This ambitious approach will represent the first continental island restoration project with a surviving lineage of now extinct, endemic megafauna. Translocation of this megafauna species could be a pragmatic and cost-effective tool to contribute to halting the ongoing extinction processes in parts of western and southern Madagascar, and would further understanding of the role of these species in pre-human Madagascan ecosystems. (C) 2012 Elsevier Ltd. All rights reserved.

3466: *-.056*

Masked owls, reputedly all of the Tasmanian race (Tyto novaehollandiae castanops) were introduced onto Lord Howe Island (LHI) in the 1920s in an attempt to control the black rat (Rattus rattus). This attempt, however, has been unsuccessful and a co-eradication of the rats and masked owls has been planned to reduce the threat to endemic species and breeding seabirds on the island. As the Tasmanian masked owl is considered endangered, translocation of LHI masked owls to Tasmania has been suggested. Before translocation is considered the ancestry of the LHI masked owl needs to be confirmed, as LHI masked owls are typically smaller and paler than individuals occurring in Tasmania. Here we sequenced three sections of mitochondrial gene regions: cytochrome b, ATP6 and ND3 to assess the provenance of the LHI masked owl and screened a suite of microsatellite loci isolated from the barn owl (Tyto alba) to assess contemporary divergence. Phylogenetic analysis revealed two clades, one exhibited by individuals from LHI and south-eastern mainland Australia and the second by those from Tasmania. Cross species amplification of microsatellite loci was successful, with 18 loci polymorphic. Genotypic data revealed significant sub-structuring between LHI, south-eastern mainland Australia and Tasmania. Data presented here indicate that the south-eastern mainland masked owl was introduced to LHI and subsequently reproduced. The genetic integrity of the LHI masked owl population is therefore questionable and as such LHI individuals may not be suitable for translocation to Tasmania. Crown Copyright (C) 2012 Published by Elsevier Ltd. All rights reserved.

3467: *+.198*

Behavioral plasticity is a strategy employed by many species to cope with both naturally occurring and human-mediated environmental variability. Such plasticity may be especially important for long-lived and wide-ranging species, such as parrots, that likely face great temporal and spatial variation within their long lifespans, and are often disproportionately affected by anthropogenic habitat change. We used radio-telemetry and roost counts to assess ranging patterns, habitat usage, and roosting behaviors of the Yellow-naped Amazon (Amazona auropalliata) at two sites in northern Costa Rica with different degrees of anthropogenic habitat alteration. We compared behaviors for residents at the two sites and for experimentally translocated individuals to test the hypothesis that this species would employ behavioral plasticity in response to habitat differences. We found that individuals in the region with dispersed vegetation recorded ranging movements and communal roosting behavior ten times larger than the region with concentrated vegetation. Translocated individuals showed flexibility in these behaviors and matched the behavioral patterns of resident birds at the release site rather than maintaining behaviors characteristic of their capture site. Our results illustrate a generalized rapid plastic response to human-induced changes in habitat for a number of behavioral traits in the Yellow-naped Amazon. Such plasticity is directly relevant to reintroduction efforts that are commonly employed as a conservation tool in parrots. Our study provides an example of how behavioral plasticity may allow some wild populations to withstand anthropogenic change. (C) 2012 Elsevier Ltd. All rights reserved.

3468: ***+.091***

Creating self-sustaining populations resilient to stochastic events is the goal of conservation reintroductions. Concern about disrupting locally co-adapted gene complexes, outbreeding depression, and hybridization has led to a "local is best paradigm" for source selection, yet this policy constrains rare plant reintroduction efforts and may not always best conserve rare species. Using progeny from controlled crosses (control, selfed, near neighbor, far neighbor and between sites) with maternal plants from two sites, we tested survival and population trajectories of US endangered Jacquemontia reclinata reintroduced in 2004 and 2005 to three sites. By 2011, survival and recruitment was greatest for mixed-population progeny, was consistent across years, and became most apparent after extreme climate events (hurricanes, drought, and exceptional cold). Populations founded from mixed sources exhibited greater resilience to stochastic disturbances than those from a single source and had positive projected population growth at two of three sites. Recipient sites most proximal to maternal origin were not those with best survival. Maximizing reintroduced population persistence calls for re-examining paradigms, using decision trees and reintroduction guidelines to guide source selection choices. The local is best paradigm may be dooming many reintroductions to failure. (C) 2012 Elsevier Ltd. All rights reserved.

3469: ***+.128***

The Eastern Hellbender (Cryptobranchus alleganiensis alleganiensis) is a large, completely aquatic salamander native to the eastern United States. Hellbender populations have experienced numerical declines and range contractions over a large geographic area, but few demographic data are available to allow biologists to diagnose specific causes of the declines. We estimated survival of Hellbenders in Indiana using radio telemetry. We monitored 21 adult eastern Hellbenders during July 2008-October 2009 and documented three mortalities. Using a known-fate model, we estimated annual survival as 0.804 (+/- 0.089 SE). This estimate is lower than expected for a long-lived species and signals the continued decline of Hellbenders in Indiana. Estimates of survivorship such as this provide baseline data for translocation programs and are useful in parameterizing population models.

3470: ***-.029***

Population viability analysis (PVA) is one of the main methods to protect endangered species. After it had been proposed, PVA is mainly used in the prediction of extinction probabilities of endangered species. With the emergence of PVA software, its application has been further extended, and the abuse of PVA has appeared. As a result a discussion about its accuracy has emerged. However, most conservation biologists believe the PVA is significant in the protection of endangered species. At present, PVA primarily apply to the conservation and management of endangered species. We summarized the application of PVA in the making out and assessment of management strategies, the evaluation of reintroduction plans, and the design and management effective assessment of the Nature Reserves. We also discussed the data collection and the selection, establishment and application of models in the use of PVA. Based on the previous studies, we suggested, in the future, the PVA could be more concerned in the use of genetic data, the development of individual-based population models, inbreeding depression and decreasing the threaten of invasive species.

3471: ***-.002***

Reports of male American Martens (Mantes americana) interacting with pre-weaned kits are

limited. During the post-release monitoring of American Martens translocated from Minnesota to northwestern Wisconsin in 2008-2010, we documented a male American Marten without a radio-collar ascending a den tree of a radio-collared female in 2011 and removing two pre-weaned kits. The female's movements immediately became unrestricted after the removal. We also documented two events where an uncollared male American Marten was at the den tree before and after the kit removal. Only female American Martens have been reported to provide care for kits. Visual inspection of the remote camera photographs suggests that all three events likely involved the same uncollared male American Marten. This is the first record of a male American Marten killing pre-weaned kits.

3472: ***+.150***

The introduction of non-native species can pose environmental and economic risks, but under some conditions, introductions can serve conservation or recreational objectives. To minimize risks, introductions should be conducted following the International Union for Conservation of Nature's guidelines and should include an initial assessment and a follow-up. In 1948, to reduce the predation pressure on Pyrenean chamois Rupicapra pyrenaica pyrenaica by golden eagles Aquila chrysaetos, the alpine marmot Marmota marmota was introduced to the Pyrenees in Western Europe. In successive introductions, about 500 marmots were released, but the fate of the released animals and their impacts on the environment remain largely unstudied. The aim of this study was to assess the success of the introduction of the alpine marmot into the Pyrenees, 60 years after the initial release, and the potential impacts of this species on Pyrenean ecosystems. We reviewed what is known about the marmot populations introduced to the Pyrenees and other populations within their native range in the Alps, particularly in terms of population structure and dynamics, habitat use and potential environmental impacts. The alpine marmot is widely distributed and, apparently, well established in the Pyrenees. Population structure and demographic parameters are similar within and outside the historical distribution range of the species, and habitat suitability is one of the main reasons for the species' success in the Pyrenees. Few researchers have investigated the impacts of alpine marmots in the Pyrenees; thus, those impacts have to be inferred from those observed in the species' native range or in other species of marmot. Introduced alpine marmots are likely to impact on Pyrenean grasslands through grazing and burrowing, have the potential to alter Pyrenean food webs and could act as vectors of parasites and disease. Although the introduction of the alpine marmot in the Pyrenees appears to have been successful, more needs to be known about the effects of the established populations on the environment before informed management actions can be taken in the Pyrenees.

3473: ***-.030***

In this study, the level of genetic diversity of captive populations of the itasenpara bitterling (Acheilognathus longipinnis) was assessed to obtain information useful for successful captive breeding and reintroduction; this analysis was performed using mitochondrial DNA (mtDNA) sequence data. Comparison of the captive and wild populations showed low levels of genetic diversity within the captive population and significant genetic differentiation among the captive populations and also between the wild and captive populations, suggesting at chance effect during the founding process for the captive population and a subsequent genetic drift. Therefore, for successful reintroduction, it is important that the reintroduced population reflects all the genetic diversity available from the captive populations, and that releasing a large number of individuals that consist of all captive populations.

3474: ***-.218***

Unionid freshwater mussels need to attach to a host fish for completion of their life cycle. It remains unclear whether the relationship between these mussels and their host fishes can be considered parasitic, mutualistic, or commensal. Herein, we studied the effects of Margaritifera margaritifera infestation on Salmo trutta, the most important host of this endangered mussel species in Central Europe. Glochidial load of host fish increased with increasing glochidial concentration, but the highest ratios of encysted glochidia to exposed glochidia were found at low concentration (15,000 glochidia L-1) during infestation. Host fish mortality occurred at infestation rates of similar to 350 glochidia per g fish weight and was highest (60 %) at the highest infestation rates (similar to 900 glochidia per g fish weight). On a sublethal level, swimming performance of hosts was inversely related to infestation rates, with infestation of similar to 900 glochidia per g fish weight reducing critical swimming speed of S. trutta significantly by similar to 20 % compared to infestation with 6 glochidia per g fish weight. The high mortality and the impaired swimming capability of highly infested hosts indicate a parasitic interaction between M. margaritifera and its host. For conservation and reintroduction of M. margaritifera via glochidia-infested S. trutta, we recommend glochidial loads of 5-100 glochidia per g fish weight, while for artificial breeding of juvenile M. margaritifera under laboratory conditions, higher infestation rates of up to 300 glochidia per g fish weight are ideal to balance high yields of mussels and welfare of host fishes.

3475: *+.014*

In light of widespread declines of houbara bustard Chlamydotis macqueenii populations across its extant range, captive breeding has emerged as a viable option for regenerating viable populations of houbaras in addition to limiting hunting pressure, habitat management and amelioration of predation pressure. Although reintroductions of captive-bred houbaras have been carried out in many regions in recent years, information on differences in ranging behavior and habitat selection between captive-bred and wild-born houbaras is lacking. In this study, we utilized radiotelemetry data spanning 13 years to assess differences in home range use and habitat selection by houbara bustards in the Mahazat as-Sayd reserve in Saudi Arabia. The mean (+/- standard error of the estimate) annual home range size, estimated using the Kernel density method, was 307.76 +/- 15.91km2, and did not differ significantly between genders. Annual home ranges of wild-born houbaras were however larger than those of their captive-born counterparts (wild-born: 423.77 +/- 62.66km2, captive-bred: 299.31 +/- 16.39km2). Rainy season home ranges were the largest (279.29 +/- 27.75km2) followed by winter home ranges (245.79 +/- 19.19km2) and summer home ranges (110.51 +/- 8.91km2) indicating larger-scale movements of houbaras when forage was available. Seasonal home ranges did not differ significantly between wild-born or captive-bred houbaras. Analysis of habitat selection patterns using the distance-based method revealed consistent patterns of habitat preferences across years and seasons and between genders, ages and whether the bird was captive-bred or wild-born. Results indicate that scrub forms the most preferred habitat for houbaras, and should be conserved for the population welfare of the houbara in Saudi Arabia.

3476: *+.124*

Aim Contemporary patterns of mammalian species richness on islands are influenced by well-understood biogeographical variables. Whether or not mammalian orders differ in their rates of persistence, however, remains uncertain. Our aims were to assess the persistence of four mammalian orders on Australo-Papuan continental shelf islands in relation to the faunas within adjacent zoogeographic provinces. We also aimed to define New Guinea's mammalian zoogeographic provinces quantitatively. Location New Guinea and 274 Australo-Papuan

continental shelf islands. Methods We compiled 4194 distributional records for 264 of New Guinea's native mammals. Records were allocated to existing mapped bioregions. We used cluster analysis to allocate bioregions to zoogeographic provinces. Using generalized linear models, we determined the persistence of insular mammals as proportions of the species present within adjacent zoogeographic provinces. Persistence rates were calculated for four major orders (Dasyuromorphia, Diprotodontia, Peramelemorphia and Rodentia). Results The classification dendrogram grouped New Guinea's bioregions into three areas corresponding to the Oceanic, Tumbanan and Austral provinces. In all but two zoogeographic provinces, the proportions of Dasyuromorphia persisting on islands were lower than other orders. Overall, species of Dasyuromorphia were much less likely to persist on Australo-Papuan continental shelf islands. Main conclusions Unlike the other orders considered, dasyuromorphs are carnivorous and insectivorous and require large home ranges relative to body size. We suggest that the resulting low population densities might expose species in this order to higher rates of extinction on islands. Translocations of threatened mammals to predator-free islands are common, but our results suggest that insurance populations of threatened dasyurids on small islands may be less secure than translocations of other taxa. Our results support calls for insurance populations of the rapidly declining dasyurid, the Tasmanian devil (Sarcophilus harrisii), to be established on mainland Australia rather than on islands alone.

3477: *+.036*

Rivers are worldwide highly fragmented due to human impacts. This fragmentation has a negative effect on fish movement and dispersal. Many artificial barriers such as river bed sills and small weirs are nowadays replaced by block ramps in order to reestablish longitudinal connectivity for fish in rivers and streams. We studied the upstream passage of several fish species on different types of block ramps with slopes between 3.6 and 13.4 %. We conducted translocation experiments in the field based on mark-recapture and on the use of PIT-tags. Temporal movement patterns were observed by an instream antenna. Hydraulic and morphological characteristics of block ramps were measured and compared with fish passage efficiency. Our results clearly showed that upstream passage efficiency differs between fish species, size classes and block ramps. We observed that brown trout (Salmo trutta fario) performed better than bullhead (Cottus gobio) and several cyprinid species on the same block ramps. Passage efficiency of brown trout and chub (Leuciscus cephalus) was size-selective, with small-sized individuals being less successful. For brown trout, size-selectivity became more relevant with increasing slope of ramp. We conclude that block ramps with slopes of > 5 % are ineffective for the small-sized cyprinid species and that vertical drops within step-pool ramps can hinder successful upstream passage of bullhead.

3478: *+.006*

Determining whether a conflict between gene trees and species trees represents incomplete lineage sorting (ILS) or hybridization involving native and/or invasive species has implications for reconstructing evolutionary relationships and guiding conservation decisions. Among vertebrates, turtles represent an exceptional case for exploring these issues because of the propensity for even distantly related lineages to hybridize. In this study we investigate a group of freshwater turtles (Trachemys) from a part of its range (the Greater Antilles) where it is purported to have undergone reticulation events from both natural and anthropogenic processes. We sequenced mtDNA for 83 samples, sequenced three nuDNA markers for 45 samples, and cloned 29 polymorphic sequences, to identify species boundaries, hybridization, and intergrade zones for Antillean Trachemys and nearby mainland populations. Initial coalescent analyses of phased nuclear alleles (using *BEAST) recovered a Bayesian species tree that strongly conflicted with the mtDNA phylogeny

and traditional taxonomy, and appeared to be confounded by hybridization. Therefore, we undertook exploratory phylogenetic analyses of mismatched alleles from the "coestimated" gene trees (Heled and Drummond, 2010) in order to identify potential hybrid origins. The geography, morphology, and sampling context of most samples with potential introgressed alleles suggest hybridization over ILS. We identify contact zones between different species on Jamaica (T. decussata x T. terrapen), on Hispaniola (T. decorata x T. stejnegeri), and in Central America (T. emolli x T. venusta). We are unable to determine whether the distribution of T. decussata on Jamaica is natural or the result of prehistoric introduction by Native Americans. This uncertainty means that the conservation status of the Jamaican T. decussata populations and contact zone with T. terrapen are unresolved. Human-mediated dispersal events were more conclusively implicated for the prehistoric translocation of T. stejnegeri between Puerto Rico and Hispaniola, as well as the more recent genetic pollution of native species by an invasive pet turtle native to the USA (T. scripta elegans). Finally, we test the impact of introgressed alleles using the multispecies coalescent in a Bayesian framework and show that studies that do not phase heterozygote sequences of hybrid individuals may recover the correct species tree, but overall support for clades that include hybrid individuals may be reduced. (C) 2013 Elsevier Inc. All rights reserved.

3479: *+.174*

Genetic markers are widely used to define and manage populations of threatened species based on the notion that populations with unique lineages of mtDNA and well-differentiated nuclear marker frequencies should be treated separately. However, a danger of this approach is that genetic uniqueness might be emphasized at the cost of genetic diversity, which is essential for adaptation and is potentially boosted by mixing geographically separate populations. Here, we re-explore the issue of defining management units, focussing on a detailed study of Galaxiella pusilla, a small freshwater fish of national conservation significance in Australia. Using a combination of microsatellite and mitochondrial markers, 51 populations across the species range were surveyed for genetic structure and diversity. We found an inverse relationship between genetic differentiation and genetic diversity, highlighting a long-term risk of deliberate isolation of G.pusilla populations based on protection of unique lineages. Instead, we adopt a method for identifying genetic management units that takes into consideration both uniqueness and genetic variation. This produced a management framework to guide future translocation and re-introduction efforts for G.pusilla, which contrasted to the framework based on a more traditional approach that may overlook important genetic variation in populations.

3480: *-.041*

The introduction of American mink (Neovison vison; hereafter mink) into Europe has had severe impacts on many native wildlife species, including the water vole (Arvicola amphibius) in mainland Britain. Although trapping has been widely used to attempt to control mink, managers have little direct evidence of its effect on mink density or distribution, particularly where immigration of mink from nearby areas is inevitable. Such evidence is needed to justify the use of lethal methods in conservation policy. During 20062010 we removed mink from the River Monnow Catchment in western Britain, using track-recording rafts to monitor continuously for mink presence, guiding a strategic trapping effort. The area monitored and trapped was increased in stages, from a core sub-catchment with 109km of water-course in 2006, to a 421-km2 catchment with 203km of water-course in 2009. In each successive sub-catchment, mink detection and capture rates declined rapidly to near-zero levels after trapping began. Detections and captures showed seasonal peaks in every year corresponding to known dispersal periods, but also declined steadily from year to year, with increasing periods in which we did not detect mink. These results

suggested that each sub-catchment was cleared of mink within a few months, with subsequent captures attributable to immigration. On average, we detected each mink 5.1 times before capture (daily probability of detection=0.059 per mink and raft), and trapped them 3.4 days after deploying traps in response. On average, mink entering the area were likely to have been present for less than 13 days before capture. Water voles had been extinct in the Monnow Catchment since the 1980s. During 20062008 (starting 6 months after mink trapping commenced), we released 700 captive-bred water voles into the treatment area to re-establish a wild population. Persistence of this population through the 4 years of the project was considered indicative of effective mink control. This study demonstrates that, even in a mainland context, a systematic trapping strategy can have a substantial impact on the density and distribution of a damaging species, in this case allowing the restoration of a native prey species. (c) 2013 The Wildlife Society

3481: *-.042*

The aim of this study was to evaluate the efficiency of releasing commercially reared individuals of the grey partridge (Perdix perdix) as a method for species recovery. This study compares the mortality risk of grey partridges depending on origin, sex and body condition, controlling for age, infection risk and release method. In total, 110 locally caught wild individuals and 75 commercially reared game-farm partridges were released and radio tracked within the same study area in the Czech Republic between 2009 and 2011. To exclude a possible effect of age on survival, only individuals in the second calendar year of life were chosen for the analysis. Commercially reared partridges had significantly higher mortality risk than wild ones. None of the commercially reared birds survived in the wild until the end of the nesting period, and none produced a fledged brood. Females from game farms showed significantly better survival than males and preferred to mate with wild males, whereas wild females avoided mating with commercially reared males. Predation was the main cause of mortality, and proportion of birds killed by raptors and mammals did not differ between wild and commercially reared birds. These results highlight the uselessness of releasing adult commercially reared partridges in an effort to establish viable populations of this species in the wild and stress the need for a change from intensive rearing methods aimed predominantly at quantity towards a more conservation breeding-oriented approach aimed at quality.

3482: *+.089*

Aim Seed transfer zones are geographically defined areas in which the interpopulation mixing of plant propagules is presumed to be genetically beneficial and unlikely to result in outbreeding depression. Crossing between individuals that differ in ploidy (the number of whole genome duplications) commonly produces sterile or mostly sterile progeny, but the landscape distribution and occurrence of polyploids is poorly known for rare plants. Seed transfer zones could provide adequate protection for rare plants with unrecognized ploidy variation provided that the distribution of ploidal variants coincides with seed zone delineations. We studied the range-wide distribution of polyploids in a threatened legume to determine whether U.S. Fish and Wildlife Service endorsed seed transfer zones have adequate protection from inadvertent mixing of individuals with non-matching ploidy. Location Pacific Northwest, USA. Methods We used genotyping with nuclear DNA simple sequence repeats and flow cytometry to screen for the presence of polyploids over the known range of Lupinus oreganus (Fabaceae). Results Genotyping (895 plants) and flow cytometry (198 plants) from 34 populations revealed that mixed-ploidy and polyploid populations were not limited to the margins of the species range. Both mixed-ploidy and polyploid populations occurred within every seed transfer zone and the nearest population (within each zone) appeared to be composed entirely of diploid individuals. Main conclusions Lupinus

oreganus seed recovery zones, assumed to be genetically safe' areas to transfer seed among populations, would not prevent the mixing of incompatible ploidal lineages. For L.oreganus, and other rare plant species with undocumented differences in whole genome duplication, interpopulation mixing without precautionary genetic screening could compromise the success of conservation projects involving interpopulation seed transfer. We recommend screening rare plant populations for variation in whole genome duplication prior to the implementation of interpopulation seed mixing projects.

3483: *+.163*

Preserving allelic diversity is important because it provides the capacity for adaptation and thus enables long-term population viability. Allele retention is difficult to predict in animals with overlapping generations, so we used a new computer model to simulate retention of rare alleles in small populations of 3 species with contrasting life-history traits: North Island Brown Kiwi (Apteryx mantelli; monogamous, long-lived), North Island Robins (Petroica longipes; monogamous, short-lived), and red deer (Cervus elaphus; polygynous, moderate lifespan). We simulated closed populations under various demographic scenarios and assessed the amounts of artificial immigration needed to achieve a goal of retaining 90% of selectively neutral rare alleles (frequency in the source population = 0.05) after 10 generations. The number of immigrants per generation required to meet the genetic goal ranged from 11 to 30, and there were key similarities and differences among species. None of the species met the genetic goal without immigration, and red deer lost the most allelic diversity due to reproductive skew among polygynous males. However, red deer required only a moderate rate of immigration relative to the other species to meet the genetic goal because nonterritorial breeders had a high turnover. Conversely, North Island Brown Kiwi needed the most immigration because the long lifespan of locally produced territorial breeders prevented a large proportion of immigrants from recruiting. In all species, the amount of immigration needed generally decreased with an increase in carrying capacity, survival, or reproductive output and increased as individual variation in reproductive success increased, indicating the importance of accurately quantifying these parameters to predict the effects of management. Overall, retaining rare alleles in a small, isolated population requires substantial investment of management effort. Use of simulations to explore strategies optimized for the populations in question will help maximize the value of this effort.

3484: *-.032*

Given the difficulties in establishing population parameters of elusive animals in the wild by traditional methods, such as trapping, much attention has been given in recent years to non-invasive genetic sampling. Our work compared estimates of population size and sex ratio derived from genetic sampling with the known number and sex of animals released during an otter reintroduction and reports on the pitfalls and opportunities that may be encountered in studies of this kind. This study makes use of 121 samples of otter spraints (faeces) collected over 7 months during a reintroduction in the Upper Thames (UK) where a total of 17 otters was released in two consecutive phases. Spraints were processed with a multiple tubes approach and seven microsatellites were used. Of all collected samples, 19 % were complete for at least five loci, the minimum required for discrimination between individuals. Six out of nine of the otters that were released in the first phase were detected, four males and two females, while none of the otters released in the second phase was detected probably due to a combination of sampling pitfalls and otter behaviour. In particular, the specific sex (mostly females) and dominance composition (lower) of the individuals in the second release group may explain our failure to detect individuals in this group. Taken together, our results add further evidence that genetic sampling approaches

represent a potentially accurate and non-invasive route to census populations of otters but that the sampling design should take into account factors like the sex ratio and dominance composition of the population in order to maximise detection and minimise error.

3485: ***+.224***

Population and conservation genetics of two freshwater fish species, Notropis heterodon and Notropis heterolepis, were evaluated in north-eastern Illinois, U.S.A., where both species have severely declined. Fishes were sampled from two remnant populations occurring in small glacial lakes (source samples) and from two man-made ponds that had been stocked with fishes from those same lakes (sanctuary samples). The goal was to obtain information that would help inform conservation programme planning to reintroduce sanctuary fishes to areas where both species are extirpated. Microsatellite data showed that the two species were genetically quite distinct and there was no evidence of hybridization in either source or sanctuary samples. Within each species, source and sanctuary samples had moderate levels of heterozygosity and were not significantly different from each other. Many alleles observed in the source samples, however, were not detected in the sanctuary samples, indicating that translocation had resulted in reduced allelic diversity of the sanctuary samples. Sibship analysis indicated that full and half sibs occurred within source-lake samples, thus reducing the effective population size of the reintroduced stock. Taken together, these results suggest that source-lake stocks rather than sanctuary stocks are more appropriate for future reintroductions of both species in their native range, unless sanctuary populations can be established with hundreds of fishes. Also, fishes should be harvested from multiple locations in source lakes to avoid over-representation of family groups. (C) 2013 The Authors Journal of Fish Biology (C) 2013 The Fisheries Society of the British Isles

3486: ***+.141***

Climate change is causing spatio-temporal shifts in environmental conditions, and species that are not able to track suitable environments may face increased risks of extinction. Assisted colonization, a form of translocation, has been proposed as a tool to help species survive the impacts of climate change. Unfortunately, translocations generally have a low success rate, a well-documented fact that is not considered in most of the recent literature on assisted colonization. One of the main impediments to translocation success is inadequate planning. In this review, we argue that by using well-known analytical tools such as species distribution models and population dynamics modelling we can maximize the success of assisted colonization. In particular, we present guidelines as to which questions should be investigated when planning assisted colonization and suggest methods for answering them. Finally, we also highlight further implementation and research issues that remain to be solved for assisted colonizations to be efficient climate change adaptation tools.

3487: ***+.103***

The plant Dianthus morisianus Vals. (Caryophyllaceae) is endemic to Sardinia. The Autonomous Region of Sardinia funded a conservation project for this species because it is one of the most threatened plant on the island. The project comprises in situ and ex situ research and experimental projects, such as the construction of protective fences and reintroduction. Juvenile plants, germinated from 200 seeds collected over 2 years and propagated without horticultural treatment, were reintroduced in November 2010. The surviving 113 plants were reintroduced 150 m from the natural population and were monitored monthly. Two years later the survival rate was > 95%, and the fruit yield per plant was higher than that recorded in the natural population. This research

emphasizes the importance of identifying an appropriate microhabitat for plant reintroduction. The use of juvenile plants aided the success of the reintroduction and reduced the mortality rate; the knowledge of the species biology, in particular the critical stage of their life cycle, is a crucial factor in plant reintroduction.

3488: *+.139*

Management interventions in small (<30 000 ha) to medium-sized (<100 000 ha) fenced reserves require careful planning to minimize alterations to predator-prey dynamics. However, when interventions result in severe changes to predator prey dynamics, learning from these outcomes can assist in informing future interventions. In small to medium-sized fenced reserves both predator and prey can adjust their behaviour rapidly following interventions. We used lion (Panthera leo) and buffalo (Syncerus caffer) interventions (lion introduction and buffalo translocation) in the Addo Elephant National Park to investigate the response of the buffalo-lion dynamic in the park. Responses did not conform to our expectations for either lions or buffalo. Rather than dominate the remaining solitary male lion, the introduced coalition split, with one of the introduced male lions (known to hunt buffalo successfully where it originated) joining with the remaining solitary male. Following fragmentation as a result of the buffalo translocation, previously large buffalo herds failed to re-form. These two outcomes resulted in a significant increase in buffalo predation primarily by the newly formed coalition of males, with the majority of buffalo killed originating from splintered herds rather than male-dominated groups as is conventionally found in large ecosystems. Ultimately, management actions can enable us to learn by monitoring the consequences of interventions, allowing us to implement what we have learned in an adaptive management framework.

3489: *-.114*

Climate change and invasive species are two major biodiversity threats expected to provoke extinctions of many species in the future. This study evaluates the joint threat posed by climate change and two invasive species: the zebra mussel (Dreissena polymorpha) and the signal crayfish (Pacifastacus leniusculus), on the distribution of two endangered freshwater species: the depressed river mussel (Pseudanodonta complanata) and the white-clawed crayfish (Austropotamobius pallipes), at the scale of Europe. We expected the native species to experience a gradual contraction over time in their geographic range size, while the invasive species would maintain or increase their spread; therefore, their overlap would increase, further threatening the conservation of the native species. To test these three hypotheses, ensemble species distribution models (SDMs) were calibrated with current distributions and projected onto present and 2050 future climatic scenarios. In agreement with our expectations, the 2050 scenarios suggested D. polymorpha may strongly benefit from climate changes (increase of 15-20% in range size), while the depressed river mussel would experience a considerable loss (14-36%), the overlap between both mussels increasing up to 24%. Although both crayfishes were predicted to be negatively affected by climate changes, the contraction was more severe for the invasive P. leniusculus (up to 32% decrease in range size). Moreover, the overlap between both crayfishes decreased by 13-16%, which may reduce the pressure upon the native A. pallipes. This study illustrates how SDMs can assist in management of endangered species over large spatial and temporal scales by identifying current and future areas of shared bioclimatic suitability and potential refugia. (C) 2013 Elsevier Ltd. All rights reserved.

3490: *+.135*

For any reintroduction it is important to maximise the probability of released individuals establishing in the target area (settling and surviving to breed). Factors influencing establishment have typically been studied at single sites, making it impossible to assess factors that vary at the site level (e.g. connectivity) or quantify unpredictable variation among sites. Using data from 14 reintroductions of the North Island robin (Petroica longipes) to native forest reserves, we show how Bayesian modelling can be used to identify general drivers of establishment and to account for site-to-site variation when making predictions for new sites. High landscape connectivity and high rat tracking rates (a density index) at reintroduction sites were key factors associated with lower individual establishment probabilities. Habitat similarity between source and release sites was also important, as robins sourced from native forest had higher establishment than those from exotic pine forest. Previous predator experience appeared to affect establishment in sites with mammalian predators, as founders sourced from sites with these predators had higher establishment than those from other sites. Our approach can be applied to a wide range of species that are being reintroduced to multiple sites, providing guidance on source and release site selection, efficacy of management interventions, and the numbers of individuals to release to achieve desired initial population sizes. The results are not only applicable to these particular species, but can be used to predict site suitability for reintroductions of species with similar dispersal behaviour or other ecological characteristics. (C) 2013 Elsevier Ltd. All rights reserved.

3491: ***+.090***

Federally threatened Scutellaria montana (Large-flowered Skullcap) is a perennial herbaceous species endemic to southeastern Tennessee and northwestern Georgia. A large population of S. montana is located at the 648-ha Tennessee Army National Guard Volunteer Training Site (VTS) in Catoosa County, GA. Due to necessary operational activities that include vegetation clearing along site boundaries to maintain security and prescribed burning and overstory clearing to reduce fuel loads as a wildfire-prevention measure, S. montana individuals and groups at the VTS may be disturbed unavoidably at times. Our objectives were to provide recommendations for land managers at the VTS and elsewhere regarding the response of S. montana to transplantation when plant rescue is necessary and to guide site selection for transplantation by elucidating the effects of pre-transplantation burning and canopy thinning on transplant survival and subsequent success. We relocated 100 S. montana individuals in spring 2010 from a site scheduled for clearing to plots that were burned (B), thinned (T), treated with a combination of burning and thinning (B+T), or not treated (C; control). Survival, growth, reproductive potential, development, and physiological measurements were used throughout the 2010 and 2011 growing seasons to evaluate the success of transplantation overall and in various relocation plots. At one year post-transplantation, 91% of the original transplants had survived relocation, and among all transplants, mean stem height and the numbers of stems, leaves, and flowers per individual significantly increased. Additionally, the percentage of total transplants that were juveniles was much lower one year post-transplantation than immediately following transplantation (5.7% vs. 27%), while the proportion that were reproductive adults was greater one year post-transplantation (37.5% vs. 22%). However, reduced survival was found in the canopy-thinned plots (84% in both plot T and plot B+T) compared to plot B (100%) and plot C (96%) one year post-transplantation. The main effects of both burning and thinning included significant increases in stem damage and in the proportion of transplants that were vegetative adults, with an associated decrease in the proportion of reproductive adults. Combined, these findings may have resulted from increased trampling and feeding activity of vertebrate herbivores in burned and thinned plots. Overall, we considered our transplantation efforts to be successful due to high survivability and continued growth and development of individuals one year post-transplantation. However, to maximize the success of S. montana relocation, we suggest that transplants be relocated into unburned, unthinned forests and that

vertebrate herbivory be subsequently controlled though the use of exclosures.

3492: ***-.005***

Populations of Neotoma floridana (Eastern Woodrat) are decreasing in parts of their geographic range in the southeastern United States, and the species is state-endangered in Illinois. Once found throughout the Shawnee Hills region of southern Illinois, woodrats were restricted to four known populations in Jackson and Union counties by the late 1980s. We used reintroductions to establish viable populations of Eastern Woodrats at previously occupied sites in Illinois. From April 2003 through March 2009, we released 422 Eastern Woodrats live trapped in Arkansas and Missouri into 5 historically occupied sites in southeastern Illinois. Recapture rate 1 month after release was 12.5%. The continued presence of woodrats at release sites, reproduction, and wide dispersal beyond reintroduction sites all suggest preliminary success of the reintroduction of this r-selected species.

3493: ***-.008***

The Socorro dove Zenaida graysoni, endemic to Socorro Island, was last reported in the wild in 1972. Fortunately, the species has been propagated in zoos in Europe and the United States, and plans are under way to re-introduce it to its native habitat. This will be the first known attempt to return a bird species extinct in the wild to its ancestral island. In order to assess the disease threats the Socorro dove may face, the avifauna of Socorro Island, with a specific focus on Socorro ground doves Columbina passerina socorroensis and mourning doves Zenaida macroura, as well as Socorro doves in captivity, were screened for blood parasites of the genera Plasmodium, Haemoproteus, Leucocytozoon, and Trypanosoma spp. We found Haemoproteus spp. in 17 (74%) of 23 Socorro ground doves, 23 (92%) of 25 mourning doves, and 3 (14%) of 21 northern mockingbirds; none of the other bird species showed infections. Here, we report the phylogenetic analysis of 19 distinct lineages of Haemoproteus spp. detected in birds of Socorro Island and compare their evolutionary relationships to parasites detected in the avifauna of the Galapagos Islands, continental Latin America, and Europe. Microscopic examination revealed 1 mourning dove infected with Plasmodium (Haemamoeba), thus underscoring the importance of using both PCR and microscopy when analyzing avian blood samples for hemosporidian parasites. The study confirms that the Socorro dove will most likely be exposed to Haemoproteus spp. that currently infect mourning doves and Socorro ground doves of Socorro Island. A monitoring program for both birds and vectors should be implemented to establish the prevalence of Plasmodium sp. and as a necessary conservation measure for critically endangered birds on the island.

3494: ***-.266***

I studied a Wood Turtle (Glyptemys insculpta) population in Vermont for 25 y and I monitored a subset of the turtles using radio-telemetry. Adults comprised 95% of the individuals I captured annually. Females ranged further from the stream than did males, and remained in the uplands for as long as 54 days. Females traveled up to 1.6 km to nest and males patrolled as much as 1.2 km of the stream. Turtles often showed fidelity to hibernacula, but moved to new locations if protective structures in the stream were lost. They also returned to upland sites. Wood Turtles often used cover on land and moved to cover when they detected my presence. Overall, 58.5% of adult turtles suffered predator injury. When combined with mechanical damage, the injury rate was 68.3%. Males were more likely than females to be missing feet (43.5% vs. 5.5%), but suffered tail loss less frequently (21.7% vs. 66.6%) than females. None of the juveniles I encountered had injuries to the limbs or tail. I documented the death of four adults during the study period. One

female was killed by a vehicle strike and another female appeared to have drowned. One male was killed by a Fisher (Martes pennanti) and I suspect that a second male was also killed by a Fisher.

3495: ***+.236***

Exploited for food, traditional medicine, and pets, many turtle populations have been over-harvested or even extirpated from historic ranges. Most turtles possess life-history characteristics that complicate conservation efforts. These characteristics include delayed sexual maturity and high embryo and juvenile predation rates. Restoration strategies include nest protection, head-starting, and translocations. We examined short-term results of these strategies on a reintroduced population of Alligator Snapping Turtles (Macrochelys temminckii) in southern Oklahoma. We released 16 hatchery-raised juveniles and 249 adult M. temminckii into pools adjacent to the Washita River near Lake Texoma on the border of Texas and Oklahoma, USA. We tracked mortality and conducted nest searches to document factors related to population sustainability. We used hoop nets to recapture individuals and track growth. We confirmed seven mortalities during 2007 and none in 2008. In 2007 we located eight nests, all of which were depredated, and 18 nests in 2008, one of which was detected before depredation and successfully protected until hatching. We compared growth rates of released juveniles and members of the same cohort that were kept in captivity. There was no significant difference in dimensional growth, but released juveniles gained more weight than those retained at the hatchery.

3496: ***+.123***

Population declines of pink (Haliotis corrugata) and green (Haliotis fulgens) abalone in southern California forced the closure of the fisheries in 1995. Overfishing was the main factor contributing to decreasing landings, and in 2004 both pink and green abalone were identified as species of concern. Translocation of adults, moving solitary abalone together to create dense aggregations, is one of the strategies that has been proposed to overcome Allee effects and to enhance spawning success and recovery. Pink (n = 35) and green (n = 113) abalone were tagged and translocated to recovery sites with preexisting pink (n = 13) and green (n = 26) abalone. Translocation and preexisting abalone were tagged and monitored to determine their long-term survival, persistence at the site, and movement at 2 islands in southern California. The mortality associated with the tagging and translocations was low (<5%) including 1 dead pink abalone and 5 dead green abalone. The density of translocated pink abalone at 2 sites remained fairly stable, decreasing slightly from 0.84 abalone/m(2) to 0.72 abalone/m(2) after 391 days and 0.56 abalone/m(2) to 0.44 abalone/m(2) after 133 days. In contrast, translocated green abalone densities at 2 sites decreased dramatically from 0.51 abalone/m(2) to 0.08 abalone/m 2 after 405 days and 0.62 abalone/m(2) to 0.01 abalone/m(2) after 388 days. Pink abalone persistence was high and immigration was rare, whereas green abalone persistence was low and immigration into the sites was common. We find that pink abalone are optimal candidates for translocation given their high rate of site fidelity, but green abalone are not. Furthermore, our results demonstrate how important species-specific behaviors are to the success of translocation efforts, supporting the use of pilot studies prior to implementation of large-scale translocation recovery actions.

3497: ***-.072***

Morphological and mitochondrial DNA characteristics were investigated for a captive population of the rosy bitterling Rhodeus ocellatus, founded from a now extinct wild population in an irrigation pond in Seto, Okayama Prefecture, Sanyo Region, Japan. The data strongly suggested that the captive population represents a pure strain of the native Nipponbaratanago, Rhodeus

ocellatus kurumeus. This is the sole confirmed recent record of this endangered subspecies in the Sanyo Region. The current critical status of the population necessitates immediate stock preservation, pending future reintroduction and/or conservation introduction to the wild.

3498: ***+.143***

The population of Gyps vultures crashed at an alarming rate in India, from 85% since 1985-86 to 0% in 1997-99. There are sporadic records of sightings, wild breeding and captive breeding of Gyps and Neophron Vultures from 2005 to 2010 from various parts of India. We noticed continued, uninterrupted successful nesting of Oriental White-rumped Vultures Gyps bengalensis in their natural habitats in Raigad District, Maharashtra from 2004 to 2011. Their breeding population steadily increased from 10 pairs in 2004-2005 to 22 pairs in 2010-2011 and the nesting success steadily increased from 30% in 2004-2005 to 70% in 2010-2011. We feel that the naturally breeding populations are a must for successful re-introduction of the juvenile captive-bred vultures in the wild, and both in situ and ex situ breeding of vultures should be coordinated till the vulture population increases to an acceptable level and stabilizes in the Indian subcontinent. Identification of natural active nest sites is the foremost requirement for safeguarding the breeding of the Oriental White-rumped Vultures in private land by winning peoples' participation.

3499: ***+.225***

At present roe deer distribution in Andalusia is limited to two population nuclei separated by the Guadalquivir River. The first one, more threatened by demographic extinction, is located in Sierra Morena, between Ciudad Real, Cordoba and Jaen provinces. The second one, relatively well conserved, is located in the mountainous zones of Cadiz-Malaga. The roe deer disappeared from many Andalusian areas mainly due to the disappearing of the suitable habitat for the species and to the increase of other wild and domestic ungulate populations. The studies carried out over the last 25 years on the Andalusian roe deer populations of Cadiz-Malaga revealed the existence of an ecotype native to the mountainous zones of these two Andalusian provinces, which presents significant differences on a morphological and genetic level with respect to other Spanish and European roe deer populations, and which was recently catalogued by the CIC (International Council for Game and Wildlife Conservation) as a singular trophy. Taking into account all these particularities, roe deer management in Andalusia is focused on two main objectives: 1) the conservation of the Andalusian ecotype native to the mountainous areas of Cadiz-Malaga, and 2) guaranteeing a good level of conservation of the current Andalusian habitat suitable for roe deer, as well as improving the potentially optimal areas for natural colonization by roe deer or potential reintroductions of the species in Andalusia.

3500: ***+.052***

In the 19th century, the red deer (Cervus elaphus) population in Sweden experienced a rapid decline in numbers and distribution. A small population was, however, remnant in the southernmost province (Skane) of the country, presumably corresponding to the nominate form of red deer (Cervus elaphus elaphusLinnaeus, 1758). After management, reintroductions, and supplementary release during the 20th century the Swedish C.elaphus population recovered. The recovery was partially uncontrolled, and included introductions of C.elaphus of continental origin. In northern central Sweden (Jamtland) the current C.elaphus population may stem from natural colonization from Norway and/or from specimens of Swedish origin that have escaped from enclosures. To evaluate the status of the current, partially separated populations, we investigated variation at microsatellite markers in 157 C.elaphus specimens from ten locations in Sweden and

Norway. Analyses suggest that the highest-likelihood phylogenetic structure among the individuals sampled is described four distinct genetic clusters: (1) animals from the province of Vastergotland in south-western Sweden; (2) deer from the southernmost province of Skane; (3) deer from the provinces Jamtland, Blekinge, and Vastmanland; and (4) Norwegian deer. Cervus elaphus from a captive herd at the Skane Zoo cluster with deer from Skane or deer from Vastergotland, depending on the method of analysis. A number of populations in Sweden may genetically match the nominate form of red deer (C.e.elaphus). The recently established C.elaphus population in Jamtland seems to stem mainly from escapees from enclosures, with a mixed ancestry from the wild remnant population in Skane and continental deer, whereas the influx from Norway is minor, if any. Our results show the need for a detailed assessment of genetic differentiation, and emphasize the value of local management plans when planning and managing introductions.(c) 2013 The Linnean Society of London, Biological Journal of the Linnean Society, 2013, 109, 4353.

3501: *+.127*

Habitat selection requires choice, which differentiates it from habitat use, and choice, in turn, is dependent upon the responses of organisms to the environmental, social, and other cues that they perceive. Habitat selection by the gopher tortoise (Gopherus polyphemus) was investigated by translocating tortoises and monitoring their movements within two sites in central Florida. The first site supported a stable preponderance of high-quality habitat, and tortoises avoided areas with a dense tree canopy cover caused by fire exclusion. The second site was badly invaded by an introduced weed, and tortoises avoided areas where the weed had formed a dense monoculture. At both sites, individuals appeared to be responding to visual cues to avoid areas that were relatively dark. In landscapes with relatively large amounts of high-quality habitat, this avoidance behavior serves the gopher tortoise well by keeping individuals within the dominant habitat type. In degraded areas, high-quality habitat often becomes increasingly uncommon, and the avoidance behavior exhibited by the tortoises will result in individuals becoming confined to small patches, causing a significant reduction in fitness and hence questioning their long-term survival in such areas. The results from our study show that in order to maintain viable tortoise populations in areas increasingly subjected to human fragmentation and degradation, it is crucial not only to suppress tree canopy cover continually and prevent invasion by exotic weeds, but also to be mindful that the avoidance behavior of the gopher tortoise could prevent individuals from fully occupying a high-quality habitat in response to restoration and management efforts.

3502: *+.116*

AIMS: To undertake disease surveillance for Chlamydia psittaci in native birds as part of a pilot study to examine pathogen diversity on Hauturu-o-Toi/Little Barrier Island. To retrospectively review the Massey University post-mortem database to determine previous cases of avian chlamydiosis in New Zealand.METHODS:Mistnetting of forest birds was conducted across an elevational gradient on Hauturu-o-Toi/Little Barrier Island. Minitip culture swabs were used to collect cloacal samples from native birds. These swabs were screened for Chlamydia family DNA using two PCR methods. Positive results were sequenced. A retrospective review of the Massey University post-mortem database of all avian cases from 1990 to 2011 was conducted.RESULTS:Ten native birds including four bellbirds (Anthornis melanura), three rifleman (Acanthisitta chloris), two hihi (Notiomyces cincta), and one whitehead (Mohoua albicilla) were sampled and one otherwise healthy female hihi was positive by both PCR screening methods for Chlamydophila. Sequencing confirmed 99100% genetic similarity to C. psittaci. A retrospective review of the Massey University post-mortem database revealed no previous

diagnoses of avian chlamydiosis in wild native New Zealand birds although it has been detected in captive parrots, and wild and captive exotic pigeons.CONCLUSIONS:This is the first report of the detection of C. psittaci from a wild native bird in New Zealand. The bird was a Passeriforme from an endangered species that was captured free-living on Little Barrier Island. The incidence of avian chlamydiosis in native birds in New Zealand appears to be very low, based on the retrospective review of the post-mortem database. CLINICAL RELEVANCE: It is unlikely that avian chlamydiosis is a significant problem for hihi population health. The detection of this organism has greater significance for other more susceptible species on Little Barrier Island and for human health, particularly for conservation workers involved in wildlife translocations. It further suggests that passerine birds may be a reservoir for C. psittaci in New Zealand ecosystems.

3503: *+.207*

Understanding the influence of environmental stressors on daily nest survival of introduced birds is important because it can affect introduction success as well as the ability to evaluate introduction programs. For long-lived birds with low annual production, adjustment to local breeding conditions can take many years. We examined nest success rates of 2 introduced bird species, whooping crane (Grus americana) and trumpeter swan (Cygnus buccinator), in Wisconsin. Both species are long-lived with low annual reproductive rates. Trumpeter swans were established in our study area approximately 10 years before whooping cranes. We predicted that trumpeter swans would show less sensitivity to environmental stressors. We used daily nest survival rates (DNSRs) as our response variable to model several environmental parameters including weather, phenology, and ornithophilic black flies (Diptera: Simuliidae). Additionally, we examined the influence of captive history, age, release method, energetics, and nesting experience on whooping crane DNSRs. Daily nest survival of whooping cranes was the most sensitive to stressors. Trumpeter swan daily nest survival showed less sensitivity to the same stressors. Daily nest survival for both species peaked later in the nesting season, after 30 April and before 30 May. We also found that the daily nest survival rate (DNSR) for whooping cranes was potentially affected by captive exposure (measured by generations removed from the wild). Our results highlight the difficulties associated with conservation of long-lived birds with low annual productivity as they adjust to local breeding conditions and that nest phenology at the source location can determine how these conditions are interfaced. We recommend that the juxtaposition of source and introduction location nest phenology be considered prior to introduction site selection. Additionally, strategically selecting offspring from captive pairs with nest phenology similar to that of sympatric species at the introduction location should be considered. Published 2013. This article is a U.S. Government work and is in the public domain in the USA.

3504: *+.219*

Survival of greater sage-grouse (Centrocercus urophasianus) has been well described in large populations across the species range. Very little published information exists, however, on survival rates of translocated sage-grouse or grouse from a long-term (>10 yr) study. Our objectives were to estimate seasonal and annual survival rates; assess differences in survival between resident and translocated, adult and yearling, and male and female sage-grouse; identify environmental and behavioral factors associated with survival; and assess the influence of mammalian predator control on survival rates of radio-marked sage-grouse in Strawberry Valley, Utah from 1998 to 2010. We used a 2-stage model selection approach using Akaike's Information Criterion corrected for sample size (AICc) with known-fate models in Program MARK to evaluate the influences of seasonal, annual, demographic, and behavioral effects on survival rates of sage-grouse. We captured and fitted 535 individual sage-grouse (male and female, resident and translocated) with

radio transmitters over a 13-year period and monitored them weekly. The top model of survival, which accounted for 22% of the AICc weight, included 3 seasons that varied by year where rates were influenced by residency, sex, and whether a female initiated a nest. A group-level covariate for the number of canids killed each year received some support as this variable improved model fit compared to identical models without it, although confidence intervals around estimates overlapped zero slightly. All other demographic or environmental variables showed little or no support. Annual estimates of survival for females ranged between 28% and 84% depending on year and translocation source. Survival was consistently highest during the fallwinter months with a mean monthly survival rate of 0.97 (95% CI=0.960.98). The lack of a control site and other potential confounding factors limit the extent of our inference with respect to predator control. Nonetheless, we suggest managers consider enhancing nesting habitat, translocating sage-grouse, and possibly controlling predators to improve survival rates of sage-grouse. (c) The Wildlife Society, 2013

3505: *+.091*

Ecosystem engineers are increasingly being reintroduced to restore ecological processes in restoration and rewilding projects. To predict and adaptively manage the impact of such species their behavioral ecology must be understood and quantified. Rooting behavior by wild boar qualifies them as ecosystem engineers due to their impact on vegetation disturbance regimes. The behavioral foraging ecology of wild boar was quantified in a fenced area in the Scottish Highlands in order to provide some of the understanding necessary to predict their ability to affect ecosystem restoration. Five wild boar were monitored within a 125ha fenced area using Global Positioning System (GPS) collars and behavioral monitoring over a 12-month period. Their activity budget, the relationship between foraging behavior and vegetation communities, and how these relationships vary between seasons was investigated. The results indicate that wild boar invested approximately four more hours daily to rooting during the autumn and winter than the spring and summer. During the spring and summer, grazing was the dominant foraging behavior (approximately 28% of foraging period) while rooting dominated in autumn and winter (approximately 76% of foraging period). Deep rooting behavior is particularly associated with bracken-dominated communities. Associations between rooting, vegetation community, and season will have a strong influence on the spatial and temporal distribution of rooting behavior. This variation could have important implications for the impacts of boar on vegetation community dynamics. These results detail some of wild boar's ecosystem engineering behaviors; however, further research is required to consider the wider impacts of a full reintroduction.

3506: *+.073*

Rewilding is emerging as a promising framework within restoration ecology to help restore ecosystem function through species reintroduction. To manage effectively such projects it is necessary to predict and quantify the interactions between the reintroduced species and their environment. To date, this has not been a priority in restoration ecology. Here, we quantify wild boar's rooting rate at a range of stocking densities to explore their potential to aid the restoration of the Caledonian pine forest in the Scottish Highlands by reinvigorating the disturbance regime. Eleven enclosures of c. 0.5ha within heather moorland, dominated by Caluna vulgaris, were used, situated on the Alladale Wilderness Reserve, 50km north-west of Inverness. Stocking densities varied between 4 and 15 boar/ha. The accumulation of rooted area was recorded weekly over a 13-week period. A cost analysis was performed to compare the use of wild boar with other ground preparation techniques. The median per capita rooting rate was 42.4m2/week (interquartile range [IQR] 45.5), but rooting rate varied temporally. Median rooting rates varied between 21.6 and

75.3m2/week (IQR 5.6) in periods that varied in suitability for rooting. Rooting rates were consistent across stocking densities. The cost of using wild boar as a ground preparation tool ranged between 184 pound and 1,961 pound per hectare depending on stocking density and rate of rooting. This experiment has direct application for managing the impact of wild boar within free range farming conditions, managed woodland regeneration schemes, rewilding projects, and in the wild.

3507: *+.099*

Reintroduction, supplemental planting for genetic rescue, and the creation of artificial seed production populations are common methods to conserve rare plant species, but empirical studies assessing the effects of artificial selection on genetic diversity are rare. I conducted a retrospective DNA genotyping study on an artificial population (hereinafter Office) of the threatened plant, Lupinus oreganus Heller, to determine whether the process of establishing the Office population facilitated genetic differentiation and if genetic diversity was maintained in the Office cohort. Genotyping indicated that uncommon maternal lineages (cpDNA haplotypes) were selected for in the artificial population and that the Office population was genetically distinct from both seed source patches. Furthermore, despite a small population size of seven individuals, cpDNA haplotype and nDNA simple sequence repeat allelic diversity was maintained in the surviving Office cohort. This study suggests that small artificial rare plant populations may be beneficial for capitalizing on the existing within-population genetic diversity, but they may also select for uncommon allelic diversity and facilitate genetic differentiation.

3508: *+.266*

We present case studies supporting management of two rare plant species in tidal wetlands of the San Francisco Estuary. We used empirical demographic analyses to identify factors to enhance population establishment and survival of Chloropyron molle subsp. molle (Orobancaceae), an annual hemiparasite, and to compare reintroduced with natural populations. Twelve years after outplanting, the reintroduced population persists but is in decline; impediments to success include the lack of adaptive management response to weed invasions and muted variance in hydrology. Transplantation of Lilaeopsis masonii (Apiaceae), a rhizomatous perennial herb, failed to meet success criteria for mitigation at local project scale, but dispersal and establishment of metapopulation patches indicated persistence at the landscape scale. This species has been found to be genetically indistinct from a widespread congener, and has few threats to persistence so long as suitable habitat is present. These two examples demonstrate the need for integrated conservation management strategies that prioritize habitat connectivity and maintain physical processes to support dispersal in response to sea level rise. For the hemiparasite, assisted colonization may sustain populations threatened by sea level rise, but only if a strong commitment to effective stewardship is realized.

3509: *+.199*

The aim of any reintroduction is to provide sufficient genetic variability to buffer against changing selection pressures and ensure long-term survival. To date, few empirical studies have compared levels of genetic diversity in reintroduced and native plant populations. Using microsatellite markers, we measured the genetic diversity within reintroduced and native populations of the threatened Cirsium pitcher (Eaton) Torrey and Gray. We found that the use of local mixed source was successful in establishing populations with significantly higher genetic diversity ($P < 0.005$) than the native populations (allelic richness is 3.39 in reintroduced and 1.84 in native populations).

However, the reintroduced populations had significantly higher inbreeding coefficients (P < 0.002) (F-IS is 0.405 and 0.213 in reintroduced and in native populations, respectively), despite having multiple genetic founders, population sizes equivalent to native populations and a positive growth rate. These results may be due to inbreeding or the Wahlund effect, driven by genetic substructuring. This suggests that the small population size of these reintroduced populations may lead to genetic issues in the future, given the low number of flowering individuals each year. This highlights the importance of considering not only the number of source individuals but the effective population size of the reintroduction.

3510: ***+.069***

The Gulf of St. Lawrence aster (SLA; Symphyotrichum laurentianum (Fernald) G.L. Nesom) is an annual plant species endemic to the Gulf of St. Lawrence region. Owing to the dynamic nature of the environment that the SLA inhabits, severe and major threats to both the aster and its habitat exist. The Committee on the Status of Endangered Wildlife in Canada listed the species as threatened in Canada in 2004. This status was assigned based on the species' limited distribution, fluctuating population size, and continued pressures on its habitat. Surveys have revealed that both site and population numbers have been further and drastically reduced on Prince Edward Island. In 2007, only one populated site of 482 individuals remained. It is possible that this species has been extirpated from Prince Edward Island. Recovery of this species on Prince Edward Island is feasible. Promising results related to seeding and the transplantation of greenhouse-grown seedlings at four in situ sites demonstrated that SLA plantlets have the potential to serve as seed stock to re-establish populations. Over the 2 years of the transplantation experiment, the pooled overall survivorship was 52.8%. Specific site manipulations that were tested may also increase the potential survivorship of the transplants and facilitate second-generation germination.

3511: ***-.009***

Covering just 2000 ha, Garry oak ecosystems (GOEs) in western Canada contain about 10% of the Species at Risk Act listed species at risk in Canada, including 30 plants listed by Committee on the Status of Endangered Wildlife in Canada as endangered. Since European settlement ca. 1840, GOE sites have been largely degraded by human disturbance, habitat fragmentation, invasive species, overgrazing, and fire suppression. A key strategy to mitigate this loss of biodiversity is to translocate rare plants to GOE restoration sites. The Garry Oak Ecosystem Recovery Team provides advice on proposed translocations but strongly encourages restoration practitioners to focus on plant populations already present on a site. There is a need for a closer look at challenges and opportunities afforded by translocation. If the approach taken is too precautionary, some rare species in this highly threatened ecosystem may be jeopardized. Current translocation efforts are being spearheaded by Parks Canada for golden paintbrush (Castilleja levisecta Greenm.), seaside birds-foot lotus (Lotus formossimus Greene), and white-top aster (Aster curtus Cronq.). Translocations like these together with further research on the genetics and ecology of rare plant species are critical to species recovery efforts within GOE and other similarly compromised ecosystems.

3512: ***+.161***

Three recent reviews of reintroduction for conservation purposes, which draw on substantial and largely nonoverlapping data sets, have come to strikingly different conclusions about its value. One concludes that "reintroduction is generally unlikely to be a successful conservation strategy as currently conducted". Another concludes that "... this review cannot conclusively comment on the

effectiveness of reintroductions..." The third concludes that there is "strong evidence in support of the notion that reintroduction, especially in combination with ex situ conservation, is a tool that can go a long way toward meeting the needs it was intended to address". The argument over the conservation value of reintroduction is of more than academic interest. It illustrates a challenge facing land managers and decision makers who may be tempted to act on stated conclusions without thoroughly understanding their underlying assumptions, methodology, and terminology. The differing conclusions can be partially explained by different criteria of what constitutes success, how to measure it, and differing time scales considered. The propriety of reintroduction is briefly discussed and focuses on the following two issues: translocation of naturally occurring individuals to new locations and introduction outside a species' naturally occurring range. Both have appropriate uses but can be used in ways that detract from the survival prospects of taxa.

3514: *-.005*

Billions of wild and semiwild animals live in captive conditions very different from their ancestral environments. Some of the potential challenges they face here, such as greater human proximity, constrained natural behaviours and altered climates, resemble those occurring during urbanization, translocation and other forms of human-induced rapid environmental change (HIREC) in the wild. These parallels between HIREC and captivity suggest that certain species could be in double jeopardy: struggling in both wild and captive environments. This raises new hypotheses for future research, including one tested in this paper: that a species' presence in captivity predicts its chances of establishment when translocated to novel natural habitats. Furthermore, understanding the mechanisms that predispose captive populations to thrive or fail can yield new insights into how animals respond to HIREC. For example, populations adjusting to captivity demonstrate rapid developmental effects. Within one generation, captive-reared animals may show beneficial phenotypic changes (e.g. smaller stress responses than F0s wild caught as adults), illustrating how adaptive developmental plasticity can help populations succeed. However, captive-reared animals also illustrate the risks of developing in evolutionarily new environments (being prone to reduced behavioural flexibility, and sometimes impaired reproduction), suggesting that disrupted ontogeny is one reason why HIREC can be harmful. Overall, analogies between captivity and HIREC are thus interesting and useful. However, captivity and HIREC do differ in some regards, captivity tending to be safer yet more monotonous; we therefore end by discussing how species-typical risk/protective factors, and the phenotypic changes induced in affected animals, may vary between the two. (c) 2013 The Association for the Study of Animal Behaviour. Published by Elsevier Ltd. All rights reserved.

3515: *+.129*

. 1. The regal fritillary butterfly, Speyeria idalia Drury 1773, was once widespread across eastern North America, but has declined significantly and rapidly over the past half-century. Although more stable in the western portion of its range, only two populations survive east of the Great Lakes, one in eastern Pennsylvania and the other in Virginia. 2. Previous studies have found that the remnant Pennsylvania population is genetically differentiated from populations in the west, and have suggested the designation of separate eastern and western subspecies. However, the historical pattern of genetic variation from which the current distinctness of the Pennsylvania population has arisen was not known, nor was the relationship with the remnant Virginia population. 3. We amplified and sequenced two mitochondrial loci (COI/II and ND4) from preserved specimens to infer historical patterns of genetic variation in this species, and we used non-lethally obtained tissue samples to assess the relationship of the two eastern remnant populations. 4. We found very consistent patterns between the two loci. Both had a very shallow

haplotype network with few mutations separating most haplotypes. At both loci, we observed distinct groups of haplotypes in the western and far eastern (i.e. New England) portions of the range; a region of transition was centred on Ohio, western Pennsylvania, and the Virginias, where both groups, and intermediate haplotypes, were represented. 5.Importantly, the extant Virginia population shared haplotypes with western populations of S. idalia and not with the extant Pennsylvania population. We discuss the implications of this result for the taxonomy and translocations/introductions of the species.

3516: *+.148*

Our view of the future of biodiversity remains limited to a restricted number of taxa, and some taxa, such as spiders, have been largely omitted. Here we provide the first assessment of effects of global change on threatened spiders using a red-listed vulnerable spider, Dolomedes plantarius (Clerck, 1757) as an example. We aim at applying this assessment to assist two conservation actions for this species, including a translocation program. We compiled all the available data on D. plantarius and modelled its current and future distributions on the basis of both climate and land cover variables at a fine resolution (0.1 degrees). We applied an ensemble modelling procedure on the basis of eight modelling techniques, and forecasted the future distribution ranges for two emission scenarios (A1 and B2) and three general circulation models. Despite uncertainty regarding the predictions, the models performed very well, and consensus emerged for models and climate scenarios to predict significant negative effects on the current distribution range of this species. In the UK, the translocated population and one out of three natural populations were predicted to remain in highly suitable environmental areas. In France, one out of six locations was predicted to remain suitable in the future. Given the phylogeographic background of this species, the predicted effects of environmental changes should be considered seriously, especially for the long-term viability of conservation programs. Our study demonstrated the importance and feasibility of studying the effects of climate change by the means of species distribution models on taxa with limited available data, such as spiders. (C) 2013 Elsevier Ltd. All rights reserved.

3517: *+.184*

Threatened populations of birds are often restored after bottleneck events by using reintroduction techniques. Whilst population numbers are often increased by using such measures, the long-term genetic effects of reintroductions and post-release management of the resulting populations are frequently overlooked. We identify an overall declining trend in population-wide estimates of genetic diversity over two decades since the initial recovery of the population from the most severe part of this species' bottleneck. Additionally, by incorporating the genotypes of known founding individuals into population viability simulations, we evaluate the genetic effects of population management under various scenarios at both the metapopulation and subpopulation levels. We reveal that whilst population augmentation has led to increased genetic homogenisation among subpopulations, significant differentiation still exists. Simulations predict that even with a low level of natural dispersal leading to gene-flow this differentiation could be ameliorated. We conclude by offering a number of key recommendations relating to post-recovery management of reintroduced bird populations which support the encouragement of individual dispersal using established management techniques such as artificial nest-site provisioning. (C) 2013 Elsevier Ltd. All rights reserved.

3519: *+.380*

The concept of "conservation reliance" offers an alternative way of thinking about recovery of

species under the Endangered Species Act (ESA) by placing endangered species along a recovery continuum. In this article, we argue that the management of species can be improved by employing the concept of a recovery continuum and extend the concept of conservation reliance to an often controversial topic: the reintroduction of species under the ESA. We examine proposals for reintroduction of a population of grizzly bears (Ursus arctos horribilis) into the American Southwest to demonstrate how the concept of conservation reliance provides an increased understanding of the management considerations underlying reintroduction efforts. Consideration of conservation reliance can aid in the planning of such efforts by evaluating the advisability of a reintroduction, and the concept can be used both as an educational tool for establishing public support and for examining the ethical implications of a reintroduction.

3520: *-.000*

Background: Population genetic studies focus on natural dispersal and isolation by landscape barriers as the main drivers of genetic population structure. However, anthropogenic factors such as reintroductions, translocations and wild x domestic hybridization may also have strong effects on genetic population structure. In this study we genotyped 351 Single Nucleotide Polymorphism markers evenly spread across the genome in 645 wild boar (Sus scrofa) from Northwest Europe to evaluate determinants of genetic population structure.Results: We show that wild boar genetic population structure is influenced by historical reintroductions and by genetic introgression from domestic pigs. Six genetically distinct and geographically coherent wild boar clusters were identified in the Netherlands and Western Germany. The Dutch Veluwe cluster is known to be reintroduced, and three adjacent Dutch and German clusters are suspected to be a result of reintroduction, based on clustering results, low levels of heterozygosity and relatively high genetic distances to nearby populations. Recent wild x domestic hybrids were found geographically widespread across clusters and at low frequencies (average 3.9%). The relationship between pairwise kinship coefficients and geographic distance showed male-biased dispersal at the population genetic level.Conclusions: Our results demonstrate that wildlife and landscape management by humans are shaping the genetic diversity of an iconic wildlife species. Historical reintroductions, translocation and recent restocking activities with farmed wild boar have all influenced wild boar genetic population structure. The current trend of wild boar population growth and range expansion has recently led to a number of contact zones between clusters, and further admixture between the different wild boar clusters is to be expected.

3521: *+.104*

The present study considers genetic diversity of 38 populations in 4 Cirsium species of the genus Cirsium Mill. (Asteraceae), occurring in different ecological regions and tries to compare degree of genetic variability among the species with wide geographical distribution versus endemic C. pyramidale showing confined geographical distribution. The results showed that the endemic species has similar value of genetic diversity parameters as the species with wider distribution. We also studied the possible admixture nature of these populations and tried to understand the relation between genetic changes, geographical distribution and polyploidy level and chromosome pairing in these species. ISSR analysis showed population difference in allele composition and frequency. Clustering and PcoA ordination produced different groupings in each species, while STRUCTURE and reticulation analyses revealed high degree of genetic admixture and gene exchange among populations as well as allelic rearrangement. No significant correlation was observed between geographical distance and genetic distance of the populations and AMOVA test revealed no significant difference among populations in each species studied. However, high amount of within population variation occurred in all 4 species indicating their cross-pollination nature and high

genetic admixture. The populations also varied in chiasma frequency and chromosome pairing as well as the occurrence of heterozygote translocations all creating more variability to be used by plants for local adaptation.

3522: -.054

We isolated and characterized 30 microsatellite loci from the critically endangered Myanmar Roofed Turtle, Batagur trivittata. Loci were screened in 9 B. trivittata samples and in the congeners the Painted River Terrapin, Batagur borneoensis, with 22 of 30 amplifying, and the Southern River Terrapin, B. affinis, with 15 of 30 amplifying. In the B. trivittata samples, the number of alleles per locus ranged from 3 to 10 and the probability of identity values ranged from 0.031 to 0.354. These new loci will provide tools for captive management and reintroduction programs for B. trivittata and the other five species of Batagur, particularly B. affinis in Cambodia.

3523: +.225

We documented survival of elk (Cervus elaphus) in the Nooksack herd of Northwest Washington, USA, 2000-2008, following a temporary harvest moratorium. To increase Nooksack elk abundance, 77 adult female elk were radiocollared and translocated from the Mt. St. Helens Wildlife Area (MSHWA) during October 2003 (n = 38) and October 2005 (n = 39). We used known-fate models to explore survival among radiocollared native adult females (n = 26), translocated adult females (n = 77), and native branch-antlered males (n = 24) in the Nooksack herd using 16 candidate models. The best model assumed similar survival for native and translocated female elk, except for a 1-year reduction in survival for the 2003 translocation cohort. The best model assumed that survival for branch-antlered Nooksack males differed during the harvest moratorium (pre-2007) and after limited permit-controlled hunting resumed in 2007. Under the best model, we estimated that annual survival for all adult female elk was 0.93 (95% CI = 0.90-0.95), except that 2003 MSHWA-translocated elk survival was 0.68 (95% CI = 0.51-0.82) during the first year post-translocation. We estimated male survival was 0.92 (95% CI = 0.76-0.99) prior to 2007 and was 0.68 (95% CI = 0.50-0.82) during 2007-2008. We did not detect a difference in mean body fat between translocated elk that died during the first year after translocation and those that survived (P = 0.39), although there were proportionally more very lean elk among those that died in the first post-translocation year. Elk that died in their first post-translocation year had marginally higher body temperatures at handling than that survived (105.3 degrees F +/- 0.6 degrees [SE; 40.7 degrees C +/- 0.3 degrees] and 104.2 degrees +/- 0.2 degrees [40.1 degrees C +/- 0.1 degrees], respectively; P = 0.07). Despite the lower first-year survival of elk translocated in 2003, we concluded that the 2 translocations contributed substantively to Nooksack elk restoration. We discuss strategic aspects of autumn translocation that appeared to promote success. (C) 2013 The Wildlife Society.

3524: -.162

Concerns regarding the potential negative impacts of regulated furbearer trapping to reintroduced Mexican gray wolves (Canis lupus baileyi), led to an executive order prohibiting trapping in the New Mexico, USA, portion of the Blue Range Wolf Recovery Area. This ban was to last for 6 months and required an evaluation of the risk posed to wolves by traps and snares legally permitted in New Mexico. We reviewed potential threats to wolves in the Blue Range Wolf Recovery Area, including threats associated with regulated furbearer trapping. One hundred Mexican gray wolf mortalities have been documented during the reintroduction effort (1998-2011). Of those mortalities with a known cause, >81% were human-caused resulting from illegal

shooting (n = 43), vehicle collisions (n = 14), lethal removal by the United States Fish and Wildlife Service (USFWS; n = 12), non-project-related trapping (n = 2), project-related trapping (n = 1), and legal shooting by the public (n = 1). Ten wolves died due to unknown causes. The remaining 17 mortalities were a result of natural causes (e.g., starvation, disease). An additional 23 wolves were permanently, but non-lethally, removed from the wild by the USFWS. Of 13 trapping incidents in New Mexico that involved non-project trappers (i.e., trappers not associated with USFWS or U.S. Department of Agriculture-Wildlife Services), 7 incidents are known to have resulted in injuries to wolves: 2 wolves sustained injuries severe enough to result in leg amputations and 2 additional wolves died as a result of injuries sustained. Foothold traps with rubber-padded jaws and properly set snares may reduce trap-related injuries to Mexican gray wolves; however, impacts caused by trapping are overshadowed by other anthropogenic impacts (e.g., illegal shooting, non-lethal permanent removal, and vehicle collisions). (C) 2013 The Wildlife Society.

3525: *+.217*

There is a paucity of basic biological information for the Sumatran rhinoceros (Dicerorhinus sumatrensis). This information is fundamental to husbandry and management practices for captive animals and for support of in situ conservation efforts. Serial blood samples were collected over an 8-yr period to evaluate patterns in hematology and serum biochemistry values among five Sumatran rhinoceroses housed at the Sumatran Rhino Sanctuary in Way Kambas National Park, Lampung, Indonesia. Understanding the basis for variance in analytes both within and between subjects can allow use of more sensitive subject-based reference values, and is particularly suitable for small populations of endangered animals. Both intra-and intersubject variability was computed for each analyte and the associated index of individuality was determined. Previously published cutoff points for index of individuality indicate where population-based reference intervals can be used with confidence (index >1.4) or with caution (0.6 < index < 1.4). Interrhino variability was small for the majority of analytes, with 12 of 19 analytes having an index of individuality greater than 1.4 and none having an index of individuality less than 0.6. With the high within-individual variability of most anayltes in the Sumatran rhinoceroses at the sanctuary, subject-based reference intervals offer little advantage over standard population-based reference intervals for monitoring the health of these endangered animals. Differences were noted (but not tested for statistical significance) in serum urea, aspartate aminotransferase (AST), lactate dehydrogenase (LDH), and cholesterol between young and old rhinoceroses, and in hematocrit, AST, alanine aminotransferase (ALT), LDH, and glucose between male and female rhinoceroses. Husbandry practices, animal management, nutrition and habitat factors may also impact hematology and biochemistry results, and these relationships deserve more careful investigation. This study represents the most comprehensive hematology and serum biochemistry comparison of Sumatran rhinoceroses held in natural rainforest conditions outside a traditional zoological setting.

3526: *+.102*

Clarias batrachus is a species of freshwater catfish widely used for human consumption. Increasing demand of this species worldwide, coupled with its ability to survive in wide range of environmental conditions, has led to its introduction in many countries. This widespread translocation and distribution of C. batrachus have resulted in the rise of wide variety of haplotypes of this species. Nevertheless, there has been constant decrement in their population density in the last two decades. Thus, to endeavour conservation of the species we employ molecular technique of DNA barcoding in solving the standing problem of crypticism and haplotype sharing of the species. A better knowhow of the genetic makeup of the unique identifier

region, that is, the 648 basepair region of COI DNA barcode will help to differentiate among closely related species and identify endemic species. In this study, a comparative analysis of C. batrachus from different regions in India and other parts of world shows presence of distinct haplotypes in different geographical locations. We also present a descriptive study of the various species of Clarias genus that have been barcoded in India till date. Our results also solve the dilemma of considering some species as synonymy of C. batrachus.

3527: ***-.056***

The conservation of tortoises poses a unique situation because several threatened species are commonly kept as pets within their native ranges. Thus, there is potential for captive populations to be a reservoir for repatriation efforts. We assess the utility of captive populations of the threatened Agassiz's desert tortoise (Gopherus agassizii) for recovery efforts based on genetic affinity to local areas. We collected samples from 130 captive desert tortoises from three desert communities: two in California (Ridgecrest and Joshua Tree) and the Desert Tortoise Conservation Center (Las Vegas) in Nevada. We tested all samples for 25 short tandem repeats and sequenced 1,109 bp of the mitochondrial genome. We compared captive genotypes to a database of 1,258 Gopherus samples, including 657 wild caught G. agassizii spanning the full range of the species. We conducted population assignment tests to determine the genetic origins of the captive individuals. For our total sample set, only 44 % of captive individuals were assigned to local populations based on genetic units derived from the reference database. One individual from Joshua Tree, California, was identified as being a Morafka's desert tortoise, G. morafkai, a cryptic species which is not native to the Mojave Desert. Our data suggest that captive desert tortoises kept within the native range of G. agassizii cannot be presumed to have a genealogical affiliation to wild tortoises in their geographic proximity. Precautions should be taken before considering the release of captive tortoises into the wild as a management tool for recovery.

3528: ***+.073***

Many island avian populations are of conservation interest because they have a higher risk of extinction than mainland populations. Susceptibility of island birds to extinction is primarily related to human induced change through habitat loss, persecution, and introduction of exotic species, in combination with genetic factors. We used microsatellite profiles from 11 loci to assess genetic diversity and relatedness in the critically endangered hawk Buteo ridgwayi endemic to the island of Hispaniola in the Caribbean. Using samples collected between 2005 and 2009, our results revealed a relatively high level of heterozygosity, evidence of a recent genetic bottleneck, and the occurrence of inbreeding within the population. Pair relatedness analysis found 4 of 7 sampled breeding pairs to be related similar to that of first cousin or greater. Pedigree estimates indicated that up to 18 % of potential pairings would be between individuals with relatedness values similar to that of half-sibling. We discuss our findings in the context of conservation genetic management suggesting both carefully managed translocations and the initiation of a captive population as a safeguard of the remaining genetic diversity.

3529: ***+.067***

Despite differences in focus, goals, and strategies between conservation biology and animal welfare, both are inextricably linked in many ways, and greater consideration of animal welfare, although important in its own right, also has considerable potential to contribute to conservation success. Nevertheless, animal welfare and animal ethics are not always considered explicitly within conservation practice. We systematically reviewed the recent scientific peer-reviewed and

online gray literature on reintroductions of captive-bred and wild-caught animals (mammals, birds, amphibians, and reptiles) to quantify the occurrence of animal welfare issues. We considered monitoring that could be indicative of the animal's welfare status and supportive management actions that could improve animal welfare (regardless of whether the aim was explicitly animal-welfare orientated). Potential welfare issues (of variable nature and extent) were recorded in 67% of 199 projects reviewed; the most common were mortality >50%, dispersal or loss of animals, disease, and human conflict. Most (>70%) projects monitored survival, 18% assessed body condition, and 2% monitored stress levels. Animal welfare, explicitly, was referred to in 6% of projects. Supportive actions, most commonly use of on-site prerelease pens and provision of supplemental food or water, were implemented in 79% of projects, although the extent and duration of support varied. Practitioners can address animal-welfare issues in reintroductions by considering the potential implications for individual animals at all stages of the release process using the decision tree presented. We urge practitioners to report potential animal-welfare issues, describe mitigation actions, and evaluate their efficacy to facilitate transparent evaluation of common moral dilemmas and to advance communal strategies for dealing with them. Currently, comparative mortality rates, health risks, postrelease stress, effectiveness of supportive measures, and behavior of individuals warrant further research to improve animal welfare in reintroductions and to increase success of such projects.

3530: ***+.339***

New Guinea is considered the most important secondary centre of diversity for sweet potato (Ipomoea batatas). We analysed nuclear and chloroplast genetic diversity of 417 New Guinea sweet potato landraces, representing agro-morphological diversity collected throughout the island, and compared this diversity with that in tropical America. The molecular data reveal moderate diversity across all accessions analysed, lower than that found in tropical America. Nuclear data confirm previous results, suggesting that New Guinea landraces are principally derived from the Northern neotropical genepool (Camote and Batata lines, from the Caribbean and Central America). However, chloroplast data suggest that South American clones (early Kumara line clones or, more probably, later reintroductions) were also introduced into New Guinea and then recombined with existing genotypes. The frequency distribution of pairwise distances between New Guinea landraces suggests that sexual reproduction, rather than somaclonal variation, has played a predominant role in the diversification of sweet potato. The frequent incorporation of plants issued from true seed by farmers, and the geographical and cultural barriers constraining crop diffusion in this topographically and linguistically heterogeneous island, has led to the accumulation of an impressive number of variants. As the diversification of sweet potato in New Guinea is primarily the result of farmers' management of the reproductive biology of their crop, we argue that on-farm conservation programmes that implement distribution of core samples (clones representing the useful diversity of the species) and promote on-farm selection of locally adapted variants may allow local communities to fashion relatively autonomous strategies for coping with ongoing global change.

3531: ***+.096***

The endangered African dwarf crocodile Osteolaemus tetraspis is distributed in Central and Western Africa. Conventionally, two subspecies were distinguished: Osteolaemus tetraspis tetraspis and Osteolaemus tetraspis osborni. The taxonomic significance of diagnostic morphological characters is still being discussed and the existence of additional species in the Osteolaemus group remains unclear. Recent molecular studies suggest the existence of three allopatric species in the genus Osteolaemus. These results supported a division of the dwarf

crocodile into a Congo Basin form (O. osborni), an Ogoou, Basin form (O. tetraspis), and a third separate evolutionary lineage from Western Africa. Several European zoos host African dwarf crocodiles. For reasons of conservation and possible reintroduction, it is important to clarify provenance of these zoo animals. Therefore, we conducted molecular and phylogenetic analyses of three mitochondrial and two nuclear gene sequences with all available samples from European zoos and museums. We also estimated the origin of the zoo animals by comparing sequences of wild animals and museum samples of known provenance. Our study strongly supports three distinct lineages of Osteolaemus as recently postulated, but also reveals a fourth evolutionary lineage. We demonstrate that, of the European zoo animals sampled, only one dwarf crocodile corresponds to the Congo Basin form (O. osborni) whereas the majority of individuals correspond to the three other forms. Four zoo animals belong to the new fourth group; but their provenance is still unresolved. The origin of these animals is probably located in an African region from which no wild animal samples are currently available. Further investigations and sampling of other regions should be completed to clarify the identity of this fourth lineage. We found potential hybrids from European zoological gardens using nuclear DNA sequences. The European Studbook will use these results for further breeding programmes to keep genetically suitable ex-situ populations as reassurance colonies for prospective reintroduction into African countries.

3532: ***-.029***

Ridgway's Hawks (Buteo ridgwayi) are critically endangered forest raptors endemic to the Caribbean island of Hispaniola, with approximate to 100 pairs remaining in the world. The species is ecologically little known yet such studies are important for understanding critical habitat needs and population dynamics. We studied the provisioning behavior of adults at 22 nests on the northeast coast of the Dominican Republic from 2005 to 2008. Mean brood size was 1.80 +/- 0.45, and the mean number of fledglings per nest was 1.10 +/- 0.97. We found that 80% of the prey items delivered to nestlings were reptiles, with lizards accounting for 65% of the prey and those in the genus Celestus accounting for nearly 35% of prey. Other prey items included snakes (14%), rats (9%), and smaller proportions of birds, frogs, and centipedes. The number of prey items and amount of biomass delivered to nestlings did not vary with brood size, but adults delivered more prey to 3- to 5-week-old nestlings and more biomass to 5-week-old nestlings. Food delivery rates did not differ between successful or failed nests, suggesting that food availability did not influence nest outcome. Given that most prey items delivered to nestlings in our study were reptiles, conservation strategies developed for Ridgway's Hawks (e.g., translocations and habitat conservation) should take into consideration their specialist reptile diet.

3533: ***+.089***

The highly endangered freshwater pearl mussel (Margaritifera margaritifera L.) has strongly declined throughout Europe and is a priority species in aquatic conservation. The complex life cycle of M. margaritifera includes an obligate development phase of glochidia larvae on a suitable host fish. Knowledge on the progression of the parasitic phase and on the factors governing excystment of juvenile mussels are particularly crucial for artificial breeding and conservation measures. The core objective of this study was to study excystment of M. margaritifera after maintaining the infested hosts under constant water temperatures between 11 and 12 degrees C and to determine the sum of daily water temperatures (day degrees) required by M. margaritifera for completion of metamorphosis. In a standardized laboratory experiment, excystment of juvenile mussels from brown trout (Salmo trutta) was found between 1700 and 3400 day degrees post infestation, indicating highly variable, development times of individual glochidia and the absence of a previously postulated threshold temperature of >= 15 degrees C for successful excystment of

living juveniles. Consequently, the parasitic phase does not seem to limit the current distribution range and reintroduction of the species into cool headwater areas, as well as the culturing under constant water temperature conditions in typical salmonid fish hatchery setups. The concept of day degrees of development may also be useful to test the ecological implications of observed genetic differences among different populations. (C) 2013 Elsevier GmbH. All rights reserved.

3534: ***+.225***

We developed an individual-based population viability analysis model (females only) for evaluating risk to populations from catastrophic events or conservation and research actions. This model tracks attributes (size, weight, viability, etc.) for individual fish through time and then compiles this information to assess the extinction risk of the population across large numbers of simulation trials. Using a case history for the Little Colorado River population of Humpback Chub Gila cypha in Grand Canyon, Arizona, we assessed extinction risk and resiliency to a catastrophic event for this population and then assessed a series of conservation actions related to removing specific numbers of Humpback Chub at different sizes for conservation purposes, such as translocating individuals to establish other spawning populations or hatchery refuge development. Our results suggested that the Little Colorado River population is generally resilient to a single catastrophic event and also to removals of larvae and juveniles for conservation purposes, including translocations to establish new populations. Our results also suggested that translocation success is dependent on similar survival rates in receiving and donor streams and low emigration rates from recipient streams. In addition, translocating either large numbers of larvae or small numbers of large juveniles has generally an equal likelihood of successful population establishment at similar extinction risk levels to the Little Colorado River donor population. Our model created a transparent platform to consider extinction risk to populations from catastrophe or conservation actions and should prove useful to managers assessing these risks for endangered species such as Humpback Chub. Received August 28, 2012; accepted March 13, 2013

3535: ***-.031***

The eastern massasauga Sistrurus catenatus catenatus is a declining species for which a captive breeding program was established in 2006. To effectively manage wild and captive populations, an understanding of genetic diversity within the species is necessary. We analyzed mitochondrial DNA sequences of 186 individuals: 109 wild snakes from 34 U. S. and Canadian counties and districts, all 52 breeding program members (23 of known and 29 of unknown origin), 18 other captives of unknown origin, and 7 outgroup representatives of desert massasauga S. c. edwardsii, and western massasauga, S. c. tergeminus. Statistical parsimony, maximum likelihood, and maximum parsimony analyses all identified eastern massasaugas as divergent from western and desert massasaugas. We found 18 different haplotypes among eastern massasaugas, comprising three geographically and genetically differentiated NADH dehydrogenase II (ND2) subunits that potentially reflect post-Pleistocene range expansion from unglaciated into formerly glaciated regions. Snakes of unknown origin could all be assigned unambiguously to these ND2 subunits. To maintain natural genetic variation, preserve diversity in captive lineages, and allow future augmentation or reintroduction, the Association of Zoos and Aquariums is managing these three geographic ND2 subunits separately within the Eastern Massasauga Species Survival Plan breeding program.

3536: ***+.152***

Migratory and non-migratory Whooping Cranes (Grus americana) historically inhabited

southwestern Louisiana until they were extirpated in 1950. Little is known about the feeding habits or dietary items of these cranes except for anecdotal evidence from local residents provided to R. P. Allen for his influential 1952 work on Whooping Cranes. Other populations of Whooping Cranes have been characterized as opportunistic omnivores, consuming small vertebrates, invertebrates, and plant material. In 2009, southwestern Louisiana was selected as a reintroduction site for Whooping Cranes. We report on four observations of reintroduced Whooping Cranes depredating at least two different species of turtles (common snapping turtle and mud turtle), which have not been previously reported as a dietary item for this species.

3538: ***-.042***

An experimental release of the European mink (Mustela lutreola) was carried out in the Salburua wetland in North Spain between 2008 and 2010. A partial removal of feral American mink (Neovison vison) was done preceding the release. The survival and the cause of death of each of 27 captive-bred minks were studied during five months after release. Only 22 % of the minks (N=6) survived during whole radio-tracking period. Predation was the most significant cause of mortality (76 %, N=16). Seven European minks (33 % of mortality) were killed by another "mink-size" carnivore-the causes of death of these individuals were of particular interest to clarify possible impact of a few remained American mink to released European mink. We used three criteria to identify the exact causes of death of these seven minks: 1. Comparison of the distances of bite marks with the inter-canine distances of small carnivores, 2. Site descriptions and signs of predators and 3. Density of carnivores within the study area. None of the criteria taken separately allowed the complete identification of the predator species. Summing up the results of all three criteria, a male American mink was found to be the most likely predator of at least six released European minks (29 % of overall mortality and 38 % of predated minks). Our results show that the presence of American mink, even if the number is estimated to be low, may seriously limit the success of reinforcement or reintroduction of the European mink.

3539: ***+.167***

The translocation of individuals or populations is a management strategy that is widely used in conservation, especially for rare or threatened species. In September 2005, an Ornate Hawk-eagle (Spizaetus ornatus) nest with a nestling was found near the newly-constructed Barra Grande dam, on the Pelotas River, in northern Rio Grande do Sul. The nest was 1.20 m above the water surface and at risk of be submerged, and both the nestling and its nest were transported to a safe location 380 m away from the original location and 30 m above the high water level of the reservoir. After translocation the nestling was monitored for 60 days, until fledging. Translocation was considered successful due to the acceptance of the translocated nestling by the adults, inferred by the observation of parental care and nest defense after translocation. The in situ management that we report may be a useful alternative for ex situ management, at least in specific cases. It also must warn us of the need to have a raptor monitoring and rescue program during the construction of hydroelectric plants.

3540: ***+.038***

After the quasi-extinction of much of the European vertebrate megafauna during the last few centuries, many reintroduction projects seek to restore decimated populations. However, the future of numerous species depends on the management scenarios of metapopulations where the flow of individuals can be critical to ensure their viability. This is the case of the bearded vulture Gypaetus barbatus, an Old World, large body-sized and long-lived scavenger living in mountain ranges.

Although persecution in Western Europe restrained it to the Pyrenees, the species is nowadays present in other mountains thanks to reintroduction projects. We examined the movement patterns of pre-adult non-breeding individuals born in the wild population of the Pyrenees (n = 9) and in the reintroduced populations of the Alps (n = 24) and Andalusia (n = 13). Most birds were equipped with GPS-GSM radio transmitters, which allowed accurate determination of individual dispersal patterns. Two estimators were considered: i) step length (i.e., the distance travelled per day by each individual, calculated considering only successive days); and ii)total dispersal distance (i.e., the distance travelled between each mean daily location and the point of release). Both dispersal estimators showed a positive relationship with age but were also highly dependent on the source population, birds in Andalusia and Alps moving farther than in Pyrenees. Future research should confirm if differences in dispersal distances are the rule, in which case the dynamics of future populations would be strongly influenced. In summary, our findings highlight that inter-population differences can affect the flow of individuals among patches (a key aspect to ensure the viability of the European metapopulation of the endangered bearded vulture), and thus should be taken into account when planning reintroduction programs. This result also raises questions about whether similar scenarios may occur in other restoration projects of European megafauna.

3542: *+.034*

Population declines of Greater Sage-Grouse (Centrocercus urophasianus) throughout the western United States have been attributed to the loss, degradation, and fragmentation of sagebrush (Artemisia spp.) habitats. Increased energy development may further fragment sagebrush habitat, isolating sage-grouse populations and resulting in genetic drift, inbreeding, local extinction, or rapid divergence. We conducted a genetic survey of 3 remote sage-grouse populations in northeastern Utah to assess mitochondrial diversity relative to other portions of the species' range. We did not detect any unusual haplotype compositions in these populations. However, haplotype composition of the Anthro Mountain and Strawberry Valley reference populations differed from haplotype compositions of other northeastern Utah populations. These populations are spatially separated by Desolation Canyon of the Green River. This canyon may constitute a geographic barrier to gene flow in this area, given low population densities and reduced dispersal potentials. This potential barrier will be an important consideration in future conservation efforts such as translocations. The halotype composition of the Anthro Mountain and Strawberry Valley reference populations may be altered by translocations subsequent to our sampling effort. The effect of these translocations on the reference halotypes and population vital rates is currently under study.

3543: *+.106*

Since the 1980s, the European eels' stocks have dramatically decreased with no sign of recovery, resulting in their classification as Critically endangered on the IUCN red list of threatened species. The European Council Regulation 1100/2007 requires that 35% of glass eels caught annually by fishing be released in European waters for restocking. However, the efficiency of this measure on population viability has never been evaluated. Here, we estimated demographic parameters of a stocked population of French eels using a multistate capture-recapture model. Using these estimates, we then estimated population size and predicted the number of future genitors obtained by stocking. We found that the stage in which eels were stocked did not influence their future survival and that the maximal number of silver eels was quickly reached, after 3years following stocking. We concluded that stocking experiments in the Mediterranean region are efficient for fast production of genitors. We suggest that further studies should assess the quality of these genitors.

3544: *-.009*

The rehabilitation of native communities by means of eradicating unwanted fish species using piscicides is an example of employing disturbance to achieve conservation successes. Such projects provide a valuable opportunity to test the efficiency of the tool and the impacts on the receiving aquatic communities, as disturbance occurs at a known time. The piscicide rotenone' has been widely used to eradicate invasive or unwanted fish species worldwide. However, there is little information regarding the impact on native fish being reintroduced to a stream after rotenone treatment. The mass depletion of aquatic invertebrates due to rotenone dosing is of particular concern, as food-limitation could negatively impact on fish growth, condition and recruitment, compromising the aims of rehabilitation. For the first time in New Zealand, rotenone was employed to eradicate brown trout (Salmo trutta) from two streams that also supported populations of banded kokopu (Galaxias fasciatus). Impacts on fish and aquatic invertebrates were studied in two treatment and two reference streams in Karori, Wellington. Analysis showed that invertebrate densities declined significantly in the treatment streams in the 2-week to 2-month period after dosing. Following reintroduction after rotenone treatment, banded kokopu condition declined significantly and levels of fish mobility were variable. One year after rotenone dosing, there was recruitment of banded kokopu juveniles in the treatment streams indicating successful reproduction, with no equivalent increase in the reference streams. Results are a positive indication for the use of rotenone as an effective conservation tool to remove unwanted fish species where they threaten native populations.

3545: *+.080*

In most metapopulation models dispersal is assumed to be a fixed species-specific trait, but in reality dispersal abilities are highly sensitive to various selective pressures. Strict isolation of a metapopulation, which precludes any influx of immigrants (and their genes) from outside and makes it impossible for emigrants to reach other localities with suitable habitat, thus reducing fitness benefits of long-distance dispersal to zero, may be expected to impose strong selection against dispersal. We tested the above prediction by comparing dispersal parameters derived with the Virtual Migration model for isolated and non-isolated metapopulations of two species of large blue Maculinea (= Phengaris) butterflies, surveyed with intensive mark-recapture. Mortality during dispersal was found to be twice (in M. teleius) to five times higher (in M. arion) in isolated metapopulations. Isolation also resulted in significantly reduced dispersal distances in isolated metapopulations, with the effect being particularly strong in M. arion females. Apart from its evolutionary and ecological consequences, dispersal depression in isolated butterfly metapopulations implied by our results has serious conservation implications. It provides a clear argument against using parameter values obtained in a different environmental setting in modelling applications, e.g., Population Viability Analyses or environmental impact assessment. Furthermore, it underlines the importance of establishing well-connected networks of suitable habitats prior to species release in areas where reintroductions are planned.

3546: *+.154*

Quaking aspen (Populus tremuloides) forests develop complex, multi-story structure and speciose plant communities, which provide habitat for ungulates and diverse wildlife species. Successfully recruiting aspen sprouts and seedlings provide important sources of structural, functional and genetic diversity vital to resilient aspen forests. Chronic ungulate browsing of regenerating aspen can degrade aspen community structure and diversity. This simplifies food webs and can have negative implications for ecosystem resilience. This paper explores how patterns of ungulate

herbivory in aspen forests are influenced by and affect bottom-up and top-down forces in aspen ecosystems. We outline management strategies aimed at decreasing ungulate and livestock impacts on aspen and increasing sprout survival and recruitment. The body of aspen research indicates that herbivory is more heterogeneous in areas that contain human hunters, predators, or fire on the landscape. The complexities of ungulate herbivory and fire on aspen ecosystems, especially in relation to scale, are imperfectly understood. Wildlife agencies responsible for elk (Cervus elaphus) and deer (Odocoileus spp.) populations should consider management strategies that use ungulate herbivory impacts on ecosystems such as aspen as indicators of sustainable herd densities. To increase aspen resilience in the face of current and future environmental change, we recommend a multi-faceted approach that involves enhancing bottom-up forces while decreasing top-down impacts from ungulates. (C) 2013 Elsevier B.V. All rights reserved.

3547: ***-.036***

Genetic variation in the major histocompatibility complex (MHC) is known to affect disease resistance in many species. Investigations of MHC diversity in populations of wild species have focused on the antigen presenting class II molecules due to the known polymorphic nature of these genes and the role these molecules play in pathogen recognition. Studies of MHC haplotype variation in the turkey (Meleagris gallopavo) are limited. This study was designed to examine MHC diversity in a group of Eastern wild turkeys (Meleagris gallopavo silvestris) collected during population expansion following reintroduction of the species in southern Wisconsin, USA. Southern blotting with BG and class II probes and single nucleotide polymorphism (SNP) genotyping was used to measure MHC variation. SNP analysis focused on single copy MHC genes flanking the highly polymorphic class II genes. Southern blotting identified 27 class II phenotypes, whereas SNP analysis identified 13 SNP haplotypes occurring in 28 combined genotypes. Results show that genetic diversity estimates based on RFLP (Southern blot) analysis underestimate the level of variation detected by SNP analysis. Sequence analysis of the mitochondrial D-loop identified 7 mitochondrial haplotypes (mitotypes) in the sampled birds. Results show that wild turkeys located in southern Wisconsin have a genetically diverse MHC and originate from several maternal lineages.

3548: ***+.180***

Translocation of mountain quail (Oreortyx pictus) to restore viable populations to their former range has become a common practice. Because differences in post-release vital rates between animals from multiple source populations has not been well studied, wildlife and land managers may arbitrarily choose the source population or base the source population on immediate availability when planning translocation projects. Similarly, an understanding of the optimal proportion of individuals from different age and sex classes for translocation would benefit translocation planning. During 2006 and 2007, we captured and translocated 125 mountain quail from 2 ecologically distinct areas: 38 from southern California and 87 from southwestern Oregon. We released mountain quail in the Bennett Hills of south-central Idaho. We radio-marked and monitored a subsample of 58 quail and used them for a 2-part survival analysis. Cumulative survival probability was 0.23 +/- 0.05 (SE) at 150 days post-release. We first examined an a priori hypothesis (model) that survival varied between the 2 distinct source populations. We found that source population did not explain variation in survival. This result suggests that wildlife managers have flexibility in selecting source populations for mountain quail translocation efforts. In a post hoc examination, we pooled the quail across source populations and evaluated differences in survival probabilities between sex and age classes. The most parsimonious model indicated that adult male survival was substantially less than survival rates of other mountain quail age and sex

classes (i.e., interaction between sex and age). This result suggests that translocation success could benefit by translocating yearling males rather than adult males, perhaps because adult male breeding behavior results in vulnerability to predators. (c) 2013 The Wildlife Society.

3549: ***+.029***

In this issue of Molecular Ecology, Neuwald & Templeton (2013) report on a 22-year study of natural populations of Collared Lizards (Crotaphytus collaris) that evolved on isolated on rock outcrops (glades') in the Ozark Mountains in eastern Missouri. This ecosystem was originally maintained by frequent fires that kept the forest understory open, but fire-suppression was adopted as official policy in about 1945, which led to a loss of native biodiversity, including local extinctions of some lizard populations. Policies aimed at restoring biodiversity included controlled burns and re-introductions of lizards to some glades, which began in 1984. Populations were monitored from 1984-2006, and demographic and genetic data collected from 1679 lizards were used to documents shifts in meta-population dynamics over four distinct phases of lizard recovery: 1-an initial translocation of lizards drawn from the same source populations onto three glades that were likely part of one meta-population; 2-a period of isolation and genetic drift associated with the absence of fires; 3-a period of rapid colonization and population increase following restoration of fire; and 4-stabilization of the meta-population under regular prescribed burning. This study system thus provides a rare opportunity to characterize the dynamics of a landscape-scale management strategy on the restoration of the meta-population of a reintroduced species; long-term case studies of the extinction, founding, increase, and stabilization of a well-defined meta-population, based on both demographic and population genetic data, are rare in the conservation, ecological, and evolutionary literature.

3550: ***+.089***

Until the early 1980s the only surviving population of the greater one-horned rhinoceros Rhinoceros unicornis in Nepal was in Chitwan National Park. Between 1986 and 2003 87 rhinoceroses from Chitwan were translocated into Bardia National Park and Suklaphanta Wildlife Reserve in the western terai region to establish founder populations and reduce the threat of local extinction from natural catastrophic events, disease and/or poaching. The founder populations increased in number through births but a rise in poaching during the period of civil strife in Nepal during 1996-2006 resulted in a dramatic decline in the populations, including in Chitwan. In 2001 the Terai Arc Landscape programme was initiated to connect 11 protected areas in Nepal and north-west India and facilitate dispersal of megafauna and manage them as metapopulations. Corridors that were restored under the programme and that connect Bardia and Suklaphanta with protected areas in India are now used by the greater one-horned rhinoceros. The successes and failures of the last 2 decades indicate that new paradigms for protecting rhinoceroses within and outside protected areas are needed, especially with reference to managing this species at a landscape scale.

3551: ***-.061***

The giant tortoises (Aldabrachelys or Dipsochelys) of the Indian Ocean Islands have been in decline since the first human settlement of the islands. They retain only a single natural population on Aldabra Atoll (and possibly Ile aux Cerfs, where tortoises are descendants of a mixture of indigenous and imported animals). Several additional wild populations are known, resulting from reintroductions to the historic range and introductions outside of that range. The historical distribution of tortoises in Seychelles is summarized, with reliable tortoise records from only 4

coralline islands and 23 granitic islands and the status of all the wild populations reviewed. This includes the first census of the Fregate Island population. In the granitic islands, only 9 islands support tortoises today and the wild population of these islands is estimated at 500-550 adults. In the coralline islands, tortoises are now present on 11 islands, with a total population of over 100,000 (almost all on Aldabra). Climate change impacts over the next 100 yrs are expected to be severe in low-lying areas of the Seychelles Islands attributable to sea-level rise and storm impacts on coastal erosion. These are projected to result in the loss of many populations and significant declines in the Aldabra population. As a result, the species should be regarded as Vulnerable by IUCN Red List Threat Criteria. Reintroduction to more of the high granitic islands could offset some of these projected declines, and it is recommended that such reintroductions be included in future conservation programs to restore ecosystem function.

3552: *+.141*

We present the outcome of a century of post-bottleneck isolation of a long-lived species, the little spotted kiwi (Apteryx owenii, LSK) and demonstrate that profound genetic consequences can result from protecting few individuals in isolation. LSK were saved from extinction by translocation of five birds from South Island, New Zealand to Kapiti Island 100 years ago. The Kapiti population now numbers some 1200 birds and provides founders for new populations. We used 15 microsatellite loci to compare genetic variation among Kapiti LSK and the populations of Red Mercury, Tiritiri Matangi and Long Islands that were founded with birds from Kapiti. Two LSK native to D'Urville Island were also placed on Long Island. We found extremely low genetic variation and signatures of acute and recent genetic bottleneck effects in all four populations, indicating that LSK have survived multiple genetic bottlenecks. The Long Island population appears to have arisen from a single mating pair from Kapiti, suggesting there is no genetic contribution from D'Urville birds among extant LSK. The N-e/N-C ratio of Kapiti Island LSK (0.03) is exceptionally low for terrestrial vertebrates and suggests that genetic diversity might still be eroding in this population, despite its large census size.

3553: *+.140*

Corals in the Gulf I withstand summer temperatures up to 10 degrees C higher than corals elsewhere and have recovered from extreme temperature events in 10 years or less. This heat-tolerance of Gulf corals has positive implications for the world's coral populations to adapt to increasing water temperatures. However, survival of Gulf corals has been severely tested by 35-37 degrees C temperatures five times in the last 15 years, each time causing extensive coral bleaching and mortality. Anticipated future temperature increases may therefore challenge survival of already highly stressed Gulf corals. Previously proposed translocation of Gulf corals to introduce temperature-adapted corals outside of the Gulf is assessed and determined to be problematical, and to be considered a tool of last resort. Coral culture and transplantation within the Gulf is feasible for helping maintain coral species populations and preserving genomes and adaptive capacities of Gulf corals that are endangered by future thermal stress events. (C) 2012 Elsevier Ltd. All rights reserved.

3554: *-.016*

It has been recognized the need for studies to determine the long-term viability of populations of endangered species, such as the Tehuantepec jackrabbit (Lepus flavigularis) populations. Currently the total population is estimated at less than 1000 individuals. We developed a viable population analysis to determine the extinction risk of the Tehuantepec jackrabbit population of Santa Maria

del Mar, Oaxaca. A total of 31 scenarios were modeled in order to test independent and combined effect of flooding, predation by domestic dogs and poaching; also we tested the effect of inbreeding depression and a hypothetical reintroduction program. The results show that the Tehuantepec jackrabbit population is at high risk of extinction. The population survived over the 500 years of simulation only in the model which involved the hunt, besides the base model. The effects of the three catastrophic scenarios in combination, as well as the inbreeding, increased the risk of extinction up to 100% and an average of 41.60 [plus or minus] 25.88 years. Based on our results, we propose that conservation and management strategies should include the elimination of threats that affect the Tehuantepec jackrabbit, as well as improvement of habitat quality. Also, assess the relevance of a translocation program with individuals from other populations.

3555: *-.025*

To infer the population genetic structure and genetic diversity of Itasenpara bitterling (Acheilognathus longipinnis), a cyprinid species endemic to Japan and distributed in only three specific regions, we investigated mitochondrial DNA variation. The distribution of the haplotypes among the three regions showed distinct geographic structure, and no common haplotypes were observed among regions. Analysis of molecular variance revealed a significant proportion of the genetic variance was partitioned among regions (93.1 %, P < 0.001), and pairwise estimates of D-A and I broken vertical bar(ST) between regions also revealed strong population structure. Given the strong genetic structure and low genetic diversity within regions, we strongly suggest that each region should be treated as a separate unit in any conservation program and any inter-regional translocations should be avoided.

3556: *+.059*

Noble crayfish (Astacus astacus L.), the most highly valued freshwater crayfish in Europe, is threatened due to a long-term population decline caused mainly by the spread of crayfish plague. Reintroduction of the noble crayfish into restored waters is a common practice but the geographic and genetic origin of stocking material has rarely been considered, partially because previous genetic studies have been hampered by lack of nuclear gene markers with known inheritance. This study represents the first large scale population genetic survey of the noble crayfish (633 adults from 18 locations) based on 10 newly developed microsatellite markers. We focused primarily on the Baltic Sea area (Estonia, Finland and Sweden) where the largest proportion of the remaining populations exists. To allow comparisons, samples from the Black Sea catchment (the Danube drainage) were also included. Two highly differentiated population groups were identified corresponding to the Baltic Sea and the Black Sea catchments, respectively. The Baltic Sea catchment populations had significantly lower genetic variation and private allele numbers than the Black Sea catchment populations. Within the Baltic Sea area, a clear genetic structure was revealed with population samples corresponding well to their geographic origin, suggesting little impact of long-distance translocations. The clear genetic structure strongly suggests that the choice of stocking material for re-introductions and supplemental releases needs to be based on empirical genetic knowledge.

3557: *+.062*

Current understanding of genetic variation in lions (Panthera leo) is inadequate to guide many management decisions necessary for conservation of the species. We studied sequence variation in the mitochondrial cytochrome-b (cyt-b) gene of 75 lions and nuclear variation at 11 microsatellite loci of 480 lions across 8 range states (Cameroon, Uganda, Kenya, Zambia, Zimbabwe, South

Africa, Botswana, and Namibia) and 13 Lion Conservation Units (LCUs) plus two other unassigned sites (Cameroon and Zimbabwe). A total of 11 cyt-b haplotypes were found, whose variation follows an isolation-by-distance model. In combination with previously known sequences, the haplotypes document the close relationship, derived position, and limited variability of Asian and West and Central African lions relative to other extant lions. Both phylogenetic analyses and substitution networks identify two clades in Eastern and Southern Africa-one restricted to Namibia and South Africa and the other more widespread across the region. However, these analyses are equivocal on which of these is closest to the ancestor of modern lions. Microsatellite analyses showed high levels of variation within and among populations, subdivision among most LCUs, and evidence of isolation by distance. While rates of gene flow are generally low, admixture among lions in northern Botswana, Caprivi Strip (Namibia) and Zambia is apparent from STRUCTURE analyses. Conservation management plans should incorporate information on genetic variability and gene flow in delimiting management units and in guiding translocations of lions to minimize inbreeding and to control problem animals.

3558: *+.057*

Picconia azorica (Tutin) Knobl. (Oleaceae) is an endangered species, endemic to the Azores. Samples from 31 populations in 8 islands were genotyped using 8 newly developed nuclear microsatellite markers. From the amplified loci, 81% were polymorphic across all populations and the species showed a relatively high total genetic diversity (H-T = 0.7). Several populations were close to Hardy-Weinberg equilibrium while others presented positive F-IS values (0.02-0.2). The largest proportion of genetic variation (98%) occurred within populations and the level of differentiation between populations, was generally low, although 27% of the population pairwise comparisons showed relatively high differentiation values (0.25 <= R-ST <= 0.65). Relatively high levels of gene flow were also found among most populations. Using the Bayesian clustering method implemented in STRUCTURE we found a particular genetic pattern in Corvo samples, and also similarities between Santa Maria, Sao Miguel and Flores populations. Considerable levels of genetic admixture within P. azorica populations might have resulted from: (i) fruit dispersal by native birds; and/or (ii) human mediated dispersal between islands. Our results revealed the existence of some genetically depauperate populations needing specific conservation measures, and indicate that arbitrary translocation of individuals between islands should be avoided. (c) 2013 Elsevier Ltd. All rights reserved.

3559: *+.175*

1. Translocation of individuals from healthy source populations to newly colonize or recolonize suitable habitat is a vital tool for the conservation of a species. Demographic, genetic and landscape factors, but also acoustic signals and cultural factors, will all affect translocation success. 2. We investigated variation in song, and response to song, of the endangered North Island kkako Callaeas wilsoni (Bonaparte 1850) in New Zealand in two translocated populations and their source population. 3. We found significant vocal variation between the source population and both translocated populations, the latter of which had reduced repertoire sizes and increased repertoire sharing, as well as structurally different song elements of higher frequency and shorter duration. 4. Despite the song divergence and clear variability in the nature and level of response among populations, we did not find any evidence for discrimination against nonlocal song in our reciprocal playback experiments. 5. Synthesis and applications. Vocal divergence and reduced variability in translocated populations suggest founder effects or reduced social interaction rates. The variation could be viewed as cultural erosion and may undermine translocation success.

Persistence of response to playback, despite vocal divergence, suggested that social restrictions on gene flow require at least a few decades of separation after translocation. The decision to translocate individuals of threatened species is becoming a more common tool for species conservation world-wide. We argue that it is important to take vocal variation into account during such management decisions as it may affect success of establishment and persistence of translocated populations.

3560: -.022

Identifying factors that affect demographic parameters and how those factors act is vital for understanding population dynamics, especially of endangered species. Moreover, specific ideas in the population dynamics of large herbivores underpin the management of the critically endangered black rhinoceros (Diceros bicornis). We studied an expanding black rhinoceros population since its establishment in 1986 in the Great Fish River Reserve, South Africa, through 2008 when managed removals interrupted natural dynamics. During the study, only 13 animals died, of which nine were subadults. In a linear modelling context, we used information-theoretic methods to evaluate the influence of independent variables expected to affect demographic parameters. For females, age at first reproduction (AFR) increased with abundance, but there was no effect of abundance on adult fecundity as measured by inter-birth intervals (IBIs). We evaluated these results in the theoretical context of population dynamics of large herbivores, in particular, Eberhardt's proposal of a specific sequence in which demographic parameters first respond to increasing density. Our observations are consistent with Eberhardt's prediction that immature individuals are impacted before adults, but the relative timing and magnitude of density effects on immature individuals was unclear. Rainfall did not influence AFR or IBIs. Maternal age influenced IBIs but much of the observed variation in IBIs was not accounted for by structural variation. Studies of populations more nearly approaching a stable age distribution and carrying capacity are needed to resolve remaining uncertainties and ambiguities in the life history of the black rhinoceros in particular and megaherbivores in general.

3561: +.004

Translocations are an important tool for wildlife conservation, although progress in the field of reintroduction biology has been hindered by the ad hoc and opportunistic nature of many translocations. We used an experimental translocation to elucidate the role of raccoon roundworm (Baylisascaris procyonis) and inbreeding depression in the decline of the Allegheny woodrat (Neotoma magister), an endangered species. We translocated woodrats from genetically diverse populations in the core of the species range to 4 previously occupied sites (reintroductions) and 2 sites supporting genetically depauperate populations (reinforcements) in Indiana (U.S.A.). In 2 reintroduction sites and 1 reinforcement site, we distributed anthelmintic baits to passively deworm raccoons and reduce the risk of woodrat exposure to roundworms. The remaining sites served as controls. We used raccoon latrine surveys and fecal flotation to monitor temporal variability in roundworm prevalence and effect of treatment. We used live trapping and microsatellite genotyping to monitor the demographic and genetic response of translocated populations over the following 54 months. At the conclusion of the study, 4 of 6 translocations were successfully maintaining abundance through local recruitment. The distribution of anthelmintic baits reduced levels of roundworm contamination, but levels of contamination were also low in 2 of 3 control sites. Reintroductions failed at control sites, one of which was due to high roundworm exposure. The other failed control reintroduction was likely attributable to demographic stochasticity and limited reproductive potential following initial mortality within the first 4 months. In both control and treatment reinforcements, increases in both allelic richness and

heterozygosity were accompanied by increases in abundance, which is suggestive of genetic rescue. Our results demonstrate that mitigation of roundworm exposure through the distribution of anthelmintic baits can facilitate woodrat recovery and that diversity within genetically depauperate populations can be restored through the introduction of a limited number of individuals.

3562: *+.156*

The extinction of large herbivores, often keystone species, can dramatically modify plant communities and impose key biotic thresholds that may prevent an ecosystem returning to its previous state and threaten native biodiversity. A potentially innovative, yet controversial, landscape-based long-term restoration approach is to replace missing plant-herbivore interactions with non-native herbivores. Aldabran giant (Aldabrachelys gigantea) and Madagascan radiated (Astrochelys radiata) tortoises, taxonomically and functionally similar to the extinct Mauritian giant tortoises (Cylindraspis spp.), were introduced to Round Island, Mauritius, in 2007 to control the non-native plants that were threatening persistence of native species. We monitored the response of the plant community to tortoise grazing for 11 months in enclosures before the tortoises were released and, compared the cost of using tortoises as weeders with the cost of using manual labor. At the end of this period, plant biomass; vegetation height and cover; and adult, seedling, flower, and seed abundance were 3-136 times greater in adjacent control plots than in the tortoise enclosures. After their release, the free-roaming tortoises grazed on most non-native plants and significantly reduced vegetation cover, height, and seed production, reflecting findings from the enclosure study. The tortoises generally did not eat native species, although they consumed those native species that increased in abundance following the eradication of mammalian herbivores. Our results suggest that introduced non-native tortoises are a more cost-effective approach to control non-native vegetation than manual weeding. Numerous long-term outcomes (e.g., change in species composition and soil seed bank) are possible following tortoise releases. Monitoring and adaptive management are needed to ensure that the replacement herbivores promote the recovery of native plants.

3563: *+.139*

1. Freshwater unionoids are one of the most threatened animal groups worldwide and the freshwater pearl mussel Margaritifera margaritifera is currently listed as critically endangered in Europe. The EC Habitats Directive requires that EU Member States monitor the distribution and abundance of this species and report regularly on its conservation status.2. The pearl mussel meta-population in Northern Ireland was surveyed to assess temporal population trends in Special Areas of Conservation (SACs) and mussel reproduction throughout its range.3. Mussels occurred in six rivers and numbers within three SAC designated sites remained stable between 2004-2007 and 2011. The discovery of more than 8000 previously unknown individuals in the Owenreagh River contributed to an overall increase (+56.8%) in the total known population. All populations actively reproduced during 2010 with approximately half of all individuals gravid. Moreover, suitable salmonid hosts occurred at all sites with 10.7% of salmon and 22.8% of trout carrying encysted glochidia. Populations were composed entirely of aged individuals with little evidence of recent recruitment.4. It is inferred that the break in the life cycle must occur during the juvenile stage when glochidia metamorphose and settle into the interstitial spaces within the substrate. Water quality parameters, most notably levels of suspended solids, exceeded the recommended maximum thresholds in all rivers.5. It is posited that the deposition of silt may be the main cause of juvenile mortality contributing to a lack of recruitment. Consequently, all populations were judged to be in unfavourable' conservation status. Catchment-level management plans are urgently needed to reduce siltation with the aim of improving recruitment. These results have implications

for the success of ex situ conservation programmes; specifically, the size at which captive-bred juveniles are released into the wild. Further research is required to assess the vulnerabilities of early life-stages of M. margaritifera to siltation. Copyright (c) 2012 John Wiley & Sons, Ltd.

3564: *-.031*

In Europe, the species Sympetrum depressiusculum is classified as vulnerable with a high risk of extinction in the wild. It is a habitat specialist, the presumed main reason for its vulnerability is the destruction of its natural habitats. Other causes of its general extinction are unknown. Published information regarding dispersal rate and philopatry is not available, although these are evolutionary strategies that can play key roles in susceptibility to environmental change. We compared the rate of philopatry in S. depressiusculum and three other related, abundant but not endangered species of the same genus (S. sanguineum, S. striolatum, S. vulgatum). We collected data in a very isolated site in the Czech Republic, more than 100 km distant from another known population of the species. Using exuviae collection (total of 6,157 exuviae) and capture-mark-recapture (total of 2,188 adults marked) methods, we acquired data allowing us to compare the numbers of emerged individuals and adults returning to the maternal site. We found a difference of nearly an order of magnitude between the philopatry of S. depressiusculum and the three other species. While in S. depressiusculum philopatry was almost 100 %, in the other species it was < 10 %. We suggest the high rate of philopatry can influence the vulnerability of S. depressiusculum in landscape altered by humans. Strict protection of the natal sites is very important for preserving species having this evolutionary strategy, and reintroductions and translocations should also be undertaken to reduce the extinction risk of this endangered species.

3565: *+.001*

Translocations are frequently used to increase the abundance and range of endangered fishes. One factor likely to affect the outcome of translocations is fish movement. We introduced embryos from five Westslope Cutthroat Trout Oncorhynchus clarkii lewisi populations (both hatchery and wild) at five different locations within a fishless watershed. We then examined the movement of age-1 and age-2 fish and looked for differences in movement distance among source populations and among introduction sites; we also examined the interactions among age, population, and introduction site. At age 1, most individuals (90.9%) remained within 1,000m their introduction sites. By age 2, the majority of individuals (58.3%) still remained within 1,000m of their introduction site, but considerably more individuals had moved downstream, some more than 6,000m from their introduction site. We observed a significant interaction between age and source population (F-4,F- 1077= 15.45, P < 0.0001) as well as between age and introduction site (F-41,F-1077 = 11.39, P < 0.0008), so we presented results in the context of these interactions. Within age-groups, we observed differences in movement behavior among source populations and among donor populations of Westslope Cutthroat Trout. We discuss these findings in light of previous research on juvenile salmonid movement.

3566: *+.052*

We conducted a telemetry study of Shoal Bass Micropterus cataractae in the lower Flint River, Georgia, during 2010. Our objectives were to (1) characterize Shoal Bass migration from communal spawning habitats and (2) evaluate the effects of translocation on survival of adult Shoal Bass and determine its value as a viable restoration and management tool. Twenty-seven adult Shoal Bass (TL 305mm) were fitted with hydroacoustic tags. Thirteen fish (control group) were released at the site of capture, and the remaining 14 individuals were transported 75 river

kilometers (rkm) downstream prior to release (translocated group). Telemetry data suggested that spawning congregations are composed of fish from local (<3 rkm) and distant (>3 rkm) home ranges. Initially, within 14 d, translocated fish remained near their release site significantly longer than control fish remained at the original capture site. After 90 d at large, the distance that control Shoal Bass dispersed from the release site was similar to distances dispersed by the translocated group, 1.6-23.0 rkm and 2.6-28.6 rkm, respectively. After 90 d, translocated Shoal Bass had not returned to the same river reach that was occupied by control fish. No sex-specific differences were found between groups. Postrelease survival after 90 d was 92% for both translocated and control groups. Telemetry results indicated that Shoal Bass in the Flint River undergo substantial migrations to spawning habitats. Stockpiling of Shoal Bass from live-release tournaments may occur in the short term, and relocation away from home ranges may occur in the long term, thus affecting adult Shoal Bass distribution. The high survival rates and the eventual dispersal of most translocated fish observed in this study suggest Shoal Bass can cope with translocation.

3567: *+.098*

High propagule pressure is arguably the only consistent predictor of colonization success. More individuals enhance colonization success because they aid in overcoming demographic consequences of small population size (e.g. stochasticity and Allee effects). The number of founders can also have direct genetic effects: with fewer individuals, more inbreeding and thus inbreeding depression will occur, whereas more individuals typically harbour greater genetic variation. Thus, the demographic and genetic components of propagule pressure are interrelated, making it difficult to understand which mechanisms are most important in determining colonization success. We experimentally disentangled the demographic and genetic components of propagule pressure by manipulating the number of founders (fewer or more), and genetic background (inbred or outbred) of individuals released in a series of three complementary experiments. We used Bemisia whiteflies and released them onto either their natal host (benign) or a novel host (challenging). Our experiments revealed that having more founding individuals and those individuals being outbred both increased the number of adults produced, but that only genetic background consistently shaped net reproductive rate of experimental populations. Environment was also important and interacted with propagule size to determine the number of adults produced. Quality of the environment interacted also with genetic background to determine establishment success, with a more pronounced effect of inbreeding depression in harsh environments. This interaction did not hold for the net reproductive rate. These data show that the positive effect of propagule pressure on founding success can be driven as much by underlying genetic processes as by demographics. Genetic effects can be immediate and have sizable effects on fitness.

3569: *+.335*

Improving our understanding of resources required by threatened small mammals is directly relevant to the success of habitat restoration and species reintroduction programs. In a case study based on southern brown bandicoots (Isoodon obesulus obesulus; Mammalia: Peramelidae) occupying a remnant of open forest with a sclerophyllous shrub understory, we investigated microhabitat composition using multivariate analysis, and disproportional use of these habitats using a variety of techniques, including principal canonical correlation vectors, chi-square test, compositional analysis (CA), and nonparametric multiplicative regression. Spool-and-line tracking of bandicoots enabled floristic and structural parameters to be recorded from sites of activity and compared with sites randomly located within 5-ha grids centered over each trapping transect. Each of the 4 methods applied contributed useful interrogation of the potential resources required by I.

o. obesulus, with most disproportional use of microhabitats across activities detected using CA. Analyses supported fine-scale preference for Xanthorrhoea semiplana-dominated microhabitat across all activities, nesting in Banksia ornata-dominated microhabitat, moving and foraging in Allocasuarina muelleriana subsp. muelleriana microhabitat, impartial use of and increased reliance on burrows for shelter in Eucalyptus cosmophylla open forest with Melaleuca decussata understory microhabitats, and avoidance of Cyperaceae-dominated microhabitat and mixed heath. These results show that within broadly suitable vegetation communities, I. o. obesulus differentially utilizes a mosaic of microhabitats for a range of activities associated with shelter and foraging. Hence, the success of reintroduction and habitat restoration programs may be improved by considering the availability of preferred microhabitats (or alternative structures in suboptimal habitats, e.g., burrows), and protecting or revegetating indicator and associated species of known preferred microhabitats, respectively.

3570: *+.180*

Decision making in guidance of reintroduction efforts is made challenging by the substantial scientific uncertainty typically involved. However, a less recognized challenge is that the management objectives are often numerous and complex. Decision makers managing reintroduction efforts are often concerned with more than just how to maximize the probability of reintroduction success from a population perspective. Decision makers are also weighing other concerns such as budget limitations, public support and/or opposition, impacts on the ecosystem, and the need to consider not just a single reintroduction effort, but conservation of the entire species. Multiple objective decision analysis is a powerful tool for formal analysis of such complex decisions. We demonstrate the use of multiple objective decision analysis in the case of the Florida non-migratory whooping crane reintroduction effort. In this case, the State of Florida was considering whether to resume releases of captive-reared crane chicks into the non-migratory whooping crane population in that state. Management objectives under consideration included maximizing the probability of successful population establishment, minimizing costs, maximizing public relations benefits, maximizing the number of birds available for alternative reintroduction efforts, and maximizing learning about the demographic patterns of reintroduced whooping cranes. The State of Florida engaged in a collaborative process with their management partners, first, to evaluate and characterize important uncertainties about system behavior, and next, to formally evaluate the tradeoffs between objectives using the Simple Multi-Attribute Rating Technique (SMART). The recommendation resulting from this process, to continue releases of cranes at a moderate intensity, was adopted by the State of Florida in late 2008. Although continued releases did not receive support from the International Whooping Crane Recovery Team, this approach does provide a template for the formal, transparent consideration of multiple, potentially competing, objectives in reintroduction decision making. (c) 2013 The Wildlife Society.

3571: *-.027*

Population variation in life history can be important for predicting successful establishment and persistence of reintroduced populations of endangered species. The Laysan duck (Anas laysanensis) is an endangered bird native to the Hawaiian Archipelago that was extirpated from most islands after the introduction of mammalian predators. Laysan ducks were restricted to a single remote island, Laysan Island (4.1km(2)), for nearly 150 years. Since the species is not known to disperse between distant Hawaiian Islands today, 42 wild birds from Laysan Island were translocated to another mammalian predator-free low-lying atoll (Midway Atoll; 6.0km(2)) to reduce extinction risk. We explored how variation in demography influences establishment and

longer-term retention of genetic diversity (rare alleles) for reintroductions of this species. We observed dramatic differences in population growth between the source (=1.18) and reintroduced (=3.28) population. The number of eggs hatched at Midway Atoll was greater than at Laysan Island, however, we found no difference in hatching success (proportion of clutch hatched) between populations. Adult females produced 3 times as many fledglings per breeding year on Midway Atoll compared to Laysan Island. We estimated population abundance of both populations until 2010 and applied a Gompertz model with a Bayesian approach to infer density dependence, process variation, observation error, and carrying capacity for the Laysan Island and Midway Atoll populations. The carrying capacity from the Gompertz model for Midway Atoll (K=883 +/- 210 SD) was estimated to be greater than that of Laysan Island (K=598 +/- 76 SD). Translocations with small numbers of founders and no immigration can create population bottlenecks, leading to loss of genetic variation over time, and potentially reducing the reintroduced population's viability or its potential to serve as a source for future translocations. Therefore, we also assessed the probability of retaining rare alleles in an isolated reintroduced Laysan duck population using life history parameters observed from the Laysan Island and Midway Atoll populations; we concluded that additional founders are needed under scenarios using demographic estimates from both Laysan Island and Midway Atoll to retain either 90% or 95% of source population genetic diversity. (c) 2013 The Wildlife Society.

3572: ***+.244***

Reintroduction can be necessary for recovering populations of threatened species. However, the success of reintroduction efforts has been poorer than many biologists and managers would hope. To increase the benefits gained from reintroduction, management decision making should be couched within formal decision-analytic frameworks. Decision analysis is a structured process for informing decision making that recognizes that all decisions have a set of componentsobjectives, alternative management actions, predictive models, and optimization methodsthat can be decomposed, analyzed, and recomposed to facilitate optimal, transparent decisions. Because the outcome of interest in reintroduction efforts is typically population viability or related metrics, models used in decision analysis efforts for reintroductions will need to include population models. In this special section of the Journal of Wildlife Management, we highlight examples of the construction and use of models for informing management decisions in reintroduced populations. In this introductory contribution, we review concepts in decision analysis, population modeling for analysis of decisions in reintroduction settings, and future directions. Increased use of formal decision analysis, including adaptive management, has great potential to inform reintroduction efforts. Adopting these practices will require close collaboration among managers, decision analysts, population modelers, and field biologists. (c) 2013 The Wildlife Society.

3573: ***+.221***

Restocking and potential stock enhancement of natural abalone stocks by seeding hatchery-reared juvenile abalone into suitable habitat is currently being investigated or implemented in a number of countries, including Japan, Australia, New Zealand, Mexico, and South Africa. A series of experiments has been conducted to investigate the feasibility and development of the technology required to do this in the Sultanate of Oman. Wild seed were collected between Mirbat and Ras Atian on the Dhofar coast and kept in a hatchery for 7 wk. Qualitative and observational data were used to develop seeding site selection criteria and a seeding density of 10 juveniles/m(2). The juveniles were relocated and seeded in 18 different sites spread over a distance of 72 km. Sites selected with an abundance of boulders with a diameter of less than 50 cm yielded the highest recovery rates. Different categories of habitat availability and selection by seeded juveniles for

specific habitat categories varied significantly among sites, which was reflected in the range of recovery rates. Boulder habitats less than 50 cm in diameter supported the most recovered juveniles (P = 0.002 and P = 0.005). Survival rates over a period of 30, 60, and 90 days ranged from 0%-80% and proved highly site specific (P = 0.04). A positive correlation was found between average seed size and increased recovery rates (P = 0.015, R-2 = 0.25). Dispersal was limited in sites with high recovery rates. Site selection was shown to be vital. A simple seeding mechanism comprising a PVC tube proves successful as an alternative to seeding by hand.

3574: ***+.201***

The ideal conservation planning approach would enable decision-makers to use population viability analysis to assess the effects of management strategies and threats on all species at the landscape level. However, the lack of high-quality data derived from long-term studies, and uncertainty in model parameters and/or structure, often limit the use of population models to only a few species of conservation concern. We used spatially explicit metapopulation models in conjunction with multi-criteria decision analysis to assess how species-specific threats and management interventions would affect the persistence of African wild dog, black rhino, cheetah, elephant, leopard and lion, under six reserve scenarios, thereby providing the basis for deciding on a best course of conservation action in the South African province of KwaZulu-Natal, which forms the central component of the Maputaland-Pondoland-Albany biodiversity hotspot. Overall, the results suggest that current strategies of managing populations within individual, small, fenced reserves are unlikely to enhance metapopulation persistence should catastrophic events affect populations in the future. Creating larger and better-connected protected areas would ensure that threats can be better mitigated in the future for both African wild dog and leopard, which can disperse naturally, and black rhino, cheetah, elephant, and lion, which are constrained by electric fences but can be managed using translocation. The importance of both size and connectivity should inform endangered megafauna conservation and management, especially in the context of restoration efforts in increasingly human-dominated landscapes.

3575: ***+.145***

The Japanese crested ibis is an internationally conserved, critically threatened bird. Captive-breeding programs have been established to conserve this species in Japan. Since the current Japanese population of crested ibis originates only from 5 founders donated by the Chinese government, understanding the genetic diversity between them is critical for an effective population management. To discover genome-wide single nucleotide polymorphisms (SNPs) and short tandem repeats (STRs) while obtaining genotype data of these polymorphic markers in each founder, reduced representation libraries were independently prepared from each of the founder genomes and sequenced on an Illumina HiSeq2000. This yielded 316 million 101-bp reads. Consensus sequences were created by clustering sequence reads, and then sequence reads from each founder were mapped to the consensus sequences, resulting in the detection of 52,512 putative SNPs and 162 putative STRs. The numbers of haplotypes and STR alleles and the investigation of genetic similarities suggested that the total genetic diversity between the founders was lower, although we could not identify a pair with closely related genome sequences. This study provided important insight into protocols for genetic management of the captive breeding population of Japanese crested ibis in Japan and towards the national project for reintroduction of captive-bred individuals into the wild. We proposed a simple, efficient, and cost-effective approach for simultaneous detection of genome-wide polymorphic markers and their genotypes for species currently lacking a reference genome sequence.

3576: ***+.306***

Habitat evaluation is considered an essential step for assessing the potential for an area to support a viable reintroduced population. Remote sensing techniques can support such investigations, by greatly enhancing the temporal and spatial coverage of habitat assessments. This study makes use of freely available earth observation data to evaluate the suitability of the Ouadi Rime-Ouadi Achim Game Reserve in central Chad for the reintroduction of the Scimitar-horned Oryx. The reserve was the last stronghold of the species within its historical range, prior to extinction in the wild in the 1980s, providing a basis for reintroduction. Results show that since the 1980s, there has been a steady increase in average annual precipitation and satellite-derived estimates of primary production. The spatial downscaling of the average trend in primary production showed that the north of the protected area exhibited a drying trend over the period 1982-2008, while the south was associated with intense greening. As a result, the subdesert transition zone preferred by oryx is currently narrowing. If this is correct, this implies a potential reduction of favourable habitat for the oryx, which could have detrimental effects on the success of establishing a self-sustaining reintroduced population.

3577: ***+.100***

The reintroduction, recovery, and management of gray wolves (Canis lupus) in the western United States has long been a source of controversy and acrimonious public debate. I discuss 3 factors that perpetuate confusion and conflict over wolves and their management: 1) the role of science and politics in wildlife management, 2) rhetoric and political actions of elected officials and political appointees, and 3) justification of wolf harvest and specific practices employed during hunting and trapping seasons. Wildlife professionals could reduce the controversy surrounding wolves by 1) clearly delineating policy decisions from the scientific input used to inform these decisions; 2) clearly articulating scientific concepts when they are used in agency communications; 3) employing collaborative public-involvement processes designed to reduce conflicts (e.g., collaborative learning, alternative dispute resolution); and 4) setting hunting and trapping regulations with public input concerning the timing of seasons, and methods and locations of take. Longitudinal studies designed to assess how human attitudes and behaviors change in response to changing policies would be particularly valuable for understanding and potentially reducing conflicts over wolves (and other wildlife) in the future. Such data could be collected through collaborations between social scientists, who possess expertise in relevant research methods and theoretical knowledge, and state Cooperative Fish and Wildlife Research units, who possess experience working with state agencies. (C) 2013 The Wildlife Society.

3578: ***+.058***

We translocated and released a total of 90 (55 F and 35 M) wild American martens (Martes americana) from Minnesota to northern Wisconsin, USA, during 2008-2010. Our objective was to evaluate the short-term results of this translocation project by comparing marten dispersal, time to residency, and survival by release method, sex, and age categories. On average, translocated martens took 18 days (range = 1-64 days) and traveled 4.6 km (range = 0.4-45.7 km) from release sites before establishing residency. Although survival probabilities for adults and males were 0.84 and 0.79 and juveniles and females were 0.66 and 0.71, respectively, they were not statistically different. Translocated adult and juvenile survival was similar to resident adult and juvenile survival reported in Wisconsin and elsewhere. Predation (primarily by other carnivores) was the main cause (85%) of observed mortality for translocated animals, but it did not appear to be a major limiting factor for adults or juveniles. Contrary to some studies, we found no significant

difference between release methods for any analyzed parameter, but we observed increased injuries to slow-released individuals. We concluded there was no benefit resulting from slow-release or an acclimation period for translocation of American martens and that long-termmonitoring of the population is needed to evaluate species recovery in Wisconsin. (C) 2013 The Wildlife Society.

3579: *-.033*

By the 1970s, government-supported eradication campaigns reduced red wolves to a remnant population of less than 100 individuals on the southern border of Texas and Louisiana. Restoration efforts in the region were deemed unpromising because of predator-control programs and hybridization with coyotes. The U. S. Fish and Wildlife Service (USFWS) removed the last remaining red wolves from the wild and placed them in a captive-breeding program. In 1980, the USFWS declared red wolves extinct in the wild. During 1987, the USFWS, through the Red Wolf Recovery Program, reintroduced red wolves into northeastern North Carolina. Although restoration efforts have established a population of approximately 70-80 red wolves in the wild, issues of hybridization with coyotes, inbreeding, and human-caused mortality continue to hamper red wolf recovery. We explore these three challenges and, within each challenge, we illustrate how research can be used to resolve problems associated with red wolf-coyote interactions, effects of inbreeding, and demographic responses to human-caused mortality. We hope this illustrates the utility of research to advance restoration of red wolves.

3580: *+.057*

Topsoil transfer is an important tool in ecological restoration, but as a technique for re-locating woodland habitats displaced by development works it would appear highly damaging to geophytes present in the field layer. The effect of moving soil using different handling techniques was simulated in pot experiments with two common geophytes, Hyacinthoides non-scripta (L.) Chouard ex Rothm. and Anemone nemorosa L., while longer term changes in Hyacinthoides populations were also followed at an active woodland translocation field site in South East England, UK. In the pot experiment, artificially damaging Hyacinthoides bulbs restricted their performance, as did planting at sub-optimal depths and orientations, but Anemone rhizomes were little affected either by damage or displacement. Provided that they were able to produce leaves and thus re-allocate biomass to their perennating organs, recovery of both species was rapid in the following season. In the field, Hyacinthoides bulb densities also eventually recovered to levels reported in semi-natural woodlands in autumn-translocated soil profiles, although bulb biomass was still significantly lower in spring-moved treatments after three growing seasons. The parent bulbs rapidly adjusted their orientation and depth in the soil profile and were enhanced by natural seedling recruitment. The results suggest that, provided careful soil handling protocols are followed, these woodland geophytes have the capacity to recover if soil translocation is restricted to their dormant period. In the longer term, the sudden change from woodland to open conditions may be detrimental and may increase competition from non-woodland species.

3581: *+.012*

Aim To explore the effects of the introduction of exotic and translocated species and possible future extirpation of native species on the functional diversity (FD) of freshwater fish assemblages. Location Japanese archipelago. Methods We examined spatio-temporal changes in species richness, FD, functional richness (the number of trait-based functional groups), and the functional group composition between historical and current fish assemblages for 27 eco-regions, and

compared the relative effects of the introduction of exotic and translocated species on FD. We also used a null model approach to determine the assembly patterns and the extent of functional redundancy. Finally, we determined the effect of the loss of endangered species on FD by comparing the observed losses with simulated random loss. Results Through the introductions of non-native species, the species richness, FD and functional richness of the fish assemblages increased 2.4-, 1.6- and 2.1-fold, respectively. The functional group composition also changed largely through the additions of new functional groups. Exotic species had a significantly greater effect size than translocated species, but there were no differences in the overall net effects of exotic and translocated species. Null modelling approaches showed that the observed FD was higher than expected by chance (i.e. trait divergent) in both historical and current assemblages. There was also low functional redundancy. In our simulation, FD decreased in proportion to the loss of species, independent of whether the species were endangered. Main conclusions We demonstrated that both exotic and translocated species may change FD and functional group composition, which might have dramatic consequences for ecosystem processes. We suggest that the future extirpation of even a few native species can cause a substantial loss of FD. Our findings emphasize the need to improve conservation strategies based on species richness and conservation status, and to incorporate translocated species into targets of the management of non-native species.

3582: ***+.177***

Reintroductions are conducted to re-establish a self-sustaining population of a species and contribute to ecosystem restoration. The brown treecreeper (Climacteris picumnus) reintroduction into two nature reserves in the Australian Capital Territory in south-eastern Australia failed to meet its predetermined criteria for success. This occurred despite prior habitat restoration within the reserves where reintroduction occurred. Low survival of reintroduced brown treecreepers, particularly due to predation by native predators, has previously been highlighted as a key factor in the failure of the programme. We compared bird behaviour and habitat characteristics between the reintroduction reserves and the sites where brown treecreepers were sourced (which support stable brown treecreeper populations). We did not identify an indication of significantly higher predation pressure in the reintroduction reserves in comparison with the source sites. However, our results revealed that reintroduced individuals may be more vulnerable to predation because of an increased flight time to reach a refuge area. This was a result of a significantly lower number of refuge areas in logs and trees and a higher number of shrubs (which may obstruct escape paths and hinder detection of predators) in the reintroduction reserves compared with the source sites. We identified a lower ground foraging habitat quality in the reintroduction reserves because of lower numbers of ant mounds and lower areas of forageable ground. However, brown treecreepers were able to disperse extensively throughout the reserves and settle in areas with generally higher-quality foraging habitat. Therefore, the negative effect of low ground foraging habitat quality would have been most pronounced immediately after release. This study emphasizes the inherent complexities of species reintroductions and ecosystem restoration. Despite experimental restoration activities within the reintroduction reserves, there were still deficiencies in habitat quality. We emphasize that further habitat restoration is required within these reserves to achieve more complete restoration.

3583: ***+.066***

Restoration programs need to increasingly address both the restitution of biodiversity and ecosystem services and the preparation of habitats for future climate change. One option to adapt habitats to climate change in the temperate zone is the translocation of southern populations to

compensate for climate change effectsan option known as assisted migration (AM). Although AM is widely criticized for endangered species, forest managers are more confident that tree populations can be translocated with success because of previous experiences within native ranges. Here, we contend that translocations of tree populations are also subject to uncertainties, and we extract lessons for future programs of AM within species ranges from a well-documented failed case of population translocation of Pinus pinaster Ait. in Europe. The failure of these translocations originated from the unawareness of several unpredictable ecological and social events: cryptic maladaptation of the introduced populations, underestimation of climate variability differences between the source and target sites, and complexity in the management schemes, postponing decisions that could have been undertaken earlier. Under the no-analog conditions that are expected with climate change, management decisions need to be made with incomplete data, implying that a certain degree of maladaptation should always be expected when restoring plant populations from local or external seed sources.

3584: *+.109*

The white-clawed crayfish (Austropotamobius italicus), a cornerstone of Spain's aquatic ecosystems, was once widely distributed throughout much of the country. Unfortunately, its populations have suffered very strong declines over the last 40 years due to the spread of introduced species (red swamp and signal crayfishes), diseases, habitat loss and other anthropogenic impacts. The present work examines the genetic variation in 23 Spanish and four Italian populations of white-clawed crayfish via the analysis of microsatellite loci. The data show genetic variation in the Spanish populations to be affected by drastic and successive bottlenecks. Notwithstanding, the diversity of these Spanish populations in terms of observed heterozygosity is similar to or even higher than that recorded for other European populations studied using these same markers. North-central Spanish populations are clearly differentiated from the country's remaining populations; they should be considered distinct management units. Processes occurred in historical and recent times, such as genetic drift and translocations, contribute greatly to this genetic structure. These data provide useful information for conservation of this species, since the preservation of its population structure and genetic variability should be goals for management decisions.

3585: *+.115*

Re-introduction is an important tool for recovering endangered species; however, the magnitude of genetic consequences for re-introduced populations remains largely unknown, in particular the relative impacts of historical population bottlenecks compared to those induced by conservation management. We characterize 14 microsatellite loci developed for the Seychelles paradise flycatcher and use them to quantify temporal and spatial measures of genetic variation across a 134-year time frame encompassing a historical bottleneck that reduced the species to similar to 28 individuals in the 1960s, through the initial stages of recovery and across a second contemporary conservation-introduction-induced bottleneck. We then evaluate the relative impacts of the two bottlenecks, and finally apply our findings to inform broader re-introduction strategy. We find a temporal trend of significant decrease in standard measures of genetic diversity across the historical bottleneck, but only a nonsignificant downward trend in number of alleles across the contemporary bottleneck. However, accounting for the different timescales of the two bottlenecks (similar to 40 historical generations versus <1 contemporary generation), the loss of genetic diversity per generation is greater across the contemporary bottleneck. Historically, the flycatcher population was genetically structured; however, extinction on four of five islands has resulted in a homogeneous contemporary population. We conclude that severe historical bottlenecks can leave a

large footprint in terms of sheer quantity of genetic diversity lost. However, severely depleted genetic diversity does not render a species immune to further genetic erosion upon re-introduction. In some cases, the loss of genetic diversity per generation can, initially at least, be greater across re-introduction-induced bottlenecks.

3586: ***-.040***

Shifts in disturbance regime have often been linked to invasion in systems by native and nonnative species. This process can have negative effects on biodiversity and ecosystem function. Degradation may be ameliorated by the reinstatement of the disturbance regimes, such as the reintroduction of fire in pyrogenic systems. Modeling is one method through which potential outcomes of different regimes can be investigated. We created a population model to examine the control of a native invasive that is expanding and increasing in abundance due to suppressed fire. Our model, parameterized with field data from a case study of the tree Allocasuarina huegeliana in Australian sandplain heath, simulated different fire return intervals with and without the additional management effort of mechanical removal of the native invader. Population behavior under the different management options was assessed, and general estimates of potential biodiversity impacts were compared. We found that changes in fire return intervals made no significant difference in the increase and spread of the population. However, decreased fire return intervals did lower densities reached in the simulated heath patch as well as the estimated maximum biodiversity impacts. When simulating both mechanical removal and fire, we found that the effects of removal depended on the return intervals and the strategy used. Increase rates were not significantly affected by any removal strategy. However, we found that removal, particularly over the whole patch rather than focusing on satellite populations, could decrease average and maximum densities reached and thus decrease the predicted biodiversity impacts. Our simulation model shows that disturbance-based management has the potential to control native invasion in cases where shifted disturbance is the likely driver of the invasion. The increased knowledge gained through the modeling methods outlined can inform management decisions in fire regime planning that takes into consideration control of an invasive species. Although particularly applicable to native invasives, when properly informed by empirical knowledge these techniques can be expanded to management of invasion by nonnative species, either by restoring historic disturbance regimes or by instating novel regimes in innovative ways.

3587: ***+.058***

Dispersal is a significant life-history trait of vagile species that affects the distribution and genetic structure of populations. Natal dispersal in birds is the movement of an individual from its hatch site to a new location where its first reproductive effort occurs. To examine the ongoing recovery of the Peregrine Falcon (Falco peregrinus) in the upper Midwest, U.S.A., we assessed the influence of sex, hack-status (hacked or wild-fledged), and hatch site (cliff or human-made) on natal dispersal distance, and we evaluated directional trends of dispersal in the midwestern Peregrine Falcon subpopulation. We found that mean dispersal distance of female peregrines was >2 times farther than that of males. Dispersal distance did not differ between hacked females and wild-fledged females; however, hacked males dispersed significantly farther than wild-fledged males. Dispersal distance among urban-hatched females and cliff-hatched females did not differ, nor did dispersal distance of urban-hatched males and cliff-hatched males, probably because the sample size for cliff-hatched birds was small. As a whole, the direction of dispersal in midwestern peregrines was nonuniformly distributed and skewed to the northwest and southeast. This report may benefit future studies of peregrine demographics and population viability analyses by providing wildlife managers with information about the patterns and natal movements of Peregrine

Falcons in the midwestem U.S.

3588: ***+.067***

Knowledge of dispersal patterns and survival rates is essential to understand population dynamics and demography, and to develop effective long-term management strategies for species of conservation concern. In New England, Peregrine Falcons (Falco peregrinus) were extirpated as a breeding species in the 1960s. Following a captive breeding and release program, the population subsequently underwent a rapid, dispersal-based expansion into its former range, particularly during the last two decades. Use of buildings, bridges, and other human-made structures for nesting has become widespread in urban areas, where the species only infrequently nested prior to reintroduction. We analyzed encounters of Peregrine Falcons banded as nestlings in the six New England states between May 1990 and June 2009 to determine: (a) differences in dispersal patterns (distance and direction) by sex; (b) differences in movement and natal dispersal among birds from cliff and artificial nest sites; (c) causes of mortality; and (d) effects of sex, age, and natal habitat type on survivorship. Of 986 Peregrine Falcons banded, 24% were encountered again at least once by December 2009. Although most encounters (76%) occurred within the study area, 24% were outside New England in eight other eastern states, three Canadian provinces, Cuba, and Nicaragua. Five percent of the marked population was later confirmed at breeding territories in the eastern U.S.A., primarily in New England. Females dispersed greater distances (natal dispersal = 152.6 km; range = 70.2- 853.5 km; n = 28) than males (88.0 km; range = 0.03-1009.7 km; n = 22). New England peregrines showed a strong tendency to settle at nest types similar to those on which they were raised (rural cliff vs. urban structures); however, we documented movement from urban to rural habitats and vice versa in equal proportions. The causes of mortality for 122 recovered birds included unknown (61%), collisions with aircraft (11%), collisions with stationary objects (8%), falling from nest site (8%), collisions with vehicles or trains (7%), gunshot wounds (2%), entanglement in fishing gear (1%), and poisoning (1%). Most deaths occurred among first-year (68%) and second-year (11%) birds, with first-year peregrines experiencing significantly higher mortality than other age classes. The estimated annual survival rate for second-year and adult falcons combined was 81%, whereas our estimate for first-year birds was only 9%; however, the latter rate likely is a significant underestimate. We found no effect of natal habitat or sex on survival.

3589: ***+.252***

The Owens Pupfish Cyprinodon radiosus represents many of the challenges of managing threatened or endangered species in fragmented refuge populations. All six extant populations of the endangered Owens Pupfish were examined to assess how management practices, including serial translocations and founder events, have influenced the genetic diversity of the species and to make recommendations for future management. Four populations were sampled twice with 3-4years intervening; two additional populations were sampled once. Populations were genotyped at nine microsatellite loci; estimated effective population sizes ranged from 34.2 to 347.8 individuals based on the linkage disequilibrium method and from 10 to 48 using the sibship assignment method. All of the populations were estimated to have undergone severe bottlenecks, and statistically significant pairwise F-ST values increased during the period between sampling. From this data we infer that the individual refuge populations have differentiated and lost genetic diversity and that without intervention they will continue to do so. For the long-term persistence of this species, we recommend founding new populations composed of 30-50 founders from each of the extant populations, regularly translocating up to 10 migrants per generation among stable populations, and maximizing habitat area and quality. Received April 13, 2012; accepted May 21,

2013

3590: *+.135*

Multiple species translocations to the same site are becoming common practice in New Zealand restoration programmes. With every new translocation, the risk of parasite transmission between populations can increase. The translocation of Hoplodactylus duvaucelii and Oligosoma smithi provided the opportunity to 1) test for Salmonella and Cryptosporidium occurrences; and 2) compare this Salmonella test prevalence with results from avian translocations at one release site, Tiritiri Matangi Island. Of the six reptile species tested, three skinks (O. aeneum, O. moco and O. smithi) and one gecko (Woodworthia maculatus) tested positive for Salmonella (n=274). This is the first record of Salmonella enterica subspecies IV 40:g,t:- in New Zealand reptiles. Test prevalences between reptiles and birds were similar, suggesting that Salmonella prevalence may be naturally low in the areas sampled.

3591: *+.045*

The status of the California red-legged frog (Rana draytonii), a federally listed threatened species, has long been uncertain in the sierra Nevada range in eastern California, USA. We examined museum collections and historical records, and conducted 213 field surveys at 151 sites over 21 years to evaluate the status of this frog in the sierra Nevada. We documented only 20 sierra Nevada localities and one Cascades Mountains locality where R. draytonii occurred between 1916 and 1975, extending from Tehama County southeast about 405 km to Madera County. the elevation range of most of the historical localities was 200-900 m (about 40 km from lower to upper elevation), but three apparently extirpated populations that may have originated from deliberate translocations occurred at 1,500-1,536 m elevation in Yosemite national Park. We surveyed directly or within 5 km of 20 of the 21 historical sierra Nevada/Cascades R. draytonii localities and found that at least one of these historical populations persists today, in large numbers. We also discovered or confirmed six new sierra Nevada R. draytonii populations and individual frogs at three additional new sites, for a total of seven recent populations and three recent single-specimen occurrences extending from Butte County southeast about 275 km to Mariposa County. Historically, R. draytonii in the sierra Nevada probably bred in stream pools, which tend to be small with limited forage and thus may have constrained the historical size and number of sierra Nevada R. draytonii populations. since the 1850's, manmade ponds sometimes capable of supporting large R. draytonii populations have supplemented stream pool breeding habitat. excluding the southernmost and Yosemite historical localities, the current range of sierra Nevada R. draytonii differs little from the historical range, and further surveys may reveal additional surviving sierra Nevada R. draytonii populations. sierra Nevada R. draytonii are threatened primarily by habitat modification and loss related to human population increase.

3592: *-.063*

Disturbance caused by habitat restoration or urbanization can threaten populations of sensitive wildlife species. We examined the effects of habitat disturbance on the ecology of an urban population of Texas Horned Lizards (Phrynosoma cornutum), a species of conservation concern in several states as a result of range-wide declines. We quantified changes in spatial distribution, survival rates, and population size and density over 9 yr (2003-2011) for a P. cornutum population on an urban reserve. The project was divided into three, 3-yr stages based on level of habitat disturbance. Spatial analyses did not support the hypothesis that disturbance associated with restoration activities affected the spatial ecology of P. cornutum on our study site. However, these

results were not entirely conclusive due to the logistical constraints of working on a single site with an uncommon species. Survival (n = 147 lizards) was affected by season (inactive-season survival was higher), stage (declining survival in later stages with more disturbance), an interaction of season and stage, and disturbance (covariate of proportion of an individual's home range in disturbed areas for a given year; small negative effect). Major causes of mortality included depredation and anthropogenic activity. We estimated a population size of 33 +/- 5 (95% CI of 28-49) individuals (excluding hatchlings) with a corresponding density of 2.68 lizards/ha in 2011, which represented a 38% decline since 2005. This decline was likely a consequence of two factors: the 2008 translocation of 17 adult lizards from an area adjacent to our study site impacted by housing development and a decrease in the annual survival rate of adults over time. Our case study addressed recent calls to examine specific mechanisms by which habitat loss and degradation affect herpetofauna and revealed classic responses of an isolated population to stochastic events and anthropogenic activities.

3593: *+.132*

Reducing the time that birds' eggs are exposed during incubation in the wild is a management strategy with the potential to reduce nest predation rates, enhance breeding success and increase the population size of endangered species. We tested whether manipulation of clutches through artificial incubation of Lesser Elaenia Elaenia chiriquensis eggs and subsequent reintroduction of new-born nestlings to their original nest, and the use of adoptive parents, were efficient ways of increasing the population size of this species. We evaluated the financial cost and benefit of three different management strategies of artificial egg incubation with reintroduction of nestlings to the original nest. We searched for nests and manipulated eggs during the breeding seasons of 2009 and 2010 in a savanna reserve in central Brazil. Real eggs were replaced by artificial eggs and artificially incubated. The following breeding parameters were monitored: hatching rate, fledgling productivity, daily survival rate of nestlings and nest success. The effect of nest monitoring frequency (daily or every 3-4 days) on breeding parameters was also tested. Hatching rate was much higher amongst artificially incubated eggs than naturally incubated eggs. Artificially incubated clutches presented higher rates of fledgling production and apparent nest success than non-manipulated clutches. Clutch manipulation did not interfere with nestling daily survival rate. Daily monitoring did not have negative effects. The clutch manipulation methodology we used proved to be viable and is a potential tool for increasing population size.

3594: *+.065*

Oyster populations in Chesapeake Bay, USA, declined precipitously over the past three decades, and on-going efforts to restore the native oysters to former abundance were considered to be ineffective. Maryland and Virginia natural resource agencies proposed the introduction of a non-native Asian oyster (Crassostrea ariakensis) that is resistant to diseases affecting the native oyster and well adapted to the Chesapeake Bay environment. Numerous stakeholders raised concerns about potential adverse consequences of an introduction of a non-native species into a new environment. In response, state and federal agencies determined that an Environmental Impact Statement (EIS) should be prepared to address the environmental consequences of such an introduction as well as of seven other oyster restoration alternatives, including several involving only the native oyster. Preparation of an Ecological Risk Assessment (ERA) of the proposed action as well as all alternatives was an integral element of EIS preparation. This series of articles describes several different analyses that contributed to and collectively comprised the ERA conducted as input to the EIS. The final article of this series in HERA describes how the ERA and EIS findings were taken into account in the final decision on the preferred restoration alternative

by state and federal agencies.

3595: *-.050*

The Critically Endangered Fijian crested iguana, Brachylophus vitiensis, occurs at extreme density at only one location, with estimates of >10,000 iguanas living on the 70 hectare island of Yadua Taba in Fiji. We conducted a mark and recapture study over two wet seasons, investigating the spatial ecology and intraspecific interactions of the strictly arboreal Fijian crested iguana. This species exhibits moderate male-biased sexual size dimorphism, which has been linked in other lizard species to territoriality, aggression and larger male home ranges. We found that male Fijian crested iguanas exhibit high injury levels, indicative of frequent aggressive interactions. We did not find support for larger home range size in adult males relative to adult females, however male and female residents were larger than roaming individuals. Males with established home ranges also had larger femoral pores relative to body size than roaming males. Home range areas were small in comparison to those of other iguana species, and we speculate that the extreme population density impacts considerably on the spatial ecology of this population. There was extensive home range overlap within and between sexes. Intersexual overlap was greater than intrasexual overlap for both sexes, and continuing male-female pairings were observed among residents. Our results suggest that the extreme population density necessitates extensive home range overlap even though the underlying predictors of territoriality, such as male biased sexual size dimorphism and high aggression levels, remain. Our findings should be factored in to conservation management efforts for this species, particularly in captive breeding and translocation programs.

3596: *-.050*

The Critically Endangered Fijian crested iguana, Brachylophus vitiensis, occurs at extreme density at only one location, with estimates of >10,000 iguanas living on the 70 hectare island of Yadua Taba in Fiji. We conducted a mark and recapture study over two wet seasons, investigating the spatial ecology and intraspecific interactions of the strictly arboreal Fijian crested iguana. This species exhibits moderate male-biased sexual size dimorphism, which has been linked in other lizard species to territoriality, aggression and larger male home ranges. We found that male Fijian crested iguanas exhibit high injury levels, indicative of frequent aggressive interactions. We did not find support for larger home range size in adult males relative to adult females, however male and female residents were larger than roaming individuals. Males with established home ranges also had larger femoral pores relative to body size than roaming males. Home range areas were small in comparison to those of other iguana species, and we speculate that the extreme population density impacts considerably on the spatial ecology of this population. There was extensive home range overlap within and between sexes. Intersexual overlap was greater than intrasexual overlap for both sexes, and continuing male-female pairings were observed among residents. Our results suggest that the extreme population density necessitates extensive home range overlap even though the underlying predictors of territoriality, such as male biased sexual size dimorphism and high aggression levels, remain. Our findings should be factored in to conservation management efforts for this species, particularly in captive breeding and translocation programs.

3597: *+.136*

Reintroduction or reinforcement (RorR) of wild populations is a common conservation strategy. Many conservation projects involve the release of individuals of poorly studied species. This may lead to inefficient results or negative impacts on the conservation efforts. Here, we provide new insights into the conservation implications and potential consequences of a skew in the sex ratio of

released birds and of the number of birds supplemented for the demography of a long-lived dimorphic bird species, the Andean condor (Vultur gryphus). We demonstrate that a RorR conservation program may be less effective in conserving a species if the sex ratios of the releases and the recipient populations are not considered. We also show that releases can reduce population declines but only if carried out over long periods (i.e., several decades). This can mean high costs for release programs and the added challenge of maintaining programs over time. If RorR programs are to be implemented, bearing in mind the importance of properly assessing their effectiveness, we urge conservation researchers and managers to consider the implications of sex ratio biases for wild populations, and particularly for dimorphic species with sexually despotic behaviour.

3598: *-.030*

The common spadefoot (Pelobates fuscus) is an amphibian species threatened by extinction with a negative population development in North Rhine-Westphalia. Apart from conservation measures taken at the spawning areas and the optimization of terrestrial habitat for the first time conservation breeding was conducted in 2011 and 2012. Spawning of the adult toads and breeding of tadpoles was carried out in tanks with a rotund-shape and a capacity of 6000 1. Not more than 300 tadpoles were kept in one tank after they had reached their maximum size. Tadpoles were fed predominantly with algae and wild herbs. The development of tadpoles was successful with a high rate of tadpoles reaching metamorphosis of far more than 90 percent. Thus almost 2500 larvae or metamorphosed toads could be reintroduced.

3599: *+.082*

Changes in habitat quality, destruction and fragmentation of the remaining habitats are the main causes of population and species extinction. Patch occupancy decreases with the turnover of the metapopulation dynamics and therefore the population prone to extinct. We have been examining the occurrence of endangered and strictly protected Hungarian root vole subspecies (Microtus oeconomus mehelyi) within the framework of Hungarian Biodiversity Monitoring System Program in Kis-Balaton. We analyzed the dynamics of root vole site occupancy based on 12-year-long period data. From a conservation aspect we investigated how root vole site occupancy dynamics differed in the area under NATURA 2000 protected marshland habitat management and in the privatized marshland under regular perturbation, compared to the initial, disturbance-free conditions. We applied multi-season occupancy models and we took into consideration the effects of perturbance, such as burning and mowing, and natural disturbances such as water cover and weather (wetter, drier weather period). Based on monitoring data there were 397 successful root vole captures and we identified 262 individuals. The results of both detection and occupancy estimates showed that the comprehensive mowing affecting the entire privatized area appeared as a disturbing effect, causing the emigration of the root voles. Most important factor in the loss of root vole is the additive effects of burning and natural aridity. Disturbances and perturbations of certain areas induce movements and spatial translocations which play role in the exchange of genes and individuals, ensuring to maintain the genetic variability of the remaining relict population. (C) 2013 Elsevier GmbH. All rights reserved.

3600: *+.073*

The survival of many species may be dependent on their ability to exist in human-altered landscapes within metapopulations; in turn, metapopulation persistence is dictated by the ability of individuals to move effectively among patches to promote recolonization. The Taylor's

checkerspot butterfly (Euphydryas editha taylori) is a species that does not naturally occur in fragmented landscapes, yet it is now restricted to a handful of small isolated prairie habitats. Current recovery plans aim to establish a stable metapopulation; however, to date little is known about the species' ability to move across the landscape. In 2010 and 2011, we conducted marking, tracking and boundary surveys to explore the movement dynamics of adults within two sites in Oregon, USA. Over the survey period, we marked 136 male butterflies, tracked 174 individuals and observed the behavior of 1,576 individual butterflies at site boundaries. Our study revealed a significant sex-bias in the movement dynamics of the Taylor's checkerspot in both suitable habitat and surrounding matrix. Males were highly motile, whereas females appeared sedentary, rarely moving from their natal site. The limited dispersal behavior of females indicates that populations cannot persist naturally in a metapopulation and thus are at high risk of extinction. Based on our findings, we recommend that managers take proactive measures to increase or enable dispersal (including translocation) to existing and/or restored sites.

3601: *+.233*

About half a million rabbits are translocated in southwestern Europe every year for conservation and hunting purposes. However, the success of traditional rabbit restocking is generally extremely low, and this has been attributed to short-term predation by mammalian carnivores. Hence, recent recovery programs have tackled the problem of terrestrial predators with the use of exclusion fences, but no additional measures have been employed to avoid aerial predation. In this study, we have therefore conducted a field experiment to test the short-term effect of total predation exclusion in rabbit restocking enclosures, comparing rabbit abundance in plots which are only accessible to raptors (top-open plots) and plots which are accessible to neither carnivores nor raptors (top-closed plots). The results showed that the top-closed plots had higher rabbit abundance in the short term, and the highest difference in rabbit abundance between the two kinds of fences was attained in the first 2 weeks. We therefore conclude that the top-closed plots were an effective tool to increase rabbit abundance during the first weeks after release through the exclusion of raptor predation.

3602: *+.105*

We investigated the implications of clonality for translocation of Wilsonia backhousei, a threatened, outbreeding, saltmarsh plant with tidally-dispersed fruit. Eight microsatellite loci were used to characterise samples from three estuaries in New South Wales, Australia, and to determine the size and distribution of genetically distinct individuals (genets). Within-population diversity was compared to the presence or absence of seed production using the t test. Ordinal logistic regression was used to investigate the relative influence on seed yield of soil characteristics (soil moisture, salinity, pH) and the number of clonal lineages within a 5 and 10 m radius. Principal coordinate analysis, analysis of molecular variance and Bayesian analysis were used to investigate the extent of gene flow within and among the three estuaries. We found individual genets could cover extensive areas (up to 225 m(2)) and apparently large populations could consist of only a few individuals. Populations that failed to produce seed had significantly less genetic diversity than populations that produced seed ($P = 0.001$). Seed yield showed a significant positive response to both increasing soil moisture content ($P = 0.003$) and increasing genetic diversity in a 5 m radius ($P = 0.003$). Gene flow was found to occur chiefly within estuaries though occasional longer-distance gene transfer was evident. To maximise adaptive potential in translocated populations of W. backhousei, we recommend sourcing propagules from multiple populations and planting representatives of the different populations in close proximity to facilitate sexual reproduction. These findings are likely to be applicable to other outbreeding clonal saltmarsh

plants with tidally-dispersed fruit or seed.

3603: *+.216*

Movement of animals is a key process affecting population dynamics. Information on factors that affect pathway use is essential for identifying and protecting pathways, and important for maintaining connectivity among populations. We present an innovative, non-invasive, approach for predicting pathways of reintroduced Asiatic wild ass (Equus hemionus) in Israel, which is based on understanding the effects of landscape factors on pathways use. The approach includes: Predicting pathways, by employing a least cost pathway (LCP) GIS models based on several landscape factors, so as to efficiently direct a field survey and explore the wild ass's general preferences of pathway types; Collecting empirical data by surveying the dung density of wild ass along each of the predicted pathways and using the data as an index of pathway use; Evaluating the predicted pathways against the empirical data collected, to estimate the general pathway preferences of the wild ass; and Developing and evaluating alternative generalized linear models, according to a priori hypotheses based on empirical data so as to quantify the effect of different landscape factors on pathway use. The analyses were conducted for the entire landscape, and then for two distinct landscape types, open landscape and landscape-barriers (mountain ridges), as subsets of the entire landscape. There were clear differences in the mean number of faeces counts between the LCPs, indicating that the wild ass prefers certain pathway types as a function of landscape features. We further found that the factors affecting E. hemionus pathway usage-vegetation; slopes; canyons; and 4-wheel drive routes-varied largely between the two major landscape types studied, demonstrating the importance of studying space use patterns at different landscape terrains. This information can be applicable to landscape planning measures that aim to enhance protection of the species. This approach provides a framework for studying animal space-use patterns of a variety of species, including elusive species, in a heterogeneous landscape.

3604: *+.114*

This project aimed to estimate a species' adaptations in nature and in captivity, assess welfare, suggest environmental changes, and find species characteristics that underlie welfare problems in nonhuman animals in the zoo. First, the current status of zoo animal welfare assessment was reviewed, and the behavioral ecology approach was outlined. In this approach, databases of species characteristics were developed using (a) literature of natural behavior and (b) captive behavior. Species characteristics were grouped in 8 functional behavioral ecological fitness-related categories: space, time, metabolic, safety, reproductive, comfort, social, and information adaptations. Assessments of the strength of behavioral adaptations in relation to environmental demands were made based on the results available from the literature. The databases with literature at the species level were coupled with databases of (c) behavioral observations and (d) welfare assessments under captive conditions. Observation and welfare assessment methods were adapted from the animal on the farm realm and applied to zoo species. It was expected that the comparison of the repertoire of behaviors in natural and captive environments would highlight welfare problems, provide solutions to welfare problems by environmental changes, and identify species characteristics underlying zoo animal welfare problems.

3605: *+.124*

Translocation is the movement of a group of individuals from one site to another. Conservationists and wildlife managers around the world use translocation to new and/or newly safe habitats as a tool for preserving and propagating threatened species whose populations are surviving at only

few and vulnerable localities. The success of translocations is typically defined as the establishment of a self-sustaining population. However, this definition overlooks the genetic consequences of translocations at the metapopulation scale, especially when maintaining genetic diversity is one of the specific aims of immediate and/or long-term management goals for the translocated population. We evaluated the potential effects of translocation on the total genetic diversity of a metapopulation in an increasingly common scenario: a small island as the source site, and a nearby predator-proofed, large island as the target site. Specifically, we tested the counterintuitive hypothesis that translocation and subsequent migration between an expanding, recently established population and the original population might actually result in the suppression of genetic diversity in the metapopulation relative to the temporal course of genetic drift in the small island population without translocation (control). Our simulations confirm that the directional genetic consequences of translocations are complex and depend on the combination of parameter estimates used for the modelling. Critically, however, under a lower rate of migration, lower rate of growth and higher carrying capacity on the translocation site, and smaller initial size of the translocated population, the total genetic diversity of the metapopulation may become suppressed following a translocation, relative to the control. At the same time, when translocations are carried out under a broader set of conditions, the metapopulation genetic diversity will typically exceed that of the control. Our approach is also informative about the genetic consequences of natural re-/colonisation events between small source and nearby large target sites and the resulting metapopulation. Overall, these results confirm the importance of translocation as a potentially effective and successful conservation genetic tool.

3606: ***+.060***

Eurasian lynx (Lynx lynx) reintroduction to the Dinaric Mountains is considered one of the most successful reintroductions of a large predator. Six reintroduced animals founded the population, which rapidly expanded from Slovenia, through Croatia, and all the way to Bosnia and Herzegovina. However, a decrease of the population size has been observed during the last 10-15 years. Considering that possible inbreeding depression would be additive to threats like poaching, traffic mortality and prey base depletion, another extinction of this species from the Dinaric Mountains is a real possibility. We analyzed 204 samples collected between 1979 and 2010 using twenty microsatellite loci and 900-bp mitochondrial DNA control region sequence to evaluate conservation genetics aspects of this endangered population. Both markers confirmed low genetic variability of the Dinaric lynx population, and considerable effective inbreeding (0.3) compared to the source Carpathian population. Our analysis of effective population size and microsatellite variability supported field observations of decreasing population number. As a natural recolonization is a very remote possibility, we recommend population augmentation from a large source population.

3607: ***+.022***

Endemic and endangered species are highly vulnerable to habitat perturbations and may be subject to variations in their population size. Management plan for these species is crucial to avoid population decline, loss of genetic variability, inbreeding and ultimately extinction. The sand lizard, Liolaemus lutzae, is endemic to a habitat of sandy coastal plain (restinga). Its geographical distribution extends for only 200 km stretch of the coast of Rio de Janeiro state, Brazil, one of South America's most densely populated regions. Extensive development and degradation of the beaches where the species inhabits, have led to the species becoming critically endangered. We used mitochondrial DNA sequences and microsatellite loci to resolve patterns of population connectivity and genetic variation within the species in order to provide a platform for a species

management plan. Our results indicate the existence of three main populations, separated from each other by the Guanabara Bay and by the Arraial do Cabo Peninsula. The low microsatellite genetic variation and heterozygosity witnessed in each of the three populations, together with high levels of inbreeding and low effective population sizes suggest that the species is in urgent need of intensive management. Based on the results of this study we propose strong measures to protect existing restinga fragments and the implementation of programmes of captive breeding and reintroduction of individuals from the heavily threatened regions to protected refugia. Such measures may be the only way of ensuring the continuity of the species.

3608: *+.091*

In March 2009, 23 dorcas gazelles (9 males and 14 females) were reintroduced in the Katane enclosure, a 440 ha fenced-in area in the North Ferlo Fauna Reserve (Senegal). In the enclosure, the dorcas gazelle live with other reintroduced (the mohor gazelles and the scimitar-horned oryx) and native ungulate species (the Red-fronted gazelle), as well other native mammals. Seven habitat types were characterized in the enclosure. Habitat preference of dorcas gazelles was studied using presence and abundance of gazelle signs (tracks, latrines, fecal deposits) and direct observations. Seasonal data were collected along a 6-km long transect in the enclosure. The presence of dorcas gazelles is significantly dependent on the type of habitat in the Katane enclosure and they prefer open habitats (plateaus) to habitats with less visibility. This preference did not change according to season and the number of fecal deposits increased with proximity to the fence. (C) 2013 Elsevier Ltd. All rights reserved.

3609: *-.066*

The Iberian lynx (Lynx pardinus) has suffered severe population declines in the twentieth century and is now on the brink of extinction(1). Climate change could further threaten the survival of the species(2), but its forecast effects are being neglected in recovery plans(3,4). Quantitative estimates of extinction risk under climate change have so far mostly relied on inferences from correlative projections of species' habitat shifts(5). Here we use ecological niche models coupled to metapopulation simulations with source-sink dynamics(6,7) to directly investigate the combined effects of climate change, prey availability and management intervention on the persistence of the Iberian lynx. Our approach is unique in that it explicitly models dynamic bi-trophic species interactions in a climate change setting. We show that anticipated climate change will rapidly and severely decrease lynx abundance and probably lead to its extinction in the wild within 50 years, even with strong global efforts to mitigate greenhouse gas emissions. In stark contrast, we also show that a carefully planned reintroduction programme, accounting for the effects of climate change, prey abundance and habitat connectivity, could avert extinction of the lynx this century. Our results demonstrate, for the first time, why considering prey availability, climate change and their interaction in models is important when designing policies to prevent future biodiversity loss.

3610: *+.175*

Managers of African lions (Panthera leo) on reserves where they have been reintroduced increasingly face challenges associated with ecological regulation, genetic degradation and increased susceptibility to catastrophic events. The Lion Management Forum (LiMF) was formed in 2010 to define these challenges and explore possible solutions with the view to developing appropriate management guidelines. LiMF bases its recommendations on the ecologically sound premise that managers should, as far as possible, mimic natural processes that have broken down in reserves, using proactive rather than reactive methods, i.e. management should focus on causal

mechanisms as opposed to reacting to symptoms. Specifically, efforts should be made to reduce population growth and thus reduce the number of excess lions in the system; disease threats should be reduced through testing and vaccination whenever animals are translocated; and genetic integrity should be monitored. The latter is particularly important, as most of these reserves are relatively small (typically <1000 km(2)). An adaptive management framework is needed to implement the guidelines developed here on reserves across the country, with regional nodes addressing more local genetic issues, within an overall national plan. Ongoing monitoring and scientific assessment of behavioural, population and systemic responses of lion populations and responsive modification of the guidelines, should improve management of lions on small reserves in South Africa. This approach will provide a template for evidence-based conservation management of other threatened species. Ultimately 'National Norms and Standards' must be established and a 'National Action Plan' for lions in South Africa developed.

3611: *+.167*

Wildlife populations are often influenced by multiple political jurisdictions. This is particularly true for wide-ranging, low-density carnivores whose populations have often contracted and remain threatened, heightening the need for geographically coordinated priorities at the landscape scale. Yet even as modern policies facilitate species recoveries, gaps in knowledge of historical distributions, population capacities, and potential for genetic exchange inhibit development of population-level conservation priorities. Wolverines are an 8-18 kg terrestrial weasel (Mustelidae) that naturally exist at low densities (similar to 5/1000 km(2)) in cold, often snow-covered areas. Wolverines were extirpated, or nearly so, from the contiguous United States by 1930. We used a resource selection function to (I) predict habitat suitable for survival, reproduction and dispersal of wolverines across the western US, (2) make a rough estimate of population capacity, and (3) develop conservation priorities at the metapopulation scale. Primary wolverine habitat (survival) existed in island-like fashion across the western US, and we estimated capacity to be 644 wolverines (95% CI= 506-1881). We estimated current population size to be approximately half of capacity. Areas we predicted suitable for male dispersal linked all patches, but some potential core areas appear to be relatively isolated for females. Reintroduction of wolverines to the Southern Rockies and Sierra-Nevadas has the potential to increase population size by >50% and these regions may be robust to climate change. The Central Linkage Region is an area of great importance for metapopulation function, thus warranting collaborative strategies for maintaining high survival rates, high reproductive rates, and dispersal capabilities. Our analysis can help identify dispersal corridors, release locations for reintroductions, and monitoring targets. The process we used can serve as an example for developing collaborative, landscape-scale, conservation priorities for data-sparse metapopulations. (C) 2013 Elsevier Ltd. All rights reserved.

3612: *+.053*

Subfossil remains indicate that the Laysan Duck (Anas laysanensis) formerly occurred throughout the Hawaiian archipelago, but for more than 150 years it has been confined to a single, small atoll in the northwestern chain, Laysan Island. In 2004-2005, 42 ducks were reintroduced from Laysan to Midway Atoll, where they exhibited variation in life history never observed on Laysan. On Laysan, females have never been observed to breed successfully at age 1 year and few attempt it, whereas on Midway, females routinely raised young at <1 year of age. Mean (+/- SD) clutch size on Midway (7.0 +/- 1.1, n = 41) was larger than the maximum clutch size of six eggs observed on Laysan. On Midway, renesting following nest failure (0.55 probability, n = 27) and double brooding (0.50, n = 54) were routine, and two instances of triple brooding were observed, whereas on Laysan, renesting and double brooding are rare (0.05 probability for both during our study; n =

21 and 19, respectively) and triple brooding has never been observed. Other novel life history on Midway included early cessation of parental care to renest. Altered life history on Midway is likely related to better feeding conditions and low population density compared with Laysan. An especially intriguing possibility is that the phenotypic plasticity observed represents exposure of hidden reaction norms evolved when the species inhabited a range of environments, but several alternative explanations exist. Future reintroductions of this species may provide opportunities to test hypotheses about mechanisms underlying phenotypic plasticity.

3613: *+.267*

After a steady decline in the early 20th century, several terrestrial carnivore species have recently recovered in Western Europe, either through reintroductions or natural recolonization. Because of the large space requirements of these species and potential conflicts with human activities, ensuring their recovery requires the implementation of conservation and management measures that address the environmental, landscape and social dimensions of the problem. Few examples exist of such integrated management. Taking the case of the otter (Lutra lutra) in Switzerland, we propose a multi-step approach that allows to (1) identify areas with potentially suitable habitat, (2) evaluate their connectivity, (3) verify the potentiality of the species recolonization from populations in neighbouring countries. We showed that even though suitable habitat is available for the species and the level of structural connectivity within Switzerland is satisfactory, the level of connectivity with neighbouring populations is crucial to prioritize strategies that favour the species recovery in the field. This research is the first example integrating habitat suitability and connectivity assessment at different scales with other factors in a multi-step assessment for species recovery.

3614: *-.145*

Managers of reintroduced wildlife commonly encounter behavioral problems post-release that have been linked to physiological condition and elevated stress hormone concentrations. However, there is uncertainty about the generality of a stress response among populations, factors influencing the intensity of the response and the amount of time needed to physiologically acclimatize. We evaluated the relationship of temporal, climatic and social factors to stress hormone concentrations in five African elephant (Loxodonta africana) populations following reintroduction. We determined fecal glucocorticoid metabolite concentrations (FGMs) in 1567 fecal samples collected from elephants reintroduced to five fenced reserves with differing reintroduction histories in South Africa during 2000-2006. Variation in FGMs across the five reserves was best explained by the number of years that elapsed since initial release. Compared with FGMs 1 year after release, FGMs were 10% lower 10 years after release and 40% lower 24 years after release. Across all reserves, FGMs were consistently highest in the dry season, although daily and monthly temperature and rainfall were not as important as other factors. FGMs did not vary solely in relationship to reserve size or elephant density. Our findings suggest that regardless of reintroduction site conditions, elephants and likely other species subject to reintroduction require an extended period of time to physiologically acclimatize to their new surroundings. Managers should prepare for prolonged behavioral and physiological consequences of long-term elevated stress responses following reintroduction, such as restricted space use and aggressive behavior.

3615: *+.112*

The giant panda (Ailuropoda melanoleuca) is a rare endangered species listed on the

IUCN(International Union for Conservation of Nature and Natural Resources) Red List of Threatened Species and in the Appendices of CITES (the Convention on International Trade in Endangered Species of Wild Fauna and Flora). The giant panda has encountered risks of gene loss and local extinction of metapopulations due to habitat loss as a result of both biogeological and anthropologenic disturbance. One way to save endangered species is to reintroduce captive animals to sparsely populated regions of their historical distribution area. However, in order for reintroduction to succeed, captive animals must be able to select habitat that ensures their survival in the wild, a crucial skill that can be honed during pre-release training. We conducted research involving pre-release training of a giant panda in an enclosure in an area of natural habitat at the China Conservation and Research Center for the Giant Panda (CCRCGP) in Wolong Nature Reserve. We found that the panda selected areas with a high density of Fargesia robusta bamboo shoots (means standard deviation of habitat plots and control plots: 2.68[plus or minus]1.14 vs. 1.58[plus or minus]0.66), but avoided areas with a high density of adult bamboo (9.91[plus or minus]2.51 vs. 12.18[plus or minus]4.68) dead bamboo (2.52[plus or minus]0.86 vs. 3.39[plus or minus]1.33) and areas with higher bamboo calms ((4.57[plus or minus]1.09) m vs. (4.98[plus or minus]0.66) m). The panda was found in areas near water ((1.59[plus or minus]0.67) m vs. (2.19[plus or minus]0.87) m) and shelter ((5.37[plus or minus]2.14) m vs. (8.35[plus or minus]7.76) m) more often than other areas, but avoided areas close to trees ((3.09[plus or minus]0.69) m vs. (2.70[plus or minus]0.42) m) and areas with higher canopy cover (1.85[plus or minus]0.57 vs. 2.10[plus or minus]0.47) ($P < 0.05$). Logistic regression showed that the density of new bamboo shoots was the main factor predicting habitat use by the pre-released giant panda. The pre-released giant panda had similar habitat selection patterns as its mother, suggesting that it may be able to learn skills from its mother, an important factor that can impact the success of the reintroduction. The pre-released giant panda also displayed similar habitat selection patterns as wild giant pandas in Wolong Nature Reserve, suggesting that it may be prepared for foraging in the wild. The fact that the pre-released giant panda selected areas close to the shelter suggests it might have learned predator avoidance skills from pre-release training, which could have a positive effect on its survival in the wild. Home range and home range,core areas were 9.21 hm2 and 1.93 hm2, about 51.95% and 10.89% of the pre-release training enclosure respectively, while the home range was only 1.4%-2.4% of home ranges of wild giant pandas in Wolong Nature Reserve. It is essential to enrich the bamboo species richness of the pre-release training enclosure (such as by including Bashania faberi in the enclosure) in the future.

3616: *-.055*

Animals in many freshwater habitats are experiencing decreased recruitment due to declines in reproductive health. Both subspecies of a long-lived aquatic salamander, (Cryptobranchus alleganiensis alleganiensis and C.a. bishopi) have experienced severe population declines characterized by low recruitment. For many states throughout their geographic range, captive propagation and translocation are the only remaining form of management given the severity of declines. These captive rearing programs should rely on techniques to assess male reproductive health, which are currently lacking. In this study, we compared the sperm health (motility, viability, and concentration) of male hellbenders from declining and stable populations. Sperm motility and viability were similar among populations, but sperm concentrations (sp/ml) were significantly lower in declining Missouri populations than in hellbenders from populations with higher recruitment in the southeast. Sperm from Ozark hellbenders was successfully cryopreserved but with low post thaw motilities. This method for assessing male reproductive health provides the first baseline comparative study among populations of this cryptic species in decline and has broad implications for use in monitoring male health across the distribution of the eastern hellbender.

3617: *-.123*

Population restoration and reintroduction are critical aspects of many plant conservation efforts. However, factors affecting the earliest life stages, critical to the establishment of new individuals, are often poorly understood. I investigated the influence of seed characteristics and manipulations of the field environment on seedling emergence and growth in Asclepias meadii (Mead's milkweed), a federally threatened tallgrass prairie species. Seeds of known mass and maternal plant were reared in a greenhouse and also in experimental restoration plots with combinations of pre-planting soil disturbance and spring burning treatments. In both the greenhouse and field, seed mass was positively correlated with emergence but not growth. Maternal relationships with emergence and growth were observed in the greenhouse but were generally undetectable in the field. Lower emergence was associated with field plot soil disturbance and burning, although there was no statistically significant effect of either treatment. Seedling growth did not appear to be affected by soil disturbance, but burning had a significant negative effect. Mass may be a useful metric for evaluating restoration seed stocks and the quality of seeds produced in maturing restoration populations. Pre-emergence manipulations of the restoration site did not facilitate emergence or growth and may have even been detrimental to restoration efforts. High survivorship of seedlings during their first year of growth and overwintering suggests that direct sowing of seeds into the field is an effective restoration technique for A. meadii.

3618: *+.373*

Modelling metapopulation dynamics is a potentially very powerful tool for conservation biologists. In recent years, scientists have broadened the range of variables incorporated into metapopulation modelling from using almost exclusively habitat patch size and isolation, to the inclusion of attributes of the matrix and habitat patch quality. We investigated the influence of habitat patch and matrix characteristics on the metapopulation parameters of a highly endangered lizard species, the New Zealand endemic grand skink (Oligosoma grande) taking into account incomplete detectability. The predictive ability of the developed zxmetapopulation model was assessed through cross-validation of the data and with an independent data-set. Grand skinks occur on scattered rock-outcrops surrounded by indigenous tussock (bunch) and pasture grasslands therefore implying a metapopulation structure. We found that the type of matrix surrounding the habitat patch was equally as important as the size of habitat patch for estimating occupancy, colonisation and extinction probabilities. Additionally, the type of matrix was more important than the physical distance between habitat patches for colonisation probabilities. Detection probability differed between habitat patches in the two matrix types and between habitat patches with different attributes such as habitat patch composition and abundance of vegetation on the outcrop. The developed metapopulation models can now be used for management decisions on area protection, monitoring, and the selection of translocation sites for the grand skink. Our study showed that it is important to incorporate not only habitat patch size and distance between habitat patches, but also those matrix type and habitat patch attributes which are vital in the ecology of the target species.

3619: *+.305*

Intentional release of wild-caught individuals has been widely used to establish new populations of the commercially valuable but threatened reef gastropod Trochus niloticus in oceanic islands. Is this also a viable strategy to enhance depleted populations of this species and other marine invertebrates? We monitored growth and survival of 765 translocated individuals and 486 in their original habitat for 5-9 months. Individuals translocated to a severely overexploited reef (mainland

Palawan) grew 2-3times faster than those at Tubbataha Reefs Natural Park, Phillipines. Despite variations in growth between the three sites, survival probabilities were consistently high, ranging between 0.77 and 0.92. So translocation is feasible, and sites at which a species has previously been found are likely to be suitable for their growth and survival. If site management can control over-fishing, this approach is likely to be a valuable tool for enhancing field populations of a large invertebrates like Trochus that have a short lived planktonic larva.

3620: *+.212*

Translocation, sea ranching, and assisted migration are under scrutiny as methods to augment populations so that harvests can be increased or populations can better adapt to changing environmental conditions. Understanding the ecological effects of any such environmental manipulation is critical to its successful application. One potential ecological effect of any type of stock enhancement is the displacement of either resident or released groups such that finding shelter or foraging habitat is adversely affected. This study examined behavioral interactions of resident and translocated Jasus edwardsii rock lobster after an introduction of 1,961 small pale phenotypic morphs to an area populated by the resident large red phenotypic morph. This translocation was an experimental stock enhancement conducted as part of a larger study to increase the yield and value of the fishery. Most translocated individuals established a home range within a couple of days of release (generally <2), and these ranges were generally less than 1.0 ha in size. Home-range kernels and foraging ranges overlapped between the two morphs, and there was no evidence of avoidance (Jacob's cohesion index 0.01, $Z = 1.06$, $p = 0.28$). This case of translocation for stock enhancement between ecotypes had no detectable adverse effect on either the resident or the translocated population, and in this species, stock enhancement could become part of an integrated conservation and harvest optimization strategy.

3621: *+.156*

Introducing species to areas outside their historical range to secure their future under climate change is a controversial strategy for preventing extinction. While the debate over the wisdom of this strategy continues, such introductions are already taking place. Previous frameworks for analysing the decision to introduce have lacked a quantifiable management objective and mathematically rigorous problem formulation. Here we develop the first rigorous quantitative framework for deciding whether or not a particular introduction should go ahead, which species to prioritize for introduction, and where and how to introduce them. It can also be used to compare introduction with alternative management actions, and to prioritise questions for future research. We apply the framework to a case study of tuatara (Sphenodon punctatus) in New Zealand. While simple and accessible, this framework can accommodate uncertainty in predictions and values. It provides essential support for the existing IUCN guidelines by presenting a quantitative process for better decision-making about conservation introductions.

3622: *+.061*

The southern rock lobster, Jasus edwardsii, shows clear phenotypic differences between shallow water (red coloured) and deeper water (pale coloured) individuals. Translocations of individuals from deeper water to shallower waters are currently being trialled as a management strategy to facilitate a phenotypic change from lower value pale colouration, common in deeper waters, to the higher value red colouration found in shallow waters. Although panmixia across the J. edwardsii range has been long assumed, it is critical to assess the genetic variability of the species to ensure that the level of population connectivity is appropriately understood and translocations do not have

unintended consequences. Eight microsatellite loci were used to investigate genetic differentiation between six sites (three shallow, three deep) across southern Tasmania, Australia, and one from New Zealand. Based on analyses the assumption of panmixia was rejected, revealing small levels of genetic differentiation across southern Tasmania, significant levels of differentiation between Tasmania and New Zealand, and high levels of asymmetric gene flow in an easterly direction from Tasmania into New Zealand. These results suggest that translocation among Tasmanian populations are not likely to be problematic, however, a re-consideration of panmictic stock structure for this species is necessary.

3623: *-.015*

From May 2007 to May 2013, we recorded the 54 died individuals of the Crested Ibis (Nipponia nippon) of a reintroduced population in Ningshan County, Shaanxi Province using banding, radiotelemetry and field surveys. Of the 54 died individuals, there were 26 nestlings, 8 juveniles and 20 released adults. Our results showed that the causes of death of the Crested Ibis included sibling competition, negligence of parents, maldevelopment, predation, shortage of food, flight strike, electrocution of power lines, bad weather, etc. According to the above-mentioned causes, we proposed some reasonable measures of protection and management recommendations.

3624: *-.054*

Introduction: Reintroduction of endangered animals as part of conservational programs bears the risk of importing human pathogens from the sanctuary to the natural habitat. One bacterial pathogen that serves as a model organism to analyze this transmission is Staphylococcus aureus as it can colonize and infect both humans and animals. The aim of this study was to evaluate the utility of various biological samples to monitor S. aureus colonization in great apes and lemurs.Methods: Mucosal swabs from wild lemurs (n=25, Kirindy, Madagascar), feces, oral and genital swabs from captive chimpanzees (n=58, Ngamba and Entebbe, Uganda) and fruit wadges and feces from wild chimpanzees (n=21, Ta National Parc, Cote d'Ivoire) were screened for S. aureus. Antimicrobial resistance and selected virulence factors were tested for each isolate. Sequence based genotyping (spa typing, multilocus sequence typing) was applied to assess the population structure of S. aureus.Results: Oro-pharyngeal carriage of S. aureus was high in lemurs (72%, n=18) and captive chimpanzees (69.2%, n=27 and 100%, n=6, respectively). Wild chimpanzees shed S. aureus through feces (43.8, n=7) and fruit wadges (54.5, n=12). Analysis of multiple sampling revealed that two samples are sufficient to detect those animals which shed S. aureus through feces or fruit wadges. Genotyping showed that captive animals are more frequently colonized with human-associated S. aureus lineages.Conclusion: Oro-pharyngeal swabs are useful to screen for S. aureus colonization in apes and lemurs before reintroduction. Duplicates of stool and fruit wadges reliably detect S. aureus shedding in wild chimpanzees. We propose to apply these sampling strategies in future reintroduction programs to screen for S. aureus colonization. They may also be useful to monitor S. aureus in wild populations.

3625: *-.040*

Background: Multi-level fission-fusion societies, characteristic of a number of large brained mammal species including some primates, cetaceans and elephants, are among the most complex and cognitively demanding animal social systems. Many free-ranging populations of these highly social mammals already face severe human disturbance, which is set to accelerate with projected anthropogenic environmental change. Despite this, our understanding of how such disruption affects core aspects of social functioning is still very limited.Results: We now use novel playback

experiments to assess decision-making abilities integral to operating successfully within complex societies, and provide the first systematic evidence that fundamental social skills may be significantly impaired by anthropogenic disruption. African elephants (Loxodonta africana) that had experienced separation from family members and translocation during culling operations decades previously performed poorly on systematic tests of their social knowledge, failing to distinguish between callers on the basis of social familiarity. Moreover, elephants from the disrupted population showed no evidence of discriminating between callers when age-related cues simulated individuals on an increasing scale of social dominance, in sharp contrast to the undisturbed population where this core social ability was well developed.Conclusions: Key decision-making abilities that are fundamental to living in complex societies could be significantly altered in the long-term through exposure to severely disruptive events (e.g. culling and translocation). There is an assumption that wildlife responds to increasing pressure from human societies only in terms of demography, however our study demonstrates that the effects may be considerably more pervasive. These findings highlight the potential long-term negative consequences of acute social disruption in cognitively advanced species that live in close-knit kin-based societies, and alter our perspective on the health and functioning of populations that have been subjected to anthropogenic disturbance.

3626: *-.069*

A small or sparse population may suffer a reduction in fitness owing to Allee effects. Here, we explored effects of plant density on pollination, reproduction and predation in the alpine herb Pedicularis rex over two years. We did not detect a significant difference in the pollination rate or fecundity (fruit set and the initial seed set) before predation between sparse and dense patches in either year, indicating no pollination-driven Allee effect. However, dense patches experienced significantly fewer attacks by predispersal seed predators in both years, resulting in a significantly decreased realized fecundity (final seed set), suggesting a component Allee effect driven by predispersal seed predation. Predation-driven Allee effects have been predicted by many models and demonstrated for a range of animals, but there is scant evidence for such effects in plants. Our study provides strong evidence of a component Allee effect driven by predation in a plant species.

3627: *+.041*

Biological invasions and introgressive hybridization are major drivers for the decline of native freshwater fish. However, the magnitude of the problem across a native species range, the mechanisms shaping introgression as well as invader's dispersal and the relative role of biological invasions in the light of multiple environmental stressors are rarely described. Here, we report extensive (N=665) mtDNA sequence and (N=692) microsatellite genotypic data of 32 Northern Adriatic sites aimed to unravel the invasion of the European Barbus barbus in Italy and the hybridization and decline of the endemic B.plebejus. We highlight an exceptionally fast breakthrough of B.barbus within the Po River basin, leading to widespread introgressive hybridization with the endemic B.plebejus within few generations. In contrast, adjacent drainage systems are still unaffected from B.barbus invasion. We show that barriers to migration are inefficient to halt the invasion process and that propagule pressure, and not environmental quality, is the major driver responsible for B.barbus success. Both introgressive hybridization and invader's dispersal are facilitated by ongoing fisheries management practices. Therefore, immediate changes in fisheries management (i.e. stocking and translocation measures) and a detailed conservation plan, focussed on remnant purebred B.plebejus populations, are urgently needed.

3628: ***+.062***

In January 2000, the last Pyrenean wild goat, Capra pyrenaica pyrenaica, died in Ordesa National Park in the Spanish Pyrenees. Since that time, there has been an intense debate over the possibility of using individuals from other extant subspecies to restore the Iberian wild goat C. pyrenaica in the Pyrenees. In the late 1990s, some Iberian wild goats of the hispanica subspecies escaped from an enclosure in Guara Nature Park, also in the Spanish Pyrenees. Between 2006 and 2012, four annual counts were conducted to quantify the demographics of the population. This expanding but isolated population numbered at least 86 free-living Iberian wild goats in 2012, reproducing in the wild with a positive increasing trend (lambda = 1.067). Given the small number of original animals that escaped, new releases are necessary to insure the genetic variability of the small population, but only if a clear decision on its conservation is finally made. In addition, the population is sympatric with a population of several hundred feral goats, C. hircus, which should be monitored closely, in order to detect any problems with competence or hybridization, although the latter has not been demonstrated in the wild.

3629: ***+.137***

Ancient trees remaining in natural areas from once widespread forest due to overexploitation and habitat modification would naturally regenerate to form small isolated populations after the halting of disturbance. The genetic processes and consequence of this regeneration are generally unknown, which prevents the effective conservation and management of these populations. One such population, an Erythrophleum fordii population, is located in Dinghushan National Nature Reserve (China). Owing to the remarkably huge trunk of the sole ancient tree (KG) relative to the other individuals in the population, the local people and local tourism administration presume that this population has developed solely from KG. We found, using genetic diversity analyses, that contrary to this presumption, the population might have regenerated from several founders with diverse genetic backgrounds. Severe degradation of genetic diversity within the population did not take place, rather, it possessed the potential ability to maintain and recover gradually with population development. According to the results of simulations, we found that the longevity of tree species with iteroparity offers the potential to maintain genetic diversity in small isolated populations. The results from this study will benefit the restoration and conservation of the endangered species E. fordii, and of other congener species and tree species with similar life history traits.

3630: ***+.312***

Iran's Persian onager populations are critically endangered. This study of their natural history in Qatrouyeh National Park provides insights for enhancing their conservation. The population as a whole is greatly affected by weather. Wind, rain and cold drive populations from the plains to the valleys of hill-valley habitats. Vegetation features and water also influence habitat use, but differently for different sex and reproductive classes. Females with juveniles use plains with high-quality vegetation, whereas females without young and solitary territorial males choose those of intermediate quality. Females with young foals are also found closest to watering points. Future translocation of Persian onagers will only succeed if prospective habitats have sufficient hill-valley refuges and enough plains with winds to moderately hot conditions. Sufficient plains supporting high-quality vegetation near water for lactating females must co-exist with plains of moderate-quality vegetation that attract females without young, so reducing crowding and competition.

3631: ***+.118***

This study investigated the conservation status of Marsilea quadrifolia, an endangered fern found in paddy fields, irrigation ditches and ponds. An evaluation at the European level based on IUCN criteria showed that the extent of occurrence (EOO) of M. quadrifolia has decreased from 5,930,000 km(2) to 5,774,000 km(2) during the past decade, whereas its area of occupancy (AOO) has decreased from 620 to 400 km(2) (approximately 35.5%). These findings allowed the species to be upgraded from the IUCN classification of Near Threatened to Vulnerable. The agricultural chemical treatments seem to be the main extinction cause of M. quadrifolia, therefore we performed toxicological with 7 most common rice herbicides. Young plantlets were incubated for 96 h with each herbicide at three different concentrations: TQ (Tale Quale, chosen in accordance with the suggested dose for rice fields described on the product label), 1:100 and 1:1.000. Results suggested that survival of the plantlets depended on the herbicide and concentration used, and ranged between 0 and 80%, and no survival at ambient concentrations for 4 (Aura, Aura + Dash, Clincher and Viper) out of the 8 chosen herbicides. We conclude that herbicides represent one of the principal threats to the survival of this species. Finally, a DNA analysis using the AFLP approach was employed to identify the most suitable genetic pool for plant reintroduction efforts. The data show that the analysed populations of M. quadrifolia suffered from low genetic variability (Nei's gene diversity varied from 0.025 to 0.036). However, the analysis of the distribution of genetic variability suggested that 4 populations were characterised by different genetic traits that are useful in defining a genetic pool for plant conservation. This study highlights a strategy for implementing a plan of action for species growing in agro-ecosystems based on an integrated approach that is able to clarify the species conservation status, the principal threat factor and the genetic pool to be used for species conservation and reintroduction. (C) 2013 Elsevier B.V. All rights reserved.

3632: *+.079*

The Amur leopard is at the point of extinction. At present there are fewer than 35 in the wild. Their natural habitat ranges from China to the North Korean peninsula to Primorsky Krai in Russia. A reintroduction plan has been proposed to increase the population in the wild; however, this proposed plan still has many questions to be answered as to how effective it will be. The main objective is to reintroduce animals from a select group within the Far Eastern leopard programme or the Species Survival programme, which consist of leopards from select populations in the Northern Hemisphere. Zoos are central to the success of this plan, providing suitable breeding pairs to breed animals for reintroduction and also raising much needed funds to finance the project. Zoos are also central in educating the public about the critical status of the Amur leopard and other endangered animals of the world. Veterinary surgeons, by the very nature of their professional skills, are at the forefront of this seemingly endless battle against extinction of thousands of species that are critical to maintaining the balance of our fragile ecosystem. Veterinarians can analyze the health risks and health implications of reintroduction on the animals to be reintroduced and also on the native population. A world without large cats is a world hard to imagine. If we look closer at the implications of extinction, we see the domino effect of their loss and an ecosystem out of control. (C) 2013 Published by Elsevier Inc.

3633: *+.007*

Population viability might become compromised by the loss of genetic diversity and the accumulation of inbreeding resulting from population decline and fragmentation. The Iberian lynx (Lynx pardinus) provides a paradigmatic example of a species at the verge of extinction, and because of the well-documented and different demographic histories of the two remaining populations (Donana and Andujar), it provides the opportunity to evaluate the performance of

analytical methods commonly applied to recently declined populations. We used mitochondrial sequences and 36 microsatellite markers to evaluate the current genetic status of the species and to assess the genetic signatures of its past history. Mitochondrial diversity was extremely low with only two haplotypes, alternatively fixed in each population. Both remnant populations have low levels of genetic diversity at microsatellite markers, particularly the population from Donana, and genetic differentiation between the two populations is high. Bayesian coalescent-based methods suggest an earlier decline starting hundreds of years ago, while heterozygosity excess and M-ratio tests did not provide conclusive and consistent evidence for recent bottlenecks. Also, a model of gene flow received overwhelming support over a model of pure drift. Results that are in conflict with the known recent demography of the species call for caution in the use of these methods, especially when no information on previous demographic history is available. Overall, our results suggest that current genetic patterns in the Iberian lynx are mainly the result of its recent decline and fragmentation and alerts on possible genetic risks for its persistence. Conservation strategies should explicitly consider this threat and incorporate an integrated genetic management of wild, captive and re-introduced populations, including genetic restoration through translocations.

3634: *-.085*

This paper presents veterinary management strategies and diagnostic findings in the reintroduction of the endangered whooping crane (Grus americana). Between 2005 and 2010, 63 (27 male, 36 female) hatchling whooping cranes were assigned to a reintroduction project involving autumn release of costume-reared chicks in Wisconsin. Veterinary care included preventive measures and comprehensive pre-release evaluations to improve fitness and reduce translocation of potential disease agents to native habitats. A total of 44 clinically normal birds were released (70% of assigned individuals). Cases of morbidity were classified according to primary body system affected. Musculoskeletal disorders were described in 57 birds (90%); five birds were removed from the project prior to release (8%), all for abnormalities that prevented normal function. Fourteen birds died or were euthanized prior to release (22%); pre-release mortality was attributed to developmental abnormality, predation, trauma or infectious disease. Chronic respiratory aspergillosis, diagnosed in seven birds (11%), was the most common infectious disease of concern. Predation and trauma were primary causes of post-release mortality; no evidence of infectious disease of captive origin was detected in the study population by the end of 2010. The assessment of data accumulated by this project helped to outline successful health management strategies, as well as identify and mitigate ongoing risks to captive whooping cranes that impede reintroduction efforts and achieving management goals for species recovery. (C) 2013 Wiley Periodicals, Inc.

3635: *+.031*

Because survival in captivity is a significant determinant of birds available for release and reinforcement of wild populations, we aimed to identify sources of variation in mortality to assess potential impacts of management on chick productivity. We analyzed characteristics of Black-bellied Sandgrouse eggs collected from the wild and produced by captive pairs. Wild laid-eggs and pulled captive-laid eggs were incubated artificially and all chicks were hand-reared until seven weeks of age. Wild-laid eggs were significantly bigger, heavier, and denser than captive-laid eggs which showed a higher variability in size. Fertility, embryo mortality, and fertile egg hatchability were similar for wild-laid and captive-laid eggs (67.92% vs. 68%; 15.62% vs. 15.7%, and 80.55% vs. 84.44%, respectively). There were significant positive relationships between egg weigh/volume and chick hatch weight. Mortality of chicks hatched from wild-laid eggs was much lower than that of chicks from captive-laid eggs (19.44% vs. 60.5%) during the first week after hatching, but

decreased and being nil from the third week. Heavier hatchlings from captive-laid eggs exhibited higher survival rates which is not the case of hatchlings from wild-laid eggs. These latter hatchlings had higher survival rates increasing with the age of eggs in relation with the period of natural incubation. The recommended age at which wild-laid eggs could be collected is at least 13 days for full chick survivability. We concluded that in our experimental captive breeding program of the Black-bellied Sandgrouse, productivity of viable hatchlings was much better from wild-laid eggs and as later as these were collected. (C) 2013 Wiley Periodicals, Inc.

3636: *+.288*

Reintroductions and translocations of northern river otters have been a common management practice throughout the United States from the 1970s to the 2000s. Though many reintroductions have been successful, populations are not always monitored or evaluated post-release. From 2009 through 2012, we translocated 27 radio-marked otters into the Provo River watershed in northern Utah. Our objective was to determine what factors influenced the translocation-related mortality of otters. We developed a series of a priori models and used logistic regression to determine the most influential factors. We used Akaike's information criterion to evaluate relative model support. We found that the univariate model including body mass bore the most model weight and that body mass was the most important factor influencing the initial survival of translocated otters. Model-averaged beta estimates indicated that otters at the high end of body mass were 4 times more likely to survive the translocation than otters at the low end of body mass. Sex was the next most important factor influencing survival, as odds ratios indicated that males were more likely to survive the translocation than females. We urge ecologists and managers to delay the trapping and translocating of otters until young-of-the-year are likely large enough to have a high probability of survival. We further recommend female-biased translocations, as females were less likely to survive translocations.

3637: *+.042*

Big Spring spinedace (Lepidomeda mollispinis pratensis) is a cyprinid whose entire population occurs within a section of Meadow Valley Wash, Nevada. Other spinedace species have suffered population and range declines (one species is extinct). Managers, concerned about the vulnerability of Big Spring spinedace, have considered habitat restoration actions or translocation, but they have lacked data on distribution or habitat use. Our study occurred in an 8.2-km section of Meadow Valley Wash, including about 7.2 km in Condor Canyon and 0.8 km upstream of the canyon. Big Spring spinedace were present upstream of the currently listed critical habitat, including in the tributary Kill Wash. We found no Big Spring spinedace in the lower 3.3 km of Condor Canyon. We tagged Big Spring spinedace >= 70 mm fork length (range 70-103 mm) with passive integrated transponder tags during October 2008 (n = 100) and March 2009 (n = 103) to document movement. At least 47 of these individuals moved from their release location (up to 2 km). Thirty-nine individuals moved to Kill Wash or the confluence area with Meadow Valley Wash. Ninety-three percent of movement occurred in spring 2009. Fish moved both upstream and downstream. We found no movement downstream over a small waterfall at river km 7.9 and recorded only one fish that moved downstream over Delmue Falls (a 12-m drop) at river km 6.1. At the time of tagging, there was no significant difference in fork length or condition between Big Spring Spinedace that were later detected moving and those not detected moving. We found no significant difference in fork length or condition at time of tagging of Big Spring spinedace = 70 mm fork length that were detected moving and those not detected moving. Kill Wash and its confluence area appeared important to Big Spring spinedace; connectivity with these areas may be key to species persistence. These areas may provide a habitat template for restoration or

translocation. The lower 3.3 km of Meadow Valley Wash in Condor Canyon may be a good candidate section for habitat restoration actions.

3638: ***+.098***

Spatially explicit metapopulation models are being used with increasing frequency to forecast changes in species' abundance in response to future climate and other environmental changes. However, to date, they have not quantified shifts in the margins of the metapopulation range - an important dynamic for understanding species responses to climate change. Here we describe a method for calculating shifts in a metapopulation's range-margin based on the geographical centroid of spatially distributed patches, where the population abundance of each patch or each landscape grid cell is used to weight its geographical (X-Y) coordinate. We evaluated our approach against a detailed virtual example and two real-world applications (threatened mountain hare in Britain and invasive European rabbits in Australia). We also investigated smoothing techniques to better portray overall trends in range changes through time. These procedures were implemented in a new user-friendly software tool, which can process the output file of the popular RAMAS Metapop software. We develop a scenario analysis to show how our weighted-centroid approach can be used to recommend species management options that are most important to long-term population viability (e.g., to choose between increasing connectivity, habitat quality or translocation) under different demographic scenarios. We show that calculating a smoothed time series of weighted centroids from a spatially explicit metapopulation model provides: (i) a useful way to identify the demographic momentum, or momentum of population shift, of the metapopulation (rather than just spatial aggregation or individual-patch behaviour) of a species through geographic space in response to climate change; and (ii) an informative metric of range movement that complements predictions of change in range area or total population size, and extirpation or founding of patches. (C) 2013 Elsevier B.V. All rights reserved.

3639: ***+.035***

Augmentative biocontrol aims to control established pest populations through enhancement of their indigenous enemies. To our knowledge, this approach has not been applied at an operational scale in natural marine habitats, in part because of the perceived risk of adverse non-target effects on native ecosystems. In this paper, we focus on the persistence, spread and non-target effects of the sea urchin Evechinus chloroticus when used as biocontrol agent to eradicate an invasive kelp from Fiordland, New Zealand. Rocky reef macrobenthic assemblages were monitored over 17 months in areas where the indigenous algal canopy was either removed or left intact prior to the translocation of a large number of urchins (> 50 ind. center dot m(-2)). Urchin densities in treated areas significantly declined similar to 9 months after transplant, and began spreading to adjacent sites. At the end of the 17-month study, densities had declined to similar to 5 ind. center dot m(-2). Compared to controls, treatment sites showed persistent shifts from kelp forest to urchin barrens, which were accompanied by significant reductions in taxa richness. Although these non-target effects were pronounced, they were considered to be localised and reversible, and arguably outweigh the irreversible and more profound ecological impacts associated with the establishment of an invasive species in a region of high conservation value. Augmentative biocontrol, used in conjunction with traditional control methods, represents a promising tool for the integrated management of marine pests.

3640: ***+.204***

Ecological niche models (ENM) have become a popular tool to define and predict the "ecological

niche" of a species. An implicit assumption of the ENMs is that the predicted ecological niche of a species actually reflects the adaptive landscape of the species. Thus in sites predicted to be highly suitable, species would have maximum fitness compared to in sites predicted to be poorly suitable. As yet there are very few attempts to address this assumption. Here we evaluate this assumption. We used Bioclim (DIVA GIS version 7.3) and Maxent (version 3.3.2) to predict the habitat suitability of Myristica malabatica Lam., an economically important tree occurring in the Western Ghats, India. We located populations of the trees naturally occurring in different habitat suitability regimes (from highly suitable to poorly suitable) and evaluated them for their regeneration ability and genetic diversity. We also evaluated them for two plant functional traits, fluctuating asymmetry an index of genetic homeostasis, and specific leaf weight an index of primary productivity, often assumed to be good surrogates of fitness. We show a significant positive correlation between the predicted habitat quality and plant functional traits, regeneration index and genetic diversity of populations. Populations at sites predicted to be highly suitable had a higher regeneration and gene diversity compared to populations in sites predicted to be poor or unsuitable. Further, individuals in the highly suitable sites exhibited significantly less fluctuating asymmetry and significantly higher specific leaf weight compared to individuals in the poorly suitable habitats. These results for the first time provide an explicit test of the ENM with respect to the plant functional traits, regeneration ability and genetic diversity of populations along a habitat suitability gradient. We discuss the implication of these resultsfor designing viable species conservation and restoration programs

3641: *+.047*

Translocation of wild animals often seems appealing and may be attempted for conservation, educational, commercial, scientific or compassionate reasons. Close examination of the actual and potential problems involved, however, casts doubt on the real value of translocation in many cases. Such problems may revolve around the following factors: cost, capture, transport, release, health, habitat carrying capacity, hunting, hybridisation, social disruption of residents, and ecological disruption by translocated animals. It is argued that translocation should not be attempted except for: (1) the reintroduction of a rare species to its former habitat if such species had been eliminated or reduced to non-viable numbers, provided that it can be guaranteed long-term protection; or (2) the translocation of animals to where they will not come into contact with a viable population of conspecifics and where the recipient ecological community is of no major conservation value.

3642: *+.442*

Species distribution models (SDMs) are increasingly proposed to support conservation decision making. However, evidence of SDMs supporting solutions for on-ground conservation problems is still scarce in the scientific literature. Here, we show that successful examples exist but are still largely hidden in the grey literature, and thus less accessible for analysis and learning. Furthermore, the decision framework within which SDMs are used is rarely made explicit. Using case studies from biological invasions, identification of critical habitats, reserve selection and translocation of endangered species, we propose that SDMs may be tailored to suit a range of decision-making contexts when used within a structured and transparent decision-making process. To construct appropriate SDMs to more effectively guide conservation actions, modellers need to better understand the decision process, and decision makers need to provide feedback to modellers regarding the actual use of SDMs to support conservation decisions. This could be facilitated by individuals or institutions playing the role of translators' between modellers and decision makers. We encourage species distribution modellers to get involved in real decision-making processes that will benefit from their technical input; this strategy has the potential to better bridge theory

and practice, and contribute to improve both scientific knowledge and conservation outcomes.

3643: *+.234*

Reducing coyote (Canis latrans) predation can be an important management objective. Here, we evaluated the efficacy of electronet fencing for excluding coyotes from focal areas on black tailed prairie dog (Cynomys ludovicianus) colonies, measured the effect of fencing on wild-born black-footed ferret (Mustela nigripes) kit survival, and modeled costs and benefits of fencing. From 27 July to 2 October 2010 in north-central Montana, USA, we erected and maintained 7.7 km of electronet that enclosed 108 ha on portions of 2 prairie dog colonies. We monitored 2 female ferrets and 6 kits inside exclosures and 3 females and 12 kits outside of exclosures. Percent of coyote sightings in the protected areas was 6 times less than expected during the exclosure period (42% pre-exclosure, 7% exclosure, 47% post-exclosure). We conclude that the electronet fencing was effective for dramatically decreasing coyote activity in focal areas where black-footed ferret litters were being raised. We found evidence that survival of kits living primarily in protected areas was 22% higher, but we qualify this finding because of low sample sizes and because our monitoring activity on the study site may have influenced coyote activity. We estimated one-time costs for fencing to be US$4,464/km and operation and/or maintenance costs for the 68 days of fence operation to be US$641/km. If fencing increased survival by 20-30%, then total cost per ferret kit not lost to coyote predation would range between US$5,400 and $3,600, or US$ 2,550 and $1,700 if fence set-up-take-down labor and use of an all-terrain vehicle were donated. Published 2013.

3644: *+.085*

Reintroductions are increasingly utilized in conservation, but the experiences and environments to which animals are exposed prior to release can significantly impact the success of such efforts. We investigated differences in the diet of reintroduced juvenile alligator snapping turtles (Macrochelys temminckii) relative to juveniles from a wild population. In addition, we compared the diet of adult female Ouachita map turtles (Graptemys ouachitensis) to reintroduced M. temminckii with whom co-occurrence was recently reestablished and wild juvenile M. temminckii in a river system where the species have enjoyed long-term sympatry. There were differences in diversity and richness of the diet of M. temminckii at the two study sites that could be due to dissimilarity in foraging experience. There was greater overlap in diet of M. temminckii and G. ouachitensis at the site of reintroduction than at the site of long-term sympatry that corresponded to variation in composition of diet. Such a difference could indicate that partitioning of food resources has not taken place at the site of reintroduction to the degree that it has at the site with long-term sympatry.

3645: *+.016*

The eastern barred bandicoot, Perameles gunnii, has undergone a dramatic decline in distribution and abundance on the mainland of Australia during the twentieth century. In 1988 a captive breeding program was initiated to reduce the chance of extinction. With the extinction of the last wild mainland population in the early 1990s, reintroductions from captive-bred P. gunnii have met limited success, and currently only two extant populations persist in predator proof enclosures in the State of Victoria. With 20 years of breeding, there are concerns that the genetic diversity within the breeding program has declined and may inhibit current and future success of the program. We have used ten nuclear microsatellite loci and sequencing of two partial mitochondrial genes (cytochrome oxidase I and ATPase 6) to determine genetic diversity within current Victorian

P. gunnii. These diversity estimates are compared with historic samples from the captive breeding program dating back to 1995, historic samples from the last wild mainland population found at Hamilton in 1992 and contemporary Tasmanian wild populations. Results indicate that the captive P. gunnii population in the State of Victoria has lost significant genetic diversity through time. Genetic diversity is also reduced in populations at Hamilton Community Parklands and Mount Rothwell. Samples from the last wild population at Hamilton collected in 1992, along with samples from Tasmanian P. gunnii, had significantly greater genetic diversity than contemporary mainland populations. The results are discussed with reference to management options for maintaining genetic diversity within Victorian P. gunnii, including crossing Victorian and Tasmanian P. gunnii to increase genetic diversity, adaptability and evolutionary potential.

3646: *+.230*

Climate change is affecting the spatio-temporal distribution of environmental conditions, forcing species to shift their range in response. Species not capable of dispersing naturally may benefit from conservation translocations. A key aspect of translocation planning is release site selection: under the 2012 IUCN guidelines for reintroductions and other conservation translocations, selected sites are expected to match the biotic and abiotic needs of the candidate species now and in the future. Here we present a methodological framework to identify optimal translocation sites under climate change. Our method is the first to explicitly combine statistical and predictive population modelling to understand the relationship between climate, climate change and population dynamics, in order to perform robust habitat suitability analyses for conservation decision-making. We use the hihi Notiomystis cincta, a bird endemic to New Zealand, as a case study. We focus on the population of Tiritiri Matangi Island, which has been provided with ad libitum supplementary food since 1996. This offers the unique opportunity to study the direct impact of climate and future change in climatic conditions on a population free of confounding constraints. Climate is found to drive hihi population dynamics, even though they are not limited by the availability of food. Thus, despite the current management of the species, climate change remains a major threat to its long-term persistence. Moreover, under predicted climate change for the country, hihi suitable habitat will shift southward: the two current largest hihi populations will face unsuitable climatic conditions in the coming decades, and habitat that was not part of the species' historical range may become suitable. Synthesis and applications. Assisted colonization is increasingly being considered as an adaptation tool for species threatened by climate change. Justifying the use of this extreme conservation action, however, requires robust evidence that it is necessary and clear guidance on where to translocate individuals of threatened populations. We show how both requirements can be met using habitat suitability modelling if knowledge of the relationship between climate, climate change and the species' population dynamics is systematically used to guide the modelling process.

3647: *+.173*

The stone crayfish, Austropotamobius torrentium, is one of the oldest freshwater crayfish species in Europe. Most Carpathian populations are in Romania, with a distribution clustered in 2 compact metapopulations in the western part of the country. Our goal was to understand if this pattern is the result of a coincidence or a complex set of restrictive circumstances. Romania is an ideal place to analyze crayfish distributions because they have not been disturbed by species translocations or massive loss of populations. We recorded the presence/absence of crayfish and measured 15 habitat variables at 428 randomly chosen headwater sampling sites, and analyzed the crayfish distribution pattern with a boosted regression tree model. Our results show that most of the Romanian territory is ecologically suited to support stone crayfish under current conditions. The

most important influences on probability of presence were water velocity, conductivity, altitude, river size, and dissolved O-2. When included as a supplementary predictor in the model, the distance from karst became the strongest variable accounting for the probability of presence and explaining the current distribution of the stone crayfish. We propose the hypothesis that at least one cycle of Pleistocene glaciation shaped the current distribution pattern by causing massive extinction in the Carpathians, except in karstic underground water bodies that offered ecological refuges. After the glaciations, stone crayfish expanding from these refuges competed with colonizing noble crayfish Astacus astacus, restricting stone crayfish to insular areas.

3648: *+.096*

Helianthemum gonzalezferreri and Kunkeliella subsucculenta, two endangered endemic species from the Canary Islands, were analysed to determine the levels and structure of genetic diversity in their natural populations. The mean value of Shannon's diversity index for H. gonzalezferreri and K. subsucculenta were I = 0.315 and I = 0.331, respectively. AMOVA and Bayesian analysis showed high genetic differentiation between populations in H. gonzalezferreri (F (ST) = 0.297). This genetic differentiation was graphically shown with the principal component analysis (PCA), in which the majority of individuals were distributed into two groups. Contrarily, K. subsucculenta populations showed a considerable degree of gene exchange revealed by Bayesian cluster analysis and PCA. The genetic differentiation between the two H. gonzalezferreri populations suggests different management strategies for each population. Translocation between the two natural populations of K. subsucculenta, to increase genetic variation, would not mean outbreeding depression, since both populations share the same genetic pool.

3649: *+.178*

We assess population genetic structure and variability in the endangered Azorean endemic Viburnum treleasei Gand., an evergreen shrub or small tree, occurring in eight out of nine islands of the archipelago. We combine RAPD and ISSR markers in eight populations and four islands covering the three subgroups of islands that compose the archipelago, and one population of V. tinus from the Portuguese mainland. Most of the genetic variability was found within populations, which is in accordance with the bi-parental reproductive strategy favored by the taxon. Gene flow estimations for the combined RAPD and ISSR markers suggest that the main cause for population variability between the studied populations is genetic drift. In accordance with the genetic structure indicators obtained, conservation measures should consider that translocation of individuals between islands must be avoided. In specific cases, the analysed populations may require the implementation of augmentation strategies due to their depleted state. With this study, a genetic background is now available to better define conservation measures for the taxon.

3650: *+.212*

The aim of this 2008-2012 study was to prove the hypothesis that the Three Gorges Reservoir (TGR) and the upper free-flowing section can offer a habitat for restocking the Chinese sturgeon, Acipenser sinensis. Thus, 498 cultured A.sinensis (including 28 sub-adults tagged with ultrasonic transmitters) were released into the TGR and the adjacent free-flowing section of the Yangtze River. The distribution, movement and growth of the released fish were collected by acoustic telemetry and bycatch information. Results indicated that only three sub-adults migrated upstream to the free-flowing section where they remained for the three-year observation (limited by the longevity of the transmitter battery). However, most sub-adults migrated downstream after release, with a migration distance of 15-877km as well as a tracking duration from 19 to 6063h. The

downstream traveling speed in the free-flowing section (mean=3.70kmh(-1), range=1.51-8.25kmh(-1)) was significantly faster than that in the reservoir section (mean=1.05kmh(-1), range=0.12-1.82kmh(-1); p<0.05). Only three sub-adults migrated to within 3km of Three Gorges Dam (TGD) and stayed in the reservoir. Sub-adults stayed obviously deeper within the reservoir (mean=11.47m) than in the free-flowing section (mean=3.63m; p<0.05). Juveniles were recaptured from 45-810km downstream their release sites with a mean traveling speed of 1.05kmh(-1) (range=0.13-1.82kmh(-1)), lower than that of the sub-adults released in the free-flowing section. Twelve percent of juveniles released in one of the tributaries of the Yangtze River found the entrance and migrated downstream to the reservoir within the first 30days. Average body weight of the recaptured juveniles increased by 10.3% (range=2.3%-15.3%) and some crustaceans (e.g. river shrimp) were found in the stomach and gut of two juveniles. The results demonstrate that the TGR and its adjacent upstream free-flowing Yangtze River section can serve as reintroduction sites to support a certain quantity of Chinese sturgeon to remain and mature there.

3651: *+.272*

Rabbit populations in Iberia remain at low densities in several areas in which their endangered predators still coexist, and the recovery of these populations is therefore urgent if the integrity of Iberian Mediterranean ecosystems is to be maintained. The enhancement of wild rabbit populations has been attempted through the use of in situ extensive rabbit captive breeding enclosures (restocking plots), which reduce mortality caused by terrestrial predators and dispersal movements and permit the breeding of young individuals which can then naturally disperse to settle in the surrounding areas. However, their effectiveness, the role of its size, the optimal habitat management that should be promoted around them and the habitat features remains uncertain. Here, we show results from a four year study of an ambitious rabbit restocking plan on a landscape scale. We measured rabbit abundance in a vast area in which thirty-two restocking plots were built to create an initial rabbit population for further dispersion, in addition to an intensive habitat management program. We also compared rabbit abundance between managed and unmanaged UTM cells of 2.5 km x 2.5 km. Our results showed that rabbit abundance was three times higher in managed cells, but four years after restocking, rabbit abundances had only reached the threshold needed to support stable Iberian lynx presence (at least 10 latrines per km 1) in 9 of the 23 managed cells. Rabbit abundance was strongly affected by habitat treatment and scrub coverage. The increase of shelter was useful in low cover areas but ineligible in places with high scrub cover, where the increase of refuge plus scrub clearing to create pastures improve rabbit abundance more effectively. In the light of our results, restocking plots should be built only in places with suitable habitat, whereas pastures should be created in dense scrublands and refuge in low cover areas. (C) 2013 Elsevier GmbH. All rights reserved.

3652: *+.214*

The population of eastern hellbenders (Cryptobranchus alleganiensis alleganiensis) in the Blue River, Indiana has undergone a dramatic decline over the last decade. Recruitment in these declining populations has been negligible, and populations are now composed almost entirely of older age classes (upwards of 20 years old). Given this dramatic decline, it is imperative to assess the impacts of these demographic patterns on population growth and long-term stability. Therefore, we developed a stage-structured, life-history model to examine the effects of varying levels of egg, juvenile, and adult survivorship on abundance, recruitment, and long-term population projections. We performed a sensitivity analysis of the model and determine which life-history parameters have the greatest potential to increase/stabilise hellbender population growth. Finally, we conducted a population viability analysis to determine the probability of extinction

associated with varying management strategies. For eastern hellbender populations in Indiana, adults (especially females) are the most important component of long-term population viability. Sensitivity and elasticity analyses of the Lefkovitch matrix revealed that survival of adult and egg/larvae life-history stages are the most important for focused management efforts. Indeed, adults had the highest elasticity and reproductive value in the matrix model. Increasing survival by as little as 20% corresponded to the turning point at which the population ceased to decline and increased abundance (28% survival of egg/larvae). The importance of the transition from subadult to adult (transitional matrix element) was identified as an additional factor in maintaining abundance based on the relatively long period spent in this life-history stage (seven years for females). A population viability analysis was conducted to assess the likelihood and projected time frame of extinction for this population under no management (similar to 25 years to complete extirpation; probability of extinction = 1) and if management efforts such as captive rearing and headstarting are undertaken (probability of extinction <0.2 at 25-30% survival of egg/larvae). Adult females had the greatest effect in reducing growth rate and population abundance when removed in exploitation simulations (91.3% versus 51.8% reduction in population growth rate), indicating translocation efforts should be designed to maintain females in the breeding pool. These models indicated that conservation management strategies aimed at ensuring the presence of adult females while concomitantly ameliorating survival at early life stages (population augmentation, translocations, introduction of artificial nest structures) are needed to stabilise the Indiana population of eastern hellbenders. This stage-structured model is the first to model eastern hellbenders and has broad implications for use across the geographic range where populations of eastern hellbenders are monitored and vital rates can be estimated. (C) 2013 Elsevier GmbH. All rights reserved.

3653: *+.095*

In many areas, the management of overexploited populations of brown hare (Lepus europaeus) is based on annual restocking. While in some cases exotic hares are introduced, in some others hares are captured locally within protected areas and subsequently released into hunting grounds. We evaluated the genetic effects of this management regime in an Italian province where the brown hare population has recovered in the last few decades, by sequencing the hypervariable domain 1 of the mitochondrial control region and by genotyping eight autosomal microsatellites in hares sampled in both hunting and non-hunting areas. Both nuclear (H (e) = 0.68 and H (o) = 0.65) and mitochondrial variability (h = 0.853 and pi = 0.012) were in line with other European populations. When comparing our data with mitochondrial sequences retrieved from GenBank, out of the 21 detected haplotypes, 14 were private to our study area. While 4.6 % of the individuals were found to carry haplotypes attributable to past introductions, 41.5 % grouped within a well-supported lineage, previously identified with a presumed native Italian taxon, L. e. meridiei. Despite the detectable geographic partitioning of mitochondrial haplotypes across the province, no genetic structure resulted from microsatellites analysis, indicating that no reproductive barriers exist among hares carrying different mitochondrial lineages. In conclusion, the local management seems to have contributed to the recovery of the species and to a full admixture of nuclear genes in the province. However, neither the extensive translocations nor the possible introductions of exotic heads seem to have completely undermined the local mitochondrial lineages.

3654: *+.287*

Reintroductions are increasingly used to reestablish species, but a paucity of long-term postrelease monitoring has limited understanding of whether and when viable populations subsequently persist. We conducted temporal genetic analyses of reintroduced populations of swift foxes

(Vulpes velox) in Canada (Alberta and Saskatchewan) and the United States (Montana). We used samples collected 4 years apart, 17 years from the initiation of the reintroduction, and 3 years after the conclusion of releases. To assess program success, we genotyped 304 hair samples, subsampled from the known range in 2000 and 2001, and 2005 and 2006, at 7 microsatellite loci. We compared diversity, effective population size, and genetic connectivity over time in each population. Diversity remained stable over time and there was evidence of increasing effective population size. We determined population structure in both periods after correcting for differences in sample sizes. The geographic distribution of these populations roughly corresponded with the original release locations, which suggests the release sites had residual effects on the population structure. However, given that both reintroduction sites had similar source populations, habitat fragmentation, due to cropland, may be associated with the population structure we found. Although our results indicate growing, stable populations, future connectivity analyses are warranted to ensure both populations are not subject to negative small-population effects. Our results demonstrate the importance of multiple sampling years to fully capture population dynamics of reintroduced populations.

3655: ***+.193***

Knowledge of mobility is essential for understanding animal habitat use and dispersal potential, especially in the case of species occurring in fragmented habitats. We compared within-patch movement distances, turning angles, resting times, and flight-related morphological traits in the locally endangered butterfly, the dryad (Minois dryas), between its old populations occupying xerothermic grasslands and newly established ones in wet meadows. We expected that the latter group should be more mobile. Individuals living in both habitat types did not differ in their body mass and size, but those from xerothermic grasslands had wider thoraxes and longer wings, thus lower wing loading index (defined as body mass to wing length ratio). The majority of movements were short and did not exceed 10 m. Movement distances were significantly larger in males. However, there was no direct effect of habitat type on movement distances. Our results suggest that the dryads from xerothermic grasslands have better flight capabilities, whereas those from wet meadows are likely to invest more in reproduction. This implies that mobility is shaped by resource availability rather than by recent evolutionary history. Lower female mobility may have negative implications for the metapopulation persistence because only mated females are able to (re)colonise vacant habitat patches efficiently. Conservation efforts should thus be focused on maintaining large habitat patches that prevent stochastic local extinctions. Furthermore, the recommendation of promoting the exchange of individuals among patches through improving matrix permeability, as well as assisted reintroductions of the species into suitable vacant habitats should also improve its conservation.

3656: ***+.189***

Wildlife translocations, the deliberate movement of wild individuals from one part of their distribution to another, are increasingly being used as a conservation tool. Despite the popularity of translocations as a conservation technique, translocations are often not successful as a result of excessive movement, poor release site fidelity, and low survival. This study compares the movement patterns, site fidelity, and survival probability of resident and hard-released musk turtles (Sternotherus odoratus) in a complex of patchy distributed wetlands. Our results are different from most translocation studies as the majority of translocated turtles had movement (minimum convex polygon area, total distance moved, number of wetlands used, and the number of movement shifts between wetlands), release site fidelity, and wetland fidelity patterns that were similar to resident turtles. In addition, the survival probabilities of resident and translocated turtles were both high.

We believe the combination of poor overland movement capabilities and the patchy distribution of wetlands surrounded by a strong boundary matrix of terrestrial habitat, potentially increased the costs of leaving the wetland. The high costs of travelling overland to more distant wetlands may have constrained the translocated turtles dispersal from the release site and increased release site fidelity. Our study suggests that hard-release translocations may be an effective conservation method for highly aquatic species unlikely to leave the wetland and travel long overland distances.

3657: ***+.199***

Conservation of metapopulations requires managing extirpated sites, particularly with current threats of increased fragmentation and displacement from global warming. Determining the habitat requirements of threatened species and how they relate to defining characteristics of occupied and unoccupied sites is key to managing suitable habitat in extirpated patches. Due to habitat destruction and degradation, the endangered Ohlone tiger beetle (Cicindela ohlone) is found in only five sites of a once more extensive metapopulation in Santa Cruz County, California. To determine the role of habitat quality in classifying sites, I measured vegetation and ground cover as well as plant and soil composition in sites in which C. ohlone are present, extirpated, and absent. I used conditional inference trees to determine what habitat factors significantly predicted the different sites types. I also analyzed habitat characteristics within present sites to determine factors that predicted egg-laying habitat. As isolation has been shown to be an important driver of metapopulation patch extirpation, I tested the spatial autocorrelation of C. ohlone occupancy to determine if extirpated patches were significantly isolated. Habitat characteristics successfully differentiated nearly 90 % of extirpated plots, which were not isolated from occupied sites. Sites in which C. ohlone are currently present were classified as having at least 10 % cover of bare ground, high forb cover, low litter cover and depth, and high soil bulk density, characteristics that extirpated sites lacked. I illustrate how the defining characteristics could be used to manage habitat in extirpated and absents sites for potential recolonization or translocation, which is vital for metapopulation persistence.

3658: ***+.138***

Translocations for conservation often involve species limited to relict distributions. However, uncertainty can exist regarding the ability of source individuals to acclimatise following a shift to a distant location. We investigated the ability of captive-reared juvenile tuatara (Sphenodon punctatus) of Cook Strait stock (41 degrees S) to adjust to outdoor, predator-protected pens within Orokonui Ecosanctuary (45 degrees S). We examined potential basking and within burrow temperatures, the influence of temperature on emergence, and growth rates in comparison with other locations. Tuatara at Orokonui reached their preferred temperature when basking over summer, and burrows provided protection from freezing over winter. Emergence was temperature-dependent and essentially ceased during winter. Growth rates of Orokonui-held juveniles were within the range for four other captive-rearing facilities and faster than for wild juveniles from a Cook Strait population. As all Orokonui-held juveniles have survived and grown we conclude that the climate at this southern location is suitable to consider a free-release.

3659: ***+.074***

Testudo hermanni hermanni is becoming seriously endangered throughout its range. It has a scattered distribution, with a small number of residual populations found in Spain, France and Italy. In this study we sampled a population of T. h. hermanni from Southern Tuscany (Massa Marittima, Grosseto), composed of native and introduced individuals (recognizable due to residual

signs of previous marking on the carapace). Overall, 95% of the captured individuals were adults and the sex ratio was slightly, but not significantly, biased in favour of females. Population density was relatively high in comparison with other Italian populations, although it was doubled by previous reinforcement. Genetic analysis performed on six polymorphic microsatellite loci revealed a high level of genetic variability and heterozygosity, with no evidence of current inbreeding processes. Moreover, introduced individuals presented genotypes similar to those of the native individuals, thus suggesting that the reinforcement intervention did not cause a significant change in the original genetic pool. Nevertheless, long-term monitoring of the population is necessary to ensure its stability and vitality. Furthermore, to preserve the genetic identity of the local population in the future, uncontrolled translocation events should be avoided.

3661: ***+.011***

European white stork are long considered to diverge to eastern and western migration pools as a result of independent overwintering flyways. In relatively recent times, the western and northern distribution has been subject to dramatic population declines and country-specific extirpations. A number of independent reintroduction programs were started in the mid 1950s to bring storks back to historical ranges. Founder individuals were sourced opportunistically from the Eastern and Western European distributions and Algeria, leading to significant artificial mixing between eastern and western flyways. Here we use mitochondrial and microsatellite DNA to test the contention that prior to translocation, eastern and western flyways were genetically distinct. The data show a surprising lack of structure at any spatial or temporal scale suggesting that even though birds were moved between flyways, there is evidence of natural mixing prior to the onset of translocation activities. Overall a high retention of genetic diversity, high N-ef, and an apparent absence of recent genetic bottleneck associated with early 20th century declines suggest that the species is well equipped to respond to future environmental pressures.

3662: ***+.192***

A quarter of all lagomorph species worldwide are threatened with extinction. Captive breeding programs, such as that developed for the Columbia Basin (CB) pygmy rabbit (Brachylagus idahoensis), sometimes are implemented as emergency conservation measures to restore small, genetically distinct populations. However, small source populations also may have low genetic diversity, which may influence attributes related to fitness, including growth, survival, and reproduction. We used mixed-effects regression models to explore the influence of genetic pedigree (% CB) on pairing success, growth, and survival during the 10-year captive breeding program at Washington State University, which included controlled pairings and outbreeding with pygmy rabbits from Idaho. Pairing success, juvenile growth, and juvenile survival declined with increasing CB pedigree of 1 or both parents, suggesting inbreeding depression among the small number of related founders. Demographic variables such as age, sex, and previous pregnancies, and environmental variables such as month and temperature at birth also were associated with production of pygmy rabbits. Our study illustrates the difficulty of retaining a unique genome of a small source population while simultaneously producing enough rabbits for restoration into natural habitat as part of endangered species recovery programs.

3663: ***-.009***

In this study, we analysed the distribution of the t(1;29) Robertsonian translocation to determine whether this polymorphism contributes to the low reproduction efficiency observed in five endangered Andalusian (Spain) cattle breeds: Berrenda en Negro (BN), Berrenda en Colorado

(BC), Cardena Andaluza (CA), Pajuna (PA) and Negra Andaluza (NA). All these breeds were reared exclusively in reproductive isolation under grazing conditions with natural mating. In total, we analysed the distribution of the rob(1;29) translocation in 714 animals: 192 BN, 235 BC, 156 CA, 56 PA and 75 NA. We also examined the translocation frequencies, F statistics and deviations from Hardy-Weinberg equilibrium among different herds and breeds and characterised the influence of geographical location and sex. The F-ST values ($P < 0.05$) revealed differences among the breeds and herds in BC, BN and CA. There were no significant geographical differences, except in the Cardena breed ($P < 0.001$). In addition to reproductive isolation, the differences observed among the herds might reflect the sporadic movement of bulls belonging to flocks with a high frequency of translocation, genetic drift and anthropic selection. The rob(1;29) frequency was reduced in some breeds, potentially reflecting the effects of human selection and breeding strategies implemented through official control programmes for this anomaly. In other breeds, the translocation remains present at high frequencies, reflecting crossbreeding with the Retinta breed, which has a high frequency of rob(1;29). No significant deviation in the expected percentage of heterozygotes was detected in any breed. The differences observed in the rob(1;29) frequencies in the endangered Andalusian cattle might be more attributable to internal factors than to differences between the breeds. Further studies regarding the mobility of sires among herds are warranted to determine the origin of the variations in the rob(1;29) frequencies observed in endangered Andalusian breeds. (C) 2013 Elsevier B.V. All rights reserved.

3664: *+.126*

In the Moctezuma river, Central Mexico we recorded thirty five species of fish, corresponding to a gamma diversity of 2.5. The river basin is located in the biological transition zone between both America's bioregions. The highest alpha diversity was found in the ecotope Jalpilla (H' = 1.58), inside the section of the river Matlapa. Between sub-basins, the greatest beta diversity was located in the river Huichihuayan (H* = 2.36). Cichlasoma cyanoguttatum, Cichlasoma pantostictum, Cichlasoma steindachneri, Gambusia marshi, Ictalurus australis, Poecilia formosa and Poecilia reticulata were new records for the basin. Fish distribution was classified in six meta-basins. The most diverse family was Poeciliidae (14 species). Poecilia mexicana was the most abundant species followed by Astyanax mexicanus. Fifty one percent of the species were Neotropical, 20% Nearctic ones; 25.7% were native; only 2.8% were deliberately introduced. Chirostoma jordani and Notropis sallei were recent introductions from the Lerma river. Changes in habitat, channelization for agriculture, water transfers between rivers Lerma and Moctezuma, and the deliberate or accidental introduction and translocation of eight species, were the main reasons of the displacement and reduction in the distribution of the endemic species. Three of the recorded species are protected by Mexican environmental laws: Cichlasoma labridens, C. steindachneri and I. australis. Action plans to preserve fish community and the ecological processes at different territorial and time scales (meta-basin, basin and sub-basin) are proposed.

3665: *+.068*

amphibians are declining globally at an alarming rate. for the sustainable management of amphibians, the development of reproductive technologies and in particular field-friendly methods for cryopreservation of sperm are required. successful cryopreservation preserves genetic material, provides for the transfer of genes from wild populations to captured stock, and assists in dispersing genetic material among populations in nature. Collecting spermatophores of salamanders and their subsequent cryopreservation could be an effective method of perpetuating genetic variation and provide for ex situ propagation and reintroduction efforts. this study investigates the practicality of using spermatophores from the Axolotl, Ambystoma mexicanum, to

assess the effects of cooling rates, thawing rates, and cryodiluents on the post-thaw viability of sperm within salamander spermatophores. in a first experiment, overall post-thaw sperm viability was 64.7% +/- 29.5% (mean +/- 1 sD; as assessed by a live/dead stain) while in the second experiment, overall post-thaw sperm viability was 86.7% +/- 8.0%. there were no significant effects of cryodiluents, cooling rates, thawing rates or the interaction effects on sperm viability ($P > 0.05$). the unique aspects of this study - cryopreserving sperm within spermatophores - potentially influenced the cooling and thawing process and likely affected the ability of water and cryodiluents to enter sperm cells. a model species such as the axolotl (critically endangered in nature) that represents a number of Ambystoma species with similar breeding habitats, may prove useful for developing field cryopreservation protocols for these and other endangered species.

3666: *+.102*

We tested the degree of habitat selection at two spatial scales by captive-raised oregon spotted frog (Rana pretiosa) individuals released at two sites in british Columbia to inform translocation and habitat-based recovery actions for this highly imperiled species. telemetry of captive-raised adults during the post-breeding season (2009 and 2010) suggests that oregon spotted frogs selected for herbaceous and shrub macrohabitats (delineated from high-resolution aerial imagery) that form continuous floating mats or mats interspersed with water. at the microhabitat level, frogs consistently selected for taller vegetation (= 122.9 +/- 8.0 cm) and thicker submerged vegetation (= 23.5 +/- 2.4 cm), based on comparisons with paired random locations (= 99.2 +/- 9.4 cm, and = 13.8 +/- 1.9 cm, respectively). Within two study wetlands, microhabitats with < 50% cover of semi-open herbaceous or shrub vegetation were also associated with higher frog presence. these results provide support for the hypothesis that oregon spotted frogs at these sites select for taller, less dense vegetation, irrespective of the floristic composition at the microhabitat scale, but not at the macrohabitat scale. these results from captive-reared animals corroborate findings from habitat-selection studies that used wild-collected animals. Differences in selection at the macrohabitat scale between years suggest that a wide range of wetland types could be considered as candidates for reintroduction efforts. our results emphasize the need for methods that transcend site-specific floristic differences among wetlands to inform potential reintroduction sites and guide habitat restoration activities.

3667: *-.217*

As wildlife populations decline or disappear, wildlife professionals are using management tools such as translocation to maintain viable populations, often with mixed results. Wild-to-wild translocations are often more successful than when captive animals are released, raising concerns that captivity may have deleterious effects on animals. although the effects of captivity have been documented on a generational time-scale, effects within the lifetime of an individual have received much less attention. here we examine how time in captivity affects foraging behavior of wild-caught ratsnakes (Elaphe obsoleta). The longer ratsnakes had been in captivity, the less successful and slower they were to react to prey in a simple laboratory discrimination task. snakes that had been captive for a year or more performed no better than expected by chance. Captivity-induced degradation of ecologically important behaviors provides a potential mechanism underlying the poor performance of animals that are released into the wild following prolonged captivity. our results also suggest that research using captive snakes may not always document behaviors representative of wild snakes.

3668: *+.191*

Many Indian tribes consume several locally growing medicinal plants as part of their diets. Genus Caralluma is one of them. Caralluma adscendens var. Fimbriata (Wall.) Gray. & Mayur. belonging to family Asclepiadaceae is succulent, perennial herb commonly called makad shing. It is an ancient Indian plant used for centuries for its ability to suppress appetite and enhance endurance. It is eaten in numerous forms; cooked as a regular vegetable, preserved like chatneys and pickles. The plant is characterized by peregnane glycosides, flavone glycosides, megastigmane glycosides and sponins. Due to over exploitation and lack of organized cultivation, the wild population has declined fast: Therefore there is and urgent need to conserve this genus. Earlier reports show a very little work on multiplication by organogenesis. But no reports were available on ex-situ or in-situ conservation techniques of this important endemic medicinal plant. Hence, ex-situ conservation was attemped through seed and vegetative propagation for reintroducing and establishment of new populations in similar habitat. An extensive survey was carried out. Plant material was collected and tried for propagation and ex-situ conservation. Propagation through seed shows 90% germination when soaked in warm water for overnight. Effect of growth regulators and different strengths of Auxins and Cytokines were tried for induction of root in vegetative stem cutting. Low concentration of auxin were found effective in root initiation. Seedlings and cuttings of two months old were used for reintroduction in similar natural habitat. Survival percentage was found 70% in newly introduced site. As the Caralluma has food potential, it is tried for the preparation of preserved food products like pickles and chatneys.

3669: ***+.112***

The success of species reintroduction programmes depends on many factors, including habitat quality and predator occurrence at release locations. For the critically endangered eastern barred bandicoot Perameles gunnii in Australia, successful releases have been achieved only in the absence of predation by the introduced red fox Vulpes vulpes. However, few fox-free sites exist, but those that are fox free may have low habitat suitability, potentially leading to reintroduction failure. We studied a reintroduced population of eastern barred bandicoots at Mt Rothwell, a 420-ha fox-free reserve which appeared to have a degraded foraging range and a lack of nesting material due to overgrazing by marsupial herbivores. We quantified habitat use and measured several variables representing movement distances and nest use. Bandicoots foraged in open grassy areas and nested at sites containing high values of litter, lateral cover and concealment cover. Mean (+/- SE) home ranges (37.2 ha +/- 11.8), daily movement distances (494.6 m +/- 32.5) and distances between consecutive nests (122.6 m +/- 29.0) were large for males. On average, the length of stay at each nest (2.8 days +/- 0.4) was longer than previously recorded values for males, and 13.2% of the nests were used by at least two individuals, suggesting that nesting opportunities were limited. Despite behaviour indicating a low resource base, bandicoots appeared to be surviving and reproducing in our study area, indicating a capacity to live in structurally simple habitat in the absence of predators. Our results are consistent with predator exclusion being a critical factor for successful bandicoot reintroduction.

3670: ***+.143***

Translocation of captive-reared animals is widely used as a tool for endangered species recovery. Frequently, translocated populations have relatively low initial productivity, requiring management intervention. A translocated population of whooping cranes Grus americana in central Wisconsin is such a case. We examined chick mortality for this population and used daily chick survival rates as our response variable to model several parameters including phenology, chick age, energetics and parent age and experience. We also developed and evaluated adoption techniques using sandhill cranes Grus canadensis to mitigate the effects of high chick mortality

and increase the probability of fledging. Our results illustrate the challenges that translocated populations can face as they encounter novel breeding conditions. We found that whooping crane daily chick survival was relatively low and most mortality events occurred within the first 20 days. Our results indicated that variables related to age of the parents as well as the pair's previous chick rearing experience were useful for predicting daily chick survival. We found that sandhill crane foster parents readily accepted replacement chicks. We also demonstrated adopted chicks acceptance of foster parents and that the chicks' source (captive-born vs wild-born) did not affect success of the adoption. Chick adoption provides several management options that could be used to bypass the period when chicks experience the greatest mortality. Reducing chick mortality and developing techniques to increase the number of fledged chicks is paramount for whooping crane recovery as well as the recovery of other endangered bird species.

3671: *+.000*

Seasonal foraging habitats, prey species and foraging frequency of Japanese Crested Ibis Nipponia nippon in farmland were studied one year after its reintroduction on Sado Island, Japan. Paddies were the main foraging habitat in spring, early summer, autumn and winter. In late summer, ibises often used levees around paddies, grasslands, uncropped paddies and abandoned paddies. Small invertebrates were important prey components in terms of frequency (70-90%) throughout the year. Ibises took Misgurnus loaches (less than 20% of prey captured) throughout the year; the capture rate of loaches in ditches was especially high in winter. Ibises depended on terrestrial invertebrates, including earthworms, particularly in late summer. No clear correlations between the rate of capture for each prey species and the proportion of habitat use in each season were detected. However, abandoned and uncropped paddies provided suitable habitats for the ibis because human disturbance was relatively low and the rate of capture for each prey type was relatively high throughout the year. Management of grass height in grasslands and on levees around the paddies will be effective in ensuring the availability of foraging habitats in late summer. Seasonal changes in the availability of foraging habitats in farmland ecosystems should be considered when designing effective management strategies for the establishment of Japanese Crested Ibis populations.

3672: *+.126*

Reduced movement across a landscape due to habitat loss and fragmentation is considered one of the primary reasons for species' population declines. Gliding mammals, such as the northern flying squirrel (Glaucomys sabrinus), are expected to be particularly sensitive to large non-forested gaps and therefore have been used as umbrella species in planning for landscape connectivity. We tested the gap-crossing decisions of the northern flying squirrel in a forested landscape in southern New Brunswick, Canada. We translocated 35 flying squirrels across non-forested gaps (50-960 m) with varying detour efficiency (distance to return home across the gap divided by the forested detour distance) and recorded the individual movement paths to return home. We found that 69% of flying squirrels took the forested route home and avoided crossing gaps. Detour efficiency was the only significant landscape predictor of gap-crossing; for every 1% increase in detour efficiency the odds of flying squirrels detouring were 15% higher. Northern flying squirrels were much more likely to take forested routes than to cross open canopy gaps, even when the direct distance was 6.8 times shorter. In addition, flying squirrels took substantially longer to return home if gaps in forest cover exceed a threshold of 335 m. Such threshold responses by flying squirrels could partly explain observed drops in flying squirrel occurrence in small, isolated patches of forest. Avoidance of gaps when detours are cheap suggests that there is a cost associated with crossing gaps. This provides support for the importance of maintaining functional connectivity in forested landscapes.

(C) 2013 Elsevier Ltd. All rights reserved.

3673: *+.067*

In this study, we evaluated the reinforcement of Pulsatilla vernalis, a rare and endangered plant species which strongly declined in Central Europe during the last decades. Actually, only nine remnant populations of the Spring Pasque flower still exist. These were subjected to population reinforcement by the governments of the Upper Palatinate and Lower Bavaria in a recovery programme since 1999. Therefore, plants were grown from local seed material and planted out in the respective populations.Here, we investigated the success of this population recovery programme and analysed whether potential bottlenecks during population reinforcement caused by seed collection, germination and plant propagation affected fitness and genetic variation of P. vernalis. For this reason, we compared morphological traits, germination and genetic variation of wild and planted individuals from each population. The total number of individuals rose from 290, when the recovery programme started to 1368 in 2012 (4.7-fold). Wild and planted individuals did not differ in most morphological traits. However, wild individuals were slightly larger than planted ones, due to their higher age. Seeds of P. vernalis germinated well and germination success of wild and planted individuals was comparable. Genetic variation within wild and planted individuals from each population did not differ significantly and we observed in only three populations slight genetic variation between wild and planted individuals. Considering the results of our study, population reinforcement of P. vernalis was a success. Increasing population sizes had no negative impact on fitness and genetic variation. Although reintroduction was recently challenged as a successful tool in plant conservation, population reinforcement is a glimmer of hope for the long-term conservation of this highly endangered species. (C) 2013 Elsevier Ltd. All rights reserved.

3674: *+.145*

Dispersal from release areas is a critical problem for reintroductions. Reliable methods are therefore needed for analysing post-release monitoring data to guide further releases. Radio tracking can greatly improve data quality by distinguishing dispersal from mortality. However, fates of animals continue to be uncertain if transmitters have short battery life and detection range, as is typically the case with small animals. We present an approach for simultaneously modelling probabilities of fidelity (remaining in release area), survival, detection and transmitter failure from post-release monitoring data, and illustrate how it was applied to translocations of North Island robins (Petroica longipes) to 17 forest fragments (5-56 ha) over 5 years. The modelling showed that fidelity probability depended on the sex (higher in females) and translocation date (higher in winter than autumn), and that variation among fragments was well explained by the "cost distance to nearest neighbour" (an index reflecting the amount of pasture and shrubland needing to be crossed to reach another forest area) and the area of the release fragment (higher in larger fragments). Combined with survival, the estimated probability of a bird remaining in its release fragment the next breeding season ranged from 0.02 to 0.39. As these estimates were refined, they could be used to assess suitability of fragments for further releases and numbers of each sex needing to be released to compensate for dispersal. The Bayesian framework underlying the approach potentially allows application to any amount of data by using informative priors derived from previous translocations or expert opinion. (C) 2013 Elsevier Ltd. All rights reserved.

3675: *+.078*

Brown-eared pheasant (Crossoptilon mantchuricum) is an endemic and first-class protected bird in China, which only distributes separately in North China. In order to understand the genetic

diversity of C. mantchuricum populations and the phylogenetic relationships between the C. mantchuricum populations and between the individuals, the polymerase chain reaction (PCR) and sequencing were adopted to determine the sequence of the mitochondrial DNA control region of 20 brown-eared pheasants from the Pangquangou Nature Reserve and Taiyuan Zoo in Shanxi Province of China, respectively. In comparing with the known structure of mitochondrial DNA control region of the other pheasants, the structure of the mitochondrial DNA control region of brown-eared pheasant was analyzed, and the hyper-variable region III, medium conservative region II, and conservative region I were identified. The phylogenetic trees were constructed with neighbor-joining (NJ) method and maximum-parsimony (MP) method. The genetic distances of the individuals between the two populations were from 0 to 0. 009, with a mean value of 0. 002. The genetic flow between the two populations (N-m) was 4. 67. Overall, the structure of the mitochondrial DNA control region of the brown-eared pheasant was similar to that of the other pheasants. The genetic distances between the two populations and between the individuals were smaller, and the genetic relationships were closer. This study could provide scientific evidence for the reasonable management of captive brown-eared pheasant and for the eventual reintroduction of the captive brown-eared pheasant to the wild.

3676: *+.002*

The common carp (Cyprinus carpio) is a species of primary importance for commercial inland fisheries of Anatolia, where it is native to some areas but has become almost ubiquitous due to historical translocations. Yet, there are concerns that this 'semi-naturalised' species can ultimately pose a threat to aquatic ecosystems, especially when present at high biomass levels. For estimating the latter, knowledge of length-weight relationship (WLR) and condition factor (K) parameters is needed, and these also represent useful indicators of fish population dynamics in general. In this study, WLR and K parameter values from published literature data were comprehensively reviewed for 68 and 75 common carp stocks, respectively, from 52 water bodies across Anatolia, which were surveyed between 1953 and 2011. Overall, Anatolian common carp stocks were characterised by slightly negative allometric growth, which became more pronounced in some over-exploited stocks and/or under critical conditions of pollution and water quality. This tendency to negative allometric growth was unlike other stocks world-wide, which showed isometric growth. Also, no altitudinal gradients were detected in the parameters under study and only slight differences amongst waterbody types (i.e. man-made lakes, natural reservoirs and water courses) were revealed (possibly a result of biassed sampling). It is argued that the level of nativeness present in Anatolian common carp stocks may be responsible for the observed patterns and differences relative to domesticated/feral common carp stocks introduced to non-native areas world-wide. This finding should be accounted for when evaluating ecological benefits vs economic losses of intervention measures for control.

3677: *+.256*

Climate change leads to rapid, differential changes in phenology across trophic levels, often resulting in temporal mismatches between predators and their prey. If a species cannot easily adjust its timing, it can adapt by choosing a new breeding location with a later phenology of its prey. In this study, we experimentally investigated whether long-distance dispersal to northern breeding grounds with a later phenology could be a feasible process to restore the match between timing of breeding and peak food abundance and thus improve reproductive success. Here, we report the successful translocation of pied flycatchers (Ficedula hypoleuca) to natural breeding sites 560 km to the Northeast. We expected translocated birds to have a fitness advantage with respect to environmental phenology, but to potentially pay costs through the lack of other locally

adapted traits. Translocated individuals started egg laying 11 days earlier than northern control birds, which were translocated only within the northern site. The number of fledglings produced was somewhat lower in translocated birds, compared to northern controls, and fledglings were in lower body condition. Translocated individuals were performing not significantly different to control birds that remained at the original southern site. The lack of advantage of the translocated individuals most likely resulted from the exceptionally cold spring in which the experiment was carried out. Our results, however, suggest that pied flycatchers can successfully introduce their early breeding phenotype after dispersing to more northern areas, and thus that adaptation through dispersal is a viable option for populations that get locally maladapted through climate change.

3678: *+.118*

Animal conservation practices include the grouping of captive related and unrelated individuals to form a social structure which is characteristic of that species in the wild. In response to the rapid decline of wild African lion (Panthera leo) populations, an array of conservational strategies have been adopted. Ex situ reintroduction of the African lion requires the construction of socially cohesive pride structures prior to wild release. This pilot study adopted a social network theory approach to quantitatively assess a captive pride's social structure and the relationships between individuals within them. Group composition (who is present in a group) and social interaction data (social licking, greeting, play) was observed and recorded to assess social cohesion within a released semi-wild pride. UCINET and SOCPROG software was utilised to represent and analyse these social networks. Results indicate that the pride is socially cohesive, does not exhibit random associations, and the role of socially influential keystone individuals is important for maintaining social bondedness within a lion pride. These results are potentially informative for h structure of lion prides, in captivity an in the wild, an could have implications for captive and wild-founder reintroductions.

3679: *-.175*

Suspended sediment (SS) loadings in freshwater habitats have increased over the past century and SS is now a significant environmental stressor. Greater tolerance to environmental stressors has been proposed as a factor in the success of aquatic invasive species. Further, parasites may interact with environmental stressors to increase host susceptibility to loss of fitness and mortality. We compared the effects of SS exposure on the gill structure and aerobic scope of the endangered white-clawed crayfish (Austropotamobius pallipes), and the invasive signal crayfish (Pacifastacus leniusculus), and assessed impacts in relation to parasite burden. SS caused gill fouling and reduction in aerobic scope in both species, though A. pallipes was more susceptible than invasive P. leniusculus. The parasite Branchiobdella astaci, a crayfish worm that infests the gills, interacted with the sediment to affect gill structure whereas infection with the microsporidian parasite Thelohania contejeani had no effect on crayfish response to SS. Juvenile P. lenisuculus had a higher standard metabolic rate than A. pallipes, which may be linked to competitive advantages such as higher growth rate and behavioural dominance. Conservation of A. pallipes often involves relocation of threatened populations to isolated stillwaters; our findings suggest that SS levels should be assessed before relocation.

3681: *+.031*

Background This report follows on from a pine marten Martes martes Expansion Zone Survey conducted in 2012 (Croose et al., 2013), which provided information on current patterns of pine marten distribution in Scotland following population recovery and range expansion since the 20th

century. The 2012 survey confirmed that the pine marten's range has spread from the Highland stronghold documented by surveys in the 1980s (Velander, 1983) and 1990s (Balharry et al., 1996) and the population has re-colonised many southern and eastern parts of its former range in Scotland. The current survey encompassed areas of southern Scotland that had not previously been surveyed: this comprised areas south of the Central Belt to the Scottish/English border, excluding those parts of Dumfries and Galloway that were included in the 2012 survey. The survey methodology involved surveying 1km transects in woodland for pine marten scats (faeces), using DNA analysis to confirm their species of origin. Records of pine martens were also collected from other sources in addition to the field survey. Main findings Pine marten scats, verified via DNA analysis, were found in five (4%) of the 117 hectads searched during the field survey. An unusually high proportion (61%) of scats was not determined to species level by DNA analysis. This is likely to have resulted in the occurrence of 'false negatives', whereby experienced surveyors believed pine marten scats were collected but identification was not confirmed by genetic analysis. Recent pine marten records, provided by recording and conservation organisations and naturalists, were collected from 13 hectads (11% of hectads) within the survey area. Pine marten presence was not detected in 97 hectads (83% of hectads surveyed) within the survey area. Pine marten presence was confirmed at four widely-spaced sites in southern Scotland and appears to be centred on three areas: immediately south and west of Glasgow; the Upper Tweed Valley; and in Annandale and Eskdalemuir in eastern Dumfries and Galloway. Pine marten populations in southern Scotland are likely to have originated from both natural spread from the core population and translocations and releases. It is likely that pine martens will re-colonise the majority of suitable habitats in southern Scotland over time.

3682: ***+.047***

Study of impact assessment of beaver reintroduction in valleys of small rivers on amphibians was carried out. The work took place on the territories of the state nature reserves: Rdeyskiy, Prioksko-Terrasny and Privolzhskaya Lesostep. Small rivers of the area under study have been inhabited by beavers in different years, so degree of beavers' impact on valley ecosystems differed. This fact allowed us to study amphibians in different conditions. The main factors of beavers' impact on amphibians were modifications of lighting and water regimes. Loss of trees due to beaver activities had increased lighting that made water heating more intense in spring. Because of damming a lot of standing water bodies appeared and diversity of habitats increased. Those facts were beneficial to spawning and metamorphosis of widespread species of amphibians Rana spp., Bufo bufo. After a long-term influence of beaver activities in river valleys, the vast majority of suitable habitats for amphibians became associated with beavers, so species diversity within river valleys was increased, for example Pelophylax spp. appeared in beaver ponds. If beaver populations were young or unstable, reintroduction of beavers had small influence on amphibians, and in some cases their impact was negative. Factor of water regime had importance only in large beaver ponds, where beaver dams maintained stable water level. When beavers left ponds, especially in spring - a mass death of eggs and larvae was observed.

3683: ***+.032***

Though fairly widely distributed and common in certain parts of Eastern Europe, the Common spadefoot (Pelobates fuscus) is in decline in its northwestern range. In the Netherlands, habitat loss and degradation were the main causal factors for their extirpation, leaving the remaining ones in geographical isolation. Despite conservation efforts (habitat restoration) their numbers and distribution did not change or more importantly improve. Some populations were lost during this period despite the fact that their biotope seemed to be in good condition. To date there are 38

extant populations known to exist in the Netherlands; their future however, remains uncertain. With the exception of just a few, population sizes are probably extremely low. Most of them are completely isolated from one another and in many cases located outside protected nature areas. Also, in many cases only one or sometimes two breeding ponds are used, making these populations and species at great risk of local extinction; especially if you factor in the possibility of unforeseen climatic events. Because of small population sizes and the isolation aspect, extinction due to genetical problems may also play an important part in the decline of this species. Inbreeding for example, might result in less viable specimens and lower reproductive success. To mike an extra effort to save the Common spadefoot in the Netherlands, RAVON (Reptile, Amphibian, Fish Research the Netherlands) and Artis Royal Zoo began a preliminary study into the reintroduction of the Common spadefoot in two nature areas and the supplementation of specimens to one nature area in Noord-Brabant province. The actual reintroduction and supplementation started in 2012. From March to May, portions of egg strings were taken from nine natural source populations. Tadpoles from different localities were reared in separate tanks at Artis Royal Zoo. In consideration that survival rates in captivity are much higher than in the wild this head starting techniques allowed for the mass rearing required to make a significant impact on the re-establishment of this species across the Netherlands. Between 2012 and 2014 a total of 51.814 larvae were reared of which 24.373 have been released in the source localities where they were originally collected as eggs. A total of 25.621 larvae were used for actual reintroduction and supplementation purposes. In 2014 750 specimens were also released in their juvenile stage. The project will last until 2016 so that a natural situation can establish with all different generations present. Monitoring on the long term will determine if the reintroduction and supplementation is successful.

3684: *+.059*

Post - released monitoring, of hog deer, Eld's deer and sambar deer in Salakphra grassland, Salakphra Wildlife Sanctuary, Kanchanaburi Province was procedure during August 16th, 2011 and April 2013. The objectives were to monitor the released hog deer, Eld's deer and sambar deer. Radio tracking, camera traping and direted observing were used to investigate the adaptability of the animal. In the case of habitat preparing, grassland improvement covered an area of 10 ha approximately by prescribe burning was done. Ten artificial salt lick sites and 21 water sources were also done in the released area. Thirty-one hog deer, 19 males and 12 females, were translocated in to the area, 4 males and I female were tagged with radio collar. Six Eld's deer, 3 males and 3 females were translocated. Four of them, 2 males and 2 females were tagged with radio collar. Ten sambar deer, 4 males and 6 females were translocated. Two of them, 1 male and 1 female were tagged with radio collar. The monitoring results from radio telemetry all year round found that the male hog deer had 124.44 ha avearge home range size. The size of the female was 79.16 ha. The average home range size of male Eld's deer was 114.69 ha. Whereas those of the female was 92.55 ha. The overall home range of the female sambar deer was 153.10 ha. The result from camera trapping during 2011 and 2012 found that 19 wildlife spececies inhabited in the area. The occurance of sambar deer had positive coreration with other 4 wildlife species significantly. Whereas the occurance of hog deer and Eld's deer had positive correlation with other I wildlife species significantly. Dhole and guar that had never been occured in the area were also photographical recorded in 2012. The result from fecal pellet group count method showed ecological density of sambar deer around the area was 0.13 individuals/ha whereas those of the barking deer was 0.03 individuals/ha. The released hog deer gave 10 newborns during the study period. Nevertheless 11 of the released individuals were died, 7 of them due to translocation and ear tag

3685: *+.103*

The yellow-spotted mountain newt Neurergus microspilotus (Caudata: Salamandridae) is listed as Endangered by the International Union for Conservation of Nature. In spite of its conservation status and laws protecting it, the species continues to decline in the wild. A captive breeding program was successfully established to support the species' recovery. To learn more about the species' ecology and winter mortality, 24 juveniles reared in captivity were released on 4 occasions in a small spring enclosure from July to September 2012 and monitored until December, when they disappeared for overwintering. In 12 visits to the site before and after overwintering, a total of 31 individuals were identified. Based on an average diurnal detection probability for this newt (0.61 +/- 0.19 SD), the observed newts during the pre-overwintering period were estimated to represent 6.5 to 41.0% of the reintroduced newts. The newts observed after overwintering were 13 to 28% of the reintroduced newts. This study demonstrates that post-metamorph captive-bred N. microspilotus released into the wild can survive to the second growing season, and provides a choice of life stage for a reintroduction plan.

3686: *+.324*

Recent ecological studies have shown that oxidative status could have a significant impact on fitness components in wild animals. Not only can oxidative status reflect the environmental conditions that animals experience, but it can also predict their chances of reproduction and survival in the future in their natural habitat. Such important characteristics make markers of oxidative status informative tools to evaluate a priori individual perspectives of reproduction and survival as well as to assess a posteriori the effect of human activities on the fitness of species of conservation concern and wildlife in general. Markers of oxidative status may therefore help conservation practitioners to identify conservation threats to animal populations and to maximize the success of wildlife management. Despite these potential benefits for animal conservation programmes, up to now markers of oxidative status have only been reported anecdotally in conservation studies. The aim of this review is therefore to raise awareness by conservation practitioners of the use of markers of oxidative status. Towards this end, we first describe how environmental disruptions due to human activities can translate into variation in oxidative status. Second, we show how individual and population variation in oxidative status may contribute to the success or the failure of reintroduction or translocation programmes. Finally, we emphasize the technical features specific to the measurement of markers of oxidative status in conservation programmes, which may help investigators with the interpretation of their results. Such prior knowledge about markers of oxidative status may encourage conservation physiologists to use them in order to enhance the success of conservation programmes and wildlife management.

3688: *+.138*

Background: Knowledge of dispersal movement of birds and their habitat preference during the post-fledging period is fundamental to the understanding of their ecological and evolutionary processes. The Crested Ibis is now being reintroduced to protected sites within its historical range, with the goal of establishing a self-sustaining population that may eventually qualify the species for delisting.Methods: We carried out an ecological study of post-fledging dispersal and habitat use of a reintroduced population of the Crested Ibis (Nipponia nippon) from 2008 to 2012 in Ningshan County, China, by using banding and radio-telemetry methods.Results: In about two weeks (an average of 14.3 days) after fledging, the activities of the fledglings were concentrated in a range of about 100 m around their natal sites, such as the oak-pine forest patches at the edge of open habitats. During this period, fledglings were still partially dependent upon parental care and

fed typically on a daily basis. Siblings increasingly became independent by mid-August and then gradually moved away from their natal sites to post-fledging dispersal locations. During the period of the post-fledging dispersal process, most juveniles moving southwest were concentrated at the mean direction (mu = 254.6 degrees, s = 70.5 degrees) with a mean dispersal distance of 5.1 km. It took an average of 56.4 days to disperse from the natal territory to the first wintering area. Also, forging habitats for juvenile ibis varied with time and local conditions. For example, paddy fields were used most frequently among all habitat types, while shallow rivers just from August to October. Masson pine (Pinus massoniana) was often regarded as the roosting tree species preferred by the Crested Ibis, with the highest utilization rate among all the roosting habitat types. The juveniles of the wild population dispersed four times as far as that of the reintroduced population, but the overall pattern of post-fledging dispersal is similar for the reintroduced and wild populations.Conclusions: Our results are very useful for us to predict the distance and direction of dispersal under various landscape conditions in other released sites. The project is a good example for reintroducing endangered species to their former ranges, and will be valuable for the protection of reintroduced populations of this critically and other endangered species.

3689: *+.459*

Enrichment, broadly the provision of stimuli to improve the welfare of captive animals, is known to be important in husbandry practice and in the success of ex situ conservation and reintroduction programs. Practical evidence of the importance of enrichment exists for a number of taxa, yet amphibians are poorly represented. There is no reason to assume a priori that amphibians would not benefit from enrichment and, given their increasing prominence in captive programs, their requirements in captivity beyond basic husbandry should be the focus of more intense study. We review the existing body of research on enrichment for amphibians, as well as that for fish and reptiles, which may be regarded as behaviorally and neurologically broadly similar to amphibians. We also briefly discuss mechanisms by which enrichment might affect amphibian fitness and, therefore, reintroduction success. Our review supports the contention that there may be important consequences of enrichment for both captive welfare and ex situ conservation success in amphibians and that amphibian enrichment effects may be highly variable taxonomically. In the face of increasing numbers of captive amphibian species and the importance of ex situ populations in ensuring their species level persistence, enrichment for amphibians may be an increasingly important research area.

3690: *+.161*

Defining habitat selection features and predicting the distribution of species are important for conservation management, and habitat suitability models can provide the statistical framework linking environmental variables to occurrence locations. However, the reliability of such models is restricted for datasets that have limited presence data, which is problematic when population size is low and population dynamics are transient, such as in reintroductions. We characterized and projected nest-site suitability for a black vulture Aegypius monachus population reintroduced in the Grands Causses, France. We performed temporal validations with subsample datasets based on chronological establishment, to assess whether first nests are able to predict subsequent nests. We compared these results to a spatial validation to ensure robustness. Predictions were reliable even with only 10 nest locations. The black vultures reintroduced in the Grands Causses selected pine trees and steep slopes, similar to natural populations elsewhere in Europe. Although our projections were made from only a small number of founders, they are conservative and indicate that a large area in this region is available for nesting and thus availability of breeding habitat is not currently a limiting factor for the species. Our findings will aid the modelling of habitat

suitability for further reintroductions. We propose conservation management strategies for this region that integrate socio-economic constraints with the prediction of sites suitable for nesting. Where habitat is the most suitable for black vultures, logging and human activities need to be reduced during the critical breeding stages.

3691: *+.052*

The Near Threatened Eurasian black vulture Aegypius monachus is considered highly threatened in Europe, and the species was reintroduced in France between 1992 and 2004. A total of 53 individuals were released, using two methods: immatures were released from large aviaries at the reintroduction site after a stay of several months (the aviary method), whereas juveniles were placed on artificial nests until fledging (the hacking method). The survival rates of released birds were compared to the survival of wild-born offspring through a multi-event capture-recapture analysis accounting for tag loss. Survival rates were higher in adults than in juveniles and immatures (0.98 +/- SE 0.02 vs 0.85 +/- SE 0.03) and were constant over time. Overall there were no differences in post-release survival between the two release methods: immatures released by the aviary method had a similar survival to juveniles released by the hacking method or born in the wild. Immatures can breed before juveniles, so releasing immatures by the aviary method could accelerate reintroduction settlement and increase population viability. Accurate estimates of post-release survival are essential to improve the reliability of viability analysis of reintroduced populations and the management of such populations.

3692: *+.124*

The Hermanns tortoise (Testudo hermanni) is an endangered land tortoise distributed in disjoint populations across Mediterranean Europe. We investigated its genetic variation by typing 1 mitochondrial locus and 9 nuclear microsatellites in approximately 300 individuals from 22 localities. Our goal was to understand the relative impact of natural and human-mediated processes in shaping the genetic structure and to identify the genetic priorities for the conservation of this species. We found that 1) all geographic areas are highly differentiated, mainly as a function of their distance but with a clear genetic discontinuity (F-st values larger than 0.4) between the Eastern and the Western subspecies; 2) the contact zone between subspecies is located farthest to the west than previously believed, and it probably coincides with the delta of the largest Italian river; 3) extinction events due to climatic conditions in the Upper Palaeolithic and subsequent human-mediated translocations in the Neolithic possibly explain the unexpected similarity among Spain, Sicily, and Corsica. For conservation purposes, the large majority of genetic pools appears native although hybridization among subspecies, related to extensive 20th century trade of tortoises across Europe, is observed in Spain and some Italian samples. Most populations do not seem at immediate risk of low genetic variation, except the French population, which has very low nuclear genetic diversity (heterozygosity 0.25) and where 50 out of 51 sampled animals shared the same mitochondrial sequence. In general, restocking and reintroduction plans should carefully consider the genetic background of the individuals.

3693: *+.019*

We explored multiple linkages among grey wolves (Canis lupus), elk (Cervus elaphus), berry-producing shrubs and grizzly bears (Ursus arctos) in Yellowstone National Park. We hypothesized competition between elk and grizzly bears whereby, in the absence of wolves, increases in elk numbers would increase browsing on berry-producing shrubs and decrease fruit availability to grizzly bears. After wolves were reintroduced and with a reduced elk population, we hypothesized

there would be an increase in the establishment of berry-producing shrubs, such as serviceberry (Amelanchier alnifolia), which is a major berry-producing plant. We also hypothesized that the percentage fruit in the grizzly bear diet would be greater after than before wolf reintroduction. We compared the frequency of fruit in grizzly bear scats to elk densities prior to wolf reintroduction during a time of increasing elk densities (1968-1987). For a period after wolf reintroduction, we calculated the percentage fruit in grizzly bear scat by month based on scats collected in 2007-2009 (n=778 scats) and compared these results to scat data collected before wolf reintroduction. Additionally, we developed an age structure for serviceberry showing the origination year of stems in a northern range study area. We found that over a 19-year period, the percentage frequency of fruit in the grizzly diet (6231 scats) was inversely correlated (P<0001) with elk population size. The average percentage fruit in grizzly bear scats was higher after wolf reintroduction in July (03% vs. 59%) and August (78% vs. 146%) than before. All measured serviceberry stems accessible to ungulates originated since wolf reintroduction, while protected serviceberry growing in a nearby ungulate exclosure originated both before and after wolf reintroduction. Moreover, in recent years, browsing of serviceberry outside of the exclosure decreased while their heights increased. Overall, these results are consistent with a trophic cascade involving increased predation by wolves and other large carnivores on elk, a reduced and redistributed elk population, decreased herbivory and increased production of plant-based foods that may aid threatened grizzly bears.

3694: ***+.250***

Spatial, phenotypic and genetic diversity at relatively small scales can buffer species against large-scale processes such as climate change that tend to synchronize populations and increase temporal variability in overall abundance or production. This portfolio effect generally results in improved biological and economic outcomes for managed species. Previous evidence for the portfolio effect in salmonids has arisen from examinations of time series of adult abundance, but we lack evidence of spatial buffering of temporal variability in demographic rates such as survival of juveniles during their first year of life. We therefore use density-dependent population models with multiple random effects to represent synchronous (similar among populations) and asynchronous (different among populations) temporal variability as well as spatial variability in survival. These are fitted to 25years of survey data for breeding adults and surviving juveniles from 15 demographically distinct populations of Chinook salmon (Oncorhynchus tshawytscha) within a single metapopulation in the Snake River in Idaho, USA. Model selection identifies the most support for the model that included both synchronous and asynchronous temporal variability, in addition to spatial variability. Asynchronous variability (log-SD=055) is approximately equal in magnitude to synchronous temporal variability (log-SD=067), but much lower than spatial variability (log-SD=111). We also show that the pairwise correlation coefficient, a common measure of population synchrony, is approximated by the estimated ratio of shared and total variance, where both approaches yield a synchrony estimate of 059. We therefore find evidence for spatial buffering of temporal variability in early juvenile survival, although between-population variability that persists over time is also large. We conclude that spatial variation decreases interannual changes in overall juvenile production, which suggests that conservation and restoration of spatial diversity will improve population persistence for this metapopulation. However, the exact magnitude of spatial buffering depends upon demographic parameters such as adult survival that may vary among populations and is proposed as an area of future research using hierarchical life cycle models. We recommend that future sampling of this metapopulation employ a repeated-measure sampling design to improve estimation of early juvenile carrying capacity.

3695: ***+.054***

We have studied metric variability of two indigenous and five invasive populations of the raccoon dog (Nyctereutes procyonoides ussuriensis) by using 21 dental and cranial size-adjusted measurements. Material consisted of 532 the raccoon dog skulls. Sexual size dimorphism (SSD) in total was statistically significant for 12 characteristics. Males were larger than females by up to 7 %. In some populations, SSDs were not explicit. A relatively low SSD is a result of life strategy and characteristics of the raccoon dog as monogamous species with wide ecological niche. Females have more obviously interpopulation structure than males due to 10 significantly different measurements while males differ by eight measurements. In addition, females where correctly classified by discriminatory analysis in populations totalling 60.4 % and males in 52 % of cases. Introduced populations are characterised by bigger measurements in skull width in relation to indigenous ones. Raccoon dogs from Europe show increase of general length of the skull. Our study does not support definite geographical or temporal variation of the raccoon dog because its linear size did not correlate with geoclimatic parameters. The one exception is a dental measurement of females which depends on temperature and latitude predictors. Size variation of the raccoon dog in areas of origin and colonised regions should be discussed from different points of view. The main factors to shape spatial and temporal skull variation in the raccoon dog are net primary production of ecosystems, adaptability to ecologically different regions and stochastic factors such as founder effect, isolation of populations due to by human activity. Observed metric variability is not deep morphological segregation and stands mainly in frames of ancestral subspecies.

3696: *+.131*

Conservation of rare plants can be accomplished by the restoration practice of reintroduction, but subsequent management is often required. In species with narrow habitat requirements, it is especially difficult to predict which management methods will be successful at new locations. Marsilea villosa is an endangered endemic Hawaiian fern with only seven remaining populations in ephemerally flooding drylands. Among its uncommon traits are long-lived sporocarps, a requirement of flood and drought to complete its sexual life cycle, and the potential for extensive vegetative growth. An experiment was performed to determine which restoration techniques might best facilitate growth of outplanted M. villosa. The following effects were tested in a split-plot factorial design: flooding (once/none), light (50% shade/full sun), weeding (bi-monthly/none), and all interactions. We hypothesized that flooding would have the largest single-factor effect and that there would be interactions among treatments. As hypothesized, flooding had the greatest positive effect on percent cover and sporocarp production. However, shade also increased cover over full sun when the plants began to experience drought. There was an interaction of lightxflooding because M. villosa grew best in flooded, shaded plots. Weeding had no significant effect except in combination with flooding. Beyond protected status, current management of M. villosa only includes weed management at some populations. This study shows that if reintroducing new populations, the need for labor-intensive weed management might be reduced if M. villosa is planted under conditions of flooding and moderate shade.

3697: *+.365*

Over the last few decades, primate sanctuaries have become more numerous, particularly in Africa. Sanctuaries play an obvious and vital role in the battle against the illegal trade in wildlife and provide opportunities for local people to learn about the importance of protecting habitat and laws governing wildlife trade. Given the multi-disciplinary role of sanctuaries, the Pan African Sanctuary Alliance provides mechanisms to exchange best practices and establishes links to other conservation partners. In April 2011, the managers of the 22 Pan African Sanctuary Alliance

members were surveyed in order to collect detailed information on the conservation activities of each sanctuary. The majority of the 22 sanctuaries conducted both on- and off-site education activities, engaging more than 429,000 people in education activities per year. Sanctuaries reported that they provided employment for over 550 local community members across Africa, as well as resources for community education and infrastructure, with an economic impact over $3 million per year. Sanctuaries were also involved in activities that promote law enforcement and believed that the activities they supported have led to better protection of primate habitats. The results of the survey demonstrate that sanctuaries have moved towards supporting and implementing community development activities aimed at poverty reduction, while conducting conservation activities. While Pan African Sanctuary Alliance sanctuaries were initially established to provide care and housing for orphaned, confiscated and displaced primates, this paper demonstrates how sanctuaries have combined ex-situ with in-situ initiatives to support social, economic, and environmental progress in primate range countries in Africa.

3698: *+.000*

For plants capable of both sexual and clonal reproduction, the relative frequency of these reproductive modes is influenced by genetic and ecological factors. Acacia carneorum is a threatened shrub from the Australian arid zone that occurs as a set of small, spatially isolated populations. Sexual reproduction appears to be very rare: despite regular flowering, only two populations set seed. It is not known whether this reflects an ancient pattern, or results from rapid land use changes following arrival of Europeans in the region 150 years ago. We assessed genotypic variation throughout the range of A. carneorum using AFLP markers, to elucidate the relative importance of clonal and sexual reproduction in this species' history. Clonal diversity (CD) within populations ranged from 0 to 0.820 (mean CD = 0.270, SE = 0.094), but the relative abundances of genets were typically highly skewed. On average, the two fruiting populations had higher CD (mean CD = 0.590, SE = 0.265) than non-fruiting populations (mean CD = 0.179, SE = 0.077) (t = 2.315, p = 0.049), but most populations contained multiple genets. All genets were population-specific, and there was substantial divergence among populations (I broken vertical bar (ST) = 0.690), implying a long history of isolation. We conclude that clonality has predominated in A. carneorum populations, with occasional sexual recruitment, and that current failure of most populations to set seed likely reflects both a long history of asexual reproduction and effects of habitat disturbance. Conservation of this species may benefit from translocations to increase genotypic diversity within populations.

3701: *+.446*

Wildlife reintroduction programs are a type of conservation initiative that seek to re-establish viable populations of a species in areas from which they have been extirpated or become extinct. Past efforts to improve the outcomes of reintroduction have focused heavily on overcoming ecological challenges, with little attention paid to the potential influence of leadership, management, and other aspects of reintroduction. This 2009 survey of reintroduction practitioners identified several key areas of leadership and management that may deserve further study, including: (i) the potential value of reintroduction partnerships for improving programmatic outcomes; (ii) the potential management value of autonomy vs. hierarchy in organizational structure; (iii) gaps in perceptions of success in reintroduction; and (iv) the need for improved evaluations of reintroduction programs and outcomes.

3702: *+.132*

Genetic marker studies can assist restoration practice through selection of seed sources that conserve historical levels of gene diversity and population genetic differentiation. We examined genetic variation and structure within and among mainland and island populations of Elymus glaucus, a perennial bunchgrass species native to western North American grasslands that is targeted for grassland restoration. Island populations of E. glaucus represent sensitive sites and potentially distinctive seed sources for reintroduction, and little is known of their genetic composition. Genetic diversity and structure were estimated using amplified fragment length polymorphism markers for 21 populations and 416 individuals distributed across two coastal California mainland locations and three California Channel Islands. Eight primer combinations resulted in 166 markers, of which 165 (99.4 %) were polymorphic. The number of polymorphic bands was significantly greater among mainland populations relative to island sites, and locally common alleles were present for each sampled island and mainland location. Population structure was high (62.9 %), with most variation (55.8 %) distributed among populations, 7.1 % between mainland and island locations, and the remainder (37.1 %) within populations. Isolation by distance was only apparent among islands. Using marker data to recommend appropriate seed sources for restoration, E. glaucus seeds are best derived within islands with collections representing a large number of individuals from matching environments. Given the limited gene flow and prior evidence of adaptive divergence among populations of this species, regional collections are recommended in all cases to maintain diversity and to avoid long-distance introductions of highly differentiated plant material.

3703: *-.133*

The critical question for the success of all captive breeding and release programs (CBRPs) is the same: will the benefit of augmenting or reestablishing a population with captive animals outweigh the loss of taking individuals from the wild? Yet, few studies have simultaneously evaluated the impact of removal of animals for captive breeding on the source population and the potential contribution of the released animals to the augmented populations. We used the endangered Key Largo woodrat (Neotoma floridana smalli, KLWR) as a model system to simultaneously examine the effect of animal removal, captive breeding, and reintroduction on the dynamics and persistence of a wild population. We used mark-recapture and telemetry data, as well as zoo records from a recent CBRP for the endangered KLWR to parameterize a matrix population model and to simulate the response of the KLWR population to alternative captive breeding and release strategies. Our results suggest that a CBRP as practiced previously would not contribute to KLWR recovery; instead, removal of wild KLWR for captive breeding could harm the population. Captive breeding programs will not contribute to the recovery of KLWR unless survival of released animals and breeding success of captive individuals are improved. Our study provides a framework for simultaneous consideration of animal removal from the wild, breeding success in captivity and survival of released animals for a comprehensive evaluation of captive breeding programs. (C) 2013 Elsevier Ltd. All rights reserved.

3704: *+.235*

Indonesian birds are especially threatened both by habitat loss and trapping for the cage-bird trade. This paper describes recent zoo-supported work at Cikananga Wildlife Center, Java, for the conservation breeding of a number of threatened passerine birds. The founder populations of these birds were obtained from local private bird keepers. Zoos and similar-minded conservation organizations have backed this work through both funding and technical support. Conservation breeding has been highly successful for the Black-winged starling Sturnus melanopterus and Sumatran laughingthrush Garrulax bicolor. A more recently initiated programme for Javan green

magpie Cissa thalassina has already resulted in breeding success and another programme has recently been initiated for the Rufous-fronted laughingthrush Garrulax rufifrons. Reintroduction trials have already been initiated for Black-winged starling and others are at the planning stages.

3705: *-.046*

In Mexico, the Scarlet macaw Ara macao cyanoptera has been extirpated from the states of Tamaulipas, Veracruz, Oaxaca (with the possible exception of a subpopulation of c. 50 birds in the Chimalapas region, although there have been no recorded sightings of these birds in the last decade), Tabasco and Campeche. At the time of writing, the wild population comprises only 150-250 birds mainly located in the Lacandon Jungle in Chiapas. The drastic reduction in the Scarlet macaw population in Mexico is related to an accelerated loss of their habitat and anthropogenic factors, such as illegal trade. It is evident that the Scarlet macaw is currently subject to very high pressures with clear indications that wild populations will disappear from the Jungle in the next 10 years. In 1993, Xcaret began a programme of Scarlet macaw reproduction in captivity, developing and using the best protocols for hand rearing, and establishing new procedures to facilitate parental rearing of the chicks without human intervention. The aim is to rear captive-bred macaws that will adapt to the wild successfully and not require post-release supplemental feeding. Endoscopy techniques have been used to determine gender and match the pairs in more than 100 enclosures. Since 2007 reintroduction procedures, developed in partnership with Acajungla A. C., Aluxes Ecoparque Palenque and some scientists of the Universidad Nacional Autonoma de Mexico, have culminated in the release of 36 Scarlet macaws in 2013 in three events, on 21 April (17), 29 June (11) and 11 August (8).

3706: *-.047*

The Alagoas curassow Pauxi mitu (Linnaeus, 1766), Crax mitu and Mitu mitu at different periods in the past, has been considered Extinct in the Wild since 1979. Endemic from a specific region in the north-eastern states of Alagoas and Pernambuco, Brazil, this bird was locally extinct mainly as a result of loss of habitat caused by the sugar and alcohol industry. Nowadays this same industry helps to preserve the last remaining areas of original native forest, in preparation for the reintroduction of the Alagoas curassow, which may occur within 3 years. Briefly, this article describes the work being carried out by CRAX Brazil, a non-profit non-governmental organization, on the captive breeding of P. mitu for reintroduction purposes, and demonstrates how a persistent joint effort by several cooperating parties can help to take a seriously threatened avian species away from the immediate risk of total extinction.

3707: *+.135*

Phylogenetic relationships among Malaysia's long-tailed macaques have yet to be established, despite abundant genetic studies of the species worldwide. The aims of this study are to examine the phylogenetic relationships of Macaca fascicularis in Malaysia and to test its classification as a morphological subspecies. A total of 25 genetic samples of M. fascicularis yielding 383 bp of Cytochrome b (Cyt b) sequences were used in phylogenetic analysis along with one sample each of M. nemestrina and M. arctoides used as outgroups. Sequence character analysis reveals that Cyt b locus is a highly conserved region with only 23% parsimony informative character detected among ingroups. Further analysis indicates a clear separation between populations originating from different regions; the Malay Peninsula versus Borneo Insular, the East Coast versus West Coast of the Malay Peninsula, and the island versus mainland Malay Peninsula populations. Phylogenetic trees (NJ, MP and Bayesian) portray a consistent clustering paradigm as Borneo's

population was distinguished from Peninsula's population (99% and 100% bootstrap value in NJ and MP respectively and 1.00 posterior probability in Bayesian trees). The East coast population was separated from other Peninsula populations (64% in NJ, 66% in MP and 0.53 posterior probability in Bayesian). West coast populations were divided into 2 clades: the North-South (47%/54% in NJ, 26/26% in MP and 1.00/0.80 posterior probability in Bayesian) and Island-Mainland (93% in NJ, 90% in MP and 1.00 posterior probability in Bayesian). The results confirm the previous morphological assignment of 2 subspecies, M. f. fascicularis and M. f. argentimembris, in the Malay Peninsula. These populations should be treated as separate genetic entities in order to conserve the genetic diversity of Malaysia's M. fascicularis. These findings are crucial in aiding the conservation management and translocation process of M. fascicularis populations in Malaysia.

3708: *+.562*

Age and structure of local vegetation (habitat complexity) are commonly assumed to be indicators of habitat quality for breeding birds, but for many species these relationships are poorly understood. The hihi (stitchbird Notiomystis cincta), an endangered New Zealand cavity-nesting passerine that only survives on mammalian predator-free islands or within fenced areas, has been the focus of intensive conservation management and research. Between 1992 and 2004 we examined the fledging success of 347 nests from four island populations. Habitat quality was improved at the two scrub/regenerating sites and one of the two mature/climax sites through management using supplementary feeding, nest-box parasite control or both. At two sites (one mature, one regenerating) management was stopped during the study allowing us to measure fledgling success with and without habitat quality improvement through management. At the population level, the number of chicks fledged per nest increased as management intensity increased and habitat quality increased. The positive effect of management was greatest for populations in lower quality habitats. To assess the relationship between fledging success and local habitat variables around the nesting site we used a height-frequency vegetation survey method sensitive to changes in vertical structural complexity at the two mature/climax sites. For 36 natural nests, a cross-validated regression-tree analysis (R-2 = 0.69) predicted that as habitat complexity increased, so did fledging success, which was generally higher for nests in trees with larger diameters (present in older forests). Because these habitats are free from nest predators, our results suggest that habitat age and complexity are proxies for habitat quality through effects on nestling food availability and/or nest-chamber characteristics. Our results support the current management approach of providing supplementary food to translocated hihi populations and suggest that supplementary food can be used to overcome resource deficiencies for this species in poorer quality habitats.

3709: *+.350*

Mulgaras (Dasycercus cristicauda and D. blythi) are protected by state and commonwealth environmental statutes; as a consequence, land developers and mining companies have an obligation to avoid, mitigate or minimise impacts on these species when they occur in their area of operation (i.e. to implement trapping and translocation programs). Here we assess the effectiveness of searching and trapping programs for mulgaras in four case studies and provide management recommendations to improve outcomes for these species.

3710: *+.108*

Bandicoots in the family Peroryctidae in New Guinea are widespread and relatively abundant, but

little is known of their ecology. We present the first detailed study on the ecology of the kalubu bandicoot (Echymipera kalubu) and observations on Raffray's bandicoot (Peroryctes raffrayanus), from mid-montane forest in Papua New Guinea. Both species were primarily nocturnal and utilised a range of habitats including those modified by human activity, although Raffray's bandicoot was more frequently encountered in less disturbed areas. Male kalubu bandicoots were larger than females, with larger animals having larger short-term home ranges and evidence for intrasexual territoriality. Mean short-term home-range size was 2.8ha (MCP, n=10), with an estimated population density of similar to 85 animals km(-2) in the study area. Female kalubu bandicoots attained sexual maturity at similar to 400g and 67% of mature females were reproductively active with an average of 1.5 young per litter. Both species were hunted, but their density, rate of reproduction and use of modified habitats suggest that they were able to withstand current hunting levels.

3711: *+.309*

Reintroducing orangutans (Pongo spp.) into the wild requires a suitable, secure habitat. To identify acceptable areas for their reintroduction and define priority conservation sites, we analysed the tree species composition in the Bukit Tigapuluh ecosystem in Jambi, Sumatra. We used this information to determine the distribution patterns of those species that represent an essential part of the diet of reintroduced orangutans. Important orangutan food tree species showed significant differences in composition, frequency and abundance among topographic forest types and recovered selectively logged and unlogged forests. Riparian forests and recovered selectively logged areas offered a vegetation composition and forest structure most suitable for the reintroduction of orangutans and showed numerous important tree species that serve as indicator species, i.e. species growing predominantly or exclusively in a specific forest type. (C) 2014 S. Karger AG, Basel

3712: *+.251*

A morphometric analysis of the leaf traits of Macedonian Oak (Q. trojana) in its north-westernmost range was conducted, including in total 130 individuals from 13 natural populations in Bosnia and Herzegovina and Montenegro. The purpose of the study was to use the analysis of variance for the morphological characteristics of leaves from small and markedly fragmented populations to determine if there are any patterns of population differentiation and detect the morphological characteristics associated with such differentiation. The Principal Components Analysis (PCA) did not identify the existence of discrete groups but rather of a continuous gradient in the variation of morphological characteristics and the absence of geographical population patterns. The morphological characteristics most closely related to population differences displayed relatively low to moderate values with PCs whose maximum correlation values were <= 0.554. The results of a cluster analysis conformed to the PCA results, indicating two population groups, which equally display no geographical pattern. The results of descriptive and univariate statistics pointed to marked variability of morphological leaf characteristic between populations and the presence of significant differences among individuals.Finally, it should be noted that the study of populations of Macedonian Oak in its north-westernmost range identified a high degree of variability in the morphological leaf characteristics studied, and indicate that many different factors impact on the patterns of the small and fragmented populations of this species in Bosnia and Herzegovina and Montenegro. Further research on a larger sample, using molecular markers, will contribute to a better and more accurate knowledge of Macedonian Oak variability in this part of its natural range. Since this is a sub-Mediterranean region, and Macedonian Oak is a very important tree species from ecological point of view, thus conservation by in situ method in

all open and preserved areas is recommended, with particular attention to the origin of the forest reproductive material for reintroduction to degraded habitats, as suggested by the results obtained.

3713: ***+.256***

The paper provides an overview on the distribution of recently discovered Scarce Large Blue (Phengaris (Maculinea) teleius) in northern Serbia (Selevenj Sands, Ludas Lake and Subotica Sands). Mapping of the butterfly and its habitat has shown that most of the suitable habitats are limited to protected areas where at least some of the wet meadows with Sanguisorba officinalis host plant are suitably managed and regularly mown. Given the known maximum dispersal distances of P. teleius, the suitable habitat patches possibly support two separate meta-populations. Fragmentation and isolation of remaining colonies represent the main threats to long term survival of the species in Serbia. Based on IUCN criteria for regional red lists, the species qualifies as Endangered (EN) in Serbia and requires immediate conservation actions. Our results suggest that mowing is of high importance for maintaining suitable habitat. Until more is known about local ecological requirements of the species, general mowing recommendations should be followed with avoidance of mowing between mid June and mid September and providing a mosaic of different mowing regimes.

3714: ***+.250***

Rehabilitation is an important part of sea turtle conservation, and post-release tracking of the animals is both a way of obtaining information on their movements and behaviour and, at least temporarily, monitoring the success of the rehabilitation process. Two green turtles, Chelonia mydas, and one loggerhead turtle, Caretta caretta, were rehabilitated after long periods in captivity, and their movements followed by satellite tracking after their release near the south of Portugal. Location data were obtained for an average of 688 days after release. All three animals showed a directional movement and the last observed locations coincided with known feeding areas for both species, near the coasts of Mauritania and the state of Ceara, Brazil, for the green turtles, and near Cuba for the loggerhead. Bathymetry, surface currents, wind, sea surface temperature, chlorophyll-a concentration and geomagnetic field were analysed as spatial and environmental variables potentially affecting movement. Only bathymetry, sea surface temperature and geomagnetic variables showed significant association with the path choice. This project supports the notion that sea turtles have the ability to survive in the wild after long periods in captivity and to return to the species' known feeding areas, justifying the effort in their rehabilitation and post-release tracking.

3715: ***+.101***

The North American river otter (Lontra canadensis) was considered to have been extirpated from New Mexico during the second half of the twentieth century. From 2008-2010, the species was reintroduced to the state; since then, there has been no formal study to evaluate the success of the new population. We conducted informal surveys in 2012-2013 in northern New Mexico in an attempt to ascertain basic demographics of the reintroduced population. We observed four otters, an adult and three juveniles, on two occasions during the summer of 2013 on the Rio Grande in Orilla Verde Recreation Area (Taos County). We photographed the adult and one juvenile on one of these occasions. This represents the first confirmed evidence of river otter reproduction since their reintroduction to the state. Additional research will be required to clarify range extents, dietary habits, ecosystem impacts, and other information important to local wildlife managers.

3716: ***+.269***

The use of translocation of animals to an ecosystem to which they are not native as a conservation strategy is controversial, but may be the only choice where in situ intervention is not possible. This strategy has been used to establish conservation refuge site populations for three important species of rare freshwater fishes in Scotland. Eleven translocations have been initiated over the last four decades in Scotland, five of these have resulted in the successful establishment of conservation refuges populations of Arctic charr, powan and vendace. The outcome of the remaining six is not yet certain. The approach taken has enabled the protection of, not only important species, but also of the considerable and discrete between-population diversity in phenotype and genotype that is found in these species.

3717: ***+.010***

Invasive house mice (Mus musculus) have detrimental effects on biodiversity, but their impacts can be difficult to detect and are often unquantified. We measured their effects on survival of a translocated population of an endangered lizard in New Zealand. Twelve captive-reared Otago skinks (Oligosoma otagense) were translocated to a 0.3-ha area of grassland/shrubland cleared of invasive mammals and surrounded by a mammal-resistant fence. Sixteen more skinks were released 2 years later but this was followed by an incursion of mice for c. 160 days. Peak mouse density was at least 63 per hectare, and they were seen attacking adult skinks (> 25 cm in length), which is previously undocumented for this lizard species. Using photo/re-sight methods and Program MARK, we estimated skink survival (phi) and detectability (p) in the presence of mice (second cohort: phi = 0.15 per annum, 95% Confidence Interval (CI) 0.01 - 0.48; p = 0.28, 0.20 - 0.38) and in their absence (first cohort: phi = 0.44 p.a., 95% CI 0.03 - 0.82; p = 0.29, 0.22 - 0.39). Survival of skinks from the first cohort during the mouse incursion was unaffected, presumably because they were already established and had access to familiar or more optimal refugia. Their survival over the entire 3 years of monitoring (0.83, 95% CI 0.60 - 0.93) compared favourably with published estimates for viable populations in the wild, protected from all invasive mammals. This suggests it may be feasible to re-establish captive-reared lizards in the wild, but mice should be considered a limiting factor, at least during the initial translocation phase.

3718: ***+.058***

The blue claw yabby Cherax destructor is a native of the Murray Darling drainage basin in the interior of south-eastern Australia. In New South Wales (NSW) the species naturally occurs west of the Great Dividing Range but recently, it has become established in eastern parts of NSW, outside of its natural range. The potential threats and translocation of this species into eastern NSW was first documented at 20 sites by Coughran et al. (2009). This paper builds on their initial work and documents a further 52 translocation sites (Table 1) recorded over the last four years. In an effort to further our understanding of the threat, we present information on the dispersal of this species together with observational information on interactions with freshwater crayfish (Parastacidae) species and suggest recommendations to help slow the translocation process.

3719: ***+.069***

Effective management of Pacific salmon requires an accurate understanding of both population genetic diversity and structure. Spring Chinook Salmon Oncorhynchus tshawytscha from the upper Willamette River (UWR), Oregon, are listed as threatened under the U. S. Endangered Species Act, and although this evolutionarily significant unit is recognized to be distinct from other Columbia River stocks, genetic relationships among its constituent hatchery and wild populations remain obscure. We used genotypic data from 13 microsatellite loci to test whether

hatchery populations of UWR spring Chinook Salmon are most similar to wild populations within the same subbasin, or whether hatchery populations from different subbasins are more similar to each other than to local wild populations. We also tested for differences between the genetic diversities of hatchery and wild populations, as measured through heterozygosity and allelic richness. Our results suggest that populations are weakly structured among subbasins and, in all cases, hatchery populations are genetically most similar to local wild populations. We also found heterozygosity to be higher (P = 0.009) in hatchery populations (median, 81.5%) than in wild populations (median, 75.2%), but observed no significant difference with respect to allelic richness (P = 0.406). We conclude that hatchery-origin UWR spring Chinook Salmon represent genetically appropriate founder populations for ongoing reintroduction programs and recommend that the conservation and recovery of this stock proceed through management actions developed specifically for each subbasin. We further recommend that current restrictions on hatchery stock transfers among UWR subbasins be continued to preserve extant population genetic structure.

3720: *+.135*

Phylogenetic relationships among Malaysia's long-tailed macaques have yet to be established, despite abundant genetic studies of the species worldwide. The aims of this study are to examine the phylogenetic relationships of Macaca fascicularis in Malaysia and to test its classification as a morphological subspecies. A total of 25 genetic samples of M. fascicularis yielding 383 bp of Cytochrome b (Cyt b) sequences were used in phylogenetic analysis along with one sample each of M. nemestrina and M. arctoides used as outgroups. Sequence character analysis reveals that Cyt b locus is a highly conserved region with only 23% parsimony informative character detected among ingroups. Further analysis indicates a clear separation between populations originating from different regions; the Malay Peninsula versus Borneo Insular, the East Coast versus West Coast of the Malay Peninsula, and the island versus mainland Malay Peninsula populations. Phylogenetic trees (NJ, MP and Bayesian) portray a consistent clustering paradigm as Borneo's population was distinguished from Peninsula's population (99% and 100% bootstrap value in NJ and MP respectively and 1.00 posterior probability in Bayesian trees). The East coast population was separated from other Peninsula populations (64% in NJ, 66% in MP and 0.53 posterior probability in Bayesian). West coast populations were divided into 2 clades: the North-South (47%/54% in NJ, 26/26% in MP and 1.00/0.80 posterior probability in Bayesian) and Island-Mainland (93% in NJ, 90% in MP and 1.00 posterior probability in Bayesian). The results confirm the previous morphological assignment of 2 subspecies, M. f. fascicularis and M. f. argentimembris, in the Malay Peninsula. These populations should be treated as separate genetic entities in order to conserve the genetic diversity of Malaysia's M. fascicularis. These findings are crucial in aiding the conservation management and translocation process of M. fascicularis populations in Malaysia.

3721: *+.218*

Seabirds are highly vagile yet many have restricted gene flow owing to physical barriers (e.g. land or ice) or non-physical barriers (e.g. philopatry), which often results in population divergence. Identification of distinct units is important for defining conservation status, guiding restoration of populations and coastal ecosystems, and managing the effect of anthropogenic activities (e.g. fisheries by-catch, customary harvesting). We collected DNA samples from 390 Grey-faced Petrels (Pterodroma macroptera gouldi) at 13 colonies across their New Zealand breeding range to examine population genetic structure. We sequenced part of the mitochondrial control region and genotyped 12 microsatellite DNA loci. We found high diversity in mitochondrial DNA in all colonies. Analyses showed a lack of genetic structure in Grey-faced Petrels that we propose is a

result of high levels of gene flow. Although, we found no genetically distinct populations we suggest that any translocations for conservation should be done with caution and with some consideration of the proximity of a restoration site to a natal Grey-faced Petrel colony. Also, the high levels of gene flow we found suggest that the method of using acoustic attraction and natural behaviour to establish new colonies offers a useful addition, or alternative, to translocations of chicks. These results provide a genetic basis for conservation and restoration efforts for the Grey-faced Petrel.

3722: *+.194*

The Eurasian beaver (Castor fiber) represents an uncommon example of an endangered species in which the restoration programs proved a spectacular success and led to enormous spatial and demographic expansion. Documented reintroduction of beavers in Poland has been conducted using animals of the eastern European origin, most likely derived from the eastern mtDNA lineage. However demographic and spatial expansion of beavers from Germany, which represent the western lineage, may have led to admixture of these two genetically distinct entities in Poland. We detected significant genetic differentiation between the populations from W and NE Poland both in mitochondrial DNA control region and microsatellites, but also substantial admixture including apparent first-generation migrants between regions. Our results indicate that beavers from the western mtDNA lineage have contributed considerably to the Polish population, particularly in W Poland. As there have been no adequately documented translocations of beavers from the western European populations to Poland, the observed situation appears to result from natural migration or range expansion from the west. In contrast to previous findings we detected a substantial diversity in mtDNA control region, which indicates that either the variation in relict populations has been underestimated, or that additional relict beaver populations survived at the end of the 19th century in Poland and Germany as indicated by considerable similarity of ancient and extant mtDNA haplotypes. The implications of our findings for beaver conservation and management are discussed. (C) 2014 Deutsche Gesellschaft fur Saugetierkunde. Published by Elsevier GmbH. All rights reserved.

3723: *+.133*

Reintroductions of plant species are increasingly popular in conservation practice. Steppe grasslands contain many rare and endangered plant species that are potential objects for such reintroductions. Most reintroduction projects, however, can only target a restricted number of species, which raises the question of how species should be prioritised. Here, we present a method to select priority species for reintroduction based on species' characteristics that are widely used in conservation practice. We first determined the local species pool containing those vascular plant species that occurred both in our target region (Thuringia, Germany) and target habitat (steppe grasslands), yielding 369 species. With the help of an a priori filter that selected currently endangered species with limited distribution, 136 potential target species were determined. These potential target species had experienced stronger decline, had a narrower phytosociological amplitude and were more likely to be species of the Festuco-Brometea class and the Festucetalia valesiacae order than non-target species. Potential target species were then ranked by a points system based on ten conservation-relevant characteristics of the species from the categories 'threat and protection status', 'distribution and decline', and 'habitat affiliation'. In the ranking, six steppe grassland plant species (Astragalus exscapus, Bothriochloa ischaemum, Prunella laciniata, Pulsatilla pratensis subsp. nigricans, Scorzonera purpurea, and Seseli hippomarathrum) achieved the highest scores. An additional seven species not specifically characteristic for steppe grasslands also scored highly. A post hoc evaluation of these 13 highest scoring species based on additional

conservation criteria left five species (Astragalus exscapus, Linum leonii, Orchis mono, Pulsatilla pratensis subsp. nigricans and Scorzonera purpurea) as species with highest priority for reintroductions and another five species as highly suitable for reintroductions. Associations between the ranking order and different ranking criteria revealed that a species' threat and rarity in Thuringia and its protection status had the highest representation in the ranking, followed by threat in Germany, regional decline and habitat affiliation. In contrast, international threat and responsibility of Thuringia for its conservation had only low representation in the ranking, probably because these characteristics applied to only a few species. The ranking list gives a selection of species for reintroductions, which combined with additional information based on comprehensive local and floristic knowledge, allows the identification of the species with the highest priority. Our method can be transferred to other regions or habitat types.

3724: *+.300*

In order to assess the efficacy of reinforcement as a conservation tool for rehabilitated elongated tortoises Indotestudo elongata (Blyth, 1854) we released 10 adults equipped with radio tracking transmitters to the Kulen Promtep Wildlife Sanctuary in northern Cambodia. Based on short-term success indicators for reintroduction studies on reptiles we studied the tortoises' adaptation to the new environment for five months. The tortoises successfully settled in the new environment without showing adverse effects regarding their behaviour, condition index (CI) and physical appearance. The annual survival rate was 76%. Based on individual locations collected, we analysed the tortoises' movement patterns including daily displacement, five-month and seasonal home range sizes using minimum convex polygons (MCPs) and fixed kernel density estimators (KDEs).

3725: *-.201*

The present study focused on the importance of translocation as stressful event in an endangered cervid species causing fatal consequences during capture operations. Fourteen free-ranging Sardinian red deer (Cervus elaphus corsicanus) have been captured for restocking propose in a protected area of southwestern Sardinia. The cervids were chemically immobilized, transported to a restocking area and released in the wild. Sixteen hours after the release, a pregnant hind was found dead and a complete necropsy was performed. Post-capture blood samples showed increased levels of creatine kinase, lactate dehydrogenase, cortisol and potassium and were highly indicative of stress-linked muscle damage. The macro-and microscopic lesions consisted of muscular and cardiac degeneration, and renal injury. In the kidneys, the presence of myoglobin in intratubular casts, detected by immunohistochemistry assay, suggested an acute renal failure associated with myoglobinuric nephrosis as a consequence of rhabdomyolysis. The pathological findings were indicative of capture myopathy in a Cervus elaphus corsicanus. This condition has never been reported in the endangered Corsican red deer. This report underlines that mortality from capture is a risk that must be considered during restocking programs. Further studies are needed to minimize stress to preserve wildlife from the risk of fatal consequences due to human interactions.

3726: *-.361*

Conflicts between humans and wild animals are a serious problem in many parts of the world and Himalayan Black bear-Human conflict is a major concern through the western Himalayas and particularly in Kashmir valley of Jammu and Kashmir state. Due to increasing human population and changing land use practices, much of the wildlife habitats have been lost to human habitations,

expansion of agriculture/horticulture lands and developmental activities. The damage and destruction caused by black bear to human property and sometimes to human life is a real and significant danger to many human communities. And with the bear often killed, captured or otherwise harmed in retaliation, these conflicts are one of the main threats to the continued survival of many species. Himalayan black bear numbers are decreasing in many areas due to large scale habitat degradation, poaching of gall bladder and skins and control to reduce crop depredation. In order to strengthen the bear conflict management activities of the Department of Wildlife Protection, J & K State, a fully equipped well trained and motivated conflict management team comprising of wildlife staff, veterinarians, staff of related departments or institutions, and wildlife NGO's should be formed at the wildlife division level to respond to conflict situations, including bear rescue, translocation and monitoring. The long term conservation of Himalayan black bear depends on adequately protecting the species and their habitats, reducing habitat degradation, strictly controlling poaching and illegal trade of gall bladder and skin and in reducing bear-human conflicts.

3727: *+.054*

The Cactaceae is a representative group of the national flora of Mexico that faces conservation problems due to overexploitation and illegal trade at the national and state levels. In this paper the present distribution of three cactus species protected by Federal Law (Ferocactus histrix, Mammillaria bombycina and M. perezdelarosae) occurring in the state of Aguascalientes is updated and their potential distribution estimated using ecological niche modeling techniques. Most populations of F. histrix were found in the western half of the state of Aguascalientes at elevations between 1750 and 2473 m, in canyons of difficult access or low hills. The populations of M. bombycina were located in southwestern Aguascalientes at elevations between 1847 and 2417 m, in canyon walls and in horizontal terrain close to the cliffs. Finally, populations of M. perezdelarosae were located in the southwestern and central regions of the state on exposed bedrock at elevations between 2024 and 2417 m. This study contributes to our knowledge of the distribution and the populations of all three species, which may be used for future reintroduction and conservation efforts of these species in the state of Aguascalientes.

3728: *+.096*

The Sumatran rhino (Dicerorhinus sumatrensis) is very close to extinction in Indonesia. Three major ad hoc meetings, not two as generally reported, to discuss ways to save the Sumatran rhino were held in 1984, 1993 and 2013. Their targets have never been achieved. Despite the great efforts of the participants and other parties, the world population of D. sumatrensis has collapsed during the last 30 years from over 800 to fewer than 100. Besides worldwide phenomena like habitat loss and poaching, other specific causes lie behind this tragedy. The status of the Sumatran rhino has been optimistically overestimated. Precious time is being wasted in finding theoretical solutions rather than implementing the recommendations of these meetings. Political will to save the habitat and protect the species is lacking. After carefully evaluating the present Sumatran rhino conservation status, a breeding project greater than those so far managed has to go ahead as soon as possible to support the ongoing in situ programme and form a viable population in controlled environments for future reintroductions into the wild.

3730: *+.148*

Gillnetting and radiotelemetry were used to evaluate the reintroduction of Paddlefish Polyodon spathula into Allegheny Reservoir, New York-Pennsylvania. Gillnetting produced 86 total captures

over 6,943 h of effort in the spring and early summer of 2008-2011. Forty-four of these individuals received implanted radio transmitters and were tracked from May 2008 to August 2011. Paddlefish showed a strong preference for reservoir sections where depths ranged from 6 to 18 m; only one individual was located in water greater than 18 m in depth. One sexually mature Paddlefish was identified, but telemetry data showed no evidence of reproduction, with only one individual making use of available riverine habitat that was predicted to be ideal for spawning. Paddlefish were susceptible to passage through Kinzua Dam: 46% of radio-tracked individuals left the reservoir during the study period. Continued efforts to evaluate Paddlefish reintroduction in Allegheny Reservoir should now focus on (1) assessing natural reproduction and (2) determining movement and survival through Kinzua Dam.

3731: *-.033*

An abrupt decline in the number of Tasmanian bettongs (Bettongia gaimardi) was observed as part of a study investigating population declines in the eastern quoll (Dasyurus viverrinus). Seven remote camera surveys were undertaken at a monitoring site between February 2012 and October 2013. An 11% reduction in bettong detections was observed immediately following the first appearance of feral cats (Felis catus) (at least three individuals) at the site. Within four months, bettong detections had fallen by 58% and by 100% within six months. No bettongs were detected in subsequent surveys undertaken 10, 12 and 16 months after cats were first observed. Cat predation and toxoplasmosis are discussed as mechanisms possibly responsible for the local disappearance of bettongs from this site, together with implications for the future management and conservation of the species.

3732: *+.164*

The eastern quoll (Dasyurus viverrinus), while still relatively abundant in Tasmania, is now threatened by the recently introduced European red fox (Vulpes vulpes). Due to a lack of demographic information on eastern quolls, molecular data become a crucial surrogate to inform the management of the species. The aim of this study was to acquire baseline genetic data for use in current and future conservation strategies. Genetic variation, at seven microsatellite loci, was lower in Tasmanian eastern quolls than in quoll species from the Australian mainland. Within Tasmania, genetic variation was greater in central than peripheral populations, with the lowest levels detected on Bruny Island. Significant genetic population structure, consistent with regional differentiation, appears related to geographic distance among populations. Levels of gene flow appeared moderate, with genetic admixture greatest among central populations. Therefore, eastern quolls from genetically diverse central Tasmanian populations will become an important source for conservation initiatives if widespread declines begin to occur. Ongoing genetic monitoring of existing populations will allow conservation strategies to be adaptive. However, in order for translocations to be successful, managers must not only consider the genetic composition of founding individuals, but also habitat-specific adaptations, disease and threatening processes at translocation sites.

3733: *+.092*

The water mouse, Xeromys myoides, is currently recognised as a vulnerable species in Australia, inhabiting a small number of distinct and isolated coastal regions of Queensland and the Northern Territory. An examination of the evolutionary history and contemporary influences shaping the genetic structure of this species is required to make informed conservation management decisions. Here, we report the first analysis undertaken on the phylogeography and population genetics of the

water mouse across its mainland Australian distribution. Genetic diversity was assessed at two mitochondrial DNA (Cytochrome b, 1000bp; D-loop, 400bp) and eight microsatellite DNA loci. Very low genetic diversity was found, indicating that water mice underwent a recent expansion throughout their Australian range and constitute a single evolutionarily significant unit. Microsatellite analyses revealed that the highest genetic diversity was found in the Mackay region of central Queensland; population substructure was also identified, suggesting that local populations may be isolated in this region. Conversely, genetic diversity in the Coomera region of south-east Queensland was very low and the population in this region has experienced a significant genetic bottleneck. These results have significant implications for future management, particularly in terms of augmenting populations through translocations or reintroducing water mice in areas where they have gone extinct.

3734: *+.127*

Reintroduction is an increasingly important tool to restore local extinctions and ecological interactions. Evaluating the success of reintroduction projects allows conservationists to learn from previous experience. Here we report on the reintroduction of agoutis, Dasyprocta leporina, to a Brazilian Atlantic Forest reserve in order to (1) determine the short-term status of the reintroduction; (2) describe and evaluate the management procedures that contributed to reintroduction success; and (3) identify the fruits and seeds consumed and buried by the agoutis, as an indication of their role in restoring ecological processes. We captured and tagged 21 adult individuals from a semi-captive population and reintroduced four males and seven females. One male died and almost all individuals lost weight (range=0-620 g; n=11) during quarantine (median=133 days; range=67-243 days; n=20). Six males and three females died, but the others gained weight during acclimatization (range=150-260 g; n=5). Individuals abandoned the food supplement up to 87 days after release, establishing home-ranges at least three times larger than in natural populations of agoutis. The estimated annual survival rate was 0.83, and 10 nature-born cubs were observed. The reintroduction was considered successful in the short-term. Among the main recommendations for future reintroductions, we suggest the reduction of quarantine and the maintenance of acclimatization periods, with structural improvements for both. Agoutis were seen eating fruits and seeds of 10 species and burying seeds of three of them. The buried seeds are from zoochoric large-seeded trees, thus enhancing recruitment in a disperser-impoverished forest.

3735: *+.096*

There are 17 extant species of macaws in the Neotropics, most of them found in South America. Two subspecies of scarlet macaw (Ara macao) are distinguished in Mesoamerica: Ara macao cyanoptera, from Mexico to central Nicaragua, and Ara macao macao, from southern Nicaragua to South America. Habitat loss, hunting, and in particular illegal traffic have resulted in the local and regional extinction of this macaw within its historical range. In Mexico, the scarlet macaw has disappeared from about 98% of its indigenous range; it is extinct in El Salvador and occurs in very low numbers in Guatemala, Belize, Honduras, and Nicaragua. The IUCN recommends two tactics for population restoration: Reintroduction and Reinforcement. In this paper we report the design and first-year progress of a project to reintroduce the scarlet macaw (A. macao cyanoptera) in the tropical rainforests of Palenque, Mexico, where this macaw has been extinct for more than 70 years. The project is spearheaded by Aluxes Ecopark Palenque, bringing together Xcaret Ecopark (as donor of captive-bred scarlet macaws) and the Institute of Biology of Universidad Nacional Autonoma de Mexico (UNAM; provider of the scientific platform for the project). The design of the project adheres to the IUCN/SSC guidelines for reintroductions. A soft-release protocol was developed as a major axis of the reintroduction of the scarlet macaw. This includes a preparation

phase to enhance survival in the wild and a long-term post-release monitoring program. Broad social support was considered essential for the success of the project, and a program was implemented to include the local inhabitants as partners in this initiative. Between April 2013 and June 2014, we conducted six successful releases of a total of 92 macaws. Survival to August 2014 was 92%. The reintroduction of the scarlet macaw in the tropical rainforests of Palenque will restore a seed-and fruit-eating avian species with important consequences for ecosystem functions and processes, in a project that reconnects people with their natural heritage.

3736: *+.232*

We developed a population viability analysis (PVA) for wild Greater Rheas (Rhea americana) in central Argentina, linking spatial data to a non-structured metapopulation model, to evaluate the effectiveness of rhea translocations as a conservation management tool in agricultural landscapes. We simulated the expansion of the agricultural area for a 10-year period and recorded abundance, number of populations over time, expected minimum population size and the likelihood of the metapopulation to fall below the threshold of 30 individuals, under two simulation scenarios: "with translocation" (artificial movement of individuals between populations) and "without translocation." We compared the results with field population data collected from the same study area. At the end of the simulation period, the habitat suitable for Greater Rheas was fragmented and reduced by 84%. We observed a decrease in population size in both scenarios, but the extinction probability was 85% higher in the "without translocation" model. This result was supported by the observed abundance of Rheas in the field, which was closer to values predicted in the "with translocation" than those in the "without translocation" scenarios. Therefore, translocation might be used as an efficient conservation tool for this species.

3737: *+.054*

The Cactaceae is a representative group of the national flora of Mexico that faces conservation problems due to overexploitation and illegal trade at the national and state levels. In this paper the present distribution of three cactus species protected by Federal Law (Ferocactus histrix, Mammillaria bombycina and M. perezdelarosae) occurring in the state of Aguascalientes is updated and their potential distribution estimated using ecological niche modeling techniques. Most populations of F. histrix were found in the western half of the state of Aguascalientes at elevations between 1750 and 2473 m, in canyons of difficult access or low hills. The populations of M. bombycina were located in southwestern Aguascalientes at elevations between 1847 and 2417 m, in canyon walls and in horizontal terrain close to the cliffs. Finally, populations of M. perezdelarosae were located in the southwestern and central regions of the state on exposed bedrock at elevations between 2024 and 2417 m. This study contributes to our knowledge of the distribution and the populations of all three species, which may be used for future reintroduction and conservation efforts of these species in the state of Aguascalientes.

3738: *+.221*

Knowledge of breeding ecology is required for many conservation interventions. The Seychelles Black Parrot Coracopsis barklyi, endemic to the island of Praslin, is vulnerable to extinction. We aimed to improve understanding of C. barklyi breeding ecology to aid conservation planning. We present the results of four years of research, including nesting cavity characteristics and availability, reproductive success, breeding parameters, parental behaviour and reproductive strategy. Thirty-six breeding attempts were studied over the four seasons. Nests were mainly located in Coco de Mer palms Lodoicea maldivica. Deeper cavities with more canopy cover were

preferred. There may be a shortage of high-quality nesting cavities in intensive breeding seasons. Average clutch size was 2.2 eggs, incubation period was c. 15 d and egg fertility was 71%. Rats were key nest predators, causing the failure of up to 33% of breeding attempts. The probability of nest success was 53%. At least 57% of fledglings survived their first year. This species breeds cooperatively and practices a highly unusual side-by-side copulation. We discuss the implications of the results in the context of former, ongoing and potential conservation measures for C. barklyi including translocation, invasive species management, nest box provisioning, habitat restoration and further research.

3739: *+.127*

Comprehensive studies on the genetic diversity and structure of endangered species are urgently needed to promote effective conservation and management activities. The big tree rhododendron, Rhododendron protistum var. giganteum, is a highly endangered species with only two known endemic populations in a small area in the southern part of Yunnan Province in China. Unfortunately, limited information is available regarding the population genetics of this species. Therefore, we conducted amplified fragment length polymorphism (AFLP) analysis to characterize the genetic diversity and variation of this species within and between remaining populations. Twelve primer combinations of AFLP produced 447 unambiguous and repetitious bands. Among these bands, 298 (66.67 %) were polymorphic. We found high genetic diversity at the species level (percentage of polymorphic loci = 66.67 %, h = 0.240, I = 0.358) and low genetic differentiation (Gst = 0.110) between the two populations. Gene flow between populations (Nm) was relatively high at 4.065. Analysis of molecular variance results revealed that 22 % of the genetic variation was partitioned between populations and 78 % of the genetic variation was within populations. The presence of moderate to high genetic diversity and low genetic differentiation in the two populations can be explained by life history traits, pollen dispersal and high gene flow (Nm = 4.065). Bayesian structure and principal coordinate analysis revealed that 56 sampled trees were clustered into two groups. Our results suggest that some rare and endangered species are able to maintain high levels of genetic diversity even at small population sizes. These results will assist with the design of conservation and management programmes, such as in situ and ex situ conservation, seed collection for germplasm conservation and reintroduction.

3740: *+.081*

With the transport of plants around the globe, exotic species can readily spread disease to their native relatives; however, they can also provide genetic resistance to those relatives through hybrid breeding programmes. American chestnut (Castanea dentata) was an abundant tree species in North America until its decimation by introduced chestnut blight. To restore chestnut in North America, efforts are ongoing to test putative blight-resistant hybrids of Castanea dentata and Chinese chestnut (Castanea mollissima), but little is known about the ecology of C. mollissima. In a forest in northeastern USA in which C. mollissima has become established, we explored questions of stand dynamics, health and genetic relationships of C. mollissima offspring to an adjacent parent orchard. We found that C. mollissima was adapted and randomly distributed among native species in this relatively young forest. The genetics of the C. mollissima population compared with its parents indicated little effect of selection pressure as each of the parent trees contributed at least one offspring. The ease with which this exotic species proliferated calls to question why C. mollissima is rare elsewhere in forests of North America. It is likely that a time window of low animal predation allowed seedlings to establish, and the shallow soil at this site limited the maximum forest canopy height, permitting the characteristically short-statured C. mollissima to avoid suppression. Our results indicate that because C. mollissima exhibited pioneer

species characteristics, hybrids between C. mollissima and C. dentata have the potential to be successful pioneer species of future forests in North America, and we challenge the paradigm that exotic tree species are wholly detrimental to native biodiversity. We contend that exotic tree species should be assessed not only by their level of threat to native species, but also by their potential positive impacts on ecosystems via hybrid breeding programmes.

3741: *+.124*

In December 2011, 59 adult Seychelles warblers Acrocephalus sechellensis were translocated between two islands in the Seychelles. Birds were captured on Cousin Island and translocated to Fregate Island using a hard release method, with minimum time in captivity. Fregate had been previously identified as a suitable host for a substantial population of Seychelles warblers, although the presence of the species had never been confirmed on this island. It was estimated that Fregate currently has the potential to support about 500 Seychelles warblers, rising to over 2,000 after habitat regeneration. All birds survived the translocation and were released unharmed at the new site within 24 hours of capture. Close monitoring of both the new and source population was undertaken over a period of 18 months. By June 2013, the Fregate population had increased to 80 individuals, which included 38 of the original translocated birds and 42 birds which had hatched on Fregate. There was also evidence that multiple generations had already hatched on the island. This shows that the Seychelles warbler responded well to a hard release translocation, with observed population growth on Fregate comparable to previous warbler translocations. The source population on Cousin recovered to carrying capacity within a single breeding season. This is the fourth translocation of this species, fulfilling the species action plan requirement of five populations of this endemic island passerine.

3742: *+.380*

We developed a distribution model of Iberian ibex (Capra pyrenaica) using the maximum entropy method (Maxent) based on information from the database of the Spanish Ministry of Agriculture, Food and Environment. The goal was to study the usefulness of such models to determine potential areas for reintroduction or natural colonization of the species. To validate the model, we used data generated from known densities of the Iberian ibex in 107 protected areas: 26 areas where the species is present, and 81 where it is not present. Findings showed that the preferred habitat for the species has steep slopes, altitude over 1,000 m, and seasonal variation in precipitation and temperature. We detected a significant relationship between the densities obtained in the study areas and their relation to the preferred habitat. Our results indicate these models can be useful in species management planning to determine areas for reintroduction of the species.

3743: *+.024*

The cactus genus Uebelmannia includes 3 narrow endemic species associated with rocky savanna habitats in eastern South America. Because of their rarity and illegal over-collection, all of these species are endangered. Taxonomic uncertainties resulting from dramatic local variation in morphology within Uebelmannia species preclude effective conservation efforts, such as the reintroduction or translocation of plants, to restore declining populations. In this study, we developed and characterized 18 perfect, dinucleotide simplesequence repeat markers for U. pectinifera, the most widely distributed species in the genus, and tested the cross-amplification of these markers in the remaining congeneric species and subspecies. All markers were polymorphic in a sample from 2 U. pectinifera populations. The effective number of alleles ranged from 1.6 to

8.7, with an average per population of 3.3 (SE +/- 0.30) and 4.5 (SE +/- 0.50). Expected heterozygosity ranged from 0.375 to 0.847 and 8-10 loci showed departures from HardyWeinberg equilibrium in the analyzed populations. Based on the observed polymorphism level of each marker, as well as the analysis of null allele presence and evidence of amplification of duplicate loci, a subset of 12 loci can be used as reliable markers to investigate the genetic structure, diversity, and species limits of the Uebelmannia genus.

3744: ***+.053***

Cracids are medium to large frugivorous birds that are endemic to the Neotropics. Because of deforestation and overhunting, many species are threatened. The conservation of several species has relied on captive breeding and reintroduction in the wild, but captive populations may be inbred. Microsatellite tools can permit the construction of genetic pedigrees to reduce inbreeding, but only a few loci are available for this group of birds. Here, we present 10 novel polymorphic microsatellite loci and the cross-amplification of these and of 10 additional loci available in the literature in a panel of 5 cracid species, including 3 species with high conservation concern. We provide the first polymorphic loci for the jacutinga, Aburria jacutinga (N = 8), and red-billed curassow, Crax blumenbachii (N = 9), and additional loci for bare-faced curassow, C. fasciolata (N = 8), Alagoas curassow, Pauxi mitu (N = 5), and razor-billed curassow, P. tuberosa (N = 5). The average number of alleles was 2.9 for A. jacutinga, 2.7 for C. blumenbachii, 3.5 for C. fasciolata, 2.6 for P. mitu, and 5.7 for P. tuberosa. The mean expected heterozygosities were 0.42, 0.40, 0.48, 0.37, and 0.59, respectively. The average probabilities that the set of loci would not exclude a pair of parents of an arbitrary offspring were 2.9% in A. jacutinga, 1% in C. blumenbachii, 0.5% in C. fasciolata, 0.4% in P. mitu, and 0.002% in P. tuberosa suggesting that these loci may be adequate for parentage analysis and to implement ex situ genetic management plans.

3745: ***+.276***

Eight cold-water streams in Lake and Geauga Counties, Ohio were evaluated to revise a predictive model for assessing streams for future introduction of threatened native Ohio brook trout (Salvelinus fontinalis). A 15-month study was conducted in streams where brook trout were previously introduced during the years 1997-2002. Fifty percent of the 16 original reintroduction streams failed to support the establishment of self-sustaining populations of brook trout, indicating there were additional factors contributing to their success and failure. Of the eight streams included in this study, four streams were designated successful, two variable, and two failed in terms of the brook trout's ability to establish self-sustaining populations as defined by the Ohio Department of Natural Resources (ODNR). Multivariate statistical methods including principal component analysis (PCA) and agglomerative hierarchical cluster analysis (AHCA) identified the most important characteristics in brook trout stream selection. Factors found to be statistically significant for brook trout success include: 1) stream velocities within the range of 1.4 to 4.7 cm/s; 2) high hydraulic conductivity of the headwater bedrock aquifer (K>4.7 x 10(3) cm/s); 3) lighter average delta O-18 parts per thousand (-9.8 to -10.4); 4) either high percent canopy cover (40 percent to 55 percent) or high percent instream cover (18 to 37 percent); 5) abundant cold-water adapted benthic macroinvertebrate taxa (10 to 16 species); 6) yearly average hyporheic water temperature of 4.6 degrees C to 17.2 degrees C; and 7) average surface water turbidity of 7 to 31 NTU. Long-term surface water temperature and dissolved oxygen monitoring over both winter and summer seasons is recommended prior to brook trout introduction to ensure their sustainability.

3746: ***+.083***

In an attempt to save the species from extinction, the last remaining 18 black-footed ferrets (Mustela nigripes) were trapped up from the wild to initiate a captive breeding program. Nearly 30 years later more than 8,000 black-footed ferrets have been produced in captivity and approximately 4,100 animals have been reintroduced into 20 sites in eight US states (Arizona, New Mexico, Utah, Colorado, Kansas, Wyoming, South Dakota and Montana), Mexico and Canada. However, full recovery of the species has yet to be achieved, mainly due to limited viable habitat, disease and reduced fecundity. This chapter will highlight the advances in the black-footed ferret recovery program over the last 10 years including: (1) adaptive management techniques employed for the captive population; (2) development of new reintroduction sites and associated challenges facing wild black-footed ferrets; and (3) optimization of assisted reproductive techniques to secure the future of this rare species.

3747: ***+.088***

Due to hunting, the distribution of Eurasian beaver Castor fiber Linnaeus, 1758 has declined in the whole of Europe until the end of the XIXth century. In France, legal protection since 1909 had lead to a natural increase of population on the Rhone water system (south-east France). Since the 1960's, a program of about 20 translocations involving more than 300 individuals have favoured the still ongoing recovery of the species. Currently the beavers occur in five out of the six main water systems of France, on more than 8000 km of waterways. Population monitoring and management is ensured by a special network, led by the national game and wildlife agency, and including NGOs and other national or local institutions. This network monitors the evolution of beaver distribution by recording its signs of presence. Owing to its diet, the beaver can create damage to trees and crops near the riverbanks. Nonetheless damage is still quite rare. The beaver network works with local communities to prevent damage by different types of effective measures. Thanks to the work of several institutions the beaver has recovered its place in most French aquatic ecosystems.

3748: ***-.154***

Many mammals are able to perceive and respond to predation risk from signals produced by their predators, even in the absence of an ecological or evolutionary experience, or both. However, it has been proposed that captivity may affect such behavior, probably due to the unnecessary costs related to preserving the antipredatory responses in that condition. In the present work we analyzed the response of captive anteaters (Myrmecophaga tridactyla; n=7) exposed to olfactory and acoustic stimuli from a natural predator (jaguar, Panthera onca). We compared the behavioral response of anteaters exposed to jaguar feces and vocalizations with those displayed when exposed to maned wolf (Chrysocyon brachyurus) and sheep (Ovis aries) signals. The maned wolf represents a sympatric carnivorous species that does not predate on anteaters, and the sheep represents an allopatric herbivorous non-predator species. The results showed that the anteaters in captivity retain the capability to respond to and discriminate between the different treatments, mainly when exposed to vocalizations. Despite this, the antipredatory behaviors may become altered, incomplete or even lacking after large periods of isolation. Even though these are preliminary results, the experiment supports the hypothesis that captive anteaters are able to retain some predator recognition ability, and this result could be relevant when de ning strategies for the reintroduction of captive-reared animals and the restoration of ecosystems that hold populations of this species.

3749: ***+.130***

Successful salmonid restoration efforts depend upon an understanding of the evolutionary processes that historically shaped population diversity, as well as the realities of currently available, altered river systems. Habitat alterations over the past century have dramatically changed the ecological forces that shaped salmonid speciation and evolution, bringing formerly separate and distinct populations into contact and in some cases leading to hybridization. Hybridization can threaten the genetic diversity within salmonid species and may affect the outcomes of restoration efforts. Here we use the San Joaquin River Restoration as a case study to discuss some of the genetic challenges of Chinook salmon restoration in a newly reopened habitat. We discuss a range of genetic management strategies-from passive reintroduction to tightly managed, active reintroduction-and the strengths and weaknesses of each approach.

3750: *+.220*

Plants are a vital part of global biodiversity, a veritable chemical goldmine and a major life supporting system for all life forms. Conservation and sustainable utilization of plant genetic resources is essential to meet the future requirement of natural resources. Medicinal plants play a vital role in the treatment of a number of diseases, and form the mainstay of 75-80% of the world population, mainly in the developing countries. Tropical countries of the world supply the bulk of medicinal raw materials in demand, resulting in depletion of the population and genetic diversity. India, one among the 12 major megabiodiversity regions of the world, is a major centre of origin and diversity of medicinal plants. In India, Western Ghats is one of the major repositories of medicinal plants, harbouring about 4,500 species of higher plants of which 10% are threatened and 30% are endemic. It is also recorded that 30% of the medicinal raw material collected comprises plant roots as compared to other parts. Tropically most of the endangered plants of Western Ghats belong to the tuber yielding medicinal plant species, due to their destructive extraction from the wild. Advances in biotechnology have generated new opportunities for genetic resource conservation and utilization. These include (i) ex situ conservation of RET species through micropropagation, reintroduction and restoration (ii) bioproduction of plant specific compounds in culture systems to avoid collection from nature (iii) bioprospecting to identify better classes of compounds and (iv) cryopreservation of shoot tips, seeds, embryo, etc. This paper addresses some of the recent developments in these areas on conservation of tuber yielding medicinal plants of Western Ghats and their sustainable utilization.

3751: *+.292*

Species reintroduction is an increasingly important tool for species recovery programs and habitat restoration initiatives worldwide. Roe deer Cap reolus capreolus (Linnaeus, 1758) densities are very low in central west Portugal (the Freita, Arada, and Montemuro mountains). This area is inhabited by the endangered Iberian wolf Canis lupus sign atus Cabrera, 1907, whose numbers have dramatically decreased since the 20th century An important step in a roe deer reintroduction program is to establish suitable reintroduction sites. The aim of the study was to identify such sites in central Portugal. An Analytical Hierarchy Process (AHP) in combination with a GIS was applied to develop a habitat suitability model, which integrated empirical models and expert knowledge. The variables used in the model included land use, hydrographic network, asphalted roads, population/villages, and relief. Three reintroduction sites suitable for roe deer were identified as potential habitats for their future natural expansion. Those sites were considered as preliminary ones. Finally, future goals and actions are discussed in relation to the promotion of the ecological and social conditions that would favour the survival of roe deer and Iberian wolf in central Portugal.

3752: ***+.111***

In 1971, the native population of the Oriental white stork became extinct from the wild in Japan. Soon after, a captive-breeding and reintroduction program was started in the Toyooka Basin, the last habitat for this large bird. The Oriental white stork usually feeds on a range of small wetland animals, such as fish, frogs, and insects, particularly in paddy fields and their surrounding areas, serving as a biodiversity indicator in such rural areas. Land consolidation projects have deteriorated paddy ecosystems, resulting in drier conditions, fragmented water systems, and wider application of agrochemicals. Therefore, alternative and balanced rice farming schemes, combining nature conservation and economic activities, to restore the paddy ecosystem were essential for successful reintroduction of the Oriental white stork. In this chapter, the authors describe the role of the white stork-friendly farming method and related techniques in the reintroduction of this species, including its effects on local ecosystems.

3753: ***+.318***

The Sumatran orangutan is currently listed by the IUCN as critically endangered and the Bornean species as endangered. Unless effective conservation measures are enacted quickly, most orangutan populations without adequate protection face a dire future. Two main strategies are being pursued to conserve orangutans: (i) rehabilitation and reintroduction of ex-captive or displaced individuals; and (ii) protection of their forest habitat to abate threats like deforestation and hunting. These strategies are often mirrored in similar programs to save other valued and endangered mega-fauna. Through GIS analysis, collating data from across the literature, and combining this information within a modelling and decision analysis framework, we analysed which strategy or combination of strategies is the most cost-effective at maintaining wild orangutan populations, and under what conditions. We discovered that neither strategy was optimal under all circumstances but was dependent on the relative cost per orangutan, the timescale of management concern, and the rate of deforestation. Reintroduction, which costs twelve times as much per animal as compared to protection of forest, was only a cost-effective strategy at very short timescales. For time scales longer than 10-20 years, forest protection is the more cost-efficient strategy for maintaining wild orangutan populations. Our analyses showed that a third, rarely utilised strategy is intermediate: introducing sustainable logging practices and protection from hunting in timber production forest. Maximum long-term cost-efficiency is achieved by working in conservation forest. However, habitat protection involves addressing complex conservation issues and conflicting needs at the landscape level. We find a potential resolution in that well-managed production forests could achieve intermediate conservation outcomes. This has broad implications for sustaining biodiversity more generally within an economically productive landscape. Insights from this analysis should provide a better framework to prioritize financial investments, and facilitate improved integration between the organizations that implement these strategies.

3754: ***+.427***

Other terms (used in one or two publications) are: adaptation assisted migration, assisted afforestation, assisted ecosystem migration, assisted population migration, assisted range expansion, assisted reintroduction, assisted species relocation, facilitated translocation, forestry assisted migration, human aided translocation, human assistance of dispersal, human assisted colonisation, human assisted establishment, human assisted migration management, human assisted relocation, managed migration, migration management, managed reintroduction, planned invasions process, plant refuge translocation, species rescue assisted migration, and trans situ

conservation.

3755: *.000*

*=two separate releases.

3756: *+.357*

For individuals that were opportunistically recaptured after completion of the study, the maximum time Known to Be Alive (KTBA) is included.*=Date of translocation to the on-site containment pen, access to the rest of Northern Paddock occurred three weeks later.+=All females had pouch young when checked 7-9 weeks after release.

3757: *-.052*

Interaction among species through competition is a principle process structuring ecological communities, affecting behavior, distribution, and ultimately the population dynamics of species. High competition among large African carnivores, associated with extensive diet overlap, manifests in interactions between subordinate African wild dogs (Lycaon pictus) and dominant lions (Panthera leo) and spotted hyenas (Crocuta crocuta). Using locations of large carnivores in Hluhluwe-iMfolozi Park, South Africa, we found different responses from wild dogs to their two main competitors. Wild dogs avoided lions, particularly during denning, through a combination of spatial and temporal avoidance. However, wild dogs did not exhibit spatial or temporal avoidance of spotted hyenas, likely because wild dog pack sizes were large enough to adequately defend their kills. Understanding that larger carnivores affect the movements and space use of other carnivores is important for managing current small and fragmented carnivore populations, especially as reintroductions and translocations are essential tools used for the survival of endangered species, as with African wild dogs.

3758: *+.063*

The gilt darter (Percina evides) is an intolerant species that is currently recognized as threatened in Wisconsin, but no targeted surveys to provide a comprehensive overview of its status in Wisconsin or monitor trends in its abundance have been conducted previously. We conducted backpack electrofishing index sampling in riffle habitat preferred by gilt darters to evaluate their current status in areas of known occurrence and provide a baseline against which to assess future abundance trends. Index sampling provided repeatable results, consistently ranking sites sampled multiple times within a season. Within the St. Croix River drainage, gilt darters were rare in upstream reaches and more abundant downstream. We also evaluated the outcome of a restoration project that was conducted during 1997 but not thoroughly evaluated. Gilt darters were moved from the St. Croix River to the Namekagon River above the Trego Dam, where they had not been detected since 1982. Sampling during 2009-2010 indicated that gilt darters were not present in the restoration area; therefore, this translocation approach to mitigation is not recommended. Non-wadeable reaches in the St. Croix River were sampled with a bottom trawl, and this sampling confirmed the presence of gilt darters. Gilt darters were present at low abundance in the Chippewa River drainage and the Black River, where habitat fragmentation may restrict distribution. This species appears secure in the St. Croix River where monitoring wadeable reaches with backpack electrofishing will be sufficient to detect large changes in abundance in preferred habitat.

3759: *+.108*

The cumulative benefits derived from historic small-scale, opportunistic interventions for the Hawaiian monk seal Monachus schauinslandi were assessed using multiple methods. The analysis focused on interventions undertaken to enhance survival of individual seals by reducing or eliminating immediate mortality risks. These interventions included dehookings, disentanglements, removing seals from high predation zones, medical interventions, and related activities. A total of 885 interventions occurred range-wide from 1980 to 2012. These included 645 interventions classified as mitigating medium-to high-risk threats, involving 532 different seals. In the Northwest Hawaiian Islands, where most of these interventions took place, we found a significant relationship between the number of interventions conducted annually and duration of field effort. The survival and reproduction of the intervention seals were tracked through multiple generations, using (1) known survival and reproduction of intervention seals, and (2) expected survival and reproduction as determined using demographic rates estimated for the population at large. This analysis indicated that 17-24% of the 2012 population comprised either intervention seals or descendants of intervention seals. If seals included in a multiyear (1984-1992) re habilitation and captive care effort are also included, this proportion increases to 32%. These findings demonstrate the important link between the sustained population assessment field effort, the number of interventions that are enabled in association with those efforts, and the current status of the monk seal population. In contrast to a metaphorical 'silver bullet' whereby a result is achieved through a single (or a few) highly impactive tools, we liken our success in applying multiple interventions to a fusillade of many silver BBs(1).

3760: *-.048*

The Glenelg spiny freshwater crayfish Euastacus bispinosus is a large endangered freshwater invertebrate of southeastern Australia that has suffered major population declines over the last century. Disjunct populations in the state of South Australia are in a particularly critical condition, restricted to a few isolated rising-spring habitats and in an ongoing state of decline. We assessed genetic diversity and gene flow within E. bispinosus across its current range using allele frequencies from 11 nuclear microsatellite loci and DNA sequence data from a single mitochon drial locus (cytochrome oxidase subunit I). Populations were characterized by low levels of genetic diversity and found to be highly structured, with gene flow restricted both within and across catchments, highlighting the species' vulnerability to further habitat fragmentation and the importance of managing environmental threats on local scales across its current natural range. South Australian populations were characterized by critically low levels of genetic diversity generally, highlighting their potential vulnerability to localized extinction. Holistic conservation efforts are necessary to conserve populations, including local habitat management and, potentially, translocations to increase genetic diversity and evolutionary potential, and reduce possible inbreeding effects and the threat of extinction.

3761: *+.074*

With the number of threatened species in rescue centres rising, scientific reports on the functioning and success of such centres is essential. Compassionate conservation tries to bridge the gap between animal welfare advocates and conservation biologists, recognising the benefits of preserving a species and its individuals. A case in point is that of Indonesia's threatened slow lorises Nycticebus spp., where illegal trade is decimating wild populations of these primates. We present 4 yr of data from Ciapus Primate Centre in Indonesia, which received 180 slow lorises between 2008 and 2011. We show that >85% of these primates were unsuitable for reintroduction; 23 slow lorises that were deemed suitable for reintroduction were released to the wild between 2010 and 2013 and were followed with radio tracking. Eleven of them died (on average 76 d post-

release), 1 was recaptured (148 d post-release), 6 are no longer being monitored (after, on average, 263 d post-release) and their status is unknown, and 5 are still being monitored (average 226 d post-release, as of December 2013). The challenges posed by work with slow lorises in the Ciapus Primate Centre over these 4 yr, with release success highly variable, show that even with concerted effort, rescue centres need to consider alternative options. We review such options, considering the pros and cons of euthanasia, life in captivity and reintroduction to the wild. We conclude that in today's global conservation crisis, it can only be beneficial to combine the expertise of animal welfare practitioners and conservation biologists.

3762: ***-.110***

Naive captive-bred animals often make excessive movements when released into the wild, which may increase vulnerability to predation, take animals into unsuitable habitat and/or increase the probability of conflict with humans. We analysed the post-release movements of captive-bred European mink Mustela lutreola reintroduced to Hiiumaa Island, Estonia. We tested the effect of pre-release enclosure type, sex, generations in captivity, pregnancy and age on post-release movements, investigated the settlement process and explored the relationship between post-release movements and survival. We found no effect of enclosures on post-release movements in the first 2 wk following release (except that individuals from large naturalistic enclosures spent less time close to water than did animals from standard zoo enclosures) but some evidence of a slight effect at 1 mo post-release. Males moved further at 3 d (but not 2 wk) post-release than did females, and juveniles appeared to move further in the first 3 d post-release than did 1 yr olds. We were unable to detect a relationship between post-release movements and survival. The largest known cause of mortality was predation, but it was not clear why mink were vulnerable to predation, and the locational data presented here were unable to shed light on this issue. Individual variation also made it difficult to define patterns. These are common problems in reintroductions, and we suggest that in future releases of captive-bred animals, more detailed post-release behavioural observations and investigation of personality types might be insightful.

3763: ***-.108***

From 2009 to 2012 thirteen wild-born pygmy slow lorises Nycticebus pygmaeus (in this paper referred to as pygmy lorises), confiscated from illegal trade, were radio-collared and re leased into secondary forest in South Vietnam. Pygmy lorises were monitored until death, recapture, or loss of collar; the longest monitoring period was 73 d. The mean (+/- SD) distances between consecutive sleeping sites were recorded for 324 consecutive days and averaged 122 +/- 108.0 m. Mean distances between sleeping sites for males and females were similar at 110.7 +/- 92.6 m for males and 128.8 +/- 116.7 m for females, with the greatest distance covered by a female (793 m). Mean height of the sleeping sites was 8.54 +/- 4.46 m (n = 60), in trees with a mean diameter at breast height of 75.2 +/- 58.4 cm (n = 225). Mean height of the trees where lorises slept was 20.2 +/- 9.0 m (n = 230). The pygmy lorises slept mostly in the >8 m band, the area of highest tree connectivity. Of the pygmy lorises studied 38% (5/13) were found dead, 7% (1/13) were returned to captivity due to severe loss of condition and for 23% (3/13) the outcome is unknown due to early collar loss. Causes of death included hyperthermia and natural predation. The remaining 30% (4/13), 2 males and 2 females, were in good condition when last tracked before premature collar drop-off, up to 73 d after release. From this limited data set, a 'soft' release, wet season release and consideration of predator density at the release site are recommendations for increasing chances of survival.

3764: ***-.093***

The African penguin Spheniscus demersus has an Endangered conservation status and a decreasing population. Following abandonment, 841 African penguin chicks in 2006 and 481 in 2007 were admitted to SANCCOB (Southern African Foundation for the Conservation of Coastal Birds) for hand-rearing from colonies in the Western Cape, South Africa, after large numbers of breeding adults commenced moult with chicks still in the nest. Of those admitted, 91% and 73% respectively were released into the wild. There were veterinary concerns about avian malaria, airsacculitis and pneumonia, feather-loss and pododermatitis (bumblefoot). Post-release juvenile (0.32, s.e. =0.08) and adult (0.76, s.e. =0.10) survival rates were similar to African penguin chicks reared after oil spills and to recent survival rates recorded for naturally-reared birds. By December 2012, 12 birds had bred, six at their colony of origin, and the apparent recruitment rate was 0.11 (s.e. =0.03). Hand-rearing of abandoned penguin chicks is recommended as a conservation tool to limit mortality and to bolster the population at specific colonies. The feasibility of conservation translocations for the creation of new colonies for this species using hand-reared chicks warrants investigation. Any such programme would be predicated on adequate disease surveillance programmes established to minimise the risk of disease introduction to wild birds.

3765: ***-.018***

Invasive species may rapidly spread throughout new areas once introduced, which may potentially lead to serious damage to local fauna and flora. Information on geographical origins, introduction routes, and biology in native regions of such invasive species is of critical importance in identifying means of transport, preventing reintroduction, and establishing control/eradication methods. The plataspid stinkbug Megacopta cribraria, known as kudzu bug, recently invaded North America and now has become not only an agricultural pest of soybean but also a nuisance pest. Here we investigate the geographical origin of the invasive M. cribraria populations. Phylogeographical analyses based on 8.7 kb mitochondrial DNA sequences of the introduced and East Asian native Megacopta populations identified a well-supported clade consisting of the introduced populations and M. punctatissima populations in the Kyushu region of Japan, which strongly suggests that the invading M. cribraria populations are derived from a M. punctatissima population in the Kyushu region. Therefore, the region is proposed as a promising source of natural enemies for biological control of the invasive pest. Based on the phylogenetic information, relationship and treatment of the two Megacopta species are discussed.

3766: ***+.132***

This one file dataset contains the information on the Long-haired rats (Rattus villosissimus) used in this study, i.e. data that was collected between October 2011 and May 2013. It contains the exact date (Date) for when a rat was released (Trip_type Release, Trip_number 0) or trapped (Trip_type = Seasonal Trapping, Trip >/= 1) in each of the two enclosures (Enclosure = Enclosure I or Enclosure II), as well as the treatments (Treatment regarding the access of cats into the enclosure: ‘high_fence’ (no access for cats) or ‘low_fence’ (access for cats)), including information on a rat’s gender (Sex = ‘M’ (for male) or ‘F’ (for female)), a rat’s weight (Animal_weight measured in g), body condition (Body_condition theoretically ranging from ‘1’ (emaciated) to ‘5’ (obese), but only categories 2 (underconditioned), 3 (well-conditioned) and 4 (overconditioned) were scored) and individual identification (PIT.ID) as well as whether they had been recaptured (New_firsttripcap_recap indicating whether the animal was ‘new’ = released/ caught the very first time, was a ‘firsttripcap’ = captured before, but first captured during a trapping session, or a ‘recap’ = recaptured during the same trip).

3767: ***+.043***

We used heat-in-motion cameras (Reconyx PC800 Hyperfire, Holmen, Wisconsin, USA) around the outside of the perimeter fences to detect predators. At least four (but up to six and always the same number of cameras at a time) cameras were placed as one camera installed at each side on the outside of the fences of each enclosure. Cameras were unbaited, to avoid attracting predators. This one file dataset contains the information on the presence/absence data of cats and dingoes on each day. 'Site' indicates the enclosure the camera was attached to ('Enclosure_I' or Enclosure_II'), 'Camera number' indicates which site the camera was on. Note that between October 2011 and April 2012, Enclosure II had two additional cameras (one facing the front gate and one additional monitoring the lower half of the back fence of the enclosure) which resulted in a total of six cameras for during that time. 'Date' indicates the date the photo(s) was/were taken, 'Photos_recorded' whether the camera was operational or photos were retained (e.g. one SD-cards was lost). And columns 'Dingo' and 'Cat' indicate whether these animals were present that day or not (na = no photos recorded, 0 = not present that day, 1 = present that day).

3768: ***-.102***

This one file dataset contains the information on the Long-haired rats (Rattus villosissimus) used in this study, i.e. data that was collected between October 2011 and December 2012. It contains the enclosure in which a radio-collared rat was released and tracked (Enclosure = 1 or 2), the treatment it was given (Cats = yes or no), the exact date (Date) for when a rat was released with a collar (collared_released), the last time it was recorded (last-time-rec), the time period in months over which the collar frequency was detected (time-collar-detected), the fate of the animal (Fate = unknown, dead or alive), the last location change detected (last_loc_change), based on the latter, the estimated time a rat was assumed alive (estimated_time_alive)), the last time a signal was detected from the collar (last_signal detected), the date of the last time an animal was trapped (last_trapped), whether dead remains were found (dead_remains_found = na, yes, or no) and whether the collar was found(collar_found = na, yes, or no).

3769: ***+.106***

Surveys were distributed to New Zealand land users in 1998 and 2008 to acquire information about New Zealand frogs with the aim of compiling and mapping their distribution and inferred population trends without costly and time-consuming field surveys. The overall frog population trend was reported as declining, with possible causes reported as an increase in agriculture, an increase in the distribution of predatory fish and disease. The resultant maps could be used for four main purposes: 1) to identify regions where Litoria populations are known to occur, which can be eliminated when considering suitable regions for translocation of Leiopelma; 2) to identify growing or stable populations of Litoria species, which may assist future disease surveys, population monitoring and to identify sources of genetic material that may serve as an Ark for declining Australian populations; 3) to highlight populations that are in decline to enable effective targeting of detailed disease studies; and 4) to approximate the stability of amphibian populations in the absence of more accurate, but costly, scientific monitoring.

3770: ***+.253***

There is abundant evidence that the probability of successful establishment in novel environments increases with number of individuals in founder groups and with number of repeated introductions. Theory posits that the genotypic and phenotypic variation among individuals should also be important, but few studies have examined whether founder diversity influences establishment independent of propagule pressure, nor whether the effect is model or context

dependent. I summarize the results of 18 experimental studies and report on a metaanalysis that provides strong evidence that higher levels of genotypic and phenotypic diversity in founder groups increase establishment success in plants and animals. The effect of diversity is stronger in experiments carried out under natural conditions in the wild than under seminatural or standardized laboratory conditions. The realization that genetic and phenotypic variation is key to successful establishment may improve the outcome of reintroduction and translocation programs used to vitalize or restore declining and extinct populations. Founder diversity may also improve the ability of invasive species to establish and subsequently spread in environments outside of their native community, and enhance the ability of pathogens and parasites to colonize and invade the environment constituted by their hosts. It is argued that exchange of ideas, methodological approaches, and insights of the role of diversity for establishment in different contexts may further our knowledge, vitalize future research, and improve management plans in different disciplines.

3771: ***+.107***

Amphibians support symbiotic bacterial communities on their skin that protect against a range of infectious pathogens, including the amphibian chytrid fungus. The conditions under which amphibians are maintained in captivity (e.g. diet, substrate, enrichment) in ex situ conservation programmes may affect the composition of the bacterial community. In addition, ex situ amphibian populations may support different bacterial communities in comparison to in situ populations of the same species. This could have implications for the suitability of populations intended for reintroduction, as well as the success of probiotic bacterial inoculations intended to provide amphibians with a bacterial community that resists invasion by the chytrid fungus. We aimed to investigate the effect of a carotenoid-enriched diet on the culturable bacterial community associated with captive red-eyed tree frogs (Agalychnis callidryas) and make comparisons to bacteria isolated from a wild population from the Chiquibul Rainforest in Belize. We successfully showed carotenoid availability influences the overall community composition, species richness and abundance of the bacterial community associated with the skin of captive frogs, with A. callidryas fed a carotenoid-enriched diet supporting a greater species richness and abundance of bacteria than those fed a carotenoid-free diet. Our results suggest that availability of carotenoids in the diet of captive frogs is likely to be beneficial for the bacterial community associated with the skin. We also found wild A. callidryas hosted more than double the number of different bacterial species than captive frogs with very little commonality between species. This suggests frogs in captivity may support a reduced and diverged bacterial community in comparison to wild populations of the same species, which could have particular relevance for ex situ conservation projects.

3772: ***-.123***

Conservation of at-risk species requires multi-faceted and carefully-considered management approaches to be successful. For arthropods, the presence of endosymbiotic bacteria, such as Wolbachia (Rickettsiales: Rickettsiaceae), may complicate management plans and exacerbate the challenges faced by conservation managers. Wolbachia poses a substantial and underappreciated threat to the conservation of arthropods because infection may induce a number of phenotypic effects, most of which are considered deleterious to the host population. In this study, the prevalence of Wolbachia infection in lepidopteran species of conservation concern was examined. Using standard molecular techniques, 22 species of Lepidoptera were screened, of which 19 were infected with Wolbachia. This rate is comparable to that observed in insects as a whole. However, this is likely an underestimate because geographic sampling was not extensive and may not have included infected segments of the species' ranges. Wolbachia infections may be particularly

problematic for conservation management plans that incorporate captive propagation or translocation. Inadvertent introduction of Wolbachia into uninfected populations or introduction of a new strain may put these populations at greater risk for extinction. Further sampling to investigate the geographic extent of Wolbachia infections within species of conservation concern and experiments designed to determine the nature of the infection phenotype(s) are necessary to manage the potential threat of infection.

3774: *+.097*

1. With the global population of beavers (Castor spp.) increasing, and reintroductions widespread, it is crucial to be able to predict potential impacts on flora and fauna based on defined foraging behaviours.2. Nymphaea alba (white water lily) is regularly consumed by beavers and provides a model system to test selective foraging behaviour and quantify potential impacts on aquatic resources in standing-water habitats.3. Using biometric relationships within N.alba pads, we accurately reconstructed the size and weight of consumed pads, demonstrating that beavers (Castor fiber) selected pads that were significantly larger and heavier than unselected pads. By selecting larger leaves, beavers may also avoid chemical defences associated with anthocyanin pigments that dominate in smaller leaves. Grazing was concentrated in shallow depths (55.710.7cm) close to the shore (2.950.62m) relative to ungrazed plots (100.5 +/- 9.2cm; 4.79 +/- 0.68m). The level of selectivity was unchanged with increasing distance from a central feeding place.4. Beavers removed 24-50% of pads within grazed areas, but relative to the whole N.alba leaf pad resource, the impact of this foraging was low (0.38-1.23% loss). Plant species diversity was unaffected by foraging, and there was no evidence of indirect effects on non-targeted N.alba pads or flowers.5. When foraging in the aquatic environment, beavers are highly selective and can have a minor effect on food resources whilst feeding optimally and opportunistically. Since beavers demonstrate adaptive foraging strategies depending on their foraging environment, this knowledge should be incorporated into future decisions on further reintroduction or habitat restoration programmes.

3775: *+.307*

To better inform conservation efforts, we initiated work on the demography, habitat selection, and movements of hirola (Beatragus hunteri) in Ijara District in 2011. As part of this effort, we captured nine adult females (>3 years old) from herds at the periphery of this species' historic geographic range in Arawale and the Burathagoin grazing fields of Ijara District. From August to December 2012, we fitted GPS collars on nine females from seven different herds (mean herd size = 7 [plus or minus] 2 SE, range = 5-11) to relocate associated individuals and to estimate demographic parameters. GPS radiocollars (Vectronic Aerospace) are set to record one location every three hours for the next 3 years. Iridium satellite communication permits us to track herds within 24 hours of movement. Once per month, we are relocating animals visually from the ground to record survival, recruitment, and age structure; we are comparing these data to those from herds occupying 1) a predator-proof sanctuary in Ishaqbini Community Conservancy; and 2) areas with higher-quality range than Arawale and Burathagoin. This effort will enable us to better understand the relative influence of predation and range quality in driving hirola population dynamics, and will provide insight into historic declines and contemporary lack of recovery. Additionally, the data we generate on habitat selection and movements can be used to identify sites suitable for any future reintroduction efforts.

3776: *+.006*

The genetic variation of the critically endangered Corfu killifish (Valencia letourneuxi), an endemic freshwater fish species of the western Balkans, was assessed for nine populations sampled in eight water systems in western continental Greece, the Peloponnese and the Ionian Island of Corfu, using mitochondrial and microsatellite markers. The analyses were based on data from three mtDNA regions (D-loop, COI and 16S rRNA sequences) and 14 microsatellite loci. Samples from the congeneric species Valencia hispanica and the phylogenetically closely related species Aphanius fasciatus were also used in the study as outgroups. Both the mitochondrial and the microsatellite analyses revealed three distinct population groupings associated with the geographical distribution of the populations: one southern group occupying rivers draining to the Patraikos Gulf, the second one including the populations flowing into the Amvrakikos Gulf and the third, more northern group, including the other populations from rivers in Corfu Island and Epirus flowing into the Ionian Sea. Within these groupings there is limited genetic differentiation between populations; in addition, there is reduced intrapopulation genetic variation, evidenced by low heterozygosity values, number of alleles and haplotype diversity. In terms of taxonomic implications and appropriate management actions for conservation, our data suggest that the major population groups should be regarded at least as three distinct conservation units (CUs), with translocation and restocking actions to take place only within the geographical range of the CU concerned.(c) 2013 The Linnean Society of London, Biological Journal of the Linnean Society, 2014, 111, 334-349.

3777: *-.033*

Restoring prairie on formerly cultivated land begins by selecting propagule seed sources and the diversity of species to reintroduce. This study examined the effects of dominant grass propagule source (cultivar vs. non-cultivar) and sown propagule diversity (grass:forb sowing ratio) on plant community structure. Two field experiments were established in Kansas and Illinois consisting of identical split plot designs. Dominant grass source was assigned as the whole-plot factor, and sown dominance of grasses (five levels of seeded grass dominance) as the subplot factor. Species density, cover, and diversity were quantified for 5 years. The effect of dominant grass source on the cover of focal grasses, sown species, and volunteer species was contingent upon location, with variation between dominant grass sources observed exclusively in Kansas. Species density and diversity showed regionally convergent patterns in response to dominant grass source. Contrary to our hypotheses, total species density and diversity were not lower in the presence of grass cultivars, the grass source we had predicted would be more competitive. Sown grass dominance effects on the cover of the focal grass species were contingent upon location resulting from establishment corresponding better to the assigned treatments in Illinois. All other cover groups showed regionally convergent patterns, with lower cover of volunteers and higher cover of sown forbs, diversity, and species density in the lowest sown grass dominance treatment in both sites. Thus, decisions regarding the diversity of propagules to reintroduce had more consequence for plant community structure than cultivar or non-cultivar source of dominant grasses.

3778: *+.106*

Climate change is recognized as a major threat to biodiversity. Multidisciplinary approaches that combine population genetics and species distribution modelling to assess these threats and recommend conservation actions are critical but rare. Combined, these methods provide independent verification and a more compelling case for developing conservation actions. This study integrates these data streams together with field assessments and spatial analyses to develop future genetic resource management recommendations. The study species was Callistemon teretifolius (Needle Bottlebrush), a shrub species endemic to the Mount Lofty and Flinders

Ranges, South Australia, and potentially vulnerable to climate change. Chloroplast microsatellite and Amplified Fragment Length Polymorphism data were combined with species distribution modelling (MaxEnt), spatial analysis and field assessment to evaluate climate change vulnerability. Two major genetic groups were identified (Mount Lofty and Flinders Ranges). Populations in the Flinders Ranges, especially the Southern Flinders Ranges exhibited the highest genetic diversity, indicating a possible genetic refugium. Lower genetic diversity to the south in the Mount Lofty Ranges and north in the Gammon Ranges may be due to post-glacial expansion into these areas from the Flinders Ranges or loss of alleles. Low levels of contemporary gene flow were identified, which suggests Callistemon teretifolius may have a limited capacity to respond to climate change through migration. Range restrictions were predicted for all future climates, especially in the north. It is likely that C.teretifolius will be adversely affected by climate change, due to limited gene flow, predicted range restriction and loss of suitable habitat. The Southern Flinders Ranges should be a priority for conservation because it contains the highest number of individuals and genetic diversity. We recommend monitoring and adaptive management involving restoration in the Southern Flinders Ranges, potentially incorporating genetic translocations from other areas to capture diversity, to assist C.teretifolius to adapt to climate change.

3779: *+.330*

A critical component of a species reintroduction is assessment of contemporary habitat suitability. The robust redhorse, Moxostoma robustum (Cope), is an imperilled catostomid that occupies a restricted range in the south-eastern USA. A remnant population persists downstream of Blewett Falls Dam, the terminal dam in the Pee Dee River, North Carolina. Reintroduction upstream of Blewett Falls Dam may promote long-term survival of this population. Tillery Dam is the next hydroelectric facility upstream, which includes a 30rkm lotic reach. Habitat suitability indices developed in the Pee Dee River were applied to model suitable habitat for proposed minimum flows downstream of Tillery Dam. Modelling results indicate that the Tillery reach provides suitable robust redhorse habitat, with spawning habitat more abundant than non-spawning habitat. Sensitivity analyses suggested that suitable water depth and substrate were limiting physical habitat variables. These results can inform decisions on flow regulation and guide planning for reintroduction of the robust redhorse and other species.

3780: *+.199*

Numerous factors influence fitness of free-ranging animals, yet often these are uncharacterized. We integrated GPS habitat use data and genetic profiling to determine their influence on fitness proxies (mass, length, and body condition) in a threatened population of grizzly bears (Ursus arctos) in Alberta, Canada. We detected distinct genetic and habitat use (ecotype) clusters, with individual cluster assignments, or genotype/ecotype, being correlated (Pearson $r=0.34$, $P<0.01$). Related individuals showed evidence of similar habitat use patterns, irrespective of geographic distance and sex. Fitness proxies were influenced by sex, age, and habitat use, and homozygosity had a positive effect on these proxies that could be indicative of outbreeding depression. We further documented over 300 translocations occurring in the province since the 1970s, often to areas with significantly different habitat. We argue this could be unintentionally causing the pattern of outbreeding, although the heterozygosity correlation may instead be explained by the energetic costs associated with larger body size. The observed patterns, together with the unprecedented human-mediated migrations, make understanding the link between genotype, ecotype, and phenotype and mechanisms behind the negative heterozygosity-fitness correlations critical for management and conservation of this species.

3781: *+.103*

Where a translocation program is used to reinforce an existing population of an endangered species, the response of the introduced individuals to cues from conspecific residents will have an important impact on the success of the translocation. If those cues induce the translocated individuals to stay at the release site the translocation is more likely to succeed than if the cues cause individuals to move away. We used conspecific models of the endangered Australian pygmy bluetongue lizard to identify behavioural parameters relevant to translocation success, that change when the visual conspecific cues are presented. Pygmy bluetongue lizards typically remain in or at the entrance of their refuge burrows. In the presence of conspecific models, introduced lizards significantly increased, and nearly doubled, the number of movements out of their burrows (mean (SE) number of movements with models = 0.44 (0.03); without models = 0.25 (0.03); P=0.012) and more than doubled the number of movements away from the release area (mean (SE) number of movements with models = 0.28 (0.03); without models = 0.08 (0.02); P=0.003), suggesting they would be less likely to remain within a resident population where they were released. We found that, by the end of the first day of experimental trials 11 of 16 lizards in treatments with models present had occupied burrows that did not have a model nearby, and that number increased to 14 of 16 lizards by the fourth day. The results suggest that cues from conspecifics will not encourage translocated lizards to stay at a release site. (C) 2013 Elsevier B.V. All rights reserved.

3782: *+.297*

Local extirpations of Pacific salmon Oncorhynchus spp. and steelhead O. mykiss, often due to dams and other stream barriers, are common throughout the western United States. Reestablishing salmonid populations in areas they historically occupied has substantial potential to assist conservation efforts, but best practices for reintroduction are not well established. In this paper, we present a framework for planning reintroductions designed to promote the recovery of salmonids listed under the Endangered Species Act. Before implementing a plan, managers should first describe the benefits, risks, and constraints of a proposed reintroduction. We define benefits as specific biological improvements towards recovery objectives. Risks are the potential negative outcomes of reintroductions that could worsen conservation status rather than improve it. Constraints are biological factors that will determine whether the reintroduction successfully establishes a self-sustaining population. We provide guidance for selecting a recolonization strategy (natural colonization, transplanting, or hatchery releases), a source population, and a method for providing passage that will maximize the probability of conservation benefit while minimizing risks. Monitoring is necessary to determine whether the reintroduction successfully achieved the benefits and to evaluate the impacts on nontarget species or populations. Many of the benefits, especially diversity and the evolution of locally adapted population segments, are likely to accrue over decadal time scales. Thus, we view reintroduction as a long-term approach to enhancing viability. Finally, our review of published salmonid reintroduction case studies suggests that large uncertainties remain in the success of reintroduction in establishing self-sustaining populations, particularly for programs employing active methods. Received September 10, 2012; accepted August 30, 2013

3783: *-.016*

Our objective was to identify and evaluate mitochondrial diversity of wild pigs in the United States (U.S.). We obtained tissue samples from 81 individual pigs in 30 U.S. states and amplified a 403 base-pair region of mitochondrial DNA. We then downloaded overlapping sequences (n=904) from public repositories to create a global reference. We used parsimony and Bayesian techniques

to evaluate phylogenetic relationships, and we used origins of published sequences from Eurasian wild boar to establish a phylogeographic reference. We then compared gene and nucleotide diversity measures for introduced pigs in North America with those of domestic swine, Eurasian wild boar, and feral pigs within broad-scale geographic groupings. We identified 15 haplotypes for introduced pigs, representing wild and domestic animals from >30 countries spanning the indigenous range of S. scrofa. Mitochondrial diversity measures and phylogenetic relationships indicated a strong association between introduced pigs and European domestic breeds, reflecting the known history of human colonization, trade, and settlement in the United States. Based on the geographic distribution of haplotypes in North America, we found that range expansion is a product of translocation from historical populations and introduction from new genetic sources. Finally, haplotype network analyses provided evidence of past demographic expansions within lineages, contributing to observed mtDNA variation among introduced pigs in North America. (c) 2014 The Wildlife Society.

3784: *+.142*

Studies have revealed an unsuspected complexity in social systems within a few lizard species, including group living, long-term monogamy and individual recognition of partners or offspring. Comparisons among these species and their relatives could provide valuable insights, allowing us to investigate traits that are shared across social systems and identify general principles relating to the evolution of sociality. The endangered pygmy bluetongue lizard, Tiliqua adelaidensis, is a member species in the Egernia group, but is thought to show a more solitary social structure than other members in this group. Within this study we used microsatellite markers to determine the mating system of T. adelaidensis. Unlike many other species in the Egernia group, we found a predominately promiscuous mating system in T. adelaidensis. We detected multiple paternity in 75 % of litters. Of the 70 males identified as having fathered juveniles, only 5 were identified as mating with the same female in more than 1 year and only 3 were identified as the father of juveniles with the same female in consecutive years. The genetic evidence suggested that partners were chosen randomly with respect to the level of relatedness among neighbouring lizards. However, mated lizards were geographically closer to each other than expected by random chance. Multiple paternities rely on the opportunity for males to encounter multiple females during the period when they are receptive to mating, and this may depend on population densities. Drivers for the polygamous mating system may be the single occupancy burrow and the central place territorial defence of those burrows in T. adelaidensis. We propose a fourth mating system for the Egernia group: polygyny within stable non-social colonies.

3785: *-.013*

Genetic factors such as decreased genetic diversity and increased homozygosity can have detrimental effects on rare species, and may ultimately limit potential adaptation and exacerbate population declines. The Gulf and Atlantic Coastal Plain physiographic region has the second highest level of endemism in the continental USA, but habitat fragmentation and land use changes have resulted in catastrophic population declines for many species. Astragalus michauxii (Fabaceae) is an herbaceous plant endemic to the region that is considered vulnerable to extinction, with populations generally consisting of fewer than 20 individuals. We developed eight polymorphic microsatellites and genotyped 355 individuals from 24 populations. We characterized the population genetic diversity and structure, tested for evidence of past bottlenecks, and identified evidence of contemporary gene flow between populations. The mean ratios of the number of alleles to the allelic range (M ratio) across loci for A. michauxii populations were well below the threshold of 0.68 identified as indicative of a past genetic bottleneck. Genetic diversity

estimates were similar across regions and populations, and comparable to other long-lived perennial species. Within-population genetic variation accounted for 92 % of the total genetic variation found in the species. Finally, there is evidence for contemporary gene flow among the populations in North Carolina. Although genetic factors can threaten rare species, maintaining habitats through prescribed burning, in concert with other interventions such as population augmentation or (re)introduction, are likely most critical to the long term survival of A. michauxii.

3786: *+.100*

Due to severe declines in abundance throughout southern California, the green abalone (Haliotis fulgens Philippi 1845) became protected under a state-sponsored fishery moratorium in 1997 and was declared a NOAA NMFS Species of Concern in 2004. Recently, H. fulgens was chosen for possible stock restoration via translocation of wild adults to depleted habitat and supplementation through releasing cultured individuals. Before a management plan could be developed, however, an understanding of the species' natural population genetic structure was needed. We used a genomic technique called restriction site associated DNA sequencing (RADSeq) to address the issue. RADSeq enabled discovery of 1,209 single nucleotide polymorphisms theoretically spread genome-wide in H. fulgens. Analyses suggested the species may be panmictic throughout our sampled range, with an effective population size (N-e) of 1,100-3,600. Hence, limitations to management, such as requiring local broodstock and restricting translocation potential, might be unnecessary. Sites with larger populations may be suitable sources for restoration of depleted sites (e.g. the Palos Verdes Peninsula), although the extent of local adaptation remains unknown. Despite this potential for restoration, results gathered on a sample of cultured H. fulgens illustrated how quickly genetic diversity can be lost through captive breeding. To help mitigate a drop in Ne due to hatchery supplementation, we recommend collection and replacement of >= 100 wild abalone per generation for broodstock and close management of the proportion of cultured individuals in the wild. Successful implementation will depend on operational capacity and the resilience of the source populations to broodstock collection.

3787: *+.167*

Pityopsis ruthii is an endangered species endemic to the Hiwassee and Ocoee Rivers in Tennessee. As part of a recovery effort focused on P. ruthii, vegetative propagation and in vitro multiplication and seed germination techniques were developed. Plants were vegetatively propagated using greenhouse stock plants and wild-collected stems. Rooting occurred with and without auxin treatments but was greatest when 0.1% indole-3-butyric acid (IBA) talc was applied to the vegetative cuttings; rooting was lowest when flowering stems were used. Pro-Mix BX substrate provided the most consistent rooting. In vitro multiplication was accomplished by the removal of lateral shoots from in vitro-grown plants that were rooted on Murashige and Skoog (MS0) basal medium with 270 clones produced from a single individual after 4 months. Nineteen clones were transplanted and secured with bonded fiber matrix into their natural habitat and 14 survived for 1 year. To avoid genetic swamping of native populations with the introduction of large numbers of genetically identical individuals through clonal propagation, seed-based propagation efforts were explored. Open-pollinated seeds were collected, disinfested and germinated, and seedlings established on MS medium. Seeds were submersed in 70% ethanol for 1 minute and briefly flamed. Seeds were surface-sterilized in a range [10% to 50% (v/v)] Clorox (R) bleach solutions with vigorous shaking for 20 minutes, rinsed three times in sterile water, and germinated on MS0. Removal of pappus from seeds was required for successful disinfestations, but the bleach concentration was not critical. Successful propagation is a step toward the conservation and recovery of P. ruthii and should allow future reintroduction projects.

3788: ***+.492***

Insect conservation in the southern hemisphere lags substantially behind developments in parts of Europe and North America, where the relatively small faunas are better documented, and where a historical culture of natural history has enabled conservation needs to be assessed and addressed by many sympathetic supporters. We contrast this scenario with the much more embryonic knowledge and capability available in Australia, southern Africa, southern South America and New Zealand, all regions with large and incompletely documented insect faunas, but an equivalent array of threats to their survival. While a few individual 'flagship species' (mainly within Lepidoptera, Orthoptera and Coleoptera) have been critical in promoting wider interests, in general insects do not signify highly on regional conservation agendas. We offer a perspective of the major needs to counter this.

3789: ***+.089***

Despite increasingly sophisticated microbiological techniques, and long after the first discovery of microbes, basic knowledge is still lacking to fully appreciate the ecological importance of microbial parasites in fish. This is likely due to the nature of their habitats as many species of fish suffer from living beneath turbid water away from easy recording. However, fishes represent key ecosystem services for millions of people around the world and the absence of a functional ecological understanding of viruses, prokaryotes, and small eukaryotes in the maintenance of fish populations and of their diversity represents an inherent barrier to aquatic conservation and food security. Among recent emerging infectious diseases responsible for severe population declines in plant and animal taxa, fungal and fungal-like microbes have emerged as significant contributors. Here, we review the current knowledge gaps of fungal and fungal-like parasites and pathogens in fish and put them into an ecological perspective with direct implications for the monitoring of fungal fish pathogens in the wild, their phylogeography as well as their associated ecological impact on fish populations. With increasing fish movement around the world for farming, releases into the wild for sport fishing and human-driven habitat changes, it is expected along with improved environmental monitoring of fungal and fungal-like infections, that the full extent of the impact of these pathogens on wild fish populations will soon emerge as a major threat to freshwater biodiversity.

3790: ***+.114***

We documented the temporal and demographic recovery of a black bear (Ursus americanus) population following a 2-year translocation program that substantially reduced both male and female bears. We estimated black bear population abundance prior to (2003), during (2004), and following (2007 and 2010) translocations in a 1,368-km(2) study area in western Interior Alaska, USA. We estimated population size of independent black bears using removal models during 2003 and 2004 and mark-resight techniques during 2007 and 2010. The pre-translocation 2003 estimate of 83-109 (95% CI) independent bears was reduced by 96% to 0-13 (95% CI) independent bears by late spring 2004, when translocation efforts ceased. By 2007 the population of independent bears increased to 56-84 (95% CI), and by 2010 the population recovered to >= pre-translocation levels (96-162 [95% CI]). The proportion of male and female bears in the post-translocation population was similar during 2007 (50% F) and 2010 (59% F), indicating both sexes rapidly reoccupied the study area. We concluded that Interior Alaska black bear populations can recover in 46 years from substantial numerical reductions and recovery in our study area was likely facilitated by its relative small size, high-quality habitat, and large surrounding environment consisting of a lightly harvested bear population. (C) 2013 The Wildlife Society.

3791: *-.035*

We planted 308 star cacti (Astrophytum asterias) in southern Texas on private ranches where existing populations were reduced or destroyed. Overall mortality was 48% with herbivory accounting for 34% of the mortality (50 of 148). Mortality varied among planting sites but was not influenced by initial size, even when cacti killed by herbivory were excluded. Mortality was highest during times with low precipitation or high temperatures. Surviving cacti on two sites increased in average size. Reintroduction through planting is a viable strategy for restoring populations of star cacti.

3792: *-.008*

We introduced 250 alligator snapping turtles (Macrochelys temminckii) originating from a turtle farm in Arkansas into six pools adjacent to the Washita River in Johnston, Bryan, and Marshall counties, Oklahoma. Additionally, we released 16 captive-bred and reared juvenile turtles. We used radiotelemetry and mark-recapture to monitor dispersal of turtles, selection of microhabitat, and patterns of movement. We placed transmitters on 16 adult turtles from Arkansas and 16 captive-bred juveniles 2-4 years old. We recorded 198 locations of 32 individuals by radiotelemetry between May 2007 and August 2008. We recaptured 45 turtles one-five times using hoop nets employed for 501 trap-nights. We compared movement and selection of habitat between sexes and age classes for the parameters water depth, bottom temperature, turbidity, and canopy cover. Adults and juveniles chose shallower depths with more canopy than available randomly. Additionally, adults chose greater depths than did juveniles, and juveniles chose areas with more canopy than did adults. There was no difference in selection of habitat between sexes. Adults utilized a larger linear home range than did juveniles.

3793: *-.010*

Overharvesting and habitat loss resulted in the extirpation of river otters (Lontra canadensis) across much of Kentucky and efforts in the mid-1990s by the Kentucky Department of Fish and Wildlife Resources have led to a resurgence of otter populations across the state. Since recovery, limited data on the biology of these animals have been collected, including levels of infection by endoparasites. We performed necropsies on 170 river otters obtained during the 2006-2009 harvest seasons. We found nematode parasites present in the fascial layers of the hind leg of 5 juvenile river otters for a minimum infection level for the Kentucky population of 0.0294. Infected animals were recorded in the Green, Kentucky, and Mississippi River watersheds. All nematodes were females of the genus Dracunculus, which cannot be assigned to the species level based on morphological characteristics. These observations are the first documented case of Dracunculus infection in river otters in Kentucky.

3794: *-.073*

The primary freshwater fish Pseudobarbus burchelli (Smith 1841) occurs across four presently isolated river systems in the south-western cape floristic region of South Africa. Mitochondrial DNA cytochrome b (701 base pairs) and control region (601 base pairs) genes were sequenced to assess the evolutionary history of P. burchelli and evaluate the role of climatic and landscape changes in shaping patterns of genetic variation in this species. We identified three historically isolated lineages in P. burchelli: a widespread lineage that occurs across three isolated river systems and two geographically restricted lineages. The results were evaluated against predictions of the confluence of river systems during low sea levels of the last glacial maximum. Occurrence

of the widespread Breede lineage in the Duiwenhoks River system is consistent with reconstructed palaeoriver systems. However, the occurrence of this lineage in the Goukou river system that formed part of the eastern Gourits-Goukou palaeoriver system can only be explained by translocation or a recent river capture or episodic inundation of low drainage divides. Extreme ecological gradients or the potential presence of instream physical barriers could have prevented an exchange of lineages between the Breede and Heuningnes river systems.

3795: *+.020*

Spatial and temporal variation in recovery of hatchery-released red drum (Sciaenops ocellatus) was assessed in nine bays and estuaries along the Texas coast. Sixteen nuclear-encoded microsatellites were used to identify a total of 167 hatchery-released fish among 4325 red drum sampled between the fall of 2006 and the spring of 2009; these were added to 41 recovered hatchery-released fish identified in a prior study of 1332 red drum sampled from Galveston and Aransas bays in the fall of 2005 and spring of 2006. Recovery of hatchery releases across bays and years ranged from 0.28% to 17.65%. Of the 208 recovered hatchery-released fish, 158 were age 0-1,46 were age 1-2, and four fish were age 2-3. The distribution of recovered hatchery-released fish and wild fish differed significantly across sampling periods within and among bays or estuaries. Allelic richness in recovered hatchery-released fish was significantly lower than in both hatchery brood fish and wild fish. In addition, recovered hatchery-released fish were produced by fewer dams, sires, and dam x sire combinations in 2005 and 2006 and by fewer dams and sires in 2007 than would be expected if reproductive success was distributed randomly among brood fish. The spatial distributions of recovered hatchery-released and wild fish deviated from random expectations for samples from the Upper Laguna Madre in the fall of 2006 and spring of 2007 and from West Matagorda Bay in the spring and fall of 2008. Correlation and principal components analysis identified a significant, positive relationship between annual average CPUE and relative annual mortality. No relationship was observed between the number of fish released per hectare and the percentage of recovered hatchery-released fish. Comparisons to other studies of red drum stock enhancement are discussed. (C) 2013 Elsevier B.V. All rights reserved.

3796: *+.087*

Last seen in 1958, the Ascension Island endemic fern, Anogramma ascensionis, was listed as extinct on the 2003 IUCN Red List. However, a 2009 survey rediscovered four plants on Green Mountain. Spores were collected and cultured in vitro at the Royal Botanic Gardens, Kew, where a living collection of thousands of gametophytes and hundreds of sporophytes has been developed. To gain further insights into the biology of this species and the potential implications of in vitro multiplication for conservation purposes, samples were characterized from the karyological point of view. Chromosome analysis of root tips has confirmed that the species is tetraploid, and flow cytometry assessments have revealed that haploid gametophytes produce diploid sporophytes, which confirms natural fertilization. In addition, an rbcL sequence from A. ascensionis has been generated and compared with those published for other Anogramma spp., suggesting a close relationship with A. chaerophylla from Brazil. Further surveys of Green Mountain have reported the presence of 40 A. ascensionis sporophytes in total. Vegetation community analyses have suggested that the present population may be confined to suboptimal habitats. We therefore propose that, prior to the dramatic transformation of the vegetation on the island as a result of the invasion of alien species (particularly Adiantum spp.), A. ascensionis may have flourished in more humid and shaded parts of the mountain. A multidisciplinary approach involving in vitro culture, invasive species clearance and controlled translocation is discussed as the future roadmap for the conservation of this critically endangered fern. Our experiences have also highlighted lessons

more broadly applicable to the conservation of extremely rare species elsewhere in the world, especially on remote island systems. (c) 2014 The Linnean Society of London, Botanical Journal of the Linnean Society, 2014, 174, 461-477.

3797: *+.136*

Recolonization by native species following reintroduction can affect resident species through a variety of processes. We examined the effects of natural recolonization by coho salmon Oncorhynchus kisutch on sculpin (Cottus rhotus and Cottus gulosus), small benthic fishes, in a small forest stream in Western Washington, USA. Provision fish passage around a small dam allowed coho access to habitat, which had been inaccessible for over 100years. We found that density (gm(-2) and number m(-2)) was unchanged, and body condition (the slope of the relationship between length and weight) of sculpin tended to increase from before relative to a 5-year period following recolonization. The proportion of sculpin comprising the total fish assemblage decreased after coho colonization relative to before but remained stable for a 5-year period after coho reintroduction, whereas coho density increased over fivefold. Additionally, we used Akaike's information criteria to evaluate the relative importance of physical and biological variables to predict sculpin density in pool habitats during the initial coho recolonization period. Physical microhabitat variables had little support for predicting sculpin density, whereas there was a significant support for stream temperature; cutthroat trout (Oncorhynchus clarkii) density and year were the most important predictors of sculpin density. Coho density was not significant in any model. Our results indicate coho introduction and subsequent recolonization have to date had minimal individual or population level effects on sculpin, therefore demonstrating that species reintroductions into their native range can have no measurable effect on resident organisms. Published 2013. This article is a U.S. Government work and is in the public domain in the USA.

3798: *+.129*

Etheastoma sitikuense (Citico Darter), a federally protected fish endemic to the southeastern United States, was extirpated from Abrams Creek in Great Smoky Mountains National Park in 1957. The species was reintroduced from 1993-2001, but recovery efforts have thus far achieved only partial success, due in part to limited knowledge of Citico Darter habitat use. After distribution of the reintroduced population was established, we monitored Citico Darters in a 4-km section of Abrams Creek using underwater observation. We evaluated macro- and microhabitat use over four summers using principal components analysis to determine macrohabitat variables influencing Citico Darter distribution, and used classification tree methods to analyze microhabitat use. We analyzed dispersal using linear regression to compare historical stocking data with current Citico Darter distribution data. We identified percentage of pools and cobble/small boulder substrates as the most significant macrohabitat variables influencing Citico Darter presence. This species most often occupied microhabitats away from riffles under intemediate-sized cover rocks. Dispersal of reintroduced Citico Darters was limited in Abrams Creek. Results of this study can be used to identify additional reintroduction zones and assist in further conservation efforts.

3799: *+.206*

The cornerstone of the recovery plan for the critically endangered Puerto Rican parrot (Amazona vitatta) is an actively managed, long-term reintroduction program. One captive population distributed across two aviaries in Puerto Rico is the sole source for release but its ability to persist as a managed resource has not been evaluated since 1989. We conducted an assessment for sustainable management of the aviary population while harvesting for release. To assess

demographic rates such as population growth, vital rates, and age/sex structure, we compiled a studbook database on all living, dead, and released individuals in the aviary population. Using an individual-based risk assessment model we applied population specific data based on the management period from 1993 to 2012 to simulate future aviary population dynamics and evaluate future potential production. We modeled four potential management strategies to harvest parrots for proposed releases; these scenarios vary the number of parrots and the life stage. Our simulations revealed that the aviary population can be simultaneously managed for sustainability and harvesting of parrots for release. However, without cautious management, overharvesting can jeopardize sustainability of the aviary population. Our analysis of the aviary breeding program provides a rare opportunity to review progress relative to conservation program objectives after four decades of active management. The successful growth of the aviary population and its ability to serve as a sustainable source for reintroductions supports the 1973 decision to build a breeding program from a small population of 13 parrots. Zoo Biol. 33:89-98, 2014. (c) 2014 Wiley Periodicals Inc.

3800: ***+.249***

The ability to propagate and successfully reintroduce rare plant species is an important component in the conservation biologist's toolbox. Unfortunately for many species, propagation methods and details about the species' life histories, which can potentially inform reintroduction efforts, are often unknown. We describe for the first time the pseudoannual life cycle of rough-leaved loosestrife (Lysimachia asperulifolia Poiret [Primulaceae]) and techniques that we have used to vegetatively propagate and reintroduce this federally endangered species endemic to the Atlantic Coastal Plain of the United States. Using simulated dormancy and controlled soil temperatures, we increased the number of collected viable rhizomes by approximately 76% over one growing season. At two translocation sites, stem numbers increased an average of 318 +/- 145 SD % in five of seven test plots between 2004 and 2010. Using dormant-season-harvested rhizomes and/or reducing competition prior to transplanting via mowing or using herbicides greatly improved translocation success. Only plots receiving neither competition-reduction treatment and outplanted with summer harvested rhizomes showed reductions (73 +/- 21 SD %) in the number of stems. Although limited flowering and no capsule production was detected at the translocation sites, a similar lack of sexual reproduction is common in many natural populations of L. asperulifolia. Overall, our results will allow the United States Fish and Wildlife Service and their conservation partners to more confidently pursue recovery goals for the species.

3801: ***+.116***

Przewalski's horse went extinct in the wild in the mid 1960s. Starting in 1985, individuals were brought from western zoos to two centers in China and breeding programs were initiated. With the increasing size of captive populations, two reintroduction projects were launched in the northwestern China in 2001 and 2010. Knowledge on genetic diversity in China's horse populations is limited, but would help improve the genetic management and assess the success of the reintroduction. Accordingly, one reintroduced and two captive populations were examined with 10 microsatellite loci together with pedigree data. The results showed higher level of diversity within the captive populations than the reintroduced population, indicating some alleles may have been lost during reintroduction. Genetic differentiation was detected among populations (F-ST = 0.09 +/- 0.05, Rhos(ST) = 0.05 +/- 0.02) and Bayesian clustering supported the presence of three subpopulations. The highest genetic differentiation was observed between the captive and reintroduced populations, and inbreeding coefficients were generally higher in the reintroduced population. Temporal estimates of both pedigree and microsatellite data showed a high, but

decreasing level inbreeding. Through simulations, we estimated that the reintroduced population needs more than 100 individuals to retain approximately 90% of its current, already depauperate, genetic diversity. We have provided recommendations for the management program concerning introgressed genes from domestic horse and the number and origin of individuals for future reintroductions. (C) 2014 Elsevier Ltd. All rights reserved.

3802: *+.096*

The effectiveness of fauna reintroduction programs has been limited by the availability of source animals and the lack of follow up monitoring to assess whether viable populations have been successfully established, particularly in terms of conserving genetic diversity. Here we present genetic assessment of the translocation of golden bandicoots (Isoodon auratus) from a large source population on Barrow Island off the north-west coast of Western Australia to two other island sites and a mainland fenced enclosure. We assessed the genetic diversity of animals translocated to each site and their wild-born progeny, and whether wild-born animals showed evidence of genetic bottlenecks or genetic drift from the source population. Encouragingly, we found no significant loss of genetic diversity in any of the wild-born populations compared to the source population and no significant increase in inbreeding or relatedness amongst wild-born individuals compared to founder populations two years post-translocation. However, we detected an approximately 10-fold reduction in effective population size between founding and wild-born populations. We found no apparent differentiation between wild-born populations and the original source population, or between wild-born animals and their respective founders. Population viability modeling predicts that each of the translocated populations is susceptible to loss of genetic diversity over time. Taken together these results suggest that the golden bandicoot reintroduction program has been initially successful as a result of large founding sizes and high reproductive rates; however, ongoing augmentation will be required to prevent genetic erosion and maintain evolutionary potential in the long-term. Crown Copyright (C) 2014 Published by Elsevier Ltd. All rights reserved.

3803: *+.170*

Protected area management agencies often struggle to reliably reconstruct grazer assemblages due to a lack of historical distribution data for their regions. Wrong predictions of grazing assemblages could potentially affect biodiversity negatively. The objective of the study was to determine how well grazing herbivores have become established since introduction to the Mkambati Nature Reserve, South Africa, how this was influenced by facilitation and competition, and how indigenous grazer assemblages can best be predicted for effective ecological restoration. Population trends of several grazing species were investigated in in order to determine how well they have become established since introduction. Five different conceivable grazing assemblages reflecting a range of approaches that are commonly encountered during conservation planning and management decision making were assessed. Species packing was used to predict whether facilitation, competition or co-existence were more likely to occur, and the species packing of the different assemblages were assessed using ANCOVA. Reconstructing a species assemblage using biogeographic and biological information provides the opportunity for a grazer assemblage that allows for facilitatory effects, which in turn leads to an ecosystem that is able to maintain its grazer assemblage structure. The strength of this approach lies in the ability to overcome the problem of depauperate grazer assemblages, resulting from a lack of historical data, by using biogeographical and biological processes, to assist in more effectively reconstructing grazer assemblages. Adaptive management of grazer assemblage restoration through reintroduction, using this approach would further mitigate management risks.

3804: +.019

Chinese goral (Naemorhedus griseus) are a threatened species in Thailand and the focus of captive breeding for possible reintroduction. However, little is known of their biology or what factors in the captive environment affect welfare. Our objective was to determine the impact of gender, season, and management on goral adrenal activity. We hypothesized that differences in fecal glucocorticoid concentrations would be related to animal density. Fecal samples were collected 3 days/week for 1 year from 63 individuals (n = 32 males, 31 females) at two facilities that house the majority of goral in Thailand: Omkoi Wildlife Sanctuary (Omkoi), an off-exhibit breeding center that houses goral in individual pens (16 pens; n = 8 males, 8 females) and in small family groups (8 pens; n = 8 males, 8 females); and the Chiang Mai Night Safari (NS), a zoo that maintains 31 goral (n = 17 males, 14 females) in one large pen. Glucocorticoid metabolite concentrations were higher in male than female goral at Omkoi throughout the year, and there was a seasonal effect on adrenal activity ($p<0.05$). Goral at Omkoi and NS were used to test the effect of animal density on fecal glucocorticoid excretion of goral housed in similar-sized enclosures. Overall, the highest levels were found at NS (n = 31 adults/pen; 27 m(2) per animal) compared to Omkoi (n = 2 adults/pen; 400 m(2) per animal) ($p<0.05$). Overall findings support our hypothesis that animal density and aspects of the captive environment impact adrenal steroid activity in captive goral. In addition, gender and season also had significant effects on glucocorticoid metabolite production. Potential stressors pertaining to the welfare of this species were identified, which will guide future efforts to improve management and create self-sustaining and healthy populations of this threatened species.

3805: +.142

We tested the hypotheses that species with greater mobility and/or higher reproductive rates are less sensitive to habitat loss than species with lower mobility and/or reproductive rates by conducting a meta-analysis of wetland vertebrate responses to wetland habitat loss. We combined data from 90 studies conducted worldwide that quantified the relationship between wetland amount in a landscape and population abundance of at least one wetland species to determine if mobility (indexed as home range size and body length) and annual reproductive rate influence species responses to wetland loss. When analyzed across all taxa, animals with higher reproductive rates were less sensitive to wetland loss. Surprisingly, we did not find an effect of mobility on response to wetland loss. Overall, wetland mammals and birds were more sensitive to wetland loss than were reptiles and amphibians. Our results suggest that dispersal between habitat patches is less important than species' reproductive rates for population persistence in fragmented landscapes. This implies that immigration and colonization rate is most strongly related to reproduction, which determines the total number of potential colonists.

3806: +.320

This study describes the early epifaunal succession associated with an artificial reef constructed to regenerate damaged biogenic habitats formed by Modiolus modiolus (Linnaeus, 1758). Clumps of live M. modiolus were translocated onto three treatments: flattened cultch, elevated cultch, and directly onto the sea floor. Photographic surveys were carried out 1, 6, and 12 months after completion of the experimental array to test the hypothesis that the artificial reef would enhance habitat complexity thus increasing biodiversity and accelerating faunal community succession. These effects were predicted to be greater on elevated cultch due to higher level of protection and greater accessibility to food compared to sea floor treatments. Univariate analysis indicated that after 12 months the artificial reef had developed a significantly richer and more diverse community

compared to 1- and 6-month stages. Multivariate analysis revealed a significant temporal shift in species composition from mobile taxa to sessile and interstitial macroinvertebrates as the artificial reef settled. Reef elevation offered no significant advantages for the development of the epifaunal assemblage. Although further regular monitoring is advisable, this study demonstrated that translocation of a foundation species can help restore marine benthic habitats through the development of a diverse community in a relatively short time.

3807: *+.207*

Translocations have become an increasingly popular tool in threatened macropod conservation in Australia. Although previous evaluations of Australian macropod translocations have been published, the number of contemporary translocation programmes awaiting analysis, and new data regarding historic translocations, required a new assessment of macropod translocation programmes. We aimed to assess trends in the way macropod translocations were conducted during the period 1969-2006, determine the number of successful translocations and identify factors common to successful translocations. Data regarding macropod translocations were obtained from a wide variety of sources, including peer-reviewed journals, grey' literature and popular interest publications. Questionnaires were also sent to translocation managers to acquire detailed information. Specific aspects of macropod translocation methodology were analysed, and classification tree analysis was conducted to identify methodological and environmental factors common to successful translocations. We identified 109 macropod translocations for which sufficient data could be collected to permit analysis. Using the presence of a population on 1 January 2007 as a simple criterion, 61% of translocations were successful. Of these translocations, 66% were also considered successful by Short etal.' s criteria (population persisted for five years and is deemed likely to continue to persist); the remainder could not be assessed due to lack of data or insufficient elapsed time since release. Classification tree analysis suggested methodological and environmental factors common to successful translocations; the overriding factor determining success was the absence of cats and foxes at the release site. Although Australian macropod translocation proponents are faced with myriad methodological options when designing a translocation protocol, the primary consideration should be whether or not cats or foxes are present at the release site. Managers should be aware that there may be no safe population level of such predators for some translocation candidate species. Ignoring this fact will inevitably lead to a repeat of past translocation failures.

3808: *+.235*

Establishing new populations is essential for preventing the extinction of critically endangered plant species. However, defining the range of environmental conditions suitable for the most severely endangered species is challenging, since few wild populations remain for study. Experimental reintroductions of these species can achieve multiple conservation goals by improving our understanding of habitat and management requirements while simultaneously establishing new populations. We demonstrate this with Arenaria paludicola, a critically endangered wetland plant species now known from a single wild population in coastal California. Before transplanting, we tested salinity tolerance in the greenhouse, and found tolerance of a broader range of soils than expected based on the current distribution. We then transplanted A. paludicola in three different habitat types, with and without neighbor removal. Success of A. paludicola transplants differed dramatically between the three habitat types, indicating the importance of variation at the habitat and microhabitat level. The best practices for transplant management are context-dependent: neighbor removal may promote the growth of A. paludicola, but neighbors may also facilitate transplant establishment in unstable substrates. After one year, A.

paludicola continued to thrive in habitats dominated by Oenanthe sarmentosa with open canopies and moist soil. This habitat differs from that of the remaining wild population. Our discovery of an additional habitat type suitable for A. paludicola will allow more effective selection of future transplant sites.

3809: *+.199*

Research addressing the effects of habitat fragmentation on species, assemblages or ecosystems has been fraught with difficulties, from its conceptual foundation to statistical analyses and interpretation. Yet, it is critical to address such challenges as ecosystems are rapidly being altered across the world. Many studies have concluded that effects of habitat loss exceed those of fragmentation per se, that is, the degree to which a given amount of habitat is broken apart. There is also evidence from different biomes and taxa that habitat configuration, that is, the spatial arrangement of habitat at a given time, may influence several landscape processes such as functional connectivity, edge and matrix effects, and thus population viability. Instead of focusing attention on the relative influence of either habitat loss or fragmentation, we must identify portions of the gradient in habitat amount where configuration effects are most likely to be observed. Here, we suggest that all species are, to a certain degree, sensitive to landscape change and that, assuming a homogeneous matrix, habitat configuration will have a higher influence on species at intermediate values of habitat amount, where configuration has potentially the greatest variability. On the basis of empirical studies and simulations, we expect that species that are relatively tolerant to fragmentation of their habitat will exhibit a wider band where amount and configuration interact compared to species less tolerant to fragmentation. Synthesis and applications. Reducing habitat loss should be a top priority for conservation planners. However, researchers should also investigate the indirect impacts of habitat loss on biodiversity through fragmentation effects. This research aims to identify windows of opportunity where habitat configuration can mitigate to some extent the effects of habitat loss, particularly through the maintenance of functional connectivity. Reducing habitat loss should be a top priority for conservation planners. However, researchers should also investigate the indirect impacts of habitat loss on biodiversity through fragmentation effects. This research aims to identify windows of opportunity where habitat configuration can mitigate to some extent the effects of habitat loss, particularly through the maintenance of functional connectivity.

3810: *+.056*

Podarcis filfolensis is an endemic lizard from the Maltese archipelago. There is evidence of human-mediated decline and even extirpation of some insular populations of this species. However, information about the intraspecific genetic diversity and phylogeographic patterns of this species is limited. Here we analyze genetic markers from a multi-locus dataset (mtDNA, 2,533 bp; nuclear c-mos gene, 353 bp; 11 microsatellites) for individuals from extant populations of P. filfolensis. Despite generally low genetic variability, two main mitochondrial groupings were clearly identified. In general, individuals from the main island of Malta were genetically distinct from those from Gozo, Comino, Cominotto and Small Blue Lagoon Rock, and also from Linosa and Lampione individuals. Three genetic clusters were detected based on microsatellite data: one was found at higher frequency on Malta, while the other two included samples from the remaining islands, showing some concordance with the mtDNA pattern. A time-calibrated Bayesian tree for the principal mitochondrial lineages indicated strong statistical support for two P. filfolensis lineages that originated in the Pleistocene (105.4-869 Ka). We show that these lineages largely meet the criteria for recognition as evolutionary significant units despite some recent admixture (possibly due to recent translocations between islands). Human disturbance, low genetic

variability, evidence of bottlenecks and extirpation on one island indicate that a thorough review of the current conservation status of P. filfolensis would be timely.

3811: *+.121*

Reconstructing the phylogeographic history of a species can aid in defining areas of conservation priority. For freshwater species, historical river structure plays a significant role in explaining genetic differentiation and population structure. However, human-induced translocations can erase the natural genetic structure, especially for species of commercial interest such as the noble crayfish (Astacus astacus). Our aim was to reconstruct the current genetic structure of the endangered noble crayfish in central Europe to identify refugial areas that are hotspots of genetic diversity. We analysed a fragment of the mitochondrial cytochrome oxidase subunit I, and the 16S rRNA from 540 noble crayfish specimens from 156 sampling sites distributed around five European sea basins. Additionally, we conducted a microsatellite analysis of 289 individuals from 22 sites. Both mitochondrial and nuclear markers revealed genetically relatively homogenous populations in central Europe that had been influenced by anthropogenic translocations. However, some areas (Eider catchment in northern Germany and Rhineland-Palatinate in south-western Germany) show a distinct genetic structure with endemic haplotypes and private alleles indicating (i) that these areas were refugia for A.astacus in central Europe and (ii) that these populations have not been subject to anthropogenic translocations. Further, we found the highest genetic diversity in the Black Sea basin and particularly high differentiation between populations from the western Balkans and the remaining Black Sea populations. The split between Western Balkan and the remaining European populations is estimated to have occurred approximately 700k years before present, whereas remaining differentiations occurred within the last 450k years. Using migration modelling, we detected that the North Sea basin and the Baltic Sea basin were colonised independently via different colonisation paths from the eastern Black Sea basin, while the western Balkans did not contribute to this colonisation. Our results suggest the existence of at least two refugial areas in south-eastern Europe. To conserve maximum genetic diversity, conservation priorities for noble crayfish should focus on the south-eastern European genetic hotspots and on populations in central Europe that hold an autochthonous genetic structure (e.g. Langsee in the Eider catchment area). We further propose that each river catchment should form a separate management unit to reduce anthropogenic genetic homogenisation.

3812: *+.080*

Understanding local adaptation and population differentiation is vital to the success of re-introduction initiatives. As other mammals living on islands, Arabian gazelles (G.arabica) show reduced body size on the Farasan archipelago, which we corroborated in this study through morphometric analyses of skulls. In the light of the steep population decline on the Arabian Peninsula - but stable population development on the archipelago - we tested the potential suitability of Farasan gazelles as a source for re-introductions on the mainland. We therefore investigated genetic differentiation between Farasan and mainland populations using eleven nuclear microsatellite loci and detected a distinct genetic cluster exclusively present on the archipelago, which we inferred to be separated from the mainland cluster for less than 2000years. About 30% of sampled individuals from Farasan Islands showed assignment to a mainland cluster with signs of ongoing introgression. Analyses using the isolation-with-migration model confirmed recent (probably human-induced) bidirectional exchange of gazelles between mainland and island populations. Hence, the surprisingly uniform island dwarfism most likely reflects phenotypic plasticity, that is, altered morphology as a direct consequence of harsh environmental conditions and resource limitation on the archipelago. Should a further decline of Arabian gazelles on the

mainland necessitate restocking in the future, Farasan gazelles may thus become an additional source for captive breeding programmes.

3813: *+.100*

Reintroduction of native species to unoccupied portions of their historical range is a common management strategy to enhance the future viability of animal populations. This approach has met with mixed success, due to unforeseen impacts caused by human or other factors. Some of these impacts could potentially be mitigated through the use of anticipatory modeling coupled with appropriate management strategies prior to release. As part of an ongoing restoration program, we evaluated a portion of the former range of the tule elk (Cervus elaphus nannodes) in the Central Valley of California for potential reintroduction of a free-ranging herd. We used a new spatially explicit population model (HexSim) to analyze four different elk release scenarios. Each scenario corresponded to a different release location, and the model was used to compare simulated elk movement and population dynamics 25 years into the future. We also used HexSim to identify likely locations of human-elk conflict. Population forecasts after the 25-year period were highest (mean female population size of 169.6 per iteration) and potentially harmful barrier interactions were lowest (mean 8.6 per iteration) at the East Bear Creek site. These results indicate the East Bear Creek site release scenario as the most likely to result in a successful elk reintroduction, producing the most elk and generating the fewest human conflicts. We found HexSim to be a useful tool for this type of reintroduction planning and believe that other reintroduction efforts could benefit from this type of anticipatory modeling.

3814: *+.020*

When dealing with remediation projects in zones affected by mining activities, the risk posed by the ingestion of the plants by fauna is often forgotten. The purpose of this study is the assessment of arsenic assimilation by the natural vegetation in these areas. To study the transfer to the trophic chain two mammals, the sheep and the vole are selected. The risk analysis is founded on the contribution of these natural plants to the ingestion. Soil samples and the same number of plants (165) growing in the soils were collected in an old mining area in the southeast of Spain. Physico-chemical properties were calculated by means of the usual procedures. To determine the arsenic content, the soil samples and plant materials were digested by means of a microwave system and the arsenic concentration was determined using atomic fluorescence spectrometry with automated continuous flow hydride generation (HG-AFS). A semiquantitative estimation of the mineralogical composition of the samples was made by X-ray diffraction analysis. The mineralogy and As content of the soils studied depends on the materials related with the mining activity. The descriptive statistical analysis of the population of plants studied points to an As range of 0.31-150 mg/kg in roots, although the concentration in shoots was lower (0.21-83.4 mg/kg). Bioconcentration (BCF) and transfer factors (TF) were studied for each plant species and soil type on which it grew. The results show that As transfer depends on the plant species and the characteristics of the soil. The potential risk of As entering the food chain through the plant species was evaluated. The exposure pathway considered was oral ingestion, calculating the contribution of the plant to the daily dose based on the arsenic concentration in the shoots of the plants analysed. In the samples studied, the levels of As in roots were higher than in shoots, and increased with the As concentration in the soil. The BCFs were generally very low, and the TFs while slightly higher, seldom exceeded unity. When undertaking with the phytoremediation of contaminated sites, the contribution of the As level in plants to the daily diet of animals should be used as an indicator for the screening of the vegetal species to be used.

3815: *-.033*

Social network analysis is being increasingly used in epidemiology and disease modeling in humans, domestic animals, and wildlife. We investigated this tool in describing a translocation network (area that allows movement of animals between geographically isolated locations) used for the conservation of an endangered flightless rail, the Takahe (Porphyrio hochstetteri). We collated records of Takahe translocations within New Zealand and used social network principles to describe the connectivity of the translocation network. That is, networks were constructed and analyzed using adjacency matrices with values based on the tie weights between nodes. Five annual network matrices were created using the Takahe data set, each incremental year included records of previous years. Weights of movements between connected locations were assigned by the number of Takahe moved. We calculated the number of nodes (i(total)) and the number of ties (t(total)) between the nodes. To quantify the small-world character of the networks, we compared the real networks to random graphs of the equivalent size, weighting, and node strength. Descriptive analysis of cumulative annual Takahe movement networks involved determination of node-level characteristics, including centrality descriptors of relevance to disease modeling such as weighted measures of in degree (k(i)(in)), out degree (k(i)(out)), and betweenness (B-i). Key players were assigned according to the highest node measure of k(i)(in), k(i)(out), and B-i per network. Networks increased in size throughout the time frame considered. The network had some degree small-world characteristics. Nodes with the highest cumulative tie weights connecting them were the captive breeding center, the Murchison Mountains and 2 offshore islands. The key player fluctuated between the captive breeding center and the Murchison Mountains. The cumulative networks identified the captive breeding center every year as the hub of the network until the final network in 2011. Likewise, the wild Murchison Mountains population was consistently the sink of the network. Other nodes, such as the offshore islands and the wildlife hospital, varied in importance over time. Common network descriptors and measures of centrality identified key locations for targeting disease surveillance. The visual representation of movements of animals in a population that this technique provides can aid decision makers when they evaluate translocation proposals or attempt to control a disease outbreak. Analisis de Redes de Poblaciones de Takahe Translocadas para Identificar Objetivos de Supervivencia a EnfermedadesResumen El analisis de redes sociales actualmente se usa cada vez mas en la epidemiologia y el modelado de enfermedades en humanos, animales domesticos y vida silvestre. Investigamos esta herramienta en la descripcion de una red de translocacion (un area que permite el movimiento de animales entre localidades aisladas geograficamente) usada para la conservacion de un calamon no volador en peligro de extincion, el Takahe (Porphyrio hochstetteri). Cotejamos registros de translocaciones de Takahe dentro de Nueva Zelanda y usamos los principios de las redes sociales para describir la conectividad de la red de translocacion. Esto quiere decir que las redes se construyeron y analizaron usando matrices de proximidad con valores basados en los pesos de union entre nodos. Se crearon cinco matrices de redes anuales usando el juego de datos del Takahe, cada ano incremental incluyo registros de anos previos. Se asigno el peso de los movimientos entre localidades conectadas dependiendo del numero de Takahe que se movian. Calculamos el numero total de nodos (i(total)) y el numero de uniones (t(total)) entre los nodos. Para cuantificar la caracteristica de pequeno mundo de las redes, comparamos al azar a las redes verdaderas con graficas de tamano equivalente, peso y fuerza de nodo. El analisis descriptivo del movimiento acumulativo anual de Takake involucro la determinacion de las caracteristicas del nivel del nodo, incluyendo descriptores de centralidad de relevancia para el modelado de enfermedades como medidas valoradas de grado interno (k(i)(in)), grado externo (k(i)(out)) y entre-distancia (B-i). Los factores determinantes se asignaron de acuerdo a la medida mas alta de nodo de Key k(i)(in), k(i)(out) y B-i por red. Las redes incrementaron en tamano a lo largo del marco de tiempo que se considero. La red tuvo cierto grado de caracteristicas de pequeno mundo. Los nodos conectados

por los pesos de union acumulativos mas altos fueron los del centro de reproduccion en cautiverio, y las Montanas Murchison y dos islas. La parte determinante fluctuo entre el centro de reproduccion en cautiverio y las Montanas Murchison. Las redes acumulativas identificaron al centro de reproduccion en cautiverio cada ano como el centro de la red hasta la red final en 2011. La poblacion silvestre de las Montanas Murchison fue constantemente el vertedero de la red. Otros nodos, como las islas y el hospital de vida silvestre, variaron en importancia con respecto al tiempo. Los descriptores comunes de red y las medidas de centralidad identificaron localidades claves para enfocarse en la supervivencia a la enfermedad. La representacion visual de los movimientos de los animales en una poblacion que esta tecnica proporciona puede ayudar a los tomadores de decisiones cuando tengan que evaluar propuestas de translocacion o intenten controlar un brote de enfermedad.

3816: ***-.025***

Response to habitat fragmentation may not be generalized among species, in particular for plant communities with a variety of dispersal traits. Calcareous grasslands are one of the most species-rich habitats in Central Europe, but abandonment of traditional management has caused a dramatic decline of calcareous grassland species. In the Southern Franconian Alb in Germany, reintroduction of rotational shepherding in previously abandoned grasslands has restored species diversity, and it has been suggested that sheep support seed dispersal among grasslands. We tested the effect of rotational shepherding on demographic and genetic connectivity of calcareous grassland specialist plants and whether the response of plant populations to shepherding was limited to species dispersed by animals (zoochory). Specifically, we tested competing dispersal models and source and focal patch properties to explain landscape connectivity with patch-occupancy data of 31 species. We fitted the same connectivity models to patch occupancy and nuclear microsatellite data for the herb Dianthus carthusianorum (Carthusian pink). For 27 species, patch connectivity was explained by dispersal by rotational shepherding regardless of adaptations to zoochory, whereas population size (16% species) and patch area (0% species) of source patches were not important predictors of patch occupancy in most species. [Correction made after online publication, February 25, 2014: Population size and patch area percentages were mistakenly inverted, and have now been fixed.] Microsite diversity of focal patches significantly increased the model variance explained by patch occupancy in 90% of the species. For D. carthusianorum, patch connectivity through rotational shepherding explained both patch occupancy and population genetic diversity. Our results suggest shepherding provides dispersal for multiple plant species regardless of their dispersal adaptations and thus offers a useful approach to restore plant diversity in fragmented calcareous grasslands. Efectos del Pastoreo Rotacional sobre la Conectividad Genetica y Demografica de Plantas de Pastizales CalcareosResumen La respuesta a la fragmentacion del habitat puede no estar generalizada entre las especies, en particular para las comunidades de plantas con una variacion de metodos de dispersion. Los pastizales calcareos son uno de los habitats con mayor riqueza de especies en Europa Central, pero el abandono del manejo tradicional ha causado una disminucion dramatica de las especies en estos pastizales. En el Alb Franconio del Sur en Alemania, la reintroduccion del pastoreo rotacional en pastizales previamente abandonados ha restaurado la diversidad de especies, y ha comenzado a sugerir que los borregos apoyan la dispersion de semillas entre los pastizales. Probamos los efectos del pastoreo rotacional sobre la conectividad genetica y demografica de las plantas especialistas de los pastizales calcareos y si la respuesta de las poblaciones de plantas al pastoreo estaba limitada a especies dispersadas por animales (zoocoria). En especifico, probamos modelos de competencia de dispersion y propiedades de fragmento focales y de origen para explicar la conectividad de paisaje con la informacion de ocupacion de fragmento de 31 especies. Ajustamos los mismos modelos de conectividad a la ocupacion de fragmento y a la informacion de microsatelites nucleares para la

hierba Dianthus carthusianorum. Para 27 especies, la conectividad de fragmento se explico por la dispersion por pastoreo rotacional sin importar las adaptaciones a la zoocoria, mientras que el tamano de poblacion (16% de especies) y el area de fragmento (0% de especies) de fragmentos de origen no fueron importantes para predecir la ocupacion de fragmento de la mayoria de las especies. [Correction made after online publication, February 25, 2014: Population size and patch area percentages were mistakenly inverted, and have now been fixed.] La La diversidad de micrositios de los fragmentos focales incremento significativamente el modelo de varianza explicado por la ocupacion de fragmento en el 90% de las especies. Para D. carthusianorum, la conectividad de fragmento por medio del pastoreo rotacional explico tanto la ocupacion de fragmento como la diversidad de genetica de poblaciones. Nuestros resultados sugieren que el pastoreo proporciona dispersion para muchas especies de plantas sin importar sus adaptaciones a la dispersion y por lo tanto ofrece un acercamiento util para restaurar la diversidad de plantas en pastizales calcareos fragmentados.

3817: *+.226*

Understanding and evaluating the factors that influence the persistence of small populations and establishment of new populations are basic goals of conservation biology. Genetic effects due to genetic drift and inbreeding can have important impacts on the success of new populations. Many bighorn sheep populations in western North America have had low numbers and many have gone extinct. Here, the possible effects of genetic drift and inbreeding are evaluated in three populations of desert bighorn sheep initiated in the 1970s from translocations. One of these has no molecular genetic data but has substantial demographic data (Aravaipa Canyon), one has both extensive demographic data and some molecular genetic data (Red Rock), and one has limited demographic data and some molecular genetic data (Tiburon Island). Overall, either from theoretical pedigree analysis and population genetic estimates from demographic history (Aravaipa, Tiburon) or from molecular data (Red Rock, Tiburon), it appears that the levels of genetic drift and inbreeding are substantial in all of these populations. This impact was larger when higher variance in male reproductive success was assumed. In other words, it appears that genetic factors are and will be important in the establishment and persistence of these populations. These examples in long-term monitored bighorn sheep populations are relevant to many endangered species in similar situations where demographic data are available but there is little or no historical molecular genetic data.

3818: *+.162*

Managers of reintroduced lion (Panthera leo) populations in small reserves (<1000 km(2)) in South Africa are challenged by high rates of population increase and how best to control them. We combined data from 14 small, fenced reserves to evaluate growth rate parameters and compared them to those in larger and/ or open reserves. Growth rates of lions in small fenced reserves were only matched by those in Nairobi National Park (NP), which is relatively small and where the majority of the subadults emigrated away from the park. Initially, South African managers unconsciously mimicked this system by removing subadults to control population numbers, but increasingly chose euthanasia and hunting in the past decade, as the demand for wild lions for translocation decreased. They have, however, expressed a desire to use other methods of population control and mimic other open systems such as Kruger NP and Serengeti NP. Kruger NP had older ages of first reproduction and longer inter-birth intervals that could be mimicked through selective contraception. Alternatively, Serengeti NP had smaller litter sizes and lower cub survival, which could be mimicked through surgery to reduce litter sizes and, less attractively as it still involves lethal management and raises serious ethical concerns, selective culling of cubs. Mimicking Kruger NP may be more desirable as it is more ecologically similar to the small

reserves than SerengetiNP. This understanding of the current situation, and howit could be altered to more closely mimic natural systems, will facilitate the development of a metapopulation-based management plan for lions in small reserves in South Africa.

3819: +.115

The definition of conservation units is crucial for the sustainable management of endangered species, though particularly challenging when recent and past anthropogenic and natural gene flow might have played a role. The conservation of the European grayling, Thymallus thymallus, is particularly complex in its southern distribution area, where the Adriatic evolutionary lineage is endangered by a long history of anthropogenic disturbance, intensive stocking and potentially widespread genetic introgression. We provide mtDNA sequence and microsatellite data of 683 grayling from 30 sites of Adriatic as well as Danubian and Atlantic origin. We apply Bayesian clustering and Approximate Bayesian Computation (ABC) to detect microgeographic population structure and to infer the demographic history of the Adriatic populations, to define appropriate conservation units. Varying frequencies of indigenous genetic signatures of the Adriatic grayling were revealed, spanning from marginal genetic introgression to the collapse of native gene pools. Genetic introgression involved multiple exotic source populations of Danubian and Atlantic origin, thus evidencing the negative impact of few decades of stocking. Within the Adige River system, a contact zone of western Adriatic and eastern Danubian populations was detected, with ABC analyses suggesting a historical anthropogenic origin of eastern Adige populations, most likely founded by medieval translocations. Substantial river-specific population substructure within the Adriatic grayling Evolutionary Significant Unit points to the definition of different conservation units. We finally propose a catalog of management measures, including the legal prohibition of stocking exotic grayling and the use of molecular markers in supportive- and captive-breeding programs.

3820: +.123

The water quality of the River Rhine has improved and might again suit the critically endangered European sturgeon A. sturio L, which was extirpated from the river by 1950. This study describes the tracking of 43 juvenile hatchery-reared A. sturio, in the Dutch part of the Lower Rhine and Delta, originating from an ex situ measure of the River Gironde population. Observing in situ juvenile downstream migration could help to identify essential habitats and potential threats, before actual stocking. Fish were implanted with transponders of the NEDAP TrailA (R) system and released in two batches, in May (n = 13) and June 2012 (n = 30). Detections collected (n = 26) exhibited no upstream movement. Test-fish moved downstream with the flow. Because the historic estuary is disconnected from the North Sea by a sea lock "Haringvlietdam", the migration of the fish followed the re-directed river discharge into the Port of Rotterdam (161 km). 96 % (n = 19) of the detections was collected from the harbor in brackish water, where fish presumably acclimatized to higher salinities. 14 % (n = 6) of the sturgeons were recaptured in coastal waters by beam trawlers, five within 1 month after release. It is concluded that sustainable coastal fisheries is a key-condition for rehabilitation of the European sturgeon. Adapted management of the sea lock will reconnect the estuary to the North Sea and create more favorable conditions for the species in the Lower Rhine and Delta.

3821: +.096

Sustained demographic studies are essential for early detection of species decline in time for effective management response. A paucity of such background data hindered the potential for

successful conservation during the global amphibian decline and remains problematic today. The current study analysed 6 years of mark-recapture data to determine the vital demographic rates in three habitat precincts of the threatened frog, Litoria aurea (Hylidae) and to understand the underlying causes of variability in population size. Variability in population size of L. aurea was similar to many pond-breeding species; however this level of fluctuation is rare among threatened amphibians. Highly variable populations are at greater risk of local extinction and the low level of connectivity between L. aurea populations means they are at a greater risk of further decline due to stochastic extinction events and incapacity to recolonize distant habitat. We recommend that management of this species should encourage recolonization through creation of habitat corridors and reintroduction of L. aurea to areas where stochastic extinction events are suspected.

3822: ***+.139***

Burrowing, herbivorous mammals play important roles as ecosystem engineers and keystone species of grassland ecosystems around the world, but populations of many species have declined dramatically because of myriad threats from human activities. Prairie dogs (Cynomys spp.) play important roles in shaping the central grasslands of North America, and have declined by about 98% across their range, with consequent losses in associated species and grassland habitat. This has prompted much interest in restoring their populations to protected areas. Managers lack a clear understanding of the long-term success of reintroductions, however, and how success may vary across different species of prairie dogs and their widespread geographic ranges. We reintroduced over 1,000 Gunnison's prairie dogs (C. gunnisoni) to a semiarid grassland ecosystem in the southern portion of their range in central New Mexico, USA, and used standard capture-recapture methods to study their population dynamics over a period of 8 years. Mean adult survival was 27% over the course of the study, with precipitation identified as the primary driver of survival. Estimated survival was below 12% during severe drought periods and during the first few years following initial reintroduction, the latter likely because of high predation. Consequently, multiple releases of animals were required to prevent extirpation, and the long-term sustainability of this population remains questionable. Over the 8 years of our study, our site experienced 4 severe droughts during spring, the key period for prairie dog mating, pregnancy, and lactation. Production of offspring at the site was low, likely because of the dry and variable conditions that occurred. We show that prairie dog restoration in semi-arid grassland environments that are typical of the lower elevations and southern extent of their range may not succeed in producing viable colonies, and that dedicated management for multiple years is needed to counteract periods of slow or negative population growth. Our findings underscore the importance of maintaining and expanding existing colonies wherever possible in these more arid regions, and suggest that reintroductions should be treated as a secondary management strategy. Our work also reveals the high vulnerability of prairie dog population extinction due to drought, which has important implications for Gunnison's prairie dog conservation under a warming and drying climate. (C) 2014 The Wildlife Society.

3823: ***+.204***

European settlement led to extirpation of native Audubon's bighorn sheep (formerly Ovis canadensis auduboni) from North Dakota during the early 20th century. The North Dakota Game and Fish Department subsequently introduced California bighorn sheep (formerly O. c. californiana) that were indigenous to the Williams Lake region of British Columbia, Canada, and Rocky Mountain bighorn sheep (O. c. canadensis) that were indigenous to the Sun River region of Montana. Although California bighorn sheep are no longer recognized as a distinct subspecies, they are smaller and adapted to a milder climate than either the native bighorn sheep of North Dakota or introduced bighorn sheep from Montana. Because reintroductions still play a key role in

the management of bighorn sheep and because local adaptation may have substantial demographic consequences, we evaluated causes of variation in recruitment of bighorn sheep reintroduced in North Dakota. During 2006-2011, Montana stock recruited 0.54 juveniles/adult female (n = 113), whereas British Columbia stock recruited 0.24 juveniles/adult female (n = 562). Our most plausible mixed-effects logistic regressionmodel (53% ofmodel weight) attributed variation in recruitment to differences between source populations (odds ratio = 4.5; 90% CI = 1.5, 15.3). Greater recruitment of Montana stock (fitted mean = 0.56 juveniles/adult female; 90% CI = 0.41, 0.70) contributed to a net gain in abundance (r = 0.15), whereas abundance of British Columbia stock declined (fitted mean = 0.24 juveniles/adult female; 90% CI = 0.09, 0.41; r = -0.04). Translocations have been the primary tool used to augment and restore populations of wild sheep but often have failed to achieve objectives. Our results show that ecotypic differences among source stocks may have long-term implications for recruitment and demographic performance of reintroduced populations. Published 2014. This article is a U.S. Government work and is in the public domain in the USA.

3824: *+.002*

The black-footed ferret, Mustela nigripes, is an endangered carnivore endemic to the grasslands of North America. We present the first investigation of ectoparasites associated with black-footed ferrets since reintroduction. We sampled more than 200 individuals from one of the largest and most successful reintroduced populations located in the Conata Basin of South Dakota, USA. We compared our findings with ectoparasite assemblages of sympatric carnivores and historic ferret records. We collected more than 1,000 ectoparasites consisting mainly of three flea and tick species, two of which were known historically from South Dakota. Despite our extensive sampling efforts, we did not detect any lice. This is notable because a putative host-specific louse, Neotrichodectes sp., was presumed to have one extinct when black-footed ferrets were extirpated from the wild. The ectoparasite assemblage on black-footed ferrets comprised only generalist parasites, particularly those found on their prey such as prairie dogs (Cynomys sp.). Oropsylla hirsuta was the most abundant ectoparasite, representing 57% of all ectoparasites detected; a flea vector important in the persistence and transmission of plague. Black-footed ferrets like other endangered species undergo repeated parasite removal and vaccination efforts to facilitate population recovery, which may have unintentionally contributed to their depauperate ectoparasite community.

3825: *+.048*

Sixty (19 male, 41 female) free-ranging adult eastern bettongs (Bettongia gaimardi) were captured in Tasmania and translocated to the Australian Capital Territory between July 2011 and September 2012 for reintroduction into fenced, predator-proof reserves. The bettongs were anesthetized for physical examination and screened for selected diseases during translocation. Reference ranges for hematologic and biochemical parameters were determined. Two bettongs had detectable antibodies to the alphaherpesviruses macropodid herpesvirus 1 and macropodid herpesvirus 2 by serum neutralization assay. A novel gammaherpesvirus was detected, via PCR, from pooled swabs collected from the nasal, conjunctival, and urogenital tract mucosa of four other bettongs. Sera from 59 bettongs were negative for antibodies to Toxoplasma gondii as assessed by both the modified agglutination test and the direct agglutination test (n=53) or by the modified agglutination test only (n=6). Rectal swabs from 14 bettongs were submitted for bacterial culture and all were negative for Salmonella serovars. Ectoparasites identified on the bettongs included fleas (Pygiopsylla zethi, Stephanocircus harrisoni), a louse (Paraheterodoxous sp.), mites (Guntheria cf. pertinax, Haemolaelaps hatteni, a suspected protonymph of Thadeua sp.,

Cytostethum tasmaniense, Cytostethum intermedium, Cytostethum thetis, Cytostethum wallabia), and ticks (Ixodes cornuatus, Ixodes trichosuri, Ixodes tasmani). An intraerythrocytic organism morphologically consistent with a Theileria species was identified in blood smears from four bettongs. These data provide baseline health and disease information for free-ranging eastern bettongs that can be used for the conservation management of both the source and translocated populations.

3826: *+.174*

During the past 150 years forest management has dramatically altered in Central European woodlands, with severe consequences for biodiversity. Light forests that fulfilled variable human demands were replaced by dark high forests that function solely as wood plantations. In the Alps, by contrast, open woodlands are still present because the traditional land use as wood pasture has remained and physiographical conditions favour natural dynamics. The aim of our study was to investigate the effects of succession on the Orthoptera communities of alluvial pine woodlands in the northern Alps. Orthoptera showed a clear response to succession, with each successional stage harbouring a unique assemblage. The influence of succession on species richness and abundance were identical: The values were highest in the intermediate and lowest in the late seral stage. The diversity and abundance peak in the mid-successional stage probably reflects a trade-off between favourable ambient temperatures for optimal development and sufficient food, oviposition sites and shelter against predators. Food shortage and easy access for predators seemed to be limiting factors in the early successional stage. In contrast, in the late successional stage adverse microclimatic conditions probably limit Orthoptera occurrence. Although all three successional stages of the pine woodlands are relevant for conservation, the early and mid-successional stages are the most important ones. Conservation management for Orthoptera in this woodland type should aim at the reintroduction of cattle grazing and the restoration of the natural discharge and bedload-transport regimes of the alpine rivers.

3827: *+.102*

The Mercury Islands tusked weta, Motuweta isolata (Orthoptera: Anostostomatidae), survived only on 13 ha Ahu or Middle Island, a mammal-free island in the Mercury Group, New Zealand. Between 2000 and 2009, 567 individuals were translocated in nine releases to six nearby islands from which mammals had been removed. These translocations occurred to reduce the chance of accidental extinction of the Middle Island population of only a few hundred adults and to contribute to the restoration of the other islands. All translocated insects originated from the captive-bred progeny of one male and two females collected from Middle Island between 1998 and 2001. Their establishment on Double and Red Mercury Islands, after their releases in 2000 and 2001 respectively, was confirmed by searching plots, and by using footprint tracking tunnels on Red Mercury Island between 2008 and 2012. Tracking tunnels provided better data and proved more cost effective than searching plots for detecting large tusked weta. Tracking tunnels demonstrated that the population on Red Mercury expanded outwards from the release sites by 50-100 m each year between 2009 and 2012. These weta are now estimated to be present over more than half the Island. Tusked weta have also survived on Stanley, Korapuki and Ohinau Islands after releases in 2007, but they remain within 100 m of the release sites. No confirmed progeny of the weta released on Cuvier Island in 2008 and 2011 were detected. No tusked weta were detected on Middle Island using tracking tunnels on eight occasions between 2009 and 2012, suggesting this species is likely to be locally extinct. Despite possible failure on one island, these translocations have resulted in a significant conservation success outcome.

3828: ***+.183***

The use of population modelling has become an increasingly common tool in reintroduction planning and assessment. Although initial reintroduction success is often measured by quantifying post-release survival and reproduction, longer-term success is best assessed through measurements of population viability. Here we develop a population model capable of providing useful results for influencing management of a reintroduction programme for a long-lived and slow-reproducing primate, the western lowland gorilla Gorilla gorilla gorilla. We used post-release monitoring data from two reintroduced populations in the Bateke Plateau region of Congo and Gabon, complemented with published data on wild and captive populations, to develop a population model using Vortex. Sensitivity testing illustrated that the model was highly sensitive to changes in the input parameters for annual birth rates, the number of lethal equivalents, and for female annual mortality rates, especially for adults. The results of the population viability analysis suggested that the reintroduced gorilla populations have a reasonable chance of persistence (>90% over 200 years) but illustrated that reinforcement of the populations could significantly improve probabilities of population persistence and retention of genetic diversity. Equally, catastrophic events could have significant negative impacts. Continued monitoring of the populations should allow refinement of the model, improving confidence in its predictions and its relevance to decision-making.

3829: ***-.046***

Many endemic species, particularly those on remote islands, have been driven to extinction or near extinction by anthropogenic influences. The short-tailed albatross Phoebastria albatrus once numbered in the millions but was thought to be extinct by the mid 20th century. Albatrosses, of the family Diomedeidae, are among the most threatened birds globally as a result of commercial exploitation, introduced predators, and mortality in commercial fisheries. We applied an experimental approach over 5 years to evaluate the translocation and hand-rearing of albatross chicks by comparing growth, physiological health indices, post-fledging survival, and migration patterns with a control group of naturally reared chicks in the source population. Hand-reared chicks had comparable or superior health and similar rates of immediate post-fledging mortality (15%), with mortality strongly female-biased in both groups. Hand-reared birds had longer post-fledging drift periods before attaining sustained flight (also female-biased) but comparable, albeit somewhat wider ranging, migration patterns to naturally reared chicks during their first 6 months at sea. Recruitment to the translocation site of a breeding pair that included a hand-reared bird occurred within 5 years of the first translocation. Success will ultimately depend on continued recruitment and breeding over the coming decades, given delayed breeding in these long-lived species. The results to date, however, have exceeded initial expectations and can inform potential reintroductions of other long-lived, migratory avian species with strong natal philopatry, and reintroductions of native species to former breeding islands.

3830: ***+.113***

North American elk (Cervus elaphus L., 1758) are an important component of Canada's natural ecosystems. Overhunting and habitat decline in the 19th century led to the near eradication of Rocky Mountain elk (Cervus elaphus nelsoni Bailey, 1935) and Manitoban elk (Cervus elaphus manitobensis Millais, 1915) within Alberta. Though elk populations have been restored within provincial and national parks, it is unknown to what degree historic population declines affected overall genetic diversity and population structuring of the two subspecies. This study targeted 551 bp of mitochondrial D-loop DNA from 50 elk remains recovered from 16 archaeological sites

(2260 BCE (before common era) to 1920 CE (common era)) to examine the former genetic diversity and population structure of Alberta's historic elk populations. Comparisons of ancient and modern haplotype and nucleotide diversity suggest that historic population declines reduced the mitochondrial diversity of Manitoban elk, while translocation of animals from Yellowstone National Park in the early 20th century served to maintain the diversity of Rocky Mountain populations. Gene flow between the two subspecies was significantly higher in the past than today, suggesting that the two subspecies previously formed a continuous population. These data on precontact genetic diversity and gene flow in Alberta elk provide essential baseline data integral for elk management and conservation in the province.

3831: *+.168*

Appropriate definitions of species limits are critical for scientific study and conservation management. Many subspecies of birds are defined on the basis of phenotypic variation. However, it has been suggested that following close examination many of these subspecies will be recognised as good species. North Island (NI) (Philesturnus rufusater) and South Island (SI) saddlebacks (P. carunculatus) differ in plumage and apparent vulnerability to introduced predators and, until recently, were considered subspecific. Here, we formally compare vocalisations, morphology and mtDNA between NI and SI saddlebacks. We show significant differences in male song and chatter calls which are fundamental saddleback vocalisations. A discriminant analysis correctly allocated 90% of birds based on significantly larger tarsus, weight and wing measurements in SI saddlebacks relative to NI saddlebacks. Finally, molecular data show c. 5% sequence divergence in mtDNA between the two groups. We concur with previous work elevating NI and SI saddleback to full species.

3832: *+.035*

Arable weeds have severely declined due to intensification of farming during the past decades, and some of them are close to extinction in large parts of western and central Europe. Recent conservation strategies stggest reintroduction of rare arable weeds to suitable types of land use, for example organic fields. To test the feasibility of this conservation option we investigated the effects of spring wheat and a common weed (Stellaria media) on growth and reproduction of a rare weed (Legousia speculum-veneris) in a semi-open glasshouse experiment. Additionally, the impact of the weeds on crop yield was assessed. Aboveground biomass and seed production of L. speculum-veneris were significantly reduced under competition with the crop and the common arable weed. With increasing competition L. speculum-veneris allocated disproportionally more resources to seeds. Wheat yield was not affected by the arable weeds. We conclude that L. speculum-veneris is a subordinate species that is negatively affected by more competitive plants. In the presence of wheat the addition of other weeds does not further harm the species. Enhanced allocation to reproduction might be a specific strategy of the species to maintain populations under competition. Reduced crop densities would help reintroducing the species, while yield losses due to reintroduction of that rare arable weed are unlikely. (c) 2014 Elsevier B.V. All rights reserved.

3833: *+.040*

Ancient plant species surviving in isolated small populations are particularly vulnerable to extinction, therefore understanding their population dynamics is necessary for conservation. The iteroparous perennial relic endemic Ferula sadleriana Ledeb. (Apiaceae) is restricted to seven distant localities in the Carpathian Basin, where it inhabits rocky hills. We monitored the species' largest population on the Pilis Hill, Hungary, over 14-19 years (depending on trait) between 1979

and 2010, and relationships were sought between climatic properties and population attributes. The population of 4000 +/- 1509 emergent individuals underwent large interannual fluctuations, with the vegetative stage displaying sevenfold and the reproductive stage twenty-eight-fold differences. Spring and early summer precipitation had a marked influence on abundances and seed set. Alternating years of high and low counts of reproductive plants suggest costs of reproduction that most probably incur prolonged dormancy and retrogression to the vegetative stage. Seed set was positively influenced by number of reproductive plants over years and by plant size within a year. Ungulates nullify yearly reproductive output by grazing on reproductive individuals. This is particularly intense in dry summers, when reproductive output is already low. The strong precipitation response of abundance, absence of clonal propagation and soil seed bank, and geographical isolation of the populations place F.sadleriana at considerable risk under an increasingly variable and extreme climate. Management should seek to maintain the species' original habitat mosaic (potentially compensating for climate variation), minimize grazing damage and anthropogenic disturbance, and establish ex situ conservation programs to provide propagules for eventual reintroduction.

3834: *+.152*

Understanding how the environmental context modifies the strength of trophic interactions within food webs forms a central challenge in community ecology.Here, we demonstrate the necessity of considering the influence of climate, landscape heterogeneity and demographics for understanding trophic interactions in a well-studied food web in Yellowstone National Park, USA. We studied riparian willow (Salix spp.) establishment and stem growth reconstructed from tree rings on the northern range of Yellowstone over a 30-year period that included the reintroduction of a top predator, the grey wolf (Canis lupus). We used climate variables (annual precipitation, stream flow and growing season length), herbivore abundance and landscape descriptors (elevation and topographic wetness index) to predict establishment and growth processes through time before and after the reintroduction of wolves. We fitted Bayesian hierarchical models to establishment data and time series of individual stem heights from 1980 to 2008. Explaining variability in establishment required models with stream flow, annual precipitation and elk abundance.Climate, trophic and landscape covariates interacted with stem age to determine stem height and growth rate through time. Growth rates of most stems ages (2+) declined after the reintroduction of wolves. However, stem growth rates naturally declined with age, and the decline we observed was coincident with faster growth rates for the youngest stems. Mean stem heights at age have remained relatively stable through time for most age classes. Estimated effects of landscape topography had approximately the same magnitude of effect on stem growth rate at age as elk abundance.Synthesis. We show that the effects of modification of a food web cannot be predicted by studying trophic dynamics in isolation. No single driver explained patterns of willow establishment and growth over the past three decades in Yellowstone. Instead, interactions among trophic forces, interannual climate variability and landscape topography together shaped how the ecosystem responded to perturbations. Top-down effects of ungulates on riparian woody vegetation must be considered in the context of plant age, and climate and landscape heterogeneity.

3835: *+.176*

AimRetrospective genetic monitoring, comparing genetic diversity of extant populations with historical samples, can provide valuable and often unique insights into evolutionary processes informing conservation strategies. The Yellow marsh saxifrage (Saxifraga hirculus) is listed as 'critically endangered' in Ireland with only two extant populations. We quantified genetic changes

over time and identified genotypes in extant populations that could be used as founders for reintroductions to sites where the species is extinct.LocationIreland. MethodsSamples were obtained from both locations where the species is currently found, including the most threatened site at the Garron Plateau, Co. Antrim, which held only 13 individuals during 2011. Herbarium samples covering the period from 1886 to 1957 were obtained including plants from the same area as the most threatened population, as well as three extinct populations. In total, 422 individuals (319 present-day and 103 historical) were genotyped at six microsatellite loci. Species distribution modelling was used to identify areas of potentially suitable habitat for reintroductions. ResultsLevel of phenotypic diversity within the most threatened population was significantly lower in the present-day compared with historical samples but levels of observed heterozygosity and number of alleles, whilst reduced, did not differ significantly. However, Bayesian clustering analysis suggested gradual lineage replacement over time. All three measures of genetic diversity were generally lower at the most threatened population compared with the more substantial extant populations in Co. Mayo. Species distribution modelling suggested that habitat at one site where the species is extinct may be suitable for reintroduction. Main conclusionsThe dominant genetic lineage in the most threatened population is rare elsewhere; thus, care needs to be taken when formulating any potential reintroduction programme. Our findings highlight both the need for genetic monitoring of threatened populations, but also for its swift implementation before levels of diversity become critically low.

3836: *+.375*

About a quarter of Chinese wild orchid species are used in traditional medicine or as health food supplements. The market demand for some species, such as those in the epiphytic genus Dendrobium, has diminished many wild populations to local extinction or dangerously small numbers. Conservation of these heavily exploited orchids currently relies on a two-pronged approach: establishing nature reserves and encouraging massive commercial cultivation in artificial settings. We argue that these measures are not sufficient to restore or maintain healthy wild populations, and augmentation and reintroduction of these species in natural forests are needed. We argue for an unconventional reintroduction approach, in which populations planted in natural forests are allowed to be sustainably harvested (restoration-friendly cultivation). Because Dendrobium orchids are epiphytic, restoration-friendly cultivation of these species will not be at the expenses of other native plants. In addition, market premiums on wild-collected medicinal plants will generate incentives for farmers who participate in restoration-friendly cultivation to preserve natural forests. With proper policy and oversight, the restoration-friendly cultivation of medicinal Dendrobium orchids will facilitate the conservation of these threatened species, encourage protection of natural forests, and benefit marginalized rural communities. Adding this restoration-friendly cultivation into the current mix of conservation approaches has the potential to turn deeply-entrenched traditional uses of orchids from a conservation challenge to a conservation success.

3837: *+.161*

The recent decline in pollinator biodiversity, notably in the case of wild bee populations, puts both wild and agricultural ecosystems at risk of ecological community collapse. This has triggered calls for further study of these mutualistic communities in order to more effectively inform restoration of disturbed plant-pollinator communities. Here, we use a dynamic network model to test a variety of translocation strategies for restoring a community after it experiences the loss of some of its species. We consider the reintroduction of extirpated species, both immediately after the original loss and after the community has reequilibrated, as well as the introduction of other native species

that were originally absent from the community. We find that reintroducing multiple highly interacting generalist species best restores species richness for lightly disturbed communities. However, for communities that experience significant losses in biodiversity, introducing generalist species that are not originally present in the community may most effectively restore species richness, although in these cases the resultant community often shares few species with the original community. We also demonstrate that the translocation of a single species has a minimal impact on both species richness and the frequency of community collapse. These results have important implications for restoration practices in the face of varying degrees of community perturbations, the refinement of which is crucial for community management.

3838: ***+.154***

The genus Leontodon L. (Asteraceae) comprises approximately 50 species with a natural distribution area covering North America, Europe, northern Africa, and western Asia. Two of these species are endemic to the Azores Archipelago: Leontodon filii and Leontodon rigens. Although both species were targeted with several taxonomic revisions, so far no studies into their genetic diversity have been carried out. In this research, the population genetic structure and diversity of both taxa were assessed using five newly developed SSR markers. Four hundred and thirty-seven individuals collected throughout the archipelago were included in the study. A total of 98 alleles (25-12 per locus, average = 19.6) and an overall excess of homozygotes (multilocus F (is) = 0.37, range 0.16-0.53) were found for L. rigens populations. For L. filii, 52 alleles in total (8-13 per locus, average = 10.4) were found, overall near the HW equilibrium (multilocus F (is) = 0.07, range -0.25 to 0.57). The two species showed an equivalent proportion of rare alleles (L. rigens 80.6 %; L. filii 76.9 %). Both a Principal Coordinate Analysis and a Bayesian analysis proposed the existence of two well-defined groups, but pooled L. filii populations from Faial Island with L. rigens populations. The largest proportion of genetic variability was found within populations (L. rigens 72.6%; L. filii 78.9 %). The highest values of gene flow were obtained for L. filii within the central group of islands. Our results update the current distribution given for the Azorean Leontodon taxa, clearly indicating that conservation measures should be applied to several populations. The results also reveal that a revision of the Azorean Leontodon should be carried out to clarify species delimitation.

3839: ***+.280***

Amitostigma hemipilioides is an endangered terrestrial orchid endemic to China. In the Southwestern China, the species is found restricted growing in the karst limestone or rock in the edge of farmland, moist hillsides or river. In the present study, the genetic diversity and differentiation was estimated within and among habitats, populations and groups of this species by ISSR markers. Using 13 polymorphic primers, an intermediate level of genetic diversity was found at the species level and population level with the percentage of polymorphic bands (P) of 64.7 and 50.9 %, Shannon index of diversity (I) of 0.3873 and 0.2949, respectively. The analysis of molecular variance (AMOVA) showed that the high level of population differentiation was presented with 45.63 % relative to the total genetic variation residing among eight populations. It was noteworthy that as much as 69.17 % of the total diversity was most likely attributed to the difference among the populations in fragmentation habitat conditions, while 16.32 % of the total diversity could be attributed to the difference among the populations in stable and favorable habitat conditions. The in situ conservation is a top strategy, thus the mycorrhizal fungi and pollinators are protected by protecting habitat and avoiding fragmentation which is helpful to the cycle of this endangered orchid species and recovery of its wild populations. In addition, to maintaining the germplasm bank of this species, the ex situ conservation by habitat simulation and

reintroduction has been considered in the living collection at Kaili University ecological garden and Yuntai Mountain scenic spot using the plants collected in this study.

3840: *+.062*

A number of phylogeographic studies have revealed the existence of multiple ice age refugia within the Balkan Peninsula, marking it as a biodiversity hotspot. Greece has been reported to harbor genetically differentiated lineages from the rest of Balkans for a number of mammal species. We therefore searched for distinct red deer lineages in Greece, by analyzing 78 samples originating from its last population in Parnitha Mountain (Central Greece). Additionally, we tested the impact of human-induced practices on this population. The presence of 2 discrete mtDNA lineages was inferred: 1) an abundant one not previously sampled in the Balkans and 2) a more restricted one shared with other Balkan populations, possibly the result of successful translocations of Eastern European individuals. Microsatellite-based analyses of 14 loci strongly support the existence of 2 subpopulations with relative frequencies similar to mitochondrial analyses. This study stresses the biogeographic importance of Central Greece as a separate Last Glacial Maximum period refugium within the Balkans. It also delineates the possible effects that recent translocations of red deer populations had on the genetic structuring within Parnitha. We suggest that the Greek red deer population of Parnitha is genetically distinct, and restocking programs should take this genetic evidence into consideration.

3841: *+.060*

Robertsonian (Rb) translocations, in particular centric fusions, are thought to play a primary role in evolution and speciation of the Bovidae family. However, Rb fusions are often polymorphic within species, being suggested as phylogenetically uninformative characters. This work studies chromosome variation in 72 captive individuals of 6 species of Alcelaphini (Antilopinae): The hartebeest (genus Alcelaphus), hirola (Beatragus), black and blue wildebeests (Connochaetes), and the topi and bontebok (Damaliscus). We infer the phylogenic relationships among Alcelaphini species and determine patterns of chromosomal evolution using G-banded karyotypes and complete mitochondrial genome sequences. The molecular phylogeny showed an early divergence of Connochaetes, followed by the split of Alcelaphus plus Beatragus Damaliscus as sister taxa. Mitochondrial and chromosomal phylogenies only differed in the position of the critically endangered Beatragus, likely due to homoplasic chromosome characters. Patterns of chromosome evolution, reconstructed using a probabilistic approach, suggest that chromosome changes leading to speciation in Alcelaphini do not exclusively involve consecutive reduction of diploid number through centric fusion but also the losses and reversions of Rb translocations in Beatragus and Damaliscus lineages. Our results provide evidence that complex scenarios of chromosomal rearrangements can be detected in relatively recent-diverged bovids, as in this group of antelopes.

3842: *+.205*

Translocations are an increasingly common tool in conservation. The maintenance of genetic diversity through translocation is critical for both the short- and long-term persistence of populations and species. However, the relative spatio-temporal impacts of translocations on neutral and functional genetic diversity, and how this affects genetic structure among the conserved populations overall, have received little investigation. We compared the impact of translocating different numbers of founders on both microsatellite and major histocompatibility complex (MHC) class I diversity over a 23-year period in the Seychelles warbler (Acrocephalus sechellensis). We found low and stable microsatellite and MHC diversity in the source population

and evidence for only a limited loss of either type of diversity in the four new populations. However, we found evidence of significant, but low to moderate, genetic differentiation between populations, with those populations established with fewer founders clustering separately. Stochastic genetic capture (as opposed to subsequent drift) was the main determinant of translocated population diversity. Furthermore, a strong correlation between microsatellite and MHC differentiation suggested that neutral processes outweighed selection in shaping MHC diversity in the new populations. These data provide important insights into how to optimize the use of translocation as a conservation tool.

3843: *+.100*

Hares in the northern Iberian Peninsula have been managed by local authorities primarily as a game species for hunting. Over the last 4 decades, populations have been restocked by means of translocation from other regions. We examined the genetic structure of wild populations of 2 species living in the northern Iberian Peninsula: the brown hare (Lepus europaeus) and the Iberian hare (L. granatensis) to assess their genetic uniqueness and distinctiveness, and thus determine if these populations have been altered by anthropogenic translocations. We analyzed 215 sequences of the mitochondrial DNA (mtDNA) control region and 6 microsatellite markers in 157 individuals from northern Iberia. We detected 3 distinct mtDNA types in L. europaeus (timidus, granatensis, and europaeus mtDNA lineages) and 2 mtDNA types in L. granatensis, (timidus and granatensis lineages). The high and homogeneous prevalence of a north European timidus mtDNA lineage in both L. europaeus and L. granatensis may be the result of an ancient introgression during the last glacial period. The homogeneous distribution of the granatensis mtDNA lineage in L. europaeus may also be the result of gene flow between species in a contact zone. In contrast, both the low prevalence of the europaeus mtDNA lineage and its highly localized presence in the center of Guipuzcoa are strongly indicative of the recent incorporation of non-Iberian L. europaeus into the local population. The data derived from microsatellite markers revealed that L. europaeus populations from the center of Guipuzcoa had a much higher assignment probability to eastern European populations than the rest of northern Iberian populations, which presented high assignment probabilities to the populations from protected areas of Cantabria and Huesca. Strict adherence to International Union for Conservation of Nature (IUCN) criteria strongly suggests that these translocations may not be beneficial, in that they compromise the genetic integrity of the native population of L. europaeus in the northern Iberian Peninsula. For this reason, we strongly recommend 1) translocations of L. europaeus individuals from non-Iberian populations into Iberian populations be avoided; 2) restocking the center of Guipuzcoa with autochthonous L. europaeus individuals; 3) carrying out periodic genetic surveys of L. europaeus throughout the study area; and 4) increasing our knowledge of the genetic structure of L. granatensis under natural conditions. (C) 2014 The Wildlife Society.

3844: *+.378*

The ecological processes that are crucial to an animal's growth, survival, and reproductive fitness have energetic costs. The imperative for an animal to meet these costs within the energetic constraints of the environment drives many aspects of animal ecology and evolution, yet has largely been overlooked in traditional ecological paradigms. The field of 'ecological energetics' is bringing comparative physiology out of the laboratory and, for the first time, is becoming broadly accessible to field ecologists addressing real-world questions at many spatial and temporal scales. In an era of unprecedented global environmental challenges, ecological energetics opens up the tantalising prospect of a more predictive, mechanistic understanding of the drivers of threatened species decline, delivering process-based modelling approaches to natural resource management.

3845: +.106

Bottlenecks, founder events, and genetic drift often result in decreased genetic diversity and increased population differentiation. These events may follow abundance declines due to natural or anthropogenic perturbations, where translocations may be an effective conservation strategy to increase population size. American black bears (Ursus americanus) were nearly extirpated from the Central Interior Highlands, USA by 1920. In an effort to restore bears, 254 individuals were translocated from Minnesota, USA, and Manitoba, Canada, into the Ouachita and Ozark Mountains from 1958 to 1968. Using 15 microsatellites and mitochondrial haplotypes, we observed contemporary genetic diversity and differentiation between the source and supplemented populations. We inferred four genetic clusters: Source, Ouachitas, Ozarks, and a cluster in Missouri where no individuals were translocated. Coalescent models using approximate Bayesian computation identified an admixture model as having the highest posterior probability (0.942) over models where the translocation was unsuccessful or acted as a founder event. Nuclear genetic diversity was highest in the source (A(R)=9.11) and significantly lower in the translocated populations (A(R)=7.07-7.34; P=0.004). The Missouri cluster had the lowest genetic diversity (A(R)=5.48) and served as a natural experiment showing the utility of translocations to increase genetic diversity following demographic bottlenecks. Differentiation was greater between the two admixed populations than either compared to the source, suggesting that genetic drift acted strongly over the eight generations since the translocation. The Ouachitas and Missouri were previously hypothesized to be remnant lineages. We observed a pretranslocation remnant signature in Missouri but not in the Ouachitas.

3846: +.051

Sequence variation of the mtDNA D-loop region was analyzed in order to investigate the intraspecific evolution and the population genetic structure of the critically endangered European eel Anguilla anguilla. An additional attempt was made to collect information on the genetic characteristics of groups of eels representing naturally recruited eels to Lithuania and Latvia and introduced into Lithuanian lakes. A total of 148 eels were investigated, and 107 different haplotypes attributed to 39 haplogroups were determined during the study. A dataset comprising 229 sequences was created using the data from both this study and from earlier studies (n = 81). Analysis of this dataset revealed that haplotype diversity was 0.99567, the average number of nucleotide differences was 12.50544, there were 145 polymorphic sites and nucleotide diversity was 0.02426. No significant genetic differentiation was detected between different combinations of samples. However, the population genetic structure of this species could be characterized as a genetic mosaic formed due to the existence of reproductively isolated groups. The existence of a genetic mosaic in this species could be explained by the different evolutionary lineages found in the eel population.

3847: +.024

The Spix's macaw (Cyanopsitta spixii) is the rarest parrot on earth. The remaining captive population consists of 79 individuals. Captive propagation is ongoing to increase the number of individuals for future reintroduction back into the wild. Unfortunately, from 2004 to 2012, only 33 chicks hatched from 331 eggs. Semen evaluation and assisted reproduction might help to overcome this problem. Therefore, a recently developed electro-stimulated semen collection technique was used in Spix's macaws. Semen collection was successful in 39 of 78 attempts in 10 out of 17 males. Examination of the semen included evaluation of volume, color, consistency, contaminations and pH, as well as determination of motility, viability, morphology, concentration,

and total count of spermatozoa. The median volume of semen samples was 5.6 mu l. On average, 34.7 +/- 21.9% (median 30%) of the sperm were motile and 23.1 +/- 22.1% (median 16.5%) were progressively motile. In addition to spermatozoa, round cells were detected in the samples. Median sperm concentration was 15,500/mu l (range 500-97,500/mu l) and median viability was 50% (range 5-87%). Morphological examination revealed in 26.5% normal spermatozoa, high numbers of malformations of the head (50.2%) and tail region (20.5%), with 29% of all sperm showing multiple abnormalities. Artificial insemination was performed in three females; two eggs laid after artificial insemination had spermatozoa present on the perivitelline layer, suggesting the possible success of the insemination technique. Although no fertilization could be demonstrated, these preliminary results are promising, as they indicate that assisted reproduction might be a tool for species conservation in the Spix's macaw. (C) 2014 Wiley Periodicals Inc.

3848: *+.123*

We examined the contribution of three types of side channels based on their hydrologic connectivity (seasonally disconnected, partially connected, and connected) to production of juvenile anadromous salmonids. Juvenile steelhead Oncorhynchus mykiss and Chinook Salmon O. tshawytscha were found in all three of these side channel types and in each year of the study. Upon connection with the main stem at high flows, the seasonally disconnected side channels experienced an emptying out of the previous year's fish while filling with young-of-year fish during the 2- to 4-month period of hydrologic connection. There were no differences between the densities of juvenile steelhead and Chinook Salmon and the rate of smolts produced among the three types of side channels. Recently reintroduced Coho Salmon O. kisutch had sporadic presence and abundance in partially and connected side channels, but the smolt production rate was over two times that of steelhead and Chinook Salmon in seasonally disconnected side channels. Within seasonally disconnected side channels, young-of-year salmonids in deep pools (>= 100cm) had greater survival than those in shallow pools (<100cm). Densities of juvenile steelhead in all side channel types were similar to those in tributaries and were higher than in main-stem lateral margins. Juvenile Chinook Salmon densities were higher in side channels than in both tributary and main-stem lateral margins. Our results suggest that improving quality of pool habitat within seasonally disconnected side channels can result in improved survival for juvenile anadromous salmonids during the period of disconnection. Habitat improvement in these seasonally disconnected side channels should be recognized as a worthy restoration strategy, especially when full connectivity of side channels may not be a feasible target (e.g., through lack of water availability) or when full connectivity may present too high a risk (e.g., flooding, stream capture, bank destabilization). Received August 30, 2013; accepted December 9, 2013

3849: *+.129*

The comeback of the Eurasian beaver (Castor fiber) throughout western and central Europe is considered a major conservation success. Traditionally, several subspecies are recognised by morphology and mitochondrial haplotype, each linked to a relict population. During various reintroduction programs in the 20th century, beavers from multiple source localities were released and now form viable populations. These programs differed in their reintroduction strategies, i.e., using pure subspecies vs. mixed source populations. This inhomogeneity in management actions generated ongoing debates regarding the origin of present beaver populations and appropriate management plans for the future. By sequencing of the mitochondrial control region and microsatellite genotyping of 235 beaver individuals from five selected regions in Germany, Switzerland, Luxembourg, and Belgium we show that beavers from at least four source origins currently form admixed, genetically diverse populations that spread across the study region. While

regional occurrences of invasive North American beavers (n = 20) were found, all but one C. fiber bore the mitochondrial haplotype of the autochthonous western Evolutionary Significant Unit (ESU). Considering this, as well as the viability of admixed populations and the fact that the fusion of different lineages is already progressing in all studied regions, we argue that admixture between different beaver source populations should be generally accepted.

3850: *+.209*

ex-situ conservation is one of the most important approaches to save the endangered species. When the population size of captive giant panda increase steadily and can be sustained by themselves, some of them should be released to the wild to rescue small populations and enlarge the distribution areas. Importantly, the first step is to find a suitable habitat for them. Huayingshan is the historical distribution area of giant panda and the fossil of A. melanoleuca baconi was found in 1993. Plentiful food resources (530 kiloton bamboos at present with 99 kiloton net gain per year) for giant panda was observed in this area which are enough for a small population. The government and villagers greatly support the project that sending giant panda back to Huayingshan, and the gentler slope of which will make monitoring work easier to perform. However, the human activity's interference was significant in this area, and some places are far from water resources, the vegetation type is less, and infection rate of the canine parvovirus was high. These questions can be solved after the reserve was built. In another side, the temperature is high in summer, and the qualities of habitats are different from the giant panda's natural habitat, in addition to the undetected impacts on the releasing of giant panda several works (e.g. wireless monitor) should be taken throughout all steps of the reintroduction to promote the giant panda's inhabit in Huayingshan.

3851: *+.253*

Forked spleenwort,Asplentrlonale (L) Hoffm., is mainly a petrophilous fern species of European mountains and very rare in Estonia,a country with flat topography and few topography and few rocky habitats. The single extant population is very small,occupies a restricted area,and is threatened seasonally by adverse human activity. An introduction rroject of the soecies was prepared according to the A, septentrionale Management Plan. The goal of the project is to introduce new populations of the species in new protected sites from ex situ propagated young sporophytes. The aim of this study was to obtain detailed data on the species ecolegy and provide inforнation for selecting suitable introduction sites. We carried out a factorial pot experiment on light avalability,soil pH and soil biota gradient in order to ascertain optimal growth and development conditions for A. septentrionale. Soil and light conditions must be considered the most important factors affecting species grown in abiotic conditions closely emulating the natural habit were the most successful and may, therefore,represent the optimal establishament potential. The survival of plants was greater, and plants gres larger with a higher number of longer leaves in acides soil (pH 4.9) than in neutral soil. Plants performed best under high, approximately 75% total available illumination. Deep shade (90%) and well as full daulight had storng negative effect on the plants. Nopositive effect of "home-soil" biota was detected. (C) 2014 Elsevier GmbH. All rights reserved.

3852: *+.247*

When a great need, the right people, and the right tools come together, history is sometimes made. From the late 1970s through the late 1980s that happened in California. At that time there was a need to capture elk, then deer and pronghorn, then bighorn sheep-the "big game species"- in

previously unprecedented numbers. The need focused primarily on translocation to re-establish populations in areas of historic range and to consolidate gains in lands available for wildlife conservation. These efforts also advanced wild ungulate research and management. The tools were helicopters, dart guns and new drugs, various ways to physically capture wildlife including net guns, and other advances in capture technology. The right people were a small group of California Department Fish and Game employees, contract pilots, graduate students, and a host of other agency personnel, friends and volunteers. The history they made lives on in the mountains, savannahs, deserts, and grasslands of California as a wildlife legacy of more elk, deer, pronghorn and bighorn that, with continued conservation, will pass from generation to generation of future Californians.

3853: *+.113*

We tested the hypothesis that brown bears were translocated from the Romanian Carpathians to Bulgaria via air transportation during the communist regime in the 1970s and 1980s. Microsatellite analysis was performed on 199 bear samples from Bulgaria and Romania. Assignment and admixture tests revealed the existence of seven genotypes (=2.8 %) in Bulgaria that were assigned with high probabilities to the Romanian population, supporting the translocation and successful establishment of Carpathian bears in Bulgaria. While we cannot rule out the possibility that active long-distance dispersal contributed to the observed pattern, the spatial distribution and sex ratio of the detected Romanian genotypes strongly favor the translocation hypothesis.

3854: *+.082*

Nine polymorphic microsatellite loci have been used to infer population genetic diversity and structure of the threatened Australian freshwater fish, Macquarie perch, across three tributaries of the Murrumbidgee River in south-eastern Australia. This investigation has revealed a high level of divergence among all three populations, along with contrasting patterns of genetic diversity. The Cotter Reservoir, which is a stronghold population for the species, has typically higher diversity and effective population size than nearby riverine populations. This suggests that the reservoir population is unlikely to have undergone a genetic bottleneck during and following dam construction. Genetic diversity estimates were comparable with one riverine site but were significantly higher than a population sampled from the Queanbeyan River. This comparison revealed significantly less heterozygotes in the Queanbeyan River and lower estimates of effective population size. Options and considerations for stock replenishment of this population are discussed.

3855: *+.220*

Conservation practitioners widely agree that optimal conservation strategies will maximize the amount of genetic variation preserved in target taxa, but there is ongoing debate about how that variation should be distributed through restoration and mitigation activities. Here, we evaluate the impacts of similar to 10 years of mitigation on the population genetic structure of Limnanthes vinculans, a state- and federally-listed endangered plant species restricted to ephemeral vernal pool wetlands in the Santa Rosa Plain of California. Using microsatellite loci to estimate patterns of neutral molecular variation, we found that created pools support similar levels of variation in L. vinculans as natural pools. Habitat creation and seed translocation have not disrupted the largest-scale patterns of population structure across the species range, but a concentration of mitigation activity towards the range center has reduced the extent of isolation-by-distance operating in this

region and shifted the location of at least one genetic boundary. Patterns of genetic variation among populations in remnant vernal pools reveal that gene flow has historically occurred beyond the scale of individual pools at the center of the species range, while small genetic populations have differentiated around the range margins. On average, L. vinculans in created pools exhibit less cover and more restricted local distributions than those in remnant pools, but these patterns were driven by two particularly productive natural sites rather than consistent differences between natural and created sites. We conclude that mitigation activities have changed the historical patterns of gene flow within the species range to a moderate degree, that these changes will likely impact remnant pools through gene flow, and that current created sites provide less heterogeneous habitat for L. vinculans than do natural pools. Studies that track individual plants will be needed to determine if the changes in gene flow due to mitigation will have positive or negative impacts on the demographic and microevolutionary trajectories of L. vinculans. More generally, this study provides a retrospective analysis of the outcome of managing an endangered plant species through intensive mitigation, and yields several insights to inform future conservation strategies.

3856: *+.122*

Understanding and monitoring the population status of endangered species is vital for developing appropriate management interventions. We used noninvasive genetic analyses to obtain ecological and genetic data on the last remaining Far Eastern leopard population in the world. During seven winters from 2000-2001 to 2007-2008, we collected feces, hair, and saliva from most of the leopard habitat. Of the 239 leopard samples collected during the study period, 155 were successfully genotyped at 13 microsatellite loci and 37 individuals (18 males and 19 females) were identified. Population size estimates based on the Capwire model were 28 (95 % CI 19-38) in 2002-03 and 26 (95 % CI 13-33) in 2007-2008. The leopard population had a low level of genetic diversity (expected and observed heterozygosity = 0.43; average number of alleles per locus = 2.62), and effective population size was estimated to be low (N (e) = 7-16) by two genetic-based methods. We observed little improvement in the genetic diversity during the study period and did find an indication of allele loss compared with individuals from the mid-1990s, suggesting that the remaining population will continue to suffer loss of genetic diversity. Given the small population size and the low genetic diversity, with little expectation of replenishment of the genetic variation by natural immigration, successful expansion of available habitat and development of a second population based on captive individuals may be crucial for persistence of this leopard subspecies in the wild.

3857: *+.274*

In this study, we used a maximum entropy (MaxEnt) approach to model the distribution of the rare European amphibian Pelobates fuscus insubricus, with the final goal of identifying suitable areas for its conservation. We generated the model starting from a dataset of all locations where this species' presence was confirmed for the region of piedmont in 2004-2010, which consisted of only 15 occurrence records. To verify the working hypothesis that population survival is higher in areas where Maxent identifies higher distribution probability values, we used suitability indexes generated by the model to compare the "historical" (before 1980) and "recent" (1980-1996) distributions of P. f. insubricus populations in the piedmont region. The average area-under-the-curve value (0.878, s = 0.075) of the Maxent model proved significantly informative. Using the Bonferroni confidence interval, we demonstrated that surviving populations occupy geographic areas characterized by significantly higher potential suitability ($p < 0.05$), and we selected areas accordingly. We therefore conclude that, in our case study, modelling the distribution of rare species may represent a useful strategy to select areas where these species are likely to persist. To

further evaluate this approach, we suggest testing it on the study of other rare species.

3858: +.225

The dispersal behavior of a species is critical for the stability and persistence of its populations across a landscape. How population density affects dispersal decisions is important for predicting these dynamics, as the form of density-dependent dispersal influences the stability and persistence of populations. Natal habitat experience often has strong impacts on individual dispersal behavior as well, but its influence on density-dependent dispersal behaviors remains unexplored. Here we address this conceptual gap in two experiments separately examining habitat selection and emigration from recently colonized patches for two species of flour beetle Tribolium sp. We found that interactions between the quality of habitat experienced during natal development and current habitat for dispersal capable adults can strongly affect the form of density dependence, including reversing the direction of nonlinearities (accelerating to decelerating), or even negating the influence of population density for individual dispersal decisions. Across heterogeneous landscapes, where individuals from different populations may experience different natal habitats, this altering of density-dependent relationships is predicted by theory to fundamentally influence regional population dynamics. Our results indicate that species which occur across heterogeneous environments, such as during conservation reintroductions, or as invasive species spread, have much potential for natal experience to interact with density dependence and influence local and regional population dynamics.

3859: -.025

A new set of 16 microsatellite markers was isolated and characterized for the threatened Australian freshwater fish Murray hardyhead Craterocephalus fluviatilis (Atherinidae) using a next generation sequencing approach. Seventy-eight fish from wild and captive populations were genotyped at all markers. All markers were polymorphic, with average allelic diversity of 5.7 and heterozygosity of 0.46. These markers will benefit substantially the ongoing conservation program of a critically endangered lineage of C. fluviatilis that includes captive breeding, relatedness and paternity analyses, reintroduction, and landscape genetics.

3860: -.226

Consequences of predation may be particularly heavy on small populations of herbivores, especially if they are threatened with extinction. Over the 2006-2010 period, we documented the effects of the spontaneous return of the endangered snow leopard on the population of the vulnerable Himalayan tahr. The study area was an area of central Himalaya where this cat disappeared c. 40 years before, because of persecution by man. Snow leopards occurred mainly in areas close to the core area of tahr distribution. Tahr was the staple (56.3 %) of snow leopards. After the arrival of this cat, tahr decreased by more than 2/3 from 2003 to 2010 (mainly through predation on kids). Subsequently, the density of snow leopards decreased by 60% from 2007 to 2010. The main prey of snow leopards in Asia (bharal, marmots) were absent in our study area, forcing snow leopards to specialize on tahr. The restoration of a complete prey spectrum should be favoured through reintroductions, to conserve large carnivores and to reduce exploitation of small populations of herbivores, especially if threatened.

3861: -.016

Ensuring the persistence of populations of endangered species requires an understanding of, and

response to, the causes of population declines. Species occurring in small populations are vulnerable to stochastic problems that are environmental, demographic, or genetic in nature and can reduce survival as much as the deterministic threats of habitat degradation. Critically endangered black rhinoceros (Diceros bicornis) populations declined throughout Africa since 1960, with numbers steadily increasing at a continental level, but remaining lower than three generations ago. However, size, demographics, trends, and factors affecting these, are poorly known. We used 18 years (1990-2008) of long-term sightings data from Ithala Game Reserve, KwaZulu-Natal, South Africa, to determine population estimates, growth rate and fecundity over time, as well as sex and age structure and age-specific probabilities of survival. Calf survivorship between the ages of 0 and 1 year was 74% for females and 94% for males. Age-specific survivorship for both sexes was significantly higher from yearling to subadult age-classes (1-6 years) than for adults (7-30 years). The most frequent cause of mortality was attributed to unknown causes while fighting injuries was recorded as the second most common cause of mortality, particularly among subadult and adult males. There was no significant difference in the sex ratios at birth, although the proportion of females in the population was 0.58. There was strong evidence for density-dependent regulation, with density in conception year a key driver of population performance (birth rate). The population does not appear to be at ecological carrying capacity; however, social effects are delaying conception. To mitigate density-dependent social effects, we recommend an adaptive management strategy of pre-selecting individuals for removal from the reserve, so as to maintain stability in the social organization of the population.

3862: *+.000*

Human disturbance and climate change have increased the risk of extinction for rare and endangered wild plant species. One effective way to conserve these rare and endangered species is through reintroduction. In this review, we summarize the advances in wild plant reintroduction from five perspectives: the establishment of reintroduction biology as an important tool for biodiversity conservation; the importance of genetic diversity in reintroduction; reintroduction under global climate change; recruitment limitation in reintroduction; and reintroduction and ecological restoration. In addition, we consider the future of plant reintroduction strategies.

3863: *+.202*

After near-extirpation in the early 20th century, beaver populations are increasing throughout many parts of North America. Simultaneously, there is an emerging interest in employing beaver activity for stream restoration in arid and semi-arid environments (collectively, drylands'), where streams and adjacent riparian ecosystems are expected to face heightened challenges from climate change and human population growth. Despite growing interest in reintroduction programmes, surprisingly little is known about the ecology of beaver in dryland streams, and science to guide management decisions is often fragmented and incomplete. This paper reviews the literature addressing the ecological effects and management of beaver activity in drylands of North America, highlighting conservation implications, distinctions between temperate and dryland streams, and knowledge gaps. Well-documented effects of beaver activity in drylands include changes to channel morphology and groundwater processes, creation of perennial wetland habitat, and substantial impacts to riparian vegetation. However, many hypothesized effects derived from temperate streams lack empirical evidence from dryland streams. Topics urgently in need of further study include the distribution and local density of beaver dams; consequences of beaver dams for hydrology and water budgets; and effects of beaver activity on the spread of aquatic and riparian non-native species. In summary, this review suggests that beaver activity can create substantial benefits and costs for conservation. Where active beaver introductions or removals are

proposed, managers and conservation organizations are urged to implement monitoring programmes and consider the full range of possible ecological effects and trade-offs. Copyright (c) 2014 John Wiley & Sons, Ltd.

3864: +.342

Animals translocated for conservation purposes may be sourced from multiple locations which may exhibit inter-site variability in reproductive behaviours. The influence that these differences may have on the propensity of pair formation, and the ultimate impact this may have on the success of the translocation, is unknown due to the low sample sizes of many translocations. We address this knowledge gap by collating an existing 18-year data set documenting multisource translocations of the endangered North Island Kokako, Callaeas wilsoni, a duetting New Zealand species with known population-specific vocalizations. We found a strong, statistically significant tendency for Kokako to pair assortatively with respect to source population (like with like) following release. Population-specific differences in sexually selected behaviours that are important in mate choice decisions, such as bird song, are likely the proximate explanation for such reproductive decisions. Accounting for the tendency to pair assortatively following translocation may be particularly important when managing highly vocal animals like Kokako that produce vocal duets and cooperatively defend territories as mated pairs. Consequently, careful consideration of behavioural variation between translocated individuals should be made, which will appropriately inform decisions relating to release-cohort composition. Failure to consider such variation may negatively impact the success of a translocation as the effective population size of the founder group may be lower than intended. Our findings make an important contribution towards understanding the impact that behavioural variation can have on the conservation of endangered species, and highlight the value of combining long-term data from multiple sources.

3865: +.185

Many reintroduction projects for conservation fail, and there are a large number of factors that may contribute to failure. Genetic analysis can be used to help stack the odds of a reintroduction in favour of success, by conducting assessment of source populations to evaluate the possibility of inbreeding and outbreeding depression and by conducting postrelease monitoring. In this study, we use a panel of 306 SNP (single nucleotide polymorphism) markers and 487-489 base pairs of mitochondrial DNA control region sequence data to examine 321 individuals from possible source populations of the Eurasian beaver for a reintroduction to Scotland. We use this information to reassess the phylogenetic history of the Eurasian beavers, to examine the genetic legacy of past reintroductions on the Eurasian landmass and to assess the future power of the genetic markers to conduct ongoing monitoring via parentage analysis and individual identification. We demonstrate the capacity of medium density genetic data (hundreds of SNPs) to provide information suitable for applied conservation and discuss the difficulty of balancing the need for high genetic diversity against phylogenetic best fit when choosing source population(s) for reintroduction.

3866: +.192

Reintroduction programs are often initiated to restore the viability of endangered plant populations, whose decline is usually caused by loss of suitable habitats. Ligularia sibirica is a species associated with wetlands. It is endangered in Europe and has declined considerably in Estonia since the cessation of traditional management and, in addition, drainage leading to the overgrowth of habitats. The purpose of this work was to estimate the extent of the impact of competition from neighbouring plants and habitat change caused by overgrowing on the survival

of transplants of L. sibirica and thereby assess the effectiveness of reinforcement. Laboratory-grown transplants were planted back into their original populations in plots arranged in a two x two (vegetation intact or removed x open or overgrown habitat) factorial experimental design, and their survival was followed for three years. The survival differed notably among populations, but the percentage of surviving plants per plot was on average higher in plots with intact vegetation and in open habitats. The latter indicates that overgrowing indeed decreases habitat quality for this species, despite the fact that plants of L. sibirica can often be found in forested habitats. The lower survival in plots where vegetation had been removed can largely be explained by increased damage caused by animals. In intact plots, by contrast, neighbouring plants provide shelter and protection. Our results stress the importance of restoration and preservation of habitat quality for the protection of this rare species, for which reinforcement may be effective.

3867: *+.170*

Understanding factors that influence recruitment can improve wildlife conservation. Endangered blackfooted ferrets (Mustela nigripes) rely on prairie dogs (Cynomys spp.) for food and on prairie dog burrows for shelter. We hypothesized that younger female ferrets with greater densities of prairie dogs in their core use area and fewer adult ferrets in their respective prairie dog colony, would produce more kits due to age-dependent productivity, increased food resources, and decreased competition. We used generalized linear mixed-effects regression and Akaike's information criterion adjusted for sample size (AICc) to rank models relating adult female black-footed ferret litter size (range 1-7 kits, n = 24 litters) to female age, core area density of prairie dogs, and adult ferret densities from 3 sites in the USA, 2005-2008. We included year and site as random effects in all models. We observed great model uncertainty; the null model was most supported and received 44% of model weight (w). The next best-supported model included ferret density only (AAIC, = 1.55, w = 0.20). Ferret density may not have been great enough to negatively affect prey acquisition and litter sizes. Mean litter size did not vary among female ages, but inference was limited because only one individual was >3 years old (F = 2.13 years, SD = 0.99). All adult females produced kits, suggesting that the observed minimum prairie dog density in ferret core use areas (12.3 individuals ha-l-) was above a threshold of minimal prey abundance for reproduction. Our findings support previous selections of reintroduction sites as those meeting minimum resource needs of individual ferrets for reproduction. Future selections of reintroduction sites may become more difficult if the number of areas with the minimum necessary prairie dog density decreases due to disease and reductions in habitat availability.

3868: *+.115*

The genus Ctenomys present a wide 2n and fundamental number (FN) variation in Uruguay and throughout their distribution across the south of the Neotropical region. In this study we analyze chromosome variation of this genus in Uruguay using G and C bands. G-band comparison makes clear that this chromosome variation is reduced if we take into account entire chromosomes and chromosome arms homologies. This could be explained as an adaptation to chromosome variation (2n and FN) without changing gene order or linkage groups. On the other hand, C-banding showed a high variability in amount and localization in the karyotypes analyzed here. These differences could be due to differential amplification of the repetitive DNA (RPSC) characteristic of this genus. Nevertheless, other mechanisms could be involved as repetitive DNA interchange at the chromocenters in the meiosis pachytene, where non homologous chromosomes meet. In this scenario, chromosome rearrangements such as Robertsonian translocations and whole chromosome conservation are of particular interest since these provide a way to conserve gene order. Why Ctenomys chromosomes are prone to rearrangements is not clear, albeit several

mammal species present a similar picture. This kind of chromosome change could be involved in the maintenance of chromosome order at the nuclear level. In this paper, we present evidence showing that chromosome and genetic changes are not unavoidably related in Ctenomys, which questions the hypothesis about speciation based on chromosomal changes.

3869: *-.011*

Captive propagation of Pacific salmon is routine, but few captive breeding programs have been conducted to successfully re-establish extirpated wild populations. A captive breeding program for endangered Sakinaw Lake sockeye salmon was established from 84 adults between 2002 and 2005, just prior to extirpation of the wild population. After several years of absence, sockeye salmon released from captivity returned to spawn in Sakinaw Lake in 2010 and in all years thereafter. Freshwater survival rates of released hatchery fry and naturally produced progeny of reintroduced sockeye salmon have not limited abundance of the reintroduced population. In contrast, marine survival rates for Sakinaw sockeye salmon have been <1%, a level that precludes population restoration in the absence of supplementation. Genetic diversity commensurate with the number of parental founders has been maintained in captivity. The 517 adult second-generation captive fish that spawned in Sakinaw Lake in 2011 produced a smolt emigration of almost 28,000 juvenile fish with an effective population size of 132. Allelic richness and gene diversity levels in the smolts were similar to those observed in captivity. This indicates genetic contributions from all or most founding parents have been retained both in captivity and in the nascent reintroduced natural population.

3870: *+.047*

The Paint Rock River (PRR) drainage in northern Alabama and southern Tennessee historically supported 58 freshwater mussel species. This study semi-quantitatively examined the mussel assemblage at 42 sites in the Paint Rock mainstem and 5 sites in Estill Fork. a headwater tributary. A total of 1825 live mussels were collected over 78.9 person-hours, with an overall catch per unit effort (CPUE) of 23.1 mussels/person-hour. Forty-one species were collected live and/or fresh dead, including federally protected Epioblasma triquetra (Snuffbox), Fusconaia cor (Shiny Pigtoe), Lampsilis abrupta (Pink Mucket), Lampsilis virescens (Alabama Lampmussel). Pleuronaia dolabelloides (Tennessee Pig-toe). Quadrula cylindrica cylindrica (Rabbitsfoot), and Toxolasma cylindrellus (Pale Lilliput). The river system continues to support a high diversity of mussels (48 species collected in the past 25 years). The survey also identified several sites in the basin suitable for the reintroduction of extirpated species.

3871: *+.196*

Reintroduction can be enhanced by data from long-term post-release monitoring, which allows for modeling opportunities such as population viability analysis (PVA). PVA-relevant data were gathered via long-term monitoring of reintroduced red-billed curassows at the Guapiacu Ecological Reserve (REGUA), located in Rio de Janeiro, Brazil, over 25 months. In the present article, we (1) assess the robustness of the reintroduction plan, (2) evaluate the viability of the current reintroduced population, and (3) examine mitigation options to increase the viability of this population. VORTEX indicates that the initial plan, fully implemented, was likely to establish a viable population at REGUA. The current population is unviable; the best mitigation strategies are to eliminate hunting altogether, or at least reduce it by half, and to supplement ten immature pairs in 2015. A positive long-term outcome at REGUA is still possible; we encourage the Brazilian government and private stakeholders to consider population supplementation, both to achieve

success at REGUA and to improve the evidence base for future reintroductions. (C) 2014 Associacao Brasileira de Ciencia Ecologica e Conservacao. Published by Elsevier Editora Ltda.

3872: -.052

Interaction among species through competition is a principle process structuring ecological communities, affecting behavior, distribution, and ultimately the population dynamics of species. High competition among large African carnivores, associated with extensive diet overlap, manifests in interactions between subordinate African wild dogs (Lycaon pictus) and dominant lions (Panthera leo) and spotted hyenas (Crocuta crocuta). Using locations of large carnivores in Hluhluwe-iMfolozi Park, South Africa, we found different responses from wild dogs to their two main competitors. Wild dogs avoided lions, particularly during denning, through a combination of spatial and temporal avoidance. However, wild dogs did not exhibit spatial or temporal avoidance of spotted hyenas, likely because wild dog pack sizes were large enough to adequately defend their kills. Understanding that larger carnivores affect the movements and space use of other carnivores is important for managing current small and fragmented carnivore populations, especially as reintroductions and translocations are essential tools used for the survival of endangered species, as with African wild dogs.

3873: +.034

Compared to their main competitors, African wild dogs (Lycaon pictus) have inferior competitive abilities and interspecific competition is a serious fitness-limiting factor. Lions (Panthera leo) are the dominant large carnivore in African savannah ecosystems and wild dogs avoid them both spatially and temporally. Wild dog young are particularly vulnerable and suffer high rates of mortality from lions. Since lions do not utilize all parts of the landscape with an equal intensity, spatial variation in lion densities can be exploited by wild dogs both during their general ranging behaviour, but more specifically when they are confined to a den with vulnerable young. Since patches of rugged terrain are associated with lower lion densities, we hypothesized that these comparatively safe habitats should be selected by wild dogs for denning. We investigated the relationship between the distribution of 100 wild dog den sites and the occurrence of rugged terrain in four wild dog populations located in Tanzania, Zimbabwe and South Africa. A terrain ruggedness index was derived from a 90 m digital elevation model and used to map terrain ruggedness at each site. We compared characteristics of actual and potential (random) den sites to determine how wild dogs select den sites. The distributions of wild dog dens were strongly associated with rugged terrain and wild dogs actively selected terrain that was more rugged than that available on average. The likelihood of encountering lions is reduced in these habitats, minimizing the risk to both adults and pups. Our findings have important implications for the conservation management of the species, especially when assessing habitat suitability for potential reintroductions. The simple technique used to assess terrain ruggedness may be useful to investigate habitat suitability, and even predict highly suitable denning areas, across large landscapes.

3874: +.032

Managers of threatened species often face the dilemma of whether to keep populations separate to conserve local adaptations and minimize the risk of outbreeding, or whether to manage populations jointly to reduce loss of genetic diversity and minimise inbreeding. In this study we examine genetic relatedness and diversity in three of the five last remaining wild populations of dama gazelle and a number of captive populations, using mtDNA control region and cytochrome b

data. Despite the sampled populations belonging to the three putative subspecies, which are delineated according to phenotypes and geographical location, we find limited evidence for phylogeographical structure within the data and no genetic support for the putative subspecies. In the light of these data we discuss the relevance of inbreeding depression, outbreeding depression, adaptive variation, genetic drift, and phenotypic variation to the conservation of the dama gazelle and make some recommendations for its future conservation management. The genetic data suggest that the best conservation approach is to view the dama gazelle as a single species without subspecific divisions.

3875: ***+.122***

Reintroduction programs for threatened species often include elaborate release strategies designed to improve success, but their advantages are rarely tested scientifically. We used a set of four experiments to demonstrate that the influence of release strategies on short-term reintroduction outcomes is related to both intrinsic and extrinsic factors. We compared different reintroduction strategies for three mammal species in an arid environment where exotic mammalian predators were removed. Wild greater stick-nest rats selected vegetation shelter sites with greater structural density than captive-bred rats, travelled further from the release site and experienced lower rates of mortality. In comparison, there was no difference in mortality or movement between wild and captive-bred greater bilbies. Burrowing bettongs and greater bilbies were also subjected to immediate and delayed release strategies and whilst no difference was detected in bilbies, bettongs that were subjected to delayed releases lost less weight and took less time to establish burrows than those that were immediately released. Interspecific differences in treatment response were attributed to predation risk, the nature of the release site, and behavioural traits such as shelter investment and sociality. Our varied results highlight the inadequacies of review articles focusing on optimum release protocols due to their attempt to generalise across species and release sites. We provide an example of a predictive model to guide future release strategy experimentation that recognises the range of intrinsic and extrinsic factors influencing reintroduction outcomes. We encourage researchers to treat programs experimentally, identify individual site and species characters that may influence release strategies and record data on movements, mortality, weight dynamics, and settling times and distances. The inherent issues of small sample size and low statistical power that plague most reintroduction experiments suggests there is also a need for increased standardisation and publication of data sets to enable appropriate meta-analyses to occur.

3876: ***+.034***

Genetic structure can be a consequence of recent population fragmentation and isolation, or a remnant of historical localised adaptation. This poses a challenge for conservationists since misinterpreting patterns of genetic structure may lead to inappropriate management. Of 17 species of reptile originally found in Mauritius, only five survive on the main island. One of these, Phelsuma guimbeaui (lowland forest day gecko), is now restricted to 30 small isolated subpopulations following severe forest fragmentation and isolation due to human colonisation. We used 20 microsatellites in ten subpopulations and two mitochondrial DNA (mtDNA) markers in 13 subpopulations to: (i) assess genetic diversity, population structure and genetic differentiation of subpopulations; (ii) estimate effective population sizes and migration rates of subpopulations; and (iii) examine the phylogenetic relationships of haplotypes found in different subpopulations. Microsatellite data revealed significant population structure with high levels of genetic diversity and isolation by distance, substantial genetic differentiation and no migration between most subpopulations. MtDNA, however, showed no evidence of population structure, indicating that there was once a genetically panmictic population. Effective population sizes of ten

subpopulations, based on microsatellite markers, were small, ranging from 44 to 167. Simulations suggested that the chance of survival and allelic diversity of some subpopulations will decrease dramatically over the next 50 years if no migration occurs. Our DNA-based evidence reveals an urgent need for a management plan for the conservation of P. guimbeaui. We identified 18 threatened and 12 viable subpopulations and discuss a range of management options that include translocation of threatened subpopulations to retain maximum allelic diversity, and habitat restoration and assisted migration to decrease genetic erosion and inbreeding for the viable subpopulations.

3877: ***+.159***

Wildlife managers are challenged to manage spatially structured populations efficiently and effectively, therefore dispersal and gene flow are vital to understand and manage, particularly for a harvested species. We used a genetic approach to describe the metapopulation structure of Rocky Mountain elk (Cervus elaphus) in Idaho to assess past patterns of population distribution and influences of harvest. We used elk tissue and DNA samples (n = 216) to examine genetic dissimilarity between 7 regions and 9 elk management zones throughout Idaho using microsatellite loci (n = 11). Using 5 approaches, including pairwise F-ST-values, assignment tests, and a Bayesian model based clustering of genotypes, we examined the distribution of genetic variation. The distribution of genetic variation between elk populations indicated low levels of genetic differentiation among regions (expected heterozygosity [H-E] = 0.55-0.61, overall F-ST = 0.011) and elk management zones (H-E = 0.54-0.60, overall F-ST = 0.017). Assignment tests and migration rates indicated directional gene flow between elk populations. A patchy metapopulation best describes the distribution of genetic variation among Idaho elk populations because likely enough individual interchange occurs between geographically separated populations. The elk populations we sampled could be part of a geographically larger patchy metapopulation potentially stretching from Yellowstone National Park through Idaho into western Canada. Because of historical translocations of elk from Yellowstone National Park, insufficient time may have passed to detect differences in genetic variation. Subtle differences in the distribution of genetic variation were observed in 2 of the 9 elk management zones within 2 different regions of the state. Our findings indicate management of Idaho elk populations and dispersal are maintaining sufficient gene flow. Metapopulation structure of a harvested species based on the distribution of genetic variation is an indicator of potential genetic consequences of harvesting and sustainable harvest levels.

3878: ***+.017***

In the past, an indigenous chamois population inhabited Velebit Mountain, but the nature of its taxonomic status is unknown. This indigenous population was exterminated at the beginning of the 20th, century, probably because of such phenomena as poaching, disturbance by livestock and sheepdogs, and possibly diseases. The chamois was re-introduced to Northern Velebit twice. The first time came in 1974, when 10 individuals were introduced from Mt Prenj in Bosnia and Herzegovina (R. rupicapra balcanica), and a second time in 1978, when 5 individuals were introduced from Kamniske Alpe in Slovenia (R. rupicapra rupicapra). A genetic analysis of the recent chamois population on the Northern Velebit showed a hybrid genetic structure, containing genes of both subspecies. Historical data confirm that the presence of the chamois in the Paklenica National can be traced back to at least the year 1985. The presence of the northern chamois (Rupicapra rupicapra) in Paklenica was investigated in the period 2008-2012. The objective was to present the population status and the shift in its distribution since 1985. It is likely that part of the population may move between the Park and the neighbouring hunting areas.

3879: +.135

The distribution of the endemic threatened plant Camellia changii Ye (Family Theaceae) is restricted to a small area in southern China, and little else is known about its status in the wild. To provide information for the conservation of C. changii we investigated its distribution, population size and structure, and habitat, and assessed its conservation status. Surveys confirmed that the species grows in a narrow band along both sides of a 4 km long segment of a stream in Ehuangzhang Nature Reserve, under the discontinuous canopy of a secondary evergreen broad-leaved forest on well-drained, acidic sandy loam soil. We found a total of 1,039 individuals of C. changii. The population has a high flowering rate but a low seed-setting rate. The population appears to be in decline because no seedlings and few young plants were evident. Our findings indicate that C. changii should be categorized as Critically Endangered on the IUCN Red List. We have recommended an integrated species-conservation plan for the species that includes patrolling the Ehuangzhang Nature Reserve to prevent plant removal, establishing an ex situ living collection that contains the entire wild genetic diversity (accomplished by grafting of short cuttings from all wild individuals), facilitating propagation for commercial use, and implementing reintroduction to augment the wild population.

3880: -.033

Wenchengia alternifolia (Lamiaceae) is a plant species endemic to Hainan Island, China. Since the 1930s it was known from only four collections and was believed to be extinct until a remnant population was rediscovered in 2010. We conducted further field surveys during 2010-2012 but located only one population, with 66 individuals. W. alternifolia is restricted to the c. 1,500 m(2) Shuangximu Valley, in a harsh microenvironment surrounded by plantations. As the population comprises < 50 mature individuals, we propose that W. alternifolia be categorized as Critically Endangered on the IUCN Red List, based on criterion D. We recommend that the remnant population and its habitat need urgent protection and monitoring and that ex situ conservation, for future reintroduction, should be implemented.

3881: +.168

In a translocation programme, social interactions among released individuals can influence the tendency for the individuals to remain at the site where they have been released. Dispersal away from the region of release may reduce the success of a translocation because dispersing individuals may move into less suitable habitat, or become separated from potential reproductive partners. In soft releases, where individuals are confined together for periods of time at the release site, before ultimate release, social interactions among the confined individuals may promote subsequent dispersal. In this study we investigated how variation in the abundance and distribution of a fundamental habitat resource, refuge burrows, influenced behaviour and possible subsequent dispersal of newly released individuals of the endangered Australian pygmy bluetongue lizard, Tiliqua adelaidensis, in simulations of translocation releases. Our aim was to determine if there was an optimal distribution of burrow resources that would minimise behaviours likely to lead to dispersal from the release site. There were more lizard movement around burrows 3.73 (SE 0.02) and fewer burrow changes 0.06 (SE 0.006) in low burrow densities than in high burrow densities (1.88 (SE 0.02) movements; 0.50 (SE 0.008) burrow changes per day). When lizards were released in burrows near to each other (50 cm) they changed burrows more often (0.97 (SE 0.01)) and had more agonistic interactions (0.04 (SE 0.004)) than when released in burrows further apart (150 cm; 0.22 (SE 0.009) burrow changes; 0.003 (SE 0.001) agonistic interactions). These results suggest changes in behaviour can be induced by altering the way resources are distributed at a release site.

We suggest that understanding the social organisation of any endangered species, and whether it can be manipulated, will be an important component of planning a translocation release programme. (C) 2014 Elsevier B.V. All rights reserved.

3882: *+.048*

Musk deer are an important economic wildlife resource, and long-term over-use has resulted in a sharp population decrease in the wild. Farming of musk deer is important to prevent the shrinking wild population from being hunted for their musk. Musk deer farming has a history of more than 60 years in China, but many problems persist. Musk deer are alert, timid and solitary in the wild, and the captive environment cannot satisfy their natural needs, leading to problems in musk deer farming. Understanding the biological characteristics of musk deer may help to identify ways for improving the welfare of farmed musk deer. In addition to gaining musk, musk deer farming can play a role in the reintroduction of musk deer, which complicates things as musk production requires tame domesticated musk deer whereas successful reintroduction requires untamed and less domesticated ones. In this article, we compare captive musk deer and wild musk deer for morphology, behavior, physiology, genetics, and nutrition, and discuss how to organize farming environment and management to satisfy biological needs of musk deer in order to improve their welfare and increase the population of farmed musk deer. (C) 2014 Elsevier B.V. All rights reserved.

3883: *+.157*

The reintroduction of threatened and endangered species is now a common method for reestablishing populations. Typically, a fundamental objective of reintroduction is to establish a self-sustaining population. Estimation of demographic parameters in reintroduced populations is critical, as these estimates serve multiple purposes. First, they support evaluation of progress toward the fundamental objective via construction of population viability analyses (PVAs) to predict metrics such as probability of persistence. Second, PVAs can be expanded to support evaluation of management actions, via management modeling. Third, the estimates themselves can support evaluation of the demographic performance of the reintroduced population (e.g., via comparison with wild populations). For each of these purposes, thorough treatment of uncertainties in the estimates is critical. Recently developed statistical methods (namely, hierarchical Bayesian implementations of state-space models) allow for effective integration of different types of uncertainty in estimation. We undertook a demographic estimation effort for a reintroduced population of endangered Whooping Cranes with the purpose of ultimately developing a Bayesian PVA for determining progress toward establishing a self-sustaining population, and for evaluating potential management actions via a Bayesian PVA-based management model. We evaluated individual and temporal variation in demographic parameters based upon a multi-state, mark-recapture model. We found that survival was relatively high across time and varied little by sex. There was some indication that survival varied by release method. Survival was similar to that observed in the wild population. Although overall reproduction in this reintroduced population is poor, birds formed social pairs when relatively young, and once a bird was in a social pair, it had a nearly 50% chance of nesting the following breeding season. Also, once a bird had nested, it had a high probability of nesting again. These results are encouraging, considering that survival and reproduction have been major challenges in past reintroductions of this species. The demographic estimates developed will support construction of a management model designed to facilitate exploration of management actions of interest, and will provide critical guidance in future planning for this reintroduction. An approach similar to what we describe could be usefully applied to many reintroduced populations.

3884: ***+.165***

An important factor in reintroductions is the amount of genetic diversity captured in the introduced individuals. Introduced populations are initially small, and thus vulnerable to genetic drift and stochastic events. The level of genetic diversity maintained is important for the long-term persistence of populations and their evolutionary potential to react to, for example, climate changes. The national extinction of many butterfly species has been pronounced in many European countries. The globally Vulnerable large blue butterfly (Maculinea arion) went extinct in the UK in 1979 and was later reintroduced from Oland in Sweden. We investigated the genetic diversity of reintroduced large blues nineteen generations after translocation on five sites in the UK, and seven sites on Oland, including the source population. We found similar levels of genetic diversity in the reintroduced and source populations, but the UK and Swedish populations were genetically differentiated; we also found significant genetic differentiation among reintroduced UK populations only a few kilometres apart. The reintroduced populations had several private alleles not found in the source population in 2011, and thus may already represent a unique subset of genetic diversity of the north-western populations of M. anion. Our results show that the IUCN and other protocols followed in the 1990s for translocating and maintaining the maximum available genetic diversity during reintroductions were largely adequate for this species, and hence will be valuable for informing the growing use of reintroductions as a strategy for the conservation of endangered species of insect. (C) 2014 Elsevier Ltd. All rights reserved.

3885: ***+.120***

Nabis pseudoferus Remane and N. palifer Seidenstucker are predators that feed on a wide range of insect pests. To reveal their current potential habitats, the effects of climate change and their future distribution in various areas of Iran we used maximum entropy modeling (Maxent). To produce the models, samples were collected from 218 areas of Iran resulting in discovering 271 points where the nabids were found. The accuracy and performance of distribution models were also evaluated by the area under receiver operating characteristic curve and jack-knife analysis. In the Maxent model, the climatic, elevation and land cover layers were the major bases for the current models. In modeling future distribution, the land cover layer was excluded. The distribution of N. pseudoferus was independent of the type of vegetation while the distribution of N. palifer varied according to differences in type of vegetation. Using jack-knife analysis, the land cover and precipitation were the most effective predictors driving the two Nabis species range expansion. From 2013 to 2050 the impacts of climate change on N. pseudoferus distribution was predicted to have a negative impact but have a positive effect on N. palifer range expansion. Results could be used in preparation of predators' conservation, translocation and reintroduction programs and application in pest management strategies.

3886: ***+.237***

The aim of any reintroduction program is to create a viable population that is self-sustaining in the long term. Thus, in the short term, it is important to evaluate the acclimation of reintroduced individuals in order to assess the potential success of the project. In this study, we radio-tracked 30 European pond turtles (Emys orbicularis) reintroduced in the Estagnol Nature Reserve (southern France) in 2008 and 2009. We analyzed each individual's dispersal over the site, its home range, and its pattern of displacement for 2 yrs after its release. About 80% of the released animals were still detected on the site 8 mo after the release operation. Home-range size was highly variable among individuals but was typical of what is known for the species. Home ranges were, however, larger in the first year after release than in the following year, probably because individuals

explored the site immediately after release. The released individuals exhibited a typical displacement pattern over the first year, with a larger displacement during spring and summer that was earlier for males (April-May) than for females (June-September). All of these results strongly indicate the success of the acclimation phase of the reintroduction operation. To evaluate the success of reintroduction programs of long-lived species, we recommend, in addition to a long-term demographic study, a fine-scale study of space-use modalities, which allows the assessment of the acclimation phase of the individuals at the new site in the short term. In this way, a rapid reevaluation of the project can be made if failure at this stage is identified, allowing appropriate management actions to be taken at the site.

3887: *-.055*

Mojave desert tortoises (Gopherus agassizii) have been translocated for decades, and research-oriented translocations recently have been recommended as a tool to help recover this threatened species. However, avoiding negative genetic impacts from wildlife translocations has been widely cautioned. Populations of the Mojave desert tortoise within a 200-276-km straight-line radius of each other (249-308 km measured around topographic barriers) tend to be genetically correlated and may be considered single genetic units for management purposes. When planning translocations among wild populations, releasing tortoises at recipient sites within a straight-line distance of 200 km from the source population would most conservatively maintain historic genetic population structure. However, the risk of causing outbreeding depression by inadvertently translocating Mojave desert tortoises between more distant populations or those of unknown provenance is low.

3888: *+.005*

Ungulate browsing and lack of overstory disturbance have historically prevented aspen regeneration on the Northern Yellowstone Winter Range (NYWR). Aspen clones regenerate if sprouts are produced that grow into recruitment stems (>2 m tall) and replace the mature overstory. Beaver reintroduced in 1991 to Eagle Creek on the NYWR facilitated aspen restoration by removing overstory trees and increasing sprouting. However, intense ungulate browsing, primarily from the Northern Yellowstone elk herd, was preventing aspen recruitment in Eagle Creek as of 2005. Since 2005, wolf predation has contributed to a 56% decrease in this elk herd. We investigated the effects of beaver reintroduction, ungulate herbivory, and predator-mediated declines in elk numbers on aspen regeneration in Eagle Creek from 1997 to 2012. Aerial photos of Eagle Creek in 2005 and 2011 showed that the aspen overstory has not been replaced 21 years after beaver reintroduction ($p > 0.05$). Sprouting and recruitment were investigated using 4-m radius circular plots ($n = 31$) established throughout Eagle Creek in 1997 and monitored annually until 2012. Beaver activity stimulated sprouting in 71% of these plots. In 2012, 77% of the plots had >= 1 recruitment stem and 75% of the paired plots associated with exclosures ($n = 16$) had aspen stems with an average height >= 2 m. Recent increases in aspen recruitment in Eagle Creek indicate that aspen communities are regenerating. This has likely resulted from decreased ungulate browsing pressure on aspen saplings from 2005 to 2012. These findings are consistent with the predictions of a density-mediated trophic cascade following wolf reintroduction.

3889: *+.231*

Endocrinology is an indispensable tool in threatened species research. The study of endocrinology in threatened species not only advances knowledge of endocrine mechanism but also contributes to conservation efforts of studied species. To this end, endocrinology has been traditionally used to

understand reproductive and adrenocortical endocrine axes by quantifying excreted steroid metabolites. From these studies a large body of knowledge was created that contributed to the field of endocrinology, aided conservation efforts, and created a template by which to validate and conduct this research for other species. In this regard noninvasive hormone monitoring has become a favored approach to study the basic endocrinology of wildlife species. Due to the increased understanding of endocrine physiology of threatened species, breeding rates of captive population have improved to levels allowing for reintroduction of species to restored natural ecosystems. Although these approaches are still employed, advances in biochemical, molecular, and genomic technologies are providing inroads to describe lesser known endocrine activity in threatened species. These new avenues of research will allow for growth of the field with greater depth and breadth. However, for all approaches to endocrinology, limitations on resources and access to animals will require innovation of current methodologies to permit broad application for use in threatened species research. Crown Copyright (C) 2014 Published by Elsevier Inc. All rights reserved.

3890: *+.095*

Relocating snakes is used to reduce potential snake-human conflict and to re-establish or augment populations. Relocation may be unsuccessful if snakes attempt to home back to their capture locations or otherwise alter their behavior in ways that reduce fitness. To better understand the conditions under which the technique is likely to be successful, we conducted two types of relocation (repatriation and short-distance translocation) using Eastern Massasaugas (Sistrurus c. catenatus) in Ontario. For the repatriation experiment, 27 snakes were captive-born, raised for four years, and released into a nature reserve previously known to host massasaugas. Other than being relatively sedentary, snakes behaved normally upon release in that they engaged in reproductive behavior. Survival (70%) was relatively high until hibernation (19 weeks). However, none of the snakes that did hibernate (n = 19) survived into the following active season. In a preliminary assessment of the effects of short-distance translocation, snakes that we moved 200 m from capture locations (n = 4) did not return, nor did they exhibit abnormal movement or basking behavior relative to non-translocated controls (n = 7). The different outcomes of our two relocations could indicate that the success of relocation depends on the extent of displacement and the source of relocated individuals, although corroborating evidence is needed before these results can be used to support management strategies.

3891: *+.325*

The Sumatran orangutan is currently listed by the IUCN as critically endangered and the Bornean species as endangered. Unless effective conservation measures are enacted quickly, most orangutan populations without adequate protection face a dire future. Two main strategies are being pursued to conserve orangutans: (i) rehabilitation and reintroduction of excaptive or displaced individuals; and (ii) protection of their forest habitat to abate threats like deforestation and hunting. These strategies are often mirrored in similar programs to save other valued and endangered mega-fauna. Through GIS analysis, collating data from across the literature, and combining this information within a modelling and decision analysis framework, we analysed which strategy or combination of strategies is the most cost-effective at maintaining wild orangutan populations, and under what conditions. We discovered that neither strategy was optimal under all circumstances but was dependent on the relative cost per orangutan, the timescale of management concern, and the rate of deforestation. Reintroduction, which costs twelve times as much per animal as compared to protection of forest, was only a cost-effective strategy at very short timescales. For time scales longer than 10-20 years, forest protection is the

more cost-efficient strategy for maintaining wild orangutan populations. Our analyses showed that a third, rarely utilised strategy is intermediate: introducing sustainable logging practices and protection from hunting in timber production forest. Maximum long-term cost-efficiency is achieved by working in conservation forest. However, habitat protection involves addressing complex conservation issues and conflicting needs at the landscape level. We find a potential resolution in that well-managed production forests could achieve intermediate conservation outcomes. This has broad implications for sustaining biodiversity more generally within an economically productive landscape. Insights from this analysis should provide a better framework to prioritize financial investments, and facilitate improved integration between the organizations that implement these strategies.

3892: *+.007*

The Bolson tortoise (Gopherus flavomarginatus) is the first species of extirpated megafauna to be repatriated into the United States. In September 2006, 30 individuals were translocated from Arizona to New Mexico with the long-term objective of restoring wild populations via captive propagation. We evaluated mtDNA sequences and allelic diversity among 11 microsatellite loci from the captive population and archived samples collected from wild individuals in Durango, Mexico (n = 28). Both populations exhibited very low genetic diversity and the captive population captured roughly 97.5% of the total wild diversity, making it a promising founder population. Genetic screening of other captive animals (n = 26) potentially suitable for reintroduction uncovered multiple hybrid G. flavomarginatusxG. polyphemus, which were ineligible for repatriation; only three of these individuals were verified as purebred G. flavomarginatus. We used these genetic data to inform mate pairing, reduce the potential for inbreeding and to monitor the maintenance of genetic diversity in the captive population. After six years of successful propagation, we analyzed the parentage of 241 hatchlings to assess the maintenance of genetic diversity. Not all adults contributed equally to successive generations. Most yearly cohorts of hatchlings failed to capture the diversity of the parental population. However, overlapping generations of tortoises helped to alleviate genetic loss because the entire six-year cohort of hatchlings contained the allelic diversity of the parental population. Polyandry and sperm storage occurred in the captives and future management strategies must consider such events.

3893: *+.001*

The reintroduction of wolves (Canis lupus) to Yellowstone provided the unusual opportunity for a quasi-experimental test of the effects of wolf predation on their primary prey (elk - Cervus elaphus) in a system where top-down, bottom-up, and abiotic forces on prey population dynamics were closely and consistently monitored before and after reintroduction. Here, we examined data from 33 years for 12 elk population segments spread across southwestern Montana and northwestern Wyoming in a large scale before-after-control-impact analysis of the effects of wolves on elk recruitment and population dynamics. Recruitment, as measured by the midwinter juvenile: female ratio, was a strong determinant of elk dynamics, and declined by 35% in elk herds colonized by wolves as annual population growth shifted from increasing to decreasing. Negative effects of population density and winter severity on recruitment, long recognized as important for elk dynamics, were detected in uncolonized elk herds and in wolf-colonized elk herds prior to wolf colonization, but not after wolf colonization. Growing season precipitation and harvest had no detectable effect on recruitment in either wolf treatment or colonization period, although harvest rates of juveniles: females declined by 37% in wolf-colonized herds. Even if it is assumed that mortality due to predation is completely additive, liberal estimates of wolf predation rates on juvenile elk could explain no more than 52% of the total decline in juvenile: female ratios in wolf-

colonized herds, after accounting for the effects of other limiting factors. Collectively, these long-term, large-scale patterns align well with prior studies that have reported substantial decrease in elk numbers immediately after wolf recolonization, relatively weak additive effects of direct wolf predation on elk survival, and decreased reproduction and recruitment with exposure to predation risk from wolves.

3894: *+.184*

Intentional moving of species threatened by climate change is actively being discussed as a conservation approach. The debate, empirical studies, and policy development, however, are impeded by an inconsistent articulation of the idea. The discrepancy is demonstrated by the varying use of terms, such as assisted migration, assisted colonisation, or managed relocation, and their multiple definitions. Since this conservation approach is novel, and may for instance lead to legislative changes, it is important to aim for terminological consistency. The objective of this study is to analyse the suitability of terms and definitions used when discussing the moving of organisms as a response to climate change. An extensive literature search and review of the material (868 scientific publications) was conducted for finding hitherto used terms (N = 40) and definitions (N = 75), and these were analysed for their suitability. Based on the findings, it is argued that an appropriate term for a conservation approach relating to aiding the movement of organisms harmed by climate change is assisted migration defined as follows: Assisted migration means safeguarding biological diversity through the translocation of representatives of a species or population harmed by climate change to an area outside the indigenous range of that unit where it would be predicted to move as climate changes, were it not for anthropogenic dispersal barriers or lack of time. The differences between assisted migration and other conservation translocations are also discussed. A wide adoption of the clear and distinctive term and definition provided would allow more focused research on the topic and enable consistent implementation as practitioners could have the same understanding of the concept.

3895: *+.080*

The rate of biodiversity loss is not slowing despite global commitments, and the depletion of animal species can reduce the stability of ecological communities. Despite this continued loss, some substantial progress in reversing defaunation is being achieved through the intentional movement of animals to restore populations. We review the full spectrum of conservation translocations, from reinforcement and reintroduction to controversial conservation introductions that seek to restore populations outside their indigenous range or to introduce ecological replacements for extinct forms. We place the popular, but misunderstood, concept of rewilding within this framework and consider the future role of new technical developments such as de-extinction.

3896: *-.012*

The Arabian Gazelle is a globally threatened antelope (Vulnerable) in Saudi Arabia. Small relict populations remain in limited areas, while historically Arabian gazelles occurred in Mahazat as-Sayd protected area in central Saudi Arabia but were exterminated by anthropogenic and other pressures, including habitat loss and hunting. Important habitat has been lost to agricultural developments, fencing of pasture for livestock and the construction of human settlements and roads. The reintroduction of Arabian Gazelles was undertaken in Mahazat during 2011-2014 to bring back this locally extinct species study its ecology and biology in a fenced protected area. We released a total of 49 (12 males, 37 females) animals. A year after release animals started breeding

and six calves have been recorded so far with more to come. The gazelles prefer to use more rocky areas where shrubs and acacia trees occur in the reserve, and do not move long distances except for one individual that moved more than 50km. Mahazat is fenced, which prevents local people from entering the reserve to poach or otherwise disturb animals. Management lessons include the need for continued monitoring of reintroduced populations. Interactions between Arabian and Sand Gazelles (Gazella subgutturosa marica) and Arabian Oryx (Oryx leucoryx) were also studied.

3897: *-.157*

Aujeszky's disease (AD) causes significant economic losses in the Spanish pig sector due to import trade restrictions imposed by disease-free countries. Most regions of Spain have achieved 'low AD prevalence' status as a result of an intensive national AD eradication programme involving vaccination and other measures. However, to achieve AD-free status that would eliminate trade restrictions, vaccination must be stopped. For this final stage of eradication, up to date and reliable estimates of the risk of AD reintroduction are essential. Here, we propose an approach based on spatio-temporal scan statistics that the assesses risk of AD reintroduction in a disease-free territory by analysing the two most frequent risk pathways: movement of live domestic pigs and contact with wildlife reservoirs. The approach is illustrated using the case of Navarre, one of the first Spanish regions which plan to stop vaccination. Moreover, direct contacts among pig farms in Navarre were used to evaluate the potential spread of AD in the event of reintroduction. Areas at highest risk of AD reintroduction were in the southern part of the region during the second half of the year through pig movements and in the western and east-central parts of Navarre through contact with wild boars. Northern Navarre, despite having the highest density of pig farms, seems to be at low risk of AD reintroduction. Analysing the network of pig movements within Navarre revealed distinct northern and southern compartments that may be used in preventive compartmentalization strategies to reduce potential risk of AD re-infection in the scenario without vaccination. The approach described here may be extended to other regions and may be useful for guiding risk-based measures that reduce the risk of AD re-infection in a more cost-effective manner. Such analysis in Spain may allow authorities to stop vaccination in the safest possible way.

3898: *-.007*

Re-introduction of rare species to parts of their historical range is becoming increasingly important as a conservation strategy. Telfair's Skinks (Leiolopisma telfairii), once widespread on Mauritius, were until recently found only on Round Island. There it is vulnerable to stochastic events, including the introduction of alien predators that may either prey upon it or compete for food resources. Consequently, skinks have been introduced to Ile aux Aigrettes, another small Mauritian island that has been cleared of rats. However, the island has been invaded by Asian Musk Shrews (Suncus murinus), a commensal species spread by man well beyond its natural Asian range. Our aim was to use next-generation sequencing to analyse the diets of the shrews and skinks to look for niche competition. DNA was extracted from skink faeces and from the stomach contents of shrews. Application of shrew-and skink-specific primers revealed no mutual predation. The DNA was then amplified using general invertebrate primers with tags to identify individual predators, and then sequenced by 454 pyrosequencing. 119 prey MOTUs (molecular taxonomic units) were isolated, although none could be identified to species. Seeding of cladograms with known sequences allowed higher taxonomic assignments in some cases. Although most MOTUs were not shared by shrews and skinks, Pianka's niche overlap test showed significant prey overlap, suggesting potentially strong competition where food resources are limited. These results suggest

that removal of the shrews from the island should remain a priority.

3899: *-.091*

Most species face multiple anthropogenic disruptions. Few studies have quantified the cumulative influence of multiple threats on species of conservation concern, and far fewer have quantified the potential relative value of multiple conservation interventions in light of these threats. We linked spatial distribution and population viability models to explore conservation interventions under projected climate change, urbanization, and changes in fire regime on a long-lived obligate seeding plant species sensitive to high fire frequencies, a dominant plant functional type in many fire-prone ecosystems, including the biodiversity hotspots of Mediterranean-type ecosystems. First, we investigated the relative risk of population decline for plant populations in landscapes with and without land protection under an existing habitat conservation plan. Second, we modeled the effectiveness of relocating both seedlings and seeds from a large patch with predicted declines in habitat area to 2 unoccupied recipient patches with increasing habitat area under 2 projected climate change scenarios. Finally, we modeled 8 fire return intervals (FRIs) approximating the outcomes of different management strategies that effectively control fire frequency. Invariably, long-lived obligate seeding populations remained viable only when FRIs were maintained at or above a minimum level. Land conservation and seedling relocation efforts lessened the impact of climate change and land-use change on obligate seeding populations to differing degrees depending on the climate change scenario, but neither of these efforts was as generally effective as frequent translocation of seeds. While none of the modeled strategies fully compensated for the effects of land-use and climate change, an integrative approach managing multiple threats may diminish population declines for species in complex landscapes. Conservation plans designed to mitigate the impacts of a single threat are likely to fail if additional threats are ignored.

3900: *+.084*

The intentional translocation of animals is an important tool for species conservation and ecosystem restoration, but reported success rates are low, particularly for threatened and endangered species. Publication bias further distorts success rates because the results of successful translocations may be more likely to be published than failed translocations. We conducted the first comprehensive review of all published and unpublished translocations of herpetofauna in New Zealand to assess publication bias. Of 74 translocations of 29 species in 25 years, 35 have been reported in the published literature, and the outcomes of 12 have been published. Using a traditional definition of success, publication bias resulted in a gross overestimate of translocation success rates (41.7% and 8.1% for published and all translocations, respectively), but bias against failed translocations was minimal (8.3% and 6.8%, respectively). Publication bias against translocations with uncertain outcomes, the vast majority of projects, was also strong (50.0% and 85.1% for published and all translocations, respectively). Recent translocations were less likely to be published than older translocations. The reasons behind translocations were related to publication. A greater percentage of translocations for conservation and research were published (63.3% and 40.0%, respectively) than translocations for mitigation during land development (10.0%). Translocations conducted in collaboration with a university were more frequently published (82.7% and 24.4%, respectively). To account for some of this publication bias, we reassessed the outcome of each translocation using a standardized definition of success, which takes into consideration the species' life history and the time since release. Our standardized definition of translocation success provided a more accurate summary of success rates and allows for a more rigorous evaluation of the causes of translocation success and failure in large-scale reviews.

3901: *+.139*

Genetic, demographic, and environmental processes affect natural populations synergistically, and understanding their interplay is crucial for the conservation of biodiversity. Stream fishes in metapopulations are particularly sensitive to habitat fragmentation because persistence depends on dispersal and colonization of new habitat but dispersal is constrained to stream networks. Great Plains streams are increasingly fragmented by water diversion and climate change, threatening connectivity of fish populations in this ecosystem. We used seven microsatellite loci to describe population and landscape genetic patterns across 614 individuals from 12 remaining populations of Arkansas darter (Etheostoma cragini) in Colorado, a candidate species for listing under the U.S. Endangered Species Act. We found small effective population sizes, low levels of genetic diversity within populations, and high levels of genetic structure, especially among basins. Both at- and between-site landscape features were associated with genetic diversity and connectivity, respectively. Available stream habitat and amount of continuous wetted area were positively associated with genetic diversity within a site, while stream distance and intermittency were the best predictors of genetic divergence among sites. We found little genetic contribution from historic supplementation efforts, and we provide a set of management recommendations for this species that incorporate a conservation genetics perspective.

3902: *+.123*

Ribes echinellum (Coville) Rehder (Miccosukee gooseberry; Grossulariaceae) is a Federally Threatened species known from only two localities: Jefferson County (Florida, FL) and McCormick County (South Carolina, SC). This perennial shrub, ca. 1 m tall, is deciduous, and reproduces both vegetatively (clonal growth) and sexually (seed production). Recent surveys of the FL population revealed a dramatic decline in plant numbers. To assist in conservation and management of this species in FL and SC populations, microsatellite genetic markers were used to identify genotypes and assess the genetic structure of R. echinellum. We genotyped seven microsatellite loci in 102 individuals: 74 collected in FL and 28 in SC. Unbiased heterozygosity was between 0.28 and 0.53. All seven loci were polymorphic, showing a range of 1.52-2.13 effective number of alleles per locus (mean = 1.75). The two populations of R. echinellum show low genetic diversity, especially in SC. Clonality was not widespread, but was higher in the SC population. Both populations show signatures of bottlenecks but isolation by distance was not evident. We found significant deviation from HW equilibrium, with higher number of heterozygotes than expected. However when HW test was done for the combined populations as two separate groups, only FL showed a significant HW test and for SC the test was non-significant. Bayesian analysis and F-ST values suggest high genetic divergence between the populations. These results are important for developing a recovery plan and an ex situ and reintroduction conservation programs.

3903: *-.035*

Background and Aims: Przewalski's horses have been imported from the western zoos to China since 1985. Yet the genetic diversity in China's populations has not been studied, thus lacking of such knowledge inevitably affects this population's management. The aim of this study was to assess genetic diversity in Chinese population of Przewalski's horses via mitochondrial DNA (mtDNA) control region and pedigree analysis. Materials and methods: Two captive and one reintroduced populations were examined based on mitochondrial DNA control region variation via fecal sampling from 2010 to 2012, together with pedigree analysis. Results: Amplification success rates of fecal mtDNA were as high as 96.2% (93.8%-100%), and were higher for sample in winter

than in summer and autumn. Two haplotypes were identified and shared among three populations, but the proportion of individuals with each haplotype varied among the three populations (F-ST = 0.10874, p = 0.00978). Haplotype diversity in the released population (0.153) was much lower than that in the two captive populations (0.4011 and 0.4966), in accordance with the direction of increase in probability of identity at the dam lines. Conclusion: Future concerns in Przewalski's horse population management should emphasize on strict reproduction control to minimize inbreeding in captivity, followed by long-term genetic diversity guidelines and non-invasive monitoring in the reintroduction programmes.

3904: *+.072*

Utah prairie dogs (Cynomys parvidens) have been extirpated in 90% of their historical range. Because most populations in Utah occur on private land, this threatened species is continually in conflict with landowners. The Utah Division of Wildlife Resources has been relocating Utah prairie dogs from private to public land since the 1970s, but relocations have been largely unsuccessful because of high mortality. Utah prairie dogs are highly social animals, but they are usually relocated without regard to their family group, or coterie. We predicted that relocating Utah prairie dogs with other coterie members would improve their survival rate and result in post-release behavior similar to non-relocated animals. We chose to investigate release-site retention as a measure of relocation success because we were unable to separate emigration from mortality. We relocated Utah prairie dogs from the Cedar Ridge Golf Course in Cedar City, Utah to 2 prepared sites near Bryce Canyon National Park, Utah in 2010 and 2011. We relocated prairie dogs as groups of coterie members or in a control group of individuals trapped with no regard to relatedness. Two months after relocation, we set traps to recapture animals to estimate release-site retention. We quantified activity budgets prior to and following relocation on study animals as well as from a previously established relocated prairie dog population on public land. The best predictor of release-site retention and recapture rate was the animal's weight at initial capture. Larger animals had high retention but low recapture rates. We found no differences in site retention or behavior between prairie dogs relocated with coteries and controls. Relocated individuals behaved more like prairie dogs on public lands than animals in the urban source population, but behaviors were still different from prairie dogs on public lands. We recommend relocating large, adult Utah prairie dogs rather than juveniles or relocating juveniles later in the trapping season to increase relocation success rate. We also suggest that future research should focus on developing additional release methods to reduce dispersal and increase site retention. (c) 2014 The Wildlife Society.

3905: *+.077*

For many threatened vertebrates, captivity may be the only option for species survival. Maintaining species in captivity prior to reintroduction presents many challenges, including the need to preserve genetic diversity and mitigation of disease risks. Recent studies suggest that captivity can alter the suite of symbiotic microbes that play important roles in host health. The Panamanian golden frog (Atelopus zeteki) has not been seen in its native habitat in Panama since 2009. Along with habitat loss and illegal collecting, the lethal disease chytridiomycosis, caused by the fungal pathogen Batrachochytrium dendrobatidis (Bd), is responsible for the severe decline of this species. Prior to the spread of Bd into golden frog habitat, conservation organizations collected golden frogs and placed them in captive survival assurance colonies. The skin of amphibians is host to a diverse resident bacterial community, which acts as a defense mechanism in some amphibians to inhibit pathogens. We characterized the cutaneous bacterial community from wild and F1 captive golden frogs originating from the same population with Illumina

sequencing to assess how long-term captivity has affected this community. We found that species richness, phylogenetic diversity, and community structure of the skin microbiota was significantly different between wild and captive golden frogs. However, after approximately eight years of living in captivity, the offspring of the original captive golden frogs still shared 70% of their microbial community with wild frogs. These results demonstrate that host-associated microbial communities can be significantly altered by captive management, but most of the community composition can be preserved. (C) 2014 Elsevier Ltd. All rights reserved.

3906: *+.060*

Reintroductions are commonly used to restore the local biological diversity and/or save threatened taxa. In human-altered landscapes, we may expect that reintroduced species affect taxa already present. In Abruzzo, Lazio and Molise National Park (central Apennines, Italy), a 30% decline in the abundance of 'vulnerable' Apennine chamois (2005: c.650 individuals, 2010: c.450 individuals) has been recorded, whereas red deer (reintroduced in 1972-1987: 81 individuals) have greatly increased (2010: > 2500 individuals). We investigated space and diet overlaps between red deer and Apennine chamois, and their effects on foraging behaviour of the latter. We also compared the composition of grasslands with that recorded when the former were absent. In 2010-2011, we found out: (1) a great space (> 75%) and diet (> 90%) overlap between deer and chamois; (2) a significant increase of unpalatable plant species and a decreasing trend of the nutritious, most grazed species by chamois, in respect to when deer were absent; (3) irrespective from vegetation type, a significantly reduced bite rate of adult female chamois in patches used also by deer, compared with areas without deer. Our results suggest a negative effect of red deer on the availability of nutritious plant species in summer-autumn, possibly because of grazing and physical damage on the grassland caused by trampling. Environmental conditions and access to high-quality forage in the warm season influence the winter survival of offspring of mountain ungulates. Our results indicate that interspecific overlap in resource use with an increasing, reintroduced population can threaten rare taxa. Reintroductions of potentially competing species should be avoided in areas where populations of threatened taxa exist.

3907: *+.131*

In the present article, the results of the first-stage of monitoring, following restoration works on a small Mediterranean wetland (Lake Stagnone, Capraia Island, Tuscan Archipelago), are reported. The recent spread of Typha and Phragmites in the lake changed diversity and composition of the plant communities. Nine years after their first monitoring (2009), a rarefaction of hydrophytes and small helophytes of conservation interest was detected. In 2010, the restoration started with the aim to remove (or at least reduce) the populations of the large, expansive helophytes. In 2012, the first post-actions monitoring were carried out using the same methods as previously, analysing the plant presence/absence and their cover value recorded in the same 15 plots selected in 2000 and 2009. The rise and fall of the populations of the various flora and vegetation types during this invasion process and the following restoration were statistically analysed. One year following the restoration, some recovery (replacement) had occurred of autochthonous hydrophytes and small helophytes. Many of these species are of conservation interest. Some aquatic plants, present on the site until the more or less recent past, were once more recorded. Given the rapid recovery of populations of many autochthonous species, the results are reasonably encouraging, rendering planned reintroductions unnecessary at the moment. On the other hand, because of the short time elapsed since restoration, the current community structure cannot in any way be considered an "equilibrium" one. Continued and regular monitoring is required to allow the reestablishment of the large expansive helophytes populations.

3908: ***+.064***

Conservation of endangered sympatric species must be carefully executed to ensure that the protection of one species does not negatively impact the survival of another. Critically endangered Pecos gambusia Gambusia nobilis and Leon Springs pupfish Cyprinodon bovinus, which are endemic to a threatened desert spring habitat, exemplify one such scenario. Recently, this habitat has been expanded, and captively bred C. bovinus were released to repopulate an area historically known to contain this species. A previous study suggested that G. nobilis are detrimental to C. bovinus populations, due to their tendency to congregate near spawning pairs and feed on their eggs. Whether G. nobilis are attracted to territorial C. bovinus, regardless of spawning behavior, remains unclear. To determine this, the number of G. nobilis in occupied and unoccupied breeding territories of both wild and captively bred C. bovinus was measured. Gambusia nobilis densities were similar between unoccupied and occupied territorial sites, suggesting that they do not show a preference for C. bovinus territories. Regardless of habitat location or prior captivity, territorial C. bovinus significantly excluded G. nobilis within their direct vicinity (5 cm), but not from their entire territory. Decreased G. nobilis density within the habitat may allow C. bovinus to defend their breeding territories more readily during the summer spawning season. This study provides empirical evidence of captively raised individuals behaving similarly to wild individuals upon reintroduction to their natural habitat and support for conservation methods that focus on common limiting factors between endangered species

3909: ***+.114***

Background: Big-leaf mahogany (Swietenia macrophylla King) is the woody species with the highest economic value in Latin America. Currently, it is subject to intensive exploitation, diminishing its natural populations. Due to this decline, the species is a preferred species for reforestation and establishment of commercial tropical plantations. Mycorrhizal symbiosis is a biotic factor scarcely studied in the ecology of this species. Therefore, the objective of this research was to identify the diversity of arbuscular mycorrhizal fungi (AMF) species associated with the rhizosphere of seedlings and mature trees of big-leaf mahogany growing in its natural habitat, a tropical rain forest in Los Tuxtlas, Veracruz, Mexico. Soil samples from a 20-cm depth were taken from the rhizosphere of big-leaf mahogany seedlings and mature trees. Additionally, spores from the rhizosphere soil were propagated on Sorghum vulgare, isolated and identified. The percentage of AMF colonization was also evaluated.Results: Twenty-three AMF morphospecies belonging to four genera were registered: 11 corresponded to Glomus, 10 to Acaulospora, one to Gigaspora and one to Ambispora. Ambispora gerdemannii, Acaulospora spinosa, A. scrubiculata, A. foveata, Septoglomus constrictum, Claroideoglomus etunicatum, Glomus tenebrosum, Sclerocystis sinuosum, Diversispora aurantium, and Rhizophagus fasciculatus were identified to species level. We report for first time the presence of G. tenebrosum and C. etunicatum in natural areas of the humid Mexican tropics. The rhizosphere soil of the trees harbor more morphospecies than soil from seedlings (21 and 11 morphospecies, respectively). Sorghum plants inoculated with rhizosphere soil from big-leaf trees showed higher percentages of total mycorrhizal colonization, arbuscules and hyphae ($P < 0.01$) compared with plants inoculated with rhizosphere soil from seedlings.Conclusions: Twenty-three AMF morphospecies included in the genera Glomus, Acaulospora, Gigaspora and Ambispora were found associated with rhizosphere soil of mahogany trees growing in its natural habitat. The diversity of AMF genera and species found was around two times greater in mature trees than in seedlings. Some AMF species were only detected when trap-plants culture methods were employed, stressing the importance of this technique. This information has great potential for biotechnological application when performing reintroductions or reforestation with the tropical tree mahogany.

3910: ***+.091***

1. Ex situ programmes for endangered species commonly focus on two main objectives: insurance against immediate risk of extinction and reintroduction. Releases influence the size of captive and wild populations and may present managers with a trade-off between the two objectives. This can be further complicated when considering the costs of the captive population and the possible release of different life stages.2. We approached this decision problem by combining population models and decision-analytic methods, using the reintroduction programme for the southern corroboree frog Pseudophryne corroboree in Australia as an example. We identified the optimal release rates of eggs and subadults which maximized the size of the captive and reintroduced populations while meeting constraints. We explored two scenarios: a long-term programme for a stable age-distributed captive population and a short-term programme with non-stable age distribution and limited budget. We accounted for uncertainty in the estimated vital rates and demographic stochasticity.3. Assuming a stable age distribution, large proportions of individuals could be released without decreasing the captive population below its initial size. The optimal strategy was sensitive to the post-release survival of both life stages, but subadult releases were generally most cost-effective, producing a large wild population and requiring a cheaper captive population. Egg releases were optimal for high expected juvenile survival, whereas mixed releases of both life stages were never optimal.4. In the short-term realistic scenario, subadult releases also produced the largest wild population, but they required a large increase in the size and cost of the captive population that exceeded the available budget. Egg releases were cheaper but yielded smaller numbers in the wild, whereas joint releases of both life stages provided more wild individuals, meeting budget constraints without depleting the captive population.5. Synthesis and applications. Optimal release strategies for endangered species reflect the trade-offs between insurance and reintroduction objectives and depend on the vital rates of the released individuals. Although focusing on a single life stage may have practical advantages, mixed strategies can maximize cost-effectiveness by combining the relative advantages of releasing early and late life stages.

3911: ***+.032***

Monitoring of the ecological efficiency of different restoration and mitigation measures is important to inform decision-making but can be challenging, especially in remote and low-resource settings. Species composition of the vegetation is sensitive to environmental variation, and can thus be used in restoration assessment, but this requires statistical approaches that can accommodate multivariate responses. We use principal response curves (PRC) to assess the efficiency of post-hydropower mitigation measures installed to secure the reintroduction of an extinct-in-the-wild amphibian back into its only native habitat. The endemic ovoviviparous Kihansi spray toad Nectophrynoides asperginis is only known from a wetland in the Lower Kihansi River Gorge in the Eastern Arc Mountains in Tanzania. River flow was diverted from the gorge for hydropower production in 1999, causing the spray wetland to desiccate, consequently threatening the toad and other plant and animal species dependent on the spray-zone habitat. To mitigate the toad population collapse, a sprinkler system was installed over a limited section of the original spray-zone wetlands to mimic the waterfall spray and toads were taken to the USA for ex situ breeding. The decline, extinction, ex situ breeding, and planned reintroduction of the species has driven substantial research on Kihansi spray toad biology. In contrast, the efficiency of the mitigation measures in restoring the spray-zone wetland habitat required for its successful reintroduction has not been formally evaluated.Here, we analyze re-sampled vegetation data from the spray-zone wetland over a period of eight years by means of principal response curves to investigate if the post-hydropower mitigation measures have successfully restored the pre-

hydropower ecosystem. The results show that the spray-zone vegetation is recovering. The wetland flora and especially species important to the Kihansi spray toad have increased and the restored ecosystem has stabilized, favoring the reintroduction of the Kihansi spray toad to its native habitat. However, the wetland ecosystem is not restored entirely and continued mitigation measures are needed. Continued monitoring is essential to support evidence-based restoration, and we conclude that assessment based on vegetation monitoring coupled with principal response curve analyses provides a cost-effective and efficient monitoring tool for such projects.

3912: *+.193*

Wild species reintroduction is a highly recommended tool when favors ecosystem functionality. It constitutes a long-term process and requires the evaluation of the different ecological aspects involved in the reintroduction process. In the case of large wild herbivores one important aspect that should be known is foraging habits. In the present study, diet at the species level was determined for the guanaco (Lama guanicoe) population reintroduced in the Quebrada del Condorito National Park (Cordoba, Argentina). Fresh faeces samples were collected within the territory occupied by reproductive groups in the Park, between February and August 2009. Diet botanic composition was determined by microhistological analysis. The diet of reintroduced guanacos was composed mainly by short grasses and sedges, characteristic plants of grazing lawns. Despite the high plant diversity in the study area, only five species represented 71% (for the cold and dry months) to 93% (for the warm and wet months) of the diet consumed during the study period in the Park: Sorghastrum pellitum, Chascolytrum subaristatum, Carex fuscula, Eleocharis pseudoalbibracteata y Lachemilla pinnata. The former four species were consumed in a proportion significantly higher than their abundance in the field, which is indicating that the reintroduced guanacos are behaving as selective consumers. Additionally, a small but consistent seasonal difference was detected in plant species consumption, with higher consumption of the tussock grasses D. hieronymi and P. stuckertii, dominant in tussock grasslands, and of woody plants, during the dry and cold season. Probably this was caused by the productivity decrease during cold and dry season months in the study area.

3914: *+.220*

European white stork (Ciconia ciconia) populations have been object to several conservation measures such as reintroduction programs, habitat improvement or supplementary feeding in the last decades. Although recent white stork censuses revealed an upward trend of most of the western populations, evaluations of the relative importance of certain conservation measures are still scarce or even lacking. In our study we analyzed the effect of supplementary feeding on the reproductive success of white storks in conjunction with other factors such as weather or nest site characteristics. We present data of 569 breeding events at 80 different nest sites located in variable distances to an artificial feeding site at Affenberg Salem (south-western Germany) collected from 1990-2012. A multilevel Poisson regression revealed that in our study population (1) reproductive success was negatively affected by monthly precipitation in April, May and June, (2) pairs breeding on power poles had a lower reproductive success than pairs breeding on platforms or trees and (3) reproductive success was significantly higher in pairs breeding in close distance to the feeding site. The number of fledglings per nest decreased by 8% per kilometer distance to the feeding site. Our data suggest that supplementary feeding increases fledgling populations which may be a tool to attenuate population losses caused by factors such as habitat deterioration or unfavorable conditions in wintering habitats.

3915: *+.086*

Global climate changes during the Quaternary reveal much about broader evolutionary effects of environmental change. Detailed regional studies reveal how evolutionary lineages and novel communities and ecosystems, emerge through glacial bottlenecks or from refugia. There have been significant advances in benthic imaging and dating, particularly with respect to the movements of the British (Scottish) and Irish ice sheets and associated changes in sea level during and after the Last Glacial Maximum (LGM). Ireland has been isolated as an island for approximately twice as long as Britain with no evidence of any substantial, enduring land bridge between these islands after ca 15 kya. Recent biogeographical studies show that Britain's mammal community is akin to those of southern parts of Scandinavia, The Netherlands and Belgium, but the much lower mammal species richness of Ireland is unique and needs explanation. Here, we consider physiographic, archaeological, phylogeographical i.e. molecular genetic, and biological evidence comprising ecological, behavioural and morphological data, to review how mammal species recolonized western Europe after the LGM with emphasis on Britain and, in particular, Ireland. We focus on why these close neighbours had such different mammal fauna in the early Holocene, the stability of ecosystems after LGM subject to climate change and later species introductions. There is general concordance of archaeological and molecular genetic evidence where data allow some insight into history after the LGM. Phylogeography reveals the process of recolonization, e.g. with respect to source of colonizers and anthropogenic influence, whilst archaeological data reveal timing more precisely through carbon dating and stratigraphy. More representative samples and improved calibration of the 'molecular clock' will lead to further insights with regards to the influence of successive glaciations. Species showing greatest morphological, behavioural and ecological divergence in Ireland in comparison to Britain and continental Europe, were also those which arrived in Ireland very early in the Holocene either with or without the assistance of people. Cold tolerant mammal species recolonized quickly after LGM but disappeared, potentially as a result of a short period of rapid warming. Other early arrivals were less cold tolerant and succumbed to the colder conditions during the Younger Dryas or shortly after the start of the Holocene (11.5 kya), or the area of suitable habitat was insufficient to sustain a viable population especially in larger species. Late Pleistocene mammals in Ireland were restricted to those able to colonize up to ca 15 kya, probably originating from adjacent areas of unglaciated Britain and land now below sea level, to the south and west (of Ireland). These few, early colonizers retain genetic diversity which dates from before the LGM. Late Pleistocene Ireland, therefore, had a much depleted complement of mammal species in comparison to Britain. Mammal species, colonising predominantly from southeast and east Europe occupied west Europe only as far as Britain between ca 15 and 8 kya, were excluded from Ireland by the Irish and Celtic Seas. Smaller species in particular failed to colonise Ireland. Britain being isolated as an island from ca. 8 kya has similar species richness and composition to adjacent lowland areas of northwest continental Europe and its mammals almost all show strongest genetic affinity to populations in neighbouring continental Europe with a few retaining genotypes associated with earlier, western lineages. The role of people in the deliberate introduction of mammal species and distinct genotypes is much more significant with regards to Ireland than Britain reflecting the larger species richness of the latter and its more enduring land link with continental Europe. The prime motivation of early people in moving mammals was likely to be resource driven but also potentially cultural; as elsewhere, people exploring uninhabited places introduced species for food and the materials they required to survive. It is possible that the process of introduction of mammals to Ireland commenced during the Mesolithic and accelerated with Neolithic people. Irish populations of these long established, introduced species show some unique genetic variation whilst retaining traces of their origins principally from Britain but in some cases, Scandinavia and Iberia. It is of particular interest that they may retain genetic forms now absent from their source populations. Further species introductions, during the Bronze and late Iron Ages, and Viking and Norman invasions, follow the same pattern but lack the time for genetic divergence from their

source populations. Accidental introductions of commensal species show considerable genetic diversity based on numerous translocations along the eastern Atlantic coastline. More recent accidental and deliberate introductions are characterised by a lack of genetic diversity other than that explicable by more than one introduction. The substantial advances in understanding the postglacial origins and genetic diversity of British and Irish mammals, the role of early people in species translocations, and determination of species that are more recently introduced, should inform policy decisions with regards to species and genetic conservation. Conservation should prioritise early, naturally recolonizing species and those brought in by early people reflecting their long association with these islands. These early arrivals in Britain and Ireland and associated islands show genetic diversity that may be of value in mitigating anthropogenic climate change across Europe. In contrast, more recent introductions are likely to disturb ecosystems greatly, lead to loss of diversity and should be controlled. This challenge is more severe in Ireland where the number and proportion of invasive species from the 19th century to the present has been greater than in Britain. (C) 2014 Elsevier Ltd. All rights reserved.

3916: *+.161*

Dispersal is an important component in the demography of animal populations. Many animals show seasonal changes in their tendency to disperse, reflecting changes in resource availability, mating opportunities, or in population age structure at the time when new offspring enter the population. Understanding when and why dispersal occurs can be important for the management of endangered species. The pygmy bluetongue lizard is an endangered Australian species that occupies and defends single burrow refuges for extended periods of time, rarely moving far from the burrow entrance. However, previous pitfall trapping data have suggested movement of adult males in spring and of juveniles in autumn of each year. In the current study we compared behaviours of adult lizards each month, over the spring-summer activity period over two consecutive field seasons, to provide deeper understanding of the seasonal dispersal pattern. We released adult pygmy bluetongue lizards into a central area, provided with artificial burrows, within large enclosures, and monitored the behaviour and movements of the released lizards over a four day period. There was a consistent decline in time spent basking, amount of movement around burrow entrances, and rates of dispersal from the central release area from early spring to late summer. Results could be relevant to understanding and managing natural populations and for any translocation attempts of this endangered lizard species.

3917: *-.251*

The threatened status (both ecologically and legally) of Caribbean staghorn coral, Acropora cervicornis, has prompted rapidly expanding efforts in culture and restocking, although tissue loss diseases continue to affect populations. In this study, disease surveillance and histopathological characterization were used to compare disease dynamics and conditions in both restored and extant wild populations. Disease had devastating effects on both wild and restored populations, but dynamics were highly variable and appeared to be site-specific with no significant differences in disease prevalence between wild versus restored sites. A subset of 20 haphazardly selected colonies at each site observed over a four-month period revealed widely varying disease incidence, although not between restored and wild sites, and a case fatality rate of 8%. A tropical storm was the only discernable environmental trigger associated with a consistent spike in incidence across all sites. Lastly, two field mitigation techniques, (1) excision of apparently healthy branch tips froma diseased colony, and (2) placement of a band of epoxy fully enclosing the diseased margin, gave equivocal results with no significant benefit detected for either treatment compared to controls. Tissue condition of associated samples was fair to very poor; unsuccessful mitigation

treatment samples had severe degeneration of mesenterial filament cnidoglandular bands. Polyp mucocytes in all samples were infected with suspect rickettsia-like organisms; however, no bacterial aggregates were found. No histological differences were found between disease lesions with gross signs fitting literature descriptions of white-band disease (WBD) and rapid tissue loss (RTL). Overall, our results do not support differing disease quality, quantity, dynamics, nor health management strategies between restored and wild colonies of A. cervicornis in the Florida Keys.

3918: *+.084*

The North America freshwater mussel fauna has suffered an inordinately high recent extinction rate, and the small size and isolation of many remaining populations portends a continued diminishment of this fauna. Causes of extinction and imperilment are varied but revolve around massive habitat loss, deterioration, and fragmentation. The National Strategy for the Conservation of Native Mussels, published in 1997, has guided efforts to address this crisis. Considerable progress has been made toward several of the Strategies' goals, particularly increasing our knowledge of mussel biology, promoting mussel conservation, and development of techniques for captive mussel propagation. However, mussel conservation should focus more directly on reducing fragmentation through bold and aggressive habitat restoration. In addition to dam removal, improvement in dam tailwater flows, and restoration of channelized streams, identification of factors that eliminated mussels from many otherwise intact streams is critical. Translocation and captive propagation will be key elements in reestablishing mussel assemblages in restored habitats, but these techniques should be used with caution and primarily to increase the occurrence of a species throughout its historical range. Conserving mussel diversity in an ever-changing world is dependent on promoting the natural, long-term sustainability and evolutionary potential of mussel populations.

3919: *+.006*

Quantifying the genetic composition of founder populations is important to the success of reintroduction programmes, especially for bottlenecked and/or specialized species, such as island endemics. By implementing admixture schemes based on genetic variability, captive breeding programmes can minimize the detrimental effects of bottlenecking, inbreeding depression, outbreeding depression, etc. Particular attention has been given to genes within the major histocompatibility complex (MHC) due to their direct correlation to an individual's immunity. However, isolating and amplifying MHC haplotypes remains difficult owing to the high diversity and paralogous nature. We describe a method of MHC I haplotype isolation based on an iterative process of primer design for the endangered island endemic, the Laysan duck (Anas laysanensis). Ultimately, haplotype-specific primers allow for direct genotyping after gel electrophoresis based on the presence/absence of their respective amplicons. Using the developed techniques, a total of eight unique haplotypes were isolated and assayed across 21 Laysan duck individuals from Laysan Island (N = 10) and Midway Atoll (N = 11). The presence/absence of seven haplotypes were variable across individuals with three haplotypes present in 95% of individuals, three in 38% of individuals, and one in 90% of individuals. The protocols described herein provide a simple, cost-effective method for isolating haplotypes and monitoring existing MHC variation in Laysan ducks, and the general approach can be applied to other molecular markers and species with low genetic diversity.

3920: *+.092*

We present a protocol for the transplantation of founding queens of the harvester ant Messor

barbarus (LINNAEUS, 1767), monitoring their survival at six months, one year and 18 months. Once established, these ants are expected to have a positive impact on vegetation restoration via their ability to disperse seeds harvested by workers, thus accelerating the rehabilitation and restoration already undertaken using conventional civil engineering. The transplantations were performed on two sites currently undergoing ecological restoration, one previously degraded by intensive fruit growing (AOA) and the other destroyed by an industrial accident (OLA), and compared with natural colonisation on a reference steppe site. Founding-queen transplantation was also performed at the reference steppe site. We report here the first steps of the protocol, with results on transplantation success and on natural colonisation.Short-term rates of survival of the transplanted founding queens are encouraging: 15% at the AOA site (after one and a half years) and 35% at the OLA site (after one year). It will take a few years longer to assess any significant impact on composition, species richness and distribution of the different plant populations characteristic of the steppe. However, we demonstrate that the density of natural nests is significantly lower at the degraded site (AOA) than at the reference steppe. It also appears that natural recolonisation by Messor barbarus at the destroyed site (OLA) would be difficult without the creation of a favourable habitat. These two results attest to the value of transplantation operations and confirm our hypothesis that for successful establishment the suitability of host habitats for founding queens is a more limiting factor than dispersal during the nuptial flight.

3921: *+.070*

The Bohemian Forest harbours one of the largest lynx populations in Central Europe, which arose from animals reintroduced in two adjacent national parks. Despite an increasing number of population modelling approaches, the differences between potential and realised lynx distributions urgently need to be explored. We used lynx monitoring data from 2005 to 2010 from 530 municipalities in eastern Bavaria and spatial estimates of roe deer densities to test the predictions that the probability of lynx occurrence (confirmed or unconfirmed) increases with (1) decreasing distance to the national park area, (2) increasing forest cover, (3) increasing proportion of state-owned forests, (4) increasing roe deer density and (5) decreasing human activity. Using a flexible additive boosting model, we identified the distance to the national parks as the dominant factor, with positive effects on lynx probability only up to 70 km from the centre of the two national parks. Moreover, forest cover and roe deer density were correlated with increasing lynx occurrence. The probability of unconfirmed lynx occurrence increased with the proportion of state-owned forest within a municipality. The most probable mechanism behind the distance variable is illegal killing outside of the national parks. We concluded that despite the small size of protected areas in Central Europe, they still provide important source areas for this large predator. Moreover, the results supported conclusions of previous modelling approaches on the exchange among existing sub-populations in Central Europe, and indicated that lynx currently might not be able to colonise the next suitable areas. (C) 2014 Elsevier Ltd. All rights reserved.

3922: *+.211*

The reintroduction and recolonization of species extirpated from former ranges are key components of species conservation. Resource availability affects recolonization success and resulting distribution patterns, but top-down processes may also play a role through density-mediated or trait-mediated indirect effects that may exclude a species from otherwise suitable habitat. We predicted that the spatial distribution of recolonizing sea otters, Enhydra lutris, on the Canadian Pacific coastline was explained by resources as well as interspecific interactions - spatial segregation from pinnipeds, the preferred prey of killer whales Orcinus orca. We surveyed the summer occurrence of sea otters and pinnipeds on Vancouver Island, Canada. We quantified

coastline density and bathymetry at multiple spatial scales as indices of habitat complexity and foraging habitat availability. We used generalized linear model selection to test hypotheses about sea otters' spatial relationship to resources and heterospecifics. Pinniped presence negatively predicted sea otter presence, even after accounting for complexity and foraging habitat. Sea otters may segregate from pinnipeds due to trait-mediated indirect effects of predation, leading us to hypothesize apparent competition between sea otters and pinnipeds. Research is needed to test this hypothesis; if true, refuge from apparent competitors may be a key component of recolonization habitat for sea otters. Species distribution models should quantify resource landscapes but also species-scapes: the spatial plane of species interactions that combines with resources to drive species distributions. Conservation plans based on recolonization models that include only resources may overestimate available habitat, carrying capacity, and recolonization success. (C) 2014 Elsevier Ltd. All rights reserved.

3923: *+.161*

Przewalski's horse reintroductions to Xinjiang, China were initiated in 1985. Here, we present the first data on population development and current problems of the Przewalski's horse in both captive and released populations in Xinjiang. From 1985 to 2005, a total of 24 captive Przewalski's horses (14 males and 10 females) were brought from western zoos to the Jimsar Wild Horse Breeding Center (WHBC) in Xinjiang. In 1988, the first foal was born. Since then, a total of 285 foals have been born and the number of animals in the captive population continues to increase. In August 2001, the first group of horses was released into semi-wild conditions in the Kalamaili Nature Reserve (KNR). Released horses were allowed to range freely from spring to fall, but were driven into a winter coral to allow for supplemental feeding and to increase winter survival, and to reduce competition with domestic horses from local herdsmen who use the KNR as winter pasture. By December 2013, a total of 89 horses (32 males and 57 females) in 14 groups had been transferred to semi-release; and within two years after the first release, the first foal was successfully born in the wild. By 2013, the reintroduced animals had formed into 16 groups (127 individuals, 13 breeding and 3 bachelor group) in 5 sites. To date, this is the most comprehensive and successful Przewalski's reintroduction effort in China. (C) 2014 Elsevier Ltd. All rights reserved.

3924: *+.172*

Reintroducing locally extinct species is an increasingly used conservation tool. However, many reintroductions fail to establish self-sustaining populations. The quality of the individuals released may crucially affect reintroduction success, but it is largely unknown which quality traits are important and how they might be improved, especially in captive-reared animals. We aimed at increasing survival of captive-bred grey partridges (Perdix perdix) after release. We experimentally tested the effects of prenatal and postnatal unpredictable food supply on post-release survival of two captive strains (two or >30 generations in captivity). 691 Full-grown birds representing all eight strain x prenatal x postnatal treatment combinations were released in autumn in 28 social groups (coveys) and followed in the field for six months by radio tracking and visual observations. Data were analysed with multistate capture-recapture models including several random effects. Post-release survival was higher in birds having encountered postnatal unpredictable food supply and decreased drastically with later release dates. Also, coveys strongly affected an individual's survival prospects. Our results suggest that post-release survival of captive-bred grey partridges can be increased significantly through simple, inexpensive pre-release measures in captivity, i.e. exposing them to an unpredictable environment. Similar measures might be successful in other species and could ultimately enhance reintroduction success. (C) 2014

Elsevier Ltd. All rights reserved.

3925: *-.002*

Fennoscandian calcareous wooded meadows have high conservation value due to very high diversity which has been maintained by consistent mowing, but undergo species loss when this management is abandoned. We compared species richness and composition of regularly mown and abandoned wooded meadows in Estonia and established species groups with respect to their response to abandonment. These meadows were very species rich with a maximum of 43 species per 0.25 m(2). Species whose populations are maintained by mowing constituted > 60 % of the floristic diversity of the mown wooded meadows. Abandonment suppressed species with a preference for dry infertile open habitats. Response to abandonment was related to clonality, height and growth form. The greatest negative response was associated with low graminoids, short-lived and non-clonal species, rosette and semi-rosette growth form. Weak competitors with low height and light seeds exhibited a slow decline; perennial life span and clonality enable a delay in local extinction of these species. Response groups had similar persistence at a national level, revealing that earlier assessment of species dynamics at larger spatial scales could underestimate the number of threatened species. Application of restoration measures could avoid local extinctions of many mowing-supported species, whereas some of the mowing-dependent species likely need reintroduction.

3926: *+.028*

Malcolmia littorea (Brassicaceae) is a threatened species growing in the coastal sandy dunes of the west-Mediterranean basin. In this study, the seed germination and seedling emergence requirements of this species were investigated in the only remaining native population in Italy. The highest germination percentage was achieved in darkness with scoring under safe green light at 5-10 degrees C. Seedling emergence was highest when seeds were buried between 1 and 10 mm in depth. The results suggest that germination and seedling emergence are adapted to Mediterranean coastal habitats by employing a common mechanism of light-inhibited germination and by germinating at cooler temperatures before the onset of the summer drought. Seeds were also collected from plants cultivated at a botanical garden and from plants reintroduced by sowing or by transplanting. For those populations, germination was maximal between 10-25 degrees C, suggesting that the thermal germination behaviour may be affected by the maternal environment of seed production within one generation. It is suggested to use seeds produced in the same environment to which they will be used for the reintroduction of this species. (C) 2014 SAAB. Published by Elsevier B.V. All rights reserved.

3927: *+.011*

The Crocodile Lizard Shinisaurus crocodilurus Ahl, 1930 is a monotypic species, with a distribution range restricted to small and isolated areas in southern China and northern Vietnam. Habitat destruction and illegal poaching are the main causes of alarming population declines and even extinction of some wild populations in China. While the Chinese population was estimated to comprise only 950 individuals in 2004, the existing status of the Vietnamese population remains unknown, since its discovery in 2002. Our work provides the first estimation of the population size of S. crocodilurus in Vietnam, which is essential baseline data for future conservation strategies. Our field research revealed a dramatically small population size of less than 100 mature individuals. This value falls substantially below published threshold sizes of several thousand individuals, required for the long-term persistence of a species. Our research highlights the urgent

need to improve the conservation activities for this species in its natural habitats and suggests means for a translocation program to restore (minimum viable sizes of) the wild populations in northern Vietnam.

3928: *+.140*

White birch (Betula papyrifera) is an open pollinate species that is, dominant in the Northern Ontario after land reclamation. In fact, this species represents 65% of all trees in the region. We hypothesized that the exchange of genetic information between fragmented populations by range-wide paternal introgression is possible in wind-pollinated species such as B. papyrifera. On the other hand, the effects of heavy metal contamination from the mining activities on plant growth and population dynamics are well documented. The main objectives of this study were (1) to assess the level of genetic variation, gene flow, and population sustainability of B. papyrifera after land reclamation; and (2) to determine the level of phytoavailable metals in soil and their accumulation in trees. We found that B. papyrifera is a Ni and Zn accumulator with a translocation factor of 6.4 and 81, respectively, and an indicator of Cu and Pb. The level of polymorphic loci, Shannon index, Nei's genetic diversity, observed number of alleles, and gene flow were determined for the fragmented populations within the targeted region. The percent of polymorphic loci ranged from 28% to 56%; the gene flow was also low with a value of 0.89, and the population differentiation was very high with a value of 0.36. Two population-diagnostic ISSR markers were identified. They were cloned, sequenced, and converted to SCAR markers. Overall, the fragmented populations of B. papyrifera in Northern Ontario are genetically sustainable based on the moderate level of intrapopulation variability.

3929: *+.017*

Species extinction and invasion concurrently affect the composition and properties of ecological communities, yet their effects have largely been studied separately, and with more focus on species and ecological functional groups than the whole-community level, We adopted a dynamic ecological network approach to compare the effects of simultaneous single-species primary extinction and invasion on a set of ecosystem metrics to the effects of extinction and invasion in isolation. We also investigated the relationship between the impact and reversibility of extinction or invasion through reintroduction or eradication, respectively. We used Monte Carlo simulations of bioenergetic ecological network models that combined trophic and mutualistic interactions, contained either prey-dependent or ratio-dependent trophic functional responses, and incorporated either white or pink environmental stochasticity. As the separate extinction or invasion impact increased, the simultaneous extinction invasion impact increased but was decreasingly additive of the two separate impacts, across all ecosystem metrics. Greater extinction or invasion impact was associated with lower reversibility for most model types and ecosystem metrics. There were also systematic differences between models with prey- and ratio-dependent functional responses. These results highlight the importance of considering the combined effects of extinction and invasion in ecological studies, management and restoration.(C) 2014 Gesellschaft fur okologie. Published by Elsevier GmbH. All rights reserved.

3930: *+.152*

Understanding the behavior of species threatened with extinction is important for conservation planning and for solving problems facing species in captivity and the wild. We examined diurnal activity budgets and habitat use of the extinct in the wild Kihansi spray toad to provide insights into ongoing conservation initiatives for this species. Observations on eight target behaviors were

made each morning and evening for 14 days, in two subpopulations at Kihansi and University of Dar es Salaam captive breeding centers. There were significantly more bouts of resting than calling, amplexing, hunting, walking, climbing, or feeding. There was no difference in mean time spent in each activity between the two subpopulations. The use of habitat was variable between age classes, subpopulations and sampling time. Young toads spent significantly more time resting at the top of vegetation and on walls while adults rested more on logs. Further, adults foraged more on the walls and vegetation in the morning and on the ground in the evening. Contrastingly, young toads foraged more on the ground in the morning and switched to elevated patches during evening. The similarity of the toads' behavior suggests that important biological traits are still maintained in captivity and retained across toad generations. Furthermore, temporal and spatial variations in the use of habitat structures between age groups suggest fine-scale resource partitioning to reduce competition in this gregarious species. These results highlight the importance of maintaining diverse habitat structures in captivity and are useful for planning species reintroduction and future restocking programs. (C) 2014 Wiley Periodicals, Inc.

3931: *-.024*

Global biodiversity loss has prompted diverse efforts to stem or reverse declines for many species. Such efforts are often implemented before the efficacy of alternative management actions is quantified. Here, we use matrix models to compare the effectiveness of two supplementation strategies, head-starting early life stages and captive breeding for reintroduction, at reducing extinction risk of declining amphibians. We use the imperiled Oregon spotted frog (Rana pretiosa) as a case study and find that when supplementation occurs after metamorphosis, captive breeding is more effective at reducing extinction risk than head-starting, but the difference declines with increasing supplementation effort. We also find that captive breeding with release as larvae yields similar reductions in extinction risk, and is two orders of magnitude more effective at reducing extinction probabilities than head-starting the same stage. Our results highlight that even basic demographic data can be leveraged to assess tradeoffs among alternative supplementation strategies.

3932: *+.094*

Efforts are underway to return the American chestnut (Castanea dentata) to eastern forests of North America following its decline due to the introduction of the chestnut blight (Cryphonectria parasitica). Approaches include developing blight-resistant chestnut lines through breeding programs and via genetic engineering. Reestablishment of resistant chestnut to eastern forests would produce one of the most extensive ecological restoration transformations ever attempted. However, this undertaking is costly and optimization of reintroduction methods is needed. We used the computer program NEWGARDEN to model whether some patterns of founder placement (regular vs. random spacing at differing densities) produce more rapidly expanding populations across a range of gene dispersal distance conditions (via both offspring and pollen). For a simulated introduction project employing 169 founders, placing founders randomly in a square of side 0.85 km produced higher rates of predicted population growth compared with larger or smaller squares under near gene dispersal conditions; this side distance was 1.0 km under far gene dispersal conditions. After 100 population bouts of mating and under near gene dispersal conditions, the trial with founder placement producing the greatest population expansion rate exhibited a 314% increase in census size compared with the founder pattern yielding the slowest expansion. Neither loss of alleles nor inbreeding or subdivision was significantly increased under the founder placement patterns yielding the most descendants. Exploring different numerical and geometrical founding scenarios using NEWGARDEN can provide first estimates of founding

patterns or stand manipulations that will return the most descendants produced per founder planted in restoration projects.

3933: ***+.047***

The pearl mussel was abundant in the Sudety Mountains until the late 19th century. No live mussels were found within the present borders of Poland since the early 20th century. In 2006-2007 we did a detailed survey of 50 streams and rivers within the former range of the pearl mussel to verify its current status and assess the habitat conditions. No live mussels were found. On the whole, the rivers and streams were found to be degraded. Only four streams met the habitat requirements of the species. Well-preserved shells with a nacre layer were found at the site of the last known population in the Koci Potok stream. To determine whether the shells represented an extinct population or rather indicated the presence of the last survivors, we placed Unio crassus shells in the stream within the historical range of occurrence. Those shells dissolved at the rate of approximately 20% per year, indicating that empty shells would not have remained intact within the channel. Possibly the earlier-found shells had been preserved in the banks above the water level. The possibility that they represent the last survivors can not be excluded. Further search for the last survivors, extended to cover northern Poland, is recommended.

3934: ***+.381***

Translocation, or the purposeful movement of organisms from one location to another for conservation, is currently being used to bolster populations of the endangered greater prairie-chicken (Tympanuchus cupido). We used radiotelemetry to compare survival between 58 resident birds and 54 newly translocated greater prairie-chickens that were sourced from a location more than 325 km away. Model averaged survival estimates were lower in translocated birds (0.42; 95% CI: 0.17-0.66) than in resident prairie-chickens (0.65; 95% CI: 0.46-0.79) through the breeding season. Habitat, sex and year were each included in at least 1 of the top 4 models, but the model averaged confidence intervals for each parameter encompassed zero. Survival of both resident and translocated prairie-chickens increased throughout the breeding season. Both translocated and resident prairie-chickens selected for core prairie habitat over agriculture, and birds tended to avoid surrounding private grasslands and wooded areas. We suggest that future translocation projects account for reduced survival of translocated birds when determining the appropriate release cohort sizes and sex ratios. We also recommend that future management for greater prairie-chicken habitat focus on the expansion of core protected patches of prairie to promote elevated survival and better chances of conservation success. (C) 2014 Elsevier GmbH. All rights reserved.

3935: ***+.017***

In northern Yellowstone National Park, quaking aspen (Populus tremuloides) stands were dying out in the late 20th century following decades of intensive browsing by Rocky Mountain elk (Cervus elaphus). In 1995-1996 gray wolves (Canis lupus) were reintroduced, joining bears (Ursus spp.) and cougars (Puma concolor) to complete the guild of large carnivores that prey on elk. This was followed by a marked decline in elk density and change in elk distribution during the years 1997-2012, due in part to increased predation. We hypothesized that these changes would result in less browsing and an increase in height of young aspen. In 2012, we sampled 87 randomly selected stands in northern Yellowstone, and compared our data to baseline measurements from 1997 and 1998. Browsing rates (the percentage of leaders browsed annually) in 1997-1998 were consistently high, averaging 88%, and only 1% of young aspen in sample plots were taller than 100 cm; none were taller than 200 cm. In 2012, browsing rates were much lower at 44%, and

young aspen were taller on average with 34% >100 cm and 5% >200 cm. Most (62%) of the variation in height of young aspen in 2012 was explained by browsing intensity. Furthermore, in 2012, 25% of stands had at least five aspen saplings tall enough to escape elk browsing (?,200 cm spring height), a condition that has not occurred for decades and happened despite a recent drought. Sapling recruitment did not increase until browsing decreased, following substantial changes in elk density and distribution, and was not significantly related to stand productivity or climate fluctuations. These results suggest that large carnivore restoration, through effects on prey, may aid aspen recovery where aspen have been suppressed by elk. (C) 2014 Elsevier B.V. All rights reserved.

3936: *+.250*

High quality oak savanna communities were once abundant in the North American Midwest, but have become exceedingly rare. Where remnant savannas remain, fire suppression and resulting woody encroachment have dramatically altered vegetative structure, resulting in reduced understory light levels and precipitating declines in herbaceous understory diversity. Restoration of fire suppressed oak savannas generally involves the reintroduction of fire, but questions remain regarding the necessity and impact of mechanical woody vegetation reduction in addition to fire. We report here on initial short-term results of a long-term experiment in a remnant fire-suppressed oak savanna in Southern Michigan to compare a gradient of oak savanna management intensities including; (1) unmanaged reference plots, (2) burning alone (low management intensity), and (3) progressive mechanical thinning combined with burning (high management intensity). We measured several metrics of restoration success: understory, shrub, and canopy cover, understory light levels, understoiy floral resources, and flowering forb diversity, in the first two growing seasons after initiation of restoration treatments. We found that increasing management intensity largely corresponded with increased understory light availability, abundance of floral resources (i.e. forb bloom abundance and diversity), and with decreased shrub and canopy cover. Low management intensity did not increase light availability, or decrease vegetative or canopy cover relative to unmanaged references, while high management intensity achieved both management goals. Both burning alone and thinning + burning generally increased diversity of flowering forbs, where the effect was greatest with high intensity management. These increases were most pronounced in the second growing season after burning, when we saw sharp increases in richness and abundance of flowering forbs. In restored plots, the flowering forb community consisted of pre-existing shade tolerant species, native and exotic ruderals, as well as savanna indicator species. In sum, low-intensity management can achieve some restoration objectives over the short-term; however, we show a clear initial advantage of coupling thinning with burning, relative to burning alone. We suggest that these differing intensities of oak savanna restoration may be appropriate under different temporal, financial, and ecological scenarios. Our work highlights the potential for restoration of understory forb communities by low or high intensity approaches, where relict populations and/or viable seedbanks exist. (C) 2014 Elsevier B.V. All rights reserved.

3937: *+.034*

Continued persistence of black rhinoceros (Diceros bicornis) will likely depend on the cooperation of many reserves and the application of metapopulation models to manage across reserves. The suitability of any reserve, however, depends on factors that promote and constrain occupancy. Constraining factors, particularly human disturbance, are of concern in small reserves because constraints have potentially greater effects, relative to reserve size, than in large reserves. We investigated landscape use by black rhinos at Zululand Rhino Reserve. South Africa, as a function of elevation, slope, patch type, areas burnt and factors associated with disturbance (distances to

nearest water point, human settlement, boundary fence, and roads). Estimated home ranges consistently demonstrated avoidance of human settlements, fragmentation of home ranges and sometimes multi-modal core areas. Resource selection functions confirmed that use of areas increased with greater distance from human settlements (log-odds = 1.3831 +/- 0.4623 [95% CI]) and from perennial water points (2.2859 +/- 0.8261). Space use was greater for thicket (1.0072 +/- 0.5775) and closed savanna (0.8656 +/- 0.6153) than for other patch types. Managers who plan reintroductions of black rhinos should consider availability of forage and cover, disturbances that might restrict access to resources, and effects of reserve size on those disturbances.

3938: *+.130*

Reintroductions of African wild dogs (Lycaon pictus) in the KwaZulu-Natal Province, through the managed metapopulation approach, promoted a population expansion from one pack in Hluhluwe-iMfolozi Park in 1997 to nine packs in three reserves by 2009. Consequently, the likelihood of dispersing wild dogs leaving their natal ranges from within fenced reserves also increased. Land outside these reserves could potentially be utilized to expand wild dog distribution and provide connectivity between the geographically isolated subpopulations. We used Maximum Entropy Modelling (Maxent) to characterize habitat niche selection of transient wild dogs outside of resident reserves, and to identify potential dispersal linkages between subpopulations. A habitat suitability model indicated four variables (elevation, land cover, road density and human density) best predicted probability of presence for transient wild dogs. Elevation (AUC > 0.80) and land cover (AUC > 0.75) were the two most influential variables when considered independently. Transient wild dogs preferred lower lying locations (130-330 ma.s.l.) covered by woodland or bushland; habitat indicative of the preferred prey of wild dogs. Considerable habitat exists for subpopulation linkages; however, the majority of wild dog movements between subpopulations required mitigation of potential or real game or livestock losses. Development of formal linkages and wild dog management between subpopulations will require a sustained approach to improving tolerance towards wild dogs, clarity on financial obligations and management responses to pack and prey population dynamics.

3939: *+.168*

Understanding the dynamics of genetic structures which arise during a population's range expansion can be applied to the conservation of recovering species and species that are shifting their range. Theoretical models, supported by several empirical findings, have indicated that fine-scaled genetic structure can arise at the wave front of a spatially expanding population. We explored the development of population genetic structure in the reintroduced Asiatic wild ass (Equus hemionus) in Israel, four generations after the onset of reintroduction, during which the population experienced demographic growth and range expansion over a complex landscape. Blood samples of the 'founding-population' and fecal samples of the 'wild-population', collected throughout the range of distribution were analyzed using mtDNA markers. Fecal samples were delimitated to 'subpopulations' according to their geographical locations. The "East" subpopulation, at the wave front of the wild population's distribution, was found to be significantly different than the rest of the population (AMOVA, I broken vertical bar(ST) = 0.13, P = 0.04). These findings were supported by an F-ST-test, Spatial-AMOVA and a Barrier analysis. The "East" region is characterized by high quality habitat patches and low landscape connectivity to the rest of the area, which possibly led to its relative isolation. The "East" subpopulation was probably initiated following a founder-effect of dispersers from the release area, which remained in the 'new area', due to its high habitat quality. This genetic structure, though it might diminish over time due to gene flow and additional range expansion, has the potential of facilitating

adaptive evolution and thereby affecting the population's long term persistence.

3940: *-.028*

Acute stressors can be costly, often requiring alteration of normal physiological processes to mitigate their effects. Animal translocation may be a very stressful event and result in a reduced ability to maintain homeostasis. The impacts of translocation on the thermal ecology of ectothermic vertebrates, which may rely on preferred habitats for thermoregulation, are currently unknown. In this study, 22 adult male Northern Pacific rattlesnakes (Crotalus oreganus oreganus) were implanted with automated temperature loggers and radio-tracked. Snakes were assigned to one of three treatments: translocation, handling control, and undisturbed control. Short-distance translocation (SDT) and handling treatments were applied weekly for 6 weeks. Hourly body temperature (T-b) was recorded during the course of the study. Mean T-b was impacted in a time-dependent fashion, where translocated snakes had lower mean T-b than handled controls during the first week of the study only, especially the first 24hr after translocation. Separating the dataset into day and night revealed that this effect was localized to T-b variation during the day only. Variance in temperature was not impacted by translocation or handling. Snake body mass and time of year were the major factors influencing the thermal profiles of these rattlesnakes. Thermal ecology in male rattlesnakes is resilient to SDT, suggesting that they quickly resume normal behaviors following repeated bouts of acute capture stress and disturbance of their spatial ecology. This study provides support for SDT as a safe measure for mitigating human-snake interactions and facilitating conservation practices regarding male snakes, which are the most frequently encountered sex. J. Exp. Zool. 321A: 442-449, 2014. (c) 2014 Wiley Periodicals, Inc.

3941: *+.047*

Bighorn sheep (Ovis canadensis) often die from respiratory disease after commingling with domestic sheep. From 2000 to 2009, we observed commingling between domestic and reintroduced bighorn sheep in 3 populations in UT, USA. We investigated how commingling affected survival of radio-collared female bighorns that were released initially (founder) and those that were subsequently released (augmented). We predicted that the proportion of young surviving to their first winter and population growth would be lower after observed commingling with domestic sheep. We observed groups of bighorns year-round on 2,712 occasions and commingling between domestic sheep and bighorns in 6 instances. On Mount Timpanogos, survival rates were best modeled as constant for females (n = 57) before and after observed commingling with domestic sheep. Survival rates of female bighorns, however, decreased significantly in Rock Canyon (n = 21) and on Mount Nebo (n = 22) for founder, but not augmented bighorns after observed commingling with domestic sheep. Also, the proportion of young surviving to their first winter was almost 3 times lower and population growth was reduced for bighorns after observed commingling with domestic sheep in Rock Canyon and on Mount Nebo. Commingling between domestic and bighorn sheep reduced population parameters in 2 of 3 bighorn populations we studied; however, on Mount Timpanogos, interactions between those 2 species were not fatal for radio-collared female bighorns. Wildlife biologists should manage for spatial separation of these 2 species and consider the location of hobby farms and trailing operations of domestic sheep near release sites for bighorns.

3942: *-.064*

Wildlife diseases pose an increasing threat to biodiversity and are a major management challenge. A striking example of this threat is the emergence of chytridiomycosis. Despite diagnosis of

chytridiomycosis as an important driver of global amphibian declines 15 years ago, researchers have yet to devise effective large-scale management responses other than biosecurity measures to mitigate disease spread and the establishment of disease-free captive assurance colonies prior to or during disease outbreaks. We examined the development of management actions that can be implemented after an epidemic in surviving populations. We developed a conceptual framework with clear interventions to guide experimental management and applied research so that further extinctions of amphibian species threatened by chytridiomycosis might be prevented. Within our framework, there are 2 management approaches: reducing Batrachochytrium dendrobatidis (the fungus that causes chytridiomycosis) in the environment or on amphibians and increasing the capacity of populations to persist despite increased mortality from disease. The latter approach emphasizes that mitigation does not necessarily need to focus on reducing disease-associated mortality. We propose promising management actions that can be implemented and tested based on current knowledge and that include habitat manipulation, antifungal treatments, animal translocation, bioaugmentation, head starting, and selection for resistance. Case studies where these strategies are being implemented will demonstrate their potential to save critically endangered species.

3943: ***+.221***

Successful reintroduction of ecologically extinct bivalve species into anthropogenically impaired urban estuaries is problematic when employing existing management tools used in estuaries where bivalves are present (GIS-based restoration models, expanding existing shellfish beds, placement of shell substrate, physical oceanographic parameters). A significant management challenge is appropriate site selection. We are proposing the inclusion of a biological parameter (evaluation of tissue histopathology) in an inexpensive and rapid site selection model to inform management decision making and identify sites with the greatest potential for reintroduction success. Use of biological biomarkers is not a new concept, but it is important that they be included in a multitiered management approach to bivalve reintroduction. This Case Study tested adult Eastern Oysters (Crassostrea virginica Gmelin) from locations that supported comparable short-term survival rates by evaluating growth and tissue health and/or disease. Biomarkers indicated oyster tissues at one site were normal, the female:male sex ratio was 50:50, and female oysters were in spawning condition. Conversely, oyster tissues at the second site exhibited multiple abnormalities, samples were 100% male, and the incidence of disease was high. Using the biomarker tool, we evaluated 4 additional sites where oysters exhibited short-term (1 year) survival. At 2 locations, we observed chronic health impacts that would preclude reintroduction, including samples from one site where a wild population was surviving. We also analyzed tissue and shell heavy metal contents. Soft tissue metal concentrations in Meadowlands samples were at the high range of scientific literature values, averaging 1.1% of total body weight, whereas tissue metal concentrations at the Keyport site were within acceptable ranges. Although initial survival and growth rates at both locations were comparable, site-specific urban stressors reduced oyster fitness at 1 of the 2 locations. We are proposing an Estuarine Reintroduction Site Selection Model, which includes a biological in situ parameter, to increase the probability of successfully managing a sustainable oyster reintroduction before commencing expensive large-scale restoration activities. Integr Environ Assess Manag 2014;10:555-565. (c) 2014 SETACKey Points Existing physiochemical bivalve restoration management tools are inadequate in urban estuaries where original native populations are "ecologically extinct." Addition of an in situ biological evaluation parameter can aide management decision making when selecting potentially successful bivalve reintroduction sites in urban estuaries. The Estuarine Reintroduction Site Selection Model, which includes an in situ biological evaluation parameter, can be used to rank reintroduction site suitability. Use of the Estuarine Reintroduction Site Selection Model can reduce costs associated

with inappropriate bivalve reintroduction site selection.

3944: *+.005*

Fences that exclude alien invasive species are used to reduce predation pressure on reintroduced threatened wildlife. Planning these continuously managed systems of reserves raises an important extension of the Single Large or Several Small (SLOSS) reserve planning framework: the added complexity of ongoing management. We investigate the long-term cost-efficiency of a single large or two small predator exclusion fences in the arid Australian context of reintroducing bilbies Macrotis lagotis, and we highlight the broader significance of our results with sensitivity analysis. A single fence more frequently results in a much larger net cost than two smaller fences. We find that the cost-efficiency of two fences is robust to strong demographic and environmental uncertainty, which can help managers to mitigate the risk of incurring high costs over the entire life of the project.

3945: *-.024*

Upper respiratory tract disease (URTD) in the gopher tortoise (Gopherus polyphemus) is highly contagious and has been implicated in the reduction of populations throughout the range. With the exception of a few limited studies, the prevalence of URTD in Georgia, USA tortoise populations is poorly known. We found that exposure to Mycoplasma agassizii and Mycoplasma testudineum, associated with URTD, varied geographically among 11 Georgia tortoise populations. The prevalence of antibodies to M. agassizii in individual populations was either very low (0-3%, n=7 populations) or very high (96-100%, n=4 populations), whereas there was variation in the prevalence of antibodies to M. testudineum among populations (20-61%, n=10) with only one site being negative. Five sites had tortoises with antibodies to both pathogens, and these were the only sites where we observed tortoises with clinical signs consistent with URTD. We did not find tortoises with clinical signs of URTD at sites with tortoises with antibodies only to M. testudineum, which provides evidence that this organism may be of limited pathogenicity for gopher tortoises. Collectively, these data indicate that both M. agassizii and M. testudineum are present in Georgia populations of gopher tortoises and that clinical disease is apparent in populations where both pathogens are present. Additional research is needed to better understand the role of these two pathogens, and other potential pathogens, in the overall health of tortoise populations, especially if future conservation efforts involve translocation of tortoises.

3946: *-.054*

1. Endangered species subjected to reintroduction programmes often occur as small and isolated populations with local high density and depressed fecundity. Variation in territory quality may lead to this low fecundity owing to increasing occupation of suboptimal territories as population density grows, known as the habitat heterogeneity hypothesis (HHH). In this context, food supplementation in poor territories may be used to produce extra young which could be allocated to reintroduction programmes.2. We analyse the density-dependent fecundity pattern and the underlying mechanism in a small population of bearded vultures Gypaetus barbatus in Arag on (northeast Spain). We then use population simulations to examine the viability of a hypothetical reintroduction programme using extra young produced by supplementary feeding on poor-quality territories and the effect on the donor population. We also compare the economic cost of such a reintroduction programme in relation to the cost of a traditional captive breeding programme.3. The wild population showed clear negative, density-dependent fecundity regulation driven by the HHH mechanism. Simulations showed that extractions for translocations had no relevant long-

term effects on the donor population viability, but a marked population reduction during the extraction period. However, the implementation of supplementary feeding to produce extra young for translocation lessened significantly this expected initial population reduction.4. Analyses showed that the annual budget of a captive breeding programme for this species could be seven times more expensive than the translocation of extra young produced by food supplementation.5. Synthesis and applications. Reintroduction programmes based on translocation of wild-reared individuals, after a supplementary feeding programme oriented to poor-quality territories, provide a source of young at least seven times cheaper than those from captive breeding programmes. The use of this approach would decrease initial effects on donor population avoiding public criticism. Increasing the number of young released during the first years of the reintroduction decreases total financial cost and increases the final population size in the new area.

3947: *+.047*

The captive genetic management of threatened species strives to preserve genetic diversity and avoid inbreeding to ensure populations remain available, healthy, and viable for future reintroduction. Determining and responding to the genetic status of captive populations is therefore paramount to these programs. Here, we genotyped 19 microsatellite loci for 240 captive giant pandas (Ailuropoda melanoleuca) (similar to 64% of the captive population) from four breeding centers, Wolong (WL), Chengdu (CD), Louguantai (LGT), and Beijing (BJ), and analyzed 655 bp of mitochondrial DNA control region sequence for 220 of these animals. High levels of genetic diversity and low levels of inbreeding were estimated in the breeding centers, indicating that the captive population is genetically healthy and deliberate further genetic input from wild animals is unnecessary. However, the LGT population faces a higher risk of inbreeding, and significant genetic structure was detected among breeding centers, with LGT-CD and WL-BJ clustering separately. Based on these findings, we highlight that: 1) the LGT population should be managed as an independent captive population to resemble the genetic distinctness of their Qinling Mountain origins; 2) exchange between CD and WL should be encouraged because of similar wild founder sources; 3) the selection of captive individuals for reintroduction should consider their geographic origin, genetic background, and genetic contribution to wild populations; and 4) combining our molecular genetic data with existing pedigree data will better guide giant panda breeding and further reduce inbreeding into the future.

3948: *-.002*

Relaxed selection following extirpation of predators or as a consequence of captive breeding may result in the loss of adaptive antipredator behavior. We propose that it is equally possible for relaxed selection to result in the loss or reduced effectiveness of parasite-defense behaviors such as grooming. In both cases, the reintroduction of captive-bred animals into a predator- or parasite-rich area could have disastrous consequences for the survival of the population. Tick infestation is a powerful force for the evolution of adaptive tick-defense grooming behavior in the wild. The regulation of anti-tick grooming has been well-studied in ungulates and consists of endogenous and exogenous mechanisms, corresponding to the programmed and stimulus-driven grooming models. These models predict that (1) smaller animals will groom more frequently than larger ones, (2) breeding males will groom less than females, (3) animals will groom more during the high-tick season, and (4) animals will groom more in tick-rich vs. tick-sparse habitat. We studied the grooming behavior of Pere David's deer (Elaphurus davidianus), which has experienced genetic bottlenecks and possible relaxed selection as a result of hundreds of years in captivity and was recently reintroduced into the wild in China. Our results indicate that most but not all patterns of adaptive anti-tick grooming behavior have been retained, indicating that some aspects of

parasite defense have not undergone relaxed selection even after many years of captivity. Nonetheless, we propose that plans to reintroduce captive-bred animals should take into consideration the historical tick exposure and present grooming behavior of the species in selecting suitable habitat for reintroduction. (C) 2014 Elsevier Ltd. All rights reserved.

3949: ***+.302***

Appropriate decision-making in conservation programmes requires an understanding of the population genetic structure of various indigenous species. However, in lake and river ecosystems rated as biodiversity conservation priority areas in the Philippines, the diversity of many species remains uncharacterized. To address the existing gap, genetic diversity of two native freshwater fishes, Glossogobius celebius (Valenciennes, 1837) and Glossogobius giuris (Hamilton, 1822) was measured. Genetic structure and estimates of genetic variation for each species was determined using isozyme analysis. A higher level of genetic variation was observed within G. celebius populations compared with G. giuris. Genetic structure indicated that the observed genetic variation in G. giuris is due to the variation among the populations. The results suggest the presence of geographical barriers even in adjacent aquatic ecosystems that could restrict gene flow among G. giuris populations. This study supports the need for immediate conservation interventions for both species as they show a low level of genetic diversity in addition to being threatened in their own environment. Possible measures include translocation and establishment of a refuge population of the species studied to be complemented with location-specific strategies that would enhance their habitat conditions. In addition, education and enhancing the awareness of the local community regarding habitat protection are strongly recommended. Copyright (c) 2014 John Wiley & Sons, Ltd.

3950: ***+.186***

The flora of the Hawaiian Islands has one of the highest rates of endemism in the world, and over half of all taxa are at risk of endangerment or extinction. When in situ management alone cannot protect plant populations, maintaining viable germplasm using ex situ storage methods will prevent species extinctions. Germplasm collections with high conservation value are genetically diverse, representative of taxa and populations, and have a well-documented history in cultivation. Ex situ facilities and conservation agencies were surveyed to determine if existing ex situ capacity was sufficient to represent Hawai'i's species of conservation importance (SCI) and to identify limiting factors. SCI were defined and their representation in ex situ collections quantified, the number of wild plants and populations were estimated, and the attempted ex situ methods were recorded. There are 724 SCI, 528 of which are located in at least one facility. Sixty-four percent of the secured taxa are represented by collections from only 10% or fewer of the wild plants. Seed banks have secured more SCI, and with better in situ representation, than any other ex situ method. Seventy-eight percent of SCI have seeds with long-term storage potential. Existing seed storage facilities are currently inadequate for representing all SCI and should be expanded. SCI with low long-term potential in conventional seed storage can be represented in cryopreservation, micropropagation facilities, nurseries, and botanical gardens. Recommendations include establishing a network to coordinate collections, improve data management, and draft conservation plans with ex situ collection goals. This type of assessment can be applied to other regions that do not have a unified and consistent method of tracking ex situ representation.

3951: ***+.087***

The temperate forests in southern Argentina are affected by different factors, among them the

invasion of exotic species, over-grazing and the exploitation of wood species. Ecological restoration can contribute to the recuperation and conservation of these degraded ecosystems, and of their scientific, productive and cultural values. Therefore, the objectives of this study were: 1) to estimate a restoration value for the native species in an area of degraded forest inside the Andean Northern Patagonia Biosphere Reserve, based on the sum of ecological and etlmobotanical parameters, so as to establish an order of priority for their reintroduction; and 2) to propose a criterion for the use of native and exotic species. First, the diversity of plant species was analyzed, and then for each species the ecological parameters of biogeographic, life form, capacity for vegetative reproduction and dependence on birds for dispersal, were established. In a parallel manner, a bibliographic study was carried out to estimate the value of total use of each species. It is recommended to reintroduce Nothofagus dombeyi (Mirb.) Oerst. (Nothofagaceae) and Austrocedrus chilensis (D. Don) Pic. Semi. & Bizzarri (Cupressaceae), because of their function as system architects and for presenting forced sexual reproduction. Other native trees and shrubs were signaled to be priority because of their potential in passive restoration, as a result of fostering the connectivity of existing fragments and from their offer of timber-yielding and non-timber-yielding forest products. The exotic species with high values of use will be promoted to be used by populations adjacent to the reserve. The purpose is that this strategy for restoration and use contributes in the conservation efforts of austral temperate forests, and the construction of bridges between restoration as a discipline and its practice integrated into society.

3952: *+.084*

A pot experiment was conducted to evaluate the effects of two arbuscular mycorrhizal fungi (AMF), Dentiscutata heterogama and Rhizophagus manihotis on the growth and nutrition of Juglans venezuelensis Manning. This species is currently considered a threatened tree species and the successful restoration of its populations depends on an increased understanding of the ecological and physiological aspects of its response to inoculation with AMF. In general, shoot and total dry weight, and leaf area were significantly higher in seedlings inoculated with AMF than in non-inoculated ones. Differences in height and leaf number between the inoculated and non-inoculated treatments become apparent after 30 days of plant growth. Inoculated plants had a greater leaf area as the result of the higher allocation of resources to leaf biomass (leaf mass ratio, LMR). The fraction allocated to the roots (RMR) was not significantly different between treatments. Differences between vital stain (SDH) and non-vital trypan blue stain (TB) showed that the D. heterogama colonization was almost entirely active compared to the R. manihotis colonization. The relative responsiveness (RR) of J. venezuelensis to inoculation with D. heterogama and R. manihotis was 21.8 and 25.4 % respectively, but colonization values were never greater than 45 %, despite low P content in the soils used. The growth and physiological responses of J. venezuelensis to inoculation with two AMF species indicate that these microorganisms should be employed when propagating this threatened species for its subsequence reintroduction into its natural habitat.

3953: *+.194*

Leucorrhinia dubia (White-faced Darter) is a small dragonfly especially associated with bog-pools in peatlands. In England, there are only three areas (including one in Cumbria) where it has strong populations, most of its former sites having been lost due to habitat changes, mainly human-induced. A project in Cumbria aims to restore a population that once existed and was lost through afforestation in the mid 20th century. Approved by landowners and conservation bodies in 2008, an annual programme based on IUCN guidelines has been implemented from 2010 onwards to restore a colony at Cumbria Wildlife Trust's Foulshaw Moss Reserve near Morecambe Bay. The

donor population, Scaleby Moss, is in north Cumbria. Translocation of larvae has been the main methodology, though experimentation with obtaining eggs began in 2013. Monitoring the populations at both sites, mainly by collecting exuviae at the breeding pools, has been the main measure for ensuring that the population at Scaleby Moss has not been adversely affected and for judging the efficacy of the translocations to Foulshaw Moss. Early years of the project were marred by poor summers but good numbers emerged at Foulshaw Moss in both 2013 and 2014, with clear evidence of on-site breeding. However, it is too soon to say whether a sustainable colony has been established. The genetic diversity of the new population has yet to be assessed and a current University-based research study on the genetics of the species in the UK may prove helpful in this regard. The long-term management of the donor site remains a concern and reinforces the need for the current project, which will continue for several more seasons. A parallel re-introduction project in Cheshire commenced translocations in 2013.

3954: ***+.265***

Since several years, translocations of reptiles are increasingly, but in most cases also uncritically, applied in nature conservation as compensation for habitat interferences. However, there is great uncertainty regarding their prospects of success. We here highlight the problems associated with translocations from a population biological point of view using the common wall lizard (Podarcis muralis), the sand lizard (Lacerta agilis) and the smooth snake (Coronella austriaca) as examples of species that are most often affected in Germany. We provide a species- and case-specific decision guide. Furthermore, we illustrate deficits in objectively evaluating prospects of the translocation success. Due to the multiple lack of knowledge dealing with the issue Translocations of reptiles", we should increasingly prioritize the preservation of populations within their original habitat. If a translocation is inevitable, a long-term monitoring for at least two generations should be mandatory. Its results should be supplied to a nationwide database in order to accumulate knowledge that in the future will provide a more reliable basis for the evaluation of the potential success of reptile translocations.

3955: ***+.281***

Capsule Sex-biased dispersal and an age-dependent effect in survival rate accounted for the pattern of first settlement and reproduction in a newly reintroduced Osprey population. Aims We estimate the survival of translocated individuals, describe juvenile movements and evaluate the success of first breeding events to document the re-establishment of an Osprey breeding population. Methods Between 2006 and 2010, 32 fledgling Ospreys were reintroduced via hacking techniques in Maremma Regional Park, Italy. We evaluated the effects of age on survival through multistate capture-mark-recapture analyses. Movements were investigated by radiotracking and using records of resightings. Results Survival was high for juveniles after the release (0.87), markedly decreased during the first winter (0.26), and improved again in subsequent years (annual apparent survival of 0.69 for immatures and 0.93 for adults). Mean distance covered in initial dispersal was greater for females (246.2 km) than for males (38.7 km). Conclusion Our results provided information on dispersal and survival rate of reintroduced Ospreys in a Mediterranean area. Despite low apparent survival in the first year, the high survival rates found in immatures and adults suggested favourable conditions for this new population. The study of demographic parameters is important for calibrating management actions aimed at the establishment of a self-sustaining Osprey population.

3956: ***+.019***

The Double-spurred Francolin Francolinus bicalcaratus ayesha is a critically endangered galliform subspecies in Morocco. To improve the viability of this threatened population, 300 captive-bred francolins were released into the Didactic Lot of the Royal Moroccan Federation of Hunting, and post-release monitoring was conducted. In this study, we used playback call counts to assess differences in habitat use and temporal variations in vocal activity of Double-spurred Francolins. The number of male calls per point count was significantly higher in the wooden matorral (WM) than in the non-wooden matorral (MT). The male responses per point count also increased depending on date, reaching a maximum in the first 10 days of March. The pattern was similar in the two habitats, although the maximum average call rates were significantly different [WM=1.575 (95% CI: 1.394-1.780), MT=0.481 (95% CI: 0.393-0.589)]. We suggest that call counts collected during this period could be used to index the annual change of the released population in that area. Further researches are, however, needed to (1) estimate the current population size of the released francolins and (2) characterize the habitats used within this protected area.

3957: *+.048*

To date, there is no information on gonadal steroidogenic activity of female goral (Naemorhedus griseus), a threatened species of Thailand. Captive goral populations have been established to produce animals for ex situ conservation and reintroduction, but as yet none are self-sustaining. The objectives of the present study were to (1) determine the influence of season on ovarian steriodogenic function; and (2) examine the relationship between gonadal hormone excretion and sexual behaviors throughout the year. Fecal samples were collected 5 to 7 days/wk for 15 months from 8 adult females housed at Omkoi Wildlife Breeding Center in Thailand and analyzed for ovarian steroid metabolites using validated enzyme immunoassays. Observations of sexual behaviors and mating were conducted each morning for 30 min/session. Based on fecal estrogen and progestagen metabolite concentrations, the overall estrous cycle length was about 21 days, with a 2- to 3-day follicular phase and an 18- to 20-day luteal phase. Sexual behaviors, most notably tail-up, increased for 2 to 3 days during the time estrogens were elevated during mating. Fecal progestagens were elevated during luteal phases and increased further during gestation, which lasted approximately 7 months. The lactation period was 5 months, and females were anestrus for 2 to 5 of those months, with the exception of one that cycled continuously throughout. Two females conceived around 2 months postpartum and so were pregnant during lactation. Birth records over the past 21 years indicated young are born throughout the year. This combined with the hormonal data suggests that female gorals are not strongly seasonal, at least in captivity, although there was considerable variation among females in estrogen and progestagen patterns. In conclusion, fecal steroid metabolite monitoring is an effective means of assessing ovarian function in this species and will be a useful tool for breeding management and planned development of assisted reproductive techniques such as artificial insemination and embryo transfer. (C) 2014 Elsevier Inc. All rights reserved.

3958: *-.063*

The African penguin Spheniscus demersus has an 'Endangered' conservation status and a decreasing population. Following abandonment, 841 African penguin chicks in 2006 and 481 in 2007 were admitted to SANCCOB (Southern African Foundation for the Conservation of Coastal Birds) for hand-rearing from colonies in the Western Cape, South Africa, after large numbers of breeding adults commenced moult with chicks still in the nest. Of those admitted, 91% and 73% respectively were released into the wild. There were veterinary concerns about avian malaria, airsacculitis and pneumonia, feather-loss and pododermatitis (bumblefoot). Post-release juvenile (0.32, s.e. = 0.08) and adult (0.76, s.e. = 0.10) survival rates were similar to African penguin

chicks reared after oil spills and to recent survival rates recorded for naturally-reared birds. By December 2012, 12 birds had bred, six at their colony of origin, and the apparent recruitment rate was 0.11 (s.e. = 0.03). Hand-rearing of abandoned penguin chicks is recommended as a conservation tool to limit mortality and to bolster the population at specific colonies. The feasibility of conservation translocations for the creation of new colonies for this species using hand-reared chicks warrants investigation. Any such programme would be predicated on adequate disease surveillance programmes established to minimise the risk of disease introduction to wild birds.

3959: *+.039*

Restoration of extirpated species via captive breeding has typically relied on population viability as the primary criterion for evaluating success. This criterion is inadequate when species reintroduction is undertaken to restore ecological functions and interactions. Herein we report on the demographic and ecological outcomes of a five-decade-long population restoration program for a critically endangered species of "ecosystem engineer": the endemic Espanola giant Galapagos tortoise (Chelonoidis hoodensis). Our analysis of complementary datasets on tortoise demography and movement, tortoise-plant interactions and Espanola Island's vegetation history indicated that the repatriated tortoise population is secure from a strictly demographic perspective: about half of tortoises released on the island since 1975 were still alive in 2007, in situ reproduction is now significant, and future extinction risk is low with or without continued repatriation. Declining survival rates, somatic growth rates, and body condition of repatriates suggests, however, that resources for continued population growth are increasingly limited. Soil stable carbon isotope analyses indicated a pronounced shift toward woody plants in the recent history of the island's plant community, likely a legacy of changes in competitive relations between woody and herbaceous plants induced by now-eradicated feral goats and prolonged absence of tortoises. Woody plants are of concern because they block tortoise movement and hinder recruitment of cactus-a critical resource for tortoises. Tortoises restrict themselves to remnant cactus patches and areas of low woody plant density in the center of the island despite an apparent capacity to colonize a far greater range, likely because of a lack of cactus elsewhere on the island. We conclude that ecosystem-level criteria for success of species reintroduction efforts take much longer to achieve than population-level criteria; moreover, reinstatement of endangered species as fully functioning ecosystem engineers may often require large-scale habitat restoration efforts in concert with population restoration.

3960: *+.011*

A new population of the invasive American Eastern grey squirrel (Sciurus carolinensis) has recently settled in central Italy from an accidental release in Perugia, Umbria in the early 2000s. The grey squirrel is known to compete with and exclude native red squirrels (S. vulgaris) in the British Isles and Northern Italy, so it represents a potentially important new conservation threat to the red squirrel subspecies of south and central Italy, S. vulgaris italicus and S. v. meridionalis, which are endemic to peninsular Italy. The grey squirrel population range in Perugia is currently expanding at a rate of about 0.29 km/year (SD 0.19), slower than grey squirrel invasions elsewhere in Europe. Nuclear DNA analysed at 12 different microsatellite loci revealed that the grey squirrels in Perugia have extremely low genetic diversity, consistent with a small founder size. Genetic assignment tests indicate that the Perugia population was founded by translocations from an established population in Piedmont, Italy. No genetic substructure is evident yet in the Perugia population. These results together have serious consequences for the management of the grey squirrel invasion in Perugia and the conservation of the red squirrel subspecies: the Perugia grey

squirrel population should be eradicated if politically feasible; otherwise new releases of grey squirrels, especially from sources other than the Piedmont population, should be prevented because such introductions could increase genetic diversity, thereby potentially increasing population range expansion rate to the much higher levels seen for more diverse grey squirrel populations elsewhere in Europe.

3961: *+.133*

Currently, the distribution of the European native white-clawed crayfish (Austropotamobius pallipes) is restricted and fragmented over its range. Many European countries have active programmes for conservation of the species, including reintroduction programmes. Here, we have studied four brooks that were restocked with crayfish coming from population rescue from the Brissionnieres after a drought in summer 2009. The aim was to validate the use of Ephemeropteran communities as potential bioindicators to select brooks for crayfish restocking. Restocked brooks were surveyed in 2010 and 2012. All brooks showed chemical and physical parameters in accordance with A. pallipes requirements. Three brooks which had high Ephemeropteran richness including Leptophlebiidae species (Habrophlebia lauta and Paraleptophlebia submarginata) have been restocked successfully. No crayfish were observed during the survey in Le Peu, which had only two Ephemeropteran species tolerant of reduced water quality and had no Leptophlebiidae species. Ecological characteristics of Ephemeropteran species and the limitations of their use as bioindicators to select brook for restocking are discussed. (C) 2014 Elsevier Ltd. All rights reserved.

3962: *+.069*

Premise of the study: Reintroductions may be essential to prevent extinction of many critically endangered species. Ideally, reintroduction efforts rely on adjacent source populations, but limited source material may necessitate crossing individuals from different and possibly distant populations. To determine the consequences of integrating multiple populations in reintroductions, we investigated levels of inbreeding depression, outbreeding depression, and heterosis for populations of Schiedea kaalae (Caryophyllaceae), an endangered species endemic to the Wai'anae and Ko'olau Mountains of O'ahu, Hawai'i. The possibility of gene flow among plants was explored through pollinator observations.Methods: Individuals from ex situ living collections of nine populations were hand-pollinated with pollen from the same plant, plants from the same population (for three populations only), or plants from different populations. Progeny were outplanted into two common gardens, one in each mountain range on O'ahu. Cumulative fitness was estimated using several independent life history stages. Key results: Inbreeding depression was minimal, and no outbreeding depression was detected. In contrast, strong heterosis was evident in progeny from between-population crosses, which had higher relative fitness than progeny from self-pollinations or within-population crosses. Observations of floral visitors provided the first evidence that biotic pollination may be important for this species.Conclusions: Results demonstrate the ability to conduct genetic rescue of rare species and suggest that reintroductions may be most successful using heterotic individuals from crosses between populations and at sites where pollinators are present and promote outcrossing.

3963: *-.080*

The American marten (Martes americana), the only state-listed endangered mammal in Wisconsin, has undergone serial reintroductions within the state. Recovery has been slower than anticipated and the limiting factors remain unidentified. The lack of basic knowledge on marten foraging in

the Great Lakes Region led us to quantify the diet of martens inhabiting the Chequamegon-Nicolet National Forest (CNNF) in northern Wisconsin using a dual approach of scat and stable isotope analyses. We collected marten scat at winter rest sites within the CNNF from 2000-2011. We identified prey items based on morphological characteristics of indigestible prey remains and calculated percent occurrence for each prey item. We sampled marten hair from museum specimens, and opportunistically from carcasses within the CNNF for isotopic analysis. We collected hair and feather samples from potential prey species in the CNNF in 2010-2013. Our concurrent analyses revealed that shrews and deer were most important to the diet of martens in Wisconsin. These findings contrast with studies conducted elsewhere that report voles as the principle diet items, and shrews as one of the least used prey items. Consequently, such a strong reliance on secondary, less profitable or high-risk prey could be contributing to the delayed recovery of martens in Wisconsin. (c) 2014 The Wildlife Society.

3964: ***+.254***

The eastern wild turkey (Meleagris gallopavo silvestris) is an economically important upland game bird that has been successfully reintroduced in Wisconsin, USA, and now occurs across the entire state. Although populations have become relatively stable across much of the state, recent concerns have been expressed regarding a plateau in total harvest and declines in local abundance across some areas. Current ecological and demographic parameters are needed to develop biologically justifiable and efficient management strategies. To address these concerns, we used demographic data collected during recent studies on wild turkey survival and productivity in 2 contrasting landscapes with differing proportions of forest and open cover. We then used the properties of deterministic matrix models to identify parameters predicted to have the greatest effect on the finite rate of population change () in both landscapes. Our analyses projected population decline for turkeys in predominately forested landscapes (=0.782) and population increases in more open landscapes (=1.112). Variance-scaled sensitivities suggested that was most sensitive to variation in nest and poult survival for populations in both landscapes. A life-table response experiment further indicated that higher in open landscapes was largely attributable to greater rates of yearling and adult female survival during the nesting and brood-rearing period. Simulated variations suggest manipulations in female harvest during the fall either-sex season had less impact on than did alterations in components of fecundity. Our model results indicated that wild turkey population growth varies across landscapes in southwest and west-central Wisconsin. Management practices that address factors influencing components of fecundity likely have the greatest effect on in both landscapes. Additionally, populations in forested landscapes may require more conservative harvests to enhance female survival if population stability or increase is to be realized. (c) 2014 The Wildlife Society.

3965: ***+.143***

Primers targeting two non-neutral major histocompatibility complex (mhc) II genes were developed and assayed across several disjoint Sacramento perch Archoplites interruptus sampling locations. Variability at the two mhc loci among sampling stocks strongly correlated to previous estimates with neutral markers, suggesting that the effect of genetic drift was not limited to neutrally evolving regions of the genome. The novel mhc primers will help develop admixture schemes in A. interruptus captive breeding programmes and will increase the success of future reintroductions of this species of concern.

3966: ***+.061***

This study examined patterns of mortality and determinants of survival among elk recently restored to four sites in Ontario, Canada (1998-2005). We predicted that: (1) elk located in release sites closer to the core of their historic range would have higher survival; (2) survival would increase as an animal's time and experience on the landscape increased; and (3) survival rates would decline as animals moved farther away from the release site. During the study, 443 elk were radiocollared and released; 218 mortalities were documented. Predation by wolves was the most important proximate cause of mortality, followed by death due to injuries from translocation and/or capture myopathy, accidents, emaciation, poaching, and Parelaphostrongylus tenuis infection. Overall, annual survival of elk across Ontario ranged from 0.45 (0.37-0.53) to 0.81 (0.66-0.90), with rates being lowest in the years immediately following release and highest in the final years of the study; this pattern was due to high initial mortality from translocation injuries and/or capture myopathy and possibly lack of familiarity with novel habitat. Model-averaged hazards further support this finding, as the most important factor influencing elk survival was the length of holding period, with elk released after limited holding being less likely to survive than those held for longer periods. Our results suggest that mortalities caused by capture myopathy and transportation-related injuries are important sources of risk for translocated elk. The method of introduction to the novel landscape and behavior in the first year should be accommodated via soft-release and appropriate release areas.

3967: *-.033*

Over a century has passed since elk were extirpated in eastern North America. During that time, numerous attempts to reintroduce elk into eastern North America have resulted in varying degrees of success and failure. An overview of restoration efforts during the last 100 years is presented here with emphasis on the differences in rates of population change among regions and differences in major causes of elk mortality during both the pre- and post-acclimation periods. Approximately 40% of recorded elk reintroduction attempts in eastern North America resulted in failure with the majority of these having occurred in the first half of the 20th century. Although rates of population change in elk were highly variable, they were not related to founding population size. Major causes of mortality varied among regions and should be considered in future reintroduction attempts.

3968: *+.293*

The emerging field of ecological genomics contains several broad research areas. Comparative genomic and conservation genetic analyses are providing great insight into adaptive processes, species bottlenecks, population dynamics and areas of conservation priority. Now the same technological advances in high-throughput sequencing, coupled with taxonomically broad sequence repositories, are providing greater resolution and fundamentally new insights into functional ecology. In particular, we now have the capacity in some systems to rapidly identify thousands of species-level interactions using non-invasive methods based on the detection of trace DNA. This represents a powerful tool for conservation biology, for example allowing the identification of species with particularly inflexible niches and the investigation of food-webs or interaction networks with unusual or vulnerable dynamics. As they develop, these analyses will no doubt provide significant advances in the field of restoration ecology and the identification of appropriate locations for species reintroduction, as well as highlighting species at ecological risk. Here, I describe emerging patterns that have come from the various initial model systems, the advantages and limitations of the technique and key areas where these methods may significantly advance our empirical and applied conservation practices.

3969: *-.009*

Background: African wildlife experienced a reduction in population size and geographical distribution over the last millennium, particularly since the 19th century as a result of human demographic expansion, wildlife overexploitation, habitat degradation and cattle-borne diseases. In many areas, ungulate populations are now largely confined within a network of loosely connected protected areas. These metapopulations face gene flow restriction and run the risk of genetic diversity erosion. In this context, we assessed the "genetic health" of free ranging southern African Cape buffalo populations (S.c. caffer) and investigated the origins of their current genetic structure. The analyses were based on 264 samples from 6 southern African countries that were genotyped for 14 autosomal and 3 Y-chromosomal microsatellites.Results: The analyses differentiated three significant genetic clusters, hereafter referred to as Northern (N), Central (C) and Southern (S) clusters. The results suggest that splitting of the N and C clusters occurred around 6000 to 8400 years ago. Both N and C clusters displayed high genetic diversity (mean allelic richness (A(r)) of 7.217, average genetic diversity over loci of 0.594, mean private alleles (P-a) of 11), low differentiation, and an absence of an inbreeding depression signal (mean F-IS = 0.037). The third (S) cluster, a tiny population enclosed within a small isolated protected area, likely originated from a more recent isolation and experienced genetic drift (F-IS = 0.062, mean A(r) = 6.160, P-a = 2). This study also highlighted the impact of translocations between clusters on the genetic structure of several African buffalo populations. Lower differentiation estimates were observed between C and N sampling localities that experienced translocation over the last century.Conclusions: We showed that the current genetic structure of southern African Cape buffalo populations results from both ancient and recent processes. The splitting time of N and C clusters suggests that the current pattern results from human-induced factors and/or from the aridification process that occurred during the Holocene period. The more recent S cluster genetic drift probably results of processes that occurred over the last centuries (habitat fragmentation, diseases). Management practices of African buffalo populations should consider the micro-evolutionary changes highlighted in the present study.

3970: *+.259*

In recent years, increasing efforts have been directed to the restoration of natural communities affected by human activities. However, restoration success is often reduced because of the lack of dispersal-limited species in the restored site. Numerous rare species are dispersal-limited, having low probabilities to re-colonize restored sites and to establish viable populations. A high species richness enhances the ability of ecosystems to maintain multiple functions. It has been shown that in highly diverse ecosystems such as alpine meadows, tropical forests and coral reefs, the most distinct combinations of functions are supported predominantly by rare species. Thus, more emphasis should be given to support rare species in restoration actions. I suggest that well-known techniques for increasing plant diversity in restoration projects should be complemented with techniques to re-introduce dispersal-limited animals. Re-establishing ecological connectivity might be of benefit for certain species in the long term. However, for the majority of dispersal-limited animal species, translocations of founder individuals might be the only tool to re-establish populations in restored sites.

3971: *+.221*

Dispersal is essential for population persistence in transient environments. While costs of dispersal are ubiquitous, individual advantages of dispersal remain poorly understood. Not all individuals from a population disperse, and individual heterogeneity in costs and benefits of dispersal underlie

phenotype-dependent dispersal strategies. Dispersing phenotypes are always expected to maximize their fitness by adaptive decision making relative to the alternative strategy of remaining philopatric. While this first principle is well acknowledged in theoretical ecology, empirical verification is extremely difficult, due to a plethora of experimental constraints. We studied fitness prospects of dispersal in a game theoretical context using the two-spotted spider mite Tetranychus urticae as a model species. We demonstrate that dispersing phenotypes represent those individuals able to maximize their fitness in a novel, less populated environment reached after dispersal. In contrast to philopatric phenotypes, successful dispersers performed better in a low density post-dispersal context, but worse in a high density philopatric context. They increased fitness about 450% relative to the strategy of remaining philopatric. The optimization of phenotype-dependent dispersal, thus, maximizes fitness.

3972: ***+.095***

Estimating and monitoring adult and juvenile survival are vital to understanding population status, informing recovery planning for endangered species, and quantifying the success of management. We used mark-recapture models to estimate apparent annual survival of the Puaiohi (Myadestes palmeri), an endangered thrush endemic to the Hawaiian island of Kauai, from 2005 to 2011. Our sample included 87 wild birds and 123 captive-bred birds that were released at various ages. Survival was higher for wild adult males (0.71 +/- 0.09) than for wild adult females (0.46 +/- 0.12). Survival of wild juveniles (0.23 +/- 0.06) was lower than that of wild adults of both sexes, indicating that recruitment may limit population growth. Captive-bred birds released when,1 yr old had survival (0.26 +/- 0.21) comparable with that of wild juveniles, but captive-bred birds released at 1-3 yr old had very low survival (0.05 +/- 0.06). Only 8 of 123 (7%) captive birds were seen again after release. Two wild birds resighted five years after marking are the oldest known individuals, being at least six years of age. Malarial infection did not affect survival of wild Puaiohi, unlike many Hawaiian forest birds. The difference between adult male and adult female survival is consistent with rat (Rattus spp.) predation of females on the nest as a major source of mortality. As such, attempting to reduce nest predation by controlling rats may be the best available management option. Releasing captive-bred birds has had little effect on the wild population in recent years.

3973: ***-.112***

Captive breeding is a high profile management tool used for conserving threatened species. However, the inevitable consequence of generations in captivity is broad scale and often-rapid phenotypic divergence between captive and wild individuals, through environmental differences and genetic processes. Although poorly understood, mate choice preference is one of the changes that may occur in captivity that could have important implications for the reintroduction success of captive-bred animals. We bred wild-caught house mice for three generations to examine mating patterns and reproductive outcomes when these animals were simultaneously released into multiple outdoor enclosures with wild conspecifics. At release, therewere significant differences in phenotypic (e.g. body mass) and genetic measures (e.g. Gst and F) between captive-bred and wild adult mice. Furthermore, 83% of offspring produced post-release were of same source parentage, inferring pronounced assortative mating. Our findings suggest that captive breeding may affect mating preferences, with potentially adverse implications for the success of threatened species reintroduction programmes.

3974: ***+.425***

Amphibian biology is intricate, and there are many inter-related factors that need to be understood before establishing successful Conservation Breeding Programs (CBPs). Nutritional needs of amphibians are highly integrated with disease and their husbandry needs, and the diversity of developmental stages, natural habitats, and feeding strategies result in many different recommendations for proper care and feeding. This review identifies several areas where there is substantial room for improvement in maintaining healthy ex situ amphibian populations specifically in the areas of obtaining and utilizing natural history data for both amphibians and their dietary items, achieving more appropriate environmental parameters, understanding stress and hormone production, and promoting better physical and population health. Using a scientific or research framework to answer questions about disease, nutrition, husbandry, genetics, and endocrinology of ex situ amphibians will improve specialists' understanding of the needs of these species. In general, there is a lack of baseline data and comparative information for most basic aspects of amphibian biology as well as standardized laboratory approaches. Instituting a formalized research approach in multiple scientific disciplines will be beneficial not only to the management of current ex situ populations, but also in moving forward with future conservation and reintroduction projects. This overview of gaps in knowledge concerning ex situ amphibian care should serve as a foundation for much needed future research in these areas. (C) 2014 Wiley Periodicals, Inc.

3975: *+.151*

Ftithia humilis is an endangered succulent plant species. Its distribution range overlaps with the coal fields of Mpumalanga and it is therefore threatened by opencast mining activities in particular. One of the 11 known populations of F. humilis was translocated from a licensed coal mining site to three suitable receptor sites within the species' distribution range. A long-term monitoring programme was initiated to track the progress of the newly established populations. Temporal trends in population demography, size classes, and fecundity were recorded. Population numbers of size classes fluctuated annually. However, flower frequency increased over time and seedling recruitment contributed significantly to population growth. Receptor sites with similar geological conditions to the donor site had more persistent cohorts, which suggest that such sites should receive priority during the translocation of endangered edaphic specialists. This study not only confirms that a Frithia humilis population can successfully establish after a translocation, but also serves as an important baseline for future comparative purposes to gauge the long-term success of the translocation effort.

3976: *+.121*

Khulan (Equus hemionus) are a first class, nationally protected animal in China. From April to November 2013, 28 infrared cameras were set up at 13 watering holes to study the activity rhythms of this species in the Mount Kalamaili Ungulate Nature Reserve, Xinjiang, China. The results showed that activity frequency was greatest in autumn (2,679 identification photos), then summer (1,990), and lowest in spring (294). Average aggregation of E. hemionus to watering holes was greater in daylight hours than at nights. Daily activity at watering holes peaked between 0:00-1:00, declined rapidly between 7:00-9:00, was lowest between 12:00-13:00 and 16:00-17:00 and rose rapidly between 21:00-22:00. Studying the activity of Equus hemionus around watering holes will provide a basis for effective protection and management of desert ungulates in northern Xinjiang. Additionally, it provides a baseline for the sympatric reintroduction of Equus przewalskii.

3977: *+.062*

Leptodactylus fallax, commonly known as the mountain chicken frog, is a large terrestrial frog currently found on two islands in the Caribbean. Habitat destruction, overhunting and disease outbreaks have contributed to declining population numbers. In order to identify appropriate conservation strategies, the historic geographic distribution of this frog must first be determined. Because no archeological evidence exists, this was accomplished by reviewing historical documents and inspecting museum collections. Inaccuracies in location and species names were identified in documents as well as in the mislabeling of museum specimens. Two means for natural immigration (dispersal and vicariance) and the artificial introduction by humans were considered. The authors concluded that the Amerindians transported L. fallax to eight islands throughout the Lesser Antilles as potential food resources as they colonized this area. The implication that 75% of the historical distribution is currently unoccupied by this species is considered in light of future reintroduction projects.

3978: ***-.311***

The use of human-assisted colonization for species affected by climate change is now being adopted by some conservationists but remains controversial. In order to deter extreme translocation actions, a decision framework is proposed that incorporates a biogeographical approach.

3979: ***+.263***

Bakersfield cactus (Opuntia basilaris var. treleasei) is a succulent' perennial in the cactus family (Cactaceae) and is endemic to Kern County, California. Due to habitat conversion and fragmentation, competition from non-native plants, and ongoing habitat degradation, Bakersfield cactus is listed as federally and state endangered. We tested a technique for establishing new populations of this taxon by translocating Bakersfield cactus pads (i.e., stem segments) and clumps (i.e., intact plants) to two sites within the historic range. Translocated clumps were more successful than pads in terms of survival, growth, and flowering. However, removal of clumps may constitute more of an impact to source populations. Cattle guards were effective in preventing damage from cows. Strategies such as supplemental water during dry summer weather and propagation of pads into small plants prior to translocation are recommended to increase the success of pads. Translocation could contribute significantly to conservation and recovery efforts for Bakersfield cactus.

3980: ***+.034***

Survival, reproductive and recruitment rates, along with health status, of translocated and resident individuals should be evaluated. However, gathering this information poses logistical constraints and requires long-term studies. Considering the urgent nature of many species' situations where translocation would be appropriate, fast-assessment techniques should be tested. We assessed the immediate response to translocation of Hermann's tortoises (Testudo hermanni hermanni) directly from captivity to the wild. Individuals were maintained in captivity 2 to 8 years before being released in spring 2013 into a natural population impacted by fire. During the critical 3 months post-release period, we radio-tracked translocated individuals (N = 12) and resident tortoises in spring 2013 (N = 14), plus another batch of resident tortoises in spring 2012 (N = 9). Movements, behaviours, body condition and body temperature were regularly recorded. All translocated tortoises acclimated well to their novel environment. We found no differences in movement, thermoregulation and body condition between translocated and resident tortoises. Body condition of all tortoises increased rapidly in spring. We found no sign of perturbation in resident tortoises.

Contrarily, resident males mated with translocated females. Translocations should be further tested on larger spatial and time scales to improve population restoration programmes, especially in threatened species with limited dispersal ability.

3981: *-.165*

Conservation fences have been used as a tool to stop threatening processes from acting against endangered wildlife, yet little is known of the impacts of fences on non-target native species. In this study, we intensively monitored a pest-exclusion fence for 16 months to assess impacts on a reptile community in south-eastern Australia. We registered 1052 reptile records of six species along the fence. Encounters and mortality were greatest for eastern long-necked turtles (Chelodina longicollis), whereas impacts on lizards (Tiliqua rugosa, Tiliqua scincoides, Pogona barbata, Egernia cunninghami) and snakes (Pseudonaja textilis) were more moderate. We recorded several Chelodina longicollis recaptures at the fence and many of these were later found dead at the fence, indicating persistent attempts to navigate past the fence. We conservatively estimate that the fence resulted in the death of 3.3% and disrupted movements of 20.9% of the turtle population within the enclosure. Movement disruption and high mortality were also observed for turtles attempting to enter the nature reserve, effectively isolating the reserve population from others in the wider landscape. Of 98 turtle mortalities, the most common cause of death was overheating, followed by predation, vehicular collision, and entanglement. Turtle interactions were clustered in areas with more wetlands and less urban development, and temporally correlated with high rainfall and solar radiation, and low temperature. Thus, managers could focus at times and locations to mitigate impacts on turtles. We believe the impact of fences on non-target species is a widespread and unrecognized threat, and suggest that future and on-going conservation fencing projects consider risks to non-target native species, and where possible, apply mitigation strategies that maintain natural movement corridors and minimize mortality risk. (C) 2014 Elsevier GmbH. All rights reserved.

3982: *+.215*

In order to preserve endangered plant populations and recover their evolutionary potential and ecological behavior, some restoration measures generally involve the reinforcement of the population size in existing natural populations or the reintroduction of new populations. Genetic monitoring of both natural and restored populations can provide an assessment of restoration protocol success in establishing populations that maintain levels of genetic diversity similar to those in natural populations. The highly threatened Spanish species Silene hifacensis (Caryophyllaceae) has only three natural reduced mainland populations in the Iberian Peninsula, following decline and extinction that occurred during the late 20th century. Preterit restoration strategies were essentially based on the implantation of new populations and reinforcement of certain existing populations using transplants mostly cultivated in greenhouses. In the present contribution, levels and patterns of genetic variability within natural and restored populations of Silene hifacensis were assessed using the molecular technique AFLP. Our results pointed out significant genetic diversity differences across the three existing natural populations though their population fragmentation and progressive loss of individuals have not had an impact on the global genetic diversity of this species. For restored populations, their levels of genetic diversity were similar and even higher than in natural populations. As a result, the past restoration protocols were successful in capturing similar and even higher levels of genetic diversity than those observed within natural pools. However, inbreeding processes have been detected for two restored populations. Finally, the main source of plant material for the long-time restored transplants appears to be the natural population of Cova de les Cendres. This study demonstrates, once again,

how genetic markers are useful tools to be taken in consideration for endangered plant species conservation plans. (C) 2014 Elsevier GmbH. All rights reserved.

3983: ***-.064***

The Desertas Islands (Madeira, Portugal) are the sole home of one of the largest and rarest wolf spider species, Hogna ingens (Blackwall 1857) (Araneae, Lycosidae). Despite its size, it inhabits a single valley in the North of the Deserta Grande Island, Vale da Castanheira, currently invaded by the herb Phalaris aquatica. This invasive species competes with the native flora and was subject to several eradication experiments, namely through fire and chemicals. The objectives of this work were to: (1) estimate the current distribution and abundance of H. ingens and respective trends; (2) evaluate the impact of the invasive plant and eradication methods on the spider population; (3) suggest future measures for the recovery of the species; and (4) evaluate its conservation status according to the IUCN criteria. The current distribution of H. ingens covers 23 ha, a recent reduction from its original 83 ha, corresponding to the entire Vale da Castanheira. A total of 4447 and 4086 adults and 71,832 and 24,635 juveniles were estimated to live in the valley during 2011 and 2012, respectively. We found a significant negative impact of P. aquatica cover on the presence and abundance of H. ingens and that chemical treatment specifically directed towards the invasive plant species may be the only way to effectively recover the spider's habitat. We suggest (1) regular monitoring; (2) extend chemical treatments; (3) ex-situ conservation with future reintroduction of adults. Based on the current area of occupancy (AOO) of H. ingens and its recent decline in both AOO and number of individuals, it was recently classified as Critically Endangered by IUCN and we suggest its urgent inclusion in the Habitats Directive species lists. (C) 2014 Elsevier GmbH. All rights reserved.

3984: ***+.065***

Large-scale poisoning events are common to scavenging bird species that forage communally, many of which are in decline. To reduce the threat of poisoning and compensate for other persistent threats, management, including supplemental feeding, is ongoing for many reintroduced and endangered vulture populations. Through a longitudinal study of lead exposure in California condors (Gymnogyps californianus), we illustrate the conservation challenges inherent in reintroduction of an endangered species to the wild when pervasive threats have not been eliminated. We evaluated population-wide patterns in blood lead levels from 1997 to 2011 and assessed a broad range of putative demographic, behavioral, and environmental risk factors for elevated lead exposure among reintroduced California condors in California (United States). We also assessed the effectiveness of lead ammunition regulations within the condor's range in California by comparing condor blood lead levels before and after implementation of the regulations. Lead exposure was a pervasive threat to California condors despite recent regulations limiting lead ammunition use. In addition, condor lead levels significantly increased as age and independence from intensive management increased, including increasing time spent away from managed release sites, and decreasing reliance on food provisions. Greater independence among an increasing number of reintroduced condors has therefore elevated the population's risk of lead exposure and limited the effectiveness of lead reduction efforts to date. Our findings highlight the challenges of restoring endangered vulture populations as they mature and become less reliant on management actions necessary to compensate for persistent threats. Patrones Espaciotemporales y Factores de Riesgo por Exposicion a Plomo en Condores de California Durante 15 Anos de ReintroduccionResumen El envenenamiento a gran escala es comun en especies de aves carroneras que forrajean comunalmente, muchas de ellas en declinacion. Para reducir la amenaza del envenenamiento y compensar otras amenazas persistentes, se realizan acciones de manejo,

incluyendo la suplementacion de alimento, con muchas poblaciones de buitres reintroducidas y en peligro. Mediante un estudio longitudinal del envenenamiento por plomo en condores de California (Gymnogyps californianus), mostramos los retos de conservacion inherentes a la reintroduccion de una especie en peligro cuando las amenazas principales no han sido eliminadas. Evaluamos patrones en los niveles de plomo en sangre de 1997 a 2011 y evaluamos un amplio rango factores putativos de riesgo demografico, conductual y ambiental por exposicion a niveles elevados de plomo en condores de California reintroducidos en California (E.U.A.). Tambien evaluamos la efectividad de las regulaciones para el uso de municiones de plomo en el rango de distribucion de Condores mediante la comparacion de niveles de plomo en la sangre antes y despues de la implementacion de las regulaciones. La exposicion a plomo fue una amenaza constante para los condores de California a pesar de las regulaciones que establecen el uso de municiones sin plomo. Adicionalmente. Los niveles de plomo incrementaron significativamente a medida que aumentaba la edad y la independencia de manejo intensivo, incluyendo el incremento del tiempo lejos de sitios de liberacion, y el decremento en la dependencia en el aprovisionamiento de alimento. Por lo tanto, una mayor independencia en un mayor numero de condores reintroducidos, a la fecha ha incrementado el riesgo de exposicion a plomo en la poblacion y limitado la efectividad de los esfuerzos para la reduccion de plomo. Nuestros resultados resaltan los retos para el restablecimiento de poblaciones de condores a medida que maduran y se vuelven menos dependientes de las acciones de manejo necesarias para compensar las amenazas persistentes.

3985: ***+.052***

With globalization, agriculture and aquaculture activities are increasingly affected by diseases that are spread through movement of crops and stock. Such movements are also associated with the introduction of non-native species via hitchhiking individual organisms. The oyster industry, one of the most important forms of marine aquaculture, embodies these issues. In Europe disease outbreaks affecting cultivated populations of the naturalized oyster Crassostrea gigas caused a major disruption of production in the late 1960s and early 1970s. Mitigation procedures involved massive imports of stock from the species' native range in the northwestern Pacific from 1971 to 1977. We assessed the role stock imports played in the introduction of non-native marine species (including pathogens) from the northwestern Pacific to Europe through a methodological and critical appraisal of record data. The discovery rate of non-native species (a proxy for the introduction rate) from 1966 to 2012 suggests a continuous vector activity over the entire period. Disease outbreaks that have been affecting oyster production since 2008 may be a result of imports from the northwestern Pacific, and such imports are again being considered as an answer to the crisis. Although successful as a remedy in the short and medium terms, such translocations may bring new diseases that may trigger yet more imports (self-reinforcing or positive feedback loop) and lead to the introduction of more hitchhikers. Although there is a legal framework to prevent or reduce these introductions, existing procedures should be improved. Ciclo de Retroalimentacion Positiva entre la Introduccion de Especies Marinas No-Nativas y el Cultivo de Ostras en EuropaResumen Con la globalizacion, las actividades de agricultura y acuacultura son afectadas cada vez mas por enfermedades que se extienden por medio del movimiento de cultivos y ganado. Dichos movimientos tambien estan asociados con la introduccion de especies no-nativas por medio de organismos individuales que viajan como pasajeros en otros organismos o mercancia. La industria de las ostras, una de las formas mas importantes de acuacultura marina, encarna estos problemas. En Europa, los brotes de enfermedades que afectaron a las poblaciones cultivadas de la ostra naturalizada Crassostrea gigas causaron grandes perturbaciones al final de la decada de 1960 y al inicio de la decada de 1970 y los procedimientos de mitigacion involucraron una importacion masiva de un stock de la extension nativa de la especie en el Pacifico noroeste de

1971 a 1977. Evaluamos el papel que tuvieron las importaciones de stocks en la introduccion de una especie marina no-nativa (incluyendo patogenos) desde el Pacifico noroeste hacia Europa por medio de una valoracion critica y metodologica de datos registrados. La tasa de descubrimiento de especies no-nativas (un representante de la tasa de introduccion) de 1966 a 2012 sugiere una actividad continua de vectores a lo largo de todo el periodo. Los brotes de enfermedades que han afectado a la produccion de ostras desde 2008 pueden ser un resultado de importaciones del Pacifico noroeste, y dichas importaciones se estan considerando una vez mas como una respuesta a la crisis. Aunque es un remedio exitoso en terminos de corto y mediano plazo, dichas reubicaciones pueden traer consigo nuevas enfermedades que pueden activar aun mas importaciones (auto-reforzantes o ciclo de retroalimentacion positiva) y llevar a la introduccion de mas especies que viajan como pasajeros. Aunque hay un marco de trabajo legal para prevenir o reducir estas introducciones, se deben mejorar los procedimientos existentes.

3986: *+.235*

Time is of the essence in conservation biology. To secure the persistence of a species, we need to understand how to balance time spent among different management actions. A new and simple method to test the efficacy of a range of conservation actions is required. Thus, we devised a general theoretical framework to help determine whether to test a new action and when to cease a trial and revert to an existing action if the new action did not perform well. The framework involves constructing a general population model under the different management actions and specifying a management objective. By maximizing the management objective, we could generate an analytical solution that identifies the optimal timing of when to change management action. We applied the analytical solution to the case of the Christmas Island pipistrelle bat (Pipistrelle murrayi), a species for which captive breeding might have prevented its extinction. For this case, we used our model to determine whether to start a captive breeding program and when to stop a captive breeding program and revert to managing the species in the wild, given that the management goal is to maximize the chance of reaching a target wild population size. For the pipistrelle bat, captive breeding was to start immediately and it was desirable to place the species in captivity for the entire management period. The optimal time to revert to managing the species in the wild was driven by several key parameters, including the management goal, management time frame, and the growth rates of the population under different management actions. Knowing when to change management actions can help conservation managers' act in a timely fashion to avoid species extinction. Determinar Cuando Cambiar el Rumbo en las Acciones de ManejoResumen El tiempo es de suma importancia en la Biologia de la Conservacion. Para poder asegurar la persistencia de una especie necesitamos entender como equilibrar el tiempo utilizado en diferentes acciones de manejo. Se requiere un metodo novedoso y simple para evaluar la efectividad de una gama de acciones de conservacion. Por esto disenamos un marco de trabajo teorico y general para ayudar a determinar si se debe evaluar una accion nueva y cuando se debe detener una prueba y revertirla a una accion existente si la nueva accion no tuvo un buen desempeno. El marco de trabajo involucra construir un modelo poblacional general bajo las diferentes acciones de manejo y especificar un objetivo de manejo. Al maximizar el objetivo de manejo, podemos generar una solucion analitica que identifica la oportunidad optima de cuando cambiar la accion de manejo. Aplicamos la solucion analitica al caso del murcielago de la Isla Navidad (Pipistrelle murrayi), una especie para la cual la reproduccion en cautiverio puede haber prevenido su extincion. Para este caso, usamos nuestro modelo para determinar si iniciabamos un programa de reproduccion en cautiverio o cuando detendriamos un programa de reproduccion en cautiverio y revertiriamos al manejo en vida libre de la especie, dado que el objetivo de manejo es maximizar la posibilidad de obtener un tamano de poblacion silvestre determinado. Para el murcielago, la reproduccion en cautiverio empezaria de inmediato y lo deseado seria mantener a la

especie en cautiverio durante el periodo de manejo. El tiempo optimo para revertirnos a manejar a la especie en vida libre fue conducido por varios parametros clave, incluidos el objetivo de manejo, el marco de tiempo del manejo y las tasas de crecimiento de la poblacion bajo diferentes acciones de manejo. El saber cuando cambiar las acciones de manejo puede ayudar a quienes manejan la conservacion a actuar oportunamente para evitar la extincion de una especie.

3987: *+.208*

Forested ecosystems of south-eastern Australia now differ physically, compositionally and functionally from their condition prior to European settlement. Understanding these changes, and how native species and entire ecosystems have responded, is crucial for biodiversity conservation and ecosystem management. Here I argue that a combination of limited historical information and a knowledge base biased towards modern ecological studies has resulted in a distorted perception of ecosystem condition, hindering the instigation of effective biodiversity conservation measures. This argument is based on recently obtained information about changes to the non-volant mammal community, which reveals relatively recent but underreported ecological changes, including major declines in species distribution and abundance, shifts in niche utilization and associated disruption of ecosystem functions. Ultimately, many mammal species are being denied the capacity to function to the extent they did historically. Following this re-assessment, it is evident that current forest management does not adequately address contemporary conservation dilemmas posed by detrimental ecosystem changes. This is especially salient when most of the factors responsible for causing changes to the mammal community are still active and include forest management and utilization activities. Therefore, additional conservation measures are essential to meet forest stewardship and biodiversity conservation obligations. For the health, functionality and sustainability of forested ecosystems, native mammal species must be capable of functioning to their ecological potential and occupy their original niche. This will be facilitated by the suppression of threatening processes (primarily exotic species), ensuring ecologically sensitive fire regimes and the reintroduction/translocation of missing species. The recovery or restoration of forest functionality based on mammal conservation should have wide-scale benefits for biodiversity conservation.

3988: *+.112*

Many lake sturgeon (Acipenser fulvescens Rafinesque, 1817) populations in the Great Lakes have not recovered from previous threats, whereby stocking can increase population abundances. Stocking in Oneida Lake, New York used two approaches: single-year stocking using sturgeon from the Des Prairies River and multi-year stocking using sturgeon from the St. Lawrence River. Stocked A.fulvescens were sampled and assigned to their corresponding stocking strategy based on age. Samples were analyzed at 12 microsatellite loci to evaluate how a single year of stocking (N=273) and multiple years of stocking (N=100) affected genetic diversity and effective population size (N-e). Single-year stocking resulted in lower genetic diversity, likely due to a small number of parents. Multi-year stocking retained most of the genetic diversity of the source population, compensating for the few parents available in a single year. Although multi-year stocking resulted in a higher N-e, the N-e:N ratio was lower (0.34) compared to single-year stocking (0.65), likely due to unequal sex ratios and family size variance across years. It is recommended that stocking of Lake Sturgeon take place over several years. However, consistent numbers need to be stocked each year and family size should be equalized across cohorts in order to maximize N-e.

3989: *+.116*

Iterative domestication processes by humans such as selection, translocation and cultivation are known to affect the morphological and genetic diversity of tree species. Since many of these species occur also in human-created homegardens (HG) this type of agroecosystem therefore represents an ideal setting to study such domestication processes. The threatened indigenous fruit tree Ziziphus spina-christi occurs in HGs and forests of the Nuba Mountains, Sudan, and was therefore selected as a model species. Five locations were sampled and the geographical position of 250 trees determined. Each location was subdivided into HG and forest sites. The diversities of morphological traits and amplified fragment length polymorphisms were assessed to study variation within and among locations and sites. Diversity of dendrometric parameters and fruit morphometries was high and differed significantly among locations. Environmental parameters affected dendrometry and fruit size, but applied regression models were of low explanatory power. Although statistically not significant, mean fruit measures were continuously larger in HGs than in forests. Higher genetic diversity was observed in HG samples. Larger dendrometric and fruit morphometric traits are likely to result from better growing conditions in HGs and/or human selection of germplasm. This is in line with the higher genetic diversity in HGs which may be a consequence of the admixture of germplasm from different origins planted in HGs. Z. spina-christi underwent incipient steps of domestication. High genetic diversity in HGs suggests those as valuable spots of improved germplasm and for on-farm conservation purposes.

3990: ***+.242***

Headstarting is a conservation technique for improving survival of species with high juvenile mortality by accelerating growth rate and increasing body size of captive-born young. With reptiles, headstarts are often kept active year-round to achieve body size goals and increase survival, omitting overwintering (brumation). As brumation is part of the life cycle of reptiles, there may be tradeoffs related to temperature response post-release when reptiles are kept active. Upon release into habitats, reptiles are either soft released, where acclimation is provided with in situ enclosures, or hard released without acclimation, directly into habitat. Soft releases have resulted in greater survival and site fidelity than hard releases, but evaluations with snakes are rare. We used a comparative approach to examine effects of brumation versus year-round activity on prerelease growth and survival of smooth green snake Opheodrys vernalis headstarts. We estimated short-term post-release daily survival rates of headstarts and compared movements of hard and soft released snakes. Despite decreases in body mass during brumation, prerelease body size, growth rate and survival did not differ among brumation treatments. Brumated headstarts exhibited rapid compensatory growth, attaining the size of active headstarts within 2 months of brumation. We observed qualitative evidence of reproductive potential in brumated snakes with the production of spermatozoa and unfertilized eggs, which was absent in active headstarts. The short-term survival rate of all headstarts during post-release tracking was 0.83 (+/- 0.01), but we lacked power to examine differences in survival among release treatments. Daily movements did not differ among release treatments. Soft releases had slightly greater recaptures, facilitating monitoring. Although brumation comparisons produced equivalent prerelease growth and survival, as a precautionary measure for post-release survival, we recommend incorporating brumation into headstarting efforts. While further study with other reptiles is warranted, we recommend a comparative framework in planning headstarting efforts with additional species.

3991: ***+.363***

Many reptile translocations fail, and they fail largely because the translocated individuals are faced with low habitat quality at the recipient site and/or disperse away from it. At least some failures may be forestalled by developing clear goals and success criteria, having a better understanding of

the species' biology and the threats it faces, ensuring the quality of the site to which individuals are being moved and following through with long-term monitoring. We incorporated these considerations into a translocation that moved more than 500 individuals of the threatened Florida sand skink Plestiodon reynoldsi Stejneger from a site slated for mining to an apparently suitable, but unoccupied site. Translocated individuals were placed into enclosures at the recipient site at densities similar to those at the donor site. Environmental conditions were varied within the enclosures in a replicated design, to tease out the conditions that promote successful colonization by translocated individuals. Overall success of the translocation will be judged by population viability, but we also set two benchmarks (initial survival and reproduction; completion of the life cycle through subsequent recruitment of offspring) to monitor progress. So far, we have monitored the translocated individuals long enough to determine that they have survived and are reproducing. Modeling of the influence of the environment on successful establishment suggests that soil and light conditions are important influences, but in a complex way that implicates the overriding importance of habitat heterogeneity. The Florida sand skink appears to be a resilient species that exploits the available habitat heterogeneity to find suitable microhabitats, and we suggest that these characteristics of the habitat and species contributed to its successful meeting of the first benchmark. These characteristics may contribute to successful translocation of xeric reptiles in general.

3992: ***+.276***

Suitable habitat for translocated animals should meet the candidate species' total abiotic and biotic needs through space and time and for all life stages. An important aspect of habitat assessment for reptile translocations is the thermal suitability of the release site. Here we provide a strategic framework for evaluating the thermal suitability of a release site for egg-laying reptiles with temperature-dependent sex determination (TSD). We apply the framework to a case study of tuatara Sphenodon punctatus reintroduced to the south-eastern South Island of New Zealand. Our approach allows the assessment of the thermal suitability of future translocation sites for tuatara, and can be readily modified for any translocation of a reptile species with TSD.

3993: ***+.246***

In wildlife translocations, it is important to identify reliable monitoring methods that can provide accurate population estimates for post-release management. We compared density and abundance estimates of distance sampling and repeated counts with complete counts (census data) to determine accuracy (percentage relative bias) and precision (coefficient of variation) for monitoring populations of blue iguanas restored to protected areas on Grand Cayman. We conducted incomplete counts (survey data) of blue iguanas at 12 transects of unequal length (range = 322.1 to 431.7 m) visited 36 times per year, yielding 405 detections in March 2010 and 443 detections in March 2013. Distance sampling and repeated counts provided accurate (range of percentage relative bias = -9.52% to 0.00%) and precise (range of coefficient of variation = 0.10 to 0.15) estimates of iguana density and abundance, and yielded an estimate of the proportion of individuals that were available for detection in the study area during the surveys ((P) over cap (a) = 0.88, se = 0.03). The use of distance sampling and repeated count methods, in carefully designed surveys with representative coverage and adequate replication, can lead to more reliable monitoring of extant wild and translocated rock iguana populations on other Caribbean islands, where individual marking may not be practical, and the proportion of individuals that is available for detection is unknown.

3994: ***+.023***

Wildlife managers consider animal translocation a means of increasing the viability of a local population. However, augmentation may disrupt existing resident disease dynamics and initiate an outbreak that would effectively offset any advantages the translocation may have achieved. This paper examines fundamental concepts of disease ecology and identifies the conditions that will increase the likelihood of a disease outbreak following translocation. We highlight the importance of susceptibility to infection, population size and population connectivity a characteristic likely affected by translocation but not often considered in risk assessments - in estimating outbreak risk due to translocation. We then explore these features in a species of conservation concern often translocated in the presence of infectious disease, the Mojave Desert tortoise, and use data from experimental tortoise translocations to detect changes in population connectivity that may influence pathogen transmission. Preliminary analyses comparing contact networks inferred from spatial data at control and translocation plots and infection simulation results through these networks suggest increased outbreak risk following translocation due to dispersal-driven changes in contact frequency and network structure. We outline future research goals to test these concepts and aid managers in designing effective risk assessment and intervention strategies that will improve translocation success.

3995: *+.112*

Translocation is an essential conservation tool often used to re-establish reptile populations following anthropogenic extirpation, but is not always successful. One factor potentially limiting success is dispersal of individuals from the release site immediately after translocation and consequent non-overlap of ranges. 'Penning' involves the use of an enclosure to restrict dispersal of translocated animals for a pre-determined period of time, with the aim of habituating animals to the release site so that they will establish a breeding population. We evaluated the utility of penning for limiting post-translocation dispersal of jewelled geckos (Naultinus gemmeus) by simultaneously tracking 19 geckos that had either been translocated into a pen for 9-10 months prior to the pen's removal (n = 10) or were translocated to a nearby site with no physical barrier to dispersal (n = 9) over a 3-week period. The area occupied by penned geckos did not increase following removal of their pen, despite suitable habitat being available outside the pen area. In contrast, un-penned geckos moved distances of up to 40 m outside of their release area, and effectively increased the area that they were occupying as a group 4.4-fold over the 3-week period. We suspect that when Naultinus geckos are released without time in a pen, some individuals may disperse too far to contribute to a breeding population and, consequently, the likelihood of population establishment and rate of population growth may be diminished. Our hypothesis is supported by a survey we conducted the following summer in which all four adult female geckos found at the penned site were gravid, but neither of the females resighted at the un-penned site was gravid. We believe that the potential advantages of penning (e.g. restricting initial dispersal, increased ease of monitoring) may outweigh the disadvantages (e.g. cost) for many herpetofauna translocations.

3996: *+.031*

Many species with restricted ranges or poor dispersal ability will occupy suboptimal habitats under climate change. The practice of translocating populations to more suitable areas outside the known historical range (assisted colonization) has been advanced as a solution to reduce the risk of extinction in the wild. Due to the high-risk and bureaucratic complexities of assisted colonization, it is imperative that a systematic process is used to select release sites that have a reduced likelihood of translocation failure. Here we demonstrate how a spatially explicit, three-stage multiple criteria analysis (MCA) can be used to identify potentially suitable sites for assisted

colonization of an endangered species. We employ this method as an initial screening process, prior to final selection of sites for assisted colonization of the critically endangered western swamp tortoise (Pseudemydura umbrina). This species occurs naturally in two small reserves in southwestern Australia, and is currently threatened by a shift to a drier climate and consequent changes to hydrological regimes. A literature review, characteristics of remnant P. umbrina habitat, and expert knowledge were used to create a composite index of habitat suitability, mapped across the entire south-west bioregion of Australia. The most suitable sites were 150 to 250 km south of the known range of P. umbrina, in areas of high landscape connectivity and low human population density. A subset of sites were examined in further detail and ranked using weighted summation. Careful use of MCA, taking into account data uncertainties and differences in expert opinion, can be a valuable tool when evaluating novel habitats for threatened species.

3997: *-.101*

A high immediate mortality rate of released animals is an important cause of translocation failure (release cost'). Post-release dispersal (i.e. the movements from the release site to the first breeding site) has recently been identified as another source of local translocation failure. In spite of their potential effects on conservation program outcomes, little is known about the quantitative effects of these two sources of translocation failure and their interactions with environmental factors and management designs. Based on long-term monitoring data of captive-bred North African houbara bustards Chlamydotis undulata undulata (hereafter, houbara) over large spatial scales, we investigated the relative effects of release (e.g. release group size, period of release), individual (e.g. sex and body condition) and meteorological (e.g. temperature and rainfall) conditions on post-release survival (n=957 individuals) and dispersal (n=436 individuals). We found that (i) rainfall and ambient air temperature had, respectively, a negative and a positive effect on houbara post-release dispersal distance; (ii) in interaction with the release period, harsh meteorological conditions had negative impact on the survival of houbara; (iii) density-dependent processes influenced the pattern of departure from the release site; and (iv) post-release dispersal distance was male-biased, as natal dispersal of wild birds (although the dispersal patterns and movements may be influenced by different processes in captive-bred and in wild birds). Synthesis and applications. Our results demonstrate that post-release dispersal and mortality costs in translocated species may be mediated by meteorological factors, which in turn can be buffered by the release method. As the consequences of translocation programs on population dynamics depend primarily upon release costs and colonization process, we suggest that their potential interactions with meteorological conditions must be carefully addressed in future programs (i) through monitoring of short-term post-release mortality to understand its link with environmental conditions, (ii) by carefully choosing the season of release to minimize exposition of inexperienced individuals to harsh conditions and (iii) by generalizing the use of long-term weather forecast to adapt release effort and staggering releases over several years to buffer meteorological effects. Our results demonstrate that post-release dispersal and mortality costs in translocated species may be mediated by meteorological factors, which in turn can be buffered by the release method. As the consequences of translocation programs on population dynamics depend primarily upon release costs and colonization process, we suggest that their potential interactions with meteorological conditions must be carefully addressed in future programs (i) through monitoring of short-term post-release mortality to understand its link with environmental conditions, (ii) by carefully choosing the season of release to minimize exposition of inexperienced individuals to harsh conditions and (iii) by generalizing the use of long-term weather forecast to adapt release effort and staggering releases over several years to buffer meteorological effects.

3998: *+.058*

Small mammal species are declining across northern Australia. Predation by feral cats Felis sylvestris catus is one hypothesised cause. Most evidence of cat impacts on native prey comes from islands, where cat densities are often high, but cats typically occur at low densities on mainland Australia. We conducted a field experiment to measure the effect of predation by low-density cat populations on the demography of a native small mammal. We established two 125-ha enclosures in tropical savanna in the Northern Territory. Each enclosure was divided in half, with cats allowed access to one half but not the other. We introduced about 20 individuals of a native rodent, Rattus villosissimus, into each of the four compartments (two enclosuresxtwo predator-access treatments). We monitored rat demography by mark-recapture analysis and radiotracking, and predator incursions by camera surveillance and track and scat searches. Rat populations persisted over the duration of the study (18months) in the predator-proof treatment, where we detected no predator incursions, but declined to extinction in both predator-accessible compartments. In one case, cat incursions were frequently detected and the rat population was rapidly extirpated (<3months); in the other, cat incursions were infrequent, and the population declined more gradually (c. 16months) due to low recruitment. We detected no incursions by dingoes Canis dingo, the other mammalian predator in the area. Synthesis and applications. This is the first study to provide direct evidence that cats are capable of extirpating small mammals in a continental setting, in spite of their low population densities. This finding supports the hypothesis that predation by feral cats is contributing to declines of small mammals in northern Australia. The conservation management of native small mammals in northern Australia may require intensive control of cat populations, including large cat-free enclosures. This is the first study to provide direct evidence that cats are capable of extirpating small mammals in a continental setting, in spite of their low population densities. This finding supports the hypothesis that predation by feral cats is contributing to declines of small mammals in northern Australia. The conservation management of native small mammals in northern Australia may require intensive control of cat populations, including large cat-free enclosures.

3999: ***+.007***

Chinese Giant Salamanders (Andrias davidianus) have experienced an 80% population decline since the 1950s. Captive salamander farms in China might provide a source population for reintroduction projects involving this species. To provide recommendations on rearing individuals for reintroduction, we studied the effect of water temperature on the growth of captive-reared juvenile (2-6 yr) A. davidianus and compared their body condition with wild specimens. We reared 240 salamanders in two groups, exposed them to different water temperatures for 7 mo, and monitored their growth over the next 3 yr. Subjects that were exposed to lower water temperatures (0.8-19.4 degrees C) were lighter and shorter than those maintained at higher temperatures (7.8-19.1 degrees C). Furthermore, subjects maintained at lower temperatures had a lower growth rate than those maintained at higher temperatures, except for the interval between the last two measurements. Captive-reared salamanders had lower body-condition indices than wild animals having the same body length during the study period. Captive subjects were 16% and 19% lighter than wild individuals of the same length when maintained in the high-and low-temperature groups, respectively. Our study indicates that water temperature affected juvenile growth, and captive-reared A. davidianus older than 5 yr had a body-condition index approaching that of individuals in the wild.

4000: ***+.216***

The objective of this study is to inventory the current genetic diversity of the bison quarantine feasibility study (BQFS) herd originating from Yellowstone National Park (YNP) using previously

described microsatellite, mitochondrial and nuclear DNA markers with the aim to determine the degree, if any, of cattle DNA introgression in this herd. This work can provide an important tool in monitoring and managing bison genetic diversity as brucellosis-free reintroduced herds are re-established throughout the US for conservation purposes. The BQFS composed of 89 Bison bison from YNP that were quarantined and tested to qualify as free of brucellosis in 2006-2007. Understanding genetic diversity of the herd is important to determine if any genetic characteristics such as cattle DNA introgression or low genetic diversity may threaten the herd's protected status. We evaluated genetic diversity at 42 microsatellite loci representing each of the nuclear chromosomes in the bison genome. We found no detectable evidence of cattle DNA introgression in this herd through nuclear markers and mitochondrial DNA analysis. Parentage analysis of the BQFS herd indicated that the majority of mature adults were actively breeding and contributing off spring. Genetic diversity levels in the quarantined herd were high and comparable to the YNP parent herd, suggesting a low risk of genetic loss in the near future. Based on these findings, the genetic diversity currently available within the BQFS herd will provide a strong foundation for bison reintroduced herds and for the preservation of the species.

4001: ***+.119***

From 2001 to 2012, the Whooping Crane Eastern Partnership released 196 costume-reared juvenile whooping cranes Grus americana in the eastern United States in an effort to reintroduce a migratory population of this endangered species. Techniques included leading juveniles from Wisconsin to wintering areas by ultralight aircraft or direct release of juveniles in Wisconsin prior to their first autumn migration. With few exceptions, ultralight-led cranes released on the Florida Gulf Coast wintered in inland freshwater habitats in subsequent winters. Wintering of the population occurred in four general regions: Florida-southern Georgia, coastal Carolina, the Mid-South (primarily Tennessee and northern Alabama), and the North (Indiana, Illinois, and Kentucky). Releases of ultralight-led juveniles resulted in the majority of the population returning to winter in Florida during the early years of the reintroduction. Later direct autumn releases and shortstopping by ultralight-led birds increased numbers in the Mid-South. Winter climate played a large role in wintering in the North. Drought resulted in changes in wintering locations, especially in Florida. Other factors influencing changes in distribution included habitat degradation and associations with other whooping cranes, especially new mates and birds released by different techniques. Breeding pairs and direct autumn-released birds exhibited greater winter site fidelity than did nonbreeders or ultralight aircraft-led birds, but fidelity was low for all groups. Causes of mortality differed across the winter range, with predation being most prevalent in Florida and gunshot accounting for the majority of mortalities north of Florida. Because most pairing occurred in central Wisconsin, the widespread winter distribution had no apparent negative effect on pair formation. There was no clear relationship between winter region and subsequent incubation success. The widespread distribution of the population poses minimal risk to the outcome of the reintroduction in comparison to reproduction problems in the core reintroduction area of central Wisconsin.

4002: ***+.226***

Elucidation of genetic mechanisms underpinning migratory behavior could help predict how changes in genetic diversity may affect future spatiotemporal distribution of a migratory species. This ability would benefit conservation of one such declining species, anadromous Pacific lamprey (Entosphenus tridentatus). Nonphilopatric migration of adult Pacific lamprey has homogenized population-level neutral variation but has maintained adaptive variation that differentiates groups based on geography, run-timing and adult body form. To investigate causes for this adaptive

divergence, we examined 647 adult lamprey sampled at a fixed location on the Columbia River and radiotracked during their subsequent upstream migration. We tested whether genetic variation [94 neutral and adaptive single nucleotide polymorphisms (SNPs) previously identified from a genomewide association study] was associated with phenotypes of migration distance, migration timing, or morphology. Three adaptive markers were strongly associated with morphology, and one marker also correlated with upstream migration distance and timing. Genes physically linked with these markers plausibly influence differences in body size, which is also consistently associated with migration distance in Pacific lamprey. Pacific lamprey conservation implications include the potential to predict an individual's upstream destination based on its genotype. More broadly, the results suggest a genetic basis for intrapopulation variation in migration distance in migratory species.

4003: *+.133*

The number of Osprey (Pandion haliaetus) nesting pairs in Europe, northern Africa, and the Middle East has reached between 9500 and 11 500 in the early 21st century. Compared to numbers from the 1980s (ca. 5500 pairs), the population has almost doubled. The increase is most obvious in countries like Germany and the United Kingdom. The largest and most important European populations in Sweden, Finland, and Russia seem to be stable. In contrast, Portugal, mainland Spain, and Turkey lost their last breeding pairs in the 1980s and 1990s. Negative trends are also reported from Poland due to persecution and from southeastern Europe and northern Africa, where only very few pairs remain. Reintroductions in England, Spain, and Italy have resulted in a few new breeding pairs in recent years.

4004: *+.078*

Elephant populations at high density commonly transform their habitat, but a low density population would not be expected to have a marked impact. Re-introduction of elephants into the Venetia-Limpopo Nature Reserve (320 km(2)) in the early 1990s established a low density population for the period of survey (0.16-0.33 individuals km(-2)). Accordingly, their impact on the composition and structure of the woody vegetation of three riparian and nine dryland vegetation types was measured between 1997 and 2010 using 148 permanent transects. Riparian habitat showed a greater change in composition and diversity, and also a greater decline of species richness, density of tall trees or total basal area, than dryland habitat. Change of dryland Commiphora Woodland was comparable to changes of riparian types. These conspicuous changes were a consequence primarily of severe use by elephants. Some species within these vegetation types declined markedly in abundance. Vegetation types dominated by Colophospermum mopane showed an increase in total basal area and relatively minor change in composition or structure, resulting mainly from the impact of moisture stress. Vegetation types that were severely impacted by elephants constituted <10% of reserve area; lightly impacted dryland C. mopane types constituted >70% of area. Some uncommon, selected dryland species were heavily impacted by elephants. A number of species may therefore be trending toward local extirpation. It was concluded that the coexistence of elephants and some plant species in this medium-sized, contained reserve was not possible. (C) 2014 Elsevier Ltd. All rights reserved.

4005: *+.108*

Habitat restoration, including revegetation of linear strips and enlargement of remnant patches, may benefit native fauna in highly fragmented landscapes. Such restoration has occurred around the world, even though the relative importance of strips and patches of vegetation remains

controversial. Using reptile communities from south-eastern Australia, we assessed the conservation value of revegetation in strips and alongside remnant patches compared with remnant vegetation and cleared roadsides. We also examined the distance that reptiles occurred from remnant patches into linear vegetation. We found that reptile species richness and counts did not substantially differ between revegetated, remnant and cleared habitats, or between linear strip and patch treatments. This may indicate that species sensitive to land clearing have already been lost from the landscape. These results imply that if specialist species have already been lost, we may be unable to measure the effects of agriculture on biodiversity. Furthermore, revegetation with the expectation that fauna will recolonize may be unrealistic and translocations may be necessary. Unexpectedly, we recorded higher species richness and counts of rare reptile species in remnant linear strips as distance from remnant patches increased. Ground-layer attributes were important for increasing reptile species richness and counts and in structuring reptile communities, explaining approximately three times as much variation as remnant shape or vegetation type (remnant, revegetated, cleared). Management agencies should protect and effectively manage remnant linear strips if rarer reptiles are to be retained, paying particular attention to ground-layer attributes. The decision to include ground layers in future revegetation activities will be more important than the shape of restored areas.

4006: ***+.045***

In Mexico City, native and exotic amphibians are commonly sold through the pet trade. This study investigates the presence of Batrachochytrium dendrobatidis (Bd) in native amphibians being sold at two commercial markets and at a herpetarium inMexico City. A total of 238 individuals (6 genera and 12 species) were tested for Bd using real-time polymerase chain reaction (PCR) analysis. There were 197 Bd-positive individuals (prevalence 82%) from five species of amphibians. Hyla eximia from the markets had very high Bd prevalence (100%; 76/76 and 99%; 88/89) but those from the herpetarium were Bd negative (0/12). Ambystoma mexicanum from the herpetarium also had a high Bd-positive prevalence (80%; 28/35). Though A. mexicanum is nearly extinct in the wild, a commercial market continues to flourish through the pet trade. Now that captive colonies of A. mexicanum are currently used for reintroduction programs, the authors recommend quarantine to reduce spread of Bd via movement of infected animals in the trade and between colonies and via disposal of wastewater from captive collections.

4007: ***+.045***

Many studies have investigated the possible impact of climate change on the distributions of plant species. In the present study, we test whether the concept of potential distribution is able to effectively predict the impact of climate warming on plant species. Using spatial simulation models, we related the actual (current species distribution), potential (modelled distribution assuming unlimited dispersal) and predicted (modelled distribution accounting for wind-limited seed dispersal) distributions of two plant species under several warming scenarios in the Sagarmatha National Park (Nepal). We found that the two predicted distributions were, respectively, seven and nine times smaller than the potential ones. Under a +3 degrees C scenario, both species would likely lose their actual and predicted distributions, while their potential distributions would remain partially safe. Our results emphasize that the predicted distributions of plant species may diverge to a great extent from their potential distributions, particularly in mountain areas, and predictions of species preservation in the face of climate warming based on the potential distributions of plant species are at risk of producing overoptimistic projections. We conclude that the concept of potential distribution is likely to lead to limited or inefficacious conservation of plant species due to its excessively optimistic projections of species preservation.

More robust strategies should utilize concepts such as "optimal reintroduction", which maximizes the benefit-cost ratio of conservation activities by limiting reintroduction efforts to suitable areas that could not otherwise be reached by a species; moreover, such strategies maximize the probability of species establishment by excluding areas that will be endangered under future climate scenarios. (C) 2014 Elsevier B.V. All rights reserved.

4008: *+.108*

Human-induced gene movement via afforestation and restoration programs is a widespread phenomenon throughout the world. However, its effects on the genetic composition of native populations have received relatively little attention, particularly in forest trees. Here, we examine to what extent gene flow from allochthonous plantations of Pinus pinaster Aiton impacts offspring performance in a neighboring relict natural population and discuss the potential consequences for the long-term genetic composition of the latter. Specifically, we conducted a greenhouse experiment involving two contrasting watering treatments to test for differences in a set of functional traits and mortality rates between P. pinaster progenies from three different parental origins: (i) local native parents, (ii) exotic parents and (iii) intercrosses between local mothers and exotic fathers (intraspecific hybrids). Our results showed differences among crosses in cumulative mortality over time: seedlings of exotic parents exhibited the lowest mortality rates and seedlings of local origin the highest, while intraspecific hybrids exhibited an intermediate response. Linear regressions showed that seedlings with higher water-use efficiency (WUE, delta C-13) were more likely to survive under drought stress, consistent with previous findings suggesting that WUE has an important role under dry conditions in this species. However, differences in mortality among crosses were only partially explained by WUE. Other non-measured traits and factors such as inbreeding depression in the relict population are more likely to explain the lower performance of native progenies. Overall, our results indicated that intraspecific hybrids and exotic individuals are more likely to survive under stressful conditions than local native individuals, at least during the first year of development. Since summer drought is the most important demographic and selective filter affecting tree establishment in Mediterranean ecosystems, a potential early selective advantage of exotic and hybrid genotypes would enhance initial steps of introgression of non-native genes into the study relict population of P. pinaster.

4009: *-.138*

Niche germination breadth may determine species occurrence under distinct environmental conditions. We choose Euterpe edulis and Lytocaryum weddellianum to evaluate germination niche breadth under light or dark at water potential of 0 MPa, and in either decreasing water potentials (-0.4 and -0.8 MPa) or flooded. In contrast to L. weddellianum, E. edulis showed high seed germination at both light conditions and expressive seed germination at low water potentials and flooding. E. edulis had wide seed germination niche breadth and seed addition of the same geographic region might be used for the reintroduction of populations of this threatened palm in different types of habitats of the Atlantic Rainforest. L. weddellianum, however, is unlikely to be able to germinate under altered conditions due to narrower germination niches and might be more vulnerable to extinction if the restricted germination cues no longer occur. Thus, ex situ initiatives might be used for the species conservation. (C) 2014 Associacao Brasileira de Ciencia Ecologica e Conservacao. Published by Elsevier Editora Ltda. All rights reserved.

4011: *-.048*

As anthropogenic change continues to fragment terrestrial habitats, conservation biologists are

increasingly concerned with how wild animals move through fragmented landscapes. Experimental translocations have recently gained popularity as a technique to determine landscape permeability by wild animals in fragmented landscapes. In experimental translocations, researchers capture individuals - usually adults - and release them elsewhere in order to determine whether they are able to cross the landscape and return to their original location. We argue that most experimental translocations have two inherent confounding factors - age of the individual and homing ability - and that the narrow spatiotemporal scale of the technique may give it limited ability to address the most important conservation and management questions in fragmented landscapes. We discuss three alternative techniques (telemetry, capture-mark-recapture, and landscape genetics), and recommend that experimental translocations only be undertaken if: 1) they avoid confounding factors; 2) they are validated by other techniques; and 3) no other options are available for obtaining the data. We stress that researchers that do proceed with experimental translocations must acknowledge that they are using an indirect proxy to quantify natural animal movement.

4012: *+.204*

Conservation of European ground squirrel (Spermophilus citellus) has been supported by the LIFE - Nature project "Conservation of Falco cherrug in NE Bulgaria, Hungary, Romania and Slovakia" during the period 2010-2014. The project activities were usually focused on EGS reintroduction programmmes with the aim of ensuring the food base for selected species of birds in areas where EGS were the primary historical prey and have become extinct. Reintroduction programmes were carried out at two sites in Slovakia: Piesocna (Moravsky Svaty Jan) and Pod Okruhlou skalou (Tisovec). Together 1 74 individuals were released at Pod Okruhlou skalou and 284 individuals at the Piesocna target site. Monitoring of both repatriated colonies focused on the natality and distribution area. At both target sites successful hibernation and reproduction have been confirmed. The repatriation date was strongly influenced by weather conditions, especially during the rainy season. It was necessary to cut the grass several times per season, in order to ensure appropriate conditions for individuals' survival. It was also necessary to protect the individuals against predators. House cats tend to hunt squirrels, which can be considered as the most serious threat at the Piesocna site. Squirrels at the Pod Okruhlou skalou site were overly attacked by foxes. Intensive care (especially appropriate management of sites and additional feeding) ensured good stability of colonies, their survival and increase.

4013: *+.104*

Since the mid-20th century, reintroduction and conservation programmes for the formerly critically endangered European beaver (Castor fiber) were established in many parts of Europe. Austrian populations, formerly extinct in 1869, were re-established by reintroductions since the 1970ies. The beaver is present in all nine federal states today, with a total population estimate of about 5,000. Information on regional distribution patterns and population sizes is available for most federal states, but was lacking for the 2nd largest Austrian federal state, Styria. This paper presents the results of the first comprehensive beaver survey in Styria. During the winter seasons of 2012/2013 and 2013/2014, beaver activity signs were collected within 65 field days along 413 km of riversides in the basins of Mur, Lafnitz and Raab (Fig 2). Single-/pair and family territories were identified by distribution patterns of activity signs, and the total beaver population was estimated by multiplying the number of single/pair territories with the factor 1.5 and family territories with 5. Furthermore, the importance of several aquatic and terrestrial parameters for the habitat suitability were analysed by establishing a generalized linear mixed model (GLMM). Based on available spatial data, the total colonisable area of the beaver was modeled using

geographic information system (GIS) tools. European beavers are present along the rivers Mur (upstream as far as Mellach near Graz), Lafnitz, Feistritz, Raab, Sulm, Lassnitz, Kainach and some of their tributaries. The Lafnitz river is colonised up to 600 m a.s.l.. Only two beaver records originate from north of the alpine water divide: near Hieflau (Enns river) and from the river Salza near Mariazell. A total of 2,331 activity signs indicate the presence of 63 beaver territories (23 along Mur river, 33 in the Lafnitz basin, 7 along Raab river; Figs 5-18). The current population estimate is 220. The territory length varies between 0.3 and 6.5 km, the mean distance between territories is 4.2 km. Territories of the Mur river are significantly longer and the distance between territories is larger than of the Lafnitz river. The GLMM shows that the probability of the presence of a territory center increases with increasing width of the riparian forest and that riverside obstructions reduce the likelihood of the presence of a territory center. The GIS-model of the potential future range of the beaver in Styria shows, that almost all lowland parts of Styria offer suitable habitats for this riverine keystone species (Fig 19). The results of this paper should be the basis for a future beaver monitoring programme in Styria and because of the high conflict potential concering human utilisation claims for a sustainable beaver management.

4014: ***+.036***

The Christmas Island White-eye Zosterops natalis occurs naturally only on the 135 km(2) Christmas Island. Between 1888 and 1900 (remarkably soon after it was first discovered and described), it was introduced to the 1 km2 Horsburgh Island in the Cocos (Keeling) Islands group. There has been limited subsequent documentation of the fate of this translocated population. Based on transect sampling in 2013, we estimate the population size to be 1084 individuals (with 95% confidence limits of 731 to 1716). This represents a substantially larger estimate than the previous estimate (of 400 individuals in 1941), and indicates a greater abundance than the most recent (1982) non-quantitative record of its status. In contrast to previous documentation, this species is widespread on Horsburgh Island, and abundant in a mosaic of natural and modified vegetation (dominated by coconut Cocos nucifera plantation with Scaevola taccada shrub layer). Contextualising the conservation significance of this introduced population is difficult because there is no reliable estimate of the population size on Christmas Island, but it is plausible that the Horsburgh Island subpopulation now comprises c. 5% of the species' total population size. However, more importantly, its significance lies in the provision of a second location that may reduce extinction risk.

4016: ***+.193***

Background There is general agreement that as well as providing a range of ecological benefits in certain locations, wild beavers can have a negative impact on a range of land and water uses through the alteration of water levels, burrowing and foraging activity. This report focusses on the range of beaver management techniques, in particular to mitigate any negative impacts including flooding of forestry and agricultural land, weakening flood protection banks, threatening infrastructure such as roads, obstructing passage for migratory fish and altering the ecological condition of protected species and/or habitats. Scottish Ministers are due to decide on the future of wild beavers in Scotland in 2015. Part of this consideration is likely to include an evaluation of what steps could be put in place to mitigate any negative consequences for land, water and infrastructure managers, should beavers be formally reintroduced. The purpose of this report is to assist SNH in developing its advice to Scottish Ministers on the question of beaver reintroduction. SNH will use the information on practical management techniques, including their animal welfare implications, costs and efficacy, to undertake a study of the risks surrounding the use of each potential management technique and any legal implications. It may also form the basis for a future

manual for the management of wild beavers in Scotland. It is likely that the efficacy, costs and legal considerations surrounding the use of any technique will change over time. This report assumes that beavers, and their breeding sites and resting places, will receive strict protection under the Habitats Regulations 1994 (as amended in Scotland), but reference has been made to derogations associated with beaver management by some European countries. Main findings - The Eurasian beaver is formerly native to Britain before being hunted to extinction. The beaver has been reintroduced to over 24 European countries and it is generally accepted to provide a range of ecological benefits. This species can lead to negative impacts, particularly in intensively modified landscapes, and can conflict with modern land uses. - Scottish ministers are set to decide on the future of wild beavers in Scotland in 2015. The presence of wild beavers is already requiring management considerations on a local scale. To date, all indications are that Eurasian beavers will survive in a Scottish environment and could restore to population densities comparable to other European countries. Similar conflict concerns are likely to arise, with comparable management solutions. - A re-establishing beaver population cannot be excluded from any given part of a river system where no significant barrier exists, once it is present. 'Beaver-free zones', whilst accessible to other colonising animals, would require constant removal effort. More cost-effective solutions may include tolerating the presence of beavers using a variety of control measures. - Beaver-related conflicts have been previously documented and tend to be focused within agriculture, forestry, fisheries and infrastructure (particularly gardens and water management systems). - A range of techniques for the effective management of beaver impacts is now well developed across Europe and North America. These range from non-lethal methods, including tree guards, dam mitigation and bank protection, to lethal control - with varied results, costs and implications for animal welfare. It is likely that the efficacy, costs and legal considerations surrounding the use of any technique will change over time. - Specific species and habitats of conservation concern, such as freshwater pearl mussel and riparian aspen stands, require further monitoring to determine whether potential impacts of beavers and their activities could be of concern and require intervention. - Lack of education on beaver ecology, unclear instructions on practicable and permitted mitigation methods, overly-bureaucratic licencing systems, financial burden and unclear benefits of beaver presence to landowners are likely to all lead to increased actual and perceived negatives impacts of beaver presence. - It is highly likely that any future programme of beaver management in Scotland would be an evolving process as populations establish and expand, and if applications for further releases are approved. Early and reactive intervention, according to a proactive management plan developed ahead of any significant increase in population densities and associated colonisation of sub-optimal areas for beavers, is recommended to reduce conflicts. - Long-term land use planning to incorporate land buffer zones along freshwater bodies, and to allow the natural regeneration of these areas with native riparian plant and tree species, is likely to reduce the majority of beaver conflicts. Where this is not practicable or desirable, it is likely that removal of beavers will be necessary. Translocation of 'problem' individuals and families is a viable, but a time-limited management option. As populations rise, targeted culling will likely become an essential beaver management tool, to reduce conflicts in areas where non-lethal management methods prove to be ineffective or too expensive. - Further monitoring and scientific investigation will be required to determine the role beavers can play in the Scottish environment, including the impact on migratory fish and agricultural land, and their role in habitat management and flood alleviation planning.

4017: ***+.176***

Background This report has been published to help inform the Scottish Government on its decision on the future of beavers within Scotland (see Gaywood, 2015). Beavers are widely considered to be 'ecosystem engineers'. This term is reserved for those species that have a large

impact on an environment, fundamentally changing ecosystems, and creating highly unusual habitats, often considered unique. This review investigates the evidence that beavers act as ecosystem engineers, and includes a meta-analysis of published studies. The impact on a wider range of species groups were considered, including plants, invertebrates, fish, amphibians, birds and mammals. We then explored how beavers will impact the biodiversity of Scotland. In particular, the effect of a beaver reintroduction on protected or vulnerable species and habitats is examined. Distribution maps of species of conservation interest were overlaid with potential beaver habitat to identify the extent and likelihood of any interaction, and ensuing influences. Main findings A meta-analysis of the literature showed that, overall, beavers have an overwhelmingly positive influence on biodiversity. Their ability to modify the environment through felling trees and impounding watercourses means that beavers not only create unique habitat but fundamentally increase habitat heterogeneity, fully justifying their description as ecosystem engineers. However, in Scotland there are some specific species and habitats which have the potential to be adversely affected, and in the event of any formal beaver reintroduction these would need to be closely monitored, and appropriate management put in place if necessary. Beaver herbivory, impoundment, and associated behaviours influence ecosystems through the creation of a variety of features: construction of unique structures such as dams and lodges, important habitat features such as standing dead wood (after inundation), an increase in woody debris, and a graded edge between terrestrial and aquatic habitats rich in structural complexity. Beaver ponds also have unique successional stages such as beaver meadows. These features give rise to riparian ecosystems supporting a diversity not often found in undisturbed areas. The impact of beavers is expected to be particularly beneficial for some plants, aquatic invertebrates, fish, amphibians and birds on a landscape scale. Some species of conservation importance will benefit. For instance, beaver ponds should benefit otter (Lutra lutra) and great crested newt (Triturus cristatus) populations. Water vole (Arvicola amphibious) habitat will be improved due to an increased abundance of slow moving water bordered by structurally complex vegetation with an open canopy. The greatest impact on overall woodland quality is likely to arise from the interaction of beaver and deer browsing - at relatively high densities deer may prevent the regeneration of woodland. Hence, careful management of deer in areas colonised by beavers will improve the availability of diverse woodland ecosystems. Beavers cause disturbance, and while this is an important influence on ecological landscapes it is likely to reduce the extent of older growth riparian woodland communities. This can have a negative impact if the affected habitat type is rare, and a large proportion is impacted. However, a lack of woodland regeneration caused by high deer abundance can lead to habitat degradation/loss. Hence, vulnerable habitats and species, such as Atlantic hazelwood and aspen (Populus tremula), need to be closely monitored where they are isolated and in close proximity to riparian areas. This is of particular importance due to the variety of associated dependent species of conservation interest, such as lichen communities on Atlantic hazelwood.

4018: ***+.095***

Background Argyll Fisheries Trust undertook surveys of fish populations and salmonid fish spawning sites as one of a number of monitoring projects investigating the effects of beaver activities on the natural environment during the Scottish Beaver Trial. Following the collection of baseline information in 2008 and 2009 and monitoring in 2010 and 2011, more intensive surveys were undertaken at six locations during 2012 and 2013. These surveys have increased data resolution at sites where beaver activity may interact with fish populations in the future. This report marks the end of a five-year trial period. Main findings. -In 2012 and 2013, surveys of fish populations and spawning activity were undertaken at two locations where recent beaver activity (tree felling and dam construction) may have affected the passage of fish between habitats. At

these locations, the study primarily focused on the migration of adult trout from freshwater lochs into streams used for spawning and the recruitment of juveniles in the nursery habitat. Despite some temporal variation, the surveys in streams in 2012 and 2013 (after dams were constructed) found no significant change to the composition of fish species or their number compared with that found in previous surveys (2008 to 2011). -Over a similar time period, surveys of fish populations and spawning activity were also undertaken at four locations where no recent beaver activity was known to potentially affect fish habitat. These surveys also found no significant change to the fish species composition or the number of individuals at these sites during the study period. -The following conclusions were reached: The surveys undertaken during the trial period found no significant change to the species or number of fish found at sites where beaver have recently become active in tree felling and dam building and also at sites where no beaver activity had been recorded. If beaver are retained after the trial period at Knapdale, future monitoring of sites where beaver are active may be necessary to assess potential beaver and fish interaction and inform management.

4019: *+.010*

Some species of animals related to forest habitats have disappeared or greatly decreased in the first half of the last century. However, during the second half of the last century the forest areas have increased and many forests have been protected. This produced conditions for a re-colonization or reintroduction of extinct forest animal species. The present study is a complete overview of records of extinct and still living Black Woodpecker Dryocopus martius, European green Woodpecker Picus viridis, Middle spotted Woodpecker Dendrocopos medius and Lesser spotted Woodpecker Dendrocopos minor in Sicily and Calabria (Southern Italy). This study was carried out to show that these woodpeckers, or at least some of them, may have existed in Sicily and are possible candidate for re-introduction projects in the island. This explains the reasons of a historical and contemporary survey on the presence of the above mentioned species in Calabria, that could be the place where these birds could be recovered for possible reintroduction projects in Sicily.

4020: *+.062*

For the recovery and conservation of endangered species such as the southern water vole Arvicola sapidus, it is important to understand the effects of predation on their populations. The impact of a predator on a prey species depends on the dynamics and structure of their populations, the abundance of both species and their relationships with other species. When considering broad geographic areas, the analysis of the trophic dimension can provide an estimate of the effect of predatory pressure on the prey. To determine the potential impact of predators on the southern water vole, we used information provided in 74 studies concerning the diet of predators in the geographical range of the prey species. The trophic spectrum was determined based on faeces, stomach contents or pellets in which the arvicoline was present. Based on the percentage of biomass consumed in each of nine trophic categories, the potential impact posed by predators was linked with dietary profile, trophic diversity, intensity of southern water vole consumption and availability of preferred prey. In the localities studied, southern water voles are not the preferred prey of any of the predators we considered, but represent an alternative prey for some of them, especially when their main prey species are scarce. The predators with the greatest potential impact on southern water vole populations are Lutra lutra and Ardea cinerea; those with a moderate impact are some mesocarnivores (Felis silvestris, Genetta genetta, Neovison vison, Vulpes vulpes and Martes foina), a bird frequenting aquatic environments (Ciconia ciconia), and the raptors Aquila fasciatus and Hieraaetus pennatus. The results obtained may be useful in the

planning of measures for the conservation of the southern water vole, such as translocations or reintroductions, and in the design of strategies to reduce predatory pressure with the aim of promoting the success of these programmes.

4021: ***+.041***

The Bearded Vulture Gypaetus barbatus occurs throughout its range in small and dwindling population fragments with limited genetic differentiation between populations, suggesting that the species might be managed as a single entity. The numbers of East and Southern African Bearded Vultures included in previous studies were small, so we determine the genetic variation within, evolutionary placement of and connectivity among sub-Saharan African populations. Mitochondrial DNA fragment analyses detected little or no differentiation between populations in Ethiopia and Southern Africa, with reduced haplotype diversity in Southern Africa compared with populations in the Northern Hemisphere. The results inform conservation management of this species globally and locally, and offer guidelines for translocations should populations continue to decline.

4022: ***+.103***

Competitive interactions with non-native species can have negative impacts on the conservation of native species, resulting in chronic stress and reduced survival. Here, juvenile Atlantic salmon (Salmo salar) from two allopatric populations (Sebago and LaHave) that are being used for reintroduction into Lake Ontario were placed into semi-natural stream tanks with four non-native salmonid competitors that are established in Ontario streams: brown trout (S.trutta), rainbow trout (Oncorhynchus mykiss), Chinook salmon (O.tshawytscha) and coho salmon (O.kisutch). Brown trout and rainbow trout reduced the survival and fitness-related traits of Atlantic salmon, whereas Chinook salmon and coho salmon had no impact on these traits. These data support theories on ecological niche overlap and link differences in observed aggression levels with competitive outcomes. Measurements of circulating hormones indicated that the Atlantic salmon were not chronically stressed nor had a change in social status at the 10-month time point in the semi-natural stream tanks. Additionally, the Sebago population was better able to coexist with the non-native salmonids than the LaHave population. Certain populations of Atlantic salmon may thus be more suitable for some environments of the juvenile stream phase for the reintroduction into Lake Ontario.

4023: ***-.001***

The co-extinction of parasitic taxa and their host species is considered a common phenomenon in the current global extinction crisis. However, information about the conservation status of parasitic taxa is scarce. We present a global list of co-extinct and critically co-endangered parasitic lice (Phthiraptera), based on published data on their host-specificity and their hosts' conservation status according to the IUCN Red List. We list six co-extinct and 40 (possibly 41) critically co-endangered species. Additionally, we recognize 2-4 species that went extinct as a result of conservation efforts to save their hosts. Conservationists should consider preserving host-specific lice as part of their efforts to save species.

4024: ***+.071***

Translocation of threatened or vulnerable species is a tool increasingly used for conservation and management. However, in some species, homing and movement behaviors may undermine the

success of translocation efforts. For the federally protected Agassiz's desert tortoise (Gopherus agassizii), translocation is a strategy used to manage declining populations, yet homing behavior in this species is poorly understood. To explore homing behavior and movement patterns after translocation, we radio tracked 80 tortoises during a 2-phase experimental translocation. Phase 1 included 40 tortoises that were translocated, then monitored for a period of 37 days (21 Sep-28 Oct 2009), and phase 2 included a different group of 40 tortoises that were translocated and then monitored for 186 days (13 Apr-20 Oct 2010). In both phases, we assigned tortoises randomly to 1 of 3 treatment groups: translocated (displaced 2, 5, or 8km from their source location), handling control, or control. After translocation, 20% of the translocated tortoises were able to navigate to their source location, and translocation distance had an effect on their ability to navigate home. We found 44% of tortoises in the 2-km translocated group returned home; 1 tortoise in the 5-km group, and no tortoises in the 8-km translocated group returned. The time required to reach home ranged from 5 to 37 days for the 2-km group, and 34 days for the 5-km group. We deemed tortoises to have homed successfully if they returned to their source location within 37 days of translocation as this reflected the duration of phase 1 and allowed for a balanced comparison between the 2 phases. We found that translocated tortoises moved at least 1.5 times more overall than the control groups, with some individuals moving >10km from the translocation site. These patterns persisted even after accounting for seasonal and sex differences in distance traveled. By identifying homing behaviors and quantifying post-translocation movement patterns, this experiment addressed a key data gap in tortoise behavior that may limit the efficacy of tortoise translocation efforts. Our results point to the need to account for behavioral responses of tortoises to minimize risk to translocated individuals and maximize the success of translocation projects. (c) 2014 The Wildlife Society.

4025: ***+.129***

Baselines rooted in historical records or concepts of previous conditions are necessarily used to identify and generate recovery goals for endangered species. However, strict adherence to various spatial, temporal, and genetic baselines can limit endangered species recovery actions, success, and the broader conservation of biodiversity. Recent approaches that deviate from historical baselines such as assisted colonization and intentional hybridization have been used to facilitate recovery but lack broad acceptance and an underpinning conceptual framework to guide their use in practice. We here present a novel framework for addressing when baseline-abandoning approaches should be implemented that requires both scientific input and management-defined thresholds. We submit that in cases where species face extreme endangerment and managers have little chance of reducing or ameliorating future threats within a species' historical range, it is better to embrace a more flexible recovery model that includes taking action that deviates from historical baselines. Embracing this reinterpretation of management baselines not only has the potential to advance endangered species recovery but could have important cascading effects on ecosystem-based approaches to conservation. Further, rethinking adherences to baselines can affect our broader social-psychological relationship with wildlife conservation and management. Overall, although historical data on baseline conditions will remain vital to the initial setting of recovery goals, many situations will require more dynamic interpretations of paths to recovering endangered species. (c) 2014 The Wildlife Society.

4026: ***+.028***

Invasive alien species present a global threat to biodiversity, particularly where pathogens and symbionts are involved. Branchiobdellidans are clitellate annelids with an obligate ectosymbiotic association primarily on astacoidean crayfish. There are several examples of branchiobdellidan

species adopting a geographically exotic host where endemic and exotic crayfishes cohabit the same water body. The first records of a western North American branchiobdellidan, Xironogiton victoriensis, adopting the eastern North American crayfish, Procambarus clarkii, in 2 river basins in Spain provide further evidence of the ectosymbionts' tolerance to adopt an exotic host. Given worldwide translocations of these and other commercial crayfish species, limnologists and agency managers need to be alert for further introductions of X. victoriensis and other branchiobdellidans. Impacts of these exotic ectosymbionts on habitat and biota at a new location are unknown, as are their consequences on native biodiversity.

4027: *-.123*

The goal of captive breeding programmes is often to maintain genetic diversity until re-introductions can occur. However, due in part to changes that occur in captive populations, approximately one-third of re-introductions fail. We evaluated genetic changes in captive populations using microsatellites and mtDNA. We analysed six populations of white-footed mice that were propagated for 20 generations using two replicates of three protocols: random mating (RAN), minimizing mean kinship (MK) and selection for docility (DOC). We found that MK resulted in the slowest loss of microsatellite genetic diversity compared to RAN and DOC. However, the loss of mtDNA haplotypes was not consistent among replicate lines. We compared our empirical data to simulated data and found no evidence of selection. Our results suggest that although the effects of drift may not be fully mitigated, MK reduces the loss of alleles due to inbreeding more effectively than random mating or docility selection. Therefore, MK should be preferred for captive breeding. Furthermore, our simulations show that incorporating microsatellite data into the MK framework reduced the magnitude of drift, which may have applications in long-term or extremely genetically depauperate captive populations.

4028: *+.161*

Identification of units within species worthy of separate management consideration is an important area within conservation. Mitochondrial DNA (mtDNA) surveys can potentially contribute to this by identifying phylogenetic and population structure below the species level. The American crocodile (Crocodylus acutus) is broadly distributed throughout the Neotropics. Its numbers have been reduced severely with the species threatened throughout much of its distribution. In Colombia, the release of individuals from commercial captive populations has emerged as a possible conservation strategy that could contribute to species recovery. However, no studies have addressed levels of genetic differentiation or diversity within C.acutus in Colombia, thus complicating conservation and management decisions. Here, sequence variation was studied in mtDNA cytochrome b and cytochrome oxidase I gene sequences in three Colombian captive populations of C.acutus. Two distinct lineages were identified: C.acutus-I, corresponding to haplotypes from Colombia and closely related Central American haplotypes; and C.acutus-II, corresponding to all remaining haplotypes from Colombia. Comparison with findings from other studies indicates the presence of a single northern lineage (corresponding to C. acutus-I) distributed from North America (southern Florida), through Central America and into northern South America. The absence of C.acutus-II haplotypes from North and Central America indicates that the C.acutus-II lineage probably represents a separate South American lineage. There appears to be sufficient divergence between lineages to suggest that they could represent two distinct evolutionary units. We suggest that this differentiation needs to be recognized for conservation purposes because it clearly contributes to the overall genetic diversity of the species. All Colombian captive populations included in this study contained a mixture of representatives of both lineages. As such, we recommend against the use of captive-bred individuals for conservation

strategies until further genetic information is available.

4029: ***+.265***

Species with specialized ecological interactions present significant conservation challenges. In plants that attract pollinators with pollinator-specific chemical signals, geographical variation in pollinator species may indicate the presence of cryptic plant taxa. We investigated this phenomenon in the rare sexually deceptive orchid Drakaea elastica using a molecular phylogenetic analysis to resolve pollinator species boundaries, pollinator choice experiments and a population genetic study of the orchid. Pollinator choice experiments demonstrated the existence of two ecotypes within D.elastica, each attracting their own related but phylogenetically distinct pollinator species. Despite the presence of ecotypes, population genetic differentiation was low across populations at six microsatellite loci (F-ST=0.026). However, Bayesian STRUCTURE analysis revealed two genetic clusters, broadly congruent with the ecotype distributions. These ecotypes may represent adaptation to regional variation in pollinator availability and perhaps the early stages of speciation, with pronounced morphological and genetic differences yet to evolve. Resolution of the taxonomic status of the D.elastica ecotypes is required as this has implications for conservation efforts and allocation of management funding. Furthermore, any reintroduction programmes must incorporate knowledge of ecotype distribution and pollinator availability to ensure reproductive success in restored populations.(c) 2014 The Linnean Society of London, Botanical Journal of the Linnean Society, 2015, 177, 124-140.

4030: ***+.063***

Wildlife managers are challenged with the task of deciding whether a management measure is appropriate or not, and furthermore they have to convince others about the merits of their decision. Population decline of some hare species (genus Lepus) has resulted in their Red Listing and conservation measures are being undertaken. Release or restocking is a frequent measure in some countries, and thousands of hares are released annually, mainly for hunting purposes. These hares can be obtained by either intensive or extensive rearing or translocation of the wild animals. Each method entails difficulties and different survival rates in the wild. Survival rates in the wild are low for hares intensively reared in cages but are higher for hares reared extensively in enclosures and wild translocated hares. The benefits of the hare release practice are significantly lower than the action's implementation cost. Hare releases have not increased significantly the wild hare population or the hunting harvest in areas where the practice has been applied. The risk of genetic and evolutionary degradation and pathogen transmission is possible in wild populations. The need for wise management of this practice is evident and the term 'Permitted Transferring Units' should be introduced to denote regions where hares should not be transferred for rearing and release.

4031: ***+.141***

Rare plant reintroductions are designed to increase the number of individuals in the wild, but can also improve genetic diversity of populations, reducing both short-term and long-term extinction risks. We used microsatellites developed for the genus Pseudophoenix H. Wendl. ex Sarg. to determine how reintroduced plants of the endangered Pseudophoenix sargentii H. Wendl. ex Sarg. planted in the Florida Keys in the early 1990s contributes to the population genetic structure of the species. We sampled 108 individuals representing wild and reintroduced populations in the Florida Keys and from the ex situ collection at Fairchild Tropical Botanic Garden. The wild stand on Elliott Key and the reintroduced individuals on Long Key displayed evidence of genetic drift, inbreeding, and decreased gene flow. In contrast, the ex situ plants and reintroduced individuals on

Elliott Key displayed low inbreeding and higher heterozygosity. All populations deviated significantly from Hardy-Weinberg equilibrium. All pairwise FST and GST values were significant except comparisons between certain age classes on Elliott Key. Analysis of molecular variance partitioned 87.8% of the genetic variation within populations. Overall, reintroduced plants have contributed to greater heterozygosity of the stand on Elliot Key. Finally, our work shows that the ex situ collection includes wild offspring of individuals that are now extirpated, supporting the ongoing importance of ex situ collections in botanic gardens.

4032: *+.123*

Premise of research. Genetic variation for ecologically important traits is necessary for populations to adapt to environmental change. Many authors have called for a greater emphasis on directly measuring quantitative genetic variation in rare species, which are expected to have reduced amounts of genetic variation due to genetic drift in small populations. The extent of among-population differentiation for quantitative traits may also help to evaluate the likelihood that genetic rescue/translocation will be a successful conservation strategy. Despite these merits, relatively few studies measure quantitative genetic variation for ecologically important traits as a function of population size.Methodology. Sixteen populations of the endangered plant Hypericum cumulicola were sampled, capitalizing on previous work that has estimated relative effective population sizes and demonstrated minimal migration between populations. This context allows more direct inference about the role of drift in small populations on quantitative genetic variation, the focus of this study. Using controlled pollinations and a greenhouse common garden, quantitative genetic variation within populations and differentiation among populations were estimated for six putatively ecologically important traits. Pivotal results. There were few significant estimates of genetic variation for most traits irrespective of population size. There was a positive correlation between population size and genetic variation for anther-stigma distance, a floral trait expected to influence the degree of self-fertilization. There was also limited differentiation among populations for all traits.Conclusions. Limited genetic variation for ecologically important traits in these populations could slow or limit adaptive responses to future environmental change, possibly increasing extinction risk. The smallest populations will be particularly sensitive to environmental/habitat changes that result in reduced pollinator visitation. Limited phenotypic differentiation, combined with previous evidence of strong heterosis in crosses between small populations, suggests little risk of outbreeding depression if genetic rescue efforts become necessary to preserve this species.

4033: *+.354*

Hundreds of rehabilitant great apes have been released into the wild, and thousands await release. However, survival rates after release can be as low as 20 %. Several factors influence individuals' survival rates, one of which is the capacity to obtain an adequate diet once released. Released individuals are faced with a mixture of familiar and novel foods in an unfamiliar forest; therefore, it is important to understand how they increase acceptance and consumption of novel foods. This is especially vital for omnivorous species, such as wild great apes, which consume several hundred species of different foods. We assessed the effects of repeated exposures and sociality (i.e. co-feeding in the presence of one or more other individuals) on the acceptance and consumption of novel foods by captive orangutans (Pongo sp). Repeated exposures of food (novel, at first) did not cause an increase of acceptance of food; in other words, the orangutans did not start to eat a food item after being exposed to that food more often, but repeated exposures of food increased consumption (i.e. quantity). After repeated exposures, the orangutans also became gradually more familiar with the food, decreasing their explorative behaviour. The presence of co-feeding

conspecifics resulted in an increased acceptance of the novel food by orangutans, and they ate a larger amount of said foods than when alone. Repeated exposure and sociality may benefit rehabilitant great apes in augmenting and diversifying diet and, once practiced before release, may accelerate an individuals' adaptation to their new habitat, improving survival chances. Great ape rescue, rehabilitation and reintroduction require large financial and logistic investments; however, their effectiveness may be improved at low cost and low effort through the suggested measures.

4034: *+.233*

Conservation biology has had a long-standing debate about the relative importance of genetic processes in increasing the risk of extinction in threatened species. We assume that priority should be given to securing a species from extinction by stopping significant declines in numbers and then managing the secured populations to recovery by creating opportunities for population growth. This two-prong approach endorses the importance of ameliorating the agents that are causing populations to decline and then understanding the genetic issues that can arise once populations become small but stable or slowly recovering. This starting point was the initiator for the research we commenced in the mid-1990s and continue to this day. The review covers six sections: (1) We identified inbreeding depression (using pedigree relatedness and molecular loci) in takahe (Porphyrio hochstetteri), robins (Petroica australis and P. longipus), and kakapo (Strigops habroptilus), and both negative and positive effects of relatedness on fitness in the highly inbred Chatham Island black robins (Petroica traversi (2) We found no relationship between pedigree inbreeding, molecular heterozygosity and fitness (HFC) in the genetically depauperate takahe, but we did find such effects in the more diverse robins; (3) We found that all threatened species in New Zealand had lost genetic diversity over time, compared with more recent bottlenecks associated with reintroductions; (4) AlleleRetain employs user-specified parameters to simulate demography, allele retention and inbreeding in animals with overlapping generations, and is particularly useful for identifying the maximum retention of allelic diversity of a reintroduced population. DOC is currently developing a website to assist with simulation in AlleleRetain to assist both DOC and community-led reintroductions; (5) A meta-analysis of 109 populations indicated that loss of neutral and functional diversity was correlated during prolonged bottlenecks, but overall loss of MHC diversity is 15% greater than neutral diversity. We found support for this pattern when we investigated the loss of genetic diversity from historical museum to contemporary samples in saddlebacks and robins. Further prioritising of individuals for targeted breeding on the basis of adaptive immune alleles in yellow-fronted parakeets (Cyanoramphus auriceps) and 16161)6 may allow the maintenance of important functional genetic diversity over a species' recovery; (6) We described the diversity of toll-like receptor (TLR) genes that mediate innate immune responses in avian species, and note they may be simpler to use than the more complicated MHC immunity genes. We found evidence of episodic positive selection in the evolution of most avian TLRs, but within 10 bottlenecked species more evidence of genetic drift occurred than balancing selection at TLR loci.

4035: *-.049*

This study reports new records of Procambarus clarkii Girard, 1852 in Southeastern Brazil and investigates its possible pathways of introduction. The Brazilian crayfish pet trade was surveyed with regard to species' availability and price, reporting its persistence, even though illegal. An invasion risk analysis was performed by applying a protocol developed specifically to invasive freshwater invertebrates (FI-ISK). Procambarus clarkii has confirmed occurrences from urban and non-urban areas in the state of Sao Paulo, including the Jaragua State Park, an Atlantic Forest conservation area. The pet trade is an important introduction vector but some subsequent

translocation can also be due to the release of live bait by anglers. Besides passive dispersal, these newly discovered populations, especially those located in non-urban areas, are likely to naturally disperse further through active spread and river drift of juveniles. Although confirmed colonized areas are all located in the state of Sao Paulo, the invasive risk analysis showed that P. clarkii is a potential highly invasive species for Brazil. These introductions have unknown consequences; however, many impacts are associated with this species establishment around the world, underlining the urgency to understand the invasion process in Brazil and develop efficient management techniques.

4036: ***+.039***

To investigate the extent and causes of recent quaking aspen (Populus tremuloides) recruitment in northern Yellowstone National Park, we measured browsing intensity and height of young aspen in 87 randomly selected aspen stands in 2012, and compared our results to similar data collected in 1997-1998. We also examined the relationship between aspen recovery and the distribution of Rocky Mountain elk (Cervus elaphus) and bison (Bison bison) on the Yellowstone northern ungulate winter range, using ungulate fecal pile densities and annual elk count data. In 1998, 90% of young aspen were browsed and none were taller than 200 cm, the height at which aspen begin to escape from elk browsing. In 2012, only 37% in the east and 63% in the west portions of the winter range were browsed, and 65% of stands in the east had young aspen taller than 200 cm. Heights of young aspen were inversely related to browsing intensity, with the least browsing and greatest heights in the eastern portion of the range, corresponding with recent changes in elk density and distribution. In contrast with historical elk distribution (1930s-1990s), the greatest densities of elk recently (2006-2012) have been north of the park boundary (similar to 5 elk/km(2)), and in the western part of the range (2-4 elk/km(2)), with relatively few elk in the eastern portion of the range (<2 elk/km(2)), even in mild winters. This redistribution of elk and decrease in density inside the park, and overall reduction in elk numbers, explain why many aspen stands have begun to recover. Increased predation pressure following the reintroduction of gray wolves (Canis lupus) in 1995-1996 played a role in these changing elk population dynamics, interacting with other influences including increased predation by bears (Ursus spp.), competition with an expanding bison population, and shifting patterns of human land use and hunting outside the park. The resulting new aspen recruitment is evidence of a landscape-scale trophic cascade in which a resurgent large carnivore community, combined with other ecological changes, has benefited aspen through effects on ungulate prey.

4037: ***+.276***

Reintroductions of flagship species, including charismatic wild ungulates, are becoming increasingly frequent to protect endangered species or stimulate conservation awareness. However, indicators that go beyond the target species are still needed to ensure the ecological sustainability of past and future reintroductions. Here, we examined woody vegetation to assess the ecological sustainability 25 years after the reintroduction of a wild ungulate, the Iberian ibex or Iberian wild goat (Capra pyrenaica). We used browsing intensity, foraging preferences, regeneration probability and pellet counting to estimate sustainable population densities. Currently, almost one quarter of the woody species showed unsustainable levels of browsing. Threatened woody species were more vulnerable to ungulate damage, with 50% lacking natural regeneration. Current density estimations (47 ind km(-2)) represent the highest densities ever recorded for this wild ungulate species and should be reduced by an effective population control. Predicted probability on tree regeneration reveals that a 50% reduction in current densities would increase the probability of successful regeneration by nearly 60%. Browsing was found to be an

efficient indicator to assess the ecological sustainability and integrate management practices. Additionally, certain woody species that are both widely distributed and highly-preferred by ungulates could work as key indicators of unsustainability. We highlight that surveying browsing and successful regeneration in woody plant communities are essential monitoring tools to provide an early warning of ungulate overpopulation and to assess the suitability of a particular area to hold new ungulates. Thus, we recommend surveying woody vegetation before and after ungulate reintroductions to ensure the ecological sustainability of highly valuable woody ecosystems. (C) 2014 Elsevier Ltd. All rights reserved.

4038: ***+.065***

Genetic structure and its artificial disturbance in the endangered Japanese bitterling Acheilognathus cyanostigma were examined based on mitochondrial cytochrome b gene sequences. The haplotype network included three major clades: the dominant clade consisted of haplotypes found from the Lake Biwa-Yodo River system and other sites, including a known site of introduction; the other two clades consisted of haplotypes found exclusively or almost exclusively from the Ise Bay area, and the Yura and Kako River systems, respectively. Their divergence times, estimated using teleost molecular clocks, yielded results concordant with geological events such as uplifting of the Suzuka Mountains during the early Pleistocene. The widespread haplotypes were suggested to be introduced from the Lake Biwa-Yodo River system during stocking of Ayu from this system. The Lake Biwa-Yodo River population is critically endangered in its original habitats, yet is invasive in habitats where it has been introduced as it hybridizes with or replaces indigenous populations and competes with native bitterlings. Utilizing introduced populations for reintroduction and genetic rescue of the original population may be possible after careful examination for factors that could negatively impact the local community.

4039: ***+.127***

Comprehensive studies on the genetic diversity and structure of endangered species are urgently needed to promote effective conservation and management activities. The big tree rhododendron, Rhododendron protistum var. giganteum, is a highly endangered species with only two known endemic populations in a small area in the southern part of Yunnan Province in China. Unfortunately, limited information is available regarding the population genetics of this species. Therefore, we conducted amplified fragment length polymorphism (AFLP) analysis to characterize the genetic diversity and variation of this species within and between remaining populations. Twelve primer combinations of AFLP produced 447 unambiguous and repetitious bands. Among these bands, 298 (66.67 %) were polymorphic. We found high genetic diversity at the species level (percentage of polymorphic loci = 66.67 %, h = 0.240, I = 0.358) and lowgenetic differentiation (G(st) = 0.110) between the two populations. Gene flow between populations (N-m) was relatively high at 4.065. Analysis of molecular variance results revealed that 22 % of the genetic variation was partitioned between populations and 78 % of the genetic variation was within populations. The presence of moderate to high genetic diversity and low genetic differentiation in the two populations can be explained by life history traits, pollen dispersal and high gene flow (Nm = 4.065). Bayesian structure and principal coordinate analysis revealed that 56 sampled trees were clustered into two groups. Our results suggest that some rare and endangered species are able to maintain high levels of genetic diversity even at small population sizes. These results will assist with the design of conservation and management programmes, such as in situ and ex situ conservation, seed collection for germplasm conservation and reintroduction.

4040: ***+.080***

The Ballinderry River, Co. Tyrone (SAC), is one of only six rivers in Northern Ireland that still supports a population of the globally endangered freshwater pearl mussel, Margaritifera margaritifera. Fewer than 1000 individuals still exist in the river; without intervention it is predicted the Ballinderry pearl mussel will be extinct by 2098. This paper aims to identify key catchment pressures on remaining mussel stocks and prioritise tributaries within the catchment for remediation work to ensure effective targeting of limited resources. A combination of redox assessment, river surveys and SCIMAP modelling was used to rank tributaries, taking into consideration their size and proximity to the main mussel population at the "sanctuary site". Diffuse and point sources of sediment were identified within the prioritised catchment. The Tulnacross tributary was selected as the highest priority tributary and has been used in a pilot remediation study. A number of different hard and soft engineering techniques have been used for remediation as well as replacing cattle drinkers with pasture pumps and fencing alongside the river. Initial observations suggest river substrate is cleaner. This study is an example of how to tackle problems within a large-scale catchment with limited resources with participation of local stakeholders and riparian landowners. (C) 2014 Elsevier GmbH. All rights reserved.

4041: ***+.100***

Restoration is an important tool for reducing extinction risk of endangered plants. Population viabilities of few plant restorations have been modeled over decadal time periods and linked with genetic and ecological factors that drive restoration processes. We modeled viability of restored populations of Mead's milkweed (Asclepias meadii, Asclepiadaceae), a self-incompatible perennial herb of eastern tallgrass prairie (TGP), federally listed as threatened in the U.S. From 1994 to 2004, we planted >600 seeds and >800 juvenile plants representing >50 genotypes across seven TGP sites. Propagule type, genotype, seed source, restoration site, precipitation and fire management significantly affected establishment, growth and viability. Plants established from seed had greater mortality and greater genetic and demographic attrition than did juveniles. Seedling growth rates also projected 20-30 yrs to reach flowering stage, and their survivorship provided a metric of site suitability for life cycle completion. Seed germination and juvenile plant size were greater in burned habitat, and juvenile size was also positively correlated with spring precipitation. Seed production required presence of multiple genotypes among flowering plants. Seedlings demonstrated a heterosis effect, with greater germination among seeds derived from inter-population crosses. However, cumulative growth of planted juveniles as well as population growth (lambda) on sub-optimal habitat conditions tended to be lower for propagules derived from inter-population crosses, demonstrating outbreeding depression. Although flowering occurred at multiple sites, positive population growth (lambda > 1) occurred at only a single site, where increasing fire frequency decreased extinction probability. These results suggest that restoration of viable Mead's milkweed populations is possible in optimal habitat. However, restoration of this species is constrained by high demographic attrition and the long period (20 or more yrs) required to complete its life cycle. Crossing among populations to increase genetic diversity and compatible mating types may result in tradeoffs, with heterosis at early life history stages, but outbreeding depression expressed in older stages. Fire and precipitation are also critical interactive processes driving Asclepias meadii growth and reproduction. They may be most effective when precipitation, a stochastic process, results in greater than average post-burn rainfall. These constraints may have implications for restoration of other late-successional plant species.

4042: ***+.050***

To understand density-dependent processes in a reintroduced free-ranging Milu deer population, we monitored the Milu population in the Hubei Shishou Milu National Nature Reserve (SMNNR)

from 1993 to 2013. We collected data on vital rates, including the mortality, survival and birth rate in the population (SMNNRP) using direct divisional counting. We used these data to explore how and if density-dependence regulates the SMNNRP. Our results showed: (1) Based on the annual rate of change of population size, the SMNNRP's development can be divided into five stages, that is, the stable growth stage (1993-1997), the rapid growth stage (1998-2006), the slow growth stage (2007-2009), the rapidly declining stage (2010) and the population restoration stage (2011-2013). (2) From 1993 to 1997, the population growth rate was 16.60 +/- 3.10 (%), and the mortality rate was 4.34 +/- 0.93 (%). From 1998 to 2006, the population growth rate was increased to 28.98 +/- 3.62 (%), and the mortality rate was 4.35 +/- 2.31 (%). From 2007-2009, the population growth rate decreased to 7.36 +/- 1.64 (%), and the mortality rate increased to 6.32 +/- 2.85 (%). In 2010, an infectious disease caused a significant decrease in population size. From 2011 to 2013, the population growth rate increased to 10.95 +/- 4.04 (%), while mortality rate decreased to 5.7 +/- 2.03 (%). (3) In SMNNRP, population density was negatively related with population growth rate (r=-0.612, P=0.005<0.01), but was not positively related with the mortality rate (r=0.425, P=0.062>0.05). (4) Throughout all stages, SMNNRP adult and fawn survival rates were relatively stable, except in 2010. In 2010, a disease outbreak caused a spike in deaths, and the survival rates of adults and fawns were 65.05% and 0 respectively. Before and after the disease in 2010, adult survival was 95.40 +/- 1.56 (%) and 96.67 +/- 0.92 (%) respectively, and fawn survival was 95.79 +/- 1.80 (%) and 94.04 +/- 2.20 (%) respectively. Survival rates before 2010 did not differ from those after 2010 for either life stage (adult: t=-0.503, df=8, P=0.628>0.05; fawn: t=0.558, df=8, P=0.592>0.05). (5) Neither adult nor fawn survival rates were related to population density in SMNNRP (adult: r= -0.493, P= 0.124>0.05; fawn: r= -0.411, P=0.209>0.05). But there was a negative relationship between the population density and the birth rate (r=-0.902, P=0.000.<0.01). Our results implied that density dependence had affected SMNNRP through decreasing birth rates since 2003. The factors regulating this population were classified into density-dependent and environment factors, including flood, disease and human interference. Our study provides information that is useful for the protection and management of free-ranging Milu populations.

4043: ***+.408***

The pace of habitat destruction and loss of biological diversity globally exceeds the current capacity of societies to restore functioning ecosystems. Working with prison systems to engage inmates in habitat conservation and ecological science is an innovative approach to increase our ability to reestablish habitat and at-risk species, while simultaneously providing people in custody with opportunities for reciprocal restoration, education, therapeutic activities, safer conditions, and lower costs of imprisonment. We present the benefits of working with prisons to conduct habitat conservation through nursery production of plants and captive rearing of animals, combined with educational experiences, and provide an overview of the Sustainability in Prisons Project Network. Examples of projects with prisons in Washington and Oregon include nursery production of Wyoming big sagebrush (Artemisia tridentata Nutt. ssp. wyomingensis) for restoring habitat of the greater sage-grouse (Centrocercus urophasianus), nursery production of early blue violet (Viola adunca) to support conservation of threatened Oregon silverspot butterflies (Speyeria zerene hippolyta), captive rearing programs for Oregon spotted frogs (Rana pretiosa) and endangered Taylor's checkerspot butterflies (Euphydryas editha taylori), and nursery production of over 60 plant species for restoration of native prairies. Including incarcerated people in conservation and science could tap into the positive potential of over 2 million inmates at over 4000 prisons and jails in the United States and create new partnerships to support large-scale habitat restoration and ecological research.

4044: ***+.088***

Changes in habitat conditions in the area of Eastern Poland (Polesie Podlaskie), often associated with anthropopressure, cause a reduction in the number of locations and population size of valuable and rare bog plant species, including a Pleistocene boreal relict, Salix myrtilloides. The aim of this study was to determine the current conditions of occurrence of this species based on the physico-chemical parameters of peatland piezometric groundwater. The results confirm the declining trend in the number of locations and abundance of this species in the past few decades, but at the same time they confirm the wide range of amplitude of the many investigated factors. Among the groundwater parameters studied, the low level of nitrogen fractions, phosphorus fractions, cations, and DOC can be considered to be a set of conditions promoting the preservation of the Salix myrtilloides population. The higher concentrations of phosphates, sulfates, Na, Ca, and Mg as well as higher pH and electrolytic conductivity can be considered to be presumably unfavorable. The lack of significant differences in the values of the piezometric water factors investigated for the sites with different population sizes of the studied species requires the identification of other limiting factors and the implementation of programs for its active protection in Poland.

4045: *+.102*

Shoal basses are a cryptic clade composed of Micropterus spp. restricted to the Apalachicola River system and three southeastern Atlantic slope river drainages in the southeastern United States. This reciprocally monophyletic clade includes the Shoal Bass M. cataractae (endemic to the Apalachicola River system), the Chattahoochee Bass M. chattahoochae, and two undescribed forms from the Altamaha, Ogeechee, and Savannah River drainages. Members of the shoal bass clade can be distinguished from all other species of Micropterus basses using 20 diagnostic characters (characteristic attributes) found in mitochondrial DNA (NADH dehydrogenase subunit 2) gene sequences. Each member of the clade additionally possesses unique characteristic attributes, which along with morphological and meristic characters can be used to diagnose this cryptic biodiversity. Biologists and managers have previously regarded the shoal basses in the Chattahoochee, Savannah, Altamaha and Ogeechee River systems as belonging to a single taxon synonymous with the Redeye Bass M. coosae, which is endemic to the Mobile River drainage. With these and previous analyses (including description of the Shoal Bass), we now recognize that what was once considered a single taxon actually comprises seven species, each of which is endemic to a single southeastern drainage. Recognizing and documenting the actual diversity of Micropterus spp. provides important information for managers who may wish to avoid stocking or translocations that could compromise the genetic integrity of native bass populations. Introductions of nonnative basses, including Alabama Bass M. henshalli, Spotted Bass M. punctulatus, and Smallmouth Bass M. dolomieu currently threaten the integrity of native shoal bass species in streams of the Chattahoochee, Altamaha, Ogeechee, and Savannah River systems.

4046: *+.023*

In grassland ecosystems livestock grazing is one of the main activities that modify habitat and which can lead to positive or negative effects on birds. In the Sierras Grandes of Cordoba, Argentina, livestock grazing began early in the 17th century, causing severe soil erosion. To restore the grasslands, livestock were excluded from a large area in 1997. However, the impact of grazing exclusion on endemic birds is not clear. We evaluated the effect of grazing exclusion on density and habitat attributes of populations of Long-tailed Meadowlarks (Sturnella loyca obscura). The highest density was recorded in ungrazed sites. Fitted models explained up to 75% of the variability in density of Long-tailed Meadowlarks. Litter depth, percentage of bare soil (with positive effects), soil compaction (estimated as impedance) and percentage of moss cover

(with negative effects) were the most important variables explaining the variation in the estimated density of birds. We concluded that 11 years of grazing exclusion favoured Meadowlark populations, providing increased availability of resources, mainly those related to soil quality. Hence, we recommend promoting alternative management practices, such as reintroduction of large native herbivores where possible and livestock grazing at reduced stocking rates, to counterbalance the cost of soil loss and the benefit that large herbivores provide to the system.

4047: *+.190*

In an effort to promote population recovery, translocations of the endemic Mottled Petrel (Pterodroma inexpectata) from remote offshore islands to the mainland are planned within New Zealand. To optimise these efforts we studied chick growth, adult provisioning and meal size for this species and report the results of a simulated translocation undertaken to assess how translocated chicks, fed an artificial diet, performed in comparison with handled and non-handled controls that were provisioned by their parents. Our results found that wing-chord length and chick mass did not differ between translocated and control chicks, with all chicks fledging at approximately the same mass and wing size ranges. There was no difference in the timing of emergence from burrows before fledging between control and translocated chicks. The probability of a chick receiving a meal each night declined as chicks approached fledging and average meal size did not change as chicks approached fledging. From these data we now have the basis with which to make recommendations to increase the success of future translocation efforts for Mottled Petrels. It is important to share any knowledge of the biology and ecology of gadfly petrels, and any successes or failures in translocation practices in order to promote the best possible chance of future conservation of these species.

4048: *+.126*

Attempts to establish seabird colonies at restoration sites using artificial visual and auditory social cues have had varying success rates, differing between sites and species. The biological mechanisms responsible for this variation are poorly understood. We used experimental call playback to test the attraction of three sympatric procellariid species to auditory social cues in northern New Zealand. To test whether the size of nearby breeding colonies affected the level of response to call playback, audio recordings were broadcast from three similar locations with varying densities of breeding conspecifics within 1km. Grey-faced Petrel (Pterodroma gouldi) were attracted to conspecific vocalisation playbacks at all three sites and also to playbacks of other species. Fluttering Shearwater (Puffinus gavia) were attracted to playback at only two locations. Flesh-footed Shearwater (Puffinus carneipes) were not attracted to playbacks, broadcast from only one location. For Grey-faced Petrels and Fluttering Shearwaters, response to call playback increased with increasing densities of nearby breeding conspecifics, suggesting there may be a relationship between attraction and the size of nearby potential source populations. For some procellariid species call playback represents a cost-effective alternative to other active restoration approaches, such as translocation. However, we caution that its effectiveness for individual species at different sites should be assessed at the outset of restoration initiatives.

4049: *+.244*

The rainbow trout (Oncorhynchus mykiss) is probably the most widely introduced fish species in the world. Since the first translocation outside of the range of its natural distribution, the species has been introduced into at least 99 countries and has established reproducing populations in many different parts of the world. The present review aims to synthesize the existing information on

these translocations, with special emphasis on self-sustaining populations in Europe, where continuous introductions have in general not led to naturalization. Our survey produced a list of more than 130 confirmed or potential self-sustaining populations across 16 European countries. The highest abundance of such populations was observed in the Alpine foothills of central Europe where naturalization is not limited to modified waters less suitable for native salmonids but also occurs commonly in pristine and near-natural waters. There is no consensus on the reasons for the absence of self-sustaining populations of rainbow trout across much of Europe, partly because knowledge of the mechanisms involved is limited, while the data collected here shed new light on the invasion biology of the species.

4050: *+.010*

Following establishment of Myxobolus cerebralis (the parasite responsible for salmonid whirling disease) in Colorado, populations of Rainbow Trout Oncorhynchus mykiss experienced significant declines, whereas Brown Trout Salmo trutta densities increased in many locations across the state, potentially influencing the success of M. cerebralis-resistant Rainbow Trout reintroductions. We examined the effects of Brown Trout removal on the short-term (3-month) survival and movement of two crosses of reintroduced, M. cerebralis-resistant Rainbow Trout in the Cache la Poudre River, Colorado. Radio frequency identification passive integrated transponder tags and antennas were used to track movements of wild Brown Trout and stocked Rainbow Trout in reaches where Brown Trout had or had not been removed. Multistate mark-recapture models were used to estimate tagged fish apparent survival and movement in these sections 3 months following Brown Trout removal. A cross between the German Rainbow Trout and Colorado River Rainbow Trout strains exhibited similar survival and movement probabilities in the reaches, suggesting that the presence of Brown Trout did not affect its survival or movement. However, a cross between the German Rainbow Trout and Harrison Lake Rainbow Trout exhibited less movement from the reach in which Brown Trout had been removed. Despite this, the overall short-term benefits of the removal were equivocal, suggesting that Brown Trout removal may not be beneficial for the reintroduction of Rainbow Trout. Additionally, the logistical constraints of conducting removals in large river systems are substantial and may not be a viable management option in many rivers.

4051: *+.207*

The Humpback Chub Gila cypha, a large-bodied, endangered cyprinid endemic to the Colorado River basin, is in decline throughout most of its range due largely to anthropogenic factors. Translocation of Humpback Chub into tributaries of the Colorado River is one conservation activity that may contribute to the expansion of the species' current range and eventually provide population redundancy. We evaluated growth, survival, and dispersal following translocation of approximately 900 Humpback Chub over a period of 3 years (2009, 2010, and 2011) into Shinumo Creek, a tributary stream of the Colorado River within Grand Canyon National Park. Growth and condition of Humpback Chub in Shinumo Creek were consistent among year-classes and equaled or surpassed growth estimates from both the main-stem Colorado River and the Little Colorado River, where the largest (and most stable) Humpback Chub aggregation remains. Based on passive integrated tag recoveries, 53% (= 483/902) of translocated Humpback Chub dispersed from Shinumo Creek into the main-stem Colorado River as of January 2013, 35% leaving within 25 d following translocation. Annual apparent survival estimates within Shinumo Creek ranged from 0.22 to 0.41, but were strongly influenced by emigration. Results indicate that Shinumo Creek provides favorable conditions for growth and survival of translocated Humpback Chub and could support a new population if reproduction and recruitment occur in the future. Adaptation of translocation strategies of Humpback Chub into tributary streams ultimately may refine the role

translocation plays in recovery of the species.

4052: *+.222*

Botanical exploration on ultramafic sites in Palawan, Surigao and Zambales has resulted in the discovery of a new hypernickelophore species (nickel (Ni) concentration >1%) of Phyllanthus (Phyllanthaceae). This paper reports in detail the Ni uptake of populations of P. erythrotrichus in Candelaria, Masinloc and Santa Cruz, Zambales, and confirms the status of P. securinegoides in Taganito, Surigao del Norte, which had been analysed only through herbarium specimens, and these were compared with a known hypernickelophore, P. balgooyi, which was collected in Narra and Puerto Princesa, Palawan. Nickel content of the dried leaves, stems and root tissues was quantified using atomic absorption spectrophotometer. P. erythrotrichus and P. securinegoides both had more than 10000 mu gg(-1) Ni in the leaves, whereas the roots had 1195 mu gg(-1) and 4636 mu gg(-1). P. balgooyi accumulated 6319 mu gg(-1) of Ni in the leaves, whereas the roots had a higher Ni concentration of up to 8116 mu gg(-1), respectively. All three species had values of translocation factor and enrichment factor of >1.0, implying that all species have great potential in phytoremediation, specifically, phytoextraction of Ni. These three species of Phyllanthus are prominent in ultramafic scrub communities and, hence, should be used in ecological restoration of mined-out Ni lateritic areas. The implications of the unique adaptation of these species are also discussed in relation to a conservation strategy for their natural populations.

4053: *+.151*

Concerns regarding the long-term viability of threatened and endangered plant species are increasingly warranted given the potential impacts of climate change and habitat fragmentation on unstable and isolated populations. Orchidaceae is the largest and most diverse family of flowering plants, but it is currently facing unprecedented risks of extinction. Despite substantial conservation emphasis on rare orchids, populations continue to decline. Spiranthes parksii (Navasota ladies' tresses) is a federally and state-listed endangered terrestrial orchid endemic to central Texas. Hence, we aimed to identify potential factors influencing the distribution of the species, quantify the relative importance of each factor and determine suitable habitat for future surveys and targeted conservation efforts. We analysed several geo-referenced variables describing climatic conditions and landscape features to identify potential factors influencing the likelihood of occurrence of S. parksii using boosted regression trees. Our model classified 97 % of the cells correctly with regard to species presence and absence, and indicated that probability of existence was correlated with climatic conditions and landscape features. The most influential variables were mean annual precipitation, mean elevation, mean annual minimum temperature and mean annual maximum temperature. The most likely suitable range for S. parksii was the eastern portions of Leon and Madison Counties, the southern portion of Brazos County, a portion of northern Grimes County and along the borders between Burleson and Washington Counties. Our model can assist in the development of an integrated conservation strategy through: (i) focussing future survey and research efforts on areas with a high likelihood of occurrence, (ii) aiding in selection of areas for conservation and restoration and (iii) framing future research questions including those necessary for predicting responses to climate change. Our model could also incorporate new information on S. parksii as it becomes available to improve prediction accuracy, and our methodology could be adapted to develop distribution maps for other rare species of conservation concern.

4054: *-.099*

Population monitoring of beavers in Croatia and Serbia is being continuously run since the first release of beavers in 1996. in Croatia(Grubesic iKrapinec, 1998, Grubesic, 2014). Serbia has started with monitoring in 1999. When the first beak has been registered in the north of Vojvodina and it has been intensified since 2004, when they started with inhabitation - reintroduction (Cirovic, 2010). As a part of monitoring beavers in Croatia and Serbia since their reintroduction, beaver losses are being recorded by place and time of death, cause, sex and age of individuals. Information is being gathered with help of a network of associates, and by evidence of events. Based on the analysis of gathered information on beaver killings in the past 18 years a growth of killed beavers has been noticed, especially after the population growth and territorial expansion of beavers, and 10 years after the release in Croatia. In the observed period in Croatia a total of 111 beaver losses have been registered, while in Serbia this number is significantly lower and amounts to 36 individuals. Based on registered beaver losses a significant rise in beaver losses in the past 7 years has been noticed on the territory of Republic of Croatia. Actually the number of killed individuals has risen significantly when the beaver population has stabilized and increased its numbers and after 10 years since the inhabitation. In Serbia, despite the stabilization and territorial expansion, registered losses are relatively small, and stagnation or slight drop in killed or died animals has been noted. The main factor of mortality in Croatia and Serbia has been traffic. About one third of beavers (50 individuals) have been killed in traffic accidents: Traffic share in beaver mortality is equal or even somewhat smaller when compared to results from some parts of Germany, where this share is from 50 % to even 86,5 % (Pokorny and associates 2014., Muller 2014). Strangulation in fishing nets has been the second most significant beaver loss in populations on the territory of Posavina and Podravina (22 beavers - 15 %). Autopsy unquestionably proved that 17 beavers (11.6 %) died from illness. For 33 of them (22,4 %), due to untimely findings or delivery to autopsy, a precise cause of death could not have been determined. When we look at beaver loss cause's on the territory of Republic of Croatia. traffic absolutely dominates, followed by unknown causes, and in third place illegal hunting and fishing (especially gillnets). In Serbia,alongside unknown causes significant influences have diseases. From all 147 losses, only one beaver has been killed underneath a tree. Even though it has been noted he has been "killed at work" the position of the beavers body and tree points that the killing was not a consequence of knocking down the tree in question (Picture 1 and 2), but that the tree fell on the beaver as it has been passing by, most likely as a consequence of wind blowing the bitten tree down or exceptionally that an another individual knocked down the tree and it fell on the beaver passing by. Mostly adult individuals are being killed (Croatia 39, Serbia 14 beavers) which is linked to their increased activity in search for food and in moving about in general. Reasearch of other authors also shows that adult individuals are mostly being killed and mainly females (Pokorny and associates 2014). Sub adult individuals are being killed when exploring the territory (new locations) or when they inhabit a zone where the get killed easily, especially in traffic. This age group has mostly been killed in Serbia (15 beavers). In relation to sexual structure in Croatia and in Serbia a larger number of males were killed than females. Unfortunately, for half of killed beavers we were not able to tell the sex. If we look at beaver killing on a seasonal level, then two periods stick out, mostly spring then autumn. In spring beavers move more, especially the sub adult ones in search for foo and exploration of new habitats, while in autumn when they are more active in agricultural lands, they are being killed in traffic or in illegal hunting (this influence is most likely significantly bigger than the recorded one, but the prefix "illegal" points to unavailability of information and data on individuals killed this way). Illness for now are not a more significant cause of population loss, but in the future, especially in areas overcrowded by beaver, the might have a more significant role in the reduction of population, considering that we are dealing with a species (rodents) that are sensitive to certain diseases (leptospirosis, tularemia) (Parker and ass. 1951, Hopla 1974, Hornfeldti sur. 1986, Morneri sur., 1988, Wolli sur. 2012). Certain problems or flaws in delivering information on losses emerge due to associates not being

educated; these are accidental findings by people who have seen this species most likely for the first time. Apart from that some information has been delivered without details that would give a clearer picture in determining a cause of death, age and sex. As a measure of protecting beavers first in line is respecting the legislation connected to illegal hunting and fishing, and the traffic killing might be decreased or somewhere completely prevented by setting up a wire fence alongside roads in troubling crosses. Also losses can be successfully prevented in watercourse construction in territories inhabited by beavers, that the personnel that is carrying out the construction is being educated and pointed to beaver protection measures while carrying out the work (lodge and animal protection from excavators). So called "dangerous" objects in which beavers can fall into and get hurt, can be adjusted in a way that beavers are enabled to get into such spaces but also to ensure the possibility of getting out from such a space. Beaver protection is being carried out by good media presentation and informing the public of beavers and their way of life, protection measures and citizen education. Such direct communication enables and simplifies information gathering on beavers, also including information on killed or died individuals.

4055: ***+.146***

The endemic Philippine crocodile Crocodylus mindorensis is one of the most Critically Endangered crocodilian species in the world. Four major captive-breeding programmes for the Philippine crocodile have greatly contributed to our knowledge about the biology and natural history of the species. In situ conservation actions, such as reintroduction, conservation breeding in large semi-wild areas with no supplementary feeding and head-starting programmes, could result in increasing the numbers of extant wild and semi-wild populations. If concerted efforts are directed at in situ and ex situ conservation, and locating quality habitats in which to establish new conservation sites for Philippine crocodiles, the species is likely to become widespread in the next few decades. Conservation measures that address species-specific issues promoting healthy viable populations in natural habitats are presented in this paper.

4056: ***+.142***

In order to stop the decline of Hungarian meadow viper Vipera ursinii rakosiensis, in 2004 MME BirdLife Hungary together with national parks and Budapest Zoo started a complex conservation programme, supported by the European Union LIFE-Nature fund. The Hungarian Meadow Viper Conservation Centre was established with 16 adult individuals, collected from six different populations. By 2013 the number of vipers bred reached c. 1700 individuals. First reintroductions took place in March 2010, with 30 adult snakes released into a reconstructed habitat in Kiskunsag National Park. By 2013, a total of 240 snakes had been released into three locations. Snakes were released by relocating the animals in the artificial burrows they used in the semi-natural terrariums at the Hungarian Meadow Viper Conservation Centre. At the release sites vipers were recorded 255 times during post-release monitoring, and 69 individuals were identified. Eighteen of the observed ff were gravid, and ten juvenile or subadult individuals were documented. In order to develop a remote-tracking method, pre-programmed radio-tags with a detection range of 200-300m were surgically implanted into the abdomens of 16 vipers. These tags also operated as temperature loggers, recording data every 5 minutes for one year. Zoos play an important role in communicating the results of this captive-breeding and release programme. Exhibits of live Hungarian meadow vipers are located at Budapest Zoo and Schonbrunn Zoo in Vienna, Austria, and there are information points about the species located in all Hungarian zoos.

4057: ***+.187***

Many West Indian rock iguanas Cyclura spp comprise small restricted island populations that are threatened by habitat conversion and degradation, free-ranging domestic animals and invasive species. In the 1980s, concerted conservation efforts were initiated for Caribbean iguanas, using a combination of captive-breeding programmes and head-starting of wild-collected hatchlings for reintroduction, and habitat protection. Zoological facilities have been involved in the conservation efforts from the start, providing expertise, resources and extensive funding for various aspects of the conservation programmes, and by providing space to house ex situ groups of iguanas as assurance populations. Health assessments of wild and captive iguanas, and databases related to the biology and health of the species have benefited not only the wild populations but also those being bred and maintained in captivity. Data compilation and analysis through the use of population-management software have made it possible to manage the genetic diversity of the individuals being captive bred for release. The involvement of zoological facilities has been fundamental to the efforts that have gone into bringing the Grand Cayman blue iguana Cyclura lewisi and the Jamaican iguana Cyclura collei back from the brink of extinction. A review of the conservation efforts for West Indian iguanas, including the role played by zoos, is presented.

4058: *+.263*

The aim of this study was to determine birth weights, body measurements, and phenotypic correlations in order to characterize the gazelle population raised in Sanliurfa Province, Turkey. For this purpose, body measurements of 93 adult gazelles (54 female, 39 male) and 41 young gazelles (17 female, 24 male) were carried out, and correlations between the measurements were calculated. Additionally, birth weights of 47 newborn gazelle calves (22 female, 25 male) were determined. Mean body weights of adult and young females and males were 13.86 +/- 0.76 kg and 19.39 +/- 0.92 kg ($P < 0.001$) and 8.83 +/- 0.14 kg and 10.74 +/- 0.17 kg ($P < 0.01$), respectively. In all groups highly positive correlations between body weight and chest circumference were detected. Birth weights of female and male newborn calves were found to be 1.84 +/- 0.02 kg and 1.95 +/- 0.04 kg, respectively. Differences in birth weights of female and male newborn calves were not statistically significant. The results indicated that the gazelles reared in Turkey were similar to both G. s. subgutturosa and G. s. marica. In order to develop an efficient conservation program, further genetic studies are required for determining the taxonomical status of the gazelle population studied.

4059: *+.146*

Translocation is occasionally suggested as a last resort strategy for dealing with 'unavoidable' loss of Growling Grass Frog Litoria raniformis habitat in urbanising landscapes. However, examples of attempts to translocate an entire population of L. raniformis are rare and their success (or lack of success) is poorly documented in the literature. In this study, we detail the translocation of a population of L. raniformis from a farm dam being destroyed for residential development to a purpose-built wetland 480 m away. The population was translocated between November 2010 and May 2011. We used mark-recapture to estimate the number of frogs in the population prior to translocation. Visual counts of L. raniformis at the dam indicated a maximum of 39 adult frogs to be present while 355 frogs were marked over the course of a single season (November 2010 to March 2011). Translocation of 156 frogs and unassisted colonisation by 32 frogs resulted in an estimated 70% of adults marked at the dam moving to the wetland and 91% of those remained there for the duration of the translocation study period. The population and two measures of habitat quality (aquatic vegetation cover and water quality) were monitored for three active seasons post-translocation. Successful breeding was demonstrated for the first year only. A decline in breeding success was attributed to a reduction in habitat quality at the wetland, particularly the

loss of submergent and floating vegetation due to the presence of Common Yabby Cherax destructor, a species that did not occur originally in the dam. We believe that colonisation of the wetland by this crustacean was due to the wetland being constructed on-line. An attempt to control the C. destructor population and re-establish the aquatic vegetation was unsuccessful. We encourage the publication of all successes and failures in future attempts to establish translocated bell frog populations. If further experimental translocations have low success rates, then translocations should be reconsidered as a conservation strategy for L. raniformis in urbanising landscapes and greater emphasis placed on in situ habitat protection.

4060: *+.151*

Concerns regarding the long-term viability of threatened and endangered plant species are increasingly warranted given the potential impacts of climate change and habitat fragmentation on unstable and isolated populations. Orchidaceae is the largest and most diverse family of flowering plants, but it is currently facing unprecedented risks of extinction. Despite substantial conservation emphasis on rare orchids, populations continue to decline. Spiranthes parksii (Navasota ladies' tresses) is a federally and state-listed endangered terrestrial orchid endemic to central Texas. Hence, we aimed to identify potential factors influencing the distribution of the species, quantify the relative importance of each factor and determine suitable habitat for future surveys and targeted conservation efforts. We analysed several geo-referenced variables describing climatic conditions and landscape features to identify potential factors influencing the likelihood of occurrence of S. parksii using boosted regression trees. Our model classified 97 % of the cells correctly with regard to species presence and absence, and indicated that probability of existence was correlated with climatic conditions and landscape features. The most influential variables were mean annual precipitation, mean elevation, mean annual minimum temperature and mean annual maximum temperature. The most likely suitable range for S. parksii was the eastern portions of Leon and Madison Counties, the southern portion of Brazos County, a portion of northern Grimes County and along the borders between Burleson and Washington Counties. Our model can assist in the development of an integrated conservation strategy through: (i) focussing future survey and research efforts on areas with a high likelihood of occurrence, (ii) aiding in selection of areas for conservation and restoration and (iii) framing future research questions including those necessary for predicting responses to climate change. Our model could also incorporate new information on S. parksii as it becomes available to improve prediction accuracy, and our methodology could be adapted to develop distribution maps for other rare species of conservation concern.

4061: *-.008*

Desert springs, which harbor diverse and endemic invertebrate assemblages, are often used as refuge habitats for protected fish species. Additionally, many of these springs have been colonized by invasive fish species. However, the potential impacts of recently established fish populations on invertebrate communities in desert springs have been relatively unexplored. We conducted a mesocosm experiment to assess the impact of both protected and invasive fish on community structure of spring-dwelling invertebrates focusing on zooplankton. Experimental populations of spring zooplankton communities were established and randomly assigned to one of three treatments, (1) invasive western mosquitofish (Gambusia affinis); (2) endangered Mohave tui chub (Siphateles bicolor mohavensis); and (3) fishless control. Final populations of zooplankton and fish were sampled, sorted, identified and counted. The treatment differences of zooplankton communities were analyzed by comparing the densities of six major zooplankton taxa. Further, we performed nonmetric multidimensional scaling (NMDS) to visualize the patterns of zooplankton

community assemblages. Four zooplankton taxa, crustacean nauplii, Cladocera, calanoid and cyclopoid copepods had significantly lower densities in fish treatments compared to fishless control. Overall, invasive mosquitofish caused a 78.8% reduction in zooplankton density, while Mohave tui chub caused a 65.1% reduction. Both protected and invasive fish had similar effects on zooplankton except for cladocerans where tui chub caused a 60% reduction in density, whereas mosquitofish virtually eliminated cladocerans. The presence of fish also had a significant effect on zooplankton community structure due to population declines and local extirpations presumably due to fish predation. This work shows that conservation-translocations undertaken to conserve protected fish species may impact spring-dwelling invertebrate communities, and such impacts are similar to impacts due to colonization by invasive fish species.

4062: *+.295*

New Zealand is home to a small but strange set of land-birds and is world-renowned as a hotspot of sea-bird diversity. This unique assemblage of species is the result of the fact that the NewZealand landmass is continental in origin but island-like in nature, being isolated fromother countries by a large extent of ocean. A lack ofmammalian predatorsmeans that many New Zealand birds have evolved remarkable features, including gigantism, flightlessness, ground-nesting habits and physiology which protects them from aerial avian predators. Unfortunately, these features have made New Zealand birds highly susceptible to the impacts of introduced mammalian predators and many species are nowthreatened. The high levels of extinction and rates of decline have spurred the development of highly innovative and revolutionary conservation techniques. New Zealand conservation efforts have paved the way for international conservation projects, in particular with regard to the use of offshore islands, pest controlmethodologies, species translocation and breeding manipulation. The use of these interventions has led to the recovery of numerous highly endangered species and the discipline continues to evolve, with New Zealand scientists contributing to ongoing development and discussion of conservation methods. While New Zealand is home to one of the largest public conservation organisations in the world, dwindling governmental and financial support is putting many successful conservation projects at risk and private initiatives are becoming increasingly vital for the ongoing protection of New Zealand's unique species.

4063: *+.300*

Red-tailed phascogales (Phascogale calura) were reintroduced to Wadderin Sanctuary in the eastern wheatbelt of Western Australia in April 2009 with individuals sourced from remnant native vegetation on farmland some 180 km to the west. Their establishment was monitored initially by radio-telemetry and trapping, and subsequently by the checking of nest boxes both within and outside the sanctuary. Translocated phascogales established well and bred successfully in their first season. Phascogales remain extant at Wadderin more than five years after release and appear to be abundant and to occupy all available habitat. They have spread beyond the fenced sanctuary to adjoining woodland and to shrubland and woodland habitat in a remnant 1.4 km away. Comparison with two other reintroductions of this species (one apparently successful, one not) suggests management and habitat factors that may have contributed to the outcomes.

4064: *+.068*

The endemic Australian greater bilby (Macrotis lagotis) is a vulnerable and iconic species. It has declined significantly due to habitat loss, as well as competition and predation from introduced species. Conservation measures include a National Recovery Plan that incorporates several captive

breeding programs. Two of these programs were established within 12 months of one another (1997/98), with the same number and sex ratio of founding individuals, but executed different breeding strategies: (1) unmanipulated mating in semi-free range natural habitat versus (2) minimising mean kinship in large enclosures, with the supplementation of new individuals into both populations. This study evaluates the long-term genetic impact of these programs and examines the congruency between the pedigree studbook estimates of diversity and molecular data. Our data demonstrate that genetic diversity was maintained in both populations, with the supplementation of new individuals contributing to the gene pool. The studbook estimates of diversity and inbreeding are not consistent with the microsatellite data and should not solely be relied upon to evaluate the genetic health of captive populations. Our analyses suggest that captive breeding programs may not require costly and intensive management to effectively maintain long-term genetic diversity in a promiscuous species.

4065: ***+.277***

An understanding of the factors that influence the distribution of the woylie (Bettongia penicillata ogilbyi) at local and regional scales has been identified as a key knowledge gap, because such knowledge may assist in the recovery of this endangered species. We aimed to investigate the seasonal home-range size and habitat use of woylies to update current knowledge of the species in the context of a substantial decline. Specifically, we examined the home range and habitat use of woylies reintroduced into a sanctuary free from invasive predators and compared these data to those from an external reference site. Eight woylies inside the sanctuary and seven outside were radio-tracked in autumn 2011. The average home-range size was 65.4 (+/- 8.2, s.e.) ha. There was little evidence to suggest any difference in home-range size between woylies inside and outside the sanctuary. Woylies were more likely to be found in the slope and low-lying valley habitats, which have greater water-holding capacity and sandier soils. These relatively large seasonal home ranges, compared with previously published estimates for the species, may be accounted for by low population density, lower seasonal food availability and clustered food distribution. Monitoring the home-range size of woylies within the sanctuary may assist in identifying the carrying capacity of the sanctuary, which has implications for how this population is managed.

4066: ***+.071***

The critically endangered Annam leaf turtle Mauremys annamensis faces extinction in nature. Because of that, the conservation value of the population kept in European zoos becomes substantial for reintroduction programmes. We sampled 39 specimens of M. annamensis from European zoos and other collections (mainly founders, imports and putatively unrelated individuals), and also four specimens of Mauremys mutica for comparison. In each animal, we sequenced 817 bp of the mitochondrial ND4 gene and 940 bp of the nuclear R35 intron that were used as phylogenetic markers for Mauremys mutica-annamensis group by previous authors. The sequences of the R35 intron, which are characteristic for M. annamensis and which clearly differ from those characteristic for M. mutica and/or other Mauremys species, were mutually shared by all of the examined M. annamensis. They also possessed mitochondrial haplotypes belonging to the annamensis subclades I and II, distinctness of which was clearly confirmed by phylogenetic analyses. Thus, both nuclear and mitochondrial markers agreed in the unequivocal assignment of the examined individuals to M. annamensis. Although no obvious hybrids were detected within the founders of the captive population, further careful genetic evaluation using genom-wide markers is required to unequivocally confirm this result.

4067: ***-.070***

Context. The arctic ground squirrel (Urocitellus parryii) comprised 17% of the biomass of herbivores in the Yukon boreal forest during the summer months from 1987 to 1996 and was responsible for 23% of the energy flow at the herbivore level. By 2000, ground squirrel populations in this region collapsed to nearly zero and have remained there.Aims. We summarise the population monitoring (since 1975) and recent experimental work that has been done on this key herbivore in the Kluane area of the southern Yukon to test one mechanistic hypothesis as the possible explanation for this population collapse and subsequent lack of recovery: predation.Methods. Ground squirrels are the preferred summer prey of bird and mammal predators when snowshoe hare (Lepus americanus) populations are declining. We used translocations into formerly occupied habitat and radiotelemetry to determine movements and causes of death from 2009 to 2014. We surveyed 158 sites between 2008 and 2013 to measure the disappearance of colonies in alpine and forest habitats over 25 000 km(2). Key results. Ground squirrels from 2000 to 2013 comprised a small fraction of the herbivore biomass in the boreal forest zone, down from 17% earlier. Most forest populations (similar to 95%) are currently extinct, whereas just over half (65%) of low-elevation meadow populations are locally extinct. One hypothesis is that ground squirrels in the forest have been driven into a predator pit from which they cannot recover. They remain abundant in alpine tundra (93% occupancy rate) and around airport runways and human habitations (97% occupancy), but there is no apparent dispersal from alpine areas down into the boreal forest.Conclusion. The predator pit hypothesis is a likely explanation for the initial collapse and sustained decline in population size from 2000 to 2013. Recent attenuation of the hare cycle and milder winter climate have allowed shrubs to expand throughout the forest, thereby reducing visibility and increasing predation risk. This conclusion will be tested in further research using reintroductions to formerly occupied sites.Implication. If the loss of this herbivore from the boreal forest is not reversed, predator pressure on the other major herbivores of the montane forest zone is likely to change significantly.

4068: ***-.047***

Conservation of genetic biodiversity in endangered wildlife populations is an important challenge to address since the loss of alleles and genetic drift may influence future adaptability. Reintroduction aims to re-establish species to restored or protected ecosystems; however, moving a subset of individuals may result in loss of gene variants during the management-induced bottleneck (i.e. translocation). The endangered Laysan teal Anas laysanensis was once widespread across the Hawaiian archipelago, but became isolated on Laysan Island (415 ha) from the mid-1800s until 2004 when a translocation to Midway Atoll (596 ha) was undertaken to reduce extinction risks. We compared genetic diversity and quantified variation at microsatellite loci sampled from 230 individuals from the wild populations at Laysan (1999 to 2009) and Midway (2007 to 2010; n = 133 Laysan, n = 96 Midway birds). We identified polymorphic markers by screening nuclear microsatellites (N = 83). Low nuclear variation was detected, consistent with the species' insular isolation and historical bottleneck. Six of 83 microsatellites were polymorphic. We found limited but similar estimates of allelic richness (2.58 alleles per locus) and heterozygosity within populations. However, 2 rare alleles found in the Laysan source population were not present in Midway's reintroduced population, and a unique allele was discovered in an individual on Midway. Differentiation between island populations was low (FST = 0.6%), but statistically significant. Our results indicate that genetic drift had little effect on offspring generations 3 to 6 yr post-release and demonstrate the utility of using known founder events to help quantify genetic capture during translocations and to inform management decisions.

4071: ***+.250***

Salix myrtilloides L. is an endangered species whose western limit of range runs through Poland. The main aim of the study was to increase the knowledge on the ecology and biology of S. myrtilloides populations in the Polesie Lubelskie region (Eastern Poland) in order to create an effective protection program. An 80% decrease in its population was found in this area. Our study was conducted to identify the mechanisms responsible for the process of withdrawal of this species from its natural stands by determining whether the processes of generative reproduction (pollen viability and germinability, seed germination ability and dynamics) in the populations occur properly and by characterizing within- and among-population genetic diversity of S. myrtilloides, using ISSR (Inter-Simple Sequence Repeat) primers. The results confirmed that S. myrtilloides pollen viability was high (84.17 +/- 8.67), and so were the seed germination ability (76% of the total number of sown seeds/24 h) and dynamics. The within-population genetic diversity was quite high for this species and the inter-population genetic variation was of medium value (Phi(PT)=0.148). The condition of two populations, their genetic diversity and sex ratio as well as the correctness of the investigated reproductive cycle stages altogether give prospects for their survival. It seems to be very important to start conservation involving the reintroduction and reconstruction of S. myrtilloides populations in the Polesie Lubelskie region. The proposed method for restoration of S. myrtilloides resources would be possible if ex situ cultivation and in vitro methods were used.

4072: *-.075*

The paper provides an overview of molecular approaches in gene pool conservation, population genetic monitoring of captive breeding and reintroduction of the Russian endemic and world rare species Siberian crane (Grus leucogeranus Pallas). The following issues are discussed: the development of views on the position of the crane in the taxonomical system of the Gruidae family; the estimation of the levels of genetic diversity, differentiation and relatedness in the captive population; the individual genetic passportization and identification by microsatellite loci; the DNA-analysis of paternity of birds obtained in the result of artificial insemination; the problems of inbreeding and its prevention in captivity; the genetic identification of interspecific hybrids, and the molecular sex determination by specific DNA-markers. Key directions of the further research are suggested: broadening the scale of genetic monitoring in the captive population and analysis of genetic diversity of the Siberian crane in the wild.

4073: *+.250*

The Sevilleta Gunnisonas Prairie Dog (Cynomys gunnisoni) Restoration project examines keystone consumer (herbivore) effects on grassland in concert with ecological restoration of a aoespecies of greatest conservation need in New Mexicoa (NMGandF Comprehensive Wildlife Conservation Strategy, 2007). SevLTER partners directly with Sevilleta National Wildlife Refuge, New Mexico Game and Fish, USFS Rocky Mountain Research Station and non-profit Prairie Dog Pals on this ambitious effort to re-establish Gunnisonas prairie dogs to blue grama dominated (Bouteloua gracilis) Great Plains grassland at the foothills of the Los Pinos Mountains on Sevilleta. While engaged in wildlife management aimed at translocation of approximately 3000 individual prairie dogs, ultimately establishing 5-6 colonies over a 500 ha area, SevLTER is focusing resources on monitoring population dynamics of reintroduced prairie dogs and their effects on vegetation production and diversity, soil disturbance and grasshopper community composition. In this experiment, prairie dogs act as the treatment on a grassland site where the species was extirpated 40 years ago. The long term nature of the project lies in the course of re-establishing prairie dogs combined with the ultimate research goal of describing the functional role of Gunnisonas prairie dogs in an arid grassland ecosystem: first we are challenged to develop

and document an economical and efficient management strategy which maximizes reintroduction success and colony survival; second we are tasked with monitoring prairie dog dynamics and their effects on the grassland throughout re-establishment and into a future state, when presumably management intervention will have subsided and we characterize the ecosystem as a~restoreda a both in the face of highly variable abiotic inputs such as precipitation and temperature and biotic impacts such as predation.

4074: *+.107*

Prairie dogs (CynomysAspp.) are burrowing rodents considered to be ecosystem engineers and keystone species of the central grasslands of North America. Yet, prairie dog populations have declined by an estimated 98% throughout their historic range. This dramatic decline has resulted in the widespread loss of their important ecological role throughout this grassland system. The 92,060 ha Sevilleta NWR in central New Mexico includes more than 54,000 ha of native grassland. Gunnisonas prairie dogs (C.Agunnisoni) were reported to occupy ~15,000 ha of what is now the SNWR during the 1960as, prior to their systematic eradication. In 2010, we collaborated with local agencies and conservation organizations to restore the functional role of prairie dogs to the grassland system. Gunnisonas prairie dogs were reintroduced to a site that was occupied by prairie dogs 40 years ago. AThis work is part of a larger, long-term study where we are studying the ecological effects of prairie dogs as they re-colonize the grassland ecosystem. With this project, we would like to further investigate the impact that Gunnisonas prairie dogs have on the landscape. A Gunnisonas prairie dog monitoring data has been collected from the beginning of the reintroduction project, but little information has been collected on how grassland species respond to the sudden presence of prairie dogs on the refuge. This project will help determine if the prairie dog reintroduction has had positive impacts on the grassland ecosystem. A Prairie dogs benefit grasslands in many ways, but their role as ecosystem engineers directly impacts other species by creating new habitat that would not be present without prairie dogs. A We have documented physical landscape changes, but we have not specifically documented benefits to other grassland species. A This work will help determine if the reintroduced prairie dog populations on Sevilleta NWR are now acting as a keystone species in a grassland ecosystem by monitoring small mammal populations to see if species richness, diversity, and density are different on prairie dog colonized areas versus non-colonized areas.

4075: *+.107*

Prairie dogs (CynomysAspp.) are burrowing rodents considered to be ecosystem engineers and keystone species of the central grasslands of North America. Yet, prairie dog populations have declined by an estimated 98% throughout their historic range. This dramatic decline has resulted in the widespread loss of their important ecological role throughout this grassland system. The 92,060 ha Sevilleta NWR in central New Mexico includes more than 54,000 ha of native grassland. Gunnisonas prairie dogs (C.Agunnisoni) were reported to occupy ~15,000 ha of what is now the SNWR during the 1960as, prior to their systematic eradication. In 2010, we collaborated with local agencies and conservation organizations to restore the functional role of prairie dogs to the grassland system. Gunnisonas prairie dogs were reintroduced to a site that was occupied by prairie dogs 40 years ago. AThis work is part of a larger, long-term study where we are studying the ecological effects of prairie dogs as they re-colonize the grassland ecosystem. With this project, we would like to further investigate the impact that Gunnisonas prairie dogs have on the landscape. A Gunnisonas prairie dog monitoring data has been collected from the beginning of the reintroduction project, but little information has been collected on how grassland species respond to the sudden presence of prairie dogs on the refuge. This project will help determine if the prairie

dog reintroduction has had positive impacts on the grassland ecosystem. A Prairie dogs benefit grasslands in many ways, but their role as ecosystem engineers directly impacts other species by creating new habitat that would not be present without prairie dogs. A We have documented physical landscape changes, but we have not specifically documented benefits to other grassland species. A This work will help determine if the reintroduced prairie dog populations on Sevilleta NWR are now acting as a keystone species in a grassland ecosystem by monitoring small mammal populations to see if species richness, diversity, and density are different on prairie dog colonized areas versus non-colonized areas.

4076: ***+.243***

This 345-page book in English titled "Wildlife Conservation on Farmland-Managing for Nature on Lowland Farms" is volume 1 in its series. This book contains 16 individually-authored chapters. Each chapter is extensively referenced. Topics covered include weed reservoir to wildlife resource, invertebrate biodiversity, small mammals on lowland farmland, agri-environment schemes and the future of farmland bird conservation, organic farming optimizing farming systems for society and the environment and landscape-scale conservation of farmland moths. Also discussed habitat use by vesper bats, local and landscape-scale management of Odonata, impacts of land use and management on the biodiversity of rivers and ditches, local and landscape-scale impacts of woodland management on wildlife, improving reintroduction success of the grey partridge using behavioral studies and water vole restoration. This book includes a list of the contributors and their respective institutions. This book will be of interest to all those working or studying in the fields of biodiversity and wildlife management.

4077: ***+.165***

Despite a vigorous reintroduction program between 1985 and 2010, numbat populations in Western Australia are either static or declining. This study aimed to document the population ecology of numbats at two sites that are going against this trend: Scotia Sanctuary in far western New South Wales and Yookamurra Sanctuary in the riverland of South Australia. Scotia (64 659 ha) and Yookamurra (5026 ha) are conservation reserves owned and managed by the Australian Wildlife Conservancy and where numbats were reintroduced in 1999 and 1993 respectively. Both sites have large conservation-fence-protected introduced-species-free areas where there are no cats (Felis catus) or red foxes (Vulpes vulpes). Numbats were sourced from both wild and captive populations. From small founder populations, the Scotia numbats are now estimated to number 169 (113-225) with 44 at Yookamurra. Radio-collared individuals at Scotia were active between 13 and 31 degrees C. Females had home ranges of 28.3 +/- 6.8 ha and males 96.6 +/- 18.2 ha, which leads to an estimated sustainable population or carrying capacity of 413-502 at Scotia. Captive-bred animals from Perth Zoo had a high mortality rate upon reintroduction at Scotia due to predation by raptors and starvation. The habitat preferences for mallee with a shrub understorey appear to be driven by availability of termites, and other reintroduced ecosystem engineers appear to have been facilitators by creating new refuge burrows for numbats. This study shows that numbats can be successfully reintroduced into areas of their former range if protected from introduced predators, and illustrates the difficulties in monitoring such cryptic species.

4078: ***+.168***

The western saw-shelled turtle is listed as threatened globally, nationally, and within the Australian state of New South Wales. Although nearly all of the geographic range of the species lies within New South Wales, little information has been available on the distribution, abundance

and structure ofNewSouth Wales populations. Through a survey of 60 sites in 2012-15, I established that M. bellii is much more widely distributed in New South Wales than has previously been recognised, comprising four disjunct populations, including two in the New South Wales portion of the Border Rivers basin. It occurs mainly in larger, cooler rivers upstream of barriers to dispersal of the Macquarie turtle, Emydura macquarii macquarii. Although M. bellii is locally abundant, its populations are greatly dominated by large adults and recruitment appears to be low. Eye abnormalities are common in some populations but do not necessarily impair body condition or preclude long-term survival. The species is threatened by competition with E. macquarii, which appears to be expanding its range through translocation by humans, and possibly by predation, disease and drought. Long-term monitoring of M. bellii is needed to assess population trends and responses to threats, and active management to restrict the further spread of E. macquarii is probably required to ensure the persistence of M. bellii throughout its current range.

4079: *+.166*

Most invasive alien vertebrate populations on the Channel Islands of California have been eradicated over the past 30 years. Unfortunately, removal of these introduced herbivores or predators came too late for some native flora and fauna, and numerous populations are now extinct. Here, we describe a systematic approach to reintroducing extirpated native taxa as a means for rebuilding natural communities and enhancing the resiliency of island ecosystems. Reintroduction efforts typically focus on a single species or site. In contrast, we propose that if reintroduction is a shared conservation goal of managers across the islands, the associated planning, implementation, and monitoring should be conducted as a cross-island initiative for the archipelago. A coordinated effort based on best practices in reintroduction biology could accrue programmatic efficiencies and economies of scale, more quickly advance ecosystem and species conservation goals, and create unique opportunities to test hypotheses in basic and applied ecology and evolution. The philosophical and technical approaches developed through this program may apply to other island and mainland systems and could be adapted to develop conservation strategies for species that may be candidates for assisted colonization in the face of climate change.

4080: *-.049*

Santa Catalina Island was home to an estimated 1342 adult island foxes (Urocyon littoralis catalinae) in 1990. Nine years later, fox sightings declined and reports of dead or dying foxes increased. An island-wide trapping effort was initiated after a fox carcass tested positive for Canine distemper virus (CDV). In 1999, only 10 foxes were captured east of the Two Harbors isthmus during 1046 trap-nights. A multifaceted conservation plan was implemented in 2000 to conserve the Santa Catalina population of island fox. Initial recovery actions took place from 2000 to 2005 and resulted in the translocation of 22 juvenile foxes from the unaffected West End of the island to the depopulated eastern portion, the production and release of 37 pups from the captive breeding facility, and the vaccination of >80% of the wild fox population against CDV. Since 2006, fox recovery activities have included an annual island-wide population survey, vaccination of 300 foxes per year, weekly mortality monitoring of 50 radio-collared individuals, blood sampling to monitor the prevalence of CDV, veterinary treatment of injured foxes, and public outreach. Low mortality rates, successful breeding in the wild, and mitigation of the original cause of decline allowed for Catalina's fox population to grow to an estimated 1115 adults by 2012 and to be considered biologically recovered. The outbreak of another virulent canine disease on Santa Catalina Island, such as CDV or rabies, continues to be the greatest threat to the long-term survival of U.l. catalinae due to the species' restricted distribution and small population size, as well as the

continued presence of domestic dogs on the island.

4081: *+.069*

Brown trout display great phenotypic and genetic variability. Use of mitochondrial DNA (mtDNA) variation has allowed the definition of seven different lineages in this species to date. One of them, the Duero lineage (DU), was initially detected in the inner section of the Duero River in Spain, where it showed a parapatric distribution with the more widely distributed Atlantic lineage (AT). Later mtDNA-RFLP (restriction fragment length polymorphism) studies detected the DU lineage in northern Spanish basins (Galicia). The aim of this work was to ascertain the origin and variability of these DU populations outside the Duero drainage. Using complete mtDNA control region sequencing, 11 novel DU haplotypes were identified. Several of them could be assigned to an endemic group in Galicia consistent with the long-time presence of the DU lineage outside the Duero River, and excluding a recent origin by human translocations. The DU haplotype group observed in north-western Iberian basins was estimated to diverge from that of the Duero River more than 100000 years ago. We therefore advocate for conservation strategies at regional and local scales rather than focussed in a single ESU as proposed in earlier works.

4082: *+.068*

Fish translocations are fundamentally different to those of other vertebrate groups, with orders of magnitude more individuals usually released, and these individuals usually having high subsequent mortality. Translocation programs for Australian freshwater fish have changed considerably in the last 30+ years, moving from wild-to-wild translocations to hatchery stockings for recreational fisheries. Hatchery stocking and wild-to-wild translocations are now also used for conservation purposes. Translocations are among the four most-common management interventions employed for threatened Australian freshwater fish, with the frequency of wild-to-wild translocations, stockings and rescues increasing significantly during the recent Millennium Drought (1997-2010). We document 99 translocations of Australian freshwater fish conducted for conservation purposes since the late 1980s. These were all reintroductions (releases to sites formerly occupied by the species) or reinforcements of existing populations, except for one conservation introduction. Excluding cases where it is too early to assess the outcome, or where remnant populations were present when the reintroduction occurred, 38% of the translocations claim full or partial success. However, for 16% the outcome is unknown, raising concerns about the adequacy of monitoring. Monitoring programs associated with fish translocations are often short-term, under-resourced and, for long-lived fishes, monitoring often only encompasses the first milestone of success (survival for some period after release), with subsequent milestones (breeding and establishment) left unmeasured. Case studies review new insights into fish translocation such as: optimal stocking strategies; the survivorship and dispersal of on-grown individuals (i.e. hatchery-reared beyond the juvenile stage); the use of captive maintenance and subsequent release; and the use of artificial habitats ('natural hatcheries') to increase production numbers. Future freshwater fish translocations require increased levels of investment and will likely benefit from combined approaches of stocking and wild-to-wild translocation, along with consideration of further conservation introductions.

4083: *+.067*

Since 1971, fauna translocations have been used in Western Australia (WA) as a tool to improve the conservation status of threatened vertebrates and, more recently, to reconstruct faunal ecosystems. Over that period, there have been 232 translocations recorded, involving over 12 000

individuals of 43 different species. Predation by exotic predators and inadequate post-release monitoring were significant issues identified from a review of the translocation history in WA. Offshore islands have played an important role as secure translocation sites for threatened species. Changes to how translocations are now undertaken in WA include the development of a state-wide Fauna Translocation Strategy, the use of meaningful success criteria, and use of genetic and population viability analysis (PVA) tools to assess long-term viability of translocated populations. The WA Department of Parks and Wildlife continues to recognise the important role that translocations can play in threatened fauna recovery programs.

4084: ***+.337***

The last two decades have seen a proliferation of privately run (including community-led) conservation management programs in selected reserves in both Australia and New Zealand. Many of these reserves have been termed 'sanctuaries' in recognition of the removal or control of exotic mammalian predators within them, making them 'safe' places for native wildlife. Bringing valued wildlife back to locations accessible to the public has been a strong motivation for these sanctuaries, in addition to restoring ecosystem processes and improving conservation status. In Australia, two large private sanctuary networks dominate the sector (Australian Wildlife Conservancy and Bush Heritage Australia; although there are several other private conservation agencies also), whereas in New Zealand there is a loose network of independent, mostly community-led sanctuaries. In both countries, sanctuaries are now key sites for wildlife reintroductions. In Australia, most translocations have been of rare mammals reintroduced into areas where they had become regionally extinct. In New Zealand, rare birds and reptiles have been the most common translocations to sanctuaries, including species returned to mainland sites after absences of many decades. Australia and New Zealand already have a long history of translocations to pest-free islands (Chapter 19), but in recent years, there have been more translocations to sanctuaries than to islands. The success of these reintroductions has been high so far in both countries, but requires sustained restriction of pest mammal populations within the sanctuaries. The current trends towards both enhanced competition and cooperation within the broad conservation sector, including sanctuaries, should lead to innovative and cost-effective management solutions, including for reintroduction practice.

4085: ***+.123***

The rationale for most reintroductions is that: (1) habitat at the reintroduction site has been adequately restored through management or natural regeneration; and (2) natural colonisation is unlikely due to isolation from potential source populations. Therefore, reintroduction is usually preceded by restorative actions such as revegetation or control of exotic predators. However, where habitat has become fragmented, metapopulation theory suggests that absences of species from some sites could be due to isolation alone. That is, local extinctions may occur by chance in habitat fragments, and these are not recolonised if isolated. Therefore, in theory, reintroductions could be used to reverse declines of some species in fragmented landscapes without accompanying habitat restoration. We discuss the evidence necessary to assess whether this is a sensible strategy, noting that it will usually be impossible to obtain such evidence without doing the reintroductions. We then review three Australasian case studies where experimental reintroductions were done for this purpose. In all cases, isolation among fragments was found to be a significant barrier to dispersal, and reintroductions led to establishment and breeding in at least some fragments. However, these sub-populations ultimately declined to extinction. In the most extensive study, monitoring throughout the landscape revealed inter-fragment variation in adult survival that could account for fragments being unoccupied. This variation in habitat quality was subtle, because it

was not associated with measures of vegetation structure or predator density. Metapopulation modelling suggested that improving connectivity (through reintroduction or corridors) would actually negatively impact the metapopulation by facilitating movement from source to sink fragments. Long-term research from the other case studies also suggested that absences from fragments followed declines due to habitat degradation, with isolation only being important in the final stages. Given the challenges involved in demonstrating that reintroduction to unrestored habitat fragments is a sensible strategy, we suggest that habitat restoration such as predator control should continue to be considered a necessary prerequisite for reintroduction in most circumstances.

4086: *+.023*

The Tasmanian devil is threatened by a transmissible cancer, devil facial tumour disease (DFTD), which has induced a decline of greater than 80% in the wild population. An insurance population has been established with the goals of maintaining an effective population of > 500 devils for 50 years that is DFTD-free, is genetically representative, is able to sustain a harvest for wild release, maintains a suite of associated flora and fauna (commensal, symbiotic and parasitic), and maintains wild behaviours. The insurance metapopulation now includes more than 550 individuals, from 128 founders, secured at 28 institutions in a combination of intensive captive-breeding enclosures, managed environmental enclosures, free-ranging enclosures, and a population introduced to an island outside the species' known historic range (i.e. a conservation introduction). In the next few years, the insurance metapopulation will incorporate wild-living, DFTD-free Tasmanian devil populations secured within their current range. Translocation of individuals occurs between multiple facilities, locations, Australian states and nations, and is influenced by a disease risk categorisation. The metapopulation is managed by generating recommendations for founder animals, breeding, translocation and harvesting, based on pedigree and genetic data using SPARKS and PMx software, supported by population modelling using VORTEX software. To achieve an effective population size (N-e) of 500, the minimum metapopulation size will need to build to between 1500 and 5000 individuals in order to maintain gene diversity at >= 95% for 50 years, while also replacing lost diversity. However, through biosecurity, risk categorisation and quarantine procedures, it is feasible to harvest from, and incorporate, diseased wild populations should they persist, permitting a lower metapopulation size.

4087: *+.087*

Small population size and isolation impact genetic diversity through random genetic drift and inbreeding, and can lead to reduced population fitness and an increased risk of extinction. Threatened species often exist as small populations that are highly fragmented and thus prone to the effects of random genetic drift and losses of genetic variation. Conservation strategies therefore need to consider how genetic diversity can be maintained or enhanced when undertaking translocations, both when restoring populations and when introducing them outside their current range (conservation introduction). Here we discuss the genetic impacts of small population size on the persistence of populations and species. We then consider how genetic diversity can be maintained in reintroductions. This depends primarily on sourcing the right number of founders and increasing the effective population size soon after the animals are released or developing reinforcement regimes to preserve genetic diversity. Finally, we discuss the use of different genetic translocations as a way of assisting gene flow in small, isolated populations to reduce inbreeding depression and increase evolutionary potential and resilience. We also discuss conservation introductions and translocations in the context of climate change.

4088: ***+.213***

Assisted colonisation - the translocation of organisms with release in areas outside their indigenous range in response to threats such as climate change - was presented in the scientific literature only a few years ago as a new tool for species conservation. The idea of planned introductions for conservation is a controversial issue, prompting vigorous, and sometimes ill-informed, debate in the scientific literature. The broad consensus was that this represented a bold new direction that had merit but carried great risk. Unacknowledged by most commentators, assisted colonisation (by other names) was already taking place, and in Australia and New Zealand was even a long-accepted part of the conservation management tool kit. In 2013, the IUCN recognised assisted colonisation as a legitimate, if inherently risky, conservation translocation, and set out a comprehensive set of guidelines for its application. We review the history of assisted colonisation, with a focus on Australian and New Zealand projects moving species in response to threats within the indigenous range. We review the current status of assisted colonisation in Australia and New Zealand and present two case studies to illustrate the application of new approaches for assisted colonisation planning: Australia's western swamp tortoise (Pseudemydura umbrina), and New Zealand's hihi (Notiomystis cincta). We conclude by considering future directions in the specific application of translocations for climate change mitigation in the region.

4089: ***+.184***

The use of predictive modelling to plan reintroductions and manage reintroduced populations has increased steadily since the mid-1990s, with Australia and New Zealand at the forefront. We overview the types of models commonly used for species reintroductions, focusing on Australian and New Zealand examples. Site selection, release strategies (e.g. number, composition and timing), population viability and management impacts are the key issues often investigated. We discuss how to deal with uncertainties associated with parameter estimation, stochasticity, model selection, integrating modelling approaches and decision making. Finally, we highlight three promising modelling approaches for planning species reintroductions and modelling reintroduced populations: decision theory and optimisation, individual-based modelling and Bayesian hierarchical modelling.

4090: ***+.266***

The aim of any trial or experimental reintroduction is to consider key questions relevant to improving the likelihood of population establishment and persistence. These questions relate to the ecology of the focal species and the suitability of the release environment. Although trial reintroductions provide insight, and generate hypotheses relating to an organism's persistence and establishment, experiments (controlled manipulations with replicates) allow clearer conclusions. However, given that reintroductions often involve threatened species, experiments may be impossible due to small sample sizes and few opportunities for replication. As such, trial reintroductions are often the only option available to get an indication of which factors influence establishment success, habitat use and requirements, tolerance of threats, competition for resources, and social and spatial requirements. Thus trials remain a valuable tool for conservation managers, provided they are interpreted cautiously, with clear understanding of the parameters documented for each trial. Trials can also be combined strategically within an adaptive management framework. Here we outline the roles of trials and experiments, provide some case studies, and offer some advice for handling the limitations inherent in both reintroduction experiments and trials.

4091: ***+.268***

Dispersal behaviour is a critical factor that can influence reintroduction success across multiple stages. To date, most attempts to deal with this issue have focused on either reducing or mitigating dispersal using various management tools, and solutions have focused on initial post-release dispersal without fully recognising or acknowledging the effects ongoing dispersal may have. We advocate for careful consideration of proposed sites for reintroduction, taking into account characteristics of the species (dispersal propensity and probability of mortality for dispersers), and assessments of both the release site and the surrounding landscape, including connectivity, linkages and invasive species management at the landscape scale. In some cases, existing knowledge may enable us to predict the effect of interactions between these species and site characteristics, enabling informed decisions to be made before reintroduction. We emphasise the need for reintroduction planning to incorporate an integrated landscape approach, viewing dispersal as a natural component of a species life history. Under integrated landscape management, the wider landscape beyond reintroduction sites could be managed, with the long-term goals of establishing self-sustaining populations and providing safe opportunities for dispersal and gene flow to recreate natural metapopulations.

4092: ***+.249***

Despite decades of research into predator control, predation by exotic predators is the leading cause of reintroduction failure in Australia and New Zealand. A variety of methods are used to deal with the threat of predation, including the exclusion and control of predators and improved prey responses. Fenced reserves and islands are becoming increasingly popular reintroduction sites and generally have high reintroduction success. However, in this chapter, we argue that the current emphasis on predator exclusion is short-sighted and does not consider the underlying issue of prey naivety. We call for a new paradigm focusing on improving the ability of prey to co-exist with exotic predators one that acknowledges behavioural plasticity and natural selection.

4093: ***-.003***

The release of individuals at a chosen location is the defining feature of reintroduction programs. However, the choice of an adequate release protocol for translocated individuals can be complicated, particularly for programs with multiple objectives, such as maximising release numbers while minimising impacts on the source population. Limited resources and the biology of species can also generate trade-offs, such as where older individuals have greater survival but are more expensive to translocate or breed. Uncertainty will surround many of these aspects: yet decisions must be made, often within strict time frames. This chapter illustrates how a structured decision-making framework can be used to guide the choice of release strategies in complex reintroduction programs. This approach focuses on clearly specifying objectives, comparing the available actions by their expected outcomes and explicitly considering uncertainties and trade-offs. An example is provided using the release program for the endangered southern corroboree frog Pseudophryne corroboree. For a 10-year release program for this species, mixed releases of eggs and sub-adults are expected to maximise the persistence of both wild and captive populations, while meeting budget constraints. Decision-analytic methods can help managers design transparent and effective release strategies, making rational decisions in the face of uncertainty.

4094: ***+.125***

Reintroductions have become an integral part of conservation management for a variety of threatened species in Australia and New Zealand. This popularity largely reflects the dramatic impact that exotic species have had on the indigenous fauna of these countries. With control and eradication of several of the most detrimental exotic species from defined areas, reintroductions can be initiated in the absence of the pressures that caused the original extinction. Despite the volume of reintroductions being undertaken, the probability that a project will achieve the re-establishment of a viable population is not guaranteed. Many of the difficulties associated with reintroductions relate to the inherent challenges animals are exposed to throughout the translocation process and following release. 'Release strategies' are components of the reintroduction process that can be deliberately designed to manage these problems. They can, therefore, improve post-release establishment probabilities. We review here several release strategies that are commonly implemented in Australasian fauna reintroductions, summarise the ecological theory underlying their design, and provide examples to highlight their influence on post-release establishment. The selected release strategies include the design of the composition and size of the release group, the timing and number of release events and the selection of release protocols (delayed versus immediate releases).

4095: ***+.126***

This 328-page book in English titled "Advances in Reintroduction Biology of Australian and New Zealand Fauna" is a volume in its series. This book contains 20 individually-authored chapters. Each chapter is extensively referenced. Specific chapter topics include: reintroduction biology, influence of predator and prey naivety on reintroduction success, disease risk management, restoration planning, modeling, and genetic diversity. This book includes a list of contributors and their respective institutions. This book will be of interest to all those working or studying in the fields of ecology, wildlife management, population genetics and computational modeling.

4096: ***+.147***

The Iberian Peninsula has a rich fauna of freshwater fish, with many endemics that are threatened. One of the threat causes is the introduction of exotic species or translocation of natives to new basins. In this paper, we review the ecological impacts of these introductions, from the genetic and individual to the community and global levels, primarily using studies from the Peninsula. Although the accumulated evidence is considerable, the ecological impact of most species and introductions has barely been studied and is probably much greater than is known.

4097: ***+.130***

Translocation of individuals across a barrier which hampers natural colonisation is a potentially important, but debated, conservation tool for a variety of organisms in a world altered by anthropogenic influences. The apollo Parnassius apollo is an endangered butterfly whose distribution retracted dramatically during the 1900s across Europe. In Finland the apollo currently occupies only a fraction of the range of its suitable habitat and is apparently unable to re-colonise other areas. Using eggs collected from wild-caught females from the species' current Finnish stronghold, a population was reared in order to translocate larvae into an unoccupied, but highly suitable, part of the Finnish archipelago where the species historically occurred until its national decline in the 1950s. In 2009 a restricted number of larvae (1 larva/10 host plants) were released on 25 islands in the inner, middle and outer archipelago zones. In 2010, nine islands situated in all three archipelago zones were (re) stocked with a high density of larvae (1/host plant). In 2011, apollo larval populations were found only on islands in the outer archipelago zone, which were

then restocked. The species remained present here in the following two years (2012, 2013) and was hence able to sustain multi-annual population establishment without restocking. Our findings demonstrate that empty suitable habitat may in reality consist of only a few sites where population establishment is possible. Hence, starting the introduction in many sites, which are putatively suitable based on biotic and abiotic criteria derived from species' existing populations, but then "zooming in" on a smaller set of promising sites showing evidence of successful establishment was key to the success of this translocation.

4098: *+.070*

Elucidating the status of populations of endangered species of unclear origin may have important implications for conservation management. In September 2013, a population of white-clawed crayfish was discovered outside of the native range in a small artificial lake in the River Neckar catchment in southwestern Germany. White-clawed crayfish comprise two distinct lineages of yet unresolved taxonomic status, of which only the western lineage (Austropotamobius pallipes s. str.) is native to Germany. To clarify the taxonomic identity and origin of the newly discovered population, we evaluated diagnostic morphological characters and sequences of two mitochondrial genes (for 16S rRNA and cytochrome c oxidase subunit I) from two crayfish specimens. Both analyses concordantly assigned the crayfish to the southern lineage (A. 'italicus'), with the closest matching haplotypes originating from northwestern Italy, southeastern Switzerland, and Lake Plansee in Austria, where an abundant introduced population of this lineage is present. The artificial lake in Germany was reportedly stocked with freshwater mussels from this Austrian lake. It thus appears likely that A. 'italicus' was introduced intentionally or accidentally during the process. Austropotamobius 'italicus' does not naturally occur north of the Alps and thus represents a non-native taxon for Germany, a fact to be considered in its management.

4099: *-.165*

The Carpentarian grasswren (Amytornis dorotheae) is a small, shy passerine patchily distributed through Triodia systems in the central and southern parts of Australia's tropical savannas. Population decline has been reported in the Northern Territory, presumably due to mismanaged fire. The species is considered Endangered in the Northern Territory and Near Threatened in Queensland, but it is not listed Federally. Here, we present the results of over 3000 surveys conducted between 2008 and 2013. We show that Carpentarian grasswrens are divided into four populations, although the northernmost one (Borroloola) now appears to be extinct. The Area of Occupancy for the southernmost population appears to have declined by 28%, while only small numbers of isolated birds now occur at the two intervening populations. Our data suggest that the four populations appear to be at different stages on an extinction pathway, from population decline, to fragmentation and isolation, to extinction, and this seems to be related to worsening fire patterns as one moves northwards. We suggest that the Carpentarian grasswren be listed as Vulnerable at the State and Federal level, and that urgent investment in long-term regional fire management using prescribed burning is required to reverse the declines in the extant populations. For the presumed extinct Borroloola population, restoration will probably need to involve translocation coupled with effective fire management.

4100: *+.204*

Wildlife reintroduction strategies aim to establish viable long term populations, promote conservation awareness and provide economic benefits for local communities. In Portugal, the Eurasian red squirrel (Sciurus vulgaris) became extinct in the 16th century and was reintroduced in

urban parks in the 1990s, mainly for aesthetic and leisure purposes. We evaluated the success of this reintroduction in two urban parks and here described the critical steps. We assessed habitat use, population density and abundance, and management steps carried out during reintroduction projects. Reintroductions have been successful to some extent given squirrels are present 20 years after release. However, populations in both parks are declining due to the lack of active management and poor quality habitat. Successful reintroduction of Eurasian red squirrel in areas without competition of alien tree squirrels involves three critical main stages. The pre project stage includes studies on habitat quality, genetic proximity between donors and closest wild population, and health of donor stocks. In the release stage, the number of individuals released will depend on resource variability, and the hard release technique is an effective and economically viable method. Post release activities should evaluate adaptation, mitigate mortality, monitor the need for supplementary feeding, provide veterinary support, and promote public awareness and education.

4101: ***-.038***

Captive-bred brush-tailed rock-wallabies (BTRW) were reintroduced into the Grampians National Park, Australia, during 2008-12. Two release strategies (methods) were examined: 'Small release with supplementation' (Strategy 1) and 'Larger release, no supplementation' (Strategy 2). Of the 39 animals released, 18% survived. Thirty-six percent of all mortality occurred within the first 100 days. Under Strategy 1, 22 animals were released in five groups. Twenty deaths occurred across 48 months, with predation estimated to account for 15% of mortalities. Under Strategy 2, 17 individuals were reintroduced across one month. Twelve deaths occurred in the five months following release, with predation estimated to account for 83.4% of mortalities. Of the independent variables tested for their relationship to survival time after release, release strategy was the only significant predictor of survival time after release with the risk of death 3.2 times greater in Strategy 2. Independent variables tested for their relationship to predation risk indicated that release strategy was also the only significant predictor of predation risk, with the risk of death associated with predation 10.5 times greater in Strategy 2. Data suggested that fox predation was the main factor affecting BTRW establishment. Predation risk declined by 75% during the first six months after release. A significant positive relationship was also found between predation risk and colony supplementation events. We conclude that predation risk at Moora Moora Creek is reduced in releases of fewer animals, that it declines across time and that disturbing BTRW colonies through the introduction of new animals can increase predation risk. We recommend that future reintroductions should employ diverse exotic predator control measures at the landscape scale, time releases to periods of lowest predator activity, and limit colony disturbance to maintain group cohesion and social structure. Furthermore, the preferred method of population establishment should be single, small releases over multiple sites without supplementation. Further testing of the reintroduction biology of this species is urgently required.

4102: ***-.162***

Predation, along with competition and disease transmission from feral domestic cats (Felis catus), poses the key threat to many in situ and reintroduced populations of threatened species globally. Feral cats are more challenging to control than pest canids because cats seldom consume poison baits or enter baited traps when live prey are readily available. Novel strategies for sustainably protecting threatened wildlife from feral cats are urgently required. Emerging evidence suggests that once they have successfully killed a challenging species, individual feral cats can systematically eradicate threatened prey populations. Here we propose to exploit this selective predation through three targeted strategies to improve the efficacy of feral cat control. Toxic collars and toxic implants, fitted or inserted during monitoring or reintroduction programs for

threatened species, could poison the offending cat before it can effect multiple kills of the target species. A third strategy is informed by evidence that consumption of prey species that are relatively tolerant to natural plant toxins, can be lethal to more sensitive cats. Within key habitats of wildlife species susceptible to cat predation, we advocate increasing the accessibility of these toxins in the food chain, provided negative risks can be mediated. Deliberate poisoning using live and unaffected 'toxic Trojan prey' enables ethical feral cat management that takes advantage of cats' physiological and behavioural predilection for hunting live prey while minimising risks to many non-targets, compared with conventional baiting.

4103: ***+.113***

The purpose of our research was testing techniques to create artificial populations of rare and endangered plants species and monitoring of them in the Mari El Republic conditions. The reintroduction of the regional Red Books species was started in the Republic at first. The objects of research were the following species: Acorus calamus L., Dianthus superbus L., Laser trilobum (L.) Borkh., Salvia tesquicola Klok. et Pobed., Serratula coronata L. The reintroduction of the studied species was carried by live plants. The propagation and production of planting material was held at the introduction nursery of the Botanical garden-institute (BGI). The seeds of Laser trilobum and Serrattula coronata were brought from natural habitat areas on the territory of the republic. The seeds of Dianthus suberbus were collected from plants grown in BGI from seeds were raised earlier from the natural population. The seeds of Salvia tesquicola were collected from plants seized from natural habitats. Cuttings of Acorus calamus were taken from the plants of BGI collection. The planting was carried in 2010-2013 in four administrative republic districts to the natural habitats where the studied species used to grow before but later their populations were reduced. The monitoring researches of newly created artificial populations were conducted in order to receive the experimental data and use them for restoration of the studied species population. The plants survival of the studied species for the next year after planting had the following values: Acorus calamus 33 %, Dianthus superbus 11 %, Laser trilobum 41 to 93 % in 4 sites with different ecological conditions, Salvia tesquicola 95 %, Serrattula coronata 70 %.

4104: ***-.236***

Living organisms, including birds, are influenced by many natural and anthropogenic factors. Apart from habitat degradation and loss, direct factors causing mortality, such as hunting, play an increasing role. Hunting has many negative consequences, including population decline, disturbance, lower availability of resting and foraging sites, changes in behavior and physiology, direct injury and lead poisoning. At present a total of 13 bird species are regarded as game species in Poland. Some of them, for example Grey Partridge Perdix perdix, experience population declines. To reduce negative effects of hunting on birds we propose to exclude the most important SPA Natura 2000 areas from hunting, to designate refuges where hunting would be prohibited, to prohibit the use of lead shots, to implement a hunting moratorium or other forms of protection of five game species (Common Pochard Aythya ferina, Tufted Duck A. fuligula, Eurasian Coot Fulica atra, Eurasian Teal Anas crecca, Grey Partridge), and to prohibit bird reintroductions for hunting purposes.

4105: ***+.067***

The ecological reserve "La Otra Opcion" is located in the Biosphere Reserve of Los Tuxtlas in Veracruz State in Mexico. Since 2010 this reserve is breeding a captive herd of white-lipped peccaries with reintroduction purposes. In four years the initial herd of ten individuals (two males,

six adult females and two juvenile females) have increased to 24 (six males, 12 females and six juveniles). This is a report on breeding success and mortality rate during the first four years of this effort to breed this species in captivity for future reintroduction goals.

4106: *+.122*

In contrast to biological invasions, translocations of individuals within a species range are understudied, due to difficulties in systematically detecting them. This results in limited knowledge about the corresponding processes and uncertainties regarding the status of extant populations. European larch, a forest tree whose fragmented native distribution is restricted to the Alps and to other Central European mountains, has been massively planted for at least 300 years. Here we focus on the genetic characterization of translocations having taken place within its native range. Microsatellite variation at 13 nuclear loci and sequence data of two mitochondrial DNA fragments were analyzed on the basis of a comprehensive range-wide population sample. Two complementary methods (Geneclass and Structure) were used to infer translocation events based on nuclear data whereas mitochondrial data were used for validation of these inferences. Using Geneclass, we found translocation events in a majority of populations. Additional cases of translocation and many instances of admixture were identified using Structure, thanks to the clear-cut ancestral genetic structure detected in this species. In particular, a strong divide between Alpine and Central European populations, also apparent at mitochondrial markers, helped uncover details on translocation events and related processes. Translocations and associated admixture events were found to be heterogeneously distributed across the species range, with a particularly high frequency in Central Europe. Furthermore, translocations frequently involved multiple geographic sources, some of which were over-represented. Our study illustrates the importance of range-wide investigations for tracing translocations back to their origins and for revealing some of their consequences. It provides some first clues for developing suitable conservation and management strategies.

4107: *+.162*

The Australian lungfish is a unique living representative of an ancient dipnoan lineage, listed as vulnerable to extinction under Australias Environment Protection and Biodiversity Conservation Act 1999. Historical accounts indicate this species occurred naturally in two adjacent river systems in Australia, the Burnett and Mary. Current day populations in other rivers are thought to have arisen by translocation from these source populations. Early genetic work detected very little variation and so had limited power to answer questions relevant for management including how genetic variation is partitioned within and among sub-populations. In this study, we use newly developed microsatellite markers to examine samples from the Burnett and Mary Rivers, as well as from two populations thought to be of translocated origin, Brisbane and North Pine. We test whether there is significant genetic structure among and within river drainages; assign putatively translocated populations to potential source populations; and estimate effective population sizes. Eleven polymorphic microsatellite loci genotyped in 218 individuals gave an average within-population heterozygosity of 0.39 which is low relative to other threatened taxa and for freshwater fishes in general. Based on FST values (average over loci = 0.11) and STRUCTURE analyses, we identify three distinct populations in the natural range, one in the Burnett and two distinct populations in the Mary. These analyses also support the hypothesis that the Mary River is the likely source of translocated populations in the Brisbane and North Pine rivers, which agrees with historical published records of a translocation event giving rise to these populations. We were unable to obtain bounded estimates of effective population size, as we have too few genotype combinations, although point estimates were low, ranging from 29 - 129. We recommend that, in

order to preserve any local adaptation in the three distinct populations that they be managed separately.

4108: *+.138*

Rehabilitation of animals followed by reintroduction into the wild can benefit conservation by supplementing depleted wild populations or reintroducing a species in an area where it has been extirpated or become extinct. The western lowland gorilla (WLG, Gorilla g. gorilla) is persistently poached; infants are often illegally traded and used as pets. Some are confiscated and rehabilitated, then kept in sanctuaries or reintroduced into the wild. Prior to reintroduction, the ability of the orphans to survive independently in their environment needs to be assessed. Here, we performed a multivariate analysis, including diet composition, activity-budget, and pattern of strata using of a group of five juvenile WLG in the process of rehabilitation and distinguished three sub-periods of ecological significance: the high furgivory period, the Dialium fruits consumption period, and the high folivory period. The consequences of these variations on their well-being (play behaviour) and the group cohesion (spatial proximity and social interactions) were examined. Like wild WLGs, diets shifted seasonally from frugivorous to folivorous, while the same staple foods were consumed and large amounts of Dialium fruits were seasonally gathered high in trees. When succulent fruit intake was the highest, thus providing high energy from sugar, juveniles spent less time feeding, more time playing and group cohesion was the highest. Conversely, the cohesion decreased with increasing folivory, individuals spent more time feeding and less time playing together. Nonetheless, the group cohesion also decreased after the death of one highly social, wild-born orphan. This may underscore the importance of skilled individuals in the cohesion and well-being of the entire group and, ultimately, to rehabilitation success. This study evaluates the rehabilitation success with regards to the methods used and highlights the need to consider a set of individual and environmental factors for enhancing rehabilitation while preserving the local biodiversity and individual well-being.

4109: *+.112*

The evolutionary history of Mexican ichthyofauna has been strongly linked to natural events, and the impact of pre-Hispanic cultures is little known. The live-bearing fish species Allotoca diazi, Allotoca meeki and Allotoca catarinae occur in areas of biological, cultural and economic importance in central Mexico: Patzcuaro basin, Zirahuen basin, and the Cupatitzio River, respectively. The species are closely related genetically and morphologically, and hypotheses have attempted to explain their systematics and biogeography. Mitochondrial DNA and microsatellite markers were used to investigate the evolutionary history of the complex. The species complex shows minimal genetic differentiation. The separation of A. diazi and A. meeki was dated to 400-7000 years ago, explained by geological and climate events. A bottleneck and reduction of genetic diversity in Allotoca diazi was detected, attributed to recent climate fluctuations and anthropogenic activity. The isolation of A. catarinae occurred ~1900 years ago. No geological events are documented in the area during this period, but the date is contemporary with Purhepecha culture settlements. This founder effect represents the first evidence of fish species translocation by a pre-Hispanic culture of Mexico. The response of the complex to climate fluctuation, geological changes and human activity in the past and the future according to the ecological niches predictions indicates areas of vulnerability and important information for conservation. The new genetic information showed that the Allotoca diazi complex consist of two genetic groups with an incomplete lineage sorting pattern: Patzcuaro and Zirahuen lakes, and an introduced population in the Cupatitzio River.

4110: *+.311*

Understanding how animals utilize available space is important for their conservation, as it provides insight into the ecological needs of the species, including those related to habitat, prey and inter and intraspecific interactions. We used 28 months of radio telemetry data and information from 200 kill locations to assess habitat selection at the 3rd order (selection of habitats within home ranges) and 4th order (selection of kill sites within the habitats used) of a reintroduced population of cheetahs Acinonyx jubatus in Phinda Private Game Reserve, South Africa. Along with landscape characteristics, we investigated if lion Panthera leo presence affected habitat selection of cheetahs. Our results indicated that cheetah habitat selection was driven by a trade-off between resource acquisition and lion avoidance, and the balance of this trade-off varied with scale: more open habitats with high prey densities were positively selected within home ranges, whereas more closed habitats with low prey densities were positively selected for kill sites. We also showed that habitat selection, feeding ecology, and avoidance of lions differed depending on the sex and reproductive status of cheetahs. The results highlight the importance of scale when investigating a species habitat selection. We conclude that the adaptability of cheetahs, together with the habitat heterogeneity found within Phinda, explained their success in this small fenced reserve. The results provide information for the conservation and management of this threatened species, especially with regards to reintroduction efforts in South Africa.

4111: *-.086*

Commercial trade, almost always for pets, represents a major threat to bird species and subspecies in Sumatra, Kalimantan, Java and Bali, Indonesia. Thirteen species-Silvery Woodpigeon Columba argentina, Javan Hawk-eagle Nisaetus bartelsi, Helmeted Hornbill Rhinoplax vigil, Yellow-crested Cockatoo Cacatua sulphurea, Scarlet-breasted Lorikeet Trichoglossus forsteni, Javan Green Magpie Cissa thalassina, Black-winged Myna Acridotheres melanopterus, Bali Myna Leucopsar rothschildi, Straw-headed Bulbul Pycnonotusieylanicus,Javan White-eye Zosterops flavus, Rufous-fronted Laughingthrush Garrulax rufifrons, Sumatran Laughingthrush Garrulax bicolor and Java Sparrow Lonchura oryzivora-are identified as at greatly elevated risk of global extinction from trade pressures, plus the nominate Javan race of Crested Jay Platylophus galericulatus, the races tricolor, hypolizus, opisthochrus, rnelanurus, omissus and bawrbouri of White-romped Shama Copsychus malabaricus, race jalla of Asian Pied Starling Gracupica contra, races miotera, robusta and (extralimital) venerata of Hill Myna Gracula religiosa, and races rookmakeri andlaurinae of Silver-eared Mesia Leiothrixargentauris. Scarlet-breasted Lorikeet Trichoglossus forsteni race djampeanus, White-rumped Shama Copsychus malabaricus races opisthochrus, omissus and nigricauda and Hill Myna Gracula religiosa race miotera may already be extinct. However, this is a conservative list because (a) some candidates simply lack information to indicate trade as a threat, (b) taxonomic revision will probably increase the number of full species at risk from trade, and (c) taxonomically undifferentiated populations were not included in this review. As certain favoured species disappear, others are targeted as next-best substitutes (e.g.Grey-cheeked Bulbul Alophoixus bres for Straw-headed Bulbul Pycnonotus zeylanicus), and commercial breeders may hybridise taxa for better effects (e.g. non Indonesian subspecies of Asian Pied Starling Gracupica contra with Indonesian race jalla). Law enforcement, public awareness campaigns, in situ management, conservation breeding, conversion of trappers to wardens and field, market and genetic surveys are all needed, but commercial breeding, while attractive in theory, presents difficulties that are probably insurmountable in practice.

4112: *+.136*

Finescale dace (Phoxinus neogaeus) were historically numerous in the Black Hills of South Dakota, but the population has decreased substantially over the past 125 years. By 2003, the only finescale dace populations in South Dakota occurred in Cox Lake and Crow Creek. In 2004, 30 adult dace were moved from Cox Lake to Mud Lake in Lawrence County in an attempt to establish another self-sustaining population. Mark-recapture population estimates in 2014 indicated 7,022 adult finescale dace in Mud Lake, with 95% confidence limits of 5,152 and 9,407 fish. The successful establishment of the Mud Lake population indicates the feasibility of transplantation to conserve this state-endangered species. New sites for additional populations should be considered due to the successful re-introduction of the finescale dace in Mud Lake.

4113: *+.395*

Knowing the extent and structure of genetic variation in an endangered species is essential for establishing efficient conservation practices. However, the proper use of this information requires understanding the role of habitat-specific selection in genetic structuring. We present a study of population differentiation in an endangered species that utilizes guidelines of recently a proposed quasi in situ conservation approach, i.e. taking into account the scale and spatial pattern of local adaptation since if local adaptation is important, the introduced genotypes must be matched to the local biotic/abiotic conditions. Following this approach, we examined the extent and structure of genetic (AFLP) and phenotypic variation and tested for adaptive significance of this variation in critically endangered Iris atrofusca growing in Israel and Jordan. From these results we propose a sampling design that would (i) preserve species adaptive potential and (ii) insure environmental match of the plant material for relocation, reintroduction or enhancement.

4114: *+.162*

The Italian hare is a species of hare endemic to central and southern Italy and to Sicily. It has been classified as a vulnerable species by the International Union for Conservation of Nature (IUCN), as it is considered to have a high risk of extinction in the next decade. Despite its endangered status, little is known about its feeding habits. In the present study, the seasonal pattern of diet composition of a population of Italian hare occupying a semi-natural landscape was estimated by using the micro-histological technique of faecal analysis. The results showed that hares had a diversified diet, consuming plant parts from over 70 species. Like other Lepus sp., the Italian hare consumed a large amount of herbaceous plants (e.g. Brachypodium sylvaticum, Trifolium pratense, Allium subhirsutum and Festuca arundinacea), although it complemented its diet seasonally with fruits of Prunus spinosa, Pyrus piraster and Malus sylvestris. Analysis of similarities (ANOSIM) evidenced significant differences among seasons, as a consequence of the seasonal occurrence of the various food items. Spring and autumn (R=0.7482, P=0.001), as well as spring and winter (R=0.7398, P=0.001), showed low diet similarities; these results were supported by similarity percentage analysis (SIMPER, average dissimilarity: 71% between spring and autumn; 69% between spring and winter) with taxa like P. spinosa, Cirsium strictus, T. pratense and Rosa canina making the greatest contributions to these differences. Higher similarities were instead found when comparing other seasons. This seasonal pattern of diet composition was clearly depicted in the graph from nonmetric multidimensional scaling (n-MDS) ordination. Our results highlight the importance of some plant taxa in the diet of the Italian hare and could be useful in managing reintroduction programs.

4115: *+.294*

The dataset contains records of Green Parrot breeding success and survival rates per nesting site,

including number of eggs laid, number of chicks hatched and number of chicks fledged. Records of sex composition are recorded as well as records of parental attendance and any nesting disturbance. For information on study site coordinates (restricted data) for this species, please contact the dataset author.

4116: -.089

For some highly endangered species there are too few reproductively capable animals to maintain adequate genetic diversity, and extraordinary measures are necessary to prevent their extinction. Cellular reprogramming is a means to capture the genomes of individual animals as induced pluripotent stem cells (iPSCs), which may eventually facilitate reintroduction of genetic material into breeding populations. Here, we describe a method for generating iPSCs from fibroblasts of mammalian endangered species.

4117: -.056

In April 2014, the Committee on the Status of Endangered Wildlife in Canada (COSEWIC) reviewed the status of caribou in the western mountains of Canada, in keeping with the ten-year reassessment mandate under the Species at Risk Act. Assessed as two ` nationally significant' populations in 2002, COSEWIC revised the conservation units for all caribou in Canada, recognising eleven extant Designatable Units (DUs), three of which --Northern Mountain, Central Mountain, and Southern Mountain --are found only in western Canada. The 2014 assessment concluded that the condition of many subpopulations in all three DUs had deteriorated. As a result of small and declining population sizes, the Central Mountain and Southern Mountain DUs are now recognised as endangered. Recent declines in a number of Northern Mountain DU subpopulations did not meet thresholds for endangered or threatened, and were assessed as of special concern. Since the passage of the federal Species at Risk Act in 2002, considerable areas of habitat have been managed or conserved for caribou, although disturbance from cumulative human development activities has increased during the same period. Government agencies and local First Nations are attempting to arrest the steep decline of some subpopulations by using predator control, maternal penning, population augmentation, and captive breeding. Based on declines, future developments and current recovery effects, we offer the following recommendations: 1) where recovery actions are necessary, commit to simultaneously reducing human intrusion into caribou ranges, restoring habitat over the long term, and conducting short-term predator control, 2) carefully consider COSEWIC's new DU structure for management and recovery actions, especially regarding translocations, 3) carry out regular surveys to monitor the condition of Northern Mountain caribou subpopulations and immediately implement preventative measures where necessary, and 4) undertake a proactive, planned approach coordinated across jurisdictions to conserve landscape processes important to caribou conservation.

4118: +.061

The distribution and abundance of woodland caribou (Rangifer tarandus caribou) have declined dramatically in the past century. Without intervention the most southern population of caribou in eastern North America is expected to disappear within 20 years. Although translocations have reintroduced and reinforced some populations, approximately half of caribou translocation efforts fail. Translocations are resource intensive and risky, and multiple interrelated factors must be considered to assess their potential for success. Structured decision-making tools, such as Bayesian belief networks, provide objective methods to assess different wildlife management scenarios by identifying the key components and relationships in an ecosystem. They can also

catalyze dialogue with stakeholders and provide a record of the complex thought processes used in reaching a decision. We developed a Bayesian belief network for a proposed translocation of woodland caribou into a national park on the northeastern coast of Lake Superior, Ontario, Canada. We tested scenarios with favourable (e.g., good physical condition of adult caribou) and unfavourable (e.g., high predator densities) conditions with low, medium, and high numbers of translocated caribou. Under the current conditions at Pukaskwa National Park, augmenting the caribou population is unlikely to recover the species unless wolf densities remain low (< 5.5/1000 km(2)) or if more than 300 animals could be translocated.

4119: *+.109*

Information associating the tillage disturbance and its effect over soil nematode communities in different tillage systems has not been thoroughly discussed, especially in Asian countries. We investigated the effect of three tillage systems, i.e., moldboard plow/rotary harrow (MP), rotary cultivation (RC), and no-tillage (NT), and three cover crop treatments (fallow, rye, and hairy vetch) with two manure applications (0 and 1 Mg ha(-1)) on nematode communities and degree of surface soil translocation (DTL). This study was conducted in Japanese Kanto region (Andosols) in 2009-2011. We calculated nematode community indices, including channel index, enrichment index, and structure index (SI), based on the composition of nematode assemblages, to infer soil ecosystem condition. DTL was calculated from data on the variation of vertical soil distribution of radioactive cesium-137 deposited following the Fukushima Daiichi nuclear power plant accident in 2011. Tillage system influenced nematode abundance of all feeding groups. Cover crop also affected abundance of bacterial feeders (BAC), fungal feeders and facultative root feeders (FFR), predators (PRD), and obligatory root feeders (ORF). SI was bigger in NT, which was 22% and 47% higher than in MP and RC, respectively. DTL negatively correlated with abundance of BAC, omnivores, ORF, total number of nematode species, and SI, but positively correlated with the abundance ratio of FFR to FFR + BAC. Our results suggested that tillage inversion exerted stronger effects on nematode community and structure of soil ecosystem than cover crop treatment and manure application. (C) 2014 Elsevier B.V. All rights reserved.

4120: *+.252*

Moving animals between locations to increase production is routine in terrestrial systems but has rarely been attempted in marine systems. Data on the biological impacts of translocating lobsters, Jasus edwardsii, from regions around Tasmania Australia with high density but slow growth to faster growth areas have been collected over 10 years documenting increases in growth, yield and value. We used a bioeconomic model to evaluate the benefits and costs of improving yield per recruit from commercial scale operations. Simulations were based on commercial scale operations, which move 100,000 lobsters per year within the range of natural larval dispersal. Translocation was combined with a catch quota and fishers could harvest both translocated and resident lobsters. Improvement in total biomass and egg production was comparable to what would otherwise require a 4.5% reduction in catch. Catch rates increased due to higher density, thus reducing fishing costs. This led to higher future rents, with the estimated NPV (i = 7.5%) of the fishery increasing by 7.4%. Commercial scale translocations have been adopted on the basis of our results and are now funded and managed by the commercial industry. (C) 2014 Elsevier B.V. All rights reserved.

4121: *+.092*

Determining whether isolated populations of a species are native or introduced is important for

conservation, as non-native occurrences are likely to be of lower priority for conservation organisations with limited resources. The great crested newt Triturus cristatus is an important wetland flagship species in the UK, and recent evidence suggested that putatively introduced isolated occurrences around Inverness (Scottish Highlands) might be of native origin. Here, we use six microsatellite loci and mtDNA sequence information (ND4 region) to genetically characterise eight Highland populations, comparing them with two populations from central Scotland (the northern limit of the species' continuous UK distribution) and central England (Leicestershire). Highland populations were characterised by low amounts of genetic variation at high degrees of differentiation, which can be best interpreted by demographic instability and isolation at the periphery of the species' range. We found no evidence for population bottlenecks in the last decades. All studied individuals possessed a single mtDNA haplotype previously described for British T. cristatus. Taken together, these results suggest that T. cristatus is native to the Scottish Highlands, with important implications for its local conservation status.

4122: *-.122*

Simian immunodeficiency viruses (SIVs) have been discovered in over 45 primate species; however, the pathogenic potential of most SIV strains remains unknown due to difficulties inherent in observing wild populations. Because those SIV infections that are pathogenic have been shown to induce changes in the host's gut microbiome, monitoring the microbiota present in faecal samples can provide a noninvasive means for studying the effects of SIV infection on the health of wild-living primates. Here, we examine the effects of SIVgor, a close relative of SIVcpz of chimpanzees and HIV-1 of humans, on the gut bacterial communities residing within wild gorillas, revealing that gorilla gut microbiomes are exceptionally robust to SIV infection. In contrast to the microbiomes of HIV-1-infected humans and SIVcpz-infected chimpanzees, SIVgor-infected gorilla microbiomes exhibit neither rises in the frequencies of opportunistic pathogens nor elevated rates of microbial turnover within individual hosts. Regardless of SIV infection status, gorilla microbiomes assort into enterotypes, one of which is compositionally analogous to those identified in humans and chimpanzees. The other gorilla enterotype appears specialized for a leaf-based diet and is enriched in environmentally derived bacterial genera. We hypothesize that the acquisition of this gorilla-specific enterotype was enabled by lowered immune system control over the composition of the microbiome. Our results indicate differences between the pathology of SIVgor and SIVcpz/HIV-1 infections, demonstrating the utility of investigating host microbial ecology as a means for studying disease in wild primates of high conservation priority.

4123: *+.162*

Hybridization presents a unique challenge for conservation biologists and managers. While hybridization is an important evolutionary process, hybridization is also a threat formany native species. The endangered species recovery effort for the red wolf Canis rufus is a classic system for understanding and addressing the challenges of hybridization. From 1987. 1993, 63 red wolves were released from captivity in eastern North Carolina, USA, to establish a free-ranging, non-essential experimental population. By 1999, managers recognized hybridization with invasive coyotes Canis latrans was the single greatest threat to successful recovery, and an adaptive management plan was adopted with innovative approaches for managing the threat of hybridization. Here we review the application and results of the adaptive management efforts from 1993 to 2013 by comparing: (1) the numbers of wolves, coyotes, and hybrids captured, (2) the numbers of territorial social groups with presumed breeding capabilities, (3) the number of red wolf and hybrid litters documented each year and (4) the degree of coyote introgression into the wild red wolf gene pool. We documented substantial increases in the number of known red wolves

and red wolf social groups from 1987-2004 followed by a plateau and slight decline by 2013. The number of red wolf litters exceeded hybrid litters each year and the proportion of hybrid litters per year averaged 21%. The genetic composition of the wild red wolf population is estimated to include <4% coyote ancestry from recent introgression since reintroduction. We conclude that the adaptive management plan was effective at reducing the introgression of coyote genes into the red wolf population, but population recovery of red wolves will require continuation of the current management plan, or alternative approaches, for the foreseeable future. More broadly, we discuss the lessons learned from red wolf adaptive management that could assist other endangered species recovery efforts facing the challenge of minimizing hybridization

4124: *+.158*

Some central and eastern populations of fisher Pekania [Martes] pennanti are expanding their ranges following historic range contractions, while many western populations have yet to do so. We investigated whether expanding fisher populations are benefiting from a mesopredator release following reductions in their carnivore predator communities. This hypothesis posits that local extinctions of the largest predators release' mesopredator populations from direct predation and competition, leading to an increase in their abundance, expansion of their range and potentially to shifts in their morphology and ecological niche. Our comparison of the conservation status and predator communities of fishers across four geographic regions of their range supports the mesopredator release hypothesis, especially in their eastern range. Our meta-analysis of fisher diet also suggests that released fisher populations may benefit by complementing their diverse diets with more large-bodied prey species, whereas those with more specialized diets (e.g. northwestern populations) or diverse diets with small amounts of large-bodied prey (e.g. populations within California) have experienced little range expansion. Further, measurements of museum specimens suggest that individuals within released populations have evolved a larger body size since the time of their most contracted range, which may help them hunt larger prey species that are expected to be more available in the absence of larger carnivores. Collectively, these data support the hypothesis that a reduced predator community is contributing to the geographic variation in modern fishers' range expansion. In addition to harvest restrictions, habitat protection and translocations, future conservation plans should consider the potential effects of the predator community, emphasizing the need to quantify fisher mortality sources and fisher-predator interactions.

4125: *-.025*

Interspecific hybridization occurs in nature but can also be caused by human actions. It often leads to infertile or fertile hybrids that exclude one parental genome during gametogenesis, escaping genetic recombination and introgression. The threat that genome-exclusion hybridization might represent on parental species is poorly understood, especially when invasive species are involved. Here, we show how to assess the effects of genome-exclusion hybridization and how to elaborate conservation actions by simulating scenarios using a model of nonintrogressive hybridization. We examine the case of the frog Pelophylax ridibundus, introduced in Western Europe, which can hybridize with the native Pelophylax lessonae and the pre-existing hybrid Pelophylax esculentus, maintained by hybridogenesis. If translocated from Southern Europe, P.ridibundus produces new sterile hybrids and we show that it mainly threatens P.esculentus. Translocation from Central Europe leads to new fertile hybrids, threatening all native waterfrogs. Local extinction is demographically mediated via wasted reproductive potential or via demographic flow through generations towards P.ridibundus. We reveal that enlarging the habitat size of the native P.lessonae relative to that of the invader is a promising conservation strategy, avoiding the difficulties of

fighting the invader. We finally stress that nonintrogressive hybridization is to be considered in conservation programmes.

4126: ***-.256***

An epidemic of the disease chytridiomycosis, caused by the pathogenic fungus Batrachochytrium dendrobatidis, induced a massive decline of populations of the common midwife toad (Alytes obstetricans) inhabiting the Pealara Massif (Guadarrama National Park, Central Spain) in the years 1997-2001. The disease outbreak caused the disappearance of about 90 % of populations, leaving only eight remnant breeding populations. In response to the disease-induced population decline, a captive breeding program was started in 2008. Populations were kept separate to minimize possible outbreeding depression. Here, we examined indices of genetic diversity and population structure in these remnant populations to inform future reintroductions. Analysis of ten microsatellite loci showed strong genetic structure between breeding sites suggesting little genetic exchange and relatively low global genetic diversity. In accordance with the demographic bottleneck observed in the last years we found strong evidence for a reduction in genetic diversity. Our results suggest that the captive breeding program should mix animals from multiple sites from the Guadarrama Mountain Range, but avoid the genetically most divergent populations.

4127: ***+.139***

Conservation translocations are an important tool to circumvent extinctions on oceanic islands. A thorough understanding of all components of a species' biology, including genetic diversity and structure, can maximize their likelihood of success. The Maui Parrotbill (Pseudonestor xanthophrys) is an endangered Hawaiian honeycreeper endemic to the island of Maui. With a population of approximately 500 individuals restricted to 50 km(2) of habitat, this species is at high risk of extinction. Using nuclear and mitochondrial DNA, this study quantified the levels of genetic diversity and structure in wild and captive parrotbill populations, and compared these genetic patterns to those observed within levels of contemporary and historical nuclear diversity derived from 100-year old museum samples. Substantial differences in the effective population sizes estimated between contemporary and historical parrotbill populations highlight the impact that introduced disease had on this species just before the turn of the century. Contemporary parrotbill diversity was low (global F (st) = 0.056), and there has been a 96 % reduction in genetic effective population size between contemporary and historical samples. This should not eliminate a conservation translocation (or reintroduction) as a viable recovery option. Measures of population differentiation (pairwise F (st) and R (st)) between different sections of the current population on either side of the Koolau Gap suggest that current genetic structure may be the result of this topographic barrier to gene flow. These data can enable the design of a conservation translocation strategy that is tailored to the patterns of genetic structure across the species' range.

4128: ***+.060***

Reintroduction of imperiled native freshwater fish is becoming an increasingly important conservation tool amidst persistent anthropogenic pressures and new threats related to climate change. We summarized trends in native fish reintroductions in the current literature, identified predictors of reintroduction outcome, and devised recommendations for managers attempting future native fish reintroductions. We constructed random forest classifications using data from 260 published case studies of native fish reintroductions to estimate the effectiveness of variables in predicting reintroduction outcome. The outcome of each case was assigned as a success or failure on the basis of the author's perception of the outcome and on whether or not survival,

spawning, or recruitment were documented during post-reintroduction monitoring. Inadequately addressing the initial cause of decline was the best predictor of reintroduction failure. Variables associated with habitat (e.g., water quality, prey availability) were also good predictors of reintroduction outcomes, followed by variables associated with stocking (e.g., genetic diversity of stock source, duration of stocking event). Consideration of these variables by managers during the planning process may increase the likelihood for successful outcomes in future reintroduction attempts of native freshwater fish. Identificacion de Correlaciones de Exito y Fracaso de Reintroducciones de Peces de Nativos Agua DulceResumen La reintroduccion de peces nativos de agua dulce que se encuentran en peligro se esta convirtiendo cada vez mas en una herramienta importante de conservacion frente a las presiones antropogenicas persistentes y nuevas amenazas relacionadas con el cambio climatico. Resumimos las tendencias encontradas en la literatura actual sobre la reintroduccion de peces nativos, identificamos pronosticadores de resultados de la reintroduccion e ideamos recomendaciones para administradores que intenten reintroducciones de peces nativos en el futuro. Construimos clasificaciones de bosque aleatorio a partir de datos de 260 estudios de caso publicados sobre la reintroduccion de peces nativos para estimar la efectividad de las variables en la prediccion del resultado de la reintroduccion. El resultado de cada caso fue asignado como un exito o un fracaso con base en la percepcion del autor a partir del resultado y dependiendo de si se documento o no la supervivencia, el desove o el reclutamiento durante el monitoreo posterior a la reintroduccion. Abordar inadecuadamente a la causa inicial de la declinacion fue el mejor pronosticador del fracaso de la reintroduccion. Las variables asociadas con el habitat (p. ej.: calidad del agua, disponibilidad de la presa) tambien fueron buenos pronosticadores de los resultados de la reintroduccion, seguidas por las variables asociadas con el stock (p. ej.: la diversidad genetica de la fuente del stock, duracion del evento de stock). Que los administradores consideren estas variables durante el proceso de planeacion puede incrementar la probabilidad de resultados exitosos en futuros intentos de reintroduccion de peces nativos de agua dulce.

4129: *+.096*

Mitigation translocation of nuisance animals is a commonly used management practice aimed at resolution of human-animal conflict by removal and release of an individual animal. Long considered a reasonable undertaking, especially by the general public, it is now known that translocated subjects are negatively affected by the practice. Mitigation translocation is typically undertaken with individual adult organisms and has a much lower success rate than the more widely practiced conservation translocation of threatened and endangered species. Nonetheless, the public and many conservation practitioners believe that because population-level conservation translocations have been successful that mitigation translocation can be satisfactorily applied to a wide variety of human-wildlife conflict situations. We reviewed mitigation translocations of reptiles, including our own work with 3 long-lived species (Gila monsters [Heloderma suspectum], Sonoran desert tortoises [Gopherus morafkai], and western diamond-backed rattlesnakes [Crotalus atrox]). Overall, mitigation translocation had a low success rate when judged either by effects on individuals (in all studies reviewed they exhibited increased movement or increased mortality) or by the success of the resolution of the human-animal conflict (translocated individuals often returned to the capture site). Careful planning and identification of knowledge gaps are critical to increasing success rates in mitigation translocations in the face of increasing pressure to find solutions for species threatened by diverse anthropogenic factors, including climate change and exurban and energy development. Problemas con la Mitigacion por Traslocacion de HerpetofaunaResumen La mitigacion de animales molestos por traslocacion comunmente se usa como una practica de manejo enfocada a la resolucion del conflicto humano-animal por remocion y liberacion de un animal. Considerada durante mucho tiempo un proyecto

razonable, especialmente por el publico en general, ahora se sabe que los sujetos traslocados se ven afectados negativamente por la practica. La mitigacion por traslocacion se emprende tipicamente con organismos adultos y tiene una tasa de exito mucho mas baja que la ampliamente practicada traslocacion para la conservacion de especies amenazadas y en peligro. Sin embargo, el publico y muchos practicantes de la conservacion creen que como las traslocaciones de conservacion a nivel de poblacion han sido exitosas entonces la mitigacion por traslocacion puede aplicarse satisfactoriamente a una amplia variedad de situaciones de conflicto humano-vida silvestre. Revisamos la mitigacion de reptiles por traslocacion, incluido nuestro propio trabajo con tres especies de larga vida: monstruo de Gila (Heloderma suspectum), tortugas del desierto de Sonora (Gopherus morafkai) y la serpiente de cascabel (Crotalus atrox). En general, la mitigacion por traslocacion tuvo una baja tasa de exito al ser juzgada por los efectos sobre los individuos (en todos los estudios revisados exhibieron incremento en movimiento y mortalidad) o por el exito de la resolucion de los conflictos humano-animal (los individuos traslocados a menudo regresaban al sitio de captura). La planeacion cuidadosa y la identificacion de los vacios de conocimiento son criticos para incrementar las tasas de exito en mitigacion por traslocacion de cara a una presion creciente para encontrar soluciones para las especies amenazadas por diversos factores antropogenicos, incluidos el cambio climatico y el desarrollo de energia exurbano.

4130: *+.028*

Human-caused biodiversity loss is a global problem, large carnivores are particularly threatened, and the tiger (Panthera tigris) is among the world's most endangered large carnivores. The South China tiger (Panthera tigris amoyensis) is the most critically endangered tiger subspecies and is considered functionally extinct in the wild. The government of China has expressed its intent to reintroduce a small population of South China tigers into a portion of their historic range as part of a larger goal to recover wild tiger populations in China. This would be the world's first major tiger reintroduction program. A free-ranging population of 15-20 tigers living in a minimum of 1000 km(2) of habitat was identified as a target. We assessed summer and winter habitat suitability of two critical prey species, wild boar (Sus scrota) and Sika deer (Cervus nippon), using GIS spatial models to evaluate the potential for tiger reintroduction in one likely candidate site, the 1100 km(2) Hupingshan-Houhe National Nature Reserve complex in Hunan and Hubei Provinces, China. Our preliminary analysis estimates that for wild boar, potential summer and winter habitat availability is 372-714 km(2) and 256-690 km(2), respectively, whereas for Sika deer, potential summer and winter habitat availability is 443-747 km(2) and 257-734 km(2), respectively. Our model identifies potential priority areas for release and restoration of prey between 195 and 790 km(2) with a carrying capacity of 596-2409 wild boar and 468-1929 Sika deer. Our analysis suggests that Hupingshan-Houhe could support a small population of 2-9 tigers at a density of 1.1-1.2 tigers/100 km(2) following prey and habitat restorations. Thus, current habitat quality and area would fall short of the target recovery goal. We identify major challenges facing a potential tiger reintroduction project and conclude that restoring the habitat and prey base, addressing concerns of local people, and enhancing coordination across park boundaries are significant challenges to meeting the broader goals of supporting a reintroduced wild tiger population. Tiger range states have committed to doubling the world's wild tigers by 2022. The results of this study have implications for China's commitment to this goal and for the future of tiger and other large carnivore reintroduction efforts in Asia and globally. (C) 2014 The Authors. Published by Elsevier Ltd.

4131: *+.015*

Astrophytum is one of most collected genera in the cactus family. Around the world several

species are maintained in collections and yearly, several plants are taken from their natural habitats. Populations of Astorphytum capricorne are found in the northern Chihuahuan desert, Mexico, and as many endemic cactus species, it has a highly restricted habitat. We conducted a demographic study from 2008 to 2010 of the northern populations found at Cuatro Cienegas, Mexico. We applied matrix population models, included simulations, life table response experiments and descriptions of the population dynamics to evaluate the current status of the species, and detect key life table stages and demographic processes. Population growth rate decreased in both years and only 4% individual mortality can be attributed to looting, and a massive effort is needed to increase seedling recruitment and reduce adult mortality. The fate of individuals differed between years even having the same annual rainfall mainly in accentuated stasis, retrogression and high mortality in all size classes, which coupled with low seed production, no recruitment and collection of plants are the causes contributing to population decline, and hence, increase the risk in which A. capricorne populations are found. Reintroduction of seedlings and lowering adult mortality are urgently needed to revert the alarming demographic condition of A. capricorne populations. (C) 2015 Elsevier Masson SAS. All rights reserved.

4132: ***+.118***

Metric craniological traits of Eurasian beaver (Castor fiber L. 1758) skulls from eleven autochthonous and reintroduced populations (944 specimens) from Eastern Europe to the Russian Far East were investigated. The significant influence of the origin and geographical variability on morphological peculiarities of skull was discovered. No significant size sexual dimorphism in beaver skulls was found. Among the factors of population polymorphism strongly affecting the skull morphology, the following three were delimited: origination from an autochthonous population, geographical, and macroclimatic. Only six macroclimatic factors of 21 were found to render a significant influence on morphological variability, integrating the values of precipitation and temperatures. Such results are discussed from a viewpoint of the concept of thermal exchange, adaptive biomechanics of the jaw apparatus, food supply base, as well as genetic factors in the course of population development. Our results indicate both an epigenetically adaptive and a hereditary transmission of skull size variability in autochthonous and reintroduced beaver populations.

4133: ***+.258***

Fire is a natural, dynamic process that is integral to maintaining ecosystem function. The reintroduction of fire (e.g., prescribed fire, managed wildfire) is a critical management tool for protecting many frequent-fire forests against stand-replacing fires while restoring an essential ecological process. Understanding the effects of fire on forests and wildlife communities is important in natural resource planning efforts. Small mammals are key components of forest food webs and essential to ecosystem function. To investigate the relationship of fire to small mammal assemblages, we live trapped small mammals in 10 burned and 10 unburned forests over 2 years in the central Sierra Nevada, California. Small mammal abundance was higher in unburned forests, largely reflecting the greater proportion of closed-canopy species such as Glaucomys sabrinus in unburned forests. The most abundant species across the entire study area was the highly adaptable generalist species, Peromyscus maniculatus. Species diversity was similar between burned and unburned forests, but burned forests were characterized by greater habitat heterogeneity and higher small mammal species evenness. The use and reintroduction of fire to maintain a matrix of burn severities, including large patches of unburned refugia, creates a heterogeneous and resilient landscape that allows for fire-sensitive species to proliferate and, as such, may help maintain key ecological functions and diverse small mammal assemblages.

4134: ***+.036***

Phylogenetic relationships among the 6 species of quolls (Dasyurus) are resolved using DNA sequences from 4 mitochondrial and 5 nuclear loci (approximately 15 kb) sampled from 1 to 29 individuals per species. Our estimate of quoll phylogeny concurs with previous DNA-based estimates in placing Dasyurus hallucatus as sister to the remaining species, and D. maculatus as sister to a clade containing D. viverrinus, D. albopunctatus, D. geoffroii + D. spartacus. We also provide the first formal description of penis anatomy in the northern quoll (D. hallucatus), documenting it as the only species of Dasyurus lacking an appendage to the penis. This appendage thus appears to constitute a morphological synapomorphy for the clade of 5 species that excludes D. hallucatus. The sequence from our single specimen of bronze quoll (D. spartacus) nested within a clade of 3 western quoll (D. geoffroii) sequences, suggesting that the species boundary between these groups (if it exists) is not yet reflected in reciprocal monophyly of mitochondrial haplotypes. Any genetic differences found between eastern and western forms of D. geoffroii would have implications for translocations of western animals into other parts of the species range.

4135: ***+.087***

The long-tailed goral, Naemorhedus caudatus, is an internationally endangered species. This species is distributed throughout Northeastern Asia including Northeastern China, the Russian Far East and the Korean peninsula. The population size of long-tailed gorals is currently decreasing in South Korea, and thus effective conservation of the animal is urgently needed. Although the evolution and phylogeny of this animal have been studied, population genetic studies are needed to design effective conservation and management strategies. To evaluate the present status of genetic diversity and genetic structure of long-tailed gorals in South Korea, we investigated genetic variability among 68 goral individuals from different regions, including 11 captive zoo animals, at 12 microsatellite loci. The level of genetic diversity was moderate in wild goral populations, but lower in the captive group. The goral population from the lower northeast region of South Korea was distinct from the upper northeast population, probably due to the natural climatic and geographic conditions. The genetic characteristics of the captive group were more similar to those of the upper northeast population than the lower northeast, confirming that the zoo animals originated in the Seorak Mountain range. Direct translocations between the upper and lower northeast populations are not currently recommended considering the natural population structure and the moderate levels of genetic diversity in the two populations. This study highlights the importance of genetic information in designing effective conservation strategies and translocations of endangered animals, including the Korean goral.

4136: ***+.311***

Understanding how animals utilize available space is important for their conservation, as it provides insight into the ecological needs of the species, including those related to habitat, prey and inter and intraspecific interactions. We used 28 months of radio telemetry data and information from 200 kill locations to assess habitat selection at the 3rd order (selection of habitats within home ranges) and 4th order (selection of kill sites within the habitats used) of a reintroduced population of cheetahs Acinonyx jubatus in Phinda Private Game Reserve, South Africa. Along with landscape characteristics, we investigated if lion Panthera leo presence affected habitat selection of cheetahs. Our results indicated that cheetah habitat selection was driven by a trade-off between resource acquisition and lion avoidance, and the balance of this trade-off varied with scale: more open habitats with high prey densities were positively selected within home ranges, whereas more closed habitats with low prey densities were positively selected for kill sites.

We also showed that habitat selection, feeding ecology, and avoidance of lions differed depending on the sex and reproductive status of cheetahs. The results highlight the importance of scale when investigating a species' habitat selection. We conclude that the adaptability of cheetahs, together with the habitat heterogeneity found within Phinda, explained their success in this small fenced reserve. The results provide information for the conservation and management of this threatened species, especially with regards to reintroduction efforts in South Africa.

4137: *+.062*

Top predator loss is a major global problem, with a current trend in biodiversity loss towards high trophic levels that modifies most ecosystems worldwide. Most research in this area is focused on large-bodied predators, despite the high extinction risk of small-bodied freshwater fish that often act as apex consumers. Consequently, it remains unknown if intermittent streams are affected by the consequences of top-predators' extirpations. The aim of our research was to determine how this global problem affects intermittent streams and, in particular, if the loss of a small-bodied top predator (1) leads to a 'mesopredator release', affects primary consumers and changes whole community structures, and (2) triggers a cascade effect modifying the ecosystem function. To address these questions, we studied the top-down effects of a small endangered fish species, Barbus meridionalis (the Mediterranean barbel), conducting an enclosure/exclosure mesocosm experiment in an intermittent stream where B. meridionalis became locally extinct following a wildfire. We found that top predator absence led to 'mesopredator release', and also to 'prey release' despite intraguild predation, which contrasts with traditional food web theory. In addition, B. meridionalis extirpation changed whole macroinvertebrate community composition and increased total macroinvertebrate density. Regarding ecosystem function, periphyton primary production decreased in apex consumer absence. In this study, the apex consumer was functionally irreplaceable; its local extinction led to the loss of an important functional role that resulted in major changes to the ecosystem's structure and function. This study evidences that intermittent streams can be affected by the consequences of apex consumers' extinctions, and that the loss of small-bodied top predators can lead to large ecosystem changes. We recommend the reintroduction of small-bodied apex consumers to systems where they have been extirpated, to restore ecosystem structure and function.

4138: *+.132*

Island endemics are typically differentiated from their mainland progenitors in behavior, morphology, and genetics, often resulting from long-term evolutionary change. To examine mechanisms for the origins of island endemism, we present a phylogeographic analysis of whole mitochondrial genomes from the endangered island fox (Urocyon littoralis), endemic to California's Channel Islands, and mainland gray foxes (U. cinereoargenteus). Previous genetic studies suggested that foxes first appeared on the islands > 16,000 years ago, before human arrival (similar to 13,000 cal BP), while archaeological and paleontological data supported a colonization > 7000 cal BP. Our results are consistent with initial fox colonization of the northern islands probably by rafting or human introduction similar to 9200-7100 years ago, followed quickly by human translocation of foxes from the northern to southern Channel Islands. Mitogenomes indicate that island foxes are monophyletic and most closely related to gray foxes from northern California that likely experienced a Holocene climate-induced range shift. Our data document rapid morphological evolution of island foxes (in similar to 2000 years or less). Despite evidence for bottlenecks, island foxes have generated and maintained multiple mitochondrial haplotypes. This study highlights the intertwined evolutionary history of island foxes and humans, and illustrates a new approach for investigating the evolutionary histories of other island endemics.

4139: ***+.023***

Inter-specific hybridization may be especially detrimental when one species is extremely rare and the other is abundant owing to the potential for genetic swamping. The Cuban crocodile (Crocodylus rhombifer) is a critically endangered island endemic largely restricted to Zapata Swamp, where it is sympatric with the widespread American crocodile (C. acutus). An on-island, C. rhombifer captive breeding program is underway with the goals of maintaining taxonomic integrity and providing a source of individuals for reintroduction, but its conservation value is limited by lack of genetic information. Here we collected mtDNA haplotypic and nuclear genotypic data from wild and captive C. rhombifer and C. acutus in Cuba to: (1) investigate the degree of inter-specific hybridization in natural (in situ) and captive (ex situ) populations; (2) quantify the extent, distribution and in situ representation of genetic variation ex situ; and (3) reconstruct founder relatedness to inform management. We found high levels of hybridization in the wild (49.1%) and captivity (16.1%), and additional evidence for a cryptic lineage of C. acutus in the Antilles. We detected marginally higher observed heterozygosity and allelic diversity ex situ relative to the wild population, with captive C. rhombifer exhibiting over twice the frequency of private alleles. Although mean relatedness was high in captivity, we identified 37 genetically important individuals that possessed individual mean kinship (MK) values lower than the population MK. Overall, these results will guide long-term conservation management of Cuban crocodiles for maintaining the genetic integrity and viability of this species of high global conservation value.

4140: ***+.109***

Release of captive bred individuals into the wild is now a conservation management approach to increase the depleted natural wildlife populations. Such method has been initiated for conservation of wild fish population on Qinghai Tibetan Plateau (QTP), a region of high level of species endemism. Platypharodon extremus is an endemic schizothoracine fish species and a dominated species in the plateau water ecosystem of the QTP in the past, it is now enlisted as an endangered species. However, phylogenetic and genetic information have not been previously considered in the ongoing stock enhancement program. In this study, genetic and demographic processes of the populations across the distribution of this species were assessed, using the mitochondrial DNA control region and Cyt-b. We found that all populations displayed low nucleotide diversity suggesting severe historical bottleneck events and high haplotype diversity indicating recent population expansion. Both the phylogenetic tree and the minimum spanning tree showed no significant genealogical structures corresponding to sampling locations. Population histories including bottleneck, exponential expansion and the absence of population structure indicate that demographic processes highly influenced genetic variation within and among populations of P. extreums. The data presented is consistent with a historical bottleneck and recent population expansion for all four populations based on several complementary analyses of the genetic data and inferred from demographic history (0.26 to 0.36 Mya). This genetic variation and demographic processes should become the baseline information for evaluating genetic effects of stock enhancement program and ongoing conservation decision making.

4141: ***+.023***

Inter-specific hybridization may be especially detrimental when one species is extremely rare and the other is abundant owing to the potential for genetic swamping. The Cuban crocodile (Crocodylus rhombifer) is a critically endangered island endemic largely restricted to Zapata Swamp, where it is sympatric with the widespread American crocodile (C. acutus). An on-island,

C. rhombifer captive breeding program is underway with the goals of maintaining taxonomic integrity and providing a source of individuals for reintroduction, but its conservation value is limited by lack of genetic information. Here we collected mtDNA haplotypic and nuclear genotypic data from wild and captive C. rhombifer and C. acutus in Cuba to: (1) investigate the degree of inter-specific hybridization in natural (in situ) and captive (ex situ) populations; (2) quantify the extent, distribution and in situ representation of genetic variation ex situ; and (3) reconstruct founder relatedness to inform management. We found high levels of hybridization in the wild (49.1%) and captivity (16.1%), and additional evidence for a cryptic lineage of C. acutus in the Antilles. We detected marginally higher observed heterozygosity and allelic diversity ex situ relative to the wild population, with captive C. rhombifer exhibiting over twice the frequency of private alleles. Although mean relatedness was high in captivity, we identified 37 genetically important individuals that possessed individual mean kinship (MK) values lower than the population MK. Overall, these results will guide long-term conservation management of Cuban crocodiles for maintaining the genetic integrity and viability of this species of high global conservation value.

4142: ***+.112***

Allis shad Alosa alosa and twaite shad Alosa fallax are two related anadromous European clupeid species which have undergone a sharp decline in the past decades. We describe the development of 13 microsatellite markers for both species, based on 454 pyrosequencing technology. The number of alleles per locus ranged from 2 to 11 in A. alosa and from 4 to 13 in A. fallax. Gene diversity ranged from 0.364 to 0.800 for A. alosa and 0.226 to 0.849 for A. fallax. These markers will help in conservation genetics studies such as assessing the extent of population decline, defining appropriate conservation units and monitoring reintroduction programs.

4143: ***+.001***

The Sao Paulo marsh antwren (Formicivora paludicola) is a critically endangered bird endemic to marshes in the metropolitan region of Sao Paulo city, Brazil. The total population is estimated to be around 300 individuals, distributed among 15 small (< 50 ha) fragments, suggesting that loss of genetic variability may affect the long-term viability of this species. To develop genetic tools for gaining information on effective population sizes, inbreeding and gene flow between populations, we describe nine polymorphic microsatellite loci isolated from a F. paludicola library using next-generation sequencing. We report on levels of variation in these novel microsatellites and eight additional heterologous loci in these birds. Expected (H (E)) and observed (H (O)) heterozygosities averaged 0.72 and 0.70, respectively, and the number of alleles per locus ranged from 3 to 10. These loci will permit evaluation of whether artificial translocations are necessary for long-term viability of this rare bird.

4144: ***+.097***

A switchgrass (Panicum virgatum L.) consensus map was developed that combined data from two mapping populations and integrated recombination data from both parents of this largely obligate outcrossing species. The consensus map consisted of 1,321 loci and spanned 2,122 cM. An analysis of the distribution of genic and genomic markers across the length of a linkage group showed that genic markers were relatively overrepresented in distal regions, while genomic markers were relatively overrepresented in pericentromeric regions. Furthermore, genic markers located in distal and pericentromeric regions identified orthologs in the genome of the closely related species foxtail millet (Setaria italica (L.) P. Beauv.) at a similar rate, but fewer orthologs

were found for genomic markers that mapped to pericentromeric regions compared to distal regions. Because cross-species sequence conservation is largely limited to genes, our data suggest that genomic markers that map to distal regions are more likely to be derived from genic regions than those that map to pericentromeric regions. A comparative analysis between loci on the switchgrass consensus map and their orthologs in the foxtail millet genome showed that the two species varied by at least nine inversions and one reciprocal translocation. Extending the comparative analysis to sorghum demonstrated that the majority of the rearrangements occurred in the foxtail millet genome in the past 13 million years. The reduction in chromosome number from 10 in the Andropogoneae to 9 in the Paniceae was achieved through a complex set of rearrangements involving three ancestral chromosomes orthologous to sorghum chromosomes 6, 8, and 9 and chromosomes III and VII in the lineage leading to switchgrass and foxtail millet. Upon insertion of ancestral chromosome 9 into ancestral chromosome 8, the centromere of the recipient chromosome was lost, and this was accompanied by a loss of repetitive DNA. The switchgrass-foxtail millet comparative map provides a guide of the rearrangements that need to be taken into account when using foxtail millet as a surrogate for switchgrass in genetic analyses.

4145: ***+.197***

Despite rapid growth in the field of reintroduction biology, results from scientific research are often not applied to translocations initiated when human land-use change conflicts with the continued persistence of a species' population at a particular site. Such mitigation-driven translocations outnumber and receive more funding than science-based conservation translocations, yet the conservation benefit of the former is unclear. Because mitigation releases are economically motivated, outcomes may be less successful than those of releases designed to serve the biological needs of species. Translocation as a regulatory tool may be ill-suited for biologically mitigating environmental damage caused by development. Evidence suggests that many mitigation-driven translocations fail, although the application of scientific principles and best practices would probably improve the success rate. Lack of transparency and failure to document outcomes also hinder efforts to understand the scope of the problem. If mitigation-driven translocations are to continue as part of the growing billion-dollar ecological consulting industry, it is imperative that the scale and effects of these releases be reported and evaluated.

4146: ***+.051***

Wildlife management to reduce the impact of wildlife on their habitat can be done in several ways, among which removing animals (by either culling or translocation) is most often used. There are, however, alternative ways to control wildlife densities, such as opening or closing water points. The effects of these alternatives are poorly studied. In this paper, we focus on manipulating large herbivores through the closure of water points (WPs). Removal of artificial WPs has been suggested in order to change the distribution of African elephants, which occur in high densities in national parks in Southern Africa and are thought to have a destructive effect on the vegetation. Here, we modeled the long-term effects of different scenarios of WP closure on the spatial distribution of elephants, and consequential effects on the vegetation and other herbivores in Kruger National Park, South Africa. Using a dynamic ecosystem model, SAVANNA, scenarios were evaluated that varied in availability of artificial WPs; levels of natural water; and elephant densities. Our modeling results showed that elephants can indirectly negatively affect the distributions of meso-mixed feeders, meso-browsers, and some meso-grazers under wet conditions. The closure of artificial WPs hardly had any effect during these natural wet conditions. Under dry conditions, the spatial distribution of both elephant bulls and cows changed when the availability of artificial water was severely reduced in the model. These changes in spatial

distribution triggered changes in the spatial availability of woody biomass over the simulation period of 80 years, and this led to changes in the rest of the herbivore community, resulting in increased densities of all herbivores, except for giraffe and steenbok, in areas close to rivers. The spatial distributions of elephant bulls and cows showed to be less affected by the closure of WPs than most of the other herbivore species. Our study contributes to ecologically informed decisions in wildlife management. The results from this modeling exercise imply that long-term effects of this intervention strategy should always be investigated at an ecosystem scale.

4147: *+.161*

We studied home range size and maximum dispersal distance from hibernacula in Northern Pinesnakes (Pituophis m. melanoleucus) at a 1418-ha preserve in Cumberland County, New Jersey, USA, between 1993 and 2003. We discovered 22 different winter hibernacula that were used by 39 Northern Pinesnakes. Of the 10 snakes monitored in hibernacula for 3-5 yr, shifting was observed by 8 individuals, and 2 females showed hibernacula philopatry for five consecutive years. The average minimum convex polygon home range of 14 radio-tracked Northern Pinesnakes was 105.51 ha (located 30-108 times/snake), whereas the average kernel density estimator home range was 50% isopleth = 38.99 ha and 90% isopleth = 133.15 ha. There were no differences in home range as a function of sex, but the number of years snakes were radio-tracked affected home range size. An adult male had the largest home range of 258 ha. The average distance traveled by radio-tracked Northern Pinesnakes from their winter hibernacula was 1321.05 m, with a maximum distance of 2146.91 m. Of all snakes followed, 27.3% (n = 3) traveled <1000 m, 18.2% (n = 2) traveled 1000-1100 m, 18.2% (n = 2) traveled 1100-1200 m, and 36.4% (n = 4) traveled >1200 m. The average number of hibernacula available per home range was 3.2. Snakes that were monitored for >= 2 yr had larger home ranges than snakes that were only radio-tracked for 1 yr. Thus, radio-tracking several adult snakes over a 3-5-yr period is the most effective method to determine home ranges, locate hibernacula sites of meta-populations, and reveal an understanding of their ecology, behavior, and conservation requirements.

4148: *+.200*

The imperiled Clark's crayfish, Euastacus clarkae Morgan (1997), was described from a handful of juvenile specimens collected from one location in 1981. The Australian Crayfish Project recently completed an intensive field survey project to better define its distribution, habitat, biology and conservation status. Euastacus clarkae is restricted to headwater reaches of highland streams feeding the Hastings and Forbes rivers, at elevations ranging from 670-1150 m. The entire Hastings River catchment (3846 km(2)) was surveyed and established the Extent of Occurrence for E. clarkae at 200 km(2). The distribution was almost entirely located within Werrikimbe National Park where the species was locally abundant. We recommended conservation down listing from Critically Endangered to Endangered and present information to support future conservation efforts and allow specific management plans to be drafted for this rare, highland species. To assist with identification we provide a key to this and other Euastacus found in the Hastings and adjoining drainages.

4149: *+.313*

A total of 37 agave species are extracted from forests of Mexico for producing mescal. This activity has caused decline of numerous populations, and their sustainable management is indispensable for preventing species extinctions. Our study analyzed demographic information about Agave potatorum in the Tehuacan Valley with the goal of developing proposals for

sustainable use for agaves in general. Westudied protected populations in two contrasting environments, and through prospective analyses and real data about extraction and reforestation rates, we simulated different scenarios of actions. Our analyses indicate that the populations' growth rates (lambda) in conserved populations are 0.9903 +/- 0.062 and 1.021 +/- 0.062, but viability analyses suggest that even those unmanaged populations would decrease 30% to 90% in 30 years. Survival and growth of early agave plant stages contribute most to.; adult stages and fecundity have low contribution but their conservation is crucial for population recovery. Based on a successful management experience with A. cupreata, we suggest that at least 30% of reproductive plants should be left to ensure seed provision for natural and assisted populations' recovery. The reintroduction of plants at two early stages of growth is recommended, particularly 1-2-year-old plants, the size categories with the highest contribution to lambda. Current efforts by local people to promote cattle exclusion from forest areas, seed collection, and their propagation in nurseries, and actions for recovery and conservation of populations are strategies of high value. Our research contributes to optimizing the effectiveness of such actions and aids in the conservation of other agave species.

4150: *+.200*

Coevolved species should avoid competition through resource partitioning, but human-induced alteration of plant/animal communities may facilitate the onset of competitive interactions. In herbivores, access to high-quality forage in the warm months, that is, during nursing and weaning, influences growth and survival of offspring. In turn, resource exploitation by a reintroduced, superior competitor should affect offspring survival of the inferior one, by decreasing foraging efficiency and diet quality of mothers and young. We assessed the negative effects of reintroduced red deer Cervus elaphus on grassland, on foraging behavior of female Apennine chamois Rupicapra pyrenaica ornata (July-October 2012-2013) and on winter survival of chamois kids, across 3 study sites with different deer densities (great/intermediate/extremely low). The size of bare soil patches was positively associated with deer density and, in areas with deer, it increased throughout July-October. The volume of nutritious plants (i.e., legumes) in the diet of female chamois was lower and decreased faster between summer and autumn, in areas with deer than in that with an extremely low deer density. Feeding intensity (bite rate) of female chamois was significantly lower and their food searching (step rate) was greater in areas with deer. Chamois kids showed a significantly greater winter mortality, with a lower proportion of younger individuals, in areas with deer than in that with an extremely low deer density. In human-altered ecosystems, unpredictable consequences can follow interspecific interactions within restored animal communities. In turn, patterns of ecological relationships among ecosystem components may be modified, with an increase of the potential for competitive interactions.

4151: *+.066*

Many species listed under the US Endangered Species Act (ESA) face continuing threats and will require intervention to address those threats for decades. These species, which have been termed conservation-reliant, pose a challenge to the ESA's mandate for recovery of self-sustaining populations. Most references to conservation-reliant species by federal agencies involve the restoration of population connectivity. However, the diverse threats to connectivity faced by different species have contrasting implications in the context of the ESA's mandate. For species facing long-term threats from invasive species or climate change, restoration of natural dispersal may not be technically feasible in the foreseeable future. For other species, restoration of natural dispersal is feasible, but carries economic and political cost. Federal agencies have used a broad definition of conservation reliance to justify delisting of species in the latter group even if they

remain dependent on artificial translocation. Distinguishing the two groups better informs policy by distinguishing the technical challenges posed by novel ecological stressors from normative questions such as the price society is willing to pay to protect biodiversity, and the degree to which we should grow accustomed to direct human intervention in species' life cycles as a component of conservation in the Anthropocene Epoch.

4152: *+.072*

Two groups of pygmy marmoset (Cebuella pygmaea) were rescued along the left bank of the Madeira River during the formation of Santo Antonio Hydroelectric Dam reservoir in the state of Rondonia, Northern Brazil. Reintroduction of both groups occurred in areas of open Tropical rainforest located within the project's Permanent Preservation Area. A post-release monitoring was conducted for three months using radio-telemetry. Individuals of each group remained together and settled in stable home ranges near their respective release sites. The mortality rate of translocated animals was about 7%. This seems to be the first report documenting the complete group translocation of C. pygmaea and the first to successfully employ radio-telemetry techniques in monitoring this species. This study demonstrated the feasibility of translocation and the use of radio-telemetry in monitoring C. pygmaea.

4153: *+.100*

This study was conducted in Dez and Karkheh regions in southwestern Iran to model habitat suitability of the Persian fallow deer Dama dama mesopotamicus and assessing trend of habitat changes since 1989. A total of 22 confirmed presence records of the species were collected from 1988 to 2003. MaxEnt approach was used to develop habitat suitability model with regards to nine environmental variables, including density of farmlands, forests, and disturbed forests, density of livestock, vegetation, residential areas and distance to the forest edge, residential areas, protected areas and frequency of surface water bodies. After validation of the model for all parameters used for 1989, relative developed map was extrapolated to the habitat conditions in 2007. Comparing the habitat suitability maps developed for 1989 and 2007 reveals that area of suitable habitats for the Persian fallow deer has declined by 30% within 18 years. A total of 1000 ha of forests (within suitable habitats for the species) has been degraded. About 100 ha of these habitats were converted to farmlands and less than 0.5 ha was altered to the residential areas. In addition, area of about 1000 ha has lost the suitability without any land use changes. Currently, reintroduction of the Persian fallow deer to the area is under consideration by Department of Environment of Iran. Therefore, results of this study are recommended for choosing reintroduction sites based on the predicted suitable maps to enhance the probability of survival for reintroduced individuals.

4154: *-.165*

In New Zealand, wild deer and feral pigs are assumed to be spillover hosts for Mycobacterium bovis, and so are not targeted in efforts aimed at locally eradicating bovine tuberculosis (TB) from possums (Trichosurus vulpecula), the main wildlife host. Here we review the epidemiology of TB in deer and pigs, and assess whether New Zealand's TB management programme could be undermined if these species sometimes achieve maintenance host status. In New Zealand, TB prevalences of up to 47% have been recorded in wild deer sympatric with tuberculous possums. Patterns of lesion distribution, age-specific prevalences and behavioural observations suggest that deer become infected mainly through exposure to dead or moribund possums. TB can progress rapidly in some deer (<10%), but generalised disease is uncommon in wild deer; conversely some infected animals can survive for many years. Deer-to-deer transmission of M. bovis is rare, but

transmission from tuberculous deer carcasses to scavengers, including possums, is likely. That creates a small spillback risk that could persist for a decade after transmission of new infection to wild deer has been halted. Tuberculosis prevalence in New Zealand feral pigs can reach 100%. Infections in lymph nodes of the head and alimentary tract predominate, indicating that TB is mostly acquired through scavenging tuberculous carrion, particularly possums. Infection is usually well contained, and transmission between pigs is rare. Large reductions in local possum density result in gradual declines (over 10 years) in TB prevalence among sympatric wild deer, and faster declines in feral pigs. Elimination of TB from possums (and livestock) therefore results in eventual disappearance of TB from feral pigs and wild deer. However, the risk of spillback infection from deer to possums substantially extends the time needed to locally eradicate TB from all wildlife (compared to that which would be required to eradicate disease from possums alone), while dispersal or translocation of pigs (e.g. by hunters) creates a risk of long-distance spread of disease. The high rate at which pigs acquire M. bovis infection from dead possums makes them useful as sentinels for detecting TB in wildlife. It is unlikely that wild deer and feral pigs act as maintenance hosts anywhere in New Zealand, because unrestricted year-round hunting keeps densities low, with far less aggregation than on New Zealand farms. We conclude that active management of wild deer or feral pigs is not required for local TB eradication in New Zealand.

4155: ***+.138***

Rehabilitation of animals followed by reintroduction into the wild can benefit conservation by supplementing depleted wild populations or reintroducing a species in an area where it has been extirpated or become extinct. The western lowland gorilla (WLG, Gorilla g. gorilla) is persistently poached; infants are often illegally traded and used as pets. Some are confiscated and rehabilitated, then kept in sanctuaries or reintroduced into the wild. Prior to reintroduction, the ability of the orphans to survive independently in their environment needs to be assessed. Here, we performed a multivariate analysis, including diet composition, activity-budget, and pattern of strata using of a group of five juvenile WLG in the process of rehabilitation and distinguished three sub-periods of ecological significance: the high furgivory period, the Dialium fruits consumption period, and the high folivory period. The consequences of these variations on their well-being (play behaviour) and the group cohesion (spatial proximity and social interactions) were examined. Like wild WLGs, diets shifted seasonally from frugivorous to folivorous, while the same staple foods were consumed and large amounts of Dialium fruits were seasonally gathered high in trees. When succulent fruit intake was the highest, thus providing high energy from sugar, juveniles spent less time feeding, more time playing and group cohesion was the highest. Conversely, the cohesion decreased with increasing folivory, individuals spent more time feeding and less time playing together. Nonetheless, the group cohesion also decreased after the death of one highly social, wild-born orphan. This may underscore the importance of skilled individuals in the cohesion and well-being of the entire group and, ultimately, to rehabilitation success. This study evaluates the rehabilitation success with regards to the methods used and highlights the need to consider a set of individual and environmental factors for enhancing rehabilitation while preserving the local biodiversity and individual well-being.

4156: ***+.123***

End of 2013 the EPRC kept in total 174 Primates in 15 species, seven of them currently in no other facility in the world. This includes 174 langurs, 18 gibbons and 19 lorises. During the year 27 individuals were confiscated and arrived at the center, 21 were born, and 14 died. End of 2014 the total number of Primates was nearly stable, and included 129 langurs, 18 gibbons and 26 lorises. In cooperation with the Forest Protection Authorities 23 individuals were confiscated and

arrived the center, 20 individuals were born and 42 died. A number of confiscated individuals arrived at the center in a critical stage of health and subsequently died. With the increasing number of animals at the EPRC, the staff of animal keepers increased from 21 in 2013 to 26 in 2014. The EPRC has already a long tradition to support research and scientific work. More than 20 Masters and PhD studies have been completed at the EPRC or via EPRC-led field projects. Also in 2013 and 2014 research activities continued with an analysis of Primates in the illegal wildlife trade, a study about the effect of parasitological treatment on the Primates and the continuation of monitoring for Delacour's langur populations in several areas. Activities for the reintroduction of Primates continued. The monitoring of released Delacour's langurs in Van Long Nature Reserve continued and a second reintroduction was carried out in 2013. In preparation of a reintroduction project for Hatinh langurs, the EPRC carried out field surveys in several potential areas for reintroduction. Suitable conditions were found in Ke Go Nature Reserve and an agreement for the reintroduction of Hatinh langurs into Ke Go Nature Reserve was discussed and signed between the Management Board of the reserve and EPRC. In September 2014 the EPRC started a reintroduction project for pygmy lorises in Cuc Phuong National Park. On 8th to 11th October 2013 the third international primate conference "Conservation of Primates in Indochina" was organized from the EPRC in Cuc Phuong National Park. About 80 conservationists and primatologists from 12 countries attended the conference. The year 2014 brought special "primate events" for Vietnam: in August the 25th Congress of the International Primatological Society was held in Hanoi, and in connection with this a Pre-Congress Training Program for students at the Endangered Primate Rescue Center, Cuc Phuong National Park.

4157: ***+.016***

Introduction: This paper provides a summary of results from a recent comprehensive review of the conservation status of all Australian land and marine mammal species and subspecies. Since the landmark date of European settlement of Australia (1788), at least 28 of the ca. 272 Australian endemic land mammal species have been rendered extinct. Results and Discussion: Extinctions have occurred at a more or less consistent rate of one to two species per decade since the 1840s, with that rate continuing unabated. A further 55 species from that original fauna are now threatened, and an additional 42 are Near Threatened. Although many factors have contributed to these declines and extinctions, and the array of threats varies amongst individual species, the threat that has had (and is continuing to have) most detrimental impact upon terrestrial mammal species is predation by the introduced cat Felis catus and European red fox Vulpes vulpes. There has been some successful broad-scale management of the fox, but the threat posed by feral cats remains largely unabated. For the 55 species occurring in Australian marine waters, the information base is mostly too meagre to assess conservation status other than as Data Deficient. For the Australian mammal fauna generally, the current conservation management effort is insufficient, with ongoing trends for decline in many species - for example, of 49 species whose conservation status changed over the period 1992-2012, 38 had deteriorating conservation status whereas only 11 had improving status.

4158: ***-.058***

Small populations are prone to loss of genetic variation and hence to a reduction in their evolutionary potential. Therefore, studying the mating system of small populations and its potential effects on genetic drift and genetic diversity is of high importance for their viability assessments. The traditional method for studying genetic mating systems is paternity analysis. Yet, as small populations are often rare and elusive, the genetic data required for paternity analysis are frequently unavailable. The endangered Asiatic wild ass (Equus hemionus), like all equids,

displays a behaviourally polygynous mating system; however, the level of polygyny has never been measured genetically in wild equids. Combining noninvasive genetic data with stochastic modelling of shifts in allele frequencies, we developed an alternative approach to paternity analysis for studying the genetic mating system of the re-introduced Asiatic wild ass in the Negev Desert, Israel. We compared the shifts in allele frequencies (as a measure of genetic drift) that have occurred in the wild ass population since re-introduction onset to simulated scenarios under different proportions of mating males. We revealed a strongly polygynous mating system in which less than 25% of all males participate in the mating process each generation. This strongly polygynous mating system and its potential effect on the re-introduced population's genetic diversity could have significant consequences for the long-term persistence of the population in the Negev. The stochastic modelling approach and the use of allele-frequency shifts can be further applied to systems that are affected by genetic drift and for which genetic data are limited.

4159: *+.019*

In Australia, many species of freshwater fish have rapidly declined following European settlement in the late eighteenth century. The freshwater catfish (Tandanus tandanus) is listed as threatened in Victoria and accordingly, broodstock management and a captive breeding program to facilitate the reintroduction of hatchery bred fish into depleted populations have been suggested. Little work has been conducted on Victorian populations of T. tandanus, despite its threatened status. This study assessed the genetic diversity and genetic structure of T. tandanus in Victoria, using mitochondrial and microsatellite markers. Genetic diversity of T. tandanus in Victoria varied greatly between sites, with the Mallee containing the highest diversity at both markers. Sites where T. tandanus had been introduced in the past typically contained lower measures of genetic diversity, with the exception of the Wimmera site for microsatellite markers. Populations could be assigned to one of four broodfish zones in Victoria, based on levels of genetic differentiation; (1) Mallee, Little Murray, Wimmera, Gum Lagoon and Safe Lagoon; (2) Loddon and Avoca; (3) Turners Lagoon and Phyland Lagoon; and (4) Goulburn. These results will assist managers with implementing broodstock management and a captive breeding program by identifying which sites should be sourced for broodstock and where to reintroduce their progeny, without compromising genetic variation or structure in T. tandanus populations in Victoria.

4160: *+.263*

One of the major challenges facing conservation biology is characterizing the genetic variation underlying adaptation to different environments. Gene expression is the process whereby genomic information is converted into phenotype and quantitative variation in gene expression is linked to phenotypic variation. Identifying gene transcription profiles that provide fitness benefits in specific environments would promote more effective species reintroduction and conservation practices. In this study, we developed a custom oligonucleotide microarray for Atlantic salmon (Salmo salar) and used this microarray to measure gene transcription in gill tissue for two Atlantic salmon strains currently being reintroduced into Lake Ontario: LaHave (anadromous) and Sebago (landlocked). We measured gene transcription in juvenile salmon from each strain that had been reared under the same conditions and identified genes differentially expressed between the two strains. We used the normalized transcription data and microsatellite genotype data to partition the variance into effects of selection versus genetic drift. We found that although there was little genetic differentiation (F (ST) = 0.038) between the two strains, 21 genes were significantly differentially expressed between the two strains, and in all cases the difference was consistent with divergence by selection. We use this analysis to predict the Sebago strain will be more likely to be successfully reintroduced, highlighting how the combination of population genetics with gene

expression can help to guide reintroduction efforts.

4161: ***+.133***

Habitat fragmentation is one of the main threats to biodiversity. Reintroductions or translocations may mitigate its effects by allowing species with limited dispersal ability to exploit otherwise inaccessible habitat patches. Despite the fact that reintroductions are among the most effective conservation measures, they are rarely used for invertebrates. In this study we investigate the potential of reintroductions as a conservation measure for beetles, and present the first genetic results for an endangered veteran tree specialist. After translocation of 10 adults in 1987, a population of the Great Capricorn beetle reappeared in Hluboka nad Vltavou (Czech Republic) in 1990s. Using population genetic analyses of 79 individuals based on nine microsatellite loci and 82 individuals based on the mitochondrial COI gene we assessed the origin of this population, and compared its genetic variation, population structure and demography to the alleged source population (southern Moravia) and to the closest autochthonous population (TA (TM) eboAsko). Although the reintroduced and the closest autochthonous populations are geographically close (24 km), their mutual genetic distance was much higher than that between each of them and the geographically distant (> 150 km) potential source population in southern Moravia. The genetic diversity of the reintroduced population was the lowest from the three studied populations and represented a subset of the alleged source population suggesting its establishment due to a translocation from southern Moravia. Despite the lower genetic variation at the reintroduced site, our results suggest that reintroductions could serve as a highly effective measure in biodiversity conservation and in some cases it may be the only chance to prevent extirpation of many endangered populations.

4162: ***+.153***

As the largest extant cat species in west Asia, the leopard (Panthera pardus) shows high morphological variation, which has led to the description of seven different subspecies in the region. Different investigations have tried to clarify its phylogenetic structure; however, sample size and spatial distribution insufficiently represent the Iranian population, the largest remaining bulk of the Persian leopard (P. p. saxicolor) in the Middle East that probably functioned as a source for the subspecies' range. We examined sequence variation in the mitochondrial NADH-5 gene for 25 leopards from different parts of Iran. Also, we examined 49 adult male skulls to understand the morphological variation of the Iranian leopard population. Our craniometrical results revealed that while no differentiation is seen based on size or shape characteristics from different parts of Iran, larger individuals normally belong to the northern range. Time-calibrated Bayesian phylogenetic analysis suggested that the Iranian female lineage is a monophyletic group that diverged from a group of Asian leopards in the second half of the Pleistocene. Three closely related haplotypes were identified for the entire country: one commonly found haplotype throughout Iran, south Caucasus and Turkmenistan and two localized haplotypes were sequenced from southern Zagros and eastern Alborz. Accordingly, the Persian leopard population in Iran as well as in neighbouring countries can be protected as a large management unit through large-scale conservation planning. Moreover, the available captive stock of the Persian leopard represents an invaluable source for reintroduction for countries interested in restoring their locally extinct population.(c) 2015 The Linnean Society of London, Biological Journal of the Linnean Society, 2015, 114, 721-736.

4163: ***+.051***

Several squirrel species are biological invaders and their establishment in an area is often marked by ecological and economic costs to native species and forest crops. The eastern grey squirrel (Sciurus carolinensis Gmelin 1788) has been intentionally introduced multiple times outside of its native range but its success in establishing and spreading has not been consistent. An intensive live-trapping programme was designed to investigate the demography and population dynamics of populations of this species on the invasion frontier in the Republic of Ireland, a region marked by the slow but steady invasion of the grey squirrel. Low densities and high breeding rates distinguished these frontier populations. These results were placed in context with other frontier and established grey squirrel populations throughout their introduced and native ranges. As expected, variations in invasion speed and impact severity between regions were reflected in population demography. The highest densities, survival rates and breeding rates were recorded in Britain where the grey squirrel invasion has been most damaging. Careful comparative demographic study of invading populations could improve management outcomes, indicate differential invasibility of invaded communities, and offer clues to enhance the design of conservation reintroduction projects.

4164: *+.037*

Maintaining genetic diversity is a crucial goal of intensive management of threatened species, particularly for those populations that act as sources for translocation or re-introduction programmes. Most captive genetic management is based on pedigrees and a neutral theory of inheritance, an assumption that may be violated by selective forces operating in captivity. Here, we explore the conservation consequences of early viability selection: differential offspring survival that occurs prior to management or research observations, such as embryo deaths in utero. If early viability selection produces genotypic deviations from Mendelian predictions, it may undermine management strategies intended to minimize inbreeding and maintain genetic diversity. We use empirical examples to demonstrate that straightforward approaches, such as comparing litter sizes of inbred vs. noninbred breeding pairs, can be used to test whether early viability selection likely impacts estimates of inbreeding depression. We also show that comparing multilocus genotype data to pedigree predictions can reveal whether early viability selection drives systematic biases in genetic diversity, patterns that would not be detected using pedigree-based statistics alone. More sophisticated analysis combining genomewide molecular data with pedigree information will enable conservation scientists to test whether early viability selection drives deviations from neutrality across wide stretches of the genome, revealing whether this form of selection biases the pedigree-based statistics and inference upon which intensive management is based.

4165: *+.186*

The Antillean manatee Trichechus manatus manatus was once widespread from the south-eastern coast of Brazil to Central America and the Caribbean. In Brazil habitat destruction and overhunting severely reduced and fragmented the wild population, restricting extant sub-populations to the north and north-east coast. In response to these threats an ambitious government-led programme was initiated in 1994, with the aim of rehabilitating orphaned manatee calves and releasing them into the southernmost subpopulation. The programme is unique within Brazil, and has invested unprecedented resources in post-release monitoring. So far 30 manatees have been released at three sites, with a high rate of success (> 75%). Time in captivity appears to be a key variable determining post-release success: too long or too short a time in captivity decreasing the probability of survival. We describe the main features of this long-term programme and identify six key lessons learnt: (1) close monitoring, health assessments and rescues can

significantly increase the success of releases, (2) combining different monitoring techniques results in high-quality data and reduces tracking costs, (3) long-term studies are needed to effectively evaluate the results, (4) releasing manatees at c. 5 years of age can increase chances of success, (5) soft-release is important to aid acclimatization, and (6) the programme has been effective in raising awareness among the general public, supporting education and fund-raising.

4166: *+.082*

High post-release survival, low dispersal and the recruitment of captive-reared individuals into the wild population are critical to the success of any reintroduction programme. Reintroducing a migratory species poses an additional challenge as success also depends on the return of captive-reared individuals to breeding grounds in subsequent years. We investigated the effects of seven husbandry and management factors on the return rate of captive-reared eastern loggerhead shrikes Lanius ludovicianus migrans and documented the recruitment of returning individuals. During 2004-2010, 564 juveniles were released in Ontario, Canada, as part of a field propagation and release programme and there were 27 confirmed sightings of returning birds during 2005-2011. Returning birds were significantly more likely to have been released in large groups of juveniles (9-10 birds) at 5.5 weeks post-fledging from the Carden field propagation site. Comparisons of the number of young fledged and survival to 2 weeks post-fledging revealed similar results for pairs comprising one captive-reared and one wild-reared individual and pairs comprising two wild individuals. These results highlight the contribution of captive-reared shrikes to the recovery of the wild population and the importance of monitoring outcomes and evaluating techniques.

4167: *-.054*

During 1997-2012 we conducted a nationwide camera-trapping survey and assessed the availability of prey and habitat for the clouded leopard Neofelis nebulosa in Taiwan. We surveyed 1,249 camera-trap sites over 113,636 camera-trap days, from the seashore to an altitude of 3,796 m and covering various types of vegetation. No clouded leopards were photographed during 128,394 camera-trap days, including at 209 sites in other studies, confirming the presumed extinction of clouded leopards in Taiwan. Assessment of the prey base revealed altitudinal distribution patterns of prey species and prey biomass. Areas at lower altitudes and with less human encroachment and hunting supported a higher prey biomass and more of the typical prey species of clouded leopards. Habitat analysis revealed 8,523 km(2) of suitable habitat but this was reduced to 6,734 km(2) when adjacent areas of human encroachment were subtracted. In the absence of hunting and large mammalian carnivores the major prey of clouded leopards in Taiwan, such as Formosan macaques Macaca cyclopis, Reeves's muntjacs Muntiacus reevesi, Formosan serow Capricornis swinhoei and sambar Rusa unicolor, could become over-abundant. Thus, it is important to address the cascading effect of the disappearance of top-down predator control. Our assessment indicated that, with proper regulation of hunting, habitat restoration and corridor improvement, it may be possible to reintroduce the clouded leopard.

4168: *+.122*

Marble trout (Salmo marmoratus) is an extensively managed salmonid taxon threatened by historical introductions of non-native brown trout (Salmo trutta) of different origins into habitats in the northern part of its distribution, namely the Po river system and the Slovenian part of the Adriatic watershed. Hybridization between marble trout and brown trout results in fertile offspring, creating an extensive zone of genetic mixing, with varying amount of introgression. A rehabilitation programme for marble trout has been undertaken in Slovenia for two decades,

dependent upon eight non-introgressed marble trout populations inhabiting isolated streams in the upper Soca river valley. MtDNA and a new marker system based on nuclear DNA, designed for identification of trouts and their hybrids in the genus Salmo, were used to determine the extent of introgressive hybridization between marble and brown trout across a large area of the Adriatic watershed in Slovenia. Individual admixture coefficients, the posterior probability of each individual belonging to one or other of the parental species and user-defined categories of admixture were determined along with basic population genetic parameters, revealing variable intensity and patterns of individual introgression throughout the zone of hybridization. In most of the populations analysed, hybridization of native marble trout with the introduced Atlantic lineage of brown trout was observed. In the upper sica river system a high proportion of genetically non-introgressed (pure) marble trout was found, enabling selection of these fish for supplementary material in the reintroduction programme. Conclusions regarding management of marble trout in Slovenia can be inferred from the patterns observed in the present study: (1) transfer of pure populations to fishless or fished-out streams is recommended, (2) to sustain genetic diversity of the species, non-introgressed individuals sorted out from the zone of hybridization should be used to produce juveniles for supplementation stocking in fish farms, and (3) extension of the marble trout repopulation programme to highly introgressed populations in small unmanaged streams, which represent a potential source for downstream genetic 'contamination'. (C) 2015 Elsevier Ltd. All rights reserved.

4169: *+.189*

An understanding of genetic diversity within and among populations of rare plant species is a prerequisite to develop effective conservation management strategies and reintroduction programs. Allium munzii is a narrow endemic species distributed in western Riverside County, California, USA and known from 18 extant element occurrences. We sampled 119 individuals from 11 element occurrences and investigated within and among population genetic diversity using two variable chloroplast markers (rpL32-trnL intergenic spacer and rpoC1 intron). Of the total genetic variation detected in A. munzii, 87.65% was due to differences among occurrences. Furthermore, our results revealed that most of the element occurrences are strongly genetically differentiated. There are low levels of gene flow between occurrences, not due to isolation by distance but possibly resulting from habitat fragmentation. Non-significant values of Tajima's D and Fu's Fs were found in all occurrences suggesting no demographic expansion in A. munzii. Ex situ seed and bulb conservation is recommended to enable introduction of individuals to occurrences with low abundance and genetic diversity. (C) 2015 Elsevier Ltd. All rights reserved.

4170: *+.084*

Reintroduction is an effective tool for restoring endangered populations. There is increasing concern, however, that demographic restoration may not equate with genetic restoration. We examine the demographic-genetic contrast in the context of one of the world's most successful carnivore population restorations. Beginning in 1982, a total of 835 river otters Lontra canadensis were reintroduced to Missouri, USA, more than 50 years after extirpation. Most otters were translocated from Louisiana, USA, and released at 43 sites across the state. An estimated population of 11000-18000 otters existed by 2000, and density estimates for Missouri otters are now similar to those reported for populations across the continent, indicating demographic recovery. We used microsatellite genotyping and mitochondrial sequence analysis of DNA extracted from fecal samples from eight southern Missouri rivers, in conjunction with mitochondrial DNA (mtDNA) analyses from several native Louisiana otter populations, to evaluate the genetic diversity and population structure of otters within Missouri as compared with

Louisiana. The Missouri population showed moderate to high heterozygosity and allelic diversity, similar to that of the source populations, but low mtDNA haplotype diversity. We detected five distinct genetic clusters distributed throughout the eight rivers, with no evidence of isolation by distance. These data collectively suggest that 30 years after restoration efforts commenced, Missouri river otters have retained genetic diversity levels similar to those of the source populations, but that genetic structure has not reached an equilibrium between migration and genetic drift. Thus, the Missouri otter population has made a robust recovery despite retaining the genetic signature of the reintroduction.

4171: *+.125*

Captive breeding to augment wild animal populations is an important tool in the recovery of imperiled species, but low post-release survival and substantial program expenses require methodologies that maximize utility. We evaluated post-release survival of captive-reared Allegheny woodrats (Neotoma magister) introduced into a declining metapopulation in southern Indiana. We hard-released 16 captive-reared woodrats in 2011 and soft-released 14 woodrats in 2012, while monitoring parallel samples of 16 and 17 wild-born individuals in each year, respectively. We obtained Kaplan-Meier estimates of survivorship for both groups and used Cox proportional hazards regression to estimate the association between survival and rearing environment (wild or captive), release strategy, sex, body condition and age at release. We quantified movement patterns and related the results of survival models to behavioral tendencies. Survivorship of wild-born individuals was consistently higher than captive-reared individuals. Patterns of survival were best explained by rearing environment and its interaction with time, although disparities in survival between wild-born and captive-reared animals decreased over time. Higher mortality among captive-reared individuals may have been due to more exploratory behavior, as captive-reared woodrats used more dens and traveled farther among successive relocations than wild-born conspecifics. Soft-released individuals had higher initial survival rates and tended to choose dens similar to those of wild individuals, providing evidence that this strategy eased the transition to a wild environment. We recommend future programs use soft-release techniques but also explore other pre-release preparatory strategies such as anti-predator conditioning and environmental enrichment to increase survival.

4172: *+.203*

Movement behaviors attributed to spawning, dispersal, or altered habitat availability are essential to the ecology of many lotic fishes and, although considerable research has described movements of sport fish, little is known about the movement patterns of nongame species. Streams and rivers, wherein plains topminnow Fundulus sciadicus are prevalent, occur in a nonequilibrium state in which habitat patches are sporadically created and lost due to hydrologic variability. This results in regular extirpation and need for recolonization of many plains fishes, including plains topminnow. Species persistence, therefore, is dependent on tolerances to fluctuating habitat conditions and life-history traits that allow dispersal over large areas. To better understand the regional distribution of plains topminnow, we monitored large-scale dispersal patterns and habitat use of two introduced populations in Nebraska. In 2011 and 2012, plains topminnow were marked using visible implanted elastomer (VIE) marks and released at the center of two 3000-m study reaches. Populations were sampled monthly from April to November in 2011 and April through September in 2012 to describe movement patterns and habitat use. Plains topminnow were highly mobile, consistently associated with select habitat features, and the estimated individual home range exceeded standard sampling reach distances by nearly four times. The movement of plains topminnow occurred at greater rates and to distances further than known for similar species. These

large-scale movements likely help maintain connectivity among populations within stream drainages and facilitate recolonization of regularly extirpated habitat patches. In this context, species movement may be critical to the maintenance and potential recovery of populations of this and other rare lotic fishes.

4173: *+.172*

The Australian lungfish is a unique living representative of an ancient dipnoan lineage, listed as 'vulnerable' to extinction under Australia's Environment Protection and Biodiversity Conservation Act 1999. Historical accounts indicate this species occurred naturally in two adjacent river systems in Australia, the Burnett and Mary. Current day populations in other rivers are thought to have arisen by translocation from these source populations. Early genetic work detected very little variation and so had limited power to answer questions relevant for management including how genetic variation is partitioned within and among sub-populations. In this study, we use newly developed microsatellite markers to examine samples from the Burnett and Mary Rivers, as well as from two populations thought to be of translocated origin, Brisbane and North Pine. We test whether there is significant genetic structure among and within river drainages; assign putatively translocated populations to potential source populations; and estimate effective population sizes. Eleven polymorphic microsatellite loci genotyped in 218 individuals gave an average within-population heterozygosity of 0.39 which is low relative to other threatened taxa and for freshwater fishes in general. Based on F-ST values (average over loci = 0.11) and STRUCTURE analyses, we identify three distinct populations in the natural range, one in the Burnett and two distinct populations in the Mary. These analyses also support the hypothesis that the Mary River is the likely source of translocated populations in the Brisbane and North Pine rivers, which agrees with historical published records of a translocation event giving rise to these populations. We were unable to obtain bounded estimates of effective population size, as we have too few genotype combinations, although point estimates were low, ranging from 29 - 129. We recommend that, in order to preserve any local adaptation in the three distinct populations that they be managed separately.

4174: *-.054*

Translocations are an important conservation tool used to restore at-risk species to their historical range. Unavoidable procedures during translocations, such as habitat disturbance, capture, handling, processing, captivity, transport and release to a novel environment, have the potential to be stressful for most species. In this study, we examined acute and chronic stress (through the measurement of the glucocorticoid corticosterone) in a rare reptile (the tuatara, Sphenodon punctatus). We found that: (i) the acute corticosterone response remains elevated during the initial translocation process but is not amplified by cumulative stressors; and (ii) the long-term dynamics of corticosterone secretion are similar in translocated and source populations. Taken together, our results show that translocated tuatara are generally resistant to cumulative acute stressors and show no hormonal sign of chronic stress. Translocation efforts in tuatara afford the potential to reduce extinction risk and restore natural ecosystems.

4175: *-.191*

To address the ongoing debate over the impact of invasive species on native terrestrial wildlife, we conducted a large-scale experiment to test the hypothesis that invasive Burmese pythons (Python molurus bivittatus) were a cause of the precipitous decline of mammals in Everglades National Park (ENP). Evidence linking pythons to mammal declines has been indirect and there are reasons

to question whether pythons, or any predator, could have caused the precipitous declines seen across a range of mammalian functional groups. Experimentally manipulating marsh rabbits, we found that pythons accounted for 77% of rabbit mortalities within 11 months of their translocation to ENP and that python predation appeared to preclude the persistence of rabbit populations in ENP. On control sites, outside of the park, no rabbits were killed by pythons and 71% of attributable marsh rabbit mortalities were classified as mammal predations. Burmese pythons pose a serious threat to the faunal communities and ecological functioning of the Greater Everglades Ecosystem, which will probably spread as python populations expand their range.

4176: ***-.191***

To address the ongoing debate over the impact of invasive species on native terrestrial wildlife, we conducted a large-scale experiment to test the hypothesis that invasive Burmese pythons (Python molurus bivittatus) were a cause of the precipitous decline of mammals in Everglades National Park (ENP). Evidence linking pythons to mammal declines has been indirect and there are reasons to question whether pythons, or any predator, could have caused the precipitous declines seen across a range of mammalian functional groups. Experimentally manipulating marsh rabbits, we found that pythons accounted for 77% of rabbit mortalities within 11 months of their translocation to ENP and that python predation appeared to preclude the persistence of rabbit populations in ENP. On control sites, outside of the park, no rabbits were killed by pythons and 71% of attributable marsh rabbit mortalities were classified as mammal predations. Burmese pythons pose a serious threat to the faunal communities and ecological functioning of the Greater Everglades Ecosystem, which will probably spread as python populations expand their range.

4177: ***-.065***

Human-wildlife conflict occurs when human requirements encroach on those of wildlife populations, with potential costs to both humans and wild animals. As top predators in most inland waters, crocodilians are involved in human-wildlife conflicts in many countries. Here we present findings of a 5-year survey on human-crocodile conflict on the island of Sri Lanka and relate the results to improving management practices. We aimed to quantify and understand the causes of human-crocodile conflict in Sri Lanka, and propose solutions to mitigate it. Visual encounter surveys were carried out to estimate the population size of Saltwater Crocodiles. We recorded 778 sightings of Saltwater Crocodiles at 262 of 400 locations surveyed, and estimate the total population to comprise more than 2000 non-hatchlings and to have increased at an average rate of 5% p.a. since 1978. We propose four crocodile vigilance zones within the wet zone and one crocodile vigilance zone within the dry zone of the country. Specific threats to Saltwater Crocodiles identified in crocodile vigilance zones were: habitat destruction and loss; illegal killing and harvesting (17 killings out of fear, (similar to)200 incidents of killing for meat and skins, (similar to)800 eggs annually for consumption); unplanned translocations; and, interaction with urbanization (10 incidents of crocodiles being run over by trains/vehicles and electrocution). Additionally, 33 cases of crocodile attacks on humans were recorded [8 fatal, 25 nonfatal (minor to grievous injuries)] and more than 50 incidents of attacks on farm and pet animals.

4178: ***+.178***

Using virtually range-wide sampling for three pond turtle taxa (Emys orbicularis galloitalica, E.o.hellenica, E.trinacris), we analyse gene flow across their southern Italian contact zone. Based on population genetic analyses of 15 highly polymorphic microsatellite loci and a mitochondrial marker, we show that the general genetic pattern matches well with the current taxon delimitation.

Yet, single individuals with conflicting genetic identity suggest translocation of turtles by humans. In addition, we identify in south-western France and the vicinity of Rome populations being heavily impacted by introduced turtles. Cline analyses reveal that the major genetic break between E.o.galloitalica and E.o.hellenica corresponds well with the currently accepted intergradation zone in southern Italy. However, introgression is largely unidirectional from E.o.galloitalica into E.o.hellenica. In the distribution range of the latter subspecies, genetic footprints of E.o.galloitalica are evident along most of the Italian east coast. Our results corroborate that E.o.galloitalica was introduced long ago in Corsica and Sardinia and naturalized there. Gene flow between E.orbicularis and E.trinacris is negligible, with the Strait of Messina matching well with the narrow cline centre between the two species. This contrasts with other Mediterranean freshwater turtle species with extensive transoceanic gene flow. Compared to the two subspecies of E.orbicularis, the Sicilian E.trinacris shows an unexpectedly strong population structuring, a finding alsoof some relevance for conservation. The differences between the two taxon pairs E.orbicularis/E.trinacris and E.o.galloitalica/E.o.hellenica support their current taxonomic classification and make them attractive objects for follow-up studies to elucidate the underlying mechanisms of speciation by comparing their properties.

4179: *+.146*

Habitat networks are often advocated as an effective measure for adaptation to climate change, while intensification of land use is a possible response to threats to food security. We examined the question of whether woodland networks are likely to help promote species range shift, and tried to disentangle the influence of land use change, as mediated by land managers' choices, climate change and dispersal ability. Using Scotland as the study area, we considered species types with different dispersal abilities and, with the help of an Agent-Based Model, constructed four stylised scenarios in with different levels of woodland planting and different land managers' choices. We then modelled range expansion of broadleaved woodland species having increasing dispersal abilities. Woodland networks could help range shift for species with dispersal distance (DD) of more than 2 km, but would be no panacea if rapid range shift were needed to preserve population viability. In particular, land use choices influenced most the movements of species with DD between 2 and 5 km. Therefore for such species potential disequilibrium between climate and distribution can be mitigated by increasing stepping stones thus improving landscape permeability to movement. Species that had DD a parts per thousand currency sign2 km moved very slowly in our simulations, and this is consistent with paleo-ecological estimates. For populations of species with short DD that might need to shift their distribution to remain viable, translocation could be a more effective conservation option than creating woodland networks.

4180: *+.181*

We summarize the results from a long-term gibbon reintroduction project in Phuket, Thailand, and evaluate its benefits to conservation. Between October 2002 and November 2012, eight breeding families of white-handed gibbons (Hylobates lar) were returned to the wild in Khao Phra Thaew non-hunting area (KPT). Wild gibbons were extirpated from Phuket Island by the early 1980s, but the illegal wildlife trade has continued to bring young gibbons from elsewhere to the island's popular tourist areas as pets and photo props. The Gibbon Rehabilitation Project (GRP) has rescued and rehabilitated confiscated and donated captive gibbons since 1992 and aims to repopulate the island's last sizable forest area. Following unsuccessful early attempts at translocation in the 1990s, GRP has now developed specific methods for gibbon reintroduction that have led to the establishment of a small independent, reproducing population of captive-raised and wild-born gibbons on Phuket. Eleven infants have been born wild within the reintroduced

population, including a second generation wild-born gibbon in September 2012. Benefits of the GRP project include restoration of the gibbon population on Phuket, rescue of illegally kept gibbons, public education, training of personnel in gibbon conservation work, and gaining experience which may prove useful in saving more severely threatened species. It is unlikely that gibbon (and other large primate) translocations will make a significant contribution to conservation of the species as a whole, and primate translocation projects should not be judged solely by this criterion. Am. J. Primatol. 77:492-501, 2015. (c) 2015 Wiley Periodicals, Inc.

4181: *+.104*

Norway rats (Rattus norvegicus) were introduced to the Falkland Islands and are detrimental to native passerines. Rat eradication programmes are being used to help protect the avifauna. This study assesses the effectiveness of eradication programmes while using this conservation practice as a natural experiment to explore the ecological resistance, resilience and homeostasis of bird communities. We conducted bird surveys on 230 islands: 85 in the presence of rats, 108 that were historically free of rats and 37 from which rats had been eradicated. Bird detection data were used to build occupancy models for each species and estimate species-area relationships. Count data were used to estimate relative abundance and community structure. Islands with invasive rats had reduced species richness of passerines and a different community structure than islands on which rats were historically absent. Although the species richness of native passerines was remarkably similar on eradicated and historically rat-free islands, community structure on eradicated islands was more similar to that of rat-infested islands than to historically rat-free islands. The results suggest that in the Falkland Islands, species richness of passerines is not resistant to invasive rats, but seems to be resilient following their removal. In contrast, community structure seems to be neither resistant nor resilient. From a conservation perspective, rat eradication programmes in the Falkland Islands appear to be effective at restoring native species richness, but they are not necessarily beneficial for species of conservation concern. For species that do not recolonize, translocations following eradications may be necessary.

4182: *+.218*

One reintroduced population of Iberian ibex was monitored between 2000 and 2007 in the Sierra de Guadarrama National Park (Central Spain) using the distance sampling method. The densities obtained from three samplings show a significant increase between 2000 (6.57 ind./km(2)) and 2007 (33.16 ind./km(2)) despite a range extension. After an initial period of balance, the sex ratio became unbalanced over time in favor of females. The age pyramid also changed after a dramatic population increase. The birth rate oscillated at relatively high values for the species (0.69-0.99 kids/female). The group size reduced significantly over time. Females with kids dispersed from the release area significantly less than males and mixed groups.

4183: *+.091*

Understanding how changes in the sizes of large carnivore populations affect the attitudes of the public is vital in order to mitigate social conflicts over large carnivore management issues. Using data from two Swedish postal surveys in 2004 and 2009, we examined the probable social effects of a continued increase in the Swedish populations of bear and wolf by comparing levels of direct experience of bears and wolves with public attitudes towards these animals. We report an increase in direct experience of bears and wolves, lower levels of acceptance of the existence of these animals, and a lower degree of support for the policy goals of both species in 2009 compared to 2004. We also find that these changes are more prominent in areas with local carnivore

populations than in other areas of Sweden. Our results imply that attitudes towards bears and wolves are likely to become more negative as populations continue to grow. The uneven distributions of the carnivore populations are likely to generate more frequent social conflicts in the future as they could cause an increase in the attitudinal divide between those members of the Swedish public who have had direct experiences of carnivores and those who have not.

4184: *+.103*

Southern Ground-Hornbills Bucorvus leadbeateri are Endangered in South Africa and there is a concerted effort to reverse their population decline. They live in groups year round, with only the alpha pair breeding, raising at most one chick per year. Each group has a home range of 50-100 km(2), but there are few data for their spatial habitat use within this range. Understanding the factors affecting Southern Ground-Hornbill movement patterns is useful to assess habitat management options for the species, to select sites for artificial nests and to identify suitable areas for reintroduction initiatives. We report daily and seasonal patterns of habitat use by four groups in the Associated Private Nature Reserves, north-east South Africa, based on data from GPS-satellite tags. Daily travel distances averaged 7.4 +/- 2.2 km d(-1) but were greater during the breeding season, although birds were constrained to forage close to their nest, and were lower in winter, when birds ranged more widely. Hourly travel distances were affected by time of day, season, temperature and group. Birds travelled farthest per hour in the morning, decreasing in the afternoon in winter. However, in summer hourly travel distances were bimodally distributed, with a minimum during the middle of the day when ambient temperatures exceed 25 degrees C. Acacia-dominated vegetation and riparian habitats were favoured disproportionately during the heat of the day in summer, presumably because they offer more shade than other habitats. Optimal habitat configurations for ground-hornbills include a mosaic of habitat types, including open areas for foraging and dense trees for shade.

4185: *-.085*

Biological invasions are a principal threat to global biodiversity. Omnivores, such as crayfish, are among the most important groups of invaders. Their introduction often results in biodiversity loss, particularly of their native counterparts. Managed relocations of native crayfish from areas under threat from invasive crayfish into isolated 'ark sites' are sometimes suggested as a conservation strategy for native crayfish; however, such relocations may have unintended detrimental consequences for the recipient ecosystem. Despite this, there have been few attempts to quantify the relative impacts of native and invasive crayfish on aquatic ecosystems. To address this deficiency we conducted a meta-analysis on the effects of native and invasive crayfish on nine ecosystem components: decomposition rate, primary productivity, plant biomass, invertebrate density, biomass and diversity, fish biomass and refuge use, and amphibian larval survival. Native and invasive crayfish significantly reduced invertebrate density and biomass, fish biomass and amphibian survival rate and significantly increased decomposition rates. Invasive crayfish also significantly reduced plant biomass and invertebrate diversity and increased primary productivity. These results show that native and invasive crayfish have wide-ranging impacts on aquatic ecosystems that may be exacerbated for invasive species. Subsequent analysis showed that the impacts of invasive crayfish were significantly greater, in comparison to native crayfish, for decomposition and primary productivity but not invertebrate density, biomass and diversity. Overall, our findings reconfirm the ecosystem altering abilities of both native and invasive crayfish, enforcing the need to carefully regulate managed relocations of native species as well as to develop control programs for invasives.

4186: *+.032*

There is global interest in restoring populations of apex predators, both to conserve them and to harness their ecological services. In Australia, reintroduction of dingoes (Canis dingo) has been proposed to help restore degraded rangelands. This proposal is based on theories and the results of studies suggesting that dingoes can suppress populations of prey (especially medium-and large-sized herbivores) and invasive predators such as red foxes (Vulpes vulpes) and feral cats (Felis catus) that prey on threatened native species. However, the idea of dingo reintroduction has met opposition, especially from scientists who query the dingo's positive effects for some species or in some environments. Here, we ask 'what is a feasible experimental design for assessing the role of dingoes in ecological restoration?' We outline and propose a dingo reintroduction experiment-one that draws upon the existing dingo-proof fence-and identify an area suitable for this (Sturt National Park, western New South Wales). Although challenging, this initiative would test whether dingoes can help restore Australia's rangeland biodiversity, and potentially provide proof-of-concept for apex predator reintroductions globally.

4187: *+.084*

The paper presents the first Polish bibliography on mouflon that includes papers published in the years 1945-2014. Forty seven publications show diversified interest in mouflon depending on its population size. Three different periods of research activity and publishing were distinguished. The first one (1945-1960) called the 'opening balance' was the time of assessment of mouflon in Poland after the World War 2. At that time, mouflon status as an alien species was not taken into account. After the first unsuccessful attempts of introduction, 'period of ambivalence' in the research and publishing activity occurred. In years 1961-1990 only nine publications, mainly popular ones, about the biology and occurrence of the species in Poland, and the history of its introduction were published. Along significant increase in number in the early 1990s, there was a significant increase in interest in this species among scientists. Majority of Polish research on mouflon has been published in the 'renaissance' phase (since 1991). Papers deal with genetic testing, parasitology or telemetry, but knowledge about the role of mouflon in the ecosystem is still insufficient. Preliminary analysis of the number of publications and their subject (figs. 1 and 2) clearly shows that despite its 70-year history in Poland, the mouflon is still little-known species. Well-documented history of the reintroductions and their development is accompanied by almost complete lack of knowledge about the ecology. In terms of hunting management, protection or elimination of this species from Poland, there are currently no any reliable information, which could be useful for decision-makers. Mouflon has been a species of small numbers, not causing severe damage to forests and fields, and apparently not influencing negatively other ungulates in the areas of its occurrence. Not being a source of hunting successes or environmental problems it still remains outside the interest of scientists. At first mouflon was promoted, but later on, sometimes after many years, researchers tried to answer basic matters. Currently priority should be given to the research on i) the genetic condition of all local populations and evaluate their stability, ii) the interaction between mouflon and other wild ungulates, iii) its dietary composition in terms of damage to forests and fields, iv) parasites in isolated populations and v) assessment of the future of the species in Poland in the time of significant increase in wolf piopulation.

4188: *+.114*

The evolutionary history of Mexican ichthyofauna has been strongly linked to natural events, and the impact of pre-Hispanic cultures is little known. The live-bearing fish species Allotoca diazi, Allotoca meeki and Allotoca catarinae occur in areas of biological, cultural and economic

importance in central Mexico: Patzcuaro basin, Zirahuen basin, and the Cupatitzio River, respectively. The species are closely related genetically and morphologically, and hypotheses have attempted to explain their systematics and biogeography. Mitochondrial DNA and microsatellite markers were used to investigate the evolutionary history of the complex. The species complex shows minimal genetic differentiation. The separation of A. diazi and A. meeki was dated to 400-7000 years ago, explained by geological and climate events. A bottleneck and reduction of genetic diversity in Allotoca diazi was detected, attributed to recent climate fluctuations and anthropogenic activity. The isolation of A. catarinae occurred similar to 1900 years ago. No geological events are documented in the area during this period, but the date is contemporary with P'urhepecha culture settlements. This founder effect represents the first evidence of fish species translocation by a pre-Hispanic culture of Mexico. The response of the complex to climate fluctuation, geological changes and human activity in the past and the future according to the ecological niches predictions indicates areas of vulnerability and important information for conservation. The new genetic information showed that the Allotoca diazi complex consist of two genetic groups with an incomplete lineage sorting pattern: Patzcuaro and Zirahuen lakes, and an introduced population in the Cupatitzio River.

4189: *+.113*

The evolutionary history of Mexican ichthyofauna has been strongly linked to natural events, and the impact of pre-Hispanic cultures is little known. The live-bearing fish species Allotoca diazi, Allotoca meeki and Allotoca catarinae occur in areas of biological, cultural and economic importance in central Mexico: Patzcuaro basin, Zirahuen basin, and the Cupatitzio River, respectively. The species are closely related genetically and morphologically, and hypotheses have attempted to explain their systematics and biogeography. Mitochondrial DNA and microsatellite markers were used to investigate the evolutionary history of the complex. The species complex shows minimal genetic differentiation. The separation of A. diazi and A. meeki was dated to 400-7000 years ago, explained by geological and climate events. A bottleneck and reduction of genetic diversity in Allotoca diazi was detected, attributed to recent climate fluctuations and anthropogenic activity. The isolation of A. catarinae occurred [approximately] 1900 years ago. No geological events are documented in the area during this period, but the date is contemporary with P'urhepecha culture settlements. This founder effect represents the first evidence of fish species translocation by a pre-Hispanic culture of Mexico. The response of the complex to climate fluctuation, geological changes and human activity in the past and the future according to the ecological niches predictions indicates areas of vulnerability and important information for conservation. The new genetic information showed that the Allotoca diazi complex consist of two genetic groups with an incomplete lineage sorting pattern: Patzcuaro and Zirahuen lakes, and an introduced population in the Cupatitzio River.

4190: *+.208*

A detailed understanding of the population dynamics of many amphibian species is lacking despite concerns about declining amphibian biodiversity and abundance. This paper explores temporal patterns of occupancy and underlying extinction and colonization dynamics in a regionally imperiled amphibian species, the Northern leopard frog (Lithobates pipiens) in Alberta. Our study contributes to elucidating regional occupancy dynamics at northern latitudes, where climate extremes likely have a profound effect on seasonal occupancy. The primary advantage of our study is its wide geographic scale (60,000 km(2)) and the use of repeat visual surveys each spring and summer from 2009-2013. We find that occupancy varied more dramatically between seasons than years, with low spring and higher summer occupancy. Between spring and summer, colonization

was high and extinction low; inversely, colonization was low and extinction high over the winter. The dynamics of extinction and colonization are complex, making conservation management challenging. Our results reveal that Northern leopard frog occupancy was constant over the last five years and thus there is no evidence of decline or recovery within our study area. Changes to equilibrium occupancy are most sensitive to increasing colonization in the spring or declining extinction in the summer. Therefore, conservation and management efforts should target actions that are likely to increase spring colonization; this could be achieved through translocations or improving the quality or access to breeding habitat. Because summer occupancy is already high, it may be difficult to improve further. Nevertheless, summer extinction could be reduced by predator control, increasing water quality or hydroperiod of wetlands, or increasing the quality or quantity of summer habitat.

4191: *+.122*

In contrast to biological invasions, translocations of individuals within a species range are understudied, due to difficulties in systematically detecting them. This results in limited knowledge about the corresponding processes and uncertainties regarding the status of extant populations. European larch, a forest tree whose fragmented native distribution is restricted to the Alps and to other Central European mountains, has been massively planted for at least 300 years. Here we focus on the genetic characterization of translocations having taken place within its native range. Microsatellite variation at 13 nuclear loci and sequence data of two mitochondrial DNA fragments were analyzed on the basis of a comprehensive range-wide population sample. Two complementary methods (GENECLASS and STRUCTURE) were used to infer translocation events based on nuclear data whereas mitochondrial data were used for validation of these inferences. Using GENECLASS, we found translocation events in a majority of populations. Additional cases of translocation and many instances of admixture were identified using STRUCTURE, thanks to the clear-cut ancestral genetic structure detected in this species. In particular, a strong divide between Alpine and Central European populations, also apparent at mitochondrial markers, helped uncover details on translocation events and related processes. Translocations and associated admixture events were found to be heterogeneously distributed across the species range, with a particularly high frequency in Central Europe. Furthermore, translocations frequently involved multiple geographic sources, some of which were over-represented. Our study illustrates the importance of range-wide investigations for tracing translocations back to their origins and for revealing some of their consequences. It provides some first clues for developing suitable conservation and management strategies.

4192: *+.121*

We describe a new population viability tool: Spatial PVA. Spatial PVA is an individual-based spatially-explicit PVA application which employs a novel stochastic dispersal algorithm that models how animals move through habitat patches. It also includes a non-random breeding algorithm that simulates pedigrees and inbreeding depression. The model repeatedly steps through annual cycles of chance environmental, dispersal and demographic events for a specified time period. We provide a case study to demonstrate how one can compare simulated kinship coefficients with sampled genetic data to test model assumptions and inputs. We also provide a translocation example for an Australian rangelands species, the Yellow-footed Rock-wallaby (Petrogale xanthopus xanthopus). (C) 2015 Elsevier Ltd. All rights reserved.

4193: *+.329*

Both natural animal populations and those in captivity are subject to evolutionary forces. Evolutionary changes to captive populations may be an important, but poorly understood, factor that can affect the sustainability of these populations. The importance of maintaining the evolutionary integrity of zoo populations, especially those that are used for conservation efforts including reintroductions, is critical for the conservation of biodiversity. Here, we propose that a greater appreciation for an evolutionary perspective may offer important insights that can enhance the reproductive success and health for the sustainability of captive populations. We provide four examples and associated strategies that highlight this approach, including minimizing domestication (i.e., genetic adaptation to captivity), integrating natural mating systems into captive breeding protocols, minimizing the effects of translocation on variation in photoperiodism, and understanding the interplay of parasites/pathogens and inflammation. There are a myriad of other issues that may be important for captive populations, and we conclude that these may often be species specific. Nonetheless, an evolutionary perspective may mitigate some of the challenges currently facing captive populations that are important from a conservation perspective, including their sustainability.

4194: *+.047*

The Apennine chamois (Rupicapra pyrenaica ornata) is a geographically isolated subspecies of chamois currently living only in limited areas of Central Italy. It is included in the Red List of the International Union for Conservation of Nature (IUCN). To assure long-term conservation of the species, the number of individuals needs to be increased creating a consistent and viable population. Accordingly, relocation for reintroduction programs is being planned. However, this could lead to the introduction of infectious diseases. Thus, knowledge on the health status of the Apennine chamois is important for the management of this species. In the absence of existing data, retrospective investigation is useful. A serological retrospective survey on 119 Apennine chamois, captured for routine marking or reintroduction operations from 1990 to 2008 at the National Park of Abruzzo, Lazio, and Molise (PNALM), was performed to detect antibodies against pestiviruses, bovine parainfluenza 3 virus (PI-3), bluetongue virus (BTV), bovine herpesvirus type 1 (BHV-1), Brucella spp., Chlamydophila spp., Coxiella burnetti, and Leptospira spp. Serums were negative for PI-3, BTV, BHV-1, Brucella spp., Chlamydophila spp., C. burnetti, and Leptospira spp., while three animals in 1992 and five in 2008 were positive for pestivirus antibodies. Although the number of samples per year was often limited, inference to evaluate the infectious status of the overall population in the PNALM was attempted. Better planning of sampling activities and a specific surveillance program for infectious diseases are needed, especially in a small and limited population like the Apennine chamois, in which reintroduction programs are considered strategic for conservation and risk factors, like sharing of grazing with wild and domestic animals, exist.

4195: *+.234*

Although significantly more money is spent on the conservation of tigers than on any other threatened species, today only 3200 to 3600 tigers roam the forests of Asia, occupying only 7% of their historical range. Despite the global significance of and interest in tiger conservation, global approaches to plan tiger recovery are partly impeded by the lack of a consensus on the number of tiger subspecies or management units, because a comprehensive analysis of tiger variation is lacking. We analyzed variation among all nine putative tiger subspecies, using extensive data sets of several traits [morphological (craniodental and pelage), ecological, molecular]. Our analyses revealed little variation and large overlaps in each trait among putative subspecies, and molecular data showed extremely low diversity because of a severe Late Pleistocene population decline. Our results support recognition of only two subspecies: the Sunda tiger, Panthera tigris sondaica, and

the continental tiger, Panthera tigris tigris, which consists of two (northern and southern) management units. Conservation management programs, such as captive breeding, reintroduction initiatives, or trans-boundary projects, rely on a durable, consistent characterization of subspecies as taxonomic units, defined by robust multiple lines of scientific evidence rather than single traits or ad hoc descriptions of one or few specimens. Our multiple-trait data set supports a fundamental rethinking of the conventional tiger taxonomy paradigm, which will have profound implications for the management of in situ and ex situ tiger populations and boost conservation efforts by facilitating a pragmatic approach to tiger conservation management worldwide.

4196: *-.050*

Philotheca sporadica (Rutaceae) is a vulnerable species restricted to a small geographic area, with very few populations protected in reserves. Environmental impact surveys identified some populations that will be impacted by gas pipeline construction. This study aimed to determine the genetic diversity of P. sporadica to advise an offset planting program. P. sporadica was found to have high population genetic diversity but all populations were genetically similar except two isolated genetically depauperate populations. Genetic diversity decreased with decreasing population size and increasing population isolation. Impacted populations were significant for the species viability, being among the largest and most genetically diverse, although a high percentage of populations were reproductively active and not inbred. A congeneric species P. difformis ssp. difformis that grows in the nearby vicinity was shown to be genetically distinct but some evidence of hybridisation was found suggesting that offset populations should not be located near known populations of this species to prevent further hybridisation.

4197: *+.039*

The island of Mauritius has experienced five reptile extinctions since the 1600s. Approximately half of the remaining herpetofauna has been restricted to offshore islets, resulting in small populations at high risk of extinction. Under the combined pressures of invasive species, habitat loss and fragmentation and climate change, translocations are considered a powerful tool in conservation of threatened and endangered species. The Bojer's skink, Gongylomorphus bojerii, on the offshore island on Ilot Vacoas represents the remnant population of the species in the southeast of Mauritius. Given the geographic isolation and its genetic distinctiveness, individuals were translocated to the neighbouring island of Ile aux Fouquets in order to re-establish historical range, minimize extinction risk and maintain genetic variation within the species. Using fifteen microsatellite loci, we assessed the genetic structure of the population on Ilot Vacoas in relation to a northern offshore population (on Round Island) and evaluated the genetic consequences of the translocation. Results revealed that the population on Ilot Vacoas exhibits significantly lower levels of genetic variation and strong differentiation ($F_{(ST)} = 0.16$) compared to the northern population. The inbreeding coefficient was low and no recent bottleneck event was detected from its genetic signature. The translocation on Ile aux Fouquets did not provide evidence of negative genetic effects. The newly established population retained much of the source's genetic material, though the effective population size was found to be relatively small. These findings confirmed the importance of incorporating genetic management and continuous monitoring to detect changes in the long-term survival of translocated populations.

4198: *+.087*

Translocations are an effectual management strategy for the reestablishment and reconnection of endangered populations and species. However, knowledge about the evolution and ecology of the

populations and species of interest are critical so that informed decisions can be made about source populations and reestablishment areas. We employed 614 base pairs of the mitochondrial control region and 15 microsatellite loci to investigate genetic variation, contemporary connectivity, and interspecific hybridization in the two remaining populations of the endangered Columbian white-tailed deer (Odocoileus virginianus leucurus) through comparisons with the closest subspecies, O. v. ochrourus. Our data revealed the dubious taxonomic status of O. v. leucurus, and that O. virginianus in the Pacific Northwest originated from a single historic gene pool. Further the results identified that populations are currently genetically isolated and depauperate, and uncovered historic introgression with O. hemionus columbianus. These results suggest that translocations are a viable approach for reestablishing populations throughout the historic range to increase genetic diversity in the fragmented populations. Despite the taxonomic ambiguity, our study revealed the presence of unique genetic variation within each population which supports ongoing conservation efforts.

4199: ***-.011***

Captive breeding of mammals in zoos is the last hope for many of the best-known endangered species and has succeeded in saving some from certain extinction. However, the number of managed species selected is relatively small and focused on large-bodied, charismatic mammals that are not necessarily under strong threat and not always good candidates for reintroduction into the wild. Two interrelated and more fundamental questions go unanswered: have the major breeding programs succeeded at the basic level of maintaining and expanding populations, and is there room to expand them? I used published counts of births and deaths from 1970 to 2011 to quantify rates of growth of 118 captive-bred mammalian populations. These rates did not vary with body mass, contrary to strong predictions made in the ecological literature. Most of the larger managed mammalian populations expanded consistently and very few programs failed. However, growth rates have declined dramatically. The decline was predicted by changes in the ratio of the number of individuals within programs to the number of mammal populations held in major zoos. Rates decreased as the ratio of individuals in programs to populations increased. In other words, most of the programs that could exist already do exist. It therefore appears that debates over the general need for captive-breeding programs and the best selection of species are moot. Only a concerted effort could create room to manage a substantially larger number of endangered mammals. Los Limites para la Reproduccion en Cautiverio de Mamiferos en Zoologicos AlroyResumen La reproduccion en cautiverio de mamiferos en zoologicos es la ultima esperanza para muchas de las especies en peligro mejor conocidas y ha sido exitosa en el rescate de la extincion segura de algunas especies. Sin embargo, el numero de especies seleccionadas para el manejo es relativamente pequeno y esta enfocado en mamiferos grandes y carismaticos que no estan necesariamente bajo una fuerte amenaza de extincion y que no siempre son buenos candidatos para la reintroduccion a la vida silvestre. Dos preguntas interconectadas y mas fundamentales siguen sin respuesta: Han tenido exito la mayoria de los programas de reproduccion en el nivel basico de mantener y expandir poblaciones y hay espacio para expandirlas? Use conteos publicados de nacimientos y muertes desde 1970 hasta 2011 para cuantificar las tasas de crecimiento de 118 poblaciones de mamiferos reproducidos en cautiverio. Estas tasas no variaron con la masa corporal, contradiciendo las fuertes predicciones hechas en la literatura ecologica. La mayoria de las poblaciones de mamiferos con mayor manejo se expandio consistentemente y muy pocos programas fallaron. Sin embargo, las tasas de crecimiento declinaron dramaticamente. La declinacion fue predicha por los cambios en la proporcion del numero de individuos dentro de los programas al numero de especies de mamiferos dentro de la mayoria de los zoologicos. Las tasas disminuyeron conforme a la proporcion de individuos en los programas a la de individuos en las poblaciones. En otras palabras, la mayoria de los programas que podrian existir ya existen. Por

esto parece que los debates sobre la necesidad general de programas de reproduccion en cautiverio y una mejor seleccion de especies son irrelevantes. Solamente un esfuerzo coordinado podria crear el espacio para manejar un numero sustancialmente mayor de mamiferos en peligro.

4200: *+.133*

The evolutionary clade comprising Nanger, Eudorcas, Gazella, and Antilope, defined by an X;BTA5 translocation, is noteworthy for the many autosomal Robertsonian fusions that have driven the chromosome number variation from 2n = 30 observed in Antilope cervicapra, to the 2n = 58 in present Eudorcas thomsoni and Eudorcas rufifrons. This work reports the phylogenetic relationships within the Antilopini using comprehensive cytogenetic data from A. cervicapra, Gazella leptoceros, Nanger dama ruficollis, and E. thomsoni together with corrected karyotypic data from an additional nine species previously reported in the literature. Fluorescence in situ hybridization using BAC and microdissected cattle painting probes, in conjunction with differential staining techniques, provide the following: (i) a detailed analysis of the E. thomsoni chromosomes, (ii) the identification and fine-scale analysis the BTA3 orthologue in species of Antilopini, and (iii) the location of the pseudoautosomal regions on sex chromosomes of the four species. Our phylogenetic analysis of the chromosomal data supports monophyly of Nanger and Eudorcas and suggests an affiliation between A. cervicapra and some of the Gazella species. This renders Gazella paraphyletic and emphasizes a closer relationship between Antilope and Gazella than what has previously been considered.

4201: *-.148*

Species loss can result in changes in assemblage structure and ecosystem function through ecological cascades. Australian vertebrate assemblages changed significantly following European colonisation, which resulted in the establishment of invasive vertebrates and the loss of native marsupials, many of which consume invertebrates. Conservation focusses on the removal of invasive carnivores and the reintroduction of regionally extinct species to fenced sites, resulting in what could be considered a reconstruction of pre-European vertebrate assemblages. In semi-arid Australian spinifex mallee ecosystems, we asked: (1) what is the effect of reconstructed pre-European vertebrate assemblages on native arachnid assemblages? and (2) what direct or indirect mechanisms (predation, disturbance and/or competition) could plausibly be responsible for these effects? We compared sites with reconstructed vertebrate assemblages with paired control sites. Arachnids were sampled using pitfall trapping and direct searching. Hypotheses regarding mechanisms were tested using scat analysis (predation) and by comparing burrow depth (disturbance) and scorpion mass (competition) between control and reconstructed sites. The dominant dune scorpion, Urodacus yaschenkoi, was less abundant and a wolf spider (Lycosa gibsoni species group) more abundant in reconstructed sites. Differences in spider assemblage composition were marginally non-significant. Scat analysis confirmed native vertebrate predation on scorpions and we found no evidence that competition or disturbance affected scorpions. We, thus, suggest that changes in spider assemblages may have resulted from ecological cascades via decreases in dune scorpions. The loss of omnivorous mammals and other changes associated with the invasion of carnivores may, therefore, have had broad-reaching consequences for native arachnid assemblages in Australian ecosystems.

4202: *+.086*

Reintroduction projects usually attempt to re-establish a self-sustaining population of endangered species within their historical ranges through the release of captive-bred individuals into the wild.

We studied the breeding biology and nesting success of a reintroduced population of Crested Ibis Nipponia nippon in Ningshan County on the south slope of the Qinling Mountains of Shaannxi Province, China. From May 2007 to October 2011, 56 captive-bred individuals, composed of 26 females and 30 males, were released into the area and monitored using radio-telemetry. The average age of individuals at release was 5.2 +/- 2.5 years for females and 6.4 +/- 2.9 years for males. Mean clutch size was 3.14 +/- 1.06 (range 1-5). Mean number of fledglings per active nest was 1.57 +/- 1.03 and mean number of fledglings per successful nest was 2.00 +/- 0.87. Most of the fledglings survived to complete their post-fledging dispersal. Mean annual survival rate was 0.552 +/- 0.064 for all released birds, 0.815 +/- 0.054 for breeders, and 0.515 +/- 0.058 for first year fledglings. The average number of young produced/year/pair was 1.57 +/- 1.03 and the growth rate () of the reintroduced population was 1.2193. We confirmed that starvation and predation by King Rat Snake Elaphe carinata were the main causes of death of released individuals, nestlings and fledglings. This is the first attempt to reintroduce captive-bred Crested Ibis to former natural habitat. The ability of captive-bred ibis to survive and breed successfully bodes well for future releases of this and other endangered species.

4203: ***+.071***

The success of translocation programs aiming to boost the effective population size of a declining species is conditioned by the ability of released individuals to survive and breed effectively while they share the ecological niche of wild individuals. Although ecological niche models are increasingly used as tools for conservation translocations, the realized niche of released and wild populations is seldom compared. In this study, we used data from long-term monitoring of a reinforced population of Houbara Bustards in Morocco. We compared the patterns of use of ecological space in the wild by 446 captive-hatched individuals which settled for breeding with the patterns shown by the wild population from the monitoring of 233 wild-hatched adult breeders. We measured the between-sex similarity of the ecological niches and of the geographical distributions. We found that wild-hatched and captive-hatched individuals shared a large part of the ecological space, suggesting a successful integration of captive-hatched mature individuals. The wild-hatched individuals used a broader range of environmental conditions, driven by females that were, on average, older than captive-hatched females and dispersed toward more arid environments. Although the wild- and captive-hatched males had different geographical distributions, they had statistically equivalent ecological niches. This finding suggests that the released Houbaras succeeded in settling new lekking grounds within a similar ecological niche over a larger spatial extent. Such feedback from post-release monitoring is still rare and appears to be a critical validation step for the subsequent use of ENM in translocation programs to assess the quality of release sites. (C) 2015 Elsevier Ltd. All rights reserved.

4204: ***+.205***

Ecotourism has motivated efforts to reintroduce lions (Panthera leo) to landscapes where they were not previously common. In 2002, four lions were reintroduced into the fenced Tembe Elephant Park, South Africa to improve ecotourism opportunities, but lions potentially compete for habitat with humans and endemic herbivores of conservation concern. We developed a population-level resource selection function to map the relative probability of lion occurrence throughout Tembe Elephant Park to predict the spatial distribution of potential conflicts. In winter, high relative probability of lion occurrence spatially overlapped with Muzi reedbeds/hygrophilous grassland habitat, which is where humans gather natural resources. Comparatively, we found no spatial overlap with sand forest habitat used by endemic herbivores. The results were opposite in summer, with lion occurrence overlapping sand forest habitat and no predicted overlap with Muzi

reedbeds/hygrophilous grassland habitat. During spring and autumn, the highest relative probability of lion occurrence spatially overlapped both Muzi reedbeds/hygrophilous grassland and sand forest habitats. These results show that lions might compete with humans in winter, spring and autumn and with endemic herbivores in all seasons but winter. Despite the success of reserve fencing in limiting human-lion conflicts, we show that communities that reintroduce carnivores continue to balance relative reward, associated with ecotourism, and risks to human safety and species of conservation concern. We discuss the importance of dynamic management practices that ensure temporal segregation between humans and lions within specific habitat types.

4205: *+.065*

Even in heavily impounded river ecosystems, aquatic populations and communities retain limited connectivity via movement through dams. This connectivity has the potential to influence population dynamics but has been infrequently characterized. We used 1995-2008 paddlefish mark-recapture data to perform the following: (i) quantify rates of movement through dams and (ii) examine the influence of dam discharge on fish passing dams. We found that there are substantial one-way (upstream to downstream) population connections maintained via fish passing dams and that dam discharge is a key driver of downstream fish movement. Results of our study suggest that population connections maintained by fish passing dams can play an important role in population dynamics depleting upstream populations and subsidizing downstream populations, particularly in years with high flow events. We suggest that the influence of hydrology on maintaining populations in fragmented ecosystems is an increasingly important consideration for conservation and management of aquatic ecosystems in the face of predicted hydrological changes from climate change. Copyright (c) 2014 John Wiley & Sons, Ltd.

4206: *+.196*

The current distribution of the bighorn sheep in Mexico represents a reduced proportion of its original area. Previous population genetics studies conducted in Mexico have only included data from Tiburon Island in the Gulf of California and few individuals from the continent. The aim of this article was to describe aspects of the population genetics of Mexican bighorn sheep in order to aid in the management and conservation of the species. We analyzed 117 samples from the states of Sonora and Baja California Sur using 91 intersimple sequence repeat loci. Our results indicated that the Mexican samples of bighorn sheep have relatively low levels of genetic diversity (H approximate to 0.26) and low genetic differentiation (theta approximate to 0.07) that may be the result of the recent colonization and origin of the populations in Mexico. The individuals from Southern Baja California are genetically different from the Sonoran sample, but this genetic differentiation is low, perhaps due to the low levels of genetic variation of the Mexican populations. The results obtained in this study are relevant for population management of the bighorn sheep in Mexico in order to design translocation plans and management strategies to maintain genetic diversity and, in consequence, the health and future survival of the populations.

4207: *+.247*

When a great need, the right people, and the right tools come together, history is sometimes made. From the late 1970s through the late 1980s that happened in California. At that time there was a need to capture elk, then deer and pronghorn, then bighorn sheep-the "big game species"- in previously unprecedented numbers. The need focused primarily on translocation to re-establish populations in areas of historic range and to consolidate gains in lands available for wildlife conservation. These efforts also advanced wild ungulate research and management. The tools were

helicopters, dart guns and new drugs, various ways to physically capture wildlife including net guns, and other advances in capture technology. The right people were a small group of California Department Fish and Game employees, contract pilots, graduate students, and a host of other agency personnel, friends and volunteers. The history they made lives on in the mountains, savannahs, deserts, and grasslands of California as a wildlife legacy of more elk, deer, pronghorn and bighorn that, with continued conservation, will pass from generation to generation of future Californians.

4208: *+.096*

Introduced species have contributed to extinction of native vertebrates in many parts of the world. Changes to vertebrate assemblages are also likely to alter microbial communities through coextinction of some taxa and the introduction of others. Many attempts to restore degraded habitats involve removal of exotic vertebrates (livestock and feral animals) and reintroduction of locally extinct species, but the impact of such reintroductions on microbial communities is largely unknown. We used high-throughput DNA sequencing of the fungal internal transcribed spacer I (ITS1) region to examine whether replacing exotic vertebrates with reintroduced native vertebrates led to changes in soil fungal communities at a reserve in arid central Australia. Soil fungal diversity was significantly different between dune and swale (interdune) habitats. Fungal communities also differed significantly between sites with exotic or reintroduced native vertebrates after controlling for the effect of habitat. Several fungal operational taxonomic units (OTUs) found exclusively inside the reserve were present in scats from reintroduced native vertebrates, providing a direct link between the vertebrate assemblage and soil microbial communities. Our results show that changes to vertebrate assemblages through local extinctions and the invasion of exotic species can alter soil fungal communities. If local extinction of one or several species results in the coextinction of microbial taxa, the full complement of ecological interactions may never be restored.

4209: *+.138*

Reintroduction is a common management tool for conserving imperiled species, but many reintroductions have included little or no postrelease assessment of project success. The alligator snapping turtle (Macrochelys temminckii) is a long-lived species that has experienced significant declines throughout its range, although suitable habitat remains. We report the findings of a reintroduction effort that was initiated in 2008 near the northwestern limit of the species' range. Two hundred forty-six M. temminckii were released into the Caney River and its tributary, Pond Creek, from 2008 to 2010. All turtles exhibited measurable growth by their first recapture 1-3 yrs after release, and no decline in body condition was observed, either in comparison to prerelease body condition or to the condition of animals in the same cohorts that remained in captivity. Apparent survival and recapture probabilities increased with age. Apparent survivorship values were higher for turtles released in the main channel of the Caney River, but recapture probabilities were higher in its tributary. Ultimately, survivorship values may have been influenced by low recapture rates and emigration, in addition to mortality.

4210: *+.122*

Nonlethal deterrents against carnivores are important components to protecting livestock and conserving carnivore populations. However, the performance of the visual deterrent called fladry, a historical tool used to defend livestock from carnivores, is often hindered by design flaws that eventually reduce its effectiveness. Our purpose was to identify a fladry design that reduces

coiling (i.e., wrapping of individual flags tight to the rope from which they hang) and maintains free movement of the deterrent in the wind. We created 6 new designs, replicated designs using 2 materials (nylon and marine vinyl), and compared them with the design most commonly used today-where flags were sewn directly onto the line along which they are strung. We conducted the study during January-February 2014 at the U.S. Department of Agriculture, Wildlife Services, Predator Research Facility in Millville, Utah, USA. Fladry made of marine vinyl and attached via 2 of our 6 designs showed the least amount of coiling, were relatively easy to construct, and did not result in significant additional costs. The 2 designs were shower curtain, where the flags are attached via circular links, and knotted, where a knot is tied in the flag below its point of attachment. We suggest users of nylon fladry modify it to one of these designs and advise new users to consider a heavier (e.g., marine vinyl) material. (C) 2015 The Wildlife Society.

4211: *+.037*

Age-specific life-history data are needed to understand animal ecology and inform conservation strategies. We compared telemetry and noninvasive genetic sampling (NGS) as methods for monitoring survival and dispersal of juvenile pygmy rabbits (Brachylagus idahoensis) reintroduced to central Washington, USA. During summer 2012, we released 104 juvenile rabbits, 85 of which were fitted with glue-on radiotransmitters and located 2-4 times/week while transmitters were retained ((x) over bar = 15 days). We tracked and recovered 63 transmitters, while signals were lost from 22. Most rabbits remained near the release site, with 9 dispersing > 1 km, and only 2 moving > 3 km. During winter, we surveyed nearly 9 km 2 around the release site and collected 117 fecal samples for genetic analysis. Forty-two individuals were identified, 38 from the summer releases (37% survival) and 4 born in the wild from parents released in 2011. Using NGS, we identified rabbits 1) released without transmitters, 2) with undetected transmitter signals, 3) presumed dead, and 4) produced in the wild. Short-termdispersal behavior was best gathered with telemetry, but information was limited, and we were unable to estimate survival probabilities because of the short duration of transmitter retention. The information on dispersal, survival, and reproduction provided by NGS allowed us to evaluate longer term reintroduction success, but was limited by the area we were able to search. We compare the results, costs, benefits, and limitations of each method for addressing specific monitoring objectives. (C) 2015 The Wildlife Society.

4212: *+.016*

From 1995 to 1997, black bears (Ursus americanus) involved in conflicts with humans in southeastern Colorado, USA, were radiocollared, translocated, and monitored by the Colorado Division of Wildlife to evaluate translocation as a management tool for problem black bears. Specific objectives were to 1) determine postrelease movement patterns of relocated black bears, and 2) estimate cumulative incidence and survival functions. Subadults did not move as far after translocation as adults and less frequently oriented toward the capture site (29% of subad vs. 51% of ad). No subadults returned to the vicinity of capture, whereas 33% of adults did. We used a cause-specific hazards model with a constant age effect across the cause-specific hazards to estimate annual survival rate for translocated adult bears (0.50, 95% credible interval CI = 0.36-0.65) and for subadult bears (0.28, 95% CI = 0.12-0.48). The annual probability of dying due to repeat conflict behavior was slightly lower (0.22 [95% CI = 0.13-0.33] and 0.32 [95% CI = 0.19-0.47]) for adults and subadults, respectively, compared with nonconflict mortalities (0.28 [95% CI = 0.17-0.40] and 0.40 [95% CI = 0.25-0.56]). Based on bears that were not involved in known repeat human-bear conflicts, translocation success was 0.64 (95% CI = 0.49-0.78) and 0.58 (95% CI = 0.42-0.73) for adults and subadults, respectively. Translocation of problem bears had mixed

success relative to repeat nuisance activity in Colorado, but should remain a viable management option. Managers should make decisions on the appropriateness of translocation based on the characteristics of the bear, identification of an adequate release site, potential effect of the translocation on the release-site bear population, and other available options. (C) 2015 The Wildlife Society.

4213: *+.035*

Livestock predation by large carnivores and their persecution by local communities are major conservation concerns. In order to prevent speculations and reduce conflicts, it is crucial to get detailed and accurate data on predators' dietary ecology, which is particularly important in human dominated landscapes where livestock densities are high. This is the case of the endangered Iberian wolf in Portugal, an endemic subspecies of the Iberian Peninsula, which has seen its population distribution and abundance decline throughout the 20th century. Accordingly, the diet of the Iberian wolf was analyzed, using scat analysis, in a humanized landscape in central Portugal. From 2011 to 2014, a total of 295 wolf scats were collected from transects distributed throughout the study area, prospected on a monthly basis. Scat analysis indicated a high dependence of Iberian wolf on livestock. Domestic goat predominated the diet (62% of the scats), followed by cow (20%) and sheep (13%); the only wild ungulate present in the scat analysis was the wild boar (4% of the scats). Our results show that even though livestock constitute most part of wolves diet, different livestock species may represent different predation opportunities. We conclude that the high levels of livestock consumption may be a result of low diversity and density of wild ungulates that settles livestock as the only abundant prey for wolves. Our findings help on the understanding of the Iberian wolf feeding ecology and have implications for conflict management strategies. Finally, management implications are discussed and solutions are recommended.

4214: *-.005*

The newly discovered endemic maple Acer yangbiense of China has only five individuals left in the wild, and thus has been classified as a plant species with extremely small populations (PSESP). PSESP species call for emergency protection procedures, such as ex-situ conservation and reintroduction. Our objectives were to examine the genetic diversity of A. yangbiense and to evaluate former conservation strategies from a genetic point of view. Our results suggested that A. yangbiense was not genetically depauperate, but its genetic loss at a species level was obvious. A parentage analysis indicated a high selfingrate in A. yangbiense and suggested the existence of a previously unknown wild individual. Former conservation strategies did not include all genetic variations of the wild population, and gene diversity of the ex-situ conserved seedlings is lower than that of the wild population. From our findings, we make suggestions to guide the subsequent protection of this species. (C) 2015 Elsevier Ltd. All rights reserved.

4216: *+.136*

The geographically referenced data on aboriginal and reacclimatized local populations of the steepe woodchuck, Mannota bobak, in Mordovia are presented. The number and density of the populations are estimated. The rates and priority directions of the Marmota bobak colonization are determined. In 28 years after the reacclimatization of steepe woodchucks in this region, they are spread, on the average, over 19.5 km from the release sites; the mean rate of their dispersal is 0.7 km per year. The size of family areas of these animals varies from 0.4 to 11.5 ha; the mean value is 2.3 ha per a family. The following factors determine the dynamics of developing the local

populations: optimal environmental conditions, the time of existence of reacclimatized colonies, and poaching.

4217: ***+.038***

Dysosma pleiantha is an endangered and endemic species in China. We have reported the flowering phenology, breeding system and pollinator activity of the species distributed in Tianmu Mountain (Zhejiang Province) nature reserves. Flowering occurred during the months of early April to late May, with the peak in the middle of the April, and was synchronous across all four subpopulations. The anthesis of an intact inflorescence lasted from sixteen to twenty-three days with eight to eleven days blossom of an individual flower. In D. pleiantha, the morphological development of flowers and fruit leading to the development of mature seeds takes place over a period 3-5 months from flowering. The average of pollen-ovule ratio (P/O) was 18 898.7. The pollen transfer in this species was mainly performed by flies, Hydrotaea chalcogaster (Muscidae). Controlled pollination experiments indicated D. pleiantha was obligate xenogamyous and self-incompatible, and pollination was pollinator-dependent. Controlled pollination experiments showed that the mean fruit set (%) under the natural condition (17.1%) was markedly lower than that of manual cross-pollination (75.6%). It was concluded that pollen-limitation and mate limitation were responsible for the low fruit set of D. pleiantha in the field. Thus, the identification and translocation of compatible mating types to create reproductively viable populations were essential for the recovery of the rare species.

4218: ***-.118***

Head-starting is an integral component of many chelonian conservation initiatives. However, the release of captive-raised individuals into wild populations carries the inherent risk of transmitting disease-causing microorganisms between the two populations, possibly with devastating consequences. Therefore, an essential component of any head-starting initiative is a preventive medicine program to identify and mitigate risks associated with disease transmission, and should include quarantine of captive animals as well as thorough health assessments of both captive and wild populations. Despite such efforts it is impossible to eliminate the risk of disease transmission, so prior to initiating head-starting projects a thorough risk assessment should be performed to ensure that the benefits of the project outweigh this risk.

4219: ***+.221***

Few data are available on the head-starting of the European Pond Turtle (Emys orbicularis) and some seem to indicate unique features during first year of development. A slow growth rate might have important consequences on the usefulness of head-starting in this species. We head-started 12 E. orbicularis galloitalica hatchlings for eight months. We individually marked hatchlings and kept them in an aquarium equipped with UV-b light tubes. Water temperature was kept at 24 degrees C and we provided them a basking site at 30 degrees C. We first fed hatchlings small fresh items, then shifted to larger prey. We measured and weighed hatchlings weekly and released them as a part of a reintroduction project. The survival rate of released individuals was 83% after one year. Released turtles started breeding when they were six years old, and attained an adult body size similar to turtles from natural populations very quickly. Our results suggest that E. orbicularis head-starting can be successful.

4220: ***+.075***

Viability models of turtle populations have shown that after adult survivorship, juvenile survivorship is the most influential parameter affecting population persistence. This suggests that increasing juvenile survivorship, such as through head-starting, might be a useful management strategy. Little is known about survivorship and ecology of juveniles of most turtle species, including even well-studied species such as the Gopher Tortoise (Gopherus polyphemus). Limited data on the fate of headstarted tortoises further constrains attempts to evaluate head-starting as a management tool. We summarize our experiences head-starting Gopher Tortoise hatchlings as part of reintroduction efforts at Savannah River Site (SRS), South Carolina, USA, and St. Catherines Island (SCI), Georgia, USA, and compare survivorship of head-started hatchlings with juveniles manipulated using other techniques. Hatchlings exhibited nearly 100% survivorship during the captive head-start period, but survivorship during the first year post-release varied among cohorts: 17 of 32 (53.1%) 2001 SRS hatchlings, seven of seven (100%) 2005 SCI hatchlings, and one of 32 (3.1%) 2006 SCI hatchlings. For two cohorts, head-started hatchlings performed as well as older non-head-started juvenile tortoises. At least 20.0% of St. Catherines Island neonates that we released into temporary predator-proof cages shortly after hatching (i.e., without head-starting) were known to have survived through their first winter dormancy. Survivorship for all manipulated hatchlings (regardless of treatment) was lowest during the first year post-release. The potential role of head-starting as a management tool merits further investigation. We recommend that future studies include an experimental component to allow critical evaluation of the techniques implemented.

4221: *+.243*

We reintroduced Blanding's Turtles (Emydoidea blandingii) to Assabet River National Wildlife Refuge, Massachusetts, USA, evaluating the relative benefits and risks of using various life stages of Blanding's Turtles collected from a donor population within the same watershed, including direct-release hatchlings (released in autumn shortly after hatching), head-started hatchlings (raised in captivity for 9 mo), juveniles, and adults. We developed a simple population model to evaluate which of several release strategies was most likely to result in a stable population at the recipient site while minimizing negative impacts to the donor site. Model results suggested that annual releases consisting largely of head-started hatchlings were most likely to achieve our goal. We released 81 direct-release and 161 head-started hatchlings at the refuge in 2007-2011. Head-started hatchlings were larger (mean = 62.7 mm carapace length, 46.6 g) compared to direct-release hatchlings (mean = 36.3 mm carapace length, 8.8 g). Simultaneous radio-tracking of 12 translocated sub-adults has provided useful information on habitat preferences that we used to select two sites within the refuge for future releases. We also released six head-started hatchlings with radio transmitters (one in 2009 and five in 2010): one was found dead a year after release. We plan to continue monitoring efforts to assess survivorship, growth, and site fidelity of all released Blanding's Turtles and to compare results among the head-started and direct-release hatchlings. We will update our models and reintroduction efforts based on monitoring data.

4222: *-.044*

Kemp's Ridley (Lepidochelys kempii) is the most endangered of the sea turtles. Most nesting is on the Gulf of Mexico coastline from Texas, USA, through Veracruz, Mexico, with greatest numbers near Playa de Rancho Nuevo (RN), Tamaulipas, Mexico. The Mexican government began protecting nesters, eggs, and hatchlings at RN in 1966, but annual numbers of nests continued to decline. In January 1978, the U.S. National Park Service (NPS), Fish and Wildlife Service (FWS), and National Marine Fisheries Service (NMFS), the Texas Parks and Wildlife Department (TPWD), and the Instituto Nacional de Pesca (INP) of Mexico implemented a bi-national Kemp's

Ridley restoration and enhancement program (KRREP) for the NPS Padre Island National Seashore (PAIS) near Corpus Christi, Texas, and RN. Its planned goals were to reintroduce Kemp's Ridley to PAIS, which included head-starting, and to enhance protection of Kemp's Ridley nesters, eggs, and hatchlings at RN. This paper summarizes collecting, transporting, and incubating eggs, attempted imprinting of eggs and hatchlings, transporting hatchlings, tracking nesters, and documenting nestings in the wild. Through 2014, 20 Padre Island imprinted head-started turtles (n = 69 nests) and 39 RN imprinted head-started turtles (n = 64 nests) were recorded nesting in Texas (n = 125 nests) and near RN (n = 8 nests).

4223: ***+.071***

Kemp's Ridley (Lepidochelys kempii) is the most endangered sea turtle and is found in the Gulf of Mexico and North Atlantic Ocean. It nests in greatest numbers near Playa de Rancho Nuevo (RN), Tamaulipas, Mexico. Historically, nesting also occurred on beaches that, in 1962, became part of Padre Island National Seashore (PAIS) near Corpus Christi, Texas, USA. Kemp's Ridley was headed toward extinction when the Mexican government began protecting clutches of eggs (i.e., nests) and hatchlings at RN in 1966, but the population continued to decline. In 1974, the U.S. National Park Service (NPS) proposed reintroduction of Kemp's Ridley to PAIS. Further planning by NPS, U.S. Fish and Wildlife Service (FWS), National Marine Fisheries Service (NMFS), Texas Parks and Wildlife Department (TPWD), and Mexico's Instituto Nacional de Pesca (INP) ensued in 1977 and led to the bi-national Kemp's Ridley Restoration and Enhancement Program (KRREP) implemented in January 1978. Its goals were restoration of Kemp's Ridley through enhancement of nesting success and survival at RN, and reestablishment of a breeding population at PAIS. At the time, head-start (i.e., captive-rearing to sizes thought capable of avoiding most natural predators at sea) was considered essential to the second goal. Tagging and marking were necessary for identification after release, and mass-tagging hatchlings was not feasible. The NMFS Galveston Laboratory, Galveston, Texas, and collaborators head-started, tagged, and released the turtles into the Gulf of Mexico. NPS and collaborators documented nestings. We review head-start and its relationships to the KRREP and reintroduction of Kemp's Ridley to PAIS.

4224: ***+.157***

Both injured and uninjured box turtles (Terrapene spp.) are admitted to wildlife rehabilitation centers where they are treated and/or released. However, nothing is known of their movements, activity, and site fidelity following release. Studies of other reptiles suggest site fidelity and survival following release may be poor. We translocated 17 adult, two juvenile, and 20 hatchling Ornate Box Turtles (Terrapene ornata ornata) from a wildlife rehabilitation center in Lubbock, Texas to four sites varying in degree of urbanization. Forty percent of hatchlings remained at the original release sites, but only 24% of adults (all females) did so. Adults and hatchlings displayed roughly similar bimodal activity patterns related to time of day, and activity related to median ambient temperature range during the study period. Hatchlings were significantly more active than adults over a wider range of relative humidity and at higher relative humidity, however. Translocated hatchling home range size did not differ significantly between urban and natural release sites. Translocation of hatchling turtles may be a viable conservation strategy, though mortality of these age cohorts (25%) should be considered when planning translocations. Our data suggest translocations of adults are not likely to be successful in most cases.

4225: ***-.027***

The scarlet macaw (Ara macao cyanoptera) is an endangered species in Mesoamerica due to

illegal traffic, habitat loss, and hunting. In Mexico, its range has been reduced by 98%. Between April 2013 and June 2014, a population of 96 individuals of A. m. cyanoptera was reintroduced (six releasing events), in the tropical rainforests of Palenque, southeast Mexico, where this macaw had been extinct for the last 70 years. This study documents the use of wild foods and range use by the reintroduced macaws for the rainy season period June to November, 2014. The macaws used 140 trees of 31 species (19 families; 84% native species) as a source of food. Seeds and fruit accounted for 70% of their diet. The remaining 30% consisted of bark, stems, leaves, insect galls, flowers and shoots. A subset of five tree species was highly dominant in their diet (regarding number of trees used, months used and feeding records). Spatial data showed that food trees used by the macaws were dispersed over 36 ha and had a highly clumped distribution. The macaws used an additional 23ha for non-feeding activities. The dietary diversity and breadth (as indicated by Levin's Index) of the reintroduced macaws closely approaches that of wild macaws. The capacity of the reintroduced macaws to use wild foods, a very low mortality in the released population (9%), and the occurrence of nine successful nesting events, attests to a short-term success of the reintroduction. We discussed the observed patterns of use of wild foods and habitat by the reintroduced scarlet macaws in the context of the soft-release protocol used and of behavioral flexibility, accumulated social learning and a high cognitive capacity typical of psittacines, aspects essential for a successful adaptation to the wild.

4226: *+.045*

Knowledge of genetic structure and patterns of connectivity is valuable for implementation of effective conservation management. The arid zone of Australia contains a rich biodiversity, however this has come under threat due to activities such as altered fire regimes, grazing and the introduction of feral herbivores and predators. Suitable habitats for many species can be separated by vast distances, and despite an apparent lack of current geographical barriers to dispersal, habitat specialisation, which is exhibited by many desert species, may limit connectivity throughout this expansive region. We characterised the genetic structure and differentiation of the great desert skink (Liopholis kintorei), which has a patchy, but widespread distribution in the western region of the Australian arid zone. As a species of cultural importance to local Aboriginal groups and nationally listed as Vulnerable, it is a conservation priority for numerous land managers in central Australia. Analysis of mitochondrial ND4 sequence data and ten nuclear microsatellite loci across six sampling localities through the distribution of L. kintorei revealed considerable differentiation among sites, with mitochondrial F-ST and microsatellite F'(ST) ranging from 0.047-0.938 and 0.257-0.440, respectively. The extent of differentiation suggests three main regions that should be managed separately, in particular the southeastern locality of Uluru. Current genetic delineation of these regions should be maintained if future intervention such as translocation or captive breeding is to be undertaken.

4227: *+.227*

Wildlife reintroductions and translocations are statistically unlikely to succeed. Nevertheless, they remain a critical part of conservation because they are the only way to actively restore a species into a habitat from which it has been extirpated. Past efforts to improve these practices have attributed the low success rate to failures in the biological knowledge (e.g., ignorance of social behavior, poor release site selection), or to the inherent challenges of reinstating a species into an area where threats have already driven it to local extinction. Such research presumes that the only way to improve reintroduction outcomes is through improved biological knowledge. This emphasis on biological solutions may have caused researchers to overlook the potential influence of other factors on reintroduction outcomes. I employed a grounded theory approach to study the

leadership and management of a successful reintroduction program (the Sea Eagle Recovery Project in Scotland, UK) and identify four critical managerial elements that I theorize may have contributed to the successful outcome of this 50-year reintroduction. These elements are: 1. Leadership & Management: Small, dedicated team of accessible experts who provide strong political and scientific advocacy ("champions") for the project. 2. Hierarchy & Autonomy: Hierarchical management structure that nevertheless permits high individual autonomy. 3. Goals & Evaluation: Formalized goal-setting and regular, critical evaluation of the project's progress toward those goals. 4. Adaptive Public Relations: Adaptive outreach campaigns that are open, transparent, inclusive (esp. linguistically), and culturally relevant.

4228: *+.168*

For the endangered crested Ibis (Nipponia nippon), a great deal of conservation efforts both in situ and ex situ, has been successfully undertaken in China, which make it possible for further expanding of distribution. On 3 June, 2013, a breeding pair (H12 female and B747 male) with 3 nestlings has been found in Fengliu village (32 degrees 58'N, 108 degrees 31'E, alt. 526 m), Hanyin County, Shaanxi Province. The breeding pair continued to breed and fledged four nestlings successfully in the breeding season of 2014 (Fig. 1). On 12 January, 2014, we found a wintering flock which composed of the breeding pair (H12 female and B747 male), and their 3 offsprings, one individual (J41) from the wild population of Yangxian County, and two unidentified individuals. Our observation showed that gene exchange occurred between the reintroduced and the wild population since the pair formation of the individuals from the two populations in 2011. The reintroduction program carried out in Ningshan County has been facilitated the dispersal of the wild population (source population) to eastern areas of Qinling Mountains and might be in favor of the establishment of the reintroduced population (satellite population).

4229: *+.018*

Due to a restriction of the distributional range of European red deer (Cervus elaphus L.) during the Quaternary and subsequent recolonization of Europe from different refugia, a clear phylogeographical pattern in genetic structure has been revealed using mitochondrial DNA markers. In Central Europe, 2 distinct, eastern and western, lineages of European red deer are present; however, admixture between them has not yet been studied in detail. We used mitochondrial DNA (control region and cytochrome b gene) sequences and 22 microsatellite loci from 522 individuals to investigate the genetic diversity of red deer in what might be expected to be an intermediate zone. We discovered a high number of unique mtDNA haplotypes belonging to each lineage and high levels of genetic diversity (cyt b H = 0.867, D-loop H = 0.914). The same structuring of red deer populations was also revealed by microsatellite analysis, with results from both analyses thus suggesting a suture zone between the 2 lineages. Despite the fact that postglacial recolonization of Central Europe by red deer occurred more than 10 000 years ago, the degree of admixture between the 2 lineages is relatively small, with only 10.8% admixed individuals detected. Direct translocations of animals by humans have slightly blurred the pattern in this region; however, this blurring was more apparent when using maternally inherited markers than nuclear markers.

4230: *+.178*

The reintroduction of beaver (Castor canadensis) into arid and semi-arid rivers is receiving increasing management and conservation attention in recent years, yet very little is known about native versus non-native fish occupancy in beaver pond habitats. Streams of the American

Southwest support a highly endemic, highly endangered native fish fauna and abundant non-native fishes, and here we investigated the hypothesis that beaver ponds in this region may lead to fish assemblages dominated by non-native species that favour slower-water habitat. We sampled fish assemblages within beaver ponds and within unimpounded lotic stream reaches in the mainstem and in tributaries of the free-flowing upper Verde River, Arizona, USA. Non-native fishes consistently outnumbered native species, and this dominance was greater in pond than in lotic assemblages. Few native species were recorded within ponds. Multivariate analysis indicated that fish assemblages in beaver ponds were distinct from those in lotic reaches, in both mainstem and tributary locations. Individual species driving this distinction included abundant non-native green sunfish (Lepomis cyanellus) and western mosquitofish (Gambusia affinis) in pond sites, and native desert sucker (Catostomus clarkii) in lotic sites. Overall, this study provides the first evidence that, relative to unimpounded lotic habitat, beaver ponds in arid and semi-arid rivers support abundant non-native fishes; these ponds could thus serve as important non-native source areas and negatively impact co-occurring native fish populations.

4231: *+.137*

When protected carnivores harm people's livelihoods, conservationists often seek non-lethal mitigation strategies. Large carnivore translocation is one such strategy but it has shown limited success. Many reported examples used methods that likely contributed to their failure. We conducted six leopard (Panthera pardus) translocations (three males, three females) within Namibia to test specific criteria for improved protocols. We moved leopards 402.7 km (SD = 279.6 km, range 47-754 km). Overall translocation success, using strict criteria, was 67 % and increased to 83 % when post-release conflict was not considered in this assessment. Four individuals successfully established new territories after exploratory periods of < 2 months. One female died in a road accident shortly after release and a male resumed killing livestock that were illegally herded within a protected area. Both surviving females produced cubs-the ultimate sign of success. When compared with resident leopards (six males, six females), translocated individuals showed no significant difference in range behaviour, survivorship or likelihood of conflict. At their capture sites, livestock depredation ceased for a minimum of 16 months, thus at least temporarily alleviating conflict. We used our successful protocol to develop a translocation suitability model for determining appropriate release sites. For Namibia, this model predicts potential recipient habitat of 117,613 km(2), an area sufficient to support up to 87 leopard translocations. Where alternative conservation strategies have failed and managers decide to proceed with translocations, we recommend the application of our conservative protocol to identify the most suitable recipient locations. Our study demonstrates the potential value of translocation under specific circumstances and as part of a larger conflict management repertoire. Our findings are useful for management of other large carnivores and conflict wildlife.

4232: *+.035*

To date, leopards (Panthera pardus) in Peninsular Malaysia have been overlooked by large carnivore researchers. This is in part due to the country's unique population of individuals that are almost all melanistic, which makes it nearly impossible to identify individuals using camera traps for estimating leopard density. We discovered a novel modification to infrared flash camera traps, which forces the camera into night mode, that allows us to consistently and clearly see the spots of a melanistic leopard. The aim of this project was 1) to determine the feasibility of identifying melanistic leopards with confidence using infrared flash camera traps, and 2) to establish a density estimate for the leopard population in a wildlife corridor in Malaysia using maximum likelihood and Bayesian spatially explicit capture-recapture (SECR) models. Both SECR approaches yielded

a leopard density of approximately 3 individuals/100km(2). Our estimates represent the first density estimate of leopards in Malaysia and arguably, the world's first successful attempt to estimate the population size of a species with melanistic phenotypes. Because we have demonstrated that melanistic leopards can be monitored with confidence using infrared cameras, future studies should employ our approach instead of relying on scars or body shape for identification. Ultimately, our approach can facilitate more accurate assessments of leopard population trends, particularly in regions where melanistic phenotypes largely occur. (c) 2015 The Wildlife Society.

4233: ***+.213***

Animal reintroductions are important tools of wildlife management to restore species to their historical range, and they can also create unique opportunities to study population dynamics and genetics from founder events. We used non-invasive hair sampling in a systematic, closed-population capture-mark-recapture (CMR) study design at the Big South Fork (BSF) area in Kentucky during 2010 and Tennessee during 2012 to estimate the demographic and genetic characteristics of the black bear (Ursus americanus) population that resulted from a reintroduced founding population of 18 bears in 1998. We estimated 38 (95% CI: 31-66) and 190 (95% CI: 170-219) bears on the Kentucky and Tennessee study areas, respectively. Based on the Tennessee abundance estimate alone, the mean annual growth rate was 18.3% (95% CI: 17.4-19.5%) from 1998 to 2012. We also compared the genetic characteristics of bears sampled during 2010-2012 to bears in the population during 2000-2002, 2-4 years following reintroduction, and to the source population. We found that the level of genetic diversity since reintroduction as indicated by expected heterozygosity (H-E) remained relatively constant (H-E(source,H- 2004)=0.763, H-E(BSF,H- 2000-2002)=0.729, H-E(BSF,H- 2010-2012)=0.712) and the effective number of breeders (N-B) remained low but had increased since reintroduction in the absence of sufficient immigration (N-B(BSF,N- 2000-2002)=12, N-B(BSF,N- 2010-2012)=35). This bear population appears to be genetically isolated, but contrary to our expectations, we did not find evidence of genetic diversity loss or other deleterious genetic effects typically observed from small founder groups. We attribute that to high initial genetic diversity in the founder group combined with overlapping generations and rapid population growth. Although the population remains relatively small, the reintroduction using a small founder group appears to be demographically and genetically sustainable. (c) 2015 The Wildlife Society.

4234: ***-.272***

Macacine herpesvirus 1 (MaHV1; B virus) naturally infects macaques (Macaca spp.) and can cause fatal encephalitis in humans. In Peninsular Malaysia, wild macaques are abundant, and translocation is used to mitigate human macaque conflict. Most adult macaques are infected with MaHV1, although the risk for transmission to persons who handle them during capture and translocation is unknown. We investigated MaHV1 shedding among 392 long-tailed macaques (M. fascicularis) after capture and translocation by the Department of Wildlife and National Parks in Peninsular Malaysia, during 2009-2011. For detection of MaHV1 DNA, PCR was performed on urogenital and oropharyngeal swab samples. Overall, 39% of macaques were shedding MaHV1 DNA; rates of DNA detection did not differ between sample types. This study demonstrates that MaHV1 was shed by a substantial proportion of macaques after capture and transport and suggests that persons handling macaques under these circumstances might be at risk for exposure to MaHV1.

4235: ***+.008***

Reintroduction success depends in part on the release strategy used. Benefits are attributed to particular release strategies but few studies have tested these assumptions. We examined the effect of delayed release (a form of so-called soft release) on the survival of a threatened passerine, the New Zealand hihi Notiomystis cincta, for up to 7 months after translocation. Birds were captured at the source site and then held in captivity for disease screening. They were then taken to the release site, where 30 were released immediately and 28 were held for a further 2-4 days in an on-site aviary. Twenty-four birds were fitted with radio-transmitters. A 1,300 ha area around the release site was searched fortnightly, and survival was analysed using a multi-state model that accounted for the effect of transmitters on detection probability. Our results indicated that delayed release had a negative effect on long-term survival, but no effect was apparent in the first 6 weeks. Survival probability from 6 weeks to 7 months post-release was 0.77 +/- SE 0.20 for immediate-release birds and 0.04 +/- SE 0.06 for delayed-release birds. Our results suggest that there is a misconception about the benefits of delayed-release strategies during translocation of wild animals. Studies that have demonstrated a benefit of delayed release in other bird species used captive-bred individuals, and we suggest that wild individuals perceive captivity differently. We recommend that biological context is considered before delayed release is used in translocations.

4236: *+.148*

Reintroduction of rare and threatened species often fails to yield quantifiable conservation benefits because insufficient attention is focused on the species' habitat requirements and biology. We demonstrate the value of such data in informing a recovery plan for Alectryon ramiflorus S.Reyn. (Sapindaceae), a tree species endemic to a region on the southern coast of Queensland, Australia. When the species was categorized as Endangered on the IUCN Red List in 1997 the total known population consisted of only 26 adult plants, in five disjunct populations in remnant patches of native vegetation. Analysis of vegetation type, soil chemistry and composition data comparing remnant patches with and without A. ramiflorus revealed that the species is not restricted to a specific soil type but prefers sites with relatively fertile soil and a more complex vegetation structure. The species is cryptically dioecious, displays asynchronous flowering between individuals, and requires insect-vectored pollination. The low rate of seedling production recorded within individual patches was attributed to the scarcity of trees of both genders, asynchronous flowering of individual trees and, in smaller patches, a sparse population of pollinating insect species. Successful reintroduction of A. ramiflorus will require consideration of these aspects of demographic success. The findings highlight the importance to species recovery plans of the knowledge of habitat requirements, interspecific relationships and critical dependencies, as well as species reproductive biology.

4237: *+.087*

Malladas de El Saler is a mosaic of dune slacks scattered along a coastal sand bar in the Mediterranean region. Active populations of the fairy shrimp T. stagnalis are exclusively present in one of these ponds. We combined field observations and laboratory experiments to find the ecological requirements of T. stagnalis that determine this habitat exclusiveness and the possibility of future establishment in nearby ponds. Conductivity revealed as a determinant ecological constraint. In the field, the species was restricted to conductivity levels below 1.28 mS cm(-1) and in experimental microcosms, hatchability, survival and reproductive potential were detrimental from 2.9 mS cm(-1). The observation of life history characteristics from the experimental individuals confirmed the importance of the unpredictable character of Mediterranean temporary ponds in shaping life history strategies, as they hatched early, matured rapidly and produced a high number of cysts. We discuss the essential steps during colonization that may explain the presence

or absence in our ponds, and revealed a harsh future for the species if the current conditions of habitat deterioration persist. We suggest several conservation measures, including a cyst bank storage or/and adult translocations to nearby suitable ponds. These measures will ensure (1) the preservation of genetic diversity and (2) healthy metapopulation dynamics with recolonization rates compensating for local extinctions.

4238: *+.103*

Aylacostoma Spix, 1827, contains species that are the subject of focused conservation efforts under the auspices of the 'Aylacostoma Project', the only ex situ conservation programme for freshwater gastropods in South America. Two species from the High Parana River (Argentina-Paraguay) are included in this programme (Aylacostoma chloroticum Hylton Scott, 1954 and Aylacostoma brunneum Vogler & Peso, 2014), as their habitats have disappeared as a consequence of the filling of the Yacyreta Reservoir in the 1990s. At present, A. chloroticum is restricted to only one known wild population in a small and fragile habitat, and wild populations of A. brunneum are presumed to have gone extinct. We used partial sequences of the cytochrome oxidase subunit I gene to provide the first phylogeographical perspective on these species from a limited dataset containing representatives of several wild populations that are successfully being bred in captivity. We found low genetic diversity and two haplotypes in A. chloroticum, and absence of variation with one haplotype in A. brunneum. The reservoir's entry zone was identified to be of great interest for conservation, and is where we suggest re-introductions and translocations should be targeted, to preserve the future evolutionary potential of the extant diversity. (C) 2015 The Linnean Society of London.

4239: *-.134*

1. Infectious diseases are widely recognized to have substantial impact on wildlife populations. These impacts are sometimes exacerbated in small endangered populations, and therefore, the success of conservation reintroductions to aid the recovery of such species can be seriously threatened by outbreaks of infectious disease. Intensive management strategies associated with conservation reintroductions can further compound these negative effects in such populations.2. Exploring the sublethal effects of disease outbreaks among natural populations is challenging and requires longitudinal, individual life-history data on patterns of reproductive success and other indicators of individual fitness.3. Long-term monitoring data concerning detailed reproductive information of the reintroduced Mauritius parakeet (Psittacula echo) population collected before, during and after a disease outbreak was investigated.4. Deleterious effects of an outbreak of beak and feather disease virus (BFDV) were revealed on hatch success, but these effects were remarkably short-lived and disproportionately associated with breeding pairs which took supplemental food. Individual BFDV infection status was not predicted by any genetic, environmental or conservation management factors and was not associated with any of our measures of immune function, perhaps suggesting immunological impairment. Experimental immunostimulation using the PHA (phytohaemagglutinin assay) challenge technique did, however, provoke a significant cellular immune response.5. We illustrate the resilience of this bottlenecked and once critically endangered, island-endemic species to an epidemic outbreak of BFDV and highlight the value of systematic monitoring in revealing inconspicuous but nonetheless substantial ecological interactions. Our study demonstrates that the emergence of such an infectious disease in a population ordinarily associated with increased susceptibility does not necessarily lead to deleterious impacts on population growth and that negative effects on reproductive fitness can be short-lived.

4240: *+.123*

Large carnivores, such as brown bears (Ursus arctos), are flagship species for the conservation of biodiversity and their reintroduction represents a strong challenge. However, the results of reintroductions have only recently been documented in the literature. Given the global decline of large carnivores, documenting the results of such attempts is crucial for future conservation management. Here we examined the reintroduction of brown bears into the Italian Alps. The majority of bears released (10 individuals) adapted well to the release area and this resulted in the increase of the brown bear population. At the end of 2012, the area with a stable presence of females was around 1250 km(2) (minimum density = 3 bear/100 km(2)). Between 2002 and 2012, 34 reproductive events occurred and a total of 74 cubs were born, thus reaching a minimum population size of 47 individuals. No less than 21 young males dispersed into adjacent Italian regions or into other countries, such as Switzerland, Austria, and Germany. However, despite a high mortality rate and at least two cases of illegal killing reported in the last 2 years (2013 and 2014), a remarkable population growth rate (current level of 15.6%) has been observed. The damages correlated with bear population size ($F = 17.922$, $p < 0.01$) were primarily inflicted on beehives (39%) and livestock (26%), with an economic relevance of (sic) 41,374 per year for compensation and (sic) 23,527 per year for prevention. The only case of injury to humans ever since the beginning of the project was recorded in 2014, when a female defending her two cubs inflicted minor physical injuries to a man. And yet, public opinion changed radically, from a widespread acceptance of bears at the beginning of the project to an opposition to their presence, the increase of their population, and the new translocations aiming at replacing problem bears that had previously been removed. According to the present trend, the population may reach a range of 60-94 individuals in 2017. Therefore, a proper management of problem bears should be considered a key factor for the restoration and improvement of the social acceptance of this species. (C) 2015 Elsevier GmbH. All rights reserved.

4241: *-.016*

Populations of epiphytic orchids in disturbance-prone environments rarely reach stable-stage equilibrium. We characterized the post-disturbance, transient dynamics of two epiphytic orchids, Broughtonia cubensis, and the leafless Dendrophylax lindenii, comparing the following indices: reactivity/first-time attenuation, maximal amplification/attenuation, and amplified/attenuated inertia. We also assessed the effects of reintroducing only seeds or only adults, by examining the elasticity of the inertia on the vital rates. For 2006-2010, the stochastic growth rate of D.lindenii was (s)=0.94, or a 6 percent decrease per annum. First-time step attenuation indicates that in 1year, the population could decrease by an additional 16 percent, and in the worst-case scenario could decrease by almost half, relative to the stable-stage distribution, in 10years. Broughtonia cubensis had a (s)=1.03; reactivity and first-time step attenuation indicates that in 1year, the population should not change by more than 14 percent of the estimated stable-stage distribution. However, the worst-case scenario projected a reduction of 40 percent relative to the stable-stage distribution within 8years. A comparison of reintroduction strategies assessed by elasticity of the population momentum showed that adults performed better when relocated to new habitats. Resumen Las poblaciones de orquideas epifitas en ambientes propensos a perturbaciones rara vez alcanzan el estado de equilibrio estable. Comparamos la dinamica poblacional post-huracan de dos orquideas epifitas, una afila, Dendrophylax lindenii y otra con hojas, Broughtonia cubensis a traves los siguientes indices: reactividad/atenuacion al primer ano; la amplificacion y atenuacion maxima y amplificacion/atenuacion en la inercia. Tambien evaluamos los efectos potenciales de una reintroduccion con solo semillas o con solo los adultos, a traves de la elasticidad de la inercia en las tasas vitales. Para el periodo 2006-2010, la tasa de crecimiento estocastico de D. lindenii era

(s) = 0.94, equivalente a una disminucion del 6 por ciento anual. La medida de la atenuacion al ano indica que la poblacion podria disminuir en un 16 por ciento adicional y, en el peor de los casos, se reducira a casi la mitad en relacion a la distribucion estable en 10 anos. Broughtonia cubensis mostro un (s) = 1,03; la reactividad y la atenuacion indican que en 1 ano la poblacion no debe disminuir o aumentar en mas del 14 por ciento en relacion a la estructura estable. Sin embargo, en el peor de los casos, se predice una reduccion del 40 por ciento con respecto a la distribucion establea los 8 anos. Una comparacion de las estrategias de reintroduccion evaluadas por la elasticidad de la inercia demografica mostro que los adultos tuvieron un mejor desempeno cuando fueron trasladados a nuevos habitats.

4242: *+.110*

From 2005 to 2011, the federally endangered freshwater mussel Epioblasma capsaeformis (oyster mussel) was reintroduced at three sites in the upper Clinch River, Virginia, using four release techniques. These release techniques were (1) translocation of adults (site 1, n = 1418), (2) release of laboratory-propagated sub-adults (site 1, n = 2851), (3) release of 8-week-old laboratory-propagated juveniles (site 2, n = 9501), and (4) release of artificially infested host fishes (site 3, n = 1116 host fishes). These restoration efforts provided a unique research opportunity to compare the effectiveness of techniques used to reestablish populations of extirpated and declining species. We evaluated the relative success of these four population restoration approaches via monitoring at each release site (2011-2012) using systematic 0.25-m(2) quadrat sampling to estimate abundance and post-release survival. Abundances of translocated adult and laboratory-propagated sub-adult E. capsaeformis at site 1 ranged 577-645 and 1678-1700 individuals, respectively, signifying successful settlement and high post-release survival. Two untagged individuals (29.1 and 27.3 mm) were observed, indicating that recruitment is occurring at site 1. No E. capsaeformis were found at sites where 8-week-old laboratory-propagated juveniles (site 2) and artificially infested host fishes (site 3) were released. Our results indicate that translocations of adults and releases of laboratory-propagated sub-adults were the most effective population restoration techniques for E. capsaeformis. We recommend that restoration efforts focus on the release of larger (>20 mm) individuals to accelerate augmenting and reintroducing populations and increase the probability for recovery of imperiled mussels.

4243: *+.358*

Metapopulation viability depends upon a balance of extinction and colonization of local habitats by a species. Mechanisms that can affect this balance include physical characteristics related to natural processes (e.g. succession) as well as anthropogenic actions. Plant restorations can help to produce favorable metapopulation dynamics and consequently increase viability; however, to date no studies confirm this is true. Population viability analysis (PVA) allows for the use of empirical data to generate theoretical future projections in the form of median time to extinction and probability of extinction. In turn, PVAs can inform and aid the development of conservation, recovery, and management plans. Pitcher's thistle (Cirsium pitcheri) is a dune endemic that exhibited metapopulation dynamics. We projected viability of three natural and two restored populations with demographic data spanning 15-23 years to determine the degree the addition of reintroduced population affects metapopulation viability. The models were validated by comparing observed and projected abundances and adjusting parameters associated with demographic and environmental stochasticity to improve model performance. Our chosen model correctly predicted yearly population abundance for 60% of the population-years. Using that model, 50-year projections showed that the addition of reintroductions increases metapopulation viability. The reintroduction that simulated population performance in early-successional habitats had the

maximum benefit. In situ enhancements of existing populations proved to be equally effective. This study shows that restorations can facilitate and improve metapopulation viability of species dependent on metapopulation dynamics for survival with long-term persistence of C. pitcheri in Indiana likely to depend on continued active management.

4244: *+.100*

A review of elk vital rates in eastern North America is warranted given continued interest in restoring the species to the region and the variation in habitat conditions, anthropogenic influence on population dynamics and predator fauna between eastern and western North America. We reviewed 23 studies of elk demographics from populations in eastern North America and summarized adult (>2 years old), subadult (1-2 years old) and juvenile (0-1 years old) annual survival and fecundity. We also reviewed and compared studies in which causes of mortality in eastern and western North American elk populations were reported. Annual survival of adult and subadult elk in populations in eastern North America was similar to those reported for western North American elk populations. Annual juvenile survival [S=0.76, standard error (SE)=0.07] in eastern North American elk populations was 53% higher than that in western North American populations. Fecundity rates in elk populations in eastern North America were low (0.75 juveniles per female per year, SE=0.06) compared with fecundity rates typical of western North American populations. Juvenile survival and adult fecundity were lower in recently restored populations (S=0.65, SE=0.09; 0.62 juveniles per female per year) than in established populations (S=0.83, SE=0.07; 0.80 juveniles per female per year) in eastern North America. Brainworm Parelaphostrongylus tenuis infection, vehicle collisions and nuisance culling were important factors contributing to mortality in eastern North American, but not in western North American, elk populations. Eastern North American elk populations have the potential to exhibit high population growth rates (=1.02-1.29). Variation in vital rates between recently restored and established elk populations highlights the need for continued long-term monitoring of translocated elk populations.

4245: *-.015*

Chromosomal translocations play a fundamental role in the evolution and speciation of antelopes (Antilopinae, Bovidae), with several species exhibiting polymorphism for centric fusions. For the past 35 years, the San Diego Zoo Global (SDZG) captive population of Soemmerring's gazelles has revealed complex karyotypes resulting from chromosomal translocations with diploid numbers ranging from 34 to 39. Poor reproductive performance of this species in captivity and elevated mortality the first month of life (perinatal) has been attributed to this chromosomal dynamism. We have extended the studies of karyotypic variation in the SDZG Soemmerring's gazelle population and analyzed the effect of chromosomal and genetic variation upon perinatal mortality. Karyotypes from 149 captive Soemmerring's gazelles were evaluated revealing two unreported autosomal combinations, now constituting a total of 15 distinct karyotypes for the 3 Robertsonian centric fusions originally described for this population. Among SDZG founders, distinct chromosomal variation and nuclear and mitochondrial genetic structure were detected corresponding to the institution of origin of the founders. Low levels of genetic distance and nucleotide diversity among individuals, in addition to high relatedness values, suggested that outbreeding is less of a concern than inbreeding for maintaining a sustainable captive population. Finally, analysis of karyotypes of offspring born into the SDZG Soemmerring's gazelle herds, in conjunction with the maternal karyotype showed association of chromosomal makeup with perinatal mortality. This supports the importance of continuing cytogenetic screening efforts, particularly to evaluate the presence of deleterious chromosomal rearrangements in stillborns. Zoo Biol. 34:374-384, 2015. (c) 2015

Wiley Periodicals Inc.

4246: *+.137*

Grass snakes (Natrix natrix)were monitored for nine years on a site in eastern England restored for an amphibian reintroduction. Counts of snakes increased between 2004 and 2012 from 1.25 to 3.83 snakes per survey visit. Grass snake counts were positively correlated with the number of common frog spawn clumps each year and peak counts of pool frogs. During surveys and incidental encounters 137 adult males, 161 adult females, 131 juveniles and 44 hatchlings were captured and individually photographically identified. Captures of hatchlings were erratic and recapture rates were low, so they were excluded from the analysis. Annualised capture data were analysed in the capture-recapture programme MARK, using the Cormack-Jolly-Seber model. The top ranked model gave an apparent annual survival rate of 0.66 (95% Cl=0.543-0.755) and an individual detection rate of 0.17 (0.118-0.245). Population estimates based on this model ranged from 53 (95% Cl=37-76) to 576 (95% Cl=400-831) over the nine years of study. Grass snake population estimates were equivalent to densities of 4.8 to 52.4 individuals ha(-1). Nevertheless, it is unlikely that these snakes were permanently resident within the study area, and annual survival may therefore be underestimated. A more plausible explanation for the large population estimates is that the snakes were temporarily resident within a patch of high quality habitat and moved through home ranges that included the study site.

4247: *+.084*

Population demographics for amphibian larvae are rarely estimated due to marking technique limitations on small body size, morphological change (metamorphosis), and the associated habitat changes (aquatic to terrestrial environments). A technique that may meet some of these limitations is visible implant elastomer (VIE) tagging. In this study, we report on the efficacy of VIE tagging a tree frog (Hylidae) at the tadpole stage for cohort identification across metamorphosis to the adult stage, in a field environment. During our preliminary captive trial, post-metamorphosis tag retention was 100% over three months, with no adverse effects observed on survival, growth or time to metamorphosis. During our field study tag retention in recaptured Litoria aurea was 95% for tadpoles and 88% across metamorphosis. By 200 days post-tagging, retention declined to 75% in the adult stage and stabilised around 50% by 300 days. Post metamorphosis the retention rate was less reliable and dependent upon sex and life-stage. Females showed the highest retention rate (max. 62%, 760 days post tagging), followed by juveniles (max. 45%, 400 days post tagging) and males (max. 20%, 760 days post tagging). We conclude that VIE tagging is a viable method for studying cohort larval movements and population demographics of amphibians up to a 50 day post-metamorphosis stage.

4248: *+.196*

Despite the worldwide increase of rhinoceros calf numbers, the growth of the population of white and black rhinoceros is slowing down mainly due to anthropogenic causes, such as poaching and habitat loss. Assisted reproduction is one of the methods of preserving the valuable genomes of these animals from being lost, and assists in breeding them in captivity to maintain the specie(s) numbers and provide an option for possible reintroduction into the wild. Since wild rhinoceros are difficult to handle and examine clinically, most of the current information available on their reproductive characteristics has been gained from captive rhinoceros populations. Nevertheless, very little is known about rhinoceros reproduction. Since the rhinoceros belongs to the odd-toed ungulates (Perissodactyls) group, like the horse and the tapir, the horse has been proposed as a

suitable model to study reproduction and artificial reproductive techniques in the rhinoceros. In this review, the current knowledge of the reproduction of the rhinoceros is summarized.

4249: *+.100*

Microsatellite markers are valuable tools for determining amount and distribution of genetic diversity and differentiation within and between populations. In this study we examined the level of microsatellite variability within and among five populations of Lobelia villosa, a rare Hawaiian endemic lobeliad on the island of Kaua'i. Populations of L. villosa were sampled from two regions on Kaua'i: two populations at the Kilohana Lookout area and three populations from Alaka'i Swamp. Nineteen microsatellite DNA primers were developed for L. villosa, 12 of which demonstrated polymorphism and were subsequently multiplexed and labeled for genotyping. An overall moderate degree of genetic differentiation was found within and between populations. Pairwise F-ST data showed population structure, and analysis with Structure software indicated two genetic clusters (K = 2) corresponding to the two sampled geographic regions. Although L. villosa exhibits moderate diversity, which exceeds that of other Hawaiian endemics with restricted distributions, measurements of F-IS were positive across 10 out of 12 loci, suggesting that inbreeding is occurring at the population level.

4250: *-.049*

CASE HISTORY: Salmonellosis was suspected as the cause of death in eight wild animals on Tiritiri Matangi Island, in the Hauraki Gulf of New Zealand, between November and September 2011, including three hihi (Notiomystis cincta), a tuatara (Sphenodon punctatus), a masked lapwing (Vanellus miles novaehollandiae), and a saddleback (Philesturnus carunculatus). An outbreak investigation to identify the source and distribution of infection was undertaken over the summer of 2011-2012. CLINICAL AND LABORATORY FINDINGS: Surveillance of five species of forest bird (n=165) in December 2011 returned a single positive result for Salmonella spp. Environmental sampling of 35 key water sources and hihi supplementary feeding stations conducted in December 2011 and March 2012 returned isolates of S. enterica subspecies houtenae and S. enterica serovar Saintpaul from a stream, a dam and a supplementary feeding station. The same serotypes were identified in tissue samples collected from post mortem specimens of the affected birds, and their similarity was confirmed by pulsed-field gel electrophoresis.DIAGNOSIS: Mortality in wildlife associated with infection with S. enterica subspecies houtenae and S. enterica serovar Saintpaul. CLINICAL RELEVANCE: This is the first detection of these Salmonella spp. from wild birds in New Zealand. Our study highlights how active surveillance in response to observed disease emergence (here mortalities) can provide important insight for risk assessment and management within populations of endangered species and inform risk assessment in translocation planning.

4251: *-.146*

Acquiring water is essential for all animals, but doing so is most challenging for desert-living animals. Recently Przewalski's horse has been reintroduced to the desert area in China where the last wild surviving member of the species was seen before it vanished from China in the 1960s. Its reintroduction placed it within the range of a close evolutionary relative, the con-generic Khulan. Determining whether or not these two species experience competition and whether or not such competition was responsible for the extinction of Przewalski's horses in the wild over 50 years ago, requires identifying the fundamental and realized niches of both species. We remotely monitored the presence of both species at a variety of water points during the dry season in

Kalamaili Nature Reserve, Xinjiang, China. Przewalski's horses drank twice per day mostly during daylight hours at low salinity water sources while Khulans drank mostly at night usually at high salinity water points or those far from human residences. Spatial and temporal differences in water use enables coexistence, but suggest that Przewalski's horses also restrict the actions of Khulan. Such differences in both the fundamental and realized niches were associated with differences in physiological tolerances for saline water and human activity as well as differences in aggression and dominance.

4252: ***-.014***

Wide-ranging large carnivores pose myriad challenges for conservation, especially in highly fragmented landscapes. Over a 13-year period, we combined monitoring of radio collared pumas (Puma concolor) with complementary multi-generational genetic analyses to inform puma conservation in southern California, USA. Our goals were to generate survivorship estimates, determine causes of mortality, identify barriers to movement, and determine the genetic and demographic challenges to puma persistence among >20,000,000 people and extensive urban, suburban, and exurban development. Despite protection from hunting, annual survival for radio collared pumas was surprisingly low (55.8%), and humans caused the majority of puma deaths. The most common sources of mortality were vehicle collisions (28% of deaths), and mortalities resulting from depredation permits issued after pumas killed domestic animals (17% of deaths). Other human-caused mortalities included illegal shootings, public safety removals, and human-caused wildfire. An interstate highway (I-15) bisecting this study area, and associated development, have created a nearly impermeable barrier to puma movements, resulting in severe genetic restriction and demographic isolation of the small puma population (n similar to 17-27 adults) in the Santa Ana Mountains west of I-15. Highways that bisect habitat or divide remaining "conserved" habitat, and associated ongoing development, threaten to further subdivide this already fragmented puma population and increase threats to survival. This study highlights the importance of combining demographic and genetic analyses, and illustrates that in the absence of effective measures to reduce mortality and enhance safe movement across highways, translocation of pumas, such as was done with the endangered Florida panther (P. c. coryi), may ultimately be necessary to prevent further genetic decline and ensure persistence of the Santa Ana Mountains population.

4253: ***+.205***

The success of species reintroduction often depends on predation risk and spatial estimates of predator habitat. The fisher (Pekania pennanti) is a species of conservation concern and populations in the western United States have declined substantially in the last century. Reintroduction plans are underway, but the ability of the species to establish a self-sustaining population is affected by predation from its primary predator, the bobcat (Lynx rufus). To develop a habitat model that incorporates both habitat of the focal species and the spatial patterning of predator habitat. To locate areas of densely aggregated habitat that would be suitable for reintroduction. Using camera survey data, we modeled the association between bobcat presence and environmental features using a classification tree. We applied this model to a spatial analysis of fisher habitat and identified reintroduction areas in the southern Washington Cascade Range. The classification tree predicted bobcat detection based on elevation and mean tree diameter. The final model identified fisher reintroduction locations primarily in or near existing wilderness areas. Fisher habitat areas identified considering both habitat and predation risk differed from those identified without considering predation. Our spatial approach is unique among fisher reintroduction plans by accounting for both resource requirements and risk of predation. It can be

used as a template for future reintroduction efforts in other regions and for other species. Using similar models to refine population management and reintroduction should improve the probability of successful population establishment and stability.

4254: *+.139*

Habitat fragmentation can exacerbate the impacts on population persistence in species with rapid life histories and specialized habitat requirements. We investigated genetic structuring across the range of the federally endangered (USA) Perdido Key beach mouse (PKBM), Peromyscus polionotus trissyllepsis, using 16 microsatellite loci. Between 2010 and 2012 we sampled the three core habitat areas that are separated by 3-5 km of developed area. One location GSP was re-established in 2010 with a release of 48 captive bred mice. The other two parks had a reticulate recent history, including extirpations, translocations and, a recent (2009) natural re-colonization. Our objectives were to document the level of genetic drift associated with the post-release phase at GSP over a 2-year period, and to test the hypothesis that PKBM dispersal connectivity is restricted between the three parks. The three populations were significantly genetically differentiated in 2010 and 2012 (pairwise F (ST) ranged from 0.135 to 0.276). However, Bayesian clustering, assignment tests and hybridization analyses revealed inter-park dispersal and reproduction in 2012 that was absent in 2010. The detection of dispersal was an important step in understanding the regulation of connectivity in this fragmented system. We propose that a combination of recent apparent population increases across the island combined with the concurrent re-establishment of beachfront vegetated sand berms encouraged inter-park dispersal detected in 2012. These results provide important information for the future conservation of beach mice inhabiting northern Gulf of Mexico and Atlantic barrier islands in light of continued development pressure.

4255: *+.191*

Freshwater mussels (Bivalvia: Unionoida) are highly imperiled with many species on the verge of local extirpation or global extinction. This study investigates patterns of genetic structure and diversity in six species of freshwater mussels in the central Great Lakes region of Ontario, Canada. These species vary in their conservation status (endangered to not considered at risk), life history strategy, and dispersal capabilities. Evidence of historical genetic connectivity within rivers was ubiquitous across species and may reflect dispersal abilities of host fish. There was little to no signature of recent disturbance events or bottlenecks, even in endangered species, likely as a function of mussel longevity and historical population sizes (i.e., insufficient time for genetic drift to be detectable). Genetic structure was largely at the watershed scale suggesting that population augmentation via translocation within rivers may be a useful conservation tool if needed, while minimizing genetic risks to recipient sites. Recent interest in population augmentation via translocation and propagation may rely on these results to inform management of unionids in the Great Lakes region.

4256: *+.039*

Freshwater eel-tailed catfishes (Tandanus spp.) have a complicated phylogenetic structure including several undescribed cryptic species in eastern Australia. This naturally complex phylogeny is further complicated by their phylogeography, with the existence of numerous populations reported to have been subject to historic translocations. The Murray-Darling Basin (MDB) population of Tandanus tandanus is listed as endangered and is locally extinct over large parts of its historical range. Conservation actions such as captive breeding for release into the wild, or translocations, will be necessary components of a recovery plan for the MDB population.

To further understand the phylogeography of Tandanus populations in south-eastern Australia and to provide additional support for the taxonomic position of previously proposed cryptic species, we sequenced a 408 bp fragment of the mtDNA control region from 240 fish collected from 26 catchments. We identified three distinct clades within New South Wales; two from coastal catchments and a third from the MDB. Phylogeographic structure was clearly affected by previous translocations, with the presence of both entirely non-endemic populations, and endemic populations subjected to introgression with fish from non-endemic source populations. Most coastal catchments possessed unique haplotypes resulting in a high degree of genetic structure among catchments, while few MDB sub-catchments possessed unique haplotypes. The phylogenetic structure was consistent with the greater hydrological connectivity of MDB sub-catchments, allowing much greater gene flow compared to coastal catchments that are hydrologically isolated. The complicated phylogenetic and phylogeographic structure observed in T. tandanus has implications for future stocking or translocations.

4257: ***+.088***

Hunting and habitat loss led to the extinction of the Formosan sika deer Cervus nippon taiouanus population in the wild in 1969. A sika deer restoration program has been in place in Kenting National Park (KTNP), Taiwan since 1984. Human deer conflicts have increased following wild deer population growth. Understanding residents' attitudes toward reintroduced sika deer is vital to management policies. To investigate the local residents' attitudes, we collected 228 questionnaires through personal interviews in 2010. A majority of the respondents agreed that they would be happy to encounter a wild sika deer (78.1%), and a majority supported the restoration program in the KTNP (75.4%). However, 59.1% of the respondents knew little concerning the restoration program. Approximately half of the respondents (47.8%) thought that sika deer caused damages to crops, and 18.4% of the respondents actually suffered crop damages from deer. The farmers and people living within the deer's range were more vulnerable to deer damage; therefore, they were more aware of deer damage to their crops and livelihoods than non-farmers and people living outside of the deer's range. In addition, most respondents (87.2%) considered sika deer as an attractive tourism resource and were supportive of the development of ecotourism (87.3%). We recommend that the KTNP should improve public participation, environmental education, and communication with the local people. The development of community-based ecotourism would increase the benefits of the reintroduction of sika deer and would help to mitigate human-deer conflicts.

4258: ***+.244***

When species face extinction, captive breeding may be appropriate. However, captive breeding may be unsuccessful, while reducing motivation and resources for insitu conservation and impacting wild source populations. Despite such risks, decisions are generally taken without rigorous evaluation. We develop an individual-based, stochastic population model to evaluate the potential effectiveness of captive-breeding and release programmes, illustrated by the critically endangered Ardeotis nigriceps Vigors great Indian bustard. The model was parameterized from a comprehensive review of captive breeding and wild demography of large bustards. To handle uncertainty in the standards of captive-breeding performance that may be achieved, we explored four scenarios of programme quality: full range' (parameters sampled across the observed range), below average', above average' and best possible' (performance observed in exemplary breeding programmes). Results are evaluated examining: (i) the probability of captive population extirpation within 50years and (ii) numbers of adult females subsequently established in the wild following release, compared to an alternative strategy of insitu conservation without attempting

captive breeding. Successful implementation of captive breeding, involving permanent retention of 20 breeding females and release of surplus juveniles, required collection of many wild eggs and consistent best possible' performance across all aspects of the programme. Under full-range' and above-average' scenarios, captive population extirpation probabilities were 73-88% and 23-51% respectively, depending on egg collection rates. Although most (73-92%) best possible' programmes supported releases, re-establishment of free-living adults also required effective insitu conservation. Incremental implementation of effective conservation measures over the initial 10years resulted in more free-living adults within 35years if eggs were left in the wild without attempting captive breeding. Synthesis and applications. For the great Indian bustard Ardeotis nigriceps, rapid implementation of insitu conservation offers a better chance to avoid extinction than captive breeding. Demographic modelling of threatened species should be used to examine whether captive breeding will bring net benefits to conservation programmes. For the great Indian bustard Ardeotis nigriceps, rapid implementation of insitu conservation offers a better chance to avoid extinction than captive breeding. Demographic modelling of threatened species should be used to examine whether captive breeding will bring net benefits to conservation programmes.

4259: ***+.066***

Both species of North American sage-grouse (Centrocercus spp.) have experienced declines in distribution and abundance. Translocation of adult birds from a stable population to a small or declining population has been a management tool used by wildlife managers to support population persistence in these areas. Captive rearing chicks and releasing them into wild surrogate broods is an untested alternative to augment declining populations of sage-grouse. We developed techniques to successfully rear sage-grouse chicks in captivity, evaluated explanatory variables that could influence hatch and captive-rearing success, and estimated the survival of domestically hatched (DH) chicks to 28 days of age following introduction to a surrogate wild brood. We collected 304 eggs from radiomarked female greater sage-grouse (C. urophasianus) during 2004-2007 in 3 study areas in northwestern Colorado. Estimated hatching success of collected eggs was 0.745 (SE=0.022, 95% CI=0.700-0.786) and was negatively influenced by the number of days an egg was stored and the percent egg weight loss that occurred during storage and incubation. We monitored 175 DH chicks in captivity for 1-10 days before introduction and adoption into surrogate wild broods. Model-averaged captive-rearing success was 0.792 (SE=0.045, 95% CI=0.686-0.865) across years, and was positively influenced by initial chick mass at hatch and daily weight gain in captivity but negatively influenced by the number of days the egg was stored and advancing hatch date. We were able to radiomark and monitor 133 DH chicks adopted into surrogate wild broods until 28 days of age. Eighty-eight percent of DH chicks were successfully adopted within 24hours. Our overall estimate of DH chick survival to 28 days (0.423; 95% CI=0.257-0.587) was comparable to published wild-hatched chick survival. Predation and exposure-related deaths accounted for 26.3% and 25.6% of the known fates, respectively. Our captive-rearing protocols and techniques were successful for collecting greater sage-grouse eggs, hatching and rearing chicks in captivity, and releasing chicks into wild surrogate broods. This success further implies that captive rearing and release can be a potential management strategy to demographically and genetically reinforce or augment small populations of sage-grouse. (c) 2015 The Wildlife Society.

4260: ***+.142***

Knowledge about species' responses to habitat restoration can inform subsequent management and reintroduction planning. We used repeated call-response surveys to study brown-headed nuthatch (Sitta pusilla) patch occupancy at the current limits of its apparently expanding range in an area

with active habitat restoration. We fit a probit occupancy model that accounted for spatial autocorrelation using restricted spatial regression. Nuthatch occupancy was related to patch-level vegetation structure and range-extension context, i.e., latitude, but not prescribed fire history. Latitude and percent tree stocking had a negative relationship with occupancy (coefficients and 95% credible intervals: -1.07 [CI: -1.63, -0.67] and -0.63 [CI: -0.97, -0.350]). The density of recently killed and well-decayed snags had positive associations with occupancy (coefficients and 95% credible intervals: 0.57 [CI: 0.17, 1.16] and 0.37 [CI: 0.05, 0.72]). Neither grassy herbaceous cover nor percent of stocking in pine were associated with occupancy. We found that restoration efforts created suitable stand structure for brown-headed nuthatches, but many restored sites in the range-extension zone appeared to be vacant. Occupied habitats in the range-extension zone had fewer snags, less frequent fire, and more shrub cover than occupied sites where the species was established. Release from conspecific competition may have permitted nuthatches in the range-extension zone to exploit habitats that would otherwise have been marginal. Alternatively, nuthatches may be restricted to such sites although there are more suitable sites tens of kilometers away. Experimental translocations and reintroductions could determine how habitat structure and nuthatch density affect the quality of restored sites in the range-extension zone and enable those sites to achieve their biodiversity potential. Published 2015. This article is a U.S. Government work and is in the public domain in the USA.

4261: *+.071*

Understanding population dynamics is critical for meta-population management, especially of endangered species, and also for megaherbivore ecology. We employed complete individual life records to construct census data for a reintroduced black rhinoceros population over 22 years since its founding and investigated its dynamics. Akaike's information criterion applied to scalar models of population growth based on the generalized logistic unambiguously selected an exponential growth model (r = 0.102 +/- 0.017), indicating a highly successful reintroduction. No evidence of density dependence was detected, and thus, we could not confirm the threshold model of density dependence that has influenced black rhinoceros meta-population management. Our analysis supported previous work contending that the generalized logistic is unreliable when fitted to data that do not sample the entire range of population sizes. A stage-based matrix model of the exponential population dynamics exhibited mild transient behaviour. We found no evidence of environmental stochasticity, consistent with our previous studies of this population that found no influence of rainfall on demographic parameters. Demographic stochasticity was present, principally reflected in annual sex-specific recruitment numbers that differed from deterministic predictions of the matrix model. Demographically driven process noise should be assumed to be a component of megaherbivore population dynamics, as these populations are typically relatively small, and should be accounted for in managed removals and introductions. Increase in age at first reproduction with increasing population size, as manifested in the study population, may provide a warning of possible density feedback prior to detectable slowing of population growth rate for megaherbivores.

4262: *-.016*

The use of restocking of animals is common practice in the management of populations subject to hunting or recreational fishing. This practice encompasses the release of large numbers of individuals in an area where the species already exists, and thus it can have detrimental genetic impacts on the target populations, especially when captive-reared animals are involved. To better understand this practice and its conservation implications, we chose as a model the wild rabbit (Oryctolagus cuniculus), a species of high economic and ecological importance, and often under

intense management for hunting or conservation purposes, particularly after the large decline caused by rabbit hemorrhagic disease in the 90's. We studied the genetics of rabbit populations in an area where restocking with captive, wild-domestic hybrids was common. We collected a total of 503 samples from 15 hunting estates that had experienced differing restocking levels, as well as from five locations with no historical restocking and five game farms. All samples were analyzed to determine the mitochondrial DNA (mtDNA) lineage typical of the two European wild rabbit subspecies and domestic rabbit. Game farms and never restocked populations were very different in their haplotypic composition. In restocked areas, the proportion of the domestic lineage was higher when releases were recent and repeated, but this declined rapidly over time, in part due to selective removal by hunting. The extended use of this practice, considering the pronounced genetic and genotypic differences between domestic and wild rabbits, represents a potential danger to natural populations, especially given the marked decrease in wild rabbit numbers experienced in recent years in its original distribution range.

4263: *+.114*

Less than 200 wild individuals of the critically endangered Western Derby eland (Taurotragus derbianus derbianus) live in the Niokolo Koba National Park (NKNP) in Senegal. A semi-captive breeding programme was established in 2000 with six founding individuals (one male, five females) transferred from the NKNP. In 2013, the population consisted of 92 individuals living in seven separate herds in the two fenced reserves of Bandia and Fathala in Senegal. Because of the low number of founding individuals in the breeding programme and the resulting high kinship, we compared the results from genealogical and genetic approaches to assess the level of genetic diversity. We used the data from the founder, F1 and F2 generations. In F1, the founder contribution was highly biased towards the only founding male, which sired all the offspring. In F2, the founder contributions were more balanced, as the male descendants of founding females entered the reproduction. This resulted in higher genetic diversity and lower inbreeding (based on pedigree data) in F2 than in F1. Results of molecular analysis using microsatellite loci confirmed the highest level of heterozygosity and lowest level of inbreeding in the founder generation; however, the implementation of a management strategy was not reflected in the empirical results. The results differed for F2, where empirical values of heterozygosity continued to decrease and inbreeding continued to increase. However, the allelic richness corresponded with the results of pedigree analyses, reflecting the more equalized founder contributions. We conclude that the overall results for genetic parameters were comparable with other breeding programmes for endangered ungulates. Nevertheless, we suggest the use of comprehensive molecular data to refine the studbook and to correct relatedness of founders and assign the missing paternities. Our suggestions correspond with the Western Derby Eland Conservation Strategy and confirm the need to introduce new founders into the semi-captive population, in order to minimize the risk of inbreeding depression and improve genetic diversity and suitability for potential reintroduction.
(C) 2015 Deutsche Gesellschaft fur Saugetierkunde. Published by Elsevier GmbH. All rights reserved.

4264: *+.055*

Coreius guichenoti is an endemic fish that has been seriously threatened by dam construction in the upper reach of the Yangtze River, and conservation of this species has been a major concern. With a large portion of its spawning grounds disappearing due to dam construction, hatchery release is proposed as a primary strategy for conservation of this species. Genetic information for C. guichenoti is limited, and previous studies are insufficient for robustly detecting genetic structure among reproductively distinct populations. In the present study, the genetic structure of

C. guichenoti was investigated using 609 base pairs of the cytochrome c oxidase I gene from drifting larvae and juveniles sampled in three different years. Our results detected two substantial genetic populations of C. guichenoti, with low levels of diversity and strong unidirectional gene-flow between the genetic populations. Based on the results of this study, releasing of juveniles from artificial reproduction should respect genetic differentiation between populations in C. guichenoti: inter-population captive breeding and translocations between the populations should be avoided. Our results suggest that it is crucial to reveal population genetic structures when considering conservation of endemic fish species.

4265: ***-.214***

The risk of predation can cause strong antipredator behaviors and marked stress-induced changes in physiology. In mothers, predator-induced stress can reduce reproductive fitness and alter offspring phenotypes. Acting via these generational, maternal stress effects, predation risk may continue to influence the demography of prey populations even when the predators are no longer present. The 10-year snowshoe hare cycle is the classic top-down predator-driven example in nature and is caused both by direct mortality and by indirect risk effects. During the decline phase, virtually, all hares die because they are killed and simultaneously hares exhibit pronounced stress effects caused by high predation risk. However, the rapidity of the decline phase varies among cycles. When the decline is extremely rapid, we expect that the risk experienced by hares is much greater than when the decline is prolonged. The enigma of these cycles is the low phase following the decline, when there is little or no population growth in spite of the absence of predators and ample food. Previously, we have shown that predator-induced maternal stress decreases reproduction and compromises the offspring stress axis. Here, we examine how the severity of predation risk during six separate population declines is related to the length of the subsequent low phase. We show that the more severe the decline, as indicated by the greater rate of loss of hares, the longer the subsequent low phase. These results support the hypothesis that the greater the degree of risk, the longer the generational impact on population demography (the longer the low phase of the hare population). Our findings have broad applicability to conservation and management efforts; even when a stressor (predator, human disturbance) is removed or when exposure may be short term (drought, fire or translocation), the signature of the stressor may be evident over several future generations.

4266: ***+.182***

After stocking with larvae from the Gironde-Garonne-Dordogne population, in 2013, three young-of-the-year Allis shad Alosa alosa, probably originating from natural reproduction, were documented for the first time in a period of nearly 100years in the River Rhine. In 2014, a further increase was observed when 57 juveniles and eight adults were caught; seven of these eight adults were derived from stocking, indicating the success of stocking measures within the framework of the EU-LIFE project.

4267: ***+.047***

Successful management of reintroduced populations requires recognizing that ecological conditions may have changed between extirpation and reintroduction. For example, characterizing dietary patterns of generalist apex predators in the past and present can help to define how their functional role may change as translocated populations grow. We identified prey remains collected from Bald Eagle (Haliaeetus leucocephalus) nests and used carbon (delta C-13) and nitrogen (delta N-15) stable isotope analysis to quantify diet composition of the recently reintroduced Bald Eagle

population on the Channel Islands off southern California, USA. We collected >6,000 prey items from recently occupied nests on Santa Catalina, Santa Rosa, Santa Cruz, and Anacapa islands in 2010 and 2011. Prey identification and stable isotope analysis yielded similar results and showed that eagles on Santa Catalina Island consumed a high proportion (similar to 60%) of marine fish and a lower proportion (25-30%) of seabirds, while their counterparts on the Northern Channel Islands consumed equal proportions (similar to 40-45%) of these prey types. Terrestrial resource use was low with the exception of eagles from one nest on Santa Catalina Island, where eagles primarily consumed ground squirrels and freshwater fish. We suggest that a combination of natural and anthropogenic factors is responsible for the interisland differences in Bald Eagle diet. Bald Eagle interactions with a robust recreational fishery off Santa Catalina Island may enhance access to fish species that are not available to eagles on the Northern Channel Islands, where the availability of breeding seabirds is far greater. The proportion of seabirds consumed by eagles on the Northern Channel Islands today is similar to that consumed by eagles from this region historically and prehistorically. This suggests that the restoration of breeding seabirds on the Channel Islands will benefit the long-term viability of eagle populations in the northern archipelago.

4268: *+.126*

Linkage disequilibrium (LD) is the nonrandom association of alleles at two markers. Patterns of LD have biological implications as well as practical ones when designing association studies or conservation programs aimed at identifying the genetic basis of fitness differences within and among populations. However, the temporal dynamics of LD in wild populations has received little empirical attention. In this study, we examined the overall extent of LD, the effect of sample size on the accuracy and precision of LD estimates, and the temporal dynamics of LD in two populations of bighorn sheep (Ovis canadensis) with different demographic histories. Using over 200 microsatellite loci, we assessed two metrics of multi-allelic LD, D, and (2). We found that both populations exhibited high levels of LD, although the extent was much shorter in a native population than one that was founded via translocation, experienced a prolonged bottleneck post founding, followed by recent admixture. In addition, we observed significant variation in LD in relation to the sample size used, with small sample sizes leading to depressed estimates of the extent of LD but inflated estimates of background levels of LD. In contrast, there was not much variation in LD among yearly cross-sections within either population once sample size was accounted for. Lack of pronounced interannual variability suggests that researchers may not have to worry about interannual variation when estimating LD in a population and can instead focus on obtaining the largest sample size possible.

4269: *+.311*

Worldwide concerns about declining eel stocks have resulted in the increased use of conservation stocking (i.e. the translocation of early eel life-stages from areas of high recruitment to areas experiencing recruitment declines) as a recovery tool. North Atlantic eels have complicated and incompletely understood life-history strategies with major differences in life-history parameters across the species' broad distributions, and these differences may be important for migrating and spawning successfully. Sex ratio, growth rate and size and age at maturation of American eels (Anguilla rostrata) stocked into the St. Lawrence River basin from donor areas over 2000km away with differing life-history characteristics were compared with those of naturally recruited eels to assess the effectiveness of a potential stocking programme in maintaining a sub-population with unique life-history characteristics (largest from across the species range, and exclusively female). Stocked eels exhibited faster annual growth, had a different sex ratio, and matured and

outmigrated at smaller sizes and ages than naturally recruited eels. Conservation stocking should be applied with caution, as stocked eels appear to be following life-history patterns comparable with conspecifics in the geographic range of the donor streams where they were collected. These findings cast doubt on the generally accepted hypothesis that the mechanisms driving eel life-history variation are environmentally induced, and suggest that more care should be taken in assessing and matching the life-history characteristics of donor and recipient sub-populations if conservation stocking is expected to be an important recovery option for eel restoration. (c) 2014 Her Majesty the Queen in Right of Canada. Aquatic Conservation: Marine and Freshwater Ecosystems (c) 2014 John Wiley & Sons, Ltd.

4270: *+.063*

Reintroduction of game species and augmentation of declining populations were carried out in many areas of Europe during the last century. In some cases, such actions have threatened the diversity of remnant endemic taxa, thus raising concerns for their conservation. The Italian peninsula hosts relict populations of native roe deer, which include the subspecies Capreolus capreolus italicus. Recent genetic investigations and historical information suggest that roe deer populations in Italy have different origins (native, introduced, and admixed). Here, we conducted genetic analyses in 3 areas of central Italy (provinces of Arezzo, Pisa, and Parma), each characterized by subareas containing roe deer with different genetic ancestries and zones of contact among them. We analyzed mitochondrial (control region) and nuclear (microsatellites) data for a total of 304 individuals. In all 3 study areas, both marker systems revealed a decreasing frequency of the C. c. italicus lineage from south to north. We found the highest degree of admixture in contact zones, but local patterns varied on the basis of population history and landscape features. Notably, we found relatively high genetic differentiation between the extremes of each sampling area, despite the limited spatial scale studied and time elapsed since introductions. Further, our analyses of a population in the Apennine Mountains reveal that this region may maintain previously undetected native diversity. Finally, the correspondence between data from mitochondrial and nuclear genomes is consistent with the absence of sex-biased dispersal in roe deer. Further investigations are needed to elucidate which conditions may favor the persistence of native diversity.

4271: *+.158*

The cutthroat trout (Oncorhynchus clarkii) is a popular sport fish that is native to the waters of western North America. Cutthroat trout comprises many subspecies, each of which has experienced range reduction resulting from anthropogenic activities. Hence, there is a general interest from management agencies to assess the genetic structure of managed populations of cutthroat trout subspecies to ensure that proper conservation plans are implemented. Herein, we utilize microsatellite data to characterize the genetic composition of 2 populations of Bonneville cutthroat trout in Great Basin National Park: Mill Creek and South Fork Big Wash. Mill Creek was used as a source population for reintroduction into South Fork Big Wash in the year 2000, and there is concern that South Fork Big Wash may have experienced a population bottleneck after, or during, the stocking effort. We found that both populations exhibit low genetic diversity, and that the source population, Mill Creek, exhibited mixed signals of having undergone a recent population bottleneck. Structure analysis revealed 4 distinct groups, but those groups did not segregate geographically, although a significant pairwise FST (0.06727, $P < 0.00001$) between Mill Creek and South Fork Big Wash populations suggests that some genetic differentiation has occurred.

4272: ***+.263***

The use of captive animals for population re-establishment or augmentation can be an important part of conservation efforts, but practitioners need experimentally derived evidence to guide the best strategies and inform whether such practices could be successful. Here, we examined how several manipulations to captive-rearing practices influence the performance of the Common Watersnake, Nerodia sipedon sipedon, during their first year in the wild. Following release, snakes that had experienced a period of enrichment during captivity to better simulate natural environments did not differ from conspecifics reared in more simplistic conditions on any measure of post-release behavior or performance. Moreover, captive snakes in both treatments exhibited habitat use, movement, thermoregulatory, and seasonal activity behaviors largely indistinguishable from resident conspecifics at the release site, and ultimately performed similarly in maintenance of body condition and survivorship. These results are in contrast to earlier releases and suggest that using older and larger individuals that have undergone a period of simulated winter dormancy may improve success during the early phase of establishment. However, captive snakes grew only one third as fast as wild native snakes, suggesting they experienced difficulties foraging in the wild. Further studies testing the effectiveness of translocation programs using captive animals as a management tool are urgently needed, but our findings do point to some success.

4273: ***-.044***

Two pervasive and fundamental impacts of urbanization are the loss and fragmentation of natural habitats. From a genetic perspective, these impacts manifest as reduced genetic diversity and ultimately reduced genetic viability. The growling grass frog (Litoria raniformis) is listed as vulnerable to extinction in Australia, and endangered in the state of Victoria. Remaining populations of this species in and around the city of Melbourne are threatened by habitat loss, degradation and fragmentation due to urban expansion. We used mitochondrial DNA (mtDNA) and microsatellites to study the genetic structure and diversity of L. raniformis across Melbourne's urban fringe, and also screened four nuclear gene regions (POMC, RAG-1, Rhod and CRYBA1). The mtDNA and nuclear DNA sequences revealed low levels of genetic diversity throughout remnant populations of L. raniformis. However, one of the four regions studied, Cardinia, exhibited relatively high genetic diversity and several unique haplotypes, suggesting this region should be recognized as a separate Management Unit. We discuss the implications of these results for the conservation of L. raniformis in urbanizing landscapes, particularly the potential risks and benefits of translocation, which remains a contentious management approach for this species.

4274: ***+.014***

In the Netherlands, Burbot Lota lota have severely declined due to aquatic system modifications and agricultural intensification. The aim of this study was to evaluate the species' distribution and population trends and to interpret its decline for conservation and management-planning. Historic and present distributions were examined by GIS analysis, while the decline was quantified using binary logistic regression of presence-absence data. Records suggest that at the beginning of the twentieth century the overall Burbot population was relatively stable but reached a turning point from a positive to a negative population phase around 1950. Today, only two areas with spawning populations remain. Recently, increasing numbers of Burbot have been recorded in one of these areas, viz. the lakes in the confluence area of the rivers Vechte and IJssel. This increase could be attributed to annual stockings in German reaches of the River Vechte since 2001, with stock material originating from the rivers Elbe and Weser. Because of the low numbers in which Burbot populations were present and the risks of introduction of non-local stock, Burbot requires

imperative tools and action plans for recovery and conservation in the Netherlands.

4275: ***+.036***

Border and isolated plant populations represent an interesting target for ecological and conservation issues. We analysed the ecological constraints and the conservation status of the eastern population of Helianthemum caput-felis Boiss. (Cistaceae), located in Sardinia. The distribution of H. caput-felis was verified via field surveys; ecological data, morphological and reproductive traits, were recorded in 40 permanent plots randomly established; the human trampling effects on plant density, plant size and plant performance were analysed. Plant density was higher in bedrock and lowland areas, in garrigue and maquis habitats; however, the differences among plants growing in different ecological conditions were not statistically significant; only human trampling intensity significantly affected plant density and lowest values were observed in areas with intense trampling pressure. All ecological variables analysed had a statistically significant effect on plant size and on the number of fruits per plant. In particular, larger plants were found in areas with the following ecological features: presence of structured soil, on the slopes, in the maquis habitat, and in areas with intensive human trampling. Conversely, plants displayed a higher fruits output per plant in deep and structured soil, in lowland areas, and in the garrigue and maquis habitats; the mean fruits output per plant increased as human trampling intensified. Human-induced threats are the main hazards threatening the remaining Sardinian population. In particular, the major threats are linked to tourism and other outdoor activities (i.e. human trampling), followed by the expansion of agricultural activities; all of these threats result in the disappearance of small localities and in reduced population size due to habitat loss and fragmentation. Our study indicates that H. caput-felis should be considered as Critically Endangered (CR) at the regional level. Urgent measures should be undertaken to protect the remaining H. caput-felis population in Sardinia and a possible integrated strategy for the conservation and management consists of a combination of in situ and ex situ measures. In particular, greater emphasis should be given to minimizing the negative impacts of unsustainable tourism and recreation use, in order to exclude human trampling and to facilitate the plant recruitment process and population renewal. In addition, an ex situ conservation strategy must be implemented and the seeds collected could be used for future translocations in suitable areas. Moreover, considering the threats observed, a long-term monitoring programme must be developed to reveal changes in the species conservation status.

4276: ***+.146***

Endangered giant otters, Pteronura brasiliensis, are found along the Amazon and Orinoco rivers and most of their tributaries. Hunting in the mid-1970s pushed giant otter populations to the brink of extinction. We studied population structure and genetic diversity of giant otters from Colombia's Orinoco basin using analyses of partial mitochondrial DNA control region sequences obtained from scat material. We collected and analyzed 54 scat samples from 22 latrines, 2 tissue samples primarily from captive giant otters and 2 from hunted animals near Puerto Carreno and Puerto Inirida (Colombian Orinoco), as well as one tissue sample from Puerto Leguizamo (Colombian Amazon). Thirty-nine partial control region sequences were obtained (258 bp), corresponding to 15 unique haplotypes. Most of these haplotypes, found in samples collected around Puerto Carreno, defined one phylogeographic group (phylogroup) not previously described. Higher genetic diversity in the Colombian Orinoco populations than in other South American populations suggests that this newly described phylogroup, as well as a second phylogroup defined from a few Colombian Orinoco and Amazon samples, should be considered distinct genetic management units. National conservation programs, particularly those aiming to

establish protected areas, should manage these independently. Current Colombian confiscated animal reintroduction and captive reproduction programs should also consider such differentiation when determining reintroduction locations or improving husbandry practices.

4277: *-.068*

The freshwater fish Piaractus brachypomus is an economically important for human consumption both in commercial fisheries and aquaculture in all South American countries where it occurs. In recent years the species has decreased in abundance due to heavy fishing pressure. The species occurs in the Amazon and Orinoco basins, but lack of meristic differences between fishes from the 2 basins, and extensive migration associated with reproduction, have resulted in P. brachypomus being considered a single panmictic species. Analysis of 7 nuclear microsatellites, mitochondrial DNA sequences (D-loop and COI), and body shape variables demonstrated that each river basin is populated by a distinct evolutionarily significant unit (ESU); the 2 groups had an average COI divergence of 3.5% and differed in body depth and relative head length. Historical connection between the 2 basins most probably occurred via the Rupununi portal rather than via the Casiquiare canal. The 2 ESUs will require independent fishery management, and translocation of fisheries stocks between basins should be avoided to prevent loss of local adaptations or extinction associated with outbreeding depression. Introductions of fishes from the Orinoco basin into the Putumayo River basin, an Amazon basin drainage, and evidence of hybridization between the 2 ESUs have already been detected.

4278: *+.063*

Aldrovanda vesiculosa is an aquatic carnivorous plant native to nutrient impoverished wetland systems in Australia, Africa, Asia, and continental Europe that has declined dramatically throughout its native range in the last century. A strong reliance upon carnivory generally limits its occurrence to specific, nutrient-poor, island-like microhabitats. Remaining native populations are generally small and fragmented, and empirical population ecology data for the species arelacking. Developing an understanding of the constraints to growth, establishment and reproduction in A.vesiculosa is crucial to conservation of the species. In contrast with the decline of the species throughout its native range, a number of large A.vesiculosa populations have become naturalised in North America. This study examines the population ecology of A.vesiculosa at one of these naturalised sites and assesses the species' potential invasiveness in terms of its ecological characteristics. Transect and quadrat surveys were used to determine the response of morphology, fecundity and spatial distribution to putative biotic and environmental variables, with glasshouse trials and a bird feeding experiment employed to test the persistence of seeds in the seed bank and after transport in bird guts. Although A.vesiculosa is capable of becoming locally abundant (up to 1260 individuals m(-2)) in wetland areas where biotic and abiotic conditions are optimal, it appears to compete poorly with floating and emergent macrophytes and is limited predominantly to specific microhabitats. The ecological characteristics of A.vesiculosa suggest that it poses a low invasion risk. The species' growth and reproductive potential are highest in shallow areas harbouring loose vegetation assemblages, with over two thirds of all individuals recorded from water 10-50cm in depth and in areas with <50% native macrophyte cover. Seeds are unlikely to play a significant role in seasonal persistence or dispersal, with poor floral success (c. 10%), few seeds produced, low seed viability (<50%) throughout the study area and no seeds recovered from the sediment seed bank or recovered from bird faeces following gut transport. The stenotopic ecology of A.vesiculosa, and the continuing decline of dystrophic freshwater wetlands globally, indicate that remaining natural populations are highly sensitive and are likely to decline rapidly without adequate management. In addition to wetland management at the catchment scale to

mitigate processes such as eutrophication, future conservation and reintroduction initiatives for A.vesiculosa must focus particularly on the identification, maintenance and restoration of optimal shallow humic microsites harbouring loose, open assemblages of emergent and floating macrophytes.

4279: ***-.015***

Arboreal squirrels of the Asiatic genus Callosciurus have shown high likelihood of establishment from few released animals, in particular, C. erythraeus has established wild populations in Argentina, Belgium, France, Hong Kong, Japan, and The Netherlands. We report the invasion process of C. erythraeus in Argentina in the last four decades and suggest management actions for each foci. Between February 2011 and November 2014 we conducted field surveys and interviews in nine sites in central Argentina to confirm the presence of C. erythraeus, describe their history of introduction, and estimate range expansion and squirrel relative abundance. We report a two decades lag-phase until the onset of translocations of C. erythraeus within national boundaries that resulted in a constant increase of the cumulative number of releases. We confirm nine new release events between 1995 and 2012 and six new invasion foci that yields a total of 13 deliberate releases and 10 invasion foci established in rural and urban areas of Argentina. Spread rate ranged from 0.12 to 0.66 km/year. An intermediate relative density of squirrels (2-7 ind/ha) was found close to release sites except in one case. All introduction events involved squirrels translocated from the first, 40 years old invasion focus, occasionally involving illegal trade. The rate of introduction events in the last decades and the translocation-lag phase described in this study should call the attention in all countries dealing with charismatic, introduced species. Translocation disruption requires urgent attention to slow down the invasion of this and other species.

4280: ***+.279***

AimConservation biologists use various approaches to augment imperilled populations in order to supplement genetic variation and restore ecological function. However, understanding genotypic, phenotypic and ecotypic variation is critical in determining the most suitable sources to conserve historical and functional variation. Bighorn sheep (Ovis canadensis) provide a classic example of restoration biology, where management programmes have re-established extirpated populations via translocations. Through this process, translocated individuals may now face novel environments, including new bioclimatic conditions and an opportunity to interbreed with historically isolated and distinct genetic units. Our goal was to integrate genetic and ecological analyses to assess some of the evolutionary ramifications of this important management practice. LocationWestern North America, but focused on the Great Basin and northern Mojave deserts. MethodsWe quantified genetic variation across 55 Herds of bighorn sheep using 16 microsatellite loci (N=347) and a mitochondrial gene (N=110). We used ordination, Bayesian clustering and phylogenetic analyses to delineate evolutionary units. We used multivariate ordination for 26 ecologically relevant variables to characterize niche-based differences among genetic clusters and then tested whether repatriated populations occupy conditions similar to source areas. ResultsWe document genetic differentiation among three traditional management units of bighorn sheep that now occupy the Great Basin and northern Mojave deserts, but also identify limited hybridization among these groups. Niche-based analyses revealed that translocated populations now occur in conditions incongruent with source ranges. Main conclusionsThis study highlights the importance of considering both genetic variation and ecological differences when implementing translocations. Early consideration of these variables may help minimize the potential for hybridization among distinct groups and mitigate challenges of managing populations

experiencing novel environments. More broadly, results from this study have implications for future restoration efforts in this iconic system, but also for similar translocation-based conservation programmes.

4281: *+.008*

Dams have contributed to the decline of migratory fishes by blocking access to historical habitat. The active transport (trap and haul) of migratory fish species above existing dams can sometimes support population recovery when the use of fish ladders or dam removal is infeasible. However, little is known about the efficacy of trap and haul conservation strategies. Here we used genetic parentage assignments to evaluate the efficacy of reintroducing adult Chinook salmon (Oncorhynchus tshawytscha) above Cougar Dam on the South Fork McKenzie River, Oregon, USA, from 2008 to 2011. We found that mean reproductive success (RS) declined as adults were released later in the spawning season in 2009 and 2010; however, release location did not affect RS. In 2010 and 2011, we tested for RS differences between hatchery and natural origin (HOR and NOR, respectively) adults. HOR males were consistently less fit than NOR males, but little evidence for fitness differences was apparent between HOR and NOR females. Interestingly, the effect of origin on RS was not significant after accounting for variation explained by body length. Our results indicate that release date and location have inconsistent or no effect on the reproductive success of reintroduced adults when active transport strategies are employed for migratory fishes.

4282: *+.060*

Genetic diversity is a primary component of adaptive evolution, and its loss or reduction can decrease the long-term survival probability of populations. Utilization of cryopreserved semen may be considered a perfect tool to improve genetic diversity, reduce inbreeding, and avoid animal translocation for breeding. The present study aimed at finding a reliable epididymal sperm freezing protocol for the critically endangered onager (Equus hemionus onager). Six testicles from three animals were processed postmortem. The effects of two transportation temperatures (22 degrees C and 4 degrees C; testicles submerged in saline), two cryopreservation techniques (conventional liquid nitrogen vapor freezing in straws and directional freezing in 8-ml HollowTubes (TM)), and two postthaw incubation temperatures (22 degrees C and 37 degrees C; evaluated after 0.5, 1, 2, and 3 hr) were tested in a 2 x 2 x 2 experimental design. Sperm samples were evaluated for motility, viability, acrosome integrity, and sperm morphology. The resulting optimal freezing protocol includes transportation of testicles at 4 degrees C, cryopreservation by directional freezing, and, if needed, postthaw incubation at 22 degrees C. With this combination of transportation temperature and cryopreservation technique, the authors obtained the following postthaw values normalized to prefreezing values: 60.3 +/- 8.8% motility, 60.7 +/- 13.3% viability, 75.3 +/- 9.5% acrosome integrity, and 94.7 +/- 2.9% normal morphology (excluding defects due to the epididymal origin of the sperm). After incubation at 22 degrees C, motility values for the above combination were 40 +/- 5.7%, 30.3 +/- 5.2%, 28.3 +/- 4.4%, and 16.7 +/- 4.4% for 0.5, 1, 2, and 3 hr, respectively. In conclusion, with this protocol, good quality semen can be stored for future use in artificial inseminations when and where needed.

4283: *+.200*

Contrasting movements and habitat use may occur among snakes, and these differences may reflect important local responses to habitat variation and/or signify that broad, unimodal approaches to species conservation are too coarse. Fine-scale differences in these behaviors (e.g.,

between neighboring conspecifics) may be underappreciated because studies either focus exclusively on one (sub) population, and/or the animals are not compared across relevant ecological boundaries. We report on a striking dichotomy of seasonal migration behavior between Rattlesnakes (Crotalus oreganus) using two neighboring den sites. The snakes at one site undertook long-distance (2.9 +/- 0.57 km [mean +/- SD], highly directional migratory behavior that was consistent across years and individuals. These movements carried them out of the habitat normally associated with these animals in this region (grassland steppe) and into higher elevation forests, a habitat not previously reported for these animals. Conversely, snakes at another den only 21 km away were nonmigratory (0.97 +/- 0.54 km) and remained in grassland habitat throughout the year. Landscape and other environmental factors may have been responsible for the difference in the behavior of these two denning groups, suggesting that our knowledge of what dictates the migratory behavior of animals such as snakes is far from complete. Stereotyping the habitat use and behavior of any snake population is potentially misleading, and more thorough exploration is needed on how these animals alter their local movement patterns in response to changes in landscapes and habitat configurations, including potential climate change effects.

4284: ***+.025***

For small, isolated populations 2 common conservation concerns relate to genetic threats: inbreeding and negative consequences associated with loss of genetic diversity due to drift. Mitigating these threats often involves conservation actions that can be controversial, such as translocations or captive breeding programs. Although such actions have been successful in some situations, in others they have had undesirable outcomes. Here, we estimated the effective population size (N (e)) of the Sable Island horses to assess the risk to this population of these genetic threats. We found surprising consistency of N (e) estimates across the 5 different methods used, with a mean of 48 effective individuals. This estimate falls below the 50 criterion of the "50/500 rule," below which inbreeding depression is a concern for population viability. However, simulations and knowledge of population history indicate that this population is still in its early stages of approaching equilibrium between mutation, drift, and genetic diversity; and no negative consequences have been identified that could be associated with inbreeding depression. Therefore, we do not recommend taking management action (such as translocations) at this stage. Rather, we propose continued monitoring of genetic diversity and fitness over time so that trends and any substantial changes can be detected. This represents one of the few unmanaged horse populations in the world, and therefore these data will not only alert us to serious concerns regarding their conservation status, but will also provide a wealth of information about how natural processes drive patterns of reproduction, mortality, and population growth over time.

4285: ***+.225***

Coccothrinax jimenezii M.M. Mejia & R.G. Garcia (Fig. 1) is assigned the IUCN's Critically Endangered conservation category. Extensive field work performed in 2015 confirmed the existence of a population of 43 individuals, mostly juvenile, in Haiti (previously reported in 1995). These field studies also corroborated that the species (18 individuals, mostly adults) is restricted to only a single site in the Dominican Republic. None of the trees was in reproductive stage during our visits. Two major conservation challenges include: (1) a current trend for water levels to increase in the hypersaline Lago Enriquillo and (2) harvest of palm leaves for making brooms in Haiti. Recommendations are made to collect seed for ex situ conservation and future species reintroductions in its original habitat.

4286: ***-.017***

Gunnison sage-grouse (Centrocercus minimus) are distributed across southwestern Colorado and southeastern Utah, United States. Their distribution has decreased over the past century and the species has been listed as threatened by the U.S. Fish and Wildlife Service. Reduced genetic diversity, small population size, and isolation may affect Gunnison sage-grouse population persistence. Population augmentation can be used to counteract or mitigate these issues, but traditional translocation efforts have yielded mixed, and mostly unsuccessful, results. Captive-rearing is a viable, although much debated, conservation approach to bolster wild conservation-reliant species. Although there have been captive-rearing efforts with greater sage-grouse (C. urophasianus), to date, no information exists about captive-rearing methods for Gunnison sage-grouse. Therefore, we investigated techniques for egg collection, artificial incubation, hatch, and captive-rearing of chicks, juveniles, subadults, and adults for Gunnison sage-grouse. In 2009 we established a captive flock that produced viable eggs. From 2009-2011, we collected and artificially incubated 206 Gunnison sage-grouse eggs from 23 wild and 14 captive females. Our hatchability was 90%. Wild-produced eggs were heavier than captive-produced eggs and lost mass similarly during incubation. We produced 148 chicks in captivity and fed them a variety of food sources (e.g. invertebrates to commercial chow). Bacterial infections were the primary cause of chick mortality, but we successfully reduced the overall mortality rate during the course of our study. Conservationists and managers should consider the utility in developing a captive-rearing program or creating a captive population as part of a proactive conservation effort for the conservation-reliant Gunnison sage-grouse. Zoo Biol. 34:438-452, 2015. (c) 2015 Wiley Periodicals, Inc.

4287: ***+.125***

We investigated the survival and breeding success of common pheasants Phasianus colchicus of two origins and in two predator densities. We translocated hand-reared and wild pheasant hens to southern Finland (60 degrees N, 24 degrees E) and hand-reared ones to central Finland (63 degrees N, 27 degrees E). Both groups of birds were treated similarly before release and translocated to areas with no local pheasant populations. Both areas appeared similar, the only major difference being the amount of predators. The red fox Vulpes vulpes was the major predator of pheasants present in the southern study, where it was abundant, whereas it was almost non-existant in central Finland. In accordance with earlier studies, the wild birds survived much better than the hand-reared ones in the area with a high red fox density. The hand-reared birds located in the low red fox density area survived better than the hens in the area of high red fox density. However, no significant difference was observed in the survival of the hand-reared birds in the low fox density area and wild birds in the high fox density area. Interestingly, after the first two weeks, the survival of pheasants in different groups was equal. We additionally found no significant differences between the bird-groups in terms of hatching success when comparing hens that managed to initiate nesting. No difference was also observed between the hand-reared birds in the low fox density area and the wild in the high fox density area in brood survival to the age of six weeks. We conclude that even hand-reared pheasants can succeed in brood production in an area with low fox densities. We furthermore suggest that pheasants that survive the two first weeks after translocation have good chances of producing a brood whether they are wild or hand-reared.

4288: ***+.137***

Given that 29% of seabird species are threatened with extinction, protecting seabird colonies on offshore islands is a global conservation priority. Seabirds are vulnerable to non-native predator invasions, which reduce or eliminate colonies. Accordingly, conservation efforts have focused on predator eradication. However, affected populations are often left to passively recover following

eradications. Although seabirds are highly mobile, their life history traits such as philopatry can limit passive recolonization of newly predator-free habitat. In such cases, seabird colonies can potentially be re-instated with active restoration via chick translocations or social attraction methods, which can be risky and expensive. We used biogeographic and species-specific behavioral data in the Hauraki Gulf, New Zealand, a global hotspot of seabird diversity and predator eradications, to illustrate the use of geographic information systems multi-criteria decision analysis to prioritize islands for active seabird restoration. We identified nine islands with low observed passive recovery of seabirds posteradication over a 50-year timeframe, and classified these as sites where active seabird management could be prioritized. Such spatially explicit tools are flexible, allowing for managers to choose case-specific criteria such as time, funding, and goals constrained for their conservation needs. Furthermore, this flexibility can also be applied to threatened species management by customizing the decision criteria for individual species' capacity to passively recolonize islands. On islands with complex restoration challenges, decision tools that help island restoration practitioners decide whether active seabird management should be paired with eradication can optimize restoration outcomes and ecosystem recovery.

4289: *-.117*

Wild canids were historically abundant in Korea; however, the gray wolf, dhole, and red fox were extirpated during the twentieth century. The causes varied. "Pest control" during the Japanese occupation, ecological destruction during wars, disease epizootics, and "vermin control" after the Korean War contributed to the complete demise of wolves. The fox had succumbed to unregulated hunting, rodenticides, habitat loss, and disease epizootics. The dhole was naturally rare; its extirpation from northeastern Asia including Korea is not established. Although the wolf and fox are extirpated, the Korean government still lists both as endangered species to facilitate the recently implemented restoration programs. Restoration will face the challenges of importing genetically diverse populations and the critical loss, fragmentation, and alteration of peri-urban habitats. The overall social support for these efforts is not clear: it may be low because of changes in social mores or simply an unintended consequence of land and water use choices and policies that people may not perceive in everyday life. In this critical analysis, we postulate that the current restoration programs are misdirected toward inappropriate species and likely employ outdated techniques. We propose that a reallocation of restoration efforts and resources to populations of existing rare or threatened species would be more ecologically beneficial with higher probabilities of success. We recognize that there can be good reason to restore the upper trophic levels, especially keystone species, but are concerned that the impetus is more about focusing on charismatic megafauna rather than pragmatic choices more likely to be effective.

4290: *+.035*

Nepeta rtanjensis (Lamiaceae) is an endemic and critically endangered plant species that grows only on Rtanj Mountain in the southeastern Serbia. Due to its endemic character and distribution separated from the other Nepeta species in Mediterranean Basin, N. rtanjensis has been considered as a relict. It has been noticed that number of individual plants in natural habitat rapidly decreases. Hence, the project of reintroduction of this plant was successfully conducted. However, many aspects of its biology still remain unknown and necessary to investigate. In that sense, epiphytic and endophytic Nepeta rtanjensis phyllosphere fungi were studied at three sites in Serbia: Javor-Locus classicus, with autochthonous population and with reintroduced plants, and Belgrade with micro-propagated specimens in experimental field. The sites in Javor are located in the area of the Mountain Rtanj. From both, the surface and the interior of leaves, a total of 49 taxa of microfungi were identified, belonging to 35 genera. Alternaria spp. was reported on all studied sites with the

highest isolation frequency (>75 %). Similarity between epiphytic and endophytic fungal assemblage was moderate. The highest fungal diversity was detected on site with the autochthonous Nepeta rtanjensis population. Phyllosphere mycobiota show the presence of a large number of viable propagules, with a low frequency of occurrence of symptoms, due to the specific anatomy of the leaf and a well-developed indumentum.

4291: *+.032*

The Central American river turtle (Dermatemys mawii) is an endangered species due to its illegal capture for consumption and modification of its natural habitat. As an alternative to those threats, captive management has been considered for its conservation and exploitation. This turtle is bred in farms for its commercial use, and otherwise, with the ultimate goal to recovery of wild populations by releasing captive born animals. Breeding of D. mawii is based in traditional knowledge and implies techniques based in breeders experience, mainly in Tabasco, Veracruz and Campeche, Mexico. Recent studies in captivity showed negative effects on their health associated to breeding practices, which suggest that breeding practices must be improved. Conservation arguments are discussed related to its imperative recovery, including aspects such as health, genetic integrity, and financial support. Captive management could be a conservation tool for an endangered species when reintroduction of captive bred animal to natural habitat is considered; however, this plan do not exist in the case of D. mawii. This situation is addressed constructively analyzing whether to make a stop on the current strategy of captive management of the species by reformulating goals, actions and conservation efforts.

4292: *-.030*

Objective. Determine the infection status with pathogenic Leptospira of one Saguinus oedipus and nine Saguinus leucopus at the Cali Zoo that had been confiscated in Colombia from illegal trade. Materials and methods. A full physical examination, blood work, urinalysis were conducted in all individuals during the reception health check-up, in addition to running the microagglutination test with a pool of 19 serovars, with a starting dilution of 1: 50. Results. A high positive titer (>= 1:3200) to Leptospira alexanderi serovar manhao in an asymptomatic S. oedipus was detected. All S. leucopus tested negative or less than 1: 50. Conclusions. Captive locations have been documented to artificially enhance opportunities to come into contact with contaminated bodily fluids from peridomestic rodents. However, infectious diseases acquired during the illegal transport of wildlife to major metropolitan centers are rarely considered a wildlife conservation or public health threat. Infection with zoonotic pathogens should also be considered an additional threat to endangered wild primates involved in illegal trade, which could hamper reintroduction efforts or other population management procedures for primate species with restricted and fragmented distributions.

4293: *+.051*

Long-term demographic data are central for the evaluation of endangered species recovery plans. We present the demography and dynamics of a managed African wild dog (Lycaon pictus) metapopulation in South Africa, based on life histories of 553 individually known animals collected between 1998 and 2006. This metapopulation, distributed across nine sites (and not including Kruger National Park), was established through reintroductions (n = 9 events) and maintained by periodic augmentation (n = 10 events) and translocation (n = 20 events). In total, 66 founders were used to establish subpopulations and mean founder group size was 9.6 animals. The metapopulation grew from 17 individuals in 1998 to a peak of 202 in 2005. Mean annual

population density was 3.3 (S.E. 0.44) wild dogs/100 km(2), approaching the upper limit of densities reported from unmanaged populations. Mean size of breeding packs was 11.0 (S.E. 0.76), comparable to pack sizes in Kruger National Park (Kruger), the only viable unmanaged population in South Africa. Fecundity was lower than in Kruger - particularly in the older age classes. Pup survival to adulthood was 45% - nearly three times the survival rate for pups in Kruger. Mean annual population growth rate (lambda = 1.08, S.E. 0.13) was higher than in unmanaged populations (range 1.000-1.038), with implications for population viability and management.

4294: *+.234*

The African wild dog (Lycaon pictus) is an endangered species and South Africa has only one remaining viable population. Reintroduction of this species in South Africa is of high conservation priority and knowledge of the species' ecology is crucial for success. In this study, important information was gained regarding the reintroduction process although it ultimately failed. The current conservation plan for wild dogs incorporates such existing knowledge, but results from reintroductions (including failures) are needed to strengthen the strategy and its success. The main purpose of this study was to quantify home range area and habitat selection patterns of a wild dog pack unsuccessfully reintroduced into Mkhuze Game Reserve (MGR). A total of 847 radio-tracking fixes of the pack were collected. The home range area of the pack was estimated to be 383.9 km(2) (100% minimum convex polygon method) or 377.9 km(2) (95% fixed-kernel method), each value being larger than the area of the game reserve (360 km(2)). Significant monthly variations in home range areas of the pack were detected, with a maximum monthly home range area reached at similar to 10 months after tracking began (19 months after the pack was reintroduced). Independent of season, the pack consistently selected Acacia nilotica low-closed woodland and consistently avoided riverine forest habitats. Small differences in home range areas of the pack were found between wet and dry seasons. The deaths of some wild dogs were the result of snares, and others may have been due to predation or hunting injuries. We present evidence that factors including game reserve area, availability of preferred habitat and climate may have contributed to the distribution patterns and ultimately the failure to maintain viable numbers during this wild dog reintroduction. The reintroduction of wild dogs to MGR was successful in that the introduced wild dogs bred, owing to the release of a pregnant female, but was considered unsuccessful since none of the dogs from the reintroduced pack survived or remained in MGR for more than two years. However, two wild dogs returned shortly after radio-tracking sessions ceased and were soon joined by another female from an adjacent game reserve.

4295: *+.011*

The distribution of the brown hyaena (Hyaena brunnea) in southern Africa overlaps widely with commercial livestock ranching. As a direct result, both perceived and confirmed conflict with farmers occurs and hyaenas are trapped for lethal control or translocation. We studied the outcomes of a conflict-related brown hyaena translocation in Central Namibia involving a subadult female - the first reported GPS-monitored translocation of this species. The animal was moved 63 km from the conflict site and after exploratory movements settled into a new home range incorporating resident conspecifics. The hyaena caused no further conflict and did not return home to its original capture site where livestock depredation ceased. The hyaena was killed in a road accident five months after release. We assess and review our results (and brown hyaena translocations in general) with respect to species ecology, previous translocations as well as monitoring data from resident conspecifics. We provide supporting information that individual hyaenas can be translocated successfully but emphasize that decisions need to be made case-specifically considering the age, sex and social status of the animals. We highlight the importance

of brown hyaena sociality when considering translocation as a management tool.

4296: *+.072*

Micropterus cataractae (Shoal Bass), an enigmatic fluvial specialist, has experienced range-wide declines because of habitat fragmentation and other negative effects of impounded rivers. In addition to these concerns, anglers often translocate Shoal Bass from riverine habitats to impoundments following tournament weigh-ins. To investigate the potential effects of this practice, we translocated adult Shoal Bass from riverine habitats to a downstream impoundment and assessed their movements, homing abilities, and eventual fates. All fish rapidly evacuated the impoundment in favor of lotic habitats, and the majority of translocated fish returned upstream within about 3 weeks. Half of our translocated fish also displayed homing to within 1 river km of their original capture site. Our results demonstrate that fluvial-specialist Shoal Bass can survive translocation into impoundments, but the differential effects of translocation associated with fishing tournaments should also be considered in the management of Shoal Bass fisheries.

4297: *+.070*

Delimitating species boundaries could be of critical importance when evaluating the species' evolving process and providing guidelines for conservation genetics. Here, species delimitation was carried out on three endemic and endangered Cycas species with resembling morphology and overlapped distribution range along the Red River (Yuanjiang) in China: Cycas diananensis Z. T. Guan et G. D. Tao, Cycas parvula S. L. Yang and Cycas multiovula D. Y. Wang. A total of 137 individuals from 15 populations were genotyped by using three chloroplastic (psbA-tmH, atpl-atpH, and tmL-rps4) and two single copy nuclear (RPB1 and SmHP) DNA sequences. Basing on the carefully morphological comparison and cladistic haplotype aggregation (CHA) analysis, we propose all the populations as one species, with the rest two incorporated into C. diannanensis. Genetic diversity and structure analysis of the conflated C. diannanensis revealed this species possessed a relative lower genetic diversity than estimates of other Cycas species. The higher genetic diversity among populations and relative lower genetic diversity within populations, as well as obvious genetic differentiation among populations inferred from chloroplastic DNA (cpDNA) suggested a recent genetic loss within this protected species. Additionally, a clear genetic structure of C. diannanensis corresponding with geography was detected based on cpDNA, dividing its population ranges into "Yuanjiang-Nanhun" basin and "Ejia-Jiepai" basin groups. Demographical history analyses based on combined cpDNA and one nuclear DNA (nDNA) SmHP both showed the population size of C. diannanensis began to decrease in Quaternary glaciation with no subsequent expansion, while another nDNA RPB1 revealed a more recent sudden expansion after long-term population size contraction, suggesting its probable bottleneck events in history. Our findings offer grounded views for clarifying species boundaries of C. diannanensis when determining the conservation objectives. For operational guidelines, the downstream populations which occupy high and peculiar haplotypes should be given prior in-situ conservation. In addition, ex-situ conservation and reintroduction measures for decades of generations are supplemented for improving the population size and genetic diversity of the endemic and endangered species.

4298: *+.095*

Increasingly, renewable energy comprises a larger share of global energy production. Across the western United States, public lands are being developed to support renewable energy production. Where there are conflicts with threatened or endangered species, translocation can be used in an

attempt to mitigate negative effects. For the threatened Mojave desert tortoise (Gopherus agassizii), we sought to compare habitat-and space-use patterns between short-distance translocated, resident, and control groups. We tested for differences in home range size based on utilization distributions and used linear mixed-effects models to compare space-use intensity, while controlling for demographic and environmental variables. In addition, we examined mean movement distances as well as home range overlap between years and for male and female tortoises in each study group. During the first active season post-translocation, home range size was greater and space-use intensity was lower for translocated tortoises than resident and control groups. These patterns were not present in the second season. In both years, there was no difference in home range size or space-use intensity between control and resident groups. Translocation typically resulted in one active season of questing followed by a second active season characterized by space-use patterns that were indistinguishable from control tortoises. Across both years, the number of times a tortoise was found in a burrow was positively related to greater space-use intensity. Minimizing the time required for translocated tortoises to exhibit patterns similar to non-translocated individuals may have strong implications for conservation by reducing exposure to adverse environmental conditions and predation. With ongoing development, our results can be used to guide future efforts aimed at understanding how translocation strategies influence patterns of animal space use.

4299: ***+.018***

The number of threatened species continues to increase due to a range of anthropogenic disturbances, and many species continue to decline increasing their risk of extinction. Translocation is a widely used management technique to establish new populations to reduce the risk of extinction. There are, however, a range of issues to be considered. For example, for some species the donor population may be impacted by translocation, for other species it must be decided whether to translocate adults or juveniles to establish new populations. The question then becomes who do you move? The endangered Macquarie perch in south-eastern Australia is continuing to decline, with the recent Millennium Drought (1997-2010) and associated events (e.g., bushfires) contributing to dramatic local declines and the need for emergency responses. Successful historic translocations of this species involved adult fish, however the removal of significant numbers of adult fish may now impact source populations and alternative translocation approaches needed investigating. The use of sub-adult or juvenile fish, that would be expected to experience higher mortality, may be an approach to establishing new populations which would have less severe impacts on source populations. However, the number of fish required, frequency of translocation and likelihood of population establishment are unknown. This study outlines the development of a population model to assist in trialling translocation scenarios for establishing new populations of Macquarie perch. The model predicts that translocations of young-of-year fish (age 0+) is unlikely to be successful unless similar to 600 females are released annually for five years. If translocating yearling (age 1+) fish, annual translocations of >100 females is required to achieve success, with stocking for at least five consecutive years required. If the frequency of recruitment failure or magnitude of Allee effects increases, then translocations of increased numbers of yearlings or prolonged stocking (10 years) is required to achieve success. The addition of small numbers of adult fish in combination with yearlings decreases the number of yearlings required, and increases the chance of success under more stressful scenarios. Crown Copyright (C) 2015 Published by Elsevier B.V. All rights reserved.

4300: ***+.165***

Most reintroduction and restocking programs consist of releasing captive-raised juveniles. The

usefulness of these programs has been questioned, and therefore, quality control is advisable. However, evaluating restocking effectiveness is challenging because mortality estimation is required. Most methods for estimating mortality are based on tag recovery. In the case of fish, juveniles are tagged before release, and fishermen typically recover tags when fish are captured. The statistical models currently available for analyzing these data assume either constant mortality rates, fixed tag non-reporting rates, or both. Here, instead, we proposed a method that considers the mortality rate variability as a function of age/size of the released juveniles. Furthermore, the proposed method can disentangle natural from fishing mortality, analyzing the temporal distribution of the captures reported by fishermen from multiple release events. This method is demonstrated with a restocking program of a top-predator marine fish, the meagre (Argyrosomus regius), in the Balearic Islands. The estimated natural mortality just after release was very high for young fish (m(0) = 0.126 day(-1) for fish 180 days old), but it was close to zero for large/old fish. These large/old fish were more resilient to wild conditions, although a long time was needed to achieve a relevant reduction in natural mortality. Conversely, these large/old fish were more vulnerable to fishing, creating a trade-off in survival. The release age that maximizes the number of survivors after, for example, one year at liberty was estimated to be 1,173 days. However, the production cost of relatively old fish is high, and only a few fish can be produced and released within a realistic budget. Therefore, in the case of the meagre, increasing the number of released fish will have no or scarce effects on restocking success. Conversely, it is advisable implement measures to reduce the high natural mortality of young juveniles and/or the length of time needed to improve fish resilience.

4301: ***+.046***

Background0pc?>Quercus oleoides Cham. and Schlect., tropical live oak, is a species of conservation importance in its southern range limit of northwestern Costa Rica. It occurs in high-density stands across a fragmented landscape spanning a contrasting elevation and precipitation gradient. We examined genetic diversity and spatial genetic structure in this geographically isolated and genetically distinct population. We characterized population genetic diversity at 11 nuclear microsatellite loci in 260 individuals from 13 sites. We monitored flowering time at 10 sites, and characterized the local environment in order to compare observed spatial genetic structure to hypotheses of isolation-by-distance and isolation-by-environment. Finally, we quantified pollen dispersal distances and tested for local adaptation through a reciprocal transplant experiment in order to experimentally address these hypotheses. ResultsHigh genetic diversity ismaintained in the population and the genetic variation is significantly structured among sampled sites. We identified 5 distinct genetic clusters and average pollen dispersal predominately occurred over short distances. Differences among sites in flowering phenology and environmental factors, however, were not strictly associated with genetic differentiation. Growth and survival of upland and lowland progeny in their native and foreign environments was expected to exhibit evidence of local adaptation due to the more extreme dry season in the lowlands. Seedlings planted in the lowland garden experienced much higher mortality than seedlings in the upland garden, but we did not identify evidence for local adaptation.ConclusionOverall, this study indicates that the Costa Rican Q. oleoides population has a rich population genetic history. Despite environmental heterogeneity and habitat fragmentation, isolation-by-distance and isolation-by-environment alone do not explain spatial genetic structure. These results add to studies of genetic structure by examining a common, tropical tree over multiple habitats and provide information for managers of a successional forest in a protected area.

4302: ***+.288***

Habitat selection by the 1st reintroduced population of giant anteaters (Myrmecophaga tridactyla) was studied at Ibera Nature Reserve (northeastern Argentina), a subtropical region of wetlands, grasslands, and forests, with properties dedicated to nature conservation or livestock production. Eighteen animals were released and radiotracked between 2007 and 2012 for periods of 6-46 months, producing 1,181 locations. The effect of land use was assessed using chi-square tests. Resource selection functions were used to assess habitat selection at 2 spatial scales using as covariates habitat type (grassland, open savanna, closed savanna, and hygrophilous forest), distance to forest edge, and distance to a main road. Habitat selection was modeled for different ages (juveniles and adults), activity (resting or active), and seasons (summer, transition, and winter) and was validated with individuals previously excluded from our analysis. Anteaters strongly selected areas dedicated to nature conservation. Adults showed higher model fit. Hygrophilous forest was positively selected and open savanna was avoided. Anteaters rested almost exclusively in forests. While active they increased the use of other habitats, except for open savanna. Grasslands were most used during the winter season, probably because they provide a constant food source and better cover than savannas. Even though <4% of the landscape was covered by forests, this habitat proved to be important for giant anteaters. Deforestation and traditional cattle management may have caused the local extinction of the species at Ibera. Establishment of strict conservation areas in nonflooded areas combined with proper management of forests and livestock may be essential for the long-term survival of reintroduced populations in Ibera and similar regions. (C) 2015 American Society of Mammalogists, www.mammalogy.org

4303: *+.255*

American ginseng, Panax quinquefolius L., is a long-lived medicinal understory herb, which has been heavily harvested since the 1700s. Because of the economic value of the root, and the increasing rarity of this plant, P. quinquefolius is often reintroduced across its range. Land managers and hobby growers recommend using 'associate species' as a way to determine ideal site conditions for reintroduction. However, the accuracy of these putative indicator species in identifying sites that will maximize growth of this rare herb has not been tested. Using a long-term ecological dataset of 26 populations, we evaluated if 20 putative indicators (herbs, shrubs, and trees) could predict P. quinquefolius performance, as measured by the relative growth rate of the leaf area, at the population and microsite level. Of the indicators, only one tree species was able to predict positive performance. If a P. quinquefolius was within 10 m of a Liriodendron tulipifera L., the plant would have increased growth, in terms of leaf area, as compared to plants that were not within 10 m of this tree. Surprisingly, the presence of most putative indicator species was found to be unreliable as a site quality measure. At the population level, four putative indicators, Aralia nudicaulis L., Acer rubrum L., Betula lenta L., and Lindera benzoin (L.) Blume, were actually contra-indicators, as their presence at a site implied lower P. quinquefolius performance. If Podophyllum peltatum L was absent from a site, but B. lenta present, P. quinquefolius had reduced growth as compared to plants present in other combinations of P. peltatum and B. lenta. The results from this study have important implications for in situ conservation strategies of this rare medicinal plant. Planting P. quinquefolius in sites that increase performance can help ensure that reintroduction projects likely have a greater chance of success, effectively reducing the waste of time, money, and resources spent on projects that have lower levels of success. (C) 2015 Elsevier Ltd. All rights reserved.

4304: *+.377*

Translocations have been advocated as a conservation tool helping species adapt to climate and land-use change, but well-documented examples of invertebrates' translocations are rare. The

paper describes a successful translocation of the threatened Clouded Apollo butterfly (Parnassius mnemosyne) in Finland, compares this to a specific failed translocation, and presents conclusions for conservation planning as to factors contributing to the success. Two apparent key characteristics of the successful translocation were greater abundance of larval resources and less open landscape. The successful site was surrounded by forest, which strongly restricted emigration, crucially supporting the survival of the small initial population. Based on 20 mated females' translocation in 2000, the local population increased slowly, reaching 600 butterflies in 2011. A large translocation patch together with host-plant abundance enabled successful establishment of the local population. Availability of other suitable grassland patches sufficiently nearby was an additional key characteristic, facilitating the Clouded Apollo's expansion. However, the expansion rate was low; it took seven years for the butterflies to colonise the five nearest patches, only 10-200 m from the translocation patch. By 2013, they had colonised all suitable semi-natural grassland patches within 2 km from the translocation site and established a seemingly viable metapopulation with 11 subpopulations. The results point to the significance of local habitat area and landscape quality, along with conditions restricting emigration, in determination, of suitable translocation sites. (C) 2015 Elsevier Ltd. All rights reserved.

4305: *+.182*

Migration in the European white stork (Ciconia ciconia) has historically described a predictable annual cycle. The white stork is extensively distributed across continental Europe, which diverges to eastern and western wintering flyways. Within the western European population, some ring recovery and anecdotal information suggests that birds are giving up their traditional wintering grounds in the Sahel and are remaining in alternative sites in the Iberian Peninsula. Here we report on long-term satellite tracking of juvenile and adult stork collected between 2000 and 2011, whose natal site is in Belgium in the northern range of the western migration path. We identified three distinct migration patterns, two of which diverged from traditional expectations. Juvenile birds showed unique migration profiles both individually across migration cycles and when compared to one another, whereas adults showed consistent migrations but failed to migrate to Africa in any of the years surveyed. Stopover and wintering locations within Iberia were strongly associated with refuse sites or modified agricultural land. Overall, non-traditional migration movements appeared to be most strongly linked to artificial food sources rather than alternate drivers such as climate or habitat.

4306: *-.052*

As an endemic species of freshwater gastropods in China, Bellamya quadrata plays an important role in ecosystem service provision and commercial importance. However, the species is overharvested and its natural habitats are under severe threat due to fragmentation and loss. To estimate the genetic diversity and population structure of B. quadrata, 285 individuals from eight lake populations across middle and lower Yangtze River were sampled. Seven microsatellite loci were genotyped. Our results showed that (i) the genetic diversity of B. quadrata was high in most of the studied populations, yet effective population sizes appear to be rather small in some populations; (ii) low levels of genetic differentiation exists among populations but gene flow was generally high; (iii) no clear geographic or genetic structure was observed in the studied region, implying mechanisms (zoochoric dispersal and anthropogenic translocations) that enhance dispersal and gene flow have promoted population connectivity. However, the comparatively high genetic diversity of B. quadrata could be attributed to a lag phase, suggesting that the genetic diversity of this species may be lost in the future and the priorities for conservation of B. quadrata are necessary.

4307: *-.036*

Breeding programs for endangered species increasingly use molecular genetics to inform their management strategies. Molecular approaches can be useful for investigating relatedness, resolving pedigree uncertainties, and for estimating genetic diversity in captive and wild populations. Genetic data can also be used to evaluate the representation of wild population genomes within captive population gene-pools. Maintaining a captive population that is genetically representative of its wild counterpart offers a means of conserving the original evolutionary potential of a species. Okapi, an even-toed ungulate, endemic to the Democratic Republic of Congo, have recently been reclassified as Endangered by the IUCN. We carried out a genetic assessment of the ex-situ okapi (Okapia johnstoni) population, alongside an investigation into the genetic structure of wild populations across their geographic range. We found that while levels of nuclear (12 microsatellite loci) genetic variation in the wild, founder and captive okapi populations were similar, mitochondrial (833 bp of Cyt b, CR, tRNA-Thr and tRNA-Pro) variation within captive okapi was considerably reduced compared to the wild, with 16 % lower haplotype diversity. Further, both nuclear and mitochondrial alleles present in captivity provided only partial representation of those present in the wild. Thirty mitochondrial haplotypes found in the wild were not found in captivity, and two haplotypes found in captivity were not found in the wild, and the patterns of genetic variation at microsatellite loci in our captive samples were considerably different to those of the wild samples. Our study highlights the importance of genetic characterisation of captive populations, even for well-managed ex-situ breeding programs with detailed studbooks. We recommend that the captive US population should be further genetically characterised to guide management of translocations between European and US captive populations.

4308: *+.113*

The ability to accurately determine the original source of invading species offers several powerful applications in invasive species ecology and management and may enable important information on the invading species in its native habitat. Lake Storsjoen in South-Central Norway was recently found to have been subjected to an illegal translocation of the European smelt (Osmerus eperlanus). The main aim of this study was to infer the most likely source (s) of the invading smelt by using microsatellite markers, and subsequently to infer its introduction history. The results indicated that the smelt is most likely a result of introduction from the large Lake Mjosa, and that the translocated smelt comprise a large number of individuals. The smelt in Lake Storsjoen showed no significant genetic bottleneck effect. However, a corresponding significant test for a recent population expansion indicates that the smelt has had a high reproductive success and population growth in its new environment. The results from this study illustrate the usefulness of applying multilocus genetic markers for inferring origin of translocated populations, demographic events and introduction histories comprising an effective tool for assessment of invasive species.

4309: *+.095*

The reintroduction of a species into its historic range is a critical component of conservation programmes designed to restore extirpated metapopulations. However, many reintroduction efforts fail, and the lack of rigorous monitoring programmes and statistical models have prevented a general understanding of the factors affecting metapopulation viability following reintroduction. Spatially explicit metapopulation theory provides the basis for understanding the dynamics of fragmented populations linked by dispersal, but the theory has rarely been used to guide reintroduction programmes because most spatial metapopulation models require presence-absence

data from every site in the network, and they do not allow for observation error such as imperfect detection. We develop a spatial occupancy model that relaxes these restrictive assumptions and allows for inference about metapopulation extinction risk and connectivity. We demonstrate the utility of the model using sixyears of data on the Chiricahua leopard frog Lithobates chiricahuensis, a threatened desert-breeding amphibian that was reintroduced to a network of sites in Arizona USA in 2003. Our results indicate that the model can generate precise predictions of extinction risk and produce connectivity maps that can guide conservation efforts following reintroduction. In the case of L. chiricahuensis, many sites were functionally isolated, and 82% of sites were characterized by intermittent water availability and high local extinction probabilities (084, 95% CI: 064-099). However, under the current hydrological conditions and spatial arrangement of sites, the risk of metapopulation extinction is estimated to be <3% over a 50-year time horizon. Low metapopulation extinction risk appears to result from the high dispersal capability of the species, the high density of sites in the region and the existence of predator-free permanent wetlands with low local extinction probabilities. Should management be required, extinction risk can be reduced by either increasing the hydroperiod of existing sites or by creating new sites to increase connectivity. Synthesis and applications. This work demonstrates how spatio-temporal statistical models based on ecological theory can be applied to forecast the outcomes of conservation actions such as reintroduction. Our spatial occupancy model should be particularly useful when management agencies lack the funds to collect intensive individual-level data. This work demonstrates how spatio-temporal statistical models based on ecological theory can be applied to forecast the outcomes of conservation actions such as reintroduction. Our spatial occupancy model should be particularly useful when management agencies lack the funds to collect intensive individual-level data.

4310: *+.028*

I examined the winter diets of a reintroduced population of bison (Bison bison) in a mountainous region of northwestern Canada. The impetus of this study was to examine the winter diets of bison in this ecological region and to test for sexual and seasonal variation in the winter diet. Microhistological analyses of fecal samples from animals of known sex- and age-classes were examined to determine winter diets. I evaluated the hypothesis that sexual segregation in bison may be due to differences in diet. In addition, diet composition between early and late winter was examined to test for seasonal differences. Overall, bison winter diets were diverse, including at least 31 genera of vascular plants, but dominated by sedges (Carex). Nine species of Carex were identified in the winter diet. Diets of adult males in early winter differed in percentages of 7 forage classes used from those in late winter. The proportion of shrubs increased in late-winter diets, while that of sedges decreased. No evidence was found to support hypotheses of sexual segregation in bison based on differences in diet, at least for this population during late winter. This study shows that bison diets in winter are more diverse than previously thought based on studies in lowland boreal environments, where dietary niche breadth was comparatively narrow. Dietary flexibility is important within the context of choosing new sites for reintroducing bison, or predicting colonization of vacant habitats as existing populations grow.

4311: *-.012*

Acacia trees are keystone species of drylands. The recruitment of Acacia seedlings is regulated by large herbivores. The study objective was to investigate the effect of several large herbivore species on Acacia germination and recruitment. These species include the addax (Addax nasomaculatus), Arabian oryx (Oryx leucoryx), Asiatic wild ass (Equus hemionus), African wild ass (Equus africanus), and dorcas gazelle (Gazella dorcas). Feces of these herbivore species were

collected in a safari reservation in the hyper-arid Arava Valley, where Acacia raddiana and Acacia tortilis trees are widespread. The study was implemented in a net house, where the feces were laid in small piles on the ground and irrigated. Two batches of the feces were studied: one in the winter and one in the spring of 2012. The winter batch encompassed only the feces of the addax, oryx, and Asiatic ass, and it was found that the addax feces generated a significantly greater number of seedlings than the feces of other species. Of this batch, only 13 % of seedlings were alive similar to 250 days later. The spring batch encompassed all five species and revealed a germination rate in the feces according to the following order: addax > oryx > Asiatic ass > gazelle > African ass. Of this batch, 46 % of seedlings were alive similar to 200 days later. This study provides new knowledge regarding the effect of each of the ungulate species on recruitment capacity of new trees to the Acacia population. The results propose that the two most significant factors affecting recruitment are rumination and size of herbivores.

4312: *-.037*

Fear of predation can have major impacts on the behaviour of prey species. Recently the concept of the ecology of fear has been defined and formalised; yet there has been relatively little focus on how these ideas apply to large carnivore species which, although not prey sensu stricto, also experience fear as a result of threats from humans. Large carnivores are likely also subject to a Landscape of Fear similar to that described for prey species. We argue that although fear is generic, 'human-caused mortality' represents a distinct and very important cause of fear for large carnivores, particularly terrestrial large carnivores as their activities overlap with those of humans to a greater degree. We introduce the idea of a 'Landscape of Coexistence' for large carnivores to denote a subset of the Landscape of Fear where sufficient areas of low human-caused mortality risk are present in the landscape for long term coexistence of large carnivores and humans. We then explore aspects of terrestrial large carnivore behavioural ecology that may be best explained by risk of human-caused mortality, and how the nature of a Landscape of Coexistence for these large carnivores is likely to be shaped by specific factors such as habitat structure, wild and domestic prey base, and human distribution and behaviour. The human characteristics of this Landscape of Coexistence may be as important in determining large carnivore distribution and behavioural ecology as the distribution of resources. Understanding the Landscape of Coexistence for terrestrial large carnivores is therefore important for their biology and conservation throughout large parts of their remaining ranges.

4313: *+.255*

Amphibians are facing an extinction crisis, and conservation breeding programmes are a tool used to prevent imminent species extinctions. Compared to mammals and birds, amphibians are considered ideal candidates for these programmes due to their small body size and low space requirements, high fecundity, applicability of reproductive technologies, short generation time, lack of parental care, hard wired behaviour, low maintenance requirements, relative cost effectiveness of such programmes, the success of several amphibian conservation breeding programmes and because captive husbandry capacity exists. Superficially, these reasons appear sound and conservation breeding has improved the conservation status of several amphibian species, however it is impossible to make generalisations about the biology or geo-political context of an entire class. Many threatened amphibian species fail to meet criteria that are commonly cited as reasons why amphibians are suitable for conservation breeding programmes. There are also limitations associated with maintaining populations of amphibians in the zoo and private sectors, and these could potentially undermine the success of conservation breeding programmes and reintroductions. We recommend that species that have been assessed as high

priorities for ex situ conservation action are subsequently individually reassessed to determine their suitability for inclusion in conservation breeding programmes. The limitations and risks of maintaining ex situ populations of amphibians need to be considered from the outset and, where possible, mitigated. This should improve programme success rates and ensure that the limited funds dedicated to ex situ amphibian conservation are allocated to projects which have the greatest chance of success.

4314: *+.111*

The female giant panda Zhangxiang (pedigree number 826) was born on August 20, 2011 in Wolong Nature Reserve, China. On November 6, 2013, Zhangxiang was transported into the acclimatization enclosure in the Liziping Nature Reserve. Before Zhangxiang left the enclosure into the wild, we conducted the first study to compare microhabitats and foraging strategies between Zhangxiang in the enclosure and giant pandas in the wild. Compared with the latter, microhabitats of Zhangxiang in the enclosure are characteristic of gentler slope, more trees, higher canopy, smaller tree DBH, and lower density of living bamboos. Diet composition and foraging behaviors significantly differed between Zhangxiang and wild giant pandas, perhaps reflecting the combined consequence of environmental conditions (e.g., bamboo species) and individual status (e.g., age, mastication ability, etc.). The difference in microhabitats and foraging strategies between Zhangxiang and wild giant pandas implied that after being released into the natural habitat in the reserve, Zhangxiang will have to adapt to the environmental conditions once again. For future reintroduction, the enclosure can be extended to the Bashania spanostachya forest in the reserve, and captive giant pandas for release can thus normally transit into the wild without human intervention during acclimatization period. For other acclimatization enclosures to be constructed in the future, ecological environment inside, including topography, forests, and bamboos as well, should as possible as can match the habitat that the giant panda to-be-reinforced populations inhabit.

4315: *+.269*

Captive management of ex situ populations of endangered species is traditionally based on pedigree information derived from studbook data. However, molecular methods could provide a powerful set of complementary tools to verify studbook records and also contribute to improving the understanding of the genetic status of captive populations. Here, we compare the utility of single nucleotide polymorphisms (SNPs) and microsatellites (MS) and two analytical methods for assigning parentage in ten families of captive African penguins held in South African facilities. We found that SNPs performed better than microsatellites under both analytical frameworks, but a combination of all markers was most informative. A subset of combined SNP (n=14) and MS loci (n=10) provided robust assessments of parentage. Captive or supportive breeding programs will play an important role in future African penguin conservation efforts as a source of individuals for reintroduction. Cooperation among these captive facilities is essential to facilitate this process and improve management. This study provided us with a useful set of SNP and MS markers for parentage and relatedness testing among these captive populations. Further assessment of the utility of these markers over multiple (>3) generations and the incorporation of a larger variety of relationships among individuals (e.g., half-siblings or cousins) is strongly suggested.

4316: *+.151*

The karyotype of the threatened ant species Atta robusta is described so as to establish the evolutionary relationships of this taxon with other leafcutter ants. Standard Giemsa staining, C-

banding, NOR banding, fluorochromes CMA(3)/DAPI, Hsc-FA technique and Fluorescence in situ Hybridization (FISH) using 18S rDNA probe were conducted on a population from Aracruz, state of Espirito Santo, Brazil, allowing for comparisons with data available on Atta and other fungus-growing ant species. The diploid chromosome number observed for A. robusta was 2n = 22, and the karyotypic formula was 18m + 2sm + 2st. Heterochromatic blocks were observed in the centromeric region of most chromosomes, where one pair of metacentric chromosomes is characterized by a GC-rich heterochromatic band in the interstitial region of its long arm. The detection of 18S rDNA using FISH confirmed the presence of single NOR for A. robusta. This is the first report of rDNA 18S detection using FISH for leafcutter ants. The cytogenetic results of this study confirm the information available for Atta and allow us to confirm the conserved chromosome number, morphology and banding pattern within the genus for the taxa studied to date, which included species from three out of the four groups of Atta indicated by molecular data. The accumulation of cytogenetic data on fungus-growing ants enhances the understanding of the genomic evolutionary patterns of Atta, since it belongs to a group of recent origin between the most well studied ants. Cytogenetic data does not indicate restrictions in relocation or reintroduction in areas where populations were extinct due to the conserved karyotype. This study allows for cytogenetic comparison of A. robusta with other ants of Atta, emphasizing the importance of chromosomal information for species conservation. (C) 2015 Academie des sciences. Published by Elsevier Masson SAS. All rights reserved.

4317: *+.046*

For endangered species that are hunted, the establishment of mixed conservation areas including both hunting zones and sanctuaries to complement translocation actions (i.e. reinforcement) can improve both hunting yields and population sustainability. However, the effects of this type of management on the demography of the exploited species are not well understood. We used multi-event capture-recapture modelling in a population of captive-bred houbara bustards Chlamydotis undulata translocated into a mixed conservation area in Morocco. The specific management practice of our system (hunting regime varying in time and space) led to a quasi-experimental situation that allowed the differentiation of natural' from hunting-induced' mortality and movement between areas. The analysis uncovered strong asymmetries in both movement and survival that were not only due to direct hunting effects. Firstly, movement probabilities were higher from the sanctuary to the hunting areas than vice versa, even in years without hunting. Secondly, in addition to a direct effect of hunting on mortality in hunting areas, our results uncovered permanent differences in both areas (even outside the hunting period). Overall, our results were consistent with predictions under a source-sink dynamic model but illustrated that mixed conservation areas should not merely be treated as homogeneous systems with spatially heterogeneous hunting pressure but rather as fully heterogeneous systems. The patterns observed may be related to (1) the choice and design of hunting and sanctuary areas by managers, which might not be neutral with respect to habitat quality, or (2) indirect consequences of hunting via an effect on local growth rate and density.

4320: *-.011*

Despite recent efforts to develop the science of reintroduction biology, there is still no general and broadly accepted definition of reintroduction success. We investigate this issue based on the postulates (1) that successful reintroduction programs should produce viable populations and (2) that reliable assessments of ultimate success require that populations have reached their regulation phase. We assessed if the viability of these reintroduced populations could be evaluated using the same criteria as for remnant populations, such as the Internation Union for Conservation of Nature

(IUCN) Red List criteria. Using modeling, we projected the viabilities of theoretical populations with various life history and environmental characteristics and we tested whether population sizes (criterion D of the IUCN) and other potential predictors are relevant proxies of the risk of extinction (criterion E of the IUCN) in the case of remnant populations with an unknown past history and in the case of reintroduced populations that have reached their carrying capacity. We found that, as for remnant populations, population size can be used as a relevant indicator (although subject to considerable uncertainty) of the viability of reintroduced populations. However, the results demonstrate the importance of the reintroduction failure filter, that is, the fact that the reintroduced populations that have successfully reached their carrying capacity are those with the highest and more stable growth rates, especially if populations have been reintroduced with a few individuals. As a consequence, the general relationship between the current size of a population and its projected viability will, most likely, differ considerably between remnant and reintroduced populations. Overall, our results demonstrate that there are no theoretical limitations on the application of some of the criteria widely used for remnant populations to define reintroduction success, although these criteria are very conservative for reintroduced populations and might be rescaled to account for the demographic filter that early extinction constitutes for these populations. Read the Commentaries on this Feature Paper: ; ; and the Response from the authors:

4321: *+.191*

Previous studies show that conservation actions have prevented extinctions, recovered populations, and reduced declining trends in global biodiversity. However, all studies to date have substantially underestimated the difference conservation action makes because they failed to account fully for what would have happened in the absence thereof. We undertook a scenario-based thought experiment to better quantify the effect conservation actions have had on the extinction risk of the world's 235 recognized ungulate species. We did so by comparing species' observed conservation status in 2008 with their estimated status under counterfactual scenarios in which conservation efforts ceased in 1996. We estimated that without conservation at least 148 species would have deteriorated by one International Union for Conservation of Nature (IUCN) Red List category, including 6 species that now would be listed as extinct or extinct in the wild. The overall decline in the conservation status of ungulates would have been nearly 8 times worse than observed. This trend would have been greater still if not for conservation on private lands. While some species have benefited from highly targeted interventions, such as reintroduction, most benefited collaterally from conservation such as habitat protection. We found that the difference conservation action makes to the conservation status of the world's ungulate species is likely to be higher than previously estimated. Increased, and sustained, investment could help achieve further improvements.

4322: *+.161*

Conservation biologists are generally united in efforts to curtail the spread of non-native species globally. However, the colonization history of a species is not always certain, and whether a species is considered non-native or native depends on the conservation benchmark. Such ambiguities have led to inconsistent management. Within the Tongass National Forest of Alaska, the status of American marten (Martes americana) on the largest, most biologically diverse and deforested island, Prince of Wales (POW), is unclear. Ten martens were released to POW in the early 1930s, and it was generally believed to be the founding event, although this has been questioned. The uncertainty surrounding when and how martens colonized POW complicates management, especially because martens were selected as a design species for the Tongass. To

explore the history of martens of POW we reviewed other plausible routes of colonization; genetically and isotopically analyzed putative marten fossils deposited in the late Pleistocene and early Holocene to verify marten occupancy of POW; and used contemporary genetic data from martens on POW and the mainland in coalescent simulations to identify the probable source of the present-day marten population on POW. We found evidence for multiple routes of colonization by forest-associated mammals beginning in the Holocene, which were likely used by American martens to naturally colonize POW. Although we cannot rule out human-assisted movement of martens by Alaskan Natives or fur trappers, we suggest that martens be managed for persistence on POW. More generally, our findings illustrate the difficulty of labeling species as non-native or native, even when genetic and paleo-ecological data are available, and support the notion that community resilience or species invasiveness should be prioritized when making management decisions rather than more subjective and less certain conservation benchmarks.

4323: *+.202*

This communication reports some evidence of the reintroduced Chinese water deer surviving in the Nanhui Wildlife Sanctuary, Shanghai, China. Deer were still present in the sanctuary in 2014 and evidence of breeding was also recorded. Historically, the deer was last recorded in Shanghai in 1890. The Chinese Water Deer Reintroduction Project was started in Shanghai in 2006, in order to restore the native Chinese water deer population.

4324: *+.360*

For the past 50 years, the endangered Persian fallow deer (Dama mesopotamica) have been translocated to various sites throughout Iran. To better understand the varying degrees of success at the translocation sites, population growth rates were measured for all the sites, and factors believed to affect the growth rate, such as initial population structures of the translocated herds and habitat characteristics, were identified and modeled. The population growth rate was used as a proxy for translocation success. Quantitative ecological data for Persian fallow deer is scarce, but expert knowledge was readily available to inform and enhance fallow deer management options. We integrated the available quantitative data and qualitative information in a Bayesian Belief Network (BBN) model to predict Persian fallow deer translocation success. The BBN model was tested using scenarios based on previous translocations to 13 sites in Iran. It correctly predicted the success of translocated populations in 11 out of the 13 sites. This model may be used as a decision support tool for future translocations, and can assist in designing reintroduction programs of the Persian fallow deer. Moreover, it should be adapted to incorporate new knowledge as evidence of translocation successes and failures emerge. Although the BBN model was developed specifically for the translocation of Persian fallow deer, this approach can clearly be applied to design and assess the success of translocation programs of other endangered species, and may be extended to design and assess alternative conservation management strategies.

4325: *+.011*

Translocations, especially assisted colonizations, of animals are increasingly used as a conservation management tool. In many cases, however, limited funding and other logistic challenges limit the number of individuals available for translocation. In conservation genetics, small populations are predicted to rapidly lose genetic diversity which can deteriorate population survival. Thus, how worried should we be about the loss of genetic diversity when introducing small, isolated populations? Historical species introductions provide a means to assess these issues. Here we review 13 studies of "assisted colonization-like" introductions of animals, where

only a small known number of founders established an isolated population without secondary contact to the source population. We test which factors could be important in retaining genetic diversity in these cases. In many cases, loss in heterozygosity (-12.1%) was detected, and more seriously the loss in allelic richness (-27.8 %). Number of founders seemed to have an effect but it also indicated that high population growth rate could help to retain genetic diversity, i.e. future management actions could be effective even with a limited number of founders if population growth would be enhanced. On the contrary, translocated organisms with longer generation times did not seem to retain more genetic diversity. We advocate that, where possible, future studies on translocated animals should report the loss of genetic diversity (both heterozygosity and allelic richness), which is essential for meta-analyses like this one for deepening our understanding of the genetic consequences of assisted colonization, and justifying management decisions [Current Zoology 61 (5): 827-834, 2015].

4326: *+.052*

A translocation of wild-bred and captive-bred Little Penguin Eudyptula minor fledglings was trialled to reinforce (augment) an urban population of this species in Sydney Harbour, Australia. Over three breeding seasons (2004/05 to 2006/07), a total of 44 wild-bred fledglings from nearby Lion Island and 19 captive-bred fledglings from local zoos were translocated to Store Beach, North Harbour, within Sydney Harbour. All translocated birds were implanted with a numbered microchip before release. During the same period, the North Harbour colony produced at least 327 fledglings, of which 113 were implanted with microchips prior to fledging. The North Harbour colony was monitored between 2005 and 2013 to identify any returning translocated or locally wild-bred birds. Three translocated wild-bred birds and 12 locally wild-bred birds are known to have returned to North Harbour, but no translocated captive-bred birds. One translocated wild-bred bird returned to Store Beach while the remaining two returned to adjacent headlands. The number of pairs of Little Penguin breeding on Store Beach, where threats are intensively managed, has increased from one in 2004 to nine in 2013. This study demonstrated that translocation of wild-bred fledglings is feasible. However, because of naturally low return rates, translocations of Little Penguins need to involve a large number of individuals, preferably taken from a sizeable donor colony, to be effective in reinforcing a threatened or declining population. The effectiveness of releasing captive-bred Little Penguins has yet to be demonstrated.

4327: *+.233*

As a native species in history, Chinese water deer were reintroduced to Shanghai in 2006 and were released into the wild, at Nanhui East Shoal Wildlife Sanctuary, in 2010. In order to study the dispersal, we used radio telemetry to track 12 released deer's movements. The research suggests that all the bucks showed clear dispersal behaviour in the initial period, and their dispersal occurred earlier and lasted longer than that of the does. Most bucks' dispersing movement occurred from the third day to the fifth day after release, and the dispersing does' movement occurred from the eighth to the ninth day. The first dispersal of the bucks lasted for 4-5days, while this was only 1-2days for the does. The mean dispersal distance for the bucks was 623m in the initial period of relocation, and that of the does was significantly smaller. The dispersal distance for sub-adults was also considerably smaller than that of the adults. Reed wetland was the main habitat the deer liked to select. After the initial period, the mean dispersal distance was 857m and bucks still dispersed significantly farther than does did. Most surviving individuals displayed a new dispersal during early December, but the adult does showed a tendency of retraction from the following January. Unfamiliarity with new environments might explain why the deer did not immediately disperse after release. After becoming familiar with food distribution and shelter

conditions, they dispersed further in order to avoid predation risk. In order to improve the survival rate of reintroduced deer, we suggest releasing the adult individuals of robust physique into the wild in future programmes, and more attention should be paid to the initial 9days after releasing the species. Increasing the local experience of the captive-born animals and helping them become familiar with the new wild habitat will improve the survival rate in future reintroductions.

4328: ***-.099***

Small populations with restricted geographic ranges such as rhinoceros (Rhinoceros unicornis) are prone to extinction due to anthropogenic factors. The identification of factors underpinning the survival of such species is of critical importance for population persistence. We used VORTEX population viability analysis (PVA) to assess rhino population viability in Nepal. We simulated deterministic single-population models under different scenarios to assess viability of two distinct rhino populations in Nepal: a source population in Chitwan National Park and an augmented population in Bardia National Park. The impacts of poaching on the populations and the potential for rhino translocation from one population to another were assessed under the PVA framework. Population and demographic data were obtained from censuses and from published literature. The model output suggested that the Chitwan population is stable and capable of supplying at least 10 rhinos every 3 years for translocation provided poaching is restricted (15animals per 3years). However, the Bardia population is more vulnerable and unable to persist without supplementation even at the lowest poaching rate (2animals per year). Supplementation of at least 10 animals every 3years for 30years is crucial for establishing a viable population of rhinos in Bardia. This level of supplementation can withstand the poaching rate of 2animals per year. Our study demonstrates that poaching is the major factor determining rhino population viability in Nepal. The supplementation of the Bardia rhino population with animals from the Chitwan population and increased effort to reduce poaching are expected to enhance the viability of rhino populations in Nepal.

4329: ***+.057***

Przewalski's horses (PHs, Equus ferus ssp. przewalskii) were discovered in the Asian steppes in the 1870s and represent the last remaining true wild horses. PHs became extinct in the wild in the 1960s but survived in captivity, thanks to major conservation efforts. The current population is still endangered, with just 2,109 individuals, one-quarter of which are in Chinese and Mongolian reintroduction reserves [1]. These horses descend from a founding population of 12 wild-caught PHs and possibly up to four domesticated individuals [2-4]. With a stocky build, an erect mane, and stripped and short legs, they are phenotypically and behaviorally distinct from domesticated horses (DHs, Equus caballus). Here, we sequenced the complete genomes of 11 PHs, representing all founding lineages, and five historical specimens dated to 1878-1929 CE, including the Holotype. These were compared to the hitherto-most-extensive genome dataset characterized for horses, comprising 21 new genomes. We found that loci showing the most genetic differentiation with DHs were enriched in genes involved in metabolism, cardiac disorders, muscle contraction, reproduction, behavior, and signaling pathways. We also show that DH and PH populations split 45,000 years ago and have remained connected by gene-flow thereafter. Finally, we monitor the genomic impact of 110 years of captivity, revealing reduced heterozygosity, increased inbreeding, and variable introgression of domestic alleles, ranging from non-detectable to as much as 31.1%. This, together with the identification of ancestry informative markers and corrections to the International Studbook, establishes a framework for evaluating the persistence of genetic variation in future reintroduced populations.

4330: ***+.058***

Przewalski's horses (PHs, Equus ferus ssp. przewalskii) were discovered in the Asian steppes in the 1870s and represent the last remaining true wild horses. PHs became extinct in the wild in the 1960s but survived in captivity, thanks to major conservation efforts. The current population is still endangered, with just 2,109 individuals, one-quarter of which are in Chinese and Mongolian reintroduction reserves [1]. These horses descend from a founding population of 12 wild-caught PHs and possibly up to four domesticated individuals [2-4]. With a stocky build, an erect mane, and stripped and short legs, they are phenotypically and behaviorally distinct from domesticated horses (DHs, Equus caballus). Here, we sequenced the complete genomes of 11 PHs, representing all founding lineages, and five historical specimens dated to 1878-1929 CE, including the Holotype. These were compared to the hitherto-most-extensive genome dataset characterized for horses, comprising 21 new genomes. We found that loci showing the most genetic differentiation with DHs were enriched in genes involved in metabolism, cardiac disorders, muscle contraction, reproduction, behavior, and signaling pathways. We also show that DH and PH populations split similar to 45,000 years ago and have remained connected by gene-flow thereafter. Finally, we monitor the genomic impact of similar to 110 years of captivity, revealing reduced heterozygosity, increased inbreeding, and variable introgression of domestic alleles, ranging from non-detectable to as much as 31.1 %. This, together with the identification of ancestry informative markers and corrections to the International Studbook, establishes a framework for evaluating the persistence of genetic variation in future reintroduced populations.

4331: ***+.091***

Small populations of endangered species can be impacted by genetic processes such as drift and inbreeding that reduce population viability. As such, conservation genetic analyses that assess population levels of genetic variation and levels of gene flow can provide important information for managing threatened species. The Sao Paulo Marsh Antwren (Formicivora paludicola) is a recently-described and critically endangered bird from Sao Paulo State (Brazil) whose total estimated population is around 250-300 individuals, distributed in only 15 isolated marshes around Sao Paulo metropolitan region. We used microsatellite DNA markers to estimate the population genetic characteristics of the three largest remaining populations of this species all within 60 km of each other. We detected a high and significant genetic structure between all populations (overall F-ST = 0.103) which is comparable to the highest levels of differentiation ever documented for birds, (e.g., endangered birds found in isolated populations on the tops of African mountains), but also evidence for first-generation immigrants, likely from small local unsampled populations. Effective population sizes were small (between 28.8-99.9 individuals) yet there are high levels of genetic variability within populations and no evidence for inbreeding. Conservation implications of this work are that the high levels of genetic structure suggests that translocations between populations need to be carefully considered in light of possible local adaptation and that remaining populations of these birds should be managed as conservation units that contain both main populations studied here but also small outlying populations which may be a source of immigrants.

4332: ***-.028***

Background: The Tasmanian devil (Sarcophilus harrisii) has undergone a recent, drastic population decline due to the highly contagious devil facial tumor disease. The tumor is one of only two naturally occurring transmissible cancers and is almost inevitably fatal. In 2006 a disease-free insurance population was established to ensure that the Tasmanian devil is protected

from extinction. The insurance program is dependent upon preserving as much wild genetic diversity as possible to maximize the success of subsequent reintroductions to the wild. Accurate genotypic data is vital to the success of the program to ensure that loss of genetic diversity does not occur in captivity. Until recently, microsatellite markers have been used to study devil population genetics, however as genetic diversity is low in the devil and potentially decreasing in the captive population, a more sensitive genotyping assay is required.Methods: Utilising the devil reference genome and whole genome re-sequencing data, we have identified polymorphic regions for use in a custom genotyping assay. These regions were amplified using PCR and sequenced on the Illumina MiSeq platform to refine a set a markers to genotype the Tasmanian devil insurance population.Results: We have developed a set of single nucleotide polymorphic (SNP) markers, assayed by amplicon sequencing, that provide a high-throughput method for monitoring genetic diversity and assessing familial relationships among devils. To date we have used a total of 267 unique SNPs within both putatively neutral and functional loci to genotype 305 individuals in the Tasmanian devil insurance population. We have used these data to assess genetic diversity in the population as well as resolve the parentage of 21 offspring.Conclusions: Our molecular data has been incorporated with studbook management practices to provide more accurate pedigree information and to inform breeding recommendations. The assay will continue to be used to monitor the genetic diversity of the insurance population of Tasmanian devils with the aim of reducing inbreeding and maximizing success of reintroductions to the wild.

4333: *+.030*

Adaptive management methods are best for ensuring management goals are met when implementing prescribed fire to areas of fire exclusion. Prior to intense logging and subsequent fire suppression efforts of the early 1900s, longleaf pine (Pinus palustris) woodlands dominated the southeastern United States. As a pyrogenic forest type, natural wildfires occur with a 2-15 year fire return interval. Remaining longleaf forests are predominantly second-growth, with current vegetation structure reflecting decades of fire exclusion resulting from continued suppression efforts. Research in longleaf savannas has demonstrated that hardwood encroachment, reduced floristic diversity, and reduced longleaf yearling establishment occurs in the absence of fire. However, few studies have examined the potential role of prescribed fire management in moderating longleaf pine regeneration in second-growth xeric sandhill assemblages. Over a seven year period of fire reintroduction, we examined the effects of prescribed fire on longleaf pine demographics and structure in a forest that experienced a significant reduction in fire frequency during the 20th century. We observed no difference in overstory composition or hardwood density between burned and adjacent unburned areas. Understory woody stem density (DBH < 2 cm) was dominated by juvenile longleaf plants, which likely caused greater variability in light reaching the forest floor in unburned vs. burned areas. Significant shifts in demographic structure were evident in juvenile longleaf plants that initiated height growth following the onset of prescribed fire treatments. Longleaf yearling density (<1 year old) averaged 5 plants m(-2) (unburned <1 plant m(-2)) and was positively correlated with fire frequency. Greater accumulation of leaf litter in unburned (10 cm) vs. burned areas (5 cm) likely influenced yearling recruitment, as expected given the species' need for contact with bare mineral soil for germination. Unlike pine savannas where reduction of hardwoods is often the target of management, intraspecific interactions (i.e. negative density dependence) likely play a greater role in successful longleaf recruitment and population demography in this sandhill assemblage. This study further highlights the utility of implementing and observing a range of fire applications when reintroducing fire to long unburned systems. (C) 2015 Elsevier B.V. All rights reserved.

4334: *+.093*

We explored possible interactions among gray wolves (Canis lupus), Rocky Mountain elk (Cervus elaphus), and thinleaf alder (Alnus incana spp. tenuifoli) in northern Yellowstone National Park. We developed an alder age structure based on annual growth rings for plants growing along six streams in areas accessible to ungulates on the northern range. Alder stems (n = 412) along the six streams originated only after wolf reintroduction. By 2013, 80% of the sampled alders along these streams were taller than 2 m, in contrast with a historical pattern of height suppression by ungulate herbivory. This pattern of alder recruitment is consistent with a trophic cascade whereby new alder growth occurred across all study streams within several years after wolf reintroduction. Although declines in elk density since wolf reintroduction likely contributed to the release of alder from herbivory, the immediate onset of new alder recruitment following wolf reintroduction indicates that behavioral responses to predation may also have been an important component in the resulting trophic cascade. These results suggest that predator conservation could play a role in the management and ecological restoration of riparian areas. (C) 2015 Elsevier B.V. All rights reserved.

4335: *+.020*

Following dramatic range and population declines, the cheetah is Africa's most endangered large felid. In Namibia, private land managers still trap cheetahs but increasingly consider moving animals instead of killing them. Across Africa, managers have translocated perceived conflict carnivores for decades, but rarely evaluated their actions. We analyse the outcomes of 15 cheetah translocations (for 23 adults and 10 dependent offspring) into free-range environments in Namibia. We released cheetahs at an average distance of 419.6 km +/- 216.1 km SD (range: 71-816 km) after captive periods ranging from 1-1,184 days (350.6 days +/- 439.0 days SD). An individual's ability to survive the first year predominantly determined the overall translocation success of 40%. Post-release conflict and homing had less impact on success. Cheetah survival was lowest in the first three months after release. Human persecution (50% of deaths) and spotted hyaenas (29% of deaths) had the highest effect on survival. The degree of habituation to humans acquired during captivity significantly influenced chances of survival. Cheetahs surviving the initial post-release period (similar to 90 days) often settled into ranges and females reproduced successfully. However, all individuals exhibited extensive movements, frequently roaming >4,000 km(2) in the first six months after release (with a maximum of 19,743 km(2) in 112 days), resulting in low release site fidelity. Soft release and larger recipient area size did not improve site fidelity. Based on these outcomes, we evaluated which unfenced conservation areas in Namibia could potentially receive cheetahs. We found that there are currently few public and/or private reserves large enough to contain the movement profiles we observed in this study. This suggests that most translocations will result in cheetahs re-entering farmlands where they face a high risk of persecution. In conclusion, translocations into unconfined areas can successfully conserve individual cheetahs. Due to high mortality and unpredictable outcomes, however, conservation efforts need to focus on improving tolerance of cheetahs in commercial livestock and game farming areas in order to reduce the number of indiscriminately trapped animals.

4336: *+.332*

The Wildlife Trust of India has taken a long term responsibility to identify a suitable habitat for the threatened families of Eastern Hoolock Gibbon from a village called Dello in Arunachal Pradesh to a nearby forested area which was the earlier home of this species. There is an ongoing successful rescue and translocation programme since November, 2011 in which four Eastern Hoolock Gibbon families comprising 11 individuals were translocated in three different habitat types in and around the forested area of the Mehao Wildlife Sanctuary. Post-release monitoring is

an obvious and required technique to study the rescued families of Eastern Hoolock Gibbons after translocation to confirm their post-release survival and better livelihood. The regular monitoring of the activity patterns has helped to understand the habitat utilization and resource use in the newly released sites. Along with the rescue operation, there is an additional task to find out the potential habitats to define as ideal release sites for gibbons. The post release monitoring was studied through the instantaneous scan sampling method to collect the information mostly about their activity patterns. The present study describes the overall activity patterns and resource use in the released gibbons on the basis of utilization of different habitat types. It was observed that the ranging pattern was mostly influenced by the resource availability and forest type. The gibbon family released in the denser forest habitat developed a general food habit whereas the family from the thinner forest area became the specialist consumer. However, further detailed study with sufficient data is required to comment on their general ecology.

4337: ***-.060***

Potential risks posed to domestic animals and human beings by zoonotic diseases in reintroduced animals can reduce the acceptability of reintroductions. The authors investigated the role of endangered water voles, Arvicola amphibius, as a host for leptospirosis, a waterborne zoonosis affecting a range of mammals. Based on samples from 112 individuals from across the UK, a 6.2 per cent exposure rate was found (7 animals were microscopic agglutination test (MAT) positive for serum antibodies), with 4 of 11 sites having positive animals. No individual was actively excreting leptospires in urine (PCR urine test, 0 per cent positive). The acquisition of Leptospira species by a cohort of 'clean' captive-bred voles reintroduced to one site in the wild was then examined. By four months postrelease the maximum exposure prevalence (by either MAT or culture) was 42.9 per cent. Thirty-five per cent were actively excreting leptospires. The rapidity of leptospire acquisition and comparatively high prevalence of infectious individuals is notable, exceeding expectation based on wild voles. One possible explanation is a lack of immunocompetence in reintroduced voles. Analyses of haematological parameters from reintroduced voles suggest a link between prior condition and disease acquisition. There may be potential to select the fittest animals before release to maximise reintroduction success.

4338: ***+.235***

Interactions among species, which range from competition to facilitation, have profound effects on ecosystem functioning. Large carnivores are of particular importance in shaping community structure since they are at the top of the food chain, and many efforts are made to conserve such keystone species. Despite this, the mechanisms of carnivore interactions are far from understood, yet they are key to enabling or hindering their coexistence and hence are highly relevant for their conservation. The goal of this review is thus to provide detailed information on the extents of competition and facilitation between large carnivores and their impact in shaping their life histories. Here, we use the example of spotted hyaenas (Crocuta crocuta) and lions (Panthera leo) and provide a comprehensive knowledge of their interactions based on meta-analyses from available literature (148 publications). Despite their strong potential for both exploitation and interference competition (range and diet overlap, intraguild predation and kleptoparasitism), we underline some mechanisms facilitating their coexistence (different prey-age selection and scavenging opportunities). We stress the fact that prey abundance is key to their coexistence and that hyaenas forming very large groups in rich ecosystems could have a negative impact on lions. We show that the coexistence of spotted hyaenas and lions is a complex balance between competition and facilitation, and that prey availability within the ecosystem determines which predator is dominant. However, there are still many gaps in our knowledge such as the spatio-

temporal dynamics of their interactions. As both species' survival becomes increasingly dependent on protected areas, where their densities can be high, it is critical to understand their interactions to inform both reintroduction programs and protected area management.

4339: *+.295*

Mitigating the threat of habitat loss requires actions such as restoring and creating new habitat. In order to effectively achieve this, species habitat requirements and use patterns need to be understood. While many studies have been conducted on the habitat choice of species, these generally focused on habitat use during periods of high activity and detection probability without considering seasonal shifts in habitat use. Understanding habitat selection by frogs during the winter season of low activity may be crucial since it may differ from that used during the summer and may be overlooked as important for population success. We describe the microhabitat use of the threatened green and golden bell frog (Litoria aurea) using radio tracking methods during winter when detection is low and knowledge is limited. We followed 26 individuals between May and July, 2011 to determine whether they selected specific overwintering microhabitats and related this to levels of individual exposure to predators, distance from the edge of the water and temperature of microhabitats. We found that overwintering bell frogs inhabited reeds and rock gabions more frequently than expected and that females used a reduced subset of microhabitats compared to males. Additionally, microhabitats used were more likely to conceal an individual from view, and the majority of overwintering sites were located within 5m of the edge of the water which may be important for reducing the risk of predation and desiccation. Rock gabions had significantly warmer (1.2 degrees C-1.8 degrees C) mean temperatures than the other microhabitats used. The information presented here can be used in habitat creation and reintroduction programmes to provide habitat which is suitable during both the breeding and non-breeding season for the conservation of other populations.

4340: *+.092*

Hawai'i has served as a model system for studies of nutrient cycling and conservation biology. The islands may also become a laboratory for exploring new approaches to forest restoration because of a common history of degradation and the growing number of restoration projects undertaken. Approximately half of the native ecosystems of Hawai'i have been converted to non-native conditions. Many restoration projects have focused on intensively managed out plantings of native plants with emphasis on threatened and endangered species. While these projects have been effective in stabilizing plant populations, this model is often prohibitively expensive for restoration at the scale needed to protect watersheds and provide habitat for rare bird species. Here we suggest ways of rethinking ecological restoration that are applicable across the tropics, particularly on islands and fire-prone grasslands. First, we suggest making use of non-native, non-invasive species to help reclaim degraded or invaded sites or as long-term components of planned restoration outcomes. Second, we suggest incorporating remote sensing techniques to refine where restoration is carried out. Finally, we suggest borrowing technologies in plant production, weed control, and site preparation from industrial forestry to lower restoration costs. These suggestions would result in ecosystems that differ from native reference systems in some cases but which could be applied to much larger areas than most current restoration efforts while providing important ecosystem services. We also stress that community involvement is key to successful restoration, as a major goal of almost all restoration projects is to re-connect the community with the forest.

4341: *+.158*

Habitat fragmentation is a fundamental cause of population decline and increased risk of extinction for many wildlife species; animals with large home ranges and small population sizes are particularly sensitive. The Louisiana black bear (Ursus americanus luteolus) exists only in small, isolated subpopulations as a result of land clearing for agriculture, but the relative potential for inter-subpopulation movement by Louisiana black bears has not been quantified, nor have characteristics of effective travel routes between habitat fragments been identified. We placed and monitored global positioning system (GPS) radio collars on 8 female and 23 male bears located in 4 subpopulations in Louisiana, which included a reintroduced subpopulation located between 2 of the remnant subpopulations. We compared characteristics of sequential radiolocations of bears (i.e., steps) with steps that were possible but not chosen by the bears to develop step selection function models based on conditional logistic regression. The probability of a step being selected by a bear increased as the distance to natural land cover and agriculture at the end of the step decreased and as distance from roads at the end of a step increased. To characterize connectivity among subpopulations, we used the step selection models to create 4,000 hypothetical correlated random walks for each subpopulation representing potential dispersal events to estimate the proportion that intersected adjacent subpopulations (hereafter referred to as successful dispersals). Based on the models, movement paths for males intersected all adjacent subpopulations but paths for females intersected only the most proximate subpopulations. Cross-validation and genetic and independent observation data supported our findings. Our models also revealed that successful dispersals were facilitated by a reintroduced population located between 2 distant subpopulations. Successful dispersals for males were dependent on natural land cover in private ownership. The addition of hypothetical 1,000-m- or 3,000-m-wide corridors between the 4 study areas had minimal effects on connectivity among subpopulations. For females, our model suggested that habitat between subpopulations would probably have to be permanently occupied for demographic rescue to occur. Thus, the establishment of stepping-stone populations, such as the reintroduced population that we studied, may be a more effective conservation measure than long corridors without a population presence in between. Published 2015. This article is a U.S. Government work and is in the public domain in the USA.

4342: ***-.063***

Orphaned bears have been captive-reared and released back to the wild for more than 3 decades, often without a clear understanding of their fates because post-release monitoring is not a common practice. As a result, management agencies lack efficacy data on post-release success rates and are often reluctant to encourage increased use of this technique. We evaluated the potential management and conservation implications of releasing captive-reared bears by documenting post-release survival, cause-specific mortality, human conflict activity, movements, and reproduction for 550 American black, brown and Asiatic black bears reared in 12 captive-rearing programs around the world. Survival rates in these programs ranged from 0.50 to 1.00 and were similar among the 3 species. The primary causes of mortality were sport hunting and road kills for American black bears, intra-specific predation and illegal kills for brown bears, and natural mortalities and illegal kills for Asiatic black bears. Although American and Asiatic black bears were involved in conflicts post-release, the majority of released bears (94%) were not documented in conflict situations. Movement patterns of captive-reared American black and brown bears showed no homing tendencies toward their rearing facility. Twenty captive-reared bears produced 21 litters. Our analyses reduce many of the uncertainties surrounding the fate of bears released as yearlings and provide evidence that releasing captive-reared bears is a defensible management alternative. (c) 2015 The Wildlife Society.

4343: ***+.178***

Greater sage-grouse (Centrocercus urophasianus) and Columbian sharp-tailed grouse (Tympanuchus phasianellus columbianus) have declined substantially in Washington, USA, primarily because native shrub-steppe has been converted to agriculture. In response, state and federal agencies have acquired and restored habitat, and augmented and reintroduced grouse to suitable areas. We examined how sympatric, translocated sage-grouse and sharp-tailed grouse used space and selected habitats within their spring-summer home ranges and at nest sites within remnant shrub-steppe surrounded by a matrix of cropland in eastern Washington. Because their life-history requirements differ, we expected extensive habitat partitioning between species. Using radiolocations of 43 birds of each species, we found that sage-grouse had larger spring-summer home ranges than sharp-tailed grouse, and the composite of home ranges for sharp-tailed grouse fell almost completely within the composite of home ranges for sage-grouse. By creating resource utilization function models using radiolocations of 53 birds of each species, we found that areas of highest predicted intensity of use for both species overlapped by >50%, even at the top 5% quantile. Both species used restored fields and areas farther from trees and roads or distribution lines more intensely. Sage-grouse used less rugged areas more intensely, and both species used 3 levels of shrub cover equally. To compare selection of nest sites relative to available sites for nesting in both species, we created resource selection function models for 30 birds of each species and found that sage-grouse selected areas farther from distribution lines, whereas sharp-tailed grouse selected restored fields. When we examined vegetation characteristics used by female sage-grouse and sharp-tailed grouse at nest sites using a case-control, use versus non-use design for 26 birds of each species, we found sage-grouse used areas with greater shrub cover, lower annual forb cover, and taller perennial grasses, whereas sharp-tailed grouse used areas with greater perennial grass cover and taller perennial grasses and forbs. When we compared habitat features measured at nest sites between species, we found sage-grouse used areas with greater moderate and dense shrub cover, lower sparse shrub cover, less restored fields, higher patch diversity, and areas farther from distribution lines than sharp-tailed grouse. These differences resulted in only 38% overlap of areas within the top quartile of relative selection values for nest sites by the 2 species, and <10% at the top 5% quantile. Because many western states are highly fragmented by cropland, understanding how populations of species with different life-history characteristics, such as sage-grouse and sharp-tailed grouse, coexist within remaining tracts of shrub-steppe at different spatial scales is important for effectively conserving and managing shrub-steppe communities. (c) 2015 The Wildlife Society.

4344: ***+.096***

The potential for interspecific competition for food resources is a key consideration when newly introduced ungulates occupy a shared range with resident native species. Management plans in Yukon, Canada, for reintroduced bison (Bison bison) and introduced elk (Cervus canadensis) called for an assessment of competition for food resources between these species and resident populations of caribou (Rangifer tarandus), thinhorn sheep (Ovis dalli), and moose (Alces americanus). To assess potential competition among ungulates, we examined dietary overlap of 7 ungulate species in southwestern Yukon, Canada. We compared diet composition at the forage class level and composite diet indices (diversity, evenness, and niche breadth) among species found at 2 elevation classes (high [1,200m ASL] and low [<1,200m ASL]) during summer and winter, using data derived from microhistological analyses of feces. Composite indices and percent of key forage classes in diets differed among ungulates during summer and winter and at both high and low elevations. Potential dietary overlap for most species pairs was low to moderate (0.07-0.60). However, for bison and thinhorn sheep at high elevations, and bison and semi-feral horses (Equus ferus caballus) at low elevations, it was high (0.82-0.97) during both summer and winter, indicating potential for food competition. Dietary overlap between species appeared to be

based primarily on morphophysiological classification (i.e., browser-intermediate-grazer), rather than body size. Bison, horses, and sheep were all grazers. Intermediate species, such as introduced elk, had only moderate dietary overlap with several species. We concluded that the potential for competition for food resources based on dietary overlap between bison and sheep and bison and horses may be high in our study area, depending on forage availability, but for all other species pairs in our study area the potential for forage competition is low to moderate. (c) 2015 The Wildlife Society.

4345: *+.211*

Reproduction is timed so that parturition coincides with optimal conditions for offspring survival, with strong fitness implications in northern regions. However, in the southern United States the breeding season of white-tailed deer (Odocoileus virginianus) is relatively heterogeneous over short distances. To test the hypothesis that genetic differences account for heterogeneity in breeding dates, we compared the degree of mitochondrial (mtDNA) and microsatellite DNA differentiation among 6 pairs of adjacent populations with breeding dates differing by an average of 35 days (DBD group) and 4 pairs of populations displaying similar breeding dates (SBD group) differing by no more than 2 days. Average mtDNA differentiation between pairs in the DBD group (F-ST=0.408, SD=0.190) was larger (P=0.03) than for the SBD group (F-ST=0.140, SD=0.092). The average differentiation at biparentally inherited microsatellite loci within the SBD group (F-ST=0.028, SD=0.021) did not differ from that observed for the DBD group (F-ST=0.047, SD=0.024; P=0.200). The similarity at biparentally inherited loci suggests that there are no cryptic barriers and populations are connected by male dispersal. The greater differences in mtDNA lineages between geographically proximate populations in the DBD group imply a maternal genetic effect on the timing of breeding, likely maintained by female philopatry. We hypothesize that this difference is a genetic legacy of restocking efforts and the recovery of remnant localized populations. Translocation efforts require careful consideration of differences between source and recipient populations. Breeding dates that differ among some proximate southern deer populations may be a fixed trait that must be considered when establishing management objectives and harvest criteria. (c) 2015 The Wildlife Society.

4346: *+.194*

Translocation to areas free of exotic predators, habitat degradation, or disease may be the most viable restoration option for many endangered species. We report on a successful translocation of the critically endangered St. Croix ground lizard, Ameiva polops, extirpated from St. Croix, U.S. Virgin Islands, Caribbean, by predation from introduced mongooses (Herpestes auropunctatus). We translocated 57 adult A. polops from Green Cay to Buck Island in May 2008. We placed 4 females and 3 males each in eight, 100 m(2), enclosures on Buck Island for 71 days, then the enclosures were opened. During the enclosure period, 20 individuals were identified and 32 others were seen. The average number sighted per survey was only 5.28 (range = 2-10). One hatchling was sighted in an enclosure, indicating a translocated female successfully nested. Body condition of the translocated individuals increased significantly by the end of the enclosure period. Population monitoring surveys at 61 sites across Buck Island showed that 5 years after the initial translocation in June 2013, the new population had grown to an estimated 1,473 individuals and occupied 58.9% of the island. We attribute eradication of mongoose, life history of the species, large propagule size, condition of habitat, soft-release, use of adults, interagency collaboration, and systematic assessment as primary factors that facilitated this successful translocation. Our findings provide meaningful insights on factors that enhance the potential for successful translocations, and point to new strategies aimed at restoring populations of endangered reptiles in

their native ranges.

4347: ***+.435***

Only a quarter of reintroduction programs succeed in restoring a self-sustaining population of an extirpated species. Optimal source population selection for restoration efforts can increase the fitness of translocated individuals and improve reintroduction success. Here, we describe the support for two strategies for selecting source populations: pre-existing adaptation and adaptive potential. The pre-existing adaptation strategy focuses on source populations with a high frequency of genotypes that confer adaptations, and within this strategy we detail the ancestry matching approach and environment matching approach. The adaptive potential strategy focuses on source populations with high heritable genetic variation that confer the potential to adapt, and within this strategy we detail the single source population approach and multiple source population approach. We review empirical tests of the different approaches, and find stronger support for the pre-existing adaptation strategy than the adaptive potential strategy. We provide a framework for source population selection based on the two strategies, highlighting the importance of gathering information on key environment features in the source and restoration locations, as well as detail the knowledge gaps. Filling these knowledge gaps is important for validating and potentially revising our proposed framework, and ultimately improving the success rate of restoring extirpated populations.

4348: ***+.131***

Translocations are increasingly used in species conservation, but success rates can be low. Experimental approaches are needed to compare the outcomes of translocation strategies, including those involving head-starting (the rearing of juveniles in captivity before release). Here, we studied the reintroduction of head-started and wild-caught juveniles of a distinctive reptile, the tuatara Sphenodon punctatus, on the South Island of New Zealand. We compared morphometric, ecophysiological, behavioural and spatial aspects for juveniles from three groups (wild-caught from a warmer climate, head-started from a warmer climate and head-started from the local climate), focusing on the first 5 months of summer following release. Group did not affect body condition at recapture, but wild-caught juveniles, despite being the only animals with ticks at release, grew faster and had higher inferred body temperatures than individuals head-started in a warmer climate. Wild-caught juveniles were less frequently seen emerged than juveniles head-started at the release site, and no animals had ticks at the summer's end. Mean dispersal distance and home-range size did not differ significantly between groups, and all groups had high survival (96.4-100% over first summer; at least 66.7-73.3% after the first winter following release). We conclude that, for most metrics, post-release performance of head-started and wild-caught juvenile tuatara was similar, and that head-started groups can differ between each other as much as between captive and wild. Our results emphasize the species-specific nature of translocation outcomes.

4349: ***-.052***

Bellamya aeruginosa is a widely distributed Chinese freshwater snail that is heavily harvested, and its natural habitats are under severe threat due to fragmentation and loss. We were interested whether the large geographic distances between populations and habitat fragmentation have led to population differentiation and reduced genetic diversity in the species. To estimate the genetic diversity and population structure of B.aeruginosa, 277 individuals from 12 populations throughout its distribution range across China were sampled: two populations were sampled from the Yellow River system, eight populations from the Yangtze River system, and two populations

from isolated plateau lakes. We used seven microsatellite loci and mitochondrial cytochrome oxidase I sequences to estimate population genetic parameters and test for demographic fluctuations. Our results showed that (1) the genetic diversity of B.aeruginosa was high for both markers in most of the studied populations and effective population sizes appear to be large, (2) only very low and mostly nonsignificant levels of genetic differentiation existed among the 12 populations, gene flow was generally high, and (3) relatively weak geographic structure was detected despite large geographic distances between populations. Further, no isolation by linear or stream distance was found among populations within the Yangtze River system and no signs of population bottlenecks were detected. Gene flow occurred even between far distant populations, possibly as a result of passive dispersal during flooding events, zoochoric dispersal, and/or anthropogenic translocations explaining the lack of stronger differentiation across large geographic distances. The high genetic diversity of B.aeruginosa and the weak population differentiation are likely the results of strong gene flow facilitated by passive dispersal and large population sizes suggesting that the species currently is not of conservation concern.

4350: *+.094*

In 1988-1989, 32 bobcats Lynx rufus were reintroduced to Cumberland Island (CUIS), Georgia, USA, from which they had previously been extirpated. They were monitored intensively for 3years immediately post-reintroduction, but no estimation of the size or genetic diversity of the population had been conducted in over 20years since reintroduction. We returned to CUIS in 2012 to estimate abundance and effective population size of the present-day population, as well as to quantify genetic diversity and inbreeding. We amplified 12 nuclear microsatellite loci from DNA isolated from scats to establish genetic profiles to identify individuals. We used spatially explicit capture-recapture population estimation to estimate abundance. From nine unique genetic profiles, we estimate a population size of 14.4 (SE=3.052) bobcats, with an effective population size (N-e) of 5-8 breeding individuals. This is consistent with predictions of a population viability analysis conducted at the time of reintroduction, which estimated the population would average 12-13 bobcats after 10years. We identified several pairs of related bobcats (parent-offspring and full siblings), but similar to 75% of the pairwise comparisons were typical of unrelated individuals, and only one individual appeared inbred. Despite the small population size and other indications that it has likely experienced a genetic bottleneck, levels of genetic diversity in the CUIS bobcat population remain high compared to other mammalian carnivores. The reintroduction of bobcats to CUIS provides an opportunity to study changes in genetic diversity in an insular population without risk to this common species. Opportunities for natural immigration to the island are limited; therefore, continued monitoring and supplemental bobcat reintroductions could be used to evaluate the effect of different management strategies to maintain genetic diversity and population viability. The successful reintroduction and maintenance of a bobcat population on CUIS illustrates the suitability of translocation as a management tool for re-establishing felid populations.

4351: *+.202*

Inheriting behavioral patterns culturally (i.e. by learning from parents) rather than genetically is considered an integral part of individual development for many bird and mammal species. I discuss the possibility that in some cases, particularly when only heavily modified habitat remains available, such transmission might have a negative effect on the individual's adaptability and chances of survival. Instead, animals deprived of normal parental care may be better suited for survival in novel environments. I describe this possible scenario with captive-reared Whooping Cranes (Grus americana) released in southwestern Louisiana, primarily in the context of human-

modified habitats used by this reintroduced population. Captive-rearing techniques based on this approach may be beneficial for other threatened species, particularly those that have little or no nonmodified habitat left and are amenable to alternative habitats if cultural transmission is interrupted.

4352: *+.023*

In most urban and agricultural landscapes, remnants of native vegetation are surrounded by an inhospitable matrix. Although vagile species come and go, many reptiles, amphibians and small mammals are effectively stranded and declining towards local extinction. In the same landscapes, other areas where these species are absent are improving in habitat quality, both through natural regeneration and active restoration efforts. So, for many species in many domesticated landscapes, there are too many individuals in some patches of decreasing quality and no individuals in patches of increasing quality. One solution to this situation is to move animals from those areas where there are plenty to suitable areas where there are none. These targeted translocations apply lessons learned from revegetation to dispersal-limited animals to in-fill distributional ranges, increase population size and improve both demographic and genetic connectivity, pushing nonequilibrial metapopulations away from extinction via an imposed mass effect. In contrast to conventional reintroduction schemes expensive, reactive interventions involving highly-trained specialists and captive-raised endangered species these inexpensive, proactive, community-driven initiatives aim to avert future declines by keeping common species common. Having introduced the wildlife restoration vision, we use two scenarios to illustrate the benefits of the approach to species, ecosystem function, ecological understanding, restoration practise and public engagement. As well as adhering to best-practise reintroduction techniques to ensure animal welfare is not compromised and avoid detrimental effects to source populations or release sites, we emphasize community participation, data quality and long-term accessibility as paramount to maximize learning opportunities. (C) 2015 Elsevier Ltd. All rights reserved.

4353: *-.252*

Restoring missing ecological interactions by reintroducing locally extinct species or ecological surrogates for extinct species has been mooted as an approach to restore ecosystems. Australia's apex predator, the dingo, is subject to culling in order to prevent attacks on livestock. Dingo culling has been linked to ecological cascades evidenced by irruptions of herbivores and introduced mesopredators and declines of small and medium sized mammals. Maintenance of dingo populations is untenable for land-managers in many parts of Australia owing to their depredations on livestock. However, it may be possible to fill the apex predator niche with the Tasmanian devil which has less impact on livestock Devils once occurred throughout Australia, but became extinct from the mainland about 3000 years ago, but are now threatened by a disease epidemic in Tasmania. To explore the feasibility of reintroducing devils to mainland Australia we used species distribution models (SDMs) to determine if suitable climatic conditions for devils exist and fuzzy cognitive mapping (FCM) to predict the effects of devil reintroduction. Based on devils' current distribution, our SDM indicates that suitable areas for devils exist in south-eastern Australia. Our FCM examined ecosystem responses to predator-management scenarios by manipulating the abundances of devils, dingoes and foxes. Our FCMs showed devils would have cascading effects similar to, but weaker than those of dingoes. Devil introduction was linked to lower abundances of introduced mesopredators and herbivores. Abundances of small and medium sized mammals and understorey vegetation complexity increased with devil introduction. However, threatened species vulnerable to fox predation benefited little from devil introduction. Our study suggests that reintroducing ecological surrogates for apex predators may yield benefits

for biodiversity conservation. (C) 2015 Elsevier Ltd. All rights reserved.

4354: ***-.115***

We investigated threats to the California condor (Gymnogyps californians), a flagship endangered species, using individual data on survival during a 20 year period of intensive recovery efforts. Over the two decades of reintroductions, condors in California had an estimated median survival time of 7.8 years suggesting that 50% of condors are expected to survive in the wild long enough to contribute to recruitment In general, annual mortality rates exceeded levels necessary for a stable population; however, mortality declined, reaching levels needed for population stability, during the second decade of re-establishment. Intensive management practices, including utility pole aversion training and clinical interventions to prevent lead-related deaths likely contributed to the decrease in mortality rates. Utility line collision and/or electrocution was an important factor causing mortality over the two decades; though, this threat has largely been mitigated through management and targeted efforts in high-risk areas. In the past, wildfires were not considered a major threat to survival of free-flying condors. However, our analyses suggest that forest fires are significantly linked to the hazard of death, and increased wildfire activity in California highlights this population's vulnerability to catastrophic losses from forest fire. Lead poisoning, which was a major driver in the population's decline, was a leading cause of death accounting for the greatest adult mortality, and lead exposure remains the most significant threat. Recent lead ammunition reduction efforts in the condor range in California hold promise for improving the recovery potential for this population. (C) 2015 Elsevier B.V. All rights reserved.

4355: ***-.046***

Introduced predators have been implicated in the decline of many fauna populations around the world and are the main factor responsible for the failure of numerous fauna reintroduction programs. As a result, control of introduced predators is a significant management action implemented in wildlife protection programs, particularly in Australia, New Zealand and on islands. Individual predators are seldom targeted in conservation programs, which usually conduct broad-scale, non-specific predator control based on the assumption that the removal of each individual predator is equally important. In contrast, predator management programs initiated by human wildlife conflict typically use profiling or specific control techniques to target 'problem' predators. We investigated whether individual domestic cats vary in the magnitude of their predation threat to wildlife by first reviewing published and anecdotal information on incidences where feral cats have had significant impacts on wildlife protection or translocation programs. We concentrated on prey species that were likely to be more challenging to cats based on their size, novelty or behavior. We then used the results from this review to create a profile of cats that were most likely to cause significant problems for challenging prey, and tested this during a translocation of a threatened mammal species. Both the review and translocation suggested that large male cats 3.5 kg or heavier were disproportionally responsible for predation events on challenging prey and possibly implicated in the failure of many protection or reintroduction programs of mammals greater than 1 kg. Some cats within this demographic profile were responsible for multiple prey deaths suggesting that both demography and prior experience may define predators capable of 'catastrophic predation' that threatens prey populations. Current control programs for feral cats and foxes that do not target particular predator profiles may inadvertently avoid controlling individuals most likely to specialize on threatened prey. We call for the application of crime-fighting forensic and aggregate profiling techniques in wildlife protection programs to determine the profile of predators likely to prey on focal wildlife species and to guide the development of control methods that specifically target these individuals. (C) 2015 Elsevier

B.V. All rights reserved.

4356: *+.093*

Reintroductions have been increasingly used for species restoration and it seems that this conservation tool is going to be more used in the future. Consequently a better knowledge of consequences of this kind of management is needed. Several authors have found differences in dispersal distances among wild and reintroduced individuals; although no common explanation for this general trend (Le. released animals moving farther than wild ones) has been proposed. Here we compared the dates of dispersal and the distribution of maximum distances during juvenile dispersal between a natural high density population and an alternative situation where young Spanish imperial eagles (Aquila adalberti) were reintroduced in a new area with a very low intra-specific density, and ad libitum feeding until the onset of dispersal. Results showed that maximum dispersal distances were longer in translocated (mean = 205 km) than in control juvenile eagles (mean = 119.70 km), and the shape of the distribution changed from leptokurtic right-skewed to quasi-normal. According to our results, for reintroduced young fed ad libitum, we can predict that effective dispersal distances will be longer in reintroduced young during juvenile dispersal than in natural populations. But dispersal distances might also depend on the distance from the release area to the nearest breeding population of the species. For reintroductions close to other existing populations, immigration of released young into existing populations would be greater than expected. (C) 2015 Elsevier Ltd. All rights reserved.

4357: *+.058*

The Amur leopard (Panthera pardus orientalis) is highly elusive, rare species, critically threatened with extinction worldwide. In this study, we conducted camera-trap surveys of an Amur leopard population in Jilin Province, northeast China. We estimated population abundance and density distribution, and explored the effects of prey population densities and biomass of prey, habitat and anthropogenic factors on the spatial distribution of Amur leopard density. Our results suggested that Amur leopard density was 0.62 individuals/100 km(2) and 16.58 individuals might live within the study area. The spatial distribution of Amur leopard density exhibited different responses to the population densities of different prey species. We found that two ecological thresholds existed in maximum responses of Amur leopard distribution to elevation and prey biomass. Vegetation and anthropogenic factors also showed significant effects on leopard population distribution. In general, there was a combination of habitat factors including, not only prey assembly and biomass, but also vegetation, anthropogenic and geographical factors driving the spatial distribution of Amur leopard population. These insights informed us that comprehensive adaptive landscape and prey conservation strategies should be conducted for saving this critically endangered predator. (C) 2015 Elsevier Ltd. All rights reserved.

4358: *+.172*

In an increasingly human-dominated world, conservation requires the mitigation of conflicts between large mammals and people. Conflicts are particularly problematic when resources are limited, such as at wintering sites. Such conflicts have fragmented many large mammal populations, making reintroductions in suitable sites necessary. Broad-scale habitat suitability mapping can help to identify sites for species' reintroductions. The European bison is a good example of a large mammal that is restricted to only a fraction of its former range. The goal of our study was to identify and assess potential habitat for European bison in the Caucasus Mountains, which is a part of its former range and has the potential to harbor larger populations. Specifically,

we used seasonal presence data from four reintroduced European bison populations and two sets of predictor variables to: (i) map habitat suitability for summer and winter, (ii) characterize habitat based on management-relevant categories that capture the potential for conflicts with people, and (iii) identify candidate sites for reintroductions. We found substantial areas of suitable habitat. However, areas of potential conflicts with people were widespread and often near highly suitable areas. We identified 69 potential reintroduction sites (10 230 km(2), 1.8% of the ecoregion) that have suitable summer and winter habitat with relatively low risk of human-wildlife conflict. These results can guide conservation efforts in establishing a viable European bison metapopulation in the Caucasus ecoregion. More broadly, our results highlight the need to map large mammal habitat suitability for different seasons in order to derive meaningful conservation recommendations. (C) 2015 Elsevier Ltd. All rights reserved.

4359: *+.051*

The leopard (Panthera pardus) is heavily persecuted in areas where it predates livestock and threatens human well-being. Attempts to resolve human-leopard conflict typically involve translocating problem animals; however, these interventions are rarely informed by genetic studies and can unintentionally compromise the natural spatial genetic structure and diversity, and possibly the long-term persistence, of the species. No significant genetic discontinuities were definable within the southern African leopard population. Analysis of fine-scale genetic data derived from mitochondrial and nuclear DNA revealed that the primary natural process shaping the spatial genetic structure of the species is isolation-by-distance (IBD). The effective gene dispersal (sigma) index can inform leopard translocations and is estimated to be 82 km for some South African leopards. The importance of adopting an evidence-based strategy is discussed for supporting the integration of genetic data, spatial planning and social learning institutions so as to promote collaboration between land managers, government agency staff and researchers. (C) 2015 Academie des sciences. Published by Elsevier Masson SAS. All rights reserved.

4360: *+.113*

Extinction and colonization dynamics are critical to understanding the evolution and conservation of metapopulations. However, traditional field studies of extinction-colonization are potentially fraught with detection bias and have rarely been validated. Here, we provide a comparison of molecular and field-based approaches for assessment of the extinction-colonization dynamics of tidewater goby (Eucyclogobius newberryi) in northern California. Our analysis of temporal genetic variation across 14 northern California tidewater goby populations failed to recover genetic change expected with extinction-colonization cycles. Similarly, analysis of site occupancy data from field studies (94 sites) indicated that extinction and colonization are very infrequent for our study populations. Comparison of the approaches indicated field data were subject to imperfect detection, and falsely implied extinction-colonization cycles in several instances. For northern California populations of tidewater goby, we interpret the strong genetic differentiation between populations and high degree of withinsite temporal stability as consistent with a model of drift in the absence of migration, at least over the past 20-30 years. Our findings show that tidewater goby exhibit different population structures across their geographic range (extinction-colonization dynamics in the south vs. drift in isolation in the north). For northern populations, natural dispersal is too infrequent to be considered a viable approach for recolonizing extirpated populations, suggesting that species recovery will likely depend on artificial translocation in this region. More broadly, this work illustrates that temporal genetic analysis can be used in combination with field data to strengthen inference of extinction-colonization dynamics or as a stand-alone tool when field data are lacking.

4361: ***+.141***

Melanistic Eurasian red squirrels Sciurus vulgaris are commonly found on the Danish island of Funen. They are thought to represent native Danish squirrel types and are presently under threat from admixture with introduced red squirrels. In response, a conservation program was started in 2009 that involves the translocation of melanistic squirrels from Funen to the squirrel-free island of Langeland. Using mitochondrial DNA of 101 historical and modern samples from throughout Denmark, we assess for the first time population structure and mitochondrial genetic diversity of Danish squirrels compared to its larger pan-Eurasian distribution. We find that Danish squirrels have low levels of genetic diversity, especially melanistic individuals. Bayesian skyline reconstructions show that Danish squirrels have most probably experienced a severe bottleneck within the last 200 years. Also, fine-scale genetic structure was found between squirrels from the regions of Funen, Zealand and Jutland, which mimics the insular geography of Denmark. Additional nuclear DNA analyses will be required to determine the precise admixture levels between original Danish and introduced squirrels and to locate unmixed candidate populations for specific conservation efforts.

4362: ***+.161***

Species of conservation concern characterized by small and declining populations greatly benefit from proactive management approaches such as population translocations. Because they often show intra-specific genetic and phenotypic variation, which can result from drift or differential selective pressures between habitats, understanding the distribution of such variation and its underlying processes is a prerequisite to develop effective management guidelines. Indeed, translocations among genetically differentiated populations potentially locally adapted are discouraged in order to avoid outbreeding depression, while translocations among populations characterized by high gene flow with no evidence for local adaptation are encouraged. Here, we first test whether 2 recognized subspecies, the North Island kaka (Nestor meridionalis septentrionalis) and South Island kaka (Nestor meridionalis meridionalis) of New Zealand fit a scenario of allopatric subspeciation following the separation of the North and South Islands at the end of the Pleistocene using 1 mtDNA (n = 96) and 9 microsatellite markers (n = 126). We then test whether morphological differences among the 2 subspecies support a pattern of local adaptation, comparing phenotypic divergence (P-ST) and the level of divergence by drift alone (F-ST) among populations. We find little population structure between islands, ruling out allopatric subspeciation in kaka. Further, P-ST exceeds F-ST, supporting an adaptive latitudinal size cline consistent with Bergmann's rule. These results therefore suggest that using neutral genetic diversity alone can be misleading when identifying management units and that the nature of phenotypic variation should be considered in translocations efforts. We finally discuss North and South Island management units but suggest that cross-island translocation be allowed.

4363: ***+.067***

Information on the parentage of captive reared clutches is vital for conservation head-starting programs. Molecular methods, such as genotyping individuals with hyper-variable markers, can elucidate the genealogical contribution of captive-reared, reintroduced individuals to native populations. In this study, we used 12 polymorphic microsatellite loci to infer parentage of a clutch of 18 eastern hellbenders collected from a single nest from Buffalo Creek, West Virginia, subsequently reared in captivity, and used for translocations in Indiana. Collectively, these markers successfully detected the presence of multiple parentage for this species of conservation concern presently used in captive management programs in zoos across many states. This study highlights

the need for genetic analysis of captive reared clutches used in translocations to minimize the loss of genetic diversity and potential for genetic swamping at release sites. (C) 2015 Wiley Periodicals, Inc.

4364: *+.035*

Background and Purpose: In the early 20th century, two native North American poeciliid species Gambusia holbrooki and Gambusia affinis were introduced to Europe as a mosquito control agent. The first introduction to Istria from Italy in 1924 was followed by several independent introductions and massive translocations. Presently, the distribution of these two species in Croatia and Bosnia and Herzegovina (Bosnia and Herzegovina) is still largely unknown and they are often confused. The purpose of this study was to determine the taxonomic status of individual Gambusia populations and to shed more light on the distribution and phylogeographic patterns of these invasive species in Croatia and Bosnia and Herzegovina. Materials and Methods: All new and literature data were plotted together to obtain the overall distribution of Gambusia sp. in Croatia and Bosnia and Herzegovina. The samples collected across this range were analysed meristically and by means of mitochondrial cytochrome b (cytb) gene sequence analysis to ascertain the true taxonomic status of the populations. For phylogeographic analysis, the cytb sequences from this study were combined with previously published data. Results and Conclusions: The methods only confirmed the presence of G. holbrooki. G. holbrooki is continuously distributed across the entire Mediterranean biogeographical region in Croatia and Bosnia and Herzegovina. Two different mitochondrial cytb haplotypes were found: the widely spread Holl haplotype and haplotype Hol5 that in Europe was previously found only in France and Greece. This suggests that G. holbrooki populations in the region originated from different stocks.

4365: *+.157*

Native fishes continue to decline in abundance and distribution. One common practice to ensure native fish persistence has been to introduce fish into new habitats. Though these introductions reduce the risk of extinction, often the introduced populations are not considered in the Endangered Species Act (ESA) listing status in the same manner as extant populations. One instance where they were considered was when Least Chub Iotichthys phlegethontis were introduced into 23 locations within the Bonneville Basin between 2005 and 2013. In 2014, 10 of these populations were considered successful and were evaluated with the six remaining extant populations by the U.S. Fish and Wildlife Service. In this instance, Least Chub were removed from the candidate list in part due to these introduced populations, which increased the resiliency, redundancy, and representation for this species to persist now and into the foreseeable future. The creation of introduced fish populations is a conservation practice that needs to be considered as a potential tool for fisheries managers, not only to ensure persistence, but also to preclude the need for federal listing under ESA.

4366: *+.139*

There is increasing evidence of large carnivore presence outside protected areas, globally. Although this spells conservation success through population recoveries, it makes carnivore persistence in human-use landscapes tenuous. The widespread distribution of leopards in certain regions of India typifies this problem. We obtained information on leopard- human interactions at a regional scale in Karnataka State, India, based on systematic surveys of local media reports. We applied an innovative occupancy modelling approach to map their distribution patterns and identify hotspots of livestock/human depredation. We also evaluated management responses like

removals of 'problem' leopards through capture and translocations. Leopards occupied around 84,000 km(2) or 47% of the State's geographic area, outside designated national parks and wildlife sanctuaries. Their presence was facilitated by extent of vegetative cover-including irrigated croplands, rocky escarpments, and prey base in the form of feral and free-ranging dogs. Higher probabilities of livestock/human attacks by leopards were associated with similar ecological features as well as with capture/removals of leopards. Of the 56 cases of leopard removals reported, 91% did not involve human attacks, but followed livestock predation or only leopard sightings. The lack of knowledge on leopard ecology in human-use areas has resulted in unscientific interventions, which could aggravate the problem rather than mitigating it. Our results establish the presence of resident, breeding leopards in human-use areas. We therefore propose a shift in management focus, from current reactive practices like removal and translocation of leopards, to proactive measures that ensure safety of human lives and livelihoods.

4367: *+.064*

Wild populations of endangered Hawaiian tree snails have declined precipitously over the last century due to introduced predators and other human impacts. Life history traits, such as very low fecundity (<5 offspring per year across taxa) and maturity at approximately four years of age have made recovery difficult. Conservation efforts such as in situ predator-free enclosures may increase survival to maturity by protecting offspring from predation, but no long-term data existed prior to this study demonstrating the demographic and genetic parameters necessary to maintain populations within those enclosures. We evaluated over 20 years of evidence for the dynamics of survival and extinction in captive ex situ populations of Hawaiian tree snails established from wild-collected individuals. From 1991 to 2006, small numbers of snails (<15) from fifteen species were collected from the wild to initiate captive-reared populations as a hedge against extinction. This small number of founders resulted in a severe bottleneck in each of the captive-reared populations. We identified key demographic parameters that predicted population recovery from this bottleneck. Species with captive populations that produced between two and four offspring per adult per year and had 20-50% of those offspring survive to maturity recovered to numbers above 100 individuals, and maintained viable populations following a decline that occurred between 2009 and 2014. Those populations that had less than two offspring per adult per year and less than 20% survival to maturity did not reach 100 individuals in captivity, and many of these populations died out during the recent decline. We suggest that small reductions in fitness may contribute to extirpation in taxa with inherently low fecundity, by keeping populations below a threshold number essential to long-term recovery. Future ex situ populations should be founded with no less than 15 adults, and maintained in conditions closely approximating the temperature and humidity of source locations to optimize fitness. Permanent translocations of wild populations for conservation purposes will be more likely to succeed with greater than 100 adults, and should be limited to locations with a similar climate to source locations.

4368: *+.221*

Background: The Osprey (Pandion haliaetus) is one of only six bird species with an almost world-wide distribution. We aimed at clarifying its phylogeographic structure and elucidating its taxonomic status (as it is currently separated into four subspecies). We tested six biogeographical scenarios to explain how the species' distribution and differentiation took place in the past and how such a specialized raptor was able to colonize most of the globe.Results: Using two mitochondrial genes (cyt b and ND2), the Osprey appeared structured into four genetic groups representing quasi non-overlapping geographical regions. The group Indo-Australasia corresponds to the cristatus ssp, as well as the group Europe-Africa to the haliaetus ssp. In the Americas, we

found a single lineage for both carolinensis and ridgwayi ssp, whereas in north-east Asia (Siberia and Japan), we discovered a fourth new lineage. The four lineages are well differentiated, contrasting with the low genetic variability observed within each clade. Historical demographic reconstructions suggested that three of the four lineages experienced stable trends or slight demographic increases. Molecular dating estimates the initial split between lineages at about 1.16 Ma ago, in the Early Pleistocene.Conclusions: Our biogeographical inference suggests a pattern of colonization from the American continent towards the Old World. Populations of the Palearctic would represent the last outcomes of this colonization. At a global scale the Osprey complex may be composed of four different Evolutionary Significant Units, which should be treated as specific management units. Our study brought essential genetic clarifications, which have implications for conservation strategies in identifying distinct lineages across which birds should not be artificially moved through exchange/reintroduction schemes.

4369: ***-.103***

The removal of eggs or chicks from wild populations to create captive populations, reinforce free-ranging populations or reintroduce species into the wild is a restoration tool that requires an assessment of potential detrimental effects upon the donor population. This is an absolute prerequisite when wild donor populations are scarce and small. Here, we forecast the population trend of the largest European population of the bearded vulture (Gypaetus barbatus) over the next 30 years under different demographic and management scenarios (removal of eggs, chicks or fledglings). Projections derived from the combination of a PDP model (Population Dynamic P-system) and a Box-Behnken design would lead to a decline in 77% of all 57 scenarios analysed. Among the 13 scenarios predicting a population increase, only 4 seem realistic in terms of growth rate (0.04%-1.01%), at least if current age at first breeding and productivity would remain constant over time. Our simulations thus suggest that most extraction scenarios would have detrimental effects on the demography of the donor population. Release of captive-born young or removal of only the second hatched chick for subsequent captive rearing and translocation into the wild appear to represent much better supplementation and reintroduction options in this threatened species.

4370: ***+.060***

Biodiversity is rapidly declining globally. One strategy to help to conserve species is to breed species in captivity and release them into suitable habitats. The way that reintroduced animals explore new habitats and/or disperse from the release site is rarely studied in detail and represents key information for the success of reintroduction projects. The European bison (Bison bonasus L. 1758) was the largest surviving herbivore of the post-glacial mega-fauna in Europe before it became extinct in the wild, surviving only in captivity since 1919. We investigated the exploration behavior of a herd of European bison reintroduced into the Rothaargebirge, a commercial forest in low range mountain intensively used and densely populated by humans, in the first six months after release. We focused on three questions: (1) how did the European bison move and utilize the habitat on a daily basis, (2) how did the animals explore the new environment, and (3) did their habitat preferences change over time. The European bison dispersed away from their previous enclosure at an average rate of 539 m/month, with their areas of daily use ranging from 70 to 173 ha, their movement ranging from 3.6 km to 5.2 km per day, and their day-to-day use of areas ranged between 389 and 900 m. We could identify three major exploration bouts, when the animals entered and explored areas previously unknown to them. During the birthing phase, the European bison reduced daily walking distances, and the adult bull segregated from the herd for 58 days. Around rut, roaming behavior of the herd increased slightly. The animals preferred spruce

forest, wind thrown areas and grassland, all of which are food abundant habitat types, and they avoided beech forest. Habitat preference differed slightly between phases of the study period, probably due to phenological cycles. After six months, the complete summer home range was 42.5 km(2). Our study shows that a small free-ranging herd of European bison can live in an area intensively used by humans and describes in detail the initial roaming behavior and habitat utilization of the animals.

4371: *+.016*

Bird species may exhibit unexpected population structuring over small distances, with gene flow restricted by geographic features such as water or mountains. The Bahama Oriole (Icterus northropi) is a critically endangered, synanthropic island endemic with a declining population of fewer than 300 individuals. It now remains only on Andros Island (The Bahamas), which is riddled with waterways that past studies assumed did not hinder gene flow. We examined 1,858 base pairs of mitochondrial DNA sequenced from four gene regions in 14 birds (roughly 5% of the remaining population) found on the largest land masses of Andros Island (North Andros and Mangrove Cay/South Andros). We sought to discern genetic structuring between the remaining subpopulations and its relationship to current conservation concerns. Four unique haplotypes were identified, with only one shared between the two subpopulations. Nucleotide and haplotype diversity were higher for the North Andros subpopulation than for the Mangrove Cay/South Andros subpopulation. Analysis of molecular variance (AMOVA) yielded a Wright's fixation index (F-st) of 0.60 (P-Fst = 0.016), with 40.2% of the molecular variation explained by within-population differences and 59.8% by among-population differences. Based on the mitochondrial regions examined in this study, we suggest the extant subpopulations of Bahama Oriole exhibit significant population structuring over short distances, consistent with some other non-migratory tropical songbird species.

4372: *+.118*

Reintroductions are used to reestablish populations to historical habitats from which they were extirpated. The long-term success of these efforts will depend on genetic diversity and the ability of reintroduced individuals to adapt to ecological change. We examined variation at circadian clock (OtsClock1b and OmyFbxw11) and reproductive timing (Ots515NWFSC)-associated genes in two threatened spring-run Chinook salmon (Oncorhynchus tshawytscha) populations that are undergoing restoration to historical habitats above dams. We also tested for an association between the genes and individual variation in arrival time to the spawning grounds. Our findings indicate that levels of genetic diversity in reintroduced individuals are similar to those found in previously studied spring, summer, fall and winter-run Chinook salmon populations. Captive-rearing programs established following dam construction and the more recent reintroduction efforts thus appear to have maintained diversity at these genes. We observed temporal (between run-years) and spatial (between populations) patterns of genetic differentiation, but little evidence that selection underlies these differences. However, there was a relationship between the circadian-associated gene, OmyFbxw11, and arrival time to the spawning grounds, and in one year of the study, "early" and "late" arrivers to the spawning grounds were more differentiated at the gene than at neutral markers. Taken together, these findings suggest that reintroduced salmon may be capable of an evolutionary response to ecological shifts that alter the adaptive landscape between fitness and arrival timing to the spawning grounds.

4373: *+.068*

We studied the population structure and historical demography of the last remaining core population of the threatened southeastern beach mouse (SEBM; Peromyscus polionotus niveiventris) located on a federally protected barrier island complex at the Kennedy Space Center (KSC), Merritt Island National Wildlife Refuge (MINWR) and Cape Canaveral Air Force Station (CCAFS) in Florida, USA. Beach mice (N = 171) were collected from 33 trapping locations along 30 km of coastline on KSC/MINWR/CCAFS and were genotyped using 10 microsatellite loci. We found four genetic clusters of mice that likely form a metapopulation. Gene flow among clusters, assessed using assignment tests, was limited suggesting that human development can serve to inhibit dispersal of beach mice. However, when the presence of roads were examined as possible barriers to movement, gene flow appeared to be facilitated suggesting that removal of thick vegetation along roadsides increases movement. We used approximate Bayesian computation (ABC) to estimate divergence time among clusters and effective population sizes for each cluster and for the pre-divergence population. Results of ABC analyses suggest that barriers to movement likely formed following the construction of the John F. Kennedy Space Center beginning in the 1960s but that this has not heavily impacted the effective size of populations. Pre-divergence and contemporary effective sizes are similar, thus, population sizes likely remained relatively large over the last 50-100 years. The population of SEBM on KSC/MINWR/CCAFS appears to be a genetically diverse core population and individuals from this population will most likely be good candidates for any future reintroduction and translocation programs.

4374: ***+.125***

The Delacour's langur (Trachypithecus delacouri) is one of the three primate species endemic to Vietnam, and listed as 'Critically Endangered' (IUCN 2015). In addition, this species has been continuously included in the biennial list of "The World's 25 Most Endangered Primates" (Mittermeier et al. 2000; 2012), since the lists inception in 2000. For an assessment of the current status of the species, 20 surveys were carried out in all nine areas where populations of the species remain. The results of the surveys show a similar pattern to surveys undertaken over the last decade: a continued dramatic decrease in many subpopulations due to poaching. Only the population at Van Long Nature Reserve is the exception, growing steadily following a complete hunting ban supported by a community based protection unit, and intensive work by the Management Board. Van Long Nature Reserve has high awareness and support in the surrounding communes as a result of a 15 year long conservation project supported by Frankfurt Zoological Society. The surveys showed that eight subpopulations of the Delacour's langur have been eradicated during the last decade. From data gathered during the latest surveys, the current total population is now estimated at 234-275 individuals in 8 subpopulations. The two largest populations occur in Van Long Nature Reserve and in a planned extension area of the reserve. As the reserve and the planned extension area are administrated by two separate provincial authorities, the langurs occurring here have been identified as occurring in two separate populations; however they do in fact form one population. All other subpopulations surveyed are already too small to survive long-term. Thus the most important conservation action for the species would be the extension of Van Long Nature Reserve. The size of the extended nature reserve and the existing habitat conditions could support a large viable population. An improvement of protection for all subpopulations outside Van Long Nature Reserve in order to secure their survival is not feasible, due to the lack of adequate protection or political will to improve protection. The translocation of groups or individuals from very small populations is also not a feasible option. Translocation requires a very high personal resource, technical and financial investment has little proven success and would need to be undertaken in areas with very difficult access and terrain. The Endangered Primate Rescue Center runs a successful captive breeding program for the species. The goal is the reintroduction of captive bred animals into a safe habitat.

A pilot project has already been successfully undertaken by the EPRC, with the first reintroduction of captive bred individuals into Van Long Nature Reserve. The establishment of a second viable population of the species would be a priority for conservation. There also exists a possibility for another reintroduction into the recently established World Heritage Site "Trang An Scenic Landscape Complex" in Ninh Binh Province. These surveys showed that the priority for a conservation intervention should be the extension of Van Long Nature Reserve and a feasibility study for the reintroduction of captive bred individualsinto the "Trang An Scenic Landscape Complex", thus establishing a second population in an area with higher protection. Conservation activities for the species are recommended, and the development of a National Action Plan for one of the Vietnamese endemic primate species would provide the basis to support the survival of the species. A further decrease of the number of individuals with extinction in foreseeable future would be a depressing shame for the country and a great and irrecoverable loss for the world.

4375: ***+.093***

There is widespread concern regarding the impacts of anthropogenic activities on connectivity among populations of plants and animals, and understanding how contemporary and historical processes shape metapopulation dynamics is crucial for setting appropriate conservation targets. We used genetic data to identify population clusters and quantify gene flow over historical and contemporary time frames in the Diamondback Terrapin (Malaclemys terrapin). This species has a long and complicated history with humans, including commercial overharvesting and subsequent translocation events during the early twentieth century. Today, terrapins face threats from habitat loss and mortality in fisheries bycatch. To evaluate population structure and gene flow among Diamondback Terrapin populations in the Chesapeake Bay region, we sampled 617 individuals from 15 localities and screened individuals at 12 polymorphic microsatellite loci. Our goals were to demarcate metapopulation structure, quantify genetic diversity, estimate effective population sizes, and document temporal changes in gene flow. We found that terrapins in the Chesapeake Bay region harbour high levels of genetic diversity and form four populations. Effective population sizes were variable. Among most population comparisons, estimates of historical and contemporary terrapin gene flow were generally low (m approximate to 0.01). However, we detected a substantial increase in contemporary gene flow into Chesapeake Bay from populations outside the bay, as well as between two populations within Chesapeake Bay, possibly as a consequence of translocations during the early twentieth century. Our study shows that inferences across multiple time scales are needed to evaluate population connectivity, especially as recent changes may identify threats to population persistence.

4376: ***+.108***

The northern Aplomado Falcon (Falco femoralis septentrionalis) inhabited the inland and coastal grasslands of Texas, New Mexico, and Arizona until about 1930, when records of aplomados in the United States decreased. In 1986, the species was classified as endangered under the Endangered Species Act. Among other recovery efforts, 102 birds were released from 2006 through 2011, in its former range in New Mexico at the Armendaris Ranch in the south-central portion of the state. To promote their survival, an extended supplemental feeding program was conducted. From 2006 through 2008, supplemental food was provided daily, whereas from 2009 through 2011 food was provided every other day. Providing food once daily corresponded with an increase in the known survival of the aplomados, where known survival was obtained from the recorded observations of falcons at feedings, and the establishment of nearby nesting pairs. Unfortunately, this increase in known short-term survival and reproduction did not seem to lead to long-term survival or retention. This may be attributable to a lack of available prey throughout the

Chihuahuan Desert as a result of ongoing drought, significant brush encroachment caused by historic over-grazing by cattle, the eradication of prairie dogs, and decreased summer and increased winter precipitation, as well as a possible increase in predation influenced by brush encroachment and the fact that the Armendaris Ranch sits at the northernmost edge of the aplomados' historical range. If the reintroduction on the Armendaris Ranch, and other areas with similar levels of prey, is to continue, our research supports the incorporation of an extended daily supplemental feeding program and efforts to improve access to prey, possibly by removing brush and restoring grasslands.

4377: ***+.168***

The European roe deer (Capreolus capreolus) is the most abundant and widespread cervid species in Europe. Despite being the subject of extensive research elsewhere, knowledge of the roe deer in Portugal is scarce. Here we review and summarize the available information on its distribution in Portugal, with the emphasis on: (i) historical distribution, (ii) current distribution and abundance, and (iii) main conservation/management problems. The roe deer is native to Portugal and its populations always persisted in a few patches to the north of the Douro river until the 1990s, when a series of reintroduction programmes restored this species to central and south Portugal. Currently, a natural expansion of the original and reintroduced populations is taking place. The roe deer is present and well established in mountain ranges in north Portugal and is naturally expanding its range towards the border with Spain (west-central Portugal). A number of threats to the species such as potential interspecific competition with the red deer and livestock, along with inadequate management, human disturbances (both roads and human settlements) and climate change have been identified. It is therefore imperative to identify research and monitoring gaps, and finally to draw conclusions under a holistic framework. This will ensure that informed decisions concerning the roe deer management are made at the national level, taking into account that changes in land use occur continuously, possibly affecting the deer abundance.

4378: ***+.182***

The conservation of rare plant species as living collections in botanic gardens and arboreta has become an established tool in the battle against worldwide species' extinctions. However, the establishment of ex situ collections with a high conservation value requires a sound understanding of the evolutionary processes that may reduce the suitability of these collections for future reintroductions. Particularly, risks such as fitness decline of cultivated plants over time, trait shifts and loss of adaptation to the original habitat due to changes in selection regimes have rarely been addressed so far. Based on a literature review and results of our own project we show that genetic drift can lead to fitness decline in ex situ cultivated plants, but these drift effects strongly depend on the conditions and cultivation history in the ex situ facility. Furthermore, we provide evidence that shifts in traits such as germination and flowering time, and a decrease in stress tolerance to drought and competition can reduce the conservation value of ex situ collections. These threats associated with ex situ conditions require more attention by researchers, curators and conservationists. We need to increase knowledge on traits that are subject to novel selection pressures in ex situ collections, and to define population sizes that prevent genetic drift. Establishing conservation networks with replicated collections across gardens and balancing the seed contribution of mother plants to the next generation within a collection are suggested as first steps to increase the conservation value of ex situ plant collections. (C) 2015 Elsevier Ltd. All rights reserved.

4379: ***-.085***

Rapid range-wide extinction of whole populations due to introduced disease would be expected to lead to significant loss of genetic diversity, with impacts being greater for a species that is geographically restricted and with disjunct populations. We investigated the genetic consequences of extirpation in the rare endemic woody shrub, Banksia brownii, following the introduction of the pathogen Phytophthora cinnamomi. B. brownii shows a pattern of disease driven population extinction typical of significant numbers of threatened plant species within southwestern Australia. Based on microsatellite genotyping of material from extinct (ex situ seed collections) and extant populations we estimated that 38.2% (gene diversity) and 36.7% (allelic richness) of total genetic diversity, based on contributions of within population variation and differentiation, have been lost from Banksia brownii due to P. cinnamomi. Furthermore, a loss of genetic diversity was reflected in the loss of 16 out of 55 and 6 out of 33 private alleles respectively from two geographically disjunct genetically distinct population groups. We found significant genetic structure within B. brownii with three geographically disjunct population assemblages that should be regarded as separate units for conservation. These findings have been important in developing reintroductions to disease free sites for each conservation unit. Crown Copyright (C) 2015 Published by Elsevier Ltd. All rights reserved.

4380: *+.101*

Large-scale human-mediated translocation of plants is a widespread phenomenon throughout the world. In this study, we examine the extent to which exotic gene flow from conspecific plantations of mesic origin impacts pre- and post-dispersal fitness, seedling phenotype, and quantitative genetic variation of the relict southernmost native population of Scots pine (Pinus sylvestris var. nevadensis Christ). For this purpose, we conducted controlled pollinations to obtain native, exotic and intraspecific hybrid progenies for testing seed viability and seedling performance under experimental common-garden conditions mimicking the severe summer drought in the native population's environment. Proportion of aborted seeds was markedly higher in exotic (>60%) than in intraspecific hybrid (similar to 24%) and native (18%) progenies. Seed germination time and rate were similar across genetic crosses. Hybrid and exotic seedlings died on average 1.2 and 0.8 days earlier than local progenies when exposed to the drought treatment. Cumulative mortality rate was positively associated to biomass allocation in needles, suggesting that higher investment in needles is maladaptive in dry environments for P. sylvestris, probably because of increased total transpiration. Native progenies harbored higher genetic variation than exotic progenies, but similar to hybrids for most phenotypic traits, suggesting low impact of exotic gene flow on the levels of heritable genetic variation of the relict population. To the extent that our results held in natural conditions, they suggest that natural selection in the relict's dry natural environment could potentially hamper the effective establishment of exotic genes via seed and pollen dispersal from the plantations, and that exotic hybridization would not significantly affect the potential of early recruits in response to selection. (C) 2015 Elsevier B.V. All rights reserved.

4381: *+.180*

Conspecific attraction can prevent occupancy of restored or created habitats by limiting dispersal to unoccupied areas. This may cause problems for threatened taxa where habitat restoration and creation programmes are implemented as part of species recovery plans. Studies on birds have found that the introduction of artificial communication cues such as calling can increase occupancy of restored habitat. The endangered green and golden bell frog (Litoria aurea) has a number of behavioural traits which suggest conspecific attraction occurs via a vocal mechanism, including aloud conspicuous call and large chorusing aggregations. To date, attempts to repopulate restored and created habitat through natural immigration and active translocation of tadpoles and

juveniles have been met with limited success for this species. We used L. aurea to determine if distribution could be manipulated via conspecific attraction using artificial communication cues. We placed speaker systems in uninhabited areas of five inhabited ponds across two locations and broadcast calls of L. aurea to see if we could manipulate distribution into previously unoccupied pond areas. Surveys undertaken before and after broadcast indicate that we successfully manipulated L aurea distribution for adults increasing both occupancy and calling around the speaker locations. This occurred in four of five replicate ponds over three months of experimental treatment, but controls remained low in abundance. We suggest that manipulation of distribution via conspecific attraction mechanisms could be a useful conservation tool for endangered amphibian habitat restoration and creation programmes, resulting in increased occupancy and programme success. (C) 2015 Elsevier B.V. All rights reserved.

4382: *+.366*

Success rates for conservation translocations of species are low and there is a need for increased understanding of how this activity is best applied. Here, using static species distribution models and a spatially-explicit dynamic simulation model, RangeShifter, we examine the impacts of habitat cover in recipient landscapes, allocation of individuals into multiple sites and species trait characteristics on the long-term fate of hypothetical translocations of a grassland specialist butterfly, Maniola jurtina, in Finland. While persistence of populations introduced to climatically suitable locations northwards of the current range can be increased by selecting sites with increasing habitat cover and by allocation of individuals to multiple release sites, local population growth rateis shown to be the key parameter in determining likely translocation success. We conclude that the long-term persistence of translocated habitat specialist butterflies, particularly with low growth rates, appears to be uncertain in modern-day fragmented grassland networks and that translocation activities should prioritize management that improves local growth rate. (C) 2015 Elsevier Ltd. All rights reserved.

4383: *+.202*

There are high rates of regional and global extinctions among freshwater species and few chances for recovery. We report here on the rediscovery after 30 years of a small fish, the southern-purple spotted gudgeon (Mogurnda adspersa), once widespread in the southern Murray-Darling Basin of south-eastern Australia. The rediscovery was in a region, the Lower Murray, where temperate riverine and wetland habitats are modified by a broad spectrum of changes including intensive flow regulation and diversions. There was some doubt whether the rediscovered population was a true remnant or a recent introduction, particularly as there was a translocated population in a nearby artificial habitat. Fortunately, a non-government organisation acted to rescue into captivity about 50 specimens as the remaining wetland habitat dried completely, soon after rediscovery, as a consequence of a decade-long drought and water diversions. We describe the habitat and ecology of fish in the rediscovery site, and provide genetic data, both nuclear (50 allozyme loci) and mtDNA (1141 base pairs; two genes), to show that they were true remnants of the regional native population. This information allows clear planning for future recovery including reintroductions, and is a case study that provides strategies, and hope, for conservation and management concerning other modified habitats. Specifically, it highlights the need for a rapid response to conserve threatened species, the recognition of remnant natural values in altered environments, and the treatment of new finds as native until there is alternate evidence. (C) 2015 Elsevier Ltd. All rights reserved.

4384: *+.149*

The success of captive and supportive breeding programs is often determined by abundance criteria but it is also necessary to consider genetic characteristics of reintroduced or supplemented populations as genetic diversity loss can reduce population viability. Genetic analysis of the parent pools is often used to determine whether captive or supportive breeding programs conserve adequate levels of genetic diversity and maximize the effective population size (Ne). This practice assumes that released cohorts reflect the genetic characteristics of parents. Here we provide a case study of how post-release mortality can alter the genetic composition of released cohorts in a supportive breeding program for an endangered population of white sturgeon. Data from ongoing genetic monitoring of wild broodstock in the Kootenai River white sturgeon conservation aquaculture program are combined with multi-year post-release abundance monitoring of captive bred juveniles to reveal high variability in recapture among families. We found that genetic monitoring of broodstock used in supportive breeding overestimates genetic diversity conservation in most year classes due to differential post-release mortality among families. Ne was reduced in most year classes when post-release mortality was considered due to reduced parental representation in released cohorts. Although rarely performed, our results indicate that post-release genetic monitoring is necessary to accurately characterize the genetic composition of released cohorts altered by post-release mortality and should be considered when designing a captive or supportive breeding program. (C) 2015 Elsevier Ltd. All rights reserved.

4385: *-.031*

The South China tiger (Panthera tigris amoyensis), although listed by the IUCN as critically endangered, is probably extinct in the wild. This leaves captive-born animals as the only stock available for reintroductions. Because reintroduced tigers will not survive in the wild unless they hunt proficiently, we aimed to determine whether captive-born tigers were able to hunt free-ranging prey and to evaluate their hunting performance as a criterion for reintroduction. The effect of other variables on subsequent hunting success, such as the availability of stalking cover and the upbringing history of tigers while they were cubs were also explored, given their relevance in reintroduction programs. Twelve tigers over two years of age were fitted with GPS collars and placed individually in 100 ha enclosures to determine their kill rate of blesbuck (Damaliscus pygargus), as a measure of their hunting performance. All tigers but one successfully hunted blesbuck, although kill rate varied substantially amongst individuals, ranging from one blesbuck every 3.14 days to no blesbuck. Tigers also killed other species indicating plasticity in their hunting behavior, and showed higher kill rates in the enclosure where cover was more abundant, confirming the importance of stalking cover in hunting success for this species. Results showed that the presence of the mother during cub development was not necessary for cubs to hunt later in life, although it had a positive effect on kill rate. Our study represents the first empirical evidence that captive-born tigers can successfully hunt free-ranging prey adequately to meet their energetic demands, validating the use of captive animals to recover wild populations, should other reintroduction criteria be met. Moreover, that tigers adapted to the African veld ecoregion suggests they should be able to adapt back to southern China where opportunities for stalking and ambush are more numerous. (C) 2015 Elsevier Ltd. All rights reserved.

4386: *+.030*

The Indus River dolphin (Platanista gangetica minor) is a freshwater cetacean that occurs only in the Indus River system in Pakistan and India. This review provides a comprehensive summary of issues surrounding Indus dolphin conservation, and includes a description of their distribution, the threats they face and a discussion of conservation and research activities, options and priorities. Information was gathered from published current and historical literature, newspaper articles, and

unpublished reports. Prior to construction of the Indus Irrigation system dolphins occurred in 3500 km of the Indus River system. Their range has now declined by 80% due to fragmentation of river habitat by barrages and large-scale diversion of water for irrigated agriculture. The subspecies was estimated to number approximately 1450 individuals in 2011, and occurs in 6 subpopulations, three of which are probably too small to persist. There is an urgent need to evaluate whether dolphins move through barrages, as this has the potential to extirpate upstream subpopulations. Pre-requisites for dolphin translocations to combat this problem are outlined. Mortality in fishing gear and high levels of chemical pollution in water courses are both increasing threats exacerbated by depleted flows. Research and conservation priorities include maintenance of river flows, mortality monitoring, canal rescues and community-based conservation. Conservation and research action is urgently needed to prevent the Indus dolphin from succumbing to the same fate as the baiji. (C) 2015 Elsevier B.V. All rights reserved.

4387: ***-.165***

One of the most endangered species is the red wolf, Canis rufus. Reintroduction of the red wolf began in 1987, but in 1993 hybridization between coyotes (Canis latrans) and wolves was documented. To,reduce genetic introgression, coyotes and coyote-wolf hybrids were captured, sterilized, and released as "placeholders". Placeholders held territories until either displaced or killed by a wolf, or management personnel removed them before releasing a wolf. We evaluated the placeholder concept by examining the number of animals sterilized and released, likelihood of displacement by a wolf, factors influencing displacements, territory fidelity of placeholders, and survival rates and causes of mortality of placeholders and wolves. Of the 182 placeholders, 125 were coyotes and 57 were hybrids. From 1999 to 2013, 51 placeholders were displaced or killed by wolves, and 16 were removed by management personnel. Thus, 37% of the placeholders were displaced leading to occupancy by a wolf. Most displacements occurred in winter (43%) and were always by the same sex. Males were more likely to be displaced than females. Home range characteristics influencing the probability of displacement included home-range size (i.e., more placeholders displaced from larger home ranges) and road density (i.e., more placeholders displaced from home ranges with lower road density). Annual survival of placeholders was higher than wolves in 12 of 14 years, with cause-specific mortality similar among wolves and placeholders. Placeholders provided territories for wolves to colonize, yet reduced the production of hybrid litters, thereby limiting genetic introgression to <4% coyote ancestry in the wolf population. Published by Elsevier Ltd.

4388: ***+.040***

A major challenge of using multiple-source populations in reintroductions for restoration and species conservation is to adequately assess the trade-off between the benefits of counteracting inbreeding depression via heterosis, and the risks of maladaptation and reduced fitness through outbreeding depression. In the 1990s, populations of the perennial plant Arenaria grandiflora rapidly declined and were nearing extinction in the Fontainebleau forest, France. This was likely due to inbreeding depression and/or fixation of deleterious alleles. To restore A. grandiflora in the Fontainebleau forest, a reintroduction experiment was conducted in 1999 using transplants from both local and non-local populations. Eight and twelve years later, we carried out a genetic study using microsatellite markers to assess the temporal dynamics of the genetic composition of reintroduced populations. We show that genetic diversity increased significantly in the reintroduced populations compared to the local-source population and that this increased diversity has been maintained for over ten years, highlighting the benefits of mixing individuals of multiple-source populations for restoration of small and inbred populations. Our results also suggest that

the level of individual admixture between local and non-local genetic sources might affect individual fitness, which is influenced by the opposing effects of heterosis, inbreeding and outbreeding depressions and local adaptation. (C) 2015 Elsevier Ltd. All rights reserved.

4389: *+.166*

Understanding the spreading patterns of an invading or expanding species is necessary for ecological theory and conservation. The return of the Eurasian beaver throughout Europe is considered one of the biggest conservation success stories. In general, the process of the spreading beaver population has been described in detail, however several aspects regarding the principles of colonisation have not been mentioned. We propose that the process of colonisation should follow Skellam's model for the case of first occupation by a pioneer beaver during the establishment of population growth. We retrospectively reconstructed the continuous colonisation of Eurasian beavers in six catchments in Western Bohemia in the Czech Republic. The extracted data from published resources were analysed to reveal (in detail) the progress of the colonisation front, in addition to considering current population densities in the catchments. All of the catchments were settled from one possible source, but were reached by pioneer beavers regardless of the distance or the considered source of the spread. However, an increased distance from the source of spread delayed the start of rapid population growth. The barrier effect among the divides of the watershed was not substantial because beavers regularly crossed the divides. The progress of the colonisation front followed Skellam's diffusion model as densities spread from sites with higher values into areas with lower values. The model did not, however, accurately predict the pattern of the first pioneer occupancy. (C) 2015 Deutsche Gesellschaft fur Saugetierkunde. Published by Elsevier GmbH. All rights reserved.

4390: *+.169*

Translocation is a popular conservation tool, but the outcomes are variable. Many tactics can be used to improve the probability of success, but a comprehensive summary of these does not exist. This increases the risk that valuable tactics will be overlooked, and inhibits effective communication. We assess the diversity of translocation tactics' used in mammal and bird translocations, by reviewing the IUCN/SSC Guidelines for Reintroduction and other Conservation Translocations, 195 peer-reviewed articles and 73 case studies from the IUCN/SSC Global Reintroduction Perspectives Series. We recorded descriptions of every technique used to influence the post-release performance of translocated wildlife. We developed the Translocation Tactics Classification System (TTCS) which defines a collection of 30 tactics and organize them into an ecologically relevant framework. We also assess the occurrence of tactics within the Guidelines, the primary literature and the case studies to evaluate how tactics are communicated within these mediums. Our results indicate that the Guidelines are a valuable resource, but do not exhaustively cover tactics, and that detailed methodological accounts are rarely made publicly accessible. This highlights the need to develop context-specific resources to support the Guidelines, and to develop and exploit mediums that facilitate recording of methodological detail, the tactical rationale behind the design and evaluations of effectiveness. Although some forms of grey literature address this issue, the general lack of information limits the ability to investigate the relationship between tactics and translocation success. Synthesis and applications. The Translocation Tactics Classification System (TTCS) provides a checklist which ensures that the full diversity of tactics is considered when developing translocation processes. Standardizing the communication of tactics, and encouraging detailed accounts of applied methodologies to be recorded, along with the tactical reasoning behind the design, will provide operational models and the data required to conduct broad-scale meta-analyses. The Translocation Tactics Classification System (TTCS) provides a

checklist which ensures that the full diversity of tactics is considered when developing translocation processes. Standardizing the communication of tactics, and encouraging detailed accounts of applied methodologies to be recorded, along with the tactical reasoning behind the design, will provide operational models and the data required to conduct broad-scale meta-analyses.

4391: *+.065*

Understanding the extent of morphological variation in the wild population of Aldabra giant tortoises is important for conservation, as morphological variation in captive populations has been interpreted as evidence for lingering genes from extinct tortoise lineages. If true, this could impact reintroduction programmes in the region. The population of giant tortoises on Aldabra Atoll is subdivided and distributed around several islands. Although pronounced morphological variation was recorded in the late 1960s, it was thought to be a temporary phenomenon. Early researchers also raised concerns over the future of the population, which was perceived to have exceeded its carrying capacity. We analyzed monthly monitoring data from 12 transects spanning a recent 15-year period (1998-2012) during which animals from four subpopulations were counted, measured, and sexed. In addition, we analyzed survival data from individuals first tagged during the early 1970s. The population is stable with no sign of significant decline. Subpopulations differ in density, but these differences are mostly due to differences in the prevailing vegetation type. However, subpopulations differ greatly in both the size of animals and the degree of sexual dimorphism. Comparisons with historical data reveal that phenotypic differences among the subpopulations of tortoises on Aldabra have been apparent for the last 50years with no sign of diminishing. We conclude that the giant tortoise population on Aldabra is subject to varying ecological selection pressures, giving rise to stable morphotypes in discrete subpopulations. We suggest therefore that (1) the presence of morphological differences among captive Aldabra tortoises does not alone provide convincing evidence of genes from other extinct species; and (2) Aldabra serves as an important example of how conservation and management insitu can add to the scientific value of populations and perhaps enable them to better adapt to future ecological pressures.

4392: *+.370*

Conservation breeding programmes have become an increasingly important tool to save endangered species, yet despite the allocation of significant resources, efforts to create self-sustaining populations have met with limited success. The iconic giant panda (Ailuropoda melanoleuca) embodies the struggles associated with ex situ species conservation. Here we show that behavioural mate preferences in giant pandas predict reproductive outcomes. Giant pandas paired with preferred partners have significantly higher copulation and birth rates. Reproductive rates increase further when both partners show mutual preference for one another. If managers were to incorporate mate preferences more fully into breeding management, the production of giant panda offspring for China's reintroduction programme might be greatly expedited. When extended to the increasing numbers of species dependent on ex situ conservation breeding to avoid extinction, our findings highlight that mate preference and other aspects of informed behavioural management could make the difference between success and failure of these programmes.

4393: *+.000*

The Amami Islands in southern Japan host many endemic species, including Amami Jay (Garrulus lidthi). Following the introduction of Small Indian Mongoose (Herpestes auropunctatus) in 1979,

populations of Amami Rabbit (Pentalagus furnessi), Amami Woodcock (Scolopax mira), Amami Ishikawa's Frog (Odorrana splendida), and other endemic species declined in the 1990s, as the mongoose expanded its population. A mongoose control project since 2000 successfully reduced the mongoose population to a very low density by 2014, resulting in the initial recovery of severely affected animal populations. Amami Jay was removed from Japan's endangered species list in 2008. However the population and range of Amami Jay are still small (ca. 700 pairs, sometimes with other family member(s), in 700 km 2), and other invasive species, such as cats and black rats, continue to damage the native ecosystem. Additionally there is a constant threat of new invasions to the Amami Islands. Our research shows that Amami Jay breeding success depends on the dominant oak (Castanopsis sieboldii) acorn production as much as predation. Therefore conservation plans should consider both short-and long-term effects and potential interaction by both invasive species and other natural factors. Understanding the Amami Jay population dynamics and sustainability from the multiple view points of, such as, population ecology, disease ecology, genetics, and also with ex situ conservation efforts as they relate to invasive species demands, will sophisticate our ecosystem research and management. We discuss how this endeavor of ours might develop as a model activity for maintaining biodiversity under threat from multiple invasive species.

4394: *+.218*

Prior to the reintroduction of a species, managers need an understanding of the expected behavior of the species in the new habitat. How a species uses its habitat and how much space individuals require are particularly important when conservation lands are limited. Critically endangered Maui Parrotbills (Kiwikiu, Pseudonestor xanthophrys) once occupied a variety of habitats on the Hawaiian islands of Maui and Moloka`i, but, due to habitat loss and disease, are now restricted to a fraction of their former range. To prevent their extinction, reintroducing parrotbills to historically occupied native, mesic forest on the leeward slopes of Haleakal(Sic) is considered a critical recovery action. Managers have selected Nakula Natural Area Reserve (NAR) as the site of translocation and restoration efforts are currently underway to support this goal. In addition, other species, including endemic Maui 'Alauahio (Maui Creeper, Paroreomyza montana), may recolonize these forests naturally as the habitat improves. However, estimates of the home range sizes of focal species are needed so that managers can estimate how many individuals might be able to occupy new habitats. Our objective therefore was to estimate the home range sizes of parrotbills and 'alauahio at three sites within their current ranges to provide estimates of typical habitat and space use patterns. Using resightings of color-banded birds from 2007 to 2014, we calculated home ranges using minimum convex polygons and kernel density estimators. Depending on estimation technique, parrotbill home ranges were estimated to encompass 9.29 +/- 1.29 (SE) ha or 9.63 +/- 1.51 ha, and pairs occupied ranges of 11.8 ha or 14.5 ha. 'Alauahio home ranges were 0.85 ranges of 11.8 ha or 14.5 ha. 'Alauahio home ranges were 0.85 +/- 0.09 ha or 0.87 +/- 0.08 ha in size. +/- 0.09 ha or 0.87 +/- 0.08 ha in size. Home range sizes varied among study sites for both species, likely reflecting the influence of local habitat attributes and quality on movement patterns and space use. Although we do not know how these species will behave in the new habitat, our estimates of home range size provide guidance for managers planning the reintroduction of parrotbills to Nakula NAR.

4395: *+.112*

Translocations and reintroductions are key elements for the population management of the critically endangered black rhino (Diceros bicornis, Linnaeus, 1758). In this study, we investigated the postrelease behaviour and habitat preferences of a black rhino starter group (n = 4) on the

individual level. The animals were reintroduced to a fenced game reserve (87 km 2) in North-Central Namibia 1 year prior to our study. We used camera traps and very high frequency (VHF) radiotelemetry to examine the animals' temporal and spatial behaviour over a period of 4 months at transition between wet and dry seasons. Our results underline a peak in drinking activity and waterhole visits occurring between 7 p.m. and 8 p.m. We found a shift in intensity in drinking activity during the period of the study. Satellite-based woody cover estimations only suggest positive correlations between the density of woody cover and favoured black rhino habitat types. Although the area seems suitable to facilitate breeding success of this starter group, it does not support a self-sustaining population. However, black rhinos were already successfully reintroduced to several additional fenced reserves in this region. The selective opening of fences in the future could help to enable genetic exchange between currently isolated groups of rhinos.

4396: *+.015*

Generalist predators characteristically switch prey in response to resource pulses. Ungulate population reinforcement through translocation provides stressed and vulnerable prey, and apex predator reintroduction provides carrion, both resources that can be exploited by generalist predators. We investigated the dietary response of black-backed jackal to two management interventions in the Karoo National Park, South Africa, namely the reinforcement of the springbok population, and then the reintroduction of lions. By analysing jackal diets from scats collected before and after each management intervention, we show that the relative per cent occurrence and biomass consumed of springbok increased in the diet following the population reinforcement of springbok. In contrast, large ungulates were more prevalent in the diet following apex predator reintroduction. These results suggest that jackals took advantage of a potentially vulnerable abundant springbok population following their population reinforcement, and then switched to foraging on carrion provided by reintroduced lions. These results provide insights into the dietary response of a generalist mesopredator to management interventions. A key lesson from the study is that the diet of generalist predators is context-dependent and should be interpreted in that light.

4397: *+.003*

The reintroduction of ex situ conserved individuals is an important approach for conserving threatened plants and reducing extinction risk. In this study, we elucidated the effects on the genetic diversity of wild populations of Vincetoxicum pycnostelma Kitag. [=Cynanchum paniculatum (Bunge) Kitag.] by modelling the genetic consequences of reintroducing plants using the germinated seeds of herbarium specimens. This semi-natural grassland herb is threatened in Japan. First, we tested the germinability of seeds from herbarium specimens collected from nine sites in Kinki and Tokai districts, Japan (one specimen per site, total 206 seeds). Next, we analysed the genetic diversity and structure of germinated seedlings and the current wild individuals using nine polymorphic microsatellite markers. Germination was observed for 38 seeds (18.4 %) from four specimens collected 3-18 years prior to the study. Although the genetic diversity of the specimens' seeds was lower than that of the wild population because of the small sample size, the seedlings from specimens taken from three sites had unique alleles that did not exist in the wild populations. Consequently, viable herbarium specimens' seeds with unique alleles could be useful resources for recovering the genetic diversity in threatened wild plant populations.

4398: *+.207*

The Puerto Rican parrot (Amazona vittata) has become an iconic and high-profile conservation species. The cornerstone of the recovery plan for this critically endangered species is an active

captive breeding program, management of the wild population, and a long-term reintroduction program. In 2002, 40 adult Puerto Rican parrots that had not produced viable offspring were selected for reproductive assessment at 2 aviary populations in Puerto Rico (Iguaca and Rio Abajo), which are the only sources of parrots for release. The goal was to enhance reproductive potential and produce productive pairings in an attempt to augment the population growth and provide ample individuals for reintroduction. Seven Hispanolian Amazon parrots (Amazona ventralis) that were used as surrogate parents for the Puerto Rican parrots were also included in the study. This assessment included physical examination, endoscopic evaluation, hematologic and plasma biochemical profiles, viral screening, and hormonal assays. Results of general physical examination and hematologic and plasma biochemical testing revealed overall good health and condition of this subset of the population of Puerto Rican parrots; no major infectious diseases were found. Endoscopic examination also revealed overall good health and condition, especially of females. The apparent low fertility of male birds warrants further investigation. The findings helped to define causes of reproductive failure in the selected pairs and individual birds. New pairings resulting from the assessment helped to augment reproduction of this critically endangered species.

4399: *+.265*

We assessed the current status of plant conservation translocation efforts in China, a topic poorly reported in recent scientific literature. We identified 222 conservation translocation cases involving 154 species, of these 87 were Chinese endemic species and 101 (78%) were listed as threatened on the Chinese Species Red List. We categorized the life form of each species and, when possible, determined for each case the translocation type, propagule source, propagule type, and survival and reproductive parameters. A surprisingly large proportion (26%) of the conservation translocations in China were conservation introductions, largely implemented in response to large-scale habitat destruction caused by the Three-Gorge Dam and another hydropower project. Documentation and management of the translocations varied greatly. Less than half the cases had plant survival records. Statistical analyses showed that survival percentages were significantly correlated with plant life form and the type of planting materials. Thirty percent of the cases had records on whether or not individuals flowered or fruited. Results of information theoretic model selection indicated that plant life form, translocation type, propagule type, propagule source, and time since planting significantly influenced the likelihood of flowering and fruiting on the project level. We suggest that the scientific-based application of species conservation translocations should be promoted as part of a commitment to species recovery management. In addition, we recommend that the common practice of within and out of range introductions in nature reserves to be regulated more carefully due to its potential ecological risks. We recommend the establishment of a national office and database to coordinate conservation translocations in China. Our review effort is timely considering the need for a comprehensive national guideline for the newly announced nation-wide conservation program on species with extremely small populations, which is expected to stimulate conservation translocations for many species in the near future.

4400: *+.260*

Intensive deer management (IDM) is fundamentally changing how one of the most important game species in North America is being managed, but little is known about how wildlife conservation professionals view these changes. The IDM approach encourages privatization of deer (Odocoileus spp.) through practices including feeding, high fencing, artificial insemination and markets in deer semen, and translocation. To evaluate support for IDM practices, we surveyed 208 registrants of the 2010 Southeast Deer Study Group Meeting held in San Antonio, Texas,

USA. Specifically, we evaluated support for IDM practices using state-agency wildlife biologists, private wildlife managers, and academics, and we evaluated how geographic region and employment type are related to opinions about IDM. Using Principal Components Analysis, we created 3 new scales that measured respondents' opinions about deer management, deer husbandry, and deer hunting. We detected strong opposition to IDM among respondents, with respondents from universities having the strongest opposition, followed by state-agency employees from Texas, and private consultants from Texas (the latter having the greatest support for IDM). Our study highlights the need for critical and empirical evaluation of the articulation between IDM and the North American Model of Wildlife Conservation, particularly the tenets that assert wildlife are held in the public trust and advocate elimination of markets for wildlife. (C) 2015 The Wildlife Society.

4401: *-.001*

Alpine mammals are predicted to be among the species most threatened by climate change, due to the projected loss and further fragmentation of alpine habitats. As temperature or precipitation regime's change, alpine mammals may also be faced with insurmountable barriers to dispersal. The slow rate or inability to adjust to rapidly shifting environmental conditions may cause isolated alpine species to become locally extirpated, resulting in reduced biodiversity. One proposed method for mitigating the impacts of alpine species loss is assisted migration. This method, which involves translocating a species to an area with more favourable climate and habitat characteristics, has become the subject of debate and controversy in the conservation community. The uncertainty associated with climate change projections, coupled with the thermal sensitivity of many alpine mammals, makes it difficult to a priori assess the efficacy of this technique as a conservation management tool. Here we present the American pika (Ochotona princeps) as a case study. American pikas inhabit rocky areas throughout the western US, and populations in some mountainous areas have become locally extirpated in recent years. We review known climatic and habitat requirements for this species, and also propose protocols designed to reliably identify favourable relocation areas. We present data related to the physiological constraints of this species and outline specific requirements which must be addressed for translocation of viable populations, including wildlife disease and genetic considerations. Finally, we discuss potential impacts on other alpine species and alpine communities, and overall implications for conserving alpine biodiversity in a changing climate.

4402: *+.342*

To understand the habitat components that contribute to the presence of populations of a rare butterfly, we examined the abundance of critical plant-components of old fields that support some of the last remaining Eastern Speyeria idalia (Regal Fritillary Butterfly) subpopulations at Fort Indiantown Gap (FTIG), a National Guard training facility in south-central Pennsylvania. We compared densities of larval-host plants (Viola spp. [violets]), adult-nectar plants (Asclepias spp. [native milkweeds] and Cirsium spp. [thistles]), and native, tussock-forming, warm-season bunch grasses that provide protective resting and pupation sites in fields occupied by the butterfly and in nearby fields that were unoccupied. We found no significant difference in violet density among sites. Fields with Regal Fritillary Butterfly populations had significantly more nectar-plant flowering structures and greater bunch-grass percent cover. Grassland habitat occupied by Regal Fritillaries was characterized by a violet density of at least 1.55 plants/m(2) and particular varieties of flowering nectar-plants available throughout the June-September flight period. Bunch grasses were also important to persistence of Regal Fritillaries; occupied sites had 20-45% bunch-grass cover and tussock formation. Understanding the habitat needs of this rare butterfly in Pennsylvania

is vital to its restoration and reintroductions of the eastern form in the mid-Atlantic and northeastern US.

4403: *+.153*

Lake acidification is a major problem in northeastern US lakes that can control fish presence or absence. We examined the history of fish populations in Lake Minnewaska, in eastern New York. We examined historical documents and found that Lake Minnewaska was fishless from 1922-2008 because of high lake-acidity. Following 30 years of recovery from acidic conditions, Notemigonus crysoleucas (Golden Shiner), a small minnow species, was introduced in 2008 and quickly proliferated, peaking at similar to 15,000 individuals in 2013. In 2012, the piscivorous species Micropterus salmoides (Largemouth Bass) was introduced, and the minnow population was effectively removed by 2014. We present a conceptual model of the history of fish in Lake Minnewaska as fish disappeared and reappeared over 100 years as a consequence of acid rain and human introductions.

4404: *+.302*

One of the depleted endemic fish species of the Great Lakes, Acipenser fulvescens (Lake Sturgeon), has been the target of extensive conservation efforts. One strategy is reintroduction into historically productive waters. The St. Regis River, NY, represents one such adaptive-management effort, with shared management between New York and the St. Regis Mohawk Tribe. Between 1998 and 2004, a total of 4977 young-of-year Lake Sturgeon were released. Adaptive management requires intermediate progress metrics. During 2004 and 2005, we measured growth, habitat use, and survivorship metrics of the released fish. We captured a total of 95 individuals of all stocked ages. Year-class minimal- survival rates ranged from 0.19-2.1%. The size-at-age and length/biomass relationships were comparable to those reported for juveniles in other Great Lakes waters. These intermediate assessment metrics can provide feedback to resource managers who make restoration-program decisions on a much shorter time-scale than the time-frame in which the ultimate goal of a self-sustaining population can be attained.

4405: *+.136*

The Western capercaillie (Tetrao urogallus) is a specific bird species, which, despite its very broad distribution and large global population size, is highly endangered in many Western and Central European countries. According to the species situation, in many countries (including Poland), breeding and reintroduction programmes have been started. One of the most complex and large-scale reintroduction programmes was started in Bory Dolnoslaskie Forest, and the Capercaillie Breeding Centre in Wisla Forest District was used as one of the sources of individuals for reintroduction. As genetic tools provide essential knowledge about species biodiversity, which is crucially important during the breeding process and reintroduction, both captive and reintroduced grouse populations were genetically analysed. We were particularly interested in genetic diversity of the individuals in both populations and the genetic relationship between them, as well as between them and other capercaillie representatives from their current range. To fulfil these goals we determined nine microsatellite loci along with a fragment of the mitochondrial control region. Genetic diversity parameters were moderate to high compared to populations from other Central and Western European countries. Both populations were clustered into three distinct genetic clades based on microsatellites. Phylogenetic analysis placed all mitochondrial haplotypes we revealed in the Eurasian clade. The present results will play an important role as they will help to preserve and maximize genetic diversity in captive populations, and will provide a basis for future monitoring

of the reintroduction process.

4406: *+.218*

We provide an account of maintaining a captive population of the Critically Endangered mantellid frog Mantella aurantiaca at a breeding facility near Andasibe, Madagascar, reporting novel observations on behaviour, fecundity, reproduction, temperature tolerance, age at maturity, and survivorship. In April of 2012, 25 breeding groups were established from founder stock collected at three natural breeding sites located on the footprint of the Ambatovy nickel and cobalt mine. Over a two-year period, 469 breeding events were recorded. Breeding activity was highly seasonal and aligned with average monthly temperatures, with peak breeding activity observed during the austral summer months of December and January. An average of 7 egg clutches per female was recorded over the two years, with the mean clutch size being 74 eggs (193 max/24 min). Tadpoles completed metamorphosis between 53 and 139 days, with 441 individuals from 22 clutches of eggs surviving to one year of age. Males were recorded vocalizing 4 months after completing metamorphosis, and the first fertile eggs were produced at 11 months. Reproduction in the F-1 generation was captured on video and we provide a detailed description of this behaviour, including an observation of males 'pulsating' femoral glands on the dorsum of a female during reproduction. Based on these data and observations, we discuss the importance of record keeping for captive amphibians, potential conservation implications of creating new breeding sites for reintroducing M. aurantiaca, as well as the advantages of running captive breeding programmes within the native range of a species.

4407: *+.250*

The Sevilleta Gunnisonas Prairie Dog (Cynomys gunnisoni) Restoration project examines keystone consumer (herbivore) effects on grassland in concert with ecological restoration of a aoespecies of greatest conservation need in New Mexicoa (NMGandF Comprehensive Wildlife Conservation Strategy, 2007). SevLTER partners directly with Sevilleta National Wildlife Refuge, New Mexico Game and Fish, USFS Rocky Mountain Research Station and non-profit Prairie Dog Pals on this ambitious effort to re-establish Gunnisonas prairie dogs to blue grama dominated (Bouteloua gracilis) Great Plains grassland at the foothills of the Los Pinos Mountains on Sevilleta. While engaged in wildlife management aimed at translocation of approximately 3000 individual prairie dogs, ultimately establishing 5-6 colonies over a 500 ha area, SevLTER is focusing resources on monitoring population dynamics of reintroduced prairie dogs and their effects on vegetation production and diversity, soil disturbance and grasshopper community composition. In this experiment, prairie dogs act as the treatment on a grassland site where the species was extirpated 40 years ago. The long term nature of the project lies in the course of re-establishing prairie dogs combined with the ultimate research goal of describing the functional role of Gunnisonas prairie dogs in an arid grassland ecosystem: first we are challenged to develop and document an economical and efficient management strategy which maximizes reintroduction success and colony survival; second we are tasked with monitoring prairie dog dynamics and their effects on the grassland throughout re-establishment and into a future state, when presumably management intervention will have subsided and we characterize the ecosystem as a~restoreda a both in the face of highly variable abiotic inputs such as precipitation and temperature and biotic impacts such as predation.

4408: *-.002*

The niche breadth of a species reflects its ability to inhabit different conditions, and to use

different resources, hence, species with wider niche are expected to be more resilient to anthropogenic derived climate change. We estimated the niche breadth of all species of the genus Astrophytum from macro-environmental variables and measures of local habitat uses, in order to evaluate whether species having wider niche breadths are less prone to experience unsuitable conditions projected by the A1B and A2 scenarios of the IPCC for 2020 and 2050, and analyzed the implications of projections for the conservation of the genus Astrophytum. Our analysis suggests that most of populations of the four species will experience increasingly unsuitable conditions due to the increase of temperature and reduction in precipitation. The species less affected were those with wider niche breadth and situated in the middle of the latitudinal range and in the middle or lower extreme of the precipitation range for the genus (A. capricorne and A. myriostigma). Although the main threats for Astrophytum species come from the destruction of their habitats and activities as illegal extraction, climate change may reduce the chances for the regeneration of populations and the success of reintroduction programs. (C) 2015 Elsevier Ltd. All rights reserved.

4409: *+.124*

Sixteen Apennine chamois Rupicapra pyrenaica ornata (10 females, 6 males) were released into a protected area, the Sibillini Mountains National Park, Central Apennines, Italy, and monitored using global positioning system radio tags during 2008-2010. Founders caught in the wild (n=8) and those reared in large enclosures (n=8) differed in movement frequency (inter-fix distance per hour) and maximum distance covered (from the release site) in the first 5 months after release: both were significantly greater in wild individuals, males moved significantly more than females, wild individuals shifted their home ranges significantly more often than captive ones, and no differences were observed between the sexes or age classes. A mixed strategy of selection of wild and captive founders has proven successful in preventing large movements in the initial stages of release yet still providing sufficient opportunity to avoid inbreeding depression.

4410: *+.044*

With global biodiversity in decline, there is now an urgent requirement to take ameliorative action for endangered species in the form of re-introductions. For the highly diverse orchid family, many species face imminent extinction. Successful re-introductions that result in self-sustaining populations require not only an understanding of existing threats, but an in-depth understanding of species ecology. Increasingly, translocations, ranging from re-introductions to assisted colonisation, are being adopted as recovery actions. Do these translocations mitigate threatening processes and account for the two key ecological attributes for orchid survival; pollinator and mycorrhizal presence? Here, we conducted a literature review identifying the known threats to orchid survival and their necessary mitigation strategies. Next, we evaluated the success of 74 published international orchid translocations on 66 species against a consideration of orchid ecological attributes. Lastly, we empirically tested an additional 22 previously unpublished re-introductions on 12 species undertaken since 2007 against a re-introduction process that accounts for identified threats and orchid ecological attributes. We identified habitat destruction, weed invasion, herbivory, illegal collection, pollinator decline, pathogens and climate change as critical threats to orchid survival. In our global review based on published translocations, the average survival rate, 1-year post translocation was 66 % yet only 2.8 % of studies reported natural recruitment in field sites. Although survival of translocated orchids is clearly being achieved, these programmes did not relate orchid growth and development to key ecological requirements of orchid population resilience, pollinator and mycorrhizal ecology. Ensuring pollinator and mycorrhizal presence shows that these two factors alone are key factors influencing survival and

persistence in an Australian review of 22 previously unpublished orchid re-introductions. In the Australian review flowering in the year following, out-planting was observed for 81 % of the re-introductions with seed set occurring in 63 % of re-introductions within the length of the study. Recruitment was observed in 18 % of the Australian re-introduced populations indicating a degree of population resilience. As orchid re-introductions will be a major strategy for wild orchid conservation in the future, we present a framework for orchid re-introductions, including criteria for success. We recommend symbiotic propagation and, for specialised pollination syndromes, the study of pollinator interactions prior to site selection and re-introduction of plants.

4411: ***+.116***

Big South Cape Island (Taukihepa) is a 1040 ha island, 1.5 km from the southwest coast of Stewart Island/Rakiura, New Zealand. This island was rat-free until the incursion of ship rats (Rattus rattus) in, or shortly before, 1963, suspected to have been accidentally introduced via local fishing boats that moored at the island with ropes to the shore, and were used to transport the mutton birders to the island. This incursion was reported by the muttonbirders - local Iwi who harvest the young of titi (sooty shearwater, Puffinus griseus) to the then New Zealand Wildlife Service (via the New Zealand Department of Lands and Survey). Investigation into the reports found ship rats had reached the island and had decimated the local land bird populations. Brian Bell and Don Merton attempted some of the first translocations of South Island saddleback (Philesturnus c. carunculatus), Stewart Island snipe (Coenocorypha aucklandica iredalei) and Stead's bush wren (Xenicus longipes variabilis) with only the saddleback being successful. Extinctions of the snipe, wren and greater short-tailed bat (Mystacina robusta) were recorded. This was the first time rats were definitively recognised as the cause of extinction of native land birds and directed further debate into the impacts of rats and how to deal with them.

4412: ***+.210***

Many countries have conservation plans for threatened species, but such plans have generally been developed without taking into account the potential impacts of climate change. Here, we apply a decision framework, specifically developed to identify and prioritise climate change adaptation actions and demonstrate its use for 30 species threatened in the UK. Our aim is to assess whether government conservation recommendations remain appropriate under a changing climate. The species, associated with three different habitats (lowland heath, broadleaved woodland and calcareous grassland), were selected from a range of taxonomic groups (primarily moths and vascular plants, but also including bees, bryophytes, carabid beetles and spiders). We compare the actions identified for these threatened species by the decision framework with those included in existing conservation plans, as developed by the UK Government's statutory adviser on nature conservation. We find that many existing conservation recommendations are also identified by the decision framework. However, there are large differences in the spatial prioritisation of actions when explicitly considering projected climate change impacts. This includes recommendations for actions to be carried out in areas where species do not currently occur, in order to allow them to track movement of suitable conditions for their survival. Uncertainties in climate change projections are not a reason to ignore them. Our results suggest that existing conservation plans, which do not take into account potential changes in suitable climatic conditions for species, may fail to maximise species persistence. Comparisons across species also suggest a more habitat-focused approach could be adopted to enable climate change adaptation for multiple species. (C) 2015 Elsevier Ltd. All rights reserved.

4413: ***+.078***

The Utah prairie dog (Cynomys parvidens), listed as threatened under the United States Endangered Species Act, was the subject of an extensive eradication program throughout its range during the 20th century. Eradication campaigns, habitat destruction/fragmentation/conversion, and epizootic outbreaks (e.g., sylvatic plague) have reduced prairie dog numbers from an estimated 95,000 individuals in the 1920s to approximately 14,000 (estimated adult spring count) today. As a result of these anthropogenic actions, the species is now found in small isolated sets of subpopulations. We characterized the levels of genetic diversity and population genetic structure using 10 neutral nuclear microsatellite loci for twelve populations (native and transplanted) representative of the three management designated recovery units, found in three distinct biogeographic regions, sampled across the species' range. The results indicate (1) low levels of genetic diversity within colonies (H-e=0.109-0.357; H-o=0.106- 0.313), (2) high levels of genetic differentiation among colonies (global F-ST=0.296), (3) very small genetic effective population sizes, and (4) evidence of genetic bottlenecks. The genetic data reveal additional subdivision such that colonies within recovery units do not form single genotype clusters consistent with recovery unit boundaries. Genotype cluster membership support historical gene flow among colonies in the easternmost West Desert Recovery Unit with the westernmost Pausaugunt colonies and among the eastern Pausaugunt colonies and the Awapa Recovery unit to the north. In order to maintain the long-term viability of the species, there needs to be an increased focus on maintaining suitable habitat between groups of existing populations that can act as connective corridors. The location of future translocation sites should be located in areas that will maximize connectivity, leading to maintenance of genetic variation and evolutionary potential.

4414: *-.099*

An endemic subspecies of Buff-banded Rail (Gallirallus philippensis) is restricted to the Cocos (Keeling) Islands, a group of 27 islands, with total area of similar to 15km(2), in the north-eastern Indian Ocean. Human settlement led to marked environmental degradation of the 26 islands in the southern atoll of the group. The Cocos Buff-banded Rail declined severely, with the last confirmed record from islands in the southern atoll in 1991. The subspecies has persisted, however, with a population of similar to 800 individuals, on a single island, Pulu Keeling, 24km north of the southern atoll. A recovery plan for this endangered subspecies recommended reintroduction to a suitable island in the southern atoll. This paper provides a brief overview of the history and status of the subspecies, and describes an April 2013 reintroduction of 39 rails from Pulu Keeling to the 1-km(2) Horsburgh Island in the southern atoll. This program has had at least short-term success, with monitoring showing successful recruitment in the reintroduced population, and its increase to similar to 54 individuals by October 2014 and similar to 121 individuals by June 2015. Much of the world's loss of biodiversity has been from, and continues to occur on, islands: this project demonstrates that well-considered mitigation of threats and translocation programs can provide solutions to this challenge.

4415: *+.122*

Gunnison sage-grouse (Centrocercus minimus) distribution in North America has decreased over historical accounts and has received federal protection under the Endangered Species Act. We investigated captive-breeding of a captive-flock of Gunnison sage-grouse created from individuals reared in captivity from wild-collected eggs we artificially incubated. We also introduced wild-reared individuals into captivity. Our captive-flock successfully bred and produced fertile eggs. We controlled the timing and duration of male-female breeding interactions and facilitated a semi-natural mating regime. Males established a strutting ground in captivity that females attended for mate selection. In 2010, we allowed females to establish eight nests, incubate, and hatch eggs.

Females in captivity were more successful incubating nests than raising broods. Although there are many technical, financial, and logistic issues associated with captive-breeding, we recommend that federal biologists and managers work collaboratively with state wildlife agencies and consider developing a captive-flock as part of a comprehensive conservation strategy for a conservation-reliant species like the Gunnison sage-grouse. The progeny produced from a captive-rearing program could assist in the recovery if innovative approaches to translocation are part of a comprehensive proactive conservation program. Zoo Biol. 35:70-75, 2016. (c) 2015 Wiley Periodicals, Inc.

4416: *-.131*

High levels of genetic diversity and high propagule pressure are favoured by conservation biologists as the basis for successful reintroductions and ensuring the persistence of populations. However, invasion ecologists recognize the 'paradox of invasion', as successful species introductions may often be characterized by limited numbers of individuals and associated genetic bottlenecks. In the present study, we used a combination of high-resolution nuclear and mitochondrial genetic markers to investigate the invasion history of Reeves' muntjac deer in the British Isles. This invasion has caused severe economic and ecological damage, with secondary spread currently a concern throughout Europe and potentially globally. Microsatellite analysis based on eight loci grouped all 176 introduced individuals studied from across the species' range in the UK into one genetic cluster, and seven mitochondrial D-loop haplotypes were recovered, two of which were present at very low frequency and were related to more common haplotypes. Our results indicate that the entire invasion can be traced to a single founding event involving a low number of females. These findings highlight the fact that even small releases of species may, if ignored, result in irreversible and costly invasion, regardless of initial genetic diversity or continual genetic influx.

4417: *+.279*

Adaptive management is a framework for resolving key uncertainties while managing complex ecological systems. Its use has been prominent in fisheries research and wildlife harvesting; however, its application to other areas of environmental management remains somewhat limited. Indeed, adaptive management has not been used to guide and inform metapopulation restoration, despite considerable uncertainty surrounding such actions. In this study, we determined how best to learn about the colonization rate when managing metapopulations under an adaptive management framework. We developed a mainland-island metapopulation model based on the threatened bay checkerspot butterfly (Euphydryas editha bayensis) and assessed three management approaches: adding new patches, adding area to existing patches, and doing nothing. Using stochastic dynamic programming, we found the optimal passive and active adaptive management strategies by monitoring colonization of vacant patches. Under a passive adaptive strategy, increasing patch area was best when the expected colonization rate was below a threshold; otherwise, adding new patches was optimal. Under an active adaptive strategy, it was best to add patches only when we were reasonably confident that the colonization rate was high. This research provides a framework for managing mainland-island metapopulations in the face of uncertainty while learning about the dynamics of these complex systems.

4418: *-.001*

The increasing fragmentation of fire-prone forests of Australia has made the remaining populations of koalas (Phascolarctos cinereus) more vulnerable to extinction. We examined the

movement patterns of koalas in remnant forest of Port Stephens following a major wildfire. Each koala (n = 55) was monitored regularly by radio-tracking for up to 35 months. The movements of koalas showed a wide variation in patterns, from highly localised movements to long-range dispersal over 20 km. Within the first 12 months, 35% of tracked koalas moved from their release location to new areas where they established home ranges. Daily movement distances of males (mean = 278.3 m) was higher than for females (141.0 m). Monthly displacement ranged from less than 10m to more than 5 km, and was higher for koalas that subsequently died. Home ranges of males (95% kernel) were significantly larger than those of females (mean for males = 58.9 +/- 10.5 ha; mean for females = 25.7 +/- 8.6 ha), and this sex difference was also evident for core areas (50% kernel). There were no differences in the movement patterns or home-range sizes of rehabilitated koalas compared with wild koalas. This study has shown that resource depletion from wildfire is short term for koalas because their mobility allows rapid recolonisation of the burnt forest, and they can maintain home ranges within sites regenerating from fire. The reintroduction of rehabilitated koalas into burnt forest may also assist in the recovery of populations in fragmented and isolated habitat.

4419: ***-.035***

The Tasmanian devil (Sarcophilus harrisii) is a carnivorous marsupial threatened with extinction from the emergence of Devil Facial Tumour Disease. The establishment of ex situ populations is a key management action for the species. We examined the initial survival, movement pattern, home range, and habit use of six devils from a total of 15 individuals translocated to Maria Island (south-east Tasmania). A total of 14 devils (93%) survived the initial monitoring phase within this study (122 days after translocation). The maximum and minimum distance recorded during one night was 21.73 km (range = 14.12-25.40 km) and 1.94 km (range = 0.07-7.71 km), respectively, while the average nightly distance travelled varied significantly (range = 7.24-13.07 km) between individuals. Short-term home-range size (90% kernel) varied from 936 to 3501 ha, with an average of 2180 (+/- 836) ha for all devils. The habitat preference of devils on Maria Island shows a positive association with agricultural and urban habitats, and an avoidance of wet eucalypt forest. The home range and habitat associations may change as competitive pressures increase with population growth; however, this initial research indicates that translocation as a management action is a powerful tool for the establishment of ex situ populations, assisting in the continued conservation of this species.

4420: ***+.164***

Vital-statistics data concerning population viability were gathered for four of the rarest orchids in Western Australia using surveys to define population sizes and habitat areas and annual measurements of plant demographics. These orchids were Caladenia melanema, C. graniticola, C. williamsiae and Drakaea isolata from the wheatbelt of Western Australia. This agricultural area has a Mediterranean climate with unreliable rainfall, and is >80% cleared of native vegetation. Surveys with 10-30 volunteers increased population-size estimates by up to 10 times and provided spatial data to define core habitat areas. These areas included most of the individuals of a species, but were only 2-10 ha in size. Within these areas, orchids were often highly aggregated in patches a few metres wide, potentially resulting in a high degree of intraspecific competition. Vital statistics were obtained using 4-m wide and 30-50-m-long transects to measure rates of emergence, flowering, grazing and seed-set for each orchid. Plants emerging at the same position in different years were considered to be the same individual, but most emerged in new positions. Many plants emerged just once in 4 years, and 2-3 years of dormancy was common. Emergence frequencies were used to provide estimates of population sizes that were two or three times larger

than suggested by data from a single year. Seed production was typically very low. Grazing by kangaroos and rabbits was most severe for C. melanema, but was greatly reduced by fencing. Severe drought prevented flowering of C. graniticola in the driest year, whereas other species were more resilient. These orchids are likely to persist as long as there are some years where rainfall is sufficient for flowering and seed set followed by a year with adequate rain for seed germination. Populations of all these orchids were stable or increasing, but they are still at high risk of extinction because of the impacts of increasing soil salinity or fire on their habitats. These species are unlikely to spread elsewhere in the highly cleared and fragmented wheatbelt. Intervention by hand-pollination, grazing protection and translocation to new locations is required to mitigate these risks. Results were summarised in vital statistics report cards with thresholds set to inform conservation management for these species. Core habitat maps and vital-statistics report cards should also be valuable new tools for terrestrial-orchid conservation in other biomes.

4421: *+.099*

Stocking of young eel is widely practised, as a measure, to meet the management target of the EU eel recovery plan. The target of the recovery plan is to increase the escapement to 40% silver eel biomass, relative to pristine conditions. The scientific information to predict the outcome in silver eel biomass from stocking is limited and may depend on whether translocation of wild glass eel or yellow eel is used, or if the stocked eels used are yellow eel from aquaculture. We evaluated the yield from stocking two different sizes, 3 and 9 g eels from aquaculture. A professional fishery recaptured 12.7% of the 3 g and 9.4% of the 9 g eels, originally stocked. Growth rate and mortality rate were different for the two stocked sizes, favouring the small eels. Brutto yield per recruit (YPR) was 13 and 9.2 g and netto YPR was 9.8 and 0.31 g for 3 and 9 g eel, respectively. We conclude that there seems to be no advantage in using larger 9 g eels compared with small 3 g eels for stocking.

4422: *+.168*

Under the European Eel Regulation EG 1100/2007, Member States exhibiting natural habitats for the European eel (Anguilla anguilla) on their territory are obliged to prepare Eel Management Plans (EMP) containing appropriate measures to safeguard the escapement of a river system specific silver eel target biomass. Stocking is one management option to reach this target. We used various methodical approaches to study population parameters in a large lowland river under the application of a multi-annual intense stocking programme. The approaches were used to further enhance modelling of stock dynamics and silver eel escapement, in particular. Parameterizing the German Eel Model III (GEM III) with values and functions obtained for recruitment, growth, and mortality resulted in an annual escapement estimate of roughly 32 000-64 000 silver eels from 2010 to 2012. Escapement estimates based on a mark-recapture study conducted in parallel revealed somewhat lower values (11 000-25 000) for the same years. In view of the small number of natural recruits, such values are only contingent if stocking had a profound effect on silver eel production. Results from modelling annual silver eel escapement values indicate that escapement targets set in the EMP for this tributary cannot be reached without stocking. This constellation is likely to apply to other Eel Management Units with low current natural immigration values as well, and might be considered a key dilemma in eel management in such catchments due to the current confusion whether translocation of recruits yields a net benefit to the panmictic stock of the European eel.

4424: *+.025*

Context Reintroduction of endangered species potentially places them back in contact with putative factors of historical decline, inadvertently providing the opportunity to evaluate their impact. Aims To monitor the long-term progress of a population of western barred bandicoot reintroduced to mainland Australia and to assess factors involved in its eventual local extinction. Methods Bandicoots were reintroduced from offshore Dorre Island to the nearby mainland peninsula of Heirisson Prong in 1995. The narrow neck of the peninsula was fenced to exclude foxes and feral cats from a 1200ha area, but the area was subject to periodic incursions. There was parallel management of a confined but unsupported population in an in situ 17-ha predator refuge. Bandicoots were assessed for abundance, body condition and reproduction two to four times annually between 1995 and 2010. In addition, perceived threatening processes (drought, disease and the abundance of cats, foxes and rabbits) were monitored. Key results Bandicoots became well established at the site, spreading to all available habitat. Numbers fluctuated strongly, peaking at similar to 250 in 1999 and then declining to apparent local extinction (with subsequent re-establishment from the refuge), and at similar to 470 animals in 2006, followed again by extinction. Conclusions Predation by feral cats was implicated as the primary cause of both free-range extinctions and the eventual elimination of all bandicoots from the predator refuge. Other contributing factors in one or more of the declines were a reduction in reproduction and recruitment in bandicoots during a one-in-100-year drought, the impact of overabundant European rabbits on vegetation used by bandicoots for nesting shelter and brief fox incursions at key times. Implications Existing methods of control of feral cats are rendered ineffective in the presence of abundant and diverse native fauna and abundant exotic species (particularly European rabbits). In addition, episodic drought in arid Australia intensifies the impact of predation by restricting reproduction of prey species. These factors hamper the attempts of conservation managers to re-establish vulnerable species at sites other than those with the infrastructure and/or management intensity to largely exclude exotic predators (and preferably European rabbits) over the long-term.

4425: ***+.104***

Context Reintroduction is a popular tool for conserving endangered species, yet many attempts fail. Soft-release measures, including acclimatisation, have been used for many species around the world, based on the reasoning that gradual and supported reintroductions should improve the success of animals released into an unfamiliar wild environment. However, experimental testing of soft-release methods is rare. Aims To experimentally test the effect of a soft-release method versus a hard-release method on the initial reintroduction success of the eastern barred bandicoot (Perameles gunnii). Methods We released 12 captive-bred eastern barred bandicoots into a predator-proof reserve using two methods: soft-release (7 days of on-site acclimatisation with supplementary food before release) and hard-release (no acclimatisation and no supplementary food). We monitored the bandicoots intensively via radio-tracking and live-trapping for 4 weeks after release. Compared with hard-release bandicoots, we predicted that soft-release bandicoots would have (1) reduced movement (first night dispersal, site fidelity and activity range), (2) more directed patterns of habitat selection, (3) improved bodyweights and (4) improved survival. Key results There was no detectable difference in habitat selection, overall weight change and survival between the soft-release and hard-release groups. There was moderate evidence that, compared with the hard-release group, soft-release bandicoots moved less, demonstrated lower individual variation in all measures of movement, and lost weight more gradually after release. In most cases, effect sizes were moderate to large but had large standard errors owing to both small sample size and high variance. Consequently, statistical testing failed to detect significant differences at the 5% level. Conclusions Despite evidence that the release method influenced some of the monitored behaviours, soft-release did not confer a consistent and substantive advantage for captive-bred eastern barred bandicoots at our site. We conclude that soft-release is unlikely to improve overall

reintroduction success for this species at fenced predator-free sites. Implications The present study suggests that the preferred option for reintroductions of eastern barred bandicoots to fenced sites is a hard-release, information that is now being used to guide reintroductions of this species. Similar experiments should be undertaken to improve reintroduction practice for other endangered species.

4426: *+.064*

Avian malaria, caused by Plasmodium spp., has been reported as a cause of morbidity and mortality in New Zealand bird populations. The prevalence of Plasmodium lineages in the Waimarino Forest was evaluated in NZ robins (Petroica longipes), other passerines, blue ducks (Hymenolaimus malacorhynchos), and brown kiwi (Apteryx mantelli), using nested PCR. The presence of P. sp. lineage LINN1, P. (Huffia) elongatum lineage GRW06 and P. (Novyella) sp. lineage SYAT05 was demonstrated; Plasmodium (Haemamoeba) relictum lineage GRW4 was not found. The highest prevalence of infection was found in introduced European species (80.5%), followed by native (19%) and endemic species (3.5%), with a significant difference between these groups. All detected Plasmodium lineages have previously been identified in New Zealand and introduced species have been suggested as an important reservoir of infection. The results of this study will aid New Zealand conservation managers with disease risk management during bird translocations from the Waimarino forest.

4427: *+.014*

Conflicts in conservation may arise if two or more threatened species are involved in prey-predator interaction. Predators may have a profound effect on small prey populations, thus conservation actions must consider inter-specific interactions involving threatened species. Here we report nest predation events on a wild population of the Endangered red-billed curassow Crax blumenbachii Spix, 1825 by a group of the Critically Endangered yellow-breasted capuchin monkeys Sapajus xanthosternos Wied-Neuwied, 1820 in the Brazilian Atlantic Forest. This is the first study to report egg predation of an threatened gamebird by an threatened primate. We recommend that systematic conservation planning for these threatened species consider interactions, especially considering upcoming reintroduction programs indicated in the National Action Plans for these species conservation.

4428: *+.003*

Urbanization, habitat degradation, fragmentation, and invasive species have led to the severe decline or extirpation of many endemic southern California freshwater fish species, including the Arroyo Chub Gila orcuttii, which has declined precipitously in recent years. Classified by the California Department of Fish and Wildlife as a species of high concern, the Arroyo Chub is native to the Los Angeles, San Gabriel, San Luis Rey, Santa Ana, and Santa Margarita rivers and Malibu and San Juan creeks. To examine Arroyo Chub population structure and genetic diversity within the species' native range, we used 10 microsatellite markers to genotype 259 individuals. We observed moderate to high genetic diversity and population differentiation both between and within drainages; Bayesian clustering supported eight distinct clusters of Arroyo Chub corresponding to eight isolated populations. Of these populations, the Big Tujunga Creek population (Los Angeles River) was the least genetically differentiated (genetic differentiation index F-ST = 0.048-0.208) and also had the highest genetic diversity (observed heterozygosity H-o = 0.890). Populations in Malibu Creek, Pacoima Canyon (Los Angeles River), and the Santa Margarita River were the most genetically differentiated (F-ST = 0.163-0.400), had the lowest genetic diversity (H-o = 0.556-0.680), and showed evidence of past bottlenecks. Arroyo Chub at

these localities are at risk for continued loss of genetic diversity due to drift and small population sizes; therefore, we suggest that in the event of extirpation, translocations from the most closely related source populations should be considered. However, we recommend that management efforts focus on improving habitat quality and habitat area for Arroyo Chub in order to maximize population genetic diversity and adaptive potential over time.

4429: *+.162*

Imperiled fish species are often managed by establishing refuge populations as a hedge against extinction, but suitable sites are often at a premium. Thus, managers may wish to consider novel strategies, such as establishing multispecies refuges that already include undesirable species. To determine the suitability of multispecies refuges, we established experimental communities that included allopatric and sympatric communities of three fish species: the endangered Pahrump Poolfish Empetrichthys latos, the Amargosa Pupfish Cyprinodon nevadensis, and the invasive Western Mosquitofish Gambusia affinis. Mosquitofish juvenile production was not significantly affected by the presence of the other species (mean +/- SE: 50 +/- 18 in allopatry, 33 +/- 6 with poolfish, and 38 +/- 7 with both poolfish and pupfish). Similarly, pupfish persisted in sympatry with both poolfish and mosquitofish, but pupfish had higher juvenile production when maintained in allopatry (557 +/- 248) and in the presence of poolfish (425 +/- 36) than in the presence of both poolfish and mosquitofish (242 +/- 32). By contrast, poolfish juvenile production was high in allopatry (123 +/- 17) but significantly lower in the presence of pupfish (6.6 +/- 1.2) andmosquitofish (1.0 +/- 0.5) individually and in a community of all three species (0.5 +/- 0.4). This suggests that translocated pupfish can coexist in refuges containing nonnative mosquitofish but that endangered poolfish are not compatible with the other species and the current management of poolfish in single-species refuges is appropriate. Consequently, our results indicate that multispecies refuges are suitable for some endangered species, which will give managers more latitude in the management of these species.

4430: *+.073*

Timing can be critical for many life history events of organisms. Consequently, the timing of management activities may affect individuals and populations in numerous and unforeseen ways. Translocations of organisms are used to restore or expand populations but the timing of translocations is largely unexplored as a factor influencing population success. We hypothesized that the process of translocation negatively influences reproductive rates of individuals that are moved just before their birthing season and, therefore, the timing of releases could influence translocation success. Prior to reintroducing fishers (Pekania pennanti) into northern California and onto the Olympic Peninsula of Washington, we predicted that female fishers released in November and December (early) would have a higher probability of giving birth to kits the following March or April than females released in January, February, and March (late), just prior to or during the period of blastocyst implantation and gestation. Over four winters (2008-2011), we translocated 56 adult female fishers that could have given birth in the spring immediately after release. Denning rates, an index of birth rate, for females released early were 92% in California and 38% in Washington. In contrast, denning rates for females released late were 40% and 11%, in California and Washington, a net reduction in denning rate of 66% across both sites. To understand how releasing females nearer to parturition could influence population establishment and persistence, we used stochastic population simulations using three-stage Lefkovitch matrices. These simulations showed that translocating female fishers early had long-term positive influences on the mean population size and on quasi-extinction thresholds compared to populations where females were released late. The results from both empirical data and simulations show that the

timing of translocation, with respect to life history events, should be considered during planning of translocations and implemented before the capture, movement, and release of organisms for translocation.

4431: *+.124*

Among the different anthropogenic processes that affected the current distribution of the European pond turtle (Emys orbicularis), the timing of the human-mediated translocations is rarely known. Here we present data derived from an archaeological specimen of E. orbicularis obtained at the Roman site of Pollentia (Mallorca, Balearic Islands) using direct radiocarbon dating. These remains correspond to the early Roman period and represent the first reliable evidence for the ancient introduction of this turtle species in a Western Mediterranean Island.

4434: *+.178*

The Chinese water deer was once widely spread in Liaodong Peninsula, North China Plain and both banks of the Yangtze River and the Korean peninsula. Due to long-term environmental changes and influence of human development, its wild population in China has rapidly declined, both in abundance and distribution. As one of the native species in the history of Shanghai, Chinese water deer was introduced to Shanghai for captive breeding in 2006 and were released into the wild in 2010. The present study was conducted in Nanhui East Shoal Wildlife Sanctuary. The reintroduction of Chinese water deer was carried out separately in June and October 2010. So as to study the movement of the deer after release, 12 Chinese water deer (sex ratio 1:1) were tagged with radio-collars. We successfully used radio-telemetry to track 10 deer, and used the minimum convex polygon (MCP) and fixed kernel estimation (FKE) methods to calculate their home range. The results showed that using the MCP method, the mean home-range size of Chinese water deer was estimated to be 671 ha (range 245-1559 ha), while using the 95% FKE method, the mean was estimated to be 262 ha (range 43-435 ha). The mean home-range size of a buck was smaller than that of a doe by both MCP and FKE. The mean home-range size of an adult female was smaller than that of a subadult female. The largest seasonal home-range size (MCP, 275 ha) occurred during the winter of the first year, which then kept on shrinking in spring and summer. Home-range overlap was found among the home range of each individual. The mean overlap size was 303 ha. The mean overlap size was 135 ha in bucks, 422 ha in does and 270 ha between the buck and the doe. The study reflects that the seasonal food change is probably the main factor for the change of home-range size. Oestrus may also result in the enlargement of home range in winter. As an attempt to reintroduce large mammals to cities, we hope to provide useful experience for future wildlife management and conservation.

4435: *+.071*

Pere David's deer (Elaphurus davidianus) and sika deer (Cervus nippon) occupy two contrasting types of niches in eastern Asia: Pere David's deer is a swamp deer adapted to wetlands, while the sika deer mainly live in forested areas. Both Pere David's deer and sika deer have been hunted since the early days of the hunting and gathering civilisation; however, these two deer have undergone different population histories. As human society entered the era of agriculture civilisation, Pere David's deer gradually lost its habitats to farmlands, and the population was greatly reduced until, finally, it became extinct in the wild in 1900. Fortunately, after 30 years of restoration and introduction, more than 4000 Pere David's deer thrive in nature reserves, zoos and safari parks in China, and more than 500 Pere David's deer could be found in the wild in 2014. Populations of wild sika deer were reduced as well due to hunting and deforestation, and were

restricted in the forest patches in the mountains in eastern Asia. Nevertheless, the sika deer in China and Japan have different fortunes. Sika deer in China did not escape the prevalent tragedy of domestication, in that wild sika deer was endangered or extirpated in its original habitats, while the farmed sika deer thrived since late 1950s. Sika deer populations in Japan also remained at low density in the mid-1950s due to over-hunting, including poaching. After one-quarter of a century of in situ protection, sika populations are still small in China and some populations were found to leave the nature reserve to areas with high human densities, while the wild sika deer in Japan have markedly increased in numbers and extended their geographic distribution during the past few decades. Accordingly, the management strategies in China and Japan were completely different. The Chinese government is seeking all the efforts on the conservation of sika deer, while the Japanese government is revising laws to harvest and to reduce sika deer population.

4436: *+.017*

Chinese water deer (Hydropotes inermis) was endangered in China and vanished from Shanghai early in the 20th century. The deer was reintroduced from 2006 onward, so as to re-establish a wild population in Shanghai. We obtained the deer from the breeding stock and released them. The deer numbers increased from 21 in 2007 to 299 in 2013. There has been a total area of 30.62 km(2) of free-range deer release from 2010 and this will be enlarged in the future.

4437: *+.129*

The range of the Amur leopard (Panthera pardus orientalis) has decreased dramatically over the last 100 years. This species is still under extreme risk of extinction and conservation efforts are rigorous. Understanding the long-term dynamics of the population decline would be helpful to offer insight into the mechanism behind the decline and endangerment and improve conservation perspectives and strategies. Historical data collection has been the challenge for reconstructing the historical distribution. In China, new gazetteers having systematic compilation and considerable local ecological data can be considered as an important complementary for reconstruction. Therefore, we have set up a data set (mainly based on the new gazetteers) in order to identify the historical range of the Amur Leopard from the 1950s to 2014. The result shows that the Amur leopard was historically widely distributed with large populations in Northeastern China, but it presented a sharp decline after the 1970s. The decline appeared from the plains to the mountains and northeast to southwest since the 1950s. Long-term historical data, mainly from new gazetteers, demonstrates that such resources are capable of tracking species change through time and offers an opportunity to reduce data shortage and enhance understanding in conservation.

4438: *+.276*

Context. In the past 200 years, many carnivores have experienced a widespread decline in numbers and range reductions. Conservation interventions include the use of small, fenced reserves that have potential restoration benefits for conservation. Over the past 25 years, the Eastern Cape province of South Africa has seen the establishment of many small (<= 440 km(2)) game reserves, and the reintroduction of the larger, indigenous wildlife that had been extirpated by the early 20th century, including brown hyaenas (Hyaena brunnea). These game reserves have restored the environment to a more natural state but little information exists concerning the benefits and implications of introducing elusive animals that are seldom seen after reintroduction. Fenced reserves have the potential to provide surplus animals that can be relocated for restoration purposes (where applicable) or serve as a buffer to the extinction of naturally occurring populations, but careful management is required to monitor populations appropriately, so as to

avoid the costs of rapid population increase.Aims. The reintroduction of brown hyaenas to the Eastern Cape has provided a case study to assess the role of small reserves and their potential to contribute to conservation, by determining the persistence and population growth of brown hyaenas in a small, enclosed reserve.Methods. Estimates of brown hyaena density were calculated using a capture-recapture approach from individually identifiable images captured during a 3-month camera trapping survey. Key results. After a single decade, the brown hyaena population increased by at least 367%, from six individuals to a minimum of 28 individuals. These results suggest that this brown hyaena population has the highest density ever recorded for the species in southern Africa. Conclusions and Implications. Because brown hyaena populations were high relative to natural unfenced populations, high fences may provide two utilities for their conservation. Fenced reserves may provide surplus animals to support reintroductions and provide protected populations to buffer the risk of species extinction.

4439: *+.028*

To protect Australian amphibian biodiversity, we have identified and prioritised frog species at an imminent risk of extinction from chytridiomycosis, and devised national management and research priorities for disease mitigation. Six Australian frogs have not been observed in the wild since the initial emergence of chytridiomycosis and may be extinct. Seven extant frog species were assessed as needing urgent conservation interventions because of (1) their small populations and/or ongoing declines throughout their ranges (southern corroboree frog (Pseudophryne corroboree, New South Wales), northern corroboree frog (Pseudophryne pengilleyi, Australian Capital Territory, New South Wales), Baw Baw frog (Philoria frosti, Victoria), Litoria spenceri (spotted tree frog, Victoria, NewSouth Wales), Kroombit tinkerfrog (Taudactylus pleione, Queensland), armoured mist frog (Litoria lorica, Queensland)) or (2) predicted severe decline associated with the spread of chytridiomycosis in the case of Tasmanian tree frog (Litoria burrowsae, Tasmania). For these species, the risk of extinction is high, but can be mitigated. They require increased survey effort to define their distributional limits and to monitor and detect further population changes, as well as well-resourced management strategies that include captive assurance populations. A further 22 frog species were considered at a moderate to lower risk of extinction from chytridiomycosis. Management actions that identify and create or maintain habitat refugia from chytridiomycosis and target other threatening processes such as habitat loss and degradation may be effective in promoting their recovery. Our assessments for some of these species remain uncertain and further taxonomical clarification is needed to determine their conservation importance. Management actions are currently being developed and trialled to mitigate the threat posed by chytridiomycosis. However, proven solutions to facilitate population recovery in the wild are lacking; hence, we prioritise research topics to achieve this aim. Importantly, the effectiveness of novel management solutions will likely differ among species due to variation in disease ecology, highlighting the need for species-specific research. We call for an independent management and research fund of AU$ 15 million over 5 years to be allocated to recovery actions as determined by a National Chytridiomycosis Working Group of amphibian managers and scientists. Procrastination on this issue will likely result in additional extinction of Australia's amphibians in the near future.

4440: *+.033*

The last century has seen an unparalleled movement of species around the planet as a direct result of human activity, which has been a major contributor to the biodiversity crisis. Amphibians represent a particularly vulnerable group, exacerbated by the devastating effects of chytrid fungi. We report the malicious translocation and establishment of the alpine newt (Ichthyosaura alpestris) to its virtual antipode in North Island of New Zealand. We use network analysis of mitochondria!

DNA haplotypes to identify the original source population as I. a. apuana from Tuscany, Italy. Additionally, a population in southern France, presumed to be introduced, is identified as I. a. alpestris from western Europe. However, the presence of two differentiated haplotypes suggests a mixed origin. This type of analysis is made possible by the recent availability of a phylogenetic analysis of the species throughout its natural range. We discuss the particulars of both introductions.

4441: *+.169*

Many endangered captive populations exhibit reduced genetic diversity resulting in health issues that impact reproductive fitness and quality of life. Numerous cost effective genomic sequencing and genotyping technologies provide unparalleled opportunity for incorporating genomics knowledge in management of endangered species. Genomic data, such as sequence data, transcriptome data, and genotyping data, provide critical information about a captive population that, when leveraged correctly, can be utilized to maximize population genetic variation while simultaneously reducing unintended introduction or propagation of undesirable phenotypes. Current approaches aimed at managing endangered captive populations utilize species survival plans (SSPs) that rely upon mean kinship estimates to maximize genetic diversity while simultaneously avoiding artificial selection in the breeding program. However, as genomic resources increase for each endangered species, the potential knowledge available for management also increases. Unlike model organisms in which considerable scientific resources are used to experimentally validate genotype-phenotype relationships, endangered species typically lack the necessary sample sizes and economic resources required for such studies. Even so, in the absence of experimentally verified genetic discoveries, genomics data still provides value. In fact, bioinformatics and comparative genomics approaches offermechanisms for translating these raw genomics data sets into integrated knowledge that enable an informed approach to endangered species management.

4442: *+.111*

The home range and characteristics of Cottus koreanus were investigated using passive integrated transponder (PIT) telemetry in the Gulji Stream, Korea, where the target species was artificially translocated. After release, tagged individuals moved up to 78 m away from the release site in search of proper habitats. The average distance moved until settlement was 17.1 m. The observed home range of settled individuals had longitudinal sections of 9.9 +/- 3.6 m and surface areas of 7.2 +/- 2.7 m(2). This is comparable to congeneric species that inhabit similar ecological habitats. Once individuals had settled, they rarely moved from that site except during the spring season. The typical microhabitat characteristics of the sites where the released individuals settled are: water depth of 5-10 cm, water velocity of 0.1-0.3 m s(-1), and the size of boulders and cobbles of 10-20 cm in diameter. This study of translocated C. koreanus individuals provides detailed information about habitats that can be used for effective habitat restoration and successful translocations attempts of this species.

4443: *+.132*

Captive rearing can be a successful short-term strategy for protecting species threatened with extinction, by bolstering existing populations, or establishing new ones. Improving the success of captive rearing requires specific prior ecological knowledge, for example habitat and climatic requirements. Here, we report on the captive rearing of the critically endangered Canterbury knobbled weevil Hadramphus tuberculatus (Pascoe, 1877). Only a single population of H.

tuberculatus is known, with an estimated population size of fewer than 100 individuals; as such, captive rearing provides an opportunity to augment the remaining population and potentially to allow the establishment of new populations, if suitable sites can be found. We successfully reared two F1 generation adults and two live larvae were recovered at the end of the project. We use a qualitative spatial analysis to determine the location of possible sites for reintroduction of H. tuberculatus. The spatial analysis suggests that there is little suitable habitat remaining for new H. tuberculatus populations. Our study provides guidelines for future captive breeding programmes and highlights the risks of removing individuals from an already vulnerable, small population.

4444: *+.196*

The spiny rats of the genus Proechimys have a wide distribution in the Amazon, covering all areas of endemism of this region. We analyzed the karyotype and cytochrome b (Cyt b) sequences in Proechimys goeldii from 6 localities representing 3 interfluves of the eastern Amazon. A clear separation of P. goeldii into 2 monophyletic clades was observed, both chromosomally and based on Cyt b sequences: cytotype A (2n = 26 female/27 male, NF = 42) for samples from the Tapajos-Xingu interfluve and cytotype B (2n = 24 female/25 male, NF = 42) for samples from the Xingu-Tocantins interfluve and east of the Tocantins River. The karyotypes differ in a pericentric inversion and a centric fusion/fission and an average nucleotide divergence of 6.1%, suggesting cryptic species. Meiotic analysis confirmed the presence of a XX/XY1Y2 multiple sex chromosome determination system for both karyotypes. The karyo-types also vary from the literature (2n = 24, NF = 42, XX/XY). The autosome translocated to the X chromosome is different both in size and morphology to P. cf. longicaudatus, which also has a multiple sex chromosome determination system (2n = 14 female/15 female male/16 female/17 male, NF = 14). The Xingu River is a barrier that separates populations of P. goeldii, thus maintaining their allopatric nature and providing an explanation for the molecular and cytogenetic patterns observed for the Xingu River but not the Tocantins River. (C) 2016 S. Karger AG, Basel

4445: *+.211*

Background and aims - Field elms (Ulmus minor Mill.) can easily reproduce vegetatively by root suckers or sprouting. They also have a long history of propagation and planting in the Netherlands. Both natural vegetative reproduction and cultivation may significantly influence the genetic structure of U. minor populations and insight in these phenomena is of utmost importance for appropriate conservation management of this species. In this study we examined the presence and extent of clonality and patterns of genetic variability within and among field elm populations in the Netherlands. Methods - We used microsatellites (SSRs) to describe the clonal diversity and structure and to calculate genetic diversity parameters in the Dutch U. minor populations. Additionally, we compared Dutch populations with two U. minor reference collections from Belgium and France. Key results - We found high levels of clonality in the Dutch field elm populations. Out of the 159 Dutch trees analysed for clonal structure only 66 multilocus genotypes were identified. Clonal richness (R = 0.06-0.96) and diversity (D = 0.44-1.0) varied considerably among locations. Six genotypes were shared between locations indicating human-mediated translocations. We revealed a low to moderate genetic diversity in the populations (H-e = 0.483-0.628 and A(r) = 2.4-2.9). At four locations some individuals were found that differed in assignment probabilities based on the STRUCTURE clustering analysis including parental species, suggesting that these might be hybrids or at least not pure U. minor specimens. This also indicates that morphological identification is difficult. When omitting these individuals genetic structure analyses still indicated the presence of two genetic clusters. Conclusions - However artificial establishment has played a major role in the distribution of the species and its current

genetic diversity in the Netherlands. These findings help facilitate Dutch gene conservation management programs for U. minor, in particular, the identification of high priority clones for ex situ conservation and efforts to restore remnant populations and hedgerows.

4446: *+.140*

Topeka Shiners (Notropis topeka) representing two genetically distinct and geographically separated Kansas populations have been under continuous culture and study in 0.045-ha experimental ponds at the University of Kansas Field Station since 2002. Experiments in earthen ponds undertaken from 2002 to 2009 established that Topeka Shiners are capable of surviving and reproducing in static artificial habitats, which at the field station are akin to isolated stream pools or farm ponds typical in this part of the Great Plains. Variables included the addition of various substrate types, sizes, and their location within ponds, and also the numbers of adult Topeka Shiners stocked at the beginning of each spawning season. Behavioral and predatory associations with several other fish species, which sometimes included the addition of Orangespotted Sunfish (Lepomis humilis), Green Sunfish (L. cyanellus), and Largemouth Bass (Micropterus salmoides), also were examined. The work demonstrated that while Topeka Shiners can be maintained and will reproduce in ponds under certain conditions, their behavior, net reproduction, and survival can be substantially influenced by the other fish species. Although formal experiments ended in 2009, both populations continue to be maintained separately in 12 ponds with only minimal care. One of the populations, taken originally from Willow Creek (Wallace County, KS), is now considered extirpated in the wild, thus its remaining members likely exist only at the field station. With respect to the state recovery plan for this species, should authorities resolve to re-introduce this relict population somewhere into its former and highly restricted range in the upper Smoky Hill River watershed, the task will be complicated by factors including severe declines in surface water flow and availability leading to loss of habitat there and in other portions of western Kansas in recent decades.

4447: *+.208*

The proliferation of private land conservation areas (PLCAs) is placing increasing pressure on conservation authorities to effectively regulate their ecological management. Many PLCAs depend on tourism for income, and charismatic large mammal species are considered important for attracting international visitors. Broad-scale socioeconomic factors therefore have the potential to drive fine-scale ecological management, creating a systemic scale mismatch that can reduce long-term sustainability in cases where economic and conservation objectives are not perfectly aligned. We assessed the socioeconomic drivers and outcomes of large predator management on 71 PLCAs in South Africa. Owners of PLCAs that are stocking free-roaming large predators identified revenue generation as influencing most or all of their management decisions, and rated profit generation as a more important objective than did the owners of PLCAs that did not stock large predators. Ecotourism revenue increased with increasing lion (Panthera leo) density, which created a potential economic incentive for stocking lion at high densities. Despite this potential mismatch between economic and ecological objectives, lion densities were sustainable relative to available prey. Regional-scale policy guidelines for free-roaming lion management were ecologically sound. By contrast, policy guidelines underestimated the area required to sustain cheetah (Acinonyx jubatus), which occurred at unsustainable densities relative to available prey. Evidence of predator overstocking included predator diet supplementation and frequent reintroduction of game. We conclude that effective facilitation of conservation on private land requires consideration of the strong and not necessarily beneficial multiscale socioeconomic factors that influence private land management.

4448: *+.040*

The distributions of most native trout species in western North America have been severely reduced, and conservation of many of these species will require translocation into vacant habitats following removal of nonnative species. A critical question managers have is "Does it matter which donor sources are used for these translocations?" We present a case study that addressed this question for a large native trout translocation project in Montana. We introduced embryos from five source populations of Westslope Cutthroat Trout Oncorhynchus clarkii lewisi to a large, fishless watershed in Montana following removal of nonnative fish with piscicides. Source populations providing embryos for translocations were three nearby (< 120 km) wild populations, the state of Montana's captive Westslope Cutthroat Trout hatchery conservation population (initiated 32 years ago using fish from wild populations located > 350 km from the translocation site), and a population in captivity for one generation comprised of individuals from the three wild populations used as single sources for this project, which were variably crossed (59% within populations and 41% between populations) to provide embryos. We used remote-site incubators at six different sites to introduce approximately 35,000 embryos from 400 genotyped parents. We later resampled and genotyped 1,450 of these individuals at age 1 and age 2. Juvenile survival for the more genetically diverse Montana Westslope Cutthroat Trout conservation population was twice as high as for other source populations, even though these other source populations were geographically closer to the translocation site than populations used to make the Montana Westslope Cutthroat Trout conservation population. Body weight for progeny from the two captive populations was higher than for progeny from wild source populations, and some differences were observed in body condition among source populations. Continued monitoring over several generations will be necessary to determine the eventual contributions of each source population and the relevance of these initial findings.

4449: *+.047*

There is an urgent need to consider more aggressive and direct interventions for the conservation of freshwater fishes that are threatened by invasive species, habitat loss, and climate change. Conservation introduction (moving a species outside its indigenous range to other areas where conditions are predicted to be more suitable) is one type of translocation strategy that fisheries managers can use to establish new conservation populations in areas of refugia. To date, however, there are few examples of successful conservation-based introductions. Many attempts fail to establish new populations-in part because environmental factors that might influence success are inadequately evaluated before the translocation is implemented. We developed a framework to assess the feasibility of rescuing threatened fish populations through translocation into historically unoccupied stream and lake habitats. The suitability of potential introduction sites was evaluated based on four major components: the recipient habitat, recipient community, donor population, and future threats. Specific questions were then developed to evaluate each major component. The final assessment was based on a scoring system that addressed each question by using criteria developed from characteristics representative of highly suitable habitats and populations. This framework was used to evaluate the proposed within-drainage translocation of three Bull Trout Salvelinus confluentus populations in Glacier National Park, Montana. Our results indicated that within-drainage translocation is a feasible strategy for conserving locally adapted populations of Bull Trout through the creation of new areas of refugia in Glacier National Park. The framework provides a flexible platform that can help managers make informed decisions for moving threatened fishes into new areas of refugia for conservation and recovery programs.

4451: *+.015*

Joint impacts of anthropogenic disturbance and climate change are of pressing concern for modern conservationists. Climate change patterns have various diminishing effects on the biodiversity of an ecosystem, requiring an understanding of a species' ability to adapt. Agricultural practices are expanding at an altitudinal gradient on the Indonesian island of Java, forcing endemic species to range at increased elevation with lower temperatures, and in human-populated areas. One example is the Critically Endangered Javan slow loris (Nycticebus javanicus), which finds itself increasingly restricted to montane regions with extreme climate patterns and habitat disturbance. We observed wild N. javanicus in a highly fragmented, montane agroforest area to determine if climate variables and forest connectivity influence activity budget and behavior. Lorises ranged at different altitudes (1275 m above sea level (asl)-1570 m asl) and were observed for six months in Cipaganti, West Java. Using multinomial regression analyses, we found loris individuals were most likely to engage in increased foraging, feeding and travelling behavior than resting when relative humidity increases and in habitats with greater forest connectivity. Regression analyses found effects of relative humidity and forest connectivity to be the most significant predictors of N. javanicus foraging behavior ($P = 0.001$, $P = 0.030$). We suggest that future-climate shifts and increased anthropogenic disturbance will detrimentally influence wild populations of N. javanicus, requiring immediate plans for mitigation in conserving these already scarce wild populations. We also suggest the altering of reintroduction protocols in relation to climate and geographic region.

4452: *+.183*

Resource managers use habitat restoration to offset estuarine habitat loss; however, there is limited information about how functionally successful restorations have been, particularly with respect to their use by mobile marine predators. Restoration monitoring efforts typically use point-of-capture metrics to assess fish community recovery and habitat use, but this provides little insight into how fish habitat use changes through time. Using translocation experiments, we integrated the movements of California Halibut Paralichthys californicus, a conservation target species, into a point-of-capture monitoring program in a restored tidal creek estuary. Large halibut (>25 cm) were captured more frequently in the main stream channel, while small ones (<25 cm) were typically caught in the innermost marsh creeks. We actively tracked these fish (n = 20; size range = 26.6-60.5 cm TL) acoustically to identify their preferred habitats and challenged these habitat associations by means of translocations to a different habitats. Large fish tended to have small localized convex hull activity spaces, remaining in areas with high water flow and sandy substratum near eelgrass Zostera marina beds. Individuals that were translocated to marshes returned to the channel and exhibited movements over long distances from their initial locations to their last tracked positions; however, fish that were translocated from marshes to the channel remained in channel habitat and moved smaller distances between their first and last tracked points. Large halibut likely selected the channel because higher water flow would lead to higher concentrations of prey. Small halibut used marshes more frequently, likely because marshes have temperatures thought to maximize growth rates. Our study can serve as a proof of concept that linking point-of-capture and tracking data provides valuable information for habitat restoration, including the fact that California Halibut utilize estuaries in a size-segregated manner based on environmental conditions. This suggests that tidal creek estuaries with a variety of channel types and morphologies-like our study site-are well-suited to support this species.

4453: *+.006*

Radio-tracking is a key technique for monitoring threatened species during ecological research and reintroduction programs. In the case of the endangered eastern barred bandicoot (Perameles gunnii), it has not been possible to radio-track for extended periods (3 months) due to difficulties

in reliably and safely attaching radio-transmitters. In this study we compared eight attachment methods. Transmitters weighing 1.2-28g were either mounted with adhesive, attached to a collar or implanted into the peritoneum. Intraperitoneal transmitters were superior in terms of attachment duration, but were considered inferior overall as they could not be fitted in the field and had a very short detection range once implanted (50m). Retention times for external transmitters differed greatly between methods, ranging from 1 to 102 days. One tail-mount attachment technique caused minimal adverse effects but another caused tail amputation in one animal, and both had short retention times (3-33 days). Neither of the glue-on transmitter methods resulted in substantial periods of attachment (2-30 days) and flank-mounted transmitters also caused severe skin trauma. Radio-collars were generally retained for longer periods (42-102 days) but resulted in limb entanglement when they were fitted too loosely or subconjunctival haemorrhages when they were too tight. Cable tie collars are recommended as the most suitable attachment technique for bandicoots, as when fitted correctly they cause minimal impact to individuals and permit long retention times, but precise fitting is essential.

4454: *+.163*

Australia has one of the worst mammal extinction rates in the world, with translocations to refuge locations increasingly being advocated to help address problems of species decline. Offshore islands can function as these refuges, removing species from threatening processes and providing a source of animals for reintroduction. Historically, the focus of many island translocations in Australia has been the conservation of a single species, with data on long-term translocation success and population dynamics after release generally lacking. Here we examine the results of a multispecies translocation onto Wedge Island, off the South Australian coast 30-40 years ago. Fewer than a dozen individuals of three species - southern hairy-nosed wombat (Lasiorhinus latifrons), black-footed rock-wallaby (Petrogale lateralis pearsonii), and brush-tailed bettong (Bettongia penicillata) - were released. All three species have shown substantial population increase and wombat activity across the island has increased exponentially with >700 burrows detected. Substantial levels of co-use of wombat burrows by rock-wallabies and bettongs were observed, providing clear evidence for interspecies interactions. Rock-wallabies showed a significant preference for wombat-active burrows (45% co-used), whereas bettongs showed a significant preference for wombat-inactive burrows (10% used). This study suggests that islands have significant potential for long-term threatened species conservation and that translocation of an ecosystem engineer may increase habitat complexity and help improve habitat suitability for multiple species and thus increase the overall conservation benefit.

4455: *+.041*

Broad-scale Australian mammal declines following European settlement have resulted in many species becoming regionally or globally extinct. Attempts to reintroduce native mammals are often unsuccessful due to a suboptimal number of founders being used, high rates of predation and a lack of knowledge of the reintroduction biology for the species concerned. We trialled predator swamping and supplementary feeding in an attempt to offset predation and improve reintroduction success for the burrowing bettong (Bettongia lesueur) in arid South Australia. We compared population longevity of a large release group (1266 animals) with five releases of smaller groups (similar to 50 animals at each). We compared release sites with (n=5) and without (n=1) supplementary food to determine whether site fidelity, body condition and reproduction were affected, and whether these traits aided population establishment. Predator swamping did not facilitate reintroduction success, with no bettongs detected more than 122 days after release. While supplementary food increased site fidelity and persistence at release sites, bettongs failed to

establish successfully at any site. Neither predator swamping nor supplementary feeding enhanced reintroduction success at our sites but results suggested that supplementary feeding should be explored as an aid to reintroduction success for Australian mammals.

4456: ***+.237***

Background: Translocating plants for conservation purposes can be a useful tool to enhance existing populations, restore lost populations or create new ones, but has rarely been done for bryophytes, especially liverworts.Aims: Here, the leafy liverwort Herbertus hutchinsiae, a representative species of oceanic-montane liverwort-rich heath, was translocated to unoccupied habitat within its current range, to establish whether its restricted distribution is due to habitat or dispersal limitation.Methods: Feasibility of establishing new populations outside the current distribution range was assessed, to test the suitability of the species for assisted colonisation. Furthermore, transplants were grown at degraded sites where the species had declined to assess potential for restoration.Results: Although maximal growth rates occurred within-range, transplants grew at all sites, indicating that the species could be dispersal limited; a conclusion supported by distribution modelling.Conclusions: Assisted colonisation is thus an option for this species to overcome dispersal limitation and to track future climate space. Reinforcement of populations at degraded sites is only recommended if the pressure causing the degradation has been removed. These findings provide an evidence base for practical conservation management.

4457: ***+.356***

Study of the population structure of mammal species inhabitate natural ecological systems directed on justify the conservation of biodiversity, sustainable use of biological resources and providing of the environmental legislation. In 1997 - 2014 ecosystem studies of southern taiga were conducted within Kaiskiy-Unzhinskiy forest with different level of modes of protection and use of natural resources. As a result of the translocation in a single natural complex since 1954 for a 60-year period it was created a population group of beaver that inhabits unevenly in reserve and neighboring areas. Using the natural differentiation of the territory and its animal population as a basis for comparative methods of knowledge allowed to indicate that the micro-population group of beavers is at the stage of maximum development. To control the complex use of natural resources and regulation of beaver populations, to increase the productivity of their groups, ensure the conservation and reproduction of habitats, the sustainable use of resources, environmental security, schemes of forest planning, wildlife management and other activities in partnership with the reserve are need. It is necessary as they are located in a natural integral territorial complex. Now it is necessary to carry out next regular activities and control: the state monitoring of objects of fauna and their habitats. It is necessary to regulate the population of beavers also in all hunting and urban areas.

4458: ***+.192***

Owing to a severe decline in its abundance, Pinus dabeshanensis has been listed as an endangered species by the International Union for the Conservation of Nature. Although several restoration events have been undertaken since the 1960s, the natural population genetic structure of this species remains to be investigated. Herein, we examined the level of genetic diversity and structure of two native and two non-native populations using 10 microsatellite loci. A relatively high level of genetic variation (H-O = 0.586 +/- 0.039) and a low level of population differentiation (F-ST = 0.016 +/- 0.011) were revealed. For forensic investigation, an assignment test was performed. To better understand the genetic differentiation between the native and non-

native populations, the individuals in the transplanted and cultivated populations may have derived from populations that were not surveyed in this study. In light of our results, we discuss the real problems faced by all four populations and provide useful information for management decision-making.

4459: ***+.007***

For many threatened vertebrates, captivity may be the only option for species survival. Maintaining species in captivity prior to reintroduction presents many challenges, including the need to preserve genetic diversity and mitigation of disease risks. Recent studies suggest that captivity can alter the suite of symbiotic microbes that play important roles in host health. The Panamanian golden frog (Atelopus zeteki) has not been seen in its native habitat in PanamA since 2009. Along with habitat loss and illegal collecting, the lethal disease chytridiomycosis, caused by the fungal pathogen Batrachochytrium dendrobatidis (Bd), is responsible for the severe decline of this species. Prior to the spread of Bd into golden frog habitat, conservation organizations collected golden frogs and placed them in captive survival assurance colonies. The skin of amphibians is host to a diverse resident bacterial community, which acts as a defense mechanism in some amphibians to inhibit pathogens. We analyzed skin swabs from wild and F1 captive golden frogs originating from the same population with Illumina sequencing of the 16S rRNA gene to characterize the cutaneous microbial community and to assess how long-term captivity has affected this community.

4460: ***+.163***

Capsule: The endangered Gran Canaria Blue Chaffinch Fringilla teydea polatzeki has been bred in captivity with the aim of reinforcing wild populations. We released and monitored 26 males and 15 females between 2010 and 2012. Survival and reproductive success were similar between the reinforced population and a stable reference population, suggesting that the process could be useful for the conservation of the species.

4461: ***+.255***

The eastern grey kangaroo is a common and iconic species of Australia. Its specialised behaviour and reproduction have evolved as adaptations to the Australian environment, allowing the species to survive and flourish, despite wide climatic and seasonal variations in habitat. Across its range, the eastern grey kangaroo is harvested and subjected to population management for a variety of reasons, including localised over-abundance, livestock competition, crop grazing, native habitat conservation, animal welfare and direct threats to human safety. Population management of kangaroos is most commonly undertaken by shooting, although other methods such as reproductive control, translocation and repellents may also contribute successfully to management. Kangaroo harvesting and population control are controversial and divisive, because the kangaroo is perceived as both a national icon and as a pest species. Although a limited number of surveys have been undertaken on attitudes towards kangaroos and their management, the socio-political aspects affecting these issues are yet to be systematically investigated. Within this review we discuss the relevance of culture and language to species management and conservation, as well as the importance of scrutiny of stakeholder perceptions, motivations and values. Future directions should examine human dimensions that influence kangaroo-management decisions and conservation. The following three key aspects are recommended as research and management priorities: (1) experimental determination of whether gaps exist between actual and perceived impacts of kangaroo populations, (2) empirical investigation of how stakeholder language, culture,

identity and values influence perceptions of kangaroos and their management, and (3) where population control is determined to be necessary, an incorporation of stakeholder differences within decision making to ensure best outcomes for both species conservation and population management.

4462: *-.098*

Context. The value of captive breeding for recovery programs of endangered carnivorous mammals is often questioned because of low post-release survival reported for founder animals following translocation.Aims. The aim of the present study was to test the effect of rearing method on survival and body mass of captive-raised Tasmanian devils (Sarcophilus harrisii) following release on an offshore island. We also compared the post-release diet of these devils with the diet of wild devils on mainland Tasmania, where a similar array of diet items is available.Methods. Twenty-eight captive-raised devils were released onto the island; 19 had been raised in intensive captive-management facilities (IC) and nine in free-range (22 ha) enclosures (FRE). Survival and body-mass change were compared between IC and FRE for up to 440 days post-release. Devil diet was assessed via scat analysis. Key results. A high proportion (96%) of the founders survived 1 year post-release. Pre-release captive-rearing method had no effect. Released devils gained an average of 14% of their original body mass, irrespective of captive-rearing method. There was very little difference in the diet of captive-reared devils released onto Maria Island relative to wild mainland devils: Tasmanian pademelon, Thylogale billardierii, was the primary food item for both.Conclusions. The intensity of captive rearing did not affect the survival of devils released onto Maria Island. This suggests that even devils held in IC facilities retain the innate behaviour required to scavenge and hunt prey, and therefore maintain bodyweight post-release. The lack of any threatening processes on the island is also likely to have contributed to the high survival rate 2 years post-release.Implications. Our study provided preliminary evidence that the release of captive-raised Tasmanian devils onto off-shore islands is a viable conservation action. Captive-breeding programs and captive-raised founders can play a viable and valuable role in the conservation action plans for recovery programs of endangered carnivorous mammals.

4463: *+.105*

The present paper highlights problems associated with the currently-accepted taxonomy of brown bear, Ursus arctos, and their consequences for conservation at the European level. The enormous morphological variability within Ursus arctos is not acknowledged in current taxonomy and conservation practice. Seven major clades are recognized in Ursus arctos by molecular researchers, and although Western Europe maintains most of the populations belonging to the relict Clade 1 brown bear lineage, no reference to this is made in current conservation policy. Furthermore, the tiny population of Apennine brown bears, characterized by unique skull morphology, is not even recognized as a distinct ESU (evolutionari significant unit) by current European legislation, nor is it included in the IUCN Red List. This may have serious consequences as brown bear conservation in Western Europe has been mainly based on restocking and reintroduction programs.

4473: *-.105*

A mass mortality was detected in the downstream section of one of the most extensive French populations of the endangered white-clawed crayfish, Austropotamobius pallipes, on June 26, 2013. This population occupied a 12 km stretch of the La Lucelle brook, with an estimated size of around 150 000 individuals. The presence of the crayfish plague pathogen was quickly diagnosed

as the cause of the mortality, and monitoring was carried out to follow the spread of the disease from 15 July 2013 for one year. Results showed that after a fast spread between 15 and 25 July 2013 (upstream progression of mortality for about 4 km), the mortality front was limited to a stretch of a few hundred meters from August until December 2013. During winter, mortality was always observed, confirming that disease remained active. In April 2014, the mortality front was halted by a large dam in the brook (2 m high), 0.56 km from brook source. Two months later, 30 live crayfish were observed above the dam. On the 30 August 2014, no crayfish were found above the dam. Infected individuals analysed for microsatellite markers confirmed the Pacifastacus leniusculus strain of Aphanomyces astaci at the origin of this outbreak. Before the crayfish plague spread upstream of the large dam, a sample of 576 individuals was collected from upstream of the dam and translocated to another stream in the same French department. In July 2014, observations by night confirmed the presence of translocated white-clawed crayfish in the receiving brook.

4474: *-.005*

Trumpeter Swans Cygnus buccinator were extirpated from Ontario in 1886 as a result of unregulated subsistence and market hunting. Between 1982-2006 inclusive, 584 captive reared Trumpeter Swans were released in southern Ontario, to re-introduce the species to the region. However, no empirical analysis of the size of the breeding range has occurred since the reintroduction programme commenced. Observational data recorded from 1,394 captive-released and wild-hatched swans marked with uniquely identifiable patagial tags therefore were analysed, using a kernel density spatial framework, to infer changes in breeding distribution. The breeding range increased 16 fold between 1991 (301,938 ha) and 2010 (4,817,904 ha). A linear effect of year best explained breeding range expansion from 1991-2010. However, visual inspection of the relationship suggests that the breeding range did not increase after 2004, which coincided closely with the end of the reintroduction programme in 2006. Migration distances calculated for adult male and female, captive-released and wild hatched swans from 1982-2010 showed that most swan breeding and wintering locations were close to release sites (median migration distance = 4.6 km, range = 01,299 km) and 40% of swans were non-migratory (wintering and breeding locations were the same). The model that best explained migration distance included a quadratic fit of year, sex, and status (captive-released vs. wild-hatched birds). Migration distance declined until about year 2000 and then increased thereafter. Migration distance was shorter for females than for males and shorter for captive released than wild-hatched swans (26.4 vs. 47.9 km for females; 34.3 vs. 60.7 km for males). However, migration distances of captive-released and wild-hatched swans were similar by the year 2010. It is suggested that re-introduction of swans into new areas, density-dependence and observer bias in re-sighting rates are the mechanisms leading to the patterns of breeding range expansion and increase in migration distances we detected for Trumpeter Swans in southwest and central Ontario.

4475: *+.263*

This 382-page book is a series on reintroduction of fish and wildlife populations. This book is organized into 15 chapters. The first chapter deals with animal reintroduction in the anthropocene. The second chapter deals with reintroduction and other conservation translocations-history and future developments. The third chapter deals with a conservation paleobiology perspective on reintroduction-concepts, variables, and disciplinary integration. The remaining chapter deals with human dimensions insights for reintroductions of fish and wildlife populations, the reintroduction landscape-finding success at the intersection of ecological, social, and institutional dimensions, setting objectives and defining the success of reintroductions, demographic modeling for reintroduction decision-making, genetic issues in reintroduction, accounting for potential

physiological, behavioral, and community-level responses to reintroduction, why you cannot ignore disease when you reintroduce animals, release considerations and techniques to improve conservation translocation success, effective and purposeful monitoring of species reintroductions, management of reintroduced wildlife populations, outreach and environmental education for reintroduction programs, the future of animal reintroduction. The book highlights a list of contributors under respective institutions. Each chapter contains a list of references. The text is written in English and indexed by subject with tables and figures. Users of this book will include zoologists, wildlife biologists, and ecologists.

4476: ***+.097***

Nonnative Rainbow Trout Oncorhynchus mykiss have displaced native Brook Trout Salvelinus fontinalis in many southern Appalachian Mountains streams. We monitored the population recovery of Brook Trout following Rainbow Trout eradication at 10 sites in seven allopatric Rainbow Trout streams located in Great Smoky Mountains National Park, USA. Rainbow Trout were successfully eradicated by electrofishing or Fintrol (also known as antimycin-A), and Brook Trout were reintroduced at low densities (39-156 fish/km) from streams located within the park. Within 2 years after reintroduction, the density and bio-mass of adult Brook Trout recovered to levels comparable to the prerestoration density and biomass of Rainbow Trout. Spawning in the first autumn after reintroduction was assumed by the presence of young-of-the-year fish in seven out of nine sites surveyed during the following summer. Brook Trout density and biomass 3-5 years after restoration did not significantly differ from those in natural allopatric populations within the park in young-of-the-year fish but were significantly lower in adults. Individual body size of adult and young-of-the-year fish were density dependent after restoration, indicating that Brook Trout populations had recovered to a point that habitat saturation triggered intraspecific competition. We conclude that Rainbow Trout removal has been a viable management technique to restore Brook Trout populations in the park.

4477: ***+.312***

Population introductions and reintroductions have become a common tool for conserving threatened species, but oftentimes introduced populations have reduced the genetic diversity compared with the source population they were founded from. Population introductions played an important role in the recovery of the Oregon Chub Oregonichthys crameri, a small floodplainminnow found in western Oregon. Unlike many introduction efforts, introduced populations of Oregon Chub were founded using large numbers of individuals (hundreds in many cases) and each population had a unique introduction history (e.g., number of founders, source populations selected, duration of the introduction effort). We used microsatellite loci to examine 13 introduced populations and their respective sources to evaluate how well the introduction program captured genetic diversity present in the wild populations. Genetic variation was reduced by roughly 25% in one introduced population, and three introduced populations showed evidence of a genetic bottleneck due to heterozygote excess. Populations introduced from multiple sources had greater genetic diversity than populations from a single source. When multiple source populations were used, all source populations contributed genetic material to the introduced population, though the proportional contribution from each source population varied. Using correlation analyses and general linearmodels, we explored the relationship between introduction history variables and genetic diversity. Our top-ranked models included genetic diversity in the source population, and this variable had the highest variable importance weight (0.999), but the number of founders and the number of source populations were also important. Overall, the Oregon Chub introduction program was highly successful at capturing the genetic variation

observed in natural populations. Results of this study will be useful for planning future population introductions for Oregon Chub and other species of conservation concern.

4478: *+.285*

Understanding and monitoring life history traits is often important in endangered species conservation. Populations of the endangered mussel Cumberlandian combshell Epioblasma brevidens have continued to decline in the Powell River, USA. Understanding and modeling mussel growth is critical for effective reintroduction of this endangered species. In this study, 2 yr old E. brevidens that were produced in our laboratory were released to the Powell River in 2009 to augment this declining population. A mark-recapture monitoring approach using passive integrated transponder (PIT) tags was used to assess the survival and growth of the released mussels. Hierarchical Bayesian growth models incorporating individual growth variations, periodic growth and growth cessations, along with multiple release occasions were developed and compared to the classic von Bertalanffy growth model. Our results showed that the hierarchical model that incorporated individual growth variation gave the best estimates of model parameters, yielding the lowest deviance information criterion value. Mussels exhibited different growth rates (K), including 0.015, 0.026, 0.110 and 0.050 (mo(-1)), corresponding to the duration of laboratory culture (ages 2, 3 and 4 yr old) and a growth cessation (GC) for 5.98 mo, respectively. The other parameters of asymptotic length (L-infinity) and age at zero length (t(0)) were 51.36 mm and -0.648 mo. The flexible structure of Bayesian hierarchical models allowed us to examine growth characteristics of E. brevidens in a changing environment to better understand the details of its growth and lifespan, thus providing useful data for conservation management.

4479: *-.034*

Geographically separated populations may show high levels of genetic differentiation, depending on the levels of current and historical isolation. In the ancient landscape of the Pilbara region, there are few plant species with restricted distributions, and one such species, Aluta quadrata Rye & Trudgen, is restricted to three separate locations on the southern edge of the Hamersley Range. We investigated genetic diversity and differentiation among geographically isolated locations of A. quadrata, using 10 microsatellite loci to assess contemporary genetic structure, and sequences of seven chloroplast gene regions to infer historical isolation. Nuclear genetic diversity was moderate, with moderate to high genetic differentiation among the three locations, and low differentiation among populations within locations. In contrast, there was no detected variation in the chloroplast genome. The high genetic differentiation is consistent with limited contemporary connectivity among the geographically separated locations, although lack of chloroplast haplotype variation indicates that limited connectivity has occurred more recently and is not due to historical isolation. The level of differentiation suggests use of local seed sources for augmentation or establishment of populations within gene flow distance of existing populations, whereas an experimental translocation established on more distant sites could use mixed seed sources to maximise genetic diversity.

4480: *+.017*

The critically endangered Wollemia nobilis W.G. Jones, K.D. Hill & J.M. Allen is endemic to Wollemi National Park north of Sydney (Australia). All known wild individuals are restricted to four sites in a single canyon system. W. nobilis can reproduce sexually but at all sites individual clumps can be multi-stemmed from a common base. In the first genetic study of this species, no genetic variation was found across multiple genetic marker types representing hundreds of nuclear

loci, indicating this species is characterised by very low genetic variation. In this study we searched for variation across the chloroplast using shotgun sequencing, bioinformatic extraction of chloroplast DNA and variant detection. Six chloroplast single nucleotide polymorphisms were detected, producing three chlorotypes. Chlorotype 1 is found in every individual surveyed at Sites 1 and 3, and in individuals from Sites 2 and 4. Chlorotype 2-the most distinct chlorotype-was found in two individuals from Site 4. Chlorotype 3 consists of a single difference from Chlorotype 1 and may represent a somaclonal mutant. These findings will guide management and translocation of this critically endangered species. This study provides a practical template that is highly informative and easily applicable to other taxa in similar circumstances.

4481: ***+.160***

The Hermann's tortoise is a strictly European species found mostly in areas with Mediterranean and sub-Mediterranean climes and, especially the subspecies T. hermanni hermanni, has to deal with various threats which reduced its geographic distribution to only a few isolated populations. Thus, with the objective of recovering natural populations of the species, numerous reintroduction projects are being carried out throughout its historical and prehistorical geographic distribution. The aim of our study is to evaluate the success of the T. hermanni reintroduction project carried out in the Albufera de Valencia Natural Park since 2011, through the analysis of movements, home range sizes and habitat use in a new environment. The recorded activity pattern presents two peaks, one in spring and another in August, and the hibernation period (from December to February) is the same as observed in other populations. We did not find significant differences in home range sizes between sexes, but a tight relation between home range sizes and body sizes with larger individuals having larger home ranges. This relation was not observed for core areas (Kernel at 50%). Considering the habitat use, we did not find differences between the groups of individuals we defined, but different types of habitats were used differently. "Scrub" was the most frequented (similar to 50%), followed by "dunes" (similar to 20%), "mallaes" (similar to 20%) and "forest" (<20%). Our results can be used to improve the success of future reintroduction projects by directing ideal habitat, timing and carapace size of released Hermann's tortoises.

4482: ***+.113***

During the last decade, the population of wild white rhinoceroses has been in steady decline, mainly because of increased poaching incidents and habitat loss. Therefore, more data are necessary on reproduction of this endangered species in order to improve captive breeding, which is at present not very successful. Currently, ~20,000 Southern white rhinoceroses are remaining of which the majority are privately owned. The aim of this study was to create reference values of several reproductive parameters for future white rhinoceros breeding. In previous studies, only low numbers of animals have been observed, often in captive settings. In this study performed between 2008 and 2016, reproductive performance was analysed in 1300 animals kept in a geographically identical, confined free-roaming environment. Analyses were performed in R (R Development Core Team, 2008) using the lme4 and fixed package to model the number of animals born (family=Poisson) and sex ratio (family=binomial). Females had a median age of 83.2 months at first calving (interquartile range: 72.9-110.7) and intercalving intervals of 29.2 (interquartile range: 24.6-34.8) months. Fertility records were excellent with 38% adult females calving per year when compared to previous research, in which first reproduction occurred between 78 and 138 months of age with an intercalving period of 3 years average. A clear seasonal calving pattern was seen with a significant increase of calvings during December-April when compared to April-December. In contrast to the Trivers-Willard hypothesis, our results did not show any significant skewed progeny sex ratios. Weather observations showed no significant influence of rain or season on sex

ratios of the calves. Furthermore, translocations of animals did not seem to interfere with reproductive success when looking at intercalving periods or age at first calving. In the free roaming environment of over 10,000ha, this captive population showed an average annual population growth (%) of 18±0.07 (minimum 5 to maximum 26). As such, comparable breeding management systems can increase population numbers and contribute to increase dwindling population numbers of the wild white rhinoceros. This is the first study to describe reproductive performances in the white rhinoceros at such large scale, indicating that confined free-roaming populations can be used for captive breeding of white rhinoceros to contribute to white rhinoceros conservation.

4483: *+.395*

Knowing the extent and structure of genetic variation in an endangered species is essential for establishing efficient conservation practices. However, the proper use of this information requires understanding the role of habitat-specific selection in genetic structuring. We present a study of population differentiation in an endangered species that utilizes guidelines of recently a proposed quasi in situ conservation approach, i.e. taking into account the scale and spatial pattern of local adaptation since if local adaptation is important, the introduced genotypes must be matched to the local biotic/abiotic conditions. Following this approach, we examined the extent and structure of genetic (AFLP) and phenotypic variation and tested for adaptive significance of this variation in critically endangered Iris atrofusca growing in Israel and Jordan. From these results we propose a sampling design that would (i) preserve species adaptive potential and (ii) insure environmental match of the plant material for relocation, reintroduction or enhancement.

4484: *+.269*

To date, there have been only limited attempts to conceptually unify ex situ and in situ approaches as parts of an integrated conservation methodology. This paper is an attempt of such conceptual integration of existing approaches for the efficient conservation of rare and endangered plant species. My integration of available plant conservation biology literature is based on the idea that ecologically significant species genetic variation is of primary importance for plant conservation. This idea is used for providing guidelines about inventory of existing populations, sampling and propagating sampled material, and use of this material in species recovery actions.

4485: *+.268*

The conservation of plant species in situ is a complex and multifaceted procedure which involves both the maintenance and management of protected areas and actions targeted at the species and population level. Most effort has been aimed so far at the occurrence and persistence of species in protected areas as a measure of conservation. However, species-level actions such as conservation or recovery plans have been undertaken for only a small percentage of threatened plant species, mostly by a few countries. The reasons for this are complex and involve scientific, social and political considerations. The planning of targets for biodiversity conservation in situ by the Convention on Biological Diversity suffers from a failure to coordinate area-based and species-based actions leading to overlap and confusion. A set of recommendations is given to help remedy the neglect of targeted species conservation.

4486: *+.030*

Despite the fundamental importance of the family Diplomystidae for understanding catfish

evolution, its species are poorly known and most of them endangered. Diplomystes camposensis, restricted to a single river basin in southern Chile, is perhaps the most vulnerable species due to its small geographic range and imminent habitat alterations by dam constructions. Using mitochondrial DNA sequences, we describe the genetic diversity across its entire distribution in the Valdivia basin and test hypotheses related to the impact of glacial cycles on the genetic diversity and structure. We found that Diplomystes camposensis has low genetic diversity and structure across the entire Valdivia basin along with a pattern of decreasing nucleotide and haplotype diversity from West to East. Demographic analyses showed evidence of population expansion in agreement with the glaciated history of the basin. Analyses of population structure showed no evidence of population subdivision. However, coalescent analyses indicated that very recent subdivision (in the last 50 years) cannot be ruled out. Low genetic diversity and genetic structure across the entire basin suggest that the species might be highly vulnerable to habitat fragmentation. Thus, the imminent construction of hydropower dams represents a serious threat to its conservation. Our results suggest that the low genetic diversity can be the product of the glaciated history of the basin, although the influence of species-specific biological traits may also add to this condition. Despite the overall low genetic diversity, higher diversity was found in the central portion of the basin suggesting high priority of conservation for this area as it might be used as a source population in case translocations are required among potential management plans.

4487: *+.178*

Interest in bison (Bison bison, B. bonasus) conservation and restoration continues to grow globally. In Canada, plains bison (B. b. bison) are threatened, occupying less than 0.5% of their former range. The largest threat to their recovery is the lack of habitat in which they are considered compatible with current land uses. Fences and direct management make range expansion by most bison impossible. Reintroduction of bison into previously occupied areas that remain suitable, therefore, is critical for bison recovery in North America. Banff National Park is recognized as historical range of plains bison and has been identified as a potential site for reintroduction of a wild population. To evaluate habitat quality and assess if there is sufficient habitat for a breeding population, we developed a Habitat Suitability Index (HSI) model for the proposed reintroduction and surrounding areas in Banff National Park (Banff). We then synthesize previous studies on habitat relationships, forage availability, bison energetics and snowfall scenarios to estimate nutritional carrying capacity. Considering constraints on nutritional carrying capacity, the most realistic scenario that we evaluated resulted in an estimated maximum bison density of 0.48 bison/km2. This corresponds to sufficient habitat to support at least 600 to 1000 plains bison, which could be one of the largest 10 plains bison populations in North America. Within Banff, there is spatial variation in predicted bison habitat suitability and population size that suggests one potential reintroduction site as the most likely to be successful from a habitat perspective. The successful reintroduction of bison into Banff would represent a significant global step towards conserving this iconic species, and our approach provides a useful template for evaluating potential habitat for other endangered species reintroductions into their former range.
Copyright: CC BY

4488: *+.260*

There is growing concern about mitigation-driven translocations that move animals from anthropogenic threats at donor sites because of their failure rate and lack of application of scientific principles and best practice. We reviewed all known lizard translocations in New Zealand between 1988 and 2013 and identified 85 translocations of 30 lizard taxa to 46 release sites. Most translocations (62%) were motivated by conservation goals for the species or the

release site, and one-third were mitigation-driven translocations, typically motivated by habitat loss due to development. Mitigation-driven translocations began in 2003, and since that time have equalled the number of conservation-motivated translocations. Conservation-motivated translocations usually released lizards on islands without mammalian predators, whereas mitigation-driven translocations usually relocated lizards to mainland sites with introduced predators. Long-term monitoring has been sparse and often rudimentary. Eight lizard translocations have recorded population growth, including one mitigation-driven translocation that was into a fenced reserve. Research on commonly used management techniques to mitigate human-related impacts is recommended to establish whether these techniques benefit lizards in the long term. Copyright: CC BY-NC-ND

4489: *+.073*

This study investigated the ecological and social potential for wolf (Canis spp.) recovery in Nova Scotia, Canada. Reintroduction potential was considered through a GIS-based analysis of land cover, human population density, land ownership, prey density, and road density. Two disconnected areas of adequate habitat for wolves were identified. Qualitative interviews were conducted with seven identified groups on public attitudes towards the wolf and its potential recovery in the province. Opinions ranged from 'love' to a strong dislike of wolves, and many interviewees associated wolves with fear and expressed concern that they would come into contact with wolves on or near their properties. It would likely not be advisable to introduce an active wolf reintroduction program in NS at this time, due to the absence of effective habitat connectivity between the two identified areas of suitable habitat, and the public unease about wolf proximity. However, a proactive public education initiative is recommended in case of future reintroductions or natural immigrations of wolves and other top carnivores from nearby populations.

4490: *+.199*

In 2015 the Federal Government Funded Institution "Orenburg Reserves" started the Programme of Establishing of a Semi-Free Population of the Przewalski Horse at the Orenburg Reserve on the territory of the steppe area "Pre-Ural Steppe". In 2014-2015 a pilot research was carried out to select the location for construction of the Centre of the re-introduction of the Przewalski horse. To evaluate the fodder stocks in the selected area we developed criteria for evaluating the vegetation condition under the influence of grazing. The results have shown that within the boundaries of the "Pre-Ural Steppe" plot it is most rational to place the Centre of the re-introduction of the Przewalski horse at the natural landmark Kursay. In 2015, the fodder stocks here ranged from 14 dt/ha (decitonne per hectare) in April to 30 dt/ha in September. Moreover, the grass cover vegetation here mostly consisted of species, well-grazed by Przewalski horse, particularly grasses, a significant number of legumes and well-grazed forbs. In the future, this area requires constant monitoring of the vegetation condition under the influence of the Przewalski horse grazing, with the following evaluation criteria: 1) the overall productivity of the plant mass; 2) the combination of well-grazed and ungrazed plant species in dynamics.

4491: *+.204*

File List HMS_Migration_Data. txt -- Comma-delimited ASCII text file of the complete migration records for the period 1934-2010. The file consists of 42000 entries with each row comprising 44 cells. No compression was used. Description In most northern temperate regions diurnal birds of prey, or raptors, migrate seasonally between their breeding and wintering grounds. Although their populations can be logistically difficult to survey and monitor because they are largely secretive

and wide-ranging, most raptors are obligate or facultative soaring migrants that congregate along major thermal and orographic updraft corridors during their seasonal movements. Hawk Mountain Sanctuary (41° N, 75° W), which straddles the Kittatinny Ridge, the southernmost ridge in the Appalachian Mountains in eastern Pennsylvania, witnesses large numbers of migrating raptors during autumn migration. The Sanctuarys long-term migration-count database is the oldest detailed archive on the timing and magnitude of migratory raptors in the world. Records comprise daily (1934-1965) or hourly (1966-present) counts of 18 North American species of raptors migrating past the Sanctuary, as well as detailed weather data recorded from the lookout during autumn migration (15 August to 15 December). The long-term data set has been valuable in understanding raptor migration and population trends. Because top predators such as raptors are sensitive bio-indicators of ecosystem changes, variations in the numbers of individual species also may reflect changes in the health of the environment. Thus, monitoring population trends of raptors can provide crucial information on environmental changes and threats facing wildlife species. Indeed, the Sanctuarys database has been key in identifying the now well-known example of organochlorine contamination of top predators and has helped the conservation, management, and reintroduction of many species such as Osprey (Pandion haliaetus), Bald Eagle (Haliaeetus leucocephalus), and Peregrine Falcon (Falco peregrinus). This database is of prime significance to conservation ecology and long-term fluctuations in numbers of migratory birds. Key words:bird counts; bird migration; Hawk Mountain Sanctuary; long-term studies; Pennsylvania (USA); population census; raptor. Copyright: CC-0

4492: ***+.215***

This repository contains supplementary information regarding the paper "Walking on their own legs: unassisted population growth of agoutis reintroduced to restore seed dispersal in an Atlantic Forest reserve", authored by Caio Fittipaldi Kenup, Raissa Sepulvida, Catharina Kreischer and Fernando Antonio dos Santos Fernandez Contained here are: R Scripts and Functions used to carry out the analyses (.R) Full carried-out analyses (.RData) Raw data files used as input (.csv). Supplementary Tables as Published (.xlsx) Abstract from the paper: Reintroduction of locally extirpated species is an increasingly popular conservation tool. However, few initiatives focus on the restoration of ecological processes. In addition, many reintroductions fail to conduct post-release monitoring, hampering both assessment of their success and implementation of adaptive management actions. In 2009 a reintroduction effort was initiated to reestablish a population of the red-rumped agouti Dasyprocta leporina, a scatter-hoarding rodent known to be an important disperser of large seeds, with the aim of restoring ecological processes at Tijuca National Park, south-east Brazil. To assess whether this reintroduced population established successfully we monitored it using mark-resighting from November 2013 to March 2015. Population size and survival were estimated using a robust design Poisson-log normal mixed-effects mark-resight model. By March 2015 the number of wild-born individuals fluctuated around 30 and overall growth of the population was positive. As the reintroduced population is capable of unassisted growth, we conclude that the reintroduction has been successful in the medium term. We recommend the cessation of releases, with efforts redirected to continued monitoring, investigation and management of possible threats to the species persistence, and to quantification of the reestablishment of ecological processes. Reintroduction of D. leporina populations can be a cost-effective tool to restore ecological processes, especially seed dispersal, in Neotropical forests. Disclaimer: For data protection reasons, the photographic records . csv sheet has been truncated to two stations and 12 days of sampling, providing only a toy example. Nevertheless, the summarised data and analysis present on the . RData files represent the full set of records. MIT License Copyright (c) [2013] [Caio Fittipaldi Kenup] Permission is hereby granted, free of charge, to any person obtaining a copy of this software and associated documentation files (the "Software"), to

deal in the Software without restriction, including without limitation the rights to use, copy, modify, merge, publish, distribute, sublicense, and/or sell copies of the Software, and to permit persons to whom the Software is furnished to do so, subject to the following conditions: The above copyright notice and this permission notice shall be included in all copies or substantial portions of the Software. THE SOFTWARE IS PROVIDED "AS IS", WITHOUT WARRANTY OF ANY KIND, EXPRESS OR IMPLIED, INCLUDING BUT NOT LIMITED TO THE WARRANTIES OF MERCHANTABILITY, FITNESS FOR A PARTICULAR PURPOSE AND NONINFRINGEMENT. IN NO EVENT SHALL THE AUTHORS OR COPYRIGHT HOLDERS BE LIABLE FOR ANY CLAIM, DAMAGES OR OTHER LIABILITY, WHETHER IN AN ACTION OF CONTRACT, TORT OR OTHERWISE, ARISING FROM, OUT OF OR IN CONNECTION WITH THE SOFTWARE OR THE USE OR OTHER DEALINGS IN THE SOFTWARE.

4493: *+.166*

aTswalu previously had Kruger origin lions introduced in 1996. They completely replaced them with Kgalagadi origin lions in 2001.*From original source populations. From Kruger NP directly, or from neighbouring reserves that are now open with Kruger NP (but were not at the time of translocation)-exact details are not clear for most of these reserves. All other introductions were via another reserve. Reintroduction history including genetic provenance and sampling. Copyright: CC BY

4494: *+.070*

Relatedness results from Coancestry between two female lions (F1 and F2) and two male lions (M1 and M2). The first number is the Wang statistic, the second is the Queller and Goodnight statistic. Relatedness testing for translocation decisions. Copyright: CC BY

4495: *+.178*

Background Re-introductions are increasingly being used in conservation biology as a valuable tool in species recovery programmes. This technique was used to establish a population of whitetailed eagles (Haliaeetus albicilla) in Scotland, where the species went extinct in 1917. Three release phases have taken place, of which the first two (1975-1985 and 1993-1998) were on the west coast and the third (2007-2012) on the east coast of Scotland. All three phases have used birds sourced from Norway. In 2014, there were 98 territorial pairs of white-tailed eagles in Scotland, of which 90 were confirmed to have bred. For this report, a conservative approach was used, and only the 90 sites with confirmed nests were considered territories. Overall, the re-introduction programme has been deemed a conservation success; however, the species may potentially come into conflict with land-use interests, including sheep farming, forestry and renewable energy. Therefore it is important to get a better understanding of how fast the population of white-tailed eagles will increase numerically and where the population will expand into. This is important for both mediating conflicts and for predicting how white-tailed eagles might be affected by other land-uses, such as forestry and renewable energy production (e.g. wind farms). There are also concerns that white-tailed eagles might be victims of persecution, but the impact of such illegal activity on the whitetailed eagle population is currently unknown. The best available predictions of future population increase used data on breeding success and age-specific survival up to and including 2007. This report presents predicted population growth using estimates of breeding success and survival up to 2014. Additional mortality, potentially caused by illegal killing or collisions with wind turbines, was also incorporated in some modelled scenarios.

Finally, models of predicted geographical range expansion in the next 25 years (i.e. up to and including the year 2040) are presented, based on habitat associations and nearest-neighbour distances. Main findings The number of breeding white-tailed eagle pairs has continued to grow almost exponentially, and wild-bred eagles now greatly outnumber released eagles. There has been a continued increase in both the proportion of white-tailed eagle nests fledging young (i.e. "breeding success") and the number of chicks fledged per breeding attempt (i.e. "productivity") since the first breeding attempt in 1983. This is probably because the proportion of birds in the population with extensive breeding experience has increased, as breeding performance improves with experience. However, since 2006, the number of chicks fledged per breeding attempt appears to have remained relatively constant at an average of 0.67 chicks fledged per territorial pair. When the updated estimates of breeding success and age-specific survival were used in density-independent predictive models, the results suggest that the white-tailed eagle population could continue to grow to over 200 pairs by 2025 and almost 900 pairs by 2040, but obviously the long-term predictions are far less certain than the short-term. When modelling the impact of additional mortality, potentially caused by illegal killing and collisions with wind farms, population growth was reduced, but not to the extent of causing a population decline. Overall, the results presented here suggest a continued exponential population growth of white-tailed eagles in Scotland in the short-term. However, density-dependence in demographic rates would need to be considered in modelling population growth over the longer term. The associations between white-tailed eagle breeding sites and habitat, landscape and topographical variables were explored statistically by comparing the locations of real nest sites and random points in the landscape. The final model suggested that white-tailed eagles were positively associated with length of coastline, area of inland water and the area of forest (all within 1 km from the nest) and also with flat topography (within 3 km from the nest). This model explained 25% of the variation in occupancy status. Information on how far away from existing pairs new white-tailed eagle pairs settle was compiled, and it was found that most newly established territories were located between 6 and 10 km from the nearest other active white-tailed eagle nest. By integrating eaglehabitat associations and the nearest-neighbour distances for newly established pairs, future range expansion was modelled spatially. The results suggested that range expansion is most likely to occur mainly along the west coast of Scotland, the Great Glen and in central and eastern Scotland where birds from the east coast release start to breed. The geographical range expansion models presented in this report can inform stakeholders of likely white-tailed eagle range expansion in the next 10-25 years, but the exact settlement order is difficult to predict. In addition, occasional long-distance dispersal events could lead to the establishment of new pairs outside the range predicted here. In the long-term, these pairs might result in new population centres, which could expand the geographical range of white-tailed eagles even further.

4496: ***+.338***

New Zealand's diverse lizard fauna is under threat, particularly from predation by invasive mammals and ongoing habitat destruction. Advances in genetic techniques have greatly enhanced our understanding of the lizard fauna. Previously sparse island populations of species have recovered strongly following eradication of pest mammals and translocations of lizards to newly mammal-free islands have boosted security for some species. Recent success with intensive predator control and predator-proof fencing on the mainland shows promise for lizard populations in easily accessible sites. However, the plight of many species whose range does not include pest-free islands remains precarious and taxonomic uncertainty means we do not yet understand the full suite of species in need of protection. Further advances in predator control techniques and increased funding for lizard conservation management are needed to prevent species extinctions.

4497: ***+.111***

Trophic rewilding is an ecological restoration strategy that uses species introductions to restore top-down trophic interactions and associated trophic cascades to promote self-regulating biodiverse ecosystems. Given the importance of large animals in trophic cascades and their widespread losses and resulting trophic downgrading, it often focuses on restoring functional megafaunas. Trophic rewilding is increasingly being implemented for conservation, but remains controversial. Here, we provide a synthesis of its current scientific basis, highlighting trophic cascades as the key conceptual framework, discussing the main lessons learned from ongoing rewilding projects, systematically reviewing the current literature, and highlighting unintentional rewilding and spontaneous wildlife comebacks as underused sources of information. Together, these lines of evidence show that trophic cascades may be restored via species reintroductions and ecological replacements. It is clear, however, that megafauna effects may be affected by poorly understood trophic complexity effects and interactions with landscape settings, human activities, and other factors. Unfortunately, empirical research on trophic rewilding is still rare, fragmented, and geographically biased, with the literature dominated by essays and opinion pieces. Wehighlight the need for applied programs to include hypothesis testing and science-based monitoring, and outline priorities for future research, notably assessing the role of trophic complexity, interplay with landscape settings, land use, and climate change, as well as developing the global scope for rewilding and tools to optimize benefits and reduce human-wildlife conflicts. Finally, we recommend developing a decision framework for species selection, building on functional and phylogenetic information and with attention to the potential contribution from synthetic biology.

4498: ***+.003***

The decline of the patrimonial rheophilic nase, Chondrostoma nasus (Linnaeus, 1758) populations was mainly caused by construction of dams and hydroelectric power-plants, together with the straightening and artificialization of the river banks and water pollution. In this study, we tested the hypothesis whether the translocation of few adult nase individuals from a river stretch to another upstream may be a credible way to accelerate the recolonization process of the species in the Ambleve River (Southern Belgium). In February and March 2011, just before their spawning period, eight adult nases (462-509 mm; 1546-2002 g; presumed males and females) were captured in the lower part of the River Ambleve. Fin clip samples were stored in alcohol for further genetic analysis. They were equipped with a 14 g radio transmitter and translocated upstream in a 18 km river stretch, where the species had disappeared since decades due to river anthropization. They were manually located two to five times/week using mobile receivers until maximum June 2012 (n = 977 locations). River temperature and flow were hourly recorded during the entire tracking period. The tagged nase individuals displayed various mobility patterns, exploited different areas of the river stretch, occupied longitudinal home ranges from 3.4 to 36.1 km (one individual finally left the new river stretch) and travelled total distances from 12.2 to 186.6 km. The tagged individuals were most of the times apart from one to another, but most individuals grouped together in potential spawning areas in late March-early April 2011, suggesting an attempt to reproduce. In September 2011, electric fishing in two potential detected spawning sites allowed to capture 16 juvenile (0(+)) nases, demonstrating the existence of spawning activity in the newly occupied river stretch. Individual genetic characterization was performed in 2014 in order to reveal a possible direct lineage between juveniles and adults. Allelic distribution of 22 microsatellite markers unambiguously identified the 16 juveniles as full-sib progeny descending from two of the translocated adults. This demonstrated that the adult nases succeeded to find spawning areas and that progeny found raised-up from the translocated individuals.

4499: *+.130*

Piroplasms, especially those in the genera Babesia and Theileria, have been found to naturally infect rhinoceros. Due to natural or human-induced stress factors such as capture and translocations, animals often develop fatal clinical piroplasmosis, which causes death if not treated. This study examines the genetic diversity and occurrence of novel Theileria species infecting both black and white rhinoceros in Kenya. Samples collected opportunistically during routine translocations and clinical interventions from 15 rhinoceros were analysed by polymerase chain reaction (PCR) using a nested amplification of the small subunit ribosomal RNA (18S rRNA) gene fragments of Babesia and Theileria. Our study revealed for the first time in Kenya the presence of Theileria bicornis in white (Ceratotherium simum simum) and black (Diceros bicornis michaeli) rhinoceros and the existence of three new haplotypes: haplotypes H1 and H3 were present in white rhinoceros, while H2 was present in black rhinoceros. No specific haplotype was correlated to any specific geographical location. The Bayesian inference 50% consensus phylogram recovered the three haplotypes monophyleticly, and Theileria bicornis had very high support (BPP: 0.98). Furthermore, the genetic p-uncorrected distances and substitutions between T. bicornis and the three haplotypes were the same in all three haplotypes, indicating a very close genetic affinity. This is the first report of the occurrence of Theileria species in white and black rhinoceros from Kenya. The three new haplotypes reported here for the first time have important ecological and conservational implications, especially for population management and translocation programs and as a means of avoiding the transport of infected animals into non-affected areas.

4500: *+.025*

In Bulgaria, chamois (Rupicapra rupicapra balcanica) are protected by law and it has been recommended to assess their genetic diversity, level of inbreeding, and extent of introgression by Alpine chamois (R. r. rupicapra) that were released in the last century in the Rhodope Mountains. Chamois (n = 81) collected between 2009 and 2013 at the "Izvora State Hunting Reserve" (ISHR, western Rhodopes, a major source for chamois translocations in Bulgaria; census size: 200-250) were screened for allelic variability at 12 microsatellite loci and compared to chamois (R. r. rupicapra, n = 135) from six populations in the Austrian Alps. Indices of genetic variability were significantly lower for the ISHR population than for the Austrian populations. A significant bottleneck signal was observed for the ISHR population, but no distinct inbreeding signal due to non-random mating. F-statistics, absolute genetic distances, Bayesian STRUCTURE and assignment analyses, and a factorial correspondence analysis concordantly indicated distinct differentiation between the ISHR and Austrian chamois. STRUCTURE identified some few ISHR chamois as possibly introgressed by Alpine chamois. ONeSAMP revealed effective population size estimates of Ne a parts per thousand 50 for the ISHR population. A reduction of shooting quotas, a reinforcement of poaching control, and exchanging chamois with other Bulgarian populations should increase its Ne to prevent further loss of genetic variability. Screening for introgression should be extended to other Bulgarian populations, particularly to areas where Alpine chamois were released in the past; and it should accompany all translocations, to prevent possible dissemination of introgressed chamois.

4501: *+.177*

The protection of freshwater biodiversity has become a priority task for conservation practices, as freshwater ecosystems host high levels of cryptic diversity, while also record similarly high rates of extinction. The Italian white-clawed crayfish Austropotamobius italicus is an endemic

freshwater crustacean, threatened by several anthropogenic impacts such as habitat fragmentation, pollution, invasion of exotics, and climate change. Previous phylogenetic studies conducted in Italy pointed out a complex phylogeographic framework for the species, with four different subspecies currently recognized. Conservation efforts, particularly when involving restocking and reintroduction, require a detailed knowledge of their population genetics. In this study we describe the genetic structure of A. italicus populations in northern Italy (Lombardy Alpine foothills and northern Apennines) by using the informative mitochondrial marker cytochrome c oxidase subunit I, in order to assess their current evolutionary diversity and past phylogeographic history from a conservation perspective. Our results contribute to the mapping of the contact area among A. i. carsicus and A. i. carinthiacus in the Orobie Larian Prealps. More interestingly, we highlight the existence of two deeply differentiated evolutionary lineages within A. i. carsicus, showing alternative phylogeographic patterns and past demographic trends. We propose to consider these two clades as distinct molecular operational taxonomic units for the conservation of this endangered crayfish.

4502: *-.069*

Many populations are threatened or endangered because of excessive predation resulting from individuals' inability to recognize, avoid, or escape alien predators. Such prey naivete is often attributed to the absence of prior experience and co-evolution between native prey and introduced predators. Many reintroduction programs focus on reducing predation rate by excluding introduced predators, a focus which ignores, and indeed exacerbates, the problem of prey naivete. We argue for a new paradigm in reintroduction biology that expands the focus from predator control to kick-starting learning and evolutionary processes between alien predators and reintroduced prey. By exposing reintroduced prey to carefully controlled levels of alien predators, insitu predation could enhance reintroduction success by facilitating acquisition of learned antipredator responses and through natural selection for appropriate antipredator traits. This insitu predator exposure should be viewed as a long-term process but is likely to be the most efficient and expedient way to improve prey responses and assist in broadscale recovery of threatened species.

4503: *+.094*

1. Modiolus modiolus reefs have disappeared from most of their historical distribution range in Strangford Lough (Northern Ireland) while remnant populations are highly fragmented. 2. This study investigated morphological differences in Strangford Lough and Irish Sea M. modiolus with an aim to provide sound scientific advice to managers involved in shellfish reef restoration programmes including translocation. 3. By characterizing shape ecophenotypes within target and source areas it is possible to select morphologically similar source stocks to increase survival and self sustainability of the restored population. 4. Linear and geometric shell shape analyses identified significant differences among M. modiolus populations from the Irish Sea and Strangford Lough. Three different phenotypes within the Strangford Lough metapopulation were identified. It is hypothesized that substrate type, tidal current strength and population thinning caused by mobile fishing gear influenced such morphological differences. 5. This investigation confirmed, for the first time, phenotypic variability in M. modiolus which could affect future supplementation programmes aiming to restore degraded populations. Copyright (c) 2014 John Wiley & Sons, Ltd.

4504: *+.157*

Establishing translocated populations is a common process to preserve and maintain genetic diversity of threatened species. In 2001, three translocated populations of noble crayfish (Astacus astacus) were established in the Czech Republic, founded by either adult or juvenile individuals from three particular source populations. We assessed genetic diversity at seven microsatellite loci after one decade (assumed three generations) from establishment. Although the translocated populations exhibited a slight but non-significant reduction in genetic diversity (A (R) = 2.2-5.0; H (O) = 0.11-0.31), the most striking result was generally very low genetic diversity in source populations (A (R) = 3.0-5.3; H (O) = 0.15-0.38). Similarly, a high degree of inbreeding (F (IS) = 0.36-0.60) demonstrates the nature of source populations, already affected by isolation and small size. In spite of that, based on the results of this study, the establishment of new translocated noble crayfish populations was successful, since there is no significant decline in genetic variability and all populations are still viable. Although source populations did not exhibit high genetic diversity, their distinctiveness makes them possible to use for conservation purposes. Continued monitoring is necessary to track the long-term progress of the translocation program, including other parameters describing the state of the population, such as the occurrence and frequency of diseases or morphological changes.

4505: ***+.080***

Reintroduction is often the only remaining option for recovery of extirpated species. According to the U.S. Endangered Species Act, species should be reintroduced to suitable habitats within their probable historical range. However, accurately defining historical range often proves difficult, especially for taxa with limited historical information, and may represent a significant impediment for successful recovery. Here, we combine ecological modeling methods with morphometric and phylogenetic data from museum specimens to define a more biologically realistic historical distribution. We apply this approach to the Mexican wolf (Canis lupus baileyi), the most endangered and genetically distinct wolf subspecies in the New World. Our model substantially increases the potential geographic range of the Mexican wolf to include areas in southern California and Baja California, areas not previously recognized as part of the historical range. Motivated by this prediction, we reanalyzed morphometric data and genetically typed the only historical specimen known from southern California, which was previously assigned to another wolf subspecies. We found that the specimen was in fact of pure Mexican wolf ancestry and fell within our predicted range for this subspecies. Our findings provide an impetus for reconsidering reintroduction sites for the Mexican wolf and highlight how critical taxonomic assignment can be to reintroduction programs and species recovery. Re-analysis of potential range in other extirpated species that have ranges defined by antiquated taxonomic approaches used on a limited number of specimens could enhance the success of future reintroduction programs and restore historical processes such as admixture that can preserve the adaptive legacy of endangered species. (C) 2015 Elsevier Ltd. All rights reserved.

4506: ***+.065***

Red foxes were absent or rare in the southeastern United States until the late 1800s. Their origins potentially include natural population increase/expansion, translocations from Europe, and, eventually, 20th century fur farming. Previous studies have found no European haplotypes in North America, but few samples were sourced from the Atlantic coastal plain, closer to the source of putative introductions. Through analysis of mitochondrial DNA in 584 red foxes from this region, we identified indigenous haplotypes in a parts per thousand yen 35% of foxes, 1 of 2 European haplotypes in 17% of foxes and fur farm haplotypes in a parts per thousand yen 13% of foxes; another 35% of foxes had haplotypes potentially indigenous or native. In contrast, only 3 of

135 (2%) male foxes carried a single European Y chromosome haplotype. Most European and fur farm haplotypes were found near the densely human-populated coastal plain and Hudson River lowlands; most red foxes of the Appalachians and Piedmont had native eastern haplotypes. Our findings suggest that the more remote, upland populations primarily reflect indigenous red fox matrilines, whereas urban-associated populations in and around the mid-Atlantic coastal plain and Hudson lowlands reflect an admixture of native and nonnative maternal sources. Autosomal markers are needed to further elucidate the extent of European and fur farm introgression in the Appalachians and further west.

4507: ***-.039***

The White-headed Vulture Trigonoceps occipitalis (WhV) is uncommon and largely restricted to protected areas across its range in sub-Saharan Africa. We used the World Database on Protected Areas to identify protected areas (PAs) likely to contain White-headed Vultures. Vulture occurrence on road transects in Southern, East, and West Africa was adjusted to nests per km(2) using data from areas with known numbers of nests and corresponding road transect data. Nest density was used to calculate the number of WhV nests within identified PAs and from there extrapolated to estimate the global population. Across a fragmented range, 400 PAs are estimated to contain 1893 WhV nests. Eastern Africa is estimated to contain 721 nests, Central Africa 548 nests, Southern Africa 468 nests, and West Africa 156 nests. Including immature and nonbreeding birds, and accounting for data deficient PAs, the estimated global population is 5475 - 5493 birds. The identified distribution highlights are alarming: over 78% (n=313) of identified PAs contain fewer than five nests. A further 17% (n=68) of PAs contain 5 - 20 nests and 4% (n=14) of identified PAs are estimated to contain >20 nests. Just 1% (n=5) of PAs are estimated to contain >40 nests; none is located in West Africa. Whilst ranging behavior of WhVs is currently unknown, 35% of PAs large enough to hold >20 nests are isolated by more than 100km from other PAs. Spatially discrete and unpredictable mortality events such as poisoning pose major threats to small localized vulture populations and will accelerate ongoing local extinctions. Apart from reducing the threat of poisoning events, conservation actions promoting linkages between protected areas should be pursued. Identifying potential areas for assisted re-establishment via translocation offers the potential to expand the range of this species and alleviate risk.

4508: ***+.145***

Conspecific attraction plays an important role in habitat selection of several taxa and can affect and determine distribution patterns of populations. The behaviour is largely studied and widespread among birds, but in amphibians, its occurrence seems limited to breeding habitats of adults and gregarious tadpoles. The Australian green and golden bell frogs (Litoria aurea) have suffered considerable shrinking of their original distribution in south-eastern Australia since the 1970s. Currently, with only about 40 populations remaining, the species is considered nationally threatened. In natural conditions, these frogs are aggregated in the landscape and do not seem to occupy all suitable ponds within the occurrence area. To date, studies focusing on the frogs' habitat have failed in finding a general habitat feature that explains current or past occupancy. This led us to the hypothesis that social cues may play a key role in habitat selection in this species. Using two choice experiments, we tested the preference of juvenile green and golden bell frogs for habitats containing cues of conspecifics of similar size versus habitats without conspecific cues. Tested frogs did not show a preference for habitats containing only scent from conspecifics but did prefer habitats where conspecifics were present. Our results show that conspecific attraction is a determining factor in juvenile green and golden bell frog habitat selection. To our knowledge, this is the first time the behaviour is shown to occur in juvenile frogs in the habitat selection context.

From a conservation management point of view, the behaviour may help to explain the failure of reintroductions to areas where the frogs have been extinct, and the non-occupation of suitable created habitats in areas where they still inhabit and develop appropriated management strategies.

4509: ***+.371***

Aim Maintaining genetic diversity and evolutionary processes are important goals in plant conservation. Genetic studies are increasingly undertaken but results from such studies are still rarely implemented as management actions in the field. We address this 'research-implementation gap' by developing a plain-language genetic assessment approach for population-level conservation prioritization based on measurement of key genetic parameters. Our aim was to improve understanding between conservation researchers and practitioners, enabling practitioners to incorporate genetic information into conservation actions and conservation genetic researchers to address research explicitly resulting in conservation action. Location Applicable globally. Methods We derived a decision-making framework that identifies appropriate management strategies for threatened populations based on the level of genetic differentiation (F-ST), genetic diversity (expected heterozygosity, H-E) and inbreeding (F-IS), characterized as 'high' or 'low' in comparison with a reference benchmark. We demonstrate the application of the framework in two case studies of threatened plants and more broadly from the literature. Results Applying the decision framework, we found that for Prostanthera eurybioides, the population of conservation concern does not currently require specialized genetic management and mitigation of ecological threats should be prioritized instead. For Allocasuarina robusta, we found connectivity was high and strategies should be put in place to maintain gene flow. In both cases, genetic information was important for designing restocking strategies accounting for the genetic structure and genetic diversity of source and recipient populations. From the literature, key examples of species types that fit each of the genetic management scenarios are given. Main conclusions We find that the application of our simplified genetic assessment framework helps to clarify management actions based on conservation genetic information for threatened flora, and should assist in bridging the gap between researchers and conservation practitioners for integrated conservation outcomes. Our framework could equally apply to fauna conservation with appropriate consideration of animal-specific management issues.

4510: ***+.280***

Urbanization and exotic species are major threats to the conservation of forest-dependent wildlife species. Some emblematic species, indicators of habitat quality for the conservation of other species, might successfully be reintroduced within cities when habitat restoration and pest management programs are combined. We studied the landscape resource selection of juvenile kaka Nestor meridionalis tracked with Global Positioning System (GPS) units and released into the predator-free reserve of Zealandia in Wellington city, New Zealand. Kaka moved beyond the predator exclusion fence into urban suburbs. The home range size and areas of high use estimated using local convex hull (a-LoCoH) ranged from 20 to 240 ha and 2 to 21 ha, respectively. Using resource selection functions and model selection we found that native forest patches and urban areas close to the reserve were selected by kaka to establish their home ranges. At a lower scale of selection (i.e., selection of habitats within home ranges), kaka selected the same habitat, but not necessarily those close to the reserve. Native forest patches throughout the city can facilitate the dispersal of individuals, while the reserve provides protection and opportunities for supplementary feeding. Urban areas might have been selected due to the placement of feeders in private backyards. Survival of forest-dwelling species in cities requires careful urban planning and management to provide the necessary habitat patches, refugia, and food sources.

4511: ***+.258***

Plant translocation has become a widely used tool to improve the conservation status of threatened plants. Dianthus morisianus (Caryophyllaceae) is a narrow endemic plant which only grows on the Portixeddu coastal dune (South-West Sardinia). Its natural habitat has been strongly modified, and it is currently considered one of the most threatened plants of Sardinia. In a conservation effort, a translocation of reproductive plants was planned. Plants were obtained from seeds collected in the natural population and cultivated at the Botanic Gardens of Cagliari University. The following two suitable areas near the natural population were identified: the first is located in a fenced site which is managed by public administration, and the second is located in an unprotected site. In November 2010, 113 plants were reintroduced in site one, and in February 2011, 25 plants were reintroduced in site two; all plants were regularly monitored. The aim was to analyse the effect of different management activities (i.e. the herbivore and human exclusion) on transplanted plants. The following consistent differences between sites with different management types were found: the survival and growth of D. morisianus were enhanced by reducing herbivory and human disturbance; in particular, fences positively enhanced the plant's long-term survival, reproductive success and seedling recruitment. This study highlights that management activities (i.e. erection of fences) should be incorporated into translocation design since they contribute to translocation success. Our experience can serve as a model for further translocations of the threatened plants of Sardinia and, more widely, of the Mediterranean islands.

4512: ***+.205***

The success of plant translocations rely heavily on obtaining suitable baseline information on the species to correctly identify the critical factors that condition population viability of the species, as well as to know how to successfully conduct the translocation. This baseline information is normally not available at the time translocations are conceived and often the timeframe to obtain this information is very short. In 2000, it was urgent to translocate a newly discovered population of a little known autumnal plant (Narcissus cavanillesii A. Barra & G. Lpez) in Portugal threatened by the construction of the Alqueva dam. Thirteen years after the translocation and annual monitoring, it was found that the translocated population has recovered the number of mature individuals available before the translocation. The aim of this study was to critically review the baseline information gathered for the translocation of N. cavanillesii and to assess the relevance of the different components. The systematically planned acquisition of baseline information made in a short period of time was in great part responsible for the successful translocation.

4513: ***+.275***

Conservation of Leucojum aestivum, a wetland-dependent species distributed in Europe and west Asia, should aim to reduce the fragmentation of wild stands, through the establishment of new populations. However, density-dependent dynamics occur in L. a estivum. For instance, fruit set and seed set increase with increasing plant density. In this study, we evaluate the effect of plant density on translocation success of two recently established populations of L. a estivum. Twenty-six populations of L. a estivum were investigated in northern Italy to find out differences in population traits (size, density, age structure, and reproductive performance) between populations from different habitats. Data obtained were used to establish two new populations of the species differing for population density (high H, mirroring the typical plant density of a wild population in Salix alba woods and low L, in which plant density was halved compared to H), to evaluate the role of density-dependent dynamics on the translocation success. 4 years after the translocation, H

produced seedlings, while L did not. Moreover, H produced a significantly higher number of fruits per fruiting plant and higher fruit set. Seed set was also greater in H than in L, while mortality was greater in L than in H, but differences were not significant. Our results suggest that population density is an important factor to account for in newly established populations, especially in those species showing density-dependent population dynamics. Moreover, the imitation of successful within-population dynamics occurring in natural stable populations may increase the translocation success.

4514: *+.154*

The existing literature on plant translocations focuses on post-translocation outcome while still overlooking issues related to the preparation phases. Yet, plant translocation programmes face significant pre-translocation challenges. In the present study, we want to share our pre-transplant experience on four rare plant species (Arnica montana, Campanula glomerata, Dianthus deltoides and Helichrysum arenarium), highlighting aspects we need to focus on while planning plant translocations. We emphasize some issues that need to be overcome before any translocation is undertaken during the four steps of translocation preparation, i.e. the selection and profiling of the target species, the seed collection, the development of propagation protocols and the assessment of plant fitness of the populations used as seed source. We discuss the implications of our results for designing translocation protocols. Our findings on A. montana show that if local seed sources are constrained to small remnant populations, seed quality may be poor. Preliminary tests using different kinds of growing medium provided valuable information for optimizing plant propagation protocols. Although it is attractive to establish propagation protocols using seeds obtained via Index Seminum (to avoid wasting collected source seeds), the results obtained were not always reproducible on the seeds collected in the wild source populations. Differences in pre-translocation plant fitness were also detected between seed source populations, which might reflect genetic diversity and maternal effects. As the translocated plants should capture as much genetic diversity as possible to ensure a high adaptive potential and improve establishment success, multisource reintroductions can be recommended.

4515: *+.165*

As the world's biodiversity is increasingly threatened by destruction and/or climate change, rare species reintroductions may be necessary to conserve species threatened with extinction. When ecological processes change or a species' range is heavily fragmented, it may be difficult to determine microsite characteristics needed for a successful reintroduction. In such cases, experimental reintroductions can be used to learn more about the species' reproductive niche while establishing new self-sustaining populations. We reintroduced the Florida endangered species, Tephrosia angustissima var. corallicola, to examine native microsite requirements and feasibility of reintroduction into nearby suitable habitat. We transplanted Tephrosia into three microsites with varying light and substrate and characterized their wet and dry season soil moisture, soil bulk density, and light conditions. We monitored transplant and recruited seedling growth, reproduction, and survival and seedling germination in each microsite for 6 years. The microsite supporting greatest survival changed across developmental stages and time. Although no original transplants survived to 2009, the reintroduced population persists in 2015 through recruitment. Highest recruit growth, flowering, and survival occurred in shady, dry microsites. Recruits germinated more in the wettest microsite, reproduced most in the driest, and persisted longest in the wettest. Recruits germinated in shadier locations than where adults were planted. Although assessing whether a reintroduction is self-sustaining will require decades of monitoring, this experimental reintroduction elucidated the necessity for heterogeneous microsites at recipient sites

and the efficacy of planting reproductive individuals for rapid next generation recruitment. Determining appropriate microsites for species requiring human assisted migration may also benefit from these techniques.

4516: ***-.022***

At Makauwahi Cave Reserve, on the south shore of Kaua`i, translocation decisions have been guided to a unique degree by the richly detailed fossil record of biota of recent centuries, which occurs on the site. To evaluate the efficacy of this strategy, ecological conditions and individual life histories for 3388 translocated native plants of 81 species have been monitored since 2005. Many species were selected on the basis of their prevalence as subfossils in the adjacent late Holocene cave sediments. Most of these species no longer occur on or near the abandoned farmlands and mine spoil used as a substrate for transplanted individuals. Records for each plant included location, date outplanted, flowering, fruiting, and, if applicable, mortality, including known or inferred cause. Also recorded was unaided recruitment, survival of transplanted recruits, and quantity of seed collected. Plant species selected for reintroduction on the basis of present occurrence near the site (many of which also occur there as fossils), and species not present but selected solely on the basis of fossil occurrence before European arrival, both show high survival rates in most cases. Species that fit neither of these criteria, but are judged suitable on the basis of their occurrence elsewhere on the island in similar habitats, generally showed lower survival rates. Primary mortality factors for nursery stock not surviving outplanting included transplant shock, irrigation failure, and human error (accidental cutting, pulling, or trampling). Much lower mortality rates were linked to insect damage, disease, and pig disturbance. Phenological records show that 80 % of translocated native species have flowered and 70 % produced seed. Unaided recruitment was observed for 43 % of the species with some rare species producing large numbers of volunteer seedlings. Translocated volunteer seedlings showed high survival rates. Insights from the fossil record have provided perspective on the site's potential and limitations and enriched interest in a restoration by almost doubling the list of plant species used in restoration programs and adding a living history element to the interpretation of the site through the juxtaposition of the fossil evidence and the translocated native species.

4517: ***+.120***

The conservation and translocation of threatened holoparasitic flowering plants provide added challenges due to their complete host dependency and often large knowledge gaps of their autecology. Here, we present the first successful, quantified field trial to establish from seed populations of dactylanthus (Dactylanthus taylorii, Mystropetalaceae), a threatened New Zealand endemic root-holoparasitic angiosperm. Establishment was monitored at four sites at Waipapa, Pureora Forest Park. The impact of two different sowing methods (broad- and central-sown), canopy state (as a proxy for soil moisture levels) and three dominant host species were tested. Establishment of dactylanthus was confirmed in 22 out of 24 plots 10 years after sowing, with earliest emergence after 4 years. Average and maximum inflorescence numbers per plot were similar to those of protected wild populations. The only open-canopy site performed worse in comparison with a closed-canopy site sharing the same dominant host species; differences in root availability and survival of the desiccation-sensitive seeds were regarded as the most likely explanations. Host species dominance had a significant impact on inflorescence numbers, indicating host preference in the species despite a wide host range. In contrast to longer-established wild populations, most of which are male-biased, female inflorescences strongly outnumbered males, considered as evidence of environmental sex determination and sex-switching of individuals. Findings from this study have increased our knowledge of the biology of

dactylanthus, confirmed translocation as an effective tool in the conservation of the species and should be applicable for the protection of threatened parasitic plants species elsewhere in the world.

4518: *+.263*

The Chinese flora occupies a unique position in global plant diversity, but is severely threatened. Although biodiversity conservation in China has made significant progress over the past decades, many wild plant species have extremely small population sizes and therefore are in extreme danger of extinction. The concept of plant species with extremely small populations (PSESPs), recently adopted and widely accepted in China, lacks a detailed description of the methodology appropriate for conserving PSESPs. Strategies for seed sampling, reintroduction, protecting PSESP locations, managing interactions with the local human population, and other conservation aspects can substantially differ from those commonly applied to non-PSESPs. The present review is an attempt to provide a detailed conservation methodology with realistic and easy-to-follow guidelines for PSESPs in China. Copyright (C) 2016 Kunming Institute of Botany, Chinese Academy of Sciences. Publishing services by Elsevier B.V. on behalf of KeAi Communications Co., Ltd.

4519: *+.261*

The successful reintroduction and restocking of the European Bison demands a reliable knowledge of the biology of this species. Yet little is known to date about the European bison, and empirical data remains insufficient to set up a reliable plan ensuring the reintroduction, maintenance and survival of populations in habitats that have been largely modified by human activity. Studies of the ecology, social behaviour and management of bison are therefore crucial to the conservation of this species and its cohabitation with humans. To meet these challenges, we focused on movement patterns and space use in a semi-free-ranging herd of European bison living in the Reserve Biologique des Monts-d'Azur (France). Bison spend over 80% of their time foraging and resting; foraging mainly occurs around the artificial feeding sites (i.e., hay racks) or in meadows. The time of day and the presence of snow have no influence on the time budget allocated to each activity. Animals, however, spend more time at the food racks in winter. Bison also spend most of their time in small groups of individuals, confirming the occurrence of both fission-fusion dynamics and sexual segregation in this species. Bison seem to follow a Levy walk pattern of movement, which is probably related to the geographical distribution and size of food patches in the reserve. The conclusions of this study provide a better understanding of the sociality, life habits and habitat use of bison, and also describe how the provision of hay affects all these behaviours. These results could be useful in the development of tools to select the most suitable habitats for the reintroduction, management and conservation of bison populations.

4520: *+.140*

Quaking aspen (Populus tremuloides) is the most widespread tree species in North America and has supported a unique ecosystem for tens of thousands of years, yet is currently threatened by dramatic loss and possible local extinctions. While multiple factors such as climate change and fire suppression are thought to contribute to aspen's decline, increased browsing by elk (Cervus elaphus), which have experienced dramatic population increases in the last similar to 80 years, may severely inhibit aspen growth and regeneration. Fires are known to favor aspen recovery, but in the last several decades the spatial scale and intensity of wildfires has greatly increased, with poorly understood ramifications for aspen growth. Here, focusing on the 2000 Cerro Grande fire in

central New Mexico - one of the earliest fires described as a "mega-fire" - we use three methods to examine the impact of elk browsing on aspen regeneration after a mega-fire. First, we use an exclosure experiment to show that aspen growing in the absence of elk were 3x taller than trees growing in the presence of elk. Further, aspen that were both protected from elk and experienced burning were 8.5 x taller than unburned trees growing in the presence of elk, suggesting that the combination of release from herbivores and stimulation from fire creates the largest aspen growth rates. Second, using surveys at the landscape level, we found a correlation between elk browsing intensity and aspen height, such that where elk browsing was highest, aspen were shortest. This relationship between elk browsing intensity and aspen height was stronger in burned (r = -0.53) compared to unburned (r = -0.24) areas. Third, in conjunction with the landscape-level surveys, we identified possible natural refugia, microsites containing downed logs, shrubs etc. that may inhibit elk browsing by physically blocking aspen from elk or by impeding elk's ability to move through the forest patch. We did not find any consistent patterns between refuge elements and aspen size or canopy cover suggesting that natural refugia are not aiding in aspen recruitment and that all young aspen were susceptible to browsing. In much of their normal range, aspen are not growing to large size classes, which threatens the future of this iconic species and calls into question the ability of ecosystems to recover from mega-fires. Our results highlight the importance of considering multiple interacting factors (i.e. fire and increased elk browsing) when considering aspen management and regeneration. (C) 2015 Elsevier B.V. All rights reserved.

4521: *+.191*

Interest in bison (Bison bison, B. bonasus) conservation and restoration continues to grow globally. In Canada, plains bison (B. b. bison) are threatened, occupying less than 0.5% of their former range. The largest threat to their recovery is the lack of habitat in which they are considered compatible with current land uses. Fences and direct management make range expansion by most bison impossible. Reintroduction of bison into previously occupied areas that remain suitable, therefore, is critical for bison recovery in North America. Banff National Park is recognized as historical range of plains bison and has been identified as a potential site for reintroduction of a wild population. To evaluate habitat quality and assess if there is sufficient habitat for a breeding population, we developed a Habitat Suitability Index (HSI) model for the proposed reintroduction and surrounding areas in Banff National Park (Banff). We then synthesize previous studies on habitat relationships, forage availability, bison energetics and snowfall scenarios to estimate nutritional carrying capacity. Considering constraints on nutritional carrying capacity, the most realistic scenario that we evaluated resulted in an estimated maximum bison density of 0.48 bison/km2. This corresponds to sufficient habitat to support at least 600 to 1000 plains bison, which could be one of the largest 10 plains bison populations in North America. Within Banff, there is spatial variation in predicted bison habitat suitability and population size that suggests one potential reintroduction site as the most likely to be successful from a habitat perspective. The successful reintroduction of bison into Banff would represent a significant global step towards conserving this iconic species, and our approach provides a useful template for evaluating potential habitat for other endangered species reintroductions into their former range.

4522: *-.153*

Confiscated and displaced mammals are often taken to sanctuaries, where the explicit goal may be reintroduction to the wild. By inadvertently collecting animals from different source populations, however, such efforts risk reintroducing individuals that have not been in genetic contact for significant periods of time. Using genetic analyses and 44 years of data from Camp Leakey, an orang-utan rehabilitation site on Borneo, we determined the minimum extent to which orang-utans

representing non-native, geographically and reproductively isolated taxa were reintroduced into the surrounding wild population. We found two reintroduced females were from a non-native subspecies, and have since produced at least 22 hybridized and introgressed descendants to date, of which at least 15 are living. Given that Bornean orang-utan subspecies are thought to have diverged from a common ancestor around 176,000 years ago, with marked differentiation over the last 80,000 years, we highlight the need for further evaluation of the effects of hybridizing orang-utans of different taxa-particularly in light of the similar to 1500 displaced orang-utans awaiting urgent reintroduction. As endangered mammals are increasing in number in sanctuaries worldwide, we stress the need for re-examination of historical reintroductions, to assess the extent and effects of de facto translocations in the past.

4523: ***+.277***

Introduction of nonnative cultured fish is one of the most important threats to native salmonid populations. In brown trout, more than a century of stocking practices has led to a large hybridization between initially geographically isolated lineages, threatening native populations and thereby intraspecific diversity. In the French region of Haute-Savoie, managers and scientists implemented together three management strategies (genetic refuge, direct translocation of wild spawners and stocking with native fry) on 19 test sites for more than 15 years, in the aim to recover pure or nearly pure native populations. Here we propose an assessment of the different management strategies based on a synthetic analysis of the evolution of the introgression rate. While none of the implemented strategies completely achieves the initial objective to restore pure native populations, they differ in their efficiency: introgression rates tend to decrease quickly when direct translocation of native spawners of stocking with native fry strategies are used. The genetic refuge strategy shows slower and more heterogeneous changes of introgression rates. In general, pure nonnative fish are efficiently removed but at the cost of an increased presence of hybrids. Our results imply that intraspecific dynamics react quickly to management practices and that these changes are probably fueled by evolutionary feedbacks that are not yet well understood.

4524: ***+.332***

Our study was undertaken to better understand how to increase the success rates of recovery plantings of a rare hemiparasite, golden paintbrush (Castilleja levisecta-Orobanchaceae). This species is endemic to westernWashington and Oregon, USA, and southwestern British Columbia, Canada. Over 5000 golden paintbrush plants were outplanted as plugs in 2007 at six different native prairie sites that were considered to be suitable habitat, based on general evaluations of vegetation and soil conditions. Outplantings were installed at regular intervals along transects up to 1 km long to include a range of conditions occurring at each site. All plantings were re-examined five years later. The patchy distribution of surviving plugs and new recruits within each reintroduction site suggested success is strongly influenced by microsite characteristics. Indicator species analysis of taxa growing in microsites around outplanted golden paintbrush identified species that were positively or negatively associated with paintbrush survival. Species such as Festuca roemeri, Eriophyllum lanatum, and Viola adunca were strong indicators at some sites; non-natives such as Hypochaeris radicata and Teesdalia nudicaulis tended to be frequent negative indicators. Overall, higher richness of native perennial forbs was strongly correlated with both survival and flowering of golden paintbrush, a pattern that may reflect interactions of this hemiparasite with the immediately surrounding plant community. Topographic position also influenced outcomes, with greater survival occurring on mounds and in swales, where soils generally were deeper. Our findings suggest that assessments of site suitability based on vegetation alone, and coarser, site-level assessments that do not characterize heterogeneity at the microsite

scale, may not be strong predictors of restoration success over the longer term and in sites with variability in vegetation and soils. By identifying suitable microsites to focus rare species plantings, survival and efficiency may be significantly enhanced.

4525: *+.138*

The study was aimed at learning lessons from historical translocations of the European native oyster, Ostrea edulis and contributing to the debate on best practice for restoration projects. An extensive literature review of over 100 documents spanning 200 years was conducted to look at translocations of Ostrea edulis and investigate temperature related reproduction. Differences among geographical locations were assessed by multivariate analysis of reproductive data. Translocations of hundreds to millions of Ostrea edulis have taken place over the past 200 years, mainly for commercial purposes. Movements were either single actions or regular events over many years. Whilst 75 separate records of Ostrea edulis movements from within European waters were documented, it is likely that many more took place. Introductions have also been made outside Europe for aquaculture; translocations back to European waters, have led to the introduction of pathogens. The timing and duration of reproductive periods and spawning temperature thresholds of Ostrea edulis in the middle region of its distribution range were similar. Cluster analysis of documented periods of reproduction indicated that introduced and restocked populations clustered with their putative donor populations. Whilst the Irish production areas clustered together, reproductive cycles in Lough Foyle in the northwest of the island of Ireland showed greater similarity to the now extinct deeper water English Channel beds. Historically, the ability of oysters to breed after translocation was not considered important. Successful reproduction and recruitment is however fundamental to conserving the species. Where translocation of stock is used to restore Ostrea edulis in areas where it has been extirpated, this study suggests that restocking should be at high densities and carried out over several years and that harvesting should be restricted to increase the chances of establishing self-sustaining populations. (C) 2016 Elsevier Ltd. All rights reserved.

4526: *+.088*

The largest extant New Zealand gecko, Hoplodactylus duvaucelii (Duvaucel's Gecko), is a nocturnal, viviparous species of conservation concern. Hoplodactylus duvaucelii, once widespread throughout New Zealand, is now confined to offshore islands, the majority of which are free from all introduced mammalian predators (mice, rats, cats, mustelids, brushtail possums). A single H. duvaucelii, caught within a fenced reserve on North Island in 2010 was genotyped to determine whether it represents a recent introduction or a previously unknown native relict population. Genotypes from seven nuclear loci and a minimum spanning network of mtDNA haplotypes revealed two clusters representing southern (Cook Strait) and northern island populations. This genetic structure is concordant with variation between these two groups observed in body size, color pattern, and scalation. The mainland specimen was found to possess a mixture of morphological character states typical of northern and southern island populations. Although the individual possessed a unique mitochondrial haplotype, high heterozygosity, and a private nuclear allele, it was no more genetically distinct than conspecifics from isolated island populations. Comparisons with live captive geckos failed to provide evidence that the aberrant specimen represented a recent translocation. We infer that H. duvaucelii has survived naturally on North Island at very low population densities since the human-mediated introduction of novel predators 800 years ago. Our findings suggest a novel conservation priority, which should be prioritized for additional study in the immediate future.

4527: ***+.227***

Managing species with intensive tools such as reintroduction may focus on single sites or entire landscapes. For vagile species, long-term persistence will require colonization and establishment in neighboring habitats. Therefore, both suitable colonization sites and suitable dispersal corridors between sites are required. Assessment of landscapes for both requirements can contribute to ranking and selection of reintroduction areas, thereby improving management success. Following eradication of invasive American Bullfrogs (Lithobates catesbeianus) from most of Buenos Aires National Wildlife Refuge (BANWR; Arizona, United States), larval Chiricahua Leopard Frogs (Lithobates chiricahuensis) from a private pond were reintroduced into three stock ponds. Populations became established at all three reintroduction sites followed by colonization of neighboring ponds in subsequent years. Our aim was to better understand colonization patterns by the federally threatened L. chiricahuensis which could help inform other reintroduction efforts. We assessed the influence of four landscape features on colonization. Using surveys from 2007 and information about the landscape, we developed a habitat connectivity model, based on electrical circuit theory, that identified potential dispersal corridors after explicitly accounting for imperfect detection of frogs. Landscape features provided little insight into why some sites were colonized and others were not, results that are likely because of the uniformity of the BANWR landscape. While corridor modeling may be effective in more-complex landscapes, our results suggest focusing on local habitat will be more useful at BANWR. We also illustrate that existing data, even when limited in spatial or temporal resolution, can provide information useful in formulating management actions.

4528: ***+.267***

Reintroductions are an important tool for re-establishing or reinforcing populations of threatened species, and thus to restore ecosystems. However, predicting how reintroduced populations will spread is difficult, and past reintroductions often lacked a thorough assessment of habitat availability and connectivity. Using the case of reintroduced European bison (Bison bonasus L.) in the northern Carpathians, we show how habitat suitability models in combination with connectivity assessments based on circuit theory can remedy such shortcomings, and identify potential habitat patches and corridors between these. European bison were reintroduced in our study area in the 1960s, and against prior expectation, have not spread along the Carpathian ridge, but instead expanded their range towards human settlements. Our analyses provide an explanation for this pattern. Although we identified a network of suitable habitat patches along the Carpathian ridge, the functional connections between them were limited due to a number of major barriers to movement. To avoid future conflicts between European bison and people, and to achieve the long-term goal of a viable bison metapopulation in this region, conservation action should focus on establishing connectivity between habitat patches through the creation and legal protection of corridors and wildlife passages, which would benefit Carpathian wildlife in general. Our study emphasizes the importance of landscape-scale connectivity analyses to guide restoration efforts, and of adaptive management to ensure the success of reintroduction projects. (C) 2015 Elsevier Ltd. All rights reserved.

4529: ***-.095***

The reconstruction of invasion history is the goal or foundation of many investigations of biological invasions. This study applies DNA profiling to investigate the sources and vectors of new propagules, to detect illegal human-mediated translocations and to improve the management of invasions by identifying invasion pathways that can be targeted for more stringent control. We

use the European invasion of the American Eastern grey squirrel as a focal example. Many human-mediated translocations of this species have occurred in Europe since the 19th century. We built a genotype database of 1421 individuals from 59 locations across Europe and one in the USA, with each individual genotyped at 12 highly polymorphic microsatellites. DNA from newly detected populations and individuals of unknown sources were compared with this database. Our results indicated illegal human-mediated translocations in Aberdeen, the Isle of Skye and Northumberland in the UK, and revealed precise details of illegal squirrel trade in Italy. We recommend that multi-locus genotype databases be set up for all major invasive species considered attractive or ornamental, as these are often subject to illegal translocations through human actions such as the pet trade. (C) 2016 The Authors. Published by Elsevier Ltd.

4530: ***+.012***

Coastal Louisiana has suffered from dramatic coastal land loss. Following translocations to Louisiana in the late 1960s, Brown Pelican (Pelecanus occidentalis) colonies were annually surveyed between 1971 and 2010 using aerial methods. The goals of this study were to describe long-term Brown Pelican colony dynamics, investigate physical changes to nesting islands via satellite imagery, and relate colony dynamics to physical island changes. Thirty Brown Pelican colonies were found, with a mean colony persistence of 5.5 years. Following exponential growth up to 2000, nesting plateaued, declined sharply in 2006 primarily due to land losses following the intense 2005 hurricane season, and stabilized at lower levels until 2010. From 1998 to 2010, island size decreased by a mean of 68.7%, with 10 of the islands vanishing entirely. Colony size was positively correlated to island size. Colonies did not shift between State regions, but appeared to shift within regions. To persist in Louisiana, Brown Pelicans will need to continue adjusting to a changing coastline, as well as planned coastal restoration projects. Future monitoring of Brown Pelicans in Louisiana may provide insight into their adaptive responses to changing availability and suitability of nesting island habitat.

4531: ***+.138***

The historic persecution and decline of European raptor populations precipitated the use of reintroduction as a species restoration tool in the late twentieth century. One of the key requirements of the World Conservation Union reintroduction guidelines concerns the need for social feasibility studies to explore the attitudes of local human populations toward restoration and reintroduction proposals. Ahead of any formal proposals to reintroduce white-tailed sea eagles to Cumbria, United Kingdom, we conducted a baseline public attitudinal survey (n = 300). We identified broad public support for this reintroduction, which transcended differences in the demographic, geographic, and employment profiles of the study cohort. There was public recognition that white-tailed sea eagles could deliver a broad range of socioeconomic and environmental benefits with few detrimental impacts. Although the value of attitudinal surveys of this nature has been questioned, we would argue that they provide a useful baseline snapshot ahead of a more structured and focused reintroduction consultation. These results reinforce the emergence of public interest in the restoration of European raptors in the late twentieth and early twenty-first century.

4532: ***-.004***

Invasive species are a threat to aquatic ecosystems worldwide. Aquatic snails have a limited ability for an active dispersal. Therefore, their ability to survive to transport in non-aquatic media may help explain their spread across unconnected habitats. We assessed the ability of New

Zealand Mud Snail (NZMS) (Potamopyrgus antipodarum) to survive attached to different materials. Two studies were conducted: (1) a laboratory study to assess the tolerance of a laboratory population of NZMS to non-aquatic media attaching snails to leaf litter, sediment and clay and (2) a field-laboratory study to assess the survival of an invasive field population after being left imbedded in clay in the laboratory and subsequently transferred to a new river reach. All laboratory animals died after 3 days in leaf litter, while in the case of sediment and clay all snails died after 5 days. After being imbedded in clay and subsequently transferred to the river, the survival of the field population was lower than that of the laboratory populations. We conclude that NZMS can be dispersed by mechanisms which imply spending some time in non-aquatic media, and that this species has a relatively high tolerance to translocation between reaches with contrasting environmental properties. (C) 2016 Elsevier GmbH. All rights reserved.

4533: *+.035*

We tested whether captive breeding and release is a feasible restoration strategy for the Harpy Eagle (Harpia harpyja) where suitable unoccupied habitat remains within its former range. From 1987 through 2006, 18 Harpy Eagles participated in a captive breeding program started in Boise, Idaho, and continued in Panama from 2001. From 131 eggs laid, 44 eagles were fledged. Most young were produced by just three females in the program, and at a higher annual rate after the birds were moved from Boise to Panama. Re-laying induced by collecting eggs for artificial incubation increased the number of viable eggs laid per female each breeding season up to six, but may have reduced female reproductive lifetime. Including rehabilitated eagles hatched in the wild, we released 49 eagles from 1998 through 2009. When the last released eagle with a functioning radio transmitter died in 2011, 63% were known or presumed to be dead, 31% were missing and possibly alive, and 6% were back in captivity. Shooting (44%) was the primary cause of death. Behavior interpreted as aggression toward humans was sufficiently frequent (23% of released eagles) in captive-raised and wild-rehabilitated eagles after release to be a concern for public safety and a potential cause of shooting deaths. This study demonstrated that it is feasible to breed Harpy Eagles in captivity at high rates needed for species restoration. It is possible to release captive-reared and rehabilitated Harpy Eagles to the wild, and is most cost effective (i.e., resulting in the highest survival to hunting-independence) when eagles are released close to the age of independence. Preventing shooting and other kinds of human persecution, and protecting remaining forest habitat, are the most urgent conservation needs for the Harpy Eagle.

4534: *+.045*

The ferruginous duck (Aythya nyroca) is a medium-sized chestnut-colored diving duck that inhabits wetlands of Europe and Asia. In recent years, this species has been declining throughout much of Europe-a decline that is attributed mainly to destruction of natural habitats, and to hunting and pollution. The ferruginous duck is listed as "near threatened" by the International Union for Conservation of Nature, and as a critically endangered nesting species in Israel. In 2009, a captive-breeding/reintroduction program was established in Israel, aiming to increase the species' population. The objective of this study was to collect data on normal hematology and plasma biochemistry analytes of ferruginous ducks in order to promote the species' conservation. Blood was collected from 49 birds, and 27 analytes were quantified. Compared to most other anseriformes studied, the ferruginous ducks in this study had lower white blood cell counts, which were dominated by heterophils rather than by lymphocytes.

4535: *+.066*

Worldwide, there are only a handful of reintroduction programs for threatened salamander species, and very few have conducted postrelease studies to examine survival, habitat selection, and dispersal. Limitations in postrelease monitoring are primarily due to size constraints of amphibians and to dimensions of the radiotransmitters available for implantation. However, due to the large size of the critically endangered Chinese giant salamander (Andrias davidianus), these animals make optimal candidates for surgical implantation of radiotransmitters prior to reintroduction or translocation. The objective of this study was to develop an anesthetic protocol using tricane methanesulfonate (MS-222) and test a surgical procedure for coelomic implantation of radiotransmitters for this species. A total of 32 Chinese giant salamanders from two age groups (Group A: 4.7 yr old, n = 16; Group B: 2.7 yr old, n = 16) were implanted with 4-g radiotransmitters designed for underwater monitoring of fish. Group A was held 16 wk before release while Group B was held 6 wk before release, and the salamanders' survival and postoperative complications recorded for the first month postrelease. Group A animals took longer to reach a surgical plane of anesthesia than did Group B animals, and this was directly correlated to mass of the animals. Postsurgery, one animal from Group B died of dehiscence before release while 83.9% animals survived after the first month in the wild. All of the animals that died postrelease were from Group B; three animals experienced dehiscence of the suture site and died while another two animals expired from trauma and fungal infection, respectively. Improvements for future studies include use of alternative suture material for closure after implantation and additional healing time of the incision.

4538: ***+.195***

Stochastic simulation models requiring many input parameters are widely used to inform the management of ecological systems. The interpretation of complex models is aided by global sensitivity analysis, using simulations for distinct parameter sets sampled from multidimensional space. Ecologists typically analyze such output using an "emulator"; that is, a statistical model used to approximate the relationship between parameter inputs and simulation outputs and to derive sensitivity measures. However, it is typical for ad hoc decisions to be made regarding: (1) trading off the number of parameter samples against the number of simulation iterations run per sample, (2) determining whether parameter sampling is sufficient, and (3) selecting an appropriate emulator. To evaluate these choices, we coupled different sensitivity-analysis designs and emulators for a stochastic, 20-parameter model that simulated the re-introduction of a threatened species subject to predation and disease, and then validated the emulators against new output generated from the simulation model. Our results lead to the following sensitivity analysis-protocol for stochastic ecological models. (1) Run a single simulation iteration per parameter sample generated, even if the focal response is a probabilistic outcome, while sampling extensively across the parameter space. In contrast to designs that invested in many model iterations (tens to thousands) per parameter sample, this approach allowed emulators to capture the input-output relationship of the simulation model more accurately and also to produce sensitivity measures that were robust to variation inherent in the parameter-sampling stage. (2) Confirm that parameter sampling is sufficient, by emulating subsamples of the sensitivity-analysis output. As the subsample size is increased, the cross-validatory performance of the emulator and sensitivity measures derived from it should exhibit asymptotic behavior. This approach can also be used to compare candidate emulators and select an appropriate interaction depth. (3) If required, conduct further simulations for additional parameter samples, and then report sensitivity measures and illustrate key response curves using the selected emulator. This protocol will generate robust sensitivity measures and facilitate the interpretation of complex ecological models, while minimizing simulation effort.

4539: ***+.285***

During inundation of the Nam Theun 2 Hydropower Project a wildlife rescue programme was conducted to respond to animal rescue needs. Based on a series of field surveys conducted in 2007, priority species were identified for rescue and translocation. Once filling of the Nam Theun 2 Reservoir commenced and islands started to form, animals trapped on these islands were captured by various means and translocated to the Nakai - Nam Theun National Protected Area managed by the NT2 Watershed Management and Protection Authority (WMPA) on the north shore of the reservoir. Various data was collected on these animals and a number of these animals were radiotracked after translocation to assess their survival. Such rescue efforts are often considered a mere act of animal welfare or a public relation exercise. The experience of the Nam Theun 2 wildlife rescue programme shows however that a carefully conducted wildlife rescue programme can provide not only invaluable scientific and ecological insights but significantly contribute to conservation of the biodiversity in the area.

4540: ***+.123***

In Europe, wild ungulates have undergone major expansion and population growth during recent decades. In certain cases, the high density achieved by these populations has led to excessive pressure on the environment, which eventually becomes a limiting factor for the population itself. One of these reintroductions was performed with the Iberian ibex (Capra pyrenaica Schinz, 1838) in the Sierra de Guadarrama National Park (Spain). This reintroduced population was monitored during six field seasons (2000, 2003, 2005, 2007, 2010, and 2014) by direct observation of the animals along transects using the distance sampling method to determine the degree of expansion over the years and the use of different habitats according to different seasons. The abundances obtained for each field season showed a significant increase from 4.16 to 8.65 individuals/km, showing a linear relationship between abundance and extent of the area occupied by the species. We observed that differences between habitat availability and use were significant for all seasons. Our data can be used as an example of the colonization process of a population of wild ungulates and their impact on vegetation to better manage future reintroductions.

4542: ***+.199***

The systematics, morphology, phylogeny, distribution, biology, economic importance and conservation of the Fars tooth-carp, Aphanius farsicus of Iran are described, the species is illustrated, and a bibliography on this endemic, critically endangered species is provided. Iran and central Anatolia show the highest diversity of Aphanius, and 14 extant (including Aphanius farsicus, an endemic species in the endorheic Lake Maharlu basin, South-western Iran) and one fossil species are known to occur in Iran based on data derived from fish morphometry and meristics, otoliths, scales and mtDNA sequences. Human-induced disturbance or anthropogenic activities, including hydrological alteration, introduction of exotic species, intensive aquaculture industry, water pollution, rapid sedimentation, natural disturbance (climate change and drought) and also limited distribution, have forced Aphanius farsicus populations to the edge of extinction. Aphanius farsicus can benefit from a combination of reintroduction, assisted colonization and capture release which potentially increase success rate of a conservation management plan. Encouraging the local communities, NGOs and media to involve voluntarily in longterm conservation programs is highly recommended.

4543: ***+.114***

Background: In 2009, anadromous threespine stickleback (Gasterosteus aculeatus) were stocked in Cheney Lake, Alaska, following treatment with the ichthyocide rotenone to remove invasive northern pike (Esox lucius), but which also extirpated native species. This introduction provides an opportunity to directly observe the evolutionary transition of oceanic stickleback to a freshwater-adapted population.Objective: Rear offspring of F-1 Cheney Lake stickleback under two common garden treatments alongside fish from Rabbit Slough, their anadromous source population, to test for rapid morphological evolution and contributions of phenotypic plasticity to morphological divergence.Methods: We reared 198 Cheney Lake and 209 Rabbit Slough stickleback under two conditions designed to simulate the habitat structure and diets of complex, shallow lake and simple, open-water habitats. We preserved stickleback at four time intervals to account for allometric growth effects. We made linear measurements of several traits and used geometric morphometrics to characterize variation in body shape.Results: Both phenotypic plasticity between experimental treatments and evolutionary divergence between populations accounted for variation in the sample, and these effects were independent of one another. Morphological differences between treatments interacted with stickleback age group, indicating an effect of rearing condition on allometric growth. Thus stickleback morphology in Cheney Lake has evolved in a single generation, but phenotypic plasticity is likely to strongly influence morphological variation.

4544: *+.124*

Background: Marine or anadromous threespine stickleback have colonized many northern Holarctic lakes after glacial recession, and their freshwater descendants have diverged in characteristic ways. Such divergence begins within a few generations, but previous studies have sampled only one generation or initiated sampling several generations after the populations were founded. Rotenone treatment of two Cook Inlet lakes to exterminate invasive northern pike also eliminated native threespine stickleback, creating an opportunity to introduce anadromous stickleback and observe their adaptation to freshwater conditions for multiple generations, beginning immediately after we founded the populations.Methods: In 2009 and 2011, we released about 3000 reproductively mature anadromous threespine stickleback into each lake. We sampled the source population and made annual samples from the two introduced populations. These samples have been preserved for future analysis. We estimated annual variation in relative abundance, made preliminary morphological observations, and assessed parasite diversity.Results: Anadromous stickleback released into the lakes produced abundant progeny, many of which survived and became reproductively mature the following spring. Both populations experienced demographic bottlenecks in 2013 and 2014 and began to recover in 2015. Preliminary observations indicate that stickleback in both lakes resemble anadromous stickleback, but by 2015 about 20% of the specimens in one population had a highly heritable, freshwater phenotype. One lake population had roughly twice as many parasite species and a much higher prevalence of a large metazoan parasite than either the anadromous ancestor or the other lake population. Our preliminary observations indicate that substantial evolution occurred during the first few generations, our existing samples can be used to study contemporary evolution, and sampling should be continued.

4545: *+.110*

The article provides a unique opportunity to study a large white-clawed crayfish mitigation exercise on the River Wansbeck, Northumberland in North East England. The translation was necessary to allow the construction of a dam on the river to reduce flood risk to the town of Morpeth. The study provides an assessment of the methodology used for the capture and

translocation activities, and provides an insight into the speed of colonisation and population establishment in a 300-m diversion channel and the structure of translocated populations. Over 3500+white-clawed crayfish were caught when the natural channel was dewatered, with 5400 animals removed the following year when the diversion channel was closed. Average density based on the number of white-clawed crayfish caught was estimated as in excess of 5 per 1 m(2). The study demonstrates the importance of the River Wansbeck for white-clawed crayfish.

4546: ***+.005***

Hawaiian land snails in the endangered, endemic genus Achatinella have experienced major declines in population and distribution over the last 100 years. Threats to Achatinella today include invasive, non-native predators (Euglandina rosea, Rattus rattus and Trioceros jacksonii), habitat degradation due to human disturbance and possibly climate change, and historically, collection by humans. The O' ahu Army Natural Resources Program (OANRP) is required to stabilize select remaining populations of A. mustelina. Stabilization goals are to maintain 300 mature snails at eight managed sites and control threats within sites. This report describes OANRP efforts to combat invasive predators by means of predator-free and -proof snail enclosures. A couple of prior attempts at excluding predatory snails were marginally successful but the identification of additional predators required substantial additional barriers. The design and construction of the enclosure at Pu'u Hapapa is used as a case study. This report includes detailed information on the physical development of predator-proof barriers, construction and costs. Additional needs for monitoring and maintenance, predator removal, Achatinella reintroduction, Achatinella population monitoring, and habitat improvement were also developed.

4547: ***+.043***

The Endangered Allen Cays Rock Iguana (Cyclura cychlura inornata) is endemic to the Allen Cays, a tiny cluster of islands in the Bahamas. Naturally occurring populations exist on only two cays (<4 ha each). However, populations of unknown origin were recently discovered on four additional cays. To investigate patterns of genetic variation among these populations, we analyzed nuclear and mitochondrial markers for 268 individuals. Analysis of three mitochondrial gene regions (2,328 bp) and data for eight nuclear microsatellite loci indicated low genetic diversity overall. Estimates of effective population sizes based on multilocus genotypes were also extremely low. Despite low diversity, significant population structuring and variation in genetic diversity measures were detected among cays. Genetic data confirm the source population for an experimentally translocated population while raising concerns regarding. other, unauthorized, translocations. Reduced heterozygosity consistent. with a documented historical population decline due to overharvest. This study provides the first range-wide genetic analysis of this subspecies.. We suggest strategies to maximize genetic diversity during ongoing recovery. including. additional translocations to establish assurance populations and additional protective measures for the two remaining natural populations.

4548: ***+.149***

The success of a reintroduction program is determined by the ability of individuals to reproduce and thrive. Hence, an understanding of the mating system and breeding strategies of reintroduced species can be critical to the success, evaluation and effective management of reintroduction programs. As one of the most threatened crocodile species in the world, the Orinoco crocodile (Crocodylus intermedius) has been reduced to only a few wild populations in the Llanos of Venezuela and Colombia. One of these populations was founded by reintroduction at Cano

Macanillal and La Ramera lagoon within the El Frio Biological Station, Venezuela. Twenty egg clutches of C. intermedius were collected at the El Frio Biological Station for incubation in the lab and release of juveniles after one year. Analyzing 17 polymorphic microsatellite loci from 335 hatchlings we found multiple paternity in C. intermedius, with half of the 20 clutches fathered by two or three males. Sixteen mothers and 14 fathers were inferred by reconstruction of multilocus parental genotypes. Our findings showed skewed paternal contributions to multiple-sired clutches in four of the clutches (40%), leading to an overall unequal contribution of offspring among fathers with six of the 14 inferred males fathering 90% of the total offspring, and three of those six males fathering more than 70% of the total offspring. Our results provide the first evidence of multiple paternity occurring in the Orinoco crocodile and confirm the success of reintroduction efforts of this critically endangered species in the El Frio Biological Station, Venezuela.

4549: *+.018*

Ninety-four subadult and adult white rhinoceroses (Ceratotherium simum) were captured between February and October, 2009-11, in Kruger National Park and placed in holding bomas prior to translocation to other locations within South Africa. A simple three-category system was developed based on appetite, fecal consistency/volume, and behavior to assess adaptation to bomas. Individual animal and group daily median scores were used to determine trends and when rhinoceroses had successfully adapted to the boma. Seventeen rhinoceroses did not adapt to boma confinement, and 16 were released (1 mortality). No differences in boma scores were observed between rhinoceroses that adapted and those that did not, until day 8, when the first significant differences were observed (adapted score=13 versus nonadapted score=10). The time to reach a boma score determined as successful adaptation (median 19 d) matched subjective observations, which was approximately 3 wk for most rhinoceroses. Unsuccessful adaptation was indicated by an individual boma score of less than 15, typically during the first 2 wk, or a declining trend in scores within the first 7-14 d. This scoring system can be used for most locations and could also be easily adapted to other areas in which rhinoceroses are held in captivity. This tool also provides important information for assessing welfare in newly captured rhinoceroses.

4550: *+.094*

Introduction of non-indigenous taxa by anthropogenic activities may lead to the generation of hybrid forms and cause genetic pollution of native species. Populations of different Salmo species are threatened in Italy by hybridization and introgression caused by allochthonous lineages introduced since historical times. In particular, Salmo marmoratus is currently sympatric with domestic lineages of Salmo trutta in most of its native geographical range and reproductive interfecundity between the two taxa is seriously threatening the genetic purity of the endemic species. To fulfill conservation purposes and fisheries management, an investigation based on single and multilocus DNA fingerprinting was carried out both to assess marble trout genetic diversity and the method's amenability to restocking practices. RFLPs (Restriction Fragments Length Polymorphisms) and SNPs (Single Nucleotide Polymorphisms) in mitochondria) 16S rDNA, D-loop, and nuclear LDH-C1* sequences were genotyped in more than 350 samples collected from different hatcheries in Northern Italy. The combination of the three markers allowed the selection of putative pure individuals of S. marmoratus to be submitted to additional highly polymorphic AFLPs (Amplified Fragment Length Polymorphisms) analyses. Additional benefits of AFLPs over other techniques emerged in connection with their potential power for fish stock identification. In fact, 52% of all analyzed samples were potentially pure marble trout, 4% were pure Atlantic trout and 44% were hybrids showing different combinations of haplotypesigenotypes. The combined approach demonstrated improved resolution to reveal

hybridization not detected by classical diagnostic markers, and to select breeders for reintroduction programs. (C) 2015 Elsevier B.V. All rights reserved.

4551: ***+.302***

In coastal and estuarine systems, foundation species like seagrasses, mangroves, saltmarshes or corals provide important ecosystem services. Seagrasses are globally declining and their reintroduction has been shown to restore ecosystem functions. However, seagrass restoration is often challenging, given the dynamic and stressful environment that seagrasses often grow in. From our world-wide meta-analysis of seagrass restoration trials (1786 trials), we describe general features and best practice for seagrass restoration. We confirm that removal of threats is important prior to replanting. Reduced water quality (mainly eutrophication), and construction activities led to poorer restoration success than, for instance, dredging, local direct impact and natural causes. Proximity to and recovery of donor beds were positively correlated with trial performance. Planting techniques can influence restoration success. The meta-analysis shows that both trial survival and seagrass population growth rate in trials that survived are positively affected by the number of plants or seeds initially transplanted. This relationship between restoration scale and restoration success was not related to trial characteristics of the initial restoration. The majority of the seagrass restoration trials have been very small, which may explain the low overall trial survival rate (i.e. estimated 37%). Successful regrowth of the foundation seagrass species appears to require crossing a minimum threshold of reintroduced individuals. Our study provides the first global field evidence for the requirement of a critical mass for recovery, which may also hold for other foundation species showing strong positive feedback to a dynamic environment. Synthesis and applications. For effective restoration of seagrass foundation species in its typically dynamic, stressful environment, introduction of large numbers is seen to be beneficial and probably serves two purposes. First, a large-scale planting increases trial survival - large numbers ensure the spread of risks, which is needed to overcome high natural variability. Secondly, a large-scale trial increases population growth rate by enhancing self-sustaining feedback, which is generally found in foundation species in stressful environments such as seagrass beds. Thus, by careful site selection and applying appropriate techniques, spreading of risks and enhancing self-sustaining feedback in concert increase success of seagrass restoration. For effective restoration of seagrass foundation species in its typically dynamic, stressful environment, introduction of large numbers is seen to be beneficial and probably serves two purposes. First, a large-scale planting increases trial survival - large numbers ensure the spread of risks, which is needed to overcome high natural variability. Secondly, a large-scale trial increases population growth rate by enhancing self-sustaining feedback, which is generally found in foundation species in stressful environments such as seagrass beds. Thus, by careful site selection and applying appropriate techniques, spreading of risks and enhancing self-sustaining feedback in concert increase success of seagrass restoration.

4552: ***+.202***

European rivers are highly degraded and restoration efforts are becoming more frequent. However, only few restoration projects have been rigorously evaluated so far. We investigated the response of fish assemblages to hydromorphological restoration measures including river widening, creation of instream structures, flow enhancement, remeandering and side-channel reconnection. We sampled 15 rivers with pairs of degraded and restored sites and calculated the effect sizes (i.e., restored-degraded) for species richness, species diversity, fish density and habitat traits. We analysed the following factors potentially affecting restoration success: (1) length of the restored river stretch, (2) time after restoration and (3) hydromorphological quality of restoration. While species diversity and density did not respond to restoration, proportion of small rheophilic fish

increased and eurytopic decreased. Short-term (< 3 years) and long-term effects (> 12 years) of restoration measures have a stronger effect on fish assemblages than mid-term effects. Furthermore, the hydromorphological quality and the length of the restored section are relevant for the restoration effects on the fish community. Future restoration projects should focus on more dynamic, self-sustaining habitat improvements extending over several kilometres and should be coupled with other measures such as restoring the river continuity and species reintroductions.

4553: ***+.449***

River otters (Lontra canadensis) were reintroduced from 1994-1996 into parts of Illinois where the species was extirpated due to over harvest and habitat loss. At the time of reintroduction, managing for the persistence of the population through habitat was very important and research was conducted to determine which watersheds had quality habitat and which needed increased management and protection. In a study conducted in the mid-1990s, biologists used pattern recognition (PATREC) modeling to identify high and low quality habitat for river otters at the subunit level (i.e., divided watershed), based on specific habitat attributes including wooded area, sinuosity, and wetland edge. We compared the habitat quality ratings of subunits with river otter use at 112 bridge sites from 2012-2014 to determine if river otters have distributed themselves according to previously determined habitat quality. We found that the PATREC model was a poor predictor of river otter use when sites were located close to the otter reintroduction points. The PATREC model was most likely a poor predictor of river otter use due to an over-emphasis on the importance of woody vegetation to habitat suitability for river otter in the model. We recommend that future work on the assessment of habitat suitability for river otter use, and accuracy of this assessment, be conducted at a local spatial scale and over a shorter temporal scale. We also recommend that watershed policies and habitat assessments consider changes to land-cover and follow an adaptive management approach to assess habitat suitability for reintroduced species.

4554: ***-.058***

In International Union for Conservation of Nature (IUCN) Red List assessments, extent of occurrence (EOO) is a key measure of extinction risk. However, the way assessors estimate EOO from maps of species' distributions is inconsistent among assessments of different species and among major taxonomic groups. Assessors often estimate EOO from the area of mapped distribution, but these maps often exclude areas that are not habitat in idiosyncratic ways and are not created at the same spatial resolutions. We assessed the impact on extinction risk categories of applying different methods (minimum convex polygon, alpha hull) for estimating EOO for 21,763 species of mammals, birds, and amphibians. Overall, the percentage of threatened species requiring down listing to a lower category of threat (taking into account other Red List criteria under which they qualified) spanned 11-13% for all species combined (14-15% for mammals, 7-8% for birds, and 12-15% for amphibians). These down listings resulted from larger estimates of EOO and depended on the EOO calculation method. Using birds as an example, we found that 14% of threatened and near threatened species could require down listing based on the minimum convex polygon (MCP) approach, an approach that is now recommended by IUCN. Other metrics (such as alpha hull) had marginally smaller impacts. Our results suggest that uniformly applying the MCP approach may lead to a one-time down listing of hundreds of species but ultimately ensure consistency across assessments and realign the calculation of EOO with the theoretical basis on which the metric was founded. El Impacto de las Medidas Alternativas sobre los Estimados de Extension de la Presencia para la Evaluacion del Riesgo de Extincion En las evaluaciones de la Lista Roja de la Union Internacional para la Conservacion de la Naturaleza (UICN), la extension de la presencia (EDP) es una medida clave del riesgo de extincion. Sin

embargo, la forma en la que los evaluadores estiman la EDP a partir de los mapas de la distribucion de las especies es inconsistente entre las evaluaciones de diferentes especies y entre los principales grupos taxonomicos. Los evaluadores continuamente estiman la EDP a partir del area de la distribucion mapeada, pero estos mapas generalmente excluyen areas que no son habitats de manera idiosincratica y que no son creadas con la misma resolucion espacial. Evaluamos el impacto de la aplicacion de diferentes metodos (poligono convexo minimo, casco alfa) para la estimacion de EDP sobre las categorias de riesgo de extincion de 21, 763 especies de mamiferos, aves y anfibios. En general, el porcentaje de las especies amenazadas que requirio el cambio de categoria a una de amenaza mas baja (tomando en cuenta otros criterios de la lista roja a los cuales calificaron) abarco 11-13% de todas las especies combinadas (14-15% de los mamiferos, 7-8% de las aves y 12-15% de los anfibios). Estos cambios a una categoria mas baja resultaron de estimaciones mas grandes de la EDP y dependieron del metodo de calculo de la misma. Cuando usamos a las aves como ejemplo, encontramos que 14% de las especies amenazadas o casi amenazadas podrian requerir un cambio a una categoria mas baja con base en la estrategia del poligono convexo minimo (PCM), una estrategia que ahora es recomendada por la UICN. Otras medidas (como la de casco alfa) tuvieron impactos marginalmente menores. Nuestros resultados sugieren que la aplicacion uniforme de la estrategia del PCM puede llevar al cambio unico a una categoria mas baja para cientos de especies, pero al final asegurar la consistencia en todas las evaluaciones y realinear el calculo de la EDP con las bases teoricas sobre las que se fundamento la medida.Resumen

4555: ***+.105***

Captive breeding and reintroduction remain high profile but controversial conservation interventions. It is important to understand how such programs develop and respond to strategic conservation initiatives. We analyzed the contribution to conservation made by amphibian captive breeding and reintroduction since the launch of the International Union for Conservation of Nature (IUCN) Amphibian Conservation Action Plan (ACAP) in 2007. We assembled data on amphibian captive breeding and reintroduction from a variety of sources including the Amphibian Ark database and the IUCN Red List. We also carried out systematic searches of Web of Science, JSTOR, and Google Scholar for relevant literature. Relative to data collected from 1966 to 2006, the number of species involved in captive breeding and reintroduction projects increased by 57% in the 7 years since release of the ACAP. However, there have been relatively few new reintroductions over this period; most programs have focused on securing captive-assurance populations (i.e., species taken into captivity as a precaution against extinctions in the wild) and conservation-related research. There has been a shift to a broader representation of frogs, salamanders, and caecilians within programs and an increasing emphasis on threatened species. There has been a relative increase of species in programs from Central and South America and the Caribbean, where amphibian biodiversity is high. About half of the programs involve zoos and aquaria with a similar proportion represented in specialist facilities run by governmental or nongovernmental agencies. Despite successful reintroduction often being regarded as the ultimate milestone for such programs, the irreversibility of many current threats to amphibians may make this an impractical goal. Instead, research on captive assurance populations may be needed to develop imaginative solutions to enable amphibians to survive alongside current, emerging, and future threats. Avances en la Reproduccion de Anfibios en Cautiverio y en los Programas de Reintroduccion La reproduccion en cautiverio y la reintroduccion permanecen como intervenciones de conservacion de alto perfil pero controversiales. Es importante entender como dichos programas se desarrollan y responden a las iniciativas estrategicas de conservacion. Analizamos la contribucion para la conservacion que han hecho la reproduccion en cautiverio de anfibios y su reintroduccion desde la creacion del Plan de Accion de Conservacion de Anfibios

(PACA) de la Union Internacional para la Conservacion de la Naturaleza (UICN) en 2007. Ensamblamos datos sobre la reproduccion en cautiverio de anfibios y su reintroduccion a partir de una variedad de fuentes, que incluyeron la base de datos Arca de Anfibios y la Lista Roja de la UICN. Tambien realizamos busquedas sistematicas de bibliografia relevante en Web of Science, JSTOR y Google Scholar. En relacion con los datos colectados desde 1966 y hasta 2006, el numero de especies involucrado en los proyectos de reproduccion en cautiverio y reintroduccion incremento en un 57% en los siete anos desde el lanzamiento del PACA. Sin embargo, ha habido relativamente pocas reintroducciones nuevas en este periodo; la mayoria de los programas se han enfocado en asegurar poblaciones de garantia en cautiverio (es decir, especies puestas en cautiverio como precaucion contra las extinciones en libertad) y en investigaciones relacionadas con las conservacion. Ha habido un cambio hacia una mayor representacion de ranas, salamandras y cecilias dentro de los programas y un enfasis creciente sobre las especies amenazadas. Ha habido un incremento relativo en las especies dentro de los programas de America Central, America del Sur y el Caribe, en donde la diversidad de anfibios es alta. Aproximadamente la mitad de los programas involucra a zoologicos y acuarios con una proporcion similar representada en las instalaciones especializadas a cargo de agencias gubernamentales y no gubernamentales. A pesar de que se tiene a la reintroduccion exitosa como el hito para dichos programas, lo irreversible de muchas de las amenazas actuales para los anfibios puede hacer de esta una meta poco practica. En su lugar, es probable que se requiera de investigacion sobre las poblaciones de garantia en cautiverio para desarrollar soluciones imaginativas que permitan que los anfibios sobrevivan a las amenazas actuales, las emergentes y las futuras.Resumen

4556: ***+.090***

Anthropogenic threats often impose strong selection on affected populations, causing rapid evolutionary responses. Unfortunately, these adaptive responses are rarely harnessed for conservation. We suggest that conservation managers pay close attention to adaptive processes and geographic variation, with an eye to using them for conservation goals. Translocating pre-adapted individuals into recipient populations is currently considered a potentially important management tool in the face of climate change. Targeted gene flow, which involves moving individuals with favorable traits to areas where these traits would have a conservation benefit, could have a much broader application in conservation. Across a species' range there may be long-standing geographic variation in traits or variation may have rapidly developed in response to a threatening process. Targeted gene flow could be used to promote natural resistance to threats to increase species resilience. We suggest that targeted gene flow is a currently underappreciated strategy in conservation that has applications ranging from the management of invasive species and their impacts to controlling the impact and virulence of pathogens. Los Efectos de la Resistencia de la Cuerda para Pescar sobre la Severidad de los Enredos de Ballenas Grandes Las amenazas antropogenicas frecuentemente imponen una seleccion fuerte sobre las poblaciones afectadas, lo que causa respuestas evolutivas rapidas. Desafortunadamente, estas respuestas adaptativas se usan rara vez para la conservacion. Sugerimos que los manejadores de la conservacion presten atencion cuidadosamente a los procesos adaptativos y a la variacion genica, con miras a usarlos para los objetivos de conservacion. Reubicar a individuos pre-adaptados a poblaciones receptoras actualmente se considera una herramienta de manejo potencialmente importante de frente al cambio climatico. El flujo genico dirigido, que involucra mover individuos con caracteres favorables a areas en las que estos caracteres se habrian considerado un beneficio, podria tener una aplicacion amplia en la conservacion. A lo largo de la extension de las especies puede haber una variacion geografica de larga duracion en los caracteres o la variacion podria haberse desarrollado rapidamente en respuesta a un proceso de amenaza. El flujo genico dirigido podria usarse para promover la resistencia natural a amenazas para asi incrementar la resiliencia de las especies.

Sugerimos que el flujo genico dirigido es actualmente una estrategia poco apreciada en la conservacion y que tiene aplicaciones que van desde el manejo de las especies invasoras y sus impactos hasta el control del impacto y la virulencia de los patogenos.Resumen

4557: *+.172*

Genetics can provide information that is critical for planning translocations for conservation, such as levels of diversity and divergence of target and source populations. For clonal plants, assessing population characteristics (size, diversity, mortality, gene flow) that influence conservation values also requires identification of different genetic individuals. We used 12 microsatellite markers to guide germplasm source recommendations for augmentation and introduction translocations to conserve the critically endangered Eucalyptus cuprea that occurs in fragmented populations in the semi-arid shrublands of Western Australia. Ramet clumps with identical multilocus genotypes were identified in all populations but clonal richness (R = 0 - 0.86) and heterogeneity (D = 0 - 0.98) varied among populations. Genetic diversity was low to moderate in all populations (mean H (o) = 0.61, mean A = 3.78) and did not differ significantly between localities. There was evidence of inbreeding in some populations but outcrossing (t (m) = 0.495) in the small number of families available for study (N = 4) and genotypic diversity of the larger extant populations suggest the generation of novel genotypes is a component of the reproductive strategy. Most diversity was within populations and differentiation among populations was moderate (F (ST) = 0.100) suggesting mixing of source population for translocation is unlikely to lead to outbreeding depression. Principal Co-ordinate and Bayesian analyses indicated the Northern population is distinct from Central/Southern populations. We recommend use of mixed germplasm to conserve the moderate diversity characterising larger remnant populations and to enable the production of recombinants through sexual reproduction. But given seed availability and the distinction of the Northern population, an initial precautionary approach to a translocation proposed for south of the geographical range may be to source germplasm from the Central/Southern locality.

4558: *+.121*

The recently evolved genus Tolpis Adans. has its major center of diversity located in Macaronesia. Although recent advances have been made to understand the relationships of Tolpis species within Macaronesia, little is still known about the genetic patterns and genetic diversity of the Azorean and Madeiran Tolpis populations. To achieve this, a set of 8 microsatellite loci (SSR) was applied to 478 individuals of Tolpis azorica and T. succulenta. Genetic structure analysis, in addition to a spatial analysis, confirmed the existence of geographically circumscribed genetic patterns allied to a barrier effect by the sea in the Azorean T. azorica and T. succulenta. A detailed analysis of T. azorica revealed three different genetic groups, each group being particular to a different Azorean sub-archipelago, while the analysis conducted with T. succulenta confirmed the occurrence of a differential grouping between individuals from Azores and Madeira populations. The impact of catastrophic volcanic events and intense humanization of the habitats is discussed, in view of the present genetic diversity and structure of the species. In general, T. azorica populations showed high F (is) values and some populations of T. succulenta both in Azores and in Madeira also showed signs of putative inbreeding. Conservation actions such as the eradication of invasive plant and animal species are advised but translocations of plants or diaspores between islands or between populations of a same island should not be attempted.

4559: *+.280*

AimAvian communities in human-modified landscapes exhibit varying patterns of local

colonization and extinction rates, determinants of species occurrence. Our objective was to model these processes to identify habitat features that might enable movements and account for occupancy patterns in habitat matrices between the Guanica and Susua forest reserves. This knowledge is central to conservation design, particularly in ever changing insular landscapes.LocationSouth-western Puerto Rico. MethodsWe used a multiseason occupancy modelling approach to quantify seasonal estimates of occupancy, and colonization and extinction rates of seven resident avian species surveyed over five seasons from January 2010 to June 2011. We modelled parameters by matrix type, expressions of survey station isolation, quality, amount of forest cover and context (embedded in forest patch). ResultsSeasonal occupancy remained stable throughout the study for all species, consistent with seasonally constant colonization and extinction probabilities. Occupancy was mediated by matrix type, higher in reserves and forested matrix than in the urban and agricultural matrices. This pattern is in accord with the forest affinities of all but an open-habitat specialist. Puerto Rican Spindalis (Spindalis portoricensis) exhibited high occupancy in the urban matrix, highlighting the adaptability of some insular species to novel environments. Highest colonization rates occurred when perching structures were at 500m. Survey stations with at least three fruiting tree species and 61% forest cover exhibited lowest seasonal extinction rates. Main conclusionsOur work identified habitat features that influenced seasonal probabilities of colonization and extinction in a human-modified landscape. Conservation design decisions are better informed with increased knowledge about interpatch distances to improve matrix permeability, and habitat features that increase persistence or continued use of habitat stepping stones. A focus on dynamic processes is valuable because conservation actions directly influence colonization and extinction rates, and thus, a quantitative means to gauge their benefit.

4560: ***+.226***

Understanding the limiting factors of recovery is essential for guiding sound management of endangered species. The Wyoming toad (Anaxyrus baxteri) is a critically endangered amphibian whose cause of decline and inability to reestablish breeding populations despite early life stage reintroductions remains unknown; habitat degradation and the pathogen Batrachochytrium dendrobatidis (Bd) are 2 potential contributing factors. In 2013, we experimentally tested the effects of habitat factors under food supplemented and predator-protected conditions (i.e., mesh field enclosures) on time to metamorphosis, the proportion of tadpoles that metamorphosed, tadpole and toadlet size, the proportion of toadlets remaining in enclosures at release (approx. 1 month post-metamorphosis), and Bd prevalence in early life stages of Wyoming toads at Mortenson Lake National Wildlife Refuge. In 2014, we tested the effects of small-scale application of vegetation height management on toadlet size, the proportion of toadlets that remained, and Bd prevalence until 1 month post-metamorphosis. In 2013, median time to metamorphosis (25.5 days) was shorter in warmer water temperatures and proportion of tadpoles that metamorphosed was 0.70. In 2013, toadlet size was positively related to forb cover up to 35% and although overall treatment effect was not significant, mid-vegetation height treatments (10-30 cm) had fewer small toadlets at release than short (010 cm) and tall (>30 cm) vegetation heights. In 2014, vegetation height treatment (11.49-31.6 cm) had marginal support for estimating larger size at release. Batrachochytrium dendrobatidis was not detected in water samples or on post-metamorphic toadlets. Our results suggest that in mesh field enclosures, time to metamorphosis is shorter in warm water. In addition, vegetation heights of 10-30 cm and up to 35% forb cover within terrestrial mesh enclosures could increase Wyoming toad post-metamorphic size, which could increase overwinter survival rates. Using mesh field enclosures for soft-release may improve the effectiveness of early life stage reintroduction efforts, but predator attraction and density-dependent growth need to be considered. Habitat management can also influence growth and

survival of early life stages of amphibians and may benefit reintroduction efforts for other species. (C) 2015 The Wildlife Society.

4561: ***-.052***

Asian Houbara Chlamydotis macqueenii numbers are declining as a result of unsustainable levels of hunting and poaching, with the main conservation response being population reinforcement through the release of captive-bred birds. We assessed the contribution of captive breeding to the species' conservation by examining the fates of 65 captive-bred birds fitted with satellite transmitters and released during spring (March-May) and autumn (August) into breeding habitat in Uzbekistan. Of the released birds, 58.5% survived to October, the month favoured by Emirati hunters in Uzbekistan, but only 10.8% of those released survived the winter to return as subadults next spring. To mitigate and compensate for the loss of wild adults to hunting, the number of released birds needs to be an order of magnitude higher than hunting quotas (with a release of between 1640 and 1920 required for a hypothetical quota of 200), indicating that releases may be costly and do not remove the need for a biologically determined sustainable hunting quota.

4562: ***+.058***

The survival of captive-bred individuals from release into the wild to their first breeding season is crucial to assess the success of reintroduction or translocation programmes, and to assess their potential impact of wild populations. However, assessing the survival of captive-bred individuals following their release is often complicated by immediate dispersal once in the wild. Here, we apply Lindberg's robust design model, a method that incorporates emigration from the study site, to obtain true estimates of survival of captive-bred Mallards Anas platyrhynchos, a common duck species released on a large scale in Europe since the 1970s. Overall survival rate from release in July until the onset of the next breeding season in April was low (0.18 +/- 0.07 se) and equivalent to half the first-year survival of local wild Mallards. Higher overall detectability and temporary emigration during the hunting period revealed movements in response to hunting pressure. Such low survival of released Mallards during their first year may help prevent large-scale genetic mixing with the wild population. Nevertheless, by combining our results with regional waterfowl counts, we estimated that a minimum of 34% of the Mallards in the region were of captive origin at the onset of the breeding season. Although most released birds quickly die, restocking for hunting may be of sufficient magnitude to affect the wild population through genetic homogenization or loss of local adaptation. Robust design protocols allow for the estimation of true survival estimates by controlling for permanent and temporary emigration and may require only a moderate increase in fieldwork effort.

4563: ***+.108***

1. Choosing an optimal strategy to sustain imperilled wild populations is challenging, and many methods may be implemented before reaching an effective strategy. A review of previous conservation efforts used to maintain an endangered desert pupfish (Cyprinidon bovinus) are presented here. 2. Conservation strategies for C. bovinus have included removal of fish hybridizing with a non-native species, reintroduction of captive-bred C. bovinus into their historic range, and habitat restoration and expansion. In general, these methods have successfully increased the C. bovinus population over a 15-year period, with habitat expansion appearing to be the most critical intervention method. 3. Habitat restorations that increased the available breeding habitat for C. bovinus and the total number of breeding males were also associated with a downward trend in individual male reproductive behaviour. 4. Since habitat expansion resulted in

unexpected consequences for reproductive ecology, it emphasizes the importance not only of monitoring species abundance but also of evaluating individual reproductive behaviour when implementing conservation strategies. Copyright # 2015 John Wiley & Sons, Ltd.

4564: *+.152*

Understanding reproduction and mating systems is important for managers tasked with conserving vulnerable species. Genetic tools allow biologists to investigate reproduction and mating systems with high resolution and are particularly useful for species that are otherwise difficult to study in their natural environments. We conducted parentage analyses using 19 nuclear DNA microsatellite loci to assess the influence of population density, genetic diversity, and ancestry on reproduction, and to examine the mating system of pygmy rabbits (Brachylagus idahoensis) bred in large naturalized enclosures for the reintroduction and recovery of the endangered distinct population in central Washington, USA. Reproductive output for females and males decreased as population density and individual homozygosity increased. We identified an interaction indicating that male reproductive output decreased as genetic diversity declined at high population densities, but there was no effect at low densities. Males with high amounts (>50%) of Washington ancestry had higher reproductive output than the other ancestry groups, while reproductive output was decreased for males with high northern Utah/Wyoming ancestry and females with high Oregon/Nevada ancestry. Females and males bred with an average of 3.8 and 3.6 mates per year, respectively, and we found no evidence of positive or negative assortative mating with regards to ancestry. Multiple paternity was confirmed in 81% of litters, and we report the first documented cases of juvenile breeding by pygmy rabbits. This study demonstrates how variation in population density, genetic diversity, and ancestry impact fitness for an endangered species being bred for conservation. Our results advance understanding of basic life history characteristics for a cryptic species that is difficult to study in the wild and provide lessons for managing populations of vulnerable species in captive and free-ranging populations.

4565: *+.139*

Sonoran pronghorn (Antilocapra americana sonoriensis), an endangered subspecies of American pronghorn, are of great conservation concern in the southwestern U. S. Following a dramatic population decline in 2002, the U.S. Fish and Wildlife Service (USFWS) began a captive breeding program that has subsequently been used to supplement the wild population. Additionally, in 2009 the USFWS proposed to establish another, self-sustaining population outside of their range at that time. We modeled Sonoran pronghorn population dynamics based on time-series of abundance and conducted a population viability analysis (PVA) to evaluate the benefit of these management actions. We found that rates of change in the Sonoran pronghorn population were closely tied to the amount of precipitation in the area but that viability was greatly enhanced by conservation actions including the maintenance of a captive population, as well as the establishment of two additional populations outside the current range. (C) 2016 Elsevier Ltd. All rights reserved.

4566: *-.102*

Global amphibian decline and extinction has been associated with the spread of the pathogenic chytrid fungus (Batrachochytrium dendrobatidis, Bd). Despite extensive research, there have been no examples of effective management abating the ongoing impact of this pathogen in the wild. The endangered alpine tree frog (Litoria verreauxii alpina) has been extirpated from 80% of its former range because of Bd. We directly tested whether source population or host site influenced the efficacy of a reintroduction of L. v. alpina. We captive reared and released 1241 individuals

from three different populations, two with a history of Bd exposure and one that was Bd-naive, into two sites where they had historically occurred, and two sites where the species currently persists. Between 6 and 9 months post-release, we recaptured 4.83% of the released animals, and observed breeding at all sites. Both released and extant animals had similar susceptibility to infection; both groups increased in Bd infection prevalence and infection intensity throughout the breeding season. We did not detect any effect on survival by site of release; however, population of origin had a relatively large impact (omega = 0.454), and animals from one Bd-exposed population were recaptured significantly more than the animals from the other Bd-exposed population and the Bd-naive population. Population exposure history to the disease of reintroduced amphibians may be used to increase post-release fitness and conservation success. Selection for mechanisms of resistance should be further explored to help mitigate the impact of chytridiomycosis during reintroduction programmes.

4567: *-.075*

Pityopsis ruthii is a federally endangered plant, endemic to riparian areas of the Hiwassee and Ocoee rivers in southeastern Tennessee. The population size and spatial distribution of this species along the Ocoee River has been documented since 1985, yet about 90% of P. ruthii plants are recorded within a portion of the Hiwassee River. Our ongoing population census of these localities delineates 57 discrete site occurrences and constitutes the first population baseline for P. ruthii along the Hiwassee River. Evidence indicates that P. ruthii populations are either stable or increasing along the Ocoee River. Augmentation of natural flow and subsequent invasion of competing vegetation are frequently cited as a threat to the species; however, historical aerial photography suggests significant portions of P. ruthii habitat are resistant to succession. We discuss recent evidence that a number of natural and anthropogenic stressors are challenging population sustainability, including: invasive plants, insect pests, plant pathogens, genetic incompatibility, hybridization, inbreeding depression, and habitat disruption. It remains unclear how these stressors are currently affecting plant populations.

4568: *+.035*

Elk (Cervus canadensis) historically inhabited Southern Quebec and central Ontario, but, by the early 1900s, the species was extirpated from this region. Attempts to re:establish an Elk population in Ontario during the first half of the 20th century had limited success. We reviewed historical documents, population census records, and a previous study pertaining to Elk reintroduced to Ontario in the early 1900s for clues to the cause(s) of their limited population growth. After an apparent rapid population increase in the 1940s followed by unregulated hunting during the subsequent 3 decades, Elk abundance in Ontario had not appreciably changed from 1970 to 1997, most likely because of the small founding population, unsustainable hunting, and accidental mortality. After the abolition of legal hunting in 1980, natural mortality appeared to be the main limiting factor. A limited sample of pregnancy and calf recruitment rates, body measurements, and physical condition parameters collected in 1993-1997, suggested that adults were healthy, reproducing successfully, and not limited by food availability; thus, it was concluded that remnant Elk populations could be augmented by introducing additional animals. A renewed Elk restoration effort, conducted from 1998 to 2001, imported 443 Elk from Elk. Island National Park in Alberta to 4 release areas across central Ontario (Lake of the Woods, Lake Huron North Shore, Nipissing/French River, and Bancroft/North Hastings), resulting in a provincial population of about 800 Elk by 2013.

4569: *+.170*

The Italian hare is a species of hare endemic to central and southern Italy and to Sicily. It has been classified as a 'vulnerable' species by the International Union for Conservation of Nature (IUCN), as it is considered to have a high risk of extinction in the next decade. Despite its endangered status, little is known about its feeding habits. In the present study, the seasonal pattern of diet composition of a population of Italian hare occupying a semi-natural landscape was estimated by using the micro-histological technique of faecal analysis. The results showed that hares had a diversified diet, consuming plant parts from over 70 species. Like other Lepus sp., the Italian hare consumed a large amount of herbaceous plants (e.g. Brachypodium sylvaticum, Trifolium pratense, Allium subhirsutum and Festuca arundinacea), although it complemented its diet seasonally with fruits of Prunus spinosa, Pyrus piraster and Malus sylvestris. Analysis of similarities (ANOSIM) evidenced significant differences among seasons, as a consequence of the seasonal occurrence of the various food items. Spring and autumn (R=0.7482, P=0.001), as well as spring and winter (R=0.7398, P=0.001), showed low diet similarities; these results were supported by similarity percentage analysis (SIMPER, average dissimilarity: > 71% between spring and autumn; > 69% between spring and winter) with taxa like P. spinosa, Cirsium strictus, T. pratense and Rosa canina making the greatest contributions to these differences. Higher similarities were instead found when comparing other seasons. This seasonal pattern of diet composition was clearly depicted in the graph from nonmetric multidimensional scaling (n-MDS) ordination. Our results highlight the importance of some plant taxa in the diet of the Italian hare and could be useful in managing reintroduction programs.

4570: ***+.130***

Red deer (Cervus elaphus) have played a key role in human societies throughout history, with important cultural significance and as a source of food and materials. This relationship can be traced back to the earliest human cultures and continues to the present day. Humans are thought to be responsible for the movement of a considerable number of deer throughout history, although the majority of these movements are poorly described or understood. Studying such translocations allows us to better understand ancient human wildlife interactions, and in the case of island colonizations, informs us about ancient human maritime practices. This study uses DNA sequences to characterise red deer genetic diversity across the Scottish islands (Inner and Outer Hebrides and Orkney) and mainland using ancient deer samples, and attempts to infer historical colonization events. We show that deer from the Outer Hebrides and Orkney are unlikely to have originated from mainland Scotland, implying that humans introduced red deer from a greater distance. Our results are also inconsistent with an origin from Ireland or Norway, suggesting long-distance maritime travel by Neolithic people to the outer Scottish Isles from an unknown source. Common haplotypes and low genetic differentiation between the Outer Hebrides and Orkney imply common ancestry and/or gene flow across these islands. Close genetic proximity between the Inner Hebrides and Ireland, however, corroborates previous studies identifying mainland Britain as a source for red deer introductions into Ireland. This study provides important information on the processes that led to the current distribution of the largest surviving indigenous land mammal in the British Isles.

4571: ***-.009***

Reintroduction of endangered fishes to historic habitat has been used as a recovery tool; however, these fish may face competition from other fishes that established in their native habitat since extirpation. This study investigated the physiological response of tidewater goby, Eucyclogobius newberryi, an endangered California fish, when competing for food with threespine stickleback, Gasterosteus aculeatus, a native species, and rainwater killifish, Lucania parva, a non-native

species. Survival, growth and physiological indicators of stress (i.e. cortisol, glucose and lactate concentrations) were assessed for juvenile fish held for 28 days in two food-limited conditions. When fed a 75% ration, survival of E. newberryi was significantly lower when held with G. aculeatus. In all fish assemblages, weight and relative condition decreased then stabilized over the 28 day experiment, while length remained unchanged. Whole -body cortisol in E. newberryi was not affected by fish assemblage; however, glucose and lactate concentrations were significantly higher with conspecifics than with other fish assemblages. When fed a 50% ration, survival of E. newberryi decreased during the second half of the experiment, while weight and relative condition decreased and length remained unchanged in all three fish assemblages. Cortisol concentrations were significantly higher for all fish assemblages compared with concentrations at the start of the experiment, whereas glucose and lactate concentrations were depressed relative to concentrations at the start of the experiment, with the magnitude of decrease dependent on the species assemblage. Our findings indicate that E. newberryi exhibited reduced growth and an elevated generalized stress response during low food availability. In response to reduced food availability, competition with G. aculeatus had the greatest physiological effect on E. newberryi, with minimal effects from the non-native L. parva. This study presents the first reported cortisol, glucose and lactate concentrations in response to chronic stress for E. newberryi.

4572: ***+.081***

The Italian bleak, Alburnus albidus, is a freshwater species endemic of southern Italy, that was recently classified as vulnerable in the IUCN Red List, due to the impact of introduced species. In this study, a combined genetic (mitochondrial cyt b and nuclear ITS1 DNA markers) and morphometric approach was applied to five populations from the whole range of A. albidus. Data were compared with those for the alborella A. arborella, an introduced species native to northern Italy. The results indicate a dramatic threat to A. albidus, as 39% of the specimens examined are the result of hybridization with other introduced cyprinids, mainly A. arborella. Hybrid individuals suggest bi-directional hybridization, but introgressed individuals exclusively show A. albidus nuclear genomes and A. arborella mitochondrial haplotypes. Morphological data discriminate the two parental Alburnus but not their hybrids or introgressed individuals. Genetic variability between the species is greater than the morphological variability. Ofanto River shows the most compromised situation suggesting the occurrence of multiple introductions, while Agri and Trigno populations are the most conserved, and deserve priority conservation actions. Given this, an update of the conservation status is recommended.

4573: ***-.093***

Isolation of wildlife into fragmented populations as a consequence of anthropogenic-mediated environmental change may alter host-pathogen relationships. Our understanding of some of the epidemiological features of infectious disease in vulnerable populations can be enhanced by the use of commensal bacteria as a proxy for invasive pathogens in natural ecosystems. The distinctive population structure of a well-described meta-population of a New Zealand endangered flightless bird, the takahe (Porphyrio hochstetteri), provided a unique opportunity to investigate the influence of host isolation on enteric microbial diversity. The genomic epidemiology of a prevalent rail-associated endemic commensal bacterium was explored using core genome and ribosomal multilocus sequence typing (rMLST) of 70 Campylobacter sp. nova 1 isolated from one third of the takahe population resident in multiple locations. While there was evidence of recombination between lineages, bacterial divergence appears to have occurred and multivariate analysis of 52 rMLST genes revealed location-associated differentiation of C. sp. nova 1 sequence types. Our results indicate that fragmentation and anthropogenic manipulation of populations can

influence host-microbial relationships, with potential implications for niche adaptation and the evolution of micro-organisms in remote environments. This study provides a novel framework in which to explore the complex genomic epidemiology of micro-organisms in wildlife populations.

4574: *+.077*

Spatial transfers of species distribution models (SDMs) are often applied in the study of land use and climate-change effects, spread of invasive species and conservation planning. However, model transferability and risk of error are rarely evaluated prior to predicting species distribution to different regions. We aim to assess spatial transferability of SDMs for stream fish and to evaluate the effect of model types and habitat heterogeneity on transferability. We developed SDMs for 21 fish species and made predictions of occurrence of these species among five pairs of river catchments (i.e. model transfers). Forty eight transfers were made for each of three modelling approaches (Lasso-regularised logistic regression (LLR), boosted regression trees (BRT) and MaxEnt) after incorporating spatial autocorrelation. In addition to internal and external evaluation of discrimination power, we assessed the cross-catchment consistency of variable selections, fish-habitat relationships and predicted probabilities of species presences. Approximately half of 144 spatial transfers of SDMs had moderate to high discrimination power. Discrimination power was low for the rest of the models. Incorporating spatial autocorrelation could not improve discrimination power in the model transfers. Friedman test showed that BRT did not differ significantly from LLR but it outperformed MaxEnt in terms of AUC in the model transfers. BRT and LLR models tended to have high overall accuracy and specificity, whereas MaxEnt tended to have high sensitivity in the model transfers. The degree of model transferability varied among species, and was asymmetric when reciprocal transfers were made between paired catchments. Ranks of variable importance in BRT models differed among catchments for most species. Temperature, base flow index, altitude and habitat condition index were more often ranked as the most important predictors in the five study river catchments, although the functional forms of their effects on fish presence were sometimes inconsistent between paired catchments. Compared to published transferability of some terrestrial taxa SDMs, spatial transferability of stream-fish distribution models was limited, reflecting the natural barriers to dispersal among catchments, and the necessity of evaluating model transferability in conservation applications (e.g. evaluation of climate- or landscape-change effects, invasive species risk assessments and species reintroduction planning). We suggest the following strategies to enhance spatial transferability: (i) match the range and location of the habitat predictors between the model region and prediction region, or alternatively choose a model training region with a large extent and a wide range of environmental gradients; (ii) use presence-absence models such as BRT and LLR and (iii) include habitat features with a sound ecological basis such as temperature and hydrology.

4575: *+.166*

Indigo Snakes (Drymarchon; with five currently recognized species) occur from northern Argentina, northward to the United States in southern Texas and eastward in disjunct populations in Florida and Georgia. Based on this known allopatry and a difference in supralabial morphology the two United States taxa previously considered as subspecies within D. corais (Boie 1827), the Western Indigo Snake, D. melanurus erebennus (Cope 1860), and Eastern Indigo Snake, D. couperi (Holbrook 1842), are currently recognized as separate species. Drymarchon couperi is a Federally-designated Threatened species by the United States Fish and Wildlife Service under the Endangered Species Act, and currently being incorporated into a translocation program. This, combined with its disjunct distribution makes it a prime candidate for studying speciation and genetic divergence. In this study, we (1) test the hypothesis that D. m. erebennus and D. couperi

are distinct lineages by analyzing 2411 base pairs (bp) of two mitochondria] (mtDNA) loci and one single copy nuclear (scnDNA) locus; (2) estimate the timing of speciation using a relaxed phylogenetics method to determine if Milankovitch cycles during the Pleistocene might have had an influence on lineage diversifications; (3) examine historical population demography to determine if identified lineages have undergone population declines, expansions, or remained stable during the most recent Milankovitch cycles; and (4) use this information to assist in an effective and scientifically sound translocation program. Our molecular data support the initial hypothesis that D. melanurus and D. couperi should be recognized as distinct species, but further illustrate that D. couperi is split into two distinct genetic lineages that correspond to historical biogeography and sea level changes in peninsular Florida. These two well-supported genetic lineages (herein termed Atlantic and Gulf lineages) illustrate a common biogeographic distributional break previously identified for other plants and animals, suggesting that these organisms might have shared a common evolutionary history related to historic sea level changes caused by Milankovitch cycles. Our estimated divergence times suggest that the most recent common ancestor (MRCA) between D. melanurus and southeastern United States Drymarchon occurred ca. 5.9 Ma (95% HPD = 2.5-9.8 Ma; during the late Blancan of the Pleistocene through the Hemphillian of the Miocene), whereas the MRCA between the Atlantic and Gulf lineages in the southeastern United States occurred ca. 2.0 Ma (95% HPD = 0.7-3.7 Ma; during the Irvingtonian of the Pleistocene through the Blancan of the Pliocene). During one or more glacial intervals within these times, these two lineages must have become separated and evolved independently. Despite numerous Milankovitch cycles along with associated forming of physical barriers (i.e., sea level fluctuations, high elevation sand ridges, clayey soils, and/or insufficient habitats) since their initial lineage diversification, these two lineages have likely come in and out of contact with each other many times, yet today they still illustrate near discrete geographic distributions. Although the Atlantic and Gulf lineages appear to be cryptic, a thorough study examining morphological characters should be conducted. We believe that our molecular data is crucial and should be incorporated in making conscious decisions in the management of a translocation program. We suggest that source populations for translocations include maintaining the integrity of the known genetic lineages found herein, as well as those coming from the closest areas that currently support sizable Drymarchon populations. Published by Elsevier Inc.

4576: ***+.135***

Dams, utilized for hydroelectric or flood control purposes, obstruct organism dispersal and have contributed to the decline of many migratory fish populations. For threatened Chinook salmon (Oncorhynchus tshawytscha) from the Willamette River Basin in Oregon, human-assisted reintroductions are being used to facilitate dispersal to historical habitats located above dams. However, little is known about the reproductive outcomes of reintroduced individuals or the efficacy of reintroductions towards the goal of population demographic viability. Using genetic parentage assignments to 3-, 4-, and 5-year-old adult recruits, we estimated the fitness of hatchery and wild Chinook salmon reintroduced above Foster Dam on the South Santiam River, a tributary of the Willamette River. Our parentage assignments indicated that the fitness of reintroduced salmon was highly variable, with individuals producing a range of 0-40 adult progeny. We also detected a possible trend towards reduced fitness in mate pairs composed of hatchery versus wild salmon. For each of three brood years (2007, 2008, 2009), adult offspring recruitment achieved or exceeded population replacement. We observed the highest cohort replacement rate in 2009, the first year that managers aimed to release wild salmon solely above the dam. Taken together, our results suggest that human-assisted reintroductions of mature adult salmon to historical spawning habitats are a promising method of restoring natural production in populations affected by dams. Moreover, the continued used of wild fish in reintroduction operations may improve population

productivity and the prospect of recovery within the South Santiam River.

4577: ***+.149***

Translocations have been recommended to reestablish, augment, and sustain genetic diversity in declining wildlife populations, including greater sage-grouse (Centrocercus urophasianus; sage-grouse). Characteristics of successful sage-grouse translocations include suitable contiguous sagebrush (Artemisia spp.), seasonal habitats surrounded by geomorphic barriers, a residual resident population, and pre-nesting releases. From 2009 to 2010, we studied vital rates of 60 translocated and 15 resident radio-marked female sage-grouse and their broods on Anthro Mountain, in the Ashley National Forest, northeastern Utah, USA to determine whether translocations could augment a declining meta-population that inhabited suitable breeding habitats in a small spatially isolated landscape. Survival rates, and nest and brood success estimates for the resident and translocated sage-grouse we studied were lower than reported range-wide averages. Nest success was similar for resident and translocated birds (survival estimate: 0.468, 95% CI=0.288-0.648) and we calculated weak, yet positive relationships to grass height and grass cover. Daily survival rates for chicks 0-19 days of age for resident females in 2009 (0.904, CI=0.875-0.940) and in 2010 (0.910, CI=0.888-0.945) were higher than for chicks raised by translocated females (0.883, CI=0.850-0.915; 0.892, CI=0.856-0.936, respectively), although differences were small and all 95% confidence intervals had substantial overlap. In 2009 and 2010, daily survival rates for chick 20-50 days of age were slightly higher for chicks reared by resident females (0.980, CI=0.963-0.994; 0.998, CI=0.978-1.000, respectively) than chicks reared by translocated females (0.877, CI=0.623-0.959; 0.988, CI=0.945-0.993, respectively). Chick survival for both groups was weakly correlated with grass cover. Although most translocated birds remained on the study area (82%), the low overall survival rates we report indicate that managers need to consider factors affecting survival of adult females, nests, and chicks when planning future translocations to augment sage-grouse meta-populations that occupy spatially isolated and space-limited habitats. (c) 2016 The Wildlife Society.

4578: ***+.120***

Large avian scavengers are among the most vulnerable vertebrates, and many of their populations have declined severely in recent decades. To help mitigate this marked reduction in abundance, supplementary feeding stations (SFS; colloquially termed "vulture restaurants") have been created worldwide, often without consideration of the scientific evidence supporting the suitability of the practice. SFS have been effective and important tools for conservation and reintroduction of avian scavengers. However, negative consequences can result from large aggregations of individual birds, disrupting intraguild processes and promoting density dependent decreases in productivity. At the community level, SFS favor the congregation of predators (ie facultative scavengers), increasing predation risk on small-and medium-sized vertebrates in the vicinity of the SFS. These feeding stations might also affect processes of natural selection and even render populations maladapted to their natural environments. We also examine future scenarios for avian scavengers in relation to ecosystem services, to changes in agro-grazing economies and in land uses, and ultimately to rewilding landscapes where SFS play a controversial role.

4579: ***-.091***

The "landscape of fear" model, recently advanced in research on the non-lethal effects of carnivores on ungulates, predicts that prey will exhibit detectable antipredator behavior not only during risky times (i.e., predators in close proximity) but also in risky places (i.e., habitat where

predators kill prey or tend to occur). Aggregation is an important antipredator response in numerous ungulate species, making it a useful metric to evaluate the strength and scope of the landscape of fear in a multi-carnivore, multi-ungulate system. We conducted ungulate surveys over a 2-year period in South Africa to test the influence of three broad-scale sources of variation in the landscape on spatial patterns in aggregation: (1) habitat structure, (2) where carnivores tended to occur (i.e., population-level utilization distributions), and (3) where carnivores tended to kill ungulate prey (i.e., probabilistic kill site maps). We analyzed spatial variation in aggregation for six ungulate species exposed to predation from recently reintroduced lion (Panthera leo) and spotted hyena (Crocuta crocuta). Although we did detect larger aggregations of ungulates in "risky places," these effects existed primarily for smaller-bodied (<150 kg) ungulates and were relatively moderate (change of <= 4 individuals across all habitats). In comparison, ungulate aggregations tended to increase at a slightly lower rate in habitat that was more open. The lion, an ambush (stalking) carnivore, had stronger influence on ungulate aggregation than the hyena, an active (coursing) carnivore. In addition, places where lions tended to kill prey had a greater effect on ungulate aggregation than places where lions tended to occur, but an opposing pattern existed for hyena. Our study reveals heterogeneity in the landscape of fear and suggests broad-scale risk effects following carnivore reintroduction only moderately influence ungulate aggregation size and vary considerably by predator hunting mode, type of predation risk, and prey species.

4580: *-.061*

As evidence mounts that the feral Cat (Felis catus) is a significant threat to endemic Australian biodiversity and impedes reintroduction attempts, uncertainty remains about the impact a residual population of cats following control will have on a mammal reintroduction programme. Also, behavioural interactions between cats and their prey continue to be an area of interest. Within the framework of an ecosystem restoration project, we tested the hypotheses that successful reintroductions of some medium-sized mammals are possible in locations where feral cats are controlled (but not eradicated) in the absence of European Red Fox (Vulpes vulpes), and that hare-wallabies that dispersed from their release area are more vulnerable to cat predation compared with those that remain at the release site. We used radiotelemetry to monitor the survivorship and dispersal of 16 Rufous Hare-wallabies (Lagorchestes hirsutus spp.) and 18 Banded Hare-wallabies (Lagostrophus fasciatus fasciatus) reintroduced to four sites within Shark Bay, Western Australia. Nearly all foxes were removed and feral cats were subject to ongoing control that kept their indices low relative to prerelease levels. All monitored hare-wallabies were killed by cats within eight and 10 months following release. Significant predation by feral cats was not immediate: most kills occurred in clusters, with periods of several months where no mortalities occurred. Once a hare-wallaby was killed, however, predation continued until each population was eliminated. Animals remaining near their release site survived longer than those that dispersed. The aetiology of predation events observed offers new insights into patterns of feral cat behaviour and mammal releases. We propose a hypothesis that these intense per capita predation events may reflect a targeted hunting behaviour in individual feral cats. Even where feral cats are controlled, the outcome from consistent predation events will result in reintroduction failures. Managers considering the reintroduction of medium-sized mammals in the presence of feral cats should, irrespective of concurrent cat control, consider the low probability of success. We advocate alternative approaches to cat-baiting alone for the recovery of cat-vulnerable mammals such as hare-wallabies.

4581: *-.041*

Translocation of the koala (Phascolarctos cinereus) has been a controversial measure that has been

utilised for over a century in the southern states of Australia. Recently, translocation has been suggested as an option in the management of some declining northern koala populations. Infectious diseases present within donor and recipient populations are important factors that must be considered when planning any wildlife translocation. In the koala, infectious diseases, caused by the bacterial pathogen Chlamydia pecorum, are one of the key threats to koala conservation. In recent years, significant progress has been made in understanding the biology and epidemiology of koala C. pecorum infections revealing complex patterns of infection and disease and the potential for 'spill-over' from C. pecorum infected livestock. Here, in light of this new epidemiological data, we provide a discussion of risk assessment and management in the context of enzootic chlamydial infections. We conclude that without careful investigation and management, significant risk of pathogen transfer is likely, especially for larger and more distant translocations. Thus, for such a programme to be appropriate, they must: 1) perform adequate molecular screening methods for Chlamydia at both donor and recipient sites; 2) implement risk mitigation strategies that avoid transmission of Chlamydia genotypes that are not enzootic to the recipient site; and 3) assess and mitigate risk associated with potential transmission between koalas and livestock Standardised and comprehensive veterinary procedures are crucial in the assessment and management of disease and infection transmission risk, and telemetric monitoring is essential for the post-release monitoring of both translocated and resident koalas at the recipient site and subsequent evaluation of programme success. (C) 2016 Elsevier Ltd. All rights reserved.

4582: *+.191*

Human dimension is an important component of large carnivore management and conservation. Here, we focus on the human-wildlife conflict related to depredation of livestock by Pyrenean brown bears (Ursus arctos), despite the population being among the smallest in the world. Two reintroductions were performed in the past to ensure the survival of the population, yet its conservation status remains critical due to small size, heavy inbreeding and disagreements over its management. We investigated the often-neglected spatial variations in attitude towards predator presence to improve our understanding of the human dimensions surrounding this conflict. We used a questionnaire to assess the drivers explaining the attitude of the local human population (n = 577) of the Pyrenees towards bear presence. Our results show that spatial variables (place of birth and county of residence of the respondent) are strong predictors of attitude. The residents of two counties in particular (Haute-Garonne and Pyrenees-Atlantiques) displayed a positive attitude, while the residents of the Hautes-Pyrenees county had the most negative attitude. People born outside of the Pyrenees also showed a more positive inclination towards bear presence than people born and raised in France's southwestern mountain range. Both these results may imply a link between the history of local communities with predator presence and their current attitude. Accounting for small-scale spatial heterogeneity in social-ecological studies of human-wildlife conflicts will prove useful to get a more accurate mapping of attitudes and inform subsequent management decisions. (C) 2016 Elsevier Ltd. All rights reserved.

4583: *+.003*

Interspecific interactions are among the key factors influencing the structure of animal communities and have high relevance for conservation. However, managers, conservationists and decision-makers rarely consider the potential side-effects of single-species carnivore management for the conservation of other carnivores. We studied how management of protected brown bears (Ursus arctos) affected interspecific interactions with an endangered apex predator, the Eurasian lynx (Lynx lynx) in Slovenia. Due to large body size and superb olfactory abilities, bears are one of the most important dominant scavengers and regularly usurp kills from other large predators, a

process known as kleptoparasitism. At the same time, bears throughout the world are usually actively managed through zone-specific culling regimes, supplemental feeding, and translocations. This can considerably alter bear densities and activity patterns and in turn influence interactions among carnivores. Overall, we observed that bear scavenging pressure resulted in substantial energetic losses for Eurasian lynx. The probability of lynx losing kills to bears ranged from 8 to 74% and strongly depended on local bear densities and monthly bear movement rates. Kleptoparasitic interaction intensity differed almost 3-fold between different bear management zones. Furthermore, the presence of a bear feeding site increased the odds of lynx losing kills by 5-fold compared to areas >1000 m from these sites. We suggest that existing bear-feeding regimes should be reconsidered in order to reduce unwanted side-effects of this controversial practice on endangered apex predators. We also call attention to the importance of considering impacts of interspecific interactions in wildlife management and conservation. (C) 2016 Elsevier Ltd. All rights reserved.

4584: *+.261*

Maximizing survival in reintroduced, captive bred animals requires evaluation to identify best practices. This is particularly true for critically endangered species like the Vancouver Island marmot, endemic to British Columbia, Canada. From 2003 to 2010, 301 captive bred marmots were implanted with transmitters and released at extinct colony locations and other potentially preferred sites to bolster wild populations and establish new colonies. We evaluated release success based on three criteria: (1) site fidelity in the first summer, (2) survival to hibernation in fall, and (3) survival through winter. We used generalized linear mixed models and information theory to estimate the influence on release success of sex, age, and release practices, as well as local and landscape level habitat attributes. Our results suggest that overwinter survival most limited release success in the first year postrelease. In all years, overwinter survival was lower for newly released captive bred marmots than for wild or previously released marmots. Release date best predicted overall success, and was positively related to site fidelity and survival to hibernation but negatively related to overwinter survival. Our findings suggest that focused attempts to optimize release dates are likely to maximize long term reintroduction success.

4585: *+.062*

Combining radio-telemetry with direct observations, we followed 22 released juvenile captive-bred capercaillies throughout the day to assess if their seasonal and daily patterns of activity, movements, and diet are in accordance with published information on wild birds. Day length was the most important factor determining birds' mobility and activity. Capercaillies were active for 46 +/- 2% of daytime, during which they mostly foraged (30 +/- 2%). The average distance travelled per day was 0.93 +/- 0.14 km. The time budget of capercaillies was mostly allocated to activities on the ground but they spent at least 20% of the daytime in trees. They fed primarily on blueberries, cowberries and pine needles. The mean daily feeding time in fall and winter was 3 h, but it increased to 6 h in spring and summer. We conclude that released birds behave similarly to wild birds and reintroduction of captive-bred capercaillies can be successful if the initial mortality is reduced.

4586: *+.180*

This study investigated factors affecting transplantation success of native forest understory vegetation on metal-contaminated soils. One year after transplantation along a gradient of historical Cu-Ni smelter pollution in Sudbury, Canada, community-level characteristics of the

transplanted plots and short-term indicators expressing the performance of selected species were assessed. Thirty 16-m(2) plots were studied, each established with 45 transplanted 0.56x0.64-m turfs 10 cm thick, extracted from mixed-hardwood forests slated for development. Species richness, diversity, and evenness were not affected by environmental conditions but short-term responses of root growth and sexual reproduction of selected species to environmental variables indicate a need for long-term monitoring. Root growth of Clintonia borealis (Aiton) Raf. and Gaultheria procumbens L. was positively related to soil temperature. Root growth of G. procumbens correlated negatively with soil Ni availability, but in all plots, root growth was comparable with the literature values from unaffected forests. Flowering frequency of G. procumbens correlated negatively with soil pH and positively with tree cover, corresponding to ecological requirements of this species. Soil SO4-2 had a negative effect on sexual reproduction of C. borealis. Unexpectedly, fruiting of C. borealis responded positively to Ni, and fruiting of Maianthemum canadense Desf. positively to As, possibly due to interdependencies among soil variables. The results are encouraging with respect to transplant success, as effects of smelter-related variables were relatively minor, but species-specific responses of the selected species to environmental factors indicate that species performance is dependent on site-specific conditions, potentially influencing long-term success of the transplants.

4587: ***+.220***

AIMS: To identify network measures with relevance to disease spread in a network of movements derived from the Department of Conservation (DOC) translocation records from 1970 to mid-2014, and to identify conservation sites that should be prioritised for surveillance activities and improvements to data collection to make the best use of network analysis techniques in the future.METHODS: Data included the source and destination of translocated specimens, the species and the dates the translocations were expected to occur. The data were used to construct a directed, non-weighted network in which a translocation event represented a tie in the network. Network density, in-degree (movements entering a node of interest) and out-degree (movements leaving a node of interest) and reciprocity were calculated.RESULTS: The data analysed consisted of 692 unique translocations between 307 sites, with the majority (518; 73%) being for birds. The constructed network for bird, reptile and frog translocations comprised 260 nodes, with 34/260 (13%) having two-way movements and 47/260 (18%) non-reciprocal movements. The median degree score (sum of in- and out-degree) was two (min 0, max 36) with a mean of 3.5 in a right skewed distribution. Most sites acted as receivers or senders of consignments with only a few having both high in- and high out-degree, and thus had characteristics that made them sites of interest for surveillance activities. These included the National Wildlife Centre at Mount Bruce, Tiritiri Matangi Island and Te Kakahu (Chalky Island).CONCLUSIONS: The presence of linking sites that join larger clusters within the network creates the potential for rapid disease spread if a pathogen were to be introduced. The important sites that supply or receive specimens for translocations are already well recognised by those performing translocations in New Zealand, and this paper provides further information by quantifying their role within the network.

4588: ***+.108***

To enlarge the population size and distribution area of crested ibis (Nipponia nippon), this species was reintroduced to Tongchuan, Shaanxi province, north of Qinling Moutains. The activity and breeding behaviors of the reintroduced N. nippon were monitored by combined using of color marking, radio tracking and satellite tracking in two breeding seasons of 2014-2015. Finally, 8 nests were recorded. The clutch size was 2.5[plus or minus]0.9, and the reproductive success rate was 80%, which was higher than that of wild population. The nest tree dominated by Populus

canadensi (n=5) and Robinia pseudoacacia (n=2) were distributed within 300 m to the main foraging river. The nest characterizes were similar with those of wild individuals. The home ranges of two satellite tracking individuals in breeding season were 262.4 km2and 20.1 km2, respectively, which were much larger than those of wild individuals. The relative low-graded foraging habitat might restrict the reintroduced population persistence in Tongchuan, and feasible wetland restoration and food supplementation were suggested to improve the breeding condition of the reintroduced crested ibises.

4589: ***+.219***

The fisher was extirpated from much of the Pacific Northwestern United States during the mid- to late-1900s and is now proposed for federal listing as a threatened species in all or part of its west coast range. Following the translocation of 90 fishers from central British Columbia, Canada, to the Olympic Peninsula of Washington State from 2008 to 2010, we investigated the landscape-scale habitat selection of reintroduced fishers across a broad range of forest ages and disturbance histories, providing the first information on habitat relationships of newly reintroduced fishers in coastal coniferous forests in the Pacific Northwest. We developed 17 a priori models to evaluate several habitat-selection hypotheses based on premises of habitat models used to forecast habitat suitability for the reintroduced population. Further, we hypothesized that female fishers, because of their smaller body size than males, greater vulnerability to predation, and specific reproductive requirements, would be more selective than males for mid- to late-seral forest communities, where complex forest structural elements provide secure foraging, resting, and denning sites. We assessed 11 forest structure and landscape characteristics within the home range core-areas used by 19 females and 12 males and within randomly placed pseudo core areas that represented available habitats. We used case-controlled logistic regression to compare the characteristics of used and pseudo core areas and to assess selection by male and female fishers. Females were more selective of core area placement than males. Fifteen of 19 females (79%) and 5 of 12 males (42%) selected core areas within federal lands that encompassed primarily forests with an overstory of mid-sized or large trees. Male fishers exhibited only weak selection for core areas dominated by forests with an over story of small trees, primarily on land managed for timber production or at high elevations. The amount of natural open area best distinguished the use of core areas between males and females, with females using substantially less natural open area than males. Although sex-specific selection has been suspected for fishers, we identified factors that distinguish the selection of core areas by females from those of males, information which will be valuable to managers planning reintroductions or providing suitable habitat to promote fisher recovery in the Pacific Northwest. (C) 2016 Elsevier B.V. All rights reserved.

4590: ***+.101***

Present global plant extinction rates are 100-1000 times greater than pre-human levels and this is especially true on oceanic islands. There is a great need to model the distributions of endangered plants for reintroduction on oceanic islands, however, there are still questions concerning what is the most appropriate spatial scale and which environmental metrics should be included in order to guide restoration efforts. We examine the impact of spatial scale (1 km, 250 m, 10 m), environmental metrics (climate, topography, soils), and species overlap for 11 rare and endangered species in the dry forest of Oahu, Hawaii, which is one of the world's most endangered ecosystems, and contains some of the highest resolution data on species locations and environmental metrics for an oceanic island. At all spatial scales, the species distribution models reliably differentiated between occupied habitat and background for all 11 species (AUC >= 0.92). The relative importance of the environmental metrics did not vary across spatial scales with soil

great group contributing most to the models followed by elevation, and mean precipitation of the driest quarter. The percent of the total island area with niche overlap for two or more species did not show any pattern with grain size, however, the 10 m model contained the largest areas of niche overlap for two or more species. There were 1292 10 m pixels on Oahu where models predict niche overlap for eight endangered species, however, only 1.2% of the total area is currently in protected areas. Results suggests that species distribution models are useful for predicting habitat suitability at all scales (1 km, 250 m,10 m), environmental metrics do not change across scales but high resolution data on soils, topography, and precipitation are needed, and 10 m resolution data are the best for informing restoration decisions for the endangered species on Hawaii and other oceanic islands. (C) 2016 Elsevier B.V. All rights reserved.

4591: ***+.186***

Most hard corals have broad distributions, and historically this was attributed to their capability for extensive dispersal leading to high evolutionary and demographic inter-dependence among regions. More recently there has been a paradigm shift in the understanding of coral dispersal, driven largely by genetic evidence, which has put greater emphasis on self-recruitment and larval retention. There is now a growing body of evidence that morphologically cryptic species exist within many recognized 'species' of stony corals. Here, we characterise levels of genetic divergence within and between five species of Acropora sampled from disparate populations spanning the Indo-Pacific Oceans. We find that strong regional genetic differentiation corresponding to the separation of the Indian and Pacific Ocean basins is a consistent pattern in three of the five species examined. Furthermore, the extent of allopatric divergence within species is similar to that observed between species, implying negligible contemporary gene flow between regions in four of the five species examined. This is consistent with the presence of numerous morphologically cryptic allopatric subspecies or incipient Acropora species. If this is confirmed, the conservation status of several species, which are already demonstrably threatened, would require re-evaluation so that risks including silent extinctions and inappropriate translocations are properly managed.

4592: ***+.111***

We studied genetic variation in four endangered animal taxa in the largest freshwater spring complex in the southwestern U.S.A., Comal Springs (TX): Eurycea salamanders, Heterelmis riffle beetles, Stygobromus amphipods and Stygoparnus dryopid beetles. They inhabit a spring complex with nearly stable conditions, which is threatened by climate change and aquifer withdrawals. The four taxa vary in their habitat affinities and body sizes. We used genotyping-by-sequencing to obtain hundreds to thousands of genetic markers to accurately infer the demographic history of the taxa. We used approximate Bayesian computation to test models of gene flow and compare the results among taxa. We also looked for evidence that would suggest local adaptation within the spring complex. An island model (equal gene flow among all subpopulations) was the most probable of the five models tested, and all four taxa had high migration rate estimates. Small numbers of single nucleotide polymorphisms (SNPs) in each taxon tested were associated with environmental conditions and provide some evidence for potential local adaptation to slightly variable conditions across habitat patches within Comal Springs. We discuss how the results of this study can add to the habitat conservation plan for Comal Springs. If part of the spring system dries, migrants may recolonise from elsewhere within the spring complex. However, genetic variants affecting survival in particular habitat patches could be lost during such droughts.

4593: ***+.127***

Kemp's ridley (Lepidochelys kempii) is the most endangered of the sea turtles. Its female population in the Gulf of Mexico suffered a major setback sometime between the ends of nesting seasons in 2009 and 2010. Prior to that, annual nests (i.e., clutches laid by multiple year-classes of nesters) at the female population's index beach in Tamaulipas, Mexico were increasing exponentially, the result of more than 4 decades of cumulative conservation efforts on land and at sea. Annual nests dropped 35.4% in 2010 and remained well below predicted levels through 2014, and annual hatchlings released (both sexes combined) also were lower in 2010-2014 compared with those in 2009. We conducted novel analyses of an available 1966-2014 time series of annual nests and annual hatchlings released on the index beach. We examined 1) the relationship between time-lagged annual nests during years 1986-2014 and cumulative hatchlings released by years 1976-2004, respectively, assuming female minimum age at maturity of 10 yrs, and 2) the time-series of time-lagged annual nests during 1986-2014 divided by cumulative hatchlings released by 1976-2004, respectively, under the same assumption. Both metrics showed extraordinary downward departures in 2010-2014, instead of expected increases. Although causes of the population's setback have not been determined with certainty, we suggest that the most expedient way to restore this population's growth would be to translocate more clutches to protective corrals, leaving fewer in situ where their survival is reduced. It could take at least 10 yrs before results of such a change in conservation practice become evident.

4594: ***+.297***

Agassiz's desert tortoise, Gopherus agassizii, faces threats from climate change. With limited mobility to move long distances to more-suitable habitat as climate change advances, whether protecting tortoises in situ or translocating them out of harm's way, a critical conservation task is identifying refugia, lands that will remain suitable under the current climate and the projected, end of the 21st Century warming and drying. While researchers have modeled tortoise habitat suitability, they have done so at coarse scales and did not identify climate refugia that may become apparent only with a fine-scale approach. It is at that scale that managers can implement measures that will foster habitat protection for tortoises throughout their current range. In this case study, we employed fine-scale habitat suitability modeling to identify current habitat and climate refugia within and surrounding the Marine Corps Air Ground Combat Center (MCAGCC) at Twentynine Palms, California. We modeled nearly 284,000 ha of currently suitable tortoise habitat within an 858,800-ha study area. Projected maximum end-of-the-century summer temperatures could reduce the area of tortoise habitat 55% to 127,650 ha; however, almost 115,800 ha would overlap current tortoise habitat and would serve as climate refugia. Applied elsewhere, where tortoise protection must be balanced with other land uses, this approach could increase the efficacy of conservation for this threatened species. Nevertheless, until validated with field studies, habitat suitability models represent hypotheses as to current and future distributions of appropriate tortoise habitat. These hypotheses should foster additional research identifying whether tortoise densities and demographic structure are more secure and whether tortoises can adapt to shifting climates more effectively within than outside modeled refugia.

4595: ***+.200***

The transient stage prior to definitive recruitment, known as juvenile dispersal, is thought to be under great evolutionary pressure and subject to a trade-off between associated costs and long-term benefits for fitness. Conspecific attraction has been shown to be an adaptive mechanism driving dispersal behaviours that may lead to negative density-dependent dispersal patterns. However, conspecific attraction can be scarcely discernible from imprinting to the natal area in wild populations. Reintroductions in the absence of settled individuals can be used as alternative

colonization-like contexts to investigate the relative role of conspecific attraction in juvenile dispersal behaviours. We examined the spatiotemporal development of dispersal movements in reintroduced juveniles of a long-lived species with deferred maturity, the Spanish imperial eagle, Aquila adalberti, in comparison with nonmanipulated juveniles from a nearby population. We found that reintroduced birds started dispersal earlier and were initially more philopatric, probably encouraged by the advantageous competitive environment in the release area. Conversely, they revealed a more expansive strategy as they matured and approached the time when settlement decisions would be made, especially in females. They returned less frequently, increased exploratory movements and dispersal ranges, and visited breeding areas, probably as a consequence of the relatively lower reproductive prospects in the release area than in nearby populations. Therefore, the singular social cueing in reintroductions may eventually lead to juvenile wandering behaviours characteristic of colonization contexts in this territorial long-lived species. Such dispersive strategies relying on conspecifics may have important consequences for population dynamics and management. They may hinder the initial settlement phase in reintroductions, although behaviours such as longer returns may enhance recruitment prospects. Translocation programmes should consider specific dispersal scenarios, as well as postrelease monitoring to increase philopatry and success probabilities. (C) 2016 The Association for the Study of Animal Behaviour. Published by Elsevier Ltd. All rights reserved.

4596: *-.005*

Threatened organisms may act as host to a suite of dependent organisms, which are potentially cothreatened, yet management is rarely coordinated between host and dependent species. Here, we test the congruency of patterns of genetic structure between two critically endangered interacting taxa; the feather-leaf banksia (Banksia brownii R.Br.), and its host-specific herbivorous plant-louse Trioza barrettae Taylor & Moir, to establish whether conservation actions should be implemented jointly for both species. We also examine the role of host population size and fire history on the density of psyllids on host plants. We show that the patterns of mtDNA variation in T. barrettae and microsatellite variation in both species support the presence of at least two conservation units across each species, with the microsatellites also showing a high evolutionary congruency between plant and insect populations. The extinction of divergent B. brownii populations, therefore, is likely to have resulted in the extinction of divergent plant-louse populations. Larger populations of host plant (>150) and more recent fire history (<20 years since fire) are important factors in maintaining T. barrettae densities. High molecular congruency indicates the importance of considering patterns of genetic diversity of source material from both host and dependent organisms for ex situ conservation, in situ supplementations and reintroductions. As dependents such as T. barrettae are often lost to extinction before their host, considering the conservation of dependent biota in the early stages of species management is paramount. (C) 2016 Elsevier Ltd. All rights reserved.

4597: *+.261*

Fewer than 250 Asian elephants remain in China, occupying fragmented habitats of Yunnan Province. One such fragmented population of 18-23 individuals occupies the Nangunhe Nature Reserve Area in Lincang City, Yunnan Province, China. The greatest threat to the survival of this population is the loss and fragmentation of habitat. In this study, we applied an ecological niche factor analysis (ENFA) model to evaluate the habitat suitability of Lincang City for Asian elephants based on geographical factors, vegetation type, and human disturbance. Optimal, relatively suitable, and marginal habitat accounted for 0.16% (38.45 km(2)), 0.61% (150.00 km(2)), and 3.34% (817.26 km(2)) of the total study area, whereas non-suitable habitat accounted

for 95.89% (23,463.29 km(2)) of this area. The marginality of Asian elephant habitat in Lincang was 1.954, indicating nonrandom selection of various eco-geographical variables in the environment. The primary factor affecting Asian elephant habitat quality was vegetation type, followed by geographical factors and human disturbance. A habitat quality map for the total distribution of Asian elephants remaining in China (i.e., Yunnan Province: Xishuangbanna, Lincang, and Pu'er) based on our current and previous study showed that just 1400.57 km(2) and 2689.62 km(2) relatively suitable and optimal habitat is available, owing to significant deforestation. In addition to reintroduction programs, conservation strategies should focus on improving the quality of marginal habitats for elephants, in parallel to placing ecological corridors through non-suitable habitat to connect all suitable habitats for this and other extremely small elephant populations in China to reduce genetic isolation and secure long-term survival for the species. (C) 2016 Elsevier Ltd. All rights reserved.

4598: ***+.259***

The 1288-km(2) Great Smoky Mountains National Park (the Park) is one of the largest protected temperate forest ecosystems east of the Rocky Mountains. Because of its global ecological importance, the Park was designated an International Biosphere Reserve in 1976 and a World Heritage Site in 1983. The Park's rich biodiversity is the result of a deeply dissected landscape ranging in elevation from 259 m (850 ft) to 2026 m (6643 ft), high primary productivity, and diverse plant communities. These conditions, and the occurrence of several species at or near their southern range limit, favor high mammal diversity in the Park. Herein, I present the first comprehensive update on the Park's mammals in over 20 years. Since that 1995 publication, several new species have been recorded, additional distribution and other ecological data collected, 3 reintroductions (2 successful, 1 unsuccessful) transpired, and numerous taxonomic revisions have occurred. To the extent that data is available, the distribution, food, reproduction, pelage, hibernation, predation, parasites, measurements, and location of specimens are given for all 68 mammals in the Park. Four additional mammal species are listed as "probable" based on their known distribution in regions surrounding the Park. The Literature Cited along with the additional listed references provided in Appendix 1 together comprise the most complete bibliography ever assembled relating to mammals found in the Park.

4599: ***-.125***

A common strategy for recovering endangered species is ensuring that populations exceed the minimum viable population size (MVP), a demographic benchmark that theoretically ensures low long-term extinction risk. One method of establishing MVP is population viability analysis, a modeling technique that simulates population trajectories and forecasts extinction risk based on a series of biological, environmental, and management assumptions. Such models also help identify key uncertainties that have a large influence on extinction risk. We used stochastic count-based simulation models to explore extinction risk, MVP, and the possible benefits of alternative management strategies in populations of Roanoke logperch Percina rex, an endangered stream fish. Estimates of extinction risk were sensitive to the assumed population growth rate and model type, carrying capacity, and catastrophe regime (frequency and severity of anthropogenic fish kills), whereas demographic augmentation did little to reduce extinction risk. Under density-dependent growth, the estimated MVP for Roanoke logperch ranged from 200 to 4200 individuals, depending on the assumed severity of catastrophes. Thus, depending on the MVP threshold, anywhere from two to all five of the logperch populations we assessed were projected to be viable. Despite this uncertainty, these results help identify populations with the greatest relative extinction risk, as well as management strategies that might reduce this risk the most, such as increasing

carrying capacity and reducing fish kills. Better estimates of population growth parameters and catastrophe regimes would facilitate the refinement of MVP and extinction-risk estimates, and they should be a high priority for future research on Roanoke logperch and other imperiled stream-fish species.

4600: *+.158*

Conservation translocations, anthropogenic movements of species to prevent their extinction, have increased substantially over the last few decades. Although multiple species are frequently moved to the same location, current translocation guidelines consider species in isolation. This practice ignores important interspecific interactions and thereby risks translocation failure. We model three different two-species systems to illustrate the inherent complexity of multispecies translocations and to assess the influence of different interaction types (consumer-resource, mutualism, and competition) on translocation strategies. We focus on how these different interaction types influence the optimal founder population sizes for successful translocations and the order in which the species are moved (simultaneous or sequential). Further, we assess the effect of interaction strength in simultaneous translocations and the time delay between translocations when moving two species sequentially. Our results show that translocation decisions need to reflect the type of interaction. While all translocations of interacting species require a minimum founder population size, which is demarked by an extinction boundary, consumer-resource translocations also have a maximum founder population limit. Above the minimum founder size, increasing the number of translocated individuals leads to a substantial increase in the extinction boundary of competitors and consumers, but not of mutualists. Competitive and consumer-resource systems benefit from sequential translocations, but the order of translocations does not change the outcomes for mutualistic interaction partners noticeably. Interspecific interactions are important processes that shape population dynamics and should therefore be incorporated into the quantitative planning of multispecies translocations. Our findings apply whenever interacting species are moved, for example, in reintroductions, conservation introductions, biological control, or ecosystem restoration.

4601: *+.404*

Translocations of large carnivores like tiger to restore extirpated populations are increasingly becoming important conservation tools in the face of persistent anthropogenic pressures and relentless habitat loss. Reintroduction success in Panna Tiger Reserve, India was variously attributed to high genetic diversity, less anthropogenic disturbance and stress, efficient execution and technological integrations. Here, we establish parentage relationships and explore the potential of mate choice and appropriate adult sex ratio to explain the successful reintroduction in Panna. We used a combination of radio-telemetry and genetic analyses to understand mating interactions of translocated tigers and their outcomes. Surprisingly, relatedness and parentage analyses of nine cubs born in 2010-2012 suggested that five cubs (55.5%) were not sired by the single translocated male tiger, emphasizing the difficulty of assessing the persistence of tiger presence over a large geographic area. Genotype data of tigers originally from Panna prior to the present reintroduced population revealed that four cubs were sired by an original Panna tiger which possibly ranged transiently within the released tigers' home ranges. Presence of additional male for mate choice can be a strong factor for successful reintroduction of tigers in Panna. Although number of females released is a good indicator of reintroduction success, the number of males released is also a very important factor for success. We hypothesize that while tigers live in female-dominated population structure within male territories, mate selection strategies operate strongly between sexes. Translocation programs should therefore deliberate on this aspect of mate selection and

accordingly consider founder compositions to achieve desirable results. This also reinforces the need for post-release monitoring and thorough investigations into intraspecific interactions and behavior which will facilitate proper management and revival of tiger populations.

4602: *+.356*

We used a species distribution model to characterize habitat use by red wolves, Canis rufus, on the Albemarle Peninsula of North Carolina, USA. Using more than 4,000 VHF telemetry locations of 178 individual animals from 1999-2008, we quantified habitat use and modeled potential habitat suitability of red wolves. Areas of agriculture where secondary road density was high (up to 1 km/km(2)) and human population density was low (less than 1.67 individuals/km(2)) were most suitable. Our study supports the baseline knowledge of red wolf suitable habitat, and shows that red wolves will use habitats altered by humans and occupied by humans at low densities. This research represents the use of the most comprehensive red wolf VHF telemetry dataset for habitat suitability modeling to date, and the results should contribute to the growing knowledge of suitable red wolf habitat. This knowledge is critical to identifying future reintroduction sites and protecting the future of this species.

4603: *+.411*

Physiological colour change is rare in insects. Unusually, both the males and females of Spesbona angusta (Odonata: Platycnemididae), Red Listed as Endangered, are capable of rapid and reversible colour change. There is only one known population of this species, which occurs in a unique habitat in the Cape Floristic Region, South Africa. Appreciation of this unusual phenomenon of distinct physiological colour change helps us appreciate that we need to conserve phenomena in the insect world as well as the species themselves. Using controlled experiments, we evaluated the importance of ambient temperature as the possible primary cue for physiological colour change. We found that S. angusta responds rapidly to short-term changes in ambient temperature, even in the absence of additional environmental stimuli and without the body temperature matching the ambient temperature. Colour change is reversible when temperature returns to its earlier level. The reason why S. angusta shows this rapid and reversible colour change may be a combination of reproductive enhancement, competitive advantage and thermoregulation. This colour change appears to have strong selective advantage in a very particular habitat type, meaning that careful conservation of its habitat in all respects is important, and must be considered in any possible future translocations.

4604: *+.005*

Mammalian reintroduction programmes frequently aim to reconnect isolated sub-populations and restore population viability. However, these long-term objectives are rarely evaluated due to the inadequacy of post-release monitoring. Here, we report the results of a unique long-term telemetry-based monitoring programme for rehabilitated Antillean manatees Trichechus manatus manatus reintroduced into selected sites in north-east Brazil with the aim of reconnecting isolated relict populations. Twenty-one satellite-tagged rehabilitated manatees, 13 males and 8 females, were released into the wild from two sites between November 2008 and June 2013. Individual accumulation curves were plotted and home ranges were calculated through the fixed kernel method using 95% of the utilization distribution. The number and size of the centres of activity (COAs) were calculated using 50% of the utilization distribution. Manatees displayed a dichotomous pattern of movement, with individuals either characterized by sedentary habits or by much more extensive movements. Moreover, home-range size was not significantly influenced by

gender, age at release or release site. COAs were strongly associated with sheltered conditions within reefs and estuaries, and also by the presence of freshwater and feeding sites. Our data confirm that manatee reintroductions in Brazil have the potential to reconnect distant sub-populations. However, pre-release identification of potential long-distance migrants is currently unfeasible, and further analysis would be required to confirm genetic mixing of distant sub-populations.

4605: *+.317*

In 2003, a reintroduction program was initiated at Badlands National Park (BNP), South Dakota, USA, with swift foxes (Vulpes velox) translocated from Colorado and Wyoming, USA, as part of a restoration effort to recover declining swift fox populations throughout its historical range. Estimates of age-specific survival are necessary to evaluate the potential for population growth of reintroduced populations. We used 7 years (2003-2009) of capture-recapture data of 243 pups, 29 yearlings, and 69 adult swift foxes at BNP and the surrounding area to construct Cormack-Jolly-Seber model estimates of apparent survival within a capture-mark-recapture framework using Program MARK. The best model for estimating recapture probabilities included no differences among age classes, greater recapture probabilities during early years of the monitoring effort than later years, and variation among spring, winter, and summer. Our top ranked survival model indicated pup survival differed from that of yearlings and adults and varied by month and year. The apparent annual survival probability of pups (0.47, SE = 0.10) in our study area was greater than the apparent annual survival probability of yearlings and adults (0.27, SE = 0.08). Our results indicate low survival probabilities for a reintroduced population of swift foxes in the BNP and surrounding areas. Management of reintroduced populations and future reintroductions of swift foxes should consider the effects of relative low annual survival on population demography. (C) 2016 The Wildlife Society.

4606: *+.267*

There is growing concern about mitigation-driven translocations that move animals from anthropogenic threats at donor sites because of their failure rate and lack of application of scientific principles and best practice. We reviewed all known lizard translocations in New Zealand between 1988 and 2013 and identified 85 translocations of 30 lizard taxa to 46 release sites. Most translocations (62%) were motivated by conservation goals for the species or the release site, and one-third were mitigation-driven translocations, typically motivated by habitat loss due to development. Mitigation-driven translocations began in 2003, and since that time have equalled the number of conservation-motivated translocations. Conservation-motivated translocations usually released lizards on islands without mammalian predators, whereas mitigation-driven translocations usually relocated lizards to mainland sites with introduced predators. Long-term monitoring has been sparse and often rudimentary. Eight lizard translocations have recorded population growth, including one mitigation-driven translocation that was into a fenced reserve. Research on commonly used management techniques to mitigate human-related impacts is recommended to establish whether these techniques benefit lizards in the long term.

4607: *+.221*

Ex situ conservation strategies for threatened species often require long-term commitment and financial investment to achieve management objectives. We present a framework that considers the decision to adopt ex situ management for a target species as the end point of several linked

decisions. We used a decision tree to intuitively represent the logical sequence of decision making. The first decision is to identify the specific management actions most likely to achieve the fundamental objectives of the recovery plan, with or without the use of ex-situ populations. Once this decision has been made, one decides whether to establish an ex situ population, accounting for the probability of success in the initial phase of the recovery plan, for example, the probability of successful breeding in captivity. Approaching these decisions in the reverse order (attempting to establish an ex situ population before its purpose is clearly defined) can lead to a poor allocation of resources, because it may restrict the range of available decisions in the second stage. We applied our decision framework to the recovery program for the threatened spotted tree frog (Litoria spenceri) of southeastern Australia. Across a range of possible management actions, only those including ex situ management were expected to provide > 50% probability of the species' persistence, but these actions cost more than use of in situ alternatives only. The expected benefits of ex situ actions were predicted to be offset by additional uncertainty and stochasticity associated with establishing and maintaining ex situ populations. Na " ively implementing ex situ conservation strategies can lead to inefficient management. Our frameworkmay help managers explicitly evaluate objectives, management options, and the probability of success prior to establishing a captive colony of any given species.

4608: ***+.099***

1. Conservation biology faces the challenge of ensuring species persistence in increasingly modified landscapes. Agriculture covers a large proportion of the Earth's surface, but the degree to which crop production is compatible with species use of the landscape is still uncertain, particularly for woodland carnivores with large territories. Here, we focus on the Iberian lynx Lynx pardinus, an emblematic endangered species that has coexisted for centuries with human-modified Mediterranean mosaics, as a case study to unravel habitat and dispersal preferences in heterogeneous landscapes.2. We estimated species resource selection from approximate to 40 000 telemetry locations for 48 GPS-collared individuals covering all the current Iberian lynx range, including more fragmented areas where the species was reintroduced from 2009. We differentiated GPS locations within home ranges (to estimate habitat suitability) and those corresponding to dispersal or exploratory movements (to estimate landscape permeability). We built mixed conditional logistic regression models with 12 land cover classes, terrain slope and roads as predictors.3. We found that lynx response to agriculture largely depends on the crop type and on the presence of natural vegetation remnants. Lynx largely avoided intensive cultivation areas such as irrigated arable lands when establishing home ranges, but frequently selected permanent crops (olive groves) and/or heterogeneous agricultural lands, which were used with smaller differences to the most preferred shrubland or forest covers than reported in previous studies.4. Such differences further narrowed down when lynx moved outside home ranges, with some agricultural covers being as permeable as shrublands for lynx dispersal. The species dispersal plasticity was also evidenced by a much weaker avoidance of roads and steep terrain when dispersing than when selecting territories.5. Synthesis and applications. We conclude that (i) the widespread consideration of all agricultural lands within a single (and usually regarded as unsuitable) class for the study and management of woodland or forest species is not supported and that (ii) the ability of woodland species to use fragmented and heterogeneous agricultural landscapes may have been underestimated, which may mislead conservation measures due to a priori assumptions that do not relate to the actual species responses to heterogeneous land covers. We suggest that Iberian lynx conservation and reintroduction may be successful in a wider set of more heterogeneous areas than previously thought, including mainly well-conserved Mediterranean woodlands but also some extensive agricultural lands with permanent crops and natural vegetation remnants.

4609: +.117

The mainland subspecies of Eastern Barred Bandicoot is extinct in the wild due to fox predation and habitat loss, but has been successfully reintroduced into several predator-fenced reserves within its former range. In the 27 years since the Eastern Barred Bandicoot Recovery Team was formed, there have been reintroduction successes and failures. Importantly, there have been lessons learnt, and now this multi-agency recovery team has an ambitious plan to move Eastern Barred Bandicoot conservation from simply preventing extinction to recovering and down-listing the mainland subspecies. Over the next five years the recovery team is planning to roll out projects involving fences, islands and guardian dogs. Some projects are novel and may not succeed, but if two are successful, we stand a good chance of recovering this subspecies from near extinction and removing the Eastern Barred Bandicoot from the threatened species list.

4610: -.001

This paper provides a broad international and national context for the symposium's focus on the conservation of the Victorian mammal fauna. As with Australia generally, the Victorian mammal fauna has suffered rates of extinction that are unusually high by global standards. The main factors that have caused loss of Australia's land mammals are predation (by the introduced feral Cat and Red Fox) and changed fire regimes, although other factors are also implicated in declines for some species. There are reasonable grounds for hope that the ongoing decline in Victoria's mammal fauna can be stemmed and reversed, especially because it is plausible to return, with intensive management, many of the 14 mammal species that have been extirpated from Victoria but have persisted elsewhere.

4611: +.085

Captive rearing and reintroduction / translocation are increasingly used as tools to supplement wild populations of threatened species. Reintroducing captive-reared Chinese giant salamanders may help to augment the declining wild populations and conserve this critically endangered amphibian. We released 31 captive-reared juvenile giant salamanders implanted with VHF radio transmitters at the Heihe River (n = 15) and the Donghe River (n = 16) in the Qinling Mountains of central China. Salamanders were monitored every day for survival from April 28th 2013 to September 3rd 2014. We attempted to recapture all living individuals by the end of the study, measured their body mass and total body length, and checked for abnormalities and presence of external parasites. Two salamanders at the Heihe River and 10 animals at the Donghe River survived through the project timeline. Nine salamanders were confirmed dead, while the status of the other 10 animals was undetermined. The annual survival rate of giant salamanders at the Donghe River (0.702) was 1.7-fold higher than that at the Heihe River (0.405). Survival increased as individuals were held longer following surgery, whereas body mass did not have a significant impact on survival rate. All salamanders recaptured from the Donghe River (n = 8) increased in mass (0.50 +/- 0.13 kg) and length (5.5 +/- 1.5 cm) after approximately 11 months in the wild, and they were only 7% lighter than wild animals of the same length (mean residual = -0.033 +/- 0.025). Our results indicate that captive-reared Chinese giant salamanders can survive in the wild one year after release and adequate surgical recovery time is extremely important to post-release survival. Future projects may reintroduce older juveniles to achieve better survival and longer monitoring duration.

4612: +.133

The giant panda is an icon of conservation and survived a large-scale bamboo die off in the 1980s in China. Captive breeding programs have produced a large population in zoos and efforts continue to reintroduce those animals into the wild. However, we lack sufficient knowledge of their physiological ecology to determine requirements for survival now and in the face of climate change. We measured resting and active metabolic rates of giant pandas in order to determine if current bamboo resources were sufficient for adding additional animals to populations in natural reserves. Resting metabolic rates were somewhat below average for a panda sized mammal and active metabolic rates were in the normal range. Pandas do not have exceptionally low metabolic rates. Nevertheless, there is enough bamboo in natural reserves to support both natural populations and large numbers of reintroduced pandas. Bamboo will not be the limiting factor in successful reintroduction.

4613: *+.083*

In the European North of Russia the Eurasian beaver had been extirpated over two hundred years ago. Owing to active introduction and dispersals from the 1930s to the 1950s, beavers have recolonized the natural ecosystems in the European North. Furthermore, Finland and Russia (Karelia, Leningrad and Arkhangelsk provinces) are now co-inhabited by two beaver species - the North American (Castor canadensis Kuhl) and the Eurasian (C. fiber L.) beaver. North American beavers, which have colonized a major part of Finland, Karelia and the Karelian Isthmus in the Leningrad Province, descended from the seven animals brought to Finland from the USA in the 1930s (Linnamies, 1956; Siivonen, 1956; Lahti, 1968). Subsequent intraregional translocations of these animals took place in both Finland and Russia (n=270). The main origins of Eurasian beavers introduced in provinces adjacent to Karelia (Arkhangelsk, Vologda and Leningrad provinces) (n=1349) were the Voronezh Province (26.5% of all releases in the study area), Republic of Belarus (20.8%), Mari El Republic (12.5%), Smolensk Province (6.2%), Bryansk Province (5.3%), Ryazan Province (3.6%), Komi Republic (1.5%), and other regions (5.6%). Intraregional translocations of the Eurasian beaver were also conducted (18%). In total, more than 1800 Eurasian beavers were introduced in the European North of Russia (Murmansk, Leningrad, Novgorod, Pskov, Arkhangelsk and Vologda provinces). The present-day North American beaver population is estimated at 12000 animals in Karelia and 1000 in the Karelian Isthmus, Leningrad Province (Danilov et al., 2007, 2012). Eurasian beaver numbers are estimated at 4000 animals in Karelia (Danilov & Fyodorov, unpublished data), with fewer than 40 beavers in the Murmansk Province (Kataev, 2015), 23000 in the Leningrad Province, 25000 in the Novgorod Province, 17600 in the Pskov Province, 21000 in the Arkhangelsk Province, and 32600 animals in the Vologda Province (Borisov, 2011). Presently, Eurasian beavers live in those areas in southern Karelia that were inhabited by North American beavers since they were released there at the end of the 1960s, i.e. one species has been replaced by the other (Danilov et al., 2007, 2011; Danilov & Fyodorov, 2015a). In southern Karelia, the closest distance between colonies of different beaver species is 10 km. Conversely, in north-eastern Karelia (Kemsky District), North American beavers penetrated into the Arkhangelsk Province and are colonizing areas inhabited by the Eurasian beaver. In 2015, they were spotted in the Arkhangelsk Province, 70 km east of the administrative border with Karelia.

4614: *+.012*

Eurasian beaver (Castor fiber) has been reintroduced in Switzerland between 1956 and 1977. Individuals from the refugium population in France (C. f. galliae) were released in the Rhone catchment area and in tributaries of the Rhine catchment area in Western Switzerland. Individuals from the refugium populations from Norway (C. f. fiber) and Russia (C. f. orientoeuropaeus) were

released in tributaries of the Rhine catchment in Eastern Switzerland. In the Rhine basin beavers of different origins came into contact. This study provides a first evaluation of the reintroduction program of beaver in Switzerland and gives implications for the post-release genetic management of the Swiss beaver population. We report on the genetic monitoring of the beaver population in Switzerland, based on the analysis of 251 dead found individuals collected from 1998 to 2014 and a combination of mitochondrial and nuclear genetic markers. We found no evidence of the presence of North American beaver (Castor canadensis) and we observed three mitochondrial DNA haplotypes, assigned to the refugium populations in France (C. f. galliae), Norway (C. f. fiber) and Germany (C. f. albicus). Based on the analysis of seven microsatellite loci, we inferred that the beaver population in Switzerland consists of two genetic clusters and we found evidence of a zone of secondary contact. We observed low levels of genetic diversity and we could show that individuals separated by distances up to 50 km were closely related.

4615: *+.341*

The swift fox (Vulpes velox) is a small grassland canid native to the North American Great Plains. A reintroduced swift fox population in Canada and northern Montana appears to be isolated from those existing in the central and southern Great Plains. We developed a swift fox habitat suitability model for southeastern Montana, the region between the 2 populations. The resulting model indicated that 67.9% of the study area consisted of highly suitable habitat. We conducted a least-cost path analysis to evaluate the connectivity of swift fox habitat in the study area to existing swift fox populations in the region. We identified a potential dispersal corridor through southeastern Montana that could facilitate movement between swift fox populations in northern Montana and northern Wyoming and identified 4 prairie dog complexes in Rosebud, Custer, and Powder River Counties, Montana, that could serve as potential swift fox reintroduction sites. Each site comprised several prairie dog colonies in close proximity and encompassed = 95 km2. We evaluated the effect that swift fox populations established in each potential reintroduction site could have on population connectivity. Our results as well as future surveys could inform swift fox management and reintroduction programs in Montana.

4616: *+.133*

Exposure of individuals to novel environmental conditions generally favors locally adapted phenotypes and can influence the likelihood of successful dispersal or the success of translocation efforts. We used geometric morphometrics to characterize American marten (Martes americana) skull morphology for descendants of animals reintroduced to the Upper Peninsula of Michigan, USA (similar to 44,000 km(2)) from genetically distinct source populations. Using univariate and multivariate analyses, we quantified associations between interindividual variation in skull shape and size, genealogical relationships, past introduction history, local harvest density of competitors, and contemporary landscape features we hypothesized would be related to diet. Effects of other sources of variation including shared ancestry (source population, geographic distance, and coancestry among descendants), sex, and age were also evaluated. Descendant skull shape was related to progenitor source population, sex, and age. In contrast to strong associations between spatial genetic structure based on neutral molecular markers and measures of landscape effects on dispersal, variation in skull shape among descendants was not associated with geographic distance or landscape features. Our study addressed a critical issue regarding a widely used conservation prescription. Specifically, when individuals are transplanted to reestablish extirpated species, do their descendants retain the features of the source population(s), or do they adapt to local conditions? Marten skull shape following reintroduction events is most likely determined by a combination of differences in morphology of source populations and geographic variation in

habitat, rather than being influenced by a single factor.

4617: *-.019*

In recent years numerous studies have documented the effects of a changing climate on the world's biodiversity. Although extreme weather events are predicted to increase in frequency and intensity and are challenging to organisms, there are few quantitative observations on the survival, behaviour and energy expenditure of animals during such events. We provide the first data on activity and energy expenditure of birds, Eurasian cranes Grus grus, during the winter of 2013-14, which saw the most severe floods in SW England in over 200 years. We fitted 23 cranes with telemetry devices and used remote sensing data to model flood dynamics during three consecutive winters (2012-2015). Our results show that during the acute phase of the 2013-14 floods, potential feeding areas decreased dramatically and cranes restricted their activity to a small partially unflooded area. They also increased energy expenditure (+15%) as they increased their foraging activity and reduced resting time. Survival did not decline in 2013-14, indicating that even though extreme climatic events strongly affected time-energy budgets, behavioural plasticity alleviated any potential impact on fitness. However under climate change scenarios such challenges may not be sustainable over longer periods and potentially could increase species vulnerability.

4618: *+.049*

African elephants (Loxodonta africana) are ecosystem engineers in that they substantially alter the environment through their unique foraging and feeding habits. At high densities, elephants potentially have negative impacts on the environment, specifically for large trees. Because of this, recent increases of elephants in the Associated Private Nature Reserves (APNR) on the western boundary of the Kruger National Park (KNP), South Africa, have caused concern regarding the survival of several tree species. Our objective was to assess the effectiveness of wrapping protective wire netting around the trunk of the tree for preventing and reducing bark stripping, branch breaking, and felling by elephants. We assessed 2668 trees -1352 Sclerocarya birrea (marula), 857 Acacia nigrescens (knobthorn), and 459 Lannea schweinfurthii (false marula) -for elephant impact in the APNR, 1387 (52%) of which had previously been wrapped in protective wire netting (789, 548 and 50, respectively). Wire netting was effective in reducing the severity of bark stripping and the relative proportion of trees that were bark stripped. In addition, wire netting had an effect on the level of impact, with a higher relative frequency of wire-net-protected trees found in lower impact categories compared with unprotected trees. Since tree mortality has been attributed to high levels of elephant impact, the use of wire netting could serve to maintain individual trees or populations particularly vulnerable to elephant impact in areas with locally high densities of elephants. Conservation implications: Since wire netting is a relatively low cost and ecologically unobtrusive strategy, it could be used to reduce elephant impact in problem areas. This method focuses on protecting trees rather than some other strategies such as environmental manipulation, translocation, contraceptives, and culling that instead focus on reducing elephant numbers.

4619: *+.019*

The Northern Bald Ibis is one of the rarest bird species, extinct in Europe for 400 years and critically endangered worldwide. The European Union-co-financed LIFE+ project "Reason for Hope - Reintroduction of the Northern Bald Ibis in Europe" aims to reintroduce the species in Europe (Germany, Austria, Italy). In order to obtain information on the genetic diversity within zoo colonies and the reintroduced population, 15 polymorphic microsatellite markers, specific for

the Northern Bald Ibis, Geronticus eremita (Linnaeus, 1785), have been isolated from next-generation sequencing (Illumina MiSeq) and are described here. The microsatellite primers were tested in 30 individuals and measures of genetic variability were calculated. Values for the observed heterozygosity ranged from 0.393 to 0.867, while expected heterozygosity ranged from 0.573 to 0.718. Ten out of 15 loci were in Hardy-Weinberg equilibrium and only one showed indication for the presence of null alleles. The newly developed PCR primers can be used to examine population genetic parameters, e.g. for future conservation genetic studies of this critically endangered bird species.

4620: *+.155*

Freshwater mussel populations have declined, in part, from changes in host communities. However, it is unknown if fish from adjacent catchments could be used to augment mussel populations in the Great Lakes inland rivers, and if so, whether this association would be impacted by known genetic structure in mussels and fish. This study tested how host fish origin (i.e. catchment) impacts the transformation of the endangered unionid Epioblasma triquetra from larval into juvenile life stages while concurrently considering potentially genetically distinct populations of mussels and hosts. We quantitatively determined if Percina caprodes and Percina maculata from the Lake Michigan, Erie and Huron basins are laboratory-successful hosts for E. triquetra. Experiments were performed in autumn and spring to document any seasonal effects on transformation. Percina caprodes was reconfirmed to be a successful host for E. triquetra, and for the first time, P.maculata was also determined to be a successful host in the Great Lakes region. Results suggest no differences in juvenile transformation with allopatric and sympatric fish and mussel pairings based on Great Lakes basin origin; therefore, transformation success may not parallel differences in genetic structure. In addition, results suggest seasonal differences in the developmental stages of E. triquetra juveniles. Knowing the most efficient strategy to optimise juvenile transformations can make reintroductions, augmentation and overall conservation efforts of E. triquetra successful. These data will help in developing recovery strategies for E. triquetra in the Laurentian Great Lakes by understanding variation in host use and nuances in this host-parasite relationship.

4621: *+.145*

Habitat modification is one of the largest threats to global terrestrial biodiversity, yet management tools such as translocations often fail because of insufficient knowledge about species' habitat suitability and requirements in release areas. Resource selection is a useful technique that can address this issue. In 2014, we investigated resource selection of Hochstetter's frogs (Leiopelma hochstetteri) inmature (>30 year) exotic pine (Pinus radiata) plantations and regenerating native broadleaf forests in Torere Forest (Bay of Plenty, North Island), New Zealand, where harvesting of pines threatens this population. We then determined whether key habitats were present at the proposed target translocation site, Orokonui Ecosanctuary (Dunedin, South Island), New Zealand. In Torere Forest, we conducted daytime searches for frogs along streams and recorded microhabitats (e.g., cobbles) in used and random available plots. In Orokonui, we recorded microhabitats in randomly distributed plots because Hochstetters's frogs are presently absent. We used averaged mixed (binomial) models to identify important resources in pine plantations and native forests and to compare resource use between habitats. Finally, we predicted habitat use in Orokonui and Torere Forest to gain an indication of habitat suitability. Frogs predominantly selected cobble microhabitats in pine plantations and native forests but also partially decayed logs >= 15 cm in diameter within pine plantations. In contrast, high gravel cover and sand-silt were avoided by frogs. In response to limited cobble cover in pine plantations, frogs were more

generalized in selecting resources (primarily logs) than in native forests. Habitat suitability was slightly lower in pine plantations compared to native forests, but mature pine plantations do provide suitable habitat for Hochstetter's frogs, at least in the short term prior to pine harvesting. Our study suggests that Orokonui provides suitable habitat for the proposed translocation of Hochstetter's frogs. (C) 2016 The Wildlife Society.

4622: *+.322*

A species' dispersal capability is difficult to quantify but important for a general understanding of a species' ecology and for applied conservation and management efforts. One approach is to use the information from individual genotypes to estimate recent dispersal rates. These genetic methods differ in the way they use the genotype data, their assumptions, and the information they give, but choosing one method over another is complicated by the lack of work that compares these methods on simulated or real data sets. We collected detailed, spatially resolved, individual data on the greater white-toothed shrew (Crocidura russula) in western Switzerland for which past studies have found an unusual female sex-biased dispersal. We analyzed the movement from 1 cohort of juvenile shrews with 7 published methods (i.e., mark-recapture, parentage analysis; genetic assignment; hierarchical F-statistics; and the programs BayesAss, IMIG, and STRUCTURE) and used a binomial test to make quantitative comparisons between the results of the methods. Our study indicates that the methods are broadly consistent, but parentage analysis appears the most powerful method for analyzing fine-scale dispersal patterns. In a conservation context, where the evaluation of long-term translocation success is critical for species management, the species studied and spatial scale considered will dictate which is the best suited method to estimate dispersal. (C) 2016 The Wildlife Society.

4623: *+.051*

Establishing a health screening protocol is fundamental for successful captive breeding and release of wildlife. The aim of this study was to undertake a parasitological survey focusing on the presence of trypanosomes in a cohort of Regent Honeyeaters, Anthochaera phrygia, syn. Xanthomyza phrygia (Aves: Passeriformes) that are part of the breeding and reintroduction programme carried out in Australia. We describe a new blood parasite, Trypanosoma thomasbancrofti sp.n. (Kinetoplastida: Trypanosomatidae) with prevalence of 244% (20/81) in a captive population in 2015. The sequence of the small subunit rRNA gene (SSU rDNA) and kinetoplast ultrastructure of T. thomasbancrofti sp.n. are the key differentiating characteristics from other Trypanosoma spp. T. thomasbancrofti sp.n. is distinct from Trypanosoma cf. avium found in sympatric Noisy Miners (Manorina melanocephala). The SSU rDNA comparison suggests an intercontinental distribution of T. thomasbancrofti sp.n. and Culex mosquitoes as a suspected vector. Currently, no information exists on the effect of T. thomasbancrofti sp.n. on its hosts; however, all trypanosome-positive birds remain clinically healthy. This information is useful in establishing baseline health data and screening protocols, particularly prior to release to the wild.

4624: *+.099*

Central America hosts a diverse, unique, and imperiled amphibian fauna, and for decades Central America been a major epicenter of research into amphibian decline and conservation. In this critical and quantitative review, we synthesize current knowledge regarding amphibian decline and conservation in the seven countries that constitute Central America. There are 495 currently recognized amphibian species known from the region, distributed among the three extant orders,

16 families, and 69 genera-though description of new species continues to occur at a rapid pace. Central America's amphibian fauna is unique: 251 species are restricted to the region, and amphibian diversity varies among the major biogeographic provinces and climatic zones found in Central America. We use data generated by the International Union for the Conservation of Nature (IUCN) to evaluate trends in extinction risk among Central American amphibians. As of 2014, there are 207 amphibian species considered threatened by the IUCN, and threat status varies according to taxonomic groupings, biogeographical association, elevation, and life history variables. Major threats to Central American amphibians include both conventional threats (habitat modification, habitat fragmentation, overharvesting, and invasive species) as well as emerging threats that operate on large spatial scales (pollution, emerging infectious diseases, UV-B radiation, and climate change). We conducted a quantitative literature review to document conservation research and to show trends in research activity. While the number of published studies on amphibian conservation increases each year, there are pronounced biogeographic biases in the distribution of published research, and most research is conducted by scientists at institutions outside of Central America with limited involvement of host-nation biologists in amphibian research. We synthesize empirical studies of conservation impacts to amphibians in Central America from habitat modification and fragmentation, overharvesting, invasive species, pollution, UV-B radiation, chytridiomycosis and other amphibian pathogens, climate change, and synergistic interactions among these threats. Much research in the past decade has focused on chytridiomycosis and the amphibian chytrid fungus (Batrachochytrium dendrobatidis), with far fewer studies on habitat modification, other amphibian pathogens, or climate change impacts to amphibians. We describe ongoing conservation actions for amphibians in the region, including monitoring, protected areas, captive assurance programs, protection of relict populations, reintroductions, and development of in-country capacity for research and conservation programs. We conclude with a list of priorities in research and conservation action for amphibians in the region.

4625: ***+.114***

Translocation is a conservation tool used with increasing frequency to create additional populations of threatened species. In addition to following established general guidelines for translocations, detailed planning to account for unique circumstances and intensive post-release monitoring to document outcomes and guide management are essential components of these projects. Recent translocation of the critically endangered Nihoa millerbird (Acrocephalus familiaris kingi) provides an example of this planning and monitoring. The Nihoa millerbird is a passerine bird endemic to Nihoa Island in the remote Northwestern Hawaiian Islands. The closely related, ecologically similar Laysan millerbird (Acrocephalus familiaris familiaris) went extinct on Laysan Island in the early 20th century when the island was denuded by introduced rabbits. To reduce extinction risk, we translocated 50 adult Nihoa millerbirds more than 1000 km by sea to Laysan, which has recovered substantially in the past century and has ample habitat and a rich prey-base for millerbirds. Following five years of intensive background research and planning, including development of husbandry techniques, fundraising, and regulatory compliance, translocations occurred in 2011 and 2012. Of 11 females in each cohort, 8 (2011 cohort) and II (2012 cohort) produced at least one brood of fledglings during their first year on Laysan. At the conclusion of monitoring in September 2014, 37 of the translocated birds were known to survive, and the population was estimated at 164 birds. The reintroduction of millerbirds to Laysan represents a milestone in the island's ongoing restoration. Published by Elsevier Ltd.

4626: ***+.125***

Conservation of endangered species increasingly envisages complex strategies that integrate captive and wild management actions. Management decisions in this context must be made in the face of uncertainty, often with limited capacity to collect information. Adaptive management (AM) combines management and monitoring, with the aim of updating knowledge and improving decision-making over time. We provide a guide for managers who may realize the potential of AM, but are unsure where to start. The urgent need for iterative management decisions, the existence,of uncertainty, and the opportunity for learning offered by often highly controlled captive environments create favorable conditions for AM. However, experiments and monitoring may be complicated by small sample sizes, and the ability to control the system, including stochasticity and observability, may be limited toward the wild end of the spectrum. We illustrate the key steps to implementing AM in threatened species management using four case studies, including the management of captive programs for cheetah (Acinonyx jubatus) and whooping cranes (Grus americana), of a translocation protocol for Arizona cliffroses Purshia subintegra and of ongoing supplementary feeding of reintroduced hihi (Notiomystis cincta) populations. For each case study, we explain (1) how to clarify whether the decision can be improved by learning (i.e. it is iterative and complicated by uncertainty) and what the management objectives are; (2) how to articulate uncertainty via alternative, testable hypotheses such as competing models or parameter distributions; (3) how to formally define how additional information can be collected and incorporated in future management decisions. (C) 2016 Elsevier Ltd. All rights reserved.

4627: ***+.236***

Conservation strategies require multifaceted approaches to monitor and protect primate populations, many of which are rapidly declining around the world. We propose that microbial ecology and next-generation microbiome analyses offer valuable perspectives and tools for investigating and monitoring primate health and improving conservation efforts. The microbial communities inhabiting primates and other taxa profoundly affect host health, nutrition, physiology, and immune systems, through relationships that range from commensal and mutualistic to pathogenic. Recent advances in DNA sequencing now make it feasible and-economically viable to identify microbiomes among and within hosts. Herein, we highlight several examples in which microbial analyses of primates can aid conservation approaches that are broadly applicable across other taxa. First, we highlight evidence for clear spatial variation (e.g. biogeographic niche specificity, both within the anatomical regions of the host body, as well as in the geographic location of the host) and temporal (e.g. seasonal, ontogenetic) patterns in microbial distribution. We emphasize that microbial communities are sensitive to alterations in the external environment and that microbial diversity correlates with habitat quality, imposing direct health consequences. Incorporating microbial host and biogeographic variation holds great potential for forest corridor assessments and for reintroduction efforts. Finally, microbial pathogens transmitted between humans and wild primate populations carry both direct and indirect conservation implications. Principally, we argue that phylogenetic analyses of infectious pathogens (e.g., Ebola, dengue, Borellia, and Treponema) can aid our understanding of modes of disease transmission and aid conservation disease abatement efforts. The application of microbial analyses to conservation is currently in its infancy but holds enormous potential. To date, no conservation policy or legislation includes microbiome assessments. Integrating new understanding of the patterns of microbial diversity and early signs of impending microbial disruption offer valuable tools for informing conservation strategies and monitoring and promoting primate (including human) health. (C) 2016 Elsevier Ltd. All rights reserved.

4628: ***+.006***

The translocation of predators believed to be preying on livestock is often perceived as a more humane and desirable method of removal than lethal control. However, the survival of translocated predators and the effectiveness of translocation in reducing conflict at the removal site are often not documented. We assessed farmers' perceptions of the efficacy of translocation at reducing livestock and game-stock losses in Botswana, and determined the post-release survival of translocated cheetahs Acinonyx jubatus, the most threatened large felid in Africa. Eighteen percent of translocated cheetahs survived. year (n = 11). The low survival rate was thought to be related to homing behaviour and wide-ranging movements post release. The majority of farmers who had translocated a problem predator from their farm within the.. months prior to the study perceived that the translocation was ineffective at reducing stock losses, both in the short (59.1%) and long term (63.6%, n = 22). At least one of the monitored cheetahs continued to predate livestock after release. In light of the low survival, significant financial costs and failure to reduce stock losses, we conclude that the translocation of problem cheetahs in Botswana should no longer be conducted, and that conflict mitigation methods should focus on techniques that promote coexistence of predators and humans.

4629: *+.128*

Magnolia sinica, a Critically Endangered tree endemic to Yunnan, China, is one of the 20 plant species with extremely small populations approved by the Yunnan government for urgent rescue action before 2015 Information on the geographical distribution and population size of this species had not previously been reported, hindering effective conservation. We therefore carried out a survey of the literature and of herbarium specimens, followed by a detailed field survey and morphological measurements and observations of surviving individuals. We located 52 individuals in the wild, in eight localities. Two distinguishing morphological characters (tepal colour and tepal number) were revised based on observations of all remaining wild individuals that produced flowers and on one 30-year-old flowering plant in Kunming Botanical Garden. The survival rate of individuals propagated from seed for ex situ conservation at the Garden was 100 % over. years; of 100 individuals transplanted to each of two reinforcement sites, 20 and 18, respectively, were alive after. years. We propose two groups of measures to protect M. sinica: (1) in situ conservation, population monitoring, and public engagement, and (2) ex situ conservation with reinforcement or reintroduction.

4630: *+.143*

The distribution of the Critically Endangered tree Manglietia longipedunculata, of which there are only 11 known wild individuals, is restricted to the Nankunshan Nature Reserve in South China. The species is threatened with extinction because of its small number of individuals and the impediments to its reproduction (a combination of protogyny, a short period of stigma receptivity, and a lack of efficient pollinators). To reduce the risk of extinction we conducted two conservation translocation trials: one to augment the sole extant population, and the other 202 km north of the current range. The latter trial was a conservation introduction in which the goals were to increase the population and to buffer against the effects of climate change. We used emerged and grafted seedlings as translocation materials. We compared the survival, growth, and eco-physiological properties of emerged and grafted seedlings at the two sites. The survival rate and growth were higher for grafted seedlings than for emerged seedlings at both sites. Eco-physiological data indicated that grafted seedlings at both sites were as efficient or more so in light and water usage than wild individuals, whereas emerged seedlings were less efficient. Grafted seedlings attained the flowering stage sooner than emerged seedlings. Our study suggests that grafting can facilitate the augmentation and establishment of new populations of M. longipedunculata and perhaps of

new populations of other threatened species facing reproductive difficulties and climate change.

4631: *+.303*

Worldwide species relocations are increasingly becoming an important part of species recovery programmes and ecosystem restoration initiatives. Monitoring reintroduced populations after release, in addition to understanding which factors affect translocations, is fundamental to understand the reintroduction process and to maximize the success of future interventions. By using boosted regression trees we evaluated the contribution of roe deer Capreolus capreolus reintroductions (1971-2008) to the current distribution of this species in Catalonia (Spain), and a partial least square regression approach was used to evaluate the influence of some variables as key for the roe deer reintroduction success. Our results show that roe deer currently occupies 85% of Catalonia territory, which represents an almost six-fold increase since the beginning of the 90s. Proximity to the nearest reintroduction nuclei was identified as one of the main drivers positively associated to the current distribution of roe deer in Catalonia, whereas the number of years after the first reintroductions and the number of animals released were important to the success of the reintroductions. We recommend the reintroduction of roe deer to release sites that contain large and suitable patches of habitat and that are sufficiently close to allow population continuity. (C) 2016 Deutsche Gesellschaft fur Saugetierkunde. Published by Elsevier GmbH. All rights reserved.

4632: *+.115*

Approximately one-third of all conservation translocations have failed to establish a self-sustaining population. Despite this historically low success rate, reintroduction is increasingly used in species recovery programmes in accordance with IUCN guidelines. Two commonly used methods of reintroduction involve `hard' or 'soft' releases. A 'hard' release means immediate release from captivity into the wild, whereas 'soft' release involves individuals being kept for a period at a pre-release site and/or an extended period under care at the release site. In general, in recent reintroduction programmes, soft release is preferred over hard release because studies have shown that soft release can increase translocation success by encouraging animals to stay near the release point thereby utilizing supplementary food and delaying dispersal. The Crested Ibis Nipponia nippon used to be widespread in Japan, but became extinct in the wild during the early 1980s. In order to re-establish a wild population, a reintroduction programme has been implemented on Sado Island since 2003. Ten ibises were hard-released in 2008, and 20 were soft-released in 2009. In this paper, we quantify the differences in post-release movements by comparing the results of 2008 and 2009 releases, to determine whether release methods influence post-release behaviour. Linear mixed-effects modelling indicates that the post-release movements of birds varied depending on their gender, style of release, and season. In the reintroduction programme for the Crested Ibis on Sado Island, Japan, soft release appears to encourage birds to remain near the release site and to form a flock immediately after release.

4633: *+.261*

A pioneering project to reintroduce the Cirl Bunting Emberiza cirlus was carried out on the Roseland Peninsula in southern Cornwall between 2006 and 2011. It involved the captive rearing and release of 376 young birds, taken from nests in south Devon. A breeding population is now established in the release area and at least 52 pairs were present in 2015. With further targeted habitat management through agri-environment schemes it is hoped that the population will continue to increase and spread into the surrounding landscape. This paper describes the development of the work, from the initial planning stages, through to the adaptive management

required to help to ensure that it was ultimately successful. Working with a small passerine requires a rather different approach from that for the larger, more robust, species that are more familiar as subjects for reintroduction. The lessons learnt may be useful for future projects involving the translocation of small passerines in Britain or farther afield.

4634: ***+.100***

Translocation is an important conservation management tool. However, not all individuals are equally suited to translocation, and temperament traits (e.g., boldness, reactivity, exploration, sociability, and aggression) are likely to influence survival in a new environment. A few empirical studies have examined the consequences of personality differences on captive-bred translocated animals, but this has not been done for wild-caught animals. We compared behavioral responses to trapping, processing, holding, and release for 56 wild common brushtail possums (Trichosurus vulpecula). Twenty individuals were captured twice, once to attach radio-tracking collars, the second time (2 weeks later) for the translocation. Consistency of behavioral responses was compared between capture events and radio-tracking allowed estimates of pretranslocation home range, rest site selection, and foraging behavior. Survivors (n = 10 survivors, 5 months later) were individuals showing the most fear or emotional reactivity during holding (less likely to have slept, eaten, defecated, or nested) and those that had the smallest home ranges and selected the safest den sites in their original habitat. Conversely, the greatest increase in body mass was recorded for individuals that had demonstrated "unsafe" behavior in their original habitat. To our knowledge, this is the first time this type of behavioral screening during handling and holding prior to release as part of a translocation has been undertaken. These methods have broad applicability for screening potential translocation candidates and are easily translated to a range of threatened and vulnerable animal species.

4635: ***+.020***

The survival of a number of birds rely on captive breeding and reintroduction into the wild, but captive populations are often small and can be exposed to the negative effects of inbreeding and genetic drift. Then, managers are concerned not only with producing as much offspring as possible, but also with the retention of the maximum genetic variability within and between populations. The Black-fronted Piping Guan, Aburria jacutinga, is an endangered cracid endemic to the Atlantic Forest of southeastern South America. Because of its conservation status and functional importance, a captive breeding program started independently, mainly in three aviaries, in the decade of 1980. Although they have supplied animals for reintroductions, genetic variability aspects have never been considered. Here we addressed levels of genetic variability within and between these aviaries. Bayesian clustering analyses revealed two lineages. Inbreeding was not detected, although we found evidences for a recent bottleneck in one of the aviaries. Then, our main management recommendations are: i) reintroducing the species in areas where it has been extinct is more prudent than supplementing natural populations, as it could involve risks of disrupting local adaptive complexes; ii) as far as inbreeding can be avoided, the captive groups should be managed separately to minimize adaptation to captivity; iii) crossbreedings in pre-release generations could improve reintroduction success; and iv) a studbook should be implemented. As populations of Black-fronted Piping Guan from conservation units are progressively declining, these captive genetic repositories may gain importance in a near future.
(C) 2016 Wiley Periodicals, Inc.

4636: ***-.018***

Euryodendron excelsum H. T. Chang is a critically endangered plant from the family Theaceae that is endemic to China. It is now present in only one remnant population in a very narrow range and with a highly isolated and fragmented distribution pattern. The species is distributed close to the local villages and faces extinction because of the high level of anthropogenic disturbance. Thus, conservation and restoration of this species is urgent. The species reproduces by seed, but its germination requirements and seedling emergence are rarely understood. In this study, the germination requirements, desiccation tolerance and seedling emergence of the species were studied under controlled laboratory conditions. The results indicated that seeds of E. excelsum were non-dormant. Optimal temperatures for germination were 15 degrees C to 25 degrees C; the germination percentage decreased and the mean germination time increased at high temperature. Seed germination was inhibited in the dark, suggesting that the seeds were positive photoblastic. Water stress also significantly inhibited germination percentage; no seeds germinated at 15% polyethylene glycol 6000. The fresh seeds had moderate moisture content of 28.6% and showed strong tolerance of dehydration. Thus, the seeds of E. excelsum tended to be orthodox. Seeds on the soil surface had the highest emergence percentage, which declined with depth of burial. Soil types significantly affected seedling emergence; seeds had higher emergence percentage in sandy soil than in mixed soil and clayed soil. Our study demonstrated that ex situ conservation and reintroduction of E. excelsum using seedling propagation from seeds is feasible.

4637: *+.141*

Predation is a key determinant of community structure and function, and thus should play a central role in successful ecological restoration strategies. The bay scallop, Argopecten irradians, was once abundant in the coastal bays of Virginia, U.S.A., until the complete loss of their eelgrass habitat, Zostera marina, in the 1930s. With the successful restoration of Z. marina in these coastal bays, attention has turned to reintroducing A. irradians with the intent of producing a self-sustaining population. The success of this effort requires an understanding of the sources and degree of natural mortality that A. irradians experiences throughout their ontogeny. The objectives of this study were to: (1) quantify predatory mortality during two successive life history stages of A. irradians, in both spring and fall spawns and (2) identify possible predators of A. irradians in the Virginia coastal bays. We conducted tethering experiments to quantify the proportional losses due to predation, and used otter trawls and suction samples to characterize the predator community over two consecutive years. Losses due to predation ranged from 4 to 80% per day, with smaller juveniles (<15 mm shell height) experiencing greater mortality in 2013, and larger juveniles (>20 mm shell height) in 2014, which we infer is driven by the absence and presence of adult blue crabs in 2013 and 2014, respectively. We propose that managers should look toward relatively inexpensive predator surveys to best judge both when and at what size restored species should be introduced into the wild.

4638: *+.124*

Background: As increasingly fragmented and isolated populations of threatened species become subjected to climate change, invasive species and other stressors, there is an urgent need to consider adaptive potential when making conservation decisions rather than focussing on past processes. In many cases, populations identified as unique and currently managed separately suffer increased risk of extinction through demographic and genetic processes. Other populations currently not at risk are likely to be on a trajectory where declines in population size and fitness soon appear inevitable.Results: Using datasets from natural Australian mammal populations, we show that drift processes are likely to be driving uniqueness in populations of many threatened species as a result of small population size and fragmentation. Conserving and managing such

remnant populations separately will therefore often decrease their adaptive potential and increase species extinction risk.Conclusions: These results highlight the need for a paradigm shift in conservation biology practise; strategies need to focus on the preservation of genetic diversity at the species level, rather than population, subspecies or evolutionary significant unit. The introduction of new genetic variants into populations through in situ translocation needs to be considered more broadly in conservation programs as a way of decreasing extinction risk by increasing neutral genetic diversity which may increase the adaptive potential of populations if adaptive variation is also increased.

4639: *+.192*

Translocation can reduce extinction risk by increasing population size and geographic range, and is increasingly being used in the management of rare and threatened plant species. A critical determinant of successful plant establishment is light environment. Wollemia nobilis (Wollemi pine) is a critically endangered conifer, with a wild population of 83 mature trees and a highly restricted distribution of less than 10 km(2). We used under-planting to establish a population of W. nobilis in a new rainforest site. Because its optimal establishment conditions were unknown, we conducted an experimental translocation, planting in a range of different light conditions from deeply shaded to high light gaps. Two years after the experimental translocation, 85% of plants had survived. There were two distinct responses: very high survival (94%) but very low growth, and lower survival (69%) and higher growth, associated with initial plant condition. Overall survival of translocated W. nobilis was strongly increased in planting sites with higher light, in contrast to previous studies demonstrating long-term survival of wild W. nobilis juveniles in deep shade. Trans location by under-planting may be useful in establishing new populations of shade-tolerant plant species, not least by utilizing the range of light conditions that occur in forest understories.

4640: *+.214*

Mitigation to offset the impacts of land development is becoming increasingly common, with reintroductions and created habitat programs used as key actions. However, numerous reviews cite high rates of poor success from these programs, and a need for improved monitoring and scientific testing to evaluate outcomes and improve management actions. We conducted extensive monitoring of a released population of endangered green and golden bell frogs, Litoria aurea, within a created habitat, as well as complementary surveys of a surrounding wild population. We then compared differences between the created habitat and natural ponds where extant frogs either bred or didn't breed in order to determine factors that contributed to the breeding failure within the created habitat. We evaluated differences of L. aurea abundance, abundance of other fauna, vegetation, water quality, habitat structure, invasive fish, and disease between the three pond types (created habitat, breeding ponds, non-breeding ponds). We discovered that vegetation and invertebrate diversity were low within the created habitat, potentially reducing energy and nutritional resources required for breeding. Also, a greater proportion of frogs in the created habitat were carrying the chytrid fungal pathogen, Batrachochytrium dendrobatidis, compared to the wild populations. In addition to causing the potentially fatal disease, chytridiomycosis, this pathogen has been shown to reduce reproductive functioning in male L. aurea, and subsequently may have reduced reproductive activities in the created habitat. Conspecific attraction, pond hydrology, and aquatic vegetation may also have had some influence on breeding behaviours, whilst the presence of the invasive mosquitofish, Gambusia holbrooki, and heterospecific tadpoles were unlikely to have deterred L. aurea from breeding within the created habitat. Through the use of scientific testing and monitoring, this study is able to make recommendations for future

amphibian breed and release programs, and suggests planting a diversity of plant species to attract invertebrates, creating some permanent ponds, connecting habitat with existing populations, trialling artificial mating calls, and following recommendations to reduce the prevalence of disease within the population.

4641: *+.183*

Genetic variation plays a pivotal role in species viability and the maintenance of population genetic variation is a main focus of conservation biology. Threatened species often show reduced genetic variation compared to non-threatened species, and this is considered indicative of lowered evolutionary potential, compromised reproductive fitness, and elevated extinction risk. The southern purple-spotted gudgeon, Mogurnda adspersa, is a small freshwater fish with poor dispersal potential that was once common throughout the Murray-Darling Basin (MDB) and along the central east coast of Australia. Its numbers and distribution have shrunk dramatically in the MDB due to flow alteration, degradation of habitat, decreasing water quality, and introduction of alien species. We used microsatellite DNA markers to assess population structure and genetic variation at both large (i.e. across basin) and fine (i.e. within river catchments) spatial scales using a substantial sampling effort across the species range (n = 579 individuals; 35 localities). The results consistently indicated very low levels of genetic variation throughout, including along the east coast where the species is relatively common. At the broader scale, three highly differentiated groups of populations were found, concordant with previously reported genealogical distinctiveness. Hence we propose each group as a distinct Evolutionarily Significant Unit. We also inferred a minimum of 12 management units in M. adspersa, with no appreciable gene flow between them. Our study discloses findings relevant for both long- and short-term management, as it informs on the geographic context in which conservation priorities should be defined and specifies biological units for population monitoring and translocations.

4642: *+.086*

Genetic pollution through introgressive hybridization of local species by exotic relatives is a major, yet neglected aspect of biological invasions, particularly in amphibians where human introductions are frequent. In Western Switzerland, crested newts make an interesting case: the Italian species Triturus carnifex was introduced at least a century ago within the range of the native and threatened T. cristatus. To understand the genetic consequences of this introduction and inform wildlife management authorities, we conducted a genetic survey on the remaining northern crested newt populations known in the area, using newly-developed species-diagnostic nuclear (microsatellites) and mitochondrial (control region) DNA markers. We documented massive nuclear introgression by the T. carnifex genome, which has completely replaced T. cristatus in most populations, especially in the Geneva area where the introduction was originally reported. However, many of these individuals retained the ancestral T. cristatus mtDNA, which could be explained by asymmetric introgression between the two species, stemming from demographic and/or selective processes. Analyses of genetic diversity support multiple events of T. carnifex releases, most-likely of proximate North Italian origin. We pinpointed the last indigenous populations in the region and recommend to prioritize their protection. Our study demonstrates the invasive potential of introduced taxa through introgressive hybridization, alerts about the underestimated rate of illegal amphibian translocations, and emphasizes the need for genetic analyses to monitor such invasions.

4643: *+.114*

Recent assertions in the literature (e.g., Keller et al. 2015) suggest that landscape genetic research has been infrequently applied by practitioners. We were interested to test this assertion, which is difficult to assess, since applications may not be detectable through searches of peer-reviewed literature. Producing publications may not be a goal of practitioners. We developed a method to search the internet for evidence of research applications and evaluated 25 different research fields in the natural sciences. We found that fields with more publications also had more applications, but the field of landscape genetics was less applied than expected based on the number of peer-reviewed publications-only about 4 % of landscape genetics articles were applied. In fact, all research fields in genetics or evolutionary biology were under-applied compared to 'whole organism', ecological research fields. This result suggests the lack of applications in landscape genetics may be due to a systemic under-application of genetics research, perhaps related to a lack of understanding of genetics by practitioners. We did find some evidence of landscape genetic applications however, which we sorted into 5 categories: (1) identification of evolutionarily significant units for conservation, (2) managing pathogens and invasive species, (3) natural heritage systems planning, (4) assessing population status, and (5) restoration of populations.

4644: ***+.065***

Aim Some threatened species are now recovering after a period of serious decline. Understanding and predicting the spatio-temporal recolonization of these species in a heterogeneous landscape are important for their conservation planning. We aimed to predict the range expansion of the endangered Asian crested ibis Nipponia nippon as it recovers from near-extinction to guide its in situ conservation and plan possible reintroductions. Location Central China. Methods We used a presence-only ecological niche model to predict breeding habitat suitability and a newly developed, spatially explicit and individual-based dynamic modelling platform to simulate range expansion. We performed a sensitivity analysis to assess the effects of uncertainty in demographic and dispersal parameters on the simulation of range dynamics. The impact of human-induced mortality risk was also investigated. Results Predictions showed that the Asian crested ibis population and the range extent would continue to increase over the next 50 years, and the species would recolonize parts of its historical range. However, the majority of the population would still be restricted to a relatively small region, and some potential suitable regions might not be recolonized for decades by natural dispersal. Moreover, the simulated range dynamics were sensitive to life history trait parameters, among which adult survival probability and the proportion of long-distance dispersal events showed the strongest effects. High human-induced mortality risks had a significant negative effect on population growth and range expansion. Main conclusions This study demonstrates how hybrid modelling can inform conservation management of threatened species as they recolonize former habitat. The findings enable prioritization of management efforts, highlight the need for long-term monitoring of the key life history parameters and provide evidence to guide the selection of potential reintroduction sites for the long-term survival and recovery of target species.

4645: ***+.331***

Increasing knowledge of post-release survival and habitat requirements of translocated animals is critical to improve success of conservation programs. We estimated survival of reintroduced captive-bred Asian houbara bustards (Chlamydotis macqueenii) in reserves of western United Arab Emirates where plantations exist as supplementary feeding sites. We explored factors influencing short- (3 months after release) and long-term (tri-monthly periods after third month of release) survival rates of released birds. We modeled life histories of individually tracked houbara using Program MARK. Mean short-term survival probability (0.76 +/- 0.14SD) was lower than

mean long-term survival (0.86 +/- 0.03SD), and observed group size and the age of released birds positively correlated with short-term survival. We hypothesize that higher quality habitat (plantations) affected survival; larger groups occurred in plantations and older birds might be better able to maintain access to plantations. Long-term survival was negatively influenced by subsequent release events. Releasing more individuals increases local houbara density. This may lead to food depletion, increase in density-dependent mechanisms between individuals, or both. Short- and long-term survival rates suggest that food availability at the release sites, together with intraspecific interactions, may influence survival of newly released and established individuals. To improve the management of translocated animals, the impact of managed food resources should be quantified to assess how it might affect population vital rates. (c) 2016 The Wildlife Society.

4646: ***+.057***

Modeling survival of reintroduced populations is critical for understanding population dynamics and therefore making appropriate management decisions. We analyzed survival data collected over the first 2 years after a reintroduction of hihi (Notiomystis cincta), an endangered New Zealand forest bird, to Bushy Park, a conservation reserve in New Zealand enclosed by a predator-exclusion fence. We constructed a set of candidate models representing different hypotheses about the effects of age, sex, and post-release acclimation on survival, and used model averaging to obtain parameter estimates reflecting the relative support for the models. In combination with fecundity data, we constructed a stochastic population model incorporating uncertainty in parameter estimation, and used this to project population dynamics over the next 10 years. The survival analysis indicated that female survival was unusually low over a 6-month acclimation period; hence, this initial low survival was not reflected in the estimates of long-term survival obtained through model averaging. The resulting projections showed that although population growth was highly uncertain, there was a negligible probability of extinction over the next 10 years, therefore indicating that the existing management regime (i.e., supplementary feeding and nest box maintenance) should be continued. In contrast, if post-release effects had not been accounted for, the projections would have shown a high probability of decline under this management regime. (c) 2016 The Wildlife Society.

4647: ***+.039***

Parasitic species, which depend directly on host species for their survival, represent a major regulatory force in ecosystems and a significant component of Earth's biodiversity. Yet the negative impacts of parasites observed at the host level have motivated a conservation paradigm of eradication, moving us farther from attainment of taxonomically unbiased conservation goals. Despite a growing body of literature highlighting the importance of parasite-inclusive conservation, most parasite species remain understudied, underfunded, and underappreciated. We argue the protection of parasitic biodiversity requires a paradigm shift in the perception and valuation of their role as consumer species, similar to that of apex predators in the mid-20th century. Beyond recognizing parasites as vital trophic regulators, existing tools available to conservation practitioners should explicitly account for the unique threats facing dependent species. We built upon concepts from epidemiology and economics (e.g., host-density threshold and cost-benefit analysis) to devise novel metrics of margin of error and minimum investment for parasite conservation. We define margin of error as the risk of accidental host extinction from misestimating equilibrium population sizes and predicted oscillations, while minimum investment represents the cost associated with conserving the additional hosts required to maintain viable parasite populations. This framework will aid in the identification of readily conserved parasites that present minimal health risks. To establish parasite conservation, we propose an extension of

population viability analysis for host-parasite assemblages to assess extinction risk. In the direst cases, ex situ breeding programs for parasites should be evaluated to maximize success without undermining host protection. Though parasitic species pose a considerable conservation challenge, adaptations to conservation tools will help protect parasite biodiversity in the face of an uncertain environmental future. Resumen Las especies parasitas, aquellas que dependen directamente de las especies hospederas para sobrevivir, representan una gran fuerza regulatoria dentro de los ecosistemas y un componente significativo de la biodiversidad de la Tierra. Aun asi, los impactos negativos de los parasitos que se han observado a nivel del hospedero han motivado un paradigma de conservacion enfocado en la erradicacion, lo que nos aleja cada vez mas de alcanzar objetivos de conservacion sin sesgos taxonomicos. A pesar de la creciente bibliografia que resalta la importancia de la conservacion incluyente de los parasitos, la mayoria de este tipo de especies sigue siendo poco estudiada, mal financiada y poco valorada. Argumentamos que la proteccion de la diversidad parasitaria requiere de un cambio en el paradigma de la percepcion y valoracion de su papel como especies consumidoras, similar al de los depredadores primarios a mediados del siglo XX. Mas alla de reconocer a los parasitos como reguladores troficos vitales, las herramientas existentes disponibles para quienes practican la conservacion deberian reconocer explicitamente las amenazas unicas que enfrentan las especies dependientes. Partimos de conceptos de epidemiologia y economia (p. ej.: umbral de densidad de hospedero y analisis de costo-beneficio) para disenar medidas novedosas del margen de error y la inversion minima para la conservacion de parasitos. Definimos el margen de error como el riesgo de extincion accidental del hospedero a partir de una mala estimacion del tamano de poblaciones en equilibrio y de los pronosticos de oscilacion, mientras que la inversion minima representa el costo asociado a la conservacion de los hospederos adicionales requeridos para mantener viables a las poblaciones de parasitos. Este marco de trabajo ayudara en la identificacion de los parasitos conservados inmediatamente que presentan un riesgo sanitario minimo. Para establecer la conservacion de parasitos, proponemos una extension del analisis de viabilidad poblacional para los conjuntos de hospedero-parasito y asi evaluar el riesgo de extincion. En los casos mas urgentes, se deberian evaluar programas de crianza ex situ para maximizar el exito sin debilitar la proteccion al hospedero. Aunque las especies parasitas presentan un reto considerable para la conservacion, las adaptaciones de las herramientas de conservacion ayudaran a proteger la diversidad de parasitos de frente a un futuro ambiental incierto.

4648: ***-.041***

The European mink is a critically endangered mustelid with only three declining and isolated populations. Based on particular interpretation of genetic studies, the origin of SW population (France and Spain) and its conservation measures were recently questioned, and considered an example of managed relocation. The four published genetic studies show similar results, revealing a bottleneck structure. The SW population was probably established by very few individuals, which can be explained by dispersal of some-long distance migrants or by human introduction. The historical records evidence a long-distance dispersal capacity of the species and the temporal trend expansion in SW Europe is spatially collated with the idea of an expanding population. On contrast, most support for the introduction hypothesis comes from critics to other hypotheses, while it lacks of structure to stand on its own as hypothesis: no ecological barrier preventing natural expansion identified, no reports or reasons for captivity breeding or translocation programs, and lacks of supporting documents among others. Arguing shifting baseline syndrome in European mink conservation has weak basis and can result perilously misleading for a species in the brink of extinction.

4649: ***+.185***

Despite the propensity of species introductions to disrupt ecosystems through community disassembly, the use of species translocations is becoming more widely accepted. In this paper, we examine ethical investigations into human migration in an attempt to evaluate how translocation may be justified. Previous attempts to make the analogy between human and species migration have been prone to black and white thinking. We argue that the disagreement between nativist and cosmopolitan approaches to introduced species can be defused by extending the analogy through the migration ethics literature. Additionally, by extending the discussion to the special status of refugees, we are able to develop a theoretical framework for species migrations that acknowledges the risk of species introduction while recognising that special obligations towards endangered species may necessitate the use of translocations.

4650: *+.209*

For several species, refuges (such as burrows, dens, roosts, nests) are an essential resource for protection from predators and extreme environmental conditions. Refuges also serve as focal sites for social interactions, including mating, courtship, and aggression. Knowledge of refuge use patterns can therefore provide information about social structure, mating, and foraging success, as well as the robustness and health of wildlife populations, especially for species considered to be relatively solitary. In this study, we construct networks of burrow use to infer social associations in a threatened wildlife species typically considered solitary-the desert tortoise. We show that tortoise social networks are significantly different than null networks of random associations, and have moderate spatial constraints. We next use statistical models to identify major mechanisms behind individual-level variation in tortoise burrow use, popularity of burrows in desert tortoise habitat, and test for stressor-driven changes in refuge use patterns. We show that seasonal variation has a strong impact on tortoise burrow switching behavior. On the other hand, burrow age and topographical condition influence the number of tortoises visiting a burrow in desert tortoise habitat. Of three major population stressors affecting this species (translocation, drought, disease), translocation alters tortoise burrow switching behavior, with translocated animals visiting fewer unique burrows than residents. In a species that is not social, our study highlights the importance of leveraging refuge use behavior to study the presence of and mechanisms behind non-random social structure and individual-level variation. Our analysis of the impact of stressors on refuge-based social structure further emphasizes the potential of this method to detect environmental or anthropogenic disturbances. Adaptive and social behavior that affects fitness is now being increasingly incorporated in the conservation and management of wildlife species. However, direct observations of social interactions in species considered to be solitary are difficult, and therefore integration of behavior in conservation and management decisions in such species has been infrequent. For such species, we propose quantifying refuge use behavior as it can provide insights towards their (hidden) social structure, establish relevant contact patterns of infectious disease spread, and provide early warning signals of population stressors. Our study highlights this approach in a long-lived and threatened species, the desert tortoise. We provide evidence toward the presence of and identify mechanisms behind the social structure in desert tortoises formed by their burrow use preferences. We also show how individuals burrow use behavior responds to the presence of population stressors.

4651: *+.061*

Habitat fragmentation is one of the most severe threats to biodiversity as it may lead to changes in population genetic structure, with ultimate modifications of species evolutionary potential and local extinctions. Nonetheless, fragmentation does not equally affect all species and identifying which ecological traits are related to species sensitivity to habitat fragmentation could help

prioritization of conservation efforts. Despite the theoretical link between species ecology and extinction proneness, comparative studies explicitly testing the hypothesis that particular ecological traits underlies species-specific population structure are rare. Here, we used a comparative approach on eight bird species, co-occurring across the same fragmented landscape. For each species, we quantified relative levels of forest specialization and genetic differentiation among populations. To test the link between forest specialization and susceptibility to forest fragmentation, we assessed species responses to fragmentation by comparing levels of genetic differentiation between continuous and fragmented forest landscapes. Our results revealed a significant and substantial population structure at a very small spatial scale for mobile organisms such as birds. More importantly, we found that specialist species are more affected by forest fragmentation than generalist ones. Finally, our results suggest that even a simple habitat specialization index can be a satisfying predictor of genetic and demographic consequences of habitat fragmentation, providing a reliable practical and quantitative tool for conservation biology.

4652: *+.198*

The importance of transient dynamics of structured populations is increasingly recognized in ecology, yet these implications are not largely considered in conservation practices. We investigate transient and long-term population dynamics to demonstrate the process and utility of incorporating transient dynamics into conservation research and to better understand the population management of slow life-history species; these species can be theoretically highly sensitive to short-and long-term transient effects. We are specifically interested in the effects of anthropogenic removal of individuals from populations, such as caused by harvest, poaching, translocation, or incidental take. We use the sandhill crane (Grus canadensis) as an exemplar species; it is long-lived, has low reproduction, late maturity, and multiple populations are subject to sport harvest. We found sandhill cranes to have extremely high potential, but low likelihood for transient dynamics, even when the population is being harvested. The typically low population growth rate of slow life-history species appears to buffer against many perturbations causing large transient effects. Transient dynamics will dominate population trajectories of these species when stage structures are highly biased towards the younger and non-reproducing individuals, a situation that may be rare in established populations of long-lived animals. However, short-term transient population growth can be highly sensitive to vital rates that are relatively insensitive under equilibrium, suggesting that stage structure should be known if perturbation analysis is used to identify effective conservation strategies. For populations of slow life-history species that are not prone to large perturbations to their most productive individuals, population growth may be approximated by equilibrium dynamics. (C) 2016 Elsevier Ltd. All rights reserved.

4653: *+.102*

Given the rapid pace of renewable energy development, there is need to assess impacts of mitigation-driven translocations on sensitive species, including federally protected Mojave desert tortoise (Gopherus agassizii) distributed widely across a global solar energy hotspot. We monitored 215 tortoises over 3 years to evaluate trans location effects on temperature, condition, growth, and mortality of tortoises adjacent to the world's largest solar thermal plant located in southern California. On the basis of generalized estimating equation mixed models, maximum daily tortoise temperature increased with environmental temperature then leveled off at highest environmental temperatures for all three groups (translocated, resident, and control) likely due to behavioral thermotegulation. This pattern was consistent among groups, but translocated tortoises had higher average maximum daily temperature and durations above 35 degrees C than resident and control tortoises in the first month post-translocation, with smaller effects in the second and

fourth months, and no differences in subsequent years. During the first year, adult translocated males had higher temperatures than females. Average percent changes in body condition and carapace length increased post-translocation for all groups and there were no differences in mortality probability among groups or years. Based on the relatively short-term thermal effects, and lack of negative effects on condition, growth, or mortality, our study suggests relatively minimal impacts following short-distance translocation releases in spring. Our study also serves as a unique example and first step for science-based, mitigation-driven translocations implemented to evaluate and reduce effects of translocation from solar energy development on sensitive species. (C) 2016 Elsevier Ltd. All rights reserved.

4654: *+.173*

The complex parasite communities of animals contribute to biodiversity, yet the conservation strategies that aim to preserve individual threatened species often overlook their parasite communities. Deeper understanding of parasite communities and how they are affected by management is important to the ultimate success of biodiversity conservation. Here we examine the dynamics between the coccidian parasite Eimeria and the threatened brush-tailed rock-wallaby (Petrogale penicillata, BTRW) to determine how parasite communities respond to the conservation management practices of captive breeding and translocation. Three BTRW population categories (wild, captive bred or supplemented) were analysed for Eimeria prevalence and infection intensity and a metagenomic assessment performed to examine community structure. Eimeria prevalence was 92% in 117 faecal samples. DNA amplicons from purified oocysts were sequenced with the Illumina MiSeq platform and the resulting sequences assigned to 28 Eimeria operational taxonomic units (OTUs). Pairwise identity between OTUs was 89.9% and 25 of the Eimeria OTUs formed a highly supported phylogenetic Glade with marsupial specific Eimeria species, indicating strong host specificity and genetic diversity within Eimeria in BTRWs. Supplemented populations had the greatest OTU diversity with eleven unique OTUs and had a greater overlap with captive bred (9 shared OTUs) versus wild populations (6 shared OTUs). There was no significant effect of population category on infection intensity ($p = 0.965$), OTU composition ($p = 0.51$) or richness ($p = 0.490$), suggesting that Eimeria community structure is maintained under the management processes applied to the BTRW. Our approach can be applied to other parasite communities in hosts under conservation management. (C) 2016 Elsevier Ltd. All rights reserved.

4655: *-.141*

Predators affect ecosystems not only through direct mortality of prey, but also through risk effects on prey behavior, which can exert strong influences on ecosystem function and prey fitness. However, how functionally different prey species respond to predation risk and how prey strategies vary across ecosystems and in response to predator reintroduction are poorly understood. We investigated the spatial distributions of six African herbivores varying in foraging strategy and body size in response to environmental factors and direct predation risk by recently reintroduced lions in the thicket biome of the Addo Elephant National Park, South Africa, using camera trap surveys, GPS telemetry, kill site locations and Light Detection and Ranging. Spatial distributions of all species, apart from buffalo, were driven primarily by environmental factors, with limited responses to direct predation risk. Responses to predation risk were instead indirect, with species distributions driven by environmental factors, and diel patterns being particularly pronounced. Grazers were more responsive to the measured variables than browsers, with more observations in open areas. Terrain ruggedness was a stronger predictor of browser distributions than was vegetation density. Buffalo was the only species to respond to predator encounter risk, avoiding areas with higher lion utilization. Buffalo therefore behaved in similar ways to when

lions were absent from the study area. Our results suggest that direct predation risk effects are relatively weak when predator densities are low and the time since reintroduction is short and emphasize the need for robust, long-term monitoring of predator reintroductions to place such events in the broader context of predation risk effects.

4656: ***+.103***

Estuaries are among the most important habitats of coastal areas. However, they are significantly affected by human activities worldwide, threatening their resources and the commercial uses depending on them. Fisheries and aquaculture are some of the main factors affecting the estuarine ecosystems today. In this work, we pursue the aims of genetically identifying the cultured and harvested oyster and razor clam species inhabiting Asturian estuaries (Eo, Ribadesella and Villaviciosa) and describing the genetic diversity and patterns of genetic connectivity between those species in these estuaries. The results revealed the almost complete dominance of the introduced Crassostrea gigas as the species that supports the oyster's production in Asturias with a striking diversity of gene pools in wild environments (Ria de Villaviciosa and Ribadesella) which significantly exceeds the diversity found in the hatcheries seeds commonly used for culturing. Moreover, we detected two different species of Ensis sp. inhabiting the Asturian estuaries: Ensis ensis inside Ria del Eo and Ensis directus, an invasive species, in Ria de Villaviciosa. Significant genetic differentiation between estuaries for the exotic oyster C.gigas and also for the native razor clam Solen marginatus were found. These results suggest Asturian estuaries are not a single management unit for some species and thus prevention must be taken for avoiding intentional or human-mediated translocations among them. Biodiversity monitoring for discovering newcomer invasive species, measures for environmental recovery of these ecosystems and strict controls to avoid increasing of harvesting pressures are also a necessity to improve the management of these relevant ecosystems.

4657: ***-.088***

Human-wildlife conflict is increasing as urbanization expands and wildlife species adjust to living near people. Translocation is often used to manage human-wildlife conflict because it is considered to be humane, yet fates of translocated animals are largely unknown. As an urban adapter, woodchucks (Marmota monax) are a common source of human-wildlife conflict due to their burrowing, foraging, and scent-marking behavior. We examined survival and movements of 27 nuisance woodchucks captured by a nuisance wildlife operator in the Chicago metropolitan area, radiomarked with internal transmitters, and translocated to exurban release sites mimicking typical practices. We also captured and radiomarked 16 resident woodchucks from the release landscape for comparison. Translocated woodchucks moved farther than residents immediately post release with no evidence of homing and most left the release site. Annual survival did not differ between translocated and resident woodchucks. However, survival was extremely low (0.18) compared to previous estimates for woodchucks, primarily due to high predation by coyotes (Canis latrans). Translocation should be used only when other nonlethal methods are ineffective (e.g., exclusion, removing food sources, selecting unpalatable plants for gardens). When necessary, the practice of translocation could be improved by reducing predation risk for translocated animals, either by selecting release sites with low predation risk, or by using soft-release methods, such as acclimation enclosures or artificial burrows.

4658: ***+.142***

Characterising the habitat use of released captive-bred birds is required to help optimise future

avian reintroduction programmes. The critically endangered Double-spurred Francolin Francolinus bicalcaratus ayesha is endemic to north-west Morocco, where it inhabits forests of cork oak Quercus suber. To improve the viability of this threatened population, 300 captive-bred francolins were released into a game reserve, and post-release monitoring was conducted. This study aimed to identify habitat variables determining the habitat selection of the Double-spurred Francolin. Auditory detection was used during transect surveys of calling males to locate birds and their habitat occupation. Comparison of occupied and random plots showed that this bird is found mostly in flat topography with high cover of shrubs and dense cork oak trees, and close to the release site and water points. Conservation of Double-spurred Francolin depends on the choice of the release point within the cork oak forest, which should be in proximity to suitable cover of cork oak trees, shrubs and water points. Such choices would allow a rapid adaptation to prevailing conditions within release sites. Further multi-scale studies are needed to improve our understanding of the effects of ecological factors on the processes of habitat selection by this endemic subspecies.

4659: *+.183*

The white-clawed crayfish is endemic to western and southern Europe and its populations have decreased over recent decades. Spanish populations are generally poorly represented in scientific reports and are usually studied only with a single molecular marker. Here, we use two mitochondrial markers (cytochrome oxidase subunit I and rDNA 16S genes) to examine levels and patterns of genetic structure across the range of the species' distribution in Spain. Data reveal the existence of two main genetic groups of white-clawed crayfish in Spain with the Ebro basin as a possible contact zone. Processes occurred in historical and recent times, such as genetic drift and translocations, contribute greatly to this genetic structure. Levels of genetic variability and genetic structure of Spanish populations together with demographic inferences suggest that the species established in the Iberian Peninsula, at least since the Late Pleistocene. Knowing the true origin of the Spanish populations is crucial when deciding upon the management policies that should be followed. Given the lack of any clear evidence against its indigenous status, we propose that current protection and conservation measures should be maintained. From a management point of view, we suggest that Spanish population should be considered as a single evolutionary significant unit (ESU) with two management units (MUs) corresponding with the genetic clusters detected in the present study.

4660: *+.155*

Wildlife is a multi-million dollar industry in South Africa ranging from intensively farmed animals on small properties to free-roaming animals in large functional ecosystems. Specific concerns surrounding the conservation value of the intensively managed wildlife have been raised. Increasingly it is recognised that genetics must be considered when developing conservation policy and management practices. Minimal genetic data are available to assist with decisions within the industry. Microsatellite markers have been developed for some species and are currently used for individual identification, parentage assessment and to inform translocation decisions. However, validation for standardized application in wildlife management has not been completed. Common species for which conservation management decisions have to be made include: Cape buffalo (Syncerus caffer), blue wildebeest (Connochaetes taurinus taurinus), common impala (Aepyceros melampus) and sable antelope (Hippotragus niger). Using existing microsatellite markers for domestic and wild species, we have developed multiplex panels for use in these species. We discriminated between 751 buffalo (four pairs were not distinguishable), 1307 blue wildebeest (three pairs were not distinguishable), 580 impala and 493 sable antelope. We

determined parentage in four case studies using a combination of microsatellite and demographic data. We also tested microsatellite markers on roan antelope (Hippotragus equinus), gemsbok (Oryx gazella) and eland (Tragelaphus oryx) and report preliminary data. Further research to improve this validation includes: extensive sampling from more diverse areas, targeted or full genome sequencing to identify additional microsatellite loci, development of sampling kits and training of veterinarians, as well as expansion of the species tested.

4661: *+.198*

Herbivores are major drivers of ecosystem structure, diversity, and function. Resilient ecosystems therefore require viable herbivore populations in a sustainable balance with environmental resource availability. This balance is becoming harder to achieve, with increasingly threatened species reliant on small protected areas in increasingly harsh and unpredictable environments. Arid environments in North Africa exemplify this situation, featuring a biologically distinct species assemblage exposed to extreme and volatile conditions, including habitat loss and climate change-associated threats. Here, we implement an integrated likelihood approach to relate scimitar-horned oryx (Oryx dammah) and dorcas gazelle (Gazella dorcas) density, via dung distance sampling, to habitat, predator, and geographic correlates in Dghoumes National Park, Tunisia. We show how two threatened sympatric ungulates partition resources on the habitat axis, exhibiting nonuniform responses to the same vegetation gradient. Scimitar-horned oryx were positively associated with plant species richness, selecting for vegetated ephemeral watercourses (wadis) dominated by herbaceous cover. Conversely, dorcas gazelle were negatively associated with vegetation density (herbaceous height, litter cover, and herbaceous cover), selecting instead for rocky plains with sparse vegetation. We suggest that adequate plant species richness should be a prerequisite for areas proposed for future ungulate reintroductions in arid and semi-arid environments. This evidence will inform adaptive management of reintroduced ungulates in protected environments, helping managers and planners design sustainable ecosystems and effective conservation programs.

4662: *+.261*

Increased human activity and environmental disturbances are currently eroding the high biodiversity of the tropics. Insights from the fossil record have the potential to inform conservation decisions in these regions. Conservation paleontology has played a transformative role in defining appropriate conservation goals in temperate systems, and recent applications in the tropics showcase the broad applicability of this field. Here, we highlight paleontology's role in the conservation of the tropics. We show that community-level and species-level responses commonly detected by paleontologists, such as range shifts, inform present-day population trajectories and functional diversity changes. Furthermore, advances in techniques such as ancient DNA recovery make querying ancient tropical diversity more tractable. These analyses have direct applications to policies ranging from reserve design to species reintroductions. Despite these breakthroughs, much work still remains for conservation paleontology to reach its full potential in the tropics. Increased regional coverage is necessary for areas such as Southeast Asia and the Amazon, two biogeographically important regions with highly threatened faunas. Taxonomic sampling biases must be eliminated, and we call for additional data representing amphibians and birds, which are underrepresented in paleontological data sets of terrestrial vertebrates. We also recommend further analyses of existing reptile collections, which are abundant but underutilized because of a dearth of comparative collections. We highlight recent contributions to conservation paleontology in these taxonomic groups and regions whenever possible, and showcase successful collaborations between paleontologists, neontologists, and conservationists outside of academia to illustrate the

influence paleontologists can exert in conservation efforts. Citation for this article: Kemp, M. E., and E. A. Hadly. 2016. Rocking Earth's biodiversity cradle: challenges, advances, and prospects for conservation paleontology in the tropics. Journal of Vertebrate Paleontology. DOI: 10.1080/02724634.2016.1179640.

4663: *+.063*

Public opinion can be an influential factor in wildlife management decisions. Evaluating public opinions can help legitimize, or delegitimize, management and facilitate long-term conservation goals. This is especially true for the controversial issues surrounding the management of predators. We surveyed Montana, USA, residents during summer of 2013 to measure public opinion regarding economic and ecological impacts of the gray wolf (Canis lupus), and current management of this species. Although opinions were polarized in some areas, a greater percentage of Montanans think that wolves negatively affect the economy, but impact tourism (which contributes to the economy) positively. These differences may reflect the belief that rancher economic losses from wolf predation of cattle is greater than overall tourism gains related to wolves (e.g., wolf-watching), in addition to the perception of wolves negatively affecting big game (e.g., elk [Cervus canadensis]). Results also show that a slightly greater percentage of Montanans feel that wolves positively rather than negatively affect the ecosystem. Regarding specific practices, more Montanans than not have a positive opinion of maintaining wolves on the landscape and also support hunting of wolves. More Montanans hold negative rather than positive opinions, however, regarding wolf trapping. This result was most evident in western Montana as assessed by a spatial distribution of opinions by county and has implications for current wolf management and nontarget species. Results of ordinal regression analyses revealed that big game hunters, males, and those who held negative opinions of the effect of wolves on the Montana ecosystem and economy were significantly more likely to support both hunting and trapping practices. Living in western Montana predicted positive opinions of hunting, but alternatively, negative opinions of trapping. These results provide an understanding of public opinion of wolf management by county as well as statistical inferences that can be useful for informing more regionally oriented management practices. (c) 2016 The Wildlife Society. Montana resident opinions regarding wolf conservation and management practices showed that more Montanans than not believe wolves positively impact the ecosystem and tourism, but negatively impact the economy. In addition, more Montanans than not have negative opinions of trapping, but positive opinions of hunting, a result that is particularly evident in western Montana as evidenced by a spatial distribution analysis.

4664: *+.482*

In recent decades, community groups have transformed habitat restoration, pest control and species translocations in New Zealand. Large areas of wild New Zealand benefit hugely from ongoing management by community-based restoration groups. Areas near cities and towns have especially good access to pools of keen volunteers. Community groups are involved in monitoring progress with their work, as well as monitoring biodiversity changes in general at their project sites. New tools powered by modern technologies are creating the opportunity for New Zealand's community volunteers to play a transformative role in biodiversity monitoring for either purpose. These tools are reducing the resources and expertise required for species detection and identification. Smartphones with cameras, GPS, audio recorders and data apps make it easier than ever to record species observations. Crowd-sourced identification of species in photographs and sounds loaded onto NatureWatch NZ allow volunteers to make observations of a much wider range of taxa than just common birds and trees. Realising this potential requires community

groups, scientists and their institutions to collaborate in building and maintaining simple, accessible monitoring systems that (i) require and promote standard monitoring methods, (ii) provide efficient data entry in standard formats, (iii) generate automated results of use to community groups and (iv) facilitate public sharing of data to contribute to regional, national and global biodiversity monitoring. Some New Zealand monitoring systems developed recently to assist community-based restoration groups with monitoring mammalian predator control are good examples of this approach. Making this happen at a large scale across many community groups and taxa requires increased and coordinated long-term institutional support for monitoring systems and training.

4665: *+.189*

Increasing our understanding of personality, at an individual and group level, is crucial to the pre-release assessment of social species within ex situ reintroduction programs. We conducted the first exploration into the personality of a captive-origin pride of African lions (Panthera leo), assessing behavioural variations and consistencies in daily activity, social and hunting behaviour, and boldness. Data were collected via direct observations, while a species-specific protocol for testing boldness, using playbacks, was developed. Differences in sex, age and session time for the activity budget were evaluated using Pearson correlations and repeated-measures ANOVA, while social interactions were analysed using social network analysis. Spearman's correlations were conducted to assess for associations between boldness scores, activity and sociality. The two boldness tests provided a range of scores per lion, indicating that the test was effective. Correlations and variations in individual behaviour indicated that adults and sub-adults have specific roles within pride behaviour. Correlations between boldness and activity and social behaviours provided information on the role of individuals, allowing investigation into the behaviour of a dominant and a social keystone. Our study indicates that evaluating various aspects of behaviour in conjunction with boldness has the potential to assist the pre-release assessment of a pride within an ex situ reintroduction program.

4666: *+.059*

In order to investigate the feasibility of reintroducing the South China tiger (Panthera tigris amoyensis) in the Jiangxi Matoushan National Nature Reserve, field surveys were conducted to assess prey distribution in the reserve. Twelve permanent transects were set in three distinct functional zones from February to April 2012 and May to July 2013. A total of 112 ungulate signs were recorded on these transects. In addition, 20 camera traps were used to survey ungulates and predators in 2012, while the following year we extended the survey site by using 30 cameras. Overall, 6641 capture events on 2930 camera days were obtained, presenting a variety of ungulate species: muntjak (Muntiacus muntjak), tufted deer (Elaphodus cephalophus), serow (Capricornis sumatraensis) and wild boar (Sus scrofa). Population structure and composition of ungulates was compared in different functional zones using a single factor of variance analysis in SPSS software. Significant differences in the distribution of ungulates were recognized between the core zone and experimental zone, but not in other zones due to differences in habitat types and management practices of the nature reserve. Using ArcGIS analysis and Salford Predictive Modeler software, we ran several predictive models to understand which areas are most suitable for ungulates. We conclude that muntjac and wild boar are mainly distributed in the experimental zone, serow are more common in the core zone, while tufted deer are located evenly in the three functional zones. Finally, suggestions for effective and feasible management strategies and techniques for Matoushan National Nature Reserve were recommended based on the results and analysis in this study.

4667: +.044

We examined the indirect effects of reintroduced black-tailed prairie dogs (Cynomys ludovicianus) on resident kangaroo rat (Dipodomys) populations. We used the Giving-up Density theory to quantify kangaroo rat foraging on a black-tailed prairie dog colony vs. foraging near the colony edge or in the surrounding native habitat. This approach allowed us to assess the influence of black-tailed prairie dogs on kangaroo rat foraging activity. Our results showed a greater foraging preference off-colony in most seasons. Kangaroo rats visited off-colony feeding trays more frequently and collected a greater mean mass of seed as well. This indicated that kangaroo rats perceived the area off the prairie dog colony as having a lower foraging cost than the on-colony or colony edge locations. Our data suggest that from the perspective of the seed-eating kangaroo rat, the colony is not viewed as high quality habitat. Both prairie dogs and kangaroo rats have been described as keystone modifiers in grassland ecosystems. What impact the reintroduction and management of one keystone species might have on another keystone species deserves additional consideration as we attempt to restore arid grassland ecosystems.

4668: -.006

We evaluated the release of rehabilitated, orphan black bears (Ursus americanus) in northern New Hampshire. Eleven bears (9 males, 2 females; 40-45 kg) were outfitted with GPS radio-collars and released during May and June of 2011 and 2012. Bears released in 2011 had higher apparent survival and were not observed or reported in any nuisance behavior, whereas no bears released in 2012 survived, and all were involved in minor nuisance behavior. Analysis of GPS locations indicated that bears in 2011 had access to and used abundant natural forages or habitat. Conversely, abundance of soft and hard mast was lower in 2012, suggesting that nuisance behavior, and consequently survival, was inversely related to availability of natural forage. Dispersal from the release site ranged from 3.4-73 km across both years, and no bear returned to the rehabilitation facility (117 km distance). Rehabilitation appears to be a valid method for addressing certain orphan bear issues in New Hampshire.

4669: +.053

Background: Wildlife repatriation represents an opportunity for parasites. Reintroduced hosts are expected to accumulate generalist parasites via spillover from reservoir hosts, whereas colonization with specialist parasites is unlikely. We address the question of how myxozoan parasites, which are characterized by a complex life-cycle alternating between annelids and fish, can invade a reintroduced fish species and determine the impact of a de novo invasion on parasite diversity. We investigated the case of the anadromous allis shad, Alosa alosa (L.), which was reintroduced into the Rhine approximately 70 years after its extinction in this river system. Methods: We studied parasites belonging to the Myxozoa (Cnidaria) in 196 allis shad from (i) established populations in the French rivers Garonne and Dordogne and (ii) repatriated populations in the Rhine, by screening the first adults returning to spawn in 2014. Following microscopical detection of myxozoan infections general myxozoan primers were used for SSU rDNA amplification and sequencing. Phylogenetic analyses were performed and cloned sequences were analyzed from individuals of different water sources to better understand the diversity and population structure of myxozoan isolates in long-term coexisting vs recently established host-parasite systems. Results: We describe Hoferellus alosae n. sp. from the renal tubules of allis shad by use of morphological and molecular methods. A species-specific PCR assay determined that the prevalence of H. alosae n. sp. is 100 % in sexually mature fish in the Garonne/Dordogne river systems and 22 % in the first mature shad returning to spawn in the Rhine. The diversity of SSU

rDNA clones of the parasite was up to four times higher in the Rhine and lacked a site-specific signature of SNPs such as in the French rivers. A second myxozoan, Ortholinea sp., was detected exclusively in allis shad from the Rhine. Conclusions: Our data demonstrate that the de novo establishment of myxozoan infections in rivers is slow but of great genetic diversity, which can only be explained by the introduction of spores from genetically diverse sources, predominantly via straying fish or by migratory piscivorous birds. Long-term studies will show if and how the high diversity of a de novo introduction of host-specific myxozoans succeeds into the establishment of a local successful strain in vertebrate and invertebrate hosts.

4670: *+.111*

The domestic cat (Felis catus) is an invasive exotic in many locations around the world and is thought to be a key factor driving recent mammal declines across northern Australia. Many mammal species native to this region now persist only in areas with high topographic complexity, provided by features such as gorges or escarpments. Do mammals persist in these habitats because cats occupy them less, or despite high cat occupancy? We show that occupancy of feral cats was lower in mammal- rich habitats of high topographic complexity. These results support the idea that predation pressure by feral cats is a factor contributing to the collapse of mammal communities across northern Australia. Managing impacts of feral cats is a global conservation challenge. Conservation actions such as choosing sites for small mammal reintroductions may be more successful if variation in cat occupancy with landscape features is taken into account.

4671: *+.016*

Although small populations are at high risk of extinction, there are regular reports in the scientific literature of purported small, isolated, persistent populations. One source of evidence of the viability of small populations comes from the alleged successful introduction of species to areas outside their original range from introductions of few individuals. We reviewed the examples from introduction compendia on deliberate translocations of birds, and the original sources, to identify and evaluate purported examples of successful establishments from small introductions. We found 23 purportedly successful introductions from few (<30) individuals. After assessing original sources, we found that two of the claims were substantiated; the rest were ambiguous or could be rejected as examples, primarily due to a lack of evidence in original sources of the number of birds released and because of supplemental individuals from other releases, releases in nearby regions, and the possibility of natural invasion. Our results suggest that reports of successful establishment of birds from introductions of few individuals have been overstated. These results strengthen the relationship previously reported between propagule pressure and likelihood of establishment, and support the lack of viability of small populations presumed by population theory. We suggest that analyses of introduction failure and success would benefit from excluding studies where introduction effort is unknown or unreliably documented.

4672: *+.088*

Reintroduction of the tiger (Panthera tigris) has become imperative to address the extinction crisis and, it also provides new knowledge of the species biology as to how these animals explore and utilize new environments. We studied six reintroduced tigers and three of their offsprings in Panna Tiger Reserve, central India, focusing on exploration strategy, movement characteristics and spatio-temporal home range patterns. It was found that the release site had no influence on home range selection by the reintroduced tigers, regardless of the release method (soft or hard release) and origin (wild caught or raised in captivity). Although there was a high rate of initial movement,

these animals exhibited strong site fidelity and territoriality subsequently. Mean (+/- SD) annual home ranges of male and female tigers were 132.7 km(2) +/- 9.0 and 73.6 km(2) +/- 9.6, respectively, and did not differ significantly across seasons. The home range sizes of males were among the largest in India and was also marginally larger for females. Comparison with previous telemetry study on historic tiger population in the same site suggests that the reintroduced animals behaved almost exactly the same way as that of native populations, offering support for reintroduction strategies which look to restore not only the species population but also ecosystem functions. The exploratory strategy and subsequent home range establishment by the reintroduced tigers offer novel insights on species behaviour in a new environment, with implication for future conservation strategies that consider translocation-based recovery of tiger populations in the range countries.

4673: *+.063*

Reintroductions inherently involve a small number of founders leading reintroduced populations to be prone to genetic drift and, consequently, to inbreeding depression. Assessing the origins as the genetic diversity and structure of reintroduced populations compared to native populations are thus crucial to foresee their future. Here, we aim to clarify the origins of the Alpine marmots reintroduced in the Pyrenees and to evaluate the genetic consequences of this reintroduction after almost 30 years without monitoring. We search for the origins and compare the genetic structure and the genetic variability of three reintroduced Pyrenean and eight native Alpine populations using pairwise genetic distances, Bayesian clustering method and multivariate analyses. Our results reveal that the Alpine marmots reintroduced in the Pyrenees originated both from the Northern and the Southern Alps, and that, despite these multiple origins, none of the current Pyrenean marmots are admixed. The reintroduction led to a strong genetic differentiation and to a decrease in genetic diversity. This pattern likely results from the small number of founders and the low dispersal capacities of Alpine marmots and thus, highlight the necessity to consider both genetic characteristics and natural history when reintroducing a species.

4674: *+.091*

The Arabian oryx (Oryx leucoryx) historically ranged across the Arabian Peninsula and neighboring countries until its extirpation in 1972. In 1963-1964 a captive breeding program for this species was started at the Phoenix Zoo (PHX); it ultimately consisted of 11 animals that became known as the 'World Herd'. In 1978-1979 a wild population was established at the Shaumari Wildlife Reserve (SWR), Jordan, with eight descendants from the World Herd and three individuals from Qatar. We described the mtDNA and nuclear genetic diversity and structure of PHX and SWR. We also determined the long-term demographic and genetic viability of these populations under different reciprocal translocation scenarios. PHX displayed a greater number of mtDNA haplotypes (n = 4) than SWR (n = 2). Additionally, PHX and SWR presented nuclear genetic diversities of = 2.88 vs. 2.75, = 0.469 vs. 0.387, and = 0.501 vs. 0.421, respectively. Although these populations showed no signs of inbreeding (ae 0), they were highly differentiated (= 0.580; P < 0.001). Migration between PHX and SWR (Nm = 1, 4, and 8 individuals/generation) increased their genetic diversity in the short-term and substantially reduced the probability of extinction in PHX during 25 generations. Under such scenarios, maximum genetic diversities were achieved in the first generations before the effects of genetic drift became predominant. Although captive populations can function as sources of genetic variation for reintroduction programs, we recommend promoting mutual and continuous gene flow with wild populations to ensure the long-term survival of this species.

4675: ***+.092***

In the southwestern United States (US), the Rio Grande chub (Gila pandora) is state-listed as a fish species of greatest conservation need and federally listed as sensitive due to habitat alterations and competition with non-native fishes. Characterizing genetic diversity, genetic population structure, and effective number of breeders will assist with conservation efforts by providing a baseline of genetic metrics. Genetic relatedness within and among G. pandora populations throughout New Mexico was characterized using 11 microsatellite loci among 15 populations in three drainage basins (Rio Grande, Pecos, Canadian). Observed heterozygosity (H-O) ranged from 0.71-0.87 and was similar to expected heterozygosity (0.75-0.87). Rio Ojo Caliente (Rio Grande) had the highest allelic richness (A(R) = 15.09), while Upper Rio Bonito (Pecos) had the lowest allelic richness (A(R) = 6.75). Genetic differentiation existed among all populations with the lowest genetic variation occurring within the Pecos drainage. STRUCTURE analysis revealed seven genetic clusters. Populations of G. pandora within the upper Rio Grande drainage (Rio Ojo Caliente, Rio Vallecitos, Rio Pueblo de Taos) had high levels of admixture with Q-values ranging from 0.30-0.50. In contrast, populations within the Pecos drainage (Pecos River and Upper Rio Bonito) had low levels of admixture (Q = 0.94 and 0.87, respectively). Estimates of effective number of breeders (N (b)) varied from 6.1 (Pecos: Upper Rio Bonito) to 109.7 (Rio Grande: Rio Peasco) indicating that populations in the Pecos drainage are at risk of extirpation. In the event that management actions are deemed necessary to preserve or increase genetic diversity of G. pandora, consideration must be given as to which populations are selected for translocation.

4676: ***+.090***

The New Zealand long-tailed bat (Chalinolobus tuberculatus) is an endemic species threatened with extinction. Since the arrival of humans, massive deforestation has occurred and invasive mammalian predators were introduced. As a result, C. tuberculatus' distribution shrank dramatically and became fragmented. To aid the management of the remaining populations, two Evolutionary Significant Units (ESUs) were designated: one on each of New Zealand's main islands. We utilised mitochondrial sequence data (cytb, 703 bp) and 10 nuclear DNA microsatellite loci to reconstruct the demographic history of this species, to characterise the level of genetic diversity in remaining populations, and to assess the current connectivity between them. Our results indicate that the North Island, with the highest genetic diversity, served as a glacial refuge, with a loss of diversity following the path recolonization to the south of the South Island. However, our data are also consistent with continued, or at least very recent, genetic exchange between colonies across the species distribution. The only exception is the Hanging Rock colony on the east coast of the South Island, which appears to be isolated. Thus, there was no support for the previously designated ESUs. Signatures of past population declines were found in three colonies, the most extreme of which was found in Hanging Rock. Consequently, we recommend that it be genetically rescued via translocation from a donor population. In general, future management priorities should treat Chalinolobus tuberculatus as a single unit, focusing on maintaining connectivity between remaining populations, together with continued roost protection and pest control.

4677: ***+.096***

Protecting genetic diversity throughout the range of a species is important for conservation, as doing so provides for long-term evolutionary potential and persistence under a changing environment. Conservation of diversity at the intraspecific level requires identification of all genetically distinct population segments within species; i.e., conservation units (CUs). Silene

spaldingii occurs in grasslands of the Columbia Plateau region of western North America and is listed as threatened under the Federal Endangered Species Act. The recovery plan identified five physiographic regions across the range of the species to use as surrogates for genetic CUs. We collected leaf samples from an average of 26 plants from each of 19 of the largest populations across all five physiographic regions and used variable microsatellite and chloroplast DNA markers to determine how genetic variation is distributed across the range of the species and how well physiographic regions reflect population structure within this species. Results of several multivariate analyses clustered our samples into four genetic groups which did not correspond well with the physiographic regions. We observed little genetic differentiation among populations in the main range of the species which encompasses nearly all of four contiguous physiographic regions. However, three other distinct genetic groups were identified: two in the disjunct northeast corner and one at the southeast edge of the main range. Modification of the CUs to reflect the genetic groups rather than the physiographic regions would result in CUs which better reflect historical patterns of population structure. Moreover, use of the genetic units to inform translocation and genetic rescue efforts could improve our ability to mimic natural patterns of gene flow. Our results suggest that physiographic regions may not always be an accurate reflection of population structure for threatened or endangered species.

4678: ***+.098***

Research in reintroduction biology has provided a greater understanding of the often limited success of species reintroductions and highlighted the need for scientifically rigorous approaches in reintroduction programs. We examined the recent genetic-based captive-breeding and reintroduction literature to showcase the underuse of the genetic data gathered. We devised a framework that takes full advantage of the genetic data through assessment of the genetic makeup of populations before (past component of the framework), during (present component), and after (future component) captive-breeding and reintroduction events to understand their conservation potential and maximize their success. We empirically applied our framework to two small fishes: Yarra pygmy perch (Nannoperca obscura) and southern pygmy perch (Nannoperca australis). Each of these species has a locally adapted and geographically isolated lineage that is endemic to the highly threatened lower Murray-Darling Basin in Australia. These two populations were rescued during Australia's recent decade-long Millennium Drought, when their persistence became entirely dependent on captive-breeding and subsequent reintroduction efforts. Using historical demographic analyses, we found differences and similarities between the species in the genetic impacts of past natural and anthropogenic events that occurred in situ, such as European settlement (past component). Subsequently, successful maintenance of genetic diversity in captivitydespite skewed brooder contribution to offspringwas achieved through carefully managed genetic-based breeding (present component). Finally, genetic monitoring revealed the survival and recruitment of released captive-bred offspring in the wild (future component). Our holistic framework often requires no additional data collection to that typically gathered in genetic-based breeding programs, is applicable to a wide range of species, advances the genetic considerations of reintroduction programs, and is expected to improve with the use of next-generation sequencing technology. Un Marco de Referencia Holistico Novedoso para Programas de Reproduccion en Cautiverio Basada en Genetica y de Reintroduccion Investigaciones sobre biologia de la reintroduccion han proporcionado un mejor entendimiento del, a menudo, exito limitado de las reintroducciones de especies y han resaltado la necesidad de aproximaciones rigorosas cientificamente en los programas de reintroduccion. Examinamos la literatura reciente sobre reproduccion en cautiverio basada en genetica y reintroduccion para exhibir la subutilizacion de los datos geneticos. Disenamos un marco de referencia que obtiene la mayor ventaja de los datos geneticos mediante la evaluacion de la composicion genetica de las poblaciones antes

(componente pasado del marco de referencia), durante (componente presente), y despues (componente futuro) de eventos de reproduccion en cautiverio y de reintroduccion para entender su potencial de conservacion y maximizar su exito. Aplicamos nuestro marco de referencia empiricamente con dos especies de peces pequenos: Nannoperca obscura y N. australis. Cada especie tiene un linaje adaptado localmente y aislado geograficamente endemico de la muy amenazada Cuenca Baja Murray-Darling, Australia. Las dos poblaciones fueron rescatadas durante la reciente Sequia del Milenio que duro diez anos en Australia, cuando su persistencia se volvio totalmente dependiente de esfuerzos de reproduccion en cautiverio y subsecuente reintroduccion. Mediante analisis demograficos historicos, encontramos diferencias y similitudes entre las especies en los impactos geneticos de eventos naturales y antropogenicos pasados que ocurrieron in situ, como el asentamiento europeo (componente pasado). Subsecuentemente, el mantenimiento exitoso de la diversidad genetica en cautiverio - no obstante la contribucion de reproductores sesgada - fue posible por el manejo cuidadoso de la reproduccion basada en genetica (componente presente). Finalmente, el monitoreo genetico revelo la supervivencia y reclutamiento de crias obtenidas en cautiverio liberadas en el medio silvestre. Nuestro marco de referencia holistico a menudo no requiere de datos adicionales a los obtenidos tipicamente en programas de reproduccion basados en genetica, es aplicable a un rango amplio de especies, es un avance en las consideraciones geneticas de los programas de reintroduccion, y mejorara con el uso de tecnologia de secuenciacion de ultima generacion.Resumen

4679: *+.195*

The use and importance of reintroduction as a conservation tool to return a species to its historical range from which it has been extirpated will increase as climate change and human development accelerate habitat loss and population extinctions. Although the number of reintroduction attempts has increased rapidly over the past 2 decades, the success rate is generally low. As a result of population differences in fitness-related traits and divergent responses to environmental stresses, population performance upon reintroduction is highly variable, and it is generally agreed that selecting an appropriate source population is a critical component of a successful reintroduction. Conservation genomics is an emerging field that addresses long-standing challenges in conservation, and the potential for using novel molecular genetic approaches to inform and improve conservation efforts is high. Because the successful establishment and persistence of reintroduced populations is highly dependent on the functional genetic variation and environmental stress tolerance of the source population, we propose the application of conservation genomics and transcriptomics to guide reintroduction practices. Specifically, we propose using genome-wide functional loci to estimate genetic variation of source populations. This estimate can then be used to predict the potential for adaptation. We also propose using transcriptional profiling to measure the expression response of fitness-related genes to environmental stresses as a proxy for acclimation (tolerance) capacity. Appropriate application of conservation genomics and transcriptomics has the potential to dramatically enhance reintroduction success in a time of rapidly declining biodiversity and accelerating environmental change. El Papel de la Genomica y los Transcriptomas en la Seleccion de Poblaciones Fuente para Reintroduccion El uso y la importancia de la reintroduccion como herramienta de conservacion para regresar a las especies a su extension historica de la que han sido extirpadas incrementaran conforme el cambio climatico y los establecimientos humanos aceleren la perdida de habitat y la extincion de poblaciones. Aunque el numero de intentos de reintroduccion ha incrementado en las ultimas dos decadas, la tasa de exitos es generalmente baja. Como resultado de las diferencias poblacionales en los caracteres relacionados con la adecuacion y las respuestas divergentes a los estreses ambientales, el desempeno poblacional tras la reintroduccion es altamente variable y es un acuerdo general que la seleccion de una poblacion fuente adecuada es un componente critico de la

reintroduccion exitosa. La genomica de la conservacion es un campo emergente que se enfoca en los retos de larga duracion que enfrenta la conservacion. El potencial para utilizar estrategias novedosas de genetica molecular para informar y mejorar los esfuerzos de conservacion es alto. Ya que el establecimiento exitoso y la persistencia de las poblaciones reintroducidas es altamente dependiente de la variacion genetica funcional y la tolerancia al estres ambiental de la poblacion fuente, proponemos la aplicacion de la genomica de la conservacion y los transcriptomas para guiar las practicas de reintroduccion. Especificamente, proponemos usar loci funcionales a nivel genoma para estimar la variacion genetica de las poblaciones fuente. Este estimado puede usarse para predecir el potencial de adaptacion de la poblacion. Tambien proponemos usar evaluaciones por perfiles de transcripcion para medir la respuesta de expresion a los estreses ambientales de los genes relacionados con la adecuacion como sustitutos de la capacidad de aclimatacion (tolerancia). La aplicacion adecuada de la genomica de la conservacion y los transcriptomas tiene el potencial de mejorar dramaticamente el exito de reintroduccion en tiempos de declinacion critica de la biodiversidad y de cambios ambientales acelerados.Resumen

4680: ***+.139***

Prey may have ontogenetic experience, evolutionary experience, or both types of experiences with their predators and how such experiences influences their ability to identify their predators is of great theoretical and applied interest. We capitalized on predator-free exclosures containing populations of native burrowing bettongs (Bettongia lesueur) and introduced rabbits (Oryctolagus cuniculus) that ensured we had knowledge of our subjects' ontogenetic experiences with predators and asked whether evolutionary experience influenced their visual predator discrimination abilities. Rabbits evolved with red foxes (Vulpes vulpes) and wolves (Canis lupus) but had less than 200 years of prior exposure to dingoes. The rabbit population we studied had been exposed to dingoes (Canis dingo) and foxes 8 months prior to our study and had heightened responses to red fox models, but not dingo/dog (Canis dingo/Canis familiaris) models. The insular burrowing bettong population had no ontogenetic exposure to mammalian predators, brief evolutionary exposure to domestic dogs and possibly dingoes, and a deeper evolutionary history of exposure to thylacines (Thylacinus cynocephalus)-another large mammalian predator with convergent body morphology to dingoes/dogs but no evolutionary or ontogenetic exposure to foxes. Bettongs showed a modest response to the dingo/dog model and no response to the fox model. These results are consistent with the hypothesis that deep evolutionary history plays an essential role in predator discrimination and provides support for the multipredator hypothesis that predicts the presence of any predators can maintain antipredator behavior for other absent predators. Prey may have ontogenetic experience and or evolutionary experience with their predators. How such experiences influence prey species' ability to identify their predators is of significance to theory on the evolution of antipredator response and to improve the success of translocations and reintroductions for conservation purposes which often fail because of predation on predator na < ve prey. Here, we show that prey recognition for two prey species with limited or no ontogenetic exposure to predators, rabbits, and burrowing bettongs was greatest toward the predator to which they had the longest period of coevolution. The results are consistent with the hypothesis that evolutionary history plays an essential role in predator discrimination and provides support for the multipredator hypothesis that predicts the presence of any predators can maintain antipredator behavior for other absent predators.

4681: ***+.211***

Understanding the factors driving dispersal behaviour and habitat selection in reintroduced populations can be critical to reintroduction success. Social factors in particular can influence

habitat selection, for example through conspecific attraction, and this can have both positive and negative effects on reintroduction success, particularly where multiple releases occur. In addition, little is known about how habitat selection differs between natal and post-release dispersal within species. Often it is assumed that information from a species' natal dispersal preferences and patterns can act as a guide for predicting post-release dispersal behaviour, but no studies to date have examined this. We examine the factors influencing habitat selection during both natal and post-release dispersal in a reintroduced hihi (stitchbird, Notiomystis cincta) population using species distribution models. We demonstrate a strong social effect in habitat selection of natal dispersers bred at the release area (largely the offspring of founders), yet find no social effect in habitat selection of juveniles translocated 2years after the first releases occurred. In addition, we establish that environmental variables are important in habitat selection in both groups. We suggest (1) that consideration of social effects and conspecific attraction should play a role in planning reintroduction release strategies, especially if reinforcement releases are considered necessary, and (2) that it may not always be appropriate to assume post-release dispersal in reintroduced populations will be driven by the same factors that influence natal dispersal.

4682: *+.130*

Limonium perplexum Saez and Rossello is an herbaceous-perennial to annual-, triploid apomictic halophyte which only lives on a small outcrop (40 sq. m.) of a low coastal cliff in Serra d'Irta (Peniscola, Castellon, Valencian Community, Spain). The population has been observed to fluctuate between 19 and 383 individuals, and the site is affected by collapse risk caused by marine storms. To save this species, 9 new populations-neopopulations-have been planted close by-0.12 to 7.35 km-, with 4,198 individuals planted throughout 2005-2014. The fluctuation of the native population shows a correlation between the number of individuals and the maximum daily rainfall of the second quarter-April to June. Survival of the founder individuals on available microhabitats fluctuates between 36 and 77 %. New populations show a strong initial decline during the first 2 years, but in that time an initial pool of seeds is generated, which can yield the first recruitment of seedlings. The establishment of new generations is not apparently restricted by seeds, but by the need to grow in safe conditions-mainly on rock crevices holding soil and being protected against the effects of excessive wind and sun radiation. Similarity between the inter-annual changes in censuses of the native population and neopopulations is taken as a measurement of the establishment success. Apparently no former similar works have been published with endangered species of Limonium, although this genus holds more than 400 species.

4683: *+.019*

Translocation is an increasingly common conservation tool used to augment declining populations or to remove nuisance animals from areas of human conflict. Studies show that venomous snakes translocated long distances may wander and experience increased mortality. However, potential sub-lethal physiological effects on translocated snakes remain unknown. We conducted an experimental study on free-ranging rattlesnakes to test the hypothesis that long distance translocation is stressful. The glucocorticoid response to translocation was variable among snakes. There was some evidence that translocation may be stressful, as baseline corticosterone levels in most snakes rose following translocation, whereas levels remained consistent in control snakes. Interestingly, testosterone levels rose dramatically following translocation, possibly reflecting effects of interaction with new environmental cues and/or resident snakes, or effects of navigation in a new environment. Corticosterone and testosterone were positively correlated. Our study shows that long distance translocation can affect steroid hormone concentrations in rattlesnakes, a result that should be taken into consideration when managing nuisance snakes or repatriating

animals to the wild. (C) 2016 Elsevier Inc. All rights reserved.

4684: ***+.170***

The establishment and subsequent spread of invasive species is widely recognized as one of the most threatening processes contributing to global biodiversity loss. This is especially true for marine and estuarine ecosystems, which have experienced significant increases in the number of invasive species with the increase in global maritime trade. Understanding the rate and mechanisms of range expansion is therefore of significant interest to ecologists and conservation managers alike. Using a combination of population genetic surveys, environmental DNA (eDNA) plankton sampling and hydrodynamic modelling, we examined the patterns of introduction of the predatory Northern Pacific seastar (Asterias amurensis) and pathways of secondary spread within southeast Australia. Genetic surveys across the invasive range reveal some genetic divergence between the two main invasive regions and no evidence of ongoing gene flow, a pattern that is consistent with the establishment of the second invasive region via a human-mediated translocation event. In contrast, hydrodynamic modelling combined with eDNA plankton sampling demonstrated that the establishment of range expansion populations within a region is consistent with natural larval dispersal and recruitment. Our results suggest that both anthropogenic and natural dispersal vectors have played an important role in the range expansion of this species in Australia. The multiple modes of spread combined with high levels of fecundity and a long larval duration in A. amurensis suggests it is likely to continue its range expansion and significantly impact Australian marine ecosystems.

4685: ***+.058***

In South Africa, African buffaloes (Syncerus caffer) are one of the wildlife maintenance hosts for bovine tuberculosis (BTB) and play a key role in the spread of the disease to other wildlife species and potentially back to cattle. We report a trace-back investigation following the diagnosis of BTB in a previously BTB-free provincial game reserve, founded in the early 1990s in the North West Province of South Africa (SA). Using the intradermal tuberculin and interferon gamma tests, we detected Mycobacterium bovis infection in captured African buffaloes intended for sale. Detection of M. bovis was confirmed by culture and PCR. Molecular typing of M. bovis isolates from three African buffaloes revealed spoligotype SB0140 and a variable number of tandem repeat genotypes which had been previously isolated from wildlife in the KwaZulu-Natal Province of SA. Diagnosis of BTB in a previously uninfected buffalo population provides evidence that the disease can be introduced into an ecosystem through the translocation of untested plains game species. We further illustrate how BTB can remain unnoticed for considerable periods of time in free-ranging wildlife populations and emphasize the need for validated diagnostic tests for application in suitable and practical monitoring programs. This is especially important for species with maintenance host potential and those in high demand at game auctions.

4686: ***+.102***

Diversity in life history tactics contributes to the persistence of a population because it helps to protect against stochastic environments by varying individuals in space and time. However, some life history tactics may not be accounted for when assessing the demographic viability of a population. One important factor in demographic viability assessments is cohort replacement rate (CRR), which is defined as the number of future adults produced by an adult. We assessed if precocial resident males (We found that 9 +/- 5% of offspring with an unassigned parent remained unexplained after accounting for sources of human error. Using grandparentage assignments, we

identified 31 precocial resident males and 48 probable adfluvial Chinook salmon produced by anadromous mate pairs from 2007 to 2012. Previously published CRR estimates for the 2007 and 2008 reintroduced adults, based on only anadromous returning adult offspring, were 0.40 and 0.31, respectively. By incorporating adfluvial females, we found CRR estimates increased by 17% (CRR: 0.46) and 13% (CRR: 0.35) for the 2007 and 2008 cohorts, respectively.

4687: *+.207*

In Scotland, UK, beavers became extinct about 400years ago. Currently, two wild populations are present in Scotland on a trial basis, and the case for their full reintroduction is currently being considered by Scottish ministers. Beavers are widely considered ecosystem engineers'. Indeed, beavers have large impacts on the environment, fundamentally change ecosystems, and create unusual habitats, often considered unique. In this review, we investigate the mechanisms by which beavers act as ecosystem engineers, and then discuss the possible impacts of beavers on the biodiversity of Scotland. A meta-analysis of published studies on beavers' interactions with biodiversity was conducted, and the balance of positive and negative interactions with plants, invertebrates, amphibians, reptiles, birds, and mammals recorded. The meta-analysis showed that, overall, beavers have an overwhelmingly positive influence on biodiversity. Beavers' ability to modify the environment means that they fundamentally increase habitat heterogeneity. As beavers are central-place foragers that feed only in close proximity to watercourses, their herbivory is unevenly spread in the landscape. In addition, beaver ponds and their associated unique successional stages increase habitat heterogeneity both spatially and temporally. Beavers also influence the ecosystems through the creation of a variety of features such as dams and lodges, important habitat features such as standing dead wood (after inundation), an increase in woody debris, and a graded edge between terrestrial and aquatic habitats that is rich in structural complexity. In Scotland, a widespread positive influence on biodiversity is expected, if beavers are widely reintroduced. For instance, beaver activity should provide important habitat for the otter Lutra lutra, great crested newt Triturus cristatus and water vole Arvicola amphibious, all species of conservation importance. Beavers are most likely to have detrimental impacts on certain woodland habitats and species of conservation importance, such as the Atlantic hazelwood climax community and aspen Populus tremula woodland. A lack of woodland regeneration caused by high deer abundance could lead to habitat degradation or loss. These are also of particular importance due to the variety of associated dependent species of conservation interest, such as lichen communities in Atlantic hazelwoods.

4688: *-.021*

The American burying beetle (Nicrophorus americanus) is a federally listed endangered beetle and since 1993 multiple organizations have collaborated to reintroduce this species to Nantucket Island, Massachusetts, USA. We present evidence that despite very successful reintroduction methods, the reintroduced population is not self-sustaining and requires human assistance for long term maintenance. Beetles were reintroduced from captive stock and each year we augmented the population by trapping wild beetles, pairing males and females, and supplying the pairs with carrion. Long term monitoring of this population has shown that, when provided with carrion, N. americanus on Nantucket have an over winter survival rate of 15 % and a reproductive success rate of 54 %. After seeing the number of beetles captured between 2007 and 2011 double, we modified protocols to determine if the established population would be self-sustaining and we have seen a drastic decline. We suggest that a lack of natural carrion is the main reason for this decline.

4689: ***+.049***

The arms race between Maculinea butterflies and Myrmica host ants leads to local host-parasite adaptations. In our study, we assessed whether sympatric and allopatric Myrmica scabrinodis populations exhibit behavioural differences towards Maculinea teleius larvae during the adoption-period when butterfly larvae need to be taken inside the Myrmica nest. The second aim was to assess the butterfly survival rate inside ant colonies from different populations. We used one sympatric host population and three allopatric populations: one infested by M. teleius and two uninfested populations. We found that ants from the sympatric population showed a higher number of positive behaviours toward M. teleius larvae during adoption than ants from the allopatric populations. There were no differences in the number of inspection or negative behaviour events. The survival of butterfly larvae was highest inside sympatric host colonies and differed from the survival of M. teleius reared by ants from the allopatric, uninfested populations. No difference was found for the survival rate of M. teleius raised by infested, allopatric host colonies compared to sympatric host populations. Our results suggest the lack of behavioural counter-adaptations of local hosts of M. teleius that more easily adopt and rear butterfly caterpillars compared to naive M. scabrinodis colonies. Our results may also have implications for Maculinea butterfly conservation, especially for reintroduction programmes. We suggest that the existence of behavioural host defences should be checked for the source host population, as well as for the Myrmica population from the reintroduction site. It may also be reasonable to introduce several Myrmica host colonies from the source butterfly host population.

4690: ***+.019***

The restoration of endangered relict populations is challenging in conservation biology because they require specific environmental conditions within an inhospitable regional climate. Urothemis edwardsii Selys is the most endangered dragonfly in the Mediterranean with only one known relict small population (Lac Bleu) left in Northeast Algeria. With the absence of successful (re-)colonization over the last two decades, the restoration of the species became a top priority. To improve the status of the species in Northeast Algeria, we carried out a reintroduction and translocation scheme during 2011-2015 and assessed the changes in distribution and population size. Our restoration plan led to the emergence of three populations of which one was restored (Lac Noir), one resulted from successful translocation (Lac Tonga Northeast), and one established after successful colonization (Lac Tonga Southwest). In three localities (Lac Noir, Lac Tonga Northeast, and Lac Tonga Southwest), signs of population growth were observed, whereas no significant trend in the source population (Lac Bleu) was detected. A new population (El Graeate) was also recorded in 2015, but its origin is uncertain. Capture-mark-recapture on adults conducted in 2015 in two sites (Lac Bleu and Lac Noir) showed low recapture rates and no sign of dispersal between the two sites. Dispersal capacity of the species and conservation implications of adult distribution are discussed. This study highlights the importance of using biological indicators in selecting host habitats for the restoration of critically threatened populations.

4692: ***+.130***

The Bokikokiko or Christmas Island Warbler (Acrocephalus aequinoctialis) is found only on Kiritimati (Christmas) and Teraina (Washington) Islands in the Republic of Kiribati. The population on Kiritimati Island is threatened by habitat degradation, sea level rise, and predation from feral cats (Felis catus), Pacific rats (Rattus exulans), and recently arrived black rats (Rattus rattus). There is scant information about distribution and abundance of the Bokikokiko. From 2007 to 2012, we conducted surveys with song playbacks at 83 points on 12 transects in the northern

half of Kiritimati Island to measure abundance of the Bokikokiko and begin monitoring for possible declines associated with the spread of rats, and we collected habitat data to investigate factors that influenced Bokikokiko abundance. Song playbacks resulted in a 263% increase in detection rate over passive listening. We detected an average (+/- 1 SE) of 0.63 +/- 0.11 birds per point using playbacks. Average population density was 0.36 +/- 0.06 birds per hectare, but abundance varied among regions, and no birds were detected in some areas with apparently suitable habitat. Range of the Bokikokiko encompassed an area of 14,180 ha but was fragmented by many lagoons and bare ground, and only about half that area was actually occupied. Estimated population size was 2,550 425 birds. Bokikokiko were more abundant in areas with taller Heliotropium trees and taller Scaevola shrubs, and less abundant in areas with more Suriana shrubs, bare ground, and grass. Conservation actions needed for the Bokikokiko include ongoing removal of rats from islets within the lagoons of Kiritimati Island, protection of preferred habitat from development and fires, and translocations to create additional populations on rat-free atolls.

4693: *+.170*

A population of Eurasian lynx Lynx lynx was established by reintroductions in the Bohemian Forest Ecosystem in the 1970s and 1980s. The most recent information on the population status indicates that the distribution has stagnated since the late 1990s, for unknown reasons. We assessed the availability of suitable habitat along the Austrian-German-Czech border, and hypothesized that the Bohemian-Bavarian lynx population is not in equilibrium with habitat suitability. Based on global positioning system data from 10 radio-collared lynx, we used a maximum entropy approach to model suitable habitat. Variables reflecting anthropogenic influence contributed most to the model and were negatively associated with the occurrence of lynx. We evaluated the model prediction using independent records of lynx from monitoring in Bavaria, Germany. Using our habitat approach we estimated the area of potential habitat, based on a mean annual home range of 445 km(2) for males and 122 km(2) for females. Our results indicated there were 12,415 km(2) of suitable habitat, distributed among 13 patches, for a potential population of c. 142 (93-160) resident lynx. We assessed connectivity via least-cost paths and found that all suitable patches could be reached by the lynx. A comparison with the current distribution of lynx, however, confirms that a significant proportion of suitable habitat is not occupied, which indicates that the distribution is limited by factors other than habitat, with illegal killing being the most likely cause. Our study provides crucial information for the development of a conservation strategy and regional planning for the Bohemian-Bavarian lynx population.

4694: *+.054*

The eastern bettong Bettongia gaimardi, a potoroid marsupial, has been extinct on the Australian mainland since the 1920s. Sixty adult bettongs were reintroduced from the island of Tasmania to two predator-free fenced reserves on mainland Australia. We examined baseline health parameters (body weight, haematology and biochemistry, parasites and infectious disease exposure) in a subset of 30 (13 male, 17 female) individuals at translocation and again at 12-24 months post-reintroduction. The mean body weight increased significantly post-reintroduction but there were no significant differences in body weight between the two reintroduction sites or between the sexes in response to reintroduction. Differences were evident in multiple haematological and biochemical variables post-reintroduction but there were few differences between the two reintroduced populations or between the sexes in response to reintroduction. Ectoparasite assemblages differed, with five of 13 species failing to persist, and an additional four species were identified post-reintroduction. None of the bettongs had detectable antibodies to the alphaherpesviruses Macropodid herpesvirus 1 and 2 post-reintroduction, including one individual

that was seropositive at translocation. Similarly, the novel gammaherpesvirus potoroid herpesvirus. was not detected by polymerase chain reaction (PCR) in any of the bettongs post-reintroduction, including one individual that was PCR-positive at translocation. None of the bettongs had detectable antibodies to Toxoplasma gondii either at translocation or post-reintroduction. Our data demonstrate changing baseline health parameters in eastern bettongs following reintroduction to the Australian mainland are suggestive of improved health in the reintroduced populations, and provide additional metrics for assessing the response of macropodoids to reintroduction.

4695: *-.112*

Reintroductions are used to re-establish populations of species within their indigenous range, but their outcomes are variable. A key decision when developing a reintroduction strategy is whether to include a temporary period of confinement prior to release. Pre-release confinement is primarily used for the purpose of quarantine or as a delayed-release tactic to influence the performance or behaviour of founders post-release. A common difference between these approaches is that quarantine tends to be conducted in ex situ captivity, whereas delayed releases tend to involve in situ confinement at the release site. Although these practices are commonly viewed independently, it may be possible for a single confinement period to be used for both purposes. We tested whether temporarily holding wild eastern bettongs Bettongia gaimardi in ex situ captivity for 95-345 days prior to release (delayed release) influenced their body mass, pouch occupancy or survival during the first 1.5 years post-release, compared to founders released without confinement (immediate release). Our results suggest that exposing founders to captivity did not alter their body mass or performance post-release, despite being heavier and having fewer pouch young when released. We conclude that, for this species, ex situ captivity does not represent a tactical opportunity to improve post-release performance but can be used for quarantine without affecting the probability of establishment.

4696: *+.102*

Reintroductions aim to re-establish species within their historical ranges through the release of wild- or captive-bred individuals following extirpation (or extinction) in the wild. There is no general agreement on what constitutes a successful reintroduction but the probability of the population achieving long-term persistence should be addressed. Here we review a 10-year trial reintroduction of the great bustard Otis tarda, a globally threatened bird species, to the UK and assess the long-term population viability. Despite changes in rearing and release strategy, initial post-release survival probability remained consistently low, with only 11.3% of bustards (n = 167) surviving from release to. year post-release. Nineteen breeding attempts were made by eight females; however, only one chick survived > 100 days after hatching, and no wild juveniles have recruited into the population. Using demographic rates from the UK population and wild populations elsewhere, and stochastic population modelling, we investigate the viability of this reintroduced population by predicting population size over the next 10 years. Under current demographic rates the population was predicted to decline rapidly. Self-sufficiency was predicted only using the highest estimates from the UK population for first-year and adult survival, and recruitment rates from wild populations elsewhere. Although changes have been made in rearing, release strategies, habitat management and release sites used, these changes appear to have a modest effect on long-term viability. Substantial improvements in survival rates and productivity are necessary to establish a viable great bustard population in the UK, and we consider this unlikely.

4697: *-.013*

Among European amphibians, the great crested newt (Triturus cristatus) is an endangered species and listed in the Habitats Directive of the European Union on appendix II and IV. Irrespective of its high protection status, a population of crested newts in the Greiffenhorstpark in Krefeld (Germany) representing one of the species' so far largest single subpopulation with an estimated census size of 8,000 newts, has been heavily disturbed by human-induced reconstruction measures. In order to reestablish the historical character of the Greiffenhorstpark betonit-layers were inserted on the ground of the main water body so that a year-round permanent water level without desiccation could be achieved. In the course of these reconstruction measures 4,390 crested newts were trapped with an amphibian drift fence and relocated into a smaller man-made pond inside a golf course nearby. By applying an individual based capture-mark-recapture approach we estimated sizes of 27 sites of crested newts in the Greiffenhorstpark and the area of the Latumer Bruch over a period of 7 years (2004-2011) and determined for each monitored water body also the habitat-suitability for crested newts. Altogether, 5,424 crested newts could be trapped and individually recognised using the program Amphident. Additionally, we analysed the genetic population structure of crested newts for 17 polymorphic microsatellite loci on the basis of 2,500 individuals. Our monitoring demonstrated the devastating effects of the construction measures on the crested newt population. The population of the main water body in the Greiffenhorstpark crashed from initially 8,000 individuals before the construction measures to less than 10 individuals in 2011. The main reason for this die off is the establishment of stable fish populations due to the lack of periodicity with desiccation events of the water body. With few exceptions, almost all 26 monitored sites showed strong declines in estimated population size. Though stable -with estimated population sizes between 200-300 individuals -the water body on the golf course only houses a small subset of the individuals that were translocated from the main water body. As a result, the translocation of thousands of crested newts obviously failed. Our data are alarming and indicate that almost all measures undertaken to stabilise the population of the crested newt in the Latumer Bruch has so far failed. In order to prevent a complete die off of the whole population of crested newts in the near future in this area, we strongly suggest to undertake measures that effectively mimic the periodicity of the water level so that the fish populations are decimated effectively to enable the return of the crested newts to this important central water body that functions as a source population for the whole Latumer Bruch.

4698: *+.178*

Populations of the Orinoco crocodile (Crocodylus intermedius) have not recovered from past exploitation, and current abundances cannot be used for assessing the suitability of habitats they occupy. Growth constitutes an alternative way of assessing habitat quality. Since 1990, more than 9000 captive-reared Orinoco crocodiles have been released into the Venezuelan Llanos. In the present study, the growth rates of 127 recaptured crocodiles from different regions were compared. All individuals from Middle Cojedes and the Aeolian savannahs grew slower than expected by the von Bertalanffy model, whereas individuals from Canos in the Apure floodplains grew faster than expected. These inferences are corroborated with growth rate measurements for crocodiles under four years of age, which were lowest in Middle Cojedes (average of 14.1 cm/year) and highest at Canos (43.3 cm/year). Low growth rates can be explained by habitat deterioration due to human activities, and high growth rates in the Canos support that the lower reaches of whitewater rivers offer favourable conditions for the species. Crocodiles in high quality habitats may reach sexual maturity in six years, whereas more than a decade is required in poor-quality habitats.

4699: *+.137*

Recovery of an imperiled plant species may require augmentation of existing populations or creation of new ones. Hundreds of such projects have been conducted over the last few decades, but there is a bias in the literature favoring successes over failures. In this paper, we evaluate a series of introductions that experimentally manipulated microhabitat and fire in an adaptive introduction framework. Between 2002 and 2012, we (and our collaborators) carried out ten introductions and augmentations of Florida ziziphus Pseudoziziphus (Condalia, Ziziphus) celata, a clonal shrub limited to very small populations and narrowly endemic to pyrogenic central Florida sandhills. Six of the introductions were designed as experiments to test hypotheses about how demographic performance was affected by microhabitat, fire, and propagule type. Introduced transplants had high survival (<90% annually), inconsistent growth, and little transition to reproduction, while introduced seeds had low germination and survival. Transplants were more efficient than seeds as translocation propagules. Shaded (vs. open) sites supported generally higher transplant and seedling survival and seed germination percentages, but growth responses varied among experiments. Supplemental irrigation increased transplant survival and seed germination, but otherwise seedling and plant survival and growth were not significantly affected. Contrary to expectations based on wild populations, introduced propagules have not been more successful in unshaded sites, suggesting that Florida ziziphus has broader microhabitat preferences than hypothesized. Compared to wild plants, introduced plants had similar survival and responses to fire, slower growth, and more delayed flowering. Introduced plants had no clonal spread. While no introduced population has demonstrated a capacity for long-term viability, one augmented population has flowered and produced viable fruits. Given that Florida ziziphus genets are long-lived, low levels of sexual reproduction may be adequate for the establishment of viable populations. Thus, after many translocations over more than a decade, it is premature to characterize any single translocation as a success or a failure, underscoring the need for a long view of translocation success. Copyright (C) 2016 Kunming Institute of Botany, Chinese Academy of Sciences. Publishing services by Elsevier B.V. on behalf of KeAi Communications Co., Ltd.

4700: ***-.063***

Of the genus Craigia, widespread in the Tertiary, only two relict species survived to modern times. One species is now possibly extinct and the other one, Craigia yunnanensis, is severely endangered. Extensive surveys have located six C. yunnanensis populations in Yunnan province, southwest China. Using fluorescent amplified fragment length polymorphism (AFLP), the genetic diversity and population structure of these populations were examined. It was found that genetic diversity of C. yunnanensis was moderate at the species level, but low at regional and population levels. Analysis of population structure showed significant genetic differentiation between Wenshan and Dehong regions, apparently representing two geographically isolated for long time refuges. There are also clear indications of isolation between populations, which, together with anthropogenically caused decline of population size, will lead to general loss of the species genetic variation with subsequent loss of adaptive potential. To conserve the genetic integrity of C. yunnanensis, we recommend that ex-situ conservation should include representative samples from every population of the two differentiated regions (e.g. Wenshan and Dehong). The crosses between individuals originated from different regions should be avoided because of a high risk of outbreeding depression. As all the extant populations of C. yunnanensis are in unprotected areas with strong anthropogenic impact, there is no alternative to reintroduction of C. yunnanensis into suitable protected locations. Copyright (C) 2016 Kunming Institute of Botany, Chinese Academy of Sciences. Publishing services by Elsevier B.V. on behalf of KeAi Communications Co., Ltd.

4702: ***+.112***

Red panda (Ailurus fulgens) is threatened across its range by detrimental human activities and rapid habitat changes necessitating captive breeding programs in various zoos globally to save this flagship species from extinction. One of the ultimate aims of ex situ conservation is reintroduction of endangered animals into their natural habitats while maintaining 90 % of the founder genetic diversity. Advances in molecular genetics and microsatellite genotyping techniques make it possible to accurately estimate genetic diversity of captive animals of unknown ancestry. Here we assess genetic diversity of the red panda population in Padmaja Naidu Himalayan Zoological Park, Darjeeling, which plays a pivotal role in ex situ conservation of red panda in India. We generated microsatellite genotypes of fifteen red pandas with a set of fourteen loci. This population is genetically diverse with 68 % observed heterozygosity (H-O) and mean inbreeding (F-IS) coefficient of 0.05. However population viability analysis reveals that this population has a very low survival probability (< 2 %) and will rapidly loose its genetic diversity to 37 % mainly due to small population size and skewed male-biased sex ratio. Regular supplementation with a pair of adult individuals every five years will increase survival probability and genetic diversity to 99 and 61 % respectively and will also support future harvesting of individuals for reintroduction into the wild and exchange with other zoos.

4703: *+.123*

Increasing urbanisation and growth of many wild animal populations can result in a greater frequency of human-wildlife conflicts. However, traditional lethal methods of wildlife control are becoming less favoured than non-lethal approaches, particularly when problems involve charismatic species in urban areas. Eurasian badgers (Meles meles) excavate subterranean burrow systems (setts), which can become large and complex. Larger setts within which breeding takes place and that are in constant use are known as main setts. Smaller, less frequently occupied setts may also exist within the social group's range. When setts are excavated in urban environments they can undermine built structures and can limit or prevent safe use of the area by people. The most common approach to resolving these problems in the UK is to exclude badgers from the problem sett, but exclusions suffer a variable success rate. We studied 32 lawful cases of badger exclusions using one-way gates throughout England to evaluate conditions under which attempts to exclude badgers from their setts in urban environments were successful. We aimed to identify ways of modifying practices to improve the chances of success. Twenty of the 32 exclusion attempts were successful, but success was significantly less likely if a main sett was to be excluded in comparison with another type of sett and if vegetation was not completely removed from the sett surface prior to exclusion attempts. We recommend that during exclusions all vegetation is removed from the site, regardless of what type of sett is involved, and that successful exclusion of badgers from a main sett might require substantially more effort than other types of sett.

4704: *+.000*

Wildlife reintroduction is an increasingly used strategy to reverse anthropocene defaunation. For the purpose of ecosystem restoration, in 2007 the guanaco (Lama guanicoe) was reintroduced to the Quebrada del Condorito National Park, situated in the mountains of central Argentina. With the aim of developing management recommendations, the project included permanently monitoring the population to evaluate its dynamics and the ecological response of the individuals released into the area. Nine years later and after two releases of guanacos (113 individuals in 2007 without and 25 in 2011 with a pre-adaptation period), only 24 individuals, which conform three reproductive groups, and one group of solitary males were settled in the Park. Here I modeled a population viability analysis to evaluate extinction risk, using VORTEX software. Initial population structure, specified age distribution, mortality and reproductive rates, and mate

monopolization recorded during field work were used in the model, whereas the remaining used demographic parameters, such as age of first offspring, maximum number of broods per year, mean foaling rate, and length of fecundity period, were taken from the literature. Each of the three different scenarios (without supplementation of individuals, and with a realistic and optimistic supplementation) and two possible catastrophic events (fires and food shortage) covering 100 years was repeated 1000 times. Even though the guanaco reintroduction project can be considered to have been partially successful since its start, the model predicts that the current reintroduced population could be extinct in the next few decades if no reinforcements occur, and that only a continuous supplementation can reach the probability that the population survives over the next 100 years. I conclude that, so far, the current population is at a high risk of extinction if further supplementation of individuals is discontinued.

4705: *+.249*

Background: In this paper we review the conservation genetics of African savannah elephants, aiming to understand the spatio-temporal research trends and their underlying factors. As such, we explore three questions associated to the conservation genetics and molecular ecology of these elephants: (1) what are the research trends concerning the conservation genetics of Loxodonta africana? (2) Do richer countries conduct more research on the genetics of African elephants? (3) Which attributes influence where scholars conduct their research? Materials and Methods: We examined available peer-reviewed publications from 1993 to 2014 in complementary online databases, including the ISI/Web of Science (WoS), Scopus and Google Scholar (GS), and searched for publications in scientific journals as well as in the reference section of these publications. We analyzed the annual trend of publications in this field of research, including the number of authors, levels of collaboration among authors, year of publication, publishing journal and the countries from where genetic samples were collected. Additionally, we identified main research clusters, authors, and institutional collaborations, based on co-citation and co-occurrence networks.Results: We found that during the study period there was a positive trend in the number of publications and a reduction in the number of authors per paper. Twenty-five countries contributed, with the majority of publications authored by researchers in the USA, Kenya and South Africa. The majority of samples were collected in Kenya, Tanzania and South Africa. Research outputs are associated with the existence of long-term conservation/research projects and research potential as measured by the literacy rate and the number of higher education institutions in a country. Five research clusters were identified, focusing on the origin and evolution of the species, methodological issues and the relatedness among elephant species.Conclusions: Research in this field should be expanded to additional countries harboring elephant populations to enable a more comprehensive understanding of the population structure and genetic differentiation of the species, and to cope with challenges associated with the conservation of the species such as illegal hunting, habitat fragmentation, species reintroduction and climate change.

4706: *-.025*

Background: Restoring a viable population by reintroduction is the ultimate goal of a large number of ex situ conservation projects for endangered animals. However, many reintroductions fail to establish a population in the wild, partly because released animals cannot acclimate to the native environment of the release site, resulting in very low survival rates. Acclimation training is a technique to resolve this problem, although it does not have positive results in all species. We tested whether acclimation training and soft-release could improve the reintroduction success for captive-bred Cabot's Tragopan (Tragopan caboti), an endangered pheasant in southern China.Methods: Reintroduction of captive-bred Cabot's Tragopan was carried out in the

Taoyuandong National Nature Reserve, China from 2010 to 2011. We built a soft-release enclosure for acclimation training in the typical montane habitat of this pheasant. Nine birds were acclimated to the environment of this release site in this cage for more than 50 days before release ("trained birds"), while 11 birds remained only in the cage for 3 days prior to release ("untrained birds"). Released birds were tagged with a collar radio-transmitter.Results: Post-release monitoring revealed that the survival rate of trained birds was higher than that of untrained birds after 50 days (trained: 85.7%; untrained: 20.0%). Cox regression analysis showed that there was a significant difference in the mortality rates between the trained and untrained birds. In addition, a survey of the habitat of the experimental and the control groups showed significant differences in habitat selection between the groups.Conclusion: Our study suggests that pre-release acclimatization training is an important factor that can lead to improved survival and habitat selection of captive-bred reintroduced tragopans.

4707: *+.155*

Rates of hybridization and introgression are increasing dramatically worldwide because of translocations, restocking of organisms and habitat modifications; thus, determining whether hybridization is occuring after reintroducing extirpated congeneric species is commensurately important for conservation. Restocking programs are sometimes criticized because of the genetic consequences of hatchery-bred fish breeding with wild populations. These concerns are important to conservation restocking programs, including those from the Australian freshwater fish family, Percichthyidae. Two of the better known Australian Percichthyidae are the Murray Cod, Maccullochella peelii and Trout Cod, Maccullochella macquariensis which were formerly widespread over the Murray Darling Basin. In much of the Mtirrumbidgee River, Trout Cod and Murray Cod were syrnpatric until the late 1970s when Trout Cod were extirpated. Here we use genetic single nucleotide polymorphism (SNP) data together with mitochondrial sequences to examine hybridization and introgression between Murray Cod and Trout Cod in the upper Murrumbidgee River and consider implications for restocking programs. We have confirmed restocked riverine Trout Cod reproducing, but only as inter-specific matings, in the wild. We detected hybrid Trout Cod Murray Cod in the Upper Murrumbidgee, recording the first hybrid larvae in the wild. Although hybrid larvae, juveniles and adults have been recorded in hatcheries and impoundments, and hybrid adults have been recorded in rivers previously, this is the first time fertile F1 have been recorded in a wild riverine population. The F1 backcrosses with Murray cod have also been found to be fertile. All backcrosses noted were with pure Murray Cod. Such introgression has not been recorded previously in these two species, and the imbalance in hybridization direction may have important implications for restocking programs.

4708: *+.086*

For the first time we have reviewed historic data throughout Europe to assess how European larch has been artificially distributed from the 17th until the mid-20th century. Over this period, larch genetic resources have been translocated with varying intensity. Especially Alpine plant material was transferred outside the native range across Europe, while genetic resources originating from the Sudetes were mainly spread to northeastern Germany, northwestern Poland, and to the Sudetes outside of the species' native range. Polish larch was mainly translocated within Poland. Genetic resources from the Carpathian Mountains (Tatras, eastern and southern Carpathians) were not used for long-distance transfer. While native larch populations in the Alps and in Poland were not significantly affected by allochthonous plant material, the native gene pool of larch in the Sudetes and Carpathians Mountains was strongly altered by Alpine plant material. We provide several maps illustrating these translocations over time and space. These findings are of special

importance, as genetic data tracking the original seed source are presently not available. (C) 2016 Elsevier B.V. All rights reserved.

4709: *+.016*

As part of a reintroduction project in Senegal, 23 (9.14) captive-born Dorcas gazelles were released into a 440-ha fenced-in area in Katane (North Ferlo Fauna Reserve, Senegal) in March 2009. After 4 years of seasonal monitoring, the gazelles showed progressive adaptation of their behavior to semi-wild living conditions. Breeding gradually became seasonal, and 53.8% of births occurred during the rainy season (July to September). Gazelle group size and composition varied seasonally. Groups were smallest during the dry season (2.29 +/- 1.72) and largest at the beginning of the rainy season (4.18 +/- 2.73). Social group composition also showed seasonal variation. There were always a larger proportion of solitary males than solitary females and mixed couples were observed throughout the year. All-male groups were found the least. The proportion of adult females with subadults and juveniles decreased during the early rainy season, while mixed adults, subadults and juveniles groups increased during this period. The mortality rate during the first weeks after release was 13%. Four years of monitoring after release, demographic traits of this released population reveal its adaptation from captive to natural-living conditions.

4710: *-.042*

1. In poorly dispersing species gene flow can be facilitated when suitable habitat is widespread, allowing for increased dispersal between neighbouring locations. The Poweshiek skipperling [Oarisma poweshiek (Parker)], a federally endangered butterfly, has undergone a rapid, recent demographic decline following the loss of tallgrass prairie and fen habitats range wide. The loss of habitat, now restricted geographic range, and poor dispersal ability have left O. poweshiek at increased risk of extinction.2. We studied the population genetics of six remaining populations of O. poweshiek in order to test the hypothesis that gene flow was historically high despite limited long-distance dispersal capability. Utilising nine microsatellite loci developed by PacBio sequencing, we tested for patterns of isolation by distance, low population genetic structure and alternative gene flow models.3. Populations from southern Manitoba, Canada to the Lower Peninsula of Michigan, USA are only weakly genetically differentiated despite having low diversity. We found no support for isolation by distance, and Bayesian estimates of historical gene flow support our hypothesis that high levels of gene flow previously connected populations from Michigan to Wisconsin.4. Prairie grasslands have been reduced tremendously over the past century, but the low mobility of O. poweshiek suggests that rapid loss of populations over the past decade cannot be simply explained by fragmentation of habitat.5. As a species at high risk of extinction, understanding historical processes of gene flow will allow for informed management decisions with respect to head-starting individuals for population reintroductions and for conserving networks of habitat that will allow for high levels of gene flow.

4711: *+.066*

Programs to conserve native fauna in invaded ecosystems often aim to reduce the impacts of alien predators. This approach can lead to unexpected outcomes in the native and the remaining invasive components of restored ecosystems. In New Zealand, suppression and eradication of invasive mammalian predators are well-established conservation strategies, particularly on offshore islands and in mainland ecosanctuaries. Predator control has achieved important conservation gains over increasingly large areas but these can be offset by the ecological release of other uncontrolled pest species. In addition, novel ecosystems created by selective predator control

and reintroductions of locally extinct or depleted native species may have unexpected trajectories as they evolve. Effective conservation requires new techniques for controlling entire suites of invasive predators over large areas, routine monitoring of the conservation outcomes of predator control, and better understanding of how modified, and in some cases reconstructed, seminatural ecosystems change when invasive predators are removed.

4712: *+.300*

Restoration efforts can be improved by understanding how variations in life-history traits occur within populations of the same species living in different environments. This can be done by first understanding the demographic responses of natural occurring populations. Population viability analysis continues to be useful to species management and conservation with sensitivity analysis aiding in the understanding of population dynamics. In this study, using life-table response experiments and elasticity analyses, we investigated how population-specific life-history demographic responses contributed to the metapopulation viability of the Federally threatened Pitcher's thistle (Cirsium pitcheri). Specifically, we tested the following hypotheses: (1) Subpopulations occupying different environments within a metapopulation have independent demographic responses and (2) advancing succession results in a shift from a demographic response focused on growth and fecundity to one dominated by stasis. Our results showed that reintroductions had a positive contribution to the metapopulation growth rate as compared to native populations which had a negative contribution. We found no difference in succession on the contribution to metapopulation viability. In addition, we identified distinct population-specific contributions to metapopulation viability and were able to associate specific life-history demographic responses. For example, the positive impact of Miller High Dunes population on the metapopulation growth rate resulted from high growth contributions, whereas increased time of plant in stasis for the State Park Big Blowout population resulted in negative contributions. A greater understanding of how separate populations respond in their corresponding environment may ultimately lead to more effective management strategies aimed at reducing extinction risk. We propose the continued use of sensitivity analyses to evaluate population-specific demographic influences on metapopulation viability. In understanding the underlying causes of the projected extinction probabilities of each population and identifying broad-scale contributions of different populations to the metapopulation, the process of pinpointing target populations is simplified. More detailed analyses can then be applied to the target populations to increase population viability and consequently metapopulation viability. Based on our research, we suggest that the best approach to improve the overall metapopulation viability is to manage the contributions to population growth for each population separately.

4713: *+.174*

Hybridization poses a complex problem for biodiversity conservation but there has been little discussion of strategies and guidelines designed to address it from a management perspective. In this article I review management approaches aimed at addressing hybridization while highlighting outstanding needs. Despite real-world efforts to manage systems in which hybridization is a concern, there has been little effort to develop broadly applicable guidelines or best management practices. Management programs would be enhanced by the characterization of the patterns and processes of hybridization in nature and integrating those into planning and policy. Doing so facilitates the development of holistic strategies that balance the importance of hybridization in many biological systems while addressing situations in which human-facilitated gene flow causes concern. The shift from reactive management to emphasizing measures designed to prevent hybridization from becoming a threat has been a positive development Examples include assessing

the genetic characteristics of populations used in translocation programs. Overlooked, however, has been the management of stable hybrid systems in which the goal is not necessarily preventing intermixing but protecting stabilizing mechanisms through policy and land use management. When hybridization emerges as a legitimate threat, activities such as culling, spatial isolation, targeted harvest, and de-introgression can protect threatened genomic units. Further experimentation of these techniques and collaboration among scientists and managers will provide lessons for establishing general guidelines for the conservation community. I hope this review stimulates discussion about approaches useful for addressing hybridization and promotes further development of new techniques and frameworks. Published by Elsevier Ltd.

4714: *+.130*

Habitat suitability is a critical aspect for the successful establishment of a translocated population. Past studies have identified multiple factors that contribute to habitat suitability, including resource availability, presence of invasive species, landscape connectivity and climate. However, visual camouflage - coloration that conceals individuals from predators or prey - is another important ecological requirement that has been largely overlooked. We provide a case study to illustrate how color patterns of a prey species can change from that of the source site following translocation. Shore skinks (Oligosoma smithi) were moved from a coastal sand dune ecosystem to an offshore island beach that differed in substrate and color. Within one year following release, the translocated population's color pattern variation had reduced to mostly one pattern type. The high match in color patterns between the skinks and release site (including a new substrate type) may have contributed to the observed color pattern shift. This reduced variation in color pattern implies that not all founders survived, potentially decreasing the genetic diversity in the population. Our study highlights the importance of considering a species' camouflage requirements when selecting habitat for release, not only to maximize founder survival and establishment success, but also to maintain phenotypic and genotypic diversity in the long-term. (C) 2016 Elsevier Ltd. All rights reserved.

4715: *+.117*

Reintroduction of locally extinct species is increasingly applied as a conservation tool for re-establishing species within their historical ranges. Thus far, this option has however not been investigated for fungi other than lichens. A large fraction of wood-inhabiting fungal species have declined because of forest loss and fragmentation, in addition to a decrease in dead wood. Here, we show the results from an experiment carried out in southern Finland, which demonstrates that inoculation is an effective method for reintroducing threatened wood-inhabiting fungi. All selected red-listed fungal species successfully established in the inoculated logs as mycelia, and three out of the seven produced fruit-bodies. Success rate was greater when the strains were inoculated in early-decay logs, including species that usually fruit in late decay stages. Inoculation can provide an effective tool for reintroducing fungal species, as the source populations remain intact and it is possible to produce massive amounts of inoculation-units with relatively low cost. Reintroductions of fungi should however be preceded by a risk assessment of the species to be reintroduced, by using source populations from nearby localities, and they should be considered complementary to the primary target of increasing the amount of their habitat. Our results suggest that the reintroduction of threatened fungi via inoculation in combination with other conservation measures can have important bearings for forest conservation and restoration. (C) 2016 Elsevier Ltd. All rights reserved.

4716: *+.219*

In this study, we examined cannibalistic behavior, growth, metamorphosis, and survival in larval and post-metamorph endangered yellow spotted mountain newts Neurergus microspilotus hatched and reared in a captive breeding facility. We designed a 2 x 2 factorial experiment, crossing two levels of food with two levels of density including high food/high density, high food/low density, low food/high density, and low food/low density. The level of cannibalistic behavior (including the loss of fore and hind limbs, missing toes, tail, gills, body damage, and whole body consumption) changed as the larvae grew, from a low level during the first 4 weeks, peaking from weeks 7 to 12, and then dropped during weeks 14-52. Both food level and density had a significant effect on cannibalism. The highest frequency of cannibalism was recorded for larvae reared in the low food/high density and lowest in high food/low density treatments. Growth, percent of larval metamorphosed, and survival were all highest in the high food/low density and lowest in low food/high density treatment. Food level had a significant effect on growth, metamorphosis, and survival. However, the two levels of density did not influence growth and metamorphosis but showed a significant effect on survival. Similarly, combined effects of food level and density showed significant effects on growth, metamorphosis, and survival over time. Information obtained from current experiment could improve productivity of captive breeding facilities to ensure the release of adequate numbers of individuals for reintroduction programs. (C) 2016 The Authors. Zoo Biology published by Wiley Periodicals, Inc.

4717: ***+.181***

Many large carnivores are recolonizing range as a result of improved management and conservation policy, habitat restoration, and reintroduction programs. American black bears (Ursus americanus) are projected to recolonize portions of theUnited States, but few studies have characterized or provided practical methods for monitoring this process. We used noninvasive hair sampling at 4 proximal study areas along the Kentucky-Virginia, USA, border during 2012-2013 to estimate demographics and population genetics, and investigate recolonization patterns of an American black bear population that was founded by 55 bears reintroduced to a fragmented mountainous landscape during the 1990s and subjected to harvest 6 years post-reintroduction. Using spatially explicit capture-recapture (SECR) models, we estimated a density of 0.26 bear/km(2), or minimum abundance of 482 bears, distributed among 2 primary core areas previously identified by occupancy analysis:a southern and northern core area. The southern core area was established by a founder adult female that exhibited post-release dispersal, but moderate asymmetrical gene flow (N-m = 6 bears) from the northern core areamitigated deleterious genetic consequences typical of such founder events. Effective number of breeders (N-B = 62 bears) was similar to the number of founders, suggesting that genetically, the population remains mostly the product of reintroduction. Despite limited connectivity with other populations in the region, genetic diversity (H-E = 0.78) was retained because of rapid population growth during the 16 years post-reintroduction (lambda = 1.14/year). This bear population exhibited demographic characteristics indicative of continued recolonization, including a significantly female-biased sex ratio (0.53M:1.00F) and female density decreasing with increasing distance from the reintroduction release areas in the northern core. Few bear detections at 2 peripheral study areas and results fromSECRmodel detectionfunction transformation suggested recolonization may continue to the southwest and northeast along prominent linear mountain ridges. Although the population has grown and is genetically stable, because of relatively low population density and recolonization direction, we suggest monitoring demographic vital rates to evaluate harvest sustainability and population viability. Our study demonstrates the utility of noninvasive genetic sampling in conjunction with SECR models to characterize and monitor recolonizing bear populations, which may also be useful for management of expanding populations of other large carnivores. (C) 2016 The Wildlife Society.

4718: ***+.129***

The integrity of wetlands is of global concern. A common approach to evaluating ecological integrity involves bioassessment procedures that quantify the degree to which communities deviate from historical norms. While helpful, bioassessment provides little information about how altered conditions connect to community response. More detailed information is needed for conservation and restoration. We have illustrated an approach to addressing this challenge using structural equation modeling (SEM) and long-term monitoring data from Rocky Mountain National Park (RMNP). Wetlands in RMNP are threatened by a complex history of anthropogenic disturbance including direct alteration of hydrologic regimes; elimination of elk, wolves, and grizzly bears; reintroduction of elk (absent their primary predators); and the extirpation of beaver. More recently, nonnative moose were introduced to the region and have expanded into the park. Bioassessment suggests that up to half of the park's wetlands are not in reference condition. We developed and evaluated a general hypothesis about how human alterations influence wetland integrity and then develop a specific model using RMNP wetlands. Bioassessment revealed three bioindicators that appear to be highly sensitive to human disturbance (HD): (1) conservatism, (2) degree of invasion, and (3) cover of native forbs. SEM analyses suggest several ways human activities have impacted wetland integrity and the landscape of RMNP. First, degradation is highest where the combined effects of all types of direct HD have been the greatest (i.e., there is a general, overall effect). Second, specific HDs appear to create a "mixed-bag" of complex indirect effects, including reduced invasion and increased conservatism, but also reduced native forb cover. Some of these effects are associated with alterations to hydrologic regimes, while others are associated with altered shrub production. Third, landscape features created by historical beaver activity continue to influence wetland integrity years after beavers have abandoned sites via persistent landforms and reduced biomass of tall shrubs. Our model provides a system-level perspective on wetland integrity and provides a context for future evaluations and investigations. It also suggests scientifically supported natural resource management strategies that can assist in the National Park Service mission of maintaining or, when indicated, restoring ecological integrity "unimpaired for future generations."

4719: ***+.444***

Sensory-based conservation harnesses species' natural communication and signalling behaviours to mitigate threats to wild populations. To evaluate this emerging field, we assess how sensory-based manipulations, sensory mode, and target taxa affect success. To facilitate broader, cross-species application of successful techniques, we test which behavioural and life-history traits correlate with positive conservation outcomes. We focus on seabirds, one of the world's most rapidly declining groups, whose philopatry, activity patterns, foraging, mate choice, and parental care behaviours all involve reliance on, and therefore strong selection for, sophisticated sensory physiology and accurate assessment of intra- and inter-species signals and cues in several sensory modes. We review the use of auditory, olfactory, and visual methods, especially for attracting seabirds to newly restored habitat or deterring birds from fishing boats and equipment. We found that more sensory-based conservation has been attempted with Procellariiformes (tube-nosed seabirds) and Charadriiformes (e.g. terns and gulls) than other orders, and that successful outcomes are more likely for Procellariiformes. Evolutionary and behavioural traits are likely to facilitate sensory-based techniques, such as social attraction to suitable habitat, across seabird species. More broadly, successful application of sensory-based conservation to other at-risk animal groups is likely to be associated with these behavioural and life-history traits: coloniality, philopatry, nocturnal, migratory, long-distance foraging, parental care, and pair bonds/monogamy.

4720: ***+.012***

The Perdido Key beach mouse (Peromyscus polionotus trissyllepsis) is an endangered rodent endemic to a barrier island at the Florida-Alabama border. Land development, tropical storms, and non-native predators have caused mouse populations to be extirpated from some areas, including Gulf State Park where mice have been absent since 1997. Because wild populations were too small to be donors, we used captive-born beach mice to reestablish a population at Gulf State Park. We released 48 mice into soft-release pens in March 2010. We monitored the fate and movements of 28 mice with radio-collars, and assessed changes in the population size through live trapping. During the first week, we found 15 radio-collars at a red fox (Vulpes vulpes) den and after two weeks the total number of known surviving mice had declined to 13. Many of these mice used the pens for food and shelter and remained in the area of their release (<100 m movements). After 12-weeks, 17 wild-born mice had been captured. Knowing that captive-born individuals can be introduced into vacant habitat and successfully rear offspring provides biologists with an alternate management option for conserving endangered beach mice when populations of wild mice are too low to allow translocations.

4721: ***+.082***

Corridors are usually delineated as areas of minimum cumulative resistance to movement through a resistance surface and characterized by their effective distance (accumulated resistance along the least-cost path). The results of these assessments depend on resistance values, which are typically derived from the inverse of habitat suitability models or from presence data of individuals within their home ranges, rather than from data on dispersal or exploratory movements. Evaluate the extent to which corridor delineation and effective distance estimates may vary depending on whether home range locations or dispersal data are used to characterize species habitat selection and landscape resistance to movement. We analyzed a large telemetry dataset (GPS collars) for the endangered Iberian lynx. We modeled corridors and effective distances three ways: (1) considering only GPS locations within home ranges, (2) considering only locations in dispersal or exploratory movements outside home ranges, and (3) considering all locations together. Delineated least-cost corridors followed similar trajectories and sometimes overlapped in the three models. The estimated effective distances were 42 % lower in the dispersal-based model than in the model based solely on home range use. Models derived exclusively from locations within home ranges may provide lower connectivity estimates than models derived from dispersal locations, affecting estimates of resistance to move between habitat areas, even when the most likely movement routes are similar. Although dispersal data are costly to gather, they potentially provide more realistic assessments of the actual isolation of populations in heterogeneous landscapes.

4722: ***+.014***

Maintaining genetic diversity within captive breeding populations is a key challenge for conservation managers. We applied a multi-generational genetic approach to the captive breeding program of an endangered Australian freshwater fish, the southern pygmy perch (Nannoperca australis). During previous work, fish from the lower Murray-Darling Basin were rescued before drought exacerbated by irrigation resulted in local extinction. This endemic lineage of the species was captive-bred in genetically designed groups, and equal numbers of F1 individuals were reintroduced to the wild with the return of favourable habitat. Here, we implemented a contingency plan by continuing the genetic-based captive breeding in the event that a self-sustaining wild population was not established. F1 individuals were available as putative breeders from the subset of groups that produced an excess of fish in the original restoration program. We

used microsatellite-based parentage analyses of these F1 fish to form breeding groups that minimized inbreeding. We assessed their subsequent parental contribution to F2 individuals and the maintenance of genetic diversity. We found skewed parental contribution to F2 individuals, yet minimal loss of genetic diversity from their parents. However, the diversity was substantially less than that of the original rescued population. We attribute this to the unavoidable use of F1 individuals from a limited number of the original breeding groups. Alternative genetic sources for supplementation or reintroduction should be assessed to determine their suitability. The genetic fate of the captive-bred population highlights the strong need to integrate DNA-based tools for monitoring and adaptive management of captive breeding programs.

4723: *+.018*

The endangered fountain darter Etheostoma fonticola is found only in the Comal and San Marcos rivers in the Guadalupe River basin in central Texas, USA. Comal River fountain darters were believed to be extirpated following a severe drought in the 1950s and were reintroduced in the early 1970s using 457 darters from the San Marcos River. In this study we used 23 microsatellite loci to describe and evaluate the genetic diversity, population structure and effective population size (N (e)) of fountain darters. We also evaluated the genetic effect of the Comal River reintroduction and the influence of low-head dams (dams) on dispersal in both rivers. Bayesian analysis of individual genotypes and Analysis of Molecular Variation supported two distinct populations concordant with the two rivers. Estimates of N (e) were much smaller (< 10 %) than census size (N (c)) in both rivers but did not indicate the populations are at risk of an immediate and rapid loss of genetic diversity. Coalescent-based estimates of the genetically effective number of founders (Nf) for the Comal River averaged about 49 darters and, together with the indices of genetic diversity and the bottleneck test (heterozygosity excess) results, were consistent with a founder event following the reintroduction in the Comal River. Finally, our results regarding the influence of dams on fountain darter dispersal were equivocal and did not support a conclusion. We recommend this issue be examined further as part of the fountain darter recovery program.

4724: *+.040*

Distinguishing the relative influence of historic (i.e. natural) versus anthropogenic factors in metapopulation structure is an important but often overlooked step in management programs of threatened species. Biotas in freshwater wetlands and floodplains, such as those in the Murray-Darling Basin (MDB)-one of Australia's most impacted ecosystems, are particularly susceptible to anthropogenic fragmentation. Here we present a comprehensive multilocus assessment of genetic variation in the threatened southern pygmy perch Nannoperca australis (578 individuals; 45 localities; microsatellite, allozyme and mitochondrial DNA datasets), an ecological specialist with low dispersal potential. We assess patterns of spatial structure and genetic diversity in populations spanning the highly fragmented MDB and test whether recent anthropogenic modification has disrupted range-wide connectivity. We detected strong and hierarchical population structure, very low genetic diversity and lack of contemporary gene flow across the MDB. In contrast, the apparent absence of pronounced or long-term phylogeographic structure suggests that observed population divergences generally do not reflect deeply historic natural fragmentation. Coalescent-based analyses supported this inference, revealing that divergence times between populations from the upper and lower MDB fall into the period of European settlement. It appears that the observed contemporary isolation of populations is partly explained by the severe modification of the MDB post-dating the onset of European settlement. Our integrated approach substantially improves the interpretation of how fragmentation impacts present-day biodiversity. It also provides novel contributions for risk-assessing management actions in the context of captive breeding and

translocations of small freshwater fishes, a group of increasing global conservation concern.

4725: *+.025*

Freshwater pearl mussel (Margaritifera margaritifera) populations are declining in Northern Ireland to the extent that a captive breeding programme was established on the Upper Ballinderry river in 1998. Previous genetic analysis of the hatchery broodstock and their first cohort of offspring showed significant levels of inbreeding (F (IS) = 0.166). The broodstock, which currently numbers ca. 90 individuals, was supplemented with new individual mussels, whilst in 2013, a previously unknown population was discovered on the Lower Ballinderry river. The aim of the present study was to determine whether the rotation of the broodstock has led to a decrease in the levels of inbreeding in the second cohort of juveniles, and to determine whether the new population found in the Lower Ballinderry was genetically distinct from the captive bred population and populations from the Upper Ballinderry, which represent the source of the hatchery broodstock. Genotyping using eight microsatellite markers indicated that levels of inbreeding in the second cohort of captive-bred mussels were high, (F (IS) = 0.629), and were comparable to those sampled from the original cohort and the hatchery broodstock (F (IS) = 0.527 and 0.636 respectively). Bayesian analysis of population structure indicated that the newly discovered Lower Ballinderry population was genetically distinct from the broodstock and its source populations on the Upper Ballinderry. The observed differentiation was primarily due to differences in allele frequencies, and was most likely a result of genetic drift. The occurrence of ten alleles, albeit at low frequency, in the Lower Ballinderry population, including four private alleles, suggests that this new population could be incorporated into the broodstock with the aim of decreasing levels of inbreeding in the future.

4726: *+.278*

Conservation actions, such as habitat protection, attempt to halt the loss of threatened species and help their populations recover. The efficiency and the effectiveness of actions have been examined individually. However, conservation actions generally occur simultaneously, so the full suite of implemented conservation actions should be assessed. We used the conservation actions underway for all threatened and near-threatened birds of the world (International Union for Conservation of Nature Red List of Threatened Species) to assess which biological (related to taxonomy and ecology) and anthropogenic (related to geoeconomics) factors were associated with the implementation of different classes of conservation actions. We also assessed which conservation actions were associated with population increases in the species targeted. Extinction-risk category was the strongest single predictor of the type of conservation actions implemented, followed by landmass type (continent, oceanic island, etc.) and generation length. Species targeted by invasive nonnative species control or eradication programs, ex situ conservation, international legislation, reintroduction, or education, and awareness-raising activities were more likely to have increasing populations. These results illustrate the importance of developing a predictive science of conservation actions and the relative benefits of each class of implemented conservation action for threatened and near-threatened birds worldwide.

4727: *+.087*

The use of conservation translocations to mitigate human effects on biodiversity is increasing, but how these efforts are allocated remains unclear. Based on a comprehensive literature review and online author survey, we sought to determine the goals of translocation efforts, whether they focus on species and regions with high threat and likelihood of perceived success, and how success

might be improved. We systematically searched the ISI Web of Knowledge and Academic Search Complete databases to determine the species and regions of conservation translocations and found 1863 articles on conservation translocations in the United States, Canada, Mexico, Central America, and Caribbean published from 1974 to 2013. We questioned 330 relevant authors to determine the motivation for translocations, how translocations were evaluated, and obstacles encountered. Conservation translocations in North America were geographically widespread (in 21 countries), increased in frequency over time for all animal classes (from 1 in 1974 to 84 in 2013), and included 279 different species. Reintroductions and reinforcements were more common in the United States than in Canada and Mexico, Central America, or the Caribbean, and their prevalence was correlated with the number of species at risk at national and state or provincial levels. Translocated species had a higher threat status at state and provincial levels than globally (International Union for Conservation of Nature Red List categorization), suggesting that translocations may have been motivated by regional priorities rather than global risk. Our survey of authors was consistent with these results; most translocations were requested, supported, or funded by government agencies and downlisting species at national or state or provincial levels was the main goal. Nonetheless, downlisting was the least reported measure of success, whereas survival and reproduction of translocated individuals were the most reported. Reported barriers to success included biological factors such as animal mortality and nonbiological factors, such as financial constraints, which were less often considered in the selection of release sites. Our review thus highlights discrepancies between project goals and evaluation criteria and between risk factors considered and obstacles encountered, indicating room to further optimize translocation projects. La Alineacion de la Amenaza, el Esfuerzo y el Exito Percibido en las Translocaciones para Conservacion en America del Norte El uso de las translocaciones para conservacion y mitigar los efectos humanos sobre la biodiversidad esta incrementando, pero aun no es claro como se asignan estos esfuerzos. Con base en una revision integral de literatura y una encuesta de autor en linea buscamos determinar los objetivos de los esfuerzos de translocacion, ya sea que se enfoquen en especies y regiones con una amenaza mayor y una probabilidad de exito percibido y como el exito puede mejorarse. Buscamos sistematicamente las bases de datos completas de la Red de Conocimiento y Busqueda Academica ISI para determinar las especies y regiones de las translocaciones para conservacion y encontramos 1,863 articulos sobre translocaciones para conservacion en los Estados Unidos, Canada, Mexico, America Central y el Caribe, publicados entre 1974 y 2013. Consultamos a 330 autores relevantes para determinar los motivos de las translocaciones, como fueron evaluadas y los obstaculos a los que se enfrentaron. Las translocaciones para conservacion en America del Norte estaban dispersas geograficamente (en 21 paises), incrementaron su frecuencia con el tiempo para todas las clases animales (de una en 1974 a 84 en 2013) e incluyeron a 279 especies diferentes. Las reintroducciones y los refuerzos fueron mas comunes en los Estados Unidos que en Canada, Mexico, America Central o el Caribe, y su prevalencia estuvo correlacionada con el numero de especies en riesgo a nivel nacional, estatal o provincial. Las especies reubicadas tenian un mayor estado de amenaza a nivel estatal y provincial que a nivel global (categorias de la Lista Roja de la Union Internacion para la Conservacion de la Naturaleza), lo que sugiere que las reubicaciones podrian haber estado motivadas por prioridades regionales en lugar del riesgo global. Nuestra encuesta a los autores fue consistente con estos resultados ya que la mayoria de las reubicaciones fueron solicitadas, apoyadas o financiadas por agencias del gobierno y bajar la categoria de la especie a nivel nacional, estatal o provincial era el objetivo principal. Sin embargo, bajar la categoria fue la medida de exito menos reportada. Los obstaculos para el exito reportados incluyeron factores biologicos, como la mortalidad animal, y factores no biologicos, como las restricciones financieras, que fueron menos considerados en la seleccion de los sitios de liberacion. Nuestra revision resalta asi las discrepancias entre los objetivos del proyecto y los criterios de evaluacion y entre los factores de riesgo considerados y los obstaculos enfrentados, lo que indica que existe espacio para optimizar aun mas los proyectos

de reubicacion.Resumen

4728: *+.203*

Semi-natural pastures have rich plant and animal communities of high conservation value which depend on extensive management. As the area of such land decreases, abandoned semi-natural grasslands have been restored to re-establish biodiversity. Restoration schemes, which include thinning of woody plants and reintroduction of grazing, are mainly designed according to the responses of well-studied groups (such as vascular plants and birds). Weevils (Curculionidae) are a very diverse phytophagous beetle family. Here, we evaluated the restoration success of pastures for weevils (Curculionidae), by comparing their species diversity in abandoned, restored, and continuously grazed semi-natural pastures on 24 sites in central Sweden. Weevils were sampled by sweep-netting. We recorded 3019 weevil individuals belonging to 104 species. There was no statistically significant difference in species numbers between the pasture management treatments. However, weevil species composition of abandoned pastures differed from those in restored and continuously managed pastures, but there was no significant difference in community composition between restored and continuously grazed pastures. The abandoned sites tended to be dominated by polyphagous species, whereas the grazed sites contained more monophagous and oligophagous species. The number of weevil species was positively related to understory vegetation height and connectivity to other semi-natural grasslands and negatively related to the cover of trees and shrubs in the pastures. We conclude that restoration of abandoned semi-natural pastures is a good approach to restore weevil communities. To maintain a species rich weevil community, pastures should be managed to be relatively open, but still have patches of tall field-layer vegetation. Restoration and conservation measures should primarily be targeted on regions and landscapes where a high proportion of semi-natural grassland still remains.

4729: *+.149*

The three surviving 'brush-tailed' bettong species-Bettongia gaimardi (Tasmania), B. tropica (Queensland) and B. penicillata (Western Australia), are all classified as threatened or endangered. These macropodids are prolific diggers and are recognised as important 'ecosystem engineers' that improve soil quality and increase seed germination success. However, a combination of introduced predators, habitat loss and disease has seen populations become increasingly fragmented and census numbers decline. Robust phylogenies are vital to conservation management, but the extent of extirpation and fragmentation in brush-tailed bettongs is such that a phylogeny based upon modern samples alone may provide a misleading picture of former connectivity, genetic diversity and species boundaries. Using ancient DNA isolated from fossil bones and museum skins, we genotyped two mitochondrial DNA (mtDNA) genes: cytochrome b (266 bp) and control region (356 bp). These ancient DNA data were combined with a pre-existing modern DNA data set on the historically broadly distributed brush-tailed bettongs (similar to 300 samples total), to investigate their phylogenetic relationships. Molecular dating estimates the most recent common ancestor of these bettongs occurred c. 2.5 Ma (million years ago), which suggests that increasing aridity likely shaped their modern-day distribution. Analyses of the concatenated mtDNA sequences of all brush-tailed bettongs generated five distinct and well-supported clades including: a highly divergent Nullarbor form (Clade I), B. tropica (Clade II), B. penicillata (Clades III and V), and B. gaimardi (Clade IV). The generated phylogeny does not reflect current taxonomy and the question remains outstanding of whether the brush-tailed bettongs consisted of several species, or a single widespread species. The use of nuclear DNA markers (single nucleotide polymorphisms and/or short tandem repeats) will be needed to better inform decisions about historical connectivity and the appropriateness of ongoing conservation measures such as translocations and captive breeding.

4730: ***+.115***

Re-establishing salmonid populations to areas historically occupied has the substantial potential for conservation gains; however, such interventions also risk negatively impacting native resident stocks. Here, we assessed the success of the hatchery-assisted reintroduction of anadromous sockeye salmon (Oncorhynchus nerka) into Skaha Lake, British Columbia, Canada, and evaluated the genetic consequences for native kokanee, a freshwater-obligate ecotype, using single nucleotide polymorphism genotypic data collected from the reference samples of spawning Okanagan River sockeye and Skaha Lake kokanee presockeye reintroduction, along with annual trawl survey and angler-caught samples obtained over an eight-year period. Significant differentiation was detected between sockeye and kokanee reference samples, with >99% stock assignment. Low proportions of sockeye and hybrids were detected within 2008 and 2010 age-0 trawl samples; however, by 2012, 28% were sockeye, rising to 41% in 2014. The number of hybrids detected rose proportionally with the increase in sockeye and exhibited an intermediate phenotype. Our results indicate that the reintroduction of anadromous sockeye to Skaha Lake is succeeding, with large numbers returning to spawn. However, hybridization with native kokanee is of concern due to the potential for demographic or genetic swamping, with ongoing genetic monitoring necessary to assess the long-term effects of introgression and to support interactive fisheries management.

4732: ***+.280***

Due to their direct dependence on wildlife products for subsistent use, people living in poverty are often viewed as being in conflict with wildlife conservation. We studied the attitudes of local people towards mountain nyala (Tragelaphus buxtoni) in Munessa, Ethiopia. A household survey (n = 214) was used to examine the socio-economic characteristics and beliefs of local people from three peasant associations and one village. Generally, the study revealed that local people had positive attitudes towards mountain nyala, its conservation and population increase, which is consistent with our predictions. For example, a greater percentage of the respondents had positive (62.5%) rather than negative (37.5%) attitudes towards mountain nyala and its conservation. Moreover, the larger proportion of the respondents had positive (66.67%) rather than negative (33.3%) attitudes towards an increase in mountain nyala population. However, compared to socio-economic characteristics, beliefs towards mountain nyala were more powerful and consistent predictor of attitudes and explained much of the variances of the two groups of the dependent variables. Thus, informing local communities about the value of mountain nyala (e.g. recreational and economic) through conservation education, advocating the need for sustainable utilization and introducing an economic benefit sharing may improve positive attitude and increase participation of local people in conservation and management of the mountain nyala population in Munessa.

4733: ***+.026***

The once 'Critically Endangered' Seychelles Magpie-robin was down to just 12 individuals in 1960 on one island of the granitic Seychelles. In 2015, due to intensive long-term management the population stands at around 280 birds on five islands, marking a significant success for this species. Translocations to the islands of Cousin and Cousine have led to population saturation and stability, a translocation to Denis Island has resulted in a continuing population increase and the founder population on Fregate Island is likewise increasing. The latest translocation to Aride Island in 2002 resulted in population increase then stability but is now showing a steep decline throughout 2014 into 2015. Reasons for this decline are yet unknown though disease, lack of recruitment, the impacts of social conflict and the possibility of genetic issues are discussed. This

report summarises the history of management for this species, compiling all available published and unpublished information, to provide a comprehensive account of the Seychelles Magpie-robin recovery.

4734: *-.063*

Exotic pathogen invasions can change host eco-evolutionary interactions and possibly create an evolutionary trap when the pathogen generates mismatches between developmental phenology and reproductive cues. Taylor's checkerspot butterfly (Euphydryas editha taylori), is an endangered species of western North America with 80 % of the extant populations dependent on an exotic host, Plantago lanceolata. Survey of occupied, recently extinct, and unsuccessful butterfly reintroduction sites spanning 4 degrees of latitude revealed widespread disease on P. lanceolata caused by Pyrenopeziza plantaginis. This fungal pathogen, new to North America, reduces the standing crop of P. lanceolata foliage throughout the winter post-diapause larval feeding period. However, disease is absent when adult butterflies and pre-diapause larvae are active. Diseased plants were frequent in Taylor's checkerspot populations with a history of persistence, but >90 % of the host plants in these populations had initiated new leaves within the first few weeks of post-diapause larval feeding. Conversely, host plants in recently extinct and unsuccessfully reintroduced populations were severely diseased, >66 % mean foliage necrosis/plant, and <23 % had initiated new leaves. Feeding choice trials with 25 larvae indicated that new leaves were strongly and consistently preferred by post-diapause larvae over all other available leaf types, both diseased and non-diseased. Because the influence of disease on post-diapause larval food resources is developmentally disassociated from oviposition, P. plantaginis invasion appears to have triggered an evolutionary trap for Plantago-dependent populations of Taylor's checkerspot.

4735: *+.141*

Dams contribute to declines in fish abundance, in part, by blocking access to historical habitat. When fish ladders are infeasible, fish can be trapped below a dam and transported above to provide access to habitat. However, this conservation strategy has received little attention in the literature, and many questions regarding efficacy remain unanswered. Here, we used a genetic parentage approach to evaluate cohort replacement rate (CRR), defined as the number of future spawners produced by a spawner, for a spring Chinook salmon Oncorhynchus tshawytscha trap and transport reintroduction program. We used CRRs to determine if the population can sustain itself in the absence of immigration, which is important when assessing demographic viability. We also evaluated the effects of release locations and dates on the fitness of reintroduced fish. Counts of adult offspring per spawner were used to estimate fitness of salmon reintroduced in 2007 and 2008. We found that fitness decreased slightly as adults were released later in the spawning season in 2007, but not in 2008. Release location did not affect fitness in either year. We also found a seasonal decline in the likelihood that a Chinook salmon collected at the trap and transport facility was produced above the dam. Finally, 2007 and 2008 CRRs were both well below one, indicating that improvements are needed to achieve demographic stability. We demonstrate that genetic monitoring of a reintroduction program helped to inform demographic viability assessments, and suggest that our approach may be broadly applicable to other philopatric species.

4736: *-.086*

Ex situ (captive) management can facilitate species recovery, but this approach is invasive and is not universally appropriate. Evidence-based approaches are critical to determining whether ex situ approaches will be effective, but in many cases biological and ecological data from threatened

populations are scarce. We generated a structured set of general biological and social/logistical criteria required for captive management to benefit threatened, free-ranging populations. We illustrate how these criteria can be applied using a case study where populations are severely threatened, but demographic data are scarce (white-nose syndrome in Canadian bats). Using (1) population viability modelling (PVA), (2) a survey of Canadian zoos and wildlife rehabilitators, and (3) literature reviews, we identified two of our five initial target species as potential candidates for captive management. PVA revealed that sustainable captive colonies require high adult survivorship relative to free-ranging populations. Our survey and literature reviews showed that Canadian zoos and wildlife rehabilitators are enthusiastic about bat conservation. However, none could currently maintain the target species, due to limited infrastructure and/or knowledge gaps related to husbandry and reintroduction of captive bats to the wild. Captive management is unlikely to stabilize target populations because released bats risk re-infection. We conclude that ex situ management is not an appropriate tool in our case study, and would represent ineffective use of available conservation resources. However, development of captive husbandry and re-introduction methods for hibernating, insectivorous bats would contribute to our global capacity to conserve similar species.

4737: ***-.091***

This review provides the first historical account of the ecology and biology of common carp Cyprinus carpio in the Danube and Adriatic basins of Croatia, and emphasises the species' economic relevance and management implications. As a semi-native species that is native to the Danube but translocated across the Adriatic Basin, carp plays an important role for aquaculture, recreational and artisanal fisheries. However, original strains have now disappeared, and because of genetic pollution in inland waters there is an increasing demand for restoring populations of the now rare and threatened wild carp, making conservation measures a priority. Translocations of carp across water bodies of the Adriatic Basin mostly for food supply did not prove successful in the long term, as the resulting ecological impacts may have been higher than the expected economic advantages. Measures for the prevention of further (uncontrolled) carp re-stocking are therefore necessary and this will require closer collaboration between scientists and environmental managers.

4738: ***+.138***

The riparian brush rabbit Sylvilagus bachmani riparius is restricted to areas of dense cover along rivers and tributaries in the Central Valley of California, USA, and is considered endangered under the U.S. Endangered Species Act. An approved recovery plan for this subspecies recommended establishment of three additional self-sustaining populations within its historical range. Concurrent with habitat restoration efforts that included revegetation of levees and provision of flood refugia, we reintroduced 325 captive-bred individuals to the San Joaquin River National Wildlife Refuge between July 2002 and July 2005, and monitored their survival with radiotelemetry. Using available survival estimates and estimated reproductive parameters, we developed a population viability analysis model to determine the risk of extinction of the reintroduced population under a series of alternate scenarios including different release strategies. All model scenarios resulted in high probabilities of extinction over 20 y in the absence of either ongoing supplementations or significant improvements to vital rates. Extinction risks over 20 y appeared independent of initial release strategies. The risk of extinction remained high (>99%) even when the initial founder population was supplemented with captive-bred brush rabbits for up to 10 y. Supplementing the population annually for the entire 20-y period would be required for the population to persist given current estimates of vital rates. These results suggest that the reintroduced population is not self-

sustaining and continued releases will not provide the anticipated long-term benefits. Additionally, our understanding of the integral components of habitat and potential limiting factors should be refined to address their influence on demographics and the cause of brush rabbit declines at the release site in a comprehensive way. These results underscore the importance of viability analyses and developing testable hypotheses about potential limiting factors early in reintroduction efforts to more efficiently guide data-gathering and monitoring criteria, identify data deficiencies, and refine reintroduction techniques in the early stages of management.

4739: *+.024*

Red-cockaded Woodpeckers (Leuconotopicus borealis) were extirpated from Tall Timbers Research Station in the early 1980s. To help meet conservation goals established for this imperiled species in north Florida, we attempted to reintroduce the woodpecker to the research station by constructing artificial cavities and translocating 27 subadult woodpeckers from 2006 to 2010. Successful nesting occurred during the breeding season following the initial translocation of four male-female pairs. Translocations were suspended in 2011 when breeding groups occupied 6 of 12 available clusters of cavity trees. The population increased steadily after 2011 and, in 2015, totaled 28 adults distributed among nine breeding groups plus a single territorial male. The 2015 population included 22 individuals produced at Tall Timbers, an immigrant female, and five birds originally translocated as subadults. Seven breeding groups in 2015 also had non-breeding helpers. New milestones documented during this reintroduction attempt included recruitment of locally produced birds into the breeding population, excavation of natural cavities, two immigration events, and natural expansion into an unoccupied area. We also documented the threat that heavy rains may pose to small populations. Expenses totaled $211,000 during the first 5 yr when translocations and cavity construction were the primary activities. After translocations were suspended, recurring management expenses were similar to$6500 annually. Because our founding population was small (N = 12), intermittent translocations will likely be needed in the future to offset the deleterious effects of inbreeding.

4741: *+.068*

Globally, one in five reptile species is thteatened with extinction, with invasive species a leading cause of extinction risk. Translocations could alleviate the risk of extinction through the establishment of populations in locations from which invasive predators have been removed. But do translocations represent a viable strategy for reptile conservation? We investigate the numerical and genetic outcomes of translocations of reptiles as reintroductions to islands cleared of introduced mammals around New Zealand: These reintroductions included nine populations of tuatara (Sphenodon punctatus), ten populations of six species of geckos and 24 populations of 12 species of skinks on a total of 24 islands. Reintroduced populations are often relatively small, which exposes them to associated demographic and genetic problems. We compared criteria for success based on abundance with available genetic data for four species of reintroduced reptiles. Three populations of skinks showed some loss of genetic heterozygosity but have nonetheless met most criteria for numerically viable populations. Whether loss of genetic variability might have long-term consequences for persistence is unclear because the genetic basis for population viability is still debated. We found that the success of reintroduction can be influenced by complex interactions between numerical, genetic and administrative constraints on project design. We thus suggest that obtaining data on the outcomes of reptile recovery will require managers to avoid mixing populations for pre-emptive genetic rescue, and a commitment to long term ecological and genetic studies. (C) 2016 Elsevier Ltd. All rights reserved.

4742: *+.082*

An important step in species conservation is to identify populations that significantly contribute to it. Considering both in situ and ex situ populations provides an integrated approach to the preservation of a species' evolutionary potential. The joint use of molecular and environmental analyses allows conservation schemes to be implemented when reintroducing captive populations, and wild populations to be prioritized for conservation purposes. We used genetic data and environmental analyses to select candidate areas for the reintroduction of a captive population of the Mexican prairie dog, Cynomys mexicanus, and prioritize wild populations for the conservation of this endangered endemic species. We estimated the levels of genetic diversity and differentiation of the captive population and compared them with those of six wild populations. We used species distribution modeling (SDM) to perform forecasts under future climate change scenarios and identify areas with suitable environmental conditions for the populations to persist in the medium to long term. The captive population showed high levels of genetic diversity (H-d = 0.692, H-E = 0.52), but was genetically differentiated from the wild populations. The genetic structure of wild populations should therefore be considered when reintroducing captive Mexican prairie dogs. In the wild populations, we found a correlation between colony area and nuclear genetic diversity, suggesting that genetic drift and/or inbreeding have been stronger in smaller colonies. The occupied climate space was well differentiated among wild colonies. The impact of agriculture and roads was stronger in the northeastern area of the species range, where SDM forecasts suggest that environmental conditions may remain suitable in the future. Finally, we identified three colonies as conservation priorities based on both genetic and ecological criteria. (C) 2016 Elsevier Ltd. All rights reserved.

4743: *+.083*

Translocation of 'nuisance' snakes is frequently employed on a large scale in densely populated areas in order to mitigate human-wildlife conflict. However, the methods used are often applied haphazardly and are rarely evaluated, especially in tropical Asia. The objective of this study was to assess the effects of long-distance translocation on the white-lipped pit viper (Trimeresurus albolabris) in South China, where they are routinely removed from urban areas or homes and translocated into national parks. Using radio telemetry, we compared the ecology and biology of 'resident' and 'translocated' snakes to determine if long-distance translocation (>3 km) is a viable conservation option. Translocation extended the period over which predation occurred and significantly decreased survival. Translocated snakes made unidirectional movements away from points of release, yet these movements were not oriented towards points of origin and there was no evidence for homing behavior. Trans location significantly increased frequency of movements and distances-moved by female snakes, but no such differences were observed for male snakes. In contrast with resident snakes, translocated snakes did not show synchrony in the onset of brumation, and translocation appeared to negatively affect reproduction in both males and females. Our results suggest that long-distance translocation is not the best management approach to handle nuisance individuals of this species. Findings of this study can be used to prescribe improvements for current strategies to deal with nuisance snakes in Hong Kong and in the region. (C) 2016 Elsevier Ltd. All rights reserved.

4744: *-.212*

Although the transfer of wild animals to captivity is crucial for conservation purposes, this process is often challenging because some species or individuals do not adjust well to captive conditions. Chronic stress has been identified as a major concern for animals held on long-term captivity.

Surprisingly, the first hours or days of captivity have been relatively overlooked. However, they are certainly very stressful, because individuals are being transferred to a totally novel and confined environment. To ensure the success of conservation programmes, it appears crucial to better understand the proximate causes of interspecific and interindividual variability in the sensitivity to these first hours of captivity. In that respect, the study of stress hormones is relevant, because the hormonal stress response may help to assess whether specific individuals or species adjust, or not, to such captive conditions ('the stress response-adjustment to captivity hypothesis'). We tested this hypothesis in rock pigeons by measuring their corticosterone stress response and their ability to adjust to short-term captivity (body mass loss and circulating corticosterone levels after a day of captivity). We showed that an increased corticosterone stress response is associated with a lower ability to adjust to short-term captivity (i.e. higher body mass loss and circulating corticosterone levels). Our study suggests, therefore, that a low physiological sensitivity to stress may be beneficial for adjusting to captivity. Future studies should now explore whether the stress response can be useful to predict the ability of individuals from different populations or species to not only adjust to short-term but also long-term captivity.

4745: ***+.147***

Historically, Polyodon spathula (American Paddlefish) occurred within the Mississippi River Basin's large rivers, traversing hundreds of kilometers to complete their life cycle. However, populations declined in response to the installation of lock and dam (L/D) structures, declining water quality, and the loosely regulated harvest of the species. By the late 1800s, American Paddlefish were extirpated from many portions of their historic range. In response, federal and state agencies sought to restore inter-jurisdictional populations of this fish. Our objective was to assess reintroduction efforts in the northeastern-most portion of its range. Using a combination of gill nets, boat electrofishing, and larval drift nets, populations were sampled in Pennsylvania and New York over a 10-year-period, post-stocking. Although American Paddlefish were at large in navigable waters of the upper Ohio River Basin, free-flowing and tail-water areas below L/D structures appeared to hold more fish than impounded reaches. The presence of L/D structures and fixed-crest dams may inhibit upstream passage of fish and reduce the availability of forage, as reflected in reduced condition-index scores. Although gravid fishes were captured in Pennsylvania and New York, little evidence exists to suggest the presence of self-sustaining populations, as only one larval American Paddlefish was captured. The recovery of this species to its historic northeast range may hinge on continuing stocking efforts, development of fish-passage structures, conservation lockages, and improvement and protection of suitable spawning habitats.

4746: ***+.204***

Global amphibian declines have been attributed to a number of factors including disease, invasive species, habitat degradation, and climate change. Reintroduction is one management action that is commonly used with the goal of recovering imperiled species. The success of reintroductions varies widely, and evaluating their efficacy requires estimates of population viability metrics, such as underlying vital rates and trends in abundance. Although rarely quantified, assessing vital rates for recovering populations provides a more mechanistic understanding of population growth than numerical trends in population occupancy or abundance. We used three years of capture-mark-recapture data from three breeding ponds and a Cormack-Jolly-Seber model to estimate annual apparent survival for reintroduced populations of the federally threatened Chiricahua Leopard Frog (Lithobates chiricahuensis) at the Buenos Aires National Wildlife Refuge (BANWR), in the Altar Valley, Arizona, USA. To place our results in context, we also compiled published survival estimates for other ranids. Average apparent survival of Chiricahua Leopard Frogs at BANWR

was 0.27 (95% CI [0.07, 0.74]) and average individual capture probability was 0.02 (95% CI [0, 0.05]). Our apparent survival estimate for Chiricahua Leopard Frogs is lower than for most other ranids and is not consistent with recent research that showed metapopulation viability in the Altar Valley is high. We suggest that low apparent survival may be indicative of high emigration rates. We recommend that future research should estimate emigration rates so that actual, rather than apparent, survival can be quantified to improve population viability assessments of threatened species following reintroduction efforts.

4747: *+.225*

Many declining populations of the imperiled Allegheny woodrat (Neotoma magister) function as nonequilibrium metapopulations in which rates of subpopulation extirpation exceed recolonization. Quantifying and maximizing survival rates thus becomes critical for the conservation of these spatially structured populations. We used encounter histories of individually marked woodrats from subpopulations in Indiana, monitored annually from 2005 to 2013, to 1) estimate apparent annual survival rates while accounting for imperfect detection, 2) evaluate differences in apparent survival between unaugmented subpopulations and subpopulations reestablished or restored through translocation efforts, and 3) describe the effect of genetic diversity on survival. From Cormack-Jolly-Seber models developed in a Bayesian framework, apparent survival was greater for adults than for juveniles, greater for females than males, and there was a modest negative effect of density dependence. Although heterozygosity rates at 11 microsatellites increased among reinforced subpopulations following translocations, we observed no effect of heterozygosity on apparent survival. However, after translocations, average apparent survival was approximately 14% greater among recipient subpopulations than remnant subpopulations. This suggests that viability of recipient subpopulations was limited by low connectivity and the absence of genetic benefits conveyed by immigration or the potential for patches to be recolonized following local extinction. Under conditions of reduced connectivity, translocation among subpopulations to replicate natural gene flow may be appropriate to facilitate the long-term persistence of this and perhaps other nonequilibrium metapopulations.

4748: *+.079*

In Alberta, wild bison, Bison bison (Linnaeus 1758) occurring outside protected areas are assumed to have originated from the free-ranging bison populations within Wood Buffalo National Park (WBNP). As bison in WBNP are infected with 2 diseases, it was assumed that neighboring bison outside WBNP were also diseased. However, the neighboring Harper Creek and Ronald Lake bison populations have not tested positive for either disease, suggesting limited contact with WBNP herds. To resolve this, we analyzed microsatellite genotypes from all free-ranging bison groups in Alberta and one from the Northwest Territories (NWT), Canada. In the WBNP area where bison have persisted, we recover a genetically panmictic population comprising all possible subpopulations within the park, plus the adjacent Wentzel Lake herd, all of which are diseased. In contrast, but consistent with their disease-free status, we find the Ronald Lake and Harper Creek bison herds to be genetically differentiated from this larger WBNP herd, despite their close proximity. This highlights the importance of the Ronald Lake and potentially the Harper Creek herds for bison recovery. We also document the genetic impacts of translocation on the species, as the Mackenzie, NWT, and Elk Island National Park (EINP), Alberta, bison populations founded in the 1960s from WBNP animals are now genetically differentiated, but the more recently founded Hay Zama bison population is not differentiated from its source population, the EINP wood bison. Interestingly, factorial correspondence analysis, FST, and its high private allelic richness suggest that the Harper Creek population is significantly differentiated from all other bison populations

studied. Though they are most closely related to WBNP populations, more study of this population is warranted.

4749: ***+.105***

AimsReintroduction has become an important tool for the management of endangered plant species. We tested the little-explored effects of small-scale environmental variation, genotypic composition (i.e. identity of genotypes) and genotypic diversity on the population survival of the regionally rare clonal plant Ranunculus reptans. For this species of periodically inundated lakeshores, genetic differentiation had been reported between populations and between short-flooded and long-flooded microsites within populations. MethodsWe established 306 experimental test populations at a previously unoccupied lakeshore, comprising either monocultures of 32 genotypes, mixtures of genotypes within populations or mixtures of genotypes between populations. In 2000, three years after planting out at the experimental site, a long-lasting flood caused the death of half of the experimental populations. In 2003, an extreme drought resulted in the lowest summer water levels ever measured. Important FindingsDespite these climatic extremes, 27% of the established populations survived in December 2003. The success of experimental populations largely differed between microsites. Moreover, the success of genotype monocultures depended on genotype and source population. Genetic differentiation between microsites played a minor role for the success of reintroduction. After the flood, populations planted with genotypes from different source populations increased in abundance, whereas populations with genotypes from single source populations and genotype monocultures decreased. In 2014, several small patches of Ranunculus clones were still present, but plants were strongly intermingled, which precluded their assignment to the original treatments. We conclude that sources for reintroductions need to be selected carefully. Moreover, mixtures of plants from different populations appear to be the best choice for successful reintroduction, at least in unpredictably varying environments.

4750: ***+.116***

The Great Crested Newt (Triturus cristatus) is considered threatened throughout Europe; consequently, the species and its breeding habitat are protected in many countries. Translocation of a population is a conservation tool used when habitat occupied by a species is scheduled to be destroyed by human development. The outcome of these translocations is rarely monitored. This study describes and discusses a translocation of T. cristatus in south-central Sweden (Orebro), which occurred because of planned destruction of breeding habitat associated with development of a shopping and industrial area. We provide quantitative data concerning numbers of relocated amphibians and subsequent monitoring in both the pond being destroyed, which is serving as the source of newts to be translocated, and the pond that received the translocated newts. The translocation exemplifies how difficult it is to determine size and conservation value of a population without thorough initial investigations. A large part of the translocated population seemed to disappear at the receiving area, which initially indicated that the translocation was ineffective. Nevertheless, longer term monitoring indicated that a population was established and reproduced in the new habitat. We argue that translocation should never be a first choice to make human development possible but one should always strive for preservation of an existing habitat. However, if a translocation is unavoidable, an appriopriate assessment of the affected population should be performed and a detailed analysis of habitats in the potential receiving areas should be carried out to select an area best fitted for the species in question.

4751: ***+.148***

Kemp's Ridley Sea Turtle (Lepidochelys kempii) conservation practices permitted by the National Marine Fisheries Service (NMFS) and U.S. Fish and Wildlife Service (USFWS), under authority of the U. S. Endangered Species Act of 1973, include translocations in which eggs or turtles are taken into captivity for various reasons and intervals, and turtles are later released into coastal waters of the Gulf of Mexico (GoM) or the Northwest Atlantic Ocean (NWAO). In 2013, the IUCN Species Survival Commission defined conservation translocation as the deliberate movement of organisms from one site for release in another, with the intention that it must yield a measurable conservation benefit at the levels of a population, species or ecosystem, and not only provide benefit to translocated individuals. Translocations of Kemp's Ridley Sea Turtles that are found injured, ill, or otherwise debilitated, then rescued, resuscitated if necessary, rehabilitated, and released into the GoM or the NWAO have not been evaluated to determine whether they qualify as conservation translocations. We refer to them as rescue, resuscitation, rehabilitation, and release (i.e., RRRR) translocations. Captivity and human care, by altering behavioral and physiological fitness of RRRR translocated Kemp's Ridley Sea Turtles, have the potential to influence post-release survival, growth, navigation, foraging, migration, maturation, natal beach homing, and reproduction. We recommend that NMFS and USFWS develop a plan for hypothesis-driven research and modeling aimed at determining if and how RRRR translocations contribute to Kemp's Ridley Sea Turtle population recovery. Similar evaluations of RRRR translocations are also needed for other sea turtle species.

4752: *-.099*

The full course of new parasite introductions in wild animals is difficult to accurately trace. We documented and analysed the invasive blood-sucking nematode Ashworthius sidemi (Trichostrongylidae) introduction and spread in European bison (Bison bonasus) from the initial phase of its progression. In the Polish part of the Bialowieza Primeval Forest (BPF) the parasite was first found in 2000. From 2002 to 2015, 165 culled bison were investigated. The prevalence and intensity of A. sidemi Schulz, 1933 infection increased over the following years, reaching 100% of investigated bison four years after introduction and a maximal median intensity of 8200 nematodes per animal in the winter of 2008/2009. Afterwards, a significant decline of median infection intensity was observed to the minimum value of 410 nematodes per animal. Between 2011 and 2014 prevalence varied from 89 to 100%. Among the factors analysed, the number of years since introduction, herd size, age and sex proved to significantly influence infection intensity. A higher infection intensity was recorded in sub-adults compared to juveniles and adults. Males had significantly lower infection intensity than females, but this was the case for adults only. The highest infection intensities were recorded in the biggest bison herds, where the winter supplementary feeding of bison is intense. Moreover, the longer the parasite was present in the host population, the more important herd size became as a factor. Our study indicates that it is not solely biological factors that determine the spread of a newly detected parasite in wildlife, but that management practices can also have a strong influence. This is especially important in endangered species under intensive human care as the management practices may pose a threat to the species. (C) 2016 The Authors. Published by Elsevier Ltd on behalf of Australian Society for Parasitology.

4753: *+.255*

Captive breeding and reintroduction programs remain a powerful but divisive tool for management of threatened species, with a proven potential to avoid extinction, but low long-term success rates and high resource requirements. Monitoring the results of reintroductions is critical to be able to assess short- and long-term success, adjusting management decisions as new information becomes available. In this study, we assessed the first 15 years of the captive breeding and restocking

program for the European pond turtle Emys orbicularis in Liguria, northern Italy. We estimated survival of released turtles by modelling mark-recapture monitoring data. We then used those estimates to update our prior expectations about long-term outcomes, and to adjust management decisions about the age of individuals to release. Modelling results suggest released turtles had sufficiently high survival, matching prior expectations, such that local extinction has been averted in the short-term. Survival was similar among caridand age classes for releases, suggesting the release of younger individuals can provide positive outcomes while reducing management costs. On the other hand, survival varied among sites, indicating the need for ongoing in-situ habitat management to ensure long-term persistence. Moreover, the late onset of sexual maturity in the species means reproduction of released animals cannot yet be determined with certainty. Captive breeding and reintroduction programs normally require long-term efforts; therefore, focused monitoring that is clearly linked to decision-making is necessary to continually refine and adjust management strategies. (C) 2016 Elsevier Ltd. All rights reserved.

4754: *+.055*

Food availability is one of the most important factors influencing reproduction in mammals. Reproductive success of some species can be negatively affected when body reserves are depleted during long periods of adverse weather conditions. We investigated the relationship of forage availability and weather variables on reproduction by black-tailed prairie dogs (Cynomys ludovicianus) and the effects of black-tailed prairie dog reproduction on reproduction by black-footed ferrets (Mustela nigripes), a prairie dog specialist predator. Prairie dogs draw on stored energy reserves to support reproduction (i.e., capital breeding), while ferrets likely rely on availability of prey during the reproductive period (i.e., income breeding). We expected that productivity of prairie dogs would positively correlate with precipitation during the previous summer and availability of spring forage and that harsh winter conditions would negatively affect reproduction. We also expected that productivity of ferrets would be positively correlated with productivity of prairie dogs because of the net increase in available prey during the ferret's litter-rearing season and because female ferrets might selectively prey on juvenile prairie dogs. At 2 sites in South Dakota during 2008-2010, reproduction by prairie dogs was most strongly influenced by precipitation received during the previous year and especially by winter severity. Harsh winter conditions resulted in a marked decline in reproduction during 2010. Although reproduction by ferrets varied little across years of our study, the success of long-term conservation and reintroduction strategies for the endangered black-footed ferret could be influenced by climate-driven changes in prairie dog reproduction.

4755: *-.385*

The level of stress that animals endure during capture, handling, transportation, and release processes is a major concern of animal reintroduction projects. Animals under chronic stress are more susceptible to disease and other deleterious issues that could reduce their survival in a new environment. Northern river otters (Lontra canadensis) have been reintroduced in 22 states in the United States and may be susceptible to developing chronic stress during the reintroduction process. We assessed stress levels in five river otters captured from wild populations in Washington, held in captivity for up to 21 days, and then transported to New Mexico for reintroduction. Glucocorticoid levels in fecal samples of all otters tested decreased from when they were held captive in Washington to the time of release. This outcome suggests that habituation to captivity before transport and release may serve to minimize the likelihood of an otter being released while experiencing a potentially burdensome level of stress.

4756: *-.009*

We report a previously unknown population of Poeciliopsis occidentalis occidentalis in La Barge Creek, a tributary stream to the Salt River in Arizona. The population inhabits a slick rock canyon with four permanent pools of water. Previous translocation efforts for P. o. occidentalis occurred more than 7 km upstream of the described location in Charlebois Spring; however, this species has not been observed at the translocation site recently. It is possible that the LaBarge Creek population emigrated from Charlebois Spring. Additional sampling is necessary to determine the source of this population.

4757: *-.119*

Currently the Mississippi delta stands as a highly degraded and threatened coastal ecosystem having lost about 25% of coastal wetlands during the 20th century. To address this problem, a $50 billion, 50-year restoration program is underway. A central component of this program is reintroduction of river water back into the deltaic plain to mimic natural functioning of the delta. However, opposition to diversions has developed based on a number of perceived threats. These include over-freshening of coastal estuaries, displacement of fisheries, perceived water quality problems, and assertions that nutrients in river water leads to wetland deterioration. In addition, growing climate impacts and increasing, scarcity and cost of energy will make coastal restoration more challenging and limit restoration options. We address these issues in the context of an analysis of natural and artificial diversions, crevasse splays, and small sub-delta lobes. We suggest that episodic large diversions and crevasses (>5000 m(3) s(-1)) can build land quickly while having transient impacts on the estuarine system. Small diversions (<200 m(3) s(-1)) that are more or less continuously operated build land slowly and can lead to over-freshening and water level stress. We use land building rates for different sized diversions and impacts of large periodic inputs of river water to coastal systems in the Mississippi delta to conclude that high discharge diversions,operated episodically will lead to rapid coastal restoration and alleviate concerns about diversions. Single diversion events have deposited sediments up to 40 cm in depth over areas up to 130-180 km(2). This approach should have broad applicability to deltas globally. (C) 2016 Elsevier Ltd. All rights reserved.

4758: *+.109*

The lake minnow (Eupallasella percnurus) is critically endangered. In this paper we characterize the genetic properties of this fish over its range of occurrence in Poland and propose the use of this knowledge in its active protection. Twelve populations of lake minnow from across its range in Poland were investigated. 13 microsatellite loci were investigated to evaluate genetic variation and distance among populations. The magnitude of the genetic bottleneck or founder effects was investigated. In the studied populations, the allelic diversity and heterozygosity showed that genetic variation in this species is low. At most loci, only 2-3 alleles per population were detected. The average number of alleles detected across all loci was 35, and ranged from 24 to 53. The average observed heterozygosity (Ho) across all investigated loci was 0.38 (range 0.21-0.59); the average expected heterozygosity (He) was 0.36 (range 0.18-0.55). The populations remained in Hardy-Weinberg equilibrium. The average Garza-Williamson M index value for all populations was low (0.47), suggesting a reduction in genetic variation due to a founder effect or a genetic bottleneck. Genetic distance among populations was high or very high (FST range: 0.20-0.64; delta mu(2) range: 1.32-16.98); this was likely a consequence of low gene flow among isolated populations, a founder effect or other genetic bottleneck, and strong genetic drift. The large genetic differences among the investigated lake minnow populations are likely to also exist among

other populations of this species, and knowledge of these differences should inform active protection programs based on translocation of wild or cultivated fish of this species. The method presented here can potentially be applied to any population of lake minnows or closely related species.

4759: *+.072*

Koala retrovirus (KoRV) is currently undergoing endogenisation into the genome of koalas in Australia, providing an opportunity to assess the effect of retrovirus infection on the health of a population. The prevalence of KoRV in north eastern Australia (Queensland and New South Wales) is 100%, whereas previous preliminary investigations in south eastern Australia (Victoria) suggested KoRV is present at a lower prevalence, although the values have varied widely. Here we describe a large study of free ranging koalas in Victoria to estimate the prevalence of KoRV and assess the clinical significance of KoRV infection in wild koalas. Blood or spleen samples from 648 koalas where tested for KoRV provirus using PCRs to detect pol and env genes. The prevalence of KoRV in these Victorian koalas was 24.7% (160/648) (95% confidence interval [CI]: 21.3, 28.1%). KoRV-A was detected in 141/160 cases but KoRV-B, a genotype associated with neoplasia in captive koalas, was not detected. Detection may have been precluded by genomic differences between KoRV in Victoria and type strains. Factors associated with KoRV infection, based on multivariable analysis, were low body condition score, region sampled, and 'wet bottom'(a staining of the fur around the rump associated with chronic urinary incontinence). Koalas with wet bottom were nearly twice as likely to have KoRV provirus detected than those without wet bottom (odds ratio = 1.90, 95% CI 1.21, 2.98). Our findings have important implications for the conservation of this iconic species, particularly in regards to translocation potential.
