# Supplementary material for: Sentiment Analysis of Conservation Studies Captures Successes of Species Reintroductions
Source: Patterns (N Y). 2020 Mar 20;1(1):100005. doi: 10.1016/j.patter.2020.100005 (PMC7660424; doi:10.1016/j.patter.2020.100005)
Supplement: Document S2. Article plus Supplemental Information [file mmc5.pdf]

Patterns

Article

# Sentiment Analysis of Conservation Studies Captures Successes of Species Reintroductions

## Graphical Abstract

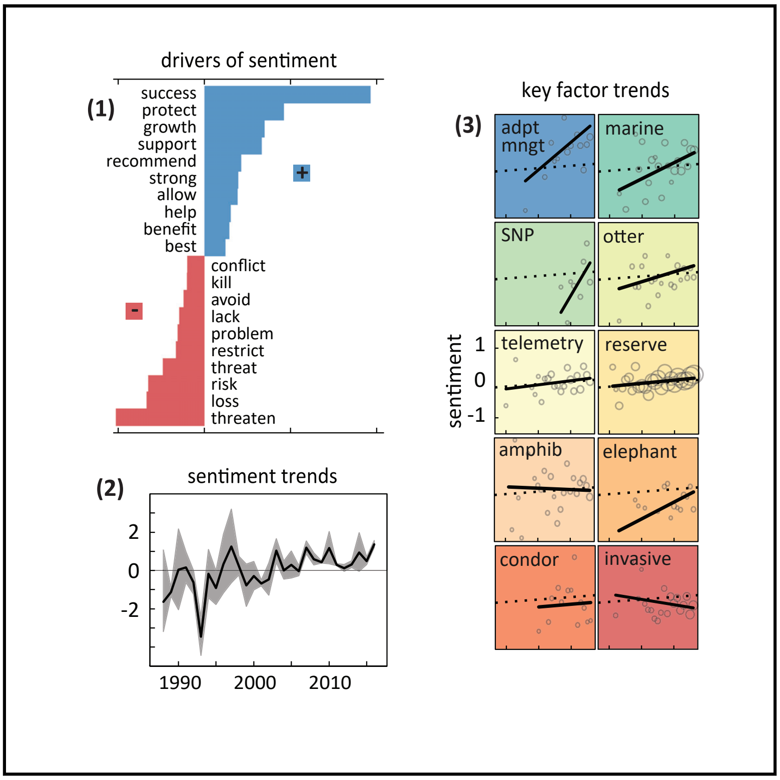


## Authors

Kyle S. Van Houtan, Tyler Gagne, Clinton N. Jenkins, Lucas Joppa

## Correspondence

kvanhoutan@mbayaq.org

## In Brief

Science and technology are increasingly integrated into our everyday lives. A key aspect of science is that the community learns through verified, published findings. Online archives and publications have vastly increased the volume of published science, affording greater access to research results while also presenting new challenges. This study uses established methods in artificial intelligence to assess whether reading scientific papers can be automated. The results are promising, although technical disciplines with specific vocabulary will require special considerations.

## Highlights

- The volume of science is rapidly growing, challenging learning within the community
- Machine learning can automate the process of extracting data from human language
- We applied five natural language processing models to over 4,300 scientific abstracts
- The approach captured broad trends and known lessons, offering future promise

Check for updates

Van Houtan et al., 2020, Patterns *1*, 100005
April 10, 2020 © 2020 The Author(s).
https://doi.org/10.1016/j.patter.2020.100005

CellPress

Patterns

CellPress
OPEN ACCESS

Article

# Sentiment Analysis of Conservation Studies Captures Successes of Species Reintroductions

Kyle S. Van Houtan,[1,2,5,*] Tyler Gagne,[1] Clinton N. Jenkins,[3] and Lucas Joppa[4]
[1]Monterey Bay Aquarium, 886 Cannery Row, Monterey, CA 93940, USA
[2]Nicholas School of the Environment, Duke University, Box 90328, Durham, NC 27708, USA
[3]IPÊ - Instituto de Pesquisas Ecológicas, Rod. Dom Pedro I, km 47, Caixa Postal 47, Nazaré Paulista, São Paulo 12960-000, Brazil
[4]Microsoft, One Microsoft Way, Redmond, WA 98075, USA
[5]Lead Contact
*Correspondence: kvanhoutan@mbayaq.org
https://doi.org/10.1016/j.patter.2020.100005

**THE BIGGER PICTURE** The volume of peer-reviewed published science is increasingly growing, presenting new opportunities for growth in research on research itself, also known as meta-analysis. Such research operates by (1) acquiring a body of scientific texts from public archives, (2) extracting the desired information from the texts, and (3) performing analyses on the extracted data. While such analyses hold great value, they may require substantial resources and manual effort throughout the project pipeline. Here, we detail how much of the process of scientific meta-analysis may be automated using a type of machine learning known as natural language processing (NLP). We apply this technique to a specific problem in environmental conservation, show how off-the-shelf NLP models perform, and offer recommendations for future improvements to the process. Such investments may be critical for advances from research, perhaps especially to ensure that scientific productivity meets practical progress.

1 2 **3** 4 5 **Development/Pre-production:** Data science output has been rolled out/validated across multiple domains/problems

## SUMMARY

Learning from the rapidly growing body of scientific articles is constrained by human bandwidth. Existing methods in machine learning have been developed to extract knowledge from human language and may automate this process. Here, we apply sentiment analysis, a type of natural language processing, to facilitate a literature review in reintroduction biology. We analyzed 1,030,558 words from 4,313 scientific abstracts published over four decades using four previously trained lexicon-based models and one recursive neural tensor network model. We find frequently used terms share both a general and a domain-specific value, with either positive (success, protect, growth) or negative (threaten, loss, risk) sentiment. Sentiment trends suggest that reintroduction studies have become less variable and increasingly successful over time and seem to capture known successes and challenges for conservation biology. This approach offers promise for rapidly extracting explicit and latent information from a large corpus of scientific texts.

## INTRODUCTION

The sheer volume of scientific literature challenges the goal of capturing knowledge from the published body of peer-reviewed science. A recent review[1] of species reintroductions, for example, manually extracted information from 361 published articles. While this was admittedly a small fraction of the total literature on the topic, it still required months of effort from highly trained experts just to obtain the raw data, which they then had to analyze. However, because population reintroductions are an effective means to accomplish an elusive task—to recover species and restore ecosystems[2–4]—understanding what determines their success or failure is considered broadly important (S.L. Becker, T.E. Nicholson, K.A. Mayer, M.J. Murray, and K.S.V.H., unpublished data).[5] Therefore, such narrated lessons from established evidence are critical for conservation practices and management decisions.[1,6] How can we lower the barrier to learning them?

Natural language processing (NLP) is a branch of artificial intelligence, or machine learning, which analyzes strings of human language to extract usable information. One goal of NLP is to automate the processing of large volumes of text with minimal human supervision,[7,8] yet crucially in a manner that approximates the performance of a human reader. As applied here, sentiment analysis (SA) parses different affective states of sentiment to capture either single or combined emotions, attitudes, or traits.[9,10] While an array of methods exists, the basic principle of


This is an open access article under the CC BY license (http://creativecommons.org/licenses/by/4.0/).

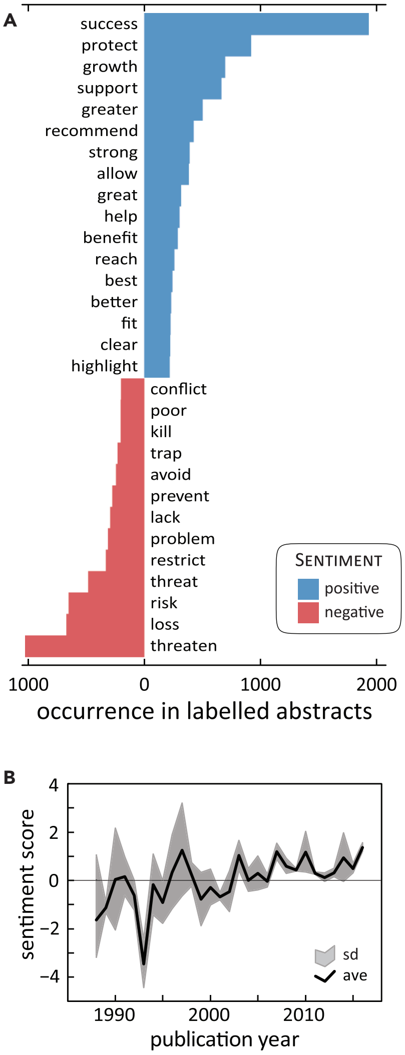


**Figure 1. The Estimated Sentiment of Reintroduction Abstracts Is Associated with Meaningful Terms**

(A) The most frequent terms occurring in 4,313 labeled scientific abstracts, associated with either positive (blue) or negative (red) sentiment as determined from the AFINN lexicon. "Success" drives positive sentiment, and words challenging reintroduction successes (e.g., "threaten," "loss," and "risk," among others) occur with negative sentiment.

(B) Ensemble (black line) of five independent models, pooled annually, of annual sentiment shows a robust increase in sentiment with an accompanying decrease in uncertainty (gray confidence interval) over three decades. This indicates that words and sentences associated with positive sentiment are increasing over time, which, given (A), suggests that reintroduction science is achieving positive results and becoming more successful over the last 30 years. ave, ensemble model average; sd, standard deviation.

SA is to use a trained set of text that has previously been attributed with a sentiment score to define the sentiment for a separate body of unlabeled text. Although SA has been developed to describe more complex and sophisticated human emotions (e.g., empathy, greed, trust, and fear), such results are, perhaps expectedly, variable.[11] By comparison, a more simplistic score of negative to positive sentiment, in the form of a weighted polarity, is far more robust in capturing basic attitudes across various types of texts and fields of study.[11,12] Aside from labeling raw texts, the lexicon-based and machine learning-based SA models have additional value as they are resilient to various structures of text strings (e.g., letter case, punctuation, and stop words) and require little to no text preprocessing. Although NLP is a dynamic and rapidly advancing research area with extensive scientific and commercial applications, and there are more sophisticated approaches to NLP than SA,[13–17] the SA approach we deploy here can produce straightforward and robust results with broad and intuitive interpretative value.

In this study, we explore the use of supervised SA to facilitate a new meta-analysis of the species reintroduction literature with a goal of understanding effectiveness and identifying what determines success. We query public databases to build a robust corpus, numbering in the thousands of scientific abstracts and use existing or "off-the-shelf" lexicons and NLP models to identify the terms that drive sentiment and the domain concepts associated with success. This basic yet novel application shows the potential to enable a more rapid understanding of the growing volume of scientific literature. Such research on research, or meta-analyses, can produce results important for research journal practices[18] as well as the topical domain itself.[19]

## RESULTS AND DISCUSSION

Since we are applying language models created for general use to extract information specifically from scientific abstracts, it is important to evaluate how the terms contained in our abstracts correlate with sentiment scores. Figure 1A shows the contribution of frequent terms from our reintroduction abstracts to sentiment scores using the AFINN lexicon.[20] The most common terms associated with positive sentiment are success, protect, growth, support, help, benefit, and others. The most negative influences are from threaten, loss, risk, threat, problem, and kill. As the term success drives sentiment more than any other term (either positively or negatively), and as this list seems to capture terms that genuinely reflect how authors communicate successes and failures of population metrics as well as reintroduction programs,[2] the abstract-level sentiment score serves as a rational proxy to capture the lessons learned from the studies. Figures S5 and S6 provide both sentence- and abstract-level sentiment scores from a range of abstracts highlighting a range of polarity values across the collected corpus.

Figure 1B summarizes the annual multi-model ensemble of sentiment of reintroduction abstracts over three decades. Though there is some variation between the five models (see Results and Discussion in Figure S4), the models converge on a general trend of decreasing variation and increasingly positive sentiment through time. To this point, uniquely over the study period, the ensemble, including the confidence interval, is positive from 2007 to 2016, encapsulating the final 10 years of the study. Taken with Figure 1A, this suggests that reintroduction studies have become more positively framed, having emerged from an earlier period of negative sentiment and significant challenges when the methods and the discipline itself were just beginning.

Our results seem to capture the terms and broad trends of successful reintroductions;[1,3,21] whether it captures the success associated with specific settings, however, is of interest. Figure 2A shows the frequency and sentiment trends of ten issues

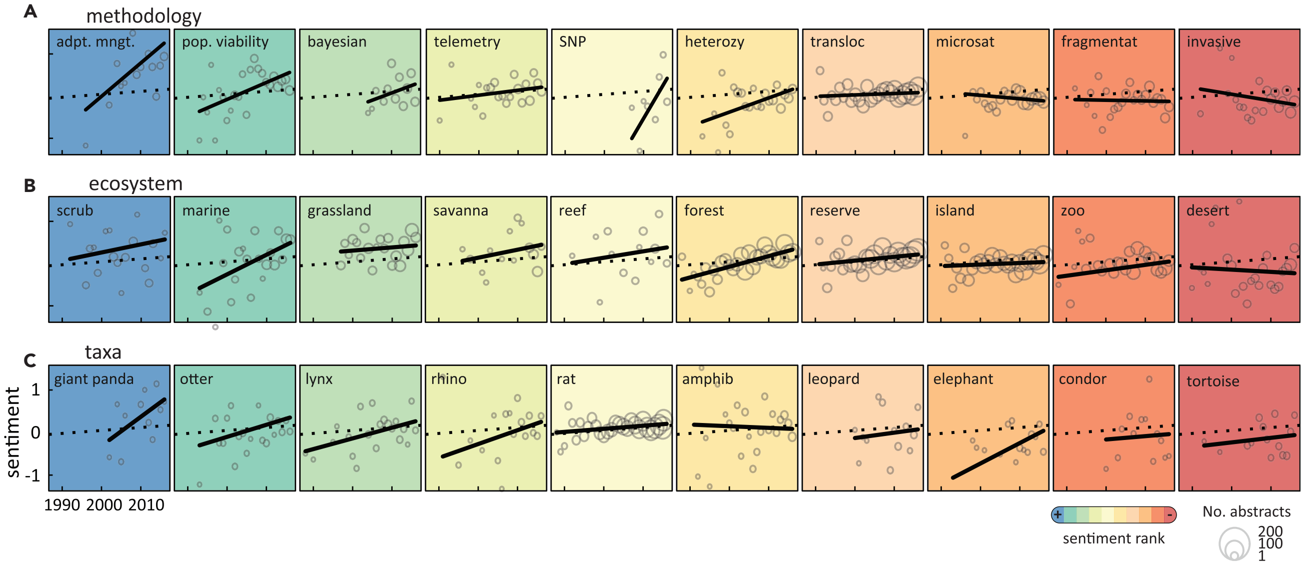


**Figure 2. Sentiment Analysis Captures the Known Successes and Failures of Key Reintroduction Factors**

Sentiment of scientific abstracts associated with reintroduction (A) methods, (B) ecosystems, and (C) taxonomic groups. Panels describe a linear model (black line) of sentiment from the annual sample of abstracts (open circles) for each extracted term. Panels are color coded and ranked left to right by the final model value. The linear model (dotted line) for all abstracts throughout the study is a baseline reference in all panels. Studies using adaptive management or population viability analyses, studies set in scrub, marine, and grassland ecosystems, and studies reintroducing giant panda, otters, and lynx have increasingly positive sentiment. By contrast, our models suggest that reintroduction studies dealing with habitat fragmentation and invasive species, set in islands or deserts, or reintroducing condor or tortoises seem to have persistent challenges.

in reintroductions, ranked by their final modeled sentiment score. Abstracts containing terms that reference methods known to be effective, such as adaptive management and population viability analysis,[2,3,22] have increasing sentiment throughout the study period. More recently deployed yet successful methods, such as Bayesian statistics and SNPs (single-nucleotide polymorphisms), also unsurprisingly demonstrate positive sentiment.[23,24] Conversely, reintroduction studies dealing with fragmented populations[25,26] and invasive species[2,27,28] have negative sentiment, indicating that barriers to success are likely impactful.

Figures 2B and 2C repeat this approach but for ecosystem and taxa contexts. Although many studies address rat depredation and island environments, neither set shows a clear signal of success, likely owing to the many conservation challenges associated with both.[2,29] Likewise, the management of small populations (such as with condors and tortoises) are challenging[30–32] while other heavily studied species with improving conservation status (such as giant pandas and river otters)[29,33,34] have increasing sentiment. Although there is variability between years, reintroduction studies in scrub, grassland, and savanna habitat, in the ocean and in coral reefs, and in forests reflect a positive sentiment that is either stable or increasing through the study. By contrast, studies involving zoo collections have persistently negative sentiment. This result may simply reflect that zoo-based captive breeding programs are often supporting species that are critically endangered in the wild[2,35,36] and face extreme challenges. Lastly, commonly applied techniques such as telemetry[37,38] and translocation may not depart from the overall trend simply because they are so widespread, and thus their trend dominates the overall corpus.

## Conclusions

While text-mining algorithms are broadly applied in a range of powerful applications,[7,8] they have yet to find a regular use in conservation science. Here, we developed a novel use of NLP by using SA to review and extract knowledge from a large body of scientific literature on population reintroductions. Our approach provided several lessons and recommendations. To begin, although the underlying models are trained on words and sentences from a general domain,[20,39,40] our application here captures the specific terms (Figure 1A) as well as the broad success and failures associated with specific management settings (Figure 2) for conservation biology more generally and reintroductions more specifically.

Next, this application is a modest proof of concept toward automating meta-analyses of the scientific literature. Future analyses will achieve greater success by using lexicons and other NLP models that are trained for the specific domain, that compare word- versus sentence-level methods of scoring, and when possible are benchmarked and validated against specific empirical metrics of interest (see Supplemental Information). Such investments may be particularly important for recursive neural tensor network (RNN) models that incorporate syntax on top of word significance, as syntax (more so than individual words) may transfer less easily from a general to an applied domain (see Figure S4). Therefore, while the significance of individual words (see Figure 1A) may retain significance across applications and word-based lexicons may find transdisciplinary applications apt, RNN models may more require domain-based treatments.

These improvements need to be weighed against the logistical cost of model training and validation, but the proposed value of automation and generation of latent information and novel

syntheses[41] is well known from other scientific disciplines. For reintroduction biology, for example, such approaches may pinpoint whether polarity from SA models correlates with quantitative data on reintroduction successes reported within the studies themselves, or more reflects the overall conservation status of the population in question. However, popular metrics within information theory, such as TF*IDF scores,[42] though useful in locating clusters of uncommon terms and key phrases, may have limitations for the purposes of training domain-specific lexicons for SA (see Figure S3).

Lastly, aside from innovating the analytical methods, greater access and data sharing from publishing groups and popular indexing services[43] will provide significant advances. Here, we manually accessed and extracted abstracts from a single repository of published literature. Although this presented a substantial body of information, future analyses will be improved by the development of public application programming interfaces and automated and open access to entire journal articles, not simply the condensed abstracts. Such advances will facilitate the intended greater good of science[1,6] by making more scientific articles, and more of each article, freely available to the public, which may advance future analyses through facilitating increased meta-analyses.

## EXPERIMENTAL PROCEDURES

We developed our corpus by searching the Web of Science indexing service, a representative database[43] of the peer-reviewed scientific literature. We used the Boolean operators AND, OR to retrieve studies containing all of the terms species, conservation, population, and either reintroduction or translocation in their abstracts. As translocation also indicates chromosomal transfer, we used the NOT operator to remove studies with the terms protein, yeast, *Arabidopsis*, *Drosophila*, *Saccharomyces*, and *Escherichia* most associated with the non-target term use and thus generating false positives. We removed studies lacking abstracts, and excluded studies without English abstracts.[44] This resulted in a corpus of 4,313 studies (Figure S1) published from 1987 to 2016 with searchable abstracts containing 1,030,558 words.

From this body of text, we estimated abstract-level sentiment with an ensemble model. We built sentiment scores from four lexicon-based models (AFINN, Bing, NRC, and Syuzhet) and one trained RNN model, the Stanford coreNLP.[45] The lexicons classify sentiment in text strings from the accumulation of sentiment scores of individual words, which each lexicon labels via some form of crowdsourcing. The RNN model is also derived from labeled training text (in this case over 10,000 scored film reviews) and models sentiment not of each individual word but rather by considering the syntax at the sentence level. We used the R packages Syuzhet, coreNLP, and NLP to generate a polar score (negative to positive) of mean sentiment for each abstract for each model. From these single polarity scores of each model for each abstract, we derived a multi-model ensemble average. We employ this approach, as NLP[46] and climate studies[47,48] show that such ensembles are reliable and perform better than individual models alone. We, however, also calculate the standard deviation between model outputs to inform on the uncertainty of the ensemble. Published studies[9,20,39,40,45] provide more information on the individual lexicons, classification methods, crowdsourcing, and R packages. An additional Supplemental Information file with annotated code (R markdown at https://osf.io/f4dc7/) also provides further details.

Now possessing a corpus of domain-specific abstracts with modeled sentiment, we performed a series of analyses to explore the patterns associated with positive and negative sentiment. We first plot the broad sentiment trends over time and then extract the terms most associated with sentiment polarity. This is a key step, as we are transferring lexicons trained from a more general domain in order to understand a particular domain—this scientific corpus. As a result, combining term frequency with sentiment polarity will reveal sentiment drivers relative to the previously unlabeled corpus, and perhaps the new domain more broadly. To understand the various factors broadly associated with successes and failures of reintroduction programs, our core aim in this study, we curated a list of methods, ecosystems, and taxonomic groupings. We extracted the abstracts containing these terms, averaged their sentiment scores in each calendar year, plotted the results across the duration of the study, and fitted simple trend models to the series. All analyses and visualizations were generated in the R environment.[49]

## ETHICS STATEMENT

Our study did not require human or animal subjects.

## DATA AND CODE AVAILABILITY

All the raw data used in this project are available at the Web of Science in a third-party project repository (https://osf.io/f4dc7/), at Github (https://bit.ly/2S6dahD), or included in Supplemental Information.

## SUPPLEMENTAL INFORMATION

Supplemental Information can be found online at https://doi.org/10.1016/j.patter.2020.100005.

## ACKNOWLEDGMENTS

J. Alt, S. Pimm, M. Cunha, J. Moxley, and two anonymous reviewers made improvements to earlier versions of the manuscript. This study was made possible from generous contributions to the Monterey Bay Aquarium.

## AUTHOR CONTRIBUTIONS

All authors conceived of the study and reviewed the manuscript. K.V. gathered the data, T.G. developed and ran the models, and both generated the figures. K.V. wrote the manuscript with contributions from T.G.

## DECLARATION OF INTERESTS

The authors declare no competing interests.

Received: September 21, 2019
Revised: December 16, 2019
Accepted: January 14, 2020
Published: March 19, 2020

## REFERENCES

1. Taylor, G., Canessa, S., Clarke, R.H., Ingwersen, D., Armstrong, D.P., Seddon, P.J., and Ewen, J.G. (2017). Is reintroduction biology an effective applied science? Trends Ecol. Evol. *32*, 873–880.
2. Groom, M.J., Meffe, G.K., and Carroll, R. (2006). Principles of Conservation Biology (Sinauer Associates).
3. Sarrazin, F., and Barbault, R. (1996). Reintroduction: challenges and lessons for basic ecology. Trends Ecol. Evol. *11*, 474–478.
4. Mayer, K.A., Tinker, M.T., Nicholson, T.E., and Murray, M.J. (2019). Surrogate rearing a keystone species to enhance population and ecosystem restoration. Oryx *53*, https://doi.org/10.1017/S0030605319000346.
5. Van Houtan, K.S., Halley, J.M., van Aarde, R.J., and Pimm, S.L. (2009). Achieving success with small, translocated mammal populations. Conserv Lett. *2*, 254–262.
6. Sutherland, W.J., Pullin, A.S., Dolman, P.M., and Knight, T.M. (2004). The need for evidence-based conservation. Trends Ecol. Evol. *19*, 305–308.
7. Aggarwal, C.C., and Zhai, C. (2012). Mining Text Data (Springer Science & Business Media).
8. Feldman, R., and Sanger, J. (2007). The Text Mining Handbook: Advanced Approaches in Analyzing Unstructured Data (Cambridge University Press).

CellPress
OPEN ACCESS

9. Liu, B. (2012). Sentiment analysis and opinion mining. Synth. Lectures Hum. Lang. Technol. *5*, 1–167.

10. Pang, B., and Lee, L. (2008). Opinion mining and sentiment analysis. Found. Trends Inf. Retriev. *2*, 1–135.

11. O'Connor, B., Balasubramanyan, R., Routledge, B.R. and Smith, N.A. (2010). From tweets to polls: Linking text sentiment to public opinion time series. In Proceedings of the Fourth International AAAI Conference on Weblogs and Social Media (AAAI Press), pp. 122–129.

12. Nasukawa, T. and Yi, J. (2003). Sentiment analysis: Capturing favorability using natural language processing. In Proceedings of the 2nd International Conference on Knowledge Capture (Association for Computing Machinery), pp. 70–77.

13. Chopra, S., Auli, M. and Rush, A.M. (2016). Abstractive Sentence Summarization with Attentive Recurrent Neural Networks. In Proceedings of the 2016 Conference of the North American Chapter of the Association for Computational Linguistics: Human Language Technologies, K. Knight, A. Nenkova, and O. Rambow, eds. (Association for Computational Linguistics), pp. 93–98.

14. Zhao, W., Peng, H., Eger, S., Cambria, E. and Yang, M. (2019). Towards scalable and reliable capsule networks for challenging NLP applications. In Proceedings of the 57th Annual Meeting of the Association for Computational Linguistics, A. Korhonen, D. Traum, and L. Màrquez, eds. (Association for Computational Linguistics), pp. 1549–1559. https://doi.org/10.18653/v1/P19-1150.

15. Afzal, N., Mallipeddi, V.P., Sohn, S., Liu, H., Chaudhry, R., Scott, C.G., Kullo, I.J., and Arruda-Olson, A.M. (2018). Natural language processing of clinical notes for identification of critical limb ischemia. Int. J. Med. Inform. *111*, 83–89.

16. Deng, L., and Liu, Y. (2018). Deep Learning in Natural Language Processing (Springer).

17. Gardner, M. ., Grus, J., Neumann, M., Tafjord, O., Dasigi, P., Liu, N.F., Peters, M., Schmitz, M., and Zettlemoyer, L. (2018). AllenNLP: a deep semantic natural language processing platform. In Proceedings of Workshop for NLP Open Source Software, E.L. Park, M. Hagiwara, D. Milajevs, and L. Tan, eds. (Association for Computational Linguistics), pp. 1–6. https://doi.org/10.18653/v1/W18-2501.

18. Couzin-Frankel, J., and Marcos, E. (2018). Journals under the microscope. Science *361*, 1180–1183.

19. de Vrieze, J. (2018). The metawars. Science *361*, 1185–1188.

20. Nielsen, F.Å. (2011). A new ANEW: evaluation of a word list for sentiment analysis in microblogs. Proceedings of the ESWC2011 Workshop on 'Making Sense of Microposts': Big Things Come in Small Packages, M. Rowe, M. Stankovic, A.-S. Dadzie, and M. Hardey, eds., pp. 93–98.

21. Seddon, P.J., Armstrong, D.P., and Maloney, R.F. (2007). Developing the science of reintroduction biology. Conserv. Biol. *21*, 303–312.

22. Brook, B.W., O'Grady, J.J., Chapman, A.P., Burgman, M.A., Akçakaya, H.R., and Frankham, R. (2000). Predictive accuracy of population viability analysis in conservation biology. Nature *404*, 385–387.

23. Clark, J.S. (2005). Why environmental scientists are becoming Bayesians. Ecol. Lett. *8*, 2–14.

24. Morin, P.A., Luikart, G., and Wayne, R.K. (2004). SNPs in ecology, evolution and conservation. Trends Ecol. Evol. *19*, 208–216.

25. Laurance, W.F., Camargo, J.L.C., Luizão, R.C.C., Laurance, S.G., Pimm, S.L., Bruna, E.M., Stouffer, P.C., Williamson, G.B., Benítez-Malvido, J., Vasconcelos, H.L., et al. (2011). The fate of Amazonian forest fragments: a 32-year investigation. Biol. Conserv. *144*, 56–67.

26. Fahrig, L., and Merriam, G. (1994). Conservation of fragmented populations. Conserv. Biol. *8*, 50–59.

27. Van Houtan, K.S., Smith, C.M., Dailer, M.L., and Kawachi, M. (2014). Eutrophication and the dietary promotion of sea turtle tumors. PeerJ *2*, e602.

28. Clavero, M., and García-Berthou, E. (2005). Invasive species are a leading cause of animal extinctions. Trends Ecol. Evol. *20*, 110.

29. Seddon, P.J., Griffiths, C.J., Soorae, P.S., and Armstrong, D.P. (2014). Reversing defaunation: restoring species in a changing world. Science *345*, 406–412.

30. Pimm, S.L. (1991). The Balance of Nature? (University of Chicago).

31. Pimm, S.L., Jones, H.L., and Diamond, J. (1988). On the risk of extinction. Am. Nat. *132*, 757–785.

32. Rideout, B.A., Stalis, I., Papendick, R., Pessier, A., Puschner, B., Finkelstein, M.E., Smith, D.R., Johnson, M., Mace, M., Stroud, R., et al. (2012). Patterns of mortality in free-ranging California Condors (*Gymnogyps californianus*). J. Wildl. Dis. *48*, 95–112.

33. Morell, V. (2008). Into the wild: reintroduced animals face daunting odds. Science *320*, 742–743.

34. Swaisgood, R.R., Wang, D., and Wei, F. (2018). Panda downlisted but not out of the woods. Conserv. Lett. *11*, e12355.

35. Beck, B.B., Rapaport, L.G., Price, M.S., and Wilson, A.C. (1994). Reintroduction of Captive-Born Animals. In Creative Conservation, P.J.S. Olney, G.M. Mace, and A.T.C. Feistner, eds. (Springer), pp. 265–286.

36. Snyder, N.F.R., Derrickson, S.R., Beissinger, S.R., Wiley, J.W., Smith, T.B., Toone, W., and Miller, B. (1996). Limitations of captive breeding in endangered species recovery. Conserv. Biol. *10*, 338–348.

37. Bograd, S.J., Block, B.A., Costa, D.P., and Godley, B.J. (2010). Biologging technologies: new tools for conservation. Introduction. Endang. Species Res. *10*, 1–7.

38. Cooke, S.J. (2008). Biotelemetry and biologging in endangered species research and animal conservation: relevance to regional, national, and IUCN Red List threat assessments. Endang. Species Res. *4*, 165–185.

39. Jockers, M.L. (2015). Syuzhet: extract sentiment and plot arcs from text. https://rdrr.io/cran/syuzhet/.

40. Mohammad, S.M., Kiritchenko, S. and Zhu, X. (2013). NRC-Canada: building the state-of-the-art in sentiment analysis of tweets. In Second Joint Conference on Lexical and Computational Semantics (*SEM), Volume 2: Seventh International Workshop on Semantic Evaluation (SemEval 2013), S. Manandhar and D. Yuret, eds. (Association for Computational Linguistics), pp. 321–327.

41. Tshitoyan, V., Dagdelen, J., Weston, L., Dunn, A., Rong, Z., Kononova, O., Persson, K.A., Ceder, G., and Jain, A. (2019). Unsupervised word embeddings capture latent knowledge from materials science literature. Nature *571*, 95–98.

42. Zhang, W., Yoshida, T., and Tang, X. (2011). A comparative study of TF*IDF, LSI and multi-words for text classification. Expert Syst. Appl. *38*, 2758–2765.

43. Falagas, M.E., Pitsouni, E.I., Malietzis, G.A., and Pappas, G. (2008). Comparison of PubMed, Scopus, Web of Science, and Google Scholar: strengths and weaknesses. FASEB J. *22*, 338–342.

44. Feinerer, I., et al. (2013). The textcat package for n-gram based text categorization in R. J. Stat. Softw. *52*, 1–17.

45. Socher, R., Perelygin A., Wu J.Y., Chuang J., Manning C.D., Ng A.Y., Potts C. (2013). Recursive Deep Models for Semantic Compositionality Over a Sentiment Treebank. In Proceedings of the 2013 Conference on Empirical Methods in Natural Language Processing, D. Yarowsky, T. Baldwin, A. Korhonen, K. Livescu, and S. Bethard, eds. (Association for Computational Linguistics), pp. 1631–1642.

46. Xia, R., Zong, C., and Li, S. (2011). Ensemble of feature sets and classification algorithms for sentiment classification. Info Sci. *181*, 1138–1152.

47. Giorgi, F., and Mearns, L.O. (2002). Calculation of average, uncertainty range, and reliability of regional climate changes from AOGCM simulations via the "reliability ensemble averaging"(REA) method. J. Clim. *15*, 1141–1158.

48. Tebaldi, C., and Knutti, R. (2007). The use of the multi-model ensemble in probabilistic climate projections. Philos. Trans. R. Soc. A Math. Phys. Eng. Sci. *365*, 2053–2075.

49. R Core Team. (2018). R: A Language and Environment for Statistical Computing (R Foundation for Statistical Computing).

**PATTER, Volume *1***

## Supplemental Information

# Sentiment Analysis of Conservation Studies Captures Successes of Species Reintroductions

**Kyle S. Van Houtan, Tyler Gagne, Clinton N. Jenkins, and Lucas Joppa**

**SUPPLEMENTAL INFORMATION FOR**

**"SENTIMENT ANALYSIS OF CONSERVATION LITERATURE CAPTURES SPECIES REINTRODUCTION SUCCESSES"**

**BY KYLE S. VAN HOUTAN, TYLER O. GAGNE, CLINTON N. JENKINS, AND LUCAS JOPPA**

Enclosed in the complete supplemental files are a series of figures (Figures S1-S6). The raw data scrape from the Web of Science service (Data S1), the results of each sentiment analysis (SA) and the ensemble score (Data S2), and the polarity breakdown of all abstracts (Data S3) are available as separate files.The figures in this supplement provide additional detail and supporting information to explain or contextualize the methods, results and discussion in the main text file.

Figure S1 for example calculates the total number of abstracts in each annual bin across the study. This helps to explain both the variability of sentiment in the early years, as well as, the popularity of the subject in the literature over the last decade. Figure S2 plots a simple histogram indicating the sentiment of all abstracts in the study lean towards positive sentiment. Figure S3 provides some transparency on a common metric called TF*IDF used in NLP. To emphasize infrequent terms over time, we broke this down in the multi-year bins. Though we do not employ this local density method in our study (preferring the more straightforward term frequency in Figure 1a) we report on it here as experts in the field may have interest. Figure S4 is a composite plot for each model of sentiment time series that highlights the trends over time and the differences between word- and sentence-level models. The word-based lexicons largely agree with one another, while the RNN (sentence-level) model generates a different set of results. As we average and report the results all of the models, the aim of our study is to provide the broad patterns that emerge from the multi-model ensemble.

Figures S5-S6 are a subset of ten abstracts tagged with either positive (S5) or negative sentiment (S6) determined by the Syuzhet lexicon. A complete graphic containing all 4,313 abstracts is too large to display here (1,771 pages) and is available as a separate downloadable and searchable file (Data S3). Though the Syuzhet lexicon scores individual words, both figures visually highlight the sentence-level average score as either positive (light green) or negative (pink) and provide the abstract's total sentiment score. Sentences in these abstracts with neither a significant positive or negative sentiment are not highlighted and have no coloration. It is important to note in our analyses that the SA models do not necessarily model the success of a reintroduction program, as the abstracts themselves do not explicitly model the success of a reintroduction program. As mentioned in the main text, our analysis therefore is more about positive or negative sentiment in published abstracts that nominally mention and therefore are associated with reintroduction programs.

Figure S5ba (sentiment = +0.9) is a case study that examines the habitat available and site suitability for a giant panda reserve in Huayingshan, China. The abstract reports sufficient food resources and community support for the project and suggest post release monitoring is feasible, however, several factors bound the authors' optimism. Figure S5b (sentiment = +1.1) narrates the ultimately successful history of golden lion tamarin reintroductions in the coastal Atlantic Forest in Brazil. Though the wild population of tamarins and their habitat reached historic bottlenecks in the 1970s, coordinated efforts to protect habitat and repurpose zoo collections for captive breeding demonstrate the value of public campaigns for conservation. Figure S5c (sentiment = +1.1) profiles the availability and uncertainty surrounding habitat for lynx reintroductions in Europe. The study provides an iterative plan for this process, even in the face of limited information, and concludes that habitat connectivity must be considered to identify release locations. Figure S5d (sentiment = +1.4) uses population viability analyses, or PVA, to evaluate reestablishing fisher populations in the western USA. The models trained with empirical data provide six practical recommendation for managers seeking to

successfully reintroduce carnivore populations. This abstract overall has a positive sentiment, though certain sentence scores are negative due to the sentiment of words therein (e.g., *endangered*, *threatened*, *failure*). Figure S5e is the highest scoring abstract in our complete set (sentiment = +2.6) due at least in part to the consistent use of the term *excellence* throughout. The study is conceptual and recommends a modification of evidence based conservation approaches so that they more closely follow established practices in the business settings to ensure practical program success.

Switching to examples of negative sentiment, figure S6a (sentiment = -1.6) develops a screening tool for a highly infectious viral disease in parrots and uses this to detect the disease in a translocated parakeet population in New Zealand. The abstract is almost entirely negatively coded, as it describes the various pathologies of the disease and its population impacts. Figure S6b (sentiment = -0.9) is a review of the impacts of alien introductions into wild amphibian populations and argues this underscores the need for more stringent genetics considerations in species translocation programs. Figure S6c (sentiment = -0.7) profiles conflicts between human and saltwater crocodile populations in Sri Lanka. The survey includes crocodile attacks to humans and vice versa quantifies the various threats humans pose to wild saltwater crocodile populations. The authors propose a monitoring scheme to keep track of the multiple and seemingly entrenched struggles. Figure S6d (sentiment = -0.4) discusses the low genetic diversity of Mojave desert tortoises and how management practices may have chronically and inadvertently exacerbated the problem. Figure S6e (sentiment = -0.3) is a review of two decades of mortality cases of reintroduced California condors. The abstract overwhelmingly identifies human factors as the leading causes of death, and then argues that these are imminently manageable and must be corrected to attain self-sustaining condor populations in the wild.

These abstracts represent some transparency into how the SA models encode sentiment to scientific texts, and the full diagram of all such polarity plots is available in the attached file (polarity_of_abstracts.pdf). The abstracts highlighted are chosen to represent a variety of sentiment scores as well as to capture some of the well-known species and issues that are in part represented in Figure 2. The abstracts in Figure S5-6 highlight some of the strengths as well as growth areas of SA as applied here. These are primarily rooted in that specific words used in the abstracts may generate sentiment scores independent of their contextual use or their correlation to objective quantitative metrics in the studies. The negatively-encoded sentences in Figure S5d, for example, likely happened due to the usage of *endangered*, *threatened*, *failure*, and *contracted* even though those sentences could be read either as having neutral or slightly positive sentiment.

This highlights the possibility that at the word, sentence, or even abstract level there may be disagreement between the SA model outputs and trained experts in the field. The point of such automated analyses is that such discrepancies are not a factor at the aggregate level, when large volumes of words and sentences are analyzed. Here we analyzed 4,313 abstracts comprising 1,030,558 words. At a reasonable pace of scientific reading – 100 words per minute – this would take 172 hours (over four, 40-hour work weeks) just to read the entire corpus. That time is just for the reading of the texts themselves without any additional manual encoding, attribution or subsequent thought or analysis on the texts. Here we ran five separate models, analysing all the words and abstracts in the corpus, in just a few hours using open access software that ran on an above-average laptop computer.

Manual analysis by trained experts will remain the gold standard for reading and extracting knowledge from scientific articles. However, there remain lessons to be learned from automation, and furthermore, the scale of the published literature suggests that such innovative methods for reviewing the scientific literature are needed. Having used lexicons and models developed for a general domain, at the present stage we are attempting to show the potential for automating and batch-processing the process of meta-analyses of large volumes of scientific texts. Further advances will be made not in

isolation from trained experts in specific topical domains, but with models supervised and trained for the specific questions of interest and importantly curated by experts to perform specific tasks. The role that NLP in general and SA specifically can play will be greatly enhanced by domain-specific lexicons.

**FIGURE CAPTIONS**

**Figure S1. Abstract count over time shows a steady increase in the volume of published studies in the reintroduction literature.** Though our raw Web of Science™ query resulted in 4,759 results, we went through a filtering process which resulted in a cleaned database of 4,313 abstracts published from 1988-2016. As reintroduction becomes a more established technique, and subsequently as scientists published studies on their results, the volume of literature increases annually roughly on a log-linear scale. From 2011 onwards, more than 300 papers satisfying our query are published annually. However, the cumulative sample did not reach a total of 300 abstracts until 1995, and there were fewer than 10 studies each year before 1991. As having a large body of sample texts for interpretation is a key for NLP processes, all sentiment analysis results in early years should be viewed in light of the small sample of scientific literature in this initial period.

**Figure S2. Histogram of pooled abstract sentiment across the entire study.** Ensemble sentiment scores from the multi-model average of all $n$ = 4,313 abstract assessed in this study. The form of the data follow a normal distribution (ave = 0.261, sd = 0.428). This indicates that most abstracts have a positive sentiment, perhaps owing to the general scientific bias to present positive (and not negative) study results. Data are accumulated in 0.1 bins.

**Figure S3. The term frequency - inverse document frequency (TF*IDF) metric across time for the 4,313 scientific abstracts reviewed.** TF*IDF is one widely-used metric in information theory to capture the significance of terms by, specifically elevating rare word occurrences, across the entire body of text being analyzed. Within the time bins here, the application of the metric in our setting seems to capture a variety of scientific (e.g., *nervosa*, *bakeri*, *garderi*) and common species names (e.g., palila, char, Blackburn's) as well as taxonomic groups (e.g., moth, snakes, tigers). Habitat settings are less prominent (e.g., *Kryvyi*, *Yampa*, *Yakima*), though also significant. This method does not capture broad use across all abstracts, but rather local clusters or occasional spikes in usage, sometimes considered key phrases. We do not employ this metric in our analyses here, mainly as it does not capture terms of value that would be relevant for lexicon training or applicable to SA here.

**Figure S4. Calculated sentiment from four lexicons, one RNN model, and their ensemble average.** The sentiment trends of individual abstracts converges among the four lexicon approaches (AFINN, Bing, NRC, Syuzhet) characterized by an initial steep decline from 1987-1990, followed by a subsequent increase until 2016. These lexicon approaches label the sentiment of each word, regardless of how they are arranged in a sentence. The Stanford coreNLP is an RNN that calculates sentiment at the sentence-level, considering syntax in addition to individual word scores. This approach values word context, such that positive words like *funny* and *witty* may together be used in phrase which has negative sentiment – e.g. "This movie was actually neither that funny, nor witty." Unlike the word-based lexicons, this particular RNN approach, scores the abstracts as having negative sentiment throughout the study period, as well as, declining from the outset. This may highlight word usage in a sentence context, and the need for domain specific lexicons and more supervised training particularly for RNN models. The initial high score variability (and later relative low variability) for the ensemble (Figure 1b) is likely a function of the sample size through time (Figure S1), which should be heavily weighed when interpreting the trends. Nonetheless, sentiment over time gradually increases, especially after 2000. The Stanford coreNLP model, suggests a flatter trend from 1990 to 2016.

**Figure S5. Examples from a range of labelled abstracts from the corpus with positive sentiment determined from the Syuzhet lexicon.** A complete file with all scored abstracts is available as a separate file. Here the Syuzhet lexicon scores individual words, and we plot the average word score at the sentence level. Positive sentiment is highlighted in green, negative sentiment is red, neutral sentiment has no color. The abstract number and the abstract-level sentiment are listed in the upper left.

**Figure S6. Examples from a range of labelled abstracts from the corpus with negative sentiment determined from the Syuzhet lexicon.** As stated for in Figure S4, a complete file with all scored abstracts is available as a separate file. Here the Syuzhet lexicon scores individual words, and we plot the average word score at the sentence level. Positive sentiment is highlighted in green, negative sentiment is red, neutral sentiment has no color. The abstract number and the abstract-level sentiment are listed in the upper left.

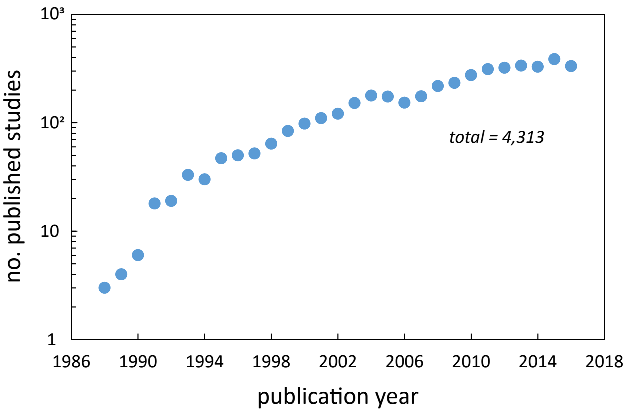

10³
10²
10
1
no. published studies
total = 4,313
1986
1990
1994
1998
2002
2006
2010
2014
2018
publication year

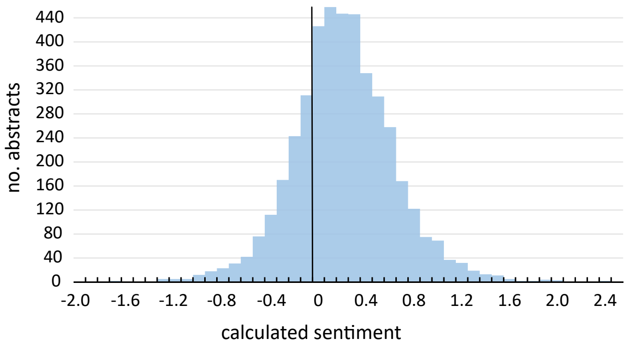

440
400
360
320
280
240
200
160
120
80
40
0
no. abstracts
-2.0
-1.6
-1.2
-0.8
-0.4
0
0.4
0.8
1.2
1.6
2.0
2.4
calculated sentiment

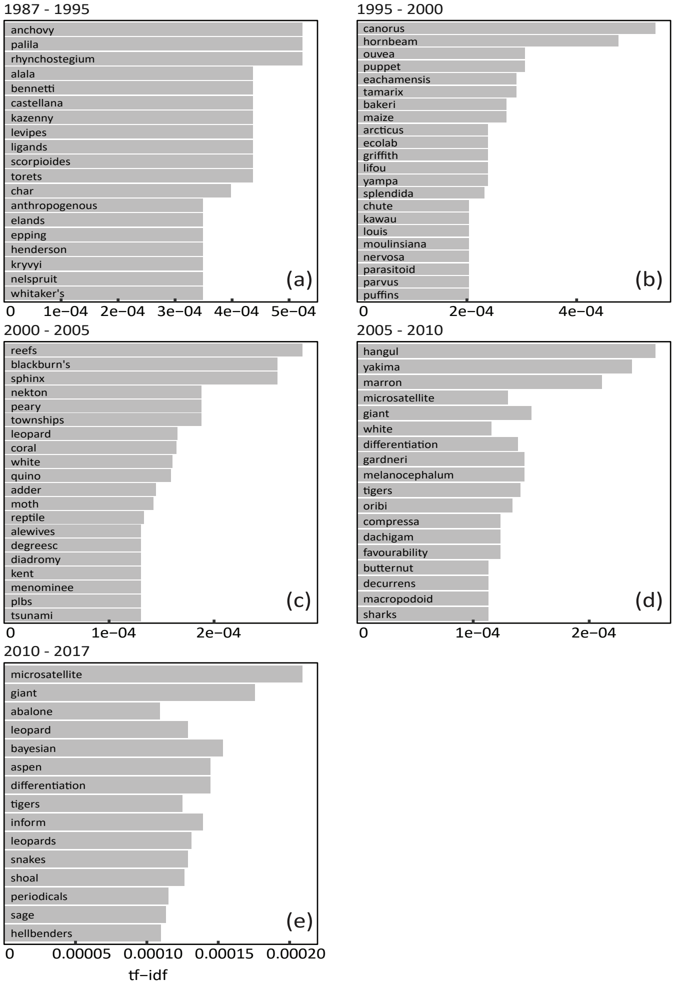

1987 - 1995
anchovy
palila
rhynchostegium
alala
bennetti
castellana
kazenny
levipes
ligands
scorpioides
torets
char
anthropogenous
elands
epping
henderson
kryvyi
nelspruit
whitaker's
(a)
0
1e−04
2e−04
3e−04
4e−04
5e−04
1995 - 2000
canorus
hornbeam
ouvea
puppet
eachamensis
tamarix
bakeri
maize
arcticus
ecolab
griffith
lifou
yampa
splendida
chute
kawau
louis
moulinsiana
nervosa
parasitoid
parvus
puffins
(b)
0
2e−04
4e−04
2000 - 2005
reefs
blackburn's
sphinx
nekton
peary
townships
leopard
coral
white
quino
adder
moth
reptile
alewives
degreesc
diadromy
kent
menominee
plbs
tsunami
(c)
0
1e−04
2e−04
2005 - 2010
hangul
yakima
marron
microsatellite
giant
white
differentiation
gardneri
melanocephalum
tigers
oribi
compressa
dachigam
favourability
butternut
decurrens
macropodoid
sharks
(d)
0
1e−04
2e−04
2010 - 2017
microsatellite
giant
abalone
leopard
bayesian
aspen
differentiation
tigers
inform
leopards
snakes
shoal
periodicals
sage
hellbenders
(e)
0
0.00005
0.00010
0.00015
0.00020
tf−idf

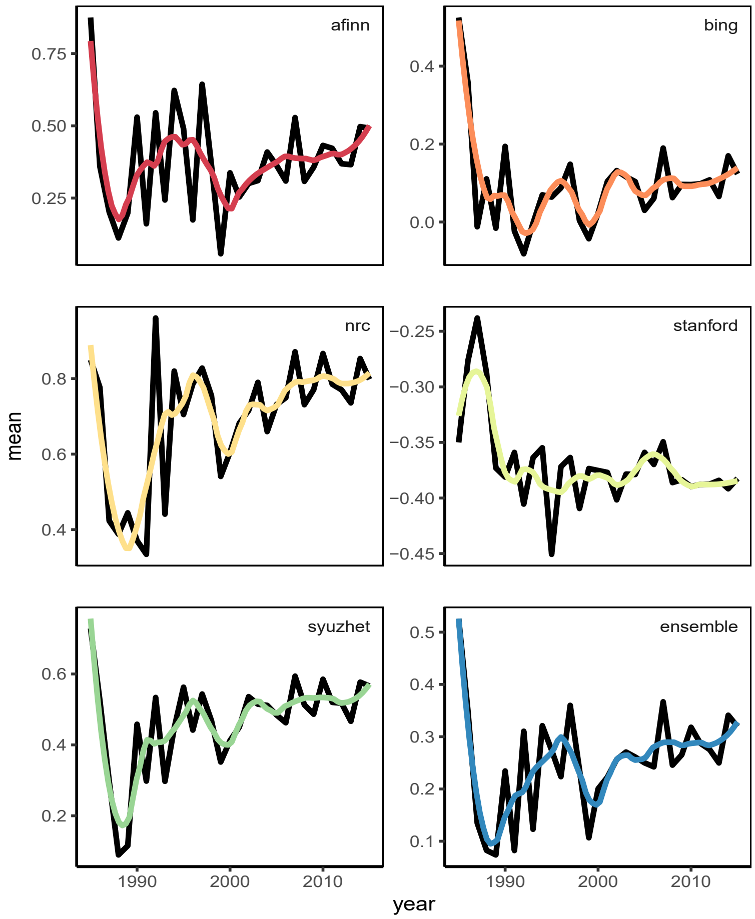
afinn
bing
nrc
stanford
syuzhet
ensemble
mean
year
0.75
0.50
0.25
0.4
0.2
0.0
0.8
0.6
0.4
−0.25
−0.30
−0.35
−0.40
−0.45
0.6
0.4
0.2
0.5
0.4
0.3
0.2
0.1
1990
2000
2010
1990
2000
2010

**A - Abstract #3850, score 0.9**

Ex-situ conservation is one of the most important approaches to save the endangered species. When the population size of captive giant panda increase steadily and can be sustained by themselves, some of them should be released to the wild to rescue small populations and enlarge the distribution areas. Importantly, the first step is to find a suitable habitat for them. Huayingshan is the historical distribution area of giant panda and the fossil of A. melanoleuca baconi was found in 1993. Plentiful food resources (530 kiloton bamboos at present with 99 kiloton net gain per year) for giant panda was observed in this area which are enough for a small population. The government and villagers greatly support the project that sending giant panda back to Huayingshan, and the gentler slope of which will make monitoring work easier to perform. However, the human activity's interference was significant in this area, and some places are far from water resources, the vegetation type is less, and infection rate of the canine parvovirus was high. These questions can be solved after the reserve was built. In another side, the temperature is high in summer, and the qualities of habitats are different from the giant panda's natural habitat, in addition to the undetected impacts on the releasing of giant panda several works (e.g. wireless monitor) should be taken throughout all steps of the reintroduction to promote the giant panda's inhabit in Huayingshan.

**B - Abstract #357, score 1.1**

The lion tamarin was first described in the wild over 475 years ago, and there are records of the species being kept as pets as early as the mid-1750s. With such a long captive history, it is a sad reflection on our knowledge that, as recently as the early 1970s, so little success had been recorded with the maintenance and husbandry of Leontopithecus in captivity and the species seemed certain to disappear from zoos as well as from the wild. After a ban on exportation of the species from Brazil, the captive population of L. rosalia outside of Brazil decreased from a total of 102 in 1968 to 82 in 1976. Zoos rarely bred a second generation, and with no prospects of further imports from the wild, by 1977, the likelihood of the captive population becoming genetically unviable and going extinct was high. Fortunately, thanks to the development of a professionally coordinated and scientifically managed captive population and the research efforts of many individuals and institutions, both in Brazil and elsewhere, the future of the captive population has been ensured. The international efforts to save the golden lion tamarin through the collaborative work of the GLTCP represents a classic example of what can be achieved when various disciplines working for the conservation of a species cooperate and coordinate their activities in pursuit of a common goal. The majority of authorities now recognize that the future of animals in captivity will increasingly rely on national and international cooperation; much of this will depend on the integrity and goodwill of the people concerned. The development of international strategies to safeguard species survival increasingly relies on field workers, conservationists, educationists, academics and zoo professionals cooperating wholeheartedly. As KLEIMAN et al. (1986) have suggested, such cross-institutional collaborative efforts with L. rosalia represent a model for international conservation projects in the future. Since the June, 1990 Leontopithecus Population Viability Analysis (PVA) Workshop held in Belo Horizonte, Minas Gerais, Brazil (IUCN/SSC/CBSG 1991), the four International Management Committees for the Lion Tamarin genus have been recognised by Federal Brazilian law as technical advisors to IBAMA on conservation, research, and management of both the captive and wild populations of Leontopithecus. Nearly 100 per cent of the tamarins that come under the committees' jurisdiction are the property of the Brazilian people through IBAMA; this transfer of title to IBAMA involved a renunciation of ownership of specimens housed at zoos internationally. For L. rosalia, ownership was turned over in 1991. We believe that this may be the first case of the international zoo community returning ownership of legally-held animals to the country of origin while continuing to maintain responsibility for their management. With the reintroduction programme for L. rosalia having resulted in an increase of protected Atlantic coastal forest, in the State of Rio de Janeiro, by about 38 per cent (2,300 ha). And with our growing understanding of the science of conservation today, the significance of the GLTCP's work with this endangered species (RYLANDS) highlights how well the adoption of 'flagship' species and publication of the plight of remnant populations in depleted environments can promote public attention and associated habitat.

**C - Abstract #980, score 1.1**

Conservation biologists often must make management decisions based on little empirical information. In Germany biologists are concerned that the recovery and reintroduction of Eurasian lynx (Lynx lynx) may fail because the remaining suitable habitat may be insufficient to sustain a viable population. However, no comprehensive study ad dressing this concern has been made that not only considers distribution of suitable habitat, but also connectivity to other populations. The aims of this study were (1) to quantify the amount and location of potentially suitable lynx habitat in. Germany, (2) to estimate the connectivity between patches of suitable habitat, and (3) to evaluate lynx conservation programs. Habitat preferences of lynx were described in a rule-based model based on the availability of forest cover (defined by patch size) and the spatial structure of the habitat. Rules were implemented in a geographic information system to predict locations of suitable habitat. Optimal connections among patches were modeled using a cost-path analysis based on. habitat-specific probabilities of lynx crossing patches. Results indicated wide variation in the size of patches of suitable habitat, with 10 areas each sufficiently large to sustain >20 resident lynxes. Overall, a total of 380 lynxes could be sustained by the 10 areas. Uncertainty analyses of model parameters and assumptions revealed little variation in predicted habitat, primarily because results were constrained by the actual distribution of forest habitat. Our. analyses suggest that lynx reintroduction programs should emphasize large, connected areas and consider broad-scale habitat connectivity in the landscape. Our approach also demonstrates how biologically plausible rules can be applied in conservation to identify areas in which success is most likely, even when few empirical data are available.

**D - Abstract #3141, score 1.4**

Translocations are frequently used to restore extirpated carnivore populations. Understanding the factors that influence translocation success is important because carnivore translocations can be time consuming, expensive, and controversial. Using population viability software, we modeled reintroductions of the fisher, a candidate for endangered or threatened status in the Pacific states of the US. Our model predicts that the most important factor influencing successful reestablishment of a fisher population is the number of adult females reintroduced (provided some males are also released). Data from 38 translocations of fishers in North America, including 30 reintroductions, 5 augmentations and 3 introductions, show that the number of females released was, indeed, a good predictor of success but that the number of males released, geographic region and proximity of the source population to the release site were also important predictors. The contradiction between model and data regarding males may relate to the assumption in the model that all males are equally good breeders. We hypothesize that many males may need to be released to insure a sufficient number of good breeders are included, probably large males. Seventy-seven percent of reintroductions with known outcomes (success or failure) succeeded; all 5 augmentations succeeded; but none of the 3 introductions succeeded. Reintroductions were instrumental in reestablishing fisher populations within their historical range and expanding the range from its most-contracted state (43% of the historical range) to its current state (68% of the historical range). To increase the likelihood of translocation success, we recommend that managers: 1) release as many fishers as possible, 2) release more females than males (55-60% females) when possible, 3) release as many adults as possible, especially large males, 4) release fishers from a nearby source population, 5) conduct a formal feasibility assessment, and 6) develop a comprehensive implementation plan that includes an active monitoring program.

**E - Abstract #2658, score 2.6**

The current shortfall in effectiveness within conservation biology is illustrated by increasing interest in "evidence-based conservation," whose proponents have identified the need to benchmark conservation initiatives against actions that lead to proven positive effects. The effectiveness of conservation policies, approaches, and evaluation is under increasing scrutiny, and in these areas models of excellence used in business could prove valuable. Typically, conservation programs require years of effort and involve rigorous long-term implementation processes. Successful balance of long-term efforts alongside the achievement of short-term goals is often compromised by management or budgetary constraints, a situation also common in commercial businesses. "Business excellence" is an approach many companies have used over the past 20 years to ensure continued success. Various business excellence evaluations have been promoted that include concepts that could be adapted and applied in conservation programs. We describe a conservation excellence model that shows how scientific processes and results can be aligned with financial and organizational measures of success. We applied the model to two well-documented species conservation programs. In the first, the Po'ouli program, several aspects of improvement were identified, such as more authority for decision making in the field and better integration of habitat management and population recovery processes. The second example, the black-footed ferret program, could have benefited from leadership effort to reduce bureaucracy and to encourage use of best-practice species recovery approaches. The conservation excellence model enables greater clarity in goal setting, more-effective identification of job roles within programs, better links between technical approaches and measures of biological success, and more-effective use of resources. The model could improve evaluation of a conservation program's effectiveness and may be used to compare different programs, for example during reviews of project performance by sponsoring organizations.

**A - Abstract #2191, score -1.5**

Psittacine beak and feather disease (PBFD) is a highly infectious and potentially fatal viral disease of parrots and their allies caused by the beak and feather disease virus (BFDV). Abnormal feather morphology and loss of feathers are common clinical symptoms of the disease. PBFD also damages the lymphoid tissue and affected birds may die as a result of secondary bacterial or fungal infections. The disease is therefore of concern for conservation biologists and wildlife managers, as it is immunosuppressive and can become an additional threatening factor among critically endangered psittacines. We conducted a PCR-based screening for BFDV in a wild population of the Red-fronted Parakeet (Cyanoramphus novaezelandiae) on Little Barrier Island, New Zealand, during a translocation of this species. Fifty-four parakeets were captured and feather samples collected for molecular screening. We detected BFDV DNA from 15 individuals, but only two showed external signs attributable to PBFD, namely abnormal feather morphology or colouration, loss of feathers and haemorrhagic feathers. Our survey represents the first positive identification of BFDV in wild New Zealand endemic psittacines and confirms the risk of spread of the virus between wild populations within this global hotspot of endemic psittacine diversity.

**B - Abstract #1707, score -0.9**

Although less than other animal groups, amphibians are sometimes concerned by the problems related to the introduction of alien specimens into natural populations. They may be victims of such introductions (especially of amphibians, fishes and other aquatic predators), or cause problems to other species through introduction outside their range. The problems posed by introductions, reintroductions and population reinforcements are discussed in a more general way. Introductions of alien species outside their range (faunistic pollution), or of alien specimens into other populations of the same species or of another interfertile species (genetic pollution), beside creating ecological problems, hinder or impede subsequent study of the history and evolution of these populations. For evolutionary biologists, they amount to a destruction of their object of study. Furthermore, such operations carry an optimistic but misleading message to the public, according to which destructions of the environment caused by human activities would be reversible. It is urgent that the main concepts of genetics and taxonomy be given more weight in decisions regarding reintroductions of animals into threatened populations or habitats.

**C - Abstract #4177, score -0.7**

Human-wildlife conflict occurs when human requirements encroach on those of wildlife populations, with potential costs to both humans and wild animals. As top predators in most inland waters, crocodilians are involved in human-wildlife conflicts in many countries. Here we present findings of a 5-year survey on human-crocodile conflict on the island of Sri Lanka and relate the results to improving management practices. We aimed to quantify and understand the causes of human-crocodile conflict in Sri Lanka, and propose solutions to mitigate it. Visual encounter surveys were carried out to estimate the population size of Saltwater Crocodiles. We recorded 778 sightings of Saltwater Crocodiles at 262 of 400 locations surveyed, and estimate the total population to comprise more than 2000 non-hatchlings and to have increased at an average rate of 5% p.a. since 1978. We propose four crocodile vigilance zones within the wet zone and one crocodile vigilance zone within the dry zone of the country. Specific threats to Saltwater Crocodiles identified in crocodile vigilance zones were: habitat destruction and loss; illegal killing and harvesting (17 killings out of fear, (similar to)200 incidents of killing for meat and skins, (similar to)800 eggs annually for consumption); unplanned translocations; and, interaction with urbanization (10 incidents of crocodiles being run over by trains/vehicles and electrocution). Additionally, 33 cases of crocodile attacks on humans were recorded [8 fatal, 25 nonfatal (minor to grievous injuries)] and more than 50 incidents of attacks on farm and pet animals.

**D - Abstract #3887, score -0.4**

Mojave desert tortoises (*Gopherus agassizii*) have been translocated for decades, and research-oriented translocations recently have been recommended as a tool to help recover this threatened species. However, avoiding negative genetic impacts from wildlife translocations has been widely cautioned. Populations of the Mojave desert tortoise within a 200-276-km straight-line radius of each other (249-308 km measured around topographic barriers) tend to be genetically correlated and may be considered single genetic units for management purposes. When planning translocations among wild populations, releasing tortoises at recipient sites within a straight-line distance of 200 km from the source population would most conservatively maintain historic genetic population structure. However, the risk of causing outbreeding depression by inadvertently translocating Mojave desert tortoises between more distant populations or those of unknown provenance is low.

**E - Abstract #3016, score -0.3**

We document causes of death in free-ranging California Condors (Gymnogyps californianus) from the inception of the reintroduction program in 1992 through December 2009 to identify current and historic mortality factors that might interfere with establishment of self-sustaining populations in the wild. A total of 135 deaths occurred from October 1992 (the first post-release death) through December 2009, from a maximum population-at-risk of 352 birds, for a cumulative crude mortality rate of 38%. A definitive cause of death was determined for 76 of the 98 submitted cases, 70% (53/76) of which were attributed to anthropogenic causes. Trash ingestion was the most important mortality factor in nestlings (proportional mortality rate [PMR] 73%; 8/11.), while lead toxicosis was the most important factor in juveniles (PMR 26%; 13/50) and adults (PM R 67%; 10/15). These results demonstrate that the leading causes of death at all California Condor release sites are anthropogenic. The mortality factors thought to be important in the decline of the historic California Condor population, particularly lead poisoning, remain the most important documented mortality factors today. Without effective mitigation, these factors can be expected to have the same effects on the sustainability of the wild populations as they have in the past.
