# Supplementary material for: Sentiment Analysis of Conservation Studies Captures Successes of Species Reintroductions
Source: Patterns (N Y). 2020 Mar 20;1(1):100005. doi: 10.1016/j.patter.2020.100005 (PMC7660424; doi:10.1016/j.patter.2020.100005)
Supplement: Document S1. Supplemental Experimental Procedures and Figures S1–S6 [file mmc1.pdf]

PATTER, Volume *1*

Supplemental Information

Sentiment Analysis of Conservation Studies Captures Successes of Species Reintroductions

Kyle S. Van Houtan, Tyler Gagne, Clinton N. Jenkins, and Lucas Joppa

SUPPLEMENTAL INFORMATION FOR

"SENTIMENT ANALYSIS OF CONSERVATION LITERATURE CAPTURES SPECIES REINTRODUCTION SUCCESSES"

BY KYLE S. VAN HOUTAN, TYLER O. GAGNE, CLINTON N. JENKINS, AND LUCAS JOPPA

Enclosed in the complete supplemental files are a series of figures (Figures S1-S6). The raw data scrape from the Web of Science service (Data S1), the results of each sentiment analysis (SA) and the ensemble score (Data S2), and the polarity breakdown of all abstracts (Data S3) are available as separate files.The figures in this supplement provide additional detail and supporting information to explain or contextualize the methods, results and discussion in the main text file.

Figure S1 for example calculates the total number of abstracts in each annual bin across the study. This helps to explain both the variability of sentiment in the early years, as well as, the popularity of the subject in the literature over the last decade. Figure S2 plots a simple histogram indicating the sentiment of all abstracts in the study lean towards positive sentiment. Figure S3 provides some transparency on a common metric called TF*IDF used in NLP. To emphasize infrequent terms over time, we broke this down in the multi-year bins. Though we do not employ this local density method in our study (preferring the more straightforward term frequency in Figure 1a) we report on it here as experts in the field may have interest. Figure S4 is a composite plot for each model of sentiment time series that highlights the trends over time and the differences between word- and sentence-level models. The word-based lexicons largely agree with one another, while the RNN (sentence-level) model generates a different set of results. As we average and report the results all of the models, the aim of our study is to provide the broad patterns that emerge from the multi-model ensemble.

Figures S5-S6 are a subset of ten abstracts tagged with either positive (S5) or negative sentiment (S6) determined by the Syuzhet lexicon. A complete graphic containing all 4,313 abstracts is too large to display here (1,771 pages) and is available as a separate downloadable and searchable file (Data S3). Though the Syuzhet lexicon scores individual words, both figures visually highlight the sentence-level average score as either positive (light green) or negative (pink) and provide the abstract's total sentiment score. Sentences in these abstracts with neither a significant positive or negative sentiment are not highlighted and have no coloration. It is important to note in our analyses that the SA models do not necessarily model the success of a reintroduction program, as the abstracts themselves do not explicitly model the success of a reintroduction program. As mentioned in the main text, our analysis therefore is more about positive or negative sentiment in published abstracts that nominally mention and therefore are associated with reintroduction programs.

Figure S5ba (sentiment = +0.9) is a case study that examines the habitat available and site suitability for a giant panda reserve in Huayingshan, China. The abstract reports sufficient food resources and community support for the project and suggest post release monitoring is feasible, however, several factors bound the authors' optimism. Figure S5b (sentiment = +1.1) narrates the ultimately successful history of golden lion tamarin reintroductions in the coastal Atlantic Forest in Brazil. Though the wild population of tamarins and their habitat reached historic bottlenecks in the 1970s, coordinated efforts to protect habitat and repurpose zoo collections for captive breeding demonstrate the value of public campaigns for conservation. Figure S5c (sentiment = +1.1) profiles the availability and uncertainty surrounding habitat for lynx reintroductions in Europe. The study provides an iterative plan for this process, even in the face of limited information, and concludes that habitat connectivity must be considered to identify release locations. Figure S5d (sentiment = +1.4) uses population viability analyses, or PVA, to evaluate reestablishing fisher populations in the western USA. The models trained with empirical data provide six practical recommendation for managers seeking to

successfully reintroduce carnivore populations. This abstract overall has a positive sentiment, though certain sentence scores are negative due to the sentiment of words therein (e.g., *endangered*, *threatened*, *failure*). Figure S5e is the highest scoring abstract in our complete set (sentiment = +2.6) due at least in part to the consistent use of the term *excellence* throughout. The study is conceptual and recommends a modification of evidence based conservation approaches so that they more closely follow established practices in the business settings to ensure practical program success.

Switching to examples of negative sentiment, figure S6a (sentiment = -1.6) develops a screening tool for a highly infectious viral disease in parrots and uses this to detect the disease in a translocated parakeet population in New Zealand. The abstract is almost entirely negatively coded, as it describes the various pathologies of the disease and its population impacts. Figure S6b (sentiment = -0.9) is a review of the impacts of alien introductions into wild amphibian populations and argues this underscores the need for more stringent genetics considerations in species translocation programs. Figure S6c (sentiment = -0.7) profiles conflicts between human and saltwater crocodile populations in Sri Lanka. The survey includes crocodile attacks to humans and vice versa quantifies the various threats humans pose to wild saltwater crocodile populations. The authors propose a monitoring scheme to keep track of the multiple and seemingly entrenched struggles. Figure S6d (sentiment = -0.4) discusses the low genetic diversity of Mojave desert tortoises and how management practices may have chronically and inadvertently exacerbated the problem. Figure S6e (sentiment = -0.3) is a review of two decades of mortality cases of reintroduced California condors. The abstract overwhelmingly identifies human factors as the leading causes of death, and then argues that these are imminently manageable and must be corrected to attain self-sustaining condor populations in the wild.

These abstracts represent some transparency into how the SA models encode sentiment to scientific texts, and the full diagram of all such polarity plots is available in the attached file (polarity_of_abstracts.pdf). The abstracts highlighted are chosen to represent a variety of sentiment scores as well as to capture some of the well-known species and issues that are in part represented in Figure 2. The abstracts in Figure S5-6 highlight some of the strengths as well as growth areas of SA as applied here. These are primarily rooted in that specific words used in the abstracts may generate sentiment scores independent of their contextual use or their correlation to objective quantitative metrics in the studies. The negatively-encoded sentences in Figure S5d, for example, likely happened due to the usage of *endangered*, *threatened*, *failure*, and *contracted* even though those sentences could be read either as having neutral or slightly positive sentiment.

This highlights the possibility that at the word, sentence, or even abstract level there may be disagreement between the SA model outputs and trained experts in the field. The point of such automated analyses is that such discrepancies are not a factor at the aggregate level, when large volumes of words and sentences are analyzed. Here we analyzed 4,313 abstracts comprising 1,030,558 words. At a reasonable pace of scientific reading – 100 words per minute – this would take 172 hours (over four, 40-hour work weeks) just to read the entire corpus. That time is just for the reading of the texts themselves without any additional manual encoding, attribution or subsequent thought or analysis on the texts. Here we ran five separate models, analysing all the words and abstracts in the corpus, in just a few hours using open access software that ran on an above-average laptop computer.

Manual analysis by trained experts will remain the gold standard for reading and extracting knowledge from scientific articles. However, there remain lessons to be learned from automation, and furthermore, the scale of the published literature suggests that such innovative methods for reviewing the scientific literature are needed. Having used lexicons and models developed for a general domain, at the present stage we are attempting to show the potential for automating and batch-processing the process of meta-analyses of large volumes of scientific texts. Further advances will be made not in

isolation from trained experts in specific topical domains, but with models supervised and trained for the specific questions of interest and importantly curated by experts to perform specific tasks. The role that NLP in general and SA specifically can play will be greatly enhanced by domain-specific lexicons.

FIGURE CAPTIONS

Figure S1. Abstract count over time shows a steady increase in the volume of published studies in the reintroduction literature. Though our raw Web of Science^{TM} query resulted in 4,759 results, we went through a filtering process which resulted in a cleaned database of 4,313 abstracts published from 1988-2016. As reintroduction becomes a more established technique, and subsequently as scientists published studies on their results, the volume of literature increases annually roughly on a log-linear scale. From 2011 onwards, more than 300 papers satisfying our query are published annually. However, the cumulative sample did not reach a total of 300 abstracts until 1995, and there were fewer than 10 studies each year before 1991. As having a large body of sample texts for interpretation is a key for NLP processes, all sentiment analysis results in early years should be viewed in light of the small sample of scientific literature in this initial period.

Figure S2. Histogram of pooled abstract sentiment across the entire study. Ensemble sentiment scores from the multi-model average of all n = 4,313 abstract assessed in this study. The form of the data follow a normal distribution (ave = 0.261, sd = 0.428). This indicates that most abstracts have a positive sentiment, perhaps owing to the general scientific bias to present positive (and not negative) study results. Data are accumulated in 0.1 bins.

Figure S3. The term frequency - inverse document frequency (TF*IDF) metric across time for the 4,313 scientific abstracts reviewed. TF*IDF is one widely-used metric in information theory to capture the significance of terms by, specifically elevating rare word occurrences, across the entire body of text being analyzed. Within the time bins here, the application of the metric in our setting seems to capture a variety of scientific (e.g., *nervosa*, *bakeri*, *garderi*) and common species names (e.g., palila, char, Blackburn's) as well as taxonomic groups (e.g., moth, snakes, tigers). Habitat settings are less prominent (e.g., *Kryvyi*, *Yampa*, *Yakima*), though also significant. This method does not capture broad use across all abstracts, but rather local clusters or occasional spikes in usage, sometimes considered key phrases. We do not employ this metric in our analyses here, mainly as it does not capture terms of value that would be relevant for lexicon training or applicable to SA here.

Figure S4. Calculated sentiment from four lexicons, one RNN model, and their ensemble average. The sentiment trends of individual abstracts converges among the four lexicon approaches (AFINN, Bing, NRC, Syuzhet) characterized by an initial steep decline from 1987-1990, followed by a subsequent increase until 2016. These lexicon approaches label the sentiment of each word, regardless of how they are arranged in a sentence. The Stanford coreNLP is an RNN that calculates sentiment at the sentence-level, considering syntax in addition to individual word scores. This approach values word context, such that positive words like *funny* and *witty* may together be used in phrase which has negative sentiment – e.g. "This movie was actually neither that funny, nor witty." Unlike the word-based lexicons, this particular RNN approach, scores the abstracts as having negative sentiment throughout the study period, as well as, declining from the outset. This may highlight word usage in a sentence context, and the need for domain specific lexicons and more supervised training particularly for RNN models. The initial high score variability (and later relative low variability) for the ensemble (Figure 1b) is likely a function of the sample size through time (Figure S1), which should be heavily weighed when interpreting the trends. Nonetheless, sentiment over time gradually increases, especially after 2000. The Stanford coreNLP model, suggests a flatter trend from 1990 to 2016.

Figure S5. Examples from a range of labelled abstracts from the corpus with positive sentiment determined from the Syuzhet lexicon. A complete file with all scored abstracts is available as a separate file. Here the Syuzhet lexicon scores individual words, and we plot the average word score at the sentence level. Positive sentiment is highlighted in green, negative sentiment is red, neutral sentiment has no color. The abstract number and the abstract-level sentiment are listed in the upper left.

Figure S6. Examples from a range of labelled abstracts from the corpus with negative sentiment determined from the Syuzhet lexicon. As stated for in Figure S4, a complete file with all scored abstracts is available as a separate file. Here the Syuzhet lexicon scores individual words, and we plot the average word score at the sentence level. Positive sentiment is highlighted in green, negative sentiment is red, neutral sentiment has no color. The abstract number and the abstract-level sentiment are listed in the upper left.

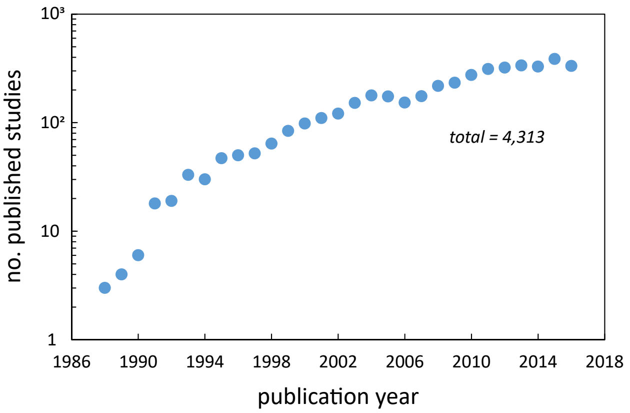

no. published studies
publication year
total = 4,313
10^3
10^2
10
1
1986
1990
1994
1998
2002
2006
2010
2014
2018

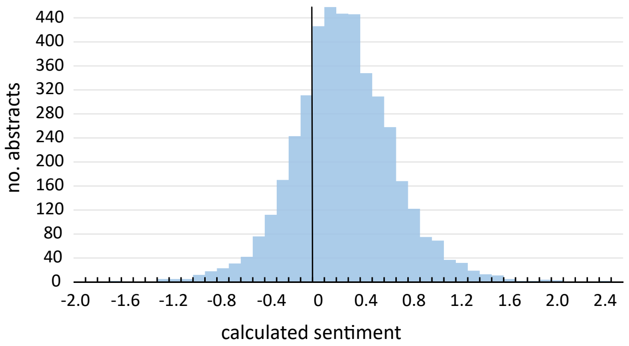

440
400
360
320
280
240
200
160
120
80
40
0
no. abstracts
-2.0
-1.6
-1.2
-0.8
-0.4
0
0.4
0.8
1.2
1.6
2.0
2.4
calculated sentiment

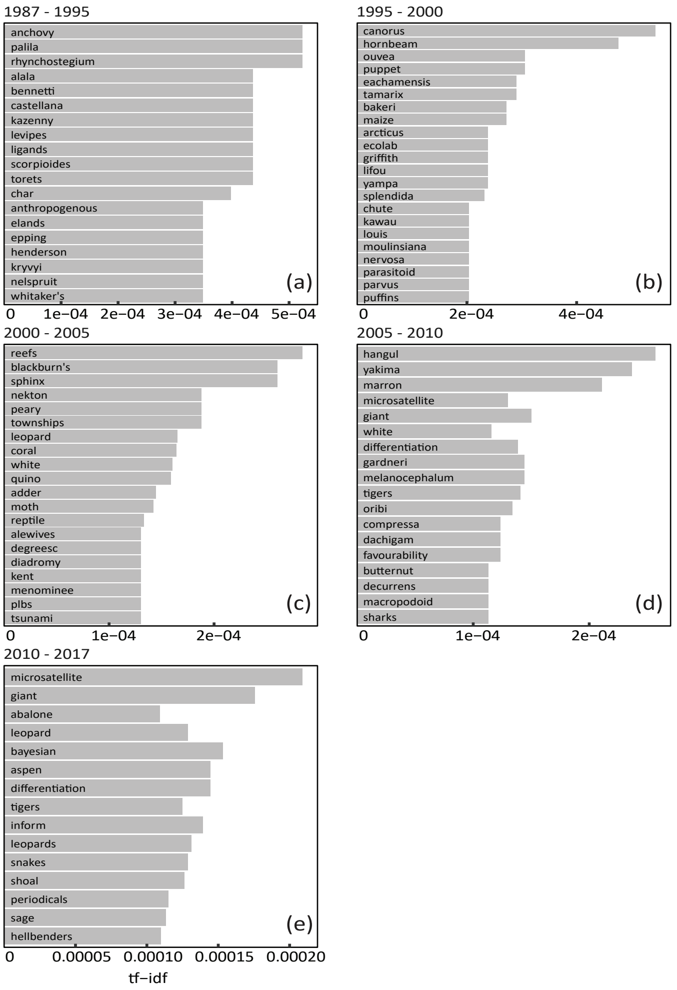
1987 - 1995
anchovy
palila
rhynchostegium
alala
bennetti
castellana
kazenny
levipes
ligands
scorpioides
torets
char
anthropogenous
elands
epping
henderson
kryvyi
nelspruit
whitaker's
(a)
0
1e−04
2e−04
3e−04
4e−04
5e−04
1995 - 2000
canorus
hornbeam
ouvea
puppet
eachamensis
tamarix
bakeri
maize
arcticus
ecolab
griffith
lifou
yampa
splendida
chute
kawau
louis
moulinsiana
nervosa
parasitoid
parvus
puffins
(b)
0
2e−04
4e−04
2000 - 2005
reefs
blackburn's
sphinx
nekton
peary
townships
leopard
coral
white
quino
adder
moth
reptile
alewives
degreesc
diadromy
kent
menominee
plbs
tsunami
(c)
0
1e−04
2e−04
2005 - 2010
hangul
yakima
marron
microsatellite
giant
white
differentiation
gardneri
melanocephalum
tigers
oribi
compressa
dachigam
favourability
butternut
decurrens
macropodoid
sharks
(d)
0
1e−04
2e−04
2010 - 2017
microsatellite
giant
abalone
leopard
bayesian
aspen
differentiation
tigers
inform
leopards
snakes
shoal
periodicals
sage
hellbenders
(e)
0
0.00005
0.00010
0.00015
0.00020
tf−idf

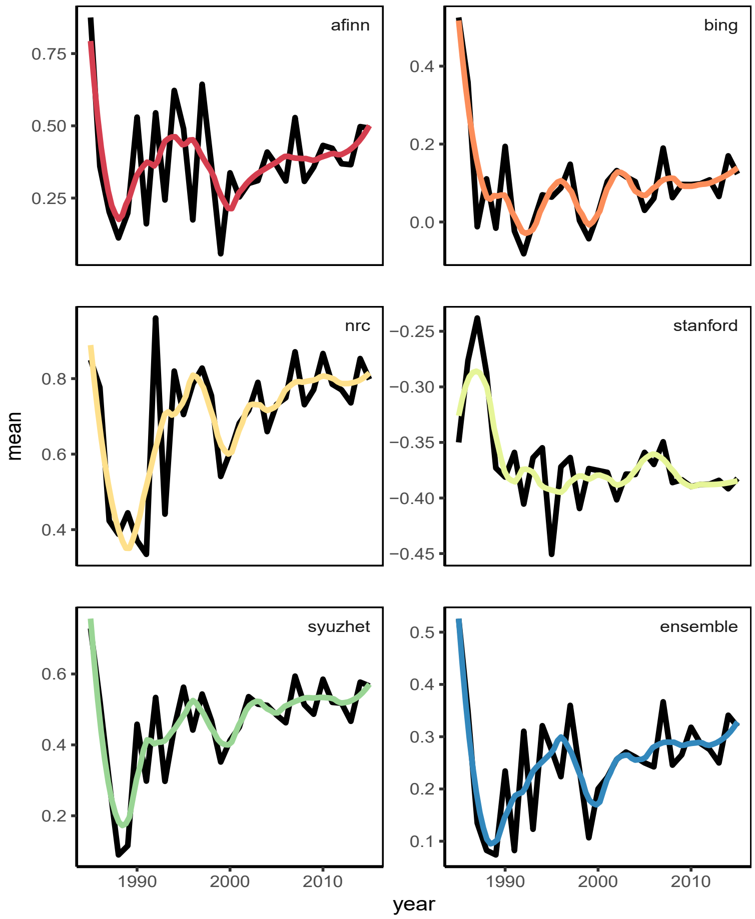

afinn
bing
nrc
stanford
syuzhet
ensemble
0.75
0.50
0.25
0.4
0.2
0.0
0.8
0.6
0.4
−0.25
−0.30
−0.35
−0.40
−0.45
0.6
0.4
0.2
0.5
0.4
0.3
0.2
0.1
1990
2000
2010
mean
year

A - Abstract #3850, score 0.9

Ex-situ conservation is one of the most important approaches to save the endangered species. When the population size of captive giant panda increase steadily and can be sustained by themselves, some of them should be released to the wild to rescue small populations and enlarge the distribution areas. Importantly, the first step is to find a suitable habitat for them. Huayingshan is the historical distribution area of giant panda and the fossil of A. melanoleuca baconi was found in 1993. Plentiful food resources (530 kiloton bamboos at present with 99 kiloton net gain per year) for giant panda was observed in this area which are enough for a small population. The government and villagers greatly support the project that sending giant panda back to Huayingshan, and the gentler slope of which will make monitoring work easier to perform. However, the human activity's interference was significant in this area, and some places are far from water resources, the vegetation type is less, and infection rate of the canine parvovirus was high. These questions can be solved after the reserve was built. In another side, the temperature is high in summer, and the qualities of habitats are different from the giant panda's natural habitat, in addition to the undetected impacts on the releasing of giant panda several works (e.g. wireless monitor) should be taken throughout all steps of the reintroduction to promote the giant panda's inhabit in Huayingshan.

B - Abstract #357, score 1.1

The lion tamarin was first described in the wild over 475 years ago, and there are records of the species being kept as pets as early as the mid-1750s. With such a long captive history, it is a sad reflection on our knowledge that, as recently as the early 1970s, so little success had been recorded with the maintenance and husbandry of Leontopithecus in captivity and the species seemed certain to disappear from zoos as well as from the wild. After a ban on exportation of the species from Brazil, the captive population of L. rosalia outside of Brazil decreased from a total of 102 in 1968 to 82 in 1976. Zoos rarely bred a second generation, and with no prospects of further imports from the wild, by 1977, the likelihood of the captive population becoming genetically unviable and going extinct was high. Fortunately, thanks to the development of a professionally coordinated and scientifically managed captive population and the research efforts of many individuals and institutions, both in Brazil and elsewhere, the future of the captive population has been ensured. The international efforts to save the golden lion tamarin through the collaborative work of the GLTCP represents a classic example of what can be achieved when various disciplines working for the conservation of a species cooperate and coordinate their activities in pursuit of a common goal. The majority of authorities now recognize that the future of animals in captivity will increasingly rely on national and international cooperation; much of this will depend on the integrity and goodwill of the people concerned. The development of international strategies to safeguard species survival increasingly relies on field workers, conservationists, educationists, academics and zoo professionals cooperating wholeheartedly. As KLEIMAN et al. (1986) have suggested, such cross-institutional collaborative efforts with L. rosalia represent a model for international conservation projects in the future. Since the June, 1990 Leontopithecus Population Viability Analysis (PVA) Workshop held in Belo Horizonte, Minas Gerais, Brazil (IUCN/SSC/CBSG 1991), the four International Management Committees for the Lion Tamarin genus have been recognised by Federal Brazilian law as technical advisors to IBAMA on conservation, research, and management of both the captive and wild populations of Leontopithecus. Nearly 100 per cent of the tamarins that come under the committees' jurisdiction are the property of the Brazilian people through IBAMA; this transfer of title to IBAMA involved a renunciation of ownership of specimens housed at zoos internationally. For L. rosalia, ownership was turned over in 1991. We believe that this may be the first case of the international zoo community returning ownership of legally-held animals to the country of origin while continuing to maintain responsibility for their management. With the reintroduction programme for L. rosalia having resulted in an increase of protected Atlantic coastal forest, in the State of Rio de Janeiro, by about 38 per cent (2,300 ha). And with our growing understanding of the science of conservation today, the significance of the GLTCP's work with this endangered species (RYLANDS) highlights how well the adoption of 'flagship' species and publication of the plight of remnant populations in depleted environments can promote public attention and associated habitat.

C - Abstract #980, score 1.1

Conservation biologists often must make management decisions based on little empirical information. In Germany biologists are concerned that the recovery and reintroduction of Eurasian lynx (Lynx lynx) may fail because the remaining suitable habitat may be insufficient to sustain a viable population. However, no comprehensive study ad dressing this concern has been made that not only considers distribution of suitable habitat, but also connectivity to other populations. The aims of this study were (1) to quantify the amount and location of potentially suitable lynx habitat in. Germany, (2) to estimate the connectivity between patches of suitable habitat, and (3) to evaluate lynx conservation programs. Habitat preferences of lynx were described in a rule-based model based on the availability of forest cover (defined by patch size) and the spatial structure of the habitat. Rules were implemented in a geographic information system to predict locations of suitable habitat. Optimal connections among patches were modeled using a cost-path analysis based on. habitat-specific probabilities of lynx crossing patches. Results indicated wide variation in the size of patches of suitable habitat, with 10 areas each sufficiently large to sustain >20 resident lynxes. Overall, a total of 380 lynxes could be sustained by the 10 areas. Uncertainty analyses of model parameters and assumptions revealed little variation in predicted habitat, primarily because results were constrained by the actual distribution of forest habitat. Our. analyses suggest that lynx reintroduction programs should emphasize large, connected areas and consider broad-scale habitat connectivity in the landscape. Our approach also demonstrates how biologically plausible rules can be applied in conservation to identify areas in which success is most likely, even when few empirical data are available.

D - Abstract #3141, score 1.4

Translocations are frequently used to restore extirpated carnivore populations. Understanding the factors that influence translocation success is important because carnivore translocations can be time consuming, expensive, and controversial. Using population viability software, we modeled reintroductions of the fisher, a candidate for endangered or threatened status in the Pacific states of the US. Our model predicts that the most important factor influencing successful reestablishment of a fisher population is the number of adult females reintroduced (provided some males are also released). Data from 38 translocations of fishers in North America, including 30 reintroductions, 5 augmentations and 3 introductions, show that the number of females released was, indeed, a good predictor of success but that the number of males released, geographic region and proximity of the source population to the release site were also important predictors. The contradiction between model and data regarding males may relate to the assumption in the model that all males are equally good breeders. We hypothesize that many males may need to be released to insure a sufficient number of good breeders are included, probably large males. Seventy-seven percent of reintroductions with known outcomes (success or failure) succeeded; all 5 augmentations succeeded; but none of the 3 introductions succeeded. Reintroductions were instrumental in reestablishing fisher populations within their historical range and expanding the range from its most-contracted state (43% of the historical range) to its current state (68% of the historical range). To increase the likelihood of translocation success, we recommend that managers: 1) release as many fishers as possible, 2) release more females than males (55-60% females) when possible, 3) release as many adults as possible, especially large males, 4) release fishers from a nearby source population, 5) conduct a formal feasibility assessment, and 6) develop a comprehensive implementation plan that includes an active monitoring program.

E - Abstract #2658, score 2.6

The current shortfall in effectiveness within conservation biology is illustrated by increasing interest in "evidence-based conservation," whose proponents have identified the need to benchmark conservation initiatives against actions that lead to proven positive effects. The effectiveness of conservation policies, approaches, and evaluation is under increasing scrutiny, and in these areas models of excellence used in business could prove valuable. Typically, conservation programs require years of effort and involve rigorous long-term implementation processes. Successful balance of long-term efforts alongside the achievement of short-term goals is often compromised by management or budgetary constraints, a situation also common in commercial businesses. "Business excellence" is an approach many companies have used over the past 20 years to ensure continued success. Various business excellence evaluations have been promoted that include concepts that could be adapted and applied in conservation programs. We describe a conservation excellence model that shows how scientific processes and results can be aligned with financial and organizational measures of success. We applied the model to two well-documented species conservation programs. In the first, the Po'ouli program, several aspects of improvement were identified, such as more authority for decision making in the field and better integration of habitat management and population recovery processes. The second example, the black-footed ferret program, could have benefited from leadership effort to reduce bureaucracy and to encourage use of best-practice species recovery approaches. The conservation excellence model enables greater clarity in goal setting, more-effective identification of job roles within programs, better links between technical approaches and measures of biological success, and more-effective use of resources. The model could improve evaluation of a conservation program's effectiveness and may be used to compare different programs, for example during reviews of project performance by sponsoring organizations.

A - Abstract #2191, score -1.5

Psittacine beak and feather disease (PBFD) is a highly infectious and potentially fatal viral disease of parrots and their allies caused by the beak and feather disease virus (BFDV). Abnormal feather morphology and loss of feathers are common clinical symptoms of the disease. PBFD also damages the lymphoid tissue and affected birds may die as a result of secondary bacterial or fungal infections. The disease is therefore of concern for conservation biologists and wildlife managers, as it is immunosuppressive and can become an additional threatening factor among critically endangered psittacines. We conducted a PCR-based screening for BFDV in a wild population of the Red-fronted Parakeet (Cyanoramphus novaezelandiae) on Little Barrier Island, New Zealand, during a translocation of this species. Fifty-four parakeets were captured and feather samples collected for molecular screening. We detected BFDV DNA from 15 individuals, but only two showed external signs attributable to PBFD, namely abnormal feather morphology or colouration, loss of feathers and haemorrhagic feathers. Our survey represents the first positive identification of BFDV in wild New Zealand endemic psittacines and confirms the risk of spread of the virus between wild populations within this global hotspot of endemic psittacine diversity.

B - Abstract #1707, score -0.9

Although less than other animal groups, amphibians are sometimes concerned by the problems related to the introduction of alien specimens into natural populations. They may be victims of such introductions (especially of amphibians, fishes and other aquatic predators), or cause problems to other species through introduction outside their range. The problems posed by introductions, reintroductions and population reinforcements are discussed in a more general way. Introductions of alien species outside their range (faunistic pollution), or of alien specimens into other populations of the same species or of another interfertile species (genetic pollution), beside creating ecological problems, hinder or impede subsequent study of the history and evolution of these populations. For evolutionary biologists, they amount to a destruction of their object of study. Furthermore, such operations carry an optimistic but misleading message to the public, according to which destructions of the environment caused by human activities would be reversible. It is urgent that the main concepts of genetics and taxonomy be given more weight in decisions regarding reintroductions of animals into threatened populations or habitats.

C - Abstract #4177, score -0.7

Human-wildlife conflict occurs when human requirements encroach on those of wildlife populations, with potential costs to both humans and wild animals. As top predators in most inland waters, crocodilians are involved in human-wildlife conflicts in many countries. Here we present findings of a 5-year survey on human-crocodile conflict on the island of Sri Lanka and relate the results to improving management practices. We aimed to quantify and understand the causes of human-crocodile conflict in Sri Lanka, and propose solutions to mitigate it. Visual encounter surveys were carried out to estimate the population size of Saltwater Crocodiles. We recorded 778 sightings of Saltwater Crocodiles at 262 of 400 locations surveyed, and estimate the total population to comprise more than 2000 non-hatchlings and to have increased at an average rate of 5% p.a. since 1978. We propose four crocodile vigilance zones within the wet zone and one crocodile vigilance zone within the dry zone of the country. Specific threats to Saltwater Crocodiles identified in crocodile vigilance zones were: habitat destruction and loss; illegal killing and harvesting (17 killings out of fear, (similar to)200 incidents of killing for meat and skins, (similar to)800 eggs annually for consumption); unplanned translocations; and, interaction with urbanization (10 incidents of crocodiles being run over by trains/vehicles and electrocution). Additionally, 33 cases of crocodile attacks on humans were recorded [8 fatal, 25 nonfatal (minor to grievous injuries)] and more than 50 incidents of attacks on farm and pet animals.

D - Abstract #3887, score -0.4

Mojave desert tortoises (*Gopherus agassizii*) have been translocated for decades, and research-oriented translocations recently have been recommended as a tool to help recover this threatened species. However, avoiding negative genetic impacts from wildlife translocations has been widely cautioned. Populations of the Mojave desert tortoise within a 200-276-km straight-line radius of each other (249-308 km measured around topographic barriers) tend to be genetically correlated and may be considered single genetic units for management purposes. When planning translocations among wild populations, releasing tortoises at recipient sites within a straight-line distance of 200 km from the source population would most conservatively maintain historic genetic population structure. However, the risk of causing outbreeding depression by inadvertently translocating Mojave desert tortoises between more distant populations or those of unknown provenance is low.

E - Abstract #3016, score -0.3

We document causes of death in free-ranging California Condors (Gymnogyps californianus) from the inception of the reintroduction program in 1992 through December 2009 to identify current and historic mortality factors that might interfere with establishment of self-sustaining populations in the wild. A total of 135 deaths occurred from October 1992 (the first post-release death) through December 2009, from a maximum population-at-risk of 352 birds, for a cumulative crude mortality rate of 38%. A definitive cause of death was determined for 76 of the 98 submitted cases, 70% (53/76) of which were attributed to anthropogenic causes. Trash ingestion was the most important mortality factor in nestlings (proportional mortality rate [PMR] 73%; 8/11.), while lead toxicosis was the most important factor in juveniles (PMR 26%; 13/50) and adults (PM R 67%; 10/15). These results demonstrate that the leading causes of death at all California Condor release sites are anthropogenic. The mortality factors thought to be important in the decline of the historic California Condor population, particularly lead poisoning, remain the most important documented mortality factors today. Without effective mitigation, these factors can be expected to have the same effects on the sustainability of the wild populations as they have in the past.
